# Genome-Wide Analysis of Long Non-Coding RNAs in Potato and Their Potential Role in Tuber Sprouting Process

**DOI:** 10.3390/ijms19010101

**Published:** 2017-12-29

**Authors:** Xiaodong Hou, Yongmei Du, Xinmin Liu, Hongbo Zhang, Yanhua Liu, Ning Yan, Zhongfeng Zhang

**Affiliations:** Tobacco Research Institute of Chinese Academy of Agricultural Sciences, Qingdao 266101, China; houxiaodong@caas.cn (X.H.); duyongmei@caas.cn (Y.D.); liuxinmin@caas.cn (X.L.); zhanghongbo@caas.cn (H.Z.); liuyanhua@caas.cn (Y.L.); zhangzhongfeng@caas.cn (Z.Z.)

**Keywords:** *Solanum tuberosum* L., tuber, sprouting, morphology, lncRNAs

## Abstract

Sprouting is a key factor affecting the quality of potato tubers. The present study aimed to compare the differential expression of long non-coding RNAs (lncRNAs) in the apical meristem during the dormancy release and sprouting stages by using lncRNA sequencing. Microscopic observations and Gene Ontology (GO) and Kyoto Encyclopedia of Genes and Genomes (KEGG) enrichment analyses revealed the changes in the morphology and expression of lncRNAs in potato tubers during sprouting. Meristematic cells of potato tuber apical buds divided continuously and exhibited vegetative cone bulging and vascularisation. In all, 3175 lncRNAs were identified from the apical buds of potato tubers, among which 383 lncRNAs were up-regulated and 340 were down-regulated during sprouting. The GO enrichment analysis revealed that sprouting mainly influenced the expression of lncRNAs related to the cellular components of potato apical buds (e.g., cytoplasm and organelles) and cellular metabolic processes. The KEGG enrichment analysis also showed significant enrichment of specific metabolic pathways. In addition, 386 differentially expressed lncRNAs during sprouting were identified as putative targets of 235 potato miRNAs. Quantitative real-time polymerase chain reaction results agreed with the sequencing data. Our study provides the first systematic study of numerous lncRNAs involved in the potato tuber sprouting process and lays the foundation for further studies to elucidate their precise functions.

## 1. Introduction

Potato (*Solanum tuberosum* L.), a perennial herbaceous plant with edible tubers, belongs to the Solanaceae family [1]. Potato is the fourth most important food crop worldwide, after rice, wheat, and corn, and an important raw material source for food and industrial processing [2,3]. Potato tubers contain large quantities of starch, which is the main energy source of edible potatoes, and their proteins have high nutritional quality, since they are easily digested and absorbed [4]. The tubers also contain 18 amino acids, including essential ones that the human body cannot synthesize, and various vitamins and minerals, which have positive effects on human growth, development, and metabolism. Furthermore, potato tubers are rich in dietary fibre and potassium and represent an alkaline food that is beneficial to human health [4].

Sprouting is the key factor affecting the quality of potato tubers. First, sprouting results in high respiration rate and depletion of potato tuber compounds such as starch, proteins, and vitamins, and it leads to the accelerated loss of water, which results in lower tuber quality and mass [5]. Second, sprouting causes a significant increase in the contents of glycoalkaloids (e.g., α-solanine and α-chaconine), which affect potato tubers’ palatability and food safety [6,7]. When the contents of glycoalkaloids in potato tubers reach 0.10–0.15 mg/g fresh weight (FW), obvious bitterness is noted, and levels exceeding 0.2 mg/g FW cause food poisoning in humans and animals that consume the tubers [8,9]. Recently, Liu et al. [10] found that superoxide anion and hydrogen peroxide accumulated in tuber buds, and that antioxidant compounds and enzymes showed important changes during potato tuber sprouting.

Various techniques and methods have been used to identify differentially expressed genes in potato tubers from dormancy to sprouting. For instance, Bachem et al. [11] used amplified fragment length polymorphism-based transcript profiling to analyze transcript-derived cDNAs during potato tuber dormancy and sprouting. Kloosterman et al. [12] used expressed sequence tags generated using large-scale sequencing technologies to identify genes related to potato dormancy and sprouting. Liu et al. [13] used the suppression subtractive hybridization approach to identify differentially expressed genes related to dormancy release in potato tubers. Liu et al. [2] investigated gene expression changes during the dormancy release of potato tubers by using expressed mRNAs associated with dormancy, dormancy release, and sprouting tubers obtained from Illumina^®^ RNA sequencing technologies. Campbell et al. [14,15,16] also assessed the transcriptional changes in potato meristems during various stages of dormancy or dormancy release. The aforementioned studies mainly focused on coding mRNAs from dormancy to sprouting, and only a few of them considered the non-coding RNAs associated with potato sprouting.

Unlike mRNAs, long non-coding RNAs (lncRNAs; greater than 200 nt in length) are widely present in mammals and plants, do not contain evident open reading frames (ORFs), and do not code for proteins [17,18]. However, lncRNAs often possess structural characteristics of mRNA, including the cap structure and poly-A tail [17]. Initially, lncRNAs were identified as a class of “hidden substances” that did not attract substantial attention from scientists and were considered noise associated with gene transcription that was of no biological significance [18]. However, in recent years, several studies have confirmed the involvement of lncRNAs in DNA methylation, histone modification, chromatin remodelling, and other biological processes. Furthermore, these molecules have been shown to interact with DNA, RNA, and proteins to regulate the expression of target genes [19]. Notably, lncRNAs were found to play an important role in rice sexual reproduction [20], cotton fibre development [21], and chickpea flower development [22]. Therefore, lncRNAs are critical to the regulation of plant growth and development [23].

Although various techniques and methods have been used to identify differentially expressed genes in potato tubers from dormancy to sprouting [2,11,12,13], lncRNA sequencing has not been used to reveal the role of these molecules in potato tuber sprouting. Therefore, the present study aimed to compare the differential expression of lncRNAs in the apical meristem of potato dormant tubers (DTs) and sprouting tubers (STs) by using lncRNA sequencing. Gene Ontology (GO) and Kyoto Encyclopedia of Genes and Genomes (KEGG) enrichment analyses were then performed on the differentially expressed lncRNAs to identify their possible functions in dormancy and sprouting. Our study provides the first systematic study of numerous lncRNAs involved in the potato tuber sprouting process and lays the foundation for further studies to elucidate their precise functions.

## 2. Results

### 2.1. Observation and Identification of Potato Morphological Changes during Tuber Dormancy Release

**Figure 1:**
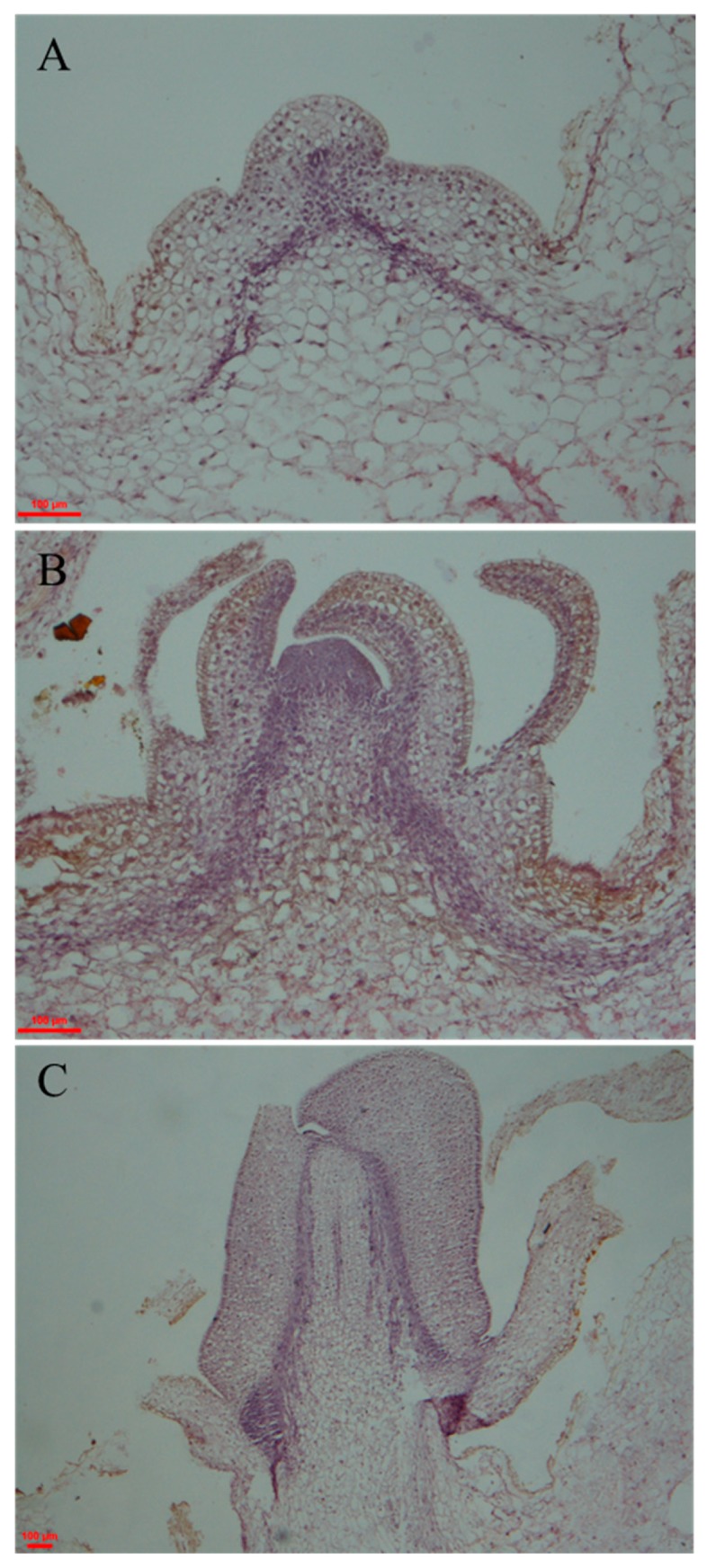
Observation and identification of the morphological changes in potato dormant tubers (**A**), dormancy release tubers (**B**), and sprouting tubers (**C**). Scale bar = 100 μm.

The right phase (DT, dormancy release tuber (DRT), and ST) of potato tuber was confirmed by monitoring the apical meristem by observing paraffin sections under a microscope (Figure 1). The apical meristem of DTs consists of (from outside-in) periderm, epidermis, cortex, vascular cambium, and pith. The periderm contains 2–3 layers of epidermal cells underneath, which are small, irregular in shape, and tightly packed. Parenchyma cells of the cortex and pith have a larger size, and the cortex and pith are separated by 3–4 layers of vascular cambium cells (Figure 1A). The meristematic cells of the apical buds of DRTs divide continuously, causing the flat vegetative cone to bulge. Furthermore, the nuclei of the cortical parenchyma cells in the vicinity are associated with an enlarged vascular cambium and dense cytoplasm. As the bud primordial cells continue to differentiate, their nucleoli disappear, and they elongate and differentiate into tracheary elements. As the apical bud grows, adjacent tracheary elements join to form xylem, which extends into the bud (Figure 1B). When the apical bud reaches 2–3 mm in length, the tuber is defined as having sprouted (Figure 1C).

### 2.2. Quality Assessment of Sequence Data

The distribution of error rates in the sequencing process reflects the quality of the sequencing data [24]. Generally, the sequencing error rate of a single base should be less than 1%. In the present study, the sequencing error rates for DTs (DT_1, DT_2, and DT_3) and sprouting tubers (ST_1, ST_2, and ST_3) were 0.03%, 0.02%, and 0.02%, and 0.02%, 0.02%, and 0.02%, respectively (Table 1), which are far below 1%. Detailed data quality assessments of the sequencing output are shown in Table 1. The quality of subsequent data analysis was ensured by filtering raw DT and ST reads to remove reads containing adapters and poly-N regions and those having low quality. Thus, clean DT_1, DT_2, and DT_3 reads were 12.19 G, 14.18 G, and 14.09 G, respectively, and clean ST_1, ST_2, and ST_3 reads were 13.29 G, 15.25 G, and 13.06 G, respectively, suggesting that clean bases obtained from DT and ST sequencing were all above 12.00 G.

### 2.3. Sequence Alignment

The TopHat2 [25] algorithm was used to perform a comparative analysis of the filtered reads against the reference genome to compare the whole read segments to genome exons, and then read segments were separately aligned to two exons from the genome. Detailed statistics associated with the alignment of reads to the reference genome are shown in Table 2. Notably, the number of proper-paired reads mapped to different chromosomes was zero for both DT and ST samples.

### 2.4. Characteristic Features of Potato LncRNAs

**Figure 2:**
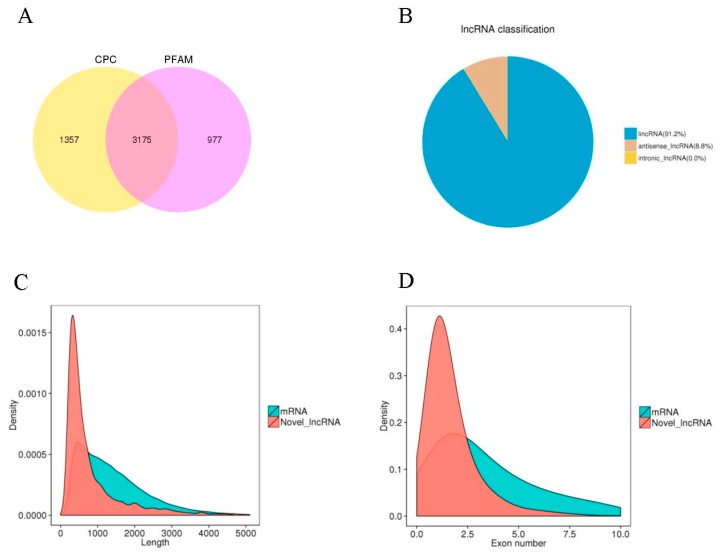
Features of potato lncRNAs and comparative analysis with mRNAs: (**A**) lncRNA screening results; (**B**) lncRNA classification; (**C**) length distribution of lncRNA and mRNA transcripts; (**D**) distribution of exon numbers in lncRNA and mRNA transcripts.

The results of transcriptome assembly and the structural characteristics of lncRNAs and their functional feature of not encoding proteins were used to establish strict screening criteria, including selection of transcripts with exon numbers, ≥2; lengths, >200 bp; and Fragments Per Kilobase of transcript per Million fragments mapped (FPKM), ≥0.5. Furthermore, transcripts with overlaps within the annotated exon regions of the database were removed. Two potential coding analysis methods, Coding Potential Calculator (CPC) and protein families database (PFAM) were jointly used to screen the assembled transcripts. The intersection of the transcripts lacking coding potential based on both methods was selected as the projected lncRNA database for this analysis [26]. In this study, the non-coding transcripts identified using CPC and PFAM were statistically counted and plotted in a Venn diagram, and the intersection of CPC and PFAM prediction results was used as the projected lncRNA database in this analysis (Figure 2A). In all, 3175 transcripts were identified from the apical buds of potato tubers (Supplementary Table S1) and designated as LNC_000001 to LNC_003175 (Supplementary Data 1). After all the screening steps, statistical analyses were mainly performed on the following three types of lncRNAs: long intergenic lncRNAs (lincRNAs), anti-sense_lncRNAs, and intronic_lncRNAs. The results indicated that there were 2897 lincRNAs (91.2%), 278 anti-sense_lncRNAs (8.8%), and 0 intronic_lncRNAs (Figure 2B).

The lengths of potato lncRNAs ranged from 201 to 20,818 bp, with the vast majority (90%) having lengths shorter than 2000 bp. The average length of potato lncRNAs was 895 bp (Figure 2C), which was significantly higher than that reported for rice (800 bp) and chickpeas (614 bp) [20,22]. In addition, the average length of potato lncRNAs was lower than that of mRNAs (Figure 2C). In this study, 2038 potato lncRNAs had one exon; 628, two exons; 249, three exons; and 260, between four and eighteen exons. The exon numbers associated with potato lncRNAs were lower than those associated with mRNAs (Figure 2D).

### 2.5. Analysis of Differentially Expressed LncRNAs

**Figure 3:**
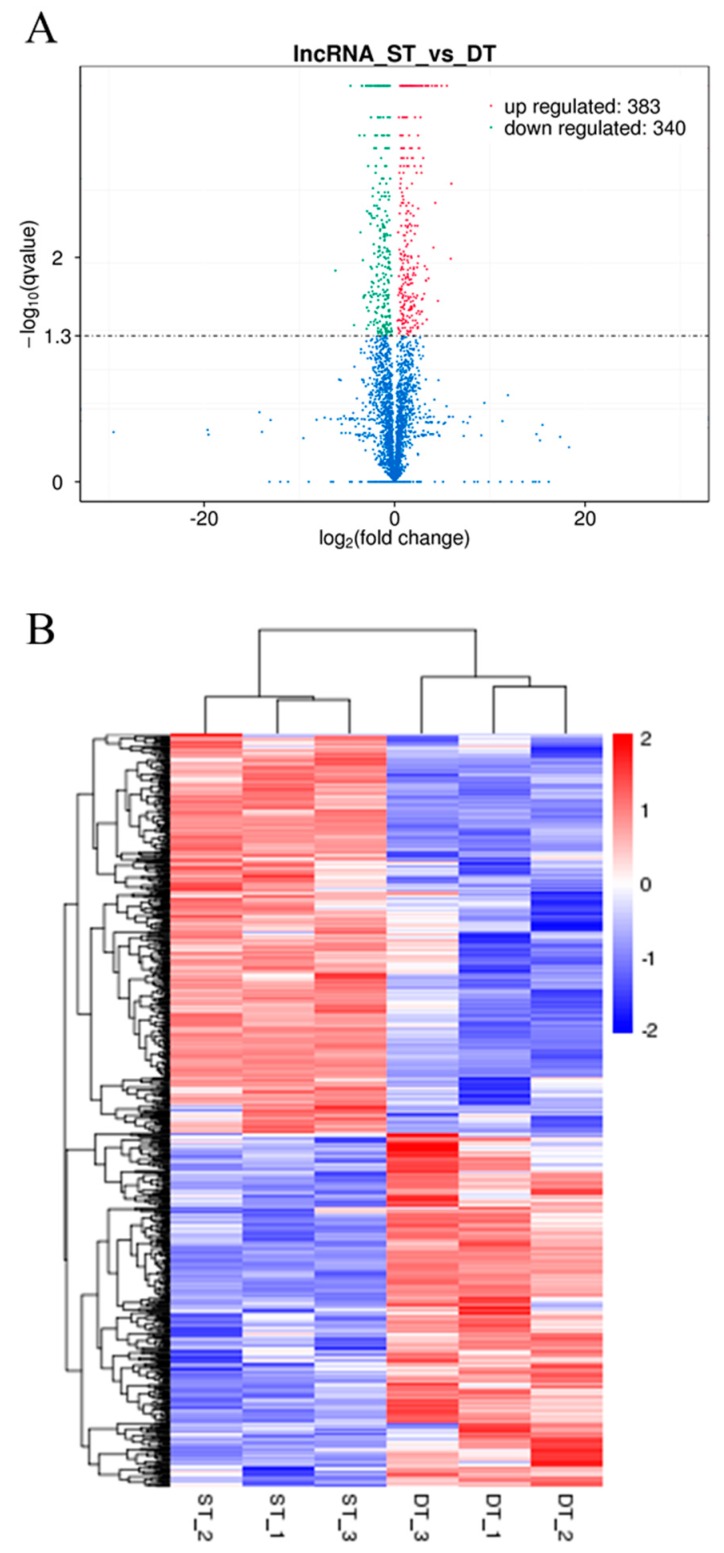
Differentially expressed lncRNAs between potato dormant tubers (DTs) and sprouting tubers (STs). (**A**) Volcano plot of differentially expressed lncRNAs between DT and ST samples. The mean expression values of log_10_ (adjusted *p* value (*q* value)) are plotted along the ordinate, and the log_2_ (fold change) values are plotted along the abscissa. Red and green dots represent significantly upregulated and downregulated lncRNAs, respectively. (**B**) Hierarchical clustering analysis of differentially expressed lncRNAs between DT and ST samples. The colour scale indicates the log_10_ (FPKM + 1) values. Red and blue indicate high and low expression of lncRNAs, respectively.

The FPKM considers the effects of both sequencing depth and gene length when determining the fragment count, and this is currently the most commonly used method for the estimation of gene expression levels [27]. After potato lncRNAs were screened, we performed quantitative analysis on the selected transcripts by using Cuffdiff software [28]. In order to explore the possible role of lncRNAs in potato tuber sprouting, we used lncRNA sequencing to investigate the expression patterns of lncRNAs in the DT and ST samples and generated a volcano plot revealing the overall distribution of differentially expressed lncRNAs (Figure 3A). Compared to the levels observed in DT samples, 12.1% (383 of 3175) and 10.7% (340 of 3175) of the lncRNAs were upregulated and downregulated in ST samples, respectively (Figure 3A; Supplementary Table S2).

Clustering analysis was used to determine the expression patterns of differentially expressed transcripts under various experimental conditions. Clustering of genes with identical or similar expression patterns led to the identification of the function of unknown transcripts and new functions of known transcripts, because the same class of transcripts might possess similar functions or participate in the same metabolic process or cellular pathway [27]. In the present study, hierarchical clustering analysis was performed using the log_10_(FPKM + 1) value of differentially expressed lncRNAs in potato DT and ST samples, with red indicating high lncRNA expression levels, blue indicating low levels, and a transition from red to blue indicating high to low expression level changes (Figure 3B). The results of the hierarchical cluster analysis of 723 differentially expressed lncRNAs between potato DT and ST samples were shown in Figure 3B. We predicted the transcription factor binding to the promoter region of these differentially expressed lncRNAs. The hierarchical cluster analysis results of these predicted transcription factors were shown in Supplementary Figure S1. Meanwhile, a series of transcription factors were predicted to bind to the promoters of differentially expressed lncRNAs target genes (Supplementary Table S3), the hierarchical cluster analysis results of which were shown in Supplementary Figure S2.

Compared to the levels observed in DT samples, 5248 and 4999 genes were upregulated and downregulated in ST samples, respectively (Supplementary Figure S2A). The results of the hierarchical cluster analysis of 10,247 differentially expressed genes in potato DT and ST samples were shown in Supplementary Figure S3B. In this study, 10,427 differentially expressed genes between DT and ST samples were divided into 8 subclusters (1113, 2153, 2097, 2631, 1563, 434, 86, and 170 genes) via hierarchical clustering (Supplementary Figure S3B).

### 2.6. GO Enrichment of Differentially Expressed LncRNAs

The GO enrichment analysis is based on the Wallenius non-central hyper-geometric distribution, which has different probabilities for selecting an individual from a category and another from outside that category, compared to the ordinary hyper-geometric distribution. This difference in probability is estimated based on the preference associated with the length of a gene, thereby allowing the accurate calculation of the probability of a GO term enriched by differentially expressed lncRNAs [29]. Because lncRNAs function through the regulation of mRNAs, we can predict their biological functions based on the co-location and co-expression relationships with protein-coding genes. In this study, the mRNAs in potato DT and ST samples that were co-located and co-expressed with the differentially expressed lncRNAs were subjected to GO enrichment analysis. The 15 significantly enriched GO terms associated with differentially expressed lncRNAs between potato DT and ST samples are shown in Table 3. In short, GO enrichment analysis revealed that sprouting mainly leads to changes in the expression of lncRNAs associated with cellular components of potato apical buds (e.g., cytoplasm and organelles) as well as biological processes such as cellular metabolic processes.

### 2.7. KEGG Functional Enrichment of Differentially Expressed LncRNAs

**Figure 4:**
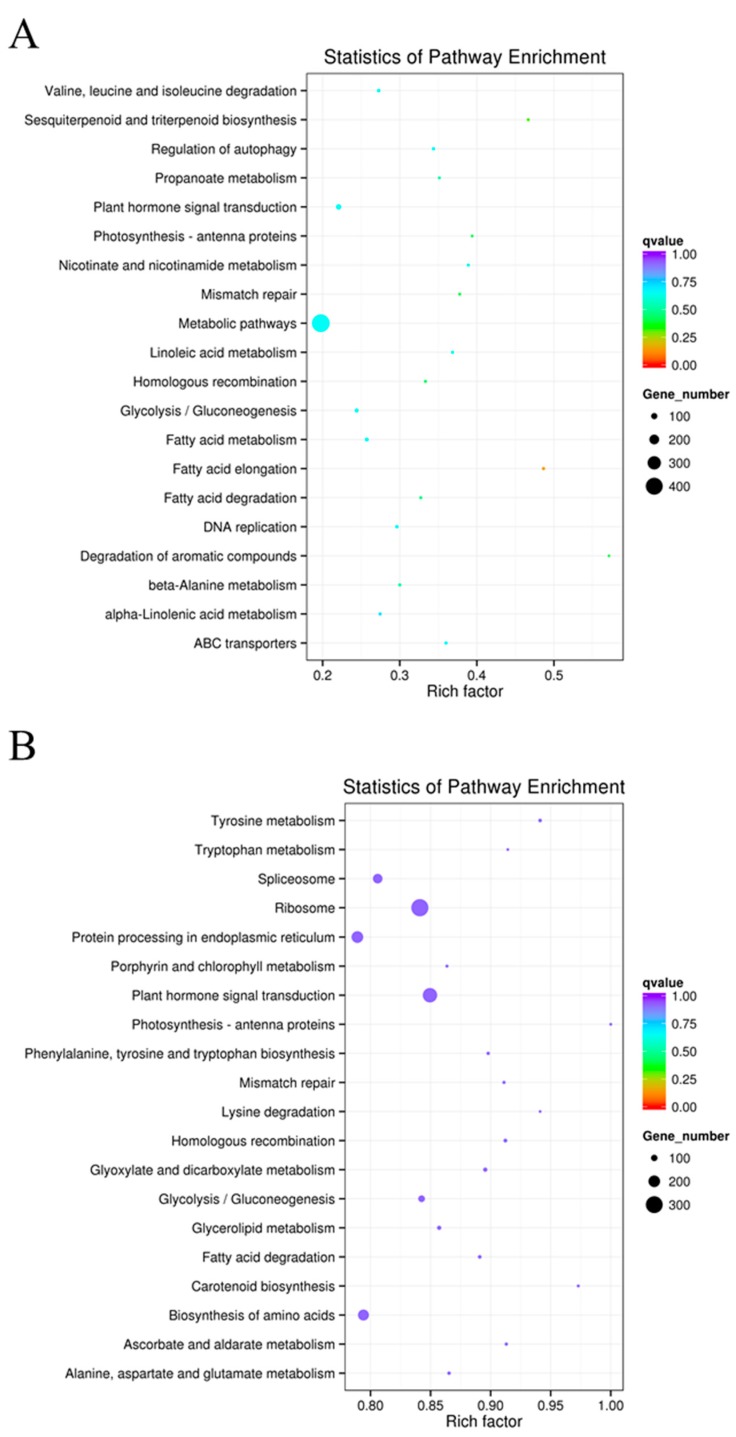
Statistics of Kyoto Encyclopedia of Genes and Genomes (KEGG) pathway enrichment analysis of the mRNAs co-located (**A**) and co-expressed (**B**) with differentially expressed lncRNAs between potato dormant tubers (DTs) and sprouting tubers (STs). The KEGG pathways are plotted along the ordinate, and the enrichment factor (rich factor) is plotted along the abscissa. The colours of points represent *q* values, and the sizes of points represent the number of mRNAs related to differentially expressed lncRNAs mapped to the reference pathway. Legends for the *q* value colour scale and the size scaling of the number of mRNAs related to differentially expressed lncRNAs are shown on the right side of the plot.

Different genes coordinate in vivo to perform their biological functions. The significant enrichment of specific genes in pathways can thus be used to identify the major biochemical metabolic and signal transduction pathways in which they participate. The KEGG public database focuses on genomes, biological pathways, etc. [30] and, in the present study, the mRNAs co-located and co-expressed with differentially expressed lncRNAs in potato DT and ST samples were subjected to a KEGG enrichment analysis (Figure 4). The degree of KEGG enrichment is measured by rich factor, *q* values, and the number of genes enriched in the pathway. Among these, the rich factor is the ratio between the number of differentially expressed lncRNAs and all genes annotated in the pathway. The greater the rich factor, the greater is the degree of enrichment. The *q* value is the corrected *p* value after multiple rounds of hypothesis testing, and it ranges between 0 and 1. The closer this value is to zero, the more significant is the enrichment.

In this study, we selected the following 20 pathways with the greatest degree of enrichment of the mRNAs co-located with differentially expressed lncRNAs: ‘fatty acid elongation’ (18); ‘sesquiterpenoid and triterpenoid biosynthesis’ (14); ‘mismatch repair’ (17); ‘degradation of aromatic compounds’ (8); ‘photosynthesis-antenna proteins’ (13); ‘homologous recombination’ (19); ‘fatty acid degradation’ (18); ‘β-Alanine metabolism’ (21); ‘propanoate metabolism’ (13); ‘regulation of autophagy’ (11); ‘DNA replication’ (16); ‘ABC transporters’ (9); ‘fatty acid metabolism’ (27); ‘nicotinate and nicotinamide metabolism’ (7); ‘metabolic pathways’ (409); ‘valine, leucine, and isoleucine degradation’ (18); ‘linoleic acid metabolism’ (7); ‘glycolysis/guconeogenesis’ (31); ‘plant hormone signal transduction’ (66); and ‘α-Linolenic acid metabolism’ (14) (Figure 4A). Furthermore, we selected the following 20 pathways with the greatest degree of enrichment of the mRNAs co-expressed with differentially expressed lncRNAs: ‘ribosome’ (307); ‘plant hormone signal transduction’ (254); ‘photosynthesis-antenna proteins’ (33); ‘tyrosine metabolism’ (48); ‘carotenoid biosynthesis’ (36); ‘homologous recombination’ (52); ‘glyoxylate and dicarboxylate metabolism’ (60); ‘glycolysis/gluconeogenesis’ (107); ‘ascorbate and aldarate metabolism’ (42); ‘lysine degradation’ (32); ‘mismatch repair’ (41); ‘fatty acid degradation’ (49); ‘phenylalanine, tyrosine, and tryptophan biosynthesis’ (44); ‘tryptophan metabolism’ (32); ‘glycerolipid metabolism’ (60); ‘spliceosome’ (162); ‘alanine, aspartate, and glutamate metabolism’ (45); ‘biosynthesis of amino acids’ (189); ‘protein processing in endoplasmic reticulum’ (202); and ‘porphyrin and chlorophyll metabolism’ (38) (Figure 4B). In particular, the following metabolic pathways appeared in both Figure 4A,B: ‘plant hormone signal transduction’; ‘photosynthesis-antenna proteins’; ‘fatty acid degradation’; ‘homologous recombination’; ‘mismatch repair’; and ‘glycolysis/gluconeogenesis’.

### 2.8. LncRNAs as Potential Regulators of miRNAs

**Figure 5:**
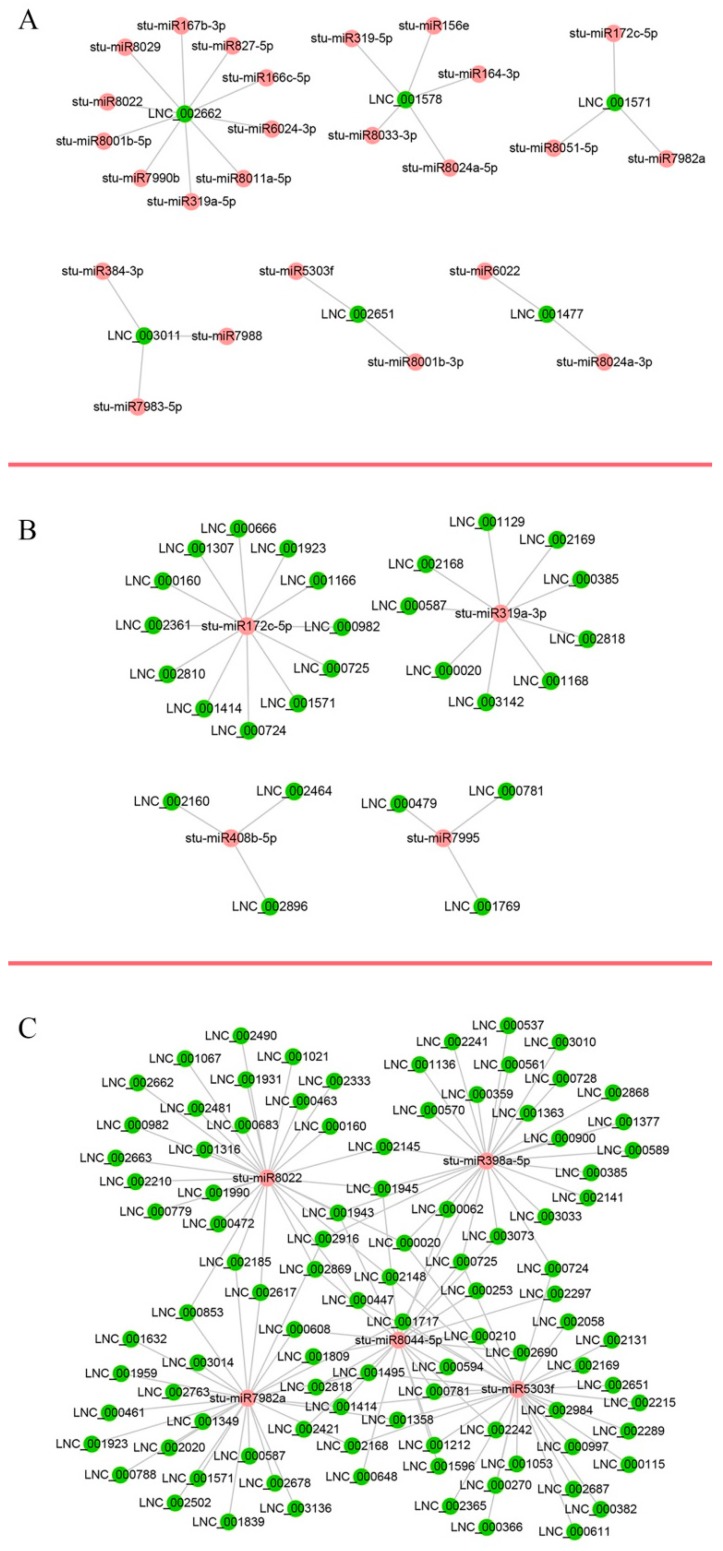
LncRNA-miRNA interaction network. The network includes lncRNAs and miRNAs. Red circle nodes represent miRNAs, and green circle nodes represent lncRNAs. Examples of one lncRNA to many miRNAs (**A**), many lncRNAs to one miRNA (**B**), and many lncRNAs to many miRNAs (**C**) interactions are shown.

The lncRNAs interact with miRNAs to regulate biological processes such as plant growth, development, and reproduction [20,21,22]. In this study, differentially expressed lncRNAs during potato tuber sprouting were used for miRNA target sites by using psRNATarget [31]. In all, 386 differentially expressed lncRNAs during sprouting were identified as putative targets of 235 potato miRNAs (Supplementary Table S4). Multiple interaction patterns such as one lncRNA to many miRNAs (Figure 5A), many lncRNAs to one miRNA (Figure 5B), and many lncRNAs to many miRNAs (Figure 5C) were recognised. This resulted in a total of 2306 lncRNA-miRNA pairs (Supplementary Table S4). The majority of miRNAs involved with lncRNAs were from miR156, miR171, miR172, miR1886, miR319, miR482, miR5303, miR7984, miR8007, and miR8011 (Supplementary Table S4).

### 2.9. Validation of the Differentially Expressed LncRNAs by Using Quantitative Real-Time PCR

**Figure 6:**
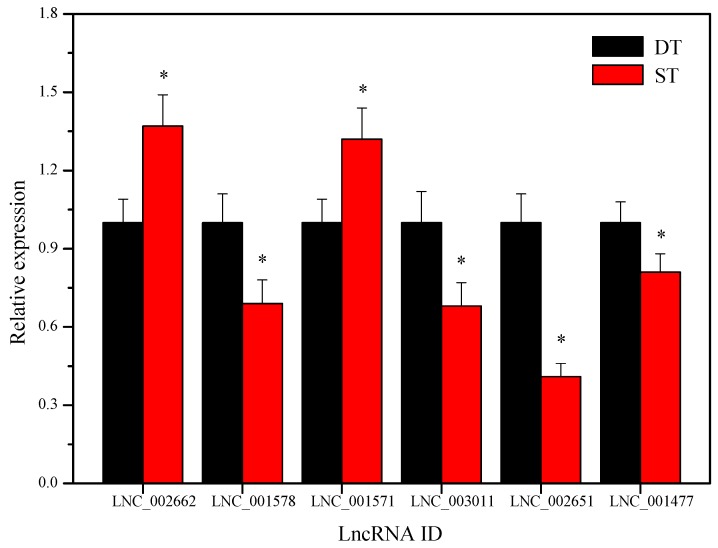
The verification of the expression level of the six differentially expressed lncRNAs (LNC_002662, LNC_001578, LNC_001571, LNC_003011, LNC_002651, and LNC_001477) between potato dormant tubers (DTs) and sprouting tubers (STs) by using qRT-PCR. The qRT-PCR data are represented as the mean ± standard deviation (SD). Columns with the asterisk (*) are significantly different at *p* < 0.05.

To validate the differentially expressed lncRNAs during potato tuber sprouting, we selected six lncRNAs (LNC_002662, LNC_001578, LNC_001571, LNC_003011, LNC_002651, and LNC_001477) shown in Figure 5A for quantitative real-time PCR (qRT-PCR) (Figure 6). The expression levels of LNC_002662 and LNC_001571 were higher in ST than in DT (Figure 6). However, the expression levels of LNC_001578, LNC_003011, LNC_002651, and LNC_001477 were lower in ST than in DT (Figure 6). These results are consistent with the lncRNA sequencing results, which revealed the higher expression of LNC_001578, LNC_003011, LNC_002651, and LNC_001477 in DT than in ST (Supplementary Table S5). Thus, differential expression analyses results coincided with lncRNA sequencing results, providing a reliable validation for the lncRNA sequencing data.

## 3. Discussion

### 3.1. Differentiation and Development of Potato Bud Primordia

From the date of harvest, life activities such as metabolism and synthesis are not completely dormant in potato tubers [32]. Upon dormancy release and bud sprouting, the apical meristematic cells of potato tubers start to divide, and the flat vegetative cone begins to bulge. The location of meristematic cells as well as their morphology is altered. The cells first become increasingly elongated from the top down, and they gradually become rectangular until apical buds form (Figure 1). As the apical meristematic cells of potatoes divide, the cortex parenchyma cells form xylem via de-differentiation and programmed cell death [33]. The xylem takes up water and inorganic nutrients from the cortex and pith to fulfil the growth needs of the buds. When the apical buds of potato tubers reach 2 mm in length (Figure 1C), the dormancy stage is considered finished, and the tubers are called sprouting tubers [34].

### 3.2. Effects of Sprouting on LncRNA Expression in Potato Tubers

The structural and functional characteristics of lncRNAs suggested that the 3175 lncRNAs identified from the apical buds of potato tubers included 2897 lincRNAs and 278 anti-sense_lncRNAs (Figure 2), which exceeded the 1113 lincRNAs previously identified from potato stems [35]. In the present study, sprouting induced differential expression in 723 lncRNAs, including 383 up- and 340 downregulated lncRNAs (Figure 3). The GO enrichment analysis indicated that sprouting mainly leads to the changes in the expression of lncRNAs that are associated with the cellular components of potato apical buds (e.g., cytoplasm and organelles) as well as biological processes such as cellular metabolic processes (Table 3). The differentially expressed cell cycle associated genes had been observed during various stages of dormancy or dormancy release in potato tubers [14,15,16]. The KEGG enrichment analysis of differentially expressed mRNAs showed that sprouting mainly changed the following metabolic pathways: ‘ribosome’; ‘photosynthesis-antenna proteins’; ‘ascorbate and aldarate metabolism’; ‘porphyrin and chlorophyll metabolism’; ‘zeatin biosynthesis’; ‘glycerolipid metabolism’; ‘spliceosome’; ‘biosynthesis of amino acids’; ‘glyoxylate and dicarboxylate metabolism’; ‘arginine and proline metabolism’; ‘plant hormone signal transduction’; ‘alanine, aspartate, and glutamate metabolism’; ‘protein processing in endoplasmic reticulum’; ‘phenylalanine, tyrosine, and tryptophan biosynthesis’; ‘carotenoid biosynthesis’; ‘glycerophospholipid metabolism’; ‘steroid biosynthesis’; and ‘limonene and pinene degradation’. However, glycoalkaloids biosynthesis was not significantly changed during the dormancy release of potato tubers, which might due to that the potato tubers used in this study were stored away from light [6,7]. Liu et al. [2] showed similar results since they found that, during the dormancy release of potato tubers, the genes with significantly altered expression were mainly involved in carbohydrate, protein, lipid, and phytohormone metabolism; cell division/cycle; and photosynthesis activation. Unlike GO and KEGG enrichment analyses in the present study, the MapMan pathway and PageMan programs were used to explore the putative functions of the differentially expressed genes identified by RNA-seq in the study of Liu et al. [2]. Liu et al. [2,3] analyzed the transcriptomic and proteomic changes during the dormancy release of potato tubers; however, the present study investigated the changes in the expression of lncRNAs occurring between dormancy and sprouting. A major challenge in deducing lncRNAs function resides in the fact that these molecules do not encode proteins [36]. Gene expression, specifically stage-specific expression, may help reveal the potential function of lncRNAs. Pairwise comparisons between DTs and STs revealed that 22.8% (723 of 3175) of the lncRNAs were differentially expressed and highly stage specific (Figure 3), suggesting that these molecules are subject to active transcriptional regulation. Most notably, many lncRNAs, such as LNC_002733, LNC_002475, and LNC_002579 (Supplementary Table S2), were highly and specifically expressed in STs (Figure 3). The precise regulation of lncRNAs in STs suggested these might play important functions during sprouting in potato tubers.

Plant lncRNAs are crucial in a wide range of biological processes participating in various mechanisms [37]. The interest and attention that plant researchers have focused on lncRNAs is largely due to the relationship found between lncRNAs and flowering. Flowering Locus C (FLC) is a key protein that regulates vernalisation in plants. In recent years, at least two classes of lncRNAs have been pointed out to be involved in the regulation of the *FLC* gene. In *Arabidopsis* sp., the first class of lncRNAs is the FLC antisense transcripts NAT lncRNA COOLAIR and the second class is the lncRNA COLDAIR sense transcripts, which originating from a region within the first intron of *FLC* [38,39]. The expression of COOLAIR results in the removal of activated histone-methylation markers on *FLC* by attracting relevant proteins, thus silencing the *FLC* sense transcript; COLDAIR results in the repressive histone methylation of *FLC* chromatin by binding with PcG protein complexes, leading to the silencing of *FLC*. Thus, both classes of lncRNAs participate in the regulation of vernalisation through *FLC* silencing, and affect the transition from vegetative to reproductive development and flowering time [38,39]. In maize, the lncRNA Zm401, which is specifically expressed in stamens, regulates key genes involved in anther development, namely Zm3-3, ZmMADS2, and ZmC5, thereby affecting the development of the tapetum, which provides nutrients for the growth and development of microspores. Therefore, the reduced expression of Zm401 results in male sterility in maize [40]. In Chinese cabbage, the lncRNA BcMF11 participates in the regulation of pollen development and stamen fertility. This lncRNA is expressed during all stages of pollen development, and its reduced expression results in delayed tapetal degeneration, which prevents microspores from reaching maturity. However, there is no impact on the vegetative growth phase [41,42]. In particular, lncRNAs were confirmed to play important roles in sexual reproduction in rice [20], cotton fibre development [21], and chickpea flower development [22]. Currently, the results of this study may form the basis for further investigations on the important regulatory role of lncRNAs in potato sprouting. Therefore, the differentially expressed lncRNAs in DT and ST samples might be candidate genes for genetic modification and target selection in potato breeding.

In our study, 386 lncRNAs that were differentially expressed during potato tuber sprouting were identified as putative targets of 235 potato miRNAs, such as one lncRNA for many miRNAs, many lncRNAs for one miRNA and many lncRNAs for many miRNAs (Supplementary Table S4; Figure 5). Wu et al. [43] predicted and validated numerous similar lncRNAs in *Arabidopsis* and *Oryza sativa* that bind to miR160, miR166, miR156, miR159 and miR172, individually. Similarly, Zhang et al. [20] predicted numerous similar lncRNAs in *O. sativa*, and two of them were proven to bind to miR160 and miR164, individually, thus playing a role in the regulation of reproductive development. LncRNAs regulate the growth and development of plants in a variety of ways [23] and future studies should elucidate the mechanisms of potato sprouting regulation by lncRNAs.

## 4. Materials and Methods

### 4.1. Plant Materials

The ‘Favorita’ potato cultivar (introduced from Holland in 1980) used in this study was grown in Ximen Village of Jiaoxi Town, Qingdao City, Shandong Province, China (altitude, 46.9 m; latitude, 36°14’26″ N; and longitude, 119°51’13″ E). The potatoes were planted on 13 July 2016, and tubers were harvested on 26 October 2016. The harvested tubers were stored for 15 days in an incubator at 12 °C (away from light) to allow full ripening and promote wound healing. Undamaged tubers of uniform size (5–6 cm in diameter) at consistent stages of maturity were stored in an incubator at 20 °C (away from light) to form dormancy released tubers. Fully mature tubers at the 0th day of dormancy release were defined as DTs, whereas those at the 45th day of dormancy release were defined as DRTs. Furthermore, those with bud lengths of 2–3 mm were designated as STs. Sixty tubers were used per sample. The DT, DRT, and ST designations were confirmed by observing paraffin sections under a microscope.

### 4.2. Observations of Potato Morphological Changes during Tuber Dormancy Release

#### 4.2.1. Dehydration, Transparency, Wax Dipping, and Embedding

1. Dehydration: Potato tuber apical buds fixed with FAA fixative (5% formaldehyde, 5% acetic acid, 45% ethanol, and 45% distilled water) were sequentially immersed for 90 min in 50, 70, 85, 95 and 100% ethanol to dehydrate and harden the samples for optimal paraffin infiltration.
2. Transparency: After a 30-min intermediate processing with anhydrous ethanol and xylene (volume ratio of 1:1), samples were processed with pure xylene for another 30 min to remove alcohol that infiltrated during the dehydration process and to facilitate wax dipping and embedding.
3. Wax dipping: Xylene was removed from the apical buds of potato tubers, and the tubers were then placed in 100% paraffin for 90 min in an incubator at 56–60 °C.
4. Embedding: The apical buds of potato tubers processed with 100% paraffin were embedded in cassettes by using a Leica embedding station (Leica EG 1160; Leica Biosystems, Nussloch, Germany) for sectioning.

#### 4.2.2. Sectioning and Staining

1. Sectioning: The wax blocks formed were trimmed with a blade and sectioned using a Leica RM 2145 microtome (Leica Biosystems) to a thickness of 8 μm. These sections were then placed on glass slides and heated for 60 min at 65 °C on a Leica HI 1220 slide warmer (Leica Biosystems).
2. Dewaxing: Slides were immersed in xylene twice for 50 min each for dewaxing, and then processed in decreasing ethanol concentrations (100, 95, 80, 50 and 0%) for 5 min each.
3. Staining: Slides were stained in haematoxylin for 8 min and rinsed with tap water for 5 min. They were subsequently immersed in and stained with eosin for another 2 min and rinsed with distilled water, following which they were subjected to rapid gradient dehydration in increasing ethanol concentrations (50, 80, 95 and 100%) for 2 min each. Samples were rendered transparent in xylene for 5 min.
4. Paraffin section sealing and microscopic imaging: Stained paraffin sections were preserved in neutral gum and organised before observation and imaging by using a Nikon Eclipse 80i microscope (Nikon Corporation, Tokyo, Japan).

### 4.3. RNA Isolation, Library Preparation, and Sequencing

#### 4.3.1. RNA Quantification and Qualification

Total RNA was extracted from DT and ST apical buds by using the methods described by Liu et al. [2]. RNA degradation and contamination were monitored on 1% agarose gels, and RNA purity was checked using a NanoPhotometer^®^ (IMPLEN, Westlake Village, CA, USA). RNA concentrations were measured using a Qubit^®^ RNA Assay Kit and Qubit^®^ 2.0 Fluorometer (Life Technologies, Carlsbad, CA, USA). RNA integrity was assessed using the RNA Nano 6000 Assay Kit and a Bioanalyzer 2100 system (Agilent Technologies, Santa Clara, CA, USA).

#### 4.3.2. Library Preparation for RNA Sequencing

Three micrograms of total RNA per sample was used as input material for RNA sample preparations. First, rRNA was removed using an Epicentre Ribo-zero™ rRNA Removal Kit (Epicentre, Madison, WI, USA), and rRNA-free residue was cleaned by ethanol precipitation. Sequencing libraries were then generated from rRNA-depleted RNA by using an NEBNext^®^ Ultra™ Directional RNA Library Prep Kit (NEB, Ipswich, MA, USA) for Illumina^®^ (Illumina Inc., San Diego, CA, USA) following manufacturer’s recommendations. Briefly, fragmentation was performed using divalent cations under elevated temperatures in NEBNext^®^ First Strand Synthesis Reaction Buffer (5×). First-strand cDNA was synthesised using random hexamer primers and M-MuLV Reverse Transcriptase (RNase H-; NEB). Second-strand cDNA synthesis was subsequently performed using DNA Polymerase I and RNase H (both from NEB). In the reaction buffer, dTTPs were replaced by dUTPs, and remaining overhangs were converted to blunt ends via exonuclease/polymerase activities. After the 3′ ends of DNA fragments were adenylated, an NEBNext^®^ Adaptor with a hairpin loop structure was ligated for hybridisation. Preferential cDNA fragments (approximately 150–200 bp in length) were selected by purifying library fragments by using the AMPure XP system (Beckman Coulter, Beverly, MA, USA). Next, 3 μL USER Enzyme (NEB) was combined with size-selected, adaptor-ligated cDNA at 37 °C for 15 min, followed by 5 min at 95 °C before performing PCRs by using Phusion High-Fidelity DNA polymerase, universal PCR primers, and index (X) primers (NEB). Finally, products were purified using the AMPure XP system, and library quality was assessed using the Agilent Bioanalyzer 2100 system.

#### 4.3.3. RNA Sequencing

The clustering of index-coded samples was performed on the cBot Cluster Generation System by using a TruSeq PE Cluster Kit v3-cBot-HS (Illumina Inc.) according to manufacturer’s instructions. After cluster generation, libraries were sequenced on an Illumina^®^ HiSeq 2500 platform, and 125 bp paired-end reads were generated.

### 4.4. Quality Control, Mapping to the Reference Genome, and Transcriptome Assembly

#### 4.4.1. Quality Control

Raw data (raw reads) in the FASTQ format were first processed using in-house Perl scripts, in which clean data (clean reads) were obtained by removing reads containing adapters and poly-N regions and those with low quality from the raw data. During this process, Phred quality scores of 20 (Q20) and 30 (Q30) and GC content values associated with the clean data were calculated. Thus, all downstream analyses were based on high-quality clean data.

#### 4.4.2. Mapping to the Reference Genome

Reference genome [44] and gene model annotation files were downloaded from ftp://ftp.ensemblgenomes.org/pub/release-23/plants/fasta/solanum_tuberosum/. Indices of the reference genome were constructed using Bowtie v2.0.6, and paired-end clean reads were aligned to the reference genome by using TopHat v2.0.9 [25].

#### 4.4.3. Transcriptome Assembly

The mapped reads of each sample were assembled using both Scripture (β2) [45] and Cufflinks (v2.1.1) [28] by using a reference-based approach. Scripture uses a statistical segmentation model to distinguish expressed loci from experimental noise and spliced reads to assemble expressed segments and reports all statistically expressed isoforms of a given locus [45]. Conversely, Cufflinks uses a probabilistic model to simultaneously assemble and quantify the expression levels of minimal isoform sets that provide a maximum likelihood explanation of the expression data at a given locus [26,28]. Scripture was run with default parameters, and Cufflinks was run with ‘min-frags-per-transfrag = 0’ and ‘--library-type’ parameters. Other parameters were set as default.

### 4.5. Coding Potential Analysis, Conservative Analysis, and Target Gene Prediction

#### 4.5.1. Coding Potential Analysis

CPC and PFAM-scans were used to predict transcripts; those with coding potential were filtered out and designated as the candidate lncRNA set. The CPC (0.9-r2) mainly assesses the extent and quality of ORFs in a transcript, and it searches sequences by using a known protein sequence database to distinguish coding and non-coding transcripts [46]. We used the NCBI eukaryote protein database and set the e-value to ‘1 × 10^−10^’ in our analysis. We translated each transcript for all three possible reading frames and used PFAM Scan (v1.3) to identify occurrences of known protein family domains documented in the PFAM database (release 27; used both PFAM A and PFAM B) [47]. Any transcript with a PFAM hit was excluded in the following steps, and PFAM searches used default parameters of ‘-E 0.001’ and ‘--domE 0.001’.

#### 4.5.2. Conservative Analysis

The Phast (v1.3) software package contains several statistical programs that are used in phylogenetic analysis [48], and phastCons uses conservation scoring and an identification program of conserved elements. We used phyloFit to compute phylogenetic models for conserved and non-conserved regions among species, and then applied the model and HMM transition parameters to phyloP to compute a set of conservation scores for lncRNAs and coding genes.

#### 4.5.3. Target Gene Prediction

The cis role refers to the acting of lncRNA on neighbouring target genes. We searched coding genes 100 kb upstream and downstream of lncRNAs, and then analysed their functions. The trans role refers to the identification of lncRNAs based on the expression levels. Although there were no more than 25 samples, we calculated the expressed correlation between lncRNAs and coding genes by using custom scripts.

### 4.6. Quantification of Gene Expression Levels and Differential Expression Analysis

#### 4.6.1. Quantification of Gene Expression Levels

Cuffdiff (v2.1.1) was used to calculate fragments per kilobase of exon per million fragments mapped (FPKM) of both lncRNAs and coding genes in each sample [28]. Gene FPKMs were computed by summing the FPKMs of transcripts in each gene group. FPKM was calculated based on the length of the fragments and read counts mapped to each fragment.

#### 4.6.2. Differential Expression Analysis

Cuffdiff provides statistical routines for determining differential expression of digital transcripts or gene expression data by using a model based on a negative binomial distribution [28]. For the analysis of biological repetition, we do not set the standard of the fold change value. In this study, the RNA sequencing experiments were performed in triplicate. Transcripts with adjusted *p* value (*q* value) of <0.05 were designated as differentially expressed.

### 4.7. Enrichment Analysis of Differentially Expressed LncRNAs

#### 4.7.1. GO Enrichment Analysis

GO enrichment analysis of differentially expressed lncRNA target genes was performed using the GOseq R package, in which gene length bias was corrected [29], and GO terms with corrected *p* values of <0.05 were considered significantly enriched by differentially expressed lncRNAs.

#### 4.7.2. KEGG Enrichment Analysis

The KEGG database [30] is a resource used to understand high-level functions and biological system utilities (e.g., those associated with the cell, organism, and ecosystem) based on molecular-level information, especially large-scale molecular datasets generated by genome sequencing and other high-throughput experimental technologies (http://www.genome.jp/kegg/). We used KOBAS software to test the statistical enrichment of differentially expressed lncRNAs.

### 4.8. Identification of Differentially Expressed LncRNAs as Targets of miRNAs

Differentially expressed lncRNAs during potato tuber sprouting were analysed for miRNA target sites by using psRNATarget webserver [31]. The potato miRNA sequences were those from Zhang et al. [49]. The miRNAs and differentially expressed lncRNAs were submitted to psRNATarget, and interaction was calculated using the following parameters: maximum expectation = 5 and allowed maximum energy to unpair the target site (UPE) = 25. LncRNA-miRNA interaction network was drawn using the Cytoscape software (Agilent Technologies Co., Santa Clara, CA, USA).

### 4.9. Validation of the Differentially Expressed LncRNAs by Using Quantitative Real-Time PCR

To validate the differentially expressed lncRNAs during potato tuber sprouting, we isolated total RNA from DT and ST apical buds. Total RNA samples were checked for quality and quantity as described previously [2]. Gene-specific primers were designed using Primer Express (Version 3.0) software, and qRT-PCR analysis was performed using an ABI 7500 Real-time system (Applied Biosystems, Foster City, CA, USA) by using three biological replicates and three technical replicates (for each biological replicate) of each sample as described. The following amplification program was used: 50 °C for 2 min, 95 °C for 10 min, 40 cycles at 95 °C for 15 s, followed by 60 °C for 1 min. The expression of elongation factor 1-a (EF1a) was used as internal control gene for normalisation [2]. The primer sequences used for qRT-PCR are shown in Supplementary Table S6.

### 4.10. Statistical Analysis

All experiments were repeated three times. Results of the qRT-PCR data were presented as the mean ± standard deviation (SD). Statistical analyses were performed using software SPSS 18.0 (SPSS Inc., Chicago, IL, USA). Differences were regarded as significant at *p* < 0.05.

### 4.11. GenBank Accession Code

The sequence data generated for this work are accessible via the NCBI Sequence Read Archive under accession number (SRA: SRP118158).

## 5. Conclusions

Sprouting is a key factor affecting the quality of potato tubers. This study revealed changes in the morphology and expression of lncRNAs during the sprouting of potato tubers. As apical meristematic cells continue to divide, a bulged vegetative cone and xylem are formed in apical buds. In all, 3175 lncRNAs were identified from the apical buds of potato tubers. Sprouting results in the up- and down-regulation of 383 and 340 lncRNAs, respectively, in the apical buds of potato tubers. The GO enrichment analysis revealed that sprouting mainly leads to changes in the expression of lncRNAs associated with cellular components of potato apical buds (e.g., cytoplasm and organelles) as well as biological processes such as cellular metabolic processes. In addition, 386 differentially expressed lncRNAs during sprouting were identified as putative targets of 235 potato miRNAs. However, the experimental validation of the biological functions of these lncRNAs and their roles in potato tuber sprouting requires further confirmation. Nevertheless, this study might provide an extensive resource for lncRNAs in potato and allow further studies to gain insights into the regulatory aspects of potato sprouting. Further experiments focused on individual lncRNAs are needed to elucidate their exact function.

## Figures and Tables

**Table 1:** Summary of potato dormant tubers (DTs) and sprouting tubers (STs) transcriptome sequencing results.

Sample Name ^a^	Raw Reads	Clean Reads	Clean Bases (G)	Error Rate (%)	Q20 (%) ^b^	Q30 (%) ^c^	GC Content (%)
DT_1	88,474,758	81,270,030	12.19	0.03	95.28	88.53	44.91
DT_2	101,834,712	94,506,478	14.18	0.02	97.04	92.28	46.02
DT_3	98,802,096	93,941,306	14.09	0.02	96.85	91.91	44.12
ST_1	93,598,076	87,083,084	13.06	0.02	97.04	92.49	45.34
ST_2	95,140,804	88,600,856	13.29	0.02	97.19	92.80	45.39
ST_3	109,587,878	101,669,792	15.25	0.02	95.98	90.32	45.13

^a^ DT_1, DT_2, and DT_3 are dormant tuber samples. ST_1, ST_2, and ST_3 are sprouting tuber samples; ^b^ Q20 corresponds to a base calling accuracy of 99.0%; ^c^ Q30 corresponds to a base calling accuracy of 99.9%.

**Table 2:** Sequence alignment results of reads mapped to the reference genome.

Sample Name	DT_1	DT_2	DT_3	ST_1	ST_2	ST_3
Total reads ^a^	81,270,030	94,506,478	93,941,306	87,083,084	88,600,856	101,669,792
Total mapped ^b^	38,206,584 (47.01%)	44,961,000 (47.57%)	48,397,116 (51.52%)	44,253,083 (50.82%)	44,551,383 (50.28%)	49,531,693 (48.72%)
Multiple mapped ^c^	1,803,952 (2.22%)	2,019,548 (2.14%)	2,434,047 (2.59%)	2,227,833 (2.56%)	2,004,069 (2.26%)	1,967,805 (1.94%)
Uniquely mapped ^d^	36,402,632 (44.79%)	42,941,452 (45.44%)	45,963,069 (48.93%)	42,025,250 (48.26%)	42,547,314 (48.02%)	47,563,888 (46.78%)
Read-1 ^e^	18,880,028 (23.23%)	21,725,955 (22.99%)	23,421,450 (24.93%)	21,534,853 (24.73%)	21,645,064 (24.43%)	25,291,412 (24.88%)
Read-2 ^f^	17,522,604 (21.56%)	21,215,497 (22.45%)	22,541,619 (24%)	20,490,397 (23.53%)	20,902,250 (23.59%)	22,272,476 (21.91%)
Reads map to ‘+’ ^g^	18,602,502 (22.89%)	22,024,764 (23.31%)	23,379,973 (24.89%)	21,728,282 (24.95%)	22,113,656 (24.96%)	24,617,535 (24.21%)
Reads map to ‘−’ ^g^	17,800,130 (21.9%)	20,916,688 (22.13%)	22,583,096 (24.04%)	20,296,968 (23.31%)	20,433,658 (23.06%)	22,946,353 (22.57%)
Non-splice reads ^h^	25,490,872 (31.37%)	30,801,757 (32.59%)	32,635,041 (34.74%)	30,406,191 (34.92%)	30,621,306 (34.56%)	33,776,061 (33.22%)
Splice reads ^i^	10,911,760 (13.43%)	12,139,695 (12.85%)	13,328,028 (14.19%)	11,619,059 (13.34%)	11,926,008 (13.46%)	13,787,827 (13.56%)
Reads mapped in proper pairs	27,006,682 (33.23%)	33,520,536 (35.47%)	34,778,164 (37.02%)	32,012,422 (36.76%)	32,201,808 (36.34%)	34,863,014 (34.29%)

^a^ Total reads: the statistical count of reads in the filtered sequencing data (clean data). ^b^ Total mapped: the statistical count of reads that could be mapped to the reference genome. ^c^ Multiple mapped: the statistical count of reads mapped to multiple loci in the reference sequence. ^d^ Uniquely mapped: the statistical count of reads mapped to a single locus in the reference sequence. ^e,f^ Read-1 and Read-2: the statistical count of each paired-end clean reads mapped to a single locus in the reference sequence. ^g^ Reads mapped to ‘+’ and Reads mapped to ‘−’: the statistical count of reads mapped to the positive and negative strands of the reference genome, respectively. ^h^ Non-splice reads: the statistical count of reads with the entire segment mapped to a single exon. ^i^ Splice reads: the statistical count of reads with segments separately mapped to two distinct exons.

**Table 3:** Fifteen statistically enriched Gene Ontology (GO) terms associated with differentially expressed lncRNAs between potato dormant tubers (DTs) and sprouting tubers (STs).

GO ID	GO Terms	Type ^a^	Corrected *p*-Value	Test ^b^	Ref. ^c^
GO:0005737	Cytoplasm	P	3.43 × 10^−62^	2551	3137
GO:0044444	Cytoplasmic part	P	5.97 × 10^−58^	2299	2819
GO:0005622	Intracellular	P	1.79 × 10^−50^	4071	5388
GO:0005623	Cell	P	8.00 × 10^−50^	4400	5864
GO:0044464	Cell part	P	8.00 × 10^−50^	4400	5864
GO:0044424	Intracellular part	P	1.45 × 10^−48^	3954	5236
GO:0005575	Cellular component	P	4.21 × 10^−41^	5755	7878
GO:0043226	Organelle	P	2.10 × 10^−39^	3355	4460
GO:0043229	Intracellular organelle	P	2.10 × 10^−39^	3353	4457
GO:0044422	Organelle part	P	4.96 × 10^−29^	1362	1685
GO:0044446	Intracellular organelle Part	P	4.96 × 10^−29^	1362	1685
GO:0043227	Membrane-bounded organelle	P	7.31 × 10^−26^	2904	3905
GO:0043231	Intracellular membrane-bounded organelle	P	8.76 × 10^−26^	2902	3903
GO:0009987	Cellular process	F	2.49 × 10^−25^	6970	9831
GO:0044237	Cellular metabolic process	F	2.37 × 10^−23^	5612	7863

^a^ GO ontology type: “F” represents ‘biological process“, and ”P“ represents ”molecular function“. ^b^ Number of mRNAs related to differentially expressed lncRNAs between potato dormant tubers and sprouting tubers. ^c^ Total number of transcripts belonging to each GO term.

## Supplementary Materials

The following are available online at www.mdpi.com/1422-0067/19/1/101/s.

## References

[1] Yan N., Liu Y., Gong D., Du Y., Zhang H., Zhang Z. (2015). Solanesol: A review of its resources, derivatives, bioactivities, medicinal applications, and biosynthesis. Phytochem. Rev..

[2] Liu B., Zhang N., Wen Y., Jin X., Yang J., Si H., Wang D. (2015). Transcriptomic changes during tuber dormancy release process revealed by RNA sequencing in potato. J. Biotechnol..

[3] Liu B., Zhang N., Zhao S., Chang J., Wang Z., Zhang G., Si H., Wang D. (2015). Proteomic changes during tuber dormancy release process revealed by iTRAQ quantitative proteomics in potato. Plant Physiol. Biochem..

[4] Zaheer K., Akhtar M.H. (2016). Potato production, usage, and nutrition—A review. Crit. Rev. Food Sci. Nutr..

[5] Sonnewald S., Sonnewald U. (2014). Regulation of potato tuber sprouting. Planta.

[6] Koffi G.Y., Remaud-Simeon M., Due A.E., Combes D. (2017). Isolation and chemoenzymatic treatment of glycoalkaloids from green, sprouting and rotting *Solanum tuberosum* potatoes for solanidine recovery. Food Chem..

[7] Uri C., Juhász Z., Polgár Z., Bánfalvi Z. (2014). A GC-MS-based metabolomics study on the tubers of commercial potato cultivars upon storage. Food Chem..

[8] Knuthsena P., Jensenb U., Schmidta B., Larsenb I.K. (2009). Glycoalkaloids in potatoes: Content of glycoalkaloids in potatoes for consumption. J. Food Compos. Anal..

[9] Mensinga T.T., Sips A.J., Rompelberg C.J., van Twillert K., Meulenbelt J., van den Top H.J., van Egmond H.P. (2005). Potato glycoalkaloids and adverse effects in humans: An ascending dose study. Regul. Toxicol. Pharmacol..

[10] Liu B., Zhao S., Tan F., Zhao H., Wang D., Si H., Chen Q. (2017). Changes in ROS production and antioxidant capacity during tuber sprouting in potato. Food Chem..

[11] Bachem C., van der Hoeven R., Lucker J., Oomen R., Casarini E., Jacobsen E., Visser R. (2000). Functional genomic analysis of potato tuber life-cycle. Potato Res..

[12] Kloosterman B., De Koeyer D., Griffiths R., Flinn B., Steuernagel B., Scholz U., Sonnewald S., Sonnewald U., Bryan G.J., Prat S. (2008). Genes driving potato tuber initiation and growth: Identification based on transcriptional changes using the POCI array. Funct. Integr. Genomics.

[13] Liu B., Zhang N., Wen Y., Si H., Wang D. (2012). Identification of differentially expressed genes in potato associated with tuber dormancy release. Mol. Biol. Rep..

[14] Campbell M.A., Gleichsner A., Alsbury R., Horvath D., Suttle J. (2010). The sprout inhibitors chlorpropham and 1,4-dimethylnaphthalene elicit different transcriptional profiles and do not suppress growth through a prolongation of the dormant state. Plant Mol. Biol..

[15] Campbell M.A., Gleichsner A., Hilldorfer L., Horvath D., Suttle J. (2012). The sprout inhibitor 1,4-dimethylnaphthalene induces the expression of the cell cycle inhibitors KRP1 and KRP2 in potatoes. Funct. Integr. Genomics.

[16] Campbell M., Suttle J., Douches D.S., Buell C.R. (2014). Treatment of potato tubers with the synthetic cytokinin 1-(α-ethylbenzyl)-3-nitroguanidine results in rapid termination of endodormancy and induction of transcripts associated with cell proliferation and growth. Funct. Integr. Genomics.

[17] Hung T., Chang H.Y. (2010). Long noncoding RNA in genome regulation: Prospects and mechanisms. RNA Biol..

[18] Mercer T.R., Dinger M.E., Mattick J.S. (2009). Long non-coding RNAs: Insights into functions. Nat. Rev. Genet..

[19] Chekanova J.A. (2015). Long non-coding RNAs and their functions in plants. Curr. Opin. Plant Biol..

[20] Zhang Y.C., Liao J.Y., Li Z.Y., Yu Y., Zhang J.P., Li Q.F., Qu L.H., Shu W.S., Chen Y.Q. (2014). Genome-wide screening and functional analysis identify a large number of long noncoding RNAs involved in the sexual reproduction of rice. Genome Biol..

[21] Wang M., Yuan D., Tu L., Gao W., He Y., Hu H., Wang P., Liu N., Lindsey K., Zhang X. (2015). Long noncoding RNAs and their proposed functions in fibre development of cotton (*Gossypium* spp.). New Phytol..

[22] Khemka N., Singh V.K., Garg R., Jain M. (2016). Genome-wide analysis of long intergenic non-coding RNAs in chickpea and their potential role in flower development. Sci. Rep..

[23] Liu X., Hao L., Li D., Zhu L., Hu S. (2015). Long non-coding RNAs and their biological roles in plants. Genom. Proteom. Bioinform..

[24] Jiang L., Schlesinger F., Davis C.A., Zhang Y., Li R., Salit M., Gingeras T.R., Oliver B. (2011). Synthetic spike-in standards for RNA-seq experiments. Genome Res..

[25] Kim D., Pertea G., Trapnell C., Pimentel H., Kelley R., Salzberg S.L. (2013). TopHat2: Accurate alignment of transcriptomes in the presence of insertions, deletions and gene fusions. Genome Biol..

[26] Cabili M.N., Trapnell C., Goff L., Koziol M., Tazon-Vega B., Regev A., Rinn J.L. (2011). Integrative annotation of human large intergenic noncoding RNAs reveals global properties and specific subclasses. Genes Dev..

[27] Yan N., Du Y., Liu X., Zhang H., Liu Y., Shi J., Xue S.J., Zhang Z. (2017). Analyses of effects of α-cembratrien-diol on cell morphology and transcriptome of *Valsa mali* var. mali. Food Chem..

[28] Trapnell C., Williams B.A., Pertea G., Mortazavi A., Kwan G., van Baren M.J., Salzberg S.L., Wold B.J., Pachter L. (2010). Transcript assembly and quantification by RNA-Seq reveals unannotated transcripts and isoform switching during cell differentiation. Nat. Biotechnol..

[29] Young M.D., Wakefield M.J., Smyth G.K., Oshlack A. (2010). Gene ontology analysis for RNA-seq: Accounting for selection bias. Genome Biol..

[30] Kanehisa M., Goto S., Sato Y., Kawashima M., Furumichi M., Tanabe M. (2014). Data, information, knowledge and principle: Back to metabolism in KEGG. Nucleic Acids Res..

[31] Dai X., Zhao P.X. (2011). psRNATarget: A plant small RNA target analysis server. Nucleic Acids Res..

[32] Wen Y., Liu B., Lu W., Zhang N., Si H., Wang D. (2013). Observation and identification of potato morphological changes during tuber dormancy release. Chin. Potato J..

[33] Teper-Bamnolker P., Buskila Y., Lopesco Y., Ben-Dor S., Saad I., Holdengreber V., Belausov E., Zemach H., Ori N., Lers A. (2012). Release of apical dominance in potato tuber is accompanied by programmed cell death in the apical bud meristem. Plant Physiol..

[34] Eshel D., Teper-Bamnolker P. (2012). Can loss of apical dominance in potato tuber serve as a marker of physiological age?. Plant Signal. Behav..

[35] Kwenda S., Birch P.R., Moleleki L.N. (2016). Genome-wide identification of potato long intergenic noncoding RNAs responsive to *Pectobacterium carotovorum* subspecies *brasiliense* infection. BMC Genomics.

[36] Kang C., Liu Z. (2015). Global identification and analysis of long non-coding RNAs in diploid strawberry *Fragaria vesca* during flower and fruit development. BMC Genomics.

[37] Zhang Y.C., Chen Y.Q. (2013). Long noncoding RNAs: New regulators in plant development. Biochem. Biophys. Res. Commun..

[38] Helliwell C.A., Robertson M., Finnegan E.J., Buzas D.M., Dennis E.S. (2011). Vernalization-repression of Arabidopsis FLC requires promoter sequences but not antisense transcripts. PLoS ONE.

[39] Heo J.B., Sung S. (2011). Vernalization-mediated epigenetic silencing by a long intronic noncoding RNA. Science.

[40] Ma J., Yan B., Qu Y., Qin F., Yang Y., Hao X., Yu J., Zhao Q., Zhu D., Ao G. (2008). Zm401, a short-open reading-frame mRNA or noncoding RNA, is essential for tapetum and microspore development and can regulate the floret formation in maize. J. Cell. Biochem..

[41] Song J.H., Cao J.S., Yu X.L., Xiang X. (2007). BcMF11, a putative pollen-specific non-coding RNA from *Brassica campestris* ssp. *chinensis*. J. Plant Physiol..

[42] Song J.H., Cao J.S., Wang C.G. (2013). BcMF11, a novel non-coding RNA gene from *Brassica campestris*, is required for pollen development and male fertility. Plant Cell Rep..

[43] Wu H.J., Wang Z.M., Wang M., Wang X.J. (2013). Widespread long noncoding RNAs as endogenous target mimics for microRNAs in plants. Plant Physiol..

[44] Potato Genome Sequencing Consortium (2011). Genome sequence and analysis of the tuber crop potato. Nature.

[45] Guttman M., Garber M., Levin J.Z., Donaghey J., Robinson J., Adiconis X., Fan L., Koziol M.J., Gnirke A., Nusbaum C. (2010). Ab initio reconstruction of cell type-specific transcriptomes in mouse reveals the conserved multi-exonic structure of lincRNAs. Nat. Biotechnol..

[46] Kong L., Zhang Y., Ye Z.Q., Liu X.Q., Zhao S.Q., Wei L., Gao G. (2007). CPC: Assess the protein-coding potential of transcripts using sequence features and support vector machine. Nucleic Acids Res..

[47] Punta M., Coggill P.C., Eberhardt R.Y., Mistry J., Tate J., Boursnell C., Pang N., Forslund K., Ceric G., Clements J. (2012). The PFAM protein families database. Nucleic Acids Res..

[48] Siepel A., Bejerano G., Pedersen J.S., Hinrichs A.S., Hou M., Rosenbloom K., Clawson H., Spieth J., Hillier L.W., Richards S. (2005). Evolutionarily conserved elements in vertebrate, insect, worm, and yeast genomes. Genome Res..

[49] Zhang R., Marshall D., Bryan G.J., Hornyik C. (2013). Identification and characterization of miRNA transcriptome in potato by high-throughput sequencing. PLoS ONE.

