# Supplementary material for: Genome-Wide Analysis of Long Non-Coding RNAs in Potato and Their Potential Role in Tuber Sprouting Process
Source: Int J Mol Sci. 2017 Dec 29;19(1):101. doi: 10.3390/ijms19010101 (PMC5796051; doi:10.3390/ijms19010101)
Supplement: Supplementary file 1 [file ijms-19-00101-s001.zip › Supplementary Materials/Supplementary Data S1.doc]

**Supplementary Data S1: Sequences of predicted lincRNAs**

>LNC_000001

TGTAGAGGTTTTCGCAAGGATCGTCGTGGTATAATCTGTTTGGGTTGGAAGGGTCTCGTTTTTCCAGAAATGGGTGATCAAGGGGCTGCTAGTGTTAATTGTTGTGGCTGATTTGCGATGTTTTAGGCATCTGTTTGTGCTAAATTTTGGGGTATTAGTTTAGTAAGGTATTTGGGACGTTTCCACGGATTCGTTTTCTGGATTCCAATTGTGGGCCCCACAATCCCGTTTTAGACCCGATTTTGGGTCCGTCTCCG

>LNC_000002

CCCGGTTAGAGTCCGTGGTCCAGCTTACTTATGTCCAGGCTTAGCTACAGTACCCGGTTAGAGTCCGTGGTCCAGCTTACTTATGTCCAGGCTTAGCTACAGTACCCGGTTAGAGTCCGTGGTCCAGCTTACCTGTGATCTGGTTTTGGCTACAGTACCCGGTTCGAGTCCGCGGTCCAGCCCACCCGTGCCCGACCTTTGGCTACAGCACCCGGTTCGAGTCTGTGGTCCAGCCCATGTAGGTTGACTCATTAGCTATAGTACCCGGTTCGAGTCTGTGGTCCAGC

>LNC_000003

TGACAGCATGACTCTTGATATTATTGAACATTCATACCTTGTGGTGTATATGATAATATTGATTTGAAGTATGTTTGGCCTTAGTCTAGGCTTAGACTAGTGTTGCCATAGACTTGCGAGGTTTCGGATGTCGTTGGGCAGTAGAACTTTTTCCGACGTCGTTTCGGACGGTCAGCTCCTTGTAGACTGATATAGCAGACTTGAGTCTGATAATCTGAGCCTTAGCTAGGCGGTAACTCAGCTTAT

>LNC_000004

TATCGCATGTGGAGCTTAAGTTTCGCAGGGACTGACATCTTTCTAGGTGGAGCATCACCTTCTGTTTTCCCGTACATGCTTTACCTTGGGTTGTCTGCCCTATATACCCAAAAAGGTTCATGCATGCTTTATGGCTTATGATGAGTGTCCCTTACACTATGAGCTAATTGCTTTTTAGTTTTTTTAATGTATTTGATTAGAATTTTGAATAATATGTTTTTTCACTCTTAACGTAGGATTTGTAGCATAGTATTTAGTAAGTTGTATTAGAC

>LNC_000005

TGACACAGGAACTGAAATAGATTTAAAGATTACTTCCGTAGAGGAGAGGGGAAGGCTGCTTAGATAGGGCGATAGCCCGGTTTTTATTGTGTATCCTTTCCTGCGCTTGTCTTGTATTGAGGTATACCGAAGAGATTCGTAAAAGTAGGTAACAGAAGGGGAGGGATTGCTGTTTTTATTAGTGAGGACAAGCCGCTTTCATTTTCAACAATCTAATGCCACACCAACCCTTTAAAGAGTAGGCGGTTCGTATCTTTCTATGACCCCCAAGAAGTAGGAGGATAGGCAGAGCAAGAGCTCTTGAAGTCTAC

>LNC_000006

TCGGTACGGAGACGTCTGTACTTATCTTCGAGAGGCTATGGTGACTTTTAGGACAGATCTTTATTACTTGATTCTCTATCGTGCGTCCTTGGTCCGATCTAATTCTTATCACTTCACTCTGTTCTCTCTCTCATTGTAATGACTCATGGTTGGTCCTGCGTGGGTGTGAGTTTAGGTGTCATCGCGTGATTTTAGTGTTAGATCTGGTTTTGATGCGGAGGTGATAGGCTTGTGGAAAGGAAGTGTGATCAGACATGGAATTCTCTTC

>LNC_000007

GCGATTGGGTGAGACCCATATCGATCGTGGAATGCTTCAATGGAAGGAGGAAGGACCATAATTCAAAATTTTGGAAGCAATGATGTGTTTCTAAGAATGACTGGATACTAGTGGCCGTCTGAGGGTAAAGTAACTGGCCGGTGTGACTATGATGTTCTGGTAGTATTGACATTGTTGGCTTTAATGGATTATTGTAAAGAGTATGAGAAAATGGATAAATTGAGTATGTCTATGAATGTTATGAGGCCGGACTGGCTGATTGTGAAGTCAGAC

>LNC_000008

CACGTCTTCATTTTTCTTCTTCTAGCAATGAGCAAATCGAAACCTAGAACCCAGTCCAGCGTACCAGGCGAACTGAATCGATCCAGTCCCGCATCCGTCGTCCTCTCCTCTCCCTTATCTACTCCGTATGGGAATAATCTAGGTGTCGAAATCTCATTGCCTCTTCGACCTTCATCTCCAAATCGGACGACTCTCCTCTCTTTTCCGTTAGAAATTCGTACTAATTCCAGCTTTCAGACTTATCTTCAAAGGAATTCGTATTTCAAATTTTAAATTTCATCAAAATTTCTCCTGCTTTCCCCCCATTTTCAGCTTTAGTTTCCTCTATAAAAGCCCCTCTTTGCCTTCGGTTTAAGGTTGGAAAAAATAGAGTTAGAAAGTTTGGGGGAAAGAGTTGGAATCAGTTGGCTTTAGAGAAGAAAAGCATAATAAAATTACTGGTATACACTGTCTTAGTCGATCGAAAAATATATAGAAATAGGTTCCAAACGATAAGAATTTGGGAGTTTAGCTTTGGGTTTCTTCTTTCTTGCTTTCTGCTGGAATATCACTTGTGGTTACATGATGCTCTCCAACTCGTGGTTCCCAGTTCGCTGCCCAGAAGAAGAATTGGAAGTGGAAACACAGGGCTGGAAAACAGAAAAATGCCGATCTTTGGAGCG

>LNC_000009

GTTGCAACTTCTGAGCAAACCCAAGTTAAAGCAAAACATGTACATTCCAATAGAGAATATCGAGCTTCATATGATGTAAACTTCTTGCTCATGTAATATATCACCTGCTCCTTCTTTCTTGTTTCATCATGTTGCCCCAAAATGTAGCTGAAGGCATTATCCAATACCGATATGTACAACAACAATGGCCTACCAGATTCGGGTGGAACCAACATAAAATTTTCTTTCTTCCTCCGTTCATACGTATTTCATACATTCCAAATATGGAGTTGGGTAAAATTTCATGTGATTCGGTAAACAAAATAGAAAAGCCATCATTGCTAGATCATTTATCTAATCTATCTAATTAGATGCAGAATGAACCAATAGTGGGATTTTTTGCTAAATGGGAAAT

>LNC_000010

AGTTTATGTGATCTTGTGATGATCTTATGTAAAGTGGAGAGCATTGTGATTTAAAGTATGATTTTCTCATGATGTATATATGAAATGATGTTGGATTATGAAGTATCCTTAAATGAAAATGTTGTGACTTGGTTGGTAGACTAAAGTGTCCCTCCTTGTTACTTAAAGTCTTGAATGCATGAATGTGTGAAATGGGCAAAACTAAGAAATGGTGCCCTTTCTATAAATGCTAAAAGAGGAATTCTAGGCCAAGACTTGAATCGGCAAAACTAAGAAATGGTGCCCTTTCTATA

>LNC_000011

GAAATAGAAAGAGTAAATACTATTTTCAATACTTTGCTTATGTACAACTACAAAGTACCAAGCATTCTAATTGGATTAGATTAATATCAATGGATCCTTTATCAGTCATTCATTGAATGATAAATAACTACAAGTGACGGAGACAGACGATTTAAATAACGGAAAATCAAAAGAAAATAGTATCGAGAATGACTGGAAAGGTGGAAACAAGACCAGATCAAATTTGATCATTATGAACAAATCCAAAATCTTTATAGATAGAGCCAATCATTAATTCCCAACCGTGGGGTGAATGGAATCCGATACATAAATCAGTTAACAAAAGAATAGAAAAAGCTTTGACTAAAGAAGGCCGATTCCCCTAAGCAATTAAAGGGCTACGGCTTTCGCGGGCAATCGCTTCTTCGAGACCAATTCCAACAATATCGAGATTGGAATTAAATTCTAGTTATACCCCGGCCTCTTTCTTGGAACTAATTGATAGGCACTTTGTTTTCTATAGAACAAGCGGGCGATATGCAGCATCAATCAAGGTAAAAGGGTAGTCACGGCCTGTGCTATAATGCACAAAAGCCCTCCACCCCACCAAAAAGTAAAGAACCCGTGGTGCCTGCTTGTGACACATAGGGCTTGCTTTTCGTGATTACTATGAAATAGATGGCGGACTCAATTCATCAGGAACGGGAAGGACGACATCTCTCTCAGAAAAAAGGGAAATAAAAGACGAGAGAATCCTGCCTTTATTTTGACTACCCTAGGTT

>LNC_000012

TGGAAGACTAGTGCATCCAAGCACAGTGCAGCCAAGAGGGTCCAAGATTGCTTCGCCGAATGGGACGGACATCGATCTTCGCTTCTACAAGGCCTGGTCGACTCTTTGTAGGAGCCTTCATTTATCAAGGGGCGCAGCGGGCTTCCTTTCCTTTACCTTTAAACAGTTAGAGCCTCTTCTCTTATCAGTCCTTTCAGTCAGTTCCTTTTCGGTTAGCTACTTAGGATGAATCACACAGAGGCGATCTCAAACATAAGAATACAATATTTTTCAAGAGACAGAAGGCCTAAGAGAGATGGGGAACTTCCC

>LNC_000013

GGTAGCTCAGCCGTTGCTTGTTCTGAATGAAAGTGAATACAGACGCACCTTTCTTTAGTGCAGAGCCGGGTTCATAATTGCGGATTGCGCAGACTGACTTCTCCTAATGGCATATAGAAACGTGGTAGGGGAGGTTAGCTCAGGGAAGGAAGCTAAGTAAGGGCGAGCTACAACAAACACAAATGAAAGGTCCGCTTCAGGATCATTTGAATAATGTGAAGATACAGACGAAGCAGCTAAAGCCGAATCAGAATATTCCGCTGAAGCAGAAACGATAGGATCGTAAGAAGAAAAAGGCTATACACCTA

>LNC_000014

TGTTATTATTGATGGTTTTAAGAAAGGAAATTCCATGAACCCATGTCTTTGCAAATTTCCAAAAGTTGGCATATGAAGTGGGATTATTGTTTGTGATTGAATGATGATAGAATTATGCTTAAATGGAGTTGAATAGTTGATTTTTATTATTAGCTATGCTTATCTATTGTGAATTATTGGAGAGTTGAGGAATGATGATTGTGGCTTTGGAAAAGGGTAAGTGGACCTAACTTCATTGTTAAGGTGGAATTGATATAGAATTGTGATAGATTGATGTGTGATCCACTTATGGTGGAGGTGTCTACTTATGGAATGTATTATGATTGAGGCCATGAAGGGCATTATATGAGTGTTATTGTATTGTTATATTATTATATATGAATGAGGCTATATGTAAAGTCCTATATGAAGCTATATGTAAAGTTTTATGTGAAGCTATATGTGAAGGTATGATGTGAATGAAGATAATGTGATCATGTTATGAATGTAAAGGTGTTGTTGTAGGTTGTTCACTGGTTTGGTTGTATAATTACTACCCTATTTTCCATGCTCTTTGATTCCATTACATGCCTTCAAGGTGTTTGACAAAATGTCCAACTATGGAGAATTGATGAATCGATGTTTTAAAGCTTGAGTTATGAGATGTATGGAAATTCCATAGATGTGTTGATGACACTCATGAATGTTAATGATAGAATATGAAGGATTCTTCAATATAAAGTATACCTTGAATTGGTAGGGCTTATGCATCCTTTTCATGTATAGATGATTATATTGTTGATTGTTGAATCCTTGGATCGGTAGGCTAATGGCACCCTTCCACACATGCTTATAATGTACCTCGGATCGGCAGGCTTATGGCACCCTTCCATTAATGAAAAGTAATGAACCTTGGAATCGGCAGGCTTATGGCACCCTTTCATGTCTGATAATGTCAAAGTAATGAACTATTCTATGGGAATGTAGGCTAAGCACCGAGCGGATTTGGTTAGATGGAAACTCCCCCGACAGTTAGGCATGAGTTTCAATGATCGTCTACCTTATCCCATAAACTATGTGCCCGCATAGGAAGCTAATGGATCCATTAAGCTATATATACCGGTCTTCCTTAGGCAAGTAGACCACCTCTTTTCGGTGTGGGGACTCATGACACCGGATTCCATGACAAGCTCTCATGGTCTATGTCGGTTAAATACTTACTTCCCATCATGTGAGATTTCATTATGGTTTCTTGACAGGTTCTACAAAGTGTAGGTAGTAGTATGGGATGCTACCTATA

>LNC_000015

GTTATTGATTGCTTGATTTTCAGTAAACCTCTAGAAATATTGAGTTGTCTGCATGGACATACGAGCTGTCACTTCATATCGTTCATTCTTTAAAGTAAAATTGTCTATTTTAATTTGGTTGGGTTTTTATTTTAGTTTAGGTGAGAGACCTGGCAATAATTATTCAGGTTCACAAGGACTGCATTAGACAAAGAGAACAATTTTAAGCTAGATGTATGAGTGTGGTTAGTGTGAGTCGATGAGATCAACAGTGGGCCCGTGGAGAAGAATGCAGCATACCAGAACATCACTCTTTCTTGCCCCAAGGTTGCAGATTGGTTCTTTGCCTTGTAGAAGTCCCAAAAAAAGAATGATTGTATTGCAAATTGATTATCTGGTTATAGATCACAGGATATTTTTTCTCCATGTGCATCCTAATATCTTTTGTCCACAATCAAGGACTGAGCAAGGATAGCTGAGCCCTGCAATTACATGACTTGTCCTTGCCCAGTTCATTGCTTGTAACCAAAGCTTTGGACGCTGTGCAATGTTACTCACTACTAATTTTGATAAACTTTATTTAAGGAGAGAGAATGTTTAGCTGCAAAGCTATCTTCTTCAGACGTGGATATTTTCTAGGTCTTTGGTTTCCCTTCTCATGCATTTCCTTTTGGGTGCTATATGTGTCCATAAGCGGCGGAGTACGGTCATCTGATTGATCCAGAGTTGAAACAAGCATATGTCGACATTGGAGGGATCTCAAGTCTCTCATGC

>LNC_000016

CAATTTAATATTCTTTGGCATGAATTCCTATATTTCTGGTACCAACAAAGCAAGCTTTCTAAATAATCCTTGTTGTTAAAGATATGTTGTGATGGTGTATAATCTCTAATTTATTTAATATCCTTCTACTGCCACGGTTTCACAAATGTGTCAAACAACTCCAATAGGTGTTCATTATCCTATATCGATGTTGTAATACTTTCAAGTACG

>LNC_000017

ATACAATAGCTTAGTTTTCATTTTTTGAAGCAAAAAAAGAACTTCCTTTTCATTCTGCTTTTATACTACTCCAGTTCTAAAGAAGTTTCTTTTTCTTATACAAAACAGTAGCACAAGACAACTTAATGGTTGAATTAAGGAAGAATATTAGGGCACAAAATCCAAACTTTGAGTCATTCCATCACTGACAAGCCATCATTTATCACATTCTAATCCTCAAAACACATAGAAAAGTTCCAAAATCTCTAAATGGGTTCACTTACATTGAAAAGTGGAAAATGACAAACGAAATTAGAACCCTAATTTTTGGGGAAAAATTCAAAAATCAATGTGTTAAGCTAACAAATACAATCATACCGTGAATTGCAGGATGAAACTGCAGCAATCTTATGTATCCCTTCGGGTATGAAGAAGACCCTCCTAGGGAGCTTGGAGCTTGGAG

>LNC_000018

CTGAAAAACACCGACCCAATGCCACCGCCTTTGAAATATTCTTTCTTAGTCTAAATATACATCAACCCTATAAATATTCTTTCTTGGTCCAAAAACCCCGTTCGTTGATTGTTTTTGAGGGTATACGCAGTGTTTGCTTGTTCATATGGGTAACTTTTGTTGATCAAGACATGGGTAATTACAGTGCCTTATAGTTATTCTTGATCTGGGTAGCTATTGTTTATCAAGATACAGGCCAAATATTGTATAATCAAGATAGTGGATAGTTATTGTTTGATCAAGATACTGGTAATTATTGTTGATCAATATTGTGGGTAACTATTGTTTGGTCAAGATACTGGGTAATTATTGTGCTCCTTATTGTTAATCTGGATACTTATTGTTGAGCAAGATAGCGGGTAACTATTGTTGATCAGGATAATGGGTAATAATGTTTGATCAAGATATGGGTAGTTATTGTAGATTATTGTATATGATTTGGTCTTCTTTCGTTGGTATCTTGAGGATATAAGCAGTGTTTGATTGTTCATATGGTTTATTGTTGATCAAGATGATGGGTTATTATTGTTTGATCAAGATGATGGGTTATTGTTGTTGATCAACACGTGGGTAATTATTGTTTGATCAAGATATGGGTATGTCTTCGTTTGGGGAGGCGACTGGTTGGGTTTTTGATTTCTTGGCCATCTTAGAATTATATACAATCTCAAGTCATGTACTTTTGAATCTTGGTTTTCAAAGATATCAATATTTAGGGAGCCATTGCTTAGGTTTCTGATTTGCAACTCTTTGATCTTCAATGTTAGGGGAGTTCTTGACTTTTTAGTGTGATTCAATTTGCAAATAGTTCAGTCTTTTTTGGCTAAATTATTGATTAATATTTGTTGTTTGTTTGATCAGGTATGGGTAATGATGATAGGAGACTGGTGGTAGTTTCTCAATTAGGACATTATTTTCACCAATGTGTGTTGTATTCCCTATTGACTCCTTTGTCTTTTATGGTAGTTTCTCAATTAGGACATTATTTTATTTTCACACATAGTTTCTCAATTAGGACATTATTTTCACCAATGTGTGATGTACTCTGCTGCCATGAAGTTCTTAGTTTAGATGCTTATGTTCTTATGGTGATCCAATTGATAAGTTCTCTTATGGTGATAAGGTCTCTTACTCTCTTATGGCTTTGGATACCTGAGAAGTACTTGACCAATTCTAGATCTTTTGGTAGTGATGTAAAATATTTCGATAGTGGCATAAAATACTTCTCAACTTTTGTTCTTTTAGCTACTTATCTTCTCTGCTTTCATTTTTTTTTGTCATCCCACTAATTTTGGTTGAATGGATGTGTAGACCATGTGGTGACACTGTTATGGACGTTCAGATTTGGAAATTTAAAATCTTGGTTAACCAACATGTGATCCTTTGCCTCCTTTTGGTCTTTTTGGTGCTTGCATTGCAAGTTGGATGTTTTTTGGCTCTGACAAAAGGGGGGGTTTCAGTCTTGTCAATGTAATCTCCAGTGATTACTCAGGTGGTTTGGGGTTTGTCAGGTTTTCTCTGAACCGCTCCCTTCTTTCTGTTCTTGCAGCCATTGCTTACAGAGCAGTGAATTCTTCTTTTCACGGAGCGGCTATGTTTTGTTGGTTTGCTTGAGATTTCTTTAGTCAAGTCTCTTATAATTATGGATATTTTGGATTCAATTTTCTTCCATAATGGGCCTTTGTCTTTGTCAAAACAAGTGTTGTATGAAGTTTCTCAACTAGGGCATTATTTTTCACCGGTGTGTGTTGTATTCTGCTGTCGTGAAGTTCAGTCCAGACACTTATGTTCTTTGATCATCCAATTGATGAGGTCTTCTATGGCTATAGATGCTTGATAAGTACTCGGCCAATTCTAGATCTCATTTGATAGTGGCGTAAAATACTTCTTTACTTTAATTCTCTAGCTACTTAGTTATCTTCTCTGTTTCCATAGGTTTTTCGTTATCACACTGATTTGTTTTGGTTGAATGGATGTGTAGTAGATCATATGATGACACTGTTATGGACGTTCAAAATTTAGAAATTTCAAAGCTTGGTTTATCAACATGTGATCCTCTGCCACCTTTTGGTCTTTTTGGTGCTTGCACAACAATCTGGATATTGTCCGGGCTTCGACAGAAGGGGGTTTTCAGTCTCTTCTATGGCATTTCTAGTGATTACTTAGGTGATTTGGGGTTCCTCAGATTTGCACTTAACTGTTCCCTTCTTTCTGTTCATGCAGCCATTGGTTGCAGGGCAGTGAATTATTCTTTCCATGGAGTGGCCGTGTTTGCCAGTTTGTGTTGAGGTTTCTTTAGTTTAAGTTTTTATGAGTATCAAGATTTTCGATTCATTTTTCTTCAGTAGTTTCTCGACTAAGATAATATCTTTCACTAGTGTGTGTTGTTATTCTGCCGCCGTGAAGTTTTTAATGCAGACACTATGTTCTTCGATGATCCAACTGATAAGGTCTCTTATGGCTATGCACACTTGATAAGTACTCATCCAATTTTGGATCTTTTCTTTAGACAACGCCATAAATGCTCCTCTACATCTGGTCTTCTAGGTACAGATATTTTGGTTATCACAACTTGTTTTTTTGTTTGGATGCATGGATGTGCAGACTATATTGTGACAGTAATAGAGGTTAAGATTTGGAGATTTTAAAGGTTGATATCCAGTATGGTATCTCTGTATACTCTTGGTCTTTTTGGTTCTTGCACTGCAATTTAGATATATTGGTCAGGGCTTCAACAAAAAAAGGGTTTTAAATCTTGTCTATATGGCATCTCTAGTGATTTCTTGGATGATATGGGTTTGTATCTTCTTTTTGTACTTGCAACCATTGCATCTTATAGAGCTATGAAATATTCTTTTCATGGAGCGGCTTTCTTTGTCCATTTATGAGGTTTCCTTGGTTCAGGCTTTTATGATTTCTGATATTTTCAATTAAAATAGTTATTTTAACGTTGGTGTGTGTTGTATTCTACTGCCATTTTTAGTGTTCAGTCCAGATGCAATGGCCCAATTTGATGAGATTCTTGATGGTTGATGGTTATGGTCCCTTGGACACCTGACAAGAACTCAATTTTGGATCTTTAATGGTCTTCTCTTTTGGTGTCCTCACTGCTCATTTTCTCTGCTTCATTCTTCGTGTATTACGCTAGCTTGATTTTGTTTTGAGAGGTTATGACTTGGAAGATTTTGAAGGTTGGTGTCCAGTGTGCATTCCTGTGCACCCTGTTTCTTGGCCATGTTGGTGCTTGCACTACAATCTAGAGATTATCAAGCTTCGAAGGTGGTTCTCCATTTTGTTTGGTATTTGTGGTGAATTCTGAGGTGGTACGGTCTTCACTAGATCTGCACTTGAACTGTTCCATTTTTCTGTGCTTGTAGCTGATGGGTCTTACAGAGGGATAGGTTCTTCTTTTCATGGACCGGCATGGTCTGTCAATTTTGTGTTGAGATTTTTTGTTCGATGCTTCTAATGATTATTGAGATCTTCAATTCAGTTTTCTTCCCTAATCAGAAAGGTGCCCTTCTCTCCACTGCTTCACTAGGGTGTATTTTGCTGCCATATTTGTTTTTCAATAGTCCAGATGCTTATGTTCTTTGGTGATCCAACTGATGAGATCTCCATGGTTATGGGGACAACATATGATAAGTGTATTTAAACTCAATCGTGCTCTTTTGATGGCCCTGTAATGCACTCTTGTGCTTTTCATGTTCTCGCT

>LNC_000019

CTGAAAAACACCGACCCAATGCCACCGCCTTTGAAATATTCTTTCTTAGTCTAAATATACATCAACCCTATAAATATTCTTTCTTGGTCCAAAAACCCCGTTCGTTGATTGTTTTTGAGGGTATACGCAGTGTTTGCTTGTTCATATGGGTAACTTTTGTTGATCAAGACATGGGTAATTACAGTGCCTTATAGTTATTCTTGATCTGGGTAGCTATTGTTTATCAAGATACAGGCCAAATATTGTATAATCAAGATAGTGGATAGTTATTGTTTGATCAAGATACTGGTAATTATTGTTGATCAATATTGTGGGTAACTATTGTTTGGTCAAGATACTGGGTAATTATTGTGCTCCTTATTGTTAATCTGGATACTTATTGTTGAGCAAGATAGCGGGTAACTATTGTTGATCAGGATAATGGGTAATAATGTTTGATCAAGATATGGGTAGTTATTGTAGATTATTGTATATGATTTGGTCTTCTTTCGTTGGTATCTTGAGGATATAAGCAGTGTTTGATTGTTCATATGGTTTATTGTTGATCAAGATGATGGGTTATTATTGTTTGATCAAGATGATGGGTTATTGTTGTTGATCAACACGTGGGTAATTATTGTTTGATCAAGATATGGGTATGTCTTCGTTTGGGGAGGCGACTGGTTGGGTTTTTGATTTCTTGGCCATCTTAGAATTATATACAATCTCAAGTCATGTACTTTTGAATCTTGGTTTTCAAAGATATCAATATTTAGGGAGCCATTGCTTAGGTTTCTGATTTGCAACTCTTTGATCTTCAATGTTAGGGGAGTTCTTGACTTTTTAGTGTGATTCAATTTGCAAATAGTTCAGTCTTTTTTGGCTAAATTATTGATTAATATTTGTTGTTTGTTTGATCAGGTATGGGTAATGATGATAGGAGACTGGTGGTAGTTTCTCAATTAGGACATTATTTTCACCAATGTGTGTTGTATTCCCTATTGACTCCTTTGTCTTTTATGGTAGTTTCTCAATTAGGACATTATTTTATTTTCACACATAGTTTCTCAATTAGGACATTATTTTCACCAATGTGTGATGTACTCTGCTGCCATGAAGTTCTTAGTTTAGATGCTTATGTTCTTATGGTGATCCAATTGATAAGTTCTCTTATGGTGATAAGGTCTCTTACTCTCTTATGGCTTTGGATACCTGAGAAGTACTTGACCAATTCTAGATCTTTTGGTAGTGATGTAAAATATTTCGATAGTGGCATAAAATACTTCTCAACTTTTGTTCTTTTAGCTACTTATCTTCTCTGCTTTCATTTTTTTTTGTCATCCCACTAATTTTGGTTGAATGGATGTGTAGACCATGTGGTGACACTGTTATGGACGTTCAGATTTGGAAATTTAAAATCTTGGTTAACCAACATGTGATCCTTTGCCTCCTTTTGGTCTTTTTGGTGCTTGCATTGCAAGTTGGATGTTTTTTGGCTCTGACAAAAGGGGGGGTTTCAGTCTTGTCAATGTAATCTCCAGTGATTACTCAGGTGGTTTGGGGTTTGTCAGGTTTTCTCTGAACCGCTCCCTTCTTTCTGTTCTTGCAGCCATTGCTTACAGAGCAGTGAATTCTTCTTTTCACGGAGCGGCTATGTTTTGTTGGTTTGCTTGAGATTTCTTTAGTCAAGTCTCTTATAATTATGGATATTTTGGATTCAATTTTCTTCCATAATGGGCCTTTGTCTTTGTCAAAACAAGTGTTGTATGAAGTTTCTCAACTAGGGCATTATTTTTCACCGGTGTGTGTTGTATTCTGCTGTCGTGAAGTTCAGTCCAGACACTTATGTTCTTTGATCATCCAATTGATGAGGTCTTCTATGGCTATAGATGCTTGATAAGTACTCGGCCAATTCTAGATCTCATTTGATAGTGGCGTAAAATACTTCTTTACTTTAATTCTCTAGCTACTTAGTTATCTTCTCTGTTTCCATAGGTTTTTCGTTATCACACTGATTTGTTTTGGTTGAATGGATGTGTAGTAGATCATATGATGACACTGTTATGGACGTTCAAAATTTAGAAATTTCAAAGCTTGGTTTATCAACATGTGATCCTCTGCCACCTTTTGGTCTTTTTGGTGCTTGCACAACAATCTGGATATTGTCCGGGCTTCGACAGAAGGGGGTTTTCAGTCTCTTCTATGGCATTTCTAGTGATTACTTAGGTGATTTGGGGTTCCTCAGATTTGCACTTAACTGTTCCCTTCTTTCTGTTCATGCAGCCATTGGTTGCAGGGCAGTGAATTATTCTTTCCATGGAGTGGCCGTGTTTGCCAGTTTGTGTTGAGGTTTCTTTAGTTTAAGTTTTTATGAGTATCAAGATTTTCGATTCATTTTTCTTCAGTAGTTTCTCGACTAAGATAATATCTTTCACTAGTGTGTGTTGTTATTCTGCCGCCGTGAAGTTTTTAATGCAGACACTATGTTCTTCGATGATCCAACTGATAAGGTCTCTTATGGCTATGCACACTTGATAAGTACTCATCCAATTTTGGATCTTTTCTTTAGACAACGCCATAAATGCTCCTCTACATCTGGTCTTCTAGGTACAGATATTTTGGTTATCACAACTTGTTTTTTTGTTTGGATGCATGGATGTGCAGACTATATTGTGACAGTAATAGAGGTTAAGATTTGGAGATTTTAAAGGTTGATATCCAGTATGGTATCTCTGTATACTCTTGGTCTTTTTGGTTCTTGCACTGCAATTTAGATATATTGGTCAGGGCTTCAACAAAAAAAGGGTTTTAAATCTTGTCTATATGGCATCTCTAGTGATTTCTTGGATGATATGGGTTTGTATCTTCTTTTTGTACTTGCAACCATTGCATCTTATAGAGCTATGAAATATTCTTTTCATGGAGCGGCTTTCTTTGTCCATTTATGAGGTTTCCTTGGTTCAGGCTTTTATGATTTCTGATATTTTCAATTAAAATAGTTATTTTAACGTTGGTGTGTGTTGTATTCTACTGCCATTTTTAGTGTTCAGTCCAGATGCAATGGCCCAATTTGATGAGATTCTTGATGGTTGATGGTTATGGTCCCTTGGACACCTGACAAGAACTCAATTTTGGATCTTTAATGGTCTTCTCTTTTGGTGTCCTCACTGCTCATTTTCTCTGCTTCATTCTTCGTGTATTACGCTAGCTTGATTTTGTTTTGAGAGGTTATGACTTGGAAGATTTTGAAGGTTGGTGTCCAGTGTGCATTCCTGTGCACCCTGTTTCTTGGCCATGTTGGTGCTTGCACTACAATCTAGAGATTATCAAGCTTCGAAGCTGATGGGTCTTACAGAGGGATAGGTTCTTCTTTTCATGGACCGGCATGGTCTGTCAATTTTGTGTTGAGATTTTTTGTTCGATGCTTCTAATGATTATTGAGATCTTCAATTCAGTTTTCTTCCCTAATCAGAAAGTGCTATCTGGATATTGGATATCACCAGGCATCAAGAAAAGGGTGCTTTGCAATCTTATCTTGTTTGGCATATCTAGTGATCTCTCCTGCGTGGTATGGGTTTCCACTTACCTGTTATGTTCTTTTTGTGCATGTAACCGTTGGGTCTTCTAGAGCATGAACTTCAGTTCAATTTTTGTTCCTAATCAACGAGTCCCTTTTCTCTTTGTCAATTATTTTTTTGGGATGATCCCCTTTTCTCTTTGTCAATTATTTTTTTTGGGATGATCCAAATGGTGAGATCCCTGAGTTTCATGTGCACCTGAGAAGTACCCAAACTCAATTTTAGATCCTCTTATGGTCCCAAGAGTTCCTCTACTTTGGTGTTCTTGCTGCTCTTATGCTTGTCATGCCACTTGATTTTGTTTGTTTGGATGCATGTTTGTAGATTACATCAGATTTGGTAATTTCGAAGTTTGATTTCAGTGTGTACCCTGTTCCTTTGCCATATTGGTACTTGTGCTTGCACTGCAATTTGGATATCATCAGGCTCTGTCAAGGGAGTTATGCAATCTTGTCTGGCATCTCTAGTGATCTCTTCTATGTGGCATGGGTTTCGCAGCCTGCGCTTTAACTGTTCCCTTCTTTTGTGCTTGTGTTGTTGTATCTTTCAAGATCATTGAAGTCTTCCTTTCATGGAGCAGCCTTGTTTGTCTTAGTGTTGAAGTTTCGTGAGTTCAGTCCTTGTATTAGTATCTAACTTTTTGATTCAGTTTTCTTCCCTGGTCAGAGAGATCCCCTTATCTGGTTGTCAGAAGGGCTGTTAAATGGAAGTTTCAATTACGTTAACTCTCTCCACAGGTGCATTTTCAGCTTTCAGTCCAGATGCTTTATATTTTGAGATGATCCAACTGATGAGGTCTCTTATGTTGGAGATCTCTCCCCTACTATTTTTTTGAAACATGTATATTATTTTCAGATATTCAATCCTTTGAGGAGTTGCTTCAGACTATTAGCTATTATGTAGAACTATAATATAAAAAGAGAGTTCCTGCACACTTTTTTCCTCCGTAAAACATTGTCATTTGAACAGTGTTTAGCACCAAAGACCGAGCTTATCCATTGAATCTCGAACAAATGAAGTAAAAAACTTGGCTCATCATTTGTGTAACATGCCATTTACACTCTTTATGTTATTTATATCAACACAGAGGTAACTATTTTTTTTTGTGTAAAATATTTTCTGATGAGATATCCTTGTAAATCCATTACATGTCAGATCATCATGCAAGTTGGGAAGTTTATCAATGGATCTTGTTCTTCTAAGTACTCTTCTGAGTTAAGTTTTGTACTAATTTTCTTAATACTATTGAAATTGCTATTGTTTATAGAACATTGTTGTTCTGTCATAGGCCATAGTGATCCGGGAGTCCCGTGTGAAGGGCTCTCGCTCAACGGATCAAAGGTACGCCAGTGATAACAGGCTGATGAATCCAAAGAGCTCTTACCGACGAAGTCGTTTGGCACCTCGGATTTCTAAACATCTTATTATCATATAACACTAGAACATAGAACAATTAAATGAAACGAAAAGAAAAGAAAGAGATGACAGAGAATCCATTGAGAAGTTTACCTGTATCCAAGGTATTTATTCTCCATGTTCTTGCTATATCGTTGATGTATTATTAGCCAACCGTGTTCCTAATATGATTAGTCAACCGTGTTCCTAATATGTCACTAAACAGATAATTTGTTCATTCAAATGTCACTTCACAGATCTGCACTACCAGCTATAATTCGTTTCTTATTTCAGGAATTGTTCTTTGCTCCTTGGTTTTCTTGTATTGGTGATCTATTAGTAACCTTGCACAAGTTAAAAGTGATCTTCTAAAAAGCCCTAACTAGACCAAGTTTCGACAGTTTTGAAAAATGATGTTGTGTTTTCCTTCATTCATCAAATATAGCTCTCCATTCACCAGTTGAACATGCCTTTTCGGCATTTGGAAGGTATTTTGGTCATTTTCTGTAAGATGGAATAATCTGATAAGTTTGAAAAGAATCTTTCTGTAGCTATTATGGGAAGTTTAGATGTGTATTGCCTAAATTATCCTCTCATCCTTCGTCATTGTACTTAATTCTATCAGTTCCGCATGTGAGTTAGTGGGGACCAGAAAACCTTTTAACTGTTAAGTATTATCTCACTTTTTCCCCTCACTATATCATTGGTTGTTCATGAAGAATTTCTCCAATAATTATAAATTGTAGTAATAATTAATACTTTAAAATATTTAAAGAATATACTAAAATTTTAGTTAACTTTCAAAATTTTATGTGTGTCACATAAATTAGGACAAAGGAAATAACATATATTATTTGAAAATTATGTTAAAAGAATATTATAGATAACAATATTAACAACTTAAAATATTCAAAAATCATATAAAATTTGGTTAACTTTCCTAACTTCATTTGTGCTTATATTAAAGTGAGACAAATCGTAGGCTCTTGTGTCTAGTACTTGAGGTATTGTATGGTTATAGAGGGGTTTTCCATTTTTATCATTTTCCCTGATTCTCTGTTGCATCATTAGGAAATAACATTTTGGTAAAGAGAGATCCAACTATGATTTCAGTCATTCAGCCTACTTAGGAGTTCTCTTGGGAGTCTTTGTGTAGTCAGGTTACGGTTAGACCTATCTTCTTTCTTCCCTCCCCCTAATGTGGGTAAAGCTGTTCGTTAACTTCCTCTGCATGATGGTTAGAGATGTTTATGGTGGATCAACGTGGGCAAGATTTTAATTAAGTTTTGCATAAAGTGATAATGAGAATGTTCTTACGTGTTTCACAAAGTTGCAACTGTCTTTGGTGGTTTGGAGTTGATACCTCTACTTTAATGTCATACTCATTTATTTCATCCCGCTGCCCTCTATAAACATCCCTTCTTGGTCGTAATGGGGAGGGCTGGCAACTCGAGTTGTTTTCTCCTTCCCGTTGTCTTATTTGATCCGGTGGGATTAATTTTGTCCCCGATGCGGGTTTCTGCTCCTTGGTTGTGGCTATTGGAAGCTTGGGCGGAGGGGTCAAGGAGGTTTTTTTTTTGAGGTATGGAATATGAGCAGATCTGTGAGTTGCAAAAAAGAAAGTCTGGCAGAATGCTTGGCCTCCTGTGCAGGAAGTGAGACCTGAAGGGTGAGCTTATGGTGAAGAGTATCTATTTTTGGGTTTTGCAGATTTATTTTGAAGTTTGACTTGGAGGTAAGTTCTCACTTTTGTTGGTTTCTTGAGGCATCCAAGGACTTCCTGTTTAGGTTATGGCAATTCCTTTCTGGTTCGACCCGAGGTAATGATGGTAAGGTTATAGACCATGCAGTTTCTATTTTTGGATATCAATAATCTTTGGAACGAAGTAAACCTCGTCTCTGCTACCTGCAACAAATTATCTTTTTTTGCATCTTCTCCGCTGGAACACTATTCAGTAGAAGTTTTTGAGCCTTCCCTTGTAGATGTTGCTCTCTATATTCCATTTTGTTAGGCGATTGACTCGTTCAAAATCACGAATAGGTCTCCCTCTGATGTCTTCAGGTTGTCTTTATCTACCCATAAGCTTACAAGTCTTTATTTTCACAGTTATATTGTATTGTCCACCCCGTTAGATGGGTTCCTGCACCCAGCATGATAAGGGGACAATATCCTATCTTCAAAAATGTCAACGGGGTGCGTGTCAAATTCGCTAAAAGTACTGTATTTTAGGATAATCCGACATGGGTGTGGCATCGAAAGTGAAGAGTCCGTGCAACTTAGACAATATCAAGGAGTTTCTGAGAAGCAGTCTGTTAAAGTCATTTACAGTGGTTTTGATAATGTTTGACTTTGGTATTTAGAGCATTTGTTGTATTTGCTGATTTTGATCTCTTACCTGTAAAACAGCACTACAGATGTCAGTGTACTATTTTTTAAGTTGAGGAAGGTATTAGCATCTTGAAGTAGCTAGGATTCTTTGGCTTCAGTTTGTTGTGGTATTTCTTTTAAAATAAGGCCACTGAATGGGTATTTCTGTTTTCTCTAAGTTCCAGTTGTCCAAATTTGTCCAAATGTGCCAAATAATTTGAGTGGGGTATTTCACTTTAAACTCCAGTTGTCCAATCTCGCCAGATAAAGAATGTTCCTCTTTTCTCTCAAGTCCTTGTAGCTATTTTGATAGCGCTGGGATTGAAGGACTTATTTTTTTTTTGTGGCTAAAATACACAAATTCAGTAACATATAAACGGATTTGCTCTCCTAAAGTGGAGAGAAGTTGGGAGGATGGTAAATCAGTTGTTGAGGAGCATGTTATATAGAAAAGGTCAGTATGATGTGGATTATGTTAAACAGAAAAAGGTCCTGGTATGATCACTTGGATTCCTGTTTTTACATTGACCTGTTTTAGTCATCTTTTGCATGTTCAAAGTATTATAATATCTATTTTGGATTAACATTTTCGGATTAACATTACAAGTTCTTCTGGTTATATTGAGTGGGGGTGTGTTCAGCTACACTCGAGCGTTCTAGTCACTCAGTAGTATCTAATGTGCTTTCTCCTAGAATGTGTAGTAATTTATTAATCTATGTGCACCTAGCAGTGTCGGAGATCCAGTTTTAATATGAGTAAGAACGGAATGTTCTCAAAATAATTGTCTCATCTCACTTGTGCAAAATGCAGTTACTGAATATAGATGGTCTATAGGTAATATTTCAACTAGTATAGGTGTGAAGTAGCTTTCTGTTCGGGTTTTTATGATGAGTTGGGTATCTCTTTCTTTGGCTTATCTCACATTCTTATTTGGTTTTTATTGGTTTGGGATTGGTATCTGTGGATTAGTATCTTGGATGTGATAAAGCTTGAGTTACGGGAAAGAGAGGAAATACTTTTTCTCAAAACATGAACTAATGGAAGTTTAACTCTGGAAGAAGCACTTGATGAAGCATTGCGAAATTTGTCCTTATTGTTATTCTTGATCTGGGTAACTATTGTTGATCAAGATAGTGGATAGTTATTGCTTGATCAAGATACTGGTAATTATTGTTGATCTAGATTGTGGGTAACTATTGTTTGGTCAAGATATTGGGTAATTATTGTGCCTTATCGTTATTTTTAATCCGGGTAATTATTGTTGATCAACACAGCGGGTAACTATTGTTGATCAGGATAATGGGTAGTAATGTTTGATCAAGAAATGGGCTGTTATTGTATATGATTTGGTATTCTTTTTTTAGTATCTTGAGGATATAGGCAGTGTTTGATTGTTGATATGGTTTGTTGTTGATCAAGATATGGGTAATTATTGTTTGATCAAGATATGAGCAATTATTGTATATGATGCCTTCGTTTGGGGAGCGATTGGTTGGGTTTTGATTTCTTGGCCATCTCAGAATTATATAAATCTCAAGTCATGTACTTTTGAATTTTGTTTTTCGAAGATACTGATATTTGGGGAGCGATTGGTTATGTTTCTTATTTGCAACTCTTTGATCACATCTCTGTAGAGAAGGAATGTTGGGGGTTTTGGGAGTTCTTGACTTTTTAGTGTGATTCAGTTTGCAATTAGCTCTGTTTTTTGGGGCTAATTTATTTATTCGTTTGCAATTAGTTCTATTCTTTTCCTAATTTATTGATTAATTATTGTTGTTTGTTTGATCAGGTATGGGTAATGATGATAGGAGACCGATTCTCCTTCTACACTTGCTGTTACCTTTGTCTCAATGTTAATTCTGAGGTTTTTGTTGTGAGAATTCATGTGTTGGTGTCACCCTGGTTACTACTCTGAAACCGAGCAATTTCCTTTTGTTGAATCACAACGCAAGTAGAATTGGAAATTTGGCTCTCTAGGCATGGATCTTCTATTCTAGACATGTGTGTCACAACCATATGGGAGCTATCAGTTTGGATTTGATATCTTGACTTCCATCTTAATGTTAATTTTGATGCTAATAATGTGTCGCATCAACTTCTTTACCTTGTTATTTTGTTTGGTCAAGAATACAGAGATGTGCATCTCTAGGTGGAAGTAGTTCTACAGTAACAAGTGTCTTGCTTCGTTGTTTGTGGTCGGGAAAGATCCAGCATTTTCCCAGGTCTGTGACTAAAGGTATTGGATTTTTCAGTATCTCATAACTTTGTTCAAAACTGTTTTGATATGGAAGTTGTCAACTATGATAATTCTTTTCACAAGTGTGTATAGCGTTTTCTGCTGCCAGTTAGTTTTAGTTGTTAGTTCAAATGCTCGTGTTCTTTGATGATTCTGGTGTATTTTATGGTGTTATGGGCAGTACCGGACGAAGTCAAAAGTCAATTCTGGATCTTTGATAGTGCTGCAAAGTGCTCCTCTGCTACTTATCTTCTCGCCACTTTCCTTGGTTATCACGCCAGATTGATTTTTGATTATTGATGAATGAATGTATAGACCACATGGTGACAGTAACAGAGGGTTAGTTTGGAAACTTCTAAGGCTTGTCCGGTATGCATCCCTGTGCACCTTTTTTCTTGGTCTTCTTGGTGAATGAATGTGTAGACCACATGGTAATGTGCTTCAATAAAAATGGGGGTTCTCAATCTTGTCTCTTGCATCTCTAGTGATTTCTTAGGTGATATGGGTTTTTTCAGCACATAACCGGTCCCTTCTGTTTGTGCTTGCAGTTGGGATTTACCGAGCAGTGAATTATTGTTTTGATTGAGGAGGCTTTGTTTGGCCATTTGTGTTGAGGTTTAATGAGTACTGAGTTACTCAAATACTCATTTCACTATTTTCCCCCAATCAGAGTGGTCCCTCCTCTCTTTGTTAAAAATTGCTGATGTATGGAACTTGCCAGCTAAAATAGTTTCTCTTGCACTTGAGTGTGTTGTATTCCGCTATCGTTTCTCGTTTTTAGTCCAGATGTGTATGTTCTTCGATGATCCAGTTGATGAGGTTCCTTGTGGTTGATGGTTATATGGTCTTATCGACACCCTGATAAATACTCAAAACTCAATCCTGAATCTTTTGATAGCTCGGTCAAGTGGGTCCTTGTTAGACCCCCCTCTTTTGGTGTTCTCTCTGCCTTAACTTCTCTGCTTCCTTTTTCTTGCATCACCCAAGCTTGATTTTGTTTGTTGGATGGATGACAGTTGAGAGGAAACTTGGGAAAATTTTGAAGGTTGATGCCAGTGTCCGTTCCTGTGCACGCTGTTTCCTTGCCTTTTTTGTGCTTGCACTACAATCTGAATATCATCAGGCTTCGATAGGGGGGTTTTCCAATCTTGTTTTTTTGCCATTTTTGTGCTTGCATTGCAATCTGAATATTTTCAAGCTTCGATAGGTTTTTTTTTTCAATCTTATCTGTAGCATTTTCTAGTGGGGTTTGCCAGCTTTGCACGTAACTCTTCCCTTCATTCCTTTGCTTGCAGCCATTGGGTCTTACAAAGCATTGAATTCTTCTTTTCATGGAGGTACCTTATTTTTGTCCATATGTGTTGAGGTCTTGTTCGCCAATCTTTTACAAGTATAGATAATTTTCAATGATTCTTCCCTATTGACTCCTTTGTCTTTGTCATAGCTACTAATGTATGGTAGTTTCTCAATTAGGATATTATTTTCACTAATATGTGTTGTATTATGCTGCCATGAAGTTCATAGTTCAGATGTTTATGGTGTTATGGTGATCCAATTGATAAGGTCTCTTATGGTGATGAGGTCTCTTACTCTCTTATGGCTATGGATACCTGATAAGTACTTGACCAATTCTAGATCTTTCAGTAGTGCCGTAAAATATTTCGATAGTGGCGTAAAATACTTCTCAACTTTTGTTCTTTTAGCTACTTATATTATCAAACTGCTTTCATTGTTTTTGTTATCACACTAGTTTTTTTTTTGTTGTATGGATGTGTAGACTATGTGGTGACACTGTTATGGACGTGCAGATTTGGAAATTTCAAAGCTTGGCTAACCAACATGTGATCCTTTGCCGTCTTTTGGTCTTTTTTGTGCTTGCATTGCAATCTGGATATTGTCTAGGCTTTGACAAAGGGTTTTTAGTCTCGTCTATGGCATCCCTTGTGATTATTTAGGTTTGCTCTGAACTATTCCCTTCCTTCTGTTCTTGCAGCCATTGCTTACAGGGCCGTGAATTCTTCTTTTCATGGAGTGGCCGTGTTTTTGTACGTTTGCGTTGAGGTTTCTTTAGTCATTTTATATGAGTATGGAGATTTTCGTTTCAATTTTCTTCCCTAAATGGCCCTTTGTCTTTATCGGATCTTTGTCAAAACTAGTGTTGTATGGAAGTTTCTCACGAGGACATTATTTTTCACTGGCGTGTATCGTATATGCTGTCATGAAGTTGTAATGACCCGTTAACTCTTTCTTTCATAAAAGACCTTTTTTCGTATAGTTTAAATGAAAATTTTGAACTATTCGTTTAACTACGGTGAATTAGTTGATGGGTATTTGTTAAATAATTAAATTAGTTGGTGTTAATAGGTCAAGTCCATAATTTAATACTCTATTCAATTTAACCTATGTATTGAAATAAATTAAATGGAATTTAACAATTAATAAGTTATGCTCAAAAAGATGTCGCGGCAGTATTCAAGAGCGACATTCTCTTCCAGGTAACAACACATTAAATCTCCAACTTCCTCCATGTGTATATGAAATTGTAAGATGCTTCATTTACATTAATTAGTATAATAATATATATTTTTTTGGAAGAAGAAACAAAGCCATTGAGTTATTGATGTTCCAAAAGCTGCTGGCCAAAAATAGTGGGCAAATCAATTTATATTCATCAATTTATGTGTTGATTACAATATTGAAGAGGAATTAATAATGGACATGTTGAGTTAATCATTTGGAATGATGTAACAATATGTTGCATTTTTGTATAATAATAGTCTGTTGCATATTGATTTTTGTTACCGAAGGTTAGTGATGGCTTAATCTGGTTTGAGTTGGAAAATTTCTTAGGGTTATCAATAACAAATCAAGTTTTGTCATATTAAGACAACTATTATATGAATACCATTAGATTTATAATATTTTGATAAATTGACACTTAACCCTTGGCTTAATATTTAGGAATATAAGACTTGACATATTTAGTTGGCACCAAGATTAATGAATTTAATTATAGATTTAGGTGAGTTTTGGGTCTAGGCTAGACATATAGTAATATATGTGTCTACAAACTCATTTGTTTATGAATATTGCATATTTTCTAGATTGGGAACATTTTGAAGCTTTACGAAAGGGAAAGGAAAAATCTAGACGATTCGTGACACGTGTTCTGCACTTAAGGTGCCATCACGACTTGTGATTTGGGTCGTGACAGAAGTTTAGTCCAGATGCTTATGATGCTTATGTTCTTCGATCATCCAATTGATGAGGTCTTCTATGGCTATGGACACTTGATAAGTACTCGGCCAATTCTAGATCTCATTTGATAGTGGGGTAAAATACTACTGCTTCACTTTTATTTTCTAGCTACTTATCTTCTCTGCTTCCATATATTTTTTGTTACCACACTGTTTTTTCTTGGCTGGATGGATCTGTAGTAAGCAGTGTGGTGGCACTGTCATGGACGTTCAGATTTGGAAATTTCAAAGCTTGGTTTACCAACATGTGATCCTCTTCCACCTTTTGGTCTTTTTGGTGCTTACACATCAATCTGGATATTGTCCGGGCATCGACAAAAGGGGGTTTTCAGTCTCGTGTATGGCCATCTCTAGTGATTACTTAGCTGATTTGGAGTTTGTCAGATTTGCTCTTAACTGTTCCCTTCTTTCTGATCATGCAGCCATTGGTTACAGGGCAGTGAATTATTCTTTCCATGGAGTGGTCGTGTTTGTTCGTTTGTGTTGAGGTTTCTTTAGTTCAAGTTTGTAAGATTATTAAAACTTCCGATTCAATTTTCTTCGGAAGTTTCTGGAAGATCGTAGTTTTCACTAGTGTGTGTTGTTGTTCTACTGCCGTGATGTTTTTCATCCAGACAATATGTTCTTCGATGATCCAATTGATGAGGTCTCCCATGGCTATGGACACTTGATAAGTACTTGAGCCAAATTGTGGATATTTTCTTTTGACAGCGACATAAATGCTCCCCTACATCTGGTCTTCTAGCTACAAATATTTTGGTTATCACATGTGTTTTTTGTTTGGATGGATGTGTAGACTATATGGT

>LNC_000020

CTGAAAAACACCGACCCAATGCCACCGCCTTTGAAATATTCTTTCTTAGTCTAAATATACATCAACCCTATAAATATTCTTTCTTGGTCCAAAAACCCCGTTCGTTGATTGTTTTTGAGGGTATACGCAGTGTTTGCTTGTTCATATGGGTAACTTTTGTTGATCAAGACATGGGTAATTACAGTGCCTTATAGTTATTCTTGATCTGGGTAGCTATTGTTTATCAAGATACAGGCCAAATATTGTATAATCAAGATAGTGGATAGTTATTGTTTGATCAAGATACTGGTAATTATTGTTGATCAATATTGTGGGTAACTATTGTTTGGTCAAGATACTGGGTAATTATTGTGCTCCTTATTGTTAATCTGGATACTTATTGTTGAGCAAGATAGCGGGTAACTATTGTTGATCAGGATAATGGGTAATAATGTTTGATCAAGATATGGGTAGTTATTGTAGATTATTGTATATGATTTGGTCTTCTTTCGTTGGTATCTTGAGGATATAAGCAGTGTTTGATTGTTCATATGGTTTATTGTTGATCAAGATGATGGGTTATTATTGTTTGATCAAGATGATGGGTTATTGTTGTTGATCAACACGTGGGTAATTATTGTTTGATCAAGATATGGGTATGTCTTCGTTTGGGGAGGCGACTGGTTGGGTTTTTGATTTCTTGGCCATCTTAGAATTATATACAATCTCAAGTCATGTACTTTTGAATCTTGGTTTTCAAAGATATCAATATTTAGGGAGCCATTGCTTAGGTTTCTGATTTGCAACTCTTTGATCTTCAATGTTAGGGGAGTTCTTGACTTTTTAGTGTGATTCAATTTGCAAATAGTTCAGTCTTTTTTGGCTAAATTATTGATTAATATTTGTTGTTTGTTTGATCAGGTATGGGTAATGATGATAGGAGACTGGTGGTAGTTTCTCAATTAGGACATTATTTTCACCAATGTGTGTTGTATTCCCTATTGACTCCTTTGTCTTTTATGGTAGTTTCTCAATTAGGACATTATTTTATTTTCACACATAGTTTCTCAATTAGGACATTATTTTCACCAATGTGTGATGTACTCTGCTGCCATGAAGTTCTTAGTTTAGATGCTTATGTTCTTATGGTGATCCAATTGATAAGTTCTCTTATGGTGATAAGGTCTCTTACTCTCTTATGGCTTTGGATACCTGAGAAGTACTTGACCAATTCTAGATCTTTTGGTAGTGATGTAAAATATTTCGATAGTGGCATAAAATACTTCTCAACTTTTGTTCTTTTAGCTACTTATCTTCTCTGCTTTCATTTTTTTTTGTCATCCCACTAATTTTGGTTGAATGGATGTGTAGACCATGTGGTGACACTGTTATGGACGTTCAGATTTGGAAATTTAAAATCTTGGTTAACCAACATGTGATCCTTTGCCTCCTTTTGGTCTTTTTGGTGCTTGCATTGCAAGTTGGATGTTTTTTGGCTCTGACAAAAGGGGGGGTTTCAGTCTTGTCAATGTAATCTCCAGTGATTACTCAGGTGGTTTGGGGTTTGTCAGGTTTTCTCTGAACCGCTCCCTTCTTTCTGTTCTTGCAGCCATTGCTTACAGAGCAGTGAATTCTTCTTTTCACGGAGCGGCTATGTTTTGTTGGTTTGCTTGAGATTTCTTTAGTCAAGTCTCTTATAATTATGGATATTTTGGATTCAATTTTCTTCCATAATGGGCCTTTGTCTTTGTCAAAACAAGTGTTGTATGAAGTTTCTCAACTAGGGCATTATTTTTCACCGGTGTGTGTTGTATTCTGCTGTCGTGAAGTTCAGTCCAGACACTTATGTTCTTTGATCATCCAATTGATGAGGTCTTCTATGGCTATAGATGCTTGATAAGTACTCGGCCAATTCTAGATCTCATTTGATAGTGGCGTAAAATACTTCTTTACTTTAATTCTCTAGCTACTTAGTTATCTTCTCTGTTTCCATAGGTTTTTCGTTATCACACTGATTTGTTTTGGTTGAATGGATGTGTAGTAGATCATATGATGACACTGTTATGGACGTTCAAAATTTAGAAATTTCAAAGCTTGGTTTATCAACATGTGATCCTCTGCCACCTTTTGGTCTTTTTGGTGCTTGCACAACAATCTGGATATTGTCCGGGCTTCGACAGAAGGGGGTTTTCAGTCTCTTCTATGGCATTTCTAGTGATTACTTAGGTGATTTGGGGTTCCTCAGATTTGCACTTAACTGTTCCCTTCTTTCTGTTCATGCAGCCATTGGTTGCAGGGCAGTGAATTATTCTTTCCATGGAGTGGCCGTGTTTGCCAGTTTGTGTTGAGGTTTCTTTAGTTTAAGTTTTTATGAGTATCAAGATTTTCGATTCATTTTTCTTCAGTAGTTTCTCGACTAAGATAATATCTTTCACTAGTGTGTGTTGTTATTCTGCCGCCGTGAAGTTTTTAATGCAGACACTATGTTCTTCGATGATCCAACTGATAAGGTCTCTTATGGCTATGCACACTTGATAAGTACTCATCCAATTTTGGATCTTTTCTTTAGACAACGCCATAAATGCTCCTCTACATCTGGTCTTCTAGGTACAGATATTTTGGTTATCACAACTTGTTTTTTTGTTTGGATGCATGGATGTGCAGACTATATTGTGACAGTAATAGAGGTTAAGATTTGGAGATTTTAAAGGTTGATATCCAGTAATGGCTATGGACACTTGATAAGTACTCATCCAATTTTGGATTTTTTCTTTAGACAGCACCATAAGTGGTCATCTACATCTGGTCTTCTAGGTACAGATATTTTGGTTATCACAACTTATTTTTTTGTTTGGATTCATGGATGTGCAGACTATATTGTGACAGTAATAGAGGTTAAGATTTGGAGATTTTAAAGGTTGATATCCAGTATGGTATCTCTGTATACTCTTGGTCTTTTTGGTTCTTGCACTGCAATTTAGATATATTGGTCAGGGCTTCAACAAAAAAAGGGTTTTAAATCTTGTCTATATGGCATCTCTAGTGATTTCTTGGATGATATGGGTTTGTATCTTCTTTTTGTACTTGCAACCATTGCATCTTATAGAGCTATGAAATATTCTTTTCATGGAGCGGCTTTCTTTGTCCATTTATGAGGTTTCCTTGGTTCAGGCTTTTATGATTTCTGATATTTTCAATTAAAATAGTTATTTTAACGTTGGTGTGTGTTGTATTCTACTGCCATTTTTAGTGTTCAGTCCAGATGCAATGGCCCAATTTGATGAGATTCTTGATGGTTGATGGTTATGGTCCCTTGGACACCTGACAAGAACTCAATTTTGGATCTTTAATGGTCTTCTCTTTTGGTGTCCTCACTGCTCATTTTCTCTGCTTCATTCTTCGTGTATTACGCTAGCTTGATTTTGTTTTGAGAGGTTATGACTTGGAAGATTTTGAAGGTTGGTGTCCAGTGTGCATTCCTGTGCACCCTGTTTCTTGGCCATGTTGGTGCTTGCACTACAATCTAGAGATTATCAAGCTTCGAAGGTGGTTCTCCATTTTGTTTGGTATTTGTGGTGAATTCTGAGGTGGTACGGTCTTCACTAGATCTGCACTTGAACTGTTCCATTTTTCTGTGCTTGTAGCTGATGGGTCTTACAGAGGGATAGGTTCTTCTTTTCATGGACCGGCATGGTCTGTCAATTTTGTGTTGAGATTTTTTGTTCGATGCTTCTAATGATTATTGAGATCTTCAATTCAGTTTTCTTCCCTAATCAGAAAGGTGCCCTTCTCTCCACTGCTTCACTAGGGTGTATTTTGCTGCCATATTTGTTTTTCAATAGTCCAGATGCTTATGTTCTTTGGTGATCCAACTGATGAGATCTCCATGGTTATGGGGACAACATATGATAAGTGTATTTAAACTCAATCGTGCTCTTTTGATGGCCCTGTAATGCACTCTTGTGCTTTTCATGTTCTCGCTACTTACCTTCTCTGAGTTCGTATAGCCTACCAGTTGATTTAGTTTGGTTGGATGAATGTCTGTAGGCTACATGGTGGTAGTATAGCCTACCAGTTCGTATAACTTTTCATGTTCTGTTACTTATTTGGATATTTTGTAGTTTGGTTTCGGTGTGCATCCTGTTGATCATGTCTCTTGCCATTTTGATGCTTGCAGTGCTATCTGGATATTGGATATCACCAGGCATCAAGAAAAGGGTGCTTTGCAATCTTATCTTGTTTGGCATATCTAGTGATCTCTCCTGCGTGGTATGGGTTTCCACTTACCTGTTATGTTCTTTTTGTGCATGTAACCGTTGGGTCTTCTAGAGCATGAACTTCAGTTCAATTTTTGTTCCTAATCAACGAGTCCCTTTTCTCTTTGTCAATTATTTTTTTGGGATGATCCCCTTTTCTCTTTGTCAATTATTTTTTTTGGGATGATCCAAATGGTGAGATCCCTGAGTTTCATGTGCACCTGAGAAGTACCCAAACTCAATTTTAGATCCTCTTATGGTCCCAAGAGTTCCTCTACTTTGGTGTTCTTGCTGCTCTTATGCTTGTCATGCCACTTGATTTTGTTTGTTTGGATGCATGTTTGTAGATTACATCAGATTTGGTAATTTCGAAGTTTGATTTCAGTGTGTACCCTGTTCCTTTGCCATATTGGTACTTGTGCTTGCACTGCAATTTGGATATCATCAGGCTCTGTCAAGGGAGTTATGCAATCTTGTCTGGCATCTCTAGTGATCTCTTCTATGTGGCATGGGTTTCGCAGCCTGCGCTTTAACTGTTCCCTTCTTTTGTGCTTGTGTTGTTGTATCTTTCAAGATCATTGAAGTCTTCCTTTCATGGAGCAGCCTTGTTTGTCTTAGTGTTGAAGTTTCGTGAGTTCAGTCCTTGTATTAGTATCTAACTTTTTGATTCAGTTTTCTTCCCTGGTCAGAGAGATCCCCTTATCTGGTTGTCAGAAGGGCTGTTAAATGGAAGTTTCAATTACGTTAACTCTCTCCACAGGTGCATTTTCAGCTTTCAGTCCAGATGCTTTATATTTTGAGATGATCCAACTGATGAGGTCTCTTATGTTGGAGATCTCTCCCCTACTATTTTTTTGAAACATGTATATTATTTTCAGATATTCAATCCTTTGAGGAGTTGCTTCAGACTATTAGCTATTATGTAGAACTATAATATAAAAAGAGAGTTCCTGCACACTTTTTTCCTCCGTAAAACATTGTCATTTGAACAGTGTTTAGCACCAAAGACCGAGCTTATCCATTGAATCTCGAACAAATGAAGTAAAAAACTTGGCTCATCATTTGTGTAACATGCCATTTACACTCTTTATGTTATTTATATCAACACAGAGGTAACTATTTTTTTTTGTGTAAAATATTTTCTGATGAGATATCCTTGTAAATCCATTACATGTCAGATCATCATGCAAGTTGGGAAGTTTATCAATGGATCTTGTTCTTCTAAGTACTCTTCTGAGTTAAGTTTTGTACTAATTTTCTTAATACTATTGAAATTGCTATTGTTTATAGAACATTGTTGTTCTGTCATAGGCCATAGTGATCCGGGAGTCCCGTGTGAAGGGCTCTCGCTCAACGGATCAAAGGTACGCCAGTGATAACAGGCTGATGAATCCAAAGAGCTCTTACCGACGAAGTCGTTTGGCACCTCGGATTTCTAAACATCTTATTATCATATAACACTAGAACATAGAACAATTAAATGAAACGAAAAGAAAAGAAAGAGATGACAGAGAATCCATTGAGAAGTTTACCTGTATCCAAGGTATTTATTCTCCATGTTCTTGCTATATCGTTGATGTATTATTAGCCAACCGTGTTCCTAATATGATTAGTCAACCGTGTTCCTAATATGTCACTAAACAGATAATTTGTTCATTCAAATGTCACTTCACAGATCTGCACTACCAGCTATAATTCGTTTCTTATTTCAGGAATTGTTCTTTGCTCCTTGGTTTTCTTGTATTGGTGATCTATTAGTAACCTTGCACAAGTTAAAAGTGATCTTCTAAAAAGCCCTAACTAGACCAAGTTTCGACAGTTTTGAAAAATGATGTTGTGTTTTCCTTCATTCATCAAATATAGCTCTCCATTCACCAGTTGAACATGCCTTTTCGGCATTTGGAAGGTATTTTGGTCATTTTCTGTAAGATGGAATAATCTGATAAGTTTGAAAAGAATCTTTCTGTAGCTATTATGGGAAGTTTAGATGTGTATTGCCTAAATTATCCTCTCATCCTTCGTCATTGTACTTAATTCTATCAGTTCCGCATGTGAGTTAGTGGGGACCAGAAAACCTTTTAACTGTTAAGTATTATCTCACTTTTTCCCCTCACTATATCATTGGTTGTTCATGAAGAATTTCTCCAATAATTATAAATTGTAGTAATAATTAATACTTTAAAATATTTAAAGAATATACTAAAATTTTAGTTAACTTTCAAAATTTTATGTGTGTCACATAAATTAGGACAAAGGAAATAACATATATTATTTGAAAATTATGTTAAAAGAATATTATAGATAACAATATTAACAACTTAAAATATTCAAAAATCATATAAAATTTGGTTAACTTTCCTAACTTCATTTGTGCTTATATTAAAGTGAGACAAATCGTAGGCTCTTGTGTCTAGTACTTGAGGTATTGTATGGTTATAGAGGGGTTTTCCATTTTTATCATTTTCCCTGATTCTCTGTTGCATCATTAGGAAATAACATTTTGGTAAAGAGAGATCCAACTATGATTTCAGTCATTCAGCCTACTTAGGAGTTCTCTTGGGAGTCTTTGTGTAGTCAGGTTACGGTTAGACCTATCTTCTTTCTTCCCTCCCCCTAATGTGGGTAAAGCTGTTCGTTAACTTCCTCTGCATGATGGTTAGAGATGTTTATGGTGGATCAACGTGGGCAAGATTTTAATTAAGTTTTGCATAAAGTGATAATGAGAATGTTCTTACGTGTTTCACAAAGTTGCAACTGTCTTTGGTGGTTTGGAGTTGATACCTCTACTTTAATGTCATACTCATTTATTTCATCCCGCTGCCCTCTATAAACATCCCTTCTTGGTCGTAATGGGGAGGGCTGGCAACTCGAGTTGTTTTCTCCTTCCCGTTGTCTTATTTGATCCGGTGGGATTAATTTTGTCCCCGATGCGGGTTTCTGCTCCTTGGTTGTGGCTATTGGAAGCTTGGGCGGAGGGGTCAAGGAGGTTTTTTTTTTGAGGTATGGAATATGAGCAGATCTGTGAGTTGCAAAAAAGAAAGTCTGGCAGAATGCTTGGCCTCCTGTGCAGGAAGTGAGACCTGAAGGGTGAGCTTATGGTGAAGAGTATCTATTTTTGGGTTTTGCAGATTTATTTTGAAGTTTGACTTGGAGGTAAGTTCTCACTTTTGTTGGTTTCTTGAGGCATCCAAGGACTTCCTGTTTAGGTTATGGCAATTCCTTTCTGGTTCGACCCGAGGTAATGATGGTAAGGTTATAGACCATGCAGTTTCTATTTTTGGATATCAATAATCTTTGGAACGAAGTAAACCTCGTCTCTGCTACCTGCAACAAATTATCTTTTTTTGCATCTTCTCCGCTGGAACACTATTCAGTAGAAGTTTTTGAGCCTTCCCTTGTAGATGTTGCTCTCTATATTCCATTTTGTTAGGCGATTGACTCGTTCAAAATCACGAATAGGTCTCCCTCTGATGTCTTCAGGTTGTCTTTATCTACCCATAAGCTTACAAGTCTTTATTTTCACAGTTATATTGTATTGTCCACCCCGTTAGATGGGTTCCTGCACCCAGCATGATAAGGGGACAATATCCTATCTTCAAAAATGTCAACGGGGTGCGTGTCAAATTCGCTAAAAGTACTGTATTTTAGGATAATCCGACATGGGTGTGGCATCGAAAGTGAAGAGTCCGTGCAACTTAGACAATATCAAGGAGTTTCTGAGAAGCAGTCTGTTAAAGTCATTTACAGTGGTTTTGATAATGTTTGACTTTGGTATTTAGAGCATTTGTTGTATTTGCTGATTTTGATCTCTTACCTGTAAAACAGCACTACAGATGTCAGTGTACTATTTTTTAAGTTGAGGAAGGTATTAGCATCTTGAAGTAGCTAGGATTCTTTGGCTTCAGTTTGTTGTGGTATTTCTTTTAAAATAAGGCCACTGAATGGGTATTTCTGTTTTCTCTAAGTTCCAGTTGTCCAAATTTGTCCAAATGTGCCAAATAATTTGAGTGGGGTATTTCACTTTAAACTCCAGTTGTCCAATCTCGCCAGATAAAGAATGTTCCTCTTTTCTCTCAAGTCCTTGTAGCTATTTTGATAGCGCTGGGATTGAAGGACTTATTTTTTTTTTGTGGCTAAAATACACAAATTCAGTAACATATAAACGGATTTGCTCTCCTAAAGTGGAGAGAAGTTGGGAGGATGGTAAATCAGTTGTTGAGGAGCATGTTATATAGAAAAGGTCAGTATGATGTGGATTATGTTAAACAGAAAAAGGTCCTGGTATGATCACTTGGATTCCTGTTTTTACATTGACCTGTTTTAGTCATCTTTTGCATGTTCAAAGTATTATAATATCTATTTTGGATTAACATTTTCGGATTAACATTACAAGTTCTTCTGGTTATATTGAGTGGGGGTGTGTTCAGCTACACTCGAGCGTTCTAGTCACTCAGTAGTATCTAATGTGCTTTCTCCTAGAATGTGTAGTAATTTATTAATCTATGTGCACCTAGCAGTGTCGGAGATCCAGTTTTAATATGAGTAAGAACGGAATGTTCTCAAAATAATTGTCTCATCTCACTTGTGCAAAATGCAGTTACTGAATATAGATGGTCTATAGGTAATATTTCAACTAGTATAGGTGTGAAGTAGCTTTCTGTTCGGGTTTTTATGATGAGTTGGGTATCTCTTTCTTTGGCTTATCTCACATTCTTATTTGGTTTTTATTGGTTTGGGATTGGTATCTGTGGATTAGTATCTTGGATGTGATAAAGCTTGAGTTACGGGAAAGAGAGGAAATACTTTTTCTCAAAACATGAACTAATGGAAGTTTAACTCTGGAAGAAGCACTTGATGAAGCATTGCGAAATTTGTCCTTATTGTTATTCTTGATCTGGGTAACTATTGTTGATCAAGATAGTGGATAGTTATTGCTTGATCAAGATACTGGTAATTATTGTTGATCTAGATTGTGGGTAACTATTGTTTGGTCAAGATATTGGGTAATTATTGTGCCTTATCGTTATTTTTAATCCGGGTAATTATTGTTGATCAACACAGCGGGTAACTATTGTTGATCAGGATAATGGGTAGTAATGTTTGATCAAGAAATGGGCTGTTATTGTATATGATTTGGTATTCTTTTTTTAGTATCTTGAGGATATAGGCAGTGTTTGATTGTTGATATGGTTTGTTGTTGATCAAGATATGGGTAATTATTGTTTGATCAAGATATGAGCAATTATTGTATATGATGCCTTCGTTTGGGGAGCGATTGGTTGGGTTTTGATTTCTTGGCCATCTCAGAATTATATAAATCTCAAGTCATGTACTTTTGAATTTTGTTTTTCGAAGATACTGATATTTGGGGAGCGATTGGTTATGTTTCTTATTTGCAACTCTTTGATCACATCTCTGTAGAGAAGGAATGTTGGGGGTTTTGGGAGTTCTTGACTTTTTAGTGTGATTCAGTTTGCAATTAGCTCTGTTTTTTGGGGCTAATTTATTTATTCGTTTGCAATTAGTTCTATTCTTTTCCTAATTTATTGATTAATTATTGTTGTTTGTTTGATCAGGTATGGGTAATGATGATAGGAGACCGATTCTCCTTCTACACTTGCTGTTACCTTTGTCTCAATGTTAATTCTGAGGTTTTTGTTGTGAGAATTCATGTGTTGGTGTCACCCTGGTTACTACTCTGAAACCGAGCAATTTCCTTTTGTTGAATCACAACGCAAGTAGAATTGGAAATTTGGCTCTCTAGGCATGGATCTTCTATTCTAGACATGTGTGTCACAACCATATGGGAGCTATCAGTTTGGATTTGATATCTTGACTTCCATCTTAATGTTAATTTTGATGCTAATAATGTGTCGCATCAACTTCTTTACCTTGTTATTTTGTTTGGTCAAGAATACAGAGATGTGCATCTCTAGGTGGAAGTAGTTCTACAGTAACAAGTGTCTTGCTTCGTTGTTTGTGGTCGGGAAAGATCCAGCATTTTCCCAGGTCTGTGACTAAAGGTATTGGATTTTTCAGTATCTCATAACTTTGTTCAAAACTGTTTTGATATGGAAGTTGTCAACTATGATAATTCTTTTCACAAGTGTGTATAGCGTTTTCTGCTGCCAGTTAGTTTTAGTTGTTAGTTCAAATGCTCGTGTTCTTTGATGATTCTGGTGTATTTTATGGTGTTATGGGCAGTACCGGACGAAGTCAAAAGTCAATTCTGGATCTTTGATAGTGCTGCAAAGTGCTCCTCTGCTACTTATCTTCTCGCCACTTTCCTTGGTTATCACGCCAGATTGATTTTTGATTATTGATGAATGAATGTATAGACCACATGGTGACAGTAACAGAGGGTTAGTTTGGAAACTTCTAAGGCTTGTCCGGTATGCATCCCTGTGCACCTTTTTTCTTGGTCTTCTTGGTGAATGAATGTGTAGACCACATGGTAATGTGCTTCAATAAAAATGGGGGTTCTCAATCTTGTCTCTTGCATCTCTAGTGATTTCTTAGGTGATATGGGTTTTTTCAGCACATAACCGGTCCCTTCTGTTTGTGCTTGCAGTTGGGATTTACCGAGCAGTGAATTATTGTTTTGATTGAGGAGGCTTTGTTTGGCCATTTGTGTTGAGGTTTAATGAGTACTGAGTTACTCAAATACTCATTTCACTATTTTCCCCCAATCAGAGTGGTCCCTCCTCTCTTTGTTAAAAATTGCTGATGTATGGAACTTGCCAGCTAAAATAGTTTCTCTTGCACTTGAGTGTGTTGTATTCCGCTATCGTTTCTCGTTTTTAGTCCAGATGTGTATGTTCTTCGATGATCCAGTTGATGAGGTTCCTTGTGGTTGATGGTTATATGGTCTTATCGACACCCTGATAAATACTCAAAACTCAATCCTGAATCTTTTGATAGCTCGGTCAAGTGGGTCCTTGTTAGACCCCCCTCTTTTGGTGTTCTCTCTGCCTTAACTTCTCTGCTTCCTTTTTCTTGCATCACCCAAGCTTGATTTTGTTTGTTGGATGGATGACAGTTGAGAGGAAACTTGGGAAAATTTTGAAGGTTGATGCCAGTGTCCGTTCCTGTGCACGCTGTTTCCTTGCCTTTTTTGTGCTTGCACTACAATCTGAATATCATCAGGCTTCGATAGGGGGGTTTTCCAATCTTGTTTTTTTGCCATTTTTGTGCTTGCATTGCAATCTGAATATTTTCAAGCTTCGATAGGTTTTTTTTTTCAATCTTATCTGTAGCATTTTCTAGTGGGGTTTGCCAGCTTTGCACGTAACTCTTCCCTTCATTCCTTTGCTTGCAGCCATTGGGTCTTACAAAGCATTGAATTCTTCTTTTCATGGAGGTACCTTATTTTTGTCCATATGTGTTGAGGTCTTGTTCGCCAATCTTTTACAAGTATAGATAATTTTCAATGATTCTTCCCTATTGACTCCTTTGTCTTTGTCATAGCTACTAATGTATGGTAGTTTCTCAATTAGGATATTATTTTCACTAATATGTGTTGTATTATGCTGCCATGAAGTTCATAGTTCAGATGTTTATGGTGTTATGGTGATCCAATTGATAAGGTCTCTTATGGTGATGAGGTCTCTTACTCTCTTATGGCTATGGATACCTGATAAGTACTTGACCAATTCTAGATCTTTCAGTAGTGCCGTAAAATATTTCGATAGTGGCGTAAAATACTTCTCAACTTTTGTTCTTTTAGCTACTTATATTATCAAACTGCTTTCATTGTTTTTGTTATCACACTAGTTTTTTTTTTGTTGTATGGATGTGTAGACTATGTGGTGACACTGTTATGGACGTGCAGATTTGGAAATTTCAAAGCTTGGCTAACCAACATGTGATCCTTTGCCGTCTTTTGGTCTTTTTTGTGCTTGCATTGCAATCTGGATATTGTCTAGGCTTTGACAAAGGGTTTTTAGTCTCGTCTATGGCATCCCTTGTGATTATTTAGGTTTGCTCTGAACTATTCCCTTCCTTCTGTTCTTGCAGCCATTGCTTACAGGGCCGTGAATTCTTCTTTTCATGGAGTGGCCGTGTTTTTGTACGTTTGCGTTGAGGTTTCTTTAGTCATTTTATATGAGTATGGAGATTTTCGTTTCAATTTTCTTCCCTAAATGGCCCTTTGTCTTTATCGGATCTTTGTCAAAACTAGTGTTGTATGGAAGTTTCTCACGAGGACATTATTTTTCACTGGCGTGTATCGTATATGCTGTCATGAAGTTGTAATGACCCGTTAACTCTTTCTTTCATAAAAGACCTTTTTTCGTATAGTTTAAATGAAAATTTTGAACTATTCGTTTAACTACGGTGAATTAGTTGATGGGTATTTGTTAAATAATTAAATTAGTTGGTGTTAATAGGTCAAGTCCATAATTTAATACTCTATTCAATTTAACCTATGTATTGAAATAAATTAAATGGAATTTAACAATTAATAAGTTATGCTCAAAAAGATGTCGCGGCAGTATTCAAGAGCGACATTCTCTTCCAGGTAACAACACATTAAATCTCCAACTTCCTCCATGTGTATATGAAATTGTAAGATGCTTCATTTACATTAATTAGTATAATAATATATATTTTTTTGGAAGAAGAAACAAAGCCATTGAGTTATTGATGTTCCAAAAGCTGCTGGCCAAAAATAGTGGGCAAATCAATTTATATTCATCAATTTATGTGTTGATTACAATATTGAAGAGGAATTAATAATGGACATGTTGAGTTAATCATTTGGAATGATGTAACAATATGTTGCATTTTTGTATAATAATAGTCTGTTGCATATTGATTTTTGTTACCGAAGGTTAGTGATGGCTTAATCTGGTTTGAGTTGGAAAATTTCTTAGGGTTATCAATAACAAATCAAGTTTTGTCATATTAAGACAACTATTATATGAATACCATTAGATTTATAATATTTTGATAAATTGACACTTAACCCTTGGCTTAATATTTAGGAATATAAGACTTGACATATTTAGTTGGCACCAAGATTAATGAATTTAATTATAGATTTAGGTGAGTTTTGGGTCTAGGCTAGACATATAGTAATATATGTGTCTACAAACTCATTTGTTTATGAATATTGCATATTTTCTAGATTGGGAACATTTTGAAGCTTTACGAAAGGGAAAGGAAAAATCTAGACGATTCGTGACACGTGTTCTGCACTTAAGGTATGTTAAGACTTTCATACTTCATTGAAGGAAGTTTTTCTAATTAAAGAGAAAAAATGGAATAGACAATGGCTAAGGGCGAAAGATGGGAGTCTCGTACTAATGGTATGGCGGACATTATAAAGGGAAAGTTTATTACTATAGATTGACATATTGTAATCTGAGAATATCAAAGCATATGGGCATTAGCTGGTGTATTATTGACTTGGTTTTTTTGTGTTTTTGTGTTTCTTTATTGAACCCGTATATTTGTGATAATTGAGGTGATTATATATCATACCAGTATTTGGTTGATTGAGATGCATCATCATCCCCTTTTATTGAAATCATATTGTGCACATGCATTGACTTGAGATTGAGTATAAGTTGGGCATGGGGAGATCGTCTGTGATGGGGATTGTGAGATTTTATTACTATAAGTTGGGCACGTGGAGATCATTCGTGTCGGGGATTGTTTGATTTTATGATAGTGCGTTGAGATCGTCCGCACAAACACGTGGAGATTGTCTGTGTCGGTATATGGACCTCGCGAGTCCCCCAAGGGTCATGAACTCTCGATGTGTTTCCGAGGAGTATCATGTATATACGGTTGAGAGAGTAATTGGCATCTGAGGCATATTATTTCATGGTGTCACATTGCATTACATCTCATTACATCCTTCATTCTTGTTTGAGTATGTGCTTTATTGATGTTGGATATTACGTGATATTTGATTATCTCTAATGATGAAAACTTGGTTGATAAGTATAATGTAGACTTGTGAAATTAAAGGATGGTTTTCTTATATACTCCTGTCACTACTTCTTTGTTGTCGGTCTATGAGATATAGTGGGTACACGTGATTTCGTACTCATACTACACTTGCCACACTCTTTTGTGGTGTAGATTGGATTCAAATTCGAGCACATTTCTTGGAGTTTTGGTGAGCTGCTTGGATATTATGCGGCCCACACCTTTCTCTATCTCTATCTATTAGGTTGATTTGGTATTCCGATAGGATATTTCTTATGTCTAGACTTGTCAGAAATTTTTGTATGGACTTTTAAAAGTACATCTTAGGGTGGAATTTCAGTTTTTCCGCAATTTATTTAAATGACTTAGTATTTAAGATATTTGATTGCTACTTACTTTTCCACTTTTTAAGTTGAATTGGTTACCTTACTGGGTTGGCTTACCTATTGGTTGGGAATTTAGGTGCCATCACGACTTGTGATTTGGGTCGTGACAGAAGTTTAGTCCAGATGCTTATGATGCTTATGTTCTTCGATCATCCAATTGATGAGGTCTTCTATGGCTATGGACACTTGATAAGTACTCGGCCAATTCTAGATCTCATTTGATAGTGGGGTAAAATACTACTGCTTCACTTTTATTTTCTAGCTACTTATCTTCTCTGCTTCCATATATTTTTTGTTACCACACTGTTTTTTCTTGGCTGGATGGATCTGTAGTAAGCAGTGTGGTGGCACTGTCATGGACGTTCAGATTTGGAAATTTCAAAGCTTGGTTTACCAACATGTGATCCTCTTCCACCTTTTGGTCTTTTTGGTGCTTACACATCAATCTGGATATTGTCCGGGCATCGACAAAAGGGGGTTTTCAGTCTCGTGTATGGCCATCTCTAGTGATTACTTAGCTGATTTGGAGTTTGTCAGATTTGCTCTTAACTGTTCCCTTCTTTCTGATCATGCAGCCATTGGTTACAGGGCAGTGAATTATTCTTTCCATGGAGTGGTCGTGTTTGTTCGTTTGTGTTGAGGTTTCTTTAGTTCAAGTTTGTAAGATTATTAAAACTTCCGATTCAATTTTCTTCGGAAGTTTCTGGAAGATCGTAGTTTTCACTAGTGTGTGTTGTTGTTCTACTGCCGTGATGTTTTTCATCCAGACAATATGTTCTTCGATGATCCAATTGATGAGGTCTCCCATGGCTATGGACACTTGATAAGTACTTGAGCCAAATTGTGGATATTTTCTTTTGACAGCGACATAAATGCTCCCCTACATCTGGTCTTCTAGCTACAAATATTTTGGTTATCACATGTGTTTTTTGTTTGGATGGATGTGTAGACTATATGGT

>LNC_000021

CTGAAAAACACCGACCCAATGCCACCGCCTTTGAAATATTCTTTCTTAGTCTAAATATACATCAACCCTATAAATATTCTTTCTTGGTCCAAAAACCCCGTTCGTTGATTGTTTTTGAGGGTATACGCAGTGTTTGCTTGTTCATATGGGTAACTTTTGTTGATCAAGACATGGGTAATTACAGTGCCTTATAGTTATTCTTGATCTGGGTAGCTATTGTTTATCAAGATACAGGCCAAATATTGTATAATCAAGATAGTGGATAGTTATTGTTTGATCAAGATACTGGTAATTATTGTTGATCAATATTGTGGGTAACTATTGTTTGGTCAAGATACTGGGTAATTATTGTGCTCCTTATTGTTAATCTGGATACTTATTGTTGAGCAAGATAGCGGGTAACTATTGTTGATCAGGATAATGGGTAATAATGTTTGATCAAGATATGGGTAGTTATTGTAGATTATTGTATATGATTTGGTCTTCTTTCGTTGGTATCTTGAGGATATAAGCAGTGTTTGATTGTTCATATGGTTTATTGTTGATCAAGATGATGGGTTATTATTGTTTGATCAAGATGATGGGTTATTGTTGTTGATCAACACGTGGGTAATTATTGTTTGATCAAGATATGGGTATGTCTTCGTTTGGGGAGGCGACTGGTTGGGTTTTTGATTTCTTGGCCATCTTAGAATTATATACAATCTCAAGTCATGTACTTTTGAATCTTGGTTTTCAAAGATATCAATATTTAGGGAGCCATTGCTTAGGTTTCTGATTTGCAACTCTTTGATCTTCAATGTTAGGGGAGTTCTTGACTTTTTAGTGTGATTCAATTTGCAAATAGTTCAGTCTTTTTTGGCTAAATTATTGATTAATATTTGTTGTTTGTTTGATCAGGTATGGGTAATGATGATAGGAGACTGGTGGTAGTTTCTCAATTAGGACATTATTTTCACCAATGTGTGTTGTATTCCCTATTGACTCCTTTGTCTTTTATGGTAGTTTCTCAATTAGGACATTATTTTATTTTCACACATAGTTTCTCAATTAGGACATTATTTTCACCAATGTGTGATGTACTCTGCTGCCATGAAGTTCTTAGTTTAGATGCTTATGTTCTTATGGTGATCCAATTGATAAGTTCTCTTATGGTGATAAGGTCTCTTACTCTCTTATGGCTTTGGATACCTGAGAAGTACTTGACCAATTCTAGATCTTTTGGTAGTGATGTAAAATATTTCGATAGTGGCATAAAATACTTCTCAACTTTTGTTCTTTTAGCTACTTATCTTCTCTGCTTTCATTTTTTTTTGTCATCCCACTAATTTTGGTTGAATGGATGTGTAGACCATGTGGTGACACTGTTATGGACGTTCAGATTTGGAAATTTAAAATCTTGGTTAACCAACATGTGATCCTTTGCCTCCTTTTGGTCTTTTTGGTGCTTGCATTGCAAGTTGGATGTTTTTTGGCTCTGACAAAAGGGGGGGTTTCAGTCTTGTCAATGTAATCTCCAGTGATTACTCAGGTGGTTTGGGGTTTGTCAGGTTTTCTCTGAACCGCTCCCTTCTTTCTGTTCTTGCAGCCATTGCTTACAGAGCAGTGAATTCTTCTTTTCACGGAGCGGCTATGTTTTGTTGGTTTGCTTGAGATTTCTTTAGTCAAGTCTCTTATAATTATGGATATTTTGGATTCAATTTTCTTCCATAATGGGCCTTTGTCTTTGTCAAAACAAGTGTTGTATGAAGTTTCTCAACTAGGGCATTATTTTTCACCGGTGTGTGTTGTATTCTGCTGTCGTGAAGTTCAGTCCAGACACTTATGTTCTTTGATCATCCAATTGATGAGGTCTTCTATGGCTATAGATGCTTGATAAGTACTCGGCCAATTCTAGATCTCATTTGATAGTGGCGTAAAATACTTCTTTACTTTAATTCTCTAGCTACTTAGTTATCTTCTCTGTTTCCATAGGTTTTTCGTTATCACACTGATTTGTTTTGGTTGAATGGATGTGTAGTAGATCATATGATGACACTGTTATGGACGTTCAAAATTTAGAAATTTCAAAGCTTGGTTTATCAACATGTGATCCTCTGCCACCTTTTGGTCTTTTTGGTGCTTGCACAACAATCTGGATATTGTCCGGGCTTCGACAGAAGGGGGTTTTCAGTCTCTTCTATGGCATTTCTAGTGATTACTTAGGTGATTTGGGGTTCCTCAGATTTGCACTTAACTGTTCCCTTCTTTCTGTTCATGCAGCCATTGGTTGCAGGGCAGTGAATTATTCTTTCCATGGAGTGGCCGTGTTTGCCAGTTTGTGTTGAGGTTTCTTTAGTTTAAGTTTTTATGAGTATCAAGATTTTCGATTCATTTTTCTTCAGTAGTTTCTCGACTAAGATAATATCTTTCACTAGTGTGTGTTGTTATTCTGCCGCCGTGAAGTTTTTAATGCAGACACTATGTTCTTCGATGATCCAACTGATAAGGTCTCCCATGGCTATGGACACTTGATAAGTACTTGAGCCAAATTGTGGATATTTTCTTTTGACAGCGACATAAATGCTCCCCTACATCTGGTCTTCTAGCTACAAATATTTTGGTTATCACATGTGTTTTTTGTTTGGATGGATGTGTAGACTATATGGT

>LNC_000022

CTTTCTTGGTCCAAAAACCCCGTTCGTTGATTGTTTTTGAGGGTATACGCAGTGTTTGCTTGTTCATATGGGTAACTTTTGTTGATCAAGACATGGGTAATTACAGTGCCTTATAGTTATTCTTGATCTGGGTAGCTATTGTTTATCAAGATACAGGCCAAATATTGTATAATCAAGATAGTGGATAGTTATTGTTTGATCAAGATACTGGTAATTATTGTTGATCAATATTGTGGGTAACTATTGTTTGGTCAAGATACTGGGTAATTATTGTGCTCCTTATTGTTAATCTGGATACTTATTGTTGAGCAAGATAGCGGGTAACTATTGTTGATCAGGATAATGGGTAATAATGTTTGATCAAGATATGGGTAGTTATTGTAGATTATTGTATATGATTTGGTCTTCTTTCGTTGGTATCTTGAGGATATAAGCAGTGTTTGATTGTTCATATGGTTTATTGTTGATCAAGATGATGGGTTATTATTGTTTGATCAAGATGATGGGTTATTGTTGTTGATCAACACGTGGGTAATTATTGTTTGATCAAGATATGGGTATGTCTTCGTTTGGGGAGGCGACTGGTTGGGTTTTTGATTTCTTGGCCATCTTAGAATTATATACAATCTCAAGTCATGTACTTTTGAATCTTGGTTTTCAAAGATATCAATATTTAGGGAGCCATTGCTTAGGTTTCTGATTTGCAACTCTTTGATCTTCAATGTTAGGGGAGTTCTTGACTTTTTAGTGTGATTCAATTTGCAAATAGTTCAGTCTTTTTTGGCTAAATTATTGATTAATATTTGTTGTTTGTTTGATCAGGTATGGGTAATGATGATAGGAGACTGGTGGTAGTTTCTCAATTAGGACAGGACATTATTTTCACCAATGTGTGTTGTATTCCCTATTGACTCCTTTGTCTTTTATGGTAGTTTCTCAATTAGGACATTATTTTATTTTCACACATAGTTTCTCAATTAGGACATTATTTTCACCAATGTGTGATGTACTCTGCTGCCATGAAGTTCTTAGTTTAGATGCTTATGTTCTTATGGTGATCCAATTGATAAGTTCTCTTATGGTGATAAGGTCTCTTACTCTCTTATGGCTTTGGATACCTGAGAAGTACTTGACCAATTCTAGATCTTTTGGTAGTGATGTAAAATATTTCGATAGTGGCATAAAATACTTCTCAACTTTTGTTCTTTTAGCTACTTATCTTCTCTGCTTTCATTTTTTTTTGTCATCCCACTAATTTTGGTTGAATGGATGTGTAGACCATGTGGTGACACTGTTATGGACGTTCAGATTTGGAAATTTAAAATCTTGGTTAACCAACATGTGATCCTTTGCCTCCTTTTGGTCTTTTTGGTGCTTGCATTGCAAGTTGGATGTTTTTTGGCTCTGACAAAAGGGGGGGTTTCAGTCTTGTCAATGTAATCTCCAGTGATTACTCAGGTGGTTTGGGGTTTGTCAGGTTTTCTCTGAACCGCTCCCTTCTTTCTGTTCTTGCAGCCATTGCTTACAGAGCAGTGAATTCTTCTTTTCACGGAGCGGCTATGTTTTGTTGGTTTGCTTGAGATTTCTTTAGTCAAGTCTCTTATAATTATGGATATTTTGGATTCAATTTTCTTCCATAATGGGCCTTTGTCTTTGTCAAAACAAGTGTTGTATGAAGTTTCTCAACTAGGGCATTATTTTTCACCGGTGTGTGTTGTATTCTGCTGTCGTGAAGTTCAGTCCAGACACTTATGTTCTTTGATCATCCAATTGATGAGGTCTTCTATGGCTATAGATGCTTGATAAGTACTCGGCCAATTCTAGATCTCATTTGATAGTGGCGTAAAATACTTCTTTACTTTAATTCTCTAGCTACTTAGTTATCTTCTCTGTTTCCATAGGTTTTTCGTTATCACACTGATTTGTTTTGGTTGAATGGATGTGTAGTAGATCATATGATGACACTGTTATGGACGTTCAAAATTTAGAAATTTCAAAGCTTGGTTTATCAACATGTGATCCTCTGCCACCTTTTGGTCTTTTTGGTGCTTGCACAACAATCTGGATATTGTCCGGGCTTCGACAGAAGGGGGTTTTCAGTCTCTTCTATGGCATTTCTAGTGATTACTTAGGTGATTTGGGGTTCCTCAGATTTGCACTTAACTGTTCCCTTCTTTCTGTTCATGCAGCCATTGGTTGCAGGGCAGTGAATTATTCTTTCCATGGAGTGGCCGTGTTTGCCAGTTTGTGTTGAGGTTTCTTTAGTTTAAGTTTTTATGAGTATCAAGATTTTCGATTCATTTTTCTTCAGTAGTTTCTCGACTAAGATAATATCTTTCACTAGTGTGTGTTGTTATTCTGCCGCCGTGAAGTTTTTAATGCAGACACTATGTTCTTCGATGATCCAACTGATAAGGTCTCCCATGGCTATGGACACTTGATAAGTACTTGAGCCAAATTGTGGATATTTTCTTTTGACAGCGACATAAATGCTCCCCTACATCTGGTCTTCTAGCTACAAATATTTTGGTTATCACATGTGTTTTTTGTTTGGATGGATGTGTAGACTATATGGT

>LNC_000023

CTTTCTTGGTCCAAAAACCCCGTTCGTTGATTGTTTTTGAGGGTATACGCAGTGTTTGCTTGTTCATATGGGTAACTTTTGTTGATCAAGACATGGGTAATTACAGTGCCTTATAGTTATTCTTGATCTGGGTAGCTATTGTTTATCAAGATACAGGCCAAATATTGTATAATCAAGATAGTGGATAGTTATTGTTTGATCAAGATACTGGTAATTATTGTTGATCAATATTGTGGGTAACTATTGTTTGGTCAAGATACTGGGTAATTATTGTGCTCCTTATTGTTAATCTGGATACTTATTGTTGAGCAAGATAGCGGGTAACTATTGTTGATCAGGATAATGGGTAATAATGTTTGATCAAGATATGGGTAGTTATTGTAGATTATTGTATATGATTTGGTCTTCTTTCGTTGGTATCTTGAGGATATAAGCAGTGTTTGATTGTTCATATGGTTTATTGTTGATCAAGATGATGGGTTATTATTGTTTGATCAAGATGATGGGTTATTGTTGTTGATCAACACGTGGGTAATTATTGTTTGATCAAGATATGGGTATGTCTTCGTTTGGGGAGGCGACTGGTTGGGTTTTTGATTTCTTGGCCATCTTAGAATTATATACAATCTCAAGTCATGTACTTTTGAATCTTGGTTTTCAAAGATATCAATATTTAGGGAGCCATTGCTTAGGTTTCTGATTTGCAACTCTTTGATCTTCAATGTTAGGGGAGTTCTTGACTTTTTAGTGTGATTCAATTTGCAAATAGTTCAGTCTTTTTTGGCTAAATTATTGATTAATATTTGTTGTTTGTTTGATCAGGTATGGGTAATGATGATAGGAGACTGGTGGTAGTTTCTCAATTAGGACATTAGGACATTATTTTCACCAATGTGTGTTGTATTCCCTATTGACTCCTTTGTCTTTTATGGTAGTTTCTCAATTAGGACATTATTTTATTTTCACACATAGTTTCTCAATTAGGACATTATTTTCACCAATGTGTGATGTACTCTGCTGCCATGAAGTTCTTAGTTTAGATGCTTATGTTCTTATGGTGATCCAATTGATAAGTTCTCTTATGGTGATAAGGTCTCTTACTCTCTTATGGCTTTGGATACCTGAGAAGTACTTGACCAATTCTAGATCTTTTGGTAGTGATGTAAAATATTTCGATAGTGGCATAAAATACTTCTCAACTTTTGTTCTTTTAGCTACTTATCTTCTCTGCTTTCATTTTTTTTTGTCATCCCACTAATTTTGGTTGAATGGATGTGTAGACCATGTGGTGACACTGTTATGGACGTTCAGATTTGGAAATTTAAAATCTTGGTTAACCAACATGTGATCCTTTGCCTCCTTTTGGTCTTTTTGGTGCTTGCATTGCAAGTTGGATGTTTTTTGGCTCTGACAAAAGGGGGGGTTTCAGTCTTGTCAATGTAATCTCCAGTGATTACTCAGGTGGTTTGGGGTTTGTCAGGTTTTCTCTGAACCGCTCCCTTCTTTCTGTTCTTGCAGCCATTGCTTACAGAGCAGTGAATTCTTCTTTTCACGGAGCGGCTATGTTTTGTTGGTTTGCTTGAGATTTCTTTAGTCAAGTCTCTTATAATTATGGATATTTTGGATTCAATTTTCTTCCATAATGGGCCTTTGTCTTTGTCAAAACAAGTGTTGTATGAAGTTTCTCAACTAGGGCATTATTTTTCACCGGTGTGTGTTGTATTCTGCTGTCGTGAAGTTCAGTCCAGACACTTATGTTCTTTGATCATCCAATTGATGAGGTCTTCTATGGCTATAGATGCTTGATAAGTACTCGGCCAATTCTAGATCTCATTTGATAGTGGCGTAAAATACTTCTTTACTTTAATTCTCTAGCTACTTAGTTATCTTCTCTGTTTCCATAGGTTTTTCGTTATCACACTGATTTGTTTTGGTTGAATGGATGTGTAGTAGATCATATGATGACACTGTTATGGACGTTCAAAATTTAGAAATTTCAAAGCTTGGTTTATCAACATGTGATCCTCTGCCACCTTTTGGTCTTTTTGGTGCTTGCACAACAATCTGGATATTGTCCGGGCTTCGACAGAAGGGGGTTTTCAGTCTCTTCTATGGCATTTCTAGTGATTACTTAGGTGATTTGGGGTTCCTCAGATTTGCACTTAACTGTTCCCTTCTTTCTGTTCATGCAGCCATTGGTTGCAGGGCAGTGAATTATTCTTTCCATGGAGTGGCCGTGTTTGCCAGTTTGTGTTGAGGTTTCTTTAGTTTAAGTTTTTATGAGTATCAAGATTTTCGATTCATTTTTCTTCAGTAGTTTCTCGACTAAGATAATATCTTTCACTAGTGTGTGTTGTTATTCTGCCGCCGTGAAGTTTTTAATGCAGACACTATGTTCTTCGATGATCCAACTGATAAGGTCTCTTATGGCTATGCACACTTGATAAGTACTCATCCAATTTTGGATCTTTTCTTTAGACAACGCCATAAATGCTCCTCTACATCTGGTCTTCTAGGTACAGATATTTTGGTTATCACAACTTGTTTTTTTGTTTGGATGCATGGATGTGCAGACTATATTGTGACAGTAATAGAGGTTAAGATTTGGAGATTTTAAAGGTTGATATCCAGTAATGGCTATGGACACTTGATAAGTACTCATCCAATTTTGGATTTTTTCTTTAGACAGCACCATAAGTGGTCATCTACATCTGGTCTTCTAGGTACAGATATTTTGGTTATCACAACTTATTTTTTTGTTTGGATTCATGGATGTGCAGACTATATTGTGACAGTAATAGAGGTTAAGATTTGGAGATTTTAAAGGTTGATATCCAGTATGGTATCTCTGTATACTCTTGGTCTTTTTGGTTCTTGCACTGCAATTTAGATATATTGGTCAGGGCTTCAACAAAAAAAGGGTTTTAAATCTTGTCTATATGGCATCTCTAGTGATTTCTTGGATGATATGGGTTTGTATCTTCTTTTTGTACTTGCAACCATTGCATCTTATAGAGCTATGAAATATTCTTTTCATGGAGCGGCTTTCTTTGTCCATTTATGAGGTTTCCTTGGTTCAGGCTTTTATGATTTCTGATATTTTCAATTAAAATAGTTATTTTAACGTTGGTGTGTGTTGTATTCTACTGCCATTTTTAGTGTTCAGTCCAGATGCAATGGCCCAATTTGATGAGATTCTTGATGGTTGATGGTTATGGTCCCTTGGACACCTGACAAGAACTCAATTTTGGATCTTTAATGGTCTTCTCTTTTGGTGTCCTCACTGCTCATTTTCTCTGCTTCATTCTTCGTGTATTACGCTAGCTTGATTTTGTTTTGAGAGGTTATGACTTGGAAGATTTTGAAGGTTGGTGTCCAGTGTGCATTCCTGTGCACCCTGTTTCTTGGCCATGTTGGTGCTTGCACTACAATCTAGAGATTATCAAGCTTCGAAGGTGGTTCTCCATTTTGTTTGGTATTTGTGGTGAATTCTGAGGTGGTACGGTCTTCACTAGATCTGCACTTGAACTGTTCCATTTTTCTGTGCTTGTAGCTGATGGGTCTTACAGAGGGATAGGTTCTTCTTTTCATGGACCGGCATGGTCTGTCAATTTTGTGTTGAGATTTTTTGTTCGATGCTTCTAATGATTATTGAGATCTTCAATTCAGTTTTCTTCCCTAATCAGAAAGGTGCCCTTCTCTCCACTGCTTCACTAGGGTGTATTTTGCTGCCATATTTGTTTTTCAATAGTCCAGATGCTTATGTTCTTTGGTGATCCAACTGATGAGATCTCCATGGTTATGGGGACAACATATGATAAGTGTATTTAAACTCAATCGTGCTCTTTTGATGGCCCTGTAATGCACTCTTGTGCTTTTCATGTTCTCGCTACTTACCTTCTCTGAGTTCGTATAGCCTACCAGTTGATTTAGTTTGGTTGGATGAATGTCTGTAGGCTACATGGTGGTAGTATAGCCTACCAGTTCGTATAACTTTTCATGTTCTGTTACTTATTTGGATATTTTGTAGTTTGGTTTCGGTGTGCATCCTGTTGATCATGTCTCTTGCCATTTTGATGCTTGCAGTGCTATCTGGATATTGGATATCACCAGGCATCAAGAAAAGGGTGCTTTGCAATCTTATCTTGTTTGGCATATCTAGTGATCTCTCCTGCGTGGTATGGGTTTCCACTTACCTGTTATGTTCTTTTTGTGCATGTAACCGTTGGGTCTTCTAGAGCATGAACTTCAGTTCAATTTTTGTTCCTAATCAACGAGTCCCTTTTCTCTTTGTCAATTATTTTTTTGGGATGATCCCCTTTTCTCTTTGTCAATTATTTTTTTTGGGATGATCCAAATGGTGAGATCCCTGAGTTTCATGTGCACCTGAGAAGTACCCAAACTCAATTTTAGATCCTCTTATGGTCCCAAGAGTTCCTCTACTTTGGTGTTCTTGCTGCTCTTATGCTTGTCATGCCACTTGATTTTGTTTGTTTGGATGCATGTTTGTAGATTACATCAGATTTGGTAATTTCGAAGTTTGATTTCAGTGTGTACCCTGTTCCTTTGCCATATTGGTACTTGTGCTTGCACTGCAATTTGGATATCATCAGGCTCTGTCAAGGGAGTTATGCAATCTTGTCTGGCATCTCTAGTGATCTCTTCTATGTGGCATGGGTTTCGCAGCCTGCGCTTTAACTGTTCCCTTCTTTTGTGCTTGTGTTGTTGTATCTTTCAAGATCATTGAAGTCTTCCTTTCATGGAGCAGCCTTGTTTGTCTTAGTGTTGAAGTTTCGTGAGTTCAGTCCTTGTATTAGTATCTAACTTTTTGATTCAGTTTTCTTCCCTGGTCAGAGAGATCCCCTTATCTGGTTGTCAGAAGGGCTGTTAAATGGAAGTTTCAATTACGTTAACTCTCTCCACAGGTGCATTTTCAGCTTTCAGTCCAGATGCTTTATATTTTGAGATGATCCAACTGATGAGGTCTCTTATGTTGGAGATCTCTCCCCTACTATTTTTTTGAAACATGTATATTATTTTCAGATATTCAATCCTTTGAGGAGTTGCTTCAGACTATTAGCTATTATGTAGAACTATAATATAAAAAGAGAGTTCCTGCACACTTTTTTCCTCCGTAAAACATTGTCATTTGAACAGTGTTTAGCACCAAAGACCGAGCTTATCCATTGAATCTCGAACAAATGAAGTAAAAAACTTGGCTCATCATTTGTGTAACATGCCATTTACACTCTTTATGTTATTTATATCAACACAGAGGTAACTATTTTTTTTTGTGTAAAATATTTTCTGATGAGATATCCTTGTAAATCCATTACATGTCAGATCATCATGCAAGTTGGGAAGTTTATCAATGGATCTTGTTCTTCTAAGTACTCTTCTGAGTTAAGTTTTGTACTAATTTTCTTAATACTATTGAAATTGCTATTGTTTATAGAACATTGTTGTTCTGTCATAGGCCATAGTGATCCGGGAGTCCCGTGTGAAGGGCTCTCGCTCAACGGATCAAAGGTACGCCAGTGATAACAGGCTGATGAATCCAAAGAGCTCTTACCGACGAAGTCGTTTGGCACCTCGGATTTCTAAACATCTTATTATCATATAACACTAGAACATAGAACAATTAAATGAAACGAAAAGAAAAGAAAGAGATGACAGAGAATCCATTGAGAAGTTTACCTGTATCCAAGGTATGGAATATGAGCAGATCTGTGAGTTGCAAAAAAGAAAGTCTGGCAGAATGCTTGGCCTCCTGTGCAGGAAGTGAGACCTGAAGGGTGAGCTTATGGTGAAGAGTATCTATTTTTGGGTTTTGCAGATTTATTTTGAAGTTTGACTTGGAGGTAAGTTCTCACTTTTGTTGGTTTCTTGAGGCATCCAAGGACTTCCTGTTTAGGTTATGGCAATTCCTTTCTGGTTCGACCCGAGGTAATGATGGTAAGGTTATAGACCATGCAGTTTCTATTTTTGGATATCAATAATCTTTGGAACGAAGTAAACCTCGTCTCTGCTACCTGCAACAAATTATCTTTTTTTGCATCTTCTCCGCTGGAACACTATTCAGTAGAAGTTTTTGAGCCTTCCCTTGTAGATGTTGCTCTCTATATTCCATTTTGTTAGGCGATTGACTCGTTCAAAATCACGAATAGGTCTCCCTCTGATGTCTTCAGGTTGTCTTTATCTACCCATAAGCTTACAAGTCTTTATTTTCACAGTTATATTGTATTGTCCACCCCGTTAGATGGGTTCCTGCACCCAGCATGATAAGGGGACAATATCCTATCTTCAAAAATGTCAACGGGGTGCGTGTCAAATTCGCTAAAAGTACTGTATTTTAGGATAATCCGACATGGGTGTGGCATCGAAAGTGAAGAGTCCGTGCAACTTAGACAATATCAAGGAGTTTCTGAGAAGCAGTCTGTTAAAGTCATTTACAGTGGTTTTGATAATGTTTGACTTTGGTATTTAGAGCATTTGTTGTATTTGCTGATTTTGATCTCTTACCTGTAAAACAGCACTACAGATGTCAGTGTACTATTTTTTAAGTTGAGGAAGGTATTAGCATCTTGAAGTAGCTAGGATTCTTTGGCTTCAGTTTGTTGTGGTATTTCTTTTAAAATAAGGCCACTGAATGGGTATTTCTGTTTTCTCTAAGTTCCAGTTGTCCAAATTTGTCCAAATGTGCCAAATAATTTGAGTGGGGTATTTCACTTTAAACTCCAGTTGTCCAATCTCGCCAGATAAAGAATGTTCCTCTTTTCTCTCAAGTCCTTGTAGCTATTTTGATAGCGCTGGGATTGAAGGACTTATTTTTTTTTTGTGGCTAAAATACACAAATTCAGTAACATATAAACGGATTTGCTCTCCTAAAGTGGAGAGAAGTTGGGAGGATGGTAAATCAGTTGTTGAGGAGCATGTTATATAGAAAAGGTCAGTATGATGTGGATTATGTTAAACAGAAAAAGGTCCTGGTATGATCACTTGGATTCCTGTTTTTACATTGACCTGTTTTAGTCATCTTTTGCATGTTCAAAGTATTATAATATCTATTTTGGATTAACATTTTCGGATTAACATTACAAGTTCTTCTGGTTATATTGAGTGGGGGTGTGTTCAGCTACACTCGAGCGTTCTAGTCACTCAGTAGTATCTAATGTGCTTTCTCCTAGAATGTGTAGTAATTTATTAATCTATGTGCACCTAGCAGTGTCGGAGATCCAGTTTTAATATGAGTAAGAACGGAATGTTCTCAAAATAATTGTCTCATCTCACTTGTGCAAAATGCAGTTACTGAATATAGATGGTCTATAGGTAATATTTCAACTAGTATAGGTGTGAAGTAGCTTTCTGTTCGGGTTTTTATGATGAGTTGGGTATCTCTTTCTTTGGCTTATCTCACATTCTTATTTGGTTTTTATTGGTTTGGGATTGGTATCTGTGGATTAGTATCTTGGATGTGATAAAGCTTGAGTTACGGGAAAGAGAGGAAATACTTTTTCTCAAAACATGAACTAATGGAAGTTTAACTCTGGAAGAAGCACTTGATGAAGCATTGCGAAATTTGTCCTTATTGTTATTCTTGATCTGGGTAACTATTGTTGATCAAGATAGTGGATAGTTATTGCTTGATCAAGATACTGGTAATTATTGTTGATCTAGATTGTGGGTAACTATTGTTTGGTCAAGATATTGGGTAATTATTGTGCCTTATCGTTATTTTTAATCCGGGTAATTATTGTTGATCAACACAGCGGGTAACTATTGTTGATCAGGATAATGGGTAGTAATGTTTGATCAAGAAATGGGCTGTTATTGTATATGATTTGGTATTCTTTTTTTAGTATCTTGAGGATATAGGCAGTGTTTGATTGTTGATATGGTTTGTTGTTGATCAAGATATGGGTAATTATTGTTTGATCAAGATATGAGCAATTATTGTATATGATGCCTTCGTTTGGGGAGCGATTGGTTGGGTTTTGATTTCTTGGCCATCTCAGAATTATATAAATCTCAAGTCATGTACTTTTGAATTTTGTTTTTCGAAGATACTGATATTTGGGGAGCGATTGGTTATGTTTCTTATTTGCAACTCTTTGATCACATCTCTGTAGAGAAGGAATGTTGGGGGTTTTGGGAGTTCTTGACTTTTTAGTGTGATTCAGTTTGCAATTAGCTCTGTTTTTTGGGGCTAATTTATTTATTCGTTTGCAATTAGTTCTATTCTTTTCCTAATTTATTGATTAATTATTGTTGTTTGTTTGATCAGGTATGGGTAATGATGATAGGAGACCGATTCTCCTTCTACACTTGCTGTTACCTTTGTCTCAATGTTAATTCTGAGGTTTTTGTTGTGAGAATTCATGTGTTGGTGTCACCCTGGTTACTACTCTGAAACCGAGCAATTTCCTTTTGTTGAATCACAACGCAAGTAGAATTGGAAATTTGGCTCTCTAGGCATGGATCTTCTATTCTAGACATGTGTGTCACAACCATATGGGAGCTATCAGTTTGGATTTGATATCTTGACTTCCATCTTAATGTTAATTTTGATGCTAATAATGTGTCGCATCAACTTCTTTACCTTGTTATTTTGTTTGGTCAAGAATACAGAGATGTGCATCTCTAGGTGGAAGTAGTTCTACAGTAACAAGTGTCTTGCTTCGTTGTTTGTGGTCGGGAAAGATCCAGCATTTTCCCAGGTCTGTGACTAAAGGTATTGGATTTTTCAGTATCTCATAACTTTGTTCAAAACTGTTTTGATATGGAAGTTGTCAACTATGATAATTCTTTTCACAAGTGTGTATAGCGTTTTCTGCTGCCAGTTAGTTTTAGTTGTTAGTTCAAATGCTCGTGTTCTTTGATGATTCTGGTGTATTTTATGGTGTTATGGGCAGTACCGGACGAAGTCAAAAGTCAATTCTGGATCTTTGATAGTGCTGCAAAGTGCTCCTCTGCTACTTATCTTCTCGCCACTTTCCTTGGTTATCACGCCAGATTGATTTTTGATTATTGATGAATGAATGTATAGACCACATGGTGACAGTAACAGAGGGTTAGTTTGGAAACTTCTAAGGCTTGTCCGGTATGCATCCCTGTGCACCTTTTTTCTTGGTCTTCTTGGTGAATGAATGTGTAGACCACATGGTAATGTGCTTCAATAAAAATGGGGGTTCTCAATCTTGTCTCTTGCATCTCTAGTGATTTCTTAGGTGATATGGGTTTTTTCAGCACATAACCGGTCCCTTCTGTTTGTGCTTGCAGTTGGGATTTACCGAGCAGTGAATTATTGTTTTGATTGAGGAGGCTTTGTTTGGCCATTTGTGTTGAGGTTTAATGAGTACTGAGTTACTCAAATACTCATTTCACTATTTTCCCCCAATCAGAGTGGTCCCTCCTCTCTTTGTTAAAAATTGCTGATGTATGGAACTTGCCAGCTAAAATAGTTTCTCTTGCACTTGAGTGTGTTGTATTCCGCTATCGTTTCTCGTTTTTAGTCCAGATGTGTATGTTCTTCGATGATCCAGTTGATGAGGTTCCTTGTGGTTGATGGTTATATGGTCTTATCGACACCCTGATAAATACTCAAAACTCAATCCTGAATCTTTTGATAGCTCGGTCAAGTGGGTCCTTGTTAGACCCCCCTCTTTTGGTGTTCTCTCTGCCTTAACTTCTCTGCTTCCTTTTTCTTGCATCACCCAAGCTTGATTTTGTTTGTTGGATGGATGACAGTTGAGAGGAAACTTGGGAAAATTTTGAAGGTTGATGCCAGTGTCCGTTCCTGTGCACGCTGTTTCCTTGCCTTTTTTGTGCTTGCACTACAATCTGAATATCATCAGGCTTCGATAGGGGGGTTTTCCAATCTTGTTTTTTTGCCATTTTTGTGCTTGCATTGCAATCTGAATATTTTCAAGCTTCGATAGGTTTTTTTTTTCAATCTTATCTGTAGCATTTTCTAGTGGGGTTTGCCAGCTTTGCACGTAACTCTTCCCTTCATTCCTTTGCTTGCAGCCATTGGGTCTTACAAAGCATTGAATTCTTCTTTTCATGGAGGTACCTTATTTTTGTCCATATGTGTTGAGGTCTTGTTCGCCAATCTTTTACAAGTATAGATAATTTTCAATGATTCTTCCCTATTGACTCCTTTGTCTTTGTCATAGCTACTAATGTATGGTAGTTTCTCAATTAGGATATTATTTTCACTAATATGTGTTGTATTATGCTGCCATGAAGTTCATAGTTCAGATGTTTATGGTGTTATGGTGATCCAATTGATAAGGTCTCTTATGGTGATGAGGTCTCTTACTCTCTTATGGCTATGGATACCTGATAAGTACTTGACCAATTCTAGATCTTTCAGTAGTGCCGTAAAATATTTCGATAGTGGCGTAAAATACTTCTCAACTTTTGTTCTTTTAGCTACTTATATTATCAAACTGCTTTCATTGTTTTTGTTATCACACTAGTTTTTTTTTTGTTGTATGGATGTGTAGACTATGTGGTGACACTGTTATGGACGTGCAGATTTGGAAATTTCAAAGCTTGGCTAACCAACATGTGATCCTTTGCCGTCTTTTGGTCTTTTTTGTGCTTGCATTGCAATCTGGATATTGTCTAGGCTTTGACAAAGGGTTTTTAGTCTCGTCTATGGCATCCCTTGTGATTATTTAGGTTTGCTCTGAACTATTCCCTTCCTTCTGTTCTTGCAGCCATTGCTTACAGGGCCGTGAATTCTTCTTTTCATGGAGTGGCCGTGTTTTTGTACGTTTGCGTTGAGGTTTCTTTAGTCATTTTATATGAGTATGGAGATTTTCGTTTCAATTTTCTTCCCTAAATGGCCCTTTGTCTTTATCGGATCTTTGTCAAAACTAGTGTTGTATGGAAGTTTCTCACGAGGACATTATTTTTCACTGGCGTGTATCGTATATGCTGTCATGAAGTTGTAATGACCCGTTAACTCTTTCTTTCATAAAAGACCTTTTTTCGTATAGTTTAAATGAAAATTTTGAACTATTCGTTTAACTACGGTGAATTAGTTGATGGGTATTTGTTAAATAATTAAATTAGTTGGTGTTAATAGGTCAAGTCCATAATTTAATACTCTATTCAATTTAACCTATGTATTGAAATAAATTAAATGGAATTTAACAATTAATAAGTTATGCTCAAAAAGATGTCGCGGCAGTATTCAAGAGCGACATTCTCTTCCAGGTAACAACACATTAAATCTCCAACTTCCTCCATGTGTATATGAAATTGTAAGATGCTTCATTTACATTAATTAGTATAATAATATATATTTTTTTGGAAGAAGAAACAAAGCCATTGAGTTATTGATGTTCCAAAAGCTGCTGGCCAAAAATAGTGGGCAAATCAATTTATATTCATCAATTTATGTGTTGATTACAATATTGAAGAGGAATTAATAATGGACATGTTGAGTTAATCATTTGGAATGATGTAACAATATGTTGCATTTTTGTATAATAATAGTCTGTTGCATATTGATTTTTGTTACCGAAGGTTAGTGATGGCTTAATCTGGTTTGAGTTGGAAAATTTCTTAGGGTTATCAATAACAAATCAAGTTTTGTCATATTAAGACAACTATTATATGAATACCATTAGATTTATAATATTTTGATAAATTGACACTTAACCCTTGGCTTAATATTTAGGAATATAAGACTTGACATATTTAGTTGGCACCAAGATTAATGAATTTAATTATAGATTTAGGTGAGTTTTGGGTCTAGGCTAGACATATAGTAATATATGTGTCTACAAACTCATTTGTTTATGAATATTGCATATTTTCTAGATTGGGAACATTTTGAAGCTTTACGAAAGGGAAAGGAAAAATCTAGACGATTCGTGACACGTGTTCTGCACTTAAGGTGCCATCACGACTTGTGATTTGGGTCGTGACAGAAGTTTAGTCCAGATGCTTATGATGCTTATGTTCTTCGATCATCCAATTGATGAGGTCTTCTATGGCTATGGACACTTGATAAGTACTCGGCCAATTCTAGATCTCATTTGATAGTGGGGTAAAATACTACTGCTTCACTTTTATTTTCTAGCTACTTATCTTCTCTGCTTCCATATATTTTTTGTTACCACACTGTTTTTTCTTGGCTGGATGGATCTGTAGTAAGCAGTGTGGTGGCACTGTCATGGACGTTCAGATTTGGAAATTTCAAAGCTTGGTTTACCAACATGTGATCCTCTTCCACCTTTTGGTCTTTTTGGTGCTTACACATCAATCTGGATATTGTCCGGGCATCGACAAAAGGGGGTTTTCAGTCTCGTGTATGGCCATCTCTAGTGATTACTTAGCTGATTTGGAGTTTGTCAGATTTGCTCTTAACTGTTCCCTTCTTTCTGATCATGCAGCCATTGGTTACAGGGCAGTGAATTATTCTTTCCATGGAGTGGTCGTGTTTGTTCGTTTGTGTTGAGGTTTCTTTAGTTCAAGTTTGTAAGATTATTAAAACTTCCGATTCAATTTTCTTCGGAAGTTTCTGGAAGATCGTAGTTTTCACTAGTGTGTGTTGTTGTTCTACTGCCGTGATGTTTTTCATCCAGACAATATGTTCTTCGATGATCCAATTGATGAGGTCTCCCATGGCTATGGACACTTGATAAGTACTTGAGCCAAATTGTGGATATTTTCTTTTGACAGCGACATAAATGCTCCCCTACATCTGGTCTTCTAGCTACAAATATTTTGGTTATCACATGTGTTTTTTGTTTGGATGGATGTGTAGACTATATGGT

>LNC_000024

GAGAGATCGGTTTGGTTTTTGATTTTTGGGCTATCTCAGAATTATATAAATTTCAAGTTACCTACTTTCGTGACTTTTGAATCTTTTAATATGGGGTGTAGGTTTCTGATTTTAAAATCTTTGATCATCTCTGTAGACAAAAGGTCGGAGGAGGAATGGAATGTTGGAGGTTTTGGGAGTTCTTGACTTTTTTTTTTGGCCAATTTATTGATTTGTTATTGTTGTTTGTTTGATCAGGTATGAGTAATAATGCTAGGGGAATGATTATAGGTACCTTCCAGTGTATTTCATGATTCTCCTTCTATGCTTGTTACCTTCTACCAGTTACCTAATATTCATATCACTGCTTTCCTTTAATGTATTGTTTATTTATTTATTTGTTCCCATTTTCTTGTGTATTATAGTAAGAGTTTATAATTGCAATCCTTTTGATTAATCCATGATATGAGTACACAGAAGATTCATGAGCAAGAGTGTATTCATTATTCAACGAACAACTACATCCATTTTTACTGGGGAATTTTCATATGAGACTATTCTTCCTTTTAATCTACTATATAAAGGTTTTAAATTGGCCCTCAATATTAAAGATAATTACATTCTTGAAAGGATCAAAGACTCTACACCTTCGTGGCTTTTTGCTTGTTAGGACTGGAAATTTAACTCCCCAAGCATGCCTACTTGGATCTCCTATTGTAGACTTATGTGTCACAACCATTCATGTGCACCTTGTTTCTTGGCCTTTTTTTTTTTGCTTTCAACTCCAAGATGGAAATTGTTAGGCTTCGACCAAAATTGGGGTTTTCAATCTTGTCTCTTGCATCTCTAGTGATTTCTTAGGTGATATGGATTATCTCAGCTTTGCACTTAACTGGTCCCTTCTTTTTGTGCTTGCAGTTGGGTATTGCATAGCAGTGAATTATTCTTTTTATGGAGGGGATTTGTTTGGCCACTTGTGTTGAGCTTTCTTTTTTTCAGGTATATTGTCCTACACATGGTAAATACTCAATACTCAAATCCTGGATCTCTTGACAGCCCCGTCAAGTGGGTCTTTATTACTCTCTCACTTTTGTTGAGAGGATCAGATTTGGAAAATTTCAAAGATTGATGATGTGATCAGGCTTTGACTGTTAGTCTATTGGTTACAGGGTAGTGAATTATTCATTTCATGGAGAGGTCGTGTTTGTTCGTTTGTTGTTGATGCTTCTTTGGTTAAGTCTTGTTTTGAGTATCCAGATTTTCGATTCAGTTTTTTTGCCTAATGACTCCTTGTGTTTGTGAAAACTATTGTTGTATGGAAGTTTCTCAAATAAGACATTAATTTCCACCTGAATGTTTTGTATTTGCTGCCATGAAGTTTTATGTCCAGACACTTATGTTCTTCAATGATCCAGTTGATAGGTTTCCTATGGTTATGGACACTTGAAAAGTACCAGCCGATTCTAGACCTTTTGATAGTGGCATAAAATACTTCTTCTCTTCTATTCTTGTAGCTACTTACTTATCTTCTCTGCTTCCATTTCTTTTTTGTTATCACACTAGTTTTTTTTTTTTTGGTTGGATGGATGTGTAGATCCTGTGGTGACACTGTTATGGATGTTTAGATTTGGAAATTTCATAGCTTGGTTAACCAACATGTGATCCTTTGCCACCTTTTAGTCTTTTTGGTGCATGCCCTGCTATCTGGATATTGTCCATGCTTCAACAAAAGATGATTTTCACTCTCATCTATGGCATCTCTAGTGATCATTTAGGTGGTTAGGGGTTCGTCAGATTTGCACTTGATTGTTCCCTTCTTTCTGTTCTTGCAGCCATTGGTTACAGGGCAGTGAATTATTCTTTTTATCGAGAGGTCGTGTTTGTCTGTTTGTTGTTGAAGTTTCTTTAGTCACGTCTTTTATTAGTATTGTGATTTTTGATTCAATTTTCTTCCCTAATGGCTTCTTTGTGCATGAGGGTTTTTAGTCCAAGCGCCTTATGTTCTTTGATGATCCAATTGTCTATAAGTACTCGGCCAATTTTAAATCTCTTTATAGTGGTGTAAGATACTTGTTCAGTTATATTCTTGTAGCTACTTGCTTATCTTCTCTGCTTCCATTTCTTTGTCATTGTTACACTATTTTTTTTGGTTGGATGGATGTGTAGATCTTGTGGTGATACTGGTATGGATGTTCAGATTTGGGAATTTCAAAGTGAGGTTGATCAACAGGTGATCCTCTGCCACCTTTTGGTCTTTTTGGTTCTTGCAATGCAATCTGGATATTGTCCAAGCTTTTACAAAAGGGGTCTTTTAGTTTTCATCTATGGAATCTCTAGTGATTACTTGGGTGGTTTGGGGTTTGTCGGATTTGGACGTAACTGTTCCCTACTTTCTGTTCTTGCAGCCATTGGTTATAGGGAAGTGAATTTTTGTTTTCATGGAGCGGCCATGTTTGTCTGCTTGTGTTGAGGTTTCTTAGTTAAGTCTTTTATGAGTATCGAGATTATGGATTCAGTTTTCTTCCTTATAACTAAGGGTCTTGTATGGAAGTTCTCAACTAAGATATTAATTTTCTCCGGCATGTGTTGTAGTTTGCTGCCATGAGGTTTTTAGTCTATACGACTTATGTTCTTCGATGATCCAATTGATGAGGTCTCATATGGCTATCGACACTTGATAAGTACTCGGCCAATTGTAGATCTTTTGATAGTTGCGTAAAATACTTCTTCAGTTTTATTATTGCAGCTACTTATCTTCTCCACTTCCATTTCTTTTTCGTTATAGTTATTTTTGGTTGGATGGATGTGTAGACCATGTGGTGACACTGTTATGGACGTTAGATTTGGAAATTTCAAAGCTTGGTTAACCAACATGTGATCCTCTGCCACCTTTTGTCTTTTTTTTTATGCTTGCACGACAATCTTGATGTTATTTGAGTTTTGACCAAAGGTGGTTTGTTCGTTAGATTTTCTCTCAATTGTTCCCTTCTTTCTATTCTTGCACCCATTGGTTTACAGGCAGTGGATTATTCTTTTGACGGAGCGGCTGTGTTTGTCCGTTTGTGTCGAGGTTTTTTTAGTAAAGTCTTTTATGAATATTGAGATTTCGTTTCAGATATGTTTCCTAATGGCTCTTCTGTCTTTGTCAAAACTACTGTTGTATGGATGTTTCTCAAATACGACATTATTTTTCACCGGAGTGTGTAATCTGCTGCCATAAAGTTTTAAGTCCAGATGCTTTTGTTCTTCGATGATCCAATTAATGACATCTCCTATGACTATGGACACTTGATAAGTACTCAGCCAATTCTAGATCATCTGATAGTGGCGAAAAGTACTTTTTTACTTTTTTTCTTCTATTATCTTCTATGCTTCCATATATTTTTCGTCATCACACAAGTTTTTTCTGGTTGAATGGACCATGTGGTGACATTGTTTTGGACGTTCAAATTTTGCAATTTCAAAGCTTGGTTTACCAATATGTGATTCTTTGCCATCTTTTGGTTTTTTTGGACGTTCAGATTTGGATAATTCAAATCTGGATATTGTCTGAGCTTTGACAAAGGGGTGTTTTTAGTGTCGTCTATGGCATTTCTAGTGATTACTTAGGTGGTTTGGGGTTCGTCAGATATGCACTTAACTATTCCCTTCTTCTGTTCTTGCAATCATTGGTTACATTGCAATGAATTGTTATTTTCATGGAGTGGTCGTGTTTGTCTGTTTGTGTTGAGGTTTCTATAGTTCAAGTCTTTTATGAGTATCGATATTTTCGATTCAGTTTTCTTCTCTTATGGCTCCTGTTATACTATAGAAAGTACTGTTGTATGGAAGTTTCTCGGCTAAGATAATATTTTTCACCAGTGTGTGTTGTGTTTTGCTGCCATGAAGTTTTTAATCCAGACATTATGTTCTTCGATGATCCAATTGACGAGGTTTTCTATGGCTATGGATGCATGATAAGTACTCAGCCAATTCTGGATCTTTTCTTTTGACAGTACCATAAAGGCTGCTCTACTTCTGGTCTTCTAGCTATATATATTCTCTGTTTCCATCTTCTTTTAGTTATCACAACTTGTTTTCTTTTGTTTGGATGGATGTGTAAACTATTGGTTACAGTAATAACGGTTAAGATTTGGAGATTTCAAAGTTTGATATCCAGTATGGCATCTCAGTACCCTCTTGTTCTTTTTGGTTCTCAGGGCTTCAACAAAAGGGGGTTTTAAATCTTGTCTATGTGGTATCTCTAGTGGTTTCTTAGATGGTATGCGTTTCGTCAGCTTTTGGACTTAATTGACCCTTCTTTTTGTGCTTGCAACCATTGGGTCTTACAGAGCAATTTTTCTTTTCATGGCGCGGATTTCTTTGTCCATTTATGTCGAGGTTTCCATGGCTCAAGCTTTTATGAGTTTTGACATTTTCAACTAAAATAGTTCTTTTTTTCATAAGTATGTGTTGTTCTACTACCATGTTTAGTTTTTAGTCTAGATGCTTATTTTCTTCAATAATCCAATGATGAGATTCCTGATGGTTGATGGTTATTGTCTCATGGACATCTGACAAGTACTCAATTTTGGATCTTTTGAAAGCCTCGTAAAATGGTAGTCTCTTTTGGTGTTCTCGCTGCTCATCATTTGTGCTTTCTTTTTCTTGTATTATGCTTGCTTGATTTTGTTTGTTGGATGTATGTATGGTGACAGTTGAGAGGAATATGACTTGGAAGATTTTGAAGGTTGGTGTCAAGTTTGCATTTCTGTGCACCTTCTTTCTTGGCCATGTTGGTGCTTGCATTACAATCTGGAGATTATCAGGTTTCGAAAGAAGGTGGTTCTCAATCTTGTCTGACGTTTGTGGTGAATTCCTAAGTGGTATGATTTTCACCAGCTTTGCACTTAAGAGTGTTCCATTCTTTCTCTGCTAGCAGCTGCTGGGTCTTACAGAACCATAGATTCTTCTTTTCATGGAGCGGCGTGGTCTGTCAATTTTGTGAGGTTTTTTGGTCAATGCTTCTAATGATTATTGATATCATCAATTCAGTTTTCTTCCCTAATCAGTATGGTTTCATTCTTCCTTATCAACACTGCTTCATTTGTGTGTATTTTGTTGCCATACATGTTTTTTAATAGTCCAGATGTTTATGTTCTTTGGTGATCCAACTGTTGAGATCTCCATGGTTATGGACAACACATGATAAGTGTATTCAAGCTCAGTCGTGGATCTTTTGATGGGTCTTTTAATGGTCCTGTAACACTGTTCTCTAGTTTTCGTGTTCTCGCTACTTACATTCTCTGATTTCATTTTGCCTACCTGTTTATTTAGTTTGGTTGGATGAATTTTTTTAGGCAACATGGGTAGTAAGAGGATAAGATTTGGAAATTTTGTAGTTTGGTTTCAGTGTGCATCCCTGTGGACCATGTTGGCCATTTTGGTGCTTACACTGCAATTTGGATATTGGATTGACCATTCCTCCAGAACAGGGCACTTTACAATCTTATCTTGCTTGGCATCTCGAGTGATCTTTCCTGCGTGGTATGGTTTTCCACTTACCTGTTACGTTCTTTTTGTGCATGTAACCATTGGGTTTTCCAGAGCATGGACTTCAATTCAGTTGTCTTCCTATTCAGTGGGTCCCTTTTCTCTTGTGGAAGTTCTCAACAATGATAATATGTTCCACTAGTGTGCATTGTACGCTGCAGCCATGAAAGTTTTTACTCTAGATGCTTATTTTCTTGGATGATCCAAATGATGAGATCCCTGATTGTTATGGACACCTGAGAAGTACCCAGACTCAATATTCCCAAGGGCTCCTCTACTTCGAAGTTCTTGGTACTTATCTTCTCTGCTTCTATTTTCTTATGTTTATCATGACACTTAATTATGTTTGGTTAGATGCATGTTTGTAGATCACATGGTGACCGTGAGGGGGATTGAATTGGTAATTTTGAAGTATGGTTTCGTCCTTCCCTTGTGCACACTATTTCTTTTTCCATTTTGGTGCTTGTGCTTGCACTGCAATTTGGAAATCACCAGGCACTGGCAGGGGAATTGTTGGGTCTGGCATCTCTAGTGATCTGTTCTATGTGGTATGGGTTTGGCTTCTAGCACTTAACCTGTTAACTTCTTTTTGTACTTGTTGTTGTTAGGTCTTTCAAGATTAGCGAAGTCTCCCTTTCATGGAGTAGCCTTGTTTCTTGTAGTGTTGAAGTTTCTTAGTTCAGGCATTGTATTAGTATCTAGCTCTTTGATTCAGTTTTCTTCCCTGGACATAAAGAGTCCTTTACTTCGTTGTCAAAAGTGTTGTTACATGGATGTTTTCAACTACGATAACTCTTTCCACAAGTGCATTTTCAGCTTTTAGTCCAGATGATTATGTTTTGCAATGATCCAACTGGTGAGGGCCTCTTATGGGTGTGGATATCTGGTATGTACTCCCTCAGTTCTGGATCTTTTCACAGCCCAGTAAAAATGCTCATCTATCTTTTGTGTTCTTGCTGCTCATCTTTGTGGCGTCCATTTTCTTTTTGCTTATCACATCAGTTTGAGTTTTGTGTGTTGGAAGGATGTATATACCCTACATCGTGAACTGTATTGGAGGATTAGAGTTGGAAATTTTAAAGGTTAGTGTTCAGTATGCTGGTTATTTAGGTGCTTCCACTGCAATCTTGATATCGTTTTAGGCCTTCGAGAAAAAGGGGGTTTCGAAATATTAATGATTTGGAAATTTTAAAGGTAGTTCAATAAGCTGGTCGTTTAGGTGCTTGCACTGCAATCTGGATATCTTTTTAGTGCTTCGAGAAAAAGGGCATTTCTAAATATTATCTACGTCATTTCTCATGATTTCCCAAGCAGTTTGCGGTGGCCAGCTTTTCACTTAACTGGTCCTTCTTTCTGTGCTTGCAGTTGTGGTTTTAAAGATTTAATTTCTTCTTTTTGTGGGGCAGCCTTTGTTTGTTGATTTATGTTGAGGTTTCATTTGGTTCAAGCTCCTGTGAGTGTCTGGTATTCAGTTCAGTGTTCTCTTCTCTAATCAGTATGATATTTTCTGAACCACTCTCTATTCAAAGTGGTGCTCTTCTAGCAGTTAGTTTAACTGAAAATTTTGCATGTCAATGGTGGTTACAGTACATAGTCATTTTCATTCAATTTCTGGTTCATTATTGGGTGCAGTATCTACAAGCCATAATCACTACTATTTTCAGACTAATTGTTGTTAGGTAAATAAAGCGCTCTTTTTTATTTTTGGAGATCTCTCCCCTATCTTCTTCTTTTTTCCTTGAAACATGTGTATTATTTTCAGATATCCAATCATTTGACAAGTTGCTTCAGACTGTAAGCTATTATGTATAACTTTAATATAAAAAGAGAGCTCCTGCACACTTTTTTCCTTGATAAACACCATTTGAACTGTCTTTGCAATTCATTGAGTCTAGAACAGATCAAGTGAAAAACTTGCTCATCTTTGTATGACAGGTCATGTACACTCTTTATGTATTTATATATATCACAAAGATATTCTTTTTTCTGCGTAAAAGATCTTCCTTGCCATTACGTATTAGATCATTGTGCAAGTTAGCACCCTGTTTCTTGGCCATGTTGGTGCTTGCACTACAATCTGGAGATTATCAGGCTTCGAATGAAGGCGGTTCCCAATCTTGTCTGGAATTTCTAATGAATTACCAGGTTGTACCTAGTTCACTAGCATTGCACTTACTGTTCCATTCCTTATGTGCTTGCAGCCGCTGGGTCTTTTTTTGTTTATGCTCCGCCTTGTTCCGCCCAACATGAACCCGCCCCACATAAACGAACCCTGCGTAATTTTTGTTTAAAAGTAATGTAACAATTATCAATCTACTGTTAATATTGAAATAATATATGTGTCTTCATTTCTAAGACAAATTGTAGAATATATAGTCATTAACTGAACTAACTTTTAAGTCGTCTTCCATCTTTGCCATTTCTTTTATACATAGAGTTTAATAATTGTGTATAATTATTCAAGAGTTATTTAAGTTTGCTGAACCAGGGAGGAATGCATCTTATTTCTTATTTTTCTCTCTAATGTATACGTACAAATCCTTAATTATCATCAATTCACTGCTTTAGCCTCT

>LNC_000025

GATGTTCAGATTTGGGAATTTCAAAGTGAGGTTGATCAACAGTTGAGAGGAATATGACTTGGAAGATTTTGAAGGTTGGTGTCAAGTTTGCATTTCTGTGCACCTTCTTTCTTGGCCATGTTGGTGCTTGCATTACAATCTGGAGATTATCAGGTTTCGAAAGAAGGTGGTTCTCAATCTTGTCTGACGTTTGTGGTGAATTCCTAAGTGGTATGATTTTCACCAGCTTTGCACTTAAGAGTGTTCCATTCTTTCTCTGCTAGCAGCTGCTGGGTCTTACAGAACCATAGATTCTTCTTTTCATGGAGCGGCGTGGTCTGTCAATTTTGTGAGGTTTTTTGGTCAATGCTTCTAATGATTATTGATATCATCAATTCAGTTTTCTTCCCTAATCAGTATGGTTTCATTCTTCCTTATCAACACTGCTTCATTTGTGTGTATTTTGTTGCCATACATGTTTTTTAATAGTCCAGATGTTTATGTTCTTTGGTGATCCAACTGTTGAGATCTCCATGGTTATGGACAACACATGATAAGTGTATTCAAGCTCAGTCGTGGATCTTTTGATGGGTCTTTTAATGGTCCTGTAACACTGTTCTCTAGTTTTCGTGTTCTCGCTACTTACATTCTCTGATTTCATTTTGCCTACCTGTTTATTTAGTTTGGTTGGATGAATTTTTTTAGGCAACATGGGTAGTAAGAGGATAAGATTTGGAAATTTTGTAGTTTGGTTTCAGTGTGCATCCCTGTGGACCATGTTGGCCATTTTGGTGCTTACACTGCAATTTGGATATTGGATTGACCATTCCTCCAGAACAGGGCACTTTACAATCTTATCTTGCTTGGCATCTCGAGTGATCTTTCCTGCGTGGTATGGTTTTCCACTTACCTGTTACGTTCTTTTTGTGCATGTAACCATTGGGTTTTCCAGAGCATGGACTTCAATTCAGTTGTCTTCCTATTCAGTGGGTCCCTTTTCTCTTGTGGAAGTTCTCAACAATGATAATATGTTCCACTAGTGTGCATTGTACGCTGCAGCCATGAAAGTTTTTACTCTAGATGCTTATTTTCTTGGATGATCCAAATGATGAGATCCCTGATTGTTATGGACACCTGAGAAGTACCCAGACTCAATATTCCCAAGGGCTCCTCTACTTCGAAGTTCTTGGTACTTATCTTCTCTGCTTCTATTTTCTTATGTTTATCATGACACTTAATTATGTTTGGTTAGATGCATGTTTGTAGATCACATGGTGACCGTGAGGGGGATTGAATTGGTAATTTTGAAGTATGGTTTCGTCCTTCCCTTGTGCACACTATTTCTTTTTCCATTTTGGTGCTTGTGCTTGCACTGCAATTTGGAAATCACCAGGCACTGGCAGGGGAATTGTTGGGTCTGGCATCTCTAGTGATCTGTTCTATGTGGTATGGGTTTGGCTTCTAGCACTTAACCTGTTAACTTCTTTTTGTACTTGTTGTTGTTAGGTCTTTCAAGATTAGCGAAGTCTCCCTTTCATGGAGTAGCCTTGTTTCTTGTAGTGTTGAAGTTTCTTAGTTCAGGCATTGTATTAGTATCTAGCTCTTTGATTCAGTTTTCTTCCCTGGACATAAAGAGTCCTTTACTTCGTTGTCAAAAGTGTTGTTACATGGATGTTTTCAACTACGATAACTCTTTCCACAAGTGCATTTTCAGCTTTTAGTCCAGATGATTATGTTTTGCAATGATCCAACTGGTGAGGGCCTCTTATGGGTGTGGATATCTGGTATGTACTCCCTCAGTTCTGGATCTTTTCACAGCCCAGTAAAAATGCTCATCTATCTTTTGTGTTCTTGCTGCTCATCTTTGTGGCGTCCATTTTCTTTTTGCTTATCACATCAGTTTGAGTTTTGTGTGTTGGAAGGATGTATATACCCTACATCGTGAACTGTATTGGAGGATTAGAGTTGGAAATTTTAAAGGTTAGTGTTCAGTATGCTGGTTATTTAGGTGCTTCCACTGCAATCTTGATATCGTTTTAGGCCTTCGAGAAAAAGGGGGTTTCGAAATATTAATGATTTGGAAATTTTAAAGGTAGTTCAATAAGCTGGTCGTTTAGGTGCTTGCACTGCAATCTGGATATCTTTTTAGTGCTTCGAGAAAAAGGGCATTTCTAAATATTATCTACGTCATTTCTCATGATTTCCCAAGCAGTTTGCGGTGGCCAGCTTTTCACTTAACTGGTCCTTCTTTCTGTGCTTGCAGTTGTGGTTTTAAAGATTTAATTTCTTCTTTTTGTGGGGCAGCCTTTGTTTGTTGATTTATGTTGAGGTTTCATTTGGTTCAAGCTCCTGTGAGTGTCTGGTATTCAGTTCAGTGTTCTCTTCTCTAATCAGTATGATATTTTCTGAACCACTCTCTATTCAAAGTGGTGCTCTTCTAGCAGTTAGTTTAACTGAAAATTTTGCATGTCAATGGTGGTTACAGTACATAGTCATTTTCATTCAATTTCTGGTTCATTATTGGGTGCAGTATCTACAAGCCATAATCACTACTATTTTCAGACTAATTGTTGTTAGGTAAATAAAGCGCTCTTTTTTATTTTTGGAGATCTCTCCCCTATCTTCTTCTTTTTTCCTTGAAACATGTGTATTATTTTCAGATATCCAATCATTTGACAAGTTGCTTCAGACTGTAAGCTATTATGTATAACTTTAATATAAAAAGAGAGCTCCTGCACACTTTTTTCCTTGATAAACACCATTTGAACTGTCTTTGCAATTCATTGAGTCTAGAACAGATCAAGTGAAAAACTTGCTCATCTTTGTATGACAGGTCATGTACACTCTTTATGTATTTATATATATCACAAAGATATTCTTTTTTCTGCGTAAAAGATCTTCCTTGCCATTACGTATTAGATCATTGTGCAAGTTAGCACCCTGTTTCTTGGCCATGTTGGTGCTTGCACTACAATCTGGAGATTATCAGGCTTCGAATGAAGGCGGTTCCCAATCTTGTCTGGAATTTCTAATGAATTACCAGGTATTGGATATCACCAGGCATCAACAACAGGGAACTTTGCAATCTTATCTTGTTTGGCATCTCGAGTGATCTCTCCTGCGTGGTATGGTTTCCACTTACCAGTTACGTTTATTTTGTGCATGTAACTGTTGGGTCTTCCAGAACATGGACTTCAATTCAATTGTCTTTCTAATCAGTGGGTCCCTTTTCTCTGTCTAAATTACCGTGGAAGTTCTCAACAATGATAATATTTCCACTAGTGTGTGTTGTACTCTGCAGCCATAAAGTTTTTAGTCTAGATGCTTATTTTCTTGGATGATCCAAATGATGAGATCCCTGATTTTTATAGACACCTAAGAAGTAACCCAAACTCAATTTTAGATCTTCTTATGGCCCTAAGAGCTCCTCTACTTTGGTGTTCTTGTTGCTCTTATGTTTATCATGGCACTTGATTTTGTTTGCTTGGATGCATGCTTGTAGACCACATGGTGATCGTTTGGGGATTAGATATAGTAACACCTCGGACTTCGAGAAAGGGCTAAAAAGGATAAACGGGCAGGTCCATGAACCCAAAATGGAGGACCCTTCACGGCACTCTAGATGGGGCCGTTTAGGGGTCCTCGACCCATTAGGGCATCATGAACAACCATGTAGGACTGCCTGGGCCATAGTCACCTAGACGACACCTTAGACGGACCGTGTTGACTTGGATGACCCGTCTAGGGGTCCGTACAAGTCATCCACTAGTTTTTAAATTTTGTGTCAACTTCAAACGATCATATCTCTCAGCACAAAATGAATTAGGTTGCCCATGACCTATCAAATTAAAGGTTTTTGAGTCATCTTTCCAACACCACCAAGTTTTCCTAATTTTGAGCAAGGAGTAAAAAGTAATGCCCATTTTATTGAAGACTTGTCGGGCAGGGCAACATCACGACCCACTAGATGCGTCGTGATGCTTTTAACGGCCCGTGAAGCCCATCTTTTAAGTCACCTCGTCAGTTTTAAGTCAGGGTCGTATTGGTCTTTTCCCCTATGCGTTTGATACTTAAACTACGTCGTTTTACTTCGTCTAAGCCTAAGTAATCAGTCAAAAAAACTGCCTAACACATTCATTCTCCTAAAATCATTAGACTTAGAAAGAAAAGAAGAGAAAGGGGCGATAGTTTTCTCTCCAGAATAAGCAGAGCTCTCTTTCAAGTTCAGCCCCGAAATTCAAGGGATGTCTTTGTAGTTTTCTTCACCAGGTATGTGGGTATTCACTAAGGGTTTCGCTTGCCCTTATGCCCAAAATTTCAGACTCCTTTTTCGATTATTATTGTTTTATGAAACTAGAGTTCTTGCCCAACTATGCAGATTCTTTACAAAACTGATTCTTGTGATTACTACTTGATTCTATATTTATTTAAATGACTTTGCATGAATTCTTATGGAGATTTGAAAGGAATAGCCAGTAATTGTAGTATTTTCACATGAATTAGCTCTTCTCCAGAATTTAGCTCAGGGTGTTCCCTGGATTTTACATCATCAATAGGATAAACATTTGCTAATATATTCCCTGATTTTAAAGTTAATATGAAAAAAAGGGTCAACAATAACCCCCATTGATGATGTTTTGAACATATTTTGAATTTGAAGCAATGTAACAATGTTTGTGAATTATGCATTATGATTATTGTATCTTAACAATATGTTTATCTGAAAAATGACATCTAAACCCAAAAAAAATTAGCAATATGAATATTTGTGACCAAACTATTAGAGGACATTTTTACTGAATTTTCCACTGTTTAAGAGTATTTTTGACCCTTTTCCAGTTTTTAAACTATATTAAGATTGAATAGATATACTAGCGGCTATGTTTGCTTAGTTATGAACTTTTCCCATTATTTCCCCCTAGAGTATATAATTATGATGGATATGCAATAAATGAATTTATTATTAAAAAGAAGTTAGCTAATTAAAATGATGTGTCAACTAGGAAAGAATACATCGTAAGTATATAATTAATTTTTATTTTAAATAAATTAGCTAATTATTTTACTTTGTTCACATCAAGAATCTGGCCTAGTTTATTTCAATTCATCTCCTATTATATTCAACGAATTCGAATTATGGATGAAAATATATCAAGGAACTGGTCCTTCGATTTTGCTTCTAAATTGGATCAACCAACACTTAGCCCAAAACTTGGACTACTAATTTAGGCCCGAAATCACTCAAACCCCACGCTCCTCTCCCTTTCCTTCGTCACTCTTCATTCTCTCCTCTTCCAACGGTCCTTTTACAATTTGGAATTAGTCTCTGCAACGGCTCTTTCGGTTTGAAATTTGATATCTCCGGCACCCTACATAGCTTTATTTATTGTCCATATATATCAGCCATTGAAATTAGGGATACTTAGCTCTGGAGAAAACTTTTCTCGTCCAAGTCTTTTTCGAGTTCTCTTTTGGCTGTTTCAGTTGTTCGTGGTGGAGGGTTTGTTATCTTCGACTCGTCTAGTCTGTTTTGAGTTCTCTTTTGGCTGATTGAGTTGCTCGTGGTTGAGGGTTTGTTGTCTTCGATTCGTCGTCCCAAAACACTGATCCTAAAAAGGTATTATTATCTCAATTTTTACTTTGCTTTACGTTGAATTTGTGTATTAGCAGTTTTTTTTCTAGATTTTGCTGACTGTCTATTTTTCAATTCACATTCAATATTTCTTTGTGTATTCATTGTTTATAATGTTTAAGCTTCAAGTATGTTTAGAGATTATTGTAAGAATTTAGCCTTTCTAATTGATAACACCTGCTTCATCCTTTGTTACAACTCTTTACCTCTTTCTGTTCTAATATCTAAAAATATCTCTTTTAGCTGGATGTTTGATGTTTATTTGACTCATTATCAAAAGCTGTTTGTTTTGATGAGAATCTATTTCAATTTATTTAAGATTCACAACTATGTAATTCCTAAAAAATATAATTTTGTTTCGGTTTTAAGTTTAGATTTCAGATTGAAATTTGACTCGAGTTCATGGCTGTTTGAAATCTTATACTTGTCTAAGCTTTGGTTTATCCACTCGTTCATTCTATCTTGCTAGCTTACTTCGAAAAAAATCTGGATATGATCTCGTGATGTTGAATCTAAGGCTAGAAAGTAGTTTTTCATCGTTTATAAGCAATCTGGTCTGTTGTTTGAATCATGAATTTATTTGTCAAGCATGATTTATTTTGCTTAACCATCTATGATCACCATTGTTTCAACCTAGAATTTGGAGTGTTATATTAATTTATCAGCTCCTCTTTAATTGATTAGTGTACTTGCTAAATGAAATACATATACAAATGACCAATGTGTCCTGTTTTTGATTGTTTCCCCTAATGGCTCCGTCACGGCGTCACCTAGGTGTCTTGTCGAAATAATTTGAAAAGGTTGTTGTTTGGGTCTTGATTTTATTTTATTTTTTTGTTTGTTCATTTCCTACTGTGTTAAAATTAAGTCGATACAAGCGTAGGTTATATTTTCGAAGTTGTCTTCACCCTATCCGCCCCTTTCTTTTTTTACGTCTCCTCTCTTTGATTCAAATTTGCCTATAAGGAAGAAGTTGCAATGCATGATTGCCTAGTCTTGTGATGTATTAGCATATTTATTTCCATACACTGGCCTATCTCTGCATGTTTTTATCTGTATGATAAGTTTTGTTTCCTCTTGGATTGTGTCTTGTGGTAAAATACTTACATGTGCTATCCAGCATGTTTTTAGCATATGAGACAATTCCCTGTACATATTCATAAGATGTTGAGAAATTGAGACGTGCTTGTTTATCTCTTTCCTTATATAATTTTGATGCCAAGATTTTTAAAGACACTATTTTCTCTTTTGCGAGAGTCATCTGATTCACTGTCTAAAAAATAAGAAAGTCTTAGTCATCTATCTTGTAGTATTCTAATATGCTAGTATAGTAGTTTTGAACTTTGTCTGGCTACCTATCCTTTAGGGTATAGATTTTTTTTTTTCTTCTTTGCTATGAATTCCCATTTGCTACTGTTTTAACAAATCCCTTGAAAGAATGTTTTGTGTTCTCACTATTTCGTTTACATTTAACTATTTTTACTACTTGTTTTGAAATCTTTTGGAGATAGAAATTTCATAAAGAGTCCTGAATGTGTGCTGGTTTAAGTTATTGTCATCAGTCTTTTCGTTTGTGCTTCTCGTTTAAATGATTCCTTGCTGTAAATAGTTCGTTTTATCCTATCCAGCGATTGATTAACTAAGGCTTGAACTATGATTAAATAGGTCATAGAATATGTATGGTTTGTCTTAAATCAAGTTCTTTCCTTTTAAATGTTTGTCACAGTCTAAGATTTTAGTCGACGTGTAACTATGATATGTTTCTGCTAAAAGGCTATAGTTATATGAATTCTTGGAGATTATGGCCTTAATGTTTCCTTCTACTGTTTCATGAAACATGTTGGGTGGCATGATTTTTTATTTTTTTACTTTTGTTCAACCTTGGTAAAGCTCGTCCTGTTCTCTATTTGAATATGCCTGACCACTAGCTTACAAGAAGTCATCACTTTGTTCCAACCTTGGTAAAGCTCGTCCTGTTCTCTATTTGTTCATTTGTTTGCTATTGTTAAGGTATAACCTTTGGACGGGTATACATTTTGAGTTCTCTCCAGTCTCTCCTCTCCCAACGGTCCTTTCAGAATTTGGATCATCTCTGCAACGGCTCTTTGGGTTTGGAACTTGAAATCTCCCGCACCCTAAAAGGCTTTATTTGTTATTTATGTAATACTGTTGGAAAAACGCGG

>LNC_000026

TCTCTTTCCTCTGCTTGCATATATAATTTACAATTGCTAAGACCCAAGAGACAAAGTGCTCATTCATCTTCTTCAAATCTCTTCTTCTCCTGATGATGGTGCAAAGCTTATGGGTTACGAGCTAAATCAACTGTACTATTAAAGCTTCATTTGGCGTTGTCGATTCAACAAACACTTAAAACTTGTGATCCTTATGATTGTGAACTGAGAAGTATATAGAATTGGATCCAGGTGTATTCTGTCACATTTTCTGAAAAACAATAGCAAGGAACATAACTAAAAGATGTGGCATAGTATTTTTTTCTCAAAAACAGTTGTCCGCCACAATTATAAATGGCCAGCTTAAGTTGTGTGGAATAAGCACGACATCTATTGTACCCTATGTTTTCAAACACAATTGGCATGTAGGAGAAAGTCTTACCATGTGACCTGAATAAATTATTGTCTAATATCTACCAGTGTTTTTCTTGACATGTAAGAGTAGAAGTCACTTTGTTATTTGGGACCAAGGGATTGTTTGTCCTTCACTGAATTAGTTGAGCTATAAAAAAGAGTATCTATGAATTTTATTAAATTTTAATAAGCGGTTGGTGAGCGCCAAGAGAATCCTCATGTTCTAGGGGTTTGGTTTAACAGTAAATGTTGTGCTATGTGGAACACAAAGATGTGCTAGCTGTATCACAAGTTTGAAACTTGACTGCTAGACCAAATTTTACATTTAAGTGAAGAACAACAGTGATAAATCCATTATCTTGAAAATATTCTGGTCATAAAAAATATTTGACGAGAGAGTCAACAAGATCATGTTGTTAGGAAAATGTTCCAAGAGTCTGTGTTCTTTTGCTACTCGCTATTCTTTTCCTGTTTCGATGTTTCATTTATCTCTTCATATGAGTTTTAAAAACTTTTTCCTTGGAGATTTTCATTCTTCAGTCCTCCGATGTCGTATCTACATGTTTTATAGAGTTGCTTTTATATATTCCTCACCCTGGATTTGAAATACCAAGTGTCAGTCACTCTTAATTTTATGCTGATCTGAGATCTCCATGTTGAACATCATATTTTTACTTGTTTTGTTCATAGATTTTCCAGTTGCACCACACTTTAAAATCATTTGAATTTTATCAAATTTTAAATTATATTATATCTAGCTTTGAAGTTTTATGGAAGAAAATATTTAGGTACAGAACATATGGCACAGTAAAATCAATTTTTTTTTTATTAATTTTGACAAAGACCAAATGGAGAATTGAGATTCTGCACTCCTCTTGTGAACCATTGCGGAAGGCAAGGGTACTACAAAAAGAGAATCGTTTACCTCGGAGCCAATTTTTCAAGACCATTCCTTGTAGTAATTTTATGTCAATATGATGGAAGGCCTTGGATTGTGTAGCTCTTAGAAAGAGTTGTGTCATTCTTCAAGGATCACAAAGGTTCGATATACTTAATAAAATCTTTTTTTTAATTATTTGCTATGCTATGTAATTTTTATATCTATTTGTCACGGTTGTTGCCTTTTTAGGGGGCTATTGGTTTGAGTTAAATGACAATCATCATGTTAATAAAAAAATTCTCTCGGAATTACTTTGATAAATTATTTTAGAAGGTCATTTTATTCTTGAAATACCAATGATTGTGAGATGGATTCTTTCTCCTAAATTCTAATGATGTGTATATTGATCAATAACATCATATGGGCACAACTTAGATTTCTACTATGACTGGTATAATTTAAAAATTAAAGCCCTACCTTCTTTTACCAAGTAATGTTATTGCAAGATTTTACGCTCTCAAAGCTGACATACTACATGATCTTCTTTTTCATTGAAGGTTTGGTGTTAGATATGTCAAATAAATATATCTTTAAGTTCAACAACAACCATGATAGTAAGTGCGATATTGTTGTGTCGGAAAATGCGTATGCGAATGTTTAATATTGGTTTACATGACATAACATAAATTAAGACACAAAAATTAACATATATTGGGCCCCGTGTAACAAAATATTGACTCTTGAAAAATCTACTGCTTGTTCATTACTAATTTTTAAAAATAGTATTAAAAAATTATTGCACTCAAAATTCT

>LNC_000027

TCTCTTTCCTCTGCTTGCATATATAATTTACAATTGCTAAGACCCAAGAGACAAAGTGCTCATTCATCTTCTTCAAATCTCTTCTTCTCCTGATGATGGTGCAAAGCTTATGGGTTACGAGCTAAATCAACTGTACTATTAAAGCTTCATTTGGCGTTGTCGATTCAACAAACACTTAAAACTTGTGATCCTTATGATTGTGAACTGAGAAGTATATAGAATTGGATCCAGGTGTATTCTGTCACATTTTCTGAAAAACAATAGCAAGGAACATAACTAAAAGATGTGGCATAGTATTTTTTTCTCAAAAACAGTTGTCCGCCACAATTATAAATGGCCAGCTTAAGTTGTGTGGAATAAGCACGACATCTATTGTACCCTATGTTTTCAAACACAATTGGCATGTAGGAGAAAGTCTTACCATGTGACCTGAATAAATTATTGTCTAATATCTACCAGTGTTTTTCTTGACATGTAAGAGTAGAAGTCACTTTGTTATTTGGGACCAAGGGATTGTTTGTCCTTCACTGAATTAGTTGAGCTATAAAAAAGAGTATCTATGAATTTTATTAAATTTTAATAAGCGGTTGGTGAGCGCCAAGAGAATCCTCATGTTCTAGGGGTTTGGTTTAACAGTAAATGTTGTGCTATGTGGAACACAAAGATGTGCTAGCTGTATCACAAGTTTGAAACTTGACTGCTAGACCAAATTTTACATTTAAGTGAAGAACAACAGTGATAAATCCATTATCTTGAAAATATTCTGGTCATAAAAAATATTTGACGAGAGAGTCAACAAGATCATGTTGTTAGGAAAATGTTCCAAGAGTCTGTGTTCTTTTGCTACTCGCTATTCTTTTCCTGTTTCGATGTTTCATTTATCTCTTCATATGAGTTTTAAAAACTTTTTCCTTGGAGATTTTCATTCTTCAGTCCTCCGATGTCGTATCTACATGTTTTATAGAGTTGCTTTTATATATTCCTCACCCTGGATTTGAAATACCAAGTGTCAGTCACTCTTAATTTTATGCTGATCTGAGATCTCCATGTTGAACATCATATTTTTACTTGTTTTGTTCATAGATTTTCCAGTTGCACCACACTTTAAAATCATTTGAATTTTATCAAATTTTAAATTATATTATATCTAGCTTTGAAGTTTTATGGAAGAAAATATTTAGGTACAGAACATATGGCACAGTAAAATCAATTTTTTTTTTATTAATTTTGACAAAGACCAAATGGAGAATTGAGATTCTGCACTCCTCTTGTGAACCATTGCGGAAGGCAAGGGTACTACAAAAAGAGAATCGTTTACCTCGGAGCCAATTTTTCAAGACCATTCCTTGTAGTAATTTTATGTCAATATGATGGAAGGCCTTGGATTGTGTAGCTCTTAGAAAGAGTTGTGTCATTCTTCAAGGATCACAAAGGTTCACTAACCTTTCACAACCTCTTCATTGGCGTGGGTGGTTCAATTGAGGCACCGCTACTGGAAAATTCAGATTCAACAAATGTCGCTTTCATCTCTACCTCGTTAGCATCAAATGATTTGGCCCTTTTAATGTTCCTCTCGCTAGTGCCTGGTAGAGATACATGTTCTTTTTCCGTGTAGGACAGAACTATTAGACTTGACTGAGTAACATTAAAGTTTTTCCAGGATGACTTTCTCAGTTGAATGTGAAATAGTCTCTTCGGCAACATTTTTTGGGTCTGATCAACATGGAGAAGTTGGTTCTGGAAACAAAAGAAATCATTTGCAGAAGTATATTAAGTATATATAAACACATATTAAGTATATATAAACACAGACACTAAGAAATATAAGTAAGAGGTAACCTTGACGCAGGTTGTGTCAAATATGTCCTCTGCCTTCATGGAGAGAAGTTTTTCGTCTGGATCACCAGAAATGAAGGCACTTGTTGAACCACTGTCATCATAATGAGTTAATACTTGAAATTTTATTTGTATATATGTGATATTTTACCACAATAAATTTGTGTATATAGCATAAACGTAATACCGAGGCACCAATGTAGTGGGTCTTTGACATTTGCGCAATCAAACTG

>LNC_000028

ATCTTCTTCAAATCTCTTCTTCTCCTGATGATGGTGCAAAGCTTATGGGTTACGAGCTAAATCAACTGTACTATTAAAGCTTCATTTGGCGTTGTCGATTCAACAAACACTTAAAACTTGTGATCCTTATGATTGTGAACTGAGAAGTATATAGAATTGGATCCAGATGGAAGGCCTTGGATTGTGTAGCTCTTAGAAAGAGTTGTGTCATTCTTCAAGGATCACAAAGCC

>LNC_000029

TTTCAATTTCACTGTTGAATTTCTTTGAGTGTTGTTGTTCCATGATGAAATTTCTCTACCTATGAAGAGAAGGAAAAAAAATAAAATTTGGCTTGAAGAAAATTCCTTTGTAGATTTGATACGATTTCCTTGTTATTTTTAATGTCATTGTTGACAGAAAGTTCCAATCTAACTTTTCCTTTCTGCCAATTAAGTTGAAAGCAGAGAAACATATACTTTACATACAGTTTCCTTGCCATTTTCAGCTTCTCTTAACAGTTGATTTTTGCTCTCTTTGTAGGCGTTGTCGATTCAACAAACACTTAAAACTTGTGATCCTTATGATTGTGAACTGAGAAGTATATAGAATTGGATCCAGGTGTATTCTGTCACATTTTCTGAAAAACAATAGCAAGGAACATAACTAAAAGATGTGGCATAGTATTTTTTTCTCAAAAACAGTTGTCCGCCACAATTATAAATGGCCAGCTTAAGTTGTGTGGAATAAGCACGACATCTATTGTACCCTATGTTTTCAAACACAATTGGCATGTAGGAGAAAGTCTTACCATGTGACCTGAATAAATTATTGTCTAATATCTACCAGTGTTTTTCTTGACATGTAAGAGTAGAAGTCACTTTGTTATTTGGGACCAAGGGATTGTTTGTCCTTCACTGAATTAGTTGAGCTATAAAAAAGAGTATCTATGAATTTTATTAAATTTTAATAAGCGGTTGGTGAGCGCCAAGAGAATCCTCATGTTCTAGGGGTTTGGTTTAACAGTAAATGTTGTGCTATGTGGAACACAAAGATGTGCTAGCTGTATCACAAGTTTGAAACTTGACTGCTAGACCAAATTTTACATTTAAGTGAAGAACAACAGTGATAAATCCATTATCTTGAAAATATTCTGGTCATAAAAAATATTTGACGAGAGAGTCAACAAGATCATGTTGTTAGGAAAATGTTCCAAGAGTCTGTGTTCTTTTGCTACTCGCTATTCTTTTCCTGTTTCGATGTTTCATTTATCTCTTCATATGAGTTTTAAAAACTTTTTCCTTGGAGATTTTCATTCTTCAGTCCTCCGATGTCGTATCTACATGTTTTATAGAGTTGCTTTTATATATTCCTCACCCTGGATTTGAAATACCAAGTGTCAGTCACTCTTAATTTTATGCTGATCTGAGATCTCCATGTTGAACATCATATTTTTACTTGTTTTGTTCATAGATTTTCCAGTTGCACCACACTTTAAAATCATTTGAATTTTATCAAATTTTAAATTATATTATATCTAGCTTTGAAGTTTTATGGAAGAAAATATTTAGGTACAGAACATATGGCACAGTAAAATCAATTTTTTTTTTATTAATTTTGACAAAGACCAAATGGAGAATTGAGATTCTGCACTCCTCTTGTGAACCATTGCGGAAGGCAAGGGTACTACAAAAAGAGAATCGTTTACCTCGGAGCCAATTTTTCAAGACCATTCCTTGTAGTAATTTTATGTCAATATGATGGAAGGCCTTGGATTGTGTAGCTCTTAGAAAGAGTTGTGTCATTCTTCAAGGATCACAAAGGTTCGATATACTTAATAAAATCTTTTTTTTAATTATTTGCTATGCTATGTAATTTTTATATCTATTTGTCACGGTTGTTGCCTTTTTAGGGGGCTATTGGTTTGAGTTAAATGACAATCATCATGTTAATAAAAAAATTCTCTCGGAATTACTTTGATAAATTATTTTAGAAGGTCATTTTATTCTTGAAATACCAATGATTGTGAGATGGATTCTTTCTCCTAAATTCTAATGATGTGTATATTGATCAATAACATCATATGGGCACAACTTAGATTTCTACTATGACTGGTATAATTTAAAAATTAAAGCCCTACCTTCTTTTACCAAGTAATGTTATTGCAAGATTTTACGCTCTCAAAGCTGACATACTACATGATCTTCTTTTTCATTGAAGGTTTGGTGTTAGATATGTCAAATAAATATATCTTTAAGTTCAACAACAACCATGATAGTAAGTGCGATATTGTTGTGTCGGAAAATGCGTATGCGAATGTTTAATATTGGTTTACATGACATAACATAAATTAAGACACAAAAATTAACATATATTGGGCCCCGTGTAACAAAATATTGACTCTTGAAAAATCTACTGCTTGTTCATTACTAATTTTTAAAAATAGTATTAAAAAATTATTGCACTCAAAATTCT

>LNC_000030

CTTCCTTTCCACAAGTCCAACCATTTATCCTCTATTTTACTTCAAGTTTATGGGTCCTTATGATCATTTATGAACTCTTGAATTATGGAATTACTACTACACATCCTATTATGGTCTATTTTTGTATATTATCATGAAATGGAATGATTATATGATGTTTATGGTTTTCATGCCTCCATGATCAAATTATGAAGGTTGTTATATATGTATATATATATGTATGTTGTTGGTGGGCTGATATATATGTATTTCGGAATTGGTTGGACTTAGTACTCTTGAAACACATGATTTTATTTACATGAACAAGTAGCCCACCATATGTTTGATAAAATGCCATTTATAGAATTCTTATGAAATGGAAGGTCCAATGTACGTCCTATGAGTCGGATGATTATATAAGTATTGAAATGATGATGAAATGGATACATGAATATGATTTTCTCTATATAGTGTGTGAGTCGACCAAGCCTAGCTCGGGGGGTTGTAGGCCCGGGTAACCGTATCGAGGGGTTGGTACCTTGGTTGCCTAGTACGGGGGGTAGTAGGCCCGTATAACCGTATCGAGGGGTTGGTACCTTGGTTGCCTAGTACGGGGGGTAGTAGGCCCGTATAACCGTATCGAGGG

>LNC_000031

ATTTTTTTGAATATATGTGTTTATAAGTATATTCAAAATTGATGACATTATAGTAAGTTTTAAAAAAACCATTCTTCATAGCATAAAAAAATAAAATAATTTAATTATGATAAAAGATTCTTAAGCATGAAAATTTCTTCATAATAAACTAATATATACCTTTTAGAAAACAATAAGATCATTATTTTAGTTAAATGAATTTGATGAATTAAATAAGTCTATTCTTAAAAAAAAAGCTTCAATGACGTGGCATGCCAACTAGGAGAGAGTAAAACTTTTGAAGTCTTAAGTATATTTGGAGGAAAATAATGATAGTTGAGTGATATTTTGGTCATAAATGATATTTCAAGGCAATTCCCTAAACTTGGGTGACCATTTAGGGAATTGACTCCCCCCTATCCTAAGGCCCTGCCTTCGTCCTTGCATTTTGAGTCGAGTCAAAACCCGATTTTCTTCCCTATTGAGTCAACCTCATCAAAGGGAGCTGAAACGTTGAAGGGAAGCATCTACTGGTCCAAAGAAAGCTGAAAAAGGGACGATATGCATGCCGATATACACTGTGATACACTGTTGTATACAATCATTAAACAGTGATATAC

>LNC_000032

AAAGTGGTGGGTGCTCCTAGGGAGGGTTAAAAAATTAATAATAATAAATAAATAATGGTAATTTTAACCATTTTTTATGGTAATTTTAGTAGTATGCAAATTAACTGAGTCAACTAAATCAGCAGCTTTTGCCTACAATACTCTTTTCTGATATAGGTACAAATATGTGTTTTTGCACTAATGGTAAGATGAAGATGGAAACATGATTAGTCGACTACGTTGTTTGCGATAGTACAAGTACTGGTATTGCGCACATCGGTTTAATTTGTCATCATTTATTTCTTACTTTTCAAGTATAAGAATGACCAAGATGCCCTAGAAAAATATTGGTGAAGCAATATGTTCTGTTATTAGTGGACTGAAGTCTTTGGCCTTAATGAAATGGTGAAGCATTGTTCACCAGTGTAGTGTGCACTGCAGACA

>LNC_000033

CATATTCATAATTGATTAAATTCATATTAGATCATTATGAACTAGATTATTTGTTTATCATGTTCTCCTGTCACTACTAGCATCGTACTTTAGCGGATACTTCCTTCTTATCAGCGCCTCAACTTTGTTTACTCCCCTAAACGGCTTTCCTCGCCTACCGGTTTCAACATTAGGATGGCATAGTATAGAAGTTTTCTCCCTGTTTCCAGTGTGCTAATAAACTAGAAATCATGATTCAAATAATTCTAATACATAATAAC

>LNC_000034

TTATTTATGGAAGGAAACAAAGGTACCATTCTATTTTCAGATACTTTCTATTTTTAATTGTCCTAGAATTATAATTGTTCTCATACTAAACTCTGTGAATTGCATTTCCAACCTCTGCATCTAAAGAAGACACAATAAAAATATGTTGGCACAATTGAGTTCTGTGAAAATATTATATAAGGTATGAGTTATATCAAATCACATTGTAATGAGTATCACAAACTTTTCAACAAAAAAATATATAATTTGAAATATATGAACAGTCAATCTATTTAAATAACTTCTTTATCGTATGTAAAATGATTAGACAGTCCATGAACCTGCAAAATTTGG

>LNC_000035

TTTTGTCCTTCACGTGACAAAAGATATTGAGGGTCACCTTCTCAAGTAATAAAACATCAATGCTACTCAATGGCTCATAGCCCTATTTATACATATGCAAAATATATATATATATGTATGTTTACACACAAATATATATGTGTGTCTTTGTGTGATATTGACTAGCTAGTTCTGTCTAGCTAGCTAGTGTGTGAAATAAATAAAATTGTGTTTGAAGAATATATTACATATGTTCCTCTCGTTCTAGAGATGTATTTTTAATGTTCAATATATGTAAAATATTAGTAAAATTTACCTATTACAAAAATGATATATTTAATAGTGTGCTTTAATTATATTCTCATTCTTTTGTAGTCAACTCCTTTTTAGGTTTCTCTAGTTTTAAGATGGGCACTAACATAAATTTCAAATCTTAAATTACATTTTGGTTTCGACCCCTTCTATTACATCATGTGTTAAGCCAGTCAAGTGGACTGGGTCAACCCGGTATCAGCCCGATAAGCATGACCCAGTAATGTCCCGGCACGGCTAGGAATGGGTCGGCTCCTTT

>LNC_000036

TGCTGGTTGGTGGGTAGACTTGCCACCAATATTGGAGAAGATGAGTGCATGTCCTTATTATGTTTCTTCTCTATAGATTATTGAATTTTAATATCCTACAAATGATGTTTGTTCTAGCATCTCGACTATTACTTTAACTCAACTGCTCCTTTAATTTGGTCACTTGTGGCTGCTGGACTGTACTACATTTAAATAGTGTCTGATGAGTTGGGTCTTCTCTGTGGCTCCTCTGTAATTATTATTGTTTAGCAATTTTAAGT

>LNC_000037

GATCTTTAAAACACTTTGCAAGATTTGCTTGCTTAAAAGGGTTATCCCTCATGCTTATTTATTTAGACCTTATCAATAACTCTAAAAAGAAGTCACATTTTTATCTTAAATTACAAATGATGCCCTAAGGTGAAATGTCATTGAGTATTGTGGGGCCATTAAACTTTCCAAACTGACACATTGACATAACACTTGTCAATTGTGCTCTAATTTCCCCATATATTTTCCATTTTCATTTTAATTTGGTGAAAGATAATTATTTGTTTTATACCCAAATTAATATTTTAAAGTGTGTAAATTACCCTACTTTAATTAACTTCATATATTTTTCAGTTGAATTTGATGAAGGATTATTA

>LNC_000038

AAGAATACAAAACAATGAGACACGTAGAAGGAGAGAACTGATACACAAAAAGCCAAAAAAAGAGGGCTCTGTTTTTCACAAAAAATTCCATATTCCTCTGAAATTAAGCAAAACACAAAAACAAAAACAAAAATATTACCGACAAAAATTAACCCCATGCTTTGTCAACAACAAAACAAGACTATGATCTCTCACAAACACGCCTTTTTAATTCAAACCAAATTCATCTATCTCTATATCTTCACCAAAAAAAAATTACACAGAGAAACCAGAAAAATCTTGAACCCAGCAAAAACTTTTATTTATTTATTTGGGAAAGTTCTGGTTATGATTTTTGTGGGTGTTTGATGAATTCTAGTTCTTTGTCTTGTTGGTGAGTTCTTGCCAAATTTGTGCTTTTTGAGTACTTGTGCTCTGTTTTCTGGT

>LNC_000039

TTATGACTTATGTATGTTGAAGCTATGTTATGTATGATCATTATGAATATGGTAAGTTATGATGAAATATGATCTTATGAATGTATGCATGAAGTAGGTTGCTTAATGTGATACTTGGCTTTGAATAGGGTTATGGGGTTCTATTCTTTGCATTGCACTAGTATTGCTTGGGTTGTCTACATGTTGGGATGATATTATGTTGGTTTGGACTATGATACT

>LNC_000040

AAAAACGAGTTGGTGTGTGGGGGTATGAACCAACCAACACGAAGGATAATAAAATCCCTCAACATACAAGAAATTCAACTAAAAAACACAACCAAATCATGTCCCAAATCAACAAGGGACTTCAACAACACAATTAACAATGTCAACAACACAACCAACAACTCCAAACAACACAACCAATCTTTTCTCAATAATAAAACGAGTTTGGTGTGTGGGAGAAAGAATCAACCCATCCCTATGAACTCACATACCTGATTAGGGATCAGCCCCGACAAAAATCCACGACGATCCTTGATTGCTCTTCTCTTTCTCCTCTTCCTTCTCTTCTTTCTCTCTTCTCTCCCAAGCCCTAACTCTTTACTCTTTAAAAATGAGACAAACTGATCTAAAAAATCAGTCCAACACTTAAATATAACCAAAAGAATTGATCAGGGAAAAGACCAAAATGCCCTTAAAATTTTCGGACTAAACTGCCCTATGCGACTGTGCCAACAGCCCAACTTCCAAAGGGCATAACTCACTCATAAGAACTCGGAATCGAGCAAACTCGGTGGCGTTGGAAAGATCATTCCACGAAAATTACAACCATAACTGGAACTACACTTAACTCATCTTGAGATAGAAGTTATGACTGCTCAAAGTTGGCCAAAAAACTCATTTTTTTCCATACTTAACTAAATTTCCTGATTTGAATTCTTTCCAAAAATGATAAATTCCAATTTAACTCTTCTTGGAACTCAATGTGCTACATCTAGGGACCTTAACTTAACACTCAAGAAATCTTTATTCCTCGGTAAAATCTCACTCCACATGGACGGCTCGAGTCTTAGTTCGAAAAATTCTCTGGGGTGTTACAATATCTCCCCCTTGGAATCATTCATCCTCAAATGACAGCTAGTCTTGGTATGGAAAGGTCTACTCTTACTAAGGTCTGCGTCTAATGGTCTGCTCTTACCATATTCAAGCTTAACCAAATCCTTAGGTTCGGATTCTATACCTCTCTATAGGGAATCTTATCCCAAGACTATAACTCTTTAACACCACATTAAAGCTTAGTATTCTAACAACTCCATGGACTCTTACTAACCACTCCTTTAGATCCAACTAAGGGTTCTCATTTGAATATTTCTCATAGTAAAACCTCATCCTTTTCTTCCTCATTGTTAATAACCTTAAATGGAACTATCCCACCATCACAACACGATAAGAACATCAACCTCCTCTTTTTAACACCACTCCATTACTAAAATGACCTCTGAAAAGATATCTCACAAACTCTTTGAATTCATTACCATCTTAATCTAAAACTCTTGACTCCATTTGTCA

>LNC_000041

ATGGCTTGATATATCACTTTTAGCTTTGATGAATTATCTTGTTTCCACTATGTGATAATTAGTTGTTGAAGGTTCATTCCAGTAATGTTTATACTTGCAAAACTAGCAAACTGCCTCCATAGCCTAGTGGCTATAGGGGTTGTTAGAAAAAGATGTGACATAGTTTCCTCCTCCTTTGTTTCACAACACCAACATCTAGAGGCAATTAGAATGTGCATTCTTTTGAGGTTATCATCTGAAGCTATTTTCCTATTTCATACCCTCCACAAGAAGAAATTCACCTTAAAAGGAAGTCTTTTACACCATATAAACTCATAATCTCTTCTGCATTCCCTCTTCTGTCTCAATAAATGCCATGCAGATTTAACTGTGAAAATACCTTGAGTATTTCCCATCCACCAAGGTCTATCACTTCTATTCTCA

>LNC_000042

TGTTGAGTACATAACATCTTTCAGGGGCTGATATGAGACCTCAGTTAAAGGATGTTTGACTTTGAATCCAAGGTGAGCTACTCTTCCAGACTGTCATGGATTCCTCTACATCTTCTTGCCTATCTTTTCAGACACAAACTTTAGATTATTAGTTTATGTTTCCTAGCTTTCAGGGTTGTATCCTCATTATTGACTAAATAGAGTTTTGCTACTACGATTTTCAGTTTCTAGAGGTCTTATTTTCGTAATATTTTGGACTAACGACTGAGTTTATACAAACTCAATTCTTTAATTGTTTTAAACTATTTTCTTACTATTTATTTTCATGAGTTGAGACATTGTGTGTTGGATTAACTTTGGATGGATAGATTGTTCTCCCATCAGGTGGTTAATGTGGGTGCCACTCACGACAATAAGGTCGTAGAATATTTTAAATAATTGAGTGATATTTTTTACCTTTTCCTTTTGATACTATGTTAAATTGGGAGGTTCTTTAGCTGGTGGACCAACCACCTGCGTTGTCTTTTTTTGACTTTGAGTAATGGTATTACGTATATTGACATTGACATGAAAATGGAATATTAATTTAGTGCTTGGCTTTATTAAGACTAAGAGCTTGACTTGTTACACGAATAATGCATCGTACGTTC

>LNC_000043

TTCTTTTATTGTGCACGAGCGTACCCGTCGGGTTTATGGGAGCCCGGCGGAGTTAGTTAGATTGCTTGTCTTTGTTGCCTGTACGCTCGTGTACATACCCCCATTTACTACTCTTTGCACTTGATGTGACCCTTTATTTGCATTTCAGTTTACTTTTGCTTATCTTGCTCAGTCGGCCGATGATGCCTACTGGGTACCTGTTGTTTTGGTACTCATAC

>LNC_000044

GTTTATACCTCATCTCTCAAATCTACTCTCGGTGGGTTGAATCCCACAATGGGATTAATACCACCACCTGAGTTTAGATCTTAAAGAGAGGTATTGACTTATTAAATCGTACAGGAAATTCAAGAATTCAAATCTTGAGTCTGACTCGACGAGTAGAACAGTTGAAAAGCTCGGGAATTTCTTTGACATCCGATCCCTTGGCTTGATCAGTTGCTGTTTTGCCAAAGACTTGGTTGACCCAATCCTTAGTTGTTTCCTTTGTTTGTATCTCCTCCCCTGTCTCTTTACGCTCATGTACATTGGCTGTAATAACTGGA

>LNC_000045

GGCAAGGGGTAATATGTTAGAAAGTTCAGAGTTTAGATGGAATGCTTGCTATCAGTGCATTAGATGTCCTAGTTGTCCAGTACCAACCCGCTTTGGAGGTTCAGTGTGCCTTCGTTCCTGTCATTCATCCAGTCTCTTGCAAGTGCTATCAAATCCTCTGTGAGTGAGGATACTCTTTATTTTGGGAGTCCTACAGGAGCTGCTCTCGAATGCGCTTACCTTGCCTTGTCGTCGATTACCTTCTGACTCTTGTGGGACTTCATCCCATCTCGTGAGGGGATCTTTGATGCGGCATTACCGGTGTCGGATAGGAAAAAAGGAGGTACAAGCGCAAGAGCAAAGATAGCTATTCCTCCCATCTTCCCTTTTCAATTTCCATTGCTTATCAAAGAAAGAATTCTAGAACTAGTGGTAGGTGGTCCCGTGGAGCTTTCAAAGGTAGTTTACTTACTTTAGTTTAGAGCGGGCAACAAGCCTTTTTTTTAGTTAGTCGGGTACTAACGGTTGGCAACCTCTATGA

>LNC_000046

GAAGCACTCGGCTGAGATTGTTGGAAAAGAGAGGCAGGGCCCGAGGCACTTGCTAACGAGCAGGCGGGACAAGATTGACTTGAAAAGACGCTCTACTTGAAAGGCAATGGCTTTGAGGGTACTAACTGTGATATATGGGTAAGGAAAAAAAAGAGTAGCTCAGGCTCTCGAGGCATACTAGCGGGGCTTGACTAGAATCGTAGAAAGAAGCTGGGGAGAAAGAT

>LNC_000047

TTTTGCTCCAAGAATGCTGTGATGCATCACTTGTTGGTCTTCTTTGAGTGATAAATGGTTGTCCAAAAGCTACAAACAATGTATTTGTCATTTGATAAAGTCAATCTTGGCATGACCTTCTTTCATCCCTTGTCATGAAATTTTTTACTTTCATGTGCTAGTGTGTGTGCATTTTGTTTAATAATGGTGGTATCCAGAAGCAACCTATGCAAACCTCGATTATTTCAC

>LNC_000048

AAGAAACCCAGAACGTACAACAGTAGCCGACAGAGACCCGACCCTTGCGTATCTTCTTCTTCTTCTTCACGCGAACACCAATCTAGGGAGAAAATTGCTCTGCAAACTTGGAAATTTCACTCAACTACTCTGCAAAACCCATAACCCTAGCAACCTAATCCATCACCCTCACGTCCCTTTTCCCCCTTTTTTTTTGAGATTTTCTGAATCCATTCACTGTTGCCTCGAAAAGAAACGGGCAGATTCAAAATTCAAACGGATTCTACCCTCTAAATCATCCATCTTCCCCTATAAATAATCCTTTACATTCAAGTGATTCGGGGGAAAAAAAAAAGTTTTGGAAATTGGGAGAAAACATGTTTTAGAGCTTGGTTCTTGGACAATACCCATTTTACATTCAAGGAATTTTTAAAACGATTTTCTAATTCTAAGTTTTGAGAAAGGTTTCATTTGAAGTTACTTTGAAATTCTTGGTTTGTTGCTCGAGTCGAAGTTGTGTTCAAGCTCGTTGTCGTCCAGTCGCTGCCCCGCAACAGGTGATCTTCGTCCCCTTACTCTTACTTCTATTATTGTCTTTGTATAAATGTTATGCCTGTTGTAAGAAAAATCATGTGTCAAGAATAATTCTCAGTTGTTTAGCTATCATGAAAGTGATGTTTTTTTTAT

>LNC_000049

TTAGATTTATACTTTTTTAAAAACAAATTATGGTTATTTTAATAAAAGAACAACTTACCAATACATTATTTACCAAATACATAAATCTTTATTATAAGTTTCAACTCTTTTATCCGAACACGTAATCACACCATTTTTTAGGCATAATACATAAATGTGTTTTTTAACTTGGCCTCAACGGGCATCTACACGCTTCAATTTTGAATGTGCACAAGTACACACTTAAACTAATATAACATTGAACAAGTAGACACATGTGTCCTATGTGGCATAATACACGTAGGATTTTTAAGACACTGATAAAGC

>LNC_000050

AAGAAACATCATTTGTGGGCTTAACATAGAGAAGAGAAATAAATTAAATGCCTTTCTTTCTTCAATTTGTGCACTCCAAACTTCCAGCCACTAGTGCACTTTTTGAAAAAAACTGTAAAACTTTGGACCTTAACGATTTGCACTGAAATCGGGCCGAAACTTTGTAATATTTCATTACAATTATTTATTTGCTGAATTTAGATTCATTTGTGAAACAAGTACACTAGTAGCTTTCTCATGATATGGCAAACAGAGTGTACTCTCTCACTGCTTGCTCGCCATTATCGGACCCTTTTCAGAACTTGCGCTAGATGTTCAGCTTTGGACGTTGGTAAGAAGCTTCATGCTTCCATCGTCACCACAGGTCTTGTAACTTGTCAAAATACATTTCTCCGTAATGCTATTCTCCAC

>LNC_000051

TGTTTTTATTGGGGCTAATCCCATTTAGTCATTATGTTTATTATTTTGTAAGAGTTTAGGACAGGTGAAAAATTGGGCCTAAGGCCCAAAGAAAGATATTTATTTACGTTAGTTTAGGACAAGTGTAGGTGACTCAAATGGGTCTAAGGCCCGAAACGAAATGGGCCCAAGTATTGGTCCAGGCGGACAGAAAACCAAACTTGGGCCCGAGTTTCTTTCCCTCTTTATTTTATTGTATTTGCGTGCTTGATAATATTTGCCGCTAATCTTAAAGTTGAAAAATTCAAATCCCC

>LNC_000052

TATAAATGAGTCAAGTATAAAATTTATTTAACACCCTAGCCCATCTGTAAAATATGTACTAAGATCAAGAGACAAATACTGAAAGAAGATGAATTTTAAAGTGGATAAGATTTGGGAATGACATAACTGTAAGGAACAAAACTTATGGTATCACGATTCAAGACTACCCCTAGTCGTAACACAATGCGCTTCATAAGTGACCCCAAGC

>LNC_000053

CAGAGACAGCCAACACACTTTAAGGAAATTTAACGATGGTTTAAATCCATACCAAGCTTCAAAAGGAGTTCGGTCTTTCACTACCTTTGTGGGAAGCCTATTTTGAAGAAAAACTTATGTATTTCCTGCCTCAACCCAAAATTTCTTTGGCAAGTTCTTCTCGTGCAACATGCATTTGGTCATCTCTATAATGTATATGTTCCTTCTCTCACTTACTCCATTTTGTTGTGGAGTATAGGGAGTAGTATGTTGATGATGAATCCCCGCTTCCTCACATAACATGTTGAACTGCTGAGAAGTGTATTCCCTTCCATTATATGTTCTTACGAACTAGATTTTATAGTCAGC

>LNC_000054

TATTAGTTGGTGAACTAACAATTAATTTATGATCCATATTTTCAAGACGTCCATAAAGTTTCATCCCTTTAGAATATTGAAGGAAAAATATATCGTTGAGACATTTATTTGTTAGACTAATTTATGGTTAGTAAACATCTTTTATTGAACTTGAAACCCCGATGGTTCTCTATTTAATTAAGGTAGAACAAAAAATGAGAGGGTATATGCAGGTTTGGACAATGCGATGAGATGTGTTGTTAGAGTCCTGTTCTGAAGGATCAACAATGGAGTGTTTATATTGTTCGTGCCCCTGGCTCTACTTGCTATTGTGAGGTTTACCATCTCTCCATTTTATCACTTTGAATTTATTATATGTATCGTTCCACACTCTTTGAAAGTGTTAAATTTTTGTATTGATGTTGTGTTGCCATGACTCTATGTTTATTGATGCTCTTCTTATGGC

>LNC_000055

CTTCTCCTTCCTTCCGCCTCCTCCCTTCCTCTTCTCCGGCGAGCTCGAGCAGCACCCAGGCGAGCGAACCAGAGCTCCGGCCAGCAGCACCAGGCGAGGCAGTGCAGCAAGCAGCAACCAGCACTGTCTTCTCTCCACCAGCAACAGCAGCAACCAGACGACGCCACCAGCAAACCTCCATCGCTGACAGCAGTTCCGACCAGCAACAGCAGCAGCCGACGAAGACAGCTGGCAGCAGCTCCAGATCCAGCCAGCAACACCAACGCCACCATCAGCTCCAGCCGGCGACAAAAGCAGCAGCTCCACGCACTGGCAACAGCAGCAAGGTCCGCTGCTTTCTCTCCCTCTTCTAGTCCTCAGCGAGCAGCGACGACAACAACAACAACTCCGGCGAAGTCAGCTCGAGGTCCTAGTCATCTGAGAAAACAGATCTAAACAGATCTGTGTGGGTTTTGAACTTGAAGTTCAAGTTGAAGAGTTACCATGAGACAAGATTATTTAATTTATGTATTTGGAATTCGCGCTTATTATTTATTTTTGATTTGTAATATTTGTATAACTCTTGAACTATCTCTATATGATATAACTTGGGGGATCGTCTAAAGGGGGAGGGGTTTACATGCTACTTGTTATATTATCACAACTTGTTAGAGAACAAGCTCATAGGAGTGTGATTTAATTGTGTGGTCATTCCTTGCTCTAAATTTACTTAAAATAAAATGGTCTTGCCGATATTTAATTGCTTTAAGTTGTAAATACGTTCTATGTTGACCATTTCATGTTAACTAATTATATCTAAAATAAGTAATAAACTTGTTATAATGCAATCTTAGCATGTAATAATAAATAATTAGTGTGACCTTTACAAGTTGATCTATGTTACCGTGTTGGTGAACCACAATTTTCTTGTATAAAATGTATAAAACTGGTAAAATAAATAATTGATTTGCTCAATATGTCACATATGTGATTTAACTTAGAAGCCATGCCTATAGGATTGAATCAAATATTTGCATGTCAATAAAATGCCCGTAAGTGTTCATAAATTTGTTTACATCTAGAAATCATGCCTATAGGTTCCTGAAATATATTATGTCTTACATAGAAATCATGTATATAGGTTTATAAATCCGTTTTTCAGCATGTTAATTAATTAAATAGCATTTTGTTTTAAAATTTTGCCACTGTTCAATGGCATAATTGTTTTCTGGCCATTTAGAATAAACATTGATAATTGACTATTGCTTTTCTGCCCATATATTTCTATCTAAACTAATCTGCCCGGATAAAATCAGCTACTAATAATCTATTACATATGCTACTTGCTACTCTAATTCAACTCCGCTTTAAATATTAATCTCTGCATCATAATTTTGCCACTTAGAATCAGCCAATGATTAATTCATGCAGCTATAAACTGATTGCTACTTAAAATAAATATTTGTATTAATAAGTTGACCTGCTACTGCTCTAAAAATTATTTCCTGTATCTGGGATACCCCTTGCCACTGTTTAAATCAGTTTGCAACTAAAATCTGCCTTATGTCATAGTTATCTGCCCTTCATTAATTAACTGTTATATTGTTGTGTTGCTTGTTGCCTGTCTTTATATTTGCACACACTCGCATGACACATTTCACTTTATATGTGTTCGCTAGCATGTTTAAATTAAAATCTAGAGGCCAATATGAGACCCTACATGCTAAATGCTCGTCCAAATTGTTTGACTTGGTGTCTAGGTGGGCCTATTATATTTTTGTTTTGTCTAAATTCACCCTAATAGCAGTCCAATGCCTCTTGGACCTTACGTGGGGACGCTAGACACCTTTTAGAGACGAAAAGCATGCGGTGGTGGGGTAGGGATAGTCTAGGCCCTATGGAGATGATGACATCGACTCGGTCTTAATGACAAACTTTGGTTTTGTCTGTCCCTACGAGAAACCGACCGAAATAAAGAAAGTCGAGTGCTGATCCTAAAGTCCTTCAGAGTCTCCCTTTCCTTTTCTCCCTTTACCTGTTATAATTATTTGGGGTAGTTTAATAAAAAATAAAAGTGTTTATTCTATCTAATTGTGCAAATTTCTATCCCCTCGCTCATCTTTGTATTTATTGTGTAATATTTATTTATTATATTATCACTTAGTCTCGCATGCC

>LNC_000056

TTTAAAGTAGATAAGATTTGGGAATGACATAACTGTAAGGAACAAAACTTATGGTATCACGATTCAAGACTACCCCTAGTCGTAACACAATACGCTTCATAAGTGACCCCAAGCCAACTCATTGTCGGACATGACACTAAGATTCATAAAGACAATAACTGAAGATAACTGATGTAGAAACTAAATTGAGATGAATGAGCAAGAATGCAAAATTGGTGTTGTCGCCATTGCCCAATACCACCAGCACATATATTTCCAGGTAATGCATTGATGACACTCTTCCAGCATCGCCTGGATAGAAATACTCTCTGAATCGAATTTAGATGTTAAAAGGACTGTCTGGGACCGAACATGGGTACATCCTGTACCCAAAGTCAACAACCCAACCCAAAGGGATCTTGTACTTGTAATTCCAATGTCAGGATGTACCCATGTTTTGTCCCAGATAGTTCTTTTAACATCTAAATGCGATTCATATAGTATTTCTATCCAGGTGATGCTGAAAGAGTGTCATCAATGCATTACCTGGAAATATAT

>LNC_000057

TCTTAAGTTGCTAAAGTTGTGAAATTTCGATAAAGAGTTGACTTTGGTCAACATATGAGGTTCCGAGGCTCAGATTGAAATTCCGAGAGTACCGTTGGTTTCGGGAGGCGATTCTAAGTCTAGAATGAGTCTTGGTTGAATTTTTAGATGCTCCGTTTGAGTTTCGAATTTTTTTGGCGTTAAACTAGTTTCTTGGCATCTTATTGAATTTCGGGTCAAGGAGTCCTCGAATTCAAATTCCGACAGTTCCATTGAG

>LNC_000058

TATACTCAAGGAATGGTTTCATTTTATACATACTTATCATTTTGAATCAGTTTTGTTTTGATTTAAATTTACCTCATGCCTTCATTCTGCAAATGGTTTGACTAGTGTTTGCTCATTGGTTTTCTGGGAAGAAATCACTATGCTCACTGCCCACAACTCTACTCTATATCTATATTAACATATTAACTTTAGTTTAAGCTTTTTTTTTATTGCTTGTTGAAGCCTCTCTTCTATTCTGTAAATAGAGTTAAATGAGTTTATTTTTAGTTCTTTTTTT

>LNC_000059

AAAAAATTTGTTAGCTCTATGATCCTGAGATCGGGAATGAAGACGACACCATTGAAACCAATAGTAATTTATGAGACATAAACCTCAAAGACGGAACTCAGACACTACAACAATCAAAGGGGTGATGGAAAAGTATTTGATCTTCTTGATTCTGATGGAGGAGAAATAAGGGTGACTTGTTTGATCTGTCATCATCCCATTTGAAATAAGGGTGACTTGTTTTATTTTAGTTGTGGTTTAAAAATAAAATTATTTTAACACATGAAAATCTTCTTCCATCCTTATTTCTGTAAAATCCCATCGTTTTCATCCTTGTTTCTGTCTCATAAATTATCATTGGCTTCAAT

>LNC_000060

CAGAAAAGAGACCCAAAAATGACGTTCAAAGTAATCAAACGGAAAGCAGAACGCGGAACAGTACTCTGCCTCTCTTTGGCGTCTTCTTCACGTCCTTCTCACTGGCAGCAACAAAAAGCACCTGCTAGCCTGCGCTTTCGTCCTGGCCAGCGCGGGATGAAGACACGTCGAGCAGCAGGTACAAGGGAATCGATCCCAAGCGTCGATTTACCCTTTCCTCTTTTGGAATGGTTTGAAATCACAAGGAAAAGATGTTTAGCTCGAAGGATGCAAGGTTTTTTTTTTGAATTTCGAATTGGAGTTGGGCCTTGTGAATTCGGATTTTTGATCCTTTTTCCCCATTTCCGAACCCTATTTTCCCGGGGATATATATTTGCTCTTATTCACTATGAGAGAAGAGGATTTTTTGGGGAGTTTCTTGAGCAATAAATTTTTTATACTTAGAGAAAATTCAAATACAATCTTTTAGTTTTAAGTTTAGGAAGGGGTCTCTGTTCGAAGTCAGTTCGAACATTCTACTTCGTGGGTTGAGTTTTCAAATTCTTGAATCCTCCTTTAGAAGAAGTCGTTATCTCCTCTGCCGGTGCTGTCTCTGGTTTGCTGCCCCCGAAAAGGTAAACCTCACTTCGATTTTGACTCGTTTGGTTTAGTATTTGTTTATGTGAGCTGAATGTCTCTATCGCCTCCACTTTTGTTCGTTTTATTGCTTCAGCTCTCTCTAGGTTTACTGTTAATGATCGATAGAAGATTTCGTATTATGAGTTGGTGTCTTAGTTAAGTAGAGCATGAAATGCGTTTTTGCGTGTGATTTGTGCCTAAAGGGCCTTGGTTAATAGTCTGACTTATTTTTGTTTCTCATGTCCAGCAATTTGTTTCCTCTTTGTTATATCATCCTTGTCTAATTGCTACTTGTTTGGTTGTCTGAGTATCAGATGTTGTTGTTAGGTCGGTTCCTATGTCTACTTTTTTCTGCATTTTTGGGTTTATTAGTTAGTAGTTTCGAGAAACTTAGGGAATGTTTTAACTTGTCTCAGTTCATTTCTCATCTTTGGAGCGTCTTTCGAATAGATTAATGGGTTTGTTCTCCGCTATTTATGTTGGTGTGATATGACTTTATTTTATATTTTTTCCTTTAACATCTTGAAGTTTTCGAAAGTGCAGCATGTTACTGCCCAACTTGCTTTGTTTTACTCGTGTTTGTGTCTCCATTTCGCATAGAACTCTAGTCAAAGAAATGCCTAATTTCACTGTTTAAGCGGAAGCTCCTTAGCATGCTTCTTTTGACCTTGTCTTATTTCGATCTTTTGTGAAGTTCGGTATTGGTGTTTGCTCGAATATATAGTCAATCTTGCTTTAACTTAAATTCGTTACCATGATTAGCCGAATGGGTCACTTTTAAATTTACATGCTATAACCTTGTTTTAAAATTTCGGTGCATCAAGCTGTCGGCAGTGACGTGATTAACTGACCCTCGACCTGATTCAATTTTAAATAACTCGCTGTAGTTGAACTAATTGTTCCAACTTTTGATGTGATAAGGCATATGGGTAGACTAAATCATTTTAAAATATATATGTGTATATATTTAAATAACGTGAAAATGGGGCCATTAAAGCACATGGGTAGGCTGAATCATTTCAATATATATATATATATATATATATATATGTATCTGCTTCCTGTTTCTGCTTCGGGACCTGCCAGTGGTTCCCCACAATTATCCAATCCATCATTCTTTATTCTCTTAGGCCTAAGGTGTTAAGTGCTAATTTCCTTTGATGTGCATATTATTTTGTGATTGATAAATCATCGTTAAGCTATGGTGTGAATATATTGAGACTAATTTTATTTCTTTTCGTTGCATGTGAAATTTGCTCGAGTCTTCATGGACTCTACCCTTGCATCTCCTCGTTGGGCAAAATGCCCAATCTTACCTCTTATCGAGTCTATCGGCCCTGAAAATTGACGAACAGGTCTCGAAGTTGAGAGAAGCAATAGATCGGGAATTTGAAAAGATTGTCTTAATTTCAAAACAAATTGTATTTTGTTATTTTGGGCATAAGCCCACTTATTTGTTATACTTGTTAGTTCTTAGGACAGGTGAAAGAATTGGGCCTAAGGCCCAAATATCAGGTGCAAGTGATTCGAATGGGCCTTCCACCCAAAACAAGATTAGGCCCAAGTACTGGCCCAGGCGAACAGTAAACCAGACTTGGGCCCAAATTTCCTTCTTTACTTTATTTTATATCTGTTTATCTGTCGATATTTATTGTTAGTCTTAAGGTTACCACGATTAAATTCCCCAAAAGATAGTCATTCTAAACTAATCTTCTAAAATATTAACTAATTAACTACTCGCTTCCTATAATTTGAAACATAATCTATCAAATGTTTAACTTAGATAATATCGCAATACTCCTCCTTGTCCCTTTTTAAAATAAGTTCCAACTATAAAATAGTTCAATTTTGCAAGTCAAGCAAAGTTAAAATTATTTTAACCAAATAAATTAGTTGTCGGATAACCGCATTAGCGGATATTCTAGGTGCTTTAAAAACCTTCCTAGAATATTAATAAGAACCCCTGAACCCCCC

>LNC_000061

TTGAAAACCCTTCTTAATCTAACACTACATCACAAGAAATTCTTCATCCCTCAAAACTTCTCTTCAGCGACACATATCATCTTCTCTCAAACCTAATTTACAAGCTTAGCCTTCTCTGTACCCATGTAACACCACTTGGAATCTTGATTAATGGGCGAGATTTGCAGTTCGATCTATTCCAAAGAAGTGTCGAAGCTTTTATATTTGTAGAGGTTGATGGAGATGCAAAACATTGCATCTGCGATTTCGCAATTTGGAAGAAACCTTCATGTTTCTTCTATTTCACCTCTATGAAATTCTTCAGTTCACTGTGGAATTTGCGGTAGTGAGGTTTTCTGTGTGAAGATCAGCTCTCCTCCATGGGCTTGACACAAATTAGATTTGAATTTGCTTGAAATGGTTGCTGGACCTGACTCTTAAAACACGGGAACTGCTAACTGTTGGTTCTGGAATTCAAAGAGCCTAAGCAGATAAACCAACAGGACATCATTTACATTAATATTATTTGATAGAGGATGTGAATTTTTTCTTCTTTATTTTCATGAAGTTGATGTGCAAACAATGACGTGACTGATGTATTATGGTT

>LNC_000062

AAAATGACCAGCATCAATACACCAAACAACCCACTACATTTTGATCTTCTTCTTCATGCCAACAGCATCACGACCCATTGAAATTGAAACCCAGAATCTCAATCGCGAGCAGCAGCCCGAGATCAGCCCAGAAATCGACCCAAAACGAACCCGGCTTCAACGATCGTGTGAGCCCACCAGTGACCTCTTGCGTGTCCGTCCTTCTCTCGTCCCTTTCGGTTTGTACAATCGCAGCAGAAGAACAGCATGCGTCATGCCCCTTTCCTCTTCCGGAATGGGTCAATTTTCAGAGAAAATGTGTTGCCCCAAATTGGTGCAAGGTATTTGGAGTTTTGAATTTTGAATTGGGCCCGAGAATCGATTTCTTTTTAGCCCTTTCTCTCCAAGTTCGAAACCCTAGCATCCATCTATATAACCCACTACAATTTCGGTATGAAAGAGGGGGGAGATTTTTTCTAGCGGGAAATTCTTTACTAAGGCAGAGAAGAGTTGGAAAAAGAGACGTTGGCAGGAAAAATAAATTCACAGAGATAATAGTCTCAAGGCTTAAAATCGTATAAGAAATACATCTAAGAGACACTTCCTTTTTCTTGTTTCGTGGGTTGCTGGGATTCACAAGAATTCTCCAAGCTCTGGTGTGGTCGGAAAGCTCGTCCTTTCTCGTTCTCGTCTCAGTTCTCTGCCCGAAATAAGGTAACATTTCTTCCATTCTCCCCTTTTGTTACAGTCCTATATCATATGACAAAAAATACATATTCCAGTGATTTTCCTTTGTGTTTTCTTATGTGCATTTAGTATATCTCTCATTTCTTTAGCTTCCGAGTTTCATAGTTATTCTTAGGCTTCTATATGTTTAAATTTTACTCCAATCACGTTTGAGTACAGTTTAGCATAAATCAACCTTTTGATGTTTATTCCATTCCACGTGAAGTGTTTTCGCTTTAGCGGTTCGTTTTAAACTTTGTTGTTAAGATAGCCTCATGCTCACTTGTTTCAAACTAATAGCACCTCGACTATAGCACAGTTCCTGATTCCTTGGGTTATGATCTTGATGTGGGACTGCGGGATACATTCTTAAATACATGTAGTCTTTATGTTCGAGTTTTGATGTTCAATCGTTCCAATAGAGTTTATAGTATTGGTGTTTGTTTGAATGACTTGGGACACATGCTTATTTTCTCTGCTGCTTAGGGATACATTTTGATCCTAATCTAAAATTCCCTCATGCTCGTCCAACTCAATCCCCTATTTACTGTAGCATCGTTTTACTTTTTCTCTTCATTGGCAAAAATTTGGTTGCGAGTCGTTGTGATATGAGTTTAAATTTCAAGTGCTAGTTTGTTTAAAGTTCATAAATGTTTTAAGTCTCGGGTCTACCTTAGTTTCTATTCGTCTACCCACCTATTCTGTTTTGATTCGCACTACCCCGTTCTGAATTTATTTGGTATTCACTTGGTCTAGTTTAGCATCGAATTTTGTCATCTGAACCAAGTGTTCACTTGGAGCTTATTTTTTATTTTTTTTACATTAAGCCTATGTAAGCGTGGTTAATTACTAAACATTTGTCCAGCATGTTTTTTCCATTTTTTTTCCTTTGCCTAGTTGAATTCGTAAGATGATATGCCCCACTAATTTCCCTAGCTAAATTAAGCTTGTTTGGAATGCATTTTGCCATCAGTTTTGCTAGTTACTGCCTCCAGGGGGTTTATTTATTTCATTTCATTTTGGCCTGATTTGGGTGGTATTTCCTAATAGGTTGTAGTGGCATTGGTTCATTCATATGAAATTATATTCACTGTTAAAGTGACTATGTTGGTGTAGCTCCAAGTTCATTTGGTTATGTGAGATCAAAATGCTTCATTTAATATTTTTCGAAATGGATGAATATAGGATAGCAACTTTGACACTAACAGTGGTATATTATGTTGTGCACTATTCTATGGCATCGTTTTTTATTTATTTATTTGTTTATCATTTTGAATTTTTATGAACCTTGCTTACATCCATTTCTTTTGCATGTGGAATCTGCCCGAGTTCACCATGAACTCCTATTTCTTTCCCGCATTTTCGAGAGAGCCATAAAGGCTCAAGTCCTTTCCTTTCGAGTCAGCCGACAAAAAATGAAATACAGGTTTCGGAGGGTAAATCGGGGCTGGAAAAAGAGTTTTCATGTTCTGAAAAAATTGTAAAAGAGTTTATACATTTGCTGTATACAGTGATATACAGTCAAAACGCAGCGATATACAGGTCGGAAATGACTATACAGGTGCTGGAAATTTGACGTGGACCATCTATATTCAATATATGGTCCGATATACATAAAAATAGATCGATATACGGCCTATATACGTCGATATACGCCTTTGGTATACACAGGTCTTTGGGGGCGAAAATGGGCCTAAAGTCCATCAGATTTGGCCCAAATTTGGGCATCCTCTTGTTATTGCCATTACTTATTTATTTGTATTAATTCTCTAACCCATTTTATGTGATTTGGTTATTTAACTTAATTAATCCCCTAAAGATGAACCACTTTTGAATATGGTAACTTATTTCGTCTTATTTAATTAGTTTCTTTACTCGATCATTTTGGAAAAGTTTACTTTTCTTTCTAAATGATTTCAAAACATTTAAGTTAAATGATTTATTTTTGTTGCATCTTTTCTTCTCGAATTAATCTTCAAAGTTAGTTGCAAAGTCTTTTTTTTCCCCAAATTTGGGCAAAAGCCCGAGTTCAAATTTTCTCCTTTCTCCTCTTGTATTCATTTGTTATTTTAGTGACTAACTCTTTACATGAATTGGTTTTAACTTAATTAATTTTTTCGAAGATAGTCGTTTCTCTTTATATTCAAAAGTTAAAATATTTATCCT

>LNC_000063

GGGAAAAAATATTTAATGATAACAAATCAGAAGATATATTAAACCAACAATGGTATGAAATAGACCTACATGATTTAGACCTCGAAAGAATAGAATGGTATGATTTAGAAATAAATTCAGATTAAAATGAACTACGAATATTTACGATATTGGACAGAATCTATGGAAGATATAAAACTATTAGAACAATTAATGGAGGAAAATTTATATATGTATCAAAGAACCCAAGATATGTTTTATCTAGAATATATCA

>LNC_000064

AATGCTTCAATGGAAAGAGGAAGGACCATAATTCAAAATTTTGGAAGCAACGATGTGTTTCTAAGAATGACTGGATACTAGTGGCCGTCTGAGGGTAAAGTAACTGGCCGGTGTGACTATGATGTTCTGGTAGTATTGATATTGTTGGCTTTAATGGATTATTGTAAAGAGTATGAGAAAATGGATAAATTGAGTATGTCTATGATTGTTATGAGGCCGGACTGGCTGATTGTGAAGTCAGACGAAGGGGAAGGTGTGTTATGGACATCGTGGAAGGGATATCTTGGGTTGTTC

>LNC_000065

CTCCACAGTCCACATATATACTTATTCTCTTTCTCCACTTCTCAGATAATAATATAACTTGATGTTTTGTTAAAATTTTGTTCATCATTTCTGTTAACAATATGTAGTTATGTACTTATTTTTCTGATTTGCTTGACTATTTTTGTGTTGGAGAAAAGAGTTTAGGGCTAAACTTGGAGCTCTATGCCGACAATAATGGTGATTTTTCCCCCCCGGTAGTTGCTTTTCTTTTAAGAGAAAAATGCTACATGCACAGATTTTACCTGATCATAAAGGTAAAGGAAAAGGAGGTAACTTGATAGGAAGATTTCATCCCTATTATCTGCACAAATGAAACATGCAAGCGGTACGTATTTGATGCGTATAGATATAAATTCTGATTCTATTTTTTGTCTGATCTCAACTCTCAATTTTGGTAAGTTCTTTCTTCCTATCACTCTTGGCTTATTGCACTTATTTGCTATTTGTTTCTTGTTTATTGAAGTGGAATATGCATGCCCTCAAAGTGTTTGATGATATGTCCGACTCATTTTATTTGGAGTGATCAAGTTCACAAATATTATTTTAATTATTTGTAGTAAACGGGTCAACTAAATTAACCGATGTCATCGGTGATGGTTCAGCTATAGGTTTCGACCTTCATGGAATTTGTTGGGTTTGTAAAATTTTGCTCTTTATTAGGATAAGTGTCAACAAGAAAAGGGGCAAAACGATAGAGAAAAGTTTTTTCCCCTCATCGTCAAATTAGAGGTTTCTGAATGTGTTGTTCTGATTATTGTTCCTCAGTGTATTCAGCCTTCTTAACTTCGGCAATTTATCAATTTGGCTGGAGAGGCCAGACAAGCATTGCGGTTAGACAGAGCATTCCTTCCATCTCTCAATGCTTTGGCGACCCCTAAACTCGAATTTGCAATTACAGTAGTAATTAAGAACAACTTGCCAGCCACCAACAAAGTCACCGACCGCATCACTTTATTACCTTTTGCTGATACGAGTTTATGTAACCATTTGTAAATGATGTATACATAGTGATATCATGAATCTTATGTACAATCAGTATATATGTGTGTATGAATTAGTCATCTTCGGATAATTGTCACATAATCAATATGTTTAACTACTGTCATCCAATATAATATATATTGGACTAGTGCGCGCACGGGC

>LNC_000066

ACTATATCTCACAATTTTCTAACCCTAATTTCTCTCCTCCTTGCTAAAGTCGCTCTATTCTCTCAACTCTTCGTTCTACTTCTCAGCTCTTCAATGGCGAATCCATCTGTTGCTTAGTCTTTTCTGTTTGTCTACACACTCTCCATCATTCTAGAACACTCACCTTCCTCATTAGCACAAAGTAGACGCAAGATTTTCAATACAAGCATCAGTTCTTCCATTCTCTTGCTCCAACTCCAACAACAACGAATTCCAGG

>LNC_000067

TTCTTAATGGTTCTCCAATCTTATCCTTTTTCCTCTTTTTTTTTTTTTCTGATACTGAGGAATTTAGCATTCAAATTGCGAGATATTTATGTTTTTGAGGTTAACAATTGGTGCAACAAAGGTGTTTGATGGTGGAATGCTTCAATCAAATAAAAGATTTACAGATTTCTACAACATATTCCTCTGTCAATTATGAAGATTTTAGGAAACAACACTATACAACTACTATCTTTCTTTGGTGATGATGAGGGACAACCCCAGAAAATTATGCTATTAACGTCCCATTATTTGAATGAGCCAACCTAGCCAAAATTCTGGTAAGAACAGAATCGTCTCTGGCAACAACACGTGTACTTTATACGAAAACAGGTGTACTTGAAGTGTTCTTCTTCTTTTGTTAATCCCAAATCCTAATGTTGCTTGAATTTCTTTTGCACCTTTTACTTCTACTTTAGCATTTCTTGGATTTTGGGTGTGTTGGATACTCTTGCATGTGTGTGTTTTTCGCCAGAAATTCATTTGAACACCTGAGAAAGTGAAGGGCAGAGAAATGGATACACCACAGAGAAAGTAAAGTCTCCATCTTCATTTTTTTCATATCTACAATTGAAAACACAAGAAAATGAGGCCGAAAGATTATCGACCTTTGGTTTTGCTCGATTTTATCGGAG

>LNC_000068

GAGGAATTTAGCATTCAAATTGCGAGATATTTATGTTTTTGAGGTTAACAATTGGTGCAACAAAGGTGTTTGATGGTGGAATGCTTCAATCAAATAAAAGATTTACAGATTTCTACAACATATTCCTCTGTCAATTATGAAGATTTTAGGTCAGTATTGACTTTCTTTGGCTTCTGGTGGTGAGTCTAAGCTATTATATTTGAAGATAAAGGGAGATTATTATTGCACTTATACATAGTAGAAAATACTTACTTTTCTGATAAAGTTTTATTAGTGCTTTAGTGATTGATTGGAAAAACATTGGCACATTTGATGAGAGAAAACAAAAAGTTAACAGAGATGTTGGAGTTTAGACGTAGTCATTGACAATGTTGGCTTCCTTCACTACATAAGAGGGTGATAATGAGTCCAATATGGCTACCAATTAATTGCTTTTCTTAATGGTTGGAGTGGTTTCTTCAACTTTTTTCTAATCTGCTTTGGCCTTTGTGTTTTTGCCTTATATTTGATTCTGGAAGCTGCATGATCCAACCATATTGTTTTACCTATATAATGAGAAAAGAAGAAACAAACCCATCCCTTATGCTTTGAAATAAAAGAGGACTCTAATTGCTACTAAGGTTTTAATATCCATAGTAACCGAATTGTGCCTCAACATGAGTTTATTAGTAAGTCTTTGTGCTTTGAAAATAGTACATCAGATATGATAGTATCCAACTTCCTCTCCAAGGTCCAATTCAATAACCTTACGCCTTAACATAGGGGTTTCTCTTTGCAGTCTGAATTATTACAAGTCATCTTTTCCTATGTGCCAACTATTTATGCTTTGATATTGTTGAGATCCTTCACAGGGGATTACTTATGATGGACTGTTAACTTTTTATGTATGCTGCAAGTACTCTTATTACTAAAATAGTACATATATCAGTTGATGTGTTAGTTTGGGTAATTCACACTTTCAGGTAGAACCAAGATAGCTCTATGTATTGGATGAAAGGATAAGCAATTCTGAGCAATTTTTGGGTGCCATTTGATTGGTTGACTTTTCTAAAGTGTGTTTTGAGACAATTTCAAACATATATATTGTTAAATGAGATCATTTATTAGATGGACTTAGATAATAACTGTGAGATACTCTGAAGAAACTAGGAAATATGAGTCAATGTACCTGGCTCAACTTCGGTTAACTTGCTGAAGTGATGTAAATCCTGTTTTTCATGGAGAGATTACAAAACACCTAATGCGCATATGCTTCATTAAGTTGCTTTAGTACATTTTCTTATCTTTGTGATAACTATGCCTCTGACATGTTCTTTCCCATCATATTTATTTAAGTAGAAATTATTCTCAATTCCATTTCATCTCCAATGAGGATGAAAAGTTGTTAGTGTGACCTATGTTACCTGGGTCCTTTCATTTGCATTGACGTACACATGTTGAGTGGGTGAGATATGGGTGTAGGTACTTCACACAAATTATTTGAATACACAAAAGAACTACCAAAAAACTGCATGTACTCCTACTAGATACTTACACCTATATGACATCACCCTTAATCAGTCAATGTGATGATATACGATGTTACAGATATTTCTGGCTGTCTTTTGTAAGAGAATCTCAACAATGCAGTGAAAAACCAATTTGAAGAACCAACTATTTTAGTGGCATGCTTCTGTGGGTGATTCTCTTTCCCTTAGCATACATATATCTTAAGGAATTTTTCTACGACTAGCAAAATGTGCATTATCAGACTAATGAAGAATCAAATAATTACTGTTTTTTTATTCTTTGGGATAGTACTTGATAAAGAAAGCAACTGATTAGTATGCATAATTATCATTCTTGACGAGGTTCCATTATAATATTGATTTCAATTTTGATATTCTTGTCTTATTTAGAAGTGGCAGTAGGGAAGGACGAACAAGGGCAAATAAGTAATTCTAATGTTAACTTTGGGGTAAATTTGTAAATTCACTTTTCACATGTGTATCCAGTTTATGCTTTCTTTCCTTGTTCATCGGTAGAATCTACCATATGCTGTACATTTGTTCTATCACTGCCTTTTGCTCTTGGTCAGATCATGATGATCGTTTTGGATCTCAGTTTTGCCTCAAATTTTTCTTCGGTGTTGTTGTAATCTTTGGTTACACCAAGTTTTAGTTCCCCTTCTCCCCCAATTTTTCGGTAATGTGTCCAACTCTTGAAAAGAACCTATAATGGTTGCTTTTTTCCATTTTTCTTTGGATTCTTAGGTTGTGTATCATAAACTGTATTTAATTTTCCCATGCATGTTTGAAAATAGAAATAAATATGTATATAACTTTGATGTACAGAGGCTTTTGTG

>LNC_000069

CCAAGATAATCAAACCATCAATAACAGTAACGTAAGCATGAGACTTTTTGGTAGTACCGGCGAACCAAATGGCCGCGGCGATGGAATCTGGGACGGAGGACTTGTAGTATCTCTCTAGATCTGGCAAAAGCGAAAGACCCCCTAACATAATTCGAATGGAGGCTGACCGCTGAGCCCACTCTGGCCTCGCGGGGCGGGCCCCTGTGGTTGCGAGCTGGAGCTGATATAGCTTATGGC

>LNC_000070

TCTTTAAAATATGGTAGTAATATAAATCAACTTCCTAGCATTGCATATCATAGCATGTAGTTATTTATGATTTGCATTAGTGAAGTTTGCTTTAATTTTCAAGTGTGTAGTATTAATGTATCCTTGTAAGAAATCTAAATGTGTGTCCGTCGAATTCCTATAGTCATTGGGTATGAAATTAGTTTACTTTATACTATTGCTTGATCTAATTTCTTGGTTCCTCTAAATATTATGTGCTTTCCTTGATCCCTTTTGTATAACATTGATACGGTGAACTAATCATGTGCATCTTTGCCTTGTTTCTATTCTATGTATCTTGGTTGTGTTGTATAAATTTAAAATGTCCTCCATGACTAATTCTTATCTTCTTTTTGTGGTTTTTCTATGTTTGAATCCGCCTTGGGTCCTCATGGACTTTTTCCATTCCCCTTTGCATTTCGATTTGGGCCATGAAGGCCCAAACTTGCTCTCCTTGCTCGAGTTGGCCTCACTACCGGGTTAGCGAAATTGGTGTACAGGTTGAAAACAGCGAGATA

>LNC_000071

CCGGAATCCGGTGAAGTTCCGGCAACCCAAAACCCCTTTTCCCTCCATCTTCTCTGACTAATGACCATCAGAAAACCAGCTAAAACAGCACTAAGCAACAGCGAACAACAAAACAACCATAGCCGCCGGAAAATCGGCAACCAGAAACGCACCACCACTGCCTCCCCGCTTCTCCATTTTTTTCAGACCCGAAACCACACCACTTGAAACCAACCTTCAGTGTTCCCAGACCACCAGAAACAACCTCCGACTACCACCAAAACCCCTTTCTCTTTCCCCATTTTTGCCGGCGAACCAACGAGCTCAAAGCTCCGACGGACAGCGACACACCATAAAACCTGCGAGCTCAAAGCTTTAGCAGCTCCAGCGAACCGGAAACAACCAAACAAGCATCAGCTCCATCCGCAAACTCCTCCGGGCAGCAATAATAACAACAACCAACACTCCAACTCCGATATCCGGAGCTGCTTCCCATTGAAGCTATTCAACAAAGTTCGAACAATATTTTTGATTCATTATTGTATATTATTGACGATAGGCAAATCTAGGTTGTTTCTTATATTATTGTTTTATTTTATTACTTGTGTATATTGTATGTTTGTTTTATTTTTTTTTATCTCTTCCTCAATTTGAAAAAATGAACTTAGTCGGGATCCACAATTGTGGATTTCGAAGGATTTTTTTATCTCTTCCTCAATTTGAAAAAATGAACTTGGTCGGGATCCACAATTGTGGATTTCGAAGGATGCCTTACACCTTCTCTTCAGAATAACTTGAACCCCTTACCTAGAATTGATTGGTTTCGTAGACATTTTTAATTGGTTTCTTAGTTTTTCCTAAAATTAGGTGGCGATTCCTTTAAAATTCGTTTATTCTTAAATTCTTTCAAAATTGATTCAATTGTTTATTTTTTGAGTTAGCACGACGTTTCTTCCTATTTGAATAGAGTAACGATGTAAACAGTTTGATTATAATATTTGTTAGATTTCAAGAAATAATTATTATTTAGTATTGTTAGTACTATTGAACAGATAATATACAAATATATTACATATAGTTTCAATTTCGTTCT

>LNC_000072

CCGGAATCCGGTGAAGTTCCGGCAACCCAAAACCCCTTTTCCCTCCATCTTCTCTGACTAATGACCATCAGAAAACCAGCTAAAACAGCACTAAGCAACAGCGAACAACAAAACAACCATAGCCGCCGGAAAATCGGCAACCAGAAACGCACCACCACTGCCTCCCCGCTTCTCCATTTTTTTCAGACCCGAAACCACACCACTTGAAACCAACCTTCAGTGTTCCCAGACCACCAGAAACAACCTCCGACTACCACCAAAACCCCTTTCTCTTTCCCCATTTTTGCCGGCGAACCAACGAGCTCAAAGCTCCGACGGACAGCGACACACCATAAAACCTGCGAGCTCAAAGCTTTAGCAGCTCCAGCGAACCGGAAACAACCAAACAAGCATCAGCTCCATCCGCAAACTCCTCCGGGCAGCAATAATAACAACAACCAACACTCCAACTCCGTTGGTTGCATATAATGAAGAGTTTGACATATTGATCAAAACATGATGGCGGATCTACTTATAAAGGA

>LNC_000073

TTGAACGGCTTGTTGAATCATTGTTGATGTTGTATCCTGATTGTCTTGTTGTGAATTGTGCAATGTTATGAAAATGGTCATCTCTTCATTATTTGTGTGAACATGTCATTTGCATTGTTCTGAGACATGGTTGTGACAAGTGTTATGTGCATTGAGAAAGAATAAGAAAATAAAGAGGATGTACCATTTCGAGGGACGTATGGCGCGCCGCGATGGATACTATATTTC

>LNC_000074

GTTATCTCTAATAACAGTACATTCTTCATTATAATTTCTATTCTATTGACAATCTTCATTTCTATTTTTATTTATTCTTAAGAAATGGAATAGTACTAGTGTAAGAAATCTATGAAGCCATTGAAAAGATGTTGGTGACAACACATGAGAAGTAAGAATTTTTTTCATTTTCATCCATTGATCACCACTGGGGACAAAAATTGGGGTCAAGTAGCCATTACAAAAATAGGTCTGGATGAAAAAATAGAGTCCTGAATCTTCAAGAACTCACAAGCAATTTCAGTATCCGATTAATTAATGCATGCTTATTTTTAGGATGATTTGAGGTAAAACATCCAAATTATAGGCCATGATTTTGGACCTGGAGGTAATGGTTTATTGTTTCTTACACTACTAATATTCCATTTCTTAAGAATAATAGAAATGAAGATTGTCAATAGAATAGAAATTATAATGAAGAATGCAGTGTTACTGGAGATAATCATAAGAAGAAGGTTTGATATTTTCATCATTTTTCTCTTGTGATTGTTAAGGCTAATGTCTTAGATAAAATGTAAAATTATTTTATTTTATATTTATTTCAAAGTATTAAATAATTTTTGAATTATTTATTCTACAATTTATGTAATAATAATAAAATAAGTTATCTTAAATAACATTTTTTTTCCGACTATATAGAAAAGTCATGACTAGGATGATCAATGGCGATTTAGTTAATTAATAAAAACTCCCAACTTAAAAGCAATTGTACATTTTGTACAGGGAGTACAGAATTTCCAAAATAATCATAAAATTCCAGCAAATTATTATGGCCACATAGTTATCTATGCATTATTAGAAGAATAAAATAAAGCACGTGGTAACATAAAAATGTAGGGGGCATTTATGGTATGCAAAAGTTAACACAAGAGTTCTTTTATATTTGGATAAAATACAAAATTATTTTATTTTTATTTATCTGAAAATAATAGATAATTTTTGAATTATTTATTCTATAATTTATATCATAATAATAAAATAAGTTATCTTACATATGACGAG

>LNC_000075

TCTTTCTCCTCTCCTCTGTTTGTCACATGCCATTAATGCCCTTAAAGCTTCTTCATTTTTCTTCATAATTTGCAAAATCTACAAATATTTTTGCATAATATTAAATACCCATCTCATATTTCTTTGTAAAAATTTATATGGTAGCCATTGACATATATAGGGATATTGACAATTTATTCTATAATTTGGTTTTGAAATCTGGTTATTTCAGTCTCTCAAGCTCAAATTAAAGGGGAAGCATTTGGATGGAAAGATTTGCTTGAAATTGTATGTCAAAGTTGGACAAAGATCATAGTACCTTAATAGCTTCCTATTAGAAAGCTTTTGCAGGCCTCGTATGAGGCAAACGTCGCTATAGAAGGCGTCGATTGGAGAGGCTTGAAGATGGATCTTAGAAGTTCACTTATTGCTAAATTTTTTCCTGATTCTCACATTGGCTCCTCCAAGCTTAAACTTTGGTACTTGTGGTAAGATGCAAAACTCCATTAAAAAAAATTTACTTCCTTATTTTGTCTTGAATGTGTGATATGTCGAAAAAATTTAGGTCCAAGTCCTGGACAACTTCATGCTACATGCTTAGTGGGAATACGTTGGAGAATTGAGTGTTTTGAATGAGTTGATCCTAACGGAACCAAGGTAAAAAGGCAAGATTGGAAGATATGTTTGTATTAAGGGTGATAGTGTGACTAAGGGGCAGAGTGGTCTCGTATAGATGTCTAAAGGAGTTGTAGTCAAAGGTAGTCGAAAAATTACACTATATCTACATGGTCAATAGTATTTTTTGATGTATATATAGAAGATATTGAACCCCTTGACTTCTTCATATGTTTACTTCTTTCTATTTTGAATCCCCTTCGTGAAAATTCTGGTTCACCTCTTGTTGTAGTCATACATAAGCATGGAAAATTGAGCTCTCCATATAAACCTCTATTGAATATTATTATTGCAAGTGATTAATAGAGTTGCAACAACTAAGAAGAATTATTGAACACTAATAAACTATCCTAAGTAAACACGAACTGTTGAAAGATAAACGTAAGCTGACTACTGGAGTACTGAGTAGCGTTCCAATACCGTTTACTTCTTCATGTTCCTGCTGAAATTAGTAACGGTAAAGGGGCGGAGCCAGATGAGGATCAGCAGAAAATTACACTGTTTATACCTAGTTAAAATTATTTTATAGTAAATGTTGAACCCTCTTTTGCTTCTTCGTGTGTTTGCTTTTCATATTTTGAACCTCTTGGTGAAAATCCTGGCTCCACCACTGGTAACAGGGAGACATAAGTATTGCGTGAAATATATTCTTGTTGAGATACTGTTTTCTCCCCTGGTTGATTGTATTTTCGTCGATTACAAGTTGTTTCCACAAGTCTCATGTCTTGAAGAATGATTTAGTATATGCTATGTCAAAGATTTTGGTCTTGGGTACTATGTAGTATATTTCTTCCTTCCTTTTGCACTTTGTGGACGAAGTAAAAATAGAGATGGGAGGGATTTGAGAGGTTTGTTTCCCAACAGTGGTGGAGCCAGAATTTTTACTAAAGGGAAATATAAAGATGTAAACACACAAAGAAACTAAAA

>LNC_000076

ATTTTAGTTTACAAAATTTCAACTTTTTTGGTAAGATTTGTTTATTACCCTTTTCTTGTTATGGAAATATTTAGTCAGAGAGGTGAATGGAATTTAATGATGTGAATAATTTTGTCAAAATATAGAATCATATTTGCACTTATTAAAGACAACCTAGTAGTATATTCTGCTGTTTATTGACTTCTTTTCTGGGGAAATAAATGGCTACATATTCATGAATAGTTCTCCACTTATTAAAACACAAATGGTATAATTCAGCCGTGGGTCTTTCAGAAATAGACTCTCTATCTCCACGAGGGAGTGGTAAGATCGGTGTACATATCACCCTTCCTTAGATGTTACCTATGGAATTACACTTAATATGTTGTTGAAACACATGTGGTTTACCTGCTTTGTATTGATTTCTGAAAAATTAATGTATTTTCAATATTCATGATCTTTTTTACAACATTCAGGATGTAGAGTAGGTTTATCGATTTCTGAGCTGAGTATCTATAAGAACCAACCTATCTACATGTGTAATTACACTGAATATGTTGTTGTTGAGGATGTAGAGTAGAATCTAGCAGAAACATCCTTATGACCATGTTTCATGTTAGAATGTTCTGTCTTTTCGCCTCATCTTGTAATCAGTTTGTTAGTGTTAAAAGAGGATGTTTTTTTTTATCAAACCAATTTGGTTAAAGAAATTGATTCAGAATATTGTTTTGCTTTGTCACTCATTGCAGGGGAAGCATTTGGATGGAAAGATTTGCTTGAAATTGTATGTCAAAGTTGGACAAAGATCATAGTACCTTAATAGCTTCCTATTAGAAAGCTTTTGCAGGCCTCGTATGAGGCAAACGTCGCTATAGAAGGCGTCGATTGGAGAGGCTTGAAGATGGATCTTAGAAGTTCACTTATTGCTAAATTTTTTCCTGATTCTCACATTGGCTCCTCCAAGCTTAAACTTTGGTACTTGTGGTCCAAGTCCTGGACAACTTCATGCTACATGCTTAGTGGGAATACGTTGGAGAATTGAGTGTTTTGAATGAGTTGATCCTAACGGAACCAAGGTAAAAAGGCAAGATTGGAAGATATGTTTGTATTAAGGGTGATAGTGTGACTAAGGGGCAGAGTGGTCTCGTATAGATGTCTAAAGGAGTTGTAGTCAAAGGTAGTCGAAAAATTACACTATATCTACATGGTCAATAGTATTTTTTGATGTATATATAGAAGATATTGAACCCCTTGACTTCTTCATATGTTTACTTCTTTCTATTTTGAATCCCCTTCGTGAAAATTCTGGTTCACCTCTTGTTGTAGTCATACATAAGCATGGAAAATTGAGCTCTCCATATAAACCTCTATTGAATATTATTATTGCAAGTGATTAATAGAGTTGCAACAACTAAGAAGAATTATTGAACACTAATAAACTATCCTAAGTAAACACGAACTGTTGAAAGATAAACGTAAGCTGACTACTGGAGTACTGAGTAGCGTTCCAATACCGTTTACTTCTTCATGTTCCTGCTGAAATTAGTAACGGTAAAGGGGCGGAGCCAGATGAGGATCAGCAGAAAATTACACTGTTTATACCTAGTTAAAATTATTTTATAGTAAATGTTGAACCCTCTTTTGCTTCTTCGTGTGTTTGCTTTTCATATTTTGAACCTCTTGGTGAAAATCCTGGCTCCACCACTGGTAACAGGGAGACATAAGTATTGCGTGAAATATATTCTTGTTGAGATACTGTTTTCTCCCCTGGTTGATTGTATTTTCGTCGATTACAAGTTGTTTCCACAAGTCTCATGTCTTGAAGAATGATTTAGTATATGCTATGTCAAAGATTTTGGTCTTGGGTACTATGTAGTATATTTCTTCCTTCCTTTTGCACTTTGTGGACGAAGTAAAAATAGAGATGGGAGGGATTTGAGAGGTTTGTTTCCCAACAGTGGTGGAGCCAGAATTTTTACTAAAGGGAAATATAAAGATGTAAACACACAAAGAAACTAAAA

>LNC_000077

GAGCTTTTCGAGAAATTTTGCTGGAATAGGAAAGAGGGACATATGATATGTAGGGATGCTGTCCATTACACTATTAATCAATTTAACTCTTCCTCCAAAAGAAAGATACTGCATTTGCCATGTTGCTAGTCTCTTTTCAAACTTCTCAATTATTCCCGACCATATATCGTTAGCCTTGAAATTTGCACCTAAAGGCAGCCCAAGATAAGTTGTTGGAAAGGACCCTATGTTGCAGCACATGATACCAGCTAGCTCTACCAGATTCTGCACTGAGTTTACTGGATAGATCGTGCTTTTTGACATATTTGTGTGTAGTCCAGAGACAGCTTCAAAGATGTGCAAAATCAAGTTTAGCTTAATAACTTGTGATCTATCTGCCCCACAAAATAGCAGAGTATCATCCGCAT

>LNC_000078

CGATCAGCCACGGAGAAATAAGGATGGACGGTGACAAGGTTGAGGCAATTCGAGATTGGGAGGCCCCGACGAAGGTACCAGAATTGCGGTCCTTCCTTGGCCTCGCCAATTACTATCGGCGCTTCATATTTGGCTATTCTGCCATTGCTACTCCACTCACCGACTTGCTGAAAAAGAGTCGTGAGTGGGAGTGGACCGACTCATGTCGAGATGCCTTCAGAAAGCTCAAGGCTGCCATCATTGAGGAGCCTGTTTTGGCACTGCCAGATTTCAC

>LNC_000079

TGTAATTCCTGCCTTAGACCTTATTTCGGGAAAGTGTTTGATTATGACAGCATGTCTCTTGATATTATTGAATATCTATATCTTGTGGTGTATATGATAATATTGATTTGAAGTATGTTTGGCCTTAGTCTAGGCTTAGACTAGTGTTGCCATAGACTTGCGAGATTTCGGATGCCGTTGGGCAGTAGAACTTTTTTCGACGTCGTTTCGGACGGTCAGCTCCTTGTAGACTGATATAGCAGACTTGAGTCTGATAGTCTGAGCCTTAGCTAGGCGGTAACTCAGCTTATGACCTTCAATCCATAATGCCCTGTTTTTCTATCGGCTATAGTACCCGGTTAAAGTCCGTGGTCTAGCTTACTCACTATTGGATACTTCCTCGGCGATTTGGGGTATATGAGGGTTGGACTGAAATGATTATTTATGGTAATCGGTTTGGTGATATTTCCCGCGACGTATGGTTGGATATTGATTCTTAGCTACAGTACCCGGTTAGAGTCCGTGGTCCAGCTTACTTATGTCCAGGCTTAGCTACAGTACCCGGTTAGAGTCCGTGGTCCAGC

>LNC_000080

TGTCTACTTGTGCATACCAAAGTTGGAGGGTATAAATGTCAAATGAAGTCGAGTTAAATAGTGTATTTATGTGTTATGCCTTTATGTATCATGCCATGCCATATATAAATGTGTCATTTACTTTGGCTTCTACCGACATCTATGCACTCCAATTTTGGTCGTGCATGGAATAATACACGAAGGAAACAGATTAACATGTAGGATCTCATGTAAAACACATGTGTCTA

>LNC_000081

TAATCTCGGTACCTTTTCCTTGTTATATCTTACCCTTCTGTTCAGTATGTTTGTATTAGTTTAGGAATTCATGTTAGATTTTCTGTTATTTTGCATAAAAATGACAATTATATATAGTTCAAAGCTTGTGGTTTTTGCTCTATGTTTATATGTACATGAATCACTTTCCAGTATCTAAACATTCCTCCAACAACTCTAATTCTTTGTTTTCCTTTTACTCTCTATTAAACATGGCCATAGTTGGAGTCAAGAATTATATTTCTATACATATCATATGTAACTTTGAGATTCTGTTTAGAAAGTTTATAGTGGGAATAACTATCTCATTTGAATATATATGTACTTGTTTCTTAATGCTCGAATTAGTATAAACTCAATGACTACTAATGTAACACTAATGAAAAGAAATGTTTGTTTGATGTGAGTTTACAAGTCTGTGAACTTCATAATTTATTTATTATCTAGCTCACATGTTTGATCGAGAGTATACTATTTTCTAATCATTTTTATCTCGTTTATCTATTTTGCATGAACACTGGAGCCGAAGAGTTTCACTTGA

>LNC_000082

TGTGGGTTGGCAAGGTAAGAGATGATTGGTTGTATCAACTTTTTAGCCATTGGAAGGACTTTTTGCATGGTTTGAGTTGTAGCAGTTAAATACTTGTGATTGTAGAAGAGTTCGATGAAGTTTCATTTGAGAAGTTTGACTTAAGGTAAAAGGGAATATGCGGTGGATATTTTGCCAAGAAACTATAATGATAAATGATTTACGGGAAAAACAAGAGTCTTGCGTGATGGATATAAGTTAAGAGATGCGAAGGAATAAGACATCACGCGTGGATCCTTAGTTTGAAGTTTAGGATTGTGTAAATTTCTGCTTTTATGTTAGATATGACAATGCAAGGTGGACTGGTTCAGCACAAATGTATGATGTGTTATCAGCTACTTTCTGAACACTTGGTATTGGTGTTGGTGGTAAACGTTGATGTATGAGGAATTCTGCAGTTTTTGAATTGGTTTGAATTGTTCAGCTTATGGTAGGTGGCTTACGAGTGTCCAACCGGAAGAACTTATAGCTCTACATGGTTATTGAGGATTTTGAACTGGGTTGATAACTCTATTTATGGAATTATCCAGACTGTTGTAACGCGTTACATAGATCCTTGATGGCAAATAGAGGTTGTGTGCTCCTAGTAGACGAAATTATTGGAGTGACCAAGAACTGCTTCAAGTTTCGGCATGAGATATATGATGGTTTGAATAATAGTTGAAGCTGGGTAAGTAAAGGATCGCGTGTAATCGATATTGGTCGTGATTCAATAGTTCTATCTCAATGAAAAGGTACTATGACATTTAGAACTTGGATTATTAGCGATTGGGTGAGACCCATATCGATCGTGGAATGCTTCAATGGAAGGAGGAAGGACCATAATTCAAAATTTTGGAAGCAACGATGTGTTTCTAAGAATGACTGGATACTAGTGGCCGTCTGAGGGTAAAGTAACTGGCCGGTGTGACTATGATGTTCTG

>LNC_000083

CCTACGTTGTTTATTTTCGAACATTCACGTCTAAGTATATGTTTACTAATTCCTCAAAATCTGCACCCAGTGATGCTATCTATTTATGTCTTATCTCGAAAGGTCCTTTGTGAAGCATAAAATTTACTATGGCATACTTAAACGACTAGCATAATTGTATAAATGAGTTGTGTTAGTAGCTAGAAGTTGCCATCGTTCTTCCAGTTGACTGCCCGCATAGCTGAGACATTGGTGTTAATTTTTTCTTTGCTTGCTGTTAGTTGAGGTTTTCTATTTTGAGAATAGCAACAATCCCCGAATAAAATAACATGATTCCTCTACTCCTTAAAAAGTTAAGTTGCTGCCTGCTTCCTTTTATTAGATTTCTTCCCTTGGTTTCCCTAAGTCAATTATGTGAATTTAATTTTGATCAAAATCCAATAACATAAATCTGAGGCAAATTTGTTCTTTCTTTTCCAGTAGTGCTAATCCTTAATGGTTTTCCTTTTTTAATCGCTAGAAGATAATGATTTCCCCATCAGCATGCCCCTTCCATTTTAAGTTGCTTGTTGAGCACATTGGTTGCTTATTTATTTCCGTGTGAGACCTGCTCGTGTTACATTCGTATATAATCCGTCCAAGCATAATATTTATGTGTATGTCTTGAAGTTCCTCTATTCTTAGTCCCTTGACAGCGTAGTGTTAGAATTCTATTTCCTATTCATGAATTAAATTCCTCCATGCTTAGTCTCTTAAGCAAAATTAGCTTTAGAACCAATATTTCATCTTCATGTATTAAGTTCTCATCATACCAAGTTGGGTTTAGCATTAATTGGATTATGAATTGTGTCTCCC

>LNC_000084

AAAAATCTTAAACCTAACAAACCTAAACCCTATTCTAGAAAAACCCTAAACCTTAAACACTAAACCCTACACCTTGAATGACCCAAAAAAAACCTAGGAGACCTTAAATGTTCAACTCAAACCCTAAGAAACCTAAACCCTAAACCTTAGGGATAAACCCCCAACCCTAAGCCTTATGAAACTCTAAGAAGCCCTAAACCCAAAGAAATCCTAAATCCTAAGAAAATATAAACATTAAAAAACAACAAAAAAAACTTTACAAACTCTAAAC

>LNC_000085

TGTTAAGATAGCCATGATCACAAGGGGAACAACAAAAAGGGCAGAGAAAAAACATATAGCCAAGAAGAGTGTTCAAACAACACCTACTGCATCTGTGGCTAAGAGAACTAGAAGCAAAGGTAAGACTATGTTTAATGAAGCTCTTGAGAAGAGCAAAAAGAAGGGAAAGATAAGGTCTGTGTTCAAACCTGATGAATCAGATATTGCGACAATCGAAGTAAGTGATGGTGAAAAGGAGGAAGCAGAGAAGGAGGTCGAAGAAAAATCTCCTGTGGGAAGGGTGAAAAAGAAGGCTTCCCTTGCTGAGGAATTTGACAAAAGGGAAAG

>LNC_000086

TTTTCTAATTGATTTTTGATTAGTTTATTGTCAATTTTTTTTTACCTGCATGTTCTACTTTAGGATCCCTCAAATTGAGAGGACTGGTTAAAATTTAATAGGGAACTCCATTAGTTTAGTTTGATTGTGATTCTAAATTTCAAGATGGAGCTTGTTATATTGAGATGCTGAGAGTCTGTTTCCATTTGTTATCCGAGAATATTAAGCTTTACTCATTATTTGAGATGCTAAAAGTTTGAATTGAAGAAATTGTGGATTTGTTATACCGCGTATGCTCTCATGTATGGTGGTGCCCAGAATTTTTTTGCCTTTCTTCACTGGTTTTATACCTATTACTTCTAAATTTCTTTGAATGCTTGTGCCTTTTTGCTTTAATTTTAGTTTTTTACTCTTTCAGATAATCTCTTGCTAGATATATACATGCATGTCATTATGCCTGAGCTGCACATCATCCCATTTAGTGCCTTTTCC

>LNC_000087

TCTCACCTCTGCTTCTACTCTTTCTTCTCTGGTTGACAACATTGACAGCCATTTTAAACCTTCAACGGAGTTAAACTTTACTTCAGAGTCTCACTCCAACTTCTCAAGTTCTTTCAATTGTTTACCTGATAATTAATGGCTTGATGCAGTCGCATGTTTGACGGAATTTCATGTGAAGGTAAGACTTGTGTGTTATGAAGAGAGATTAGTTACAAGAGTTGATTGTTTTGGCAGATGATTGAAGGTGCTAATGAAGTTGAACGGTGGGCTTAGGTGAATAGACATTTTCCCTTATAATATCTTTTTTTCATATGGTATTTTCCCCAAAAAAATA

>LNC_000088

AATTAATAAGGAGCGAATCACGAACACTTTGAGCTGTTGCAAAGAATGCCTTAGGGAGCTCAAACCTTTCAGGAGAGAATACAGGTGTGAATTCTGCATGGTATTTGATACTTGAGGTAATAGATGCGGCATCAGGAGCAAAAGAACTCAGGTCGCTCTCACCACCTATATATATGTCAGGCAATGGAATAAACATAAATCATGTAAGTAATAAATTGGAAGGAACTAATGGAAATACAATCGTGTACAACAAGTTCAATGGAATAAACATAAATCATGTACGTAATTCCTAAGATCCATACATCAAGTTTCTGATGCTTCATTATTATATACTCC

>LNC_000089

ACAAGTTTTAGTTCTTCTGCTATTACATTCTCTAAATCAAATAATTGTTTAGAAGATCAGAGGTTCTTAGGATGTTGTCTTACGTTATAACATCTGCCAGTATGAAGACAATAATACTTGCATTAAAGGATAGGCATGAAACAAGTATCCTAGATTCTGCTATGCTGAATTTGCAATGACTTGAAGCAAGACTCTATTTATGAGTCAACTAGGTTGGCAATTTATTGTTTTGGTACTGCGTGTGTTGTTAGGTGAAATCTGGTACTGCCTGTGTTGTTAGGTGAAATCTGTGTCTGATGAGTTAGGCAAATGATGCGCCAATGACTATGAATTATTTTGGCACGAAGTTTAAGAAAGTAATATAGACTTTTGAATTTTGTGATTTTAGACTAAAAATATATATAATGTACACAATTTGATCTTACACGTCATATAAAAAGTTAAAATTAAAGAGTCACTAAAAAAGTGAAGACGCTTTTTTTTTAAACGAACTAAAAGGAAAGTAGTACAAATAAATTGAAATTGAGGGAGTAAAATATGTTCTATTACTCTGTAAGGATGTTATATTTAGCTGATTTCGGTTTGTTTGTGTCATTAACATGTGTATATGGCTGTTTTCTGCTCAATTAGCCATACAAATTTGTTACTTTATAGTTGTTATGAATATTGTCTATGTAATACTTATAACACTAATAAATTTAAATGTTGTCTATGTGACATTCCACTCGTGATTGACTTTATTTTTGTTATTATGTTTAAGTTGTTAATCAAATGGTGGTCTACAGTAACAAGTATATCTCTTTTTCCTTAGCTAAAAGATGTGTTGAATTGGAAGGAAGATAATAAAAAAGAAGAAGATTAAGTTAGAGCCATAAGATTGGACTGATTAGTTTTTGTGTAAGACGACAATATGTTGTTCCCAATTCATTTTAAATGCCCAAAGGTTCTGGTACATGATAGCATTTGTTCAATTGTTTTAAAGTGGCACATACTTCAACTTAAATGTGTTAGTTGTTTCACACAACTATCTGTGTGTACTTAAATAGCACACGTACTTTATCTACAATTTCTCAGGTATTTAATTACTTGCTTGCGACTTGCTTGTGTTCCTTATATTCCATATATACACACCATTGCTTGAGATACTATTACATGATGGAGGACTTATAAGTACAGGTAAAATAGATAACATTAGAGTAATTAAAAAGACAAATCTTCTCTCTTTTTCTTTAGACAAATAAAATATATACATGGTAATGAGTGCAACATCTGGACAAGCTGAAATGAAGAGGAGAACAAATGTTTTCCGAAGGCAATGATTAGCATCTGATTACTAATTTGGGTAAATTGATGCGTTCTTCTGGATTTATGTTAATGCTCATTGATTGTATCTGCTCGTGTTCATCTCTTTTATTATTTGATAATAATATACTATATAAACTGAAGTTCTCAGTAATTGAGCCAAACAAAAGTTCAAATTCTTTGCACATTGAATGCCAACTTTTTTTCCATCTCTGTTTCAATAACAATTTTACTACAGGCAACCAAGTAGAGCAATGTATATGGAATAACCTTTTCTGTTTTCTTTTAAGATGATTTAGCATTATAATGTTTTCCCTACTATTCAGTTCCTAAAAATATGGGGGTGGGGAATCAATTTCAAAAATGGATTGAAGAAAAATTGATCATGATTTCTTTGGGAACGCTTCTGTGATTGTTTTTTTAAATCCAATTGTTCATGCAGCTTATAGGTATTAATGATAACGATGTCTATGGAAGGTGA

>LNC_000090

ATAAACGAGGGACTACAGACATTTAAGGAAAGTTTCCATTTTTTCATACTCTTATTCGTGCAATAGATCTATTCCATAGAGACTTATTCTAACTCGTTCGTTTCTCATAATCATTCGACTACCCATCATACTAGAGGTGGTTGAAAAGCAGAGTTAGTTCTTTTTCGACGAGTTGTTCTTAGCAAGAGTTGTGAGGAAGTCATGAGACTCCAGAGGGGCTTAAGAGAAGCCCAAGAGTTAGTAAGTGTTCAGTTTCAGAGGAATGCACCATAATTCTATGAATCGTGCGTTTGAGCTAAAAGTGAGTTGTACTCTCTTCTTTAAGTCCTGTTTCAGCTAAGATCCACATGAGAAAGTATGTTTAGAGCTTGTGTAGTTTTTTCACCTAAAAAGGTAGCATTTGCTACGAGATCATGAGCAGTAGTAGTGATAGAGCCCATAGTTAGAGGAAAGTATGCAGAGATTTAGTAATCCTAGAAAGGGAGAGAGTGATGAGGCAAACTATGTTATTATAGTTATGCACCTAGTAAGTTATTAATGAAGAGTTTTCCTTTAAGGTTAGACTAAGAAGTGATGAGTAGTGAAGATTCCACCATAGAAGGTCGTGTTTTAGCTAGAAAGGAGTTTGCGATAAAGTGTCATTCTGTAGGTAATGACCTAGTGTATGTGTTGAATAAGAGAGCAGGGTATTTATGAGGTGATAGATTTAAGCTAGGCTTATGATTTTTGAGAGAGAGAGCCCATAATATTTAACGTGTAATAGAACTAATTAGGCATTGATAAATAGTGAATGAGTGAATAGTACTTATGTTGCGAAGTCGAAGGTGATAGTAATTGTAAGTATGAAGACTAAGATGAGACGATTGAGTATTTGTCATAAGGTCTTAGTGGACGAGTGCTTCTGAATTTGTATCTAGTAGTTGTAAGTTAGTATAGTAAGCTAGTAATCATATTAATATTTGGGTTTAGTAATAAACTTCAAGCTTGAATTTGAGATTAGTGTCATGATCAAAAGAATGGCAGCATGAGGGTGATGATGTTAGTCTTGTGACTTGATCTCGAAGACTTGACATAAAATAGGTGAGGATTTGAGATGTTAGCTGGAAAAGATATGAGTGGTACAAGTGAGAGACCATAACATTTAGATAAGGGCAGTGAAGTGTGGTTAAGTCACAAGAGCAACAAGATCATTAAGAGGTGTGCCTAGATAGCACAATTTAGTTGTCAATATCGAATAGGTCCATAACTTTCAAGTGTTAGCTTTAGACCTAAGGGGGGAATGATAAGATGAGAGATCAAGTCAAGTGTTTGAGAGTAAGATAGTGATGAGTTCCAAAGAGTTTGGGGGATATCTTTTTAGAGGTCCTTTTAGTAATGAAGTTGTTAGAGTACTAAGCTTGAATGTGGTGGTAAGGAGTAATAGTCTCGGGATGAGATTCCCTATAGAGAGGTATAAAATCCAAACTTAAGTATTTGGGTTAAGCTTGAATATAGT

>LNC_000091

TTTTGATTAAAGTTTAATTTGGATTGTGATTAGCACTAAAACACATCCATTGTTAGTGTTTGAATGATGATCAAACACTACCTTTGCTATTTTAAAAAGCAGAGACTTGTTCTTCACTACCTAATTCCTCCTATTTTCTCTCTTCTACAAATGTTGCATTATGTTTAACCAACTCCATATATTTCGTCTCTCTTTTTTGGGATCTGAGTTACTTTTACTTTTCCTAATCCACTAAAGCTAATCACTATTTGTGTTTTTTTCTCTCTCTGTATTTTGGATTTCAAGAAAGAGTTTGAATACTACTCACTACAATCATTTGTGTGCTTGGTTATACATTCTTTTCGTTTGGTGGGTTGCAAGGCATTAGGATATAGACCCATTTTGTGAAGTGGGTTTTTGTTTAGACTTGCTCAAATGATTGAGTTTGAGTTTCAGATTATTAGAAAGTTTTGATCTTTTTGACACAGGTATGGAAAATGTGTGTTTTTTTGGGTGTTTGATATGTGGGTTTCAGTGAAAATCTGAAAAATTGGGATTTTCAGATTTGTAGTTTTGTTATCTTTTTCTTGATTTTGTTTTGAAATTGAAATCTGGATTGGGTTGTTTGCTTGCTTGTTTGTTTTGT

>LNC_000092

AAATCAGATAATAACTAAACAGATATTCCCAATGAAAACAATCCGAAGTCCAAACCCAGATTGCTATTCCACAAGGGAATAAAAATTCCAGATGACAGCTTATGTCTATCGAGTTGAAGTACAAAGACATTTCTAGCATTCTCAACCCCATATCGGCTATGAATGATCCACTTGTAATATTAACACCTTAACTACCAAGATAGGGAACCGATATCCATGAATCTATTTCTTTAGCTTCAAGTCAATCAACTTTACAAGCCTCCCCCCTCCCCCTTTTTTCTTTTCCAACAACTCAAAAACCAAAATCAGAAACAAAAACAATACGACACAGAAAACAGTGTAAACTCATAGTCCGACAACCAGGAAAAAAATAACTTTGCTAATCTTTTGTAAGGATTCTTGAAATTATAAGTCGTTGGAGCTAAACAGACCATGTAAACTGGATTGGAAGGTCAATATGAAAGAGAGGGCATCAAATAAATCCGCCTTATAATCATGACCACAATAATTACACTTCGCTCTATCAACTCTGTTAGCATCTTTGAACTTCTAAAAATGTTCCCAAGCACTTGGTCTAGATTGTATGGTTTCTCTTTTCTTTGTACCTATACTAGTAGTTTCAACCAAATTTGTAGGTGAAGAAGTTCACTTTCAGTTAAACCCAC

>LNC_000093

AGTTACGACGAGCGTCGTTCGGATAATGTTTGAGTTTCCGGTTTGCTAAAGACGGATCTTTACGGGTTTGTTCTCTTGTTTGATTTGTTACTTAATTTATTTTAGTTTGATATTTGAACGTGTGATTTTGTGCAATATGCTCATGTGAAAATCAATGTCATATGTATCTCATACTAGGCATGTGCTACTATTTATGACTCTATGAATTTGTTGGTTTAATCCTTGTTTATATGATTTTAGACATTTGAAATAGAATGATAATCGTATGATTCACACTAATGCGGTCCTCTTTTGCTTAAGTTAATTCACATCCCTTAATATTTGCTAATCTATCTTAATTGATATAGTATATATGGTTTGATAATTGATTCCTTGACTTAATAGGATATTTAATTAATTTGTGTCTAAATGTTTATTTACCATAAAAATCGAAAGTGTTTTCTTTGCTGATTATAACCCTTTGTCATGCTAACTGATTTCAATTATTCCCTTTAACTTTCTTGTTAAATAATGACTTATTAGTTGGCTTTTCTAGGTTGACTTGCTAATAATCCTCATAATTGATTTACTTAATAACTGTGATTGACTCCTTTAATTTTAATAATCTTTAGTCTACCATAAGTCATTATAATTTGTTTAAATATGGAGGAATTTGATTGCATACTTACTGGAAATTGATTCTTTCTCTCTTGTTGAAAACTTGGTCAAATCTTGTTTACTTGTTTTATCTATGGTAAAGTATTTAATTTGATTGGCCAAGTTAGGGGTATTTACAATGCGATTACTACTGCCTTGCCATAAAATGTATCCTGTTTGTCTGATCTTGTGTGGTATCAATTAATCGGTTTTCCTTAATTGCTTTTTACTTGTGCTGCTTGCCTTAATTAAAATGAAGCAATTGTCTGTCTTGTTTTATATTTTAGTAAAATCATGTCCTTTTGGTAAAAAAGATCTTATTGATTGGTAATCTTCTTGGTAAGGAAATATAATCTCCCGTAATTGCTCTTTGCTAATTATTCTGTACTCCTCATTCACTATATAAACAGAAGTGTTCTC

>LNC_000094

TTTCTCTATTTCTAAAATAAATAAAAATAATAAATTAGATAAGGGTAAGGTAATACATATTTTTATATTTATATATATATATATTACCCTTATATATCTTTCTTTTTTTTATATATAATATTATTACCCTTATATATATATTACTGATACAACATAAATTTAATAGGCTCTCATTCAAGAACAAATTTTGCTCCCTTTCTCAGGTATTATTTCTTCCCTTTTCATCTTTTCCCTCTTCCCAGTTTCAATTCTTCTTCCAGATTCTGCTTATCACCAAAACAGAAAAAAAAAAAACACAAAATTGTAGTTATTAGTTGGAAGTCAATTACTATAGTATTACATTTGTACTGATATTGCAAATGTAAAACCCAAAGTAATAACCTCCCTTATGAGTTTCCTGTTTCATAGCTCATATTTAATGCTTTCAATAGTATTCAATCTTTCATAGATATAACATGGACCCAGGAGTATGCTTTTACCTTAATCCCCAACATCATCAAAATATCGCCACAGTAGCTGTTTTCTTTTGCTTTTTTTTTTCGCTTTCTTGTTTTTTAAATTTTAAATAGTACCATCAAAGTTTAGTTTCTATGAATAAAGTTTTTCATATTGTTTTTGCTAAATGTATAGTCATTTCACCTGAGTTTCAGAGATAATTGTTCCTCTTTTAATATTCAGCAACATTTTTTGTCCCTGTAACAGCTTTTCTGTCAAAAAATAATTGTCCAAGTGTACTTTGCACGTTAATAATGCCTGCTAACTGATTTAATGTCTTCATTGCGCTTCGCTAAAGGGCCCTAACAAATTTACACATGACTTTACTTCTACTATGACAAGACTATTGTTTTTCTTTTCATTTGTTCTTCATTTTGTTTTTGCTTGCAAAATACAAACTTTATTATCTTTCCGCTGTTTACATCTCATATCACCCAATAGTAACAAGTACAACACTGAAGTAGTATAGTTTGTTTGCTCACTTTGGCTGTAGCTCCTTTCTTTGTATCCAATTATCAAACTGCTGCTTCAACATTCTACAATTGTACAGTAGTATCTCATGACTGAAAAAGAACTAATAGCACATAAATGGTTACCCCATCAAGATAACAGCAAAAGTCAATAGTATCATACATCCTAGGTCATCTCTTATGAGTTTGTGCTGTACGTTCATTCTTTCTCCTAAGTTGCTTTTACAAG

>LNC_000095

GTTCGCTTTCCATTTCTGAATACCAAAGAGCGATTGAGAGAAGAGCAGTACGTAGGACTTAAAAAGAGGGTTCCTTCCTCTCTTCACTCTCCTAATTTCGATCCTCACCTTTCTGGTGCCAAAGGCCTACCCTTCCACTTTACAGGAAGTTCTAAAGGAAGAAGTCGAAAAGGAAAAGAAAGTCACCTTTCCTGCCAACCTCCTGGCTTTATTCTTATACTGATCGAGCTGCACTATTTTTTATTCTAATAGATTCCACTTCGCTTGCTTGCACGACTTCCCCCTTGCTTAAAAAAAAAGGGGCAAGTCAAGTAAGTAAGCAGTGATCTGAACCACTTTGAAATAGAAAGAAAGATAGAAGGAAAGCAAGAAAGAACTATCTGACTTCGCTATCTTTCTATGAGCTCCTAGGAAAGAGATCCTACTCCCAAAGAAAGGAAGCTTAAGAATAGTCATTAAGAGAGGTGACCCTCTTTTGCTTTTTCAAGCTATGAATGTAGAGATCAGGTTGAAGAGTGAAAGGGACGAAGTGGTATGAAGTTGCTTGAACCTAGCGCTCGGGAGGTTGTGCTCGACCGAAAGGGTAAGGGCAAGAATACTTGGCTGGACGTAGTTCAGGATCGATGTAATTGAGACAACTAAGTAGAGGATGGGAAGATGTGAGGCCGGCCAAAGGTGGTTCTATATAGCTCTTACGCTTTTACAACTCGTACTATTTTATTTGAAAGTGAA

>LNC_000096

GTTTTCTCCTGAGAAATCTGTCAACCGAGTCTCTTGAACTCTGTTGTCCACATCCGTGTTGCATTTTGAGTCGGCCTTCATCCCTGTTTGAATTTCGGAAGCTGAAACAGTTGAAAGAGGAGTTGCATTACAGGTCTCAAAGGCTGAAAACAGGCTGAAATACATCATGTATACACTGATATACATATCAGTGCAACAAATACGTGGCTGAAATCGGAAATACAGGTCGCAAGGGGAAGAAATGGATTGTATACACTTGGTATACGGTTCAATATACAAGAAAATTGGTCAAAATCCGACCCGTATACAAGAATATACAACAG

>LNC_000097

CTGTTTCGATGTTCTTCGTTCATGGGTTCTTTTATGCAATTCTCTTTAGTTATAGTTATTCTGTGTACTCGTCGGGCTTATGGGGGTCCGTTCGGGTTTTTATTTAACTTGCGCACGAGTGTACCTTCTGGGCTTATGGGGGTCCAGTTAGGTGTAGTTAGCTTGTAGATTACTTTAGTTAGTTCTTTCTACACTCGTGTGCATTCCCTTGTTAGTTAGATTTTAGTTCTTGACTTCGGTTTGTGTTATTCCTTCTTTTCATATTGC

>LNC_000098

AAGGACGTTCAAACATAGTACCTTCAACATCTTTAGTCTTCTATTTTTCTTCATTTCAATCACTAAAAAAATGTCTTTTAAACCAAAAGTTAGAGTTTCAATTTATTGGGATGGTGAAATTATACATGACGAAAATATCGTTTATTATAATTGTCTTTTCAAACACAATGCTAAATATTTAATTAATGTCTGATATCATAAATTTCTTTCATCAATTTATAAAAGAATGGGTATAAATAGTACTGATTATAATTTGATTATAGTCGGAAAATAACCTACTTCATTTTTGTTACAAGGACAAGTAAATTTTGGAGAGTGGATTATCTATAGTGACGAATCATTGACCGACTTTTTGAGGGTTCCAAATGACTACATAGATCAAATCAAGTTAACACTTATGATACATAATGTAGTAATTTTTTCCTTAAATAAAGCATGGAGGAGTTTTCAGAATAAAAAAATAAAAATACATAATGTAATAATTTTTTTCGTTTTTATGATATGTCAAATCAATATAATTGTATGACAAACACTGCAA

>LNC_000099

NTCGAATAGGTTTAACATACCAGATTTGCTATTTTTTTCCCTAAATGCAGTACATTTTGTCAAAAGCTGAACTATAAACTAATAACGTTTTATTGTTGTTCCCTGGATTAAAGCTAATTCTTTGGGTCCTTATTTGAATGTGAACAAGTCCTTCAAGTTCTTAAATTCAGCTCTATTTTGTCGAGTACTGCATTATAAAGTGGCTGTTGCTATTCTGCTATTTCAAAATACTTAACAGTTTACTCCCTCCAAAACACCTTTTGCATTAGTGCGACATTTTTTTCAAGTGAATCCAAAATTTGTCATTATTAGTTACTCAGTGCTCATCTCTATGATATATCATAGCTTATAACAACTAACTACATTCAACTTTATAGGGACAGACTATGTTGTGGGGGGTCATTTGAATTGCATTGAGATAATGCCAGCCTACTCCATTTTTTAAGGAGATGCCCTTTGGTGTGAAGACTCATACTTCCACAAATCTGACACAATTTTTTGCTTCTTATGGCCTCATCTTTCGAAGAGTCCGGAGGAACTGAATGAATTTCCCATGTGTACTGCAAACATCTTTTGCCATTGTTTAATTCAATAGGTGAAATTTTGGTGTGTGCCCCAATTGCACATGTCCAATCAAATACATGTATTGATAACATGTGTAAGTGATAAACTTCTTAAGCATCGTCTCTTGCCTATTCTGGTTACATTAGAGGAAATATTTGAATTGTAGTGTTTGTAACTATATGACCTCTATATAAGTAGATAAATCAACAAAACTTTGAGCTAAAATATGGATGTTGCTCTTACAAAATAAATAAATATTATCAATGGAACATGTTGGGCCGTGCAGAGCACGGCTTTCCTTATCTAGTTTCTATTATTTAGAAGCATGATGACGACCAACGACCAGTGGCAAAAAGGATAATAAATCAAATATACAAGGGCAAATTATAACTACTATACTAAAATTTATTGATATTCGTGCACTCATGCTCAGAAGTCAGACCAAGGCTCTATTTTTTTTAATACTCTAGAAGTATCTACATCTTTCTCCACTCTTCCTATGAAAAAAA

>LNC_000100

TTTATTTGCTGTCATTAGTCTGCTGAAGCTGAAAGCATTAGACCCGAACATAGCACTTGATCAAATTGCTTCTCTAGCTGCATGGGATTTTTGCATCTGCTGTTGTATCCAAGATTTCAAAGACAAAATACCCATGCTACAGGTGAAATCTGGTCAAATGGATTTTGTCTGAACCAACAGATCGTCTCTGCTGAGCCTTCTGCAGCACATTGCATGCATATATCTTCTGTTCAGCAATAATAACTAGATGACTCCAGCTACTTGGAAGTCTTCAAGCCAGTCATAATCCTTA

>LNC_000101

GGTTAGGCTCAGATCCTGAAGGATGGGCAGACTCGGAGCCTGAAGCATGGTCGGACTCAGGTCCGGACATAGACCCCTCTGAACTGGAAGCAGCCCCAGTTGGTGACCCGATCAGTATGTGCTCCTCATCAGACTGGGAGACAGTGACTACGTCGGGAAGCACCTTCCGGGGGGTGCCTCTGAGTGCAGCGCAAGAAGTCCTAGGGTTGAGAGGCACATATGTTGGATCAGTGTCGGCATCTGTGTCCTCATCAATTAACCGGAAACTAGGGGCAACAGATTTTGACTTGCCCCTCTTTGCGTAGGTGACTATCTTCTTGGGTGCCATTGTACCTGCGGGAGCGATATTAGTGCCCATGACCAACCAGAACTATTAGAAGATAAA

>LNC_000102

TTTTACGTAAGTTTTAAATAATACTACTATGTTACTCTCTCTATTCCATTTATGTAAGTTATAATACTACTATGCTACTCTCTTTATTCCATTTATGTAGCATGTATAGAATTTTGATAGTCAACTAAATTTTTACATGCCTTTTAACTTTTTAAATTATTAATTATCGCTGTATCCTAATTAACCGAATGTTTCCAAAAAAAAAATTTAATTTCCAGGAACAGGTTATTAATGCAATATTTAATTAGCAATTATAATTATCAATATAACTACAGTAAGTGTAAATTTGGCCCCACATGTCAAAAGAGATAGTCTCAAATAGGAAGCAATCACACGTGTATGGAGTACAATGCAAGTGGGCCCACATATTCAGTAAAATTGTCTACAAGCATTCAAAAGATAGACTGTAAATAATTGAAAATAAAATGACTTTTTTGTCCAAAAAAGAAACGCTACATCCATGTAAAGTTGAAGTACAAAAAAGTTAAAAGGATAAAAATTTGTGAAATACCAAAATAACCCTTGGTCGGTAAGTTGGCAAGACACCCGAGAGGTGCTTGTCAAGCCGCTTGTATTACTCCTATATATTTTACTCCTATATAGAAGTGGAGTTAATAGGACTAACAAAACAATACAATTTGTACAATAAGCTAAGAAATATGTACTTTACTCTTTACTCCTATTTAGAAATGAGAGTTTGTGAGGACCAACATTGCACTAAAGAGTTGGACTAAAAGCTTTAGAAATTGTATATTGAGGCCCACCAAAAAGATGAACTAAAGTAACTTCATCTACTTCTACTTTTTCTACACATTGGAATGTGTACTGCTTTCTGTACACGTGTTTTTT

>LNC_000103

TCCCCCCCCCCCCCCCCCCCCCCCCAGAAATTCGAACCCTGATTCTCCCCTTATATATAATCGCTTCCTGTTCAGAAAAAAGGGAGGGTGGAGAAAAATATTGGAAAGTCAGGAGTTTGTTTTTTCTTTTTGGAAAATTTTGAATCAAGGAAAAAGGATAAGACACAGGGAAATACATAAGTTTCTTTGAGGCTGAAATAGACACTAAAATCAACTAGTGTTTTAAAGAGCATCTCATTTCCAGTTTCTTCTTTCGAACACGACCCTCCCGCTGCTCGTTGGAACTCTTTAAGTTGCTGGTGGTGGAATAGAACGCTCCAAACTCGTCCTGTCCCGTTTACTGCCCTCGAGAAGGTAATATTTCATTTTTTGATTTTAGTTTCTTTCGTCGTGAGCTAATAGTTGCATAAGTTTATGGTTTGGTGTTGTAATGTTTTTGATCAGCTGATTCGAGGACTGCTTGTTTCTTGATTGTTGCGAGTCATTCGAAATGTTAGAATTTTGAGAATATTCTGACTTATTGTTGGTTGCAAATCGGTAGTGTAAACTTATTTGTCTATTCAAATGATCTTTTGGTCTTGGTGTTTCCATTTGGTATCGAGTTGACTCATATAAGCTAATATCTTTTATTTCTATGTTAATCTCATTATGTCGCCTGTGGTTCGTAGTTCCATTATGAATTTTCGGTTCAACTTTATCTGCTTCTCTTTAGCGTTTCAGTTCTTGCTTAAGGCTGAATAGCAGCGTTTGGGTTTATGTTGTTGGTTGTTATTTTATAAAGCCGAACTTATGGAATTATCAAGTTTCTTTTTAGCTATCAGTGAGCCATCTCGGGGATTGTCAATACTTGTCGCAAAATCGATTCAATATTTTGGTGTACCTCGATGCTTATCGTATCATCTTTTGCAATCAGATCTTTGAGTAGTCTTTTATTCTTAAGTTCTTCATAATGCTGACGTAGCTTTTTTTCATAGTGATTTTTATTTAGCTTTTGAATGAAAN

>LNC_000104

ATTGCATTGAAAATTGTACTACACCATTTGAGTCTAATTCACTAGAAAATAAACACCTATACTACACGTGTCCTTCTACCTTTTTCCAGTTTTCAATTATTTTGCTTTGGTTCTTTCTTTCTTCTACTTTTTCTATTTTTTATTCTCCTCATTCACACAATTGTCGGAGAAGGAGAAGAAGAACTTACAGATGGAGGAAGAGCAATTGATGGAAAATTAGAGAAGAGTGATTCGATTCCGAATTGATGCATTAATTTGGTGATGAAATTAAATTTCATATTTCACTAACGAAATTGGAGATCAAATTGCATACTGTTTTTGATGTGATAATGAGGCAAACACATGCTTTAAAGTTATATGAAGACATAGACTTCAACAAAAGCTCTTCTCGCAATTCTCAAAATTTTGTATCTCAATCTCTTATCGTGGTTAGATTTTCTTATTTTTGGTGAGTTCTATCACCCTTCTTCTTCCTTTGAGAAATATAGGA

>LNC_000105

AGTTTGTAAGAGAAGGAGTACCGCTCAAGCTTCAACTCTGCTCCTTCCCTCAGTTCTTCTTAGTACCCTTTCATGAATGGGAAGAGGAAGTGGGAAGACCTCCCTACCCAACCTTACCTAAGGTCGAGCAGTTTGCAACTCCGATCCCTAAGCCAGTTAGCCTTTGACAGGCTTTCCCATGCAAAAGAGTTCTCCCAAGCTTCTGACCATGATTCTGAAAGAAAAGATTGGTCGTACAGTCGTT

>LNC_000106

GGTTCTATGTCTATTCCTACTTCTGATGTAAGTTCTTAAAAGTTGAAACTGAAGAAAGTTAGAGAGTTGTGAAACCAATGGTTGGTAATACAGAAGATTTTAATTGGAGTATGTAGTAGTCCTATTCTTCACGGTTCAGATTGTTGACTTATGTATTTGAACATTTGGCGAAGAGAGCTTGAAAGTGACATGCAAGAAGGAAGAAATAAAATTAAACTGGAAATAAACTTGTGTTGTCTCTTTTGGTTTGATAG

>LNC_000107

TTTAAATCTAGTTCTTCTATGTTATTCGGTTTATGATGTGTTATTGGTTGTGTCTTATGTTCCGTTTATGCGTTAACTTCGTTTTTGGTCTCTATATGTTAGGATTCGGTTAATGAGTTTTAGCTAGTAACATCCTTTCTTTCTATATGAGTATTATTTGTGTATAAATTCTGTTTGTATTTGGTTTAGTGTTATTTCCGAGCATGTTCGATCTCTTCATGCTTGACTATGAATTCTCCACGGTGCAATTCTTTTATGTTGTATTATCTCATTTTCGACTTTAGTTGAATGCGAAAAGGATCTTTCTAGTCGTCTGTGGTAAATGTTCTTATCTATGGTATTTTGTTGTGTTTGGGTAAGATAATCATCTCATGGTATAGTTTCCGCTTATCTAAAGAGTGTGATTCTTTTCCTTTGTATTGTATCCTTACTTGTTCCATCTCGCCTCTTATGAGCTGGTAATATTCGCTTTAGTTTCTCTATGGCTTACTATTTTTCAGATTTCTATCCTGCGTAGGTGTCAATTTTCATGTTTGTTATTTGTAGCTTTTCTTGAGTGGTTACACTTTGTTTTCGTTCTCTTTTCTTTTCTAAATGAATAGTTAGGACCAGCAATAAGCTGCTGCCCAGAATGTAGGCATTTGGAAATTATATTTTGTTCCTAACTCGAGTCATGCCTGCTCATTCCTTATTGCGCATACCATTTACATGCTTATTGGCGGCTTGGTTTCATAGTAACTACTGCATCTGTTTGCCCTAGTATGTATATATGTATATATATATATATATA

>LNC_000108

CTTAGCTAATAGCATGATTCGGTTGGATATCTCAGATTCTAGACGTGTCTTGGCCCATATGGGAGTTCAGTCTTCCTTGCTTGACAGGATCCGTGGTTGTCAGTTTGAGGATGAGGCCTTGGTGGCCCTTAGAGATCGAGTGTTAGCGGGTGATGGTGGTCAGGCTACCTTAGATCCTGATGGAGTGTTGAGATTCGCCGGCCGCATCTGTGTTCCGAGAGTTGGGGATTTGATACAGTTGATACTTTCTGAGGGCCATGAGTCTAGATACTCTATCCATCCGGGCACAGCGAAGATGTATCGTGATCTGAGGCAGCATTACTGGTGGGGTGGCATGAGGAGAGATATTGCTAACTTTGTTTC

>LNC_000109

AGGCAAACATTTGCTAACCTCATTTCCTATCTCTTCAACTATCAGAACACAAAAGTTGTCACTGTTCTTCTCTTTAACCTAATTTTTCATCCATTCATAGCAGATCGATTGAACACAGAAGTTGGGGAGAATTGCAAAAATCTAGAGGTTTTCTTCTTCAACTGCTGGAGGTTCTCTTCTTCAATCTTTGAGGTTATAGCTGAAGAAACTACAAGTTTGGGCTTTTGAGGTTTTATTCTTGAATCTCTTCTTTTGTCTTTAATCTTTGGGTGTGGTTAGTGTATTTGATGTATTTCTCTGATTGTGGGGTTTTTTCCCATTAAATTGTAGCTATTGTGGGTTTAATGTTTTGTTGGAGTTATGATGAAATTGACTACTCTAGACCATATTTAGCTTGACATTGATGTTGTATTTCTTGTCCATTAGTTATTCAATCGGGTTTGAATACCCTTGGATTTATGATGTCTTCATTGATTTCACCTCTCACTTGAGTAGGATGTCAAAAATGCACGTTCATTATTGTTGAAGATTTACGTTAGAGCAACAGGAAGCAAGAGATACATTAACATAAAAATGGAATAAACTAAAATTTGCTATTGGTTCGAAAAGTGATCTTTTCTGTCAATCATCACCATCCAGAGCACAGCGCCATCTNN

>LNC_000110

CCCGGCGGTCTCTTGCTTTATGTCTCCTCCATCACCCCTTTTTGATGAAAAGAGACACCCTACGCCGCCTATGCCTAATCCTAATGGCCCCAAAAATCTGAAAGGTTTGATGTCGATTTGATTTTAGTTGCAGTCCTCGGTCTCCAACAATTTTTTGGATTTCAAATTTGACTTTGAAGTTAGATTTAGCCTATAAAAGGAGCAAGGAATAAAAAAAGAAGAAGAGGAGAACGAGATCGGAGAATAAATAGAAAAGAGGGAGAGGACATACTTCAAGCATTAGGTTTTCATAAAATTTCTATATTCTTGCATCTTTTCGTTTTCAGTTTTTGAATTCGAGTTCAAGGAATTTAGAAGTTAGAATCTTT

>LNC_000111

NACCGGAGCCGAAGAGTTTCACTTGAGACTCGATAAGGACGCTGATGTACAGATTCCATTCATTATCACCATCGTTCATCTCTATTTCGTTAAGACAGCCAACCTACAAAAGGAAAAATCTTTTATGGAGCTTACATGGCTCTCGATTATTTTATATTACTTGCACATCCTTACTTTTTCCTGCTTCATATTTGTTCATACATCGACTAACGGAGGAGATAATCTTTTCTTCTGTTAGAGCCAAGCATGACCTTTAATTACTTCAGCTTGTTTAATTTATTTGTGTTGATGTGATTAATGTTTTGAGTTTACTTATATGTTCTCTAATATATAACTAGTAGTTGTACTTAATTTGGAGGTTTCTTTAAGCTATGAGACATTTCCCTCATCACATAAAACTCTTATTTTGAAAACTTAAAAAATAAGTGACCTATCTTCTTCTTTTGGTAAGACCATTAATTAAAACTCGTAAAAAGACAAAATGACTTTACTATATTTTTGACTTTAAGTTGTACGCCAGTTTTCCAACTTCTTATATTGTTAATAACTAGTTCTGCCCGTGTCTTGCTCTAAATTTGATTACACACTTTCCATATAGCTGTGTCATCCTTTTTCTTATATACTAAGTATTAACTATTTTTTTTATTTTTTTAAATGGATAAATAATAAAGTAAGAAATATCAATCACTTTTCTTAGCCTAATTAATTAACATAAGTTTGGCCGGTTAACTGTTTTTAACAGATTTTAAAGAATGTCTTACTCCTTCCTTTTAATATAAATTAGAATCCTTACCTAGAATCTCAC

>LNC_000112

CCACGTGGCAGAGGAGTGGAAATCTGGAAATTTCCAGAATTTAGGACGAACTTCGTCCATTTTTTTTCCTTCAACTTCAACATCAGATTGCTCCTTCATTTTGAGTCCAAATTAGGTGCTTCAAAAGGCAAACTTCAAGAGAATTTCGAGGGGAATCTAGCGGTGATCTCGGAAACGCGAGGCAAGGCTTAATTTGAGGTAAGAAATCAATTTTAGCCACTGCCAGTGTGATTTCCGGATGGTTTTGTTATTGAATTTTGAAATGTGAGATCTTGATCATCTTAGGTCCGTTTTGAGTCATTCTTGTGGCTAACTTGTAGAGTTTTTCGCAAGGATCGTCGTGGTATAATCTGTTTGGGTTGAAAGGGTTTCGTTTTTCCGAAAATTGGTGATCAAGGGGCTGCCCTTTTTAATTATTGTGGCTGATTTGCGGTGTTGTAGGCATCTGTTTGTGCTAAAATTTTGGGATATTAGTTT

>LNC_000113

TATTAGTCTATGTGTAAGGCTAAACAAGTTCGTTACATTTTATTTATGTGCATTTATCAAACACTTCATGCAATTTTGACTTAAAATAACATAATATTGAATTGTTATTTGGATGCTCAGTTTTCAAACATATGGTTCTGCAGATACAGTTTATGGAACTTATTTTTGGATTTATTTTTAAAATTTAGTATTACCATCATATTTTACAAGTCTCAATTGGATGCGTTATTATAGTTTTCCTTTTCTTTTTTGAGAAAAAAATTGACTTATTGATCTCTAGCTTTCTTTTTACCACTTCGTTTTGTATGAAGGCAGCTACACAAGATTAGAAATGATTATATTTGAGAAAGTACAAGTGACCTAAAAATACATTGATTATGTGTTGTTATGCTGATCAAAATATTTTAAACAAAATGAGATAAGATTGAAAATTACATTAAGATTGGTTCAAG

>LNC_000114

CATGTTTATGTGTTAAATTTGAAGGATGTATACGTCATTTGGCAAAACGATGTCCCTTCAGGTAAGGAGTTTAAGAGTTTGTTTGAGTCAAAGAAGGATCAAATTATAATTGCATCTGAAGATGTTGCCATTTAAAGATGAAAAGAAATCAAACAAAAGGACCGTCCATGGAGGCATAGCTTAGGATTGTACCCCTGTGTGGGACTGATTATTTGTATCCTATTTTTCCTATTATGTATCCACATTCAGTTTTATTCATAAAGTTTGTATAAATTCAGATTTTGGAACAATGTTGTCAAATGACGTAATACATCCT

>LNC_000115

NNAGACCTTAGATGTTGACAAAAGAATTTTATGCATGTTTAAAATATGTATACAACACTTTGTTTTTGATTGCCAATGTGTAAGAGCTTCGACATTAACAAAAGAATGTGTGTCTTAGTTTAATATCTCACAAATTGTAGAGAACTGTAATTGGTGTTGCTCTCTAACTCTTCCTGCCCATATTGTTGTTAAAATATCTTGGGTGGACATATGGTACTAAGATTCAATATCGATTGAATAACAACTGATACTCTTGATTGGACTTGCAAAGTCCAAATTGTGGTCATGACTCAAGAGCGAGAAAGTTTGGAGAAGAACATCAAATTTCAAAATCTGATTTTGAAAGACGAAGAGAAGAAAGTAACAAGAGTTGTATCTGAATCTTCAGAAAAACTTGAAGAGATCCATTTCCAACATTATTGATGCAAGGTTGTCGTGATCAGAAAAGTTTACGTTTTACTAACTAATTAATTATTATAATGGAGAATTAGCATAATATGCAAAAACAGTTTAATATCAGATGACCACCACCATTTTGGTGTTCTCTTGAATACATCCAAAAGTATTCATGATGTCTAAGGGCCCTTGTAGTGTGTAGTTTCGACTTCATATTAACATTCCTTCACATCCAATTTTGTTGTGAATTTCATAGCTTAGGTATAATTGTTATGTGCATTAATCTTCTGCATCATCTTTGTAATATACTATACATGTGGCTACTACATGGGTCCGAC

>LNC_000116

NNTTGATTGTTATAGAGCATCCTGAGTTTTTTTAAGGTTATTTATATGTGTTGTCTTCATGGATAGCTTAACATGATCTTAGGATACTGTACTGCCATCGCTTTCTATGATTTTCAAATTTTATTACAGGATACTATAGTGTTATACCTCTCTGTGATTTTGAAATATGATTATTTTAGGCCAACATGAGCTTTTTCTTTGACATTTTTCATCTCTATTATATTTCTGGACAACTTGATGTCATCCTGCAGTTTCATTTCCCCCTATGACGTACCCAGGGTACATTGGTTAGTGACGAAGTTGGGCTGTAAAATCAGAACGTTGATGACACATTCACGAGGAAACAATATTATTCGATCTCCTTGCAAAACAATCTGTTTCACAGAGGCAACACAAACATTGACACTATGGAGCACAATACCGCATGTGAGTGTTTTGCTGTTTACCGTAAGGACTCCAATTTGAGCCTCTTTTTTCTAATTTGCTCATATTTTTCTTAAACTTTTATGAAATAATTGCC

>LNC_000117

NTTTCCTTTATTTCCCTTATGCTGTCTCCACTCTATTTTGGTGCTATTAATGAATCATGGTGACAATATTGTTTACTCTTCACAAAATTTTGGAGTGCATAGTCTTTTCAATTAAGGAAAAATGACCTTTAGCTGATATTTTGTACTGACATCATGTTGCTATGTATGTCTAATTACATAGCAACTATGCTTACTGCAATTTTGTTTGGTATACAATATTACTTTTCCTTTAGCTTAACTCGTGTCACCAACTAAATAGTTTCATTTTAGCAATTGTAAACATCATTAAATAGCTGCAATAACTATGTTGCTTTACTCATGTCTGAAACAACCTAATGAGGTTATGTTTTCCCTTCCCATTCTTCAAATGATCAAAACTGTCCAACACAATAATAGGAACACATTTATCCTTCACACCAACATAAACTTGTTTATATGCCTGTTCAACTACCAAATCAGCTTCAAAATATCAAAACATCTTCTATTACATAAGTTATCTTTCATTTGTCGTATTCTACAGTTTTATGGCTCCAAACACAACATATGATGTACTGATTGTAGCAACAACTACTCATTCAATCAATTGAATTTGTTCTTTATGCATACTTCCATCGAAATGTTGTACTACTTATATGATGTTTGTCATTACTCCATATTACACTGCTGCTGCCAGACGCCTCTTTTTGGTCAACTGTATTTGCTTTTCCCAN

>LNC_000118

GTGATGTGTTATCAGCTACTTTCTGAACACTTGGTATTGGTGTTGGTGGTAAACGTTGATGTATGAGGAATTCTGCAGTTTTTGAATTGGTTTGAATTGTTCAGCTTATGGTAGGTGGCTTACGAGTGTCCAACCGGAAGGACTTATAGCTCTACATGGTTATTGAGGATTTTGAACTGGGTTGATAACTCTATTTATGGAATTATCCA

>LNC_000119

NNAGAGAAAAATCGTGCATAAGCTTATACTCACTAAAACATAAGATGAGATAATATTGTTTTAAAAAAGTCATATATTCTGATTCTCTTTGATTAGTCTATCATATATATAAATTGAAAATTTATCTTGAGATCTTGTGCAATGTAAATAATAAACGGTTGATGATGAATTCATCAAGCTCCTTATATATTCTATCCAGCTAGTTTGCAACCAAACAAGGGAGGAGGTTATTTCACCTCTTAGTTTCTTTATTTGTTTGGTGCAATAGGGGAGGAAGTTATTTGTTTGGTGTAGTAGGACAGAAAAATATACTCCAGCAACAACAAGAAGCAACTCTTTCTTGTTAACACTAGGATCAGAGTCTAGCATCTCATATAGTGTCTCAACCATCTCATAATTCCTTCAAGTGATAAAGCTTAGCATTTGAAAGTGAACTTCATCAGAAAAAACACATCATGTATCTCATTATATACCATGTATGCATTTTCAAACTACTGCAACTTGTTACTTCCCTTTACACTTAAACCAAATAAGAAGAGAAACTCTCTTAGAATTAGGAATATTGTCGTCAACTCATGCAACA

>LNC_000120

TCTCAACCGAGACCAGAATTCAAATATAGAAATTAAACAATCAACTCAACACTTTTTGGGAATTTTCTGTTATTTATCAAAAACAGAGAAATGGTACTCAAACCAATAGGGAAATTCTAACTAGTAAAAGAAATGAGATTAATATTGAGCTCCATTCAAGAATTGATCTGCAGAAGAAGAAGAAGAGAGGAAAGAATCAGATTGAGATGAGATGAGCAGAGAATTTGAGAGGGAGAAAAGAGCACGAGGAAAGGGTTGAGAATTACCAGATTTTAGTTAGCCTTCTCTCTCTTCTGTGATGTGTATATATAACCATTTGTCCACGTGGCACGATCTCGTCCCTTGAGCTTAATTAACTGAAATAAATC

>LNC_000121

NNCAAACTACTTCCTAATGACATTTGCCTTCTCAATCTTATCCTATTGATAATAGTAATATCAAACTATTACTATTCTTTTGCTTTGGTATTATCTTTTGATATATGGACTGTGAGGACACAATCTCATTTTCAGTTGGGGGTGCCTCCCTTGTACTCATTTTGTTGATTGTTTCTGCATACTGTGATTGTGCCCTTGTCGGGCGTACTTTTCTTTCTTTGTGATGGATTGGTGTGCCCTTGTCGGGCTTGATGTTGTTGATGTTTCTGTTGATATTGTGTTGGATTGGTCTATTTTAGTTTTTGATTTGTAGTTTGTAGTTTCTTTGTTTATTTTTTTATAATAAGATGTGGATTGACGATGCCTGATGATGGATAGAGAGTGATGTCCTTCTTAAGGTTAAATCATCGATTTCTTGTAAGTGTTTGATTGGCGTTGATTGTGGCGAAGTTTGAGTAGGCGTTCCCGAATAGATGCATGAGGTTGCCTGATGATGGATTGGTTGACGACTTTCTTAAGGGCCAAATCATCGTCAGTTTGGGAAGGTTTTGACTTGGTTGGTACTAAGAGCAT

>LNC_000122

CTCAAACTTGAAATAGGACTTTTTCGAATTCAGTTCCCCACAATCAAGCATGATGGGGTTATGATCAGAACCTATTCTGGGCAGCAAGTTCTGTTTGATCTGAGTGAAAGCTTCATCCCATTGAGATGAATACAAAAACCTATCAATTCTGGAAGCTGAGCTATGGCCATCTCCTCTTCTCCAAGTGAAAGAGCCCCCCAAAAGAGGGGGGTCCACGAATTCCATCGCATTTATCCATTCCGTGAACTCCTGCATGTCCCCTGTTATTCTGTGCCCTTCTGATCTTTCATTTGGGTACCTTGTGACATTGAAGTCACCACAAGATACCCATGGGCCACTGCATATCTCCTTGATGTTGCTCAACTCTTGCCACAACTCTCTTCTAGTCACAGTATCACAGGAAGCATACACAGCACTAAGGAACCAAGTAAGAAC

>LNC_000123

NNTTACACCAAAAATGAAACAAAAATATATACAATATATATCTTCTGTTGAGGTGGCTACATCTTCAAAACATCACACATTCCTTTTCTTTCCTATTGTCCACCAGATGCATGTAGGAATAATTCTCCACCATCTCTTCTGCCTTACAAAATTGTCTGGATCATTCCAGCATGCTAGCAACACAGCAGTTTTCTTAATTATCCATTTGAGCCTTTCCATACTCAGTAAGAGCTGTACAGATTACAGGTAACTTTGCAATGGATAAGTAGGTGGCTACTTGTTCTATTCATTATCCATCTGAACCCTTTCATACTTGGTAAGAGCTGCCACAGAGTACTAGTAACTTTGCAATGGATAAATAGTTGGCTATTTGTTTCATTCATTATCCATTAGAGCCCTTTCATACAAGACTAAAGAGCTGCCTCAGAGTACTGGTAACTTTGCGATGGATAAATAATAGATGGCTCTTTATTTCATTTTCTCTCCACATAGAAGGCATTTAGAACACATAGAAATATTGTGCAAATGACACTTTGAGCAATGAAGGGGTAGCACNN

>LNC_000124

TGAAGAACTCATGTTTGTTGCGCGGAAGTGATTTTGTAAGAACATCAGCAACTTGATCATTTGTGCCACAAAATTTCAGTGAGATTATTCCACCAGCAACCAGATTGCGAATGAAGTGATAGCGAATGTCGATGTGCTTCGTCCTACTATGAAATGCAGGATTTTTTGTCATAGCAATCGCTGCCTTATTGTCACACCAGATTTCAGTTGAACCAATCTGCTCTTGTTGAAGATCCACTAACAATTTCCTTAACCAAATGGCTTGGCACCCGGCAGAAGTTGCAGCCATGTACTCTGCTTCAGAAGATGACAAGGCGATCGTATCTTGTTTTCTTGAACTCCATGAGATTGCTCCAGAACCAAGACTAAAGACACTCCCAGATGTGCTCCTGC

>LNC_000125

GCATAACTAATACATGCATTGCTAATGCAAGCATTACCAATACACTTTATTTAGCATTATTTCTATACACTCTACCAAACGACCCCTTACTTCGCTATTGCAGATTCTCTAGTTAATGTTGGGAACTTTTTCTAAACATTTGTGGTATCTCATGGGTGATGTCTGTTAGGGATCTGTTAGAAAGCTGGCAAGTTCAATGGGTTCCTAGTGGGCTTAAACAGATTTGGAAGACTATCCCACTCTGCATCTTCTGGGCCATCTGGCTGGCAAGGAACAATGGCTTGTTTTGAAGGGTATAGAGATCACTTTTCTAGAGTTAAAAATAAGTGTCTGCATAATCTGTAATTCTGGTGTGATAGCAATATGTTAGTTAATCTAGATCAGTACATGAATTTTCTCGAGTTAGCTAGAAAAGAGTAGCAGATTGTCTGGTCAGGTTCTGTTTTGATTCTTGTATCCTTCTTGGTACTGTTTCAATAATACTTTATTCCTTATCACCC

>LNC_000126

GTCCTGAGATGTTGGCATAACTACTCCATATCTTCAGGGTTTGGGTTACTCTCCTTGGCATCACCCACTTCAGTCCTTTTTTATTGATGAAGAGGTCCCACAGTTGGTTGGTTATCCTACAGTGAAGAAACAGGTGAGTCACCGTCTCAGCCTTTTCCCCACATAGGAAACATCTAGGGCTCAAATGGATACCCCTCTTCATGAGATTTTCCTGAGTCAAAACAGCCTGTTTAGCAAGTAGCCAAGTAAAACAAGACACTTTGTAGGGAATTTTGGCTTTCCAAATCATTTTCCAAGGCCACCCATTAATCTGGGTATTGAAAGGGTTGAACTTTTTGTAAGCCCCCTTGACACTGAATCTTCCTTGCTTGTGATCCTTCCATTCCAGGCAGTCTTGGACAGTGTTTCCTCCTCTGAATTGTTCCAGAGTTCTGTAGAATTCTACTACTCTCTGGATCTCCCAATCATTGAAGGGTCTTCTAAAGCTCAGATTCCATCCTTGGTTTGACC

>LNC_000127

CTATGCTCCTCTATTCTCAATTTCCCTCATCCAACAGTAACACACACACAACTCCAGCAACTTTCACCCAGAAAAGAACGAACCCATTCCCAAATTTTTTGCCTCTTCGATTCCCCATTTTCTCCTTCAATTCGTACGTTCAAGCGTGCTTAGCACGAGAACAACCACATTATCCTCTCAATTTCCCTTTTCTTTTGCCAACTTTCGTTCAAAAAATGGTACGAATTCGTACACTACCTAGTTGGCAATCCTTATCCGCTCAAGGATTTTTGAAACTTCTCAAGTCCCACATCGAATTCCATCTTCAAATTCAACTCGTTATTTGTTCTATATAAACCCATTTCCTCTTACTGTTCGAGGGGGGTTTTTTGGGAGGCAAAGAAAAGAGCACCCCGCTAAGTTTTTGGGAGTTC

>LNC_000128

NAGTCTGAAAACCTCACACCCACTACTCCCTTGGACTTAATAGAGGTATATATAGTTCAAGCAAAAGGCAGTGCTGTGCCTCACTAGCTGCAGAAAGAGTGGGTGCTGCAGAAGAGGTGGATTTCTTGTGGCAGGGGCGTGTTTCAGATAATGTGGTAGTCTCCTTTTGTCCCTGGCCCCTCTCTGTTCTCTCTGGGAACTTGTGTGAGGAAAAAGTATGGACATGGTTAAACATGGAGAAGTATGGAATGGGAAATACTTGTACAGGGTTGCACCAGAAAAGTGAAGTAGGTGAAGCTTATGCAATTATCTTTGACTTAGTGATCATACACGACTAGCCAATTAGTCTTTATTCTGTTTCTTCATGTTAATTGATATGGATTTTAGTAGTAGGTGATTACTGTTGGAAAGACAATTTTGCCTATTTTCTCAGATCTGTTGTTAGTCTATCTTTAGTAATATGACCCAATGGGATGCCATTTATTCAGAAAATAAAAN

>LNC_000129

NACTTTCCATCTTAAGAATCCAAAGAAAATGTGTTCACTTGTAGTATCCCGTCTACTTTATTTCCCCTCTCCTATTTTTGGTTAGCTTTATGTACCCCTGAGAAACACTGATTGAACATTTTACCTTTACAAATTGCTCTACTGATGGATTTAATTTGCCATCACGTGTTTTCTGTGCATTTAACGCTATTGCTGGTCTAAATTATTGCCCCAAACTCATCATCTTAATAGTTGCTTCTATTCCCTCATGAAACCACCACTCATGAAACCTCTTTTTATTGTTGTACGTGAAAACCATATTATAACAAGCTTTCAAGCTAGGAACTACATGCAATGTTTTAACGCATTCTCCTCTTTAGATTTTATTTGATGAAAATGGAGATAAACTGCACCTTTCACACTAGCAACAGCTGAACATTATCCTTTTTTCCCCCCAGAATGACCTA

>LNC_000130

NGATCCTAATTGTCGCAAATTTTTGCTCGATAGTTTATGTTTCATCATATAAGCAAAGTACTTGTACATTCAGTAAGATATTTTGGTTGAAGAATACTGTTGACTACTAATGACCTATGTCAGAGTAGAGAGAAAATGTAATAGCACATTTGATGATATGTAGTAATTTATGGATGATCTTTATGTACTTTGTTATAATATATAAAATTCCCGAATCAAGCACATATTGTACAGAACTATCCGTTATTATTACTTCAGTATGAATCACTATCTTGATGCAAGATATTGAAACATGATTAATATTGTTCGAAGGCATACATTATGAGGTTATCTTTTT

>LNC_000131

NATTACACCTCTAAGATTAACGACTAAGCAAATACATCATGGTTGTTCGGACTTGGCCTGTTCAACTATCTTTGTACAATGTTGAACATCTAAAGTAAACAACAGCAGAGACGGACTGTGATTCTTCAACTTCATTAACTGCGAGCAACCTTGTCATACAGATCACGAGGCCAATTCTGATTCAAATTGCTTGCGAGGAAGTTTGCGATCTGCAGAACCAAATTCACATCAGTTATCACAAGATGTTATTGTTAATTAAAAGAAGTTTGACTTAATATATGATGAGAATCATTGTCCAATATATGTTCTCAGAGATGTACTAGACAACATCTTCACTAGGCTGCTGTTTTTATCTGATTTAGTTAAAGAGATTTAGAAGCAAAAGCAGATCTGTGAACACCTGGTTCATGATAGGTCTCACATGTAATCGAAAAACTAACATTACATCTAAATAAGAGGA

>LNC_000132

AAAAAGTGAGAAATATTTTTTTAAAAGTAAGACTATCAATTTGAAATGAAAAGTATTGCTACATTATAGGTTTTATACTTGCTTAATTTTACTTTAACAATAGAGTATCAATTATCAATAAATAGTGGAAGTGTAATCATTTTTATATATTTTTTCTAGTATCTTATAAGGTGGACCCCATGACAATTTGCATTGCTGGTTTTCAGTCATCGATTTTGATGGTTCTTTTTTTTTATCTTCCAAGACAATTAATTAGTTTTAAAAAGGAAAAAAATGAAAAGAATCAAAATAGGAAACATAAAAAAAAAAAAACTTTCTTTCAGGTGGTTCAATCACACTGATATAGAAAAGAGAAAAAATCTTCCTTTTTTCTACTTTATCNN

>LNC_000133

CGAAAACTCAAATCGAAATGATCAGGAAGAAGAGAAACGCAATGCAGTAGTACTTGAAGAATGATATAACTGACCTTCTAAAATCTGGATTGGATGTCAACACCTACAATAGGGTAATTTTCTTTTGTTGGAATTGATTCATCTATGTATTCCGTTATTCTATCACTTAGTTTTATTTTATATGACCTCTTTCTTTATACTGATTTGTTGCCCCTCAAACTCTTACTTGTTTTCCTATGCTTTGGTGGTTAATTCCTTTAGTTTTTTGTATGTGCCTGTTAGTTCTCCTAAAAAATATTGTGCTTATATTTTTTTTCGTTGGACACAATTTGAAATACTCCATAATGACTATCTGGTTGTGGTATTACTTTTCAATAGCTAGACAATTAACTATCCTTCTAACGTGCACATCATATCTCTTCTTCTTGATAAAAATATTAGGTCCCCCAAA

>LNC_000134

TCAAAATATACCCTAGGTAAGTGCAAGACCTCTTCATCTCCTTTCTTCTCCCCAAAAGAAGTCACTGCCTCAAGAACATTTAGCTAAGTCTTGCTTTCTCATCATGCTTTCAGACAACTCCTGATGCTGTAACCTCAAAAGCATTCAGCTAAAGCCATCTCGATGTATCTTTGTCAAGCTTAATAGTAGTTCTGTATTGGAACCCCAAAAACTCTTAAAAGAGGTTTTCTTGTCTACAATGTTATAATG

>LNC_000135

TATATGCATGTGCAAATGAAAACACAGATTTAAAAGGCACACACAGTTTAATTTTATACTCTATGATAACTTGAGTCTCCACTTTTTCTGGCTTTCTCTGCAAATCAATAGAGCAAGTTTTCCACGTCTGTTTGTCCCACCTCTACGTACTGTCAGCTTTCTCCCCAATATTTAACTCGTTCCCACCTTCTCCATTTCACTGCACACATGTTTATGATCTTATCTAATAAAGTTGTTTAAATTGTACAACAACTACTATGAAAGCTCTTGATTTATTGGCTCTTTTCTTCCCCTACAGGAAAATAATAAATCTCCTGTTTACCATTGTTCAAAATTCCCAAGAAGACAGAAAAGGCAG

>LNC_000136

GTTAGATTCTCCAAAAGTAGTGTATATTTGAAGAACGTGAAACGGGTGCGGCATCGAAGGGTGGATAAACAACTTCAATTTTAATATGCCTTTTGCTTCAGCAATCCTTGCCAATCTAACATGACTATTTTCTTTGCTTTCAGCTAATTGAGATAGGCAACATTCATTTGCTTAGAGTATCAACTTAGTCAAATAGATAATGTATATGAAATAGCTATCATTATAGGTCTTTTTTCAAGAGTTCGACACATTACGAGGAAAAATTCATTCTCCTCTTTCCCTCTGTTAACTGTTGTATGATTATAGTTTGTTAAGGGGGGAAATATTGCTCAGTCAAGTGCAAATTTACTTATTGACAGCTCACATGAATCTCTTACATTATCTTGACAGCTCACATGAATCTATTCTCTGTTTTTCTTTGTATCATGTAGTATGTGGGGN

>LNC_000137

AGACAGCATATACACCTTTCATTCCCCAAGCTGTTCTTCCTTCTAGGTAATGTCAGCGGCAATTATGGTAATTGTCATTTAGTAAACAGAGTTACTGGCATATGAAGAACTACATAAAAAGAAACTTTTAGTGCTATGATGTGTACTCTACAGTCTCATTTTTATAGACTTAATGAATCATACACTTCATCTGTATTTGTTGCCCCACATTTTTGCTCATACCCTTCATTTATATTATGTCGTGGTAAACAGTTACACAGCCAGTGAGGTAGAAGTTGCTGCAGATAATAGTATGGAACAGCGAGCTCTCCTTGTGGAATCAAGAAGGTGAGTTAGTCTAGTTCTATTATTCAATTTGTTGTTTCTTACTAGTTTTTGCTCGTCTCTAGGTTGTTAAGACTCATTTTTATCAAATTCAGATATGCAT

>LNC_000138

CCAGAGGTTGCCAAATAGGATGAACTACTCCATCTTCCTCTCTAGCTTCCCAAACTTATAGCCTTTCCTAATAGTACCTTACTCATTCTCTCTATATGGGTTATATCTGTGACTCCATAAAAAGTGGAGGCTTAAGGTCCTTATTTGAAAAGAAAAAAGTGGAGGTCTTTCTTTAAACTAGAGGGAAGGATGTTTTGGCAAAGCTAGAGGGAGGCCTAAACCATTCACCCTGCCAGAATCTAATTACATCTGGAGATACCAAATCTGGGTGCTCGTCTGTATGTCCTAATAGTAATCTTCCTTGTCAGAAATCTTTTCAATGGGGGTTTAATCTATAGTTCAGATGTCTACACAGTTTTTTTTTCCTTTTGTAATACTTTGGATAGCTAACCTCTGTTCAAAATGAAGTTCTCATTTAGATGGATAAAA

>LNC_000139

NNTTTATTTATTTATTTATTTATGTGCTATGACCCTCTCTTTAAATTTCGTTGTGCAAAGTTTTGATCGTGGTTGGATTAGTCGGTCTTGTTGTCTCAATTTAGATAGCTCATTATAGTTTATTTAATTATTTAAAGTTTGATCTTTGTTGTATTTTGAGTGGTTTATTTTTAGATGTGCACATGAGGCTATGCAAGGCATATGAGTATGTTAGATCATTTTAACTATAATGAATAGTGCTTCATATTCTGTTTCAACTTGCCAGTTGTTCTCCATAATTATTTGATTTATTAATTTGTCTTCTCTGGCTTAAGGTGTTAAATCTAATTCCCTTTCGATGTGCATATTACTATGTGATTAATTTAATCATCGCTAAGCTACGGTTTTGAATATATTGTTATTGGAGGCTAATTTTAT

>LNC_000140

TGAATTTTGAGTCTTCCAATAAGATACATTCTATAAAGAAGAAGTAACTTGAATGGAGGATTACCAGAAATGCATTTGTGCAGACTTTTAGCTAGAGTCTGAACACTAGTATAACATTCAAATTTTACCTTTTGGTATTACTTAGAAAATAAGGAACATTGTCCTTGTCTAGAGAATATTCGATAGCACCATAAGCTATCAGTTTACATTATTCATTTATTTATTTTTTTGCAGAGAATGTGTGAAACAAATACTAAGAATTGAAAAAAGCTGATGAACCCACACACCGCTGGCAAAAAGAGTTTTGCTTTAGTCTGTACCAAATTGGTGTGTTCTCTTATTTATTATTGATTCTTGACCTGTTATTTAAGCAATTTCTGGTATTTAATTATTTTTGTGTGACTAGGAAAAA

>LNC_000141

ATGTAGGTTTGCAAAATTGCTCTCTGATAAGAAGAAACTCCGAGCAAACAAAGCGTCTTTTTAATTCAAGGTCTCGAGTTGTGTTAGTAACCTTACTATGCTAAATAATAAGCGCTTGCAATATTTATGATTGCGCGCATTTAAGTATTTCAACCTCAAAAATAAAGGAGGAGTTTGAGGGATAAATTTCAATAGAAATGTCTCTCTATGTAATGTATGTTTGGATTATGTAACCTTTTGGTTGTGTTAAATGTTTAAATAAATTATCGTAATT

>LNC_000142

NNTTGTGTGACATTGCTACTCCCACAAAATACTTGAAATTTGTAGTCAAATAATGGTAATTTTGACGAGAGGGAGTTTCTCGGATGGTAAGCACCTCTCACTTCCAACGAGTCACCACGGGAGCAAAAGTGGTGGGAGCTCCTAGAGAAGGTTAAGAAATTAATAATAATAAATAAATAATGGTAATTTTAACCATTTTTTATGGTAATTTTAGTAGTATGCAAATTAACTGAGTCAACTAAATCAGCAGCTTTTGCCTACAATACTCTTTTCTGATATAGGTACAAATATGTGTTTTGCACTAATGGTAAGATGAAGATGGAAACATGATTGGTCGACTACATTGTTTGCAATAGTACAAGTACTGGTATTGCGCACATTGGNN

>LNC_000143

GTCTGAAACAATTCAATAATATTGTTTTTCTTGCTTCTTTCCTTTTCTTCTTCTCATTTTTTTTCAATTGAATAAACAAGCGAGGACTGTAATGAAGTTGCTGGATGGTGAAAATTGGGAGGTGAGAACTGAACTTTTCATTTCTCGAAAATAATTCTGACAATATTTTCTATATCTAAGACACTACCGAACTTATGTGCATTAGTTAAACACACTTGAAGTATTCTTAGAGGGTA

>LNC_000144

AGAAATGACAAGAGCAGTACCAAACGACCCCTGCACATGCTTCTTCTTCACGCAAAAATTCCAGCAAATCCGCGAAGAAATCCAGTCGCCTCATCCCGTCCCCTCGTCCCCTTTTGTCAGAACTCACCATCCTTTCTACTGTTTGCCTTTGAAAAGAGGAACAATTTCAAAAGATTTTGAATCTTTGCTCAACAGTTTCCCCCCTAATCGATTCCATCCTCTTCCTATATATACTCTCCTTCTATTAATGTCTAAAGGGGGGACATAGAGAGAAAAAAC

>LNC_000145

CGCCATAGTTGGTTTACTATGTGAAAATCACATTTTGGTAGTTGTTATTATTGTTGTTTCGGTCTAGTGCAAAATTTGTTTTATCTTCGGAATGACGAGAGCAAAACTTGCACTTTGCTTGAAAATACAAATCTTGGATTGTTGCATTGGACATTTAAAGTGGATTTTCTAGAAAAGAATATATTTGTGAAATAGTATCTTCAGGGGTCATTTTTGTGATGACTGTGATTGGATTTGTGTTAGTACCATCTTCATAGTATTTGTGTGATTTGGGATTATAATTTGGAATCATCCAAATTCTTCAGTTTGGGTTTATATTTCATACAAAAGAATCCN

>LNC_000146

CAATTATCAAGCATTTTAATACAAATTTAAGGAAGGAGGTTTATGTCCATTTTGTTTCTGTTTCAGCTTTCAATCGAGTTTTCAAAATCATCCAAAACTTACCAATATTTTCTCTAGTTTGTTCTGCTGCATTTTATTTCTTTGCCTGCTGTCCAAGTCTATACTTCTGCAGAACAAAAAAAGGGGGACAAAACTGCCTAATGTGTTCTCGATAGGAAAAAAATATCGATGAATAATATTGTACCTTAGAGAACTTAATGATCTTTCTTGTTGTCTTTTATTTATTTAGAAGCATCAAC

>LNC_000147

NNTACAACATGTTTCAATTCCTGCTGCACCTTTGACATTGTCTCCGGATTATTCAGAAGCTCTGTCATTACCCACTCGACCGTTGTGATTGTAGAGTCAGTCCCACCAATAACAATGTCCTACAATGAAGAAACAAATATAAAGACAGGGGAAAGGAACTTAGTCATCACAGGAATTTTGTAATGCTTGATTCTACCTGTAGGAAGACGCGTATCCATTATTTTAAGTTTATAGGTTCTGAACGACAACTTTTTTTGTTTATTGGGTTCTGGATAAATATTTATATATGTTAAGTGAATTTCTTTATAGAAATACATAGTTTGGGTCAAAGCTACTGGATTCTGCGAACTCATAACT

>LNC_000148

TCTACATTAACACTTGACTAACCGTATATGAGCTTCCTCTATTCCTTTTTCCATCATAAAGTTTTATATTTCCATGTTGCTTCTAAATTAAAACTTGTATCGTTTTAATTCCAATCTTTTAACCTTTTAGGTGAACCAGGAAAATAAAAAATGTTGGATGCATAAAAGTTTTATTCCCAATGGTAAGAGTGACAATTTGACAAGGGTGGTCTTTTTTTTTCTTAAAGATTTTTTAAAATGTAGAAAATTATTTTTCTTGTTAATTCTGCTTATTTTCTTATGAGAATTTTCTGATACAACAAGTCTACAATCAGGCAGACGAAAACTCAAA

>LNC_000149

CTTTCTAGAGCTAACTGCATTAACGGAATGCATTGACGGAATTCCAGTCCAAGCACGTTTACCAAGAACGTTGACCAAGGTCAAACTTTGGCAAAACACAAAAGCTAAAACTCCTAATCGTACACACTCCAAACAAAAGCCTCGAAAACCGCACAAAACATCACACTAAACCAAAAAGAACCCTTCCGTAGCTGATGGAACTGACGGAATTTTGATCTGACACCAGAATCATCGGATGTTGACAAAAGTCATCTCTTTCATGCGTCAAGCCTCCAAAAT

>LNC_000150

TGGGCTCATCAAATGGAAGGGGAAGAGTGGTTGTTGAGCAGTGGGGAATTCAGCATTGTTGTTGGTAATCTGGTTATCTCCAAGTCAGCTATCTGACTGAAACTATTTTTGGTTGCATTGCATACTTCGTACCTCCTTTTACCAATCTCGTCTCCATTAAAAGGGGGAGAAAATAAAGAAATAGAACTTGCTTCTTTGTCGCTTCTTTCTATGATAGTGGAAAAGGATAGGGGATTGCCCATAGATAGGGGTATGTTCTTGTGAATCTTATTATTGTGGCGACTTCCATTTTTTTCTTTTTTTTTGAAGAAGGT

>LNC_000151

AAATTATATCGCCTTTATTTTTGTTATGTCGAAGGCTAATCCTTATTATCATTTTTTATTGCATGGGAAATCTGCTCGAGTTCGCCATGAACTCCCTCTTCCTCCATTCGCATCTCATTTCGGCCATGAAGGCCCAAATTTCTTTAGTTTTCGAGTCGGCCGGCCCATTAAAAAATGACGAACAGGTTCCGAAGTTGAAAGAGGCAATAAACCGAGAAATTGAAGTTTGGGCCAAAGGCCCGC

>LNC_000152

CTTCTTCTTGATAAAAATATTAGGTCCCCCAAAATTGAGGGCTTAAGGCATTTGTCTTACTTTTTATAGCATAGAGCTGACCCTGTGTTGCAATTCTCAAGCCTTCATTAGCTCTTGAAGAAGGTTAACAACCTCATATATCGCCTTCCCAGAAATATTTATGTTTAAATACACTTATATAATATACGCGCATGATGTTTTAACCATACATTCTGAAATATAGCGATAGACTCTTCTCATATGAGCTTGACATGTTTAAATATTCTTCTATTTGCTAAAAGGCTTCTTCCATGATTGTGAT

>LNC_000153

TATTTTTTTTGAAACCTTGTTTATTGCTTGTTGTTTCGGTTCATTTCGTGTCTTTCTAGGTCTATCTTAGGTTAGCTTTCCTCTACTCATTCTAGTATTATCTCTTACTAGTTGCTTATGATTGAGTTTATTTTGTTAAGAGTGATTAATCTGAGTCTTTTCCCCTTTTGTTTGATAAATTGTGACTTTAGAGATCAATCTTCATGTGTATTTATTTCTTTAGTCAAATAACATTTGTCTCGTTGGTCTCATTCAGCCTACATGGTAATCCTTTCCATAATACAAGCATGCTCCTGATTAGCGGAACAAGTTTTNN

>LNC_000154

GAAGCTCCCTGATCTTTTTTCCTCTCCTTCTCATATATGTCTCTACTCATTTTTATCTATTTTTGATTTATTACCGATGCCTAGTGACGGACTGTTGGTTTTAGATTAAATGTATCTTAGTTGTTTGAAGCATGAGACACGTTCTTGGGTTTGTTGTTGTTTTGTTTGGGAATGTCGATTCATGATCTTGGAGCTGCTTAATGAATGTTACTGTATTTGCCGTCCTTTAGGTGCAACAAACTCGTTCTTCTTTCGGTTGCGGCCATTTGTCTTAAAATCTN

>LNC_000155

ATATATTGTTGCCTTCATGTATTTGGGCTGTGGGACAACATAAATGACTATTTTCCTCCATAGCCTTTCTTACCAGAATATAGTTGTATGTGACCATAATTTATTTATTTTCACTCTTAATCTTGCTTTGCCTCACTTAGTTGATGTTATATCTTCTTACTTAGTTAAGAAGCTCATTTGATTAATTGGCTTATTTTAATCCTAATTAACTGCTGCAATTATCTCATTTCTTTGGATATAAGCCCTTCGTCTTTTGTTTATAAGTGCTTTTATTTTGCAATTATGTTCGTATCTCCTAN

>LNC_000156

CACTTTCTAGTATCTAAACATTGCTCCAACAACTCTAATTCTTTGTTTTCCTTTTACTCTCTATTAAACATGGCCATAGTTGGAGTCAAGAATTATATTTCTATACATATCATATGTAACTTTGAGATTCTGTTTAGAAAGTTTATAGTGGGAATAACTATCTCATTTGAATATATATGTACTTGTTTCTTAATGCTCGAATTAGTATAAACTCAATGACTACTAATGTAACACTAATGAAAAGAAATGTTTGTTTGATGTGAGTTTACAAGTCTGTGAACTTCATAATTTATTTATTATC

>LNC_000157

CTTGCTAAGTCTCTATCTCTTAATGCATTTTTTCATGGACTGCATTCAAGTCGGCAATCATGTGATTGTTTTCTTGTTAAGTTTTGAAGATGTGGGAATATGGTTTTGCTAATATTTCAATATTGAAAATAGGTCACAAGAGTTGTGTTGTGCTCTAATGTTAAATATACTTTTTTTTAAATGGCATGTATTACTACTGTCATTTGTTTGATTACAAGATAATATTTTCTTGCCTCTTTTTAAATATGTTTCATGGATTTCTTATCAATAAGGCCTGT

>LNC_000158

NGTACACTGACACAAGGCCTCCAACTACCAAATCCGTTGTTTATAGAAGGATCCAAGGAAGGACAACCAAGTAGTATAAGAAGAAATTGCATATTTCTCAAGGGAGTCCTCCAGTAATCATTAAGTGTTATCCTTTTTAGTTAGTTTTCGTATAATTTTCTTGAGTATTTTCCCTCTTTCTAGCAAGTGTAGTTTTAGAAACTTCTTGTAAGGCCTTTTGGATTGCCCAACTTGTTTCTATGTTTGAATTTATGAGTTTGAATCATAATATTAAGCTACAAGAATATG

>LNC_000159

NGTACACTGACACAAGGCCTCCAACTACCAAATCCGTTATTTATAGAAGGATCCAAGGAAGAACAACCTAGTAGTATAAGAAGAAATTACATATTTCTCAAGGGAGTCCTCCAGTAATAATTAAGTGTTTTCCTTTTTAGCTAGTTTTCGTATAATTTTCTTGAGTATTCTTTCTAGCAAGTGTAGTTTTAGAAACTTCTTGTAAGGCCTTTTGGATTGCCCAACTTGTTTCTACGTTTGAATTTAAGAGTTTGAATCATAATATTAAGCTACAAGAATATGT

>LNC_000160

GTTATCCATTTATTAATCTTTAGGATGCTGCTCATTTTATTTTATTTTCCAAGTATGTCTTGTGGGTGTTTTGGGAATCCTTATGGTGGTCTTGGGGCGTGGTTCAGCCTTCTCCTTTGAATATTTGACCTTGTCTTCTATTAGTGGATATTGTTCGGGTCTAGTTCGGTCGAGTTTATGTTCTAAGAATTACGAATATTTTTCTGTTGATTGTTGCTAGATGGCATGAAATCTCGATGTAGATTAACTTGACGTAATAGGCTTCGATCGAANN

>LNC_000161

GTATTTACTTTCCTTCTCTACCTGTTGGTTAAATTTCACTTTCTCTTATTTATGTGCTCTGTGGATGTAAACATCCCATTTCATATACATAATAAATCTTTACAGCAATAACATCAATTTTCCAAAACAGATAGATAAGTATTGCAAAAGAAAATGATCAATATACTATAGCTAAGGACAAAGAGATTTTGCAATTCTTTATTTTTGTCTTTTAAATGAATTGATCTTTAATTTAAGTCAATAGCATAAAAACTTATACCTACATAACATA

>LNC_000162

NNGCTGACGAAGGGGATGAGCGGAAGAGGGAGTCTACTGGACGCGGTTCAACTAGCGGAGACTCTTGGAACAGCTGGAGTAAGAAGTCCCCAAGTGAGCGTCTTATGGGGGGCCGTCAAGCACATACGGCAAGGACCAAGGGAGATCTCGTTGTTGCATAGCTCAGGTCGGAGCAAGGTGCCATCGGACGTTCAACAGGTAGTCTCACGATCGGGCACTCATGCCCCGACATTGTCATTGTATACTCCCGCGGGNN

>LNC_000163

TCAGCTGCGGCGTAATCAAAAAGTTGTTATTACATATACAATGACATCTGCATATACAAAGAAACATCTTACCCATTACTGCTGATGCTATGGTGACTGCGACTGTGAGAGATGGCGACAAGTGCGGGTAGCCGGAGAGGTGGAACAAAGGCTTTTGGTGGGCCTGCAGATAAGGGGACCATATAACAGTGCCAAACTTACCTAAATATTACCATTTACAATTTACGNN

>LNC_000164

GTTCCAATAATGGTAGCATACAGGCAAGTCATTAGACAAAGTCGATCTATGCAACACAAAAAAACCATTACATACATGCAAAGGAATAATTAAAGAAAATCGAAGATGTGATTCGTTGAAGTTCGATCTAACCAAATTTGAACATAATACCCAAATAAAAAGGAATAGAAAACAAATGAAATTACGACCATCACCTGTTACTTTGCCGCTATTGCCTTCCAATGATACTCATAGATTTACCCACAAATGTTAGTGAATTGAAAAATTAAGAGTGTGAAAGTAATTGAGCAGCAACCAAGAAACAGGGGAAAATGAAGGGAAAAAAAAGGAGGTATGAGC

>LNC_000165

NCCCATGTATCTTCAAGCTAAGCAAGTAAAGAAGAAGGCTGGATATATGTGGAACATTTCAAATTTCGAGATTCAAATAAGTTTTATCATCAACTTCATCATTATTTTTGTGGTACTTTATTTACAGTCTTCCTGGAACAGAGAACTAAATCCTAATTCAGAACATTTATATCTGCCTATTATAAACATGTTACTGTATGACGACACTATTAATTATTAAAATGAGAGCTTGCAAAAN

>LNC_000166

TGTAAATGCTTTTCAAATATAAATCAGGAACCGGTAGCACAAGGTGGTTTGCTTCCTTCATTGATGGCCAACAAAGCTGATGAAGTTTATGGGCTCAGTAGTACTAATTCTAATTCAGTTCTGCTCCAAAAGACTCCGACGGTTGGTGCAAGGCTTCCTATAGTAGGAAAAATAAGTACTTGTAATAATCAAGGGGTAAAGGTAGATGCACCTGGATTCAAACTTGATGANN

>LNC_000167

GCCAGATGATCTGCCAATTTATTTCTTCTCTGAGGATATGCTTAATAATCATTGTTCTTCCATGCATTAGTCTCCATATTTCCTCTAAAATTGTAGCAACTCGCCAGGGAACCTTCTAGCTTTTTTCTACAATGTTTTTCATTAGAAGAGAATCGATTTCAATCATCACTCTATCCATCTGTGCTGTCTTTATGAAATTTAAGGCATGTAAAATAGCCATANN

>LNC_000168

NNTTAGATGCGGGGAAGAGGGGAACGAGATGGCTGGCTAAGCTGCTCCACCAGGTCTAATTTCCGGCCGGGAGATGGTTTGTATGGATGGTTCGGGCAGCCGGTTCCGGAACCACGAGCTTCCTGAGAGAGTTTGAATTAGATTCATTGTTTGAAAACGCGGATTTGGTTCACTTTCGGTCGATTGACCGGTGGTTTGGTTGTTCTTAAGGCTAGGG

>LNC_000169

NTGCCGTTTTGTTTCTATGAGATTTTTTCCTTTGTTGTGTGAATTTCAATGTCCGTTTGGATATAAGAACTAAAACCTGAAATTCAAAATAATTGTTGAAAAAATATTTCTACTCTCAAACTGAATTTTTCAATCCAAGTCTTAAAAAAATTGAGGATCACTGATGATTCAAATATTTAAGAACTCTTTAATAAGTTTTGCTCNN

>LNC_000170

GGAATGTGGAACCTAATTCGAAATAGAAGATGAGGGCATTCTATTAATTAAATAACAAGATGAAAGGACTGCATCACCCCAAAAACGCAGCGGAACATGGGATTCAATGAGGAGAGTGCGAGCAGTCTCAATGAGATGCCTATTCTTTCTTTCCGCTACACCATTTTGTTGAGGAGTGTATGGACAAGATGTTTGGTGAATAATTCCTTGGTGAGACATAAACTCCTGAAACTGGGATGATAAGTATTCTAAGGCATTATCACTACGAAAAGTACGAACAGAAACACCAAACTGATTTTTTATTTCAACAAAGAAGCTTTTGAATATAGAAAATAACTCAGAACGATCTTTCATTAAGAAAACCCAAGTACATCTTGAATAATCATCAATGAAACTAACAAAATAACGAAATCCTAAGGTGGAACTGACTCTACTAGGACCCCAAACATATTAGAATGAACTAATGAAAAAATAGACTCCGAACGACCCTCAGTACCACGTGAAAATGTGGCACGAGTGTGTTTCCCAAGTTGACACGACTCATAATCTAATTTGGACAAACTAGATAAACTAGGCACCATCTGTTGCAGTTTAGATAGACTCGGATGTCCCAAACGTTTGTGAATGAGGTCCGGAGGATCTGTAACAGAGCATGCCGTGAAGGAATTTGAAGAGGTAAGATAGTAAAGGCCTTGTGATTCATGTCCTGTGCCAATCGTCTGTCCCATACTGCGGTCCAGCATGAGAAAAAAATCATCAAAAAAGGTTATGCCACAATGTAGGGCATGTGTCAAACGACTAACAGATGCCAGATTAAAAGGGGAACCAGGGACATAAGGGACATAAAGAACAGAGTCTAGAGTGACAGAAGATAAGGGTGTAGCTTTTCCAACCCCTTTTGGTTTTGTTTGTATCCCATTGGCCAAAGTAATAGCAGG

>LNC_000171

CTGAGTATACCCTTTAGCAACAAGGCAAGCCTTAAGCCGGTTGACTTGACCATCTGGACCAATTTTGACCGCATAAACCCAACGACAACCAATAGTAGACTTACCTGTAGGAAGGGAGACAAGCTCCCAAGTACCACTCGAATGTAAAGCAGACATCTCATCAATCATAGCTTGCCTCCATCCAGAATGAGAAAGTGCTTCTCCTGTAGTCTTAGGGATGGAAATCGAGGACAAAGATGATACAAATGCATAATGGGGTGAAGATAAACGATGATAACTCAAGAAAGTATAATGGGGATTAGTATTTCGAGTAGTTCGTATACCTTTATGAAGTGCAATCGATTGGCTAGCAGGAGGCAAGGCCACAGTGGGAGCAGGGTTCGGCACATGACATGAATCATTTGAGACTATTGCTGGACGTGGACGTCGATGATAAGTTAGGAGTGGTGGCACTATAACTGGAGATGGAGAAGATACCGTAGGTCCCTCAATAGTCGGGACAGGCAAGACTTGAGGTATGGGTAAGACCTCAGAGATATCAAGATGATCAGAAGATGTGTAGTAAGGTTGAGATTCAAAGAATGTTACATCGGCAGACATAAAATATCGGCGGAGATCATGTGAATAGCAACGATACCCTTTTTG

>LNC_000172

ATTCAACTTTAGCTTCTGAAGGAGGAATCATGTCACTTGGAATTTCGGAAAGTGAATCCTCTCTTTCAGATCCTGGCCTTGGTAGAACTTGTCTTTGATTGGGATTTCGATTGAAAAGATGTTAGGCTGGTTCCTCATGTGCCTAAGAAGCCGAGGCAATCTCGATTGAAGCCAATTCAATATTTTCCTACTCAATCTTAT

>LNC_000173

CAATTTGTACAACATTTTCCGGTCCAATTCTTTCAATAGTGCTTTCAAACAACTTGTACATTTTGGTGGAATCGGTAGATTCATTGCTAGCACCGACAGAACCAAGAAATACACTACCGATTGGAGAATTCACCAAAATATTGATGATCATTTTACCATTTCGTGCCGTCCATTTGTCCATCATAATGGAACATCCAAACTTTGTCCATTGCACTTTATGCTCATCAACGATTTTTTCCACTTTCTCCACCTCTTTTTTTAGGTGAGTAACTCTAACTTCATGAAATGTAGGAGGCTTCATTCCGGGGCCATGTTGTCCTACTGTCTCAATAAATTTATCAAATGATTTGTGATTGACACAATTAAAAGGGAGCCCCGCATCATACATCCATATCGCGAAAGCACTTACCGCACGCTCTCTTAGAATTTTCTTTGTTTCATCAATTCCTCCTCCTTTTTCAAAGCCTCCTTTTTGTGTTGATTTATGCGGAAAATAGAGATTCATAGGGCCTTTCGTCACATTCCGCGCGGTGGATGATGCACCTC

>LNC_000174

GGTCTGTTCGGGTTTTTATTTAACTTGCGCACGAGTGTACCTTCTGGGCTTATGGGGGCCCAGTTAGGTGTAGTTAGCTTATAGATTACTTTAGTTAGTTCTTTGTACACTCGTGTGCATTCCATTGCTACTTAGATTTTCGCTCTTGACCCTCGGTTTGTATTATTCCTTCTTTTCATATTGCCTTTACTTTTCAAGTTCAGTCGGCCTATGATGCCTACTGGGTACCGGTTGTTTTGGTACTCATGCTACGCTCTGCATCTATTTCGTGATGCAGGTCCGGGCACTAGTAGCCAGCGTTGATCGAGCTTGGAGCAGACTGATCCGGAGACGGGAGTGAGC

>LNC_000175

AAACTTTTTGGTAAATCAGCGTCTAGTGTTGAGGCTTTCATATTTAGTTTGTGTCATTAGGCATTTTAACTTTTAAATTTTAGCCAAAACTTGACTTTGGTCGATATTTTTGGTAAACGTGCTCAAATTAGAACTCTATCAGTGTCATTAGCTTCGAAGGGTCATTTTTGATCTAACAGGAAGGTTGGTTCAAGTTTTGAGGCTTCCGTTTTTTGTTTGTTCCGTTAGGTGTTTTAAATCTTATGTTTTGGCCAAAATTTAACTTTGGTCAATATTTTTGTGAACGTGCTCAAATGAGAATTCCG

>LNC_000176

CCGCTACATGTCTTCAATGGAACTCCCTTACTTGATTTTGTGACTTTTGCTTAATGAAATTTTTGGATTCTCAGTCGGTACAAGTTCGCTCAATAGGGAGAATGAAATGAATCGGTTTAACTTTTTGGGATGCCTTCATTATTTAGCTATCCAGGAACCCCCAACAACTCCCTATGCTATGGTTTTTTGAATGCAGAAGATTAGCTACTAAGAAGCGAACTCCGACGTCCTTTACATATGATAAACAATGACATAGTAATCTAATATGTCAAAATTGAACCATCTGTTATATTATTTGGGTGTATTACATTAGTTAGTATTTGCTGATCGTTCAAATCAACACCATACTGATGTTTGTTGTACATATTCTGAATATTTCTAATAGTATATGATATTAACCATCTTCTGCAGAAGTCGTCACCC

>LNC_000177

GGAGCTTCCGCTACATGTCTTCAATGGAACTCCCTTACTTGATTTTGTGACTTTTGCTTAATGAAATTTTTGGATTCTCAGTCGGTACAAGTTCGCTCAATAGGGAGAATGAAATGAATCGGTTTAACTTTTTGGGATGCCTTCATTATTTAGCTATCCAGGTACCCCAACAACTCCCTATGCTATGGTTTTTTGAATGCAGAAGATTAGCTACTAAGAAGCGAACTCCGACGTCCTTTACATATGATAAACAATGACATAGTAATCTAATATGTCAAAATTGAACCATCTGTTATATTATTTGGGTGTATTACATTAGTTAGTATTTGCTGATCGTTCAAATCAACACCATACTGATGTTTGTTGTACATATTCTGAATATTTCTAATAGTATATGATATTAACCATCTTCTGCAGAAGTCGTCACCC

>LNC_000178

ATTTTCAATTACAGAGCTATGTCTCAATTCCCCGTTTGCATGTGAGTATTTGATTCGTGTTTATATTCTGATTCATTCCTTTTTCGATGATCAAGTAAATATTTGTTTTTCGGAGATTATTGAGTTAGGATCGACAAATTTTCTTTGATTAACAATCTTCACTCTACCTATGAAATTATATTCATATTTATATTTTATGTTTCCTTAGTATAGTCGTCTGATTTTAAATATTTAATTTTCCACTTTCTGCATGTATTTATTCTTGGTGGATTTTATTATACATGTGGTTGTTTATTAAGTTCTTCCTGAAGAATATTCTACTTTGATGCCTTAATTGTTACAACACTTAACTAAAACATAAAGAAGAGAATAACAAGAAGAGAACATTGATGCATTCAGTATGTTTGATGAAATGCCCCAATGAAAGGAGTATAACGCGTTCCAGCAAGTTTATAAACATCTTCTATGCAATCAATCTATAGCATTGGACATTGATCCTAGGAACTCCCTTACTTGATTTTGTGACTTTTGCTTAATGAAATTTTTGGATTCTCAGTCGGTACAAGTTCGCTCAATAGGGAGAATGAAATGAATCGGTTTAACTTTTTGGGATGCCTT

>LNC_000179

TCGGAAGCCTATGCACTAGCTAGATTAGGATAAATTTTTTGAAAATTTTAAGGGATATGGCTGAGATGTTTGACAAATATTCAAAGAGAAAAGATAGATTCCATCACCAGTAATCCTCGGGTTTGCTTTTAACTGCAAGTTACAGAAAGGGATACCGACCAGTTTAGACTCAATGAACACAGAGGCTGATCAAGGTAATGTATCTCAAATTTTCCTCCTCCTAACAGTCCTTATTGAAAAGAAAAAAAAACTAATCTGAAGACTATTTACTGTTGAAATAGAACTTGCGAATTTTGTTTAAAATTTGTGTATACTCTCTCTGATTGTAAAACTTCTTTTGACAATGGTGATGGACTGAATGATCTTATCTCCTTAATTTTTTGCCCTCTATGTATCTCCAGAGTGTTGCATCCATTCTTTTATACGAACTTATTTTGTTATCAGTTAAGCGTGATAATTGAAACATTTAGCTTTAGCCGATAATAGTTTGTTGGTTTTAATTTGAAAATTCCAAGAGAAGTAGGTATTGCCACGATAATAGAGAAATTGATAGTATAGAAGCAACTAGGTTCTTTTGTTTTTTAGTTATAACACACATGAAATTGAAAAAAGATTTGAATGTTGGTTAACATATGAAGTCTCAGAGGACCTGTCCTTTTCAATTGCAATAGCAAGAAGCCAACGACGTCTACAATGGGTTAGAGTCTAGCAATTTTGTGATGATTTTTTTTCTCGTGAAAATCCTCTCTGATTTTTTATTTTTGAAAAAAACAACTTAATGAAATAGTGTAGGAATAGTTATATTGGCTGTATTTTTTTAGATGACAACTATATTGCCATT

>LNC_000180

TTTTCTTTCATCCAAACTTAAATTCAAGGCCTACACATTAACTTCATCTTCATATCTGGGTGTGCCTTCTTAGAAGACAACCAATGAGGATCTTGAGTTGGCCTTGGAAGTTGGAATCAAACAACTCAAGTATGCATGTAAGATATTGGCTTTCAAAACTCCATCTCAAGTACCATGTGCCTGAGGCATACAAAGCGAGGTAAGTTTTTATCTATTTGGTGAAAATTGGCATCTACAAATGTAGACATTTATCACAGTTCATATGCTATTATTATAATAAGTTTCAAGCTTTTTCTTTTTATATGGTTGAACTTATCATGTGTATGTTTGAGGTTTTTAAAAGTTTTATAATTGAATAAACCATTTTATAGCTATTATAAATAATTGATTTTTCAGTTTATCACTACTTACTGATATGTTTGTAGCTAACTTCTTGATTTTCTTTGTGAGATGAGGGAGTTAATTATTGACTTGAGTTGTACCTTCAACACTTTCTAACGGAGTAATTAAATTAAAGTTGAGCAATACTTTCAACAATTACACTGTTAAAACTTACCTATACTACTTCTTCCCTTTTTAAGAACAGAGGAGCTACTTTGTTGGTAGAAGCTGCTGATCATAAAAAATAAATTTGAAACAAAATGGATTACACTACTGTGTGTCAATATGTGATTTTTTGATATACTTTGCACTATTGCTCTATGGCTTTTCAGTTTTTCTTTCTTTTTTTCCCCTTGTTGTTCATTTCAGTATATAAAAGTCTGATATGGCCGACATAACATATTTATATAGGTACACAACTATAGGTAGAGTTAGAAGTGAAGGACAACCAATGACAAGGTTGTAAATATGCTCATTTTCAAGGGTAATGTATAATCGTCCTACTTAGATTTTTTAGCAAAC

>LNC_000181

CTAAGAACAACTCACCACGTTCTTTCCCGCTTGCCATATTTATTGCAAGTGGTAATAATATCGGCTGAAGATTGATGAACCCAAGGTGTTCGATGATATGCCTTGAAGAACTAAATCATTCGCTGATGATTTGTGCAGCTCAGTTAGCTTTAGATCAAGATTGGGATGGTGTAATTATCACTGAGGCAAATTATCAAGCACTTGAGGCGCTTAAACATTAGTTGATCCTAGAGGGTATTTAAGTAGCTGGAATGATAATGGTTATGGATCTTGTTCTGGTACTTGGGTCGGAATAAAATGTGCTCAAGGACAAGTTAAGTTATTCAGCTTCCTTCAAAAGGATTTGGGGGGAAAATCACTGAAAGTATTTGTCAATTTCAAGCACTCAGAAAACTTAGTCTCCATGATAATGAAATTGGTGGTTCAGTCCTTTCATATTTGGGTTTTTTACCCAATCTAAGAGGTGTTCCGTTGTACAATAACAGATTTTTTGGTTCTATTCCTGGTTCACTTGGTTTATGTCCAGTTCTTCAAACACTTGAACTTAGTAATAATTCTTTATCTGGTGCAATCCTTGATAGTCTTGTGAATTAAGCTAAGCTTTATTGGCTTAATCTTAGTTACAATTTATTGTCTGGTTATTTAGTTGCAGAAAACTGACAACATCCAGTTGATTTGAATTTGAATAGAGTCTCACCTAAACTTCATATGGGATGCTGAAATATGCCCCATAAAGAGTAATAGAGACGTAAAGAAGTGTATTCCTTGCATTTTCATCCATCTTTGTTAGGATTTCTCTTTGCCTTCTCCGCTGCTTCATCTTTCTCCCATTCTCTTTGCGATTTCCCTGCTTCTTCCACTTCATTCACTGATTTCAGGTAACAACTCCTTTAGATTTCTTGTGATTTACCCTTAATCTATAATTTCCTTCGGATAGCCTTTCTTCTTTCCTGCTTGTGTTTAATTTTTTCTCATTTATAAGCTTATTCTTGCTACTTCATATGCAAATTAATTGGTGTAGCTTCCATAGCTTTTTCCAGTTTTCC

>LNC_000182

GTTTATACTCTTCTCACCACCACACCGCCACTTCTTCAACTATGTAAACTGCAGCTACCTCATCAGATTCGAAATTGGGATATCTATATTTTAAGGTATTTTTTCCACTCCTCTCATGTCTTTGGATTTCCTCATCTTAATCTTCATAGATTGAATCTCGGATCGGAAAGTTTTGGACAAATTACTGGTTTGTTTTTATTAGGTGAGTTCGCTTTTTGTTGTCTCTATAGATGCATGGACAAATTTGTTGTACTTTTCGTGGATGAAGATTCGAAATTGTTAAATTTGAACTATGATTTAGCTACCTATGCTGGTAATTGCAAAATAATGAACTGCATTAACTACGAAATAGCATTTCCAATTTTTCTAGGAAATCTCTTTAGTTCCACTCGCAAGCAACTCTAGGAAATGAATTGCATTAACTTAGTGTGTATCTGCTGTGGCTACTTGCTTCTAGGATTGTGTTGGTGTAAATGAGAATTTAGTTTTAACGGTTAAGATGTCCCACATCGGTAGAGGAATGGAAATTTGGTCTCCTTATATGGACTTGGACAAACCTCCCCTCATGAGCTAGCTTTTGGGATTGAGTTAGGCCCAAGTGTCATATTTTTACAAGCTTTCTGGTTAAAATTGAACAGTGTGGTTAATACTACTTCTACTCGCAATTTGAGATCTATTCTCACTTGAACGTTAATTGGTGACGCCACAGGTGTCTTTGTTGTTTGACAATAGGCATAATAAGTTGTTTGTGGATATGTTTTCTCAAAGTGACTGATCCTTGTGCAGGGGATATCAAGTCTATTCCATTTATTGAAGCTTTAGGACAATTTTCATGTCGTGTAGGTAACGTTGCAAGTCAAAGTTTAAACTTTGACATCACATATACTGAATTTTCTGTCCTAGGAAATGCAATTGTTAAGTTAAGTGCTTTAATGCAATCAACATTTTTAATCAGGAATTTGAGATCATTCTACCTGTATTTTGGTGTTTTTGTTGTTTTCAGTTCAAATTCACAGTTTAATTTAGACCTTACTGAAATTTCTTGCCAAATACTCAATTACTGAACTTGCTTCTCTCGATCTGTTCAAGCGGCAAATTGCTCGCCCCGTCCTTTTCTCTATGATGTGCAATGGTAAAATTTGAATAGTTGATTCAATTCTTTTGATCTAATGTTTTACATTTATTTTTTCTGTTGATTTGTGTTTTTCCTTTCCCATATATTTTAATAACTTGATTGTTCTTTCCCAGAGTTACCAATGCATACAAAAAACCTGGTTACTTTTGAACACAGTTGCGAAGAAACTGACTAGTCGCTACTACACGGAAAAATATGCAGAAAAATTCAAGCTTGGAACAACTATATGGAAATATGCAGGAAGCTTTTTAAAAGATGTAAACTTCTCTTATCTACCAGGAACCATCAGTTTAAAGAAAAGATGATAAGCTAAAGGAAGGACTGAAACAGGATAGAGCTACAATTCGAAAGTTATTGGTGTTGGAAACCTGCCTATGACCTTGGTGGGATCAATCATAATCCAGACTCATTTTATCACTCTAAAAGGAAGAAGCATCTCAACCTGTCCAAATGGAATGCATACTGATCCAGAAAGGCGACCACTACTAGTTTAAGCTCGGGTTTGGAAAATGCTCTCAGAATTTTCTCTCTCTCTGTCACTCCACAACATCGATTAGATGAAGAGAAGAGGAGAAATCTGGTGATTTATGTTCGCAGAAGAGAAGAGAAGCTAGGTGTTGATTTTCCATGAAAGAAGAGAAGAGAAGCGAAGATTGATTGATTCTCTCTAAGCTCGCAAATTTGCTCTTCCATCGATGAATATTCTCAAAATCGTCACAGTGGTATTGCAGTATCTATGGTGATGAAGTATGCTGACAATCTGTGGAGGTCATTAACCTTGTGAAGATGGTGGAGTAGACTTCACTGACTCGGTAAGGTTGACAGTGGTAGAAGGTCGTAGC

>LNC_000183

AAAAATACAAAATAGTTTATACTCTTCTCACCACCACACCGCCACTTCTTCAACTATGTAAACTGCAGCTACCTCATCAGATTCGAAATTGGGATATCTATATTTTAAGAGTTACCAATGCATACAAAAAACCTGGTTACTTTTGAACACAGTTGCGAAGAAACTGACTAGTCGCTACTACACGGAAAAATATGCAGAAAAATTCAAGCTTGGAACAACTATATGGAAATATGCAGGAAGCTTTTTAAAAGATAGAAAAGATGATAAGCTAAAGGAAGGACTGAAACAGGATAGAGCTACAATTCGAAAGTTATTGGTGTTGGAAACCTGCCTATGACCTTGGTGGGATCAATCATAATCCAGACTCATTTTATCACTCTAAAAGGAAGAAGCATCTCAACCTGTCCAAATGGAATGCATACTGATCCAGAAAGGCGACCACTACTAGTTTAAGCTCGGGTTTGGAAAATGCTCTCAGAATTTTCTCTCTCTCTGTCACTCCACAACATCGATTAGATGAAGAGAAGAGGAGAAATCTGGTGATTTATGTTCGCAGAAGAGAAGAGAAGCTAGGTGTTGATTTTCCATGAAAGAAGAGAAGAGAAGCGAAGATTGATTGATTCTCTCTAAGCTCGCAAATTTGCTCTTCCATCGATGAATATTCTCAAAATCGTCACAGTGGTATTGCAGTATCTATGGTGATGAAGTATGCTGACAATCTGTGGAGGTCATTAACCTTGGCTGAGGTATTTGTACTTATTATTTATAGTGAAGATGGTGGAGTAGACTTCACTGACTCGGTAAGGTTGACAGTGGTAGAAGGTCGTAGC

>LNC_000184

AAAAAATACAAAATAGTTTATACTCTTCTCACCACCACACCGCCACTTCTTCAACTATGTAAACTGCAGCTACCTCATCAGATTCGAAATTGGGATATCTATATTTTAAGAGTTACCAATGCATACAAAAAACCTGGTTACTTTTGAACACAGTTGCGAAGAAACTGACTAGTCGCTACTACACGGAAAAATATGCAGAAAAATTCAAGCTTGGAACAACTATATGGAAATATGCAGGAAGCTTTTTAAAAGATAGAAAAGATGATAAGCTAAAGGAAGGACTGAAACAGGATAGAGCTACAATTCGAAAGTTATTGGTGTTGGAAACCTGCCTATGACCTTGGTGGGATCAATCATAATCCAGACTCATTTTATCACTCTAAAAGGAAGAAGCATCTCAACCTGTCCAAATGGAATGCATACTGATCCAGAAAGGCGACCACTACTAGTTTAAGCTCGGGTTTGGAAAATGCTCTCAGAATTTTCTCTCTCTCTGTCACTCCACAACATCGATTAGATGAAGAGAAGAGGAGAAATCTGGTGATTTATGTTCGCAGAAGAGAAGAGAAGCTAGGTGTTGATTTTCCATGAAAGAAGAGAAGAGAAGCGAAGATTGATTGATTCTCTCTAAGCTCGCAAATTTGCTCTTCCATCGATGAATATTCTCAAAATCGTCACAGTGGTATTGCAGTATCTATGGTGATGAAGTATGCTGACAATCTGTGGAGGTCATTAACCTTGTGAAGATGGTGGAGTAGACTTCACTGACTCGGTAAGGTTGACAGTGGTAGAAGGTCGTAGC

>LNC_000185

AAAAAATACAAAATAGTTTATACTCTTCTCACCACCACACCGCCACTTCTTCAACTATGTAAACTGCAGCTACCTCATCAGATTCGAAATTGGGATATCTATATTTTAAGAGTTACCAATGCATACAAAAAACCTGGTTACTTTTGAACACAGTTGCGAAGAAACTGACTAGTCGCTACTACACGGAAAAATATGCAGAAAAATTCAAGCTTGGAACAACTATATGGAAATATGCAGGAAGCTTTTTAAAAGATGTAAACTTCTCTTATCTACCAGGAACCATCAGTTTAAAGAAAAGATGATAAGCTAAAGGAAGGACTGAAACAGGATAGAGCTACAATTCGAAAGTTATTGGTGTTGGAAACCTGCCTATGACCTTGGTGGGATCAATCATAATCCAGACTCATTTTATCACTCTAAAAGGAAGAAGCATCTCAACCTGTCCAAATGGAATGCATACTGATCCAGAAAGGCGACCACTACTAGTTTAAGCTCGGGTTTGGAAAATGCTCTCAGAATTTTCTCTCTCTCTGTCACTCCACAACATCGATTAGATGAAGAGAAGAGGAGAAATCTGGTGATTTATGTTCGCAGAAGAGAAGAGAAGCTAGGTGTTGATTTTCCATGAAAGAAGAGAAGAGAAGCGAAGATTGATTGATTCTCTCTAAGCTCGCAAATTTGCTCTTCCATCGATGAATATTCTCAAAATCGTCACAGTGGTATTGCAGTATCTATGGTGATGAAGTATGCTGACAATCTGTGGAGGTCATTAACCTTGGCTGAGGTATTTGTACTTATTATTTATAGTGAAGATGGTGGAGTAGACTTCACTGACTCGGTAAGGTTGACAGTGGTAGAAGGTCGTAGC

>LNC_000186

CATGAGCTAGCTTTTGGGGTTGAGTTAGGCCCATGTGTCATATCTCATCCCAATTTGTTGTTCACCAATGATGGGCTCCCCATTTAGAAGTGTTCACGCTCCAGTTGAGGTCTGGGCGTGTGGGGGAGTGTTAAGATGTCCCACATCGGTAGAGGGATGGAAATTTGGTCTCCTTTTATGGACTTGGACAAACCTCCCCTCATGAGCTAGCTTTTGGGATTGAGTTAGGCCCAAGTGTCATATTTTTACATTAACAAGCTTTCTGGTTAAAATTGAACAGTGTGGTTAATACTACTTCTACTCGCAATTTGAGATCTATTCTCACTTGAACGTTAATTGGTGACGCCACAGGTGTCTTTGTTGTTTGACAATAGGCATAATAAGTTGTTTGTGGATATGTTTTCTCAAAGTGACTGATCCTTGTGCAGGGGATATCAAGTCTATTCCATTTATTGAAGCTTTAGGACAATTTTCATGTCGTGTAGTTCAAATTCACAGTTTAATTTAGACCTTACTGAAATTTCTTGCCAAATACTCAATTACTGAACTTGCTTCTCTCGATCTGTTCAAGCGGCAAATTGCTCGCCCCGTCCTTTTCTCTATGATGTGCAATGAGTTACCAATGCATACAAAAAACCTGGTTACTTTTGAACACAGTTGCGAAGAAACTGACTAGTCGCTACTACACGGAAAAATATGCAGAAAAATTCAAGCTTGGAACAACTATATGGAAATATGCAGGAAGCTTTTTAAAAGATGTAAACTTCTCTTATCTACCAGGAACCATCAGTTTAAAGAAAAGATGATAAGCTAAAGGAAGGACTGAAACAGGATAGAGCTACAATTCGAAAGTTATTGGTGTTGGAAACCTGCCTATGACCTTGGTGGGATCAATCATAATCCAGACTCATTTTATCACTCTAAAAGGAAGAAGCATCTCAACCTGTCCAAATGGAATGCATACTGATCCAGAAAGGCGACCACTACTAGTTTAAGCTCGGGTTTGGAAAATGCTCTCAGAATTTTCTCTCTCTCTGTCACTCCACAACATCGATTAGATGAAGAGAAGAGGAGAAATCTGGTGATTTATGTTCGCAGAAGAGAAGAGAAGCTAGGTGTTGATTTTCCATGAAAGAAGAGAAGAGAAGCGAAGATTGATTGATTCTCTCTAAGCTCGCAAATTTGCTCTTCCATCGATGAATATTCTCAAAATCGTCACAGTGGTATTGCAGTATCTATGGTGATGAAGTATGCTGACAATCTGTGGAGGTCATTAACCTTGGTAATCCAAAAAACTAAAATTCTACTAT

>LNC_000187

GTAAACTGCAGCTACCTCATCAGATTCGAAATTGGGATATCTATATTTTAAGGGGATATCAAGTCTATTCCATTTATTGAAGCTTTAGGACAATTTTCATGTCGTGTAGGTAACGTTGCAAGTCAAAGTTTAAACTTTGACATCACATATACTGAATTTTCTGTCCTAGGAAATGCAATTGTTAAGTTAAGTGCTTTAATGCAATCAACATTTTTAATCAGGAATTTGAGATCATTCTACCTGTATTTTGGTGTTTTTGTTGTTTTCAGTTCAAATTCACAGTTTAATTTAGACCTTACTGAAATTTCTTGCCAAATACTCAATTACTGAACTTGCTTCTCTCGATCTGTTCAAGCGGCAAATTGCTCGCCCCGTCCTTTTCTCTATGATGTGCAATGGTAAAATTTGAATAGTTGATTCAATTCTTTTGATCTAATGTTTTACATTTATTTTTTCTGTTGATTTGTGTTTTTCCTTTCCCATATATTTTAATAACTTGATTGTTCTTTCCCAGAGTTACCAATGCATACAAAAAACCTGGTTACTTTTGAACACAGTTGCGAAGAAACTGACTAGTCGCTACTACACGGAAAAATATGCAGAAAAATTCAAGCTTGGAACAACTATATGGAAATATGCAGGAAGCTTTTTAAAAGATAGAAAAGATGATAAGCTAAAGGAAGGACTGAAACAGGATAGAGCTACAATTCGAAAGTTATTGGTGTTGGAAACCTGCCTATGACCTTGGTGGGATCAATCATAATCCAGACTCATTTTATCACTCTAAAAGGAAGAAGCATCTCAACCTGTCCAAATGGAATGCATACTGATCCAGAAAGGCGACCACTACTAGTTTAAGCTCGGGTTTGGAAAATGCTCTCAGAATTTTCTCTCTCTCTGTCACTCCACAACATCGATTAGATGAAGAGAAGAGGAGAAATCTGGTGATTTATGTTCGCAGAAGAGAAGAGAAGCTAGGTGTTGATTTTCCATGAAAGAAGAGAAGAGAAGCGAAGATTGATTGATTCTCTCTAAGCTCGCAAATTTGCTCTTCCATCGATGAATATTCTCAAAATCGTCACAGTGGTATTGCAGTATCTATGGTGATGAAGTATGCTGACAATCTGTGGAGGTCATTAACCTTGGTAATCCAAAAAACTAAAATTCTACTAT

>LNC_000188

GTTTATACTCTTCTCACCACCACACCGCCACTTCTTCAACTATGTAAACTGCAGCTACCTCATCAGATTCGAAATTGGGATATCTATATTTTAAGGTATTTTTTCCACTCCTCTCATGTCTTTGGATTTCCTCATCTTAATCTTCATAGATTGAATCTCGGATCGGAAAGTTTTGGACAAATTACTGGTTTGTTTTTATTAGGTGAGTTCGCTTTTTGTTGTCTCTATAGATGCATGGACAAATTTGTTGTACTTTTCGTGGATGAAGATTCGAAATTGTTAAATTTGAACTATGATTTAGCTACCTATGCTGGTAATTGCAAAATAATGAACTGCATTAACTACGAAATAGCATTTCCAATTTTTCTAGGAAATCTCTTTAGTTCCACTCGCAAGCAACTCTAGGAAATGAATTGCATTAACTTAGTGTGTATCTGCTGTGGCTACTTGCTTCTAGGATTGTGTTGGTGTAAATGAGAATTTAGTTTTAACGGTTAAGATGTCCCACATCGGTAGAGGAATGGAAATTTGGTCTCCTTATATGGACTTGGACAAACCTCCCCTCATGAGCTAGCTTTTGGGATTGAGTTAGGCCCAAGTGTCATATTTTTACAAGCTTTCTGGTTAAAATTGAACAGTGTGGTTAATACTACTTCTACTCGCAATTTGAGATCTATTCTCACTTGAACGTTAATTGGTGACGCCACAGGTGTCTTTGTTGTTTGACAATAGGCATAATAAGTTGTTTGTGGATATGTTTTCTCAAAGTGACTGATCCTTGTGCAGGGGATATCAAGTCTATTCCATTTATTGAAGCTTTAGGACAATTTTCATGTCGTGTAGGTAACGTTGCAAGTCAAAGTTTAAACTTTGACATCACATATACTGAATTTTCTGTCCTAGGAAATGCAATTGTTAAGTTAAGTGCTTTAATGCAATCAACATTTTTAATCAGGAATTTGAGATCATTCTACCTGTATTTTGGTGTTTTTGTTGTTTTCAGTTCAAATTCACAGTTTAATTTAGACCTTACTGAAATTTCTTGCCAAATACTCAATTACTGAACTTGCTTCTCTCGATCTGTTCAAGCGGCAAATTGCTCGCCCCGTCCTTTTCTCTATGATGTGCAATGGTAAAATTTGAATAGTTGATTCAATTCTTTTGATCTAATGTTTTACATTTATTTTTTCTGTTGATTTGTGTTTTTCCTTTCCCATATATTTTAATAACTTGATTGTTCTTTCCCAGAGTTACCAATGCATACAAAAAACCTGGTTACTTTTGAACACAGTTGCGAAGAAACTGACTAGTCGCTACTACACGGAAAAATATGCAGAAAAATTCAAGCTTGGAACAACTATATGGAAATATGCAGGAAGCTTTTTAAAAGATAGAAAAGATGATAAGCTAAAGGAAGGACTGAAACAGGATAGAGCTACAATTCGAAAGTTATTGGTGTTGGAAACCTGCCTATGACCTTGGTGGGATCAATCATAATCCAGACTCATTTTATCACTCTAAAAGGAAGAAGCATCTCAACCTGTCCAAATGGAATGCATACTGATCCAGAAAGGCGACCACTACTAGTTTAAGCTCGGGTTTGGAAAATGCTCTCAGAATTTTCTCTCTCTCTGTCACTCCACAACATCGATTAGATGAAGAGAAGAGGAGAAATCTGGTGATTTATGTTCGCAGAAGAGAAGAGAAGCTAGGTGTTGATTTTCCATGAAAGAAGAGAAGAGAAGCGAAGATTGATTGATTCTCTCTAAGCTCGCAAATTTGCTCTTCCATCGATGAATATTCTCAAAATCGTCACAGTGGTATTGCAGTATCTATGGTGATGAAGTATGCTGACAATCTGTGGAGGTCATTAACCTTGGTAATCCAAAAAACTAAAATTCTACTAT

>LNC_000189

TGAACAAACACCGGCCGGCTTCTTGGCGGTGTGCGTTAGTGAAGCTAATAGTTCGGATGCGCATACGTTTTCTTGCTCCGTTTCCTTGGTACCCGAACCGGAGAACTATCCACAAGACCGCTTCATGCTCTAAACTCGAGAAGAAGCTTTCGAAGGCACGTGTACCATTACTTGTACTTGTTATTCCACTACTGATTTTTGGCAAGATCAATATATAGTGATCAGTTATGATTGCTCAAACTACGGTACATAGTTTCAAGTTAGGCCTTACTTCAGTCTGGTCTCCTCTCCCAGGTCTCAGCTTCAGGCTCCCACCGAAAAACGTTTGAAAACGGCAAGGCAAGCAAATGCACGAACGTATTGTAGGCTTTCTCTCATAAGAATCTTATTTTAATCAGTATACGCCTGATCGGCGAGTTCAGTCACACCTATAGGTCCGCTACTTCAAAGGCATCGCCTCACTTAGATCGCCGGGGTTGGAGCTTCTTCGACCTCTAGCCATGACGCTCCTCCCTTACCTTGGGCTTCAGTAGGTTTCCTATGCTAGTGAATCAGATCTTTGGCTTTACCGTGTTACTATTCGGAGAATGGAGGTGATCTCTCTCTATCTTCAGTCAGTCATTTCTACCGGCTCCGGC

>LNC_000190

GGAAAGTCAACAATAAAGAAAAAAGAAATAAATAGAATATAGAAAATCATTGAATTTGTTTGCTCCGATACAAGAGATCGGCCCCGAAGCAAGATATGGTGGGTGCCAGCAACTTTCACTTGTTAGGAGTAGGGGCTGAAAAGAGCTCTTTGTATAGGTTGGGTTTTCTGGGCTTTGATGCTTACCTTCTTATTATTAAAAGGGGAGGGTTTAATAAAGGAGCTGAAAGCCACTTGACTGTAAGGAGAGGAGGTCAATAAAAAAAAAAGAGATGTTAATTAATCAAAGGTCCAACTGATCACCACGCCTGTATTGTAAATATGCAGTTAGGAATAATCCAGCCAAAGTAATAGGAATTAGGCCTAACACGAT

>LNC_000191

TCCAAAGGAGCCTTCGGGCCAGTTTTTCAGAAAAAGAGTTATCGTTTTCTAAATGAAGCAAGGTTGGAAACTGAGATTTCCAAAAGAGCCTTCGGGCAAGTTTTTGAGTAATTATCTCATATCAGCAGGAATAAATATGTTTTAAAAACATAAGAGCCAGTACATTTTTGGGAGTAGTATCGAGCACCGAATTGGGGGAGCGTTCAAAGAACCCACAGCCCCCATAGAACCATGTTAGCCATCATGGGTAGAAAAGGATCATACTTTTTAGATGAATCCTTTTCAGATTAGACTAGTGGATCCATTAGGCAGTTCAGGTCCTATACCTTTGGCCGGGAATAGGATGCTCTGGCAGCGTGAGGTCGATCGCTGTATCATCACTATAGCTCTTATGTGATGGTTGTCGGTTAGAGAAACTCCCACAGCAGTTATTGTATTTTCATACACAGAAAGTATTGTATTTTATATACATCAGTTCATTTGTATTTCTATATACATCTCAGGCTTATTGTATCTTTGCATACACACAGAGTTTATAGCATGTTTTAAACAGTCTTTCTTTATATCGCACTTGTTTTAAATTGCCTTATATTGAAAGAAGTCAGTCAGGTTGAGTTGAGTTGAGCCAGGTAAGCTATTCAGTTTCCTCCAGATCTCCAG

>LNC_000192

CACGGAGGAGGGAGGGGTGAATTGCTAATCCCTTGTCAATCTTCCCGATGCGTTTTCTGCAATTATGCGTCACATGCTTTGTTGTCTTTGCATTGAGTTGATACAGTTACAGGGGTCTGCCTCTTCCTTCTCGGGGTGCCAAAAGACCCATTCTTACCCATCTATATTTTCACTCTATAAATAGCAAAATTACCCATTGTTTTGCTGAAAGAATTAAGGATATTTTGAGAGAAAAAAGATGTTGGTGATGGGAATAAGCTTGCAATGTTTGATGGACTTGGTTGTAGCTGGATTTTCCCTAATGATTGGGTTGGGAATTTTCGCTTTTATTGCTTCAATTTTATGCTCTGCTGCTTTCTATCAGAATGCAAAGCAACTTTCTTGAAACCCCTTTATGGATTTAGGATAGAAAAAACATAAGAAATAAAAGTTGTCGTCCTTCCACTATCTCCTACATGAATCTGGCACTCTTGCCTGCTTGTAAGCCAGGAACTGCATGGAGAGGATAAACTGAACTGATAAGTAGAGCCCATTTTAACTGTTTTGTTGTTTGTAATAGGTAGAGAAAAGTATGTGCCCTTAGAAAAAAGGCATTTGTTTTGCAATAGAGAAATGAATATGCAGTTTTGAGACAGCCAGCTATTTCCCC

>LNC_000193

CTATATTTCATTCCGACGTTTTTTCTTTCATTCTACGGGTTTCTCATTGTAACTCTCAACTATTCATCAATTTTCATCGCTCATCTAAAGGGAATCTTCAATGAAATCAATTCTTTTCGTTTGTCGAATTCGCAATGGAAAAACCTTACTTTTGGTTTGTCGATTTTTGCAGATATGTTTGAGTAAATATGAGTTCTCTTGCTGAAAACAACAACAATGAAGATTACCAGATGCTTCTGAGCCATTGTAGATTCAAATATTGCTGACCCATTGTTGGCTGAATTTGACAAAATTTACAATTAAGACAAACGTAATTCGAATGCTCAATAATTGGGACTTGCCAACGATTTGGAGCAAGGTCTGAATCGTCTTCTCTCTTACTTTGGTTTTGTTATTGTAGATCCTTACCTCACCATGTATGCATTTATGATAATAGAGGATTTTACTCCCATCAACTTCTAAGGTTTACCAAGATCCACCCTTCAAGATGAGACCTATAAAGTTAACCTGACTATTACCCTTTTGACTTCGCATGATATTGCAACATCAAATATTTCATTTAACATTGAACCTCTTCAATCGGAAGGCCTGAATTCCCTATAGGCCAAGTTAAACTTGTGTGAAATTTCATCTAAAAATAGAGTAGTTTCTCTCTGAAATGTAATTAAAATATTGTAACTAGTTCAAATGAGATTGTAATTGTTGAGACGTTTTAGTATGAGAAGGTCTAATGACTTCTTTTTTCTGAGCTAACAGTCAAGATTAAATAGACATGATAAAGAAGTTAATTGTTCCTTATTGTTTCATATGTCTTTCCTAAAACCTTTTCATACTATCCCCAATTTTCATCTGTTTACTTTAGCTTTCTCTGTTCAATAGTCTTCTCTTTTGTAGGTCTCGCTGTGTTAAGGTTAGTGTTATCTCTTTGCTTGAATTTTTGGTTCTTTTGTTTGTGATGGTGACGATTAGCCCTTTTACCTCTTCTCTGTTTGTGGTGTGTGTCGATTATATTTGGTTTCTAGCAATTTTTGCAGATTTTCTGATTGTGCTTTGGGGTTGCTCTACATGCTCGATATTTTGTCAAATTTTGTTATTAGGGATCCAAATTTTAATTGAGAATATGAGAGAATATACTTTCCTACCGTTAGATAACATTCTAAAAAATAACCTTTATGTATTATAAAAGAGTCGAATTCAATTTGTCATTATCACACATATACCATAGATTAAAAAAGAGAGATGTAAAGACAGGAGAAAAAAAACTCTTCGAGCAACAAGAAAGCTATGAAAATGAACAAAATAGAATGAAGAATATTAGAAGTTTAGAGGTGGCTAAAAAGTTAAACAGAAAAAGTGATCTGCTACGTGCTTGTTAGGTTAAAGAATGAAAAACATGTATAGTAAATAGATGAAAAAGTCTTGAATGTCGTCTACTCAATTTGTATAGGCAAAGAATTGACATGTTGAGATGAGCCAGAATGCTCCGCCAAAGAAATATCGAAAATATATATAAGTTAAGCTAATGCAGGGGCATATCTACCCGAGAGAAAGGTTGTCGAGTGACATCCCTTCGTCAAAAAATTACATTGTATATATAGGTAAAAATTTAATATAATGACTATATATAAAAGTGTTGACATATCTTGACACAAGTTGAAGGTTTAGTGTAGTGGTTAAGGGGTTGCAAATCTTGTTGAAGGTTGTGTGTTCTATTTTTGATAGCAATGTTTACTTTTTTACTTTATATTTCGACACCTCTTGATAAAAAATACTTTATATTTTGACACCCTTGATAAAAAATTTGGCTCCGCCACTGTGCTAATGTGACATTAGATATAGATTATTTATATTTCAATTTAGTTGATTAGTACATAATGTATTATACTCACCACACATTGATACCCTGACTCACAAGAAATATTGTTCTTCTACAGGTAGTGCATCTCCAATGCTACATTAGTGTCATAGCACGCTGAGTGATTTTCCTAATCTTTCTAAATACCTTTTTTAATAGTGGGACAAATTTCTTTTGCTCGGTGATTCTACAAACTCTTGCTGCTACTATACCACTGTCACTTGCTGCTCTATAAACAAGATAATGGTGGCTTCTACATTATTATTGGTTCGTCTCTTGAAGGTTGAAGGCACTGGTGGCTATTCTTAAGGTTCAGCCAAGACTATACATTCGAAGCAAGCAAGATCATAACTATATGAGGAACATTTTCAAAGTTGAAGGCACTGGTGGCTATTCTTAAGGTTCAGCCAAGACTATACATTGCCCTATGGAAGAAGAATCCCATAAAGAAATCTCTTTAGCATTTTTACATGGATCCAAGTAAATTTATGGGAAATTGAGGGGATCAAATTTGAAGGGTCGCCACAAGTCAATTTGATCTCGTTGAAGTGGATGATGAAGAGTTTTTGAACTATTGTAAAAGTTTGAGTACATTATATTTAATTTTGTGAAATATATTCATTGCTAGAATATATTGAACTATTGTAAGAGTACTTTTCGAGCTATTGTGTACACATATTTAGAATATTGCTTTATAAGTATGTGTATGTACACAAGACATACTATCTTTTGAAGTTTATTGCAAATATATACATTTTAATTACTTATTTGCTATTTTGATGCTTGAGATTATTTATTGCAAAACAATATTGGTATTTTTTAATTAGATATTGTGGCATATAAATATTTTATAGCCAAAATAATATTACCACACTTAAAAGTGTAGCTATAGGACTGTAAATTGTGGTATGAATTGATATGCTCAAATTACACCTCCATAAAGAACTATAAGCGATCGTTATCAAGTAA

>LNC_000194

CTATATTTCATTCCGACGTTTTTTCTTTCATTCTACGGGTTTCTCATTGTAACTCTCAACTATTCATCAATTTTCATCGCTCATCTAAAGGGAATCTTCAATGAAATCAATTCTTTTCGTTTGTCGAATTCGCAATGGAAAAACCTTACTTTTGGTTTGTCGATTTTTGCAGATATGTTTGAGTAAATATGAGTTCTCTTGCTGAAAACAACAACAATGAAGATTACCAGATGCTTCTGAGCCATTGTAGATTCAAATATTGCTGACCCATTGTTGGCTGAATTTGACAAAATTTACAATTAAGACAAACGTAATTCGAATGCTCAATAATTGGGACTTGCCAACGATTTGGAGCAAGGTAGTGCATCTCCAATGCTACATTAGTGTCATAGCACGCTGAGTGATTTTCCTAATCTTTCTAAATACCTTTTTTAATAGTGGGACAAATTTCTTTTGCTCGGTGATTCTACAAACTCTTGCTGCTACTATACCACTGTCACTTGCTGCTCTATAAACAAGATAATGGTGGCTTCTACATTATTATTGGTTCGTCTCTTGAAGGTTGAAGGCACTGGTGGCTATTCTTAAGGTTCAGCCAAGACTATACATTCGAAGCAAGCAAGATCATAACTATATGAGGAACATTTTCAAAGTTGAAGGCACTGGTGGCTATTCTTAAGGTTCAGCCAAGACTATACATTGCCCTATGGAAGAAGAATCCCATAAAGAAATCTCTTTAGCATTTTTACATGGATCCAAGTAAATTTATGGGAAATTGAGGGGATCAAATTTGAAGGGTCGCCACAAGTCAATTTGATCTCGTTGAAGTGGATGATGAAGAGTTTTTGAACTATTGTAAAAGTTTGAGTACATTATATTTAATTTTGTGAAATATATTCATTGCTAGAATATATTGAACTATTGTAAGAGTACTTTTCGAGCTATTGTGTACACATATTTAGAATATTGCTTTATAAGTATGTGTATGTACACAAGACATACTATCTTTTGAAGTTTATTGCAAATATATACATTTTAATTACTTATTTGCTATTTTGATGCTTGAGATTATTTATTGCAAAACAATATTGGTATTTTTTAATTAGATATTGTGGCATATAAATATTTTATAGCCAAAATAATATTACCACACTTAAAAGTGTAGCTATAGGACTGTAAATTGTGGTATGAATTGATATGCTCAAATTACACCTCCATAAAGAACTATAAGCGATCGTTATCAAGTAA

>LNC_000195

TTTCATTCCGACGTTTTTTCTTTCATTCTACGGGTTTCTCATTGTAACTCTCAACTATTCATCAATTTTCATCGCTCATCTAAAGGGAATCTTCAATGAAATCAATTCTTTTCGTTTGTCGAATTCGCAATGGAAAAACCTTACTTTTGGTTTGTCGATTTTTGCAGATATGTTTGAGTAAATATGAGTTCTCTTGCTGAAAACAACAACAATGAAGATTACCAGGTTGATTAGATCATATTATACTACAATGATCGATGCTCAAAAATATGCTTCTGAGCCATTGTAGATTCAAATATTGCTGACCCATTGTTGGCTGAATTTGACAAAATTTACAATTAAGACAAACGTAATTCGAATGCTCAATAATTGGGACTTGCCAACGATTTGGAGCAAGGTCTGAATCGTCTTCTCTCTTACTTTGGTTTTGTTATTGTAGATCCTTACCTCACCATGTATGCATTTATGATAATAGAGGATTTTACTCCCATCAACTTCTAAGGTTTACCAAGATCCACCCTTCAAGATGAGACCTATAAAGTTAACCTGACTATTACCCTTTTGACTTCGCATGATATTGCAACATCAAATATTTCATTTAACATTGAACCTCTTCAATCGGAAGGCCTGAATTCCCTATAGGCCAAGTTAAACTTGTGTGAAATTTCATCTAAAAATAGAGTAGTTTCTCTCTGAAATGTAATTAAAATATTGTAACTAGTTCAAATGAGATTGTAATTGTTGAGACGTTTTAGTATGAGAAGGTCTAATGACTTCTTTTTTCTGAGCTAACAGTCAAGATTAAATAGACATGATAAAGAAGTTAATTGTTCCTTATTGTTTCATATGTCTTTCCTAAAACCTTTTCATACTATCCCCAATTTTCATCTGTTTACTTTAGCTTTCTCTGTTCAATAGTCTTCTCTTTTGTAGGTCTCGCTGTGTTAAGGTTAGTGTTATCTCTTTGCTTGAATTTTTGGTTCTTTTGTTTGTGATGGTGACGATTAGCCCTTTTACCTCTTCTCTGTTTGTGGTGTGTGTCGATTATATTTGGTTTCTAGCAATTTTTGCAGATTTTCTGATTGTGCTTTGGGGTTGCTCTACATGCTCGATATTTTGTCAAATTTTGTTATTAGGGATCCAAATTTTAATTGAGAATATGAGAGAATATACTTTCCTACCGTTAGATAACATTCTAAAAAATAACCTTTATGTATTATAAAAGAGTCGAATTCAATTTGTCATTATCACACATATACCATAGATTAAAAAAGAGAGATGTAAAGACAGGAGAAAAAAAACTCTTCGAGCAACAAGAAAGCTATGAAAATGAACAAAATAGAATGAAGAATATTAGAAGTTTAGAGGTGGCTAAAAAGTTAAACAGAAAAAGTGATCTGCTACGTGCTTGTTAGGTTAAAGAATGAAAAACATGTATAGTAAATAGATGAAAAAGTCTTGAATGTCGTCTACTCAATTTGTATAGGCAAAGAATTGACATGTTGAGATGAGCCAGAATGCTCCGCCAAAGAAATATCGAAAATATATATAAGTTAAGCTAATGCAGGGGCATATCTACCCGAGAGAAAGGTTGTCGAGTGACATCCCTTCGTCAAAAAATTACATTGTATATATAGGTAAAAATTTAATATAATGACTATATATAAAAGTGTTGACATATCTTGACACAAGTTGAAGGTTTAGTGTAGTGGTTAAGGGGTTGCAAATCTTGTTGAAGGTTGTGTGTTCTATTTTTGATAGCAATGTTTACTTTTTTACTTTATATTTCGACACCTCTTGATAAAAAATACTTTATATTTTGACACCCTTGATAAAAAATTTGGCTCCGCCACTGTGCTAATGTGACATTAGATATAGATTATTTATATTTCAATTTAGTTGATTAGTACATAATGTATTATACTCACCACACATTGATACCCTGACTCACAAGAAATATTGTTCTTCTACAGGTAGTGCATCTCCAATGCTACATTAGTGTCATAGCACGCTGAGTGATTTTCCTAATCTTTCTAAATACCTTTTTTAATAGTGGGACAAATTTCTTTTGCTCGGTGATTCTACAAACTCTTGCTGCTACTATACCACTGTCACTTGCTGCTCTATAAACAAGATAATGGTGGCTTCTACATTATTATTGGTTCGTCTCTTGAAGGTTGAAGGCACTGGTGGCTATTCTTAAGGTTGAAGGCACTGGTGGCTATTCTTAAGGTTCAGCCAAGACTATACATTGCCC

>LNC_000196

TTTCATTCCGACGTTTTTTCTTTCATTCTACGGGTTTCTCATTGTAACTCTCAACTATTCATCAATTTTCATCGCTCATCTAAAGGGAATCTTCAATGAAATCAATTCTTTTCGTTTGTCGAATTCGCAATGGAAAAACCTTACTTTTGGTTTGTCGATTTTTGCAGATATGTTTGAGTAAATATGAGTTCTCTTGCTGAAAACAACAACAATGAAGATTACCAGATGCTTCTGAGCCATTGTAGATTCAAATATTGCTGACCCATTGTTGGCTGAATTTGACAAAATTTGTACCTCGCTTGCCCTGCTTCCCCTATTTTTACGGCATAATATTGATAAGTTTAAGGAAATTAAGACAAACGTAATTCGAATGCTCAATAATTGGGACTTGCCAACGATTTGGAGCAAGGGTAGTGCATCTCCAATGCTACATTAGTGTCATAGCACGCTGAGTGATTTTCCTAATCTTTCTAAATACCTTTTTTAATAGTGGGACAAATTTCTTTTGCTCGGTGATTCTACAAACTCTTGCTGCTACTATACCACTGTCACTTGCTGCTCTATAAACAAGATAATGGTGGCTTCTACATTATTATTGGTTCGTCTCTTGAAGGTTGAAGGCACTGGTGGCTATTCTTAAGGTTCAGCCAAGACTATACATTGCCC

>LNC_000197

TTTCATTCCGACGTTTTTTCTTTCATTCTACGGGTTTCTCATTGTAACTCTCAACTATTCATCAATTTTCATCGCTCATCTAAAGGGAATCTTCAATGAAATCAATTCTTTTCGTTTGTCGAATTCGCAATGGAAAAACCTTACTTTTGGTTTGTCGATTTTTGCAGATATGTTTGAGTAAATATGAGTTCTCTTGCTGAAAACAACAACAATGAAGATTACCAGATGCTTCTGAGCCATTGTAGATTCAAATATTGCTGACCCATTGTTGGCTGAATTTGACAAAATTTACAATTAAGACAAACGTAATTCGAATGCTCAATAATTGGGACTTGCCAACGATTTGGAGCAAGGTCTGAATCGTCTTCTCTCTTACTTTGGTTTTGTTATTGTAGATCCTTACCTCACCATGTATGCATTTATGATAATAGAGGATTTTACTCCCATCAACTTCTAAGGTTTACCAAGATCCACCCTTCAAGATGAGACCTATAAAGTTAACCTGACTATTACCCTTTTGACTTCGCATGATATTGCAACATCAAATATTTCATTTAACATTGAACCTCTTCAATCGGAAGGCCTGAATTCCCTATAGGCCAAGTTAAACTTGTGTGAAATTTCATCTAAAAATAGAGTAGTTTCTCTCTGAAATGTAATTAAAATATTGTAACTAGTTCAAATGAGATTGTAATTGTTGAGACGTTTTAGTATGAGAAGGTCTAATGACTTCTTTTTTCTGAGCTAACAGTCAAGATTAAATAGACATGATAAAGAAGTTAATTGTTCCTTATTGTTTCATATGTCTTTCCTAAAACCTTTTCATACTATCCCCAATTTTCATCTGTTTACTTTAGCTTTCTCTGTTCAATAGTCTTCTCTTTTGTAGGTCTCGCTGTGTTAAGGTTAGTGTTATCTCTTTGCTTGAATTTTTGGTTCTTTTGTTTGTGATGGTGACGATTAGCCCTTTTACCTCTTCTCTGTTTGTGGTGTGTGTCGATTATATTTGGTTTCTAGCAATTTTTGCAGATTTTCTGATTGTGCTTTGGGGTTGCTCTACATGCTCGATATTTTGTCAAATTTTGTTATTAGGGATCCAAATTTTAATTGAGAATATGAGAGAATATACTTTCCTACCGTTAGATAACATTCTAAAAAATAACCTTTATGTATTATAAAAGAGTCGAATTCAATTTGTCATTATCACACATATACCATAGATTAAAAAAGAGAGATGTAAAGACAGGAGAAAAAAAACTCTTCGAGCAACAAGAAAGCTATGAAAATGAACAAAATAGAATGAAGAATATTAGAAGTTTAGAGGTGGCTAAAAAGTTAAACAGAAAAAGTGATCTGCTACGTGCTTGTTAGGTTAAAGAATGAAAAACATGTATAGTAAATAGATGAAAAAGTCTTGAATGTCGTCTACTCAATTTGTATAGGCAAAGAATTGACATGTTGAGATGAGCCAGAATGCTCCGCCAAAGAAATATCGAAAATATATATAAGTTAAGCTAATGCAGGGGCATATCTACCCGAGAGAAAGGTTGTCGAGTGACATCCCTTCGTCAAAAAATTACATTGTATATATAGGTAAAAATTTAATATAATGACTATATATAAAAGTGTTGACATATCTTGACACAAGTTGAAGGTTTAGTGTAGTGGTTAAGGGGTTGCAAATCTTGTTGAAGGTTGTGTGTTCTATTTTTGATAGCAATGTTTACTTTTTTACTTTATATTTCGACACCTCTTGATAAAAAATACTTTATATTTTGACACCCTTGATAAAAAATTTGGCTCCGCCACTGTGCTAATGTGACATTAGATATAGATTATTTATATTTCAATTTAGTTGATTAGTACATAATGTATTATACTCACCACACATTGATACCCTGACTCACAAGAAATATTGTTCTTCTACAGGTAGTGCATCTCCAATGCTACATTAGTGTCATAGCACGCTGAGTGATTTTCCTAATCTTTCTAAATACCTTTTTTAATAGTGGGACAAATTTCTTTTGCTCGGTGATTCTACAAACTCTTGCTGCTACTATACCACTGTCACTTGCTGCTCTATAAACAAGATAATGGTGGCTTCTACATTATTATTGGTTCGTCTCTTGAAGGTTGAAGGCACTGGTGGCTATTCTTAAGGTTCAGCCAAGACTATACATTGCCC

>LNC_000198

CTATATTTCATTCCGACGTTTTTTCTTTCATTCTACGGGTTTCTCATTGTAACTCTCAACTATTCATCAATTTTCATCGCTCATCTAAAGGGAATCTTCAATGAAATCAATTCTTTTCGTTTGTCGAATTCGCAATGGAAAAACCTTACTTTTGGTTTGTCGATTTTTGCAGATATGTTTGAGTAAATATGAGTTCTCTTGCTGAAAACAACAACAATGAAGATTACCAGATGCTTCTGAGCCATTGTAGATTCAAATATTGCTGACCCATTGTTGGCTGAATTTGACAAAATTTACAATTAAGACAAACGTAATTCGAATGCTCAATAATTGGGACTTGCCAACGATTTGGAGCAAGGTAGTGCATCTCCAATGCTACATTAGTGTCATAGCACGCTGAGTGATTTTCCTAATCTTTCTAAATACCTTTTTTAATAGTGGGACAAATTTCTTTTGCTCGGTGATTCTACAAACTCTTGCTGCTACTATACCACTGTCACTTGCTGCTCTATAAACAAGATAATGGTGGCTTCTACATTATTATTGGTTCGTCTCTTGAAGGTTGAAGGCACTGGTGGCTATTCTTAAGGTTCAGCCAAGACTATACATTGCCC

>LNC_000199

AAATAAACCTCTCCTCGTCAAAATTAGGCTCTTTAAAACCCTAAATACTTTTCAATACACACTACTCTAACCCAGGTGAACCCTCTAGTTTTAGAAAGGATATTTGCGAAAAGTTTAAATTGAACACGATTTGTTTACTCACGGATTTGTTGAGCATGAAAACTACAAAAGAGAGGTTCAAGATGATAGTCCATGTTTATTCTTCACCAGTTACAAGGCAAATGGTCCAAGTTGAGTGAAGTTGATTAATGTTACACGGTGAATAGTCCAACCAGCCTCTTACACCAGAGTATGATATGTTCGTGCAGACAAGTTAATGCAAAATATGTTCAATTTTCTCCTTAGTGCCACAAAAGTGTGTATTTCCTAGACTGCCAGTTGATCTATTTTTGGATTCCAACTGATATATTAGTTGACTCAGTTTGAAATGTACTATTAGTTTGACTAGTACAACCAATATATTAGATATATAGTTGTAGAATGTGGAGTTTAAATTTTATGAATATATTAGAGGTAAGGTATGTCCCCCTCGTTGAATTTTGA

>LNC_000200

GCAGCTCCGAACCAGTGAACAAGGCGAACTCCGACGGTGCTTTTTGAAAGTTTCAAGCCAGTCAAAAGACGGATCTTTAACAGATCCGTCCGGGTGTTGAACTTAGAGTTTAAGATGTCGAAGAGTCTCCTAGAAGAGAATTCCAGACGGAGCTATTTTACTTATTTTAATGTTGTATATTTTATTCATTTGTTCTTTATTTTGGCTTGTAATATTGTGTATAACTCTAACACTATCTCTATATGATATAACTTGGGGGATCGTCTAAGGGGAGGGGATTTACGTGCTACTTGTTTATATTTTATTATTATTCAAATGTGTTAGAACATATGCTTGCAGGATTATAATTGTTTGATTATTCGTGTTTAACTTTCTTCATTTAAAATCCGGCTTAAGGTAAAATAAAAATAAAAATAAAAATATAAATTACATACCCTTAATCACCTTTTGGCTAAATACTCTAAAATGAATCACGTGTTTAATCGGTTTATTGTCTTTGTAGTTTATTTATTTGCCCCGTATGACCACTTTTAATTATGACAAGATAAAATTGTGTATATGTTTACTACTGTATTTTGTTATAAATCACCAGCTCTCCTTTCACTAAAAAATAGGGAAAGTGATTAAATAATTGCCTAGTTACTAATATGCTTTGTCTTAAAAATTTACTTTAAACATCTTGCCTATAGGATTTCACTTCAAACATATTACTTGATGTAGTAATTGCAAGTTGTATATTATCTAGACATTACGGCTATAGTTTTAAAATCATGTTGATAAAAGAGTGATTTTAATCTGTTAAGTTCCTTGTTAATTCACTCTTTAATATGGATAATTGCTAACTTTTGCATGTCTTAATCAGATGCCAACAATTATAATATCTCTTCAATTAATTAATCCTAGTAACTGTTTTGCTAATTATTACAATTCAGCGGTAAATAATTTTAGCTAACACTTAGAAATTATGCATATAGGACATAATTAATAAGTTGAGATGTCAATCAGTATATTATTAAAGAAAAGGAGTAAAATTATGTGTTAAAATTATAATCTGCTCCCTAGATCGAATCTGGCTCAAGTCAATTAGGTTCCAATCTGTTGTGCTATTGAAAATGCTAATAACTCATTCTGATCTGCTACTATTCTAAAATATTTCTTCTGCTACTTTAAACACTCGCTTGCTCAAATTACATACTGTTAATAAAAAAATATTTTTGCATTTTTGAATAACTCTGGCCACTCAAACTCAATCTAAAATTTGCTTCCTATTAATTATTTACCACCTTTTTTAAAATCAGTAATAATAATTATATGCATGTTGTTTAAACTGTTTGTTTAATATTGCTTATTCCATTCACTTCTCCTGTTCTAATGAGTGTGGTCTGTTTGTATGTCACACACTTAGCATGAAACCCTTTCACTATTATATGTGTTTGCTAGCATGTTAATTAAAATCTAGAGGCCAATATGAGACCCTACGTGCTAAATGCTCGTCTAAATTGTTTGACTTGGTGTCTAGGTGGGCCTATTATATTTTATTTTGTCTAAATTCACCCTAATAGCAGTCCAATGCCTCTTGGACCTTACGTGGGGACGCTAGACACCTGTTAGAGACGAAAAGCATGCGGTGGTGGGGTAGGGGTAGTTTAGGCCCTATGGAGATGATGACATCGACTCGGTCTAAATGACAAACCTTGGTTTTGTTTATCCCTACAAGAAACCGATCGAAAATAAAGAAAGTCGAGTGCTGA

>LNC_000201

CTCTCTCCGGCCGCCGCCTCCCCCTCTCTCTCTTCTCCTCCGACAGCCGCAACCGGCGGCAGCTCTGACCAGCGAAATCAACCGGGGAGAGATCTATCCTCTCCTCTCCGCCTCTTTTCCTCCTCTCTCCCTTCGTCCTCCTCTCCCCTTCTCCCTTCGCTCTTCCTCCTCTTCCGTCCAGCAGCCACCGGCGAGCAGGACCAGCATCAGCAGCCAGCGAGGACATCCACCAGTGACCAGCAAGCACTGGCGAGATCCAGCAGCCAGTCGACTGCCTCTTCTCCCTTCTCTTATTTTCGGACAGCAGCTCCGAACCAGTGAACAAGGCGAACTCCGGCGGTGCTTTTTGAAAGTTTCAAGCCAGTCAAAAGACAGATCTTTAACAGATCCGTCCGGGTGTTGAACTTAGAGTTTAAGATGTCGAAGAGTCTCCTAGAAGAGAATTCCAGACGGAGCTATTTTACTTATTTTAATGTTGTATATTTTATTCATTTGTTCTTTATTTTGGCTTGTAATATTGTGTATAACTCTAACACTATCTCTATATGATATAACTTGGGGGATCGTCTAAGGGGAGGGGATTTACGTGCTACTTGTTTATATTTTATTATTATTCAAATGTGTTAGAACATATGCTTGCAGGATTATAATTGTTTGATTATTCGTGTTTAACTTTCTTCATTTAAAATCCGGCTTAAGGTAAAATAAAAATAAAAATAAAAATATAAATTACATACCCTTAATCACCTTTTGGCTAAATACTCTAAAATGAATCACGTGTTTAATCGGTTTATTGTCTTTGTAGTTTATTTATTTGCCCCGTATGACCACTTTTAATTATGACAAGATAAAATTGTGTATATGTTTACTACTGTATTTTGTTATAAATCACCAGCTCTCCTTTCACTAAAAAATAGGGAAAGTGATTAAATAATTGCCTAGTTACTAATATGCTTTGTCTTAAAAATTTACTTTAAACATCTTGCCTATAGGATTTCACTTCAAACATATTACTTGATGTAGTAATTGCAAGTTGTATATTATCTAGACATTACGGCTATAGTTTTAAAATCATGTTGATAAAAGAGTGATTTTAATCTGTTAAGTTCCTTGTTAATTCACTCTTTAATATGGATAATTGCTAACTTTTGCATGTCTTAATCAGATGCCAACAATTATAATATCTCTTCAATTAATTAATCCTAGTAACTGTTTTGCTAATTATTACAATTCAGCGGTAAATAATTTTAGCTAACACTTAGAAATTATGCATATAGGACATAATTAATAAGTTGAGATGTCAATCAGTATATTATTAAAGAAAAGGAGTAAAATTATGTGTTAAAATTATAATCTGCTCCCTAGATCGAATCTGGCTCAAGTCAATTAGGTTCCAATCTGTTGTGCTATTGAAAATGCTAATAACTCATTCTGATCTGCTACTATTCTAAAATATTTCTTCTGCTACTTTAAACACTCGCTTGCTCAAATTACATACTGTTAATAAAAAAATATTTTTGCATTTTTGAATAACTCTGGCCACTCAAACTCAATCTAAAATTTGCTTCCTATTAATTATTTACCACCTTTTTTAAAATCAGTAATAATAATTATATGCATGTTGTTTAAACTGTTTGTTTAATATTGCTTATTCCATTCACTTCTCCTGTTCTAATGAGTGTGGTCTGTTTGTATGTCACACACTTAGCATGAAACCCTTTCACTATTATATGTGTTTGCTAGCATGTTAATTAAAATCTAGAGGCCAATATGAGACCCTACGTGCTAAATGCTCGTCTAAATTGTTTGACTTGGTGTCTAGGTGGGCCTATTATATTTTATTTTGTCTAAATTCACCCTAATAGCAGTCCAATGCCTCTTGGACCTTACGTGGGGACGCTAGACACCTGTTAGAGACGAAAAGCATGCGGTGGTGGGGTAGGGGTAGTTTAGGCCCTATGGAGATGATGACATCGACTCGGTCTAAATGACAAACCTTGGTTTTGTTTATCCCTACAAGAAACCGATCGAAAATAAAGAAAGTCGAGTGCTGA

>LNC_000202

TAAAGTTTGACTTTAGGCTCCCAAACTTATTGAGACCGTCCTGATCCTTTGTTGAACTCCTTAATAACTAGTTCGATTATATATCATCTTTTATGTTTTAGATGCCAACTTTATGTTAAAAACTTAAAATGGTATCCAATTTGAGAATTGGGTGAATATTTTATTTAATTTCATCTATAATACTAGTTTCTCGCTAAAAACAGTGTTTGCATGTGAAAAGATTAGTAAATTCTGCTTGTGCAACTTTAGGTAAAGAAAATAATTGTTCATGCAATAAGCCAAATCATTGAAAAGTTATGCCTATTTTTTTTTGTTTTAACATCAAAGTAACATATTTCATTGAAAAAATGAACTTATATAACCATTTTATCTCCTGTCTCATAAAGTTTTCCTTTGCCCTGCATTAATTTTTTGTGTTCTTTTTCTGGATATGCCTAAAGAATAATTTCATTTATACTATTGTCAGCCACAAAACTTTTATCATCTGGTGTTTTCATTCTAGGCATGCTTTTATCTTTTGTATCGTTGCAAGATAATGCATTTGGGGGGAAAATTTGGAAACTCTTCTAGATGTATTTCAAAAGATAAATGCCTATTCACGCTCCTTCTTAGTCAGTCCCTAGAACAATGACATCTTTCAATATTTAGAAACTATTTAATTTAAAAAATTGCATTTTACTCATTACCATACAAATATTTAGGGTGGACATGGTATGGTAGATACCGAAATCGGAATTTTTAACACTGCGGTTTGGATATTATAGTTTTTGAAATGCATTTTTAAATAGTTTGGTATTCGC

>LNC_000203

GCACCACAACTGTGAGTAGGAGGCTATGAGACGTTTTAGGAAATGTTTCCCTTCTCCTTGTTCTAAGTCGTGCTATAGAGTTGAATTAAGTTCTTCCCTAACGGTTGCTCTTTGCTATGCTAGATCATGCCTAATACAAGAGGAAGACGAGCCCGCGCACCCGAGGAACAACCGACCAATGGGGATCTTCGGGATGCCCTTAATTTATTTGCCCAAGCTATGGCTAATCAAGCCAACCATG

>LNC_000204

GTTCGTGGGTTGAGATTGCAGCTTCGGCTTGAGACCCAGTCCTTAGTTTCTGCGGGTCGATCTTTTTTAGACGTAGTTGACCATGCTAGTACTATGGAGCATCTTCGTCGCGAGGCCCAAGGGGGCAGCGAGAAGAGGGCTAGGCATGAGGGCAGCTACAGTGACTCCCGCCCCAGGTCTAGAGATTATCATGACAGGGCCGGACGACGGTTTCGACAG

>LNC_000205

CAACCATGTGAGAGGTTTATATCTCACCACCTAGGATGCTTGGGGTGTGACCAACATCAACCATGTGAGAGGTTTATATCTCACCACCTAGGATGCTTGGGGTGTGACCAACATCAACCATGTGAGGGGTTTATACCTTGCCACCAGGGTGTCCGGGTGTGAACGGCATCCCCCATCCTAAGTTGGGGTTATAGATTGGTTGGATCATGCATACACATATGATATCTACTATTAGTATAGATTTACTTAAACATGTTTTGACTTCACTTTGATCATGTATCCTCATATTGTTATTCATAATGCCTATGAAACTATTTCTTGTATTTCGATTGTGTCCTTGTTTATTGTTGTGTTGCACCCCGCATACTTAGTACATTCCAAGTACTAACGCATATTTTGCCTACATGATGTCACCATGTAGGGACCGGAGCTACTCTTGATCCTTCTCTTCGTTCACGTGGCTAGTACGGTGCTTATCCGAGTTGTTG

>LNC_000206

TGTTGCTAACTAGAAGAAGAAATGATATAACGAGTTTGATAAGGTCAATTGAAAAGGGCCAGCGTCTAACAGATGTAATAGAAGAAAAACATCTACTAGAACCACGAAATCCAGAGGTAGGAGTCCATATACAAGATGACTCAACTAAACATGTACCACAAAATCCAGAGGTAGAAGAACAAGTTCAATTGAATTCTACTACTATTGAAGTAAATGAACAATGTGAAGAACAAGGCAAGA

>LNC_000207

GTGGGTTGATTGTGGAAACCTTCCTGTCCACAAGTCCAACCATTTATCCTCTATTTTACTTCAAGTTTATGGGTCCTTATGATCATTTATGAACTCTTGAATTATGAAATTATTACTACACATCCTATTATGGTCTATTTTTGTATATTATCATGAAATGGAATGATTATATGATGTTTATGGTTTTCATGCCTCAATGATCAAATTATGAAGGTTGTTATATATGTATATATATATGTATGTTGTTGGTGGGCTGATTTATATGTATTTCGGAATTGGTTGGACTTAGTACTCTTGAAAGACATGATTTTATCTACATGAACAATAGCCCACCATGTGTTTGATAAAATGCCATTTATAGAATTCTTATGAAATGGAAGGTCCAATGTACGTCCTATGAGTCGGATGATTATATAAGTATTGAAATGATGATGAAATGGATACATGAATATGATTTTCTCTATATAGTGTGTGAGTCGATCAAGCCTAGCTCGGGGGGTTGTAGGCCCGGGTAACCACATTGAGGGGTTTGTACCTCTTTCGCCTCTCCAAGGGGGTGGTAGGCCTTGGAAAGTATTATATTGATGGTCACTCACCACACATGTACTACGTCCCACTAAATATGATTTTTATGTAAACGTCATTATATATATCATTTCATTAATGTGCTATCTATCGGGTATGATTATGCATTTTGCCTATGATGTCCATCCCTTGTTGCATTGCATTGCATCCCATATACGTAGTACATTCAAACGTACTAATGCATACT

>LNC_000208

TCTGCTACACTTCCAACAGTGTAACGTTCATCAATGGCGTGAATACAACAACGGCAGCGTGAGACACCAAGAACAGTCGCGTCGTCTTCCTCCTTCACCTCGGTAGCAGGTGTCCTCGTACGCTTAGAGCCGTCCACCTCCATTCCGTCCGATTTGTTGAGGTTAGCCACGTTCTGGTCTCTTCGGAATTCTCGCCTCATAGGTGGTGGAATCTACAACAACGCTCTTTCAAGAAAGAAAGGTAACCTCCTATATTCTCGCTTTCATCTCTTTGAATCCTTTGTTACTGTCCAAAAATCGTTTCTGTTACTGTCCCAATTTTGGATTCCCCAAATTCAGAATTAGGGTTAGGGGTTGGATAAAATAGGGAAAAAGATAGAAAAGGGCTAGAAAATACAGCTTAACCGACCTTAGTTTAGCAAATACACTAAAAGAGTTTTTTTCTTGTTCGTTGCTTGTTGGGGTTCATCGGAATTCTGAGTCGCATTTCAGTGGTGAAAGCTCGTCTTTGCTAGCTCGTTTGTTCGGCTGCCCATTATAACATAAACGCCTTTTCTTAGTTTTGCTCTGTTCAGGTCCTAAGTTATTCTATGTTTTCCGTTTAGGCGTTTGTGATTATGTTTGAGCCGTCATGTGGTAGGTTTATTCATATCAATGGTCCTCTGTTCTACTGGATGATTAGTTTATTTTATAAAAAATGTGATATAACTCTTAGTTTTTTTTTTTCGCTTCTCCAAATTTGGGGTTAACTGGTAGTTTGTTTTGCTGCATAAACTTTATGTTCCTCTTTCCGTTGGTTGACATCTTATGCTTGAGTTTATGTCTACTTGAATGGCTAATTTCTCATTTGATTTACTGATCTTACTCTAAGGGTTATGGATATGTTGCTAGTTGTTATTGTCCCAATTTCGGATTCCCCAATTCAGAGTTAGGGGTTGGATAAAATAGGGAAAAAGATAGAAAAGGGCTAGAAAATACAGCTTAACCAACCTTAGTTTAGCAAATACACTAAAAGAGGTTCTTTCTTTTTCTTGTTCGTTGCTTGTTGGGATTCATCGGAATTCTAAATTGCATTTCGGTGGTGAAAGCTCGTTGTTGCTAGCTCGTTTGTTCCACTGCCCATCCTAACATAAACACCTTTCCTTAGTTTTGCTCTGTTCAGGTCCTAAGTTATTCTATGTTTTCCGTTTAGGCGTTTGTGATTGATTATGTTTGAGTCATCATGTGTTAGGTTTATTCATATCAATTGTAGTTTTTTCCTCTGTTCTACTGGATGATTAATTTATTTTATAAAAATGTGATATAGGTCTTAGTTTTTTATTTTTTATTTTTTTTCGGTTCTCCAAATTTGGGGCTTAACTGGTAGTTTTGTTTTGCTGCATAAACTTTATGTTCCTCTTTTTGTTGGTTGACATCTTATGCTTGAGTTTATGTCTACTTGAATGGCTAATTTCTCATTTGATTTACTGATCTTCCTCTAAGGATCATGGATATGTTGCTAGTTGTTATTGTCCCAATTTCGGATTCCCCAATTCAGAGTTAGGGGTTGGATAAAATAGGGAAAAAGATAGAAAATGGCTAGAAAATACAGCTTAACGACCTTAATTTAGCAAATACACTAACAAAGGTTATTTCTTTTTCTTGTTCGTTGCTTGTTGGGGTTCATCGGAATTCTGAATTGCATTTCGGTGGTGAAAGCTCATCGTTGCTAGGTCGTTTGTTCCGCTGCCCATTCTAACATAAACACCTTTCCTTAGTTTTGCCCTGTTCAGGTCCTAAGTTCTTCTATGTTTTCCGTTTAGGCGTTTGTGATTATGTTTGAGTCGTCATGTGTTAAGTTTATTCATATCAATGGTAGTTTTTTCCTCTGTTCTACTGGATGATTAATTTATTTTATAAAAATGTGATATAGCTCTTAGTTTTTTTTTTTCCGCTTCTCCAAATTTGGGGATTAACTGGTAGTTTTGTTTTGCTGCATAAACTTTATGTTCCTCTTTCCGTTGGTTGACATCTTATGATTGAGTTTATGTCTACTTGAATGGATAATTTCGCATTCGATATACTGATCTTCCTCAAGGGTTATTGATATGTTGCTAGTTGTTATTGTCCCAATTTTGGATTCCCCAAATTCAGAGTGTTAGGTTGTGGATAAAATAGGGAAAAAGATAGAAAAGGGCCACAAAAAATACAGTTTACACTCAGAGTGATAACTGACCTTAGTTTAGTATATACACTAAAAGATGTTCTTTCTTTTTCTTGTTCGTTGCTTGTTGGGGTTCATCGGAATTCTGAGTCGCATTTCGGTGGTGAAAGCTCGTTTGTTCTGGTTTGCTGCCCATCATAACATAAATGCCTTTTCTTAGTTTTGCTCTGTTCAGGTCCTAAGTTTCCGTTTAGGCTTTTGTGATTATGTTTGAGTCGTCATGTGTTAGGTTTATTCATATCAATGGTAGTTTTTTCCTCTGTTCTACTGGATGATTAGTTTATTTTATAAAATTGTGATATAACTCTTAGTTTTTTTTTTTCTACTTCTCCAAATTTGGGGCTCAACTGGTAGTTTTGTTTTGCTGCATAAACTTTATGTTCCTCTTTCCGTTGGTTGGCATCTTATGCTTGAGTTTATGTCTAGTTGAATGGCTAATTTCTCATTTGATTTACTGTTCTTCCTCTAAGGGTTATTGATAAGATCTGAGGCTTTAAATTTACCACTGGGGGTCATCTTACATTCTATTTCGTGTTGGATTTCCAAAATATTTTGTTGTCAAATGCTGTTATGGGTTGTGTTTGCTCGTCTTAGCTTGTTCTTTGTTTTGTGGTTTAACATAAAGGGCTTATTTAACTCAAAGTAAACACCACAAGTGGATCAGATTCTTATTTTCTTAAGGTTTCCAGCTTATTGCTTGTTGTTTAAACGTAAGGATTGGGTGTTTTCTTAGTATTTCAGTTCTAAATACTGATAACACATTTTGATGGTTCAATTTCTCCCGATTCTCTTATCTTATATAAGTATGCTTAGTTGTATTCATTGCTTGTCTATTTTGAATCATTCATACCTGGTTGGTTTGAATTCTCGTTGTTGTAATCATAGTTTTCTCCCATCCTGAGTTCCCTTTCTTTTGGTTCAAAATATGCGAGGTCTTTAAATTATCGATGTTGCTATTTGGTTTGGTTTCATGAAAGTTTAGGCTTAAATGATAATTGAAGTAAGCTTTGGAAGAATAATGAATACATCAACTTTGTTACTTTAGATTCATTTACAGTGTTCAATTTAGTGAGTTATTTATTTTTCTCCTTTAAGGATGCCTCATTTTTATAGCATTTCAAGCTCCCATCTTAATCAGTAGCCTATAATTTAATTTCTTGAAGTCCTTCAAATTCTTTTTCTTGCATTGAAGTACTAATTCTCCTTTTTATTTTTGTTTGATGCATGTGAATTTGCCTTGAGTCCACTGGACTCCTCCCATAATCTTAGTAAGTAATCAGGGCTATATGCAAGTCAAAAATGGATCCCTTCCATTTGGCTTAAGGTGTATACAATAAGTCCTCTCTATGCAGCACCCTATAGACCCTGCAATGCACAAATTTGTCCAATACTTCGTGACACTGAAATTCTTTTCCTGAATGCACTGGCTACCCTATAATTGACCATGAAGTTTAAGAATTCTTTTTGTTGTTGTTGATACTTGATAAAGTTGAGTAAAGTTTAAGAAATCAGTTCATTGACAAGTATAGATCTGAAAATGTTAAGTAAGTTATGTTATAGCGTATTGAACGTAAATGATGTATAATTTCTTGTTTCTCTTTAAACATCTCAATTGTCCTTCTGATAATTTGCTTATAAATTTTCCTACTTAACCAGCTTGGTGAAAGAGGTGTACTTCCTACAAAATGCTGAATGTGCAGGGTTAGCGCCATTCGATTGTTGGCTTTGTTTGAGAGGAATAAAAAAAATTGCTCTTTGCATAGAAAAACAACAAGTTTAATTTTATTTGCCTTTTTTTGGTGGTGAAATTAGTTGTCCCACCACTCATTCTAGAAATATATATATGCCTTCCTGCTCCTTTGTTAATCTATAATTCTTGACCCTATAGATTTTCATGATTATGGCATGCATACTACTGCATTTTCATCTCTATATTGTGCAGAGAAGTAAGGGTGGTTCGTAAAATTCTGGCACATCACAGTGTTCTAACTAAATAATTTGAATATCTTGC

>LNC_000209

CCAAACTTTGTCCATTGCACTTTATGCTCATCAACAATTTTTTCCACTTTCTCCACCTCTTTGTTTAGGTGAGTAACTCTAACTTCATGGAATGTAGGAGGCTTCAATCCGGGGCCATGTTGACCTACTGTCTCAAAAAATTTATCAAATGATTTGTGATTGACACAATTAAAAGGGAGCCCCGCATCATACATCCATATTGCAAAAGCACTTACCGCACACTCTCTTAGAATTAAGGACTGAATGTTCAAAACTAAAGGTCGCTCCACCGTAGATAGGAAGGCTAGACTTCCCATACTCACTGTCTTCCGACTCAAGGCGTTCGCTACTACATTCGCCTTTCTCGGATGGTAGAGAATAAAGAGGTCATAGTCCTTTAGAAGCTCAATCCAGCGAAGCTGCCTCGAATTAAGATCCCTTTGGCTCATTATATATTGAAGACTTCTTTT

>LNC_000210

GATAGAGAGTGACGTCCTTCTTAAGGTTAAATCATCGTTTGCTTGTGAATGTTTGATTGGCATTGATTGTGTCGAAGTTTGAGTAGCCGTTCCCAAATAGATGCATGAGGTTGCCTGTTGATGGATTGGTTGACGGCTTTCTTAAAGGCAAAATCATCATCATTTTGGGAATGTTTTGACTTGGTTGGTACTAAGAGCATAGTCATGCATGTGAATCATGAGCATACCTCTCCTTTTTAAAAAGCTTGAGCATGTTATGAATGATTTGTGACAACTCTTATAACTCTTTTTGCGACTTCTGATTCGTTAGCGGTTAAAATGTCAATGCTACTTGTATTTTGAAAAGGAAGTAAGATTTTGAACCATTTTGATTGATTTGCAGGCCATGTGTGATGAGTTATTGAATAATTAATGCCTTGTGTTCGCTTTTGTCACCTAGAACTTCCCCGGCTGGTTTTGAAATGTTGGTCTTGATTGAGTTTTGTTGAAGAAATGATCTTAGGCTTTCTTTGATGTTCTTGAAGTCCACTTGGCCTAAAAATCCCACCTTTGCACTAAATATATCCTTAGTTGTCCCCTTTGAGCTTAATCTTTTTTATTGGCAACCACATTACAAGTCTTATCCTTTTTGAAAAAAGTAACCCACTTTTGAACCCTTCCCTCCTTGAACTTGAGGTTGTGAAAAAAATCGAGGCTAAAAACCTAAGTTGGTGGTGTTATGGGAAATTCATTCAAGTGATGCTTACCACATCCGCTTGATAGAATAAAAAAGAAGATAAAAAAAGAAGAGAGAAATTGAGGGTTGGATGAAAAGTCTTAAAGAAAGAAAATGAATAAATGAGACTTGAATCCTCCAAAAGTTGAAAATGGTTGTACGTGGGGGATTCTAGAGTAGAAATAAGAAAAGAATAATGAAAAATAGTGTGGAATGTGCATGAGTTTGAAAAGTGAAGAAGAGTGGAGAAAGTTGGAAATGATGAGTTGTTGTGTAGTGTTCAAGGAGGGTGTAATCACTTATACCTAAATGATATCCTAACCTTTCCTAAGCCTATATTACAAGCTTACCAAAGTCCTTTGAGATTTCTAACCAAATGGAGTTGAGTCAATAATGATTGAAAGTATGGCAAGCCAATGAATGGTGTGACATTTGTGTACATGAGATGATTCTTTGTGAGTGTGAGTATTGCATCTTTGTTGTCCCATGTCTTGTTTATATGTGTGTGCGGTTTTGGGACACTTCTTTCTTGTAAGGGCATGTAAGCTGAAAAAGTGGTGATTTTGAAAATTCCTAATTGAGTCAAGAGCATGTGAAAGTGTTAATGAGTTGAGTCACCTCTTGAGTTTGAGTTGTTGTCACTTAAATGATTCTTGGTTTTTTATAGAAGTAATGAGAATGAGAAGATTTCCTATGTGGAAAAAGTTTGCATGTGGACTATTTGTTATTACCAACCGAAGCCATTATCTTTGTGCTTAATTGAGAAAGGGAAGTTGATTTGTGGTGTATTTTGAG

>LNC_000211

GGAAGATCACATTTGCATATAGGCATCTATATTTTGTATAAACCTTGGGACTTGTACATGATCTATGTGGTGTATATTTACATGAAATTTTGAGGACTAATGTGATCATGTCACCATGGGTTGTAATATAAGTATGACTCTATGTTTTGTTCTTTTGGGGTGTGTAAGCCCAAGTCTTGTAATATGTTAAATTTACCACTAATTTT

>LNC_000212

CTTGAGTCCTTCCTGACTACTAAATCTTCGGAAGGTCTTCGTGCAAGTCAACTTGCATGTAGGGTTGTGTTCTTTTTTCAGAACAGTGGCTGTTCAGGAAAGCAACCCTTGTATCATCCACGGAGCTAGAAGCTTTGGGGTACTCATCAGATATATGCAACGGAAGCAGCTTCCAGACTGATGATGGAAGCCCTTATTTCCATGCATATGGAATTGAGAAGATCCTATGGACCTGAAGAGTCTGGTTGCCCTCAATGGATCAGATGGGATAAACTTGAACTAGGTAGTGAAAGGACAGAGCTTGGTGGATGGCTGTCACACGGGTAACACTTCTTATTTCATTTAATAGAAAGATCTATTTTTGTCACACTTCTTGTGATTAATCAATTAGTGTGTTCCAACTTACGAGAACTACTCTGCTACTTCCACTTGTGTTTTAAAATGCTAGTTGTGCTTAGATGTAAATGTTCTAGGTGATACAACCACAGAGAAATAACGTACCTTAAAAAATGAGGAACACATGATGCAGGGATCTCATAATTTTTCCCACCAATACAAGCACACACCCTTTTCATCCTAAATTTGATCTACTAGTTTAAGTCTGTCATAGTCTTCAATTATTTCTATGGACTTGAGGATATGCTGCAAATGAGTTAGTCATCTGAGTAGAGTGGACATTACATTGCAACATGAAAATAGTATGTAGTTTTGGTTAGAGTAGTATCTCCCTACAACTTTCAGTGAGCCTAGTCTTCTAGTGGCTCAACTTTGTTTGGAAGACGTAATAAGTTACTGTATAATTTCTGAATTTGTC

>LNC_000213

GAAGCTTTGGGGTCCTCTCCTGATGAATCCATTCATCAAAAGTTCTAGAAGCTTTGTGAACGCCAACTAAATATCACAGGATGGTGATGCTGAATATTCGCTGCTTGTTAACTTGAAAAGGTTGGATGATCTTCAATTGTCAAGGCCAATTTTCATCGAAAGCTTGCATAATGATCTTGCTGTGACTGCCCCCAAGGCTTAGCTCTAGTGGAAAAAGGTGGAGGATTTGTGGCTTAGGTCGCAGGTTCAAGCCCCACACCATGCAAAGCGATGCCCGGTATTTAAGTGGATCCCAAGGCTTAGCTCTAGTGGAAAAAGGTGGAGGATTTGTGGCTTAGGTCGCAGGTTCAAGCCCCACACCATGCAAAGCGAAACCCGGTATTTAAGTGGAGCCCAAGGCTTAGCTCTAGTGGAAAAAGGTGGAGGATTTGTGGCTTAGGTCGCAGGTTCAAGCCCCACACCATGCAAAGCGAAACCCGGTATTTAAGTGGAGCCCAAGGCTTAGCTCTAGTGGAAAAAGGTGGAGGATTTGTGGCTTAGGTCGCAGGTTCAAGCCCCACACCATGCAAAGCGATGCCCGGTATTTAAGTGGATCCCAAGGCTTAGCTCTAGTGGAAAAAGGTGGAGGATTTGTGGCTTAGGTCGCAGGTTCAAGC

>LNC_000214

TCTTAACGAACATTGTCGTAATTAATTATCAGGGGTGGGGAGTGGCTGTTCTTATTGGTATACCCCATAACGAAGCTGTGTTCAAGACACACCCTATGAACTTTCTAAATGAAAGGACTCTCAAAGGAACCTTCTTTGGAAACTACAAACCTCGTTCGGATATTCCTTCTGTTGTTGAGAAATACATGAATAAGGAATTTGAATTGGAGAAATTCATCACTTATACACTTCCCATATTCTGAAATCAATAAGGCTTTTGAATTAATGTTGAAGGGAGAAGGCCTTCGTTGCATCATCACCATGGAGGACTAAACTTACTGTCCTAGAAAAGGAGCTTCTAGCTAGTCGCTATTGTTTGAGAAAAGACCAAGCATTGGGTGGAACACTATGAGAAGGATCCGAAAGCTGCAATGGCTGGACTATTGAACATGATTGTTTGAGATTTTTAAATATGCTGTCTTTCTGATGAACGAGGCATCTTTTAAGCATGGTGAGAGGGAAGCTCTGAGATCTTGTATAAAAGCTTTGAACTTCTGGGCTACTGAGATTTGTTGGGAGTTACAGGACTTCACCCTCAACAAATTGAAGGGGATTGAAGATGAGCTCATTATCAACGTATCATTGTTCCAGAAATAGGCATTAGTGCAGTATGGAGAAGTGTAGTATTTAAGATGGAAAATTTCATTAAATGCTCCCACAGATGTCTGCCAAGTCTTCCTTCAGGATTTTAAATCTTCTGTCCTTCTGGTGAAAGAGGCATTTTTTCAGAATGGTGAGAAGGAAGCTCGTAGAATCTTGTGTAAAAGCATTGAACTTCTGTGCTACTGAGAGTCGTGGGGAGTTACAGGACTTCGTCCTTAACAAATCAAAGGGGATTGAAGATGAGCTCATTATCAAACTTAAATCTGCTATAATAGAAGTTGCTGTATGTGCCCTTAACAAATTGAAGGTTGTATGAACTTCAATTGTCAAGGCAAATTTCCATTGAAAGTTTGCATAAAGATCTTGCAGAGACTCTCAAAAACTTCAGAAGTATTGATGACGAGGTTTGTGTTGTTTTTTCAGAACAATGGTGGCTGTTTCAGGAAAGCAACCCTTGCATCCACAGAGCTAGAAGCTTTGGGGTACTCTCCAGATGAATCCATTCTTCAAAAGTTCTGGAAACTTTGTTAACGCCAACTACATATTCCAGATGTTTTTGAAAGCCTTGGAGCAACTTTGAATGTTGCTAAACCAACAAAAGGTTCAAGTGTGGCTATTTTTTGACTGGGGGCTGTAGGCCTTGCTGCTGCAGAAGGAGCAAGAATTGTTGGTGCTTCAAG

>LNC_000215

CTTTTAGAAAGTAAATATTGTTCTTTTGTCTAGTGAGATAGCAACTTACTTTTGTCCAATTCCATCCATAAAGTTAGATGGAACTGTATTGTATCTTTTTATTATTGGTGTAAAAAGATAGTTCCTTAGTTGTATATTCTTATTTATACAATAATCTTAACGAACATTGTCGTAATTAATTATCAGGGGTGGGGAGTGGCTGTTCTTATTGGTATACCCCATAACGAAGCTGTGTTCAAGACACACCCTATGAACTTTCTAAATGAAAGGACTCTCAAAGGAACCTTCTTTGGAAACTACAAACCTCGTTCGGATATTCCTTCTGTTGTTGAGAAATACATGAATAAGGAATTTGAATTGGAGAAATTCATCACTTATACACTTCCCATATTCTGAAATCAATAAGGCTTTTGAATTAATGTTGAAGGGAGAAGGCCTTCGTTGCATCATCACCATGGAGGACTAAACTTACTGTCCTAGAAAAGGAGCTTCTAGCTAGTCGCTATTGTTTGAGAAAAGACCAAGCATTGGGTGGAACACTATGAGAAGGATCCGAAAGCTGCAATGGCTGGACTATTGAACATGATTGTTTGAGGTAATTTGATTTTTAAATATGCTGTCTTTCTGATGAACGAGGCATCTTTTAAGCATGGTGAGAGGGAAGCTCTGAGATCTTGTATAAAAGCTTTGAACTTCTGGGCTACTGAGATTTGTTGGGAGTTACAGGACTTCACCCTCAACAAATTGAAGGGGATTGAAGATGAGCTCATTATCAACGTATCATTGTTCCAGAAATAGGCATTAGTGCAGTATGGAGAAGTGTAGTATTTAAGATGGAAAATTTCATTAAATGCTCCCACAGATGTCTGCCAAGTCTTCCTTCAGGATTTTAAATCTTCTGTCCTTCTGGTGAAAGAGGCATTTTTTCAGAATGGTGAGAAGGAAGCTCGTAGAATCTTGTGTAAAAGCATTGAACTTCTGTGCTACTGAGAGTCGTGGGGAGTTACAGGACTTCGTCCTTAACAAATCAAAGGGGATTGAAGATGAGCTCATTATCAAACTTAAATCTGCTATAATAGAAGTTGCTGTATGTGCCCTTAACAAATTGAAGGTTGTATGAACTTCAATTGTCAAGGCAAATTTCCATTGAAAGTTTGCATAAAGATCTTGCAGAGACTCTCAAAAACTTCAGAAGTATTGATGACGAGGTTTGTGTTGTTTTTTCAGAACAATGGTGGCTGTTTCAGGAAAGCAACCCTTGCATCCACAGAGCTAGAAGCTTTGGGGTACTCTCCAGATGAATCCATTCTTCAAAAGTTCTGGAAACTTTGTTAACGCCAACTACATATTCCAGATGTTTTTGAAAGCCTTGGAGCAACTTTGAATGTTGCTAAACCAACAAAAGGTTCAAGTGTGGCTATTTTTTGACTGGGGGCTGTAGGCCTTGCTGCTGCAGAAGGAGCAAGAATTGTTGGTGCTTCAAG

>LNC_000216

GATTCGTCGATATAGGGTTTCTCTCTTGGTGGGTTTCGGAAATTTTCTGATCTTTCCTTCGGTTTCTAGGGCTTACAGCAACATCAATTCACGGAGGATGAACCAGTGGTGTCGGAATCAGCAATCGTCGTATGGGCTTACAGCAACAGCAATTCTCGGAGGACTGTTGGAAACAGCGAATCTGTTGTATGAAACGAACAAGAACTCAAACTAGGGTTAAAGAAGAGCAATTACTCAGCAGTGTGAATGAGGAAGAGAGAGAAGAATCGTTCGAGGATTCTGAAAACTCTGGTGCTATTGTGGAGGAACCAGTGATGTCGGAAACAACGAATCGTCCTATGGTCACTCTCCTTTGTCAAATTCCAGAGGGTTCTAACCATAATGCTTCAGCTGCTACAGTTGGTATAATCAAGTTGTTGATTGGGCACTTTGATTTGGATCCAAACCGAGTCTTTGATATTATTTTGGAGTGTTTTGAACGTCAGCCTGGTAAATAGTATATTTTTGGACTTGATTCCCATATTTCCCAAGTCCCATGCCTCCCAGATTCTGGGGTTTAAGTTTCAATACTACCAACGCCTGGAAGTAAATGATCCTGTTCCAAGTGAACTTTATCAGCTGACGGCCTTGCTGGTGAAAAGAGACTTCATTGATGTTGATAGCATGCTAGCAAAATAGGTAGAATAATTGTTGCTGCTACTGGGAAGGATCTCATGGATGAAGAGAAGCAAGGAGATGTGATGGTGGATCTGTATGCTGCATTTGACATGGAAACAGAGGCAGTTGCTGAGCGTTCCTCGGAGCTAGAAAATAGTCAACCCCTGGGCTTGCTTATGGGATTTCTTGAACTGGATGACTGGGGTGGGGAGTGGCTGTTCTTATTGGTATACCCCATAACGAAGCTGTGTTCAAGACACACCCTATGAACTTTCTAAATGAAAGGACTCTCAAAGGAACCTTCTTTGGAAACTACAAACCTCGTTCGGATATTCCTTCTGTTGTTGAGAAATACATGAATAAGGAATTTGAATTGGAGAAATTCATCACTTATACACTTCCCATATTCTGAAATCAATAAGGCTTTTGAATTAATGTTGAAGGGAGAAGGCCTTCGTTGCATCATCACCATGGAGGACTAAACTTACTGTCCTAGAAAAGGAGCTTCTAGCTAGTCGCTATTGTTTGAGAAAAGACCAAGCATTGGGTGGAACACTATGAGAAGGATCCGAAAGCTGCAATGGCTGGACTATTGAACATGATTGTTTGAGGTAATTTGGTAAGGTTCCTTCTTAAGTGTTCTTTGTCAATGACAAATTATGTTGGTGTTGTTCCGCCATACTACTCCAGGATACTGAATTCTTTGAATGCTTATGCTGTAATCGCTAGGACGTGTTTGAAAGTTCTATGCGTGATATAACCATTGCTATGATGAGGAATTATCCACAGCTTATGCCCAAATTTATGTCTGACAAAGCAAAAATTCCCTATCTTCTTGAACTCATTGTTCATATGAACCTAGAGCTCTATTCTCTTAAAAGACAAGATTAGGTGCATTTGTGAAACCTATTACTATCATGAACTATTTATGGGATTGCTAATATGAAGTAATAATTTTTTTTTGAACTGGTAACTCAAAGCTGTTTTCTTTTGCTTTTCCAGATTTTTAAATATGCTGTCTTTCTGATGAACGAGGCATCTTTTAAGCATGGTGAGAGGGAAGCTCTGAGATCTTGTATAAAAGCTTTGAACTTCTGGGCTACTGAGATTTGTTGGGAGTTACAGGACTTCACCCTCAACAAATTGAAGGGGATTGAAGATGAGCTCATTATCAACGTATCATTGTTCCAGAAATAGGCATTAGTGCAGTATGGAGAAGTGTAGTATTTAAGATGGAAAATTTCATTAAATGCTCCCACAGATGTCTGCCAAGTCTTCCTTCAGGATTTTAAATCTTCTGTCCTTCTGGTGAAAGAGGCATTTTTTCAGAATGGTGAGAAGGAAGCTCGTAGAATCTTGTGTAAAAGCATTGAACTTCTGTGCTACTGAGAGTCGTGGGGAGTTACAGGACTTCGTCCTTAACAAATCAAAGGGGATTGAAGATGAGCTCATTATCAAACTTAAATCTGCTATAATAGAAGTTGCTGTATGTGCCCTTAACAAATTGAAGGTGACTGTGTTCTATTGATGCAGCTTGGTCAAATGTGCAATTGTTGTCGTTGCAGGGTGGTGATGGTGATGATGAATACTCACTGCTTGTTGACTTGAACAGGTTGTATGAACTTCAATTGTCAAGGCAAATTTCCATTGAAAGTTTGCATAAAGATCTTGCAGAGACTCTCAAAAACTTCAGAAGTATTGATGACGAGGTTTGTGTTGTTTTTTCAGAACAATGGTGGCTGTTTCAGGAAAGCAACCCTTGCATCCACAGAGCTAGAAGCTTTGGGGTACTCTCCAGATGAATCCATTCTTCAAAAGTTCTGGAAACTTTGTTAACGCCAACTACATATTCCAGATGTTTTTGAAAGCCTTGGAGCAACTTTGAATGTTGCTAAACCAACAAAAGGTTCAAGTGTGGCTATTTTTTGACTGGGGGCTGTAGGCCTTGCTGCTGCAGAAGGAGCAAGAATTGTTGGTGCTTCAAG

>LNC_000217

GATTCGTCGATATAGGGTTTCTCTCTTGGTGGGTTTCGGAAATTTTCTGATCTTTCCTTCGGTTTCTAGGGCTTACAGCAACATCAATTCACGGAGGATGAACCAGTGGTGTCGGAATCAGCAATCGTCGTATGGGCTTACAGCAACAGCAATTCTCGGAGGACTGTTGGAAACAGCGAATCTGTTGTATGAAACGAACAAGAACTCAAACTAGGGTTAAAGAAGAGCAATTACTCAGCAGTGTGAATGAGGAAGAGAGAGAAGAATCGTTCGAGGATTCTGAAAACTCTGGTGCTATTGTGGAGGAACCAGTGATGTCGGAAACAACGAATCGTCCTATGGTCACTCTCCTTTGTCAAATTCCAGAGGGTTCTAACCATAATGCTTCAGCTGCTACAGTTGGTATAATCAAGTTGTTGATTGGGCACTTTGATTTGGATCCAAACCGAGTCTTTGATATTGTACGTTCTTTATCCATAATGATATCGTACATTACTACTCTGTTTAATCCATTTTTTGACTTCCATTCCTTTTAAACCTTTGAAAGTTCAGTGTTCAGTTCTGTCTTTGGCCTGTGATTATACAGATTTTGGAGTGTTTTGAACGTCAGCCTGGTAAATAGTATATTTTTGGACTTGATTCCCATATTTCCCAAGTCCCATGCCTCCCAGATTCTGGGGTTTAAGTTTCAATACTACCAACGCCTGGAAGTAAATGATCCTGTTCCAAGTGAACTTTATCAGCTGACGGCCTTGCTGGTGAAAAGAGACTTCATTGATGTTGATAGCATGCTAGCAAAATAGGTAGAATAATTGTTGCTGCTACTGGGAAGGATCTCATGGATGAAGAGAAGCAAGGAGATGTGATGGTGGATCTGTATGCTGCATTTGACATGGAAACAGAGGCAGTTGCTGAGCGTTCCTCGGAGCTAGAAAATAGTCAACCCCTGGGCTTGCTTATGGGATTTCTTGAACTGGATGACTGGGGTGGGGAGTGGCTGTTCTTATTGGTATACCCCATAACGAAGCTGTGTTCAAGACACACCCTATGAACTTTCTAAATGAAAGGACTCTCAAAGGAACCTTCTTTGGAAACTACAAACCTCGTTCGGATATTCCTTCTGTTGTTGAGAAATACATGAATAAGGAATTTGAATTGGAGAAATTCATCACTTATACACTTCCCATATTCTGAAATCAATAAGGCTTTTGAATTAATGTTGAAGGGAGAAGGCCTTCGTTGCATCATCACCATGGAGGACTAAACTTACTGTCCTAGAAAAGGAGCTTCTAGCTAGTCGCTATTGTTTGAGAAAAGACCAAGCATTGGGTGGAACACTATGAGAAGGATCCGAAAGCTGCAATGGCTGGACTATTGAACATGATTGTTTGAGGTAATTTGATTTTTAAATATGCTGTCTTTCTGATGAACGAGGCATCTTTTAAGCATGGTGAGAGGGAAGCTCTGAGATCTTGTATAAAAGCTTTGAACTTCTGGGCTACTGAGATTTGTTGGGAGTTACAGGACTTCACCCTCAACAAATTGAAGGGGATTGAAGATGAGCTCATTATCAACGTATCATTGTTCCAGAAATAGGCATTAGTGCAGTATGGAGAAGTGTAGTATTTAAGATGGAAAATTTCATTAAATGCTCCCACAGATGTCTGCCAAGTCTTCCTTCAGGATTTTAAATCTTCTGTCCTTCTGGTGAAAGAGGCATTTTTTCAGAATGGTGAGAAGGAAGCTCGTAGAATCTTGTGTAAAAGCATTGAACTTCTGTGCTACTGAGAGTCGTGGGGAGTTACAGGACTTCGTCCTTAACAAATCAAAGGGGATTGAAGATGAGCTCATTATCAAACTTAAATCTGCTATAATAGAAGTTGCTGGTGGTGATGGTGATGATGAATACTCACTGCTTGTTGACTTGAACAGGTTGTATGAACTTCAATTGTCAAGGCAAATTTCCATTGAAAGTTTGCATAAAGATCTTGCAGAGACTCTCAAAAACTTCAGAAGTATTGATGACGAGGTTTGTGTTGTTTTTTCAGAACAATGGTGGCTGTTTCAGGAAAGCAACCCTTGCATCCACAGAGCTAGAAGCTTTGGGGTACTCTCCAGATGAATCCATTCTTCAAAAGTTCTGGAAACTTTGTTAACGCCAACTACATATTCCAGGTGGTTTTTG

>LNC_000218

TGAGGCGATCTCTTGCTGCGTCAATCAGTCTTCTTCTTCAGCTGCTCGTCGGAATCCTCGTCTCAGGTAAGCTGACCACCATAAATTACCTTCCAGCTTGTGTTTATAGCTTTGCTATGTCATCTTTTTCACCTAATTTGAGTAAAAAATCTGAAGCTTTGAGCCAAAATACATTTCTAGTCACCTTTAAACTCTCACCAGGTGCTATGTATTTAAATCTCGATGGAGCTCACTCCAAGCTTGTGACTTTTTCTTCACAAAATCAAGGGAATTAGATCTAACAGCTTTGAGTATCTGATTGCTTGTTGTCTTTTCCAACTCACGGGTGAAGTATTCTATGTTCATCCTCCATATGACCTTGTAGCTTGCACTTCCTGCAGTAAGATGGAATGTAATCATATTGAATCTTAA

>LNC_000219

CAAAAAAACAAACTACCCTTTTATAGTCCAAATAAATACATAGTATTTTATAGTAAACATGGTGAAGGTAGCATTGTTGGTGGTTATGTGGATTGCAGCAGTAGGTGTGATGTTAACTCCCAATGCAGAAGCAATCATCTCTTGTGGGCAGGTTATTGAGAGGCTGACTCCATGCAGCGAATTGGTGAGGGAAGGTGGTGTTCTTCCAACATAATGCGGTAGTTGAATTGAAACCCTCAATGGCGAAGCCGCCACTACTCCTAATCGTCAAGCGGCTTGTAATTGTATTAAATCTACTGCTGCAACCATCAGCCACATCAACCTCGATCTTGCTGCTACTCTCCCTAACAAAGGGTGCAGTAAGTTATGCATGTTAGCAGGATGCAGAAAATAACTATTGCCTATTGGCGTAGATGGAAGAAGCTTTGTTTTATGTTTTTCAGTCATATATATAGTTTAACAGCTGTGTTTTAGAAATTAAAT

>LNC_000220

GCATACTCTATGCCTACATGATGTCACCATGTAGGGACCGGAGCACCGCTTGACCATCCTCCTCCGCGTGGCTAATACGATTTTCTATCAAGGTTATTGGTGAGTCCTCCATTCCGGGGACGTTGCTTATGATCTTTTGCTATGTATAAGACTTTTCCTTTTTAGTTATGTTTTGACTATGAGGGTGAGCCCGGTAGTTTGTCTTGGCCCCCGC

>LNC_000221

CGAGACCTAGGATCACGAGTGACCCCAAGCTAACCTTGAGCTGGCATATCATAAGCATATTAGATCAACACCTTTAAAAGCCTAAAATCTAAGAAATCTAAAACCCCTAAGAAACCCTAAACCCTAAGAAACACTAAACCCTAAACCTTAGGGAAATCCCAAGACTTATGAAACACCAACAAACCCTAAAGCCTAAGAAATCCTAAAGCCTAAAAACCTCCTTAAAAATACTAAGCCTAAAAACACCCTTAAAACCCGTAACACCTAAAAACCCTAAATTTAAAATCCTAAGCCCTTAACCCAAAGAAACCCTAAGCCTTAAGAACCCCTAAACCGTAGGAAATCATAACAATCTTGAAAACCCCTTATAAAATCCTAAACCTAAAAAAACCCTAAACCTAAAATATTCTACAAAAACTTAAGCCCTAAACCTTAAAAACCCTTAAATAAAAACCTAGACCTTATACCCAAAGAAACCCTAAACCTTAAGAAACCCTAAGCTTTAAGAAATAAAAAAAAAGCCCTAAAACACTTTTAAAAACCGTAAACCCTAAAAACCCTATAAAATCCGTACGATCTAAGAACCCCTTAATCGTTAACAAACCATAAAAATCCTAAACACCTTAAAACCCTAAACCTAAAAATTCCTCTAAACCTAATAAAACCCTAGAAAATCTTAAGCCCAAAATCCTAAATCCTAAAAACCCCTTAAAAAAAACCAAACCCTAAGAAATCCTAAACGCTAA

>LNC_000222

AAAACCCTAAACCCAAAAAAGCACTAAGAAACCCAAAACCCTAGAAAACAATAAATTTATTTTTATAAAGTCTAACCCTAGAAAACCTCTAAAAAATCTTAAACCTAACAAACCTAAACCCTATTCTAGAAAAACCCTAAACCTTAAACACTAAACCCTACACCTTGAATGACCCAAAAAAAACCTAGGAGACCTTAAATGTTAAACTCAAACCCTAAGAAACTTAAACCCTAAAC

>LNC_000223

ATCCAAATTCTTAAAAAAACCTAAGAAACCCTAAAACATTTAAAAACCTTAATCATAAAAATCATAAACCTAAAAAAAACCATAAACTCTAAAAAACACATAACCTAAAATCCGAATCCCTAAAACCTAAACGAAGAAACCCTAAGAAACTCCTAAACCTTAAACCCTTATAAACCCTAATACCCTAAAACCTAAGATCCTAAACCCTAAACCAAAAAAAATCACTAAGAAATTCTAAACCCTAAAAATCACTAAGAAAGACTAAACCATAAAAAACCTAAAAAAAAAAAAAACTTTAAAACCCCTAAACCTAAAGAACCCTAAATCCCAAACTCTAAACCTTGAACCCTAAACCCTTAAAATCATAAGCCCAAAACCCTAACCCTAAGAAAACCCCAAACCCTAAACCCCAAAAAGCACTAAGAAACCCAAAACCCTAGAAAACAATAAATTTATTTTTATAAAGTCTAACCCTAGAAAACCTCTAAAAAATCTTAAACCTAACAAACCTAAAACCCTAAACCATAGAAAACGCCTAAACCTAAAGCACTAGACCCTAAACCCCTAGACCCTAAATGACCCAAAAAAAAAACCTAGTAGACCTTAAATGTTAAACTCACACCCTAAAAAGCTTAAACCCTAAACCTTAGGGATAAACCCCCAACCCTAAGCCTTATGAAACTCTAAGAAGCCCTAAACCCTAAGAAATCCTAAATCCTAAAAAATATAAACAATAAAAAACAACAAAAAACACTTTAAAAACTCTAAACCTAAAAAATCTTAAACCTACCAAACCTAAACCCTAAACCGTAGGAAAACCCTAAACCTAAAACACTAAACCCTAAACACCTACACCCTAAATGACCCAAAAAAAAACCTAGGAGACCTTAAATGTTAAACTCAAACCCTAAGAAACTTAAACCCTAAACCTTAGGGATAAGCCTGGACCTTAGCCTTATGAAACTCTAAGAAGCCCTAAACCCTAAGAAATCCTAAGAAGAGCTAAACAATAAAAACCTTTAAAACCCTAAAACTAAAAATTCACAAAACAAACCTTAAAAACCCTAAAAAAATTCCTTAATCTAAAACCCACCCTATGTCCTAAGCCCTTAGAACTCATATATCGTAAAAAACAATATTGATCCTAAAAAATCTTTATCAATCCTAAATTGAAAACAACTCTTGACCTAAAAAATTTTGAAAAACTTAAGCTCTAAACCCTAACCTAAAATCCCTTAAACAAAAACCTAAACTCTTATTCATCATGTTCAGATTATACTATTCTTCAATATGCTCTGTTAAGTTATATATATTTGTTCAGCTTTGTCA

>LNC_000224

CCTAGACCCTAAATGACCCAAAAAAAAAACCTAGGAGACCTTAAATGTTAAACTCAAACCCTAAGAAACTTAAACCCTAAACCTTAGGGATAAACCCCCAACCCTAAGCCTTATGAAACTCTAAGAAGCCCTAAACCCAAAGAAATCCTAAATCCTAAGAAAATATAAACATTAAAAAACAACAAAAAAAACTTTACAAACTCTAAACCTAAAAAATCTTAAACCTAACAAACCTAAAACCCTAAACCATAGAAAACGCCTAAACCTAAAGCACTAGACCCTAAACCCCTAGACCCTAAATGACCCAAA

>LNC_000225

CACGGTGACTGAAAAAGAAGAAGAGGAAATTAGTGAATCTGAAATTACTGGTGCCTACACTCTATCATAAACTTATGCACAGTCCTGGAACAATTTATATCTTCCTTAATCTCCTAGCGCAGTGCCTTGTTTGAAATACTTGAGTCCATCCTGACTACTAAATCTCTGGAAGGTCTTCGTACAAGTCAACTTTCATGTAGGGATAGTGGTGACTGTTCAGG

>LNC_000226

TCTTTCTTTGTTATGGATTGGTGTGCCCGATTTGATATTGTGGTTGTTTCTGTTGATATTGTGTTGGATTGGTCTATTTTAGTTTTTGATTTGTAGTTTGTAGCTTCTTTGTTTTTGTTTTGATAATAAGATTTGGATTGATGATGCCCGATGATGGATAGAGGGTGACAACTTTCTTAAGGATAAATCGTCGATTGCTTGTGAGTGTTTGA

>LNC_000227

GGGAATTCTCTCAGTCCTAACATGAAGCAAAGAACATCAATTATGGAAATCTTTGCTTTTTGGTAACAACTTCATCAACCTACCACACCAGGAGATTAAGAGCACAATCACTTAATTGGCAGTGGAACAGTTCTTTCAGCATTTCTCAGTCAAGAGGAGTCATTTTGCAATGGTGAAATAAGATCATGCATGTAGTTTTCAGGCTACATGATGTTGTGTGGTGAATCATCCAAAAAGCTATGTATTTTTGGAGGATACAACACGAGGGTGGCAATATTTTTGAAAGGTTTGAGGGTGGCAAGATAAATTTTAGAATAACCAGTACCTTTGACATCAAAATTGATGCTAAATCACTTTTTGGTCTTTGATTAACTCTTATTCATGTTATGTTGAATTTTCTATGAGGCCTTCATGATAAACTTCTATTTTGGACA

>LNC_000228

GGTTTAGTTAGTGAATTTAGTTGTTGGAATTAAGTCAAAACTAATGTAGTTAAAGTTCAAAAGTTGTTATGTTGTTTAAATAAGTTAAAATGAATAATGTCGGTTTAGTTAGTGAATTTAGTTGTTGGAATTAAGTCAAAACTAATGTAGTTAAAGTTCAAAAGTTGTTATGTTGTTTAAATAAGTTAAAATGAATAATGTCGGTTTAGTTAGTGAATTTAGTTGTTGGAATTAAGTCAAAACTATTG

>LNC_000229

GTGAGCATCCGGTTCGAGGTCCGGCCTCCGTACTGTCAGATTCTACTAATTAGGGTTGAGGTTCTGGTTCGATGTTCTTCGTTCATGGGTTCTTTTATGCAATTCTCTTTAGTTATAGTTATTCTGTGTACTCGTCGGGCTTATGGGGGTCCGTTCGGGTTTTTATTTAACTTGCGCACGAGTGTACCTTCTGCGCTTATGGGGGTCCAGTTAAGTGTAGTTAGCTTGTAGATTACTTTAGTTAGTTCTTTGTACACTCGTGTGCATTCCATTGTTAGTTAGATTTTAGTTCTTGACTTCGGTTTGTGTTATTCCTTCTTTTCATATTGCCTTTACTTTTCAAGTTCAGTCGGCCTATGCTGCCTACTGGGTACCGGTTGTTTTGGTACTCATGCTACGCTCTGCATCTATTTCGTGATGCAGGTCCGGGCACTAGTAGCCAGCGTTGATCGAGCTTGGAGCAGACTGATCCGGAGACGGGAGTGAGCACACGACGTTTTGTACTATTTCAGTCTCCATCTGTATATATAGACTTGTCTTTTAC

>LNC_000230

CTTTATCAACTCTTGGCTAGTTAATCATAGTATTATTGGGTTTTCATAGTTTGGAATTTAATTGTGTTGATGATTTTAGCTGAATATGTTCTTCCCATGTGTCTACTGCTATGAAGTATTGGGACATTAACATAAATATTTCTGTCCATGAATATTGAATCGGCCCTAATGCGAACTTAGTCATTGTCAGTTTCTTTTGCTAACTCTAAAATGCTCTTCTATAATGTTTTCCAATTTAATTTTCTCACCATAGTTCAGGGTCATACAAGCTTTGGCTGCCCAGATTTTACTTTTTCATCTTGTCTTGATAAATTTTACATAATAATGCATATTAATTTGTAATTTGTTGCAAGCATGGTTATAGATCGGGTCTTGTTTAAGTGTAAATTTTTTGCACCAAAGGGTAAATCTATCAAATTTGGTGTTGAGAACTTGCAATAGCTTTCTTTTCTCTTTTTATTAGTGATGGTCAAAGCTTGCTTGTTAACCTCTTGGTGATTAAGTTTATTATGGAAAGTGTAGAATAAAAGATTAGAACAAAGAATAAATCTTGCTTGCTACAGATTAGTGGTAGGTAATTT

>LNC_000231

CAGCCGGTGAAATCCCTTCGTCCTCCTCCCTTGTCTCGTTTCCGGTGAGTCCCAACAGCAACGCGGCAGCAGTGCCGGCGAGGACAGCCAGCAGCCGCTGGTCGCCTCTCCTTCTCTCCCTCTCCCTTCTTTCTTTTTCTCCGGCGAAACAGCAGCAGCGCGAGAACCAGCAGACGTTGGTCGCTTCTGCTCCAATTTCCAGTGGACAACAGCTCAGCGAGAACAGCAGCAGCAGCCGGCGAGTACAACCAGCCAACCGAGCAACTGCTCCATCCGGCGACAGCCCTAGCGCCTCCCTCCGTTCCCTTATCCGACGAGGAGAGCAGGCGATCACCACCACTCTGCCACCAGGTGGGTGACAGCAGTAGTTCCAACCAGCAGCTCCAATAGCAGCACCTCCGGCCAAGGAACTCTAGTGAATGTCGGGTTTTGATCGGAAAATAAAGGATCTCGATAGATCCGTCCGGGTGCATTCATTTAAATTATTATCTCTCTTTTAGTTTTTTTATTCTCCTTTTCATTTGCAGTCGGCTTTGATTTTATGGAAATTGCATGTATGCTTATGTGAAAGTTGATTTGCATCTTGCTTAGAGTATGCTAACTTGTTTATATTTTAGTTTAAACATGATCTAGGCTGGATTTGATTTGAATGCCTTGTTAGAACTCCATTATTCTTCTGAAATTTACTCATATTTGTAAATACGATTAGATGTTAATATGATTTGAGTTTTAATATGGGTGAAGTTTCTGACTCCTACTAATATGATATATGATTCACCACTTGCCCAAAATATGTTCTGAC

>LNC_000232

CATGGACCCATGATCTCCTTAAATCTCTAAGTTTGGCTTATGAAGTGGGTTTTATGATAATGCTTGGGTATTGATGGAATTGTACTTAGATTGAATTGTATTATTGATTCCTATTGTTATCTATATCTATTGTTGGTGGATTGTTGGTGTATGGATGAGTTGAGAATTATGGCCTTGTAGGCAAGTCATGGGTAAATTCTCTTGTGATGTTAGAATTGTTGAGAATTGGATAATGGTAATTATGGCTTTGGAGAATGGTAAGTTGACCTACTTTCATGCTATATTGAAATTGTTCTTGTTTGGATTGATCCACTTATGGTGGAGGTGTGTACTTATGGAATGTATTATGATTGAGGCCATGAAGGGCATTATATGAGTGTTATTGTATTGTTATATTATTATATATGAATGAGGCTATATGTAAAGG

>LNC_000233

GTTCAATGATGTGGGTTGTAAACCTTATTAGATACCATTGACTCTGGTATCTAATAAGTTTTACCTACTATTGGATTTAAACCAATGACTCCCGCCGTATGAAAGCAGTACTCTAACCACTGAGTTAAGTAGGTCATTTATCATCCCAAAGAGAACCAAATGAAACCCATCCTGTCGATGGATTATAAATATCATATTACTTATAAGCAATACTAATCTAAGGAATACCACTCAGAGAGATCAAAGATTCTTGATGTTGGATCATGGAATATTTCTCTTGATAAGAATTTACCTACATGATAAAATATGTATCACAAGCACTAAGGGCTATAGCTCAGTTGGTAGAGCAACTCGTTTACACGCGCGCCAA

>LNC_000234

CACAATCTGGGAAGACGTGCATGAAGAACAACAATATCAAATAGAGACGAAGCAAACAATAACAAAAGGCTATATACCAAACTATAAACAAAACTCACTGAAAAAAGAGAAATCAGTCCCAAAACTTGAAAGAATCAACAATAAAAAGCATATGAACTTGAGAAAAAAAAAATATAAAGATAGAGCTGGCCGGAGTTTGAACCCCTCCGGCCAGATCTGGAATTGAAAAGAAAAACGAAGTGAAAGAGGGGTTACCGGAGTTTGAGTTGTTGTATTTCTGGTTTGAACAGATGTTTGCAATGGGTTTCGATGTGGGTCTGGTTAGTCTGATGGCTCACTGGAAATTTCACCGGTGTTGGAATCACGATGGAGTCGTGCTTGGTTGTTGTTGTTCGAGCTTCGTGCTCCTTGCCGAAAATGGCATTTTCTGGTTGTTTTGGAGTGGTGGAGCTGTTGTTTGAGGACTGGTTTGTAGTGGTACTTTATTTTTTTGTTGTTGGTTGGTCTCGGGGTAGCGAGCAAGAAGGGGA

>LNC_000235

GGAATTCATGTATTTCCTTGTAATATGTGTTTAGGGTAATGAGACTTGGTGATGGGTTGACTTGTCCACATTGATTAATTCTAATGATGAAAAAGGGATAATAAAAGGCAATGTGATCAATTGTTTGTGTGATGTGTTGAGAATTGTTTGAAAGGCTTGTTGAATCATTGTTGATGTTGTATCCTGATTGTTTTGTTGTGAATTGTGCAATGTTATGAAAATGGTCATCTCTTCATTATTTGTGTGAACATGTCATTTGCATTGTTCTGAGACATGGTTGTGACAAGTGTTATGTGCATTGAGAAAGAATAAGAAAATAAAGAGGATGTACCATTTCGAGGGACGTATCGCGCGCCGCGATGGATACTATATTTCGAGGGATGTGTC

>LNC_000236

CTTGATCTTGTGTGTTGCTGATCTTGTGAGTGCCTTTCTATGGAACTTGTGACTGATGAATATTGAGCTTGTTGTTGAAGATATGCAATTGTTAGAGTGTTGTTGTTGAGGATATGAAACTATTGTTGTTGTTGAGGATATGAAATTGTTAGAGTGTTGTGTTGAGCTATGTGCTTTGTAAATTGTGAACTGTTAGGTTGGGCTGGTT

>LNC_000237

ACGGATGCCTGTTTTACTAGGCGCGATAGAATACTCAACCAAGCATTCAGCTCTATGGATTACTCCCGCACCAGATAGTTCTCAGCCGCAAGGTTGCCGGCATGCCCCAATGAGAAGGTACTCTCAAATCGAATATCTCGTGGCACCACATCTTTAGACAAAGGGCAGGTTTAGCTATAGCCCAGTCAACTGTTGGAGGCAAAGACAAAGAAAAAACGGAAAAGCCGAGCACACTGCATCTCCAGTGAAGCATCCTACTAAGGTTTC

>LNC_000238

TCCACTGTGTCAGTCCTACTCCCATCAGCTACCACTTTCTGTTACATTTTTCATAAAATGCTTTGTTAGGTTCTAGCAATAACTTCTATTTTTGCTTTATATAGGATTCAAGTTCATCGAAAGGGAAAAACGCTTTCTATCAACAAGTTTTTCTATTTGTAAGACAAAGAAGGAAGAACTTACTTGAACACTTTTACATGGATTATTGACATCACAATAACTCTGAAGAGTAATTAAAGCTTGATTATCCAAATCCTCAGCATAATTGTAACACTTACAATTAGCACCAATTGTCCTCTTAAATAAGGGGCAACATTTATCTATAGAG

>LNC_000239

TGTGGAGACATTACCTATATGGTGTTCATTGCGAGGTATTTACTGATCACAGAAGCCTACAGTACATATTCACTCAGCGGGATCTGAACTTGAGACAGCGTAGGTGGCTTGAGTTGCTCAAGGACTACGACATGACCATTTTGTATCACCCCGGGAAGGCCAATGTGGTGGCCGATGCCTTGAGTAGGAAGACTCCTAGTATGGGGAGTCTTGTCGCGATTAGTGTTGAGAGGCGACCTTTGGTTCGGGATGTTCAGAGGTTATTTGACAGCATGGTGAGATTTCGGGTTTCTGAGGAGAGTGGAGGTGTGTTTGCCTTCATTGAGGCCCGTTCATCTTTGATGGAGCAGATTAGAGAGCATCAGTATGAGGATGCGAAGTTATGTCTCATCCGGGACAAGGTGATGAGTGGTGAAGCCAAGAAGGCTAAGATTGACTCCGAGGGTGTATTGAGGATTGAGGACCGA

>LNC_000240

AAAAAATGGGGATTAAGGATATGGGTTCATTGCGTGAATCAATCAAAAAGAAGAAAATTTTAATGGAGAAAGAGAGGAAGCTTGAAAAGGAGAGGGGACATACGCAGAAAGAAAGGCAAAGGGCCCAGATAAAGAGTTTCGAGGGGAAGTACGCTGGTCAGCAATTCTTAAAAGCAAAAAGAGAGAACAGAAATGTGGTTCAGCCTCTCTTTCCGATGAAGCAACGATATGAAGGAAAAAGAATCAATCGCCTAAGGCTGTTAAGCAAAGAGAGAAGGAGAAAATCAAAGAGCTCAGGTGGTTGACGAGGATTTCTTTAACTAGAGAGTGTGATCTGGTAGCATCTGGTTTACCATCATTTGCAGACAAACAAAAAAATTGATCTTTGTTTGAGGTTTACAATTTTAATGTATATGGGGAAGTTCGTGAAATAAAGAAGAAATTAAGCCAAAGTGAAGAAACATTGCAGGTGACACCGAGACGTAAACCGCATATTCG

>LNC_000241

TGAAAATCCAACAATCAAAATGGAACTCCATTTCCGGCGAGGTGAGGTTTCACTTCCAAATTAACTAAAATTAATGATACTTTACTTTTTTTTGTGTGTGTTCTTGTTGATGTGTTTAGTTGTATTTCAAATGGTTTATTTTTTATTTATTTTTGTTTTAGATCTGGCCGGAGGGTTCAAACTCGGCCACCTTTATTCTTATATCTCTGCCCAAATTTAGCTACTGATGGTCTATTATTGTTGATTATTTTTAATTTTGGCAACTGCATCATCTTGTTATTAT

>LNC_000242

CTGATATACATCAGCTGTATACCAAAAACGGGTTTTGGTTAAACCCCAAAAATACCAACGAATTTGGCCTAAGAAAGGCCCAAATTCAACTTCTCTCCGTTACTCTTAAATTATTTAATTATAATTATTTATTGTTTGTTGTTTATTAACTCTAACTTTAGAATTGTTAATTAACTTAATCGGATTCCCCAAAAGATAAACTATTTCTCTGTGTTGTTTTATTTCTAACAAATCTCATTTTATC

>LNC_000243

GGGTGATGAGGAGTAGTTGTATCTATTTGACATTTTAGACTTCTATATTTTTGGAATTGTTTAATTTGATCGTATTTTAGTTTATTTTGAAGTTGGATGAAGTTTATTTTGAAATAGTTTGTATATGTTGAAAATATTGTGTTGTTTTGGTTGGATGAAGTTTATTTGGCTTTGGGTTGTTTTGGTATAAATATGCAGGTGGGCAACTAAAAATTGCAGCAATTTGCTGCCTGTTTTGCAG

>LNC_000244

CCTAAACGAAGAAACCCTAAGAAACTCTAAACCTTAAACCCTTATAAACCCTAAAACCAAAGATCCTAAACCCTAAACCATAAAAAATCACTAAGAAATCCTAAACCCTAAAAATCACTAAGAAAAACTAAACCCTAAAAAACCTAAAAAAAAAAAAACTTTAAAACCCCTAAACCTAAAGAACCGTATATTCCAAACTCTAAACCTTGAACCCTAAACCCTTAAAATCATAAGCCCAAAACCCTAACCCTAAGAAAACCCCAAACCCTAAACCCAAAAAAGCACTAAGAAACCCAAAACCCTAGAAAACAATAAATTTATTTTTATAAAGTCTAACCCTAGAAAACCTCTAAAAAATCTTAAACCTAACAAACCTAAAACCCTAAACCATAGAAAACGCCTAAACCTAAAGCACTAGACCCTAAACCCCTAGACCCTAAATGACCCAAAAAAAAAACCTAGTAGACCTTAAATGTTAAACTCACACCCTAAAAAGCTTAAACCCTAAACCTTAGGGATAAAC

>LNC_000245

CATTTTTGGCTAAAACTCCAGCCATCTTTAGCATTAACAGAAGCTCATCTCTCCATTTTCGTGCAAATCTTGAATATCCCACGACTTTTCTCACCTTTCTCTCTCCGTTGGCAGATTTGGTCATCATAAGAGGCCAAAGGACCTTCTGCGATTTCTGATGAAAGCTCAGAGACAGATTTTTGAATTCAAACGACTCTTTGATCCTTGAAATCCCGCCTAAATGTTTAGCCAATAACCCTAGTTCATTCATATAAATATTAGCATACTGCACTG

>LNC_000246

GCGAAGTCTCACCGAAATCTAATGGTGAGGATCTCAGTTAAGCGTCTGCTATCGATCCTGAGTTTATGTATTTGTATCATTAGAAGATGTAGCACCAAATGGCGTTAGAACATGAAATGTACGGTATGTAAAATAGTTGAATGAAACACTTGTTAGACTGAACATACATAATTGAGGAACAACTCAGAGAAAGAATGAATATTGGATAATTGTGTAAAGTAAAGCATGCCAATGCATTGAAAACAATAAAGCTTTACAACTTAGATATATGCAATGATATCAAATGCTAAAATAGTATACTTTACTTTTCTTGGGAGTTTCTCTAATTGACAACCATCAGTATTTGCCTCACGATGATACAACGTCTTGCCGACATTGCCAGAGTCATCCAACACTTTGTCAGGGTAAAGGACAAACTGAACTAATGGATCCAATATATAGGCCCTTACCAGGGGCTTTATGATGAGTTACCCAAATCAATGACACGATCTTATCCTACGTTAGATGCTCAATTATGGAGTTTGAGTTATCTAAGCCCTTACTCAACTCGATGCTCAATAC

>LNC_000247

TGAGCAAGTCTTTATGCGAAGTAGGGTGGTGATCATATCAATCATATAAATAGATTGCAATGCATCTGGTGGAATATGTTTGGCGCATAATCAGCGGTTGTTAGACATTATTTGAGATTTGTTACTACAATGGATTTCTGGAGGAAGAGGAGGAAATTAAGACATGGGAAAGAGCTCATTGACGTTAATATGAACTATAAAAAAAGCGCTCAAGGGAGTTGAGAACTCTTTTCCAACAGAGTGCGACACCTCCTTATTTTCTGTATACTTTTGGAGAGCACGTGATATTTTAGGAAGTATTGATGCAGGATGC

>LNC_000248

GAAAGTATTCCTTTTGTTTCAGTTTGTAGTGTTTAGTTTGATTGGATGCAGAGTTTAAGATTGTTCAGTGGAGGTGGAGTCAGAGTTTGAAGCTTATGAGTTCTGGATTCTAATCTTTTTAAGTTATTGAGTTTTAAATTAATAATTCATACATATTCGTGGATTTTTAAGACAATAATAGAGTGTTTCGACCAAAGTTATTAGGTTCGGCTGAATCTGTAACCAATACTCTAGCTCGGCCATTGCTCATGATCTCTCGGGGGTGGTGGTGGGGTTGCAACTCTAGCTCCTAATGTACCGGCAATGCAAGAGCTACATTTTGCA

>LNC_000249

AGTACGGGGGGTAGTAGGCCCGTATAACCGTATCGAGGGGTTGGTACCTTGGTTGCCTAGTACGGGGGGTAGTAGGCCCGTATAACCGTATCGAGGGGTTGGTACCTTGGTTGCCTAGTACGGGGGGTAGTAGGCCCGTATAACCATATCGAGGGGTTTGTACCTTGGTCCCTAGCTCGGGGGGTGGTAGGCCCGGGTAACCACATTGAGGGGTTTGTACCTCTTTCGCCTCTCCAAGGGGGTGGTAGGCCTTGGAAATACTATATTGATGGTCACTCACTACATATATACTATGCCCTACTAAATATGATTTTTATATAAGTATCATTATACATATCATCTCATTAATATGCTATCTATCGGGTACGATTATGCATTTTGCCTATGATGTCCATCCCTTGTTGCATTGCATTGCATCCGGTATACTTAGTACATTCAAACGTACTAACGCATACTCTATGCCTACATGATATCACCATGTAGGGACCGGAGCACCGCTTGACCCTCGTCCTCCGCGTGGCTAATACGATTTTCCATCAAGGTTATTGGTGAGTCCTCCATTCCGGGGACGTTGCTTATGATCTTTTGCTATGTATAAGACTTTTCCTTTTAGTTATGTTTTGACTATGAGGGTGAGCCCAGTTGTTTGTCTTGGCCCCCGCTAAAGTACCTATGTTTAGAGGTGGTGTTGGACAAGTTGTGTATTATGCTTGAGCTTGACTCATCTATGGTATATATGTATCATTTCTTCTTCTTTTTTTCTTGCTCCCTTAGTCCCGATTTCCC

>LNC_000250

TATATCCTTGAAACCCTTTGTTTTCTCCTCGTAGATTTCATGTGGATCCAAGCTCGTGTAGATTATGTGGAAAGAGTATTGTTGGTAATCCATCCATTAAACCTCAGGTTGTGTATGGATTTCTGGGATTTGCACTATTGTACAGGTTGTGCAAAGTATTCTGGACTTGAGCAACTTGTTAGGTTCACATTGAACCCAACCAATTTTTCTCTTCTCTTTTGGACTGATCGTTCGAAATAATCAATTTCGAAGTTATTCTGCTATGTGTATTGTGAAATAATAAACCTACTATAGATGTTAATTGACTGCTACAATCTATGAGTCTTGATTTTATACCATATGAAACACCCTAAACTCTCACCAGGTGCATGGTATATCAACCAACTGAAATTCATTCAGACATCATATAATGTGTAGTAGCTTTTATCATCTAATATAGCTTTATCAATATCATATGTAGAGTGTATTCAAATCAAACCATTTATACTCTTGTAGTGTTTGACTGCCTTATCCTCAACTGTGGTGTGCTTTGTTTATTTAAGATGATAATTCTCTTGGCCGTATCCAGAACCTCTTGGAGAGATCTAAATTCATATGTACCTGCAAAAAGAAAAACCAAGTTAGCAAAAAAATCAGCAAGAGTATTTCCCTCCCTTAAGGAGTGTTGTACTCTTACTGACATGGAATTCCTCAATCTATTGATGGAATTGACCTCCATTGTCACATTCCAAGGTATTTCCCACTCTCCCTTCAGCATATGTACCAAAACCAACGAGTCAGTTTCAATTATAATGTTTGGAAAGTTGTTTTCCAAACAACATTGAAGACCTTTTCTAATAGCAATAGCTTCAGCAACTAGATTAGTAGAATCAGGGATCCTCACCCCCTTAGCAATCACCAGCTCTCCTTCATGATTTCTGATACAAAAGGCAGCAGCACTAGGCCCAGGATTACCTCTAGAAGCCCCATCTGTATTACATTTCAACCACCCAGATGGTGGAGGATGCCATTTAACCCATTTAGAGACAAACCTAGGTCTGAAAATATCCAATAGAGCAACCATCTGAGGCCAAGTGGAGGGGACATCAGTATATTGAAACTTTAACTACATAAATTTCTGAACAGTACTATTGATGTCCCAAATCACTTTACTTGTGGTGTAATTACCACCATGAAGCACAGTATTTCTCCTTTTCCATAAGAACCATAAGATTAGATTAGGAATAGCTTGAAAAATCACCTTTTGCCTAGTATTTCCTTGAACATCCCACCATTTTTTAATAGATTGCTTCAACTGAATCCAAGGCCCTAATATTCCAGCTGCAGATGAGAAATACTGCCACACTTTGTTGGCAATTTCACCTTTCAAAATAAGTGTTCCATAGTTTCTCTTGCTGGAACAGTACAACATAGGCAAAGTTGAGACTGATTAGGGTTCCAATGAGCTATAACAGCAGCTACTGGAACTCTATTAGTCCAAACCCTCCAACTAAAGAAAGATATCTTAAAAGGAAGATCTTGCACCCATATCATCTTGCAATTTTCATTTACTTCCTTTTTTTTCCTCAAAATCTCCCATGCACTCTTAACTGAGAACTTTCCGGTGCTTGTCTTGATCCACCACGGTTTGTCTCCCATATCAGTCCGTCTAATATGATCCATATGCTGCCTAATATGTTCCACAACATGATAAGGCAGATGATCATACATACTATCATAATCCCACCCTACTTCCTTCATAAAAAACTCAATGTCCATCAAAGAGTGACAAGTTTGTACTTCAGTTTGATATGTGTAAAAAGGACCTAGCTGGGTCCAATTATCATACCAAACAGTAGAAGTTCCACCCTTAGGTTCCCACCATATAAATTTTTCTATGTCGTCCCTATTTTGTAACATATTCTTCCACAATTGGGATCCACCTTTCCATTGAACCAGAGCTGGAAT

>LNC_000251

AATATATACAAAATCATGTGGGTTGCCTAAGTGAACAGCCCACCAAATAAATAAACAAAACAATAAAAGACAACACCTACCAGCTAAGAAATATCAAAAAAAATAATTAGGAAAGTGGAAGTTGAAGAGTACTTGCAGGTGCAGCGCCTCTTCCTCGATACATCATCAAATCGGAGCTGATATTCGACTTCAATTCGGTGTAATTTTTTCTCCTCGTAGATTTCATGTGGATCCAAGCTCGTGTAGATTATGTGGAAAGAGTATTGTTGGTAATCCATCCATTAAACCTCAGGTTGTGTATGGATTTCTGGGATTTGCACTATTGTACAGGTTGTGCAAAGTATTCTGGACTTGAGCAACTTGTTAGGTTCACATTGAACCCAACCAATTTTTCTCTTCTCTTTTGGACTGATCGTTCGAAATAATCAATTTCGAAGTTATTCTGCTATGTGTATTGTGAAATAATAAACCTACTATAGATGTTAATTGACTGCTACAATCTATGAGTCTTGATTTTATACCATATGAAACACCCTAAACTCTCACCAGGTGCATGGTATATCAACCAACTGAAATTCATTCAGACATCATATAATGTGTAGTAGCTTTTATCATCTAATATAGCTTTATCAATATCATATGTAGAGTGTATTCAAATCAAACCATTTATACTCTTGTAGTGTTTGACTGCCTTATCCTCAACTGTGGTGTGCTTTGTTTATTTAAGATGATAATTCTCTTGGCCGTATCCAGAACCTCTTGGAGAGATCTAAATTCATATGTACCTGCAAAAAGAAAAACCAAGTTAGCAAAAAAATCAGCAAGAGTATTTCCCTCCCTTAAGGAGTGTTGTACTCTTACTGACATGGAATTCCTCAATCTATTGATGGAATTGACCTCCATTGTCACATTCCAAGGTATTTCCCACTCTCCCTTCAGCATATGTACCAAAACCAACGAGTCAGTTTCAATTATAATGTTTGGAAAGTTGTTTTCCAAACAACATTGAAGACCTTTTCTAATAGCAATAGCTTCAGCAACTAGATTAGTAGAATCAGGGATCCTCACCCCCTTAGCAATCACCAGCTCTCCTTCATGATTTCTGATACAAAAGGCAGCAGCACTAGGCCCAGGATTACCTCTAGAAGCCCCATCTGTATTACATTTCAACCACCCAGATGGTGGAGGATGCCATTTAACCCATTTAGAGACAAACCTAGGTCTGAAAATATCCAATAGAGCAACCATCTGAGGCCAAGTGGAGGGGACATCAGTATATTGAAACTTTAACTACATAAATTTCTGAACAGTACTATTGATGTCCCAAATCACTTTACTTGTGGTGTAATTACCACCATGAAGCACAGTATTTCTCCTTTTCCATAAGAACCATAAGATTAGATTAGGAATAGCTTGAAAAATCACCTTTTGCCTAGTATTTCCTTGAACATCCCACCATTTTTTAATAGATTGCTTCAACTGAATCCAAGGCCCTAATATTCCAGCTGCAGATGAGAAATACTGCCACACTTTGTTGGCAATTTCACCTTTCAAAATAAGTGTTCCATAGTTTCTCTTGCTGGAACAGTACAACATAGGCAAAGTTGAGACTGATTAGGGTTCCAATGAGCTATAACAGCAGCTACTGGAACTCTATTAGTCCAAACCCTCCAACTAAAGAAAGATATCTTAAAAGGAAGATCTTGCACCCATATCATCTTGCAATTTTCATTTACTTCCTTTTTTTTCCTCAAAATCTCCCATGCACTCTTAACTGAGAACTTTCCGGTGCTTGTCTTGATCCACCACGGTTTGTCTCCCATATCAGTCCGTCTAATATGATCCATATGCTGCCTAATATGTTCCACAACATGATAAGGCAGATGATCATACATACTATCATAATCCCACCCTACTTCCTTCATAAAAAACTCAATGTCCATCAAAGAGTGACAAGTTTGTACTTCAGTTTGATATGTGTAAAAAGGACCTAGCTGGGTCCAATTATCATACCAAACAGTAGAAGTTCCACCCTTAGGTTCCCACCATATAAATTTTTCTATGTCGTCCCTATTTTGTAACATATTCTTCCACAATTGGGATCCACCTTTCCATTGAACCAGAGCTGGAAT

>LNC_000252

TACTGTTTACTATGGATAGGATCCTCGACTGTGGTAGGTATGTTCCGAAGATTCACCTCAGCTTTAACAAGTCTAAGTATTAGCTCTACCAATGGTCTAGTGTTCACCATTATAGCTTAGTTTTAACCCCTTTCGACAGTGTAATTCCACTAATGACTAGGAAGTTCAAGATTTAACTCATTTTCTATTAGGAAATTAGAGTCCCGGTGATAGTTTGCTGGAGTAGTAATTTAGTTCTTAGAAAAATACTTTTCTTGTTCTCCAACTTGACTAACTTAAGCATTTTTTAAAGTATGATTCTCGTGTGCTGTCTGCTGTTTGGGAGTGTGAGGACATAGCTTCTCTTTCTTGGCAAATATGGGGCCTTGCAAAATTTAGTTCTCATTGACGATGGAAATTCTAAGAGTCAGTCTTATTAGATCTGTGATATAGATTAACTCTAATGAGCAACCTTGTGTTAATTTGTAATATTGTGTCCTTACAACAACTATGCCTTAGTTCCAA

>LNC_000253

CGTCATATACGACAACAACAATTCCAACGGCGTCAACGATTTGCGCCTCACTCGAGTCAATTTACACGCTGCCGCGTCGTCCTCGCCAGCAGGAGATTGAGCCACATCGAGCAGCAGGTACACTCAATCGATCAAAGCTGTTCGCTTCCCATTTCCTCTTTCAGAATGGGGTTGAAGATCTCAGAAAAGTATGTTTGCCTCTAATGGTGAAAAATATTTGAAGTTTGAATTTCAAATTTGGGCCTGGGTTTTGCCCAATTTTTCATCGGTTCCCTCTTTTATTTTCGGAACCCTATTCTCCTTTCTATATATTGCTCTTTGTCCACTGTAAGATTTTTTTGGTGGAGAGAAATTTAGTTGGGAGAAATTATTGGAGATTTTTTAGTTGGCGATTTTAAACCGAGAAAGAGAAATAGCACCGAAAAATAGCTATAAAATATATACTAAGTACGCTCGAGAGGAGATGAGATAGCTAGTGTCGTTCTTCTTTCCCGTTTGCCATTGTTTCCCAACATTCGTGAGTCCGAGATTCTGCCCGGATCCTCTACTCATTGTTCATATTCAAGTTTTTGTTGTGGTGGGAGGACTGTAGCTGCTGACTCGTTATTCTCCGTTCGCTGCCCGGAAAGAGGTAAATCCTTTCTTGGCTTTGTTTAATGCTCAAGGCTTCCATGTTGTTCTGTTCATGATGTGCTCTTTGCTATGTTTGTGTTCTGTTTATGTGCTAATTTTGTTTTAGTCTCCATATGTTAGGATTTGGTTAATGAATATTTGCTAGTAATATCTTTTTTCTCTATATGTGTTATCTGTGTATACACTCTAATTTTTGGAGTAGTTTAGTGTTATTTGTTAGCATATCAGATCTCATTTTGCTCGACTATAAGCTCTCCATGGTCCAATTTCTTTCTGTCATATTTGCTCATTCCGATCATAGCTAAATGTGAAAAGATTAAGATTATTTAGTTGTTTGTAGTCATTGGTTTTAGTATTTCTCTCTTAGGACATGATTCAAAATTTAGTCTTAAGTTACTCTTTTTTGCCTATATCTTTTCGATTCTTTCAGATCTTTTTTTTTCTTCCTTCTCTTTGCGTTCACCTGCCTTAATTTCATCCTAATGTGCAATATACAGTTCCTTGTATACTCTCGTGTGCTTTATTTATTTATTTTAGCCGAGGATCTATTAAAAAATGAGAAATAATTTACCTTTGGGGCTGTCCTTTAGCATTTCCGAAAGAGTCAGACTCGGACGGGTGAGAGCTTTTAACTCATGTGCATCCTATTTCTTTAATTCGTTATCTTTAGATCCACATTTGATTCGGTTGAGATAGGTTTATCGTTTTAACTTGTTTGTTGCAGCTGCCTGCTTGTTTAACTGTGGTCTGTTTTTTTTTTTTTTTTTTCAAAATCCGATCAATCTTGTCTCTGTTTTAAAGTGAATCATTCGGTTAGTCTCTTGCATGTCAAAGTCAAGTTGTGATCAGTAGGACAATTTCATAGTTGACTGTCATAAACTTGAAGTATGTTAACCTGTTCTTGCACCTTATTTGAATTGGTTTGATGCTAAGTTCGCATATTCGAAATTCTATTATGTTAAATTCTTCCCATTTTATTAATTAGTAGTCTTTGTATGATATGGATTCAGCTTTAAGTATGATGTTTAAATTAGAACATTATTTCATATTCGGTAAAAGTTTTCTATGTTTAGTTAAAATAAGAGCATGTTGTTATCAATAGTCTTTGGTCGTCTCGCTTTGCTACGTGTTTCATCATCTCGAACTGTAGAAGCCCATAATGTATCGCTAAGTGTTTATTCCCATTGTTGTCACAACTTCTTTTTTGGAGGGCCCCAAACTCTATCGCTTTGAATTATTTAGCTCTAATATGCTTCTAGATGATATAGCATATGTTGAGATTTGGTTAATTTCTACATGTTTTTTTTTCTTTTTGTTAAAAACTGAAATTGAAAAATAAAATAAAATAAAATAAATTGTGCTAGTAAGATGGGTAGTTTTCGTATTTGGGAGCTTATCATGCTGAGAATGGACATATATGTAAGTATGCCTGTATGAGCTTGCTAGTTAGATTCACTCATATTTATGGATAACATATTGCCTGTTAACTTCCCTTTATAATGGAGTAATAATTGGGTATAAGGTTCCACCTCTCTTATTAAAAAATACATATTGTATGAGGCGTGATATTTAATAAAGTTCATTGTGTTCTCATAATTGTTTCCATCAATTGATCTTTGCAGAATAGCCTTTTCTTAACGGTCTAAAACTTGTTCTTCCCCTTGTTGACTAGTTTTATTTTATTTTGTTTATTGTGAGCGATTGCGGAGAACTCCATTTGGGTCTACACCCCTTTGCATTTCGGGAGAGCCATAAAGGCTCAAATTCCCTTTCCAAGTCAGCCAGCCATAAAAAACCGACGAACAGGTCCCGAAAGAATATGCAACAGATTGGAGAGTCGAGAAATTAGGCCAAAGGCCCACTCTTTATTTTAAAATTGTATTTTGCTATTTTTGGGCCTAAGCCCTTTTATTGTAATTATTATTATCTATTAGCTATTAAGACAGGTACAAGGAGACAAGAATTGGGCCAAAGGCCCAAAGAAAAACAAATTTCAAAGATTAGTTTAGGACAGGTACTAAATATTGGGCCTTTGGTCCCGAAACAAAAATTAGGCCCAAATACTGGCCCAGGTGGACAGGAAAACCAGATTTGGGCCCAAATCTCTTTCCCTCTTCATTTTGATTATGTTTATTTACTTGTTAATATTTGTTGTTAATCTTAAGGTCGAAGAATTCAAATCCCCGAAAGACACCCATTTTGAATTAATTACTTAACTAAATTATTCATCTTTTATTCGACTTAAAAATAATAATAATAATAAAATAAACAAATAAATAAGTTTACAAAACATTTCGCTTAGATATATTTCAACATTTTCTCCTTAAAATGGTCCTAACTACAAAATAGTTCAATTTTGCAATTCAAGCTAAGTTAAGATTATTTTTAGCCAAACAATTAATCGCCGGATAACCGCGTATTAGCGGGCGTTTCGGGTGCTTTAAATCCCTTCCCAAAATGCTAATAAGAACCTCGAACCCCTTTAAAGTTTTCATGAGATTTTCTGTTTTAGTCTTTTGAAAACGATAGTTTTCTTGATTTTTCTTAAAAATTAAGTGGCGACTCTTAAACCAGTCTAAAATATATCTTTAAATAAAACAATATTATTCTCAACTGTCACACTTGCTATGTTCCTTGAATAATGC

>LNC_000254

AAAATTGTCATCAGTTACCAAAGCGGTGGTCCACATAGTAACCTTTGTATTTAACGATATCTTTATTGATGTTCAAAGATTAATATTAGTTAAGCCACCACAGCTTAGCTATTTATATATGTGAACGGAAGGAAAAGACACATTTTGCTTTTGATTAGCCATAGTTTTGAGGAAGTTGTATGAACATATAGGGAAAAGAATTACCGTTGTGCCTTTTAGGCAAGCTTCGTCTGATGAGTTTTGTACCTGAAACTTCGCAGGTAAAAGATTAACAATTCAACTAATTCCATCACAAATATCAAAATGCATCAGTATAATCCTTGCACTAACAAAATTCTATTTGAATCTATCTAATTCTCAGCTCATCAGTTTAATTAATGCATATGCACCTATAAAAGGAATAAGTGTGTTTAATGTTCGCATGGTTTGATTGTGATATTCCTCCTGTTCACATAATGCGTCACAAAGACATTTTTGTTACACTTGAAAATTATGTGTTTAGGGACTCGATTGAGTAATTTCCAAGTTTGTTTTTTCTTTTACAAAATTTAAAGTGGCTAAATAGATGGTTCATCTGTATATGTATGATGTCACCTAATGACATTGTTGAGCCACAAGGACTACACTCCTCTCTTTTCTAAAAGAGAGTGCGTAGATTATACATGTTAAGATCTGTTGTATTTATTTAATTCCGCTCTTAGTTGTGATGACATTTTCTCTGTGTAGCATACCAAAAATGAAGAGGCATAACACTTGAAGAGGCCCTACTACTAAAGTAATTCCGCATGACAAGTTTCCGTTTCATTGTTGGAATGAATTTTTTTTCCAGTTTAGAGAATATTAGTTCATTCACAGATGTATACGTGGTAGGGGTGTACATGGTCGGGTTAGTTCGGATTTTT

>LNC_000255

TTATCTTAAAAGAAAGACACATAATATAGATTAGCTAATTTGAAAGAATTTAATTTAATTTCTTACTTCAAATGCTATTACTATTCACCATATGAATACGAGGGTTTGCTCTAAAACTCTGAAACCAAAACAAAATCTGGAAATGAGTAGTGGGTGAACCAAAGTGCAGAATAGCTTGAGCTTTGAACCCTAATAATCGAAGAAATAATGGGTAAAAAGGAGAGGGCGCAAGAGAGAAGAGAGCAGAGACGCCAAGAGATCTCTCTTCTCCGTACTATTCCTTATTCTGATCAACAAAGGTCAATTCTTTGATCCATAAAAAAACTTTCTTGATATGTTTTGCATTTTGTCCTGAATTGAGTCGAACATATCCGTGCCTGAATTGAGGGTTTCTGCATTTGATTCAGAGGAAAAATGACAAGTCAAAGGGTCTAACTGTTAAGTGCTTAATTTTGCCCTGTAGCTTAAGCATTGGGTTTTAGTTAAATTAACAACAGAGGAAACTATGTGTAGATAATGGAACAGTCTCTAGCGTATTCAATTTTGGCATACTTATTAATTAAAAAAGGCATCTTCAGTTTTAGCATCTTATGCATGAGATACAAGAATATCATAGTTAAGATGACAAACTCAAAAGCAGCTCATTTTTCCTACTTTCAAATAGTTAGTATCTTAGGAAATAGTTTTAGGTTGAAGATTGTTTGTGTGTCCTTATGCTTATTGGACAAACAGCCTGCACTGCAAATGTTGCAGTTTGGATTCTAATAATGTGACCATATTCTCAAAAAAGAAATAATTTTAGGTTGAAGATTGTTTGTGTGTCCTTATGCTTATTGGACAAACAGCTCTGCAATGCAAATGTTGCAGTTTGGATTCTAATAATGTGACCATATTGTTCTGTTAGAAGCAGAGACGAATCTAGGATTTGTAAAATGTATTGAAGTGTGAATTGATCCTTAAT

>LNC_000256

TCTCTTCAAGTTTCTTCAGCTACTGCTATTGCCTCTTCAATCTTTTCAAGTACTCTTCAATTTCAAGGTAATTTTTTCTTCTTTCCCTGCCTTCCTCTTCATTTTTTTTAGATCTGTTTTCTTGTGTTAAGTTTTTTCCCTTCACATGTTTTCCTCTCGGTTCTCTTCAGAGCTTCATTGCATTTGGCTGAACCGATTTATAAAGCTTTGGTTTTAGATTTAGTTTTATGGGGTTATGAAGTTTGTGTAAAGATATGATATTTGGATCTAACTTA

>LNC_000257

ATTTTTTCTCTTTCACTTTGCTTTTTTGCTCTCTTTTTTTTCGTTGCTTTTTCATCATCTTCTTCCAGGATTTTAGGTTAAACTTTGTCCTGTTTTGTTGATTTTATGTTCCATTGTTTTGATATTGCTTTCGATTTTTGCTCCTTCATAGTTATGTGGATGTAGGTATGTTGCATGCACAAAATTCCTATTTTTAATCCACTTCTTCTTCATCTCCTATGCCCAGAAGTGTAGACCCGTCTCAATTTTGCATTGATGAGTCATATTATAAGAGAGTACAACAAAAAAATTCTTTTGCCACATACCAACAAAGACCTCAAAATTCAACT

>LNC_000258

ATTTCCAAAGGGCCTTCGAGCTAGTTTTCAGAAAAGAGTAATAGCTTTCTAAATGAAGCAAGCAGGGAAACTGAGATTTCCAAGATAGCCTTTGAGCTAAGTTTTTGAGCAATTATCTCAAATCAGCAGAAAGAAGTATGTTTTTAAAACATAAGAGCTAGTATATTTTTGGGAGTAGTATTGAGCACCGATATGGGGGAGAGTTCAGACAACTCACAACC

>LNC_000259

TAAATTTCCAAAAGAGCCTTCGAGCTAGTTTTTGAGTAATTATCTCATATCAGCAGGAATAAATATGTTTTAAAAACATAAGAGCCAGTACATTTTTGGGAGTAGTATCGAGCACCGAATTGGGGGAGCGTTCAAAGAACCCACAGCCCCCATAGAACCATGTTAGCCACCATGGGTAGAAAAGGATCATACTTTTTAGATGAATCCTTTTCAGATTAGACTAGTGGATCCATTAGGCAGTTCAGGTCTTATACCTTTGGCCGGGTATAAGATGCTCTGGCAGCGTGAGGCCGATCACTGTATCATCACTATAGCTCTTATGTGATGGTTGTCGGTTAGAGAAACTCCCACAGCAGTTATTGTATTTTCA

>LNC_000260

TGGAATTATTATGTGATGTTTATGGTTTTCATGCTTCCATGATCAAATTATGAAGGTTGATTTATATATATGTATGTTGTTGGTGGGCTGATTTATATGGATTTTGGAATTGGTTGGACTTAGTACTCTTGAAATACATGATTTTATTTACATGAACAATAGCCCACCATGTGTTTGATAAAATGCCCACTTATAGAATTCTTAGGAAATG

>LNC_000261

TATGTTGTGAAGTAACTTCCAAGAAGGGAACAAATCAGCCACATCAAATCCTCCAATAAAGAGCCATTTGTGGAACGAATAGATGAAATGAGACTTGAAAGCTCATCTTGTCGAATTCCACTAAAGGACTTGACCATCTTTGCACTAAGAAGTTCAATAATACAAATTTTACGCATTTGTCTCCAATAGTCACCATATTGGGAAAATGCAACGTCTTTATTATTGTAAAATATGATGTTTGTGGATACAAATTGTGGCCTATCAGCAAAAGCGAGGTCATGAATTTTTAAAATTTCTTGTGCAATAGTAGGTGATGATATGACCACAGCAGGAACTTGCCCGAGTTGCAAGTACATAATAGGTCCATATCTTTGGGATAAATTTCTAAGAACGCGATGTGGAAGTCCTCCTCCGATCAAATGATGAAGATTTCCAATAAAGGGAAGTCTCCATGGCCCTGGAGGCAATCTTTGTTTTTTGTTTTGACTCCATTTTCTAAGTAGAATTATTACAAGGGAGGACAAGAAGAGAAGCAATGAAACTAAGTTAAAAAGAGAAGAAAACATTTTTAGTATTGGAAACGAAGTTTTTGATGGATATTGCTTTCAACTAACATTAGCTCATCTTATAGTGGCTCCTTATTATAGCATTGCATAGTGTGATTATGTAGGAGATTCTTCGACTTCACGTGATAAGGATAACAATAG

>LNC_000262

TGTTGATTGGTTTCCTCTTCTTATATTTTTCATGTACAACCGGAGAGTTGGAGTTTCTACTTTGGAACTCGTTATTGGCCTAAGATGTTAGGCCAACAACAATTAGGATCATCGCGATGCTTGGAGTCTCGTAAATAGGAGTCTAATGTTCCTCAGCCTTCCTTTTGTTGCCCTTATGTTTTTATATGTTGACTAACTTTTACTCTTAAGTTTCATCG

>LNC_000263

GATTAGGAAGCCTTTTGAAAATAATATCCTCCGTGTCTTCTATCAAATGGGATTCATTTATTCGGCTGAATTGCAACTGCGAATTTCTAATACTAAAAGTACTTCTTTTGAAATACTGTAGAATTATTCTCAGTTAGTCGTAGATGCCTGCCTCTCTGATTTCTCAAAAGTTTCTCATTGATGGGGAAAATATTGTTGTGATGTATGTTTTCCATGGTTGAGTTCTTCTTGGTTAATGATTTAGTTGAAAATTAAACTATGAATTCCATCTTTTGGTGTCTTTATGAAATACATCATGACATCTTGAGGTATTGGCATGGATAAAGTATCACTTGTGCACACGAAATCGGACACCTGATAGTTCAAAAGCTGTAAATGTTTTAGTGTTTTTAGTGCTATGGACTCATGGACTGATTATTCATCTTTTTAAAAGCAGTTTCTTGTTGACAATACCGCC

>LNC_000264

GGTTCGACTTCATGGGGTGCCAGTTTCTATTATATCAGATCGGGGTTCTCAGTTCACGTCCAGCTTTTAGAGGACCTTTCAGGATGAGTTGGGCACTCTGGTTGACCTTAGCACAGCTTTCCACCCGCAGACGGATGGCCAGTCGGAGCGTACTATTCAGGTCCTTGAGGATATGTTGCGGGCCTGTGTTATGGATTTTGGAGGTCAGTGGGACCAGTTCTTACCTTTGGCGGAGTTTGCGTACAACAACAGCTACCACTCTAGTATTCAGATGGCCCCATTCGAGGCATTATATGGTAGGCGTTGTCGCTCTCCAGTTGGTTGGTTTGAGTCTACAGAGCCTAGGCCGCGTGGTACAGATTTGTTTCATGAGGCCCTGGATCAGGTGAGGGTGATTCAGGATAGGCTCAGGACGGCTCAGAGTAGGCACCAGAGTTATACGGATCGGAGGCGCCGACCTTTGAGATTCTCTGTTGGTGATCGGGTATTCCTCCGTGTGTCGCCCATGAAGGGCGTGATGAGGTTTGGGAGGCGGGGCAAGCTTAGCCCTAGGTACATTGGGCCTTTTGAGATACTCCGGACAGTTGGGGAGGTTGCTTATGAGTTGGCCCTACCTCCAGCATTTTCAGCTATCCACCCGGTTTTCCATGTCTCGATGTTGCGGCGATATGTTCCTGATGAGTCCCATGTTCTCCAGTATGATGCAGTCGAGTTGGATGATCGTTTGACATTTGTAGAGGAGCCAGTTGCCATTCTAGCCAGAGATGTGAGGAGATTGCGCTCGAGAGCCAT

>LNC_000265

GCGGCTTTATGCCGAGTTCTCAAAGTGCGAGTTTTGGCTTGAGTCTGTAGCCTTCTTGGGGCACGTGGTGTCCAAGGAGGGTATTAGGGTTGATCCAGCGAAGATTGAGGCTATTCGTGATTGGCACAGGCCCACCTCTGTGACTGAGATTCGTAGCTTCGTCGGATTGGCAGGCTACTATAGGCGTTTTGTTGAGGGATTTTCTACTATACCAGCGCCTTTGACTCGGTTGACTCGCCAGGATGTTCCGTTTGTGTGGTCGGAGGAGTGTGAGAGGAGCTTTCTGAGGCTTAAGGAGTTGCTTAC

>LNC_000266

CTGAAAAAACAACATGCTCCTCCTCAAATCGATATAATGAGAAACTTTTGCCTCTCATTTTTATTTTAAACATTTCTTGATATGAAGCATCCTTGATCAAGCAATATTTATCTTCAAAATTTAATTTGTAGCCTTTTTCCATCAATTGACCAACACTAAACAAGTTTTGATCTATGTCAGGTACATAAAGCACAGCAGAAACTGGTTTAGTACCTGAATGACTTGGATATCAATGGTTCCTTTCCCTCTTACAGGAAGATGGTCGTCATTATCTATTTTGAATTTGGTAATGAACGTGGTCTTCAATTCCCTTGAAGAGATACATGTCATTTGTCATGTGATTAGTGTATCCACTGTCAATCAGCCAACACTCGCTCGATCTACTGGAAAAACATGTAGCAACACATAGTTGATCTTCCTCCTCACCA

>LNC_000267

TGGAGCATATATTTTCGAGTTAGGAAAACCAATACACAACTTAACTTGCAGAAACACAAGGTCTGGCATCCACAAGAAAGAAGAAGTTGCAACGTCTAAATGTAAGACGACTTTAAGTAGTGAAAGGTTTTTGAGCATGTGGAAACCCGTAAAGCTGCATGTTGGCCTGAAAATGCAGTTGAACAGCCTCAAACTTTCTAGTTTATTTATGCCGTACAGGCAGGAAGGCAATTTATAAGGAGCATTGTTGAGAATCAGACGATCCTTGTCTAGATTCTCAAGGGTGAGATCCATGATACCGTTTCTCGACAACAAAAGCAACCATTCATCAATAACTGAGTGCTGAGAAGAAGGTATTGATGAGATATTGACGAGGAATGTCTTAATGGCTCCACAATGTTGCAACAGAATTGTATTAATGACGTCTATTATTAATGGCTTGTTGTGGCAAAAGTCAGCAGAAAATACAAGCTTGGGAATTGAAACCCAAATATGTTTCCACGGTCTAGACAAAACACTCATTCTTGCCGCTTCTTCCACAGATAAGTGCTCCTGAATCTGGTGTTTAACATTAATAGGGAGATCTGTAAGTCTATCAAGTTCGTCCCTTTCAACTTTAAGTTTCTTAAAGCAGCTACTGATCATTGC

>LNC_000268

TCTGTCAAACGCCTAATGCTTCACATTTGATATAGCCAAATTTGGTCCATATATTGATAGTCTTAAGTAATTTATCATTTTAGCGTCATATATGATTCTGCGAACTTAGCTCCTATCCCATGTATTTATTTCATCCTGCTCTAAGTATTGGACCTTCACTTGGTGTTTTACTCAGCTCCTCACTAATCTTTGTCATCAACTCCATGCTTTGCTTCAACCTTTATCAAAACTTTGACTTTAAATTTGATCAGTGTTTCATGAATGGTTGTTCTTCATGTTCAAAAGGATTGAAATATTTCTCCTGTTACTAGTAGATGACTGCC

>LNC_000269

GAAAAGGGTATCAAGAATCTTGGTGTTATTTCCATTAATTTGTGTTATAGATTGTGGGGTTACCCTGAATCTTGAGGCGACAGAAGAAATGTTATCATTAGGTTGAAAAACATAACTTATGAGAAGTCTTGGTAGATTTTGTGAAGATGTCGTGTTTGGACATTTGCAGAAAATTGGGAATTTTATGGATTGACCTATGTCAATGTCTTCTGGTACGACGGTTGGATTAACAGCCTCAACAGATTGATAAGTAGTAAGATTTTGGTATTTAGATGTTGATATACCATACATAGTGTCACCTGATTTGAAAGTGTAGTTAAAGCCAGCATAAGATATGCTACC

>LNC_000270

AAATTGCTACTGCACTCGTGCCGGATCCTTCAAAAATACATAACCTTTAGAGGATCCGACACATACCTGACAATATTTTTGAAAAGTCAGAGCAACATAATGAGATATGCAGCATGTAAGAATGGAAGACGTATTCAACTGCCATTCCACGGATAAACATCTCTTAAAGTCACAGTTATTCTTTTCTTGATATCATAAAAATAGAACTGTCCTCGACTTGTATGTCCAAAACTTATATCCATCTTTCCTTCAGCTCTTCCTGATCTTTTGGCATCTTTTGACACATTTATTTATTGCTGCCTCCTAATTACTGGCTAAATCAAGACCTTCAGCATAAAGTCCCATTGACTACTTAAGTAGATATGAAGGTTTTGCAGTGTTGGTTTTGATTAAGAATCCGGAAATTTCATCCAGGAAACTTTCTTTGTACTCAGGAAGTTAGTTTGACCAGATTACATCCAGACGCTACTTACGCCATCTTTCCTCAATCGAAAGGGGACACAGACAATCAACCATGTCCTGATATTGTTTTATTTGCGAAATTGGATGTTAAACTTGTTTTACTGACACATGGAAGTAATTTCTTTTTAGCCTCCTGATCTTTTTTGCCACATTCTTGCAGTTAACATTGGCAAGATGAGGATTGTAGGCTTGGACTTCGAAGATGCAACTGGTGATGAAGATAATAACCAGCAGAATATTTGAGCTGCAGCTCAGCTGCACTACCTTGTGTCTCTTGGGCAGTTGACTGTAACAATTGTTTCAGAATATAAATTGGGATGTATTATATAGTTACTCATGCAGTTTTAAGCTTATTATCTTGGCGTTATGTTATCTCAAAAACTGCTATGGGTTTTGCTAATTTTCATTTTAGATTAAGATGTATTACTAGTTAACCGCGTTATACTGGGGCTTCAAGCTCAATATATATATAAATCTGGTGCACAGAATCAAGCCACACTCTTTTGATTACTATTAAGCTATGTTTACAATTTCTGTGAGTTTAGAATCATTTGCTTTCTTCTTGCTAAAGGATTTAATTTCCAGTACTAGTAATAAGTATACATGTGTAAATCTGTCATCTTTTGCGACCTGAGACAGTTTTCTTCACTGCTCCAACAGACACTAAGAACTGATCCAGGGATGATAGGGTCCTCATGTCATCTGGAGGAAAATAGCTAGCAGCCTTTCCTAAGAGTGAAGTGAAGAGAAGCGAGAGAGAAGGCCGAGTCACATAGCGCTCACCCAAGAGATCGTCAAGAAACCCAAAGGCTGCTAACAAATCCGAGCTGCTTGTGTTTATTGGAGCAGCAAAATGAGCAGGGATAATTCTCTTGAATTTCCAATCTCGAACAATACTGTCAATCCAGTCTCTAACCTGAAGTTTTGAAGGAATAGACAAGATTTTTTACAAAAAGACATTTTTTTTCCAACAAGAAAAGGATCTTCCCATAAAATACTCTGTATGGGATAAAACAGTTGCATTGAGAAATGCAGCCATCGTTATAATTTGATCCTCGATTTCTGTTAATATGTTTTACAAGTATGTGCATAATTAATAGAGACAATGCGAGCTTTGTGTATCTCGCTGCCCTTTTCCTTTAATTCTTTGGGCGTTGGTCCACTTGGAAGGAAGTTATACGAGGTTA

>LNC_000271

NNAAAAAAAAAGCTGGAGTATTTAGTTCGTGTTGAGTGAAGGAAATTTTGCAACTTATCAAGACATTTGACCACTTAAGGCATCAATGGATTTATGAAACAAAATTTTATTATACTCTACATGCAGCTCCATTTAAGAATACAAACTTCTATTCTTTTGAAAAATACAGAGAACATCTGCTCAAATTTACTGATAATGATTCAAGGTATCAATAGATTTTGGAAAATGAATTTTCTTATATTTTATGATCAGCTTTATGTAAGGCTATCAACTCGAATTTACTGGTCATTTAGAAAATTTTGATGTTCATGCTACTTCAGGAACTATAGCTGCTATGCTCAACACGCCTATGGACAGTGTGAGTAGTAGTTTAGTGTTTTCAATTTTTTTGCTTCTTGATTATTTTTTCCAAATATATAAGTTGAACCTCATTGAGTTACAATAACAATATACCCAGTCTGATCCCACAAGTTGAGAATGTAGAGGGTGGGGTGCACATAGACCTTATTCCTACCTTTGCGGAATAGAGAGGATGTTTCCGATAGACCTTCGGCTCAAGAAAAACATTTTTCAACAATAGGTTTGAAAATAGTGTATTTCTTCTTCATGTGTACTATGTTTTCTCATCTTTGAACTACATATCAAAGTAGTTGTTCTTATGATGTCGGATTGTTTAATCAACCTTGATAATTCATCAAATGATTAGTATTCAGAAAATTCTCAATTGATTCAAATAAAATTAAAAAATAATTGTTGAGGAAGGACAAACAGAACATGGATGGAGTACATTTGTTTTTTAATTAAATTCCTGGTTAGCCTACCTAAGAGAGTATCATTGAGAATTTTTCTCTTCCGCCAACTGAAAGCTGGTTATAACTGTATGATTTTTCTTTTTCTGTCAGTGACAACCCACATGTTCTTTAGTGCTTGCCGAACTCTTGCAAAAATGTTCATATGTTATGTGTTAATCAAGTTAGGTTAAGACCACAACTTAAGAAAACTTAGGAACTCCACTATCTCCAAGAATAATGCTTTTGTATGTGTTTCTACTGAACTCCTTGTGAACCATTTCCATATTTTCTAATGAATTTGTTACTTTACTGACTCTGGCCAACCATGGGAGTGATCTTCAAATTAAAAGATTAAACTCAATTATGTCAAAAGATCAAGTTATGAAGCCACTGCCTGATCATCATATATTATTTTAATTCCAGAAGTAATATTTGTTCTTCATTCAATTGTATCTAGAAAAATACATAATATATACTAAAAGATATTTTCTCCTAAATGACTGATTTGATTATCTTATTCAGTTTTCATAAAGTCAGAAGGTTAGTCGTACATAAGAACTATCAATGATTATGGTTTAACCATGCTCGATACCCTGTTGGTGCACATTAATATTGTTGGGAGTTTATACTTGAAGCCTAAAAGTATTATAGTAACGATATTTTCCTCTTTTTTTTGGTCGGTATAATCAAATAATTTATTTGTTGAGTTTGAATATATGATATTTATGGTGTTTTTCACTTGTCCTTGAATATCTACAGCCACTTGGTATACTTGTAATTTCAACTTTTTTCTTGGGTTAGTGCTTTTGTTTACTTAGCAATTCTTGATTTATTATACGCTCTTTGTTCACATGATTCCTGCATTGCATGACATCCCGTCTATATAACATGTTTCANN

>LNC_000272

TGGATAAATTGAGTATGTCTATGATTGTTATGAGGCCGGACTGGCTGATTGTGAAGTCAGACGAATGGGAAGGTGTGTTATGGACATCGTGGAAGGGATATCTTGGGTTGTTCAATTTCGGTTTTGTATATTCTTATGTGACCACCTACCTCATTTCAGACATGGTTTTGGATTATGATTCTCTGTCTATGTTCAGACGTGTTTTTACTTTTGTGGGTGGATGCCTAGTGGTGAATCGGATGGACCGATTCATGCTCTCTTGGCAGAATAGAATACTTGTGTGGGTGGGCTGATTCAGAGATAGTGGGTTCGATGTGGTATTAGGGTGGTGGAAGGTTTTCGGAAGTTCTACAAGTGTCATCTTGCTTTTGGAA

>LNC_000273

AGCGATTCCAGTCCAATCACGCGTACTAGACGATCCAAATCCATCGCTGTGTTGTCTTCCTCGCTGCAACAGCAACAAAAAGCATCTGCCAGCCGTCCTACCCCCCTTTCCTCTTCCGGAATGAGTCAAATTTTAGAGAAAAAAGATGTTTATCCCAAATGGTGCAAGAAATTTATGGTTTTTGAATTTCGAATGCACTCGATTTCTACCCGAATTCCCCCCAAAACCTACCCTAATTTTCCCCTATATAACGTCTTTTATGTTCATCTGCACCAGGAGAGATTTTTTTTTGGGGTTTGGAGTTGAAATTTGAACTAAGGCAGAGAGAAAGTGAATTTTTTTTTTAGTAAATACAAAGGAGAGTTAGAGTAGAAACAGTTTGAATACACAATATACTTCGAAAATAGAGAGATAATAGTTTCGGTTTTGGGTTCAGTTTTCTGCCTAGACTTTTTGGATCATCGTTCGAGCTCGATTTTTGTGGTGAAGATCGTCGCTTTAGTTCGTCATTGTCTCGGTCGCTGCTCCTCAACAGGTATATGACTCCCAACTCTTTTACTATTAAGTTTCGGTAGAGATTTCGACTTGAGTTGTGTTCTGTATAAGTTTTCCAGTTTATTTTTATTCGGAGCATGTAAAGCTAAATGTTGTTGTTAGTATTCGTTGTGCCTTTCCTGAAATGGTTGTGAAGGTTCCGTGTTTCAAGATGATTTAGTGCCTGTTGTTAAATGTCTCTCTGTTTACATTTTTATTGAATTCATGATCATTATGCTCGCCTCAAGTCTTGGTTTGTTTCATTGGATGGCTATGATATTGTTTTTTTATATATATATATTTTCGATGAAGTCGCATTCGTGCCTTCATTATGTGTTTAGAAAGCGTCGAAAGGATTTAGGCTGCTGTACCTTTTGTTTCTTATGAATATGAGCTGATTTTTTTTTGTTGAAGTTATGTTCTTTAAATGCATGTTTATAAATTTGTCCGTGTCTAGCTTTTTTTTGAAAGAAATTTCTTTTACTCGTCCCCTTGTTCACTTGTTACTCGATATTTTTGAGTTCTAGATTGTAGCTAAGAGTATTTGTTTATCCTTCTCTGTTTATAAGGCATGGCTCGACTTACTTTAGTGTCATTTAAATGGTTTTAAAATCTGGAAATTTTAGTTATTGCCCTAGTCACCCTTCATAGTTGTAACTATTATGTGATAGAAAGCATCTCCGCAATTTGGGATTTACTATAGTGTCATGAAAACATAATTTCGTTTTGGTCCGAGTGTACTCTTAAAATATCGCTTTATGTCACCTCTAACTCGGCCATTATACCTCCGTTTGATGCTTTTGGGTGCTATTTGATGATACAAGTCTTCAATTTTAATGAATAGCATGGTTATCATAGCTTTGTTTGACCCCTGATCGATTTGAGATTATTTCGGTTTAACACGGTTTCGTCTTAAATGTAAAACCACTA

>LNC_000274

CCACTCTAGCAGCCGCCTCTCAACTCTCCTCTCCATTTTTTCCGGCGAAAATTGCCACCACTTGCTGGTGGCAGCCGTTTCTCTCTCCCTCTCCTCGTTGCTCCCCTCTCCTTCCTGGCGAGCAGCAGCCAGCAGCTCTGGCGAGCAGCAACCAGCAGCCTGGCGAGCAGCAGCCAGCAACTCCTGCGACCAGCAGCTCCAGCCGGACAGCAACTCCAGCGAAGCAGCAGCAGCCGACGAAGACAGCGAGCTCCGACTCCACCAACCAGCAGCAACAACAACTCCGGCCAATAGCTACGAAACCGATAATATCAACGACGAATTTGTTACTCCAATAGTCCGACCAAGCTGAAAACAGAGCATTTTGATAAGGACAGATCTCTACAGATCTGTCCCAGGTATTGAACATTTATTTATTTTATTTTCTAAAACCTTCATGAATATGGGCGGCGTGTTCTTGAAATTTATGTGTGACTTTTAGTTTGTGCTTAATCATATGTTAAAATTTTCATCTATGTTTGGGCAAGTTCATCTTGAATGGGTCAAGTTCCTTGTTATTTTTTATGATTTCTTTAAACTAGTGCCTTATCAAAATGTCATGCCATATTTAGTTTGATTTAAGTATATGAGTTTAGTGTATTGTGTGTTTGAATGTGGTTAGTTTTGCTGCTATTACTTGAATCATTTTGATTAACTCCAACAAGGTTTTAAGTTTTGAGTTGGGTTTATTTAATTTATCCTCTCGGATTCATAACAATTGTGGATTGTTAGTCATATAAGCATTGCTAATTGACCACTTCTACGGTATAAATATTCCCCAACTTTTGCTCCCTTTGTATATGTTAATGGGTTGATTAAGTGACGCCTATCGAATTGTCGAAATGGTAATCTTGATTACTTTCCTGAAATTACTTATCAACTTATTAATCTAGCATTATTCTTTTGCGGGACTACATGATTGATGCATCGAATTGATCCTTCTTGCCATATGTATTTCTTGATTGTTGATGTCACACAATTTAGGCATCATTATTTTGGTAAGAATCACAGTTTCACTCTGTAATTTTTTAAAGGATGACAATGCATATAATAAAAAAAATATTCCTTCCTTAACTAATTGTTTCTTTCCGCTAACTTATGTATATTATTAACATAGTAACTTCCTTAATTGATTTTCTTAGTATAATTAGGATCTAATTGTCTCTTACTTGCTTATTTTTGGTAAAATCCTTACTGTTTAATGAATTATATGACTGATTTTGTATTTTTCTTTATTTTAGAAAGCAATGATAGTCAATTGACCCCGAAATATTTATTTTCCTTTAATATGTGCCCTCCCTTTCACTATATAAAGAGACCTCTTAGTTCATTATTTGCTCACGGGCTACACACGGCAAAATAGACCTTAATCTTGAATACTTGTAAAAATAAAAATACTCTCTACTCTCATAATAATTAAGTTTTCCGGTAAACTTTAAAGCTTC

>LNC_000275

AAATATGAAATTGTCCAGATTTGTGTTTATCTAAATGTGGGCTAACATCTGATAACTTATTTTGATCCTATTTGAAAATCTAGTGATATCCATATTGTACATCAAGAACACCTTGAGTGAATGAATTCAAAGTCTTGAAGAGAAGGTGTTTTAACTTTCAAAATTGTCAATTGTTGTATTTAGCCATGGTTCAAAGTTTGAGTTCTCTTGTATTTCAAGATATACATGTTGTCAAGTTTATTTGTGTTTCCTACTCCATATAAATGACAAAAATTTTAAACTAATGAAGTTGATATTTTTTGGTTAACTCTTGCATATCTGGCTACACATAACATTTTGTTTCGTATAAAACATGCCTACAAGAATGGTCCACATGGCGGTCTTGAACTAGCTGGAATTATTCATCTTTAACTGTATGGATGAACATATAGAGTATTCTTATTTGGATGGCCATGAATTATGCATTCTGGAATTAGTAAAAGCCTCGACCACAAGTGATCAACGAGCAAGTAAAAATGGAGTGCAAGTATAAGTATTTTGACATTCTTAATAGTAAACAGAAATGTAAAGACAAGTTATGAATAGAAAAACTGTACAAATAGAGAAACTACATATATAGAGGATTTGAATAGGCTTGTCGGAGGAGCTAGTTATTGGTTGCTTTAATTGTAACTCTTCTGATTGTTGTTTTAAATATTGTGTTTGTTGAACCAAAAATAGATTACTTTAAGAGTTTTATAAATTTTGATGTGCT

>LNC_000276

AGGTGGGTTTAACTGAAAGTGAACTTCTTCACCTACAAATTTGGTTGAAACTACTAGTATAGGTACAAAGAAAAGAGAAACCATACAATCTAGACCAAGTGCTTGGGAACATTTTTAGAAGTTCAAAGATGCTAACAGAGTTGATAGAGCGAAGTGTAATTATTGTGGTCATGATTATAAGGCGGATTTATTTGATGCCCTCTCTTTCATATTGACCTTCCAATCCAGTTTACATGGTCTGTTTAGCTCCAACGACTTATAATTTCAAGAATCCTTACAAAAGATTAGCAAAGTTATTTTTTTCCTGGTTGTCGGACTATGAGTTTACACTGTTTTCTGTGTCGTATTGTTTTTGTTTCTGATTTTGGTTTTTGAGTTGTTGGAAAAGAAAAAAGGGGGAGGGGGGAGGCTTGTAAAGTTGATTGACTTGAAGCTAAAGAAATAGATTCATGGATATCGGTTCCCT

>LNC_000277

CTAAAATTTGCTATTGGTTCGAAAAGTGATCTTCTCTGTCAATCATCACCATCCAGAGCACAGCGCCATCTACTCTGGCCAGCGCTTCCTGCCTTCGGTCGAGTCCGCCATAGTCACTTGAGCTATAGCCATGGCTGTAACTTGTAACTGCTCCTCTTTTTCTTTGCATTTCTCAGTTGGATTCTTTCCGGTTTATCGATGTATATGAGCTATTGTGAAGTTCCATGGGTGGATAATTTCTTGTATATCATCATCATACTTCTGATTTTCCTCTTTTCTCCTTTACTGATTCCTTCTAATTGTTGAAGTACCTCTTCTTTTTGCTCCATTAAATTATCAAAAGTGCAATTGTTTGAC

>LNC_000278

TTTAAGAATAGTCATGCTTCTTTTGAGATTTGCATAAATTAGATGTTATTCCTCTGTCTTGATTGAGAGTTTAAGTGATATCGATTTTTGTTTTATTTTATTTCCTCATGCCTACTATTTTGCTCGTGAACAAATGCTTAGGTTTGTTCCATTTTATTAACACATGGATTTATTATCATCTTTGTTTAGCATTGTGCCGCATCTTAGGCTTAAGTGACATATATTCCTACAGTTCACCCCATTTTTGATTAATAGTTGCCACCTTATCATTTGTCTTTACCTAGATAGTTAGGCATAAACATATTACTCATTTGGTCATCACTAGTCTAATTGCCTATATGTAACCATATTTCTAGCACTTCATCTTATCATATGAGAGTCAATATAGGAGAAAGATTTATTGGATATTGTGTGCTTTCCTTCGTCACCTTTGCATAGTATTAGTATAGTGAACTAATCAATCATGTGTTTCACCTTATTATTATTATTTTTTATGCCTTAGTGATATACTTATATTGTAAAACTTTAAAGTATGATTCATTTCTAATTTCTATCTTGTTTTATTCTTTTTCTATGCATGAGCTGCCTCTTTCCGAGTCTTTGGAGGCTCCCTCCCTTTGCGTTTCGTGGATTGGGCTTCATAGGCCCAATTCTATGTTTTCCTATCGAGTCCAAAAATAGCCGACGAACAGGTTACGGGAAGTACATGGGCTGGAAGTGAATTTTTGATGTCCGAAAAGGCTGAAAAGGGACTGTATACCTGCTGTATACAGCCTTTATACACTGATATACGGGTCTAGTTCAACAAATACGGGTTTAAAAATGGAAATACAGGTTGCAGGGGTCAAAATGGATTGTATACATTTGGTATATGGTTCAAAATACAGGAAATGAGGCTGATATACGAGTCGTATGCACTGATATACGACTTGCGTATACAAAAACGGGAACTGATTTAAAATCAGCAGCTTTGGCCCAAATTTGATTACTTCCTTTCCCTCTCTTATTTATTTTATTGTATTCGACTAATACTTTGATCATCTTATGGTTCAATTTAATTAAATTCCCCCAAGAATGGTC

>LNC_000279

TTAATTTTCACAAGAGGTAACACTTAATCCTTTTTATATAGTTTTATGTCATGATTATACAAGCATTGGTTGCAATATATTGAAATTGTTAAATTTTGTATGTGTTTAAACCATTAATATTCGTTGATGTTATAATATGAAGAATGTTCAAATCTTTCATATCAACTTTATGTTTAGATATTTTATATCATTAGACTTAGTGCGAAATTGTTATAACAAACCTTATTTTATTTCAGGTTTAAAC

>LNC_000280

CTTTACTAGATTTCAGGTGAGCTTTGATTTTGATTTCTTGCCTTCTATTGCTTCATTCAAGCCATAGATCAATGGGTTCTCCTCTTCTTCACTGTAGTTCTTTCTAATCCTCCTGACGGTATTTCTTTCTGAAATTAGCAAAATAGATAGATTCAAATCTTTTGTAGTTGGATTCTCCGTATTGTACATGGATTGGCTAGCTTTTGATTTGTCGTTCTTGCTTTGGTTAAGTGTTTTCTTGATTGATCAAAGAAATCAAATTGCCGTATTTAAGGTTTGCTCCTTCTAAACCTCATCATGTTGAATTTTGCGCGAATTTGGTTATCAATTGGCCGTATTTGTAGTCTCTTCGTTCTTTACGTTTATTATGTACAAAAAATCATATTAACATTGTGGTATTCTATTCAAATAAAGTGATCGAAATTTGATAATGCTCATAAAAGGTCTATCCACTTTCCCTATTTGCAGGGTAATGATTTACACATATTTCCATCTGGGTTTGGATGGTCTTGTTTCGTATGTCTATGCTTACCACTTTTCAAAGTGATTAACTCCTTCAAAGGGTTGGGATTTTGTTGTCTCTCTTCTTCTGCTCGAGCTATTTTTTTGCAATTGGTTTATCTGAAGATATACACTTCCTC

>LNC_000281

GAGCTTCGGAGGTATGCCTTGGCATGGACCAACTGATACCCCTTAAGTTAAAAAACACCTGCCACAGTTTCTCTGTCACTCTACAATGGATGAAGAGATGGTTTGTTGTCTCTATTTCTCTTTCACAAAAAAAACATCTGGAACATAATTGTCTTCCCCTTTTCATTAGATTGTCCTGAGTTAGTACTGCTTGTCTGGCTACTAACCAGGTGAAACAAGCCACCTTGTGAGGTACTTTAACTTTCCATATCATCTTCCAAGGCCATGGTAAGAGCATTGCAGCTGGCCTATCAAGGTGTCTGTAAGCTGATTTTACTGTGAATTTCCCATTTTTGGCCTTCAACCAGTGTAGGTTGTCTTCATTAGGTTGAAAGTTGATGGCCTGCTCCAGAGTATTGTAGAAATCTGTTAGACTGTCAATCTCCCAGTCATTCAACATTCTCCTAAAAGAGAGGTTCCATCCTTGGTTGTCCCTGACATCTGCTACTGTTGCCTCC

>LNC_000282

TAAGGTTGTTTGACATGTGCAGATAAAATATGGCTGATATTATACTATTACTGTTTGATTATATTCCATTAAGAAAGCAGGCCAATCTATTATCCTGAAGGCCAGCTAATTTCAAAAGGTCTTCCTTCGGTTGTCAACGTTACGCTTAACCATACAGAGAAGCTTTGGTCCTGAAGACAAAAGCCCGGTTGCTTAGTTTGTTGTATTTGAAATCTTACATCTGTGCCAGTGATTTCTTTGCATTCCAATTCCTAGGACAACAATATAAGAAAGTAGAAATATTGCTTTAAATTTTTTTGCTTCTGCTGAAGCTATAGGATCTTTTAATTATATACATCTTATGCATTATGCATCTCTGCGATCAAATCATTACAGAACTTATATTTCGCCCTACTATTGTAAATTTCTACGTGAATTGACCGTGCTTTTATGACTTCTACCGATTAACAATCATCTTCACCACATTATCGATACAATGACTCTTTCTTTTGCTCAAGTAACTTGTTGCAGCAATAAAGTTGCACTTTTTGGGAGGTGTCCACAAAACTTGAAATGTTGAAACCACAAATTTCCTTCCCTCCTCTTGGAGAAATCAGAAGAGTACTATGAGTTTTTTTGAAAGTGAGNN

>LNC_000283

AAAAACTTTCAATAATTTCTTACCATTCAAGGTATAGTTTTATTCATGTCTTCTTCTTTTTCCCCATCTTCTTCTCGAATTTCCTTAGTTTGTGATGTACTATGGTGGCAATTCTCTGTCTACAAAGACACTGTGTTAAAGAAAAAACTAAAATTGAAATTTTGGGTTAAGAAAAGAACTTCCTTGAGGATATTTGATTTTGATTTTGATAGTTAGCTTTCCTTTTCCTTCATTTTTTTTCGAACTCAATCAGACG

>LNC_000284

ATCGGGCTTGCGAAATTGTGCATATCATTTTCGGCCGGAGACTGAAGTGCTTTGGTAATGTTTTGCAACTATTTAATGAGTGTAAATGAACAGGTTGTTTGAGTACGACCTTCTCAATGGATCTGATGTCTTCTAGTGGTGGATTTAGCGGTTGTTCATGATGTGGTACTAGTGATATATGGAAGTGGAGAAATCTGAGTTGTCAGATGTTTCAACTAATCGGGTCAGAAGGTTGATGTTGCATTCTCGATTGAGAAATCAAGTGGTTGCTACTATGAACGCTTGAGTTAAGCATTAATTATAAGGAAAGTTATAGTGGTGGATCGAGTGAATCGATCCTGTCTTGTTTCTAGGACAAGGTACGACCCTTGGGTGGTTATGATTATATTGGGAACTAATGACTTCATGATGAGTCAAAAGAAATGGGCAACAATAAGCCATGGATGAAGAAATTTTTAACAAGTATACTCGTGAAAAGGCAATTAAGGATTTTAGCGAGAAGGTACGATTAAAAAGGAATCGGGTTGGTTTTGTCAAGATGTGGGTTGGCAAGGTAAGAGATGATTGGTTGTATCAACTTTTTAGCCATTGGAAGGACTTTTGCATGGTTTGAGTTGTAGCAGTTAAATACTTGTGATTGTAGAAGAGTTCGATGAAGTTTCATTTGAGAAGTTTGACTTAAGGTAAAAGGTAATATGCGATGGATGTTTGGCCAATAAACTATAATGATAAATGATTTACGGGAAGAACAAGAGTCTTGC

>LNC_000285

ACTCAAACGACAAAAAACCCAGAAGCGTCGTACAGTAGCAGCAGCTAGAGCAGCGTGCTTTCATCCTGGTGACTGCGGGATGATGCCACGTTGCGCCCCAAGGACTAGAAAATCAATCCCAAGCATCGATTCGCCCCTTCCTTTATTCGGAATGGGTTGAAATCACAGAAAAAGTGTGTTTTCCCGAATTGGTGAAAGGTTTTGGGAGTTTGATTTTTGAATTAGGCGTTTTCTGTCCAGATTTCACTCGTTTTTCCCTCCATTCAATCTACCCTAATTTCCCCCTATATATATTCTCTTTCTATCGTGCCAAGGGGGGAGAGGAGATTTTGATTTTCTTTGAGAAAGTAGAGGGAGGAGATTTTTGATCTTCTTTGAGAAAGTAGAGGGGATTTTTTTTTGGGAGAGTTGAAAATTTTGAATGGGAGAACAGTAAAGAAAAGGGGAGAAATTATTGGAGATCTTTGACTTAGAAGATTTTGAATTAAGAAAAAAGGAACAACATAGTAACTAGCTCTTTGAACGAAATTTTGCACCTAGGAAGTCTTAGACGTCTCTTTTCTTTTTAAATTCGAAGAAGCGAGTAGTTCCCTGTTAAAAAGCTAGTTCGAAATTTGTGGTGAAAGCTTGTCATCCCCTGTTTGTTGCTCTGTTTCGTTGCATCGAAAGAGGTAATACTTCTTCCTTGGTGG

>LNC_000286

TATAGTTGTGGGGATTCTCACATTTGTTTTACCTGCTTTGTCTAGTTGTCCCTTGGTTTGGAACTGTGGATTGGTACCTCTCAAAGGCGAAGGAAATTTAGTGACCCGATAGCATGGTTGGATATTCTGTCTTACAATCTTATAGAGACCAGACCATTGTATACATTAATAATTTCAAAACTTGACACCTAAAACATAACTACTGGATCATTCTCCAGTTTCTTTTGATTATTTAGTGCTAATGATCAATTTATATCAAAGAGCATAGTTGACTTACTTTAAACTTTGCAGGTCCAGTTGGATATGATAAGGGAGAAAAAATGACTGAAGTGTATCGAGATGTTCAGAGAAGTGATACTAGTATATATCACTTTCTTTTGTGTAATTCTTTGTTGTTGTTAGTTGGCTGGAAGTTTTTTTTTGTTTTAATATTATAATTTGCAGGTCTATTTAAGTCTAAAAAGGCAAAAAAAGTGATTGA

>LNC_000287

GTTCACAGTAATACCAAAACGACCCTCTTATACGCGTTCTCCTTCACGCGAGAAACGCGTACAATACCCGACCCTATGCGTTTCTCTTCTCTTCTCCTCTGTGAGTCAACAATCCAGCGATTCCAGTTCAGTCGCGCGTACTAGATGATCCAAATCCATCGCTGTGTCGTCTTCCTCACTGCAACAGCAGCAAAAAGCATCTGCCAGCCGTCCTACCCCCCTTTCCGCTTCCGGAATGGGTCAAATTTCAGAGAAAAAGATGTTTATCCCAAATTGTGAAAGGAATTTATGGCATTTTGAATTTTGAATGGAACCGATTCCTATCCGTTTTTCACCTTCCACCCCCC

>LNC_000288

NAAAAAACGGAACCCCAGTGATGCGAGGTAGATGACTCACCTCATCATGAAGATTTCAAGAAGAAGAAAAAGCTTCTTTGTATTTCTTCGTGAATATACAATCAATGAGAATGAGTACTTATACAATGAGTCCTAAGACTAAATTAGGAAAGACAATATCACATAATCAATTCCTAGGCAATTACAATCAATTACATTCAATACCAAATCATTCTACTCCTATAATCATGCTATCAGTATCAATCAACACTTTATGTTGGACCTTTGTGTAAAATAGTTTGTTCGCCGGCGGAACATCTTCATCACAGTAATTGGACACAACCTTTCTTCCTTCATTTAAATGGAACTATGTAATTTATTTCATTCAGATGCTTGCTGGAATGAGAAAGAGATGGGTTGAAATGTTAGGAGTTTTTTCCAGATAATTTTTGGAAAAAGGAAGAAGGGAAAAATATACTTGTATTCAAACGATACATTTGGAACAAGTTCAAGTGTCTAATCATTCTGTAAGTTTATAAGGCTATCTTACAAAAGGTGTCATGTTAAAGGGGCTCCCCTATCTATGTATTTGGCCAGTCTTTTTTGTTTAAATTTCAGTGTTTGGGACTTGGTCATTATAGTTGAGGAGCATGTTTTTTCACCACAATGCATTCATGTTTCGCCAAAAAAACCAAACTGGACATTGACATTCTGATGCCCTTGATCTTTGTAGGAGAATAATCTCTACTGCCTTATAAGTTATAACCACATTTTTTGTTGGATACATCAGTGTAGCAGATATGCATCTCAGACCATCTAAGAGCGTCGCN

>LNC_000289

TGGCTCCCATAATGGTGATACCTAGTTGTCGTTTTAGCATTCGAATTTAGCTAAATGTTACTCCTCTTGTTGATAAGATATGGTGTGCTATAAATTTAGTGTAAGCTCGTTTGAATTGAATTACTATGATTTATTAAATCATTGGCGTGCAAATGTGACTCGTGTGCTAACATTAGCCAAAAATTATTTTATCTTGTTTTTATATTGTAGTTGGTGTGTATGTGTATATATATAAGTGTAAAGTTATTGTGGTCTTTTTCC

>LNC_000290

TTTTGGCTAAAATATTATATTCCCTCCAACAAAATTCACTCTCCCTCCCATTTTTATTTGGTCAAAAAAAAAAATTCATAAACAATAAAAAAATCTTTTCAAGTCTCTAAAAGAGATTCTATTTGCCCTAGATCTGGAACTTTTCAAGGGATTTTCTCCAAAATTAACAGAAATCTCTTAAAATTTTGTCGTTGTTCACCTATCGAGGGCAATGAAAGGAAAAGAGAAACGGAGGAGACTAGGCATCTGGCGGAGACAATCGACTCAA

>LNC_000291

CCCTAACAAACAGTTAACCCTAAACTTATAAAACCCTAAACCCTCAATTCAAAACCCAAATCCTAAACTAAAATCCCTAAGAAACACAAAACCCTAAACCTAAAGAAACCCTAAATCATACTAACCCTATAAATCATCTAAAAACCATTTAAAACCCCTAAAAAACCTAAGATCTAAACCCTAAATCCTAAATATCTGTAAACCCTAATTTACAAACACAAAAAACCATAAGTGTAATCCCTAAACCCAAAAAAAACCCTAAGAAACACAAAACACTTTAAAACCCTAAAAAACCCAAAAACCCATTAAGCTTGACAAACTCTTAGATCTAAACCCTA

>LNC_000292

GTGTAATTTATTATTTTTGTACATTTGTATAAATGGATATTTGAAGGTCTTAGACGCATATATAAGTTTTTCTTATACTTGTACATAACCGTTGTCCATCATTTTACTTGTGATTCATAATATTTATTTTTTCAGTTCTCTCTCAAGTCCAGGTTTCTTAGTTGATTTGTATTGACCTGTTTTTCAAGATATCTACAAATCTCCCTACGTTGTGGTTATTCAGATGCAGAGGATTGGCTACTAAGCTTAGGAATCTAGATCCAGCTCATTGTAAATGTTGAATTACAATATGTTTGTGTTGTATCTTAAAATACAAACCGTTCTTATATTTTGGAGGTATTGGGTCTATAACAATACTAGAAGATGATTCAAGTTCTAAAATTTGTATATTAAAGATGTGACAGATAATAAGAATTCTATTATCTTATTGGCTAAATGATAAGTGAGTATTGCTAAAGTGCATACCTGTCTTTTTTTGGTTAAAATTTGCCTGTGCACTTGTATACCTGTCATATTATGATGCTCACCTCAAACTCTAGATTTTCTTGACGTTCAATTGTTTCCATTTCATTAGTTGAACAGTTTATCATTTTCTATGTCCTTGTGTTTTTTCGTGTTCTTGAAATACTTTTACCACTAATATCCTTTGTTTTTTTACTCCCTCC

>LNC_000293

CCGGGAGTTCGAGAATTAGCGATATACATGGCGTACACCCCAAATATACACTTTGTGTATATCACGATCAAGGAAGATGGAAAAAGGGCCAAAGCCCAATGAATTATTACTTCTTTTTTTGCAGATTTGGAAAATTTGGGCAAAAGCCTAAATTCAATATATCCCTCTTAATTTATTTGTTTGTCTATTTGTTGATTGACTAACGTTTTATTTATTTTTTATGGTTGAATTTAATTAATTCTCAA

>LNC_000294

CTCCCTAAGTTTAACTTGATATGAAAAATAGATCCTCCCAAAGCTTTTATAAATTGTGATATCTCTCTTTTTTGACGCTAATGGTTTTCTAGCCTGCAATTAGTAGGTTTGTCGATACGTATCTAAGAACTTGCAGGCCTCTTTGAATCAACAAAAAGTTCAAGGAGATGCAAATGAAATTAATAATTATTTACGTATCGTATTTTAGGTTCTGGTGTGAGGAGAGAGAGGGCTGTCCTTCGAGTACCACCTGATGCTTTTGAAACTGCTAGTTACATATGGTATTTTAGTCTATACTCTATAGACATCATTAATGCCTTCTGTCCGTTGATAATTAATTGTTTTATATTATGTTACTTAATGGTGGCAGATGCAAGGCGTTGTAACTTTCTTTGGCCGCGTTGCAATGTTGATAGACCAGAATACCCAGGCATTTCACATGTTCATGTCGGCACTCCTTCAGGTATATGTTCACTTATTTATCCCTTGCATCCAAATAGTTCTTGAACAACCAATACCTCCTTAACTACCTTATGGAGTTCTGTATTTGCTACCCAAATACTCTGTGGAAAAGTATATAGGATGGTGA

>LNC_000295

GGTCACAGAGAATAAAGGGACACTTACTTCATTTACTTTGAGGAGCACATCCAAATCTGGATCTCTCATCACCATTCCAGTAGATGAAGAAGTCATTGCAAACACTGAATCCGAAGAAGATGGACAAACATTCTCAATTGAAGTAAAAATGTCATTCCCAAGTGACCAAAAGAGATGCTATGTTTTGGTGTCGATTTGATGTAAATCTCAAAGATAGCTCTGGCTCAACAACTGGGATGATTATGAACAAAGAAGGAGAAAAACTATTATCCCTAACTGC

>LNC_000296

NTTTCAAAATGAGGGTAGAATCTGAAAATCTTTGAGTGTGAAGTCGTATGTTGTCTTAGTTGTTGTTTGACGGGTTGTAGCAGTTGGTTTTCTCCATTTCCGAATCCCGTGATCATGCTACTGCCTGGCAGACCCTGCTTGAATCAAGTTTATATCTCCATTTTGTCTAAATTTTAGTTGAAGGTACATTGTTTATAAGTGTTCGGCTAGTCTGAGATGGTGTTCTGTTGCCTTCTTTCATTTAGTATAAACTAGAATTTATCATGACATCTTTTGTTGCCTAATGCTTCCGTATCGAGTTGGATTTCAATTCTCTATTTAGCGGTTGCATTTTCTATTCACTTTAGTTTAAATGCTAAAATCATGTTACTAGCTTTGAATTTATCTTATGTAGGGTGGAGTAGTGTTTAAGTGCTAATTCAATGAGAATGGGAGCAAGTCTAGTAGCCCTCCTTTGTTGTCAAGTCTTTTCTTGTTTGATCTGCATTAGTGTGTGATCGTCTGCCCAGTTTTTCGGCTTATTAGAGTGATTTTTTTATTTTTTTTCTTTGGAGCTGGAAGGTTTTTTAATTAATTTTCTACCCCTTTTCGGCCNN

>LNC_000297

AACAATCTTTGGAGTGAGATTACACTGGGTTTTGTTGTTGTTGTTATTGTTGTTGCGTCTGCGAGTTTGTCCGTTAAAGGAAAAAAGCTTAGCTCAGAGACAAATAATGAGGATTGCAGAGAGCAGAGGGGAACACTTCAAAGGGCTGTTGCTCCTCTTTGCAATACCTGTGATTTCAATTTTTGCTGTGAAAAAATATAAATTCAACAGCCCTTTTCAGTTGAACAACTACCAAATGACCCCTAAATCTTTGCTTGTTCTCTCGAAATTCTCAACCCCAAAGTGAGAATCAATAGTAGTAGTAATGATAATAATAATAATTGTTGCAAAAATTCTGAGGCTATGATGATGATTTTCTTCTTTCCCTGAGCAAATCGATAGCGCAGGAAAGAAATCGGACCCATATCTTGGCGGGGGCAACCTGGATTTCAGGTTACCTTGCAATCGGTGCTTGCAGCCCTGGCGGTGATTCCAGTCTCTCTTGCCATGTTGTAAGT

>LNC_000298

TATAAAACCGATTTGGTGGGATCACACGTTACCTTGTATTGTGTTGTTAGTTTAAATATAATGTTTGTTTTGATAGTACCTTAAAATTTTATTGTATTGTATCATTTAATCTTTAGGTAAGATGAAAAGTCCCATTTTGTTTAAGACCGCCTTGGTGTGATCTGTCATTCCCTTTTTTTTTCTCATTTTGTTTTTTCTTATCATATTTTATCCTTAACCATATTTTTTATGGTAACTCCACCCCATACCCTATTTTTACTTGTAGATTTATCCTTCAAATTATTGATGTGTGACATTATGTAACAACGGAAAATAATTTAATCTATCCGGTCATTGTATCCATCAAAATAATGCAATACGAGACATTATGAAACAACAACCATCCAAACAAAGTGTTAGATATTTTTTTTGAAACTGGTAACATGTAACAAAGTGTTAGATATTTGTGCTTATATTTGGCCTTGA

>LNC_000299

CCCCCCCCCCATAAAAAATACAAAAAAGAAGAAGAAAAGAAACAGAGAAAAAAGAAAATAAGGCTCTAAGATATTCTTACAGCAGATAATCTGTGAAAAATAAGGGCATGCCTCTAAGTTGCTACATGTTTCATAGTAAATATATGAGCGAGGATGCAAATAATTTCTTGTCAAAAGAAGATGTTAGTCCATTGGTTATGGAGTATGCAAGGGGAAGAAGTTAGGATTTGAGGTTTAGGAGAAATCTAAAAGACTAGGAGGTTGGAGAATTTCAAAATTTCACTGGATTTGCTTCACCAACAAAACGTTTTTATGGATATTGCTGATGTATGGAGATGGAAGACAGGAGGAAGTGGTGTTGTTTTCTCAGTGAAATCTTTTTACGAGAAACTCTTATCTAGGGAGGATTCAACTTTTCCACACAGTTCGTTATGGATCCCCAGGATACCTCGAAAATTTGTTTCTTCACTTGGTTAGCTGTGAGAGGGGTAATCTTGATGGCGGAGAATTTGAGGAAAAGGAAGATC

>LNC_000300

CATTGACCGACTTTTTTGAGGGTTCCAAATGACTACATAGATCAAATCAAGTTAACACTTATGATACATAATGTAGTAATTTTTTCCTTAAATAAAGCTTGGAGAAGTTTTCAGAATAAAAAAATAAAAATACATAATGTAATAATTTTTTTCGTTTTTATGATATGTCAAATCAATATAATTGTATGACAAACACTGCAAAAGCTTCATAAAGAGATATAATTTAATGCAAAACGTGAAAAATATTCTTCTTTGTAAAACAAATTTTTTTCTAAAACTTTATAAAGTGTAAGAAAAACTTCCACTTTACAACGTGTAAGCAAAACTTACATTTTATAAAGTGTAAGAAATAATTCTGTTATATATTAAAATATATCCGTTATGAGACTAACGGAAACATTTAGAATATTTCATAAAATAGATTTTTTTC

>LNC_000301

NTGTTTGTCTTTTTCTTTTAATTTGTGGAGGAAATCTTTCGCTTTTTTCAAAGTATCTTCTGATATAGTCCGAACGATCCCTGTGATGATCTTCCCCTTGTATTATTACTTTTATCTCTGTCCTTGGTAATCCAAGGAGGGTCCATCCTGCACACATAACATTTTGCTTTATTCGGATATATCGTAATAATCACTGTAATAATTATCACTATCGATATCCTTGGAGCTATCCTATTCATAATCATATATAGATAAATCTTCTCTAATAATATCATTAATAATTTTACATATTATAAATTCCTGCAAGTGTCCACTGTGGCATATGGCCTGTATATTTTAATACTCAA

>LNC_000302

AAATAACTTTATTTGAACTTAGTTATATATACATTAAAAGCTAATACATTGAGCTGATGTAGCTTTTAATGTATTGTAACAGAGAGCTGTTGGTATGGTAAAAAAAAAATTAATAAGTTTTACACATACAGACAACCATACACTGGTAAAGGAACATCAAAACACATAATACTATGATATAAATGAAAATACTATTTCTTATTGAGTTGAGTTTATAATTGTGAAAAGTGGAAAGTGGTAGCAAATTGTATTTTGGGGAATGGGTAGTGTTGTATCATTTAGCATTAACAATGATCACCAACATAAAGAACATGGAACATGTTAAAAAGATTGAATACTAAAGTTTGAAAGAACCTAAAACAAATCCTTCCACTGTTTATATATAGAAAATAGGTAATATCAAATAACTAATGTTAAAATGTGGTTCCATTTCACATAATGAAATAGCCTGATAATATGTTTAAAAAAATGTTAAATTATTATACCGTGAAATTGTCCATCATATGTATAGGTGT

>LNC_000303

TCTGCTATATCTTTGCAACAGAACTCACCATACCTTCTAGACCAATTTATGTTGGGATAGACTGTCATACGGAGTCTGCTAGTATCTTTAAAGACCAGGAATTTGACTCTTGCACTGCTAGACTTTTTGCCTCCATATTTTGCATATCGCCTATTCTTCTAGAGGAATATGTCTGTGAGTAATGCCCAAGGAATCTGAAAAGAATTTCCAGATATAATTGGCAAAGTGCCCAGACACAAAGACGTGATCAATTGAATATGTTCCAAGTTTGCTACAACAAGAACAAACTGCAG

>LNC_000304

AAGGTATGTTAACTTTTAGTGTTGGGTGATCCTTTCCCCCACACACACCTAGCCCCTTTTATTTAATTAAGAAAGGATTGGCTTTGTTGTTAAAGTTGTGAGTTGTTGTGGTTGAAGTTGTTGGAGTTGTTAAAGTTGTTAGTTGTTGTTGTTGTTGAAATTATTGTTGGTATTGTTGTTGATTGTGATGTTGAGATTCTTGATGATTTGGGACTTGTTTTCGGTTGGGGTTTTGAGTTGAAATCTATTGAATGTTGAGGGATTTCATGTTTCTAAGTGATTGGGGCTGGAACCCTTGAAGTTAGAGAGTTTAGAGTTGGAGACGTGAAGGAGAAAGTCGGGCAGCTTGCGAGGAGCACGTCCGCGTCGCGGACTTGTTCCCTCTGTGAACAAAAATCTTCTCCGCGTCGCGGATAGGACGCGGAC

>LNC_000305

ATCTAATTCTTATCACTTCACTCTGTTCTCTCTCTCATTGTAATGACTCGTGGTTGGTCCTGCATGGGTGCGAGTTTAGGTGTCATCGCGTGATTTTAGTGTTAGATCTGGTTTTGATGCGGATGTGATAGGCTTGTGAAAAGGAAGTGTGATCAGACATGGAATTCTCTTCGAGACGAGTGAGTAAGGTTGTGTCCAAGGGATGTGTTTTGAGTTTATGGTATTGAAAGATGAACATTTATTAAGAGACTTCAGAAACGTCTTATTTCTCCGGTCATTTATTCAACAACGAGAATAGTGCTTGTGTGACTTATACGACGGAAGTGGCTAGATAATGAAGAATGGTGTAAGTAAGAGAATGACTATGTTGAGAGTGCTGCGACTTTGGAAA

>LNC_000306

CTTGTGTGAAATCAGTTGCAGTTTGATATTTTTTATTTTAACGTCCTGGAAAGACGTATTAGCAAGTGTTCCCATTTGATCAGCCAACCTATCCCCAGTTGATTTAGGTATTTTACTAATTGAGTCGGTTAGTTTGATATAAATAGATTGTGGCATTGTATTCAGATCATGATTTCAGCTACTATTTTAACTGATCTATATACTCCATTTCAATTGATGTGGCTTAGTTTGAGTAGGTAGAGAGTTTAATAAATCAAGAAAGACTTCTCAATTTCGTGGTCCTAAACATGTTAGTAGGTTTTTTGTGGCTATAAGAATATAGTTTAAAGGGTTAAA

>LNC_000307

GAATAATGAGTAAAACTAGGGGTGGCAAAATCAAACCCAATCCGCAAACCCAGTCAAGTTTGGGTGGGTCAATAACCCGTCCGTTTAGCTCAATCTATGTCAACCCATTACAAGATTGGGTTGAATTGACTCATGAGTTATCATGTCAAAATATTTTTAGAAGACATTTTCTTTTATTTGATATGTTATTTATAGCTATAATAAAGAAGAAGAAAATTTTATTTGGTACTAAAAATTTATGAAAGAACAAATAAAGCAATTAAACTTTAGCAAGGATTGGGAGGGTTGGGTTATGACCCACATTTTAGTACGTTTTAGCCTAACCTGTTTCAGTCCAAGTAGCTTTTGACCTGCACATTTATTAATTCAGTCCAATTTTT

>LNC_000308

GATCTTGGAGTTTCACTTCATATGTGGAAGTGGAAAATCGAGAAGACACCAACACACCTCCAAAGAATAAAGAAGGTATCATCGACTGTGAAAAGTCCTACATTTCTGTGTCAGATAAAGGAACATGGCATGTCCCTTGATAACTCATGTAAAGATTTTACAATAATCTTGTTACGCTTATGTGAATTCTCTGACATTAGAGTAATGCACCCGTCGTTTCAATTGTTCTCTGGTGATTTCTTGAAACAGGAAAAATGCACAAACAAATTGTTCGTATTACAAGATTTCTGCCGACATTGTTGGAATTTGCTTCCTCTTTTCATGATTTTACAGCTGTTAATTCTTGGCCTATAAAATTCTTTTGTCTATTTTCTATGCATATCTTTTTTAGATTTTAACTATTCCTTAATTAGAAACTGTCAGGCAGCTTTTATTCAAACATTCTTCTTTTTCTATTTCAGTTATTGTATGCTCACTAACTAATGTTATTAN

>LNC_000309

TGTGATGTCTGACTCAATCCCAACACTTCATAAATCCATTGTCTCATGTTTTAATTTGGATTATTTCCTTGTATATCATTGTGCATTTCTGTTTTGTAAATGCTTTGCTAGTTTATTTGTTGATCAGAGTTGTCATAACTTATATATAACAATAGTATCTCCTCAATTCTCGTGCATCTGTTAATAATTTATAGGTAGTAAGTATCCTACTTGCTCCTTTCTTTGTTGATGAGTCACTAAGGTCAATGTTCTTTTCTAAAAATTAGCATTATTTGTTAGATTCTTGTTTAAATATTGGTGTTTAGGTCAAGATTTCAAACCTTGTTTAGTTTTGTTTGATGAATGCTTTCTTTAAAAATGGGATCATGAATGTATTTGATGGTATAATACTCCAGTAGATCATCGCTTTTAAATTTGATGGGTTTGAGTAGGATCATTAAGTAAGGTTGAACGGATGTAGGGGG

>LNC_000310

CTATCATGAACCAGGTGTTCACAGATCTGCTTTTGCTTCTAAATCTCTTTAACTAAATCAGATAAAAACAGCAGCCTAGTGAAGATGTTGTCTAGTACATCTCTGAGAACATATATTGGACAATGATTCTCATCATATATTAAGTCAAACTTCTTTTAATTAACAATAACATCTTGTGATAACTGATGTGAATTTGGTTCTGCAGATCGCAAACTTCCTCGCAAGCAATTTGAATCAGAATTGGCCTCGTGATCTGTATGACAAGGTTGCTCGCAGTTAATGAAGTTGAAGAATCACAGTCCGTCTCTGCTGTTGTTTACTTTAGATGTTCAACATTGTACAAAGATAGTTGAACAGGCCAAGTCCGAACAACCATGATGTATTTGCTTAGTCGTTAATCTTAGAGGTG

>LNC_000311

AAGAAATTTTGCTTAGCTTTCTCTACTTAATCTCCTTTGTATTCTTTTTGTTACTAATGGGAATAAACTTCTTATTTTGGTTGACTTCCATCAAAATTTGCAGGTCCATTGGAATTTGATAAGTCCAAAAAAGTGATTGAAGTGCCTCGAGTTTCCCAAAGAAGTCCGTTTGAATCTAGAAAGGCAAAAAAAGTGATTGGAGTGGCTCAAGGTTCTAAGG

>LNC_000312

AAAAGAAAAAGAAAAAGAAACATTGATGGTTGAATTGGTTGCTAAAATTCACCAGCAAGATAACCATCAGTTATAGGCATACAGGGAAATGAAATTCACCTTTGAGTTTGGTCTCTAGACAACTGATGGGTTAGTGCCACGTTAGATAGAAGACTGTTTGCTGTGGTACAGAGAAATCAGATTACAAATGAAGCTCGGGGCTTAGGGTGTTGGGTAATGAGCTTCCAATGGTGTAAAGGTTAAATTTATGTGGTGTTAGTGTTGTTTTTTGATGAAGGAGTCCATTCGGTTTCTTTGGGAGTTCAAGGGGATTGAGACAAGGAGTCCATGGATTCCTAGGGCACCGAGTAAGGTGTGCTTCTTTGTTTGGTTGGCGGCAAGGGGTGCAATTTAACTGCAGAAAATCTGTGAAAGAGGAAGATTAGCTATGTCAGTTAGTGCTTTATGTGTAAGAGTGGATGTGAAAAT

>LNC_000313

CAGAATTTGGATAAATTTTTATTGTGTATCTTCAAATAGATAATACACAATATTTTAGTAGAACATTTTGCAGTAAAAAGTGTCATATAATTTTGGGTAAGAGTAACAGACTATTAAGAAAAAACATTAGCCTAAATGGAAAAGCTTATCATACCGAGGATACAAGAAACTGCATATTCATTTTAACATTTGAAACGTATCCAAGAAGGGTTGTCAAAGAGTACCATAGCACAATTAGATATAGTTTAGCCTAAATGACAACACCAACTGTGTTGCCTCACATACGTATAGGATTCAGAGGATACATAAAAATCAAATATGATTTAATATTAACATGGTTTCCTTCTCTTCTTCTATAATCTTCTCTATTTTTTTCTCAGTTTCACAATTATTTTC

>LNC_000314

NAAAAGCATTCACCTGAAATCCTAACTTCTCTATCCTTGATAAGATCAGCAAACCTTATATCTGATTCAAAAGCCTTCAATTGTTTTAAAAAAGGTGTTCGCCAAAATATACTCGCCTAACTAAAATGAATACTTCATTCACTTATTTTAAATTTGCAGCTACAAGTCAACTCTAAAATTGTATGTGTTGTGTCTTCAGCCATCTGCCAATTTATTGTGTTTTCCATGTGACAATTGGGAAATGTTAATAGGAAGAAAGAGAACTGACACGCAGGTTAGTACTATATAATATCTGATTATGTTGCTTTACTTTTGCATATACAATTCAATCTTCTATTTTGTTGCTAAAGGCTTTCCAATCTGCAAGGAGAAATTCATGTTAGTCTTTTGACCTCTATTTGGAAGGCTCATTAGTTCATTATTACACTTGTTTGAATTCAATTAGAATAGATCAAACACGTC

>LNC_000315

CCTGTATAACTTGCTCACGGGCCAAACGATCCTAAAGGGATCCTTGGTGGCTTGTTAGTATTCGAGGGGTTTATAAATTACGGGACTCATATGATTTCCTTTTAATTTCTACTTCTGCTAGTAGTTTATGATTTTTATTTGAGATATGCTTAGTTAGTACCTTTTTCTCTGCAAGAATATTTTAAATGAACTAAGTGGACCATTGATCTTTTGTCCCCAAGTTCATCTCCATCCATCATTTGGAGGTTCTCTCAGAAGTAACAAAAGATAGTCCAAACATAGAGGGGAACTGAGAAATGCTGGATAAAAAGATAAAGGCACATCTGGTA

>LNC_000316

GCTTGGTTAGTATATAGTGTTTATATGGTATGTGGTTGATTAGTTATCATTGGAATAAATAAGATCGAATATACACATCTGTTTTTGAATTCCCTTGATAAGAGGATTTGCCTAATTAAGATAGTTGTGAGGTTGGTTTGCATGTTTCTTCTCCCAACCTGAAAAAGCTATAAATCAATAGGGCTAACAACATAATACTTCTCATTACCTTCATATGTTGAGTTACATGTTTTACGAAGTTTTGATTAAACTAACCTACTAAAAAAGAAAGAAAAGAAGCTTGAGT

>LNC_000317

GTTGGGTTGATGACGTTCTTAGCATGTTGTGTGTACTCCCATTCGTTTGCATAAGTTAGTCCCAACATCGGGTTAAAACTTTAGAGGGTCATTGGTGTTTTCCCCACTACTCTATTCGAGTTAGTTTTTTCTTGTTATGTTCTTCTTTTTGAGCCTATCTCTTGAATGCCATTGAAACCGTGTTATTTTATAATCATTGTCATCAAGCCTCTAGTGTGTTGCTTTAGTTCGTTATTGCCGACATTGTCATTTGCGTAGGTCTACGGTTTCAAGTCTAGCCTATGTGGCGAATCATATGTGATGTAAATGGTATTTGGCCCGTCAATTTAGCTTTCTCCGTCTCTCGATCTTGTTAGTATCTGTTGCTTTGAATGAAATATTTGAGTTATTGTCATGTTTTCTCTTTGCCCACTGTTTTGACATAAGTTTGTAGTA

>LNC_000318

AGCTAGGTTACATTGACTCCATTACAACATTATGAGTGCATACATGATAAATACCATACCACCAATATTCTCTATAGATACATTAATTTTTCATCCAGGCTGTAAATTTGTTATACACTTTCTTACAATATTAGGCGAATACGTCAAGGCTCTGCAAATCAAATTTTGAAACATTTGCGAAAATCAGTTATTTAAGGTTCTATTTGAAAGCCTATATGCAAGTGCTTTTGTCTACATTCAAATAGTTCATTGCACATGATATTTATCAGTTGTAAATATCATTTTTATTTCCTTACCTACATACGTTTTTCCCTTTCCTCTGCTAGCATTTTCTCAGACTATTTTCTCTTCGCAATATCTTATTATTTTTCCAATTACAATTACTATAGGTTTGGTTGAAACTTTCCATCTTAAGAATCCAAAGAAAATGTGT

>LNC_000319

CATGAGTGTCGACACGTGATAAATCTCCCCGTATTCATGTGAAGTCGAGGACATTAGATAAGAATTTATGCGAGCTAAGTGTGTCTAGTTAACTTAGTCCGCTACCAATGTGTTGGCTCAACGACTTTAACCTTCTTATGTTTGATTAAGTAGTGCAGTCATAGGAATATTTATCCCCTATGATGACGAATTGAACATTTACTTAAGCTTTCATATGTCTCTCAATTTACTTGTTGTCAAGAATTCTAAAATGCTAAGTTCAATCTCATATTTTTAGAAAACAAAATTGTTTACCCAGAATTATGTATAGCCCCTTTTATTTTGTATCTGTACTTTAGATCCTCATTCATATAACATCACCATTA

>LNC_000320

TGTGAAATTCTCGTTTTGTTATTTCATTTTATCTTTTGGTGTGATATATGAAGCCACAACACGTGAAACGAGGGTGGGTGATGATTATTTTTTTTTGTAATGAATGGCAATCTCTCGGATTGCTTTTCTGGGTTTGTTATGATATAAACTTGTCCTAAGCTATTTTTTCACCCGTGTTAGTAATTGTAATCGCAGAAATTTGTACATGCTGAAATAAGTACACGCCAGTGAATGGAAGAATACTACAATGTTATGTTCTTTCTATCGTTTCTTTGGAACAGGATTGTACATTTTTTAGTTATTTTTGTACCTTATGAGGTTTTTTGTTTTACTTTACTTGTGGAATTTCACTAGTGTGTTGTTGTGGAAGAAAACATCTCCCATTGTGTTCCTCTATTTTTACGTTGTTTGTTGGTCGTTGN

>LNC_000321

TTTTTTTTACATGGCATGTTCTCTTTCCTGTCAATGTTCTGTTAGTCGGCTTATTTTGGTATCATCTAAAGTTAACTAGTACTTATTTTGGAAATAAGCTCCTATTTTAGCATCATTCTTTGTGCCACTGTTTTTATCTTAAACTTTTAACTCATGTTTTCCGATCTGATTATTTATTGTTGCTTACCTTGTGGATTCAAGCATTTAGGTAAGTTTGATCCAAATGAATATGTTAGGCAGTAGGTACTAACATCGTACATTTCTTCCTGGATTTAACTCGTGTATTAGGAGCTTAGGATGAATAGGATAAAATCTGAAATCTTGACTAATAGATGCCCCTCGTTCGCTTATAGAGGTGTATG

>LNC_000322

NNGGCTTTTGATTTATTGAATTGCAAATCTCAGGTCAAGAGTAGTAAAGCTACATTGTTTTAATCTGGAAAGAGACGGCTCAAATCATTAATATTAAGAGTAAGGCTAATGGCAAATAGGGTTTGGCAGATCATGCACTCCATTTCTCTGTATCATCATTTTTAATTTGCTTAAACTAAGGTTAGTGTTCAATAATTTAGAAATTTTTTTTTTGGGTGCAAGTTGTTTGAGTTGTGTCAACATTTAATGTCTTCTATTTTATCTATGATTTGGTGTTGCATATAATTTGCAGATTGAATGATATACTTTATTCCTAACTTGATCATCCTGATCTTTACTAATACAAAAACATAACTTAAATAAATTATGTATGGAAAATAGATTTATCATGTTGGGCCCGTGCTGAGCACGGGCAA

>LNC_000323

GCAAAAATGACATGTAATTACAATAATTTTATAAACATATTCCAATGTAGGTAATATAAAATTACTTCCTCTGTCCCAATTTATATGATGTTGTTTGACTTGACATAGAGTTTAAGAAAGACATAATACATAAAATATGCCTTTTAACTTGGTTTTAGCTGACATCTACGCCCTCCAACTTTGAGTGTGCACAAGTAGACAATTAAACTTGTATAAAATTAAACAAGTACACATATGCGTAGGACGCCATTTAGGATGCAAAATTCCATGTATGACACCACGTTGGAAGTGCGTGTCTACCTATTCAATTTTATACAAGTTTAAGCGTCTACTTATGCACAACCAAAGTTAGAGGGCATATGTGCCAACTGAGGTCACGTTGGAGGGCATAGATACATGNN

>LNC_000324

AGCTCGTTAATTAATTAATATTAATTCATCTTATTTTAGTTTTAGCTGAATTCGCTAATTAAATTCAATTTTGCCTCCTTGCATCTCTCTTATTCGTTGGGATGAACCCAACAACTCTCCGAGTCAGCCCCCATGCCGAATGGCACGAAAGGGAAGTACAAATACAGGTCTCGAAAGCTAAAACACATGTTCAAGGAGCTGATGTACAGCTATATATGAGGGTGGAATGGGCCCATAGCCCAGAAATACATCAACAAAAGCTCACTTTTGGACAGCTGTGAATTTAGGCCCAAAGGGCTCAAATTCAACTTCTCTTTTTGACTCTTAGGGGTCGTTTGGTACAAAGATGCATAATGTAGGGATTATTAATACATGGATTAGTAATGCAGAGATTAGTAATGCAGGN

>LNC_000325

TCATAGTGATTTTTATTTAGCTTTTGAATGAAATTTTTGGCAATAGGATATTGAAACCGAATCTGGAAAAAAATAACTATTGCCTGACATATGCATGATTTGGATCGATAGCATGTAACCTCCTTAAATCTCAATCTCGTTGTAGTATCGGCTCTTATTAAGACCAGTCCATGAGAATTGATGGTTTTAATGCTCTCTGTTAACTTTCATTTTTTATGTGTATTTTTTATTTATTTATTTTCTTTTTATTTTTATTTAAAATATGCTTGGCAGTGCATTTTCATTTTTCCATAGACTTGATGTTCCTTCTATTACTTCATTTCAATTATTACTCGTGTCTTCCCTATAAGCATATGGTGTACGCTAATCTTGTTTTCCTTTCTTTTC

>LNC_000326

GAAAATAGCATGTCGTTTAATTCCTTCATTTGGTTTGGTATTACTACTCGATAGTGCTAATTCTCTCTAGATGTATTTGCTTATTTATGTTGGTTTATGCTAGTGCCCAGCATATGATAGTTGAGTCATAAATGTTCTTATTTATTTTTATTTATTTAACCATTTCTAGTCTCTTTTCTTTCCTTTGCCATCTAATGGTTTAGTATACTATTGCTCCCAACGTCTATGCTTATTTTGTCATAGAGAATTAGCATTGTTAGGTTCTATTGGAAGAGCTATTCTGACTATGGCATAAGCTATTTGAGATCATAATGTGTGGGCAATTTTTACTTGATGTCTTTTCTTTATATTAATTTATCAATGGATGATACTTATGTATTTC

>LNC_000327

ACAAAAATGCTTTCATACTTCACATTCAGTGTTTCCTCTTTTATTTCTTCGGCTTCTGCTTCAGATTTCAATTACCCAAAATATAATGCTTTATGTTCCTGCCTCTTTGCTTCTTTCCATTTGCGAATGGAATCATTTGACAGAACGCCTCACAAATCTGGTAGTTCACTGCGTTCTAGGGTGTAGGCTTAAAGAGGGTACGATAATGTTGCTCTAACATCAGACGATAACGGAAGATAATTAAAAAAACAGAAAACTCATTTGTAGCATCAATAGAAAATGGAGCCTTCATATGATAAAATTAC

>LNC_000328

AAAAAATTATGAACCTTGTTTACCTTTTTCGTTCTGTGTTTCATTCCAACAATTTTTATCCCATAGATGAGCTTCATATCGCCCAGTCCATGAGTGCCTATCAATTATTAGTCTGAAATGAACTGACCCTCCATTAAAGGAGAAAGAAAAAATATAATTGTACTAGGAAAAAAATGTCGACTAGCTGCTCTGTAACTTGAGCTGCGTTGAGGAGGTGGACCCATCAAAGGCACATAATCATGGCTGTATAGAGCAAGCTTGAACTACCAATTGCATGTGAATTGCAAAGCCCTGTTGGAGATATGAGCTGCCAGCCCTGCATAAGAGTGCTA

>LNC_000329

CTAAATATACCACTCCTATCTCTGGGAAGCCTCCAGATCAGTCCAATGTCACAGTTAGAGATGAATATGAGGTAGATAATTCAGAAGATGAATTTGATGCTGATAATCTGCCTATTAATGATAGAGATGAGGAAGATGAGATTAGTGAAGCACTAATCAAAGCTTTCAGTCCCAACAATGAGGATACCCTTGAACGAGAAATTCAACATGTCACCAACACTCAAGGCCTTTCTCCCAGAGGCATCCACTTGGACAGACTTCCCCTCCAAAAATCTGTTTCTCCCATTTCTGTGACAGCAGGAAGACCAAAT

>LNC_000330

TTTGTTTCCATTTATTATTCAATGAATTAAGTTTTACCCATTACTTGAGATGCTGAAGATCCATTTATGTTGAATAGAAGAAATTGTGAATTAGTTATGCAGCCGAAACTATTGTATGTTCATGATGGTGCCCATAACTTTTTTCGTTTTGATATGGTAATTCCCGTTGCCCCTCCCCCCACACATCCCTTTTTAAATTTTTTCTCGACTGATTTGCTTCAGTTTAGTTCTATTACTCTTTCTGCTAGATATATGCATGCATGTCATTTACAAGTTCGAACCTTTACAACAGATCTTGTTGATTGGGTATTTCGGATTTCTTGACATCGCAGGCCAAATTCGCCCATGAATTCTGCCAGAGGAAAC

>LNC_000331

NNCGCTGTTCTTTTTACTTCTCATCAACTCTTCAATGGTAAAGCCACCTGTTCTTGGCTCTTTTCTGTTTGTCTACATCTCTCTCAAGCTTTGGTGAAGGTTTCTGTTTATCTCCGACGAACTGCGCAGACTTTAAATCTGTCGCTGAAGCTTTGCTGAAGATTTCTGTTTCTCTCCTACGAGCTGCGCAGGTACTTCTTCATCGTTTAAGCGACCCTAATTTCTCTCCCTCTGAACTCTAAGTTCTACCTCTGAACTCTTCAATGGCGAATCCAT

>LNC_000332

AAAATGATGATTCACTGAAGCATGCTACCCTGCAGTTCTTGAATAGTTACTTGTCGAGAATTTTGTTGTGCAAATTCATATCCTATTTCTTGTTATGAAATGTTATTAATTGGCTTGGCCATCTGAATAGGTCAAGTTTAACATGTTCTGGTCTAAGCCTCATTCACTGGCACCACCACCTGATTCACTATCCAGAATTGAAGAATTGTAAGCTGGAGAACCTT

>LNC_000333

NNTTTTTTTAGTGAAACATACAGAGATAACTTACGTTAAATAGACAAAAATAGAGGAAAGTTTGGGGAAGAAATAGAAGAGAGCCAGATTACATTGGAAACAAAAACATACAGTAAAGATTACTAGGTGCACACAAAGTCATCTATGAAATACATAGGGAATAGAATCTGAATGATCGGAGTTTAGAGTTTAGTTTTGAGCTCACTTTCTCGTCCACTGCTTCGAGTTCGTCGGATCGTCACTTGCGGTGGT

>LNC_000334

NTCAGATCTCTCTTCTCTTTTCTTGGTTCATCCTTTGGATGGAACATTTAGTTGGAAACAGATATTTGGCTTAGGACCACAAGAGCACATGTTATAATGTCTAACTTGGTTCCACTATATTAGTAGGATCAGGTTCATTTAGTTGGAAACAGATATTTTCTTGGTTCATCCTTTTTCTAATGCCAATTGTAAACACTTTCAAAATCAACCAATTTTCGTTTTATAGATCTTCATATCCAACACACTCTACCCTCCCTAGACCCACTTGTGAGATTTTATTGGATGTGCTGTTGTAGATCTTCATAACCAATCATTTTTCCCCGAAGCTAATTACTTACAAGNN

>LNC_000335

ATGCTTCACCAGATAGTTTATGCATGCTTAAACTTATTTTGATGTGGTAGAAAACACATTCATAAGTTCTGTACTCCAAATTTAACTTATACAAGTCTAAAAAAGAAAAATGTTAATCTGTAAAAAATTTCGCCTCTAGGCACGTGAATGTTTGTGCAGGTTAACTTATATTTCGGTTAACATATGACTTCATGGATTATTTTCTCTATGCTTTATGATAATTTTACAGCCCATTTGCTAATTTTTTTTTAATCTTATTCTTTTGTACATGTACGCTCGGGAGCGCATTTGGAGTTGCTCCATTCATCTGTCCAAATCAGATTTGTGCTCACGT

>LNC_000336

NAGGCATCTGGCGGAGACAATCGACTCAATCTTCTTGGAATCTGGTGGAAGCATAAGAATTCCCAGAGACTACAATGGTGGAGAGAACGAGAAAGTTCAGAAATTGCTGGAAAACTTGGGGATAAATGAGTTCAAAGAGTACGACTAAATTTTCCTTTAAATTATTAAACAGAACAAGGAAGATAATATCCTAAAGCTTCATCACTTCAAAAGGGTTTTATCGGAATACAGTTTATCAGGTAGAAATTCTACCTTTAAAAGTTTGAATTTGTTGCATCTGAGTTTATTATGTGGGGGTGGATTTTGATTTTCTTGTGTTGTTGATTTTTGG

>LNC_000337

AAAAAAAGAGAGGAGATTTTTGGGATTTTCTTATAGTTGGAGAAGTTATGTTAGAGAAAAATATCGAAAAGTAGCTAGCGTTAAAAGAGGGAGAAATACACTCAAGCGAAGATTTCTTGAAACCATTTTGGGCTCAGTTTTTGCTCCTGTTTGCTACACTCTGTCCTTTCAAATTTCGTCGGAACCTCGCGTGGTGGTGGAAGCTCTCTTGTAATCAGTTTGTCCTGTCCCGTTCGCTGCCCGAAAAAGGTAACGCCCCCTCTTATATAATGTTCTCTTCGTATGTCTCTAAGAAGCGTTTTGAAGTTTGAAAGCTTATGTATTGCG

>LNC_000338

GTGATTTTGAATTTTGCAATGTGAATGATAGTAATACTTTCTTGAATCTATTTCCTTTAGGGCATTGAGTGAGGCATGTTCCTTTTGAATTGACATTTACTGTAACTTGTTATATGCTGAGATTCTTTGTGGTCATCTTCACCATTTTATTTTAAATTGACATATAACCTGTAATATGCCGATTCTTTGACTGTCTTCACTATTTTATTTTATTTTTGCTGTGAGTGCTACTATTTTTATGAACAGTTTATATTTAGGAACTTTCATTCCTTTCCTTGAGATATTAAGAACACC

>LNC_000339

CATTTCAGTTTGTTTTGTTGCTTCCTCGATTGACTTTGTTCTTTGCTTGGCTCATGGCTGTTGTATGATAAAAGGTTGCTTGGTTTCGAATTAATGCATATTCGTCGATTACCACTTGTTATCGTCCTTATGATTTATTACATTTTGAATAGTGCACCAGGTTACATCTACTATATGTTTTGGACGTTTTTGAGTATGTGAAATAAGTT

>LNC_000340

TGGTGTGTTACTTGGAGATCATGGCATGCATGACCGTGTACGTGAATTGCCATTCATTAATGAGTAGAAGATGACAAATATTTCTTGGTAGAGAAGTTCCGCTAGTTTTGAGGGAGGAACTTTAGCTCAATAATGCGTGTGGACTTCACATTTTTCATCGAGTAAAAAGTTATAAAGAAAGTTGATAAATTGGAAAACTTTCCCAAGTTTTGAGAGTGACGTAAATGGACCGTTTGTAGTCATTGAAGTTTTCAACGTTTTTAATTTCCTTGTGCACGTGTACCACGCAAACCTACAC

>LNC_000341

TTACCTATAAATACATCCTTACATCTTTAGCCAGGGGAGAGATTTTTTCAGCCTCCATAGAAGCCTTGGGCATTCATTTCTTTAAGAAGTTTTTCCATCGCACTATAGCCCCTTAACCACTGTTTATTTTGCTCCAAAATATCATTTTGGTTCTATTTTGATCTCGGCTAAGAACTCTAGTTTCGAGTTGGTCGTTATTCGAACGTAGGCCTTAGACCAAAGCATCTCCGTTCACTGCCCGATAACAGGTAATACTCGCCCTTTTCTTTTCCGCTGTTTTGTCTCTGTGATAAAATGTTGTGTGTGCTTTAATAAACTGATATA

>LNC_000342

GTCATATTCCAGAATGTCAGTTTAGAGAGCTCCTCAAATATTGGAAGTCTGAGAAATTCAAGGTAATTAAAATGTATTTCAGAGTCTGAAGCTGACAACTTTTTTATTTTTCTATTCTCTTCTGTGATAGTGTTGTTGTAGCTTGATTTTTTCAAGGTGATATTTGAGCGTGCGACTCTAAAGTAACCTACTTTTTCTATCATCTTTCGTTGAAGCCATATGCGATTCACATAAAAGTAGAGAGATGATGTGTATGGATCAGTATGTATCCTCCACTGATGTTCATTGAGTGGGATTGTTTTGTCTATCTCATTGTTAAGTCGAGNN

>LNC_000343

NNGGGTCTGAACATTGAAATACAGGTCTGAGGTGCAGGACACAGGTTGCAGGGGTTAAAATATATTATATACATTCGGCATACAGCCCGAGATACAGGAAACGGGGTTGATATACAAGTTGTATGCGCTGATATACACCTTGTGTATACAAAAACGCGAGTGATCTAAAGTCCTAAAATCAGAATCTTAGCCCCAAATTTGAGTTGTTTTCCCTTTGCTCTTACTTATATGTTTATTATGTTGACTAACCGCTTGTTTAATTATGGTTTAACTTAATTAGATTCCCGAAG

>LNC_000344

GGAGAACAGGAGATATTTCCTATCATTTTCACTATCAAAACTACTTTTTCAAAGGCTAAACATTCACAAGAACATCACATATTTTACACTTCAATATCATCTAACGAGTCTACTATAGTCTTTATTTGTTAATTTGTCGGAGGAACGAAGTAGATCCTTGATCGACAACCTGGTTCACTGCCCACAAAAAGGTTAGATCTA

>LNC_000345

NNTTTTCTTGTGTTGTTGATTTTTGGGATTCTAGCTTGTTGTAGTATTACAACCATCAGTTGATTTGATTACATCCTTTCGAACTTTGTTTAAGAACCTTTTTTCTACATTTTCGAGCATGTCTATTGTCAGTGAGTTCCTTTTCCATAGGGTCTGAACTGGTTAGTCGAGAATGCTTAGAACCTTTTGCACCAAGTTTGGCACATCCATGTCTAATGGACTGCTTTGATGTGAAGGGGACGATTTCTTGGTTGTACATAGTGTTGTCTTCGCTTCTCCCTCATGCTTCCTGATCTTGATTCATATTCTNN

>LNC_000346

NNATTTGAATGATGAGTTTTATTATACATTTTGCTGTCCTTAGGCGTTCTACTTCATTTCTTACTCATGCGAGCTGCATCATTGGATTTTTGGACAGTATTGTTTCTGTTTTGATTTTTGCTGATCATGAGGATACAATAGTGTGCGCAATTCAGTTTCTATCATCTTGTATTTCTTTTGTCACAATTTATGTGGCATAATTTGGTAAGTATGTAACATAAATTGGGATAGAGGGAGACCTTTTAAGTTAAACAACCAATTATGTAAGAGATCGTTTCAGCTAAGAGAAAAAAATACACGAG

>LNC_000347

CGCTGGTCTTTCGAGCTCATTCTGTCTCTGTTTGCTGCCTCAGAAAAGGTATAATTCCTCACTGATTCAAACTCGTTTTATCTGGTGCATTGTTCGGTCTTAAGTTGAGTATGTCTTAGTAGTTGTGAAGCATGAGGCGTGTTCTTATGTTCTTCGGTGGTCTTGTTTGCGAGTATTAGTCGGAATCATGGAGTTAGTTAATATGGAGCTCGAATGTTATTACATCTATTATCTGTTAAATGTAACAGTTTCTTTTCTCCATTTTGTTCAATTTTATTTATTTTTTGGCATCTGCNN

>LNC_000348

GTCATGTACAATTTTCGCAGTTTCCATGGATTTTACTCCTCTGTGCTTCGTCTCCTTGTCGGTATATTTTTTTTTCATCTTTTGGGTTTATCGTATGCATATTCTCTCAAGTGATTGCTTTGTTTTTGAGGTAATGATTTTATTTGCTCTTCACTTTTTATGCTTTATCTTCTTCGTTGTCCTCGATTCATGCTTTTATTTGAATTCTCTTTGTCTGTTTGATATTGAATCTTGCTCGAATTTGGTTATTCCCCTTTGTGTGCTTTGG

>LNC_000349

GTCTGATTTTGTTGCTATTTTATAGCTTTAAGGTCACATTGAAACATTAATGACTCTAAGGTGTTCGACAATATGCCAAAGAAAATGAACAATTTACCTGGAATATAATCTTCTTCAAACTTGTAGGGCTCTATCTATTCCATCTCTTGAGCCCCGAAGAAAACTCGCTACCCTTTGTACTCCATATATCTTTTGACTCTATATATGTGGGTATCTACGAGGGTTCACCGATGTTATGATTTTATATTCTTATGAGATTTTAGTTTGGGTCAAAGAATGAACTACA

>LNC_000350

NGATAATATTATTGGGTTCTTATTGATTTTTATTTTGGGATGACTGTCGTGTTGCCCTTCATTGAACATCTCTGTCCTCTTAAGCTAAGGAACACAAAACCTTATCTTTCCAGTCACCTAGTAAGTTATATTGAGTTCGTTGCCGACTCGAATAGTGTGCTGGTCTTATTTCTGCTCGTTGTGTTTCGTGTCCTTATGTTGTGTTGCTTCAATATTCTCTTTGTATCGTCTTGAATTGATTTCGCCTATATGCCACTTGATTTGTTTATTTTCTGTGCCCACTTGGGTC

>LNC_000351

TTGATGCAATGAGCGGCACATGGAGTCCAATAAATGAGTGGGTACACACCCATCATCATGCTTCCCGCTTTAACATTCTCACTAGCATTATCAGTGACAATTTGTACAACATTTTTCGGTCCAATTCTTTCAATAGTGCTTTCAAACAACTTATACATTTTGGTGGAATCGGTAGATTCATTGCTAGCATCAACAGAACCAAGAAATACACTACTGACTGGAGAATTCACCAAAATATTGATAATCATTTTACCATTTCGTGCCGTCCATTTGTCC

>LNC_000352

NCTTGAGTGATAACATTATCAAACATTGCACTCAAATTCCCCATAAATTGCTCTGGCTGTGAAACATTTTGTGTGTTATACAGGATCCTAGAAACCGAGGTGTCTAACATGGACGCGAAAGATACATTACAATAGCGGACCAAACACTCATCAAACCATACAATTGCCTGTTTCTTGAGCGGACATTCAAGTTGAATCCGCTCTCTTGCCACGTCTATGCAGTTTTTGCAGTCTTTAGGTACAGCGTCTCCTCTACAGAGGAATAGTCCATAAACTTTGTC

>LNC_000353

NTTTTTCTAAATTCTGTAAGAACATGAAGTTGGATCACAATCGTTTTTATTTTTCTTTACTAAATTAACCTTCTTCCTCATCTCAAATGAATGCTGAGTCTGTTACCATGGTTTTGTTTCAAGATACTTATTAGCCTGAAATATTCTAAAATGGAGCTATATGCTAAGTCGGGATTCACATGACCGACCCCAACTTGAGTTTGGGATTGAAACATAGCTATTCTTATTATATGCATTTCAAGTTCTATGGTATTACCTAATGAAATTTATTCATCGC

>LNC_000354

GCTTAATATATGATTTATTTGGGTTAAGCTTTAAGGTGGTGGCTTGCATAATATTTGAACTTGTCTCCATGGAAATGTGTTCCATCCTCATATCTAATGTCACCCTCTAATAAAACGTTATTTGAATGATATAGTAGTTTGTGTTTTAATTCCTTGCCTTCATTTATTTAAATTGCATTGGAAGAATGTTGGTTCTAACTTTCTTTATTTTTGTTT

>LNC_000355

NCTTAGCATAACTGCAGTAAAAGTGATATTTGCATTCCTTTCTCTATGTTTTATTTTGTTTCGTTTCTGTTGCTGTTGATTGGTAAATTGTTGGGCTTATTTTGAGAAAGTTATGAATCGAGAATAACTCTTTGCTTTCATTTTGACCTCTGCTCTCGTTTTAGCCACAATAAGTGGACCCTTCTGTGTATATATCTTTGTTTAGTTGGAGTCATTTAGAGGTCCAAAATCGTGTTCTTTTTTATTTAGCCACTTTGAATTAAAAGAACGAN

>LNC_000356

CTAATTCAATCGAAGTTGAAATTATCTTTGTTTTTTTCTATAGAGATTTTAGTTAATTCATTTTTTGATTTGACTTTTCTATGTTGATATATTTTCTGTCATTGGATGGATATGCTTGCTTAACTAGAAAAAGGTTGAACTTTGATTGGATAAAGACTTCCTCATAGAGATTTAACTTTGAATTTCTTAGGACTTAATTTTGGACAGATTGTGTGTAGTTCTTTATACAGATAATTGTAAGTTAAGTCGAGCCGAGTTAGTTGCGGTN

>LNC_000357

NAAGTGAAAAATGAGGGGGAGGCTGCTGGTGGCTGTGAGATGATGTTATGGTAAAGCTCTCGAGTATTTGGTTATATTTTGAAGCTACAGGGCATGTAATCTTTGCTTTCCAGAACTTTGTTATGTTGATTTCTTCAAGAAAACTTGTTTCACTCTCATTTTCCTTGTGCTATTTGTCATTTGGGTTTTCATTAACACCATGATGAATAGAAGTATGTTTTATGCTGCAAAGTAGTAGTGCGGCTTTCATTTGAGAGGGACA

>LNC_000358

CTCTCTTTGCATTTTCCAAATTGAGCCATGGAGGCTCGATTTTCTCGTAGAGTCAACCCTATCAGCAGAACGCTGAAATACGAGTTCCAAAAGCAGAATACAGATCTCGGAAAACCTAAAAACAGGCTGATATACCTACTGTATACATCGATATACAGTCAGAAAAAATCATTTACAGGTCCAAAACGTGAAAACACAGGTGAAAGTGCAAAAAAATGCGAAGTTCAAATGAAATTACAGGTCCGGGAGTTCGAGAATTANN

>LNC_000359

NNAAGCTCTAGATATGTTTTTTCAATCAATCTTGCTCATGCCTCCTTTTTTCCCCCTTCATTTTCCCCTGTTTCTTGGTTGCTGCTCAATTACTTTCACACTCTTAATTTTTCAATTCACTAACATTTGTGGGTAAATCTATGAGTATCATTGGAAGGCAATAACGGCAAAGTAACAGGTGATGGTCGTAATTTCATTTGTTTTCTATTCCTTTTTTTTTGGGTATTATGTTCAAATTTGGTTAGATCGAACTTNN

>LNC_000360

GGTTAATGACGTGCATGGTGATCTGTCCCAGATTGGTTTTGATCTGGATCAACTAAATAGGATGGTTTCTGGTCTGGTGAGCTCTTCTTTCAGCCATTGTGTGTATTTCTCCAAAAAGATTTCAAGGCTCTTTTCTTTCTGTTATATCTAAATCTGTGTGGAGTACAAGCTTTTTTCAATGGTCTTAACTGTCATCTTGGTCATTGTGCCTGTCTATGTAAAC

>LNC_000361

TTTTTCCTATTTTATATTGGGCGCTCCTTATGAAGCCTCTGAATCTAATCAGAATCATCAATTTTGTGAAAATTTGTATGAGTTTTAGTTTATTTGGATTTCATGGTATTCTAATATTGTTAAATAATTCATGGAAGAAAAATAAAAATCTTCCATATGTATTTTTTTTAGGAGAAAGTTATTGACTTCTATATATGGGTGTACATGACCGGGT

>LNC_000362

NNGATCAATTGGCCCAAAGAACTTCGAACTCAGCCAGACAGATCATTGTTCCCAAACTCTAAAGCTTTCAAGTAAGAACACTCCTCTATCCTTGTAGAGATACTTCCTGTTAGCTTATTGTTTGATAGATTAATGACTTGAAGAAGAGTTACCTTTAGTAGTTCTAATTCAGAAACAAGACTTCTAATCTTTGTTTTTAACTAGAGTAGTAGTGTAAGTTTGGTTGCACTTTCATTACTATAACATNN

>LNC_000363

TTTGGGTATTATGTTCAAATTTGGTTAGATCGAACTTCAACGAATCACATCTTCGATTTTCTTTAATTATTCCTTTGCATGTATGTAATGGTTTTTTTGTGTTGCATAGATCGACTTTGTCTAATGACTTGCCTGTATGCTACCATTATTGGAACAAATATTGTGTGATAACTCTCCTTCAGTACTATAAAAGTAGCACAAAAGTTGAATAAATGAGAAGGAAAAAAAGGATTAAAAGCTGAN

>LNC_000364

GTTTTTTCAATCAATCTTGCTCATACCTCCTTTTTTTTCCCTTCATTTTCCCCTGTTTCTTGGTTGCTGCTCAATTACTTTCACACTCTTAATTTTTCAATTCACTAACATTTGTGGGTAAATCTATGAGTATCATTGGAAGGCAATAGCGGCAAAGTAACAGGTGATGGTCGTAATTTCATTTGTTTTCTATTCCTTTTTATTTGGGTATTATGTTCAAATTTGGTTAGATCGAAC

>LNC_000365

NNAACAATTTGACCTCCATTTAATCCAATACATGCTAATACAATAGTTGCTCAATTAAATGAGCCCCATAGTACAACTTAAATTACATTAAATTACAGTTGCAGTCTGCTCAGAATCCCAACTTTCAAACCCCTCCCCCTTCCTTTTCTTACTGTCTTTATGATGTTTACAAAAAAGGGAAGTTTCAACTGATTGTTCATTTCATATTTGTACACACCAGCTGCAGCACTGATGATGCA

>LNC_000366

TGAAAGGCCAAATGAGAATGTCCCTGAACTTATAAGTGTGCTGAACTTCATTGTTGGTATGACAAAAGCTATTGATAATCACCATGCCAATTGATGAATTGATAAGATCTATGTTGCTTGTACTCTTCAAAAATGTCATTAGGTGCGTATCGAATTCTTTAAAAATAGTACATTTTGGAAGGATCTGATACAAATGTGGCAACATTTTTGAATAGTCCGAGCAACTTAGGATGGGAT

>LNC_000367

NCGCGTGAATCAGTACTAGGCTGGATTTTGTTGTCGTCTTCCTCGATGTCCCTTTCAGTTTGCAGTCGCGGCAGCGGAAAAGCAAACACGCGTTTCTCCCCTTTCCTCTTCCGGAATGGGGTTGAAACCTCAAGAAAAGATGTTTAGCTTGAATGATGAAAGGGTTTTCGAATTTGAATTTCGAAATGGGACTTTTCTGTGGGAATTTTTTGGTCCGTTTTGCCCCCNN

>LNC_000368

TGTGGACTTAAAGCAACAGAAAGCAACAGTAACAGGCTTTGTGGAGCCAAAAAAAGTGTTGAAGGCAGCACAATCCACTGGTAAAAAATGTGAGATTTGGCCATATGTGCCATATAGTATGGTGGCACATCCTTATGCTGCTGCTGTTTATGACAAAAAAGCACCTCCTAATTTTGTAAGGGCAACTGCTGATCCTTCTGTTGCTCACTTAAATCCTGTTGAAGANN

>LNC_000369

NTTTTGGGGGGAGGAGGATTTTTGGGGAGCAAAAACAGTAAGAGCAGCTTAGGCAAAAACAACCAGAACTTATAGAAATATATTCAAGAAAGGGAAGGGGCGGCTAGTTCTAGTTTGAGTATCATCCCTCTTTCTCGTTTGTCCTCATTTCCCGACGTTCGTTAGAGTTTAAAGTTCCACCCAAGTCTCCAATTTGTTGATTCATATCCGGGTCTTGGTGGTNN

>LNC_000370

NTTGGTACCACTATTTAGAAGTGGGAGTTGTGCTAGGAGCAAATTGTACTGTAGCCAAACACATTTCAAATTGTATTGTGAGGCCTCATAATTTTTGGCCCAATTTGATTAGGTATTACCACTTGGAGGCTCTTTTATTTCCACGTGTGAGCAATTGATATTGCTTCAGGTAATAGGTATAGAATTATTGTGGATTTTTCTACAAACTGTACTGCTGACTTT

>LNC_000371

GCTCAAAGGAATGCACTTAAAAATACAGCTATATCGCAGTTGTTGCAAAGTTTTTTCAGCAGGCCAAACTTCGTGGGACTAAATGATGCAAGTATGTGCCATCTCTAGCCCATACGTTATATCAATCCTTGCACCAATACAAGTTGTACTCGAGAGGGAGCTACCGCAGGATTCAGTTCTTCAATTTATCACTTTTAACATATGTATGTTCTCTAATN

>LNC_000372

NCTTTCGGACATACATTTCCATCTGCGCTTCCTTCCTTTCTTTGGTCACCGCATCATTATGGGTATGGATTTCCTTTCTTATTGGTTATTGCAGCTTCTAAATTACTACTGATTTTGTTACAGCTTATTCAATCACTAGGGATTCAAAGCAGATTATTGGTTTAGTGTTGCCTCTATTCAGTAGTATAGTTGATTAAAGTAGATCTTGACATACTGTT

>LNC_000373

CTGCATTGCTAGCATTTGTTGAAGTGATAAGCTTGTATAGTTGGTAAGTTGATGTATAAGAATAAAATGTTGCATATTCTGAGTGCAGAGTAGTGCAGGCCTGTAGCTTGCTCCTTCTAATTTGAGATTAGAGGTTAGTTCATTGATTGTGTACTCTGACGGACCAAGTAACTGTTTCTGTTTATAAATAGTTAGATACCAATAAAATGTAACANN

>LNC_000374

ATTGTTTAATTCAATAGGTGAAATTTTGGTGTGTGCCCCAATTGCACATGTCCAATCAAATACATGTATTGATAACATGTGTAAGTGATGAAATTCTTAAGCATCGTCTCTTGCCTATTTTGGTTACATTAGAGGAAATATTTGAATTGTAGTGTTTGTAACTATATGACCTCTATATAAGTAGATAAATCAACAAAACTTTGNN

>LNC_000375

NNCATACAAGTTCTACCCATCTGGAATTTCCCACTCTTGAACGAGAAATGTAGAACGTACAAACTCAACGAAAAAATTATAATCCTGCACACACATTTTGACAAGGGATCAACAGATTGTTTCAAGGAATTAAGGAAACTAAAATGATACCTAGTTATGGTATGACTTTATTTGTTTCATTTCAGTTTTTAAAAGCTTTGTCG

>LNC_000376

TGTCATTCTCCTGCATAATCTGAATATTCCGCCAGTACAAGTCTAAATTCTGTTTCTCCATAACTTGCTTGAGTTGGCCTGAAACTGGAGAAACCATTAAAAATTATGAACAGTTCTGCAAGATATATTTTGAAAACATAGCCTAACAGATTATGCAAAAAGAGAAGATGATGTCATAGTAAATTTTTCTTATCTACATATGCATGACATACGTTTATACACAGTATATTGACCAAAATATTGCAGAAAAATGATAAAAAGTTCCATAGTGATTTATTTCTATGGGAAAAATAAGATGGAATAGTAAATGTAAAACAATGAGAAATTGGATTTGAATCTAGCCCTGCTTTGTCTCATGAAATTTGAGTCATAGAAGGCAAGAGTCAGCAGATATTTTAGAGGGTACTCTCGGTACACTTAGTAAGTAACTAGCTTCAAAGAGGGGAAGCAATTTTGAGACCAATGGAAAGAAGGAAAACTAGTTTTGCAACAACACTTGAAAGTTCTTTGTGTAAGGACACTTACTTACGAATAGAGAACAGGCTCCGAACCTTAAGATTCTGAAGCTATTAGAGGGCAACTGGTTTTGGTTGGAGTCTCTTTGATATTCGTGCTTATTTATACTACTATTTTATTACTTTTACGGATGGACAAAGAAATACTAGAAGCAGCACACGAGAAAGGCACTTGTAGTTCAAGAAAGGCTCCCGAAACCAATTCGCAAAGCCCCCTTTATTCCTCCTTTATTCTCACATAAAAAACACGTCACATTTTTAGTCAAGAAGACGCTTCATCGATGATTCTGTTAGAAGGAAAGAAAATTGTTGCTCAAAGTGGTTTTTGCATGTGGATTTTGCTTGTTTAGTTATGTAAAATTAGTTATAAAGCAGTATAAAGGATGATATTCCTGAAGAAGCAAATGCTTGAGCAAAGGGAGAAATAGCATGGGAGACAGCGAGAACTAGGGGGGGTTGGGGTGAATTGTGAGATTTTGATATTCATGAGAGGAACCTGAATGTAAGTAGCAAAATAATAAGCATTCAGTGAATTTAACAGAATCAACTCATACTTTTTAAAGCTGATTTGCCTAAGCCATGAGTAGATCACAATAATTACAAGGAGTAGAATTTTGTTACTGTTAATCTGGCTAGGCTGTGTTGTTTTTGGCAATTACAATACAAGAGAATCCCTCCTTTTTTGGAACATAACTGATTAATTCATTACACCTATTAGAGAAAATAATGAAGTGGTTAACATATATGGTGTATGGTAAACTCTATTGAAAGTGAATAAAAATTAAAGAAATTGAAAGAAAATCTTGAGTAGTTAGTAAGTATGGATTCAAGTATTATTTTTGTTTTGGAGAGTTACTTGAATTCATTATGTATTACTTTCTTTGTTTTTAATTTGGAGAATATTTGAAAAACAATGTTGGAACCTAATGTTTTGTGAATTTTATCTCATTGTGACCTCTGGTCACGGGTTCAAGCCGTGGAAACAACCTCTTGCAGAAATGTGGGGTAAGGTTGCGTACAATAAACCCTTGTGGTTCAACCTTTTCCCGTACCCCGCGCATAGCGGGGGCTTTAGTGTACGGGGCTGCCCTTTAGATTTATCTCATTGTGATAACAGAAATGTCTAGACTTTCAAATTTTTCTTTGGTTTAATCATATGGTATTTGAAATTTGTTGGTCTGATTTATTTTGTATACGTGTTGGGTAAGCTAATTAAGGGAGGTAAAATGCTCTCTGTTAAGAAGTTCTTCATTCCTAGGGTCCGAACTCGAAACCTCTCGTTAAGGGAAGTTTTCATTACTGGTGACAATTGGCTCCTTCAGTACAGTACAAATATAAAAGTGGAGAAGTCGAGAAAATATGTGTAAATATTCACATTATAAAAGGGAATTTCATCATTATAATTTATATTGCTAATTATGTTTCGACTCATATAAGTTGTTGTCGGTTGTTATTGTTGTTTTACTCGTTATAGGGAACGGATTTCAAGGGCAACAAAGGCCCTTAGATAGGACTTCATCTATGGCTAGAAGAGATTTTCTTTCTATTGTTGTTTTACTCGTTATAGAGAGCGGAATTCAAGAGAAACAAAGGCCCTTAGATAGGACTTTATCTATGGGAAGTGATTTTCTTTCTATCATTGTTTTACTCGTTATTGAGAACAAAATCCGAGGGAAACAAAGGTCCTTAGATAGGACTTCATCTATGGCTAGAAGAGATTTCCTTTCTATTGTTGTTTTACTCATTATAGAGAACGGAATTCAAAGGAAACAAAGGCCCTTAGATAGTCATAGGAGTTTCATCTATGGCTAGAAGAGATTTTCTTTCTAATGGAACTCATCACTAAAGTTGTTAACTAATTCAAAATATCGAGTGCTCATAGAATCACCCTGAACTATCATGAACTCATATTAAAAAAATACTCAAGAGAAGAATATAACATGATATGTGAACATTACACTCCACATATGGACTAAATTTCAATCCATGCATGATCTATTCTATTTAATACCACCTACCAAACGGGCCCTTAAACTATTGGATGTTCATAGAAACTCCCCCTTTCCTCACCCCCAAACTACCATGAACTCATTATGTCAGGTGGCATACAGCCAAGTTTGGTTCAATTTTTAAAAAATTAGTTGTACTAATATTTCAAGCCATAATGATCACCTTAAAACGGAAATGTAATTAGTCAATGTTTAACCAATTAAAAGTTAAACTTGTAATAAATTCAATATGTAATTATATACAGCTAGAATTAATTAAAATGCAAGATATAGAGTATCAAATCTGGATAGAGATACCTTTTAGAGAAAAACAGTAACGGATCACTACATTATCACTAAACTTAATTTTGTTAATAAATTTATTTATATCCATATAAGATGGCTCATTTTAAACTCGAAATAGAACTCAATAAACGTGTGATAATAAGACAGTTAATCTACATCTTACCATTTTGCATAATAATTATTAATAGAGGCATTTAGTTAAAGCAAAGATATGATAAGCTTGAAATATATTGATGTAATTACTATTTCTAGCAAGTTGATATGATCTTCTAGTAGAACTATGAGTTGGTTGTTTGAATTTGTAAATTTTAATGCCATGGAATCATTAATTTTCCATATTTGGAG

>LNC_000377

TGTCATTCTCCTGCATAATCTGAATATTCCGCCAGTACAAGTCTAAATTCTGTTTCTCCATAACTTGCTTGAGTTGGCCTGAAACTGGAGAAACCATTAAAAATTATGAACAGTTCTGCAAGATATATTTTGAAAACATAGCCTAACAGATTATGCAAAAAGAGAAGATGATGTCATAGTAAATTTTTCTTATCTACATATGCATGACATACGTTTATACACAGTATATTGACCAAAATATTGCAGAAAAATGATAAAAAGTTCCATAGTGATTTATTTCTATGGGAAAAATAAGATGGAATAGTAAATGTAAAACAATGAGAAATTGGATTTGAATCTAGCCCTGCTTTGTCTCATGAAATTTGAGTCATAGAAGGCAAGAGTCAGCAGATATTTTAGAGGGTACTCTCGGTACACTTAGTAAGTAACTAGCTTCAAAGAGGGGAAGCAATTTTGAGACCAATGGAAAGAAGGAAAACTAGTTTTGCAACAACACTTGAAAGTTCTTTGTGTAAGGACACTTACTTACGAATAGAGAACAGGCTCCGAACCTTAAGATTCTGAAGCTATTAGAGGGCAACTGGTTTTGGTTGGAGTCTCTTTGATATTCGTGCTTATTTATACTACTATTTTATTACTTTTACGGATGGACAAAGAAATACTAGAAGCAGCACACGAGAAAGGCACTTGTAGTTCAAGAAAGGCTCCCGAAACCAATTCGCAAAGCCCCCTTTATTCCTCCTTTATTCTCACATAAAAAACACGTCACATTTTTAGTCAAGAAGACGCTTCATCGATGATTCTGTTAGAAGGAAAGAAAATTGTTGCTCAAAGTGGTTTTTGCATGTGGATTTTGCTTGTTTAGTTATGTAAAATTAGTTATAAAGCAGTATAAAGGATGATATTCCTGAAGAAGCAAATGCTTGAGCAAAGGGAGAAATAGCATGGGAGACAGCGAGAACTAGGGGGGGTTGGGGTGAATTGTGAGATTTTGATATTCATGAGAGGAACCTGAATGTAAGTAGCAAAATAATAAGCATTCAGTGAATTTAACAGAATCAACTCATACTTTTTAAAGCTGATTTGCCTAAGCCATGAGTAGATCACAATAATTACAAGGAGTAGAATTTTGTTACTGTTAATCTGGCTAGGCTGTGTTGTTTTTGGCAATTACAATACAAGAGAATCCCTCCTTTTTTGGAACATAACTGATTAATTCATTACACCTATTAGAGAAAATAATGAAGTGGTTAACATATATGGTGTATGGTAAACTCTATTGAAAGTGAATAAAAATTAAAGAAATTGAAAGAAAATCTTGAGTAGTTAGTAAGTATGGATTCAAGTATTATTTTTGTTTTGGAGAGTTACTTGAATTCATTATGTATTACTTTCTTTGTTTTTAATTTGGAGAATATTTGAAAAACAATGTTGGAACCTAATGTTTTGTGAATTTTATCTCATTGTGACCTCTGGTCACGGGTTCAAGCCGTGGAAACAACCTCTTGCAGAAATGTGGGGTAAGGTTGCGTACAATAAACCCTTGTGGTTCAACCTTTTCCCGTACCCCGCGCATAGCGGGGGCTTTAGTGTACGGGGCTGCCCTTTAGATTTATCTCATTGTGATAACAGAAATGTCTAGACTTTCAAATTTTTCTTTGGTTTAATCATATGGTATTTGAAATTTGTTGGTCTGATTTATTTTGTATACGTGTTGGGTAAGCTAATTAAGGGAGGTAAAATGCTCTCTGTTAAGAAGTTCTTCATTCCTAGGGTCCGAACTCGAAACCTCTCGTTAAGGGAAGTTTTCATTACTGGTGACAATTGGCTCCTTCAGTACAGTACAAATATAAAAGTGGAGAAGTCGAGAAAATATGTGTAAATATTCACATTATAAAAGGGAATTTCATCATTATAATTTATATTGCTAATTATGTTTCGACTCATATAAGTTGTTGTCGGTTGTTATTGTTGTTTTACTCGTTATAGGGAACGGATTTCAAGGGCAACAAAGGCCCTTAGATAGGACTTTATCTATGGCTAGAAGAGATTTTCTTTCTATTATTGTTTTACTCATTATAGAGAGCAAAATTCAAGGGAAACAAAGGCCATTAGATAGGACTTCATCTATGGTTAGAAGATATTTTCTTTCTATTATTGTTTTACTCGTTATAGAGAGCGGAATTCAAGGGAAACAAAGGCCCTTAGATAGGACTTCATCTATGGCTAGAAGAGATTTTCTTTCTATTGTTGTTTTACTCGTTATAGAGAGCGGAATTCAAGAGAAACAAAGGCCCTTAGATAGGACTTTATCTATGGGAAGTGATTTTCTTTCTATCATTGTTTTACTCGTTATTGAGAACAAAATCCGAGGGAAACAAAGGTCCTTAGATAGGACTTCATCTATGGCTAGAAGAGATTTCCTTTCTATTGTTGTTTTACTCATTATAGAGAACGGAATTCAAAGGAAACAAAGGCCCTTAGATAGTCATAGGAGTTTCATCTATGGCTAGAAGAGATTTTCTTTCTAATGGAACTCATCACTAAAGTTGTTAACTAATTCAAAATATCGAGTGCTCATAGAATCACCCTGAACTATCATGAACTCATATTAAAAAAATACTCAAGAGAAGAATATAACATGATATGTGAACATTACACTCCACATATGGACTAAATTTCAATCCATGCATGATCTATTCTATTTAATACCACCTACCAAACGGGCCCTTAAACTATTGGATGTTCATAGAAACTCCCCCTTTCCTCACCCCCAAACTACCATGAACTCATTATGTCAGGTGGCATACAGCCAAGTTTGGTTCAATTTTTAAAAAATTAGTTGTACTAATATTTCAAGCCATAATGATCACCTTAAAACGGAAATGTAATTAGTCAATGTTTAACCAATTAAAAGTTAAACTTGTAATAAATTCAATATGTAATTATATACAGCTAGAATTAATTAAAATGCAAGATATAGAGTATCAAATCTGGATAGAGATACCTTTTAGAGAAAAACAGTAACGGATCACTACATTATCACTAAACTTAATTTTGTTAATAAATTTATTTATATCCATATAAGATGGCTCATTTTAAACTCGAAATAGAACTCAATAAACGTGTGATAATAAGACAGTTAATCTACATCTTACCATTTTGCATAATAATTATTAATAGAGGCATTTAGTTAAAGCAAAGATATGATAAGCTTGAAATATATTGATGTAATTACTATTTCTAGCAAGTTGATATGATCTTCTAGTAGAACTATGAGTTGGTTGTTTGAATTTGTAAATTTTAATGCCATGGAATCATTAATTTTCCATATTTGGAG

>LNC_000378

TATCCGGTAAGCATCTGCTCAAGTATTCCCATGTGTCAACACTCCATATGTCATCAATGTAGAGAAGGTACTTCTTTCTTAACAGAAATTTCCGCAAAGCATCTGCTGGATTTTTCTCATTCTGTTGGTTAGAACATTTGACTTGATTCAAAATTCCGAGCAGCAACTTTCTTCTGTCATAAGTTTGGGAAACACAACACATAGCTTGGACGTCAAAATGACGCCGCATTTTCTTATCATGAAAAACTTTCTTTGCTAGTGTAGTTTTTCCAAGACCAGGCATCCCTACGATAGAAACAACATCCAAGTACTTTGGTCCTATAGTGAGCTG

>LNC_000379

CTTTTGTTAGAGGTATTAATATTGTTGAATGTGATACTTCTAATCAAGCAAGTGTTTTTGTTAACTTACGCGTTTATGTTCAGAGGCTATGTCACGGATCAATGTAGTGATAATGCATTTACGTGCTCGTGAACCTGAGAATGAACTTGCATATGATAGTGCTGTTTTTACCCTTGGTAAAATATTTCAGTTTCATCGAGAACTTATCGACTCAGCCCAGGTCATTCCCATTTGGTTGAATTGCCTGCCTATAAAAGGTGACCTTCCTGAAGCCAAATATGTTCATGGTCAACTATGTTCCATGGTTGAAAGGTCAGATAGAGAACTTTTGGGTACCAATTATGAACACTTCCCAAAAATTATGTCAATTTTTGCAGAGGTTATATGTGGTGGAAAACATCTTGCTACAGAAGAAACAACAAAGTGTATGATTAATCTGTTGAGGAATCTTCTTAAAGAACTACCACCAGACACTTTGACATCAGCATGGTCATTGATATTGCCTCAGCAAGAAATGGAACTAAAAGCCATTTTATTTCCTGGAATACTTCATGTGGTACGTTTTCACAACAAAACATTTTGTATGCTTGACACTGATACCAATGAAAGCTCTCCCCCTTACTCTTTACCAT

>LNC_000380

AAAACCCAGCAGTTAGACAGGTTTGGATCATCGTGATTGCTTACATTCCTGTTACTCAAGTTGGTTGCTCACAGTATGGTTTAACCAAAACACCAATGACTTCATGTTCAGAATGCTCTATATGGACTTGGGCTTTATGCAGAATATCATGGTTCTGTTTTCAAACCTTTTGTTAGAGAGGCTATGTCACGGATCAATGTAGTGATAATGCATTTACGTGCTCGTGAACCTGAGAATGAACTTGCATATGATAATGCTGTTTCTACCCTTGGTAAAATATTTCAGTTTCATCGAGAACTTATCGACTCAGCCCAGGTCATTCCCATTTGGTTGAATTGCCTGCCTATAAAAGGTGACCTTCCTGAAGCCAAATATGTTCATGGTCAACTATGTTCCATGGTTGAAAGGTCAGATAGAGAACTTTTGGGTACCAATTATGAACACTTCCCAAAAATTATGTCAATTTTTGCAGAGGTTATATGTGGTGGAAAACATCTTGCTACAGAAGAAACAACAAAGTGTATGATTAATCTGTTGAGGAATCTTCTTAAAGAACTACCACCAGACACTTTGACATCAGCATGGTCATTGATATTGCCTCAGCAAGAAATGGAACTAAAAGCCATTTTATTTCCTGGAATACTTCATGTGGTACGTTTTCACAACAAAACATTTTGTATGCTTGACACTGATACCAATGAAAGCTCTCCCCCTTACTCTTTACCAT

>LNC_000381

TAAAAACCTAACATAGGAAACTTCACCCAAAAGGTTATAAAAGCTATACGATCCTTCTAGGGGGTCCTAGTTAGTGAGGTGGCGAATTCTCCTTCAGCCGCCGTCATCGGTGTTCTCGCCGCCGGCGCCGGCATCTTAGCCACCCATTCCGCCGTCATCGATGTTTGTGCCAGTGGCGGCGGTTTCATCTATAGCCCTTTAGACTGAAAAATTACTGATTTTGAATTTTCGATGATACCCAGATGGAAGAAACATAGCTAAGAATGATTTTCGTGGATCAGATCTGTATTCATCTAATGCTGGTCTTTTCCATATTGAGAATCTCGGAGCCAATCTATTGAAGACTCCTGCAAGTGCCCAAAAAAGTATGACGTTGCTGAATGGAATCGAAAGATAAAAGCTTATTGCTCATTTGAAGGAATTGGCGCTATGCCTCTCCCTAAAACGTTTACCAGATTGTGCTGGCAGTAAGACATTATTTGTACGACCAGGGCATGTGTAAGACAAGCTGCTTTCTTATATCACAATGATCAGTCTTCGGAAATTGATGATATAGTGGTTCGGAAGATGAGGCAAAAAAAGCTTATGAAGCAACCTAGTTGAATGTTCAAATCAATTGACCACTTTTATTTGGTAATACAACTATTGCCAAGACAATGCTTCATTATAAGTGCATAACTTTTCACTTTATTGTTTGAGCATTGTGTTGATTTCTCTAACTTATTTTTCTTTGAACAAAATTGTTTTTTCATCGCGAAATGCAACACCATGCTCAGAAGTTAGAGAGTGAAATGCTATGCATTTGTAATTATATATTTTCTTGGCTAGAGTTGTATTGCCAAGTCAAGTGTTTTAATTGATTTGAACCTTCGAGTGAGTTGCTTCATGATCTTCTTATGCCTTAGCTTCAGAACTTACCACTATATCATCAATTCTCGAAGAGTGTGGCCTATCTGAATGATTTTAACCTGGAAATGAACGAGACGAGGAAACCACTTGGTGTTGTAAACCTCTAGTGTTGAGCCTTTATTTTTGCTAGTTCTTAAATATGTATATCTTAGTTTTGTAATTGATTTATGTGTGTTTAGTATATTTCTGTTGTTACCCCTTTGTAATCTTTTGGATTCAATGATGAAGTTGGTATGAAAATATTGTAGGATTTGTTGAAAAAGAGTGCCATTTGTGGATGGCTTTGAGAGTTTAACACCCCAACTATGAGATGTTCACTTCTTTTTTATTCTACCTAAATTGTCATCATTTGTTGGTTTAATTAACTTCAAGGTGTGTTTTTAATACATAGACGGTGATGGTTCAGCTAGAAAAAAAAAGAACAAGTGATAGTTAGGATATGAAACTCGCAAAGGAAGTAATAGTTACATGTTTTGACAATTAACTCATAAAAGAGCTATATAACTGAAGATTATTTTTTTGTTATGGAC

>LNC_000382

TGGAGAATGAGGAAAGCTATGGACTGGAGGCGATTCTTGTGGATTGGGTTGTTGATTGTTTTCAACAAGGAAACTTGGAAGCTTTGGTGAGAAGTGATATCGAGGCCTTGAATGACAAGAAGCAGCTCGAGAGGTTCGTGATGGTTGGTATTTGGTGCGTTCAGGAGGATCCGTTAACGAGGCCTACTATGAGGAAGGTCTGTCAAATGCTTGAAGGATCTGTTGAAGTGACAATTCCACCATGTTCATGTAATTTTAGCACAACTATTTAACATCATATGCCTCATTTACGCGCGATTTCCTCCTCTGTATAGTACTTTTGAGTGGTCTAGTTGTATTACTTTCTTTAATGTTGAGTTCACTTTAATAGTACTAATGATCTGTATTGCTCAGACTCTTCAAAAATGTCATCGGGTGCAGCTCTTCACATTTCCAT

>LNC_000383

GTTGTAGCCGCGAAATTCGTCCTTGCTCGCCTTGAGATGGAGCCATGTTGCTCGTCAGGACAAGGGAGTCGATCCCAACCGTCTATCTCACCATTTCCACTTTCAGAATGGGTTGAATCTGCAGAGAAAAGGTGTCTCACCGAATTGGTGAAAGATTTTGAGTTTTGAAATTGAAAAGGTCCTCTTCTGTTCAGATTTTGACCCGATTTCCCCCCAAAAATCTGCCCTAATATCCCTTCTATATAATTCACTGTTGAGAGAAGGTGGAAGGGGTGGAGGAGATTTCTTAGTTGGAATTTAGATTTGAGCAAAAATACACATAAATAGATTAAGCGAAAATAGCTAAAGTACCTACTAAATACTTCAAAAAAGAAAAACGGAGCAGAACTGGCTTTCGGTTATCGCCGTTCGAGTCGGAACATGTGGTGTTGGAAGTCATTCACCC

>LNC_000384

ATCTTAACTGCATGAGAGCATAAGGTGTCCACCTTTACCTCATTATTTGGAAGTTCACTGTTGTTTAAGAAGGAAAAGATTGTTTATGTGTATTACTTTGTCAAGATAAGTCAAATGTTGTTTATAGCAAACTATTTCTAGGATTTTAATTTATTTATTTTCATAGCCTCATATGTTCCGTTTTGAAGTGTAGTTATATCGTTTCCATGGGATGAGAAGCATGTCTTGACCCATTTATTTTCTATTGCTTTTATCACTAGAATATATTTGCCCTTTTAATTGTTTGTTGTTGGTTTACGGTTGGGTGTGATAGGCTTATTCAAATCCACATGACCACTAGTTGATCATGGCTTAATTATTTCAAATTGGAAATTTTAGGATGGGCTATTAATTTATCTATTGTTAAAGCTTTGCATCTGCTCAATTCTAACTATAGGCTTAGTTGTTTAAGGTCATTGAAGTGTCATTCGTGTATGGATTTAAGGC

>LNC_000385

AGCTTATTTGCCCCCATGATCGAACTCTATGAATTAGCATGTCATGTTCAAATCAAGTTCATATCTCCATTCGGTCTAAATTTTAGTCGAAAATCCTTGTTATTTGTAGTTGAGCTTTTAAAAGATGTGAAGTTCCGTTACTTTCCAGATTCTAGGCTTACTTCCATCTATTGTTTTCTTTTCGATTTACTACCTATTTTCTCTTTTGATTGTCCTCTTTTTAGTTCATCATATGGTCCAAGTTTCTTATAAGTTGTATTAACCTGCTTATGTTTTGAAATTAATCTTGATTCGGTTAAATATGTGGTAGTCGATTATATTGTGATGTTCGACTATTTGGGTTTGTTGAGGTAGTAGCTCTTGTGGTAAGAATGATTATCAGTTTAGCTGTAGTTCTTTCAGTATATGAATTTGGTTGTCTTTAAATTTTATAGTTAGTGTAGCTATACTAATCTTGCATGTTTTAAACTTAGATGAACCCTCTTTGTTGCTTTTCACTGTTTAAGGGAAGCACTTGCTAAGTCTCTATCTCTTAATGCATTTTTTCATGGACTGCATTCAAGTCGGC

>LNC_000386

TACACAAATAAATTGAATTCAGTTATACGAATCCTCATTGAGTACGTAACATGATTAAAATAGAAGGATTGAAAGTTTGACAAGTTATTCTCCTCTACTAATTACAGTATTCCATCCATAGTAATTACCCTCTAAACAAGGTAAATATCAACAAATAAGACCGTTAATTAATTTAATTAATAAGATTATCATTAAGTATGATGTAATTAGTACTGTCAGTGCTAAACCACCACCCTCTTCAGTAAATAATTACTAAAATAATAATATGTGAGGAATAAAACATTCATGAAATTCAGTTCATTTTTTGGCAGTTTCT

>LNC_000387

CTTTTCCATAATTTTAGAAACTTTAAAAAATTACGATTACCGTAAGGAATCAAGTTTTAAAAGCTTACGAGCATGAGGCATTCCACTGTCTACTAATCAGATTTTGTGCTTTACTGTCTGTCCATGTTGTGAACTTAAGTTCTAATTTCCAGCAAACTGAATCAAAGGTTTTTTTCCTTTTTGAAATCCACATGATTTCGTGTGTGTGTATATATATCATTAGGTTCTTACTATGTGTTCCTTTTTAACACCTGGCAGCTGACAGTGTCCATGAATGAGTGTATATATTTTAACTCACCAGTC

>LNC_000388

ATAAATAAATTGCTTATGTGTGTACATATTTTATTGGGCTGTACACCCACGCAAGTGAGCTCGGGTCTCAGGTGTGCAGGCAAGGTGTTTGATGAAATTCCTGAACCATGTGTTGTCTCGTATAATGCGATGCTTGATGCTTGTGGGGAAAATGGGGATATGGGTTTGGGTTTTTGATGTTTTCTCGCATGCCTTATAGAGATGTTTATTCATGGACTAGTATGATTCATGGGTACCTGAGGTTGCTGTTAAGTTTTTTCGAGAATATGATGGTACAAGAGGATGTGAAGTGTGGTGTATTGAAGCCAAATGAGGTTACTTTTGTTAGTATTTTTCTTCTTGCACGTTTTTGGATTTTGGTGTGGCGTTATATCTAGGGAGGCAAGTACATGGTAAGGAATGAGGGGTGGTTTGGTATATGCTTTGAAGGTATTTGATGTGATGGTTAGCAAGCAGGTTTGCATTGGAATGCTATGATTTCTTTCCTAGCTTTGAATGGTAGGGAGAAGCAAATGGGCTGCAACCAAATGAGGTACCTTTGTGGCAGTTCTATCAGCTTGTGCAAGTACCAAGTTTGTCAATTTCGGTTTTCAATTGGTTGAATCAGTGTCATGTGAATTCGGACTTGTAGCTAAGATGGAACATTATGGCTTCATGAAGAAGATGCCTTTTGAAGCTGATGCTACTTTCTTGGGGGATCTGATGGGTGCTTGTAGACTTCATGGAGCCATTGAGTTGGGAAACGGAGTAGCTCAACTATTGCTTAAATCATGACCGAATCATTCAGGGTGATATGTGCACTCTCAAGCATTTATGCTGGCACAGAGAGGTGGAATCACACAGTTTCCTTAAGAAAAGCAATGTTAGATGCTGGAATACAGAAGGTTCCGGCCCACAGTTTTATTTAATAGAGCTGATAACTGTAGCAGCTTTTATTTGCAAAATTGTCAATGTGCCTGCTTGGTGATGAAATCTGGACTCATCAAAATCAGCTAAAAGGATCTTTCATATTGGTTTTGCACAGAGATTGAAGCACTGCTTAATGAAAGCTGATGTCATAGCTTGACAGATCAAGGAGGGTACATTTTTTGTGAAAATGCACAGCAAACCTGGTTCCAAAGTCTGCTTGTCGAGGTATTTGATGCGATGGAGCAGGTTTGCACATGGAATGCTATGATTTCTTCCCTAGCATTAACGATGTATGTCAAGGTTGCTGAAGTGTGTGACCATCTCATCTAAAAGCTTAAGCTATTATTTCATTATATTCTCAACAAAGATGAGAGTAAAAGGGGCTGCAACCAAATGAGATTGCCTTCGTGGCAGTTCTATCAGCTTGTCTGTGTGCCAAGCTTGTCGATTTCGGGTTCTAATTGTTTGAAGCAATGTCACATGAATTTGGACTTGCAGCTAAGATGAAACGTTATGGTTGTGAGGTTGATCTACTGCAGGAAGCATACGACTTCATAAAGAAGATGCCTTTTGAAGCTGATGCTACTGTCTTGGGGGGCTCTGATGGGTGCTTGTAGACTTGGTGGAGCTATTAAGTTGGGAAATGAAGTAGCACAACTATTGCTTGAGTCGCAACAGAATCATTTTGGGCGTTATGTGCAACTCTCAAGAATTTATATTGGCGCAGAGAGGTGGAAAAGAACAATGTTAGATGCTGGAATACACAAGGTTCCAGCCCACAGTTTTAGTTAGTAGAGTTTAGTTTGTAAAATTGTCGATACGAAGTCATGACTCATCAAATTAAGCAGTTCTGCTGGATTTCTTCCTAAATGTGATATCTCTGCTAGGATTTAAACCTTAGTATCCAAGGTTGTTTCTCAAACTTTTCAACTGCTACACCAACTCTATTGTGGTCGTCGTTATTATATATCGATGAATTTGGAACTGTTTTAGGAATTTGAATGTGCTTTCTACTGTTATTTCAAACTTTTTGCTCTACTGGTGTTTCCCTTTTGATCCCTTTGCATTTTATGAACTTGTTTTTTCCTCACAATTGAAAATGCTGAATTTATTTCTACA

>LNC_000389

ATTGCTCTGGTTGCTGAGGTCAAGCAAACTAATGAACATTTGTCGATGAACGGACTCAATAAAGCTGAAAAGGATGTGAAGGTATTGGATGAAGAAAATGAGGCTTTGGAGGAATCAAGTGACACATCGATAAGGCTCTACAAGAAAACGAAGCACACACATGTTTCTTGTGGAAAAGCTTCATCTACCAAGGCCATGAGGATTGACTTTCAAGACGAAATACTTATGGACATTCTCAGTAGGTTACCCCTGTT

>LNC_000390

ATAACTGAGAGGCACTATAGCCATATAGGAGTAGTGGCAAATTTTCATAACACCTCTCTGGTTGAATATCAAAAGAAATGATAATACATCGTATGTTAATTCAGGGAGCAACAAATTTGGTTTGATAATTCATGTATTGTGGCTTTTTGGATGAAATCGGACATTGAGACTTTTTATGCTGGCAAAGTTTTTGTTTATGAAATGCTTTTACAAAAAACAAACATGGAAGAACATGTGTATTTCTCTTGAAGAAGAACGTCTTTGTATTGGTGTATTTTCCCTTTTATGTTTTGACCTTAAACATCAAAACTCATGAGCTTCTGAAATCAGTGCTCCATAAATA

>LNC_000391

CTCTTCATATATACTTGGTTTTGTGTGGATATATTTTATTCAATTACTCTGTGGCATGCTAAAGGAATTGAGATGCAAACATGGAAGCTTTCTACCTATTCAGATTCTAATGAGATGCATACCATATTGAGACATGCTCGGCATGAAATGAACCTGGAAGGGAAGCCACAGGAGCAGGAATGGAGAGAAAGCGAAAGAGGAAAGGTGTTTTCCCATTCTATTGATCAATTCAGAGTTGGTCCAAATTTTAGAGTGATGTGAAAGGTGATTTGTAGGACACAAAAGTCTTTGCAAGTTCTGGGCAACCTGAGAGATGATGACATCCGTATACTTTCCACCAGCCAATGTAAATGACAACCAAAGCAAGAAAGAAAAGATGATAATAGTTCTCACATAACTTGATGATTCTTTGAGATATTTCCTGAGTTTGTGACACAAATCTGGAGAAGAGGAAAAACTGGACAGTGAAGCAGAAGCTGGAGAAAGAAGTTAAAGCTGGATTTTTTCTAATTGGCAATCTTTGGTGTTGCTGTTGCGATGTTTTGTACATTAAGATAATAACTAGGAATGGCAATAAGTTTTGTTGGGGAGTTCTTAGGATATATGCTGTGTTTTTAGATAAATTAATGAAATGACTCTCCAGAATAGCTGTTGAAAGCTTCGTTGGCTCGACTCAATGACCAAAATGGTCATTCGGAAAAAACTTACTCGTACCTGGTATTTCCACCAAGTAAATGAATGCAAGACATGTATTTTAGTCCTATGGTTAAAATTCTTTATTTTCTGTAAGCTGATGGCTGATTTCTTTTTAGAGAGAGTTAGAAGTAGATTAGTTTAATCTTTTAAACTTTTCATAT

>LNC_000392

GGCCGCTTTCACTTTTAGTGGGATTTATTCTATCTTAAAGTAGTATGTGAACCACCCATACTCATGTCATTGCCATTCACTAAGAGTGCACCATGAAAGTGAAGAGTCCACATAGTTTAAAATAATGTTTTAATTTGTAATGTCCACATGCGTAGTTTTGTCATATATTGTAAAGAGTCTAGCATAAGAACTTTTTACCTTATTTTTGGAATTTGTTCTTTAAAAAAATTGTAACACGTGGAGAAATGGTGGGTTCCCTATTTTTGCTTAGGCCCGCATGGATTTCAAGAGACTATAGGTCCTTTGAGACAACTCTCTTTCATGTGATTTTAGGTTTACCTCGTTCTTTGTTAACACTTTCACTCGCTATGGTTTCACATTCATAAATTGACGTAGGACATGGCTAAACCATCTCAAACATCATTTCTCCCATTTTATCCTCAATGTGTGCTTTATCTAAATATTCTTAGTTGTAAAAGATTTAGTAACAATACTTTTTATATAATTTTAAATTTAGGAAAATAAAGAAATTGATGAACTGCGATAGAAAATTGAATGGTTGTACTCTCATATTCCTAACTTGTCCATTCCTTCTGAAACTGAGGGAATGTTTTCCACATTATTATTACTTTGCATCATTGTTGTCATCATCCTTAGTATTGTTACTATTCCTATTGTTATTTGTTATTTGTGATGTCATTGTAATAATTGTTATCGTTGTTAATATTTATTTATTATTATTAGTAGTATTATTATCATGATCATCTTTGTCATCGCCGTTGTCACCATTATTTTTTCTCATCCTATGTTCTCTTGATTTGTTGACTATCATCTGCAGGCTATGGATCAAGCTTCAGAGAAAGGTCTAATTTTCATTGATGGATCTGGACCCACATATGCTACTGCCACACAGCAGGCAAACCAATGTTGGCCAAACAAAAATTCGAGAACAGGGCATAGTATGCACATAACAAAGCTCAAGAAGGTGAGTCGTTGGTGAGGAGGAGTACGAG

>LNC_000393

GCCATGGCTCTTGGAGTTTGCCTACAACTCCTGTGCTTGCTGCCTTCTTTGTTCTCAAACGGTGTGATGAAGTATCAAGGTCTTATTTGGATTTTTTTTTGAAGAAATTTACTGCGGAAGTTGAATCAAATGACAAGGTTGTTTTGAATAATTGTAAGGTAAGTATGATGTGAAATGAAGTTTGACTTGCTCTATTGGTTTATTGTCTTATTTATTGTAGGTTGTGGTCACTGTCTTTATTGGTTGTGACACATGTTTTGTTCTTAAGAATAAATTGATATAGGTACAAAGGCATGCATTTTACAGTGGTCTGATCAATCATTAAAGAGTACAAAGATGGTTATGCTGATTTTGAGAATTTTTTATCGCCTCAATTTGTTGGATTTACAAGTAACCCAATGTGTCGTCTATTTTTAAGCTGTTTTGATAAAAAGAAAGGATTATATCTAAAGATGCACTTAACTCCTAATTCTTACAAAGTCTAATAAAGAACCTTTGGTATGATAATTTATCTCTTGATTCATATTTTTAGTATGGTTATTGCAGTTAGTAAAAATTGCAAGGCTTCTAAAGGCAAACACATCTTGTATTTGTGTCATATTTTCACACTTTGTCCC

>LNC_000394

GGCTCTTGGAGTTTGCCTACAACTCCTGTGCTTGCTGCCTTCTTTGTTCTCAAACGGTGTGATGAAGTATCAAGGTCTTATTTGGATTTTTTTTTGAAGAAATTTACTGCGGAAGTTGAATCAAATGACAAGGTTGTTTTGAATAATTGTAAGGTTGATGATAATCCTTCTTTGACAAATGCTTCCAAGATGTAGCATCTATTATTGCGAGAGTTGGTCTTGGGGCTCACTGCATTAGCATAGGAAGTGTCCGACATGTTTGAGCAAGCTGTTGGCAGAAAATACACTGCGAGGATATTACTAACATTGAAGAGTTCTCTCGCCATTTTGGAGCTGTTGATTTGAGGATACCTGATAAAGAGAGAACACTAACAGAGGCTTGAGCACTTACCATCTGATCTGATCCTACTAAGCAAAAGTAATCACTTGGAATATTTAGAACTGGCAAGAAGAAGACGCACTTCTTATTCTCATATTTACGCGTGACATTGTTACTCAACAAGAAATACTTGAAAATTGTAGTTAATAGATGGTGCTATCATATTAGAAAGTATTTGTTTTGTAATCTATAGCAAGAAAATCATCTGAGTTCAGTAAGTAAGGGGGATTTGCCAAAGTGTCCTCTCTTTTATGTAAGGCATCATTTTTCTTCCTGATTTTACAGATGAACTTACAAAAGGGAAGAGATTTTCAGTTCTTGGTTTCCATGGAGAACTTTTCCATCATAATTTAATTTTGACTATGCCTATTTAAGCTTCTTCTAGAATTGCTATGTTTTATTTTCAAATTGGATTTTAACATGCATGATTCCAAAAAAAAATAATGTGCAGGTACTGGTCACGTTTGTTATGGCATTTTGGTTGCTGATCGTAATGGTTGCATGGCTACTATGACCAACTTGAAGAGAATTACCACATGAACATATGTTGTTGAGATTTGTGTAATACACAAAGCAAATGCTTTTGCTTTCTCACATATATGCAATATTATCAACTTCTTCATTGCAATGACATTGTACC

>LNC_000395

GGCTCTTGGAGTTTGCCTACAACTCCTGTGCTTGCTGCCTTCTTTGTTCTCAAACGGTGTGATGAAGTATCAAGGTCTTATTTGGATTTTTTTTTGAAGAAATTTACTGCGGAAGTTGAATCAAATGACAAGGTTGTTTTGAATAATTGTAAGGTTGATGATAATCCTTCTTTGACAAATGCTTCCAAGATGTAGCATCTATTATTGCGAGAGTTGGTCTTGGGGCTCACTGCATTAGCATAGGAAGTGTCCGACATGTTTGAGCAAGCTGTTGGCAGAAAATACACTGCGAGGATATTACTAACATTGAAGAGTTCTCTCGCCATTTTGGAGCTGTTGATTTGAGGTACTGGTCACGTTTGTTATGGCATTTTGGTTGCTGATCGTAATGGTTGCATGGCTACTATGACCAACTTGAAGAGAATTACCACATGAACATATGTTGTTGAGATTTGTGTAATACACAAAGCAAATGCTTTTGCTTTCTCACATATATGCAATATTATCAACTTCTTCATTGCAATGACATTGTACC

>LNC_000396

AGCGTGACTATGGAGAACATTAGTAAGCTTTAATATTTTTCTTTTGACTGCAGAAATGAGCTTCAAATCACTATTAGCAAACCCAGAAATTTAGTTAAAGTTATTCACACCAATGTATATAAGAAACAAAAGAATATAGCTAGAATAATGTGACAACATTTTACTACTAAGGAATAGTTTGGTGTGAAGTATAAGGTATAATAATCCCGGGATAAAATGCAGGATTATTATCCTACATTTGGTTGGAGGTATTAGGTAGTCCTTGGATTATTTATCCCACCATTTATACCATAGTGATGGGATAAGTTATCCCATATACATGGTGGAATAACTTATCACAGAATAATGAATCCCTCGAAAACTTGTTCCCAACCAAACAACCCCTTAGTGGCAGAACTTGGGGTGAATAAGAGAGCCAGAAGCAC

>LNC_000397

TAATACCTTAATCTGCATTCCCTCCGGATTCAAAAAGATGAGGATATCTCTTCTTCATGTCCTCTTCGGCTTCCCAAGTGGCTTCCTCAACAAATTGGTTCCTCCAAAGGACCTTGACTGATGCCACATCTTTCGTTCTCAACCTACGAACTTGGCGATCTAAAATCTGAACCGGCACCTCCTTATAAGATAAGTTATCTTTGATCTTAACACTTCCAGTCGATAGGATCAATGAAGGATCACCTATGCACTTTTTCAACATAGAGATATGAAATACCGGATGGACCGCTGCTAGCTCTTGTGGTAGCTCTAACTCGTAAGCCACACTGCCGACCCTCTGGACTATGCGATAAGGTCCGATATACCGGGGACTAAGTTTCCCCTTCTTACCAAACCTC

>LNC_000398

CCAAAAGTGACCTGGCTCAAAACAATTAAGCAAAACAAAAATATAGGCAAGAGACTAAACATCTGCATAATTAGGAAAGTAGGTAGAAAGCTCCACCCATTCATCTTCCTCAAAAGATTTGCTCTACTGTTAACTTGGTTTTGCAAAATCCTCCTCTGCTTCGATGCTCATCCTCAACTATCTGCTCAAAGCTCCATCTCTAAGGTTTCCAAGTCTTAATAGTTAGAGCTACATATCGGAATTGGTGGATCTATTGTTTGGATACTCCTACCATTAGCACCCCAAGGTCTCACTGGGTGTAGGTGTTTGCATCCAATGGAATCACTCCGAACAGTAGCAAACTTTGTAAACATGGTCAATGTTCTACGTCTCGATTCAAACCATCTGCAACATTTTCCAGATTTACTAACAGAAACACTGAGATCACCAACTAACGGATGTAAATGCCCATAAACAGGAACTTCCAACATCTATAGTAAGTTACCCTACTGTTACTGTTTTGCATAACCCGTTACAAAAATATTGTTGTATCTTCCTTGAATTCTGATATCATGATTCTTGATTGGATCTTCTGATGTGTGGTGTTCCTTCTTTATCTAAATTTAGTATCCTTCTTTCTGTTGTTGGAATTTCACCAAAGTAATTAAATTGAAAATCACCTGCAAAATCAAAAACTAGGTTAGTAAAAAAATCAGCGAGAGCTCTTATGGAGTTGACCTCCATTGTCACACTCCATGGGACCTCCCAGTTCCCCTGAAGAATTTGCACCATAATCCAAGAATCTGATTCTATAATGATATGTTGGATAACATTGTCTTTACAGTAGAGTAAGCACTCTCTTATTGCTGAAACTTCTGCTTTAAGGTTGGTAGTGTCCTTAATTCTTGAGCCTTTTGCAACTACCAAGTCTCCTTCCCCATTTCTAATACAAAAAGCAAAAGAACTAGGTCCAGGGTTCCCTCTTGAGGCACCATCAGTGTTACCTTTAAACCAAGGATATTGGGAGGGGGAACCCATTTTACAATTCTGGTTACATAGGCAGTTCTATTGTTCTGCAAAGCATCAATAATATCTATCCATCTTCTCCCCTGTATATTTAAATTGAATCTGGCCTTAACAAACATCAAAATAGTGTCATTAACTTCCCAGTACACCTTCCTTTCGTGGTATGAGCCACCATGTAGGATTGTATTTCTTCTTTTCCACAAAAACCAGAGTAGGATGATAGGAAGTACCTTGAAAACTTCTCGTAGCTTACTATTACCTTCAATATTTCTCCACATTCTCACACTTTGCTTCAAGTTAAATCTTTGATCAATAATACCCGCTGCCCTACAGTAGTGAGTCCACACTCTATCTGCCACCTCTCCTTTGAGAAATAAATGTTCTATTGTTTTTCTATCTGGATTAGCACAACATCCACAATCTTCAGAGATATTAGGATTCCACAAATGCAAAAGAGTTGATACAGGAACCTTACCCAACCAGATTCTCCAAGAAAGAAAAGAGAATTTGAAGGTTAATCTTTTACTCCATATTGCCTTTAGATCTTCATTTACATCTGCTCTTTGCCTCAAAATTTCCCAAGCACTCTTAGTAGTGAATTTGCCAGTACTATTTTAAGGCCACCATGGCTTGTCCATTACATTCTCAAATTGAGCAAATCCCATAGTATCTCGAACATGTTCAATGACATGTTCTGGGATTACATCTTTCATTGCACCATAATCCCAACCATCATCTTTAAAGAAATCACCAACATCATTTATAGGGTGGCAAGATTGTACCTCTGTTGAATGAAGAAATATAGGACCAGAATTGGTCCAGTTATCAAACCAAACTGTTGAGGTTCCACTTCTTGGTTCCCACCACAAAAAAGACTCAATTTTTTCCCTGTTCTCCAACATTGCTTTCCACACTTGAGATCCACCTTTCCATCATACCAATGTAGGAGTCTGTTTCTTGCAGTATTTATTTCATAAATAATTAGACCACAAATTAGTTTGTGTCCTGAACCTCCATTACAACTTAGCGTACATTGCTTGAGAAACATCTTTTAAGGATCTGAACCCTAAACCCCCTTCATCCTTTGGCAAAAAAACAATATTCCATGCACACCAGTGTTTGATCCTTCCTGTCTCTTTGGAATGCCAAAAGAATTTTGCAAATATTCTTTGTAATTCTTTAAAAATACTTTTAGGAGGAACAATAGCTGAAACAACATAAATTGGAATGCTCTGTAGAACATTTTTTTAGTAAAACCTCCTTTCCTCCATATGATAAAAGCCTACCCTTCCAAGCTTGGAGTTTAGTCCTTACTCTGTCCAACAAATCATCATAGTGCTCATTCTTTTTCTTTGAATGTGTAATGGGACATCCTAGGTACTTAAGAGGAAAAGATCATTTGGCCATTCCTGTATCCAATTCCATCTGCTGCACAACCTCCATAGGAGAATTTTGATGCATATAGAAGAGACTCTTATCCTTATTCACCTTTTGCCCTGATTCCTGCTCATACTCCTGCAAAATCATCATAATCTTTCCAATAGAGTGCTTATTAGATGAGGCAAAAATGATAGTGTCATCTGCATATGCTAAGTGATTAAGGTTTGAGCTCCACTTAGGCATACCATAACCCATAAAGGCATTATCTTCAAAAAAATGATTAAGAGCTCTGGTTAGAACTTCAGCAGATAAGATAAATAAAGCAGGGAAAGGGGATCCCCTTGTTTAACCCCCCTAGTAGAATGAAAAAAACCAACGGATTGGCCATTTAACAGAATAAAGTAGTAATTATTAGATATCAATCTCCAAATCACATCTACTACCCCATTACAGAAACCCATCTTTCTAAGAATCTTCATTAAAAAAAAATCAAGATACTCGGTCATAAGCCTTGGCCATATCCAGCTTCAAGATTACATTAGAAGGTTTTCCTCTTTTCCCTAAATCAGCAACAAATTCCTGTGTTAAGAGCACATTCTCAATGATATTTCTCCCTTTGACAAAACCAGATTGGTTAGGAGAAATCAATTTGGGAAGAATATTCTCCAATCTATCATGTACAATTCTTGATAAAATCTTATTAACAAAGTTACTCAAGTTAATAGGCCTCAGGTCTGAAAAAGACTGCACATTGTCTTTTTTCGGGATGAGTACTAGATTAGTATGAGTAATAGACTTTGGGAGGGTGTTTCCTGCAAAGAACTCTGCCACCATTCTACCCACATCAACACCTACTATATCCCAGCAAGAGTGAAAAAACCTTTCTGATAATCCATCTGGACCACAAGCATTGTCACCATTTAAGCTGAAAACTGCCTTCCTTATTTCTGATTCATCTGGTATTGCCCCTAAAACTGCATTGTCTTCCTCTGTAACTAGTTCTGGAATGTGATGCAATAGTGGAAAATCAGATGCATCCCTCTCTTGAGTAAATTGTTTCTGATAATAGTCTATTGCTTCATTAACAATTTGGTCTTCTTCCTCTACCCAACAACCCTGCTCATTAAGAATTCTTGTCACCTTCAATCTATTTCTTTGCCCTTTCACTATGCTGTGGAAAAACTTAGTGTTTCTATCTCCACTTTCAAACCAATCATATCTAGCATTCTGTTGCCAGTAGCTTTCCTCCAAATGTAGATATCTGGTGTACTCAGCTTTAGCTCTTTGCATGACTGCTCTATTATCCTGTGAGGGGAACTCTTCAAATAGTTTCTCTCTCAGTTTGGCTATCTCTTCCCTGATGAGTAGTTGTTGAAAGACATCCCCATATGTCTGTCTACTCCATATAGTGAGAGCCTTTTTCACCTGCTTGATCTTCCTTTTATAGTCAAGAAAAGGGTTATTTGTAAAGCTTGAATCCCAACCCCTCCTAACCATTTCTTTGAAATCTTCATGCTCTGTCCAAAACTTTAAGAATTTGAAAGGCCTTTGGAGCCTGCTATTTCTGTTCTCACAGCTAAGTAGCAGGGGGGCATGATCTGAGCCATCTCTAGTCAGATGTTCCACTTCTAAATGAGAATACAATCCCTGAAACTCAAAGTTCGAAAAAACTCTATCAAGCCTTTCGAAGATGCAATCATCACATGCTATACCATTCCACCAAGTAAATGGGCTACCCTTATATTGAATTTTCAAAACATCACAGGCTTCAACACAATTATTGAAATCTTCAACATCAGCAGCAACCACTGGGATCCCTCCTATCTTTTCTGCACCATTCAAAACAGCATTGAAATCTCTTCCTAAGATCCAGGGCCTGCTGATTCCATTGGACAGAGAAAGAATATCGTCCCACCATCCTCAGATTTTTATCACACTTTGCATAAACAATAGGCACCAAGAAAGAAATACCAAGTGTCTGGCTGCACAAATTCAGGGATAACTGCTGCTCAGTATCACTAATCAAAGTAGCTAAGTAGCTGTAAAACTATGGTTCACAAGCAACCAAATTTTGCCATTACAGTTGTGAAACATTAAAGGCATATGTAGTCTGCTTCTGTATCTGTTTATGTGCCTAGTGTGTTGAAAAGGTTCAAGAAGTGCTATGAAAGAAAATTTATGGTATTTGTTCAACATCTGTACTCTCTGAAAAGCCTTTCGTGACTTAACATATCTAATATTCCATATAAATGCTTTCATCATTTCTTCATTGGCTGAGACCCTCTTCCTCTCTTGGGTTGCAATCTAATAGGTTGAGGAACCTCTCCATTTCCTTGCGTTTTCCCCTTTCTTGCATATTTAAGATTATTAATAACTCTTGGGGACAAATCAGCATGTCTGAGCTAATGACTCTAGTGTTGAGATACCTCCTTTCCAGTCAGTTCCTATAGTAAGAGAGTTTCCAGAAGTTTTTCCTGATGATCTACCCGGAATTCCTCCTGTGAGAGAAATAAATTTCGGCATAGATCTCATTCTAGATACTCGTCCTATCTCTATTCCGCCATATAGAATGGCACCAACAGAGTTGAAAGAGTTGAAAGAGTGATAGATACAACCCTTTGAAACTAACTTTCTCGCCTTAAGGTACGAAATAAAACGACCCTTAGGCACTGCTGAACTACTACTCCAATCTAAGACTAGCTTATTCGGAAACTGAAACTTGACTGCTCGAGTTCT

>LNC_000399

GTTTGTGAAAAGTTGAGGGACAGAAGTCAACGGCATAAACACCAGGCGAACCCGTCTCGATGCGACACTAGTGCGGGCGCGGGTGCGGGGTGTGTGTCGAATTCGGTCAATCCAATTCGGACACTTTGACCGAAGCCGAGAAAAGATTTGGGGAAGAAAAAATCAGTTTCCAACGACGTAAAGAGCACAAAGCTTGAAAAAGTACATAGAAGAGAAGAAAGAAGAGAAAATCAGTTTTCGGCGACGTAAAGAGAACAAAGCTTGAAAAAGTACAAAGAAGAGAAGAAAGAAGAGAAAATCAGTTTCTGACGACATAAAGAGGACAAAGCTTGAAAAAGTATAGAGAAGAGAAGAAAGAAGAGAAAATCAGTTTTCGGCGACGGCGACGTAAAGAGGACAAAGTTGAAAAAGTACAAAGAAGAGAAGAAAGAAGAGTTTTTTCTTGAAAGAGTAAAGGAAATACAGAGTCTTGTTTTTTATCTTTCTTGAAAATATATGTACACAATTACATACACGGTCTCTTGAAAAAAAGAAAGCTCAAAAAGTCTTCCAAGTACAGTAGCGTCAGAAAGATGACATCCCATCAGCCATCAACTAGTCCAAAA

>LNC_000400

CGAGGGGATACATAGTTGTAGCAATTGATTCTCCCATGGACACTAATATGACAACATATCGTGATGAGGAGGAAAAATCAAGAGCTGAACTTGTTGTACTATTTTCTTTCGCTGAATGCTTGGATGCTACTATTTGATTGAGGTGATCTGCCTTACTACTTTTTCATGGAAAAAAGGTGTGGGACTTGATAAAACTGGCGGATTATATCTTACAGAGAAGGAAGATATTGACTCGTCAAGGATAGAATTACTGGTGAATCACTTGGAGGTTAGGCGAAAGTACAAAAAGACATAAATCCTAGCATGCATGCTATCTTCCAGTCCTACTGCTGCATCAGATGCCAAACCTTGCAAAGTAGGTACTCTTCCAACCTATAATATCAGTTTCAAGTGTGATGAAAGAGCTGAGAAAATGAAAGAGGT

>LNC_000401

AGGTAAGATTTCCAAAGGGGTTGCAAATGGATCCGCCGGTTCACCAATCATTGATGGTTCTAAAATGGCTAAGCCTACATTACATGCAATTGTACCTAGAATTACTACTGGAAAAATATATAAAAGATCATTGGGCCATGCGGGCTCGCCAAAATAATTATGACCCATACTCTTAGCCAATTTAGCTCTTAATACAGGATCATTCAAGTCAGGTTTTTTTGTTACTCCCATAATCCATTTTCTCTTCACAAAATTCCCTTCAATCCCCCCGTTTAGTTGACATGAATGGTTAAAGGGAAATGTCCAAATCCATGTCTTATTATTTTCAATAATCCATACTTGTAGCAATGAATATAT

>LNC_000402

TTTAAGTTTTTATAGGAGTGATCGTTTGTATAATTAGCTTCTTTTCAAAGCTGTATACAAAACTGAATTCAAATTTTTTGAAAGGTAATGGCGGTAATCTCGGTTAAGAAAGGCATACAATTCCTTAATTCGCGAAATATTAAATCGTTAGACTTCATACCAAATACACCACAATTTCTTCGTACCAGCAACCCAATTTTTGAGATACTGATATCGACGACTTCAATTAAAAAACAAATCAATTGATTTCATCATTGATCAACAGGTACATAATACATTGAATTCACGTCTTTTTATCTTTTGTATTTGTATGCATAGGTATAAATCGATTGGTAATTATATAGAATTTGTTGTGTATTCGCTTCATCTTTTTG

>LNC_000403

AGGAGAGTGATTTGGCTCATAGAAGCGAGGATCTGGAAGAAAGGGTCAACTATGGCAGTGACTGAGGGGGACTTGTTCTGTGATAAACAAATGACTTCAAACCTCAGGGCATCCAAAAGAGGTGAACCTGAGAAGAGCAGTAAGAGTAAACGTTCAAGTACTGGTGCTATGACCTTAAAGGTGGAAGATCCTCCCTTGGTTCGAATCACCCTGAAT

>LNC_000404

GCGCCCATTCCCACCCTCTCCGACCAGCGCCACGCCACGATCCCCGACCAGCACCACGCCACGCCACGCCACCATCGCAGCAGCAAAAGAAGGTCGCGCGCAGCTGCAGAGTTAGAGGAAGGTTGCGCGCAGCTGCTGGTCGCACCCAGCAGCAGGAGGTCGCGCCGCCTCCATTCCGACCGGCGCCGACTTGGACTTTGGTCCTGAGGATCCAGATCAATATAAGTATTATGAGTACTAATAGTAGTTTTACCTGACTTACTTTGCTGCTGTTGTTGTGTGGAGGTTGTTGCCTTGTCCTGAGATGAGTTATTTTGTGAGCCAATATGGTGATGATCAAGTGTTTCCATCATTTATAACGTGGTATACTAATTGGGTCTACAATTCAGAA

>LNC_000405

GCGCCCATTCCCACCCTCTCCGACCAGCGCCACGCCACGATCCCCGACCAGCACCACGCCACGCCACGCCACCATCGCAGCAGCAAAAGAAGGTCGCGCGCAGCTGCAGAGTTAGAGGAAGGTTGCGCGCAGCTGCTGGTCGCACCCAGCAGCAGGAGGTCGCGCCGCCTCCATTCCGACCGGCGCCGACTTGGACTTTGGTCCTGAGGATCCAGATCAATATAAGTATTATGAGTACTAATAGTAGTTTTACCTGACTTACTTTGCTGCTGTTGTTGTGTGGAGGTTGTTGCCTTGTCCTGAGATGAGGAGATTGTAGAGTTGGAGTATTCGGGTTGGCCAATCAAAAGAATAGTACTTTTCCAATGCAAGTGGTTTGATCCAACT

>LNC_000406

TTATGATTATATTATGGTTTAATTGATGTTTTTAGGTGAAAAACTCCATGGACCCATGATCTCCTTAAATCTCTAAGTTTGGCTTATGAAGTGGGTTTTATGATAATGCTTGGGTATTGATGGAATTGTACTTAGATTGAATTGTATTATTGATTCCTATTGTTATCTATATCTATTGTTGGTGGATTGTTGGTGTATGGATGAGTTGAGAATTATGGCCTTGTAGGCAAGTCATGGGTAAATTCTCTTGTGATGTTAGAATTGTTGAGAATTGGATAATGGTGATTATGGCTTTGGAGAATGGTAAGTTGACCTACTTTCATGCTATATTGAAATTGTTCTTGTTTGGATTGATCCACTTATGGTGGAGGTGTGTACTTATGGGATGTATTATGATTGAGGCCATGAAGGGCATTATATGAGTGTTATT

>LNC_000407

CAGATACAAACTTGGGTCCAAAAGTGACCCAAGTCCCAAAAACAGAAATAGCACAGCCAAAAAGACACTCCATTAGGAAACAAAAGAAGCTCCAGCCGCAATGGCTGCTTCTTCTTCTTCTTCAAACAAATCGACGGCTCCCCTGGAAAATTCAAGCACTTCGTCATTGGAAGATCCTCCTGGCGATCCGTCTACCTCGTCATAGCCGCTGGATCGATAAAGATCCAAGTATGGGTTGTAACCACCTCAACATCAAAAATTCTCACTGATGTCATGATTCAACAACCATTGAATACTTCTTCTACTTGGATGCGGACGATCTGAACCTGGGTTATTAACAAAGCTGAAATACGCCAAAAACAATCAGTGACGGAGCGGTGTTGAACCCCATCAGGATTAATATCACTGTTTAAGGCGACACATCTTGGACGTTGTAACCAAACTATTTATGGCTCGGAGATCAATGTTGTCCATCTTGGTGAGATACTACAACATGTAATGCTTCCATTGGTGCTGATCTGCTGACATATATAGTTGTGATTTGGTATGGATCATGACATGATGGACAATCTTCTTCAGCTATTATTTGAAAAGGCGCCTGGGGCAATCTGATCAAGCATCCCTTGCAAGTCCCTACCATCCGACTCCAATGTAGCATTGTAAACAACCATTTCACCCTCCACTATGTGGTCTTCATTTTCTTTATCTTTCGAGAGTTGTACTAACCGATCTTCTCTTGCAAGCCCGTCATGATCTGCAATTGCATTCTCCTCTTCACTAATTCCTCCTTTAGCACTCTCCTTGCTTTCGTTGTTGTCTTTATGCTTTTTTGTTTCCTTCCCCTGTGTTGCAAATGCTTTTGTAATCCACTCTTTGGTTGTCTCTGGCTCACATGTTTTGGTTATTGTGTGTTGCTCATTTCCATTGACTCTCTCCATGTCCTTCCTTGAGATACCAGTTGAGGGGTGCGTGTTTCGTGTTTCATCCGCGACTGTAATTTCTTCATTAAGAGCTATAAAGGAATTGCTAATTCGCACGTCTGTAACACCTGCAATGTGTTCCTCTTGATTTTGCCTTGGGAAAGTTCTGTTGGTAGGATGCCATTTTTTAAAATGCCTTCCTATACGAAAAACTGGAGTGCCTAATTGATTATGGATCTCTGTGTCCCTGTTATCGTGTATTTCTGTATCCGCGTGAATAGGTTGTCTGAGTTCAGGGTGAAGCACCCTACACTCCTCTTCTGCATGACCCTGTAATTTACATTCTTTACAATAATTTGGCAATACGTCATACTGAATGTTAACCTTTTCCACCCTTGATGCATTTGTATCCTCATTCATTATCTCTATCTCAATGACCTTAGGGAGATCTGCCAACTTGGTCGGGTTTTGTTAATTGTTGTCGTATCAAGACGTAGTGGTTTCCCGACTGCTGAAGCAAGGGAGAAAATGGACTCCTTAACAAAAAATGTTGGTTTTAAATCCGGAAAGGAAATCCATGTCATTGCTTGAGTAGTTTCCTCATTTGTATTGAACTTTGCATCATAAATAAAAGGTCGCATCATATACGAGTAACCATCCTTTGACAATATATAGTAGACAGGCTTAGACATCATATTAATAAAATCATCTTCTCTGTCAAACCGTATGAGAATGTGTCTATTTTG

>LNC_000408

CAGATACAAACTTGGGTCCAAAAGTGACCCAAGTCCCAAAAACAGAAATAGCACAGCCAAAAAGACACTCCATTAGGAAACAAAAGAAGCTCCAGCCGCAATGGCTGCTTCTTCTTCTTCTTCAAACAAATCGACGGCTCCCCTGGAAAATTCAAGCACTTCGTCATTGGAAGATCCTCCTGGCGATCCGTCTACCTCGTCATAGCCGCTGGATCGATAAAGATCCAAGTATGGGTTGTAACCACCTCAACATCAAAAATTCTCACTGATGTCATGATTCAACAACCATTGAATACTTCTTCTACTTGGATGCGGACGATCTGAACCTGGGTTATTAACAAAGCTGAAATACGCCAAAAACAATCAGTGACGGAGCGGTGTTGAACCCCATCAGGATTAATATCACTGTTTAAGGCGACACATCTTGGACGTTGTAACCAAACTATTTATGGCTCGGAGATCAATGTTGTCCATCTTGGTGAGATACTACAACATGTAATGCTTCCATTGGTGCTGATCTGCTGACATATATAGTTGTGATTTGGTATGGATCATGACATGATGGACAATCTTCTTCAGCTATTATTTGAAAAGGCGCCTGGGAGATCTGCCAACTTGGTCGGGTTTTGTTAATTGTTGTCGTATCAAGACGTAGTGGTTTCCCGACTGCTGAAGCAAGGGAGAAAATGGACTCCTTAACAAAAAATGTTGGTTTTAAATCCGGAAAGGAAATCCATGTCATTGCTTGAGTAGTTTCCTCATTTGTATTGAACTTTGCATCATAAATAAAAGGTCGCATCATATACGAGTAACCATCCTTTGACAATATATAGTAGACAGGCTTAGACATCATATTAATAAAATCATCTTCTCTGTCAAACCGTATGAGAATGTGTCTATTTTG

>LNC_000409

CAGATACAAACTTGGGTCCAAAAGTGACCCAAGTCCCAAAAACAGAAATAGCACAGCCAAAAAGACACTCCATTAGGAAACAAAAGAAGCTCCAGCCGCAATGGCTGCTTCTTCTTCTTCTTCAAACAAATCGACGGCTCCCCTGGAAAATTCAAGCACTTCGTCATTGGAAGATCCTCCTGGCGATCCGTCTACCTCGTCATAGCCGCTGGATCGATAAAGATCCAAGTATGGGTTGTAACCACCTCAACATCAAAAATTCTCACTGATGTCATGATTCAACAACCATTGAATACTTCTTCTACTTGGATGCGGACGATCTGAACCTGGGTTATTAACAAAGCTGAAATACGCCAAAAACAATCAGTGACGGAGCGGTGTTGAACCCCATCAGGATTAATATCACTGTTTAAGGCGACACATCTTGGACGTTGTAACCAAACTATTTATGGCTCGGAGATCAATGTTGTCCATCTTGGTGAGATACTACAACATGTAATGCTTCCATTGGTGCTGATCTGCTGACATATATAGTTGTGATTTGGTATGGATCATGACATGATGGACAATCTTCTTCAGCTATTATTTGAAAAGGCGCCTGGGGCAATCTGATCAAGCATCCCTTGCAAGTCCCTACCATCCGACTCCAATGTAGCATTGCCAACCATAAGAAAATTTGCCAACCACAGCATATTGCAAATTTTCCATGATGTTCATCCTTGCCACCTCCTTTTCAGTCCAAACTACCCTTGGTACCCCGTTGTTATAACAAACTTGTTTTGTTGGAATTTGTTCCACTGTCAATGTACTGCCTTGTACCTCCTTATTGACTTTGAACAAATTAGCATAATCTGGTACTACCTTACCATTCGTGTTGTTGCGCAATTTATGAAGATCATTCGACGAACTCGTATCCTCAATTATTCCAGCAACTGTTCTAGTGTGTTGTAATTTAGGGAAAGACCTATCTGATATTAGTAATGCCGGGTCAGCATCAAACTGACCGGCGGCCACAGCCATTCCAG

>LNC_000410

CAGATACAAACTTGGGTCCAAAAGTGACCCAAGTCCCAAAAACAGAAATAGCACAGCCAAAAAGACACTCCATTAGGAAACAAAAGAAGCTCCAGCCGCAATGGCTGCTTCTTCTTCTTCTTCAAACAAATCGACGGCTCCCCTGGAAAATTCAAGCACTTCGTCATTGGAAGATCCTCCTGGCGATCCGTCTACCTCGTCATAGCCGCTGGATCGATAAAGATCCAAGTATGGGTTGTAACCACCTCAACATCAAAAATTCTCACTGATGTCATGATTCAACAACCATTGAATACTTCTTCTACTTGGATGCGGACGATCTGAACCTGGGTTATTAACAAAGCTGAAATACGCCAAAAACAATCAGTGACGGAGCGGTGTAAGATGAGTATCTTGTCTTCACTTCATTTATGCAATTTGGTACTCAGTTTTTTTATTAGTCTCATCAACAGGTTGAACCCCATCAGGATTAATATCACTGTTTAAGGCGACACATCTTGGACGTTGTAACCAAACTATTTATGGCTCGGAGATCAATGTTGTCCATCTTGGTGAGATACTACAACATGTAATGCTTCCATTGGTGCTGATCTGCTGACATATATAGTTGTGATTTGGTATGGATCATGACATGATGGACAATCTTCTTCAGCTATTATTTGAAAAGGCGCCTGGCCAATCTTACAATTTCCTTCAATGTTACATTGTGTGGGAATTTGAATGCGCAATTCATCTATCTCAGGCCAACCATAAGAAAATTTGCCAACCACAGCATATTGCAAATTTTCCATGATGTTCATCCTTGCCACCTCCTTTTCAGTCCAAACTACCCTTGGTACCCCGTTGTTATAACAAACTTGTTTTGTTGGAATTTGTTCCACTGTCAATGTACTGCCTTGTACCTCCTTATTGACTTTGAACAAATTAGCATAATCTGGTACTACCTTACCATTCGTGTTGTTGCGCAATTTATGAAGATCATTCGACGAACTCGTATCCTCAATTATTCCAGCAACTGTTCTAGTGTGTTGTAATTTAGGGAAAGACCTATCTGATATTAGTAATGCCGGGTCAGCATCAAACTGACCGGCGGCCACAGCCATTCCAG

>LNC_000411

ATGTGATCGTGATATGATTGTGATTGATGACCTGTGCATATTCATTATTCATCCCATGTGTGAACTATCTGTTGCATGAGTTCTGAGACACTGATATGAGGATGGATGGATATGAGACACAGTTGAGACTAGCTCTGGCTAGCGATTTGGATGCCGATGGGATCTGGTTCCGGCGGTGATACATGGTCCATGTGTGGCCCCCATGGGTTCTGATTTGAGTATTCAGCGCGGACTGATTACGTCAACAGATGTGTATCGTAGGATAGACATGC

>LNC_000412

CATCACGAATAGATCTTTAGTTTGAAGTTGAGGATTGTGTAAATTTCTGCTTTTATGTTAGATATGACAATGCAAGGTGGACTGGTTCAGCACAAATGTATGATGTGTTATCAGCTACTTTCTGAACACTTGGTATTGGTGTTGGTGGTAAACGTTGATGTATGAGGAATTCTGCAGTTTTTGAATTGGTTTGAATTGTTCAGCTTATGGTAGGTGGCTTACGAGTGTCCAACCGGAAGGACTTAAGCTCTACATGGTTATTGAGGATTTTGAACTGGGTTGATAACTCTATTTATGGAATTATCCAGACTGTTGTAACGCGTTACATAGATCCTTGATGGTAAATAGGGGTTGTGTGCTCATAGTATACGGAATTATTGGAGTGACCAAGAACTGCTTCAAGTTTCGGCATGAGATATATGATGGTTTGAATAATAGTTGAAGCTGGGTAAGTAAAGGATCGTGTGTAATCGATATTGGTCGTGATTCAATAGTTCTATCTCAATGAAAAGGTACTATGACATTTAGAACTTGGATTATTAGCGATCTGGTGAGACCCATATCGATCGTGGAATGCTTCAATGGAAGGAGGAAGGACCATAATTCAAAATTTTGGAAGCAATGATGTGTTTCTAAGAATGACTGGATACTAGTGGCCGTCTGAGGGTAAAGTAACTGGCCGATGTGACTTTGATGTTCTGGTAGTATTGATATTGTTGGCTTTAATGGATTATTGTAAAGAGTATGAGAAAATGGATAAATTGAGTATGTCTATGAATGTTATGAGGCCGGACTGGCTGATTGTGAAGTTAGACGAATGGGTAGGTGTGTTATGGACATCGTGGAAGGGATATCTTAGGTTGTTCAATTTCGGTTCTGTATATTCTTATGTGACCACCTACCTCATTTCAGACATGGTTTTGGATTATGATTCTCTGTCTATGTTCAGACGTGTTTTTACTTTTGTGGGTGGATTCCTAGTGGTGAATCGGATGGACCGATTCATGCTCTCTTGGCAGAATAGAATACTTGTGTGGGTGGGCCGATTCAGAGATAGTGGGTTCGATGTGGTATTGGGGTGGTGGAAGGTTTTTGGAAGTTCTACAAGTGTCATCTTGTTTTTGGAAAGGTATAGAGAGGCTACCGTATGTGACTACAGTTTTTGGCTATTTTGGTATTTATCTTGCCCCTGTATGCATGGTTCAGCTAGGTGTGGTTCGATGCTTAGGTGGCCCGTGGGCCTAGTTCTTCCGTTGCTGGAGTTGTCTACAATTACGGATCCTACTTCAGTGTTTTGGTGGCATCCTTAGAGGTTTTATATGCTTGGATGGGTCGATCATCAGTTGGATACTTGATCATTTCTATATGTGGGTTAAATAAGCGTGATTATTCCAAGATGGGTGCTCGATAGATATGCATCAGATTAGTTGACTCAGACTTGTGGAGATGTGGTAATGGGAATGGAATTTTTTTCTAGAGTCGGTGATGGTTCGTGTGTTGTGAGAGCTTGGTGATTGATTTAGGTTCATCCTTGATTTTTCTGTGGTTATTGATTCCGAAATTTCTCTCGTGGTTAC

>LNC_000413

GCCCAACAGAGGAGGCTTAATCCAAAGAAAATAATGCCAAAAAAAATAAAAGAAAAATGCATCAAAGACAAATGGACAAAAACTCATCCATCAGTAAAAAAGGAAACTTCAAGAAGCCTTCTATCAGTTCATCATCCCCCTTCATTATCGATGGAAACTCCAAGAATGTAGATTCAATCCCATGAATGGAGACACAATCCAAGAACTTCCCCCAATGGGTGACCTTCAAGTTCAGAATCCAACAAGCTTCCAGATTCGCAAGAAGCTAATACTTTGATTTGGTACTGATGGTCACCCCAAGCTCTCACCATGTGGCTGCTGGAATTCGCACAAACAGATTCAGAGTCTCCCACATTCATTTACAATTTTTTATTTTCGGTTCGACCCTCCTTATGATGTGAAGTAATGCTTATGGGGCT

>LNC_000414

TGCAGGTTGTAGTTGTGGAGGTTCGGTTGGAGTGTAAGGAGTATACGTATTCTATCCCCTTAGCTTGTGTTTAGAGGTTTATTTGCTGAGTACAGTGTGGTTTGGTATTCACCCCTTGCTTCTACATTTTTTTGTATGTTAAGAGCCCGGACCTTTGTGATATTTTTTTCTTCTTCTTGTCTAAAGCTTCTTATGGAGGTTTGTGAGGTAGCTACTTGTCATTTCAGCGGACCTTCTTTCTCCTTAATTATGATCTTGTTCTATTCTAGAAACAACGTCATTTGAAACTTGTATTTTCTTTCAATTTTGTTATACACAGATTAGAGGCTTGTACACGTG

>LNC_000415

TCCTAGTCTAAGACGTTATTTTTTATTTTCTCCTAGGATAAGCACTTCCTAGTTTGAATTTATTTTTAAAAAAATTCACATTTGCTAATAACTCACAAAAAATTTCCGGTGAAAACTGGGGTAAAATTTTTGTTTCTTTCTTTTGTTTGTGATGGTTTTCAGGTTTTTTCAAGTGAAACATGGATTTGAAATTCAAGAACAAAGTTTCAACTGTTTTCAGAATGATCAATTCCCATGATAGCCAGAATCAACTTCTTCAATGCACGTAACAGCCTGACCATCTGACATTTGCTTCCATTCAGCTACTGATCAACAAAACAAGCAGGCATTCAAGAACATTTTCAAATTGTTATTTTGAAGAGTGCCAAAAACTCACTCTAAGTGGCAAGTTAAGCGTCGAATCCATTCAAGATCAAGATACTCACCAAAATCAAGGTGATATCTCAAATTCAAGACTCAAGTGAAGCTGCATAGATAAGAGTTTGTACTTTTATTTTTCTTTTGACTATTAGCTTCTTCTTTTTTATGCAAGGATAAGAGCCGCGACCTGGAGCCTCGATGTAATCTCACTTAACTTCCAATCCATTACCTCATCTCTTTGAACTACACGTGACCTGATTCCCTTATAACCCGGGATATGTAGGATGTCCAAAATCAAAACTCGGTCTCACAGATTTTTTTCCTTCAAATACAAGTCCGGTCAAAACTTGTCACGTTATCTACTTTTTTGTCTGAAAACTCTTCGTATTTTCCGTCAAAGAGGGGCAAACTGTAGACACGTAATTTTGACCGAACTTAAAAATTTT

>LNC_000416

CCTTAACTTCCTGTAATTGAACATGTCATTGTAAAAATACAGAAGCAGTGATGGGAGAGCAGGAAGTGTTTGCATCTGGATTCCGTGGTAAGACAGCCAATTCCTTATCTTTTGGATGAACAAGCCGTTAAATGTTTATTATGTCTTCATAATGGCATATCATGTATAAAGAGTTAATCCTTTATGTATATCAGGAAATTCCTCTTGTGTTCGTGTCATGAGCATGTATATTTTTCTACTTTCCTGCTTGAAGCTTAGAACATTATTATGGCAAAAAAATAAAATAAAATCAAAATTTCTTAACTGAATAGTTGTTGTATAATCACTAATTTTTTATTTTTTATTTTTGTTTTTGAGAATGGCAACATATTATTCATTAGCACAAAGATTGTTAAATTATCTCTAGTTTTAATCATCTCCCTATTTCTCCGTAAAAGGCTTTGTCTTAATAACTTCAATTTGTTAGGACCATATACATGCCGAATGGTTTTTCCTCATATGCTTATGCAACACTTTTTAATCATGTAGGTCCGTCCAATCTAGAAGCTGAGGCTATTAGTGAGACTGCAACTTAACTTTCATACAAGTTAAATACCAAGTCAAAGCGCCTTCAGTTCAACTTTAACTTCGGTATCTCTCATTTCAACTATCATTAGCTCCTAACCATCTGTTTCCTAATGGACTGTTCAATTCCTGTTAAATTTATCATCTCACCTTACTCAAATGTTCTATAGGCCTTAAGGTCTTAGAAAAATAACTTAGCCCAGAAAAAAAGGGGTGTACTGAAAACATAAAATAATCAAATAAGCATCTTCCCTGTGTAGCAGTTTCAGTATTAGATCAGGTTGAGCCAAATGTACTCACAGTATGCATATGGATTATGAAGATGTTAGTCTACTCACTTCCGTGGTTGAAATTTGAATCAGTAAAAGATCTTATTTGTATTGATGACTCCATGCCATTAATAATGGCCTATATTTCACATTCCTACTAAGGTTGTTTGTGGTTGTGATTTCAGCAAGTATTTTTGTATTGAAGTTGATGTACACCATATTGGATTTTTCCACTACTTTTGGACAATCAGGACAACATATTGAACTAGTTATAACCAATCAATTACATGACAAGAAATGAAAAGAGATACGTTGTGTCATTCTCAAAAAAAAAGATATATGTTGTATGTCATAATAATGTAAGTAGTTCATGATTTCCAGTACTAGGCTCAGTTGATTGTTTCAGTGACTTACTGTTGATTCTTCATTTCTTAATTGAACCCGTTTGGCTCGTGTTGCAAAATTTAATGTACACAATGTCTCGCACAAATCTTCTTCATGGGGACTAACATGAACCAGCATTAGTGTTTTGGAATCATCACCTGGATATTAAGAAAGCACATGAAATTCTGGCATTATTTGTGTGACTATACTAAAGAGGAGTGAAGAAAATGTCATCTACTTGTCTAGATTTTGCATGTTAAATGATAAACGGTAAGTTTATTGGTGTGACTGTAAAAAGGAGTGAAGAAAATGTGAAATTTTCTACTAGTCTAGATTTTGCATGTTAAATGATAAATGGAAAGTGTCCCTGAGAATGTGACTCACAAGAATAATTTCTAAATGCTGCATATGAAAAACATATGTTGACAGTCCAATGAGTATGAAAAATATTTAACAAGATAAGAACTTGCTTAGTTCAAGTTACTAGAACAACCAGGAACATCAAAGAAATTGGGGTGTTCTATTTCCACGCGTTATACATCTCTCAATGATATTTTAACCTACTGACTGATGCTGGTCCATAGGTCTCCCTTCTTAGGCTCATGCTACTGGTTTCTGTTTCTAATATGCAAAGAAGACCAACAAGAACATATGTAGACATATTTATGTACAAGCATTTCAATCTCTCCGTCCTCTAAGATTGAACGTTGAGTTTTCCACTTAGCTCTTTGCCATCCTACGAGCTGCACTTACAGCAAGGTTAGATTGGAAAAGGAAAATGATGTCCACAAGAAAGGCCAGGAGATCGATTAAACAACTCCCATAAAGTAAGTCTTAGTCGTACTACCTAAGTTACGCTAGACATATGAAGAAGTCTAATTGTGAATATTTGTAGAATCTTTGATGTGCTTTACTTCAGAGCATGTTGCCGTTCTTGTTTCAGTCCGTGAATGCAGGGTATTGTTTGACTCTAGCTATAGGTCAGAGCTTCTATTGTGCTGCTTCAGAGATCGATGGTGAAGAAGCTCGAACTAAAGTAGGTCTCCTGTTTCAGTTTGACATTCATTATGCAGCTTAATTAAATGTAAAATCTATTTACATTTTTCCACTCCTCCAGTAGCATATTCAAACTCACTTCATTTGAATTCTTTGCTTTCCTCCACTTCAGCATACAATCCACTCGAGTATGCCTCTCTGTTTGCCATCAAAGTTTTCTGTGCTTCCTTTTTTTTTTTGGTTATTTCATGTTAAGTTTCTACCATAAGCATCACTGGATGTGGGACCAGCAATTTAAGTGTTCTGTTTTTGTCAATGAAGAATTCGAACGAAAATGTTATATTAAGCCATCAATATCTTTTACTTGAAGAAACTAATGTGTAACAGTGTAAGGTAGTGTACCCATTTCAGTTTCATCTTTAATCCCAACTTGCA

>LNC_000417

CTCCGTACTGTCAGATTTTACTAATTAGGGTTGAGGTTCTAATCCGTGTTCTTCGTTCTTGGGTTCGTATATGCAATTCTCTTTAGTTATAGTTATTCTGTGTACTCGTCGGGCTTATGGGGGTCCGTTCGGGTTTTTATTTAACTTGCGCACGAGTGTACCTTCTGGGCTTATGGGGGTCCAGTTAGGTGTAGTTAGCTTATAGATTACTTTAGTTAGTTCTTTGTACACTCGTGTGCATTCCCTTGTTAGTTAGATTTTAGTTCTTGACTTCGGTTTGTGTTATTCCTTCTTTTCATATTGCCTTTACTTTTCAAGTTCAGTCGGCCTATGATGCCTACTGGGTACCTGTTGTTTTGGTACTCATGCTACGCTCTGCATCTATTTCGTGATGCAGGTCCGGGCACTAGTAGCCAGTGTTGATCGAGCTTGGAGCAGACTGATCCGGAGACGGGAGTGAGCACACGACGTTTTGTACTATTTCAGTCTCCATCTGTATATATAGACTTGTCTTTTACCTTTCGAGACAGTCCAGTCTCTGTTGTCCATTTTTGGGACTTGTACTCATTTTGTTAGTAGCTCTGTACTAGTGACTTCCAGGTTCAGGGAGGGATCTTTATTTGTATTTATGTTTTGGTTTGCTTCCGCCTGTGTATATTGTTATTTGCCTACTCTTGTTTAGTTTCTACTCTCAGACCCATTACTTGTTGTTCCGGGTTACGGGTTGGCTTACCTGCTGGTGGGTTATAGTAGGTGCCATCATGACTTGAG

>LNC_000418

TGGCTTGTGATATGAAGCATGATAAGTGGTAAGTTCACATTTGGTGGCCTTAGGATGATGCTTTAGTGTGGATGGTGGTATGGGACGCTATCCATACATTGCACAAAGTGTTGTCCAAGGGTTACTTGAGGTGGATGGTTTAAGGTGAAGGAATGGGTTATGTGTGGAGTATGTGCTAAAGTGGTTAATGACTTGTATGTTGGTTATGGTTGGATATTGTGACTCTTATACTTGTATTTTTTTCATGAAGTTTTACCAAATGGCATAAAAGCATGTTTCTCCAATAAACGTCCCTTTTAGCATGTTTTTGCATGGCTTCCATACTTAGTACTT

>LNC_000419

CAAAGAAAAGACAAAATAGATGGCAAATCCGTCATCTTACTTAAAACACAAAATAGATAATTAACTTTTTCTCAACATTTCCAGAAGAATCCATTCTTTCATATCTTTGTACCAACAATGGAGAGACATCCACTCCTTCACAAGTATTCAGAAGTCTCTTTTCTCCAAACTTGAATTGAAGGCCTGCTCATTAACTTCATCTTCTCAGCTGGGTATGCATTTTTAGGTTCAACTTAAAAGCTAAGTTGGACAAACATCTCAATCTTGAATATTCATCTTCTGCAATCATTTTGTGCCTCACTTCGGAGTGTACCTTAATTTTTTCTCCATTCTTGCACCAGACTGTTCTATTATTTATGGTGAGTTTCGTTTGAATTTTGTTTCCTCTTTTGTGTTTGTATTGTATCGACTAATATGCACATGTAGAGCTATTTCTTCTTATCTCTTGCTCCGATCTTTTGTAGTTCATGTGAATTTAGTTCATTGTGGGATGAATTGAACTTCAATCTCTGAAAATAAGTGTTATCTATCTTCTTATATTTCAATACTCTATTTTAGTTGTTGTTTTCCTTTTTGGTATGTGTATGTGTTCGTTAATAAAAGTTTT

>LNC_000420

CTTAATTGTGCTAATTCCATATTTCTCTATTTTCTACTAGTGTAGGTTCCGGAAAGTGAGCGGGTTCTATAGTAAGCTTGGAACTTGAAGTTCTTCCAAGATCTTGTGGTATGTCCTCATATGTCGAGGACAAGACTTTTCTATTCTAATTTTATGATAAGGTTCTTGTAATAGACTTAATAGACTTCTTTTGATTATAAAGGGCCGTGTCCCAACTTATGTTTTGTGATTGTGTCTAAGATGGCCATGTGAGACTTAGACTTCCGCTGTGTGTTTTTAAAGTTGTTTTAATGAAG

>LNC_000421

ATTGACATAAGATCAGAAAGAGACCTAACCCTCCCAAAAAATTTGATAACATTACCTTCCATCAGAGAATACACCGTCACAACAACTTGGAGAGGAATATTGATCACATTAATGGGATGACTGCCGTGGAAGCACCCAGAAGATACCTTCTGCAGAATGATGCAGTCTCCTTACAGTTCTAGTTTCTGCACAATGGAAAAGAACATTAAAGGAGAGCGTAGCTCGTGGTATCAACTAATTCTTAAGATTTTCATGTCTACAATCATCAACTCCAAGGTACAAACACTCCAAGGTCTGGTCTGAGCTTCTAAATCGTTTGAAAGAGATAGCAAAGAGCTTGAACTGAAGGGGTGTTTTGACTCTATGCATCAAAGAAGCAACAGCAAATAAAAAAACTAAAGCAGGGATAGAACGAAATGCTTCACTTAAGCAAATGATGTCTTAAAGTTGCATCAAGGATTTCTTTCGAAATTGTGGTTGTTTTCTCAACCTCGATGAAATTCTCTATCCTATATCTAAAAGGTGTTATCAGGTATAAACTTTCACACAGCAAGAGCTCATGTTCATATTTCGTGTATTGCACTAATTTTTCATCACCTTCTTCCCCTGCCACCTCCTCCTTCCATAGCATGTCTATTAAATTAGACCATACATTATTCTTTATGGACAATGAAAACCTATGATTATTTTGGTTTGCATTGTGCATACTCCTTTGTTAGCATGTCATTTAATTCATTATTGATGATAGTTTTGTGCTGGTCATTAATTCTGTAGAGCTGTGAATTGAGATAATTCTCATGTGTGCTCTATATACTCTTCCAGTTATTTAGATTTTATTTTTAGACAAACTCTTTATAATCGTTGTTTTCCACTTTCTCCTGGTTCATGTAGCATATACTTTGTTGTGATCTTGAGTTTACATGAGAAAAAGGAAAATTGGTTCTAGTGCTGATGCATGCCTAAGCTTTACCAAGTATGTTGTTTTGATATACTGCTCCCTCATTCTTTCTGTAATATAGACAATTTTAAAATAGTTTCTCTCCATTCAAAGTCAACACGCAATATATTTTGCTTTTCTTATCTTAAATTTGTCATGATTTCTCAGCTTTCGTCAAGCTGGTGCTTCTGTCATTCAAATTTTTGTTGGTCCCCTGAGGGTAAATTCTTTGTTCTTCTCTCTGTTTGTTAGTGATCATTAAGTGTTTTGTTGTGTTCTCCCATATTTATTATTTATATTTGCTGAAAGAGTCTTGGAATACATGCTGAAAGGGTGAATATGATTGTTTATCCAATGGACAGAGGTTTACTCTTACTGTCTAATTTTGATTTTTTTAAAAAAATAGAAATTCTTACGTATGTTGTGTGTTTACTGATTATATAGAGATTTCAATTGTTGTGTAGTGACAATAATATTGTTATACTATGTTTTTCTTAGGTCATGTGGACATGATCGTTTTTTCTGAATTTCATGGTATAGAAAAAGTTGTATCACTACAGATTATTTTT

>LNC_000422

ATTGACATAAGATCAGAAAGAGACCTAACCCTCCCAAAAAATTTGATAACATTACCTTCCATCAGAGAATACACCGTCACAACAACTTGGAGAGGAATATTGATCACATTAATGGGATGACTGCCGTGGAAGCACCCAGAAGATACCTTCTGCAGAATGATGCAGTCTCCTTACAGTTCTAGTTTCTGCACAATGGAAAAGAACATTAAAGGAGAGCGTAGCTCGTGGTATCAACTAATTCTTAAGATTTTCATGTCTACAATCATCAACTCCAAGGTACAAACACTCCAAGGTCTGGTCTGAGCTTCTAAATCGTTTGAAAGAGATAGCAAAGAGCTTGAACTGAAGGGGTGTTTTGACTCTATGCATCAAAGAAGCAACAGCAAATAAAAAAACTAAAGCAGGGATAGAACGAAATGCTTCACTTAAGCAAATGATGTCTTAAAGTTGCATCAAGGATTTCTTTCGAAATTGTGGTTGTTTTCTCAACCTCGATGAAATTCTCTATCCTATATCTAAAAGGTGTTATCAGGTAAATCGACAAGCTTCTGTATTCCAATTCTAAAGGGTTTAAAAGGTATCCAAATTGATAATTTTTTATGTCTAAAAAGGCTTCTACATTATCAAGTAAGACTCAAGAAGAATCATGTGTTTGTTTGGAAGGACAAGAAGTGATTAAAATTGGGTTAATGGGACAACATCCTTGGTTATCTTTAATCAAGAGAATTTTGAATCCTTGTTTTTGTCTGCATTTATTTTCTTCTACGTTCCAATAGCTCATCATCCTTAGATTCTGAAATTCGTATATTGAGGAATAAGTTTATATTGTTGTGTCTTAATACTTGAACCTATGTTGGATATCATCTACTCGGACCATATATGCTCAGTACCTGTACATTTTCTCTATCTGGACAATGTTAAAAAATGAAAGACGAGATTGTTGCAATCGTTAAGTAAATCAATATTGGCTTCTTTAACTTATTTGACTTTTGTTTACAGGTATAAACTTTCACACAGCAAGAGCTCATGTTCATATTTCGTGTATTGCACTAATTTTTCATCACCTTCTTCCCCTGCCACCTCCTCCTTCCATAGCATGTCTATTAAATTAGACCATACATTATTCTTTATGGACAATGAAAACCTATGATTATTTTGGTTTGCATTGTGCATACTCCTTTGTTAGCATGTCATTTAATTCATTATTGATGATAGTTTTGTGCTGGTCATTAATTCTGTAGAGCTGTGAATTGAGATAATTCTCATGTGTGCTCTATATACTCTTCCAGTTATTTAGATTTTATTTTTAGACAAACTCTTTATAATCGTTGTTTTCCACTTTCTCCTGGTTCATGTAGCATATACTTTGTTGTGATCTTGAGTTTACATGAGAAAAAGGAAAATTGGTTCTAGTGCTGATGCATGCCTAAGCTTTACCAAGTATGTTGTTTTGATATACTGCTCCCTCATTCTTTCTGTAATATAGACAATTTTAAAATAGTTTCTCTCCATTCAAAGTCAACACGCAATATATTTTGCTTTTCTTATCTTAAATTTGTCATGATTTCTCAGCTTTCGTCAAGCTGGTGCTTCTGTCATTCAAATTTTTGTTGGTCCCCTGAGGGTAAATTCTTTGTTCTTCTCTCTGTTTGTTAGTGATCATTAAGTGTTTTGTTGTGTTCTCCCATATTTATTATTTATATTTGCTGAAAGAGTCTTGGAATACATGCTGAAAGGGTGAATATGATTGTTTATCCAATGGACAGAGGTTTACTCTTACTGTCTAATTTTGATTTTTTTAAAAAAATAGAAATTCTTACGTATGTTGTGTGTTTACTGATTATATAGAGATTTCAATTGTTGTGTAGTGACAATAATATTGTTATACTATGTTTTTCTTAGGTCATGTGGACATGATCGTTTTTTCTGAATTTCATGGTATAGAAAAAGTTGTATCACTACAGATTATTTTT

>LNC_000423

ATTGACATAAGATCAGAAAGAGACCTAACCCTCCCAAAAAATTTGATAACATTACCTTCCATCAGAGAATACACCGTCACAACAACTTGGAGAGGAATATTGATCACATTAATGGGATGACTGCCGTGGAAGCACCCAGAAGATACCTTCTGCAGAATGATGCAGTCTCCTTACAGTTCTAGTTTCTGCACAATGGAAAAGAACATTAAAGGAGAGCGTAGCTCGTGGTATCAACTAATTCTTAAGATTTTCATGTCTACAATCATCAACTCCAAGGTACAAACACTCCAAGGTCTGGTCTGAGCTTCTAAATCGTTTGAAAGAGATAGCAAAGAGCTTGAACTGAAGGGGTGTTTTGACTCTATGCATCAAAGAAGCAACAGCAAATAAAAAAACTAAAGCAGGGATAGAACGAAATGCTTCACTTAAGCAAATGATGTCTTAAAGTTGCATCAAGGATTTCTTTCGAAATTGTGGTTGTTTTCTCAACCTCGATGAAATTCTCTATCCTATATCTAAAAGGTGTTATCAGGTAAATCGACAAGCTTCTGTATTCCAATTCTAAAGGGTTTAAAAGGTATCCAAATTGATAATTTTTTATGTCTAAAAAGGCTTCTACATTATCAAGTAAGACTCAAGAAGAATCATGTGTTTGTTTGGAAGGACAAGAAGTGATTAAAATTGGGTTAATGGGACAACATCCTTGGTTATCTTTAATCAAGAGAATTTTGAATCCTTGTTTTTGTCTGCATTTATTTTCTTCTACGTTCCAATAGCTCATCATCCTTAGATTCTGAAATTCGTATATTGAGGAATAAGTTTATATTGTTGTGTCTTAATACTTGAACCTATGTTGGATATCATCTACTCGGACCATATATGCTCAGTACCTGTACATTTTCTCTATCTGGACAATGTTAAAAAATGAAAGACGAGATTGTTGCAATCGTTAAGTAAATCAATATTGGCTTCTTTAACTTATTTGACTTTTGTTTACAGGTATAAACTTTCACACAGCAAGAGCTCATGTTCATATTTCGTGTATTGCACTAATTTTTCATCACCTTCTTCCCCTGCCACCTCCTCCTTCCATAGCATGTCTATTAAATTAGACCATACATTATTCTTTATGGACAATGAAAACCTATGATTATTTTGGTTTGCATTGTGCATACTCCTTTGTTAGCATGTCATTTAATTCATTATTGATGATAGTTTTGTGCTGGTCATTAATTCTGTAGAGCTGTGAATTGAGATAATTCTCATGTGTGCTCTATATACTCTTCCAGTTATTTAGATTTTATTTTTAGACAAACTCTTTATAATCGTTGTTTTCCACTTTCTCCTGGTTCATGTAGCATATACTTTGTTGTGATCTTGAGTTTACATGAGAAAAAGGAAAATTGGTTCTAGTGCTGATGCATGCCTAAGCTTTACCAACTTTCGTCAAGCTGGTGCTTCTGTCATTCAAATTTTTGTTGGTCCCCTGAGGAGGTGGAAATTTCTCCATCAAATACTACCAAGTACGCAGAGAGTCTATTGATGAAAGGGACTGGGAAGGAAAATGTGGGTGATTGAATTTGAATAGTGGCCAGAAGTAAACTCGATTGTAGTGAACAATAAAGATTTTTTGTACTATTGCCATATATGTTTGGATGCA

>LNC_000424

ATTGACATAAGATCAGAAAGAGACCTAACCCTCCCAAAAAATTTGATAACATTACCTTCCATCAGAGAATACACCGTCACAACAACTTGGAGAGGAATATTGATCACATTAATGGGATGACTGCCGTGGAAGCACCCAGAAGATACCTTCTGCAGAATGATGCAGTCTCCTTACAGTTCTAGTTTCTGCACAATGGAAAAGAACATTAAAGGAGAGCGTAGCTCGTGGTATCAACTAATTCTTAAGATTTTCATGTCTACAATCATCAACTCCAAGGTACAAACACTCCAAGGTCTGGTCTGAGCTTCTAAATCGTTTGAAAGAGATAGCAAAGAGCTTGAACTGAAGGGGTGTTTTGACTCTATGCATCAAAGAAGCAACAGCAAATAAAAAAACTAAAGCAGGGATAGAACGAAATGCTTCACTTAAGCAAATGATGTCTTAAAGTTGCATCAAGGATTTCTTTCGAAATTGTGGTTGTTTTCTCAACCTCGATGAAATTCTCTATCCTATATCTAAAAGGTGTTATCAGGTATAAACTTTCACACAGCAAGAGCTCATGTTCATATTTCGTGTATTGCACTAATTTTTCATCACCTTCTTCCCCTGCCACCTCCTCCTTCCATAGCATGTCTATTAAATTAGACCATACATTATTCTTTATGGACAATGAAAACCTATGATTATTTTGGTTTGCATTGTGCATACTCCTTTGTTAGCATGTCATTTAATTCATTATTGATGATAGTTTTGTGCTGGTCATTAATTCTGTAGAGCTGTGAATTGAGATAATTCTCATGTGTGCTCTATATACTCTTCCAGTTATTTAGATTTTATTTTTAGACAAACTCTTTATAATCGTTGTTTTCCACTTTCTCCTGGTTCATGTAGCATATACTTTGTTGTGATCTTGAGTTTACATGAGAAAAAGGAAAATTGGTTCTAGTGCTGATGCATGCCTAAGCTTTACCAACTTTCGTCAAGCTGGTGCTTCTGTCATTCAAATTTTTGTTGGTCCCCTGAGGGAAAATGTGGGTGATTGAATTTGAATAGTGGCCAGAAGTAAACTCGATTGTAGTGAACAATAAAGATTTTTTGTACTATTGCCATATATGTTTGGATGCA

>LNC_000425

ATTGACATAAGATCAGAAAGAGACCTAACCCTCCCAAAAAATTTGATAACATTACCTTCCATCAGAGAATACACCGTCACAACAACTTGGAGAGGAATATTGATCACATTAATGGGATGACTGCCGTGGAAGCACCCAGAAGATACCTTCTGCAGAATGATGCAGTCTCCTTACAGTTCTAGTTTCTGCACAATGGAAAAGAACATTAAAGGAGAGCGTAGCTCGTGGTATCAACTAATTCTTAAGATTTTCATGTCTACAATCATCAACTCCAAGGTACAAACACTCCAAGGTCTGGTCTGAGCTTCTAAATCGTTTGAAAGAGATAGCAAAGAGCTTGAACTGAAGGGGTGTTTTGACTCTATGCATCAAAGAAGCAACAGCAAATAAAAAAACTAAAGCAGGGATAGAACGAAATGCTTCACTTAAGCAAATGATGTCTTAAAGTTGCATCAAGGATTTCTTTCGAAATTGTGGTTGTTTTCTCAACCTCGATGAAATTCTCTATCCTATATCTAAAAGGTGTTATCAGCTTTCGTCAAGCTGGTGCTTCTGTCATTCAAATTTTTGTTGGTCCCCTGAGGGAAAATGTGGGTGATTGAATTTGAATAGTGGCCAGAAGTAAACTCGATTGTAGTGAACAATAAAGATTTTTTGTACTATTGCCATATATGTTTGGATGCA

>LNC_000426

CAGAGCTTGGTTTTAAGAAAAATTCAAGCACTTCATTACTGGCAACTCCTCCTGGCGAGCCCTCTACCTCGATATAGCCGCTAGATCGATAAAGATTCAAGCATGGATTGAAATCACCTCCACATCAAAAAACTCTCACTGATTTCATGATTCAACAGTCATTGAAGACTTCTTATACCTAGATGCAGGCGACCTGAGCGTAGGTTATTAGCAAAGCTGAAGGATGCCAAAACAGTCAGTGATGGAACCTTGTCAAGATGAGTTTCTTGTCTTTAATTCCTTTGTGCATTTTGGTACTCAGTCTTTTGATTAATCTCCTCAAAAGGTTGAACCCCATCGAGATTAATATCACTTTTTAAGTCGACACATCGCATACGTTGTAATCAAACTATTTATCGCTTGGAAATTAGTGGTACCAAAAAATCTCCCCTTTCTCATACTGTCGTAGATACTTTATCATTCTAAACCTGTTGTAATGTATGTGCTTATCCATTGTAGCTTTTGTTTTGCACTTTGCTAATCCTCAAATTAGGTATATTTTCTTTCTCTAGTTACAATATTGTCCTAGCCTTCTGTGATAGTTCTTGAATGTTATGATATTGTTGTCTCCCAATTCCTGCAAAATGAACAAACGATTTAGTAAAAAAAATCATCTAGATTTTTTCCTTCTTTGAAAGTATGAACCACTTCCAC

>LNC_000427

CTGGCAATTTGTCTTTTCAGAGCTATCGCCCCAATAAAACAAATATTCTTGTGGTAGGCCCTGTCCCTGGTAAAAAATATAGTGAAATAACCTTCCCTATTCTTTCTCCGGACCCTGCTACTAAGAAGGATGTTCACTTCTTAAAATATCCTATATACGTAGGCGGGAACAGGGGAAGGGGTCAGATTTATCCCGACGGCAACAAGAGTAACAATACAATTTATAATGCTACAGCAGCAGGTATAGTAAGCAAAATCATACGAAAAGAAAAAGGGGGGTATGAGATA

>LNC_000428

ATTTACTAGTAGCTCTTGATTTATGAGGATGGATTATGATCCATATGGCCCCAATTTTCTTCCATTTTCTTCTCTTTCTTCTTTTGGTTGATGAGATTTTGTCATGAGTTCCAGATCAGTTGGTAAAAAACTTGTTGAGTTGCATTTATTTGAGAGGTACTTGGCCCATGCACGTGCTCAATACAAAACAAATACATGCAATAAGGCTTGTTTAAGCTTTCATTTTCAGCCTGCCTAAACTTGTCCAAGATAAGTGCTGAAGTGTGTCTACCACCTCATCTTAAAGTTTAAAATATCATAAATTTATTTTATTATGCTAAGATACCCCAATGCGTGGGCCTGACTCTTTTTTTTGTAACAAGCCTGTGGAAATGCTGTTTATTATCCGTGTAAGACTTGAACTCGTG

>LNC_000429

GTCAACATAGCATTGAATAATCTTCTAGAACACAAGTTTGGGTCACTGGACTCGCTTCTTCTTTGGACAGATTTCTGGACCAAAATCCAACTCTTATTGGTATTCAATCTGGGCCTCCAAATAGAAGTAAGACTTTCCCAAAACATCTACTTTGGTCTGGACTCTGGAGCTCTTATTCGGTCACAAAAACATGTTTTCATTGCTAATTAGAGCCTATTAAAGCTCTAGATATTCCATTGCCCATAATGAAGTTGTGCTTTCTTGAATGCTATCAACAAGTGTCTTCCACCTCATCTAATTGTGTTAGTATTCCCGAAAAGGGTTTGTAAAGGTCTTACACCATCCTTTTAACTGCCT

>LNC_000430

ATTGTTATTGATTGGTTTGATCCACCTTTGGTGGAGGTGGTTATTTGTTGTATTATTGTGAATTGAGGCCATGAAGGGCATTATATGAATGTTATTATATTGTTGTATTATTATACACATATGAGGCTATATGTAAAGTCCTATATGAAGCTATATGTAAAGTTTTATGTGAAGCTATATGTGAAGGAATGATGTGAATGAGTTTTATGTGAAGCTATATGTGAAGGAATGATGTGAATGAGTTTTATGTGAAGCTATATGTGAAGGAATGATGTGAATGAGTTTTATGTGAAGCTATATGTGAAGGAATGATGTGAATGAGTTTTATGTGAAGCTATATGTGAAGGAATGATGTGAATGAGTTTTATGTGAAGCTATATGTGAAGGAATGATGTGAATGAGTTTTATGTGAAGCTATATGTGAAGGAATGATGTGAATGAGTTTTATGTGAAGCTATATGTGAAGGAATGATGTGAATGAGTTTTATGTGAAGCTATATGTGAAGGAATGATGTGAATGAGTTTTATGTGAAGCTATATGTGAAGGAATGATGTGAATGAGTTTTATGTGAAGCTATATGTGAAGGAATGATGTGAATGAGTTTTATGTGAAGCTATATGTGAAGGTATGATGTGAATGAAGATAATGTGATCATGTTATGAATGTAAAGGTGTTGTTGTAGGTTGTTCACTGATTTGGTTGCATAATTACTACCCTATTTTCCATGCTCTTTGATTCCATTACATGCCTTCAAGGTGTTTGACAAAATGTCCAACTATGGAGAATTGATGAATCGATGCTTTAAAGCTTGAGTTATGAGA

>LNC_000431

CTGATCTTCTATCGTTCTAATCTAGGGAGCAACTCAAGAACGTAACTGAAGCACAACTTCTCTCCGATACAGGTAGAGAAATTTCAGCAAAATCTGCAAGACATTATACATACTTCTCTCAAATTCGGTTGGAGATTTGGGGATTTTCTATTTTGCCCAAATCTGTATTAACCCTAAATTTATCTAGTCGTAGGCCTCAATTTCAAATTAGGAGTGTCAGTTTGTTAGAGGACAATCAACAAACATATAAGATCAAAAGGAACAAGCTCAAGAATTTTAATATGAAATATTTATCATTCAAGAAAGTAGGGAGCAAGTTGAAGTTTGTGAGGATTTTATTAGGGGGGGTTTAAAGACTATATCTTATGTATATATGTTGCAAATTTCTTTTTTGAATTAGAAGTTGATTAAAGT

>LNC_000432

TTCTTCCTTTGAAAATGTTGTTCAAATCGGAGAAGACCACCGGAACAAAGATCTTCAACATCAACATATCGGCTCCTCTCAAAAGGAAGATTCCATGGAGGAACAATCAAGAACGTGGAGAAAATCCATTTGGTTCAAAGTGGAAGTGGAAGAGCCGACAATAACTTGTATATTATTTCCAATTTCAGATTTTAGCATTTTCTGGTCCTTTACATTTTGGATCTTGGTTAGCCAA

>LNC_000433

GTGATTATTTATGAAGTGAGTTTATTGATGATATAGGACTCGAGAATGTGAACAAGATAGCAAGATATCACCTACAAGACATTTACTGGTTTCTGTCTATAATGTTTATACCCATGGAGTGGTCGTATGCACTTATCATCACTTACCCAATCATGTATTTGTGGTATCATCACTTACCCAATCATGTATTTGTCATACGTAGCCTCT

>LNC_000434

CCAATAGCTCCGGCGAGTCGCGCGAAGCGGCAGGCTCCGAGCAGCTGCTGCCTCCTCCGTTCCCCTCTCCTCTCCCCTTTTCGACTAACGAACTCGGCGCAGCCGCACCCCTCTCTCTCTTTAGCTGCTCCATAGAATGTGACTGAAACTGTCTTCGTCTACTCTCATTTTCATCTAACTCTGGCGATTGCAAAGAGAGAAAGAGATGGGTCTGCTCACTCACAAAGTAGAAAGGAGCGAGATAGCATCAGGTGACCATATCTACACTTGGAGAACAGTCTTCACCTACTCTCATCACAGTTAGTCTCAGTCTGCAACCCCTTTTACACCATTTCAATTGTTCGTTGGTTTCAGCACACGACGAATGGTTTGGATAGCCCTCCTGTTTAAACTAGAACACATTTTAAATTTTATTAAGTAATTTTGTACAGTAGCATCTGGGCTTTAGATTAGAGAGATGATTGGGGAAATACAAACAGACCTCATTGTAAATTGTGAATGAATTTGACAGATGCACACAATTGAAATCCACAAAAAAGTTGGAGTGTTATTCCTCTTTGTGGATGTTGAAAGTGTATTTCAAAACTAAAAGTTGTTACTACTATTCTGGTCTTCCTAGCTGCATCTTGTTTAGTTTCAAACTGTTCCACTCGTCTGGTCGCATTGCTAATCAGTGTTGGGCGTTGTGTTATCAAATTTCAGCTTGCTTAAAATGCTACTGTTAGAATTTTTTTAACTATCCTTGTTAATATGTCTAATGTGATTGTAACGTCTAATGTTATTGTAATAAGTTACAGACAAGTGTTATCTCTTTTCTCTGTGAATGTGATTTAATATATCTTTATAGGTTTAAATTTGCTAGTTTGAATTCTCTATATTGGCTGAATTGATTCTAACTACAATCTCCTCCAGTTAGTACTTAATTATTCCGTAACTCTTGATTGTTTAACGATTCTAGTATATCTCATTTTAACGTCCAAAGTTCACGGGTCGGAAAACCAAAGTTTCAACTTCCAACCAAAATCAAAATTTGAAAAAGTTTAGGAGTATCACTGAAACAAAAAATACAACTCAAGCTCAACTCCAACTTAGAATAGCTTCAGTGAGAATGACTAGGAAATTTAGTGAATGGCTAGGAGAAACCTATTTTGAATTATCAAGAAAAAGAAAATGAAAAAACAACACAAACTTGTTTACTAATTGTCAACTGTTAATAATGACTGAAATCTCTCTATAATGACCACTAAAGAAATTATAAATTGTTTCTTTTCATGCTAGGCAATTGAGGAAGCTCAACATCTAAAAAAGTTGATACAAGACTAAGCGTGGCAACGGGACCGGAACCGGG

>LNC_000435

GGGTAATGCTATCGCTTAAATATGTGGTTATTGCTTTCAAAATCATGTCAGATCAGGTCATTGTTATTCACAGTCCCTTAAGATGAGTCTTTACCATTATTTGCTTAGGACTTAGTATTTAGTTTACATGATTAATGATAACTCCTCATGCCAAAGTGGTAGTCAACTATTAAGGATATATTATTAGTCATTGTTTCATGTTAAGCATTCATTGCCCACTTTTGCTATACTTTTCTGTAATTCGTAGTTTGCCATATTATCCGAAGTGTAGCTCAACTTTGGTCAAGTCCAACAAATATGTTCCTTGTTTATCCTCGTGTGTTTTCAATTTTAGTGATTGTATGCATCCGTCCAG

>LNC_000436

TAGAACAATTTAAAACATTCTCAAAAAAATGTGGTTGCCTTCACTGACACAAAACAAAACTGATAACATTGAAGCAAGAAGAGGCAGCAGTAGCCATGACAAACGATTCTGAAACTTTTTGAAGTAACTGAAGAACGAGTTAGATAACCGTGCTATTTTTACCTATTGCTTGGATTGTTTTAAATCATTGAATATGCCATGTTATTTTGAATCTTGTCATGTCTTTTCTTCTTCTGCTTGAAC

>LNC_000437

TTTTAATTCAACTTTAATTCATTCGGCAATTTATTATGTTTCTTCATTTGTTATGATTAGATTACTTGTCTGCTCTTTCGTTTGCGATTAGTTTGGATAGGTTGTTCCTACAAATAATTGAAAATATCTTTTGGAGTTTATATCTCAATTTTAAGAGGGTTTGGTTAAGTTTAGAATTGATTTTAGGAGAAATACTAGATTGAAGCACGAAAAGGATGAAGTTTATGGAACTATATCGGGT

>LNC_000438

AGAAGAGAAAATTCTATTTTCTTTTACTTTTTTTTAAATGGTTTGGATAGAATTATAAAAGTTGAAAACTGAAGTGTTTTTTTTTGTATTTTATAAAAGATTAGAAAGTTTTGAAAATGTTAATTTGGTCCTAGATTTACTTAATTACATTTTAAAATGGATTTGACCCTTTCAGTTTTCGGTTCATATTTCGTCGACCAATCCTTCCTAATTCACATCAGGAAGGGCTCGTGCAGCTGCATCAGGATTTGAAAAAGGAATTGATCGTGACTTTGAACCTGTTCTTTCCATGACCCCTCTTAATTGATATGAGACAGGAGATCCAATGCTTGAATGAAGTACAAATTACTTTGATTCAATCATACATCTTGGAATCAGCTTAAGTATTCCTTTTTTTT

>LNC_000439

CATAAATATATCCAAACAAATAAGTGCAAAACTCAACATAACCGGTTAAAGAAAACAAACTTTACCAAAGAAAAATGAAAATGGAAATAACATTGGCTTGGAGTTTGAACCCCTCCGACCATCTGAAAAAAATAACGAAGTAAAAGAGGGATTGTTTGTATACCGAAGCCGGAGAGAGATCGATTGTTTGGAGATTTGGCTCACTGGCGGTGATCTGATTCGCGATGGTCGGCGTTCTGATTTTGAAGGAATTAGAGTTGGGGTCTGATTTGACTCGCGAGATCGCTGGAACTTGCAACTTGGTGGAGCTGTTTGGACTTGCTGGTTGCAGTAATGGAGTTGCCGACTTCTAACTCGTCGGGTTTATCGCGATTTTGGAGCTGATGTTGTTGCTCACGACTGGAGCTCTTGTGGGGTTGTTTGGCTATTGTTGTTGATCGGTTAAGTGGGGTTTTGGGTCTGGTCGGAGATGGCTAATAGAAGAGGGAGCAGTGATGGTCGGCTTCTGATTTCGCCGGAGAAAGTTGTGAACGGAGGTTGTTATTTGGGGGGAGATCTGGTTCGCCGAAGAGGTAACGGGAGAGAGAAGAATGGGGTTTTCCGATGGGGGTCGTTTGAGAGTTGTACCTTAAAATTTGTACCTTCCGGATACATAAGTCACCATCCGGA

>LNC_000440

GGCGGTCACTTCTTGTCTCACTCAAGTGTGTCTTAAGGTTATATTAGGTAGAGGTCCTATGGTAATGAAATGACATGTGTTCTTTTAAAGTTAAGCATGGATTGTATATGGACATATGTTGAGTATATGTATGGTTGGCTATGGTCTTATATGTTAATGTTGACTTGTATTATGTCTCTTATACTTGTGAAGTGAAGGTTTTATCAAAATGATATGAAAGCATTTACTTTCTTGTAAATGTCCGTTGTGCATATTTTCTTGCATGGTCTCCATACTTAGTACTTAATTGTGCTAACCCCTTTCTTTCCCTTTTTGGACTAAAGTGTAGGGATTTGGAGATGATGACATGTCATGGCTTTGATAGGTTTCTAAGTGTTGAAGATGAAGACTTGGTGAGTCCTCATAGATTCGAGGACAATACCCAATATGTTCTTTTATGTCTTTTTAGTATTGTTTAAGTTGTGTACGGGATTAGTCCCGAATTTTGTACTCTAAAGTATTAGATGGTTTGAGACATCATGTATAAAGTCTAGAAAGTCTTCCGCTTGCTA

>LNC_000441

CAAAAACGTTTTCCTTCTTCCCCAGGAACCTTTCACGCCCCTCTCCCATCAATCTCTGCCGAAGAACGGCTATGAACCGTCGGAAAATGGCAATTTCTGGTGGTTTTTGGCCAGGTCCATCCGCCGTCCGTCAATCATAACCTCTTCCCATCAACTTTACATCCCTTCCATCATCTTTTATGAACCAAAACAGACCTCAAAAGCCTTCCCACCAGCACTGCCACACCTGAAAAGAAGAAAGGCATGAAGAACCATCGGATTTGGGGCTAGTCGGCCCTTTAATCTGATGGGTGTAAGGTTCCATCACTTTTTTTTTTTTTGAATGTAGTAGAGGCCCCAAATCGGCTATAAAGAGGAGAACTTTGGCTCTCCTTTGAAAAGGATGATAGAGAAAAAAAGGGATAAGAACAGCAATCAAGAATGCAACAATAGATATAAGAACATATAGAAACAATAAATTTCAAAAGGTTACAGTTTGATACACAATTTCTCTTCTCTTCTCTTTGTTTTCGATTTTTTTTTGGGGTTCGAATTCAAGTAAGATCGAGGTCAGAATCCTTGACATCAAAGCCTTAGTTCGCTGCCCTATACAAGGTCAGTAATCACCCTCGTCTTACACTTTGTTTTCATTTTTCTTTGGAAATAAGAATATAGTTTCTGTGTCGTTATCTCAATTTTGCCTATCTAAACTCATTTTGTTATAAAAATGTTAGTAAAGATTGGTGTCGATCAGAGGGGCGGTTGATTTATCAATCTGATTATATGTGTGTTTTGTTTTACTAAATGTAAATACTGGCTTGCTCTTTAGATTTTTTTTTCTTCCATTGCTTTTATATTCATCTGGATATCTTTCATAGCTCTCCGCATTTTTACTGTTTGAATGTATGGTGATCCCCTGACAACTGAGTGTCTGTTGTTGATATGAGTAAATATTGATTATATTGGGTTTGTTGGTTGTAGCATCTTGGTAACGACATCTTTGGTCGTCTAATATTTGTTTATACCTTACTCATGTTGTAGTGATGAGTTGTGCTCGGCTAAATTCACTTCGCATCTAAATAATGCTAATGTCGCCTAATTGTCAGCTTCTCGATAGTTTGGATTCAATTGTCATGTTCTTTTCACGTTAGGTCTACCTATTTGAGTTCCATAATCTGATTCTTTCCAAAATTTGGCCTGGTTGGGTTTCGCTCTTCATTATCCACGTTTAAGTTTCACATTAGGCAAACGACTAGAAGTCGTTTGGGTTCACTTGGCTTGCTACTGTCGGCATCATTTTAAATAGGCGTAGGAGTTGCAGAAACTACCTAATGTGATAGTTGTATGTGGAATTGTCTTATTGTCTTTTGGGTGTAACAACTTCAGTTTCTCTCTTCGCAGATCCCTTTGTTAAATTTAAATGACTTTTGATCCCTTTACCTATTCAGAAAATTCACAAATTAGTTGTGTGATTTGTCACTTAGTTCCCGCTATGTCCCTTTTTGGAGGTCCATAGCTCCACTGCTGCTTGTTTTGAGTTCTCAATATTCATAGCATGTCCCAATCTCTACAGATGTGTCCGATAATGCTCTTTCTGTATTTTAACTTTTCAAAATTTTGGAATGTTAATATAAAAGTTTGAAAGCGCTTAACTTTGTGTCCAAATTTTCTTCCAGTTGTCTGTCCCAACAGGCAGCCATTGTTTCAGAAATCTGGTCCGTGTTCATGTGTTGACAAAATTTAAGCTGGATACTTTGTTTTAAAGCTTGTTACTGAATGGACGGTGATTATAAATGTTAGTGTTTGCTCTGTTTGAATTGAGTTCTATCAAAAGAAATATGGCTTTGTTGTATGGAAACAAAAATACCGGGATTACTTTTAAAGAAGTTTGTCGAGTCGAAACTAGTATGCTATTATACTCTTGGATATGTGCGACCAGTTGAATATATTTTTGCCTTATCATTTCATCGTCCCTAGTTTTCTTTTATTTATTTATTTTTTCTCAAAAATCGTCAATGGGGCTGTCCTAATATTGAGCTTTCTGAGAGAAACTTTTATACTAGTAAAATAAGAGTCATTTTTGTGTTAACCAGCATGTCTTAAACTCTGCAACTTTAAGTTAATAGAAATCTGATATATTTTTCATTGGTGAATACTTAAACATGGTTTGAAAGTCATTTTGTTATGTGTACCTAGTTGTGGCTGATTGTATGCTTGCGTGGTCAATGGATGCGATCTATATAACTCGTGGCAGAATATTGTAGTCACTGATTTCAAGTTTATAACATACACTTTTTGTTTCCATTTAATTAAATCCTCTCCAGATTAGAGTGAAGTTAGTTCAATTTATAGTTCACCGTTTAAGATTCCTATTTCGTCGGAGTATGTTTATGTTCTTTTTTGAGAGATTTGTGTACCTCTATTGACTTATAGTGCATCGAACTGGTAGAATTTTGGTATGCTTCAGGAAATATTAAAAACATGATAGGACCTTCCTACCATAGACCAAAAAATAACAATCTTTTCCCTGGAACCTATAAACTTTTGTCTTTAAATCTCGTGAATATCAAGTTGGAGTTAACAAAATTTATATATGTAGAGTTAGAATTACATTGTTTCTTTTGAGATTATACTTAAACATGATATAGTTTTCAAGCATATATAAGAAGGTACAGGCACATATTTGCTCATGACTGAGGACATCGTTGTCTGGCATGCTATCACTGATCTTTGCCTATTGATATATTATGTCTAATTTTGTTCTCATTCCCTCTTTCGAAGCATATAGCGTGTATTTATTTTCACTGCAAGTTCTTCTTCTTATAAGTTTCCTCATCCTTATTTATTTAACCCATCTGGAATTAAGAC

>LNC_000442

GGTCTTATACCTTTGGCCGGGTATAAGATGCTCTGGCAGCGTGAGGTCGATCGCTGTATCATCACTATAGCTCTTATGTGATGGTTGTCGGTTAGAGAAGCTCCCACAGCAGTTATTGTATTTTCATACACAGAGATTATTGTATTTTTATATACATCAGTTCATTGTATTTCTATATACATTTCAGGCTTATTGTATCCTTGTATACACACAGAGTTCATAGCATGTTTTAAGCAATCTTTCTTTATATCGCACTTGTTTTAAATTGCCTTATATTGAAAGAAGTCAGTCAGGTTGAGTTGAGTTGAGCTA

>LNC_000443

AACCGAGTTGCAGTATTTATCAAAAATCAAGAATTTTTTTTGGATTTACGAGAAATTCATTTGATTGGACATGAGAAAGTAGAGAGACATCGACTCATTCATATCTTTAGAACAGGATTGCTCACTAATAAGCTCTTCGTCTTCTCATTCGGATTTTGCAGTATTTGGTAAGTTTTTTTTAATCTCAATTATTGCCCTGATTTTTTTCCTTCTTTTTCGTTGATAACAAGTAACAAATATTGTAGTCCTTAAGTTCAAACATGTGTTTAATGACATGACTGTGGACCATAGTCTATTTGATTTTCTCACCATCAGTTCATGTAAAGAGAATCTCTGTGAATTGCTTTCCATAATGTAGCCCTACTTGTAATTAGTAATATTGTTCCTGCATTTCTATCCCAAAGTTCTAGACCTCCCA

>LNC_000444

TATAAACTGTTCTTTGAATGTCTAGTTCTGCCACTAGAGGTCGACATGGAATTCAAGACAACTGGAGATTCAACTTTGAGAGAAGGGAACTGTAAAGTTAAGAGGACACTAGGCTTTTGAGTGCTGAGAGAGGAGTATGTTATAAAGACTGAGAGAGAAACCTATAAGACTTCACATGATAACCTTCGGTCGCCCTGTGGTTTGGGATTGGGACTCCCATGTTGGAGGTCTCAAGTTCGATACCCCATGCCAGCGAAAGCAAGGGGTTTACCTTCTGGGTTGAGCTCGTCTCACCGGGCTTTCCTAGTGCGGGTTACCTCTTCTGTGTGGTTTGTGAGCTATTGTATAGGAGTGGGGGTTTTATCATGTGCGCACCAAAAGGGTAGTGGCTGCG

>LNC_000445

ATTTTTTAGGAGGTAAGAATTTTCAAAAGGGAAAACCCTAAATTTTGTTCCTTTCCTCCTCTAAAAGAAGAGCAGCCGCACCCCTCTCTTCTCACCAAACAACTCCATCTCCTCCACCTTTCCGAGCCATTTTCCGGTGAGCTTCCACCACCCAACCAGCCGCCTGAAGCAGCTCTCTCCAACAACAGCCTAAACCTCACCCATTTCCCCTCCAAAACAACCTTCAAACACCCAACAACAACCGAAAATGACCAACACCTTCAAACACCTATTTCACTCTTTTCCGGCAACTCCATGAACTCCAAACCACCAGACCCACCTCCATAACCATCGCTGCTGCCAGCAGCTCAACACCACCAGATCTTCACCGCACCACTGATCTTCCGATGAGCTACAATAATCGAAGGTTCAAACTCCGGCGAGGAGCTCATGAACCGGAGCAACACAACAAGGTACCCCGAAGTCGCTTCTCATCGAAGCCATTCGAATAAAAGTTTGAATGTTATATTCATTATTATGTATATTGTTGACGATA

>LNC_000446

CAATTTCAAAAATATATGTTATGGTAAATAGTGTTTGTTTATTTAAGCGAAGATTTGAATAATAAGCTTAGCAAAAAATAATATTGCAGATTGTTTTGTGAATGTTCAATGTGGATCGTCATATATATCTCTTATTCACGCAAATCTATGAAGATGGTCAATGAAAATTTTTATTAGTTAAATGCAATTATGTATATATTTATTTCAATTTAATTAGGTTCAATCATGTAATTAATCTTATCTCACTAATATTACCGTATAGACAATATTTGTTTTGTATTTTCTTGTCATCTAACCAGATGTGCTTTGCATATGTATTCTACGTTAAATTGTTTAGACGTCATATGAACATGTGAAGAGAACAAATACATTTTATATATTCA

>LNC_000447

CGGGTCTATTTGAATTAAGTTCGTCTGCATTGCTGATAACTCTACTCTGGATAATCGGTTAGCTCTCCTTCATTAGTTTAAATTTCGTCTTGCATCATCTATCATGATTATCGTATGTTTGTTGTATGTTCGAAAACGAACAGAAGGAAGCATCCAGGTCAGTTTCTTATCCCTTTTGCTCTATGTGCGATTTTATGCTACCCTTGATTAGCCAGAATGCGTGTATGTCTGTTTTTCACCTATATGTTGAAATAAGTAATATTATGCGAGTATTATTGTCTTGGTGTATTGTATATACTCGTATCTTCTTCAGCAGGTTTGGACATTTTGTTCAAGTTGAAGAGTCGCCTGTTAAGAGATTTTCATGGAGTTTTTATTTTTGTTTTAAGTTAATTTTGTTGTTGTTGGGTTTATTCCTATTCGGTTCTGTAATAATCTTGTAATAACTCTCTAACTCTCTAGTACATATATATGAAATATTTGGGGGATCGCCTAAGAGGGGAGTGGGATTTATGTGCTAATTGCTTTACTTATATATGTTGAGTCTATTTGTGTTGTGATAATAACTAGCCCATAAACTGTTTTAACATGTTAGATTAAAACATATTTTATCACATTCTAAATAACTAATGGAATAAATGTGCTACTTGCCTTATCAACGCCTCAGATTATTATGCAAGCATGTTAGAAAAGATTAAATAAATGAATAAATTGCAGAATTAATCTCATTCATTAACTTTGTTAAAATCACCTACTTTCGAATTAAATTTGTTAATATTTCATGTTATTTGTATCATATATCATCTCACCTAGTTTAACAAATCTTGCATGTCAGCATGTAATTGAGCATGTCTATATTTAGAATTATACCTATAGGAACTACTTCAACATGTTAAATAAGTAGTTGATTTTAATAATACACTAGTTTCAGCATGATTGATGAATTTAAAAGGAGTAAAATCTGAATCTTTTAAGTACTGATCTGCCTTAATTGTTATCTGTTAATGCGAGCTTTATTAATAATCTCCAACATGTTCACACTTAGTTATCATGCCTATAGGTTTTTCTGAATATTACTATGATTTTCAGCATGTTCATATTTAGAAACTATGCCTATAGGAGCCATGCATACAACTTTTCAATCGCCTAGAAATCATGTTCATAGGCGTAATCAAATAATTTCCAGCATGTTAAATAATTGAAATAAAACTATGCACATTTCCTGTCCACTGGTACATAATTTCTGCAATGCAATTAATCTCTACAACGTAAAATAAAGTAAAAATGATTTTCGGTATGTTCAAAACTGATTTAAATCTGCTATACACTTTGTTGTTCACCGAGATAAGTCTGCTACGCATAGCTTCTGCCACTTAAAATAATTTAGATAGCCCTCTAAAATGTTAATAACAAAACAAAACTAACTTATTTGCATATTTCCTGCACTATGAGTGGTTAATATCTTCCCGCATTTTAAGAGTTGAACCAGCATAACAATATTTCCTGAACTAAAATCTAAACTATTGTTAACGAAAATCTGCTTTTGTCTTGTTATGCCACTTAAAATTAAGTTTCTGCATCTGCTACTTTCTGCCCCGATTAAATATTGTTCGAATTAATTTCTGCACTGTTTTGCCACTTAAGATAAGCTACTAAATCAATTTCTGCATATTAATTAACAACTTTCTGCCACTCGATCATCTAAGACTTTAAAATCAGCTCTATGCGAGATTGTTTAGTTGAATTACTTTAGCCACTGTTTTCCTCATAATATCCCTGCCTGCTCAATTTACTGTCAATACTTGCTCTTTGATCATATGTCCACACACTTAGTAATGAGACCTCTCACTAATTACGTGTGTTTGCTAGCATGCTAATAATAAAATCTAGAGGCCAACATGAGACTTTACATGCTAAATGATCGTCCAACTTGTTTTGCTTGGTGTCTAGGAGGCCTATTAGGAACATTTATTTTATTTTTTTAGTCTAAAACCTCCTTATTAATAGTCCAATGCCTCTTGAACATTAAGTCAGGACGATAAGCACCTTTTAGGACGAAAAGCATGCGGTGGTGAGGTAGGGGTAGTTTAGGCCCTATGGAGATGATGACATTGCCTCGAGCCGTAAACGACAAACATTTGTTTTGTCTGTCCCAAAATAAAAGGCCGAGTGCTGATTGGTAAAGTTGTCAGAGTCTCCCTTTCCTTCTCCCTTTTATATGTTTATAATAATTATAATTATTTGGGGTAGTTTAACAAAAAATAAAATTTGATTATTCTATTTGATTGTGCAATTTATGTCTTTTCGCTTATCTTCAATCCACCCAAAGCCCAAAACAGTTGTTAGGTCGTGCACCAAAACCCGTTTTGCAAAAAAATGGGGCGCGGCAGGGGGTCTTGTCCCAACATCCCTCTTTGTTTTAGAGGCTTCAAAAACAGTTAGTAGTTCAGTTGTCTTTACATTTCATTTCATATGTTAAGACTTGAGTTGCCATTATGGCCAAGTTGAATGTTGACCTTTAAAACATTCTAAGTTATTTTACCAAGCTATTTGAGTAAGTTCATTTGATCATTGTAATAATGTTTAGAGTTTTCCGCTGAGTTAGTAAGCCAGGCCAAGGGTTCGCTTGAAGCTAGCAATGGTCTTCGAGTGCTAGCCACGTCCAGGGTGTAGGCTCGGGGCGTGACACTGAGCCTGTCCATTTACCAATACTGAGTACCAATTATTTGCAATAATTCTCCAAATTAATCAACAACCACCACTGAAAAACCCATCTTTCTTAATACTTTCATTAAAAAAACCATGATATTCTATCATAAGCTTTTGTCATCTCAAGCTTAATGATAAGATTTGATGGTTTTCCCCTTTTTGTGATGTCAGAAGTAAATTTCCTGAGTAAGCAAAACATTTTCAATAATGTTTCTTTCCTTCACAAAACCTGACTGATTAGAGGAGATCAATCTAGGTAGCACCAGTCCTAGCATGTCATGAATAACCCTTGAGATAACTTTGTTAACAAAGTTACTCAAACTTATAGGTCGAAGATCTAAAAATGAGTGTATATCATTCTTCCTAGGCAACAACACTAGATTGGTATGGGTGATGAATTTTGGGAGAGTATTGCCTACAAAAAAGCTATCCACCATCCTATATAAATCTGATCCAATAATTTCCCAACACTTCTGATAGAAAAGACCTGTGAAGACATCAGAACCACTAGCACTGTCACCATTAAGATTAAAAACAGCTTTTTTAACCTCATCCATGGATGGCATAATTCTAATTAAAGCATTATTTTCTTCAGAAATAACATGTGGAATGTGCCTGAGAAGAGAAAAATATTCGGCATCCCTTTCCTGAGAGAACTGTTTCTATAGAATCTTATAGCTTCATCTTTTATCATCTACTTGTCCTCCGAAAGAACCCCTTGCTCATTATGAATTTTGACAAATGTAATCTCTTCCTTCTTCCTTTCACTAGACTATGAAAAAATCTCGTATTTCTTTCTCCCCTTTCAAACCAAGTAAAACCTGTTTTTTGCTTCCAATATTCTTCTTCAAAAAGTAAATACTTTTTGAATTCGGCTTGTGCTTTTTGCACCGCTGCTTTATTTAAACTACTAGGATCCTCCTCGAATAACTTCTTTTGATCTTGACAATGTCCTCTCTAATTATTAATTGCTTAAAAATATCATCATACACCTCTTTACTCCATGAAGTAAGTTAAGATTTCAGCTTCTTTAACTTCTCTTTGAAGCTAAAAAATAGATTATCAAAATCATTAGCAGCACACCAGTTTTGTCTCACAACTTGTTTGAAAGACTCATGTTCTACCCAAAAGTTAAGAAATTTAAAAGGTTTACAAATATTCACCACATGTTCTTCACAGTTTAGTAACATAGGGGCATGGTCTGATCCAATCCTAGAAAGATGTTCAACCTCGGTATGATTAAACCAATTTTGAAATTCATGGTTAATTAACACTCTATCCAGCCTTTCAAAAATACAGTCTTCTGCAGCTCTACCATTCCACCATGTGAAAGGACTACCTCTAAAAGGCACTTTTGTGAGTCACAATCTCCAATACAATTAGAAAAATCATCTACATCTGCAGGTTGTACAACAAGACCTCCTAACTTCTCCTCGGCATTTAGCACCACATTGAAATCCCCACCAATAATCCATGGAAGACTCATGGAATTATGCAACTGATAAATGTCTTCCCACAAAGCCCTTCTTTGCACACCATCACATTTTGCATAGACAAGTGTTACGAAAAAATCCTTATTCAAAATAGTATCTTGAACCTTGATAGTGATATGCTGCATCCAAAATAAAATCAACTTGAAAACCTTCTTTGACAAAAATCCAGATTTTGCCATTAAAATTGGATTTTGTAAAATGCATGCTTAATCTCCGCCTATATTTGTTGATTACACTAGCATTTTTAAAAGGTTCCATTAAAGCAATAAAATTGAATTTATGTGCTTTATTAAGCATTTGAACCCTTTGAATAGCTTTTTGTGTCTTTACCGATTTTATATTCCAAAAGAAGAATTTCATCATTAATCTTTGTTCTTCTGCAAGCCTCTTCTTGAACTTGTCCTAGTAGGCAAACTCCCCATAGAAATGCTACTATTCTTCTCTTTTTTGCCAGATTTGTTGCTTTTGAGAAAGACCTCCTCTCCATTCTTATTACTCTCATCAAATGACCCTTCTTTAGAATTGGAAGAACCATTCTGTTGTTGTACTTGTTGACCTTGAATCAAAGGTGGTGCATCGTCATCTTCGAAATTTTAATCAACTGCATCTGTACTGCTAGATAGTTGTTGTAACTGATATTTTTACATCTTATGTGGTGCCTCACTTATAGAATGTGAATCAACTTCCTCTGTGCTGCTAGAACAACTATGTTGTTGAGATTGTTTTGGATGTGATTGTGGTTGAGGAATTCTTTCAACTAATGAGTGTGAAACAATGTCCATTAGAACTTTCAAAGGAGAAATTGTTGCATTCTCTTGTGCAACAATAATTGGATATTCAACTACTGTCGGATTACATTTTTCATTATACTATTCTTGACATTGTTGTCCTGATTGTTCTGTACTATCTACCTTGTAGATTGCATTTTCATTACTCGTCTCGATGCTTTTATCATGGTGTTGCTGCTGGTTCTCCACATTCTGCTTTGAGTACGCTGGCGCCTCACCAACCTCTGACGAGACAGCTTTCTAGCTGTATGGCAAATTGCCCTAGACGAGAACACTCTGCAGAGGACCGGGTCCCTACATTTGTGAAGATCATCAAAACATCTGGAACAGTAAGACTTATCATTTGAACTGCTCCTTGTTTGCTGTTTCAACATGTATCATCTGGTGATAATATCTCGTTATTTTTAATCATCTCCTTCCAAAATTTCTTGAAATTGATAATGAGCCTTGATGATAACAATATCTCTTCATGTTTCATTTGTACTTTTTCATCTGCTAATCACACTTAACAGTAAATATCCCAGTACTAAACTATTGTATGCATATAACAAAACCACATAATATGCATAAATTCAAGTATAATGATAACAATATAT

>LNC_000448

CCAATATCCAAAAGAAATACCTATTCTACAATTAGGAAACAAAAATGAACAAATTAGGAAAGGGCCGGGTCTTCTTCTCCATGAAGCCCTAGCTACAGCTCTGCTTCACCATTGCAACCTTCATCATCCATATCCATTGCCATTAAGCGGACTCGACTAAATTGGTACATCAAGCTATGGGTGTATTTCATCTCAAATGTTAACAAGTCCCACTAACATCATGAAATCAGCCCAAACGAAGACTTCTCCAACTCAATGACATTTAGTTAATTATTCAGCTGTGATAGGTTGAGCTCAACAAGGAGCTAATACCACTGACTACAACTTTAGGACAAAGCTACAATGATCTTCTTCAGGTAATCTGCGTTTATGTGCCATTCCGTTGTGTGCCTCTGTTTATGTTATGGCTTTTTTCTCATTCTAGCATATGTTTGCAACGTCTTGATTTTTGGTATTCTGTACCACTGTAATCTGCATTTATGTGCTATTCCACTGCGTGCCTCTGTTTTTGTTATGGCTTTTTC

>LNC_000449

ATAATGATACAATTGTTTGCAAAGATTGTCAAATTGTGGTATCACCATGTTATATTGTTTCCTTAGGGGGCTTGTCTCTGGTCATTGTACTCCAAAGAGATGAATGAATAGCTCCAGTTTGTCTGACTTTATTATCTCAGTTAAATGTTCACCATAGGTAATATCTACTTGAAGTTATAATTCATTTGGAGATTATCCATAGTTAAACATGCTTCATACATAACAATCCTACCAACGGGGGCAAATTTATAGAGGAGACTATGGGGAACGTGAAACCATGGTCTCTCCGTAAAATTAGTTGGTTAATATATATATTTTTTGAAATTGGTATAATATTAACTATTGACACTCATGCTCCAAGAAGGTTGAATAGTGTTTGGTTGAAGGTTGAGTTATACCAAGAGGAATAGGGATCAATTCTCACTTGATACATTTTCC

>LNC_000450

TTTCAAATTGATCAAAACCAAAAGTCTTAATCGACTTGTCAAAACAAATATTCCATGCTCTAGATGCTTGCTTTAGTCCATAAATGGATCTCTTAAGTTTACACAACATGTGTTCATTGCCACTTTCTATGAAACCTTCTGGTTGTGCCATATAGATGCATTCATCAAGACTTCCGTTTAGAAAAGCCGTCTTGACATCCATTTGCCAAATCTCATAATTGTAATGAGCAGCAATGGACAAGAGAATCCTAATGGATTTAAGCATAGCTACCGGCGAAAAAGTTTCCTCATAATCAATTCCTTCTTTTTGAGTAAACCCTTTCGCAACAAGCCTAGCCTTAAAAGTTTTCACTCTTCCATCCACACCTCTCTTTTTCTTGAAGATCCACTTACAACCAATAGGTTTGACATCAGCAGGGTGTCCTA

>LNC_000451

CGTTCTATTTTCTTTTGGTAGTTCGATCGTGGAATTTCTTTGTTTCTGTATTTCCGGAGTGTGAGTGTGTGACTTGTTATAATTGATCCTATTGATAGTACAGAGAATGGGTCTGTTATCTTGATAGAGATGGTTCTACTTTGTCAGATATTTATTGGAAAATTTGGAACACGAAATAGATTAAGAAATATTTGAACTATGATTCATACTTAATATTCAGACCTCGTGTCCGGGCTCCAAAAAATTTTCAAACAAAGAATTTTAATTTCTAAATCGAAAGATTCTTTTCTTTCAACCCCTATTTATATTTTGACCAAAAGCAAAACCTTTCTTTGAATTTTTAGTCATTCTATTTATTCCGGGACTAAGTGATGATCCGAGGATTCTTACTCAGGGAATCCTTGATTTGATTTAGGTTAGGTTTTTTTATTGAATCATCGTGGTTCTAGTATGAATCTGAGGTTTTAATCGATTCATAGGGTCTTAACAAGAGAATTCCTATCAATAATAAAGAAAACAAATAATAAAAGCCATATTCCACAAATTCTATAAAGAAATAGGGAACAAGAGAATTCAAGAGGCCCATAAGTATCAAAATAAAGATAAAGACGACTGCGCCAACTTGATATTTTGGTATTATCGCCACAAAGAAGAGCTTTCGGATTTTCCAGAGAAGATGGGATCAGAACTTAATAAATTTCAAACTTTCTATTCCATATCCGTTGCAACTAGTATTTGGGTGTTTTTGCTTGAGCTGTACGAGATG

>LNC_000452

CGAAACCGGTCCTCGTTACCGGATTTCCTTACCGGAACCGGGTCTTACCGGAACCGGTTTTACCGGAAATTTCCAAAGGTTCCGTCCGGTCCCTTAGTTACCGGATTGGAACCGGTTTGGACCGGACCGGAACCGGTTCTTACCGGATCATTGTGTACCGGTAATTTTTTTTTATTTTTTTATTAACTTTAATTATTATATAATATTATATTATTAATTAATAAAATTATATTATTAAAAAACTTCAATTTAAAACTTCAAACTTTAAAGTTACTTTAAAGTTTCA

>LNC_000453

AACACCTAATAATATATCCAAACAAATCGAAATAGCCCAAATTCTCTTTACTCACTCGCAATAGGCAAATCCTAGCTCGTCGGTGTTCTTCGTCGATTCTTCTTCAATCTTTGCTTATGTGATATTAGTTGTGATTTCAGCTGTCACAATGTCTGAATCAAACCTTACACCATGTCTGAAACTAGTTGTGATTCAATTAGTAACTCAATGATGATGACTTGCTTTGGTGGTGAAATTGCTCGTTGTTTCACTTCAAGGACTTCATTGAACCCCGAAGAAGATTTCATAGGTGTTCTAAACCTAAGTGAAAATGGAGAATTTGAGAGAGTCGTAGAATGCGGTTAAGATTGAAAGAGACAATTTGAATATTTTTTGAGAGTATAAACTACTTCAAGGTGAACAAATCAAGAAATTTGAAAGCGAAAGTGTTGAAGTTGAAGATGTTGTGAATATTGTCATTTGTTGTGTTTGTGGTATTTATTGTTGCAATTTTCAAGTGATCTAGTTTTGTGCAAGTGTATATTTTTCTTGGTTACAATTTTCAAGTAAGGAAGAAATATTTCCTTTATTTGGTTGCAATTTTCAAGTGTAAGCAAGATATGTTCTTCAATGCAATTTTCAGGTGTTAGTTTAATCGCAATGAGTATTTTGATTAGCTAATCTTATCTTTTGAATTGGATTTTGTAATAACTGATTTGAAATCGCAATGAGTATTTTGATTAGAGAATTTTATCTTTTGAATTGGATTTTGTAAGAACAAATTAATTTTGGTACTTGTCAATTGTAATGGTTGAATTTTCTATATCTGAGATGGGATTTTTGAGGTTGTAGATTTGGTTTTTCTTTGCAACAGATAATTAATCTCAAACTAATTAACATGACCCAAAACTGTTTAAGGAAACTTATAAGTCAACATTAAAATTGCACTTTCATAAAATAGAAAATAGTGCCATAATTCTGAAACTTAAATCACTGGTATTATATTGGTGCTTGTTCAGACTATTAAATCTGTCACACTTCAGTTAGTCTAGTAAAAACATAAGTTTAGCCAATACAAGAAAGTCATCAGCCTATACAAAAACATAAGTCTAGCCTTAACATTATTAAAGGCACCAACCTATACAAAAAAGGTCATCAACACAGTTTCATCATCCCCTTCTTGACCTTAACTTTTGAACCATCTGTGCTTGTAGCTGGTTTGTAGTAATTGCATCTTTTTTCTTTCAAGTTAAGCCAATTGATAATATGGAGAGATTTGTAGGTTCATTAACACCAGATTTGTCACCTCTAAAGCTTATTACTCTAGTTCTAAGGTTCTGCATCTTTCTCAGTTTTATTAAATAAGCTTCTTCAGAAATTGTTCTTAGCCTTAGCCAAGGGTCTTCCTTAGCTATTGGATTCATGTTGACTTTCTTGTGTTGGTTGGGGGGCTGTTAAGTTGATGTCTTCCTCAGAAGCTCCTCTTCATCAATCAAATCTCTCCTAGGTTGTCTCATATTTCTCCTAATAGCTTAATTTCCCTACTTAGCGATTTGCTTCCCCTACTCAATAGCTTGTTTTTTCTGTTTTGCAATTTTTATTCATAAAATAAAATAAGAAATTAAATAATAGAATTGGATTATGATTTTGTTGTCAAATAGATTGAATAAAAATTACCTTTTGACAACCTCTTGAATTATGATTTTATTCTCCACAAGTACTACATGTCATCACTTTTCCTTTCCTTGATTGCTTCCACACTCATTGCCTCTTAATTGTTTCATTCTTTTCTTTATCCCTCAGAACCTTAGATCTTTCGACAAGCTTGACCATTTCTGGTGGTTAGAAATGGTTCAGTGACATTAAATGATCAAATTTCTAGAATGGCTCTCCCCTAACACATTGTAACTTGTGTTTGTAAAATACCCAGGATGTCTCCTTACTGTAATACTAATCAATTTTAGACCTAGGTTTCATTTTCTTCTACTTCATGGTCTTAATTGCATGTGGACAAGGAATTTCAGTGAGATCTCGTGTCCTATATGTGGTCTTTTTCTCTCCTAGATTGACAATGTGTCTATTTGCATCTTCAGCGACCTCATAGTTATTTTTCCTATTGCCGTTCACCTTACAAACTCTAAATATCATCAAATACTCATTATACAACATTTCACTTTTGGGACTAAACTCATTATCTTACGACTTCCTCACAACAGTCTCTTTTTGTTGCCAACCTCCCCATTACTTTGACCCTGATGTCTTCCAACATTTCAATGATTGACTTGTATGTTGCTACAAGTATCCAAGTATGGAAGGACTCTATAAAAGTTATTGTTCACAACATATACACATTGTATCTAAATAAGCTTTGCACCATCTTTTGGAAGGATACTTGTACAATAAATTTTGTCTAGCCTCCTAATCAACTTCCTTAATCTCTTCAAGTTGGTTTTCAAACTCCTTCGAAAATGAGGACCATGCATCCCACCACAATAATTTTTTCAGCTCACCTTAACTTCGTCTTTTGCATCAATTTGCCTCAACAGATCTTGCACAATATTTTTGATGTGCTTTAGGTAAAATTGTTCTTACTGCATCAAGCAATCCTTGAGTATCAAATAAAAATAATTAAATTAAATAAAGTAATAGTATTAAATAAAGGGACTTAAATGAATTGCAACTTAGTAGTATATTATTACTTTTTGCATATCTTATATGAATGACCTTCACCATTCTGAAGATCATGTAAATGTTGCAATTGTTGCATGAACAATGTCCAAATTCTTTTAGTCTCTTTTTCTACCACGGCCCAAGCTAGAGGTTAAAACAGTTCATGTTATCTTGACCAATAACAATCAATACTTGTCTCTTAGCTTTTTGTTTTAATGAATGTACTATCTAACCTAATAAATGGTCTTAACTCTGCCTTAAAATCCAACTGTATTGACTTGAAACAAATATACATCCTAAAAAATATTTTGTTCTCATTGGATAGTGTCTTATTAGATAAATTAATTACAATGTCAGATTCTGGATTGCAACTCCTCAATTCAGTATCATAACCCTTTAGTTTCATGTAGGAGTCTACAAAGCTACCACCCAGAGTCTCTAATATCTCCTTTTAGCTATCTTGCACTTGGCTTTACTTACATTTAACTCAAACACTCTATGTAAGCCAATCTTTATTTCTTTGACCTTGTACTTAGGGTCACTTTGCAATTTCTCCTTAAAATACTGAGTTATTTTTGTATAATCAATTAAAGAGGTTTCATATGTTGTTTCACACTCTATATGATCTACTAGTGTTTAGACACTAGCCCTATTAGTGGCCAAATCCCTTGAAATCGGGCATACAAATGGACAACCTTCAGTACCACATTTATATCTCAATCTTATTTTATTACTCTTCACTACAATCAGGTCTACCTTCTTAGTCATAGCATAAAATTTGCAGAATTATTTTACTTTTGTTATGTCCTGAAAGTTAGACCCTTAACAATCTCTTTACAATCTTGCAAACTATCAATCATATTTTTTTGTAACAAAATTTTTCAATTCATCGATATCATAATCTGAATAGCTAAACATCATTTGTGCCTATTATTATTACTTTCACTTTCACTACAGTTTGTGCCTTTATTAACTATTGGTAAAAGTAAAAATGACTCATCATGTACGACAATATCATGTACATCAATAGCTCATTCATAGTCAGCAGTAGCAAACACATGAATGGATTTATAATCTCAAGAGATAAAAGATGATAATGTCCTAACTCCTTCATCTCCTACTATTTCAAAATATTTTTCAGAAGAACCAAGTATAATAAGTTGTTGCACAAAATCTAACTTATATGTGTACTCATTGACTAAGTCAGTAAAAAATTTAGATCAGAATCATACCTTCTCCAATAATGGACCCATCTCTCATCATAGTGTGATTCTGGATTATTAAGTCACTCACCACCATATGAAAGTATATGGTAACATTAACCATTTTAATAACCTACAAAAATTTAAACAAAGTAATTATTACTACTAAATAAACTGTTAAATATCAAATATAAGTCAAACTATGAAAAGTTAACATGTCATATTACTACTACATAGCACTAAAGTTAAGTCAGCAGTTTAGGAAGAGTTAAGTATTTATGTTAATTATTCACTACTAATTAACATATAAGCTAAGTGAAATAGTTAAGTCTCAAAGCACAATGTTAGTTAGTTAGTACTCCCATTATGAAAAAAGAAGAAAAAATGGACAGTGGCGGTTTATTACATATAAGTCAGCAGTCTAGGAAGAGTTAACAAGTCATATTACATTGTTAAGTGTTTGAGTTAATTACACAATACTAATTAATAGATAAGCTAAGTGAAATAGTTAAGTCTTGAAGCATACGATTAGTTACTTAGTTAGCTAGTACTCACATTAGGAAAAAAAGAAGAAAAA

>LNC_000454

AACACCTAATAATATATCCAAACAAATCGAAATAGCCCAAATTCTCTTTACTCACTCGCAATAGGCAAATCCTAGCTCGTCGGTGTTCTTCGTCGATTCTTCTTCAATCTTTGCTTATGTGATATTAGTTGTGATTTCAGCTGTCACAATGTCTGAATCAAACCTTACACCATGTCTGAAACTAGTTGTGATTCAATTAGTAACTCAATGATGATGACTTGCTTTGGTGGTGAAATTGCTCGTTGTTTCACTTCAAGGACTTCATTGAACCCCGAAGAAGATTTCATAGGTGTTCTAAACCTAAGTGAGAATATAGTTCTCCGGAAAATGCTTTGATTGAAGTAAATTTATTGAAGTCTAAATTGGAAGTGGCTACAGTGAAAATGGAGAATTTGAGAGAGTCGTAGAATGCGGTTAAGATTGAAAGAGACAATTTGAATATTTTTTGAGAGTATAAACTACTTCAAGGTGAACAAATCAAGAAATTTGAAAGCGAAAGTGTTGAAGTTGAAGATGTTGTGAATATTGTCATTTGTTGTGTTTGTGGTATTTATTGTTGCAATTTTCAAGTGATCTAGTTTTGTGCAAGTGTATATTTTTCTTGGTTACAATTTTCAAGTAAGGAAGAAATATTTCCTTTATTTGGTTGCAATTTTCAAGTGTAAGCAAGATATGTTCTTCAATGCAATTTTCAGGTGTTAGTTTAATCGCAATGAGTATTTTGATTAGCTAATCTTATCTTTTGAATTGGATTTTGTAATAACTGATTTGAAATCGCAATGAGTATTTTGATTAGAGAATTTTATCTTTTGAATTGGATTTTGTAAGAACAAATTAATTTTGGTACTTGTCAATTGTAATGGTTGAATTTTCTATATCTGAGATGGGATTTTTGAGGTTGTAGATTTGGTTTTTCTTTGCAACAGATAATTAATCTCAAACTAATTAACATGACCCAAAACTGTTTAAGGAAACTTATAAGTCAACATTAAAATTGCACTTTCATAAAATAGAAAATAGTGCCATAATTCTGAAACTTAAATCACTGGTATTATATTGGTGCTTGTTCAGACTATTAAATCTGTCACACTTCAGTTAGTCTAGTAAAAACATAAGTTTAGCCAATACAAGAAAGTCATCAGCCTATACAAAAACATAAGTCTAGCCTTAACATTATTAAAGGCACCAACCTATACAAAAAAGGTCATCAACACAGTTTCATCATCCCCTTCTTGACCTTAACTTTTGAACCATCTGTGCTTGTAGCTGGTTTGTAGTAATTGCATCTTTTTTCTTTCAAGTTAAGCCAATTGATAATATGGAGAGATTTGTAGGTTCATTAACACCAGATTTGTCACCTCTAAAGCTTATTACTCTAGTTCTAAGGTTCTGCATCTTTCTCAGTTTTATTAAATAAGCTTCTTCAGAAATTGTTCTTAGCCTTAGCCAAGGGTCTTCCTTAGCTATTGGATTCATGTTGACTTTCTTGTGTTGGTTGGGGGGCTGTTAAGTTGATGTCTTCCTCAGAAGCTCCTCTTCATCAATCAAATCTCTCCTAGGTTGTCTCATATTTCTCCTAATAGCTTAATTTCCCTACTTAGCGATTTGCTTCCCCTACTCAATAGCTTGTTTTTTCTGTTTTGCAATTTTTATTCATAAAATAAAATAAGAAATTAAATAATAGAATTGGATTATGATTTTGTTGTCAAATAGATTGAATAAAAATTACCTTTTGACAACCTCTTGAATTATGATTTTATTCTCCACAAGTACTACATGTCATCACTTTTCCTTTCCTTGATTGCTTCCACACTCATTGCCTCTTAATTGTTTCATTCTTTTCTTTATCCCTCAGAACCTTAGATCTTTCGACAAGCTTGACCATTTCTGGTGGTTAGAAATGGTTCAGTGACATTAAATGATCAAATTTCTAGAATGGCTCTCCCCTAACACATTGTAACTTGTGTTTGTAAAATACCCAGGATGTCTCCTTACTGTAATACTAATCAATTTTAGACCTAGGTTTCATTTTCTTCTACTTCATGGTCTTAATTGCATGTGGACAAGGAATTTCAGTGAGATCTCGTGTCCTATATGTGGTCTTTTTCTCTCCTAGATTGACAATGTGTCTATTTGCATCTTCAGCGACCTCATAGTTATTTTTCCTATTGCCGTTCACCTTACAAACTCTAAATATCATCAAATACTCATTATACAACATTTCACTTTTGGGACTAAACTCATTATCTTACGACTTCCTCACAACAGTCTCTTTTTGTTGCCAACCTCCCCATTACTTTGACCCTGATGTCTTCCAACATTTCAATGATTGACTTGTATGTTGCTACAAGTATCCAAGTATGGAAGGACTCTATAAAAGTTATTGTTCACAACATATACACATTGTATCTAAATAAGCTTTGCACCATCTTTTGGAAGGATACTTGTACAATAAATTTTGTCTAGCCTCCTAATCAACTTCCTTAATCTCTTCAAGTTGGTTTTCAAACTCCTTCGAAAATGAGGACCATGCATCCCACCACAATAATTTTTTCAGCTCACCTTAACTTCGTCTTTTGCATCAATTTGCCTCAACAGATCTTGCACAATATTTTTGATGTGCTTTAGGTAAAATTGTTCTTACTGCATCAAGCAATCCTTGAGTATCAAATAAAAATAATTAAATTAAATAAAGTAATAGTATTAAATAAAGGGACTTAAATGAATTGCAACTTAGTAGTATATTATTACTTTTTGCATATCTTATATGAATGACCTTCACCATTCTGAAGATCATGTAAATGTTGCAATTGTTGCATGAACAATGTCCAAATTCTTTTAGTCTCTTTTTCTACCACGGCCCAAGCTAGAGGTTAAAACAGTTCATGTTATCTTGACCAATAACAATCAATACTTGTCTCTTAGCTTTTTGTTTTAATGAATGTACTATCTAACCTAATAAATGGTCTTAACTCTGCCTTAAAATCCAACTGTATTGACTTGAAACAAATATACATCCTAAAAAATATTTTGTTCTCATTGGATAGTGTCTTATTAGATAAATTAATTACAATGTCAGATTCTGGATTGCAACTCCTCAATTCAGTATCATAACCCTTTAGTTTCATGTAGGAGTCTACAAAGCTACCACCCAGAGTCTCTAATATCTCCTTTTAGCTATCTTGCACTTGGCTTTACTTACATTTAACTCAAACACTCTATGTAAGCCAATCTTTATTTCTTTGACCTTGTACTTAGGGTCACTTTGCAATTTCTCCTTAAAATACTGAGTTATTTTTGTATAATCAATTAAAGAGGTTTCATATGTTGTTTCACACTCTATATGATCTACTAGTGTTTAGACACTAGCCCTATTAGTGGCCAAATCCCTTGAAATCGGGCATACAAATGGACAACCTTCAGTACCACATTTATATCTCAATCTTATTTTATTACTCTTCACTACAATCAGGTCTACCTTCTTAGTCATAGCATAAAATTTGCAGAATTATTTTACTTTTGTTATGTCCTGAAAGTTAGACCCTTAACAATCTCTTTACAATCTTGCAAACTATCAATCATATTTTTTTGTAACAAAATTTTTCAATTCATCGATATCATAATCTGAATAGCTAAACATCATTTGTGCCTATTATTATTACTTTCACTTTCACTACAGTTTGTGCCTTTATTAACTATTGGTAAAAGTAAAAATGACTCATCATGTACGACAATATCATGTACATCAATAGCTCATTCATAGTCAGCAGTAGCAAACACATGAATGGATTTATAATCTCAAGAGATAAAAGATGATAATGTCCTAACTCCTTCATCTCCTACTATTTCAAAATATTTTTCAGAAGAACCAAGTATAATAAGTTGTTGCACAAAATCTAACTTATATGTGTACTCATTGACTAAGTCAGTAAAAAATTTAGATCAGAATCATACCTTCTCCAATAATGGACCCATCTCTCATCATAGTGTGATTCTGGATTATTAAGTCACTCACCACCATATGAAAGTATATGGTAACATTAACCATTTTAATAACCTACAAAAATTTAAACAAAGTAATTATTACTACTAAATAAACTGTTAAATATCAAATATAAGTCAAACTATGAAAAGTTAACATGTCATATTACTACTACATAGCACTAAAGTTAAGTCAGCAGTTTAGGAAGAGTTAAGTATTTATGTTAATTATTCACTACTAATTAACATATAAGCTAAGTGAAATAGTTAAGTCTCAAAGCACAATGTTAGTTAGTTAGTACTCCCATTATGAAAAAAGAAGAAAAAATGGACAGTGGCGGTTTATTACATATAAGTCAGCAGTCTAGGAAGAGTTAACAAGTCATATTACATTGTTAAGTGTTTGAGTTAATTACACAATACTAATTAATAGATAAGCTAAGTGAAATAGTTAAGTCTTGAAGCATACGATTAGTTACTTAGTTAGCTAGTACTCACATTAGGAAAAAAAGAAGAAAAA

>LNC_000455

AACACCTAATAATATATCCAAACAAATCGAAATAGCCCAAATTCTCTTTACTCACTCGCAATAGGCAAATCCTAGCTCGTCGGTGTTCTTCGTCGATTCTTCTTCAATCTTTGCTTATGTGATATTAGTTGTGATTTCAGCTGTCACAATGTCTGAATCAAACCTTACACCATGTCTGAAACTAGTTGTGATTCAATTAGTAACTCAATGATGATGACTTGCTTTGGTGGTGAAATTGCTCGTTGTTTCACTTCAAGGACTTCATTGAACCCCGAAGAAGATTTCATAGGTGTTCTAAACCTAAGATCTGAAAATGTGATGTTGCTTTGCTTGTTGAGCCTAGCCGGAGCTTGATTGGTTGTTCGCCGGAGATGGAGCCACGTGAAATCTGCAAAACAGAAACAACATAAAAATTGTGAAAAGGAAAGGGGAAGGGGCCTGGTGTTGTTTTCTTCTTCTTTTCTCCGGCTCATCGGAGTTTGCAACTCCTCGCCGTAGCTGCTGGTTGCATCAAACAGGGAGGTTGTAGAGGTTGAGTGATGATTCTCGCTGGAAAAAAGAGAGAAATGAATGGTGGTTTCGCGTGGCTGTTCGGAGCTTTAGTTGCTAGTTTCTGTGGTTGTTTTCCGATGGATTTTGGTGGTGGAGTGTCGCGCCCCGTTTTTTACAAAACAAGTTTTGGGTACACAACTTAACAACTCTTTTTAGGCTTAGTTTTAAAATAGGAAATAAGAGGATTCAATCTCAACCGTTAATTTTACAAGAGATGAGGGGCTGAGATCCAATCTCAAATGACGGACGCTCCAGATTAAAAGATGAAATCTTAACATCATATTGGATTTAATTTGAATTGGCCAAAATTGAATAAAAAATTGGCTAGAATTGAAATGAAAGATGGCTATAATTGAAATACAATTGGAAATTGAGGAGGCTGTAATTGAAAGGAATTAGAATAGAATAGGCTAACATTTAAACAATTTTAAGGAAAATTAAAGAAATGCTATCCAATTAAAATACTACAAATATTATAAATATTTTATATAATTAAAGTCACTTAAATTTTTAAAACATTTGAATTATTTCGAGTTTTACTAAT

>LNC_000456

CCAAATTCTCTTTACTCACTCGCAATAGGCAAATCCTAGCTCGTCGGTGTTCTTCGTCGATTCTTCTTCAATCTTTGCTTATGTGATATTAGTTGTGATTTCAGCTGTCACAATGTCTGAATCAAACCTTACACCATGTCTGAAACTAGTTGTGATTCAATTAGTAACTCAATGATGATGACTTGCTTTGGTGGTGAAATTGCTCGTTGTTTCACTTCAAGGACTTCATTGAACCCCGAAGAAGATTTCATAGGTGTTCTAAACCTAAGACACAGGTACCCCGGATTAGCATCCTGCA

>LNC_000457

ACATGTCATTTGCATTGTTCTGAGACATGGTTGTGACAAGTGTTATGTGAATTGAGAAAGAATAAGAAATTAAAGAGGATGTACCATTTCGAGGGACGTATCGCGCGCCGCGATGGATACTATATTTCGAGGGACGTGTCGCGCGCCGCGACGGATACTATATTTCGAGGGACGTATCGCGCGCCGCGATGGTTACTATTATCGAGGGTCGTATCGCGCGCCGCGATGGATGCATGGACAGATATGTCCCCCATGGGTCCCGGACTGAGAGACAACGGGTGTGTATCACA

>LNC_000458

ATAAATCAGTAAAATTGACGGAGACAGACTAGTTAAATAACGGAAAAGCCAATATTTAAAACATGTATCAAAAATTACTGGAAGGATGCAAACAAGAATAGTTATAGTTTGCTCATTCGCAACAACTCCAACCTCGTTATAGATAGAGCGTATAGCGAATTCCCAACCTGGGGGTGAATGATATCCGAGAAAAAACTCAGTTACTAGAAGAAGACACAAAGCTTTTGCTGTGTCACTTAAGTTATATAGGAATTCCTGAGCCCAAGAGTTAAGAATAACAAGTTTTTCCTTACCCAAAATTGAATACCCGCTTAGAATACCAAACCAGATGATATTTGTTGAGAAGTGCAAAATCGTATCCATACGATTCTCATTTTGTATCGTGATTAATTGGATCGTTTCTTTATGGATTCCTATCCCAAACTCTTCGAGATGTGTTTCCGAGTATTCCTTGATCATTTCGTCCAAGAAGAGGAGTTCCTCTAATTCTCTGAATTTTTCTAGAAGACTCTTTTCTTGAATATTATTCAAGACAATTTGGGATTGCCCAGTATTCCACCAATTAGTAACCCAAGATTCCAGACATTTATTAACTGAGAAAGAAATCCACCAGGGCAAAAATACTATAGATGCAAGATAGAAAAGAGGAGTGAATGCTTTCTTTTTTGCCATTTTTTTGTCTGTAAATTGTTAATCAACAAATTCGTACACTCTATACGATCGAATATTTCTTACACACATGAGTTTTATACAAGGTATCGAACTTATGAATCTATTTTCTTTGTGATTTTGTGGTTAGTCTTTGAATCTAGAAAGAATACAGAAATAGGCTAAGAATTCACTTCGAATGAAGAAATTTAGAATATTGTTTCCTGATATCTCAATTGGTCAAATTAAAAATAAAGTGTATCCTTTCGATGAATGATCGAAAGAACCACTTTAAGTAATGAATATCATTCAGATTTTGAGACGAAAGAACTCATTT

>LNC_000459

TCGAATTCATTTTTCTCCTATAAAAATCCATCTTTGCTTTCGGTTTTAGGGGAGGAAAAATTATTCTTGAACAATTGACTTTTACGCTTGAGAAATTTTGAATGCATTTTTAGTCCTAGATTTTAAAAGGGTCTTCGGCTAAGATTTGTTTGGATTTTTTATTTTATTGCTCGAGGTTTGAAGTCGGTGTTGAAGTTGTTCTTAAGCTCGTATTGGTCCCGGTTCGCTGCTCCGAAAAGGGTAACAACTCCTCTTCTCTCATTTCATTTTTATTTCAGTTAATTTGTTCTCTGTTTGGTGAAGTAGTTGCGTATCCATATTTGTTTCTTTCCGTTTGAACTCTCTTAGTGCTATAATGCATGAATTAGGATGATGTAGTAGCTAGTTTTATGGTGCAATGTCCTCATTTCTCACTGTCTGTTGTTGCTTTGGTTTGAGAGTGAGAAAACAATAGAAACTTCATGTATTTTGCTTCACACCCTTACCCCTCAACTGAAGACAAGTGTTCTTGTATGTTTATTTTGAATAGTATTTTAGCTAGATTAACTAATTTCTTGAAATTGTGATTGTTCCTTTTATTAAAATTTCCCTTAGAATTGTAGCTAAGTGTTAAGGTATGTCTTTTCTTTTCCGCAGAATGTGAAATTCTTGCTTAAAATAGTTAAATGGGTTTGTGTATAATGAGACAGCCCTAACCCCTACATTTTAATTTGTTACTATCGAAATGTCCTGAAAGCTCTCATTTAAATTCATTGTTCGAAAATCTGATCCTATTTTGAGGATTAAGTCATCAGTGATCTAGTTAATCCAAGCATGGCTTTAGGTTCTTATTTTAATATCTTATCCAATTAGGCATATTTGTGAGTGCTGAGTAGATTTAAAAAATTATGGCCTTGAGGTTTGCTATTGTAAAGGTTCCTTGTTAATCCTAGTTTGAATTCGCGCGGTAGATATATCTATCCTCTGTTTTCGATCAACTTGAGTGGTATATATCTCTTTGGATTTAAACTCATGACGGGATGCTTGTAACTCTACCATTTTAATGTGACGATTTAACTAAGATTGCTCTTATTTTCCAAAAGAATAAAAAATAAAATAGCCCTCCCCATCTTTGTCCAAAGTCTAAAAAATATCCTTCTAATTTTTAACTTGTTGTTTAACTTAGTTATTTACTCGAACGTTAATATTCAACAGTGATATGAACCTTGTTTTGTATTATGCCATTGTTTAACATATTTGCTTATGTTGCCTAGTTTAGTTATTTAAATGTAGTTATTTTCTTCCTTTGCCTATAATGAGTTAATTAGCTATTGTCTTCAATTTTGATCCTTCGATATGACAAACCGGCAACCTTAAGTCCCCATAGATAGAGCTAGTTTTGTATTGTATGAGGTGATGTTAGCTCTTTAATATATCTTTGGATCGCATCAATTATTGCTTATAAATTTTAGTCATATGTACGTATGTGTGGGCATGTGCTTTGGAAGTTTCATGTGGCTTGATTTTCATTACCTTAGTATAAGTTTTATTACCTCCTATTTTGTGTTGAACTTAGTGACCTTTTGGTCTTAATTTGGTCTAAAAGTGACTGTAGTAATATTTGCCTCTCAAAAAGTATTTAAGCCACTTTCATCTTGATTTTAAGTTACGTCTTAATTGTTCCCTTAATTCGTAC

>LNC_000460

TCAGTTGAAGTACTGAGCCTCCCGATATCGGGAGGCTCAGTACTTCAACTGAGATAATGAAAAATTATTTCACCCTTTTAGTTCTTTTTAGTTGGAACTTTAGGCGGTTCTCGAAAAAAGATAGCGAAAAAAATTATCCCTAGAGTCGAGACTAAGAGGAATGTATAAACCAATGCTTCCATAGATTCGATCGTGGTTTACAATTATAGCTTTCATACCTGTTTGTTTCTTTTTATTTCCTTTTTTTATCATCTTCCAGATAAAGAACGAACAAGAGTAAAAACGAAGTTGATTCAAATAAAGATTTCTCTGGTACCCTATGTCAATAAAAGTAAAAGAAAGAAAATAAGGGCGAAAGAAAGATACCAAAGCAATGTTGTATCAGGCTGCCTGTCTTTTTGTAGTTGGATCTCCAAGTTTTTGGAATGCTCCAAATTCGACTTGAGCATCTAAATCTGGGTCAATACCAGCAAAAACATCTCTGAACAAGGTTCTAGCACCATGCCAAATGTGTCCGAAGAAGAAGAGCAAAGCAAACGAAGCATGCCCAAAAGTAAACCAACCCCTTGGACTGCTACGAAAAACACCATCGGATTTCAAAGTAGCACGATCTAATTAAAAAA

>LNC_000461

AGATGGAGCAATCTCACAGCTTCTTCATCCTTCGCTCAGAAATCGGACGGACGCCATTCTCTATCCGTTGAGAACAAACGACATTCTCTCTGTTGTCTCGTTTTGGTGGAGAAGTCGTCTTCACTGTTAGCTCGTTTCGTCCAGTTTGCTGCCCAAAAAAAGGTAAACGTCTTTTCTTAGCTTTGTTTTTGTTGGATTCGTTGTTGTTCTATACTTTCTGTTTAAGTGTTCTTAGTCATATTTTAGTTATTGTGTGTTAGGATCGATTAAGAATCCGCTTTGTCTTGCCTAAATTTTAGTTACTATTTCATTTAAAAATGTAGAAAAGTTCAATTTTAGTTTTTGTTTTGGTGCTAGCTGATTAGTTTAAGCTATGTTGATCACATTCTGGCGCATCTTTCTCGACTTGGATTATATGGTTGTAATTTGCCTTGTGCTTTTCTTTCTAGTAGAGACCGGTTTTCCTTGGCTTAGCCAGTTTATTTACTAAAATTGATTCTCGATAACTAGCATAGTGTTTTGGTTTACTAAGTTGGTTTGAACTCGAATTTGTTGACTGTTGATTAGCTTTAAGTTCTCTTTCTTATTTTATTATTCAATTACTAGTCCTGAATATTAGTTGCTATTAAGATTAGTTATTTCCTTTTTCTGTTTATGTGTTGTTGATGTGTCTGCATGTTTTCAAGATTAGTAAGGTTATTTTCATCATTGTTTAAGTTGGTAGCTATTTCCTTTTCTTGTTCTTACATTCAAGTGAGGTGTTAATGTGTTGGGAGAGATTTTGACTAATTTTATAGTTGGCTCAAATCCTATGGGATGATTCAGCTTACTGAAGTTTTGGTCCGAAAGCTCTAGAAATTAGTTCTGTTTAGATTATATTCCTGAGTTACCATATGGCAGGCTTCTTGAGTGTTGTATTTAAATACGGGAACTTGTGGTTGTTGTTATTTGAGTTCATGTTAATGTCAATCAACTCCCTTTAACAGTGAATCCATTTCTATTTGATTTAGTTCTTTCCCTTAGTTCATATGTGTGGAGTATGAGGTCTAGCTAAAATATGGAAAGCTTCTATCTTTATTTTGTTTAACTGATAGATACTTATAGTTAAATATTTGGTATAATTTCAGCAGTTATAAAGCATGTCACTTAGAGTTTTGAGTTACTTTGTTTGCTTAGTAAGTTCTCTCGTGTGTTAATCAATTGTCTTGTGATTAAGTTAATATCATCCTAATAGTGTTAATCTTTTTCCTCTTTTTTTTTCTTTGTGCATGAACACTCTCTTGCTCGAGTCCAATTAGACTCCAACCATCTCTTGCATTCTTCTCCCTTTTGAGTCAACCTCTACAAAAGTCGTGGAAAGAGAAGCTGAAAATGATAAATTTTGGAGTCCAAGTCCAAGTAGTGGATAGCAACAATAGTCCCAAATGGCTAGAAGTATCATTTCTGGACTTAAAAAGTCTAAGAATGATATGCCAATAGCAACAACGAGGAGCGGAATAGGGCCAAAAGCCCACTTAAATTTCCAGTTCTGTATTTTGTGTAGATTGGGACATAAAGCACATATATTTTGATTATTATTCTGCTAGTCTAGGATAGGTGATGATTGAGCCTACAACCCAATGGAGAAATTAGCTTATCTTAGGATAGGTGCAAACGAGCTATAAGGCCCAAAATGAAATTAAGCCCAAGTACTGGCCCAGGCGGACAGATCCAGACTTGGGCCCAAATCTTCTTTCCCTATCTCATTTATTTGCTATTAGGTGTTTTACATGTTTATTTAATTTTAAGGTTTCCTAATCAATTCCCCAAAAGATAGTCACTTCGAAATTTTCTAAAGACTAAATAATCATGCTTATTTTTCTACTTAAATTACTTCAAAATTTTACTCAATAATATTTTAATATCTCCTTCTCCCCTAAAAAATCATTTTAAGTCTTAATTGCTCATTCCCTCTATTTAATCAAGTATGCATATTATCTTTAAAATAACTAGCCAAATAGTTAATTATTGGATAACTGCGTGTTAGCGGGCATTTCAGGTGCTTTCAAATCCTTTTCGAAATGCTAATATGAACCCCGAACCCCCTTATGATATTTTCATCAGATTCCC

>LNC_000462

AGCTTTTGGGGTGTGAGTTAGGTCTAAGACCTAATTTAACATGGTATCAGAGCAGGGCCCGTCTCACCCGATGTTGGACCCCCAAAATCAAAATTGCCCACGCACCAGATGCTAAGCACTGGGCGTGAGGTGGGGTGTTAAAGAATGACAAAAGTCCCACATCGGTGATTAATGAGATGGGTGGACTCCTTATAAGGCTTGGGCAATCCTCCT

>LNC_000463

CCATTACTGATCCCCAACACATACAATGAAGGGAAACAGATGATTCTACAAGTTTTACAAGGTTTAGAAGATCACTTGCCTCAATTCGAACACACTATCACTCCGTAACTTGAGTCTTCCCCCCTTTCAAACGGTATCTGAATCAAAGCAATCCATGGGGATTTGCCCGAAGAGTTGGATCATTTGCAGAGGTTGAAATTGATTGATGTCTCAAGCAATAACTTCACTGGTGCCATTTCAACATCTTTAAGTTAGCTTGTAGAGCTTTGCATTTTGCGCCTGTCTAGCAACCAATTTTCCGGAAAAACCCCATCTTCCCTTTCCAATCTAAAAATGATGGAAGTGTTGAAAATAAAGGAAAATTTTCTCGAAGGAGAGATCCCTCGAGAACTTGGTGATCTTCGTTACATGACTGCTTTAAACCTGGAAAGTAACCACCTTACTAGCTCAATACCACCATCAACCATGCGAGTCATTGCTGCTTCCAACAACTATATGAGACCATCTTCCAAACTTAGAAGGGCTTGCTTCACCTCTCAGAAAACATCCTAGGTGGCGTTATTCCACCAAACTTGGAAAAATGCAAAAAGCTCAAAATTTTGTCATTGTCTATCAATGAGTTCACTGGGACTATACCAAGAAAGTTAGGCAACATAACAGATCTTACAGAATTAGCTCTCACTTGGGAGGTAGTATTACATCTCTTAGCAAGAAATTTTGAATTTTCTTCTTTTTCACTCTTCCACCAATTATTGGTACTCATATATATATGAGTACCATCCCCGCAAGCATTTTCAACATGTCAGCACTGCAGATCCTAGGAATATCAGAAAACAGGTTTTCAGAATATGGACAAGATGGAATAGTATCCCCAAGCTGTGATGTCTCCGCTACGGAGAATCAGACTGTTGTGTTATCCTTTCAATTCTGCACAAGTACCGAAGATCGCGCTGTAGCATATTGGATTATCGAAAGCAAGTCATCATTTCTTGATGTATGTAGTATTTATGAAATTAATATGAAGTTACAGATGCAGAAATAATTTAAAAGGAACATCTCTGAGATATATGGTTGTTGGTATTTATAATACATAACTCTTCACAAGTCTGATATGCTAAATTGAGAAATAGCCTACTTATATGCTGTAAAAGTTTTAGTTAAAGATGTATTTTACTATTTTACTAAACTAAGCTTATAAATAAGATTTCAAATAACATATATACCAAGTTTTTGTTAGATTTTAGGTCTCTAATTGAAATTGCGCTTTAGGTCACCAATTATGTTAATCCGCACTTACCTTCCTTCCTATTTTTTGAGGAATAATACTATTATCTTGTTATTTAAAATTAAAAGAA

>LNC_000464

CCTAATTTCTCTTCTCCTTGCTAAAGTCGCTCTATTCTCTCAACTCTTCGTTCTACTTCTCAGCTCTTCAATGGCGAATCCATCTGTTGCTTAGTCTTTTCTGTTTGTCTACACACTCTCCATCATTCTAGAACACTCACCTTCCTCATTAGCACAAAGTAGACGCAAGATTTTCAATACAAGCATCAGTTCTTCCATTCTCTTGCTCCAACTCCAACAACAACGAATTCCAGGTGTGAACATTTTCATTTACTTGAGAAGGGAAGTTGACAAAGCTTCATACCCAAAAACCAATATTAACTCCTCAGAGAAACGATTATACTACAGGTACACCTAGCTTAATTTGGGGAGTTCTTATAGGAATGTGGTATCTTTTCGGGAATTCAGTTTTGTGTACTTGAAGATTCTTCTTTTTTGTTGTTAGAAGTGCAAAGGGAAGGGCAGATTCAATAAAGTAAAGGGGTCCAAGTTCCTTATTGTGTCCATTTTGATTACAATAATCAGATAGAATATAATCTCATTCATTCTGGAACTTGTGAATTTTGGGATTATTTTTTGTTGGAGATAAATTGCTGTGATTGGTCCAAAAGGGCGGGTTATAGACGGATTTCTCGGTTATCAAAAAAAAATTAGCTATATACTTTTAATATATGAAAGTATGCAATTAACTTTGTATATGATCTTTTCTAATTTTGAGACTTCTTTTAGTCAAGGGATGTTTTGTTACTTCCATAAAGAAATTGACAGATAGTACTAATACTAACACATAGGAACGTAAGAAATTATATTACCGTTTCCGACAAATTTCAGAAGGTGGTTTCTATAATTCTCTTGATTCTGTAACAACTATAGTAGGATTTTGGTTTGCAAAGTTTGTTTATGTAGTACTCAATCTTACTTTTAACAATCTGTAACTTCACTCTGGACTGAAAACTTCGAACTTCAATAATATTGAGGAAGCTAAAGTAGCAAAGATGGTTGGCTATTATATAATAAATAAAGGACAAGACGAAATATCAATAAGAATATGCTGTGAGTTCTATACTAGAGAGAGTAAGAAAGAAGTATCAAATAAATGCAACCTGTGAAACCAAACCAAAATCCAACTTGAGGAGAGTCATTCTTTGATCAGAGAAAGATGTCTTCGAGAAGGGAAAACACAATGCAATAGCAGGATGTTTTCCTTGAACGTCTGCTTTCCATCTGTTCTCTTCTTTATCCCCAAAAGAGCATACTTGATGAGGTTTCCCAGATCGAGAAGTTTGAAGGAAGCTTCTTAGCACTTATCTTAGCTTGGAACAATTTTCCCT

>LNC_000465

GTCCACTCCCACCACAAGCGTAGGTCGGAGGCGGTGGGTATAGCCCACTTGGACCTTGCTGCGGCGGCGGCGGTGGGTACACAGAAGTGGATCCCACCATCGGAGTTGGATTTAACGCCGGATTAGCAGTAACGGGGTTTTCAATTTTAGAATCAACGACAGTATTACCGGTGATCCTATCACTAAATTGATAGGAAAAAGTGAGTTGACCTTTGGGTTTACCGGAAGGTTTTCTGATTTGATAACTTACAAATTGTTTACTTCCATTACTGCTATTGGAAGATTCGAGAAGCTCTTTGATGGGTACATCAACTTGTCCAATATCTTTATCTCCAAGCGCCTTTTGACATCGGAGCTTGAAAACAAGATGCAAACGGTTTTGAACAGCAGCGTTATCATCAATGGTGAACTTGATCGGAAAATTCCAGGTTGGGTTATTGTCACCGTCATGATCAACGTGGGTTTTGGTTTTCTGATTAGATCTGTCGTCATCGGCGCCGGAGATTGAAATGACGACGTATACATCCATTTTTGTGATGAGATTGACCTTGTTTAGATCTTTACCGGAAACAACATTTATGTCTAATGTGCGATACTCCATGGTGTTTTCAGTAGTGAAAATTTGAGAAATTGAGATAGAAAATGGAGTTATGAATTGGGGAGATAAATAGAGAGGCGAGGTCAACGTGTTTGCGATACTTGGCTGGCGACTTGTTCAAGGAAGATGATGATATAAGTAACCATGGTTGTAACGTTTATCCATATATTAACTATGTATGGTTAAAGACTACTCTGAACTCTCACCCCCACTTTTGGAGGTAAAACTTACCTGCACCGGCT

>LNC_000466

CAGACATCAAATTAAAGGCTAACTTTGGTAGACCTAATACATATCACAAGGTAATGGAAGAAGTTAGTTTTACAGTCCCAGATCCTATAACATTACAGATTGAACCATCAGCAACTGTCACAGGAGAGAGTGCTCCGTGTGATTGAAAGCTAGAATATATGTTAGGATTACCTGTCATGTGATTTTCGAAGAGGATATAAGACATGCTTTTCCTGACTCAACAAGGGTAGTAACTGAAATAGATTATTTTTGTAATTTTTGATATTGTGAGAATTTTGTAAACTCTTTCGCCGAAATCAAAATTCTT

>LNC_000467

TCTATATACATTCTATAACAATGAGTATTTTCTTTTGATATTCTCAGTTTTATTTACTTATTGATTTATATACATTATTGGAAAATGTTGTATGCGTTTTTTTTATGCAACCCAGAAAAGATTTTAAAAGTACATATCTTTTAGGGCAAAGTGTGCAATTTTTGTAACTCTAGTTTATCTAGGTCCTAGGAATCACTTGGTGAAACTTAGTTCTTGTGGAATTCCTATCAAGTCAGGGTCTTGGGGAACCAAGTCAGAACCATGAGGAATCAATCAATTACGTTGAATGTGCTTAGAGATGTATCATATGATACCAGCCGACATGTCTCATGCCACCAAAATGTTGTTACCGTGTGGGTATTGGTCCAATAACTGCAGTTTCCTGAAAAGTGCCTCTTTCCAGGGCGAAGGACGTTAAGAGCTTGCAAAAAAATTGCTGAATCAATCCCTAAATAGTACTTAGAATAGAATTTCTTTTCACCGAGTAATAAGCATGAATAAAAAGTAAGCTCACGTGAATATTGAGTTATACCTGTGAGTTATTTTGTTATGAATCATGAGCTTAGATGATTAGTTGGCAAGTAAATATCAATAGCGGATGAGGTAATTACTTAGGGTTTTTATTTTGGCGCAATGTAGGTAGTCTCTACTGCATATTATTCAACTCTTGTGGAGCTCTCTCGAAGGCTTTTTTTTGTGACATGTACTTTAACTTTATGCTTATCTCAGCACTTCTAAAATTTTGGAGTTTGGACTTGTTTAGTTACGTAAATGGTTCATTTGTTCTTGATAATTCATTATACTGAAAGTCCTATACACCCAACTAAATATCCAATTTAGAGTGGTAACGAATCTTTAGAAGACCAAGATCACACTGAACCAATAAAATACCCACTACCTTTTCCAATTAGGCATCTATAACTGTGGTAATTCGGAACATTTCCAGTTAGTGAACTTTTATTCGTTCCTTTATTATTTATTCTATTCTTGTGCTCCTTAATCTTCTCTCCTCTTTGTTTTACTTGAATTTAAGCCTCCAATAGTAGTCCTGTTTCCTGTCTTGATTTTTAGTCATATTCTTCTACCCTTTCTATAGTGAGACCCAGAAATGGCTCAAGGTACTTGTGCGATTCTTAGTATGCATCGCTCCTTAACAGCCCCCCACTTTGATGCCTTGAAACCCAAGTATTCTGGTCCATATGTCAAGGATACAGCCAAGTTTGTAGCCTGTCCAACATCACACTTTGGTATTTAATAAGATCATTCTTTCAGTCATTTGTTCGCTTATCTCAACAGTAAAGTCAACTATCAATGTGATTTGATTTTCTGACTGGCTTATAAATTGCTTGATTTTCATACTAGCTTTTGTTGAGTTTAGTTGGTGATGTTATTTAGTTTGGGAAC

>LNC_000468

TAAATTCTGATGCTAAGGCCTCTTGTTCATTCTTCTTTGCAATTATACTTTTAGGTACAAATTTCCAAAGCTTATGGACTTAATTTTCTATTCAAAAGAAGTGGTGAAACATGCTCTTCTTCATCACCAAATCAGCCCACGTAGGAGTACTCATCTCCCTTTGTTTTTCTTTCGAGTCCTCATGAGCTGAGGTGTTCGATGTGACCATGGGTGACTTGATCGCTGAATTGGGTTTGAGCAACATGGCAGCCTCACTTTCTTGAATAATCTTAGCTACTTCAGGATTAGAGATATATAATGTAG

>LNC_000469

TTCTCTTGTGGAACTCTCTCTAAGGCTTTTTTTTGTGACATGTACTTTTACTTTAAGCTTATCTCAGCACTTCTAAAATTTTGGAGTTTGGACTTGCTTAGTTACATAAATGGTTCATTTGTTCTTCATAATTCATTATACTGAAAGTCCTTTACACCCAACTAAATATCTAATTCAGAGTGGTATAGAATCTTTAGAAGACCAAGATCATACTGAACCAATAAAAACCCACTACCTTTTCCAATTAGGCATCTATAACTATGGTAATTCGGGATATTTCTAGTTAGTGAACTTTGATTCGTTCCTTTATTATTGGATTCTATTATTGTGCTCCTTTATCTTCTCTCTTCTTTGTATTTCTTGAATTTAAGCCTCCAATATGTAATCCTGTTTCCTGTTCTTGATTTTGTGTCATATTCTACTACCCTTTCTATAGTGAGACACAGAAATGGCTCAAGGAACTTGTGCTATTTTTAGTATGTATCGCTC

>LNC_000470

ACGGACCTTAGAGTTTTTTTGCTCTTTTCGCCTTTGATAACACTGATTCATTAGGAAAGTCATTTTCCTAGATTATTTTTTTTTTAAAGCATTTTGACCAATCAAATATTAAAAATATGGAATAAATAGACAACACTCTCAAAAAAGAAAAAGAAAAAGTACTCAAGCACTTTGGGTAAAAATGCATAAAACCCAAAAATAGTTGGAATACATTTGCAATAACTCTGAATGAAAAACAAAGAAATTACCCCGGCAAAAATGGAAATAGTTAGAAGTATAAGGGGATTTTCGAGGAAAAAGGAAAATCACATAAAAGGGGAAAGAGATTCACATCTCACCCGATTCTCTTCTTGTCATCAATGGTTCCTGCCTTCCTGGCAGCTCCATCTTGTGCCCTCATCGGGTCGAGACTCTCTGATCACCCTTTCGGCAAATTCCTTCACTCTCCAGTGACATCACAGGGC

>LNC_000471

CATCATTGTCTGTTGTGCGATACGATGCTAGACCATTACCCATCACAATTCTGCACCCCGCTGATACATCACATGATAATTTTACACCAAAATATCTCACATGATCCTTAACACTGTTGCCTTTCACCGACCTACAAAGATGTGATCAAAGTTACAAAGAGGCCAACAACAGCTCTAGTAACATTCAGCCAGTCGTTTATTACTACCAAGATAATTAGAGAGGAGGTGCACCTTTAGGTGTCTCCAGTCTCGACATGTTTCTAATCCAAAAATTGGTCTTTCTGGATCCTAAATTACCAGCAAGATTACAGATTATTTCGTTACAACATGGTTCAGCAGTAAAATTACCACTTCTCTTACCAAATATCAGTGTATTATGGTTTCACTTTCTTGTTTGTTTATCAACAGAAGAAAAGTGAGAGAGACTCTCTTCTTTTCTTTTTTGCATCATCACAATTATGTACGGTTGTGTTTCACGTACCTCATACAGCTTTAGAAAGTTCTTGTTAGAATTTATTTTAATGTTTTGTTTTTTTCAAATTGCTTTCTCATTTGACAAATGGAAGTTGTATGATGTATCAGCATTTTTGCCATAATGCTTTATAAAATGGATCCATATGTGCCCTCAAACTTGTTATGATTTGCCATAAGCATTTTCCACTGTTTGCATTTGTAGTTTAACTTAAACTAACCGCATGAAAAGCAATTTGAAGAAATCCAGTGCATATATTTCTGCTGAGCACATGAAAGAGAAACTTTACCTGCTGCAAGAAGGATCCTCCCCAGAATTATCGCAGACCTTTTCAACTGTATAAACTAAACTTCCAAAGCCAAGATCATAAAGCCAGACCTGCAATTTCAAGATCTCACAGTCAACATAATGCAACCACAAATCAAGACAGCAAGAGGCAGAGTCACTTGAATGTGTCTAAACATAGTTCGAATGCTCACAATGAGAAAGACTATTACCATGAGTCTTTATCATATATTCATGTGTAACTTCTTAGTGCAGAAGTTACAAAAAAATACAATTATTGACAACCTTCTTTTTTTAATAAATTCATAAACACCATTTCAACATATCTTCGGTATTTACTATACTGTACAGATATAATCTCA

>LNC_000472

CAAATATCAATTCAATTTGGAAACCAAGTAATTCTCATTGAACTCACCGTAAAAAATGCTAAACATACTCTTCAATCCAGTACTACTACTCCCTGCTAGTATTTCTTTTCTTTTAATTTCTTCTTGCATCATTCCATTTACTCTTCTAATTTTTTACCACTTCAATTTTGTCAAGTTTTACATAATTTCATTCTATTAAATTATGCACTATGATAGTCCACTGCTGATATTTACTTCGACTCTTGAATGTGTGGTCTGACCCTTCCTTTGAATCTTGAAGAATGTGTGGTCTGACCCTTCCTCAGAATCTTGAATGTCTTTTTAGTAACAAACTTTTGCATGATTTATTAAGAGAGTGTGATGCACTATGAGAAGGGAAGTGGAATAGTGATAAAAGTCGGTGTGATGTGATCAGTCATGGCTTTAAGTCGTGACCAGCCTCTTGCAGACATGCAACCTAATCGTAGGAAAAAGTATTTGTACCTATATTCCAAGCTACAATTCGTAAGTCCTGCATTTAGATAAATCACATTATGCTGGTTTAAAATTCCATGTACACCATCTTTTATGTGCTAACAAGATTAGATGTGCTTTTGTTACCCAGTCATGATCATGCACCAAACTTGATGGTGGAAGGAGGAGTGTCTGTGAAAGTGGTAGATCTTCAGTCTCAAGAGTCTTGTTTGAAAGGATATTCTTTTCCGCTAATATTTACTTCACTTCTTGAATGTGTGGTCTGGCCCTTCCCCAGAAACGTGAATGACTTTTTAATAACAAAGTTTTGCATGATTTATTAAGAGAAAACGATGAACAATGATAAAAGTCGATGTCTTGTGATCAAGAGATCATGGCTTTAAGTCACGATCAGCCTCTTGCGGAGATGCAACGTAAATCTATGTATTATGACCCACTGTGGTCTGGCCCTTTCTCATACCCTGCTCATAGAGGGAGCTAAGTGTACCAGGCTGCCATTTCTGCTCTCTTTTTTTCAATCATCACTGATGCTCCTTCTTTCTCTTTAACTTCATATTTTCTGCTGGTTTCTTAATCACATAAATTGTGCTATAGAATCTTGAACAACCTTTTGTGTTTTACATTATATGCAAAATGACATGTGGATCCAATATGGGTTAGTGATTTTATCCATTACTACTATTTTTCATTACATTATTACTTTTGACTCTCATTTGAATACTTGAAATTTTCTACCAGTTTCTTTATTAGTTCAAGTGTGCTACAGAATCTTGAAAACCCTTCTGAGTTTTACATGATTCTTTTAAGATGTGCACATAATGTACTCACTAATTTGTTGTTTTGTACTCAACAACTGTGTATAATTGAGATATTCAATAATTGGATTCCACGATTATTCTGAATTGTCTCACATTCAATAATTTAGGAAATAATTTGACAAGTAAATAAGAACGAACCAAATATATGTTTATGGATATTTGGGGAAAGTTGGGAACTATATACTTTAGCTATAAAAAGATCATCACATGCCAAATATGAATATTTATAAAATCTATCTAACTCATAACACAACAGTATAAACAATTTACGCCAGACATTATAATTATGTTACAAGAAGCTAGGATTAAGCTTTTATAGAAAAAGTACTGGATATTCCTCAACAAAGATCGG

>LNC_000473

GAAAAATTTAAGACTGAAATTTAAAAAATAGGAAGAAATCTTAATCTTGCAATTGAAAAGATATTTTTAAATCATTCCATGTTGAATTTGGAAGGTTATTCAAGATCAACACGAAAGTTAGAATCATGAGTGATAATAAACAAAAGAAGTCTTAATTTCTAGATTATAAGAAGAAGTGTTTACTCCCGTTCATTTTTACTTATTTATTTTGAAATATATTAATGATTAATATGAATATTTTATAATGCTATCCATATTAATGTATCTAGAAAATGATTTTGAAAAAAGCAATTAATATTATAAATAAAGTATATGAAACTAATTATTATTATTTTTTCGTATGTTTAAAGTAACAAATAAAAATATAAATTTATATTTTAAAAATGGTAGACAAAATAAAATTGATCGGAAAGAGACTATTATTAACTCACTCGAGTAGGACCGCCGAGTCATAGAGACGGAGATCGCTGAGCGGAGAATTTAAGGTATTGTTTGTCCATATTTTAATGCTTTATTGTGGTCCTGAAGTAGTTCTTATTGCCCTGCAAGTGGTATTTTAATTTGAAAGAGATATTTTGAAAACGTTTTTGAATTTAACGTAAATTATTAGTTACATATTTTGAATTATTGACCGTTTTAAAGATACTCGTTTACTTGAGTGACTAAATTTAGATACACCTCTGACCACCCCTACCACTTGACATAGCAAGTAATCTCAAACTCTTGTATAGCATGGGACTATGTTGAGAATATAATCAACTAATAAAAGTGTGCTCTCTCTAACAGCTTAAGTTTTTAGATGAGATGGTCACACACTTCAACAGACTATTAATAAAAGGAGAGAGAAGAGTTGGAAATATCCTTAAAATTAACGAGAATTTAGGGGTGTATTTCACTCATGTTGCAAGATTGATGATGTATTTAAGTTCAGT

>LNC_000474

ATTTTTTTTGTCTTTTTTATGAAAAGTAACAAATAAAAATAAAATTTTATTTTTGAAATAATGGATAAATAAAATGAGCATAAAAAGTATCTATTAGCAAATACAAGATAAAATATTTCGAAAACATTATTTTTATATCACTAATCAAAATGACACCTTACATTGTTTTGGAAAATATTGAAAATCACGGGTGAGTAACTAATGTTAGGAATAATATCAGAATACTGATTAATCAATACTAGATATTCAACTATAATAAAATATACTAACGGTCATTAACAAGACTTCAATTATTAATTAATATATAGAAGAACAAAGACAAGAGTTTTGAGTTTTTTTTTTTTAGATATCAAAATTATTTTCCCAACACATTATACCATGTAACTATG

>LNC_000475

ATCATAACTTTTGAGGACAAAAACAAAAATTTATGAATATTTGAGGGACAATTTTCCCTTCTTTATTTGATCATACCCAAAAATTGACATATTCTTATTTGTATACGGTAAATTATTTATTCCACTTGAATCAAAGTACTGCTTTAGTTTTGCCTTTTGCAAATAATACGAGAATAATAGAAAAATAAGAACACTTCCATTTCAATAAATCGGCGGAATCATATGTTTCATTTTATTTAACAATATTGGATACGAGAGGGAATTTATTTGACAGTATTAGATAAAATAAAATAAAGTAAATAAATATATGTTATTTTTACCAACTTAAACTTTAAAATAAGATATGTTTCAATTTAACATAGTATCATAGCAGGTAATGATCGATTCTAACTCCTAAGTCCAAATCTCAAC

>LNC_000476

AGAAGATTGCTAGTTTTCTCAACCGGAATCTTAGAATATTAGGATATGAAAAACAATAAGTAAACCCTGAATGGAAAACAGAGAAGCAGGTATGTAGAAGATGAGTACAAAAGAGGTTGTCAACGACAAGGGAGAGTATAAAAAGCTGTCAGGCAAGACTAACCAGAGCAGTGTACGAAAACTGGGTCAAACAAAGAAGAAAGTATTGTACCCCAATCAGTGATGCAGTATGGGGCTGGTTTATCCCTCTGCTACTATTAGAGAAGATAATTCATGATTCTGCCTGTTTTTTTTTTTATTATAGAAGATTGCTACTTTTCCCAACCAGAATCTTAGAATACTAGGAAATGAAAGCACTGAACAATAA

>LNC_000477

GGCTTCTTCTTCGGTAGAGAACCATTTATTTTTTGAAGGTTTTTTTATTTTTTGGTATACTTCTCCATTCCTTTAGCTTGCCTTTAAGTGCTTTGAGCTTGCAAGCTAGAATATAATTAGACTTGCCTTGCAACCTCAAAAGACATTCACCATTCTCTTTTTCTGTCCACAAAACCTTCCGTGCTTAACCACCAATTCTCAAATTTGAAATAAGATTTGTTCTGTTCCCAGAGACCCATACTATCATAGTCAATTCTCTGTAGAAGTTTGATATTGCTGACCTATCATTCCACTCTTCTCTGTAGAAGTTTGATATTGCTGAACCTGTCATCTTACTCTACTCTGTAGAAGTTTGATATTGCTGAACCTGTCATCCCACTCTTCTCTGTAGAAGTTTGATATTGCTGAAACTATCATCCC

>LNC_000478

TGAATTAGGTGTGCTACTAGATACCGTAGCGAAGATCTTTAAATTATCTTTCCAACGCCGCTGAGTTTGCTAAGTTTCGAGGTCGGATGAGTGAGATATGACCTTTTTAAGATAGGCTGACCAGTTAAGGAAGGTTAGCCGAAAATAGAGAGGGGTATTTTGGTCTTTTCCTTACCAAATCAGATTTAATTCGTTTTTAGTAAGGTCTTAAGGGTCTAATCCTATTAGGTTCAGTTTTATAATCCTAGTTTACACCTAGGGTTTTAGTTGAGAGTTCAAGAACAGAAAAGAAGAAGAGAAAAGAGGA

>LNC_000479

CCAAACCCGGCCCAATTTCTTACCCACAAAAAGAACCCAGAACGTTCCAAAACATCAAATACGACATCGATTCCAAACATCAATGCTCTCACAACGTCCAGCAGTAGCAGAATATTCCAAGAGCAATTCGATTATTCCCTTCCAACGTTCACTAGCAGCAAACAGTTTCTCACGGCAATGCACGATTCCCAAATGGAAATATAAGAAAAGTAGCAGAAGCAGTGAGCAGTATGCTGCAATTTTGTTTTCTTCTCCAAACGTCCCTATCCTTATTAATGTCGTAGCAGCAGCCAAGCAAGTCGTTGGTCCCCCACTTTCCCCTTTCGGAATGGGGTTGAATTCTCAGGAAAATGTGTCTCCCCCAAATGGTGAAAGACTTTTGAGTTTGAATTTTGAATGGGGCCATTTCACCCGAATTCCCCCCAAAATCGTGCCCTAATTCCTCCTATTTAAACCCTTCTCTTCTTCTTTGTTGGGGGTGGGAAGAAAATTGGGGATATTGAGTTGGGAAAAAAGTTGGAATTTCAGTTGATACGAAATAGGTTCGAGTTTTGATTTTCGGCCTTCTTTAGATTTTTCGGTTTCCAGTAGCTAAAGACTACATACAAGAATATATTTGCAAGAAGAAAGGGATAGATACAGTTATTAAAACTGGAAATTCTGAGTCCTAGAAATCTGTTTCGAAGCTTGCATAGGAGATTTTTCATAGTAGCCTTGTTTGATTTTCATTTGTGGTGAAGTCGCTACTCCTCTGTTCGTTTTGGTCGCAGTCGCTGCCCGGAAGAAGGTAACGCTCTCTCCCTTTTAATACTTTCCTCATCTTTTCCGTTCCAGCATAATGTCTATAGTTGTGCTTTTGTTTTGTTTGCTATTGTTTGTTGTTGAGTTCTGGATGGTTTGATATAGTGTGTATTTCTTTATGCTATTTCAATGTAGTTGGATTCGTCTGTTCAAATCTATATTTTTTCGTTTTCGATGTCTTAAGATGTCAAAATGGGAACTCATTTCGCTAACGTTGATGGTATGAGCTATTTCTTCTTCAAATGAAAGATGATTTCTCAAACTGTCTAATATGGAAATTGGATGTTATATTATGTACTGTTTGAGATTAAGTCGTGGCTGCTCTCCCCTTCTTGTTATATTTGTTTGGTTTTGGCATCTCAAACCAACGTGTTGGTATTCATGGCTATTTGTTTGTCCTGGAAGTCCAGTTTTCAGTTTAACGGTATATTTTGGCCCAAATATGATTGTTTAGCTTTACTATTTGCCATGTTTTGGTGTTACTGTTTGATAGTATCATCCTCTAAGGATATTACTTTCTTCTTTTTGCCTATTTGTGTTTATATTTGCTCTATATTTTGCTATTATGTATTCCCCTGATAAGACTTGGCAACGTTTAGTTCTAACTAGTCCCCTTAGGAAAATTTTGGAAGCATATGGTATATGTTGACCAATTCTTATTTTTTTATTTGTTGTTTATAACCTCTCTAGTTATTACTTTTCTCTACTGTGCTATTCCATGCTGGGGATATTATAATTTATGCTTTGTTAGTTCTTATGTTTGGACAAATATGTATATTGTTCCTATATTTCTCTAATCACTGAAGTGTACTGTAATGAACAAGTTTTATCTTTGTTTCTCCATCTTTGTCATCTTGTATGTGTGTCATTAGTCTACATAATTATTTAAGTGCCTATAGTGTTCTCTTTATTTTTCTTTTAAAACTATTGTTGTCCATGTGCCACTGCACGATTCTCTCAAATGGGGATTATGTGTTAATTTCCACTTTCTTGGGTCAAAATGCATATTTTCCTGTAGGTTGGTTTACACTTCTTGCATCTATATCAGTTGGAATTATATATTAACTCCTTATACTCTTTTGCTTGCATGTGAAATCTGTCGGAGGTCACCACGAACTCGTTCCTTTCCCTTGCATTTCCAAGAGAGCCATAAAGGCTCAATTTCCTGTGTTTATCAAGTGGGCCAACTAAAAATTGTCGAACATGTCTCAGTTGGGAGAACACGCAGCAATTTTGAGAATTGAAAATTGGGCCTTTATTTCAGAAGACTGTATTTCGTTATTATTGGGCCTAAGCCCATTTATTCATTATGTTTGTTATTTTATAGAGTTTAGGACATGTGAAAAATTGGGCCTAAGGCCTAAAGAAAAATATTTATTTTTACGTTAGTTTAGGACAGGTGCAAGTGACTCTAATGGCCCAAGACAAAATGGACCCAAATACTGTCCCAGACGGACAGGAAAATCAGATTTGGGCCCAAATCTCTTTCCATCTTCAATTTGATTATGTTTGTGTTTGTTGTTAATCTTAAGGTCGAAAAAATTCAAATCCCCGAAAGACACCCATTTTGATTTAACAATTTAACTAAATCGGTCTTTTAATTGAACTTAAAGAATAATTTTGAGAAATATCTCACTTAGATATTAGTTCTACATTCTCCTCCTTTTCCCTTTAAAATGATTCTAATTATAAAAATTGTTCAAATCAAATTAAGTTAAATTATTTTAGCCAAATAGTTAATTGCCGGATAACCGCGTATTAGCGGACATTTCGGGTGCTTTAAAATTCTTCTCGAAATGCTAATACGAACCCTCGACCCCCTTAAATATTTTTCAATTGATTTCCTGTTTTAACCTTTTGAAAAAATAGTTTTCTTAATTTTTCTTGAAAATTAAGTGGTGACTCTTAAAACCAG

>LNC_000480

CCAACCCTTTCCATTTTTGCTTTGTTTCTGCTTTGGATGATCAAAGGATTCCATCTCCTGTTGTTGTGGATCATTTAATATTATGCTGTGAGATTCTTGCAAAGTTGGATTTCATCTATCGTTATTCATTTCTCCAGGTGGTGCTCGCCTGGGGTGCCTTGGAAGCTGCATTCCGCCACTTCCAACTGGGAATAAGTGTCTCCTCCAGGTTGACATATTGGATCTTGTTTGGATCTGGATGGGTGGTTTGGTTTTCCGTGATGATTGTTTTAGTTAGAGGTGGGAAATCCTCCGGTGGGAGGCTCCCACCGATGGTGTTGACTGTCATGTGGCCGGCGCTTTTCGTAAGGTCCCTCGTGGTCCTTCAATAACACTATTTTTCATTTTTACTCTTATTATTGATATTTTGTTATTATAATCTCGACAATCATCAGTTATCACTGTTACTTCATTTCATGATC

>LNC_000481

CAGGGTATATAATTTGTAAAGATTATGTGAATTAAATGTCATGCAGAAAATGGACTTTGAGCTTTGGTATGGTGCTCAAAGTTAAAGGGTGTAGTACCTAAGAAATGAGAATACCTCAGATGTGTTGAGCAGGCCTATTTTAGGCTGCTCCACTACATCTAAGTGAGTTTTGCTTGTCTATAAGATCTATACAATTCTAAATTTTCTTGTCATCTGATTGATTCATGCAATCCTTGTCATACGATTGGATGGCCAGCAGGTGATAAGCTAGGTCACCGTATCCATCTCCATGCTTTTTTTGCTGCCTATTATCCTTTGCCATAGTGTCTTGCCATTTTCTTGTCGAGGTAAG

>LNC_000482

GATAATTATCACCACAGAAATTTTTTGCTCATTTTTCTGTAAGAGCCTTTTCTATTCAATCTCCCATTCTGTTTTAACTTATATGCTTTCTAAGCTCGTTATACTACAAAACTGTTGCATAAGGCTATACTTTTGACTTTAGAATTCAGAACTGTAAATTCTATAGAACTGAATTAACTTTCAGCCAAGAACTCTTTGTTTCTTGGTTTTGCTGTATTCTTTTGCTTTTGTCAATTTCTCCTGTGATCTTTTGAAATTTTACTATAAAATAATTCGAACCTTGTGAAAATATGTTGATTTGGATCAAAGGTGATTTTTAATCTTGAACTATATGCTTGACACAAAAACAGTAGCGGTACCTTGAGGATGAGGTTTGAACTGATTGGCCT

>LNC_000483

GCAAAAAGAAGCTCAAAATCAGCTGTAGTTTAAGATGGGGACTTGCATAGAAAGAAAGTGTCATCTTTTAAGAAGAAAAGAAAAAGAAATTTTAAGGAAATACAGAATCTTAGCCTTACTACATAGAAAAGTCAATCCGATTGATTATTTCGATGTTTGGAAGTGGGGTTTGGAAATAATGGAATGTTCTCTGTGAGGTCTTTTATAAGAAACTATTGGTTAGGGAGGATATGACTTTTCCATGTTATGGAATATGGATCTCAAGGGGGCCGAGGAAGGTGTGCGTTGTCTTCGTTAGTTAGTGTTACATATGTAAGGAGGCAGGTGAAGATGTGGATCATCTTCTCTTACATTGCGGGCTAACCATGAGGCTATGGTGGGATAAATGTTGTATGGTTAGGCACTCCGTGAGCATTGCCA

>LNC_000484

AGCAGCTTGTGGACATTCTCTTGGATTCAAGTTTTGCTGTAAATATTAATTGAACAGTCTAATTAACTCCATTTTAGTTAAACTGCTTATTCATGTACTTCTCTATGTCCTTTTCTCTGTATCTTCACAGAGGAACATCATGTAGTAGTTCTATTTATGTTATATATTGTGATAAGGGCTATGAAAGCAATTTAAATAAGTTCTCTAGTTTTGGGCTTTCCCGTAGCACAGTTAATTTTCTGCACTAGTATCTTACAGAGATTTTAAAATACAAATTATTGCAATAGTGGCATCCGATAATGTTGTGTCCTGCATGTGATATATGAAAATCATTTGAAGGCATTGCATATCCTATGAAAATCATTAAAGGCCTTGCATATCCTATTG

>LNC_000485

TCTGTTTATTTTGGTCTTTTTTTTTAACCTCGAAAACCCCATAATTAATACACTTCCCTGTTCGCACTTTTTTTTGGGTCAATTCCTGTTAGAAGGGAAGAGCTAAACGTGGGAATTGGGTGGCTCTGAGATTTTGGATATCCGGAATATGATTAAGATATTAAGCAATGAAAAAAATAGGAGAAGTTGTTTAAGAGCTATTATATGGAGAAAACTTAGAAAGATTGAATTTTTATGGGTTCTTTTTTCACTTTTTCTAGCAATATGTGAACTATCCCATCTCCTTTGACATTATCTAAATGAGGATGAATTGTAGAACATTTAATGTTCAAATTTAAACTTGGATTTGATTTAAATATTATGTTATCTACACTATCTTATTATACCATATTTGAATATTCTCATATCTACACTATTTAATTATGTACCATAATTAACG

>LNC_000486

CCTAGATGATGTATTATTGTAACCTGGGTAGATCTACAAAAGGATCTGCACCCAATGGTGTGACCTAGTGGTCAAAGAAGCGGTTGAGAACCCTGAGGTATCATATTCAAAACTTAGTGAAGACAAGAAAAATTAGGTGATCCTTCCCATCTGTTCTAGCCTTGGTGGATAGAGTTACCTGTTGCTGGTGAGAGGTGGCAGGTATCCCGTTGAATTAGTCGAGGTCCGCGAAAGCTGGTACGAACACCACGGTCACCAAAAAATAAGAAGAAAAAAAATTCTCCAAAAGGAACTTTCTTGTACTGACATGTTATTGTGCTTGCAACTTGGACCTTTAAGAGAAGCTTGTCACTGACTAATGCTTGTGTGGTTCTATTGTGCAGTGAGTCTGATATTTATGTGATTTTTCTAATATACCGGGATAGATTCTAGGCATATAGCCTTGAGCAACATCCTAAAGTGCT

>LNC_000487

TTTGCTTTCGTCATCATGTGATCTGATGTTTGTTTTTTTTTTTTTTTTGCTTTCTTCATGACTTAAGTTGAAATTTTTTTGAAAGTAAAGCAATGGGTGATTTTAAGTCATGTCCTCAAAGTTTAACATAACAATGTTAATATTAATTAAACAATGGTAAAAATGTAAGCCAGTAATTTCAATGTTTTTCTTTTAATGATCTTGACAAATCTTAAAAGAGTAGCACATGTGTACATCTTACTCACAGACTTCTTCCCTTTCTCAAGACGCTGAATTTGATGTCTTAATGGATACAGTATAGCATAAAACAGAGGAGTTCTCTTTTTATAATTTAGATTCTTTCCTCTGTGTGAATACTTACAGCTGAAGAGTCCGGAAAGTTCACAAAAAATTAAAATTAAATTAACCTTAATGTATTTAAGTTCATGCTGCATACTATTATTGTTTTCTGATGTTAACACCCACACCAACACACACCCACCCACACACACTTGGAAATTTTAGAAATTTTGGTTAAATTTGTATCTAAGTTCAATGAGCTCATTGTCGAAATGGATAGCCGGCAATACTTCTGTTGTTCCCTATTTATTTATTTTCGAATTGGATTTCTGGATTCGCTATTGCATCTGTTGTTCCCTAAATTCTTTCAGTTCAATGTCTAAATTGATTGATCAAGGAAGCAAAATCTCTTGAGATAGAGCACTAGCTTCAGTACTCTATACTTCTGTTCTTATATGGTGTAGATTCTCTACTTTTTGGTATACCTTTAGTATATGGGAGATTTTCACGTTACTAAAATTACTTACCTTCTCAAAATATATTATTATATG

>LNC_000488

CCTAAATCTCTTTTTCTTCATTCTACTTAGTTATTAGTATTGTTTTTGTGCTTATCCTTTTTTTTAAAAAAAATATATTTTGTTTTTCTTGTTTTGTTCTTGAGTTCCCTAATTTCAGTTTATTCTGGTCACTTTTTAAAGCCTCGAAACCCCCATAATTTTTATATTTTCGTATTCGCAATTTTTTTGGGGGTCAATTCCTGTTAGAAGGAGAAGAGCTTAATGTGGGAAGAAAGTATTTAAGTGTAGGAATTGGGTGGTTTTGAGATTTTGGGTAATTGGAACATGATTAAGTTATCAAGCAATGAAAAAATTAGGAGAAATTGTTTAAGAGCTATTTTATTTTGGGTTGGAAGCTGGAAAGGGTAGATTATAGGGAGAAAACATAGAAAGATTGAATTTTGATATAGAAACTTTAACTTTGATATTATCTGTATGAGCATGAAGTGTAGAACGTTTAACTTTGACTCTTCTAATATATGAGTGGCAGATAAAAGTACCAAAGTGATTGCTAAGACGTTAGTTTGAGAGAAAAACACTGTATCCAAGTTTAATTTATATAGGGATGTAAAATTTTGGAACTTGTGAAAATTGTATAGAATAATGCCTGTCTGCCCTTATTTATATGATAATCCTACTTTGGTATGTTCTAAATGTTACACTACTCTAGTTTTTGGCATTAGTCCCATATAGCTTTCACGAATTTCAAGAAGCTAATTCCTTTTATTACTCAATAATCTCTTCTTTCTAACTTGTGAGGGTGGTTTTTCCAGTCTTCTAGGCAATACCTACAGCACCTGTAAAATTATTTAAAATGTGCAGTTAGTTAAGCCTGTTTATCAACTTTCCACTTCCTGGCAGTTTGTCCTTAAAATGACACAAGTTTCTTTTTGGGCTTATATAGATCTAGGTGGCGTTAGGTATTTATTTATTCAGTTAATTATCTATTTGTGAAAATGTTTCAAAGCGCCTCTGTGTGGTATATAGCATATCCGCGTGTGGTGCCAAATTCTATCCAATGGATAGGTT

>LNC_000489

CATAAATCAAAATATCTTATGATATTTTTTTCCAATTTGTTTAAGTCATGAGACGGTGAAAGAGTCAAAAGTTACATTCTGTGATCTAGATCCTCTTGCTGCCAACTCCAGTTCTCATGATAAAAGGAAATAGTGGATGTATGTCTCTCTGGGTATATGCCTACTTTTTACATGATGTAAACTGACCCTCTACCAAGAGAAGCTTACAGTTAGCACTTCTATAGATGGTGTATGACACTTGTATTTGATTTACTAAATCGATGAACGTACTGTTTTTATTTCAATTACTGTAGAATCTTTGGGTTGTCTTTCAAGGAACCGAGCAAATTGGTTGGTCTTTTTCATTTCCCTAAAGGAAGACCCTTGTCCCAGTTAGATGGTCCCCTAGAAAAGGATTTTTTTTTGGTATAAAATACGTG

>LNC_000490

GTCTGGATAAACTTGTCAGTGTGGATCCATGTGATAGGTGGATTATTGGTAGGTAGAAACTTTTGATATTTGTCGGGTTGTTTAAGAAAAAGAAGAGCAGCCTTTTGGCATCTGTAGGGGAATATGTGAAAAATATCTTGTGCATGTAATTGCATTGGTGATTTGTTGGTTCAGTGTGTCAGTATTGTGACTCTAATTCTAGTTTTTTGGTTCAATAATGCAGAATGACAAACCCCATCATATGTTTTGAACCTCTAACTGCTAACCTTGAC

>LNC_000491

TGGGACTAATTGCTTGTCCGGGTTGAAGGGAGTCGAACCAATCTATCGTTCAAGGTAACAGGGTATTAAGAAAATCGGCATGGGGTTCCGTACCTTATACTGTTAAAAGCGACTCTAAATTACATATAATAAGAACACGTCTTTACTTCACGTCTTTTCGGTTAGATAATGGACGACGATAAGCCACCGCCCAGTAGTGCTCTCTGAGCTTGATTGCTCCGAGCGGTAGGTCACA

>LNC_000492

CATGGTATCAGAGCAGGGCCCGTCTCACCCGATGTTGGGGCCCCCAAAATCAAAATTGTCCACGCACCAGATGCTAAGCACTGGGCGTGAGGTGGGGTGTTAAAGAATGACAAAAGTCCCACATCGGTGGTTAATGAGATGGGTGGACTCCTTATAAGGCTTGGGCAATCCTCCTCCCTTTGAGCTAGCTTTTGGGGTGTGAGTTAGGCCTAAGACCTAATTTAACA

>LNC_000493

AAGCAAGACATCACCGATGAAGTTAGAAAAAAGCAAAACTTAGAGGAAATTGTGCAACACATGCTTCTCTCCCTTGTGTTAGAGTAAAATCAGCAAAACAGAGATACACAATTGTGCATATTTCCCAGATTTGTTAGAGGAAACTTAGTAGGTAGAGATACAGACGTCGCTCTATTCCAGCTTATAAAGAGGTAATTTGGGTCTCTTTGTTTTGATCCCAGAACAACAAAATGAGGTTGTGACCTTGTTGTGATTCATATTATTCGGATGCAATCTTGAAGTTCTGTTCTCTTCAAGGTTTACATGAATCCAACAAAAATTCAGAACTCTGGAATGGCATCTATTGTCAATCCTAATAAGCATGCATACCTTTCACTTGGTGCCTTAGCAATTAGGGGACGAGGGGAAATGTTACAAGAGTAAGGGCTGAAAAAAGAATTCCAATTGACCAAAGTATGAACATTTAATGCATCAACATTATCAAAATGAGCCCATACAAAATAATATTACTGTACCTCCTGGTTTTGATCCAATTAAGAAAATGTTACCTGCTCTCCAGAAGTTCAAGTGATGCAAGATACTCAAACAAATGAAATAAGTATTTATTGTCTCAAATTGTTATCTTTGTTGTTTTTAGTTAAATGAAACTCTTGTTTATGTCTGAATTGAATTAATTTGCAGGTGCACGTGAATTTGATAAGGCAAAAAGAGTGAGAGGAACG

>LNC_000494

GTTTTAAGCGCCTTATCACGAAAGTGACCGAGCAAGCGAATGATGATGGTTCAGCCGGTAAGCTAGCAAGCTTACGAAATGAAGGAAGAGGAGACCCTTCTATTATAAGTTGAAAGCGAAGTCGATAGTCCTCGTATTGGTAATTATTATACGCTAAAAGTCTATATAAGATATAGAAGGAAAAATATGATAAAATTCAATAAATAAAGAAAAAGAAGAAGGCCCTCTCCTTACAGTCAAGTGGCTTTCAGCTCCTTTCCTTCTCCTCGCTACTGTCAAAATGTTGAATTTCTTTTTTTACATA

>LNC_000495

TGTTTGGGTTGCTCGACTCGAAGTCATTCGGTTCATTTCGCTTGTCATTGTTGGAATCATTTGAAATAGGTGTAGGAGTCTAAAAAATTAACCAATATGTTAGCTGTATATGGAATTATCTCATTGTTTTCTGAGTGCAACAACTCGATTTGATCTTATTGTTGTATTTGAATTACCTTTTATTCTAAAAATCCCACAAATCAGTTGTCATGTTGATATGTTACTTAGTCCCTGCCATAGCTTTTTTTAGATCCATAGCTACGTGTTGCTCTACGCCGTGATTTTTGCTTCTTGTTTAAGTTTTTCCTAAATTCGTAGCATGTCTCTATCTTCATTTAACTTTTCGAAATTTCGAAATGATAGTTACGATAAAAGTTTGAAAGTGCTTAACTGTGCCTTGCTGCTAGAATAGATATGCAAGTGCTGAAAAATTGTGAATCATGTTTTGTTTAAATGATAAATATTTCACTTTCATGAGAGCA

>LNC_000496

CTAAAACACTAAAACCCTATTGCTCTTGCCAAATCTCCTGCTTCCTTCTTCTACTTCTTTCTCTATTTCTCCCAAAATTCTCATTCACAAAGAGGCGCCGCAAAATCTATTTTAAGGTTACTGATGCTGAAATTTTTCATAAACAATGAATTCCTTCATTTCTTCGACGAATAATGTGATGAAATCTATAAAAGAATGGTCCCCCACACACAAGCCTCTAATGCCAACGAAAGGGACCACTACGAGCTTACCATGAAGTATGAAAAGGAGAAAGTTCAGATCTGAATCACCAAAAGCAAAAAATTAAAAGATTGGACACGTCCAATTGCACTCTAGCCACGCATCCACGCACATCGCGTGGAATGCGTGGCCACACTTGGGCAGTTGTCGACACTTGTCGC

>LNC_000497

TTTCACTTTCTCTAGAACTTTCTTGCTACCGTCTCACAATTTCGTAAGGGTTTTTGGTCGCCTCCTTCGTAAGCTCCGTCGATTTTGTCAACTCTTCTTTCCATACAGCTGAGCTCTTCAATGGCCGATCCATCTCTTCTTCAGCCTCTTCTGTTAGTCTGCAGAGTTTCAATTTCTGTCTGAAGCTTTCCTCAACATTTTCGTTTGCTTTGGATGCGCTGCGCAGGTATTTCTTTATCAGTTAACCTAACTTTCTCCGATTGCTAATTTGTCTTGCGTAGATACAGCTAGAATCAACGATTTACATGCTCAGCAAGAGAAAACTACCTAAAACTTACCACCGAAGAAAACACAAATAGCATTTCACATTTTTTCTGGTAGTGCCACAAATCGGTCAAACTGATAA

>LNC_000498

CAAATCTCTCAATCTTTCACCACCTCTAAGTCTTGCTGCCACCACCATCTTCACCGGATTCACTATTGTTGATTCCAAACAAATTTTCAAACTCATATAAAACTCTTGCAAAACCAATCAACCTAAATCAATTGAGATAAATTTACATATATTTATATCACCCCAAGATACTTTGGATCTGATTTGAGAAAAATCAATATGGGTTTACTTTAAATCCGATTGGACCAACCGAAAAAATATTTTTTTTTACTAATAGATCCTTTAATTATCTATATCAAATCTCAATCAATCAACTATATCTCAAAAATTGTCGCTATTAATCTTGTTAATCC

>LNC_000499

AATGTGTATTTGTCATGTAAATTTTAAAGAATTCCATGCTTATGCCATCCGTGCAATTTTAACCAAAAGATTGTTGGTTAAATAGTATTTCAGAGACATACTTTTAAATATGCTAATCTGGTTGATGTTTCTGGAGTGGATCTACTGATATTCTTTGATACCCAACTCTTGCATACTTTCTTGTGCCAATGTACCATTGGAGGAAGCTGTTATTAGCCAGTATCCAGAACTATGCTGTCCCAGTTCCATTAAGGTCGCTATTTGAATCTTATCCATTCTGTTCTGC

>LNC_000500

GTGCTACCTGTCATTTCTATAACCTGTTTTTGCCTCTCTGTCTGTCATTACTGTGTCCATCTTCTCCACAGCACAACACAAGCCATAGAAGACCACTCACAATTCCAACATTCTTATTATTTTCTTTTTAATCTAAAGAACTCAAATCTACTTGGAATTGTATACGGAAAATGGAGATCTGAAAAATTGAAATCAGATTTGAGGCCGAGGTTTGAGCTTCACTCAACGATTTTGGTCATAGTGGAGAAAAGCAAATATCCAAGTTAGAAGAATGCTTTACTTGTAAATACCAAATGATGAAGAGACGTATGGATGGAGGAAATTGTGTACTGGAAGTAAGGTCATGTTGGTCTACAAGATTTTGACTTCATCTAATGAAGTTTGTACAACTTAGGAAGTATAGATGTTTAATGAGAATCTTTGTTTAATTAGGTTCCTTTTCAATGTTCCTTTAAGAAATTTGATTGCAGCAGTAAATTTATAATTTTGATGTCAAAAGTACTCCAAATCACGTGTTTGGCCCGTGCTGCAGCATTAAACATCTAGTGATTATTCAGAAATAATACTAAAATTTGAAGAATAAATAATGTAATAACATTCACTTTCTCATCTGAATATAGAAATTTGTTTTTATTAAAATTAATAAATATAAACTCAAATAAAATACTTAGCAATCTAATATTATCAACAAAGTATAGAAAAATAATTGTTAGCTATGGTTTTTGGTAGTTAGTGG

>LNC_000501

GGAAGCGTCAAAACCCTAGGGTTAAGTCGGCGGCCGGGCCTTAGCCTCAACCGGCAGCATTAGCAGTGGCCGCCCTGTATTTTCAATATCTATACCCTGTATTTTCAATAGCTATAGGTATTTTTGTTTGTACTTACTTGAATATCTTGCTGCTGCTATGAAGAGAAACGAAGAAATTATCGGATTGCGGTGATGTTTTTCATCTCCGCAAGATGAGGTTCTATCTTCTATGGTTCGTATTCCCTGAGCAGATCTTCTCTTTTGTTTAGTTCGATTAAACATCTATTCATATTTTCAGTAAAAAAATTCCTTCGATATCTGTTTTTATGTGGAGTAATTTGATTGCTGAGTTTTGCTATTCATCGAAGGTCCTATGGACGAAGCGCCAATACACAATGCGCTTTCGGCCAAGTTTGCTTTCGGATATCGATTTCTGAATCCAGATCTCCGAGCCTGAATCATTCTGGTTCGGACTGCTCATAATCGGATCTGATTTCTGACATTTGATTTTTTATTTAAAAGAAGTAGATGAAAGCGAAGGTACCCAGCGGAGAGGGTAAAAACTCTTGAACGAGACCTCTCTAACACTATAAACAATACCTTCGCAGATTAAACATTTGCCGAATCGTAGGAAGCTCCAAATTTTCCATTTTGGTGCTTCTTTAACCCTTGGCTAAATTTGTACTTCCTTTATCTCTATTTTCTACTATTCATTTGCACACTTGCTTTTGAAGTTCCACTCCTTCGGTGTCTATTATGTCCTGCGCAGTGGTTCGAATCAAATACTGCATTTATCAACATGCAGTTCATTTATTTATATAAATAAACCACTTCGCAACAGTTTCTTTCAGCTTAAAGAAATAGTAATCTCAATGAGAGGAGCCCAGACAGTAATATCACGAACAAAAATCATAGCTGTCTAATGTCATGATAGCAGACTCTAAACGAACAAATTGTGAATTAGCTAAGAGATATTTGATATTTCTAATGAATGAAGGATGCTGAATATTAAGTTTGAAATTTTATCATTTTTTGATACCAATTGTTAACTCATCAATTTCAAAATCCAAAGTCATCAAGTCTGTAAGCAAATTGAAGAACTTTTGTTATTGTTTTAGTTTCTTTTAAGCAAATTAACCTAAGCAAGTGACATGAAACAACACATTTCACATATTCATAAATGATATAAGTCGATTTACATATTCACATTCGCACAATCGACTATCATTTTAGAGTCGCTTTCTGCCATGATGTCGGTTAAACCAAAAAAGGAGGGCTGAGGCCTCTGCCCATATGCTAGTGCCTTCACCTAAAACAATCGGCCTCTAGGTTTGATGTTATGAAATTGGTTGAAGGAGATTAAGGAGAGGGATCGCCTTATAAATTAGCATGCCCTTATCGATATAAATTAGCTAATTTGTTAAAGTCTCTAGTATAATTATTAAAAATATCTATATTTTGAAATAATTATTTAAAATGTCAATACTTAATAAATAATTGTATCTTAAAATAACTTCTAATTTATCTCAAATTTTTTTCTCGTCTAATTCTTTGTTCTTTAATTTTTAAATTCTCTCTCATCAATAACTGCGGATTTTTTCCCCAGAAATTGGAAAAATATATCTCAAAAGATATTCTTAACATTCTCATTGTTTT

>LNC_000502

TATTTTTAATTATTTTTTTCTTATTTATTTCCTCTCTTCTTCACAACAACATAAACCAAATCTCTCCACTGCTCACCTTCTCTAAGCTTGGATTCACCATGGAATTTCATTATTCCTCTTGATTCTAGAATTTTGATTATTCAATTTGTGCATGAAATTAGGAATTTTTTTGAAAACCAACTCTTTATTAATATTATTCAAATTGTTAATCATCATGCCTAACCACATGGTCAAACGGGTTAGAAGATTCTTATCTTGATTTTTGGTGTCCTAGTTTAATAATTTAAATTAAAGTTCGAAAAGAATTTTGGATTGTCAACGAAACTCATATGTGATTTTGAAAATAGAAATGAAAAGATCCATTGTTATATATCCATTACCTCAATTTGAAGAATTCGACAAATCCATCCATGAAAATAACTTGAGTATCAATCAAATAGTGGATGAAACGGTTAGGGATAAAAATCTCATCAGATTTTACCTTTCAAAATTGTTGTTGCGGTCATCGATAATGAAAACATGAAACATATCATCAACTCCTTCAAAACTTGAGTTTTTTTTTATATATCAAAGGTTTAGGACTTGATAATATAATGGTAGGAAGGAAGAAGTGAGTTGGGTTGATTGATAAACTTTTGATGTTTTCAAGGAAGAAGAAAATGGTGGAAAAAAGATTAATCAATGGCAAAAAATGAAAGATTATCAAAGATTTATAACTTTACCTTTCAATAATAAAACTTGAAATTTCATAACTGCACATTTCATTACTGTAAGCTCAAAACTATCATAAATAAAATTTGAATTTTTTTCTTCAATAGCAGAAAAAATTTCATCAAACAAATGTCAAGAAAAAAGAAAAAGAAAAGAAGAAACAAAAAGAAAAGAAGAAAGAGGCTGGAAAGAGAAGAAAAAAAACATAAAAGGAAAATAGAATAAAGAAAGAAATAATAAATTTTTTATTAAAAAAATAATATATATGACGTGGAAGGCGAGTGCATTCACTTTCTAACACAAATCTGGGTGTGACCTTTTAGGGGGCTATATATTCTATTTTAAAAATGTATAAGGGGGTAATAGGACCCCCGTGAAGTATAAGTGTGTAGTTGAAAATTCGGTAATAGATTAAGGGGGCATTTGACTATTTG

>LNC_000503

TTCCCGATCAATTGGTTTTTCATTATTTAATAAGAAAGATGGCAAATCCTACCATTGTATTTCCTTTTTTCATTTGGATCCTATCAACTTGGAGAGTGTCTAGATTAAGACACTTCTCGTATTCGGACTATCAAGATCGACTAAATGAGAGTGTGGCTGACATGGTAAAAGAACAAATCAGCAGAGAGATAGATCACGCCTAAAAGCTGCCATTTCTATTAAATACGATACCGCTCCTCAATCTTATTCTGTTTCCGATCCTGCTTCAGATTCTCTTTCAGTTGTGGAAACAAGCCCCTATTCCTATCGGTTGAGTAGCCCAAGCTCCTTAAAGCGTATTGTCCCCTCTCCTGTGAAAGAAAGAGGTTGATGCACCCAGCTACCCGAAGTAGAGCTTTCTTTGTCCCACCTTTCCTTACTGACCG

>LNC_000504

GAGATGTTGAGGTTTATCCATAATTGTACAAGTATTGGAATATTGTGTTTTGAAATGAGAATTATTGAGATAAAGAAGATAGCAAGAAATATTCGTATGGCAATAGGTCAAATAGAGAAGATAATGAGATGCTTTTATTCTTTAATCACGAGGGGTTGACTTGTGTCTTCAGATGGACACAAAGATTATAATGAGTCTCTGTTATGAATCAAAAGAAGCTGGTTTTGCCACTCATATATATCAAGAGCCAAGATATCAAGGTCCCACTAAATTCCCTTTTTCTATTCTGTAACAAGAGTATTTATTTTTGCCTATAAATAGGCTGCTCTAAATCACTCAATTTGTCAATCAAAACACATTTAATTAAAGATTCTTCAGGGCCCTTATTGCTGCCTCTCCCTCAAGGGCTTCTCTCCTGCATGCTTATATGTTGGGAAAGAAGCTCTAGCAGGGAGAGCCAATAACAAGTGTCTTGTTTAACAACTTATGTTGCTCTTCACAGTTCACAGGACAGTCTTTTTTCACTCTCCCTCAAAGGCTTCTGGCTTAAAATGCCTTTTGTGCTAATGCTAGAGGTCTTTCGAGTGAGAGTGAATAAATTCTGCCATGATATATATAATTGCAGAATTATGACTCAACAAAGAAACTGTTCTTCTTCAGGGCTCACGTGAACCTAATAGCCTTATATATTTAAGGTGGATCAACGCTGTACAATTGGCGGAAAAGTGCTCTACGATCACGATCAAGAGAGAGAGGAATGAGGATCTAGTTTTAACCCTGA

>LNC_000505

CCTGAGGCTACATATAGAATTTTTTGAGACTTTTTCTGGTTGGAGTATGTACTTTGCAGTAACTTTTTGCAAGTTGCGAGGAATATCCTCCACTTCCACTAGTATTTTGGACCAAGCATGGCGCACTAGGAGATTATAGTGTCGAGTGGAGTTGTGTGAGGAAGGAAATGCTATTTGAGTTTGTAGCTAATACTTCTATTTAAAAAAAAGAAAAAAAAGAAAGAAAGCCAGCAAGCTAATACTTCAATTTGAATAATGCATCATCTTTGACTATTTGGGTAGCTTAATAAGGGTTGTAGTTGCTAATGTTGGTTATATAGTTGAATTTTCTGCCGTTCTCTTCATATGCACCAGCG

>LNC_000506

AATTTTTTGAAAAATAAGGTGAAAAAAAAGAAGAAAATAATAGAAAAATTTGTCACCGTTCGATCATCCCAGGGTCATGATCGAACGAACTAGGACTCTTACAGCTTTTCAATCTCAACAGTCCGTACAACTTCCGCACCTCCTTCGCCCCCGGAACAGAGTCCTTCCAGTTTGCGCCACCTGTCTCCTCCGCATGCGGCGGCGGCTCCAGCCGTAGCGGCGACGATGCACTGTCACTCAACCGTTTTCCCTTCCCGCCGATCTTTCCCTCCGTTCCGTTTGATTGTAACGCCGTGATGACGGACTTTAGAGCTCTGCTTGGGG

>LNC_000507

CTTTATTTTACATTAATTAATCCCTAACAAATTATTAAAATTCTCTAATAATCATATCTTCTTCCAATCTAAATCCTACCCACTTTTATCCCTAATCTGACTAGAATAATTATTCATTTGAAGTTATATCACAATTCTCTTTGTCTTCTCCGGCGAAGAACAATCAACCTATCACCACCACAAACCTATCTCCATAACGTCATCCCCTCCTATTTTCCCGACAACCAACCCATCTCCAACAAAGAACAACCACCAAACAGCTACCCAAAACTCCAAATCAGTCATTGACGAACACCGAATCAAACCCCTTTACCTTACCCCTTTTCGATTTTCTTACTTTTAGTTTTTATTGCAGGTGTTTCGGCGAGCTCAAAGCTTCAACCGTGGATCTAATGATGTTTCAATGAGCTTCGTTTTTAGATCCATGACGTTTGACTAAGTTTTGTGCTTCCAAATCTAAACAACCAACAAATAAGGC

>LNC_000508

CTTTTTTTCATCTTCTTCTTTCTATTTTGAACCGGTGAAAAAATAGAAAGGTAAAAAAAAGCTCCTTGAGAGAAATGGTGTTTTCTGCAATTAAGCAGGTACAGTGCTCCATAGTTAAATCTCAATCAAAGTACACTCCGTACAAAGTATGTCCCCTCTTAAAGAATCCCTAGTCATATTTTTCTATTCCTGTGTCCCTACAATCTTGGAGCGAGCAAGCATGGGGTCAGTCATATATAATTGTGGCCTTTCGAAACCCTAGGATCAATAGCACCGTTACCTGATACAAGGCTTGAGCAAGGAGATTCCTCCACATCACTCCAGTTATCAGTGGTTTAGTTCGTCCAACTGGTTTCTTGTTCATTAGATCGCAACTGGGTCGTTCAGTTGCCAAAGCTAAGGCCCCC

>LNC_000509

ATTGGAAGTATTAGAAAGAAAGTATGGATTCACCTGATCACATCTGAGATATTATCTTTGATGGTTTCTGTTTATCTCATGTTTCGTCTTGATTGTGATTTTTATCTCCTTCAAAAAGGAGGTAGAGAGGATATCAAATATGCTGGATGTGTTGGGTGGTTGTGGAAATTATTCATTTAACATCTTACAGTATATCCATTTTCTTGTTCCTTATCCTTTTCCTATTCCTCATTTCAATTACAATGGTAGGCTTTCGAGATGGATTCCGTACTTTAGAGAAGACCTTAATTAAATTTTCCTAATAGTTCCCCTTAGCCCATATAACATTTCAAGTTTAATGAATGAAATAAAAGAGAAAAGCTAATTTTCTTGTATAAAGATGTTTTTTGTGTAGGAGGTTATTCTACTTTCATTTGGGTTTCTTTCAACTTAATACACTCCGC

>LNC_000510

GTGATTCCAGAGTAGTCTTCTTCCTAATTTAGGTGTTCAAAATCGAATTTAGACGTCGATTTGACCATTTTTAGGGTGATATTCCTCGTAATTTCTTTAAATTTTGAAAAACATTAGTAGGTGATAATTAAAGTTATCTATTGAGTGATTAAGTTTTGATTGCAAGGGTATGCAAACTATCAATTATGCTTCAATTTCAAACTAGTTGAGCTTGGCTATATAAGTGTCCTGTATCAGTTTTACTTTATTCAGACCCTTTTCATTTGATGTGCAGTATGAATAAGTTGATGGTTAAGTCTCATAATTTTGACCTTAGAATTGAAATACTTGTAATTATTTGAAATACTTGAAGTAAAGTCCTTCCAAAGGTACAAGTGAAATTGGATGAGATCAGTATTGCGGTCTTTGACTTGATACTGCTGAAGTGAGGTTGTCTTAGAAGAAGGAGATTAGGTGTGGTTAGATTAAGTCACTTCCAAAAAGTTCAAGTTATTGTGATCAGAATTAGAGTTAGACGAAATCACATTTTAATGGCCATTTCTGCATCACGAATCATGTTGTCTTATATGTGATGGGTGATAGGGTGGCAGCTGCCATATTGTTTGAATGGGTTTCCGATCCAGTTGATGTTCCGACATCCCAAATCGAGGTGGCTCTAAACTCAGGTTTCCTACCTCTCTAGTTACAGAGAGAGGAGAGGGGCGTTGAAGCCTTAATTCGAGCTAGAAATCTCGCTAGAGAACTAGCCATATCAATCTAGATTCTAGATGGTGTAAATACTAGCTTGTGCGCACCCTGAGTATTCCACCAGGTACCTCCTATCTTCCAACAACGTAGATACTAATTAGATGTAGACAAATGGAAAGAAATCACCTAATATTTTTTGCCTATGCTGGTTGTGACAATCTTATTCTGCAAAACTAGATATTGGTTTTCCTAGAAAGAAAAAGAATCAGACTTGCAACTGCCCTCCGAAACCCACTATGCCAAGAACCCCATCGATCCAGATGGGATAGTTTGTTTTTACATGCATTTTAGCTGCAAAACGAAGAGTGAAATTTTCTTGAGAATCAACTCCAATCTCCCACCCATGCTCTGTGCGCATACGAGCAGTTTGTTTAATAATTTGTTGAGATTTATCTTCTTTTGTTGGGTTAATAGTTTTTGTTCTTCCAGATGAAGCAAAGGGGTCTGACATTGTGTTAGAAGCGATACTGGAGGCAGAAGTGGCAAATAATGCCAAAGAGTTCTTCTCGCAGCCACCAAACCAATTAAAAAGTGAATGAAGAGGTACATCTGAAGAAGATTTGCGCCATTAGAGGTCAGAAATCAGCTTCATGCCAAGGGAGCCTGATGAAAGATTGCTAAAGACTGAATGATTTTAGCCTAATTCGAGTCTCTTGTTGTGGTTTTTGAGCCATGATACCTCGTTTGTTCAGATATCATAGGCATCTAAAGAACATAAGACCCGTGTTGCCACAAGGGAAACTACTCATCTTGTTTGACTGATGGTTTTCTGCAACCAGTTCTACCTATTTCTTAACCCTTCTAAATGACCCGTGTTGACAGATGCTTGGATTTTAGAGTTCTTTCATGTTTGTTATAATTTCTGAACTTTCCCTCTTCTCCGGCCAAGCCGTATATGTTGCATATGAACTTCTTTGCAATATAATTGGCAGAAAACTGTTGAACTAATATAGTAGTGTTATCCCTCAATACTAGTGTCAGATATAACTGTTAGATGGTGTAAATAATCAAAAGTCGAAAACTGAAAAAGAATGGCTAAAAGTTCTGTTTAGAAATCTAAAGAAGTTTGTTAGTTAATGGGGGAGTTACGTTTCAGTGTTTAGCAGGACGTATGATTGCTGTGTTTATGAGAGTTGCTCTTATTTCATGTTCGCATATTTATCACTTTATAACCAAATCATAGCTGTCGATGGTCGATAGTGGTCATGATTTTTACTTGAGTGTTCAA

>LNC_000511

AATAAACCTCACTTAAGGTTTGTTACCCACCGGAATGGATTGCGCTTTTCTTTCCCACCGGTTTCAGAGCGCTGTTTTCAACCCCATGAGGAGTCAGTCAATGCACGATGAGGAACTACCAACCGGTAACCAATTGACGTCGGATTCCGGTGAAGCGAAAAGCGCCTTTACCTTTAGTTGGAAATAAATATAGGTCGGATTTCCACTTCTATAAGCGAACTCAAACCACAAATGTTATCTGTCCTACCGGTGAGTTACAGTTAAACGCCCTACTCGCTATACCGTTAGTGACGCTTATCACGCTTTCAATCGG

>LNC_000512

CTGATTGTTCCTTTGCTTTCTAAGTGTAGTTTGGTCCATTTTGGTGTTACTCATTAATCAAATCTGGAGATTTTATTCCATGTTGTAGTTGATCATTTGTTATTCAATATTTTTGAATTGTCTACATTCTTGTTTGAATCTTATTTATTGTTTGCTGAAGCATAAATAAATTGTTACTATTTTCCTTATATGACTTAGTTTGAGAATAAATTGTTAGTTCTTTTTTTCCTTTAATACATATCATATGATCTTTGACGATGTTATTGATTTTAACGACTTGATTTCCTAGTCTAGTTTCCCATGTTCTTTATTTTTAGGAGCAGCTCTTAATAATTTCATATTCCATAATTTAATCTGCTTAGTGGATTATTTTTATCCATTTTTGAACACGTGTGGTTGATCCCAGGAAGTTTGGTTA

>LNC_000513

CTTTATTCCCTTATAGTAATTCTTTTGTCCTGGTAAGTGGAGACGTCCATGTTTCAGATCTCAAATTGAAGGGGCATGTTTCACCTGTTATTTTTGTTTCTATTCTCAACCACTGATTCTTAAGTTCAGAAATATAGAGTGATTACTCAAAACTAAGAAAGAGAATTATAAACTATTGTTAATTTCAAATAAATTAAGTTACTCAAAGCTAAGAAAGCAATTCTGTGTTTGTTAATTCTCTGAGAACTGGTTTGCCGGCTTCTATATGGACAATTCTGTGTAGTTGATCCCTTGATATGTCTTCTGATTGAACCTTATTGAGAAGTGATTAGTATGTTTGGTTGTTTTTGTATGGTTTATGATTTCTTATTAACAAAACTTGAATAGAATAATTTTGTTGTCCTCTCTTTTGCTTTA

>LNC_000514

TTTGGAAGGTAAGGTGACGAGGACCTGCCTCATTGAGGTGAAGCGCTTGCCTTTTTTGAATTGATGCGCCAATAAATCCAAAAAATTAAATAACTAATTGACTATATAAATAATCAAAATCTCAATAACAATAGTATATTAGCAAATATTTTAATTCAAAGAACTTGAGGACTCAAAATTCATTAGACAAGCTATACTAAATTACTTTAAAGTAAAAAGAAGCTAGAAAAAGAATTTACCGGTGCCCAATTACCAAAAACAAACTTTTTGCAACTAAAGTCTAAAGACATAGAGAACCCAATCGCAATGCGACTGAAAAAAGGTAAGTGATAATAAAACCCTAAAATCCCAAGCATCGTCTTTTTCTTTTTAAAGAAGTAGTGATTATTTTTTTTGAAAAAGGACAATGCCTTTTTGCTAGTTTTTTTCACAACTCTTGCCTTGTTGTAGAATGATGACCTAGTGATGCCTCGTCTCTCGCTTAAGGTGCTAAGGTGAGCGCCTTTGATAATGCTAGTCTAAGTTGCGCTAACTCTTCACTTTTGATGTCACACCTCTGTCAACATGACGTGGGTGTGGGATCTGTATCGGATATGGTCAACCAGTTTTGCAT

>LNC_000515

CTAGTAGATAAACCTTCACAACTATAAATGTTTAGAAATGACACCAAAACTTCTTGTGGATTCATATCAGCAAGCAACTTTTGCTACGCCTCTGCCCCAAAAGGTAGGGATTGGTTATATCAATTCCCCCTTATTCATTTCATCATCATACAATAATAAAATAAAGTAGAAACTAAGTGATCTCTAATATGGAACTGGATTACCACAGGGATCCGTAATATGACTATCACAACTGATTGTAGAGTTGATCATTCGGACTTTTCAATTCTTCATAGATGTAGATCTCGGACCTATGAATGGGGATATTTCCG

>LNC_000516

CTATGGTCTCTCGAAAACAGTCTCTCTACCTCTGTGAGGTAGTGGTAAGCCTGTGTATACTTTGTTCTCCCCATACTCAACTTATTGGATTCACTAGATATGTTGTTAGATGAAATCAATTATTATTTTCTTAATACTGCAGTTAGAGGGGGACTTAAATGGAGCAACATGGACTATGGAGATTCATATAGTAGATCCCAACTTGGTTGGGTGGATGCATTGATCTTTTTTATTCTTGTAATCAAAACTGATTTTGAATTTTGTTTTAACTTTTCTCTAATTGTTGTTCAAGTAAAACTGAAAATATGCATATGCAACCCATAATTTTGAAGTGATTTTG

>LNC_000517

GTCGGGGGTGCAAAGGCGATTCGCTTATGATCGTATAGAGACCGGCGGATCGTGCCAACACCCACGAAACGAACACACTTTTCTAGGCGAGTAGGCCCGCCCTACTTTGGTGGGCTTGCCCGCCAATTTTTGGAAGCTCTCAATTTCCATTGAGGGCTCACAAATCAATGCATAATCATACTGAATGAATCTCTACTATTACCCCATCATGTTGCTTTTGGGCCCTCTTTGCGCATGGGCCATCAAGCGGTATCTTAATTGGCTTAGA

>LNC_000518

TATAGTTCCAATATGCGCTAATACAAATCGCCATTTCACTTCACCTATTAATATATCCCCATCCGGCCACACATTGAAGTTTACGTGCGTGTTAATATCAAGAAAGAAGAAAATGGGGAACGAATTGATGGTTTTTATGATAATGGTGATTGTACAAGTTGCGTATGCCGGAATGGTAATAATATCAAAGTTAGTGATGGATGGTGGCATGAATGCTTTCGTTCAATCAGCTTATAGGCCTATTTTTGCAACTATCTCCATTGCTCCATTTGCTTTCTTCTTAGAGAGGAAAACTAGGCCCAAGATGACACGCTCTATACTTTTTCAGATATTTTTGTGTTCTATTTTCGGGGATAACAGCGAATCAAAATATATATTTCATTGGGCTAAAGAATTCAACTCCAACAATTGTCTCCGCCATTGATAATCTAATCCCAGCTTTCACCTTTATCATAGCTGTACCCTTGGGGATTGAAAAATTGGGATTAAGAAGTATAGCAGGACAAACTAAGTTTTGGGGTACAATAATATGTGTTGGAGGTGCAATGTTATTGTCATTATATCATGGAAAAGTTGTTATTGGTCAATTAGGATTTCACTGGAAATATGCAGAAAATACAAGCAAAGATGTCAATTCTGCCAATTCCAATTTCTTTTTAGGACCTTTTCTACTTATACTCTCCAGTCTCACTTATGCCATTTGGTTAATTATTCAGGCAAGGGTAAACGAGAAGTATGCAGCACCATATACAAGCACAACGTTGATGTGCCTAATGGCAAGTGTGGAGTGTGTTATCATTGGCTTTTGTGTCGTTCCAAAACTCTCTGAATGGACATTAAATCCCATTAGAGCTGTCTCAGTTGTTTATAATGTACTATTTTTCATATGCTTTTTAAATATTTATTAAAAAAGATAACCAAAAAGTATATATTTTTCATATGCGAGTATTAATTTGCTCATGATGATGAGGTTTATTTGATGTTGATTACAGGGAGCTATGGCTACGTCATTTACATATTTTCTGAGCTCGTGGTGCATTGAGAAAAAAGGCCCTTTATACGTTTCGATGTTCAATCCGTTGTTCTTAGTTATTTCTGCATTTCTCAGTTGGATATTGCTTCGTGAGAAATTATACCTTGGCGTGTAAGTATATATTTAGCCTA

>LNC_000519

AATTTGAATGCACATCTGGATGATTCTTGTCTATAGATCTGTACATTGAATGATTAAGATTCTTTGTTTTTAAACATCTGATTATGTGTAGAATTTAATGCCTCAGCCAAACGACTTATTGACAAGAGGCTAAAGGGACAACCATTATTTAGAGTCAAGGTGGGTAAGGGATTGTCTTGACCAACTCCGGTTGCCATAGTCTGGGAACCATTAGCTAACATAATAGTGGGAAGGGACTGAGAGTAAGTAATATTAGACAATAAAGATTTGTTACCAGAAATGTTATCTGAAGTGCTTAAGTCCATGATCCAATGGCCAAGTGTGGTGGACAGTAAGATACAAACAACACGATTAACATGATTAACACGATTATCAATCTGGGCAACAGAAGCTGTTGATGAAGATGGCTACTTACTCGCTCAATACCAAAGAAAGTCAGTGTATTCTACCTTAGGCAAAGAGAACAGTTGATTGGTTGGATAACCAGTGGATTCAGC

>LNC_000520

GTTCTGGTTGCCTCTAGAAAACCCAATTTCTCTTTGTTATATAGTGTTAGAGCTTGTAATATGGATTTTGAGTGGGAACTAGTTCTCCAGTCTCAGCTCTTCAGGCAGCATGCTGTTTGCAACATACGACATGCTCTCTTGCGGATAACATACATGAATATACGACTGTTATATTCATGTATCCTTCATGTCCTATGGTGTGACCCCTCTGGTGGGAAGGAACTAGTAGAGCCTGGGTGGATCCTGGCTGGGATAGTGGCACCCAATTCTTAACCAGTTCTGAAAAAAGACTCAGATCAAGAGAGAACTACT

>LNC_000521

TCTGATTCTGCTTACATAAAGCATCTTGCTTTCAAGATGGCCAAACGTGGATGGAATGTCGTTGTGAGCAATCATCGGGGTCTCGGTGGAGTGTCAGTAACTTCTGATTGCTTTTATAATGCTGGATGGACGGAAGATGCCCGAAGAGTCATTGACCATCTCCACACCCAATACCCTCAGGCTCCTTTATTTACTGTTGGCCTTAGCATTGGTGCTAATGTTCTGGTACGGCATTATATATTTTGTAATTGAATATTCTATTATTTTCCTTGTATGACCACAAAATTCTGCAACACTTATGTACTAAACAATCTTCTTTATGGTTACCTGAAAAGTGAACCTACATTTAAAGATAATGGAAGCAAATTATCATAGCTTTTTGCAGTAAATATTTTCAGAGGTTATTGTCAAAAAGGATCATATACATGAACACACAACAGGTACACAAGCATATGACCACTGTCTCGATGGATTTCATAACATTTAATTTGTCAGTTCTTCATCCTTATATACCAAATCCCTAGTGTATAGAAACATTATTACTGTTACAAAAATTTCTGCAGTAGTACAGAAATTCACATCTAAATAATATATTTTCCGTTTACTAGTTGGTCCATCTGATTTAGTTTGATATGTTACAAACCTTAAGCATTAATCAATGTGCTAATGATTCAATTTCCAATGTTTTTAAGTAAATGGGCATTCTTCTTCTAAATTTTGAGAAGCCAGTGAGATCAGGCATATGCATTATGGCATATGTTCTTAACGAAGCCAATAACTATTTTACTGACAATTACTAATTTCCAGGTAAAATATCTTGGTGAGGAAGGAGTTAATACTGCAATTGTTGGGGCAGCTGCAATATGTTCTCCTTGGGACCTTTTGGTTAGTATCTAGACCAACTAATCTGCATTATCTGACTTTTATTAAGGTATTGATGTATTGCTAATTTCTTTGGTTTCATACATGTCATTCTGTATCTGGTATTTCTTCCCACTAAATTGGATTCTACTACATATTGTTTGTATGTTGGGTCAAGGGTTATTCTGGATGGTATGATCTGTTGGATTATCATGATCTGGAAAGATTGTCTTGCCTGAAACAATTCTATGAAGATGCTAGATGAAGATAGCGATGGTGGAAGCCTAATTCAACAAAAGTCAAATTAGTCATTTCTTAAGTTTATTCTGTACCATAAGTACCTGATTTTGTTATTCTGTCCTTTGCTTAGTTGCACAAGAATTCATCCCTGCTTCTCTCTATATATCTGAGTCAAATCCACTTTTCTCTTTCTTTTCAGATTTGTGACAGGTATATTAATCGCAGGCTTGTGCAGAGAGTCTATGACAGAGCTCTGGCAATTGGATTAAGAGGTTATGCCAAGCTGCATCAGACAGTAATGTCTCGGTTGGCAGATTGGGAAGGCATTGTTAAGGCACGTTCTGTAAGAGATTTTGACCATAGTTCTACTCGTGTTCTTGCTGATCTTGAGACTGTTGATACATATTACAGGAAAGCTAGCTGTACCAGTTATATAGGAAATGTGATGGTTCCTCTCCTTTGCGTTAGTGCCTTGGATGATCCAGTTTGTACAAGGGAAGCAATTCCATGGGATGAATGTCGGGCAAACAAAAATATTGTACTAGCTACCACGCACCATGGAGGACACTTGGGATATTTTGAAGGGATGACTGCAAAAAGCCTCTGGTGGGTTAGGGCAGTTGATGAATTTCTTTGTGCTTTGAACTCCAGCTCATTGATCCATAGCAAAAAGGTTACTACAGTGCATAGTTCCCTGGAAACATCCATTGATCCACCATATGTGAATATTAGAGAAGACGGATTGGTGACTGCGTTTAGCAATGATCCATCAGGTGAAGTAGGAAGTCAGCAGAATGAGCAGCCGGTCGAAGATGACCCGGCTGGAGATAGGATTAGAGGTGTCAATGGTGCAATGCAGCTAGAAACAAATGCAACAACTCAGTTTGATCCGAGAAGTGAGAGCACCAGGTTGGAAGATGCTGGTTCGAGAGGACCTGCAAAAATGCTGGTGACTCCTGAACCAGATTTTGATGCAGTGATTGTTCCTGTTAAAAGATGTTTAAATCAGCTTTCTCGGCGTAGTAAGATTTCAATGTGGGTACTTGCATATATTGCTATCATAACCACTTGGCCAATTCTTGGTTCAGCTTCACCAATGTTCTTCAAGAAAAAGCTGCGGAACATCTTCTCACTGAAAAGATGACGATGCCATGCATGTAGCAGTGGCTAAGCTCTATTCATTAGTACTATTTAAGATACATTTATGTACAATTGCTGGGGAAGAGGGAAATAGTTAAACCCCAAAGTTGAATTCGGGAATGGCTGTACTGTTTCTTGTAACTTCAAAATGATCATATAATAACAGCCACTTGTTAAAAAGATTTTCATTGTTGCCTGATTATTTCACGTCTGCTAGACAAAACAGCCTCTCTACCTATAGGTAGAGGTAAGGTTTGCCTATACTCTACCCTCTCCAAACCTCATTTGAGAGATTACGTTGGATATGTAGTTGCTGAACAAGATCCATAACATTCTTGGAAAACAATTTGGCCAAGTGGTCAAGTCATGCATTTGTTAAATCATATATATTCCTGCAGGTTATTTTAGTCTACACCAATTTCCCAATGCATGAGGTATTGGATCTCCT

>LNC_000522

GCTAGTGACAGTAGTCGTAGTGACAATCGTTGAAGTTCCATGAATAGTTGCAGGTCATTTCTTGAGTATGGATGCTAGTGAACCCTTTTCATCAATAAAAGCAAGATTAGCGAATGACAGTTTTTGTAGTGATTTGAAGTTTCTTGTAATCGTAATGGTTGAAGTGAATGGTATGTTTCAATTTAGTTTGTAAAACTAATAGTACACTTTATGATATATTTTTTTGTTGAAATATTTTGTTATTTCTTAATACTAATAAGCC

>LNC_000523

CAAGCCAGTTCATCCCTCTGCACTTCTCCGAGGATCCACCACGAGAGCTCGCGGCGAAGCGCCGGAAAATGACGAAGCTCTTCGCGTTTGAGAAGATGCCCAGAATTGGTGGAGTGCTTCTTTCAATTCCCAGTACTTGAAAGAATGTTGGATTAACTTTCAAGGTGCACCTCGTCCAGGAGTGTCTAAAGAAAAATTTTGTAGTGGTAGATGGTGCAAATGTTGGCCTCACTAATCAACGCTTATCCTGCAGTGACAATATGTCAGCAACTTTGTCCATCTAGTCAAGAGTAAATTCTTCTCCACATTGATCAATCCACTTGCTTAAGCTAAACGGCTTGAAGCTTGTTAATTTGGCCTCGAAGC

>LNC_000524

GAAATATTAATAAGTGTCTAAATTGAATTTCGCTAAACAAAGAGTAAATATAAACAATTTACCAAGTAAGGGGTGAAATTTAATCCTTTTCTTCTATGTCCCCAGACTTGTGCGCTTTTTCGTTACGCTACCCTCTTCCTGTTGGCTGTTGCCCTCTTCTGAATTCGATAATGGAATTAGGGCTCAGGTTTTGTCTTATTCTCCGAGAATTCAGAGGAAGGCGTTGGCCTATCTTCTACTTCAAATCTAGGGTTCGCCAATCTGATTTTGATTGGTCCGATAATCAGGTCCAAAATATACGGTTCTTCCTGAAGACAAAAGGCATAAACTGTGGAAAAGGTTGAAAACTTCAGCACTTGGCTGATGAATGGATGCAATAATGGTTCTTCCCTCTCTTTGACGTGAAATTCCGCTCATCACGTAATAAGATGCAGCACTGTCGAGGCCGCTGGTTGGTTCATCAAGGAAAAGAAGTTTTGGCCGCGTTAGAATCTCCATGCAAACACTAAGTCTTCTCTTTTCTCCTCCACTAATTCCTTTATTTTCAAATCCTCCAATTCTTGTGTTCATTGCATCTTGCAACCCCATCTCCTTAATAGTCTGCTCTGCTATTTGTTTTTTCTCTGATTTTGTCATGGAAACTGGGAGTTGGAGTTGTGCAGAGTAGTAAACTGCTTCTTGAACTGTTAGTGTTGCTAACAGAGTTTCATCTTGAGTCACATATGCCTGATTTTATAGGATAAAGAACATTACCATGCATGCTAATGAAAAAAATATAGTAATGTTATTGTGAAAAAAGGAAAACAAAATGAGTAGTGCATGCGGCATTCAGCTGAAGATTTTAGTTTCTTACAGATGTTCCATACGAAAGTTTCTGTTTGTGACCATTGATCAGAATTTCCCCGCTCTGCCTCGTGCTGAAATCCAGTCGCCCTGGGAGGTGCCCATCAGCTAAGGAATCGAGAAGTGTAGATTTGCCACAGCCAGAAGGACCCATAACGGCCAAGAGCTCACTAGGTCGAGCATAGCCAGTGAGACCTTTAAGTATGGATTTGCTTCCACCTTTTTTAGTGCTAACAGTAACCCATAAGTCATTCCAAGTCAAGAAAACGCCTCCACTTTTTGATACCATTTGTCCTTTCTCAGCATTATTCAACTCTACTTCCTGTAACTCCATATCCAAATTTACAGAAGCCATGCAATACCGTGAAAATTTGTAGGAAATACAAAGACTAAAAGGTGGTTTATTTCTTTTTCATGCAAAAGGTATGGGGTATTTAAGGAGGTTGTAGTCATCTTTGTCAGCTAGGGCTTTAATGAGATAATATGTCAAAAAATATCTTACTATTACAAACAGTTCAAGGGAACCAACTCATTTATGTTTCTCTTTGGAGTCTGCATTTTCATCTATTAATGGCTCGTTCTCATCCTAACTCTCATTTTTCTTTTTTTTATTCCCTCTTGTCAACTCTTGCTTCTTTGATTTGAATATTTTTTTCTTTCATGAAGTTTGGGACCATGGTAGAGATGGTCAGATATCTCTAGTAATTCGTAACCAGTATGGAAGTCGCTTTCTTTTAATACTATGTATAATATAATAGGTTTAGCAATAACTAGCTACTACCTGTTTAAGCACACAGTTAGGCTGTTCTTAATTGATTCTTGTACTCTATGGCTAGGTATATGTAGGGGTGGCCACTTATCCGTTTAAAACAAAAACCCAGAAATATGAAACTCCACAATCAAATTAAATAGAATCTTTACTGCTTAATGAGGATGCTAACAATTAGAAATTAAATGATCGACTGAACAATACAAACCCAATTACTACACCATCTAAAAGCCTGTACTTAAACTGGCCAAAATTGAAAAGTCGAATTGAACTGAACTAATTTTGGTTCGGTTTCAATTTAAACTTTTACTGAAAATAAAAAAATTGAACCAAACTGACTGAATGTCCAGGTTAACAAAACCAGTCATGTATGCTACATAGAATTGTCATATGTGTTATTAGCGGACAACGTCATCTACTCTAAATAGAAGCAAATCCAGAATTCGTATGAGTTTGAGTTCGGATTAGTACCGCAGCTCATTCGATATACTGAACAGTGTATTTGCTCAGTGAATTTCTCAACATATTTACAAGATTTGAGCCGAAGTTATTGAGGAGTCGTCGGGGACTAGTCTAAAAACTTTTATGTGGTCGATTCATCATTAAAGACTTGATCCAAAGAGCTTGGAATAATAGAGAAAGAGAATATTTACGTCATTTCACCAAACAAGTGAACAACAAGAGTATTAATTAGCGAATAAGGAGCCCAATATAGCAAAACATAATTATTACATAAACTTAAATATCACCTGTACAAGTGTCGATAAGAACTTGATAACGGGTGAAACATATACACAGAAGAATAACATGATAAAAATAAATAAATAGCAAATGAGGTATTTAAATGATATAAATAAATATTATATCTCAAATGTGTTATTGTTTTCGTTGTATCTCACTTAATCTATTTTTACGGACGGAAAAAATAGACTTACTATAAATCTCAAAATTTCAATTATGCCACATAATTGAAATAAAATAAAAATTATAAATCTCTATACATGTGAAACTCTAAAAAAAAAAGGTTAGAAATGATTGTGATCCTACACTTTCTCGAACTCTTGATAGGGAAGTCCAACGACCATGCACCAAATATCAAGTGAACAATTTCGGTCTTAAATTAAAAATATATAAGAATGTATTAAAATGCTCTTTAATTTTGTAGTTTTAAATATATCCCGTAGAAGCTTAGAATTAAAGTGTTATTTAAAAAGTAGAGAGATATTAATTTCGAAGCAGACTAAAAAAGAAAATTAGACTAATAAATTGAAGCGGATGGTGTAATAAATAAATTAGAGTTTCAACAGTTAGAGAATGCTTTTATTAGCCAATTTCGTTTGGATTTGAAGTCTGTTACAGAGATCAATCAACAATGAATCAAACTATACAGATTTAGTTATAGAGATCAATCAATAATTAATCAACAACATGGATTTAGTTATAGAGATCAATCATTTATTAGTCAACTACATCACCACCAACCTTTTTTCTTCTATCATATATTTTAGAATTCCTTTTTTCGTACACAAAATAGAAAATCTATCAACATTTAATTCTCTCTCTCTCTATATTTTACCATATATTTTCGAATTCTTTAAAATTTTCAGATTATAGTCTATAGTATTATAAAAAATAAATAATTTGAAAATAATAATAAATGACAGTTTTATTTATTATAAAGTCTTTTAAGCGGATTTTTATCCTTTTAAAAGTAATATAGATATAGATTCTTGTACCATGGCTAGGTATATGTAGTAGGGGTTGGCACTAATCGACCGCACTGATTTTTGAACAAATTACAATACTGAAATCAAATAGAAGCTTAATAACTGATTAGTGAGAAAATTTACCATTTGGAGATCAAATGGCAACTGAACTATACAAACCTAATTATTACACCATCTAAAAGCATATAATGAGAATGGCCAAAACTGAAAAATTGAATTGAACTGAACTAATTTTGGTTCGGTTTCTATTTTAAGTTAAACCAAATTGAAAATGGAAAAATTGAACTGAATTGATCGAATGCGGAGGTTAGCATTTGTATTAGCGGACAAAATTTGTATAGGTTCAAGTTTGGAATAATAGTACCACAACTCGTTTGATTTAAGGGACAACAAAATTTGTTAATTTTTTGATATATTCTGCAATGTTTGAACTGAAGTTACTGAGTTTGGATTTACATCTTGACGCAAGTTACTTCCTCACCAATCACTATCTTAGAGGGACTGGTCTTTATGCCTTACATCGTAACGCAAGCTACTTCCTTACCAGTCACCATTTTAGAGGGGGCTAGTCTAAAAACTTTAACGTGGTAGATTCAAGACTTAATCCAAAGAGCTTGGAATTATAGAGAGATACTAATTATTTCATTTCACCAAACAACAATATTAATCAGCGAATATTAAGCCTCCCGCCCTCACCCCACCCCACTCCCAACATAAACACACCTCTACATAAATTATTAAAACATAATTAATACGTAAACTTAAATATCACCGGTACAATTGTCGATAAGAACTAAACAATACGCATTACGCGAAACAAGGAAATGTCGAGTAATTTTTAGCAATTTTATTAATATGTTTCTTATTCCCATTGCTTGCTTCTCTAATTGTATCACATTTTGGGGTGATTTTTTTTTATATTTTAGAAAAAGTCAACGAAAGATGAGCCCATACCTGATTACCAAGGATTATTTTAGAAATCACATGGGAGAAAATAAATGCTTTTGAATAAGAGGCTAAAACATAAGTTTGCTTCATTCATGGAACTTTCTCTTCACAAAATGTATTTACTATAGTTATGGGCTTTTGTGAGAGAAACACAACACTATCGATCCCGAATTAAAGAGGCCAGTCCATTAATACTACTACAACAACTCACATTAACTCTTCTTCCCAACCAATGATTCAATTCAACTTTCTTCATTTTGGATAGGTTTAGGATTATATATTATATACAAATACAAATTCAACTATTCAATGTGACAGCTCAATCTAATTTAGATTTCAGATCTCAAATACTTAATTCTATTTAATTAATGACGTCGGTAGCATTTTATTATTTTATATAGCTTCTCATTGAATTTTGGTCTATCATTGTGATTTCAAAGAGATAGTTAATAGCAATAGATTTTCGTTGTGAGAAATTTTTTAGTGGTAATTGAGTTAATGTCATTATATTCAGAGTTGTTGAAGCCTTTAGCGTCATTTAGATAAAGTGTAGGTTATAAATACCAATATTTATTATATGGCAAAAATTATATTATTGTTGATAAATTCTTTATTTGTTGTAGTGTTTTCCGAGTGACATGTTGGACATTTAGTGGTAATAGATTTTCCTTTAGTATTGTTGCGAAAATATTTAGTGGTAATTGAGTTAATGTCATTGTATTTCGAGTTGCTAAAACCTATAACCACATTTAGATAGAGTATTGATTATAAATATCAATATTTGTTATTCTCGCTAAATATATTGCTCGCTACATTTTTTATTTGTTGTAGCATGTTTTTCCATTGAAATGTTGGCAAAATTATATATAAGCGGTATCAATTTGCGACAATTTAGCTTGCAAGTGAATTTGGGTCTATCGTCGTGGTGATTTTATTTAAGATTTTACTGCTACCCAAAATTAAGAAATGGAGGAAGGATATTTATTTTGTCTTATTTTGAAATAACGTCACATCCTAGAGCGTTTCATGTGTGTACACATTTATTTATTAGAAAATTATTATTTACACACAAATATTAGAAAAAAAACTTTTGTAATGTGTGGGTCTCCGCCATTGCTTCCAGTAATTTTCTGAAGTATTCTTTTAGCTGAAACGTATTAATGAGTTTGCTAACTATATTAAGCTGATCAAATTTAATGACCAAGATATTACATTTTATATCTCATACTTTTATTTTTAGTCTTCTTAAAAAATAATGATAGTGTCATTTTGTATTAGGAAATTGTTTAACTTTAAATTTTTCATTTTTAGTTAATGACATGCTCATATATATCATAAGAAATGTCATAAGATGCCTATAAAAATTACAAGTTGCAAGTGTACATTAGATATATACTAAAGTCCTTACGTTATAAACTTGTACTTAATCAAATACTGTCATATACATGAAAGGAAGGCA

>LNC_000525

ACTTGTGCGCTTTTTCGTTACGCTACCCTCTTCCTGTTGGCTGTTGCCCTCTTCTGAATTCGATAATGGAATTAGGGCTCAGGTTTTGTCTTATTCTCCGAGAATTCAGAGGAAGGCGTTGGCCTATCTTCTACTTCAAATCTAGGGTTCGCCAATCTGATTTTGATTGGTCCGATAATCAGGTCCAAAATATACGGTTCTTCCTGAAGACAAAAGGCATAAACTGTGGAAAAGGTTGAAAACTTCAGCACTTGGCTGATGAATGGATGCAATAATGGTTCTTCCCTCTCTTTGACGTGAAATTCCGCTCATCACGTAATAAGATGCAGCACTGTCGAGGCCGCTGGTTGGTTCATCAAGGAAAAGAAGTTTTGGCCGCGTTAGAATCTCCATGCAAACACTAAATGTTCCATACGAAAGTTTCTGTTTGTGACCATTGATCAGAATTTCCCCGCTCTGCCTCGTGCTGAAATCCAGTCGCCCTGGGAGGTGCCCATCAGAGTGTGGAATCATTCTTCAATGGACCCTGACATGATTCGTCCGCGGAGAAGCAAGCCTTGTCCACC

>LNC_000526

ATATTTTTTTTGTGTTTTAGTAGAAGTAGTAGGCAATGTTTTAAAATCTGGAAAATATATAACTTCATTTTGTCTTAGTTGAAAAATAGGTTGCAATTTTTCCTTAATAATGTAACCCTCTATATTCTATACCTACAAATGTGAGTTTTGAATTTCTCTCGGCAGATTATGTTACTATATGCATGGGTATAAGTTTTCTTTAGTTTTAAAGATAAGTCATGTC

>LNC_000527

TTCGAATTGTGAGGGCGATCCTCCCGGTGAACTGACCGTACCCCAAACCGACACAGGTGAACAAGTAGAGTATACTAGGGCGCTTGAGAGAACCATGTCGAAGGAACTCGGCAAAATGACCCCATAACTTCAGGAGAAGGGGTGCTCTCCTATCTTTTGATTAGGAAAGCGGCACATACCAGGGGGTAGCGACTGTTTATTAAAAACACAGGACTCTGCTAAGTGGTAACACAATGTATAGAGTCTGACACCTGCCCGGTGCTGGAAGGTCGGAAGGAGAAGTGTTATAAGCTTTGAATGGAAGCCCCGGTAAACGGCG

>LNC_000528

CCAATTTCTCTTCTTCACTTACTTTCTTTCATTGCTAATTCCCAAAATAAACTTCTCAGTTTTTTTTTTGTTTTTCCGATGTGGGGGTTTTTCAACAGAAAGCTTTTTAGATCTGTGGTCCGGTAGTTTTCCGATGAGATCTGAGTTGATCTGATTGTCCGGCAGCAATTTTACGTGAAAGACTCGCCGGATTTTGTTTCCGCCGGCGAGGGAGATTAGTTTTGAGTTGCAGGCGGCGTAGTATGTGGGTCCAGTTTTGGTAAAGAGAATTAGGGTAAGCCGGCTTGATAGTAACTGGGCCTTGAGTTGTTTTTGGGCCAAGAAGCCCAATTGACAGCTGGAGATTATTGGTCCGGTTTGTAAGGTCCAAAACATGTGTGAATGTGCTGGATTTTAGATTGCGGTTTGGACTAATTCTAAAAAGATATTATTGGTTGTGTTTGTTTTTTTGTTGAAAATTTCTCCTCCTAAACAAAAATACACAAAAAAAAAAAAAAAAAAATCTTCGGAATTTGTTCAATAACTGGTTTTGCAAAAACCTAGTAGATTTTTTATTTTTAAAAAAAGTAAAAACTTTGGTGTGCTTGGTAGGTTGTACGAGAAAAATGTTGAATATGGAATAATAGTATAAGAAAATATACGAAATAAAGTATTAGTATTAGTAATAAAAACTAAGTTCATATATATTATTTATTTTTCTAAATTCACTTTACCAAATGACCCGATTATTGTTCAAACACAAAGAATTGATTGATACTTAAAAGTAAAAATGTTAATTTGCCAATGTTCTGTTTTAATTAATCAAGAATATAATTAGGGAGTATTAGCTGGAAATGGCATTGGTTCTACATACTATTCCGCTTATTTTTGGGGTGATAATAAATACTAGTATGAGAAAATGCATTCTTCCTTTGATTTTTGT

>LNC_000529

TTCATTTTGTTGATGTGAAAGCATAATTTTAACATTACGATTAATTCAACATTGAAAAACTTGCAAGTCGAATTCGTAAATTTTAATTTTGATTTAGTCTCTAGTTACTACCTTACGTCGATTTTATGTGACTAATTTGAATATGAGTGTCAAAATTAGGAATATTCAGAACATACATTTTGTAAGAAGTGGGTATTTACGTTGTTTTTCCTTTTTGTATTGATATGAAGCGCATGATAGTCAAAGTTAGCATAGGAATCCTAACATGAGCCAGTTTCTCGTTAATTGTGTCTATGGTGAAGTCCTAATGTGGGGTGTTGAGCCTAGCTAGTGCCTAGTGGGGATGTGATCAGGTGTGATCGAGTGCATGCAAGCTTTAGTCTGTGATTACTGATTAGTCATTATAATTTATGATTATTCACTGGGACATTATCACGTGCTTTCTGTTTGCC

>LNC_000530

TCCAGAAGTGTTATTTCCAATTTGTGTTATGTATACATTTGTCTTCTCTCTTGCTTGAAGTCAAGCGGGGCTTCATTCTTTGGATGGATAACGTTAGGTGAAATGAAGTCATGAACTGCTTGAATCAGTTAAACAATCTATTCATTTTTGTCTTAGGCTTACTTTGTGGAGGTCCAGATGGTCATTCAACTGTGGTCTCTTTTATCAGAAAGCCACTCAATTTGATTTTTAACTCAAAAGTCACTCAACTATGAGTTTTTACTTACTAAGTCACTCTACCTATTTAATTGATTTTTTTAAATTAAAATTCGCTTACATGAAATTTTTATTTCTAACCAAAATTTCAAAAAATAAAAAAATGTCCATCTAAACCATTTAATGACCCGCCCACTTAGCCCGACCCACTAAAAACGTGATCGGGATCATTGAAATTATCATCCTCTACCTCACCCACTATATTTTCTTGGACAACCTCTATTACATCATCCCTTAACTCAGTACACTATCACCTTGAACTTTTTCAGCTAGCACTATGCTTTAAAAAAATTCAAACAGTGACGTTCAAATAATAAATCCTAACTATTATATTTTGGGGGGAAAGGGTAAAATAACAGGGTCACTATTGGTATCCTTTTATGTAAGACTTTTGAAACAACTTTATTAATAACACTGCTCAAACTAGTAGGTCTTACATCTCCAAAACTGAGAAATTAGTGTGGGTGATGTATCTAGGGAGCTCCTGTACTACAAAAAATGCTTTCACCAGTTTGATTACATCTGTTTT

>LNC_000531

AATTGTTTGTGGTATCTTTAATGGTGAAGTCTCGGTCAAGATCAAATAGATTTCTGGGTTCTTTATTGTTCCTATTTTTTCTTTAAAATATACCAAAAGTTGAATGCCTGCTGTTAGAAGATTGGATGGTATTATTTCATTTTTCCTAAAATATATGAAGATTAATAATCAGTGTTTGTTGCATTCGACATAAAAGTATGCTTTCTATCTTCTTCTTTTTTGTTGATGGATTTCATAATGCGATTTCAAGATTTTCTTGATTGGAATTCAACCCTGTAATTGATATTGTTTTGCTTTAGTGGTTTATTAGGTTCAAGAGTTCACAATTTTGAAAAACTTCTGCATCTCCACTACATTTTTTGCTCTGTAAAGTTAATAAAGATACATCTGTGTTTTCGTCTACAATGTTCTCTTGTTTTCTTCAAAATATCTCGCTGATTCTCTCTTCATTCGATCCACCATATGGTTATAGTATGGAGTACTAGAGGAGCTTTGTGGAGGCCTCTTCTGTTCCAATATAACAGCAGTTTTTGAGGGATGCACTATTTCACTTCTAGGGATCATCATGGCTAAGATGTAACTTTACAATGAATAAAGGCTGGTTATGTTGGCGAGGATGCAGA

>LNC_000532

GGAGTCTTGGACCCACCTAGAATTAATTATAGGTAAAACTTGTCAAGGATAAGATATATAATTCCTTATGGTTAAACCTCCCAAGGTAAGGCTACAGAAGAACCAAAAAAGCAATAAAGCTTGCTTTGAAACTAACAAACAATTCTCATAGATAAGCTCTACTTAATAAGAACAGTAAAAGAAACAATTTTAGACTGAAAAAATTGTTCTTACCTACGAATAGAACTACTTCAACATAGAAACAAGCTTGCTCTTGCATCCTAAGACAATTGGGACTCAACAATAATCTAGGAAGCAATTAACGTACCTAAAAACCTATTAAAGAAGTTCTCTGTGGAACCACTACAAGAATACACCAAATTATCTGTCTAAAAACTTAAATAAATAAGCTGAAACCTAGATCAACTGATGAGCTGGAAATTAAGCATAAATTAAACCTCAGAAATGATAAAACATTCTTCAAATGTTCACCCTATAAGAGAGTTTCCTTGTGTTACTTGTGCCCTGCATGAGGTGCCTTGGTTATTCTGATGGAAACTGAAAGCCTTCAGCTAAAATTAACAGTTGCTTTACCAATTGTGTCTTTTAGCTTTTGCTCGACTTATGATTGCTGTGAACTGATTCTATACGCAGCTGAAATTACTATCTTAGTG

>LNC_000533

GAATCTTTTTTTTTACTATCCCATCACATGTGTTTCTTTTTTTGGGTTTAAAAATAAGAGAAGATTATTCTATTTGGGTCATTGTCCGGGTCGGGTAAAAATAAAAAAATTCCGGCAGTGACAGTGAAGAGTGGCACGCGTGTGGAGACAGACAGTGCGGTCCACGGAGTCAGGCCAACAGTAGGACCGAGTTTTGTCTGTGGTCAACCAGGCAACCTTGCCGGTACAGCTACACCCCCATGCACGTGCTCTTTCTCCCTTGCACGTGTCACCCAGCTAAGACCCCAGCCCATTTTCTTATCCTCCATTTTGTAACACACAATATTGATAAGAGGAGAGAAAAATGAGAGGGGATTACAATATAGAGAATCGAATCCAGATCAATAAGATGAGAGTTTAAGTAGTTAATTAATTAAATTATTAAGACTTTCATAAATTTAATTTAATAGATTACCTCGTCCACTTAAATTGGTTCACTTTATCAGATATGTGCCTAACCTATAAGTCCTTTAACACTCATACTTGTAAATTTAGAATTGAATATTTGTTGAAAATTGATCAGTATATTTTGTTTGTGTTTGATCAAACTAATGGATAATAC

>LNC_000534

TGAAACCATTCACAACGTTACATTGTTCCATGCCATTATTTCAATCATCATTATTATTATATAGTCCCCGCCTCTTGGAGGGTTTTCAAAAGCCATATTTAGGTCAAGCTTTTTATATGTTGTACAAATTATGAACATATGTTGAAAACTATAAATAGAACAGGTACTAGTGTTGAACCACAGAGAAAATAAAAACGCGAAAAATAGAAAGAGGAGAAGAATACGTTGATTTCGAGAATAATAATGATGATTCAAAGGTTGAAGATATGCATCGGAATGGTGAAATTGGCATTTGAGTTTGTTGCGGTGTTTGTGAAAGCAGTTGGAAGTGTCATCTCGGATGAAGAGGAGAGGAACTACGTTGCTCAAACGCCATACATCGGCCTTCTTCCATAATTTTTGTCATTTCATTTATTTACTGGTCCAACTTATTCAGATTTCATAGATATATGATAACCGTAGTTTAGCTTTTTTTCTCCATCGTGAGGCTTCATGTAAAAAAGAGCTTTCTTTGGGAGAGAGAGAGAGAGAGGTGTTAGAGGTTATTAGTTCCTTTTCCTTTTCTCTGTTGTAATTTTTGTCTAGTCTGGTCTAACAGTAGTCCTTTTGACTTGTTCCCGGCCTTCAGGATATAACAGTCTAATCATAAAGTGTAACTCAATAAATTCTGGACAAAAGTTGAGTCCAAAACTCCACAAAAAGAACACCTCCCTCCACTTAATAAAAACTCTCTTTTTGACTAACTTTATGCACTATATATTTTCTGTGTGTAAAACAAATGAAGACCACAACTATTTATAGTAGAGGAAGTCTTTCTAGTAATGAATAGGATGATAATGAGATAGTAAATAGTAAAGATAAAAAACCTTGAGGTCAGGGGCGGAC

>LNC_000535

TTTCTTTGTTCTCATATGTTATTAGAGAATGTTGATACATCAATTTAGATTTCGAGCACCAATTTCGTTACTTATATAAGCCATCTCTTCTTATATTTGATTTGCTGATATTGTAGATATATAGATGAAAGAACACACACATCTAGGTGTTGTAAGCTGGAGTTTTGTTGATTTGATCTAACTAACAGAAAAAAGGGGATCCTATCGGAGCAGCCATGGTACTGCAAAATTGTATCATCATTCATGTAAATGCTTTGTACTTCTTGCTTTGGTGATTCCTCCCTGTTCCTTCAAAAAGAGAACAAAAAGCGGAATGACATGAGAGATTTTGAAAGTCTCTTTATATGGCAAAATTCTAAGCTCTACTACAGCTTGGATGGATACCTCCATTCGAGAATAGATCATAGTAGGCAATCAAGATGTGGGACTATACTGGATATGTTATTGTTGACAGAGGTGATCAAGTTGAAAAGTGAACATATTGGTAGAGCTTTTTTCTAAAAATAATAGATGTGTTTGTACTTTGTTCATTAGAAATTGCAGGTATCAATCTCAAGTGTGGTTGCACTTCTATCCACATGTAAGTGTTGTCTCATTTGTTATCTAGAAATTTGACTTGCATAGACAAATTATTGGTATTACCACTTTATAATTGATTCGATTGTTGCTTATGATGTTAATATTTACATGTATTGCTATATTCATTAAAATTTATGTCTGGAAATAGTGGCAGACAAAGCATAAGCTTGATGGGTTCAATTGTGTCGATTATCTTCGACCCGATACATGACCCATATGTGATTTAAAGAGAGTAATGTTTTCCGTTGTAAGAACCTAAAGGATGAATCTATGAAATTTAAACATTGAATCCTCTTCTTGTGAAGGCAATCTTAACCAATACTTTTCAGTGTGTAGTAGAGATTACTCCTTACTAGGTGGTTACCATTTGAGAATTTTGGTTGGCCTTTTCCTGAAATTTTAGTGTCATATATATGAGATATGCTAGAGTGAACCTTCAAAACTTCATAATTGAAATTCATTGTTTTTCCAAGTGTGATTCCATCTATTCTACAAGTTAGCAGACAATTACAAAATGAAAAAGAAGAGGGTTATCATCAAAGTAATTCATAATTTTTCTGTTTATCTTAGAACTGCTGAAAATTTGAGTAAACATTCAACTGTTAGTACTATACCCTCCTTTGGATTGTTAATATAGTAGTTCCGACTACTGAGCTTGAATTGGGTTGCCTCTCTAATCGAGATCGTAGTCCTCAAGCACCACTAAGAGAGGCAACCCAATACAAGCTCTATCTAAGATCCTTATGATCGGATGAATAATTTTATCAGTCATTGTTTAAGTTATTTTGTTCCAATGAAGATATTCTTATAAGTAAACTTTTATGTACTTATTTGGCATACTTCTACATCAACACATCTTTAAATGCTTATAATTGTGTTGCAACTTTATCGAGCTTTTGTAATATCAGGTCATTATTTTTCATGTGCTACTTTTTCATTTTTCTTTTAATTGAACTCCTATCTTGCACCTCCAAATGATGATGTAA

>LNC_000536

TCCAAGTTATTGTGTACATTATAGACAAATAAAAGGTAACTGATTATTCCACTATTCAATAATCTATATCCAAGTTATTAGGATTTGTTGCTAACCTACCCAAATCCTAATAATCTATATCCTCCTGAAAGAAAAGAGTAAGAAATAAAGACACAAGAAAATGCATATAGCTAAATTCTTTATATCTTGCCAAGATATTAGTTTATGGTGTCTTAAAACTCAGTATCTAAACAATATCATACAAAGCAAAATACAAATAAATAGGTACAAATGCACATACACAGTAAACAACAGAACATCAACAAAGAGTAGAAAATTAATCTCATAAACTGGAAATGAAAGACAAAAGACAATACCATGTTATTGACCTAAAAAGTATTACTTGTAATTGGCTTAGAGCTTTTAGGCAATGTTGCTTCAATACTTGACATGCATATCAAATGATCTTTGAAATTATGCAACATATCTCCATTTGGTGGACATCCAAGTTGACAAAGTGAATATGAATCTACACTTCAAAAAGTTATTTCTCACTCTTTGATGAACATAGGAAAATTAGTACACCACAAAATGCCATAAATTCATAAATCTGTGTCAAATTGCAATAGAAAAAGCAACCTAATAGATGATTACAACAACCTGAGATATTGTTAAACTCAAATATCTTCCAAAATGTACTAGACTATACTAGAGCTTCTGGAATATGCTTCTTTTAAGTTTTTTCCTTAACATGTAAGATACTTCATTTGCAATCTAAACATTAGTAGAACATGTTAACCAAATAGAGAATCCTCACAGTCATAGGTCTCCTTAGCAAATAATCCTAATGTCTCATTTTGGCCTTTAGTTTTGTTATACCTGATGAAATATTTACTGAAATATACAGGTCGGGTTCTTTGTTCTCTCTTATTTATGATTGTCGAGTCATCATTATGCATTGTTTTATTAACTTGCTCTATCTTAGGATAATTTTCAGTTATTACTTCAGGCTTCTTTATTATGTTTATGAATCTTTCTTTGTTTTTGTGTTTGTTCTTGATAAATTAAGTTTATTGGGATTTCTGGAGAGAATTACACTCTTATTATGCTCATGATAGCTCTTTTGTTATTATGTTCCTTAATTGTCTTGCTGGAAGTTTGCCACGATAACAATCCTTTGAGTTTTATACCCTGACCGCCTGATGCCATCTCAATTATTTTTGAAAGAGAATTAACCTATATTAAGAAGTCTAGAACATGTGGTTTGAATCAAAGGCACATAAGAAAAGTCACATTACCAATACAAGAGTTATCTTATTAGTTGAGTTATTGATACCTTTATTTATTAAGAATATAGATATTTTTGATGAATGGAAAGCTTTTAGAAGAAAAGCAACTCTTCAGTCAAGATTATTACCAAAAGAACAAGGAGGAGTGCATCCACAAATTCATGATACAAGATCGTTGTGGAAGTTCACAACTTTGCTGTTCTGTATGTCTGTAATGACATGGATGAACATGATTATCCCTTTGTGTGCGAGTCTCTGCAACTCACAAGAAAATTATGATCAATCCTTGATCTATATGCAAGAACTACTTTAGTCAATTTTGTTAATTAAACCTTTCACGAAAGATAATTCAATTTCTTACTTGCTGATTTTTCCTTCTGGTTCATTTGGATTTTTAAGTGTCGAATGAAAACATATCTCTATTTGATAGAGGCAATCAAGATGTGGGACTATACTGGATATGTTATTGTTGACAGAGGTGATCAAGTTGAAAAGTGAACATATTGGTAGAGCTTTTTTCTAAAAATAATAGATGTGTTTGTACTTTGTTCATTAGAAATTGCAGGTATCAATCTCAAGTGTGGTTGCACTTCTATCCACATGTAAGTGTTGTCTCATTTGTTATCTAGAAATTTGACTTGCATAGACAAATTATTGGTATTACCACTTTATAATTGATTCGATTGTTGCTTATGATGTTAATATTTACATGTATTGCTATATTCATTAAAATTTATGTCTGGAAATAGTGGCAGACAAAGCATAAGCTTGATGGGTTCAATTGTGTCGATTATCTTCGACCCGATACATGACCCATATGTGATTTAAAGAGAGTAATGTTTTCCGTTGTAAGAACCTAAAGGATGAATCTATGAAATTTAAACATTGAATCCTCTTCTTGTGAAGGCAATCTTAACCAATACTTTTCAGTGTGTAGTAGAGATTACTCCTTACTAGGTGGTTACCATTTGAGAATTTTGGTTGGCCTTTTCCTGAAATTTTAGTGTCATATATATGAGATATGCTAGAGTGAACCTTCAAAACTTCATAATTGAAATTCATTGTTTTTCCAAGTGTGATTCCATCTATTCTACAAGTTAGCAGACAATTACAAAATGAAAAAGAAGAGGGTTATCATCAAAGTAATTCATAATTTTTCTGTTTATCTTAGAACTGCTGAAAATTTGAGTAAACATTCAACTGTTAGTACTATACCCTCCTTTGGATTGTTAATATAGTAGTTCCGACTACTGAGCTTGAATTGGGTTGCCTCTCTAATCGAGATCGTAGTCCTCAAGCACCACTAAGAGAGGCAACCCAATACAAGCTCTATCTAAGATCCTTATGATCGGATGAATAATTTTATCAGTCATTGTTTAAGTTATTTTGTTCCAATGAAGATATTCTTATAAGTAAACTTTTATGTACTTATTTGGCATACTTCTACATCAACACATCTTTAAATGCTTATAATTGTGTTGCAACTTTATCGAGCTTTTGTAATATCAGGTCATTATTTTTCATGTGCTACTTTTTCATTTTTCTTTTAATTGAACTCCTATCTTGCACCTCCAAATGATGATGTAA

>LNC_000537

GTTTTTGTCTTTTTACTAGCTATGTGGGATACAACTGTTATCACCTCATTTTTCATTTATTGTTACCGTACTGTGTTCCTTCTACATCTTTCTTTCTCTCAAAGTATTCTATCTACAAAATTAATGATTTCTCTGTGCTCCAAATCGCTCTTCTTCGGCGGCGTTGCTTCAAGGCTCTATGTCTTTTTATTATTGTGATGGTTAAGAGTGATCAAGTTATCCTAAAAACTTCTAGGTGAAGCACTCTGTTCTGCTTGTACCGGCGAGGACATGGGACTTCTGCTGAAATTATTGTTCACTACACCATAGGCAATGTGGAGGTCACTTTTTTGGAGATGTACATGTTTTGGGTCTTAACTGTAAAGTACCCTCCTCCTTTGGCACTCTGATCTATCTTGCACTCATACCACTAAAGAACCAAGGTAGTTTTCTCCATTTTTTGTGCTTGTAATACATAAATGTGTCTTATAGTTATGGCTATGTATGAGTTTTTAATAAAGATCAAGTGTTACAGTAGATGAACTCACTTCCTATCTATATTTGCCTATTTTTTTATTTTTTTTAAAAAAAACTAATAATAAATCAATTGACACTATCATCTAGACACAATATTATTGTGATCCATAGGTATCAAAGTTAATCTAAGTCAGAGACAACACTAAATTGAGATTCCAACTACATCTGAAAACATGTTGTTAATTCAACTACCAAGTCATGATCCTTCTGCGCTATGACCTTATGAGTTGAATGAGCACATGCGACATGAGAGCTGAAAAATTAGAAATCATTGTTTCAAAATTATGAATAGGATGTCATAATGGTATCCAAGAGAGTAACAACATATAAATGAAATAAGACTACAAGAGAGATATGTTGATCCGTTTTAATAACCGTGTTGGTAAGTCAATATGCCACTTTCTGATTTTAGTAGATATTTGCATCTATTTAGATTAGATGTCTCAGCTTATTATGATAGTTAAGAATTGATGAAAGACTACTATTTCAAATACTCTATATAGTGTTGTTATGATTGATGAGATAGTGCCGTTTTGTTTCTATGAGATTTTTTCCTTTGTTGTGTGAATTTCAATGTCCGTTTGGATATAAGAACTAAAACCTGAAATTCGGTAAAATAACTGTTGAAAAAATGTTTCTATTCTCAAACTGAATTTTTCGTTCCAAGTCTTAAAAGAAGAGAGGATCACTGATAATTCAAATATTTAAGAACTCTTTAATAAGTTTTGCTCTCTTATAATATAATAAATTCTTATTTGTACTAGGCTTTCTTCTATAGTCCTATATTACCTTGCACGCAACAATGTTATTATAATCAAAAACCTTGGAAACATATGTCATATGTTTGAGAAAGTTAATAGGGCAAGAAATGTAACAAGACAAATGAATATCAATTACTTTGTTATATTTTCTTTTGAGCATTTTATTTGTGTATTGGTAATAGTTGAATTGAGTTTGTATTGTTTGTTTTAAGCTAATTTGTATTTTCTTGCTAAAAAATTGTAGTTGTGTTAAGCATATGACTTTACTGTATTGACGTCTACTATATTAAATAAAAATCCTACCTTACTTTAGTAACTATATAACCATTCCATTTCACACGCTTACAAGTTACAGCTTTATAGTGTCATATATGTTAGTTCATGTTTCTCAGAGACAACATTTATATCCGACACTCAGCAGATAGGAAAATTAGGTGTGGCATCATAGCGGGGGCGATCATATGCTACTTCAGAGTTCCAGATTTACATGTAAGTTAATATGCCAGTTTCTGTTAAAATGTTTGAGGCAACTTCACATGAGTTTAGTTGTTCTTAGCAGTTATTTATATCAATTTACATTTGATGCTTCATCTTAGTGCGGTTGTTTCAAATTATGAAATGAAAATCGTTCTTCACTTTGATGTTAATTAAAATTTTACAGTAAAAATTGATTGGTAATACTTTTCAAGAATGGAACAAGGATTGCAACCTCTATA

>LNC_000538

CAAAATTAAAAAAAACCATCATTTTCATCTTTCTTCACAGTTCACGTTCAAAAGTTTTCTCAATCATAAAATATGATAGATTGTGATTAGTTGTTGCTCGTGCTTTGGGTTCTATTTATTTTGATTTGATTAAAAAAAATCAAGAAAACATCTTGGAAAAAATATAATTATGAGTAAATTCAAGCAATTGATCTTAAACTTATGAAAACGGTGAATGATATGTTTTCTTGCAAAGCTTTTCTTAATTTTCTATTTGATGATTATGGTGATGGGTGTACGTCAAAGAAGAAGAATATAAAAGGGG

>LNC_000539

TATAGATCTAGGCTTTTTTAACCCTAACTAATATCTGATTTATTTCTATGAAGAACTATGCCTTTGGAAAATCTTCTTCAGTGATTTTTCCAGCCAACATGTTTGTTTTCTTTATTTATGTGATAATTCTGGTGTTTCCATTGGATATTTTTAAATCCATATACTCGTATACATGGAATGTGGTAAGGGTAGTCCTAGAATGTGGTAAGGGTAGTCCTGTTGATGACTGCAATAATAAATCTCCATTTAAAGGCCAGAATAACTTATACCAAGTTTCTTCACATATGATGCAGTGTGGGTATCTGATTCCAACTTCAGATAATGCTTAATTCTTGAAGTGAGTTGGGGAGTCTGCTTTGCTAAACTATAAAGTTAAAGTTTGAACAGAGTATTGTACTATTAAATCACAATCTGAGGCGACACACTATGTTACTTTTTTTCACATCATATAATCTACAGTCATGAACTTTAAGTAGTGTGTTTATTTTATTGTGTTATAATAGAGTTAAATTCTTAATTTACATATTGCTCCGTGTTGGAGAAGTACTTTATGTACTCCCTCTGTCCCAATTTATATGACTCACTTTCCTTTTTAGTCAGTCCCAAAAAGAATGACACATTTCTATTTTAAGTAACAATTTGCCTATAAAATGTCTTTTTTACCCTTAATGAAATGATTTACAACCACACAAATTTCTATCATTCATTTTTTACCACAAGTTTTAAAAGTCTTTCCATTTTTCTTAAAGTTTGTGCCAAATCAAAGTACATCATATAAAATGGGACGGAGGGAGTATTATATTTGAAACCTATGAATAGGCGTCTTGTATTCATTGAATTGTAATATACTTTTTCTTGTCTCATTCTCTCACATGATATATGAGCCTACCTTTGTAGTTTTTTCTCGCACCCATAAGTCCACATTCAAATACATGAGTGAACCCTAGAGGTGGTTACACATGTGGAAGGTTGTTGAGTTATATAAAAGTGGTTTATAAGGTCTTTGACAACTTTATTTTTGTCTTAAGGTTTTTTTTTTTTTTTTTAAATATTTTTGTCTTAAGGTTTGCTCAAACTTTCACATGGTATCTGAACTCCTACTCCTATGATCTTGATAGACCTCAATAGGGCAGTCTTATGCTGGTTGTTGGCCTAAGTTCCTTTTCTTTTCATAATTTGCATCTTCTTGTCCTTTCTTGGCACCACTTCTTTCATGCAAAAAAATAAAATAAGGTGGAGGATGGATTATGTTCAAGGCTATTGACTCGGAAAAATTATTGTCTGTTCTTGGCAGGGATTTAAGTGAGTAATTGTTAAATTGTTCTGTAATGAGTCCTTTGTGGTGTATCAATACATAAGAATCCTTAATGTCACATTCAATCAGTCAGCTAAGTGAATAACTACGAAAGATGATTATTTTTTTCCTTAATATATTTCTACGTCTCTCTGATTGCTTAAAAATGATCATAATGAGTGAGTTACCT

>LNC_000540

CTGATTTATTTCTATGAAGAACTACTTCTTTGGAAAAATTTCTTCAGTGATTTTTCCACCCAACATGTTTATTTTCTTTATTTATGTGATGAGCCTGGTGTTTCCATTGGATATTTTTACATCCATATACTCGTATACATGGAATGTGGTAAGGGTAGTCCTGTTGATGACTGCAATAATAAATCTCCATTTAAAGGCCAGAATAACTTGTACCAAGTTTCGTCACATAGGATGCAGTGTGGGTATCTGATTCCAACTTCAGATAATGCTTAATTCTTGAAGTGAGTTGGGGAGTCTGCTTTGCTAAATTATAAAGCTAAAGTGTGAAACAGTATTGTACTATTAAATCACAATCTGAGGCGACACACTATGTTACTTTTCTTCACATCATATAATCTGCAGTCATGAACTTTAAGTAGTATGTTTATTTAATTGTGTTATAATAGAGTTAAATTCTTAATTTACATATTGCTCCGTGTTGGAGAAGTACCATATGTATCATATTTGAAACCTATAAATA

>LNC_000541

TTCTTCAGTTAACTTTCATTCTGTGTACTTATTTTCTTTATGCCCAGCCCAACATGTTGTTTGCTAAGGGTTACCAAAAGAGATATTTGATGGAATGACAACAGCATTCCTCTTTTCTGAGTAGAGGAAAAGAGATTGCCCAACTGGAATCTGGTATGATGACGGAATCCTGCGTGATTGTTGAACCCTCCGTCAACTTGCAAGTTCTTTCTGAATAGCACTATACACAAGGAAATCATTCACCGTCCAGCTATGGGAGAATCTGTAGAGCGTGGATGGTATGATCATCGCTAAAGAGGACAACCGAGTTGGAGGTTCAAAATTGGCTGTGGAGGAGGAAGAAAAAAATGAACATAAAAAATGGCATCTTTGCATATCTCAACATGATCTTGTAGTCATATAAATGTCATGAAATGTTTAAGACTGCAAATTCTGAAAGGTTTTGAAAAAGTTTGGTTGTTCGGTTCGCTGACAAAACGAGCCTTAGGGCAATTTTTGGGACTGAAGTCTAGTTTAATTACATTTGAATGTCAAGCCATTATCTGGTCAACTTCTGTGACAATTGCTAAGTTTGGTTGGAAAACTTTGAATGACCATGACTTCACTTGCAAAACACTGAACTGTTTATGTTTTTCTCTGTTCATCCTATATATGTGAATTAAATCTAGCTTAGATTTGGTGTTAAAGACAAGTGCAACTGCTAATGTGATATGGAAAAAGCCTTACTGAAGATACATAAAAGAGCAGTCTTTGTCGAGAGGTTTCATCCTACAGTCACTAGTGAGCTGCGAATCCCAGCCGATTTTCTTAGGCATATATATCAGAAGAATCACCTGATAAAGCCACG

>LNC_000542

TTTGTATCGAGGAATCGATTAAACATGCTTTATTAAGGTTCATATTTGGTGAGATAAAATCAGTCTGGTCTAGTCTCATGTCTGGAATGCTTCCAATTGGATTACTTATACAGTAGGGCCAGGGTTTCCCCGAGGATAACTTTCCCTTCAGTTTGGGGCAATGAAAGGGTGTTAAAGTTTTATCTTCGGGAAAGGGTTGGTCTACTGCTCGTTTACTAGCATGTTTTTTCCCAAACATTAATAAAATCTTTTTAATTTAGCCAAAAAAAAATCGGGAAAGTGATATCACTGTGAAACAACTGGAAAAGATTATGGGCATTCAATTTGGTAGGGAGTAATAATTGCTTGATGCATGTCTAGCTCATTGGAGAGCTTGGATGCGAGATATTTACAAGTGAGGTTCTTGTCAGCCTTCTGATCAAATGATATCTACTGTATTTCATCTGATACATTTCATTAAAGATGGC

>LNC_000543

TTTTTCTTGGCTTCTGCTTCTGGAGTTGCACTATTTACAGATTACATTAACTAAATGACAAATGGAGAAAAAAAAACTGTTGATTGGTGAAGAGAAATAGTTTGGATTACAAAAATGACAATAATGCAGAGTCCCTTTTTAGCAAACCTTAGTGCATGTTCCTTTTTTGGGACTTTTGACATGTTATGCCTCAAATCTTTTTTACAACACTGGATTTTCTCGAGAGTTCTTCATAAAATAAATGCATGTGGTAATTTATCAATGTTTCACAATTCTATTTTTGATCATTGATGTAATAAACTTAATTTTTTTGATGTTATTGTAGAATGTGATTTATTTAGCATGACACTTCAGCTAATATTCTTTCGAAAGAACTACTGAGAGTGACCGACCTAACTCTGTTCCTTGTTAGATTTCAAAGTCTGCCATCTACAATTGCTGCACTTGTGTAAAACAGTATTTCTTACATATTATTTTGAACTTGATGTGGTATATGGAAAAAGAGATATTTGATAATGATGGAAAGACAACAGCATTCCTCTTTTCTGAGTAGAGGAAAAAAAATTGCCCAACCGGAATCCAGTGTGATGACAGAATCCGGCGTGATTGTTGAACCCTCCGTCAACTTGCAAGTTCTTTCTGAATAGTACTGTAGTACTCCTACCCCCTTTGCCCTCAATCAGAGGATTTAGGTTATTTTTTTACTGGTTACTACTTTTTCCTGGTTCTTGAATTGACATTGCTGGTTCTTGAATTCTTGAATTGATGTTCTG

>LNC_000544

CTTAGAGCCAAAATGATGGATCATCTCCCTCAAATCTCTTCTTCTCCTAACGGTTCAAAGCTTATGGGTTACGAGTTAAATTAACTATACTGTTGAAAATCCCAATTTGAAATACACGACATATAAGAGGAAGAGAGCTGATAAAGCATAAGCCCCAAATATTTATGAAAGTTATCCATGTTTGGTATAGTTGCGTAGTTTCGTGGCAGAGGAGAATTGTGGATCCTTCCAATGAAGGCCTCTTTTCTTTAGTCCGAGGAGAATTGCGGATCCTTTTCTAAGGGTAAAATTGTTGCCAAGATGAGCGAAGAGGTAATTCATTTGAACCATACAACATAAGTGAGGATCTTCTAAAACTAGAAGGGCAACAAAAGAACAAATTTGATGATGAAGAGCTAGAGCATCTTGTTGAGTAAAGAAGGTAACACTATTTACCTTTTTTCTTATAGGCCATCAAGGTTGAGTGAAGAGCTGAAATATGATTCCAAGAAAAGATTACTTGATTGGGGTAGTAGAATTTTTAATTACAGACAAGAAAAGGGGATTATGCTACTATGTTTGAATGAAGGTAATGTTCTTCATGGTGACTATTTAGTATGCGGTAGATACAGATTTGCATATATGATTTTTTTCGCTTATTAATTTCTATTCAAAATAGAAGGATTTAGCTTTGACACAATGTATTCGATTTTAATTCTCGGGAGAGATGTTGATATGGATTACTTAACGGCTATTTGCAGGTATTAAAATAGCGTGGATATGAATTTCAAGTTCATTTTGGCAGCACTTAAACCAATGAAAAAAGTACTTAAATCAAGGTGCTTTTGGTGTGGTGAGTTCTCTTCTTGGAGCTCAATTCTCTTGGAGAGCTTTTCGTGAACTTTTTTTTGTTAGTGTTTGGTCTCAACAGTTTTGAAAAAGAGACTGTATCACCTATCCTTTGTTGCTAGGAGTTTAATTCTCTCAGAGAGCTTTTGGATAACTATTTTTATTAGTGTTTAGTCATAGTATTGGTCCCATGTGAAAATTTAAAAAGTTGGTATGTTTAATATTTTGTTTAAGCGATTTTATTAAGAGGAGGTATTGCAAAGAGAGAAATTGTTACTTAATATGTATTGTTGAAAATTATTGAACCTATAGAGAAATGATTTTACACTGCAATGCATATTTGCACTTTTAGTATTCATATCTTTGAAATTCCATCATACTTCTCACTATGATTCACAGAAATGATCATGCTTTATATCACGGAGCTTCTTTGTTGATGTACCATCATACGTCTAATTAAGGAATGAAGATGTTTCATGATTTTCTCAATGAACTTAAAATTGGAGTTTTCTACCGACGACTGGAAATGCAATGATCCTCTGATTAGCACGGGCCAAGTCTATCTAGTTTGAATAGAAGAATGGAATGTCCACCCATATTTCACTATGAAATTTGGAAATTAATAGCAATAAATATAAATTAAAAAAATAGTCTTTACTCGTTCATAGTTCTAATTTCCAAAAGCATTGCTACAGCATTAGGGTTTTTCCCGCCCACTCCATATAATACATACTTAGCAGCAAATCGATCAGGCAACAATTTGCCGGCGATCGGAATGGCAGAGGAGGTGGAATTCCGGTTGTACAATACAATAACGAAGTATGGGGAACTTTTTC

>LNC_000545

CTTAGAGCCAAAATGATGGATCATCTCCCTCAAATCTCTTCTTCTCCTAACGGTTCAAAGCTTATGGGTTACGAGTTAAATTAACTATACTGTTGAAAATCCCAATTTGAAATACACGACATATAAGAGGAAGAGAGCTGATAAAGCATAAGCCCCAAATATTTATGAAAGTATAGTTGCGTAGTTTCGTGGCAGAGGAGAATTGTGGATCCTTCCAATGAAGGCCTCTTTTCTTTAGTCCGAGGAGAATTGCGGATCCTTTTCTAAGGGTAAAATTGTTGCCAAGATGAGCGAAGAGGTAATTCATTTGAACCATACAACATAAGTGAGGATCTTCTAAAACTAGAAGGGCAACAAAAGAACAAATTTGATGATGAAGAGCTAGAGCATCTTGTTGAGTAAAGAAGGTAACACTATTTACCTTTTTTCTTATAGGCCATCAAGGTTGAGTGAAGAGCTGAAATATGATTCCAAGAAAAGATTACTTGATTGGGGTAGTAGAATTTTTAATTACAGACAAGAAAAGGGGATTATGCTACTATGTTTGAATGAAGGTAATGTTCTTCATGGTGACTATTTAGTATGCGGTAGATACAGATTTGCATATATGATTTTTTTCGCTTATTAATTTCTATTCAAAATAGAAGGATTTAGCTTTGACACAATGTATTCGATTTTAATTCTCGGGAGAGATGTTGATATGGATTACTTAACGGCTATTTGCAGGTATTAAAATAGCGTGGATATGAATTTCAAGTTCATTTTGGCAGCACTTAAACCAATGAAAAAAGTACTTAAATCAAGGTGCTTTTGGTGTGGTGAGTTCTCTTCTTGGAGCTCAATTCTCTTGGAGAGCTTTTCGTGAACTTTTTTTTGTTAGTGTTTGGTCTCAACAGTTTTGAAAAAGAGACTGTATCACCTATCCTTTGTTGCTAGGAGTTTAATTCTCTCAGAGAGCTTTTGGATAACTATTTTTATTAGTGTTTAGTCATAGTATTGGTCCCATGTGAAAATTTAAAAAGTTGGTATGTTTAATATTTTGTTTAAGCGATTTTATTAAGAGGAGGTATTGCAAAGAGAGAAATTGTTACTTAATATGTATTGTTGAAAATTATTGAACCTATAGAGAAATGATTTTACACTGCAATGCATATTTGCACTTTTAGTATTCATATCTTTGAAATTCCATCATACTTCTCACTATGATTCACAGAAATGATCATGCTTTATATCACGGAGCTTCTTTGTTGATGTACCATCATACGTCTAATTAAGGAATGAAGATGTTTCATGATTTTCTCAATGAACTTAAAATTGGAGTTTTCTACCGACGACTGGAAATGCAATGATCCTCTGATTAGCACGGGCCAAGTCTATCTAGTTTGAATAGAAGAATGGAATGTCCACCCATATTTCACTATGAAATTTGGAAATTAATAGCAATAAATATAAATTAAAAAAATAGTCTTTACTCGTTCATAGTTCTAATTTCCAAAAGCATTGCTACAGCATTAGGGTTTTTCCCGCCCACTCCATATAATACATACTTAGCAGCAAATCGATCAGGCAACAATTTGCCGGCGATCGGAATGGCAGAGGAGGTGGAATTCCGGTTGTACAATACAATAACGAAGTATGGGGAACTTTTTC

>LNC_000546

CTTAGAGCCAAAATGATGGATCATCTCCCTCAAATCTCTTCTTCTCCTAACGGTTCAAAGCTTATGGGTTACGAGTTAAATTAACTATACTGTTGAAAATCCCAATTTGAAATACACGACATATAAGAGGAAGAGAGCTGATAAAGCATAAGCCCCAAATATTTATGAAAGTTATCCATGTTTGGTATAGTTGCGTAGTTTCGTGGCAGAGGAGAATTGTGGATCCTTCCAATGAAGGCCTCTTTTCTTTAGTCCGAGGAGAATTGCGGATCCTTTTCTAAGGGTAAAATTGTTGCCAAGATGAGCGAAGAGGTAATTCATTTGAACCATACAACATAAGTGAGGATCTTCTAAAACTAGAAGGGCAACAAAAGAACAAATTTGATGATGAAGAGCTAGAGCATCTTGTTGAGTAAAGAAGGTATTAAAATAGCGTGGATATGAATTTCAAGTTCATTTTGGCAGCACTTAAACCAATGAAAAAAGTACTTAAATCAAGGTGCTTTTGGTGTGGTGAGTTCTCTTCTTGGAGCTCAATTCTCTTGGAGAGCTTTTCGTGAACTTTTTTTTGTTAGTGTTTGGTCTCAACAGTTTTGAAAAAGAGACTGTATCACCTATCCTTTGTTGCTAGGAGTTTAATTCTCTCAGAGAGCTTTTGGATAACTATTTTTATTAGTGTTTAGTCATAGTATTGGTCCCATGTGAAAATTTAAAAAGTTGGTATGTTTAATATTTTGTTTAAGCGATTTTATTAAGAGGAGGTATTGCAAAGAGAGAAATTGTTACTTAATATGTATTGTTGAAAATTATTGAACCTATAGAGAAATGATTTTACACTGCAATGCATATTTGCACTTTTAGTATTCATATCTTTGAAATTCCATCATACTTCTCACTATGATTCACAGAAATGATCATGCTTTATATCACGGAGCTTCTTTGTTGATGTACCATCATACGTCTAATTAAGGAATGAAGATGTTTCATGATTTTCTCAATGAACTTAAAATTGGAGTTTTCTACCGACGACTGGAAATGCAATGATCCTCTGATTAGCACGGGCCAAGTCTATCTAGTTTGAATAGAAGAATGGAATGTCCACCCATATTTCACTATGAAATTTGGAAATTAATAGCAATAAATATAAATTAAAAAAATAGTCTTTACTCGTTCATAGTTCTAATTTCCAAAAGCATTGCTACAGCATTAGGGTTTTTCCCGCCCACTCCATATAATACATACTTAGCAGCAAATCGATCAGGCAACAATTTGCCGGCGATCGGAATGGCAGAGGAGGTGGAATTCCGGTTGTACAATACAATAACGAAGTATGGGGAACTTTTTC

>LNC_000547

AAAACAGACAAACACCAAATACAGTTAAGGTAGCAATCTTTTTGAACTTCAATTTGAACAGCTTATTGCTTTGTTTGTGTCATTAAATCTACACCGTATGTCTCATTATTGCTTTGTTTCAGCTTGACTTTATGTTAAAAAGAAAAATTAGATACGTAAATTAAAAACACTGCAGGTTGAAAAGTCATGTTCAGTTTATGCCTTTAGTGTTTCACATCTATTCTCTCCATTAACATTGGAATGTAATTTTTATTTATGCCTTTCGATGGAGATGACCCATTTCAGGGTAATTAGTCTGCTTTAGTTGTGTATCCTCCGTCTCTTTCAGATCACCTAGGATTGTTATAACTATAAAATTCTATTAATGCACATATATATAAACAGTTGTACAACTAAGTGCTAATGTATTAGGAAATATTTTCACAGTAGTAGTTGTTTCCATATTATACAATACACTTTTCCTTATGTACTTTTCTGTAACATCTATTGTGAGATCTTAACATCTCTGCCTTCCACCTTCTGCAGTTTAATCAAGATGTGATCAGACCAATCACTACTTACCATCGCACGTCCTCTGTTGAAAATTGAAATTTTAAAATCAAAGGACAAACAAGTTCCCAGTTTGTTCATCCAAAAGTACCTGATGAAGAAGTTGAATCTTTCTTACAAAAATGAGGTAATTAACTTCTTGTATTTTTGGCAATTTAGTAATCAATAAATATGATTAACATTTATTTACAATCCTACCTATTGTGATTTAAGTGCTGAAAAATTATGTAGGAGCATGAATTCCTGTAGTTTATTATTCTGTTAGATGTAGTGTGGTCCTTGTACTTTTAGGACTTAGTTTTCCATGCCAAAATATTATCAGTGCAACGCCAAAAATAGTTCGCGTCTCGAATTGAATCAACGCCAAAAATAGTTCAGATCTCCAGTTGGAATGAATTACTGATGTAAAAAGTTGCTTGCGAAGCAGGTTGCCGTCGCAACATCTTGCTTCAAAGCACTGGAAACTCCTTAAGCAAAATTATACGTATTGGGTTATCTGATCAATTTTTAGTAATTCTTCGTTTATTAAAACATATGCAATTCACGCTCTCTGTTTCAATGGTGTTTGGATTTGATAACTTTTTCAGAAATCAAAATTCTTCCCCAATTGCCACAAACTGATGCTATTGATCTTTTTTGATATGACATGTACCAATGTTTTTCTAAATTCTGTAAGAACATGAAGTTGGA

>LNC_000548

TCTCTATTGTCCTAAATTTCTATTCCACGCTCTCCTTTTTCCTATTCTTTCTACTTTCCAGAACTCATGCTCTCTCCCTCACACGTTCCTCTACTCCTTCACAAGAAGAACGACGCGAACCCAATAATTCCAACGAGTCCTAGCCGTGGTTTATCCAACTTTTGGCGAGGAACATCAATTTCATATAGCTGTCCCGTTCGTCCTTTCTTATCCAGCGAGGAGTAAGAATTCAAACAATGACTCGTCCTTCTTATTTCTTGTCAATTCTTTTTCTCTCGATTTTACCCTCTCTAGTACCATTCGAGGTTTCCAAGCACTTTCTAAAAGAATTTCGAATTCACAGTAGTCCCACATCAAAAATCTTTCCAAATTTCGTGAGATTTTAGGTCCTATAAATAGGCCTTCTCTTCACTGTTCAAAGGGAGTTTTTTGGGAGGCGAGAAAAGGAACACCCAGCTAAGTGTTTTAGGAGTTCTTATTTTTCGTCACTGAAATTTTGTTGATTTGCGGCTATTTTGAGTGGGAGTGGTGTTGGAATTCGTCACGTTCAAACTTGTTGTCCGGTTCGCTTCCCAAAATCAGTCAACCTCCTTTAAGCCGATTGAAAGGAAGCTAATATTATTGGATTAAACACCAAGAAGACAGCACCAACAGTAGCCTAAAGGGCTAAAAGAGTAGCATTTTCTTGGAGTTGAAAGTTAGAAAATGGCACCAGCTGAAGCAGAGTTGCAACCGCCTCAAAAAGCTGAAAAACGAGGAGCAAAATTGGGTTAAAACCCATAATTAATGAGGCAAAGTTGCAGCAGCCTTAAAGTGCTGAATTTAGCATTTTCTTCGACTTAAAAGGTCGGAAAATAGCGCCAGAAGCAGCAGAACAAGGAGCAGATTAAGGGTCAGGAAGCCCAAATTTTATTCTCTCATTTATATTTAACTCTTTTTATATGGTGTGTTTGCTATGATCTTACTAACTCTTGTTTGTTTTGCCTTTGAACTAGATGAATCCTCACCAGAGTTATCAATGTTATTTCTTAAATCATATCTCTTGTTTCTTATCACTTGTCAAATTGTTTTAAGTTAGACAACTTAAGTTTATTAGAATAACTTAAGAGTCTTTCATGTTCTTTCATAGTTATTGAAAAGCTTTAGTCCATGTTTATGAGTTTGAATTAATTGTATAGTTCAAAAACTTGACTATGGATAAAATTTTAAGTTAGAATTATTCTTGAGATTTTGCAAGTTTCAAAAGATTTTTATCTAAGTTTTAAACAGGAATTTTTATAAGTAATTCTTTTTATTTTATAAAATTAGCCAAAACTTTTAAAATTCGTCGGTCAACCGCATGTGCGGATTCTCTAAGATGCTTAAACCCTTCCTTAGAGGATTATTTGAACCCTTACCTGATTTTGATTTTATTTAAAATATTTCCTTTCAAAAACTCCTTTTTAAAATAATTTTCTTATTTTTTCTAAAAAATTAAGTGGCGAC

>LNC_000549

TCTCTATTGTCCTAAATTTCTATTCCACGCTCTCCTTTTTCCTATTCTTTCTACTTTCCAGAACTCATGCTCTCTCCCTCACACGTTCCTCTACTCCTTCACAAGAAGAACGACGCGAACCCAATAATTCCAACGAGTCCTAGCCGTGGTTTATCCAACTTTTGGCGAGGAACATCAATTTCATATAGCTGTCCCGTTCGTCCTTTCTTATCCAGCGAGGAGTAAGAATTCAAACAATGACTCGTCCTTCTTATTTCTTGTCAATTCTTTTTCTCTCGATTTTACCCTCTCTAGTACCATTCGAGGTTTCCAAGCACTTTCTAAAAGAATTTCGAATTCACAGTAGTCCCACATCAAAAATCTTTCCAAATTTCGTGAGATTTTAGGTCCTATAAATAGGCCTTCTCTTCACTGTTCAAAGGGAGTTTTTTGGGAGGCGAGAAAAGGAACACCCAGCTAAGTGTTTTAGGAGTTCTTATTTTTCGTCACTGAAATTTTGTTGATTTGCGGCTATTTTGAGTGGGAGTGGTGTTGGAATTCGTCACGTTCAAACTTGTTGTCCGGTTCGCTTCCCAAAATCAGCCGATTGAAAGGAAGCTAATATTATTGGATTAAACACCAAGAAGACAGCACCAACAGTAGCCTAAAGGGCTAAAAGAGTAGCATTTTCTTGGAGTTGAAAGTTAGAAAATGGCACCAGCTGAAGCAGAGTTGCAACCGCCTCAAAAAGCTGAAAAACGAGGAGCAAAATTGGGTTAAAACCCATAATTAATGAGGCAAAGTTGCAGCAGCCTTAAAGTGCTGAATTTAGCATTTTCTTCGACTTAAAAGGTCGGAAAATAGCGCCAGAAGCAGCAGAACAAGGAGCAGATTAAGGGTCAGGAAGCCCAAATTTTATTCTCTCATTTATATTTAACTCTTTTTATATGGTGTGTTTGCTATGATCTTACTAACTCTTGTTTGTTTTGCCTTTGAACTAGATGAATCCTCACCAGAGTTATCAATGTTATTTCTTAAATCATATCTCTTGTTTCTTATCACTTGTCAAATTGTTTTAAGTTAGACAACTTAAGTTTATTAGAATAACTTAAGAGTCTTTCATGTTCTTTCATAGTTATTGAAAAGCTTTAGTCCATGTTTATGAGTTTGAATTAATTGTATAGTTCAAAAACTTGACTATGGATAAAATTTTAAGTTAGAATTATTCTTGAGATTTTGCAAGTTTCAAAAGATTTTTATCTAAGTTTTAAACAGGAATTTTTATAAGTAATTCTTTTTATTTTATAAAATTAGCCAAAACTTTTAAAATTCGTCGGTCAACCGCATGTGCGGATTCTCTAAGATGCTTAAACCCTTCCTTAGAGGATTATTTGAACCCTTACCTGATTTTGATTTTATTTAAAATATTTCCTTTCAAAAACTCCTTTTTAAAATAATTTTCTTATTTTTTCTAAAAAATTAAGTGGCGAC

>LNC_000550

CACAAATAGAAAGTGTGGTCGAAATGGGAAATATACCATCAGTTGTCAATGAAGTTGAAATAAAATCATGAGAGAGTTCCAATCCTAGTAGAAGCAAAAAGAAATTAGCCGATTTCTTCCAATTTGTCTAAGTTTCAGCTGACAAGATTACTACCTGGTACCTTTGGGAGGAAGATACATGCACCTAGTGGAATAGTCAAGATACATGCAAGTTGGGTTGAAGGCACCTTATTAGTCTTAGAAGTGTCAAATCAGAATATTTAGTATGATGTATCATCTGAGAACTTCCTGGGGTGTTTTTGAACACTTGTAGAACTAATTCTGTTTTCTAACCATTAGTTAACTCTATCAAAAGAATATCAAGTTGAAGCTAGTTAAGGTGTAGGTAGAATATCTATCAACCAACTTATCTTCATGTAGTTCTATTCAGTTCCATTTG

>LNC_000551

GAAAAATATTTGGAGTTTGAATTTCGAATTTGGGGCCCTCGTTTCTGTCCGTTTTGGATCCCTTTCCCCCCCATTTCCGAAACCTACTTTTCCCTCCTATATATTTGCTTTTATTCACTGTGAAGGGGTGGATTTTTCTTGGTTTTGGCTTCAAAAAAAAAATGACACATAAAAGAGACAGTGAGCAAAAATACAAGAAAGAAAAAAAACTGATATCACCAAGCATAGGCAGGAAAAAATCAAAGTAGAATTTAAAACGATAGTGTTTATATCACTGAGATTTCTGGGTGCTCTGTTTGGAATTCGAGTTTCCGTCGTAGGTTCGTCCTTGGTGGGGGAAGTCAATCCAAGTCTCGTTGTCCCAACTTCGCTGCCTTTAAAAAGTCCATTGTGGACTATTTCCCTCCTTGCATCTCGAGTCAGGTCGAAGGCCCAATTCCTCTCCTCCCGAGTCAGCCAACAAAGGGAACATATGTCCAAGGCTGAAAAACCAAAAATGC

>LNC_000552

AATAGGAATAGGTATATACAATCCTGCTTAGAATAGAAATAGGAATAGGAATAGTAATAGAAATATGAATAGTATTTACTTTCCTACTTGGTAAAGGATTGTATTGTAGTTCTATAAATAGGGTCTCCATGTAATAATATCTATACAACAATTCAATAATATTTTTTTCCAATATTTCTCACAAGAACAACTGATTGAAAAGAAAAAAAAACTACATACCTTGCGCAATCTAGAAACAGAAAGAGAAGAAGAACCAACGTAAGTATTTTTACAAAGATTCCAGCCGCAGTCGACAAAGGAGGGAAAATATTTGGTAAAATTTACGTAAGTATTTTTACAGAAATAAAGAAAAACCTAGTTAGCGCTTGTCTCTAAAAGCGAGCTTTTTCTAGCTTTTATAAAAAGCACGCTTCTCCCGTTTGGGACAGAAGCACACATTTCTTCACTATCGCGTCACTTCAAGGCATTAAAGCGACAACCTATTGCTTCGCTTCTCGCTTTAGCAAGCGCTTCAACGTTTTTAACAAGACTGGTTTGGAGCCAACCCATTGGACCAACTTGGGATATTGGTCTCCGTTTAAATACTAGATTTAGTTCACTTACGTAGAACTCAAACTAGTGTACTAAAATACAAGCCCCATGCCAATTGAGCATAGTATCTTTAACATACTTTTGGAATCTAAACTTGGAAATATAATGATTTATTTGGTTGAACCATTGAAACACACGTGTGCTAACGAACTTGGTATATGAGATTTTGCCTTCTAGGAATTAAGATGAAATTGTCCGTTGTATTGACTTTATATACTTAATTGTATTTTGGTGATTCTTTCTAATTATGTGCTTATACATAATGTCTTTGGAATGTTTCAGTGGAGATTGTGAACTTGGAATGTTTTGCTTTGGTTTCAAATGACACCAGAGCTAAATATTCCAAGTCCACATTCCCACTGAAACATTCCAAAGACATTATGTATAAGCACATAATTAGAAAAAAATAAATCAAAATGCTTCTGTAATCACCAAAATACTAAGTATATGAGTCAATACAATGGACAGTTTCATCTTAACTTTGAGAAGACAAAATTTTATATAACAAGTTCGTTGATACGCATATATGCTTCAACCTAATAAGTCGTTACATCTCTAGGTTTAGATTCAAAAAACATGATGAAAATGACTATGCTCAATTGGCAAAGGTTGGTGGTTTGTATTTTAATACAATGGTTTGAGTTCTATGTCAAGTGAACTTAATCTAGTATTTAAATGGAGTAAAGTGAGATCATCATCTTTGAGTTTTGTACTGTACATTAGCCCAAATGATTGGCTCCAGACCAATTTCTCGGTTACCAAATAAAAATATGACTACAAAACTCATCGTTACAAAAAACTTTTATCTACGAAGTTAACATGTAATTGATTAGCATAAGTTATTGCATATTGACAAATGCTACCAATTATCCTTGCAGTGTTATCAAAGGCGCACTTAAGCCTTGAAGCGAGGCTCAAAACATGTTGAGCGCTTTGTCTCGTTTAATGTGCTTCAATGTTGTCAGCAAGGTTCTAAAACATATTTTTCCTTGCCAATGAACTTCTTCTGAAGAGACGACACTAAACAATTGATATTTCATTTTATCGTAATTATTTTTCAATTTCTTTGTTCATATATTTTTCATTCATGCTTATAAGTTATTAGTCTTGGACTCAACATATATATTTTTATTTTTCTCCCTTCGCCTTTTTTTCTTTAAAGTCCACACTTTATTTGCGCTTTGTGCTTGAAGCCCCAATAGACCTTAGAGCTTTTTTGCACTTTTCGCTTTTGACTTCACTTTATCCTTGCAAAATTGAGGAAACTCATTCACTTCCAAACCCCCACCCTCCTCCTCGACTACTTTTACGTAAGCATTATATGGTGATATCTCGATTTGGAGGCAAAATGGAATGGTTAAAGAGGACTTATAGAGCTCGTTTGGATTAGCCAATAAGTTTTTAAGTGTGGAAACTGTTTTTGTAAACAAG

>LNC_000553

TGTTTTTCCATTTCTTGAATTTCCCTTGTTCAAGAGCTAGAATTATTGAGGTTAAGGAGGTGCTATTTTTAGTTGTTTATAATGTCGTCTAAGCCGTTGGACTATGAGAATTTGAATGAGAATGTGAAGAAGTGTCAATATGCTGTACGAGGTGAGCTCTATCTTCGAGCTTCTCAACTTCAGAAGGAAGGAAAGAAGATTATATTCACAAATGTAGGTAATCCTCATGCCCTCGGACAGAAGCCGCTGACGTTTCCGCGCCAGGTCATTGCTCTCTGCCAAGCTCCATTTTTTCTGGATGATCCTAATGTTGGACAATTATTCCCTGCTGATGCAATCGCAAAAGCTAAACATTATCTTTCGTTGAATTCCGGAGGTCTAGGTGCTTATAGCGACTCCCGTGGCATTCCCGGTGTAAGGAAAGAAATTGCAGATTTCATTGAGAGACGTGATGGATATCCGAGTGATCCGGAACACATATTTCTCACGGATGGTGCCAGCAAAGGAATAATGCAAATATTACATACTATCATTCGGG

>LNC_000554

GCAAAATTGTCATTGTGAGCTTGAGAATCTTACAGTGTCAGTGATAATGGAGTAGGGTACAAATGTACTGTTTAGTTGTTAATTTGGTTCTTTAATCTTTGTGATCTGAGTTGAAAGCAAAAATTTTCACTTTTTCTCTTTGCCCAATGTGTGTTTTGATTTTCAAGGCGAAAAAAAGGTAGAGAGAATGACACATTGATATATCGTTTTCATCACAATTGGGCTTTTCCTACTTTAGTTATTGTTGGTTTTGTTTGTTTTTTCTACTTCTTGCATTATTCTCCTTTTGTTTAAAAAAGAAATCAACTTTTGATCCATTGAATCTGCACATAGTTATACAGAGCCATCACTTTCT

>LNC_000555

TAAAAAATTGAGGCTGCTATCGTAGTTATATACAAGATGTAGTCACTACATGGATGGTGATGTTCTTCTATCTCATAGCTAGTCCTTCCTTTCTCTCTTTTATTCTTTCATTATGTCAAAACATCAAAACTACAATAAACACTACTAGGTTAATCTTTCTACTCCTAGTCCAAACATTCTAGGAATATTAAAAACTACAATTAATACTACTTTTATTACCATAAATTCTATCATAATAGATTTGCGTCCTTTAAATATCAATAAAAAGAACACTCATACAATCTTATCTCCGACAAAAGCGAACAACTTCTTCTCTACCTACTCAATCAACCATGAATTCAAACCAAACAAATTTTACCACAATTCCCTTAATACTAATGGACATATTGGTTGCAGCCCCCATCTGCATGACGCCGCCAATACCTAACACAAACCCATCATCTTGGCTCAAAATGGGTCTCCACGGACACTCTCTAACACTACATGTCTTGATGGAGAATCAAGAAATAATGCTAGTGATACAAAAGCTTTCCATTCTAGCTTAGATTATTTCGGAAGGGATGAGGCGAGCCATGGAGACTTACAACAGCCATCTATGGCTGTCGACAATCCTTCGGCCACCAATAAACTACCAGCTCCCGGGGACTTATTGTTCTTTAACATTCCAACAAGGGAGACACCACTGTGCACTCTCATGCACCAAACCCATCCAATTGTAGTGACAACCCCTCTATCAGGTTGCTGCGAGTTATCACTGCTATTGGATGAGTTAGTGCATGAGAGTGTACAATGGTCTCCCTTGATGGAATGTTCTTTAAGTAACCCAGGAGTTTGTAATTCATTGGTGGCTAAAGGGTTGTCGACATCCATAGATGACTGTTGTAAAACTCCATGGCTCTACTCATCCCTTTCGAAACAATCTGAGCTAGGAAGGAAGAATTTGTATCACTAGCATATTTCTTGATTCTCTATTAAGACATGCACTGTGAGAGAGTGGCCTTGGAGACCCATTTCGACCCTGGGTTTGTTTTGGGTATTGGCGACGTCATGTAGATGGGGGTTGCACCCGCAACCAATCCGTCCACTGGTATTATGGGAGTTGGTGGTAAGGTTTGTATGGCTTGGATCCATGATTGACTGAGTGATAGAGAGGAGGTTTTTCGCTTTTGTCAAAAATGAGATTGTATGGATCTTCTGGTCATTGATATTTAAAGGAGGCAAATCTATTGTGATTGAATTAATGGGAATAGAAATAGTATTTCTTGCAACTCTTAATATTCCTAGGATGTTGGAACTACGAGTAAAAGGATTAATTGC

>LNC_000556

TTCTAGTTTGCATTCTATTGTGTGAAGCATGTTCTATTTAGGGGTTGCACAATTATATTCAATAGTCGTCTCATGTGGGATACTGGGTATGTTGTTGTTGTCTCATTTAGCATTCAAATTTTATTGCTTGGCTTCCTCCATGCACTCAAGAGACATTTGTGCTCACTTAACGGGATGGTGCACATAATATTGTTCAAAGCAAATTTGCTTCAACAACATTCCTTTTTAGCCGAGGGTCTATTGGTAACAACTTCTCCCATAAAGGTAGGGGTAGGGTCTGGTTACATCCTACCCTCCCCAGGCCCCACTTTTGGGGTTACACTAGATATTTTGTTATTTATCAAAACAAATATAGATACTTGAAGTATTCATAAAAAGAGTATTATTACTTCAAGCATTCAAATGTTCTAGAATAGCTAAAATCTTATTGAAAATGTAAAAGCCTATGATTTAGATTCTACATACAAT

>LNC_000557

TTGTCTTTGATCTCGTCATCTGAACCTAGTTGTTTTATATTTGACTCATCCGTTATGATTTTTGGAGGGGGAAAAAAGAAAGAAAGAAAAAATGATCGCGATTAGTTACTTAACCATTTTCCTAAAGTTTCTCTTCAAGAGAATGAATATAACAAATCAACTTTGTTTCTGCATACCACTGGCTTACCTCTGGTAAGTGATAGTAAATGTCGGGTCGGTGGATAGGATTATGCCACGTGTATGCTCGTTATTATAAAGGGTATATGCTCTAGTTTTTGAACGACAGGTGCACCAATGTCCCACGGAGGGTATCTGCATACCATATACGATAGTTCAGGGGTATATTTGTCCTTTTTCCCTTTATTCTATTCATGTTACTACAGTGTGTTGGAATT

>LNC_000558

TTATTGTTCAAATCAGAAGGAAAGAAATGTTGGCTTGCCTTCTGATCATATCGCAGTTGGTGCAAAAGTGATATATGCTGCTGCTCCTGCAATGGGTCATAATCAGATGTCCAGAACTCATGTATGCTAAAGTTCTGAATCTCAATTCCAGTGACATTTTAGTTAAATGTCCTACAATAGGATCCTTCTCTTTCCAGGTATCATTCCTCCTTCAACATGAAACCTATTTAAATTGAGGGCATGTGCCACTTTTCGGCTGGTTGGAGATTGACAGGTTTTTATTATTAATTTGTCTATGCCTTTCCCTTTCAAAACACATCGTAGACATCTAAATCTTATTGGATCAGTCCATACAATACTTGTGCTAAATCCTAAATTCAAGACACGTTGGATCAAGTCAACATTTTCGGTCAATAAGTTCCAAATTAATGATTTAATTCAAAATTATATGACTTAAGTGAAATCCAATGTCACGACCCAAATCTGAAGGGCCGTGACCGCCACTTAGTGAGCATATACCCCACCAAGCAAACCAACTCTCTTAGTTATCATTAGGTAAGCAGTCAGAGGAAATGTTCTATTGCTCAATGGTCAAAGAATCTAACTTCAAGAATTTTAGTTTCAGGAACTCTGTCTGTCATGCCCCATTGCGCACTGTGATCTGTCTGTCTAAGTTTTCTTCCCCTGGATATGGGTGTTAAATTTGGTGTTAAATTTAATTTGGTTTGATTTGGTTTTTCTGTTTATCTTGAGACTTGAGTACCTCTATTTGTAATGCTGAGCAGCTATGCACGACCAACTAGTTAAAGTTAACATAACGAGACCGGAATGTAGAAGTCGAAAGCTTGATAGTTTTATCTTCTGTTTTAGAGAATACAAAATGTTCTCCAATTTTCTTACCTTTTTTGTTCCAATTCTATTGGATGTCCC

>LNC_000559

GAAGAAGTTGAGTGAACTGCTACAATCAGTTTCTAGTTGTACTGGCTACTTCGATCTTAATGGAATCATCAGTTTTATGTAAAGAATCGAGAACATTTGTAAAGGGTAAAGAATGTTAACAACATTAAAGTATTGAGGGAACATCGAATGCTCTTTTATGGTTCCGATGATTACTATCTTGTTATTTCGGTGTGGAGATCAGGCAGTTGAGCATATGATAATTGATAGTAGTTGCAGTGCCACCATTATAGGGGTCAAGTAGGATTTTGTTTTACTGTTCACTTTGTTTTGTAATTAACTCAATCACACAATATTGTGTATGTTCTCATTGGAAAGTTCTGAAAAATGTACAAAGTTCAATTTTTTTTTGATGTAGTCCCCTAATTTAGTTTACTTGCATGTTGATTCTTCACTTTGAGTCATACAAATTTTGTTTATTGGACCTGTTCATTGGGAAAAAAGTTTATAAATTTTAAGTGTGGGTGATTTATAATTAAAAGTTGATGCTTACTTACTCTGTCCTATGAATTGTTTGGAATTGGTCTTGCCAACTTAATTGTCT

>LNC_000560

CAATCTAGTTTTCTTTATTTTTCAACTTCAAATTCATTAATCTTGAGGAAAAGCCTTACTTTGTATGAACATGCATTATTAATTTAAATAATTGGTTGATAGATCTTGCTTTTAATTTTTCACTGTATATGTTATATATAATCGAAGTACAAAAATTAAAACAGAAAGAGAATTTCAATAAGAACAAGAAATCTGAACACAATGAAGGCAAATTTAGAAAGGATCAAGGGGGTTATTTTAAGAAGATACTATGTAACAAACAAAAGCAACAAGCTATTCGTGGTGCATAATTTATTAATTCTCCGTCATACAGTTGACTTATATCAATTAATTCTTTACTTGTTCATTTTAACAAGTAAAATGTGGTTAACATGATATAAGTCAATTGTGAGATGTAAAATTGATAAGTTGTATACCATTAATAGCTTGTTGTTATTGTTTGTTAGGTAGTTTGTTCTTGGAAAAGCACTCTTGGCCCTTTTCAGATTTGTGTTCATCGGATTCTTCAGATTTTTTTATTTTTATTGACACCCTTTTTATGTTTCAACTTTTGTAATTCGGTCATATAGAACATACATTGTGAAATACAAATAGCAATACGTATCAACAAATTATTTGAATTAATATTTCATGTTGGTACAAGAAAAGATTTATTTTGTATGATCATGAATTATTAATTTAAATAATTCGTTGATAGGTCTTGCTTTTAGCTTTTCACGGTATATGTTCTATATAATCGAAATATAAAAGTTGAAACAGAAAGAGAATATCAATAAAAACAAGAAATCTGAAGAACACACTGAAGGCAAATCTAGAAAGGAACAAGAGAGTTACCTACTTAACAAACAAAAGCAACAAGCTATTCATGGTGCATAATTAATCAATTCTACAATTAACTTGTATTAATTAACTCTTTATTTGTTCATTTTAACAAGTAAAATGGGATTGCACAATAGAAGTCAATGTAAGATGTAGAATTAATAAATTATGTACCCTGAATAGTTTGTTTCTTTTGTTTGTTAGGTAGTATCTTCTTGAAAAAACCCTCTTAGTGATTATCAAATTTGTTTTCATCGTGTTCTTCAGATTTGTTGTCCTTTTTGGCATCCTTTTTCTCTTTCAACTTTTGTAATTCGATCATATAGAACATATATTGTGAAATACAAAAGGCAAGATACATGAACGGATTATTTGAATTAATATTTCATGCTCGTACAAAGTAAATCTTTTCTTCCATACTAATGAGCTCATAGTTGAAAAAGTAAGGAAATTTTATTGACCAATTCTAGAAATCTGACAAAAGATACTTTGATATTTTTTCTTGAGATAAATTATAATAGGATAATAATCTATTTTTTCTGGTATGAATTATGAATCAAAAAGGAGTCATCATTAATCTTTGCTTAAATCAAATATAAATAGCTTGAGTATATATTCACACAAAACTATAAAATGCATACACTAAACATTGTTATATGTGTAAAATAAATCAGAATCTTAAAATAAACACAAGGAAAGAATTGGTTTACAAGGAGACAAAAAAGTGAGAACAGATAATTTTTGTGTTAAAAGGAAACAACTATATGTTTCTATACCCTTTGTGTTTGTTTGGGAGAGATGTGAGGATTCTCAATAATTTATTGTATTTAGATAAATACAATTGGGAGATAAATAAAGAAATAATAGAAAAATGAAAAATACAACAAAGGTTTTGTCAATCTTGTTATTAGTCATCCAAAATTTATTAAAATCAGAAAACAAGAGAATTCCAAATCTGACTTAACATCTTTAATAAATTAGTGACGATATACTAATTGAGCCATCAATATATAGATGAACACATGGAAAAAGAAGCAAATAAATTCATTACATGTCAAAATGAGATTTAGCTAGAATCAAATCACCATCATATAATTCAAGTCATACATTGTAGAGAACAACAATAGATTGAATATAATAAACAAATCAATATTGATTTAATTGCATTAATTTACAATAATATATAAGTGTATATGGAGAAAACTCTTTAGTCATCTAAGTTTTCAGCAAAATTGCGTAGAAGGAATAGACAAGGTGATGCAACGTACTACCACAATAGAATAAAGATGACTAGAGCTAGTGAAAATAGAAGATACATAACAAGAAAGAGAAAAATAGAAAGATATGGGTTATAGTTTGAATATGTTTTGCAAAAACTATTGACACATTTTTAAAATATAATAATATTATTAATTATGATAAATACAATTTTATTTCTGTTTATGCATAGAAAAGATCACTTTTTGTTGTTGTTGTTGTTATTACTGAGACATTCAACGGTGTTTTAGGTGTCTAACAAGGCCACCACCTTAGATAAGTAAAAATGAAAATTAGTGCCAACCAATGAACTATGTGTATGTCATAAAGCATTTGTTTGTATTTTATTTATTTTTCAGATGTTGTATTCGATAATCATACCAAAAATCAAATAATTTCAGATTTTGTATTTGAGGGAACTACTAAATGAATTATTCGTTTGTATTTACCTGGTGTATTTAATATTAATATTAGAGTCCAACTAATTTATCTAATTTATATTTTGCCACCAAGCAACACATTAGAGGGAAACACTCTGTGAAGTATTTGTTTGTATTTTATTGATTTTTTTGTCTGATTTGTTCAATATTTATATTGGAGTCCAATTGATTTAGATTTTGCCACCGAGCTTCACAATTGAGGAAAACACTCTATGAAGTATTTTATTTAATTTTTCACCTATTGTATTTAATATTTATATTAGAGTTCAAATAATTTAAATTTTGCCACCGAGCAAAGCATTCAAGGAAGCACTCCATGAAATACTTGTTTGTATTTTATTTATTTTTCATCTACTATGTTTGATATTTTTATTGAAGCCTAATTAATTTAAATTATACCGGCTAGCAACACATTGTAGGAAGTAGGGGTGTTCACGAGTTGGTTTGGATCGGTTATTGGTCAAAACCAAACCAAACCAACTTAATCGGTTTTAAAATTATCAAAACCAAACCAAACCAAATACAATATACATTTATCGGTTTGGTGATTATCAGTTTCGGTTTGGTTTGGTTCGGTTATTCGGTTATTAACCATACACAAAAATAAAAGAAACAATTCATTTAAAAATGTGAAAAAAGTGACAACAATCCATTCAAAACGAAAAATAGTTAGAATGACTTAAATTACTAAACTTTTAATCACTAAAATTTACTATCAATAAAGCTTTAACATTCACTATATTGGCCTAGAAGAAAGAGACAATAACATTTTCAGTCCCACAAATAATTGACATAGACACTCATATACATGAAATCAATAAGATAAAGTTTCATCTTGGACATTTTTGCTTTCTATAAGTAAATTATAAAGAAATTTTAATGAAAATATGTATAAGATCTTTAAAATTGTTTATAAAAATAATATATTAAGTATGTATATTAATAAAATATATGTATATAATTCATCAGTTCGGTTCGG

>LNC_000561

ATTTTTTTGTTACTTTTCTAGCATTGGTCAATCTAGTTTTCTTTATTTTTCAACTTCAAATTCATTAATCTTGAGGAAAAGCCTTACTTTGTATGAACATGCATTATTAATTTAAATAATTGGTTGATAGATCTTGCTTTTAATTTTTCACTGTATATGTTATATATAATCGAAGTACAAAAATTAAAACAGAAAGAGAATTTCAATAAGAACAAGAAATCTGAACACAATGAAGGCAAATTTAGAAAGGATCAAGGGGGTTATTTTAAGAAGATACTATGTAACAAACAAAAGCAACAAGCTATTCGTGGTGCATAATTTATTAATTCTCCGTCATACAGTTGACTTATATCAATTAATTCTTTACTTGTTCATTTTAACAAGTAAAATGTGGTTAACATGATATAAGTCAATTGTGAGATGTAAAATTGATAAGTTGTATACCATTAATAGCTTGTTGTTATTGTTTGTTAGGTAGTTTGTTCTTGGAAAAGCACTCTTGGCCCTTTTCAGATTTGTGTTCATCGGATTCTTCAGATTTTTTTATTTTTATTGACACCCTTTTTATGTTTCAACTTTTGTAATTCGGTCATATAGAACATACATTGTGAAATACAAATAGCAATACGTATCAACAAATTATTTGAATTAATATTTCATGTTGGTACAAGAAAAGATTTATTTTGTATGATCATGAATTATTAATTTAAATAATTCGTTGATAGGTCTTGCTTTTAGCTTTTCACGGTATATGTTCTATATAATCGAAATATAAAAGTTGAAACAGAAAGAGAATATCAATAAAAACAAGAAATCTGAAGAACACACTGAAGGCAAATCTAGAAAGGAACAAGAGAGTTACCTACTTAACAAACAAAAGCAACAAGCTATTCATGGTGCATAATTAATCAATTCTACAATTAACTTGTATTAATTAACTCTTTATTTGTTCATTTTAACAAGTAAAATGGGATTGCACAATAGAAGTCAATGTAAGATGTAGAATTAATAAATTATGTACCCTGAATAGTTTGTTTCTTTTGTTTGTTAGGTAGTATCTTCTTGAAAAAACCCTCTTAGTGATTATCAAATTTGTTTTCATCGTGTTCTTCAGATTTGTTGTCCTTTTTGGCATCCTTTTTCTCTTTCAACTTTTGTAATTCGATCATATAGAACATATATTGTGAAATACAAAAGGCAAGATACATGAACGGATTATTTGAATTAATATTTCATGCTCGTACAAAGTAAATCTTTTCTTCCATACTAATGAGCTCATAGTTGAAAAAGTAAGGAAATTTTATTGACCAATTCTAGAAATCTGACAAAAGATACTTTGATATTTTTTCTTGAGATAAATTATAATAGGATAATAATCTATTTTTTCTGGTATGAATTATGAATCAAAAAGGAGTCATCATTAATCTTTGCTTAAATCAAATATAAATAGCTTGAGTATATATTCACACAAAACTATAAAATGCATACACTAAACATTGTTATATGTGTAAAATAAATCAGAATCTTAAAATAAACACAAGGAAAGAATTGGTTTACAAGGAGACAAAAAAGTGAGAACAGATAATTTTTGTGTTAAAAGGAAACAACTATATGTTTCTATACCCTTTGTGTTTGTTTGGGAGAGATGTGAGGATTCTCAATAATTTATTGTATTTAGATAAATACAATTGGGAGATAAATAAAGAAATAATAGAAAAATGAAAAATACAACAAAGGTTTTGTCAATCTTGTTATTAGTCATCCAAAATTTATTAAAATCAGAAAACAAGAGAATTCCAAATCTGACTTAACATCTTTAATAAATTAGTGACGATATACTAATTGAGCCATCAATATATAGATGAACACATGGAAAAAGAAGCAAATAAATTCATTACATGTCAAAATGAGATTTAGCTAGAATCAAATCACCATCATATAATTCAAGTCATACATTGTAGAGAACAACAATAGATTGAATATAATAAACAAATCAATATTGATTTAATTGCATTTATACAATAGCTTAATGTATGTCAACTGAAATTTGCATACGCATGACAGACAAAAATATATATTTACATACACAACAGATATCAAACATATATATCACATAACATATAATAATATGCATACTTGTATATTTTTGTACGTATTTATATGTCATGCGTATGTGTATGTGTGTGTATATATATATATATGTTATGTATATGTCTAATATGTCTTGTGTATGTAAACATATATTTTTGTCTGTCATGCGGATGTAGATTACAGTTGACATACATTAATTTACAATAATATATAAGTGTATATGGAGAAAACTCTTTAGTCATCTAAGTTTTCAGCAAAATTGCGTAGAAGGAATAGACAAGGTGATGCAACGTACTACCACAATAGAATAAAGATGACTAGAGCTAGTGAAAATAGAAGATACATAACAAGAAAGAGAAAAATAGAAAGATATGGGTTATAGTTTGAATATGTTTTGCAAAAACTATTGACACATTTTTAAAATATAATAATATTATTAATTATGATAAATACAATTTTATTTCTGTTTATGCATAGAAAAGATCACTTTTTGTTGTTGTTGTTGTTATTACTGAGACATTCAACGGTGTTTTAGGTGTCTAACAAGGCCACCACCTTAGATAAGTAAAAATGAAAATTAGTGCCAACCAATGAACTATGTGTATGTCATAAAGCATTTGTTTGTATTTTATTTATTTTTCAGATGTTGTATTCGATAATCATACCAAAAATCAAATAATTTCAGATTTTGTATTTGAGGGAACTACTAAATGAATTATTCGTTTGTATTTACCTGGTGTATTTAATATTAATATTAGAGTCCAACTAATTTATCTAATTTATATTTTGCCACCAAGCAACACATTAGAGGGAAACACTCTGTGAAGTATTTGTTTGTATTTTATTGATTTTTTTGTCTGATTTGTTCAATATTTATATTGGAGTCCAATTGATTTAGATTTTGCCACCGAGCTTCACAATTGAGGAAAACACTCTATGAAGTATTTTATTTAATTTTTCACCTATTGTATTTAATATTTATATTAGAGTTCAAATAATTTAAATTTTGCCACCGAGCAAAGCATTCAAGGAAGCACTCCATGAAATACTTGTTTGTATTTTATTTATTTTTCATCTACTATGTTTGATATTTTTATTGAAGCCTAATTAATTTAAATTATACCGGCTAGCAACACATTGTAGGAAGTAGGGGTGTTCACGAGTTGGTTTGGATCGGTTATTGGTCAAAACCAAACCAAACCAACTTAATCGGTTTTAAAATTATCAAAACCAAACCAAACCAAATACAATATACATTTATCGGTTTGGTGATTATCAGTTTCGGTTTGGTTTGGTTCGGTTATTCGGTTATTAACCATACACAAAAATAAAAGAAACAATTCATTTAAAAATGTGAAAAAAGTGACAACAATCCATTCAAAACGAAAAATAGTTAGAATGACTTAAATTACTAAACTTTTAATCACTAAAATTTACTATCAATAAAGCTTTAACATTCACTATATTGGCCTAGAAGAAAGAGACAATAACATTTTCAGTCCCACAAATAATTGACATAGACACTCATATACATGAAATCAATAAGATAAAGTTTCATCTTGGACATTTTTGCTTTCTATAAGTAAATTATAAAGAAATTTTAATGAAAATATGTATAAGATCTTTAAAATTGTTTATAAAAATAATATATTAAGTATGTATATTAATAAAATATATGTATATAATTCATCAGTTCGGTTCGG

>LNC_000562

TCTTCCTTCTTTTTTTTCATCATTGTTCTAATTAACTCTATTGTTTCTCCACGTGAAAGATTTTTTTTTGTTGGAAAAATTTATTCTAGAATTTTGAATTTTCTTTTGAGTTTAAAAATGGGCTAAAAAGGAAAAGTCATTTATGGAGAGAATCAAAAGAAACTCTTCAATTTCTTACCTAGAAGATGTTTATTACTCTATTCCAAATCACACATATAGTCATTACTTATTAATAGATATCTGATATATACATTTAGTGATTCAAGATTAAATTATATTCATGTAGTGGTATCCATACATCTCACATGGGATATGAATTAATAATCATGGATCTGAAGACCGCCAAGTAGATTTGGAGAATTTTGAATACCTTAGAATATTAATGAATGAAGCATGAAGGGTTTACAATATACTAAAGTGGAAACGTAAGCAAGAGATGAC

>LNC_000563

TTTGATTACTTGTTCATAATCTAGTGTTTTTAGTTAGCAATTGACTTGTCTTTGGTTGATGGACTGGTAATTTCCTACAATGTCAGAGATCAGGTTCACATAGTAATTAAGCCTTATTGCAACATCAATGAATGTAATTCTCATATATTAAATTTGTAGATACATTTTTCTGAGCTGGGGAAAAATCTCTCTAATTAAAGAGGGGGCACAGGAATACTCTGGCTTAGACTCACGGGGAGTAGCGAGAATGTATGAAACTGGAGCAAATAATATTTGAGGGAAAATTAGGAGAGAATGTAAACACAAGAAAGGCATGCAAGCTATTAATCACTCAAACATTAAGGTCTAGCTAATCATTGGCAAATGTTGTGCCATGGATCTATTAGTAGCAAGTCTATTATTTTTCCCCGACTTTGATGGCTGGACTTGCTCTATAT

>LNC_000564

GTTGAGTTAGGCCCAAGTGTCATATCTTTACATGGTATCAAAGCCAGGCCCATCCCAATTTGTTGTTCACCGATGATGAGACCTCATTTAGAAGTGTTCACGCTCCATTTGAGGTTTGGGCGTGCGGGGGAGTGTTAGGATGTCCCACAACGGTAGAGAAAAAGGGAATTTGGTCTCCTTATATGGACTTGGACAAACCTCCCCTCATCAGCTAGCT

>LNC_000565

ACACAGTTGAGACTAGCTCCGGCTAGAGATATATGAGATGGACTAGCTCCGGCTAGCGATTTGGATGCCGATGGGATCTGGTTCCGGCGGTGATACATGGTCCATGTGTGGCCCCCATGGGTTCTGATTTGAGTATTCAGCGCGGTCTGAATTACGTCAACAGATGTGTATCTTAGGACAGACATGCATCACGACTACATGACATCATTATTGCATTTGCATCGCATTTGACTTCATCTTTGTTTGTGGTGTGTGGATTTTACCTACTTGCTCCTCTTCATGTGATATATGATCTATTTGCTCTTATATGAGGATATGAGGTTGATGTTGAGATACTGTTGGATATATCTGTTGATTATGAATTGTGTAGTATAGGTT

>LNC_000566

CTAGTAATCTGAGATCCTCTGCTGAGTTTGTGATTACCTATCAAGAGCTTGTGGATGTATCCTCTGTGGTTAAAGCTAAGGATAATCCTTGTCAAGATTCTGATGATGAGGAAGACAGTTGCAGTGATTATTATCAAGATTCTGATGACGAGGAAGACTGTTCCAGTGATTATTATTATCATGATTCTGATGATGATGAGGATAATTTTGATCAAGATTCTGATGATGATGAGGATAATACCGATCAAGATTCTGATGATGATGAGGAAGACGAGCCGTGATTATCATCAAGGTTGTAAGACCCTCGTCCAAGATTTTACCTTCAAAAGCTGAGTTGTGCAACTGTGTTAACATTCAGAACTTGTTTACTCAGGTTTGTGATGTTTGTCATTAGTACCCTGCATATCCAAACAGGGATGCTTTTGCTTAGTTGTACATAACTTGGTGCTGTCAAGTTATTAAATTACATGTCCTTGCTAAAAAACTAGAGTCCTTATTTTTTCGGATTTATTCTAGCAGACAGTTTCAGAATGTTAACATTATCAGATTTAGCCATTTTCAATCCTCACCAGAATGTTCTCATATTTTCCACTGAGTCAGAATGTTAGGAAGTCTTTTGATTTACATTCCTGTCATCACAGCCTTGCTTATATTTCTCTCTTTTTCTTCGGTACCAATGAATTGTTAGTTTTCTTGTGTTGCC

>LNC_000567

CTCTGCTGAGTTTGTGATTACCTATCAAGAGCTTGTAGATGTATCCTCTGTGGTTAAAGCTAAGGATAATCCTTGTCAAGATTCTGATGATGAGGAAGACAGTTGCAGTGATTATTATCAAGATTCTGATGACGAGGAAGACTGTTCCAGTGATTATTATTATCATGATTCTGATGATGATGAGGATAATTTTGATCAAGATTCTGATGATGATGAGGATAATACTGATCAAGATTCTGATGATGATGAGGAAGACGAGCCGTGATTATCATCAAGGTTTTAAGACCCTCATCCAAGATTACCTTCAAAAGCTGAGTTGTGCAACCGAGTTAACATTCAGCACTTGTTCACCCAGGTTTGTGATGTTTGTCATTAGTACCCTGCATATCCAAACAGGGATGCTTTTGCTTAGTTGTACATAACTTGGTGCTGTCAAGTTATTAAATTACATGTCCTTGCTAAAAAACTAGAGTCCTTATTTTTTCGGATTTATTCTAGCAGACAGTTTCAGAATGTTAACATTATCATATTTAGTCATTTTCAATCCTCACCTGAATGTTCTCATATTTTCCACTGAGTCAGAATGTTAGGAAGTCTTTTGATTTACATTCCTGTCATCACAGCCTTGCTTATATTTCTCTCTTTTTCTTCGGTACCAATGAATTGTTAGTTTTCTTGTGTTGCCTCTCAAATCATGTCTTTCTTTGACCTACTTAGGTGAAAACGTTGATAAACGATAGCCAGGAT

>LNC_000568

CTAGTAATCTGAGATCCTCTGCTGAGTTTGTGATTACCTATCAAGAGCTTGTAGATGTATCCTCTGTGGTTAAAGCTAAGGATAATCCTTGTCAAGATTCTGATGATGAGGAAGACAGTTGCAGTGATTATTATCAAGATTCTGATGACGAGGAAGACTGTTCCAGTGATTATTATTATCATGATTCTGATGATGATGAGGATAATTTTGATCAAGATTCTGATGATGATGAGGATAATACTGATCAAGATTCTGATGATGATGAGGAAGACGAGCCGTGATTATCATCAAGGTTTTAAGACCCTCATCCAAGATTACCTTCAAAAGCTGAGTTGTGCAACCGAGTTAACATTCAGCACTTGTTCACCCAGGTTTGTGATGTTTGTCATTAGTACCCTGCATATCCAAACAGGGATGCTTTTGCTTAGTTGTACATAACTTGGTGCTGTCAAGTTATTAAATTACATGTCCTTGCTAAAAAACTAGAGTCCTTATTTTTTCGGATTTATTCTAGCAGACAGTTTCAGAATGTTAACATTATCATATTTAGTCATTTTCAATCCTCACCTGAATGTTCTCATATTTTCCACTGAGTCAGAATGTTAGGAAGTCTTTTGATTTACATTCCTGTCATCACAGCCTTGCTTATATTTCTCTCTTTTTCTTCGGTACCAATGAATTGTTAGTTTTCTTGTGTTGCCTCTCAAATCATGTCTTTCTTTGACCTACTTAGGTGAAAACGTTGATAAACGATAGCCAGGAT

>LNC_000569

TTGAAATTTTGTCTTAAAAGAAGAGCATCACATAACATGATAACACATAATAGGAGTGTCACAATGATAGAATTATGTATAAACTATATATTTTCTGTATTTTGTATTATGGTTATGATTCTTTACATTTATCTTATGTTTATATTTTTAACACACACCAGCTATTTGAAGAAGTGCAATAGTTAGAAAGTCAGAATATCTTTGCATTATTGTTGAGACTTAGCAAAAAAATCTCAATTTTATGTGCCTTTCAACTTCATTCATGGTTCCCTTATGTTAATTTAGTTAATTCTTACAAGAAAAATAATTCATATTGTTTTTGTGCCATGACTAAATAGATTCATTGTCCGAGGAAAAATGAAGAATAAGGGGAAGCGATTTTGGCTAAAGGGTACCAATAGGACAAGCTTTGCGGAGAGTTTAATGGGAAGACATTTGAATGGATGAAAGTTCCTTTGCATCAACTATAGAAGATATATTTAGTTTTTGAGCTCCTGTTATTTAACTG

>LNC_000570

GACCAACATAAGATGCCAAGAGACTCTGTGATTTAGACAGAAAACTCTAGCTTCAAGCTTGTTCGAGTTTGTTATTGTTCGAATATAGACTTTGATCGAAGCAGCCCCGTTCACTGCCCTATAGCAGGTGATTTCGCCTCTTTTCCTCTAATTTGGTGTCGAGAAAGATTTTGCTTGTTCTAGCTATAATGAAGTGATGTATTTTTGTTCGGTTTAATCTTTTTAGGAATATGAAGTCATATTTCTTTCGTGTTTTAGCCATCTTGAAATTAAGGAATGACTTAGGATGTGTTTGGAATGAAGAAAAATATTTTTCGGAAAATGTTTTCCAAATCAACTCATTTTCCTCAAATGTAAGGAACATAACTTTCCTTCAAAACTTAAGGGAAACATTTTCCAAAATTCTCTTCCAACTTCAAATTATATGCTTGCCATGATCGCGCTTTATCTCGATGTATATAAATTTGAGTTTAAGTCTCTTTTGTTCCGCTGTTAGCAGCCTCCGTTCTATTCTCTGTTAGTAGAAATGTATGTATGAGTTTGTTCCTTTTCATCCTGAATATTTAATATTTGGCTATGTAAAAAAAAGTTAATGTTAAAGGATCATGGTGTGAGAAGTAAGTTTTCTTTATAGTTCAAATCTGTTGAAGTTGGAATTTAATGTTGTGTCTATAAGGCACGTATGGTTGCTTCATACTTGGCTGCTTCGAGTTTGGTTCAAAAATCGTCATGTATCTTGTCTGATCCTTGCAGTTTAATTTGTATCTCGGAATGTATTTTGTGGCGTGACTTTATTTGGCTCTATTCTTGACTTATTCAATATCGATTAAAGTTGAATTGTCTTACTAATCTCTTTACCATCTCTTTACTTGCATGAACTTTGGAGCAAAAATGGAGTCCGAATTGAGGACTCTGGCCAAAATACAGTCTCATGGACTCGCTACCATAATACCCGCATATGGAGTCTATATCTTTGACTATCCGGAATAGCCAGCTAAACGAGTGAAACTCACTTGGAGTCGCCTATATCTATTTATATCTTTAGCATTTATTTGTTTTTATTGATTTTTCTTAACATGATGAACTTACTTATGTGATTTAGATGTGTTTTTAGTTTTTTTTTTCATTCGAGATAGTCGACATAGGAAAACTGATCACGTTTAAATCTAATTACTTAGTTACCCACAACCTAATTATCGATAAACTATCTATCATATGTGAGCTTTTCCTTTTTTGAAAGAAATCTAGCATTTATTTAAGCTCAATTTGATTTTTTTTAAAGAAGCGTTTTTTTGGGGTCATAAATTAGATTTTGTATTGTGTTTTTATAGCCATGACTTTAGAGACTAATAATTTTTTTTTAAGCAAATGACTTTTTATTTTCTTCTTTTCAAGTTTTTGTCATTCTATTTCAATCAATTTTCAAATGTAGAATGTGTTTCAAGGTGCAAACAAATTTTAAGACATGCGAATTAACTTCATATACTGAAACCTTTGTTTTAGTATGCATTTTCGTACCAAATTTTGTACAAATAATTGTCGTTTTAAACAAGTGATTATCTATTGTTTTCAAAAAAGAAAAATGCAAGTCAATTCTATCCAAATATAAAATAGTTTTTCAAATGTTAAAATGTTTT

>LNC_000571

TTAAAACCTTAATTATAAAGTTAAAAAAGAAAAAAAATCTGTTTCTTCTAGGGTTTTTCTAGCTGTATATTTTTTTCTCCTCCTCAATCTCTTAGCAGTCGACTTCCTCTTGCACTTCAAGTCTGCAAGACTACAATGCTCTGTTGCTACTGCGTGCTGGATGTTGCCTGCTGGTGTACTGGTTGTTGCTGAAGCCTGAAAGATGGTCCTTTCTTGTGGTGTGTTCATTGACGAGATGGCGTCACTTGTCTCTACCGTCTCCATTCATGATACCTCGGTAAGTTCTTGGTTCCTTTTTTTTTTTTTTTTTTTTTCAAAATTCTATATACTTTGGTTTTGGTATTTGTTGATGTGCTCTCCTATTTGGCTGGTTTTAGTGGCGGAGGCAAGTTTTCCATCAAGGGGTTAAAAAAACTAAACACCCTAATAGTATCCGCAAATGAATGGGCCGGATAAAAAATGTTCAGTTTAGAGCATAATATTCTAAAAACTTACATAAATAAAACATAAATGTAGAATGCAAACATGGGGAAAATACAATCTCTTAAAGAACAAGTTTAAAGCACATATTTATAACATGCTAATAAGGAGATATTAAGCAGCACAAGTCATATTCAAATTGGAAGAAATATAAGAGAAGAGTATTATTGAGTTGTGTATCTACATTATTACATTGAGACCCTATTTCTAGATATTATATAATCCTTTTTCAAGTATGATGCTATATGATATTCATGTTCCAAGTAGAATTCTCTACTATTCATGTTTCTATTCCTATTCTAACACTCCCTCTCAAGCGGGTGTATACAAGTCTTATGTACCAAGCTTGTGACAAATATAATTGATGCGAGGACTGGTGAGAGACTTGGTGAAGATATGTGCTAGTTGATTACTCAACTTCACAAATTTTGTAACAATATTTCATGAGAATATCTTTTCTTTCACAAAGTGATAATCAATCTCACCGTGCTTAGTCCTCTCATGAAACATTGGATTTGATGCAATATGAAGGATCACTTGATTATCATACACAATTTCAATCGAATTGATTTCTACAAGTTTTACTTCTCTCAACAACTGTTTGACCAAGCTATCTCACAAGTTCCTATAGCCATTGCTCGCTATTTTGCTTCTGCACTAAATCGAGCAACCACACTCAGTTGCTTACTCTTCCATGATACCAAATTATCTTCTACTAAAATACAATATCTAACCAATCACCATTTGTATATCTAGTGATATGGACATGGTCTTGATCCTCAAAAAGTAATCTTTTACCGGGAGCTGAATTTATATATCACACAATACAAAAAATTGCATCCCAACGACTATTACAGGGGGAAGTCATAAACTTATTTACAACATTCATTGGAAAAGAGTTTCCAGGTCTAGTCACTTTGAGAAAGTTCAACTTCCTAACCAGCCGTCTCGAAAGGAATTTAGCATTCAGATCCATAAGAGTGTCAATGGGTCTACACTAATCATTCCAGTCTCTCAAGAATGTCTAAAGCATACTTGCGTTGCGAAATAACAATACTTGATCTGGACTAAGCAAACTCAAAACATAGAAAATACTTTAATCTATCAAGGTCTTTAGTTTGAAAATGTTGAAAGAGATGTTTCTTCAAATTAGTGATACCATCTTGATTATTGTTGGCGGTAACGATAATATCAAGGTAAATAACCATCTTGATCATAGATTTGGTGAAAATGCCGATAAAATACAGAGTGATCAGCTTCACTACAAGTTATGCCATAACTCATAAATTACGATTTTGAACTTCCCAAACCATGCTCGAGAAGATTGTTTCAGACCATAGATTGACATGTGCAATGGACATATAAGGCTTCTAGACTCCTCAAAATAATAAAATCAGATGGTTGCTCCATATAGATATATTCCTCAAGATCACCATGCAGAAAAAACATTTTTAATGTCCAAATAATGAAGAGACAAATGATGAATGACATTCATAGATAGAAAAAGAACATATGTTATCATAGTCTCAAGAGAGAAAGTGTCACTAGAATTTAAGCCCAAATACCGAGTATACCCGTTGGCAACAAGGTGAGCCTTATCAACCTAACTATCTAGACCAATTTTGACTGTATTAATCCAATGACAACCAATAGAAGATTTACCTACGGGAAGTGAGACTATCTCTCAAGTACCACTCGTATGTAAAGCAGACATCACATCAATCATAGTCTACCTCCATCCAGAATGAGAAAGTATTTCACCCATAGTCTTAGGGATGAAAATAAAGGACAAATGATACAAAGGCATAGTGGGGTGATGGAACTACAAGTGGAGAAGTAGAAGTAATAGTAGGTTTCTCAAACGTCGGTACATGTAAGACTTGAGAGGTATGAGTAAGACCTCAGAGACATCAGGATGATAAGAAGATGTATAGTAAGGTTGAGATTTAAAGAATGTGGCATCGTCCTACATAAGGTATCGACGAAGATAAGGTAAATAAAAACAATACCCCTTTTGAGCTGGAGAGTAACCAAGAAAGACGAATTTACGAGAATGAAGGCTTGATTTATCTTTCCTTAGGGCTAAGCTAGGTTATGAACAAAGTATCTACTCCCAAGGAAATGAGGGCGAATAGTGATAGATGTGACTGAGAAAATAGTATGCCGTGTGAAACATGTTTCTAAATAGAAGATTTCATCATTCTATTTATTGCCCTAAATCACAGTAGAACATCGGAATTAAGGAGAAGAGTGCGAGTAGTCTTAATAAAATGCCTATTCTTTTTTTCGGCTACACCGTTTTGCTGAGGAGTATACATACAAGAATTCCTTGGGTGAGTCATAAATTCCTGAAATTAAGAGGATAAGTATTCTAAGGCATTGTCATTGCAAAAAGTATGAATAGAAATGTCACTCCCCGAGGCTACCCCGAGGCATAAACACGGGACTTAGGATCACGAGTGAACCTAAGCTAACCCTGAGCTGGC

>LNC_000572

AGAAAACAAAAATTGAGAAAAAAGAGGAAAGAGAAACAAATATATCAATAAAATAATCTCTCTCACGACCACCCACCCACCTTTCCCGGACCGGCGCTGACCCCACCCATTCCCGACCGGCGCCACGCCACCATCTCCGACGGGCGCAGCCTCAGCCGCAGGTCGCAGCAGCAGAGGGCTGCAATAGCAGCGCACAGAGTTCGGCAGCAGCAGAGAGCAAAGGGCGTCGCAGCTCTCAGTCGCAGAAGCTTGCCGGCGACGCAGCTCGCCGTCGCAGAAGTCAATTTATAAGGATCAAGTGGGGGGCTAATTAATCCTCACAGTCACTAAGAGGAAGAGATTACTTGGGAAATATGTGACTTTTAACCAGTTGGCCAGCAGAGATAGCTCACCAGCAAGAACAGATGTTTGGAAATTATTGTTAGTGTAAGTATTGGGGTGCCTATACTTAGAAACTACCTTTGAATATAGTCTTAAGACTAACAAATATTGAGGCCAGCACTACAATATTGAGGCCAACACTAGATGTTTCGATAGAGGAGGTAGTTTTTACTTTTTAGTGTTATATTCAACATGTCAAGATTAAGCTTTTACAGGACCAAGTTTCTAACAATTTTGTGGAGTGGGTGTGGGGTCAAGACAGCTTCATTATAAGAAAAAAGTACAAAATGGACAAAGATTCTAATAAAAGATATACAAACAATAGTGGAAAAGGAGAAACCATAAGAGTCCACCAAAGAGAAAAAGATGCAAGTGCTATGTATGAAACCTATTTGAGAAGATAAAATACACATTATGTAGCTCATAAACAAGAAGATCTGGTAAAATCAATTACTTCTCTAACAAGAGATCGTCAAACACAAATGAGTTGCATTGTAATTTCCAAGTGATGCTTTCAAAAGTCGGAAATCTTGAATAGATGAAGGAATTAGGTGTTTACCAATTGTCAACTGTTAATAATGACTGAGATCTCTCTATAATGACCACTAAAGAAATTTTAAATTGTTTCTTTTCGTGCTAGGCAACTGAGGAAGCTCAACATCTAAAAAAGTTGATACAAGACTATATTGCTTCTTTCATCAAAGTAGGAGATAAGGTTTGGTGCTTCTAAATTTCTAAAGTTCAAGTACAATTGTTTTCTATAATGTGTTTGTGTCCACCTAATATTGATAGTTAGCTTTAATTTTATGATAATTTGTTTGTGTCCATTGAGTGTTTGTTTAGCATAAGTACCAACACTAGTTGATC

>LNC_000573

GCCAACTGCAAAGATTAGGCTAAATTCTGGTCATTAGATGTATATTGGTTCTTGAGTGAGTAGTGTTTTTTGGTAAAGATTGTGCTATGTTGGCATTACTAACATGGGAAAATAAGTAGTAGGGAGACTGCTAGGGTGAGTAATTGCTAATGGAAGGGTAGAAGTAGGGGAAGTTGTAATTGTTGTCAAGAGTTTGGTGGGGAGAAAGTGTGGGTACTTTATGGCACCTCTGGAAGTTTTGTAGCGGAGAGAAGCTAAAATATCGCAAAGTTGTTGGGTGTGCACTGTGCATGGGATGGTTTGGAACCATCATACAATTTGGAGTGATTAGACTAAGTAACAAAGAAGTATAACAGAAAAGCAGTGTAGAACAAGGATATCAGTTGTTCTTATCCCTATTTCTGAGAAAGCTTTGGATGAAGGTCTAAGTATTAGATGTCAACTATTTAATTTAGGAGCTTTAGCATTTTTACGTCAAAATAAATTATTTAAGTAGTATCGGAATTTAGTTGACACGGGGCTTATTCATCTAACTGAATTATTGCTATTTCAATTCTATTGTCCATATGATTATTGCTTTTGATTATTGTTATTTTTCTGTTATTACGAGTACATCTGTTCCTATTGTGCATTTCCTAAAACACACTTTAAATTCACTTGGTGCTTGAAGCCAGCAGACCTTGGTGCCTTCTAGCTTTTCACCTTTGAACACTGAATTATACTAATAGAGTTCCCCTACTCAAGAAAGCAGTATTTACGATGCTACTTTTTTTTTTAAATAGGTAACTTTGTATTATATCACAAAACAGTACAACCATTATATACAGATGTCTCCTTTTCCACTGGTTTCCGTCTATATTGGGTCTAAAACATCAATTACTGATACAGTGTCATTGGAGTATACTTGATTACTCCAAAAACATAACAATAAAATGTAGTTTAACTTGACTTTCTGGACATCATTCTCTATGCTCTCAAAACACCTAGAATTCCTTTCTTTTCAAATAGTCCACCATATACTAGCAGGGATAATTCTCCATCTGTTTCTGTCCTTTGCATTCACCCCTGCTGCCTCCCAACTCTTCAAAGCCTCTGATACCTTTCTGGGCATGGTCCAGGAGATGCCTTTGAGGTTCAAAAAAATCCTCCATAGTTGTTGGGTATACTTGCAGTGTAGGAACAAATGGTTCACTGTCTCTAATGCCTCCCCACAAAGGAAACAGCTATATAGCAAAGGTCAATGTTCCTTTTTTTCTTCTTTTATTGTAGGTCAAACCCATTTCAGTATTAACAGTGATACTCTTTTATAACTTTACCTTTATAAAAAAAAACAGTGATACTCTTTTATGATAATGTTTCCGGATCCATCTATTACATTTTGCCTCTGTTGCTTTAG

>LNC_000574

CTTCAACAAGTCCAGCAACGACGCTGCGAACCTCAAATCGAAAGCTCCCTGTGGTGGTTGGAATCATCCTCGAGCTCGCTATGTAGATTTGATCTTAGTTGTGGTTAATTCTCATTGGCGAAATCTCTTTGGTAGATCAACTGGTTCGATGTTGTATATGTATAACTAGTTGAATTTCTGCTTCGTTTTGCTTGATTTGATTTGGTTTCAGCTTCGAATGCATTAGCTATATAGTTCTGTTTCCTCATCGCTCGAACACTGCCCATATCTTAAATGGATGATGCTTGTTTTCTTGATTTGTTGTCTCTATTTGCTGGAAACTCGATTTAAATAATCTCCCCTGCCCTCCTACATAGTTGAATTTGAGGATTGAACTGTAATGGCGATTTTGAGTTGATTCCAAGTTTGGGTCTTTTCTGTTAGTCTCTGCCTTGACAAGTGTTAATTTTAACCCCTTCATTAGCGTCATCTAAATCACTCTAGTTCCATCTTGGCATTGAATTTCTTTATTATACTTGTGATCCCAATTATTACTTGTTTAATGTTTGGGTGTTTTGCTCACTGCCCCATTTGCACTGCTCTGGC

>LNC_000575

AAAAAGTCGAGTCTCATTTCCTATGTACAAGAATCAAATGGTGAAAAAAGTAAACATAAGCAAGAGAGATTGTTTACCCCAAGATTCGTGAATTGTCATGATAGCTTGAAGCGGGTTCAAAAGACCAACTCTATTGAGTTTTTACTGTCTATTACCATAGATGGATTTCCACAACGGGAGGTTTTTGAAACAAAAAATGAGTGGATGGTTAGGAAGACCAAGATACACAAAGGATTAGTAATTGAGATTATGTAAGTTATCCAAGAGGATATTTCTGTACATAAAAGGAATTCAAATTGGGGATTTACGTTCGTAGAAATGATCAAGAAGTACTCCCCATGATTCTGATCCAGAGTATGTTCCTATCCACTAAGTAAGTAACTAACTATCAAGAACGAAGTAATCTTTTACTTTGG

>LNC_000576

CAAATAGGAAGTTTCGAATCTAATTTGCATATTATAAAGGATTACCAAATATAACACAAAATTTCTCCACCTATTCCTTCTAGTCGCGCCTCTCGGTCTGTCATTATACCTCGAGAAGTAGAAAGAATTACAATCCCCATTCCACCTAAAATTCGCGGAAATTCGTTGATAATTAGAATAGATTCGTAGACCAGGTCGACTGATTCATT

>LNC_000577

TGTTATTATCATTGTTATTGTATTGTTAATTGTTGGAATATGAGGATGATGAAGATTGCCATGGAAAGAGGGTAAGTTGATCAAACTTGATAAATATTGTATAATTGGCATGGAATTGAGTATTGTGGCTTATGGCCTTTGAAAAGGGTGGTAAGATGGCTTGATCTCTTAGTATTAATATAATTATTATTGATTGGTTTGATCCACCTTTGGTGGAGGTGGTTATTTGTT

>LNC_000578

AATCGAAAATGGTAATAGTTAGGATTCATTAAAAAAATCGAGAACCCACTCATGGGACAAGAAACCCTTCCCGCATCAGGCACTAATAAATTTTTAACGTCTAATTAGATCGGGAATCATTCAAATTAAGAACAAAAGCTCGTTGCTTTTTCTTTACCTATAATTTAATTGAAGCCGTAGCCTTATCCATTTATTCATTCGACCCAACTTTATTTTGTTCCGTTCCAAGAATTCTAACACAGTTTTATACCCATCTAGGAACAATGAAATATTTTCAGAACTTTCCGTTGATACGACATGCTATTTTTACCATTCATTCCCTTTCAGGATCAGTCGCGGTCTTCCAAACTTTACCGAGAGTATGGACGAATCCCTCACTTCATCCATATGTGTAAAAGACACTAGCCGCACTTAAAAGCCGAGTACTCTACCGTTGAGTTAGCAACCCGAAGAAAATATCGAAAAAATTATGTAGAGCCAATTCGAAGAAAAAGAAATAACTAGAGTAGACAAGACAACCAAAAACCATTGAAAGAAGAAATCTAAAAAAATACATTTACACTTTCTAATTCATTTAAAACATAAAATAAAACTAAATAGATCCACTTCATTTATCACAATGAATTATATTTGTTCGATACACTGTTGTCAATATAAATATTGAATATTGAAAAAAAAAAACAAAATAAATTTCAATTGAAAACAATAAAAAATAGTAAAAAAAGGGCTTGTGTTGGATTGGAACTACAAATCTAATCCAAATAAAATAGATACAAAAAAGTATAGATGGGAGAATAAAAAAAAATAAGTGGAAAACAAAACTACAATTTACTTTAATTTACTTAGATTTATTGAATTCATAATGGATTCAATCAAGTTAACTGAGATAAGCAAACTTGATTTCGTTCCGAAATGCCCAAATTTAATATTTTGAAGATCAATAAAATAAGAGGTTTTGTCGTTATAGAACACGGAATTCACTGGAAGCAATGATAAATAAATACAAATAGAAAAGGAAAGGGAGGAAATACAAAAAAATAGAAGAGAAAAGTCATACAAAGTTATATACAAATCACTACCCCCTTTTTTGTATTACCTTAATTTATTTTCTTAATTGAATTTCGGTGGATTAAGACGAAGTTCCTTAAAAACCTCTGCCTTCTTTAAAATATCCTGAACAGTTTCTGTAGGTTGAGCCCCTTTTTCAAGGAAATATAAAATAGCAGGAACATTTAAATAAGTTTGATTCTTTATCGGATCATAAAAACCCACTTTCTGAAGATCTTTTCCTTCTCTTCGGGATCGAACATCAATTGCAACGATTCGATAGACGGCTCGTTGCTTTCTACCACATCGTTTCAAACGAAGTTTTACCATAACATTCCTCTAAGAACCGGTATGGAATTGATTCAATTATGGA

>LNC_000579

TTGAATTTTTGCTGAACTTTTTCCCCCCTAAATCGACCCCAAATTCCCTTTATATAAACCTCTATTTTTCTTTAGTATGGGGGTGGGGGAAAAGAGAAGAGGAATTTTGGAAGTCGTAATTTTAAATTGATGAATATAGTATCGATATTATCAAAAACCATAAAGTTGGGTCTAGATTTCTTGGAATTTCTTTTTTGTCTCATCCGAAGCTTTTAGTGGAGTTCATCAGAATTCTCGAAGTTCATCTCTTCTTTGGTGTGGAAGCTGCTTCATAGTCAAGCTCGTCGATTCCAGTCGCTGCCTGGAAAAAGGTAATACCTCTCCTTTTATTTTCCGTTTTAGAAGTCATGTACCTAGTTTGGGTTTCCTTGTTCGATTCTCACTCTGTTTCGAGTTTTTCTTTCGTTGTATGTTGAGCTTGTCC

>LNC_000580

CTTCTTTTTCGAACTCCAGATATGGAATGTTATATATAGTCAGGTAGACTAGAGAATTTAGAGAAAGGGATAGCCTTATATAGATATAAAAAAGGACAACATTCGACCATTGAATTGTGTCTGTTTTTATTATCTAGCTTATCATAATTGCAGCTACCTGTGTCTGTCTCGGCTCTCCCCCAAACTGAAAACAATATAGACGGAGAAGAACTTTTCTTTGCTGGTTATTATGAAGAGGAAACGGAAAGGTTTTTGGTGGTATGTTGGATTTCGGGGAGGGGGACAATATATGCACATGCTATCAGTATTTGGGCTACAGATATATGTTTTTAGAATATAGACCAAAAGGATTTCCAGAAATGCAATAGTGAGAACATATATAGATGGGATACCACCACGACTGTTGTTATGTTCCCA

>LNC_000581

AGCGAAGATGAGCAGAAGTGTAACGGCTCATCACGCATGCTTTTGAAATATTTCCTTTTGCCTCCGTGCTTCCATCTCTTTGGATCATCTGTTACTCTCAGTAGCATCAAGAAGATTAGCAATGGAGGTTTTGGGTGTCGTTTTCCTTTTCAAGAAGATTAGTAATGGAGATTAACAATTCAGGTTTCACAATTTTCCCTTTTCGTTCTTGAATTGAGGGGTTATACATTTAAAGTTTTGCTTTGCTCTTTGTTTTATTTCATTCTTACAATAAGCAGATGCTTTGTACCTTCACCCATTTTCCCTTTTTCTCTAATACAAAATCTCAATTTTCTTGTTCTTTTGGGTGTCGTTTCCTAAGCCAGATGTTCTTTTTTCCAACCTTAATTCAACTCGTCTGCAATCGCCATTCTTATGATAGTTAATGATTGCTTATGACTTCAGATTCAATGTGTAGCTGGGTCTGATCTTGAATCTTAAATTGTGTTCTTGGTTGAATCTTTAATTGTACAAAACTGAATTTCGTGATGCTGTAAATTGAAATCATTTCAGAGTAATTCCCTTTCTTGGCAGGTTGAGCTCACAGAGAAAAGAGGAGAGAAAAATTCAGATCCAATTGCAACTTTCACACACGATATAATTAATTCCCTTTCTAGGCAGGTTGAGCTCACAGAGAACTAATACCACTGCCAAAGAGGAGAGAAAAATTCAGATCCAATTGCAACTTTCACACACGATCTAGTACAATCTCCTTGTGTTGATCATTAGTCTTAATCAACAATTGTTTCTGGGACACAGTGGGTACCTTGCAATCATATCAGAATCATCACCTACT

>LNC_000582

CAGAAGTGTAACGGCTCATCACGCATGCTTTTGAAATATTTCCTTTTGCCTCCGTGCTTCCATCTCTTTGGATCATCTGTTACTCTCAGTAGCATCAAGAAGATTAGCAATGGAGGTTTTGGGTGTCGTTTTCCTTTTCAAGAAGATTAGTAATGGAGATTAACAATTCAGGTTTGTTGAGCTCACAGAGAAAAGAGGAGAGAAAAATTCAGATCCAATTGCAACTTTCACACACGATATAGTAAGTCTGTTTCTCTTCCCGTGAAAATCTTTGTGATTGTTGCGAGCCCTTACAGTTTCTGGCATGATATCGTTATTGTAGCATTCTGAAGTTCTCATTTTGACATCTTGTTAGCCGTATCTTGGGAATTTAATTCCGATCCATGTTTATTATAACTTTTCCTTCATTTTGGACTTCTTGTATTGAATTATATGTTAATCTTTGAGTACCTACAAAACAGAAAACTTGATTAGCAAAAAAAATCTGCCCCAAGGTACTAAAAGAGAAGTGTTGTGACATGATG

>LNC_000583

AGCTTTTAAAATTTAGTTTCGAAAACGACTTTTGAGGCAGAAGACTCTGTGACGGCTCTGCGACGCGAAACGAAAATTCCCAATTCTGGGAACCAGCTCCGCGATGCGCCACCACTGCTCGATTTTGTTCGACGAAATTTCTTCTTCGTTTTCTAGCCCCAATTCACCCAAACTCGGTTCTTTTTTTTAAAATTACTTTTAGATACATATACCCAACAAATGTACCTAAGAACCTAACTCGAAACGACTCTATAATA

>LNC_000584

GCAATTTGTCCACCGATTCTGCAATCTTGGTATCATTCACAAGTTCCTTTCTTTGAAAGAACATCATACCTGGACGATGAAAATGACCCATTCTTTTATGCCAAACTTCTGTAGAGTTGCCTGACATAAGAAAAGTTGTCTGCTCTTCTTCCAAAGGATTGAGTGGGAAATACCTCCCATCCATTTTGACCTTGAATATCTCATTGCCGGATGGATCTTCTATCACACAAGTTTTGTTTTTGAAGAACAGACTGAATCCATTCTCCATTAATTGACCAACACTGAGAAGGTTTCGACCAAGGTTTGGAACATACAAAACATTTGGGATAAGCTTAGTTCTAGAGCCACTGTAATGGCTATGGCACCCTTGCCTTTCAGAGCAAGACATTCGCCATTTCCAATTCTAACTTTCTTTGTCTCAGTGTTCCACAATTCTTTGAACAACATCTTATCAGATGTCAGTCATGTGGTTAGGAGCAAGCGCA

>LNC_000585

AAAAAAATAAAGAACTCTATATTTGGAGAGTAAAATAGAGTGTAGGGTTGGAGATGCCCTGAGAAGTACTAAATTTTGGATCTTTGAAAACCTCGTAAAATGGTCCTCTTTTTTGGTGTTCTTGCTGCTTATCTTCTCTGCTTCATTTTTCTTGTATTACGCTAGCTTGCTCTTGTTTGTTGAATGTGGATGTGTCGTGACAGTTGAGAGGAATAAACTTGGAAGATTTTGAAGGTTGGTGGCCTGTGTGCATTTCTCTGCATCCTGTTTCTTGGCCATATTGGTGCTTGCACTTCAATCTGGAGATTATCATGCTTTGAAAGAAGGCGGTTCTCAATTTTGTCTGGCATTTGTAGTGAATTCCTTGGTGGTATGGTCTTTGCCAGATTTGCACTTGAACTTCTTCCATTCTTTCTGTGCTTGCAGCTGCTGGGTCTTACAGAGCGATAGATTCTTCTTTTCATGGAGTGGCATGATCTGTCAATTTAGTGTTGAGGTTTATTGTTTGATGCTCTAATGATTGAGATCTTCAATTCAATCTTCTTCCCTAAACAGAATGGTTCACTTCTCTCTTGTCAACACTGCTTCACTAGTGTGTATTTTGTTGTGATATTATTTTTTTAATAATCCAGATGCTTATGTTCTTTGGTGATCCAACTGATGAGATCTCCATGGTTATGGACAACACTTGATAAGAGTATTCAAACTCAATCATGTTCTTTTGATGGCCCTGTACTTCTCGTGTTCTCGCTACTTACCTTCTCTGATTTCGTTTGGAGATTTTATAGTATGGTTTGGGTGTGCATCCCTCTGTGGACCATGTTTCTTGCCATTTTGGTGCTTGCACTGCAATCTGTTTATTGGATATCACCAGGCATCAAGAACAGGGCACTTTGCAATATTATCTTGTTTGGCATCTCTAGTGTTCTCTCCTGCATGGTATTTTCCACTTACCTGTTACGTTCTTTTTGTGCATGCAATGGTTGGGTCTTCCAGAGCATGAACTTCAACTCAGATTTCTTCCGAATCAGCAGGTCCCTTTTTCTTTGTCAAAACCTACGGTTGTGTAAAAGTTCTCAAGAATCATAATATTTTTCACTAGTGTGTGTTGTACTCTGCTGCCATGAAGTTTTTAGTCTAGATGCTTTTTCTTGGACTTATCCAAATGATGATGAGATCCCTGATTGTTATGGACACCTGAGAAGTACCCAAGCTCAATTTTATATCTTCTTATGGCCCGATGAGCTCCTTCTACTTCGGTGTTCATGCTACTTATCTTCTCTGTTTCTATTTTCTTATGCTTATCCCGCTACTTGATTTGGTTTGTTTGGATGCATGTTTGTAGACCACATGGTAACCATGAGGGGATCAGATTTGGTAATTTCAAAGTTTGGTCTCAGTGTGTGTCCTTCCCTTGTGCACCTTGTTTCTGTGCCATTTTGGTGCTTGCACGGCAATTTGGATATCACCAGGCACTGGCAAGGGAGTTACACAATCTTGTCTGGCACCTCTAGTGATCTCTCCTATGTTGCATGGGTTTCGCAGCTTCAACTCTATCTGTTCCCTTCTTTTTGTGCTTGCTGCTGTTGGGTCTTTCAAGATCAGTGAAGTCTTCCTTTCATGGAGCAGCCTTGTTTGTCTAAGCGTTGAAGTTTTCCTTAGTTCAGGCTTTGTATTAGTACCTAGCTCTTTGATTAAATTTTCTTCCCTAGTCATAGAGATCCCCTTATCTCGTTGGCGGAAGTGCTGTTAAGCGGAAGTTTTCAATCATGATAACTCTCTCCACAAGTGCATTTTCAGCTTTTAGTCCAGTTGCTTTTGTTTTGCGATGATCCAACTGATGAGTTATCTTATGGTGTGGATCTCTGGTAAGTACTCGCTCAGTTCTGGATTTTCCATCATCTACCTTTTGTGTTCTTGCTGCTTATCTTCTCTGCTTCCATTTTCTTTTTGCTCATCACACCAGTTTGAGTTTTTTCTGTTGGAAGGATGTATATACCACATAGTGTATGTGTATTGGAGGAGTAGATTTGGAAATTTAAAAGCTTAGTGTTCAGTAAGCTGGTCGTTTAGGTGCTTGCACTGCAATCTGGATATCGTTAGGGCTTTGAGGGAAAGGGGGTTTCTAAATCTTTGCATTTAACTGTTCTTTCTGTGCTTGTAGCTGTGTTCTTAAAGATCTAATCTCTCCTTTTTGTGGAGCAGCCTTTGTTGATTTATGTTGAGGTTTCGTTTGGTTCAAGCACCTGTAAGTGTTCGGTTTTCAGTTTGGTTCTCTTCTCTAATCAGTATTTTATCTTTTCTCACCACACTCTTTTCAAAGTGGTGATCTTCTAGTAATTCGTTAAAATTAAAATTTTACATGTTAGTGGGTTACAGTACATGATCATTTTCATTCAATTTCTGGTTCATTTATTGGGTGCAGCATCTGCACCATAGTCACTACTATTGTCAGACTAATTATTGTTAGGTAAGTGAAGCCTCTTTTTTTTTTTTTGAAGATCTCTCCCCTATCTTTTTTTTTTGGAACATGTGCATTATATTCAGACATTCAATCATTTGACGAGTTGCTTCTGAGTGTTAGCTATTATGTAAAACTTTAATATAAACTTGGAGCTCCTGCACACCTTTTTTCTCCATTAAGCATTGCCATTTGAACAGTCTTTGCACCAAAGAGGGCAATTCATTGAATCTAGAACAGATCAAGTAAAAAAACATGGCTCATCTTTTGTGTGACAGGTCATGTACAGTCTTTATGTATTTATATCACAAAAGTAACTATTGTTTTCTGTGTAGAAGATCTTCTCATGAGATACCCTTGTAAGTCCATTACATATCAGATCATTGTGCAAGTTGGGAATTTTTTTCTATGAATTTTGTTCTTCTAAATACTCCTCTGAGATGAGCTTTGTACTATTTTCTTGATATCGTTGAAATTGCCATTGATTATAGGTAACTGTTGTTCTGTCATTCTCCGTGTTCTTGATGTATGTTTAGTCAAATGTCACTTCACTGATTGCATGTTTCCTAAAATGTCACTTAACCGATAATTTGTTCAGCCAATGTCACTTCACAAATCTGTCACTGATGGTTTCTTTACTTCGTTCTCGTCATGTTCTATTACCAGCTATAATTCATTTCTTATTTCAGAAATTGTTCCTTGCTCCTTGGATTTCTTATATTGGTGAGTGATTAGTTGCACAAGTTAAAAGGGGGTCTTCTAAGAAGCCCCACCCAGACCAAGTTTAGACAGTTTTGAAAAGTGCTGCTGTCTTGGCCTTCATTCATCAAGTGCAGCTCTCCATTCAATCATTTACGGAGTTGCTTCAGACTGTTAGCCATTGTGTAAAACCGCACACTGTTTTTGCCATAAAACATTGCCATTTGAACAGTCAAATATTGAATCTAGAGCAGATCAAGTAAAAAACATGGCTCATCTTTTGTGTAACAAGTCATGTACACTCTTTTATGTTATTTATATCAACGCAACTAGTTTTGTGTTACCAGAGACGGTTGATGATTTTTGGATATTTACAGAATTTGGTAGGTCGGGATTTGTTGGTGCAGTTGTATCCGTAAGTTTTCTGATTTTGCTATGTAGGTATGGAGTATGAGCAGATCTGTGAGTTGCAGAAAAGAGTGTCTGCAGAATGCTTGGCCTCCTGTGCTGTGGAAGTGAGACCTGAAGGATGAGCTTATGGCGCAGGGAATCTCTTTCTGGGTTTTGCAATTTTATTTTGAAGTTTGAATTTGCTGTGGATTCTCACTTTTGTTTGTTTCTCGAGGCAACCAAGGACTTCCTGTTTAGGTTATGCCAATCTCTTTCTGGTTCTACCCGAGGCAATGCTGTAAACCTCATCTCTGCCAAGAGTTATCAGCCTCAAGATGTTGCGTTGGAGTAACTGTTGGTCCAGCTTTGTTCCTCTTTTCCCTCAAGCACTTGTAGCTACTTTGATAGCATTAGAATTAAAAGGGAATATATTTTTCTGCTAAAATACACGGATTCAGTTCCATATAAGTGGATTTGATCTCCAAAAGTGGTGAGAAGTTGGGAGGATTGTAAATCGGTTGTTGTGGAGCATGTTAAACAGAAAAGGACCTGGTATGCTGTGGAGCATGTTAAACAGATAACGTCCTGGTATGGTCACTTGGATTCCTATTTTTACATTGACCTGTTCTAGTCATTTTTGCATGTTGGAAAGTACTTGAATATCTGTTTTGGATTAACATTACAAGTTCTTCTTGTTATTTTGTGGGGGTGGGTGTTCAATTACACTTGAGTGTTCTAGTCGCTCAGTACTATCTAATGTGCTTTTCTCCTACAATGTGTAGTAATTATTAATCTATGTGCACCTTGCAGGATGAGTGTTCTATTAGAGCGGCAGTGTCGGTGATGGAGTTTGAACTATGAGTAAGAATTGGATGTTTTCAGAATAATTGTCTCCTCTCACTGTCTCACATGTATGAAATGCAGTTACTGAATATAAGTGGTATATAGGTACTATTCTAAAAAGTATAGGTGTGAAGTAGCTTTCTGTTTTGGGTTGTTATGATGAGTTGGGAATCTCTTTATTTGGCTCATCTCACATGCTTATTTGGTCTCTTTTGATTTGGAAGTAGTATCTTGGATTTAGTAAAGCTTGAGTTCAGGGAAAACAAGGAGATACTTTTTCTCAAAACATGGACTAATGAAAGTTTAACTCTGAAGAAGCACTTGATGAAGCATCCCGAAATTTTGATGAAGCACTTGATTCCATGTGTGTAGTGATGGAGCTGGTGCAAAAACTGATTTCAGGAAGGGCAGTGCCAAGTCTACTTAACTGCCGAGTCTTTTTGTACAGCAACAACAACAACAGGCCCAGCGCAATTCCAAAAGTGATCGCATAAGTGGGGTCTGGGAGGGAAGGACGTAAGAAGACCTTACCTCTTAACTTTGAGGGACAGAAAGGCTTCTCTGGTAGACCTTGGAGGTCACTTCCCAGTCTGTTCCTCCCTATCTTCATTTCCAAGTTGTGTACTCTTTTCTTTGTGTTTATGTTGAATACATTTATTTGAAGATTATTTTGTTAGTTCTATATGATCTCACATTGTTTCGGCACCAATAGTAGTTCTTGTGGTTTCTTTTGTAGTAGTGTTGATTTATTTGTTGCTACATTTTGTGTTTAGGTGACCCTCGTAACTCCGCCTGTTAAATCAGCCGGTCCCAAGCCCGGACTAAGGAGGAGGGGAAGGTGTTCAAGTTTGCAATTAATTGTCTGTTTTTTTTAATTAGTAGAAATGTTTCTGGAGTCGCCTATGAGCAATTATGGCATCTCTAATTACAATTATCACTTATTAGTAATGTACATCGGATGAAATGTAAAGAGATGCATTAGGGACTTGTCATGCAATTGGTAATTACAAATTGTGGCAACTAAATGCATTGCAATCTTGCAGCTGTTGCAATTTACTTATGATTTGCCTTAACACCGGAATTATTGTTTCCCTTATTGGTGTCTATAGATGGTTTCCAACACTGCATTCAACTAGGGATTTGTGTTAAGCCTGGCATGTGCCTATTCAGGAATCATCAATGTGTTTTTCTGTCTTGATTGATCACCCATTGTAAGTTGATACTCTTTTCAACTGTTTGCTTTTTGTTTGTGAGGTCGATCGTGGTTGCAGGTTAGTGTTTAGCCGATAAGTCCCTTCATGTTCCTTTGTTGAACAATGTTACAACTGTGAGATGCAACACTGGATGTAGGAGCTCCCTAATACTGTGACTTACTGTGGGAGTGTAACAAGTAATTTAACGACTCTTTAGTTTAAGGCAAAGTATGCTCTCGTCTTTTGGTCTTTAAAGGTCGATTGGAAAGACCAAAAGATGTGGTTCAAAAGCTAATGCTGCAAAAAGTTGCCATAGTTGTCTGGTAATTGAACAATGCAAGAAAAGATGCCCATTGGTTTCAGCTTCCCGATGGCATAGAAAACACCTAGAACCCTTTTTTTGAAGTTTATCATGAGTGAGACAAGTTTTCCAAACAAGAAGTCAAGTGAAGCAAACCACTTTAAAAGGAGCTCTTGCTTTCCATCTCTTCCCATGGTCATGGGTTATTGAACTGTTCCA

>LNC_000586

GAAAAAAATAAAGAACTCTATATTTGGAGAGTAAAATAGAGTGTAGGGTTGGAGATGCCCTGAGAAGTACTAAATTTTGGATCTTTGAAAACCTCGTAAAATGGTCCTCTTTTTTGGTGTTCTTGCTGCTTATCTTCTCTGCTTCATTTTTCTTGTATTACGCTAGCTTGCTCTTGTTTGTTGAATGTGGATGTGTCGTGACAGTTGAGAGGAATAAACTTGGAAGATTTTGAAGGTTGGTGGCCTGTGTGCATTTCTCTGCATCCTGTTTCTTGGCCATATTGGTGCTTGCACTTCAATCTGGAGATTATCATGCTTTGAAAGAAGGCGGTTCTCAATTTTGTCTGGCATTTGTAGTGAATTCCTTGGTGGTATGGTCTTTGCCAGATTTGCACTTGAACTTCTTCCATTCTTTCTGTGCTTGCAGCTGCTGGGTCTTACAGAGCGATAGATTCTTCTTTTCATGGAGTGGCATGATCTGTCAATTTAGTGTTGAGGTTTATTGTTTGATGCTCTAATGATTGAGATCTTCAATTCAATCTTCTTCCCTAAACAGAATGGTTCACTTCTCTCTTGTCAACACTGCTTCACTAGTGTGTATTTTGTTGTGATATTATTTTTTTAATAATCCAGATGCTTATGTTCTTTGGTGATCCAACTGATGAGATCTCCATGGTTATGGACAACACTTGATAAGAGTATTCAAACTCAATCATGTTCTTTTGATGGCCCTGTACTTCTCGTGTTCTCGCTACTTACCTTCTCTGATTTCGTTTGGAGATTTTATAGTATGGTTTGGGTGTGCATCCCTCTGTGGACCATGTTTCTTGCCATTTTGGTGCTTGCACTGCAATCTGTTTATTGGATATCACCAGGCATCAAGAACAGGGCACTTTGCAATATTATCTTGTTTGGCATCTCTAGTGTTCTCTCCTGCATGGTATTTTCCACTTACCTGTTACGTTCTTTTTGTGCATGCAATGGTTGGGTCTTCCAGAGCATGAACTTCAACTCAGATTTCTTCCGAATCAGCAGGTCCCTTTTTCTTTGTCAAAACCTACGGTTGTGTAAAAGTTCTCAAGAATCATAATATTTTTCACTAGTGTGTGTTGTACTCTGCTGCCATGAAGTTTTTAGTCTAGATGCTTTTTCTTGGACTTATCCAAATGATGATGAGATCCCTGATTGTTATGGACACCTGAGAAGTACCCAAGCTCAATTTTATATCTTCTTATGGCCCGATGAGCTCCTTCTACTTCGGTGTTCATGCTACTTATCTTCTCTGTTTCTATTTTCTTATGCTTATCCCGCTACTTGATTTGGTTTGTTTGGATGCATGTTTGTAGACCACATGGTAACCATGAGGGGATCAGATTTGGTAATTTCAAAGTTTGGTCTCAGTGTGTGTCCTTCCCTTGTGCACCTTGTTTCTGTGCCATTTTGGTGCTTGCACGGCAATTTGGATATCACCAGGCACTGGCAAGGGAGTTACACAATCTTGTCTGGCACCTCTAGTGATCTCTCCTATGTTGCATGGGTTTCGCAGCTTCAACTCTATCTGTTCCCTTCTTTTTGTGCTTGCTGCTGTTGGGTCTTTCAAGATCAGTGAAGTCTTCCTTTCATGGAGCAGCCTTGTTTGTCTAAGCGTTGAAGTTTTCCTTAGTTCAGGCTTTGTATTAGTACCTAGCTCTTTGATTAAATTTTCTTCCCTAGTCATAGAGATCCCCTTATCTCGTTGGCGGAAGTGCTGTTAAGCGGAAGTTTTCAATCATGATAACTCTCTCCACAAGTGCATTTTCAGCTTTTAGTCCAGTTGCTTTTGTTTTGCGATGATCCAACTGATGAGTTATCTTATGGTGTGGATCTCTGGTAAGTACTCGCTCAGTTCTGGATTTTCCATCATCTACCTTTTGTGTTCTTGCTGCTTATCTTCTCTGCTTCCATTTTCTTTTTGCTCATCACACCAGTTTGAGTTTTTTCTGTTGGAAGGATGTATATACCACATAGTGTATGTGTATTGGAGGAGTAGATTTGGAAATTTAAAAGCTTAGTGTTCAGTAAGCTGGTCGTTTAGGTGCTTGCACTGCAATCTGGATATCGTTAGGGCTTTGAGGGAAAGGGGGTTTCTAAATCTTTGCATTTAACTGTTCTTTCTGTGCTTGTAGCTGTGTTCTTAAAGATCTAATCTCTCCTTTTTGTGGAGCAGCCTTTGTTGATTTATGTTGAGGTTTCGTTTGGTTCAAGCACCTGTAAGTGTTCGGTTTTCAGTTTGGTTCTCTTCTCTAATCAGTATTTTATCTTTTCTCACCACACTCTTTTCAAAGTGGTGATCTTCTAGTAATTCGTTAAAATTAAAATTTTACATGTTAGTGGGTTACAGTACATGATCATTTTCATTCAATTTCTGGTTCATTTATTGGGTGCAGCATCTGCACCATAGTCACTACTATTGTCAGACTAATTATTGTTAGGTAAGTGAAGCCTCTTTTTTTTTTTTTGAAGATCTCTCCCCTATCTTTTTTTTTTGGAACATGTGCATTATATTCAGACATTCAATCATTTGACGAGTTGCTTCTGAGTGTTAGCTATTATGTAAAACTTTAATATAAACTTGGAGCTCCTGCACACCTTTTTTCTCCATTAAGCATTGCCATTTGAACAGTCTTTGCACCAAAGAGGGCAATTCATTGAATCTAGAACAGATCAAGTAAAAAAACATGGCTCATCTTTTGTGTGACAGGTCATGTACAGTCTTTATGTATTTATATCACAAAAGTAACTATTGTTTTCTGTGTAGAAGATCTTCTCATGAGATACCCTTGTAAGTCCATTACATATCAGATCATTGTGCAAGTTGGGAATTTTTTTCTATGAATTTTGTTCTTCTAAATACTCCTCTGAGATGAGCTTTGTACTATTTTCTTGATATCGTTGAAATTGCCATTGATTATAGGTAACTGTTGTTCTGTCATTCTCCGTGTTCTTGATGTATGTTTAGTCAAATGTCACTTCACTGATTGCATGTTTCCTAAAATGTCACTTAACCGATAATTTGTTCAGCCAATGTCACTTCACAAATCTGTCACTGATGGTTTCTTTACTTCGTTCTCGTCATGTTCTATTACCAGCTATAATTCATTTCTTATTTCAGAAATTGTTCCTTGCTCCTTGGATTTCTTATATTGGTGAGTGATTAGTTGCACAAGTTAAAAGGGGGTCTTCTAAGAAGCCCCACCCAGACCAAGTTTAGACAGTTTTGAAAAGTGCTGCTGTCTTGGCCTTCATTCATCAAGTGCAGCTCTCCATTCAATCATTTACGGAGTTGCTTCAGACTGTTAGCCATTGTGTAAAACCGCACACTGTTTTTGCCATAAAACATTGCCATTTGAACAGTCAAATATTGAATCTAGAGCAGATCAAGTAAAAAACATGGCTCATCTTTTGTGTAACAAGTCATGTACACTCTTTTATGTTATTTATATCAACGCAACTAGTTTTGTGTTACCAGAGACGGTTGATGATTTTTGGATATTTACAGAATTTGGTAGGTCGGGATTTGTTGGTGCAGTTGTATCCGTAAGTTTTCTGATTTTGCTATGTAGGTATGGAGTATGAGCAGATCTGTGAGTTGCAGAAAAGAGTGTCTGCAGAATGCTTGGCCTCCTGTGCTGTGGAAGTGAGACCTGAAGGATGAGCTTATGGCGCAGGGAATCTCTTTCTGGGTTTTGCAATTTTATTTTGAAGTTTGAATTTGCTGTGGATTCTCACTTTTGTTTGTTTCTCGAGGCAACCAAGGACTTCCTGTTTAGGTTATGCCAATCTCTTTCTGGTTCTACCCGAGGCAATGCTGTAAACCTCATCTCTGGTACCTGCAGCAGATCATCTTTTCTTGAATCTTCTCCTCGGGAACACTATTCTGTAGAAGTTTCTGAGCCTTCCCTAGTATATGTTGCTCTCTATATTCCATGTTGTTAGGGGATTGGCTCATTTACCTCCCAAGTCTTCTGGTTTTCCTTATCTACCCATAAGCTTTAAATGGAGTACAGGTGCTAGAGTAGTGGAATTTTAATGTGTTATCTACCATAAACACAAGTATTAGTTGGCAGTGATATCGTGTTATCCAACCAATTAGTTAGGTAGGTTCATGCAGCTAGCATGAGAAGGGATGAGATTAAGGAGTTTCTGAAAAGCGATCAGTTATAGTCTTTTACAATGGTTTTGATAATGTTAGACTCCTGTTTTTTACATTTTTGTTGTATTTGCTCATTTGGATCTCTTACCTATAAAACAGCACTACAGATGTGAGTGTACATTTTTTGAAGTTGAGGAAGGTATTAGCATCTTGAAGTAGCTAGGATAGTTTGGCTTCTGTTTGTCATGGTATTTCTTTTCTAAATATGGCCATTGAATGGGGTATTTCTGTTTTCTTTGAGCTCCAGTTCGAATTTGCGAGATAATTTGAGGGGGGTATTTCTCTTTTTTAAGCTCCAGTTGTTCAATTTCCAGATAACTCTGAATGGGGTATTTCTGTTCTTCTAAGCTCCAGTTGTCTAAATTTGCCAGCCAAGAGTTATCAGCCTCAAGATGTTGCGTTGGAGTAACTGTTGGTCCAGCTTTGTTCCTCTTTTCCCTCAAGCACTTGTAGCTACTTTGATAGCATTAGAATTAAAAGGGAATATATTTTTCTGCTAAAATACACGGATTCAGTTCCATATAAGTGGATTTGATCTCCAAAAGTGGTGAGAAGTTGGGAGGATTGTAAATCGGTTGTTGTGGAGCATGTTAAACAGAAAAGGACCTGGTATGCTGTGGAGCATGTTAAACAGATAACGTCCTGGATGAGTGTTCTATTAGAGCGGCAGTGTCGGTGATGGAGTTTGAACTATGAGTAAGAATTGGATGTTTTCAGAATAATTGTCTCCTCTCACTGTCTCACATGTATGAAATGCAGTTACTGAATATAAGTGGTATATAGGTACTATTCTAAAAAGTATAGGTGTGAAGTAGCTTTCTGTTTTGGGTTGTTATGATGAGTTGGGAATCTCTTTATTTGGCTCATCTCACATGCTTATTTGGTCTCTTTTGATTTGGAAGTAGTATCTTGGATTTAGTAAAGCTTGAGTTCAGGGAAAACAAGGAGATACTTTTTCTCAAAACATGGACTAATGAAAGTTTAACTCTGAAGAAGCACTTGATGAAGCATCCCGAAATTTTGATGAAGCACTTGATTCCATGTGTGTAGTGATGGAGCTGGTGCAAAAACTGATTTCAGGAAGGGCAGTGCCAAGTCTACTTAACTGCCGAGTCTTTTTGTACAGCAACAACAACAACAGGCCCAGCGCAATTCCAAAAGTGATCGCATAAGTGGGGTCTGGGAGGGAAGGACGTAAGAAGACCTTACCTCTTAACTTTGAGGGACAGAAAGGCTTCTCTGGTAGACCTTGGAGGTCACTTCCCAGTCTGTTCCTCCCTATCTTCATTTCCAAGTTGTGTACTCTTTTCTTTGTGTTTATGTTGAATACATTTATTTGAAGATTATTTTGTTAGTTCTATATGATCTCACATTGTTTCGGCACCAATAGTAGTTCTTGTGGTTTCTTTTGTAGTAGTGTTGATTTATTTGTTGCTACATTTTGTGTTTAGGTGACCCTCGTAACTCCGCCTGTTAAATCAGCCGGTCCCAAGCCCGGACTAAGGAGGAGGGGAAGGTGTTCAAGTTTGCAATTAATTGTCTGTTTTTTTTAATTAGTAGAAATGTTTCTGGAGTCGCCTATGAGCAATTATGGCATCTCTAATTACAATTATCACTTATTAGTAATGTACATCGGATGAAATGTAAAGAGATGCATTAGGGACTTGTCATGCAATTGGTAATTACAAATTGTGGCAACTAAATGCATTGCAATCTTGCAGCTGTTGCAATTTACTTATGATTTGCCTTAACACCGGAATTATTGTTTCCCTTATTGGTGTCTATAGATGGTTTCCAACACTGCATTCAACTAGGGATTTGTGTTAAGCCTGGCATGTGCCTATTCAGGAATCATCAATGTGTTTTTCTGTCTTGATTGATCACCCATTGTAAGTTGATACTCTTTTCAACTGTTTGCTTTTTGTTTGTGAGGTCGATCGTGGTTGCAGGTTAGTGTTTAGCCGATAAGTCCCTTCATGTTCCTTTGTTGAACAATGTTACAACTGTGAGATGCAACACTGGATGTAGGAGCTCCCTAATACTGTGACTTACTGTGGGAGTGTAACAAGTAATTTAACGACTCTTTAGTTTAAGGCAAAGTATGCTCTCGTCTTTTGGTCTTTAAAGGTCGATTGGAAAGACCAAAAGATGTGGTTCAAAAGCTAATGCTGCAAAAAGTTGCCATAGTTGTCTGGTAATTGAACAATGCAAGAAAAGATGCCCATTGGTTTCAGCTTCCCGATGGCATAGAAAACACCTAGAACCCTTTTTTTGAAGTTTATCATGAGTGAGACAAGTTTTCCAAACAAGAAGTCAAGTGAAGCAAACCACTTTAAAAGGAGCTCTTGCTTTCCATCTCTTCCCATGGTCATGGGTTATTGAACTGTTCCA

>LNC_000587

GAAAAAAATAAAGAACTCTATATTTGGAGAGTAAAATAGAGTGTAGGGTTGGAGATGCCCTGAGAAGTACTAAATTTTGGATCTTTGAAAACCTCGTAAAATGGTCCTCTTTTTTGGTGTTCTTGCTGCTTATCTTCTCTGCTTCATTTTTCTTGTATTACGCTAGCTTGCTCTTGTTTGTTGAATGTGGATGTGTCGTGACAGTTGAGAGGAATAAACTTGGAAGATTTTGAAGGTTGGTGGCCTGTGTGCATTTCTCTGCATCCTGTTTCTTGGCCATATTGGTGCTTGCACTTCAATCTGGAGATTATCATGCTTTGAAAGAAGGCGGTTCTCAATTTTGTCTGGCATTTGTAGTGAATTCCTTGGTGGTATGGTCTTTGCCAGATTTGCACTTGAACTTCTTCCATTCTTTCTGTGCTTGCAGCTGCTGGGTCTTACAGAGCGATAGATTCTTCTTTTCATGGAGTGGCATGATCTGTCAATTTAGTGTTGAGGTTTATTGTTTGATGCTCTAATGATTGAGATCTTCAATTCAATCTTCTTCCCTAAACAGAATGGTTCACTTCTCTCTTGTCAACACTGCTTCACTAGTGTGTATTTTGTTGTGATATTATTTTTTTAATAATCCAGATGCTTATGTTCTTTGGTGATCCAACTGATGAGATCTCCATGGTTATGGACAACACTTGATAAGAGTATTCAAACTCAATCATGTTCTTTTGATGGCCCTGTACTTCTCGTGTTCTCGCTACTTACCTTCTCTGATTTCGTTTGGAGATTTTATAGTATGGTTTGGGTGTGCATCCCTCTGTGGACCATGTTTCTTGCCATTTTGGTGCTTGCACTGCAATCTGTTTATTGGATATCACCAGGCATCAAGAACAGGGCACTTTGCAATATTATCTTGTTTGGCATCTCTAGTGTTCTCTCCTGCATGGTATTTTCCACTTACCTGTTACGTTCTTTTTGTGCATGCAATGGTTGGGTCTTCCAGAGCATGAACTTCAACTCAGATTTCTTCCGAATCAGCAGGTCCCTTTTTCTTTGTCAAAACCTACGGTTGTGTAAAAGTTCTCAAGAATCATAATATTTTTCACTAGTGTGTGTTGTACTCTGCTGCCATGAAGTTTTTAGTCTAGATGCTTTTTCTTGGACTTATCCAAATGATGATGAGATCCCTGATTGTTATGGACACCTGAGAAGTACCCAAGCTCAATTTTATATCTTCTTATGGCCCGATGAGCTCCTTCTACTTCGGTGTTCATGCTACTTATCTTCTCTGTTTCTATTTTCTTATGCTTATCCCGCTACTTGATTTGGTTTGTTTGGATGCATGTTTGTAGACCACATGGTAACCATGAGGGGATCAGATTTGGTAATTTCAAAGTTTGGTCTCAGTGTGTGTCCTTCCCTTGTGCACCTTGTTTCTGTGCCATTTTGGTGCTTGCACGGCAATTTGGATATCACCAGGCACTGGCAAGGGAGTTACACAATCTTGTCTGGCACCTCTAGTGATCTCTCCTATGTTGCATGGGTTTCGCAGCTTCAACTCTATCTGTTCCCTTCTTTTTGTGCTTGCTGCTGTTGGGTCTTTCAAGATCAGTGAAGTCTTCCTTTCATGGAGCAGCCTTGTTTGTCTAAGCGTTGAAGTTTTCCTTAGTTCAGGCTTTGTATTAGTACCTAGCTCTTTGATTAAATTTTCTTCCCTAGTCATAGAGATCCCCTTATCTCGTTGGCGGAAGTGCTGTTAAGCGGAAGTTTTCAATCATGATAACTCTCTCCACAAGTGCATTTTCAGCTTTTAGTCCAGTTGCTTTTGTTTTGCGATGATCCAACTGATGAGTTATCTTATGGTGTGGATCTCTGGTAAGTACTCGCTCAGTTCTGGATTTTCCATCATCTACCTTTTGTGTTCTTGCTGCTTATCTTCTCTGCTTCCATTTTCTTTTTGCTCATCACACCAGTTTGAGTTTTTTCTGTTGGAAGGATGTATATACCACATAGTGTATGTGTATTGGAGGAGTAGATTTGGAAATTTAAAAGCTTAGTGTTCAGTAAGCTGGTCGTTTAGGTGCTTGCACTGCAATCTGGATATCGTTAGGGCTTTGAGGGAAAGGGGGTTTCTAAATCTTTGCATTTAACTGTTCTTTCTGTGCTTGTAGCTGTGTTCTTAAAGATCTAATCTCTCCTTTTTGTGGAGCAGCCTTTGTTGATTTATGTTGAGGTTTCGTTTGGTTCAAGCACCTGTAAGTGTTCGGTTTTCAGTTTGGTTCTCTTCTCTAATCAGTATTTTATCTTTTCTCACCACACTCTTTTCAAAGTGGTGATCTTCTAGTAATTCGTTAAAATTAAAATTTTACATGTTAGTGGGTTACAGTACATGATCATTTTCATTCAATTTCTGGTTCATTTATTGGGTGCAGCATCTGCACCATAGTCACTACTATTGTCAGACTAATTATTGTTAGGTAAGTGAAGCCTCTTTTTTTTTTTTTGAAGATCTCTCCCCTATCTTTTTTTTTTGGAACATGTGCATTATATTCAGACATTCAATCATTTGACGAGTTGCTTCTGAGTGTTAGCTATTATGTAAAACTTTAATATAAACTTGGAGCTCCTGCACACCTTTTTTCTCCATTAAGCATTGCCATTTGAACAGTCTTTGCACCAAAGAGGGCAATTCATTGAATCTAGAACAGATCAAGTAAAAAAACATGGCTCATCTTTTGTGTGACAGGTCATGTACAGTCTTTATGTATTTATATCACAAAAGTAACTATTGTTTTCTGTGTAGAAGATCTTCTCATGAGATACCCTTGTAAGTCCATTACATATCAGATCATTGTGCAAGTTGGGAATTTTTTTCTATGAATTTTGTTCTTCTAAATACTCCTCTGAGATGAGCTTTGTACTATTTTCTTGATATCGTTGAAATTGCCATTGATTATAGGTAACTGTTGTTCTGTCATTCTCCGTGTTCTTGATGTATGTTTAGTCAAATGTCACTTCACTGATTGCATGTTTCCTAAAATGTCACTTAACCGATAATTTGTTCAGCCAATGTCACTTCACAAATCTGTCACTGATGGTTTCTTTACTTCGTTCTCGTCATGTTCTATTACCAGCTATAATTCATTTCTTATTTCAGAAATTGTTCCTTGCTCCTTGGATTTCTTATATTGGTGAGTGATTAGTTGCACAAGTTAAAAGGGGGTCTTCTAAGAAGCCCCACCCAGACCAAGTTTAGACAGTTTTGAAAAGTGCTGCTGTCTTGGCCTTCATTCATCAAGTGCAGCTCTCCATTCAATCATTTACGGAGTTGCTTCAGACTGTTAGCCATTGTGTAAAACCGCACACTGTTTTTGCCATAAAACATTGCCATTTGAACAGTCAAATATTGAATCTAGAGCAGATCAAGTAAAAAACATGGCTCATCTTTTGTGTAACAAGTCATGTACACTCTTTTATGTTATTTATATCAACGCAACTAGTTTTGTGTTACCAGAGACGGTTGATGATTTTTGGATATTTACAGAATTTGGTAGGTCGGGATTTGTTGGTGCAGTTGTATCCGTAAGTTTTCTGATTTTGCTATGTAGGTATGGAGTATGAGCAGATCTGTGAGTTGCAGAAAAGAGTGTCTGCAGAATGCTTGGCCTCCTGTGCTGTGGAAGTGAGACCTGAAGGATGAGCTTATGGCGCAGGGAATCTCTTTCTGGGTTTTGCAATTTTATTTTGAAGTTTGAATTTGCTGTGGATTCTCACTTTTGTTTGTTTCTCGAGGCAACCAAGGACTTCCTGTTTAGGTTATGCCAATCTCTTTCTGGTTCTACCCGAGGCAATGCTGTAAACCTCATCTCTGGTACCTGCAGCAGATCATCTTTTCTTGAATCTTCTCCTCGGGAACACTATTCTGTAGAAGTTTCTGAGCCTTCCCTAGTATATGTTGCTCTCTATATTCCATGTTGTTAGGGGATTGGCTCATTTACCTCCCAAGTCTTCTGGTTTTCCTTATCTACCCATAAGCTTTAAATGGAGTACAGGTGCTAGAGTAGTGGAATTTTAATGTGTTATCTACCATAAACACAAGTATTAGTTGGCAGTGATATCGTGTTATCCAACCAATTAGTTAGGTAGGTTCATGCAGCTAGCATGAGAAGGGATGAGATTAAGGAGTTTCTGAAAAGCGATCAGTTATAGTCTTTTACAATGGTTTTGATAATGTTAGACTCCTGTTTTTTACATTTTTGTTGTATTTGCTCATTTGGATCTCTTACCTATAAAACAGCACTACAGATGTGAGTGTACATTTTTTGAAGTTGAGGAAGGTATTAGCATCTTGAAGTAGCTAGGATAGTTTGGCTTCTGTTTGTCATGGTATTTCTTTTCTAAATATGGCCATTGAATGGGGTATTTCTGTTTTCTTTGAGCTCCAGTTCGAATTTGCGAGATAATTTGAGGGGGGTATTTCTCTTTTTTAAGCTCCAGTTGTTCAATTTCCAGATAACTCTGAATGGGGTATTTCTGTTCTTCTAAGCTCCAGTTGTCTAAATTTGCCAGCCAAGAGTTATCAGCCTCAAGATGTTGCGTTGGAGTAACTGTTGGTCCAGCTTTGTTCCTCTTTTCCCTCAAGCACTTGTAGCTACTTTGATAGCATTAGAATTAAAAGGGAATATATTTTTCTGCTAAAATACACGGATTCAGTTCCATATAAGTGGATTTGATCTCCAAAAGTGGTGAGAAGTTGGGAGGATTGTAAATCGGTTGTTGTGGAGCATGTTAAACAGAAAAGGACCTGGTATGCTGTGGAGCATGTTAAACAGATAACGTCCTGGATGAGTGTTCTATTAGAGCGGCAGTGTCGGTGATGGAGTTTGAACTATGAGTAAGAATTGGATGTTTTCAGAATAATTGTCTCCTCTCACTGTCTCACATGTATGAAATGCAGTTACTGAATATAAGTGGTATATAGGTACTATTCTAAAAAGTATAGGTGTGAAGTAGCTTTCTGTTTTGGGTTGTTATGATGAGTTGGGAATCTCTTTATTTGGCTCATCTCACATGCTTATTTGGTCTCTTTTGATTTGGAAGTAGTATCTTGGATTTAGTAAAGCTTGAGTTCAGGGAAAACAAGGAGATACTTTTTCTCAAAACATGGACTAATGAAAGTTTAACTCTGAAGAAGCACTTGATGAAGCATCCCGAAATTTTGATGAAGCACTTGATTCCATGTGTGTAGTGATGGAGCTGGTGCAAAAACTGATTTCAGGGTACATATTGCTCTCTATGTGCAGATACTTTGTAGTTCCAGAACTGGAGAATCTGCTTTCTGGATATTATATATATTTTTTGTTGTTGAAGGTGCTATGAAATTGTGGATGTCACGAATGCAACTTCTTCATGGTATGAGATGTATGTTGAGTTCTAAGATAGAAGTGATTTTGACGTAGTGGTCCAGTGTTTCTTTCATTGCACAATTACCCTTGGGCAATCTCATAAAAAGACTAGGACCTGTTGGAGCCATGACATTTTTATTTTTCATCTGATATGCATCTATGAAGTAATATCTTGTCATTCGAAGTCATGTTTTGCACTTGCCAGGGCCGATAGTTGTCTTTGACATTTTTACTTTTTAGCAGCGATGCATCTCTCAAGTAGTATCTTGTCATTTGAAGTCATGTCTTGCTCTTCAGGGCCCAAAAGTTGTCTATCATGTGACATCATATGTGGGAGAATTTCATTATTTGTCCAAATTAATTTGTGAGAATGCTCCATAGTATTCTAACATAGAGTCTTTTCTTCAATGAGGTTTGCAAAAATATACCAGCACTATGAGTCTGTTCGGTTCTCCAATTTTAAGTTGTGAGAATTTTACAAGATAATGGAATACAGTACTTTTCTAGCACCACGACTTAAAGAATTTTGATGACTTGTAATTTTCTCTCTCACTTCTTTCGTAGAAGGGCAGTGCCAAGTCTACTTAACTGCCGAGTCTTTTTGTACAGCAACAACAACAACAGGCCCAGCGCAATTCCAAAAGTGATCGCATAAGTGGGGTCTGGGAGGGAAGGACGTAAGAAGACCTTACCTCTTAACTTTGAGGGACAGAAAGGCTTCTCTGGTAGACCTTGGAGGTCACTTCCCAGTCTGTTCCTCCCTATCTTCATTTCCAAGTTGTGTACTCTTTTCTTTGTGTTTATGTTGAATACATTTATTTGAAGATTATTTTGTTAGTTCTATATGATCTCACATTGTTTCGGCACCAATAGTAGTTCTTGTGGTTTCTTTTGTAGTAGTGTTGATTTATTTGTTGCTACATTTTGTGTTTAGGTGACCCTCGTAACTCCGCCTGTTAAATCAGCCGGTCCCAAGCCCGGACTAAGGAGGAGGGGAAGGTGTTCAAGTTTGCAATTAATTGTCTGTTTTTTTTAATTAGTAGAAATGTTTCTGGAGTCGCCTATGAGCAATTATGGCATCTCTAATTACAATTATCACTTATTAGTAATGTACATCGGATGAAATGTAAAGAGATGCATTAGGGACTTGTCATGCAATTGGTAATTACAAATTGTGGCAACTAAATGCATTGCAATCTTGCAGCTGTTGCAATTTACTTATGATTTGCCTTAACACCGGAATTATTGTTTCCCTTATTGGTGTCTATAGATGGTTTCCAACACTGCATTCAACTAGGGATTTGTGTTAAGCCTGGCATGTGCCTATTCAGGAATCATCAATGTGTTTTTCTGTCTTGATTGATCACCCATTGTAAGTTGATACTCTTTTCAACTGTTTGCTTTTTGTTTGTGAGGTCGATCGTGGTTGCAGGTTAGTGTTTAGCCGATAAGTCCCTTCATGTTCCTTTGTTGAACAATGTTACAACTGTGAGATGCAACACTGGATGTAGGAGCTCCCTAATACTGTGACTTACTGTGGGAGTGTAACAAGTAATTTAACGACTCTTTAGTTTAAGGCAAAGTATGCTCTCGTCTTTTGGTCTTTAAAGGTCGATTGGAAAGACCAAAAGATGTGGTTCAAAAGCTAATGCTGCAAAAAGTTGCCATAGTTGTCTGGTAATTGAACAATGCAAGAAAAGATGCCCATTGGTTTCAGCTTCCCGATGGCATAGAAAACACCTAGAACCCTTTTTTTGAAGTTTATCATGAGTGAGACAAGTTTTCCAAACAAGAAGTCAAGTGAAGCAAACCACTTTAAAAGGAGCTCTTGCTTTCCATCTCTTCCCATGGTCATGGGTTATTGAACTGTTCCA

>LNC_000588

GTTAGAACAAAAATACTCAAGAAACTGATTTGAAGAATAAAAATTGAGTGAACTTATGAAATTCCTACTATGTATTAATTTGCATAAGATCTGAATTTATACAAGGAAAAACCTAACCATTTCCTATCTAGTAGCCACTAACAACCATAAAAGGGGCCACTAACAGCCATTAAAGGGGCCACTAACTTCAGAAAGTTGAGCAACTAAAGTTGACTAAGTCTAGCTTAAAAATAGGAAGCACTAAACTGAAATAAGTAAAAAACAGTAAAAATAATAAGCTGGGAAAAATGCCAATTTTCTAACAGATACTGGGTAATTATTGTTGATCAGGGTAATAATGTTTGATTAAGATGTGGGTAATTACTATTTGATCAAGATAATGGGTAATTATTGTTTGATCAAGATAATGAGTAATTATTGTTTGATCAAGATATGGGTAATTATTGTTGATTAAGATATGGGTAATTACTGTTTGATCAAGATAATCGGTAATTATTGTTGATTAAGATATAGATAATTATTGTTTGATCAAAGATAATGGGTAATTATTGAATATTGTTCTCTTCGGAAGATGAGCTGATGCCTTCCTTCGGGGAGTTCTTCGAAAGATGAGTTGATGCCTTCCTTCGGGGAGTGATCGCTTCTGCAATGATTCCGCAATTCAAAAAGTCTTTTTTGATGTGCCTGTAGTAGCATATGTGTTTGGTTTTTGATTTCTTTGCTATCTCAGAATTATATAAATCTCACGTTATCTACTTTTGAATCTATGGAAATCTTTGATCATCTCTTTAGAGAAGGAACGTTAGGGGTTTGGGGAGTTCTTGACCTTTTCAGTTTGCAATTAGTTATGTATTTTGCTAATTTATTGATTCGTTATTGTTGTTTGTTTGATCAGGTATGGGTAATGAAGCTTGCTGTTACTGTTACCTGCATCTCAATGCTAATTCTGCTGTTTTTGTTCTGAGAATTCGTGTGTTTTGTCATACTCTGAAACCTAGCAATTTCCTTTTGTTGGATCAGATACGAGTAGAATTGGAAATTTGGCTCACCAGGCATTGCCTACTTGGATTTGGATCTTCTAGTCTAGTGGAGCTATCAGTTTGAATTTGATATCTTGACTTCCATCTTTAATGCTAATTATGATCTCCTCGCATCAACTTCTTTTACCTTGTTATTTTGTTTGGTCAAGAATACATAGATGGGCATCTAGGTGCAAGTGGTTCTACAGGAACAAGTGTCTTGCTATGGTGTTTGTGGTCGGAAAGATGCGGCAGTTCCCAGGTCTGTGACTAAAGGTTTTGGAGTGTCCAGTCTCTCAAAGCTTTGCTCAAAACTGGTATGGAGTATGAGCAGATCTGTGAGTTGCAGAAAAGAGTGTCTGCAGAATGCTTGGCCTCCTGTGCTGTGGAAGTGAGACCTGAAGGATGAGCTTATGGCGCAGGGAATCTCTTTCTGGGTTTTGCAATTTTATTTTGAAGTTTGAATTTGCTGTGGATTCTCACTTTTGTTTGTTTCTCGAGGCAACCAAGGACTTCCTGTTTAGGTTATGCCAATCTCTTTCTGGTTCTACCCGAGGCAATGCTGTAAACCTCATCTCTGGTACCTGCAGCAGATCATCTTTTCTTGAATCTTCTCCTCGGGAACACTATTCTGTAGAAGTTTCTGAGCCTTCCCTAGTATATGTTGCTCTCTATATTCCATGTTGTTAGGGGATTGGCTCATTTACCTCCCAAGTCTTCTGGTTTTCCTTATCTACCCATAAGCTTTAAATGGAGTACAGGTGCTAGAGTAGTGGAATTTTAATGTGTTATCTACCATAAACACAAGTATTAGTTGGCAGTGATATCGTGTTATCCAACCAATTAGTTAGGTAGGTTCATGCAGCTAGCATGAGAAGGGATGAGATTAAGGAGTTTCTGAAAAGCGATCAGTTATAGTCTTTTACAATGGTTTTGATAATGTTAGACTCCTGTTTTTTACATTTTTGTTGTATTTGCTCATTTGGATCTCTTACCTATAAAACAGCACTACAGATGTGAGTGTACATTTTTTGAAGTTGAGGAAGGTATTAGCATCTTGAAGTAGCTAGGATAGTTTGGCTTCTGTTTGTCATGGTATTTCTTTTCTAAATATGGCCATTGAATGGGGTATTTCTGTTTTCTTTGAGCTCCAGTTCGAATTTGCGAGATAATTTGAGGGGGGTATTTCTCTTTTTTAAGCTCCAGTTGTTCAATTTCCAGATAACTCTGAATGGGGTATTTCTGTTCTTCTAAGCTCCAGTTGTCTAAATTTGCCAGCCAAGAGTTATCAGCCTCAAGATGTTGCGTTGGAGTAACTGTTGGTCCAGCTTTGTTCCTCTTTTCCCTCAAGCACTTGTAGCTACTTTGATAGCATTAGAATTAAAAGGGAATATATTTTTCTGCTAAAATACACGGATTCAGTTCCATATAAGTGGATTTGATCTCCAAAAGTGGTGAGAAGTTGGGAGGATTGTAAATCGGTTGTTGTGGAGCATGTTAAACAGAAAAGGACCTGGTATGCTGTGGAGCATGTTAAACAGATAACGTCCTGGATGAGTGTTCTATTAGAGCGGCAGTGTCGGTGATGGAGTTTGAACTATGAGTAAGAATTGGATGTTTTCAGAATAATTGTCTCCTCTCACTGTCTCACATGTATGAAATGCAGTTACTGAATATAAGTGGTATATAGGTACTATTCTAAAAAGTATAGGTGTGAAGTAGCTTTCTGTTTTGGGTTGTTATGATGAGTTGGGAATCTCTTTATTTGGCTCATCTCACATGCTTATTTGGTCTCTTTTGATTTGGAAGTAGTATCTTGGATTTAGTAAAGCTTGAGTTCAGGGAAAACAAGGAGATACTTTTTCTCAAAACATGGACTAATGAAAGTTTAACTCTGAAGAAGCACTTGATGAAGCATCCCGAAATTTTGATGAAGCACTTGATTCCATGTGTGTAGTGATGGAGCTGGTGCAAAAACTGATTTCAGGGTACATATTGCTCTCTATGTGCAGATACTTTGTAGTTCCAGAACTGGAGAATCTGCTTTCTGGATATTATATATATTTTTTGTTGTTGAAGGTGCTATGAAATTGTGGATGTCACGAATGCAACTTCTTCATGGTATGAGATGTATGTTGAGTTCTAAGATAGAAGTGATTTTGACGTAGTGGTCCAGTGTTTCTTTCATTGCACAATTACCCTTGGGCAATCTCATAAAAAGACTAGGACCTGTTGGAGCCATGACATTTTTATTTTTCATCTGATATGCATCTATGAAGTAATATCTTGTCATTCGAAGTCATGTTTTGCACTTGCCAGGGCCGATAGTTGTCTTTGACATTTTTACTTTTTAGCAGCGATGCATCTCTCAAGTAGTATCTTGTCATTTGAAGTCATGTCTTGCTCTTCAGGGCCCAAAAGTTGTCTATCATGTGACATCATATGTGGGAGAATTTCATTATTTGTCCAAATTAATTTGTGAGAATGCTCCATAGTATTCTAACATAGAGTCTTTTCTTCAATGAGGTTTGCAAAAATATACCAGCACTATGAGTCTGTTCGGTTCTCCAATTTTAAGTTGTGAGAATTTTACAAGATAATGGAATACAGTACTTTTCTAGCACCACGACTTAAAGAATTTTGATGACTTGTAATTTTCTCTCTCACTTCTTTCGTAGAAGGGCAGTGCCAAGTCTACTTAACTGCCGAGTCTTTTTGTACAGCAACAACAACAACAGGCCCAGCGCAATTCCAAAAGTGATCGCATAAGTGGGGTCTGGGAGGGAAGGACGTAAGAAGACCTTACCTCTTAACTTTGAGGGACAGAAAGGCTTCTCTGGTAGACCTTGGAGGTCACTTCCCAGTCTGTTCCTCCCTATCTTCATTTCCAAGTTGTGTACTCTTTTCTTTGTGTTTATGTTGAATACATTTATTTGAAGATTATTTTGTTAGTTCTATATGATCTCACATTGTTTCGGCACCAATAGTAGTTCTTGTGGTTTCTTTTGTAGTAGTGTTGATTTATTTGTTGCTACATTTTGTGTTTAGGTGACCCTCGTAACTCCGCCTGTTAAATCAGCCGGTCCCAAGCCCGGACTAAGGAGGAGGGGAAGGTGTTCAAGTTTGCAATTAATTGTCTGTTTTTTTTAATTAGTAGAAATGTTTCTGGAGTCGCCTATGAGCAATTATGGCATCTCTAATTACAATTATCACTTATTAGTAATGTACATCGGATGAAATGTAAAGAGATGCATTAGGGACTTGTCATGCAATTGGTAATTACAAATTGTGGCAACTAAATGCATTGCAATCTTGCAGCTGTTGCAATTTACTTATGATTTGCCTTAACACCGGAATTATTGTTTCCCTTATTGGTGTCTATAGATGGTTTCCAACACTGCATTCAACTAGGGATTTGTGTTAAGCCTGGCATGTGCCTATTCAGGAATCATCAATGTGTTTTTCTGTCTTGATTGATCACCCATTGTAAGTTGATACTCTTTTCAACTGTTTGCTTTTTGTTTGTGAGGTCGATCGTGGTTGCAGGTTAGTGTTTAGCCGATAAGTCCCTTCATGTTCCTTTGTTGAACAATGTTACAACTGTGAGATGCAACACTGGATGTAGGAGCTCCCTAATACTGTGACTTACTGTGGGAGTGTAACAAGTAATTTAACGACTCTTTAGTTTAAGGCAAAGTATGCTCTCGTCTTTTGGTCTTTAAAGGTCGATTGGAAAGACCAAAAGATGTGGTTCAAAAGCTAATGCTGCAAAAAGTTGCCATAGTTGTCTGGTAATTGAACAATGCAAGAAAAGATGCCCATTGGTTTCAGCTTCCCGATGGCATAGAAAACACCTAGAACCCTTTTTTTGAAGTTTATCATGAGTGAGACAAGTTTTCCAAACAAGAAGTCAAGTGAAGCAAACCACTTTAAAAGGAGCTCTTGCTTTCCATCTCTTCCCATGGTCATGGGTTATTGAACTGTTCCA

>LNC_000589

CTTATTCCCTTCAACCCTATAAAGACAATTGTTCTATCCCGCGTCACTTTTCCCCCCTATGATTTGGTGTTATTTCGGCCTTATTGTTGTTGATCAAGATATTGGGTATGGGTAATGAAGCTTGCTGTTACTGTTACCTGCATCTCAATGCTAATTCTGCTGTTTTTGTTCTGAGAATTCGTGTGTTTTGTCATACTCTGAAACCTAGCAATTTCCTTTTGTTGGATCAGATACGAGTAGAATTGGAAATTTGGCTCACCAGGCATTGCCTACTTGGATTTGGATCTTCTAGTCTAGTGGAGCTATCAGTTTGAATTTGATATCTTGACTTCCATCTTTAATGCTAATTATGATCTCCTCGCATCAACTTCTTTTACCTTGTTATTTTGTTTGGTCAAGAATACATAGATGGGCATCTAGGTGCAAGTGGTTCTACAGGAACAAGTGTCTTGCTATGGTGTTTGTGGTCGGAAAGATGCGGCAGTTCCCAGGTCTGTGACTAAAGGTTTTGGAGTGTCCAGTCTCTCAAAGCTTTGCTCAAAACTGGTACGAAATGGAAGTTCTCAACTGGGATAATTCTTTTCACAAGTGTGTATAGTGTGTTCTGCTGCCAGATAGTTTTAGTTGTTAGTTCAAATGCTCGTGTTCTTTGATGATCCTGGTGTCTTATGGTGTTATAGGCACTTGATAAAGTCAAAAGTCAATGCTGGATCTTTGATAGTGCTGTAATGTGCTCTTCTACTACTTATCATCTCGCTGTTTTCGCTTTGTTATCACGCCAGCTGGTTTTGTGTTCATTGGTGAATGAATGTGTAGACCACATGGTGACAGTAACAAGAGTAGTAGTTTTGGGAACTTCTAAGGATTGTATCCGGTATGCATTCATGTGCTCCTTGTTTATTGGTTTTTTTGGTCCTTGCACTCCAAGATGGATATTGTTAGGCTTCAATCAAAATGGGGGTTCTTGATCTTGTCTCTTGCATCTCTAGTGATTTCTTAGGTGATATGGGGAACTTCTAAGGATTGTATCGGTATGCATTCCTGTGCACCTTGTTTATTGGTCTTCTTGGTCCTTGCACTTCAAGATGGATATTGTTAGGCTTCAACCAAAATGGGGTTCTTAGTCCTTGTCTCTTGCATCTCTAGTGATTTCTTAGGTGATACGGGTTCTGTCAGCTTTGCACTTAACTGGTCCCTTCTTTTAGTGCTTGCAGTTGGGTCTTACCGAGCAGTGAATTTTTTTTTCATGGAGGGGCTTTGCTTGGCCATTTGTGTAGAGGTTTCTTAGTTCAGATAATTGTTAGTATTGAGGTACTCATTTCAGTTTTTTCCCCAAATCAGAGTGGTCCCTTTTCTCTTTGTCAAAAATTGCTGCTGCATGGAAATTGCCAAAAGCTAAGATAGTTTTTCCTACACACTCTGGATGCATATGTTCCTTGATGATCCAGTTGATGAGATTGCTGGTGGTTGATGGGTATATGGTCTTATTGAAACCTGATAAATACTCGGAACTCAATCCTAGATCTTTTGACAGCCCGGTCAAGTAGGTCCCTGTTGAACCCTCTCTTTTGGTGTTCTCTCTGCGTCATCTTCTCTGGTTCCTTTTTCTTGCTTCACCCCAGCTTGATTTTGTTTGTTGGATGGATGCCAGTTAAGAGGACTAGATTTGGAAAATTTTGAAGGTTGATGCTCAGTGTCCGTTCCTGTGCATGCTGTTTCCTTGCCATTTTTGTGCTTGCACTGCAATCTGAGTATCATCAGGCTTCGATAGAGGGTTTTCCAATATTTTTTCTTTGCCATTTTTTGTGCTTGCATTGCAATCTGAATATTATCAGGTCTTCGAGAGAGGGTTTTGTCAGCTTTGCATGTAACCCTTCCCTTCATTCCTTTGCTTGCAGCCATTGGGTCTTACAAAGCAGTGAATTCTTATTTTCATGGAGGTACCTTATAAACCTCCATAGGTGTTGAGGTTTCGTTAGTCAGTCTTCTATGAGTATAGATAAATTTCTCTTCCCTACTGACTCATTTGTCTTTGTCATAACTATTGTTGTATGGTAGTTTCTCAATTAGGACATTATTTTCACCAATGTGTGTTGTATTTTGCTGCCATGGAGTTCTTAATTCAGATGCCTATGTTCTTATGGTGCTACAATTGATAAGGTCTCTTATGGTGATGAGGTCTCTTACTCTCTTATGGCTATGGATGCCTGATAAGGACTTGACCAATTCTAGATCTTTCTATAGTGGCGGAAAATACTTCTGAACTTTTGTTCTTTTAGCTACTTATCAAACTGCTCTCATTTTTTTCGTTATATCACTAGTTTTTTTTGGTTGTCTGGATGTGTAGACCATGTGGTGACACTGTTATGGACGTTCAGATTTTGAAATTTCAAAGCTTGGTTAACCAACACGTGATCCTTTGCTTCCTTTTTTGGTCTTTTTGGTGCTTGCATTGCAGTCTGGATGTTTTCGGGGATTTGACAAAATGGGGTTTTCAGTCTCATTTATGGCATCTCTAGTGATTACTTAGGTGTTTGGGGTTCGTCAGGTTTGCTCTGAACTGTTCCCTTCTTTCTGTTCTTGCAGCCATTGCCCAGGGCAGTGAATTCTTTTTTTCATGGAGCGGCCGTGTGTTTTGTTGGTTTGCGTTGATGTTTCTTTTGCCAAGTCTTTTATGAGCATGGAGTTTTCGATTCAATTTTCTTCCCTAGTGGCCCTTTGTCCTTATCAAAACTAGTGTTGTATGGAAGTTTCTCAACTAGGACAACGTAATTCACCGGTGTGTGTTGTATTCTGCTGTCATGAAGTTTAGTCCAGACGCTTATGTTCTTCGATTATCCAATTGATGAGGTCTTCTATGGCATATGGACCCTTGATAAGTACTCGTCCAAGTCTAGATCTCATTTGATAGTGGCATAAAATACTTTTATACTTTTGTTCTGTAGCTACTTACTTATCTTCCCTGTAGACCATGTGGTGACACTGTTATGGACGTTCAAATTTGGAAATTTCAAAGCTTGGTTTACCAACATGTGATCCTCTTCCATCTTTTGGACTTTTTGGTGCTTGCACAACAATCTGGTTATTGTCCGGGCTTCGACATAAGGGGCTTTTCAGTCTCGTCTATGGCATCTCTAGTGATTACATAGGTGATTTGGGGTTCGTCAGATTTGCACTCAACTGTTCGTTCCCTTCTTTCTGTTCATGCAGCTATGGTTACAAGGCAATGAATTATTCTTTCCATGGAGTGGATGTGTTTGCCTGTTCGTGTTAAGGTTTTTTTCAGTTTAAGTTTTTATGAGTATCATGATTTTCGATTAAGTTTTCTTCAGGAGTTTCTTGACTAAGATAATATTTTTCACTTGTGTGTGTTGTTATTCTGCTGCCGTGAAGTTTTTAATGCAGACACTATGTTCTTCGATGATCTAATTGATAAGGTCTCCTATGGCTATGGACATTTGATAAGTGCTCATCCAATTTTGGATATTTTCCTTAGACAGCGCCATAAATGCTCCTCTACATGTGGTCTTCTAGCTACAGATATTTCGGTTATCACAACTTTAGTTTTTTTGTTTGGATGGATGTGTAGACTATATGGTGACAGTAATAGAGGTTAAGTTTTGGAGATTTCAAAGGTTGATATCCAGTATGGTATCTCTGTACCCTCTTGGTGTTTTTGGTTCCTGCACTGCAATTTTGATATATTGGTCAGAGCTTCAACAAGTTTAAAATCTTGTCCATATGGCGTCTCTAGTGATTTCTTAGACGGTATGGGTTTCATCAGTTTTGGACTTGATTGTACCCTTCTTTTTGTGCTTGCAGCCATTGGGTCTTACAGAGCAATGAATTATTCTTTTCATGGAGCGGCTCTCTTTGTCAGGTTTCTGTGGTTCAGGCTTTATGAGTTCTGGCATTTTCAACTATACTAGTTTTTTTTCTGTTAGTGTGTGTTGGTATTCTACTGCCATTTTGAGTGTTTAGTCCAGATGCTTATTTTCTTCGATGATCCAATTGATGAAATTCCTGATGGTTGATGGTTATTTATGGTCTCATGGACGACTGAGACCATCTTCAACCCACCTCTATTTTACTCTCCATTCTCTATATTTGGAGAGTAAAATAGAGAATGGGCACTCCAACCCAACTCTATATCACCCTCTATTCTTCATATTTAGAGAGTTGAATAGTAGTTCTCCAA

>LNC_000590

ATAAAAATAGTGATTAATAATGGTCTAATAAATTAGACACCGACCCAACTTGGCCGCCTTTGTAATGTCTTATTCCCTTCAACCCTATAAAGACAATTGTTCTATCCCGCGTCACTTTTCCCCCCTATGATTTGGTGTTATTTCGGCCTTATTGTTGTTGATCAAGATATTGGGTAATTAGTGTTTGATCAAAATTCTAGGTATGGGTAATGAAGCTTGCTGTTACTGTTACCTGCATCTCAATGCTAATTCTGCTGTTTTTGTTCTGAGAATTCGTGTGTTTTGTCATACTCTGAAACCTAGCAATTTCCTTTTGTTGGATCAGATACGAGTAGAATTGGAAATTTGGCTCACCAGGCATTGCCTACTTGGATTTGGATCTTCTAGTCTAGTGGAGCTATCAGTTTGAATTTGATATCTTGACTTCCATCTTTAATGCTAATTATGATCTCCTCGCATCAACTTCTTTTACCTTGTTATTTTGTTTGGTCAAGAATACATAGATGGGCATCTAGGTGCAAGTGGTTCTACAGGAACAAGTGTCTTGCTATGGTGTTTGTGGTCGGAAAGATGCGGCAGTTCCCAGGTCTGTGACTAAAGGTTTTGGAGTGTCCAGTCTCTCAAAGCTTTGCTCAAAACTGGCACTTGATAAAGTCAAAAGTCAATGCTGGATCTTTGATAGTGCTGTAATGTGCTCTTCTACTACTTATCATCTCGCTGTTTTCGCTTTGTTATCACGCCAGCTGGTTTTGTGTTCATTGGTGAATGAATGTGTAGACCACATGGTGACAGTAACAAGAGTAGTAGTTTTGGGAACTTCTAAGGATTGTATCCGGTATGCATTCATGTGCTCCTTGTTTATTGGTTTTTTTGGTCCTTGCACTCCAAGATGGATATTGTTAGGCTTCAATCAAAATGGGGGTTCTTGATCTTGTCTCTTGCATCTCTAGTGATTTCTTAGGTGATATGGGGAACTTCTAAGGATTGTATCGGTATGCATTCCTGTGCACCTTGTTTATTGGTCTTCTTGGTCCTTGCACTTCAAGATGGATATTGTTAGGCTTCAACCAAAATGGGGTTCTTAGTCCTTGTCTCTTGCATCTCTAGTGATTTCTTAGGTGATACGGGTTCTGTCAGCTTTGCACTTAACTGGTCCCTTCTTTTAGTGCTTGCAGTTGGGTCTTACCGAGCAGTGAATTTTTTTTTCATGGAGGGGCTTTGCTTGGCCATTTGTGTAGAGGTTTCTTAGTTCAGATAATTGTTAGTATTGAGGTACTCATTTCAGTTTTTTCCCCAAATCAGAGTGGTCCCTTTTCTCTTTGTCAAAAATTGCTGCTGCATGGAAATTGCCAAAAGCTAAGATAGTTTTTCCTACACACTCTGGATGCATATGTTCCTTGATGATCCAGTTGATGAGATTGCTGGTGGTTGATGGGTATATGGTCTTATTGAAACCTGATAAATACTCGGAACTCAATCCTAGATCTTTTGACAGCCCGGTCAAGTAGGTCCCTGTTGAACCCTCTCTTTTGGTGTTCTCTCTGCGTCATCTTCTCTGGTTCCTTTTTCTTGCTTCACCCCAGCTTGATTTTGTTTGTTGGATGGATGCCAGTTAAGAGGACTAGATTTGGAAAATTTTGAAGGTTGATGCTCAGTGTCCGTTCCTGTGCATGCTGTTTCCTTGCCATTTTTGTGCTTGCACTGCAATCTGAGTATCATCAGGCTTCGATAGAGGGTTTTCCAATATTTTTTCTTTGCCATTTTTTGTGCTTGCATTGCAATCTGAATATTATCAGGTCTTCGAGAGAGGGTTTTGTCAGCTTTGCATGTAACCCTTCCCTTCATTCCTTTGCTTGCAGCCATTGGGTCTTACAAAGCAGTGAATTCTTATTTTCATGGAGGTACCTTATAAACCTCCATAGGTGTTGAGGTTTCGTTAGTCAGTCTTCTATGAGTATAGATAAATTTCTCTTCCCTACTGACTCATTTGTCTTTGTCATAACTATTGTTGTATGGTAGTTTCTCAATTAGGACATTATTTTCACCAATGTGTGTTGTATTTTGCTGCCATGGAGTTCTTAATTCAGATGCCTATGTTCTTATGGTGCTACAATTGATAAGGTCTCTTATGGTGATGAGGTCTCTTACTCTCTTATGGCTATGGATGCCTGATAAGGACTTGACCAATTCTAGATCTTTCTATAGTGGCGGAAAATACTTCTGAACTTTTGTTCTTTTAGCTACTTATCAAACTGCTCTCATTTTTTTCGTTATATCACTAGTTTTTTTTGGTTGTCTGGATGTGTAGACCATGTGGTGACACTGTTATGGACGTTCAGATTTTGAAATTTCAAAGCTTGGTTAACCAACACGTGATCCTTTGCTTCCTTTTTTGGTCTTTTTGGTGCTTGCATTGCAGTCTGGATGTTTTCGGGGATTTGACAAAATGGGGTTTTCAGTCTCATTTATGGCATCTCTAGTGATTACTTAGGTGTTTGGGGTTCGTCAGGTTTGCTCTGAACTGTTCCCTTCTTTCTGTTCTTGCAGCCATTGCCCAGGGCAGTGAATTCTTTTTTTCATGGAGCGGCCGTGTGTTTTGTTGGTTTGCGTTGATGTTTCTTTTGCCAAGTCTTTTATGAGCATGGAGTTTTCGATTCAATTTTCTTCCCTAGTGGCCCTTTGTCCTTATCAAAACTAGTGTTGTATGGAAGTTTCTCAACTAGGACAACGTAATTCACCGGTGTGTGTTGTATTCTGCTGTCATGAAGTTTAGTCCAGACGCTTATGTTCTTCGATTATCCAATTGATGAGGTCTTCTATGGCATATGGACCCTTGATAAGTACTCGTCCAAGTCTAGATCTCATTTGATAGTGGCATAAAATACTTTTATACTTTTGTTCTGTAGCTACTTACTTATCTTCCCTGTAGACCATGTGGTGACACTGTTATGGACGTTCAAATTTGGAAATTTCAAAGCTTGGTTTACCAACATGTGATCCTCTTCCATCTTTTGGACTTTTTGGTGCTTGCACAACAATCTGGTTATTGTCCGGGCTTCGACATAAGGGGCTTTTCAGTCTCGTCTATGGCATCTCTAGTGATTACATAGGTGATTTGGGGTTCGTCAGATTTGCACTCAACTGTTCGTTCCCTTCTTTCTGTTCATGCAGCTATGGTTACAAGGCAATGAATTATTCTTTCCATGGAGTGGATGTGTTTGCCTGTTCGTGTTAAGGTTTTTTTCAGTTTAAGTTTTTATGAGTATCATGATTTTCGATTAAGTTTTCTTCAGGAGTTTCTTGACTAAGATAATATTTTTCACTTGTGTGTGTTGTTATTCTGCTGCCGTGAAGTTTTTAATGCAGACACTATGTTCTTCGATGATCTAATTGATAAGGTCTCCTATGGCTATGGACATTTGATAAGTGCTCATCCAATTTTGGATATTTTCCTTAGACAGCGCCATAAATGCTCCTCTACATGTGGTCTTCTAGCTACAGATATTTCGGTTATCACAACTTTAGTTTTTTTGTTTGGATGGATGTGTAGACTATATGGTGACAGTAATAGAGGTTAAGTTTTGGAGATTTCAAAGGTTGATATCCAGTATGGTATCTCTGTACCCTCTTGGTGTTTTTGGTTCCTGCACTGCAATTTTGATATATTGGTCAGAGCTTCAACAAGTTTAAAATCTTGTCCATATGGCGTCTCTAGTGATTTCTTAGACGGTATGGGTTTCATCAGTTTTGGACTTGATTGTACCCTTCTTTTTGTGCTTGCAGCCATTGGGTCTTACAGAGCAATGAATTATTCTTTTCATGGAGCGGCTCTCTTTGTCAGGTTTCTGTGGTTCAGGCTTTATGAGTTCTGGCATTTTCAACTATACTAGTTTTTTTTCTGTTAGTGTGTGTTGGTATTCTACTGCCATTTTGAGTGTTTAGTCCAGATGCTTATTTTCTTCGATGATCCAATTGATGAAATTCCTGATGGTTGATGGTTATTTATGGTCTCATGGACGACTGAGACCATCTTCAACCCACCTCTATTTTACTCTCCATTCTCTATATTTGGAGAGTAAAATAGAGAATGGGCACTCCAACCCAACTCTATATCACCCTCTATTCTTCATATTTAGAGAGTTGAATAGTAGTTCTCCAA

>LNC_000591

CTTGGCCGCCTTTGTAATGTCTTATTCCCTTCAACCCTATAAAGACAATTGTTCTATCCCGCGTCACTTTTCCCCCCTATGATTTGGTGTTATTTCGGCCTTATTGTTGTTGATCAAGATATTGGGTAATTAGTGTTTGATCAAAATTCTAGGTGATTATTGTATGATCAAGATTCTGCGTAATTATTGTGCCTTATCGTTATTGTTGATATGGGTCATTATTGTTGCCTTATTGTTATTGTTGATACTCGCTAATTTTCGTTTGATCTGGGTAACTATTGTTGATATGGGTAATTATAGTTTGTTCAAGATATGAGTAATTACTGTTTGATCAAGGTAATTGGTAATTATTGTTGATTAAGACATATGTAATTACTGTTTGATGAAGATAATGGGTAATTATTGTATATGATTCTCTTCGGAAGATGAGCTGATGCCTTCCTATGGGGAGTGATCGCTTCTGCAATGATTACATTCTGGGATTCCACACTTCGAAAAGTCTTTTTTTTTTATGTGTCTGTAGTGGCATATGTGTTTGGTTTTTGATTTCTTGGCCAGAATTATATAAATCTCATGTTATCTACTTTCAAATCTTTGTAACTCTTTGATCATCTCTTTAGAGAAGGAATGTTAGGGTTTGGGGATTTGGTGTTATTTCATATACAGTTTAATGGCTAATTAGTGTATGATCAAGATTCTGGGTAATTATTGTATGATCAAGATTCAGGTAATTATTGTATGATCAAGATACAGGTAATTATTGTTGGTCAAGGTAGTGGGCAATTATTGTATGATCAAGATTCTGGGTAATTGTTGTGCCTTGTTATTGTTGATACTGGCTAATTATTGTTTGATCCGGGTAACTGTTGTTGATCAAGACACTTGGTAATTATTGTTTGATCAAGATACTAGGTATTTATTGTTGATCAAGATAGTGGGTAGTTATTGTTTGATCAAGATAGTGGATAGTTATTGTTGTTGATCAGGATACTGTTAGAACAAAAATACTCAAGAAACTGATTTGAAGAATAAAAATTGAGTGAACTTATGAAATTCCTACTATGTATTAATTTGCATAAGATCTGAATTTATACAAGGAAAAACCTAACCATTTCCTATCTAGTAGCCACTAACAACCATA

>LNC_000592

CTTCTTCCCCATCACGCCGCCCACCTTCTTCCTCACACTACCTAAGCTAGCTAGCTTTCTCCATAGACGCCCAGGCTCCTCTCCCATCACCTTTTCCTCAAACGTCTTCTCCACAGACGCCTATGGCCCCCCCAGTCTCCTCTCCCATCGCCGCTCATGGCCGCTCCCCACGTGCTCCCCCACCCCTTTCGTCAGAAACGGCTCTGGGCCGCCGGAAAATGGCCCATCAGTCACTCTAACCTCTCAAATCGACTTGAAAAGGTCGGTTTTTGAGAGATTTTTCTTCTCCTTCATGATTTATTTGCTTTTACCGCTGGGAGCTGCGTTTCCGAGAATCCAAAATTTGAATTCTTGGAGCCCAAATCGACTATAAAAGGAGGACTTCGGTTCTCAACGAAATCATCCAAGAATTGACGAAAACATCCGAGAATCAACTAAAAGATCCAGGAACGAGCAAGAAAATACAATAATCATCTGAGAATCACAAAATAGCTCGGCTTAGTTTCCTTTACTTCTCTGTTTTTTGTTTGGCTTCGAGTTTGAGTTGAGGTTGGAATATTGACAACAAGTTCTAGTTTACTGCCCTATACAAGGGCATCATGAGTTGGCAATTGATAGACCTTATTGATAGACCTCAGCTAG

>LNC_000593

GGTTATCAGTTTGGTGATATTTCCCGCGACGTATGGTTGGATATTGATTCTTAGCTACAGTACCCGGTTAGAGTCCGTGGTCCAGCTTACTTATGTCCAGGCTTAGCTACAGTACCCGGTTAGAGTCCGTGGTCCAGCTTACCTGTGATCTGGTTTTAGCTACAGTACCCGGTTAGAGTCCGTGGTCCAGCTTACCTGTGATCTGGTTTTAGCTACAGTACCCGGTTAGAGTCCGTGGT

>LNC_000594

CAGGTAATGATTTTGCAATAAGATTCTTACTCTTTGTGAATACTCTGACATTTGTTAACTCAATGAGATGATATTGCTTCATGCATGAGTATTTCTGAAAATAGAGTAATGCATCTGTTATTTTAATTGTTCTCTACTAATTTCTTGAAACAGAAAAAATGCACAAATAAACAGTTGGTATTACAGGATTTCCATCGACTTTGTTGGGATTTGCTTCTTTTCATGATTTTACAAGTCGACAAACAATGATTATGTGCTCAAGGAACTTGAAAACATGAAGGAACAACGGCTTGCACATAGAAATATGCTATGCTATGCCATACTAGATATGCATAAATGTCACCACAAAGAAATCAACTATTTCTATTGCAAAAGCAAGCTAAAAGAGCTAAATCAAAAAGGCGGTGACTCTTGTAGAATTCAAATAATACACTATCTTTGTCACAAGTTCAGAGTCATGACAAAAATCAAAGTGTGGTTTGAACTTCCTTCATCAGCTAGAGATAAAGGTCAGAAAAGTTAAATTTCCATATAATTTTGCTTGTGTATGTATAGGTATACATACCTTCATAGATAAATTGATTTACTATCATTTATACTATTTTGTCTTCCATCATTTTGGAGGACATGCTTCCGCGGCTACACAATCAACTACTTGCACCTCAAACTTTGAAGTAGGTGAGTCGGAAGGGAATTGGAAAATTCAAAATAGAAAGACTCAATCAGATATGGTAGGTACTTTACATGGTCTGTGGTGATATTAAGTACAAATCAGTTCTATCTTCTTCCGGACATAATTACCCTCTAGATAATGTAAGAATTTAATATGATTTGTAAAAATTAGTTCTCTTTCTTTGGGACATAATTACCACCAACTAATATAAGAATGTGACTATGATTTTATCTTCCCAAATGTCAGTCACTTGGATGTAACTTGATTTCACTGGGAATATGATTTTATTTCCTCGGAGAGGTTTCAAATACATTGCTTCAGTATTTCTTATAAAAGAAGGCATATTGTTAACTTTAGCTGGTAAATATCAGATGTTAGCCTCAAATCTCAATTGAAGGGGAAATGACTAGTTCTTCTAAGCATTTTAAAAGACTATGCTATTTAGATTGATCATAACTATTGCTTTGTAAACAGATACACTGCATATGGAAAATGGAGTCTTGATTCTCAAAGTAAAATTGAAATTGGCTTCACGACAAAACTCATAAAGATCTTGATGATAATTCACAGTCTCAGTTGCGAAGAGCAAAAAATATAACTATTTTGTAAGCTCTCTTTGTTCATGCTTGTTGTTTATCCCTGAGTTTATTTCAATTTAAGACAATATTAGTCATTCATAGACTTATCAGGCTTTGCTATGAAGTTTGAAGCTTTAAGGATCGTCGTCGTTTAAGTTTTCACTTTATTTTTTTGTTGAATTCCACGACCAACTTCCTAAGGAGCTCAAGAGAAATCATATTATTGTCATTACTGGTCAGATGATGCATTCTAGGATTTTTAGGGAAGTTCTCCTCTGCCTCTGCAGTTAGCTCGATTGCATATGTAATGGTCGAATTTCGAGATGTTTATCCTAAGGTTTCAGGTTTTATCAGGTTTGACTACTAGAGTAGCTCATAGTGATAGTCTGTTACTTTCAAGTCCATTTTTAACCGTCGATAGAAGATTTTGTTACCTGCAATAGAGACCACCCGTGAGGATATCAAGATTTAGTTGTGGATCTGTGTCCAGGTAAATTTAACACCGCTTGCAATTTGATCCGTGAGATACTGGCTTGCTGTTAACCTACCCAATGTCCATGTTATCGTGCACATTATAGACAAATAAAAGGTAATTGATTATTCTACTATTAAATTTATCTTCCTACTTATCTATATTTTGCAACTAGACTTTACTTGAGCTTCTAACATATCATCTTTTAAATTTGTCTTAGACTTTACATGTAAGATAATTCATTTGCAATCGAAGCATTAGTACAACTGATTAACCAAATAGAGAATCCTCCCAGTCATAGGTCTCCTTAGCAAATAATCCTAATGACTCCTTGTGGCCTTTTGTTTTGTTATATTTGATGAAATATTTACTAAAACATGCAAGTCAGAATTCTTTGTTCTCTCTTATTTATGGTTGTCGAGTTATCACTATGCATTATTTTATTAGCTTGCTCTATCTTAGGATAATCTTCAGTCATTACTTCAGGCTTTTTATTTTATTTTATTGATAAACTATAACTTTAGGCTTCTTTATTATGTTTATGGATCTTTTTTTGTTTTGTGTTTGTTCTTGATAATTTAAGTTTATTGGAATTTTTGGAGAAAACTATACTCTTATTTTGATCATAGTTCTTTTGTTATTATGTTCCTTAAGTGTCTTCCTGGGAGTTCACCACGTTAGCAATCCTTTGAGTTTTATACCCTAGCCTCTTACGCCATCTCAATTATTTTTGCAAGAGAATTAACCTGTCTTATGAAGTTTAGAACCTGTGGTTAAACTCAAAACACATGATAGAAAGCCACATCAACAATACAAGTGTTATCTTATTATTTGAGATATTGGTACCTTTATTTATTAAGAATATAGATATTTTCGATGAATGAAAAGCTTTCATAAGAAAAGCAACTCTTCAGTGAAGATTACCAAAAGAACAAGGAGAAGTGCATCTACAAATTCATGACACAAAAGCATTGTGTTCATAGTTTGATGCTCTGTATGTTTGTAATGACATAGATGAACATGATTCTCCCTTTGTGTGCGAGTCTCAGCAACTCACTAGAAATATGGTTAATACTTGATCTATATGCAAGAATACTTTAGTAAATTTGTTATTTAAACATTTCATGAAAGATAATTCAAATTTTTACTAGCTGATTTTTCCTTTTGGTTCATTTGGATTCTTAAGTGTGGAGAGAAAACATATATATCTCTATTTGACATAGACAATCAAGTTGTGGGACTACACTGGGTATGTTGTTGTTGTTGTTGACAGAGGTGATAAAGTTGAAGAGTGAACATATTCGTGGAGCTTTTTCCAGAAATAACAGATGTGATCTTTCCCCAAACAATTTCATGGAAATTATCTCCTTGGCTGTTAAATTTGTGTGTTGTTTATCGAAATACATAAAATTTACTTGATTGTACACATTTTTTTGGATTAGGTGCTCATACTTTGATCATTAGAAATTATAGGTATCTACACATCTGGCGCTAAAAGAGTTTTGCCTCTCAAGCGTGGTTGCACTTCTCTTCACATGTGACTTCCCACAGGGGATCATTGCCCTTTGATGAAAAACAATGGTGCTTGAGGATTATTTGTGTGTTAATGGAGTCATCCTTTCAATCTTCTTAATTTTTTCATTTTCTAGGGAGTTATGCTTCAATACGTTGAGAGTTTGATCTCTAAAACTCCAAGAAATTTGATCTACAATGATTGATTTCAACTTTATCACTGGCCTGTAAATTGCAAGGGAGGATAACTTTACAAGTTGTATCTAAGACCCTTAACTATCGGATGGACAATTTTATTAGTTATTGTTAGGTTAATTTTGTTCCAATGAAGGTATTCTTAAGGGTAAACTTGTATGTACTTAGTTGAGATACTTCTACGTCAATATATCTATAAATGCTTATAGTTCTGCTGTAATTGCATCAAACAAGCTTTCGTGATATTGACTCTATGTTTTTCTTGTGCTACTTTTTCGTTTTTCTTTTAATTGAATTCCCATCTTGTGTCTCCAAACGATGATTGAAAGATTCACCATAAAAAATAGAAAATGTTGG

>LNC_000595

ATCGAATGTAAAAAGGTCCTACATTGTTGTGTTAGATGAAGGAACATGCCATGTCCTTGTATAGCTCAGGATTTCCATCGACTTTGTTGGGATTTGCTTCTTTTCATGATTTTACAAGTCGACAAACAATGATTATGTGCTCAAGGAACTTGAAAACATGAAGGAACAACGGCTTGCACATAGAAATATGCTATGCTATGCCATACTAGATATGCATAAATGTCACCACAAAGAAATCAACTATTTCTATTGCAAAAGCAAGCTAAAAGAGCTAAATCAAAAAGGCGGTGACTCTTGTAGAATTCAAATAATACACTATCTTTGTCACAAGTTCAGAGTCATGACAAAAATCAAAGTGTGGTTTGAACTTCCTTCATCAGCTAGAGATAAAGATACACTGCATATGGAAAATGGAGTCTTGATTCTCAAAGTAAAATTGAAATTGGCTTCACGACAAAACTCATAAAGATCTTGATGATAATTCACAGTCTCAGTTGCGAAGAGCAAAAAATATAACTATTTTGTGCTCATACTTTGATCATTAGAAATTATAGGTATCTACACATCTGGCGCTAAAAGAGTTTTGCCTCTCAAGCGTGGTTGCACTTCTCTTCACATGTGACTTCCCACAGGGGATCATTGCCCTTTGATGAAAAACAATGGTGCTTGAGGATTATTTGTGTGTTAATGGAGTCATCCTTTCAATCTTCTTAATTTTTTCATTTTCTAGGGAGTTATGCTTCAATACGTTGAGAGTTTGATCTCTAAAACTCCAAGAAATTTGATCTACAATGATTGATTTCAACTTTATCACTGGCCTGTAAATTGCAAGGGAGGATAACTTTACAAGTTGTATCTAAGACCCTTAACTATCGGATGGACAATTTTATTAGTTATTGTTAGGTTAATTTTGTTCCAATGAAGGTATTCTTAAGGGTAAACTTGTATGTACTTAGTTGAGATACTTCTACGTCAATATATCTATAAATGCTTATAGTTCTGCTGTAATTGCATCAAACAAGCTTTCGTGATATTGACTCTATGTTTTTCTTGTGCTACTTTTTCGTTTTTCTTTTAATTGAATTCCCATCTTGTGTCTCCAAACGATGATTGAAAGATTCACCATAAAAAATAGAAAATGTTGG

>LNC_000596

CAAAGAATTAAGAAGGTATCATCGAATGTAAAAAGGTCCTACATTGTTGTGTTAGATGAAGGAACATGCCATGTCCTTGTATAGCTCAGAAAAAATGCACAAATAAACAGTTGGTATTACAGGATTTCCATCGACTTTGTTGGGATTTGCTTCTTTTCATGATTTTACAAGTCGACAAACAATGATTATGTGCTCAAGGAACTTGAAAACATGAAGGAACAACGGCTTGCACATAGAAATATGCTATGCTATGCCATACTAGATATGCATAAATGTCACCACAAAGAAATCAACTATTTCTATTGCAAAAGCAAGCTAAAAGAGCTAAATCAAAAAGGCGGTGACTCTTGTAGAATTCAAATAATACACTATCTTTGTCACAAGTTCAGAGTCATGACAAAAATCAAAGTGTGGTTTGAACTTCCTTCATCAGCTAGAGATAAAGGTCAGAAAAGTTAAATTTCCATATAATTTTGCTTGTGTATGTATAGGTATACATACCTTCATAGATAAATTGATTTACTATCATTTATACTATTTTGTCTTCCATCATTTTGGAGGACATGCTTCCGCGGCTACACAATCAACTACTTGCACCTCAAACTTTGAAGTAGGTGAGTCGGAAGGGAATTGGAAAATTCAAAATAGAAAGACTCAATCAGATATGATACACTGCATATGGAAAATGGAGTCTTGATTCTCAAAGTAAAATTGAAATTGGCTTCACGACAAAACTCATAAAGATCTTGATGATAATTCACAGTCTCAGTTGCGAAGAGCAAAAAATATAACTATTTTGTAAGCTCTCTTTGTTCATGCTTGTTGTTTATCCCTGAGTTTATTTCAATTTAAGACAATATTAGTCATTCATAGACTTATCAGGCTTTGCTATGAAGTTTGAAGCTTTAAGGATCGTCGTCGTTTAAGTTTTCACTTTATTTTTTTGTTGAATTCCACGACCAACTTCCTAAGGAGCTCAAGAGAAATCATATTATTGTCATTACTGGTCAGATGATGCATTCTAGGATTTTTAGGGAAGTTCTCCTCTGCCTCTGCAGTTAGCTCGATTGCATATGTAATGGTCGAATTTCGAGATGTTTATCCTAAGGTTTCAGGTTTTATCAGGTTTGACTACTAGAGTAGCTCATAGTGATAGTCTGTTACTTTCAAGTCCATTTTTAACCGTCGATAGAAGATTTTGTTACCTGCAATAGAGACCACCCGTGAGGATATCAAGATTTAGTTGTGGATCTGTGTCCAGGTAAATTTAACACCGCTTGCAATTTGATCCGTGAGATACTGGCTTGCTGTTAACCTACCCAATGTCCATGTTATCGTGCACATTATAGACAAATAAAAGGTAATTGATTATTCTACTATTAAATTTATCTTCCTACTTATCTATATTTTGCAACTAGACTTTACTTGAGCTTCTAACATATCATCTTTTAAATTTGTCTTAGACTTTACATGTAAGATAATTCATTTGCAATCGAAGCATTAGTACAACTGATTAACCAAATAGAGAATCCTCCCAGTCATAGGTCTCCTTAGCAAATAATCCTAATGACTCCTTGTGGCCTTTTGTTTTGTTATATTTGATGAAATATTTACTAAAACATGCAAGTCAGAATTCTTTGTTCTCTCTTATTTATGGTTGTCGAGTTATCACTATGCATTATTTTATTAGCTTGCTCTATCTTAGGATAATCTTCAGTCATTACTTCAGGCTTTTTATTTTATTTTATTGATAAACTATAACTTTAGGCTTCTTTATTATGTTTATGGATCTTTTTTTGTTTTGTGTTTGTTCTTGATAATTTAAGTTTATTGGAATTTTTGGAGAAAACTATACTCTTATTTTGATCATAGTTCTTTTGTTATTATGTTCCTTAAGTGTCTTCCTGGGAGTTCACCACGTTAGCAATCCTTTGAGTTTTATACCCTAGCCTCTTACGCCATCTCAATTATTTTTGCAAGAGAATTAACCTGTCTTATGAAGTTTAGAACCTGTGGTTAAACTCAAAACACATGATAGAAAGCCACATCAACAATACAAGTGTTATCTTATTATTTGAGATATTGGTACCTTTATTTATTAAGAATATAGATATTTTCGATGAATGAAAAGCTTTCATAAGAAAAGCAACTCTTCAGTGAAGATTACCAAAAGAACAAGGAGAAGTGCATCTACAAATTCATGACACAAAAGCATTGTGTTCATAGTTTGATGCTCTGTATGTTTGTAATGACATAGATGAACATGATTCTCCCTTTGTGTGCGAGTCTCAGCAACTCACTAGAAATATGGTTAATACTTGATCTATATGCAAGAATACTTTAGTAAATTTGTTATTTAAACATTTCATGAAAGATAATTCAAATTTTTACTAGCTGATTTTTCCTTTTGGTTCATTTGGATTCTTAAGTGTGGAGAGAAAACATATATATCTCTATTTGACATAGACAATCAAGTTGTGGGACTACACTGGGTATGTTGTTGTTGTTGTTGACAGAGGTGATAAAGTTGAAGAGTGAACATATTCGTGGAGCTTTTTCCAGAAATAACAGATGTGATCTTTCCCCAAACAATTTCATGGAAATTATCTCCTTGGCTGTTAAATTTGTGTGTTGTTTATCGAAATACATAAAATTTACTTGATTGTACACATTTTTTTGGATTAGGTGCTCATACTTTGATCATTAGAAATTATAGGTATCTACACATCTGGCGCTAAAAGAGTTTTGCCTCTCAAGCGTGGTTGCACTTCTCTTCACATAGTGGATCTTCAAAACTTCATATTTGAAATTCATTCCTTTTCCAAGTGTGATTCCATCAATCTACAAGTTAGCAAACAGTTACAAATTGAAAAAGAAGATGGATACCATCAAAATACTTCATAAATTTACTATAAATACCTTTCTTACTGCTGAAATTTGAGTAAACAATTGATTGTCAGTACTATGTTGTGTTGTTAAATTGCTTAACTTCTCCTTTGGGTTGTTAATACAGTAGTTTCAACTTCTGAGCCTGAATTGGGTTGCCCCTCTAAACGAGATTGTAGTTTTACATGGACATTTTTACTTTTTCGTATTCAATTTTTTTGTTTGGATGGTATCTGATCTTCTTACGCCCTCGATTTTTATTCTAGGTGACTTCCCACAGGGGATCATTGCCCTTTGATGAAAAACAATGGTGCTTGAGGATTATTTGTGTGTTAATGGAGTCATCCTTTCAATCTTCTTAATTTTTTCATTTTCTAGGGAGTTATGCTTCAATACGTTGAGAGTTTGATCTCTAAAACTCCAAGAAATTTGATCTACAATGATTGATTTCAACTTTATCACTGGCCTGTAAATTGCAAGGGAGGATAACTTTACAAGTTGTATCTAAGACCCTTAACTATCGGATGGACAATTTTATTAGTTATTGTTAGGTTAATTTTGTTCCAATGAAGGTATTCTTAAGGGTAAACTTGTATGTACTTAGTTGAGATACTTCTACGTCAATATATCTATAAATGCTTATAGTTCTGCTGTAATTGCATCAAACAAGCTTTCGTGATATTGACTCTATGTTTTTCTTGTGCTACTTTTTCGTTTTTCTTTTAATTGAATTCCCATCTTGTGTCTCCAAACGATGATTGAAAGATTCACCATAAAAAATAGAAAATGTTGG

>LNC_000597

CAAAGAATTAAGAAGGTATCATCGAATGTAAAAAGGTCCTACATTGTTGTGTTAGATGAAGGAACATGCCATGTCCTTGTATAGCTCAGAAAAAATGCACAAATAAACAGTTGGTATTACAGGATTTCCATCGACTTTGTTGGGATTTGCTTCTTTTCATGATTTTACAAGTCGACAAACAATGATTATGTGCTCAAGGAACTTGAAAACATGAAGGAACAACGGCTTGCACATAGAAATATGCTATGCTATGCCATACTAGATATGCATAAATGTCACCACAAAGAAATCAACTATTTCTATTGCAAAAGCAAGCTAAAAGAGCTAAATCAAAAAGGCGGTGACTCTTGTAGAATTCAAATAATACACTATCTTTGTCACAAGTTCAGAGTCATGACAAAAATCAAAGTGTGGTTTGAACTTCCTTCATCAGCTAGAGATAAAGGTATCTACACATCTGGCGCTAAAAGAGTTTTGCCTCTCAAGCGTGGTTGCACTTCTCTTCACATGTGACTTCCCACAGGGGATCATTGCCCTTTGATGAAAAACAATGGTGCTTGAGGATTATTTGTGTGTTAATGGAGTCATCCTTTCAATCTTCTTAATTTTTTCATTTTCTAGGGAGTTATGCTTCAATACGTTGAGAGTTTGATCTCTAAAACTCCAAGAAATTTGATCTACAATGATTGATTTCAACTTTATCACTGGCCTGTAAATTGCAAGGGAGGATAACTTTACAAGTTGTATCTAAGACCCTTAACTATCGGATGGACAATTTTATTAGTTATTGTTAGGTTAATTTTGTTCCAATGAAGGTATTCTTAAGGGTAAACTTGTATGTACTTAGTTGAGATACTTCTACGTCAATATATCTATAAATGCTTATAGTTCTGCTGTAATTGCATCAAACAAGCTTTCGTGATATTGACTCTATGTTTTTCTTGTGCTACTTTTTCGTTTTTCTTTTAATTGAATTCCCATCTTGTGTCTCCAAACGATGATTGAAAGATTCACCATAAAAAATAGAAAATGTTGG

>LNC_000598

CATCAATACACCACCAAAGAATTAAGAAGGTATCATCGAATGTAAAAAGGTCCTACATTGTTGTGTTAGATGAAGGAACATGCCATGTCCTTGTATAGCTCAGAAAAAATGCACAAATAAACAGTTGGTATTACAGGATTTCCATCGACTTTGTTGGGATTTGCTTCTTTTCATGATTTTACAAGTCGACAAACAATGATTATGTGCTCAAGGAACTTGAAAACATGAAGGAACAACGGCTTGCACATAGAAATATGCTATGCTATGCCATACTAGATATGCATAAATGTCACCACAAAGAAATCAACTATTTCTATTGCAAAAGCAAGCTAAAAGAGCTAAATCAAAAAGGCGGTGACTCTTGTAGAATTCAAATAATACACTATCTTTGTCACAAGTTCAGAGTCATGACAAAAATCAAAGTGTGGTTTGAACTTCCTTCATCAGCTAGAGATAAAGGTCAGAAAAGTTAAATTTCCATATAATTTTGCTTGTGTATGTATAGGTATACATACCTTCATAGATAAATTGATTTACTATCATTTATACTATTTTGTCTTCCATCATTTTGGAGGACATGCTTCCGCGGCTACACAATCAACTACTTGCACCTCAAACTTTGAAGTAGGTGAGTCGGAAGGGAATTGGAAAATTCAAAATAGAAAGACTCAATCAGATATGATACACTGCATATGGAAAATGGAGTCTTGATTCTCAAAGTAAAATTGAAATTGGCTTCACGACAAAACTCATAAAGATCTTGATGATAATTCACAGTCTCAGTTGCGAAGAGCAAAAAATATAACTATTTTGTGCTCATACTTTGATCATTAGAAATTATAGGTATCTACACATCTGGCGCTAAAAGAGTTTTGCCTCTCAAGCGTGGTTGCACTTCTCTTCACATGTGACTTCCCACAGGGGATCATTGCCCTTTGATGAAAAACAATGGTGCTTGAGGATTATTTGTGTGTTAATGGAGTCATCCTTTCAATCTTCTTAATTTTTTCATTTTCTAGGGAGTTATGCTTCAATACGTTGAGAGTTTGATCTCTAAAACTCCAAGAAATTTGATCTACAATGATTGATTTCAACTTTATCACTGGCCTGTAAATTGCAAGGGAGGATAACTTTACAAGTTGTATCTAAGACCCTTAACTATCGGATGGACAATTTTATTAGTTATTGTTAGGTTAATTTTGTTCCAATGAAGGTATTCTTAAGGGTAAACTTGTATGTACTTAGTTGAGATACTTCTACGTCAATATATCTATAAATGCTTATAGTTCTGCTGTAATTGCATCAAACAAGCTTTCGTGATATTGACTCTATGTTTTTCTTGTGCTACTTTTTCGTTTTTCTTTTAATTGAATTCCCATCTTGTGTCTCCAAACGATGATTGAAAGATTCACCATAAAAAATAGAAAATGTTGG

>LNC_000599

TGGACTCCGTAATTTGCATGGACATAATTCTTATGTTGAACCCACTTCTTCTTAATCTCACATGCAGAAGAGTGTAGAACCATCTCAATTTTGCAGTGACAAGTCCAATTATAAGAGGGTACAACAAAAAAATTCTTCTATCACATACCTGCAAAGAACTCAAAATTCAACTGGGACTTCTACTTCATACGTGAAAGTGAAAATTTGAGAAGACATTAAGTCATCAATACACCACCAAAGAATTAAGAAGGTATCATCGAATGTAAAAAGGTCCTACATTGTTGTGTTAGATGAAGGAACATGCCATGTCCTTGTATAGCTCAGAAAAAATGCACAAATAAACAGTTGGTATTACAGGATTTCCATCGACTTTGTTGGGATTTGCTTCTTTTCATGATTTTACAAGTCGACAAACAATGATTATGTGCTCAAGGAACTTGAAAACATGAAGGAACAACGGCTTGCACATAGAAATATGCTATGCTATGCCATACTAGATATGCATAAATGTCACCACAAAGAAATCAACTATTTCTATTGCAAAAGCAAGCTAAAAGAGCTAAATCAAAAAGGCGGTGACTCTTGTAGAATTCAAATAATACACTATCTTTGTCACAAGTTCAGAGTCATGACAAAAATCAAAGTGTGGTTTGAACTTCCTTCATCAGCTAGAGATAAAGATACACTGCATATGGAAAATGGAGTCTTGATTCTCAAAGTAAAATTGAAATTGGCTTCACGACAAAACTCATAAAGATCTTGATGATAATTCACAGTCTCAGTTGCGAAGAGCAAAAAATATAACTATTTTGTGCTCATACTTTGATCATTAGAAATTATAGGTATCTACACATCTGGCGCTAAAAGAGTTTTGCCTCTCAAGCGTGGTTGCACTTCTCTTCACATGTGACTTCCCACAGGGGATCATTGCCCTTTGATGAAAAACAATGGTGCTTGAGGATTATTTGTGTGTTAATGGAGTCATCCTTTCAATCTTCTTAATTTTTTCATTTTCTAGGGAGTTATGCTTCAATACGTTGAGAGTTTGATCTCTAAAACTCCAAGAAATTTGATCTACAATGATTGATTTCAACTTTATCACTGGCCTGTAAATTGCAAGGGAGGATAACTTTACAAGTTGTATCTAAGACCCTTAACTATCGGATGGACAATTTTATTAGTTATTGTTAGGTTAATTTTGTTCCAATGAAGGTATTCTTAAGGGTAAACTTGTATGTACTTAGTTGAGATACTTCTACGTCAATATATCTATAAATGCTTATAGTTCTGCTGTAATTGCATCAAACAAGCTTTCGTGATATTGACTCTATGTTTTTCTTGTGCTACTTTTTCGTTTTTCTTTTAATTGAATTCCCATCTTGTGTCTCCAAACGATGATTGAAAGATTCACCATAAAAAATAGAAAATGTTGG

>LNC_000600

CAAAGTTGTTTATCCTCCTGCAATTTCTCTCCTTCCTGGTAATTTGCATGGACATAATTCTTATGTTGAACCCACTTCTTCTTAATCTCACATGCAGAAGAGTGTAGAACCATCTCAATTTTGCAGTGACAAGTCCAATTATAAGAGGGTACAACAAAAAAATTCTTCTATCACATACCTGCAAAGAACTCAAAATTCAACTGGGTATAATGTAAATTTCTTCGTGTTTCTTTTTTCCCCATTCATATATATCCTTTTAAAAAGTGTCAAAAATTTCATTTGTAAACCCCAAAATTTGCATACAAATATAGCAGTAGTGTATGATAATATGTCTGAAAGAACTGAATCATATTGTTTTCCTTTCTGGTTCTTCCCTTTTCAACATCTTCTTTGTATCTTCTCATTTGGTGCATTTACAAATTACAAAATATTCTTGGTTTTGTATTCTAGAGAGTAATACAATTTTTCTGCTCTTATATATTATGAGAGAGTGTTGAAATATCGATTTAGATTTTGACCATCAAGTTCTGAAATTTTACTGTATTTAATTCCAATTGATCCAGGACTTCTACTTCATACGTGAAAGTGAAAATTTGAGAAGACATTAAGTCATCAATACACCACCAAAGAATTAAGAAGGTATCATCGAATGTAAAAAGGTCCTACATTGTTGTGTTAGATGAAGGAACATGCCATGTCCTTGTATAGCTCAGGATTTCCATCGACTTTGTTGGGATTTGCTTCTTTTCATGATTTTACAAGTCGACAAACAATGATTATGTGCTCAAGGAACTTGAAAACATGAAGGAACAACGGCTTGCACATAGAAATATGCTATGCTATGCCATACTAGATATGCATAAATGTCACCACAAAGAAATCAACTATTTCTATTGCAAAAGCAAGCTAAAAGAGCTAAATCAAAAAGGCGGTGACTCTTGTAGAATTCAAATAATACACTATCTTTGTCACAAGTTCAGAGTCATGACAAAAATCAAAGTGTGGTTTGAACTTCCTTCATCAGCTAGAGATAAAGGTCAGAAAAGTTAAATTTCCATATAATTTTGCTTGTGTATGTATAGGTATACATACCTTCATAGATAAATTGATTTACTATCATTTATACTATTTTGTCTTCCATCATTTTGGAGGACATGCTTCCGCGGCTACACAATCAACTACTTGCACCTCAAACTTTGAAGTAGGTGAGTCGGAAGGGAATTGGAAAATTCAAAATAGAAAGACTCAATCAGATATGGTAGGTACTTTACATGGTCTGTGGTGATATTAAGTACAAATCAGTTCTATCTTCTTCCGGACATAATTACCCTCTAGATAATGTAAGAATTTAATATGATTTGTAAAAATTAGTTCTCTTTCTTTGGGACATAATTACCACCAACTAATATAAGAATGTGACTATGATTTTATCTTCCCAAATGTCAGTCACTTGGATGTAACTTGATTTCACTGGGAATATGATTTTATTTCCTCGGAGAGGTTTCAAATACATTGCTTCAGTATTTCTTATAAAAGAAGGCATATTGTTAACTTTAGCTGGTAAATATCAGATGTTAGCCTCAAATCTCAATTGAAGGGGAAATGACTAGTTCTTCTAAGCATTTTAAAAGACTATGCTATTTAGATTGATCATAACTATTGCTTTGTAAACAGATACACTGCATATGGAAAATGGAGTCTTGATTCTCAAAGTAAAATTGAAATTGGCTTCACGACAAAACTCATAAAGATCTTGATGATAATTCACAGTCTCAGTTGCGAAGAGCAAAAAATATAACTATTTTGTGCTCATACTTTGATCATTAGAAATTATAGGTATCTACACATCTGGCGCTAAAAGAGTTTTGCCTCTCAAGCGTGGTTGCACTTCTCTTCACATGTGACTTCCCACAGGGGATCATTGCCCTTTGATGAAAAACAATGGTGCTTGAGGATTATTTGTGTGTTAATGGAGTCATCCTTTCAATCTTCTTAATTTTTTCATTTTCTAGGGAGTTATGCTTCAATACGTTGAGAGTTTGATCTCTAAAACTCCAAGAAATTTGATCTACAATGATTGATTTCAACTTTATCACTGGCCTGTAAATTGCAAGGGAGGATAACTTTACAAGTTGTATCTAAGACCCTTAACTATCGGATGGACAATTTTATTAGTTATTGTTAGGTTAATTTTGTTCCAATGAAGGTATTCTTAAGGGTAAACTTGTATGTACTTAGTTGAGATACTTCTACGTCAATATATCTATAAATGCTTATAGTTCTGCTGTAATTGCATCAAACAAGCTTTCGTGATATTGACTCTATGTTTTTCTTGTGCTACTTTTTCGTTTTTCTTTTAATTGAATTCCCATCTTGTGTCTCCAAACGATGATTGAAAGATTCACCATAAAAAATAGAAAATGTTGG

>LNC_000601

TCAAAGTTGTTTATCCTCCTGCAATTTCTCTCCTTCCTGGTAATTTGCATGGACATAATTCTTATGTTGAACCCACTTCTTCTTAATCTCACATGCAGAAGAGTGTAGAACCATCTCAATTTTGCAGTGACAAGTCCAATTATAAGAGGGTACAACAAAAAAATTCTTCTATCACATACCTGCAAAGAACTCAAAATTCAACTGGGACTTCTACTTCATACGTGAAAGTGAAAATTTGAGAAGACATTAAGTCATCAATACACCACCAAAGAATTAAGAAGGTATCATCGAATGTAAAAAGGTCCTACATTGTTGTGTTAGATGAAGGAACATGCCATGTCCTTGTATAGCTCAGGATTTCCATCGACTTTGTTGGGATTTGCTTCTTTTCATGATTTTACAAGTCGACAAACAATGATTATGTGCTCAAGGAACTTGAAAACATGAAGGAACAACGGCTTGCACATAGAAATATGCTATGCTATGCCATACTAGATATGCATAAATGTCACCACAAAGAAATCAACTATTTCTATTGCAAAAGCAAGCTAAAAGAGCTAAATCAAAAAGGCGGTGACTCTTGTAGAATTCAAATAATACACTATCTTTGTCACAAGTTCAGAGTCATGACAAAAATCAAAGTGTGGTTTGAACTTCCTTCATCAGCTAGAGATAAAGATACACTGCATATGGAAAATGGAGTCTTGATTCTCAAAGTAAAATTGAAATTGGCTTCACGACAAAACTCATAAAGATCTTGATGATAATTCACAGTCTCAGTTGCGAAGAGCAAAAAATATAACTATTTTGTAAATTTAACACCGCTTGCAATTTGATCCGTGAGATACTGGCTTGCTGTTAACCTACCCAATGTCCATGTTATCGTGCACATTATAGACAAATAAAAGGTATCTACACATCTGGCGCTAAAAGAGTTTTGCCTCTCAAGCGTGGTTGCACTTCTCTTCACATGTGACTTCCCACAGGGGATCATTGCCCTTTGATGAAAAACAATGGTGCTTGAGGATTATTTGTGTGTTAATGGAGTCATCCTTTCAATCTTCTTAATTTTTTCATTTTCTAGGGAGTTATGCTTCAATACGTTGAGAGTTTGATCTCTAAAACTCCAAGAAATTTGATCTACAATGATTGATTTCAACTTTATCACTGGCCTGTAAATTGCAAGGGAGGATAACTTTACAAGTTGTATCTAAGACCCTTAACTATCGGATGGACAATTTTATTAGTTATTGTTAGGTTAATTTTGTTCCAATGAAGGTATTCTTAAGGGTAAACTTGTATGTACTTAGTTGAGATACTTCTACGTCAATATATCTATAAATGCTTATAGTTCTGCTGTAATTGCATCAAACAAGCTTTCGTGATATTGACTCTATGTTTTTCTTGTGCTACTTTTTCGTTTTTCTTTTAATTGAATTCCCATCTTGTGTCTCCAAACGATGATTGAAAGATTCACCATAAAAAATAGAAAATGTTGG

>LNC_000602

GCTAGCTTTTGGGGTGTGAGTTAGGCCTAAGACCTAATTTAACATGGTATCAGAGCAGGGCCCGTCTCACCCGATGTTGGGACCCCCAAAATCAAAAATTGCCCACGCACCAGATGCTAAGCACTGGGCGTGAGGTGGGGTGTTAAAGAATGACAAAAGGCCCACATCGGTGGTTAATGAGATGGGTGGACTCCTTATAAGGCTTGGGCAATCC

>LNC_000603

TGTTGAAGTTGTTAGTTGTTGTTGATGTTGTTAGTTGTGTTGTTGAAGTTATTGTTGGTTGTGGGACAGGATTGGGCTGTGTATTTGAGTTGTAATTTATTGTATGTTGAGAGATTGATGATCCTTAGTGTTGGTTGGTTTATACCCCCACACACCAACTCGTTTTTAATTCCAAGAAAATATTTATTTTGGTTGTTGAAGTTGTTGGTTCTGTTGCTGAAGTTCTGGCTGATTTGGGACAGATTTGGTTGTGTTTTTTTTTAGTGGAATCCACAATATGTTGAGGGGTTTATGACCCCAAGTGTTTGGGAAAAGAACCTTTGAAGTAAGGGTGGTTTAGAGTTGAAGAAAAGAGGGAGAAAAGTTGGGTTTTTTTCTGGGGAAAGGGCTGGCCTGGCAGAGTGCAGAGTGCCCAACAGTCCACTTTGAAGCTGAGGGCTGCTGCCTGGCAGAGTGCCCAGTAGTCCCGTTTGAAGTTGGGGGCTGGCGCCCCGCGCCTCTTAGAGCGCCAAGGACGCCAATCCTTCCCGTTACCTTCATTCCCCACTTTTTCATACATGTTCCTAGGTATTATACCTATGTTTCCTAGTTGTTTCCAACACTCCAAGGTACTTCTAAAAGTCAAGAACTCATCCGTAACATGTGAGCAAACACCTTGAATCCAAAATTCCAATTCAAGGAGAGTTAGTTAGAAGTCAAGTGAAGTTAGAAATTAAGAGTTAAGTATAGAAGTTTCAAGTTAAGTAAGCGGGTATAGAATTTCAAGTCAAGTTAAGAGTTCA

>LNC_000604

CCCAAATCAAAATAGGAGCTGCTAAACCCTATTTCCCTCTCCGTCACATTTCCACCCATAAATTGTAACTTTGTCGAGAAAAAAGTTACAATTGTAAAAAAAATCCATACAGAAAAAAGAAATCTTCAGCTTCTTCAAGAAGAAGACATGGACTGAAATGGCTAACAGAGACGCAGTTCCAAAAATTTTACCCAAAGGCCTTCATTTGGGAAACTGAAGAAACAGTTCGAAGAACTCCGACATCGTTTGGAGGAGTTGGGGAGTCTATGGTAGCGGATATGGGTTGTGGTGAAATATTTATCTGAAAGCTTGTAAATATCTCCAAAATTGTCTATTTTCACACCGA

>LNC_000605

CCTGAAATGCCTTCAAAAGTTGTTAGAATTTCCCTTAGATGTTTAAATTTGCAACACCTATGAGCTATGCTTCACAAAATATGAGGGCATCATCTGTATAAAATGGATAGGTGATTTCCAACTCATTTCCCATGCATGTTTTGACACTGAAAGCTTTTATCTAGCCATTAGTTTTTGCTATCCTGAACATATGATTCAGCCCCTCCATTGCAATTAGGAATAGGAATGGAGACAGACGATCCCCTTGTCTAAGACCTCTATGTGATTGGAAGAAATCCTCAATATTCCCATTATAATGAGAGAAAATTTCACACTTGAAATGCAAAATCTAATCCAGC

>LNC_000606

TCCTTTTGTATTGGAGTTTCCCCTTACATGCTCGTACAATCAATGTACTGACGTCATTCGACCTGCATATTTTTATGGTGCAGATACAAGTTCTTAGGGTCATCTACCAGTGTTGCATTGATACCACTTGCATTCTAGCTAGCTATGGTGAGCCTCTTTGCTTTCTGGAGGGCGTCACACTTAATTCCTGATTTTTGTTAAGTTGTCGAGGGTTCTATCCCGATATCTATATTAGTACCTAGAGTTTTCATAGATAGTTAGACGTTGACGAGTCTCATGTTATTTCCCCATTCATGTTGAGG

>LNC_000607

CCTAAATGCACTAGAGTTGCTGGACGTTTCTCTCCTCTTTGAAGAAAACTGCCAAATTTCTTCAACAATGGCAGTGCTCTGAATGCCTGACCATACCATTCTGGTAAACATATCGATCTTCACACCACAAATAGGTCAATATTCACGAAAATGGTATGCAACTTTTACCTTTGTTTTTAAATTTCGATTGTCTCCACTCCATCTTTTTCTGCTCGGATCTGCCCAGATCACGTGAAGCTTGACCAAAGAGTAATGTTTAAATTTCAAAACACATCTTTCTCAACTCAAGATCCCTCCCAAAATACAAAGCTTAGGGCTTATAATCTCATCTATAAATGTTTCCCCTGTTTTGCCTTTGAAAGGGACGTTTTCTTTGGGTTAGCTAAAAGAATCATCCGACAAGCTTTCACACTCACAAAAAGCAACACATTTATACATTTTAATATTGCTTACAAAGAATTTTCGTTTCTCCTCTTTGTTGCACTTGACTTTTTATGAAGAACGAAGATAGATTTGAGGCTGATAACGTCCCTAGTTTACTGCACTACAAAAGGTAAAGTTCAATCTTTTTGGTTTCTGACTATTTAATTAAGCATCCGTGGTTGTGTTAGAAAATAATGAGTTGGATATTTCTTTGTCCGTAGTTACATTACGACGTGTTAATAAATTGTGTACTTCCGTGATATTATAATGTCTTTTAGGGTCGTTTGAAAATCTGATCCTAATCATATGGACGGAGATATCATGTACTGCTATGTATCCTGCAAGATTTGAAACTTGAGTTGGATGGCCCTTACCTTTTTTTTTTTGTCCAAAAATAATGATAAGTTGACTACTTTGGTTTGAATAGGATATGCTAATATGTAGATAATGTGTACTTACTATGACTAAAGATTTTGAACTATTATTTTGATATAATAAAAGTGTTTAGACATATATTTATGGAGCATGTTTTGTGTATACTTAATTTGTGTCAAGTACGTATATTTAGTTTGGCTTAGATGAGAATACAACATGTTGTTGGAGTGATTACTATAACTTTCGATTGACTATTATCTTCTTTTATATAGTATCAAGAATCAATTTTCCTCATCTATTTTCTCATGTTTATTTGGCATGAAAATTGGAGCCGGCGAGTCTAGTTTTGAAACTCAACGACACCACTACTGCATAGAGCTCGCATATACCTCACATGGAATTGATCATATACATTTCTTTCACACTACATCTATTCGCTTTTCCTATTTTTAGAGACGTGTCCGGTTAAAATGAGACATTATAGTTAATTAGTTATCTAGCACATTACTTGCTGTTATGCTTACTTATTTGTTTGTCTTGTTGTTAGAATTTGCACAAATCTGCTTGAATGCCTTAGCAATTTACCTCTAGTTAGTAGCTTATTCATGTTTATAATATTTTTTTACTCCTAGATTTAATGACCTTAAATTTTTGATAATTTGTCAAATCATTTGATTTGTGATTAAAAATCAG

>LNC_000608

GAAATGGGTTTTAAAACCTATAACACGGGAACACATGCTATTACTAAGATAGGTAGTTGTCAAAAGAGCTTCTCTCCAAAGAAATTTAGGGATTCCACTTGTGAATATTAAGGTTCTAGTTACTTCCATAAGATGCCTATTTTTCCTCAATACTTCACTCCATTTTGTTGGGATGTATCATGACAAGAACTTTGGTGCACTATTCCTTGGAAGTGAAAAAATTGCCTAGTTGGTCATTAAAAAACTCTCCCATTCTCACTCCGAAATACTTGAATCTTCTCATGAAATTTTATCTCAACATAAATAGAAGCCTCCAATGTATGTTTTAATTTCCCTTTGTCTTTCAATTGGTACACCCAACTAAGTTTAGTGTGATAATATATGAAATTCAAAAACCACCTTTTTCCATTTACAATTGCTACCCTAGAAGGCCCCTAAACATCACTATGGATTAACTTAAAGGGTTTTGTGGGCATGATATTTTTGAGAAGAAAAGAATGAAAGGCGATGTTAAGCCATTTCACAAAATTTACATTGGAAAGAAACAAACTTTTATTCGTAAAGTAAAGAGGGGTTAATTTGATAGTTCTTTTCCAGCAATTGCTGAGAAGGAACCACCATCAACTCTGACTTCTCTATTTCCTGCATAAGGAGTACAAGTTGAGAGAAAATGGGTATTCCATGTCATGTGATCAGGTGCTCAAAAAAACAGATGGTACAATACCTGTTTGGGAAAAAAACAAGTGAGAGTGGAATGATCAGGTTTACTGGTTTGTAGTTGAGATTGGAGAAGTTCAGTAGCTCTAGTTGCTCCTTGGTGAATGGTGACATTTTTGGAGAAGGTTTCAACCTTGATTCTTCGAGAGCGACCTGGTACTCTAGAAGACCCAGCTGCCACTGTTGTCTTCCCCTTTACTCTTCCAGTTATGCAATTTCCCGAGGATTTTCTAGCAGGTTTCACGAGTGCCACTATATTTGCAAAAATCACACCATGACTTCTTTTTCTTGTGATTTCATTTTTCGCAACAACTAGAGCGAGTGTGGCAGTATTTTTTGCAAGGATCACACAACGACTTCTTTTTCATATCATTTTCATTTTTGTAGCAACTTGAGCAGAAGAATCTATATTTTTTGCTTCCAAATTGGAGTATGTTTTTAGCATAACATTCCTCCTTGTTTCTTCTCGCCTAATCCTTCAAGGTGGGCTGGGTCTTTTCCAAGAATTTGTGAAAGAAGTTTGTAGAATTCTTAGTTCAATCTTACTAGAAATTTTCCTTTTTCATTTCTCTTACGCTATCCTTACGACACTCCCATTCATCATTATAACATTTATCTAATTCTTTCCATATAGAAACCATCTTATTATAGTAATAACACCTCTCTCCCCCTGCCTTCCTTTCTACAATCTGACAAACATTTTCTACATCAGAGTAAGTCTCTCTAGCAGCTCCCCAAACATCTTAACAGTGGGGAGAAACAAATATGGTTTACCTATGGCGGCAGGTTCCATTGAGTTTAATAGCCAAGCGATTACCATTGAGTTTTGTGATCTCCATGCATTCATTTTCGGGTCTCTTGGTGAAGGCCTTCTTGTCTATCTAGTTAGGTGTCCAAGTTTCCGTTGACCATCAATTACCAAACAAAAAGATCGTGCCCATTCCAAATTATTCTTGCCATTAAGCTTGTGACAAGTAAGTTAAAATGATAAATGAGAGGAATCGTCACTTTGACTCATAGTTGTCTTGGTTGGAACTTTGCTGATAGAACTCTTAGCTTCTAGTGATGCTGCTAGGTGAAGTTGCCTTGTAAATGTTGTTTAGAGCCTGTTTGGATTGACTTATTTTATCAAATTTCAGCTTATGCTAAGGGCTTATAGGAACTTTTTTGATTTAAGCAAACACTCTAAAAGTGGTTAATAGTTATTTTGCTTAAAGCCACTTAAAATAAGCCAATCCAAACAGGCTCTTAATGATGAACAACACAAGAATAAAACAGCTCTGATACCATGTAAGTTATGGTACCAAGAATAAAAGTTGTTTTGATATCATATGGTATGGAAACACTATGGCAATAGAGATTACGAAATAAACTAGAAGTTTGATTCTTGATTTTAGGATTTTTGAGAATCAAAAGATGATTTAAACTAATCTAATTTCCTAAATACTTAACAACATTAGATCTGATTTTATCGGATCAATTAGATCCTACAAATTAAATCCGATTATGATCAAATATTCACAAAACAACTATATCTGCCCTTATTCG

>LNC_000609

AAATTTAGGACGATACAATAAAGGACAAAATAAGAATTTGGTATAATAATACAATTTTTTTAATTTCTCTTTAGAAAACTACAAAGTTTAGTTATCTATTCAAACAAGATATACAAATTACTAATAGATTTAATGAAATCTCAAAGAAGCCCACGTTCAAGGTATTACTCAGTAAATACATATATATCTTAACTTAAGATTGACTATTCTAGCTTAATTCAAATTGAATTTTTTTGAATCCTTTTATTTGCGAGGAGCTGGATGAGAAGAAACTCTCACATTAT

>LNC_000610

TTTAATTTATGCATTTTTTTTTTCTAAAACTGGTAAGGTTGTATTCTTCAGCATTAAGGGCATGCTGGCCACCACCAAAAAGTGTCAATTACAAACTTCCTATCAAATCTACAATTTGATCTGTACCTACTATACACAACTGTTTACACCAAAAAAGTAGAGATACTATACAATTCCATTTATGCATCTTTCGTACCTTTTTAGTTTATTTAAAAAAATACCTTTCTATATTTAGTACTCCCTCCGTCCCAATTTCTGTGACATAGTTTGACTGGACATGGAGTTTAAGAAATATGAGAAGACTTTGCAATGTTCCAAAACTACCCCCGAAGTAAATGGGTTTTTAAATAGAAGAGTACACTTTTATTAATTTCTTGCCAAATAAGTCGGATCCAACAAGGGTAAAACGTTGATAGTGTCAGTAAACACTTGCCAAATAAGGAATTGTGTCATTCTTTTTGGGAGGGACTAAACAGGGCAGTGAGAAATATAAATTGGGATAGAGGGAGTAGGTTTATAATTCCAATATTTCCATTTTATTTTTAGTGACAATCCCTTGTAAGAATGAGATGATTTGTTTAAGTCCACAACATTCAAAGGGTATTTCTGGTATATTATACACACCTTTAGTTTAATACCACAAGATTCTAAAGTCTTCTTTACATTTTAAAACTTTGTGCCTAGTCAAGTGAAGACACAAAATGAAATGGAGGGAGCCTATCTCAACACTTAACTTTAAACGGACATGTACATATTATATGCTACCATCTTGTTGCGAAAGCTATTCGTTAGTCTTGAAGTTGTTTTGTTAGGATATCGGCAAAATTATTCATTTGAATTTTGACGAAATTAATAAAAGTACATACTGATTTGATCTTTTGATAAAACTGACAATCTATCCCTATGTGTTTTGGTCTTCCGTGAAATACGGGGTATTAAACCATATGTATTGTGGTT

>LNC_000611

GTTTATACTAAAAATTAACCCTTAATATCACTTTCATTTACTCTTTTATCCTCAAATACCCAAAACTCTCTTCAGTCAAGTTTTATTTCATTCTCAAATTCCCAAATACTCAATCTCTCTTTCTCCATTACATCTGAAGCTCTGGATTCATTAGAAGCTTCTTTCCATGGCTACGATAGATTTGATCTGAAGCACGAATCATTTTTTCTTTCTTCCTGCTCGATCTAAAGCATGAAGCAAGAGGAGCTGATCGAAGTAGTGATGCGTCTTGCTGGAGATGCCTCATGACCATTCATAGTTGCTGGAGAAGTACGCCAGCCAGGCTTGAAGGCTTCATCATCATTTAAAACCTTCCTACAAGCTTCTGGTGTTGTAACAATACAATAATTAATTTAAATAAGGAAAAAATGTCAAAAAAGGAGAAAAAAAGATAAATACAAATGGAGGTCATAGAGAGGTGTCACATCGCCTATGTTTCTCCTTTATTATATATATTGATAGATAGATATAGATTATCTTTATAATATTTAAATTAAGGAGTGTTAAAATATATGATAAGAGAAAAAAACTATAATAGTAAAATATTTTTTAAATTAAAACTACCCCTTTAAAGCTTGAATCTGATTTGCTCTAGAAATTTGTTGGCTTGTATTAAATGACCGTATTTATATTAGGTGTATGTATGTGAATATATACATGTTCGAGTACTGATTATTTATATTTTCTTCTGTAAATTTTATATGCAGAGCTCTAGAAGAAATAATTATTTGTCAAAAACTCAAAAGATACAGAGCCAGATCGATATGAGAAAAACGTACTTTTTATTTGTGAATGTAAGTTTTGGTTTAATTTTGTTTTCTTCTCCTAATGTTTTGGGTTTTGTAAATAATATTTTTTATTAATTTACTTTTAATATACTCTATTTATGTGTCTTATCAAACAATGTTAACATTAACAATAAAGGAACGTTTTGGACCTCCATG

>LNC_000612

AGAAGACGAACTTAATAGCTTGAATTTATATAATAAACTACATAGATCATCCAACTACCTTATTAAAACATAAATATTAAAATAATAAACTAAACTTAATACAACCTAAATTAAAACATAATCTTCCAATAACTTTTCAAATTTGGAAGATAGCTTTAGGTGTTCTTTTCCTTCTTAATCACTTTGCGAATTTTGTTGCTGGGTTGTTTGACATTCAAATCGTCTTTAATTTCATGCCTTGTAGATTTTCTAGCAGCTTTGCTACTCTTGCAGCAGTTTTACTACTCTTGGTAGATATCTTTGTAGTTAAATCAATCACATCTTCAGTAATTAATGTACCACATTCTGAAATGCTGCTCTTGATTGGAGTCTAAATTAAAATAACATAAAGATTATAAGTACTTCAAGTGTTTTCAATCTGATTAGTAATATAGAAGATGTAACTTATCTAAAGTTAGAATACCTCAAAGAGTTTATCTTCATCATAGCTTTGCTCATATTCAAAAAGTGATTTCTGTTTGAACATTATAAACATAATTAACAATTTATACGTTACTAAAAAATTTGTATGTATAATGTAAACAAATATGTTGGTGTTTAAACACTTACATTAAGATTGTCCTGAAGGTCAGGATGACAAAATTCCTTTAAAAGAGTATCATCATCGGTGAAATTAACAACTTTATAGACATTATCTTTTTTGTTAATGTTTGAGTCCTTGATCAAAATCTTAAACATAAATTTCTTCTCCATTAAATCACCAAGTTCTGTTGGATAAGAACTTTCTTCATCTTCAAGTAATCCTTCTTTCAACTCTTTTGCAGTTTTACCAAGTAGTTGTATAGTCTCTTTATTCCAAAGAAATATTGAAATAAATGCAGTGCCATCCATAACAGACAGTTGGAGC

>LNC_000613

ATATAATCAAGGCAAAAATATATTTAAGATAATATTTTACAGAATGTTATATTTTGCTATGTAAAATACTCTATTTTTATAGCATATGTGTTATTGAAGATTTATTATATATGATTTATCTATTTTTGAAATTAGTTCACGAAGTTTGCGCACATTGATACAATTGTCAGTGTGCTGTTTAGTGTGAATTTGGTGCGAATGTTTTGGTGCAATTTTGGGGTTTGTTGACGTTGTTTTTATCGTGCATTTTTTGGTGCAGTTCCAATGTTTTTCCGGTGGTTTTTTGGGTGCAATTCTAACGTTGTTTTAGAGATGATTCTGATGCTGCTGTTTTGGTTTAATTCAAATGATGTTTTAATATTTTTATTGTGAAAATTTAGTGCAGCTTTGGATCTTTTCTGTTGCTGTTTTGGTGAAACTTTGGGGCTGTAGCATTGTATATATTTTTTGAATGAAGATTCAACATGTTATATAAAGCAAGCTCAGAAATTGATGTGGATCCACCATATTGTTCACCAAAGTGTTGCATGTTTTTTAACATATGGGAATAAGGTATGTCTCTTAAATTGCACTAACGTATTTGTAAAAATCAGCTAATAGTGTGTTGTTTAGATTTTGTATGCAGTAAAGTTAACTTTAATGATCCTTCTGTTACTAATGAATGTTAAACTATAAGTCTGAGAATATAAAATACACATTAAGACAACATG

>LNC_000614

TGAAAGGTGAGGTACCTCTTCCGAGTGTGGATCTTCGCTAAGTTTCTGAGTGATTTGTCAATGTTTGTATTGTAGATGGCGGAGAAGTTGATAATGGGAAGATTTTTTTTCTGCTAGTGTATCGATGGAAAATAGCAGTGATTATAATTAGCTAATTAAGCTTTGGATCTTTTCTGTTGCTGTTTTGGTGAAACTTTGGGGCTGTAGCATTGTATATATTTTTTGAATGAAGATTCAACATGTTATATAAAGCAAGGTACTCCTTCAACAAATTTGATAGAATTATTATAAATCATTGTTCATTATGACTACCTTTGAACTACTATATATTGGAAGAATGAAGATGAATTGCTTGGAGCTTTAGAATATCTGATTCCTATTCTGAATATATCAGCTCAGAAATTGATGTGGATCCACCATATTGTTCACCAAAGTGTTGCATGTTTTTTAACATATGGGAATAAGGTATGTCTCTTAAATTGCACTAACGTATTTGTAAAAATCAGCTAATAGTGTGTTGTTTAGATTTTGTATGCAGTAAAGTTAACTTTAATGATCCTTCTGTTACTAATGAATGTTAAACTATAAGTCTGAGAATATAAAATACACATTAAGACAACATG

>LNC_000615

AAATTTTCCAGATTTGAAAGGTGAGGTACCTCTTCCGAGTGTGGATCTTCGCTAAGTTTCTGAGTGATTTGTCAATGTTTGTATTGTAGATGGCGGAGAAGTTGATAATGGGAAGATTTTTTTTCTGCTAGTGTATCGATGGAAAATAGCAGTGATTATAATTAGCTAATTAAGTTCCAATGTTTTTCCGGTGGTTTTTTGGGTGCAATTCTAACGTTGTTTTAGAGATGATTCTGATGCTGCTGTTTTGGTTTAATTCAAATGATGTTTTAATATTTTTATTGTGAAAATTTAGTGCAGCTTTGGATCTTTTCTGTTGCTGTTTTGGTGAAACTTTGGGGCTGTAGCATTGTATATATTTTTTGAATGAAGATTCAACATGTTATATAAAGCAAGCTCAGAAATTGATGTGGATCCACCATATTGTTCACCAAAGTGTTGCATGTTTTTTAACATATGGGAATAAGGTATGTCTCTTAAATTGCACTAACGTATTTGTAAAAATCAGCTAATAGTGTGTTGTTTAGATTTTGTATGCAGTAAAGTTAACTTTAATGATCCTTCTGTTACTAATGAATGTTAAACTATAAGTCTGAGAATATAAAATACACATTAAGACAACATG

>LNC_000616

CAAAATATGAACAAAATTTTCCAGATTTGAAAGGTGAGGTACCTCTTCCGAGTGTGGATCTTCGCTAAGTTTCTGAGTGATTTGTCAATGTTTGTATTGTAGATGGCGGAGAAGTTGATAATGGGAAGATTTTTTTTCTGCTAGTGTATCGATGGAAAATAGCAGTGATTATAATTAGCTAATTAAGTGCAGCTTTGGATCTTTTCTGTTGCTGTTTTGGTGAAACTTTGGGGCTGTAGCATTGTATATATTTTTTGAATGAAGATTCAACATGTTATATAAAGCAAGGTACTCCTTCAACAAATTTGATAGAATTATTATAAATCATTGTTCATTATGACTACCTTTGAACTACTATATATTGGAAGAATGAAGATGAATTGCTTGGAGCTTTAGAATATCTGATTCCTATTCTGAATATATCAGCTCAGAAATTGATGTGGATCCACCATATTGTTCACCAAAGTGTTGCATGTTTTTTAACATATGGGAATAAGGTATGTCTCTTAAATTGCACTAACGTATTTGTAAAAATCAGCTAATAGTGTGTTGTTTAGATTTTGTATGCAGTAAAGTTAACTTTAATGATCCTTCTGTTACTAATGAATGTTAAACTATAAGTCTGAGAATATAAAATACACATTAAGACAACATG

>LNC_000617

CTGGAAAGAGACCAAAATATGAACAAAATTTTCCAGATTTGAAAGGTGAGGTACCTCTTCCGAGTGTGGATCTTCGCTAAGTTTCTGAGTGATTTGTCAATGTTGTGAGTGCTATTTCTTTCATCTCTTTAGTAAATCTTCTATTGTCTTTTCTACTATGAATGTCTTCTGCAAATATATGGTAAAGTTAATTTTAGAATTTCAACATTTGAACATGCAAATGATAACATAGCATAAAAATGACTTTCTTCAAGCAGGTTAAGATTAATATATATTGCATAAAACATTTGATAGGAGTTGTTTTCAGATCTAGTATCGTTCCGACAAAAATTCAATGTACTGTGAAATGTGTATTGTAGATGGCGGAGAAGTTGATAATGGGAAGATTTTTTTTCTGCTAGTGTATCGATGGAAAATAGCAGTGATTATAATTAGCTAATTAAGTTCCAATGTTTTTCCGGTGGTTTTTTGGGTGCAATTCTAACGTTGTTTTAGAGATGATTCTGATGCTGCTGTTTTGGTTTAATTCAAATGATGTTTTAATATTTTTATTGTGAAAATTTAGTGCAGCTTTGGATCTTTTCTGTTGCTGTTTTGGTGAAACTTTGGGGCTGTAGCATTGTATATATTTTTTGAATGAAGATTCAACATGTTATATAAAGCAAGCTCAGAAATTGATGTGGATCCACCATATTGTTCACCAAAGTGTTGCATGTTTTTTAACATATGGGAATAAGGTATGTCTCTTAAATTGCACTAACGTATTTGTAAAAATCAGCTAATAGTGTGTTGTTTAGATTTTGTATGCAGTAAAGTTAACTTTAATGATCCTTCTGTTACTAATGAATGTTAAACTATAAGTCTGAGAATATAAAATACACATTAAGACAACATG

>LNC_000618

CTGGAAAGAGACCAAAATATGAACAAAATTTTCCAGATTTGAAAGGTGAGGTACCTCTTCCGAGTGTGGATCTTCGCTAAGTTTCTGAGTGATTTGTCAATGTTGTGAGTGCTATTTCTTTCATCTCTTTAGTAAATCTTCTATTGTCTTTTCTACTATGAATGTCTTCTGCAAATATATGGTAAAGTTAATTTTAGAATTTCAACATTTGAACATGCAAATGATAACATAGCATAAAAATGACTTTCTTCAAGCAGGTTAAGATTAATATATATTGCATAAAACATTTGATAGGAGTTGTTTTCAGATCTAGTATCGTTCCGACAAAAATTCAATGTACTGTGAAATGATGGCGGAGAAGTTGATAATGGGAAGATTTTTTTTCTGCTAGTGTATCGATGGAAAATAGCAGTGATTATAATTAGCTAATTAAGTTCCAATGTTTTTCCGGTGGTTTTTTGGGTGCAATTCTAACGTTGTTTTAGAGATGATTCTGATGCTGCTGTTTTGGTTTAATTCAAATGATGTTTTAATATTTTTATTGTGAAAATTTAGTGCAGCTTTGGATCTTTTCTGTTGCTGTTTTGGTGAAACTTTGGGGCTGTAGCATTGTATATATTTTTTGAATGAAGATTCAACATGTTATATAAAGCAAGCTCAGAAATTGATGTGGATCCACCATATTGTTCACCAAAGTGTTGCATGTTTTTTAACATATGGGAATAAGGTATGTCTCTTAAATTGCACTAACGTATTTGTAAAAATCAGCTAATAGTGTGTTGTTTAGATTTTGTATGCAGTAAAGTTAACTTTAATGATCCTTCTGTTACTAATGAATGTTAAACTATAAGTCTGAGAATATAAAATACACATTAAGACAACATG

>LNC_000619

CAAAATATGAACAAAATTTTCCAGATTTGAAAGGTGAGGTACCTCTTCCGAGTGTGGATCTTCGCTAAGTTTCTGAGTGATTTGTCAATGTTTGTATTGTAGATGGCGGAGAAGTTGATAATGGGAAGATTTTTTTTCTGCTAGTGTATCGATGGAAAATAGCAGTGATTATAATTAGCTAATTAAGATCTACCATCATCTCTTCTTCGATATAAAGGATATCCTTCTTCATCAAGTGAGGTAGATGGTAAAAATCTTTTTGGAAAGTGCTTTGTACATTTGCCTTTTTGCATGCAAGGGGATGATTTCCTTATTGAACCACATGGCCCATGCATCATGAAGTTTGTAACAACCACATAATAATCCGGATCAAGTAATTTATCAGGCAATTCAGCTGAAATAATCGCATCAATGTCTCCAACATTTGGAAATTTATCATGGAGAAAAAGTAAGATATGTGCATGAGGCAATCCACGCTTTTGAAATTCAATAGTGTAAATCACTATATGAAGAGAAAACAAAATATTATTTGTAATTTATGATTTTATTTAAATAAAACAAATTATATTAACTTGAAAATGTAATATAATAAAATAAAGCAAAATAAAGTTCATACCCGCTTTTACTTCTCCAAAAACTTTATTGTCTCTTAAATCTTTTATCAAATGATCCAATTTGATTTTAAAGACTCTTGTTATAATGTCTGGGCGATCTTCTGGATGCAATCCCCTACTATGCACAAATCTCTTAATTTCAGGCCATTTGGGATTGCATGTAAATGTAATGAATAAATCAGGATATCCAGCCCATTTACATATTGCCATTGCATCTTGATAATTTTGCAACATTTAACGTGTTCCACCAGTGAAAGTAGAAGACAATATAATTCTTTGTCCTTGAGAAGATGGTTCTGTGTCTCCATGCAAAACTGCTTCTTGTAATCCTTTATATAAATGTGTTCTCAAATTTCTTTGATTTAGTCTATAAAACAATAAGCGAGAAGATTCAATCATTGTATATCCATCAACCAAGAATTGTTGAAATAATCTTTTAGCTGAAACAATTGTAGGTACTTCATTCTTCCTTTCTTGAATTTTATATGCAAAATATTCGCAAGAACTAACACGTTTTCTTCCCTTACTTGATTCATCAATTCTATTTAAAGGAATATCTTCTCTATAACCATCTTCCCCATAAGGTAGTAGTAATGGATATTGCAAACCTAAATATGCTGCATTAAGCTCATTTATTCTCTGAAGTTGTCCATTTTGACTTTCGATTATTATATCTCTGTCAGTATTTGTTGGTTCAAAATCGCCAACAACCAAAGCTGCTACCTCTGAAACTGATGGCAAGTTATATCCTCTTCCATCTGATCCTCTTCTTCCGATAAGTCTTAGTTTAACATTTGTACTTTGTTCAGCTTGAAATCTATCTCTAACCATACGAAAACTCTTTGCAAGAACATTATGTTTATCAAGCATTTCCTTCAGTTCGGTGACAATTTGAGCATGGATTTGATTGTTGCTTTCACCGCTACTACAAAGTGAATAGTAATATGTTGATAATAGTGTTTAATTACTACAGTTATAATTAATCTTACATAAAGCATAAATAAAATAAGTACACACCTGATAACATTCATTCTATTTGTTACCTCATTTTCTGTATCATAAATATATAATTGTGCAAAATTTTGGAGTAGATCCCTCATTAGGCAATAAGCTTCCAATTTTATGATAATTCTGTCCAGATAATCTGAAAGTTCTTGGCCCTTTTGTTTGATTGACAGATGCATCAATTTTTCCTCCCATTGATGTAAAAGAAAACATGGAATTATAAGTTCGGATATTCTCTCTGAAATGGTTACTTTTTTCACCTACAATAAATTATAGAAATGTAAAGTTGATAAACAAAATTATTGACGTGTATTTATATTTGTATTAAATAAATTAATTTACCTGATCCAAATAATAATTCCTTCAACATAGTAGG

>LNC_000620

GGTGATTTCTCAATTTCTTCTTCACTGATTTATTCTTTAGTCATGCTCTCTATTTTCTCTCTCCCGTGATATCTATATTCAACTACATTTAGTTCCTATGGATTTGGTGTTGTTTCAATTGCATAAATTAGGATTTTGATTTTGGTCTAATATCCGTGAGAATCTTTTAGGAAGACAAAGATTATTGTGAAACAGAAATGAGAATCTAAAATGTCGTATTTTTATGAAATTTCCTGAGTATTTGAATCTTTTCTTAACTTATTTTTCACATGCATATCTTTATTGACTGTTTTTGTGTCAATTTTATAGTCGTCAATGGCAATAAGAAAAACTCAAAGAGAATAGAGGAAGGCTATGTCAACTACATTCCTAAAGAGATATTCTTCTTCTTGCGGTTTCAATTATAATTCAACTGGAGGATCTTTAGGAGTTGCCATGGTTAAGACAATGGTTGTCAAAATATAGTGACAGGTTTCTACTCAATCAAGGTCTATCGAAGGTACAGATGCAGAGTTAAACTAGCCCTTTTGTGAGTTTGTGGCCTGTTCTCTACATCTTGCAAAATGGACAGTGTGAGATATCTCAACCATGTTGTAGATAGGAGAATCAAAGCCATTGATTTAATATGCTTCGACAGAGACAATGGTTTGAGAGATGGTTCAGTGTCCCTTAGGGAGCATGGCATATAATGCCACTTTGCGGCATATGTTGCATGTCATAAGAGAAGAACTTCAAGAGGTTTCCATTTACATGCGTTTGAGATTTCAAATGGAGATTGGTTTGAAATGGCTCAAAACAGACTTAGTATAAGTTTAGAAGATATTTTGTTTTCCACAATTGTTCGTCTTCAAGCTTCTCCCAATACTCATTTTCATCTTCAATCTTCTCCAAGTACGCTGCAACTTTGAGAACATTGTTTCAAGATTGGAAATGCAATTTCTTTGAAGTGGTAGAAGTGATTATTTGAGCATTTAATTTCATTATTTATAAGGTAAAAAACTTGCTCCAATTTGCAATTCTAATTTCTTTTTCTTTTTATGTTTGTGCATTGTTGCCATCAATTGTAACTTCATTTAACAGTATAGAAAAGTCAACTGATGATAAGCTTGATTTGTGTTACAAACTTCATCTTCTCGAGGATACAAACACAACTATCTACACAGTTTAGTTTTTGAAGCTGCTTTAGAGACGACAATAAATCAATAACTACTCCAAATTTAAGAAATTGTACTAATTAAGGACAGTTGTAGTACAAATAAATATGAATATTACTAACTTATTGTTCATGAAGCTAAGTAATAGCTTAATAGTGGCATGGGAATAAAAAAATTTGTTTTCTATTTTCCCCCCCTCTCGTTATTATTAACTAGTAATTGGAATTATGTATATTTATTCTACATATATAGTGATGATTACACTTTGCATAAGATTGGATGATTTTTTGCTTTTATGGCCAACTTGAGTTAATTGTGGTGCCAAATCGGTAAAGAAATTAAAACTTGCTTTCAAAACAACCCATTACTAAAATTAGTTTATATGGCCCCAAGCGAACCTTTGCCCTGACTCAACTCTCAATGGAAGACTTACTCAACAATAAAGGAAACTCA

>LNC_000621

TTTTGTTTCTCCTATTTTGTTCTCCATTCTCTTTCACCACTCAGAGCCAATCGTCAATGGCAATAAGAAAAACTCAAAGAGAATAGAGGAAGGCTATGTCAACTACATTCCTAAAGGTACAGATGCAGAGTTAAACTAGCCCTTTTGTGAGTTTGTGGCCTGTTCTCTACATCTTGCAAAATGGACAGTGTGAGATATCTCAACCATGTTGTAGATAGGAGAATCAAAGCCATTGATTTAATATGCTTCGACAGAGACAATGGTTTGAGAGATGGTTCAGTGTCCCTTAGGGAGCATGGCATATAATGCCACTTTGCGGCATATGTTGCATGTCATAAGAGAAGAACTTCAAGAGGTTTCCATTTACATGCGTTTGAGATTTCAAATGGAGATTGGTTTGAAATGGCTCAAAACAGACTTAGTATAAGTTTAGAAGATATTTTGTTTTCCACAATTGTTCGTCTTCAAGCTTCTCCCAATACTCATTTTCATCTTCAATCTTCTCCAAGTACGCTGCAACTTTGAGAACATTGTTTCAAGATTGGAAATGCAATTTCTTTGAAGTGGTAGAAGTGATTATTTGAGCATTTAATTTCATTATTTATAAGGTAAAAAACTTGCTCCAATTTGCAATTCTAATTTCTTTTTCTTTTTATGTTTGTGCATTGTTGCCATCAATTGTAACTTCATTTAACAGTATAGAAAAGTCAACTGATGATAAGCTTGATTTGTGTTACAAACTTCATCTTCTCGAGGATACAAACACAACTATCTACACAGTTTAGTTTTTGAAGCTGCTTTAGAGACGACAATAAATCAATAACTACTCCAAATTTAAGAAATTGTACTAATTAAGGACAGTTGTAGTACAAATAAATATGAATATTACTAACTTATTGTTCATGAAGCTAAGTAATAGCTTAATAGTGGCATGGGAATAAAAAAATTTGTTTTCTATTTTCCCCCCCTCTCGTTATTATTAACTAGTAATTGGAATTATGTATATTTATTCTACATATATAGTGATGATTACACTTTGCATAAGATTGGATGATTTTTTGCTTTTATGGCCAACTTGAGTTAATTGTGGTGCCAAATCGGTAAAGAAATTAAAACTTGCTTTCAAAACAACCCATTACTAAAATTAGTTTATATGGCCCCAAGCGAACCTTTGCCCTGACTCAACTCTCAATGGAAGACTTACTCAACAATAAAGGAAACTCA

>LNC_000622

GGTGATTTCTCAATTTCTTCTTCACTGATTTATTCTTTAGTCATGCTCTCTATTTTCTCTCTCCCGTGATATCTATATTCAACTACATTTAGTTCCTATGGATTTGGTGTTGTTTCAATTGCATAAATTAGGATTTTGATTTTGGTCTAATATCCGTGAGAATCTTTTAGGAAGACAAAGATTATTGTGAAACAGAAATGAGAATCTAAAATGTCGTATTTTTATGAAATTTCCTGAGTATTTGAATCTTTTCTTAACTTATTTTTCACATGCATATCTTTATTGACTGTTTTTGTGTCAATTTTATAGTCGTCAATGGCAATAAGAAAAACTCAAAGAGAATAGAGGAAGGCTATGTCAACTACATTCCTAAAGAGATATTCTTCTTCTTGCGGTTTCAATTATAATTCAACTGGAGGATCTTTAGGAGTTGCCATGGTTAAGACAATGGTTGTCAAAATATAGTGACAGGTTTCTACTCAATCAAGGTCTATCGAAGGTACAGATGCAGAGTTAAACTAGCCCTTTTGTGAGTTTGTGGCCTGTTCTCTACATCTTGCAAAATGGACAGTGTGAGATATCTCAACCATGTTGTAGATAGGAGAATCAAAGCCATTGATTTAATATGCTTCGACAGAGGTACTACATATAATTGTTGGTATAGTTATATAACCCCCACAATAGTTGCCAACTATTTACGCCTCTTGCTGGTTGACATTCTTCAGTTTTCTTCTTTCCCTTTTGTGTTATCTCCATTGCAACATATGTACAACAAGAGTTTTTTACAGGTTTTGAAATTTGCATTGATTACTTCGCATAAATTTTCACCTCATTTCAAACTTAAAATTAATTAATACCAGAATTTTCTATCGGTACTAAAAGCGAGTTTGAAAAGAAAATGTAATTGTTTTGATTTAATTTTTTTCTGCAGAACATTTTGTTATCCACTTTTAGGGAATAATGATCACTCCAGTTGACTTATGAATGTGGATCAAGTTTCAATACCTACACCCATGCAAATTCATGTTAAAATAATAGTTACATTATGTGTCTGAATTATTTTCCTGGTCTTTCATCTAATTTGGTAAAGTTTGTCTTTTTTTGATACGATACTTGAAGAAGTTAGCTACTCAAACATGAGTTACTGTGACTTGTTCGATATGAGCTAAACTTCAAGGTTTAAAATCTTAGCTTTTGTTGTGATGCAAGAGCTGAGGCCAAATTTTGAGGATTGTGGATACGTTTCTAAGGTAAACACCACTGAAATGTCACCTAAACTGTCATGCACAAGTATAATCTCTGCATGACGATCTTGTGTTACTGTGTCATATCGTGACTATTACTGTGTTAAGAGTTCTTAATTTCCTGGACATTCTCTTTTAGTTTCATGTCATGTTTGATTGTTTGTAGTGCATCCAAGATCATTATTTGTATGCATAGTAATTGAATAGAAAGTTCCTGTCAAATCTTTAAAGTTGCATTTTGTACATTCATGTCTGATGCTGATTTAAATCCTTCTAACGATACAATAACATATTATGAGTTATGAAGGTAGAGAGGCTATTTCCGATAAGACAATCCTTCTAATGATATATTTAGAAAATTTAATTATCTTTTATTAAATTTTAACTATTGAAAAATGCTAAAACTGTTTGAGTGATTTACTTGCATCAAATACTATAAGTGCACACATGCTTCATTTGCTACGTAGTGCCGACACATTTATGATCAAATTTAATCAGCAACATTCCACTTTAACCTCACAAACTCCACTAATAAGTACCACCACATCTTTTCCTTCAATCAACCATAAACTACCAAAAACAGAAAAAAGCTCCCATCCATAGGTCACCAACAACCACCACACAAACCCTACATCTGAGGCCTTGAATCAATTTGCACAGTCGGTGGCTCGAGATTTTGGTTAGCTTTTTTAGTTCCCGTTTAAGATTTCATCAGTATTGTCACTCCTGTTTCGCTCAATGGTCTTTTAATTGTATAAATTTTTATCTAAGTGATCAAATGAGGTCCATTTCTTTCACTTCTCTGTGAGATACTCACCCACTTCACTCATTTCATTTCCTATAAATGGATCAACTTGCTTACTATTTGTTATATGGTCTGCTTCTATTGATCTTTTTCTCAAGCATACATCCCTTTTGTTTTAATTGTGCCTTGGGTTAGTTGCTTCTCTATGCTTTACCTTCTCTGCTGTGTTTTATTTAATCTTGTGATGTGTTTTTTTTTTTAAATCCTCGATTATTAATATAAGTCCTACAAGCTTACTTTTCCACGTACGATCAAATGACATCTAACGAATGGTGTGATTCAAATTATGTGTGGTGATCCTATATTGCATAATTATTCAATAGCATTTGAAATACCACATGCGTTATCTTGACTCTTATTGTTCTTGTAAAAACACCATCAGACAATGGTTTGAGAGATGGTTCAGTGTCCCTTAGGGAGCATGGCATATAATGCCACTTTGCGGCATATGTTGCATGTCATAAGAGAAGAACTTCAAGAGGTTTCCATTTACATGCGTTTGAGATTTCAAATGGAGATTGGTTTGAAATGGCTCAAAACAGACTTAGTATAAGTTTAGAAGATATTTTGTTTTCCACAATTGTTCGTCTTCAAGCTTCTCCCAATACTCATTTTCATCTTCAATCTTCTCCAAGTACGCTGCAACTTTGAGGTAATTTTTTGTTGTCCTTCTGAATTCCTCTTCAATTTCTTTTTTAGATTTGATTCATGAAATTCTAATTTTCATATGTTATTTTTCTTGATTTTCTTGTGGGCTTCAATTATGAGTTGATGTAGTTTGATATCTGAGAAGAATCCTAAATGACATAATGAATATTCAAAGACCCATTAAAAAACAAACGGAATAGGACCAAAATACCCTGAACTATGCGAAAAGGTCTAAAAAACCCTTCATCCACAAAAATATCTTTCTAATCATCTTTTTTGTCTAAAAATATCATTTCATTCACCTTTTTGGCTCGCTCATACCTTGAAACTAGCAACCCTTTCTTTTTTTTTCTTTTTTTAAATGTTATTATGTTTCATTTTCTTATTAGATGAAATAAATTCCCCATCTCTATTAGATTTTTTC

>LNC_000623

GGTGATTTCTCAATTTCTTCTTCACTGATTTATTCTTTAGTCATGCTCTCTATTTTCTCTCTCCCGTGATATCTATATTCAACTACATTTAGTTCCTATGGATTTGGTGTTGTTTCAATTGCATAAATTAGGATTTTGATTTTGGTCTAATATCCGTGAGAATCTTTTAGGAAGACAAAGATTATTGTGAAACAGAAATGAGAATCTAAAATGTCGTATTTTTATGAAATTTCCTGAGTATTTGAATCTTTTCTTAACTTATTTTTCACATGCATATCTTTATTGACTGTTTTTGTGTCAATTTTATAGTCGTCAATGGCAATAAGAAAAACTCAAAGAGAATAGAGGAAGGCTATGTCAACTACATTCCTAAAGGTACAGATGCAGAGTTAAACTAGCCCTTTTGTGAGTTTGTGGCCTGTTCTCTACATCTTGCAAAATGGACAGTGTGAGATATCTCAACCATGTTGTAGATAGGAGAATCAAAGCCATTGATTTAATATGCTTCGACAGAGGTACTACATATAATTGTTGGTATAGTTATATAACCCCCACAATAGTTGCCAACTATTTACGCCTCTTGCTGGTTGACATTCTTCAGTTTTCTTCTTTCCCTTTTGTGTTATCTCCATTGCAACATATGTACAACAAGAGTTTTTTACAGGTTTTGAAATTTGCATTGATTACTTCGCATAAATTTTCACCTCATTTCAAACTTAAAATTAATTAATACCAGAATTTTCTATCGGTACTAAAAGCGAGTTTGAAAAGAAAATGTAATTGTTTTGATTTAATTTTTTTCTGCAGAACATTTTGTTATCCACTTTTAGGGAATAATGATCACTCCAGTTGACTTATGAATGTGGATCAAGTTTCAATACCTACACCCATGCAAATTCATGTTAAAATAATAGTTACATTATGTGTCTGAATTATTTTCCTGGTCTTTCATCTAATTTGGTAAAGTTTGTCTTTTTTTGATACGATACTTGAAGAAGTTAGCTACTCAAACATGAGTTACTGTGACTTGTTCGATATGAGCTAAACTTCAAGGTTTAAAATCTTAGCTTTTGTTGTGATGCAAGAGCTGAGGCCAAATTTTGAGGATTGTGGATACGTTTCTAAGGTAAACACCACTGAAATGTCACCTAAACTGTCATGCACAAGTATAATCTCTGCATGACGATCTTGTGTTACTGTGTCATATCGTGACTATTACTGTGTTAAGAGTTCTTAATTTCCTGGACATTCTCTTTTAGTTTCATGTCATGTTTGATTGTTTGTAGTGCATCCAAGATCATTATTTGTATGCATAGTAATTGAATAGAAAGTTCCTGTCAAATCTTTAAAGTTGCATTTTGTACATTCATGTCTGATGCTGATTTAAATCCTTCTAACGATACAATAACATATTATGAGTTATGAAGGTAGAGAGGCTATTTCCGATAAGACAATCCTTCTAATGATATATTTAGAAAATTTAATTATCTTTTATTAAATTTTAACTATTGAAAAATGCTAAAACTGTTTGAGTGATTTACTTGCATCAAATACTATAAGTGCACACATGCTTCATTTGCTACGTAGTGCCGACACATTTATGATCAAATTTAATCAGCAACATTCCACTTTAACCTCACAAACTCCACTAATAAGTACCACCACATCTTTTCCTTCAATCAACCATAAACTACCAAAAACAGAAAAAAGCTCCCATCCATAGGTCACCAACAACCACCACACAAACCCTACATCTGAGGCCTTGAATCAATTTGCACAGTCGGTGGCTCGAGATTTTGGTTAGCTTTTTTAGTTCCCGTTTAAGATTTCATCAGTATTGTCACTCCTGTTTCGCTCAATGGTCTTTTAATTGTATAAATTTTTATCTAAGTGATCAAATGAGGTCCATTTCTTTCACTTCTCTGTGAGATACTCACCCACTTCACTCATTTCATTTCCTATAAATGGATCAACTTGCTTACTATTTGTTATATGGTCTGCTTCTATTGATCTTTTTCTCAAGCATACATCCCTTTTGTTTTAATTGTGCCTTGGGTTAGTTGCTTCTCTATGCTTTACCTTCTCTGCTGTGTTTTATTTAATCTTGTGATGTGTTTTTTTTTTTAAATCCTCGATTATTAATATAAGTCCTACAAGCTTACTTTTCCACGTACGATCAAATGACATCTAACGAATGGTGTGATTCAAATTATGTGTGGTGATCCTATATTGCATAATTATTCAATAGCATTTGAAATACCACATGCGTTATCTTGACTCTTATTGTTCTTGTAAAAACACCATCAGACAATGGTTTGAGAGATGGTTCAGTGTCCCTTAGGGAGCATGGCATATAATGCCACTTTGCGGCATATGTTGCATGTCATAAGAGAAGAACTTCAAGAGGTTTCCATTTACATGCGTTTGAGATTTCAAATGGAGATTGGTTTGAAATGGCTCAAAACAGACTTAGTATAAGTTTAGAAGATATTTTGTTTTCCACAATTGTTCGTCTTCAAGCTTCTCCCAATACTCATTTTCATCTTCAATCTTCTCCAAGTACGCTGCAACTTTGAGGTAATTTTTTGTTGTCCTTCTGAATTCCTCTTCAATTTCTTTTTTAGATTTGATTCATGAAATTCTAATTTTCATATGTTATTTTTCTTGATTTTCTTGTGGGCTTCAATTATGAGTTGATGTAGTTTGATATCTGAGAAGAATCCTAAATGACATAATGAATATTCAAAGACCCATTAAAAAACAAACGGAATAGGACCAAAATACCCTGAACTATGCGAAAAGGTCTAAAAAACCCTTCATCCACAAAAATATCTTTCTAATCATCTTTTTTGTCTAAAAATATCATTTCATTCACCTTTTTGGCTCGCTCATACCTTGAAACTAGCAACCCTTTCTTTTTTTTTCTTTTTTTAAATGTTATTATGTTTCATTTTCTTATTAGATGAAATAAATTCCCCATCTCTATTAGATTTTTTC

>LNC_000624

CCATTCTCTTTCACCACTCAGAGCCAATCGTCAATGGCAATAAGAAAAACTCAAAGAGAATAGAGGAAGGCTATGTCAACTACATTCCTAAAGAGATATTCTTCTTCTTGCGGTTTCAATTATAATTCAACTGGAGGATCTTTAGGAGTTGCCATGGTTAAGACAATGGTTGTCAAAATATAGTGACAGGTTTCTACTCAATCAAGGTCTATCGAAGGTACAGATGCAGAGTTAAACTAGCCCTTTTGTGAGTTTGTGGCCTGTTCTCTACATCTTGCAAAATGGACAGTGTGAGATATCTCAACCATGTTGTAGATAGGAGAATCAAAGCCATTGATTTAATATGCTTCGACAGAGGTACTACATATAATTGTTGGTATAGTTATATAACCCCCACAATAGTTGCCAACTATTTACGCCTCTTGCTGGTTGACATTCTTCAGTTTTCTTCTTTCCCTTTTGTGTTATCTCCATTGCAACATATGTACAACAAGAGTTTTTTACAGGTTTTGAAATTTGCATTGATTACTTCGCATAAATTTTCACCTCATTTCAAACTTAAAATTAATTAATACCAGAATTTTCTATCGGTACTAAAAGCGAGTTTGAAAAGAAAATGTAATTGTTTTGATTTAATTTTTTTCTGCAGAACATTTTGTTATCCACTTTTAGGGAATAATGATCACTCCAGTTGACTTATGAATGTGGATCAAGTTTCAATACCTACACCCATGCAAATTCATGTTAAAATAATAGTTACATTATGTGTCTGAATTATTTTCCTGGTCTTTCATCTAATTTGGTAAAGTTTGTCTTTTTTTGATACGATACTTGAAGAAGTTAGCTACTCAAACATGAGTTACTGTGACTTGTTCGATATGAGCTAAACTTCAAGGTTTAAAATCTTAGCTTTTGTTGTGATGCAAGAGCTGAGGCCAAATTTTGAGGATTGTGGATACGTTTCTAAGGTAAACACCACTGAAATGTCACCTAAACTGTCATGCACAAGTATAATCTCTGCATGACGATCTTGTGTTACTGTGTCATATCGTGACTATTACTGTGTTAAGAGTTCTTAATTTCCTGGACATTCTCTTTTAGTTTCATGTCATGTTTGATTGTTTGTAGTGCATCCAAGATCATTATTTGTATGCATAGTAATTGAATAGAAAGTTCCTGTCAAATCTTTAAAGTTGCATTTTGTACATTCATGTCTGATGCTGATTTAAATCCTTCTAACGATACAATAACATATTATGAGTTATGAAGGTAGAGAGGCTATTTCCGATAAGACAATCCTTCTAATGATATATTTAGAAAATTTAATTATCTTTTATTAAATTTTAACTATTGAAAAATGCTAAAACTGTTTGAGTGATTTACTTGCATCAAATACTATAAGTGCACACATGCTTCATTTGCTACGTAGTGCCGACACATTTATGATCAAATTTAATCAGCAACATTCCACTTTAACCTCACAAACTCCACTAATAAGTACCACCACATCTTTTCCTTCAATCAACCATAAACTACCAAAAACAGAAAAAAGCTCCCATCCATAGGTCACCAACAACCACCACACAAACCCTACATCTGAGGCCTTGAATCAATTTGCACAGTCGGTGGCTCGAGATTTTGGTTAGCTTTTTTAGTTCCCGTTTAAGATTTCATCAGTATTGTCACTCCTGTTTCGCTCAATGGTCTTTTAATTGTATAAATTTTTATCTAAGTGATCAAATGAGGTCCATTTCTTTCACTTCTCTGTGAGATACTCACCCACTTCACTCATTTCATTTCCTATAAATGGATCAACTTGCTTACTATTTGTTATATGGTCTGCTTCTATTGATCTTTTTCTCAAGCATACATCCCTTTTGTTTTAATTGTGCCTTGGGTTAGTTGCTTCTCTATGCTTTACCTTCTCTGCTGTGTTTTATTTAATCTTGTGATGTGTTTTTTTTTTTAAATCCTCGATTATTAATATAAGTCCTACAAGCTTACTTTTCCACGTACGATCAAATGACATCTAACGAATGGTGTGATTCAAATTA

>LNC_000625

ATTCTTTTTAAACTAAACAAAACACATGCATTGGTGAAAATTATTCCTAGGAGGTATTATCATCTTGAAATATGTCTCATTTATCAAGTTATTATCATCTTGATATAAATATGCATTTCATTAGTTAAAGCTTCTCGTTTTGAACTGGAAACCGGTTGTGTGATTTAACGGATCAAAAGGAAGAAAAACAGCAGTTGTGTGATTTATCTGGGAAACTGTTTCTGATTTTCTGCGTGAATACCTTTTCTTTGATGATGGAAGGGAAAGTGTTCTGCTTGTTTAGCA

>LNC_000626

GAAAAAAATAGAAAAAGAAAACGAACGCTAGCTGCTGCGTTTCTTTTCCAGCGAAGGAGCAAGGAAGAGAATAAAGAGTTGAAGGGTTAGACTGTGAGGGGTGTAGGATGAGAAAGAGGTTTGAGTTTGACCAAAGGAATTCATACGGACCATTCGAGTGACGAGCTTGGTATTTCAACACGAGTACTGTGAGTAGTAATTTTACCATAATTTGTGTTTTATAACTTTCATGATTTATACATGGTTTATAAAGTGAATGATTTACCGAAATGCTCTGAACTTGCATATGAGACAAGTCTTTTACTTGATATGCTATCAAATGAGTTATTTGAGATGAACTCATTTTTCTAAGGCATGTTTATAGATACTTGAGATATGATTACCGTTATCGAAAGAGTTTAACATTTACTTGAGAAATAGTATTTTGATATATGTTTTTTATTAAAGTTTTATATACTATGATTTGATAAGAAATTAAATGTCTTATAACATTTTTAAATGTTTCTATTGATAAATGGGAGTAGACCTTGATGGATCCCGTTGCTAACGGCAGGTTCATTAGACTTGGTGAACCTAGTAGTTACAGACTACCGATAAAGCTCTTGCTAGTGGGAAGGTAGAACTATGATACCGTAACCAATATCCCTTGAGTAGGGATCTAGCGATATGAATATTTAACTATTTTATGAGGAGTTCACCATTATTGGCTACTTCTTAAAGAGAAGGTTACACTAATTACATGATTCATCCTTGGAAACCTCCTAGCTATAAGATTGATATGAAAGTATTTTTTTAAGTTTATTTCTAAAAGTTCACATTGATTTTGTTTACATGAAACAAATAAGATTGAATATTGTTACCGTTTCTGAAAGGGCATCGATTTCATCATTTTGATTATTAAACTAGCATGTTTTTTTACTTTTCCCTAATTAAAATAGTTGTGGTTAAGTATTATTACTCACTGAGCTAGCGTCTCACTCTTTGCTAAATGTTTTTACAAAGAGCTTAGGTGACATTGGAGAAGATCTCATTAATTAGAGAGTGTGGACTGATAGTATCTGGTGAGCCTCGCATTGCTTCGCGCGGCAAGAGACATATTGAATTGCTATGATTTTTTTTTTTTAAAAAAACACTCTAGAGTCGCTCCATTTGTCATTCCCTTTATTTGAGTCATGATTATTCCTTCGTATTGTAGTCCAATGACTAGTTTTGAACTTATTTTGTTGTATTTTGGGATGAATTGGTATTATTGCTTGGCAATTAGATCATCGATGAATATTCTGTCTTGGCCTATAGTTCTTATTAGTTGTGTTGTGGATTTCTGAGGTAGGGAAAATTCAGGATAGGCCTAGAAACAAAGGAAACTCTGTCCGATTTTCTATAGAATTCTGGTAAGGCTTGCTTTGGAATTCTCCCCCTTAGCGCCGGTCACGTTCCTTAATTGGGTCTTGACAAAGTTAGTATCAGAGCTTAGGTTATTCCCTTGACTTATGGAGCATACGTTAGTAGTAGAATCTCAATTATGGTTACGTTGTTGTCCATTTCTATAATCGTGATTCTGCAAAGACGTGATAGGATGTGTCTGTCTTTCTATCTCATTCCTCCTTATGTGTGCGACTCATAAGGTCAACTATATGAATCTAACATTGTTTATCTTTCTATAACACATTTTGAATTTAGTCACCCAATAACAAGAATAGTCCAAAGGTTTGGAGTCTTCAATGGTCACCGTGTAAACATCTATCTCTTCCAATTAAGGGGAGCTTACTGAAGTAGTTGAAAAGTCCAAGAAAGAGGTCGTTATGCCACTTTGAGGGATTTTGTGCACATATGTGAAATGTAATATATATCTCGTCTGGAAAGAGCACTACTCTATCACTTTAACAATATGAGAATGTTACTGTTAAGACACGTATGTTCCTGTAATGATTTAGAGATATTAATGGACATAATGAATGGAGTCAGGCACCAAATAGGTCCCAAGACCATCTTCAAGCAACTACAAAGATTCAAACTTTCAAAAAAAGTATGCTTCGGAGTTTCTAAAGATGAAACCTCCTAGATTCGTAGGTTCTAACAACTCAAAAATCCTTAGAGATTTTCGGTAAAGATTCTTGGGGTGAAATACTTAAAGCTTTATGAGCTCTAGCATGTTCCAACAAGAGTACTGTAAATTTGGCAACGTTCCGACGATGCATGACTAAATACATGACATGATATTATACTAATCAAGATACTAGTAGGGGAACACCAACAAATTGAGGCGATTCCCTAAGTTATTTATGATTTTTTTCCTTCTAGACCGTTAAAAGTGAGTAACTTGATAATAGGCTTAAGAAATTAGTTCAAAAAGATGGATTGAATTGATATATTACTCTAGATTTTTCGATTGACTAGAGTTTTTTAAGATTTAGAGATTTGTTGGAAGGTTGGTTAGCCCCTTTAAAAACCTTTTATATGAAGACGTTAGTGTACTTTGAGGTAGTTGACATTGCTAAAATGATTGGAAATATATAACGTGATTAGTGAACGATCAAACATTCATTCAAAAGACCAAAACTAAGAACCTGCTATGGGAATCTTACTAAAGAGTTCAAAATTATGAATTTTACTGGTTCCTACTCCCGTGGGATATTTTTCTTTTGTTGGAATTTTACTAAAGAGTTGAAAATTATGAATCTTTCAGTGCCAATGTTTAAATTATATCCAAGTCTATTCTCATAGCTATAATGAACACAAGGATCATCTCTGGATGGTATTTAAAAAATGACAAGAATATCAACTTTATGTCGAGTTTTGTAATTGTAAATTAGTGTATCAACGAATTGAAATTATTATGTGTACTGAAAAGATAGAATCACTTGACATTGTACTATAAATGTTTGGTGGAGTAGTTCTAAAGAATTGTTGCGTTACTGACCAAGTTATAAAAAGAAAAGAAACAAATTTCAGTAGATGTTGGAATGTGAGTAGAGCTTTATAAGCTCAAGACATATTGACCATCATACCAGTGATAGTGCTATTCTTAGCATTGGAGCATTCATGGTGCTTTGTGATGCTTTAAGGATGGAATTAGAATATGTTATTATATAAAATGGTCGTGTAATAGCTTATGTTTTGAGGAAACTAAATTGGTAGGAGCAGAACTATTCCACTCATAATTTGAAGATAATGGTAGAGGTTTAGATGTGCTATTTATACCATGAAACCGATGAGATTTTTATAGACTACAAGAGCTTTGTATTTATGTTCAAGTAGAAAGATTTGATTCTTCAATCTACACCACCAGAGGAAACTACTCGATGACTGTTAAATTCACTATTCAGTGTCATCTGGAAAATGACCACGTTAAGGCAGATGCATTGAGAAGCAAATCCATGAGCTAATTGACATATAACTCTTGCAAATATACTTTTCACCTAAGATATGCATACCCATTGAGGTCACCTAATACAAGGATGGAAATTTTGTGAATACTGAAATAAGGTTCTTGTGAGTAAAGACAAGAATATGAGTATTAACAATATGTTGCTTTTGGAGTAAGTGGTTGATTGTGATAATCGAACATAGATGGTGAGAGAGAGATTATTCTTGAGGAACCCATAACTTTAAATACATTGTAGATTCTGGATCCACTAAGGCATATCATTACCTGAAGTGATTGTAATGGTGAGAATATATAAACAAATATATTGCTTACTTTACTTCTAGTTGGTTGGCCTGTGAACTTGTTAAAGATTAGTATAAGCAACATACAAGACTATTACTGTAGTTTGAGGTTCAGGAGGTAAATTGGAAAGAGCTACTATTGATTTTATGACCGGGTTATAACCACAAACACTTAGAGGTTATGACCCTATGTGGGTATTTGTAGATCGAATTACAAAGTCAACACACTTCTTGCTGTTCAAAACTAGATAAATGGAGAAACAACTATAGAAATTTACATGACCAAAATTATTTGACTCCATGAGTTTCAGTGTCCATCATATTTGACAGAGTATTACTGTCTACTTCCTGTTGTTGTGAATTTTTCCATGAAATATTGGGTACATAAATAGATTTTATCACTACATTTGATTCGTAAACAAACGGGCAGTCTGAGCGTACTGTACAAATATTAGAGGGTGTGTTATGAGCTTGTATTCTTGATTTTAAGTGTAGTTGGGATATTTACTTACCAATAGCCGATGTTGCTACAATAATAACTTTTAGTCAAGCTTTTAGAGGTCTCTATAAGAAGCCTAGTATGCTAGAAGATGTCATTCTCCATCAAATGGTTCAAAGTTGACGTGGCTAATATATTTGAATTTGGGACTACCTAAACTTGTTACAGGAAGCTAGGGACAAGGTTCAATTAATCAGACAAAAGTTGCTCACAATTCAAGTAGGTGGAAGTCTTATACATATAAGAGGAAATGGAATTTAGAATGCATAGTTGGGGACCAAATCTTCCTAAGAGTCCCTTAGCAAATGAAGTGAAGTGATTCGAAAAAAAAAGAGAGGAGGAAAATTGAGCTCAAAATTTATAAGATCATATAAAAACTTCTTGGCCCTATGAGAGAGTGGTGTTGTGTCATTTAGCATTTCCTTTTGTGATGTATTTTACTTGCCCAGTATGTCGTGTGTGCTAAGAAAATGTATGTCAGACTCTTCTCATGTGCTTGAAGCACAAACTATACAACTTGAGGAGAAGTGGACTTATGCCGAAGAACCAGTGGCTACTATTGACAAACAAGTAAAAAAGGTTAGGATCAAAATTTATCCGTAAAAATCTTGTAGAAAAACCACACAATTAAAGAAGTTACTTGAGAGTTGGAAAATGATATGCGAGTCAAGTATCCTCTCTTGTTTCAGCCTACAGGTACAAACTTGTTTAAATTCGGGGACAAAATTTTTTAAGGTGGGTAGAATGTAATACCCTATATGTACAATTTAGCTTGAAAAGAAAAAAAGTGGAGTAGAAGTTAAGTTTCTATTGAATTGAGTAAGATCCTTTTGAATAGAAGATGCCCATGCAAAAAGATTTTTTTAGAGGAGGATAAAAAATATGCGGATTTCCCTATTGATTAACCTCTGAAGCGGAAGAGAAGAGAAAGTGGTAAAAATAAGGACAATTATGTCTTTTCTTTGAAAAAAAAGGAAGAAAGAAAGAGTAAGGGACTAGATTATTAAAAATAGAAAACAATTAAAAG

>LNC_000627

CTTTTTTCGTATTTTGGAGATGAATTAGTATTATTGCATGGAAATTAGATCATCGATGGATATTATATCTTGGCTTATAGTTGATATTAGTTGTGTTGTTGATTGAGACAGGAAAAATTCAGGATATGCCTAGAAACAAAAGAAATTTTGTCCGATTTTCTGTAGAATTCTAGTAAGGCTTGATTTGGGATTCTCCCCCTTAGCGCCGGTCACGTTCCTAAATTGGGTCGTGATAACCACCGATTTTCAGTGAATATCATGCTTAATTGTTTGCTTGATTTGGCAATGAAGATCAATGATTGAAGGTCAAAAGTGTTCATAATGCCACTTTCAACTATTGTACAAGTACAAGTTCATTTTCTTGAATTATCCATGTGTGGTCTTACAAAAAG

>LNC_000628

TGAAACTTGAATGTGAGACATAATCAAAATTTTGATCACCTCAATATGAGACTGTCCCAAAAAATTATTGATTTAACTACAATTCAACTTTGTAAAAGGTAAAAGATAATTAAGCTCTAGCTTTTTTCTGTTGACAAATCAGAACAAGAGCAGATCATGTAACCTCGCATCTTCCAAACATTTTTGTTTCAAGAAGTAATGGTATTTGAGATTTTTTATATTCTTTTTTCGTAAGTTTTTTTTATAGCAGGTAATGAAATTATGTAAGAAAACTTTTGTTGATAGTCACGTAATCTACACATGTTTTTGTAGGATTTCCTTAGTTTTTTGACAATATCTAACTCAAAGATGAAAGTTGTTAACTTTCGAATATTTGGAGGATATTTTTTTGCCAAAAAAGATATTTTAAGGATGTACTTTAAGTTTGGAGTATAGTTTAAGGATGTTTTTGACAAAAAACTCTATTTGTAAATATGATTGTGCA

>LNC_000629

CAGTATCTCAAAGCTTTGTTCAAAATTGGTACGATATGTAAGTTCTCAACTATGATAATTCTTTTCACAAGTGTGTATTGTGTGTTCTGGTGCCAGCTAGTTTTAGTTGTTAATTCAAATGCTCGTGTTCTTTGATGATTCTGGTGTCTTTTATGGTGTTATGGACACCGGATGATAGTGTTGTAAAGTGCTCCTCTGCTACTTATCTTCTCACTGTTTTCCCTTGGTTATCATGTCAGATTGATATTTGTTTATTGATGAATGAATTTGTAGACCACATGGTGACAGTGACAGAGGATTAATTTGGAAACTTCTAAGGCTTATCCGGTATGCATCCATGTGCACCACATGGTGATGGATATTGTTAGGCTCTCTTGCATCTCTAGTGATTTCTGAGTGTATGTCATTTTTAGCTGTCAAGAATACATGGATGGGCATTTAGGTGCAAGTGGTTCTACAGGAATAAGTGTCTTGCTTCGGTGTTTGTGGTTGGGGAAGATGCATCAGTTCCCGGGTCGTTTACTATAGGTATATTGGATTTTCATTCTTTCAAAGCTTTGTCTAAATGGGTACGATATGGAAGTTCTCAACTATGATAATTCTTTTCACAAGTGTGTTCTGCTGCCAGTTTTAGTTCTTAGTTCAGATGCTCACGTTCTTTAATGATCCTGATGTCTTTTATCGTGTTATGGGCATCAGACAGAGTCAAAAGTCAATTATGGATCGTAGCTAGTGCTGTAAAGTGCTCATTTGCTTTTTGTATCTAGGTACTTATCTTCTTTGTCGTTTTCCCTTGGTTATCATGCCAACTTTATTTTTTTTATTGGTGATAAATGTGTAGACCACGTGGTGACAGTTAAAGATTATTAGTTTTGGAAACTTCTATGGCGTGTATCCGTTTTGAATTCCTGTACACCTTGTTTCTTGGTCTTTTTGGTGATGGATATTGTTCTGCTTCGACCAAAGCTGTGGTTTTCAATCTTGTCTCTTGCATCTATAGTGATTTCTTAGGTATTATGGATTTTGTCAGCTTTGCACTTAACTGTTCCCTTCTTTTTGTGCTTGCAGTTGGGTCTTAATGAGCAGGGTTTTATGAGTACTGAGATTCTCATTTCAATTTTCTTCCCCAATCAAAGTGCTTCCTCTCTTTATTAAAAAATGTTGTTGTATGGAAGTTCCCAACTAAAATAGGTTTTCTTGCGCTCGTTTGTGTTGTATTCTGCTACCTTTTTTAGTTTTTATCTAGATGCTTATGTTCTTTGATGATCCAATTAATGAGATTCATTGTGGTTGATGAGTGAGTATTAAATCTCTTCCTAACGCCTTTAGGGGCTGTGAAGAGAGCCCAGTTCCAGGCCCTCTTTTAGAAAGAAAAAAATGGTACAAAAAGAGGGCTTGGGGGTATATATACTATACAAATTGATGATGGTTTATCATCATCAGAACAAAGATCAGTCTAGGTCGAGATGAATGGAAGATGCTTCTGGAACTAGTTTTTGATAGGGTCTATCTTAGCTCGAAGGAAAGAACAACCAAAGTTAGACCCCCAAAGAATATGGATTAAAATCTGTTAGTATTTGACAAAGAACCTAACGGAATTTAACAAGCAGTAACCGGAATCAAAGTTTTGCAAAATGGTGTCAACCGGTAATAGAATGTAGCTAGATAACTGATGCCACAGCATCCAAAATATGTGGGATGGAGTTCATGGTACACTAACTTAACCTTCAAGGTCAGATAGTGCTACAGTGAACATCAATCCACTATCTAGCTCAAAATTAGCCTCACGAACTACTAACACTATTATTCGCGTAGCCCTTTTTTGTTTTGTGCTATTTCATCATAGGCTGTCGGAACTTGCTTCTTAGCGCTACGTGGGAAGAGAATTTCTCTATTCAGCCCCCCCGACTTCAATTACAAACTCTTACTTCTCGGTCTAACTTGGACTAATCCCTTAGCAGGCAAGAAATTCCTACTAACCAGACAGAAAGTAAGTTTCCACTCACTACCAAAGCGCTGTCGCACCTCTACTATTTTCGGCGTAACGAGGTTTTAGTCTTTACGAGGGCACCCCCTAGCCAGATTCAATCCCAAGGGTCAATCAAGCCTTTGAAACTAGTGAAAATAGTCGTCACAGTCTGAGTTCCTGCTTTCAACGAGACTTTCTTAGCTTGTCAAACTTTCTCTCCTTCACCAGGAAAGTTATCCGGTAAGCAAGATCTTTGATTGATGTGCCTGAATAAGGTTGATCAATGGTAGCTGCTTGGAGCTTTCTCTATACCTTAATTGAACTGCCCCGCTCTTAGCTCGGGTTCTTGCTTAATGAGGCGTCCCTTGTTTGCTCTTGGATTGGACTAAGCGGCAATCCTTACTGTGGAAGCAGACCTTGTCATTCTTCTCCTTCAATGCCAGTGGATAAGTACACTAGGGCTGGAATGCTCTTTTATCATGTATGTGGATTACTTGGTCGATTACCTGATTGCACTAGTCTCATAGCCATCTCTTTAGCGTCCCGCCATTCCTTCTTGTTAACACAGGAAATCTAACTCATTCTAAGGTAGAGCTCTTAGAGTCCTACTTAAAGTGAAAAGTAGTTCTTTAGTTAAAGCAAGGAGAGCAGAAACTGAGGGAAAAAACCTATGTTCTTACGCGGGATGGGAATAAAGGCTACTAAGTAACCTTAGAATGGGGATGCCAATCTTATACAGAAAATACCGCTATCTAAAGTAACCTTGCAGGGGGGCTGCGTATAGCATTCTAAGATCTCAATACGAGAAAGAAATCAGACCTATAACCTCACTGCAGTGGCTTGTAGACTTGCTTTGCCGCGGAAAGCTCAAGTGAAAACCTATTAGAACTGTGGGCCTCACCCGTCTACTACTCGACCTTAAGTAACTACAGTTAGGAGGATCCATCCAATAGCTTAATGACTTGCTTGGGTCATCTAAGAGGTAAGGCCTCCTCCGATCGACCCTAACTTTCATTATTGAGCTAAAGGAGTTCCCTAGCCCTTCAAGTTTACACAGGTCCCCAAAGATAAAGGCTTTCATCCTTCCTGAATATTCTGGGAACTTTCTTCTATTAATTGTTTTCGAAATGCCGATTTTCCTGTCTGCGCTAAGGCGATAAGGAAAAAGAGAGGGGCCTTTAGTATGTGGAAAAAAAGGCAAAGATCACATTCCAACTAACGTTGACTGGTAATTGCCTCAATGAGGCGGATAAGGCGCAAGAACCTAAAAGGCGGACTAAGATAAGTTATTCAGCTAGGGAATGCGTTAAAGAGCCGTTATGTAGCATCTGAGAACAAGAAAGCAGACATGCACACGAAAAGACATGCACACGAAAAGCAGACATGCTTTCAAAGGGCTTTCAAAAATCAATCATGGATCTCTTGAAAGCCCTTTGAAGTGGGTCTTTGTTACTCCCTTACTTTTGGTATTTTCTCAGCTTCTTGTTTCTTGTTGCATTGTCACCCGAGCTTGATTTTGTTTGTTGGATTAGAGTTGGAATTTTTTCAAAGATTGATGATGTGATCAGGCTTTGATAGTTAGTGTTTGCTCCTATGCACCCTGTTTCTTTGCAAATTTGTTCTTTCTCACCAATCTGAATTTTGTCAGGCATTGATAGAGGGATTTTCCTATCTTGTTTCTTTGCCATTTTTATCCTTGCACTGCAATCTCAATATTATCAGGCTCCGAGAGAGGGGTTTTGCAATCTTGTCTGTGGCGTTTTGTAGTGGGTTTCATCAGCTTTGCACGTAACTCTTTCCTTCATTCCTTTGCTTGTAGCCATTGGGTCTTACAAAGCAACAAATTCTTCTTTTCATGGGATGACCTTATTCGCCCATATGTGATGAGGTTTCGTTAGTTCAGTCTTTTATGAGTAGAGATAATTTTCAATTCAGTTTTCTTCCCTAATGACTTGTTTGTCTTTGTCAAAACTTACTGGTTTATATAAGTTTCTCAATTAGGAAATTATTTTCACCAGACGGTGTGTTGTATTCTGCTGCCATGAGGTTCTAAGCCCTCACACTTATGTTCTTCGATGATCCAATTGATGAGGGATAATTTTCAATTCAGTTTCTTCCCTAGTGACTCCTTTGTTTTTGTCAAAACTACTGTTGTATGGAAGTTTCTCAATTAGGACATTATTTTCACATGTGTGTTGTATTATGCTTCCATGAATTTCTATGTCCGCATGCTTATGTTCTTCAATGATCCAATTGATGAGGTCTTTTATGACTATGGACACCTGATAAGTATTTTGGCCCCTTCTTGATCTTTTGATAGTTGCATAAAATACTTTTTTTACTTTTGTTCTTTTAGCTTCTGATCTTCTCTGCTTCCATTTTTTTCCGTTATCAGACTAGCTTTTTTGGTTGTATAGATGTGTAGACCATGTGGTAACACTGTTATGGACGTTCAAATTTGGAAATTTCAAAGTTTGGTTAACCAACATGTGATCCTCTCTCACCTTTTGGTCTTTTTGGTGCTTGCACTGCAATTTGATGCTGTCCGGGCTTTGGCAAAAGAGGGTTTTCAGCTTCGTCTATGGCATCTATAGTGATTACCATTGTGGTTTTGGGTTAGTCAAATTTGCACTTAACTGTTGCCTTTTTGTAGTTCTTTCATCCATTGGTTATATGGCAGTGAATTATTCTTTACGTGGAGAGGCTGTGTTTATCTGCTTGTTGTTGACGTTTCTTTAGTTAAGTCTTATATGAGTATCGAGATTCTCGGTTCAGTTTATTCCCCAATGACTCCTTTGTGTTTGTCAAAACTACTGTTGTATGGAAGTTTCTCAAATAGGATATTAATTTTCACCGCAATGTGTTGTATTCTACTGCCATGAAGTTTTAAGTCTAGACACTTATTTTCTTCGATGATCCAATTGATGAGGTCTCCTATGTCTATGTTTCTTGAAAAGTACTCAACTATTTCTAGACCTTTTGATAGTGGCGTAAAACACTTATTCACTTCTATTCTTGTAGTTACTTACTTATCTTCTCAGGTTCCATTTTTTTTCATTATCACACTAGTTTTTTTTTTTGTTGGGTGGATGTGTAATGGTGATTCTGTTATGGATGTTCAGATTTGGAAAATTCGAAGCGTGGTTGACCAACATGAGATCCTCTGCCACCTTTTGGTCTTTTTGGTGCTTGCACTGCAATCTGGATAATGTCCGAACTTTGACAAAAGGGAGGGGAGGGGGGGGGTTTAGTCTCATCTATGGATTGGCTAGTGATTACTTTGGTGGTTTGGGGTTCATTGGATTTGCAATTAACCTTAACTGTTCCCTTTTTTCTATTCTTGCAGCCATCGGTTACAAGGTAGTGAATTATTCTTTGATGGGGCTGCCCTATTCGTCTGTTTGTTGTTGAGGTTTCTTTAGTCAAGTCTTTTATGAGTATTCTGATTTTCGATTCAGTTTTCTTCCCTAATGAATCCTTTGACTTTGCCAAAACTATTGTTGTAGGGAAGTTTCTCAACAAAGATATTTATTTTCACTGGTATATGTTATATTTTGCTGCCATGAAGTTTTTAGTCCAGACAGTTGCATTTTTCGATGATCCAATTGATGAGGTCTCCTATGACTATGAACACTTGATAAGTACTCGTTCTAGATCTTTTTATAGTGGTGTAAAATACTTATTTCACTTTTATTCTTGTAGCTACTTATGTTTCTTCTCTGCTTCCATTTCTTTTTCGTCATCACATAGTTTTTTTTTGTGGTTGGATGTGTAGACCATGTGGTAACACTTTTATGGACATTTAGATTTGAAAATTTCAAAACTTGGTTAACAACATGTGATCCTCTGCCACTGTTTTTGTTCTTTTTGGTGCTTGCATTGCAGTTAACATATTGTGCGGTTTTGACAAAAGGGTGTTTTCAGTCTCGTCTATGGCATCTCTAGTGATTACTTAGGTGGTTTGGGGTTTGTCAGATTTGGACTTAACTGCTCCCTTCTTTCTGTTATTGCAACCATTGATTACAGGGTAGTGAATTATTCTTTTCATGAAGAGGCTATTTTTGTCCGTTTGTTGTTGAGGTTTCTTTAGTTCAGTCTTTTATGAGTATCGGAATTTTCGATTCAATCTTCTTCCCTAATGGCTCCTTTGTCTTTGTCAAAACTACTATTGCATGGAAGTTTCTCAACAAGGATATTAATTTTCACCGGTATGTGTTGTATTCTGCTGCCATGAGGTTTTTAGTTTGGACGCTAGTGTTCTTTGATGATCCAATTGTCTATGGCTATGAATACTTGATAAGTACTTCACCAATTTTATATCTTTTGATAGTGGTATAAAATACTTCTTCACTTTTATTCTTGTAGCTACTTGCTTATCTTCTTTGCTTCCATTTCGTTGTCGTTATTACACTAGTTATTTTTGGTTGGATGGATGTGTAGATCATGTGGTGATACTGTTATGGATGTTAAAATTTGGATATTTCAAAGCGTGGTTAACCAACATGTGATCCTCTGCCACCTTTTGATCTTTTTGGTGCTTGCACGACAACCTAGATATTGTCCGAGCTTTGACAAAATGGAGTTTTTAGTCACATATATGGCATCTCTAGTGATTACTTAAGTGGTTTGGGGTTTGTCGGATTTACATTTAACTGTTCCCTTCTTTCTGTTCTTGCAGCCAATGGTTATAGGGAAGTGAATTTTTGTTTTCATGGAGGGGCCGTGTTTGATTGTTTGTGTTGAGGTTTTTTTTAGTTAAGTCTTTTATGAGTATCGATATTACGGATTTAGTTTTCTTCCTTAATAGCTCCTTTGTTTTGTATGGAAGGTTTTCTCAACTAAGATATTAATTTTCACTAGCATGTGTTATATTTTGCTGCCATGAGGTTTTTAGTCCAGATGGCTTATGTTCTTCGATGATCCAATTGATGAGGTCTCATTTGTCTATCGATTCTTGCCATTGATTACAGGGCAGTGGATTATTCTTTTGACGATGCAGTCATGTTTGTCCATTTGTGTTGAGGTTTCCTTAGTCAAGTCTTTTTTTCTTAAACAGGACATTATTCTTCACTGGCGTGTGTTGTATTCTGCCGCCAAGAAGATTTAGGTCCAGATGGCTTTGTTCTTCGATGATCCAATTAATGACGTCTCCTATGACTATGGACACTTGATAACTCAACCAATTCTACATCATCTGATAGTGGCGTGAAGTACTTCTTTTACTTTTTTTCTTCTAGCCATTGGGTCTTACAGAGTGATGAATCATTCTTTTCATGGAGCGGCTTTCTTTGTCCATTTATGTCGAGGTTTCCATGGTTCAAGCTTTTATAAGTTCTAACATTTTCGAGTAAAATAGTTTTTTTCCCATTAGTATGTGTTGTTCTACTGGCATGTTTAGTTTTTAGTCCAGATGTTTGTTTTCTTCAATGATCCAATGATGAGATTCATGATGGTTGATGGTTATTGTCTCATGGACATCTGACAAGTACTCAATTTTGGATCTTTTGAAAGCCTCGTAAAATGGTTGGTGTCCAGTGTGCATTCCTGTGCACCCTATTTCTTGGGCATGTTGGTGCTTGCACTACAATCTAGAGATAGCAGGCTTCGAAAGAAGCTGCTGGGTCTTACAGAGCCATAGATTCGTCTTTTCATGGAGTGGCGTGGTCTGTCAATTTTGTGTTGAGGTTTTTTGGTTGATGCTTCTAATGATTATTGAGATCTTCAATTCAGTTTTATTCCCTAATCAGAATGGTTCCGTTCTTCCTTGTCAACAATACTTCACTGGTGTGTATTTTGTTGCCATATATGTTTCTTAATAATCCAGATGCTTATGTTCTTTGATGATCCAACTGATGAGATCTCCATGGTTATGGACAACACATGATAAGTGTAATCAAGCTCAGTCTTGGATCTTCTGATGGATCTTTTAATAGCCTTGTAACACACTTCTCTAGTTTTCGTGTTCTCTCTACTTACCTTTCTCTGATTTCATTTAACCTACCAGTTTATTTAGTTTGGTTGGATGAATTTATAATTTTGGTTTCGGTGTGCATCCTTGTGGACCATGTTGACCATTTTGGTGCTTGCACTGCAATTTGGATATTGGATATCACCATGCCTTAAGAAAAGGGCACTTTGCAATCTTATCTTGTTTGGCATCTCGAGTGATCTCTCCCGTGTGGTATGGTTTTCCACTCACCTGTTACATTATTTTTTGTGCATGTAACCATTGGGTCTTCCAGAGCATGGACTTCAAGTCAGTTGTCTTCCTAATCAGTGGGTCCCTTTTCTCTTTGTTAAAATTATTGTTATGTGGAAGTTCTCAACAATGATAATATTTTCCACTAGTGCGTGTTGTACACTGCAACCATAAAGTTTTTAGTCTAGATGCTTATTTTCTTGGATGATCTAAATGATGAGATCCCTGATTTGATATGGACACCTGAGAAGTACCCAAACTCAATTTTCCCAAGGGCTTCTCTACTTTGGTGTTCTTGGTACTTATTTTCTCTGCTTCTATTTTCTCATGTGTATCATGCCACTTGATTATGTTTGTTTGGATGCATGTTTGTAGACCACATGGTGACTAGAGGGGGATCGAATTTGGTAATTTCGAAGTATGGTTTTGTCATTCCCTTGTGCACCCTGTTTCTTTGCCATTTTGGCGATTGCGCTTGCACTGCAATTTGGATATCACTAGGCAATAGCAGGGGAGTTGTGCAATCTTTTTTGGCATCTCTAGTGATCTCTCCTATGTGGTATTGGTTTCACAGCTTGCACTTTAACTGTTACCTTCTTTTTGTGATTGCTGTTGTTGGATCTTTCAAGATCAGTAAAGTCTCCATGAACTCCATTTCATAGAGTAGACTTGTTTGTTGTAGTGTTGAAGTTTCTTAGTTCAGGCTTTGTATTAGTATCTAACTCTTCAATTCAGTTTTCTTCCCTGGTCATAGAGATTCCGTGGGTAGCCAGGTAGCCGTCGGTCTTGTCCCGACACCTATCTTCAGTTAGTAGAGGCTTCATAAATAGTCAAAGTTAATGCGTCAGTTCAGTCTTTAGTATTCAGAAGTTTTAAACATCATTATTATTATATTATTATTATTGTTATTGTTTCAGACTTTTGCATGGGATATCTTTAAGTGATTTTAAATGTTCATTTTATACGGCTTCCGCATGTATGTTTAGTCTTCCTCTCAGTAGTTAGCTAGGACAAGGGTTTGCTTGGGACCAACAGTGGGGTTCCGAGTGCCAGCACTCAGAACAGTGGGGTTTCGAGAGCCGGCCACGTCCAGGTGTAGGCTCGGGTTGTGACACAAATGATGAGGTCTCTTATGGCTATGGACACTTGATAAGTACTCAGCCAATTCTAGATCTTTTGATAGTATCATAAACTACCTTTTTACTTTTATTCTTCTAGATACTTTCTTATCTTCTCTGCTTTCTTATATTTTTCGTTATCACACTAGTTTTGTTGTTGTTGTATGGATGTGTAGACCATGTGGTGACATTGTTATGGCATTGAAAAAATGGGGTTTCAGTCTCGTCTATGGCATCTCCAGTGTTACTTAAGTGGTTTGGGGTTTTCCAGATTTGCATTTTACTATTCCCTTCTTTCTGTTCTTGCAGCCATTGGTTACAGGGCATTGAATTATTCTTTTCATGGAGTGGCTGTGTTTGTTTTTTTGTGTTAAGGTTTCTTTAGCTTGAGTCATTTATGAGTATCGAGATTTTCGATTCAGTTTTCCCTTTTCTATATCAAAACTATTGTTCTATGGCAGTTTCTCACTAAGATAATATGTTTTACCCGTATGAGGTTTTTAATTCAGACACTATTTTCTTCAATGATTCAATCGATGAGGTCTCTTACGGCTATGGATACTTGATAAGTACTCGGCCAACTCCGAATCTTTTCTTTTGACAGCGTCATAAAGTGCTCCTCTACTTCTGGTCTTCTAGCTATATTTGTTCTTTGTTGACATCTTCTTTTGGTTACAAAAACTTCTTAGTTTTTGTTTGGATGCATGTGTAGATTATATGGTGATTGTAATAGAGGTTAACATTTAGAGATTTCAAAGGTTGATATCCAATATGGTATCTCTATACCCTCTTGGTCTATTTGGGTCTTGCACTGCAATCTGGATATATTGGTCAGGGCTTGAACCAAAAACGGGTTTTAATTCTTGTCTATATAGCATCTTTAGTGATTTCTTAGATTGTATGGGTTTCGTCAACTTTGGACTTGATTTGTACCCATTTTTTTTTGCTTGAAGCCATTGGGTCTTACAGTGCAATTAATTATTCTTTTCATGGAGCGGTTCTCTTTGTCCATTTATGTCAAGGTTTCTTGGTTCAGGCTTTTATGAGTTCTGACATTTTCAACTAAAATAGCTTTTTTCCCAATAGTGTGTGTTGTATTCTACTGCCATTTTTAGTTTTTAGTCCAGATGCTTATTTTCTTCAATGACCCATTGATGAGATTCCTGATGGTTGATGGTTATGGTCTCATGGACACCTGACAAGTACTCAATTTTGGATATTTTGAGAATCTCGTATCTCTTTTTGTGTTCTCGCAGCTCATCTTCTCTGCTTCACTTTTCTTGTATTACGCTAGCCTGATTTTGTTTGTTGTATGCATTTATGGTGGCAGTTGAGAGGAATAGGACTTAGATGTTTTTGAAGGTTGGTGTCTAGTGTGCATTCCTTTGCCCCTGTTTCTTGGACATGTTGGTGTTTGCACTACAATCTGAAGATTATCAGGTTTCGAATGAAGGTGGTACCTAGTTCGCCAGCTTTGCATTTACTATTCCATTCTTTCTGTGCTTGCAGTCGCTGGGTCTTACAAAGCCATAGATTCTTCTTTCATGGAGCGGCATGGTCTGTCAATTTCGTGTTGAGGTTTTTTGGTTGATGCTTCTAATGATTATTGAGATCTTCAATTCAGTTTTGTTCCCTAAACAGAATGGTCCCCTTCTCTGTTGTCAACACTGCCTCACTAGCCTGTATTTTGTTACCATATTTGATTTTTAATAATCCAGATGCTTATATTCTTTGGTTATCCAACTGATGAGATCTCAATGGTTATGGACAACACATGATAAGTGTAATCGAACTCAATCATGGATCTTTTGATGGCCCTGTAATGCACTCCTCTACTTTTCGTGTTCTCGCTACTTGTCTTCTCTTATTTCATTTAGCCTACCATTTGATTTAGGTTGGTTGGATGAATGTTTGTAGGCCACATGGTGGTAGTAAGAGGATGAGATTAGGATATTTTGTTGTTTTGTTTCGGTGTGCATTCCTGTGGACCATGTTTCTTACCATTTTTGTGCTGGCACTACAATTTGGATATTGGATATCACCAGGAAAAGGGGGCTTTGCTATCTTATCTTGCGTGGTATGGGTTTCCACTTACCTGTTATGTTCTCTTTGTGCATGTAACCATTGGGTCTTCCAGAGCATGAACTTCGGTTTTCTTCCTAATCAACGGGTCCCTTCTTTCTTTGTCAAAACTACTGTTGTGTGGAAATTCTCAACAATGATAAAATTTTCCACTAGTGTGTTTGTACTTTGCAGCCATCAAGTTTTTAGTCTAGATGCTTATTTTCTTGGATGACCCAAATGACGAGATCCCTGATTTTTATGGACACCTGAGAAGTACCCAAACTCAAATTTAGGTCTTCTTATGGCCCTAAGAGCTCCTCTACTTTAGTGTTCTTACTGCTTTTATGTTTATCATGGCACTTTATTTTGTTTTGCTTGGATATCATATTTGGAAATTCTGAAGTTTGGTTTCAGTGTGTGTCCTTTCCTTGTGCACCCTGTTTCTTTGCAATTTTGGAGCTTGCGCTTGCACTGTAATTTGGATATCACCAGGCACTGTCAAGAGAGTTATGCAATCTTGTTTGGCACTGCAATTTCGATATCACCAAGCACTGGCAAAGGGAGTTATGCAATCTCGTATGGCATCTCTAGTGATCTCCCCTATATGGCATGGTTTCGCAGCTTGAACTTTAACTGCTCCCTTCTTTTAGTTCTTGCTGTCGTTGGGTCTTTCAAGATTAGGATAGTCTTCCTTTAATGGAGCAGCCTTGTTTATCTTAGCGTTGAAGTTTCCTTTGTTCAGGCCTTGTATTAGTATCTAGCTCTTCGATTCAGTTTTCTTCCATGGTCATAGAGATCCCCTTATCTCGTTGTCAGAAGTGCTGTTAAGTGGAAGTTTTCAACTACAATAACTCTCTCCACAAGTGTATTTTCAGCTTTTAGTCCAGATGCTTTTGTTTTGCGATGATCCAATTGATGAGGTCTCTCTTGTGGTGTGGACATCTGGTAAGTGCTCGCTCAGTTCCGGATGTTTTCATCATCTACCTTTTGTGTTCTTGCTGCTTATCTTCTCTGCTTTCATTTTATTTTTTCTTATCACAACAGGTTTTGAGTTTTGTCTATTGGAAAGATATATATTCCACATAGTGAACACTATTTGAGGATTAGATTTGGAAATTTAAAAGTGTAGTGTTCAGTAAGCTAGTCGTTTAGGTGTTTCCCCTGCAATCTGGTATCGTTTAGGGCTTCAAGATTAGGGAGTTTCTAAATATTGTCTATGTCATTTCTAGTGATTTCCCAAGCACTCTGGGTTTTGGCAGATTTGCACTTAATTGTTCATTCATTTTGTGCTTGCAGTTGTGGTCTAAACATAGTAAATTTTCTCTTTTCGTGGAGCAGCCTTTCTTTGTTGATTTGTATTGAGGTTTCGTTCGGTTCAAGCTTCTATGAGTATCCGATTTTCAGTTCAGTTCTTTTTTTTTTGTTCTCTTCTTTAATCAATATGATCTTTCTTCACCACACTCTCTTCGAGTGGTGCTCTTCTAGTAGTTAGTTTAACTGAGAATTTTGCACGTCAGTGGTGGTTACAAGCATTCAAGTTCTTAGGAGTTCTCTTGGTATTATGTGTAATCAAGTCTCGGCTCGGCCTATCTTCTTTCTCCCCCTCCCCTTAACACCTGTAGTGCTGTTGGTTGGCTGCCTCCACCTGATGGTTATGGTGGGTCAACGTGTGCAAGATTTTAACCAGGTCTTGCATAATGTGATAATGAGAATGTTCTGTCGTCGACTGTCGATAACATTCTTTTCAAAACCTCTTATAGACTAGGCTGGGATCTTATCCATTTTAGATGGTGATCTTACATGTTTCAGAAAACTGTAACTACCTTTGGTGGTATAGAGTTGATATCTCTACTTAATGTTGTACTCATTTGTTTCATCCCATTGCCCTCTATTAAATTCCTTGCTTGGTCGGAATGGGGAGGAGTGTCAACTCGAGTTCTTTTCTCCTGCCAGTTGTCTTATCTGATCCGATGGCTTTCATTTTCTCCCGGATGCAGGTTTCTGGTCTTTGATTGTGGCTGTTGGAAGTTTGGGTGGAGGGGTTAAGGATGTTTTATTATGATGTTTCTGCCATAATGAAACTATTCTTATGTTACCTGGTTGACAATTTTTGGGTATTTACAGAATTTGATAGGTCTGGATTTGTTGGTGCAGTTGTATTAAGTTTTCTGATTTTGCTATATAGGTATGGAATATGAGCAGATTTGTGAGTTGCAGAAAAGAACGTCCGTCAGAAAGCTTGGCCTCCTGTGCTCGAAGTGAGACCTGAAGGGTGAGCTTATGGCGCACGAAATCCATCTCTCGGTTTTGCAGATCTATTTTGAATTGTGAATTTGCTGTGAGTTCTCACTTTTGTTGGTTTCTCGAGGTATCCGGGGTCTTCCTGCTTAGATTATGCCAATTTCTTTCTCTGTTTTACCAGGGTAATGATTGTATGGGTATAGACAATGCAGTTTCTGTTGTCGGATGTCAATAATCTTTGTAACGAAGTAAACCTCATCTCTGGTACCTCCAACAAATCATCTTTTCTTGCGTCTTCTCTTTTGGAACACTATTCAACAGAAGTTTATGAGCCTTTCCTTGTAGATGTTGCTCTCTATATTTCATACCAGTATACGTACCCGCACGTTGCGCGGACATTATAAAATGTAATAATGTTTTAAATTTAAAATAACTAATAAGGTTTATTATTTTCTTTGTAATATATTTTTAAAATAATGTAATAAATCATTAAATTATTTATTTGAGCAAGTTCTATTAATATTTATGTACAC

>LNC_000630

CTAATTCTGAAAAGCACCCACCCAATGCCACCGCCCTTTGAAATATTGTTGCCATAAATAGACTTGCTAGGAAACAAATTTCTCAGTTACGATTTGTTTTAGCTTTAGTTCTACTATTATTTGGTGTTCATTCTTGAGGATATAAGCCGTGTTTGATTGTTGCTATGGATAGTTATTGTTGATCAAGATATGGGTAACTATTGGTATGATCAAGATGATGGTGCGGTTTTTTTTGCTAATTTATTGACTAATTATTGTTGTTTGTTTGATCAGGTATGGGTAATGATGGTAGGAGACTGATTCTCCTTCTACATTTGCTGTTACCTTAATGTCAATGTTAATTCTGAGGTTTTTGTTGTTTAGAATTCATGTGTTAGTGTCATCCTGTTTACTACTCTGAAATCTAGCAGTTTCCTTTTGTTGAATCAGAACACAAGTAGAGTTGGAAATTTGGCTCTAGGTGTGCATCTAGGTGCAAGTGGTTCTACAGGAACAAGTGTCTTGCTTCAGTGTTTGTGGTCAAGAAAGATCCAGCAGTTCCCAGGTATGCAACTAAAGGTTTTTGATTTTCAGTATCTCAAAGCTTTGTTCAAAATTGGTACGATATGTAAGTTCTCAACTATGATAATTCTTTTCACAAGTGTGTATTGTGTGTTCTGGTGCCAGCTAGTTTTAGTTGTTAATTCAAATGCTCGTGTTCTTTGATGATTCTGGTGTCTTTTATGGTGTTATGGACACCGGATGATAGTGTTGTAAAGTGCTCCTCTGCTACTTATCTTCTCACTGTTTTCCCTTGGTTATCATGTCAGATTGATATTTGTTTATTGATGAATGAATTTGTAGACCACATGGTGACAGTGACAGAGGATTAATTTGGAAACTTCTAAGGCTTATCCGGTATGCATCCATGTGCACCACATGGTGATGGATATTGTTAGGCTCTCTTGCATCTCTAGTGATTTCTGAGGTATATGTGTTTTTTCAGCACATAGTTGTTCCTTCTTTTTGTGCTTGCAGTTGAGTTTTACCGAGCAGTGAATTATTCTTTTCATGGAAGAGGCTTTGTTTGGCCATTTGTGTTGAGGTTTAATGAGTACTGGAGATACTCAATACTCATTTCAGTTTTTTTCCCCAATCAGAGTGGCCCCTCCTCTTTGTCAAAAATTGTTGTTGTATGGAACTTGCCAGCTAAAATAGTTTCTCTTGCACTCACGTGTGTTGTATGGAACTTTCCAGCTAAAAATTGATGTGTATGTTGTTCGATGATCCAGTTGATGAGATCCTGGTGGTTGATGGTTATATGGTCTTATCGACACCTGATAAATACTCAAAACTCAATCCTGGATCTTTTGACAGCCCGTACAAGTGGATCCCTGTTAGACCCTCTCTTTTGTTGTTCTCTCTGCCTTAACTTCTCAGCTTCCTTTTTCTTGCATCACCCAAGCTTGATTTTGTTTGTTGGATGGATGATAGTTGAAGGTTGATGCCCAGTGGCCGTTTCTGTGCACGCTGCTTCTTGCTATTTTTGTGCTTGGACTACAATCTGAATATAATCAGGCTTCAATAGAGGGGTTTTCCAATCTCGTTTCTTTGCCATTTTTATGTGTGCATTGCAACCTGAATATTTTCAGGCTTAGAGAGGGTTTTTTAAATTTGTCTGTGGCATTTTCTAGTAGATTTTGTCAGCTTTGCACGTAACTCTTCCCTTCATTCCTTTGCTTGCAGCCATTGGGTCTTACAAAGCAGTGAATTCTTCTTTTCATGGAGGTACCTTATTTGCCCAAATGTGTTGAGGTCTTGTTAGTCAGTCTTTTACGAGTATAGATAATTTCCAATTTTTCTTCCCTATTGATTCCTTTGTCTTTGTCATAACTTCTGTTGTATGGTAGTTTCTCAATTAGGACATTATTTTCACCAATGTGTGTTTTATTCTACTGCCATGATGTTCTTAGTTTAGATGCTTATGTTCTTATGGTGATCCATTTTTTATTTCTTGGTTATCTCAGAATTATATAAATCTCACGTTATCTACTTTCGAATCTTGGTTTACGAATCTTTTGATATGGGGTGTAGGTTTCTCATTTCTGATTTTCGAATCTTTGATTATCTCTGTAGACAAGCTAAGGTCAGAAGATGAATGTTAGGGGGGGTTTTGGGAGCTGTTGATCTTTTTTCTTGGCCAATTTATCGATTCGTTATTGTTGTTTGTTTGATTAGGTATGGGTAATGATGATAGGGGAATGGTTATAGGTACCTTCCAGTGTATTTCATGATTCTCCTTCTACACTTGTTGTTACCTTCTGTCAGTTACCAAATATTCGTATCCTTGCTTTCCTTCATGTGTAGTTTATTTATTTATTTGTTCTCATTTTCTTGGGTATTATAGTGGGAGTTTATAATTGCAATCCTTTTGATTGATTCAGAATACGTGTACACAGCAGATTCGTGAGCAAGAGTGTATTCAACGAACAACTACAATAATTTTTTCTACGGAACTTTCATATGAGTCTATTCTTCCTTTTAATCTACTATATAAAGGTTTTAAATTGACCCTTAATATTAAAGATAATTACATTCTTGAAAGGATTAAGGGTTCTACCTTCCTGACTTTTCGCTTGTTAGGACTGGAAATTTAGCTCTCCAGGCATGCCTACTTGGATCTTCTATTGTGGACTTATGTGTCACAACCATACAATTGACCTATCAGTTTGGATTTGATATCTTGACTTCCATCTTGAATGTTAATTTTGATGCTAATCACGTGTCTCCTCACATCAACTGCTTTTAGCTCCTTAGTTGGTTTGGTCATCAGTAAATGTCATTCTTAGCTGTCAAGAATACATGGATGGGGCATCTAGGTGCAAGTGGTTCTACATGAATAAGTGTCTTGCTTCGGTGTTTGTGGTTGGGAAAGATGCAGCAGTTCCCAAGCCTTTGACTATAGGTCTTGGATTTTTCAGTCTCTCAAACCTTTATTAAAACTAGTACGGTATGAAAGTTCTCAACTATGATAATTCTTTTCACAAGTGTGTATAGTGTGGTCTGCTCTGCCATTTTTAGTTTAGATGGTCATGTTTTTTGATGATCCGATATCTTCTATGGTGTTATGGGCACCAGATAAAGTCAAAAGTCAATTTGGAATCTTTGATAGTGCTTCTCTGCTTTTTGTGTCCTAGCAACTTATCTTCTCTGCCGTTTTCCTTGGTTATCATGCCAGCTTGATTTTGTATTGGTGAATGGATATGTAGACCACATGGTGATATTAATAGTGGATTAGTTTTGTTCAGAGGCTTGTATCTCGTATGCATTCTTGTACACTTTGTTTTTTGGTCTTTTTGGGGCTTGGACTCCAATATATTGTGTATTATAGTGGTAGTTTATAATTTCAATCCTTTTGATTGATTTCCAATGTTATTAGAAATTGGAAGCTGGTGACTATAAATGCAATTTCATGTTTGCTTCCATGTTTTGGGAAATTCTGAGGTCTTTGTTGTGAGAATTCATATGTTGGTGAGCATTGTTCTATCACCCTATTTACTACTATGAAGCACATCAATTTCCTTTTGTTGAATCAGAATACTGGAAATTGGCTCCCCAGACATGCCTACTTGGATCTTTTATTCTAGACCTATGTGTCACAATCATACAGTGGAGCTATCAGTTTGGATTTGATATCTTGACTTCCATCTTTAATGCTAATGCTAATCATGTCTCTCTTCGCATCAAATTCTTTTACCTTCTTATTTGCTTTGGTCATCAGTGTATGTCATTTTTAGCTGTCAAGAATACATGGATGGGCATTTAGGTGCAAGTGGTTCTACAGGAATAAGTGTCTTGCTTCGGTGTTTGTGGTTGGGGAAGATGCATCAGTTCCCGGGTCGTTTACTATAGGTATATTGGATTTTCATTCTTTCAAAGCTTTGTCTAAATGGGTACGATATGGAAGTTCTCAACTATGATAATTCTTTTCACAAGTGTGTTCTGCTGCCAGTTTTAGTTCTTAGTTCAGATGCTCACGTTCTTTAATGATCCTGATGTCTTTTATCGTGTTATGGGCATCAGACAGAGTCAAAAGTCAATTATGGATCGTAGCTAGTGCTGTAAAGTGCTCATTTGCTTTTTGTATCTAGGTACTTATCTTCTTTGTCGTTTTCCCTTGGTTATCATGCCAACTTTATTTTTTTTATTGGTGATAAATGTGTAGACCACGTGGTGACAGTTAAAGATTATTAGTTTTGGAAACTTCTATGGCGTGTATCCGTTTTGAATTCCTGTACACCTTGTTTCTTGGTCTTTTTGGTGATGGATATTGTTCTGCTTCGACCAAAGCTGTGGTTTTCAATCTTGTCTCTTGCATCTATAGTGATTTCTTAGGTATTATGGATTTTGTCAGCTTTGCACTTAACTGTTCCCTTCTTTTTGTGCTTGCAGTTGGGTCTTAATGAGCAGGGTTTTATGAGTACTGAGATTCTCATTTCAATTTTCTTCCCCAATCAAAGTGCTTCCTCTCTTTATTAAAAAATGTTGTTGTATGGAAGTTCCCAACTAAAATAGGTTTTCTTGCGCTCGTTTGTGTTGTATTCTGCTACCTTTTTTAGTTTTTATCTAGATGCTTATGTTCTTTGATGATCCAATTAATGAGATTCATTGTGGTTGATGAGTGAGTATTAAATCTCTTCCTAACGCCTTTAGGGGCTGTGAAGAGAGCCCAGTTCCAGGCCCTCTTTTAGAAAGAAAAAAATGGTACAAAAAGAGGGCTTGGGGGTATATATACTATACAAATTGATGATGGTTTATCATCATCAGAACAAAGATCAGTCTAGGTCGAGATGAATGGAAGATGCTTCTGGAACTAGTTTTTGATAGGGTCTATCTTAGCTCGAAGGAAAGAACAACCAAAGTTAGACCCCCAAAGAATATGGATTAAAATCTGTTAGTATTTGACAAAGAACCTAACGGAATTTAACAAGCAGTAACCGGAATCAAAGTTTTGCAAAATGGTGTCAACCGGTAATAGAATGTAGCTAGATAACTGATGCCACAGCATCCAAAATATGTGGGATGGAGTTCATGGTACACTAACTTAACCTTCAAGGTCAGATAGTGCTACAGTGAACATCAATCCACTATCTAGCTCAAAATTAGCCTCACGAACTACTAACACTATTATTCGCGTAGCCCTTTTTTGTTTTGTGCTATTTCATCATAGGCTGTCGGAACTTGCTTCTTAGCGCTACGTGGGAAGAGAATTTCTCTATTCAGCCCCCCCGACTTCAATTACAAACTCTTACTTCTCGGTCTAACTTGGACTAATCCCTTAGCAGGCAAGAAATTCCTACTAACCAGACAGAAAGTAAGTTTCCACTCACTACCAAAGCGCTGTCGCACCTCTACTATTTTCGGCGTAACGAGGTTTTAGTCTTTACGAGGGCACCCCCTAGCCAGATTCAATCCCAAGGGTCAATCAAGCCTTTGAAACTAGTGAAAATAGTCGTCACAGTCTGAGTTCCTGCTTTCAACGAGACTTTCTTAGCTTGTCAAACTTTCTCTCCTTCACCAGGAAAGTTATCCGGTAAGCAAGATCTTTGATTGATGTGCCTGAATAAGGTTGATCAATGGTAGCTGCTTGGAGCTTTCTCTATACCTTAATTGAACTGCCCCGCTCTTAGCTCGGGTTCTTGCTTAATGAGGCGTCCCTTGTTTGCTCTTGGATTGGACTAAGCGGCAATCCTTACTGTGGAAGCAGACCTTGTCATTCTTCTCCTTCAATGCCAGTGGATAAGTACACTAGGGCTGGAATGCTCTTTTATCATGTATGTGGATTACTTGGTCGATTACCTGATTGCACTAGTCTCATAGCCATCTCTTTAGCGTCCCGCCATTCCTTCTTGTTAACACAGGAAATCTAACTCATTCTAAGGTAGAGCTCTTAGAGTCCTACTTAAAGTGAAAAGTAGTTCTTTAGTTAAAGCAAGGAGAGCAGAAACTGAGGGAAAAAACCTATGTTCTTACGCGGGATGGGAATAAAGGCTACTAAGTAACCTTAGAATGGGGATGCCAATCTTATACAGAAAATACCGCTATCTAAAGTAACCTTGCAGGGGGGCTGCGTATAGCATTCTAAGATCTCAATACGAGAAAGAAATCAGACCTATAACCTCACTGCAGTGGCTTGTAGACTTGCTTTGCCGCGGAAAGCTCAAGTGAAAACCTATTAGAACTGTGGGCCTCACCCGTCTACTACTCGACCTTAAGTAACTACAGTTAGGAGGATCCATCCAATAGCTTAATGACTTGCTTGGGTCATCTAAGAGGTAAGGCCTCCTCCGATCGACCCTAACTTTCATTATTGAGCTAAAGGAGTTCCCTAGCCCTTCAAGTTTACACAGGTCCCCAAAGATAAAGGCTTTCATCCTTCCTGAATATTCTGGGAACTTTCTTCTATTAATTGTTTTCGAAATGCCGATTTTCCTGTCTGCGCTAAGGCGATAAGGAAAAAGAGAGGGGCCTTTAGTATGTGGAAAAAAAGGCAAAGATCACATTCCAACTAACGTTGACTGGTAATTGCCTCAATGAGGCGGATAAGGCGCAAGAACCTAAAAGGCGGACTAAGATAAGTTATTCAGCTAGGGAATGCGTTAAAGAGCCGTTATGTAGCATCTGAGAACAAGAAAGCAGACATGCACACGAAAAGACATGCACACGAAAAGCAGACATGCTTTCAAAGGGCTTTCAAAAATCAATCATGGATCTCTTGAAAGCCCTTTGAAGTGGGTCTTTGTTACTCCCTTACTTTTGGTATTTTCTCAGCTTCTTGTTTCTTGTTGCATTGTCACCCGAGCTTGATTTTGTTTGTTGGATTAGAGTTGGAATTTTTTCAAAGATTGATGATGTGATCAGGCTTTGATAGTTAGTGTTTGCTCCTATGCACCCTGTTTCTTTGCAAATTTGTTCTTTCTCACCAATCTGAATTTTGTCAGGCATTGATAGAGGGATTTTCCTATCTTGTTTCTTTGCCATTTTTATCCTTGCACTGCAATCTCAATATTATCAGGCTCCGAGAGAGGGGTTTTGCAATCTTGTCTGTGGCGTTTTGTAGTGGGTTTCATCAGCTTTGCACGTAACTCTTTCCTTCATTCCTTTGCTTGTAGCCATTGGGTCTTACAAAGCAACAAATTCTTCTTTTCATGGGATGACCTTATTCGCCCATATGTGATGAGGTTTCGTTAGTTCAGTCTTTTATGAGTAGAGATAATTTTCAATTCAGTTTTCTTCCCTAATGACTTGTTTGTCTTTGTCAAAACTTACTGGTTTATATAAGTTTCTCAATTAGGAAATTATTTTCACCAGACGGTGTGTTGTATTCTGCTGCCATGAGGTTCTAAGCCCTCACACTTATGTTCTTCGATGATCCAATTGATGAGGGATAATTTTCAATTCAGTTTCTTCCCTAGTGACTCCTTTGTTTTTGTCAAAACTACTGTTGTATGGAAGTTTCTCAATTAGGACATTATTTTCACATGTGTGTTGTATTATGCTTCCATGAATTTCTATGTCCGCATGCTTATGTTCTTCAATGATCCAATTGATGAGGTCTTTTATGACTATGGACACCTGATAAGTATTTTGGCCCCTTCTTGATCTTTTGATAGTTGCATAAAATACTTTTTTTACTTTTGTTCTTTTAGCTTCTGATCTTCTCTGCTTCCATTTTTTTCCGTTATCAGACTAGCTTTTTTGGTTGTATAGATGTGTAGACCATGTGGTAACACTGTTATGGACGTTCAAATTTGGAAATTTCAAAGTTTGGTTAACCAACATGTGATCCTCTCTCACCTTTTGGTCTTTTTGGTGCTTGCACTGCAATTTGATGCTGTCCGGGCTTTGGCAAAAGAGGGTTTTCAGCTTCGTCTATGGCATCTATAGTGATTACCATTGTGGTTTTGGGTTAGTCAAATTTGCACTTAACTGTTGCCTTTTTGTAGTTCTTTCATCCATTGGTTATATGGCAGTGAATTATTCTTTACGTGGAGAGGCTGTGTTTATCTGCTTGTTGTTGACGTTTCTTTAGTTAAGTCTTATATGAGTATCGAGATTCTCGGTTCAGTTTATTCCCCAATGACTCCTTTGTGTTTGTCAAAACTACTGTTGTATGGAAGTTTCTCAAATAGGATATTAATTTTCACCGCAATGTGTTGTATTCTACTGCCATGAAGTTTTAAGTCTAGACACTTATTTTCTTCGATGATCCAATTGATGAGGTCTCCTATGTCTATGTTTCTTGAAAAGTACTCAACTATTTCTAGACCTTTTGATAGTGGCGTAAAACACTTATTCACTTCTATTCTTGTAGTTACTTACTTATCTTCTCAGGTTCCATTTTTTTTCATTATCACACTAGTTTTTTTTTTTGTTGGGTGGATGTGTAATGGTGATTCTGTTATGGATGTTCAGATTTGGAAAATTCGAAGCGTGGTTGACCAACATGAGATCCTCTGCCACCTTTTGGTCTTTTTGGTGCTTGCACTGCAATCTGGATAATGTCCGAACTTTGACAAAAGGGAGGGGAGGGGGGGGGTTTAGTCTCATCTATGGATTGGCTAGTGATTACTTTGGTGGTTTGGGGTTCATTGGATTTGCAATTAACCTTAACTGTTCCCTTTTTTCTATTCTTGCAGCCATCGGTTACAAGGTAGTGAATTATTCTTTGATGGGGCTGCCCTATTCGTCTGTTTGTTGTTGAGGTTTCTTTAGTCAAGTCTTTTATGAGTATTCTGATTTTCGATTCAGTTTTCTTCCCTAATGAATCCTTTGACTTTGCCAAAACTATTGTTGTAGGGAAGTTTCTCAACAAAGATATTTATTTTCACTGGTATATGTTATATTTTGCTGCCATGAAGTTTTTAGTCCAGACAGTTGCATTTTTCGATGATCCAATTGATGAGGTCTCCTATGACTATGAACACTTGATAAGTACTCGTTCTAGATCTTTTTATAGTGGTGTAAAATACTTATTTCACTTTTATTCTTGTAGCTACTTATGTTTCTTCTCTGCTTCCATTTCTTTTTCGTCATCACATAGTTTTTTTTTGTGGTTGGATGTGTAGACCATGTGGTAACACTTTTATGGACATTTAGATTTGAAAATTTCAAAACTTGGTTAACAACATGTGATCCTCTGCCACTGTTTTTGTTCTTTTTGGTGCTTGCATTGCAGTTAACATATTGTGCGGTTTTGACAAAAGGGTGTTTTCAGTCTCGTCTATGGCATCTCTAGTGATTACTTAGGTGGTTTGGGGTTTGTCAGATTTGGACTTAACTGCTCCCTTCTTTCTGTTATTGCAACCATTGATTACAGGGTAGTGAATTATTCTTTTCATGAAGAGGCTATTTTTGTCCGTTTGTTGTTGAGGTTTCTTTAGTTCAGTCTTTTATGAGTATCGGAATTTTCGATTCAATCTTCTTCCCTAATGGCTCCTTTGTCTTTGTCAAAACTACTATTGCATGGAAGTTTCTCAACAAGGATATTAATTTTCACCGGTATGTGTTGTATTCTGCTGCCATGAGGTTTTTAGTTTGGACGCTAGTGTTCTTTGATGATCCAATTGTCTATGGCTATGAATACTTGATAAGTACTTCACCAATTTTATATCTTTTGATAGTGGTATAAAATACTTCTTCACTTTTATTCTTGTAGCTACTTGCTTATCTTCTTTGCTTCCATTTCGTTGTCGTTATTACACTAGTTATTTTTGGTTGGATGGATGTGTAGATCATGTGGTGATACTGTTATGGATGTTAAAATTTGGATATTTCAAAGCGTGGTTAACCAACATGTGATCCTCTGCCACCTTTTGATCTTTTTGGTGCTTGCACGACAACCTAGATATTGTCCGAGCTTTGACAAAATGGAGTTTTTAGTCACATATATGGCATCTCTAGTGATTACTTAAGTGGTTTGGGGTTTGTCGGATTTACATTTAACTGTTCCCTTCTTTCTGTTCTTGCAGCCAATGGTTATAGGGAAGTGAATTTTTGTTTTCATGGAGGGGCCGTGTTTGATTGTTTGTGTTGAGGTTTTTTTTAGTTAAGTCTTTTATGAGTATCGATATTACGGATTTAGTTTTCTTCCTTAATAGCTCCTTTGTTTTGTATGGAAGGTTTTCTCAACTAAGATATTAATTTTCACTAGCATGTGTTATATTTTGCTGCCATGAGGTTTTTAGTCCAGATGGCTTATGTTCTTCGATGATCCAATTGATGAGGTCTCATTTGTCTATCGATTCTTGGTAAGTACTCTGCTAATTTTGGATCTTTTGATAGTTGCGTAAAATACTTCTTCACTTCTATTCTTGCAGCTACTTATCTTCTCCACTTCCATTTCTTTTTCGTTATCACATTAGCTATTTTTGGTTGGATGGATGTGTAGACCACGTGGTGACACTGTTATGGACGTTCAAATTTAGAAATTTCAAATCTTGGTTAGCCAACATGTGATCCTCTGCCACCTTTTGGTCTTTTGGTGCTTTGCACTGCAATTTGGATGTTGTTTGGGTTTTGACGAAAGGGGGTTTTAGTCTCGTCTATGGCATCTATAGTGATTAATTAGGTGGTTAGTTTGTCAGATTTGCTCTCAATTGTTCCCTTCTTTCTATTCTTGTAGCCATTGATTACAGGGCAGTGGATTATTCTTTTGACGATGCAGTCATGTTTGTCCATTTGTGTTGAGGTTTCCTTAGTCAAGTCTTTTGTGAGTATCAAGATTTTTGATTCAGTTATCTTTCCTAATGGCTCCTTTGTCTTTGTCAAAACTATTGATGTGTGGAAGTTTCTTAAACAGGACATTATTCTTCACTGGCGTGTGTTGTATTCTGCCGCCAAGAAGATTTAGGTCCAGATGGCTTTGTTCTTCGATGATCCAATTAATGACGTCTCCTATGACTATGGACACTTGATAACTCAACCAATTCTACATCATCTGATAGTGGCGTGAAGTACTTCTTTTACTTTTTTTCTTCTAGGTACTTATCTTCTCTGCTTCCATATATTTTTTGTCATCACACAAGTTTTTTCTAGTTGAATGGACCATGTGGTGACACTGTTTTGGACGTTCAGATTTTGTAATTTCAAAGCTTGGTTTATCAACATGTGATTCTGTGCCACCTTTTAGTCTTTTTGGTGCTTGCACTACAATCTGGATATTGTCTGAGCTTTGACAAAGGGGTACTTTTAGTCTCGTCTATGGCATTTCTAGTGATCACTTAGGTGGTTTGGGGTTCGTCAAATATGCACTTAACTGTTCCATTTTTTCTGTTCTTGCAACCACATTGGTTACAGTGCAGTGAATTATTCTTTTCATGGAGCGGTCGTGTTTGTCTGTTTGTGTTGAGGTTTCTTTAGTTTAATCTTTTATGAGTATCGAGATTTTCGATTCAGTGTTCTTCTCTTATGGCTCCCGTTGTACTATAGAAAGTACTATTGTATGGATGTTTCTCAACTAAGATAATATTTTTCACCATTGTGTGTTGTGTTCTGCTGCCGTGAAGTTTTTACTCTAGACATTTAAGGATGGCAATGGGGCGGGGCAGGTTTAAGGCTACGGGGGGCAGGGCGGGAGGGATGCAGGGTGGGTTGAAGTGGAATTTTTAAAACTAATGCGGTGTGAGACGGGTTGCAGGGGTAAATGTGATTTTATATGGGTTCAAACTTAATTTGAGCTTTTTACATGTTATAAGAGTGACAAAACATTATTTATTAAGATACTTTCTTTAAAGCTACTAAAATATTCAATATAGTAAATCAAAATGGTTCAATAAAAAATAATACAATTTCTTACATGTTTCCCAATTGACCTCTAAAAAATAAACGTTTAAGTAATTATCTATTAAACTAATACAACTTAATGAAAAAATGAATTTATTTTTATGAGTTTTATTTTTAGTACGGAAAAGGGTCAAAATTGCCCCTGAACTATGCGAAATAGATCATTTATACCCTTCGTTACATTTTGGGATAAAAAATGTCCCTGCCATTATCCTAAGAGACCACAATTACCCTCAAGAGTTAACACCCCAAATTTTTAGTGACATGGCAAGCCACGTGGGACTAATCCTTCCACCTAAACGTTGCCAACTTGGATTCACTACAAAAGAACCAGACCATTAGCGGCGACAACAGTCGCCACAAAATCCCAAAATATTGCCACTAAAGGTTATGAGCGATTTTTAGTGGCGACTAAGGCTGCAATAACCATTTGCTAGTAATTCCTATTTTTTCAACAAAATAATATGATAATTAATTTTCGATTGCAACCTAATATTCTAAAATAAATAACTTGGACTATTAACATTAAAAACATCCAATATTATTATGAAAATCATCAAAATTACTTCTTGTTTCAAATCTCCTACTACATAATGCATCATCATACAACTTATACATTTACATATGATATAAAAATAAAACATATAGTGTCTTCACACTCCTCCTTAATCGATATCATTTTTGGTTTTTTAAAAACAATGAAGAAAAAACACGCAAATCAGAATTTATTGTTAAAAACCTTTAGTGACGATTTTCTGGGATTTTGTGGCGACTGTTGTCGCCGCTAAAGGTCTAGTTCTTTTGTAGTGAATCCTAGTTGGCAACGCTTAGGTGGAAGGATTAGTTCCCCACGTGGCTTACCATGTCACTAAAAATTTGGGGGTGTTAACTCTTGAGGGTATTTTGTGGTCTCTTAGGATAACGACAGGGGCATTTTTTATCCCAAAATGTAACGAAGGGTATAAGTGGTCTATTTCGCATAGTTCAGGGGCAATTTTGACTCTTTTCCATTTTTAGTATCAAACTTAACTAGAAAAAGAAAATGTTAAAAAAATTATGTGGGGCGGGTTGAGAATTTAAGAAGTTATGCAGGGCGGGGCAGATTAAAAAATTTGCAAGTTAAGCTTAACCCCCCGGCTTGTGCACCGCCCCGCCCCATTGCCATCCCTATAGACATTATGTTCTTCGATGATCCAATTGATAAGGTCTTCTATGGCTAAGGACACTTGATAAGTACTCGGCCAATTCTAGATCTTTTCTTTTGACAGTACCATAAAGGCTCCTCTATTTCTGGTCTTCTAGCTATATATATTATATGCTTCCATCTTCTTTTGGTTATCACAAACTTGGTTTTTCTTGTTTGGTGACAACAATAGAGGTTAAGATTTGGAGATTTCAAAGTTTGATATCCAGTATGGAATCTCTTTATCCGGTTGTTCTTTTTGGTGCTCAGGGCTTCAACAAAATGGGGTTTTAAATCTCATCTATGTGGTATATCTAGTGATTTGTTAGATGGTATGGGTTTTTCAGCTTTGGAATTGATTGACCCTTATTTTTTTATGCTTGCAGCCATTGGGTCTTACAGAGTGATGAATCATTCTTTTCATGGAGCGGCTTTCTTTGTCCATTTATGTCGAGGTTTCCATGGTTCAAGCTTTTATAAGTTCTAACATTTTCGAGTAAAATAGTTTTTTTCCCATTAGTATGTGTTGTTCTACTGGCATGTTTAGTTTTTAGTCCAGATGTTTGTTTTCTTCAATGATCCAATGATGAGATTCATGATGGTTGATGGTTATTGTCTCATGGACATCTGACAAGTACTCAATTTTGGATCTTTTGAAAGCCTCGTAAAATGGTAGTCTCTTTTGGTGTTCTCACTACTCATCATTCATGCTTCCTTTTTCTTGTATTATGCTAGTTTGATTTTGTTTGTTGGATGGCTGTATGGTGATAAGTTGAGAGGAATACGACTTGCAATATTTTGCAGGTTGGTGTCCAGTGTGCATTCCTGTGCACCCTATTTCTTGGGCATGTTGGTGCTTGCACTACAATCTAGAGATAGCAGGCTTCGAAAGAAGCTGCTGGGTCTTACAGAGCCATAGATTCGTCTTTTCATGGAGTGGCGTGGTCTGTCAATTTTGTGTTGAGGTTTTTTGGTTGATGCTTCTAATGATTATTGAGATCTTCAATTCAGTTTTATTCCCTAATCAGAATGGTTCCGTTCTTCCTTGTCAACAATACTTCACTGGTGTGTATTTTGTTGCCATATATGTTTCTTAATAATCCAGATGCTTATGTTCTTTGATGATCCAACTGATGAGATCTCCATGGTTATGGACAACACATGATAAGTGTAATCAAGCTCAGTCTTGGATCTTCTGATGGATCTTTTAATAGCCTTGTAACACACTTCTCTAGTTTTCGTGTTCTCTCTACTTACCTTTCTCTGATTTCATTTAACCTACCAGTTTATTTAGTTTGGTTGGATGAATTTATAATTTTGGTTTCGGTGTGCATCCTTGTGGACCATGTTGACCATTTTGGTGCTTGCACTGCAATTTGGATATTGGATATCACCATGCCTTAAGAAAAGGGCACTTTGCAATCTTATCTTGTTTGGCATCTCGAGTGATCTCTCCCGTGTGGTATGGTTTTCCACTCACCTGTTACATTATTTTTTGTGCATGTAACCATTGGGTCTTCCAGAGCATGGACTTCAAGTCAGTTGTCTTCCTAATCAGTGGGTCCCTTTTCTCTTTGTTAAAATTATTGTTATGTGGAAGTTCTCAACAATGATAATATTTTCCACTAGTGCGTGTTGTACACTGCAACCATAAAGTTTTTAGTCTAGATGCTTATTTTCTTGGATGATCTAAATGATGAGATCCCTGATTTGATATGGACACCTGAGAAGTACCCAAACTCAATTTTCCCAAGGGCTTCTCTACTTTGGTGTTCTTGGTACTTATTTTCTCTGCTTCTATTTTCTCATGTGTATCATGCCACTTGATTATGTTTGTTTGGATGCATGTTTGTAGACCACATGGTGACTAGAGGGGGATCGAATTTGGTAATTTCGAAGTATGGTTTTGTCATTCCCTTGTGCACCCTGTTTCTTTGCCATTTTGGCGATTGCGCTTGCACTGCAATTTGGATATCACTAGGCAATAGCAGGGGAGTTGTGCAATCTTTTTTGGCATCTCTAGTGATCTCTCCTATGTGGTATTGGTTTCACAGCTTGCACTTTAACTGTTACCTTCTTTTTGTGATTGCTGTTGTTGGATCTTTCAAGATCAGTAAAGTCTCCATGAACTCCATTTCATAGAGTAGACTTGTTTGTTGTAGTGTTGAAGTTTCTTAGTTCAGGCTTTGTATTAGTATCTAACTCTTCAATTCAGTTTTCTTCCCTGGTCATAGAGATTCCGTGGGTAGCCAGGTAGCCGTCGGTCTTGTCCCGACACCTATCTTCAGTTAGTAGAGGCTTCATAAATAGTCAAAGTTAATGCGTCAGTTCAGTCTTTAGTATTCAGAAGTTTTAAACATCATTATTATTATATTATTATTATTGTTATTGTTTCAGACTTTTGCATGGGATATCTTTAAGTGATTTTAAATGTTCATTTTATACGGCTTCCGCATGTATGTTTAGTCTTCCTCTCAGTAGTTAGCTAGGACAAGGGTTTGCTTGGGACCAACAGTGGGGTTCCGAGTGCCAGCACTCAGAACAGTGGGGTTTCGAGAGCCGGCCACGTCCAGGTGTAGGCTCGGGTTGTGACACAAATGATGAGGTCTCTTATGGCTATGGACACTTGATAAGTACTCAGCCAATTCTAGATCTTTTGATAGTATCATAAACTACCTTTTTACTTTTATTCTTCTAGATACTTTCTTATCTTCTCTGCTTTCTTATATTTTTCGTTATCACACTAGTTTTGTTGTTGTTGTATGGATGTGTAGACCATGTGGTGACATTGTTATGGCATTGAAAAAATGGGGTTTCAGTCTCGTCTATGGCATCTCCAGTGTTACTTAAGTGGTTTGGGGTTTTCCAGATTTGCATTTTACTATTCCCTTCTTTCTGTTCTTGCAGCCATTGGTTACAGGGCATTGAATTATTCTTTTCATGGAGTGGCTGTGTTTGTTTTTTTGTGTTAAGGTTTCTTTAGCTTGAGTCATTTATGAGTATCGAGATTTTCGATTCAGTTTTCCCTTTTCTATATCAAAACTATTGTTCTATGGCAGTTTCTCACTAAGATAATATGTTTTACCCGTATGAGGTTTTTAATTCAGACACTATTTTCTTCAATGATTCAATCGATGAGGTCTCTTACGGCTATGGATACTTGATAAGTACTCGGCCAACTCCGAATCTTTTCTTTTGACAGCGTCATAAAGTGCTCCTCTACTTCTGGTCTTCTAGCTATATTTGTTCTTTGTTGACATCTTCTTTTGGTTACAAAAACTTCTTAGTTTTTGTTTGGATGCATGTGTAGATTATATGGTGATTGTAATAGAGGTTAACATTTAGAGATTTCAAAGGTTGATATCCAATATGGTATCTCTATACCCTCTTGGTCTATTTGGGTCTTGCACTGCAATCTGGATATATTGGTCAGGGCTTGAACCAAAAACGGGTTTTAATTCTTGTCTATATAGCATCTTTAGTGATTTCTTAGATTGTATGGGTTTCGTCAACTTTGGACTTGATTTGTACCCATTTTTTTTTGCTTGAAGCCATTGGGTCTTACAGTGCAATTAATTATTCTTTTCATGGAGCGGTTCTCTTTGTCCATTTATGTCAAGGTTTCTTGGTTCAGGCTTTTATGAGTTCTGACATTTTCAACTAAAATAGCTTTTTTCCCAATAGTGTGTGTTGTATTCTACTGCCATTTTTAGTTTTTAGTCCAGATGCTTATTTTCTTCAATGACCCATTGATGAGATTCCTGATGGTTGATGGTTATGGTCTCATGGACACCTGACAAGTACTCAATTTTGGATATTTTGAGAATCTCGTATCTCTTTTTGTGTTCTCGCAGCTCATCTTCTCTGCTTCACTTTTCTTGTATTACGCTAGCCTGATTTTGTTTGTTGTATGCATTTATGGTGGCAGTTGAGAGGAATAGGACTTAGATGTTTTTGAAGGTTGGTGTCTAGTGTGCATTCCTTTGCCCCTGTTTCTTGGACATGTTGGTGTTTGCACTACAATCTGAAGATTATCAGGTTTCGAATGAAGGTGGTACCTAGTTCGCCAGCTTTGCATTTACTATTCCATTCTTTCTGTGCTTGCAGTCGCTGGGTCTTACAAAGCCATAGATTCTTCTTTCATGGAGCGGCATGGTCTGTCAATTTCGTGTTGAGGTTTTTTGGTTGATGCTTCTAATGATTATTGAGATCTTCAATTCAGTTTTGTTCCCTAAACAGAATGGTCCCCTTCTCTGTTGTCAACACTGCCTCACTAGCCTGTATTTTGTTACCATATTTGATTTTTAATAATCCAGATGCTTATATTCTTTGGTTATCCAACTGATGAGATCTCAATGGTTATGGACAACACATGATAAGTGTAATCGAACTCAATCATGGATCTTTTGATGGCCCTGTAATGCACTCCTCTACTTTTCGTGTTCTCGCTACTTGTCTTCTCTTATTTCATTTAGCCTACCATTTGATTTAGGTTGGTTGGATGAATGTTTGTAGGCCACATGGTGGTAGTAAGAGGATGAGATTAGGATATTTTGTTGTTTTGTTTCGGTGTGCATTCCTGTGGACCATGTTTCTTACCATTTTTGTGCTGGCACTACAATTTGGATATTGGATATCACCAGGAAAAGGGGGCTTTGCTATCTTATCTTGCGTGGTATGGGTTTCCACTTACCTGTTATGTTCTCTTTGTGCATGTAACCATTGGGTCTTCCAGAGCATGAACTTCGGTTTTCTTCCTAATCAACGGGTCCCTTCTTTCTTTGTCAAAACTACTGTTGTGTGGAAATTCTCAACAATGATAAAATTTTCCACTAGTGTGTTTGTACTTTGCAGCCATCAAGTTTTTAGTCTAGATGCTTATTTTCTTGGATGACCCAAATGACGAGATCCCTGATTTTTATGGACACCTGAGAAGTACCCAAACTCAAATTTAGGTCTTCTTATGGCCCTAAGAGCTCCTCTACTTTAGTGTTCTTACTGCTTTTATGTTTATCATGGCACTTTATTTTGTTTTGCTTGGATATCATATTTGGAAATTCTGAAGTTTGGTTTCAGTGTGTGTCCTTTCCTTGTGCACCCTGTTTCTTTGCAATTTTGGAGCTTGCGCTTGCACTGTAATTTGGATATCACCAGGCACTGTCAAGAGAGTTATGCAATCTTGTTTGGCACTGCAATTTCGATATCACCAAGCACTGGCAAAGGGAGTTATGCAATCTCGTATGGCATCTCTAGTGATCTCCCCTATATGGCATGGTTTCGCAGCTTGAACTTTAACTGCTCCCTTCTTTTAGTTCTTGCTGTCGTTGGGTCTTTCAAGATTAGGATAGTCTTCCTTTAATGGAGCAGCCTTGTTTATCTTAGCGTTGAAGTTTCCTTTGTTCAGGCCTTGTATTAGTATCTAGCTCTTCGATTCAGTTTTCTTCCATGGTCATAGAGATCCCCTTATCTCGTTGTCAGAAGTGCTGTTAAGTGGAAGTTTTCAACTACAATAACTCTCTCCACAAGTGTATTTTCAGCTTTTAGTCCAGATGCTTTTGTTTTGCGATGATCCAATTGATGAGGTCTCTCTTGTGGTGTGGACATCTGGTAAGTGCTCGCTCAGTTCCGGATGTTTTCATCATCTACCTTTTGTGTTCTTGCTGCTTATCTTCTCTGCTTTCATTTTATTTTTTCTTATCACAACAGGTTTTGAGTTTTGTCTATTGGAAAGATATATATTCCACATAGTGAACACTATTTGAGGATTAGATTTGGAAATTTAAAAGTGTAGTGTTCAGTAAGCTAGTCGTTTAGGTGTTTCCCCTGCAATCTGGTATCGTTTAGGGCTTCAAGATTAGGGAGTTTCTAAATATTGTCTATGTCATTTCTAGTGATTTCCCAAGCACTCTGGGTTTTGGCAGATTTGCACTTAATTGTTCATTCATTTTGTGCTTGCAGTTGTGGTCTAAACATAGTAAATTTTCTCTTTTCGTGGAGCAGCCTTTCTTTGTTGATTTGTATTGAGGTTTCGTTCGGTTCAAGCTTCTATGAGTATCCGATTTTCAGTTCAGTTCTTTTTTTTTTGTTCTCTTCTTTAATCAATATGATCTTTCTTCACCACACTCTCTTCGAGTGGTGCTCTTCTAGTAGTTAGTTTAACTGAGAATTTTGCACGTCAGTGGTGGTTACAAGCATTCAAGTTCTTAGGAGTTCTCTTGGTATTATGTGTAATCAAGTCTCGGCTCGGCCTATCTTCTTTCTCCCCCTCCCCTTAACACCTGTAGTGCTGTTGGTTGGCTGCCTCCACCTGATGGTTATGGTGGGTCAACGTGTGCAAGATTTTAACCAGGTCTTGCATAATGTGATAATGAGAATGTTCTGTCGTCGACTGTCGATAACATTCTTTTCAAAACCTCTTATAGACTAGGCTGGGATCTTATCCATTTTAGATGGTGATCTTACATGTTTCAGAAAACTGTAACTACCTTTGGTGGTATAGAGTTGATATCTCTACTTAATGTTGTACTCATTTGTTTCATCCCATTGCCCTCTATTAAATTCCTTGCTTGGTCGGAATGGGGAGGAGTGTCAACTCGAGTTCTTTTCTCCTGCCAGTTGTCTTATCTGATCCGATGGCTTTCATTTTCTCCCGGATGCAGGTTTCTGGTCTTTGATTGTGGCTGTTGGAAGTTTGGGTGGAGGGGTTAAGGATGTTTTATTATGATGTTTCTGCCATAATGAAACTATTCTTATGTTACCTGGTTGACAATTTTTGGGTATTTACAGAATTTGATAGGTCTGGATTTGTTGGTGCAGTTGTATTAAGTTTTCTGATTTTGCTATATAGGTATGGAATATGAGCAGATTTGTGAGTTGCAGAAAAGAACGTCCGTCAGAAAGCTTGGCCTCCTGTGCTCGAAGTGAGACCTGAAGGGTGAGCTTATGGCGCACGAAATCCATCTCTCGGTTTTGCAGATCTATTTTGAATTGTGAATTTGCTGTGAGTTCTCACTTTTGTTGGTTTCTCGAGGTATCCGGGGTCTTCCTGCTTAGATTATGCCAATTTCTTTCTCTGTTTTACCAGGGTAATGATTGTATGGGTATAGACAATGCAGTTTCTGTTGTCGGATGTCAATAATCTTTGTAACGAAGTAAACCTCATCTCTGGTACCTCCAACAAATCATCTTTTCTTGCGTCTTCTCTTTTGGAACACTATTCAACAGAAGTTTATGAGCCTTTCCTTGTAGATGTTGCTCTCTATATTTCATACCAGTATACGTACCCGCACGTTGCGCGGACATTATAAAATGTAATAATGTTTTAAATTTAAAATAACTAATAAGGTTTATTATTTTCTTTGTAATATATTTTTAAAATAATGTAATAAATCATTAAATTATTTATTTGAGCAAGTTCTATTAATATTTATGTACAC

>LNC_000631

AGCGGCTAAACTAATTCTGAAAAGCACCCACCCAATGCCACCGCCCTTTGAAATATTGTTGCCATAAATAGACTTGCTAGGAAACAAATTTCTCAGTTACGATTTGTTTTAGCTTTAGTTCTACTATTATTTGGTGTTCATTCTTGAGGATATAAGCCGTGTTTGATTGTTGCTATGGATAGTTATTGTTGATCAAGATATGGGTAACTATTGGTATGATCAAGATGATGGGTAATTATTTATATTGTTTAATCAAGATATGCGTCCCTGTTCGTTTTGGCTTTAGTTCTCTGTTTTTTAATGATTTCGTTGGTATCTTGAGGATATAGGGAGTGTTTGATTGTTGATTATTGTCGATCATAACACTGGGTAATAATTATTCGATCAAGATACTGGGTAATTATCATTGATGATGATATTGGGTAACTATTGTTTGATGAAGTTGTGGGTAATTGTTGTTGATCAACACATGGGTAATTATTGTATGGTCAAGATATAGGTAATTATTGTATGATCAAGATACAGTGTAATTATTGTATGGTCAAGATACGGGTAATTATTGTTAATCAAGATATGAGTATTATTGTATGGTCAAGATACATGTAATTATTATCTATCAAGATATGGGTATTATTGTATGATCAAGATAGGGTAATTATTGTCGATCAAGATACTGGGTAATTATTGTCAATCAAGATATGGGTAATTATTGTATATGATGCCTTCCTTAGGGGAGCTATCGGTTTGATTTATGATTTCTTGGCCATCTCAGAATTATATAAATTATCTAATTTCGCATCTTGGTTTTCGAATCTTTTAATATGGGGTGTAGGTTTCTGATTTTCAAATCTGTGGGCAAGGAAGGTTAGGGGTTTTGGGAGTTCTCGACTTTTTAGTGTGATTTAGTTTGCAATTAGTTATGTTCTTTTGGCTAATTTATTGATTTGTTTGCAATTAGTGCGGTTTTTTTTGCTAATTTATTGACTAATTATTGTTGTTTGTTTGATCAGGTATGGGTAATGATGGTAGGAGACTGATTCTCCTTCTACATTTGCTGTTACCTTAATGTCAATGTTAATTCTGAGGTTTTTGTTGTTTAGAATTCATGTGTTAGTGTCATCCTGTTTACTACTCTGAAATCTAGCAGTTTCCTTTTGTTGAATCAGAACACAAGTAGAGTTGGAAATTTGGCTCTAGGTGTGCATCTAGGTGCAAGTGGTTCTACAGGAACAAGTGTCTTGCTTCAGTGTTTGTGGTCAAGAAAGATCCAGCAGTTCCCAGACCACATGGTGACAGTGACAGAGGATTAATTTGGAAACTTCTAAGGCTTATCCGGTATGCATCCATGTGCACCACATGGTGATGGATATTGTTAGGCTCTCTTGCATCTCTAGTGATTTCTGAGGTATATGTGTTTTTTCAGCACATAGTTGTTCCTTCTTTTTGTGCTTGCAGTTGAGTTTTACCGAGCAGTGAATTATTCTTTTCATGGAAGAGGCTTTGTTTGGCCATTTGTGTTGAGGTTTAATGAGTACTGGAGATACTCAATACTCATTTCAGTTTTTTTCCCCAATCAGAGTGGCCCCTCCTCTTTGTCAAAAATTGTTGTTGTATGGAACTTGCCAGCTAAAATAGTTTCTCTTGCACTCACGTGTGTTGTATGGAACTTTCCAGCTAAAAATTGATGTGTATGTTGTTCGATGATCCAGTTGATGAGATCCTGGTGGTTGATGGTTATATGGTCTTATCGACACCTGATAAATACTCAAAACTCAATCCTGGATCTTTTGACAGCCCGTACAAGTGGATCCCTGTTAGACCCTCTCTTTTGTTGTTCTCTCTGCCTTAACTTCTCAGCTTCCTTTTTCTTGCATCACCCAAGCTTGATTTTGTTTGTTGGATGGATGATAGTTGAAGGTTGATGCCCAGTGGCCGTTTCTGTGCACGCTGCTTCTTGCTATTTTTGTGCTTGGACTACAATCTGAATATAATCAGGCTTCAATAGAGGGGTTTTCCAATCTCGTTTCTTTGCCATTTTTATGTGTGCATTGCAACCTGAATATTTTCAGGCTTAGAGAGGGTTTTTTAAATTTGTCTGTGGCATTTTCTAGTAGATTTTGTCAGCTTTGCACGTAACTCTTCCCTTCATTCCTTTGCTTGCAGCCATTGGGTCTTACAAAGCAGTGAATTCTTCTTTTCATGGAGGTACCTTATTTGCCCAAATGTGTTGAGGTCTTGTTAGTCAGTCTTTTACGAGTATAGATAATTTCCAATTTTTCTTCCCTATTGATTCCTTTGTCTTTGTCATAACTTCTGTTGTATGGTAGTTTCTCAATTAGGACATTATTTTCACCAATGTGTGTTTTATTCTACTGCCATGATGTTCTTAGTTTAGATGCTTATGTTCTTATGGTGATCCATTTTTTATTTCTTGGTTATCTCAGAATTATATAAATCTCACGTTATCTACTTTCGAATCTTGGTTTACGAATCTTTTGATATGGGGTGTAGGTTTCTCATTTCTGATTTTCGAATCTTTGATTATCTCTGTAGACAAGCTAAGGTCAGAAGATGAATGTTAGGGGGGGTTTTGGGAGCTGTTGATCTTTTTTCTTGGCCAATTTATCGATTCGTTATTGTTGTTTGTTTGATTAGGTATGGGTAATGATGATAGGGGAATGGTTATAGGTACCTTCCAGTGTATTTCATGATTCTCCTTCTACACTTGTTGTTACCTTCTGTCAGTTACCAAATATTCGTATCCTTGCTTTCCTTCATGTGTAGTTTATTTATTTATTTGTTCTCATTTTCTTGGGTATTATAGTGGGAGTTTATAATTGCAATCCTTTTGATTGATTCAGAATACGTGTACACAGCAGATTCGTGAGCAAGAGTGTATTCAACGAACAACTACAATAATTTTTTCTACGGAACTTTCATATGAGTCTATTCTTCCTTTTAATCTACTATATAAAGGTTTTAAATTGACCCTTAATATTAAAGATAATTACATTCTTGAAAGGATTAAGGGTTCTACCTTCCTGACTTTTCGCTTGTTAGGACTGGAAATTTAGCTCTCCAGGCATGCCTACTTGGATCTTCTATTGTGGACTTATGTGTCACAACCATACAATTGACCTATCAGTTTGGATTTGATATCTTGACTTCCATCTTGAATGTTAATTTTGATGCTAATCACGTGTCTCCTCACATCAACTGCTTTTAGCTCCTTAGTTGGTTTGGTCATCAGTAAATGTCATTCTTAGCTGTCAAGAATACATGGATGGGGCATCTAGGTGCAAGTGGTTCTACATGAATAAGTGTCTTGCTTCGGTGTTTGTGGTTGGGAAAGATGCAGCAGTTCCCAAGCCTTTGACTATAGGTCTTGGATTTTTCAGTCTCTCAAACCTTTATTAAAACTAGTACGGTATGAAAGTTCTCAACTATGATAATTCTTTTCACAAGTGTGTATAGTGTGGTCTGCTCTGCCATTTTTAGTTTAGATGGTCATGTTTTTTGATGATCCGATATCTTCTATGGTGTTATGGGCACCAGATAAAGTCAAAAGTCAATTTGGAATCTTTGATAGTGCTTCTCTGCTTTTTGTGTCCTAGCAACTTATCTTCTCTGCCGTTTTCCTTGGTTATCATGCCAGCTTGATTTTGTATTGGTGAATGGATATGTAGACCACATGGTGATATTAATAGTGGATTAGTTTTGTTCAGAGGCTTGTATCTCGTATGCATTCTTGTACACTTTGTTTTTTGGTCTTTTTGGGGCTTGGACTCCAATATATTGTGTATTATAGTGGTAGTTTATAATTTCAATCCTTTTGATTGATTTCCAATGTTATTAGAAATTGGAAGCTGGTGACTATAAATGCAATTTCATGTTTGCTTCCATGTTTTGGGAAATTCTGAGGTCTTTGTTGTGAGAATTCATATGTTGGTGAGCATTGTTCTATCACCCTATTTACTACTATGAAGCACATCAATTTCCTTTTGTTGAATCAGAATACTGGAAATTGGCTCCCCAGACATGCCTACTTGGATCTTTTATTCTAGACCTATGTGTCACAATCATACAGTGGAGCTATCAGTTTGGATTTGATATCTTGACTTCCATCTTTAATGCTAATGCTAATCATGTCTCTCTTCGCATCAAATTCTTTTACCTTCTTATTTGCTTTGGTCATCAGTGTATGTCATTTTTAGCTGTCAAGAATACATGGATGGGCATTTAGGTGCAAGTGGTTCTACAGGAATAAGTGTCTTGCTTCGGTGTTTGTGGTTGGGGAAGATGCATCAGTTCCCGGGTCGTTTACTATAGGTATATTGGATTTTCATTCTTTCAAAGCTTTGTCTAAATGGGTACGATATGGAAGTTCTCAACTATGATAATTCTTTTCACAAGTGTGTTCTGCTGCCAGTTTTAGTTCTTAGTTCAGATGCTCACGTTCTTTAATGATCCTGATGTCTTTTATCGTGTTATGGGCATCAGACAGAGTCAAAAGTCAATTATGGATCGTAGCTAGTGCTGTAAAGTGCTCATTTGCTTTTTGTATCTAGGTACTTATCTTCTTTGTCGTTTTCCCTTGGTTATCATGCCAACTTTATTTTTTTTATTGGTGATAAATGTGTAGACCACGTGGTGACAGTTAAAGATTATTAGTTTTGGAAACTTCTATGGCGTGTATCCGTTTTGAATTCCTGTACACCTTGTTTCTTGGTCTTTTTGGTGATGGATATTGTTCTGCTTCGACCAAAGCTGTGGTTTTCAATCTTGTCTCTTGCATCTATAGTGATTTCTTAGGTATTATGGATTTTGTCAGCTTTGCACTTAACTGTTCCCTTCTTTTTGTGCTTGCAGTTGGGTCTTAATGAGCAGGGTTTTATGAGTACTGAGATTCTCATTTCAATTTTCTTCCCCAATCAAAGTGCTTCCTCTCTTTATTAAAAAATGTTGTTGTATGGAAGTTCCCAACTAAAATAGGTTTTCTTGCGCTCGTTTGTGTTGTATTCTGCTACCTTTTTTAGTTTTTATCTAGATGCTTATGTTCTTTGATGATCCAATTAATGAGATTCATTGTGGTTGATGAGTGAGTATTAAATCTCTTCCTAACGCCTTTAGGGGCTGTGAAGAGAGCCCAGTTCCAGGCCCTCTTTTAGAAAGAAAAAAATGGTACAAAAAGAGGGCTTGGGGGTATATATACTATACAAATTGATGATGGTTTATCATCATCAGAACAAAGATCAGTCTAGGTCGAGATGAATGGAAGATGCTTCTGGAACTAGTTTTTGATAGGGTCTATCTTAGCTCGAAGGAAAGAACAACCAAAGTTAGACCCCCAAAGAATATGGATTAAAATCTGTTAGTATTTGACAAAGAACCTAACGGAATTTAACAAGCAGTAACCGGAATCAAAGTTTTGCAAAATGGTGTCAACCGGTAATAGAATGTAGCTAGATAACTGATGCCACAGCATCCAAAATATGTGGGATGGAGTTCATGGTACACTAACTTAACCTTCAAGGTCAGATAGTGCTACAGTGAACATCAATCCACTATCTAGCTCAAAATTAGCCTCACGAACTACTAACACTATTATTCGCGTAGCCCTTTTTTGTTTTGTGCTATTTCATCATAGGCTGTCGGAACTTGCTTCTTAGCGCTACGTGGGAAGAGAATTTCTCTATTCAGCCCCCCCGACTTCAATTACAAACTCTTACTTCTCGGTCTAACTTGGACTAATCCCTTAGCAGGCAAGAAATTCCTACTAACCAGACAGAAAGTAAGTTTCCACTCACTACCAAAGCGCTGTCGCACCTCTACTATTTTCGGCGTAACGAGGTTTTAGTCTTTACGAGGGCACCCCCTAGCCAGATTCAATCCCAAGGGTCAATCAAGCCTTTGAAACTAGTGAAAATAGTCGTCACAGTCTGAGTTCCTGCTTTCAACGAGACTTTCTTAGCTTGTCAAACTTTCTCTCCTTCACCAGGAAAGTTATCCGGTAAGCAAGATCTTTGATTGATGTGCCTGAATAAGGTTGATCAATGGTAGCTGCTTGGAGCTTTCTCTATACCTTAATTGAACTGCCCCGCTCTTAGCTCGGGTTCTTGCTTAATGAGGCGTCCCTTGTTTGCTCTTGGATTGGACTAAGCGGCAATCCTTACTGTGGAAGCAGACCTTGTCATTCTTCTCCTTCAATGCCAGTGGATAAGTACACTAGGGCTGGAATGCTCTTTTATCATGTATGTGGATTACTTGGTCGATTACCTGATTGCACTAGTCTCATAGCCATCTCTTTAGCGTCCCGCCATTCCTTCTTGTTAACACAGGAAATCTAACTCATTCTAAGGTAGAGCTCTTAGAGTCCTACTTAAAGTGAAAAGTAGTTCTTTAGTTAAAGCAAGGAGAGCAGAAACTGAGGGAAAAAACCTATGTTCTTACGCGGGATGGGAATAAAGGCTACTAAGTAACCTTAGAATGGGGATGCCAATCTTATACAGAAAATACCGCTATCTAAAGTAACCTTGCAGGGGGGCTGCGTATAGCATTCTAAGATCTCAATACGAGAAAGAAATCAGACCTATAACCTCACTGCAGTGGCTTGTAGACTTGCTTTGCCGCGGAAAGCTCAAGTGAAAACCTATTAGAACTGTGGGCCTCACCCGTCTACTACTCGACCTTAAGTAACTACAGTTAGGAGGATCCATCCAATAGCTTAATGACTTGCTTGGGTCATCTAAGAGGTAAGGCCTCCTCCGATCGACCCTAACTTTCATTATTGAGCTAAAGGAGTTCCCTAGCCCTTCAAGTTTACACAGGTCCCCAAAGATAAAGGCTTTCATCCTTCCTGAATATTCTGGGAACTTTCTTCTATTAATTGTTTTCGAAATGCCGATTTTCCTGTCTGCGCTAAGGCGATAAGGAAAAAGAGAGGGGCCTTTAGTATGTGGAAAAAAAGGCAAAGATCACATTCCAACTAACGTTGACTGGTAATTGCCTCAATGAGGCGGATAAGGCGCAAGAACCTAAAAGGCGGACTAAGATAAGTTATTCAGCTAGGGAATGCGTTAAAGAGCCGTTATGTAGCATCTGAGAACAAGAAAGCAGACATGCACACGAAAAGACATGCACACGAAAAGCAGACATGCTTTCAAAGGGCTTTCAAAAATCAATCATGGATCTCTTGAAAGCCCTTTGAAGTGGGTCTTTGTTACTCCCTTACTTTTGGTATTTTCTCAGCTTCTTGTTTCTTGTTGCATTGTCACCCGAGCTTGATTTTGTTTGTTGGATTAGAGTTGGAATTTTTTCAAAGATTGATGATGTGATCAGGCTTTGATAGTTAGTGTTTGCTCCTATGCACCCTGTTTCTTTGCAAATTTGTTCTTTCTCACCAATCTGAATTTTGTCAGGCATTGATAGAGGGATTTTCCTATCTTGTTTCTTTGCCATTTTTATCCTTGCACTGCAATCTCAATATTATCAGGCTCCGAGAGAGGGGTTTTGCAATCTTGTCTGTGGCGTTTTGTAGTGGGTTTCATCAGCTTTGCACGTAACTCTTTCCTTCATTCCTTTGCTTGTAGCCATTGGGTCTTACAAAGCAACAAATTCTTCTTTTCATGGGATGACCTTATTCGCCCATATGTGATGAGGTTTCGTTAGTTCAGTCTTTTATGAGTAGAGATAATTTTCAATTCAGTTTTCTTCCCTAATGACTTGTTTGTCTTTGTCAAAACTTACTGGTTTATATAAGTTTCTCAATTAGGAAATTATTTTCACCAGACGGTGTGTTGTATTCTGCTGCCATGAGGTTCTAAGCCCTCACACTTATGTTCTTCGATGATCCAATTGATGAGGGATAATTTTCAATTCAGTTTCTTCCCTAGTGACTCCTTTGTTTTTGTCAAAACTACTGTTGTATGGAAGTTTCTCAATTAGGACATTATTTTCACATGTGTGTTGTATTATGCTTCCATGAATTTCTATGTCCGCATGCTTATGTTCTTCAATGATCCAATTGATGAGGTCTTTTATGACTATGGACACCTGATAAGTATTTTGGCCCCTTCTTGATCTTTTGATAGTTGCATAAAATACTTTTTTTACTTTTGTTCTTTTAGCTTCTGATCTTCTCTGCTTCCATTTTTTTCCGTTATCAGACTAGCTTTTTTGGTTGTATAGATGTGTAGACCATGTGGTAACACTGTTATGGACGTTCAAATTTGGAAATTTCAAAGTTTGGTTAACCAACATGTGATCCTCTCTCACCTTTTGGTCTTTTTGGTGCTTGCACTGCAATTTGATGCTGTCCGGGCTTTGGCAAAAGAGGGTTTTCAGCTTCGTCTATGGCATCTATAGTGATTACCATTGTGGTTTTGGGTTAGTCAAATTTGCACTTAACTGTTGCCTTTTTGTAGTTCTTTCATCCATTGGTTATATGGCAGTGAATTATTCTTTACGTGGAGAGGCTGTGTTTATCTGCTTGTTGTTGACGTTTCTTTAGTTAAGTCTTATATGAGTATCGAGATTCTCGGTTCAGTTTATTCCCCAATGACTCCTTTGTGTTTGTCAAAACTACTGTTGTATGGAAGTTTCTCAAATAGGATATTAATTTTCACCGCAATGTGTTGTATTCTACTGCCATGAAGTTTTAAGTCTAGACACTTATTTTCTTCGATGATCCAATTGATGAGGTCTCCTATGTCTATGTTTCTTGAAAAGTACTCAACTATTTCTAGACCTTTTGATAGTGGCGTAAAACACTTATTCACTTCTATTCTTGTAGTTACTTACTTATCTTCTCAGGTTCCATTTTTTTTCATTATCACACTAGTTTTTTTTTTTGTTGGGTGGATGTGTAATGGTGATTCTGTTATGGATGTTCAGATTTGGAAAATTCGAAGCGTGGTTGACCAACATGAGATCCTCTGCCACCTTTTGGTCTTTTTGGTGCTTGCACTGCAATCTGGATAATGTCCGAACTTTGACAAAAGGGAGGGGAGGGGGGGGGTTTAGTCTCATCTATGGATTGGCTAGTGATTACTTTGGTGGTTTGGGGTTCATTGGATTTGCAATTAACCTTAACTGTTCCCTTTTTTCTATTCTTGCAGCCATCGGTTACAAGGTAGTGAATTATTCTTTGATGGGGCTGCCCTATTCGTCTGTTTGTTGTTGAGGTTTCTTTAGTCAAGTCTTTTATGAGTATTCTGATTTTCGATTCAGTTTTCTTCCCTAATGAATCCTTTGACTTTGCCAAAACTATTGTTGTAGGGAAGTTTCTCAACAAAGATATTTATTTTCACTGGTATATGTTATATTTTGCTGCCATGAAGTTTTTAGTCCAGACAGTTGCATTTTTCGATGATCCAATTGATGAGGTCTCCTATGACTATGAACACTTGATAAGTACTCGTTCTAGATCTTTTTATAGTGGTGTAAAATACTTATTTCACTTTTATTCTTGTAGCTACTTATGTTTCTTCTCTGCTTCCATTTCTTTTTCGTCATCACATAGTTTTTTTTTGTGGTTGGATGTGTAGACCATGTGGTAACACTTTTATGGACATTTAGATTTGAAAATTTCAAAACTTGGTTAACAACATGTGATCCTCTGCCACTGTTTTTGTTCTTTTTGGTGCTTGCATTGCAGTTAACATATTGTGCGGTTTTGACAAAAGGGTGTTTTCAGTCTCGTCTATGGCATCTCTAGTGATTACTTAGGTGGTTTGGGGTTTGTCAGATTTGGACTTAACTGCTCCCTTCTTTCTGTTATTGCAACCATTGATTACAGGGTAGTGAATTATTCTTTTCATGAAGAGGCTATTTTTGTCCGTTTGTTGTTGAGGTTTCTTTAGTTCAGTCTTTTATGAGTATCGGAATTTTCGATTCAATCTTCTTCCCTAATGGCTCCTTTGTCTTTGTCAAAACTACTATTGCATGGAAGTTTCTCAACAAGGATATTAATTTTCACCGGTATGTGTTGTATTCTGCTGCCATGAGGTTTTTAGTTTGGACGCTAGTGTTCTTTGATGATCCAATTGTCTATGGCTATGAATACTTGATAAGTACTTCACCAATTTTATATCTTTTGATAGTGGTATAAAATACTTCTTCACTTTTATTCTTGTAGCTACTTGCTTATCTTCTTTGCTTCCATTTCGTTGTCGTTATTACACTAGTTATTTTTGGTTGGATGGATGTGTAGATCATGTGGTGATACTGTTATGGATGTTAAAATTTGGATATTTCAAAGCGTGGTTAACCAACATGTGATCCTCTGCCACCTTTTGATCTTTTTGGTGCTTGCACGACAACCTAGATATTGTCCGAGCTTTGACAAAATGGAGTTTTTAGTCACATATATGGCATCTCTAGTGATTACTTAAGTGGTTTGGGGTTTGTCGGATTTACATTTAACTGTTCCCTTCTTTCTGTTCTTGCAGCCAATGGTTATAGGGAAGTGAATTTTTGTTTTCATGGAGGGGCCGTGTTTGATTGTTTGTGTTGAGGTTTTTTTTAGTTAAGTCTTTTATGAGTATCGATATTACGGATTTAGTTTTCTTCCTTAATAGCTCCTTTGTTTTGTATGGAAGGTTTTCTCAACTAAGATATTAATTTTCACTAGCATGTGTTATATTTTGCTGCCATGAGGTTTTTAGTCCAGATGGCTTATGTTCTTCGATGATCCAATTGATGAGGTCTCATTTGTCTATCGATTCTTGGTAAGTACTCTGCTAATTTTGGATCTTTTGATAGTTGCGTAAAATACTTCTTCACTTCTATTCTTGCAGCTACTTATCTTCTCCACTTCCATTTCTTTTTCGTTATCACATTAGCTATTTTTGGTTGGATGGATGTGTAGACCACGTGGTGACACTGTTATGGACGTTCAAATTTAGAAATTTCAAATCTTGGTTAGCCAACATGTGATCCTCTGCCACCTTTTGGTCTTTTGGTGCTTTGCACTGCAATTTGGATGTTGTTTGGGTTTTGACGAAAGGGGGTTTTAGTCTCGTCTATGGCATCTATAGTGATTAATTAGGTGGTTAGTTTGTCAGATTTGCTCTCAATTGTTCCCTTCTTTCTATTCTTGTAGCCATTGATTACAGGGCAGTGGATTATTCTTTTGACGATGCAGTCATGTTTGTCCATTTGTGTTGAGGTTTCCTTAGTCAAGTCTTTTGACATTATTCTTCACTGGCGTGTGTTGTATTCTGCCGCCAAGAAGATTTAGGTCCAGATGGCTTTGTTCTTCGATGATCCAATTAATGACGTCTCCTATGACTATGGACACTTGATAACTCAACCAATTCTACATCATCTGATAGTGGCGTGAAGTACTTCTTTTACTTTTTTTCTTCTAGCCATTGGGTCTTACAGAGTGATGAATCATTCTTTTCATGGAGCGGCTTTCTTTGTCCATTTATGTCGAGGTTTCCATGGTTCAAGCTTTTATAAGTTCTAACATTTTCGAGTAAAATAGTTTTTTTCCCATTAGTATGTGTTGTTCTACTGGCATGTTTAGTTTTTAGTCCAGATGTTTGTTTTCTTCAATGATCCAATGATGAGATTCATGATGGTTGATGGTTATTGTCTCATGGACATCTGACAAGTACTCAATTTTGGATCTTTTGAAAGCCTCGTAAAATGGTTGGTGTCCAGTGTGCATTCCTGTGCACCCTATTTCTTGGGCATGTTGGTGCTTGCACTACAATCTAGAGATAGCAGGCTTCGAAAGAAGCTGCTGGGTCTTACAGAGCCATAGATTCGTCTTTTCATGGAGTGGCGTGGTCTGTCAATTTTGTGTTGAGGTTTTTTGGTTGATGCTTCTAATGATTATTGAGATCTTCAATTCAGTTTTATTCCCTAATCAGAATGGTTCCGTTCTTCCTTGTCAACAATACTTCACTGGTGTGTATTTTGTTGCCATATATGTTTCTTAATAATCCAGATGCTTATGTTCTTTGATGATCCAACTGATGAGATCTCCATGGTTATGGACAACACATGATAAGTGTAATCAAGCTCAGTCTTGGATCTTCTGATGGATCTTTTAATAGCCTTGTAACACACTTCTCTAGTTTTCGTGTTCTCTCTACTTACCTTTCTCTGATTTCATTTAACCTACCAGTTTATTTAGTTTGGTTGGATGAATTTATAATTTTGGTTTCGGTGTGCATCCTTGTGGACCATGTTGACCATTTTGGTGCTTGCACTGCAATTTGGATATTGGATATCACCATGCCTTAAGAAAAGGGCACTTTGCAATCTTATCTTGTTTGGCATCTCGAGTGATCTCTCCCGTGTGGTATGGTTTTCCACTCACCTGTTACATTATTTTTTGTGCATGTAACCATTGGGTCTTCCAGAGCATGGACTTCAAGTCAGTTGTCTTCCTAATCAGTGGGTCCCTTTTCTCTTTGTTAAAATTATTGTTATGTGGAAGTTCTCAACAATGATAATATTTTCCACTAGTGCGTGTTGTACACTGCAACCATAAAGTTTTTAGTCTAGATGCTTATTTTCTTGGATGATCTAAATGATGAGATCCCTGATTTGATATGGACACCTGAGAAGTACCCAAACTCAATTTTCCCAAGGGCTTCTCTACTTTGGTGTTCTTGGTACTTATTTTCTCTGCTTCTATTTTCTCATGTGTATCATGCCACTTGATTATGTTTGTTTGGATGCATGTTTGTAGACCACATGGTGACTAGAGGGGGATCGAATTTGGTAATTTCGAAGTATGGTTTTGTCATTCCCTTGTGCACCCTGTTTCTTTGCCATTTTGGCGATTGCGCTTGCACTGCAATTTGGATATCACTAGGCAATAGCAGGGGAGTTGTGCAATCTTTTTTGGCATCTCTAGTGATCTCTCCTATGTGGTATTGGTTTCACAGCTTGCACTTTAACTGTTACCTTCTTTTTGTGATTGCTGTTGTTGGATCTTTCAAGATCAGTAAAGTCTCCATGAACTCCATTTCATAGAGTAGACTTGTTTGTTGTAGTGTTGAAGTTTCTTAGTTCAGGCTTTGTATTAGTATCTAACTCTTCAATTCAGTTTTCTTCCCTGGTCATAGAGATTCCGTGGGTAGCCAGGTAGCCGTCGGTCTTGTCCCGACACCTATCTTCAGTTAGTAGAGGCTTCATAAATAGTCAAAGTTAATGCGTCAGTTCAGTCTTTAGTATTCAGAAGTTTTAAACATCATTATTATTATATTATTATTATTGTTATTGTTTCAGACTTTTGCATGGGATATCTTTAAGTGATTTTAAATGTTCATTTTATACGGCTTCCGCATGTATGTTTAGTCTTCCTCTCAGTAGTTAGCTAGGACAAGGGTTTGCTTGGGACCAACAGTGGGGTTCCGAGTGCCAGCACTCAGAACAGTGGGGTTTCGAGAGCCGGCCACGTCCAGGTGTAGGCTCGGGTTGTGACACAAATGATGAGGTCTCTTATGGCTATGGACACTTGATAAGTACTCAGCCAATTCTAGATCTTTTGATAGTATCATAAACTACCTTTTTACTTTTATTCTTCTAGATACTTTCTTATCTTCTCTGCTTTCTTATATTTTTCGTTATCACACTAGTTTTGTTGTTGTTGTATGGATGTGTAGACCATGTGGTGACATTGTTATGGCATTGAAAAAATGGGGTTTCAGTCTCGTCTATGGCATCTCCAGTGTTACTTAAGTGGTTTGGGGTTTTCCAGATTTGCATTTTACTATTCCCTTCTTTCTGTTCTTGCAGCCATTGGTTACAGGGCATTGAATTATTCTTTTCATGGAGTGGCTGTGTTTGTTTTTTTGTGTTAAGGTTTCTTTAGCTTGAGTCATTTATGAGTATCGAGATTTTCGATTCAGTTTTCCCTTTTCTATATCAAAACTATTGTTCTATGGCAGTTTCTCACTAAGATAATATGTTTTACCCGTATGAGGTTTTTAATTCAGACACTATTTTCTTCAATGATTCAATCGATGAGGTCTCTTACGGCTATGGATACTTGATAAGTACTCGGCCAACTCCGAATCTTTTCTTTTGACAGCGTCATAAAGTGCTCCTCTACTTCTGGTCTTCTAGCTATATTTGTTCTTTGTTGACATCTTCTTTTGGTTACAAAAACTTCTTAGTTTTTGTTTGGATGCATGTGTAGATTATATGGTGATTGTAATAGAGGTTAACATTTAGAGATTTCAAAGGTTGATATCCAATATGGTATCTCTATACCCTCTTGGTCTATTTGGGTCTTGCACTGCAATCTGGATATATTGGTCAGGGCTTGAACCAAAAACGGGTTTTAATTCTTGTCTATATAGCATCTTTAGTGATTTCTTAGATTGTATGGGTTTCGTCAACTTTGGACTTGATTTGTACCCATTTTTTTTTGCTTGAAGCCATTGGGTCTTACAGTGCAATTAATTATTCTTTTCATGGAGCGGTTCTCTTTGTCCATTTATGTCAAGGTTTCTTGGTTCAGGCTTTTATGAGTTCTGACATTTTCAACTAAAATAGCTTTTTTCCCAATAGTGTGTGTTGTATTCTACTGCCATTTTTAGTTTTTAGTCCAGATGCTTATTTTCTTCAATGACCCATTGATGAGATTCCTGATGGTTGATGGTTATGGTCTCATGGACACCTGACAAGTACTCAATTTTGGATATTTTGAGAATCTCGTATCTCTTTTTGTGTTCTCGCAGCTCATCTTCTCTGCTTCACTTTTCTTGTATTACGCTAGCCTGATTTTGTTTGTTGTATGCATTTATGGTGGCAGTTGAGAGGAATAGGACTTAGATGTTTTTGAAGGTTGGTGTCTAGTGTGCATTCCTTTGCCCCTGTTTCTTGGACATGTTGGTGTTTGCACTACAATCTGAAGATTATCAGGTTTCGAATGAAGGTGGTACCTAGTTCGCCAGCTTTGCATTTACTATTCCATTCTTTCTGTGCTTGCAGTCGCTGGGTCTTACAAAGCCATAGATTCTTCTTTCATGGAGCGGCATGGTCTGTCAATTTCGTGTTGAGGTTTTTTGGTTGATGCTTCTAATGATTATTGAGATCTTCAATTCAGTTTTGTTCCCTAAACAGAATGGTCCCCTTCTCTGTTGTCAACACTGCCTCACTAGCCTGTATTTTGTTACCATATTTGATTTTTAATAATCCAGATGCTTATATTCTTTGGTTATCCAACTGATGAGATCTCAATGGTTATGGACAACACATGATAAGTGTAATCGAACTCAATCATGGATCTTTTGATGGCCCTGTAATGCACTCCTCTACTTTTCGTGTTCTCGCTACTTGTCTTCTCTTATTTCATTTAGCCTACCATTTGATTTAGGTTGGTTGGATGAATGTTTGTAGGCCACATGGTGGTAGTAAGAGGATGAGATTAGGATATTTTGTTGTTTTGTTTCGGTGTGCATTCCTGTGGACCATGTTTCTTACCATTTTTGTGCTGGCACTACAATTTGGATATTGGATATCACCAGGAAAAGGGGGCTTTGCTATCTTATCTTGCGTGGTATGGGTTTCCACTTACCTGTTATGTTCTCTTTGTGCATGTAACCATTGGGTCTTCCAGAGCATGAACTTCGGTTTTCTTCCTAATCAACGGGTCCCTTCTTTCTTTGTCAAAACTACTGTTGTGTGGAAATTCTCAACAATGATAAAATTTTCCACTAGTGTGTTTGTACTTTGCAGCCATCAAGTTTTTAGTCTAGATGCTTATTTTCTTGGATGACCCAAATGACGAGATCCCTGATTTTTATGGACACCTGAGAAGTACCCAAACTCAAATTTAGGTCTTCTTATGGCCCTAAGAGCTCCTCTACTTTAGTGTTCTTACTGCTTTTATGTTTATCATGGCACTTTATTTTGTTTTGCTTGGATATCATATTTGGAAATTCTGAAGTTTGGTTTCAGTGTGTGTCCTTTCCTTGTGCACCCTGTTTCTTTGCAATTTTGGAGCTTGCGCTTGCACTGTAATTTGGATATCACCAGGCACTGTCAAGAGAGTTATGCAATCTTGTTTGGCACTGCAATTTCGATATCACCAAGCACTGGCAAAGGGAGTTATGCAATCTCGTATGGCATCTCTAGTGATCTCCCCTATATGGCATGGTTTCGCAGCTTGAACTTTAACTGCTCCCTTCTTTTAGTTCTTGCTGTCGTTGGGTCTTTCAAGATTAGGATAGTCTTCCTTTAATGGAGCAGCCTTGTTTATCTTAGCGTTGAAGTTTCCTTTGTTCAGGCCTTGTATTAGTATCTAGCTCTTCGATTCAGTTTTCTTCCATGGTCATAGAGATCCCCTTATCTCGTTGTCAGAAGTGCTGTTAAGTGGAAGTTTTCAACTACAATAACTCTCTCCACAAGTGTATTTTCAGCTTTTAGTCCAGATGCTTTTGTTTTGCGATGATCCAATTGATGAGGTCTCTCTTGTGGTGTGGACATCTGGTAAGTGCTCGCTCAGTTCCGGATGTTTTCATCATCTACCTTTTGTGTTCTTGCTGCTTATCTTCTCTGCTTTCATTTTATTTTTTCTTATCACAACAGGTTTTGAGTTTTGTCTATTGGAAAGATATATATTCCACATAGTGAACACTATTTGAGGATTAGATTTGGAAATTTAAAAGTGTAGTGTTCAGTAAGCTAGTCGTTTAGGTGTTTCCCCTGCAATCTGGTATCGTTTAGGGCTTCAAGATTAGGGAGTTTCTAAATATTGTCTATGTCATTTCTAGTGATTTCCCAAGCACTCTGGGTTTTGGCAGATTTGCACTTAATTGTTCATTCATTTTGTGCTTGCAGTTGTGGTCTAAACATAGTAAATTTTCTCTTTTCGTGGAGCAGCCTTTCTTTGTTGATTTGTATTGAGGTTTCGTTCGGTTCAAGCTTCTATGAGTATCCGATTTTCAGTTCAGTTCTTTTTTTTTTGTTCTCTTCTTTAATCAATATGATCTTTCTTCACCACACTCTCTTCGAGTGGTGCTCTTCTAGTAGTTAGTTTAACTGAGAATTTTGCACGTCAGTGGTGGTTACAAGCATTCAAGTTCTTAGGAGTTCTCTTGGTATTATGTGTAATCAAGTCTCGGCTCGGCCTATCTTCTTTCTCCCCCTCCCCTTAACACCTGTAGTGCTGTTGGTTGGCTGCCTCCACCTGATGGTTATGGTGGGTCAACGTGTGCAAGATTTTAACCAGGTCTTGCATAATGTGATAATGAGAATGTTCTGTCGTCGACTGTCGATAACATTCTTTTCAAAACCTCTTATAGACTAGGCTGGGATCTTATCCATTTTAGATGGTGATCTTACATGTTTCAGAAAACTGTAACTACCTTTGGTGGTATAGAGTTGATATCTCTACTTAATGTTGTACTCATTTGTTTCATCCCATTGCCCTCTATTAAATTCCTTGCTTGGTCGGAATGGGGAGGAGTGTCAACTCGAGTTCTTTTCTCCTGCCAGTTGTCTTATCTGATCCGATGGCTTTCATTTTCTCCCGGATGCAGGTTTCTGGTCTTTGATTGTGGCTGTTGGAAGTTTGGGTGGAGGGGTTAAGGATGTTTTATTATGATGTTTCTGCCATAATGAAACTATTCTTATGTTACCTGGTTGACAATTTTTGGGTATTTACAGAATTTGATAGGTCTGGATTTGTTGGTGCAGTTGTATTAAGTTTTCTGATTTTGCTATATAGGTATGGAATATGAGCAGATTTGTGAGTTGCAGAAAAGAACGTCCGTCAGAAAGCTTGGCCTCCTGTGCTCGAAGTGAGACCTGAAGGGTGAGCTTATGGCGCACGAAATCCATCTCTCGGTTTTGCAGATCTATTTTGAATTGTGAATTTGCTGTGAGTTCTCACTTTTGTTGGTTTCTCGAGGTATCCGGGGTCTTCCTGCTTAGATTATGCCAATTTCTTTCTCTGTTTTACCAGGGTAATGATTGTATGGGTATAGACAATGCAGTTTCTGTTGTCGGATGTCAATAATCTTTGTAACGAAGTAAACCTCATCTCTGGTACCTCCAACAAATCATCTTTTCTTGCGTCTTCTCTTTTGGAACACTATTCAACAGAAGTTTATGAGCCTTTCCTTGTAGATGTTGCTCTCTATATTTCATACCAGTATACGTACCCGCACGTTGCGCGGACATTATAAAATGTAATAATGTTTTAAATTTAAAATAACTAATAAGGTTTATTATTTTCTTTGTAATATATTTTTAAAATAATGTAATAAATCATTAAATTATTTATTTGAGCAAGTTCTATTAATATTTATGTACAC

>LNC_000632

TATAAATGGCTATAGCGGCTAAACTAATTCTGAAAAGCACCCACCCAATGCCACCGCCCTTTGAAATATTGTTGCCATAAATAGACTTGCTAGGAAACAAATTTCTCAGTTACGATTTGTTTTAGCTTTAGTTCTACTATTATTTGGTGTTCATTCTTGAGGATATAAGCCGTGTTTGATTGTTGCTATGGATAGTTATTGTTGATCAAGATATGGGTAACTATTGGTATGATCAAGATGATGGGTATGGGTAATGATGGTAGGAGACTGATTCTCCTTCTACATTTGCTGTTACCTTAATGTCAATGTTAATTCTGAGGTTTTTGTTGTTTAGAATTCATGTGTTAGTGTCATCCTGTTTACTACTCTGAAATCTAGCAGTTTCCTTTTGTTGAATCAGAACACAAGTAGAGTTGGAAATTTGGCTCTAGGTGTGCATCTAGGTGCAAGTGGTTCTACAGGAACAAGTGTCTTGCTTCAGTGTTTGTGGTCAAGAAAGATCCAGCAGTTCCCAGACCACATGGTGACAGTGACAGAGGATTAATTTGGAAACTTCTAAGGCTTATCCGGTATGCATCCATGTGCACCACATGGTGATGGATATTGTTAGGCTCTCTTGCATCTCTAGTGATTTCTGAGGTATATGTGTTTTTTCAGCACATAGTTGTTCCTTCTTTTTGTGCTTGCAGTTGAGTTTTACCGAGCAGTGAATTATTCTTTTCATGGAAGAGGCTTTGTTTGGCCATTTGTGTTGAGGTTTAATGAGTACTGGAGATACTCAATACTCATTTCAGTTTTTTTCCCCAATCAGAGTGGCCCCTCCTCTTTGTCAAAAATTGTTGTTGTATGGAACTTGCCAGCTAAAATAGTTTCTCTTGCACTCACGTGTGTTGTATGGAACTTTCCAGCTAAAAATTGATGTGTATGTTGTTCGATGATCCAGTTGATGAGATCCTGGTGGTTGATGGTTATATGGTCTTATCGACACCTGATAAATACTCAAAACTCAATCCTGGATCTTTTGACAGCCCGTACAAGTGGATCCCTGTTAGACCCTCTCTTTTGTTGTTCTCTCTGCCTTAACTTCTCAGCTTCCTTTTTCTTGCATCACCCAAGCTTGATTTTGTTTGTTGGATGGATGATAGTTGAAGGTTGATGCCCAGTGGCCGTTTCTGTGCACGCTGCTTCTTGCTATTTTTGTGCTTGGACTACAATCTGAATATAATCAGGCTTCAATAGAGGGGTTTTCCAATCTCGTTTCTTTGCCATTTTTATGTGTGCATTGCAACCTGAATATTTTCAGGCTTAGAGAGGGTTTTTTAAATTTGTCTGTGGCATTTTCTAGTAGATTTTGTCAGCTTTGCACGTAACTCTTCCCTTCATTCCTTTGCTTGCAGCCATTGGGTCTTACAAAGCAGTGAATTCTTCTTTTCATGGAGGTACCTTATTTGCCCAAATGTGTTGAGGTCTTGTTAGTCAGTCTTTTACGAGTATAGATAATTTCCAATTTTTCTTCCCTATTGATTCCTTTGTCTTTGTCATAACTTCTGTTGTATGGTAGTTTCTCAATTAGGACATTATTTTCACCAATGTGTGTTTTATTCTACTGCCATGATGTTCTTAGTTTAGATGCTTATGTTCTTATGGTGATCCATTTTTTATTTCTTGGTTATCTCAGAATTATATAAATCTCACGTTATCTACTTTCGAATCTTGGTTTACGAATCTTTTGATATGGGGTGTAGGTTTCTCATTTCTGATTTTCGAATCTTTGATTATCTCTGTAGACAAGCTAAGGTCAGAAGATGAATGTTAGGGGGGGTTTTGGGAGCTGTTGATCTTTTTTCTTGGCCAATTTATCGATTCGTTATTGTTGTTTGTTTGATTAGGTATGGGTAATGATGATAGGGGAATGGTTATAGGTACCTTCCAGTGTATTTCATGATTCTCCTTCTACACTTGTTGTTACCTTCTGTCAGTTACCAAATATTCGTATCCTTGCTTTCCTTCATGTGTAGTTTATTTATTTATTTGTTCTCATTTTCTTGGGTATTATAGTGGGAGTTTATAATTGCAATCCTTTTGATTGATTCAGAATACGTGTACACAGCAGATTCGTGAGCAAGAGTGTATTCAACGAACAACTACAATAATTTTTTCTACGGAACTTTCATATGAGTCTATTCTTCCTTTTAATCTACTATATAAAGGTTTTAAATTGACCCTTAATATTAAAGATAATTACATTCTTGAAAGGATTAAGGGTTCTACCTTCCTGACTTTTCGCTTGTTAGGACTGGAAATTTAGCTCTCCAGGCATGCCTACTTGGATCTTCTATTGTGGACTTATGTGTCACAACCATACAATTGACCTATCAGTTTGGATTTGATATCTTGACTTCCATCTTGAATGTTAATTTTGATGCTAATCACGTGTCTCCTCACATCAACTGCTTTTAGCTCCTTAGTTGGTTTGGTCATCAGTAAATGTCATTCTTAGCTGTCAAGAATACATGGATGGGGCATCTAGGTGCAAGTGGTTCTACATGAATAAGTGTCTTGCTTCGGTGTTTGTGGTTGGGAAAGATGCAGCAGTTCCCAAGCCTTTGACTATAGGTCTTGGATTTTTCAGTCTCTCAAACCTTTATTAAAACTAGTACGGTATGAAAGTTCTCAACTATGATAATTCTTTTCACAAGTGTGTATAGTGTGGTCTGCTCTGCCATTTTTAGTTTAGATGGTCATGTTTTTTGATGATCCGATATCTTCTATGGTGTTATGGGCACCAGATAAAGTCAAAAGTCAATTTGGAATCTTTGATAGTGCTTCTCTGCTTTTTGTGTCCTAGCAACTTATCTTCTCTGCCGTTTTCCTTGGTTATCATGCCAGCTTGATTTTGTATTGGTGAATGGATATGTAGACCACATGGTGATATTAATAGTGGATTAGTTTTGTTCAGAGGCTTGTATCTCGTATGCATTCTTGTACACTTTGTTTTTTGGTCTTTTTGGGGCTTGGACTCCAATATATTGTGTATTATAGTGGTAGTTTATAATTTCAATCCTTTTGATTGATTTCCAATGTTATTAGAAATTGGAAGCTGGTGACTATAAATGCAATTTCATGTTTGCTTCCATGTTTTGGGAAATTCTGAGGTCTTTGTTGTGAGAATTCATATGTTGGTGAGCATTGTTCTATCACCCTATTTACTACTATGAAGCACATCAATTTCCTTTTGTTGAATCAGAATACTGGAAATTGGCTCCCCAGACATGCCTACTTGGATCTTTTATTCTAGACCTATGTGTCACAATCATACAGTGGAGCTATCAGTTTGGATTTGATATCTTGACTTCCATCTTTAATGCTAATGCTAATCATGTCTCTCTTCGCATCAAATTCTTTTACCTTCTTATTTGCTTTGGTCATCAGTGTATGTCATTTTTAGCTGTCAAGAATACATGGATGGGCATTTAGGTGCAAGTGGTTCTACAGGAATAAGTGTCTTGCTTCGGTGTTTGTGGTTGGGGAAGATGCATCAGTTCCCGGGTCGTTTACTATAGGTATATTGGATTTTCATTCTTTCAAAGCTTTGTCTAAATGGGTACGATATGGAAGTTCTCAACTATGATAATTCTTTTCACAAGTGTGTTCTGCTGCCAGTTTTAGTTCTTAGTTCAGATGCTCACGTTCTTTAATGATCCTGATGTCTTTTATCGTGTTATGGGCATCAGACAGAGTCAAAAGTCAATTATGGATCGTAGCTAGTGCTGTAAAGTGCTCATTTGCTTTTTGTATCTAGGTACTTATCTTCTTTGTCGTTTTCCCTTGGTTATCATGCCAACTTTATTTTTTTTATTGGTGATAAATGTGTAGACCACGTGGTGACAGTTAAAGATTATTAGTTTTGGAAACTTCTATGGCGTGTATCCGTTTTGAATTCCTGTACACCTTGTTTCTTGGTCTTTTTGGTGATGGATATTGTTCTGCTTCGACCAAAGCTGTGGTTTTCAATCTTGTCTCTTGCATCTATAGTGATTTCTTAGGTATTATGGATTTTGTCAGCTTTGCACTTAACTGTTCCCTTCTTTTTGTGCTTGCAGTTGGGTCTTAATGAGCAGGGTTTTATGAGTACTGAGATTCTCATTTCAATTTTCTTCCCCAATCAAAGTGCTTCCTCTCTTTATTAAAAAATGTTGTTGTATGGAAGTTCCCAACTAAAATAGGTTTTCTTGCGCTCGTTTGTGTTGTATTCTGCTACCTTTTTTAGTTTTTATCTAGATGCTTATGTTCTTTGATGATCCAATTAATGAGATTCATTGTGGTTGATGAGTGAGTATTAAATCTCTTCCTAACGCCTTTAGGGGCTGTGAAGAGAGCCCAGTTCCAGGCCCTCTTTTAGAAAGAAAAAAATGGTACAAAAAGAGGGCTTGGGGGTATATATACTATACAAATTGATGATGGTTTATCATCATCAGAACAAAGATCAGTCTAGGTCGAGATGAATGGAAGATGCTTCTGGAACTAGTTTTTGATAGGGTCTATCTTAGCTCGAAGGAAAGAACAACCAAAGTTAGACCCCCAAAGAATATGGATTAAAATCTGTTAGTATTTGACAAAGAACCTAACGGAATTTAACAAGCAGTAACCGGAATCAAAGTTTTGCAAAATGGTGTCAACCGGTAATAGAATGTAGCTAGATAACTGATGCCACAGCATCCAAAATATGTGGGATGGAGTTCATGGTACACTAACTTAACCTTCAAGGTCAGATAGTGCTACAGTGAACATCAATCCACTATCTAGCTCAAAATTAGCCTCACGAACTACTAACACTATTATTCGCGTAGCCCTTTTTTGTTTTGTGCTATTTCATCATAGGCTGTCGGAACTTGCTTCTTAGCGCTACGTGGGAAGAGAATTTCTCTATTCAGCCCCCCCGACTTCAATTACAAACTCTTACTTCTCGGTCTAACTTGGACTAATCCCTTAGCAGGCAAGAAATTCCTACTAACCAGACAGAAAGTAAGTTTCCACTCACTACCAAAGCGCTGTCGCACCTCTACTATTTTCGGCGTAACGAGGTTTTAGTCTTTACGAGGGCACCCCCTAGCCAGATTCAATCCCAAGGGTCAATCAAGCCTTTGAAACTAGTGAAAATAGTCGTCACAGTCTGAGTTCCTGCTTTCAACGAGACTTTCTTAGCTTGTCAAACTTTCTCTCCTTCACCAGGAAAGTTATCCGGTAAGCAAGATCTTTGATTGATGTGCCTGAATAAGGTTGATCAATGGTAGCTGCTTGGAGCTTTCTCTATACCTTAATTGAACTGCCCCGCTCTTAGCTCGGGTTCTTGCTTAATGAGGCGTCCCTTGTTTGCTCTTGGATTGGACTAAGCGGCAATCCTTACTGTGGAAGCAGACCTTGTCATTCTTCTCCTTCAATGCCAGTGGATAAGTACACTAGGGCTGGAATGCTCTTTTATCATGTATGTGGATTACTTGGTCGATTACCTGATTGCACTAGTCTCATAGCCATCTCTTTAGCGTCCCGCCATTCCTTCTTGTTAACACAGGAAATCTAACTCATTCTAAGGTAGAGCTCTTAGAGTCCTACTTAAAGTGAAAAGTAGTTCTTTAGTTAAAGCAAGGAGAGCAGAAACTGAGGGAAAAAACCTATGTTCTTACGCGGGATGGGAATAAAGGCTACTAAGTAACCTTAGAATGGGGATGCCAATCTTATACAGAAAATACCGCTATCTAAAGTAACCTTGCAGGGGGGCTGCGTATAGCATTCTAAGATCTCAATACGAGAAAGAAATCAGACCTATAACCTCACTGCAGTGGCTTGTAGACTTGCTTTGCCGCGGAAAGCTCAAGTGAAAACCTATTAGAACTGTGGGCCTCACCCGTCTACTACTCGACCTTAAGTAACTACAGTTAGGAGGATCCATCCAATAGCTTAATGACTTGCTTGGGTCATCTAAGAGGTAAGGCCTCCTCCGATCGACCCTAACTTTCATTATTGAGCTAAAGGAGTTCCCTAGCCCTTCAAGTTTACACAGGTCCCCAAAGATAAAGGCTTTCATCCTTCCTGAATATTCTGGGAACTTTCTTCTATTAATTGTTTTCGAAATGCCGATTTTCCTGTCTGCGCTAAGGCGATAAGGAAAAAGAGAGGGGCCTTTAGTATGTGGAAAAAAAGGCAAAGATCACATTCCAACTAACGTTGACTGGTAATTGCCTCAATGAGGCGGATAAGGCGCAAGAACCTAAAAGGCGGACTAAGATAAGTTATTCAGCTAGGGAATGCGTTAAAGAGCCGTTATGTAGCATCTGAGAACAAGAAAGCAGACATGCACACGAAAAGACATGCACACGAAAAGCAGACATGCTTTCAAAGGGCTTTCAAAAATCAATCATGGATCTCTTGAAAGCCCTTTGAAGTGGGTCTTTGTTACTCCCTTACTTTTGGTATTTTCTCAGCTTCTTGTTTCTTGTTGCATTGTCACCCGAGCTTGATTTTGTTTGTTGGATTAGAGTTGGAATTTTTTCAAAGATTGATGATGTGATCAGGCTTTGATAGTTAGTGTTTGCTCCTATGCACCCTGTTTCTTTGCAAATTTGTTCTTTCTCACCAATCTGAATTTTGTCAGGCATTGATAGAGGGATTTTCCTATCTTGTTTCTTTGCCATTTTTATCCTTGCACTGCAATCTCAATATTATCAGGCTCCGAGAGAGGGGTTTTGCAATCTTGTCTGTGGCGTTTTGTAGTGGGTTTCATCAGCTTTGCACGTAACTCTTTCCTTCATTCCTTTGCTTGTAGCCATTGGGTCTTACAAAGCAACAAATTCTTCTTTTCATGGGATGACCTTATTCGCCCATATGTGATGAGGTTTCGTTAGTTCAGTCTTTTATGAGTAGAGATAATTTTCAATTCAGTTTTCTTCCCTAATGACTTGTTTGTCTTTGTCAAAACTTACTGGTTTATATAAGTTTCTCAATTAGGAAATTATTTTCACCAGACGGTGTGTTGTATTCTGCTGCCATGAGGTTCTAAGCCCTCACACTTATGTTCTTCGATGATCCAATTGATGAGGGATAATTTTCAATTCAGTTTCTTCCCTAGTGACTCCTTTGTTTTTGTCAAAACTACTGTTGTATGGAAGTTTCTCAATTAGGACATTATTTTCACATGTGTGTTGTATTATGCTTCCATGAATTTCTATGTCCGCATGCTTATGTTCTTCAATGATCCAATTGATGAGGTCTTTTATGACTATGGACACCTGATAAGTATTTTGGCCCCTTCTTGATCTTTTGATAGTTGCATAAAATACTTTTTTTACTTTTGTTCTTTTAGCTTCTGATCTTCTCTGCTTCCATTTTTTTCCGTTATCAGACTAGCTTTTTTGGTTGTATAGATGTGTAGACCATGTGGTAACACTGTTATGGACGTTCAAATTTGGAAATTTCAAAGTTTGGTTAACCAACATGTGATCCTCTCTCACCTTTTGGTCTTTTTGGTGCTTGCACTGCAATTTGATGCTGTCCGGGCTTTGGCAAAAGAGGGTTTTCAGCTTCGTCTATGGCATCTATAGTGATTACCATTGTGGTTTTGGGTTAGTCAAATTTGCACTTAACTGTTGCCTTTTTGTAGTTCTTTCATCCATTGGTTATATGGCAGTGAATTATTCTTTACGTGGAGAGGCTGTGTTTATCTGCTTGTTGTTGACGTTTCTTTAGTTAAGTCTTATATGAGTATCGAGATTCTCGGTTCAGTTTATTCCCCAATGACTCCTTTGTGTTTGTCAAAACTACTGTTGTATGGAAGTTTCTCAAATAGGATATTAATTTTCACCGCAATGTGTTGTATTCTACTGCCATGAAGTTTTAAGTCTAGACACTTATTTTCTTCGATGATCCAATTGATGAGGTCTCCTATGTCTATGTTTCTTGAAAAGTACTCAACTATTTCTAGACCTTTTGATAGTGGCGTAAAACACTTATTCACTTCTATTCTTGTAGTTACTTACTTATCTTCTCAGGTTCCATTTTTTTTCATTATCACACTAGTTTTTTTTTTTGTTGGGTGGATGTGTAATGGTGATTCTGTTATGGATGTTCAGATTTGGAAAATTCGAAGCGTGGTTGACCAACATGAGATCCTCTGCCACCTTTTGGTCTTTTTGGTGCTTGCACTGCAATCTGGATAATGTCCGAACTTTGACAAAAGGGAGGGGAGGGGGGGGGTTTAGTCTCATCTATGGATTGGCTAGTGATTACTTTGGTGGTTTGGGGTTCATTGGATTTGCAATTAACCTTAACTGTTCCCTTTTTTCTATTCTTGCAGCCATCGGTTACAAGGTAGTGAATTATTCTTTGATGGGGCTGCCCTATTCGTCTGTTTGTTGTTGAGGTTTCTTTAGTCAAGTCTTTTATGAGTATTCTGATTTTCGATTCAGTTTTCTTCCCTAATGAATCCTTTGACTTTGCCAAAACTATTGTTGTAGGGAAGTTTCTCAACAAAGATATTTATTTTCACTGGTATATGTTATATTTTGCTGCCATGAAGTTTTTAGTCCAGACAGTTGCATTTTTCGATGATCCAATTGATGAGGTCTCCTATGACTATGAACACTTGATAAGTACTCGTTCTAGATCTTTTTATAGTGGTGTAAAATACTTATTTCACTTTTATTCTTGTAGCTACTTATGTTTCTTCTCTGCTTCCATTTCTTTTTCGTCATCACATAGTTTTTTTTTGTGGTTGGATGTGTAGACCATGTGGTAACACTTTTATGGACATTTAGATTTGAAAATTTCAAAACTTGGTTAACAACATGTGATCCTCTGCCACTGTTTTTGTTCTTTTTGGTGCTTGCATTGCAGTTAACATATTGTGCGGTTTTGACAAAAGGGTGTTTTCAGTCTCGTCTATGGCATCTCTAGTGATTACTTAGGTGGTTTGGGGTTTGTCAGATTTGGACTTAACTGCTCCCTTCTTTCTGTTATTGCAACCATTGATTACAGGGTAGTGAATTATTCTTTTCATGAAGAGGCTATTTTTGTCCGTTTGTTGTTGAGGTTTCTTTAGTTCAGTCTTTTATGAGTATCGGAATTTTCGATTCAATCTTCTTCCCTAATGGCTCCTTTGTCTTTGTCAAAACTACTATTGCATGGAAGTTTCTCAACAAGGATATTAATTTTCACCGGTATGTGTTGTATTCTGCTGCCATGAGGTTTTTAGTTTGGACGCTAGTGTTCTTTGATGATCCAATTGTCTATGGCTATGAATACTTGATAAGTACTTCACCAATTTTATATCTTTTGATAGTGGTATAAAATACTTCTTCACTTTTATTCTTGTAGCTACTTGCTTATCTTCTTTGCTTCCATTTCGTTGTCGTTATTACACTAGTTATTTTTGGTTGGATGGATGTGTAGATCATGTGGTGATACTGTTATGGATGTTAAAATTTGGATATTTCAAAGCGTGGTTAACCAACATGTGATCCTCTGCCACCTTTTGATCTTTTTGGTGCTTGCACGACAACCTAGATATTGTCCGAGCTTTGACAAAATGGAGTTTTTAGTCACATATATGGCATCTCTAGTGATTACTTAAGTGGTTTGGGGTTTGTCGGATTTACATTTAACTGTTCCCTTCTTTCTGTTCTTGCAGCCAATGGTTATAGGGAAGTGAATTTTTGTTTTCATGGAGGGGCCGTGTTTGATTGTTTGTGTTGAGGTTTTTTTTAGTTAAGTCTTTTATGAGTATCGATATTACGGATTTAGTTTTCTTCCTTAATAGCTCCTTTGTTTTGTATGGAAGGTTTTCTCAACTAAGATATTAATTTTCACTAGCATGTGTTATATTTTGCTGCCATGAGGTTTTTAGTCCAGATGGCTTATGTTCTTCGATGATCCAATTGATGAGGTCTCATTTGTCTATCGATTCTTGGTAAGTACTCTGCTAATTTTGGATCTTTTGATAGTTGCGTAAAATACTTCTTCACTTCTATTCTTGCAGCTACTTATCTTCTCCACTTCCATTTCTTTTTCGTTATCACATTAGCTATTTTTGGTTGGATGGATGTGTAGACCACGTGGTGACACTGTTATGGACGTTCAAATTTAGAAATTTCAAATCTTGGTTAGCCAACATGTGATCCTCTGCCACCTTTTGGTCTTTTGGTGCTTTGCACTGCAATTTGGATGTTGTTTGGGTTTTGACGAAAGGGGGTTTTAGTCTCGTCTATGGCATCTATAGTGATTAATTAGGTGGTTAGTTTGTCAGATTTGCTCTCAATTGTTCCCTTCTTTCTATTCTTGTAGCCATTGATTACAGGGCAGTGGATTATTCTTTTGACGATGCAGTCATGTTTGTCCATTTGTGTTGAGGTTTCCTTAGTCAAGTCTTTTGACATTATTCTTCACTGGCGTGTGTTGTATTCTGCCGCCAAGAAGATTTAGGTCCAGATGGCTTTGTTCTTCGATGATCCAATTAATGACGTCTCCTATGACTATGGACACTTGATAACTCAACCAATTCTACATCATCTGATAGTGGCGTGAAGTACTTCTTTTACTTTTTTTCTTCTAGCCATTGGGTCTTACAGAGTGATGAATCATTCTTTTCATGGAGCGGCTTTCTTTGTCCATTTATGTCGAGGTTTCCATGGTTCAAGCTTTTATAAGTTCTAACATTTTCGAGTAAAATAGTTTTTTTCCCATTAGTATGTGTTGTTCTACTGGCATGTTTAGTTTTTAGTCCAGATGTTTGTTTTCTTCAATGATCCAATGATGAGATTCATGATGGTTGATGGTTATTGTCTCATGGACATCTGACAAGTACTCAATTTTGGATCTTTTGAAAGCCTCGTAAAATGGTTGGTGTCCAGTGTGCATTCCTGTGCACCCTATTTCTTGGGCATGTTGGTGCTTGCACTACAATCTAGAGATAGCAGGCTTCGAAAGAAGCTGCTGGGTCTTACAGAGCCATAGATTCGTCTTTTCATGGAGTGGCGTGGTCTGTCAATTTTGTGTTGAGGTTTTTTGGTTGATGCTTCTAATGATTATTGAGATCTTCAATTCAGTTTTATTCCCTAATCAGAATGGTTCCGTTCTTCCTTGTCAACAATACTTCACTGGTGTGTATTTTGTTGCCATATATGTTTCTTAATAATCCAGATGCTTATGTTCTTTGATGATCCAACTGATGAGATCTCCATGGTTATGGACAACACATGATAAGTGTAATCAAGCTCAGTCTTGGATCTTCTGATGGATCTTTTAATAGCCTTGTAACACACTTCTCTAGTTTTCGTGTTCTCTCTACTTACCTTTCTCTGATTTCATTTAACCTACCAGTTTATTTAGTTTGGTTGGATGAATTTATAATTTTGGTTTCGGTGTGCATCCTTGTGGACCATGTTGACCATTTTGGTGCTTGCACTGCAATTTGGATATTGGATATCACCATGCCTTAAGAAAAGGGCACTTTGCAATCTTATCTTGTTTGGCATCTCGAGTGATCTCTCCCGTGTGGTATGGTTTTCCACTCACCTGTTACATTATTTTTTGTGCATGTAACCATTGGGTCTTCCAGAGCATGGACTTCAAGTCAGTTGTCTTCCTAATCAGTGGGTCCCTTTTCTCTTTGTTAAAATTATTGTTATGTGGAAGTTCTCAACAATGATAATATTTTCCACTAGTGCGTGTTGTACACTGCAACCATAAAGTTTTTAGTCTAGATGCTTATTTTCTTGGATGATCTAAATGATGAGATCCCTGATTTGATATGGACACCTGAGAAGTACCCAAACTCAATTTTCCCAAGGGCTTCTCTACTTTGGTGTTCTTGGTACTTATTTTCTCTGCTTCTATTTTCTCATGTGTATCATGCCACTTGATTATGTTTGTTTGGATGCATGTTTGTAGACCACATGGTGACTAGAGGGGGATCGAATTTGGTAATTTCGAAGTATGGTTTTGTCATTCCCTTGTGCACCCTGTTTCTTTGCCATTTTGGCGATTGCGCTTGCACTGCAATTTGGATATCACTAGGCAATAGCAGGGGAGTTGTGCAATCTTTTTTGGCATCTCTAGTGATCTCTCCTATGTGGTATTGGTTTCACAGCTTGCACTTTAACTGTTACCTTCTTTTTGTGATTGCTGTTGTTGGATCTTTCAAGATCAGTAAAGTCTCCATGAACTCCATTTCATAGAGTAGACTTGTTTGTTGTAGTGTTGAAGTTTCTTAGTTCAGGCTTTGTATTAGTATCTAACTCTTCAATTCAGTTTTCTTCCCTGGTCATAGAGATTCCGTGGGTAGCCAGGTAGCCGTCGGTCTTGTCCCGACACCTATCTTCAGTTAGTAGAGGCTTCATAAATAGTCAAAGTTAATGCGTCAGTTCAGTCTTTAGTATTCAGAAGTTTTAAACATCATTATTATTATATTATTATTATTGTTATTGTTTCAGACTTTTGCATGGGATATCTTTAAGTGATTTTAAATGTTCATTTTATACGGCTTCCGCATGTATGTTTAGTCTTCCTCTCAGTAGTTAGCTAGGACAAGGGTTTGCTTGGGACCAACAGTGGGGTTCCGAGTGCCAGCACTCAGAACAGTGGGGTTTCGAGAGCCGGCCACGTCCAGGTGTAGGCTCGGGTTGTGACACAAATGATGAGGTCTCTTATGGCTATGGACACTTGATAAGTACTCAGCCAATTCTAGATCTTTTGATAGTATCATAAACTACCTTTTTACTTTTATTCTTCTAGATACTTTCTTATCTTCTCTGCTTTCTTATATTTTTCGTTATCACACTAGTTTTGTTGTTGTTGTATGGATGTGTAGACCATGTGGTGACATTGTTATGGCATTGAAAAAATGGGGTTTCAGTCTCGTCTATGGCATCTCCAGTGTTACTTAAGTGGTTTGGGGTTTTCCAGATTTGCATTTTACTATTCCCTTCTTTCTGTTCTTGCAGCCATTGGTTACAGGGCATTGAATTATTCTTTTCATGGAGTGGCTGTGTTTGTTTTTTTGTGTTAAGGTTTCTTTAGCTTGAGTCATTTATGAGTATCGAGATTTTCGATTCAGTTTTCCCTTTTCTATATCAAAACTATTGTTCTATGGCAGTTTCTCACTAAGATAATATGTTTTACCCGTATGAGGTTTTTAATTCAGACACTATTTTCTTCAATGATTCAATCGATGAGGTCTCTTACGGCTATGGATACTTGATAAGTACTCGGCCAACTCCGAATCTTTTCTTTTGACAGCGTCATAAAGTGCTCCTCTACTTCTGGTCTTCTAGCTATATTTGTTCTTTGTTGACATCTTCTTTTGGTTACAAAAACTTCTTAGTTTTTGTTTGGATGCATGTGTAGATTATATGGTGATTGTAATAGAGGTTAACATTTAGAGATTTCAAAGGTTGATATCCAATATGGTATCTCTATACCCTCTTGGTCTATTTGGGTCTTGCACTGCAATCTGGATATATTGGTCAGGGCTTGAACCAAAAACGGGTTTTAATTCTTGTCTATATAGCATCTTTAGTGATTTCTTAGATTGTATGGGTTTCGTCAACTTTGGACTTGATTTGTACCCATTTTTTTTTGCTTGAAGCCATTGGGTCTTACAGTGCAATTAATTATTCTTTTCATGGAGCGGTTCTCTTTGTCCATTTATGTCAAGGTTTCTTGGTTCAGGCTTTTATGAGTTCTGACATTTTCAACTAAAATAGCTTTTTTCCCAATAGTGTGTGTTGTATTCTACTGCCATTTTTAGTTTTTAGTCCAGATGCTTATTTTCTTCAATGACCCATTGATGAGATTCCTGATGGTTGATGGTTATGGTCTCATGGACACCTGACAAGTACTCAATTTTGGATATTTTGAGAATCTCGTATCTCTTTTTGTGTTCTCGCAGCTCATCTTCTCTGCTTCACTTTTCTTGTATTACGCTAGCCTGATTTTGTTTGTTGTATGCATTTATGGTGGCAGTTGAGAGGAATAGGACTTAGATGTTTTTGAAGGTTGGTGTCTAGTGTGCATTCCTTTGCCCCTGTTTCTTGGACATGTTGGTGTTTGCACTACAATCTGAAGATTATCAGGTTTCGAATGAAGGTGGTACCTAGTTCGCCAGCTTTGCATTTACTATTCCATTCTTTCTGTGCTTGCAGTCGCTGGGTCTTACAAAGCCATAGATTCTTCTTTCATGGAGCGGCATGGTCTGTCAATTTCGTGTTGAGGTTTTTTGGTTGATGCTTCTAATGATTATTGAGATCTTCAATTCAGTTTTGTTCCCTAAACAGAATGGTCCCCTTCTCTGTTGTCAACACTGCCTCACTAGCCTGTATTTTGTTACCATATTTGATTTTTAATAATCCAGATGCTTATATTCTTTGGTTATCCAACTGATGAGATCTCAATGGTTATGGACAACACATGATAAGTGTAATCGAACTCAATCATGGATCTTTTGATGGCCCTGTAATGCACTCCTCTACTTTTCGTGTTCTCGCTACTTGTCTTCTCTTATTTCATTTAGCCTACCATTTGATTTAGGTTGGTTGGATGAATGTTTGTAGGCCACATGGTGGTAGTAAGAGGATGAGATTAGGATATTTTGTTGTTTTGTTTCGGTGTGCATTCCTGTGGACCATGTTTCTTACCATTTTTGTGCTGGCACTACAATTTGGATATTGGATATCACCAGGAAAAGGGGGCTTTGCTATCTTATCTTGCGTGGTATGGGTTTCCACTTACCTGTTATGTTCTCTTTGTGCATGTAACCATTGGGTCTTCCAGAGCATGAACTTCGGTTTTCTTCCTAATCAACGGGTCCCTTCTTTCTTTGTCAAAACTACTGTTGTGTGGAAATTCTCAACAATGATAAAATTTTCCACTAGTGTGTTTGTACTTTGCAGCCATCAAGTTTTTAGTCTAGATGCTTATTTTCTTGGATGACCCAAATGACGAGATCCCTGATTTTTATGGACACCTGAGAAGTACCCAAACTCAAATTTAGGTCTTCTTATGGCCCTAAGAGCTCCTCTACTTTAGTGTTCTTACTGCTTTTATGTTTATCATGGCACTTTATTTTGTTTTGCTTGGATATCATATTTGGAAATTCTGAAGTTTGGTTTCAGTGTGTGTCCTTTCCTTGTGCACCCTGTTTCTTTGCAATTTTGGAGCTTGCGCTTGCACTGTAATTTGGATATCACCAGGCACTGTCAAGAGAGTTATGCAATCTTGTTTGGCACTGCAATTTCGATATCACCAAGCACTGGCAAAGGGAGTTATGCAATCTCGTATGGCATCTCTAGTGATCTCCCCTATATGGCATGGTTTCGCAGCTTGAACTTTAACTGCTCCCTTCTTTTAGTTCTTGCTGTCGTTGGGTCTTTCAAGATTAGGATAGTCTTCCTTTAATGGAGCAGCCTTGTTTATCTTAGCGTTGAAGTTTCCTTTGTTCAGGCCTTGTATTAGTATCTAGCTCTTCGATTCAGTTTTCTTCCATGGTCATAGAGATCCCCTTATCTCGTTGTCAGAAGTGCTGTTAAGTGGAAGTTTTCAACTACAATAACTCTCTCCACAAGTGTATTTTCAGCTTTTAGTCCAGATGCTTTTGTTTTGCGATGATCCAATTGATGAGGTCTCTCTTGTGGTGTGGACATCTGGTAAGTGCTCGCTCAGTTCCGGATGTTTTCATCATCTACCTTTTGTGTTCTTGCTGCTTATCTTCTCTGCTTTCATTTTATTTTTTCTTATCACAACAGGTTTTGAGTTTTGTCTATTGGAAAGATATATATTCCACATAGTGAACACTATTTGAGGATTAGATTTGGAAATTTAAAAGTGTAGTGTTCAGTAAGCTAGTCGTTTAGGTGTTTCCCCTGCAATCTGGTATCGTTTAGGGCTTCAAGATTAGGGAGTTTCTAAATATTGTCTATGTCATTTCTAGTGATTTCCCAAGCACTCTGGGTTTTGGCAGATTTGCACTTAATTGTTCATTCATTTTGTGCTTGCAGTTGTGGTCTAAACATAGTAAATTTTCTCTTTTCGTGGAGCAGCCTTTCTTTGTTGATTTGTATTGAGGTTTCGTTCGGTTCAAGCTTCTATGAGTATCCGATTTTCAGTTCAGTTCTTTTTTTTTTGTTCTCTTCTTTAATCAATATGATCTTTCTTCACCACACTCTCTTCGAGTGGTGCTCTTCTAGTAGTTAGTTTAACTGAGAATTTTGCACGTCAGTGGTGGTTACAAGCATTCAAGTTCTTAGGAGTTCTCTTGGTATTATGTGTAATCAAGTCTCGGCTCGGCCTATCTTCTTTCTCCCCCTCCCCTTAACACCTGTAGTGCTGTTGGTTGGCTGCCTCCACCTGATGGTTATGGTGGGTCAACGTGTGCAAGATTTTAACCAGGTCTTGCATAATGTGATAATGAGAATGTTCTGTCGTCGACTGTCGATAACATTCTTTTCAAAACCTCTTATAGACTAGGCTGGGATCTTATCCATTTTAGATGGTGATCTTACATGTTTCAGAAAACTGTAACTACCTTTGGTGGTATAGAGTTGATATCTCTACTTAATGTTGTACTCATTTGTTTCATCCCATTGCCCTCTATTAAATTCCTTGCTTGGTCGGAATGGGGAGGAGTGTCAACTCGAGTTCTTTTCTCCTGCCAGTTGTCTTATCTGATCCGATGGCTTTCATTTTCTCCCGGATGCAGGTTTCTGGTCTTTGATTGTGGCTGTTGGAAGTTTGGGTGGAGGGGTTAAGGATGTTTTATTATGATGTTTCTGCCATAATGAAACTATTCTTATGTTACCTGGTTGACAATTTTTGGGTATTTACAGAATTTGATAGGTCTGGATTTGTTGGTGCAGTTGTATTAAGTTTTCTGATTTTGCTATATAGGTATGGAATATGAGCAGATTTGTGAGTTGCAGAAAAGAACGTCCGTCAGAAAGCTTGGCCTCCTGTGCTCGAAGTGAGACCTGAAGGGTGAGCTTATGGCGCACGAAATCCATCTCTCGGTTTTGCAGATCTATTTTGAATTGTGAATTTGCTGTGAGTTCTCACTTTTGTTGGTTTCTCGAGGTATCCGGGGTCTTCCTGCTTAGATTATGCCAATTTCTTTCTCTGTTTTACCAGGGTAATGATTGTATGGGTATAGACAATGCAGTTTCTGTTGTCGGATGTCAATAATCTTTGTAACGAAGTAAACCTCATCTCTGGTACCTCCAACAAATCATCTTTTCTTGCGTCTTCTCTTTTGGAACACTATTCAACAGAAGTTTATGAGCCTTTCCTTGTAGATGTTGCTCTCTATATTTCATACCAGTATACGTACCCGCACGTTGCGCGGACATTATAAAATGTAATAATGTTTTAAATTTAAAATAACTAATAAGGTTTATTATTTTCTTTGTAATATATTTTTAAAATAATGTAATAAATCATTAAATTATTTATTTGAGCAAGTTCTATTAATATTTATGTACAC

>LNC_000633

CTAATTTATAAATGGCTATAGCGGCTAAACTAATTCTGAAAAGCACCCACCCAATGCCACCGCCCTTTGAAATATTGTTGCCATAAATAGACTTGCTAGGAAACAAATTTCTCAGTTACGATTTGTTTTAGCTTTAGTTCTACTATTATTTGGTGTTCATTCTTGAGGATATAAGCCGTGTTTGATTGTTGCTATGGATAGTTATTGTTGATCAAGATATGGGTAACTATTGGTATGATCAAGATGATGGGTAATTATTTATATTGTTTAATCAAGATATGCGTCCCTGTTCGTTTTGGCTTTAGTTCTCTGTTTTTTAATGATTTCGTTGGTATCTTGAGGATATAGGGAGTGTTTGATTGTTGATTATTGTCGATCATAACACTGGGTAATAATTATTCGATCAAGATACTGGGTAATTATCATTGATGATGATATTGGGTAACTATTGTTTGATGAAGTTGTGGGTAATTGTTGTTGATCAACACATGGGTAATTATTGTATGGTCAAGATATAGGTAATTATTGTATGATCAAGATACAGTGTAATTATTGTATGGTCAAGATACGGGTAATTATTGTTAATCAAGATATGAGTATTATTGTATGGTCAAGATACATGTAATTATTATCTATCAAGATATGGGTATTATTGTATGATCAAGATAGGGTAATTATTGTCGATCAAGATACTGGGTAATTATTGTCAATCAAGATATGGGTAATTATTGTATATGATGCCTTCCTTAGGGGAGCTATCGGTTTGATTTATGATTTCTTGGCCATCTCAGAATTATATAAATTATCTAATTTCGCATCTTGGTTTTCGAATCTTTTAATATGGGGTGTAGGTTTCTGATTTTCAAATCTGTGGGCAAGGAAGGTTAGGGGTTTTGGGAGTTCTCGACTTTTTAGTGTGATTTAGTTTGCAATTAGTTATGTTCTTTTGGCTAATTTATTGATTTGTTTGCAATTAGTGCGGTTTTTTTTGCTAATTTATTGACTAATTATTGTTGTTTGTTTGATCAGGTATGGGTAATGATGGTAGGAGACTGATTCTCCTTCTACATTTGCTGTTACCTTAATGTCAATGTTAATTCTGAGAACACAAGTAGAGTTGGAAATTTGGCTCTAGGTGTGCATCTAGGTGCAAGTGGTTCTACAGGAACAAGTGTCTTGCTTCAGTGTTTGTGGTCAAGAAAGATCCAGCAGTTCCCAGACCACATGGTGACAGTGACAGAGGATTAATTTGGAAACTTCTAAGGCTTATCCGGTATGCATCCATGTGCACCACATGGTGATGGATATTGTTAGGCTCTCTTGCATCTCTAGTGATTTCTGAGGTATATGTGTTTTTTCAGCACATAGTTGTTCCTTCTTTTTGTGCTTGCAGTTGAGTTTTACCGAGCAGTGAATTATTCTTTTCATGGAAGAGGCTTTGTTTGGCCATTTGTGTTGAGGTTTAATGAGTACTGGAGATACTCAATACTCATTTCAGTTTTTTTCCCCAATCAGAGTGGCCCCTCCTCTTTGTCAAAAATTGTTGTTGTATGGAACTTGCCAGCTAAAATAGTTTCTCTTGCACTCACGTGTGTTGTATGGAACTTTCCAGCTAAAAATTGATGTGTATGTTGTTCGATGATCCAGTTGATGAGATCCTGGTGGTTGATGGTTATATGGTCTTATCGACACCTGATAAATACTCAAAACTCAATCCTGGATCTTTTGACAGCCCGTACAAGTGGATCCCTGTTAGACCCTCTCTTTTGTTGTTCTCTCTGCCTTAACTTCTCAGCTTCCTTTTTCTTGCATCACCCAAGCTTGATTTTGTTTGTTGGATGGATGATAGTTGAAGGTTGATGCCCAGTGGCCGTTTCTGTGCACGCTGCTTCTTGCTATTTTTGTGCTTGGACTACAATCTGAATATAATCAGGCTTCAATAGAGGGGTTTTCCAATCTCGTTTCTTTGCCATTTTTATGTGTGCATTGCAACCTGAATATTTTCAGGCTTAGAGAGGGTTTTTTAAATTTGTCTGTGGCATTTTCTAGTAGATTTTGTCAGCTTTGCACGTAACTCTTCCCTTCATTCCTTTGCTTGCAGCCATTGGGTCTTACAAAGCAGTGAATTCTTCTTTTCATGGAGGTACCTTATTTGCCCAAATGTGTTGAGGTCTTGTTAGTCAGTCTTTTACGAGTATAGATAATTTCCAATTTTTCTTCCCTATTGATTCCTTTGTCTTTGTCATAACTTCTGTTGTATGGTAGTTTCTCAATTAGGACATTATTTTCACCAATGTGTGTTTTATTCTACTGCCATGATGTTCTTAGTTTAGATGCTTATGTTCTTATGGTGATCCATTTTTTATTTCTTGGTTATCTCAGAATTATATAAATCTCACGTTATCTACTTTCGAATCTTGGTTTACGAATCTTTTGATATGGGGTGTAGGTTTCTCATTTCTGATTTTCGAATCTTTGATTATCTCTGTAGACAAGCTAAGGTCAGAAGATGAATGTTAGGGGGGGTTTTGGGAGCTGTTGATCTTTTTTCTTGGCCAATTTATCGATTCGTTATTGTTGTTTGTTTGATTAGGTATGGGTAATGATGATAGGGGAATGGTTATAGGTACCTTCCAGTGTATTTCATGATTCTCCTTCTACACTTGTTGTTACCTTCTGTCAGTTACCAAATATTCGTATCCTTGCTTTCCTTCATGTGTAGTTTATTTATTTATTTGTTCTCATTTTCTTGGGTATTATAGTGGGAGTTTATAATTGCAATCCTTTTGATTGATTCAGAATACGTGTACACAGCAGATTCGTGAGCAAGAGTGTATTCAACGAACAACTACAATAATTTTTTCTACGGAACTTTCATATGAGTCTATTCTTCCTTTTAATCTACTATATAAAGGTTTTAAATTGACCCTTAATATTAAAGATAATTACATTCTTGAAAGGATTAAGGGTTCTACCTTCCTGACTTTTCGCTTGTTAGGACTGGAAATTTAGCTCTCCAGGCATGCCTACTTGGATCTTCTATTGTGGACTTATGTGTCACAACCATACAATTGACCTATCAGTTTGGATTTGATATCTTGACTTCCATCTTGAATGTTAATTTTGATGCTAATCACGTGTCTCCTCACATCAACTGCTTTTAGCTCCTTAGTTGGTTTGGTCATCAGTAAATGTCATTCTTAGCTGTCAAGAATACATGGATGGGGCATCTAGGTGCAAGTGGTTCTACATGAATAAGTGTCTTGCTTCGGTGTTTGTGGTTGGGAAAGATGCAGCAGTTCCCAAGCCTTTGACTATAGGTCTTGGATTTTTCAGTCTCTCAAACCTTTATTAAAACTAGTACGGTATGAAAGTTCTCAACTATGATAATTCTTTTCACAAGTGTGTATAGTGTGGTCTGCTCTGCCATTTTTAGTTTAGATGGTCATGTTTTTTGATGATCCGATATCTTCTATGGTGTTATGGGCACCAGATAAAGTCAAAAGTCAATTTGGAATCTTTGATAGTGCTTCTCTGCTTTTTGTGTCCTAGCAACTTATCTTCTCTGCCGTTTTCCTTGGTTATCATGCCAGCTTGATTTTGTATTGGTGAATGGATATGTAGACCACATGGTGATATTAATAGTGGATTAGTTTTGTTCAGAGGCTTGTATCTCGTATGCATTCTTGTACACTTTGTTTTTTGGTCTTTTTGGGGCTTGGACTCCAATATATTGTGTATTATAGTGGTAGTTTATAATTTCAATCCTTTTGATTGATTTCCAATGTTATTAGAAATTGGAAGCTGGTGACTATAAATGCAATTTCATGTTTGCTTCCATGTTTTGGGAAATTCTGAGGTCTTTGTTGTGAGAATTCATATGTTGGTGAGCATTGTTCTATCACCCTATTTACTACTATGAAGCACATCAATTTCCTTTTGTTGAATCAGAATACTGGAAATTGGCTCCCCAGACATGCCTACTTGGATCTTTTATTCTAGACCTATGTGTCACAATCATACAGTGGAGCTATCAGTTTGGATTTGATATCTTGACTTCCATCTTTAATGCTAATGCTAATCATGTCTCTCTTCGCATCAAATTCTTTTACCTTCTTATTTGCTTTGGTCATCAGTGTATGTCATTTTTAGCTGTCAAGAATACATGGATGGGCATTTAGGTGCAAGTGGTTCTACAGGAATAAGTGTCTTGCTTCGGTGTTTGTGGTTGGGGAAGATGCATCAGTTCCCGGGTCGTTTACTATAGGTATATTGGATTTTCATTCTTTCAAAGCTTTGTCTAAATGGGTACGATATGGAAGTTCTCAACTATGATAATTCTTTTCACAAGTGTGTTCTGCTGCCAGTTTTAGTTCTTAGTTCAGATGCTCACGTTCTTTAATGATCCTGATGTCTTTTATCGTGTTATGGGCATCAGACAGAGTCAAAAGTCAATTATGGATCGTAGCTAGTGCTGTAAAGTGCTCATTTGCTTTTTGTATCTAGGTACTTATCTTCTTTGTCGTTTTCCCTTGGTTATCATGCCAACTTTATTTTTTTTATTGGTGATAAATGTGTAGACCACGTGGTGACAGTTAAAGATTATTAGTTTTGGAAACTTCTATGGCGTGTATCCGTTTTGAATTCCTGTACACCTTGTTTCTTGGTCTTTTTGGTGATGGATATTGTTCTGCTTCGACCAAAGCTGTGGTTTTCAATCTTGTCTCTTGCATCTATAGTGATTTCTTAGGTATTATGGATTTTGTCAGCTTTGCACTTAACTGTTCCCTTCTTTTTGTGCTTGCAGTTGGGTCTTAATGAGCAGGGTTTTATGAGTACTGAGATTCTCATTTCAATTTTCTTCCCCAATCAAAGTGCTTCCTCTCTTTATTAAAAAATGTTGTTGTATGGAAGTTCCCAACTAAAATAGGTTTTCTTGCGCTCGTTTGTGTTGTATTCTGCTACCTTTTTTAGTTTTTATCTAGATGCTTATGTTCTTTGATGATCCAATTAATGAGATTCATTGTGGTTGATGAGTGAGTATTAAATCTCTTCCTAACGCCTTTAGGGGCTGTGAAGAGAGCCCAGTTCCAGGCCCTCTTTTAGAAAGAAAAAAATGGTACAAAAAGAGGGCTTGGGGGTATATATACTATACAAATTGATGATGGTTTATCATCATCAGAACAAAGATCAGTCTAGGTCGAGATGAATGGAAGATGCTTCTGGAACTAGTTTTTGATAGGGTCTATCTTAGCTCGAAGGAAAGAACAACCAAAGTTAGACCCCCAAAGAATATGGATTAAAATCTGTTAGTATTTGACAAAGAACCTAACGGAATTTAACAAGCAGTAACCGGAATCAAAGTTTTGCAAAATGGTGTCAACCGGTAATAGAATGTAGCTAGATAACTGATGCCACAGCATCCAAAATATGTGGGATGGAGTTCATGGTACACTAACTTAACCTTCAAGGTCAGATAGTGCTACAGTGAACATCAATCCACTATCTAGCTCAAAATTAGCCTCACGAACTACTAACACTATTATTCGCGTAGCCCTTTTTTGTTTTGTGCTATTTCATCATAGGCTGTCGGAACTTGCTTCTTAGCGCTACGTGGGAAGAGAATTTCTCTATTCAGCCCCCCCGACTTCAATTACAAACTCTTACTTCTCGGTCTAACTTGGACTAATCCCTTAGCAGGCAAGAAATTCCTACTAACCAGACAGAAAGTAAGTTTCCACTCACTACCAAAGCGCTGTCGCACCTCTACTATTTTCGGCGTAACGAGGTTTTAGTCTTTACGAGGGCACCCCCTAGCCAGATTCAATCCCAAGGGTCAATCAAGCCTTTGAAACTAGTGAAAATAGTCGTCACAGTCTGAGTTCCTGCTTTCAACGAGACTTTCTTAGCTTGTCAAACTTTCTCTCCTTCACCAGGAAAGTTATCCGGTAAGCAAGATCTTTGATTGATGTGCCTGAATAAGGTTGATCAATGGTAGCTGCTTGGAGCTTTCTCTATACCTTAATTGAACTGCCCCGCTCTTAGCTCGGGTTCTTGCTTAATGAGGCGTCCCTTGTTTGCTCTTGGATTGGACTAAGCGGCAATCCTTACTGTGGAAGCAGACCTTGTCATTCTTCTCCTTCAATGCCAGTGGATAAGTACACTAGGGCTGGAATGCTCTTTTATCATGTATGTGGATTACTTGGTCGATTACCTGATTGCACTAGTCTCATAGCCATCTCTTTAGCGTCCCGCCATTCCTTCTTGTTAACACAGGAAATCTAACTCATTCTAAGGTAGAGCTCTTAGAGTCCTACTTAAAGTGAAAAGTAGTTCTTTAGTTAAAGCAAGGAGAGCAGAAACTGAGGGAAAAAACCTATGTTCTTACGCGGGATGGGAATAAAGGCTACTAAGTAACCTTAGAATGGGGATGCCAATCTTATACAGAAAATACCGCTATCTAAAGTAACCTTGCAGGGGGGCTGCGTATAGCATTCTAAGATCTCAATACGAGAAAGAAATCAGACCTATAACCTCACTGCAGTGGCTTGTAGACTTGCTTTGCCGCGGAAAGCTCAAGTGAAAACCTATTAGAACTGTGGGCCTCACCCGTCTACTACTCGACCTTAAGTAACTACAGTTAGGAGGATCCATCCAATAGCTTAATGACTTGCTTGGGTCATCTAAGAGGTAAGGCCTCCTCCGATCGACCCTAACTTTCATTATTGAGCTAAAGGAGTTCCCTAGCCCTTCAAGTTTACACAGGTCCCCAAAGATAAAGGCTTTCATCCTTCCTGAATATTCTGGGAACTTTCTTCTATTAATTGTTTTCGAAATGCCGATTTTCCTGTCTGCGCTAAGGCGATAAGGAAAAAGAGAGGGGCCTTTAGTATGTGGAAAAAAAGGCAAAGATCACATTCCAACTAACGTTGACTGGTAATTGCCTCAATGAGGCGGATAAGGCGCAAGAACCTAAAAGGCGGACTAAGATAAGTTATTCAGCTAGGGAATGCGTTAAAGAGCCGTTATGTAGCATCTGAGAACAAGAAAGCAGACATGCACACGAAAAGACATGCACACGAAAAGCAGACATGCTTTCAAAGGGCTTTCAAAAATCAATCATGGATCTCTTGAAAGCCCTTTGAAGTGGGTCTTTGTTACTCCCTTACTTTTGGTATTTTCTCAGCTTCTTGTTTCTTGTTGCATTGTCACCCGAGCTTGATTTTGTTTGTTGGATTAGAGTTGGAATTTTTTCAAAGATTGATGATGTGATCAGGCTTTGATAGTTAGTGTTTGCTCCTATGCACCCTGTTTCTTTGCAAATTTGTTCTTTCTCACCAATCTGAATTTTGTCAGGCATTGATAGAGGGATTTTCCTATCTTGTTTCTTTGCCATTTTTATCCTTGCACTGCAATCTCAATATTATCAGGCTCCGAGAGAGGGGTTTTGCAATCTTGTCTGTGGCGTTTTGTAGTGGGTTTCATCAGCTTTGCACGTAACTCTTTCCTTCATTCCTTTGCTTGTAGCCATTGGGTCTTACAAAGCAACAAATTCTTCTTTTCATGGGATGACCTTATTCGCCCATATGTGATGAGGTTTCGTTAGTTCAGTCTTTTATGAGTAGAGATAATTTTCAATTCAGTTTTCTTCCCTAATGACTTGTTTGTCTTTGTCAAAACTTACTGGTTTATATAAGTTTCTCAATTAGGAAATTATTTTCACCAGACGGTGTGTTGTATTCTGCTGCCATGAGGTTCTAAGCCCTCACACTTATGTTCTTCGATGATCCAATTGATGAGGGATAATTTTCAATTCAGTTTCTTCCCTAGTGACTCCTTTGTTTTTGTCAAAACTACTGTTGTATGGAAGTTTCTCAATTAGGACATTATTTTCACATGTGTGTTGTATTATGCTTCCATGAATTTCTATGTCCGCATGCTTATGTTCTTCAATGATCCAATTGATGAGGTCTTTTATGACTATGGACACCTGATAAGTATTTTGGCCCCTTCTTGATCTTTTGATAGTTGCATAAAATACTTTTTTTACTTTTGTTCTTTTAGCTTCTGATCTTCTCTGCTTCCATTTTTTTCCGTTATCAGACTAGCTTTTTTGGTTGTATAGATGTGTAGACCATGTGGTAACACTGTTATGGACGTTCAAATTTGGAAATTTCAAAGTTTGGTTAACCAACATGTGATCCTCTCTCACCTTTTGGTCTTTTTGGTGCTTGCACTGCAATTTGATGCTGTCCGGGCTTTGGCAAAAGAGGGTTTTCAGCTTCGTCTATGGCATCTATAGTGATTACCATTGTGGTTTTGGGTTAGTCAAATTTGCACTTAACTGTTGCCTTTTTGTAGTTCTTTCATCCATTGGTTATATGGCAGTGAATTATTCTTTACGTGGAGAGGCTGTGTTTATCTGCTTGTTGTTGACGTTTCTTTAGTTAAGTCTTATATGAGTATCGAGATTCTCGGTTCAGTTTATTCCCCAATGACTCCTTTGTGTTTGTCAAAACTACTGTTGTATGGAAGTTTCTCAAATAGGATATTAATTTTCACCGCAATGTGTTGTATTCTACTGCCATGAAGTTTTAAGTCTAGACACTTATTTTCTTCGATGATCCAATTGATGAGGTCTCCTATGTCTATGTTTCTTGAAAAGTACTCAACTATTTCTAGACCTTTTGATAGTGGCGTAAAACACTTATTCACTTCTATTCTTGTAGTTACTTACTTATCTTCTCAGGTTCCATTTTTTTTCATTATCACACTAGTTTTTTTTTTTGTTGGGTGGATGTGTAATGGTGATTCTGTTATGGATGTTCAGATTTGGAAAATTCGAAGCGTGGTTGACCAACATGAGATCCTCTGCCACCTTTTGGTCTTTTTGGTGCTTGCACTGCAATCTGGATAATGTCCGAACTTTGACAAAAGGGAGGGGAGGGGGGGGGTTTAGTCTCATCTATGGATTGGCTAGTGATTACTTTGGTGGTTTGGGGTTCATTGGATTTGCAATTAACCTTAACTGTTCCCTTTTTTCTATTCTTGCAGCCATCGGTTACAAGGTAGTGAATTATTCTTTGATGGGGCTGCCCTATTCGTCTGTTTGTTGTTGAGGTTTCTTTAGTCAAGTCTTTTATGAGTATTCTGATTTTCGATTCAGTTTTCTTCCCTAATGAATCCTTTGACTTTGCCAAAACTATTGTTGTAGGGAAGTTTCTCAACAAAGATATTTATTTTCACTGGTATATGTTATATTTTGCTGCCATGAAGTTTTTAGTCCAGACAGTTGCATTTTTCGATGATCCAATTGATGAGGTCTCCTATGACTATGAACACTTGATAAGTACTCGTTCTAGATCTTTTTATAGTGGTGTAAAATACTTATTTCACTTTTATTCTTGTAGCTACTTATGTTTCTTCTCTGCTTCCATTTCTTTTTCGTCATCACATAGTTTTTTTTTGTGGTTGGATGTGTAGACCATGTGGTAACACTTTTATGGACATTTAGATTTGAAAATTTCAAAACTTGGTTAACAACATGTGATCCTCTGCCACTGTTTTTGTTCTTTTTGGTGCTTGCATTGCAGTTAACATATTGTGCGGTTTTGACAAAAGGGTGTTTTCAGTCTCGTCTATGGCATCTCTAGTGATTACTTAGGTGGTTTGGGGTTTGTCAGATTTGGACTTAACTGCTCCCTTCTTTCTGTTATTGCAACCATTGATTACAGGGTAGTGAATTATTCTTTTCATGAAGAGGCTATTTTTGTCCGTTTGTTGTTGAGGTTTCTTTAGTTCAGTCTTTTATGAGTATCGGAATTTTCGATTCAATCTTCTTCCCTAATGGCTCCTTTGTCTTTGTCAAAACTACTATTGCATGGAAGTTTCTCAACAAGGATATTAATTTTCACCGGTATGTGTTGTATTCTGCTGCCATGAGGTTTTTAGTTTGGACGCTAGTGTTCTTTGATGATCCAATTGTCTATGGCTATGAATACTTGATAAGTACTTCACCAATTTTATATCTTTTGATAGTGGTATAAAATACTTCTTCACTTTTATTCTTGTAGCTACTTGCTTATCTTCTTTGCTTCCATTTCGTTGTCGTTATTACACTAGTTATTTTTGGTTGGATGGATGTGTAGATCATGTGGTGATACTGTTATGGATGTTAAAATTTGGATATTTCAAAGCGTGGTTAACCAACATGTGATCCTCTGCCACCTTTTGATCTTTTTGGTGCTTGCACGACAACCTAGATATTGTCCGAGCTTTGACAAAATGGAGTTTTTAGTCACATATATGGCATCTCTAGTGATTACTTAAGTGGTTTGGGGTTTGTCGGATTTACATTTAACTGTTCCCTTCTTTCTGTTCTTGCAGCCAATGGTTATAGGGAAGTGAATTTTTGTTTTCATGGAGGGGCCGTGTTTGATTGTTTGTGTTGAGGTTTTTTTTAGTTAAGTCTTTTATGAGTATCGATATTACGGATTTAGTTTTCTTCCTTAATAGCTCCTTTGTTTTGTATGGAAGGTTTTCTCAACTAAGATATTAATTTTCACTAGCATGTGTTATATTTTGCTGCCATGAGGTTTTTAGTCCAGATGGCTTATGTTCTTCGATGATCCAATTGATGAGGTCTCATTTGTCTATCGATTCTTGGTAAGTACTCTGCTAATTTTGGATCTTTTGATAGTTGCGTAAAATACTTCTTCACTTCTATTCTTGCAGCTACTTATCTTCTCCACTTCCATTTCTTTTTCGTTATCACATTAGCTATTTTTGGTTGGATGGATGTGTAGACCACGTGGTGACACTGTTATGGACGTTCAAATTTAGAAATTTCAAATCTTGGTTAGCCAACATGTGATCCTCTGCCACCTTTTGGTCTTTTGGTGCTTTGCACTGCAATTTGGATGTTGTTTGGGTTTTGACGAAAGGGGGTTTTAGTCTCGTCTATGGCATCTATAGTGATTAATTAGGTGGTTAGTTTGTCAGATTTGCTCTCAATTGTTCCCTTCTTTCTATTCTTGTAGCCATTGATTACAGGGCAGTGGATTATTCTTTTGACGATGCAGTCATGTTTGTCCATTTGTGTTGAGGTTTCCTTAGTCAAGTCTTTTGTGAGTATCAAGATTTTTGATTCAGTTATCTTTCCTAATGGCTCCTTTGTCTTTGTCAAAACTATTGATGTGTGGAAGTTTCTTAAACAGGACATTATTCTTCACTGGCGTGTGTTGTATTCTGCCGCCAAGAAGATTTAGGTCCAGATGGCTTTGTTCTTCGATGATCCAATTAATGACGTCTCCTATGACTATGGACACTTGATAACTCAACCAATTCTACATCATCTGATAGTGGCGTGAAGTACTTCTTTTACTTTTTTTCTTCTAGGTACTTATCTTCTCTGCTTCCATATATTTTTTGTCATCACACAAGTTTTTTCTAGTTGAATGGACCATGTGGTGACACTGTTTTGGACGTTCAGATTTTGTAATTTCAAAGCTTGGTTTATCAACATGTGATTCTGTGCCACCTTTTAGTCTTTTTGGTGCTTGCACTACAATCTGGATATTGTCTGAGCTTTGACAAAGGGGTACTTTTAGTCTCGTCTATGGCATTTCTAGTGATCACTTAGGTGGTTTGGGGTTCGTCAAATATGCACTTAACTGTTCCATTTTTTCTGTTCTTGCAACCACATTGGTTACAGTGCAGTGAATTATTCTTTTCATGGAGCGGTCGTGTTTGTCTGTTTGTGTTGAGGTTTCTTTAGTTTAATCTTTTATGAGTATCGAGATTTTCGATTCAGTGTTCTTCTCTTATGGCTCCCGTTGTACTATAGAAAGTACTATTGTATGGATGTTTCTCAACTAAGATAATATTTTTCACCATTGTGTGTTGTGTTCTGCTGCCGTGAAGTTTTTACTCTAGACATTTAAGGATGGCAATGGGGCGGGGCAGGTTTAAGGCTACGGGGGGCAGGGCGGGAGGGATGCAGGGTGGGTTGAAGTGGAATTTTTAAAACTAATGCGGTGTGAGACGGGTTGCAGGGGTAAATGTGATTTTATATGGGTTCAAACTTAATTTGAGCTTTTTACATGTTATAAGAGTGACAAAACATTATTTATTAAGATACTTTCTTTAAAGCTACTAAAATATTCAATATAGTAAATCAAAATGGTTCAATAAAAAATAATACAATTTCTTACATGTTTCCCAATTGACCTCTAAAAAATAAACGTTTAAGTAATTATCTATTAAACTAATACAACTTAATGAAAAAATGAATTTATTTTTATGAGTTTTATTTTTAGTACGGAAAAGGGTCAAAATTGCCCCTGAACTATGCGAAATAGATCATTTATACCCTTCGTTACATTTTGGGATAAAAAATGTCCCTGCCATTATCCTAAGAGACCACAATTACCCTCAAGAGTTAACACCCCAAATTTTTAGTGACATGGCAAGCCACGTGGGACTAATCCTTCCACCTAAACGTTGCCAACTTGGATTCACTACAAAAGAACCAGACCATTAGCGGCGACAACAGTCGCCACAAAATCCCAAAATATTGCCACTAAAGGTTATGAGCGATTTTTAGTGGCGACTAAGGCTGCAATAACCATTTGCTAGTAATTCCTATTTTTTCAACAAAATAATATGATAATTAATTTTCGATTGCAACCTAATATTCTAAAATAAATAACTTGGACTATTAACATTAAAAACATCCAATATTATTATGAAAATCATCAAAATTACTTCTTGTTTCAAATCTCCTACTACATAATGCATCATCATACAACTTATACATTTACATATGATATAAAAATAAAACATATAGTGTCTTCACACTCCTCCTTAATCGATATCATTTTTGGTTTTTTAAAAACAATGAAGAAAAAACACGCAAATCAGAATTTATTGTTAAAAACCTTTAGTGACGATTTTCTGGGATTTTGTGGCGACTGTTGTCGCCGCTAAAGGTCTAGTTCTTTTGTAGTGAATCCTAGTTGGCAACGCTTAGGTGGAAGGATTAGTTCCCCACGTGGCTTACCATGTCACTAAAAATTTGGGGGTGTTAACTCTTGAGGGTATTTTGTGGTCTCTTAGGATAACGACAGGGGCATTTTTTATCCCAAAATGTAACGAAGGGTATAAGTGGTCTATTTCGCATAGTTCAGGGGCAATTTTGACTCTTTTCCATTTTTAGTATCAAACTTAACTAGAAAAAGAAAATGTTAAAAAAATTATGTGGGGCGGGTTGAGAATTTAAGAAGTTATGCAGGGCGGGGCAGATTAAAAAATTTGCAAGTTAAGCTTAACCCCCCGGCTTGTGCACCGCCCCGCCCCATTGCCATCCCTATAGACATTATGTTCTTCGATGATCCAATTGATAAGGTCTTCTATGGCTAAGGACACTTGATAAGTACTCGGCCAATTCTAGATCTTTTCTTTTGACAGTACCATAAAGGCTCCTCTATTTCTGGTCTTCTAGCTATATATATTATATGCTTCCATCTTCTTTTGGTTATCACAAACTTGGTTTTTCTTGTTTGGTGACAACAATAGAGGTTAAGATTTGGAGATTTCAAAGTTTGATATCCAGTATGGAATCTCTTTATCCGGTTGTTCTTTTTGGTGCTCAGGGCTTCAACAAAATGGGGTTTTAAATCTCATCTATGTGGTATATCTAGTGATTTGTTAGATGGTATGGGTTTTTCAGCTTTGGAATTGATTGACCCTTATTTTTTTATGCTTGCAGCCATTGGGTCTTACAGAGTGATGAATCATTCTTTTCATGGAGCGGCTTTCTTTGTCCATTTATGTCGAGGTTTCCATGGTTCAAGCTTTTATAAGTTCTAACATTTTCGAGTAAAATAGTTTTTTTCCCATTAGTATGTGTTGTTCTACTGGCATGTTTAGTTTTTAGTCCAGATGTTTGTTTTCTTCAATGATCCAATGATGAGATTCATGATGGTTGATGGTTATTGTCTCATGGACATCTGACAAGTACTCAATTTTGGATCTTTTGAAAGCCTCGTAAAATGGTAGTCTCTTTTGGTGTTCTCACTACTCATCATTCATGCTTCCTTTTTCTTGTATTATGCTAGTTTGATTTTGTTTGTTGGATGGCTGTATGGTGATAAGTTGAGAGGAATACGACTTGCAATATTTTGCAGGTTGGTGTCCAGTGTGCATTCCTGTGCACCCTATTTCTTGGGCATGTTGGTGCTTGCACTACAATCTAGAGATAGCAGGCTTCGAAAGAAGCTGCTGGGTCTTACAGAGCCATAGATTCGTCTTTTCATGGAGTGGCGTGGTCTGTCAATTTTGTGTTGAGGTTTTTTGGTTGATGCTTCTAATGATTATTGAGATCTTCAATTCAGTTTTATTCCCTAATCAGAATGGTTCCGTTCTTCCTTGTCAACAATACTTCACTGGTGTGTATTTTGTTGCCATATATGTTTCTTAATAATCCAGATGCTTATGTTCTTTGATGATCCAACTGATGAGATCTCCATGGTTATGGACAACACATGATAAGTGTAATCAAGCTCAGTCTTGGATCTTCTGATGGATCTTTTAATAGCCTTGTAACACACTTCTCTAGTTTTCGTGTTCTCTCTACTTACCTTTCTCTGATTTCATTTAACCTACCAGTTTATTTAGTTTGGTTGGATGAATTTATAATTTTGGTTTCGGTGTGCATCCTTGTGGACCATGTTGACCATTTTGGTGCTTGCACTGCAATTTGGATATTGGATATCACCATGCCTTAAGAAAAGGGCACTTTGCAATCTTATCTTGTTTGGCATCTCGAGTGATCTCTCCCGTGTGGTATGGTTTTCCACTCACCTGTTACATTATTTTTTGTGCATGTAACCATTGGGTCTTCCAGAGCATGGACTTCAAGTCAGTTGTCTTCCTAATCAGTGGGTCCCTTTTCTCTTTGTTAAAATTATTGTTATGTGGAAGTTCTCAACAATGATAATATTTTCCACTAGTGCGTGTTGTACACTGCAACCATAAAGTTTTTAGTCTAGATGCTTATTTTCTTGGATGATCTAAATGATGAGATCCCTGATTTGATATGGACACCTGAGAAGTACCCAAACTCAATTTTCCCAAGGGCTTCTCTACTTTGGTGTTCTTGGTACTTATTTTCTCTGCTTCTATTTTCTCATGTGTATCATGCCACTTGATTATGTTTGTTTGGATGCATGTTTGTAGACCACATGGTGACTAGAGGGGGATCGAATTTGGTAATTTCGAAGTATGGTTTTGTCATTCCCTTGTGCACCCTGTTTCTTTGCCATTTTGGCGATTGCGCTTGCACTGCAATTTGGATATCACTAGGCAATAGCAGGGGAGTTGTGCAATCTTTTTTGGCATCTCTAGTGATCTCTCCTATGTGGTATTGGTTTCACAGCTTGCACTTTAACTGTTACCTTCTTTTTGTGATTGCTGTTGTTGGATCTTTCAAGATCAGTAAAGTCTCCATGAACTCCATTTCATAGAGTAGACTTGTTTGTTGTAGTGTTGAAGTTTCTTAGTTCAGGCTTTGTATTAGTATCTAACTCTTCAATTCAGTTTTCTTCCCTGGTCATAGAGATTCCGTGGGTAGCCAGGTAGCCGTCGGTCTTGTCCCGACACCTATCTTCAGTTAGTAGAGGCTTCATAAATAGTCAAAGTTAATGCGTCAGTTCAGTCTTTAGTATTCAGAAGTTTTAAACATCATTATTATTATATTATTATTATTGTTATTGTTTCAGACTTTTGCATGGGATATCTTTAAGTGATTTTAAATGTTCATTTTATACGGCTTCCGCATGTATGTTTAGTCTTCCTCTCAGTAGTTAGCTAGGACAAGGGTTTGCTTGGGACCAACAGTGGGGTTCCGAGTGCCAGCACTCAGAACAGTGGGGTTTCGAGAGCCGGCCACGTCCAGGTGTAGGCTCGGGTTGTGACACAAATGATGAGGTCTCTTATGGCTATGGACACTTGATAAGTACTCAGCCAATTCTAGATCTTTTGATAGTATCATAAACTACCTTTTTACTTTTATTCTTCTAGATACTTTCTTATCTTCTCTGCTTTCTTATATTTTTCGTTATCACACTAGTTTTGTTGTTGTTGTATGGATGTGTAGACCATGTGGTGACATTGTTATGGCATTGAAAAAATGGGGTTTCAGTCTCGTCTATGGCATCTCCAGTGTTACTTAAGTGGTTTGGGGTTTTCCAGATTTGCATTTTACTATTCCCTTCTTTCTGTTCTTGCAGCCATTGGTTACAGGGCATTGAATTATTCTTTTCATGGAGTGGCTGTGTTTGTTTTTTTGTGTTAAGGTTTCTTTAGCTTGAGTCATTTATGAGTATCGAGATTTTCGATTCAGTTTTCCCTTTTCTATATCAAAACTATTGTTCTATGGCAGTTTCTCACTAAGATAATATGTTTTACCCGTATGAGGTTTTTAATTCAGACACTATTTTCTTCAATGATTCAATCGATGAGGTCTCTTACGGCTATGGATACTTGATAAGTACTCGGCCAACTCCGAATCTTTTCTTTTGACAGCGTCATAAAGTGCTCCTCTACTTCTGGTCTTCTAGCTATATTTGTTCTTTGTTGACATCTTCTTTTGGTTACAAAAACTTCTTAGTTTTTGTTTGGATGCATGTGTAGATTATATGGTGATTGTAATAGAGGTTAACATTTAGAGATTTCAAAGGTTGATATCCAATATGGTATCTCTATACCCTCTTGGTCTATTTGGGTCTTGCACTGCAATCTGGATATATTGGTCAGGGCTTGAACCAAAAACGGGTTTTAATTCTTGTCTATATAGCATCTTTAGTGATTTCTTAGATTGTATGGGTTTCGTCAACTTTGGACTTGATTTGTACCCATTTTTTTTTGCTTGAAGCCATTGGGTCTTACAGTGCAATTAATTATTCTTTTCATGGAGCGGTTCTCTTTGTCCATTTATGTCAAGGTTTCTTGGTTCAGGCTTTTATGAGTTCTGACATTTTCAACTAAAATAGCTTTTTTCCCAATAGTGTGTGTTGTATTCTACTGCCATTTTTAGTTTTTAGTCCAGATGCTTATTTTCTTCAATGACCCATTGATGAGATTCCTGATGGTTGATGGTTATGGTCTCATGGACACCTGACAAGTACTCAATTTTGGATATTTTGAGAATCTCGTATCTCTTTTTGTGTTCTCGCAGCTCATCTTCTCTGCTTCACTTTTCTTGTATTACGCTAGCCTGATTTTGTTTGTTGTATGCATTTATGGTGGCAGTTGAGAGGAATAGGACTTAGATGTTTTTGAAGGTTGGTGTCTAGTGTGCATTCCTTTGCCCCTGTTTCTTGGACATGTTGGTGTTTGCACTACAATCTGAAGATTATCAGGTTTCGAATGAAGGTGGTACCTAGTTCGCCAGCTTTGCATTTACTATTCCATTCTTTCTGTGCTTGCAGTCGCTGGGTCTTACAAAGCCATAGATTCTTCTTTCATGGAGCGGCATGGTCTGTCAATTTCGTGTTGAGGTTTTTTGGTTGATGCTTCTAATGATTATTGAGATCTTCAATTCAGTTTTGTTCCCTAAACAGAATGGTCCCCTTCTCTGTTGTCAACACTGCCTCACTAGCCTGTATTTTGTTACCATATTTGATTTTTAATAATCCAGATGCTTATATTCTTTGGTTATCCAACTGATGAGATCTCAATGGTTATGGACAACACATGATAAGTGTAATCGAACTCAATCATGGATCTTTTGATGGCCCTGTAATGCACTCCTCTACTTTTCGTGTTCTCGCTACTTGTCTTCTCTTATTTCATTTAGCCTACCATTTGATTTAGGTTGGTTGGATGAATGTTTGTAGGCCACATGGTGGTAGTAAGAGGATGAGATTAGGATATTTTGTTGTTTTGTTTCGGTGTGCATTCCTGTGGACCATGTTTCTTACCATTTTTGTGCTGGCACTACAATTTGGATATTGGATATCACCAGGAAAAGGGGGCTTTGCTATCTTATCTTGCGTGGTATGGGTTTCCACTTACCTGTTATGTTCTCTTTGTGCATGTAACCATTGGGTCTTCCAGAGCATGAACTTCGGTTTTCTTCCTAATCAACGGGTCCCTTCTTTCTTTGTCAAAACTACTGTTGTGTGGAAATTCTCAACAATGATAAAATTTTCCACTAGTGTGTTTGTACTTTGCAGCCATCAAGTTTTTAGTCTAGATGCTTATTTTCTTGGATGACCCAAATGACGAGATCCCTGATTTTTATGGACACCTGAGAAGTACCCAAACTCAAATTTAGGTCTTCTTATGGCCCTAAGAGCTCCTCTACTTTAGTGTTCTTACTGCTTTTATGTTTATCATGGCACTTTATTTTGTTTTGCTTGGATATCATATTTGGAAATTCTGAAGTTTGGTTTCAGTGTGTGTCCTTTCCTTGTGCACCCTGTTTCTTTGCAATTTTGGAGCTTGCGCTTGCACTGTAATTTGGATATCACCAGGCACTGTCAAGAGAGTTATGCAATCTTGTTTGGCACTGCAATTTCGATATCACCAAGCACTGGCAAAGGGAGTTATGCAATCTCGTATGGCATCTCTAGTGATCTCCCCTATATGGCATGGTTTCGCAGCTTGAACTTTAACTGCTCCCTTCTTTTAGTTCTTGCTGTCGTTGGGTCTTTCAAGATTAGGATAGTCTTCCTTTAATGGAGCAGCCTTGTTTATCTTAGCGTTGAAGTTTCCTTTGTTCAGGCCTTGTATTAGTATCTAGCTCTTCGATTCAGTTTTCTTCCATGGTCATAGAGATCCCCTTATCTCGTTGTCAGAAGTGCTGTTAAGTGGAAGTTTTCAACTACAATAACTCTCTCCACAAGTGTATTTTCAGCTTTTAGTCCAGATGCTTTTGTTTTGCGATGATCCAATTGATGAGGTCTCTCTTGTGGTGTGGACATCTGGTAAGTGCTCGCTCAGTTCCGGATGTTTTCATCATCTACCTTTTGTGTTCTTGCTGCTTATCTTCTCTGCTTTCATTTTATTTTTTCTTATCACAACAGGTTTTGAGTTTTGTCTATTGGAAAGATATATATTCCACATAGTGAACACTATTTGAGGATTAGATTTGGAAATTTAAAAGTGTAGTGTTCAGTAAGCTAGTCGTTTAGGTGTTTCCCCTGCAATCTGGTATCGTTTAGGGCTTCAAGATTAGGGAGTTTCTAAATATTGTCTATGTCATTTCTAGTGATTTCCCAAGCACTCTGGGTTTTGGCAGATTTGCACTTAATTGTTCATTCATTTTGTGCTTGCAGTTGTGGTCTAAACATAGTAAATTTTCTCTTTTCGTGGAGCAGCCTTTCTTTGTTGATTTGTATTGAGGTTTCGTTCGGTTCAAGCTTCTATGAGTATCCGATTTTCAGTTCAGTTCTTTTTTTTTTGTTCTCTTCTTTAATCAATATGATCTTTCTTCACCACACTCTCTTCGAGTGGTGCTCTTCTAGTAGTTAGTTTAACTGAGAATTTTGCACGTCAGTGGTGGTTACAAGCATTCAAGTTCTTAGGAGTTCTCTTGGTATTATGTGTAATCAAGTCTCGGCTCGGCCTATCTTCTTTCTCCCCCTCCCCTTAACACCTGTAGTGCTGTTGGTTGGCTGCCTCCACCTGATGGTTATGGTGGGTCAACGTGTGCAAGATTTTAACCAGGTCTTGCATAATGTGATAATGAGAATGTTCTGTCGTCGACTGTCGATAACATTCTTTTCAAAACCTCTTATAGACTAGGCTGGGATCTTATCCATTTTAGATGGTGATCTTACATGTTTCAGAAAACTGTAACTACCTTTGGTGGTATAGAGTTGATATCTCTACTTAATGTTGTACTCATTTGTTTCATCCCATTGCCCTCTATTAAATTCCTTGCTTGGTCGGAATGGGGAGGAGTGTCAACTCGAGTTCTTTTCTCCTGCCAGTTGTCTTATCTGATCCGATGGCTTTCATTTTCTCCCGGATGCAGGTTTCTGGTCTTTGATTGTGGCTGTTGGAAGTTTGGGTGGAGGGGTTAAGGATGTTTTATTATGATGTTTCTGCCATAATGAAACTATTCTTATGTTACCTGGTTGACAATTTTTGGGTATTTACAGAATTTGATAGGTCTGGATTTGTTGGTGCAGTTGTATTAAGTTTTCTGATTTTGCTATATAGGTATGGAATATGAGCAGATTTGTGAGTTGCAGAAAAGAACGTCCGTCAGAAAGCTTGGCCTCCTGTGCTCGAAGTGAGACCTGAAGGGTGAGCTTATGGCGCACGAAATCCATCTCTCGGTTTTGCAGATCTATTTTGAATTGTGAATTTGCTGTGAGTTCTCACTTTTGTTGGTTTCTCGAGGTATCCGGGGTCTTCCTGCTTAGATTATGCCAATTTCTTTCTCTGTTTTACCAGGGTAATGATTGTATGGGTATAGACAATGCAGTTTCTGTTGTCGGATGTCAATAATCTTTGTAACGAAGTAAACCTCATCTCTGGTACCTCCAACAAATCATCTTTTCTTGCGTCTTCTCTTTTGGAACACTATTCAACAGAAGTTTATGAGCCTTTCCTTGTAGATGTTGCTCTCTATATTTCATACCAGTATACGTACCCGCACGTTGCGCGGACATTATAAAATGTAATAATGTTTTAAATTTAAAATAACTAATAAGGTTTATTATTTTCTTTGTAATATATTTTTAAAATAATGTAATAAATCATTAAATTATTTATTTGAGCAAGTTCTATTAATATTTATGTACAC

>LNC_000634

GCTAATTTATAAATGGCTATAGCGGCTAAACTAATTCTGAAAAGCACCCACCCAATGCCACCGCCCTTTGAAATATTGTTGCCATAAATAGACTTGCTAGGAAACAAATTTCTCAGTTACGATTTGTTTTAGCTTTAGTTCTACTATTATTTGGTGTTCATTCTTGAGGATATAAGCCGTGTTTGATTGTTGCTATGGATAGTTATTGTTGATCAAGATATGGGTAACTATTGGTATGATCAAGATGATGGGTAATTATTTATATTGTTTAATCAAGATATGCGTCCCTGTTCGTTTTGGCTTTAGTTCTCTGTTTTTTAATGATTTCGTTGGTATCTTGAGGATATAGGGAGTGTTTGATTGTTGATTATTGTCGATCATAACACTGGGTAATAATTATTCGATCAAGATACTGGGTAATTATCATTGATGATGATATTGGGTAACTATTGTTTGATGAAGTTGTGGGTAATTGTTGTTGATCAACACATGGGTAATTATTGTATGGTCAAGATATAGGTAATTATTGTATGATCAAGATACAGTGTAATTATTGTATGGTCAAGATACGGGTAATTATTGTTAATCAAGATATGAGTATTATTGTATGGTCAAGATACATGTAATTATTATCTATCAAGATATGGGTATTATTGTATGATCAAGATAGGGTAATTATTGTCGATCAAGATACTGGGTAATTATTGTCAATCAAGATATGGGTAATTATTGTATATGATGCCTTCCTTAGGGGAGCTATCGGTTTGATTTATGATTTCTTGGCCATCTCAGAATTATATAAATTATCTAATTTCGCATCTTGGTTTTCGAATCTTTTAATATGGGGTGTAGGTTTCTGATTTTCAAATCTGTGGGCAAGGAAGGTTAGGGGTTTTGGGAGTTCTCGACTTTTTAGTGTGATTTAGTTTGCAATTAGTTATGTTCTTTTGGCTAATTTATTGATTTGTTTGCAATTAGTGCGGTTTTTTTTGCTAATTTATTGACTAATTATTGTTGTTTGTTTGATCAGGTATGGGTAATGATGGTAGGAGACTGATTCTCCTTCTACATTTGCTGTTACCTTAATGTCAATGTTAATTCTGAGGTTTTTGTTGTTTAGAATTCATGTGTTAGTGTCATCCTGTTTACTACTCTGAAATCTAGCAGTTTCCTTTTGTTGAATCAGAACACAAGTAGAGTTGGAAATTTGGCTCTAGGTGTGCATCTAGGTGCAAGTGGTTCTACAGGAACAAGTGTCTTGCTTCAGTGTTTGTGGTCAAGAAAGATCCAGCAGTTCCCAGGTATGCAACTAAAGGTTTTTGATTTTCAGTATCTCAAAGCTTTGTTCAAAATTGGTACGATATGTAAGTTCTCAACTATGATAATTCTTTTCACAAGTGTGTATTGTGTGTTCTGGTGCCAGCTAGTTTTAGTTGTTAATTCAAATGCTCGTGTTCTTTGATGATTCTGGTGTCTTTTATGGTGTTATGGACACCGGATGATAGTGTTGTAAAGTGCTCCTCTGCTACTTATCTTCTCACTGTTTTCCCTTGGTTATCATGTCAGATTGATATTTGTTTATTGATGAATGAATTTGTAGACCACATGGTGACAGTGACAGAGGATTAATTTGGAAACTTCTAAGGCTTATCCGGTATGCATCCATGTGCACCACATGGTGATGGATATTGTTAGGCTCTCTTGCATCTCTAGTGATTTCTGAGGTATATGTGTTTTTTCAGCACATAGTTGTTCCTTCTTTTTGTGCTTGCAGTTGAGTTTTACCGAGCAGTGAATTATTCTTTTCATGGAAGAGGCTTTGTTTGGCCATTTGTGTTGAGGTTTAATGAGTACTGGAGATACTCAATACTCATTTCAGTTTTTTTCCCCAATCAGAGTGGCCCCTCCTCTTTGTCAAAAATTGTTGTTGTATGGAACTTGCCAGCTAAAATAGTTTCTCTTGCACTCACGTGTGTTGTATGGAACTTTCCAGCTAAAAATTGATGTGTATGTTGTTCGATGATCCAGTTGATGAGATCCTGGTGGTTGATGGTTATATGGTCTTATCGACACCTGATAAATACTCAAAACTCAATCCTGGATCTTTTGACAGCCCGTACAAGTGGATCCCTGTTAGACCCTCTCTTTTGTTGTTCTCTCTGCCTTAACTTCTCAGCTTCCTTTTTCTTGCATCACCCAAGCTTGATTTTGTTTGTTGGATGGATGATAGTTGAAGGTTGATGCCCAGTGGCCGTTTCTGTGCACGCTGCTTCTTGCTATTTTTGTGCTTGGACTACAATCTGAATATAATCAGGCTTCAATAGAGGGGTTTTCCAATCTCGTTTCTTTGCCATTTTTATGTGTGCATTGCAACCTGAATATTTTCAGGCTTAGAGAGGGTTTTTTAAATTTGTCTGTGGCATTTTCTAGTAGATTTTGTCAGCTTTGCACGTAACTCTTCCCTTCATTCCTTTGCTTGCAGCCATTGGGTCTTACAAAGCAGTGAATTCTTCTTTTCATGGAGGTACCTTATTTGCCCAAATGTGTTGAGGTCTTGTTAGTCAGTCTTTTACGAGTATAGATAATTTCCAATTTTTCTTCCCTATTGATTCCTTTGTCTTTGTCATAACTTCTGTTGTATGGTAGTTTCTCAATTAGGACATTATTTTCACCAATGTGTGTTTTATTCTACTGCCATGATGTTCTTAGTTTAGATGCTTATGTTCTTATGGTGATCCATTTTTTATTTCTTGGTTATCTCAGAATTATATAAATCTCACGTTATCTACTTTCGAATCTTGGTTTACGAATCTTTTGATATGGGGTGTAGGTTTCTCATTTCTGATTTTCGAATCTTTGATTATCTCTGTAGACAAGCTAAGGTCAGAAGATGAATGTTAGGGGGGGTTTTGGGAGCTGTTGATCTTTTTTCTTGGCCAATTTATCGATTCGTTATTGTTGTTTGTTTGATTAGGTATGGGTAATGATGATAGGGGAATGGTTATAGGTACCTTCCAGTGTATTTCATGATTCTCCTTCTACACTTGTTGTTACCTTCTGTCAGTTACCAAATATTCGTATCCTTGCTTTCCTTCATGTGTAGTTTATTTATTTATTTGTTCTCATTTTCTTGGGTATTATAGTGGGAGTTTATAATTGCAATCCTTTTGATTGATTCAGAATACGTGTACACAGCAGATTCGTGAGCAAGAGTGTATTCAACGAACAACTACAATAATTTTTTCTACGGAACTTTCATATGAGTCTATTCTTCCTTTTAATCTACTATATAAAGGTTTTAAATTGACCCTTAATATTAAAGATAATTACATTCTTGAAAGGATTAAGGGTTCTACCTTCCTGACTTTTCGCTTGTTAGGACTGGAAATTTAGCTCTCCAGGCATGCCTACTTGGATCTTCTATTGTGGACTTATGTGTCACAACCATACAATTGACCTATCAGTTTGGATTTGATATCTTGACTTCCATCTTGAATGTTAATTTTGATGCTAATCACGTGTCTCCTCACATCAACTGCTTTTAGCTCCTTAGTTGGTTTGGTCATCAGTAAATGTCATTCTTAGCTGTCAAGAATACATGGATGGGGCATCTAGGTGCAAGTGGTTCTACATGAATAAGTGTCTTGCTTCGGTGTTTGTGGTTGGGAAAGATGCAGCAGTTCCCAAGCCTTTGACTATAGGTCTTGGATTTTTCAGTCTCTCAAACCTTTATTAAAACTAGTACGGTATGAAAGTTCTCAACTATGATAATTCTTTTCACAAGTGTGTATAGTGTGGTCTGCTCTGCCATTTTTAGTTTAGATGGTCATGTTTTTTGATGATCCGATATCTTCTATGGTGTTATGGGCACCAGATAAAGTCAAAAGTCAATTTGGAATCTTTGATAGTGCTTCTCTGCTTTTTGTGTCCTAGCAACTTATCTTCTCTGCCGTTTTCCTTGGTTATCATGCCAGCTTGATTTTGTATTGGTGAATGGATATGTAGACCACATGGTGATATTAATAGTGGATTAGTTTTGTTCAGAGGCTTGTATCTCGTATGCATTCTTGTACACTTTGTTTTTTGGTCTTTTTGGGGCTTGGACTCCAATATATTGTGTATTATAGTGGTAGTTTATAATTTCAATCCTTTTGATTGATTTCCAATGTTATTAGAAATTGGAAGCTGGTGACTATAAATGCAATTTCATGTTTGCTTCCATGTTTTGGGAAATTCTGAGGTCTTTGTTGTGAGAATTCATATGTTGGTGAGCATTGTTCTATCACCCTATTTACTACTATGAAGCACATCAATTTCCTTTTGTTGAATCAGAATACTGGAAATTGGCTCCCCAGACATGCCTACTTGGATCTTTTATTCTAGACCTATGTGTCACAATCATACAGTGGAGCTATCAGTTTGGATTTGATATCTTGACTTCCATCTTTAATGCTAATGCTAATCATGTCTCTCTTCGCATCAAATTCTTTTACCTTCTTATTTGCTTTGGTCATCAGTGTATGTCATTTTTAGCTGTCAAGAATACATGGATGGGCATTTAGGTGCAAGTGGTTCTACAGGAATAAGTGTCTTGCTTCGGTGTTTGTGGTTGGGGAAGATGCATCAGTTCCCGGGTCGTTTACTATAGGTATATTGGATTTTCATTCTTTCAAAGCTTTGTCTAAATGGGTACGATATGGAAGTTCTCAACTATGATAATTCTTTTCACAAGTGTGTTCTGCTGCCAGTTTTAGTTCTTAGTTCAGATGCTCACGTTCTTTAATGATCCTGATGTCTTTTATCGTGTTATGGGCATCAGACAGAGTCAAAAGTCAATTATGGATCGTAGCTAGTGCTGTAAAGTGCTCATTTGCTTTTTGTATCTAGGTACTTATCTTCTTTGTCGTTTTCCCTTGGTTATCATGCCAACTTTATTTTTTTTATTGGTGATAAATGTGTAGACCACGTGGTGACAGTTAAAGATTATTAGTTTTGGAAACTTCTATGGCGTGTATCCGTTTTGAATTCCTGTACACCTTGTTTCTTGGTCTTTTTGGTGATGGATATTGTTCTGCTTCGACCAAAGCTGTGGTTTTCAATCTTGTCTCTTGCATCTATAGTGATTTCTTAGGTATTATGGATTTTGTCAGCTTTGCACTTAACTGTTCCCTTCTTTTTGTGCTTGCAGTTGGGTCTTAATGAGCAGGGTTTTATGAGTACTGAGATTCTCATTTCAATTTTCTTCCCCAATCAAAGTGCTTCCTCTCTTTATTAAAAAATGTTGTTGTATGGAAGTTCCCAACTAAAATAGGTTTTCTTGCGCTCGTTTGTGTTGTATTCTGCTACCTTTTTTAGTTTTTATCTAGATGCTTATGTTCTTTGATGATCCAATTAATGAGATTCATTGTGGTTGATGAGTGAGTATTAAATCTCTTCCTAACGCCTTTAGGGGCTGTGAAGAGAGCCCAGTTCCAGGCCCTCTTTTAGAAAGAAAAAAATGGTACAAAAAGAGGGCTTGGGGGTATATATACTATACAAATTGATGATGGTTTATCATCATCAGAACAAAGATCAGTCTAGGTCGAGATGAATGGAAGATGCTTCTGGAACTAGTTTTTGATAGGGTCTATCTTAGCTCGAAGGAAAGAACAACCAAAGTTAGACCCCCAAAGAATATGGATTAAAATCTGTTAGTATTTGACAAAGAACCTAACGGAATTTAACAAGCAGTAACCGGAATCAAAGTTTTGCAAAATGGTGTCAACCGGTAATAGAATGTAGCTAGATAACTGATGCCACAGCATCCAAAATATGTGGGATGGAGTTCATGGTACACTAACTTAACCTTCAAGGTCAGATAGTGCTACAGTGAACATCAATCCACTATCTAGCTCAAAATTAGCCTCACGAACTACTAACACTATTATTCGCGTAGCCCTTTTTTGTTTTGTGCTATTTCATCATAGGCTGTCGGAACTTGCTTCTTAGCGCTACGTGGGAAGAGAATTTCTCTATTCAGCCCCCCCGACTTCAATTACAAACTCTTACTTCTCGGTCTAACTTGGACTAATCCCTTAGCAGGCAAGAAATTCCTACTAACCAGACAGAAAGTAAGTTTCCACTCACTACCAAAGCGCTGTCGCACCTCTACTATTTTCGGCGTAACGAGGTTTTAGTCTTTACGAGGGCACCCCCTAGCCAGATTCAATCCCAAGGGTCAATCAAGCCTTTGAAACTAGTGAAAATAGTCGTCACAGTCTGAGTTCCTGCTTTCAACGAGACTTTCTTAGCTTGTCAAACTTTCTCTCCTTCACCAGGAAAGTTATCCGGTAAGCAAGATCTTTGATTGATGTGCCTGAATAAGGTTGATCAATGGTAGCTGCTTGGAGCTTTCTCTATACCTTAATTGAACTGCCCCGCTCTTAGCTCGGGTTCTTGCTTAATGAGGCGTCCCTTGTTTGCTCTTGGATTGGACTAAGCGGCAATCCTTACTGTGGAAGCAGACCTTGTCATTCTTCTCCTTCAATGCCAGTGGATAAGTACACTAGGGCTGGAATGCTCTTTTATCATGTATGTGGATTACTTGGTCGATTACCTGATTGCACTAGTCTCATAGCCATCTCTTTAGCGTCCCGCCATTCCTTCTTGTTAACACAGGAAATCTAACTCATTCTAAGGTAGAGCTCTTAGAGTCCTACTTAAAGTGAAAAGTAGTTCTTTAGTTAAAGCAAGGAGAGCAGAAACTGAGGGAAAAAACCTATGTTCTTACGCGGGATGGGAATAAAGGCTACTAAGTAACCTTAGAATGGGGATGCCAATCTTATACAGAAAATACCGCTATCTAAAGTAACCTTGCAGGGGGGCTGCGTATAGCATTCTAAGATCTCAATACGAGAAAGAAATCAGACCTATAACCTCACTGCAGTGGCTTGTAGACTTGCTTTGCCGCGGAAAGCTCAAGTGAAAACCTATTAGAACTGTGGGCCTCACCCGTCTACTACTCGACCTTAAGTAACTACAGTTAGGAGGATCCATCCAATAGCTTAATGACTTGCTTGGGTCATCTAAGAGGTAAGGCCTCCTCCGATCGACCCTAACTTTCATTATTGAGCTAAAGGAGTTCCCTAGCCCTTCAAGTTTACACAGGTCCCCAAAGATAAAGGCTTTCATCCTTCCTGAATATTCTGGGAACTTTCTTCTATTAATTGTTTTCGAAATGCCGATTTTCCTGTCTGCGCTAAGGCGATAAGGAAAAAGAGAGGGGCCTTTAGTATGTGGAAAAAAAGGCAAAGATCACATTCCAACTAACGTTGACTGGTAATTGCCTCAATGAGGCGGATAAGGCGCAAGAACCTAAAAGGCGGACTAAGATAAGTTATTCAGCTAGGGAATGCGTTAAAGAGCCGTTATGTAGCATCTGAGAACAAGAAAGCAGACATGCACACGAAAAGACATGCACACGAAAAGCAGACATGCTTTCAAAGGGCTTTCAAAAATCAATCATGGATCTCTTGAAAGCCCTTTGAAGTGGGTCTTTGTTACTCCCTTACTTTTGGTATTTTCTCAGCTTCTTGTTTCTTGTTGCATTGTCACCCGAGCTTGATTTTGTTTGTTGGATTAGAGTTGGAATTTTTTCAAAGATTGATGATGTGATCAGGCTTTGATAGTTAGTGTTTGCTCCTATGCACCCTGTTTCTTTGCAAATTTGTTCTTTCTCACCAATCTGAATTTTGTCAGGCATTGATAGAGGGATTTTCCTATCTTGTTTCTTTGCCATTTTTATCCTTGCACTGCAATCTCAATATTATCAGGCTCCGAGAGAGGGGTTTTGCAATCTTGTCTGTGGCGTTTTGTAGTGGGTTTCATCAGCTTTGCACGTAACTCTTTCCTTCATTCCTTTGCTTGTAGCCATTGGGTCTTACAAAGCAACAAATTCTTCTTTTCATGGGATGACCTTATTCGCCCATATGTGATGAGGTTTCGTTAGTTCAGTCTTTTATGAGTAGAGATAATTTTCAATTCAGTTTTCTTCCCTAATGACTTGTTTGTCTTTGTCAAAACTTACTGGTTTATATAAGTTTCTCAATTAGGAAATTATTTTCACCAGACGGTGTGTTGTATTCTGCTGCCATGAGGTTCTAAGCCCTCACACTTATGTTCTTCGATGATCCAATTGATGAGGGATAATTTTCAATTCAGTTTCTTCCCTAGTGACTCCTTTGTTTTTGTCAAAACTACTGTTGTATGGAAGTTTCTCAATTAGGACATTATTTTCACATGTGTGTTGTATTATGCTTCCATGAATTTCTATGTCCGCATGCTTATGTTCTTCAATGATCCAATTGATGAGGTCTTTTATGACTATGGACACCTGATAAGTATTTTGGCCCCTTCTTGATCTTTTGATAGTTGCATAAAATACTTTTTTTACTTTTGTTCTTTTAGCTTCTGATCTTCTCTGCTTCCATTTTTTTCCGTTATCAGACTAGCTTTTTTGGTTGTATAGATGTGTAGACCATGTGGTAACACTGTTATGGACGTTCAAATTTGGAAATTTCAAAGTTTGGTTAACCAACATGTGATCCTCTCTCACCTTTTGGTCTTTTTGGTGCTTGCACTGCAATTTGATGCTGTCCGGGCTTTGGCAAAAGAGGGTTTTCAGCTTCGTCTATGGCATCTATAGTGATTACCATTGTGGTTTTGGGTTAGTCAAATTTGCACTTAACTGTTGCCTTTTTGTAGTTCTTTCATCCATTGGTTATATGGCAGTGAATTATTCTTTACGTGGAGAGGCTGTGTTTATCTGCTTGTTGTTGACGTTTCTTTAGTTAAGTCTTATATGAGTATCGAGATTCTCGGTTCAGTTTATTCCCCAATGACTCCTTTGTGTTTGTCAAAACTACTGTTGTATGGAAGTTTCTCAAATAGGATATTAATTTTCACCGCAATGTGTTGTATTCTACTGCCATGAAGTTTTAAGTCTAGACACTTATTTTCTTCGATGATCCAATTGATGAGGTCTCCTATGTCTATGTTTCTTGAAAAGTACTCAACTATTTCTAGACCTTTTGATAGTGGCGTAAAACACTTATTCACTTCTATTCTTGTAGTTACTTACTTATCTTCTCAGGTTCCATTTTTTTTCATTATCACACTAGTTTTTTTTTTTGTTGGGTGGATGTGTAATGGTGATTCTGTTATGGATGTTCAGATTTGGAAAATTCGAAGCGTGGTTGACCAACATGAGATCCTCTGCCACCTTTTGGTCTTTTTGGTGCTTGCACTGCAATCTGGATAATGTCCGAACTTTGACAAAAGGGAGGGGAGGGGGGGGGTTTAGTCTCATCTATGGATTGGCTAGTGATTACTTTGGTGGTTTGGGGTTCATTGGATTTGCAATTAACCTTAACTGTTCCCTTTTTTCTATTCTTGCAGCCATCGGTTACAAGGTAGTGAATTATTCTTTGATGGGGCTGCCCTATTCGTCTGTTTGTTGTTGAGGTTTCTTTAGTCAAGTCTTTTATGAGTATTCTGATTTTCGATTCAGTTTTCTTCCCTAATGAATCCTTTGACTTTGCCAAAACTATTGTTGTAGGGAAGTTTCTCAACAAAGATATTTATTTTCACTGGTATATGTTATATTTTGCTGCCATGAAGTTTTTAGTCCAGACAGTTGCATTTTTCGATGATCCAATTGATGAGGTCTCCTATGACTATGAACACTTGATAAGTACTCGTTCTAGATCTTTTTATAGTGGTGTAAAATACTTATTTCACTTTTATTCTTGTAGCTACTTATGTTTCTTCTCTGCTTCCATTTCTTTTTCGTCATCACATAGTTTTTTTTTGTGGTTGGATGTGTAGACCATGTGGTAACACTTTTATGGACATTTAGATTTGAAAATTTCAAAACTTGGTTAACAACATGTGATCCTCTGCCACTGTTTTTGTTCTTTTTGGTGCTTGCATTGCAGTTAACATATTGTGCGGTTTTGACAAAAGGGTGTTTTCAGTCTCGTCTATGGCATCTCTAGTGATTACTTAGGTGGTTTGGGGTTTGTCAGATTTGGACTTAACTGCTCCCTTCTTTCTGTTATTGCAACCATTGATTACAGGGTAGTGAATTATTCTTTTCATGAAGAGGCTATTTTTGTCCGTTTGTTGTTGAGGTTTCTTTAGTTCAGTCTTTTATGAGTATCGGAATTTTCGATTCAATCTTCTTCCCTAATGGCTCCTTTGTCTTTGTCAAAACTACTATTGCATGGAAGTTTCTCAACAAGGATATTAATTTTCACCGGTATGTGTTGTATTCTGCTGCCATGAGGTTTTTAGTTTGGACGCTAGTGTTCTTTGATGATCCAATTGTCTATGGCTATGAATACTTGATAAGTACTTCACCAATTTTATATCTTTTGATAGTGGTATAAAATACTTCTTCACTTTTATTCTTGTAGCTACTTGCTTATCTTCTTTGCTTCCATTTCGTTGTCGTTATTACACTAGTTATTTTTGGTTGGATGGATGTGTAGATCATGTGGTGATACTGTTATGGATGTTAAAATTTGGATATTTCAAAGCGTGGTTAACCAACATGTGATCCTCTGCCACCTTTTGATCTTTTTGGTGCTTGCACGACAACCTAGATATTGTCCGAGCTTTGACAAAATGGAGTTTTTAGTCACATATATGGCATCTCTAGTGATTACTTAAGTGGTTTGGGGTTTGTCGGATTTACATTTAACTGTTCCCTTCTTTCTGTTCTTGCAGCCAATGGTTATAGGGAAGTGAATTTTTGTTTTCATGGAGGGGCCGTGTTTGATTGTTTGTGTTGAGGTTTTTTTTAGTTAAGTCTTTTATGAGTATCGATATTACGGATTTAGTTTTCTTCCTTAATAGCTCCTTTGTTTTGTATGGAAGGTTTTCTCAACTAAGATATTAATTTTCACTAGCATGTGTTATATTTTGCTGCCATGAGGTTTTTAGTCCAGATGGCTTATGTTCTTCGATGATCCAATTGATGAGGTCTCATTTGTCTATCGATTCTTGGTAAGTACTCTGCTAATTTTGGATCTTTTGATAGTTGCGTAAAATACTTCTTCACTTCTATTCTTGCAGCTACTTATCTTCTCCACTTCCATTTCTTTTTCGTTATCACATTAGCTATTTTTGGTTGGATGGATGTGTAGACCACGTGGTGACACTGTTATGGACGTTCAAATTTAGAAATTTCAAATCTTGGTTAGCCAACATGTGATCCTCTGCCACCTTTTGGTCTTTTGGTGCTTTGCACTGCAATTTGGATGTTGTTTGGGTTTTGACGAAAGGGGGTTTTAGTCTCGTCTATGGCATCTATAGTGATTAATTAGGTGGTTAGTTTGTCAGATTTGCTCTCAATTGTTCCCTTCTTTCTATTCTTGTAGCCATTGATTACAGGGCAGTGGATTATTCTTTTGACGATGCAGTCATGTTTGTCCATTTGTGTTGAGGTTTCCTTAGTCAAGTCTTTTGACATTATTCTTCACTGGCGTGTGTTGTATTCTGCCGCCAAGAAGATTTAGGTCCAGATGGCTTTGTTCTTCGATGATCCAATTAATGACGTCTCCTATGACTATGGACACTTGATAACTCAACCAATTCTACATCATCTGATAGTGGCGTGAAGTACTTCTTTTACTTTTTTTCTTCTAGCCATTGGGTCTTACAGAGTGATGAATCATTCTTTTCATGGAGCGGCTTTCTTTGTCCATTTATGTCGAGGTTTCCATGGTTCAAGCTTTTATAAGTTCTAACATTTTCGAGTAAAATAGTTTTTTTCCCATTAGTATGTGTTGTTCTACTGGCATGTTTAGTTTTTAGTCCAGATGTTTGTTTTCTTCAATGATCCAATGATGAGATTCATGATGGTTGATGGTTATTGTCTCATGGACATCTGACAAGTACTCAATTTTGGATCTTTTGAAAGCCTCGTAAAATGGTTGGTGTCCAGTGTGCATTCCTGTGCACCCTATTTCTTGGGCATGTTGGTGCTTGCACTACAATCTAGAGATAGCAGGCTTCGAAAGAAGCTGCTGGGTCTTACAGAGCCATAGATTCGTCTTTTCATGGAGTGGCGTGGTCTGTCAATTTTGTGTTGAGGTTTTTTGGTTGATGCTTCTAATGATTATTGAGATCTTCAATTCAGTTTTATTCCCTAATCAGAATGGTTCCGTTCTTCCTTGTCAACAATACTTCACTGGTGTGTATTTTGTTGCCATATATGTTTCTTAATAATCCAGATGCTTATGTTCTTTGATGATCCAACTGATGAGATCTCCATGGTTATGGACAACACATGATAAGTGTAATCAAGCTCAGTCTTGGATCTTCTGATGGATCTTTTAATAGCCTTGTAACACACTTCTCTAGTTTTCGTGTTCTCTCTACTTACCTTTCTCTGATTTCATTTAACCTACCAGTTTATTTAGTTTGGTTGGATGAATTTATAATTTTGGTTTCGGTGTGCATCCTTGTGGACCATGTTGACCATTTTGGTGCTTGCACTGCAATTTGGATATTGGATATCACCATGCCTTAAGAAAAGGGCACTTTGCAATCTTATCTTGTTTGGCATCTCGAGTGATCTCTCCCGTGTGGTATGGTTTTCCACTCACCTGTTACATTATTTTTTGTGCATGTAACCATTGGGTCTTCCAGAGCATGGACTTCAAGTCAGTTGTCTTCCTAATCAGTGGGTCCCTTTTCTCTTTGTTAAAATTATTGTTATGTGGAAGTTCTCAACAATGATAATATTTTCCACTAGTGCGTGTTGTACACTGCAACCATAAAGTTTTTAGTCTAGATGCTTATTTTCTTGGATGATCTAAATGATGAGATCCCTGATTTGATATGGACACCTGAGAAGTACCCAAACTCAATTTTCCCAAGGGCTTCTCTACTTTGGTGTTCTTGGTACTTATTTTCTCTGCTTCTATTTTCTCATGTGTATCATGCCACTTGATTATGTTTGTTTGGATGCATGTTTGTAGACCACATGGTGACTAGAGGGGGATCGAATTTGGTAATTTCGAAGTATGGTTTTGTCATTCCCTTGTGCACCCTGTTTCTTTGCCATTTTGGCGATTGCGCTTGCACTGCAATTTGGATATCACTAGGCAATAGCAGGGGAGTTGTGCAATCTTTTTTGGCATCTCTAGTGATCTCTCCTATGTGGTATTGGTTTCACAGCTTGCACTTTAACTGTTACCTTCTTTTTGTGATTGCTGTTGTTGGATCTTTCAAGATCAGTAAAGTCTCCATGAACTCCATTTCATAGAGTAGACTTGTTTGTTGTAGTGTTGAAGTTTCTTAGTTCAGGCTTTGTATTAGTATCTAACTCTTCAATTCAGTTTTCTTCCCTGGTCATAGAGATTCCGTGGGTAGCCAGGTAGCCGTCGGTCTTGTCCCGACACCTATCTTCAGTTAGTAGAGGCTTCATAAATAGTCAAAGTTAATGCGTCAGTTCAGTCTTTAGTATTCAGAAGTTTTAAACATCATTATTATTATATTATTATTATTGTTATTGTTTCAGACTTTTGCATGGGATATCTTTAAGTGATTTTAAATGTTCATTTTATACGGCTTCCGCATGTATGTTTAGTCTTCCTCTCAGTAGTTAGCTAGGACAAGGGTTTGCTTGGGACCAACAGTGGGGTTCCGAGTGCCAGCACTCAGAACAGTGGGGTTTCGAGAGCCGGCCACGTCCAGGTGTAGGCTCGGGTTGTGACACAAATGATGAGGTCTCTTATGGCTATGGACACTTGATAAGTACTCAGCCAATTCTAGATCTTTTGATAGTATCATAAACTACCTTTTTACTTTTATTCTTCTAGATACTTTCTTATCTTCTCTGCTTTCTTATATTTTTCGTTATCACACTAGTTTTGTTGTTGTTGTATGGATGTGTAGACCATGTGGTGACATTGTTATGGCATTGAAAAAATGGGGTTTCAGTCTCGTCTATGGCATCTCCAGTGTTACTTAAGTGGTTTGGGGTTTTCCAGATTTGCATTTTACTATTCCCTTCTTTCTGTTCTTGCAGCCATTGGTTACAGGGCATTGAATTATTCTTTTCATGGAGTGGCTGTGTTTGTTTTTTTGTGTTAAGGTTTCTTTAGCTTGAGTCATTTATGAGTATCGAGATTTTCGATTCAGTTTTCCCTTTTCTATATCAAAACTATTGTTCTATGGCAGTTTCTCACTAAGATAATATGTTTTACCCGTATGAGGTTTTTAATTCAGACACTATTTTCTTCAATGATTCAATCGATGAGGTCTCTTACGGCTATGGATACTTGATAAGTACTCGGCCAACTCCGAATCTTTTCTTTTGACAGCGTCATAAAGTGCTCCTCTACTTCTGGTCTTCTAGCTATATTTGTTCTTTGTTGACATCTTCTTTTGGTTACAAAAACTTCTTAGTTTTTGTTTGGATGCATGTGTAGATTATATGGTGATTGTAATAGAGGTTAACATTTAGAGATTTCAAAGGTTGATATCCAATATGGTATCTCTATACCCTCTTGGTCTATTTGGGTCTTGCACTGCAATCTGGATATATTGGTCAGGGCTTGAACCAAAAACGGGTTTTAATTCTTGTCTATATAGCATCTTTAGTGATTTCTTAGATTGTATGGGTTTCGTCAACTTTGGACTTGATTTGTACCCATTTTTTTTTGCTTGAAGCCATTGGGTCTTACAGTGCAATTAATTATTCTTTTCATGGAGCGGTTCTCTTTGTCCATTTATGTCAAGGTTTCTTGGTTCAGGCTTTTATGAGTTCTGACATTTTCAACTAAAATAGCTTTTTTCCCAATAGTGTGTGTTGTATTCTACTGCCATTTTTAGTTTTTAGTCCAGATGCTTATTTTCTTCAATGACCCATTGATGAGATTCCTGATGGTTGATGGTTATGGTCTCATGGACACCTGACAAGTACTCAATTTTGGATATTTTGAGAATCTCGTATCTCTTTTTGTGTTCTCGCAGCTCATCTTCTCTGCTTCACTTTTCTTGTATTACGCTAGCCTGATTTTGTTTGTTGTATGCATTTATGGTGGCAGTTGAGAGGAATAGGACTTAGATGTTTTTGAAGGTTGGTGTCTAGTGTGCATTCCTTTGCCCCTGTTTCTTGGACATGTTGGTGTTTGCACTACAATCTGAAGATTATCAGGTTTCGAATGAAGGTGGTACCTAGTTCGCCAGCTTTGCATTTACTATTCCATTCTTTCTGTGCTTGCAGTCGCTGGGTCTTACAAAGCCATAGATTCTTCTTTCATGGAGCGGCATGGTCTGTCAATTTCGTGTTGAGGTTTTTTGGTTGATGCTTCTAATGATTATTGAGATCTTCAATTCAGTTTTGTTCCCTAAACAGAATGGTCCCCTTCTCTGTTGTCAACACTGCCTCACTAGCCTGTATTTTGTTACCATATTTGATTTTTAATAATCCAGATGCTTATATTCTTTGGTTATCCAACTGATGAGATCTCAATGGTTATGGACAACACATGATAAGTGTAATCGAACTCAATCATGGATCTTTTGATGGCCCTGTAATGCACTCCTCTACTTTTCGTGTTCTCGCTACTTGTCTTCTCTTATTTCATTTAGCCTACCATTTGATTTAGGTTGGTTGGATGAATGTTTGTAGGCCACATGGTGGTAGTAAGAGGATGAGATTAGGATATTTTGTTGTTTTGTTTCGGTGTGCATTCCTGTGGACCATGTTTCTTACCATTTTTGTGCTGGCACTACAATTTGGATATTGGATATCACCAGGAAAAGGGGGCTTTGCTATCTTATCTTGCGTGGTATGGGTTTCCACTTACCTGTTATGTTCTCTTTGTGCATGTAACCATTGGGTCTTCCAGAGCATGAACTTCGGTTTTCTTCCTAATCAACGGGTCCCTTCTTTCTTTGTCAAAACTACTGTTGTGTGGAAATTCTCAACAATGATAAAATTTTCCACTAGTGTGTTTGTACTTTGCAGCCATCAAGTTTTTAGTCTAGATGCTTATTTTCTTGGATGACCCAAATGACGAGATCCCTGATTTTTATGGACACCTGAGAAGTACCCAAACTCAAATTTAGGTCTTCTTATGGCCCTAAGAGCTCCTCTACTTTAGTGTTCTTACTGCTTTTATGTTTATCATGGCACTTTATTTTGTTTTGCTTGGATATCATATTTGGAAATTCTGAAGTTTGGTTTCAGTGTGTGTCCTTTCCTTGTGCACCCTGTTTCTTTGCAATTTTGGAGCTTGCGCTTGCACTGTAATTTGGATATCACCAGGCACTGTCAAGAGAGTTATGCAATCTTGTTTGGCACTGCAATTTCGATATCACCAAGCACTGGCAAAGGGAGTTATGCAATCTCGTATGGCATCTCTAGTGATCTCCCCTATATGGCATGGTTTCGCAGCTTGAACTTTAACTGCTCCCTTCTTTTAGTTCTTGCTGTCGTTGGGTCTTTCAAGATTAGGATAGTCTTCCTTTAATGGAGCAGCCTTGTTTATCTTAGCGTTGAAGTTTCCTTTGTTCAGGCCTTGTATTAGTATCTAGCTCTTCGATTCAGTTTTCTTCCATGGTCATAGAGATCCCCTTATCTCGTTGTCAGAAGTGCTGTTAAGTGGAAGTTTTCAACTACAATAACTCTCTCCACAAGTGTATTTTCAGCTTTTAGTCCAGATGCTTTTGTTTTGCGATGATCCAATTGATGAGGTCTCTCTTGTGGTGTGGACATCTGGTATGGAATATGAGCAGATTTGTGAGTTGCAGAAAAGAACGTCCGTCAGAAAGCTTGGCCTCCTGTGCTCGAAGTGAGACCTGAAGGGTGAGCTTATGGCGCACGAAATCCATCTCTCGGTTTTGCAGATCTATTTTGAATTGTGAATTTGCTGTGAGTTCTCACTTTTGTTGGTTTCTCGAGGTATCCGGGGTCTTCCTGCTTAGATTATGCCAATTTCTTTCTCTGTTTTACCAGGGTAATGATTGTATGGGTATAGACAATGCAGTTTCTGTTGTCGGATGTCAATAATCTTTGTAACGAAGTAAACCTCATCTCTGGTACCTCCAACAAATCATCTTTTCTTGCGTCTTCTCTTTTGGAACACTATTCAACAGAAGTTTATGAGCCTTTCCTTGTAGATGTTGCTCTCTATATTTCATACCAGTATACGTACCCGCACGTTGCGCGGACATTATAAAATGTAATAATGTTTTAAATTTAAAATAACTAATAAGGTTTATTATTTTCTTTGTAATATATTTTTAAAATAATGTAATAAATCATTAAATTATTTATTTGAGCAAGTTCTATTAATATTTATGTACAC

>LNC_000635

AAATCTGCTATATATCCCTAAATATATGCTATTCCTAATTGTAATATCTCTGAAAATATGCTACATTAATCTATGCTATCTCCTATCATCATTACACAAATATTTAGAGTTATAACATAGATATGTACAGATATTTAGAATCTATCCAATCATGCTAACATCCCCCCTCAAATTGATGTCGGTGGATCAAAAAGCATCAATTTGCCTACCAAAAACTGATGACGTTGTCGTGCCATAGACTTTGTAAACACATCAGCTATTTGAAGATCACTGGATACATGTGGAAGAGTAATGACTCTCTTATCTACAGCTTCTCTGATATAATGACAATCCACTTCAATGTGTTTGGTCCTTTCATGATAAACTGGATTAGTAGCAATCTGAATAGCACTTGTATTGTCAGCATGGAGAGGAGTGGGATTAGATTGAGGAAATCCAATTTCTGTAAGTAAACCACGAAGCCAAACAATCTCAGAGCAAGCAGTAGACATTGCTCGATATTCAGATTCGGTGGAAGATTTTGATACACGATCTTGCTTCTTGCTCTTCCAAGATATCAAGGAATCTCCAAGAAACATGCACCAACCCGTGACGGAGCGACGAGTATCAGGACATCCAGCCCAATCAGAATCACTAAATGCATTAAGCTGAATCTCAGAACCACTAGGAAAGAATAATCCACGAGTAGATGTTCCCAAGAGATATCGAATGATGCGACGGACAGCCACCAAATGTAGATGGCGTGGAGCCTGCATAAATTGACTCACTTGTTGAACTGCAAAAGAGATATCAGGCCGAGTAATAGTAAGATAATTTAGGCTCCCAACTAATTGCCGATACAAAGTTGGATCAGGAAGAAGATCTCCTTCCGCACTGCAGTACTTTACATTCAATTCCAATGGAGTATTTACAGAGGAAGATTCTTGAAGACCAGCCAAAGCGATCAAGGCCTGAGTATATTTGTGTTGGTTCAAGAACACACCTAAAGAATTATAATGAACTTCTAACCCCAAAAAGTAAGTAAGAATACCAAGATCTTTCATATGAAAAGAATCCTTAAGCTGCTGCTGGAGGCTAGTGATTAGTGAAGAATCAGTTCCTGTGATAATAATGTCATCGACATACACCAAAAGAAGAACACAACCAGCAGATGTTTTTCGAAGAAACAAAGACGAA

>LNC_000636

CTCATCCTTAGACATGTATGCATGCATAATAGGAGCTCCAAAGGGTAACATCACAAGATTTTAGAATCAAAGAGATGGTCCACCTACACATGGCTTTCTTATCACTGTCACAACCATGACCCATGTAATTTATTTTGCACAATAATAATGCACACTATAGTCTATATAGAGGTCTATGTTCTTTTGTTGTAGCTAGTTGGGTCGTTCCTAAATTTTGAGGTCACATTGTAAGTGATTAAGTTTCATTCCTGGAATATAGAAATGAAGAAAGGGAGATAGCAAACAAACAATGATATTTATATTTGAATGATTTGTACGAGTTTTTTAATTCTTCTCCTCACGGTCATCAACCCAAGAGCTTTACTTTGTTTCTATCGATGAAATACTAGTTGAAACTTCACGATGAATACGGAGTCAAGTTTTAATGATCACAATTGCGGAGTTATGAAAACTTGCGGCTTACAACTTGAAGACAGAGAATATTTTGTATGCAAAAGACTTGTAGATCATCACTGAAGAGCAAAGCTTTTCTATGTGTTTTTCTTCTTTTCCTTTCCTTTATTTTGGCCTTGTGAAGAACCTTTTATATAGTTGTGAATTAGGGTTTTGGGGTATTTTAGTCCTCAAGCAAATTTAGAGGACCTTGGATTTAAAGAGCATGAGTGGGTCTATCTTGTCAACTTTAATA

>LNC_000637

CTTTCTTCTAAGAACTAGAAGAACAGGTTGAACAGAGGTCGATGCTTGTAGTCCATATTTGTTTCTTTAGGTTCAAAAAGTGGAGATTGCACTTCCTTGGTTGACCATGAATCTTGGTCTTGTCGAATCATGGGAGTGCTTTAACTGTTAAGATTTCTCCTGAAATATTTAAGTCAAGTGTAGGTTTGTCAAATGTTTAATTCATGACGCACTACCTCCCATTTACTGACTCCATAATGTGCTAGTATAGGTAGAATGAATGTAAAGCCTATCTTTGTGATTGATAGATTAAAATAGTTTGTTTAGGAAATTCTTAGTACAATTGTAATGTCCACTCAACAATTGAATAAAAAAAATAATAGAGATACTTCAAGAATTTATTAATGCCTTATTCATACTTATCCAGTCAGTTTCGAAGGTTCTATAGTTGTTTTCCACCTTTCCTTTCCTTTTCGTTTCTTCAACCCAAATGAAGCCTCTGAAGCTGACCTCTTCTGCTTTAGCTTATAAATATGATTTTTGTTCTCTTCTAAGCTATGTTACACAAATTCGTTTGTTGTACGTACCGGGTAAGGGTGTATGCAAGGATTGGGTATAGCTATATCAAATTCTTTGCTAATTATTAGTGGCCTTTGAAGCTTTGTTGCCCGCATCCTCCAAAAGTACCACCGCATTCACGGTTACTTTCTTAAAAAAAGAAAAAAGATATGCCGCCGCATTCGTGTTGGATCCTCAAAAAATGTTTTTACATTTGGAGAATCTGACACTTGACCGACAACATATTTTGAGTATCTAAGAAACAAAGTTTTGAAGGATCAGAACAAAGTATCCACATGCTTATCGCACCCATTCAACAAGTATATGTCAAGGCTAGTGAAAGATTTGTACAAAAGTGGTTCTAACTTCTAAGAACCTTCCCCCCTTGCAGAGATTGACCAATTATCTCTCACTCTTTATTAGTTTTTTCAACCCAAATGAGGCCTGAGAACTTATCTCTTGCGCTTTCATTTATGAGTATGACCTTTTATCATAAAACTGAATATGACCATTTATCAATAAAATGAATATGATCCTTAGTCCATCCAACAATGCACCTTTCTTCCTAATTGTGCAAAAATAAAATGGGGAAGAGTGAATAGAAACTAGGGTTCATTACAATAGGTAATACAGAACCAGTTTGGGTCTATGCCATGTGGATTGATATACATGGAAAGTTAAGCTTTCGTAAGAAC

>LNC_000638

CAGATCATAAAATGATCTGGTCCAAAAACAAAATACTCTGTCCAAAAACAAAATACAAAAATGAAACAACCAAAATAAAGGAAAAAGGAGTTGGTCATCTTCAAGCTTGAGCTTCCATGGAGGATGAAGACGAAGTGCTGCCTTGGAGAAGCTGCGTTCGGTTCGTTTCCATCCCATTGAATCCACACAGCTCGAACAACTTAGGTTGAGCCCCAAAGAGACTGATACCACTGCATAAGGAAGCCACATATGAGTTCCATCTCCGCATAAACAATATATTGCAATTACATCCACTTATGCAAGTATGCACGTACTACCATCGCCTTTTGATATTTTTTTGTCATCCTCTTGTTGTTGTTGTTTGTCTCAATTATGCTTGTGT

>LNC_000639

CAGATCATAAAATGATCTGGTCCAAAAACAAAATACTCTGTCCAAAAACAAAATACAAAAATGAAACAACCAAAATAAAGGAAAAAGGAGTTGGTCATCTTCAAGCTTGAGCTTCCATGGAGGATGAAGACGAAGTGCTGCCTTGGAGAAGCTGCGTTCGGTTCGTTTCCATCCCATTGAATCCACACAGCTCGAACAACTTAGATTGAGTCAGAAACCCTAACTAATACCACTGCATAGGCTAGGTTGAGCCCCAAAGAGACTGATACCACTGCATAAGGAAGCCACATATGAGTTCCATCTCCGCATAAACAATATATTGCAATTACATCCACTTATGCAAGTATGCACGTACTACCATCGCCTTTTGATATTTTTTTGTCATCCTCTTGTTGTTGTTGTTTGTCTCAATTATGCTTGTGT

>LNC_000640

TGGTAGGACCCCAGCTAGGGGTAGAGGCCGACCTCGAGGTCGTGCCAGAGCTACAGCACCAGCCCAGGACCGAGAGCAAACTTCGGAGCCGGTGATAGAGCCACCAGCAGCTCCAGCGCAGCAGCAGCCGATGGGCCCGGGACCAGCCCAGCCACCGCCCGAGCCTGTGGCAGCTCCAGGTATAAAGGCTATGCTCGTACAGATACTAGCTGCCATTGGGGGTATGCAGCAGGCCCCAGCTCCAGCCGTTCCAGTACCGCAGGGCCAGCCTACACCAGCGGTACCGGTTGTTCAGCAGCTAGCGGCACCGGTTGTTCAGCCTGACGATTTGGAGGGCGTCATGCCACTACGGG

>LNC_000641

CAGCTATCGTGCTACTGGCTTTTAACCGAATTGCAACTTTCTAGCTCTTAGCTTGACTACCTCTACCGAGAAGCTCTCTCTCGCTTCAGCCCAACCAATTCACAGACGATGAAAAGAAGTCCTCAAATCCTAAGTACACTAAGTGGGTAAAGACGAAGCAGTTGCTCGTTAGCTGGATCAACGCCACCTCCGGATGGAAAGGACAAGGAGCTGTCGGATGTCAATCTCTTTTCTTTTATGCTGCATGCAGCTACCTTCCATCGGCACATTATTCGCTCTGCTACAACGAACGAACTAAATGGCAGACCATCGAGAGTGAGTGAACGGCTAGCCAAAGCTTCTCGTTTGGAGAAAAATCGTGGTGGAAATGCGGTTGAAAGCTGAAATGAGACGACAGAACGAGTGAAAGAAGTTACTGAAAGTAAAGCCCCTTTTGTTGTGTTGGGCAACATGGGAGTGCGAACTCGGTTCCCCGTACGACACACACAACTATTAACGCTCTTTCTCAATCAGGTCTGGAACGCACAGTGTTGTTCAACC

>LNC_000642

CCGTGCCTTCTTCTGCGACCCAATATCACTCTTGGCCCATTTGCTTTTCTCAAACAAGACATCATCAGACATCTTCATCTTCTCTCTGGTGAAATTAGAACATGCTATAAATGGTTGAGAAGATGGTGTAGAAGAAGGTTGAATTCCAACATCCCCCTCAAGGTGGAGGGGAGCGGAGTGAGACCCTTACTTGCATAGAACTCCATGATGTTGCGGGCCATAAAGAGGCTTAGTGAATATGTCAGCGAGTTGCTCTTTGGAAGGAACAAAAGATAGGGAAATGAGCCCTAACAAGTACTGTTGACGGACAAAATGACAATCCAACTCAACGTGCTTCGTCCGCACAGGTACAAGAAGTTGAGGCAGAACAGAGAGATCATCTAGTAATAGAGTAAGTCAGGTTAATTCAACTACCAATCGACGCATAGATCGATACTCTGCCTCTGTGGAAGAGAGGGATATAGAGATATGTTTATTGGACTTCCAAGAAATGGGGGAACCCCCTAAACTAATGAATAAACCACTGATAGATCTCAGTGAGTCTCTACACGAAGCCCAATCCGCATCACAAAAAGCATTAAGGGAAAAGGATGGAGAAGAAGAAAAGAATAGACCCTGATGCGGATACTGTTTCAGGATCCAATGGAGAGGCAATTGGAGGAAGATGTGAGACATCAAATTCGCACAACAAATCAGAGGAAATTTGCTCTTGGTTATCTCACGAAATCTTGTACTATCAAGTTCTCCCATTCTCTTGACACCAATGGAAGCTCGAAAAGAGTGCTTCTTTATTAACGAAATGCCATGAAACATGTTTCATTTGTCAAAATTTTCACCTTGTAAGAATACTTGAATGTTCAACAACATAGACTATAATAGAAGCTCAGTTGATGGTGCATGAAATTTGTAATAGGTTGAGAACGAAAACTGAACATGTAACGAATTGAACGTGCTATAGCCTGCTCCTAATCATTCTGATGTTCTTACTCCATTGGAGGAAAAATGGAAACTTGAGTGTTATTCACAAAATAGATAAGGGTATTGCAGAACTTGAAAGAGTTGTACTTACATCGATCAGCTCCTTCCTGGCATCTGTTAACTCAACATTTGCCCGCTGCTCTTTAACAACTAGAGCTTGATTCAAAGCTTCAAGATCTTCTAGTTCTTCTTTTTTCTCATTTAGGCTCTCTTCGATCTCGTCGAATTTTTTCTTCACCTCTTGGTCATCTCACATTTGTTCAGTTACATTCTTGGCCCCTGTGAGACGTTCAATCTCCAACTCTAGCGCTTGCTTTAAGTCGAGTTTCTTTTGAAACTCCATGTTCTTCTGACGGAGAGTTTCTTTCGCCTTCTGCATGTAAAATTCTCAAGATGTCAGAGACTAACTACATAACTACAGAAATTGAGTAAACACACAGAATGAGCTTACAACCTTCTGTTCTTCTGCCAAAGACAAGACACTTTCATCATACCTCTTTTGCTCCTCTGCTGCTCTCTCATTCTTAAACACATAAAAGAGAAATTTTACAGTGATCCAATTCCCAAAATGAAGAGTAGGCCAAGACAATATCTGTATATTCGTAGACTATGCAATGACAGACAGTATCAACAAAGCTTAAGTGAGAACAAAGTTAAAAGTGGTAAAGTACTAGATTCTTGTAGCTTCATTTTTTATTAGTATATCTGCAAGGAAGAAGATTTCAGATGCATGTAACTTAAACATGGAAAAATGAGAGTGATGGATGAATGAAGCTTGTTTGAATTGACAGTAAAATCCAGATGAAGTTACTTATTCAGGTCCTTATGCCTATCCACTATCTTAGTTCTACAACATATGAGAGTATTTCTGTAACATCTTATTTTTGTACCTCATTGGTAGTAGTCTATTCCTTCATATGCTTATACCTCCATATACTTTATTTACAAACCAGTTAAGTCAAATCAGTTAAACAGCAATTCACATTCTAACTATTTCATGCTAATAATTTGGATGCCTTAGAGCATATGCTTTCTAACACAAACCATCTCCCATAGCATACATAGAGAAGTAAGATTTTCTACCCACGCGCCCAACTTGATTCAATTTCAGCAAAGTATATTGATTTGAAAGTAAATCCCTTGAATTCTTGATGAAGTTCGTTTGTATCCTTGTGGGTTGTGTTGTTGGAGTTTTCTTGAGATTGATTCACGTTTTTAGTTGTGTTTTCGAGTAGAATCTGATGTTGTTTGAGGGTATAAATAATTCTAAGTGTTTGGGAAAAGAACCAATGGAGTTTAGAGGGTTTAGAGTTGAAAAACGAAAAAGGAATTTCGTCGGGCAAAACTGGGCAGTGCCTCCGCGTCACGGACCTGCTCCCCAGATTTGGGAAAAATCGTTTCGCGTCACGGAGCGGTCACGAAGCGTTCTGCCTCAAAAGTTGTTTTCGAACCAAAAATTTAAATATTTCTCTGCGTCACGAACCCACCCTCAGATAGTAATTTCTTGATCTTTTCTCATGTTTAGCTATCTAAAATCATTCCTAAATATCATGAGATCTTTCCTATCACAAATCACAATCCTTGAATCCATAATTCAATTCAAGGAAAGTTAAGAGTCAAGTTAAAAGTTAAGAAGCAAGTCAAGAGAAATTCTTTAAAGCTTTCAAAAGTCTCTTACAAACATTTTAACTTTGTTTCAAGACTTAAAGTTCAAGTTGAGTAAAGAGTAAAGAGTAAATATTGAAGTTCATTTCTTCAAAGAAGTATATGGGGACT

>LNC_000643

CCGTGCCTTCTTCTGCGACCCAATATCACTCTTGGCCCATTTGCTTTTCTCAAACAAGACATCATCAGACATCTTCATCTTCTCTCTGGTGAAATTAGAACATGCTATAAATGGTTGAGAAGATGGTGTAGAAGAAGGTTGAATTCCAACATCCCCCTCAAGGTGGAGGGGAGCGGAGTGAGACCCTTACTTGCATAGAACTCCATGATGTTGCGGGCCATAAAGAGGCTTAGTGAATATGTCAGCGAGTTGCTCTTTGGAAGGAACAAAAGATAGGGAAATGAGCCCTAACAAGTACTGTTGACGGACAAAATGACAATCCAACTCAACGTGCTTCGTCCGCACAGGTACAAGAAGTTGAGGCAGAACAGAGAGATCATCTAGTAATAGAGTAAGTCAGGTTAATTCAACTACCAATCGACGCATAGATCGATACTCTGCCTCTGTGGAAGAGAGGGATATAGAGATATGTTTATTGGACTTCCAAGAAATGGGGGAACCCCCTAAACTAATGAATAAACCACTGATAGATCTCAGTGAGTCTCTACACGAAGCCCAATCCGCATCACAAAAAGCATTAAGGGAAAAGGATGGAGAAGAAGAAAAGAATAGACCCTGATGCGGATACTGTTTCAGGTAACTTACAACCCTCAAAGCTCCATGATAGTGATCAAGACTTGGTTTTTGCATAAATTGACCGAGAATCAAAACTGCATGACATATATTCGGTCGTGTATGAGTTGATCCAATGGAGAGGCAATTGGAGGAAGATGTGAGACATCAAATTCGCACAACAAATCAGAGGTATATTTCTTTTGACTGACTATCA

>LNC_000644

GAAGAATTGGGACTAAGGCCCAAAAGATAGAAAATACTTCTATTATGCTAGAATAGGACAGGTACAAATGACTTAAATGGGCCGAAGGCCCAAAACGAAAATGGGCCCAAATACTGGTCCAGGCGGACAGAAACCAGATTTGGGTCCAACCCTCTTTCCCTCTTTACTTTACTGTATTTGTTTTGCTTGATAGTATTTGTCGTTAATCTTAGGGTTGAAAAGTTCAAATCCCCGAAAGACGCTCA

>LNC_000645

ACTAAATAGATGCATCACTTCCCCAATGATTACACAAAGGCATGACATTCATCTCGGTACCATTGTTTGAGCATTCATTACACGTTAGAAGGGAGGGTCATCTATCTATATTGCTACGTTATGTTAAGTCAACATTCGTCTATCATTGTTTATGTCTTGAATTCCTGGCTTTTCTTTGATCTCATGTGTCCCGCTTTAGAATTTAGGGATATCGATTCTAGCCTCGGAGACCATTATTTTGGTCTATTGGCTGTCCGCGATTGTTGCTAGCAGAATACTTGCCCTTTCAAAGCTTATTGTTAATTTACTGTTTGATCTGATCGAATTTGTTTGAAATCATATGATTGCTAATTTAGTTTTGTTCTAGTTTTTGGTTTGTACTGTAGATAAAAAATATTAATTTAAAGGTATCACTTGTGTTAATTCAAAATTGTAGGTAACGTGCTCTGTCCTTGTCATAAGCTTAAAAA

>LNC_000646

TGAAACTTTCATCTCGTACAGCTCAAGCAAAAACACCCAAATACTAGTTGCAACGGATATGGAATAGAAAGTTTGAAATTTATTAAGTTCTGATCCCATCTTCTCTGGAAAATCCGAAAGCTCTTCTTTGTGGCGATAATACCAAAATATCAAGTTGGCGCAGTCGTCTTTATCTTTATTTTGATACTTATGGGCCTCTTGAATTCTCTTGTTCCCTATTTCTTTATAGAATTTGTGGAATATGGCTTTTATTATTTGTTTTCTTTATTATTGATAGGAATTCTCTTGTTAAGACCCTATGAATCGATTAAAACCTCAGATTCATACTAGAACCACGATGATTCAATAAAAAAACCTAACCTAAATCAAATCAAGGATTCCCTGAGTAAGAATCCTCGGATCATCACTTAGTCCCGGAATAAATAGAATGACTAAAAATTCAAAGAAAGGTTTTGCTTTTGGTCAAAATATAAATAGGGGTTGAAAGAAAAGAATCTTTCGATTTAGAAATTAAAATTCTTTGTTTGAAAATTTTTTGGAGCCCGGACACGAGGTCTTAATATTAAGTATGAATCATAGTTCAAATATTTCTTAATCTATTTC

>LNC_000647

GTCCGGCGAATCGGACAGCCACCAGAAACCTCACCCAGCAGACGTCGGCGAGCCACTGTGTCTCTCTGTCACTTCTCTCCGGTGGAAGATCCAAGCATGGAACTTCATTCGAAGACAAAACTTAAAATGAGAAATCCAGAACGATTAACCGGTGACTTCTTTCCTAATACTCTGATTACTTTTGTGAATTTTTATTATTTTCATTTTTAAAGCTTTATTAGAGGTTGTAATACATGTCTATCATATTGTTTGAGCCACTTTTGAATAGGCTGGGTATAATCTATTAATACTTTATGATTAACAAGTTGCTAAAACTGATTTTAATTTTTAGTTTTGTTTACTGTTGAC

>LNC_000648

TATTTGCCAAGAATATTTGAAAATCTATGATCAAACGCTAGCTTAGTATCTGCATCTATAGTGGTTTTATTTGAATTAATTTGGTGTAAAGCAGATGAATTGTCTATTATGAAGTGCTCTAACTTCAATCTATATAACAGCACCTCAGTCGCAAACAATTAGTTTTATGTAAATGCTGTACATATTTGTGTTGTATTTACGTTACTGTTTGAGCCATCTCAAGAACTTTTATTGAATATATTGCATCATTCAGTCAAATGCTGTGTTCTGGGGCTACTTTTATTTGCTAGTGTAAAAGCCATAGCTAGATTCATTTGCCGTATCCAATTGTATATGAAAGTTTTGATGATTCCTTTGCATATCCCTATTGCATGTGATAGTTTATGCACTACTCATTTCAGTGTTGAACAGAATCCCTTAGCATATTTGTTTTTCTCTGTGTTTTCTGGATCAGTTTATTGAGAAACTCGAATGAGGGAGCTAAAAGAAGATGATATACTGCATTATCAGAAGTTGATATATCTCTGGGGTCCCTCTACTTTTGTCATTTGGTAAGTTGCAATGGTTTTGTTCACGAAGGGAAGACAACTGCAAGACTATTCTGGGACACTTAACACAAACATTTGGTAAGCAGAAATGACTAAATGACCATCAAATGCTGCATTAAGTGGAGAGAATGGTATGTTCTTTTTCCAAGTGAATATTTACAGCCTCCTGTCTCCCAATGTCCAATGGAATAGGCCAGGTTGAAGATGGTATCATTAGTCTACTGATTCTATCATCATTCATCATGGTCATCACCAAAATGGCTACTCCACTTAATTGACCTTTGCACAATCCTTTTAGGTGTCTGATCATTATCTTACCAGATGCCTCATGTCCTCACTAGTGCAGACCTTGACAATGGGGTTTCCTTCTGAAGAGTCATGCTAGATGTTGATTGTACTGCGAAGTACCTAGCTTCTTCAGCTCCTGATGATGACATCCGGAATTTTTGTTTTCCCGCTTTGTATGCTTATAGATTCAGTTTTCTGATAGCTTGGTCATCTTCATTTGCTAATGATCTTCATTTATCATATCAGCTTTTACTAGGGCTTTCCGATTGAAATCTCATTCGGAAGCTGACGTCCCTAGCTGATGTAAAAGCTTTTTAATGAATCATTATTTCTTGGTAGATTTGAGTGCTGATATCTTATACAATCAAGTAGATGGTTTAATGCTTATTCCCAAGGTTCACATCAGACATCAATTATAGTTCCTTTCTATATAAAGTTGTGAATCACCTTTCACCGGACTGCTATCATACAATCCCTTTTTCATACTTGGAGTCTGGAGATAAGTATACAATCACATCAGACTGTTTTGATTTGCTTGAGCTGAGGATCGTTCAGAAACAAACTCTCTATCTCGGAGAGGTAGGCATGTGTATACTCTACCCTCCCCAGACTCCACTTTGTGGGATATCACTGGGTTTGTTGTTATTGTTGTTGTAGTCTGGAGACAAGTTTTCTCCTAGTGGAAAACTTATTTACCATGGTCTTTTGGGGATTTGGAGTGTTAAAGAAGGGCACACTTTTGTGAAGGATAATTTATATACAATTGCTGGTGTTTTGGGTGCAAATGAACAGTTCCAGTATCACTAAAGTGTTATCTGCCTTTAGGATTTGCATCTATAGTGGTTTTATTTGAATTATTTTGGTGTAAAGCAGATGAATTGTCTATTATGAAGTGTTCAATCTAGATAGCAGTGCCTTAGTCCCTAACAATTAGTTT

>LNC_000649

GATACCATTGCTTGAAATTTTGGGTTATTGTGGACAGTTAACTAGTTAGCACTTCTTTTGATATTAGATATGAAAATCTCAAAACAGGTCATCATACAGTCCTTGAATGTCAGTCGTTGGTGCCTTTATTCACCCATAAAAGAAGCTGGCTGTTATACTGGTGAAGTTATTGTCTTAACTCTTAAGCATATTATTTTCTAATGACCTATCTGAAGAAATAACGGGAAAATGCATAGGATTTAATTATTTTTCTTGACAAGTTAAATGCGGAGGACTAAAATTGAATATCAGTTGTAGAGTTGGTCATTTTTCTTTTTTGATAGACGTATACTAAGACAATTGTATAGTCCCCCAAAAATAATAAACAATCAAAAATAAAAGGAACCCTTCCTATCTACGTCTCTTCTTGCTGTTGTCTTAAGCTTCAAAAATATCTTTTGCTTCTTTTAAACATTCTTGTTTACACCAAAATAATGAGCAACTAAGCATTTCATCTTAAACTTCTAGATGTTGCAGTGCTTGAGTGACTAAGCATTTCATCTTAAACTTCTTGATGTTGCAGTGCTTGTCCCCAAAACTCCTTTGATTTCTCTTTTTCCAAACAGTGCACCATATACATGTTGGGAAATCCTCTGTCTCTCTTTCTGGTTGGTCAAATTTTCATCCGTTACGACTCGAGACTACATCCTAGTCGTAACATGGTCCTTAGAGCCACAAGTGACCCAAACTAACCCATGGCCTAGTATGACACTATGATTTATGTAAGCAACAACTTTACAGGATGTGAAAGTCAAACTGAATTAACTGACTGGTCTGAAATATGAAATTTAAATACTCATCTATCTGAAAGTCTTTACTAAGATGAGTTTCTAGGAC

>LNC_000650

TCAAACTAGAAGTTACCAAGGAACCATGCATAGCACTGAATAGGGAGCCGCCGAATACCCCAGCTACGCCTAACATGTGAAATGGGTGCATAAGGATGTTGTGCTCAGCCTGGAATACAGTCATGAAATTGAAAGTACCAGAGATTCCTAGAGGCATACCATCAGAAAAACTTCCTTGACCAATTGGGTAGATCAAGAAAACTGCGGTAGCAGCTGCAACAGGAGCTGAATATGCAACAGCAATCCAAGGTCGCA

>LNC_000651

ACAGTACCCGGTTAGAGTCCGTGGTCCAGCTTACCTGTGATCTGGTTTTAGCTACAGTACCCGGTTAGAGTCCGTGGTCCAGCTTACCTGTGATCTGGTTTTAGCTACAGTACCCGGTTAGAGTCCGTGGTCCAGCTTACTTATGTCCAGGCTTAGCTACAGTACCCGGTTAGAGTCCGTGGTCCAGCTTACCTGTGATCTGGTTTTAGCTACAGTACCCGGTTAGAGTCCGTGGTCCAGCTTACCTGTGATCTGGTTTTAGCTACAGTACCCGGTTAGAGTCCGTGGT

>LNC_000652

CATGTACAGAAAATATTTGATTATACGTGCTGTCGTTCCCAGTCAATTAGGGAATCAGAGAAATTTTACTCCCTGTACAAAACTAACAAAAATATAAAACTGAAGTTTGATGTTCATCACTGGTAGTCTCTGTATATTGGTTGTGGATTCCATTTGAGATGCAAGTTCAGTTTCCCTGATTTAACCTCAGCTAATTCGTACGAGTCTTTGTATTCACCTTCAATTAGAACCCTTTTTAAAGTCAGTATGCATTTTCCCATGAAATCCTTTCCAAATTTGTCATGGTCCCAGACTTCCAGCATTAACATGTCATGTAATCCGTCCTCAAGAACAAAGTCAAAAGTTTGATTCCATATCGGGTTTAGACTTTCATTTACAACCCTGGTTTTACTCTTGATTTGAGCCTTCTTCATGATCAGAACGACTTAGGGGTCAGCCTTCCCCCCTATATCAGCTGGGGGCAGGTCATTACCTGATACCACGGAAACAGAAAGTACCCCTCGTACTATTACCTCTCTTCTATTGTTGATTTCACTTCCGTTTGGACTAACTTCTTTTCCTTCTGCCGAGTTTTTAAGAACCCTCTCTAATGAAGTCATTGACTCATTCTGAGAAAAAAGCTTACTTAGCCCATTGTTCATGCCATTTCGGGCAGTACAATAGCTCCAAGTGCACCTGGACCCTATTTTTCTTATCTCTCTGAATTTCCAAATCTTTCACCAACTTCAACCAGATATCCTTCGCTTTACCAGGCTCAAGCTCATTCAAACAGATATGGGAACATCCAATTAGTTCAGCTGACTGAAGCCCTTCATCGTCATAGATTTTTACCACCAAGTGTTGTGTCAATGGATCTTCGACTACGAACTGAAAATGCTCATATTCCAAATTGGGTTCAAATCATTGTTAGCTCTTCTTCATTCTGTCTCGTAGAGGGCCTACATATAATACAACAAAAGGATTAGATTTTCCAATGATATCTTTATTTGTTAATTCCTTTGCTTGAACAAGTTTCATCTACAATACTCCGGTAGGCTGGTCACTATAATCCCCAAGTAAAATGGGAATAACTTTTCGTACTGGCCACATGATAGAGTCTTCAACAGCGTTCCGGATGGTTCCCTCAATTGCATCAGAAAGGCCAGGAATTGCTGTCATGTCACCACCAATTACTTAAGCGTAAAATCCAGCTTTTTCTTCTGCCTTGGAGAATAGCATACAACTCCGAAGCAAGGAAACTCATCAACAAGTGGCCTGAAGATGAGCCTGAAAATACCAGTAAACCCAATGTTTTTCACCTGCACTGATGAAGAATGTGATAAAGATTAAAGAGCGTGTTCTTGAAGAATTGTGGTGCCAGCTGAAAAGAAAGATTATATTTTTATTGTTGGTGCAGATCTAGAGACATTTTAACCTGTGCAGCTATTTCATTTTCTCAATTTTTAGGATTCGATAGATGTCGAACATGTATGTTTTATAAACTTATGTCGAACATCTATGTTATTTAGATCTTATG

>LNC_000653

AGCAGACGGCGTGTAGAGTTCCCAGCTCCAACGTTCACGAAAGGACACGGAGTCGAGACGGAGTAGACGGAAAGCATCAGTACACTACGAATTTTGTCTCCAACGTCTCTGTCCCGGCAGCGAACAGAAGCATGCTTTTGTCCCCATTTCCTCTTCTGGAATGGGGTTGAAAATCTTAGAAAAAGTATGTCCTCCTTTAATGGTGATAGAAATTTTGAATTTTGGGAGGATGGAAATTCCGATTTTACCCCAAGTTTCTATCCGTTTCCCCCCAAAATCTACCCTAATTTCCCCTATATAATCATCTCTTCTATTCATTTGGAAAGGGGGGGGGGATTTTTGGATTTTTTTTTCGGAGTTTAAAATATTCTTGTGTTAGAGAATCATTAAAGAAAGTAAAGAAATATTAAGAGGAAGTTGTTGGAATTTGGGAGGAGATACAGAAACTAAAATCAGAGAGTGAAAATTTCGGTTTGATAGAGTCTTTGCTCATTTTCTCTGGTTCGTTGTCGGTGTTCGTGGTCGTGTGGTGGAAGAGCTCGCTCCAGCTCGGCGGTCCCAGAACGCTGCTCCATAAAAGGTATACAACCTCGCCCCTCTTAAGTTCTTCCTCTGATTCTAGCTCTATTCGTC

>LNC_000654

TGTCAGTGGATCGCTCCTGCTTTTGGTTCATGGGTAGAGATTTGTCTCGAGGAATAAATTTTGGATGTTGGATCTTGAGTCTCTTCTTTTGTTTGAGGTGATATGAACAGATGACTTTTCAAGGAGATGAGTTGTCAATGACAAAACTTCAATGCCTGTTGTTGCAAGGTGTTCTCTTTCCGTCCGCATGGGACTAACCTAGAGCCACTTAATTTATACCAAGATGGTTATTTTTCTGATTTCTTGCTGCC

>LNC_000655

TTGAACTTCAAGAAACAGTTAAAGAATGAAGAGATAAGTCAGATAACATGGAGAGTGGTTGAAGTGTGGATAAGAACACTAAATACTTCCACAGAATGGCTACAACACATAAAAGATTCAATGCCATTGATGTCTTACAGGTAGAATGCAGCACTATCACTGACCCTGAACCTGAAGATATCAAATCACACATTCAAGACTTCAATCTCAAATTGTATAAGGAATCAAAAAGCTGGATACCTGATTTTATTGTGCAGGATGCCCCAGTTTTTTGTGGGGAGGAACAAGACAATGCAAAGGGAATTTGAGGAAGAGGAAGTCCTAAATTCTGTTAGATTGTGTGCAGGTGATAAGGTTTCTGGGTTTGATGGCTTTCCTATGTGTTTATCAATCCTACTGGGAGTTTCTGAGGGAGGATGTAATGGCTAATGAACACTATCCACCTCTTTCATTCCCACTATGTCTTTGGAAAAAGCTTTAATGCCAGTTATGTGGCCCTCATTCCTTAGAAGACTGGTGCAATGGAGCTTAGAGATTACAGACCAATCAATTTGGTTAGTGGGGTGTATAAAATCATAGCCAAAACGCTGACTGAGAGACTGATGATCAAATATAAAGTGACATTCATCAAAGGGAGACAAATAATGGATGTAGAACCCATTGCTAGTGACTGTGTGGACACTAGATTGAAAGG

>LNC_000656

CTTTTCTATCTATTCTTAGAATTGCAGATATTACTGTTGTTTGGAGAATTTCCTTTCAATTGAAGTTTGTACGACTACTTCTCCACCTTAGTTTCCTTTCCATTTGGTTATTTAGACCAAAAATCAAATGGAAAGAAGTGGCATGATATACTGTAAGCTAACATAAAGTTTAAGTTGACTCTAGATCCCTTTTTTGTGGTTTGCGGGGTTAGTGGTACCGAAAGACGGTGTTAAAAATGTATATCATGCGCT

>LNC_000657

CGGGAAATAGGGACTAAGCGCAAACCATTTCAAAATGACATCATAGAACAAGAGTCAAGCTCATTTACAAAATACTTGTCCAACGCACACCTCTACATACATAGATAGGACGGGGCCATGACATTCTACTGGCTCCCCTCATAGTCAAAATACAATTGCAAGCTAAAGTCTCAAAACATAGGAGTAGGAAACATAACATAGCCCTCGAATCATTGAGGACTCACCAACAACTC

>LNC_000658

AACCAAACGGCCACATAAAAATGAGCCCATCTAAACGAGCCGTGATATCTAAGCCCAATAAGAATAGATCACGGAGAAGTTGTTTACGATCTGTGAAATCACGTTTTTGTAACAGCGTTTCGTTGGAAAATTTAATTTTGCTCCGACGTTGAGCTCCGTCCGGTGGAATCTCCGATTATTTATCCTCTGTGATCACCGGTAAGCTCGTTTTCTCTCTTTCGCATTTTACTCCTTTTACATTTTTGTCACGAGAATTGAAATCCTTAGTTGTTATAAATTGGCACTTGAAATTGCGTTTAGTCCAAAATAGGGCAATACTCTGTCATTTTCCTTTAGTTGAACTGCTCTGATTATGAAATTTCTACAAATTCTATTTCTTCTAGCATTGACTCAATGTAGTCAGTCCCACAAAGTGGAGTCTGGGGAGATTGAGGTAGAGAGGTTTTAGGGAAAAAAACAATTGAAGGCAATCCAGAAAAAGTATAGCAAAGCATTCTGAACAGTTACTACGGCATAGTAATAAGATAATTGAATTACAAGAAACTACAACAACAATACTAGGACTACTGGTATGGACAGGTAACAAGACAATGCATGAACTCTCAGTGAATACGTCAAATAAAATTGGAGTGTTTAATGAGCATATATATAGTTGTGGATGAATTTCTTCTGTTTTGATGATCCACATTCAGATCCTTTTTTTTTCGAGGTTAGATGGTACAAATTAGACTCAACGAATCTCTGATAATGTGTTCTATCCGTTGAAACTTACATTAACATCAAGTGCGTAATACATAGCTAAAAATGGCAGTCCCTAGCAATCTGAATGGCGAGTATTACACATGAAAGAATTTACAAGTGAATGAAGGAAGAGAAATTAGAGTTTGAAAGTTTTATCCAAATATCCCTAGTCGTTAAGGATTGTTGAGAAAGGTCTTTTCTTTTTTACACTGTTGAGGCACTTATGGTTGTTAGCTTTTGGGAATTGGAAGGTAAAAGAAAAGGGACACATAGTTAAGTGCTAATAGTGTATTGGAATTGTTGTAACTCTACACAGAGAAAAGAATATGTGAAGGAGTTCGGGTATAGGGATTCAAAACAAAAAGGTAAAGGCTTTTGCTTGATCAAATCTACAAAGAGGGAGGATAGAGCAGAAAGAGCATAGATGGATGTGTATAGAAAGCAGCGGGTGCAGGCACACAACTCGAGTTAAATTCATTTGGAAATAGTATAGCATGACAAAGGGTGAAAAATAACCAAATATCACATGAATTTAGTGGTGAGTTAATGGATTCTAGACATATAGCTCCTGTTGTATACGTGATATTCTGTCCGTTACAACGCATAACTCTAGGTTATCCCCATTTGAATCCTTGTAATGCCTCGAGTTAGAGACTGCAGTGGTTGTGATAACTCACTAGTTAACAAAACAGTTGTTACACCATGTTTGCTACCAAGCAGTGTTATCAAAGGCACACTTGAGCCCTGAAGCAAGGCTCAAAACGTATTGAGCGCCTCGCCTCGCTTTATGTGCGCTTTAGTGTTGTCATCAAGGTTCTAATGCATATTTTTCCTTGCCAATGAGCGTCCTCGTCAGAGGTGACACTAAACAATTGATATTTCACTTTATCATAAAAAAATCAATTTCTTTGGTCATATAATTGTCATTCATGTTTATAATTATTAGTCTTGAACTAAACATATATATTTGTATTTTTTTCTCCCTTTGCGCCTTTTTTCATTAAAGCCCACGCTTCATTTGCACTTTGCGCTTAAAGCCCCAACAGACCTTAGAGCTTTTTTGTGCTTTTCGCCTTTGATAACACTGCTACCAAGTCAGTTACATTTCCACTTTGTGTAGTTTGCCTCCCAATGTGTAGAGACACTGAATTTCAGAAGCCATTAGGGGGTGTATAGGGGCTCGGCAGAGCACAATCTGTGTATTTTTATATGAATGTTGTGGAAGAAGTGAAGAAAGTCTTTGACATTTATTTTAATGCTCTTGCATAATTCATAGTGTCTTATGTTCAGTTTTTTCTCTTGGTCTCTTTTAATTTTCATTTTCTACAGATAGTTATGAACTTATGATTTTGAAATATATATACTTACCAACATGTTAAAAGGCACTGGGCTCTCATTAGGGCAATATTATCAAATCGCACCCGGGCTTTAAATTTTTTTATTCCTGTTGGCCTCTCACTGCAGACCCTGTTTGTTCGTATCTCTCTACTGACTGCTCCTTTTGTATCTCAATTTTTGGTTACCTAAGGAAAATCAATGTTAATTTGAGCTAATGTCTTATTATAGAGACTTACAAGTACACAAAATGTTTTACTGTGTGAAAGATATGGAGTAAAGGTGAAAATGACAAAATCAATGTTATGAGCTAAATATGTGTTGTAGTAAACCTTTACATCTTCAGAAAAAATATATCACTTCTGTAATTGATTTCTCCTAGAGAAGTATAATATTCTATTCAAAATTGTTCCATTTATAAGATTGTTCCCCTAACAATGAATATTTTTTTCTCCTAAAGTATCTTTTAAATATTCAAATAATATTTGATAATCCAAAATTGAAGTTCATAGGGCTTTTGCTTCTTCTTTTAGAGCTTATGCCCCACAAATGCAGAGATAAAATGCACTCCTTTTCTGTCTCAAATTTTAAGAAGGGAGGGAGAGAGGGTGCATTGAATTTGAAGCATGTTGTGATGGTTAGTCTGATAACTGTAAAATAGAAACTAGAAGCTCATGCAAATGTTTATGTCATAGCTACTAAGATAACATATATTGATATCTAGAAAATGCAAAC

>LNC_000659

CCTAAATTTATTTTCCTCTTCCATTTTCCTCCTTAGCCGCCAATACCCAAATCCCTCAACTCTATTTTTCCTCTTCCCCAAAGCCAACTGAAGCAGCCACCAAACAACCTCTTCTCCCTCACCACAAACCACCATTGCTCCACCCACCAAGCTCCAAACACAACCAACAACCACCGTCTGAAAACAACCCAACAACACTCCATTTTCGACGAAAAACGACCATCAAACAACCACTTCCAAAACCCCCCTTCGTTTTTTCTCATTTTCCTGGCAGCAACTCCGGTGAGGAGCTCAAAGCTCCGATGAATCGGAGCTAAATGAACCAGTCCCTTCCCCTCTCCCCTTTCACGATTTTTTCTCTTATTTTGCAGGTAACCTGCTGCCATTCAAATGAACTTCAAGCTCCGGCGCAACTCTGAACCAGACGCGACCTATCTCCGACGAACTTCGAGTCCAACTCCGTCAGACCCCACGTGAGCTCAGGTACCCCGAAGTCGCTTCTCATCGAAGTCATTTGAATAAAGTTCGAATGTTATATTCATTATGATATATATTGTTGACGATAGACAAATATTAGGTTGTTTCTCATATTACTTGTTTACTTTATTACTTGTGTATATTGCATGTTTGTTATATTTGTATAATATTTTTACATCCTCCTTAATTTGAAAAAATGAATTCAGTGGGGACCCACAGTTGTGGATTTCGAAGGATGCCTAACACCTTCCCTTCGAAATAACTTGAACCCCTTACTTAGAATCTAATGGTTTCGTAGATCACTTTAATTGGTTTCTTAGTTTTTAACTAAAATTAGGTGGCGACTCCTTTAAAATTCGTTTATTCTTAAATTCTTTCAAAATTGAATCAATTGTTTATTTTTCGAGTCACCGCGACGTTTCGCCCTATTTGAATAGAGTAGGGATGTAAACAGCGCCAGCCAGTTGCGCCAAATGTTGCCGGACCTTCTGAAGGGCCCGGAAGTTCGAGAGTTCGTGAATTTCTTGCCTTGAATCCTCCACAGTTTACTGGGACAGATCGCAGAGAAGACCCTCAGCACTTTGTTGATCAGCTTCATAGGATCTTCAGAGTCATGCATGCCTC

>LNC_000660

ATTTTGTACCTAGGAAGCTCAGCGACTAGTAAATCTTGTATTGCTGTATCGGGGATCATTTTCTCAACACATGCATAATACTCAGTCCACAGACTCTGTAGTAAAGTTCCCTCTTCTTGAGCTTTATAATACAATCCTGGGTTCAAAACATGGCCTGCAGCATGCAAAGGCCGATGGAGTTGGTCTGACCACCTTGCATCAATAATTTCAAACACTTTCTCATATTGCTTCTTAATTCCACGAAAACCCCGTTCAATAGTTTCTTTGGCTCTATCCATTGCTTCATAAATATAGCCCATTGGCGGTTTTTTCTCCCCATCCACCAAACGAAGCACTTTTGTCAAAGGGCCACAAATTGTAAGTGCT

>LNC_000661

CAAAAATCCAACACCCACAACGTAATTTCTAATAATTTAACAAACATTGTGTGAAACAGTGAAAATGACTATACACCGACAAGTACTGCAACGACAACATTAGAAAATTATCATCATCGTTGGAGCTGGGTTTGTTGGTGCTACTCTTTCTCATACTCTTGGGAATGTTTCTTTTCAATTTCAGTTTTTGTTGTTTAACTTACTATTTCCCCATATTTTTATTTGCAAGTTCTTTGAAAGT

>LNC_000662

ACAAACTTCAGATATCATGGATAAGCAAGAGGCAAAGGCTCTCTGCATACGTTGAGGTTTGTGGTGATATAATCAATGGGCCTTTTGATATTTTTGGTGTAAATGGTGGGTTATGCTCACGGCTTGAAGCCAGATGAAGTGTGACGATGGCACAAGAATTTGAGTGATTGATATGAATGTTGCAACAACATTGAGTTTTCTTGCAATAGGCTTTGATATTTTTTAACTTAGCTCTCTTAAGTCTTTTACACTCTTTCTACTGAATGTTGTTCTCTATTTTTTCCAGCCAAAATAGTCATTACAAGAAGCAACTCCTAATAATGTCTACTTTGCTACCTTATATAATTCATTGAGAC

>LNC_000663

GTGTATTTGCCCCTTTTCCACATCTATTTTTTCTCATCAAAATTTATGTTTATTGTACAATCCTAACCTAAAATACATGCTTTTATCCTTCAATTCCTAATGGGTATGTGTTTTCGGTTGTTGGGTCTTATCTATTAGATTTTCTGATTTTTGAATGTGCTATTGTAGGATTTACTTCTTGCATGTTCCTAAATTGATTTGAACTACTCATGGATGCTTGAAAATTGGTGTTGCCCTTCTTTTTCTTCTCACTTTTCTCATTTTTCTTTGGCAAGTTTACATTTTTGATAGTCACAATTTATTGGGTATTTTGTTTTCAGTTCTTTTATTATGCATTTTCTGCCTTTTTTAGTAGTTTATTGTAGAATTTTGTAATTTCAAGTTCCTAATTAGGCTTTATATACTCATGTATGTTTCAAAATTAGTATTTTTTTTAATGTGCAAAAATTCCCCACCTTAATTTGTATACTATCATTAGTTTGAAGCAAACGTTAATTGAGTCTCTGTGTGCTCTGTTTTAGGTCCATCTTTCCCACTCTTCCTCCATATTAGCCATCTCCATGCAGAAAACCCTCAATTGCTTCAAACTCTTGTAACCATGGCTGTCGTATCGACGCTACAAAGCTTAAGCATCACTTCTCTTCATCAAGGAGATTTGGGAATGTGAATGGCTATTCAAAATTCATTTATTTATTGGTAAGCACTCCGATTTTAGCATCTTCTATTTTGTTTCTATTTTTCATGATTTTAGAGAGTAGAGAATATTGATTTCTAAAATTGGAGAATATTGATTTTCTATTTGCCTACCGCTTTTGGAATTCACTTACCATTTTAATTTCAATTTTAATTTTTTCATTGTTGTTTGGAGTTCAATTTTTCATAATTTTTTAGGTTTTTGTTTGTTTATTGTTGTGCAGTAGTTTTCCTTCTATATTTGTTGATTTTGATATATCTTTAAATCTTGGATCATGTTAGTTATTTGGATAACAAACTTCGCTACTGTATGGAACATGTTTTTCATAAACTTAGGAACAGTCTTGAAAGTCTTCGGACTAAATTAAGGTAGGATTTGAAGGAAAATAGATTCTTGAGGGAAACTTTTGATTTTTGAAATAGAAAATCTAATGAAAAATCTTTTCATACCTATGCTCAAGGATCTTTTATTTCATCTTCTAAGCTAATAGATTACTCACTCAAATCAATTGCATGTTTTCAAGTAGTTTCGTACTTATTTCCTTGATATGTTCATGGATAATTAATCTAGCAGCCTCGACATGAGTAGAGAAATATTCAGGCTTAAAAGTATCCTTGTTCCTTATGAATTTTAAGCTGCCTCAAATGGAAGGAATTTTATTTTGAGAGTTCTATATTTGTTTGGAATTTATAGTCTATCAAAATTGATAAACGATAATTTTACTTGGTGTACCAAAGAATGGTTTCGAAGTCATATTGAGCAATCTAATGAAATCTAAGGAGAGGGGGCAGTAATATATCATATATGGGGAAATAAATTGCTCCAACAGAAGAGGTTAAGAAGGCAAAATGATTTCAAACTGTTAATTAGAGTTGACAGACCATCAAAACATCTAAAATTTCTTTCCTTCGAAATACACCAAAGATATACATAATGGAATCATGTGTCGGGTCTTCTTGATGGGGTTATCAACTCCTCCAAGACTACAATTTTTCACTACTTCTTTGACACTCATGAGTAGAATCCAATTGATGCGAGTAATTGAAATGAACATATTCCAAAGAGTTGTTGACACTGGGTAGTGTAGAAATAAACGATTCACTAATTTCAGGCTATTGAGACACATAAAACATTTGTTTACTGGCTGAAGTTTCCTCCTGTTTAGATTATTAGGGTTAGGACATCATTTATCACATATTTTTATTATTAGTTGGTTCAATTATGTCTTACCCGAAAGGTTGTTTAAGTAAATGATTCGGCAATGCATTAGCTGATGCCAATGATGAGATATTAGTTTATACTTAACTAAAACACCTTCAGAATGTAACTAGAGATAATAATGAGAGACTTTTATTTTTTTCTCAACTCCTTATGAGTGACAAATAAAGTTGTTGATATACTGACAAATATCTTAAATATATCTCAGTTTACAGCTTTATTTAGTTATTCTCCTCTTTGTTTTCTGTCCATATTTGCGTTGTCACATATTTTGCTGGTTAAAAAAAAGAAGATAAACCCACGTTTGATGGTATAAAGAATAAGAAAAAAGTTAACTTCAATTTCTCCAAGAATATTGTGGTTTAGACCTAGAGCTATCATTTTCTGATCATATAGTGATGATGAATTCAGGTGAGAAATTCCTGCAGTAAGCATTATCTTGAACCATGAAAATAGTGAAGCAACTTCTACTACCTTATCGTTATTGGCTAAGAGATGTATTCTACCTGTATGATACATGGGTTACCAACAATTACTGATTTCTCTAAGGCGCAAAGACTTGTAGTGCGCTGTCAATGTATATCACGTACATGGTGTATTCCTCAAACATGATAGATATCTATTGGGGAAGGGGTGTACGAACACTACAAAGAGAAGATGTGCAAGATATTATGTGAATTATACTATTAACGGGGGAGTGTTTCATTTGTGATTAAATTCCTTTGTCGTTCTCCTATTAGGCTATAATCATGTGTATTGCTTCTGAAAACACATGCCCTTTCTAATGTTGTTGAACTTCCATATCTACGACGATCTCTGTTGGGTACACTGAGATTGTGGATTTATCATTTGGCTAATTTCTTCTTTGTTCAAGAGTCAACTACTTTGTTACTCTGTACCAAATGACATAAAAGTTACTTGTCTGTCATATTTTTGGGATTAGTTGTTTGTACTTTTTTCATTCGAGTTTGCAGGTTTCAACATATCTCAACACTAAAATAAGTTTTGCCTCTCAAGTGTGGTTACACTTCTATGTATATGTGTAATGCATTTGTTCCAAGTAATTTTTTATTTCATTTTTATTTGTACTCTTTGAAAAGCAGACAATACTTTTTACCCAAAAAAATTAGCAGGGCCTCTCTTCAATTTGGGAGTCTTAC

>LNC_000664

CTGGAAAGAAGCTAAAGCAGCTCTCCCTTTTCATCAAAACATATTCTCCATTTCTCTCTCTTTCAAGCTTGAAACTGGTCCATCTTTCCCACTCTTCCTCCATATTAGCCATCTCCATGCAGAAAACCCTCAATTGCTTCAAACTCTTGTAACCATGGCTGTCGTATCGACGCTACAAAGCTTAAGCATCACTTCTCTTCATCAAGGAGATTTGGGAATGTGAATGGCTATTCAAAATTCATTTATTTATTGGTAAGCACTCCGATTTTAGCATCTTCTATTTTGTTTCTATTTTTCATGATTTTAGAGAGTAGAGAATATTGATTTCTAAAATTGGAGAATATTGATTTTCTATTTGCCTACCGCTTTTGGAATTCACTTACCATTTTAATTTCAATTTTAATTTTTTCATTGTTGTTTGGAGTTCAATTTTTCATAATTTTTTAGGTTTTTGTTTGTTTATTGTTGTGCAGTAGTTTTCCTTCTATATTTGTTGATTTTGATATATCTTTAAATCTTGGATCATGTTAGTTATTTGGATAACAAACTTCGCTACTGTATGGAACATGTTTTTCATAAACTTAGGAACAGTCTTGAAAGTCTTCGGACTAAATTAAGGTAGGATTTGAAGGAAAATAGATTCTTGAGGGAAACTTTTGATTTTTGAAATAGAAAATCTAATGAAAAATCTTTTCATACCTATGCTCAAGGATCTTTTATTTCATCTTCTAAGCTAATAGATTACTCACTCAAATCAATTGCATGTTTTCAAGTAGTTTCGTACTTATTTCCTTGATATGTTCATGGATAATTAATCTAGCAGCCTCGACATGAGTAGAGAAATATTCAGGCTTAAAAGTATCCTTGTTCCTTATGAATTTTAAGCTGCCTCAAATGGAAGGAATTTTATTTTGAGAGTTCTATATTTGTTTGGAATTTATAGTCTATCAAAATTGATAAACGATAATTTTACTTGGTGTACCAAAGAATGGTTTCGAAGTCATATTGAGCAATCTAATGAAATCTAAGGAGAGGGGGCAGTAATATATCATATATGGGGAAATAAATTGCTCCAACAGAAGAGGTTAAGAAGGCAAAATGATTTCAAACTGTTAATTAGAGTTGACAGACCATCAAAACATCTAAAATTTCTTTCCTTCGAAATACACCAAAGATATACATAATGGAATCATGTGTCGGGTCTTCTTGATGGGGTTATCAACTCCTCCAAGACTACAATTTTTCACTACTTCTTTGACACTCATGAGTAGAATCCAATTGATGCGAGTAATTGAAATGAACATATTCCAAAGAGTTGTTGACACTGGGTAGTGTAGAAATAAACGATTCACTAATTTCAGGCTATTGAGACACATAAAACATTTGTTTACTGGCTGAAGTTTCCTCCTGTTTAGATTATTAGGGTTAGGACATCATTTATCACATATTTTTATTATTAGTTGGTTCAATTATGTCTTACCCGAAAGGTTGTTTAAGTAAATGATTCGGCAATGCATTAGCTGATGCCAATGATGAGATATTAGTTTATACTTAACTAAAACACCTTCAGAATGTAACTAGAGATAATAATGAGAGACTTTTATTTTTTTCTCAACTCCTTATGAGTGACAAATAAAGTTGTTGATATACTGACAAATATCTTAAATATATCTCAGTTTACAGCTTTATTTAGTTATTCTCCTCTTTGTTTTCTGTCCATATTTGCGTTGTCACATATTTTGCTGGTTAAAAAAAAGAAGATAAACCCACGTTTGATGGTATAAAGAATAAGAAAAAAGTTAACTTCAATTTCTCCAAGAATATTGTGGTTTAGACCTAGAGCTATCATTTTCTGATCATATAGTGATGATGAATTCAGGTGAGAAATTCCTGCAGTAAGCATTATCTTGAACCATGAAAATAGTGAAGCAACTTCTACTACCTTATCGTTATTGGCTAAGAGATGTATTCTACCTGTATGATACATGGGTTACCAACAATTACTGATTTCTCTAAGGCGCAAAGACTTGTAGTGCGCTGTCAATGTATATCACGTACATGGTGTATTCCTCAAACATGATAGATATCTATTGGGGAAGGGGTGTACGAACACTACAAAGAGAAGATGTGCAAGATATTATGTGAATTATACTATTAACGGGGGAGTGTTTCATTTGTGATTAAATTCCTTTGTCGTTCTCCTATTAGGCTATAATCATGTGTATTGCTTCTGAAAACACATGCCCTTTCTAATGTTGTTGAACTTCCATATCTACGACGATCTCTGTTGGGTACACTGAGATTGTGGATTTATCATTTGGCTAATTTCTTCTTTGTTCAAGAGTCAACTACTTTGTTACTCTGTACCAAATGACATAAAAGTTACTTGTCTGTCATATTTTTGGGATTAGTTGTTTGTACTTTTTTCATTCGAGTTTGCAGGTTTCAACATATCTCAACACTAAAATAAGTTTTGCCTCTCAAGTGTGGTTACACTTCTATGTATATGTGTAATGCATTTGTTCCAAGTAATTTTTTATTTCATTTTTATTTGTACTCTTTGAAAAGCAGACAATACTTTTTACCCAAAAAAATTAGCAGGGCCTCTCTTCAATTTGGGAGTCTTAC

>LNC_000665

ATGAAACCCTTTAAATTTTAGCTTAGTCTAGACCAATTTCATTGGCTATAGCTGCTTTATATCCACCTGGAAAGAAGCTAAAGCAGCTCTCCCTTTTCATCAAAACATATTCTCCATTTCTCTCTCTTTCAAGCTTGAAACTGGAGATTTGGGAATGTGAATGGCTATTCAAAATTCATTTATTTATTGGTAAGCACTCCGATTTTAGCATCTTCTATTTTGTTTCTATTTTTCATGATTTTAGAGAGTAGAGAATATTGATTTCTAAAATTGGAGAATATTGATTTTCTATTTGCCTACCGCTTTTGGAATTCACTTACCATTTTAATTTCAATTTTAATTTTTTCATTGTTGTTTGGAGTTCAATTTTTCATAATTTTTTAGGTTTTTGTTTGTTTATTGTTGTGCAGTAGTTTTCCTTCTATATTTGTTGATTTTGATATATCTTTAAATCTTGGATCATGTTAGTTATTTGGATAACAAACTTCGCTACTGTATGGAACATGTTTTTCATAAACTTAGGAACAGTCTTGAAAGTCTTCGGACTAAATTAAGGTAGGATTTGAAGGAAAATAGATTCTTGAGGGAAACTTTTGATTTTTGAAATAGAAAATCTAATGAAAAATCTTTTCATACCTATGCTCAAGGATCTTTTATTTCATCTTCTAAGCTAATAGATTACTCACTCAAATCAATTGCATGTTTTCAAGTAGTTTCGTACTTATTTCCTTGATATGTTCATGGATAATTAATCTAGCAGCCTCGACATGAGTAGAGAAATATTCAGGCTTAAAAGTATCCTTGTTCCTTATGAATTTTAAGCTGCCTCAAATGGAAGGAATTTTATTTTGAGAGTTCTATATTTGTTTGGAATTTATAGTCTATCAAAATTGATAAACGATAATTTTACTTGGTGTACCAAAGAATGGTTTCGAAGTCATATTGAGCAATCTAATGAAATCTAAGGAGAGGGGGCAGTAATATATCATATATGGGGAAATAAATTGCTCCAACAGAAGAGGTTAAGAAGGCAAAATGATTTCAAACTGTTAATTAGAGTTGACAGACCATCAAAACATCTAAAATTTCTTTCCTTCGAAATACACCAAAGATATACATAATGGAATCATGTGTCGGGTCTTCTTGATGGGGTTATCAACTCCTCCAAGACTACAATTTTTCACTACTTCTTTGACACTCATGAGTAGAATCCAATTGATGCGAGTAATTGAAATGAACATATTCCAAAGAGTTGTTGACACTGGGTAGTGTAGAAATAAACGATTCACTAATTTCAGGCTATTGAGACACATAAAACATTTGTTTACTGGCTGAAGTTTCCTCCTGTTTAGATTATTAGGGTTAGGACATCATTTATCACATATTTTTATTATTAGTTGGTTCAATTATGTCTTACCCGAAAGGTTGTTTAAGTAAATGATTCGGCAATGCATTAGCTGATGCCAATGATGAGATATTAGTTTATACTTAACTAAAACACCTTCAGAATGTAACTAGAGATAATAATGAGAGACTTTTATTTTTTTCTCAACTCCTTATGAGTGACAAATAAAGTTGTTGATATACTGACAAATATCTTAAATATATCTCAGTTTACAGCTTTATTTAGTTATTCTCCTCTTTGTTTTCTGTCCATATTTGCGTTGTCACATATTTTGCTGGTTAAAAAAAAGAAGATAAACCCACGTTTGATGGTATAAAGAATAAGAAAAAAGTTAACTTCAATTTCTCCAAGAATATTGTGGTTTAGACCTAGAGCTATCATTTTCTGATCATATAGTGATGATGAATTCAGGTGAGAAATTCCTGCAGTAAGCATTATCTTGAACCATGAAAATAGTGAAGCAACTTCTACTACCTTATCGTTATTGGCTAAGAGATGTATTCTACCTGTATGATACATGGGTTACCAACAATTACTGATTTCTCTAAGGCGCAAAGACTTGTAGTGCGCTGTCAATGTATATCACGTACATGGTGTATTCCTCAAACATGATAGATATCTATTGGGGAAGGGGTGTACGAACACTACAAAGAGAAGATGTGCAAGATATTATGTGAATTATACTATTAACGGGGGAGTGTTTCATTTGTGATTAAATTCCTTTGTCGTTCTCCTATTAGGCTATAATCATGTGTATTGCTTCTGAAAACACATGCCCTTTCTAATGTTGTTGAACTTCCATATCTACGACGATCTCTGTTGGGTACACTGAGATTGTGGATTTATCATTTGGCTAATTTCTTCTTTGTTCAAGAGTCAACTACTTTGTTACTCTGTACCAAATGACATAAAAGTTACTTGTCTGTCATATTTTTGGGATTAGTTGTTTGTACTTTTTTCATTCGAGTTTGCAGGTTTCAACATATCTCAACACTAAAATAAGTTTTGCCTCTCAAGTGTGGTTACACTTCTATGTATATGTGTAATGCATTTGTTCCAAGTAATTTTTTATTTCATTTTTATTTGTACTCTTTGAAAAGCAGACAATACTTTTTACCCAAAAAAATTAGCAGGGCCTCTCTTCAATTTGGGAGTCTTAC

>LNC_000666

GTTTCCTTACTTCAAATGCTAAATTTTGTTCTTATTTTTTTTCTTTCTCTTGTTATGTGAACCTTTTTCCGAGTCCATGGTGGACTCCTTCTTTCTTGCATCCTCAAGATGAGCCATAGAGGCTTGATTTTTCTCCATCGAGTCAACCCGTCTTTATGTGGATCGTGGGGAGGCAGATTTACATGTTGAGGGCAGATTACAAGTCCCAAAATCCTAAGAAATAGGCTGTATACATATTGTATACAGCAGTATACATTCATTATACAGCAAACACAGGACAAAAAATGCGAAAATACGGAGATAAGGTAAAATACAGGTGTTCGGAAGCAAGAGATACATGGAAAGAGCTGATATACATGCTGATATACACTGATATACAGTTAATATATACAAAAACGGGAATTTGGTTAAGACCAAAATTTCCAACAAATTTGGCCCAAAAGGAGGCCCAAATTCAATTTCTA

>LNC_000667

TTTTACCCACAATTAAAAAAACCCTCCCATTTTTCATAATTTTAACCCATCCATAACCTGGTTAAAAATCCTCTTCTTCTTCCCCAATCTAACCTAACTTAAACACCCACTCGTGTAGTTCTCCCACAAATTGATTCTTTTAATTCTAGTCCTTCGAGCCCCGTGAATAACAGATGAACAAACACCATCTGATTTTACACTTCTTCGGTGTTAGCATAGCCGTCATTCACCGTTTCAGCACAAACAACTTGTGAGCTGCTTCATTCTCTAGTTTCTGTTCAATCATGGTGCTTGTTCCATGTTCCTAGAGGCATGAAAAGCTGGATATGCAATAAAAGTTAGGAAAATATCAGAAGTTGTGGAAAGTTAAAAGTGACTTTATGATGGATTGAGTGTGGGGAAGAGATCAAGATTTGGTGGTTGCTTTATAAGTGAAATTGAATTAAGGAATTTGAGTTTCATCATGTTTTTTCTATTTCATGCATTCAAAATCAAAATCTTTATGAGTGATTTCAGCCAAATTAATTGAAATATTCTACAAAGGTTTTTTGTTGAAGAAGATGAAGAATAAGAAATTGTCCAAAGGGTTTTTGAATTTTTTTTTTATCTTCTTCTTCCTTTGAAAATGTTGTTCAAATCGGAGAAGACCACCTTCAAGTGACCACCGGAACAAAGATCTTCAACATCAACATATCAGCTCCTCTCAAAAGGAAGATTTCATGGAGGAACAATCAAGAACGTGGAGAAAATCCATTTGGTTCAAAGTGGAAGTGGAAGAGCCGACAATAACTTGTATATTATTTCCAATTTCAGATTTTAGCATTTTCTAGTCCTTTACATTTTGGATTTTGGTTTAGCCAAACAACTTTATTTGGCTTATTTTGTTTTTTGATTTCATATAAATAAATACCTGGCTAGTCGGTCAAAAATTTGATTTTAAAAAAAAGTTGTTCAAATCTAAATGTTTGAAATAAGTCTTTGTTGTAGAAGTTAAAAAAAGAGGAATTTGAGAGTTTTGATTATCTTACCTAACTTCTTTTTTGGTTTTGGGTGCTTTTGGTTACAATTTGGGTTATGAATGGGTTAAAATTGTGAAAGATGGGTGGGTTTTTTTAATTATGGGTAAGAACTAAATTAAATTTGGATAGACCGTTGAGATCATTACACGTGTCCCTCCGTTTATTCTAAGGGTATATACATTCCAAAATATTAACGGTAAGGGTATACACAATCCAAAAGTATAACGAA

>LNC_000668

AGCAGCCACACATCATGTCTCCTCTTTCATTTTTTCTGGAAAATTAGCGTATTTTGTCAACAATAAGCAGAGGGTAGGTACTCCATCTTCACGCCAATGTCCTCTCAATCATGTGGCTCCTACAAATCTTATCCTAAGGGTTTCCAACGTTTGGATTTGAAGCTACTCGTCTACCCTAAAAGCTTTGAAGCTATCACACCTAGTTTTAAAGTTTTGTTCTTCATTTTGTTGCTGCAATTTTGGATTTGAACGGGTTTAACCTCGTCTTCATATTCGAGCATATCTTCGACCGAAAATGCCCCCAGTTCACTGCCCCAAAATAGGTCAATGACATCTAGTTTCTTTTTTTTATTTCCGTGAGATATGAACGAATTTGTGTTTTAATGTGTTATGGACTTACTATTAGTTTCATTTCAGC

>LNC_000669

ATTACATTGAGACCTTATTTATAGACCCTAGAATACAATCCTTTACCAAGTAGGATACTATTTAGTATTCCTATTTCTATTTCTATTCCTATTCCTGTTCTAATAGGATTGTATAAACTTATTCCTATTCTAATAGGATTGTATAAACCTATTCCTATTCTAATAGGATTGGATAAACCTATTCCTATTCTAACACTCCCCCTCAAGCTAGTGCATACAATTCATGTACCTAGCTTGTTACAAATGTAATTAACACGAGGACCGATGAGGGGCTTGGTGAGATATCTGCAAGTTGATCACTCGACTTCACAAATTTGACAATCAATCTCAATGGGCTTGGTCTTCTCATGAAATACTGAATTTGATGCATAATGCTGATAAAACAGAGAGTGATAAAATTACAGTGTTGAACTTCCCAAACCAAGCTTGCAAAGACTGTTTCAGACCATAGAGTGACTTACGTAATCAACATACAAGGCTACTAAACTCCCCCTGAGCAACAAAATTAGGTGGTTGCTCCATATAGACTTCTTCACAATGCAACGGTCGCCGAAAGTGCTCAAAATCTAGTATAAAGTGTATGGACAAGATGTTTGGTGAATAATTCCTTGGTGAGACATAAACTCCTGAAACTGGGATGATAAGTATTCTAACGCATTATCACTACGAAAAGTACGAATAGAAACACCAAACTGATTTTGTATTTCAGCAAAGAAACTTTTGAATATAGAAN

>LNC_000670

GCGCTATAACCTTCGATTTTGAATCTATGTTGTTAATGTTTTAGCTTAAATTTAGTCTCATCCTAGCCTACTGTGTGTGATTTTCGTTGGTTATTTGTTTAAGCTCATAGGCTGCCTCTTGAGTTTTACTTTTCATTTTACTCATTTTAGGTGTATTATAGGGTGTAAATCTAAGTATTCATGTTATGTTTATATTTCTCTGATCTGGGTTCTCTGATTATTAATGCCTCGTGAAGTTTTATTATGTTGAAGGATTGGTGTTTCAATC

>LNC_000671

CCGTATCAGCATGCGGTGCTTTTCGAACGAAGTGTCTGTAATAAGCTATATCACAAGCTCATGTTTCTACAATCTTAATCTGTATATATTTGAATGATGCTATAAATCTTCATCAAAGTGCTTATTTTTTTATAATTGAACAAAAGCGAGTCATCAATTGTTCTGTTTTTGTTGTGATCTATTTCTACCAAGTTACTGACTTCTTCTGTAAGAAGTCTTCAGGGGGGCTAAGGATGTAATAGAAGTGGTTAATCTCGAGGATATAGTGGGTATGAGGAGAGGTAGAGGTAGACAAAAAAGTACTGGGGATAGGTGATTAGACAAGACATGATACAACTTCTGCTTACCGAGG

>LNC_000672

ACCGTCTCCAGTTGGGGAACTTGGAATGGTAGAGATGACCCAGGCTCTGGAACTAATTGATGTTTGCTATGAGAAGGCTATTAATGGTGGCAAATGCAGGAGACTGCCGAGCAGTACTTTGCCGTAATGTCAATTGACATGTCTCAAGACCAGACTAAATTATCCATCAGAGAGGAGGCGTGTTGAAGGTGGATTTATTGATGATGGCTATCTTAATGTATACTATCAGCCCACCTTGGGGGACTGGGACATGAAGCTGCCTCGTGGCTCTGCCTCTCCTCTGATTGCAGAGCCAGAATTTCGACAGATAGTTCTGACAGAGGATGATGAATTCCTTATTAGCTTGAATCTGGGATGTCTTGTCTAGTCAACAAGCAGTTAGTTTAGTGCGTCGAGGGATAAAGACGGCATGATGATCCTGAACTGTGTGCCAAGGACCTTGTCATGGAGGCCTTGCGGCTCAATACATTTGACAATCTCACTTGTATGCTTTACTTCCCCTGATCATCAAGAGTTATCACCAATGCGTCAAAGGAGATTAAGGAGCTGCAGCTTCTCTGCAGAAGCCTTGTATAGCTTGCAGAGCTGGTTAGACAACAAAGGGAGCCATTGATGGCGGTGCAAGCTGTTTGCAGATGCCTCATTTGGGCAGAATGAAGAGCAGCTATAGTTTTGGTAGTAAGATCTTTTTTTCCTGCTTTGGTATTTGGGTGGATGTATGTTACATACCATTCGTTGGTGAGTTGGGAGCTACACTGACTTTTAGTTTATTAT

>LNC_000673

GGAAGCTGGTCGGAGACCAAAACAATCAAACTCTTGAAGCAATTCAAAGATTCAGCAGAGTGTTGAGAAACTCGTATCTCCCTTTGTTTCTCATCCTGGATCAGGAGTGAGTAAGCATGATTGATGCTTGGCAGAGGAGTGGACATTAGGATGTTGCTCCTAACACCTGAGTAAGTGTCATTCAAGCCCATGAGGAACTGAATGAGCCTACCCACTTGGTGTGACTTCAGGGTTTTGTCCTTTCCTCCACAAGAACATTCACAAGAGCAGTGATTGCAGTTCTGGAATAGAGAACGCTTTCAGCAATTTCCTTAGACAAGGAGTTGAGCAGCCAAGAAATCACCATGTCATTACAGCGACTCCAAGCAGTATAAAGAGGGGCATAGATCTGAGGTTCTAAGAAGGTGCCATCAATCAAGCCGATTTTATTCTTAGCTGAAAGAGCTATGAACATGGCCCTTCTCCATGCTCCATAGCCATTACCATTGAAGGTAGAATTCACCAAGAGCATCCCAGGGGAATCTGAAGGATGCAGGTAAAAGGGATGAGAATGGTCATTGTTGCTGGAGGTTTGAGGAGGAGGAAGAGGCTGAGTTCCTGGGAGA

>LNC_000674

GGAAGCTTCCAAAATGATGAGTCAGCTCATCCAGGTGTCTCAATCAAACTTTGAACCAACGGTCTTGATTTAATTTTATTGTCTTGTGATGGACGGTTGTGATGCACAAGTCTAAAAGGAAATAAATGTGTCTGAACCTTGTTCACTTCACACTTTGAAACTATGTCTTTCCTTTGTCCTCCGTTGTTGTCGTGAGTTTGAGCTTCTTCATCACCAACATCCCCCCTCAAGTTGGAGGGAGAGGAAGTAACCCCCAACTTATGAACATTCGATCTATGAGAAGGACCAGATAACGACTTGGTGAAAACGTCGGCGAGCTGAGAGTGAGAGGGTAGATATGAGAGGGAAATGAGACCAGCTAGAAATTGTTGCCGGACGAAGTGACAGTCGAGTTCGACGTGCTTCGTCCGTTCATGGAAAACTGGGTTATTC

>LNC_000675

GTAGAGATGACCCAGGCTCTGGAACTAATTGATGTTTGCTATGAGCAGGATGTTGGCAACTCCTGATGATTTTGATGATGATGCCATGGGTGCTGAAGATGATCGTGAGACACGAAGTTTAAACAAGCTGTCTAGGCTTCTAACTCCTCAAGTAGCAAGACCCAAGATCCCCAGCTATGCTGTCAACGCCAGAAACCGTTGTGGATGGGAAGCGCCAGATAAGTGGAGAATGGTAAAGTTGTGTACCCGTTATTCACCGAGTTCTAAACCTAGCATCACACAAGC

>LNC_000676

AGAAAATCTATTACAATAGCTGTTCTTCCTGTGGCTTTTCTATTCTTGCTTTCTTTTGTAGCTTGTACAGGACCATTGAGGTGGATTCTTCTATCAAGCTATCTCATCTACTACCTATCTTTGTCTTTATGGATTTTGGCTGCATTGCATACTACATGACATTCAATCCATCTACCAAATTGAGGGATTTTCCATCATTCACTATCTCTATTTTCTTGGTTTTGTTATCGAGGGATCAATCTCTTACTTTTTTTGTTTTCCTTCAACCTCGTTGAAAGAATCAACAATCTCGTTGTGTACTGAAGCATAAAGAGGATAATGTGTTTATATTCATTTTCAGCATGTTAATTTAAACAAACTATGTCTTCAATCTTCTCCAGCTCTTTTGGGTTGGGTTTCAATGCAGTATTGAGCAAGTCCAAGTTTTTTTCTTCTTTTTTATCTCTGGAACTTCCCAAGTTTTGTTTCAGTGACTTGCAAATGTTTGTCACTAATTAGTAGTCACTCTAACGCCGAAACTAAACTTTGATTCAGAAAATACAAGAGC

>LNC_000677

GCTGAAATATGATTCTAAGAAAAGATTACTTGATTGGGGTAGTGGGATGTTTAATTACGGACAAGAGAAGAGGATTATGCTACTATGCTTGAATGAAGGTACTTTAAAATAACACGGAATAGTCAAGAAAGATCAACCGCTTTCTCTAATATGGAAAACAATTGAATTTCAAGTTTATTCTGGCAGCACTTCAACCAATGAAAAAAGT

>LNC_000678

AATCAAATTCAAATCGCCCTCTTTGAATTACAGAGTAACGGCTTCTTCTTCGTTTTCTTGAAGAAGAGTTGATAAGGGTTTAATTTTTCGATCGTTTTACATTACAAGGAGGATATGATGACGACCCAGAATAGGTATTCCTCTGTTTCGAGTGCGGTGGTAAAGAGACAAGCAATTCATCGGGGTTGGAGAATAATCAGATCAGAGAATTGAATGTTGGAGCTTCCATAATCTGACAGATCTCGAATCATACAGCTATCTCTGCTCGCAACTCGATTTCTAATTCCTCCAAACTTGAAGGTCTTGTGATTGCATACCGGCTGCTTGAGTTATCATCAACCTGGACCCTGAAGTTTCCTCCTATCGGGTATAAGTTG

>LNC_000679

TGAAAATTTGAAAGAAGGAAGAGAAGTCAAAGGGATTGGGAGGTTTCCACACTAGCACTCATGTGGATCTTGTGGAAAGAAAGGAATAGGAGAGCTTTTGATGAAATTGAGTAAGACAATGTACATTTAAGGAATAGCCTCTCATTCCTAATTTCTTTTTGGTGAATTCATGAGGTTCCAGACTTCCAGTGCGTATAGAAGATTGGGTGGTGTTCGTTGAGAATCATATTACGTTATAGGTTTTCTACATTTTGGTATACATC

>LNC_000680

AAGCAACCACTTTTTAGCACATGTAATTGAAAAACAATAACTTTATAAAATATCAAATATTACAGGTGAAACTCCGTAACAATATCATATTTTTAAAATATATCCATTGTTAGTGGCTATTTATAAAAAGATGATTAGAAGTGGTTAGTGGATGCAAACAGGCTTATGGGTAGGAATCGGGTCGGATCATTTCTCAACCGCATCGTGGAATTGAGAAATAAAATAGGGGTCAGATTATTATGGCTTTGAGCAATCAGTGTGATTGATTGATAGAGAGAGCAGTAGAAGAAAATTGGTGTGTTGATCAAAGAGATTTAAACATGTCGTTGATGAAGGGAGAGCCATTGGTGAGGAAGATTGCGCTGATAGCTAAGTACGCAGTGTTTCCGGGAGCTATGGCTGCTGCCGTGATCTATTCTCCTCCGACTTATGCTTCTTATTATAAGCCCCAATCTTCTTCTTCAAAATAATCTGGGGATACCCTTTTGTTTTCCTCATGGGATTCTTTTTGACTTGCT

>LNC_000681

TACAGGGGTCCCAAAAGACCTATTTCAAAAGTTCCGAGTATCCAGAAAATGATACTTTATAATTCGCCAACTCTACTTGTAAAAGACGATTTATTTATTGAAAATAACTGAACTTTGTCTCAACTAGTTAATATTGCCTATATGGATCATTTGCTTCGTAGTGGTTGTTCTTGCAAGTTTTTGAATCGATTGCAGGAGAATAATGTGATTTAGGTCTATTTGATTTTGTTCGATCAACAGAATCAATCATACTTCCACTACTGTGGACCGACTTTCTTAAAGCACCTTTAGGCAGCAACTACTATTAGGATCTAATTTTGAGACTCCGATCTTTTCTATGTTATTTTCTTTATCTATCATTTCTCTTGATGCGATATAAAAGACGACTTCCAAGAGTTACTTAAGAGTTGAGACCCCTTTTAGCACTTTTGCTGAACATAGTGAATCACCAGTGGCC

>LNC_000682

GAGATTGTTGGAAAAGAGAGGCAGGGCCCGAGGCACTTGCTAACGAGCAGGCGGGACAAGACTGACTTGAAAAGACGCTCTACTTGAAAGGCAATGGCTTTGAGGGTACTAACTGTGATATATGGGTAAGGAAAAAAAAGAGTAGCTCAGGCTCTCGAGGCATACTAGCGGGGCTTGACTAGAATCGTATGTTTTTGTGTAGAAATTTCCTTATAAATTCAATTAAAA

>LNC_000683

CCCTAACCCACAATCACTGTCAATACCATTTCCATATCCCCTGCTCTTTTCTTCTTCATTTTTTTTTCATGCCAAAAAGAGACATCACTTTATTTGAAGAAATTAAACATAAATAGAACAACTTTGCGAAAATAGATTGGGCCATTTTTGGGGTCATCGCAGAAGAAAAAAGAAAAAGAAAAGTACGCATAGTCAAAAATAAATCTCAACGCATGGTCAGATCTGCAGAAAGACGATTGCGATCATGAGAAATCAAGCAAGTTTCTGTGACTTGAAGGGAAATCAAGCAAGATATGGCAACCATTGTTAGATCCACAGTAAGATGGTTGTAAAATTCATCCATGAATCAATTTGCAGGAAAATTAAGAAAACAACTTCAGCACCTTTCTCCTAAAAAATGATTATATTCGAAAGGTAAAATCCTCAAGAATCAAGACCCCTAAATTTGATGTTGGCAAGCTGATGAGGTTTATGATTATATGCAAATCCTCTAAAAATTTGTATCTAATTCTTTGCCTTCAAGAAAAGGGTGGGGTTTGGGACGTTTTCTTTTTCTTTTTATTATTTAATATTTTTTTAAAGCAAATTCATTGCTTCCACTTGCTTTTATATGTGTGACAACAAGCGCCAGATCTTACTCAGCAAAACAAATGCACATAAGCCTAGTAACCAGTCAAAGGGTTTAAAAATCTCATTTTAATTAAGTTTAAGGGTTCAGATAACAAATAGTTAAGTAAAATGATTCAAAATACAAACTGATACAAGTACAAAGGTCCACCAGACCCTTTTGCCGAAATGAATAAGGGACCACAACAATTTGTTTTTAAAAAAAAAATGAAGGATTAGTGGTCCCTTCTAAATGGTTCAATGTCCCG

>LNC_000684

TGGGATTTGGGCGTTTGTGTGTTGAGGCTGTGGGTCTGGGCATTTGTGTGCTGAGGCAGATGCTCAAAATCAAAAGGAGGAGGGAGAGTTGCCTTTTGTTCTTTGTCATTTTGATTTTCCCTTTTTGGTTTGTCTGGTTATAGCTAATCTGTATATAGGGGAAAACATCAAGCTTTGAGGGATGTTGCTATGAATCTACTTGTTTGTTAATGTAAATAATTTGAAGGTGGTGCATGTGGATGTTGTTGCTTTTCGAAAATAAGGTTTTATGTCATTTAGCTTTATCCATGTTCATCTAGTTTATTGTCTAGCATGCCATTGGTCTTAGCCTTTAACTTAGGAGGATTTGAAGAGAATATAGGATTGTGGATAAGCCTATTCCTTCTTCTTTTGTGTTGTACTTTTTGCCTTCTTTTCCTTTGTTCTGCACTTCAAAGTCAAGTTGCATCTTGTTCATTTAGACACTACAACCTTGGTAAATTGAAATAATTTAGTACTCCCTCTATTTATTTGATGCGCCATATTTTTTTAGCATGTTTAAAGAAGAATGCTATTCATATGTATTTATAGAACAGATTTATCTGCTTATGTATCCAGTTTGTCACTTGCTTACTTCTACTCTGCTTGTTCTCATTCACTTGTTTCCCCCTTTCTAATGTTTAGATCAATGGGAAATACTTTTATTTTTTCTAGAATAAAAATAGGAAGATTGACTACTTTGGTCAAAAGAAAGAGGAAGAGTCATTCCAGAGCCGAACCTGACTTATTGATGATGAATTTAAGACCATGGGAATTGGAAATAAAATATGCTTTTCATGTCGATTCCACCCATCTATTGATAACATTTGGATTATTTACGCTGATACCCCATGCCTAACAGTCTGAGGGCCTGGTTGCTGGACAATGGGTTTACCATACTGCATTTTCTGGTTTTATAGTTTACACTCCTTATTTGACTTGAGGTTCAAGAACCGAG

>LNC_000685

GGAAATACTTGACAAAAAATAATACTCAAGTGCATTATTGAAGAAGCTCTGCACCAAGTAGCTTGGAGTACCATTTCCTCCAGGACTCTGGTCAGTGGCAAGAGCACAGCTGATTAGGCGATGAGTTTGAACCATGCCACAAACAAAATCCTTGTATTTAAGTGGAGAAGGGTAGAGGGCGGGCTCATTATCCACGGAGTTTCGACCGTGCACCATTAACACTCCGGGATTTCTCGGTTATGAAAATGAAAGGTAGATTGGAGTACCATAGCGAGCAAGAAGAATGTGTGCTTAAAGATATTTGATTTCTAGTGGTTTTCTATGTGTCTTTGTAAATCTTTACCCAACAGCTTATGAGAGTAGGGGTTTTATC

>LNC_000686

GCTAAAATGACTCCAAACTACAATCGAATTTGCAGTAACACACTCATATTTTACGTGGGATCTAGTTTCCGCTGAATATTTTTTGGCATTTATCCGACATGACACAAGTCTAAACTTGATAATGGAAGAATATAGTAATTGGCGAGATTAAATTTCAAAAGATTCAACTGATTTCTTCATGGAAAAATTGAAGTGTGAAGGAGAAACTTTTCATTTTTTTCTTTTGTTTGAAGAGTATATGAGTGATAAGGATAGAACCCCTTTGTCTTTCTGCATATACATCTATGTTAAGAAAACGTGTAGTAATCTAAAACAGTAGTCATCACACTCTTTTATTTCTATAAGAAATCGAATTGAATGATCAATAATAAAGGAAAAAGACAAACGAAAACAATTAATGTTATCCATGGTAAACCTATCAAGACTAAAGATCACTAAACTAAGAAAGATTCCCTTTTTACTAACTTAAGTTAATTTAGTATCCTGTATTGAGGGAAAACTATTGTGTCCTCCTTTTGTTGGTGCACAAGGGAACTAGACTAAAAGGATTGAGTACTCGTAAGGTTGCACCATGGGGGAGAGAGTGGTTCTTGTGGAGGTAGGTGCTTGATGGTTTTTGCTGAAGGATGGTTTATGGTCCAATAAAAC

>LNC_000687

AGAGCATTCATTATTCTTACTATTAGGAATATAAACGTATTATTGAGTTAGGCTGTCAGAGTTAACACTCAATTAGGCCGTTGTCAGCTAAATGTATATTTATTGTATCTTGTATTCCATTATCATCATCTTTTACATTCATTATTTTTCCTGCTCTTATTTTCACTCGATATTAGAGTCTCTTATGATCTTGGTAGAGATTTAAATGGCTTCCACCTACTAGTACTCAGACTTATGCTGTTTGACCGACCTAAGTTTTCTTTATTTCGTAGTCTTATTACTCTTGCCTTCTTTGGTAATTGCTCTGGCGGCACAACTCCTCTTTCCAGTGATTGTTTCCGATATCTTTCTGTTGATTGTTCCGCATAACCGCTCCTCTTTCTGGTGACCGTTTTGGCAACACTGTGTCTTGTTTTGTTACTGTTTTGGCAGTACCATCTCTTTTGGCCAGTATTCTGATCATTCTTCCAGCTAATGTTACCACAACAAATTTTCTGATGAAATTCTAGTGACTGTTACTTTTGAGTCAGTCTCTCATTGGTTGGATATAAGATGTGTCTCTCACCCACCT

>LNC_000688

AAAAAGCAGAAATTGATTTTCTTTTTTGGGATTTGTTTGTTTGTCGCTAACTGCCTAACTCACTGCTTGGCATTTTAACTGTACCCTTTTTGTCTCCTAAAAGAAGAAAAGAAAGGTATATTGTCCCAGGGCTTTGGTCTAGTGGTAAGACCAGCACGTGATGTTTGGATTAGGTGCTCATCGCAGGTTAGAACCTCACTGCATGCAAAATTTTGGTATTTTAAGTGGAGAAGGATAGAGAGCTGGGCCCATTATCAGTTGAGTGTTGAACTGTGTGTGGTGATATTTTGGTTTTAAAAAATAGATAAAAGGAAAGGTATCTTG

>LNC_000689

CGTCTTCAAATGGGTAGAAATTTGTTTTCCAATAAGGGACAAGGTCCAACTGGATAGACCATTTCGCATAGTTCAGGGAAAATTTGACCATTTTTCCTTGTGAAAAATGCCCACGTGTCAGTAAATAAAGGCATATTTGGACCAATATTGTAACGACGTGGTATATTTGGCCCCACCTATTGACGGATGGCAAATTTGGACCATTTCACAAAGTTTTATGGCGCATATTTGGACCTTTTCCGTTCTAATAATAAAATACTCTTGGAGTATACACCACCCATTTGTTACCGG

>LNC_000690

TTGTTATCTTCTTCTTTTTCTACTCTTCATTCTTCCCTTTTATTCTCTCTTTGTTTTATTCTTTTTTATTAATTCTCTACATTAATGAAATAAATATAACTCCTAAAGAAATTTATTTCACTTTAACCTAATTTTTCAACTTGGGTTTCTAAGAATTCATTCAATCATCATATATTTTGTAGATGACTTAAGGGATTCAAAGTTCAACAAATGCACATAAATTATTTTGGAGTTCACATATTGTGAGGTATTGCTAATGGAATTTGATGGACATTTGTATCAGCTTTCGGTTCGTTTGGATACTTGGATTTTCTACATACATATTTCTAAGCAAGTAAAGAAGAAGGCTGGATATATGTGGAACATTTCAAATTTCGAGATTCAAATAAGTTTATCATCAACTTCGTGGTACTTAATTTACGTCTTCGTGGAAGAGAAAACTAAATCCTAATTCAGAACATTATATCTGCCTATTATAAACATGTTACTGTATCACGACACTATTAATTATTAAAATGAGAGCTTGCAAAACTGATTTAGACCAAAGAGAGCTACTTCATTTTTATTTGTTTTAGCCACCTGGTGCAAATTAGAACAATATTGAGCATTATAACATTTTTTATTTTATATTCTCCTTATTTAGAAGTGGGAGTTGTGCTAGGACGAACACAACACTAAAAATTGGTACCACTATTTAGAAGTGGGAGTTGTGCTAGGACGAA

>LNC_000691

TACAACACTGACTTTAAAAAAAAACAGGATTGGCAATTACACATATCCAGCATGCCACATGACACATGGTGATATTTTAATTGCTTTGTTTAGATCAATAATGCTCTAACACAAAACCACTACATATCGGTTCTCATTTGAACTTAAGAGTCTAAGTCATTTTTTTCTGTCTCACTTCAGAACATTCAGAAAACAAAGAGATGAATAAATGGACAAAATATAAAAGATATAACGCAACAGCACATAAGGAGGGCATACAGGGGGAAAATAAAAGCAAATTGGATTTTCTTTCTTCCGAAAGGTTTATTGAGACTCCCTGTGACTCAAACACTTGTTCTTTCTTTTGGGGGGCATTGCAATTGCTATATTGCTTGCCCTAAAATCAGAGTTTCTATCTACAGAAGATCAAACTGCGCTACAATATTACTACTTAATAACATTTTCCTCTATTCACATACTTATAACTTTTAATACAATCTCCAAACTGCAGCAAGTCATTCAATCTGGCACTGAGGTAAAACCTAAGCTAAGTAGGTGAGTCCAACCGAGATGGACCAATATTCACCATAATCTCAATCTCAATCACCATAATCCCAGAAATAAATGGGACTAACAAGGACGAAACAAATACAACATAAAATACAGTTGCAATCAGATTAGATAAACTCAATTCAGATTATAATTTCCAAAGGCAATTAGATAGACATGTATGAACTACATCCACAGAGAAAAACTGCTTACCGAGTTACTTGAGCTGCTACCCAATTTGAAGACATCTCAGCTACTGAAATTTAAAATTGATAAAGAAATTATGAATTTTTCGAAACCCAAATAAGTATCATTTTTATTTGTTTCCCTAGTTAATTTGTCAACATAGTAAAACAATTATATTAGGTATAATCCAGAGGAAAGGGACAAAATATACTAATTAAGTTTTATCAATTTTAATTACTAGTAATTTGGGAACAATAATGAACGGCCGATACCTGGAGAGTACCTTAAAACTTGTTTTGTTTCAATCCCTGCTATCATTTGCCCATAAATAGAACTTGAAAGTTTTGTCATTAACCGATCTTCTTCATCAATGCACAATGGAACTCAAAGGTTTATAAGATCTCCATTGCTGGAATTCAACGGTTTCTTCAACAAAATTCCTTTCCGCAGATCCTTTTCTTACTCTGATTGCACTAACTATAAATTAGAGTTTGATAATTTTGGGGGAAAACAACGATATCACGATCGGGTTCTTGAACAAACAAGGGGAAATTTTGTTTGCCGGAGATGAAAAACTTAGGTGAAGTGCACTTACCCTTAAAAAATCTTACTCCATCCGTCCC

>LNC_000692

GTGGGCTTTAATGAAAAAAGGCGCAAAGGGAGAAAAACATAAAATGTGTTTGTTTAGTCCAAGACTAATAATTATAACCATGAATGACAAATATATGACCAAAGAAATTGAAAAAAAAATATGATAAAGTTAAATATCAATTGTTTAGTGTCACTTCTTCATAAGAGACTCATTGACAAGGAAAAGTTTGTCTTAGAACTTTGATGACGACACTGAAGCGCACATAAAGCGAGGCGAAGCGCTCAACACGTTTTGAGCCTCGCTTTAGGGCTTAAGCGCGCTTAAAG

>LNC_000693

TCAAACTCCGGCCAATATTTTTTCCAACAATATTTTATCTTCTCCGGTGGATATTAATGTTTTGGCTTTTTGCTTGAGTTGAAACTTCATTTGTTACCTTTGTTCTATTTTATTTTTGTTGGGTTTGATTCTTATTACTGATATTTTGTTCTCTTTGTTGGTTATTATTTATTTATTATGATTGTTGAAACTGATTTGAATTATTTTCATGTTATTATTTTTGTTCAAGTAAAATAGTGTATATTATCTCATTTCAGATTTCACCGGAAAGATTAAACTTCGGTCAAACTTCATTTATAACTAGCTAACCTCTTTTCCTGTTCATTGATCTACATTATTTTATTTATTTTTATTACTATTATTTTTGTTTATTTTATTGCTGACATTTGTTTGACCACTTAACGTTATAGTTGTTGGTGTATAATTTTCCTAGATTTGCTTACCCTTATTTGTTATTATTTAATATATATATATATTTTGATGATAAACATGTTTACAAAAATAAAAAAATCTCAAGCTACTGTTCAAATGTTTCTTTTTGTATTTGTTTGCCACTTTCCAATGTTCTATTTTGTTTTGCTTATTACCGTCTTTTGATTATTTTGTTATTGTTATAATGTTAAAATCAATTACTTAGCTATCATATCTAGTTGTTAATTTTATGTTACTTTTAGACTCTTTTCCAATAATCTTTTAATGATTGTTTCTATATATATGTGGTTTGTCTTTTGATAGTTTTTGTGGTTTACTTCGTTGATTACTTTGGATCGGAATATAATCTTGCTTATTATTATTTCATTTTACCATTGCGTTATCATTGGTTTATTTGTGGATTTAATTTTTTGTTTGTTAAAAATGGTCAATGAGAAAATCTCTCCGTCGATTTTGGAGGCCTTTACATTGTAAAAGACGGAAATTTAGTTTAAGTTTATATATTTTATTTGTTAAAAATGGCCGATGAAGAAATTACCGTCGATTTCTAAGGCCTCTCATGTTAATAAGACGGATGTTTATTTTTAAGTTGTTGTCTAATTCATTTACTTTTAGTCATATTTATTTAGTACTAACACTTTTACTAAGTGTCTTTACAGGTACTCCGAAGTCACTTCTCATCAAAGTCATTCGAATAAAGTTCGAATGTTATATTCATTATGATGTATATTGTTGACGATAGACAAATATTAAGTTGTTTCTCATATTACTTGTCTACTTTATTACTTGTGTATATTGCATGTTTATTATATTTGTACATTATTTTATTCTCCTCCTCAATTTGAAAAAAAATGAATTCAGTCGGGATCCACATTTGTGGATTTCAAAGGATGCTTAACCCCTTCCCTTCGAAATAACTTGAACCCCTTACCTAGAATCTCTTGGTTTCGTAGATTATTTAATTGGTTTCCTAGTTTTCTTAAAATTAGGTGGCGACTCCTTTAAAATTCGTTTAATCTTTAAAAAAATTCAAAATTTAATTAACTGATTATTTTTTGAGTCACCGCGACGTTTCTCCCTATTTGAATAGAGTAGGGATGTAAACA

>LNC_000694

GTTTAGTTCTAAGCTCTCAAATATTACTTCCATGATTTCTTCTTGCAACTTTTGTACTGCCTTTGTGTAATTTCCCATTTTCTCCCTGCATGATGAGCTGCTTTAGGCTTTTGAAATTATATTCTTCTTGAACATTTAGAATATATGTATATCAGAAATATTTCAGAGATAAACATATTCACTTAAGAATACAAGACTCACTTGTAAAATGTGGGGTTTGATGGCCACAAATCTATCCAATTTGAGATTGGATTAGCATAGTGCTTGAGGAAGTCTCTCCAAAAGGATACTTTGTCTTTGTCATCATTTAGACTGGTACTATACCTTACGGGGTCATGAACATTTGAAGACAAGAGATGCAGTTTTTCTTCACTTGGTAAATCGAAGAACTCAATCGCATTATCTAATGCATCTTTCATGACTGACAATGGAATTCCATGGTTGACTACCTGCACACAGTCATTTATTGCATGCATGCTGTTATTCTACGTCCAGGTTGAATTAGTAAAGAACTTCTTATTCTCTTTCCTTTTAAACTGCTAAGTATATGAAATCTAGGACGTCATCAACTGAAAATAAAGGGAAGAGAGAAGGAGATAAAGGCCAGAACATTTTTTATTTACTTGGTGAGTGTATATTGTTATCTCTTAAATGGTATCAGCATGAAGAAACTTTACGTACCTGGAAAAAACCTAGTTCCTTGCAGGCCAGGTATACTTGCTGGATTATCTGAGGCCGAAGAAGAGGGTGTTGCAGTAAGGATAGATCCACAATTGGCAAGCATGTGTTGGAGTTGGACAAGGTTCCGTTGGGGCGTAGGGATGATGGAACGATGTAACGTTTGGGAATACAGGGTTGAAGGTGACGACGGCAATAATGGTTGGACGGGATGGCATTTTAAATGAAGAATTTGCATCGTTTTCACTCCAACTTGCTCAGAGATCAGTTGAATTGTAAATCTTTCTCTACTGATCATTAGTAAGTTAAATCAGTTGATTGCTCAGAGATAAGTTGATGTCTCTACTCCATAGACATGAGATGAATTAGTTTTTCATC

>LNC_000695

AATGATGGATAGCAAAGAATAATCTGTGTGTGGTGGCAAGCCTAGTGCAAGATCCGGCTCAGGACATGCTGGATAGCAATTAACGGCAATGACCTGGGAGCCTTTTGCAATGTCTTCATTTCCTTGCAGGCCAGGTATACTTGCTGGATTATCTGAGGCCGAAGAAGAGGGTGTTGCAGTAAGGATAGATCCACAATTGGCAAGCATGTGTTGGAGTTGGACAAGGTTCCGTTGGGGCGTAGGGATGATGGAACGATGTAACGTTTGGGAATACAGGTTAGTCCCTCTTCAGTAAGTGTCATGGCACTTGTAAATGAATCATTTGGTGTAGCTGAGCTCTTATCCATTTTGTTGTGTTTCAAATATGACTTGGACTCGCTATTTATATAACAGGAAAATATGAAGATATAATTATATTTTCCTTTTAAAAGTTTACTTATTTTGTTATACAATGAATATGTGTAGGAATTTGATGATACCATGACTATAAGCAATGAAAAGTATGCCTTATTGTATGCTCATAATAAATGAATTATTGTATAATAGCACTATTGTTATAAATGAAGGCAGGAAAATCTTAGATGATTTTCACAAGTTGATCCAGA

>LNC_000696

AAGAATGATGGATAGCAAAGAATAATCTGTGTGTGGTGGCAAGCCTAGTGCAAGATCCGGCTCAGGACATGCTGGATAGCAATTAACGGCAATGACCTGGGAGCCTTTTGCAATGTCTTCATGCCAGGTATACTTGCTGGATTATCTGAGGCCGAAGAAGAGGGTGTTGCAGTAAGGATAGATCCACAATTGGCAAGCATGTGTTGGAGTTGGACAAGGTTCCGTTGGGGCGTAGGGATGATGGAACGATGTAACGTTTGGGAATACAGGTTAGTCCCTCTTCAGTAAGTGTCATGGCACTTGTAAATGAATCATTTGGTGTAGCTGAGCTCTTATCCATTTTGTTGTGTTTCAAATATGACTTGGACTCGCTATTTATATAACAGGAAAATATGAAGATATAATTATATTTTCCTTTTAAAAGTTTACTTATTTTGTTATACAATGAATATGTGTAGGAATTTGATGATACCATGACTATAAGCAATGAAAAGTATGCCTTATTGTATGCTCATAATAAATGAATTATTGTATAATAGCACTATTGTTATAAATGAAGGCAGGAAAATCTTAGATGATTTTCACAAGTTGATCCAGA

>LNC_000697

GAAAAACTAGAAAATAAGAAATGCCAATGAAGAAGATCAGAGACGTAACCGCTAATTTTTCGAACCAATTTTCACTTCCCTTCGTAAGTATTTTTTTTTCCTCGAATCAGTTGAAATTTGTCCCTATTTTTGTGTAATTATAGTACTAATTAGACTGAAAGTCATGTAAATCTGCAATTTTATTTGTTTGCTATTCATATATCCCGAGATTATATGCAAATCATACACAAAATCGCTGTTTGTAATTGGAATGGACAAATTCAACTGCAAATTCAAGCTTATAGAAGTTTAATTAGTAAGTTGATGATATGATTGGTGAGCATATGCATAAACAATGTTAAATTTCTGAACTATATTGTTAATTGCTGAAGAAATTAGAAATTAGATCAATGAAGAAGCTAACTACTTAATTAACTCACTTAATAAATGATTAGTTCTCTTCTGAATAAGTCAATAGCTTTCTAAAGTTATTCGTAATGCTCTAGGCAAAATCGCTAACACAATATTTGTGATTATAGTTTTCTCTATACCTTTCTGAATGGATAAACGAAAAAATCATTTCTAAATCAAATTTCAACTTCAAGGAGAAACATGATGTTCTTATCAATATATTGGCTTGGATTTATGATTCTCTAATATGTAAGATTGAAGAAAATATTGATGTCTCTTGTTTTAGCAGTTTATCTTATTTTCTGAGGAGATCAGCTTTCATTCTATGAACAACATTGTAAATTAGCAGCTTGAATTAGGACCAATGACAATGACTTAACCATTTTATACAATTTGGTGGATAATGAATTAATTTGAAGTAAAACCAAAAGCAAATAAGATGAAGAAACTGCAGGCAATTAGAGTAGCATTAACAACAGTTCTGAATTTTCGATATTGCAAAGGAGTTAAATCATTATGATGTGAAATAGAGTCAGTTATCAATTTGATTAACAAAGGTGATGACTGCATCATTTATTCCACTAACTGCTGTAAGAAATATATGTAAAAAGAAGCAATTCAGTGTAAGAAAATATAGATAATTTGTCACGATGATTAATTAATTTGAAGGAGTTCTGAAATGATAACTGGATAGTGCTGGACCTCTGAAATTTCGCTGAGATCACCGTGATGAAAAGTTTTCTGTAGTGTTAGACTCATCATATTGACATGCCAGGCTTATGAAAATTGAGATTCCGTTATGTCTTATCTGGGGAAAAAAGAAATAAGGAAAAAGATTATAATACTAAAGTTGTTGTTATGCTGGTTAATTTGTGGGCTACTGTACAGAACAGATATTGTTGGAAGCTGATTGTATCTGTATATACTTTACAATGGTAAATCACCCTAGTGTCTTTGCAGGTCTAATTAGTTTAAGATCTTAATGAATTCTCCTTATCAATTTACTGTCAACAAAAAAATAAACACGTGTACTAGAGATGAATGTGGTATGGTTTACATTAGGATTAAGCAGGAATACCTTTTAAACAACTTGAATGCTTTAATGTGATGAGTGTATTGTAACATCTATGTGCTGAAGTCTGGTGTTAGCCAACTGAGGTCAAGCATTGGCTGAATGAAGATCCTGATGGTTCATTTTATGATGTTGACAAATCATGCTTCCATAAATCCGTCAAAATGAAAATCGTACTTAAAAATAAATGAACTGATAAACTTTGTTCCAAAATTTATTTGTCACAGTGAGATCTTGTATATGAAGAATGAAGAGACCCTCACGATTGATATTAGAGTGTGTCATAAGAAAAACAGCTACTAGTTATATGCCTGTGTATTTAAACAGTACGAACACAGAGGTTGTGCAGCATTGAATTTGAGTGTAAAGAAGAGATTTCTTGTATGCTTTGCTCTGCTCTGCTCTGCTGTCTCATTTACCATAATCTTCATGAGTTTATTTTTAATTTGTCTATGTACTTTGCTTCCACAATATCTTCTCCAGAAATGAAGTCTAGAAAATCACTGAAGCTTCCTTCTTTATAGGACAAGATATGTTGTTCATTGACAAGCTCTGATGCAGGTCTAACTTTTTTACCTAAAGCTAGACTATGAAGGCTGACAATTGAAATGCGCTTTTTTTCTGAGTTAACTGTTGCTCGGTGGACAACACTTTTGTATATCCCATTGGTCAGTACTTCCATGTGATCTCCCAGCAGAACAGTGAGAGCTCCC

>LNC_000698

GAAAAACTAGAAAATAAGAAATGCCAATGAAGAAGATCAGAGACGTAACCGCTAATTTTTCGAACCAATTTTCACTTCCCTTCGTAAGTATTTTTTTTTCCTCGAATCAGTTGAAATTTGTCCCTATTTTTGTGTAATTATAGTACTAATTAGACTGAAAGTCATGTAAATCTGCAATTTTATTTGTTTGCTATTCATATATCCCGAGATTATATGCAAATCATACACAAAATCGCTGTTTGTAATTGGAATGGACAAATTCAACTGCAAATTCAAGCTTATAGAAGTTTAATTAGTAAGTTGATGATATGATTGGTGAGCATATGCATAAACAATGTTAAATTTCTGAACTATATTGTTAATTGCTGAAGAAATTAGAAATTAGATCAATGAAGAAGCTAACTACTTAATTAACTCACTTAATAAATGATTAGTTCTCTTCTGAATAAGTCAATAGCTTTCTAAAGTTATTCGTAATGCTCTAGGCAAAATCGCTAACACAATATTTGTGATTATAGTTTTCTCTATACCTTTCTGAATGGATAAACGAAAAAATCATTTCTAAATCAAATTTCAACTTCAAGGAGAAACATGATGTTCTTATCAATATATTGGCTTGGATTTATGATTCTCTAATATGTAAGATTGAAGAAAATATTGATGTCTCTTGTTTTAGCAGTTTATCTTATTTTCTGAGGAGATCAGCTTTCATTCTATGAACAACATTGTAAATTAGCAGCTTGAATTAGGACCAATGACAATGACTTAACCATTTTATACAATTTGGTGGATAATGAATTAATTTGAAGTAAAACCAAAAGCAAATAAGATGAAGAAACTGCAGGCAATTAGAGTAGCATTAACAACAGTTCTGAATTTTCGATATTGCAAAGGAGTTAAATCATTATGATGTGAAATAGAGTCAGTTATCAATTTGATTAACAAAGGTGATGACTGCATCATTTATTCCACTAACTGCTGTAAGAAATATATGTAAAAAGAAGCAATTCAGTGTAAGAAAATATAGATAATTTGTCACGATGATTAATTAATTTGAAGGAGTTCTGAAATGATAACTGGATAGTGCTGGACCTCTGAAATTTCGCTGAGATCACCGTGATGAAAAGTTTTCTGTAGTGTTAGACTCATCATATTGACATGCCAGGCTTATGAAAATTGAGATTCCGTTATGTCTTATCTGGGGAAAAAAGAAATAAGGAAAAAGATTATAATACTAAAGTTGTTGTTATGCTGGTTAATTTGTGGGCTACTGTACAGAACAGATATTGTTGGAAGCTGATTGTATCTGTATATACTTTACAATGGTAAATCACCCTAGTGTCTTTGCAGGTCTAATTAGTTTAAGATCTTAATGAATTCTCCTTATCAATTTACTGTCAACAAAAAAATAAACACGTGTACTAGAGATGAATGTGGTATGGTTTACATTAGGATTAAGCAGGAATACCTTTTAAACAACTTGAATGCTTTAATGTGATGAGTGTATTGTAACATCTATGTGCTGAAGTCTGGTGTTAGCCAACTGAGGTCAAGCATTGGCTGAATGAAGATCCTGATGGTTCATTTTATGATGTTGACAAATCATGCTTCCATAAATCCGTCAAAATGAAAATCGTACTTAAAAATAAATGAACTGATAAACTTTGTTCCAAAATTTATTTGTCACAGTGAGATCTTGTATATGAAGAATGAAGAGACCCTCACGATTGATATTAGAGTGTGTCATAAGAAAAACAGCTACTAGTTATATGCCTGTGTATTTAAACAGTACGAACACAGAGGTTGTGCAGCATTGAATTTGAGTGTAAAGAAGAGATTTCTTGTATGCTTTGCTCTGCTCTGCTCTGCTGTCTCATTTACCATAATCTTCATGAGTTTATTTTTAATTTGTCTATGTACTTTGCTTCCACAATATCTTCTCCAGAAATGAAGTCTAGAAAATCACTGAAGCTTCCTTCTTTATAGGACAAGATATGTTGTTCATTGACAAGCTCTGATGCAGAACACTTTTGTATCTGCCATTGCTCAATACTTCCATTTGATCTCCCAATTGAACAATGAGAGCTCCTTCAATGAGTGGAACTGAATGCCACTTTTCATCTCGATCCATGATTTGCAAGCCCAGATGGTTTTGAAGAATGATTGTTAGCATACCATAATCTGTATGTGGTGGCAATCCTAGTGTAAGATCTGGCTCAGGACATGCTGGATAGCAATTAACTGCCATGACCTGGGAACCTTCTGCAATGTCTTCATTATGCAGTTGCAGGTGACATGGACACGACACGACAGTGCATGACAAGTAGTACCTGGAAGAAACCAAATTTCTTGCAAGCCAGGTGTACTTCCTGGATTATCTGAGGCCGAACAAGAGGGTGTTGCAGATAGGATAGATCCACAATTGGCAAGCATGTG

>LNC_000699

GAAAAACTAGAAAATAAGAAATGCCAATGAAGAAGATCAGAGACGTAACCGCTAATTTTTCGAACCAATTTTCACTTCCCTTCGTAAGTATTTTTTTTTCCTCGAATCAGTTGAAATTTGTCCCTATTTTTGTGTAATTATAGTACTAATTAGACTGAAAGTCATGTAAATCTGCAATTTTATTTGTTTGCTATTCATATATCCCGAGATTATATGCAAATCATACACAAAATCGCTGTTTGTAATTGGAATGGACAAATTCAACTGCAAATTCAAGCTTATAGAAGTTTAATTAGTAAGTTGATGATATGATTGGTGAGCATATGCATAAACAATGTTAAATTTCTGAACTATATTGTTAATTGCTGAAGAAATTAGAAATTAGATCAATGAAGAAGCTAACTACTTAATTAACTCACTTAATAAATGATTAGTTCTCTTCTGAATAAGTCAATAGCTTTCTAAAGTTATTCGTAATGCTCTAGGCAAAATCGCTAACACAATATTTGTGATTATAGTTTTCTCTATACCTTTCTGAATGGATAAACGAAAAAATCATTTCTAAATCAAATTTCAACTTCAAGGAGAAACATGATGTTCTTATCAATATATTGGCTTGGATTTATGATTCTCTAATATGTAAGATTGAAGAAAATATTGATGTCTCTTGTTTTAGCAGTTTATCTTATTTTCTGAGGAGATCAGCTTTCATTCTATGAACAACATTGTAAATTAGCAGCTTGAATTAGGACCAATGACAATGACTTAACCATTTTATACAATTTGGTGGATAATGAATTAATTTGAAGTAAAACCAAAAGCAAATAAGATGAAGAAACTGCAGGCAATTAGAGTAGCATTAACAACAGTTCTGAATTTTCGATATTGCAAAGGAGTTAAATCATTATGATGTGAAATAGAGTCAGTTATCAATTTGATTAACAAAGGTGATGACTGCATCATTTATTCCACTAACTGCTGTAAGAAATATATGTAAAAAGAAGCAATTCAGTGTAAGAAAATATAGATAATTTGTCACGATGATTAATTAATTTGAAGGAGTTCTGAAATGATAACTGGATAGTGCTGGACCTCTGAAATTTCGCTGAGATCACCGTGATGAAAAGTTTTCTGTAGTGTTAGACTCATCATATTGACATGCCAGGCTTATGAAAATTGAGATTCCGTTATGTCTTATCTGGGGAAAAAAGAAATAAGGAAAAAGATTATAATACTAAAGTTGTTGTTATGCTGGTTAATTTGTGGGCTACTGTACAGAACAGATATTGTTGGAAGCTGATTGTATCTGTATATACTTTACAATGGTAAATCACCCTAGTGTCTTTGCAGGTCTAATTAGTTTAAGATCTTAATGAATTCTCCTTATCAATTTACTGTCAACAAAAAAATAAACACGTGTACTAGAGATGAATGTGGTATGGTTTACATTAGGATTAAGCAGGAATACCTTTTAAACAACTTGAATGCTTTAATGTGATGAGTGTATTGTAACATCTATGTGCTGAAGTCTGGTGTTAGCCAACTGAGGTCAAGCATTGGCTGAATGAAGATCCTGATGGTTCATTTTATGATGTTGACAAATCATGCTTCCATAAATCCGTCAAAATGAAAATCGTACTTAAAAATAAATGAACTGATAAACTTTGTTCCAAAATTTATTTGTCACAGTGAGATCTTGTATATGAAGAATGAAGAGACCCTCACGATTGATATTAGAGTGTGTCATAAGAAAAACAGCTACTAGTTATATGCCTGTGTATTTAAACAGTACGAACACAGAGGTTGTGCAGCATTGAATTTGAGTGTAAAGAAGAGATTTCTTGTATGCTTTGCTCTGCTCTGCTCTGCTGTCTCATTTACCATAATCTTCATGAGTTTATTTTTAATTTGTCTATGTACTTTGCTTCCACAATATCTTCTCCAGAAATGAAGTCTAGAAAATCACTGAAGCTTCCTTCTTTATAGGACAAGATATGTTGTTCATTGACAAGCTCTGATGCAGAACACTTTTGTATCTGCCATTGCTCAATACTTCCATTTGATCTCCCAATTGAACAATGAGAGCTCCTTCAATGAGTGGAACTGAATGCCACTTTTCATCTCGATCCATGATTTGCAAGCCCAGATGGTTTTGAAGAATGATTGTTAGCATACCATAATCTGTATGTGGTGGCAATCCTAGTGTAAGATCTGGCTCAGGACATGCTGGATAGCAATTAACTGCCATGACCTGGGAACCTTCTGCAATGTCTTCATTTGCAGGTGACATGGACACGACACGACAGTGCATGACAAGTAGTACCTGGAAGAAACCAAATTTCTTGCAAGCCAGGTGTACTTCCTGGATTATCTGAGGCCGAACAAGAGGGTGTTGCAGATAGGATAGATCCACAATTGGCAAGCATGTG

>LNC_000700

AAAAAAGGGCTCAACTGTGAGCCGTCAGATATAGATAGACTCGTGGCTAACTAAAGAATCGAATTGAAAACGAATACGGGTAAAACCGGCGGAGCAGGAAATTAATCCGTTTGAAAGCCAAGCCCCCTTCCAATGAAAATTGAAAACCAGAAACTTCAATGGAGAAGAAGCAAAGCAGTAGGGGCAGAGAAGATCAGAGACGTAAAAGAAAATGCAGAGTTTAATTTTCTGAATCAATTTTTCACTTTCCTTCGTAAGTGTTTTTGCCTCGAATATGTTGATTTTTTCCTCCAATTTTTGTGTGCAAATAAAGTCTTTATTAGATATAGTGAGAATCATGTAAAACTACAATTTATTTGATGATATTCATATATTTTGCGCAAGTTTTGACCATCATGAGAGGAATCGAGTAACTTCAAGTCGCTGATTTTGGTTTAGTAACTAAATTATGTAATAGTTACTGAATAAGTTGGGATGATTTTGTGTAATAGTCGTTAAATACTTAGCATTGCGCCAT

>LNC_000701

ACCAACTTAACCCAAATTCAAGCAAAAAAAACTTAACTGAATGAACCTAAAATGGAACAAAATGACCAAATACATATCCCTACTCTGACTCTACATATGGCGTGTGACATTGTACACTAAAGGTAATGGTGCACGTGGCATCTTCATATCCTGGGTCGCTATTTGCCTATCTCTCTAGCTTTTCCCGCGACTCACTCACTCTCTTGTAGACCTCAGGTGGTCTAAACAGAAGGACCAAATAACAAGGTTTGGTACACCATCCATTGGGACATTGCAGAGGGATAGAATTTAAGATGTTTTTAAGAGTTCATAGTCCTACTCAAAAGTTGGAGTTCAAATATTAGTATCATGGGATCTCTTGCATTTTGGTGTAGGTTTGAATTTGTAAATATTGTGCCAAATGATTTGATAAAGTTCACCTAGACTTTTGGTAGAGGGATATAACTCCTTATATTTGTACTGTC

>LNC_000702

TTCTTGTTCCATTTTGAAATAAATAGTGTCGACTATAAAAGTCAGAATAAGAGTGTTATAGTTTTATTTCTCTGTAATTACCATACCACTACAATCTACAGAGTCTTTAAGTTTCTTTGCTTGAATGTTTCTCTATACGAAAAATGTCCCAAAATAATAAACATTTTTACTTTGTCATCAAAAATAATAAACATTTTTACTTTTATAAGAATACTGATGTCTCACGTTATTATGGTTTTCCTTTTCTTTTTTGAGAAAAAAATCGACTTACTGATCTATAGATTGCTTTTTACGACTTGTTTTGTATGAAGGCAACTACACAAGATTAGAAATGACACTACAAGTGACCTACAAATACATTGATTTATATGCGTTGTTATGGTGATCAAAACATTTTAAAGGAAACAAGATAAGACTTAAAATTACATTAAGATTGGTTCAAAAATGTATAATTGTTTACATAGTCTCTTCCTCTATTTAAAATTTGGTAGGCTTCTTATATAGAAAATGTCTCATAGAAATTCTATAGATGCTAAGATTTGGCAGATATTGTCAGATTACTCAATTAGAGCGAGCTAAATTAGACTTGACAAATGCCTAAGTATTTCATTATCTCTGTTTTGTAACATATTTATTGCATTTTGTCAGATCTTATTGTGATAAGGAGGAATTATTTCTTAAGAATTGTGTATCATTTTTATTTTTGATGTGATAACATTACTGTATGGTCCTAAGGAATACAAATATTATGCTTCAAAACAATTAATGTGTAACATTATAATGCCATGGTAGAAGGCTAATGAGCTTGAGGTATTGTAAAAGAGTATTTTAAAAGTATGGTATGTATATCGTGAGAATATAGCTATCTATAGAGGTTTTGTGGCCTTTCAACAGAGATCTAGGAACACATTTACACACAGATCATTGGTGTTGGAGGTATGTTAGGTTGTGCAGAGCTAAAATTGGAGGTATATTAGGTTCTCTCTTTTGTTAGGTCTTCTCTTCAATGTTGTAGAACATTTATATATATATAAATATAAGGTTCAATTGTAATGGTTTTGATACATCAGTCAATTTTATTGTTTCTTGTTCCATTTTGAAATAAATAGTGTCGACTATAAAAGTCAGAATAAGAGTGTTATAGTTTTATTTCTCTGTAATTACCATACCACTACAATCTACAGAGTCTTTAAGTTTCTTTGCTTGAATGTTTCTCTATACGAAAAATGTCCCAAAATAATAAACATTTTTACTTTGTCATCAAAAATAATAAACATTTTTACTTTTATAAGAATACTGATGTCTCACCCTAGAAACACAACGGCTCCAAGATAATTTTTTGTCTCTTTGATTCGAACCTCTATTCATTGTCATTAAATTACAAAATCCTAACTAAAATTTTCATTCTTATATCATTTAACAGTGGTAAATAGTATGAACCATGGGATGATAATATGATGTGTTGGATGGATTTTGCGTTGTGTCTTACCAGCTTTCTGTACAATCTGTTGTTTGAGTATATAAACAAGAACATATTGTTTAAAGTGGAAAAGTCTTACCTCAGATAAGTTCGAAACAGAAAGGCTGCTTAATTTACTCTAGAAAGAAAAATGTTGTAGAAATTAGACTCTTGCATCTTATACTCATTATTCATCTATATGTAAAAACTTTAACTTTCACATTTTTCTCTATTTCAGGTTACACAAAGACACAACTTGGCAGCTTGTTTGGAAGGAGGAGTTTGATGCTCCGAGGAAGGCAATTATATTAGGCTACAACCCTATTAGACCTTGGATGTTTGTAGCTCACTGATGGTCTTCAAAGCATAAAAAGAAGGAAAAATTACAGAAATTCCACATTTTAGTTTACTTATTATCACTTATCCC

>LNC_000703

ATTGAAGAAGAACACGGCAGTGAAGCAATGAAGAAAACAAAAGAAGTTAGGTCACAGTGATTAGCGGCATGGAAGTGTCAGCTGCCATTTCATTTTTCCTACTAGCTTTTGTCTGTGAATATGAACATGTAAGTAATTAAAGTAAGTGGTCAGAATTTGTTCTGACATTTGGTCAGTTTTCTTACAGCATTAAAGCCAAATAGCAGACTCCATCAGACAAACAACCTTCAGAAGAATTTCGTGATATCTACTACCATCACTTACCCACCTTTTTATCTCTTTAC

>LNC_000704

CTCTGAGCAAAATAGAAGAAAAAGGTTGGTGCTCAAGATTTAATCGATCAATAACAACTCCAGCATTCATCTTTACTTGGTAGTCACAGGGAATGTCAAGAATTTGAGTAGAGAGGGCCATGACTTCTGAAGTTGCAATGTCTAGATATACACTCTCAAAGTTGGAATGCTTACTTATTAGTCGAGGATAAATTTCAGTGTTTGTTTATATATTATGCTTTATTATCATTGTTGTTACTCTTTAAACTATTATAGTTCATACTGAAGTAAGTACACAATGTAGAGAATAGGAAGGAAGTTAATTGCTAGGAGTGCAATGGGATGATGGGAGGAATGAAAGAGACTGAAGAAATGTTAGATTTTGCACCAAAGCATTACATACGTAGCAATGGACTACATGAATACAATATTGGAACGCCTTCTGAAATCCAATGTGAAGTATAGTTTTGTGCGAGACATGGGCAAAAAAATGAACTCTATTTCATCTCAAAACTATTTTCGATTCTTGTGCATGGCAGAAGTAAGGCTTAATTTCACTATTGAATAAACACGTGTGCTACACGTGTCCTTCTACATCTTTACTTTTCAATTATTTTCCTATGGTTCTTTCACCAATCTATCACTATGTTTTCCCCCATATAGAAACTTAGAATGGAAAAGAGAGCCAACGCAAAGTCACCATACTCTCTCCTTTCAATAGCCTAAAATTTGACTCTTAAACTTCTTCTGACTGAGTTCTTTCTACCTTCTTCTTCCTCTGCTCGATTGNN

>LNC_000705

AGAAACTAATGTTTCGCCTTCCACGGCTCTGAGCAAAATAGAAGAAAAAGGTTGGTGCTCAAGATTTAATCGATCAATAACAACTCCAGCATTCATCTTTACTTGGTAGTCACAGAGAATAGGAAGGAAGTTAATTGCTAGGAGTGCAATGGGATGATGGGAGGAATGAAAGAGACTGAAGAAATGTTAGATTTTGCACCAAAGCATTACATACGTAGCAATGGACTACATGAATACAATATTGGAACGCCTTCTGAAATCCAATGTGAAGTATAGTTTTGTGCGAGACATGGGCAAAAAAATGAACTCTATTTCATCTCAAAACTATTTTCGATTCTTGTGCATGGCAGAAGTAAGGCTTAATTTCACTATTGAATAAACACGTGTGCTACACGTGTCCTTCTACATCTTTACTTTTCAATTATTTTCCTATGGTTCTTTCACCAATCTATCACTATGTTTTCCCCCATATAGAAACTTAGAATGGAAAAGAGAGCCAACGCAAAGTCACCATACTCTCTCCTTTCAATAGCCTAAAATTTGACTCTTAAACTTCTTCTGACTGAGTTCTTTCTACCTTCTTCTTCCTCTGCTCGATTGNN

>LNC_000706

GTCCCATGTTTTGTGGTTGTAAAGTTGGTTTGAGGAAAAATTGATAAAAAGTTGAGTTTTTGAGAAGTTAAGTTTTTATGAGTTAAAGTTGGTTAAAAAGTGCTTTTTCGAGTCATTTGGAGTTTCGTGACTCGGATCGGATTTCCGTCGATTCCGGCAGTTTTAGAATGTCGAAACAGGTCTGTGTGAAGTTACGGAGTTGAATTTGGAGTTGAAACGAAGAATTGAGGTCCTAAGTTGCTAAAGTTGTGAAATTTCGATAAAGAGTTGACTTTGGTCAACATATGAGGTTCCGAGGCTCAGATTGAAATTCCGAGAGTACCGTTGGTTTCGGGAGGCGATTCTAAGTCTAGAATGAGTCTTGGTTGAATTTTTAGACGCTCCGTTTGAGTTTCGAATTTTTTGGCGTTAAACTAGTTTCTTGGCATCTTATTGAATTTCGGGTCAAGGAGTCCTCGAATTCAAATTCCGACG

>LNC_000707

CTGTTCATGAGTCTGAGGATCAATGCCCATGGAAAGCAGGCGCTTTTTCAGGTGAGTATTCCACAGATTCTTTATCTCATTGTCTGTCCTCCCAGGTAATTGTGAAGCAATTGCAGCCCATCTAGATAAACTTAGTAATGATTATTCATAGTTATTGAAATATAAATGATGCTTATAGTGTTTTTTACATTACGTAAACGAGTACTAAGAAATACTAGAAGTTAAGCATGCAACTACGTAAGGCAAAAACCAACCTGTTTCCAAGAATGCCATGTAACTGTATGACAAGCTTCTGTTCTTCGGGGCTAAAAGGACCTCGTATGATGTTTGGCCTTAGATAATTCGTCCATCTAAGACGACAACTTTTCCCACAACGAAGAAGACCTGTTAGTGAAAAAGTGGAAAACAGTTGACAAGAATTCAGGCAAACTATGAGGTTATGATACCATAGAGAGCAATGTTACCATTTTATAATATTAGTCTATCATTCTAAACTCTCCTACTAACAGCATTAGTCATTAACACAATATCAGCTGCTCAAGCTGGTTACAATAACCAAAGAACTATGGTATTAGCCAGGAAACTGTAACTAGGCTGGAAACAGTGAACCAGCACGCTCGAGGCTAGTTCACTCGGGTAACATTTGGCACCTACATA

>LNC_000708

TGCTTACAAAAATCCATTTCAATGATCTTTTAATTTTACAATGGTTGCACAGATGCAAATCGTGCATTCCTTTTCTTTTTGCACTTCGTTGATTCTTACAATTACTTCTTCTCTGGTATTGTGCTCACTTATCCCAAAAAAATCATGCAGCAGTTGGATATATACTTCGTTATTCCTTACCAGAAGTAGTATTAGTTATATTCCAGCAAAAAAACTTACACCTAGGGGGGGCTAAGCCTGACCGCAGAATATAATATTTGACTGGTTAACCAAGCCAAGTCAATGCGCGAAAAAGAGTAGTACTAATTACAGGGGATCCTATTCTAAGAAAAACCATTTTAGCTAACTAAATAATTAGCTTTATCTTATAGAATTCACAGATTAGGTAGTTGACTCTTATCCAATTGATAGGATCCCTTGGCTCTTTTGGCAATGTTTGGAAGGAGTCAAAGTGCCTACCAATATCTTATTTGGAAGCATACTTAGTGTCACGACCCGAGACTACCCCTAGTTGCGAAACGGTGCTTAGAGCCACAAGTGACCCTAAGCTAACATCATGGTTTATCTGTCATGACACTAAGATAAACATGAAATAGTATCTTAAAACAACTGGATGCGGAAGCTAACTAAAATAGTCCATTATACTCAATTGTAAGGTGGATATTTAAATTTTTGTTTTTAGCCGAATCTTGGCACCCGTATCCATACATGGATCCGTATCTCCGAATCTTAAAATTTAGATCATAGAGGATCTGACCTCTAGATCCGCACCCATATCAATCACTCGCACCCGAGTCCGAACAACTTAGGGAATAACTGTATGCTTGGTCATGCAATAACTGAAAGACTGAACTATGGGAGATACTGAAATCGACATATAACTATGTGAACTACTACATGGAGTTTGATGTATAATTCCCCTATCAAAATGGCCCAATATACTTTGCTAAAGGTATAAAGGCTGATTTGAGCTAATGTGGATCCACTAAGTTAATGTCCTTAAAAGACTAAGGAGTCGGCCTCAACTAGTGAAAAAACCCCTTAAACCCTACGGTGGCACGTAGTTGTGGAACTTAGGGATTGCTACTAAATGAATCATGCCTCAATAACGGGTGAGTCT

>LNC_000709

TGTGAAATTAATCGAGGTGCCACACCAATATTATCTTAGCTTTAATTATTTATTCCGCAAATCATTTTTCAAACCCACTACTAAAGACCAATTAAAATGGAATATTAAATTGAAACGTCTTAATTTTACTTGTATTCCGTTCAATTTTCACTATTGATTTATTTTCCTCACCAAACTGGATATTTTAGACGAGTAAACGTCTCTTTGACATTTTAGCAAACACCTTGTATGCATTTTCACCAATTGAATTCATTTCCCATTCGCCTACTTGTCTGATGAGGATTAAGAGAGGGATTGATGCAGATTTGTTCAAGTAGATCCTGGAAGGAAAAATGAAAAAAGAATCAGCTTTTGGTCCTGTTGGTATTCTAGATGGCTTCAATGATCATAAAAGCACAATCTCAGCAGTAGCTCTCTACATGTTAAAATCGTAAGTGTCACATCTAGTAGACTGCTGCTGCCAGGTGATCAAAAACAAAATGAGAAGTGGACCATAATTGCAAAATTTTCAGGGGTCCCTTCTAAGGTGACTCTGAGGTTATGTCTCAAATCTTCCATTTCCGATACAGGCATAAATTGGCGTCTATGTGTATGCAATCCATAAATTTGTAAACCTTGTGAGAGAAGTCATGGTTGCATCAGTTTTCTCTGTGATTTTTCGTCTTTCATGCATTTCAGTTGATATTTTGTGGCTTCAATCAGAGGCGAATCCTACATATTTGTAAGTTGTGGATGCAAGTTCAACAACCACCACCTCTGACTCAAACTATATGCATTAAATTTTGTAAAGCATGTTAGTTGGATGCATGTGACCCATGCCTCTAACTTGAATTTAATATTTACAGGTGCAACCACTATCTTTAAATCGTAGATCAACCTCAAAGTATATCCTGTAATTTCTTGCTTAACCTAGAACTCTCACTAATTATGAGTTTCATGGATTTTAGTCGAGATACTGGAAACTTTATTTGGTGCCTTTTGATGGACCAAATATGTCTGCCACAATACCTAACTATTTTAGAAAGAAAAAGGGCTGGCCAAGAATTGAATATTTGTTTGGTCCCTACAAGTAACGAATTCTGTCAGCACTCAACAAAAGATTAATCATTAGCTTCCTTTCTTACATTGGCTCTTTTATCATTAAAACCATAACATGTCAGTTGGTAAACTGGACACCGTTATACAATATCATCCATGCTCTCTCCTATGGCCATTGTAGTTGCAACTGGAAATTTCACCTATGATATTGGATGCATAAATGGTTTTAAATATTGGTTTCTGAGATTCTGGTGTTTTAATAGATTTTGTTCTTGTGTGCTATATTTGAGGCGGATATGGCTTGTATTAGAGTAAACTTGAAACCTGGTATACCACTGTTAAACTGAAGCATGTATTGTTAGGGATTTAGAGAAGTACGTACAGAGCCAGAGCTATTCTTTGATGACTGTAAGATGAGGCGTATTTACTACAAAGTGATTC

>LNC_000710

ATTCCATCATGTATTTTTTGGTGTATTTGGAAGGAAAGGAATCTCAGATGTTTTGATGGTATCTCAACTCCAATTAACAGTTTGAAATCCTTTTGCCTTATTAGTCTCTTTCAATGGAGCAACTTATCCCCTGTAAATGATATATTACCCCTGTTAGATTTCATTAGCTCCCTCAATATGGCTTAGCGACCATCCTTTTGCTTGTGGTTAACTCCCTGATTAAATAGGTGTTTTTGTTCTCAGGAGTTAACCATATTTTTGTGTATGTAATTTGCATCTTCTTGATGCCTTTCTAATACAACATCTTACTTCATCAAAAAAAAAGAATACATGAAGTGTTGCACTGTCTAATGAATTTGTTACAGCGGGACTACGATGAGGGCGTAGCCTTATGCACTAGGGAACATGAAATATGTGGTATTATTCACCCTAAATTTGTATAGTACTGTTCAAATGAATGCAATGTTTAACCAGATAACCATGATGAAAAAGAGTTCCTTGCCTATTCGCAATCAAGCGTTATGACTTTATGTTGACTGATTAATGCTTGATCTAGTTCCCCGTTTGGAATACTATCGGCAGACCTAACTGTTCTGTGGAGGAGAGTTTTTGGTGTGGTGCTTTAGTTCCTGGCTCCTATCCAAGTCTGTGTAGAAAGGATTCAGAATTTTGTACAGTTACGAATGCTCTTTAATAGCTTCATCAATAAAAGAATGCTCTTAAATATATACTAGAAACATGATCTGTTTTTTCTTATGGAATAGAAGAACTATAAGCATCTATTGTGCAGAGTTGGTGATTATAAATCCAGGAATTTTTTCATTCCTCTATTTTTCTATCAACTAGAAAACCCGCACTTTTTTTCCTCACCAGTATCCTCTTCTGTTTTCTTCTTTATTTTCTCTTTG

>LNC_000711

AGTTTTTTTAGCTTTACTTAAAAGTCGTTATAAATGAATCATATAATCAATCATGACTAGTTTCGTATTGAGGCCTAGTTGATGGATTAATGAGATTACTGGAAATTGGAATAGTGGGTAATTAGTTTGGACGTTCTGTTACTTGTTCACTATTGTTTGACATTGTGAGAAATGTCTGACCAGTTTGGGAAGAGGATCTCATTAGTGATCTAGGATCTGTAGTTTTTTTGTTTTGCTTTACTCAAAATCCTTGATGTCGATATAAAGGATTCATATAGTCAATTTCAACTAGTTTGGGATTGAGGCCTAATTGATTGATTAATTGAGATTACTTTAATAGTGGGTAATTATTTTGGGTGTTATGTTATTTATTCACTATTTGTTGGCATTGCCAGATATATTTTGACCTGTTTGGTAAGAGGATCTCATTAGTGATTTAGAATCCGTAGGTGTTTTTACTTTACTCAAAACCCTTGACGTCGACTGCCGGTGGATCTAGTTCCTTCTCTTACTTTTCCGGACTATCAATGATGGCTAATCAGGCCATTCTGTATCTTTTGACTGCAAAATGTGCAATGTGGTGTAGTTGGATTAGTTACTATCTTTGGATTGTTGAGAAATGGTGGAAACGTAAGATTCACTCAGTTTACATTAGTTGTGGGATTTCTATGTTAGTAAATTTCTTGGATCTCTATGTGCAATGTATGTGCATAGTTATGATAAATCTAGGAATTAAAAATCAACCATCTTCTGCCCA

>LNC_000712

CCAGTTTGGGAAGAGGATCTCGTTAGTGCTTTAGAATCTGTAGGATTTTTTGCTTTACTCAAACCCTTGACGTCGATATAACGGATTCATATAATCAATCGCGACTAGTTTGGGATTGAGGTCTAGTTGATTGATTAATTGAGATTACTGTAATAGTGGGTAACTATGTTGGACGTTCTGTTATTTATTCACTATCGTTTGGCATTGTGAGAAATGTTTGACCAGTTTGGGAAGAGAATCTCGTTTGTGATTTAGTAGGTGTTTTGCCATGAAAACCAAATATTATTCACTTTATTTTGGAATTTTTAAAGTTGGAGCTGGAGTTGTGTCTGGTCATATCTTTTGCGAAGAATTTGAATGTGCTTTTTTTAAATATGATTTAGACCCCCAATTTCTAAAATTTAGCAAAATCACCC

>LNC_000713

CTATTTATTCTGGTGTGGATCACAGGCTTTTCATTTTTATTTCCATTTGATCCGATCTTGTTGGAGCATATCAGGTCTCTTCACTAGCATTTTGAGATGATCCTACATCTCCATCTTGTAATCAGTCTATCAAAGACAATTAGAATTAGTCACCTTGACAATGTACTTGTTTAAGTTATGTAGCTGGAGGCTTTTCTCAAAGAGAAACTTATGTTTGTAGGCTGACTATGTACTTCGGCAGAATATACCAATATCTTTTGTCATCTTTGTATCTTCACTCGCTGCATGAGAAAAATTTTGGTAGTTCACACTATATCTTCATGCATGATGGCATGGGCAAGGACATGTGTATCCCAGTTTTCTTGCTTTGCTTAACATTATTGC

>LNC_000714

CGGTCAGTAAGGAAAGGTGGGACAAAGAAAGCTCTACTTCGGGTAGCTGGGTGCATCAACCTCTTTCTTTCACAGGAGAGGGGACAATACGCTTTAAGGAGCTTGGGCTACTCAACCGATAGGAATAGGGGCTTGTTTCCACAACTGAAAGAGAATCTGAAGCAGGATCGGAAACAGAATAAGATTGAGGAGCGGTATCGTATTTAATAGAAATGGCAGCTTTTAGGCGTGATCTATCTCTCTGCTGATTTGTTCTTTTACCATGTCAGCCACACTCTCATTTAGTCGATCTTGATAGTCCGAATACGAGAAGTGTCTTAATCTAGACACTCTCCAAGTTGATAGGATCCAAATGAAAAAAGGAAATACAATGGTAGGATTTGCCATCTTTCTTATTAAATAATGAAAAAC

>LNC_000715

CAAAAGGCTCTCTTCTTTGTTCTTCATCCACCATTTCAAAGCTTGAATTTAGTTGTTGTATCTTCTCCAACTAAGATTGAATTTAGGGTTTGCAATTTTCTCGAATTTCATATTGAACAATCGAAATCTTCCCCAATTCGACAGCTTGATTTTTTTTCACTTTGTGTTATTAGGATTAGTAAGTAAAGATCTAACCTTTTCTCAGAAGAAATAGTAAGTAAGCTTTCCGAGCAACGTCAGTAATGGAAGATCGTAGAGAAAAGGAAGAAGAACTAGAGATTAGATTAGAAAGAAGATAGTAGAGATTAGATTAGGAAGAAGAAAGGGTCAATTAGGTTCATAAAAGGTAAAGGTAAAGGTAAAGTTTTGTGACACGTGTCAATTTGTGAGTCGTGTAAATTCATGAGGTCCACTAATATATACGTGGACA

>LNC_000716

GATCGTAGAGCACTTTTCCGCCAATTGTACAGCGTTGATCCACCTTAACTGCATAGGAATCAGAAGGCAAAAGAAAAGAGGTGAATTTCACAGTCAAATATTACTGTAACTAATAGATATTTAATAAATTTCTTAATACATATATAAGGCTATTAGGTTCACGTGAGCCCTGAAGAAGAACAGTTTCTTTGTTGAGTCATAATTCTGCAATTATATATATCATGGCAGAATTTATTCACTCTCACTCGAAAGACCTCTAGCATTAGCACAAAAGGCATTTTAAGCCAGAAGCCTTTGAGGGAGAGTGAAAAAAGACTGTCCTGTGAACTGTGAAGAGCAACATAAGTTGTTAAACAAGACACTTGTTATTGGCTCTCCCTGCTAGAGCTTCTTTCCCAACATATAAGCATGCAGGAGAGAAGCCCTTGAGGGAGAGGCAGCAATAAGGGCCCTGAAGAATCTTTAATTAAATGTGTTTTGATTGACAAATTGAGTGATTTAGAGCAGCCTATTTATAGGCAAAAATAAATACTCTTGTTACAGAATAGAAAAAGGGAATTTAGTGGGACCTTGATATCTTGGCTCTTGATATATATGAGTGGCAAAACCAGCTTCTTTTGATTCATAACAGAGACTCATTATAATCTTTGTGTCCATCTGAAGACACAAGTCAACCCC

>LNC_000717

TATATATATAGAAGCGGATTGGAAGCAGCTGGCCGTTGTCCTGCCAGCTTGCTGTCCAAGGGCTTTTTGGTCATTCCGGTAGTACGTGTCATAACGTAGCTTCAAGATGAGAAAAGAAAGTGTGCAGTTTCAACTTCTTCTATCTAATTGTTTCTGCCATATCTTTATCACCACCTTCGTTCCTGTCTTCGATTTGAGCTTCTTTGTTATCTCAGGTGAGCTTTGAATTCATTGTCAGTGTTTTGTACTAATTTAATTATTTTATTGTGATGAAGCTTGTAGAAATTAGGTTTTAAGATATGTCTGCGGATAAAAAATCGATGGTACCAACTTCTTCTTAAATTGTTGTGTTGTTTTTTTTTATAATTATTTATCTTTTTCCTCATAATGTGGTGCATGTATGCCTATTTACTTGCAGGAAAATGATTGAACATTTTGTTATTGCCTATTTACTTGCAGGAAAATGACAACATCCAGTTGATTTGAATTCTCTCTTTTCTTTCTCTGTTGCTTCATCTTCCTCCCATTCTCTCTGTGCTTTGTTCCACTTATTTCAGGTAACAACTTGGAATCTATGAAGTATGTGATGGGGCTAATCAGAATACCAGTCGAATGCAGTAAATATATATAGGACATGATTAATTCGTAACTTCTAGTCACCTTACTTTGTAAAATCCTCGTATGCAGAGAGAGTATTTAGATATTTATTTTTTGTAGTGTAACTTGGAAAATTGCATTGATATATTTAACAGAAACAAAATATGAACATTTGATAATTTTAAAAACATGTGACAGTTTGGCCGAGTGGTCTAAAGCGCCAGATTTAGGCTCTGGTCCGAAAGGATCCCACAGCTGCCATTTTTCTTTATCACTTTATCCCATCAAATTTGAGATTATTTTATCCCATCTTTTAAGTGGGATAAATTAGTTCAGAGATCATATCTTAGAATAATTTAATCCTTATATCCAATTACTCCTAATGATTTTAAAAAGAAAATGGTTTAGCTAAAAAAACATCTCAAAAAATGTTAAAATTTATCACTAATATCTATGCATATAACTTAAATCACAATATTAAACCCCTTCAAATTGTCCAGCTTTAAAAGTCAAAACTTTGGCCACGAGGATAATTTGAACTAATAAAATTACTACTTTTAATAAGTGTTACACGATATGAATTTAGGTTAATTCCACTGGCCTTTGGCAATAAGATTTAGTACCCATTTGATAATTTAAGGAATTGCTTCGTGAAATTTACCCCTCTTATTATTTTCTTATTCTTTTGGGGTTGTTGTTGTTGATGTGATTTGCATCCATGTCTACATTTGTTTAATAATCCTCGAGTTCCCTAAATTTATTTCTTTACTTTAAGTAAATTGCCTTTTATTGAAATTTTAATCACATGTTGACTACAATAGTTGTTAATGGAATTAGTACATAAATAATGCTAATACTATTACTATACTAAATCTTTTTCCTTTGTTTTATAACTTGTTAGCAAATTTCATTTAAATGCTTAAACTATGACTTATTATAATTGAGCATCTGAACACGTGATAAAGTGCTCC

>LNC_000718

GAAGAATTCTAAAAGAACTAGGGAAAGCTGTATCCCTCTATGTCATGGGAAAACCTGCCTTTGGGTAATTTCTCAAGGTATAATTTAACTTGTCATCATAGTCTATATGCTGCTTGAACTTGTTAAGCTGCTGGTGGCATAAGTTACGTGAAACTAGACGATTTGTTATCTATCACCCTTCTTCCCCTGCTTTGATGCGAAATATACTTGAAGATAGATTCCTCTTCTGAGCCATTGATCCATAGGAGTGGAGGCTTATATTGAAAAGCTTTCTGAAGTTTAATGTTGGAATCTACTGGCCGCTAGCAGCA

>LNC_000719

TTCACAAATCATACTCTGTTCTTTTGCATAGTTATTTTTCTCTGTTTTCTGGAATCAGTTTATTCAGTTTCCGCGGCATAAGTTTATTGTGTTTCGTAATGGAATTACAGTAATATGGAAGAAGAGTTGTGATAATTTGTCAGCAACAAGGGAAGTTATTTCTTTATTGATTTGTTTTATGTACAGCGGGAGAACTGGACTTTAAATTAGTAACCTATGAAATCATAATGCTACACTAAAAAAGCCAAATCTAAATTCTAGGCTTTTGTGCTTGAGAGCTAAGTTTGATCACAAACTACTCTCAGCTGCAAGATGAAGTTTGTGTGTTCTTCCTGTCCATGTTATCTATAGCAGCCTAGCTTCTTTTACTTTTGGTGCATGAAACTTAAGTGAAAAAATACTTAGCTTTTGGAACATATACACTTGAAGATTCACAAGCTTCTAAAGCTCCAGTTCCCTACCCATGGGGTGTGGGGTGGGTGGGAAATAAAATAGAAAGAGGAAGTGAACTTTAAATTGTGGCTCAGATTTTGAACATTCTGCTTTTATCATTGTTGATTCTGTTATGCCGACAGTGGTTTACAAATGC

>LNC_000720

AGCTGATCAATTCCAGGAAAGAATTGACACTAAGTACAGGAAAGAGATTAACCCAACCAAAAAGAGTAATCAAACATCTTGCTATAAAGGTAGAGATGGGAGTTGCGGTGCCATCAAAAAATCTTTTGTTCCTCTCCGTCCATAAGCACCACAAAATACAGGCTGGAACCAAAGACCAGATGTGCTTGATGGACTTCCCAACTTCCCAAGAACTCCAACACACAAAAGCCTCTCTCAATGATCTTGGCATGACCCAAGTGAGTCCAAAAAGTGAGAAGAACATGTTCCATAGGCCTGTTGCAACTGTGCAATGAAGGAACAGGTGATTTAATAGATTCTGAGTTGCACAAGCACATGTAGCATCTGTTGACTATTTGGAATTTCCTCCTCATGAGATTATTCTGAGTCAAGCAAGAGTCTTTTCGAGTAATCCAACTGAAACAAATAACTTTAGGTGGCAACTTAGTTCTCCAGATTAATTTCCATGCCCATTGATCAATTAAAGTCTTCTTGGAGCTTTGTTGAAGGTAGCATGCTTTAACTGAGTAGGATCCACCCCTGCTATATCTCCAGATAAGCTTGTCCCTGCTTTGTCTGTTGATTGCACTAGTCTGAATGGTGGCCATCAGGCTAAAATCCTCTTCCAACTCCCAGTCATTAAGATTTCTCCTTAGGATCAGATTCCACACATTGTCTTCC

>LNC_000721

TGGACCTGTAACTTCCGCTATCATAAGTGTGACCATTTCTTTTTGGCAGTATTAGTTTGTAATTTGCTTGCCTTTTAGCTCTAATGTGATATATTTATAGTACTGGTTATACCAGAAAAATAAAAGATGTGGAGAGTAATGGTTACCATTTAAAAAAAAAAAAAAAACCGGGAGAGTAAGAGTACTTTGAGATTTTTCTCTGCTATATTTGTACTACTGGTTACTTTGTGCTTAGGATGCTGACACATGACTAGAAAACTATTAACAACATTGATGCAGATGTATCATATAACTGCTCTAATTTCTGTACAATTTTCACTCAATGATAAATGGATTTTTTTAAAGGGAATTTAACCACTATACTATAAAGTGTTGTATACTAATGATAAATAGATTTCAATTCTCATCCATGTTAATTGCAGTTCGAGCAATGCAACAAGGCCATGAACATAGCATACTTCTATAGTAGCTTGACATTGGGAGAATATCCGTCCCCTTTTTATAGAATCCATCTTAATCTCC

>LNC_000722

TATATAATTACTGCTGTTGAAATCTTTCTATATGTTGGGATAATGCTTGTTCAAAGACTAGTTTAATTAGATTCTTCCAGTGTTCTAAAAGGCGATTGCCTCGTCGCCAATGGCGAGAGGCGAGGCAAGGTAACCGTTCATTGCCTTTTAGTTTTGTGGCGAGGCGATCTTCAAAAGACGACGCCATGGCGACAAAGGCGAGAGGCTAGAAAGGTGTCGCCTTTTGTATTTTCTTTAAAAAGGTGACCAATTTAGGGTTTTAAAAGTTGAAATGTAATTTTAGGATTTTCGACTAGACTCTTAAGGCTACTAGCTAGACTTTTTTGGCGACTCCAAAGTCCAACCAATACGCATTTCTTCTATCTTC

>LNC_000723

CATAAGTTACGTGAAACTAGACGATTTGTTATCTATCACCCTTCTTCTCCTGCTCTGATGCGAAACATACTTGCTTTATTATTGAATCTAAAGGGTGTGTTTCTCTGCACTGGTTTGATTGGAAAATCATGGCTCTGGCCTCTGGCTAGTTTCATTAGCTTGAAGCTGGACTTTAGATGCTCTACTTCATAGTTTGTGCACAATTCTGTTTTACTTTGATTAGGCGATTGCAAATCTTGTGCGCATGATAAGTCATTTGTTTTAACTTAATACAGACACTGATCAACTCAGAGCTTGTACTATGACC

>LNC_000724

CAGCTTTAGAAAGTTCGTATCAATTGCATTGAAAATTGTACTACACCATTTGAGTCTAATTCACTAGAAAATAAACACCTATACTACACGTGTCCTTCTACCTTTTTCCAGTTTTCAATTATTTTGCTTTGGTTCTTTCTTTCTTCTACTTTTTCTATTTTTTATTCTCCTCATTCACACAATTGCCGGAGAAGGAGAAGAAGAACTTAGAGATGGAGGAAGAGCAATTGATGGAAAATTAGAGAAGAGTGATTCGATTCCGAATTGATGCATTAATTTGGTGATGAAATTAAATTTCATATTTCACTAACGAAATTGGAGATCAAATTGCATACTGTTTTTGATGTGATAATGAGGCAAACACATGCTTTAAAGTTATATGAAGACATAGACTTCAACAAAAGCTCTTCTCGCAATTCTCAAAATTTTGTATCTCAATCTCTTATCGTGGTTAGATTTTCTTATTTTTGGTGAGTTCTATCACCCTTCTTCTTCCTTTGAGAAATATAGGAATAATATAAATAAATAAATGCAATAAATTGGTTCTTTATTAGCAAATATAAAGGACGGATTTTGAATCTGACAAATATAGATGCAATAAAATTTTGATTTGAATGTATAATCTTTGCATGTGGATCTTTGGGATTTTAAACAAAGTTCTCTAATTTTTCATTCAATCGTGTGTTCAAAGTTCATTGTGTGTTTTTATTTTGTAGCTTTCATCATTGCTCAAATTGAAAAAAAAAATCTCCTTCTTGGTTTCTCAGTTTGAGTTGCATGTCAGTACTTACCTTCGCTCTCCACTGTACCAAAAGTGTTTGATTGTATGCCTAAATGAACTTTTCCCCTTCTTTAACTGTACATTTATAGAAATTTCTACTCAAATTTCCTGCTTATTGCACTTCATTGCTGGTCTACGAAATTCTGATCTTCTCTATTTAAATGTTTGTCCTTTCTTTTTTCCTGTTTGTTGGTGAAATTTTTGTCAACTTAGGGTCACTCATTTGTTGGTAATCGATTACCTTTTTGTGGGTATACTTTTTGCAATTCTTCCTGCTCCATTTTGGTATGCGGGTTATAATTCTGTTGCATGGATTAATTAATAACTTCTCTTCACTGTTGGTCTATTGTGTTTGTTGTGCTCTCCTTCTCCTCCTGTTATGTCTATGAACAGATGCATCTTTCTCTTCTTGCAGCAACTATCCAAATATATTTTTGTTGTATGTATACATTGTCATTATCAAGACCTTAGATGTTGACAAAAGAATTTTATGCATGTTTAAAATATGTATACAACACTTTGTTTTTGATTGCCAATGTGTAAGAGCTTCGACATTAACAAAAGATGTGTGTCTTAGTTTAATATATCACAAATTGTAGAGAACTGTAATTGGTGTTGCTCTCTAACTCTTCCTGCCCATATTGTTGTTAAAATATCTTGGGTGGACATATGGTACTAAGATTCAATATCGATTGAATAACAACTGATACTCTTGATTGGACTTGCAAAGTCCAAATTGTGGTCATGACTCAAGAGCGAGAAAGTTTGGAGAAGAACATCAAATTTCAAAATCTGATTTTGAAAGACGAAGAGAAGAAAGTAACAAGAGTTGTATCTGAATCTTCAGAAAAACTTGAAGAGATCCATTTCCAACATTATTGACGCAAGGTTGTCGTGATCAGAAAAGTTTACGTTTTACTAACTAATTAATTATTATAATGGAGAATTAGCATAATATGCAAAAACAGTTTAATATCAGATGACCACCACCATTTTGGTGTTCTCTTGAATACATCCAAAAGTATTCATGATGTCTAAGGGCCCTTGTAGTATGTAGTTTCGACTTCATATTAACATTCCTTCACATCCAATTTTGTTGTGAATTTCATAGCTTAGGTATAATTGTTATGTGCATTAATCTTCTGCATCATCTTTGTAATATACTATACATGTGGCTACTACATGGGTCCGACAAAAATACTATACATGTGGCTACATATAACATGTGCGGCACAAAACCGACAAATATACTTACCCTTGACCCTTTGACAATGATTCCACATTATCTTTGAAACTTAAAACAGGGTACATTCTAAATCAGGAGCAAAACAAAAATATGGATGATTATGGAACACTAAAGAGGTAGGAAGTTTAGTTATTTCTGAATGTTACGTATCCTTACTCCTTCCTGTTTGTCATTCCGTCGTCTACCAATTCTATATTTCCATTCATTCTGTTGTTTTGTAATATCATAGTTTAAATATTTGCCTTGCAGGTGCTATGTAATACTGATAGGTTGAATGTTGTTATTTTTTCCTTCCAGCAAATACCTTTCATTCAGATTTTTGGTAATATTAGAGTTAAAATGCCCGTTTTGCTAATGTTGTATGATAATGATAAGTTGGGATCTGTTATTCTTTTTCTTTCAGCAAGCATTTCTATATTTTGCCATCACTTCATCAGATGAATGAAATCCTTTTAGTTGTTGCATTGTGCTGGTTTTGTACTTTAGAAGTATTAGAATGTCACAGATTCAACATTTACTCTCTATAGCTTATTGAAATGTGAAATAGTTACACGTGTTCAGTTTTCCTCCCATTGATTTTCTTCTAAAAAAGGAAAACTACATATCGGGGGAGAGAAGACGGATGACTCATGGGCTTAACTTCATCTACATCTACAAATTGGGACAAGCTTGATGTCATGAAACAAGCATGGGACGAACTCTGCATCACATAATTGTACACGAACAAGGAAAGTGGTTCATCAAGTTGGCTGTAATGTCTTTCACACGCAAAAGGTATGAATGTAATTGAATTTTCGTACGTTCCTTTCTTTTTACTTTCAGTGGATTTCTCCTCGCTACTGTTCTTGCTCTCGAGTTTCAAGAATGTTTGCATTTTTGTTGTTGTGTGTTCAAGCTCTAGATATGTTTTTTCAATCAATCTTGCTCATGCCTCCTTTTTTCCCCCTTCATTTTCCCCTGTTTCTTGGTTGCTGCTCAATTACTTTCACACTCTTAATTTTTCAATTCACTAACATTTGTGGGTAAATCTATGAGTATCATTGGAAGGCAATAGCGGCAAAGTAACAGGTGATGGTCGTAATTTCATTTGTTTTCTATTCCTTTTTATTTGGGTATTATGTTCAAATTTGGTTAGATCGAACTTCAACGGATCACATCTTCGATTTTCTTTAATTATTCCTTTGCATGTATGTAATGGTTTTTTTGTGTTGCATAGATCGACTTTGTCTAATGACTTGCCTGTATGCTACCATTATTGGAACAAATATTGTGTGATAACTCTCCTTCAGTACTATAAAAGTAGCACAAAAGTTGAATAAATGAGAAGGAAAAAAAGGATTAAAAGCTGAAGAAAATCAATTGATACATCTAATAAAGTGGGACACTGCCACATTGGAGAAGAAAATTGTAGATTTAGAAATAAATAGAATAAGAGTAAGAAATGGTAGTTTACTCATGAAGTGGTTATGGAGATATATTGTCAAAGAAGAGGCACTTTAGAGAAGGGTGAATATGAAAGTATGGGGTAAAAAAGGTGAATGGTGCATAAATAAGCTTATAGAGCCATATACAAATATGTATGGAAAACATATAAGAAGAATGTGGATCTTTTTGAACTAATGTACTCTTTGTTTTAGATGATGGAAGCAAAATAGTATTCTTGCATAACAGCGGATGAGGTCACCTAGTTTTATTTCAAGTTCTTATGGATTTAACTCCTTTGCTATTCAAACACATTTGATATACTTTTCAATTAATAATTTTGGATTCTTGCTCGCTTTAATAATTACGCCTCGACTAAGCTAAGGTGGCAAAAAACAATCAAGGTCTAGAGACAGAACAACAAGCAGCCTAAACTATGGGAAGGGTTAATTTCAGAATCTCTTAAATTTCAAGCCTTCAGGTACACAATTTCACAGACTTTTTTCAGGCTCTTCAAATTTTGGCCCCCTTTTCTTCAAAAAGAAACTTAATGTTCTGTTTGGTGTACTGATAATGATTAGTGACAGATTATGGTGTTGTATATAGTATTTAATAGTTGTTCTTGGATAAAAGTTACAGTTTTAGTATTGGGATACAAAACTTTAAAACACTTCACCCCTAGATTCAACTTTTGCAAGTGACCTAGATTTCTTTGGTTATTTTTGCAGTAGATAATTTAATCATATTTATAATAATGATTGAAAACAATTCTGAGATAAAGGGGATGATCATAATAGTGTGACTATTAATGGCTAAGGACAAACAAAGAGATTATTCTTTTGCCCATTGCTATTGGAAGACCAACTATTTTGCAGATTTCATGGCTAATTTTGGAGAGAATCAAAAAGGAGAAGATTTTCTTCACAGAAGCTCCGACTCTACAAAAGCCAAAGCTCCCCTGAAGAATGACTCAGACCAAGTTCCAACTTTTAGAATTAAAACTCATGTCAAGCAGTTCACATTTGTATAGGTTATGCTAATCCTTTTGCTTGAATTTTTCAGTCCCTAATATGTTTTTGGTGGTGTCCTCCAACATAGCTATAGATTTTTTTTATAGTCACTTAACTCTAGAATATGGATTATTGTGTATATTGCTGAATATTCATCCTTTTTGCAACTAGGAAAAGGTTTGGGAATTAATCTCATCCCTTCTCATCTTTCTTTATGAAATAAAAAAGTTGGGTTTGCAACCAAAAAAAATGGTAACTCTACGTGGGAATTTTCTATAGCATGTGGCCAGCACCTCTTCATTTTCATCCACTTAATGAGGTATATGTATGAATTATTTGAGACACATAATATGCATTGAAGAAGTTTTCACATTTTTGTGATTGTACCTATGGCCATGGGATAGGCTTTGCTTAAATAAATTGTTATTCGGCTTTTGATTTATTGAATTGCAAATCTCAGGTCAAGNN

>LNC_000725

TTGGACTTTGGAGTATTAATTTTCTCTCTCTATTTTGATTTTCTTCTCACAAAATTTCAGATATCTCACTATTTGCTTTTTAAAAAATAAAATTTCAATTTTGGCTCGACTGGCTTTCAAATCTCCTACTCTGATTATTCACACCTATACTAATTTTATTTGCTTTCAAATATTTTATCATCATCATCAATATTTTAAAATGACAGTTGTTGAGACGAACACAACATTCAAAAATTGTACTGAAAGCTGTCCAAATTGAACACAACACTAATTGGTTGTACGAACATCAATTTAATTCGTAATGTACAAGTGGGCTCCATTTCATTGTTAATTGAGCTTTATGGTCTTTTCTTTTCTCATTATTTTTTACTCTTATTTAGAAGTGCCACTTTTAAAGGACTAACACAGCTTAAAATAATTGTACCTTGTGTTTTAAAAGTGGCACTTCTAAAGGACTAACACTGCCTAAAATAATTGTACCTTGTGTTGAAAATGTGATGAGACAAATAAGGGCATTTTGGTATTGTGAAGATTAATTTTGGTGCATTCATTGTAATAGAGACATATGTACTCTATTTTGACAAGTGTGTTGTCTTTTTATATTTTATACTTAGCCAACCAGATATTATTACTTTACTTTAAGCCAACAAGATAGAGTACCAACACAGCTTTAGAAAGTTCGTATCAATTGCATTGAAAATTGTACTACACCATTTGAGTCTAATTCACTAGAAAATAAACACCTATACTACACGTGTCCTTCTACCTTTTTCCAGTTTTCAATTATTTTGCTTTGGTTCTTTCTTTCTTCTACTTTTTCTATTTTTTATTCTCCTCATTCACACAATTGCCGGAGAAGGAGAAGAAGAACTTAGAGATGGAGGAAGAGCAATTGATGGAAAATTAGAGAAGAGTGATTCGATTCCGAATTGATGCATTAATTTGGTGATGAAATTAAATTTCATATTTCACTAACGAAATTGGAGATCAAATTGCATACTGTTTTTGATGTGATAATGAGGCAAACACATGCTTTAAAGTTATATGAAGACATAGACTTCAACAAAAGCTCTTCTCGCAATTCTCAAAATTTTGTATCTCAATCTCTTATCGTGGTTAGATTTTCTTATTTTTGGTGAGTTCTATCACCCTTCTTCTTCCTTTGAGAAATATAGGAATAATATAAATAAATAAATGCAATAAATTGGTTCTTTATTAGCAAATATAAAGGACGGATTTTGAATCTGACAAATATAGATGCAATAAAATTTTGATTTGAATGTATAATCTTTGCATGTGGATCTTTGGGATTTTAAACAAAGTTCTCTAATTTTTCATTCAATCGTGTGTTCAAAGTTCATTGTGTGTTTTTATTTTGTAGCTTTCATCATTGCTCAAATTGAAAAAAAAAATCTCCTTCTTGGTTTCTCAGTTTGAGTTGCATGTCAGTACTTACCTTCGCTCTCCACTGTACCAAAAGTGTTTGATTGTATGCCTAAATGAACTTTTCCCCTTCTTTAACTGTACATTTATAGAAATTTCTACTCAAATTTCCTGCTTATTGCACTTCATTGCTGGTCTACGAAATTCTGATCTTCTCTATTTAAATGTTTGTCCTTTCTTTTTTCCTGTTTGTTGGTGAAATTTTTGTCAACTTAGGGTCACTCATTTGTTGGTAATCGATTACCTTTTTGTGGGTATACTTTTTGCAATTCTTCCTGCTCCATTTTGGTATGCGGGTTATAATTCTGTTGCATGGATTAATTAATAACTTCTCTTCACTGTTGGTCTATTGTGTTTGTTGTGCTCTCCTTCTCCTCCTGTTATGTCTATGAACAGATGCATCTTTCTCTTCTTGCAGCAACTATCCAAATATATTTTTGTTGTATGTATACATTGTCATTATCAAGACCTTAGATGTTGACAAAAGAATTTTATGCATGTTTAAAATATGTATACAACACTTTGTTTTTGATTGCCAATGTGTAAGAGCTTCGACATTAACAAAAGATGTGTGTCTTAGTTTAATATATCACAAATTGTAGAGAACTGTAATTGGTGTTGCTCTCTAACTCTTCCTGCCCATATTGTTGTTAAAATATCTTGGGTGGACATATGGTACTAAGATTCAATATCGATTGAATAACAACTGATACTCTTGATTGGACTTGCAAAGTCCAAATTGTGGTCATGACTCAAGAGCGAGAAAGTTTGGAGAAGAACATCAAATTTCAAAATCTGATTTTGAAAGACGAAGAGAAGAAAGTAACAAGAGTTGTATCTGAATCTTCAGAAAAACTTGAAGAGATCCATTTCCAACATTATTGACGCAAGGTTGTCGTGATCAGAAAAGTTTACGTTTTACTAACTAATTAATTATTATAATGGAGAATTAGCATAATATGCAAAAACAGTTTAATATCAGATGACCACCACCATTTTGGTGTTCTCTTGAATACATCCAAAAGTATTCATGATGTCTAAGGGCCCTTGTAGTATGTAGTTTCGACTTCATATTAACATTCCTTCACATCCAATTTTGTTGTGAATTTCATAGCTTAGGTATAATTGTTATGTGCATTAATCTTCTGCATCATCTTTGTAATATACTATACATGTGGCTACTACATGGGTCCGACAAAAATACTATACATGTGGCTACATATAACATGTGCGGCACAAAACCGACAAATATACTTACCCTTGACCCTTTGACAATGATTCCACATTATCTTTGAAACTTAAAACAGGGTACATTCTAAATCAGGAGCAAAACAAAAATATGGATGATTATGGAACACTAAAGAGGTAGGAAGTTTAGTTATTTCTGAATGTTACGTATCCTTACTCCTTCCTGTTTGTCATTCCGTCGTCTACCAATTCTATATTTCCATTCATTCTGTTGTTTTGTAATATCATAGTTTAAATATTTGCCTTGCAGGTGCTATGTAATACTGATAGGTTGAATGTTGTTATTTTTTCCTTCCAGCAAATACCTTTCATTCAGATTTTTGGTAATATTAGAGTTAAAATGCCCGTTTTGCTAATGTTGTATGATAATGATAAGTTGGGATCTGTTATTCTTTTTCTTTCAGCAAGCATTTCTATATTTTGCCATCACTTCATCAGATGAATGAAATCCTTTTAGTTGTTGCATTGTGCTGGTTTTGTACTTTAGAAGTATTAGAATGTCACAGATTCAACATTTACTCTCTATAGCTTATTGAAATGTGAAATAGTTACACGTGTTCAGTTTTCCTCCCATTGATTTTCTTCTAAAAAAGGAAAACTACATATCGGGGGAGAGAAGACGGATGACTCATGGGCTTAACTTCATCTACATCTACAAATTGGGACAAGCTTGATGTCATGAAACAAGCATGGGACGAACTCTGCATCACATAATTGTACACGAAGTTACCAAGGAAAGTGGTTCATCAAGTTGGCTGTAATGTCTTTCACACGCAAAAGGTATGAATGTAATTGAATTTTCGTACGTTCCTTTCTTTTTACTTTCAGTGGATTTCTCCTCGCTACTGTTCTTGCTCTCGAGTTTCAAGAATGTTTGCATTTTTGTTGTTGTGTGTTCAAGCTCTAGATATGTTTTTTCAATCAATCTTGCTCATGCCTCCTTTTTTCCCCCTTCATTTTCCCCTGTTTCTTGGTTGCTGCTCAATTACTTTCACACTCTTAATTTTTCAATTCACTAACATTTGTGGGTAAATCTATGAGTATCATTGGAAGGCAATAGCGGCAAAGTAACAGGTGATGGTCGTAATTTCATTTGTTTTCTATTCCTTTTTATTTGGGTATTATGTTCAAATTTGGTTAGATCGAACTTCAACGGATCACATCTTCGATTTTCTTTAATTATTCCTTTGCATGTATGTAATGGTTTTTTTGTGTTGCATAGATCGACTTTGTCTAATGACTTGCCTGTATGCTACCATTATTGGAACAAATATTGTGTGATAACTCTCCTTCAGTACTATAAAAGTAGCACAAAAGTTGAATAAATGAGAAGGAAAAAAAGGATTAAAAGCTGAAGAAAATCAATTGATACATCTAATAAAGTGGGACACTGCCACATTGGAGAAGAAAATTGTAGATTTAGAAATAAATAGAATAAGAGTAAGAAATGGTAGTTTACTCATGAAGTGGTTATGGAGATATATTGTCAAAGAAGAGGCACTTTAGAGAAGGGTGAATATGAAAGTATGGGGTAAAAAAGGTGAATGGTGCATAAATAAGCTTATAGAGCCATATACAAATATGTATGGAAAACATATAAGAAGAATGTGGATCTTTTTGAACTAATGTACTCTTTGTTTTAGATGATGGAAGCAAAATAGTATTCTTGCATAACAGCGGATGAGGTCACCTAGTTTTATTTCAAGTTCTTATGGATTTAACTCCTTTGCTATTCAAACACATTTGATATACTTTTCAATTAATAATTTTGGATTCTTGCTCGCTTTAATAATTACGCCTCGACTAAGCTAAGGTGGCAAAAAACAATCAAGGTCTAGAGACAGAACAACAAGCAGCCTAAACTATGGGAAGGGTTAATTTCAGAATCTCTTAAATTTCAAGCCTTCAGGTACACAATTTCACAGACTTTTTTCAGGCTCTTCAAATTTTGGCCCCCTTTTCTTCAAAAAGAAACTTAATGTTCTGTTTGGTGTACTGATAATGATTAGTGACAGATTATGGTGTTGTATATAGTATTTAATAGTTGTTCTTGGATAAAAGTTACAGTTTTAGTATTGGGATACAAAACTTTAAAACACTTCACCCCTAGATTCAACTTTTGCAAGTGACCTAGATTTCTTTGGTTATTTTTGCAGTAGATAATTTAATCATATTTATAATAATGATTGAAAACAATTCTGAGATAAAGGGGATGATCATAATAGTGTGACTATTAATGGCTAAGGACAAACAAAGAGATTATTCTTTTGCCCATTGCTATTGGAAGACCAACTATTTTGCAGATTTCATGGCTAATTTTGGAGAGAATCAAAAAGGAGAAGATTTTCTTCACAGAAGCTCCGACTCTACAAAAGCCAAAGCTCCCCTGAAGAATGACTCAGACCAAGTTCCAACTTTTAGAATTAAAACTCATGTCAAGCAGTTCACATTTGTATAGGTTATGCTAATCCTTTTGCTTGAATTTTTCAGTCCCTAATATGTTTTTGGTGGTGTCCTCCAACATAGCTATAGATTTTTTTTATAGTCACTTAACTCTAGAATATGGATTATTGTGTATATTGCTGAATATTCATCCTTTTTGCAACTAGGAAAAGGTTTGGGAATTAATCTCATCCCTTCTCATCTTTCTTTATGAAATAAAAAAGTTGGGTTTGCAACCAAAAAAAATGGTAACTCTACGTGGGAATTTTCTATAGCATGTGGCCAGCACCTCTTCATTTTCATCCACTTAATGAGGTATATGTATGAATTATTTGAGACACATAATATGCATTGAAGAAGTTTTCACATTTTTGTGATTGTACCTATGGCCATGGGATAGGCTTTGCTTAAATAAATTGTTATTCGGCTTTTGATTTATTGAATTGCAAATCTCAGGTCAAGNN

>LNC_000726

ATACCTGTACCGCCTCCCCAAAAGAAAAAGGGGGGAGGGGGGATGAAATATAATTTTTGTTGATCACTTCTGTATGTAGAGATGATTTGATTACTTATTCTAGCTGAAGTTGAGCTGGAACATACATATTTGTGCTTAGGTACTTTTAGCCCAGCTTATGGAGACCAACATATGTGCATGTTTCTTTCATCTTCTATCATACTTCCTTTTTGTTTGGCC

>LNC_000727

GTGTTTAACCGATTGAAAGCGTGATAAGCGTCACTAACGGTATAGCGAGTAGGGCGTTTAACTGTAACTCACCGGTAGGACAGATAACATTTGTGGTTTGAGTTCGCTTATAGAAGTGGAAATCCGACCTATATTTATTTCCAACTAAAGGTAAAGGCGCTTTTCGCTTCACCGGAATCCGACGTCAATTGGTTACCGGTTGGTAGTTCCTCATCGTGCATTGACTGACTCCTCATGGGGTTGAAAACAGCGCTCTGAAACCGGTGGGAAAGAAAAGCGCAATCCATTCCGGTGGGTAACAAACCTTAAGTGAGGTTTATTCGTCTCTA

>LNC_000728

AACAACCCGACCTTGTGCGTCTCCTTCTTCACGAAAATCCCAAAAAAATTTGGAAAATCCCAACAAATCACGGAAAAGAAGGGCACCAATCAACATCGCGTCCAATCATCCCCGTTCCCGTCCCCTTTTTCCCCTTTTTTCAGAATTTCCCATCCCCTTCACTATTGCCTTGAAAAAGAGCAAAGAGAATTCGAATTTCGAATTCAAAATCATCCGAATTTTCTGCTTGTTTCCCCCTCAATTCAGCCTATAAATACCCCTGCTCTTTCAATGTTATGGGGGGATAAGGAAGCACAATAATTTCTTACTAGATATTCAAGAATAGGCTTATATTTTCCCTCTCGTTTGCCCCTAACCGGAATTTTCCGAAGCTCATCGGAGCTTCATTTGTGGTGGTGTGTAGCTTCACGGTCAAGCTCGTAGTTTCAGTTCGCTGCCCCAGAAAAGGTAACCTTCCATTTCAGATTTTAATTTCTTTTATCATAAATTAATGGTTGCTTAGTTTCATGATTTGGAGTCATGATCTTTCCGTTCAAATTGATGTTATGGCTATTTGATTTCGTTCTCGTCTTAAATATGATTGTTTTCATTTAGTTACGAGGTTTGGTTTCTTCCTGTTAGCCCCTCTCTATTTCTAATGTTAATTTTGTTATGTTTCCTGCTGGATTTTTGAAGTGTTGTTGCTCTACGCCATGGTTTCGGCTTCTTGTTTGAGTTTTCTCATATTCATAGCATGTTTTAATATTTAGAAATTTGTCAAAGAGTGTTCCTATCTATATTTAACTTTTCGGAATGTCAAAATGATAGTTACCATGAAAGTTTAAAAGTACTTAACCTCAGAGGAAAAGCCGACGCCCAGTAGCGTTACTATTAGCTGGTATTTTATTTGGAGTAAGTTTATGATCTTTATACTTGTAGTCACCTCAAGCTTGGTTAATTTCTTGAAATCCATGCATGTCCTGCACTTGTTTTTTTTTTTTTGAAATTCTAAGTGTTGTTGTATGTTTCTTTTTCCTAAACTATGCAATATTTGTTGTTGAATAGTCACATGAATTTATGTGTAACTAAGGCATAATTGATAGCTCTCTGTTTCAACTTTAGTGCAGCCAATCTTTTGGCATTTTGCTACTAGTAAGATGACAGTAAGTTTAAATATTGTTTGAGATGAGTTTTTTTTTTCTTCCTTTAAATTGAATGCATTTTGGTGCATAATTAATATAGCGTAAATGACGTATGATTATGAATAAGATTTTCTTGATCAATTTTGTTTGCCTGGTGGTCTACAAATGTCCAATTTTCTTCATGTAAAGAGAATCTCAGTTATGCTATTTGTTTGCTTATGAATACATCTTTGTTAAAGTATTGGCCTGAGCTAATTCAATGACATGTGCTTATATGCTTTGGTCTCATTTATTGTATGTGTATACGGTATGTAAATGAATGTTCCCAAACTTGAGTCTGAGATAGAGAACAACCATTGGTCGAATTTCCCCTCCTTCATTTGTATAATTAGTCAAATTTTTCTTTGGTTTCTAGCCATATGCATTTTCATAATCCACTTTGCCTTATTAGGATAATACATATTTTCATGAAGATTTCAGTGAATGTTGTGAATTAGTTAACGTGTTTCTTTATTTATCTAAATGTTGTTTTCTTTATGCTAATATAGTTCTTTTTTCTTTTTTTCTTTTTTTGTTTATTTGTTGTGAAATCCACCTCGGATGGAGCCCAATGGACTCCCTTTTCTCTTCGCATTTTGAGAGGGTCATAAAGGCCCAAAATTCTTTTATCGAGTCATCCAACCTTGAGAAATCGAAGAACAAGTCTTTGGAGTTGACGAACAGG

>LNC_000729

AAAACAAATAGAGATTGTTTATGTTGAAAACATAGAAAATTGATGCCAAAATCTTGCTGCATGTGCACATCACCAAAAAGAAAAGACGTGTTGCAATTCTTTCCTCCGGAAAATTGTTGGTATCTGCACATCTATTCTCAATCACCGAAAAGAGATGGTGTTGCAATCTGTAGAACATCAACGTAACTTGAATTGGCAGAACAATGTTAAGACATTGGTCAGTCAGAATTCATATAGCTGGGTTTAACTTGTTTGGGATTGAGATGTAGTTGTTGTAGATGAGGGCACTCGGTCATGTAAATGATAGAAGCATCAGTTCCCAAGAGATGAATGGTGCTTAGCTATCGTAGAGAGAGGAGGGACGCTTGAACAGTCGTCCAAAGATAACGACACTAATTGCCAAAGAGA

>LNC_000730

AGAACTCAGGGAGGAGGGTTTCACGTCCACAAGCGACAGACATCACTTGCGCTATGCTGAAAGCAGCAGGAGCTCTTGTGAGCTGTGGCTTGGAGGTGTTGAGGAGCTTTTCTTCTGAAGCCAGAGGAGATTTTCTTTTAAAAGCAACTACTACTAGAGGTTTTGACTCTTCAATTTGATGTCTGTTAAAAAATCAGAATTACATTACTATCTATGTCCCTGTACAATTTTACATTTATGTCTCATTCTCCTTATGTAGTCTTGGCTGTGTAGGCACGTGTTCCACAATAAACAAATCCTAAC

>LNC_000731

ACGGCACCCAGAATGATCTTTGTACCCTACCCTTTTCCTTCCCATAACACATCGAGCGCATTATTAAGGGGTATGCTACGTCAATATTGGACGGAGAAAGTAATTAAAAGTGTAAAAGACAAATCTACTGGGAATAACAGGGATGAGGAGTCATGAAGAATATTAACTACCCTATCCCCACTGCTTAGCTAAGCAATTTGTATTCTAGTGCTGCAAATTTGCCATAAGCAAAGATCTTATAGGGAGTTCTAAAGGTGGCCTTTCTCTTTCTTTGAGCCCACAAAGGGAGTAGAAAGAAATGATCTCATGAATCTCTTTTTTTTAACAAAGCAAAAATGGAGGATCTAACAAGAAAATGCCACAAG

>LNC_000732

GTACAAAAACTACTATCAAAGTTCTTAATTTATTGGGTGTTTTGTTTATTTACAGGAAATTAATAAATCTGCTCTTTCCGATTGTTGAAATTTTTCCCAAAAACAGAGAAAGACCAAATACAGTTAAGCAAAACGACGCGTATGCCTAAGCAGCTCAAAGTTCATTCTCCCCTGCAATCTCTCTTCTTCCAGGTATGTTGCATGGACAAAATTTCTATGTTGAATCCACTTCTTCTTCGTCTCCTATGCACAAGACTGTAGACCCATCTCAATTTTGCATTCATGGGTCCTATTATATAAAAAAA

>LNC_000733

ATAAAAAAAAAGTTGAATAGTGGTTCAAGGTATTCTTTCGTAGAGTAAGTTATCTTGGCCTTCTTCCTTTATTTCCCTTTTATTTTATTTCCTTTTTTTCTGTTTCCGTGGCTTTTCCTATTAATAAATGTTGTTTGCATAGAACACCGGTGCCATAGAGTTGAGAAGGTTTGCTTTCAAGAAAATAGATCTAGAATGTACTGAACTGCTAGTACGGACAGGTGCTCCATCGAAGCTAGGAACTGTGACCAAGCTATTGGCATAGATATGTGAGAGATATAGCCATTTCAGGTATGGCTTTGTCAGAAAAAGTTATCTTAGTGTTTGCCCTTCAGTTTCCTCCTAGTTTATTACTTCTTCTCTTTCCATTTTAGGTGTTTCCATTTGGATCGTCAATTGTTTGGTGTTAAAATTTCAATTTAACTTCACATGTTTATTGATACTCTTTGGACTCTACTGACATCAACTTAATGGGGATACTGTTCTTTGTGGTATTGATGTCTTAAAACTTTGTATGGAGATGTGCTTGTTCTATCCGATCTCATTCTCGATGTCATGGTCTCTGTTGGTTTGATTTGTGCTTAATTGTTACTTTGTAATTGGGTTATACAGTAACATTATATGCTTGGTGCTGCAGTGTCGAAAAAGAGCCCTCTTCAGAACTTACTACTTGGGCTTAGGTTTGCTCACATATCTTCCATAACTAGCTAAACCTGTCATGGGCTTTTTAATTTATCACCTCCCAAATTGCTGAAACACTTTCTTTCTGCAGCATTATGATAGGCGTGGGCATTATGACTTGCTATTAAAAAATGATGAGACAAAGGAACAAATTCAAACTATTATCAAGGTATATTTGCTGATGTGTTCACCATTCATTTCTCTTACCTTTACTGCTCTTTTGTTCACTTATAGATCTAGCGACATCTATGGGTGATTTATTAAGCGGATTTTCCGAAAAAGGGTTTTTAATGAGACAATGCTTCCAACTTTCAAGTGTTGGGAAAAATAAATGTATGTGCAGATAGTCTTCAATAACTTGTTACAAATAATGTACTGATCTGTTTTTTTCCTAGTAAAGAGTGTGCTTACTTTAATCCTCAAAAAAAGAGTGTTTACTTTGGTGAGTATGCAATTGAAGTACTATAGTTGGAATCGATAAATCAAATTCTTTTGGTCTTGTTTATTATGTGGCAGTTTATAGTGTTTCAGTGGACGAGTTCCGCTTGAGCTTTGTATTGCTTCTCGGCGGTGTAGACTCCTTAGCAAGGAAGCTGGAGCTGCTTATGCTCTTTTTGATCTCTTCCATTGTTAAAGGTGAAAAGGTAAGAGCTGAATTATCAGAGGTAATACAGCCAATATGATGTGACTATGATTGACAGAATTATTACTGGAGAAATAATCTCTTCTCTTGCTATTTGGTGAAGTACAAAGGTATATGTCATTTTGTATCAGATTTTGCTTACACTTGGAATCAGTTTTTGTTTACTGTTCAAGGAATCAC

>LNC_000734

AGAAGCTCCAGAAAGGTATTGCCATAAAAAAAAAGTTGAATAGTGGTTCAAGGTATTCTTTCGTAGAGTAAGTTATCTTGGCCTTCTTCCTTTATTTCCCTTTTATTTTATTTCCTTTTTTTCTGTTTCCGTGGCTTTTCCTATTAATAAATGTTGTTTGCATAGAACACCGGTGCCATAGAGTTGAGAAGGTTTGCTTTCAAGAAAATAGATCTAGAATGTACTGAACTGCTAGTACGGACAGGTGCTCCATCGAAGCTAGGAACTGTGACCAAGCTATTGGCATAGATATGTGAGAGATATAGCCATTTCAGGTATGGCTTTGTCAGAAAAAGTTATCTTAGTGTTTGCCCTTCAGTTTCCTCCTAGTTTATTACTTCTTCTCTTTCCATTTTAGGTGTTTCCATTTGGATCGTTCATCCTCTCTATTGCATTTGAAGCGATTGTTTGGTGTTAAAATTTCAGTTTGACTTCACATGTTTTATTGATACTCTTTGGACTCTACTGACATCAACTTAAGGAAGATACTGTTCTTTCTGGTATTGATGTC

>LNC_000735

GAAAAATCATCAGAGAGACAAATAGTTGTTTTAGGTTTTTGGCTTTCCCAGAGAAAGTTTCCTTCTCCCTTCACTTCCCTTTTCTAGTATTTACCTTTTCTTTTTCTGTTTTCTTGTTCTCTTTCCATGGTTGATTTAGCTTCATGGAATTGTGATTCTCTCAACTGTTTCAGCCTTTCAGGTGTTTTTGCCCATGCCCGTCCCGTTTCACAAGTGCTCCAAGTGGTACAACCGTCGCTAGAAGCTCCAGAAAGGTATTGCCATAAAAAAAAAGTTGAATAGTGGTTCAAGGTATTCTTTCGTAGAGTAAGTTATCTTGGCCTTCTTCCTTTATTTCCCTTTTATTTTATTTCCTTTTTTTCTGTTTCCGTGGCTTTTCCTATTAATAAATGTTGTTTGCATAGAACACCGGTGCCATAGAGTTGAGAAGGTTTGCTTTCAAGAAAATAGATCTAGAATGTACTGAACTGCTAGTACGGACAGGTGCTCCATTGAAGCTAGGGAACTGTAAGCAAGCTATTGGCATAGATATGTGAGATTATAGCCATTTCAGGTATGGCTTTGTCAGAGAAAGTCGTCTTAGTGTTTTCCCTTCAGCTTCCTCTTAGTTTATTACTTCTTCTCTTTCCATGTCAGGTGTTTCCATTTGGATCGTTCATCCTCTCTATTGCATTTGAAGCGATTGTTTGGTGTTAAAATTTCAGTTTGACTTCACATGTTTTATTGATACTCTTTGGACTCTACTGACATCAACTTAAGGAAGATACTGTTCTTTCTGGTATTGATGTC

>LNC_000736

TTTCACTTTCCATGGTTGATTGAGTTCACTCATGCATGTTTCTATTTTTCTATGTTGTTTTCTAGAACATCTGTGCTATAGAATTCAACAAAATTTGCTGTCAGAAAGATGGCAATTTTAGTATGTACAAAATGCTATGTTAATACAGGCGCTCAAAAGTATGGAAACCATTGCTAGAAGTTATGAACCATTGGTGTAGCTATGTAAGATATATAGCTCCCTTCTGTTCCCTTTCTTTTATTTACTTTTATCCTTTCATATTTGATTAAGCTTTTTCAGTTGTACACAATTCTATTAGTGTATGTTGTTTTTGTATAGGTCCTACAGAATTCAGAAAAGTTTGCTATAAAGAAGAAAACAAGTCTAGGATGTATACTGCTGGGTGATCCAAGTAGAAAATTCATCACAGAGACATATAGTTGTTTTAGGGTGTTTGGCTTTCCCAGAGAAAGTTTCCTTCTCCCTTCATTTTCCTTTTCTATTATTTACCTTTTCTTTTTCTGTTTTTTTGTTCTCTTTCCATGGTTGATTCAGCTTCATACATTGTGATTCTCTCAGCTGTTTCAGGTGTTTTTGCCCATGCCTGTTTCACAGGTGCTCCAAGTGGTACAACCATCGCTAGAAGCTACAGAAAGAGTTCAACAAAATTTGCTGTCAGAAAGATGGCAAATTTAGTATGTACAAAATGCTATGTCAAGACAGGTGCTCAAAGTATGGAAACCATTGCTAGAAGTTATGAACCATTGGCGTAGT

>LNC_000737

TTTACTTGTTTCTTGATTAAAGTTGGATATTCTCATATAAAATCAATATTGGGGCATCCTGCATCCTCTTAGATGGTTGACAAAAGCATCATGGCATATGATTTCACTGCCTACTCATGAGATGTTCCACACTTTATTACAAGCCATCAACACTTGTCACCTACTCTACAGTTTGCTTCCTTACGTACTTGTCAATACACACTAGATCCACTCGATTTGTTGGTATGGAGTCATGGCCTCACCAATTTTCTGATGCTACTTTTTTCAACACTGCCACTTCAGAAGACCGCTCAACTGATCATTATAACTATTAAAGAAGTAATGTTTATTTATGTCTGGATAATTACAAGTCCCAGAAAATACCTTTCAAATTTT

>LNC_000738

GTTTCTATTGGATTTTATTTTTTTGAAACTGATAACTTGAGTTTCTATTGGAATTATTGCCTTGGAACACATACAGGAATATGATTAAAGCTACACAGTACATGTACTTGTTGATGACTTTGTGGACCTACTGTTCAGAAAAGAAATAACAATGTTGCCAATAAAAATTAATGTCGACGAACTTTATTGACAACATAAAGAAGTTCTGGGTAGATAAGAAAGGATGAACTGTGTTTGCTGACAAGTGAAATAAAAGGAAAGAGTACCAGTATTTATCACCTTTTCCACTAAGATGATTTAGTGGAGAATATAGTGTTCATCTAGTTTGAAACTCTTAAAATCTTAAGAAAAATATGCATCCTAATGTTATTTACTCGACCACTTAAATAATAATTCACTGAAGGACAAACAATAATAACAATGACACAGAGCTTATATCTCCCTTTAAGTAAAAGAATAACACGGTGGTGTGCTTGAGTGGATGGATCACCTTTGGTGAAGGTAAGTTTGGTAAAGTGCTGGCTGTTTGACTCAGTTCCTCAAAAGTTTCTCTCTTTGTGGTCTTATTGTATTTTTATGCCCCCTCTTAGACTTGACTTGGTGAAGCTATGCAGAATGAGAAGTCAAAGTACACAGTAATTCTCCGTCATTCTCTC

>LNC_000739

CAAAGCCTCTCAAAGGCTGAAAAAATAGGGTTAGGGTTTATAGTGTTAGGTGTGTTTGGTCGGTTTTTCTCTACCAAGCTAGGGTTTTCTTCCAAGAAAAAAACATGAAAATTATTACATATATATGCCCTTGTAATTTTTTATTTTTTTGTAGTAGATTTATTTTTCATCACCTTGAAATTAAGTCACTGATCTTAATGTTAGAGAAGGTGCTGTTTGTTTTCCAGGGAGGGTTAGACACAGAGATTATGTATTTTGGTAGCTAATTAAAAATTATGGTGACAAATGTACTCTTTTAGTAATTAATTGTTAGATCTTTTTATGTAATCAGCAGTGATGCTAATTAGTGACTCTGTAACTGTATTCATATTCATATATATATTATTTTTGTTTATTCTTGTTATATATATGTACTGTTTCTCACATGAGTCATGTAAATTTTGTTTTATATATACAGACTAAAAATATTTAGTTTTAATTTTATTTTGTATAACAATCGACTTTGACCTTTAACTTGTTTGAGATTGAAGCCTAGTTAATTTATAAGTATAGTTTAGTGTTTCCTCATTTTCAATTCATTACTACTTCGACGGTCAAATAATATAGTTTCTCACCACAATATTTAAAAAAAAATTGAATTCTCAATTTACATGAAACGTTAG

>LNC_000740

TGAGGTTTGAGGACTACCAGTTAGCCTTTGTTCTTTGGTCCTCTGCAGCCAGAAATGACTCCTAAAGTTGGAGCAACACTTCCAACAACCAACGCTTCTCACCCATCAACAGAAGGGTCTAGTTGCTCAGAAATTGGACAAGGAGGTGGTCAGCCAAGTATCAATGACCAACAGTGAGTATCAAACTTAACCATTTATTGTTTTACTTACTGCGTGAGTTATAAAATTCCTAATTTTGACTTCTAGACTTTCCTGACATTCTTTTTATTTCCACTGCCTAAGTTATTACTCCACAGCCACGTTTTAACCTGATAGTTTTATTCTTCTTCTGGTTACTAATTTACAGATATGTGTCTCAAAATCCTACTAAGACGTTCTTGAACAATGATCTATTGGCCTTTCCCATTCAGCATTGCATTGTACATTATGCTTATGTACTTGTGAAAGAAATATGATGCCTGTCGGATGCAGTGTACATATGGAGAAGTGAGAACCTTGTGAGATGATGTCTGTTGGGTGGGCTTTGTAACGTTGGTACCTTTGTCTTTGGTGCCTAGATTGTTATTTTTTTAATTGTCTCTGGCCTTTTTCTTTTTAAAACATTTTCTTGCTATATGTATCAGCATCCTTGGCATACTCGTATTGGTAACCTTAACATCTTTGACACTCAGTACTTTGGTATTTGTGATGATTATATCCATGTGCTTTTGCTTTGTTTATCACTTTTGTCTTTGAATGCAACAAACTGTGTCTTG

>LNC_000741

AAAAGTGCTTCTTAGAGTATCATCACCTATCCAAAGCTCATTCCAAAAATCAATCTTCACTCCATCACCCACTCTGAATGAAATATTGACGTTGAATTTTGTCCATAATCTCCTGATGGTTTTCCATACACTACACCCAAAAGTACCCAAAACTTCCTCAGTTGTCCACTGATTGAGCAGCCCATATTTTTCTGTGATGAACCTTCTCCACAGAGCCATATCTTCTATGCAAAATCTCCATAACCATTTTTGTAGTAGGCATGCATTCTGTTTGCTGAGATTCTTTATACCTAGGCCCCCTTGTTTTTTGTTGAGTGTTAGGATATCCCACTTCACCAGGTTGTACCCTCTCTTCTCCTTGTTTCCTTGCCAGATAAAAGATCTTCTCAGTTGATTGATTCTCTTTTCAATGATTTTAGGTATTGGGAAAAGACTCATCATAGAAGTGGGAAGGCCATCCAACACAAATTTAATGAGTGTCAACCTCCCTCCAAGGGATAAATATTGACTCTTCCATCTTGTAAGCTTCTTTTCACATCTTTCCAACACTTCATTCCAG

>LNC_000742

AATGATCAAAGGTTGCTTAGAATTATTTTAACAATTTAAGGATAATTTGCGAAAAATGAAACTAGCTTTTACCTGAAGTATTCTAAATCTCGAGAAGAAAAACAAAGATGAATGAGTTTAAGTTTATGATGTACCAGTTTTATTTAACAGAACTGCTTTTGAGATGTGAGCAAGCAAGTGATGAACAAATTTTGAGTTTACCCTTGGGTCTTATATTTTGTTGGAGCTGGACAATTGAGTAAATGAAAAAGAGTTAGAACTTTTACGAGGTTCTTTATACCTGCCTGGATACTTCAAAATGCACTAACAGTAACAG

>LNC_000743

GTACTTTGTTCTGGTGAATAAATGCTTCATCTATAGACTTGAGGCATACACATCATGTGATCAGGTTCTGCCGGGATTGCCTGCTTGGGCAAAATACTTGAGTATTAGAATATCTTCATTCATCCAGAAGCCCTTTGACTGTTTAACATAACGAATTTACCAATATTTTAAATAGCAGCTTATAAAGTTAAACAAACCCCTAAAACTTAAATCTGAACCTAAAGCCACCAACATGCCCCCAATATGTTCATGCAAAGGAAGTGGTATATAAGGTTTACTAGCATGCTTCAGTAACAGATGCATTGCTTTGGAGTTGGACTGTTTTTTCCTTGAGCTGAGGGTCTATCAGAAAAAGTCTCTTTACCTCCAAGGTAGTAAGGCCTGCCTACACTTGACCCTCCCAGACCCCAATATTTAGGATTACACTGGGAATATTGTTGTTGCTTCAGTTACAGATGCATTGTGCTCTCTTGTATTGTTCTGTACTGGCTTCATGTTTGCTTCACCTACTGAATTTTATGTCTATAATGTACCTATGTAATATCAAATAGGATAATTTCTAGATGGTTAATAACATCATTGCTAAGACAAAGCTGAATGTTACCAGAATTTAAGTGGAAGCATAGTTGACAGTAAAACGAGGAGGCTCTGACTGACCTTATTTAAAGTGCCTTGAGGCTTTCGGTGTTACTATGTTTTATCGTTTGTTGAGGCTCCGCCGTCTGTTTATTTAGAAAAGATGGGGTAATCCATAAAAATAGGTATTAATCAATAAGCTTTAGAAGCAAGCATCCTCTATTAAGTGCCTACTCCATATAAAAAGAAAGTGCATCCATCTTTACAATTAAGAATCCCTTGTGTTCTTCAGTTGGAGCTTTAGAAGCTTTTCTCTTTGTCCATAAAGATAGTAATGACTTTGCTGTATTACAGTAATACGTACCATTTTTTTAAACAAGAACTCTTCTGTAGTACAGTATTGCATTGCTTCCCAATCGTTCAGAGAGATCATTGTCAGCTTTGATCAGTAATGCCTGCAGCTAGCATTCATC

>LNC_000744

AAAAAAAGGAAGAAAGAGTATGTTCTTGGAGCCTCTTCCTGTGAAGGAGTCATGAAATCGATTAAAATAGAGAAAAATAACTAAATGGAGAAGAAGTGGAACTCTTGCTATTTCAAGTATGGATTTCAAGGGTGAAGTAATCAACAATGAGACTGTTTTGCGGGATCTGAAGCACCAATTTGATATAAATTGACAGATTGAGAGTCTATCATAAAACCTCGAAAGATTTGGGTATTCTGAATTTAGTATGAATTAGCCTAAACGAAGGAAGTATTACGAGACCGATATGATGTGATTATAGATTGATTGAAGGAAGTCATACAGATTGCCCAAGTGACAGATTTGAGAATTCAGGTGGTATTGAAGAGGATTTCATTAACTAGCGTGTGAGTTGGTAGCATCTGAATTGGAATGTAATATCGGATTTTCATGTCCATTCCTTTGAAAAATCTGTGTACTTCATCAAGAAGGGGCTTACAGACTCCAGGATCTGTCACTGCTGTATAATGACAATCAGGGCAAAGTTTCCGACCATCATTCAGAGTGATATACTTCAGGTCTCCTATCTACAAATAGCTGCAGCACTGAGGAGTTCCATCAGATGTATGCTTTTCACAGTAAGTTTGGCCCCAGTATGTACACCTTGACCAATCCGAGCGAATCTGTACCAAACATAAGTATCAGTATTCCGATGGTGTCAGTATGATCAAATGGAAAGC

>LNC_000745

ACTAAATATAGAAAGAGACAGTCTTTCTAGGACAGATCAAGAAGGAAAGTAAGAGCATGTTTAGATTGGCTTATTTTAAGTGTTTTAAGCACTTTTTTAGTGTTTGGGTAAAATAGTGCTTTTAAGCACTTGTGTCTAAGCCAAAAACTACAAGTCCATCCAAACAGGCTCTACGACACTTAAAATTGGGATGGAGGGAGTACTTCTTTTGTTGTGGTACTAAAGTATGTATTGCTAATACCAACATAATTATGTGTGATTCAGTGTATTATCTTATGCGGGATAGGATATGGAATAAAAATAGTAATTATCGATACTTGGATAAGAGTATGTAGAAGCATAAATTTTGCTTTTAAAGTAGACAATTATTCAGCTCATAACAATCCCTACATTGATATTCATTGCATAACACTCCTTTACCATGATCCCTGCATAAACTAGTCCGCAAACCAAATGGCCCCTCAAGGTATGAATAGGGTGTGCCTATTTCCTATACAAAGACCTTGAGGACATGTAACAAGTGGGCAGAGTAAGGCTGGCATGTCTCTTGGTCAGATGCCTCTCCAACTCTTTTAAGCCAAGTATTATCATATTCATGTTATGAATATCTCTTTTCTTGAGAATTGCTATATATGCCAAATATACTTGTCAAATTTTTTGATCACAGCAGCGGTAGATCACTTGTCATAAAAAAGATATGCCATTAATGGGAAATACTTTACTTATTTCTAAAGATAATTTCTTTAAAAAGATTGTGGTTGGATTTTGTTATGGTCTTTATCTATCTCTATCACAAACCAAGTGCAATGACTGCCCACTCTTTTAGGAGATATTGGCATATCGCATTGTCAAGCCAGTTGTACTCTTTGGTTGTTCTATCCTGTCTGATCTAATTGATGGTAATTTGCGACCCCTCAGCGGTCGCAATTATTTGCATTAGGGTGTAAAAAGCTATTGGGCCCGCCTAAGTGCGGGCCCTTGATGGCTCGCATGACCTTGTAGGAAGGTCCTTCCAATGAGTTGCAGTATGCTGCAAATCTTGTCCGCGC

>LNC_000746

CTAACAGGGTCTAATGGTTCATGGGAGAATAAGGAGAAGAAGAGGGTGGTCGTGGAGTTGTTAGGATGGTTAACGGAATCGACCATCATGCCCAAAAAGATCCGACCAATTGAAGGGGTGGTTCTTCTTCTTCCTCGTCTCAGGTGATCCTTTAATTTGTTGCCACTGTTTCCTTCAATCTTAGACAAATTAAGTTTTATGACAAAGGCCCTGCTGCTTACGGATTACGGAAATTAGGTCGTAATCTTTTTTCAAATCTTTTAGATTATGCAATTTTTGCTTCAAATATTTACGTATTTATATTAACTCTGCTAATTTAATTGTTCTGATTTTTGTTGTGTAAATTCTCTGTCCTGCAAAATCTTTTCTGATTTACCTTTAATTCGATCATTTTGGACAACTTTTCCCCCTCCGAG

>LNC_000747

CCAACCCCATTTTCCCTTTCAATTTCAACGCACACAACAGCAATACAACGACACAGACTGACCCTTGTACATCGTTCTCATTCTCCCTCACACATCAATATCCAACAACTCCTACAGCTTTCTCTGCAACAGCCTCCCTTTTTGACCTCTTCATCCGGATATCCTGGGATTTTGGGAAAAATCTAACACAAACGAACAACTTTCCCATTCCTTGGATAAAAAAAAAGTTTTGAAATTGGGGTCGAACTCAATTTTCGATTTCACCCTTTTTCCCCCAGAAAAAGTCCGAACCCCAATCCCATTTCTATAAATACCCCTTTCTTGTTCTTGATTAAGAAAAAAAAGGGAAGGAAGAAAAAGATTTTCACTATCTAAAAAAAATATATATAGAGGTTTCATACATTGAAAGGAGGAAAAATAAAGAGCTAATTTTCTTGTGTATCGATTCTTCGCTTGTCCGAGTTAAGTTCACAGTGGTGGAATCGCTTTCAAACTCGTTGTCGTCCAGTTCTCTGCCCCATAAAAGGTAATACTTGTTTTTGATTCATGCCTCTTTTGTGCCTAATGTGAGGACGATTTACTTTTGATCGCTGTGTAGTGTTTTGCTCGAAGCTTTACTAATTCTGTATGGCATTGAGTCGGTTTCTTTCTATTTTCTTCTTGTTATTCTAACTAGTATAAATCATGTGAGTTGCTTTTAAGACTAAGTTCCGTAATCCACCTGATGGTTGCTGCACCTCTAAATCTCTTATTTAATAATGTTGGTTACATATCCTTTGTTATCGGGTAATTCGAGTTAACTTGAATCAATAAATTGGTCATTTCCAAGCCTCTGGAGCATTTTTTCTTTCTTTCTTGTGCTAAACTTGCCTTATTAAAATGTTCTATTATTGCTAGTTAGTTTCTCAGTTGTACGTCCTTATCATCTTCACCCGTAAATCATGCTAAAATTGAATCTTGTCATCCGTACTCAAGTTGGTTTTGAATGTCTCTTTCTTCCGCTTCTCTCAATTGCTTGGGATTTATTATCTCATTCGTGTAACATGTCTTGTTTTAATCGAATTTATGTCTCCATTTAGTCTAAGTTTTAGTCGAAAATGCTTAAGTAAAATGTAATGGATCATTTTTTTTTTGTTTCTTATTACTAATTTTCTTATCTTGTTGGCTTTGTTGTTCAAACTCATAAAGATTACGATGTCGATAATTAGGAGGTCGTAGGATTCACTTCAACAATTATCAAAGTAGTTTATGTTTTGCTTCTGTTCTATTTTGATAGCTACTGGTTTATCTTCTCCTTTCCTTATAATTGATCTATTGTTTATTTGTTTAAAGCTTTTTAAAGTTGTGACCATAAAGTGGAAAGTTGACTAAATAAAAATGATTTTACTAACATTCCACCTGATGCCTTCCAAGATTAGTGTAAGTTTAGGATTATCTAAGTATTGTGGCCATATTAATTTTATTTGCCTCTAGTTCATCGTAGAATCTATTTTGATTTAGTCAAAGTCGTGTTGTACCATGGTTAGAATGTTAACATTATATTGAGTTTTGACCATACTGTGAATAACCTCTTTGCTAGCTTTAAATGCTATGATATTCTTATTTTAGTTGAATCAATTTGGTATCTACATTCCTCTTCTGCTAATTCTTGTTCTTTTTTAAAATTTTTATTTTATGTTTTTC

>LNC_000748

CACTGGCAAAGTATTCTGCAGACGATATTTGATACTATTAATATGAGTAGTTAAAATTAATAGGGATAATTGTACATAATGGCAAACTTTTAATGTAAAATAAATACGGTAGCTATCGTTTCATTTATTTCCTTTAAGGAGCAAAGTCTCGGTCAGCTTGCTATTAATGGACTCCACCGATTTGTATAAGTAAAAAAAAAAAAGTTGACGTTATTCTCTTCCTTTATACAATATCTTCTTCTAGTTTATATCTCTTATTTCCTTCAATTTCTCTCAAATCGTCTCCCCAAAATTAGTTTGAAATTTCAAATCTCCACCAGCACGTTCTCAATCGCGGTTTCAAAATCCCTTCAAAAATTTCAGAGTTTTCCTTCAAACAATGGATGCCTAATCTCCATCTAAAAGATTCACAAGAGGTTCAATGTCCAAATCCAACACAATGTAAGAGATCAAATCCAAATTGAAAAATAACCCAATCAGATTGGAGGGAATGAGTGCAAAATCGAAGCAATCCAACATCAAAATTGCGGAGGGAGGTAAGAAGGATCAGTCTGTTGCTGGGTCAAGTAAAAAAAGGAAAGAAAAAACAACTAATTATACAGAAGAGAAAGCAAAAAGAAAGGAAGTCGTTGTTTCTGGCTTATTGG

>LNC_000749

CTTGAACATTCAGTTTTCTTGATTTTATGAATTTCTTGATTCATGTCATGTTAACAATCTTATTCTTTATGGGCTGTCTTGGGATTTTTTTGGTGATGCAAGTTGTTTTTCAATAGATCAAGAAAGTAGTAATTGGTTTTGATTCTTTTGTTCTTATAGCTACTTGTCCATTTTTTTTAACATGAAATTCACTGCTTTATCTTGAATAGCTTTTTAATGATTCTTAATTGCTATGGAGCTTTATTAGTTGTGCTTTTTCTTTTCTTTCCGTATGACTACATAGGTTATTAGATAATCTGATTATTAAATTTATCACATGCTTGTTTTCTTGATATGCTTTACTTGAGTCGAGGGTCTATCCAGAATAGCCTCTCTACCTTTACAAGGTAGGGATAAGGCCGAGCCCCCACTTGTGGGATTATACGTTGTGTTTTGTTGTTGTTGATTTTGAAATTTAATGTACTTAGGAGATATTTTAGGCTGTGGTTTCAATACATTGCATTCTCGTGCTTGATACTGCGGTGGCTATTGATTCTCTTGCTAATTCATGTTGAACCAAAAGAAACTTAAAGAAGCTGAAACATCCCACCTTTTCTTTAAATAACCTTCTACAATCTTGAACTTCGACTAAGTCTCAGGATTGTCAATTTTTATGCTCTTTATAGGAGAAGTGTTAGAAGCAAATGAGTTTAGGCACATTCTATATCTAGGGGAAAGTTGGGATAGTTTTGTTGTTTTTGTAATCATGGGCATGTCTTTGCATAGTTACTGATGTGATTTAATTTTTTACCATATCAAGCAACTAAAAGTCTAAAACTAGTGGATAACCTTTAAGCTGCCACAAATGTTGAAGCAAGTACTTGCGGATATTGCTTGATTTATCTGGTTCCTTCATAAACTTGATTATTGTGTTGGTCAGGAGGCCGATGATAAAATTTTGCTAGTTTTTCTCGATGTAGTTAGGTGGTTAACATATCACGCAACTATAAAGTGCAACAAGTTTGTTTTGTAATTATGGCGGAGAAGAACAAAACCAATTGGCTAAAAATGTACTTCAAATAACCCTATAATTGTGAGTTTGAGCCTCTTGGAGTGGTGGATATTTGTCAAGTTCAAATTTGAATGGGTTCCCAGATTTCTTTCATGTGGATTAGATTGGGTGGTTTAAGTGGAGTGTTGGTCCACTAAAAGGTCACTGTATCACTGTTGTGTGCTCTTTTTGAACATGAATCTCGAGGGCATCATCATGTTGGGGCTTCGTGACTGGTTCTCTGCCTGAGCATTGAAAGGATGAGCAGTGGAAGGATGACAGCTGGTAGTTTGTGTCCAGTGAATGGCAGAGATTTTTGGTTCATCCAACGTATAATCACGAGGAATACACTGGTTTTATTGAAAAGCTATGTTGGGTTGAAAAAGACTGTGTTGGGTAGAAAACCTAACTTCTCTTGCTCGCGACTCCTTTATCCTTCTATCTTATTTCCATTGATTTTTTTTATTACTCCCATCTCAAGGCATTGTCGCTTTTGATTCCCTGCTGGTTTCCCTAATGCTCGAATTAGTAACACTATCTCCCATTTACCAAACAGATCACATATCATAATATCCCATGCATACTCGTTCTAGTGAATCTTTCAACGTTAACTTGAAAGTACACACAACATTCCCAGCTCCGGCAAATGAAGTTTCTTCTTGTCAAGTGCTCAAGTAGTTCCTTCTATCATCGAGATAGAATCCCCATGCTCAAATGAAGGTTCTTCTTGCCTTTATTTTAATGGAAGTGATGTTTTCTTATTGTTTCACATTTTATTTTATTCCATATAAATTTCAGAAAAAGTTGTGCCAATATTAGTTATAAAACACGTGATACTAAACTCTTGCATAACCTTTGATAAAAAGGTAACTAAAATGCTGCATTAGTAATGTTGAGATTATATTAGTAAAGAAAAGTTCTATAACGTAAAAAAATGCTGTAAGTAATGCAGAGTTTTATACTGAAATCAATTTTCCTTATTCCTCAAATGTAACCACCGCTGCTGGTTGCTGTTAAACATACTTTTATAGATAAAGTAGCACTTTTTGAGTACATAATAAATTTTACTTGTCACTTTAAGGTTGCAAATGCTCACAGTACGTTGAATGAAATTCCACTAGATCTTGAAACTTTGAAACATTCAGAGTGTTATGCTTTTCTTTTTGGTGACTTCATTTATGTGATGGATGATGCCGATTTTTATGTGCCTTAATTCTTCTCACTTGGGCTTCCAAGCGTATACATCCACTTTTTATACTTTTGGATGCATTGCATTGAGCATGTATATGTTCTTATCATAAGTTGCGTGCAATGCTTTTATTATGTACTGTGAGCATATGCAACCTTAGTGTATGGATGTCCTTCTTGCTATTGGTCCTTCTGATATAGTATAGATGTCCCAAAAGGCAAAACCTCATTTGCTGGTGATCCTTTGCTATCTATTTGATCTGTCTGATCATTTTAGATTTACCTTTTGCTTTGCAGATGAATTTCCTAACTACATCAGCTGGACTTCTTTTCCTTCTATTTTTCTTCCTCATAGGTGAGCTGAAAACTTTGAAAAAATTTCCCTGTAATTGCACTCAACTCCCAGCCTAGGATTCAGTTAGCCAAATGTAGCAACTTGCTTGTATAGCCGTGTAGTGATTAGTCAATTTTCCCTCCTGGCTTAAGGTTGAGTTAACTAAAGTAAACTTGTCTAAGAGTAGTTAATTTCTGGTCTTTGAAAGTTGACCCTATTGTAGTCTCATGTCTGGAGCTAAAAGTCAATGTTCTTCTTAGTTTGAATTGGCTGACAAAAAGAGGTGTCTGATGAATTAGTATATCTTGTCCAACTTCCCGTTTTCACAGGGTTTCGGCGGCTCTTAATGTTTCACGTGCTTAATTGTGGGTGATTTTGATGCATTGCATGTAGGAGTTGTCGGTCCTTATAACAATAGAAACATGCTCATGAATACTTATAAAATGATCCTAATGTTAAACTTTATCCGGTCAGATTTTGCATTTTCAGCATTTATTTTTTCCTATTTCGATTGGTTTGACAGCATCGTTTGGTTCACTGATCGAATTTTGGCAATATGTTGTAGTTGTGTAACCTTTATGTTATAGTTTGCAGGATAGTGCATTACTAGTATCTTGAAGCATTCAGAAAAAGAGAGTGTATGGAAGCCTTCTTAGTCTATAAGATGAGCATTCTCACAAAAAACCTGAGAATCTTACAATATTGAGTTCAAATTTCTGGAGTCATTCTTTTAAAGGGGCTCTTGAGCAGAAACGGGAAATTAAGCCATGGAACTCAAATAAAATTTCTTCCGAGTTTGATGCAGCCTTGTACAGTTCCATGAAACCAAGTCTTATTTGAGTCATCCTGGATGGGCACCAATCACAGATTTGTTAGATCTTTGGTGTTGAGTCCAGAAAGTTCTTGACCCAAAAGCTGCCTGTGGTTCATAAAAAAGTGCTTGGTGATTATTACAGCCCATGATAAATTCAACTATTCAAGAAAATCATCAAAGGGTGAAACCTACTGGTCAATGTAGTGGGAAGCGGAGGCAAAAACACCAGGAATTAATCACTCTCATATTCATGTCAAGGAGATATATACCAACCGTAAGTTTCGAGGAATTGACAAATCTGGTGTATACAAACTAAAACCCGGTGTAGGGCATTGTCCAATGGCAGGTGTTCCGTTGATATTTCCTGTCCACTCTTGGCACAAAAGGAAGAACACAATGAAGTAGGGTAAGGTTAGCTAAAGGATTTGATGGGGCTAAGGATGGTTCCAAAAAGAGCAGCTACTTTAGATGAATGCTACTGCGATTGCACTCTTTACTTGCATGTATGAAATTGACATTACATGTATCCTGTTCGTTTGAGACTTGTTGCTGCAAGACTGCCGTGAGCCATGATAAAGATGGGTGAACTAGAGATCAAAGGAATGAAAAGAAGCTGATAGCATAATACACGAGTAGTTTGGGCCAAGAATAACCTTGATCATCATGAGAACTTTCCTGACCATCCCTAAATCCCAATGAAAGCCTTTAATTGGGGCTTTGCTGGAAAAACCTGCTTAAAAAACCTTATGTTGTTCTTGAGGAAAGCTATATGGACATGTGTGAAGACCAAGAGCGATTATTCACTTCTCTTCTTCCATGGTCCAAGTGTGGGATAAATTGATTCTCCATTTGTATTTAGATTTTTGTCTTTGTATTTTCTCAATTCCAAACCCTTTTTTTAAGTCCTACCTTGAAAGGAACATACAATGAGATCGTACCTGGATAGAGATGGTGGTCTTACTCTGATGTCTCCAAGCTGGCCATAATAGAATAGCTAGGTGAAGCAGTCGATAGATAGCAGGTTGCTTACAAACTCAACCATTTTAAACACAATTGCTACTTTTTTCTTGTCATTGACTGAGTGGTAGTTTTCGTCCCTGTCAAATAAAGATGACTCCTAGAGAGTTACCAAAATATGAAGTTCTCTTCTTTCTTTTTGATACTTGGTTGACAGGGCCTTTCCCAGTGAGCGCATTCAATTCTAAAGTCCATTTCTTATTATTAGAAGCAATATAGTGGCTGCCTTTGAGGAAAAGATACTTGCTCTTGGTGTTAGCATAAATATGGAAGTCAATTAACTGGGGTTGGGGGAGC

>LNC_000750

TCTGTCTGTTTTCTTCAACTATCTCTCCTCTTCCCCTCTACATTGACGACAGTTTGCAGGATAGTGCATTACTAGTATCTTGAAGCATTCAGAAAAAGAGAGTGTATGGAAGCCTTCTTAGTCTATAAGATGAGCATTCTCACAAAAAACCTGAGAATCTTACAATATTGAGTTCAAATTTCTGGAGTCATTCTTTTAAAGGGGCTCTTGAGCAGAAACGGGAAATTAAGCCATGGAACTCAAATAAAATTTCTTCCGAGTTTGATGCAGCCTTGTACAGTTCCATGAAACCAAGTCTTATTTGAGTCATCCTGGATGGGCACCAATCACAGATTTGTTAGATCTTTGGTGTTGAGTCCAGAAAGTTCTTGACCCAAAAGCTGCCTGTGGTTCATAAAAAAGGAATTAATCACTCTCATATTCATGTCAAGGAGATATATACCAACCGTAAGTTTCGAGGAATTGACAAATCTGGTGTATACAAACTAAAACCCGGTGTAGGGCATTGTCCAATGGCAGGTGTTCCGTTGATATTTCCTGTCCACTCTTGGCACAAAAGGAAGAACACAATGAAGTAGGGTAAGGTTAGCTAAAGGATTTGATGGGGCTAAGGATGGTTCCAAAAAGAGCAGCTACTTTAGATGAATGCTACTGCGATTGCACTCTTTACTTGCATGTATGAAATTGACATTACATGTATCCTGTTCGTTTGAGACTTGTTGCTGCAAGACTGCCGTGAGCCATGATAAAGATGGGTGAACTAGAGATCAAAGGAATGAAAAGAAGCTGATAGCATAATACACGAGTAGTTTGGGCCAAGAATAACCTTGATCATCATGAGAACTTTCCTGACCATCCCTAAATCCCAATGAAAGCCTTTAATTGGGGCTTTGCTGGAAAAACCTGCTTAAAAAACCTTATGTTGTTCTTGAGGAAAGCTATATGGACATGTGTGAAGACCAAGAGCGATTATTCACTTCTCTTCTTCCATGGTCCAAGTGTGGGATAAATTGATTCTCCATTTGTATTTAGATTTTTGTCTTTGTATTTTCTCAATTCCAAACCCTTTTTTTAAGTCCTACCTTGAAAGGAACATACAATGAGATCGTACCTGGATAGAGATGGTGGTCTTACTCTGATGTCTCCAAGCTGGCCATAATAGAATAGCTAGGTGAAGCAGTCGATAGATAGCAGGTTGCTTACAAACTCAACCATTTTAAACACAATTGCTACTTTTTTCTTGTCATTGACTGAGTGGTAGTTTTCGTCCCTGTCAAATAAAGATGACTCCTAGAGAGTTACCAAAATATGAAGTTCTCTTCTTTCTTTTTGATACTTGGTTGACAGGGCCTTTCCCAGTGAGCGCATTCAATTCTAAAGTCCATTTCTTATTATTAGAAGCAATATAGTGGCTGCCTTTGAGGAAAAGATACTTGCTCTTGGTGTTAGCATAAATATGGAAGTCAATTAACTGGGGTTGGGGGAGC

>LNC_000751

TTATGAATTTCTTGATTCATGTCATGTTAACAATCTTATTCTTTATGGGCTGTCTTGGGATTTTTTTGGTGATGCAAGTTGTTTTTCAATAGATCAAGAAAGTAGTAATTGGTTTTGATTCTTTTGTTCTTATAGCTACTTGTCCATTTTTTTTAACATGAAATTCACTGCTTTATCTTGAATAGCTTTTTAATGATTCTTAATTGCTATGGAGCTTTATTAGTTGTGCTTTTTCTTTTCTTTCCGTATGACTACATAGGTTATTAGATAATCTGATTATTAAATTTATCACATGCTTGTTTTCTTGATATGCTTTACTTGAGTCGAGGGTCTATCCAGAATAGCCTCTCTACCTTTACAAGGTAGGGATAAGGCCGAGCCCCCACTTGTGGGATTATACGTTGTGTTTTGTTGTTGTTGATTTTGAAATTTAATGTACTTAGGAGATATTTTAGGCTGTGGTTTCAATACATTGCATTCTCGTGCTTGATACTGCGGTGGCTATTGATTCTCTTGCTAATTCATGTTGAACCAAAAGAAACTTAAAGAAGCTGAAACATCCCACCTTTTCTTTAAATAACCTTCTACAATCTTGAACTTCGACTAAGTCTCAGGATTGTCAATTTTTATGCTCTTTATAGGAGAAGTGTTAGAAGCAAATGAGTTTAGGCACATTCTATATCTAGGGGAAAGTTGGGATAGTTTTGTTGTTTTTGTAATCATGGGCATGTCTTTGCATAGTTACTGATGTGATTTAATTTTTTACCATATCAAGCAACTAAAAGTCTAAAACTAGTGGATAACCTTTAAGCTGCCACAAATGTTGAAGCAAGTACTTGCGGATATTGCTTGATTTATCTGGTTCCTTCATAAACTTGATTATTGTGTTGGTCAGGAGGCCGATGATAAAATTTTGCTAGTTTTTCTCGATGTAGTTAGGTGGTTAACATATCACGCAACTATAAAGTGCAACAAGTTTGTTTTGTAATTATGGCGGAGAAGAACAAAACCAATTGGCTAAAAATGTACTTCAAATAACCCTATAATTGTGAGTTTGAGCCTCTTGGAGTGGTGGATATTTGTCAAGTTCAAATTTGAATGGGTTCCCAGATTTCTTTCATGTGGATTAGATTGGGTGGTTTAAGTGGAGTGTTGGTCCACTAAAAGGTCACTGTATCACTGTTGTGTGCTCTTTTTGAACATGAATCTCGAGGGCATCATCATGTTGGGGCTTCGTGACTGGTTCTCTGCCTGAGCATTGAAAGGATGAGCAGTGGAAGGATGACAGCTGGTAGTTTGTGTCCAGTGAATGGCAGAGATTTTTGGTTCATCCAACGTATAATCACGAGGAATACACTGGTTTTATTGAAAAGCTATGTTGGGTTGAAAAAGACTGTGTTGGGTAGAAAACCTAACTTCTCTTGCTCGCGACTCCTTTATCCTTCTATCTTATTTCCATTGATTTTTTTTATTACTCCCATCTCAAGGCATTGTCGCTTTTGATTCCCTGCTGGTTTCCCTAATGCTCGAATTAGTAACACTATCTCCCATTTACCAAACAGATCACATATCATAATATCCCATGCATACTCGTTCTAGTGAATCTTTCAACGTTAACTTGAAAGTACACACAACATTCCCAGCTCCGGCAAATGAAGTTTCTTCTTGTCAAGTGCTCAAGTAGTTCCTTCTATCATCGAGATAGAATCCCCATGCTCAAATGAAGTTTGCAGGATAGTGCATTACTAGTATCTTGAAGCATTCAGAAAAAGAGAGTGTATGGAAGCCTTCTTAGTCTATAAGATGAGCATTCTCACAAAAAACCTGAGAATCTTACAATATTGAGTTCAAATTTCTGGAGTCATTCTTTTAAAGGGGCTCTTGAGCAGAAACGGGAAATTAAGCCATGGAACTCAAATAAAATTTCTTCCGAGTTTGATGCAGCCTTGTACAGTTCCATGAAACCAAGTCTTATTTGAGTCATCCTGGATGGGCACCAATCACAGATTTGTTAGATCTTTGGTGTTGAGTCCAGAAAGTTCTTGACCCAAAAGCTGCCTGTGGTTCATAAAAAAGTGCTTGGTGATTATTACAGCCCATGATAAATTCAACTATTCAAGAAAATCATCAAAGGGTGAAACCTACTGGTCAATGTAGTGGGAAGCGGAGGCAAAAACACCAGGTGATTTTTTTTTTCATTTGTTCAAGTTTTGGTGAATAGAGTTATTTGTACTTGTGCTAGCAGGTACCCCATGAAATTAGTTGAGGTGCGTGCAAGTTGATCCGTATATCACGGTTATCATAAAAAAGTACTTTGAAATGACGAGCACCACTTTAATAGTTGAGGTCAAGTATATAAATCCTTTATTATACCTGTTTTGATTTATTCAAACCCATCTATGATTCACACTTGTGTAACTTTGGATGTTTATAAGCAAACACATTTTAAAAAATCGGATCTTGATCAGTAATCTCAATTTGAAACGAACGAAATTATGATGTCCTATCGTGAAGAAACTAATGAAAGTGTGGGGTGATTTTCATGTACCTATCAACTTTAAAGTTGAATGAGATAAGAGTGTGTTTTGGAAATGCAGTCAGGTGCACGTCTTCAAGTAACTCATTCCAATTTTAGAAGACATCAACTATAGTACAAGTGTACAGAGAAGAGGCTTAGAAGTTGGAGTGTTCCAGGGGAGATAAATCTTATAAGGTGAACTGTCTGTAAGAAATGGCTTCTAATCTTCAAGAAGCAAGAAAGGAACCTTTGTTACAACTTACAATTGCTTAACATGGATTAAAAACTAGCTTCTTACAAAATTTACATTTGAATCCTTGTTCTCTCAGTACCTTAGACATTTGGGATCTTTGTAGTTTCAACAGGCAACCTATTTTGGGGATCTGTGACTATTACTGGAAATTAGGAGTATTGACGCCCTAGGAATAACAATGTGGAAAGTGAGCAATGGAGCATATATAATGTGAGGGGATTTTCCCTTCTTATATTTGCATACTAGGAGTACTTTCCACTTGTCCCATGTGACTACTGGCCTAATGAATAGTCTTCTCCTTCCTGGATTGGGTTATCTAATCTAGTCTAGAATATCATCTGCACAATCTTTGTACCAAGTGGACCCTAGTCTTCAGGGAGAAAGAAGCCTTTGTTGTACGATTGCTAGCCTGGTTGTAGAAATAATCTCTTATCCCGCAACTTTCTCTGCATCGTTGTATGTCAGGACTGTAGACATTTTGGATCTCTTAGATTTTAATCTTACCCCTATGATAGTATGTATTACTTACACCTCTGATAGTAGATCCTATATCGAGTTAGCTGGGGTTGAGACATCCTTCAATCCACTCAAGGCATTCTTTGATAGACTAACAAACTATCCTTTGATGGATTTTTGCTAAAATATTCTCATCCGGACCTTCTCCCCAGCATTGCAGATGTCTATTGTCATTTCATTCTTCTATAAATGCAATTTAAAAGAGACATTAGATCCTGGTCTCAGACTCTGAGCGCAATGGATATATAGGATTCTGATAGCAGAAGTCAACTGATTTGAAATTAAGGCTTTGTTGGTCAACTAGATTATGGATTTAGCTTATGTGAGATAATCAGATTCTAAACTCAGGCTTATCATGGCAAAATGATGGTATTACTTTTTCTGCTTGCCCACCAAATTATCTCACCTTGCAGGCAGATTCCATTAGGTTATATTCTCTAGTCGATGCATGTGGTGGAGTACAATTAAATTGTAAAGGTGAGCATTACTGGTTTTTATAACCACCTTATTTTCCAAT

>LNC_000752

CTTGAACATTCAGTTTTCTTGATTTTATGAATTTCTTGATTCATGTCATGTTAACAATCTTATTCTTTATGGGCTGTCTTGGGATTTTTTTGGTGATGCAAGTTGTTTTTCAATAGATCAAGAAAGTAGTAATTGGTTTTGATTCTTTTGTTCTTATAGCTACTTGTCCATTTTTTTTAACATGAAATTCACTGCTTTATCTTGAATAGCTTTTTAATGATTCTTAATTGCTATGGAGCTTTATTAGTTGTGCTTTTTCTTTTCTTTCCGTATGACTACATAGGTTATTAGATAATCTGATTATTAAATTTATCACATGCTTGTTTTCTTGATATGCTTTACTTGAGTCGAGGGTCTATCCAGAATAGCCTCTCTACCTTTACAAGGTAGGGATAAGGCCGAGCCCCCACTTGTGGGATTATACGTTGTGTTTTGTTGTTGTTGATTTTGAAATTTAATGTACTTAGGAGATATTTTAGGCTGTGGTTTCAATACATTGCATTCTCGTGCTTGATACTGCGGTGGCTATTGATTCTCTTGCTAATTCATGTTGAACCAAAAGAAACTTAAAGAAGCTGAAACATCCCACCTTTTCTTTAAATAACCTTCTACAATCTTGAACTTCGACTAAGTCTCAGGATTGTCAATTTTTATGCTCTTTATAGGAGAAGTGTTAGAAGCAAATGAGTTTAGGCACATTCTATATCTAGGGGAAAGTTGGGATAGTTTTGTTGTTTTTGTAATCATGGGCATGTCTTTGCATAGTTACTGATGTGATTTAATTTTTTACCATATCAAGCAACTAAAAGTCTAAAACTAGTGGATAACCTTTAAGCTGCCACAAATGTTGAAGCAAGTACTTGCGGATATTGCTTGATTTATCTGGTTCCTTCATAAACTTGATTATTGTGTTGGTCAGGAGGCCGATGATAAAATTTTGCTAGTTTTTCTCGATGTAGTTAGGTGGTTAACATATCACGCAACTATAAAGTGCAACAAGTTTGTTTTGTAATTATGGCGGAGAAGAACAAAACCAATTGGCTAAAAATGTACTTCAAATAACCCTATAATTGTGAGTTTGAGCCTCTTGGAGTGGTGGATATTTGTCAAGTTCAAATTTGAATGGGTTCCCAGATTTCTTTCATGTGGATTAGATTGGGTGGTTTAAGTGGAGTGTTGGTCCACTAAAAGGTCACTGTATCACTGTTGTGTGCTCTTTTTGAACATGAATCTCGAGGGCATCATCATGTTGGGGCTTCGTGACTGGTTCTCTGCCTGAGCATTGAAAGGATGAGCAGTGGAAGGATGACAGCTGGTAGTTTGTGTCCAGTGAATGGCAGAGATTTTTGGTTCATCCAACGTATAATCACGAGGAATACACTGGTTTTATTGAAAAGCTATGTTGGGTTGAAAAAGACTGTGTTGGGTAGAAAACCTAACTTCTCTTGCTCGCGACTCCTTTATCCTTCTATCTTATTTCCATTGATTTTTTTTATTACTCCCATCTCAAGGCATTGTCGCTTTTGATTCCCTGCTGGTTTCCCTAATGCTCGAATTAGTAACACTATCTCCCATTTACCAAACAGATCACATATCATAATATCCCATGCATACTCGTTCTAGTGAATCTTTCAACGTTAACTTGAAAGTACACACAACATTCCCAGCTCCGGCAAATGAAGTTTCTTCTTGTCAAGTGCTCAAGTAGTTCCTTCTATCATCGAGATAGAATCCCCATGCTCAAATGAAGGTTCTTCTTGCCTTTATTTTAATGGAAGTGATGTTTTCTTATTGTTTCACATTTTATTTTATTCCATATAAATTTCAGAAAAAGTTGTGCCAATATTAGTTATAAAACACGTGATACTAAACTCTTGCATAACCTTTGATAAAAAGGTAACTAAAATGCTGCATTAGTAATGTTGAGATTATATTAGTAAAGAAAAGTTCTATAACGTAAAAAAATGCTGTAAGTAATGCAGAGTTTTATACTGAAATCAATTTTCCTTATTCCTCAAATGTAACCACCGCTGCTGGTTGCTGTTAAACATACTTTTATAGATAAAGTAGCACTTTTTGAGTACATAATAAATTTTACTTGTCACTTTAAGGTTGCAAATGCTCACAGTACGTTGAATGAAATTCCACTAGATCTTGAAACTTTGAAACATTCAGAGTGTTATGCTTTTCTTTTTGGTGACTTCATTTATGTGATGGATGATGCCGATTTTTATGTGCCTTAATTCTTCTCACTTGGGCTTCCAAGCGTATACATCCACTTTTTATACTTTTGGATGCATTGCATTGAGCATGTATATGTTCTTATCATAAGTTGCGTGCAATGCTTTTATTATGTACTGTGAGCATATGCAACCTTAGTGTATGGATGTCCTTCTTGCTATTGGTCCTTCTGATATAGTATAGATGTCCCAAAAGGCAAAACCTCATTTGCTGGTGATCCTTTGCTATCTATTTGATCTGTCTGATCATTTTAGATTTACCTTTTGCTTTGCAGATGAATTTCCTAACTACATCAGCTGGACTTCTTTTCCTTCTATTTTTCTTCCTCATAGGTGAGCTGAAAACTTTGAAAAAATTTCCCTGTAATTGCACTCAACTCCCAGCCTAGGATTCAGTTAGCCAAATGTAGCAACTTGCTTGTATAGCCGTGTAGTGATTAGTCAATTTTCCCTCCTGGCTTAAGGTTGAGTTAACTAAAGTAAACTTGTCTAAGAGTAGTTAATTTCTGGTCTTTGAAAGTTGACCCTATTGTAGTCTCATGTCTGGAGCTAAAAGTCAATGTTCTTCTTAGTTTGAATTGGCTGACAAAAAGAGGTGTCTGATGAATTAGTATATCTTGTCCAACTTCCCGTTTTCACAGGGTTTCGGCGGCTCTTAATGTTTCACGTGCTTAATTGTGGGTGATTTTGATGCATTGCATGTAGGAGTTGTCGGTCCTTATAACAATAGAAACATGCTCATGAATACTTATAAAATGATCCTAATGTTAAACTTTATCCGGTCAGATTTTGCATTTTCAGCATTTATTTTTTCCTATTTCGATTGGTTTGACAGCATCGTTTGGTTCACTGATCGAATTTTGGCAATATGTTGTAGTTGTGTAACCTTTATGTTATAGTTTGCAGGATAGTGCATTACTAGTATCTTGAAGCATTCAGAAAAAGAGAGTGTATGGAAGCCTTCTTAGTCTATAAGATGAGCATTCTCACAAAAAACCTGAGAATCTTACAATATTGAGTTCAAATTTCTGGAGTCATTCTTTTAAAGGGGCTCTTGAGCAGAAACGGGAAATTAAGCCATGGAACTCAAATAAAATTTCTTCCGAGTTTGATGCAGCCTTGTACAGTTCCATGAAACCAAGTCTTATTTGAGTCATCCTGGATGGGCACCAATCACAGATTTGTTAGATCTTTGGTGTTGAGTCCAGAAAGTTCTTGACCCAAAAGCTGCCTGTGGTTCATAAAAAAGTAACACATGGAAGAAAACATTGCAATTTCAAATCTTGGGTGTCATTTCCTAAGTAATTCTTGGATTTGTAAGTTTTTATATTCAGAATTTTTCCAAGTTCTTTCTTAATTGAATTAATTTATATGATATTTGAGTTTGACTAGTCTTTGCCTTTTTATTTAATCTTTTTGCATCTACATTTATAATTTATTGGTGCAGTGGTTCTTATTACTAACAATCAATCACTAGCAACCAAGCAGTAGAAAATTATTTCTCTGTTCAGAATTCTTTTGTGCACTTATAAATTTTGTATGTCCATAAATTGCTATATAGGAGTGCATCTTATGTCCTCCAAAATCAATAATCATGGCATCTTTATGTTGTTCTGTTTTCACAGCCTTTATTGCCTTGCACCATAAGTCTTTTACCAGAAACCCTGAATTACAATTATGCTTGAGAAGATTGAACCTTAGAACAACATGAATTTGGAGCATCTTTGTTGGGGTTCTGTTTTCCAATTGTTTATTTTCCCCGAACCATAAAAACTAACAAAAGAAACTTTGAGAAACCACAAGGAACTGTTTCAACAGTAAAGAAGAACCCCCTTAAATGCCTTTTGTCTTCTCATATGTGTGCCTCGGATTACAGTTAGTACCTCAAGATGGCTTTTATTTGTTTGAGGTAAGTCAAGGAAAAAAAGTGAAAGTGAAAGAGGTGATTAAATGGGATGTCTTGCATTTTCAAAGGATAGATAATAGGTCAAATGGAATCAACAATGCTATATATTTTGGCTTTTATCATACTCAAATCTTTTTTTTTTCTTTTTCTGTTTCTTCCACATTTGCCAGTAGAGTTGTACAGTAGAATACATTTTGTATTTCCAACTACC

>LNC_000753

GTTCTAATTTTTTACTAAAAAATTCTCTGCTTATTGCTAAGAACATTCTCCGACTCCGAGGTACTTATAGGGCTGTGATTATTCACTTCAGATGGAAGAAAGCGTGGTTTGTGCCGGAGATATATGACATTCCACATCAGGGGCATAAGAGCATCCGAAAAGGGATCCCTGGAAAGTTTGACTCTGAACTTTGGAATTTCAGATCTTATCAGTATACTGAACATGCAAGCCCAACTCTGTGCTATTGCAGGGATTTCTTGGTAGTTCTATGTTGAATTTTGGAGTTCCGAACTGCTTACTTGTAAACTAGAGAGGGATCTTTTGGGAGTTAGACTTTGAACGTTGGAGTTTCAGATCTGTAAACTGAACAGACAGTCGATTGAGTTTTGTAGTTGACTGGAGATCAGGTTACAAAAATGGAAACAACGAAGAGCTCTTGAAGTCAAGCATTAGTTAAGATTTGGTACAGGCATTGACAAAACAAAGTTAAGTCTGGACTGTACAAATGGTCTTTTTTTAATAAACTTACAGAATATTGATTCCCCTGTGGCATTAAATTGTCAGCATGTGACCAAGTGTGAATTCTATTTTTTTGAATCTTAGTTACATAAATGACCCTGCTTAATTTGCATGTACTATCACTTAGAAAATAGAGTGAAGGAGCAGAGTTGCAAGTAAAATGAATTAAAAAGTTGTATTAGATGGGGGCATTCCGAGAATCGAACTCGGGACCTCTCGCACCCAAAGCGAGAATCATACCACTAGACCAAATGCCCAGTTCTTGTTGGTCCCTCAGTGGTAATAATAGTTTGAAGTTGATATTGGTTGTCTGGAGCTTGTACCATTCTGTGCAATAATTTTCTAGGTGAGATAAACATTCTCCATTGTTTGAGCCTTTGAAAAGTTAAAATCATCTTAGAGTAAATAATTTTTTTTATAAATTAAGGGGCATTCCGAGAATCGAACTCGGGACCTCTCGCACCCAAAGCGAGAATCATACCACTAGACCAAATGCCCAGTTCTTGTTGGTCCCTCAGTGGTAATAATAGTTTGAAGTTGATATTGGTTGTCTGGAGCTTGTACCATTTTGTGCAAAAATTTTCTAGGTGAGATAAACATTCTCCATTGTTTGAGCCTTTGAAAAGTTAAATCATCTTAGAGTAAATAAAAACTTTTATAAATTAAGGGGCATTCCGAGAATCGAACTCGGGACCTCTCGCACCCAAAGCGAGAATCATACCACTAGACCAAATGCCCATCGTTGACATTGTCTTATTTGAATTTTAGTCAAATGTATTTTAGCTTTGTCAGCTATTTGATCACACAATTGTCATTGCTTACTGTAATATAGAATTCAAATAATCCGTATTTTACACCATATGATCTTCATTTGCTTGCTTAAATGTTCTTGAGTACTTAATTATCAAAAAAAATGTTCTTGAGTACTTTTTATTTGGATGTGAGGTGTATATAGAAGGTTGCTTAAAATAATATGCCTCTGCAGTTCCTCAAATTACTTTTCTCGATGCCTATAGATGCTATCAGCTTTGGTTGTTACTGTCACTTGTATATTTACAATCCTATGCTATCATACTCAACCCTTTGGTCATCTGGAATCCAAAACACGTGAGATCTGTGTAGTGATTAAGAACTGTCATTGGACATGAGGCCAGGTCTCCGGCGTTAGCTGAATTAATATTTCATGTTTGAGCTTCATCGTTCTAGTTACAGCTGAAGCAAATAAAATCTCCACTAAGGATTATCCCATTGATAGTTAACCATGTGCTTTTTTCTGGGTTCTCTTACTTCCAGTTCTCTTTTGGATACCTTCTTTGTTAAGCTATATCATTATCACTTGTTTCATGGAATGGTGCAGCTCATGTTATGCGATTTTGGGTGCAAAGCAACATGGTACAATGAAGTTCATGCTTCTTTGTTTCACTCAAGTGAAATGAAAATGGCTCATGAACTGTGGATATCCGTTTCATTCCTTATTTTGACTTGAAATTCAGAGAGCTTCCTTGATTAATGATATCAGTTGTTATTGTAAACTTCCTTGACCTGTAAATGACCTTGTATCTATAATGATTCTTTGATGTTACGACAAATCCATGTACCAACTTCATTAAGTTTTGCCTGCTTTGTCAAATTGAGAATGGATGGCAAAAAAGCATATCTGAAATGATGGCTTGTGATATTTGAGATACTCGAGAACTTATCTGTCCAACATTTTATATTTAGGGATCTTTTGGGAGTTAGATGTTGAACGTTGGAGTTTCAGATCTTTAAACTGAACAGGCAAGCCCAACTCTGCACCATTGCAAGTATTCATGATGTATTATTTTAATTTTTAGCTTATAGATCTAGATTTACTCTTATTCAGCTTCAAGTGCTCATGCCATTGGGTTTATCCATTAACATGAATTAAAAGAATCTTTACCTGCAAATTTTGTTTTATGAAGACAGTCGATTGAGTTTTGTAGTTGACTGGAGATCAGCTTACAAAAATGGAAACAATGAAGAGCTCTTGAAGTCAAGCATTAGGTAAGGTTTGGTACATGCATTGACAAAAGAAAGTTAGTTTTAGACTGTACAAATGGTCTTTTTTTTAATTTACAGTATATTGATTCCCCTGTGGCATTAAATTGTCAGCATGTGACCAAGTGTGAATTCTATTTTTTTTTAATCTTAGTTCTATAAATGACTGATGAACTTGCTGCAATTTTCAGAACATCAATCTCAATGCTGATTCTTTTTACAAAATACTAAGAATCTTGCACCGATGAATCATACGACTAGACTAAGTACCCACTGTTTATGTTCCCTGTAGACAATCTTGCAATAAGGTTAACACTAAGTCATCCAAGATGACAAGGTGAGGTTGTGTGCTAGTTGAAATTAAGGTGGAGGGTAAATTATAGCATTTGTATGATAACTCCGAGACCGCAGAAAAGAAACTATGAAGTGTGTTTTCTTATATCTCTAGTCAATTGAGCTAGGTTAGCCATTTATGGCCTGCTTAATTTTGCATGTAAAAAAGTTGATATTAGTTGGGGCATTCCGAGAATCGAACTCGGGACCTCTCGCACCCAAAGCGAGAATCATACCACTAGACCAAATGCCCAGTTGTTAGTATTTTCTATCTTTGAGTTTGTCAAAATGAGTTTCCTGATGTGAGTTATTTGATCATAATTGCCATTTCTCATCTGCAATATAAAATTCTGATAGTCCTTAGTCCTTACCTCATCTGAATTGTACACTATTGATTCCCCGGTGGCATTAAATTGTCTGCATGTGACCAGATTTTAATTTATAGTTTGAATCTCAGTAAACATATATAAATGGCTGCAATTTTCAGGACATCAATTTGCTGATTGTTTTACAAAATACTACTACTTGTCCAGCTGGTACATTGTTGACTACAAGGAGATTGAATTATTTTTTGCATTAAACTT

>LNC_000754

AAGAGTTCTAATTTTTTACTAAAAAATTCTCTGCTTATTGCTAAGAACATTCTCCGACTCCGAGGTACTTATAGGGCTGTGATTATTCACTTCAGATGGAAGAAAGCGTGGTTTGTGCCGGAGATATATGACATTCCACATCAGGGGCATAAGAGCATCCGAAAAGGGATCCCTGGAAAGTTTGACTCTGAACTTTGGAATTTCAGATCTTATCAGTATACTGAACATGGATTTCTTGGTAGTTCTATGTTGAATTTTGGAGTTCCGAACTGCTTACTTGTAAACTAGAGAGGGATCTTTTGGGAGTTAGACTTTGAACGTTGGAGTTTCAGATCTGTAAACTGAACAGTCGATTGAGTTTTGTAGTTGACTGGAGATCAGGTTACAAAAATGGAAACAACGAAGAGCTCTTGAAGTCAAGCATTAGTTAAGATTTGGTACAGGCATTGACAAAACAAAGTTAAGTCTGGACTGTACAAATGGTCTTTTTTTAATAAACTTACAGAATATTGATTCCCCTGTGGCATTAAATTGTCAGCATGTGACCAAGTGTGAATTCTATTTTTTTGAATCTTAGTTACATAAATGACCCTGCTTAATTTGCATGTACTATCACTTAGAAAATAGAGTGAAGGAGCAGAGTTGCAAGTAAAATGAATTAAAAAGTTGTATTAGATGGGGGCATTCCGAGAATCGAACTCGGGACCTCTCGCACCCAAAGCGAGAATCATACCACTAGACCAAATGCCCAGTTCTTGTTGGTCCCTCAGTGGTAATAATAGTTTGAAGTTGATATTGGTTGTCTGGAGCTTGTACCATTCTGTGCAATAATTTTCTAGGTGAGATAAACATTCTCCATTGTTTGAGCCTTTGAAAAGTTAAAATCATCTTAGAGTAAATAATTTTTTTTATAAATTAAGGGGCATTCCGAGAATCGAACTCGGGACCTCTCGCACCCAAAGCGAGAATCATACCACTAGACCAAATGCCCAGTTCTTGTTGGTCCCTCAGTGGTAATAATAGTTTGAAGTTGATATTGGTTGTCTGGAGCTTGTACCATTTTGTGCAAAAATTTTCTAGGTGAGATAAACATTCTCCATTGTTTGAGCCTTTGAAAAGTTAAATCATCTTAGAGTAAATAAAAACTTTTATAAATTAAGGGGCATTCCGAGAATCGAACTCGGGACCTCTCGCACCCAAAGCGAGAATCATACCACTAGACCAAATGCCCATCGTTGACATTGTCTTATTTGAATTTTAGTCAAATGTATTTTAGCTTTGTCAGCTATTTGATCACACAATTGTCATTGCTTACTGTAATATAGAATTCAAATAATCCGTATTTTACACCATATGATCTTCATTTGCTTGCTTAAATGTTCTTGAGTACTTAATTATCAAAAAAAATGTTCTTGAGTACTTTTTATTTGGATGTGAGGTGTATATAGAAGGTTGCTTAAAATAATATGCCTCTGCAGTTCCTCAAATTACTTTTCTCGATGCCTATAGATGCTATCAGCTTTGGTTGTTACTGTCACTTGTATATTTACAATCCTATGCTATCATACTCAACCCTTTGGTCATCTGGAATCCAAAACACGTGAGATCTGTGTAGTGATTAAGAACTGTCATTGGACATGAGGCCAGGTCTCCGGCGTTAGCTGAATTAATATTTCATGTTTGAGCTTCATCGTTCTAGTTACAGCTGAAGCAAATAAAATCTCCACTAAGGATTATCCCATTGATAGTTAACCATGTGCTTTTTTCTGGGTTCTCTTACTTCCAGTTCTCTTTTGGATACCTTCTTTGTTAAGCTATATCATTATCACTTGTTTCATGGAATGGTGCAGCTCATGTTATGCGATTTTGGGTGCAAAGCAACATGGTACAATGAAGTTCATGCTTCTTTGTTTCACTCAAGTGAAATGAAAATGGCTCATGAACTGTGGATATCCGTTTCATTCCTTATTTTGACTTGAAATTCAGAGAGCTTCCTTGATTAATGATATCAGTTGTTATTGTAAACTTCCTTGACCTGTAAATGACCTTGTATCTATAATGATTCTTTGATGTTACGACAAATCCATGTACCAACTTCATTAAGTTTTGCCTGCTTTGTCAAATTGAGAATGGATGGCAAAAAAGCATATCTGAAATGATGGCTTGTGATATTTGAGATACTCGAGAACTTATCTGTCCAACATTTTATATTTAGGGATCTTTTGGGAGTTAGATGTTGAACGTTGGAGTTTCAGATCTTTAAACTGAACAGGCAAGCCCAACTCTGCACCATTGCAAGTATTCATGATGTATTATTTTAATTTTTAGCTTATAGATCTAGATTTACTCTTATTCAGCTTCAAGTGCTCATGCCATTGGGTTTATCCATTAACATGAATTAAAAGAATCTTTACCTGCAAATTTTGTTTTATGAAGACAGTCGATTGAGTTTTGTAGTTGACTGGAGATCAGCTTACAAAAATGGAAACAATGAAGAGCTCTTGAAGTCAAGCATTAGGTAAGGTTTGGTACATGCATTGACAAAAGAAAGTTAGTTTTAGACTGTACAAATGGTCTTTTTTTTAATTTACAGTATATTGATTCCCCTGTGGCATTAAATTGTCAGCATGTGACCAAGTGTGAATTCTATTTTTTTTTAATCTTAGTTCTATAAATGACTGATGAACTTGCTGCAATTTTCAGAACATCAATCTCAATGCTGATTCTTTTTACAAAATACTAAGAATCTTGCACCGATGAATCATACGACTAGACTAAGTACCCACTGTTTATGTTCCCTGTAGACAATCTTGCAATAAGGTTAACACTAAGTCATCCAAGATGACAAGGTGAGGTTGTGTGCTAGTTGAAATTAAGGTGGAGGGTAAATTATAGCATTTGTATGATAACTCCGAGACCGCAGAAAAGAAACTATGAAGTGTGTTTTCTTATATCTCTAGTCAATTGAGCTAGGTTAGCCATTTATGGCCTGCTTAATTTTGCATGTAAAAAAGTTGATATTAGTTGGGGCATTCCGAGAATCGAACTCGGGACCTCTCGCACCCAAAGCGAGAATCATACCACTAGACCAAATGCCCAGTTGTTAGTATTTTCTATCTTTGAGTTTGTCAAAATGAGTTTCCTGATGTGAGTTATTTGATCATAATTGCCATTTCTCATCTGCAATATAAAATTCTGATAGTCCTTAGTCCTTACCTCATCTGAATTGTACACTATTGATTCCCCGGTGGCATTAAATTGTCTGCATGTGACCAGATTTTAATTTATAGTTTGAATCTCAGTAAACATATATAAATGGCTGCAATTTTCAGGACATCAATTTGCTGATTGTTTTACAAAATACTACTACTTGTCCAGCTGGTACATTGTTGACTACAAGGAGATTGAATTATTTTTTGCATTAAACTT

>LNC_000755

AAAATTCTCTGCTTATTGCTAAGAACATTCTCCGACTCCGAGGTACTTATAGGGCTGTGATTATTCACTTCAGATGGAAGAAAGCGTGGTTTGTGCCGGAGATATATGACATTCCACATCAGGGGCATAAGAGCATCCGAAAAGGGATCCCTGGAAAGTTTGACTCTGAACTTTGGAATTTCAGATCTTATCAGTATACTGAACATGCAAGCCCAACTCTGTGCTATTGCAGGGATTTCTTGGTAGTTCTATGTTGAATTTTGGAGTTCCGAACTGCTTACTTGTAAACTAGAGAGGTTAGCACAAATCCATATACTACCAGCACTCGTGTTTTGTTATTTTTATCTTGTAGTTCTGAAAAGATTAGCTTTCAGAGCATCAAGTCTTGCCATTCCATTTTCTTTATTTGATAAGGATGCATCTTGGCCTAAATGTTGTTATTTGAAAAAACATGACTTTTGGCTGATTGACTTGGCTGACTTTACATCAGAATATGTACTGAACAGGAGACTGGAAAGAGCTCTGCGAGTCAAGCAATATATGTGGTTTACTAAAATACACTAGAAAGAAGAATCAGTTCTAGAACTGTTTGGATTACTAAACTAATTAATTTTAACTGTCTAAGATGAATAACCATTAATCATTGGAATCCTACCAAATTCATAAATGCATGATGTGCTTACTGCAGTTTCCAGAACACAAATCTCACTATTGAATATTCAGCAAAGTGTTAAATGGATATCCTACATTTGCCTTAAAATCCTTTATAACTATCAGGAGTACAGGCTTGTAAAAAGGCACTTAAAAATTTGGGGCATTCCGAGAATCAAACTCGGGGACCTCTCGCACCCAAAGCGAGAATCATACCACTAGACCAAATTCCCTGCTTTTATTGGTCCCCCAGTCTAAATGATAGCTGGAAGTTTCATGTTCAATTATCTGGAGTTTATACCATCCTATGAAATGTTTTTTCTTGGTGGGATGAGAATTCTCCCGTGTCTAGGTGCTCCTCCAATGCTAAAGATAATGTATACAGAGAGACATAACAATGCTATTAAAGGTGTTTTAGTTGTCGACAGTGGGAAGTTTTCAATATCTGGTTGTAATATCTGAATATACTCAACTGTAGAGTCAGCAAGAGATGAAGGAGCCCTTGGTGAGTTAAATCAACTTAGAATAAATTAAAACTTTAGCAAAATCAAGGGGCATTCCGAGAATCGAACTCGGGACCTCTCGCACCCAAAGCGAGAATCATACCACTAGACCAAATGCCCAGCTGTTTGTTGGTCTCTCAGTCTAAGAAATAGTTAGAAGTTTCGTGTCAGTTGTCTTGAGCTTGTACCATTTTTCAAAATGATTTTCTTGGAGGGATGAGCATTTTCTAGTCTCTGGGGACCTGGGGGGATCCTTCCTTGGGTCAATCGGTGTTATGGTCCTTTACGAATTTGATTTTAGTTTTATAGATATCTTCATTTTTACATATAAGAGATCTGAAAAGGTCATTTTCTTCTATTCAATTTGTAAGTAGTCATAGGAGATCATTTCTTCCCTCTCCCTCCCTCCCTTTGTTAAAATAATGTATATAGAGAGGCATACTATGCTATTAAAGGTGCTTTAATTGCAGACATTATTGAAGTAGGTTATATCTGGTTGTATCTGAACAAACTCAACTGTAGAGGAGGAAATTGTCTCCTATGGCACTGCAGAGTCAGTTACAGATGAAGGAGCATTTGGAGAGTTAAATCAACTTAAGAGTAAATAAAACTTTAGCAAAATCAAGGGGCATTCCGAGAATCGAACTCGGGACCTCTCGCACCCAAAGCGAGAATCATACCACTAGACCAAATGCCCTGCTTTTGTTGGTCCCCCAATCTAAATGATAGTTGGAAGTTTCATATTCAGTTGTCTGGAGTATCAACTTATGAAATGATTTTTAGGAGGGATGAGAATTCTCCTGTGTCTGGGGGATCCTCCAATGTAAAAATAATGTATACAGAGAGACATAACATTGGAAGTAGTTGTAGAAAGGGGGAAGTTTCCCATATCTGGTTGTAATATCTAAACATACTCAACTGTAGAATCAGCCAGAGATGAATGAGCCCTTGGAGAGTTAATTAGGTTCGAGTAAATTAAAAAATTTAGCTAAATGAAGGGGCATTCCGAGAATCGAACTCGGGACCTCTCGCACCCAAAGCGAGAATCATACCACTAGACCAAATGCCCATTGTTGGCATTGTCTTATTTGAGTTCTAGGCAAATGTTTTCCCCCCTTTGAGTTATTTGATCCCAAATGTCATTGCTTATCTGTAATGCAGAATTCAAATACTCCTTATTTTACACCAGATGATCTTCATTTCTTGCTTAAATGTTCTTGAGTACTTTGCTTATTTGGATGTGAGGTGTATATAGAAGGGTTGCTTAGAATAATATGCCTCTGCAGTTCCTCTGATTCTCACTGCACTTGATGCCTAAGACGCTCTCAGCTTTGGTTGTTACTGTCACTTGTATATTTACTATCCTATGCTATCATATTCAACCCTCTGGTCATCTGGAATCCAAAATCCATGAGATATGTCTAGTAATTAAGAACTTCCATTGGACATGAGGCCAGGTGTCTGGCATTAGCTGGATTAATATTACATGTTTGAGCTTCATAGATCGAGTTAAGTCGCAACTGAAGCAAATAAAAGCTCCAATAAGGAGTATCACATTTATAGTTCAACATGAGCTTTTTGCTGGGTTCTCTTCCAGTTCTCTTTTGGGTACCTTCTTTATTATCTATAATAGCATCATTACTTGTTTCATGGAATAGTGCAGATCATGTTAAGTAATTTTCATGCTATATAATTTTTGGGTGCAAAGCAACTTGGTACAATAATGCTCCTGCTTTTTTTTTTCTTACTAAAGTGCAATATATATCGCTCATGACCTATGGATATCGGCTTTATTCCTTATATTGACGTGAAATTTTGAGAGCTTTTGCCTTGGTTTTGCACAACACCTCTATGTCATTGATTAACGATTTCAGTTTTTACTGTTAACTTCCTTGACCCCTAAATGACCGTGTTATGGATCAAACTTAATGATTCTTTGACGTTACAACAAATCCATGTACTAACTTCACTTTTAAGTTTTGCCTGGTTTGTCAAATTTAAAATGGATGGCAAAAAAGCAAATCTGAAATGATGGCTTATGATAGAAGAGATACTTGAACTTGTCTGTTCGACATTTTATATTTAGGGATCTTTTGGGAGTTAGATGTAGAACCTTGGAGTTTCAGATCTGCTTTGTTGTAAACTGAACAGGCAAGACCAACTCTGCACTATTGCAAGTATTCATGATGTATTAATCTGTATGTTATAGATCTAGATTTACCTTGTGCAGCTTCAAGTGACTCATGCCATTTGGTTATCCATTAACATGAATTAAAAGAATCTTTACCTGCAAATATTTTTGTATGAAGACTTGAGATCAGCTTGCCAAAATGGGAACAATAAAGAGCTCTTGAAGTCAAGCATTAGGTAAGGTTTGGTACAGGCATTGACAAAGCAAAGTTAGGTCTTGACTGTACAAATGGTCCTACATCTTGATAATGAGTAAACTAAAAAAAAATTATTGATTCCCCTGTCGCATTAAACTGTCAGCATGTGACCCAGTGTGAATTCTATTTTTTGAATCTTAGTTACATAAATGGCTGATGAACTTGCTGCAATCTTCAGAACATCAATCTCAATGTTTTTTTTTTTTAACAAAATACTAAGAATCTTTCAACCCATGAATCATACAACTAGATTA

>LNC_000756

CCGACTCCGAGGTCCCAATAGGGCTGTGATTATTCACTTCAGGGATCCCTGGATAGCTTGACTCTGAACTTTGGAGTTTCAGATTTTATCAGTAAACTGAACATGCAAGCCCAACTCTGTACTGTTGCAGGGATTTCTTGGTAATTCTATGTTGAATTTTGGAGTTCCGATCGGCTTACTTGTAAACTAGAGAGGTGAGCACAAATCCATCTACTACCAGCACTCGTGCTTTGTTATTTTTATCTTGTAGTTCTGAAAAGATTAGCTTTCAGAGCATCAAATCTTGCCATTCCATTTTCTTTATTTGATAAGGATGCATCTTGGCCTAAATGTTGTTATCTGAAAAAAAATGACTTTTGGCTGATTGACTTGGCTGACTTTACATCAGAATATTTACTGAACAGGAGACTGGAAAGAGGTGCAAGTCAAGCAATATATGTGGTTTACTAAAATACACTAGAAAGAAGAATCAGTTCTAGAACTGTTTGGATTACTAAACTAATTAATTTTAACTGTCTAAGATGAATAACCATTAATCATTGGAATCCTACCAAATTCATAAATGCATGATGTGCTTACTGCAGTTTCCAGAACACAAATCTCACTATTGAATGTTCAGCAAAGTATTAAACGGATATCCTACATTTGCCTTCAAATCCTTTATAACCATCAGGAGTACAGGCTTGAAAAAAGGCACTTAAAAATTTGGGGCATTCCGAGAATCGAACTCGGGACCTCTCGCACCCAAAGCGAGAATCATACCACTAGACCAAATGCCCATTGTTGGCATTGTCTTATTTGAGTGCTAAGCAAATGTGTTTTCCCCTTTTGAGTTATTTGATCCCAAATGTCATTGCTTATCTGTAATGCAGAATTCAAATACTCCTTATTTT

>LNC_000757

CAAAGGTCATCGAGAACCATTCACAAAAATAAGAAAAACACTAGTAAGTTCAGCCTTCTTTAAAACTCTGCTTTGCAAGGCAATCAAACCTCATCCCAACGATGTTGGATGATGCAATCCTACAGGTCAACCTACAAATCAATAAAACAGATTTCAGAAAATTTTGAGAATGAAGTTTCAGTCATGAACACAGAATAATGCAGCTAAGCTATCTGCTTTAATCTTACTCTACTTTCAAGGGGAAACAAAATTGAAATGGAAGAAAAACAGTTGTGCAATCAAGAAAAGGAGCAAAGGGGACATATTCCCTCTCATAGTTAGCAGAAGATCGAAGGTGAGGGTGGAAGCAAAAAGACCTGTACTTCTACAAGCTGCCTTTGGATGTTGAGTGAAGGAATATTTCAAGAACTTAATTCTCTATCTGGTTCATGTAAATCATAGCTCTAAGCTAAAATCAGGACACGAGCTAAGGATCTCACTCCAAAAGTCATTAACAAGCTAATTGCCCGTTTCACGTTTCAAACCCACAGGTATCCCTTTGAATCCAAACCCAGAAACTTATATCAGCAATTATGAAGTAATCTAAAGATTCAAAAAAATCCCAAATTAGTGTATCCGGTTAAATCTATTCTGACCAACTTGTTTAAACTTTTGAGTAATTTAAGAGAGTGAGCACACTGTAAGGGATTAACGGAAGCTTTTATAAGCACAAGGCTTATTTAGAAAACTAATCACAATTATAGAAGGAATAATTATGTATAACTAATTCTTGGCATTAAGAAAAATAAATTATATCAAAAGTAAAAGCACTTTTCACCTTTAAGTCTCCTTTATTTCCATTTGTTCTAAGAAAAAATTACTTTGCATTCCAAGAACCTTTTTAGTTTGTCTAGACCTATCGCAGAAAACCTCTAGTATTAGGGAACTAGTAATTTGTCTTAACAGATGAACAATTGAAAATTGGATCCAAATGGCATAGAATGGATATTGATGATTCCTACTACCTAAAAAAACTAGTTTAGGAATGAGGTGCAGTAACAGTATAGTGGTTGTTGCATGGTTATATCTATTCTATCCGTAAGTAAGTGGCCTATTTTATTTTTTGCTAATAAACAAGGTGATCTTTGACTATACCATCAGTGTATGAAGTCAGTAAACAAGAGAAAGGAACTCATAAATTTGCTAGATTTTCTAAAATAAAGTAATTAATTTCGATGGGAAAAAAACAAAAAAAACTCATAAGGTAACTTTGAAAAGAAGGTAGCATGGGAAAGGAGAAAATGGAGTGGGGGGAAAAGTCAATAGGAGGTGGACTATAATTACACCTCTCTTTTTTGCTAACGTCCGCTACCAAAAACACAGATTCCCTCACTCCCTTATTTTTCTAAAATATTAACAAAATATGAATACCTAACTTTTGAGTCAGGAATTATCGAAAACGATCTTTCTATTTTATAAAGATAAAGATAAAATCTATGTATTTTTTATTCTTCTTCAATTTCACTTATAAAATTATATTAAATATTATAATTCTGTATAAATCTATAATAGAGGCTCATTCATGGTTTTAAATTTTCTAGGTTTAATTTTAAAGATTAGGCATTGAATTCATTACATTATTAGAATTCTTGGTTTGATAATATAGATAATTTTTTAAGTGATCAGTATAATTTTAACTTGTTGTGA

>LNC_000758

ACCAAACCCACCACTTTCCCCTCATTTCATTTCTCTTTCATCTTTCTTAGAAAAAAAATTCAACCTCTTCTACTCTTTTCATGGAAGAAAAATCACCAGTAGCAGAAATCATGGCTCAATCAAGGCAATCCGTCTTTGTTTTCATCCAAGTCGAAGAAGAGAGTTTCACCGCAACATCTGTGTGTGGCTATATATACCCACATTTTTTCTTTCTCATTTTTTTCTTCATTCCCATGAAATTGTTTGAGTATTTTTGTTGGTTTTGTTCGATTTTTAATTTATTTATGTAAATTTTGTAACTTATTTTCGTATTTGTATGTTGTTTAGTCTAGATTTCATCTTCTATTTATGTCTTTGTTTAATTTTTAGGTTTTCATACTAATTATGTTGAAATTTTTTGAGGATTTTGTTTGTTATGCTTTCAATTACATGTTTCATGATTTCTATTATATTATAGGATTCTAACAAAAGTAACTTTCGTGATGATGTAGAAAACTACAATAGGATCAACCACAAAATGAAGCAAAGAATTAAATGAAAATGCATAGAAATAAAAGAGGAGTATAAGGATGGAAAAAATGGAGAAGAAATTTGGAGAAAGAAGATCGAAAAAGAGGCTAGCATATGGGGAAGAAGATGGCTTTATAAAACCCTAATGACTATTAGACCCATTTAACCAGATCTCACGCTCCTCAGGTGTGTGTATTACACACCCCTTTGTTGTGTCGGCCCCATAGGATGCCAAGTTAGAAAACATGTTTAAAATATAGTGTTTCGAAGAGTTCAAGAGTTCAGTTGACAAATGATTAAGTAAAAGGGTTTAGAATACAAACTCATACAAGTAGAAT

>LNC_000759

CTCTTTTTTACATGAAGCCTCACGATGGAGAAAAAAAGCTAAACTACGGTTATCATATATCTATGAAATCTGAATAAGTTGGACCAGTAAATAAATGAAATGACAAAAATTATGGAAGAAGGCCGATGTATGGCGTTTGAGCAACGTAGTTCCTCTCCTCTTCATCCGAGATGACACTTCCAACTGCTTTCACAAACACCGCAACAAACTCAAATGCCAATTTCACCATTCCGATGCATATCTTCAACCTTTGAATCATCATTATTATTCTCGAAATCAACGTATTCTTCTCCTCTTTCTATTTTTCGCGTTTTTATTTTCTCTGTGGTTCAACACTAGTACCTGTTCTATTTATAGTTTTCAACATATGTTCATAATTTGTACAACATATAAAAAGCTTGACCTAAATATGGCTTTTGAAAAC

>LNC_000760

GGCGTCAACAGTGAGCAGAAGCAACGAAACGACAAGAAATCAATGGTGTGAACATGAAGACCGACGGAAAGCAGCGAGGAACAGAGAACGTACACTGTGATCTTTTTTGGCTCCTTATTCACGTCCCTCTCAATCCGTACAACACGCGTCCTACCCCTTTCCTCTTCCGGAATGGGGTGATTTTTCGGAAAAAGGGTGTCTCCCCGAAATGGTGAAAGGATTCAAAATTCAAATTATTGGGCTCTGTCTATCCGAAATCATCCGTTTCCCCCCAAGAATTTTGAACCCTAATTTCCGATATATATATTTCCCTCGTCCTCACTGTGAAGAGGGGGATTTTTTTTTTAGTTTAAATTTTGGTGTTAGAAAATTTATTAGGAAGGTAGAGGGGAAAATACGGCTGAGACAAAAGAAATTAGAGGCTATATAGCGTCAAAAATAAGGAATCGATAGCTCCCTGTTATTTCCTCTCATTAACCATTCATCGGAGTTCAATATTCTCGAAGTTCGCTTGTGGTGGTGGAAGAGCTCGTCCAGTTCGTTGTCTCAAATCGCTGCCTCCGAAAAGGTAATATTTCATTTCTAATTTTAGTTTTTTTTTTTCGCTGTAAGCTGATGGTTGCCTAGTTTATGGTTTGGTGTTGTAGTATTTCCGGTCAGCTGATTTGAAGACTGTTTTTGCTTGGTTGTTGCGAGTCATTCGAAAAATGTTAAAATTTTGAGAACTTTCTGACTTCTTGTTGGCTGCAAATCAGTAGTGTAAACTTGTTTGTCTATTCAAATGATTTTTTCTTTAGCTATCAATGAGTTATCTTGGGGATTGTCAATACTTGTTGCAAAATTGATTCAATACTTTGGCGTACCTCGCTTATCGTATATCTCTTGCAATCGAATCTTTGAGTAGTCTTTTATTCTTAAGTTCCTCATAATGTCTGTGTAGCTTGTTCGTAGTGTTTTTATTTAGTTTTTTGGATGAAATTTTTGGCAATAGGATATTGAAGTCAAAAAAAAAAAAAAAACTATTGCCCTACATATGCATGATTTGGATCAATAGCGTGTAACCCCCTTAGATTTCAAGCTTGTTAACTCGTTGGAGTACCTATTCTTATTAAGACCACCCCCATGGAAATTGACGACTTTAATGCTCCCTGT

>LNC_000761

GTTTTAATGGATATTCAACAAAGACTCTTAGGGTATAGTGCTTGGAGATATAACAAGTTAACAACAGGGTATAAACTTTATGGAGAGTTGTGATTCCAGAAAGTATGGTAGGGGTCAGAAGAGTTCAATGGCAACAGTAACTTCAAATTAGTGAAAAGAGGTAGAGATCATTCCTGGAAGCACAGATGGATGCCAGCTTGAGCGTTGCCTTTTCCAACCATTTTTCAGCATCTCATGTAAGTGGAGCATGATAATTCAATCAAGTGCACATGTTTGAAGATGAAAAACCTTGTGAGACCCGAGATTCAGGAGGAATTTACAAGATCTAGAGGAGAAGATGTGGGTCACTCTGTTAGTGGATTTGCGGTTGTGAGGAAGTTGTGGTGGTCAACACTAAAGGTTTATTAAGTCCGACGTCAATCCGGCAACTGTAAAGCAAATTGTTTAGTTGGATCTTCAAAAGGAGGATTACGA

>LNC_000762

CAGATTTTACTAGCGCCAAGATTAGATAATCAAAACATTCATTTCAATTTTGAAGAATCTTTAATGTTTCTTTCATTTGCTACTTCTAGCATGAGGAAATTTGCTACAAAATTGCTAAATTAGAGCTAAAGGAGGATGATTCTTCAATAAACGCAATGGTGTATTAAGGAGTAAAATTTGGGCTTTGAATGGCAATCGGCGATGACAGGAGAAGCGGGAGCGCTGGAGTTTTAGTTACAGTTCAATCAAAATTCAATCAAAATATTCCGAGACATATGATATTGCATTGATGAAGTATTGGAAGAAAAAACAAGAGCAAGAGAGGAGGAGATATAATCGATTTGGAAAATGAATTGTGTTACATGAGGGAAGCTTACTGTCACGGACTTAGAGTCTCACTAAAGCGTCGTGCCGCATTCGATAACTTACTCACTATTCTTTCAAGATCTTGTAAGCTCAAGTAAGCCTTTGAAACTAAGATCGCTAAAAGAATGTAAAGAAAG

>LNC_000763

TCGGGGCAGTAACTCTTGTGATAAGAGTGGTTATCAGTTAAGCTGTAGTTCGTTTAGCATATGAATTTGGTTGTTTTCAACTTCTACAATCAGTATAGCTGTACTACTCTTGCGCGTCTTAAACTTGGATGAACTTGCTTTGTTGTTTTCCACTGTTTAAGGGAAGAGCTTGCCAAATCTGTTTTAGACTGAGCTTAACCTCCAATTTTCCTCATTGTTGTTTCATGTTTGATTGAGTCATAAGAGTTTTGAATAGATTAATTCATTATGCTCGAATGTCTGTTCTCTCGCTCTATTTCTGAATGCATCGATTTTTTCTTTTATTTCGCATTAAAGTCGATGAGTTCGTAGCTTTGTTCTGAGAAATATTAGAATTTAGTTTGCTGATGTATACATGTCTGAAGTGGATCGTGAAGTGTCACTAAATTCTTGTCCCTTCCCTATATCTGTGCCTTGTCTTCTCATATCATTTATTCCCATGATTTTCTTATCAGTGTTCCATGTTGCTTCATTGTTGCTCGTTTCTATCGGAATTTGGTACTAATTCTCATCTGTTTTTGTTTTGTTTCCTGCCAAATCCCTTTTGAGTCCAAGTGAATTTTGTCTCGATATCTTGCA

>LNC_000764

NCTTCAATTCACCCTTCTAGGTGGGCATCAAGAATCTTGGAATGCTTGAGAGTAGACAAAGCATCATTCCTTCGACGTGCAGGTAATGTAGAGAATAAGAGAAATACGACTAAAGTTATACCATTATAACAGTCCATTCACTTCTACCTGAGTTAAATATTTACCCAAAAGATATCATCATATATGCACATTGTACTACACCTTCTGCCTTCCAAACCAAACTAATTTTTGCTTCATCTTTTGATTTCATGGAATACAGTAATATAGACATAATTTACATTTTCCTCTGCAATTGGAGTTCCCTACATTGTGATAATAATGCTAATATCACATGCCATACACATGAATTCTCTTTCAACAAAATCCAGCATATATGTGCAATATTTTTAACAATTAACTCACTATGGTTCCAGTTGTACCTTCGAAGTTGATCTTATTGTTGCCAGTACCTCGAAAACAACATGTATTTCTGGTCATCTAGCTGAAAATTTCCTCTCCTTGACAGTAGAAGAGATGCCTTTTTATCCTTGTAATTCTTCAGTGTCCAAAAGCTGCACAACATTAAATGGAAGATAATAGAAAGTGTCTACATAAAAAGTGACCACATACATCAAACAAGCCTATATCAGGAAGACCCTNN

>LNC_000765

TCTTACTTCACTGTGATGTTTGCTTTGATATTTTTTCTTCCCAATTTTCATGCTATTGCTCTCTTCAAATCTTTGAGATTCAGCCTAACTTTGATGAATATAGAAGCGTATGTTTGGGGAGGGTGAGTGCTAGCATCTTTAACAATGGAGAGAGGTATTTTGGCGAGCAGCTCAGAGGGAGTTTATACAACAATCTCTGGGTTAATCAGTTGTTAAGAAGTTTGAAACCGGAGAAGGAGGAGAGCAAGATCAAACCCGAGGTTGCTTTCTCTGTTATCGCTATACAGAATGACAAAGAGACTTTGTTACTGGAAACAAAACATTACTAGGTTCTTAGATTAATGGAACAGTCTCACTCAGTATCAGGATCATGATCAATCATTCGATACCACGGAGGATGAACTAGAGAAAATGTGTGCTACTTATTTAAGGTTTTTGCTGCCTCAGTGACCGATTTACAGATCTTCTACGTTGTAGTTATTGGGATGCAGAGGATTGACTACTAAGCTTAGGAATCTAGCTCCAGCTTATTGTAAATATTGAATTAGACTATGTTTGTGTTGTATTTGAAGATACAAATAAACCTTATATGTTTAT

>LNC_000766

TATCTCTTCTTACTTCACTGTGATGTTTGCTTTGATATTTTTTCTTCCCAATTTTCATGCTATTGCTCTCTTCAAATCTTTGAGATTCAGCCTAACTTTGATGAATATAGAAGCGTATGTTTGGGGAGGGTGAGTGCTAGCATCTTTAACAATGGAGAGAGGTATTTTGGCGAGCAGCTCAGAGGGAGTTTATACAACAATCTCTGGGTTAATCAGTTGTTAAGAAGTTTGAAACCGGAGAAGGAGGAGAGCAAGATCAAACCCGAGGTTGCTTTCTCTGTTATCGCTATACAGAATGACAAAGAGACTTTGGACTGAGGACTTTGTTGCGGCATTGGATGAGTAAAGAAAATCAAGATTTACTGGAAACAAAACATTACTAGGTTCTTAGATTAATGGAACAGTCTCACTCAGTATCAGGATCATGATCAATCATTCGATACCACGGAGGATGAACTAGAGAAAATGTGTGCTACTTATTTAAGGTTTTTGCTGCCTCAGTGACCGATTTACAGGTACCTCCAGATCTTCTACGTTGTAGTTATTGGGATGCAGAGGATTGACTACTAAGCTTAGGAATCTAGCTCCAGCTTATTGTAAATATTGAATTAGACTATGTTTGTGTTGTATTTGAAGATACAAATAAACCTTATATGTTTAT

>LNC_000767

TATCTCTTCTTACTTCACTGTGATGTTTGCTTTGATATTTTTTCTTCCCAATTTTCATGCTATTGCTCTCTTCAAATCTTTGAGATTCAGCCTAACTTTGATGAATATAGAAGCGTATGTTTGGGGAGGGTGAGTGCTAGCATCTTTAACAATGGAGAGAGGTATTTTGGCGAGCAGCTCAGAGGGAGTTTATACAACAATCTCTGGGTTAATCAGTTGTTAAGAAGTTTGAAACCGGAGAAGGAGGAGAGCAAGATCAAACCCGAGGTTGCTTTCTCTGTTATCGCTATACAGAATGACAAAGAGACTTTGTTACTGGAAACAAAACATTACTAGGTTCTTAGATTAATGGAACAGTCTCACTCAGTATCAGGATCATGATCAATCATTCGATACCACGGAGGATGAACTAGAGAAAATGTGTGCTACTTATTTAAGGTTTTTGCTGCCTCAGTGACCGATTTACAGGTACCTCCAGATCTTCTACGTTGTAGTTATTGGGATGCAGAGGATTGACTACTAAGCTTAGGAATCTAGCTCCAGCTTATTGTAAATATTGAATTAGACTATGTTTGTGTTGTATTTGAAGATACAAATAAACCTTATATGTTTAT

>LNC_000768

TTTGCTTTGATATTTTTTCTTCCCAATTTTCATGCTATTGCTCTCTTCAAATCTTTGAGATTCAGCCTAACTTTGATGAATATAGAAGCGTATGTTTGGGGAGGGTGAGTGCTAGCATCTTTAACAATGGAGAGAGGTATTTTGGCGAGCAGCTCAGAGGGAGTTTATACAACAATCTCTGGGTTAATCAGTTGTTAAGAAGTTTGAAACCGGAGAAGGAGGAGAGCAAGATCAAACCCGAGGTTGCTTTCTCTGTTATCGCTATACAGAATGACAAAGAGACTTTGGTAAAATTTAATTATTTCTCTTCTATAAGACATGTTTGCTTCAACTAATGTCACATTAGTGTGTAAATATGATGGAAATAAGTTGATATGTGTGATATGAAATATTTATTGTGAATGAAAGCGGATGCAACATTTAAGCTTGATAGGTCCAGTACATAAGATTTTTAGTGTTGAAACACACTTATGAAATTATGGCCCTGTCTTAAGGGAACTGCTAATTTCTTAAATCATTTGCAGTCGGGTAAATATTTCTCACCCCGATAGTCATCGGGATCCACAAACTCTGTCAAAATCAAAGAAAAGAGATAGGGTTCAACCTTGAATCGATCAGCTGGTTAGGCAGTTTGACTATCGCAAGCTCTTGCTACCCGAAGATTTTCTGCAAAGAGAATTTCCTACTAGAAGGCATAACATAGTATAGTTGTCCAAATCATCTCTTGCTCTTATCATTTGAGATGCAAGCTTTAATTTGCACGTTATGCAACTATTTAAGATTAAGCACCTGAGAAATCACCTGTTATGTCCAGTTAGAAGCTATTAATAGTGCAACATATTTATTAAAAATATATTTTCTATCCATTGCTAAGAAGTTTGAGGTTCTTGGAGCCATGAGCACTAACGTCTGAGATGTTTAGAGTTTCTAAATAATGACCTGTCAATGTTTTAAATATGTTTTTGTGTATATCCTTTCTCCTAGCATATTTTGCTATCGCCTTTGTCATGCTTCTTTAGTATCTTTATTAGGCTCTATATACAAATATATAATGTGTAGTCTGGAATTTTTCAGCTTTGTCTAGATTGATTCTTTTTTAGAACAGCAAGACTCTCGGAAAGAAATTTTACAACTCAACAAGTTATTTAAAT

>LNC_000769

ATGTAAATAACATAACAAATAAGTTAAATAGACCAATTATACATTAAATATGTAAATATATAGTTAGAAAATTTGAAATAGATTAGTAGACTCACCTCGTAGCATTGACTCAAATCAGAAAGATATGTAAAAGATACAACACCTTTAGACAACTCATCAGTAATAGAATGATGTTTTTGAGAACTTGTCTAGGAGAGCCTCTCATATTTATAATCACATGATGAAATCAATCTGCAAAAAAATTCATAACATATAGTTTTGATATAATTTAGAATTATTATTATTAAACAAAAGAAGAATTAACTACATTGCAAATGTTGAGAAACATGCCTAAGCAAAACTAAATAATTTTCTAACCTGTATTTGAATTCAGTAGGCTGAGGAAGATTAGGATTAATCCAGATTTTGGTTTGATACCAACAACTATGATACCAACAATTACGTACTGAGTAATTTTCTGATGATTGATATTAAACAATGTCAAAAAATTATACTAATTAAATTTGTATTTTATATACAATTGCTGCAATTACAATGTTTAAACCTCTAAATTTGTGAGCCTTCATTAATTGACAAACAACGATCAAAGGTTCATCTGCAGATCCCGTCAAATGTGGTTGTATTTGGTCCGCAAGTTCACCCCATAATGTTGTTGACAGTCTATTTTTACTGTATGAATGGAATTTAATTACATTTTAATATATAAAAAAATATATATCTATATATAAAAGGATACAATGAATTT

>LNC_000770

GCTGAATTACCTGATTCTAATGAACAATATCTTTATTCACGTGTCCTTCAACATATGATGCATGGTCCTTGTGGTGATATAAATCCTACAAATTCATGTATGGGAAAAAAGGGCTTCTGTAAATTTCAATATCCAAAAGACTTTGTTGAACATACATCAAAGGGAAAAAATTCATATCCACTTTATAAGAGACGTAATACTGGAACAACTGTATACATAAGAAAACATCATATGGACAACTCGTGGGTTGTCCCATACAACCCATACTTGCTTTGTAAATTTGATTGTCATTTAAATGTTGAAGTTTGTTCAGATATTAAAGTTGTGAAATATATTTACAAATATATTTGCAAAGGACATGATAAAATTGCATATTCTCTACATGACAATGCAGAAATAGACGAAATAAAAGAATATCGATCTGCAAGATGGGTTTCGCCACCCGAAGCAGCCTGGCGTTTATTTGCTTTTCCTATTAGTGAAATGACTCCAGCTGTGTATCATCTTCAATTACATTTAGAAGGACAACAATTTGTTTCTTTTAAAAGAACCGAAAGC

>LNC_000771

ATCAAGCTTATTGGTCTAAAGTTTTTAAGTTCCTTTGCTCCTTTCTTCTTTGGGATCAAGGCAATATAGGTGGCATTGAAACTCTTCTCAAACATTTCCTGCTCATAGAAATTATGGAAGGCCGCCATGACGTCATGCTTAAGAATTTCCCAACATTTGATGAAAAAACCCATAGTGTAACCATCAGGTCCTGGGGCTTTATCAATGGCACATAACTTCAAGCATTTATAAACTTCCTGCTTCTCAAAATTTGACCTGTAGTTGTGATCTTTCTTCCTCTGATATTGAAGGACAATTCACTAAATTTCCATCAGGTCTCCATCTTTTCTGTTTCAGAATACAGTTTCTCATAAAACTGAATGATTTCCTCCCTTACTCTGTCTGGTTCTGTGATGATCTTTCCCTCTACTTCCAACTGGTCAATATGGTTGCTTCTTTTGTGGGCATTTGCAGATTATGGAAGAATTTTGTATTGTTGTCTCCTTCCTTCAACCACAAGATTCTAGACCTTTGTC

>LNC_000772

ATAAAGTTGTACTAATAGTATAAAAAATTGTACTGTACCATTAAGCCCTATTCAATATTGAATAAACACGTGTCCTTCTACATGCTTTGGTTTTCAATTATTTGCTATGGTTGTTTCAATACATTTCGATTTTTCATTCTCATTCCTACTCTTGGCGGAGAAGTCGAAAAAGTGACTTAGAAATGAAGGTATTACATTTTCGGAGAGAAAGAGCAAATTGATGGAAAATTAGTCCCCCAACTGGAACATCAATTTGGTGATGAAATTTCTCATTTAAATTGGAGGGATTTTATTGTGAATTATAATCTTCCAAACTACTTATTTTGGCTTATCCGGGAGGTGATGGATAGATGTGAAAAAGGAAAGTTAGTGTATGACTCATGTTGGATGGAATTATCAATATTACAAACGAGTCCATGCAAACATGCCTTCGATTTAGATTGTGTCACGAGGAGTTCTCTCTGCTTACTGTAAGAGTAATTACAGAAAGAAAGAGGCAATGTATTAAGAATGTATCCAGTGCCACTTCAATCAAGAGTTTACTCTAACTACTACCATGTCATGGGAGATGAAAACTCACCAAGGATTTGATTCCTTTGTGATTCAAATATCTTTGGTATACTAACTAATTAGTACTGTTGGATATCCTTGATCACGTATAATTATGTTGTTGAGTGAGGCAAGTGATCAAGAAAAACAATCAAATTCCAGAAACAGAACAAGGAGGAGCAAAACCATGAAAAAGCTAAAAATCATTCAAATTCCAGAAACATAACAAGGAGGAGGAGGCCAAACCATGAAAAATGATTGATTTCAGATTCTCTAATTTTCAAGACTTAAGGTACAATTTCACATACCTTTTTCAGGCTTTTAAAACTGTAGGTCTCTTTTTCTTCAAAACGAACCATAGTGTTCTATTTTGAATGGTGTTGTTGTATTGTTTAGTAGTTATTCTTGGATAGAGGTTGCAGTTTTAGTATAAGATACAAAAAATTTAACATACTTCACCTGTAGATTCGACTTTTATGAGTGATCTAAATTTCTTCTGTTATTTTTGTATTGTCTGAATTGCAACTTTTGATATGATAGTGGCAAGCAATAACTCGACACCATATATAGTTTTATCATATACATAATAATAGCAGTTCTGGGACTGAGAGAATGATCATACAATAAGAG

>LNC_000773

ATAAAGTTGTACTAATAGTATAAAAAATTGTACTGTACCATTAAGCCCTATTCAATATTGAATAAACACGTGTCCTTCTACATGCTTTGGTTTTCAATTATTTGCTATGGTTGTTTCAATACATTTCGATTTTTCATTCTCATTCCTACTCTTGGCGGAGAAGTCGAAAAAGTGACTTAGAAATGAAGGTATTACATTTTCGGAGAGAAAGAGCAAATTGATGGAAAATTAGTCCCCCAACTGGAACATCAATTTGGTGATGAAATTTCTCATTTAAATTGGAGGTAATCTTTCAGTCTAATCGTAAATTCCTGTGTTTCACTCAAAGGTTAGTACTAAATATATCTGATAATCATTTTTGAAGATTTCCCTCCCCTTTTTTCTCCGCAATTTTATCCTATTGATAAGTTGGAGATTTGGCAACATTTTTACCTTTTTTTGATTCATTATTTTCTGGCATTCTCAGCTTATTTAACTAATTTTTGATTGATTAGAATCTGTTTCGTTCATTTGCATTCTCGATTTAGCTAAGTTATATTCAATTAATATGCATTTCTCCTGGTTCCATAGAGATAGAAGTGTGCAATGTGCGCTATTAGGACAGAGCTTATATGAGCATTTTTTTGTGTCTTTGTGCTCGACTCACTGGCTAAGATTGTGCATATACAAGGTCACCACACCTGTTTTGTTGAACCTCTATCCACATTCACAGGTACATCGAGTTTATTGTTAACTTGTTCCTCATAAGACTAGAAAAAGAGCTTGCTTGATCAGATTTCTCTTCAAGTGTTATAGCAGCTGCATCACAAGTTTGATGAAACTTTTCAAATATGATTGAATCCGCCAATGTTCTTTATTTGTTTCTAAAGTGAAGGCTGCAAAAGTTACCTGACCTGAAGGAGAAGTGAGTTTCTTTTCTGAAATATCATTATATGTTCACGTAGGAGAAGTAAACTATTGATGGTTGACTTGTGATTGGTGATAGTTCATAGTTCAGTGATGAAATTCAAATATCGAATACTTAAGCTGGGATTTTGATCATTGACTGTTTGTTTCTCATTTCTTGCACAATGATTGTTTGGTAGTTCCCTATTAATGAAATAATAGTGTATATGAACTTGCAGGGATTTTATTGTGAATTATAATCTTCCAAACTACTTATTTTGGCTTATCCGGGAGGTGATGGATAGATGTGAAAAAGGAAAGTTAGTGTATGACTCATGTTGGATGGAATTATCAATATTACAAACGGTTGCAAAACACTTTCAAAGTTATTCATTTATAATTATTTTTCACAATTATATCAAGTAAAAACATCTTTTGCTAAGTTTTTTTGGCAGAGTCCATGCAAACATGCCTTCGATTTAGATTGTGTCACGAGGAGTTCTCTCTGCTTACTGTAAGAGTAATTACAGAAAGAAAGAGGCAATGTATTAAGAATGTATCCAGTGCCACTTCAATCAAGTAAGTTTCACTTTTTTCTGGTGATTATTTTTATTTTATTATGTTATATGTGGAATATGGATGGAGAGATTTTATATACACCCTCTTTTTATTTGTAGACATCTTAATATATCCAGCCATTTTTTCAGGAGTTTACTCTAACTACTACCATGTCATGGGAGATGAAAACTCACCAAGGATTTGATTCCTTTGTGATTCAAATATCTTTGGTATACTAACTAATTAGTACTGTTGGATATCCTTGATCACGTATAATTATGTTGTTGAGTGAGGCAAGTGATCAAGAAAAACAATCAAATTCCAGAAACAGAACAAGGAGGAGCAAAACCATGAAAAAGCTAAAAATCATTCAAATTCCAGAAACATAACAAGGAGGAGGAGGCCAAACCATGAAAAATGATTGATTTCAGATTCTCTAATTTTCAAGACTTAAGGAAATAACAAATGGTAATGTCTGGGTACAGAAGAGTAGATATGTAGAAAATGTTGGAAATTTGATGCTCTCCAAAGTACATTAGATAGTCCAGATGTGTTGGTTGACACATTAATAGTAATGTCTGTAAATGCAGGTTGTTTCCAACAACTTTGAGCATTCTCCCTTTCTTACTTTTTGATTATTTCGTTTTGGTGTAAATTTGAGTCAATATTTTATGTGCTCTACCTTATCAATGATTTTGGAGTGAATAAATTTTATTTCCATACTTGATCATCCCTTTCGTTAATACATATACACAACTTGATTAAATTGTTTCTTTGCGATAAAAATAGCTGAATAATTTTCAGCATACTCTATTATTTATCGAGACCAAATCAACTATGTTGGACCCACGGGCCATGCCGTTCTAGTATACATATAAAAGAAAAAGGTTACCACATGTCACAAATTACACTTTCACCATTTTGTTTTGGGC

>LNC_000774

CGATTTTTCATTCTCATTCCTACTCTTGGCGGAGAAGTCGAAAAAGTGACTTAGAAATGAAGGTATTACATTTTCGGAGAGAAAGAGCAAATTGATGGAAAATTAGTCCCCCAACTGGAACATCAATTTGGTGATGAAATTTCTCATTTAAATTGGAGGTAATCTTTCAGTCTAATCGTAAATTCCTGTGTTTCACTCAAAGGTTAGTACTAAATATATCTGATAATCATTTTTGAAGATTTCCCTCCCCTTTTTTCTCCGCAATTTTATCCTATTGATAAGTTGGAGATTTGGCAACATTTTTACCTTTTTTTGATTCATTATTTTCTGGCATTCTCAGCTTATTTAACTAATTTTTGATTGATTAGAATCTGTTTCGTTCATTTGCATTCTCGATTTAGCTAAGTTATATTCAATTAATATGCATTTCTCCTGGTTCCATAGAGATAGAAGTGTGCAATGTGCGCTATTAGGACAGAGCTTATATGAGCATTTTTTTGTGTCTTTGTGCTCGACTCACTGGCTAAGATTGTGCATATACAAGGTCACCACACCTGTTTTGTTGAACCTCTATCCACATTCACAGGTACATCGAGTTTATTGTTAACTTGTTCCTCATAAGACTAGAAAAAGAGCTTGCTTGATCAGATTTCTCTTCAAGTGTTATAGCAGCTGCATCACAAGTTTGATGAAACTTTTCAAATATGATTGAATCCGCCAATGTTCTTTATTTGTTTCTAAAGTGAAGGCTGCAAAAGTTACCTGACCTGAAGGAGAAGTGAGTTTCTTTTCTGAAATATCATTATATGTTCACGTAGGAGAAGTAAACTATTGATGGTTGACTTGTGATTGGTGATAGTTCATAGTTCAGTGATGAAATTCAAATATCGAATACTTAAGCTGGGATTTTGATCATTGACTGTTTGTTTCTCATTTCTTGCACAATGATTGTTTGGTAGTTCCCTATTAATGAAATAATAGTGTATATGAACTTGCAGGGATTTTATTGTGAATTATAATCTTCCAAACTACTTATTTTGGCTTATCCGGGAGGTGATGGATAGATGTGAAAAAGGAAAGTTAGTGTATGACTCATGTTGGATGGAATTATCAATATTACAAACGGTTGCAAAACACTTTCAAAGTTATTCATTTATAATTATTTTTCACAATTATATCAAGTAAAAACATCTTTTGCTAAGTTTTTTTGGCAGAGTCCATGCAAACATGCCTTCGATTTAGATTGTGTCACGAGGAGTTCTCTCTGCTTACTGTAAGAGTAATTACAGAAAGAAAGAGGCAATGTATTAAGAATGTATCCAGTGCCACTTCAATCAAGAGTTTACTCTAACTACTACCATGTCATGGGAGATGAAAACTCACCAAGGATTTGATTCCTTTGTGATTCAAATATCTTTGGTATACTAACTAATTAGTACTGTTGGATATCCTTGATCACGTATAATTATGTTGTTGAGTGAGGCAAGTGATCAAGAAAAACAATCAAATTCCAGAAACAGAACAAGGAGGAGCAAAACCATGAAAAAGCTAAAAATCATTCAAATTCCAGAAACATAACAAGGAGGAGGAGGCCAAACCATGAAAAATGATTGATTTCAGATTCTCTAATTTTCAAGACTTAAGGTACAATTTCACATACCTTTTTCAGGCTTTTAAAACTGTAGGTCTCTTTTTCTTCAAAACGAACCATAGTGTTCTATTTTGAATGGTGTTGTTGTATTGTTTAGTAGTTATTCTTGGATAGAGGTTGCAGTTTTAGTATAAGATACAAAAAATTTAACATACTTCACCTGTAGATTCGACTTTTATGAGTGATCTAAATTTCTTCTGTTATTTTTGTATTGTCTGAATTGCAACTTTTGATATGATAGTGGCAAGCAATAACTCGACACCATATATAGTTTTATCATATACATAATAATAGCAGTTCTGGGACTGAGAGAATGATCATACAATAAGAG

>LNC_000775

TACTCTTGGCGGAGAAGTCGAAAAAGTGACTTAGAAATGAAGGTATTACATTTTCGGAGAGAAAGAGCAAATTGATGGAAAATTAGTCCCCCAACTGGAACATCAATTTGGTGATGAAATTTCTCATTTAAATTGGAGGTACATCGAGTTTATTGTTAACTTGTTCCTCATAAGACTAGAAAAAGAGCTTGCTTGATCAGATTTCTCTTCAAGTGTTATAGCAGCTGCATCACAAGTTTGATGAAACTTTTCAAATATGATTGAATCCGCCAATGTTCTTTATTTGTTTCTAAAGTGAAGGCTGCAAAAGTTACCTGACCTGAAGGAGAAGTGAGTTTCTTTTCTGAAATATCATTATATGTTCACGTAGGAGAAGTAAACTATTGATGGTTGACTTGTGATTGGTGATAGTTCATAGTTCAGTGATGAAATTCAAATATCGAATACTTAAGCTGGGATTTTGATCATTGACTGTTTGTTTCTCATTTCTTGCACAATGATTGTTTGGTAGTTCCCTATTAATGAAATAATAGTGTATATGAACTTGCAGGGATTTTATTGTGAATTATAATCTTCCAAACTACTTATTTTGGCTTATCCGGGAGGTGATGGATAGATGTGAAAAAGGAAAGTTAGTGTATGACTCATGTTGGATGGAATTATCAATATTACAAACGGTTGCAAAACACTTTCAAAGTTATTCATTTATAATTATTTTTCACAATTATATCAAGTAAAAACATCTTTTGCTAAGTTTTTTTGGCAGAGTCCATGCAAACATGCCTTCGATTTAGATTGTGTCACGAGGAGTTCTCTCTGCTTACTGTAAGGTGCATATCCCGTTTCTCCATTTGTATGAGTACATTATTTTTTGTAGAACAATAATAACAATCAATACCATAGATCCATAACTATAAGTTGCTTATTAAAAATATAGCAATAACAGAAAAGGCAAGATTCTTACCTCTTCTTTGCCAACTACTACAGACATCTCCCATATTTGATTCATGTAATTGTACCCAAGGTCAGAAACAAATGTACAAAGAACTTGAAGAACCTAACAATACTCTTATTGTTATAAACTCTATTCTTCCCCCTATTACAAGTTCGATAGCACTACAAGAAAGAGCAAAATTGTATGATCATTTGAAGAAAATAAAATATGTAGCTTTTGAATTCTGCATAAAGGATTTGTTTTTATCACTCCAATGCTTCTATGAAAATTTTCTTACTAGGAGCACCGAGCACACTAAATCTCCATCCAAAGAAATAATAGTAATGAGAATCTTAGATGAATTGTCAATTTTCTATTCTTTGAAAAGTCTAATAATTTAAGTATTTAAGAATTTTAACAAGTTTATCCTCTCTTAAAATTTTGTTGAGGACTGTTTTAACAAAGAAAAGTCTCATTGACACCGCACCTTCTTTAATAATTCTGTTTGAGCATTAAAATGAAGATATTTTTTGGATGTTAATGGAAGTCTTTAACATCCTTGCAGAGTAATTACAGAAAGAAAGAGGCAATGTATTAAGAATGTATCCAGTGCCACTTCAATCAAGTAAGTTTCACTTTTTTCTGGTGATTATTTTTATTTTATTATGTTATATGTGGAATATGGATGGAGAGATTTTATATACACCCTCTTTTTATTTGTAGACATCTTAATATATCCAGCCATTTTTTCAGGAGTTTACTCTAACTACTACCATGTCATGGGAGATGAAAACTCACCAAGGATTTGATTCCTTTGTGATTCAAATATCTTTGGTATACTAACTAATTAGTACTGTTGGATATCCTTGATCACGTATAATTATGTTGTTGAGTGAGGCAAGTGATCAAGAAAAACAATCAAATTCCAGAAACAGAACAAGGAGGAGCAAAACCATGAAAAAGCTAAAAATCATTCAAATTCCAGAAACATAACAAGGAGGAGGAGGCCAAACCATGAAAAATGATTGATTTCAGATTCTCTAATTTTCAAGACTTAAGGAAATAACAAATGGTAATGTCTGGGTACAGAAGAGTAGATATGTAGAAAATGTTGGAAATTTGATGCTCTCCAAAGTACATTAGATAGTCCAGATGTGTTGGTTGACACATTAATAGTAATGTCTGTAAATGCAGGTTGTTTCCAACAACTTTGAGCATTCTCCCTTTCTTACTTTTTGATTATTTCGTTTTGGTGTAAATTTGAGTCAATATTTTATGTGCTCTACCTTATCAATGATTTTGGAGTGAATAAATTTTATTTCCATACTTGATCATCCCTTTCGTTAATACATATACACAACTTGATTAAATTGTTTCTTTGCGATAAAAATAGCTGAATAATTTTCAGCATACTCTATTATTTATCGAGACCAAATCAACTATGTTGGACCCACGGGCCATGCCGTTCTAGTATACATATAAAAGAAAAAGGTTACCACATGTCACAAATTACACTTTCACCATTTTGTTTTGGGC

>LNC_000776

GAGAAGTCGAAAAAGTGACTTAGAAATGAAGGTATTACATTTTCGGAGAGAAAGAGCAAATTGATGGAAAATTAGTCCCCCAACTGGAACATCAATTTGGTGATGAAATTTCTCATTTAAATTGGAGGTACATCGAGTTTATTGTTAACTTGTTCCTCATAAGACTAGAAAAAGAGCTTGCTTGATCAGATTTCTCTTCAAGTGTTATAGCAGCTGCATCACAAGTTTGATGAAACTTTTCAAATATGATTGAATCCGCCAATGTTCTTTATTTGTTTCTAAAGTGAAGGCTGCAAAAGTTACCTGACCTGAAGGAGAAGGATTTTATTGTGAATTATAATCTTCCAAACTACTTATTTTGGCTTATCCGGGAGGTGATGGATAGATGTGAAAAAGGAAAGTTAGTGTATGACTCATGTTGGATGGAATTATCAATATTACAAACGAGTCCATGCAAACATGCCTTCGATTTAGATTGTGTCACGAGGAGTTCTCTCTGCTTACTGTAAGAGTAATTACAGAAAGAAAGAGGCAATGTATTAAGAATGTATCCAGTGCCACTTCAATCAAGAGTTTACTCTAACTACTACCATGTCATGGGAGATGAAAACTCACCAAGGATTTGATTCCTTTGTGATTCAAATATCTTTGGTATACTAACTAATTAGTACTGTTGGATATCCTTGATCACGTATAATTATGTTGTTGAGTGAGGCAAGTGATCAAGAAAAACAATCAAATTCCAGAAACAGAACAAGGAGGAGCAAAACCATGAAAAAGCTAAAAATCATTCAAATTCCAGAAACATAACAAGGAGGAGGAGGCCAAACCATGAAAAATGATTGATTTCAGATTCTCTAATTTTCAAGACTTAAGGTACAATTTCACATACCTTTTTCAGGCTTTTAAAACTGTAGGTCTCTTTTTCTTCAAAACGAACCATAGTGTTCTATTTTGAATGGTGTTGTTGTATTGTTTAGTAGTTATTCTTGGATAGAGGTTGCAGTTTTAGTATAAGATACAAAAAATTTAACATACTTCACCTGTAGATTCGACTTTTATGAGTGATCTAAATTTCTTCTGTTATTTTTGTATTGTCTGAATTGCAACTTTTGATATGATAGTGGCAAGCAATAACTCGACACCATATATAGTTTTATCATATACATAATAATAGCAGTTCTGGGACTGAGAGAATGATCATACAATAAGAG

>LNC_000777

GAAAATTTAAAATGTATGTCTAATGGTATTCGGTATGGTATGCTAATGGTATAATAATTTTAAAAATCGAATATTGAAATTATGATACCAAAATTTCAAATACCGTATCATACCATACCATGCCCACCCCTACTTGTTATACATCAATTTCATAGCCTAATTACCAAACAAAATTAAAGACCATTTAACTATGTTCTTTAAAAAGGCATTTAACTTCCACCCCTTTTGGCTACAATTAGGCAAAAATGTAGAAATGTATCAATTTTCAAATCTTATACATGTTGATAAAAACTGGTTCTTTAATTTCTATACAGATTAGATTACTTAGTTCCTCTTCTTTCTTTATTTCTTCATCCGATCCGATCTTCGGTATTCATTTTAGGCTTTGATTAATCCGAATTAATGCCAATAAATCCCACTTTGGGAGGTAAACTCCCAAAGCTCGAATATATA

>LNC_000778

GAGCTCTGTTGCTGCTGCTGTGCTGTGTGTAGCCGTTTCGAACTTATTCCCTATGCACATTTTGGCCCCCTTCCTCTCTTTTCTTCCTCGTAATTCGTGCACCTCGGGAAAACCCCACTTCTTAAACGCTCACAAAAATCTTCAAAACAAAGAAATAGTCTTCACTGTTTGAAAACTCAGTGTCTTTAATGGTAGTACAGGACAAATAAAGAAGAAAGAACAAAGCTTTAAGTTTTTTTTTTTTTTTTCAAGAAAAGAGAGAGAGAGAGTGAGTGTGTTAAAATCAGTTTGACTAAGTAGGGTTTGTTTGGACCAACCTTCAAGTTCTTGGCTATGGCAGTGCTGTTACTACATTTTTCAGGCAGGTGGTTTTTTACTCAAGAAAGGTTAGATAGAGAAGTAGGAGTAGAGCTTGATTCAGAAGTTTCGACGTGTTCAACAGTTAAACCTGGTTGAATCTTCTGTTCCTTGTGTTTTTCTGGAACATGTGAACTTTTGTAAGTACATTGTTCCTTAAGTAGGGAATGAAGCCATCGGTCCGCGAAGCATATCAAGCTTTGTATCATATTTTGACAATAGTAGCAATTATTCATAGTGGATGAAGTATTTTTTTTGCATATATAAGCTACTCAGGTTTATATTTTCTGAGTTTTGACCATTCAATGAAAATCTAATGCTCAATTAGCTTTAA

>LNC_000779

ATTTTCTGCCGGCAGATGCTTGGTGGGAGAAAATAGATGCTTGGTGGGAAAAAATAGAACAGCTTTATTCGATATGAGAGCTTTTCCATTCATATACTCATCTTAGTTGAGCGGACTCTTCACTTGTGATGCCACACCGCTATTGGATTCTCTGAAATTACACTACTTTGGAGAATCCGACATGTACCTATTGACATTTTAAGAGTCCGAGCAACATAGATGATACATGAAAAAGTTAATAGCAATGAAGAAATTAACAAGAGCATTCACCAAACTAAGAAAAAATCATTTTGATCATTCACGATCAATAAGTATGGACGCCATGATATACTAATACTTGTGTGGATCAAATAAGTAGAGACGTATAAGGTAATACTTCGTTTTTATTACTTTTGCATTCTGATAAATTGTGCAATCTCATCCATTGTATATCAACATTTTTATCAATTAGATCTAAGCATATGAAGTCATGTCGATTTATTTCTGCATTTCAAGTTCTCTCTTTTCTATCTGATTCTAGAATTTCTTGAGTGCTTTATGTAATATGAGATTCATATGAAATATACCACTTTTACTGCATCGTGAGCACGGGTGCACACATGTCCTTGCCTGTTCACATCTCGAAAAGAAATCTTCTTAGGAGCTTGAAGTGTGTCCTGGTCTTTTCCTTGTAATGTTTGCATGACTGTCCAGTAAAAATATTTTACCTCCAATGTGGAATTAGATAAGCTAATATGCAACTTTCTCTATATCTTTTTAAAATTAACAGCTTCTCTTATGCATCTCATTTTCTCAACTGAGGTACTTAAGGGAAAAAAGCATGAGAAAATAAAGAGGCAAAATAGTTTATTTCGTTTCTCATGAAACCAATTAGCATTCAAATACTCAAGTGTGGAGATGCATTTATGTAAGTATATATTGAAAACTCTCATTTTAATTTATGATGTGTAAACAAGACGAGTAGACGATGAGCTCAAAGGAATGAAGTTAAAAATACATCTATATCCCTATTGCTGCAGAGTTTTTTCATCAGGCCAAGCTTGGTGGGCGCAAGAGTCAATTTTTTTTTGTTGTAACTGAATGATGCAAGTGTGTGGCATATCTCTAACCCATTTTTTTATATTGTGATATTGGAAGCTAATAGCATTTGAGTGTTTCTGTTTTCTTATAGTATGATCTACTATGTGCTTTCAAGTGTGGAGGTCTTCTTCCTTCACACATTAATATATTTGATGAAAGTCGTTTTTCAAAACTTCTTAGTAAAGAATGGTTCATTGATGTCAGAAATAATTGAAATTCTGTGAACTAAATTTCAGCCTTCACAAACTAAAAGAGAAGTGTACAACTACTATATTTGATGTGGAGTATCTTAATTAGATGTGTGTTTTTGATCTTTCATATATGAATCTTAATATTTAAGGTGCAGTTATTTTGAAGTGTCGTCTCTTGATTATTCTTTGTAATGATTTTTTTTTTTGCTATAGCAAGGAACTTTTCTATGCATGAATTAAAGAAAAATGTTGATTTTTAAGTCTCTTCATTAAGCTACAGTTCATATTGTAAATCCTTTTATTGATTACCATTTTGTTGAAGTGCTTAGTTATAACAGGGACCTTAATTTGGTATGTAGGATTTGAAGAAGAAAGATGAGTCCAACCATGCAGGGGTTGCTATTGAAGAGCAATACAAATGACATTTACCTGAGAAAATTACTAAATAATTAAGGAGTATGCATAAGAGCAATTTCAAGTTTATCACCCTTCTAGCATTGATTAATTTGCAGGAACAACATATAACATTTCTCTGGGCACTGCAAAGAAAATACTTACAAAGAGTTGAAGAGTTTACGTTATTTGTGACACATTGTATACTCCAGAAGTTGTGACACCACTAGAATTTGAACGATAGATTGTTAAATCATAACGTTCCGAACAACTTCTTGAGCATTTTTCTTTTCTTTTTTTTGTCCTGTACAAGTTGTTTGAGTTGCTTTAATGCTTGAGACTTTGTTTTTAATTTTTCAGATCATTCCTTATATTAGAACATACACACAACCCGAATTAATGTAATCCTTGCAATTTGTGCTTTTATAACTGAATAATTTCCAGCAACATTTTATTTACAAAGAACAAACTAATTATGTTGGGCCCGTGCTTAGCACGGGCCATGCCCTTCTA

>LNC_000780

CATTATATAAAATAAAAAGATTCATAATGGCTACCAATGACTCTTCAATTTCAAAGCTTCCTATGAAAGAAATCCCAGGTGATTAGGGTGTCTCATGTAAAATTAAATACTTTAATACACACCTTTCTTCATAAAGGAAGTTCTGTCTTTCTTCATCCAAATAAGAAAAAGATGGGACGCTCGCTTTTAGAGCACAAGAATGACTTAGAATCGGTACCGCCATCCCGGTCACACTGGTCAGCGGCATTCATTCAATTGTCCTGTTCAGAATGAATGCTACTATTACTCTTTTTCAGCTTGAGAACCAATTTGAACACGATAATTTTACATAGAGATCGTTAGGGGTACATATCTGATGCGGAAGACATCATCACAGAGAGAAGCAAAAGCGTTAGTCGTAAATTGGTTTCTACCTCCTTGCCCTAGC

>LNC_000781

GCTTTCTTCTATCCCAGAATTACCGCCTATACTCTGTATTTTGCCCAGAGATCACATTCACCCTCCCCTGTCATCTTTCCCAAAGATCACCTCAACGATCTTCCGTTTTCCAAAAATCGGATGCCCCAGGCTCTTCTCCATGGCCGCCCCTATGTGCTCCCCCATCCCCTTCGTCAGAGAACGTCTCTGGGCCGCCGAAAAAATGCCACCATCAGTCACTCAACCCTCTCAAATCGAGCTGAAAAGGTCGATTTTTTCTCCCCCGCATGATTTCTTTGTTGTTTCCTCTGAGAGTTGCGTTTCCAAGAATTCAAATTTTGAATTCTTGGAGCCCAAATTAGCTAGAAAAGGGAACCATTTGGTTCTCATAAAAGGGATCCGAAATCATCCAAAAATAATTCAAAAATCAATCGAGTTAAAACATACTTCAATTTTTTTAGAGTTCAAAACTTTTTCAACTTTTGGTCCTTCTCTTTCGACCTGAAATTGGAGTCCGGAGAAGAGTGATCGGAATCGTTGATGTTGGAACCCTTGTTTGCTGCCCTCAGAATGGTACTGTACCTTGCCCTTATTTTGTTCAGATTCCAGTTGGTTTCCTTTTTTTGGGTTGATTTTTGACAATTAAATATGGGTTGTTAATCTGTTTGTCTCCTACTTTTATGTATAGTGAAGTTCTTGTTTCTTATATATATATATATCACCTTTGTTGTTGCTTTGATTTGGATATGATAGGTATATATGGATGGCTGAAATGTTAATATCGTTTAAGCTTTCTCGACCACAATATAGCCTAATCTAAATTCGAAAAGTGCTGGTTTAATTTGATTATGAGAAGTGTTTGTTTGATGTTGTAGTTTTAATATTTTTACTTTCTTAGTTGACTCTCCTGTCTATTCTAAATTCGTGCTCATTTGGTTTAACATTCTCAGTAGTCATGTTGCGTCGTTTCTGTGATTTTCCATTCTAAATATGTTATATTGAGTGTTTGGATAATCTATTTTCGCTGCCTATATCTTAAAATTTAACGAACTTGGTATATGAAACTTTATGGTATGTATAACAAATCAATTTCAAACAGTGAGTGAATTGAATCAGAGACACTTGTCTCATATTGTTTTTTGGCTACTTTCCATCCCAAAGTGGAAACATGTACATAGTAAAACAAAACCAGTACTATGACCCTACTTAAATTGTTTATGTCTACACCTAATTGTATTAGTCAAAATAAATTTAAATGACTTTTTTGTCCCTTTCCCCTTCTTTACTTATTTTGATTCAACAGTTTAAATGTCATGTGCCCCTTTTTACTCCAAAGGATCTAGTGGAGTCGTCTCTAAAATTTATGAAACTGTTGGTCTTATAAAAGTTGATATCAATGTCTTGTGTGTATTCATGCTTGTTTGCATATTCACATTATTGCACCATTTAATTGATCACTAATCTTTATCTTCTTTTCGTCTTCTTATGTTTCATGTACAACGGGAGAGCTGGAGTTTTTACTTTGGAACTCATTATTGGCCTAAGATATTAGGCCAACAATAATTAGGATCATCGCAACGCTTGGAGTCTCGCAAATTAGGAGTCCAATGTCTTCTTAGTCTTTCTTTCGTTGTCCTACTTATGTTGTTTTGATATGTTGGCTAACATTTACTTACAAGTTTTTGATATATTGCTTGTCTTTTATTTTGAAGATTACTTATGTGAGCTAATATTATTTGTTTATGGATTTAGTTGAAACACTTTTAGAACAAATGATACAAAAAATATTTTCTTAAAAAATGGGTTAGCATTTTTACTTATAACATTCTTTCAGATCTTTTAAGTTGAAAAGAGAAGGCTTATAGTTTAAAAGTTTCCAAATGAAGTTTCAAAGAGGATAAACTAATATTTGTTTAGGCATAATTCATCGCTTAGACCTTCAAATGAATTTGAAAATAGTTTAAAAGCTAAAGCAATTACCCTTCAGAAATATATTCAAGGCTAAGATATTTACCCTTAAAAATACATATTTTTAAAAAACAAGTAGGGAAGTAGGCC

>LNC_000782

CTTCTTCCCTTTCCCAGAAACGTTTTCCTTCTTCCCCATACGTTTCTCTTCCCATCAAACTCTTTTCCTCTCCTCTCCCATCGATCTCTCTCCTCTGCCATCGTTTCCGCCTACAAACCACCGGAAAATGGCAATTTCCGGTGGTCATCAATAGATCTCCCTACGCCGTCCATCAATCATATCCTTCTCATGTCAACTTTACGTCTCTTCCATCGTCTTTTCTGAACCATGGTAGACCTCAAAAGGCCATCCATCACTGCTACGCCTGAAAAGGTGAAAGGCATGAAGAACCATCATATTTTTGGGCTAATTGTCCCTTTAATATGATGGGTGTAAGGTTCCAACATTTATTTTTTGAACGTAGTAGAGGCTCCAAATTAGCTATAAAAGAGAAAACTTTGGCTCTCTTTCATCCATTCAGAACGGAGAGAAAAAACATTCGAAATCGTTGCGAAATCAGCGAACAAGGATCTGAAATCACCTAGGGAACAAATTGTAAGATCATCTTTTGTCCTCTGTTTTTGATTTGTTTAAATTTGAGTTGGAAGTTAGAATCTTGACGTCGAAGTTCTAGTTTGCTGCTCTACATAAGGTTAGTAATCACCATCTCCTTATGTTTTGGTTTTATTTTTGGTTTCTAAAAAATTATTCTTGCGTCGTTATCTTGATTATGCCATTTCGATTCAATTAGCTACTCTAAATAGGCCATAAGAACGATTAATAGAAATCAATGGAAATTAAAAGAACTGCTGATTAATGTATCTTATATGTGTTCGTTGAAATGCCTTTTACTGTATGCATCCCCTCTTAGATGTCTTTTTGTGCAAGAAAAGTTAATTCTAGACAGTAAGCATGATGTCCGTCACCATTTTTGTGATCTATATAATTAAAATTGGCGCTGCATCTCCTTGTTGTTAATTGCTATTTTTTTATCTGTTAAGATCCAAAACTATATAGTCAAAACAGTCATAATCTTTTCATCCTTACGCTGTCATTTTGTTTCCAACTAATTTTAATCAAATAGGCATTTTGTTATATAAAATTAATTACTGTTGCATCTCACTTAAGTTTAGTGTGAATACGAGTAGTAGAATATAATAGTAGTGGCGTGTAATTCCCTCTTGTTAATTAAGAAAATGTGTTGTTGGTTGTATGTTTATGGAAAAAATGTATTTTTGTGTCTAAAGCAATTTCATCAACTTTAGATCTGCCTCGCTTTGAATGTACATTAAATCCAGATTGTCTCCTTTGTGTACGGGTCAAAAACCATGTTTCCTGTATCCTTCCTACTATAGCTCAAACTTACCATTTCTTTAGTATGTAAAAGGGGTAAGAACCAACCCACTGTTAACTTTAAGGGGACATCAATTGATTTCTTGAAAAGAAAACATGCTTCATAAAAACTCGAATAAATCATCAGCTTGATGTTACATTTCTATTGATAGACGATATATTTTACGAAGTGACTCATTGTTTTTCTTTTCTTGGTGAATGATGTTTAGTGAAGTTTTAGAACATGTAGTACTCAACTAATGTGTTAGAATAGTGACCTTGAATTATCAAAATCTAGTTGATTGTTATGATTATTGTTGGCATTATATCTTTCTTTTCTCAAATATATGGGTATCTATGCCTCAAGAAAGATAGATAGGGAACAAGCCTCTTGTATGTTTTTTTTAAACCGTGACTTTTGTTACACTTTCCAATATAATATATGTTATGTAAAGTTATGTTAAATGCTGCTCTGTTTTGAATTTGTTATTGTTATTTGAATAGTTCCTAATCTCTTTTCCTCTTCTTATGTTTCATGTACAACCGGAGAGCTAGAGTTTCTACTTTAGAACTCGTTTTGGCCTAAAATATTAGGCCAACAGCAATTAGGATCATCACAACGCTCGGAGTCTCGCAAAATAGGAGTCTAATATTTCTTAGTCTTCCTCTTTTCAAGTCTTAATTATATTGTTTGGGCACATTGGCTAACCTTTACTTAAAAGTTTTAGATATATTGTTTGTCTTTTATTTTGAAGCTTATTTGACGTGAACTGATATTATTTGCTTATGAATTTAGTTGAGACACCTTTAGAACAAATGATACATAAGACATTTTCTTAAAA

>LNC_000783

CAATCTTTTAGCAACAACTTAATACCATCTAAGCCCAAATTCTTTATTTCTCCCTAGTCCAAACCCACTCCAAACCAGTACCCCCAACCCCTGTACTCACGTCTTCTTCCTCAATACAATGAGCATTTACCCAAAAAATTAGAGAAATAATCCGCCGAAACTCAAATCAAACCCGAATCGTAGACTAGCAGCCCAGCTCACTCGCGCGTGTCCAATCCTCCCTCTCTCCCGTGTCCTCTCCATTTTCAAAAATCATCCTTCAACATCCAGGTGTCTCAATTGTGTTGCTCCTTTGTCCTTGCTCGTCAAATCAACGATCCTCCACTCAATTCCGTTGGGAATTCGTACTAATTCAAGCTGGCAAAATTTGAATTTCAAATTTAAAATGTCATCCAAATCTGCTCGTTTTTCCCCTTATCGATTCCATTCTCCCTCTATAAATACCCCCATTCTTCTACATTGTAAAAGGGGGAGGTGAGCTAGGATTAGGGACGAAAAAGATTGGAGAAAATATTGAAACTTAACTAGCATTAGAGAGGAAGAAACGGACTCTAACCATATAGTTCTTGAAAGCATAGAGGAACCGGAAATTCCGATTTCGTTCAGTCTTTACTCTAGCTTGTTCTGTTTGTTCGTTCCAATTCTCGGAATCGTACCTTAGTGGTGGTGAAGTTGTTTCAAGCTTGTTGTCCCACGTCGCTGCTCCGAAGAAGGTAACCTCTCTTCCTCTTAAATTTGTTTTGCCATTTCGGCTGAATTCAAATGTATGTAATATGGATTTAATTTACTTTGTGGTTCGATTTGATGATTCCAATCTATGATATGCTCTTCTTGATAAAGGATGTGGTTGTGTATCGTTTTAAAGTGGTAAAATAGTAGGCAGAATCTCGAAAAGCAAATCGATGTGGAGGTTGTGTGTTGTTTCGCCTATTGTTCGTGAAATATGACAATTTGTTTTCTCCATTTCGAATCTCATGGTCATTATACTCGCTCTAAGTTTGGTTTATGTGTTTGTTGATCACTAGGACATCGTCCTGTCTCTATTTTTTGTAGTAGTTTTCTTGTTTTGGCTATGTGTTCTGAAGGTGTCAAAGAAGTTATTTTGTTTCTTTTCTTTGTATTTTCGGGTATAAATTAGCAAGTTTCGTTCACATCAGTAGAGCTCCATTTCTTTATTGTCATCTTGTAGTGTAAGTGTTTTTTCTTTTCGATATAGGACCATCCGTTATGTTCTCCTATAGCTGTTGGCTCTCTGCTTTGTTTCCTGCCAATAGTAGGTTCATCTATATTTTGTTTTATGTTTTTAAAAAATACTTTCCTTTTAGGAAGATGATGTGTTGTTTAACTGTGGTTCAAGCATTGAGTCAACTTAGTACAACATGCCTTAAATGAGTTCTTATTGCTTCTTTGGTTATGGGTTCTGCAAAGGACATCTAATCGTGTTTGTTTGCTCGTTTGCCTTGATTAATTATCGGGTTGTATGCAAAGATATTTCATTTCAATATTAATTGAGTTTAGGTTAGTTCATGAATTAAAATTGATGTATTCTATTTGACCAGAAAATTGAGTTAATTGGGTATGCCTTGAATTTCGATTGTTGAGTAGCCGCATTTTGAGCATGAAGTAGCACATTTGTTTGTGTTCTCTTATGCTTCCACTTTGGTTATTGTTTTTCCACAATACTAATTCTTTTTTTTTATTCTTATTTATGTGAAATCTGCCCGAGTTTACCATGGACTCCTCCCCTTGCATTTTCTTGTTGAGCCACAAAGGCTCAAACCCTTTCCCCGAGTCGGTCAGCAAGAAGTCGAAATACAGGTTTTCGGAGGATAAACAAAGCTGGATTTTGATTTTTTGAGCTCCGGAAAGGTCGAAAACAGGCTGTGTACCTGCTGTATACAGCAGTATACGCTGGTATACATGGCAAAATACAATATACAGGTGCAGGAAAGGGGAAATACAGGTCAAAAAGGACGGAATACAAAGTGCAAACAAAAATACAGGTTTCGCAAACCCTAAAATGGGTTGGATACGGCTCGTATACATCGGTATACACAAAGTATACACAGATTTTCAAAAGGAAATAGGCCCAAAGGCCTTGATAATTTCCAGCAGGTTAGGTGGTGCAGAAATGGGCCAAAGCCCAGTGAGAAATCAGAAAAAAAAAAGAAAATTGAAGAAGAGCAAATGGGCCAAAAGGCCAATTTAATTGGATTTGGGCCAAAGTCCAAGTCCAATTATTTCTCGTCCCTTTATTATCGTTTTTATTGTATTGATTGACTAACTTTGTTTATTCATGGTTCAATTCAATTAAATTCCCGAAGATAGCCATTTCTCTTTATATATATATCTGAATTAAAATATTTTATACTTAGCTCTGTTTTTCTTATTAGTCT

>LNC_000784

ACCAAGTAAGTGTTAATCTTTGTTTCCTTTACTTTTCATTTACATTTTGCCCTATTTTTCGTTTAAAATATGCCAAATATTAAAGAAAAATGGAGATTTTACGTTTTTTTACCGTTGTAGCTTTGTGATGTGGCAAAAAATCACTTCCTCACGCGCCTAATAGAGTGTGTTGTCAATTTCTACGCCAGGTGGACGAAAAAGGTGTACGCGACAGAGGAATAAACAAGTTCGGGGG

>LNC_000785

ATCAAATAGAAAGGCTCGTCCAGTGAGTGATGACACTAGCGAGTAAACTGCAAAAGGGTCTTGCTTGGTATAATATAAGCGGATGACTTAGTTATAATTAGCTCAGATGTGACTTCTTTGCTCGGTTTCGGTTGCTTGACTCAATAATGGCCCTTCAAAGTCATTGACTCCATATTTAGGGAACGGTTGAACTCCTTTTTTCTTTTTCAAGCTGTCGACAGTCATGTTCTTATATTTCTTTACTGT

>LNC_000786

AGTTAGTGGAAGGTTTTCTGCGTTCAGTCAATCTACGCAGAGTGAATCGGTCCCTAAGGAACCCCCGAAAGGGCTGCCGTCCGATGGGTACACGAAAGTGACGAAGTTGCTTTGACTACAGAACCATGCCTGTCTGTTGGAGCGAATTGGATGATCGGGCCGAGGGCTGCCCTCTCTTCCCCTCACTCTCCTTTCCCTAATATGAACCTTGAGTCATCAAAGCCTTTCTGACTCGGCCTGGCCCGGTCGCCCTACGCGACTGGCGCTTCAAAAGGCGAAACTCTC

>LNC_000787

CTCACACTACGCTCACTTCTTATCTCTATCTACCTCTACGAAACAATTTCGATAGTTTGCACTTTCATACATACAAAAAATAATATAAAAAAGATGAGACCATCGATACAGATGCAAACAAAATAAATCAAATAAAACACTTTATTAATTCCTCTCAGAAGATTGCCATAAATCAGTTTTTTCCAAGCTTTAATCATATAGTCTCTTATTGAAGGATCACTTCAGAGTTCTGTTTTGTACTTTTGACTTACAGATCTGCTAAGTGGTAAACATGCTTGGCAGAATCACTAATTTGAGTCTTGGCGGTGTATTTTGTTAAAGTGTTCTTGACAAAATGGTTGTGATTTCTTTGTCTTACTATTTATTAGTGCTCTTAACTTTTTCTTAAGATGGTGTAACTGAGCAAAATAGAATTTTAATAATATTATCTAGAGAGACTTTAATTTGTGAGCCGGATTTTTTTTAATGGAAAGAAGAATGGACTTTCTTTTTTCAGCGAAGAAGAATTGGTTGAAGAAAGAAGATTTGAGGGAGAAGGGCTTGCTAGGTGTCAATATAGTGGGATCCACCGTTAAATTTAACAACATTTTTTATCTTAATGGTTAGTTATAGGGGAATGGACCAAAATGATCATTTTTTACTAATCTTCAAGGACTATTTTAATAAGGTGATATATATTAAGGATCAAAATACTTTAACCCCTATACATCAAGGACCATTTTGGCCATTT

>LNC_000788

AACAATATCACCGACCAACCCACTTTTAAACCTCCCAAACCCGGCCCAATTCTATTCTAAAAAAAAAAGAAAAAGAAACTCAGCAACGTGGGCAAACAGAGGAAGCAATACACAGCAAACCCATATACCCGATCCCTGCGCATTTTCTTCTTCTTCTACGCTAATCTGCCAGTCCAGCAGCTCACGTGAACCAGACAGCTGCAGGGTGCGTTTTCTTCTCCCTCACGCGTCAATATCCAACAACTCCCACAACTTTCTCTGCAACAGCCGCCCATCTCGATTTTTTCATCCGAATTTTGGGGGATTTCGGTTGAAATTTGATACAAGCAGCAGCTTTTCCTCCCCTTGAGAAAAAAAAAAAAAATTCGAAGGAGGTTGAATCCGGATTGACGATTTTGCCCCAAGCTATTTCAAAAAATTCACCCGACTCTTTCAAATCGGGAGCTCTAGTTTTCCCCTCTATATATACTCCCTCTTTCTCCATTGTTGGGGGGATTTTTGATTTTATTTTAAAAGAGTTAGAAAGGAAGAGAAAGAAAAAGAAGCGATAGTTAACAAAGAACAGTAGACGAAAAATATAAATAGATTTTAAAGTGATAATTCCCAGGGTTCAGTGTTTAGGGAAGTTCGAGCCCTCACGGAAATATTTTGAGGTTGGTGGTGGAAGCATCTTTAGCTCGTCCTCGTCTCAAGTTCGCTGCCCAGAAAAGGTAATGTCTTCCTCTTTGTGACGTTCTTTCCACCTCTTCTTCCATTTCAGAGTGACATTCTGTTGATTGAGGCTTGCTTTTAGTTTGGTTGGTGGTTGGTTGTTAGTTGCTATTAAGTTGATATGTTATGTTACATATATATAGAGTTATTTGTTATACCAAGTTAAAGCTTTGGATATTCGTTATTACTTCTCTATTGTTGTACTTGAAGTTTTGTCTTGTCGATATCTTTCCTTGAGCTTGTTTCTCGAATGTCATCGAATGCGTTTCACTGCTTAATATTCGACACCACGGGTTTCACTCTTTCATCTCAAGTCGAAAGAATGGGCTAAGATGTCAATATTTAGAGTTTATGTGTTGTGCTCGCGACTATTCGAAATTCGTATTGTGTTTTCTGTCTGTATAAATGGCTTCATGGGTTGCAAGATGATTTTAAGTTCAAATTATATCGCGTACATGTCGCTGAACCGATTTGCTTTTGCACACATTTACTCTGTTTTTATTGTTGATACTGCATGCCCTCTGGAAAGAGCTATTTTAGTCAAGTGAAATATCACAATTTGGATAAGTTTCTAATGTGTCATTTACGGGAAGATATCCTTGTATTTAATACGTATTCATTTTGAGTGATTTTGCCTTGGTTTGTTGTTTGAGGCTTCCTGTAGTGGAGTTTTACGATTAAAAAAAAAAAACTCTTCGTTATGTTCAGTTTTACGTAACTGTTTGGTTCATGTCCTATATTTTATCGAAAAAATACTCTCTTCAGCACATAGTAACCATATATGCTTTTTACTCCACTGACTACTGAATAAAATGTACATATTTGGGTTGGGTAAATTTATTTTCTCAAACCTGTTGCTAAGTTGATAATTTATGGTTGTTTATTTATTTATTTTTATTATTTTTATGCCACCGTGCAGCGTCATTTTGGTATCACTGTTGTTGTATTGATCCATTTTGTTTGTAATAGCAGTTTGTCCATATGCCCAACTTGTTTGTTTTGTTCTAATTATTGGGAAGCATGTTGAATGTCTATTATTTTATTTTTTATTTTTATCTTAGTTTATTTACTTTACTTTATTTTATCTTATTTTTGCGTGTTTGTCATCACTCTAAATTCTACCTTCTTCTCTTGCATGTGAAATTCACCCGAATTTGCTACGAGCTTCTACCTTCTTTCCCTGCATTTCGGGAGAGCCGTGAAGGCTCAAATTCCTTCTTATCCGAGTCTATCAGCCCATAAAGTCGACGAACAGGTCCCGAAATTGAAAGGAGCAACCGATTGAGAAAATTTAAAAAATTTGGGCCAAAGGCTTACTCTTAATTCAAAAAAATTTGTATTTTGTTATTTTGGGCCTAAGCTTGTTTAAGTTTATTATTTTATTAGTATTTAGGACAGGTGAAAAATTGGGCCTAAGGCCCAAAGAAATGTCTTATATATATTAGACTAGGACAGGTGCAGATAACTCAAATGGGCCAAAGACCCAAAACGAAAAATGGGTCCAAATACTGGTCCAGGCGGACAGAAACCAGATTTGGGCCCAACTCTCTTTCCCTCTCTACTTTACTGTGATCATTTTACTTGTTAGTGTTTTGCCGCTAATTTTAAAGGTTGAACGATTCAAATCCCCAAAAGACATCCATTTTGAATTAATCGCTTAACTAAATTATTCCTCTTTTATTTGACTTAAAAATAAATAAATAATAAATAAATAAGTTTATGAAATACTTCATTTAGATAATATTTCATTATTTTTCTATTAAAATAGGTCCCAACTATAAAGTAGTTCACTTTTGCAACTCAAGATAAGTTAAATTATTTTAGCCAAGTAGTTAATCGTCGGATAACCGCATTAGTGGACATTCTAGGTGCTTTTAAAACCTTCCTAGAATGTTAATAAGAACCTCGAACCCTTTTTAAAGTTTTCATTAGATTTCTGTTTTAGTCTTTTGAAAACAACAGTTTTN

>LNC_000789

TCTAAATGTATTTACGATTTAAATTTTGGTATATTAAGAAAGGTAATTAATTATTAGGTGTATTGTATTATATTAATACCACTACAATTATGTCAATTGAAGGGATAAAGACCTAACCCTAAGCTTGAAATTTAGGAAAGTAATTTTTGGATTGAGTGAGTTTTTGGGTCTAGGCTAAACCTATAGTAATATGGGTGTTGTTAGTTTCGAAATCTTATTATGATTATGTACTTGAATTCCATCAGCCAACCTCCCCTTTCATCTTCCCCAAAAGATCACCTCAACATCTTCCTTTTTCCAGAAATTCGGACGCTCCAGGCTCCTCTCCATCACCTTCCCCACGTGCTCCCCCATCCCCTTCGTCAGAACGGCTCTGGTCGCCGGAAAAACGCCTTATCAGTCACTCAATGCTTTCAAATTCGCCTGAAAAGGCGATTCTTGGGGGATTTTTCTCCCCCTTCATCATTAGTCCTCCCGTTTTGGCTTTTCAAGAATTCAAAATTTGAATTCTTGGAGCCAATAGAGGTTGCGATTTTGGCTATAAAAGGAGCCATTTGGTTCCTTCTAAAGACGGAGAAAAAGATCCGAAAATTAGATCCGAAAAAAACAAGTTAGAAAATCGATCAACGTCCAAAGACTGGAAATCCAACACAAAATCCAAGGAAACCAACAAGGATTCGAAAATCTTTTGTTCTCTGTTTTTCGTTCTTCTTCGAGTTTGAAGTTGAAATTTCTTGACGTCAAAACTTTAGTTTGCTGCTCAACACAGGGTAAAATTCTTTTTAAACCTCAATTCTTTTTAGTTGATATGTTTGTTCACTGATGGAAATAAGTTTAGTCTTAATGATATAAACTTCTGCTCTTCTAGCATAACTTAAACCTCTTGACGTTTTGATATCATACTGTTTGTCTTCATTTAAAATCGAACGTTGTATTAATCTAAAGTTTCAAAGTAAGACTTGCTATAATCTCAATATGTATTTTTTGGATTGTTCTAGGCTTAGAAGTGAGTCTTTGCTGATTTGTATCTATCTATTCATACATACCTATATTTTAGTTGAATATGTTGAAATTTTTAATTAATGTGGGGCTGCTCGTTCTGATTCCTCTATAGAGTTACTTTTTAAGAAAATCCATCAGTTTGATGTCTGTAAGTTGCGGAAATTCACTCATGTTTGTCAATGCAAATTCAAGTTGCCTAGAAAACACAAGGCATGTTTATGCTACCGATCTTGAGTTTTAAAAAATATAATTTCGTGAAATGTTCAGCTTGAAAACTCAAGATCGGAAAACTCAAGATGTTTGAGTAGATGTGTTACAGCTTAAAAGCTGTATATGTTATCTTGTTGTTTCAAAATTCAAATTCCGCTTAAAGTTTGTGAATTCTCTATTCCAAACTTTGTTTACCTCTCCACTTGTAACTAAGTTTTTGTATATACAAAAAGATGTATTCTTAAAAGTATGAATGAAGTAGCGTTTAAAATCAGTCCTTCACCTGCTACCGTTTCAATTTTTCAATATGAAATCGATTTCCTCTCTTATGATGGTCTTCTTATGGATGGTAGTGTGGAAGTATTCAAATTAGAATATATTGTTAATATATTGGATTAGGCTACTGCTTTTCTCTCAAGAGTGTTGACACATAATAAATCTACTTTTTTTTGTTCATTAGAACTCCATATTATTTGCTAAAATAGCTAAGATATTTATAGTATTTAAGCATATTCAATACTTTAGAAGTTCTGTCAAGTTCTTGTCAACTGGCCTAGACTTCAAGTCTGTTAAAGCTCTTTAGTTAAGAGACCAACATGAAGTCTTCTCTTATTTGTTTTATTTATGTCGTTGATGTGCGATATGTGAAATCTTTCTTCTCTGTTTTGACTCTCTAAGTATAAGGTGGGGTTGTCGGCTTGTAATGAACATCTACTAGGCATTATCAGGGCTCATGTGAACTAATCGATTTAACTATTACTCTAATATGATCAATTTTGACCTGTTTCATTTTTTTTTCTTGGAGAATATGTTTGCTCTTGCTAAATAATATTGTGAGTCAGGTCATCACTCGTCATGTTTGAATGTTCTTGGCACTTCCTAGTTTCTGTTTTATGTCTGTTATGGAGTTATAACTTGACTGAAATTATATCTCGTATATTTTTAGCTCTTTACCGCATTTAATTCTATATCTTTGTCGTCAACTCCATAGTATGTTTCAGTTTTTTTTTAATCAAAACTTTGCCCTCAAATCTTGGGCAGAATGCATACATGTGTTTTTCTTGAGTGACTGTTCTGCATATTTAATAGAATAAAAAATATTTCTCCTATTGTTAGTAGAGGACTGTTGATGTATCACAATGGAAGGATTGCAGAATTCTAAACTTAATCTATCACTTTCAGCATATGATATTTGTGCATATTTATCTTCTTGGGATATCAGGCAACTTCTCTATAAATTAGAAGAGTAATAATAAAGTGAGAATCATTTTTCAGGAGTTTACCAAAGAGATGGTTAAATAAAGAAACAAATTCTTTTCTTTTGAAAATTTACTAATCTGTTAAGAGGGTTCCCAAATTTGGATTAATGTTAACCTGGGAGTAACATATGGTTTGAAACTAGGAACCTAAGGTTGAAATATACCATTTGTATGCCTAGTAATAAACCTGTAAATCATAGTTGTTCAACATGTCTTGTTTAAATGATTCTATGTATAGACTTCAATAAACTTACCTTGATAAATATTTATTGTTCCTAATTCCTCTTCTTTCTTATTATACATGAATATTTGGAGCACATTTGGAGTTGTAGCACGGCTCTGGTCTGGCTCATATCAGAAAAGCATTAGCAGCTGTATTTTTGCTCGAGTTTAGCATCCGCATTTCGTCATCGGTTCCGTCATAGAGCCGGGCTTACAATCGGCCCATAAGGAATAGGAGTCTAAGATCTTCAGTTCCTCACCTTCCTTTTACTTTAACTACATCGACTTGTCTATATATACATATATATTGTTATTATTTTTTTGACCAACTCATTTACTTATTCACCATATATTTTTCTTGGGTTATTGCTCTATGTGCGGGTTCGTAGAAAC

>LNC_000790

TCTTCTTATTCTTTTTGTTCTTCTTGGGCAAGTGGGAGGAAATGTCAAACATATTTGTTTTGAATTCATGATCGTTGTCGCTGGACTATTTTCTGGAAGGGATGCTAATATGTTTTTGGTTCTTGATTGTAGTTCGTTGTTTTGGTTGCTGGGAGAGAATAATAAGAAACAATAATCAATTTTTGTTGGGAAAAGATGTTAGATTGTTTGCTAGGAGGAAATGATAAGAAAAGATGTCAGATTCTTACATGTCACTGAATTGTTCTCAATTTCTCTAGGGTTTTCTGTGGATAGCAATGTATTGGAGAAGAAACAATGGCATCTGTGTCGATTAGCATCACCAGCCTCGTCGGCATCTGCGTTTGGAAGCTCGAATTTTACATCGTCAACTCCATCAATGGTGGCATTGATGTGTATGGAGCTATGGCATGCGTGTGTTGGATCATTAATTTCTCTGCCGAAGATGTGAGGCGACCAGATAATGGCAGCAAAACATTGAGTGAACTTAAATTTCAGGTAACACTCTGTTCATATGAGATCACTGACCTCATCCTTTTTCTTGGCATTTGGTATTGACTTGTTTCTTGCCACTGCGGATAGAAGATTACTTGGATGTGGCTATCCTGATCCAATGAGCGTTATGGCACCTGTATTGGAATTATAATTACGCAGA

>LNC_000791

CAAAATATACATTACATACTCTTTTTCTACGAGATGCTGGGAATCCCTTGTACCACATAGTATGTAGATTTTGAAGAAGAGTATTTGGCTTTCATTTATTTTGATTTAGTTTTTTTTAATTGGAGTAGTGTAACGATGTTACTATTGCCAAATTTCACTGCCATAAAACGTATCAATAGTTTTCTATTCTAAATAGAAAGAAAGTCAAATTTGTC

>LNC_000792

ACTTGCATAACACGACAGTCATAGGTGATTCAGGTAAATACAGAGGGATAACACACGCTGCTCAATTAGTTCTAATGGTAAATGTTGTTTTATTACATCCTCAAATAGAGGCCTCACATTCGAACTTGGGAAACATTAAGTCAATAAGTGCACTAAAATGAATATAGTGTGAATAGACTTCCTTCATCAATTAGCCTCGCTGAATTTGTTAAATTATCACACAAATTTTCTTAAGTTCTTGATGAGGTCTCTCTTAAGAGTTATCTCCTCTGTTTAATCCTTCAGTCTTTCATACTCGCAAATATCAATACTATGTGTCCTGAAAGAAATTGGGAGTGTAACACTACTTGGTTTGTTCTGTCATGTAACCTGAGATAATTTCTTTTCTCAGC

>LNC_000793

TGGGTAATTGAAGTCAATTTGGGAGATTCCGGCTAAATCAGGGGTATATGTGTTAAGTTTGTAGACGACGAGGATAAAAATAAGAGCATAGTTAACTAATAAATCAAAGTTGAGGAATATTTTTGACCATTTTCCTTTTTTAAATTATTTATGTGTCCCTCAAAAAGATGTATATACTCTCTGACTTGTGTGGGTCCATTTGCAACTTTAAATGCTGCTACATCTGCCCTTTTCTATTTATTTCTAAACTTAAATCTATAGTCCAATGATATTTTTTTTGGAAAAAATGACAAATATAACTTCGAACTACCATAAGTAATATGTACATATCTTCGTTATATTTTTAGAACATTAATGCTCATGTCGTTCAAAAACTCGAACATATATACCCTTCACCATTAATATTTACAATTGTTTTTTTTA

>LNC_000794

TACCTTTTCAACCCGTGTATTTTTCAACGCACAGGCTTTTCTAAATCATTTTTTAACCAAAATAATGGCAAGCAACCGTCTCTCTTTGAATGCCCAAAAAATTTCACAGACAAACTATCAAATTTGGCCTGTGAGAATAAAGTCATATTTTGAAACCTATGTGATATGTGATGTTGTGATGAAAGAAAAACCCTTACAACCTCTCTCTGCAAATCATACAAAAGTTCAGATTAAAGCACTTTCAAAAAAACTTCTACACAAGACAATGTCATTGCATGTGTTTTTGAAGAACAAAATCAAAAGAAGAAAAAGAAAAAAGAAGAAATTTTGCAAGTTCAATAACAATGAAAAAAAA

>LNC_000795

ATTCCATTAATTTTGCTCCAAATTTCCTCTCCATAAATACCCAAAGTTGTCACTAGCTTTTGCACCCAACACTCAAAACAAATACATTTGTAGTACTATATCATCCTCTACTATTTCTATCGATCTTTACTCAATTTTTGTAACAATGGAAATGTTTGGCAAAATTGCATGCTTTGTGCTTTTGTGCATGGTGGTGGTTGCACCCCATGCAGAGG

>LNC_000796

TATAAATTACATAAGTAGCATTGATAAATAAAGTCATTTTTCTGTCTTGGGCATCAAAAAAATAAATCGTGACTATTCTCTAACTCTTGAGAAGTTCAATGATTTTAGAATAAGATATTCAGAAGCATATATCTACACCACAAGAGAAATTGTTAATAAAAACAAATGAATTTAGACTACAATAAAAGTTAAACTATTACAAACTTCAACTGACGTTATTACAAGTCATATAAACTTTTTTTCGTCAAAAATACTTAAATATTAAGAAATAGTAAATCAATAAAACAAAAGGGCACTGTATATATAGTAATAACTATTTTGAAGCAAACTTTCTGACAATCTAAATTACATAAGTAGCATTGATAAATAAAGTCATTATTTTGTCTTGAGCATAAAAAACAAATCGTGACAATTCTCTAATTCTTGAGAAGTTTACTAATTTTTGAAAAGATATTTAGGAACATATATCTACATCACAAGAGAAATTGTTAACAAAAACAAATGAATTTAGACTTCATCATGTTAACTTCATATATTTGAACATTACATACTAATGTATAAACTAAGCAAAAAAAAATTATTTTATCATCAGAATTATTTTCCTCAAGTACTCGAGGTTAAGGAATATACCCTCTTAGGATAGTACAATTTACTTCATCATAATAGTGGTACCTCATATTCCGCTAAACTTCAAACTCACTTAATGACAATAAATCACACGAAATATTTTTAAATGCAAGAGGAAGAAGAGTAAAAAAAATTGTAGTTGAAAATGAGAGGAAACCCCTCTATTTATAGTCAACAAAGGGTAGTATGAACAAGTATTTATTTTTGTCTTATCTGAAAAGTCATGACCTTTCCGGGAAGTCACAACCCTTTGCAAAAGTCACAACTCTTTGGAAAAGTCACAACTCATCAAAAAATCACAACCCTTTGAAAAAAGTCACAACTTATCAAAAAAGTCACAACCTAAGTTAGGGGTATTATAGTAATTTAATATCCAAGTTAGGAGTTCATGCTTTTAAGGTAATATAGATATGTGATGTGATGAATTACATTGAATTTGAAGAATAAATACAAGCTCAAATTAATGAATAATGAGTCGATTCAACCTAAATTATTAATTGCAAGGTACATCATATATATTGTTATCAACTGAATTTCAAAAGTAAATTACTCTAACGACTTTAAATATGCAATTCTTATAATATACATCGAATATTTAACTTATTAAAAGTTTTATATAATATGTCACAATAAATAATTTAAAATGTTGGTGTCATGGTTGTAAAATGTTTCATGTCTTATAATTTTTGCCTTTCTTTTTAAGAAATAACAACCTCCACCTTCTTATAGTTGCATATATATTAAGAAGTTGTCATCAAAGGAATGTAGTATTTAATAAAAATTGTACATAAATTCGATCAACATACTTCCTTACTATGAATAGTAAATCATTATCCATTTCAC

>LNC_000797

GGCTTCCATCTTCCTCCACCTTGGTTGCTTCCTGTTCTGCTCGCTGAAGTTTACCGCTTTGCTTCTGCTTGATGTCGCTTTTTCAATTCAGATTAGTACTAGCTAACATCAGATAGGCACAATGGCAACTCTATTTGGCAATGACGTCTTGAAGTTCAAGAATCTAAAGCGTCTACTTCCATGAAAGATTTTCTAAAGTATCTATCTCCTAAAGTTTGATCGTCCACCAACTGAACAGGTAAATTGTTGAAGATGCAACAAAATCTGGACCAAGGATAGTTTCATCATTTGGTGTCCTTTTGGGTTTTCGCGTGATTTAAACACAGTAGCAACTCACGCTCTTCCCACTGACAATTACCAAGGAGAAAAAATGGGCACTCCTTCTTCCCTCTTTGCTTCCTTCTTTGGTTTGCTGTTTCTTTATCATTCTTAATTCAAACTTTTCTATTTCTTTTTATACATCAAACATCTATTGTCCACATATAGCTGGTCATAAGAGTTGTTGTCGAGGTCCTATTTTGTTTTCACCTTCATGTATTGATTTCATTTGCTTCAATTTGACTTGCTAGAGTATATATATAGCATGTTGTTGCTTTACCGTTGCGTGGTTGGTCCTACAATGAGTAGCTTTAGATCTTCCTAAAGACTAGGCTGTTTTGGTCATTTCAATAAGCCTTCTGAGGCACTAGCTGTCCTGTATCTAACTCTTAACATGGTTGTGCTTTGTTTTGTTTCTAGCTGTGTGTGTTTTGGTTTTTCT

>LNC_000798

GTACAAGCAGGGACTCTAATCGACCCACCCGCTAGGATTCAGGTTCAGCTGTGAGTTGGTAAGCTCCATCTTATCCTGGGGCTATCAGAGGATGTTTACTTTTGTTGTACAGTTTGGGTATAGTCGGGGCCTTGTCCCGACAGTGCTTATGTCACTCATAGAGGCTATTGTGGACACAATATTCTGGGTTATTTTTTGTATGTTTCAACCTCCAGCCAATAGTCTTGG

>LNC_000799

TCAGGGATATTTTTGACCCTTTTCCGAAAAATTACTTCTACTTTTTATGACTATACAGGTCCGTAATCCTAATATTGATTATTTAATATTCTTTTATGTATTTCTTTTCTTTTCTTCGTCTTTCCCTCAATTCAATCACCTTCTTCCTTCAAATTACTGTTCATTGGTGTTCTCGGTTAGATTTAATATATATATATATATTATATATATATAATTGTAACTGTTTTTACCATATTTGGGGATGCATGTTTCTTTCCTATTTGGTTATATTTTGAAAAATATTAATTAATTGAAAGAAAATCCATTATTTGGAGAGTTTGTGTTCGACACTCTTTTCAGGTTTTGATCAATATTGGCCGTATGCAGTTATAATTTATGTTTTTTTTTTTTTCTGGAATAAATTTGTGGTGATTGCTATTAAGCGTATAAGCGCACTTCACTTTGCACATCATGTATTCGATAAAAGTCCTGTTCATATTTCTAGAACAAATGTCTATTGATTCAAATCCTTAATTTCCCTACAATTGAAAGCGATGTATGTTTTAAAGTGCATGTGCTCAGAGACTTGACAGAGAATAGAGATGCAAACAGTTTCACTTTCGAGAATAAATCAAATTGGTTAGCTTTTATTGTAGCATGTAGAAGAAATGCTTGTTGGATTGTTGCTATGTCTGCTACAGTTACAATAGTTGAAGATAGACTATCAAGTATTTGAATTAATGTAGAAAAGAGACATCTATATGATTGAGAGAAGTTGATAATCAAGTTGACGTCAAAAGGTTGGTGATCAAGTGTAATATTGATACATTCAAACTTACATCTCAATTAAGAAGTACAAACGGTCGAAGTCAATTATAGAACTCAACTGGAGTTCGTCAACCCCTCCGTAGTCCCACACTTAGACAGTGGTGTCTCCCTTCACTGTTGGTCGTCACGAGCTACTAGACAGCTCGTCTTAAGCACCGGCCATGGTCCGTCTAGTGCTCCGTTGACTTCAGTTTTCACTTAGGTAGCTAGTGGTTCCACTGTCACTCTCCACGAGCCATAAGATGGCTCGTCTAATGGACCACGGCTCGTCTGGGGCCTCGTGGTGTTAGACTTGAACTCTATTCTTTCCAAATCACTCCCACGAGCATCTTCACGGCTCGTGCAACACTTCACGACTCGTGTAGCCTTTCGTATAGCTTCTTGCACTTCATTTCCCATCTTTCTTCTTGTCCTTTGCATCTAAGTGTTTCACCTGTACAACATAAAAGAAACCATCATATCCACATAAACAAGCTCGACTTCTCACACTAATCCTTAGTTTTGAGCGTCAAATGTTCTATGAGTTCACGACACATCAACAAATGATCATCATGATTCATGATTCTTATAATATCAGGGCCCGTGGAGTAGAAGTCGAGTTTATTACTGCACAACTATGCACAGAGAGTAGAGTTGGATTTTGCAGGAACTCAGTGCAAGTGTAAGTCCACGGAATAAGAAATTCAATGGTGTTGGTGTTTCTCTGGCTATTTACCTATTGACAATTGCTAATGTCATATCTTATCATATAATGTTATGCAACTATATTGATTGTATATTTGTGTCATCTTTTTAGACACTCAGATATGATGGTTGCTACCTTTTTCTTCAGTGGATCGAGCGTAAGGATACCACGAACAAGTCGTAATTCATAACAAGGGGAGAACTCAAGTTCTTTAGAGTTTCCAAGACTTGATTCGAGTACAAGGCATATGCATGCGGCTGCTTTGAGATCTTAGTTGTGAGATGCACGACATTTGATCCAATGGTGAGATACCTCTTCTGAGCTGATATGGATTCTTCCCTACCTTGTTGTTCATCTTTTCGAACATAAACATTTTAGACTTTAAATTTATGTTTTCTAGTTGGGGTTGCATTCTCTTATCCTAGATTTGTGAGATGTTTTAGTATTATGACTTTGAGGTTGTAGGGAGTTCATTCCAC

>LNC_000800

TCACCTTCTTCCTTCAAATTACTGTTCATTGGTGTTCTCGGTTAGATTTAATATATATATATATATTATATATATATAATTGTAACTGTTTTTACCATATTTGGGGATGCATGTTTCTTTCCTATTTGGTTATATTTTGAAAAATATTAATTAATTGAAAGAAAATCCATTATTTGGAGAGTTTGTGTTCGACACTCTTTTCAGGTTTTGATCAATATTGGCCGTATGCAGTTATAATTTATGTTTTTTTTTTTTTCTGGAATAAATTTGTGGTGATTGCTATTAAGCGTATAAGCGCACTTCACTTTGCACATCATGTATTCGATAAAAGTCCTGTTCATATTTCTAGAACAAATGTCTATTGATTCAAATCCTTAATTTCCCTACAATTGAAAGCGATGTATGTTTTAAAGTGCATGTGCTCAGAGACTTGACAGAGAATAGAGATGCAAACAGTTTCACTTTCGAGAATAAATCAAATTGGTTAGCTTTTATTGTAGCATGTAGAAGAAATGCTTGTTGGATTGTTGCTATGTCTGCTACAGTTACAATAGTTGAAGATAGACTATCAAGTATTTGAATTAATGTAGAAAAGAGACATCTATATGATTGAGAGAAGTTGATAATCAAGTTGACGTCAAAAGGTTGGTGATCAAGTGTAATATTGATACATTCAAACTTACATCTCAATTAAGAAGTACAAACGGTCGAAGTCAATTATAGAACTCAACTGGAGTTCGTCAACCCCTCCGTAGTCCCACACTTAGACAGTGGTGTCTCCCTTCACTGTTGGTCGTCACGAGCTACTAGACAGCTCGTCTTAAGCACCGGCCATGGTCCGTCTAGTGCTCCGTTGACTTCAGTTTTCACTTAGGTAGCTAGTGGTTCCACTGTCACTCTCCACGAGCCATAAGATGGCTCGTCTAATGGACCACGGCTCGTCTGGGGCCTCGTGGTGTTAGACTTGAACTCTATTCTTTCCAAATCACTCCCACGAGCATCTTCACGGCTCGTGCAACACTTCACGACTCGTGTAGCCTTTCGTATAGCTTCTTGCACTTCATTTCCCATCTTTCTTCTTGTCCTTTGCATCTAAGTGTTTCACCTGTACAACATAAAAGAAACCATCATATCCACATAAACAAGCTCGACTTCTCACACTAATCCTTAGTTTTGAGCGTCAAATGTTCTATGAGTTCACGACACATCAACAAATGATCATCATGATTCATGATTCTTATAATATCAGGGCCCGTGGAGTAGAAGTCGAGTTTATTACTGCACAACTATGCACAGAGAGTAGAGTTGGATTTTGCAGGAACTCAGTGCAAGTACACTCAGATATGATGGTTGCTACCTTTTTCTTCAGTGGATCGAGCGTAAGGATTACAAGGCATATGCATGCGGCTGCTTTGAGATCTTAGTTGTGAGATGCACGACATTTGATCCAATGATGAACTTATGCAGAATATCCAGGGGGAGGTGTCATGATGAATGCTATTTGCAGATGACATAATATTAATTGACAAGACGCATAATGAAGTTAATGCTAATCTAAAAGTTTGGAGAT

>LNC_000801

CACCTTCTTCCTTCAAATTACTGTTCATTGGTGTTCTCGGTTAGATTTAATATATATATATATATTATATATATATAATTGTAACTGTTTTTACCATATTTGGGGATGCATGTTTCTTTCCTATTTGGTTATATTTTGAAAAATATTAATTAATTGAAAGAAAATCCATTATTTGGAGAGTTTGTGTTCGACACTCTTTTCAGGTTTTGATCAATATTGGCCGTATGCAGTTATAATTTATGTTTTTTTTTTTTTCTGGAATAAATTTGTGGTGATTGCTATTAAGCGTATAAGCGCACTTCACTTTGCACATCATGTATTCGATAAAAGTCCTGTTCATATTTCTAGAACAAATGTCTATTGATTCAAATCCTTAATTTCCCTACAATTGAAAGCGATGTATGTTTTAAAGTGCATGTGCTCAGAGACTTGACAGAGAATAGAGATGCAAACAGTTTCACTTTCGAGAATAAATCAAATTGGTTAGCTTTTATTGTAGCATGTAGAAGAAATGCTTGTTGGATTGTTGCTATGTCTGCTACAGTTACAATAGTTGAAGATAGACTATCAAGTATTTGAATTAATGTAGAAAAGAGACATCTATATGATTGAGAGAAGTTGATAATCAAGTTGACGTCAAAAGGTTGGTGATCAAGTGTAATATTGATACATTCAAACTTACATCTCAATTAAGAAGTACAAACGGTCGAAGTCAATTATAGAACTCAACTGGAGTTCGTCAACCCCTCCGTAGTCCCACACTTAGACAGTGGTGTCTCCCTTCACTGTTGGTCGTCACGAGCTACTAGACAGCTCGTCTTAAGCACCGGCCATGGTCCGTCTAGTGCTCCGTTGACTTCAGTTTTCACTTAGGTAGCTAGTGGTTCCACTGTCACTCTCCACGAGCCATAAGATGGCTCGTCTAATGGACCACGGCTCGTCTGGGGCCTCGTGGTGTTAGACTTGAACTCTATTCTTTCCAAATCACTCCCACGAGCATCTTCACGGCTCGTGCAACACTTCACGACTCGTGTAGCCTTTCGTATAGCTTCTTGCACTTCATTTCCCATCTTTCTTCTTGTCCTTTGCATCTAAGTGTTTCACCTGTACAACATAAAAGAAACCATCATATCCACATAAACAAGCTCGACTTCTCACACTAATCCTTAGTTTTGAGCGTCAAATGTTCTATGAGTTCACGACACATCAACAAATGATCATCATGATTCATGATTCTTATGTAAGTGGTCATGTTTTTGCTGAGTATTCCGTGCTTGTGAAAAGTAGGAATTATGTCGATCTAGAACAACAAATTTCCTCTAGCTCCTGTCATACCTGAGATTATACATGATGATCATCGTGAAACAAAGGGAATAATATCAAATGTATCCTCCTCTGCTGCTTGACAGCCTAAGGAGAAGATGATCATGCAAAAAAATAGCTAGGGGAAATGTGTTGGCTCTTTAGAGGAGGGTGAAGTGTTCATGCTCCGAATTGAGATCGAACATATCTTAGATTAATAATTAGTATTAAATTCATTTTTAATCTGACATATTTTTATTGATTTTCTAAAGAATATCAGGGCCCGTGGAGTAGAAGTCGAGTTTATTACTGCACAACTATGCACAGAGAGTAGAGTTGGATTTTGCAGGAACTCAGTGCAAGTACACTCAGATATGATGGTTGCTACCTTTTTCTTCAGTGGATCGAGCGTAAGGATGAACTAGACAGAATTAATAGCCAACAACAGAAGACATTTAGAGTATATTATATTTTGTTTTTCTTGCACCACTAATGGAAGAGAAGTCTTTAGAGGAAAAGAAGTTGCAGTTAGCTGTATTATGAGATTTTTTGTTATGTTCATATTTTTGGCATAAAAATTATTGAGCTCTTTGGTTGTACATTTGCTTTATGCTCTCAGAAGACACTTGGTGCTTCCCATGAAATGATATTTATATCCTCTTTTGGTAAAGGAATTTTTTTCTAGCAATAGATAATGCTTGTAAGATCCTTGGTAAAAGTTATAAATCTTGTGCTTGGTGTATTGTGATATGTACTGATTGAGACGCTAATAAAATAAAATTAATTTTTTTTCAAATAAGAAGTTATTTATAGTCGTTGAGGTACCTACCTTTTCACTCAACTTTTAAGACTATGATCTAAAGC

>LNC_000802

AGTTTCTCTTTTAAAATTTTCCATTATAGATGCTTAATTTGTCTACTCTTATGCATGTTCTAGATAGATTTCGTTATGAGCATTAAACTAATCATGATTTGCCGATCTTATGGACTATTTCTTGTTGATTTTCTGAACTCCGGAAGCATGTGTATTTTAGCTTTATCAAAATGTCATTATATTCTGATTTGATATAAGTTTGATGTATGTTTGCTAAGCACTATTTTGGTGTTGACATGCCGTTGAATTTTTAAATTTTTGTTGATTCTTGGTTCAATTTGTTAAGTTTTGATATGACAATTGATAAATTCTCTTAGATTTGGCTGTTCTCCTATTTAAGTTTGATTATTTTTCTTCTGTGAGGATATTTTTTTATGAATTGAATTGTGATCTTTAATATCTTCTACTGATATATAATTATTGGCAGGTTTTCTTCCTCCTTATCTAGTTATGGTAAAATTTATTAGAGCAAATAAGTCTTGTGACTTGATTTGAGCTGTTATGATATTGTCTTTCCTTAAATAATAAATTGCTCCCTTTCAGCTGATGATGTGTTTGTTAGTTAACGTATTTTAGGTGCCTTATTCATGTTTAGACAGATTGTTACTTGTTTATATTTGGTAAAAAAAAAGATTACTAACAATCTGATTTGGCTCTCCTTTGAAATAAGGAAAATGTTATATTAATAGATTCC

>LNC_000803

GCAGAAGCTCCAGCTGGTGAGAACAACCAGCGAGCTTCAGAGCAGCGCCATAACCAGTGAGACAGCAGCTCCAGCCGGCGAGAAACACCAACAAGGTAGCGGCTCCGGTGAGCCCTACTCCTTTCTTCTCTCTTATTTATGGCCAGTAACTCCAGCAGATGAGCATGGTGAAATAATGATAATGGAGTTTGCTTGGACCTCATCTCTTCGGTTGGTTTTAAGACGAATCACAACAGATCCGTCTGGGCTGAATTTATTTCTGGGAGTTTGAAGAGTTGCCTGGGATGGGTCATTGGGACAAGATTTTTTAAAAATAATATTTGTATTTGCTATTATGGTTCTGTAATATTTTTTGTTGTAAACTCTAACTTTATCTATATATATATGAATAACTTG

>LNC_000804

TATCAACCATTTAGTTGATTTATGATATCCTCTTATTTATTTGTGTAGTACGTCACAGACTCTATTTATTTGTTTGTTGTTCTATGGATTCTTGGATATATAAGGATTTTTCATATCTTGGAAAGTATGCCTGGAAGTCCTTGGATACTCTATTGCCCATTCCCCGTTTACTCAGATGGTACACATCAAAGAGCGATAATATAGTTGAAGGTGATTCATTTAAGTACAAAGGAAGAAGTACGAAGATTGTGTACCCATACCTTACCCCTACTATTCGTGAGATGGAACAGAATTACATGACAACATTTAAGCCATATTAAGATGGAGTGAAGGACATGGTCATCAATGTCTTCAATACCCAAATGAAAGGTGTGACCACCCTCACTTCAAGAGTAGAGAGTGTGGATGATGAATATTTGGGTGTTCACAATACTATCCAACCTTGTGTAATTCTGTTTCTAGTAGACAAAAAAGTGTACCGAGTACTTCTAATAATGGAAATTTGGGAAATTTGAGTGATCGTGTTGTATCCCTTGAGCAATTAATGGTGGAAGTTGTTTCTTATGTACGGAAAGAAAAATTGAGTAGAATGGAAAAGAATAAAAAAAAAACAAGAGAAAGTTGATGAAGAGCTTGCAACAGTTGATGAAGACTTTGCTATAATTGATGAATATCTTACAGATAAAGTTAATGAAGTGGAAATTGATGTTGTTGATAGAGTGGCAGGTGATCTAGTTGATGAAGTTGCAATTAATGCAGTTGATAAAGTGACAGGTGATGTGGTTGATGAAGTGGCAGTTGA

>LNC_000805

TCTTGTCCGGATGTTTGAATTGTGCAAGTGAAATGCACATGAAAGGTTCCCAAAGAGTGATAACGTATGTCAAAGGAACTTTGGACTATGGTGTTAAGTTCTGTAAAAGTCAGAATTTCAAATTGTAAGGTTATTCTGATAGTGATTGGGGAGGGTCAGTTGATGACATGAAAAACACTTCAGGTTATTGTTTTAGCCTTGGTTCAGGATGTTTTTCATGGTGCTCAAGGAAGCAAGATTTTGTGGCACAGTCAACTGCAGAAGCTGAATTATAGCTGCAGCAGCAGCCGTAAATCAAGCTTGTTGGCTAAGGAAGATCTTGATTTAGAACAACAAGAAAGCATAGAAGTGTTTGTGGATAATCAGACAGCCATAGCTATTTCTCATAATCTATTTTTCATGGGAAAACCAAGCAAGTATCCG

>LNC_000806

CTTTAGTTTACTAATTATATGTAAGGCACTAACAACTAACAAGAGATGCTTCCGGTCGTAGGGCTATTTTAATAAATTCAAAGTTATTTCATTTGTTTTTGTGTTATACGTGTACGCCTGACGCGTGTATTCCCTTCAAATTTCCACAAAGGTTTTCATCTTGAATTTGAAAAACAACAAACTCATCCAAGAGTTCTTCGCCATTTTGTCTCCTTTAATCTTTTTTCAGTTATACAGTATTTTACTAGCTGAGATTTCTTGCTGAAACTTCTGTTTGACCAGTAGAAGATAAAGGAAACATCTTCATCTACTTGGCTTCACTTTACTCAGGCTCCTAGTTTTAATACTAGAGGAGATTGTTGAATCCGGAAAGATACACCTTAATCGGTGAAATATCTTTAAGGACAATGTCATAGGCACACCTCAAACTACAAAAAAATTTCCAACAATCTTAAGGATATTTATTTTATGAGTATGGAACACAGAAGCAATTATGAAATTTTCCATCTACTATCAAAGGGTTCTCTACACGATATTCATGATTAGAGAAGAGCACCTAAAAGAAGCAAAGTTATGGACGAACAAGAAAAATCAGAGGAGACATCACAAACCCACTTCATTTTAGTGTATGGAATTAGGTTGGAGTTGGCAACTTGGCATGTTAAGAACTGGATTGTGACTAAACCATATGCTTTTCCTGTCATAAGGTATGTCGATATGCTAAACATTTCTATAATTTTAATATTGAGAAATATGATTATATTATATTTAAAGCTAATGATAAAGCTTTCGAGAAAAGAGTTGAGAAAATTGACAATCCTATTAAACTAAACTAATTTGGATAAAGTAGATATTATTATTGTGGTCCTATCTCAAGTAAATATTGTGGCCAGCATGAGTGACTGGGGTGATTAACTTTATGCTACTAGGCATATTTGTATGAACAAAATAATTATGTGACTTGCACCCAAGTTAATGTAAGGAGAAGACTTTATTTATCTTAGTGACCGAAGAAATACGTTAGTTCTCGAAAAAAAAAAAGTCCTTCTCAACTCACACGTGAAAAAATATTGGCTTTAAATAAGTTCCATGTTTCTATTAGTTGAATCAATTTGATTGATGTGAAATTATTAGAAACTGTTGAAGCAAAGTTTTCTTTGCGAATGCTATGGTTGTTATACAAAAAATAATATTTGTATTGGCAAAGGATATGGTAATCATGATTTAGGTGTTTTAAAATATTTTTAGTCGTTCAATAAAATATTTTTAGTCATGAATGAAGATGTCAATACTTAATTATTTAATTTTGACATGCTAAATTGGAATCATATGAATAACTTAGAAAGTTTTATATTTTTATTTATTAATCTTTTGGTAATAAAATTCTTCTCTCTGTTAATCTTTCTTGTTTTTCAAATGTCAATGTTTAAATATGTTTTACGTGTTTATTATGAAATTGTGACGAATTTCTAGAGTATTCATGAATACTAGGACATGCACATAGCCTCGTAGAGCAAAATCACGATAACAACAAAGATTGTGCAAGTATAAAGTGATAAAATAAGACTCTG

>LNC_000807

CTTTAGTTTACTAATTATATGTAAGGCACTAACAACTAACAAGAGATGCTTCCGGTCGTAGGGCTATTTTAATAAATTCAAAGTTATTTCATTTGTTTTTGTGTTATACGTGTACGCCTGACGCGTGTATTCCCTTCAAATTTCCACAAAGGTTTTCATCTTGAATTTGAAAAACAACAAACTCATCCAAGAGTTCTTCGCCATTTTGTCTCCTTTAATCTTTTTTCAGTTATACAGTATTTTACTAGCTGAGATTTCTTGCTGAAACTTCTGTTTGACCAGTAGAAGATAAAGGAAACATCTTCATCTACTTGGCTTCACTTTACTCAGGCTCCTAGTTTTAATACTAGAGGAGATTGTTGAATCCGGAAAGATACACCTTAATCGGTGAAATATCTTTAAGGACAATGTCATAGGCACACCTCAAACTACAAAAAAATTTCCAACAATCTTAAGGATATTTATTTTATGAGTATGGAACACAGAAGCAATTATGAAATTTTCCATCTACTATCAAAGGGTTCTCTACACGATATTCATGATTAGAGAAGAGCACCTAAAAGAAGCAAAGTTATGGACGAACAAGAAAAATCAGAGGAGACATCACAAACCCACTTCATTTTAGTGTATGGAATTAGGTTGGAGTTGGCAACTTGGCATGTTAAGAACTGGATTGTGACTAAACCATATGCTTTTCCTGTCATAAGGTAAGTTCTGGCAAGTGTTATGAGGCCGAGGTCATTTCTGGCAAGTGTTATGAGGTCAGGGTCATTTTCGGCAAGTGTTATGATGTCGAGGTCATTTTCGACAAGTGTTCCAAAAGTCCTTACAAAAGCCACTTAGC

>LNC_000808

GCCTGACGCGTGTATTCCCTTCAAATTTCCACAAAGGTTTTCATCTTGAATTTGAAAAACAACAAACTCATCCAAGAGTTCTTCGCCATTTTGTCTCCTTTAATCTTTTTTCAGTTATACAGTATTTTACTAGCTGAGATTTCTTGCTGAAACTTCTGTTTGACCAGTAGAAGATAAAGGAAACATCTTCATCTACTTGGCTTCACTTTACTCAGGCTCCTAGTTTTAATACTAGAGGAGATTGTTGAATCCGGAAAGATACACCTTAATCGGTGAAATATCTTTAAGGACAATGTCATAGGCACACCTCAAACTACAAAAAAATTTCCAACAATCTTAAGGATATTTATTTTATGAGTATGGAACACAGAAGCAATTATGAAATTTTCCATCTACTATCAAAGGGTTCTCTACACGATATTCATGATTAGAGAAGAGCACCTAAAAGAAGCAAAGTTATGGACGAACAAGAAAAATCAGAGGAGACATCACAAACCCACTTCATTTTAGTGTATGGAATTAGGTTGGAGTTGGCAACTTGGCATGTTAAGAACTGGATTGTGACTAAACCATATGCTTTTCCTGTCATAAGAAATTTTTCTCCAAGTCTAAAAGAGATTGGCATGAAAGAATGGAAGAAGCTCTTTGGGCCTATAGGACTACGTATCGCACGCCGACACAAGCTACTCCTTATTCTTTTGTTTATGGAGTTGAAGCAGTCATTCCAGTTGAGCATCAAATCCCATCATTGCGGCTTGCCATTCAAGAAGGACTCACTGATGAAGAAAATACTCGGTTACACCTTGAAAAGTTGGAGGCTCTTGATGAAAGATACTAGAGGGTCAACAAAGTCTAGAATGCTATCAAGCTCGTCTAGCTGAATCTTTTAATAAGAAGGTCCATCGATCTTAGATACTTTCAAGTTAGTGAAAAAGTTCTTATTGTAAGAAGACCCATTATTACTTCTCATCAATCTGGAAGAAAGTTCTCTACTAAATGGGATGGGTCGTATGTTGTACAAGAAGTATAATCAAGTGGCGCTTACAAGTTGGTTGACTCAGAAGGATTGGGAATTGGCCCCATTAATGGGAAGTTCATAAAGAGATACTGTTGTTGAAGGAAAGAAGCACTCTCCTTAAGTTACGAGCATAAACTACATGTTACTCTCGGCCCGCAAGAGTATAAATTGTGCATGACACTCCAAAGAAATATCACTCTTTCAGAACTACATTATGACTTGATCATCTTCACTGAGCTAGGTACGTAGGCGCTTGGAGTTTTCCCCTTGATCCTCTTCACCGAGGTACGTAGGCGCTTAGACTTACATCCTAAGTTTAGTATCATAAGTTCGAAAAAAAGAATGGCATTTCATTATTTGGGCATCACAATATTTCTTTAAAATGCCATGGATAACACGTGAGTCATTCGAGTTGGTACAAATTCGCAATGCTTTCAGCCATTTTTTGTGTACATGTTATAGTTCTAAGCATCCTCTTTCAAACGGAATAAAACTTACGCCATTTCGAACTTTTCACAACGAGATATGATCTTCTTTGGCCACAACTACGCAAATCTGGATAGTCGAGCAAGGCAAAATTTAAGTTGACTTCAAAGAAACATCTGTACAAGTCTGTTAAGATTTTAGCCATGATTTTTGTGCATGTTATAGTTCTAGGAGTCTTATTTCCAATGAAAAACAAATTTCGTCGTTTCAACCTTTCCACAACAAGATTACGGTTTTTTTGTCCACAATTGTACTTAAAGTTGAGTAGTCGAGCAAGACAGAAATTAAGTCACCTTCAATGATATATCCATACAAATACGTAAAGATTTTAACCATGATTTTTGTACATGATATATTTGTAAGAGTCTTCTACCTAATGGAACAAAATTCTGATCGTTTCAACCTTTCTACAGTGAGATATGATTTTTTTTTGTCACAACTACGCAA

>LNC_000809

AAAAAATCGTTCAGCGACAAAAGAAAAGTTGAGTTCGAGGTTTTTATTCGTGTTTTCTAATTTGTGGTTTCAGTTGTGGTCTTTTCTTTGTGAATTAATTTTCACAAGAGGTATAAAATGTGAGAATTGAAGCAAGATTGATTGCAAGCTATGTGTCACCACATTATTGATACTTTGGAAGGAATTTTTACTTATATGTCTTTGTTTAGGAGTAATATTTGATATATTTTAGTTAGATTTTCTTGTCATTGGACATATTTTACTTTTATTTAAGGAGTTTGTAATGAGATATTATTCTTTAGTATAATTTTAGTTATTTTTATTATATGATCATGGGTGAATTTTGATATAAATATTTGTCATTTAATAAATTAATTCATCACAACTTGATTACTTTCAATGACAAAAAATGTGTTGTCGTAGTACC

>LNC_000810

GAGAAATGAAAACAAAAATTAAAAAGAAATCAACAAGAAAGGTATAAAATGTGAGAATTGAAGCAAGATTGATTGCAAGCTATGTGTCACCACATTATTGATACTTTGGAAGGAATTTTTACTTATATGTCTTTGTTTAGGAGTAATATTTGATATATTTTAGTTAGATTTTCTTGTCATTGGACATATTTTACTTTTATTTAAGGAGTTTGTAATGAGATATTATTCTTTAGTATAATTTTAGTTATTTTTATTATATGATCATGGGTGAATTTTGATATAAATATTTGTCATTTAATAAATTAATTCATCACAACTTGATTACTTTCAATGACAAAAAATGTGTTGTCGTAGTACC

>LNC_000811

GTTTCAATAAGGTCCATGTGGTGTCTAATTTCATTCTACTTCAACTTTTTTCTTCCATAGACATACATGTTGGGTAAATGGATTGTGAATGTATGGCATCAACCGCTACACATCTGAAATAGCTGATGCCTTGAAATATAAGGTTAGCAGAGGGATATACTATTATGATTATTTGCGTCTTTTCAATTCCATGGTGGGACCATCTCTTTGGGGTAAGTGCAGTCTACTAGGTAAAGCATGCCATTTTATTTTGTTGGAACAAACAAACAAGCAGTATGTGTTCAATGATAAGCAATGGTATGTCTTCATCTTCTTGCTCAAATTGCTTCACCAAGTTGTTTGGAATCTAGAGCTGCTAAAATTTGGAAGTTTGAAGTAGTTTTTAAAGATGGGGTGAGGAGTGATGCATGTACCCTGAGCTGTGTTGCAGTTTTAGGTTTCGTCTAAAGCAGCTTAGGGAAATGGAATTTAGTGGCCCATTCTTCTTTTATCATATGTTGTCAGAGTTGAAGGTCCCGACACTTTTTCCTGTTATGTTTGGGTTCTGTTGTACTTCTGTAGATATTTTGGTCGTTGGTCTAACTGGAATAAATATACCTGCCACCAAGTTT

>LNC_000812

CAATTTCATCTTTTCTACTTCTCTCAAGACTTCTCTCTTTTCTCTCACTATGTTATATGTTTTCCATGTAGAATTGATGTATTTCATCACTTCAAATCGGTTCTTCTTCAACGTTGGTCGATAGCTCTTCATCATTATCTTTGTACAGGAAACATGGGATTGTGAAATAGCTATTACAAATTACTGCAAATACTTCAGGATCCCCTTATGCACAACTCCAGCACAACACTGTGGTTGTTGAATTAGGATATGCCTATGATGTACTTGAAGATATGAGCAGTTATGATTTGAGTATGTTATGTCTATTGAAGAGGGGTATAATGAGAATGTCGCATTCGGTGTTGAGGATTTGATGCACAACAACCCTGCAATGAAAAAGAGACGTATCATTCAAGACCGCCTCATGGCTGAAATGTTTGCGGGCAAAAAACACTCATCCATGAATTCGACTTACATTGTTGGGGAAGTTAGATGGACTATGACTGGAGTTCAAGTGCAGTAAAGGAAAAGACTGAGAAAATATTAATTAGTGGGAGTATGAGAATTGACAGTGTAGTCAATGAGAAAGCTGGTAGATCAGCAGGACTACACGGTGGTATGTGCTAACACAAAAACAGACTCAAGATCGCATATTGGACTGCTGACACTTATGCAACAATGGCTGTTGCTTCTATTTTATTTCGTGGAACTGATCATTCGTATTCCCATAAACTTCTCAATAAATCCAAATAGGTAATGGATGTGCCAGATGTACAAGATGATAGCAGTTGAAGAAGTTGAAGAAATTATTGACAGTAGCATATGTTGTCATCTGCTTTGAGTCCTAAGTGTTAGATATTCGATAGTTAATCTTTCACTAGAGAAAGTTCAACTATTTCATTCTCAGATGTTCATCACCACATGTATTGCAGTTATTTAATTTCCCAACTCTTTTAAGTTTGTAAAAGTGAAGTGGGATCATATAAAATATCTTTATTTTGTATATGTTGCTATGATTCAA

>LNC_000813

TTCTACTTCTCTCAAGACTTCTCTCTTTTCTCTCACTATGTTATATGTTTTCCATGTAGAATTGATGTATTTCATCACTTCAAATCGGTTCTTCTTCAACGTTGGTCGATAGCTCTTCATCATTATCTTTGTACAGGAAACATGGGATTGTGAAATAGCTATTACAAATTACTGCAAATACTTCAGGATCCCCTTATGCACAACTCCAGCACAACACTGTGGTTGTTGAATTAGGATATGCCTATGATGTACTTGAAGATATGAGCAGTTATGATTTGAGTATGTTATGTCTATTGAAGAGGGGTATAATGAGAATGTCGCATTCGGTGTTGAGGATTTGATGCACAACAACCCTGCAATGAAAAAGAGACGTATCATTCAAGACCGCCTCATGGCTGAAATGTTTGCGGGCAAAAAACACTCATCCATGAATTCGACTTACATTGTTGGGGAAGTTAGATGGACTATGACTGGAGTTCAAGTGCAGTAAAGGAAAAGACTGAGAAAATATTAATTAGTGGGAGGTACGCAATATGTATTTTTATATTCATTTTTTGTATAAGGTTATATCATATGTTTCAAGTATTTAGTTTGCTTATACTTTAGGACTCTGTATACATCTTTTGATTGTAAATAATAATTTTCTCTACTTTTTGTGTATTTGATGTATACTCATTCAAAAGAGAGAGTTCATATATTTTTAAAGAAGAGACTCAACTGGACCATGTGCCGTCGTATTTTTCATTGCTTAGGAGAAAACGAAATATTCTTATTATATTTCTAGGAAAAGCACAACTGATTGGTTGGGACCTTATAAGATGTTGTTCTTGATAATTTTGAGAAGACATAGGATATATATTTGTATCTGGCGTAAAACACTTTCACAGTGTCTTTCATTGTAGGTGTCATGTCCCTTCTCAATTTGGAGGAAAGATGAGAATGTGCGGCATCTCTACTTGAACTATTTGCTATAAATAGTGGAATACTTCTCTTGATATGATATATGTAAAAACTGTATCATGTTCTAATTTTCTAATTTTAATAATTGCGAGCATCTGAATGTTGAACTAATTTTTCAAACAATATTTTTTGTTATTTTGTAAATAAAAGGGAACATTTAACCATTTTGGATCATATTGAAGAAAATGTTCGTGTGTTGTGACGAAGAATCACTGCTAAAATTGAATATTATTACTGATGAAATAGCAGCAGCTCGGACAAGCTAATGTCCGCTGAAAATATCAGTTGCTGAAGCAGTGATAGATGTATGTGAGGAAAGAACTAAAATAAAACTAGCGACTCTATCGCAGAATTAAATCTCACTCACGAAAGAAAAATCATTTCTCATTCACCTTGGAAAACACTTCATCTTTTCCATTTGAATATACCGTTGGATTCTAATGTTCATTAATATGTGGAAATTTCGATTCAAAATCTTTGTTAATTTTTTGATTTTTGATTAATTTTTTACTTGCATGTTCGTAATGGAGTTTGAGATGCCTTGAATTGAATTCATGAGTTTCATTACTTTGGATATGGTAATCCCCGTTGCTCACCCCCCATCCATTTTTTTATTATTTTCTCGACTGATTTTACACCCATTAGTTCTGCTTTTGCAAGAGTTCCTCAGTTGTTCTATTATTAAAGTCAGATATGATCATTTATCACGTTTCTATGGATTAATTTTTTAGTGTCTCTTGACATTTGTATTTGAGAGAGAGCGCTATGCTTAGGTCAGTTCCTTCAGTCAACTTTTTTGGTAAGCCAAAAGTGAACTTTAGGTAAGTAAGTAGTCTCGTATGAAATTCAGAAAACATTCATATCTGGCACTTGAGGAGGTCAATCTTAAGAGGTTGTGATTGCACAGCTACTCAATTTTGGTTTATGTTTGATCATCAATGAAGAGTCAATTATTAGTATATCAAAACCTAATTTTTTTTTTTGATGAAGTAATTAGTTCTATTGCAGGCATCAAGTAGATGCATAATAGTTACAAAAGCTTAGTAGCTAAAGGTCAGCTCTCCTTTACATTGTTAATTAAGAGTCAGGGAGCTGACAAAGTTTAGAAATGAATCAGGGGAATCTAGAGGGGATAAATAAACCCAACTATACTATACAAAAACATGAGGCAGTTAGCTTTTAAAGATTGGTAGGTAGCTGATATTCCATCAAAGCATCTCCTATTTCTTTCGTTCCAGATACTCCAAAAAATGGTTGCAAATATCATCTTCCAGATTCTCTTGATAGTGCTATCAACTTTCCAGCAACTCCAGCTTATCACAACATCCCTGATATTCTGAGGCATGACCCATGTGAGGCCAAAAAAAGCTAAAAACATACTCCACAAGTCTGCTGATTTGACATTGTAGAAATAGGTGGTTGTGTGTTTCTGCTTCCTTCAAAACCTAATTTATGCTTGTAGAAGGTGAATATCACTCTGTACTGCTGCTACTGAACTATGGTGCTGCTTTTATTTAGTAGTTTTGTTTTTATTTA

>LNC_000814

CTCAAGACTTCTCTCTTTTCTCTCACTATGTTATATGTTTTCCATGTAGAATTGATGTATTTCATCACTTCAAATCGGTTCTTCTTCAACGTTGGTCGATAGCTCTTCATCATTATCTTTGTACAGGAAACATGGGATTGTGAAATAGCTATTACAAATTACTGCAAATACTTCAGGATCCCCTTATGCACAACTCCAGCACAACACTGTGGTTGTTGAATTAGGATATGCCTATGATGTACTTGAAGATATGAGCAGTTATGATTTGAGTATGTTATGTCTATTGAAGAGGGGTATAATGAGAATGTCGCATTCGGTGTTGAGGATTTGATGCACAACAACCCTGCAATGAAAAAGAGACGTATCATTCAAGACCGCCTCATGGCTGAAATGTTTGCGGGCAAAAAACACTCATCCATGAATTCGACTTACATTGTTGGGGAAGTTAGATGGACTATGACTGGAGTTCAAGTGCAGTAAAGGAAAAGACTGAGAAAATATTAATTAGTGGGAGGTGTAAGATTCTATCAAGAAGAATTCAATGACGA

>LNC_000815

TTTCTCTCACTATGTTATATGTTTTCCATGTAGAATTGATGTATTTCATCACTTCAAATCGGTTCTTCTTCAACGTTGGTCGATAGCTCTTCATCATTATCTTTGTACAGGAAACATGGGATTGTGAAATAGCTATTACAAATTACTGCAAGATCCCCTTATGCACAACTCCAGCACAACACTGTGGTTGTTGAATTAGGATATGCCTATGATGTACTTGAAGATATGAGCAGTTATGATTTGAGTATGTTATGTCTATTGAAGAGGGGTATAATGAGAATGTCGCATTCGGTGTTGAGGATTTGATGCACAACAACCCTGCAATGAAAAAGAGACGTATCATTCAAGACCGCCTCATGGCTGAAATGTTTGCGGGCAAAAAACACTCATCCATGAATTCGACTTACATTGTTGGGGAAGTTAGATGGACTATGACTGGAGTTCAAGTGCAGTAAAGGAAAAGACTGAGAAAATATTAATTAGTGGGAGGTACGCAATATGTATTTTTATATTCATTTTTTGTATAAGGTTATATCATATGTTTCAAGTATTTAGTTTGCTTATACTTTAGGACTCTGTATACATCTTTTGATTGTAAATAATAATTTTCTCTACTTTTTGTGTATTTGATGTATACTCATTCAAAAGAGAGAGTTCATATATTTTTAAAGAAGAGACTCAACTGGACCATGTGCCGTCGTATTTTTCATTGCTTAGGAGAAAACGAAATATTCTTATTATATTTCTAGGAAAAGCACAACTGATTGGTTGGGACCTTATAAGATGTTGTTCTTGATAATTTTGAGAAGACATAGGATATATATTTGTATCTGGCGTAAAACACTTTCACAGTGTCTTTCATTGTAGGTGTCATGTCCCTTCTCAATTTGGAGGAAAGATGAGAATGTGCGGCATCTCTACTTGAACTATTTGCTATAAATAGTGGAATACTTCTCTTGATATGATATATGTAAAAACTGTATCATGTTCTAATTTTCTAATTTTAATAATTGCGAGCATCTGAATGTTGAACTAATTTTTCAAACAATATTTTTTGTTATTTTGTAAATAAAAGGGAACATTTAACCATTTTGGATCATATTGAAGAAAATGTTCGTGTGTTGTGACGAAGAATCACTGCTAAAATTGAATATTATTACTGATGAAATAGCAGCAGCTCGGACAAGCTAATGTCCGCTGAAAATATCAGTTGCTGAAGCAGTGATAGATGTATGTGAGGAAAGAACTAAAATAAAACTAGCGACTCTATCGCAGAATTAAATCTCACTCACGAAAGAAAAATCATTTCTCATTCACCTTGGAAAACACTTCATCTTTTCCATTTGAATATACCGTTGGATTCTAATGTTCATTAATATGTGGAAATTTCGATTCAAAATCTTTGTTAATTTTTTGATTTTTGATTAATTTTTTACTTGCATGTTCGTAATGGAGTTTGAGATGCCTTGAATTGAATTCATGAGTTTCATTACTTTGGATATGGTAATCCCCGTTGCTCACCCCCCATCCATTTTTTTATTATTTTCTCGACTGATTTTACACCCATTAGTTCTGCTTTTGCAAGAGTTCCTCAGTTGTTCTATTATTAAAGTCAGATATGATCATTTATCACGTTTCTATGGATTAATTTTTTAGTGTCTCTTGACATTTGTATTTGAGAGAGAGCGCTATGCTTAGGTCAGTTCCTTCAGTCAACTTTTTTGGTAAGCCAAAAGTGAACTTTAGGTAAGTAAGTAGTCTCGTATGAAATTCAGAAAACATTCATATCTGGCACTTGAGGAGGTCAATCTTAAGAGGTTGTGATTGCACAGCTACTCAATTTTGGTTTATGTTTGATCATCAATGAAGAGTCAATTATTAGTATATCAAAACCTAATTTTTTTTTTTGATGAAGTAATTAGTTCTATTGCAGGCATCAAGTAGATGCATAATAGTTACAAAAGCTTAGTAGCTAAAGGTCAGCTCTCCTTTACATTGTTAATTAAGAGTCAGGGAGCTGACAAAGTTTAGAAATGAATCAGGGGAATCTAGAGGGGATAAATAAACCCAACTATACTATACAAAAACATGAGGCAGTTAGCTTTTAAAGATTGGTAGGTAGCTGATATTCCATCAAAGCATCTCCTATTTCTTTCGTTCCAGATACTCCAAAAAATGGTTGCAAATATCATCTTCCAGATTCTCTTGATAGTGCTATCAACTTTCCAGCAACTCCAGCTTATCACAACATCCCTGATATTCTGAGGCATGACCCATGTGAGGCCAAAAAAAGCTAAAAACATACTCCACAAGTCTGCTGATTTGACATTGTAGAAATAGGTGGTTGTGTGTTTCTGCTTCCTTCAAAACCTAATTTATGCTTGTAGAAGGTGAATATCACTCTGTACTGCTGCTACTGAACTATGGTGCTGCTTTTATTTAGTAGTTTTGTTTTTATTTA

>LNC_000816

TTGTGAAATAGCTATTACAAATTACTGCAAGATCCCCTTATGCACAACTCCAGCACAACACTGTGGTTGTTGAATTAGGATATGCCTATGATGTACTTGAAGATATGAGCAGTTATGATTTGAGTATGTTATGTCTATTGAAGAGGGGTATAATGAGAATGTCGCATTCGGTGTTGAGGATTTGATGCACAACAACCCTGCAATGAAAAAGAGACGTATCATTCAAGACCGCCTCATGGCTGAAATGTTTGCGGGCAAAAAACACTCATCCATGAATTCGACTTACATTGTTGGGGAAGTTAGATGGACTATGACTGGAGTTCAAGTGCAGTAAAGGAAAAGACTGAGAAAATATTAATTAGTGGGAGTATGAGA

>LNC_000817

CAAACGTTGGCTCTCCAAACCAAACAGACAGATTCACGGCGATAGACGGAAAAGCAAGCAATTTTTGTTCTCTTCTTCCTGTCCCTTTCATTCACAGTAGGCAGAGGCACGCGATCCCCCCTTACCCCTTTCGGAATGGAGTTGAAGAGCTCGGAAAAGGGTGTTTCCCTAATTTGGTGTAAGTTTTTGTAGGATTTGAATTTCGAATTGGGCCCTCTCCATCTGGATTTTCATCTTTCCCCCCCAAATTCTACCCTAAAATTTCCCTATATATTTTCCTCTTAGTCGCGGTTGAAAAGGGGGGGATTTTTATTTTGAAAAAGAAAAAGTATTGAAAAGTTAGGGGGATATTTTTAGTTGGGAAAATTGTTAGCGAAAATACAGAGAATAGTTTGAGCCAAAGTAGTTTGAATACAAAATAAAAAAAATATATACACTCCGAATATAGAGCGGCTTGAATTCTATCCAGACCTTTGGTTCGCTGTTCGTAGTCGAGTCAGCATAGCGGTGAGGTCGTCCCCTGCTCGTTCTCGTCCCTGTCTCGCTGCCCCGAAAAAGGCCACGAAGGCCTCAGGAATTTTTATCGAGTCTGCCAGCCCTAAAAATTGACGAACAGGTCCTGAAGTCGAGAATATGCGACAAATTGGAGAGTCAAAATCCGGCCAAAGGCCCACTCTTTATTTCAAGATTGTATTTTGTTACTCTTAGGCCTAAGCCCATTTATTTGCTATTATATTATTAGAATTTAGGACAGGTGGCGAGAATTGGGCCTTTGGGCTCAAAATGAAATTAGGCCCAAGTGTTGGTCCAGGC

>LNC_000818

ACCCCAAAAATGCCCGAAAATGTGCTTCGGAAACACCTAAAATTGTAATTTCCATATATCTCAAAATCCGTGCATGATTTCGGAAATTCGACTTCATATTCTTATTCAGGGGGTCAAATTGAGTGGGAAATGAGTCTAACCCGAATTTTAGAACAACCGTATCAAAATTCGAAAATCGCAAAAAATGCGATTTTGAGGGTCAACTTTAAAGGGGCATATCTCTCAGCACACAAGGAACTGGAAGGAGCTCAAACTATCAAAATTAAAGCTCTGTGAGTTAGCTTTCCAACGCAATTTGTTTCGCCTCATTCGGAGTTCGGACGAAGAAGTTATGCCCATTTTCGTAAAGTCTGTCCAGACGGAAATGCAATTTCCAGAATCTAAAATTACTGTTCACCCGCCTCAAAAATTTTTCTAAGTGTTGGGACATTTTTATAAAAAGGGGACATATCCATACTTAGTCATTT

>LNC_000819

ATTTTTTTCAATTTTGCGCCAAAACAAACGCGGCAAACAAAATTTCCATGTAATTATTTTCCCCAAATGCACATCACTGCTAATTTAGTTCCCACTACAATTAGGTTTTACTTTTTTCTTTTGATTTGATGCCTCTTTTATGTTTTACCCAAAATCTACTTTTTCACCGATTCTTTTTTTGTTTCTTTTCTTCTTTTGATACAAGATCCAAAGCTTTTGGACTAGAAAATGTCTTAATTGGGAGTAACGACGAAGATGTAATCAAACGGAGACCCGCACAACAGACAAAGAAGGAGGTACATTCTTATAATCATACCGTCAGTGGATCATATCCTTTGTTTGGGTTTTGCACGTTCACTACATGGTGGCACCTTCAAGGTCATTTATCTCTATGTATCTCTTTCTCTTTACTCAAGTAAGTCATCTTATTAAATGATGGTTTGGTCTATTTTTTTCTTAATTCTGGATAGGAAATTTTTTTAGTATTAGGGAAGTCTAGTAGCTCATAAAACTATCATTTTCTTTTTAAATAAGAGACTCTTTTTGGGACAAACAAAAAAGGAAAGTAAGACGTGTAATAGTAACCAATGAAGTACCATTCTGATGAGGAAAATACTGGAAAGTTCAAAGTGCTCAAAAGTTCAATCAACTTTCTAGTTTTTATTTTTTCTTCTCTTTTATATTTCTATTTACTGCTTAATTTGATTATGATTTTCATACTTAGTAGGCGAAAGGTTTCGTGTATTTTCTACTTGTTTAGTAAATGGTAATACTCCTATACAACAACTCTTATGCTTGCTTTAGCTGATATGATATATAATATTAGGATCATAGTTTAGTGAATCATAAGTCTAGATATGATTGACATTAATTAATATATTTGTTGATTTTTTTTCACATGCCAACCATGTGTATATGCTTGGAAATCTAAGACTTGGATATGCAAAAAAATATTACTGTTAGAGTTCTTAGTGATAAAATATCACATGTTTAAACTTGGTTGTTACTTTAATTTGAGATATCTTTTTATCCATGATAATATTCAATACTCTCTTTATCATATTCTTTGGGGTTCATTCATTAGACAATTGTTATTGTTAAAGTTGATATTACTCCATAGAGTTGTCTTGGAGATGTCTTACTTTGTGAAGGTAAATCCAATTAGTACATTACCAGATGATGTGGTTTAGTCAGCATCTTAAGGACAATAACAGATCAAATTGAATGTCTATCTTTTTTTTTGGTTAAAATGGTTTGGCTCAAGGCCAATTTATTCAAAACAAATTATATACAAGCTATAGCTAAAAGCCCGGCCA

>LNC_000820

ATTTTTTTCAATTTTGCGCCAAAACAAACGCGGCAAACAAAATTTCCATGTAATTATTTTCCCCAAATGCACATCACTGCTAATTTAGTTCCCACTACAATTAGGTTTTACTTTTTTCTTTTGATTTGATGCCTCTTTTATGTTTTACCCAAAATCTAATCCAAAGCTTTTGGACTAGAAAATGGTATGATTTTGTTTGGGGATGCTATGGTTATTTGGAGAAGCACAAAGCTAAAGTAGATATATTCTTAGATATGCAGTCTTAATTGGGAGTAACGACGAAGATGTAATCAAACGGAGACCCGCACAACAGACAAAGAAGGAGGTACATTCTTATAATCATACCGTCAGTGGATCATATCCTTTGTTTGGGTTTTGCACGTTCACTACATGGTGGCACCTTCAAGGTCATTTATCTCTATGTATCTCTTTCTCTTTACTCAAGTAAGTCATCTTATTAAATGATGGTTTGGTCTATTTTTTTCTTAATTCTGGATAGGAAATTTTTTTAGTATTAGGGAAGTCTAGTAGCTCATAAAACTATCATTTTCTTTTTAAATAAGAGACTCTTTTTGGGACAAACAAAAAAGGAAAGTAAGACGTGTAATAGTAACCAATGAAGTACCATTCTGATGAGGAAAATACTGGAAAGTTCAAAGTGCTCAAAAGTTCAATCAACTTTCTAGTTTTTATTTTTTCTTCTCTTTTATATTTCTATTTACTGCTTAATTTGATTATGATTTTCATACTTAGTAGGCGAAAGGTTTCGTGTATTTTCTACTTGTTTAGTAAATGGTAATACTCCTATACAACAACTCTTATGCTTGCTTTAGCTGATATGATATATAATATTAGGATCATAGTTTAGTGAATCATAAGTCTAGATATGATTGACATTAATTAATATATTTGTTGATTTTTTTTCACATGCCAACCATGTGTATATGCTTGGAAATCTAAGACTTGGATATGCAAAAAAATATTACTGTTAGAGTTCTTAGTGATAAAATATCACATGTTTAAACTTGGTTGTTACTTTAATTTGAGATATCTTTTTATCCATGATAATATTCAATACTCTCTTTATCATATTCTTTGGGGTTCATTCATTAGACAATTGTTATTGTTAAAGTTGATATTACTCCATAGAGTTGTCTTGGAGATGTCTTACTTTGTGAAGGTAAATCCAATTAGTACATTACCAGATGATGTGGTTTAGTCAGCATCTTAAGGACAATAACAGATCAAATTGAATGTCTATCTTTTTTTTTGGTTAAAATGGTTTGGCTCAAGGCCAATTTATTCAAAACAAATTATATACAAGCTATAGCTAAAAGCCCGGCCA

>LNC_000821

CTATTTGAAGGTGTCACCCATGAAGGGTGTGGTTCGTTTTGGTAAGAAGGGCAAGTTGAGCCCAAGATACGTGGGTCCTTATAAGGTGATGAGGAGAATTGGCAAGGTAGCTTATGAGTTGGAATTGCCTAGCGAAATGAATTTGGTTCATCCGGTATTTCATGTGTCTATGTTGAGGAAGTGTGTGGGTGATCCAAATGCCATTGTGTCTCTTGATGTTGTGGGTGTTGTTGAAGATAATTTGACCTATGAAGAGGTTCCGGTCCAGATTTTAGATAGACAAGTGAAGAGGTTGAGAAACAAA

>LNC_000822

GTTGTGTTTGTGCAGGCTAATTTTACTTGTGATGATGATACTATTTTACATCAATATTATGGAGCTTATAGGGATTAAAGAATGTGTTTTAATTGTATTTTAAAGGTTGTATATACTCGTGAACAAGTTGAGGACCTTGATACATTGGTTGGGCTGTTTCTTGGCTAACGCACGGAGGTTTGTGTATAAACTTGTACCTGCACTATTTGAACTGCTGGTATATTTGAAGGTTCATATTGGTAGATTTGGTTACCTCTTGTCACTTTGCATTGTTGTGTTGGTATCCTTTGAGACGTTAACGACACTTGTGTAACTCGCCCACTTGTATGGATTGTTTGATCACCTAGCACTCTTGTTTGGACCTTGTCCTTCTTGTGGATGTGACTTTGTCACTCTTGGCATGCCTCTTGATTCGGATCAATTGCCATCTTGACTTTGGATTTTTAGTTCGTCTTAAGTATGGAAGTTGTCCATTTTAAGTACGAATTAGAAAGCCATTTTTAATATGGTTCTTGGTTCTCTTGATTATTGGGCTTTGACCCTTCTTGATACAGGGCGTCGACCCTTCTTGATACTTGCTTGTGCTTGGTGTGAAGACATGATGTACATATTGGGCGAGCCTTGTTATTGAATCTCTTGGAAAATGATGCTATGAGCGTTCTTGACATTTGGACCTCTAAATATCGAATTTGATACTCTTGATATATTATTTACTTGATTTATTTATTATGTTCGAGTATGTTGCCCATGACTTGAGAAAGTTTGCTTGTGAATTGGATGGCTCCGAAACTACAGTTTTACAC

>LNC_000823

GACTTTGGGAAAGTGGATATTGAAAATCGAAATGGGTAAGAATGGAGACAAGTGTTTAGCCACAGAGAATACAATGCCACTACTCAGCTCAAAGAGCTTTTCTTTTTCCTTTTTATTTTCCCACAAAAACAAAATAGGCTCTCATTAAGCTGACAAAATTGACACTCAAATCAGCCATATCGATAGGAGAGAAGTACGGCCACAATGGAGCTTCGTTTTGCTGATAGAATTTTTAATGGAGACAACTATTGACATAGATGAGAACCCCCAGCGAAGTTGATAGTATTGGTGAATCTCACATTGGTTCAAGCAAAGGGGGCCTTTATCATTTATTGTTGTGTCCTTCCTTTATAACTCGTAATATTGCTCCATTCATTAGTTTCATGTGTACTTTGTTCATCATGTTACTTATAAGTGTTCATCATAGAAAGTTAGTAAGATCATTTGTAGAATAGATTAATCAAATGAGAATACAGATAGAATATGGGTGCTCTTCAATGCATAATATTGAGAACTCCAATTGGTGATGACTTTTTTTCGAGTGCTGCTTGTGTTGTATGATTCTAGCTGAGACAGGTAAATTTGGATAGTTCTAGAAATAGGGGAAACTCTGTCAAATTTTTTTGTAAAATTTGGAGCTCTTCTCCTGGCCTTGAATTGGGTTGCTGACATTTTCCTAGTTAGCAAATTTAAAATGCACAAATAAAATTGGGATTTACATATTGGATGTAATGCTGCAAAGGTGAAGAGCAGAGTATTTTACCTGAGTTTGTTGAATATCAAGTTCTTGTGATAGAACAACATGATGATGTTGTGAAGGCAGGGTGTTAGTAGTGTATTCCATCATCAGCTATAAATCATGAGAATATTTATGTCATTTTTTCAAGAAAAATTGTGAGGCAATATGTAATGAAAGAGGCATTTTGTGAAATGATTTACCTGAATGCAATGCAGTATGCCTGAATGCTTTTGCTTGCCTATTCTCTGTTTTCTTTGAAAAATGGTGGAATGGCTTAATCATTCTATTAGATGTACATGCACTTACTTTTGGTCATTTCCTTAGTAAATTCAATGACTTTTTGGCCATTTCCTTGGTACAATAGTACCAAAATTGGAGGATTGATATGCTAGTTTTGCCTCAGGCTATTCAGTGTACAACTCTACATGACTTACATCACTTACATAATCTTTTATTGCTTGGTATTAATTCAATTGGCCACTTAACTATCTGAATCTATTGCTTGGTATTAATTATTTAATTAGACCTTATTAATCAAATGATCACTTAACTATCTGAATCTACCTACTAAAATTATGTGTTCTTTTTTTTTTAAAAAAAATGGAAAAACTTTATCAAGAGTTTTGAATAACTAAGTATTGAACTCTAATTAGTTATTCATGTATTTTAGATGTACATGCAATGCCTTTTTGCCACTTGTGTACCATTTAACTATTTCATTGGTTTAACTCTACATCACTTTTCCAGTCTTGTAGGAGTACTTGATATTTAGATTATTTTGCTGCTATGCACAAGTGTCTTACCCCCAACGATCCCAAATACTATGTCCTTTTCATTTGAAATTTCAACTTAGATTGCAAGTACACAATTAAGAAAATAACCATTCAACCACTCAAATTCTAAGTTCCCTACATAAATAGGGGAGGATCTGACCTGAACCCCTTGAGCAAAATATTACATTGTATATATAAGATAATTTTTTAATGTCTATAATATATAGATTTTTGAATTTCCTAAACACAACACTAGTGGTTAGATGAGTAGTTTAGGGGATTTAAAATCACCTTCCAAACTAATACGGCATACATGATGATTCAGTCGAGGTTGTTAACGTTTACCAACACATAGGTGTTCTAAATTTCTATAACGCAATTCCAAAAGGAGTCGGTACTTGGACATTGAATCCCTTCGGACCTGGA

>LNC_000824

AGAATAGAGACTTGACGAAAATTAATGGAAACCTTAAAAAAGAGGAGAGTTAATATTGTATGCCTACGGAAAACTAAATGAAATGTGCAAAAGGCTAGAGAGATAGGCAACACAGGATATTGATTATAGTATTTCCAGATTTATAAAACTAGGAATATTGTGAGAATTATTTTAGAACCAAGATTAAGGACAAAGTCGTAGAGGTAAAGTGAGTTGGTAATAAGATTTTCTCGTTTACTCGTGTTGGTAAAATGGAAAAACCAATATTAATAGTGCTTATACTCTACAAATTCGATTTGATGCTAAAGGTCAAACTAAATTTTGGGAAGATATTGACACCTAGGTTCAAGTGATTCTATGCAACCAACAAATTCTTAATATGGGATATCTAAATAGG

>LNC_000825

GATGATTATGCAACCACTGTGCTCTCTTTCCTTGGCATGGTGAAATAGTAAACTGTGGTCCTATGTTTTTCCATTTTTATTGGAGAAAAGTAAGACAAATGTCATGTTTGATTAAGTATGTTCACTTTTATAAGAAAATTAGAGCTTGTTGTTCTCAGTTCAATATTAGCTCTGTGGTACTGTTTGGCTTAAATGTAATGTTAAAATGCTAAATTTTCTAATCAAGATATATCTTAAAATATTCAATTTGCAAGATCTGTATCATAGATTGAAAGGTCAATTTGATTTATAAGTGTATCCCTATTTTCCT

>LNC_000826

GGGGTTCGTTCGGGTTTTTATTTAACTTGCGCACGAATGTACCTTCTGGGCTTATGGGGGTCCAGTTAGGTGTAGTTAGCTTGTAGATTACTTTAGTTAGTTCTTTGTACACTCGTGTGCATTCCATTGTTAGTTAGATTTTAGTTCTTGACCCTCGGTTTGTGTTATTCCTTCTTTTCATATTGCCTTTACTTTTCAAGTTCAGTCGGCCTATGATGCCTACTGGGTACCGG

>LNC_000827

TTTATTTCGTTAAGAGTAAGAAGCTCATTAATTAATTTGTGTTAATTCGTCTTGTTTAAGTTTTGACTAAATTCATCGATTGAATTCGTTTTGGTTACAATTGAAATTTATTTGGGCTATTACTCTTAATCAATTTCTTTCAACCCTTTGCAAGATCCAGTTTTGGGCCTTACTTCCAAATTGAGCAGCCAATTCCCAACTCAAAGAAATCCAGCCTAGCACTTTTTAAATTTAGCAGCCCACCAAAACGACCCAGTTCCAATTCAATGCAATACCCGACTCGACCTTTTTCTTCTCAAAGAACCCAGATGCTCAAGATGACAACAACAACTCGCAATCACACGATGTCCAGTTCCAGCCGGTCACGATTTAACATCGTCTTCACGTTTGTTCCAAACGGCAACTCCAAACGATGCTATCACAGAAGAAGCAGCAACACGACGTGAAAATGGCGTGAACGACAACAATAACAATATCAAACGAGGCAGGCAACGAATTGACGGTGATACACGACGTCTCAATTCCAATTCATGGCGTGAGCAAGACAAATGCAGTACATTGCAATTCTTGTCGTCTTCCTCAAACGTCCCTCTCCAAATCGTACAATTTCAGTAGCAAAAAGCACCTGCCAACATGCATTTTTCATCCTTGTGGCCTCAAGATCGAGCCACGTAGCTCCTCAAGGATGATGAGTCGATCCTAGCCCTCCACCTCACCTCCAATTCGTTCAGAATCGTGAGAATCAATTGGGATATTCTTTGATTTTGGATAGAATTCTGTCTTGGAATTCAAAAATTGTGGGCATTAAATCTGGGTTTTCACCA

>LNC_000828

CAACTTTGTTCCTATAATCAGACAAATTGGTAACCCTAATTTCTCGCCTCCTTCCTACAATAGCACTATTCTCTCAACTCTTCCTTCTACTTGTGATCTCTTCAATGGCTAATACGTCTGCTCCTTAGTATTTCCTCTTTCTCTACACACTTTCAATCTCTCTCTCAAGCCACAATTTCAATCTCTCTCTCCGCCTTTGCTGAAGATCTGGGTTTCTTTCGAACCAGCTGCGCAGGGTTCAAATCGGATTGTCAAGCTTGCCTCAACATTTCTGCTTGTTTCGGATGACCTACACAGGTATTGCGTCATCGTTTAAGCCAAATTAACATGTTTGCTGTGCCAGCTCTAACGGAAAGCCTAAATTGGTCCTTGAACAAGGTTGGTCGAAGAGCCCTTCGTATTT

>LNC_000829

CTTGTCAATCTTTTGACTTGGTATTCGATTTGGTGGGTGGATTCTGGGTGTCAAGCTGGTCATGGTAATATGTACAAATATTATATTAATGGTAGTGAATGGAATCTTCCATTGGCTTATCTCTCATGTAAGGCTTATTTTAATCTTCATTTCGCGGGCATGTCAATTTGGAACTCTCCTCCTATGGAAGTAAAATGGCTATTGCTAAAAGATGTTTGTAGTGACATCTAGTTGTGGGCTTGAGACAGTGTAAATGTTGTGAATGCAATATCTTCTTTTCTTTTTCTTTCCCCATATGTTTTGTAGGTTGTGTTTGGCCATGGTTACAAGTTGTTTTTAAATATTTGTGAGAATTTAGAGTGGAAATTATTTTTAGGTGATTCGTCCCATTTGTCCTAGCCTTGGTGGATAGAGTTTTTTGATACCTGTTGTTGGTGGGAGGTAATATGTATCCCTTGGAACTAGTCCAGGTGCGCAAAAGCTGGCCAAGTCACCACCGCCATAAAAAAAAAGAAAATAACTTTTTGATATTTTTCAAATTCCTAAAAAGTTGAATTTTGAATTTTATGACCAAATTAGTCAACTCCAAAAATATAAAAAAAAAAAAATTCAC

>LNC_000830

GCAAACTGAAAAGCATGTACCTGGATGGTCTTTACCAGTGTTGCCGGCGCTGTGCACAACGTCAAAGACTGCATAGAAAGTTTCGAGTTTTTTAGGGATGAAGGTGTTAAGGGGAAGTTGCTGAGTGGAAGTAGAAAGAAACAGAAAAATAGAGAGGAGATTGATAACGCATCGTCCTGGATGGTTGCTAGAGACTTGAGTCGGTGTTCTGCACACTACAATCGCAGCAGAGGAGAACTTTCAAAAGCATTCGAATCTGTTTTGCTATAAAAGAAGGTGTTAAGGGGAAGTTATTGGGAAGAAACAGGAAACACAGGGAGGAGATTGATAAAGCATCATACCTGAATGTTTTGTAGGGATGTGGGACGGTTTTCTGCCAACGTGAACGACATGAGAGGAGAACTTTCAAATTCAGTCGAGAAAGGTACTGAAGGAAAGATTGTTGGGAAGGAACATGAAGAACATCGGCGATTTGAACAACTTTAAAGGCTACGTATGCAAAATATCAAATTCTGCCGAAGAAGTTGTTGTATGAAAGTTGTTTGGAAGATTGTGAAAAGAGCCAAGAGGCCTTCGTCGCATCTGTTATGAAGAGTTACGTTGTATATGAAGGGAAACGACTGAGTGTGAAGTTTTGGGAGGAGGGGAAGGGTACTTGTGGAAATATATTAAAATATAAGGGAGTCCTTGCTTGTGTTGGCAC

>LNC_000831

ACTCTCTGCCCTGTTAATATTAACCAGTATACTGTTATAATATCTGCCCTCTAAATTGAATAGTGATTGCTATATTTTTGTATGTTCACACACCTAGCATGAGACCTTTCACAATTACTTGTGTTTGTCAGCATGTTTAATAAAATCTAGAGGCCAACTTGAGATTTATGTGCTAAATGCTCGTCCTATTTGTTTGACTTGGTGTCTAGGTAGGCCTATTAGGAGCCTTTATTTTATTTTTAGTCTAAAACCTCCTTATTAATAGTCCAATGCCTCTTGGACATTAAGTTGGGACGATAAGCCCCTTTTAGGACGAAATGCATGTGGAAGGTGGGGTAGGGG

>LNC_000832

GTTATGTATGATCATTATGAATATGGTAAGTTATGATGAAATATGATCTTATGAATGTATGCATGAAGTAGGTTGCTTAATGTGATACTTGGCTTTGAATAGGGTTATGGGGTTCTATTCTTTGCATTGCACTAGTATTGCTTGGGTTGTCTACATGTTGGGATGATATTATGTTGGTTTGGACTATGATGCTTACTTTGTGTGCATATTGGATTTACTTGGCAAAAAGCAT

>LNC_000833

AAAGAAGAGAACCTGCTTGTGGAAAAGCCTAAAGTCTAGAAGGTGTGGAAAAAGGCTAGAGTGTTGGACCCTGCTACTAACCCATTGATCGATGAAAAAGGAAGATACAAAGCTCAACTAGTGGAGGAAGATAAGGATGGTAAGGAACAGGTTCAGGACCAACAGTGGTCTATTACTGTAGGGAACTCTGCTAGAAGAGTAGCTTAGCCTGCACTGTAGGTAGTGCGTACTTCTAATGATTTTGAGACCCTGGACTATTATTATTACTATATATAAGAAGCTAGGGAAGCAGCTGTTCGTCAACTTGCTTGCTTCCCTAGCCAGGGGGTAATATGAGTATTTTCTTTATTATTATGCTTGATCATTAT

>LNC_000834

CATTAAACTCGTGAAAATTCTTTCTTCCCCTAAACATCATGTTAATGCCGCTTTACTTTTCTGTTAACGTTGACAAGATCTGCATGGACTTGGAATTCAACTGTTTCTTTCAGAAAGTAATGGTTCCATTGACATTACTTGAAGTTTGGTTGACCAAAATCTGGTACCATTTCTCTTGTATTTCTTACTAAAAAGAAACAAAACATAGAATGGTAAAGTTGACATTATTTGGTGTACCTAGTTAAAAGGAGAATTGAGTTCCGAAAGAAGATGCT

>LNC_000835

GAGAGTCAAGGAGGGAAAGAACATGTGATGGTGACTTCTTTCACTCTAGGTTTCATAGAATGTGGGTTAAAAAACCACTTGTGTAGGTCGGGGTTGAGAATTGGGGGGCCGCGCCCTACCCCGTCGGCCTAAGCCTCGCTATGAGAACTGACGCTTTCTAACTCTCTATGAGGAGTGAAAGCCACTTGACTGTAAGGAGAGGAGCTCCGCCCCTACTTCAATACA

>LNC_000836

CTTCTCTCTGTCTGTGTCAAGATTACTCCATATCTCTTTTTCTGGTGAATTTTCGGGTGAGTTTGGGGAAGATGCCATTGGAAAATGAGGAAAAGGAAAAAAAGTTTGTGAAAAAGAAAAGTAAGAAAGAGAAAAGGAAAAAAAAGAAAAAGAAGAAAAGGAAAATGAATAAAAAACTGGCGGCAAGGTGACATGCCATGTTGTATCCACATCATGCAAATGTTAGGCCACGTGGACAACCATTAGAAAGAGGGATATTTTTCAATACTTTGGCAGTGATAGGGGCATATTTGGCCCTATAGTATAACGAGG

>LNC_000837

CTTTCCTGAATTAATTATGATTCATGTTAAGTGCTAGCTCCAATTATTTTATTATTTAAATGTTTGATCAGCTCCTACATACGCTATTTTTGAGCTGAGAAACCCTTTGTATTCGTCTTCAAGGGCTTGTGGTGTGTCCCAATGGAATAGAGTCACAAAAGGAGTTATACCTAAGGAAAAGTTTATTAATTAAATGGATAGTATTATAAATAATTAATGAGAGATCAGGGAGCTGTAATTATATTATTACCATTAGCGATTAGCTCGTTGAACACATTATTGTAGAATTTGATGCCTTCTGGATTCACTCCTTTGCTAACCTTACCATCTTAAATAAGCACAACAAGGAAACAAAGCTTTCAGCAGATCAACTATATACATAATTTTCCTTGTAAATATATTTCACATCCATAAGTTATAAGGGTTAAAGATTAGGGAAACTGAATATATGGAACAGGCTGAATAACATGGTCCAATTATCCTGTATAACTAGAGTTAGA

>LNC_000838

ATCTTCAGACCATTCAGGAAGTTGCAAGAATAACATACGCCCGAGATTTTTGGCTATTATCAATGAAGGTAATAGAATATATTTTCGAAGAATTGATTGAGTTATTAACATGTAACCTCTTATTATTTGTGCAAAAGGAATGTTATCCCAGGCTTCTTCGATCGCTATCTATGCTTGAAACCTAGAAATCGATCACTGATCCAATTTGAGTACCTCTGCAGGATAGACCTCAACAGAAAACTGAAGAGTAACGGCAGCAAGTGATTGAGTTCAGTAGTTCCTCATATAAAATTATTGACTCTAGAGATATAGTAATATGAAGAAGACAAAATTGTTTCAAGCACCGACAGAACCGGAAGCGCCCCTTCTTTCAAAGAGAGGAGGACGGGTTATTCACATTTCATTG

>LNC_000839

CTCCTCAATTCGACACACACGAGATGAATTTCTAGAGTATGAATTGTGAGAGAAAAACTATTGAAGTACTCATTCAATTCTCTTCAGAGTTTCAAGATTTCTTAGAACTTGAGCAAAGCTGGATGATTATTAGAGCTTCAATCGCATATGTGTGTAAAGAGCCCACCATATGAAATTGATAGAAATTATGGGACAACATTTATATGTGGCATGATCTCAAACTACTTAATGGTAACTAGGAACTATGGTGCTTTAAGGAGGTGACTGTAGCTGGAACTTCTGATACCATTTGTTTCATTCAAGGATGCTGATTATGATGACTCAATTTGTGTAGTGAGTTGTATATACACTAAGTAAGTTACAGAGTGTTTTTGGTTTCAATTTCAGGGGTTTTATATTTGTCATTGTTGATATGCGAAGAGTAGGTCTCGGTTCTTTGTTGTTGGTTCTTTCCAAAGTACTACATTTTTGTAGATTGACTTTGGTAAATGAAAAAGATAGTGTTATCATTCCTTATGAAGGGATTTTTTCTTGTCAAGTATTGTAAGTTCATAATCTGATTGTAATATATTATTTTTTACCTGCC

>LNC_000840

CTCCTCAATTCGACACACACGAGATGAATTTCTAGAGTATGAATTGTGAGAGAAAAACTATTGAAGTACTCATTCAATTCTCTTCAGAGTTTCAAGATTTCTTAGAACTTGAGGTAAAATTCTCTCTCAATCTTTGTGCAAATTGTCTTAACGTCAAATTGTTTCCTACTTGTTACTGTCTTCTTTCTTCTCTATTGTTCTTTATATATTTAGTTGTTTGAGTTTTATTTGTCATGAATGAACTCAACTTACTTGACAACTTCTACTTTTGCTGACTCAAATCAGCTGAAGTATTTTGTTATTTTTTATTATTGTTGTTGCACATCACCCACAAAAATTCCAAATTGGGAAATCACAATCTAAGACGCAAGCTTCAACAATGGTGGATTTCGAAGCTTTTCATATTATTATTTTTTGATTCGGTATACAATCATAGTTAATCAAATTTTTAGGTTCCAATTTCTTCTTATGTTCCAACATTAGAGTCTCAAATGCCTACCGATAGACACGAAGCTTCTCTATTTATTAGATGAATGAGGACAAGAAGATCTTAAAAGAGGTAAAGGAAAAGACCAACTTCATTTTACTGCTTTAGGATCTAACTTGATTCTACAAGTTTTATTGGAATTGCTATAAAAATAAACTCAATTGTTTTTTTATTAACTTAACACAATTTGAGAAGAAAAAAGAAATTTTGAATGTCTGGACAATCGTCAATATCTCTGATGGTGCCTACTCCGCAAAGCGCAAAGTTGAAGGGAGCTCAACGAGTTACTTTCTAAGCAAAGCTGGATGATTATTAGAGCTTCAATCGCATATGTGTGTAAAGAGCCCACCATATGAAATTGATAGAAATTATGGGACAACATTTATATGTGGCATGATCTCAAACTACTTAATGGTGAGTCTTGATAGTATAGTATTTCAAAAAGTTACAATCAGATTATTAATTAGTACTACATATTATACACATCTAGGTTGTATAAGTGTGACAACAGATTGATATATTTTAAGATCGACTAGGGATGGAAAGTATAAGTACTTGAATAACTATAAGTGAACCCTTCTAGATGCTACAATGCAGACTTTGTTGCTCTTTTCAGCTATACGCATTAAGGGAGTCCACAACTTCAAGATTTCCATTCAAAATTTAATCTAATCTCTATACTAACACGCAAACACATCTAATTTTAGGCGAAGAATTAAACACCAAAAATGCTAGACTAAAGTCGTGTAATAGGAGAAATTGTTAAGTTGTTTTAAGTGTTTTATTTCAAAGGCATAAAGAGAGAGGTGAATGAAAGGATAATTTTCAATTTAGTGTTTTAGGATATATTTCTTGCATCTAGAGATTTTATTGAAATTTCTAGAAAACTTAACTTTGTTGCTCTGCGGTAGTCATCACAGAAATTGAGAAGGAAAAGAAATTTGATGATTTTATAATTAATTTAATGACTTGTGAGATAGTCTCTGCTACTTGACATTGATTGGATTACATTAAGCATTGATATTGTAGTGGCACTTCAAAAAATATGCATTAGAATTTGAAGTTCGTTGTAATCTCTCTTTGTTATTGTTGGTGTTTTTCCTTGGTGGACAGGTCTATTTTCTCTAAATTCTTGCAAAGTTTTGTACAAATAATTATTAATTTATGTGTACTTTCTTCATATTTTGGTATTTTTAAGATTGAATTTCAATCAGTGTGATGTAAATATTATTAGATGGGTCCAAGTTTGATTTCATTTTTACTAATTGCAGGCGATGGACTAAGAGTACCTAACATTTATGAGTATAATAATTTCAAGATATAATTTGTTTTCTCCTTCAACCTGTGATTTTCATTTCTTCATCCTATATGCTTTTATGTTTGATGCATTCTTTGTCGGTATATTTTGCCTAGCCAACATGAAAGTTTACTCACATAATGGAATCTATGTATTATATAATAACTAAATCATGAGTCAGAAAAATTTTCTTTCAATCTATTTCTATTTGTCACCGTCTCGTGAGTTCGTTCTCCCTCATCTCTCTCTCTCTTGTGCTCTTTTTCGCTCCCAGTTTTTTCTCTCTTTAAATTTTTTGTCTATATAATTGTTCTCGTACTCTCTGTTTCAGCTAACCCATGCTATTAAAATTGGAATTTTGAACATTATATAGATGCTTTAAAGGCAATCCTTATAATATGACAAATAAAATTTGAAACTTATTTCTTCTCCATGAAATATGTTATCACGTTGAAGTTGAAATTTGTTCTTTGTTTCTTCTTGAGTGTGCTTATAATTATGTTGCCTTCTTTATAGTTTTGTCTTTTTAATTATATTAATGAGAAACTCGGTCAAGAATATGCTTCTGCAATCTAATATTGGGGATACATATTCAAGAATTATGATGTTGAGGAGAGGTGCAAAATAATTAGGAAAATTACACAAAGGAAGACACTTAAATGTCTCTGCTTCACTAAGAGTTGTGCATTTTACCTCACCTTACAAAGGTTGGTTTTCTAATTTGGACATGGCGTGCATAACTAATGTTCTTTGCAGGTAACTAGGAACTATGGTGCTTTAAGGAGGTGACTGTAGCTGGAACTTCTGATACCATTTGTTTCATTCAAGGATGCTGATTATGATGACTCAATTTGTGTAGTGAGTTGTATATACACTAAGTAAGTTACAGAGTGTTTTTGGTTTCAATTTCAGGGGTTTTATATTTGTCATTGTTGATATGCGAAGAGTAGGTCTCGGTTCTTTGTTGTTGGTTCTTTCCAAAGTACTACATTTTTGTAGATTGACTTTGGTAAATGAAAAAGATAGTGTTATCATTCCTTATGAAGGGATTTTTTCTTGTCAAGTATTGTAAGTTCATAATCTGATTGTAATATATTATTTTTTACCTGCC

>LNC_000841

CTAGTATGGTCTATGTCGGTTAATGCCTATTCCCATCATGTGGGATGTGCACTCTAGTTATTGGATAGGTTCTACAAAGTGTAGGTGGTAGTATGGGATGCTATCTACACATTGCACGAGTAGGCCTAGAAGATGCTAAAGTGAGTTCCCTAAGTCTAAATGGCTATTATGTGAAAGTCCCCTAAATGCTTAAATGTCTCTTAAAGTGGTTCATTAAA

>LNC_000842

CTGATATTGGCGGCGCCGCTGAGGAAGGAACGAAGAGATACCTTTGAGCACCCGTAGACAATGGCTGAGGTAGTTCAAGACAATGTGGCGCTGTTGGGATATCATTGTGCATCTCTGTTGAAGGAGAAAAGTCATCCCAAGAAATTTTCAAATGCTGATTTTTATATTTAGAAAAAATATTGGTAAAAGGGTAACTAGTTTCAAAAAATACTACATCTCTAGAAAGATAAACTTTGGAGGTGGTCGGGTCAAAGCATTGATGACAATAATATTTATTTGAAAATCCCAAATAGACACAGGGCTTTGATTTCGAATCAAGCTTATGTTGTGCATATGGTTTTAACCATGGATAGCATAAACAACCAAAAAATTTGACGTTGTCATAATTTGGTGATTCTTGAAATAAAAGCTCGTATGGACACTTGTCTTTTAAATTTGGGGTTGTCATTCTGTTAATGAGAAAAACAGCTTGATGACATGCAAAACTCCAAAAGGTGGATGGTAAAGAGGCTTGATGCATTAGAGTTTTTGCAGTCTCGACAACATGGCGATGTCGTCTTTCAACTACAGCAACTCTTTGTGGCGTATAAGGTGGGGAAATTAAGTGTTCAATGCCCTGAGATTTCAAATAAGAAGTTAAACTCTGAAATTCACCTCCTCCGTCGGTATAAAAAGATAATATTTTTGTTTGAAACCGACGTTCAAGCAATGGATGTAGTGTTTTGAAAACATCTAAAACCTCGTGTTTGGACTGAATCGTGTGGAGCCACATGTATTTTGAAAACTGATCCACGAACAAAACATAATATCTTTTCCTATCGACGGATAAAATTGGTGACGGGCCCCACAAATCACTGTAAATAATTTGGAGAGGCATGTGACTCTGTAAAGAATTTGTAGAAAAAGATTGCCTGTGACTTTTATTTGATTGACATGAATTACAAATGGAAAGAGAGTCTTGCTTAACAGGAATGGAAAATTTATTTAAAAACAAATCTAAAGTACGACGTGTTGGATGCCCCAAACGCCGATGCCACAGATCTAAAGATGCAGCCACATTGCATTGAGGTTGAGAAGAAGAAGAAAGATTGTCTAGCGG

>LNC_000843

CAACAATACCAACCAGCGAAACTCCATTCGAACGATAGCTCCGGCCAGCGACTCAGCAAACAAGCAACATAAAATATGATAATAGAGTTCACTCAGAGCTCGATATTTTGGTTGGTTTAGGACGGATCTCAACAGATTCGTCCAGGCTGAATTTAGTTTCGGGAGTTTGAAGAGTTGCCTGGGATGGGTCATTGGGACAAGATTTCTTTTGTTTTTAGCTATAATATTTGTATTTGCTATTATGGTTTTGTAATATTTTTTTTGTAACTCTAACTCTCTCCATATATGAAATAATTTGGGGGAATTGTGTAAGGGGGAGGGGGATTCCATGCTAATTATTTTATTTATATACTTCATTATTTGTGATTGCTAAATTTATCCATA

>LNC_000844

CAATACCAACCAGCGAAACTCCATTCGAACGATAGCTCCGGCCAGCGACTCAGCAAACAAGCAACATAAAATATGATAATAGAGTTCACTCAGAGCTCGATATTTTGGTTGGTTTAGGACGGATCTCAACAGATTCGTCCAGTGTACGGGTCGGTAATTGGATTAAGTATGGATCTTCATTGTTGTTTTCTTTTACTGCATAACTGGCTGAATTTAGTTTCGGGAGTTTGAAGAGTTGCCTGGGATGGGTCATTGGGACAAGATTTCTTTTGTTTTTAGCTATAATATTTGTATTTGCTATTATGGTTTTGTAATATTTTTTTTGTAACTCTAACTCTCTCCATATATGAAATAATTTGGGGGAATTGTGTAAGGGGGAGGGGGATTCCATGCTAATTATTTTATTTATATACTTCATTATTTGTGATTGCTAAATTTATCCATA

>LNC_000845

CATCTTTCTGCCCTTTTCCCTCTTTCACTTTGCTTTTTCCGCTCTCATTTTTTCCTCTCACTTTCTCATCATCTTCTTCAATGAGCGGTGAAATCTACTGGACTTCGTAAGTCTTTTTTTGGTTTTAGATCCTATTTTGTAGATTGTATGTTAGATTGTTTTGATATTGCTTTTCATTTTGCTGTTTTGTGGGTATGTGGTTATAAGTATGTTGCATGTACATAATTCCTGTGTTAGTCCTATTCTCAATTTTGCAGTCTTGAATTTGATTATAAGAGGTAACCAGAAATTTCGATCACACACCAATGAAGACCTCAAAATTCAACTGGGTATAATGTAGATTACTTATTGTGAGATCTTCAGGTATATTTTAGGAGATATTGTTGTGCCGGGTAATATTTAGGGATATGCTTTGAACTTTTTCCTTTCTTACAATAAGTATTTTATATGTCATTTAGAGAAAAATATTTAATGTCATATATCAATAGGATCTGTATTCTCGAATTTAGAAACCGGAGTTCAAATATATTATTGTGGTTTGAACTTAGAGTTGTCATTTCCTGTTGATGTAGTAATGGGGAATTTAAGAGGGGTGTTTTCTGATAATGTTCTATGATATTGCTTTACTTGATGTGAGATAAGAGGAAAAAGTTCACAGATATGTTGGAGGTTAGACGTCTCCCTTGACAGTACTAGGGTCCTTCAAAGAAGGGAAGATTTCACCGGGGTGGAGGAAAGTGGTGAGTCCATTAAGGCTACCAATTTCTTTTCTTATGAAAAATACAAGGAGCACCTACTCAAATTTACTGATAATGAGTCTAAGGCATGAACAAATTTTGAAAAATGAATTTTGTCGTATTCTATGCGCTGCTCTATTTAAGGCTATCAAATTCTGTTCTTATGAAAAAATACGAAGAGCATTTGCTCGAATTTACTGATCATTTAGAAAGATTTATTGCTGGTTTCACTGTAGGAATTATAGATGCTATGCTCTGAATGCCTATGGACAGTAAGTAGTATTTTTATATTTTCAATCTTTTGGCTTCTTGATTTCTTTTTCTAAAAACATAAATTGAACCCCATTAAGTTGTATATTTATGTTGATTTGTACTATATTTTCTCATCTATGAACTACAACATCGAAGTAGTTGTTGTGTATGTTGTTTGTATTATTTAATTAAACTTCAAAATTCATTAAAGGACTAGATTTCAGGAATTCTCAATTGATTAAAGTGAAACAAAAAAAGCTGTCAAGGAAGGACAAACAATATATGAAGAGAATACATTTTCTTTTTGAATTAAATTTCTAAGTTAGCCTTACTTAGATGAGTAGCTTTGAAAATATATTCTCTTCCGCCAGCTGAAAGTGATTACAAACTACATGAGTTTTTCCCTAGCAATCAGTTACAACCTTGTGTTCTTGACTTTGGTAAATGACACAACATTGTGGAATGAAAATGCTGATACTTTACTATTTGAAGGTTAATATAATGAGACAAGTTATGTTTTAAATTTTAGTCTTTATATTGATTAGACATCTCTAATTAGTTTCTTTTTATTGATATATTTGCATATAATTGTACCTCAGAAAACTTAACTTTCAAGTTCTTGCGGACAATTACGACACAGTAGATGTGATGACCATATGTTCATCAAGTTATATTAAAATCAGTAAACCACAACTTTAAGACACTTAGAAAGTCATACAATCTCCAAGAGTAATGTCCTTATAGGTGTTTTTTGCTGAACTCTTCATGGACCATATTGCCTTCTATATTTTCTAACTAATGGATTTCTTCTTTTATTGACTGTGCCCAAAGCAAAGAGTGGTCTTCAAATTGATGAAAAAATTATGGCATCGGTGCAATTTTTATGCAACTAAGATGTTTGATTATATGCCTGATTTCCCTGGCTATGTCTAAAATGTGTATGTGTTTCCTCTCAACAACACTATTTTGTTGAGGGGTATAGACACAACTACTTTGATGAACAATACCAAGCTATTGTAGGAGATTGGTGAACTGAGAGTTGAAAAACTTAGTGTCATTATCTGACCTTAATGTTTTAACTATGACTCCAAACTGATTTTTGACCATGTTAAAGAACGGTTGCAATACCACAATTACTTCAGACTTTAACGGTAACAAATATAACCAAGTGAATCTAGTATAATCATCAACAATAGTTAGAAAATAATGCTTCTTATAAAGAGTAGGAATTTTATAGGAACCCCAAAGATCCATGTGTACAAGTTCAAAAGGAAATACACTTCTTGAATTACCAGTAGGAAGCTTCAATCTAGTTTGCTTAGCAATAGGGAAAATGGAACATTTATCATGTATAGAACTACTATCATGGTATTTTACAGGTGATATGTCCTTCATGATGGCTTGTGATGGATGACCCAATCTCATGTGCCACAGCTTGTATGCTTCTCCATTTCCATAAGTAGATGTTGCACTTATTGCCTCTTTCTCATTCTAGCTAACTTTATTTACCTTTAGTTTATACAAACCATCTTCCTCTCTACCAATACCCCCCCTCACTCTGCTACTGTGAAGGTCGTGAAATATACAAAAATTAGGGAAAAATTGACAGAGCAAGATAGTTCCTTGCTTAATTTCGACACTACCAAGAGATTGAACTTAAACTCAGGTACATAAAGAACATTGATAACTAGTACTTCCTTCAGTATAGTTGTTGCACCAACACGTGTCACATTTTCCTTGTCTTCTGTAAGTAAATATACCTTGTTTTTGCTAGAGTTATATACCTGCCTACCTTGTTCTAGTAGGTTTAAGCTGGATGATATATGATGTGCTGCTCCAAATTCTATAATCCACTCTCTACAAGTAACTGCTGACATTAGGCAAGTAATTGTACCTTTATGTTAGCATGTTGCTCATCTCCATCTTTGTTAAGCAGATTTACAAGGTGCTTGTATTGTTCTTCATTGAAATATGGTCATTTGTTATTAGTTGCATCATAGAGCATTTGTCCTCCTTGCTGAGACTGCATTTGGCAGCCAAGACCTGTTGATGGATTCATGACTCTGGATCCAACAACATTAGTGCCATTATCAAATTTTAC

>LNC_000846

TGTACTCTTCTTTTTCTGAAATCACTCTCTTCCTCTTCTTTTTCCAGAAAGAAGACGTCTCCCTGTACTCTTTCTCCTTCCCCAGAATTTTTTTTTTCTTTCCCAGACGCGTTTTCCTCCTTCCCCATGGCCGCTCCTCACCTCTCCCATCGCCTCAGTCAGAAAACAGCTATGGACTGCCGGAAAATGTTAATTTCCGGTGGTTCCTTGCTCTATGCCCCCATCAGTCACTCAACCCCTTTCAAATCCGTCTGAAAAGGGAGACTCTTGGAAGAAACTTTCTTCCCCTTCATCATTATTCCTCCAGATTTGGCTTCCCTAGAATTCAAAAAAAAAAAATTGAATTCTTGAGGCCCAAATGGAGGTTACGATTTGTCTATAAAAGGAACCATTTGGTTCCATTCGGGGGGAGGGAGAAGATCCGAGGAACAACCATTGGGAAATCATCAAAAACGTCCAAAGACAATCCGAAAATCTTTTGTTCTCTGTTTTCGTTTCTTTTTTTTTTGAGTTGGAGTTCGAGTTCGCAGTTGAAATTTTGACGTCAAAGCATAGTTTGCTGCTCTTGAAAAGGTACACTACTTCGCCTTTATTTTGTTCAAACTCTAGCTGTTCGTTTGGGTTGGTTTCTTGATAATTTCGAAAAGGTTTCTTAATCTGCTTGTTCAGATTTTATCTTTTATTTTTAGTACAGTTAAGTTTTCCCATTAAAAATGGTATATATTCCCTTGTTCTTGTTTGGATTTGGATATGATTTAGGATGCGCACTTATGAAATGCTGAGGATGTGTTTCTGATTTGAGTTTCTGAACTGCAATATAGCTTAATTTTTATTTTTATTTTTTATTTTTTTCGAAGTGAAAAGGCCCTGTTTAATCTATGATTAAGGAAGTTGTCCCCTCTCTGCAGTCATTTGATGCAAAGGCTATTGTTTGTTGTTTTAGATCTAAGATTAATCCTAACCTTTATGCTTGAAGTTGACCGATGTCTTTTATATATACCTAAGCAGCCCCCGATTTGTGTATTAGCTCGAACAAATTACATGAAAGAACGAAGGCTATTCTCAAGCATGGTTAGCTGCCGATAGTAGTATCATTGAAACATTCATACATCTGTTATCCGACTGTTTCTTTGGTATATTCATTGATATGAGTACCTTAATGTTGGCAACCTTTAGTTTAGTATGAGATGGACTCCTAAAGGGCATAAAACACTATGAATGCTTGATGTCCAGTTGTTCTCTTTAATTTCCTGAAAACCTCCTCCATTTTTTAATGGAATATGATAAGTTTCTCTCTTTGTTCTTACATCTCGCCTAAGTAATTAGAAGGATCCCCATGATATCTTTGTAATATATTGGATTGAACTACTGCATTTTTCTCCTTTCTCTCATGAGTGTCGACACGTGATAAATCTGCCCGTATTCATGTGAAGTCGAGGACATTAGATAGGAATTTATGCAAGCTAAGTGTGTCTAGTTAACTTAGTCCGCTACCAACGTGTTGGCTTAATGACTTTTAACTTTTTCTTATGTTTGATTAAGTAATGCAGTCATAGGAATATTTATCCCCTATGATGACGAACTGAATATTTACTTGAGCCTTCATATGTCTCTCAATTTACTGGCTGTCAAGAATTCTAAAATGCTAAGTTCAACCTCATATTGTTTTTTTTAGAAAACAAGATTGTCTACCCAGAATAATGCATAGCCCCCTTTATATTGTATTTGTACTTTAGATCCTCATGCATATAACATCACCGTTACATAGTATATAATGAAATAATATACAATATCCTCTCTTGTTCCTTAGAAACTACTTGTATTAATATTGTTTGTTTCTTTTATTGGACTCAAAAAATAAATATATATTCATGTGCTTCACCAGATAGTTCATGTATGTTTAAACTTGTTTTGATGTGGTAGAAGACACATTCATAAGTTCTGTACTCCAAATTTAACTTATACGAGTATAAGAGAAAAAAAAAATGTTGATCTGTAAAAAATTTGCCTCTAGGCACGTGAAATATTTGTCTCGTCATTCATTTATTGCTTACTTGTATTTGATGTTGTTGCTAAACTTTATGACTTATGTTTATTACTACTTTGCTGACATGATTATGGTTATTCCTAATCACTTCTTATTCTCTTCTTATATTTTTCTAATTTGTTTCTTTTTATCTTCTTATATTTCATGTACAACCGGAGAGCTGGAGTTTCTACTTGGAACTCGTTTTGGACCTAAAATATTAGGCCAGCAACAATTAGGATCACCGCAATGCTTGGAGTCTCATAAATAGGAGTCTAATGTTCCTCAGCCTTTCTTTTGTTGCCTTATGTTTTTTTTATGTTGGCTAACTTTTACTCTTAAGTTTCGTCGTCCTACTTATATTGTTTTTGATATGTTAGCTAACATTTACTCTTAGGTTTTGATACATTGCTTGTCTTCTTTTATTTTTGAAGATTACTCATATGAGCCAATATTTGTTTGCTTGTGAGTTTAGTTGAAACATTTTTAGGAAAGGGGTTAGCATTTTTACCTATAATACTCTTTTTTATGTTTTTTTTTAGTTGAGGAAAGGGGCTTATAGTTTAAGAATTTTCCAAACAAAGTTTCAAAGGTTAAAATAATACTAGTGTTTCAAAATTGGAAAAGGAAGTATATATCGGCTTAGGCACTTAAGGAATAGGAAGACTTTTTGTCTAAAACCCTCTTTGAAATTTTAAAAGGGTGAAATAATTTTCATTTAGGTATCTAAGTAGGAAAAAAATAAAATTCATGAGTTGGGACTTCCA

>LNC_000847

GCCATTTTCCCCTAACTTGAAAAATACCCATTTCTCCATTTTTAGACTTTGAGAGCTCGAAAATAGGTGATTTCAAGTGGGTTTTCGCAAGGAATTCATTGGGTAAGTTATTTCCAACTTATATCTTTGATTTCCATCGATTATTTATGGATTTTAACATCAAAATTTCAGATCTCAACTGTAAAATTTGGGGGTTTTTGCCTAATCCGAATTTAATAGAAAATTTGAGTTTGTGAACTCATTTCAACTCCTTTTTCGAAACGGTTTTCACTATTGGATTCTAAATTTCTTGGGGAACATTTTTCACTAAAAATTTCCAGATTTCGAGAATATTTTTCGGAACGAGATTTTTTTGACCATTTTACCCTTTTTCTTCAACTTTCACGATATTGGTGTTGTTAGATTCGTATTTATTGTGGGATTCCATTTATGAATAGATTGTGGTGATTTGGAGCGTGCTCGGAAGGGTAAAACTCAAATTTGAGTTACTAGTTGAAGTGTTTTGAGGCAAGTGACTTCTAAACTTTGTTAAACTCTTAGACTACACATGACTACTTTCCTAATTGTGTTGGGGAGTAATGGGGATTGAGGATGGGTTTTATTTGTTGATT

>LNC_000848

ATTCTCACTAGCATTATTGGTGACAATTTGTACAACATTTTCCGATCCAATTCTTTCAATAGTGTTTTCAAACAAGTTGTACATTTTGGTGGAATCGGTAAATTCATTGCTAGCATCAACAGAACCAAGAAATACACTACCGATTGGAGAATTCACCAAAATATTGATGATCATTCTACCATTTCGCGCCGTCCATTTGTCCATCACAATGGAACATCCAAACTTTGTCTAAAAAAATGGAACATCCAAACTTTGTCCATTGCACTTTATGCTCATCAACAATTTTTT

>LNC_000849

CCTCCCTCTTTCCTCCCCATTGCTTCTCCTTCATGTTAAAAAAAAAGAACATATAAAATGAAAGAAAAAGTCCCAATTATTATCTCTAAATTTGTGATTGAGAAGAAGAGGTACCTTTTCTTTCACAGATCCATTCTCCAATCTTCTCTCTTTCATTCTGCAATTCGATTTCACCGAAATCCGATTTGGTTTTTACTCACATCTGGTTGTAATCTGCTGATTCTGAGCTTACTTGTATGCTTCACTGTTCTTCCATCTCACTGTTGACTTTTGTGTTTGCGTTAATTTGTTATAACCTGTATTGATTTTTTGATGTTGACTGAAGTAAATA

>LNC_000850

GTCAGATTTTACTAATTAGGGTTGAGGTTCTGATTCGTGTTCTTCGTTCTTGGGTTCTTTTATGCAATTCTCTTTAGTTATAGTTATTCTGTGTACTCGTCGGGCTTATGGGGGTCCGTTCGGGTTTTTATTTAACTTGCGCACGAGTGTACCTTCTGGGCTTATGGGGGCCCAGTTAGGTGTAGTTAGCTTATAGATTACTTTAGTTAGTTCTTTGTACACTCGTGTGCATTCCATTGTTAGTTAGATTCTCGT

>LNC_000851

TTACAGATAGGGTATATGTTTCTCTTTGGAAAGTACCCACATGAAAGATCTCTTTCTACATTGATGCTACCCATCAGAATATTGATGTTCTTCCTCCATTTTTCTCCATGATGAAGCCCGATCTGCCTTGCTTGACTTAGCTCCATTTCCCCGAAGAAGGACCAACTTACGAATCTGATTTGCACCGTATTCCGCTGTGATTGAAGCCTTTTAGCTGAGTTTACTCTTTCTCGATCTACATAGCCCTGTTTCCCCATTCATTTATGTGTATACCTGAAACCTGCGAATCTATCTAGCTTTATGTATCCAGCTGTGTAATAAACTGCTATGATATGCTTCCTCATGTTTGTCAAATACTGTGTGTGATCTTATTGTTACTTTCAAGACCATTTGTGAATAGTTGATGTTCCTGGCCCTTCACAACCTTTGTTACAACTTGGTCTTGACTGATGTTAAACTTGTGTGATTTCTGGTCTGACATGATCACATTGAGGGATGATTGCGCGTGTTCTGATTCTGAGTGTAGGAATTTGTGCTTTGTCTATGTTTAATATCCTTCTTTCCTTCAATGGTAGTTCCTGGAAACAACTATATTGATGTTCTGCTTGTGATTCTATAGCTATATTAGCTAATGAATCTGCCAAACTATTACCCTCTCTAAATATGTGTGTGATCTTTGCTTTAATCTTTTCCATTATCTCTCTGATTTCTTCCACTTTTTCTGCTATTTTCCATGGAACCCTCCAAGTCTGCTGAATGATTTTCTTGAGACTGAGTGAATCTGTCTCAATAATCACCTTCTGCATTTTTCTGTTAGAACACTCTCTAAGAGCTGTTAAAATGGCAACAGTTTCTGCTTCCATATTTGTTGCAATACCAATACCTTTAGCTTTAGCATATATTAGATCCCCCTTATCATCTCTAATACAAAACCCAAAAGAGCTCAAACCAGGGTTACCTCTGCATGCTCCATCAGTGTTAGACTTCACATAATGATCATCAGGAGGTTTCCAAGTGACCCTTAAGACATGTATCTTTCGCTTATATTGATTAAGCCTTTGAATTATGGCTTGCCATGTCCATCTCATATTCTTAATCCATGGAAATTGACTCTTCACCATCTTCCTTACCACTTCAATTACCATTTCCACCATTCTTTCCCAGCTGATGCTACTGTCATGTTTGAGGGCATTCCTTCTCTTCCATAAAGTCCACATGATGATGGCTGGTATTGCTTTATATATGCCTTGCAATTTTGGTGTTGCCTCATGTTTCCACCAAGATATTATGAGTTGTTGAAGATGCATTCCATCAATGTTGATTCCTGCAAAATGAGCAAAGTACCTCCATAGCTTGTAGGTTATAGGGGCTGTTGGGAATAAATGTGTCATTGTTTCCTCCTTTTTTCTGTCACAGCACCAACATCTAGAAACAATATTGATTCTCATCTTCTTAAGATTGTCATCAGTGGCAATCCTCCTTTTCCATACTCTCCACATGAAAAAATTAATCTTAAAAGGCAATTCCTTATTCCAGATTACCTCACAGTCCCTTCTTACTTCCTGTTTGTTTCTCGTAATCTGCCAAGCTGACTTTACTGTGAATATTCCTTGTGCATTGGCCATCCACCAGACCACATCATTTCCAAACAAAGTGTTTGGAGGTGAGATGTTCTCCATAATATGATCAGTCATCTCTAGAGACAGATTCTGAAGCAACTTCTCCTTGTCCCATCCCTCTCCTGTACAAAACTCCTTAACTTCTACCTCCTCTTCCTTTGCATTTTCTTCTATATGGTACAATGCTCCTTGTTTAGTCCAATTATCAAACCAGAAACTTGAATTACCTGCCTTAATTTGCCACCAAATTTCATGCTCTACTTCCTCCCTTATTGATATCATCCTTCTCCAAACATGAGAGCTCCCATACCCCTGAGCAATAATAGGGTGATGCTTCTTACAGTATTTGTTCCACATAAATGATGCCCATAGGGTGTTTGTGGAAGTTCTGAAATTCCACCAGAGCTTGGCAAACAGTGCTTTTGAAATATCATGTAGTGATCTCCATCCCATTCCTCCTTCCCCTCTTGGATAACACATTTTATCCCAGCCCACCCAATGCTTGTTCTTGGCCCCAGCAGTGTTAGCCCAAAAGAACTTAGCAAAAATTCTATGCAGTTGGGTAATGACACAAGCTGGTGGATTCATGGCAGAAACTACATAAATTGGCAATGATTGAAGTACATTGGCTATTAGGACATATCTTCCTCCAAAAGAGAGTAACCTGTTCTGCCAGGAACTTATTCTCTTCATCACCTTTTTTATCAAACTTTCAAAGTGTGCTCTGTTCTTCCTTCCATAGAAAATAGGGCATCCTAGATAAGTGAAAGGAAAAGATCCTTGCTTAATCCCTGTTATTCTTTTAATTTTCCTGCATACACCAAATGGCACTTTTTCATGCAGATATACAAAACTCTTGTCAAGGTTTATCATCTGACCTGACACTTGTTCATATTCTCTCAACACATTCATCACTTTTTCATTGAGTATGACTGTCCTGAACAGAACAATATAGTATCATCAGCATAACTCAAATGATTAATCTCAGGGCTCCACTTTGGTAGTCCAAAACCTTTATACTGCTGATCCTTGAATAGATGATTGAGATTTCTGGCCAAGACCTCTGCTGCTATGATAAACAAAGCTGGAGAAAGTGGATCCCCTTGTTTTAGTCCTCTTGAAGATTGAAAAAATCCAAAGCTCTGCCCATTCACAATCACTGAGTACCAGTTGTTTGATATCAATCTTACCACCATGTCAATGATTCTTTCTGAACAGCCAAAACTCCTTAGCACCTTTGTGAGAAAGATCCATGACACTCTATCATATGCTTTAGCCATGTCCAATTTCACCACCACATTATGGTTTTTGTTTCTCCTATTTATATCCCTAATGATCTCTTGAGCTAGAAGCACATTCTCAGTGATACTTCTCCCTTTCACAAAAGCTGCTTGGTTCTGGGATATAATAGTAGGTAGCACTTTTACTAATCTATCATGTAACAGTCTTGAAATGATCTTGTTAATGAAAGTGCTAAGACTTATTGGTCTCAAATCACCAAAGCTATCCACCACCTCCTTTTTAGGAATAAGAACTAAATTAGTGTGAGTTATAAACCTAGGAAGCTCCTGACCACAGAAAAAGGCTCTCACCATGTTGACTATGTCCACTTTCACAATATCCTAACAACTTTGGAAAAACTGTCCAGAGAAGCCATCTGGTCCACTTGCACTATCTCCATTTAATGCAAATACCACTTCTTTGATCTCCTCTTCAGCTGGTAATCTTCCCATTTCTTCATTCTGCTCTTCTGATATCATTTTAGGTATATGGTCTAGCATTGAAAAATCTGTAGGCACATTTGTTTCCCTAAATTGGTCTTTGAACACATTTACAGCCTCTTCCCCAATATTTTGAGTGGTGGTTATGGTATCTCCTTGCCTGGTCTTTATCACTGATATATGCATCTTTCTCCTTCTCCCATTCACAAATGCATGAAAAAACTTAGTGTTTTTATCACCCTCTTGAAACCATCTCATTCCTGCTTTTTGCTTCCAATACTCTTCCTCAATGTGCATGTATCTTTTTAGGTCTGCCTCAACTCTCTTCAATTCTGTCCTGTTTTCTTCTGATGCATTAATTTCTAACTGAACTTCTTTGGCCCTTATTAAATCCTCCAATGTAGCCACCTGTTGAAAAACATTTCCAAAGGTTGTTCTGCTCCATTGTACCAAAGCTAATTTGACCCTTTTCATTTTGGTTTGTACTACTGTGAATGGTGATCATGTTGCTTCCATCTTCCATATATCCTCCACTGTTTTCTGGAAACTATGATGTTTTGTCCAGAAGTTGAGAAATCTAAAAGGCTTATATACTTGTTCCTGGCCTGTACTGCAAGTAACATGTAATGGGGCATGATCTGAACCCTCCCTGATCAGATGTTGTACTTTTGTATTCTGCAATTCAGCCATAAATGCATTATTCCCAAAGACTTTGTCCAGTCTTTTAAATATGCAATCTTGCTCTATTCTGCCATTCCACCAAGTGTAACAACTTCCAGAGAATTGTATCTCATTTAATGCACAAGAGCTTATGCATTGTACAAAGTCCTCTACTTCTACTTGAGATACAGGTAATCCTCCCAACTTTTCAGACTCATCAGTAATGGTATTGAAATCTCCCCCTACCATCCAAGGGCAATCAGTATTGCTAGCCACCTCTTCAAGGTCTTCCCACAATTCCAATCTCTCCAGGGCACTACATCTAGCATAAATTGCTATAATCTTGAAATGAGCATTGTTGATTCTATGTTTGAAAGAGACAGTAATTTGCTGCACTGAGTCAATACAATCCATCTCCTCCCACTCCTCATCCCAAAACAACCATATTTTGGAAGAACAATTAACTCTGGCATGCTGTTTCCCAAGTCTTCTCATGTATCTATCTATTTCTGAAGGACTCTGAAAAGGCTCTAAGAGAGCAATGTATGAGTAATGATGTTTTCTATTTAGATCTAGTAATCTCTCAAAAGAATTTTGAGACTTTACTGATCTGATATTCCAAAATAGAATTTTACTAATCATTGATCAAAAAGTATGGCCTTGCCTCTTCTACTCTTTGTATTAACATAAGATATGCCTAAACTGTTGTTCTTGTGAGTCCCATATCTTCCCTTCAAATGTCCTATTTGTCTTGGAGATAGATCTCCCTCTCTACTGATG

>LNC_000852

AAGATTTGAGATGATTTCGTGAAGAATTGAAGATGTTGAAAATTTGGGAGTTCTTTATGTTCTTCACGTGTTCTGGGTCTTCTTAAGGAAGAAGAAGCAAAAAGAGTACATTTGATCCAAAAATTCCAATTGAAAAGTGTTAAACATTGTTTGGGGTGCAAAAAATGGTTTTGCAAAACCGGAGTTAAAAAATGGCATGGATGAGCCTTTTCTTTAATAGCAATGACATAGATGAACCAAACTTTTAACGGATG

>LNC_000853

CAACAATACTAGCAATGTGTCTCATTCACAAATTATCCTTGCACTATTATTTGAGAGAGAAACAAAATCTACTAGTATATTATTAAATAATTTTTTTTGTGTCCCTTGGGTTATTCCAAAGGTCATATTTAGCTAAAGCTTCTTTATCTTTTTTTATGACGATGGAGGAAATTCTCCTAACTATAAATAATAACAACAGAATGTGTTTATAATATCAAGTAAAATACGCGGAAACTAGAAAAAAAGAAATTGAGAGATTTTATCATTTTAATTATTCATTATTATTCAATATGGTGATTCAAAAATTAGAGATATGCATCTCGATGGTGAAAATGGCAATAGAGATTGTAGCAGTGTTTGTAGATACAATCGTCTCTAAAGATAATTCTTCATCAACATCACACTCCAATATTCTTCCAACACCCTATGTTGGGTTTTGAGTTAACATATGAATTAATCACATAGTCCATCCAGTTTTGTTTGGGATCGAGACGTAATAGTTGGTGATAAATTCTTTCTTTTAGATGTATAATTTGTTTGTGTATTGAGTTTTGGTAGCTTTTGAGATCTCCATCATAGATGAGCTAGTTATGATTTTTTTTTTTAAATTTAATTTTATAAATTTAAGAATTCTTGTTTAAGTTTTGTAATTTGTAGCATAGTCAAAGAAATTCAAATCATGTTGCTTAATTAATAGCTTCTTCTTTGTGTTCTTCAATTTGTTGTTATTATTATTTGCAGTATAATTGAATACAATTTGGAGAATAAAGATCCAGATAAGTTGCTATACTATAAAAATTAATTGGATAATTTTAAAGTTCTTCTTCTATTTTATATACGAATTTCTTATTCCAACATATATATTCCCAACTTTTTGGATCTAGTTTCTTTTGATTTTTTATTTATTAATTTTTCTGGGTTGTGTCCCTAAATAGAGAGAGGAGAAAGAAAATGAGCTACTGCTTTTATTTGTCAAAACTAAATAATATTCATTTTTTTTTACCATTTTCACCTAGAAAGTGGTATTTGCTTTCTCACCTAGCTATTACTATATATTTTTGTATAAAAAGAAGAAACCTCACTAGTTAGGAGCT

>LNC_000854

AAGAAGAAGAAGAATGTCTGGACTAGTAGATCATATGTGGACTAATGAGGTTCGAAACAAGAAAGGTAAGGATGAAACTGAAGTACCAATTGAAACAAGAAAAGGAAGCAGTGAATTGATTAGTCAAAATTATGAAAAATGTGTCTCTTTATTGGGTAAGTTGATGAGTTACTCTGAAGCTTCCGTCTCAATGATTGTCGATTGTGTTAGCTCATGAACAACACATTTCTCTCTAATAGTGCTTCCTTTACTCACAATGATGTTGTAATAGGTTCCCCAATAACTTTGTGTACAAATACTTGCACTTAAATTCAAATTCTACAATAATGCTG

>LNC_000855

CGCAATCGCAGCTTATTCATCGAGAAGCCACTCACGGCACACTGACTAGAAATCATCTGCGAGAAGGTCTTCTGGCACAGGCATACTACTAATCGATTCTTTGGTAATTCGTGATTGAATACAACCTAGGAAGAGTTGTACGCCTACATAACAGCCCTCCCAGCCCAAAGGAAAATGCACAGATCATTTGAAAAAAAAATG

>LNC_000856

CTAGACTAAAATCGTGTGAGAAGTCGTCCTCTTCTAAGCTCATTCGTCCCAGCTCGCTGCTCCGAAAGAGGTAACTTCATCTCTTCTCCTATATCATGTTGGTTTACTTTCCATTTGATGAATTAGCTATATATTCGTTCTATTTGAGTTCGGGCTGATCTTATGATATGTGAAAATAGGATGGAAGTAGCAAGGAACATGTTTTCGTTTTGAATTTCCAGTCGCTAGTC

>LNC_000857

GTTTTCAGAATGCTTGGTTGGTTTTCACTTCTATCTGTGCATATATGCTTACTCATTTAGTGGTAGTGTTAGCATATTAAGGGATTTGGATATGCATTTAGTATATATTTTGTTGTGGACATTTTATAATGTTTACGCATTTAGTATTAACTAAAGTTGCCCTAACTTTCTTAGCCTTAACTGTTGGTGTCATTTGTTAGCATGCATTTGTTTATCAAATGGGTTGGATAGTATATGTATTATTGTGGACATTAAGCGCTAACAATAGTTTAAGTAACCATTTGTTTTATCATTAGCATAGCTTGAATCTTTAGAATTTGTGCTCC

>LNC_000858

CACGAAGGACAAATCATTTCCAGAATTCTTTGAAAGAGATCAAGAACAAGCTAGGTATCTTAACAAGGGGAAGACTCAAGTTCAATAGAGCTTCCAAGGATAGATTCCAGGTTGCTCGGGTACCACACCTGGTATGAAATATATCTCAGTTAACTCAGGTATCATGTCGTGTACCTTGGGTACCATGCCTGGTACAAAATATCTCTTGATTGGCTCGGGTACCACGCCTTGTACATATTATCAGTTGGCTTGGGTATTATGCATGATACATAATATCTCTTGATTGGCTAGAGTACTACATCTGGTATATGTTATCATTTGGCTCAAGTGTCACGTCTGGTACATTTTTTACTATTCTCTTGTGTATTGGTTCCTGGAGTGAACCGGACTTGTTTGTGATCATGTGGGACATGCTTCATATAAATGGGTAAGTCGGGATTGGTGATTGAGAGTCTAGGTGTATTGCTGACTTGGTTGATGTATGTGTTGTCTTGTCTTTACTATATTTTAGTTATGTGTTTACATTCATATCTTTGGAGCTAGACGATAATTCTACCAGTACACTGTGGTTTATGTACCCATATTGCACTTGCTCTTTCTTTGTTGAGTACAATACATATTCAGGTGGTTGTTTCGCTATCTTAATTGTGATTCCCTAGTTATTGATACTTCCTCCGGTCTGCCACGGATTCATCTTTATTTTCTTTGTCCGTCTATTTGGAGCTCAGACTCTTTCGATTTTTTCCTTGTTTTTGGGATTGCATCCTCTTATTCTAGATCATATAGAGTTTTGATACACAACCTTCTGATTCTATGGGAAATATTTCTGCAAGTGTTTTTAGACTGTGGTTTTTAGATATGAATCTTGATTTTTTTACTTAAATTGTTATTTTAATTTTGGAGTTAATTACGGGTTGATTGGTGTTGGTTTTCCCACTGATTTTAGTGTGGGTGTCACTCACGGCGAGTCGGAA

>LNC_000859

AGGACAAATCATTTCCAGAATTCTTTGAAAGAGGTTGCTCGGGTACCACACCTGGTATGAAATATATCTCAGTTAACTCAGGTATCATGTCGTGTACCTTGGGTACCATGCCTGGTACAAAATATCTCTTGATTGGCTCGGGTACCACGCCTTGTACATATTATCAGTTGGCTTGGGTATTATGCATGATACATAATATCTCTTGATTGGCTAGAGTACTACATCTGGTATATGTTATCATTTGGCTCAAGTGTCACGTCTGGTACATTTTTTACTATTCTCTTGTGTATTGGTTCCTGGAGTGAACCGGACTTGTTTGTGATCATGTGGGACATGCTTCATATAAATGGGTAAGTCGGGATTGGTGATTGAGAGTCTAGGTGTATTGCTGACTTGGTTGATGTATGTGTTGTCTTGTCTTTACTATATTTTAGTTATGTGTTTACATTCATATCTTTGGAGCTAGACGATAATTCTACCAGTACACTGTGGTTTATGTACCCATATTGCACTTGCTCTTTCTTTGTTGAGTACAATACATATTCAGGTGGTTGTTTCGCTATCTTAATTGTGATTCCCTAGTTATTGATACTTCCTCCGGTCTGCCACGGATTCATCTTTATTTTCTTTGTCCGTCTATTTGGAGCTCAGACTCTTTCGATTTTTTCCTTGTTTTTGGGATTGCATCCTCTTATTCTAGATCATATAGAGTTTTGATACACAACCTTCTGATTCTATGGGAAATATTTCTGCAAGTGTTTTTAGACTGTGGTTTTTAGATATGAATCTTGATTTTTTTACTTAAATTGTTATTTTAATTTTGGAGTTAATTACGGGTTGATTGGTGTTGGTTTTCCCACTGATTTTAGTGTGGGTGTCACTCACGGCGAGTCGGAA

>LNC_000860

AGGCAAAGGGTAGAGTACTACGACGGCCAAATTAATTCTTGTTGTAGCATAATGTCATGCTACAACACCGTCGTTGTAGCGGGATTAGGCCTCTATGGCAGGATAAGTCGCCAAAAATGCATAAACTCCCCAAAACCACCCATAATCATCCAAGAGTTTTTTGGGGAAGTGTAGGGGAGACCTAGGGTGATTTTCTCTTATTTTCAATCATTTTCATCTATGAAGGTAAGTTAATTACTATTTTAACTTACTAATTCGATTCTTAAGCCTTAAAATTTGTGGTGACAATCTTAAGGCAAAGTTTTGTTTAATTTAATGATGAAGAACTCCTGATTTGGATGGGATGATCATGAGAATGACCTAGGGTTCAGAATAGGGTACAATCCAGGTTTTTAGGCTATTGAGGTGTGATTATGTGTTGTATTGATTATTTGTAGATCAAGAACAAGCTAGGTATCTTAACAAGGGGAAGACTCAAGTTCAATAGAGCTTCCAAGGATAGATTCCAGGTTGCTCGGGTACCACACCTGGTATGAAATATATCTCAGTTAACTCAGGTATCATGTCGTGTACCTTGGGTACCATGCCTGGTACAAAATATCTCTTGATTGGCTCGGGTACCACGCCTTGTACATATTATCAGTTGGCTTGGGTATTATGCATGATACATAATATCTCTTGATTGGCTAGAGTACTACATCTGGTATATGTTATCATTTGGCTCAAGTGTCACGTCTGGTACATTTTTTACTATTCTCTTGTGTATTGGTTCCTGGAGTGAACCGGACTTGTTTGTGATCATGTGGGACATGCTTCATATAAATGGGTAAGTCGGGATTGGTGATTGAGAGTCTAGGTGTATTGCTGACTTGGTTGATGTATGTGTTGTCTTGTCTTTACTATATTTTAGTTATGTGTTTACATTCATATCTTTGGAGCTAGACGATAATTCTACCAGTACACTGTGGTTTATGTACCCATATTGCACTTGCTCTTTCTTTGTTGAGTACAATACATATTCAGGTGGTTGTTTCGCTATCTTAATTGTGATTCCCTAGTTATTGATACTTCCTCCGGTCTGCCACGGATTCATCTTTATTTTCTTTGTCCGTCTATTTGGAGCTCAGACTCTTTCGATTTTTTCCTTGTTTTTGGGATTGCATCCTCTTATTCTAGATCATATAGAGTTTTGATACACAACCTTCTGATTCTATGGGAAATATTTCTGCAAGTGTTTTTAGACTGTGGTTTTTAGATATGAATCTTGATTTTTTTACTTAAATTGTTATTTTAATTTTGGAGTTAATTACGGGTTGATTGGTGTTGGTTTTCCCACTGATTTTAGTGTGGGTGTCACTCACGGCGAGTCGGAA

>LNC_000861

CAATTCTTTGTGGGACTGAAAATGTTATTGTCTCTTCCTTCTAGGCCAATATAGTGAATTTTAAAACTTTATTGATAGTAAATTTAGTGATTGAAAGTTTAGTAATTTAAGTCATTCTAACTATTTTTCGTTTTGAATGGATTGTTGTCACTTTTTTCACATTTTAAATGAATTGTTTCTTTTATTTTTGTGTATGGTTAATAACCGAATAACCGAACCAAACCAAATCGAAACCGATAATCACCAAACCGATAAATGTA

>LNC_000862

AGTACTTCCTCCGATAGAAGCTTTTCAAAGGGTGCTTCGCTTCCAGCTCTTCTTGTTTGATACCGCTTCCCGTCCTTGTAAATATCATCTCTTACTGCATTTGTTAAGGTGTCATCAGCATATTGGAGATGTGTAATCTCTTGACTGTAATGACCTTTCACGGCTACCTCAAATCCCTTAATTTACGGGTAGTTATGATCATGTTGTTTAACCCTTCCATGTCAACTATGTAACAAAAAGGGGACAAGGGGTCATCCTGCCTCAAACCTCTCTGAGCTGGAAAAACTCCTTCTGGGGAGCCATTAATAGTGATTGAGAATCTGAC

>LNC_000863

CCGTTTCAATTTGCTTGGCCTATCTTGACTTGGCAGGGAGTTAAAGAAAATAAAGAGAACTTCTAAATCTTGTGGTCTTAAATTAAAGACATGTCAAATGTACCCAAATGCTCTTTAATCTTGGAGTCTTAAACATGTCAGGTACAAAGTTGGAATTAAAGTGTTGCTAGAAAGGGAAAGGGTCATTCTTTTTGAAATCAAGTAAAAAGGTAA

>LNC_000864

GAGAAATTTAATGATACTTTTGTATATACCACTTCTTGACGTGGCATAAATTACTAAACCGTGAAAGGAACAGTGAGCAGTAAGTGGATACCTCTAGTCATATAGTTAGACATGTCGCGAGTAGATATTTTATAGCCTAGTTGGTTGTATGTTAAATAAACAATCTTTTAAAAGCTTGCAGTTAAATATCATCTAGAAATTAATCAGGGTTAGCTCCA

>LNC_000865

AGAAAAGAAATATTTCTGCAATTGAGATTTTTGCATACATATTTTAATATTTTAAAATTATCAATTTTATTTTGACTTTATTTCCAAAAATAAATAAATTCCAATCTCCTCTTCACTGAAAAATGAGCTCATTTGAATTAGGGAAAATAGGCAGATTGAAGTAAAGCACGCCGGCGACAACTTGGATTATCACTTTAATAGAAACCTATGCAATGATTTACTTACCAGAGGTACTGTACATGCGACAGCTCCCTTTATGTGAGCCAATTAAAGCACCCTTCAACAATGGAAAAACAAAAACATATCAGTAAAAGTTGGATATATAACTACCTCGCCATCAGGAGAGTAGCAAGTAGCAGTAACCATTTCATGAAGATCTGTCCAGTCCACAACTTTCCGATCAGATACATTCCAAATTCGAACTTTTCCATCCAGTGAACCGCTGATAAAGTGGTCGTCATCTACTGGATTGAAGTGTATGCAGGTCACTGCCGCACAGAGATTCATCAGATGTAAAAATGTGTGATGGTCTGAGTCTCGATATCCCATAGCCTGACTGTCTTGTCCATTGAGGATGAAAGGAGTTGCTGCATCAAAATTTCATCTAGC

>LNC_000866

AATACAAAAAAAAAAACCTATGCCCTAAACCCTAAACCTATAAACCCCTAAAAAACCTTAACCAAAAAAGCACAAATCCCTAAGCCCTAAAAACCTAGATAAAAGCCTTAACCTAAAACTTTAAACCTAAAAAAACCCTAAGCCCTAAACCCTGAATCCAAAACCCAATTCCTAAACTCAAACCCCTAAGAAACACTAAACCCTAAACCTTAAGAAATCCTATATCGTACTAACCCTATAAAACCCCTAAAAACCATTTAAATCGTATATGCTAAACCCTATAAAACACAAAAAACTCTAAGC

>LNC_000867

CTGAGAAGGAAGAGTCTGTCAAGAAAGAGCCGGAGACTCAGTTGGCTCCTAGGAAGGCAAAGGCGTGAGCATTGAATCTTCTGCAATGCCAAAGCTGTTTTAAGTTTAATGTCCTTAGTCTCTGTTATGAAACAATTTTGGCAGCTTGAACCATTTTTCTCCTTTAATTTGTCGCATTGTTCTGCATACTCTTACTATTTCTAAAGTTGAGTTGAATGTTTACCTTAAAAAAAAAAAAAAAAAGATAATTCAATCTAATATTAACACGATTGCAAGAACTGCAGAACATTATACTGGAGCTTGCTAGAAAGGCTAAAACATTTCAGTCTTTTTTGTTTTTTCGACTAAGCCCATGGCCGTGGAAGCAGTACTACAAACTTCTTGTTTG

>LNC_000868

CTTCACTCTCATTCTTCTTCCTCAATACAACGAGCAGAACCCAGAAATGTCAACAAACAGCAGCTCCCACAGCTTTCTCTGCAACAGCCGCCCGCCTCGATCTTTTCATCCGGATTTTGGGGGATTTCGTTTGAAATCTTGTACAAGCAGCAGTCCGTTGGTCTTTGGGAAAGGAATTTGAATTGAGGATTGGATATGAATTTCCTTTTTAGCCCTTGCCCTCGTCCAAGTTTCATCCCCAAAAATCGCCCCAAGAACAGACCCCAGAATTTCCTCTATATAAACATCGCTTTTTATTCACTGAGGGGTGTGGGGGGAAATTTTTTAAGGCACCAAAAATAACTAATACTTACTAAATACACTTCAAGGAAAAGAGAGAGGAAGAAAGTGGG

>LNC_000869

TTTTGACAAAGGTAACAGTTTTGTGTATTAATATTTAGCACTAAAGGCTGGGATCCCAAATTTACATCATGGAGCTACATACAATTGCATGTACTTAAAACAAAACTAAACTAGATCCTACTTCATCTTACAAAGGACCTAGGATATCTCTAACCGCTATAGGATCATCTACATACTCCGAACTACACCAATAGCAAAAAGTGATTATGCAGTTCATTTTAATCCTATGTAAAGGACTACTACTACTCTCAAAGCATCTCATATTTCTCTCCTTCCATATGGTCCACCAAATCACTGCTGGGACAATCCTCCATCTGCTTTGGTCTGTACTGCCACTGCCTTCATTGTTCCAGCTTTCTATAGCCTGACTTGCCCTTTGTGGCATGGTCCAGTTTGTGCCCCTGAAGCTGGTAAATAAGTGCCACAGTTGTCTAACCACCTTGCAATGAAGAAACAGATGGTTGATTGTCTCTGCCTGTTGCTCAAAACAAAACATCTAGGACACATATGTATTCCCCTCTTCATTAGATTCTCCTGTGTTAGTATCGCCTCTTTAACTACCAACCAAGTAAAGACTGTCACCTTATATGGAATTTTGACTTTCCAGATAAGCTTCCAAGGCAAAAATCTGAGTTGAGTCCCCATTTGATTCAGGTTCTTGTAGGCAGAGCTAACTGTGAAGATACCTTTGCTGTGATGAATCCACCTAAGAGTGTCTTCTCCTTCTTTCAATCCTCCAAAATGGTCCAGGGTGCCATAAAATTCAGCTAGTCTATCCACCTCCCAGTCTTGTATCATCCTTCTGTATGTTAAGTTCCATCCATGGATGGACCAAACTTCTTGCACAGTGGCCTCATGTTGCTGATTCAACAGATAGATGTCTGGAAAGAGCTGTTTGAGGCTGCCATTTCCTAACCAATTATCGTCCCAAAACAAAATCTTTCTTCCATTGTTTACATTGAATCTAGTTCTTTGAAAGAAACAATGCCACAAATTTCTGATTGATCTCCATAGGCTAATACCATAGGTAGAAGTCACTTCCTTAGTTTTCCAGTGATCTATCTTCTCATACTTGGCCTGAATCACTCTAACCCAAAGGGCCTGCTCCTCTTTAGGAAATCTCCACAACCACTTCATCATAAGGCTTTTGTTCTGTTTCTTCAAGTTTCTAATGCCTAAACCACCTTGTTTCTTTGAAAGAATTACAGTCTTCCATTTGACCAAATGAAATCCTTTGTATTCTTTCTCTCCATTCCACAAAAAATTCCTCC

>LNC_000870

GCTGGAGCTTGCTTTTGTGGGGCTATGCAAGTACTTGGAGGACTTCATTAAGTTTTCTTGGAGCATCTAGGATGTTACTTTTTTTTTGGAAGTTCCCTAATTACTTGTTAAAGTTTCTCATGTACAGAGGATCCAGTGCTGAAAAGTTTATCCATACAAAATGTTATTATTGGGAATCAATTTGTTAAAACCGCTGTTCTGCTATTAAATCTTCAAGGAGTGACAATGCTATTTACTGTCATCATCGTTGATGGTTTACTTCATGTCACACCAGTTATGGATCTCTTGTAAATGAGGATTCTGTAGGTCAACGGGAAGATCTTTATGTTTGA

>LNC_000871

TAAACATTCTTCCATTGTTCTTGCTTGATCTTGATTGATGTTTTGTTCTTCCTCTGTCAAGAGCAATATAGGGTCTACAATATCCATGACATGTGCAGGCAAAGCCATTGAAACATACTTGTGAATGTTTAGACTCTCGTTAAACATCGCGTCAGTTGGTCTTCTACTGATAAATAACTCTAGCAACACTATCCCAAAGCTGTACACATCTCCGAATGTGGATGCTTGACCGCCTGATCCATACTCTGTGAGGACTCTGAGGACTCTGAGAATTGCTACTCAATGTGTCAAGGAGAAATGTTGCTAATCCAAAGTCACCAACATGAGCAGACATGTCTTCGTCGAGGAGTATGTTGCTTGGTTTTAGATCACAGTGAGCTATTGGTGTTTGGCAGTGGTTGTGGAGATAGTCAAGTGCAGAAGCAACATCTATGGCAATGTTTAGTCTTTGGATGAAACTCAGCTTCTTTGTTTGGTGTTGCTGATCATCATTTTCTGTATGCAACCAATCATGTAGGTTTCCGTTAGTCATGAACTCAAAAACTATGCATTTGAAGTCATTACCTTGGTGATCAATGCTCGAACAAGCAGTCTTGATTTTGAGAAGATTACGATGCCTAATAGTACTCAAGGTTCTGCATTCATCCAGGAAACTCCTCAAGGCACCTCTTTGTTGTAGGTTCAACACTTTCACTGCCATTATTGTATTGTCAACATCGAAATGTGCTTTGTATACTGAACCAAAATTTCCTCTACCAACCAAGTTAGCATCAGAGAATCCATTTGTCGCTCTATGTATTGCCCGATATGTAGTTCTTGGTATCTCAAACAGTTGCTCAGCATTCCAAGGTTGTGCCTTTCTTGAGTTGCTGAGTTTGTAATAAGCAGCACAGGAACATAGCACCAAAACTGATAATGCAAGAGTAACTATAACAGCTAGCAGTACTCTTGAATCAAGATGCTTGGTAGCTTTAGAGCATTCAGGCAAATGTAAGTTGGGT

>LNC_000872

TGATTACTGATATTGAAAAGGTTTTACACTTATTTGAGAAATAGTATTTTGACAGGTGATTGTTAAACTTTGAAATGTTCTGATTTGGTAAGATATAAATGTTCTATCTGATTTTGAAATGCTTCTACAGATTATAAGAAAAGTATAAATGGGAGTAGACCTTGATGAATCCCGTAGCTAACGGCGGGTTCGTTAGACCTGGTGCACCTTGTAGCTCTGATTTCTGATAAAGCCCTTGCTAGTGGGAAGTTAGAACTAGTATACTGTTATTGATATCCCTCGAGTAGGGATCTACTGATATGAATAATAACTCTTTTATAAGGAGTTCACTGTTATTGGTTACTTCTTAAAGAGAAGACCACAGTGATTACATGGTTCATCCTTGGAAACCTCCCAGCTATAAGATTTGATATGAAAATGTTCTTTTTCTGAGTTTGCTATTATGTATTTACATTGATTTTGCTTATATGAGGCACCTAAGGTTGAATATTGTTATTGTTTCTGAAAGAGCATTGTTTCATCATCATGACTATTACACTAGCATGCTTTCTGACTTGTCCCTCATTAAAATGGTTGGGGTTAAGTATTAATTACTCACTGAGCTAGCGTCTCACTCCTCGCTATTTGTTTTTTTTACAGAGAATTCAAGTGGTATTGAAGAGGATTTCATTAACTAGCGTGTGAGTTGGTAGCATCTGGTGAGCCCCGCATTGTTTCGCGTGGCAAGAGAACTATTGATTTGTTATGTTTATTTAGTTTCTTTTAAAAACTCTTAGAGTAGCTCCATTTGTCATTCTTTTGATTTGAGTCAAGATTAGTCCTTCGAATTGTAGTCAAGTGACTAGTTTGGAACTTTT

>LNC_000873

TTTTTTGCTTTAGATGTAGTGCAATGCAAATAGATCAGTTTCATTTGCTTGTCTTGGCGGCAGGGTTTTCCGGACAGTTCCTTTGTATGATTGTTCGTGATTAATGTTTCTTAAATTCCTTTGTTTAATTCAACTTCAATTTACTTTTCCTGATATTTCTGTTATCAGTTATCACTGTGTTAATGAAGTGTCTGAGCTTGCTGATAAAACGTAAGATGAGTGCATTAACACAATGCTAATCAAGTGCCTAAGATTTTAAAAAATGGTTTCCAACAATTAAACTCCAGACTCAGCATCAAAATTCCAGGTGGAACCTCAAAATTGATCAATGAGATTTTACAATAAAAAATGCAATCTTGGGTAAGGACGGGTCTGGTGTTTGGACTTTCTGCATAGTAGGGACTTCTGGCAACTCTCTTGAAAAAGTCAGGTAGTAGACCAACTCGGTTCTGAATACCGGGATATTCGGCATCCTCATCTGCTAGGTTAAAGTTGTACATGATTTTAGCGAGTACTTGTGTCAGATAGAGTTTGGCAGGAGTTTCTTTTTTTGTGGTTTAGTTTGGTTAGAATTTGTATGTCAATTCAAGGATAAGTGATTACTCTAGGAATTCTCAAAAGTATCATCTTTGCCGGGTAAAATGTTAGATCAATTGATTTGAAGAATAGTTTATGGTTGATGAAAGAACTGAATGTATATCCTTTTGAGGCTTTCAAATGATGACCAAAAGATACATTAAAATCTATACAAATTGCT

>LNC_000874

CTTTAACATATTGAAAGGTACACCTCGAGCTAGTTCCAGACAACCCATAATCGTGCATCTACCTGACAATGCAATAAGAGGTGGTCCACATTTTCTCTTGAACATCTGCACATAAAGCACTAGCTAACATATGTGATCCTCTTCTTCCTCAAGTTTCCAGAGGTCAGAACTGCTCCCTTGCTGCCAACCAAGCGAAGCACACCTTTTTACGTGTGCTAGGAATCCAAACTAATACATGCAGGAAGCCCATCCTTCCTCATCAAAAGCTTGTGGTAGTATGATTTTAAATGGAAAAACTCCATCTATCCTTGCTTCCCACCTCCACAAGTCCTATCTACTGAGTGTTGAGTTTTGTTTGTAGAGAATTCGATCAACATTTGGAAATGCATCAGCATATATACATGTATATTTACATACTTCACATCTTCCTTCTTTTTTTCTGGGACAACCTAAAGTTTTAGTATGCAAAGGCAAATTAAT

>LNC_000875

TAATCTTTCTTTGTGATCTTCTAGATATATGCCATCTATAGAGTGCTTTTTTCAACTGCCATGAATCCAGCATGTTTCAAGAAATTTAGTAGTCAATATGAGAAGGAAGAATCTATTTCTGGTTTAGTTTGTCTTTCAAATTTTCTTCTTATGTTTTTCCATAACATATCTTCTCCTCTTTTTTCAACCTCTTGGTGCATTAATTTCTTTCTTAACAGGAAATATTTAGAGTAGATGTGTCTCCTCTTACTCTGGAAATACGTGTGGTCCTCTTCCTTTTAATCAACTTGGACCATTGAGGGAAAAATAATAATTTCTTGATCATTCAATTTGACTTTTCA

>LNC_000876

CAACTAGATCCAGCTCTGCTAGCATGTGGTGGAGTATCTGGAAGGAGAGGAATAGAAGATGTTAGGGGCTAAAGAGCTCAAGTTGGAAAGTTTAATCTGATTGTATTTTATGGTCTCTTCCTGGTGTAAAATGGATGTAGTTGAGGATGCTGAAACCCTGTTACAGTTCATAGATATTTTGTATAGATGGCCAGGGTTCACCTTTCTTTTTCTCTCTCTGGATGTAATCACAACACTTTCTTAGTGCTAGTGACATCAGTAATACTTGTCAGTTAAAAAAAAAGGTGGATGGGGAAACCTAGAGAGATTGGCAACATATGTTATAAATTGTGGTATTGTGGGATAAAACTGGGAAGCAAGTGGGACTCATTGCAGTATTAGGATTTAAAGTGAAGTTGTAGAAAAAAAGAAAATTAGCGATATGATTATCTCATTAAGTTTATTGTAGGAAAAGTATGCACTCAACATCATGAGTGTCTATGTACTACAAATAGAATTAGACAAGAAAACTAAAACGAAAATTTGGGAATATAGATAGTAGGATAACCTTGGTCGAAGAGATCCCATGGGACTAGAGGCCATAGAAGGAAGGTGAATGGGAGTTGGAAGCTGATGTAGATATTGTGGGAAGTAAATGACAAGTTGCATTATGAAAGTAAAGAGAAGTCTTTGGTGCATTTTAGGGGCTTAGGACTTGGGGAGCTCGATTAGAGAAGTGTGTAAAGAGCTGTAAAATTAAAAGACAGAACAAATGGATTATGAATATCAAAAACCTTTAAAGAGTATATCAATATAAGAAGTCAGTTCCCTCATGTTCTTGTAGACCTCCATATGCACCAGTCTGTAGGTGCGGCAATATGCAGGTTGAAGGCGATAAAAGGGCACAAGCACACGAAGAAAAGTTGTCTCAAAAGATCAATACAAGCTTATGTAAGAATAAACACAATAGAATAAAAAAGATCCATATAGGCTACATTAGCTAGTTGGATTAACTGTTAAACTTAGCTTTTACACTTATGTTGTGTCCAATTTTGTGAGAAGTTCTCTTAATCGGGTTAGAGACTCCTATGTTCATGTAGAAATAAGTTTGATTTATGGAAGACCAAATGAGACGATACAATATCAATGCTTCATGATCATTAAAACTGTTTCTCTTTGATATTGTACATTCTAGCTTCTGCTACAAGTTTGAT

>LNC_000877

AGTTTATGTTGGTGTTTTTGTGACTTTTTGGTGTTTGATGTACTTTGTATTTGTCATAAATAAGCTTGTTGAGTTATTTCACTATATGCTACTGAATAAAGACAGATTTGTCATGTCAGCATGCATCAGGTACAGTTAAATGTACGTAATATCTATTTCTATGAGGATATATGTGGGTATTGATGGATGATGTTGATATTCTGGTTTTAACTCTGCTTATAGTTGAATATGATTCTCTAAGTGTTTGAATTGAGGTTTAAATGGTTAGCTCCCTTACTTTAAGTCTTTGTTTTTATCTTCTCATTCAGTTCTCGGTGCTTATTGGTTAAGATATCGGTTGAGTTCTCCTAAATAGATGACTTATGTGGCCTAAGTTGAAAACTGAAACTTAGTGGACTGACATAAAGAAGAG

>LNC_000878

TCCTGATATTACTATTGCTGTGAAATTCACTCATGATACATCTGAAACATTTATGAGAATATAGGCAAATTAAGCAAATTTGAAGATAAACAGATTTTGAGGAAATGTTGTAAAGATCACTATTTGTTTAGTTTTTGCTGCATATCCTGTATTGCAAAAGTTTAAAGTATAGGTGGAAACTCAGCAAGTGTGTTACAATGTCGATGGTGTTCGATGAATATCTGGTAGCCATTATGGTTGAAGATTTATTTGTCCCTCTACAGTATAAGGTTGTTTATTGTTGAAGATAGGGGTAAGATTTGCATATACTCGAGGATCTCCACTTGTGGCAATACACTAGCGAGGTCACTGAAGGAGGAGCCTTCCTATGCAAAGGTGAAAAAAGGGACTTCAAATGGAGGTCCTAGGGTTGTAAGAATAATACATGCCATGAACTTATGAAATTAAGATGGAGTTGAAAACTCATTTTTCTATTGCTTGTGTTTGGCCTCTATCCAGTTGGGAGGAAGATCGGATTCATAAGTTCTTATCCCGGTCTATAATTGTCTAACTGGTCGGGAGAGAGATCAGATTCGTGAGTTCCTATCCTGCTATATTGTTAGCGTTGTCTAACTTGTCGAGAGGAAGGCCAGTCATCCTCGGTATCTTGATGTAATGTATCACCTGTTTTTGTATATTAATCAAGTATCATCAGGATCGATTTCAATTGATATCAACACTTTGTGAATTGTGATGAGCCAATATGCTTTATCTGTACATTGTGTTTGAACTGCAAGATTTAATTAGTTAAAATTATTTCCTTCATGTGTTATGTTTCTATGACTCCTGAGTCCTGCTCCACTCTGAGCTGCTGAGGTCGTTTTGCTACGACACATATTGAGG

>LNC_000879

TCCTGATATTACTATTGCTGTGAAATTCACTCATGATACATCTGAAACATTTATGAGAATATAGGCAAATTAAGCAAATTTGAAGATAAACAGATTTTGAGGAAATGTTGTAAAGATCACTATTTGTTTAGTTTTTGCTGCATATCCTGTATTGCAAAAGTTTAAAGTATAGGTGGAAACTCAGCAAGTGTGTTACAATGTCGATGGTGTTCGATGAATATCTGGTAGCCATTATGGTTGAAGATTTATTTGTCCCTCTACAGTATAAGGTTGTTTATTGTTGAAGATAGGGGTAAGATTTGCATATACTCGAGGATCTCCACTTGTGGCAATACACTAGAGCGAGGTCACTGAAGGAGGAGCCTTCCTATGCAAAGGTGAAAAAAGGGACTTCAAATGGAGGTCCTAGGGTTGTAAGAATAATACATGCCATGAACTTATGAAATTAAGATGGAGTTGAAAACTCATTTTTCTATTGCTTGTGTTTGGCCTCTATCCAGTTGGGAGGAAGATCGGATTCATAAGTTCTTATCCCGGTCTATAATTGTCTAACTGGTCGGGAGAGAGATCAGATTCGTGAGTTCCTATCCTGCTATATTGTTAGCGTTGTCTAACTTGTCGAGAGGAAGGCCAGTCATCCTCGGTATCTTGATGTAATGTATCACCTGTTTTTGTATATTAATCAAGTATCATCAGGATCGATTTCAATTGATATCAACACTTTGTGAATTGTGATGAGCCAATATGCTTTATCTGTACATTGTGTTTGAACTGCAAGATTTAATTAGTTAAAATTATTTCCTTCATGTGTTATGTTTCTATGACTCCTGAGTCCTGCTCCACTCTGAGCTGCTGAGGTCGTTTTGCTACGACACATATTGAGG

>LNC_000880

ATTGACTTTCTAATGTGTCAGTTGACCATTTTCTTGGGTTCTTTCCAGCTATTCTGTTTGTAGATTTTGACGTAATACTGTCTGCAATATTTAGAGTTGAACGTACGTACATTGCATTGATTCTTTTTTTTGTTTCTCATTTCTTTGTCGTGTCCTGTAATAGAGTTGATTGTTCATTTGTGCATTATGTATTAGTTTTACTGTCTTGGCAAAAAAATGTGTTACTGGCTTAACTGTCTTAAATGTAGCTTTACTTATCTAGGTTTGTTTATCTAAGGAACACAGCAAAATAGCCCATAACAAAAGCAAAGGCGTTTACTTGTGAGAATCTGGAGATAAATAGTGTGCACGTCGATGACATGTTTTACCTCTACTGATTTCATTTGTAATTCACATGCAAAGAAAGATTTTGATTTTTTAATTGGCATGGCACTATAAGACAGCTGTAATTCCATGTCAATGTTGTCAATGTTGCTAGCTGTCTCATGATTAGATCTTGTCCAATTGCTCTGAGCTCGTTTTATGCTTAGATAAATGGTAAGTTTTAACCTTAATATTCCTTATAATTGTCACTTAACCGATACATTGTGGCACCTTGGTCTTGTCCAGCATCAAGAAAGAAAAATTGATTTATATTGAGATGGTGCTTTAATTGTCCGTGTACACAAGATAGATCTCTCTTTCATGTAAACCTGTGAGCAATTACACATGACTTGACTGATATGTGGGGCACGAGTAAAGCAGAGTATGTGCCCCATTTTTAGCTTCTTCTGGTGGTGTGTTAGTGATGGTTTAGGGGCTTATAGTTAATATGAGTCTCGTTTGTCATGCTGACAAAAAAGGATCTGGTTAGTTGTGAACATTGTTGGCTTATACTCTTTGGCATCCTGTCTTATGATATTTATTATAATATAGTTCTTTAGCTGGCAGAATTCTGAATGTCAAAAGAATTCACGGGAAGGAAGAGAAAAAAGAGAATAAGATAGACGCCACTACTTTGTTTTGTACATACAACTTATGTAGAGGAGCCTGCACAGATTCACTTGCATGCCTTTTAATTTGACAGTTGTATGCTAATAGTCAAAACTTTGTCACTAAGTATTGTGAGAATATCACT

>LNC_000881

ACAATAAGGAATAAGGAAAATAAGCAAGAGCAGAAATAATCTAATGTTATGAATCATGAAAGAGAACGAGAGGGGAGAGAGAGGTACGAGATCTTCTCTGAAGGGAGTTGTAGCTGTTGGACAAGCTCATTGCGATGAACCCCTTTTTCAAGTGCACTGAAATAAAAATAATAGAGATTACTAGCACAGCAAAATCATTAAGGAGCTTGGTAGTCATATTCTCAATCAATTACAAGCAAATAAAATACACTATCAATACTATGCTGCTTGGACCCTCCAAAAATGTTGTC

>LNC_000882

ATAATATGAAGCCTAATGAACCTAAAAAAAGGATCAAGGCCCAGCAGAAATTACTTATTTTTCGAGACCCCGTTATAAGTTCTATCCATATATCGTCTGATCGCCAAGTCATACTTTACTCGATTGCATTTTATTGGAATTGAGATAATACCCCAGAGAAATTTGACTTTACTTTTTTGTTTCCGAAGTAACTCTCATCAACTAGCACTAGTTTGAGGTGAACTAGCACTAGTTTGATGTCAACCTTTAGGAGTAATTCATCAAATTGGTTAAATCTAAAATAATAACATATTCTTTATTTAATACGATCCCACTATACATATCGATATACATATAGATTCACCGACTATATTTCAGCCA

>LNC_000883

ATTTAGTTCAACCCCTAAACTTCATTTTTTTCATATTATCTTGAAAACCCCATGAGCCATGACTAGCAAGAAAATGTTAACTATTTTGTCATTTTTATGTCTACTTCTTATGGGAGTTTTGCATTACAAAGTTCCATTTTGTGGTGCAAATATTGGAAAATTTTATAGATTCAAAGGTGATTATTCTATAAATAAGAGAGGTTATGTTAAATCTCACAACAAAGGAAGCTTCTTCTCAAGAGGAATTAATAATATTCCAAATATGGATGAGATTTTTGGTGATGATAAGAGAAAAGTTCGTACTGGTCCAAATCCTCTACACAACAAATAAGTAAGTAATTGTAGGGGAAAAAATATGAGTTTTATTTGATCTTCAACTTTTTTTTATATTTTATATTATGTTTTATTTTTATTATATGGTCTTTGAAGGAGGGAATGCTTTCAAGAAATCTTGAATTTTTGTATATGAATATTGTTGAAAAATCTTGATATTATTGTGTATATGAGATGAGAATTGTTGCACCCTCTCATGATAATGAGATTTTGTTTTGAG

>LNC_000884

AGTCTTCTCTCTTCTAAGTAGGTCGTTTTTTCATTTTCATGGCTTAGGGTTTCTGAAATTTTCATGTAGTGTTACTCTCGTAGGGATTCAAGTTGGAACATTCATTTTCATTTTCATGGATTATCTCCTCTCCCCCTAAACCTAATATCCTATTTTTCTTCTTTTCCCTTTTCTTATTAAGCAATTTCAAGATCAATTTCACCCAAAATGGGTTAGATTTTCAGTGCAATAATTTATTGAAGTTGAGGGATTCAAGTTGGAG

>LNC_000885

TTCAGCTGCTGTCCAAATTTTTCTTATCTACTGCTACTGTAATTATGTCAAAATCTGGAACATTGGATCTGGCTTCTGGGGTTGGTGGCAAAATAGAGAAGAAGGAAGTACTTTCAGCTGTTGAAAAGTACGAAAAATATCATGCTTGTGATGGAGAAGCGGAGGAAGAGAGGAAAGCTAACTACATTGATATGGTTAACAAATATTATGATCTAGCAACAAGCTTTTATGAGTATGGTTGGGGTGAATCTTTCCATTTTGCTCCTAGATGGAAAGGGGAATCACTTCGCGAAAGTATCAAACGTCATGAACACTTTCTTGCTCT

>LNC_000886

GTTAGGCCTAAGACCTAATTTAACGTGGTATCAGAGCAGGCCCCGTCTCACCCGATGTTGGGGCCCCCAAAATCAAAATTGCCCACGCACCAGATGCTAAGCACTGGGCGTGAGGTGGGGTGTTAAAAGAATGACAAAAGTCCCACATCGGTGGTTAATGAGATGGGTGGACTCCTTATAAGGCTTGGGCAATCCTCCTCCCTTTGAGCTAGCTTTTGGGGTGTGAGTTAGGCCTAAGACCTAATTTAACAG

>LNC_000887

GTATCTTCATCTCTCCACTCTCTCACTCTCCGCCATAAAATCTCATTACAATGTGAGTATCACCGATCTCCTTACTCCTCCATGGCCAAATCCTCAAGGGGCTACCCTCCATTGTTACCATCGAAACCAATCGAATTCAAACACAAACCACTACTACTCAAAATCAAACACCCAACCCCAATTAATTTTGACTCCGACTGAATCAAACCCTTCAAACGAATCTCGAATCACCCATTTTTATGAAAATTCTCTGATTTTTTTAACCAAGGGGATTGGGGATTGAAACTACATTATAGAGTTAGTTTGATCAAGGTGGAAGAGTGTGTAAATTGACTGGAAACTACATCGGAGGAATTGCAACTGGGTATATCTAAACGTCTCGCTGCTATATGGTTTCTGAGATAACAAAAGGTTGACTATTAAGTTAGAGAAACTAGAAACTATTGTTTTACGAAGGGGAAGAGCTTGTAAATAAGTTGGCAAAGTTGGAAAGAGATGTCAAAGAATAAGAAGGCGCAAGAATTGACTCTGCGACATTTTTAAAAACTATGCGCAGAGTAAGAATGTAGATATGAGCTCTTTTGGAGTATGATGCGTCTGCTCGGCATTTTATAGAAACAAAAGGACTTTCACCAACTCTTGATTTTTAATGGATAATCTAGAAAATTGTTGGCAGAAATGTTCACGTGTTATGTCTAGATTAAGTTCAAAAATACCGTGGAGTCAAACTGTACAAATGTGCATGATGGGAGGTCCGAAAAGGGCAGTAGCTTGGAAAGAAGTGAGATATCACTTATCCCTCCCTATAGCTTATACATATCCTATATTATCTTTTGTTTTTATTTTTATTGCAACTTTGAAACTTTCTTCGAATGGTATTGCTAATGAAATGCGGCTTTCATTCCATGGAATAGTAGCACAATAAGATTTTCCCATTCAATGTCATTTGGCAATGATGATGAATCATGGAGGTAACTTCACGGTTGAACAGTTTGTCAGGCTTCATTGTGAAGACATATTGCAAGTTCATTACTTTATCCTCTCCTCGGGATCCAGTTTCAAAATGTACAGAGGTTGTTGATGTACTTAAGCCGAAACTATGATGAATTTATTTATCATGCAAACTTAAAAAAGCAAGAAGCCTAAGTTCATTTGTGAAAATATTCATCGTGTATCATTTGAGTTGAATAACTTGTATTATATGAAGACAAATAACTACCGGAGGAACCAATGAAGTTACTACTATGATACAATGTTATGTTAAGAAATTTCAGCAAAAGCTTGTGATATTCTTTACTTCTGACTGTAAGTTCTTATTTCCTATTTCTTGTTGCTTCAGTAAGTAGTAAACTATATATATACCAGTAGGATAAATTCTCTGATAAACAAAAGCTTATCAATTTCACATTATTTTTTTTAGTTTACTATAAAATGAAGCTTATAAGTTGTTTGGAAAGTTGTATAATGGAGATAGAGATAATAACAGAAAATTAGAAAAACTAAAGCCAAAGCGATTATTGGA

>LNC_000888

TCTTCATCTCTCCACTCTCTCACTCTCCGCCATAAAATCTCATTACAATGTGAGTATCACCGATCTCCTTACTCCTCCATGGCCAAATCCTCAAGGGGCTACCCTCCATTGTTACCATCGAAACCAATCGAATTCAAACACAAACCACTACTACTCAAAATCAAACACCCAACCCCAATTAATTTTGACTCCGACTGAATCAAACCCTTCAAACGAATCTCGAATCACCCATTTTTATGAAAATTCTCTGATTTTTTTAACCAAGGGGATTGGGGATTGAAACTACATTATAGAGTTAGTTTGATCAAGGTGGAAGAGTGTGTAAATTGACTGGAAACTACATCGGAGGAATTGCAACTGGGTATATCTAAACGTCTCGCTGCTATATGGTTTCTGAGATAACAAAAGGTTGACTATTAAGTTAGAGAAACTAGAAACTATTGTTTTACGAAGGGGAAGAGCTTGTAAATAAGTTGGCAAAGTTGGAAAGAGATGTCAAAGAATAAGAAGGCGCAAGAATTGACTCTGCGACATTTTTAAAAACTAGTAAGAATGTAGATATGAGCTCTTTTGGAGTATGATGCGTCTGCTCGGCATTTTATAGAAACAAAAGGACTTTCACCAACTCTTGATTTTTAATGGATAATCTAGAAAATTGTTGGCAGTTGGAGTAAGTAGTTCGCGTTGAATGAAAGAAATTTTTCAGCTTATCAAGACAACTGCAGCCACTTAAGGATATCAACTTCTATTCTTTTGGAATATACAGAGAGCATCTGCTCAAATTTACTGGTAATGATTCGAGGCATTAATAGATTTTAGAAAATGAATTTTGTCATATTTGATGCGCTATCAACTTCTATTCTAATGAGAAATACAGGGAGCATTTGCTCGAAGTTATCATTTGGAAAATTTGATGTTGTTTTTAATATACTCAGTCTGATCCCACAAGTTAGGAATGTGGAGGGTGGGGCGTATGCAGATCTTACCTCTATCTTTGCAGGGTAGAGATGTTGTTTCCGGTAGATCATCGCCTCAAAAAAAGCATTTTTAAGAACAGGTTTGATAAATACAAGAGTAAATACAATGCTGAAAATCATTGTATTCGTTCTTCATGTGTACTATGTTCTCTTCTTTGAACTACAATATCAAAGTAGTTGTTCTCTGTGATGTCTGGATTATTGATTAATCTTAATAAACCGTTAAATGACTAGAAGCCAAAAAATTTCAGTTGATTAAAATGAAACTGAAAAAATTGTTTAAGTAGGACAAACAAAAATATGGATGAAGTACATATTGTTTCTAAATATTTTGGTTAGCCTAATTTAGAGAGTAGCATTGAGAATCACTCTCTTCTGGCAGCTGAAAGCTGGTTACAACTGTATGATTTTTTCTTTTGCAATTTGTGACAATTTACTTTGGTAAATGACAGACCATTGTTAAATGAAAGTGTTGATATGTTACTATTTGAAACTGAATATGATGAGGCATATAATCTTTTGAATTTTAGTCTTTACTTTACTCAGACATCTGTAATTGGTTTCTTTATCTTCATGATATATTTGCATCAAATTGGTAACTCAGTAATTTAATTTCAAGGGCTTGCTGAATACTTGCAGAAATGTTCACGTGTTATGTCTAGATTAAGTTCAAAAATACCGTGGAGTCAAACTGTACAAATGTGCATGATGGGAGGTCCGAAAAGGGCAGTAGCTTGGAAAGAAGTGAGATATCACTTATCCCTCCCTATAGCTTATACATATCCTATATTATCTTTTGTTTTTATTTTTATTGCAACTTTGAAACTTTCTTCGAATGGTATTGCTAATGAAATGCGGCTTTCATTCCATGGAATAGTAGCACAATAAGATTTTCCCATTCAATGTCATTTGGCAATGATGATGAATCATGGAGGTAACTTCACGGTTGAACAGTTTGTCAGGCTTCATTGTGAAGACATATTGCAAGTTCATTACTTTATCCTCTCCTCGGGATCCAGTTTCAAAATGTACAGAGGTTGTTGATGTACTTAAGCCGAAACTATGATGAATTTATTTATCATGCAAACTTAAAAAAGCAAGAAGCCTAAGTTCATTTGTGAAAATATTCATCGTGTATCATTTGAGTTGAATAACTTGTATTATATGAAGACAAATAACTACCGGAGGAACCAATGAAGTTACTACTATGATACAATGTTATGTTAAGAAATTTCAGCAAAAGCTTGTGATATTCTTTACTTCTGACTGTAAGTTCTTATTTCCTATTTCTTGTTGCTTCAGTAAGTAGTAAACTATATATATACCAGTAGGATAAATTCTCTGATAAACAAAAGCTTATCAATTTCACATTATTTTTTTTAGTTTACTATAAAATGAAGCTTATAAGTTGTTTGGAAAGTTGTATAATGGAGATAGAGATAATAACAGAAAATTAGAAAAACTAAAGCCAAAGCGATTATTGGA

>LNC_000889

TCTTCATCTCTCCACTCTCTCACTCTCCGCCATAAAATCTCATTACAATGTGAGTATCACCGATCTCCTTACTCCTCCATGGCCAAATCCTCAAGGGGCTACCCTCCATTGTTACCATCGAAACCAATCGAATTCAAACACAAACCACTACTACTCAAAATCAAACACCCAACCCCAATTAATTTTGACTCCGACTGAATCAAACCCTTCAAACGAATCTCGAATCACCCATTTTTATGAAAATTCTCTGATTTTTTTAACCAAGGGGATTGGGGATTGAAACTACATTATAGAGTTAGTTTGATCAAGGTGGAAGAGTGTGTAAATTGACTGGAAACTACATCGGAGGAATTGCAACTGGGTATATCTAAACGTCTCGCTGCTATATGGTTTCTGAGATAACAAAAGGTTGACTATTAAGTTAGAGAAACTAGAAACTATTGTTTTACGAAGGGGAAGAGCTTGTAAATAAGTTGGCAAAGTTGGAAAGAGATGTCAAAGAATAAGAAGGCGCAAGAATTGACTCTGCGACATTTTTAAAAACTATGCGCAGAGTAAGAATGTAGATATGAGCTCTTTTGGAGTATGATGCGTCTGCTCGGCATTTTATAGAAACAAAAGGACTTTCACCAACTCTTGATTTTTAATGGATAATCTAGAAAATTGTTGGCAGTTGGAGTAAGTAGTTCGCGTTGAATGAAAGAAATTTTTCAGCTTATCAAGACAACTGCAGCCACTTAAGGATATCAACTTCTATTCTTTTGGAATATACAGAGAGCATCTGCTCAAATTTACTGGTAATGATTCGAGGCATTAATAGATTTTAGAAAATGAATTTTGTCATATTTGATGCGCTATCAACTTCTATTCTAATGAGAAATACAGGGAGCATTTGCTCGAAGTTATCATTTGGAAAATTTGATGTTGTTTTTAATATACTCAGTCTGATCCCACAAGTTAGGAATGTGGAGGGTGGGGCGTATGCAGATCTTACCTCTATCTTTGCAGGGTAGAGATGTTGTTTCCGGTAGATCATCGCCTCAAAAAAAGCATTTTTAAGAACAGGTTTGATAAATACAAGAGTAAATACAATGCTGAAAATCATTGTATTCGTTCTTCATGTGTACTATGTTCTCTTCTTTGAACTACAATATCAAAGTAGTTGTTCTCTGTGATGTCTGGATTATTGATTAATCTTAATAAACCGTTAAATGACTAGAAGCCAAAAAATTTCAGTTGATTAAAATGAAACTGAAAAAATTGTTTAAGTAGGACAAACAAAAATATGGATGAAGTACATATTGTTTCTAAATATTTTGGTTAGCCTAATTTAGAGAGTAGCATTGAGAATCACTCTCTTCTGGCAGCTGAAAGCTGGTTACAACTGTATGATTTTTTCTTTTGCAATTTGTGACAATTTACTTTGGTAAATGACAGACCATTGTTAAATGAAAGTGTTGATATGTTACTATTTGAAACTGAATATGATGAGGCATATAATCTTTTGAATTTTAGTCTTTACTTTACTCAGACATCTGTAATTGGTTTCTTTATCTTCATGATATATTTGCATCAAATTGGTAACTCAGTAATTTAATTTCAAGGGCTTGCTGAATACTTGCAGAAATGTTCACGTGTTATGTCTAGATTAAGTTCAAAAATACCGTGGAGTCAAACTGTACAAATGTGCATGATGGGAGGTCCGAAAAGGGCAGTAGCTTGGAAAGAAGTGAGATATCACTTATCCCTCCCTATAGCTTATACATATCCTATATTATCTTTTGTTTTTATTTTTATTGCAACTTTGAAACTTTCTTCGAATGGTATTGCTAATGAAATGCGGCTTTCATTCCATGGAATAGTAGCACAATAAGATTTTCCCATTCAATGTCATTTGGCAATGATGATGAATCATGGAGGTAACTTCACGGTTGAACAGTTTGTCAGGCTTCATTGTGAAGACATATTGCAAGTTCATTACTTTATCCTCTCCTCGGGATCCAGTTTCAAAATGTACAGAGGTTGTTGATGTACTTAAGCCGAAACTATGATGAATTTATTTATCATGCAAACTTAAAAAAGCAAGAAGCCTAAGTTCATTTGTGAAAATATTCATCGTGTATCATTTGAGTTGAATAACTTGTATTATATGAAGACAAATAACTACCGGAGGAACCAATGAAGTTACTACTATGATACAATGTTATGTTAAGAAATTTCAGCAAAAGCTTGTGATATTCTTTACTTCTGACTGTAAGTTCTTATTTCCTATTTCTTGTTGCTTCAGTAAGTAGTAAACTATATATATACCAGTAGGATAAATTCTCTGATAAACAAAAGCTTATCAATTTCACATTATTTTTTTTAGTTTACTATAAAATGAAGCTTATAAGTTGTTTGGAAAGTTGTATAATGGAGATAGAGATAATAACAGAAAATTAGAAAAACTAAAGCCAAAGCGATTATTGGA

>LNC_000890

CTGACATGATACCTTAGTCACAGGGATTATCAGAGAGTTTCATTTTATTGTTGAGTTAGACATATCATGTAAATTACACTTCGACAAAGATACTATAATCTTATGTAACCATATTCTTTTTATTTGATAAGTTGAAAGTGATGTAAGTGAATCTTTTCGTTGGATCTAGATATGCGAGCTATATTTGTACTATATAGAACGATGAGATTAGTTACATGTTTGTTAAAAGTTAATAATTTCATAATTAGGGCATTTTAGACTTATACTACGTGTCTTTTTAGATGCGCAGAGTAAGAATGTAGATATGAGCTCTTTTGGAGTATGATGCGTCTGCTCGGCATTTTATAGAAACAAAAGGACTTTCACCAACTCTTGATTTTTAATGGATAATCTAGAAAATTGTTGGCAGCTTATCAAGACAACTGCAGCCACTTAAGGATATCAACTTCTATTCTTTTGGAATATACAGAGAGCATCTGCTCAAATTTACTGGTAATGATTCGAGGCATTAATAGATTTTAGAAAATGAATTTTGTCATATTTGATGCGCTATCAACTTCTATTCTAATGAGAAATACAGGGAGCATTTGCTCGAAGTTATCATTTGGAAAATTTGATGTTGTTTTTAATATACTCAGTCTGATCCCACAAGTTAGGAATGTGGAGGGTGGGGCGTATGCAGATCTTACCTCTATCTTTGCAGGGTAGAGATGTTGTTTCCGGTAGATCATCGCCTCAAAAAAAGCATTTTTAAGAACAGGTTTGATAAATACAAGAGTAAATACAATGCTGAAAATCATTGTATTCGTTCTTCATGTGTACTATGTTCTCTTCTTTGAACTACAATATCAAAGTAGTTGTTCTCTGTGATGTCTGGATTATTGATTAATCTTAATAAACCGTTAAATGACTAGAAGCCAAAAAATTTCAGTTGATTAAAATGAAACTGAAAAAATTGTTTAAGTAGGACAAACAAAAATATGGATGAAGTACATATTGTTTCTAAATATTTTGGTTAGCCTAATTTAGAGAGTAGCATTGAGAATCACTCTCTTCTGGCAGCTGAAAGCTGGTTACAACTGTATGATTTTTTCTTTTGCAATTTGTGACAATTTACTTTGGTAAATGACAGACCATTGTTAAATGAAAGTGTTGATATGTTACTATTTGAAACTGAATATGATGAGGCATATAATCTTTTGAATTTTAGTCTTTACTTTACTCAGACATCTGTAATTGGTTTCTTTATCTTCATGATATATTTGCATCAAATTGGTAACTCAGTAATTTAATTTCAAGGGCTTGCTGAATACTTGCAGAAATGTTCACGTGTTATGTCTAGATTAAGTTCAAAAATACCGTGGAGTCAAACTGTACAAATGTGCATGATGGGAGGTCCGAAAAGGGCAGTAGCTTGGAAAGAAGTGAGATATCACTTATCCCTCCCTATAGCTTATACATATCCTATATTATCTTTTGTTTTTATTTTTATTGCAACTTTGAAACTTTCTTCGAATGGTATTGCTAATGAAATGCGGCTTTCATTCCATGGAATAGTAGCACAATAAGATTTTCCCATTCAATGTCATTTGGCAATGATGATGAATCATGGAGGTAACTTCACGGTTGAACAGTTTGTCAGGCTTCATTGTGAAGACATATTGCAAGTTCATTACTTTATCCTCTCCTCGGGATCCAGTTTCAAAATGTACAGAGGTTGTTGATGTACTTAAGCCGAAACTATGATGAATTTATTTATCATGCAAACTTAAAAAAGCAAGAAGCCTAAGTTCATTTGTGAAAATATTCATCGTGTATCATTTGAGTTGAATAACTTGTATTATATGAAGACAAATAACTACCGGAGGAACCAATGAAGTTACTACTATGATACAATGTTATGTTAAGAAATTTCAGCAAAAGCTTGTGATATTCTTTACTTCTGACTGTAAGTTCTTATTTCCTATTTCTTGTTGCTTCAGTAAGTAGTAAACTATATATATACCAGTAGGATAAATTCTCTGATAAACAAAAGCTTATCAATTTCACATTATTTTTTTTAGTTTACTATAAAATGAAGCTTATAAGTTGTTTGGAAAGTTGTATAATGGAGATAGAGATAATAACAGAAAATTAGAAAAACTAAAGCCAAAGCGATTATTGGA

>LNC_000891

TTTAAATCAAAAGAATTTTTAAATACTTTTATAGTATTTGAATAAAATAAAAAATCTTTTTAAACACTTATTTATAAGTCAAAATAATAAAAAATAAAAGGTCATAATTTAGAATTCCTAACTTGTTACGGGTTATGCTTATAAAATATAGGCTATAAGCTCATTCAGGCTTGATATCTACCAAGTCATTTTATGAAATACTTGTTACCTTCATAAACATAAATACTAAATAACTCTATCGGCCAAAATGCACTTTTCGGGATATATATTTTAG

>LNC_000892

TCCAAAGGACACCCCTTCGCTGCAAAATTACAATGTGGATAGGTAAATTCTCTTTTATGTATATATACTATATATATGTTGAATTTTTTTGGCTTCTTATGTGTTTACTTCTTCATATTTCACACCCCTTAGTGAAAATCCCGGCTTGGCTACTGTCTTGAGGTATATTTATGAATATGTTATAGATATCGTCACTTAGACTGCACTTCAGTGAAGGTATCAATGCATTTAGGGAAAACCAGCAACTAGCTGATCTATGAGCTATGTCTTGAACAAAACGAGGGCACTAAACAATAACAAAATGAGATTTAGTTGTTATGCAAGACAATATCGATGGAAATTTGAGTGGTCTTGAATAGGGAATTTAAGATTAGTTTCTATACGTGATT

>LNC_000893

TTCTTTTTTACTATTTAAGTTTGTTTTGGCCAATTAATTTTATGAAAACTCTCTTTTCCTTATCCTGTCTAGTTTCTGGATTTAGAATACCAATGTATCAATCAACTATGCCTCAATCCCAGTTAGTTAGGTTGGCTATACAAATTCTCTATATCTATTACTCTAGATTTCTATTATAACTTGAAAATGTGATTATGTGGAATCAAAGAGTTGAGTCTTTCAGGCCTATTGGCTTTTAATAAGTGCTTCTAAATTACAGCCTCAACCTTAGTGTACAAGGGGTTTTAACTGTTATTTATCGAACCTGTTTTCATGTAAAGCTAGTTATGCTTGTATACTTTGCATCAAACTGACTCAAGCCATACCCCTGTTTAATGTTTGGTGACTTTTGCTGCAAGCTCAGTTATGTCTGAGTAGTCATTTTGTATGCTTGTTAATTTTTAGAGGCAAAACTTTTTTCCTCTAGTAATGTTTTTAATGTGTTATTCTACAATTGTTGAATGAAATGATTAAAAGGTATTCCTAAAACAAAATTTTCAATTGTGATTTTTTTGGTATTTTATTGGAACTCTTCTTGCTATTAAATTTTGTGCTTGTCCTAAGAATGTCACTTTTAAGCACATGTAATTTTTGCCATCTTTTTCGGCGCAACCTTATTTATGTAATAGATTCCATGTTGGACATGGTTAGAGATTTGCACAATTTTAATTTGGACTCTCTAGATTAGTGTTTCCACATTCCTTCTATAATATCTGCC

>LNC_000894

GTAGAAAGAGTTATTTTAACTGAGCCAATATGATTGGTTCTAATAAAACATACAACCACCTATTTATAGTAGTTTTTGTTTACCATCTATGTTGGGCCCAGGCACAGAAACCATTAATTTGAGATTAGATTTAAATGACATAATAGTTTCTCCTCCTAGTGGCCATATTTTCTTTCGGGGTTAATGTCTCCTTGAAAAGTAGGCTCCATCTTTTCATTTACATACACCAACTACAAAAAAGCACATCAGAATTCTATTTTGGGATATTCCTTGTATATTTAATTCCAACTTTTACCCCAAATCATTTAATTCCATTTTTCCCCGCCTTGGATAAAGCAACCCAACATATACAATGAAATGACATAAATTGACTAGTTTCCATTATTGAAAAGACAACCTTGCAATGAGCTGAATATATATTACAACAAGGGATGCAACCTGCAACCCATGGGCCAATGTAGGACTAGCTCCCACTCCCTCCCCAGTTGGAAATTTCACCAATCTCTTTTATCATCCTAGACTCTTAAAGATGTGGAAAAAAACTAGGACTCTACAAGACATTTTTGAATTTTAACCATTTGAATTCCTCCGTAATCCCAATTGAAAGACCATTAAGATGTTTCCTTAATAACTTTGTTGTAAAAGGTCACTCAAATGCTTTCTTTTATTCCTAGCTTTTAATGGTGCAATGTGAAGCATAGGTAATGAGTTATTACTGAAGAAATGTGTGTGTGAACATACAAATGAA

>LNC_000895

CTTTTACCATTTCTATCGTCCTAATTTTCAAAAATTTGTTCCTCGTACTAATAATTGCCAACATTTATAAAATAAGTCAATATAATTTCACCTATGGAAGAAAATCAAGTATATATATTGGTGAAAAACTAATTTCAACTAAAATATCCAACGGTTAGGACAACACATCTTGTTCTAAGAAAGTGCAAGATAAAAAGGGAAAAAGCATTGTGAATTGCTCTGAGACTTATCAAGATCAAGCAGTAGGTTCTTAAATAGAAAAAGATGAGGCAAAAAGGTCTAAGAGAATGAGAAGTACTAATGAGGAGGAACAAGTTAAAAGTAGCATTACTTTAGAAAAAGAGGAGGATCCTTCTTTGCTTTCGAAAATAACCACAAAAGAAAAGACGACACTTCCAATGGACACCAAATCTTGATAAGAAATTAGACGAGCTTGTTTGCGAGTTAGGAGAAAAAGGTAAGGCTTAATCTGGAAGAGTTAACCATAAACGAATCAATCACTAAATTTTTGTGGTGACTTCCG

>LNC_000896

GCGCTTCACAATTTTTTAAGCATTAAATCTCTTTTTGTTTCCATTAAAATCCCTTTCTTTTTCTTCATTCTGAATCCATAGTCAGCCGCCATGTTATTTGTACCTCTTTTGTCATTAGTTCCAGAACTTGGTCTTCTTACAGATCTGGTTCTTTTCCACCTCAACTCGAATCATTTTTGTGGTGAGATCTGGTTTTTCAAAGGAAAATAAAAATGGTGCGAATATGTGTTGGAATGCTAGGGTTTTACTCCGATTGACGAGTGACAACCGGAGAAGACGCTGGAGAAACAGAAAGCTCATCCTCATTCTCAGCTTCTGACTCATCATCCACAACCTCAGACTCGTATTCATTTCCGCTAACATTCAAAGAATCCTTCCCAAAAACCCCAATTCCCTTAAAATGATTCTTCACTAAGCACCAAAGAATTATGAGTTACCACTTACCACAGCAGAAGCCAGAAAAGGACTGAAAGAAGACACGCCATGTCTAAGACGAACATGAAAATGGAATTTTTCATTGGAGAAAGGAAGAGATAAAGGAGGGGTAGCAGTGGATCTTCGAGAGAGTTTGATCTCAG

>LNC_000897

TAATTCTTGTCCCCATAAGCAAATACATTTAATGTTTATCGACTTAATTGTGAGTAATGTTCAAGTTTAGGCGAGTTTACTTTTAAAGTGGAAGATAAATATTTGTCTTACTCTTAGCTTGCTCATTGAGTATTTACTTTTATGCCTTGCCTTAGTAATTTTAAATAATCCAACTAATTCATTATGTGTCGCTACTGTAGAGCGTAAATTCCGGCTTGCATCTTTCATTTACCAGAACAATAAATTGTTGTTAATAGTCTGAATGCCATATTCTTCTTGCTCCTGCT

>LNC_000898

CAAATAACAAGTATCATATAATATAAAATAATTAATATTATATATGGCATTAAAAGTTGTATTTGTTTTGTTCCTTGCAATGCTTTTGTTTACGATCGAAGAATATTAATGCTGAGATTGTTGGAGACTATTTACACTGTGTATTCATGATCGTGATTGCTTTATTAGCCTTCCATGTCAGGTTGGGTGTGGTGCCTATTGTGCTATACATCCATCACTTACTCTTAGTGCTACTGATAACGTAGAAATTCAAACTCGCGTTTGTAATGTTGGATGTTTTCTTGGCCATTGTTACAAATTTCTCATCAATAAATATGATCATGAAAAGTTTGGAAGGTGCATGACAAGTTGCAATGAGAACTATTGTATTAATGACAATGATAATAATATTGCTCTACCAAAAGCTTAACAAGAAGCATTAATTTTGACCACAAAGTATATATATATTGTGTCATCTATGACACTATGCTAATTAAATACTTTTGTGGTTGTAATTTTCTAAATTATGTAATCGTTATGTGTGTTGGTATTTGAATAAAAGTCTAGTGTTTCCT

>LNC_000899

TGGAGGGGGAAGGGTGGCGGACATCAAATGGATTAGAGTTTTCAAAAAACTAGAAGAGGTTCAATTCCTCTTTGATGTGGTTAAGGCAAAAGATAGATATCAGAGAATAAATTCAAAGGTAAGACTGGTTTTTTCTTAGGTCATCGGGTAAAGTTTAATAGTTCCAAGCTAAACGTACTAAACCAATTAGCTTATTACTATTGGAAAATTCCATTCATTTCTTG

>LNC_000900

AGTACAGTGACGAAAACAGAAAAAAACAACGACAGCAGCAGGCTCGGCTCGACAAAACTGCTTTCACCTTGTCCTTGCATCGTGAGATCAATACACATAGCACCTCAAGGACAAGTGAGTCGATCTAAGGCGTCGATTCACCCCTTCCTCTTCCGGAATGGGTTGGAATCGCAAGAAAATGTGTCTCTCCAAAATGGTGAAAGAGTTTTGATTTTGAATTTTGAATTTGGGCCTTGGTTTGCGAGATTTTTGTTTTCCCCCCCCCCCCATTTTTCTGAATCCTTCTTTTTCTATATAATTGCTCTTATCACTATGGAGCAGGGGGAGGGATTTTCATTTTTATTTTTTGGTAGGTTTTAAATTTTTTGAACGTTTGTTAGAAAATAGAGGGGAAAAGATTGAGAGAAAAGACTCTGATACCCGAGAAATCATACACCTCTAGATCCTAAGTCGTGATAGAGTCTTTGTTTGGGTCTGGGTCGCATCTTGTGGTCAGCTTCTTGAGATTTGGATTTGTGTGGTGGAAGAAGTTCGTCCTCTCGCTGTTGTCCCGTCTCAGCTCGCTGCCCTCGAAAAGAGGCTCAAATCTCTTTCATTATTAAGTCAGCCAGATTACCGGGCGTACAGGGCAGAATGCGAAATATAGGGGTTGGAAATTAAATTTACAGATTCTGGATAGCGGAAAAAAACAGCTTGATACAACCTTTATACACTGATATACGGTCCAGCCCAACAAATACGGGATTAAAAATGGGAATACAGGTTGCAGGAATTGAAGAGGGCTGTATACACTTGGTATACAATCCAATATACACGAAAATGGACCAAAATACAGCCCATATACAGTGGTATACGCTAAACGTATACACAGAATTTCAAGGGTAAAAAGGGAGGCGAAAATGGGCCAAAGTCCATTGTTGTTTGGGAGCAAACTTTAGTTTTAGATTTCTTGCTCTTATTATTTGTTATTATATCAACTAACATCCTTATTTCTTATGGTTTGATTTAATTAAATCCCCCAAAGATGACC

>LNC_000901

AGCAGCAGCAACCAAACAGAGTAAGAACAGCGTCTTCACGGTAGCAGGAAAACGACAGAGCCATTAAATGCTCGACGGCAGCATCGACATATACACGGCAAAGCAAACGACGTCTCACTAATGTTCATCTATTCACGCGATCCCAACCGTTTATCTCCCCTTTCCTCTTCCGGAATGGGGCGAATTTTCAAAAAAAAAAAGGTGTTTGTCCCAATTGGTGCAAGAAATTTATGGCGTTTGAATTTCGAATTCCCACCCGAATTCATCCGTTTTTCCCCCTATTTGGAACCCTAATTTCTCCCCTATAAATACTTTACCTTCTTCACTGTGAAGGGGGCCGAAAAATTGGGAGTTAAGGAACATATTGGAAAAAGAGGTTGGGAAATTTCCACAGGGATTTACAGAGAGAAGAGTCATTAAGCTATAGAATTCATATAAGAAAATACATCCTAAATATATACTTCTCTCCTCGGTTGTGGGTTACTTGGAACCTGCTGAATTTCTGAAGTTCTAAGCTGGTACCGAAGTCGCTCCAAGTTCGCCCTTGTCTCAGCTTTGCTGCCCGAAAAAAGTGAGCTCATGGAGCTCTAGTGCCCCTCTTTTCATTCTCATGGGCCATGGAGGCCCAAAATTGCATTTCCCTCGAGTCGTATTTAAAAATTCAAGTCCCAACGGACTAAGGTGTACATGTTGAAAAGGGTGAAGTTGGAAGATTTAAAACAGGGTCAAAGATGAAATACATGTTTCGGATGTTGTATACATGTGGTATACAGTGGTATACGTTGAATGTATACAAATTCCCAAAGGCAAAATAGGGGCCCAAAACCTTAAGTGATTTCCAGCAGGCTCAAATGGAAAAAAAAATGGGCCCAAAGTCCATTTGAAATGCAGCAGAATAGAGGAAAGTTTAAATGGGCCCAAGCCCATTATTTGAACCAAATTTAGACTTAGAACAGGTGAAAATTGGGCCTAAGGCCCAAATAATATTTTATTTCATGTTAGTTTAGGACAGGTACAAATGACTTAAATGGGCCAAAAGGCCCAAAACGAAAATGGGCCCAAATACTGGCCCAGGCGGACAAGAAAACCAGATTTGGGCCCAACTCCCTTTCCCTCTCTACTTTACGGTGTTCATTTTACTTGTTAGTATTTGCCGCTAATCTTAAGGTTGAACAATTCGAATCCCGAAAGACGTCCATTTCGCTTTAAGAAATTAGCTAAACCAGTCAACTCTTAACAAGACTTAAAGAAATAAATTTTGCGAGATAGCTCACTTAGATAAAGTTTAAACATTTCCCCTTTTTCCCTTTAAATAGTTTCTACTATAAAATAGTTCAATTTTGCAAATCGAGTTGAGTTAAAATTGTTTTAGCCAAATAATTAATCATCGGATAACCGCGTATTAGCGGGCATTTCGGGTGCTTTCAAACCCTTCCCGAAATGCTAATATGAACCCCGAACCCCTTTGAACATTTTCATTAGATTTCTGTTTTAATCTTTTGAAAAAATAGTTTTCTTGATTTTTTCTTAAAAATTAAGTGGCGACTCTTAAACCAGTTCAAAATATATTTTCCAAGTAAAACAGGTTGATGATAATGTATTTCCATAAATTTAGGTGTGTATAATTGATGGAATATTGCATTCCATGGATTTCTCTCAATAAATTTGTTTTTTCCCTATTAATTAGAAAATGTTAATTTTGATGATAATGTATACTTTCCATAAATTTAGGAGTGTATGTATACTCATGGGATATTTTTTTTCCTATGCATCAGAAAATTTTATTTCTCTTCCGTCTTCTCTAAGAAAGGTATCCCGATATTACTTTCTAGTATCTAAATCAATTTACCCTTTTCTAGTCTAACCAACCATCGAC

>LNC_000902

ATTCATTAGCAGCAGCAACCAAACAGAGTAAGAACAGCGTCTTCACGGTAGCAGGAAAACGACAGAGCCATTAAATGCTCGACGGCAGCATCGACATATACACGGCAAAGCAAACGACGTCTCACTAATGTTCATCTATTCACGCGATCCCAACCGTTTATCTCCCCTTTCCTCTTCCGGAATGGGGCGAATTTTCAAAAAAAAAAAGGTGTTTGTCCCAATTGGTGCAAGAAATTTATGGCGTTTGAATTTCGAATTCCCACCCGAATTCATCCGTTTTTCCCCCTATTTGGAACCCTAATTTCTCCCCTATAAATACTTTACCTTCTTCACTGTGAAGGGGGCCGAAAAATTGGGAGTTAAGGAACATATTGGAAAAAGAGGTTGGGAAATTTCCACAGGGATTTACAGAGAGAAGAGTCATTAAGCTATAGAATTCATATAAGAAAATACATCCTAAATATATACTTCTCTCCTCGGTTGTGGGTTACTTGGAACCTGCTGAATTTCTGAAGTTCTAAGCTGGTACCGAAGTCGCTCCAAGTTCGCCCTTGTCTCAGCTTTGCTGCCCGAAAAAAGATTACTGTCAAATGTGTTGTTGCCATGCCATCAAGTGTGTCGTTCCTCTTATTGAATAATCTTGCCTTATTTATGAGTTTCATAGTTATTAGTACGGATATTAATCCTTTTTCTTCCTTTTCTTGTTTCATGTGATATTGAGTGAGCTCATGGAGCTCTAGTGCCCCTCTTTTCATTCTCATGGGCCATGGAGGCCCAAAATTGCATTTCCCTCGAGTCGTATTTAAAAATTCAAGTCCCAACGGACTAAGGTGTACATGTTGAAAAGGGTGAAGTTGGAAGATTTAAAACAGGGTCAAAGATGAAATACATGTTTCGGATGTTGTATACATGTGGTATACAGTGGTATACGTTGAATGTATACAAATTCCCAAAGGCAAAATAGGGGCCCAAAACCTTAAGTGATTTCCAGCAGGCTCAAATGGAAAAAAAAATGGGCCCAAAGTCCATTTGAAATGCAGCAGAATAGAGGAAAGTTTAAATGGGCCCAAGCCCATTATTTGAACCAAATTTAGACTTAGAACAGGTGAAAATTGGGCCTAAGGCCCAAATAATATTTTATTTCATGTTAGTTTAGGACAGGTACAAATGACTTAAATGGGCCAAAAGGCCCAAAACGAAAATGGGCCCAAATACTGGCCCAGGCGGACAAGAAAACCAGATTTGGGCCCAACTCCCTTTCCCTCTCTACTTTACGGTGTTCATTTTACTTGTTAGTATTTGCCGCTAATCTTAAGGTTGAACAATTCGAATCCCGAAAGACGTCCATTTCGCTTTAAGAAATTAGCTAAACCAGTCAACTCTTAACAAGACTTAAAGAAATAAATTTTGCGAGATAGCTCACTTAGATAAAGTTTAAACATTTCCCCTTTTTCCCTTTAAATAGTTTCTACTATAAAATAGTTCAATTTTGCAAATCGAGTTGAGTTAAAATTGTTTTAGCCAAATAATTAATCATCGGATAACCGCGTATTAGCGGGCATTTCGGGTGCTTTCAAACCCTTCCCGAAATGCTAATATGAACCCCGAACCCCTTTGAACATTTTCATTAGATTTCTGTTTTAATCTTTTGAAAAAATAGTTTTCTTGATTTTTTCTTAAAAATTAAGTGGCGACTCTTAAACCAGTTCAAAATATATTTTCCAAGTAAAACAGGTTGATGATAATGTATTTCCATAAATTTAGGTGTGTATAATTGATGGAATATTGCATTCCATGGATTTCTCTCAATAAATTTGTTTTTTCCCTATTAATTAGAAAATGTTAATTTTGATGATAATGTATACTTTCCATAAATTTAGGAGTGTATGTATACTCATGGGATATTTTTTTTCCTATGCATCAGAAAATTTTATTTCTCTTCCGTCTTCTCTAAGAAAGGTATCCCGATATTACTTTCTAGTATCTAAATCAATTTACCCTTTTCTAGTCTAACCAACCATCGAC

>LNC_000903

ATTCATTAGCAGCAGCAACCAAACAGAGTAAGAACAGCGTCTTCACGGTAGCAGGAAAACGACAGAGCCATTAAATGCTCGACGGCAGCATCGACATATACACGGCAAAGCAAACGACGTCTCACTAATGTTCATCTATTCACGCGATCCCAACCGTTTATCTCCCCTTTCCTCTTCCGGAATGGGGCGAATTTTCAAAAAAAAAAAGGTGTTTGTCCCAATTGGTGCAAGAAATTTATGGCGTTTGAATTTCGAATTCCCACCCGAATTCATCCGTTTTTCCCCCTATTTGGAACCCTAATTTCTCCCCTATAAATACTTTACCTTCTTCACTGTGAAGGGGGCCGAAAAATTGGGAGTTAAGGAACATATTGGAAAAAGAGGTTGGGAAATTTCCACAGGGATTTACAGAGAGAAGAGTCATTAAGCTATAGAATTCATATAAGAAAATACATCCTAAATATATACTTCTCTCCTCGGTTGTGGGTTACTTGGAACCTGCTGAATTTCTGAAGTTCTAAGCTGGTACCGAAGTCGCTCCAAGTTCGCCCTTGTCTCAGCTTTGCTGCCCGAAAAAAGGTAACACTCCTCCCTCATCTGTATCTTATTCCGATTTCGATATCATGTGATGAACATCTTTTGTGTTCTCGAGATTTCTTTTATGTTTCTTCCTTATTTTGTCTTCTACTTTTGATTGAAGGTCTATTTGATTTAAATATGGATCGGCGGATCCGTCTCCTTCATTGTTTAGGCTTCTGGTTAATCATCTCATTATTCGCTATTTGTTCTTAGCTAAATGTAGGAGTCCAGATATCTAGATGTCGGGATTTACCCTTTGTATATGTGACGCCTTTAGTCTCGAATCTATTTATGTGAATCTTGCTATCTTGTGTCTGCTTACCTTAACTCGCCTTCAAGCATAGGCCTCCTCATTATTTGCTACTGAATATGGCACGATTCGAAATAAGTGTCGGGTCATGAAGGATAACTGTTAGGAGTTATGATTGCAAGCCTAGAAATTTTCATTTTATGCAAGACTTTACTTGATGTGCTGAAATTTGTGTTTCTGTTGGTTACTATCATTGTTTGATCTCATCGTATTGCCTATATTCCTTTTTTAAGCTTCTTTTAATCTGTTATAGTTGTTGGTATTTTATATATATATATATATATATATATTGTCACGACCCGAGAGTACCCCCTAGTCGTAACATGGCGTATTCGACCCCGAAGGGGCCTTATACGAGCCACATAGTCATTCATTGCATTCAATAATGAAAATAGTGCGGAAATA

>LNC_000904

TGGATCCCACGTAGCCCAACTCCTTTAAAAACTTTTACTTTTTGATTGAACATTCTCTTCACATCTAAGAAATATTTGTGCATATAAGTTCTTGCTTGCTACTCTAAGCCAACAAAATTATTGCATTGCATAGAAGTCGCTAAGATATTGGGAAAGTTAAATATGGGAAGTTTTGAGAAGCAAGTGAAGGAGAGAACACAAGAGTTGAAGATCATTTTCAAGAAGGGAGTAAAAATTGTGGGAGATTCTTGCAAGAAGGGTTGGTACAAGATGAAGAACATGAGGAGTTGAAATCTTTTTATTTTTCCTTAATATGAGAGGTTTTGATTATGGGAGAGAGGGTTGATTTTATGTCACTAGGTATATTTTGGTTTAATTTGCAAGCTTGGTTCTTGAAAAGATAGAAATGTAAAGAATCTTTAATTTGGTATTTCTCTAGTGTTTCTATGGAAC

>LNC_000905

ATACAGTAAAGAAATATAAGAACATGACTGTCGACAGCTTGAAAAAGAAAAAAGGAGTTCAACCGTTCCCTAAATATGGAGTCAATGACTTTGAAGGGCCATTATTGAGTCAAGCAACCGAAACCGAGCAAAGAAGTCACATCTGAGCTAATTATAACTAAGTCATCCGCTTATATTATACCAAGCAAGACCCTTTTGCAGTTTACTCGCTAGTGTCATCACTCACTGGACGAGCCTTTCTATTTG

>LNC_000906

ATTTAGTACTAACACTTTTACTAAGTGTCTTTACAGGTACCCCGAAGTCGCTTCTCATCGAAGCCATTCGAATAAAGTTCGAATGTTATATTCATTATTATGTATATTGTTGACGATAGACAAATATTAGGTCGTTTTTCATATTACTTGTTTACTTTATTACTTGTGTATATTGCATGTTTGTTATATTTGTATAATATTTTTACATCCTCCTCAATTTGAAAAAAATGAATTCAGTCGGGAC

>LNC_000907

CCTTCATCGGCTTAGTTCATTGCTTTAAGAATGATGAGAGATGACTCAGAAAAAAGAATGATGAAAGATCTCAGTTGTGATGCTGTTATAGTGTTATTTATGTAAGCATTGGTGTAAGGGCGTCTTATTTACCCCCTTGTTCTGTTTGTTAGTCTGTCTGTTTGTCTAGCACTGTCAATGGGATCTTCTTGAGCTAGCTGGAGGGCTTCTGTATCCTGCAGTTACTGTATCCTACTGGTGTGGATCTTTCCAATTGGTGTCTTTGAGTGTAATTTAGTTTTTCACTGATGTAAGCTTCATATTTTTGGTAACACCTGGCACTATATATGCTATTTCTTGCGCCTTGGGGACTTTCTCATGGAAATGGCATAGGTTTCTGTGATGTTTTTGCAAGTTTTGAGTTATTTCCCTCAATCTACACTTTTACCTGTCGAGGATTTATCAGAAACAACCTCTACCCCAGAAAAGTAGGGGTAAGGTCTGCATACATCCTACCCTCCCAGACCCTTGTGGGATTACACTGGATAAGTAGTTGTACTTAACACTTTTACCGGGGATTGCGCTTGGTTAATGAGTATATTATAGTGTTAAGGAAGAGGCTTTTTTTATTTGAGTTATTGAAATGTCATTTGGATTAGTAGCTAATATTTTAACATGAACAATCAATCATTTATTTATATTGTTGTCCTTATTATGATACTTAGAGTAGCCAATTGCTGCTTAAGTTTGGTTGTTATACTTCACATTTCTTCATTTGTATTCCTTTGC

>LNC_000908

CGTTCTTTCACGTGATGATTTGTTATCATGGTAAACTTACCATTCATCAAGATCCTAGGTCACACTATGAAGGTTTGGGAGAGGGGGGTGGAGTTGAGGGTGAGGAAGATCATGACTATTTCTGAGAACCAATTCGGATTTATGCCTGGTTGCTCGACCACAGTAGCCATTCATCTCGTAAGGAGACTGGTGGAGCAGTATAAGGAGAGGAAAGACTTACATGGTGTTCATCGCCCTAGAAAAGGTTTATGACAAAGTCCCTAGGGAGGTTCTATGGAGGTGCTTGGAGGGAGAGGGAGAGGGAGAGGTAGACGAAATAAGTATTGAGGAGAAGTGATTAGACAGTACATGACACAGTTGCAGCTAACTGATGACATGACCTTAGATAGGAAGGTGTGAAGGACACGAATAAAGGTAGAAAGTTAGTAGGTAGGAGTGCATCCATACTAGTAGGTAGGAGTGCATTGTCTTGTTG

>LNC_000909

GGTTGTGTTGCTGTATTCTTGATAACAATAGCTGTTTAGAAGTTTCATATGCAATATTTGGCTGATCATTGTGATATGACCTAGATTTCTCAAGAAATGTAAAAAATTGAATTCTCAATGACTTTTGTGCTTCAGTATACATTGGTTGAGACTATGCCAAGATAAAACTGTTTTAACTTTTGTAATGACTTAATATCTTGTTCAAATTATAGAGCGGATTGGTCTGCTTTGCTTAGCAAATTATGCTGTCTGAAACTGGTAGAGTAAATAAGCGTTATGTTAAGTGTTAACTGGGAGAGTAAATACAGGATTTCAGTGGATTTGGGGATGTTTCGTTTTTCCTTTTTCATTTATCTGAAGAGTTAAGGAAGTTGAATAGATATAAGAATAATAGCTCATTGATGAAGCTGAATGAGTTCTGAAGTGGGAGATGGAAATGTATGTAGTGGAGGTGGTTGAAAAGAAGTGCTAAGAGGACTGCTGGTTTGGAGTGGTTAAAGATGTAGCAATGCAATAAAAAACATGGAATAATCATTAACGATTGACAGAGTATAGCATGAC

>LNC_000910

GAAAATAATTAAAATAAAATAAAAATAAATTGAGAGGAAGTAATTAGATTGCATCATCTTTTTTTGGAAAATTAATTAGATTAGATCATCATAGCACAAATATTCACATCCCCATTTTTTCAATTCAGTTGGAGATTGAAAAATCTGAAAAAAAAAACACAAAAATAGAAAAAAAAAGATCAATAAAAAGCAAAAAAAAAAAAAAAACATATCTTCCTTTCCCTATTATTTTTGCAGAAGAACATATACATAAACACAAAAAAATCCCCTGCATCTTCAAATACGTCAATGGCAATTCCTCCGTTTTTCTTTGAATTCTGATTTTCTCACACACAGACACACACTCAAACTTCAATCTCTGTTACCCATTTTCAGGAATTACACTTCCCATATCTAGTTTTTTTTTTTTTTTTTTTTTATTTTTTTTTTTTTT

>LNC_000911

GAAACGTTTTTTGATTTTTTATCATCTTTTACAGAGATGTTGAAGTTGTTGAGTTGTGCATTGATTTTTTCAATGATGTTTCCGGTTACTTCAAAAGTTTCAGAAAATTTAGGGAATTTTTCTTTGGAAAATTCAGGGATTTTGAATTCTGGATTGGTTTCAATATTATGGCTAGAAGAAATAGAAGCCGGCGTTGGTCTGCCGTTGATGCTAGGTTTAATATCTTCTATTTGTCTAGAGATGATATGAAGGATTTGGTTGGAGTAATTATTTTTTTCAACTACTCGTTCAATATGTGCAGATGTGAGTAATCCACCTGTCTTATCACTTTTCTCTTTGAAAGGAGAAGCTTTAATTATTGTTCCTACAATTGAGATGTTAATTCCTTCTAGAGGAGTAGTGATATATTCAACAATTTTGTTAGAAGCAGTATACCATTTGTTAATGATAACTTGTAATGACTCAGCACAATTTTCTATCCTACCAGTCATTTCAAACCATTTGAAGAATTCTATTTCACAACCAATTCTTCTCATATCAGCCATCCACAAATCTCTAAAGGATTCTCTTTGCTTAAGAGTGTAAGAAGAAACATACCATCTTCTTTTTACCTAATTGTCAGGATGAGTCCATTGTTCCTACAGGATAGTCGGATCGGGTTCAAATGTTTTATTTACCTTAAGCATTCCTATCAACCAGGATGTCTTTTCCTTAGGAGGCTCCATTTCACTTGCAGTAGGGCTACCTGGAATATCTTGGTAAAATACTTTGGGTATGTTACCTGAAAAGTAAGATTGCCATTAATGGTGCTTACTAAATCACCATAAGTATGATTGCTATAAGGAATATTACTGATATTTCGATCTTGAATTTTTGTCCTTACTTTATCAGCAAAGAGATGAGGTAAACCGCTGATAAAGCGTTCTTTCCAGAAATCCAAATTGCAATCTGGACGAATCATAACTTTACTGAGAAAGGCTTGTTTGTAATGGATAAAGTTGTCTAGATTTGGACAACTTAAATTACTCAGAATTTGGAGACTTCTATCTTGAAATAATTTTGGTTCTCCAATAAAGTTCTTAGCTATGTGGTAAAGAAGAGTCCATTGTTCCTGCAGGATATTCGGATCGGGTTCAAAGGATTTATTTACCTTAAGCATTCCGATCTACCAGGATGGCTTTTCCTTAGGAGGCTCCATTTCACTTGCAGTAGGACTACTTGGAATATCTTGGTAAAATACTTTGGCTATGTTACCTGAAAAGTCTACTCCTGTTACTTTACCTTTGCTGTTGTCAGCAACAGAAATGATCTCATCATTATCTGCTTCCTCCGGGGTTTTTGAAGAAGAAGGTGTCCTGAGAACGGGTCTTGAGGGACACAAAGTAGAAGGGGACCAGGATGACCTTCGATTCGAGGAAGATCTACTTGCTAGATTGTCTAAGAATTTGAGTTCTATAGATCCGTCTGGAAACTAAATGACTCTTTCAATCTGAGATCTTTCAGAATGTGAAACAAAGGGTTGAGTTATGTTCTCAAAGCGCCAATCATTACTGGAGAGTATATCTTTCCATTGTAAAGTTCGAGGAACAAAGATGGAGCTATGTTCATGGTTGGGTTCCATAAGCATAGTCTCACCCTTTATGCTCTCATGACGAGCTTGAGGAGCAAGGTGTGTTTTCATGAGACGATAATATACTCGATATATAATAGCTATTTCACGAGTATTGATTTTACTGTTCAAGTTTTTGGTTTTGACATTGAGAGTCAAAGAATCCATGACATTAGGATCATTAATGTCAACAGAGAAGTTAGGATAACAAATAAAGTAAATTGGCCCATCAGCAAGATTACTCTGCAAAACTCCTAACATAGAATCATCAAAATTCAAAAACCTGCCATCTCTAAGGATGACACAAATAGGAATATCAAGGCCTTTTCTAAAGAG

>LNC_000912

AACAATACCTGATCTAGACTGAGCAACCTCAATACCAAGAAAATACTTCAATCTGCCAAGGTCTTTAGTTTTGGAAATGCTGAAAGAGATACTGCTTCAAATTAGCGATACCAACTTGATCATTGCCAGTGATAACGATATCATCAACGTAAACAACCAAATAAATACACAGATTTGGTGCTGAATGCCGATAAAACACAGAGTGATCAGCGCCACTACGAGTCATGCCAAACTTCTGAATTACTGTGCTGAACTTTCCAAACCAAGTTTGAGGAGACTGTTTCAGACCATATAGTGACCGACGCAATCGACATACAAGGCTTCTAGACTCCCC

>LNC_000913

CCAAAGATCACGTTCTTCTTCCCCATCACGCCTCTTCCTCTTCCCCAAAGAATGACGCTCCAAGCTCCTCTCCCATGGCCTCCATCACCTCCCACACGTGATCCCCCATCCCCCTTTGTCAGAGAAATGACTCTGGGCCACCGGAAAAACGCTCCCTCAATCACTCATCCTCTCAAATCGACCTGAAAAGGTCGATTCTTGAAAGATTTCCTTCCCTCTTCATTATAATTTTTCTATTCTTCCTCATACTTGGCTTCCCAAGAATTTAAAATTTGAATTCTTGAAGCCAAGTGGAAGTTTGAATTTGGCAATAAAAACAGACCTTTGGTTCTCAATCCAGGAAGGTCCAGAAATGAGTGAGAAAATCGAAGAATCGATCAAAATAAGAAAAATCGCAGCCACTTCAAGTTTTTGTTAGAGTTCGAAATTTCTTTTCACTTCTTGTCCTTCTGTTCTCTGTTTTGAATTTGAGTTGACGTCGAGTTCGAAGTTGAAATCTCTTAACGTCGAACCCCTTGTTTGTTGCTTCATACATCAGTAGTCATCATTATCTTTTCTATATTTTGACTTTCAGTTTCGGAAACTTTGTGGCTGTTTGAATATCCTTACTTTATTCAATCTTGTATTTCTAAACGGTTCCCAGAATTACCTCATTTAGCTTTGATTAAAATCAACCTTTAAATAATTTGTCGAAGTTGTGATATTTGTATCTGGTTAGAGTTGTGAATTGTGAATTCAAAAATGACTAGTGGCCTGTCTTTTATTCCGTTAACAATCAGTTAAATTTCAGATGCTTCCTTTTAAGTTATGTGGTTTCTTTATATGGACGGAATGCTGGCTCAAGACTGTTCACAGCCGATATTAAATTCGGATACTCTCTCGCATAGTCTGTTTCGCATAACCTGAAGCATATTCATCTTCATCTTCATTTTTCTATATTAATCATATCTCGTTGTCTTGTCGGTTCTACTTCGTCATCATGATTTCCCCTAAAAAATATTCACGTGTGGACTGTGATCACATACTGCCTCTATTAGATGTTAAATTTTGTTATGCCAAAATATGTAAGTCTCTTGATTAAAGTTAGTTCTGTTAACCGCCTTTAGGCCTCTTGTCTAGATACACATTTAATTCAAAGTTGTTTCTGCTGCTTGGCATAGAAAGTTTATTTTTTTTACATTTAGCTAAATATGAATAACTCGGTTTGAATAGATGTCGGTGTTTTCCTCCCTTGGAATGATTCTTAAATGATAATAATGTTATACGGAGTGAAATTCCTATATAGCTACAACAGTAGCCTGGTCTCATATTGCTTCTTTATTTAACCTAAATTCAATATCGATTTGGTTAGTGAATCTAATATTGCTTTAAGTTAATATGGCTGGCTCCTTAAATGGTTGTTTCTTTATGTTCGTGGTCCTTTGAAACTCCTTTTTTGTATCTGTCAAGTTTTAGCTTCTATATTTACTCTTTTTTTTTGTTCAATCTCGTCTCTATTGATGCCTAGTTTGAATACTTCTTTCTGGTTGGTAATTAAAGAGTAATGCTACATGATTCAATCTAGAACAACTTGACATGAATATGTTTGGATTCAAAACTGGTACCCCTTCGTTCCACCCCCCTCCCTGTTTGAAGATCTGGAAAATATAAGAGTATGGATAAATGTTTGGTCATCACATAGTTAACTAAAACGTAATCCTTCATCTAAGATATGTGAAAATTCTGGTTTATACATATCCTCCATTTGGGGGGCTGATTTTATGATTCGACAAGAACTAATCCAAAGAGTTCTCAAGTGACTAGTTTTAATCTAGTAGTGTTGCGGATTGATTTTTTGTTTAGTGATCAGTTAAAGGTGATCTCGCTTCAGTCCTTATTTTATGACTTTATAGTTAAGGTAGTACTTTACAATCCAAAATAATTTAATTGATTAGCTTTCCAGTGGCTCTTCCTTTAATTGTATACCAAGATCATAAAATAATATGAGTGTTCACCTCCTGCCCCGACTCTTCTTGTGATGTTTCTCTCTAGATTGATTAAAGTTTTTGGTATACTAATTACTTAAATATCGTCAAGGAATTGCAAGAATAGTATTATGTTGTTGTAGTTGTTGTTTTTGAGTATATTTACAGTATGCCTACGACTACATGATTCTTATTTTACATCTCTTTGATGAACCAAAAATTCAGTCGCTAATTCTATTTCTTCTTTGTTTGTCACATGTACACTCGAGAGCGCATTTGGAGTTAGTATATGACTCCATCTCTGTCCAATTCAGAGAAGCGACCAATAGCCTACATTATTTGCTCGTGTCTAGCAGCCCTCATCGCATCAATAGTGTCAACGCTTCCTTTTCAGTTCAAAGTTTTTTTACATTATATATATATGCGCGGTTACTTCGGTTAACATTTTACATATTAGTATTTAACATTTATATTTTTGATTTCTCCTATTTGTGTTTGGGTTTGTACAAACCATCTAGAATGATAGTAAGACGCAGTCATTCATCTCACATTCCTCTAATTATTGGGTTTTAGTATGACCATTTGGCTTATCACGATGCTTTCCAATGAAGTTTTCATCTATTATATCTTTTGGTTGTGAAATCTTAACGTTTCAGAAAAGTGAACTTGGAAACGACTTCCTTTTATCTCTTTAAAAGGAGAATGTATCCATTTAAGATTTGATTCAATAAGAGTTAATAACGATTTTTTTAGCAACTTAATGAATGCAGATTTTTGAGTTCCAAAAGTGCAGATTTTAGCTTGAAAATTCCAGATTTTGATAGGTAAAAAGAG

>LNC_000914

AAACAATATAAATTAGCTAGTGATATTATTTTAGCAACTTCACTCACAAATCAGACAATACTCTCTCTTCCCTCTAACCTCTCTCTGAACTTTTCTATTCTTAGACCTTAGGCGCCGCTACAAATCAGAAATATTCTCAGATTCCACACTATTCTAGGCTCGTATTTGACTATTAGGAGCTAAAATCTTCAAAAGCCCTCTCATCTGTTTGAGGGTTTGTGTGATTTTACTTGGTGTTTGGTGATTTAAGTTGGTGTTTTGTGATTTTAGCTACATCTGTGAGATCTATTGGTGGTTCTGTTTGTAGCTCGAGATGGACCTAGATTTCTTCAAGAAGTTCGATAATTTAAGGTATTTTTCTTTGATATATTTTTCTTCTTTTAAGTTTTTTCCTAAGGGGTTAGTGAAACAGTCTGTTTCTGTTGTTACATGTGCTAATTGGTTGAAACTATGAGCGGTTTTTTATTTGTTTGATTTAGGGTTATTGAAATTGTTGGATTTTGCTGGTAGTTAGTTCTATGTGTTGTAATTATTTAGTTTCATTTTTTGCATCTATGTCAATTTGTTGTTGTTAGTTATAGTATAATTGTGGTATTTTTGTACTGTATGTGCTATAGTATCTACCATTTGTGGTCTTAGTTTATTGTTGTTAGAATCACTTTTAGTAGATTGCTCAACGTAACATTGTTGGATTTCTTATTGCGTTAGGTTTTAATAAATATGGTAGTGTGTTTTTACTCTATTTTCCTTTATTTCTTGATAATAGATTATTGTGAATCAGATTTCTCTTGCCATTGATGTTGAATTCCTAAGGCATGATTTTATAAGACAAGAATTTGTGAGGCATGATTTAAACTTGTAAATTTTTTGTTTTCTAATAATAATTTATGTAATGATGTCGCATTTATCGAATGATCATGAAGTTAGATAGAGTTGTATCTACTTCTTTGTAAGAAATGATCACTTTTTCATCAAAAAAACATCAGCGCGGAATCTGTTTTCTTGATGTATCCAATGATTGGTTAATATAAACTTTACTGCTGGAGAGAAGAAGTATGTGTTTTATTAATATGTATGTGTATTTATACAAGAGAGCAAACTACAAATAAGGAAAGAATCAACTACTAAAGGATTGTGATCTTCACCTAATTATAATAGAAACTAAATCACTAATGAGATACTAAGATATGGAATATCAATCAAGAGATATCCTAATATCAATCAAGAGATATCACTAATATGCCCCCGCAAGATGGTAGATCGGTCTGATATGCCAATCTTGGATCTTAGTGCTTCGAATGGAGCTTTTGGAAGCGGCTTGGTAAGAAGATCAGCCAGCTGATCTCTGGATGAAACATGAGAAACACGCAGCAGGCCTTGTTGGCAGAGATTTCGAACAAAATGACAATCAATCTCGATGTGCTTCATTTTGGAGTGAAAAACTGGTTTAGCACACACATAGGTTGCGCCAAGATTATCACAATAGATAACGGGCGGTTCCTTGAGAGAAATAGATAACTCCTGAAGCAAATTACGGACCCAACAAATTTCAGCTGATGCTGAAGCAATAGCACGATATTCAGCTTCTGTG

>LNC_000915

AGCTTACTCTTGGGCTTCTCTTAAGCCCCTCTGAATTCTCATGACTTTCTCAAGACTATTGCTAAGAACAACTCATCGACAAAGAACTGACTCTGCTTTTCAACCACCTCTAGTATGATGGGTAGTCGAATGATTCTGAGAATGCACGAGTTAGAATAGATCTCTGTGACTTAGCTCTATTGCACGATTAAGAGTGTGAAAGAAGGGAAACTTTTCTTAAATGTTTGTAGTCCCTCATTTATAGAAGTGGGGCCCTCACCTG

>LNC_000916

ATAAAAGACGCTGGCCATTAGATCCGCTGCCCTCCTCCTCCTCCAGAATCACCACCTCCAGAGCCACCGTAGCCATTTTATCCACCGCCGCTTAAGTCACCGTATTCTCCGGCGCCACTGGAGCCATCGTATTCAGCACGGCCACCAAAACCACCGTATCCACCACCGCTATCCGGACCTCCAAATCCACCTCCGCTACGAATCAACACCGCTACTATGTACACCAATTGGAGCTCGGCTATTTGTGGTTGCTCTAATTCCGCAGCTTGACATATGTAGTAATCGTTTTCCCTAGTTGAGGTTTTGCACTTCTTATCTTGCGGAGGACGTTGATTAAGTGAAAATTGATATCTGCAAGACCTCGACTCTAATTTCTGAGATTGACCGAGTATTAATTTTCCGACAGATGCTAATTTGGTGGCAACATGTGGTGGAGACGAAGGAGAATTTATATGGAAGATTGGTTAAGGTGATTCTAGTTTAAAGTTATTAGAAGATCACAGAGATTTTGTTTAGTTTGGCTTTTAGTCCTGATGGTCAGTTGCCTTCTCCTGAAAGCATTGATGGGCATATGAGAGTGGGACACAACTTCAGGTAGTCTGAAGGGCAAACTTGAAGGTCCTAAAAGGGAAATTCAAGTGAAACATTTTCTTATGCCTAAGAAATTCATCTTTGCATGACAATTCGAGATCCAGGGGTCTGGTAACATCAAGAAGACATGGTGTTGGCTAGTGCAGAGTATCCTATTGTTTTGATATGGAATGATGGTATCCGAGAGGACACGAGGCAATGGTTTCTGAGAGGATATAGATATGTACTCTATGATGTTTAGAGATTTGATATGTGGAAGATTATTGAAATGAGAACAAGATTAGCTTTTACCTTCTAAAAAAAAGAAGATTAGCTTTTGGAATCAAGTGGAGTAATTTTTTTCATGTTATGTTGTTGACATTAAGATTATGAATGAATAGTTATTTGTTTTTGTTTATTGTGATTTTAA

>LNC_000917

ATAAAAGACGCTGGCCATTAGATCCGCTGCCCTCCTCCTCCTCCAGAATCACCACCTCCAGAGCCACCGTAGCCATTTTATCCACCGCCGCTTAAGTCACCGTATTCTCCGGCGCCACTGGAGCCATCGTATTCAGCACGGCCACCAAAACCACCGTATCCACCACCGCTATCCGGACCTCCAAATCCACCTCCGCTACGAATCAACACCGCTACTATGTACACCAATTGGAGCTCGGCTATTTGTGGTTGCTCTAATTCCGCAGCTTGACATATGTAGTAATCGTTTTCCCTAGTTGGTTGATTTTCTTTCTCAGAGGTTTTGCACTTCTTATCTTGCGGAGGACGTTGATTAAGTGAAAATTGATATCTGCAAGACCTCGACTCTAATTTCTGAGATTGACCGAGTATTAATTTTCCGACAGATGCTAATTTGGTGGCAACATGTGGTGGAGACGAAGGAGAATTTATATGGAAGATTGGTTAAGGTGATTCTAGTTTAAAGTTATTAGAAGATCACAGAGATTTTGTTTAGTTTGGCTTTTAGTCCTGATGGTCAGTTGCCTTCTCCTGAAAGCATTGATGGGCATATGAGAGTGGGACACAACTTCAGGTAGTCTGAAGGGCAAACTTGAAGGTCCTAAAAGGGAAATTCAAGTGAAACATTTTCTTATGCCTAAGAAATTCATCTTTGCATGACAATTCGAGATCCAGGGGTCTGGTAACATCAAGAAGACATGGTGTTGGCTAGTGCAGAGTATCCTATTGTTTTGATATGGAATGATGGTATCCGAGAGGACACGAGGCAATGGTTTCTGAGAGGATATAGATATGTACTCTATGATGTTTAGAGATTTGATATGTGGAAGATTATTGAAATGAGAACAAGATTAGCTTTTACCTTCTAAAAAAAAGAAGATTAGCTTTTGGAATCAAGTGGAGTAATTTTTTTCATGTTATGTTGTTGACATTAAGATTATGAATGAATAGTTATTTGTTTTTGTTTATTGTGATTTTAA

>LNC_000918

AAATGGCCTACCATTGAGTTTTCCGGTCCTTGCATGTAAGTTGGGTCATAAAGCAGCATGAATCTTGTGAAATTTAAGTAAAGAGAAATTAAATGATTCATCAACTAGGGATAGCAATCTTACTCCTGATTTGAGCAAGAATTCAAGGATCCCTTGCCTGAAACAGACAACATACCACTAGAGAAATGTTTGATTTTTTGGTAACACCTGCCATTGATCAATAACTTCATCTATCATTGTTGACATCCATGATCCATGAAATGAACCTTGTTTATGAAACTTTTCTGAAGGACAAATATCTTACTAGCTAAGTCACTTCTCATTTTGCCTACTCTTTCAAATAAACCAACGGAAAGGTGTAAAGCAAGAACACTTAAAACTTTCACTTATCATGGTTGAGATGCTCGTTGTGCTGGTCTATTAGCATAACACTGCAGATCACTTGGGGTTCTGTGTTGTATTTTCGGCCTTCATATCTGCTAAGTGGTGAATTAGCAGGGATCACTTAGGAGTTATATGATGGAGTTTGGACTTCCATAGCTGGTAAAGTTGCAGCTCATATAAGTAGGTACAGAAAACAACATGAACCTTGTGATTGCCAAAGTAAATATGGGGTTTTTTAATCTGAGATTGTACGCCAAACAGACAACATATCACTTCAGCAATCCTTGATTTTTGGGATCACTTAGAAGTTCTATGTTGGATTTTGGACTTTCAGAGCTCCGTAAAGTTGCAGATGACAAGTAGAGTATAACTCCAACGATGAAATATTAATTTGTCAATATAGTGAAGAGGTTTTTTTTAATGAAATCTCAACCACATTAATGTAAGAAATTTCCAAGAAAATAATTCAAAGCATTTAAACGTTTCTTGAACTCATCACACAGAAAATTCAAATGAAGGGTAAAAAAGGAAGAATATTTCAAAAGTAGTCTTGAAAATTGAACAATTAAGTTAATTTGAAATATGAAAAATGTCTCAACAATAATTTATGGATGGGAAAGAGTTGGAAACTACTACCCCCTCCATTTTAAAATAATTGAATTGTTGAGACATTTAATATTTCAAATTAATTTAATTGTTCAATCTTGGAGATTACTTTTAAAATATTTTTCCATTTTTACCCTTTATTTGGATTTTCTATGTGATGAGTTTCAAAAAACTTTAATTGCTTTGAATTATTTTCTTGAAAATTTCTTACATTAATGTGGCTAAAATTTTCATAATAGAAACGCTTCACTATATTGCCAAATTAATACTCCATCGCTGGAGTATATTGTACATTCCAACCTGCCTCCTTGGGACTTAGCTCTGAAGATGAATTCCCGTTAGTGATTCCATGCAGTAGCACTTGAACAAGGCAAGGATGGGAATTTATGTGAAATCTTGTTACCAATGAAGTACAGAAAAAACAAAGTTGGTGGTCCTCTTGGATGGGATGATCTGGACTGTCTTTCTAATTATTTCCTGCGTTTCAATTTGTTTGTCTTATTTTTTTTTTAACTTTACATTTTAATAAGAATGTATCTTTCCTTTTCTA

>LNC_000919

CGCATTCCGATGGAAGAATTAAATACTCAAAAATTCTATTCTCTGCAACTAACATTCTCTGCTTAGAGAAATGGCCTACCATTGAGTTTTCCGGTCCTTGCATGTAAGTTGGGTCATAAAGCAGCATGAATCTTGTGAAATTTAAGTAAAGAGAAATTAAATGATTCATCAACTAGGGATAGCAATCTTACTCCTGATTTGAGCAAGAATTCAAGGATCCCTTGCCTGAAACAGACAACATACCACTAGAGAAATGTTTGATTTTTTGGTAACACCTGCCATTGATCAATAACTTCATCTATCATTGTTGACATCCATGATCCATGAAATGAACCTTGTTTATGAAACTTTTCTGAAGGACAAATATCTTACTAGCTAAGTCACTTCTCATTTTGCCTACTCTTTCAAATAAACCAACGGAAAGGTGTAAAGCAAGAACACTTAAAACTTTCACTTATCATGGTTGAGATGCTCGTTGTGCTGGTCTATTAGCATAACACTGCAGATCACTTGGGGTTCTGTGTTGTATTTTCGGCCTTCATATCTGCTAAGTGGTGAATTAGCAGGGATCACTTAGGAGTTATATGATGGAGTTTGGACTTCCATAGCTGGTAAAGTTGCAGCTCATATAAGTAGGTACAGAAAACAACATGAACCTTGTGATTGCCAAAGTAAATATGGGGTTTTTTAATCTGAGATTGTACGCCAAACAGACAACATATCACTTCAGCAATCCTTGATTTTTGGTAGCTTTTTTCTGCCATTGACTGATATATTCGGCTGTCATTTTTTGACCTCCATGATACAAAAAAAATGAACCTTCTTTATTAAGCTTTGCTCAAGGACAAATATATTTGCTAACAGTCTCTTTGTTTGCCTACTTTTTTCGGATCGAAAACAAATGTAAAACTGCAATAACATACATTTTCTGGTTATCAATTCTTTTGTGTCTTAGGGATCACTTAGAAGTTCTATGTTGGATTTTGGACTTTCAGAGCTCCGTAAAGTTGCAGATGACAAGTAGAGTATAACTCCAACGATGAAATATTAATTTGTCAATATAGTGAAGAGGTTTTTTTTAATGAAATCTCAACCACATTAATGTAAGAAATTTCCAAGAAAATAATTCAAAGCATTTAAACGTTTCTTGAACTCATCACACAGAAAATTCAAATG

>LNC_000920

GCGCACATTCGCCCGCATTCCGATGGAAGAATTAAATACTCAAAAATTCTATTCTCTGCAACTAACATTCTCTGCTTAGAGAAATGGCCTACCATTGAGTTTTCCGGGATCACTTAGGAGTTATATGATGGAGTTTGGACTTCCATAGCTGGTAAAGTTGCAGCTCATATAAGTAGGTACAGAAAACAACATGAACCTTGTGATTGCCAAAGTAAATATGGGGTTTTTTAATCTGAGATTGTACGCCAAACAGACAACATATCACTTCAGCAATCCTTGATTTTTGGTAGCTTTTTTCTGCCATTGACTGATATATTCGGCTGTCATTTTTTGACCTCCATGATACAAAAAAAATGAACCTTCTTTATTAAGCTTTGCTCAAGGACAAATATATTTGCTAACAGTCTCTTTGTTTGCCTACTTTTTTCGGATCGAAAACAAATGTAAAACTGCAATAACATACATTTTCTGGTTATCAATTCTTTTGTGTCTTAGGGATCACTTAGAAGTTCTATGTTGGATTTTGGACTTTCAGAGCTCCGTAAAGTTGCAGATGACAAGTAGAGTATAACTCCAACGATGAAATATTAATTTGTCAATATAGTGAAGAGGTTTTTTTTAATGAAATCTCAACCACATTAATGTAAGAAATTTCCAAGAAAATAATTCAAAGCATTTAAACGTTTCTTGAACTCATCACACAGAAAATTCAAATG

>LNC_000921

GCGCACATTCGCCCGCATTCCGATGGAAGAATTAAATACTCAAAAATTCTATTCTCTGCAACTAACATTCTCTGCTTAGAGAAATGGCCTACCATTGAGTTTTCCGGGATCACTTAGGAGTTATATGATGGAGTTTGGACTTCCATAGCTGGTAAAGTTGCAGCTCATATAAGTAGGTACAGAAAACAACATGAACCTTGTGATTGCCAAAGTAAATATGGGGTTTTTTAATCTGAGATTGTACGCCAAACAGACAACATATCACTTCAGCAATCCTTGATTTTTGGTAGCTTTTTTCTGCCATTGACTGATATATTCGGCTGTCATTTTTTGACCTCCATGATACAAAAAAAATGAACCTTCTTTATTAAGCTTTGCTCAAGGACAAATATATTTGCTAACAGTCTCTTTGTTTGCCTACTTTTTTCGGATCGAAAACAAATGTAAAACTGCAATAACATACATTTTCTGGTTATCAATTCTTTTGTGTCTTAGGGATCACTTAGAAGTTCTATGTTGGATTTTGGACTTTCAGAGCTCCGTAAAGTTGCAGGTGAGCACAAATCAGCCATGTTTTATTTTTATTTTTGTCCTCTTAGATCCAGATGAGTTTACCTAGTGTTTTGAGAGTAAGAGGAATCTCATCTTACATGATGAGATACTAAAAATGAAATTTGGCTGAAAAGTTAGTTGACTACAATCACGAAGCATTCTGAATAGGAATTAAAAGAATCAACTCTAGGTTATTTACTGTTGACATGAAAGTTGCCTTTTTCTGTTATTACATCAAAAGTTGTTTTTAAAATCCAATTTTACTGAATACATAAATGCCTGGTGAGCTTTCTGCAGTTTCCAAAACATCAACCTCAATGTGATTGTTTTTCGAAACTGCTACCTCCATTTCAAAATAATTGAATTGTTGGGTATTTCACATCCTTTGATTTTAGAAGATTAGGACATAAATTAGACATACTTTTCATTTTTACCTTTATTAGTTATTATCAAATTAATCTTTAGAATAACTAAACATTAATCATAATAACTAATTCCAATACTAACTAAATTTTTATACAAAATTCAATATGCGACTCGTTAAATGACTAATTTTTTTTTTTTGTTGAAACCAAAATTTTGGAGGTAAG

>LNC_000922

TGCGCACATTCGCCCGCATTCCGATGGAAGAATTAAATACTCAAAAATTCTATTCTCTGCAACTAACATTCTCTGCTTAGAGAAATGGCCTACCATTGAGTTTTCCGGTCCTTGCATGTAAGTTGGGTCATAAAGCAGCATGAATCTTGTGAAATTTAAGTAAAGAGAAATTAAATGATTCATCAACTAGGGATAGCAATCTTACTCCTGATTTGAGCAAGAATTCAAGGATCCCTTGCCTGAAACAGACAACATACCACTAGAGAAATGTTTGATTTTTTGGTAACACCTGCCATTGATCAATAACTTCATCTATCATTGTTGACATCCATGATCCATGAAATGAACCTTGTTTATGAAACTTTTCTGAAGGACAAATATCTTACTAGCTAAGTCACTTCTCATTTTGCCTACTCTTTCAAATAAACCAACGGAAAGGTGTAAAGCAAGAACACTTAAAACTTTCACTTATCATGGTTGAGATGCTCGTTGTGCTGGTCTATTAGCATAACACTGCAGATCACTTGGGGTTCTGTGTTGTATTTTCGGCCTTCATATCTGCTAAGTGGTGAATTAGCAGGGATCACTTAGGAGTTATATGATGGAGTTTGGACTTCCATAGCTGGTAAAGTTGCAGCTCATATAAGTAGGTACAGAAAACAACATGAACCTTGTGATTGCCAAAGTAAATATGGGGTTTTTTAATCTGAGATTGTACGCCAAACAGACAACATATCACTTCAGCAATCCTTGATTTTTGGTAGCTTTTTTCTGCCATTGACTGATATATTCGGCTGTCATTTTTTGACCTCCATGATACAAAAAAAATGAACCTTCTTTATTAAGCTTTGCTCAAGGACAAATATATTTGCTAACAGTCTCTTTGTTTGCCTACTTTTTTCGGATCGAAAACAAATGTAAAACTGCAATAACATACATTTTCTGGTTATCAATTCTTTTGTGTCTTAGGGATCACTTAGAAGTTCTATGTTGGATTTTGGACTTTCAGAGCTCCGTAAAGTTGCAGGTGAGCACAAATCAGCCATGTTTTATTTTTATTTTTGTCCTCTTAGATCCAGATGAGTTTACCTAGTGTTTTGAGAGTAAGAGGAATCTCATCTTACATGATGAGATACTAAAAATGAAATTTGGCTGAAAAGTTAGTTGACTACAATCACGAAGCATTCTGAATAGGAATTAAAAGAATCAACTCTAGGTTATTTACTGTTGACATGAAAGTTGCCTTTTTCTGTTATTACATCAAAAGTTGTTTTTAAAATCCAATTTTACTGAATACATAAATGCCTGGTGAGCTTTCTGCAGTTTCCAAAACATCAACCTCAATGTGATTGTTTTTCGAAACTGCTACCTCCATTTCAAAATAATTGAATTGTTGGGTATTTCACATCCTTTGATTTTAGAAGATTAGGACATAAATTAGACATACTTTTCATTTTTACCTTTATTAGTTATTATCAAATTAATCTTTAGAATAACTAAACATTAATCATAATAACTAATTCCAATACTAACTAAATTTTTATACAAAATTCAATATGCGACTCGTTAAATGACTAATTTTTTTTTTTTGTTGAAACCAAAATTTTGGAGGTAAG

>LNC_000923

TAAATACTCAAAAATTCTATTCTCTGCAACTAACATTCTCTGCTTAGAGAAATGGCCTACCATTGAGTTTTCCGGTCCTTGCATGTAAGTTGGGTCATAAAGCAGCATGAATCTTGTGAAATTTAAGTAAAGAGAAATTAAATGATTCATCAACTAGGGATAGCAATCTTACTCCTGATTTGAGCAAGAATTCAAGGATCCCTTGCCTGAAACAGACAACATACCACTAGAGAAATGTTTGATTTTTTGGTAACACCTGCCATTGATCAATAACTTCATCTATCATTGTTGACATCCATGATCCATGAAATGAACCTTGTTTATGAAACTTTTCTGAAGGACAAATATCTTACTAGCTAAGTCACTTCTCATTTTGCCTACTCTTTCAAATAAACCAACGGAAAGGTGTAAAGCAAGAACACTTAAAACTTTCACTTATCATGGTTGAGATGCTCGTTGTGCTGGTCTATTAGCATAACACTGCAGATCACTTGGGGTTCTGTGTTGTATTTTCGGCCTTCATATCTGCTAAGTGGTGAATTAGCAGGTGAGCACAAAATAGCATGCTTTTCTGAATCATGATTTACTATTCTTATCTTTCAGATGCAGATGGGTTTAGCATAGGTGTTCTCTTTCAGATTAGGAAGAAGTCAGTTTACCTGTTGTGATAATAAAAATGTACATTAGCTGAAAGTCTTAGTTGACTTACAATTAAAGTTCATACCTAACAGAATATATAGAAAGCTCTGATAATTGAGGACTATGTATATAACAAGAAGAACTAGAAATTGTCTGCATTGGCAGTTTGGATTAAAAATATATGTTGTAGTTTGCCTTTAAAAGTTGCCTGATTTTTGTTATTGCATAAACTAATGTGTCAAAGTTGACTTTGGGTCAGATCTCTGACACTATTAAGTAATTTCTCTTTATCCCTTTAGAACTACATGCCATATAGGTATGTTCTTAGTTAAAACATACATACTTTGTTTTGCTTTTACCTTTAATGCACTAAATAGAGCATGAGTCAGAAATTCTTGCAGATAACACTTATGTTGTATCTGCACTTGTTTCACAAAGAGTAGTCAGATTTAAAGGTTAAATCTTCTTTTAAAAGGAACAAAGAATGATTGGTTGATGGTGGCATTCAATGAAACCAAGATTGTTTATTTTGACTTGTCAGATTCTAAGGTCAATTTTATTTAAATGAATTAGGAATAATTTAGATGTATATTCATTAAAAAAAAAAGAATGGGGCATTCCGAGAATCGAACTCGGGACCTCTCGCACCCAAAGCGAGAATCATACCACTAGACCAAATGCCCAGTTGTTTATGCATGTCATTTTGACTCATGATCAATGTGTTTTTCCGAATATCAGCAGTAACTCAATCATAATCATCACTGCTAAGTTGCAAAAATAGGAGCATATGTCATCTTTTACTCTTAACATTCCTCGTTTTACATATGCAAGTTGTTGCAATTAGTTGTCTCTGAATGGAAAATACATCCTTACATAAGTTAGATGTCAAACAGTCTGTTGACTTTTGTTGATTTGCGTTTTTATAGCATAAAAGTAAAAAAAATATAAGGAGAAAAAGCTGAGAGTATGTCCTAGCCTTGGTGGACAGACTTACTTGGTACTTGTTGCTGGTGGGAGGTGGCCGGTATCCCATGGAATTTGTCGAGGTGTGCGAAAGCTGGCCCGGACAATATGGTCATCAAAAAAAAACAACTGAGTAATAAATAGGAAAAAGGAGGACGAAATTCCACAGATTAATTCTTGTTTTTATTCTATTTTTCTATTGTCATAGTTGGTTTTAGATATGATCTTTGATACTAGTAAATCTAATTTCAGCCAACCCCTGTTGAACTGCTTAGTTCAAACACACAACATTTATGTTTCTCCTACCTTTAGAAGTTTAATTCTAAATAACGC

>LNC_000924

TGAGTCCATAGATGGCTTTCTTTAGACGACAGATATGGGAGGGGAACTGCACGTCTGCAAAACCCGGAGGTTGGACCATGTACACTTCTTCTTCTAAACGACCCTGCAAGAACGCGTTGTTGACATCAAGTTGGCGAAGGGGCCAATTCTGATGGACAGCAATTGAGAGCACCAAGCGCACAGTAGTTGGTTTGACAACTGGACTAAATGTGGCATGGTAGTCAACTCCAGGACGTTGTGTAAACCCCTTTGCAATAAGTCGAGCCTTGTAGCGTTCGATTGTCCCATCAGCTTTCCGTTTTAACCTGTATAACC

>LNC_000925

TGAATATTTGATTCAGTCTTTATATAATTAGGCTTTGCTCGTGAGGCTCACTAACTTAACACTCTTTTCAAAAGATGATTGAGTCTATGTGTATTTCTTACTTGTATTTGAATTTATTTTAGTGGTATGATAATAGTTTATCGTCTATTATAAAAAAAAAATTGAGAAAAACTTTAAAATTTTAAGAAGAACCTAATTTTTTTATACGAAATTGGGCTGCCAGGGCCCTTTACTAAAATGAGAAAAAGGGACCACGCCTCAAAATGACCCAGCCCAGCTAATAAATCTATCTGTTAAGACAACAAACTAAATGACAAAAAGAACAAAAGAAATAAAGATTCCTAAATCCCCAATTCCAGAATGCAGGACTCAGCTATTCTCTGCAATGGTGAGTGCTGAAAACTGGTCTACCGGTGGGTGCATTCGATTCGTCGATGGCTGCAACAAGTTGAGGTCGGGTTGTTTCCACATGAGACACTCCAAATTCTCACGAATGCCATGGAAACCTAGCCCATTGAATACACTTCACCCCAACACGAACAACTCATGTCTGACTGATTGCAGGGGCAATATCCAAGTCTCTCTTCCTTGTCACTTCTCCCACTCCCTCTGTAACTGTTACATCTCTTGTGGAATTACTTTCAATTGACAATATTTTCTCACTCCCAACTATTTGTAATGGCTCTTGCAAAGTAGCTAGTTCATCAAGAATATATTCAGGTGACTCTGTATTGACATATCTGTTTGAGCTTATTGCATGTCCTGACTGTTGTGCTAACATTAGGTCTCATTCTTTTTTCTTTAGCCTTCCTTCATCTTGCTCAACTGCTAGCAGATGCAGATTGTCATTCCTGAGGATTATTTCCAGTCCAGTAGTAGTTGCATTCAGTTTTGTTGATTTTATATGTTGTATTGTATTCCCCTCCGTTGTATCTTCCTAAATGTCAT

>LNC_000926

AGAATATTAAATAATCAATATTAGGATTACGGACCTGTATAGTCATAAAAAGTAGAAGTAATTTTTCGGAAAAGGGTCAAAAATATCCCTGAACAATTGGAAATGGATCAAATGTACCCTCCGTTTGATTTTGGGGTCAAAAATACCCTTACCGTTAGTGTATTGGCTCACTTTTACCCCTCAAAACTAACAGATGCCATGTCACCTTAAAAATCACATAAAACAATATTTTTTTTGCTGACTTGGAAATACACATGGACAAAAAGACCTAACGAGTAAATTGTAGTGAAATAGTCATGGATCTGCAAAAGAAATCACTGAAAGCAAAGATGCAAATCCAATCGAAAGTAATCTACAACATCGATGTTGCCATTGTTGTTCTCCTACCCGAAGATGATGTAGATACCATTCATAAAACTCGGCGAAACTTATTAAAAAACCGTTAAGCGTTGAAATTTTGACTCAATCGGTTGCCTTCTCATCATCTTGGCAATTTTTAATTTTCTTTTGAAATTGGAGAAGATTAATAGCATAGGAAAATATATTGTGAAGAATTGAAACTACAGGAAATTATACAATAAATAAAATTGTGCAAGGATTTTGACTTATGGAGTAATTATCCCCAGCCAATAATATTGAAACCTCTGTTGTTTTATTTTCCCTCATCAAAATTCTGAGGTTTTTTTTCCTTTATCTGCTGCCACAATGAGAATTGAAAACTTGAAATTCAAAAGAATAATGATAAAATTATTGTGTATAAGGGAGATAATTAATTTATCTTAGAGGAATAATAGAATTTTTGAAAGTATAGAAGAAGGAGAAGATAAGAAAGAAATTCAAATGAAAAAAAATGAAGAAAAATTTCTTAC

>LNC_000927

CAAAGACAAAGCAACAAAATAAATAAAATAACAAAACAAAAAACCAAAATAGGCATCTGCCTAAAATGGAAAAGGAAAAGGGAAAGTGATGTTTGGAACGGAACTTCTCGCATCTGCCACTTCAGATCGGCGGCTACTTTCCGGTGGGACTCATCCTCCATAAATGACCTCTCCACTTCGTGAAGGGTAGTGAGGAGACCAGATGCTCGTCTCGTCGTTCTCCAAATGTAATACCTCCAGCTAAGTGTATTTCCTTTCGTATAAAGAAATCTCACTTACTATTTGAAGTTTACATGTACTCCTAGGCTCTAGTAGTTAGGTTCTTGTCTTGTCTCGAGATTTCGGGTTTAATCGATGAAGGATGAAGACTGATGCTCGAGGTAATTAGTCATTGCTCTTCTTTGCTTATTCCCCAAATTACTAGATCTTACATTCATTGAAATCTGTTAGAAGATTGATTCTGATAGTCTTTAGATGATTAAAATTGTAAAGTTGAGATGAATTACAAATCTCCACTGTGTAAGGAAAATAATTTCGTAACATAGTAATCAACTAGCATCAAATATTTTCTGTATTATTAATCTTCGATAGAATGAACCTCGGAGAGGCTAATACCACACATCAAAAGATAAATTCTAGGTTCTAGATTATCTGCTAATTTACTACTAGATTACTTCTTATACTTACCTGATAACAAGTCACTATATGGCATGAATCTGCTTCTAACTCATCCCTTGACAGAATGAACCTCAAAGAGGTTAATACCACTCATCAAAAGGATTTATCGAAAACAAAGTCAATGACCATCTAGCAACTTGTTGCCCTTAAATTTGATACTACAAGATAATCACAGAGTTGCAACGAATACATAGAAGAGACGTCCCCACTACTTTGCCTAAGTTTATTACCATTGTACACCCTAAAATCTCACCAGGTGGTATAGTAATTAGGCTTTCTGAAATCACTTCAGGCAAAATTGTAGATATCAAACTCTTGTTCTGTTCCAATAGTTGCTGACTATGTGACTGAAAACCTGTGAATATACTAATCAGTTGTCTAGTTGTGTCATCCTCAAAAAAGGCATGTTTATTTTATCCATGCTGAGTATTTTCTTTGCAATTGCTGGAATATCCGGAAAATTTTCCACCATGTAAGTACCTGCAAAATTAACAGTCATGTTAGCAAAGAAATCAGCCAGAGTGCTGTCTTCCCTTAGAGTGTGTTTAACATGAATAGGGACACCTTCCCTCATTCTTTTAACAGCCTCAACCTCCATAACAACACTCCAAGGGACCTCTCATCTCTCCTCCAAAAGTTGAATCATAGTGTGAGAATTTGTTTCCACTATAAGGGGTAATAGATTTCTGTCAATACAGAACATAATGCCTTCATTTATTGCAACAGCTTCTGCTACAAGGCTAGTGGAATTTGGTATTCTTCTCCCTTTCACCACCACTATGTCTCCAAAGCAATTTTTCACATAAAATGCAGCCGAACTAGGGCCAGGATTACCCTTAGAAGCACCATCAGTATTGCATTTATACCATCCATCAGGAGGATGCATCCACCTCACAATTTTGTGTCTAAACCTAGGCTGCCATTTTTCCAGTACATTCACAATAGC

>LNC_000928

TCTCTACAATATTATTATATATTGACCTATATTAATTTATTTCCTTACCTTAACTGAATTTTGTCAGATGTTGGTGTTTCTGGTTATGGTAAATTGCCACAATTTTGATATTTTATTGAAGTGAAAGTTTAAAAGATCAAAGCTTTCATTTATGTTAAACTTTAACCATATTGCTCCTAATTGTAATCTTGAAAACTCTATTGGCACGTCAAGATGATATTTTGAAACAGTTCAGCAGTTTATGAGCTCATCCAGATCAAGGTTAGCCATAGTATACTTTAAACTATAATAATTTCATTATAGCTTGTTTAATTAAATAGAAATAGTTTGTAGCATTTAACACCTAATATATAACTATGTTTCATTTTCATGATACAGTGATCAATGAACCTTATCAAGTTATCCCTTCAGGTTGTATAAGATGGAAATCAGGACTAACTATTAAAGAAGTGCTTTTGCAATGATATTTTGAGAAGCAACCTCAATTTTATTACTTCGTTTCTTTTTAATGTTTAGTATATCTAAATTACATTTTTCAAGTTGGACCAAATTTTGTTGATGTTAATTTTTCTTTAAGGCGTATGTAATGAGATATCATCTCATGAATGTTATTTTGGTCTTTCCTAGTTAATTTATGACGGTGT

>LNC_000929

TCCGGCGAAATCGCCACCTCCTCCATCTTCGTCCAGCAGGCTCCTGAGAAACCCCGTGCCGCCTGCTCGCCGCCTCTTCTTGAAACCCCGAGCCCCTCTTCTTCCCCTCCATTCTCTCTTCTCCAGCGAAGATGGAAGCAAGCGAGGACCAAGTGAGCTCCTCATCTCCTTTGCTCCGGCGAGAACAGCCCCGAGCCACCAGCGAGACCCTTCTCCTCTCTTTTGCTTCGGCAAGAGCAGCCAACAGTCGCTGGCAAATTGGACCAACAACCATCATCAACCGGAGCGCTGCTCTGACCAGCAAGCCCTAGCGTCGTTCCTCACATTTTACGGTGAGGACGGCAAAGACGAGCACCAATAGAGTCTGGTCAACACACGGTTTGAGATTTCGAGACACAATAGAGTCTGGTCAACACACAGTTTGAGATTTCGAGACGGATCTTAACAGATTCGTGGATGATATTTTGAAACAGTTCAGCAGTTTATGAGCTCATCCAGATCAAGTGATCAATGAACCTTATCAAGTTATCCCTTCAGGTTGTATAAGATGGAAATCAGGACTAACTATTAAAGAAGTGCTTTTGCAATGATATTTTGAGAAGCAACCTCAATTTTATTACTTCGTTTCTTTTTAATGTTTAGTATATCTAAATTACATTTTTCAAGTTGGACCAAATTTTGTTGATGTTAATTTTTCTTTAAGGCGTATGTAATGAGATATCATCTCATGAATGTTATTTTGGTCTTTCCTAGTTAATTTATGACGGTGT

>LNC_000930

TTTCCGGCGAAATCGCCACCTCCTCCATCTTCGTCCAGCAGGCTCCTGAGAAACCCCGTGCCGCCTGCTCGCCGCCTCTTCTTGAAACCCCGAGCCCCTCTTCTTCCCCTCCATTCTCTCTTCTCCAGCGAAGATGGAAGCAAGCGAGGACCAAGTGAGCTCCTCATCTCCTTTGCTCCGGCGAGAACAGCCCCGAGCCACCAGCGAGACCCTTCTCCTCTCTTTTGCTTCGGCAAGAGCAGCCAACAGTCGCTGGCAAATTGGACCAACAACCATCATCAACCGGAGCGCTGCTCTGACCAGCAAGCCCTAGCGTCGTTCCTCACATTTTACGGTGAGGACGGCAAAGACGAGCACCAATAGAGTCTGGTCAACACACGGTTTGAGATTTCGAGACACAATAGAGTCTGGTCAACACACAGTTTGAGATTTCGAGACGGATCTTAACAGATTCGTGGTGATCAATGAACCTTATCAAGTTATCCCTTCAGGTTGTATAAGATGGAAATCAGGACTAACTATTAAAGAAGTGCTTTTGCAATGATATTTTGAGAAGCAACCTCAATTTTATTACTTCGTTTCTTTTTAATGTTTAGTATATCTAAATTACATTTTTCAAGTTGGACCAAATTTTGTTGATGTTAATTTTTCTTTAAGGCGTATGTAATGAGATATCATCTCATGAATGTTATTTTGGTCTTTCCTAGTTAATTTATGACGGTGT

>LNC_000931

GCCTCTACGATCTTGGTAGAAAATCAAAGAGTTTCCGCTGCCGGTGGGTGGCTATTACCCATATATGCCGCCCGTCTGGCCAATGATTATGTTAACAAAAGTAGGACAAATGATACATGCATGAACTCCATGTTCAAGGGAAGAAACTCTGCCAGACAGGTCACCATGAATCAATTTTTGGTGACTTTCTAGGGTTGTTTTGTGTCAACCCCATATCCGTTGGTATTTGTGTAGACCGTTCCATCTTCCCGGTGACTACTTTTTCATATTTAATTTTTGTAGCACCGTGTCATCATTCCGGTGACTACTTTCTCATATATAATTTGTGTAGCACCGTGCCATCCTTCGGGTGACTACCTTTTTTTTGTCATATCTGATTTGCATAGCACCGTGCATCTCTCCGGACTACTTTTCATATTTAATTTGTGTAGTACCGTGGATTTTTCCCGAACTATTTTCTCATATCTGAGTTGCATAGCATCATGTGTCTTTTCAGTGACTCCTCAAATTTGATTTGCATAGTTCCATTCG

>LNC_000932

AAATGTTTTTACTTTTCTTGAAAAGAAAATATAATATTATTTTATATTTGGTTTATTCTCTCCTTTTAGGACTCCTTTTCTAGTAGGAAAGGTTTTAGGACTCTATAAATATAGGTTTGTTTCTTCTAACACAATAACAATAACATCCGCAATGTAGTCGTTTAAGAGTCTTGTTTATGGGGAGATTTATTCTCACTAAAGTTTCAATGTTTTTTCTTTATATTAGTTTTGATATAATAATATTTTGATTTTTAGTATAAGGTTTTGTTATCTAATTTATCAACTATATGATTTGTAAATTGTAAGCTTCCGCATGACGCCGTAATCACCCTTCAGAACCCAACAGTTTTCATGTCTCACTTGTCCACCATGGGTCGATCGTTTCGTAATTGAATAACAAGCAAAATATTCCTCTGTCCTTAATTACTTATTTAATTTTCTTTTTTACATATTTATTTTGACAAATCAAGAAAAAATAATTATTTTTT

>LNC_000933

TATGAATTGTGTAGTATAGGTTGTTGGTTCGCTGTTAGAATGAAGTTTCGGTGGTTCGGTTGGGATTGAAAGGAGTTGTTTGTAGCTGCTAGTTTAGCTTAGTTTAGTGTTACTTGCGAGTACCTGTTGCTTTTGGTACTCACCCTTGCTTCTACACAGTTGTGTAGGTTGACAGCTCTATCAGATCCGGCTTATTACTCATCTTCAGATTAGAGCTTCCAGACTTACTTGAGAGGTAGCGGTTCATTCCAGACGTGCCCTCAAGTTATCTATATTTACTGTTCTGTTCTATTCTAGAACTTTACTATGAGACTTGTATATTTTATTCAGATTCCGTATTAGAGGTTTGTACATGTGACAACCAATTATGGGGTTATGTTGAGTTTATTTAAAGACTTCCGCT

>LNC_000934

ATTATATTCTAATGGCATTTTGTTTTCCAATCCTTTCTTCTACACTCTTCTACGCTCTTCTTTGCTTACAATTAACAATGCCGATGCTCTACTCAGCAAAGTACAAACTTTGAGTTCATTCTTAACTGGGTATTTTAATTGCAGTGTATTCAAGAACCGAATCCTATATGACTATATCTATAGTTTTTATGGGATTATAATAACGATTAGGGAGGCATGGCTCAAGCTCTCTCTGTCAAACAACAATGGGGAACAAAAGCAACAAATTTGTGTTAAGCTCCGGGTCACTACCATTGTTGTCGATAGATTGACGTTGGGCATCCATCTCTGAACCTCATTTGATACCAAAAATCAAATTATTTTTCCTCCTCAAGGTTCCAGTCTCAGGAGCTTATCAACGAAAAAAAAATAAAAATCTTGCACCCGCCACTTTCATAAACCCTGAAGGTACATTGGTTGAAGCTTTTTTATTACAAGCTTCCCAGTACAAGCAAATTTGCTATTTTTGACATTATGTAGGCATGGCTGACTGCTGATGAGTCAAATGGTCGTGACTCGTGAGTGATCCTAGCATTTTTTGGCTCATCTTCATGAGGTATTTCAAAACGTAACGTCTCCCTGTTATGTATTTCTGGGATAGCAAAGGGCATTGAGTGAAGGTTGTACAATTACAAACACTTGAGGCATCAATGGATTTATGAAACATAATTTTATTGCTCTACATGCAACTCCATTTAAGGATACCAACTTCTATTCTTTTGAAAAATACAGAGAGCATCTACTCAAATTTACTACTAATGATTCAAGGCATAAATAGATTTTTTAAAATGAATTTTGTCATATTCTATGATCTCTATGTAAGGTTATCCACTTCTATTCTTATGATAAATATTGAAAAGTATTTGGAAGATAGTTTGTCTGCATTGTAAAGTTGTTTGTGGAATGGGAGAAATTAGTTGTATTATTGCACTTTGAAGTTCATGTGTATAACTTATGCAATGATCTATTAGCTTTTACAATATATACTCTTTTTCGAGTTACTACGCAAATTATATACCACTTGAAAATGCTAAATGTTGGG

>LNC_000935

CAACATTCTAGTTTGGGGTATATTTCTATTATATAGAAGAGTGAAGACGACCAGACGACCAAGGGTAGTTTGGAAATTTTATTATATTCTAAGGGCATTTTGTTTTATAATCCTTTCTTCTACACTCTTCTACGCTCCTCTTTGCTTACCAATTTTTATTATATTCTAATGGCATTTTGTTTTCCAATCCTTTCTTCTACACTCTTCTACGCTCTTCTTTGCTTACAATTAACAATGCCGATGCTCTACTCAGCAAAGTACAAACTTTGAGTTCATTCTTAACTGGGTATTTTAATTGCAGGCATGGCTGACTGCTGATGAGTCAAATGGTCGTGACTCGTGAGTGATCCTAGCATTTTTTGGCTCATCTTCATGAGGTATTTCAAAACGTAACGTCTCCCTGTTATGTATTTCTGGGATAGCAAAGGTTGGCTACTAAGCTAGGGCATTGAGTGAAGGTTGTACAATTACAAACACTTGAGGCATCAATGGATTTATGAAACATAATTTTATTGCTCTACATGCAACTCCATTTAAGGATACCAACTTCTATTCTTTTGAAAAATACAGAGAGCATCTACTCAAATTTACTACTAATGATTCAAGGCATAAATAGATTTTTTAAAATGAATTTTGTCATATTCTATGATCTCTATGTAAGGTTATCCACTTCTATTCTTATGATAAATATTGAAAAGTATTTGGAAGATAGTTTGTCTGCATTGTAAAGTTGTTTGTGGAATGGGAGAAATTAGTTGTATTATTGCACTTTGAAGTTCATGTGTATAACTTATGCAATGATCTATTAGCTTTTACAATATATACTCTTTTTCGAGTTACTACGCAAATTATATACCACTTGAAAATGCTAAATGTTGGG

>LNC_000936

CAACATTCTAGTTTGGGGTATATTTCTATTATATAGAAGAGTGAAGACGACCAGACGACCAAGGGTAGTTTGGAAATTTTATTATATTCTAAGGGCATTTTGTTTTATAATCCTTTCTTCTACACTCTTCTACGCTCCTCTTTGCTTACCAATTTTTATTATATTCTAATGGCATTTTGTTTTCCAATCCTTTCTTCTACACTCTTCTACGCTCTTCTTTGCTTACAATTAACAATGCCGATGCTCTACTCAGCAAAGTACAAACTTTGAGTTCATTCTTAACTGGGTATTTTAATTGCAGTGTATTCAAGAACCGAATCCTATATGACTATATCTATAGTTTTTATGGGATTATAATAACGATTAGGGAGGCATGGCTCAAGCTCTCTCTGTCAAACAACAATGGGGAACAAAAGCAACAAATTTGTGTTAAGCTCCGGGTCACTACCATTGTTGTCGATAGATTGACGTTGGGCATCCATCTCTGAACCTCATTTGATACCAAAAATCAAATTATTTTTCCTCCTCAAGGTTCCAGTCTCAGGAGCTTATCAACGAAAAAAAAATAAAAATCTTGCACCCGCCACTTTCATAAACCCTGAAGGCATGGCTGACTGCTGATGAGTCAAATGGTCGTGACTCGTGAGTGATCCTAGCATTTTTTGGCTCATCTTCATGAGGTATTTCAAAACGTAACGTCTCCCTGTTATGTATTTCTGGGATAGCAAAGGTTGGCTACTAAGCTAGGGCATTGAGTGAAGGTTGTACAATTACAAACACTTGAGGC

>LNC_000937

GTCCACATAAACCAGATATAATTAAATGTTTGTTTACACGCGCTGCCACATAATGACTGGACCCCAGAACCCACGAATTTCCTTTAATCTTTTCATTTTCCTCTTTCTTTCTCTAAACCCTAACCTTTTTTCGATCTGACCAAATTCACCCCATTCTCTTCGATCTGAGATTTCGTTCCAATCGAAGTTTGCTGGGAAGGTTGGGGAAGGTTCGTTTAGCTTTTTTCGTTTGTTGGGTCGAAGTTTTCTCTAAGTAAAGGTTAGTTCTTTTAGTTCTTTACTAGCGATTTTGAACGATATTGATTATTGTGTTTGATTAGGGTTATGAAATTGACAAAAAAATGAAGAGCAATAACTGGGTTTTGGTATAATAGCATTCCATTGAAGAAATTTTTTGAACCATAACTATGAAATCAGAGAAATTAC

>LNC_000938

ACTCTTTAAATTGAAGGTCTGAATGAACCCTGGTTAGATTGTTGTCCATTTTGGATATGCAATTGTGGAATCGAGGATCAAATAACATAATGAGTTTGATGAATAGGGAATCTTCATGATTCCAATATCATGCTTTTGAAAATAATTAGCTCCCACTGCTTTGCATTTCCAATTATTTTTGGAAAAATCAAATTGACACAAATTAACTGAAAATTTGATATGTATTTGGTTTGGTGGAAAAAAGAACCAAACTGACATGTATGTATGTATATATTAAAACATTTGTATATTTTCTTTACCTATGTTGTTCAATTCCTTCAAAATACTTGATTTCCCTTTTCTTTCATGTTCAC

>LNC_000939

GTTTGTACATGTTTGTGAAGTAATAGTGTATGCCTCCTCAGTTATTTTCTCCCAATCAACTGAAAACATTCTGTTGCTCATCTTGACATAAAATAATTCTACTCCAGAAGGGTCAGAAACAACACATTCACGATTCTTAAAATGCAGAGAATAATTATTTTCAAGCATTTGTCCAACACTCAACAAATTTTGACTCATATCAGGTGTGTACAAAACATCAGGAATAGTTTTGATACCTGATATTGTTGAGATAGACACTGTACCTCTGCCTTTGACTTCTACTGCCTCAC

>LNC_000940

TTTTGGGAAAGGGCAGCCTATTCATCTTTCCAAGTAAACAAGCTTGGCAATCTGTCCACCGATTCTGCAATCTTGGTAGATCATTCACAAGTTCCTTTCTTTGCAAGAACATCATACCTTGATGATGAAAATGACCCATTCTTTTATGCCAAACTTCTGTAGAGTTGTCTGTCATAAGAAAAGCTGTCTGCTCTTCTTCCAAAGGATTGAGCGGGAAACACCTCCCCTCCATTTTGACCTTGAATATCTCATCGCCTGATGGATCTTCTATCACACCAGTTTTGTTTTTGAAGAACATATTGAATCCATTCTCCATTAATTGACCAACACTGAGAAGGTTTCGATCAAGG

>LNC_000941

AGTGCGGGTATGGGGCCCTTCGTCAGCAGTGGTTCGTTTAGCAAGCAGTCTTATTGCTTGAAAGCACACACAGCACACAGTAGGAGAGTTCGAGTGCGAAAGCAGTGTACTCATGCCCCTGGGATAAGGTATGCTTTTAGTGCGTTATCTAACTATAAGAGCAGGAGCAGCAAGCACTTACTTTCTTTAAGACGCTAACTTCCACAGCAGCTAAGAATTATCTCAGGTTTTCCGTTAGTGCGCTCCGGAAAGAAGGAGCAAAGGCGAAAAAGCT

>LNC_000942

GTCAAATTGAGTGGGAAATGGGTCTAACCCAAATTTTAGAGCAACCGTATCAAAATCCGAAATTTCCAAGTGAAGCCTTTTTCGAGGGTCTACTTTGAAGGGTCATATCTCCTAGCACACGAATTATTTGGGTGGCCAATAATATATCCATGGAAAGCCCTTTGAGTTAGCTATCTAACGCACTTCGTTTCACCTCATTCGGAGTTCGGACGAAGAAGTAA

>LNC_000943

AAAAAAAAGAGGAGATAAATCCAATTCTTTTAGATACTTTATCTAAGAAACTCTACCCATCTATGTTCTAGATGGGGTATATCTCGCCAAACTGGATAAAGCTATTATTCTAATCTAGACTATAAAAAAATATGCATCATTCGCATACCGGTGGCAGCTTCGAATAGGTCATATCAATTAATTAGAAATTAATTCTAGGGAAGAGAGACCCATACAAATTAATCATGTGCCAAGAACCAGATTTGAACTGGTGACACGAGGATTTTCAGTCCTCTGCTCTACCAACTGAGCTATCCCGGCTATTCCC

>LNC_000944

AAAAAATATTCATCTCTTTCTCCATAACCGCCACCCTTCATCTCTCTCATGCTCCGGCGAACAACCAGCAGCAACAACTGCTCCGACCAGCAACTCTGGCGCCTTCTTTCCCTCTCATCTCCCTCCGCCTCTCCTTCCTTTCTCCCTTTCTCTTCTCTTTTCTCTCCTCTCTTCGCAGGAACACCAGCAGCAGCAGCAGCAGCAACAGCTCCGGCGAGGCCAGCACTGTTTCTCTCCTCTTTTCTCCGACCAGCAGCTCCAGCAACAGCCAGCAACACTGGCAGCAGCAGCCTCCACGAGAACAGCAGCAACAAACAGCTTCTCCGCCTGGCGAAACAGCGAGCAACGCCAAGCAGTTGCCAGCAACACCGGACAGCAGAACCAGCAGACGCTGGCCGCCTCCACTCCTTTTTCGGTGGACAGCAGCAATGACGCTGCTCTGAGCAGCAAGCCAGCGCTGCTCTCTCCTTTTTCCGGCGAGATGGAACACAGGGGACAGTGTTTGCTCGGATCAATTGTTAAATTTTTGGCTTGTCGAAGACGGATCTTAACAGATCCGTCCGGGTTAGTTATCTTATTCGATTTTTTAAATTTTTTTTCTTAGTGTTATTATGAAATATAGGGCTATTTGTTATGTGTGTCTGAAGCATGAGCTTATTGTGATTGTGAATTCGTGGCCCACTGATTCTACATCTATGTTCTAATGCGAATTTCCTTTCTTTGGCTAAATTGGTGCTGAATCTACTCGTTAAAGTTTAATCGTTTTGTTGACTTAATTATGTCATAAACCGCTCTAAGTGCTTGATTTACGGAAATTATATGAATTTACTTGGTAATTATAGATTAATATTCACTTTGATTGATTTGTAATATTGGATTGATTTGTGTACTTAGCATGTTTAAATTTTAGTTATTGATTGAAAACTTAATTTTAAAGAATGTTTGTAGCTACTACCAATTTTACTGGTGTATCATGATTTGATTGGTCAAATTGGAATTTTTAATTTTAGGAAATCTGTAGTCATTAGTACACCTTTGATGTGGATATTTGAATTTGCAAACATGCCAAGATGTTATAATCGTGACTAATTGTTTCTTTCTTTAGTCAAGATGGTCTTCTCTTAGTGGAAAGATTCTTGAGCCGCGTGAATGTTGTTTTTCCTTTAATATAGTTGTCCTTTTGGGACTGATCTTACTTGTTTATTTTACTAATATTAATTGATTTGTCTTGCCTTAATTAAGTTGGGAATTTTTTTTTTCTTACTTGAATATATTTGGCAAAAGATTGCGACTAATATTAATTGACCCCTTTCCCAATATATTCCCGATCTTCTATATGTTGTTAATGTAGTGACTCCTTAATTGATTGTTTTAAAATTAAAATCAGCAAGCATGTGTATTTTAGGATTTCTTTTGTCTTATTTTGGGATTTTTTAGTTGATTTAGTTCTCCCATTGATTAAGGAAATCATATTTCTAATTGATTATTAATTTATTCCTTTCCTTTATTGTATGTCCTCTTACTATATAAAGAGACCCCTCTTTTATTTATACACACGGTTGAATATGGTCACACTTAGACAAACACCCCACACATTCTGACATTGTTCACACCTTACTTGAATACCAAAATTATTGCTCTACTCTTAAAAAAGAATTAAGCTTTCCGGTAAACGCATAAAAGCTCCTCTACTCGCTATTTTTTTGTTGTGTTCGTGTGGGTTATTGGACTAAGTGTGGGTCTTCATTGCTGTTAAATATATCTGCATAATTGGTTAGTTCCTCTTCGTTAGGTTAAATTTTGTTTTTGCATTATTACTTTGAATATTGTTGTATGTTGGAATGAATAAAAGGAAGCACCCGAATTAGTTTCCGTTTTTCCCTCTCTATGTGTGATAAAGTCTATGGTTATTATTTGTTTGTTGTGACTTCTTGTGTGTCGCTTATACGAAAGTAATTTGTTGCTATGAATATTTGAGGTTACGATTATTTCACCATTATATACATGTCTTACATTATCCCTTCTAGCAGGTTTTGAACCTAGAGTTCAAGGGGTTGAAGAGTCTCTTAGAAGAAATTTCCGGATGGAGCTATTATTTATTTTAGTGTTACATATTTTATTCATTTGTTCTTTAATTTGGCTTGTAATATTGTGTATAACTCCAACACTATCTCTATATGTGATATATTTGGGGGATCGTCTAAAGGGGAGGGGATATACATGCTAATTGTTTATTTTGTTTTATTTTAGTTTAGTTTGTTTTGCTATGTGTGTTTGTTATAGATAACGAGCTTGATGGAATCCCTTAAATAATCTAACTTAAAATCAATTATTTATTTATGCGCATAGTTTTAAATCAATAAATTTGTAGTGAATAACTTTAGAAGATGTTGCGGGATAATAATCGTTGGACTTTCCGTTAAATTAATTAATAAGAGTTTGATAACTGTAGTTTATACAGATCTTAGTTTTATGTGTTTAATAACTTTTTCAGCACGTTAAACTAATTAGCTTATTCATTTAGATTTTAACCTTAGCTTTATGTAAATAAATTCAGACTACTTAGAAAAGCATGCCTATAGGATTCAATTAATAAAATTGACGTTAATTAATCAGTTTAAATAATTTCAGCATATCTAGCTTATCGTTTAAATATTTGTTAGTCACTTAGATATCAAGCCTGTAGACTCTCAAATTTCTCATGCATGTTAATAACTAAAAGAATTAAAATCTGTCTCATATGCTTGCTGCCTTTTTCTTATCTGATATAATTATTTTCTGCATTTTTCTGCCACTTAAAATCAACCATTTTTTGCATGAATGTGCTATTGTAAAATACTGCCTTAATTATAAATCTGCTCGAACTATCAACTTTTGCATTAATCAACTACTGCTTAAAATAAGCTGATTCATTTTAGCATGTTAATTAATAGTAATCTAATTCGTTACTGCCATTTAAAAAAAATAAAATTTAATGATTTCCCTCTGCATGCCCATTTTAAAATTTGCCCTGTCTAGATAAAAAAATTTTAAAATTAAGTGAAGCTCTAGCTTTAAATATTATGCTCGCATTAAAATTTTGCAACCTTAAATATCTGCCGCCAGATAATTATTTATCTGCATTAAGACATTTCTCTGCCAATATTAAATAAATATTGCGATATTATTATTTTGATACTAAATCTGCTCCTGCATATTTTAATCTTAAATTCCGTCCAAATAAAATTCTGCCTGAATTTTAGTCTCTACATTATTAATAATTATCTTGCAACTTCTAATCAACTAATAATTAAATATTTCCTGTATGCTTTATAAATAAAAATAAAATAAATTTTGCATTTTGTCATAACGCTATGTCCTTGTTAATTTATTTATAACATTATTGTGAAGCTTGTGTATGCTTGTATGCTCACACACTTAGCATGAAACCTTTCACAAGTATCTGTGTTTGCTAGCATGTGAATAAACTAATCTAGAGGCCAATATGAGACCCTACGTGCTAAATGCTCGTCCTAATTGTTTGACTTGGTGTCTAGGTGGGCCTATTATATTTTATTTTGTCTAAATTCATCCTAATAGCAGTCCAATGCCTCTTGGACCTTACGTGGGGACGCTAGACACCTTTTAGAGACGAAAAGCATGCGGTGGTGGGGTAGGGGTAGTTTAGGCCCTATGGAGATGATGACATCGACTCGGTCTAAATGACAAACCTTGGTTTTGTCTGTCCCTACAAGAAACCGACCGAAATAAAGAAAGTCGAGTGCTGATCCTAAAGTCCTTCAGAGTCTCCCTTTCCTTTTCTCTCGTTACTTGTTATAATTATTTGGGGTAGTTTAATAAAAAATAAAAATTGTTTGCTTATCTAATTGTGCAAATTTCTATCCCCTCGCTCATCTCTTTATTTATTGTATAATATTTATTTATTATGTTATCACTTAGTCTCGCATGCCTCTAATTAACTAAAATAATTAAAGTGCCTTTAAATGCTTAATATCATGC

>LNC_000945

CTAATCACCTCTCCTCAATGTTTCTTCGGGGTACCTCTACCCCTCTCGGAACCATCCATAGCCAACCTCTCATACCTCTGTACTGGGGCATTCATGCATCTCCTCTTCCCCATCTTGTCATTCACTGAGGCCACGCCTACCTTGTTCCCGATATCTATTCCTATCTCTCCTAATATGCCCACATATCCATTGTAGCATCTGCATCTCTGCAACTTTTATCTCCTTAATGTGAGAGTGCTTGACTGGCCAACTCTCTTTCCTATACAACATAGTTGGTTTAACCACCACTTTGTAAAATTTGCCTTTTTACCTTTTGGTGGCACTTTCTTAGCACGCAATACTCCGAATATGAGCCTTCATTTCATTCACCCTGCACCAATACGAAATGTGACATCATCGTCTATTTCCCCATTTCCTTGGATAACTCGTGAATCAAGCCTCACTTCC

>LNC_000946

AGGAAACTAAAATTTGACAAAAGGGAAAGATGTAGGTGAAATCGGAACTTCAGCCATCGTCTTCTTCGTCTGAGCTCTTGCAGCAGAGGAAAAGCTTGACTCTTTCACCATTGGATTACAACCATTGTTGTGTAGAGGTAAGATTTGGATACAATTTGAGTTTTGGGGTATTTCAAATCGAGCATTGTAGATCCCACCAACGCCACAAAATCGTTCTCACCGGAGACTTCGCCAACTCAACTATTTCCAGCTTGAGCTCAACAAGGAACTAATACCACTGCCTAAGGATTTAGAAAGAGCGCTATATTGAACGAAACCAGGGGGAGATAATTTGTATGTACCTTTTCTGCTATTAGGTCTGAGTATGCTGCAATTTTGGGCCAACAGCAACTAGCAGCTGCTGATTGGGAACCTTCTATGTATCCTTCAATAGAACCTGATCTGAATATTTAGATGGATACCATCCAATATTGGGTCTACTATGTGCATCGGGATATGGTAAGTGGCATGTTCCAAAACATTTTGTGTATCTAGAGCCTCATTCTCTCCATGTGTCGTGTCAATCAAAGTTATTTATGTTGTGTACCCTTTGTCCATGACTTCATTCCTGGCATCCATTATTTCATCATAGTGTTCTGGTTTACTATCTCTCAGCACTATATGACATGCATTACTCTGAACATTCTATTTTACTATTTCCCTTCTTGATACGTTGTTGTGATCGCTTCCTTTTCCTTGTGCCGAGGTAGCTAGTGTTGTGTTGTCCCCTGCTATCCATTTTCCTCCTTGTTCCTTATTATGTATTAATCCTTCTTCCATAATGGCTGCTTCATCATGCTCATGTTGATTAAACACATTAAAAGAATTGTTGGTACTGATGTTCTCATGTCTTTCTACAGCCTTTTCTCCCATTATTTGTCCCGCCTTCTTTGTAAACACCCTTGTGGTGGGCTTCCATTTACTGATCACTGCTCCTTTATAAAACTACATTTTGTAAAGTTGTTGTGGTATGATTGCTTCCTTCTCGTGTTGTTTCTCCTGTTGTAATTCTGGATGCAATATCATGCACTCCTCCTCTTTGTGACCCTGAATTCTACATTTCTTGCAATATGATGGGAGTGAGTCATAAAGTATTTTAACTTTAATGATTTTAATATCCTGATTATTGTTATTTTCTATCTGCATTTGAACATATTGAGGATTGTCCACTAGCAAATCCAGTTGAACCTTCACCCTTGCACAACTTGTCCGAGTCTTGTTGATTGTTGCAAGATTTAGGTGCAATGGTTCCCCTACTATTGCCTCTAACGAGAACAATACCTCCTTCACAAAGAAAGTAGGAAGGATATCTTGGAAAGAGATCTAGGTTGTGGCTTGCGAGGTTTGTTCACTTGCCTTGAAGATTGCACCATATATGAACGGGCGCATTTGATATGCATAACCATCTTTGGCTATTATGTAATACGAGTTTTTGGATAACATACTAATGTAATCTTTAATCCTGTTAAACCTCATTAGAATGTGTCTACTCCGCAGGTGGCTGATGAGTCCACGAATTGGACTCATTTAGGGCTTTATTTTAATAAAAATAGTATCCTCAAATGCTTATTTATTCTAAATATCTGATGAAAACACTTAAGTTTCAGGTATTTGAAGTTTTAGTGGAAGCATGGACACTTAGGCGCAAGAAGGAACAAAAAGGCTGAAAAGAATGAAGAAGCTGAAGCCTGAGCATCGCCAAGCACGCTTGGCGATTTGCCGAAGGGACGCACTCCGCCCTTTGTTCCAGTGTGCTGAGCCCTGAAGGAAAGGATGAAGTCGGCAGAAAAAGGAGCAGTCGGCGCATCACCGAGTAGTTCCGTGAAGCCGTACTATGTCGCCCAATGACACAGAGCAAAACGATGTTGAAGGTTGGTGCAAGACGGCGACGAACTACACCAAAGGGCGAATCACCGAGTTAATCAGCGATTCTGACTAACCTCACCAAGCAATCCGCCAACACTATTTTCTGAAATCTATAAATACTAAACTCTTTATTAATTTTAAAAGAGAGTCGAACAATTATAATAATTTTCACATAAAATAATACTTTTTGCTATTTTTGCTCTAAGCTTGAAACGGTTTTGGAGACTTGAAGAAGAAGAGTGTTTCATCTTCTAGATTTGGAGTTGGGTCTCTTGTATTCTTTATTCTTGGTCTGTAAC

>LNC_000947

GAAAAAAGGAAACTAAAATTTGACAAAAGGGAAAGATGTAGGTGAAATCGGAACTTCAGCCATCGTCTTCTTCGTCTGAGCTCTTGCAGCAGAGGAAAAGCTTGACTCTTTCACCATTGGATTACAACCATTGTTGTGTAGAGGTAAGATTTGGATACAATTTGAGTTTTGGGGTATTTCAAATCGAGCATTGTAGATCCCACCAACGCCACAAAATCGTTCTCACCGGAGACTTCGCCAACTCAACTATTTCCAGCTTGAGCTCAACAAGGAACTAATACCACTGCCTAAGGATTTAGAAAGAGCGCTATATTGAACGAAACCAGGGTCTGAGTATGCTGCAATTTTGGGCCAACAGCAACTAGCAGCTGCTGATTGGGAACCTTCTATGTATCCTTCAATAGAACCTGATCTGAATATTTAGATGGATACCATCCAATATTGGGTCTACTATGTGCATCGGGATATGGTAAGTGGCATGTTCCAAAACATTTTGTGTATCTAGAGCCTCATTCTCTCCATGTGTCGTGTCAATCAAAGTTATTTATGTTGTGTACCCTTTGTCCATGACTTCATTCCTGGCATCCATTATTTCATCATAGTGTTCTGGTTTACTATCTCTCAGCACTATATGACATGCATTACTCTGAACATTCTATTTTACTATTTCCCTTCTTGATACGTTGTTGTGATCGCTTCCTTTTCCTTGTGCCGAGGTAGCTAGTGTTGTGTTGTCCCCTGCTATCCATTTTCCTCCTTGTTCCTTATTATGTATTAATCCTTCTTCCATAATGGCTGCTTCATCATGCTCATGTTGATTAAACACATTAAAAGAATTGTTGGTACTGATGTTCTCATGTCTTTCTACAGCCTTTTCTCCCATTATTTGTCCCGCCTTCTTTGTAAACACCCTTGTGGTGGGCTTCCATTTACTGATCACTGCTCCTTTATAAAACTACATTTTGTAAAGTTGTTGTGGTATGATTGCTTCCTTCTCGTGTTGTTTCTCCTGTTGTAATTCTGGATGCAATATCATGCACTCCTCCTCTTTGTGACCCTGAATTCTACATTTCTTGCAATATGATGGGAGTGAGTCATAAAGTATTTTAACTTTAATGATTTTAATATCCTGATTATTGTTATTTTCTATCTGCATTTGAACATATTGAGGATTGTCCACTAGCAAATCCAGTTGAACCTTCACCCTTGCACAACTTGTCCGAGTCTTGTTGATTGTTGCAAGATTTAGGTGCAATGGTTCCCCTACTATTGCCTCTAACGAGAACAATACCTCCTTCACAAAGAAAGTAGGAAGGATATCTTGGAAAGAGATCTAGGTTGTGGCTTGCGAGGTTTGTTCACTTGCCTTGAAGATTGCACCATATATGAACGGGCGCATTTGATATGCATAACCATCTTTGGCTATTATGTAATACGAGTTTTTGGATAACATACTAATGTAATCTTTAATCCTGTTAAACCTCATTAGAATGTGTCTACTCCGCAGGTGGCTGATGAGTCCACGAATTGGACTCATTTAGGGCTTTATTTTAATAAAAATAGTATCCTCAAATGCTTATTTATTCTAAATATCTGATGAAAACACTTAAGTTTCAGGTATTTGAAGTTTTAGTGGAAGCATGGACACTTAGGCGCAAGAAGGAACAAAAAGGCTGAAAAGAATGAAGAAGCTGAAGCCTGAGCATCGCCAAGCACGCTTGGCGATTTGCCGAAGGGACGCACTCCGCCCTTTGTTCCAGTGTGCTGAGCCCTGAAGGAAAGGATGAAGTCGGCAGAAAAAGGAGCAGTCGGCGCATCACCGAGTAGTTCCGTGAAGCCGTACTATGTCGCCCAATGACACAGAGCAAAACGATGTTGAAGGTTGGTGCAAGACGGCGACGAACTACACCAAAGGGCGAATCACCGAGTTAATCAGCGATTCTGACTAACCTCACCAAGCAATCCGCCAACACTATTTTCTGAAATCTATAAATACTAAACTCTTTATTAATTTTAAAAGAGAGTCGAACAATTATAATAATTTTCACATAAAATAATACTTTTTGCTATTTTTGCTCTAAGCTTGAAACGGTTTTGGAGACTTGAAGAAGAAGAGTGTTTCATCTTCTAGATTTGGAGTTGGGTCTCTTGTATTCTTTATTCTTGGTCTGTAAC

>LNC_000948

CAAAAGGGAAAGATGTAGGTGAAATCGGAACTTCAGCCATCGTCTTCTTCGTCTGAGCTCTTGCAGCAGAGGAAAAGCTTGACTCTTTCACCATTGGATTACAACCATTGTTGTGTAGAGGTAAGATTTGGATACAATTTGAGTTTTGGGGTATTTCAAATCGAGCATTGTAGATCCCACCAACGCCACAAAATCGTTCTCACCGGAGACTTCGCCAACTCAACTATTTCCAGCTTGAGCTCAACAAGGAACTAATACCACTGCCTAAGGATTTAGAAAGAGCGCTATATTGAACGAAACCAGATGCTGCAATTTGCTCTGAACCTTCC

>LNC_000949

TAAAAATAAAAACTTATAAGTGAAAAATTGAAATTAACTTTTCCAGCAAAGAAAAGGTTAGCCCAGCAAAAAAAATACAATGAGGCAAAACAGAGAGTTTAGGAATTTTCTTTACAAGGGTGGTTGCCCGTCTCTTGTTTTAGCCAATGATTTTCCAAAAAAAAAAAAAACAACAACCCTTGGTTATGTTAATTACTGGATCATTGAACTTGGAAAGCATATATATACTACTAGTTTTTTTTGTCTCTGTTAGACTATGTATGTATGTATGTTTGTTGATTTTCCCATAAAATTTGAGGCGGTGATGATGATTTCTTCGGATTCTGGGCTAGCTGAATCGGAGATGGAGACTATCCGATCCGTATCTTGGCTTGGCAAATCATTATTTTGCAGCCATGGCGTGGTCGCTTCTCTTTCGCCCCGATCTCTTTGCAGATTTGAGCCTCTAGGCTCTAAATCTTAATCTTTCGTGTTTTGCTTATTCTCTAACCTACCCAGCTGACCCCAACTTCCCCACTTTACCTTCTAATTCTTTCTTGTTTTTTTTTTCATAAATTATATATTTTCTTTTTGATTTTATTTTTCATAAATAGTACTTATTCTATTCCTAATTATTTGTCTATTTTTTAATTGACGACATAGTAAGTTTATCACTTTATCTTTTATTAATTATGAAGTGGATAATTTAAAATTTTACGGTTTTCAAGAAATTCTACATTTTTCAAAATTAATTAATGAATGGTATAATAGATAAAAATTTTTTGTCATTTCTTGATTTGTTAAAATAGATAACTAAAAAGGAAATATTGGACAAGTAATTAGAGACAGAGAGAGTAAATGGAAAAGGGCATACATTTTTTTAAATTTATTTCAAAAAGTTAGTTACACGCTTAAATTATTATCACGACATATTACACACACATCTTTACTATTTAAAAGTGAATTTAATATCACCCTTCGTTACTTAACATCACTCTCACAATATATGGTGTGTTACACTTGCTGCTACATCGGCACTGCGTAGCTGTAGCGATACATCTGAATTTATAATTTTTTATATTCTTTTTT

>LNC_000950

GAAAACTCTTAAGTTTCAGGTATTTGAAGTTTTGGTAGAAGCATGGACACTTAGGTGCAAAAAGGAACAAAAAGGCTGAAAAGAACGAAGAAGCTGAAACCTGGGGATCGCCAAGCACACTTGGCGATTCGCCGAAGGGACGCACTCCGCCTCTTGTTCCAGTACGTGAAGCCCTGAAGAAGCAGGATCAAAAAGGCGATGAAAGGAGCAGTCGACATTTCACCGAATAGACCCGCGAAGCAGTACTATATCGCCCAACAACACAGAGCATGATGATGCTGAAGGGTAGTGCAAGACGGCGATGAACTACACCAAAGGGCGAATCGCCGAGTTAATTGGCGATTCCGACTAACCTCGCCGGATGATCCGCCAACACTATTTTCTGAAGCCTATAAATACTAAACTTTTTATTAATTTTTAAGAGTCGCACATTAATTTTAATTTTCCACAATAGAATAACACTTTTTGGTATTTTTCTCTCAAGTTTGGAGAGGATTTTGGGAACTTGAAGAAATGAGTTCATCTTCCC

>LNC_000951

GATGAAACCCTTGAAGTTAGAGGGTTTAGAGTTGGTAACGTGAAGGAGAAAGTCGGGAAGCTTGCGAGGAGCATGTCCGCGTCGCGGACTTGTTCCCTCTGTGAACAAAAATCTTCTCCGCGTCGCGGACAGGACGCGGACTCCACTGTTTTCAGAAATTATGTTCGAAGATTTTCTTAAGTTTGGCTGTCCCAAATCATTCCTAAATCATTCCTAAACATCATGAGGCC

>LNC_000952

AGAAAAGCCGTTTAGGGGAGTAAACAAAGTTGAGGCGCTGATAAGAAGGAAGTATCCGCTAAAGTACGATGCTAGTAGTGACAGGAAAACATGATAAAAATAATCTAGTTCATAATGATCTAATATGAATTTAATCAATTATGAATATGATAGGAAGGAATAATTTGAAATAAAAAACCCGCATAGTAAAGAACATGACTACATATATGCATGACG

>LNC_000953

ATCCCTTTTTTATTTGTTTCTCTCTTTACTGTTCAAAGAAGAAGTGGTTTTGTTAAGTGTATACGCACTTTGTATGAGAAAGAAAGGATATAAACATAGTGGTTGTCTAACGAGATACTATTTAGAATAAGATCGTCAGGTGAGTCACATATTGCGCATTTACCGCTTTCGAATTTTTGAAATTGGATTTAGACTTTTTTAGACTTTATCGACTTATTTCATATCATGGTTCAGGCGTTAAAAATAC

>LNC_000954

TACCTCCCGAATATAGATACGAGCCAACCTCTCAGCACTGAAGGTAGTATGAACAGGAAGGAAGTGTGCTGACTTGGTCAATCGATCCACGATGACCCAAATGCTGTCAACACCTCTAGAAGTCCGAGGTAACCCCACAACGAAGTCCATAGTGATACGCTCCCACTTCCACTCCGGAATGGGTAATCTCTGGAACTCGCCACCAGGCCTCAAATGCTCGGCCTTTACCTGCTGGCAGCACAAGCAACGGGAAACAAAGTCAGCAATATCTCTCCTCATGCCACTCCACCAGTAATGCTGCCTCAAATCACGATACATCTTCGCTGTGCCCGGATGGATAGAGTATCTAGACTCATGGGCCTCAGAAAGTATCAACTGTATCAAATCTCCAACTCTCGGAACACAGATGCGGCCGGC

>LNC_000955

GAAAGATTATTTCTCAATTTGACACTCATGAATGTTGATGATAGAATATGAAGGATTCTTCAATGTAAAGTATACCTTGAATTGGTAGGGCTTATGCATCCTTTTCTTGTATAAATGATTATATTGTTGATTGTTGAATCCTTGGATCGGTAGGCTTATGGCACCCTTCCACACATGCTTATAATGAACCTTGGATCGGTAGGCTAATGGCAC

>LNC_000956

GGAAAACAATGGGGGCCTTTAGAGGAATTTGGAAGAAGTTTGAGGATGAGAGTATAAAAATGGGGAAAAGGCATCTCGTTTTCCCCTTTCAACATGAGACCGAAGGAGAAATAAGTAGATAGATTCACAGAGCAACTTACTTTCTGTTGAAGAAGAAAGAAGAAGAATCTGTAGCAGCAATACATCCAGGTTCCACAGCAGCAATACATTGCGTTGAGATCTTCATTACTTTCATCGGTAAACCTTTTCTTTTTCGTTCGTCTGCGTTATTATGCTAAGCCTGATTCAATAGTAGATAGGTGTTTATCTCTGATTGTGTGCGTATGTGTAGGTTAGTTCTATTGCGTTTTTTTTACTAGGTTAGGTGCCTATATAAATGTCTGATTTAGAGAATCCAGCTATTTGTGTGAGTTGAGTGGAACCTTAGGGCAGATTCTACAAGCAAGAGAAGATTGTTTAGGTGTTTACATCAACTGACTCAGTGGTGATGGTTAAGTAAATCAGCCTTCCTATGCTGATGTGTCTTTCAACACTTCATCTCCTATGACTCCAACATCTTCATCAAATTCTCTTGTGGTGAACTTCTATTGGACTAGCCGCTAGCTTAGTTCCAAATATGGAATCAACTCAACTTTATACTTCCTTTGATATAGGATCCCTTTACTGGATCTCAACACCCCTATCTCCAACAAGATTTTGATGCAAACCACACTGTTTCATAAGATCAATAGATCATCAACATATATCAAGATAGCTACAAAGTCTCCCTTTTTTTCTTTGTAAACAATGAATAGTTATATAAACGCTGGGAGTATCCAGCATCAAGCACATATCATTAAGTTTGATATTCCATTGACTTGATGCTTGTTTCAAACCATACAATGATTTCCCTAACTTGCCCACTTTGGTCTCACTTGTCTTGTCTCATGAATCCCTAAGGTAATTCCATGTACACTTCATGCAAGTCTGCTTGTAGGAAAGCATTGTACACATCCATTTGGTGCATGCCCACCCTTTTGATGTTGTAAGGCAATGCTAAATCTCATAGTTACCATTCTGGCACCTGGTGAGAATGTCTCATCATAGTCCAATCTTTCCTTTTGGTTGTATTCTTTCGCAACAAATCTAGCTTTAAACCTTTAACTTCCCCATTAGCCTTGAACTTTATTTTGTACATCCATTTTCAACCAATTGCATTTTATCCTTTGGGTAGATCCAAAACATCCCATGTCTTTTTATCTTCCGAAGCTTTGATCTCCAACTCCACTCCTTCAATCCACCTCTTATCTTTTAAAGCTGTTGTGAAGTTCTGTGAGTGTGAAGGTGTTGAGATTTTCCAGGAAATGCTTGTATTTTATGGAAAGTCTGTTGTATGGCAGCTAATCAGATAATGGATAGTGTTACTCCATGATTCTTCCTAGTGGTAACATAATCCTGCAACCATAAGTGTTCCTTAGCTCTTCTGTCAGATTTTTTCAATTGCTAGGACTGAGTGTCATGTCCAACAGGTTCATGCATTGGTTCAACTGGTTCTGCAAGTTTAGGTTTCATTGGTTCAACAACTTCAGGTTCAGTGGAAAGTGGTGCTCCTTCTGTTGTTATATTAGCCAACTCATCAACAGTGGAAACAGATCCAGGAACAGGGGCTACAACATCTTCTAGATCTTCTAGTAATGGTTCAGGAGAATCTAGCATTCTCCCCCTATAATGAACCATATGAGGCTAGATCACTTGTGTATGAGAAGAAACTTGAGCAAAGGGAACTAGATGCTCCTTTTTTTTTTTTGGTGAAGTAATTAAATTGCATTGGAAAGGCATCAAGAAGATGCAAAGGTTACAACTAAAAATAAAGTTGCTTCAAACATGAAAACAACATGAAGCTAACTGTGCAAAAACCTGTAAAGCAGCCATAAAGATAAACCCCCTTAAACCCTACTGTACTAGAGTTAAGGAGCTAACAAAATCCAAAAAAATTTCCATACTGTTTACAGGGGAGAGAAGGTGCCAATTATAAAGACTAACTAAACACCTAGTTTTAATGGTGCTTATGGGAGTTGATATTCCATCAAAACATCTGCAGTTTCTCTCTTTCCAAATGCTCCAAAAATTACTGTTGGGATCATCCTCCAAGTACTTTTGATGGTGTTGTCAACTCTCCTACATATCCAGCTAGCATAAGCATCTTTGATGGAGAAGGGCATAACCCATTGGAGGCCAAACAATGAGAAGAAGAAATGCCATATGCCGACTGTTGTGGGGCAGTGCAAAAAAGATGGTTGACACTTTCATTGTCTTGTTTACAGAAAAGACATCTGTTGATGACAGTGATGCCCTTTTTCCTAAGGTTGATACTCCTTAAAGATGACATCTCTGTAAAAAGAAATGATTAGTGGATATATCAAGCAACTTATAACCTATGTTGCTTGGACTCTTCAAAAATGTCAAGGGGTGTGTGTCGGATTCGTCAAAAGTAGTGTATTATTGGAGAATCCGACACGGGTGCGGTATCGAGAGTGAAGAATCCACGCAACTTAGCTTATAACCTTTGTGAGTTTCTGAATATCCCATAAAAACTACTGGTGTTCATATCCTGAAAACTTGTTCTCTCTCACCATCAGGGTGGCATAACACAAGCATCCAAAACTCTTGCGTGAGCAACTACAGGTGGTCTTCTGTACAACATTTCATAGGGTGTCTTGATATTTAAAATGATAGCAATTAGCCTAGTGATTATATAAACAACAGTGGTAATACACTCTTTTATAACTTAGTTGCCATATGTGCTTGAAACTTCAGAGCTTTGTTTGTATCTATGCTTTCTCTCTATCACTCAATTTTTGCTGGGGAGATAACCACAACTACTTTGATGTATGATTCAATAAGCATCAAACAGATGAGTGCATTGCTTATTAAGGACCTCCACTCCCATTGTCTGGTTTAATAATCTTCACTACTGTATCAAACTGTGTTCTCAACATAGAATGATAAAAATAAACTTCTGTAAGATTACAATAACATCACCT

>LNC_000957

GAGCTATTGCAGGGGTTACCCTGTGCGTACCCAAAAGGTAGCGGCTGTGGGTTTCCCTTGTCATAAAAAAAAAGGAGTATCAGAAGGATGCAGAAACACTGGGTGTGTGTGGTCAATTTTCTCATTCGCCATTGATAAATAAAACCAAACTTTTCACAGCTACACAGCTAGCGCATGGCCAGTTTCTTCTGCTCTGCTACCATGTGACTTGAGTTCGAGCTCAAGTTCTGACTATGGAACCTTAGGTCAAACCCAAATGTGTGAGAGAAGACAAGCGAGAGAGAAGAAGATATGTTGGAAAAGTAAATTGTGATTATATCATCAAATGAAGTGATTACAGTCTCTATATATACACAGCTCCTAAGCAAG

>LNC_000958

ATGCTAATTTAATTTAACAGAAGCTTAACAAAACCCAAATCTGTGCTTAGAGGAATGACAAACATTTGTTTCCTCTTGTCTTTACTGATATCTGATTCTGTGCTACACAAGTGAGGACTGGATTAGATCAAATTGGCTTCCTTTACGTACTAAATTTCACTACAGAATTTAGATGCATGCAGTTTGAGTTCAAGAAAGAAACTTACGGTGTATTTTCTTTGGGAAATCTAACATTTTAATTGCAGTATATTGTGGAGCTCCACTTCAGTGAAGCAAGACTGCATCCGGAGATAAGTGATAATCAGTCCTTGAAAGGCTTAAAGCAGCTGCGGAGGTGTAAATAGTTTTTGTTATATTAAAAACATGAACTCTCTACTTGAAAGTTAAATTACCTAAATATCCTTATAAATAATTAAATACCTTAATCATCCCAAATAGCCTAATTAAAAGCCCCAAGAAAATTACCATCACAAGTCACAACTCTTCATGCTTAACCATACCTGTCAGGCTCTCCAAAGGTTTGGAACATTTCCTC

>LNC_000959

TCCTATGTAAAGTGGATAGCATTGTGAGTTAAAGTATGATTTCTCATGATGTATATATGAAATGATGTTGGATTATGAAGTATCCTTAAATGAAAATGTTGTGACTTGGTTGGTAGACTAAAGTGTCCCTCCTTGTTACTTAAAGTCTTGAATGCATGAATGTGTGAAATGGGCAAAACTAAGAAATGGTGCCCTTTCATAAGTCTAGCTTTCCTAAGTAAATGTAAAACCATGAAATCGGTAGACCTAATGGTGGTAGCCCTTCTCATGTGTGAAATGATGAACCTTGGATCGGTAGGCCTAAGGCACCCTTCTAGGGGGATTTAATGAGCTATCCTTATGAACCTTGATTGGCCATGACTAAGAAATGGGGCCTTTCTTGGGAAGAGGTACTATGAGTTGGTTGAACTAGAAAAGGAACCTTCTCTAAGTACATATGTGATTGAATACTCTATGGGAAACAAGGCTAGCACCGAGTGGATGTGGTTAGGAAGGTGGCCTAGTTGAGTATGAGATTAGGCACAATGAACTTCCTTGGACATCCCTTAACCATGTGCCTACATGGGTTGCTTACTAGTTCTACCTTTGGCAAGTAGAACAACCTCCACGGAGTAGGATAGACTCCGGATTCCATACCTAGCTAGTATGGTCTATGTCGGTTATGCCTATTCCCATCATGTGGGATGTGCACTCTAGTTATTGGATAGGTTCTACAACGTGTAGGTAGTAGTATGGGATGCTATCTACACATGGCACGAGTAGGCTTTGAAGATACTAAAGTGAGTTCCCTAAGTCTAACGACTATTATGTGAAAGTCCCCTTATGTGCTTGAATGTCTCCAAAATGGTTTCTTAAAGAATGTTGGTCTATTGATGTTATGTTGTAAATGAAATGTCCTTATCTAAGGTAATCTTGGGGAACACTTAAGTGTGCTTGAAGAGGTTGTATGGGCGGTCACTTCTTGTCTCACTCAAGTGTGTCTTAAGGTTATATTAGGTAGAGGTCTTATGGTAATGAAATGACATGTGTTCTTTTAAAGTTAAGCATGGGTTGTATGTGGATTTATGTTGAGTATATGTATGGTTGGCTATGGTCTTATATGTTAATGTTGACTTGTATTATGTCTCTTATACTTGTAAAGGAAAGGTTTTATCAAATTGATACGAAAGCATTTATTCTTTGAAAATGTCCTTTTTGGCATGTTTTTGCATGGTCTCCATACTTAGTACTTAATTGTGCTAACC

>LNC_000960

GAAAAATAATATGGGTAGAGTCAGAATGATGGAACGACCCTAGTGAAAAAGAGAAATTATATGGATAGGGTCGGAATGATGGCACAACCCTACGCAAATCAGATTTGAGGAGTCACCAGAAAGACGAATGGAACTATGCAAATCAAATCTGAGGAGTCACCGTAAAGACACGATGTTATGCAACTCAGATATGAGAAAGTAGTTCGGAGAAATCCATGGTACTACGCAAATTAAATACGAAAAGTAGTTCGGAGAGATACACGGTGCTACGCAAATCAGATTTGCACGGTGTTACACAAATTAAATATGAAAAAGTAGTCACCAGAA

>LNC_000961

TGAGGGGCTGGGTGAGATTCTTGCAAGATGATCAGTCGACTTCACAAAATTGCCAGTCAATCTCAATGTGCTTGTCTTCTCATTAAATATTGGATTTGATGCATAATGTCAGTCAATCCCAATGAGCTTAATCTTCTCATGTAATACTGAATTTGATGCATAATGCCGATAAAACACATAGTGATAAAATTACAGTGTTGAACTTCCCAAACCAAACTTGCAAAGATTGTTTCAGGGCATAGAGTTTATTATTTTGCGCCCAAACGCACACACAAGTGTACGAAGTTGTGCAAGTAATAAAGTAGCTCTTTTTAAGAGTTGGGTATCGTACCCACACTTAAGATTAGATGTATTCAATTCTTCCAACAATTAAAATTTAGAACAAGTGTTATCAAGAAAAGTGCTATGGGATTTTGACTGATTTATTTATCTAATTATTGAAGTAAACTACTACAATTTGATTTCAATTGGATTTCAAACAATGACTTGAGGAATTTCAGGGCTGCAGCCTGTTATGGATTCATGCAAATTATCTCATTTAATGGATGTCAATCGATTATTGAATCACTGATTTCAGAGTTAAATGTATGATCATGGTGTCTCGATCTCTAATCGCCTACGGTAATTGGCAGCCTAACTACATCGTTGAGTGGGGATAGACTGAACCAATTATTGTGCATTACGATTTTGTCCTGTCTATTAACCAAGCAAGGTTTCTAGGTATTTCCTTATCCTAGATACAAATCAACCCTAAACCTAGAGAAGATCATGTTCCTTTT

>LNC_000962

CAAGCTTCAATTCTCCATTCCAATCTTCATCAAGGATTCTCCAAAGTTGAGGTATGTGGGTTGATTGTGGAAACCTTCCTTTCCACAAGTCCAACCATTTATCCTCTATTTTACTTCAAGTTTATGGGTCCTTATGATCATTTATGAACTCTTGAATTATGGAATTACTACTACACATTCTATTATGGTCTATTTTTGCATATTATCATGAAATGGAATTATTATGTGATGTTTATGGTTTGCATGCTTCCATGATCAAATTATGAAGGTTGATATATATATGTATGTTGTTGGTGGGCTGATTTATATGTATTTCGGAATTGGTTGGACTTAGTACTCTTGAAGTACATGATTTCATTTACATGAACAATAGCCCATCATGTGTTTGATAAAATGTCCATTTATAGAATCCTTAGGAAATGGAAGGTCCAATGTACATCCTATGAGTCGGATGATTATATAATTATTGAAATGATGATGAAATGGACACATGTATATGATTTTCTCTATATACGTGTTGTGAGTCGACCAAGCCTAGTACGGGGGGTAGTAGGCCCGTATAACCGTATCGAGGGGTTGGTACCTTGGTTGCCTAGTACGGGGGGTAGTAGGCCCGTATAACCGTATCGAGGGGTTGGTACCTTGGTTGCCTAGTACGGGGGGTAGTAGGCCCGTATAACCGTATCG

>LNC_000963

GTGAAGAAGAGAAAGAAGAGAGGAAGAAGAGGAGTTGGAGCTAGGGTTGAAGATTTCTTGGTGTTTCTTCTTCAAATTTCGTGGGGAATTCATCTTTAAGGTATGGGGATCCTTTATCCTTGATCATTCTTTCTTTCAAGGAGCCCAATCAAAAGGTTTTCAAGGTCTTTCAAATACACCAAAATCCTATTTCGAATTCTAAGCATGGGTCCTTGCATTAAATGATTTCCAATGATGTTTTATGTTGTTGTTAATGATTATTGATGGTTTTAAGAAAGAAATCCTCATGAACCAATGTCTTTACAAATTCTCTAAATTTGACCTAAAGTGGGTATATTGATTTTGTGTAATTGATGGACGATTATTGTGTAAATTGTTGTGGGCTGTTCATGTATGTATTTATCTATGTTGAATTGTTAAGAATTGTTATTGAATTGAAGGATGTGG

>LNC_000964

TTGGTGAGTCCTCATAGATTCGAGGGCAATAACCAACATGTTCCTTTTTTTATGTCTCTCTAGTATTTGCTTAAGTTTTGAGTGGGCTTAGTCCCAATGTTATATTCATGTATTAGATGGTTTTGAGACATATTGTATAAAGTCTAGAAAGTCTTCCGCTTGCTATTTATTGAAAGTATCTTCGTGTGTGAAGAAAGTTTTTAATTCCTTATGGTTTTGGTGTCTATGCGATGAATGAATGCTAAGAGGCTTGTATTAGAC

>LNC_000965

GCCAAAGTGTTATATGACACCGCCAACCTCTTTTTTGATTCCTGGAACATCTTCATCGAATTACTACCCACAGTCGAGATAAGTTCGTTGCTCTGATACCAATTGTTATGATCTCAATTGAGATATGGAAATTCAAATGGAAAAGAAAATAATCGAACACAATAGTTTTTGGGATTTTCTGATTTATAACTGGAAAATTAGAGACAAGGATATCTACTACATGGAAATTAAAACTACATTTAACGGAAACTAAC

>LNC_000966

AATTAAGTCAAAACTAGTTAGAGTTAAAAAGTTGATAAGTTGTTTAAATAAGTTCAAATAAATAATGTGGTTTAGTTTGTGGATTTAGTTGTTCAAATCAAGTCAAAACTAGTTAGAGTTAAAAAATTGTTAAGTTGTTTAAATAAGTTCAAATAAGTAATGTGGTTTAGTTGATGAATTTAGTTGTTGGAATTAAGTCAAAACTAGTTAGAGTTGTTTAAATAAGTTCAAATATATAATGTGGTTTAGTTGGTGGATTTAGTTAGTGGAATTAGTTCAAATCAAGTTTAATTGTAAGTGACACTATGTTTAATAACTTTATTGTTGGTGAATGTAGTAGATCTATAGAGCATAATTATGTCCAAGGTTCTAGAATATCATATATGGTTGATGATTCTTCTGGGATGCATTCTGCATAGAATATCTTAAGTGAGATATAATTATGACTCGACTAAACCTCCATAGAAAGAAGATTAAATTTTATTGTATACCTCTATGCAAGATGTTATGTGCATATTGAGTTGTGCTTAATTAGGCTTTCAAATTCTTTTAGTTAGATTCTGGATGAATATCTGCAACAATGCTCATCCAGTTACTTTATGTTCTGGACTTCTAATTCATTCACCAAATAGTTTGGATCTGAGATGTAGTAGTTGATTAGATTATCTTGACATAACTGTACTAGTAATTCTAGTTTGGTACTTGTAGCTTCTAGCACAACCCTGACACTAGAGAGTATCTGAGTTTGTTATTGCGGAGTTGGTGTTTGTAAACTAAATCTCTCTTCTGCATATTCTATATTTGACTTCAGCAAACCTAAGAAGAATGTGAAAGTTTTAGTTTGACAGTCATATGAACAGTTTATTGTATGAACAATAAACCTATAATTGCAGGTAAGTATTGTACTTTCGACGTGTAGTGCGCTGTTCACATGCTCGTCATTTTCATGAGGTGCCCTAAATTAAGAAATGACCAAAATGTTTCTAGAGCGTGTATTCATACATGTTCCCAGGGGAAATGTTTGAGAGCAACAAAACCAATAGAACTATTAGAATCGATTTCCCCATCAACATTAACATGAATTAAACCATCTTGTTCTAGGGAAATTATTTTTCGAGCCCAATCTAATTCTTTCTCTGAAATTGCGATGTGGACTGCTTCTTTTCTTCGCAAATTCGAGGAATTGTTGGACGAGCTATCTGTGATCTGCTTGCTTATTACTTGAATATGTGAATTTTCAATAGTGACTTGCTTTGTCGGAGTTATTCCATGAATATAATTGTCAAGTTTTTGTTGAAGCAATCATATATCTTCCTTTTTTTTATTTTCCTTCTAGAAAAATTGGCACATTTATGTATTTAATGAAATGTTAAGTTTTGTATTTCGTAAAGTATCAAAATAATCAATTTAATGTTTTTTCTCAGGCTAACATCATCAAGAGGACTGGAACTACCGTAGTTGACACAAATAAAAAGAAAATTTTTAAGGAAATTGAGGTCAAGTATCGACTTCAACATGCTCGACTTTTGGATTGATGATTTCATAAAGATTTATTGCAAATTTACTAGAGATTTTAATTCCATTTGAATTAGGAGTTAATTAGTTGGTTTTATGTTGAGATTATTGCATTTAGTTAGATCTCAATTTTTCTTGACATAGAAAAAATAATTTGTTTTGACATTGTTTACTTTTACAGAAAAATTATTTTGCTATTTGTTCTTCAATTATAATATTAGAAAATATTTGCTTTACATAATATTTCTTGCTTGTCATGAAGTAGATAGTTTTGAATGTACGAATTGTTTGTATGTATTTGTGTCTTGAGAAAAACTATATATAGAATTTTTTGGGATGTCTAAAGAAACTTGGTAGCGACATTAGAATATGAATATGACATTTAGCAAACTATAAAATAATGTCACCAATTATATGACATTCAATAATAGCATTTGTTGACATTAACTAAAGTGTCAGAAATTTTAATGTATAAATGTCACAAGTATCTTGAAATTTGTTTTACCA

>LNC_000967

GCAAAGTTCATCAATATTAACAGCCTTCTCCCAGATTTCTTCCTTAGGTTCCTTGTAACTATCCATGACTCTTTTGTAAATTTTGGAGATGGCTAGCATAGCCCTGAATGCTGAGGAATACAACTTTTACCAATTTCAAAAAACTAAAGTCACAAATATTTTCACGAATTGAAAAGACATTCGTATAGAGAATTGGCATGGTCTAGGCATAGCATAACGAATTTACTCATATTACTCGGATCAGAAAACTCTGCAAACAAAATTGTGGCAGCCATTGGATTTTCAAAAAAAAAAAAAAAAAAAATTTCAAAAAAAAATTGTGGTAAGCCATTGGTCTGTTCTCTTTGTAGAAGATTTTGCAATTCTGGTTGCTCTTCATCTTGCGAGACTCGGTGAGTTCATCTATCTCTTCTCATATTTTAAATTTAGTTCTAATATGTGTTACTCGAGTCGAGGGGTAAATAGTACTAGGCAAGTCCCATGAACGAGCTCGATCTTGAAA

>LNC_000968

CTAAGCCCTCTCCAAAATTCCACCAAGATCTTCAACCAAATTCAAACATCTCAACCTCTAATTCCGGATTCAAGACTCAATCTCAAGCTTCAATTCTCCATTCCAATCTTCATCAAGGATTCTCCAAAGTTGAGGTATGTGGGTTGATTGTGGAAACCTTCCTTTCCACAAGTCCAACCATTTATCCTCTATTTTACTTCAAGTTTATGGGTCCTTATGATCATTTATGAACTCTTGAATTATGGAATTACTACTACACATCCTATTATGGTCTATTTATGCATATTATCATGAAATGGAATTATTATGTGATGTTTATGGTTTTCATGCTTCCATGATCAAAATATGAAGGTTGATATATATATGTATGTTGTTGGTGGGCTGATTTATATGTATTTCGGAATTGGTTGGACTTAGTACTCTTGAAGTACATGATTTCATTTACATGAACAATAGCCCATCATGTGTTTGATAAAATGCCCATTTATAGAATCCTTAGGAAATGGAAGGTCCAATGTACGTCCTATGAGTCGGATGATTATATAAGTATTGAAATGATGATGAAATGGATACATGTATATGATTTTCTCTATATACGTGTTGTGAGTCGACCAAGCCTAGTACGGGGGGTAGTAGGCCCGTATAACCGTATCGAGGGGTTGGTACCTTGGTTGCCTAGTACGGGGGGTAGTAGGCCCGTATAACCGTATCGAGGGGTTGGTACCTTGGTTGCCTAGTACGGGGGGTAGTAGGCCCGTAT

>LNC_000969

CTTCGTCTTCATCGTTCACTTCTTTCTTCTTTGATTTCGATGCACTATAAAACTCAACACCCCCCTTCAAGTTGGAGGGGAGGGAACTGATACCCAACTTGGACAGAATCATGTGGTGAGAAGGTCCAGCCAATGGTTTAGTAAAGAGATCAGCTAGCTGAGCCGATGAAGGGACGAAGGATAAAGAAATCAAGCCCGCAAGGAATTGTTGTCGGACAAAATGGCAATCAAGTTCCACATGTTTAGTGCATTCGTGAAACACCGGGTTACGAGCAATATAAATAACAGCTTTTCTATCAGAGAAGACAGGAATAGGTAGAGTCTGTTGTAGAGATAGGTCCTTGAGCAATCGAGTCAACCAGGTGAGTTCAGTAGTAACCCGACACATCGATCTATACTCTGATTCAGCTTAGGAGAGGGAAATGGAAGCTTATTTCTTGGATTTCCAAGAAATAGGTGAGCCGCCAAGACTGATGAAAAATCCACTAACCGATCTGCGCGAGTCTTTACAAGAGGCCCAATCCGCATCACAAAAGGCAATGAGTGACAAAGAGGAGGAAGCGTTTAAAAACAAACCTTGGTCTGGATTAGATTTCAAATATCGCAACACTCTGAGTCCGGCATTATAATGACCAACACACGAGGTTTGCGTATATTGGCTAAGACAAAGCACAACATAAGACAAGTCCGGGCGAGTGTGAGTGAGGTAATTAAGCTTACCAACAAGCCGTCGATACATTGTGGGATTTGCCAGGAGAGGACCAGCAGAAGTCGATAGCTTTTCAGAAGGAGCAAAAGGTGAAAAAACAGGCGGCAGATGTGAGCAATCAAACTCCTCTACGAGTTCGAAGGTGATACAATTGCTCCATTGGATTGACCAAACCTCTGCTCCAGATCTGTCCAAAGATCCTTCGCAGTTCTTGAATAGATGACAGAATCTGCAATATCTTTGGACAAGGAATTAAGGATCCAAGAGGTGACCATATCATTACATCTACTCCAAGCTGGATAGGTCTGATCATCAGGCTTGGGTTGTGGACTGACTCCATTGATGAAGCTTAACTTATTTTTGGCTGAAAGTGCAATAAGCACAGATCTCCTCCATCCCTGATTCTGCTGTTTTGTACATTCTACAATGGACTAAACTGTAAATAAAAAAAGGTTTCCTAACCGACCTATACCTAATTTGTACACATATACGTATTCTAGAATATTCTACAGAAAGATACTACACTATATCATGTATATAAGTATATCTAATGTATGTACAAATTATTGACGCTTGTATCTATATGTAATTGGGCCATCATTGGATTTTTTTATTTGATTAAATGTCTTCCCATTCTGTTGTGCAGGCCCACTATCGATATGGTCAAGACCCAATGTTTTAACTTCAGTAATGTTCA

>LNC_000970

TTGAAAATCCATTTTACACCAATCACCTTGCGATTTCTAGGTCTGTCAACAAACTGCCAGGTTCCGTTCTTCTCGATCATGTCTAGTTCATCTTGCATAGCTCTCCTCCATGCCTGAGAGTCTTGTGCTTCAGCATAGCTTGTTGGTTCAGAGGTAACCAAGTTACACCGCTGATAGACATCTTTTAAGGATCGAGTTCCCCTCACTGGTACATCATCCACTAGTTCATCTTCTGAAAGTTGAGGCTCCTCTTTTGAAAATAAAT

>LNC_000971

GCTAGCTTTTGGGGTGTGAGTTAGGTCTAAAACCTAATTTAACATGGTATTAGAGCAGGGCCCGTCTCACCCGATGTTGGGGCCCCCAAAATCAAAATTGTCCACGCACCAGATGCTAAGCACTGGGCGTGAGGTGGGGTGTTAAAGAATGACAAAAGTCTCACATCGATGGTTAATGAGATGGGTGGACTCTTTATAAGGCTTGAACAATCCTCCTTCCTTTGAGCTAGCT

>LNC_000972

TCAGTCAACATCAAAAAATCAATACAGGTTATAACAAATTAACGCAAACACAAAAGTCAACAGTGAGATGGAAGAACAGTGAAGCATACAAGTAAGCTCAGAATCAGCAGATTACAACCAGATGTGAGTAAAAACCAAATCGGATTTCGGTGAAATCGAATTGCAGAATGAAAGAGAGAAGATTGGAGAATGGATCTGTGAAAGAAAAGGAGAAGCAATGGGGA

>LNC_000973

TGTCGATACCATATGGCATCCAAGACCAACTCCTTGAAGTAACAGGTGTTGCTTGTAATAAATTGAAGGATACCGAATGGATTTCTAAATTCCGTACTCGTACATGTACAGGATATTATTCCAGTGGAAGGTCTCAACCACGATGATGCAGTCCCAGGAGTTTATACAATTCACTGTCTACCCTTGAGGTTGGTTCACCTACCAGGTGCATCCTCTTCGAGTGATCATGTCAAGACATCTGTGATACTTTTCATGGATAAAAGAATACGTTAAGTTATGTGAATGCAGAATTTCCAAGAACTTAAAATGTACTAGATTCCTTTAGCTTGTCTACCCATGGGATTATGTATATTTTGTTCCGGGTACAAATATGAATGTTGAG

>LNC_000974

TTTTCCCCCACACACCTAGCCCCTTTTTCTTATCAAGAAAGAATTGGCTTTGTTGATAAAGTTGTTAGTTGTTGTGGTTGAAGTTGTTAGTTGTTGTGGTTTAAGTTGTTGGAGTTGTTAAAGTTGTTAGTTGTTAGTTGTTGTTGTTGTTGGAATTATTGTTGGTTGTGTATCATGATTGGGTTGGGGTTTTGAGTTGAAATCTATTGAATGTTGAGGGATTTCATGTTTCTAAGTGATTGGGGCTGGAACCCTTGAAGTTAGAGAGTTTAGAGTTGGAGACGTGAAGGAGAAAGTCGGGCAGTTTGCGAGGAGCACGTCCGCGTCGCGGACTTGTTCC

>LNC_000975

TTTGGCTTATGACGACACTTTCGAATTTAGTTTTCATTTTGTTACTTACCATATCTTTTGGTCACGAAAGCTTAACATTTAAAAGAAAGTGAACTTGAGAGGAACCCATTTTTTAGAGTATAACGTATTCATTTTAGATCTGTTTTAATAATAAAATAAGAGTTAAAAACAATTGTGTAGCAATTTTATAAAGTGCAGTTTTTTTTTTTTGAGGTGCCAACGTACAGATTTTATCCTGAAAATTGCAGCATTTTGAGGGGTAAGAACTGCAGCTTTAAACAGCCCAGCAGTGCAGATTTTTGGTGCAAAATATATACATTTTTGGAGTAAAATGCTATGTTATGAGAGTTTTTTTTTTTGATGCGGCATTTTGGGTTCAAAAAATGATGCATTTTCAGTCTCAAAAGAGTGCTGAATTTGAG

>LNC_000976

GGAAAATCAATTATGTTGTCAACTCCCATTGTCTTTCGAAGGAAATCTTAGAATCCCCTGCGACTCAATCTTGCATTCCCTTAACACTAGTTATACAGATTATTTCTTGAAGATTTTTTAGTCCTATCTAAATGAATCTATTTGGCCTTGGACTTGCTTGTTAAGATCTGTTAGTGCTGAAATTTGTGTCAAGCTGAGTATGTTCATTGTGTTGATAAACCATGGTTCGTCTAAGTCTCCGTAACAACATACATATTTTCGTTTGATAGTAGTTTCTTTTAAAATCC

>LNC_000977

CGTTATTTGTTGTCAACATGATTCAAGGCTAAGTTGAGTTTAGGATAAAAGTTAGTATCTGATGGAGCTTGTAGGACTTTATAAAAGGATCCATTCCAACTTATTTTCTGGTTGTTTGTTAAACATATTACATTTATATCCTTGAATTAAGCTACTCTGGATTAGTTATTCACTAAAATGATCAGATTCAAGAGTCTTATATTGGTTGTCTGGTTTTTCTCTTTGATTTCCTGAAGACTTTCCTCCATTTTTCTAAATAAAATTTGGGTTAGTTTCTCTCTTTGTTCTCCTATCTCGACTAAGTGGTTAGAAGAATTCT

>LNC_000978

ACCTTCTCAATGGAATTGGACCTATCTTCAAAATTTCTACTATTTCTTTCTTTCCATACTGTCCACCAAATGCAATGTGGAATTATCCTCCACCATGCCTTTTGACTCTTGCTCCCCCCTCTTCTTATCCAACAACTCAACAAATCTGCAGTATGCTCGGGCATGGTCCAGTTTGTTTTTGTGAGACTAAGGAAGATGGCCCAAATTTGTGTTGTAAAGTTGCAATGCAAGAATAGATGCTGGTTGGTCTCTGCTGCGTTCCCGCATAATGAACACCAAGATACTATAATCTTTCCTTTTCTCCTCAGCACTTCATGTGTCAGGCAAGCCCTTCTAGCAACCAAC

>LNC_000979

GAAACATAAACCTCAGAATTCTCTCTTTTGATCCCAAAAATATATTAGGTTTTGTTAAAAGGAGTGGACTTTCTGCCAAAATTCTTTCAATTTGTCGATTTGGAGCCGATGCAATCTCAATCTGCGATGAAGGACCCATTTTCGCAACCATTTTACATCAAAGTAAGCCATTTTGGTTTTGAACTGTTTGGTTGGTGGATTCTATTGTGAACTACAAAAGAAGAAGAAAAGAAAATCAACTTTCTGATCATTTTCAACCTGTCTGCTCGTCGGCATCAGTAGTTGCACAAAGGAACTAGCATGCTTATTTCCTGCTCATCTAGGCATGTTTCTGCAGTTTTAACCATCAATTGCAGTTGTGGAGCGCAGAGTTTGTCTGATCATAAATTTACTCTCTATGTTTTAAGTAGGGTTCTTTTTACATGGTTTTCTATCTACTTGTATTAATATTTTTGTCTGCTTGTGTTATTAAACTTAGAAGATATTTGGTGGTATCTTTGATTGAAAATAAAGTTGATGAATGATTGTGATTATTGAATAATTTTGTTAATCCTATCTGTCATTCTAGAGCTAAACTAAGTATTTTTTTGCACCGAGAGTGGCAGGCAGTGATGCTTTTGAATTTAAAGAGAAAGATGACTAATTCTATTTCAATTGGAGGATTCTAAGACTATGAACTAGATAAGAAATAAATATTTATGTGGTCAAAACAAAACTTTATTTCTGATGTAATAGTGTATCTAATGTAATACTATGAAATAGATGAACCTTTGTTCTACACATACACATTTAGTATGTTGCATGGTTAAAAAATTATATGATTGAAAAACAAAGAGTAAGATTGTTTAGGCAACAAAAGAAAATGCTATAGAGAGAGCAAACTTACTATGCATTTGGAGTGAAAGAAATTAGAAAATTGCAAACACCCAACAAAAGACAATTTGTTTTATGATGTCTGGTGGAGATCTTCACCCTCAAAATATCCACTAATAATGTAAAGAATTACGGGACACATTCGAGCTATACTTGATGGGAATCCTATTTCTGTGAGGACTACGTAAACTAACAGGACATAACTAATGAATTTCTGGTTTAACTGTTTAGAAGTATAACGGATTTCCTCATTAAAGAATGTTCAAGTGGTGTGCTTTTAAGACAATGTTGTTCATTATATAGTAGAGAAGATGTGCAATACATGTTAGACATTACAGTGAGTTGAAACTATAATGAAATATATGTAGTTTCTAATTTTGAATTTTTATGTTGTACTGGTACAAAAGTTGTTTGCATAAAGATTTGTATTATCCTTTTGTGCTGAGTTTCTTGACAATCCTTGTATATTCCTTTCTCCATTTTCATTAGTAGTTTAATTTTAGTTTTGAAGTGTTTGCATGAAGCAAAAATCACATGTACAGAGGTTTTGTTTTCCCTGTTCCATGAACTGAATATATTTATTAACTGATTTGATGAAGACGTGATGAATAAGCCATTGCTGCAGCTTTTTCTTATGATCTGGAGCAATATCAAGTGGTTTTATAACTTATTCAGCTCATGAGAACTTTAGTGCAAATAAAGTTTCTGTACATACCGATTTCATTGCCACCTGTTGTTTCCCCTCTTGAACATTATAGTATGTTACTTTTTGTCAGCTTGTTATGTTTATTCTTATTCTTATCCTAATTTTCACGAGTCAAGTACTGCATATTATGCATCCTATGCCTTAGATACAAGTTCAATTTTTATTTTCAGTAACTACTTTCATACAAATATGAAAATTAATTTTAACATGTTGACGAATTCTCAGTGATATTTTTATGATTAGGTAAGATAGAGTTATCTTGAGACAGGGGTGTTTCGGAAACAACCTCTCTATCTTCATGAGGTAGTGGTACGGTCTGCAACACTCTATCATCTACAAAACCCCACTTAGTGAGATTATACCGGGTATGTTGTTGTTTTTTTTTTTATTATTTTATGATAATTTCTTTTTGACTTGATTGAAGTTTGGATAAAGAATGTATAAAATCAAGTTAAGGGGAATGAATTCAAAAAGATGCAGAATGTTAAGTTGTATGAGTTGCTATTACTATACAATTTTATATATTTCTCAAGAATTTGATTTAAATTCTCTTTCATATAGGTTCTTTAGGAACTGAACCTCTATTTGTTGTGATATGTCGTCATTCCAATGTTGTTTCTACAAGATTTGTTTATGCAACATTAATAGTTCGTATAGTATAAGAATGTTGTCTTATCCAAAGAAGACAAGAGAGTTGGTTTCATATTATCATGGGGCAAAGAGTTGGCTTTGCATACTAAGGCGGCTAGTTGTGATTATAATTATTATTTAGTACTTGCCATTATTAGCACTAACCTGAAAAGGAATAATGTCTTTTCGAGTTGTGACAAGAGAGAGGCTCACGTGACTCTTTATTTGGGACATGTATAAAATTTTAAGACATGTTTAATACTCTTTATATATGAGAACATTTAGTTTCATATTTAACCAAAAAAAGAGGAGAAAAAGAATTGTAGTCACTGCCTAATGAGATGATCAGCATTGAGCTACAACAATTTAATATCAATGATACTGCAGAAAGGAATCAGAGAACTCAGTGGCTGTTGACTTATACTAAACAACCAAGTTCTTATAATTAACTCATCAACTTAGTTATTGGAACTTTTTATCCTTATAAGAACTACCTTTTTTCTTCAAACTTAAAGCTATGCAGAGCTTGGTATCTTCTCATTCTTCTAGGATTATTTTCAATCCTTAGTTGGTGAATAAAATATAAAGCTAAGATTTTCAAGCATGACTACCATCAAGCTTATCTCTTGCCAATTTCTGCGTTTCTCGGGTATAACAGCTTAAAGACAAGGTGTCAGTTCTTGAATTGTGTGTGAATAACTGATCTGTAATCTTAAGTCGCTAGAGAGACACTTTATTTACTCTTAGTTACATTTAAGTAGATTATCTCTTGTTCTCATGTGCAAACTTGATCTTTTCATATGTCAAATACGTAAAATACTTTCTTGATAATAGGTGGTAGTGACACTTAAAACTTAGGACCTCTACTTACTTTGTTATTATGTTGAATTGTGCCTTATATTTTTAATGGTTCAAAATTTTAAATGAGGTTGATTAAACAATTTAGCACTAATATATCTTCCTTTAACTTCTTGTAATGTTGAATTTAAAAGGAAAACTTCTTGTAGAGTAGGATATGATATACTTACAAACCTTGATCCTTAATGTTGCTCAAGTGTCAAATCAAAACTTTTTTCATTTCTTTTTTTATTGATTGTGAAGCGTATAACTTTTCATTTTAATTATTGTAGATAGTAGTGTTGGAGTTCATTGTTGATATATGTTGTTATGGGGAGTTCGTGAGGGCTTGTTATTGGCCTGAATCTCCTTGTGAAGTATGTTGATGATGGTAGGCTTGATAAAAAGTGAGGCATTAGCGTATATGATTATCATGATGTTGACACATAAACAGAAAGATTGCCTCTAATCTGAACTACTTGCTCTTGGAATTTGTGATTCAATTATAACTTGTTGAAACATGCTTCTTGCAATACCCCTATTATATTCCTTAGGTATCAATAAAAGAGAAGAAAAATCAAATGACAGGGACGTATACAATGACTCCACTAGTGTGACTCCATACTAGTGTAATTGTTCACTTCTTATTACATAGCTTTTGTTGTGATAGTCCAGGCTTCTATATTCAAATAGGACTTTTCTTACTCAAAAAAAGAAGAGAAAAACTCTCTTTGGGCAGTTCCATTCAACTAAATTAAGTTCTCTTGTTGAATAAATTTAATTGGATAGTCATAAAGATGTATTTATTTTCTTATTGATTTATTTTGATTTTGCAGAAAAAACTAGCATCTGTTACTAAACTAACATGAACTTTTTCTTTTTACAGATGTTAAAATTTGGAGTCTTAACAATCCACAAATAAAGTGCAGATGATCAAGTCTAACTAAAAATATCGAAGAACAAGTTGAAGACCAGAGACACAATTTTTATTTTTTTTTGGAGTTGTTAAATGTGTTTCCATTGCTACCTAATTGTTTATATTTTATGTGTTTAATAAATATTATAAAGTTGATAGATATACTTTTGGTAATATGATAATTTAGTTCTTTGGAATTTACTATGAAACTTTATATTAATACAAATATTTCTCGTAAACTTTTAATGTATTTTTAAAAAATATATTTACAACAATAATCATATAATAATTAGACTTTGAGTTAGAACTTACAAACAATTAACAAATATAATCTTATTTCACTAGAAGAACTGATAAGGAGC

>LNC_000980

GAGAAACATAAACCTCAGAATTCTCTCTTTTGATCCCAAAAATATATTAGGTTTTGTTAAAAGGAGTGGACTTTCTGCCAAAATTCTTTCAATTTGTCGATTTGGAGCCGATGCAATCTCAATCTGCGATGAAGGACCCAGTAAGCAAGCATCTTAATTACATCTATGGAGATTGAGGGCCGAAGATTACCCTAAAACCTAATAACGTGATTCAGTGCGTTGAATATGGGTAAGATAGAGTTATCTTGAGACAGGGGTGTTTCGGAAACAACCTCTCTATCTTCATGAGGTAGTGGTACGGTCTGCAACACTCTATCATCTACAAAACCCCACTTAGTGAGATTATACCGGATGTTAAAATTTGGAGTCTTAACAATCCACAAATAAAGTGCAGATGATCAAGTCTAACTAAAAATATCGAAGAACAAGTTGAAGACCAGAGACACAATTTTTATTTTTTTTTGGAGTTGTTAAATGTGTTTCCATTGCTACCTAATTGTTTATATTTTATGTGTTTAATAAATATTATAAAGTTGATAGATATACTTTTGGTAATATGATAATTTAGTTCTTTGGAATTTACTATGAAACTTTATATTAATACAAATATTTCTCGTAAACTTTTAATGTATTTTTAAAAAATATATTTACAACAATAATCATATAATAATTAGACTTTGAGTTAGAACTTACAAACAATTAACAAATATAATCTTATTTCACTAGAAGAACTGATAAGGAGC

>LNC_000981

GAGAAACATAAACCTCAGAATTCTCTCTTTTGATCCCAAAAATATATTAGGTTTTGTTAAAAGGAGTGGACTTTCTGCCAAAATTCTTTCAATTTGTCGATTTGGAGCCGATGCAATCTCAATCTGCGATGAAGGACCCATTTTCGCAACCATTTTACATCAAAGTAAGCCATTTTGGTTTTGAACTGTTTGGTTGGTGGATTCTATTGTGAACTACAAAAGAAGAAGAAAAGAAAATCAACTTTCTGATCATTTTCAACCTGTCTGCTCGTCGGCATCAGTAGTTGCACAAAGGAACTAGCATGCTTATTTCCTGCTCATCTAGGCATGTTTCTGCAGTTTTAACCATCAATTGCAGTTGTGGAGCGCAGAGTTTGTCTGATCATAAATTTACTCTCTATGTTTTAAGTAGGGTTCTTTTTACATGGTTTTCTATCTACTTGTATTAATATTTTTGTCTGCTTGTGTTATTAAACTTAGAAGATATTTGGTGGTATCTTTGATTGAAAATAAAGTTGATGAATGATTGTGATTATTGAATAATTTTGTTAATCCTATCTGTCATTCTAGAGCTAAACTAAGTATTTTTTTGCACCGAGAGTGGCAGGCAGTGATGCTTTTGAATTTAAAGAGAAAGATGACTAATTCTATTTCAATTGGAGGATTCTAAGACTATGAACTAGATAAGAAATAAATATTTATGTGGTCAAAACAAAACTTTATTTCTGATGTAATAGTGTATCTAATGTAATACTATGAAATAGATGAACCTTTGTTCTACACATACACATTTAGTATGTTGCATGGTTAAAAAATTATATGATTGAAAAACAAAGAGTAAGATTGTTTAGGCAACAAAAGAAAATGCTATAGAGAGAGCAAACTTACTATGCATTTGGAGTGAAAGAAATTAGAAAATTGCAAACACCCAACAAAAGACAATTTGTTTTATGATGTCTGGTGGAGATCTTCACCCTCAAAATATCCACTAATAATGTAAAGAATTACGGGACACATTCGAGCTATACTTGATGGGAATCCTATTTCTGTGAGGACTACGTAAACTAACAGGACATAACTAATGAATTTCTGGTTTAACTGTTTAGAAGTATAACGGATTTCCTCATTAAAGAATGTTCAAGTGGTGTGCTTTTAAGACAATGTTGTTCATTATATAGTAGAGAAGATGTGCAATACATGTTAGACATTACAGTGAGTTGAAACTATAATGAAATATATGTAGTTTCTAATTTTGAATTTTTATGTTGTACTGGTACAAAAGTTGTTTGCATAAAGATTTGTATTATCCTTTTGTGCTGAGTTTCTTGACAATCCTTGTATATTCCTTTCTCCATTTTCATTAGTAGTTTAATTTTAGTTTTGAAGTGTTTGCATGAAGCAAAAATCACATGTACAGAGGTTTTGTTTTCCCTGTTCCATGAACTGAATATATTTATTAACTGATTTGATGAAGACGTGATGAATAAGCCATTGCTGCAGCTTTTTCTTATGATCTGGAGCAATATCAAGTGGTTTTATAACTTATTCAGCTCATGAGAACTTTAGTGCAAATAAAGTTTCTGTACATACCGATTTCATTGCCACCTGTTGTTTCCCCTCTTGAACATTATAGTATGTTACTTTTTGTCAGCTTGTTATGTTTATTCTTATTCTTATCCTAATTTTCACGAGTCAAGTACTGCATATTATGCATCCTATGCCTTAGATACAAGTTCAATTTTTATTTTCAGTAACTACTTTCATACAAATATGAAAATTAATTTTAACATGTTGACGAATTCTCAGTGATATTTTTATGATTAGGTAAGATAGAGTTATCTTGAGACAGGGGTGTTTCGGAAACAACCTCTCTATCTTCATGAGGTAGTGGTACGGTCTGCAACACTCTATCATCTACAAAACCCCACTTAGTGAGATTATACCGGATGTTAAAATTTGGAGTCTTAACAATCCACAAATAAAGTGCAGATGATCAAGTCTAACTAAAAATATCGAAGAACAAGTTGAAGACCAGAGACACAATTTTTATTTTTTTTTGGAGTTGTTAAATGTGTTTCCATTGCTACCTAATTGTTTATATTTTATGTGTTTAATAAATATTATAAAGTTGATAGATATACTTTTGGTAATATGATAATTTAGTTCTTTGGAATTTACTATGAAACTTTATATTAATACAAATATTTCTCGTAAACTTTTAATGTATTTTTAAAAAATATATTTACAACAATAATCATATAATAATTAGACTTTGAGTTAGAACTTACAAACAATTAACAAATATAATCTTATTTCACTAGAAGAACTGATAAGGAGC

>LNC_000982

GAGAATATTGTCTCTATCCCTTAGTTTTTTGCCAAACTGTAATTTTCATTCAACCAAATAGCATATATACACCTCTAGTTCATTTTGGACTCTAGAACTAGTCCTTAAGAGATCAGCTAACTATTTATATGTTATATTTACAACAATTTTTTTTATCTAGACAGGGTAAACATTCAAATGATCAAGAATTGGCTTCCACTTCAGATTTAACTGTCCTTTGGTGTGCAGTTGGAGACAAATTTCCTTTAACAACTTGGCAGCAAGTGTGCACTGTTGTGAGAATCGTCTTCATGAATTCCTTTAATTCCATATATGGTAGAAACTTGTTGCGTACATTCAGCCCAGTATTTCTGCGAGCGGTCCACCTTTATGGCCTCTATTTAACACCCATTGTAGTTCATCCTCCCAACTTCCTATATGTCTAGGCCCTTTTATCCATATCAAGAGTGAATCCCATATGTTGGCGGATTGGAGTATTCACACTCAAAAAAAAGATGGTAGAATAGTTCCGTCGAGCCTGTATTACATAATATGTACCCAGCCGAAACAATTATACCTCATTTGCTTAGTCTATCAATTGTGGCAAGTCTTTGGTGAACTGGTGCTTTCGCAACATGCCTTTCCCAGTGTGAGAAACTTTCAGGGAACTTTAGAGAATTGTGGTAGGAATGACTTGTACATTCTCTTGATACTGAACACTCATTGATATGTACACTTATGGAACTCCTCAACCATATCAAATTCATAAGCCATTTTCGAGCCTCGAGGATCTTCCTAACAAACCAACTTGTTTAGGGATTGCTATTTCATCCAGATGTGTTTTTTTTTATGTAGAAGCTGTGAATCCAGTGAACCCAAAAATTCTCTTTCTTAGAAGCGACGACCCATAAGAGTTTACATATAACTGTGTTGTTCCATAAGTGGTAGTCTATCAAGCTCAATCCTCCAACAGATTTGGGCTTACACAAGGTTTTTCAAGCCACCAAAGCTCTTCTAAACGTCTCATTGCTCCCCATCCATAAGAAGGTTCAATTCTTGGAGTTACACGGATGCAAGATAAGTACTTTACTTGATAGTCTTTCACTATGTTGAAATTATATTTGTGGTATTTGTTCTAATCACTCTCATATTGGGGTACTTGAAGATAAGTAATGACTCATCCGATCGTTTTGCATTTTGGAGCCTCTTCCTTCTAAAAGATGCTTTCTATATCCTTTAAATTGTGATTTATGAATTTTGAGGATGCGTGATACAATTTATTAAAGCACAATGATTTATGGATCTACATTCAAAATCACGTGACGACACAATAATCTTTGGATCTACATTCACGATCATGTGAAGTTTTGATAATTAATAAACTCTTTATTAAGTGTCAATCCATATGTTGAAGATTGAAGAAAAAAGTATAACGAACCAAATTCTCAAAGATCTCCAAGAAGAAGTTAATCTACTAGCAACAGTATTAGAGAAATTTATTCGAGAACTTGTTCTCCTAAGCAGGGAAGGAGACTGCTAAAGATAAAAAAGTGATTTTTTTATTTGTGCACCAAGATTCCTTGGAGAATTGCAATATGGGAGAAGCCAATTCAACAAGAAACTGAAAAGTTATACAAAATAGGCTATCAGAAACTTGCCTATGAAATTAATTTTAAAAAAGGAGATAAAGAAGCTCATGCACATCAAAAAGAAGAACCAGGGGCGGCTCTAACACTACAAGAAAAACAGTCTCTAATTAGGAAGGGAAATATAGTCCCTGAAAGTTCAAATCTGGTCTCAAATGAGAAACTGAGACCAGAAAAATTTGGTCGCCACCCGTCCTAGATACTTTCGTCGCTAGTGGTCAGTAAGGATAGGGGAAAAAATATGGTCCCTGAATTGTTTTGGGACGGGAAAAACCCAGGTCGTGAACTCCTTTTAGCGACTACAAATCTAGTCTCATATAGACCATATATCTTAGTATTCAGGTCGTTAAACCTCTTTTGCGACCTGCCGTAACCTGGTCCATGTAAATTTTTAGAGACGAGTGTACCAGCTGGTCCTAAAATAGAGTTAGAGACCATTAACAATCTGCTCCTAGAATAGCTTTAGCGACCTTAACAAACTGGTCCTAGAGTAGTTTTAAAGATGAACAACAAGCTGGTCCTAAAATAGCTGTATAGGCTAGCATCATTTCTAGTCCTAGCTTCTTTATTAGAGACGAGCAACAATTCTAGTCCTAGATTGACCTTTAAAGACGATCAATAATTGTGGTATCTAACTGACCACCATTGATCCTAGAGCCATTAATATCACAAAAAATCTAAATTCATCAACAAATATCAAATTAATTACTACTAAAAACCTGAATACAAAAAGATGATAAAGAGAGAGGGGGAAAGAAGATAGAGATAAGAGTGCGATTGATTACAATAAATTGGTTAACATCCCATCACTACAAGAAAAGCTGTCAGTTAAGACCAACATTTAGGGGCGAGTTAGTAATCACGTATCTAAAGATATATGTATTAGAATGATATTATATTCCGGTCCTTATTTATATATTTTATTGTGAAGATACTAAATTCAGTCCCTAAAGAACTTATTAATTGGTCCCAAATTCTAATTGAAGGACTTAAGCGGGAAAATATACTTCTAGCCAATTAATTTTAATACTTAAAAAGAAAAGCTTTTTTTTTCACCAAAATTTAGGCGCCAAATATTTTTAATAGAAGCCAAAAAAAGAAAAAATCTGCTCGACTTCTGAAAAGTAAAAACAAAAAAGGAGAAATCTTTAATTTCAGGAATTTGGTCGCAACTTGTAATAGCTTTCTCTTCAAATCAGAACTGACGCTCTTTCATAGTATTTCCATCGAAACTCTGCCCTAATCTCAACACCAGCGGATTTCCATAGCAGCGGCGTCTTCTTGTTGTTATGTAGTTGCTGCTTATATTAGCTGCTCGGTGAAAAACATAGGTTTACTTCATCTTCTAATTAAATTGAGTTGCTTGTTTCTTTCATAAACTGTATTGTTTTCTTGTTTTATTCAAGTTACTCTTCTTCTTCTTCCTTTTTTCTAACATTAAATAGGTTTAAAGTTTCATAATCTACTTTGTTTTCTTTTGTACAATCAGTAAAAGATTATTGGTTTGAGCTTTATTATAGTTGACTCGATTTAGTGAAGTGTATTATGAGTCTGAATATTAAGCAAGAGAAATATGGGCGTGTGATATTTGTCGCTTCGTCATATTTAACGACGAAGGATCATGATGAACCTAGATAAATTTTTTTTTCTCTATTTGTTATTTGAATTTAATCTTATATGAAATCAATGGGTGCCTTAGATGAAAATATTGATCTTTTTGATGATTTTTCGTGTAAATTTTCATACAGTAGGAGTTAGGATTCGCATCATCTCGTTTTCACGGCTAACTTTCCACCATCTCGTTCTGGGCACAAAAGGTTAAGTAGGCTGCTTTGACATAGTTTAAAAAATCAGCTTTTTGTCCCATGTTAATCTATTTGAAGTCTCATCATTGGCTGTGCAATTCCCCAACTTTTCCAGTATATTATGTCACGACCCAAGGGTACCCTTTAGATGTAACATGGCACATAGGACCCCGAAAGGAACCACATAAGCCGCTTAGCATATCAAAACATAAGTAAAGAACAATAGAGAGTAAAACACATTTCGAGTCTTAAGAAATTTTTCATAAAAACAAAGCAAAAGTCTAGACTTGTCTCAATTAACCATCTATTACAATCAAAGGAAAAAAATGGTCACAGCCCTTAACAACCAAATCCGAAAATGAAAGTACAAGAAGGAAACTACTAAATGACAATATTCGTCCTTGAACCATGAGGACTCACCAACTAGCAAGGAGAAAGATCAATTCCAAGCTCTAGCCACAATGGGAACCACCTTGAGAACTAGACCCTACACTTGGGAAAAATGTAGAAAATATGGGTTAGTACAAAAATGCACTAAGTATGATAGTCATGCAAGAAATCATGAAGTCATGCTAAAAAGGACATTTTAGTTTAAAACCATACACTTTACCAAATTTGAACTCATCATGAATACATTGGAAAGCATAAGTCATAAACCATATTATCATGCATAGGTCATCATATCATAAGGCAACATATCATAAGTACCTTCAAAGTCTACTTGTGCAATGCATGAGTAAGATCCCATAATCCTACCCACACTAAGTAAAGCTTCTTAAATAACTATGGTTCATAAATTATATTCTTGTTCATATTAACATATTAGGGAACGAGCCTCAATCGACATAGACCATGTGAGCTAAACATGGAATCCGGTGTCTACCCCACACCGAAAAGGGTTTGTCCTACTTGCCTAAGGTAGAACCATATTCTTAGCTTAGGTGGATCCACTAGCTAAAGACCTATGTGGGCACATAGTTACAGGATAGGGAGATTGATACTAGAAACCCGGACTCAACTATCGTATAAGCTTTCATCTCATATTCAAATCCACTCGGTGCTAAGCATTGATTCCCACATTTTTCATATTAAGTTTTAACTTAAGTTCATAATCATGGAAGAATGTCTTGACATTCAATCCAACATAATCATACTACAGCTCCTAGATTCATTAGGTGAGACTAGTTTTCCAACCTTAGAATCATTACATGTGACAATCCTTTCACATCATATCATATTCATGAATGAGTGTGTACAAGAGAACAACCTCCAACAACACAACAACTAGGTCATAATCATAATCACTTTACTCTCAAGTGTTCATAAAACATTTACATAAATATCATAATCATGCATAAATCTCATACTTTGTCCTTCAAGGATCATAAATCATACTTATCATTAACAAATCTAGA

>LNC_000983

TCGTTTGAAATCTTGTGCAAGCAGCAGTTCCTTGGTCTTGGGAAAAAATTTGAATGGAAGATTAGACGAGAATTCCCGTTTCTACCCTCACCCTCGTCCAATTTTCATCCCCAACTCCCCGTCTATATAAACCCCTTTCTGCTTATTTGTTGGGGGAGGGAGAAAAATATTAAGAGTTAGCTAAGGGTTGAGAGGAAGAAAAATATCTAAACCAATTTTTTTTTAGTTGTTCGGATCAGTCCCGCTCGTTTTTTGTCTCGTTCATTCGAGATTTTCGGAGTCATGTTTCGGTGGAAGAATCGCTGTCCTCCTCAGCTCGTTTGTCCTAGTCTCGCTGCTCCGAAGAAGGTAATATCCTTTTCTTTTATAATGATGTATATTCCATGATGGGTTCTTCCTCAATAGTCATTCTCTGTAAGATTTTTGTTTCCTTTGTGATAGTTGTGTCCCTTAGTATGGTCTAATTATGAGTTTGAATATCATCATCTCTCTAAATAAATAAACTGATGTGATCGGTTTAGTGTCGTTGCTTTACGTATTGATCAATTTGAATGCTTCCTATACTCTCGATTCGGAGGTCATTATCTCGATTTTATATAGCAACCCGAATCGGCATTTTGTTCTCTCTTGCGAAAATTCGAAGCCTTATGTTCATTAGTTGAGATCATTTTATAGCTTGTGCTTGTTAGATTTCTCAAGTGTTTGGTGATTAAATGCTATCGGGACTCTTATCCCCTTCTAGTTTGTACACTGTTTTGCTATGGATAAAAAATAAAATAAAAATAATAACCGATTTTAAGCCTAGATTTAGTAAATCAACTTAGTTACGTTTAATGTTTTAATCTTTAGTTGATGACCCATCTGTGCCATTATTATTTTTTTAAACTTATATCAAACGGATATTGACTTTAATTTAAACTATTAGTTGGTTGCTCTACTACTTTTGATCCTACCTTGTTAATATTTTAGCTTGAATTCATTCTTGGTTAGGCATATGATTTCTTCAGTCGGCTATCTGTTTAAAAGATTAATGATGGAATTTGTCTCATTCAAAATTAGCTATGGTTTCATTTTGTTTAAAATTTGGAAGAATTCTCAGCTTTCTTTCTCCTTTTAGCACTAGTATGTTTGCCTCCCTTAACCTTGAATTGTTAATTATTCATGGTAGAGATTTATTTGTATGGTGTACTTTCTTTGCTCTGTTATATTTAACTAGATGCAATTCGGTATAGTCAAAGTAAAATTTGACAATCTTGGTTTAGTTTTTTTTATTTAAAGTTCCCAGTGAATATTTAATGGGATAAGACCTTTCAAATATTTTCAAATCCTTTTTGTCGTAACTTGAGTGATGATTATTTTCAGTTGTTGAAGGTCTTCGTTTTGCACGTTGTTAATTTGATTGAGAACCTGTTCTCTTATGATAACGCATCACCCTGCTTCGTTTAGATTTCATGTCTTTAAGACCATATGTTTTTTTTTCTGTTCTTCAAAGTGAGAAAAAATGTATGTTTCAAGTCATTGGTTGTTAGGATTTTCATGACTTTGGTTTGGAATTTTTAAGAAAAAGGGTTGCTGGTTAATTATTGTTACAAAAATGTGAGTTAATTTTAATCAAATGCTAAATCTTGTTTCCTCCAATTATTTGGGTATATAGTAATGTTCTGTTTTGTGTGTCTCCCATTGTTCTATTAATCTGAATGTTCACCAGTAGCCCAATATAGTTATGAAATTTTATTTTATTTTACTTTGAGAAATTCATCGATTACCCTTTGTGTGTTTTTTTTTAATTAATTCCTTTTGTTTCTTTTATTGCATGAACTCCTTCAACGAATCCAATCGGCTCCATCTATGCTTTGCATCATCTCGTTGGGCCAAAGGCCTAATATTATCGAGTCGACCTCATCATTCCAAGAATTAAAAGTTGACTCACAAGGCGGGATATTGAATTCTCAAAGTCCCACAAGCCTAAAAAGGGGTTGTATACAAGTTGTATACAACTGTATACACTCATTATGCAGCGATATACAGGTCAAAAAATATGAAAATACAGGGGTGAGGCGAAATACAGGTGTTCGGAAGCAAGAGATACATGAAAAGAGCTGATACATACGCTCATATACACTGATATATGTTCAGTGTATACGCAAAAATGGGAATTGGATTAGCTCAAAATTTGCAGCGAAA

>LNC_000984

GTCTTTTGGGCCTCAGGTGGTTAACGATCTCAGCCAACAGTGGGCTGAAGTATTATAAACTAACCTACTTCTACAAGTTAACTACAAATAGGACAATACATATAATGTTAGGATTAGTTACATGTTAGGAGTCTTTTCCTTGCTGTTCGTAAACTGTTAGGAGTCTTTCTCTTGCTGTATATATACAAGGCATTGTACAATTGAAAACATCGACCA

>LNC_000985

CTCTAAACAACATTACAATGGTGACCTTAATTTTTATGAGACTATCAGTAATGTTAGTAATGTTTGATAGATTAATTGTGAACACAAAATAGATTCTTTGCGTAAATGCATTTGAGTAATGGATTACTGTATGTTTTATATGTCTAATGGCAAAGTTTATTTAATCTTTCTTACACTACTTTTGTATTAACAATTTCCTTCTCAGTCTAATAGATGCAGGTTAAACTATGGGCTTCTTTGTTTCCGTATATAATGTAGTATTCTTGTTGAATTGAAAAAAGTATTTTGATGAAAATTAACTGGATCCATATTGAAATAGCCGCAGATCATCTTTGCATTAACTTAATGTACTATTCTTGATATGGTTCTATTTCTTTCATATTCAAATTTACACATATGTAGATTTCCATGGTTGCATCAAGTTCCAAAGGTGATTTTGATCAAAGATGGTCCAGAAGATCCAAGCATGAAAAATGCATATATTTTTGTCTAATTTAAAAAAGTATGTAAATCTATGTAATTAGCACTCCCACATATGGAAATGTTAAACTATGTAAGTATCTCAGTTCAACAATCAGTAGTATGACATTCACATGAATATGTAATGAACAAATTTGGCAGCTATAATGTGTTTAAATTAGTAATTTTCTCATTGCAATATAATTTAAGTCCATTATAAATTATACGTCGCAACCGTGCGGTTACTCCATCCATTACTGC

>LNC_000986

TAATTATTTAATTTCCTTGTGGCTAATTGTGTATAATCTATCATTAACTTTTTTCTTCTTAATTATAGTAATCCGATTTCTTACTTGTTTTATATTTGGTCAAAAAATAATTTTGTGAAAACTTTGGGCTCTCCTTTGAAATAAGGAAAATGTCATATTAATGGATTCCTTAAATTATTTATTTCCCTTATATGTGCCCCTCTTCTACTATATTATTGACCCCTCCTCCCTTAATTCTTGTCCAAAATAGATTCTTACTTGTTTATTTATGGTAATTTAGTATTAGGTGGTTATTTGACTTAGAAAATTAATACTTTTATGTGCCGGTCATTGCTCTTGGCTTACCGTTTTATAATTGATGATCCTTGTCTTATTTAGCTACTCTTATATTTGGTAAAAATAAGTTAAAAAATAAATCTGATTTTGACTCCTTAAAATAAAGAAAACATTGTAATTGATCCCTCTTCCCTTAATTACATACTAGACTGATTTCTTACTTGCTTATTTTAGGAAAATTAATTTTACAGATTTAGCTCTCTTTGTTAAGGAAATCTTTTAATCTGTAATTGCTCCTTATTAATTATTTTTCCTTTAGCATGATCCTTTCCTATCACTATAAAAGAGGACTCTCTCATTCTTTTGATCGAGGACAGACACACTTTTACACTTTCTGACCCACGGTGGTACACATTCATACATTTTACTCACGTCCTAACTTGCTCACATATTCTAATTCTAAGCAAAATAATATTGCTCTCTATACTATGAAAAAGAATTAAGATCTTCCGGTTAATTAAAGTTCATCTCTGTGTTGTTGTTTTGCTGTGTTCGGGTCATTCTCTGGGTTAAGTTTGTCTCCATTGCTGATTTAATAAACTGCTTAATCGGTTAGTATATTTCTATACATTTAATTTTCTGTTTTGCATCAATATTATTTACTTGTATGCATAAATCCAAATGGAATTTAATAAATAAAGGAAGCATACGAGTCAGTTTTCTATTCCTTTCCTATAAGTGTTTACGTATGTGTGATTGGTTGTTACAATTTCTGTCTAAATATGTAAATGGTTGCTTTGTGTTGTTACTTCTAGTGTATTGTTCTTATCAATTAACAGGTTTCAAACTTGAAGTTTCAAGTTGAAGAATCGCCAAGAGACTAGATCATTTAATTTTATGTATTTTTGGATTTGTATTTATTACTTATTTCAGTTGTGTAATATTTGTATAGTACTCTAACTCTATATATAAATATAATTTGGGGATCGTCTAAAGGGGGGGAGGGATCTACATGCTACGTGTTTACTTTATTGCAACTTAATTGTGTAGAAAGTATGCACATAGGTTGTGATCATTTATCCGCTCATTGTTTGTTAATTTGTCTTTTAGGTTAATTGATCACTTAAATCCTTGTTTAAATATCTACCATCGTTTTTCTTTATTTGTCTGTATTAATGAATGCCTTGACATTTTAGCATATAATAATGTAAAATAAATCTCTTACATGTCACCTGCCTTTGTTGATTCTGGACCACCTTTATGTATGAGAGCTAATTTATTTAAATGTTATTTAGTAATCGATCTACTAAGTAAATTAGAAAATACGCCCATAGGAATTACTTTAAGCATATTCAATAATTAAATAAATTTGTTTTGAATATGCCTTGCTCACTAATAAATGACTTTTGTTCTCTTATATATATAAAGTATAAGTATAGTAAAATAAAGGTAGAAAGCATGCTTATAGGATCTAAATAATTTTAAAGTGCAGTATGTATGCATAACGGCGTAACTCTGTTACAAAAATACCATGCCTATAGGATTTAATAATTTGTTTAATTGAAATAGATGTCTTGCCTTAATTTTGATAATTTTTAGCATGTTTACACTTAGAAATCATGCATAGAAGACTTAATTAATAAGTTGAGACGTCAATCATCATGTTAATAAATGAAAGAGTGATTTTCTGTTTTAAAATAAAAATCTGCTTCCTGAATTGAATCTAGCTCAAGCTAATTAATTTCTACTTTATTTGCTACTGAAAATATGCCAATAAGTCATTCTGTTTTGCTACATGCTTTGTAAAATAAAATAAAGTAATTTAAAATAAGATAAAAAATAAAATAAAAAATATTATGTCTTGTATTTTGTGATCTATTTTCTAATCACCTAAAATAATATTTATGTCTTGTATTTTGTGATCTACTTTCTAATCACCTAAAGTTAATTAATAATTATATTAAGCATGTCTTTTAAAATTTGCTACTGCTTTAAATGTTTCTGCCACAGGTTTTCTCATTTTTTTTTAGCTAATCTACTCTAATTAATTTGGTCAACAAATAGTTAAAATCTGCCTTATAATAACTTTCTACCACTCTTTTAAGTATTCATGCGTTGTATTGCTTGCTATAATGCTCGTTTGTCCACATACTTAGTATGAATCCTTTCACTTTATTTGTGTATGTCT

>LNC_000987

ACAAGTTCTTACTTTGCTTTTCATCTGCTCAAAGCTCTCTCATTTTCAATTTTTGGAAAGTGTTGCTGCAATTCAAGTTTTTACTTTGCTCTTCATCTACTCAAAGCTCTTCTCATCTTCAATTTTCGGAGAGTGTTGCTTCAATTTCAAGATGGATCTTTGATGTTGTAAGCGGGTGCTTACTATTGGTAAGTGGTGAGTAGCGGTTGAATTCTCTTTGTCCTTTCTTTTTATCTGATTTTCGTTTTCGTCGAATGTAGTTTGTTGAAAATTTTAATTCTAATTCTTTGTCCACGATTTCTGGTTAATTTATTGCCACTAATAAAAGTTCAGACTTACAAGTACAATGCTAATAGGGAACTCAATTAGTTTAGCTTGATCATCATGATACTATGTTTCGAAAAAGAGCCTGTATATTTAGTTTTTAAAAATTTGCCTTATTTAAGATACTCAAGGAACTATTATGTTGATAAATGCAGAAATTGAGGATCTGTTGGATTGCAGTTAATGTAACATGCCTAATAGTGCCCTAAAAATATTCTTTGTAAAAGATACTTTTCTCTGCCTATTCTCTTCCATTTTTTACTCTTTGGATTTTTTCCTTTGTTTTTCATGCCTCTCTTACATCTCTTGCTAAATTCAACAAAGTGCGTCATAGTTTTCTTACTTTGATATATGTATATCGTAAAACCAACTCTAACTTTGTTTGTGCACAGTGGGTGTCGAGGCCAAATTTCAGGTTCCTTTTCTTCTATCTTACGCTCAAATCTTTTTCAGTTTGAATTTTATTTTAGGCTTTCTCCTATTTGATGTAATTTTGGTTTCTACTTGATAAGTGGAAAAATATATGCATATAGAACATGATTCATCTTTTTTGCATGTTTGTAATAGTTGTTGGCTGCTGTTACTAACATCAGTTTGGTACTACTATATTCATGATTTTAACTTTGAGTTTTCAAAGCCCTTCCATTGCTTTGATGGACCAAAAAATTAGAGGGCATAGAAACATGTGATTCTATCTTTTTAAGAAAATCTCAATGTATGATCTGCGTCCATTCTTTTGCATTTTTCTTCCTTTTCTTTTCTCTGTGTTAAACTTTTTCTTTGTGAATTTTGTACTACATGGTTGTAGTTGATTGTTTAAACTCTTCTTCAGAACTAACTTTGATTGATTAAATCAGAAAGGAAAAATATAAATGAAAAGAGAAGAACTTTGATGCAGCAAATGTGTTTGACGATATGCATCAAGTACAAATTATTCTTTTATTTATTTTTTAAATTAGGCATTCTAAAGTAGTTGTGTGAGATCCATAGGAATAAATTGCTGGTATTCTGTGGTAATAGGAAGTTCTGCCCTAGAGTTTATCTATCACACCTTCTATCCTATTAACCATTTTTCGTAAGTGCATCTAATGTCATCTTACTATGTTTACATGGAGTAGGTAGCGGAAGATGTAACAATAATATATATGAAAGAGCCTATATGTGAAGACACGATATTTCGTGAGCTTACTTTCTTTAATCATCTCACTATTACATTTGCCTTATCATGTGAACAAGCAAAATGTTGAAATTTTGTCTTAAAAGAAGAGCATCACATAACATGATAACACATAATAGGAGTCTCACAATGATAGAATTATGTACAAACTATATATTTTCTGTATTTTGTATTATGGTTATGATTCTTTACATTTATCGTATGTTTGTATTTTTAACACACACCAGCTATTTGAAGAAGTGCAATAGTTAGAAAGTTGGAATATCTTTGTATTATTGTTGAGACTTAGCAAAAAAATCTCAATTTTATATGCCTTTCAACTTCATTCATGGTTCCCTTATGTTAATTTGGTTATTCTTACAAGAAAAATAATTCATATTGTTTTTGCCATGT

>LNC_000988

TGTTGATTTAAATTGGCAGCATTAAAGCAAGTATCTTAATTAGATTTACCAAGGATAAGAGCTATATCATAAATCATTAAAAGAAACAGGAATTAATGATATAAAAAATGAATTAAGAACAATATAATTAATAATAGGGTGGCACTGGGCTGTAATTGTGTATATCCCTGAAAAGTATTGTTCATAAAAAAATTAATATGTAAAATTGAATTAAAAACTAATTACTTGC

>LNC_000989

CCCCACTTTGCTTTGTTCTTCTTCACGCGCAAACGCAGAAACCCAGAAACTCGGACAGCGACCCCACCCCATACTTGCCTTCGTCTTCTTCACGCTAAATCAACCCGGAAATTGCGTCTCCTACCTTGCCAGAACCCATACCCTGCATACCCATCACCCTCACGTCCCCTTTCCCCCATTTTAAGGAATTTTTTCAAAGCTTTCTACTGTTGTCTTGAAAAGGGGCAGAATGAATTCAAATTTGGAATTCAATTTTTCACGCGATTTCCCCCCTAATTTCCATCTATATATCCATCTATTTTAATCACTGTGAGGGAGTTCTTTTTTTTTCGAAGTTTGAGTTTTCAGCAAATAAGCACTTTACACTTTAGAATTTCGCATTTAATCCTTCGATTCTAAGTTCCGATTCTCTTGGCTTGCTGTTCATATTGAGATTTCGTGATAGAAGAATCGCTGCTCCCAGATCGTCTTATTTTAATCACTGTGAGGGAGTTCTTTTTTTTTCGAAGTTTGAGTTTTCAGCAAATAAGCACTTTACACTTTAGAATTTCGCATTTAATCCTTCGATTCTAAGTCCCGATTCTCTTGGCTTGCTGTTCACATTGAGATTTCGTGGTAGAAGAGTCGCTGCTCTCAGCTCGTTTGTCCTAGTCTCGCTGCCTCAACAAAGGTAATATCCATTACATTTGATATTCTGTTTGTTTCAATCTCTATTATGTGATAAAATTTGTTTCAATTGTTTGATGCTTTCTTATGGATTTTTGCTATTTTCTTATGTTGATATGTCTTTGCCATCAAATCTAGGTCTTGTTGAACAAATATCTTGGTTGTTTAATAGGTGTAGCTATTGATCAAATCTCCTCTTCTCTTTTATGCTTGATCAAAGAATGTTTATTTGTCGTTTGGTTTCATATTAACTACTGCTTCAATTCAGTTTCTTTTGGCTAATACTGTATGAGCAAGTTCCTTCACATGTCATTCGAATTCAATGCATTTGTTAGCTTTTACGTGTTTCTTCTTAAATTTGATTGCTCGTAGTTAGTTATTTCGATCCCTTCTAGGTTTCAGGTCGTTTACTGTTTGTTTTTAAGAGTGACTTGGTTTAGATATGAATATGTGGTGGTTCGTTCATAAAGATTCATGGAGGTTTAAATTTCATTTGGTATATGATTAAGCCTCGATTTAGTACTTGTTTAGTTCTAACCCCTCTTAATTCATATTTTTGTGTATCGGTCATGGCTTTGCTGAAATTTAACCTTGTCGAATATAGTTCGCTTTTACGTAAAATCGATTGGGCGCTTGTGTAATATATTTATCGTGGCTTGAGTACAACGGTTAAGTGTCATGTGGTGTTCGACTATTTTGGGTTTCATTAAGTCAGTAACTTTCCAATAAAATTGACTTTGTTAAGATAATTGTGTTTTTTCTTGTTCTACAAATCATTTTTTATCTTTGAAAGACTAGGATGATAGATTACAATAATAGTTCTCACAATGGTGTATCCGTTTAATTTTTGTATTTCATTTATTTATAACAACAATCCTAGAGTTAATAGCTAAACCTGCATTTAATTTGTTTACTGGAAAATTACGTCTATGGTCTATTTTTTGTTCAAATCTTTACCCACTTTCCCTGTTTTACATAAATGGTTAAGAAATCATGATAATGTTATTGTAATAATCAAGTTCCTTGGTTTGATGGTGGTATGTGTTCTTTTCTTTTACTAAAATTTTAGAGTTGTACAAGTGCTTTGTTTACCATGATAATTTGGGTGTTTAGTTTGTTTTGATTTGTTCTAAACTAGTGCTCCCATCCTAAGAGATTGGTCTCCTATGATTGACCTAAATGTAGCTCTTTGTCATATAATCTATAATTTGTTAGCTGCCCAGTTTCTTTATAATCCATTAGTTATGACAACTTTGGTTTGCCGTGATTTGTGTTCCCTTTAACTATCTTATGTGCTTCTTTAGTCAAAGTGATTGTTTAGTTAGTCATGCCTACTGTTTAATTGTTCATCTAATGAATAAATTCGTTTTCCTAATCCTTGTTGTCCTTTCTCTTTGCATGAACTCTATTTCGGGATGAGTCCAATATGACTTTGTTTTCCTTGCGTTATTTCGTTGGATCAAATCCAACAATACCCATACTTGAGTTATCCCCACCAATGTCCGAAGAAATTGAAAGCTGAAACAGGTCTCGGAAAGCGAGAAAAAGGGCTGTATACATCAGATATACACTAATCTACATCCTGTGTATACAAAAACGGGATTGGTTTAAAGGCCAAAATTTTCAGCAAAATTGGCCCAAAAAGGGGGCCCAAATTC

>LNC_000990

TTTTATGATAATTGTTTTCAAGTATATAAAAACTATTTGTATGAGTGGTACCTTTTCATCATGTAAAATCATCTCCAAAGAGTAAGGTATTTCTGGTTTGAATTTATCAGGAACTATCCACAATCGAATAACACGAACTTTTAAGTTCCAGTGCATTGATTTAGATGTAATTTCTGAGATTGTGTGAAAGTGCAAAGCCATCTATATTACTGAATATCGCAGAACAATCGACGAACAATGTAAAGGTATGTAAAGTTATTACTGAAAGATGTCTATT

>LNC_000991

TGAGTGTTGAATGATGAAATATTTGTTGTTAGTAATGAGCTGAATCCTATATAGTTTGAGCCTAAGTTTTCTCTTCATCGTAGTCTTTCCGAAGCTATCATATGTATTTCATTTTCAAAATAGCTTTATCGCCTGTTTGATCTGCTTTTCTCTGGTTATGGTTCTTCTCGAGTTTTTCTTGGTTGAGTTGGGCTCATTTCGATTTTCTGTTTACTTATTCGTTAATCATCTTAATACTACTCCGTCCATACTTGTCGAAATAGAGTATGTGCTTTCTTGTCATGTGTTTGGAGGACGCGAATGACCTAAGCGGTTATGTTAAGCTATTTTCGAATACGAGTTATCCGAGTTGCAAGTAGT

>LNC_000992

GTTAAGAGTTTTAGTTTTGCAATTTGAATGGACTGTTACTGACTGGGTGAATTGCTATAATGGATGTTTTACCTTGAAAATGCCCATACACATACGTAAAACTTACTAGTTGGGCTATTCTGGAAAGTTCTCCTTAAAATTGATTTTAACTGTGAAAAGGTTAGCAGAACTTGTGATGTGTGTTATTACAACCTTTCATATTGTTAATGGTGTGCCCTTGCTTTCCTTTTCAAATTAAATTCATCCTCATATTTATTGGTCTTATTGGCTGCTATTTTTTCTATGAACAAAATTTGTTGTTGCAAATAAAAAGTATTTAGACTTGGAAAACTCTCTGTGATGTATGACTGAAGATCAAGCTGAGAAAGATAGGATAGTAGATGTATTGAAGCATAAGAAGCAATAATCAAATCTCAAATCATGATTTTCCAATATCCGTTGTTACCTCAATTGATAAAGTAATTTTAATGGTCATACGAACAAAATGCTGTAATCATTAACTTAGTGAATAAGAAGGATGGTTTTATGAGTATGTGACTGACTACTGATAATTGAATAAGGTGATGATCAAGAAGATTTAGGATCTATACTTATAGGACTGCAGACTGCTTGATTTGTGCTAATCCTTGTTTGTTAAACTACTTGAGATATGCTATTAAACCTTTCAACAGATGCATGCCATTTTTTTTCTTAGTTCTCCCATTTTCTCAATATTATAACACTGATAATTAACTTAATTTTCAGACCTTCAATGATAACTAATAACAAAACTTCATATGTTGTCAATTAGTTGTACTTAACATGGCAACTGTAACTATCACACTAATGCAAATGCAACTTCCATGATTTAAGAAAGTGGTCCTGCCACTATTTCTTTCCTTTTGCATTCCATATAGTTTGCTGTTTGCATGTAGTACTAGATCTAGAGCTAATATTGAATCAAGTAGCAACCACATTCCATTTGTCATTCCATGGTAAGAACTGATGATTAAAACTTGTGTGGTTTTGTCTAGTTTATAGTCGTTCCAGCACAAGTTAAAGACTAGTAATTATATATTTGTATCTGAAAAATGCATTGAGTAAAAAGTTCATGTTTGGGAGGATGCACCTAACTGCTGCTTGAAATACACAATTTTGCTGAAATCTCCATGGTACTTCATAGGGAACATAAAATTATTCGCTACTAGTGTTGTGTCTTTCAGTTTTAGGTGTAATTGGATGTATTTGTTGGAATATGAATGGAACAAAAAGAAGACAAGGATAAGTTGAGGGGAAAGAAAGATATGTCATGTCACATCCTGGTTGGAGAAAAAAGATTTTGAAAGGACTGAAAATAATATGTCTCATTTGGAGAACAAGGTTTTTTCCACCATTCACCTAACCAATCAACCTGCTGATGACCCGAGAGAGTTAATGCAATAACTTTTCATGTTATGAGTCTCCATATAGCATATATTGATGTCTTTTGTCATGAATCTTCTATTGGAATATCATTTTATTGATTGAAGCTTGTTTGATTTGAAAACATCCCCTTTTCTTTTTTCTCTATGAAGAGATTTGAAGATGCCCCTCTTATTCCCTTTGATGCGGGGCACATATAGGAAGTGTCCAACCTTTTAATTCACTTGGAGCTAATTTTAAAGCAA

>LNC_000993

CCTACTCCAGCTAGATAGCATATCTAGTCTATTCATTGGCATTGTCCAGCTCAAACCAATCATTGCTAGAAATGTATCCCACTGTTGAGAAGTCACCTTACAGCGCAAAGAAAGATGACAATTTGTTTCTGCATTTCTTCCAAAATAGATGGCAGCTAGAGGAAAGGTGTATTCCCCTTCTCTTCAATCTTGTCTTGCGTCAAGCGAGCCTTCTTAACTACCAACCAAACAAAACATGCGACTTTGTATGGTATCTTGGCCTTCCATAATTGCTTCCAGGGCCATTGCACACTAAGATTATTGTTATGGTTCCTCGTATGATATGTTGTCTTGACTAAAATAGCTCATGAACTGTCTCTCAATTGCCATTATCTATCTTGCTCTAGTGAGACTCCATTGAATAGGTTGAGAGTGTTGAGGAGTCGTGTGAATCTGTCCATCTCCCAATTATTTAAGAGCCTTCTGAAACTGAGGTCCCAACCTTGATTATTCCAGACTTCAGTAATATTAGCTCTTTGTTGTTGATTCAGTAAGAAAACATCTGGGTATAGAAGTCTGAGGGGGCCAGATCCTTACCAGTTGTCTTCCCAAAGAGAAATTTTGTGACCATCCAATGTTGTTACTGAAAATATGCCATAGATTTCTAACAGTTTTCCAGACACTGACCCCAAATACAGTGTTTACTGTTTTGGTCTTCCATGCATCATCTTCTCCATACTTCTCTTTAATCACCCTCTTCGAGAGTGATTTGTTTTCATTTGAATATCTCCATAGCCACTTCATGAGCAAACTTTGATTCTGCTTCCTTAGATTCTTTATACCAAGCCGCCCTTGTTGTTTGTTGAGTGTCAG

>LNC_000994

GCTCTATTTCATAATATGATATTTTTATCTCACGGTCAATAAGATAAAACATTCAAGTCGCTTCATGGATGGCATTCATGTCATTGTAGGCAACTTAGACCATGTGTTGAAGCTATGACAGAAGAAAAATGAAGGGAATCTTTTACAATGTTCTCCTCTTTCTTTGTTTTGTTTTAAAGTGCTGGAAGGAAAATATAAGAGGGAAATCTTTCTTACCTTAGTGTTTTACAATTTAGTCTAGTATTATTAAAAGCAAAAAGCGCAAAAAAGATCTTAGCCTGTATAGTCTTAGCCTTATGGAAAGAAAGAAATGCTAGATGTTTTGAGGATAAGAGCAACAACATGGAGAAGATCAGAATGAATAGCCTTAGC

>LNC_000995

GCCGCGTCTTCCTCGCTCAACAGCAGCAAAAAACATCTGTCAGCCGCGTCTCCCGTCCTTGGGGGTGTGAGATCGCTCCGCGTGGCACGACAAGGACGGGAGTTCGATCCCAGTCGTCGATTCCCATTTCCTCTTCCGGAATGAGGTTGGAGATCTCAGAAAAGTATGTTTGCCTCTAATGGTGAATTTTTTTGAAGTTTGAATTGAGATGGGCCTTATGCCTATCCGAATTTCATCCCTTTTCCCCCAAAAAAAATTCCGAACCCTAGTTCCTTGCTATATATTTGCTCCTGTTCACTGTGGAAAGGGGAGGCGAATTTTTTTGGAAAATTTATTAAAAGAAATCAGTTTAGGTGAGAATAGGGATGTACTAAATACACTAAAGAAATCAGAGGAGAAGAGGAGATAACTAATTCTAGTCCAAGTTCCATTCCTCCATTTTTGCCTGCCACAAGTTCGTTCGAGATTCCACCTAGTTCTTTGGTTCGCTGTTGGTGTTCGTGGTCGTGTGGTGGAAGAGTTCGTTCTGCTCGTTGCTGTCCAGTTTGCTGCACTCCAAAACGTAATATTCATTTCTGTTCGTATCCTTAGTTTCACGATGCGCTGGTCATATGAATTTATTGTATGAGACATTCAAATGTTGAGATTTTCAGATTGTTTCGACTTGTTTTGACTGCAAGTCAATGATGTAATTGGCCTATTTTGAATGTTGGTTGTTTGGTTGTTTGCTTGAAGTCTTGATGAGTCCGTTTGGTCTCTAGTTCATCCCTCCCCATGTCTGTTTTTGGACTCTCTTCTCCTTTTTTTCTTGGATCTTGTTGATTCTGACTCTCACATATTTGTATTGATTTCATCTTCAAGATGTTGTCTTGTTAAGCTTAATTTTGAAATTTCGGATTGATCACAATCGCTTGTCATTATAATTAATTCCAACTCGTTAAGACTATCGAAACTACATTTGCCTGAGCTTTACTCGAACTATGTATTTGATTTGTTTCCAAAATATGCTATTTTATAAAATAAAGAAAAGAAAAATTAATTAAATTACATCTACTGGTTTATTTCTATGCGTTATTAGGAGCCTGTAAATTGACCATTTATTTTATTTTTCATATGTACATGTATGTGTCTTGAATTTTTCCTAAGCTTAGACTTTGATAGTTTAGTGCTAGAATTTCATTTTTTCTAACCATGGTTTTTTTTTTTTTAAAATAAAATCCTCCATGCTTAGTTTCTTAGTATTGATAAAAATAAAATGAACCATAATTGTTTGTTTGATACATAGTTATTCATCTCTTTTATTTTAGCCATGGATGAATTGGTCAACAGTGACTTTGTTCAATTATATATATGCAAGCATGTTGCTAGTGTCTCCTCAAATGAACAATGCTCTTTCATTCTTGAATACCATCACTGTTAATCACTCTCTTTGTCATAAATTAAAAAATGTCATATCGTATATTTTATGCACTACCATTAATCTTTTTCCTTCTTCTCTTGCATGAACCCCCTCGGTTTGAGTCCATCGGACTCCCTTTGTTACTCTCTTTGCATCACGGGTTGGGTAAAAACCCAACATCTTCTCCATCGAGTCAACAATCATTTTATACGAGCTGGAAATTGAGAACAGGTGTACGGAAAGTCCGAAAACAGGCAGTATACATGCTGTATACAACAGTATACACCGGTATACATGGTGAATATTGATATACAGGTCCAAAAAATCAAAATACATGTGTTGAAAGTTGAAGTGGACTGCATACG

>LNC_000996

AAGCTTCTCGCTAGTGCTTTACCCCACCACTGAACATTGCGTTGCTATGGTCAATAAACAGCCTTAAAGTGGTTCCGAATGACCTTGAGGAGGCCCCGGCGCAGCACCTTTCTCGCTCGAAGGATCTATGAAAAGAACGTCCCACTACGAATTCCTATCCCATTAATAAAGTACAGATAAATAATATATTCCAAGTCTGACTTTTAAAGCATACGCATTGAAAAAAACCTTTGGCTTTGGACTTTCTGCTTTGATAGATCTTTCCTCTCAAAATAGGTTTTACCTAACCCACTATGTCCTTTGTTTAATACCCATTTTCAGGAGCGGGAATCTTGAATTGAAAACCCTTTCTTCTGCCCTCGCCACTTCTTTCGCTGACCCTATCCACCTCCTTTGCGGAATACCAGAAAAAGAGATGAATTAGCGGATCATAGGGCTAAGTCGAAGCATGCCTGAACCTTCTTCGTCGGAGACTGAGTTAATATACAATCCCACTTTGGAGACTTCCCTTCGGGATAGGTAGGATATGGTGCCACAAATTGTAGTCTACTCGGCATGCGTCGATTCTTTGCTGGAGAAGCCTAGATCACTAGTTTTGGACTATCGAGAAACCCCCCTCTGCCCGCATCCCTGGAACTTGCTTTGCTTTTTTGCTTTAAAGACCCCGCCTGCTTCACACCTTCTATGATAGGGGAAATACTTCCGCACGAGTACTAATTTGATTAGCATAAATGAGCCTCTTCTGAGCCCTTGAGATTTTACATATCAGCTTCAGAAGATTCAATTGGATCATTCTTGGCACAGAAGAATGAGGAAGCAAAGGTAGAGTGTGCTCTGGAACTCCAACACTTGGGTTG

>LNC_000997

GAACAGTAGCCGACCCCTGCACATCTTCTTCTTCTACACAAATCTGCCAGTCCAACAACTCCCACAGCTTTCTCTGCAACAGCCGCCCACCTCGATCTTTTCATCCGAATTTTGGGGGATTTTGTTTGAAATTTGATACAAGCAACAGCTTTGAGGGAGAAAAATTCAAAAGAGGTTGAATCCGGATTGACGATTTTGCCCCAAGCTACTCCAAAAAATTCACCCGTCTCTTTCAAATCATGAGCTCTAGTTTTCCCCTATATATATCCCCTCCTTCTCCATTGTTCAAGGTGGGATTTTGGGGGAACCAAAAAGAACCTAAAGAATTCAAGCAAAAATCGTAAAATACACACTAAACATATTCGAAACAAATAATAGATCAGTGTGATCATAGTGTGATTCCCCTGCTCATTTGTAGGAGTCTTTGTAATCTCCTTTTGGTGGTGAAATCGTCTTCGTGGCTGCTCGTCTCACCCCTGTTTCGCTGCTCTCTCAAAGGTTATAGGCAGTTGAACGAATACACAAAGGCTGGAAACCCAGATACAAGCCTCCAAAAGTCCTAAGATGGACTATATACATCCATTATACACTGTATACATTCAGTATACGTCAATATACAGGCCAAAAACGCAGGGATACAGGGCTGATGCGAAATACATGAGTTTGGAAGAAAGATATACATGAAAAGGGCTGATATACATGCCGATATACACTGATCTACATCAAGTGTATACAAAAACGGGATTGAGTAAAATTCCCAAAAACAGTAGCGAATTTGGCCCAAGAAAGGCCCAAATTCAATTTTCTCCCTTACGCTTTAATTATTTAATTGCGATTACTTATTATTTGTTGTTTAACTCTAACTTTAGAATTTTTAATTAATTTAGTTGAATCCCCCAAAAGATGAACCATATTCTTTATGTTAGTTTCTTTAAAAATAATTCATTTTATCAACTCTTATGCTTATTCAAATGTTTTAGTCAAAACTTTCACT

>LNC_000998

CTGTGAGAAATATAGGAAAAGAATATTATTGAATTGTTGTTGTGTCTACATTATTACATTGAGACCCTATTTATAGACCCTAGAATACAATCCTTTACCAAGTAGGATACTATTTAGTATTCCTATTTCTATTTCTATTCCTATTCCTATTCTAATAGGATTGTATAAACCTATTCCTATTCTTATAGGATTGTATAAACCTATTCCTATTCTAATAGGATTGTATAAACCCTATTCATATTCTAACACTCCCCCTCAAGCTAGTGCATACAATTCATGTACCTAGCTTGTTACAAATGTAATTAACACGAGGACCGATGAGGGGATTGGTGAGACATCTGTGAGTTGATCACTCGACTTCACAAAATTGACAATAAATTTCAATGTGCTTGATCTTCTCATGAAATACTGAATTTGATGCATAATGCCGATAAAACACAGAGTGATAAAATTACAGTGTTGGAACTTCCCAAACCAAGCTTGCAAAGACTGTTTCAGACCATAGAGTGACTTGTGTAATCAACATACAAGGCTACTAAACTCCCCCTGAGCAACAAAATTAGGTGGTTACTCCATATAGACTTCTTCACAATGCAGCGAACATTGTAAGTGCTCAAAATCTAGTATAAAGTACATGGTTCATGTTGGAATCAATAGACAGTCGATATTAAACAAGTCACCGAAAAACTGCCCGAAACATGCTCATATGCCGGAGTATTAGATTTGATGGGTAAAAGTGGTCACAATCTAAGAAATAAAATTATATGGGTAGGGTCGGAATGATGTCACGACCCAACTAGAAAATAGAAATTATATAGGATAGTTCGAATTGATGGAACGACCCCATTGAAAAAGAGAAATAATATGGGTAGGGTCGGAATGATGGCACGACCCTACTGAAAAAGAAAAATAATATGGGAAGGGTCGGAATGATGGCATGACCCTACTGAAAAAGAAAAATTATACGGGTAGGGTTGGAATGATGGCACAACCCTACGCAAATCAGATTTGAGGAGTCACCGGAAAGACGAATGGAACTATGCAAATCAAATCTGAGGAGTCACTGAAAAGACACATGATGCTATGCAACTCAGACATGAGAAAATAGTTCGAAAAAATCCAC

>LNC_000999

CAAAAATTATTAGCATAATACATAATCATGACCCTTAACTTGTCTTTACCGAACAACTATTACCTCTAACTTTAAGTGTGAACAAGTAGACATTTAAACTTGTATAAAATTGAACAAGTAGAAACATGTATCCTACATGTTAGAATACACGTAGGACGCCACGTAGAACAAAAAATTGTCATGTAAGGTGTCATGTGGGATGAACGTGTCTATTTGTTCAATTTTATTTAAGTTTAAGTGCCTAATTGTGCAAACACAAAGTTAAAGATCATAGTTGATAACTGACGTCAAGTTAAGGGTAATATTTATGTATTATGCCAAAAAAATTCTCTGGTTTTGGC

>LNC_001000

CACTGTTCAAGGGACGGATTTTTGAGACTTGAGAGTACCCCTAAAAGGTTTGGGAATTCTCGGTTCAATAGTTTAAAATTTTGTGGATTTTGTGGTCGTTCGAGTTGAAAGAGGTGGTGGAATCGTCTCCGTCAAACTCGTCGTTCCGATTCGCTGCCCAGAACCAGGTAATTTTCTTTCTTTTCTTGAGTAATTTTTATCTCTGTTTCTTTCATTTTATCATTCATGTGTGTTGCTTATTTTTGTAATAGTTTATTTACTTTTTAGCACCTTAAATGTGAATGTTTCCTTCTTCTTTTGTTTAGTATATTGCAGCTCTTGCCTTACTGTTGTTTTCTTTAGTCACTATTAATGTATGCTACCTCACTGTGGTCATGATTGAAAGTTTAAGTCTCTCTTGAATGTCTTAACGTTTTCTTTTATTCCTTT

>LNC_001001

NTGAGCGGCAGGCAATTATGTTAGGGATGCTATTGTAGCAGTCTCCTTTGAGAAGGAGTACTTCAACGGGTCGACCTGTTTGAGGCGTGTCTGTCCATTAGGTTCAACGATTCTCTATTCTGCTTATTGAACCACGATTTCCATGTAAGCTTCTTCTAAAATTGTTAATCTCCTACATTCCTTGTTTCTTTATTTCTCACATACCTCTGTAAAGTTAGTCTACTGCAATATATCACAAATTATTCAAACGTTTCGTTTGTTTTGCACGTTTATCACTTAATCCTGGAATGTAGTACTCTTCATTACATAGTATAATGGAAGTTGTTAAGTAGTGTGGTTCTTGAATAAGATAACTACTCATTCTCTTCAAATATTTTATTGGCATAAAATTTCCTATTCAATTTTCAGGGGACAGATTGGAACATTTTGCATCACTAGTACTTATCTTCTGGAGACTCTGATTATATGGTGTTTGAGATTTTTTTCAAAGTTGTATAGTATAAAATTCGCAAGGCATAAGTTCAGATTGTATATTTGCAAGGCATAAGATCGGAATATGCTTTAAGTTGTATAGTATATAATTCGAATGATAGGAAACGTTTCTCCTCAAATAGAATTCTATACAACACAAATTCAAATTGATCAATTTCCTACAAGTCACAAATCCAAATTGGTCGGTCTCCAATGCAGGTACCAACACACCGAATGAGGAAAAAAAAATGGGTTGAAATAAGGGGAAATTGTGCATAATGACAAACTGTTAATTTAAAATAAATAATACAGCTATTGTTTCATTTATTTCCTTTGAATAGCTACAGTTTCACATATATTGCTATTGACGGATCCCACGAATTTGTATAAGTAAATAACGGAAGTTATTCCTTTTATACAATTCACTTCATCTCCTTCCTCTCTCCCATACCCTTCAATTTCTCCTAAATTCTCTCCACAAAATTAGTTTGAATTTCAAATCTTCACCAGTACGCTCTCAATCGTGACTTCAAACGTTTCAGGTATTGCCAAAACCTATCTAAATCGATTTTTTTTTCAATTTTTCAGATTCAAGTTTGAAA

>LNC_001002

TGGTACTTAACTTGATAACCATGAGCGTTAACCGCAATGATGTGACTTTTTAGGTTTGACTGTAAATTCAGGTGTTAGATTTATATCGTTTACTAGTACTGTTTATCTACCTGGAATCTCGTGATGCAACAGAAACCGAAGTGATGCGAGTTGAAGTATCAAAACTGATTTGCATGAGAAAATTTGATCAATGAATTTCTGTTGCATATATTCCTGCTAGTAGAGATACCTGATCTGTTTTACTTAGTCTGGACTTGGAATTGCCAATGCAGCAGATGTGGT

>LNC_001003

AAGGGTACCGAAGTTAGCGGCTTTAGAGATAGGGGCGAGGGCGGCGGGACATAGACTCTGAGTCCTAAATCTAAGTTCCTGTTCCCAGTGCGAATGCATTATGCCCGGTTTAATCAGTTACACCAGCTAACATAACAACACTCAACACTCCGAGTGGCATAGGCGCGGGAAGACCAGTTAAGGTCTCGTAAGATAGAGTGACCCACGCACAGCGGCTTCAGGTTCTAAAC

>LNC_001004

GTTTTTTTTCCTCTCAATTCCATTTTTTCACTTCAAAAAATGGAGAGAATTAAAGAAGAATGTAATTGTAGTTTTTTTCAAAGATCAAAACCTTATATAGCTATGATTTCTTTACAATTTGGTTATGCAGGAATGAATATTATAACCAAAGTTTCACTTAATAGAGGCATGAGTCATTATGTTCTTGTTGTCTATAGAAATGCCTTTGCTACTATAGTCATTGCTCCTTTTGCTCTCATCCTTGAAAGAAAAATCAGGCCAAAGATGACATTGATGATGTTCTTGCAAATATTTG

>LNC_001005

ATGGATATATCAATTCTAGCAATTTTCAAATAGATCAGGATCTCTGAATGGCTGAAATATAGTCGGTGAATCTATATGTATATCGATATGTATAGTGGGATCGTATTAAATAAAGAATATGTTATTATTTTAGATTTAACCAATTTGATGAATTACTCCTAAAGGTTGACATCAAACTAGTGCTAGTTCACCTCAAACTAGTGCTAGTTGATGAGAGTTACTTCGGAAACAAAAAAGTAAAGTCAAATTTCTCTGGGGTATTATCTCAATTCCAATAAAATGCAATCGAGTAAAGTATGACTTGGCGATCAGACGATATATGGATAGAACTTATAACGGGGTCTCGAAAAATAAGTAATTTCTGCTG

>LNC_001006

CTTCACGCAACAACAACAAAAACCAGCGAACAAATCGCGTTGCTAACCCAACGAAATCGCGGAAATTCAGCAGCAAATCACGACCTCGAGCACAACGCCTACCTCTCCATTATCAATCCGTTGAAAATTGTTGAGATGGGACAATTGACAGCTTCAACCTTATCTCTTAAGAGGGAGTTGTCGTTCCTCTGCTCGTTCCTGTCCCGATTCGCTGCCCGGAAAGAGGTAAAAATCTTTCTCACGTTCTGTTTTGTTTGATCTTTAGTTGTTGAGCTTTATGTTTTCTCAATTATGTCTTGGTGTTTTCTTTATGATTGGAATTGAGTGGAATCTATAATGCTTAGCTGAATTTCTTTTAGTTGATTTTTTTACTGTTTGGTTGTTCTCCGAAGTATGAATGTATAGTATTTCGCTCCTTCTTTGGTCACTGTTAACCTTCCATGGTTTTATAAATGTTGAACTTTTCCGTCCATTCAGTTCATCATCTCGTTGTTCATCAGATTGTGTTCTTAAAGTCGGCTAAGCCTTGGCTTGGTATGGCTTTGCTTTGTTTTCCATTACTCCGATTCGATTTTTTTCTTTGGTTCAACCTAAGTTCATGGATTGTTGTTTAATATTATATTGTTTTGCAACCACTTGCTAATGTTCAACTTTGGATCAATTAGCTTAATTTTCTCTAGATATGATTTATGTCTTCCGTGTCTAGACTTTAAAGCTTATATACATGTGTTTCCCCTGTTAGTGTCAATCCATGAGTTGTTTAGTAACCAAGTGATTCTTTAATGCGAATTTGTTCATGTACTGATCTATATTCCAGCATATGATTTGTTCAAGTTTAAATCTGTTGGTTTGTAGCTAATTTCCTCCTTTGTTTGTATAAGTTTAATTTTTGCATGATTTATTAAAATTCTTTCCCTTTTATGCTTAAATTATTTGGGCAATGTGTTGACCACTCAAAAATAATTTTGGTTACTGCCTATATGTTTAAGTTTGCTGTTTTTTCTTATAAGTTAATTGGATTCAGAAGTCTAAAGTTGAATATGCTCTTAACTTTGCTAATTTAATTCCTTTTGTTCTGCTGCAAATGTCCTCCCGAGTCCAATGGACTCTCGTCCCATTGCATTTCCGATTTTGGGGCCAAGGTGGCCTAATGATCGTTTTTCTCGAGTCTGTCTCGAAATGGATCAAGAAGC

>LNC_001007

ATCAGCAGTAGCAATACACCAAACAAACCAGACGACCCCACTGCCTCATCTTCTTCACGCAACAACAACAAAAACCAGCGAACAAATCGCGTTGCTAACCCAACGAAATCGCGGAAATTCAGCAGCAAATCACGACCTCGAGCACAACGCCTACCTCTCCATTATCAATCCGTTGAAAATTGTTGAGATGGGACAATTGACAGCTTCAACCTTATCTCTTAAGAGGGAGCCGCTTGCAAAACTCATCGGAATCTCGTTGTGGTAGCGAAGTTGTCGTTCCTCTGCTCGTTCCTGTCCCGATTCGCTGCCCGGAAAGAGGTAAAAATCTTTCTCACGTTCTGTTTTGTTTGATCTTTAGTTGTTGAGCTTTATGTTTTCTCAATTATGTCTTGGTGTTTTCTTTATGATTGGAATTGAGTGGAATCTATAATGCTTAGCTGAATTTCTTTTAGTTGATTTTTTTACTGTTTGGTTGTTCTCCGAAGTATGAATGTATAGTATTTCGCTCCTTCTTTGGTCACTGTTAACCTTCCATGGTTTTATAAATGTTGAACTTTTCCGTCCATTCAGTTCATCATCTCGTTGTTCATCAGATTGTGTTCTTAAAGTCGGCTAAGCCTTGGCTTGGTATGGCTTTGCTTTGTTTTCCATTACTCCGATTCGATTTTTTTCTTTGGTTCAACCTAAGTTCATGGATTGTTGTTTAATATTATATTGTTTTGCAACCACTTGCTAATGTTCAACTTTGGATCAATTAGCTTAATTTTCTCTAGATATGATTTATGTCTTCCGTGTCTAGACTTTAAAGCTTATATACATGTGTTTCCCCTGTTAGTGTCAATCCATGAGTTGTTTAGTAACCAAGTGATTCTTTAATGCGAATTTGTTCATGTACTGATCTATATTCCAGCATATGATTTGTTCAAGTTTAAATCTGTTGGTTTGTAGCTAATTTCCTCCTTTGTTTGTATAAGTTTAATTTTTGCATGATTTATTAAAATTCTTTCCCTTTTATGCTTAAATTATTTGGGCAATGTGTTGACCACTCAAAAATAATTTTGGTTACTGCCTATATGTTTAAGTTTGCTGTTTTTTCTTATAAGTTAATTGGATTCAGAAGTCTAAAGTTGAATATGCTCTTAACTTTGCTAATTTAATTCCTTTTGTTCTGCTGCAAATGTCCTCCCGAGTCCAATGGACTCTCGTCCCATTGCATTTCCGATTTTGGGGCCAAGGTGGCCTAATGATCGTTTTTCTCGAGTCTGTCTCGAAATGGATCAAGAAGC

>LNC_001008

GGAAGAGTGGAGCTAGAAGAAGAAGCCACAATGCGTGGAATGTAACTCCTCTTGCTCTAATGTGGGTCATTTGGAAAGAGAGGAATATGAGAGCTTTTGAAGGGGTGGAGATGAGCTTTGCTCAATTGAGAAGTAGTCTCCGATCCTTATTTTCTTTTGGTGCACCCATGTAGTTCTTATTTGTATCTGTGTTGTCAAAGGCGCGCTTAAACCCAGAAGCGAGGCTCAAAACACGTTGAGCGCTTCGACTTGCTGAATGTGCGTTACAGTGTCGTCATCAAGGCTCTACGACATACTTTTTTCTTGCGAATGAGCATTTCCTGAAGAGGTATGTTGTACTGAAATGTAGCTATTTTGGGAGTTATAAA

>LNC_001009

TTATGATTAGCTGTAGAGAGTAAAATGTTGGACTTCTAATGTGAAAATTGAATATGGACTGATTTGGTATTCACATGATTTATTTTGGTCTTGTTCCAGTGAAGAAAATAGTAAGAATTAATGCTCCTACTTGTTTAATTTGTTTTCGTCATTTAATTGTCTCTTAATTTTAAGTCCAATAGGTAGTGTAGCACTAAATTACAATTGATTCTTGGTGCTCTACAATTCATTTCTCTTGTTGCATTGTTCTTTTTCTATCTTGGGCAGTAAGATCATGATAATGACTTCATGGATGATGTGTTGTTTTATTTTTTAACATTTGTACA

>LNC_001010

GGGGTTTACAGTTCTCGACCCTTTGTTTAATCAGTTCTTGCTATAATTGATAGGCGAGATAACTGGTGAAGAATGTAGGTGTTGATGCTATTCTCCTTTTTTTTTCTTGATAGCGAATAAATCTTTTGTGTAGGTTTCACATATAAGTTGATGGCATCCTTGCTCAAACGTGCAAGGGAGCAGTCTCACCTCTTTCAACTTCCATCCTGAACGGGAAAGGGGCTCAACGCAAGAAAATAATCCTGGAGGTGCAACACTTCCTTGAGGAAAGAGAAGGTCAATTGGCTCTAAGTTGGGTGGTGGGAGAAAGGGAGCCGAGGGTCTATTGAAAATAGTCTCTCTATCTCTACGAGGTAGAGTTAAGGGCGGTTTGGGATTACACTGGGTACGTTGCTGTTGTTGCAATGGGGATGTTTCATGTAGTATTCAACGAATGATGAAGTCTTCCTTCTCAAAAAGATACCCCGACAAGTGAAAGGAGAAGAAGCGCTGTAATGGCTGGTCACTTGGCTGATGAGGGACAGCTTCCTAATAAGTTTAAAGCAAAAAAATCAAATATTTTGTGGTCAGGTACGTACTGC

>LNC_001011

CCCGACCACCTAGACCTAGACATATGTTAATACTAACATATGCCCTCATTGTAACCATATTATATTAAAATATAGTAATAATAGTAGTAAATAAAATACTCAACTAAGAAGCAAAATAAACACAACCAAACTTCAATAATAAAAAAAAAACCGTCATTCATCAACCATTGAATTTACTCTTCAACTTTCTCTGGTTCCTCAGATACAGGGGCAGCTTCCTCTGCTTTCTCTGCTTCCGGTTCCACAACTGGGACTGCTTCTGGTTCAGCTTCGGGTGCTGCAGCCTCCTCAACGGGTGCTGCAGCCACCGGTTCCTCAGCTGCGGCTGCAGGTTCTTCTGCAGCCGCAGGTTCCTCAACTGGGGCTGCAACAGGTTCTACTGCAGCTGCCACTGCGGCTGCAGGTTCTGCAGCTGCAGGCTCTTCAGATACAAGGGCTGCTGCTTCCTCCG

>LNC_001012

ATCTGTCTTTTTAAGAAAATCATATTAATATATATCATTCTTCAAAAGATTTTGTTGGTGAACAAAATATGATAATATCATCTTCTTGAGGCAACTTAAAAGTAACTTTCTTCTTCAATTTCCCACTTTGTGTTCTATTCATCAACCTTTCTTCTTCTTCTTCTTCTTCTTTTTCTTCACCAATAATATTAATCTCCTCAATTTTTCTAACTCTTTCTGAAAAACTAATTCTTGGAGTACCAAACAAGTCCTTATTCATCATAGCTTCGGGTGATGAAAAACTAATTCTTGGTGTATAAAAATTGGAATTATTCACTTCATGACATGGTGAAGATACATAAATCCTTTTAGTAGTAGTTTTAGGGTTATTTATGTAATTTGGTGAATCCAAAAAACATTGGTGTAAAGATTTTTCTTGATGAAGAAGAGTACTAGTATTATTTTTAGTATTTGGTTGATGAATTTCTGAATCTTTTTGGTTGAGACAACAAAGATTTGAACCCATAATTTGAGATAATATGCTTTATCCGTTTTAGTGGTGTACATGGAGTAATCGTCTTGTTGCTGATAGATATGACTATGTGATCTCGAATTTAGAGTATCATACTTTCACGCAACTCACATGGTTCAACAAGCGATAAAGATAGAATACAAAGAATAAATAAATTACAAAATTGTCATATGGTGGATATTAAGGGTGAGAAATGAGGTGAATTAGATTTT

>LNC_001013

TGTCGAAACACTGGTGACTTGATACACTAACCACTCATCTTCCTCCTCTTGGCTTGTAGAACTGGGAGGTGCATAAAAGAATGGTGTAGTCTCTGAAAATACCACATCACTTGACACAAGATATTTCCCAAGTTGAGTAGAATAACATCGATACCCTTTTTGAAGGCGAGAATAGCCTAGAAAAACACACTTCAATGCCTTGGGATCTAACTTGGTAACAAATGGTCGAACATCTCGAACATAGCATGTGCTTCCAAATATCTTGGGTTCAACAGGAAATAGTGACTTGTTTGGAAAAAGAACAGTGTAAGGCCTATTACCAACAAGCACAGTAGATGGCATGCGATTAATCAAAAAACAAGTCGTGGAGACCGCATCTGCCCAAAACTGTTTAGGAACCTTCATTTGGAACAAAAGTGCCCGAGCTGTCTCAAGTAGATGCCTATTCTTCCTCTCAGCAACTCCATTTTGAGAGGGTGTATCAACACATGAAGACTGGTGGAGAATACCACGTTGTCTCATGTATGTCTGAAACAGTTCTGACATATATTCTTTGGCATTGTCGCTTCTTAAAATACGTACCGAAGCATTGAACTGAGTCTTAACTTCAGCATAGAAGGCACAAAAGTGAGTAAACACTTCAGAACGACTCTTCATAAAATAAATCCAAGTCATCCGTGAGAAATCATCCACAAAAGTGACAAAATACTTATGCCCAGTTTTGGAAACGACAGGACACGGTCCCCAAACATCAGAATGAACTAACTCAAAAGCNN

>LNC_001014

CAACAAGTCTCAAATTGTAACGAAGGAAGAAGACCCAAATAAGTCACAATTTGATCTGAACAGGGAGTCCACCAAAGATAGTTTCTAACACAGGCAGCCTCCTAAATATTACTTCAGAAACATAGAACAGAACAACCTATAAGAAACTCAAACCTGACCAACAAAGTAGCAGCAGAATCTGGTCTGAAGATTTGTTTATTTATTTATTTATTTCAAAATAATTTTTCCCTTCGTCAAACGTGGTTATATGATCTGAAACCACCACAAAGCAACCAAAAAATATCAAGGCAACCACCTCCAACAGTTAGGCAACTACCGGAAAAGTTGTCAATTTCCCGGTGGAGTTGCTGACCCAATACGGGCGAATCAGTAGCTTGGCTCTGATACCATGTAACTTATAAGGTAGGAAGAATAACTTGAATAACTTATTCATTACTCAACAAATACAATGATATAAATACATAAGGAGACTAAGTTAACTATGGTAACTAGATAGCCAAATCCCTATAAATCAGCTATGCTAATAAATACACA

>LNC_001015

TAACATCATAGGTGTGGGGATGGTTACCTGCTCCTTGAGGCTAATGAGAAGTTTTTTTCCATAAAACAGATGGCACTGTGACTTGTCAATTGGGATTGAAGACGAAAGCTCCATTGCTAGAGACCTGATATGCGAGACCTGGTGATCGGTGGGGCAGCAGAACAGGGATTGATGCATTTTCTTGATAGGACTTCCCAGCTACCTCATTTATGACAATGTTTCATGTGTATAAGATCTTGTGAAGAGGATTAATCCATGCAAGATACTTGTAGCTCAGACGTTAAGTGACACTGACTATCTGCATCAACTTCTCGGAAAAATTAAAATCAGACTTCTAACTACCAAAAACACGAGTGTAAAATGAAAAGAAAAATCTCAGTCACCACGTAACTGCCACATGCATGTACTTTGTTAGATTTGCAGCTGAAAATGGTGTTAGGACATGCATTCAACACCCTTTTTTTATTTTTCGATCAAAATTTCTCAGTTTCTCTCAACAATATTAACTACAATTAGTCTTTTACCCAATTCTTCGATAACAATTTCCC

>LNC_001016

TGGGAAGAGAGGTAGAGGATGAGCACTCACATCCTCTGTTTTCATCTTATTCCTAAAATATTAATAATAATAACTATATAGCTATATACACCTCTTTAGATCTCTGGTTATAATTTCTACCATTTCCCCAATTTGGGTTTTTTCTTGAAAAAAATGTTCAATCTTTACAAATGATACATTGTATTGTTTTTTGATTGATGGTACAAGGATCCATGTTCTTGGATTGAATCCAACACTTTCCCCTTGCTGTTTGATCAGTATTCCCACCTGGAGTCTACCTGATTTGCTAGATAAGGTCTGTTTCTTGTTTTGTTAATCCTTTTGTTTATCCACATTTTTGGATCTTTTTATAAAAAAGGAAAAAAAACTGGGGTATG

>LNC_001017

GAGACAACTAAATCTAAGGTGAAACCCGAAGAATCTGAACCTTGCAACCTGGGCTTGGTCTTATAAGTTGTTGTCAACACTTTTTAGGAAAGAAAACTTGGGGAGTTCTTTAAACCAACAGTACATATAAGTGTCTCATTTTAGGCTTCGTTACCTAGCTCATCACAAAAGTTTGGCCCAATTTGGTAGCCCATTGGTGCATTTTTTGAATTTTGACAACTAGTCTGGCATCAGTTTGCAAGTCAAGCAAATGTCACTCCTTCATTCTGTTCATTTATTTGTGCATTATGCTTTATAGCAGCATTTTTTTACTCCGTGTTGTTAGAGAAAATCCACGAC

>LNC_001018

AAATTTTTATTTTTTTTGTCAATGATGATGGTGAGACGTGAATGTAGTATCTCTATTTGTTTTAATACTATATCGAATTGTGTGATTATTCATGTGTTATCAAATTAATGAAAGGATAAAAAAGGGGTAGGTTTAGGACATGAAGTGAACTTTGCCTGTCTCTTGCAACAACTTTCCTTACGTGCAATACACAAACCTAAATAATTCTTTCATGCAAGTTACTTGTCTTCCACAACCAAATCTCCACTTGGAATATGATATGCATGCCTTTTCACAAATTACTTTTTCCAATATTTTGAATCAAGAAAGGAAACTAAAAAGGGAGACTTGGCGTAATTGATACCGTTGTTGCCATGTGATTAGAGGTTATGGGTTCGAGTAATTGTGTGAACAACTTCTTGCAAAAATACATGGTAAGCTTAATACTTGATATTTGTGCTAGTGGGAGATAGTTGTTGGTATCTGGTGGAATAATCAAGGTATGCACAAGCTGACTAAAAAGAACTAAATTGAAAATGGCTATAAATAATTAAATTGAATTACACATGCATGCATCTAAAAGGTCACTTGTTAAACAAAAGCTTCATTAGAAAGAAAATGGGAGGATCCTTTCTTGTTGTGTGTGTATTGTCAATCTTGTGATAAGTGTATATAAGAAATTGCATGTGAAGATCCAAGATTTATAATGGAACTGTGAATACCACTAGGTGTTAATTTTATTTTAATAGCATGACTTGTTAGGACTTTGCCTCCACTAAAAGACATTTGAAATTCTCACCACTATATTTATAAAAGAATATTGGTACGTTATATTATATTGTATGTGTGGCTTACATGTCACACGTCTTCTTTAACTCATCATATGTTCAATCAAATATTTTCACATAAAATGTAATGACGGGAATAAGAAAAAGGTGATGTACAATTCAAATTAAAGACCTAACTTAATTAAAAGGTCAATGCTAGCTAATCAACCAACTCTAATGGACCGT

>LNC_001019

CGCTGATCCTCCACTTCTGCATATTTAAGACTTATGTTCAGAAAACTAAACAAAAATCAGATTCATCGCCTACTGTATCATTGATTACAAGCTATATATAAGAGAAGTGGCATGGTTGACCTCTGAAAACTTCAGCTCATCTAGAGTAGGAATGGTATCATCATCCTCTCTTTGGTTGTCTGGTCGTTTTTTCGACAATTTTTCCTCGAGGGATGTGACTTGCTTCAAGCAGTTCTCTTTCTCGAATTGAAGGGATTTTATTTGCCACAGAAGTTGGCTATTTGCTCTTCTAAGCTGGCAAAGCTCATTTTTCAGCTCGGAAACTTTGGTACACATTACTTCTTTTGCAGTTAAGTCTCCTTCCAGCCTCAGAACTTTCTCCTCTAGTGCAATTGTCTTGTATTTGCATTTTTCTGCTTCCGCCACTTCCTTCTGCATGCAGGTGATTTCCTGAAGTAATGATGCCTTCTCTTCCTTCAACTCTTCACAATCTCCATATTTCAACAACAGTGTAACTTGTGTTCTCTCACTTTCAAACTTCATCCCAGATAGTGCTTCTTTAAGATACAAAACTTCCTCCTGAAGTAATGGTAATGTATGCAGTTGAATCTTTAGGCTAGAATTCTCTTCAGTCATTTGCAGCATTTGACATTCAGAAGACAGTAGTTTTGACTCAAGCTCATTAATGGTGCATTTAAGCTTTGCTTCACTGGATACAAGATGTTCCATCATCCTCAGTAGCTTATCACAATTAGCTGCCAAAGTTCCCTCCTTGGCTTTGGAAGTACCAAGTTCACTCATAAGATCTGACATCTCTACCATCAGGGATTGTACCTGTTCTTGCAACTTTTCGACTTCAACTGTGTTCTCCGGAGACATTTCACGCAGAGTGCACCCTTCTACAAGAAATTTTTCTCCATGCTCTTTAGTCTGTAGGTGGAGATCGTCAAGTTTGAACTTAAGGATCTTCTCTTTTGATTCAATCTCTTCCATCACTGAAGAAAAGTTAGCTTCTAAAGACTCTATCCTTTTCAAACAGGTGGAGAAAGAATGCTGAGATTTCCTCAATTCTGCTTCCAAAACATTATGGCTCACATGTAATTTTAATTTTTGTTGCCTTAGTTCTGCATTCAAGTTTTGAAGCAAACAACACTCTTCCATAAGACTTTCTGTTGTTGCTTGGAGCTTTGGATTTACTTTCTTCAGATACTCGCACTCTTCTTGAGCATCTAACCATCGCTTTTGCATCTCCATCAACTGTTCTTTCATATCGAGCTTTTGTGACTCCTTTTGATGTTCTAATTTTCTAATCTTGCTCTCAAGGCTCACAACACGATTACCAGAATGCTGC

>LNC_001020

TAAAAAAGTACTTTGTACTGACGGACCTAATTGTGCATCTTCATCTTCACAGAAGGCCTTGGATGTCCGGTGAAACTTTAGTCATTCTTCCACCTTCTTCTTTCAATCGTTGATGTTTAGGTTTTTTTCCACTATCCTCATAAGTTTTGTCTCGAAAATTTAAGTGGTACTTTGATTTTATTTTTCGTTTTGATTTTAGGTCTTATTTTGTCCACTTATTATTCGAATGGTTTGATTTTTCATTCAATTTGTTGATCTATTCGGTTTCTGTGTTGTAGTTATGTTTCATGCACTGAATTTAGATTTAATTTGATGTTGTTTTGATCCTTCGTATTTTGCAGGGATGAAGCGTAGGAACTTCATATTCAAAATATCTTCCCAATTTCTCCATTTCTCATAGGGAATGTTGACAAAAAAATTGAGAGTGTTGCATGCCTTGAAGGAAGGTCACAGGTTCTATCCTTTTTGCTAAAAAGTTTGGTTCCCACTGTTTGGGTTAGTTTCTGAACTATCTTCATGACTTCTCCCTGTTTTTCTCACTTTTTCAAAGTGTCAAATATCCTGTTGACGGATATCTGATGGTAGTTTATGGCTTGAGAGCCCTAAGACTTTTAATTGGTCTCTCTAATTTCACTAATGTAATCGCTAGATAGTAATTTACAACAATGAATATACACTTCAATCATCTTAAAAGTCTTAAACAATTACCTGAACAAGTTGGTAATTTGTTTTAAACACAAAAACTATAATTAATGTTGATAATAGGGATGATCGAAACAGTATATTAGATACCATATATTCTAACTTAAAAGGTCACAGTTGATTCGCTATTACAAGGTATTCAATAGTGTTTGTCTTAGCTGAAAATACATCTATTTACTTGTAACAATAGCTCACGTTTTCTGTCTCGCTGTTTATATAATACTCCCCCTGTTTCAATTTGTTTGTCCTATTTTGACTTGACATGAAGTTTAAAAAATATAAAAGAATTTTGAGTCTTGTGGTCTTAAATTAAAAGTTATGTCAAATGGACCAAAATGTCATCTAATCTTGTGTTTTTAAACATTTCATGTAAAAAATTGAAATTGAAGAGTTGCTTAAAAAAGGAAAGAGACATTCTTTTTGGGACTAAAAGGAAAGTAAGACAAATAAATTCTACCGGATGGAGCAATTCTTCTTTGTAAGTATCACTTATGTATTAATAACTGTTATGTTATTTATTTGATGGTAAAGAGGCATGAAGCATAAGGGAGATTTCATAAAGTTACTCTAATTTAGAGAGGAATCGTAACTTTAGTTATTTTGTTACCAATAGGCTTACAGATTGAACAAAAGAATTTATAAAATGATGATGATGATAGTCGAGATAATATTTTTTGGATATCTGAGTGCTTTTTATGGAAGAGGAAGAAATTATTGTGAAAAAATTTTACATCTTATTTCTAATATATAGAGACGTGGTAACACAAAAAGAGGAAAAAAATATAACTCTTTGAAAAGCTTGAATCTTTAAGCATACTCTTGGCAACTTTTCTAACTGATTCAATTAACTCTTGAAATGTTCCAATTGTTAAAAAAATAAATAAGGGAAGAAAAGGTCAAAAATTTACTATTATTTAAGTCCGGAATTGTTGTGTAAGATCGGTGAGTGGGCTTCCATGTTGTTTGTCCTCTGTGGAAGTTCTACCCTCAAGTTTCCACGTTAGGTTCATGTTCCCTAGAATAGAAACTAGAAAGACATAATTGGATATAGTTGGTCATTTTTGTATTCTATGGTGATACAAAGCTTACATTTTAATCTGCTTTTTATTCATCTTGATTCAGTATTTTCACATTGACAGGTGATATGATATGCTCTGCTTTTATTTCTACAAAATCATTCATCCATAATTGCATTTAAGGTCATTTGAATTTTCGTGGAGTTGATGTGAAAAATGTAGCAATAACATAGAGGAAAGAACATGCTGATGAAAACACCTCTGCAGTATGAGCTTAGTTTTCTTAAGAATCTCCCCGATACATTTGTGTTTTCTAAGACATTTATAATATTTCGTTTGAAGCTTTCTTTGGCAATGCCAACATAAATCGCCTTGGATTCCTTCGATTATTTGATGTATGTTTCTCCCTATCTCTTCTCAAATAGGATACCTTTTATCACATGTTTCTAACAATATATTAGCTACAAGTTACATGTCTTTTAAATAGACAAATATTTTTATCTAGC

>LNC_001021

TAAAAAAGTACTTTGTACTGACGGACCTAATTGTGCATCTTCATCTTCACAGAAGGCCTTGGATGTCCGGTGAAACTTTAGTCATTCTTCCACCTTCTTCTTTCAATCGTTGATGTTTAGGGATGAAGCGTAGGAACTTCATATTCAAAATATCTTCCCAATTTCTCCATTTCTCATAGGGAATGTTGACAAAAAAATTGAGAGTGTTGCATGCCTTGAAGGAAGGTCACAGGTTCTATCCTTTTTGCTAAAAAGTTTGGTTCCCACTGTTTGGGTTAGTTTCTGAACTATCTTCATGACTTCTCCCTGTTTTTCTCACTTTTTCAAAGTGTCAAATATCCTGTTGACGGATATCTGATGGTAGTTTATGGCTTGAGAGCCCTAAGACTTTTAATTGGTCTCTCTAATTTCACTAATGTAATCGCTAGATAGTAATTTACAACAATGAATATACACTTCAATCATCTTAAAAGTCTTAAACAATTACCTGAACAAGTTGGTAATTTGTTTTAAACACAAAAACTATAATTAATGTTGATAATAGGGATGATCGAAACAGTATATTAGATACCATATATTCTAACTTAAAAGGTCACAGTTGATTCGCTATTACAAGGTATTCAATAGTGTTTGTCTTAGCTGAAAATACATCTATTTACTTGTAACAATAGCTCACGTTTTCTGTCTCGCTGTTTATATAATACTCCCCCTGTTTCAATTTGTTTGTCCTATTTTGACTTGACATGAAGTTTAAAAAATATAAAAGAATTTTGAGTCTTGTGGTCTTAAATTAAAAGTTATGTCAAATGGACCAAAATGTCATCTAATCTTGTGTTTTTAAACATTTCATGTAAAAAATTGAAATTGAAGAGTTGCTTAAAAAAGGAAAGAGACATTCTTTTTGGGACTAAAAGGAAAGTAAGACAAATAAATTCTACCGGATGGAGCAATTCTTCTTTGTAAGTATCACTTATGTATTAATAACTGTTATGTTATTTATTTGATGGTAAAGAGGCATGAAGCATAAGGGAGATTTCATAAAGTTACTCTAATTTAGAGAGGAATCGTAACTTTAGTTATTTTGTTACCAATAGGCTTACAGATTGAACAAAAGAATTTATAAAATGATGATGATGATAGTCGAGATAATATTTTTTGGATATCTGAGTGCTTTTTATGGAAGAGGAAGAAATTATTGTGAAAAAATTTTACATCTTATTTCTAATATATAGAGACGTGGTAACACAAAAAGAGGAAAAAAATATAACTCTTTGAAAAGCTTGAATCTTTAAGCATACTCTTGGCAACTTTTCTAACTGATTCAATTAACTCTTGAAATGTTCCAATTGTTAAAAAAATAAATAAGGGAAGAAAAGGTCAAAAATTTACTATTATTTAAGTCCGGAATTGTTGTGTAAGATCGGTGAGTGGGCTTCCATGTTGTTTGTCCTCTGTGGAAGTTCTACCCTCAAGTTTCCACGTTAGGTTCATGTTCCCTAGAATAGAAACTAGAAAGACATAATTGGATATAGTTGGTCATTTTTGTATTCTATGGTGATACAAAGCTTACATTTTAATCTGCTTTTTATTCATCTTGATTCAGTATTTTCACATTGACAGGTGATATGATATGCTCTGCTTTTATTTCTACAAAATCATTCATCCATAATTGCATTTAAGGTCATTTGAATTTTCGTGGAGTTGATGTGAAAAATGTAGCAATAACATAGAGGAAAGAACATGCTGATGAAAACACCTCTGCAGTATGAGCTTAGTTTTCTTAAGAATCTCCCCGATACATTTGTGTTTTCTAAGACATTTATAATATTTCGTTTGAAGCTTTCTTTGGCAATGCCAACATAAATCGCCTTGGATTCCTTCGATTATTTGATGTATGTTTCTCCCTATCTCTTCTCAAATAGGATACCTTTTATCACATGTTTCTAACAATATATTAGCTACAAGTTACATGTCTTTTAAATAGACAAATATTTTTATCTAGC

>LNC_001022

CGAATATTTATTTTTAAGTTATTGTTTAATTCATTTACTTTTAGTCATATTTATTTAGTACTAATACTTTTACTAAGTGTTTTTACAGGTACTCCGATGTCACTTCTCATCGAAGTCATTCGAATAAAGTTCGAATGTTATATTCATTATGATGTATATTGTTGACGATAGACAAATATTAGGTTGTTTCTCATATTACTTGTTTACTTTATTACTTGTGTATATTGCATGTTTGTTATATTTGTATAATATTTTTTAC

>LNC_001023

GCTGAGACGAAAACGAGAGGGTGAGGGTGCGAGGATCTAATCATGGCGGCGAAAATGGTGATGGTGTGCGACGGAGATGAAGGCGCAAATGGTGATGGTGTTCGGCTAGATTTGGTGTGGTGGCTTAGGTTGTTGGTTTTGTGAGGTTGGAGAAGGAAAGGAAAATGGTCGTGACGTGTGGCGGAGATGAAGGCAAGTAAAGGAAGGAATATGAGTGATTTCGCC

>LNC_001024

TTTTCCTATAAGTTGCTTAGCTTTCACTCTCATCAATCATCTACACTACAACATAAAGAAAAATCTCAAACAAGAGAAACAACAATTCTCATATATATAGATATAGAGAGATGTCAAAGCAAGTGATTAGTAGTAGTGCTTGTTGGGGAAAAGGTGAAAAAGTGCCACAAATTTGTAGGAAGACAGAGAGGAAGTTGAGCTTTGAATTGAAGTTGGAAACAATTGAAGAAGAAAGTGATCAAAAATATGGTGGCCAATTTTGCAAAATGTCACACTCATCTTACAAGCTAGCTACTACTTCTTCTAATATTAGTAAGGGCTAGCATATCTTGTGTATCACAAAATTGGCTAATAATTCAACTTTTCTTGCTTTATTTTGCAATTATAAGAGGTTATATGTGCAATATTCCCACCCCACTACACTTCTTATTTTAATTGTTTATTTATTTTGGAGCTCAACTAATTCAAATTTGCAAT

>LNC_001025

CAACTTTCAAGAACTTTGTGGAAAACATCATGAAGTATGTGAAGCTATACAAATCCCATGGAAGCCAAGAAGATGAATTGTCAAGTGCTAGCACTACTCAGAAGAATAATGAGTTAACAGATGATCATGACAAGCAGAAGGTTAAAAGATTCAAAGCAGCCATGCAGAAAGTGATGTTAAAATTTGAGTCCAAAAAATCTCAAG

>LNC_001026

CAGAAATTTAAAAAAAAAAAGGGCTGAAAGCCCTAACAGAAGCAGCAGCAACGCTGCTACTTCAGGGATCAAAAGAAGAAAAAAAAAAGGACTCTTCTTGTTCTATAGTGAGCGAACGAACGAGGAGAAACATAAATTTAAATTGAAAGCCTTAGCTCTCAAACTAAAGAGAGAGGTGGAAGAAGAAGAAGAACAAGAAGAAGGTAAGTTTCTTAACTTAGTCTAAAGGTTGAATTTGTATCTTCCATGACTATAAGATTGGGAATTGGGAAGTTGAGAAACATGGGTTTGGAAGAAAAAGAGGAAATTTATTTCGTTCTTATGAACCAAGTGAATATAGTCAATGATTTGAACTATAAACTTTGGTATTGGGCTTATATGCAATAAGAGTAATTGGGCAGTAAGGTTAGGTTGATCTTTTTACAGCGAGAATTGACCAAGAAGAGACTAATGTGTGTGATTAAAGCACTAATTAAGTGATTGGGCAGATTTCGAATCAGTCATGAAACCTCTAG

>LNC_001027

CTCTTAGAGTTGCTCCATTTGTCATTCTTTTGATTGAGTCAATATTAGTCCTTCGAATTGTAGTCAAGTGACTAGTTTAGAACTTTTTTTCCGAAATTTGGAGATGAATTGGTTTTATTGCGCGACAATTTAATCATTGATGGATATTAAATGGTGGTTTATAGTTGCTATTAGTTGTGTTGTGAAATTCTGAGACAGGAAAAATCCAGGATAGGCCTAAAAACAAAGGAAACTCTGCCCGATTTTCTGTAGAGTTCTGGTGAGGCTTGCTTTGGGATTCTCCCCCTTAGCGCCGGTCATGAT

>LNC_001028

GTATAACCAAACATAACCATCTTCAACTCTAACTTCATAAATTCCAAATGAAGTGAAAAATATTTGAACCTATGGCCAAATGCCTATTAAATTATAGTAATTGAAAGCTAATCCATGAATCATAGTGATATATAAATATTTCAATCACATCGGTCACGGGTTTGAGCCCTATACCAAGCAAATTAAGCCTAGTATTTAAGTGGAGAAAGATAAAACAATGGATCTAGGTTTATATTGGATAATCGGGAGTTCCGGATCATCCCAAAGTTCTGAAAATTACGATTAGGATTGGTTCTTTCAAGTGCTGATGATAGATTCTTGAGCTATGATGAGATGGAGATAGTGGAAGTAGTTTCTGATCAGGTGTCTGTGGTTTTGTCCCATGCCACTGTTCTTGAAGAGTTCCAGACAATGAGGGAGA

>LNC_001029

GGTGAGTTGATTCTGTGTTGACAAAGGTGATGGTAAAACCTTTTGAGGCAAGTTTCATGGCAAGATGTGTGAAGGGATTTATATGGCCTTGAAGAGGAACTGGTATCAGTACTGCATGTGGCTGTATGTGTTCTTTTAGCTCCATTTTAGTCCTAAAATATAGCAGAATGTAGTTACTTTTTTTGTAGTATTAATATAGCACTTTGGAGTTTGGACTACATGCAAGAAGTATATAGGAGATGATTGATTATTTATATTAGTTCTTTGATGCAGAAGTGTGTCTAAGTTGGTGTTCTGCTGTATAGCTGCACTTGTGTGAATTTTGGAGCCTCTCCTAGGCTACCTCTTAGGAAATTATGGGCCATTATGTCATGCAAGGCACGTGTCTTGCATTTATTTGTCTGATGTAAAACACCGGCTTGTGTCCTGCATTCGTTTTTGTCTGTGTGTAAAAACC

>LNC_001030

CCCATTTGCTTCCACCAACCTCCGGCCGGCCACCACCACCGCCGCCACTGCCGCCGGCGGCCACCACCCCTTGGCCGGAAAAATCTTTAAATTTTTACCACTTCTTCCTTTCATCCTCCTCTACACAAATCCGCAAACCATTCATTCATATTCATAGCCAAACTCCGTAGATCTAAGAAAACCAAACATAAATTTTCACCTTTTTCTTTAGATTTACGAAATTTCGGCCATTCCCGTGCTATATCCTTTACATAAAAGTGTAGAGCTCTTCAAGGGCTATCCTTTGATGTAAAGTTTATGTCCAAATCCGTCAAAAAAAATTCCGGCGAAATTTTCCGGCGACCTCCAATCGTCCGAAAATTTATGTCAACCATTTCCCCCTCTCATCCTCTATCCAAATCCGTCATTATCACATTTTTA

>LNC_001031

TAGCTCTTCTCAAATTTTCTCCTAACCCCCCTCTCTGATGAAGGCAGTGGGGGTTTATATATAGGGGAATTTGGGGTCGATTTAGAGGGGAAATCGGGTTGAAAAGATGGATCCGAATCCAATTTGAAAAATCAAAATCTCGAAGAGATCAGAAATAAATTTTCCATTGTACCAATTTTCGACGGAAAAGAGGGGAGAGGGGAAGTGGAGGACGCGATTTGCTGCGGGATTTCCGGTCTGAGCGACTGTCGATGTTGACGTTTACTAGCCTTCTGGGTTTCGTCTGGAAGAAGAAAACGAGCATGGGCAGTGGGGTCGGGGTGTTGTTGGAATCGCTGATTTGCAGCGAGGAAGACGAAGTTGGGCGGTGGGATACTGTGTATATACGTAT

>LNC_001032

TATAACTCTAACAATAAGTTAATATGTGAATGAAATAGATGCAACTAAGGCTTGGATTATACCAAAACATAAACAATTGGCAAGTAATAACACTCCCTTCCCCTAATATCCTTAAGCAAAGATCAGTATTGAAATTGATTAAGATTAGGGGCTACTATAAGCTCGTTCACTATGCCTAATTTACGTGTACATCCCTGCTCCGATCCAACATGTTAGGTTCGACCTTGTCTCGTTACATCACTTTTAATTTATTCATTCTCACATATTGAGATTTGTAACTTGTTTTCTATCTTACTTGTGGGTGCTTTATGCTATTGCCTTTTGAAGCAAAAATGCCTGAAAATACAGACAATAGTCCCAAAAACAAAGGTAGGTTCTTGGTTTAGCTGCAACGTAGTTGGATTGAACAAAACAAAGGACACTCTTCTTCTCACCTTCTCTCATATAGAGCTGTTGTTT

>LNC_001033

TCAACCTTTAGGAAGAATGACATGATAGATGAAAATATAACACATAATTAACATAGATGGTTGACATTGAAGAGTGCTACAGGAGTGGCTTGCTAATAAATATGCAACAAAGTGAAATGTGAGTTCCATAGAATGAATGTGAGAGTAGTAATATTATGTGGGAGTTGAAGCCTCAGCACGTGCACGGGATATATTAGCTTCTGGGATGCATCCCAAATTGTGTGAGTCAGTATGTTGAAGTATCCTGTATTCCAAGTACTAATTGAGTGTTACTTTTAATAAGTAAGAGGTTAGTGTATAATTTGAGTTTATGAGGCAAAGTATTTATCTGTGAGCAAAGAACTGGCAAAGTTGGAATGATCTATGAATTTGGCTTCATGATCTATAATTTGTGCATCTCTTGAAACTTGCCCTTTCCATTTTTAGAATTTTAGGATTAAAGATGACAACATAATAAATGTAAAACTAGATGTGCC

>LNC_001034

CTTCTTGAAGATGAATGCACCTGCTTGTATTCATAGTTAGTTGTCTGATATGCTATTTGGTAACTAGTGTCTTAATTTTATTTTATTCTGGTTGAGATTTCCTCTCATTTAGCGCCAATAGACATTTGGTTTGAATGCTTAGGTCTTGTGAGAGCATTAATGTTATGGTATTACTATATTGTTGTTCCCTTATGCGTTTTAAACACAATGACATCTCTCGTTGAGAAATAGGATTAAAGCATTGGTCCTTATCAAGTTTCGTTCTTAATTTTATTAAGGAATATCTAAATTTCATTTACCAAGTTTAGTAGCTTCAAATTTGCTGCTCTATTATAATGAGTTTGAAATCTTTGGACAACTGCCTCATGTCTTTTTTTTCAAAAATAAAATAAAATTTTTATACGAGAAGTGTAAATCTATGTAGTGTTTAATATGTGGCTATTTGTTAGCATGTGATCATTACGTGTCTCTTTAAAATTTTGATTTAGTTTCATTATCAATCTTAACAAGATATCTATATGTTATTCAATATATGTTAGTTATTAACATTTTAATTATTCTTACCTTCTATTTAATGCTAAATCTGTCCAGTTTCGATTAATTACTTCTCTTTCTTTTCTCTTTTCATGAAAACTCTATCCGAGTCCCTTTTGGGGGCCCACGGTTCCCTCGCATTTTCTCGTTGGGCCATGGAGGCCTAACAATCCCTCATCGAGTCAACCGGCCATTGAAAATTGACGTACAGATCCCAAAGTTGAAAGGCGCAACAAATCGAGAAACTGAAAAGATTGGGCCAAAGGCTCACTCCTAA

>LNC_001035

TGTATGGTCTCATGTGCTTTTGGTTAAGAAATCTCCATACCCAATAAAATCATGCATCAAAAGATTTCTCTAACTGCTGGAATGTTTGGCTGTTTGTAAAAGTAAGTATGCTATTGCTATATACACATAGTCAATACATTTGGTATAGTATAAGATTCACCTGTCCTGAATAAATCCAAAAATTGTTGAATTAGACAAGTGATTCCATCGTTTCTGGCTCAATAAGAGACAAATTGTCCGGACATAGTTTTTCAAATGAGGAATCAAGCCATTACTTTGTAACTGCTTTATATAAGTGAAGAATTTCTTGCCTGCATTGGCGACATGCAAGTGGAGAGCATCATTAAATAGAAGAAAAATTAAGTGTCCAATAATTATGTGACGGTTCAGAGCAGTTATGTGACTTCTGAAAAAAGTGTTGATGTAAGTAGTGTGGATACAAGCACACATCAGAAGTTATGTTGAAGATAGAAGATGGATGACATGCATACATGACCTAGAAGGCATCTATTAGCTGCTGAGCTGAAATGGAACAGTAAGGCTTATTCAATCACGATGGTTTGAGCCAAAGATGTGATTTACTGTCTAATTAGAGCTACCAATCTGAGAGATTTCAGGACTTCAACTTGTCAAGCTGTAAGTTGACTTCTTAGCTCACATTATAATCTATCAATCTCAAAGAAGTTGTCCCTTTCATAGTGGCCACGTGTGGGAGAAGGGTGGTGAAAGAGCATTATATCCATGATTGGAACCATTTAAACAAAATAGTTGATTGCTTTACTTGTAGGAAGGACAAAGTGTTTGATGCATCTATGTTGTAGATATATTTTGGCGTGAATATATGTACAATGAGATTAAAGCAAGGATAAAAAACACTTTTTCTATAGTAACAGAAAGAACAAAGGTAGAAATTAATGTGGAAGTTCTAGCAAAAGAAAAGGTAAATATTCAGAACTTTA

>LNC_001036

CACAATCATCTCATCTAGTTTTGAAGATCATGCAACAAAAGTCTAATCGAAATACATCAGCATGCTCGGATATGTCTTGTACTCAAGAGTTATATTTGTATAACCTCCCAAAAAGAATCGATTCAAATGGGGTTGAATAATATCTCGTCTCCACTTTGTCAATATATACCTTTCATCAATTTTATCAATCTTCTTGTGTGTCAAAATCTTGAGTATATGACAGCAAAGTATACCTCTAGACTCAAATCATTTGCAACTACAAATAATCACCACTAGGCATATATTCATTTGTATACTCTACTGAATTTCCACCATTTTTATGCATAATTGAATAGTTACTAATTACGAATTTCTCAACACCGAAGATAGCTTTATGATGGGAGGGGGTGCTGACTTCACAATGATACAATTTTTTAAATTGTGTCTTGAAAAATTTATTTACCGTGTGAGTGTAACACTGAATTACTTGTTCCTCTCACTTGAACAATGACTCACTTA

>LNC_001037

AAACAATAGTACCCGACCCCACCCCTTGTGTTTTCTTCTTCTTCCTTGCAAATTCAGAAAATTCCAAAAAAAATCAGTAAAAGTCAATACCAAGAACCTTAATTTCTTCATCCAACTACTCCACCCATCACCATTGATAATCCCTCCCTCCATATTCAAATATTGCAGACCTCAAGCTTGATTTGTTGACTTTGGATTAGATAAAAATCTCACCCAAATCTGATGCAAATCAGAGCTATGACATCAAGTCCGGA

>LNC_001038

ACTTGTTGCTGCTTATGTTGGCCCTTACCACTAAACCCTATTGCTTGTTGCTGCTTATGTTGGTCCTTACCATTCTGTTATACAGAGAAACCGCTGTCGAGTCTGATAAAAAAATTTGGTCTTCTCATGCTCCACAGCGTTTCTCTGTATAACCAAATGTTTTAGGGTCATTATTCTTTTCTGGATCCTCAACCCTTTGCAACTTTTTTGACGTCCTGGTAATGGTATCATTAAGAAAAAACATTCTTTTTTCTCAATTGCTGTATGTTTACTTGCTTTCGATTGAAATCTTGAACACTAGTGTGATGCTATTTTCATATGCATATGATATTTATTCTGTTATTAGTCCATTTTTGTATATTAATCCTAGGGGCAGCAGCATTAGGTATCTTTGCTGACTATTCAGCAATAGCCTGTTGAAATACACCATAGCTTTACCCTGTGGGAAGCGGCTACCGATTTCCCTTTGTCATAACAAAAAAATACACCAATAGAAGTAATGTAGTATGATTTAGCCCAAAACAGGATTTTCTGAATAACTGTTAGGCAGGGATGATCACTGGGTTCTACAGTCCTGTGGACTTATTAAATTTTAACTTTTAGTATACCATAAGTTGGATGACTCAACTGTATGATAATTGATCCTTTGACAGTTTAAGCCTTTATGTTGCACTGGCTATAGAACTTCTGCAAAGAATGATTGGGAAATTGCAAGGTATTAATTCCAACCTTACCATAGAATGCTCCCTTTGGGGTATCATGAGTAGTTAAGGTAGGTCTTCAACATGTTGGAAATCAAGAACATTCTTTGCATAAATGAAAGAATCTAAGCATTGAAACCGTGGATAAATAGCAAGTCTAGAGTCTTCAGTGCTGCACTGAGCATTATTGCTGCAAATGTAGCCACTTAAAGAATATTTAAGTCTGTTGCCACTCAATTACTCAACTATTGTAACAAGCATGGAGGTTCTATACTTAAATTTGATGACTCACCTTATGGAAGGAGGAACAGTAGAAAAAAAGGTTTGATGAAATAGTAAGGGATAACTCAAGCAGCACGTGTGGATTGCTGTACACTTAGATGAACATTGCAATGCACTGTAATCGTTTGTATCCTAAGATTCAGGAATGATAATGGTGTATATGACCAAAACTCTCTACCTCAATAGAACTTGAAAAAAGAAAGTGTGGTGGTTCTTATTTCCGGGGAAGGATTCGTCAAGGTTATGGGTGGAGATGTATGCAATTGCAGGATTGGGGAGGGGTAGATATGGAATGTTAATTGGCATATTTGGGAAGAATGGATTCATATTCTGAGTTTATTGACTTTAAGAAGGTACAAATGATTTCTTAACATGAGAGGATGCACTATAGTCCAAGTATTTTGCACTTTGGGCGAAGGAAACAAAACGAAAGTCTGGTCAAAATACGAGAGTCCCAAGAGGATGATGCAAAACTAGTGGCAGAATTCAATGTTTGTGAGTTTAGCTGCTACGGAATACTGTGTTTAAATCTCTAAAGTTAGAACTCTTATCATTTGTACAGCAACAGTTCCAATAATTTAACAGTTTTCCAAATTGTACTGTACTTATCTTATGTTGAAAGAAGTTCAGTAACGCATTGAGATTCACAATTCGAGTTTCTTATTGATATACAACTAACAACTTTTCACTTTTTCTTCTGGTCTTAGACAAGAAGTGACTCCTTTATGTGGATCCTTATTTGTTTCAGAAAGAAGAATCTAGCAGCAGATGGGTCATATTTGTGGACGAGGCTTCCTAAGTATATTAAGACTGATGGACATGCTCGTTAGACCTGTCTCTGGAAAGAACAGAAGAAAGAGAAACATTATCTTCAACAAACATGCTTGTATGCAGAAAGTGTCCCTAGCTGGGTAGTCTTTGGACATATTTTGGAATATAGTAAGTAATAGTTATATAGCATTTACTCGGGAAGATGTCGAAAAAGACATGCTCTGCAGGCCCAGCCCTTAAGGGTCCTGGGGAGGCGGGGGTCAGGACCTGATAAACATTAAAGAACTCTTTCTACAGATTGAATACAAAGCCTGTAGGTATGAATTAAGTCTGCACTTCAACTTTTAGTGATTCCATGGATTCATGGGCATTCAGGACACATTTCAGTACAACTTAGCTATTTTGGGGACCACAAAATCAATGGCAAATATTTCCTCAGAATATCAGATCAACCTTCTGGTTTGACTGCACTTGATAAAGGACTGTCTTGGTATCGGAAGAAGTGCTTGGACCTAAAATTTTCTGGTTGACGAATAGAGCGAAGAAGGTCATAGATTCCCTGGGTGCATGTTCTTGCACAAATTACCATAATCCAGCTCCAGATGTACAATATTACACTAAGATTATCCACACATGCTACATCAGTGAATTGCAATGCTCGAGGTTGCTTAGCTTTGCTAGTATTGAACCAGTGGTCGTTTCTGTATCGGTGTACTTGAGCTTGTAGCAACTGCAGTTTTTAACACAGCTGAAATATTTGAATTCCTTAATGATCTGAAGGTCAGTCCTTTCATTATCATTTGTTATGATTTCAGTTTTCTTTTTGGTTCTTTTTGCTGTGAATTAATGGGTTAAGCAATAATTGACCTAAATTTGCACTTGCAACCTTTTGATTATAACTGCAAAATGCTTTTGCATTTTAAATTACTTATTGAAACA

>LNC_001039

GGACGAGGCTTCCTAAGTATATTAAGACTGATGGACATGCTCGTTAGACCTGTCTCTGGAAAGAACAGAAGAAAGAGAAACATTATCTTCAACAAACATGCTTGTATGCAGAAAGTGTCCCTAGCTGGGTAGTCTTTGGACATATTTTGGAATATAGTAAGTAATAGTTATATAGCATTTACTCGGGAAGATGTCGAAAAAGACATGCTCTGCAGGCCCAGCCCTTAAGGGTCCTGGGGAGGCGGGGGTCAGGACCTGATAAACATTAAAGAACTCTTTCTACAGATTGAATACAAAGGACACATTTCAGTACAACTTAGCTATTTTGGGGACCACAAAATCAATGGCAAATATTTCCTCAGAATATCAGATCAACCTTCTGGTTTGACTGCACTTGATAAAGGACTGTCTTGGTATCGGAAGAAGTGCTTGGACCTAAAATTTTCTGGTTGACGAATAGAGCGAAGAAGGTCATAGATTCCCTGGGTGCATGTTCTTGCACAAATTACCATAATCCAGCTCCAGATGTACAATATTACACTAAGATTATCCACACATGCTACATCAGTGAATTGCAATGCTCGAGGTTGCTTAGCTTTGCTAGTATTGAACCAGTGGTCGTTTCTGTATCGGTGTACTTGAGCTTGTAGCAACTGCAGTTTTTAACACAGCTGAAATATTTGAATTCCTTAATGATCTGAAGGTCAGTCCTTTCATTATCATTTGTTATGATTTCAGTTTTCTTTTTGGTTCTTTTTGCTGTGAATTAATGGGTTAAGCAATAATTGACCTAAATTTGCACTTGCAACCTTTTGATTATAACTGCAAAATGCTTTTGCATTTTAAATTACTTATTGAAACA

>LNC_001040

TCTCAACCTCTGCATACGCAAAACTTGATCTATATAGGCAGTGCTGTATTCAAACATCAATTTGAAGCAGTCTTGTGGATTTTCCGCCTACGAATCAAAGATAAACACTATCCTTGTTGTAGTATTTTGGTTTTTCCCCATAAAATAGTTAAAGTAAATTTCCTACAAAGTTGCACATTCCATAGCATTCTCTTTATTCTATGATGATAAAATTTACCGTTCCAAAACGAAAAAAAGAGAACTTACCAATGAGATCATCA

>LNC_001041

CTTTTTGGAAGTCATGACAGACGGACCCGTTTTGAGTTCGCTTCCTGTTTGGCTTGACTTTTCCTTCTTCCGATGGTTGCTCCTATAACTCGGGCTTGAAGCTTCTATTAGCGCTGAGGTGAGATATGAGATAGTATAGAAGGGGAGTGTTGAGTCCCATCCAGCGGTAATCCTTCGTCATTTCCAATTTCTTAGCCGATAGGCGAACCATTGAGTCTTGGTTTAGCTTCTTTTTAACTTCACTTAACGAGTCAATGATTGCTGTTGAGCCCATTTATTCGGGTCACCTCTTAGAAACGAAATCTGTCTGGCTCTCCT

>LNC_001042

AATATTTTTAGAGTTTCATTTATCGATAAAAAGGTCATAAATCATGCTATGTTTCGGGTCAAAGAAGAGCAAAACCAACCAGTGTCTACTTGTGTTGTTCCATTGCTAAGCATGTGTGGACTCAACACTATTTTGTAGGAAACAACCAAGAGAACATAGCCTGCAAAGTATAGACGATTATCTCTAATCCATATGAATAACATGTTGTGCTGACTTAAATTTGTCAAGAAAATCAATTGACTCAGTGCTCCTTGAATTGTTTAGTTTATATAAGGAGGATAATGAGTTGAATTTAGATAGCACTTTTTTATAATGACAGTCTACATTCATTCCAATACAACCAACATTATGGTCCTGTATCGCCTAGTGTACTAAGACAAGATATTTTCGTTTGTATGTTGGATTCGTAGATAGATCATCTAGCCTTAAAGTTATGAGTTTGCTTGAATTCAACAACAACAACATATCTAGTGAAATTCCACAAAGATGGGTCTGGGGAGGGTAGAGTGTACGCAAGACCTTACCACTACCTCATGGAGGTAGAGATGCTATTTTCGAAAGACACTCAAATCAAGCCTTGGGTTGTATCCAAAGAGGATGGGTAATGATGGGTAACCAATAGGACTCCAGTCACCTCATCTAGTACAGTTGGCAATGGGGACAATGACCATCAAATCTGGATTGTACCTTAAGAGGATGGGTTTGTTCCTCACAAGATAGTTATTGAAGGCATTGCTAGTTGGATTCGTAGTAAATTTGAATTGGCTAGACCCTTATGAAAGAAGTTTCCAGAATCTACTCATGATATGTGGTTTGAGGAACTCAAGATTATGTACAATCGTTGGTGACGTTGGATTCTGTGCAAGGAATTGGAGAGAATAACTTGTATCTTATTCAATAGGTGAACCTGTATAAATACAATAATATTAGAAGCTTACTAAGGACAGAAATAAAGAGG

>LNC_001043

TTAAAACTCTTGTGGCTTTGTGTTTGTTTTGCTTGACTACAGTGGTCAGTATTTGTGATCTAATGCTATTTATGTTTCTCCCCTGTTCATGTGTGGCTTAATATTCTCTTTATGCTTACATACACATGTTCTGTGTGGCCCAAGGCCCTGAATTCTTTATTGCCTTGCATTTAACTTGAATATGTTTACTGCTTCACCTTTTCAATGATGTCAAAAGGGGGAAGTTTTGTTGTTAAGTTGG

>LNC_001044

GTATCTTCATCTCTCCACTCTCTCACTCTCCGCCATAAAATCTCATTACAATGTGAGTATCACCGATCTCCTTACTCCTCCATGGCCAAATCCTCAAGGGGCTACCCTCCATTGTTACCATCGAAACCAATCGAATTCAAACACAAACCACTACTACTCAAAATCAAACACCCAACCCCAATTAATTTTGACTCCGACTGAATCAAACCCTTCAAACGAATCTCGAATCACCCATTTTTATGAAAATTCTCTGATTTTTTTAACCAAGGGGATTGGGGATTGAAACTACATTAAAGAGTTAGTTTGATCAAGGTGGAAGAGTGTGTAAATTGACTGGAAACTACATCGGAGGAATTGCAACTGGGTATATCTAAATGTCTCGCTGCTATATGGTTTCTGAGATAACAAAAGGTTGACTATTAAGTTAGAGAAACTAGAAACTATTGTTTTACGAAGGGGAAGAGCTTGTAAATAAGTTGGCAAAGTTGGAAAGGGATGTCAAAGAATGAGAAGGCGCAAGAATTGACTCTGCGACATTTTTAAAAACTGTAAGCTCTTGAACTCTTTTTGATAATTTTTATCCTAAATAGCAGTAGATTTCTTAATATTTGTTTAGGGTACTTCATTTTTGCAACTGGTTGTTGAAATAAATCTCTTATATCTCAACCGCTTGCTGATTATTTGATTTTTATGGCTCTCATTGTTAATATTGTCTTTGTGAATTATGTTGTTCCTCTCTTTTATAATTGAAGATATTGAGAGTGGTAATCAAACTTGTTATAAATTGTATCTCACAATCTTTTCTTTGATCTCATATGAACAGTTCTCCTTTGCACTTTATCGGGTGTGGTTCTCCTTGTAGAAAATTGATATGTTTATAACATGTGACCGATAAAAACAACTTTAGTACACTGGATAGACAACATGCAAAACTATAAGTTACCTATTAGAGATACTGCTTCTCCTCCCTTCAAATCTATTGTCCGAAACTTTAATTTTATCCAATTTCTATTGTATAACTAAAAAGAATATTTCAGGAAGTTCCTTCAAGTATAAGAACACTCAGTTATTACAAGTGTTATTCGACAAC

>LNC_001045

GTATCTTCATCTCTCCACTCTCTCACTCTCCGCCATAAAATCTCATTACAATGTGAGTATCACCGATCTCCTTACTCCTCCATGGCCAAATCCTCAAGGGGCTACCCTCCATTGTTACCATCGAAACCAATCGAATTCAAACACAAACCACTACTACTCAAAATCAAACACCCAACCCCAATTAATTTTGACTCCGACTGAATCAAACCCTTCAAACGAATCTCGAATCACCCATTTTTATGAAAATTCTCTGATTTTTTTAACCAAGGGGATTGGGGATTGAAACTACATTAAAGAGTTAGTTTGATCAAGGTGGAAGAGTGTGTAAATTGACTGGAAACTACATCGGAGGAATTGCAACTGGGTATATCTAAATGTCTCGCTGCTATATGGTTTCTGAGATAACAAAAGGTTGACTATTAAGTTAGAGAAACTAGAAACTATTGTTTTACGAAGGGGAAGAGCTTGTAAATAAGTTGGCAAAGTTGGAAAGGGATGTCAAAGAATGAGAAGGCGCAAGAATTGACTCTGCGACATTTTTAAAAACTATGCGCAGAGTAAGAATGTAGATATGAGCTCTTTTGGAGTATGATGCGTCTGCTCGGCATTTTATAGAAACAAAAGGACTTTCACCAACTCTTGATTTTTAATGGATAATCTAGAAAATTGTTGGAAGGTAAGAACTGTAGCTTTAGTAAATAAATCTTTTTTGTCCATGTCTTATTATGTTTCTAATATTTGAATCTTGTTTTGTGTTGGTGTCTTCACTGTTTCTTATTTCTCTTCGAGTGTATTTTTATGAACTGATTTTATTTTCAAGTTTTTATTCCTTCCATGAAATATTAAAGTTGGAGTAAGTAGTTCGCGTTGAATGAAAGAAATTTTTCAGCTTATCAAGACAACTGCAGCCACTTAAGGATATCAACTTCTATTCTTTTGGAATATACAGAGAGCATCTGCTCAAATTTACTGGTAATGATTCGAGGCATTAATAGATTTTAGAAAATGAATTTTGTCATATTTGATGCGCTATCAACTTCTATTCTAATGAGAAATACAGGGAGCATTTGCTCGAAGTTATCATTCGGAAAATTTGATGTTGTTTCTAATATACTCAGTCTGATCCCACAAGTTGGGAATGTGGAGGGTGGGGCGTACGCAGATCTTACCTCTATTTTTGATGTTGTTTCCGGTAGATCCTCAGCTAAAAAAAAGCATTTTTAAGAACAGGTTTGATAAATACAAGAGTAAAAAGGCTATGCTGAAAATCATTGTATTCGTTCTTCATGTGTACTATGTTCTCTTCTTTGAACTACAATATCAAAGTAGTTGTTCTCTGTGAATGTCTGGATTATTGATTAATCTTAATAAACCGTTAAATGACTAGAAGCCAAAAAATTTCAGTTGATTAAAATGAAACTGAAAAAATTGTTTAAGTAGGACAAACAAAAATATGGATGAAGTACATATTGTTTCTAAATATTTTGGTTAGCCTAATTTAGAGAGTAGCATTGAGAATCACTCTCTTCTGGCAGCTGAAAGCTGGTTACAACTGTATGATTTTTTCTTTTGCAATTTGTGACAATTTACTTTGGTAAATGACAGACCATTGTTAAATGAAAGTGTTGATATGTTACTATTTGAAACTGAATATGATGAGGCATATAATCTTTTGAATTTTAGTCTTTAGTTTACTCAGACATCTGTAATTGGTTTCTTTATCTTCATGATATATTTGCATCAAATTGGTAACTCAGTAATTTAATTTCAAGGGCTTGCTGAATACTTGCAGAAATGTTCACGTGTTATGTCTAGATTAAGTTCAAAAATACCGTGGAGTCAAACTGTACAAATGTGCATGATGGGAGGTCCGAAAAGGGCAGTAGCTTGGAAAGAAGTGAGATATCACTTATCCCTCCCTATAGCTTATACATATCCTATATTATCTTTTGTTTTTATTTTTATTGCA

>LNC_001046

CTTCATCTCTCCACTCTCTCACTCTCCGCCATAAAATCTCATTACAATGTGAGTATCACCGATCTCCTTACTCCTCCATGGCCAAATCCTCAAGGGGCTACCCTCCATTGTTACCATCGAAACCAATCGAATTCAAACACAAACCACTACTACTCAAAATCAAACACCCAACCCCAATTAATTTTGACTCCGACTGAATCAAACCCTTCAAACGAATCTCGAATCACCCATTTTTATGAAAATTCTCTGATTTTTTTAACCAAGGGGATTGGGGATTGAAACTACATTAAAGAGTTAGTTTGATCAAGGTGGAAGAGTGTGTAAATTGACTGGAAACTACATCGGAGGAATTGCAACTGGGTATATCTAAATGTCTCGCTGCTATATGGTTTCTGAGATAACAAAAGGTTGACTATTAAGTTAGAGAAACTAGAAACTATTGTTTTACGAAGGGGAAGAGCTTGTAAATAAGTTGGCAAAGTTGGAAAGGGATGTCAAAGAATGAGAAGGCGCAAGAATTGACTCTGCGACATTTTTAAAAACTATGCGCAGAGTAAGAATGTAGATATGAGCTCTTTTGGAGTATGATGCGTCTGCTCGGCATTTTATAGAAACAAAAGGACTTTCACCAACTCTTGATTTTTAATGGATAATCTAGAAAATTGTTGGAAGCTTTGAAACTTTCTTCGAATGGTATTGCTAATGAAATGCGGCTTTCATTTCATGGAATAGTAACACAATAAGATTTTCCCATTCAATGTCATTTGGCAATGATGATGAATCATGGAGGTAACTTCACGGTTGAACAGTTTGTCAGGCTTCATTGTGAAGACATATTGCAAGTTCATTACTTTATCCTCTCCTCGGGATCCAGTTTCAAAATGTACAGAGGTTGTTGATGTACTTAAGCCGAAACTATGATGAATTTATTTATCATGCAAACTTAAAAAAGCAAGAAGCCTAAGTTCATTTGTGAAAATATTCATTGTGTATCATTTGAGTTGAATAACTTGTATTATATGAAGACAAATAACTATCGGAAGAACCAATGAAGTTACTACTATGATACAATGTTATGTTAAGAAATTTCAGC

>LNC_001047

ACTTTCCATCCCTGGCATTCAAAAGAAAGGGGGGAAAGGGAGAGTGAAAAGCACCCTGTAGATAAAGATCGAGAGCCTGTGGACCGGGGTTGAACCATATCCCCCTTCTGTTAATCGAGAGTGTGACGAAGTGAACCGAAAATATTGTGCTGTAAACTGATGTTGTGAGGCGGAGATAGCTGCACTTCTTGTTCTTTTTTCC

>LNC_001048

GTTGTTTTTACCATGACATGTTAATACAGTTTTTATATTGCTTTTGCAATATTGTATATGCTAATGTGCTCTATTTCCCTTCAAAAGTTATGGTGTTTAATTTGGTAAAACTGCAGGTATTGTCTTTGGTACCTCCTTTATTTGACCGATTGATACTCATGGTGAATGGTTTTGTTAGTTAAATCTTTCTTGAGAATGTTTTACTTGTGAATCCTAGGGCTATACCTGGATGGAAACATGATTTCCTTTTTTGCTGATAAAATCTATTTGTTTAGGTTATTGATGGTCTTCCAGCTAAATTCGTGCTTGGGAAAGACGGTGAACCTCCACCAAAGTCTTTCAAGGAAGCTATGCCATATACTCAATCATTATTGACTAAAGGAAGAGCAGTTGGGTATCATGCAAAGGCTGCACCTCCTACTGTCACAATGCCTCACTTGAAGGCAATGGTTTCTGGTGCAGTTAGGGGATTTTTGGATGTTGCCTTCAATTTCAACACGTAAACTTTGCTGGATGACAACATAATTAGTTAGTTGGAAAATGTTCTTAAAGTTATATATGATAAACAGAAAGTCCCTTCTAGTTAATAGCTTGTCAATTGATGCCTTGATATGACCCGTGATTTCTCTTCCTGTCACAGTTCAATTATTAAAAGTTGGTTGGAAACTTGTGATGCATGGTGATGACACATGGCTCAAGTTGTTTCCTGGAATGTTTTCTAGGCATGATGGAGTTAGCAGTTTCTTTGGATGATGGATCTGAGAGGACAAGAGGTTACAGTAGTCTGGAAGAGACCTTTTGCTGTTTGTATATGAAGGCTGCAGATCTTCACAAATCTTGGAAATCTGGGGAAGAGAAAAGGTGGTTGATGGTAAAAGACATTGTCCAAGTAGATCAAAATGTGTCTCGACATTTGGTTGATGAACGTAGTCGCGCTGACTGGACTCTGGAGTCATCTGATTCTTCATTATCTTGGCTTGGATCATGTTGGACATCTTGGTGGGCGCAATAGAGTATTCCGATGACCCCCAAACTCGGAGAGATGGATGAAGTGATCAAGACGATCGATCTAAATTCTTTACCTACCAACAATAATGATCAAGGAAGGACACTCTTGTTGGTAGTAAGTGACCGTGGAATGACTGAAACGGAAAATCATGGGAGGATCATCATTTGAGGAAAATGACTCTTTGGCACTTTTTATTGGTCCTACAAATTTTGGAAGTACATCACTCCTAATAAAGCCAACCAGCGTGGTAGCATTTTGAGGCCGTTCGGAAAGGAAAAACTCCGAAGGAGTGATTTTGGAGCGCGCATGATCGGCCTACAGTTGACCAACAACAAGTTATTGGAGTTGAATTCCCGAGAATTGCTTAGGCTCCTTGAAGTGCAACTACCTGGTTTAGTATGTGAAAACTTCTTATGCGACAACTTGAGGGATGATGGATCTGAGAGGACAAGAGGTCGCAGCAGTTTGGAAGAGACCATTTGTTGTTTGTATATGAAGGCTGCAGATCTTGGAAGTCTGGGGAAGAGCCAATTTGACATATGTTTCCTCAAAACTTTTATTTGGGGAAAATGCTAATTCTTGTATTGTTTTCCATGTAGATCTGCTTGTGAGGAAAATTGTTACAGTACTATTATGGCAGCATATTATAATTTCGTGAGTGGCTAATCTCATAGAGCAACTGACATTTGCTCAATTTTTGAGAGGATGGCATCAAAGGTGGTGTTAATTGGACTAATTTTTCAAAATGGTTAAGAGCAAGTGGGAAGAACTTATATAAAGTTGTTCCAGCTGGTTTCAGTGATCATCCTAATCAATGTAAGCTTAGTTTCTCTTATGTGGTCTAGACGGTCAGAAAAGAACTTTATGACCATAGTTTCCTTGATGCAATTGTTCCCAGGGTGTCAGATTTTACATTATATCACTAAATATCAAGATGTTGCAGGTAGTTATGATGCTACTTTATGGCTCAGATAGTTTATGTTGTCCTAGGTTTCTGTTCAACGACTATTGTGTTGTGCCATGGTGTATACTTTCCAA

>LNC_001049

AGCATGATTTGAGTTGGAGGAGGAAATTCCTCCAATGAAATGGAATATGTCTAAGTTGCATGCATATTGAGTTCGTAGAGGTAGTCCTATTCTCTCATGTTGTATGATGTGTGTTTTAAATGGAGATTTTTTTTTTTCCTAAGTTGTGCATTGTGTGTAATATGTCATGCTTGAATGTGTTCTTATGAAATGGAATGTTAGCTTCTTATGTGGTGGAAACTTTCCTAAAGAGTAGAGGAGGTAGTTCCTCCCATGTTTATGTGAATTAATGAGGTTTGCGCATTTCCATAAAAACTTGTGTTTTTGAAGAAAGATGAGTTGTATGAGTTGTTTCCCTCATGTTGTAGTGCATTGGTTATAAGTGATCTATTGTACTTCATGAAAATGAGGTATGAGCATGTCTTAGTAAG

>LNC_001050

CTACTGGCACCATCCGTGTTACACATAATTTTTCCTTTTGGTGGAGGTTGCCACAGTACCTTCTTGTAGTGCATAATGGGTTTGAGTTTGTTCAGGAAGTCAATAACTTATGGCCAGTGTATTGGTACGTTTTTTAGAGTTGAGAACTTAGTGTTTATCAGTAGCCAAATATCAATATTTACTTGGTACACTAGTTTATTGAAACTTATCATGCCTTCATATATACTTGAAAGTATTCCTCATTTTCCATAATTCTCATATGATTTTACTAGGGATAGCCTTCATTATCTACTATAGTTTTGTGTGCCCATGTATAGTCCACCACTTGGTAATTAGTTGTATCAGTTGTCCTTCTGGGTTGATACCTGCACAAAAAGTAAAAGTTCATCAAATCTTTGCAGCTGTAGGAGAGGTTAGAAAAACATGTGTCATGGTCTTCCTTTTTGGATTAGAGTAGCAGTAACATCTAGAAGAGAAGCTGATACCCATTCTTTGCACCACTTCATATATGGGCAACCTAAACTTCCAAGCCCTCCATAAGCCTCCGGTGATACCAACCCACCTCTCAAGATGTATTTTAGAAGAAAATATCGACAAAAGAATAAAGAGTTGAATTCTGGACAAACAACTGCTACAACAGTTGTCTAGCAGTTGTCTAGCAGTTATTATTCCTAGTTTATAGTTTATAGTGTCAAGTCTTATCCTTTTACGTTCTCTATATATATGTAATTAGGATTAGTTATTGGTATTATGTAATTAAGAATCAAAACTACTATTTTCTGGAGTCTTGTCCTCTCCATACGGACAACTTTATCTGTATAGTTGTGCCAC

>LNC_001051

CTAAAGTTAAGCATGGGTTGTATATGGATTTATGTTAAGTATATGTATGGTTGGCTATGGTCTTATATGTTAATGTTGATTTGTATTTTGTCTCTTATACTTGTAAAGGAAAGGTTTTATCAAAGGATATGAAAGCATACATTTCTTGTAAATGTCCGTTGTGCATATTTTCTTGCATGGTCTCCATACTTAGTACTTAATTGTGCTAACCCCTTTCTTTCGCTTTTTGACTAAAGTGTAGGGATTTGGAGATGGTGATATGTCATGGCTACTTGATAGGTTTTTAAGAGTTGAAGATGAAGACTTGGTGAGTCCTCATAGATTCGAGGGCAATAACCAACATGTTCTTTTTTATGTCTCTCTAGTATTTGCTTAAGTTTTGAGTGGGCTTAGTCCCAATGTTATATTCAAGTATTAGATGGTTTTGAGACATATTGTATAAAGTCTAGAAAGTCTTCCGCTTGCTATTTATTGAAAGTATCTTCGTGTG

>LNC_001052

CGACAACAACTAAAGCAAAAGCAAATTCAACCTTAGCCGATTAGAAGGCTAGCGTAATAGCTAAAGGAAAAGGCTATGGTGATAGACCCCAAAGAAGATACCCTTGCTCACACCTTAGCCGGATTCAAAACCAAAAGCCATTGCGCCTTTAGCCGACTGGGAGGACTCCCTAGAAATTCCTTGTTTAGAGAAAGCTGTACAATCAATGAAAAGAGCGGACAATACGATTACTGAGGGTGAGCAGTCTCACATTCTCGAGGAAAAGCAGATGGTGAAAGGCCTGGCAAGCACATAAGCTCGATCGGGCCTTACTCACTCAACAAGGGCAGAAACTTCACTCGCCGAGGAAAGAAAATAAGGAATTTTTAGGGAGAAGGATTTGGCCCTGACTGATAAACTAACAAAAGGAAAACGAAATGCAACTCATCCGAATAAGACCAATTGGCATAGGCCATAGACAGAGAGGATGAAAGAAAGACATAGGAATTTCCCCCTGAAGCTAAAGGGGCCTCTCTCCATCCATAAAGGAAGAAGAAGGCAAGACTAAGTCATTTAGTTGTTTTCTATTTCCATTCTGCCTTCACCATGCCTACCCTGTAAAAAGGATTCTCCAACTGATGATTTTTCTGTTTCGGATGTGAAGGCTTAGGCTTCCATTTTTGTTCTAGGGTTTCTTTATTCGCCGGGGCGCTCAGTTCCTTCCTGTTCCCAGAGCTGAGGCATTGC

>LNC_001053

GGTGAAAGGGTTTTTCCCTTTTAACGTTAAAAGAGGGTTTCTTTCTCCAGATGAAACTCGCTCACTCTCTCTCTTATTCAAAATTCAAATTAAATACAAATGGGTTCTCTTGTAGATTTTGAATCACAGATGTTCTTTGGATCGTGTTCTGTGCGAGTGTGAAAAGCATGGCTACCAGGCGATCCGTTATCCAGTTGCAACCACCGGTGCCGGAAATATCGATGCGAATTCTTGCTGACTCATGTACCACAGGCAATCCTAAGGGTTAGCACCATTCAATTATTAGCTTTGTTTTAGAGGAATCAAAACAATGGCAGAAAATGCTTCCACCGTCTTCAATGTTCACGTGCCGGAGTTTGTGGATGCTATTTGGGTTGCTCTGAGGGATCCAACGTTGGCTGTTCGGGAGAAGGCTGTTGAGACATTGTGTGCCTGCGTTCGTGTTATTGAAAAGTGTGAGACAAGATGGTGTGTTCAGTGGTATTATTGAATGGTTAAGCCTACCCAAGATGGATCGGGTTGAAACGCTCATGTTCATAGTATACATTGATCTTTTCTCGCAGTTGGGGAGCTGCTAAGGAACACAGGGGAGTTCATTATGTCAAGATACAGGGAGGTTGCTGAAATTGTTCTAAGATACCTGGAGCACCGAGATCGCCTAGTTCGCCTCAGCATAACTTCTTTACTTCCTCGAATTACCCATTTCCTGCGTGATCGATTTGTACTAACTATTTAACGAATGTGCCAGTGGGTTCCTTGCTCTTGGGGAGATGGCAAGTTCTCTAGATGGTGAACTCATTAACTATTTGGCGACAATAACCTCTCACTTGGGTGATGTGGAGGGGGGCTGCATTGGTTTTGAGGCAGCTTGTAGAGGAAGAAGCTTGTGATCTTAGTGGAGAAGGTTTTGCTCATTTTATGGATCATCTATATGAACGCATTACCACATTTCTTGATAGTAATGAAGTTTCTGAGAATCTCGGTGCATTGAGGGATATTGACGAGATAATAGATGGCACCATCAGCGAAAATGCATCAAAAGTGACACAATTCTCCAATTACATTCGCGTTGCTTTTGAAACAAAGTGTGATCCTGAAATCTTGGTCCTTTGCTAGGTTCTTGGTCAAAAGTTCAGGGAGTTCCTCGTATTCTCTTTTGATATTTGCACTGTGAGAACACAATCTCATTTTCTGTTGGGGGTGCCTCCCTTGTAGTCATTTAGTTGATTGTTTCTGCATATAGTGATTGTGCCCTTGTCGGACGTACCTTTCTTTCTTTGTTATGGATTGGTGTGCCCTTGTCAGGATTTGATATTGTGAATGTTTATGTTGATATTGTGTTGGATTGGTCTATTTTAGTTTTGATACCTAGTTTGTAGCTTCTTTGTTTTTGTTTTGATAAAAAGATTTGGATTGACGATGCCCGATGATGGATAGAGCGTGACGACTTTCTTAAGGGTAAATCGTCGATTGCTTGTGAGTATTTGATTGACGTTGATTTGAGTAGTTCACGAATAGATGCATGAGGTTGTCTTTTTGTAACTTTTGATTCGTTAGCAGTTAAAATGTTCAATGCTACTTGTATTTTCAAAAGGAAGTAAGATTTTGAACCGTCTTGATTGATTTGCAGGCCATGTGTGGTGAGTTATTGCATAATGCCTTGTGTTTGCTTTTGTCACCTAGAACTTGTCCGGTTGGTCTTGAAATATTGGTCTTGAATGAATTTGGTTGGAGAAATGATCTTAGGCTTTCTTTAATATCTTGGAGTCCACTTGGCCTAAAAATCTCACCTTTGTACTAAATATATCCTTAGTTGTCCCCTTTCAGCCTAATCCTTTTTATCGGCAACCATATTACAAGCCTTATCCTTTTTGAAAAGGTAACCCTCCTTTGAACCCTTCCCTCCTTGAACTTGAGGTTGTGAAAGTTGAGTCTAAAAGCCTAAGTTGTGGGCGTTATGGAAAATTCGTTGAAGGGATGCTTACCAAATGAGGGCATGGTGTAAAAAAAAAAATATAAAGAAGAGAGAAATTGAGGGATGGATGGAAAGTTTTAAAGAAAGAAAAAGTTTTGAAAAAGAAAGAATGAAAATGAATAAATGAGTCTTGAATCCTCCAAAATTTGAAAATGGTTGCATGTGGAGCATCTAAAGTGGAAATAAGAAAAGAAAAAATGAAAAATAGTATGGAATGTGCATGGGTTTTAAAAGTGAAGAAAAGTGGAAAAAGTTGGAAATGATGAGTCGTTGTGTAGTGTTCAAGGAGGGTGTAGTCACTTATACTTAAATGATATCCTAACCTTCCCTGAGCCTATATTACAAGCTTAACAAAGTCCTTGGAGATTTCTAACCAAATGGAGTGGAGTCAATATTGATAGAAAATAGGTGACATTTGTGTGCATGAGATGATTTTTAGTGAGTGCGAGTGTTGCATCTTTGTCCTCGTATTGTTTGTATGTGTGGTTTCGGGATACTTCTTTCTTGTGAGGGCATGCAAGTTGAAATATTAATGATTTTTCTTAATTGAGTCAAGTGAGCTAATGAGTTGAGTCATTTCTTGAGTTTGAGTTGTTGTCACTTAAATGATTCTTGATTTTTTATAATGTTTCATGTGAATGAGAGGATTTCTTTTGTGGAAAGTTTTCATGTGGACTATTTGTTGGTACCAACCGAGGCCATTACCTTTGTGCTTAATTGAGAAATGGAAATTGATTTGTGGTGTATCTTGAAATAAGAATCTCTTATGTTTTTTGATTTTCTTGACTTGATGAAAAACAAACTTTCTAAGTTGGGGTGTTGATTTGTGGTAGATTTCTACTCATTTGAGTCTTTTTTTCGTAGTTATTTTGGCTTTTAGTTTATAATTGTTGCTTTTTGATGTGGTTTTGCACGAAAATTAAATGGAGAGTTCGTACAAAATGTTGAACGTGCAGGGTTAGCACCATTCGATTGTTGGCTTTGTTTTAGAGGAACCAAAACAATGGAAGAAAATGCTTCTACCGTCTTCAATGTTTCACGTGCCGGAGTTTGTGGATGCTATTTGGGTTGCTCTGTGGGATCCAATGTTGGCTGTTCGGGAGAAGGTTGTTTAGACATTGCGTGCCTGCCTTCTTGTTATTGAAAAGTGTGAGACACGACGGCGTGTTCAGTGGTATTATCGAATGTTTGAGGCTACCCAAGATGGATGGCGGAGAATCGCTCCTGTTCATAGTATAATTGGATCTCTTCTCGCAGTCGGGGAGCCGCTAAGGAAGTAAAAAGGAGATTGAAATAGAAAGAACGCAGAATGCTGCACTTTACTTTTGGGCACATCTATTTTTATTTGGTTTTGTGTGGAAGAAGATCAGCAGATATACTCCTAAGTCTTTCTTCAGTTTCAGCCTTAGAAGGATGGGTCTGCATTGGCTTTGAGGCGGCTTGTAGAGGTAGAAGCTCGTGATCTTAGTGGAGAAGGAACACAGGGGAGTTCATGATGTCAATATACAGGGAGGTTGCTGAAATTGTTCTATACCTGGAGCACCAAGTACTTGAAATCGAGCTTCATGTTGCATCACGGGATAAACAACTACTTCAATCACAAACATCTAGCTAAAACAGCTATACATAGAGGCGTTGACTCTTGCAACATTCAGAAAATACATTGTCTTTGTCTGAAGTTGAGAGTCACGACAAAAACGAAAGTGTGCTTCCAAGTTCTGATCCTTTGTTAGCTAAATGAAAATCATAAGAAGTCAAATTTTCATATCAACATCAAGTAGATGTGATGTAAGCAGAAAAGTTTTTTCCACCACACTGATTACTTCAAATAGAGGTAAAAAAAGGATTTGAAATCAATTAACAGAGTGGTAATCTTTTGCCAAAGAAAGACTCTGCACTCCGCAACAAGAAAAGGAAGTCATCTTTCCACCAACAGTGGGAAAAGAAAGAGGGAAGGGGAACAACATCTTATTCTCTTCGTGAGAACTTCCGGATAGTAGAAGCATTCAGAACGGGCTCCGCATTCATTTCGTTGGCAGAAACGATCCATTCCATTCGGGGCTTCGGGGAACCAAAAAATTGGACTTGAGAATCAAGGATAGATAGACAAGAGATAGATAAACGATATATATATTTCTCACAAAAAAATTGTGAGATAAAGAGCAGATGATGAGAAAATAAAAAAAGTCAAGCCTCGAAGCACAAGTGAGATAGCGTTGGAACCATATGATGTTCTACTCGATTATTCAAGCTTTGGATTGAAAATTGAATCATCCACACCAGTAAAAGGGCTATAAAAGGTCGACTTCTTAGATTGCTCCCATAGAGGCAGACTCTCACTTGAGGCTCTAGCATGTGTCGGACATATTGCTAAAGCAATGGGACCAACCATGGAGCCTCATGTTCGTGGTCTCTTGGATCCTATGTTTTCTACTAGGTTTTCCGTAACACTGGTTGATTCGTTAGAGTTACTATCTGAAAGCATTCCACCTTTGTTGCTGACCATTCACAATCGGTTGTTTGAATGTATCTCAGCAATCCTTTCAAGATCTCATCATCCAATGTCAAGACAATCAGTTGCTTTGAGTCGAGGGCATCTTGCTACAATTACCCCCCAAGTACCAGTACTGAGTGGTTCTGTGCTAGTTCAACTTACTTTGCAAACTCTTGCTCGTTTTAATTTCAAGGGGCATGATCTTCTTGAGTTTGCAAGGGAGTCTGTTGTTATATATTTAGAAGATGAGAATGGAGCTACACAGAAGGATGTTGCGTTGTGTTGCTGCAAACTCATAGCAAATTCTTTTTTGGTGATGTCTTCTACGCAATTTAGTCCTTGTATAATCAATCACGCAAATGGAAAGCAACGTCGACTAGTTGAAAGAAGTAACTTGTTATTGCGTGGAATTGGGAGTCACAAATGGCAAGGATACCGAACTCAGTTGCAAATTCTTTAGAGATCAACCTCTCGTTACTCCTTGGATCATCAGACCCTGATGAAAATTACTTGTCCTTCATGGTTAAGTATGGTTCTTTCCTTTTCGTTGAGCTGATTATCTTTTAGTGGATCAAAATACTAACTCAATCTGATGATGTGGTTTGTTGTAGAAATGCTTTTTCGGTGTTCGAGAATGCGGTAATCTTAAAATATTCAGAGACAAAAGGCGCCTTGACTCACATAATCAGGACATGTGCTACCAATTACCAAATACCACTATGCTGCACAATCATGTGTTTCAATTTTGCATCTGGTTCAAAAATATGATTTTGCAGTTTCCCATTTGGCTGATACAGTTGCTTTGGGTTGAAAAGAAGTATGCTGATGGTAGCATGGGTTCTTCTCTTATCAGAGAGATAGGAAGGACAGCCCCCGAAAGATTATGTGATGGATACAATAGGGGCCGAAAAGGTTGGGCGCTTTCTTGTAGAGTTTGCTGATAGATTGCGTAAGTTGGTCTCAACTAATATTGGCATATTAATCCCACATTTTGGAGTTGAATCTTATTAGATGAGAAATGTTCTTGTTGGGATGTTGGGTAAGTTGGTCGTGAAGGCTTTTGATGATAGTGAAGTTAAAGTGAGTTCTAAATCGATTCGTCTCTGAACCACACAGGCCATGTTGGAAATCTTGTTTGTAGGGATGTGTCAGCCTATGCAAGGAGTCGAGTGCTTCAGGTGTGGGCTAAATTATGTGAAGAGTATTCAGTTTCATTTGGCACGTGTAATATGTGAGGTTGCAGAAGTAGCGGTTGGGAGGTCAGAGGAAAAGAGTGCAATTGTCAGAAAATCAACACTGAATTTACTTATTATGATGTTGTTGTTGTAACCACCGGTGCCGGAAATATCAATGCTCTCAATAGAGTTCTTGTCCACTTGTGCACCAGAGGCAATCCTAAGGATGGGGCTGCATTGACTTTGAGGCGGCTTGTAGATAAAGAAGCTTGTGATCTTAGTGGAGAAGATTTTGCTCGTTTTATGGATCATCTATATGAACGCATTACCACATTTCTTGATAGTAATGAAGTTTCTGAAAATCTAGGAGCG

>LNC_001054

ACAAAACAAAACATCACTTCCGTCTTTTCCAGACTTATCTCCATCATCGTCTAAACACTCCTGCTTCTCGGTCCAAGAGAATTCCGACCATCTTACTTTTTGATGGAAAATCAGCCAAACGGTTACCGTGAAAATTGGATTGCAGTTGCAAGTGACCGTAGCCTTTGATGGTTATGATTATTTGAGCTTTTCATATTAACTTCAAACAAGGTCCAAACCAGCTTCTCACTCCATGTCTAGTCCTTCTCTCCTTACACAAGAACAAACACGCTTGAGCTAAATCAACAGGTCGATTGTTACTCAAATGAAGCACTTAAACAAAGCAAAAACAAAATATCAGTGAAATCTCAGAAACAGCCACAGGTGGATTGATAACTATCATGCTTTCTACTTCTAAGGAGGATAACCACTCATTTTATGAAGCAAGCGACATTTCCAATTTCTAGATGGAGCAAGGCCCTTAAACCTATCCCAGTTGCAGTTTTTCTTCTGGCAGATTTGGAGGATGAGGTTATTTGGAATAACTTGGTTGTCGG

>LNC_001055

CAAAACAAAACATCACTTCCGTCTTTTCCAGACTTATCTCCATCATCGTCTAAACACTCCTGCTTCTCGGTCCAAGAGAATTCCGACCATCTTACTTTTTGATGGAAAATCAGCCAAACGGTTACCGTGAAAATTGGATTGCAGTTGCAAGTGACCGTAGCCTTTGATGGTTATGATTATTTGAGCTTTTCATATTAACTTCAAACAAGGTCCAAACCAGCTTCTCACTCCATGTCTAGTCCTTCTCTCCTTACACAAGAACAAACACGCTTGAGCTAAATCAACAGGTCGATTGTTACTCAAATGAAGCACTTAAACAAAGCAAAAACAAAATATCAGTGAAATCTCAGAAACAGCCACAGGTGGATTGATAACTATCATGCTTTCTACTTCTAAGGAGGATAACCACTCATTTTATGAAGCAAGCGACATTTCCAATTTCTAGATGGAGCAAGGCCCTTAAACCTATCCCAGTTGCAGACCGTGGTTTACCACACACGATCAAAGGGCGCTCTTTCCTCTCTTTCAAGTGAGAAACACAAAGGAAAGATGAGGATGACTAATGATAA

>LNC_001056

AACAAAACATCACTTCCGTCTTTTCCAGACTTATCTCCATCATCGTCTAAACACTCCTGCTTCTCGGTCCAAGAGAATTCCGACCATCTTACTTTTTGATGGAAAATCAGCCAAACGGTTACCGTGAAAATTGGATTGCAGTTGCAAGTGACCGTAGCCTTTGATGGTTATGATTATTTGAGCTTTTCATATTAACTTCAAACAAGGTCCAAACCAGCTTCTCACTCCATGTCTAGTCCTTCTCTCCTTACACAAGAACAAACACGCTTGAGCTAAATCAACAGGTCGATTGTTACTCAAATGAAGCACTTAAACAAAGCAAAAACAAAATATCAGTGAAATCTCAGAAACAGCCACAGGTGGATTGATAACTATCATGCTTTCTACTTCTAAGGAGGATAACCACTCATTTTATGAAGCAAGCGACATTTCCAATTTCTAGATGGAGCAAGGCCCTTAAACCTATCCCAGTTGCAGATGGTCGGCTGGTTTTTTTGCAATCTAAGGCTAACGTGATCATGTCATTTGCTATCCTTCCTTTGCACCCAGTGTTCCACAGTGTTTCTCTAATTCTATAACCTGCAAACCTTCTTTGAATGCTAATTCTAGGAC

>LNC_001057

GAAATGTACCAATATATAAGTGCTCACCCAACTCCATTAGTATGAGGCCTTTTGGGATGTGCCCAAAAACAAATCCGTGAGGGCTTGACCCAAAGCGGACAATATCATACTAATGTGGAGTTAGGGTGGTGGTACGTGCGGTCCCAACATGTGGTATCAGAGCCCAGGTTCGGGTGTGTCGGAGTAGTGGACTGCGTTTTGTGAGAAGTTCCCGATGTGACAACAAGACCAGAGATCTGCAGGGGTGCCTTGTGTCGAAAGTCTTCCTGGCAAAGTGGTGGTCCAGGCGGCGTGTCCCGTTATGTGATAATGGCGGTGCAAGATGATTTGGCAGATCAGATGGTGTGACCTAG

>LNC_001058

AATTATCAGATATCAGAAAAGTATCATACTTCAATTTGAATTTTGAATTGAATAGAAAATTGAAATTATGTTTTCGGAGAGCACAACCTTTGTTACCCCGAATAGAACATAAAACGACAATAAAACAATAAAAGAGAAGAAAGACAGATATTATAGATATCTCCACACGTGTTTAAGCCATACAAACCTTCTTTTCTTATACACTTCACAATCGTATCTACTCAAAAATCCGTAAACAAAATATCTCTCTTCTCTGTGAATCA

>LNC_001059

CGTCTACATACCCCACTTCTCCCGTCCGTTTGGAACAACAAACACTAATATTGTCAAATTTCTGGGTTTGTTCATTTTGTTGTTAGAGAAATCAGGGGCACGATCTACACAATCTACAGGTGAATCAGCTCTCCTCTACAGTTAGAGAAAATTCAACAGCTTGGAGGATATGGTACTTCTCTCTAGTTCTGTTAGAGGTTTGGGGATTCTCGATTACCATCTTCCCACATTTTATCAGCCGTACGTCTCTCTCAGTTCAAAATAATGAGCTTTAGTTTCTGGAGGAAAATTAACAAGAAGATAAGATCCAAAGCTTGCATCAATTTCTAGGTTTGTTGAGAAAAATTAGTCATGGGTTTCTCAGTTCCATCTATTTAAGGAAATCATGTATCTCTATTTTGATTGCAGAACACATTCTAATTTGTGGTAAAGAGAATCGTTCTTGTCTTCTTACTTCTAGTTGGCTTATGGGTCTGTTGGATGATTGAGTATTCTTCTCTTTGGGGCAAGTTTATGCTATTTTTACTTTGAATTGCTTTGTGGAATAATTAATTTTTCCAAATTCTGTAGTTTCCCTGATTTTTCAGTTTCCATTTAGGTTTGTTTTTTTCTCTTTTTAACTGAAAACGTTTTTCTGCTTTAAGCACTAGTGTAGTTTAGTCATATCCTTAATTAATGTGTGAACTGAATCCTTTAATATTTCTGCATCCATGTTTTTTCGATTTCTTTTTGTATGGTTGAGGTCATAGAAAAACATAGTTGTCTCACTTCTTCTTTTGATAAAGTAAAGGATAAAACTGTGTTAAGTATAATATGATTTGTTTGTACAGACTAGAGTTAACTTCTTTCTCTAGCTCTTAACAAATATTTATTTAGCTTATTCTTGCTCGACTTAACGTGTAACCTAATTGTGATTCACTGCATCCGAGTTCACCTTCAAAGTTACCATTTTGACTAGTTTGTTGGCAAGCATCAATCTGAAAAAAGGATAGAGCTTGTACATTCTTTTAACTTTGTCTTATTTGACGTTTGAATCTTGTCTAATCTCTATGGATAAGTGTGTCGTGATACTTGAATAGAAATTTTCTTGATGCTATTGTTTGTTTACAATGATGTCATCAAATCTCTTCATTCATGTGCCTCTCTTTGACAATGATGTACTCGATATTTGCTTGAGATAGAATCCATCTTGTCTTATAACTTTATTATGTAGGTGCCAAGGAGCTAAAAATTGACTTTTAGAAGAATCAAAATCACCTAGTTTTGAAATATGTTTTCATAGTATGTACTGAAGATCCATTATGGAAACATTCTCTAGTTCAATAGCTATCTATCATAAGAAGTCTGTCCTGTAGCTATCTGTGTTTTTTTGAAGTTTGCTTTTACTTGAAAGCCAAATAACAATATATATAAATCAACCCATCATTACATATTTTGGCTATTCATTTTACAAGGTAACAAAATTGATAACGATTTCATTTTGTTTGCACTGTACATAAGTTTAGTATTTTTGAATGAAAATAGGGAAGGCAACAAGAAAGTTATTAATCTCTAGCCTTAACTTTAATTATAAGTCCCTGTCCCTGTAAAATCTAGTCATAGGTCACTATGTATATCCTTGCAACAACTATTCCCTGAATATGATTGTCTCTGCTTAGAATTGGTTCATGTATTAACTGGTTTTAGGATTCTCTAAGACAACCAAATCACTTCTTAGTAGATGGTTATTGTTTCCTTATCTGATGCATAGTGGATTCTCAAAGATGACCACAATTATAACTTATATTGTCGTGTTAAATTGACAGCTCCTCTTCATACAACTCATCTTCACCTTGCTGCGACTAATAATCTGGAAACATTCTTGAAGCTTCTCTTCATACTGTTCATCTTTACCTTGCTGAGACTAATAATCTGGACACATTCTTGGAATTTCCATCTCT

>LNC_001060

TTTGGAACAACAAACACTAATATTGTCAAATTTCTGGGTTTGTTCATTTTGTTGTTAGAGAAATCAGGGGCACGATCTACACAATCTACAGGTGAATCAGCTCTCCTCTACAGTTAGAGAAAATTCAACAGCTTGGAGGATATGGTACTTCTCTCTAGTTCTGTTAGAGGTTTGGGGATTCTCGATTACCATCTTCCCACATTTTATCAGCCGTACGTCTCTCTCAGTTCAAAATAATGAGCTTTAGTTTCTGGAGGAAAATTAACAAGAAGATAAGATCCAAAGCTTGCATCAATTTCTAGCTCCTCTTCATACAACTCATCTTCACCTTGCTGCGACTAATAATCTGGAAACATTCTTGAAGCTTCTCTTCATACTGTTCATCTTTACCTTGCTGAGACTAATAATCTGGACACATTCTTGGAATTTCCATCTCTTTAAAGGTATTTGAAGTTTTGGTGGAATCATGGACACTTAGGCGCAGAAAGGAACAAAAAGGCTGAAAAGAACGAAGAAGCTGAAGCCTGCGTATCGCCAAGCACACTTGGTGACTCACCGAAAGTATGCACTCCGCCCTTTGTTCCAGTCACGAAGCACTGAAGGAGAAGGAGAAGGATAAAAAAGGCGATGAAAGGAGCAATCGACGCTTCACCGAATACTTTTGCGAAGCAGTACTATACCACCCAATGACACAGAGCACGACGACGCTAAAGGCTAGTGCAAGATAGTGACAAACTACACCAAAGGGCGAATCGCCGAGTTAATCGGCGATTCCGACTAACCTCGCCGAAAGATTCGCCAGCACTATTTTCTGAAGTCTATAAATACTAAACTTGTTATTAATTTTTAAAGAGTTATTATTATAATTTTCCGAATAGAATAGTAGTTTTTAGGATATTTTTCTCTCAAGTTTGGAGAGGGTTTTGTTGCAGACTTGAAGAAAGAAGAATTTCATTTTCCCCAAGTTGAAAGTGGGTCTTCTCAATTCTTCTTCTTTTGCTTAAATCAAGGTTTAATTTCTTATACCGATTTGATCTTATTATCATTTTTGGTGTATTTGTTATTTCTTGATGTGTGGCTAAAACCCCCATTCTTGGGGTGTGATTGAGCAAATATGCGTTGATATTGTTGTTGGG

>LNC_001061

TTTGAGTAGGAAAGTAATTATGCCAATGATTGGGATAATGAGCTAATGATGGATGGAAAAGGATAATTTGCTAATTGGCATTCTGCATATAATTTAGTTTTGTTGTTTCTTATTTTTATGGTTGAAACTTCTAATTTCTCTTTTAACTAGATTTCGAGTTAGAATTTTTCATATGATATCACATTATGATTTATGTTTTAGTATATTAAGATAGGTAATTAAATGTTAGGTGTATTTTATTACATAGGTACCATTAGAATTATGATATCCGATAAGTTGGAACTTAACCGTAAGCTTGAAATTTGAAAAATAAAATAAAATATGAGATTTGACGTATCAATTGGCATTAAGTTCAAGAACTTGATTATAGATTTGAGTGAGTTTTTGGGTCAAGGCTACACATATAGTAATATGGGTGTGGTTAGTTTCAAAATCTTATTGTGATTATTTATTTGAATAAATTGTTTTGATTTGGAAGCACAACAAAAGGGGAATGCTCAAGTCCCAAAGTGATTGTTCGATTAATTGAGATAAGTGGATTTTCTAAACCCTTGCTAAGTGTATAGAATCGTGTATTTCCTTGTGGTATGTGTTGGGAAGTAATGAGACTTGGTGATGGGTTGACTTGTCCATATTGAATTATTTTAATGATGAAAAAGGGGTAATAAAAGGCAATGTGATTTATCATTAGTGTATGATATGTTGAGAATGGTTTGAAAGACTTATTGATTCATTGTTGATGTTGTATCACGATTGTGTTGTTGTGAATTGTGCATTATTATGAAAATGACCATCTCATAATTATTTGT

>LNC_001062

TTTGCCTTTGCTAGATTGGGCTCCAAGATTGATTGTGATAACTTGATCTGCCTCAATTCTCTTAAATTTCATTATTATCCTTAGAAGTTTTAATAATTTATGCTTTTAACAGACAACACTAGAAAGTAGTAGACCACCATCATATTTCCTTTAATGCGTCCTTTCATTTCCCCTTTATTTGGGAACAATAGATTGACTGTTCAGGACAGGAGTTATTTCTCAGTGGTAACTGATATCTGTTTTGCTGGAATTCATATTTTCTTAGCTGCTGCACGGAACTTCTCTTGCAGGATCTCATATACTCTATATGAATTTCGTCGTTGACATTGAGTGAAGTTATGAACATTCATGCTTCTCGAATGCAGTTTCAAACTTCTGAAATTGAGTTCGGCCCAAATTTTCATTTTCTTTACATGTTAATTGAACCCCAACATCCCTATTTTGGTTAACCCAATGCTTCACCACCAATCTTATATTGTCCCGGCTTTTAAATGTCCTGCTCCTGCCTGCTGACGAAGTATTAAAATATCCACATTGGTTGAGGGAAGGGTTATCGTATCCCTATATGCTTCTGGACATCTTCA

>LNC_001063

CAGTGGATATTTTAGACATTGTTACCCATTCTCAACTGGTTTTAGGTGGAAGAGTTTTCTCCACTGTCTGTCTTTGCTGGCATGGCAGAGATTCCCTGTTTTGCTTTTAGGACTCCCTTAGCTGTGCAGAATGTAGAGTAAAGAGAAGGGGAGAAGAGCTTATCCGATCTACCAGATGTAAATGTAAAATATCTTTAATTGGTTGGACCAAAATATACTCTGAACTTTGTGGGAAGACTAATAGATTGAGAATCCAACTTTAAATTATCTGTCAAGGAACCACCTCGATTGCATGTTGTGTTTTGATTGCCGTGTTTCATATTTTTGTACGGGAACTTCTATGGTGTTATACTCAAGAAGATTGTACTAGTTACCTCATTGTTGGTGCCCAATAATTATTCTTCATCTGCTGTAACACCTAATGATGGCATCTGGCTTTTAATCAAATATCTGGCTTTCTTTTAGGCATTACAGATTCTTCACATCATAGTATCTCTC

>LNC_001064

CTCAAAGATAATTCTGGAAACATCCTAAGGGGATTCGCGAGAATTCAATATTAATTCCTTTATCGTTCTTCATCATTTGAGTTATCGTCATTTCGTTGCAATTTCAGGTTAGTGTTTCTTTCAAATTTTCTCCTAATTCAGTTTCAATTCTCTTCAAATATCCCAATTTCATGATTGTTATCCTTACTGTTCATTTTTATTTCGTGCTTGATATTTGTTTCAATCGTATGTGTTTATTTTTCTTCAAAATTATACATTGTTTCACCTTCTTTCTAAATACAGAAAGATTCATCAGTATTCTCAGTTCTCTTCATCCACTCTCCA

>LNC_001065

CTAATTTCATATATTCCTGAATATCATATATATGATTGAATAAATTTATGTTAGTTATTATGTATTAGAATTATTTGAATCATGATTTCTAGTTTATTAGCACACTGGAAACAGGGAGAAAACTTCTATACTATGCCATCCTAATGTTGAAATCGGTAGGCGAGGAAGCCGTTTAGGGGAGTAAACAAAGTTGAGGCGCTGATAAGAAGGAAGTATCCGCTAAAGTACGATGCTAGTAGTGACAGAAAAACATGATAAAGATAATCTAGTTCATAATGATCTAATATAAATTTAATC

>LNC_001066

GCTATGGCTATTCATTTGAGTATTCAGGTAATGAATAGTCAATGAGCGAAGGGCGCAGTTACCACAGCTATGAGTTCAAGCGGCTACCCTATAGTAGCAGTTCATTGAGGCTCGACAGACCTGGGCTAAGGAGATGATCTGGGTAAACCCCCTTTTTTGTGAGGTAAGGCACCTCCCACTTTCTGCTAGAGCAAAAATGAAAGAAATCGTGTATGTAATCCGCCCTCAAAGAGAGCTCAGCTCAAGAGGACTACATTCAACAGAAGAACGAATGAAAAAGGAATTCCTACAGGGAAAGGCAAATTGAAGG

>LNC_001067

TATACCTCTGAGTTGAATATCTTATTATTGAGTTGAGCTATGTTTCTTTTGAGTTGAGTGAGTTGAGAAGAGGTAAGTATGTTTCCTTTTTATCAAAGTTCAAGCTTATGTTTATGCTTTAGAATTCCTCTTACATGCTCGTACATTCCCCGTACTGAGCCATTTGGCCTGCATCTTTTCATGATACAGACATGGGTATTCAGGGTCATCAACAGGAGCGTCGTTGATACATCCAGAAGTTTGAGTTAGCTATGGTGAGCCTCCTTGTTTCCAGAGGATTTCATTTACTTTTTAGCTTAGTCAGGATGTCGTGGGTCTTGTCCCGACTTCTATCTTTAACAGTTAGAGGCTTCATAGATAGTCGGTATAGTTGAAAAGTTTGTATCATTTATTTTGTTAAATGTTTAAAGACTTAAGTTGCCTATTTTATGACGAGTTGAATATTATTTTTATATTCAGAGTTTTTCATTTGAGTTAAAGTTTTG

>LNC_001068

TAAAACTCAAAAAAAATTCTCAACAAAATAAATATCACCCTTTTTATTTATTTCTTCTTCACAAATTTCTAGTATTCAAGATATCTATATTATGCTTAAAAAAACCCAAAGATCAACGAAGAAGAAAAAAAATATACGACATGAACCTCTTTATTTCTTTTCATATTTATACTCATAACATATCTTTAAAAAAAAAACAAAGAAACTTCCCAAATTCCTGGTCGAATTCAAGGTGTTCATGTGGTACTTTCGTTCTCAGATTAGATGGCATAAAATATGAGATTCAATTTTATGATGAGCCTAATCTTTTCAATTTATATCAGGTTAGTATAATCAGTGTTACACTTCAAGGAATTACTATGCAAAATCATGGAGTAATTAACCTGGCTCTGATGCCAATGATAGAATTTAAACAACGTTTGGTACAAGATTTGTGTACCTTTGATTGTTGCAGCGGAATGAAATTCACGAACTGAATTATTTGCTCCAAATCCTTCTTTGAATCACTAGGAAACAAAGATAGTGTTTTACAAATTAAATGCTTTTTGTTGTGTCTTTAATCCAAGTCTTTTTGAGTTTTTGTTGTTCTTCCTGTTTTTCTTTTCTGAAGCGTTCAAGAAAAAAACAAAAAAAAACTTAATTTAAAAAGGAGAGAATAGTAATTGTTCCCACCCCTTTTTAAATTAATAT

>LNC_001069

CTCATGACATATTCTGTCTGTAATCATAAATTCTAAAAAAGCCTTTTTTATGTATATTTACATGCCCAAAGTTAAAGCTTTGGCTAGTCCACTTGTGGTATGTTTCATTGTTTTTTCCTTGTATCATCTCATCTTTTTTATTTTGTGGGACAATGATTTATCCCTTTTTCTGATCATTTTATATCAGTCATGCACAAAGGAACAACTTTTGCCATTTTAACTCATGATCAATGGCAAACTAACTTTGATCCTTTGTCATGATTCTAGTTATTGATGAGTTTGAATGTTGTAATTGTAATTGATTGGTAAACTGATAAGAGAGAATTAAAGCCTGAGATGATGGTCCATGACTCATGCACTCCAATTATCCTTGTGTCTTGGAATAAAAAAGCTAACACATTAAAGAAAGAAAACAACTACCATTAAACAGGTGGTAAGATAGATAAGATCACTTTATTAATGAACGATT

>LNC_001070

TCTATTCTAGGCCCAAAAGGGATTCTAATTCCTGATTCTTAAATAGAAGTCCTTATACATTGTTGGCTAAATCAGTGACTTTTGATGTTGAGAAAAGTAATTGAAAGAGAATTAGTTTGACTTAATCAGGATGCAAGTTAGATTATCTGGGAATATAAGATGCATTCTTTCTACCGTACTATAAATTCATGTCTGCTGGTTGTTTACATCTAAAGTCAGATGATTCTTTCACATTGTCTTGTAGGTTAATATCCTTAGGCATTGGCCTTCCCATTGCTTAGTATTAGATGGATTTACTGGCTGATCTGAAAAAAACTAGTGAGAATTATTTAGGAGGTTTTGAGAGAGCCTGATGTGAAGATACCTCTGTTTTACCCCCTTATGTTTTTGATTTATCATGGAAGATTGACATGGTGTGCATCTTAGAAATTGAGAATTCTATCATCTATAAATAGATGTTTGCTTCTTTAGTGGTTATGGTCCTGACTTGAATTATTGCATCTTGCTTCACCTGCAACTTTTGAGTTGGTCCTAGCTAATGACTGAGAAAGGAACATCTGGTTATTTTGGATGCACCTATGAATGTCTTATAATGCACTGTTTTTTGATTTCTCCATTTATAACAAAGTTAGGTATGACTACTATATAGTAATGAGTGAGGAAATTAAGTCCATTTCATCATTACTGTTGCTCATAGTTTATTCTGGACAAAAGAAGCTGCTACTCTTGCCATTATATCCAGC

>LNC_001071

GGGAATTATCGGGTAACTGTGAGCATGCTTTGTGTTATTAACGGACCCTCCTCGCTATGTGGAGTGCTTGCATGCTTAATTACTGTTTATTTGAAGCATGTTGGACCTTAGCTTAGGTTTGACTAGGGCTTGCCTTAGAAAATACATGATTCGGATCCAATAGGCCTTAGTTTTGCCCCGACGTCGCTCGGCCGGCTTAGATCCCTGTAGACTGGTGTAGCAGACTTGAGTCTGATAGTTTGGGC

>LNC_001072

CTTCTATGAAGTTCTTCACTTCTTCAGTGAAAGGACCGAATGTCCAAGCTTTTCCTTTCTCTGTTCTGCGACTCTCTCAGTTCGTTCTTCTATGAGTTTTTCTTTCACCATTCTCTCTGTTTGCGCTCTCCCGTGATTTCTATCTGCGATAACATTCCGTTCTAATGGATGTGTTGTTTTAGTTCCATAAATTAGGGTTTAGTTCTGCTCTAATATCGTTGTGAATGGCATTACGAAAACTCGAAGAGCATAGAGGAAGGCTATGTCAGCAAAATTTTCAAAGATAAGTACGTAATACACTTATATTTTATCAATTTGTGAAAGTCATTCGTTTTTCGCTTTCCTTCGACTTTTCCCCTCTTGGTATAAGTTCACTACATAAACCTCTTTGTGCTAAAATTAAATGAAAGGAGAAGGGCTCAATGATTTAAGTAGACTAAGTAATTTAGGATTTTTGTGATGTTTACTTTGCCGTTATACTTTTTCTTGATGAATTTGCAGGACTTATTTTCAAATTTATGGTGAGTGTGTTTTCTGTTTTAAAAAATATTGGTTAGTGTTAATCAAATAAACAATAAAGGTAATGCTTAAATTGCAAGCTTTAGTCAAAATACAGACAAATTCTCGAGTCAACTAATTATTTATACAATAAAAGTTGCACAGTTTTTCCTCTTGCGGTTTCAACTATAACTTCGAGGAGCATATTAAAAGGAGCTTTAAGATTTTTCATGGAAAAGACGATGGTTTTCAAAACATAGTGACCTGTTTCATTCTATAAAGGACTATCGAAGGTGATTGTTACTCAATTTTTAATATTAGACTGAGTTGTATTTGCCGTTTTAGATCGTTAAGCTTTCTGTACTTTCATGTTACTATTGAAAGTTATCGAATAATACAAATTACGCCTAAAATTCAAATAATTGAGAAACGATATATTGTTTATCAAGAATTAGGTTGTTGTTGATCATTATTATATAATTAATCTTATGTAAAAATACACTACAAAAAATGAAGAAAAAGAAGAATGAAGTGTTCTAGGTGATGAGAAACATGTCATTAGAGGTAAAGTGCAGGAAATTCAACGCACCATCTATTCCTATGAGTCAACGACTTTGGTGGAAAGGACACTGAAGTTGAAGTGGTAAATGATGTAATTGACACAAATCACATAATAAAAAGAATTGTAATTTGTAATCAGAATCTTGCACAATGTGATGGTAAACCTGTATCGTGGTGAACAGATACTATCTCCGTTGGACTACATTATAGTACGTCTGTCCCAAGTTTTGCTCCTGCTGTCTTTAAGCCTTTTAATGAAACAAGTTGATTGAAGAGATTCAACGAAGGTAACTTCGAAGCTTGAGGCATTTTCCCATTAGACGTAGAACTCCTTCCATTGCTTGAGGGAAAAACACTGCGCCCCAACTGCAAGACAGCTGATGGAGATATGATGTGTTTGGATGAATGTGTTTGAAGAACTCAATTGCATTTCATTTAGGCCTTTCTTTGACAACTTCCACCAAACGTGGCGTGTAAAGAGAGATGGAGGAAATGGAAACAACATTTTACCACTACAATCTTGCTTTGGTGATGATGCGGGACAACCCCAGAAAATTATGCTATTGACGTCCCATTATTTGAATAAGCAAACCTAGCCAAAATAGTGGAATTTACAGGTACTTGCA

>LNC_001073

CTTCTATGAAGTTCTTCACTTCTTCAGTGAAAGGACCGAATGTCCAAGCTTTTCCTTTCTCTGTTCTGCGACTCTCTCAGTTCGTTCTTCTATGAGTTTTTCTTTCACCATTCTCTCTGTTTGCGCTCTCCCGTGATTTCTATCTGCGATAACATTCCGTTCTAATGGATGTGTTGTTTTAGTTCCATAAATTAGGGTTTAGTTCTGCTCTAATATCGTTGTGAATGGCATTACGAAAACTCGAAGAGCATAGAGGAAGGCTATGTCAGCAAAATTTTCAAAGATAAGTACGTAATACACTTATATTTTATCAATTTGTGAAAGTCATTCGTTTTTCGCTTTCCTTCGACTTTTCCCCTCTTGGTATAAGTTCACTACATAAACCTCTTTGTGCTAAAATTAAATGAAAGGAGAAGGGCTCAATGATTTAAGTAGACTAAGTAATTTAGGATTTTTGTGATGTTTACTTTGCCGTTATACTTTTTCTTGATGAATTTGCAGGACTTATTTTCAAATTTATGGTGAGTGTGTTTTCTGTTTTAAAAAATATTGGTTAGTGTTAATCAAATAAACAATAAAGGTAATGCTTAAATTGCAAGCTTTAGTCAAAATACAGACAAATTCTCGAGTCAACTAATTATTTATACAATAAAAGTTGCACAGTTTTTCCTCTTGCGGTTTCAACTATAACTTCGAGGAGCATATTAAAAGGAGCTTTAAGATTTTTCATGGAAAAGACGATGGTTTTCAAAACATAGTGACCTGTTTCATTCTATAAAGGACTATCGAAGGTGATTGTTACTCAATTTTTAATATTAGACTGAGTTGTATTTGCCGTTTTAGATCGTTAAGCTTTCTGTACTTTCATGTTACTATTGAAAGTTATCGAATAATACAAATTACGCCTAAAATTCAAATAATTGAGAAACGATATATTGTTTATCAAGAATTAGGTTGTTGTTGATCATTATTATATAATTAATCTTATGTAAAAATACACTACAAAAAATGAAGAAAAAGAAGAATGAAGTGTTCTAGGTGATGAGAAACATGTCATTAGAGGTAAAGTGCAGGAAATTCAACGCACCATCTATTCCTATGAGTCAACGACTTTGGTGGAAAGGACACTGAAGTTGAAGTGGTAAATGATGTAATTGACACAAATCACATAATAAAAAGAATTGTAATTTGTAATCAGAATCTTGCACAATGTGATGGTAAACCTGTATCGTGGTGAACAGATACTATCTCCGTTGGACTACATTATAGTACGTCTGTCCCAAGTTTTGCTCCTGCTGTCTTTAAGCCTTTTAATGAAACAAGTTGATTGAAGAGATTCAACGAAGGTAACTTCGAAGCTTGAGGCATTTTCCCATTAGCTGCTATGTGAGCTCCTGGTTCCATATGGTTTGTCGAAAAGCTCATTTGATTTGGAAAGTTCGGATAAAGGCTACGTATCCTCTTAAGTACATCATGTCTATCAAGAACTTCTTCCATGGAGCTTGCCATTCATTAGTTCTTGATTTTGGTGTTTGTACGAATTTTGCTTAGGATCTTCCGTGAATCTCATGTTCATGTAGACGTAGAACTCCTTCCATTGCTTGAGGGAAAAACACTGCGCCCCAACTGCAAGACAGCTGATGGAGATATGATGTGTTTGGATGAATGTGTTTGAAGAACTCAATTGCATTTCATTTAGGCCTTTCTTTGACAACTTCCACCAAACGTGGCGTGTAAAGAGAGATGGAGGAAATGGTTCATCTCTAGCACACAGATTCATTTCACAAATTGTCCCTTTTTGTTAAAATTAACTTGTGACCTTAACTAATTCCTTTGATTTTGCTAAATCTAAACAGGAAACAACATTTTACCACTACAATCTTGCTTTGGTGATGATGCGGGACAACCCCAGAAAATTATGCTATTGACGTCCCATTATTTGAATAAGCAAACCTAGCCAAAATAGTGGAATTTACAGGTACTTGCACAATAAAGGAAAGGAAAGAAATTGAAAGATGCGATGCAGGGAACATTGAGCCTAATTCTAAAAATATAACCATTATCAGGGTGTTGAAGTATCAGCACACAACTAGGAACACAAAATAGCTATCCACCATCTTCTTGAAGTTGAACACTTGCTCAGGTGAATTATGCAGTCTCACTCGATAGTGGGGATCGATTGTTAGGAACCAGGAAGAATGAATTAGCAAAGATGATATGCTGAGCTAGACTATAGCAACCTCCTTTTGGATTATTCTCTTCCAAAAAACTTGATCTCCTAATTTAAAATTGGAAAAAAATCCAAAAAATAGAATTGTATGTGTCAAATTGATATGAATTGTGGCCTTACATGTACTTCAACAACGGGATAATATTGAAATGGTTTGTTTCCTACTGTGCTTAATTACCTTTTTGAACGATTCTGGAAATATCACGGGGCTTACAAGGTCTTTAAAGGACTCTTGAATAAGTATGGTCCTGACAGAGTCATTGATATACCAATTATTGAGGTTGTTTTCATTCATCATCAATGTTCTATAATCTTACTTAATTCCCTTCCATTTTTAGTCACCGGAATATAATCAATTTGCATCCTTTATTTTAATTCTCTTTTAGCCATCATGACACGTAGATTTTTTATTCTAGCAATATCTTGAAATCTTTTCAGCCGCTCTGCAATACATTACATTATAACTGGTGATGTAATCTCAGGCACACGAAAAGAGAGCAATTTGATTGTTGC

>LNC_001074

CTTCTATGAAGTTCTTCACTTCTTCAGTGAAAGGACCGAATGTCCAAGCTTTTCCTTTCTCTGTTCTGCGACTCTCTCAGTTCGTTCTTCTATGAGTTTTTCTTTCACCATTCTCTCTGTTTGCGCTCTCCCGTGATTTCTATCTGCGATAACATTCCGTTCTAATGGATGTGTTGTTTTAGTTCCATAAATTAGGGTTTAGTTCTGCTCTAATATCGTTGTGAATGGCATTACGAAAACTCGAAGAGCATAGAGGAAGGCTATGTCAGCAAAATTTTCAAAGATAAGTACGTAATACACTTATATTTTATCAATTTGTGAAAGTCATTCGTTTTTCGCTTTCCTTCGACTTTTCCCCTCTTGGTATAAGTTCACTACATAAACCTCTTTGTGCTAAAATTAAATGAAAGGAGAAGGGCTCAATGATTTAAGTAGACTAAGTAATTTAGGATTTTTGTGATGTTTACTTTGCCGTTATACTTTTTCTTGATGAATTTGCAGGACTTATTTTCAAATTTATGGTGAGTGTGTTTTCTGTTTTAAAAAATATTGGTTAGTGTTAATCAAATAAACAATAAAGGTAATGCTTAAATTGCAAGCTTTAGTCAAAATACAGACAAATTCTCGAGTCAACTAATTATTTATACAATAAAAGTTGCACAGTTTTTCCTCTTGCGGTTTCAACTATAACTTCGAGGAGCATATTAAAAGGAGCTTTAAGATTTTTCATGGAAAAGACGATGGTTTTCAAAACATAGTGACCTGTTTCATTCTATAAAGGACTATCGAAGGTGATTGTTACTCAATTTTTAATATTAGACTGAGTTGTATTTGCCGTTTTAGATCGTTAAGCTTTCTGTACTTTCATGTTACTATTGAAAGTTATCGAATAATACAAATTACGCCTAAAATTCAAATAATTGAGAAACGATATATTGTTTATCAAGAATTAGGTTGTTGTTGATCATTATTATATAATTAATCTTATGTAAAAATACACTACAAAAAATGAAGAAAAAGAAGAATGAAGTGTTCTAGGTGATGAGAAACATGTCATTAGAGGTAAAGTGCAGGAAATTCAACGCACCATCTATTCCTATGAGTCAACGACTTTGGTGGAAAGGACACTGAAGTTGAAGTGGTAAATGATGTAATTGACACAAATCACATAATAAAAAGAATTGTAATTTGTAATCAGAATCTTGCACAATGTGATGGTAAACCTGTATCGTGGTGAACAGATACTATCTCCGTTGGACTACATTATAGTACGTCTGTCCCAAGTTTTGCTCCTGCTGTCTTTAAGCCTTTTAATGAAACAAGTTGATTGAAGAGATTCAACGAAGGTAACTTCGAAGCTTGAGGCATTTTCCCATTAGGTAAGGGGTTCTTGAAGTCTTCTTTTAGTAATGGCACCTCAAAATCTGCCTTCTTATGTTTAACTAAATTATTCTTAGCTGCTATGTGAGCTCCTGGTTCCATATGGTTTGTCGAAAAGCTCATTTGATTTGGAAAGTTCGGATAAAGGCTACGTATCCTCTTAAGTACATCATGTCTATCAAGAACTTCTTCCATGGAGCTTGCCATTCATTAGTTCTTGATTTTGGTGTTTGTACGAATTTTGCTTAGGATCTTCCGTGAATCTCATGTTCATGTAGACGTAGAACTCCTTCCATTGCTTGAGGGAAAAACACTGCGCCCCAACTGCAAGACAGCTGATGGAGATATGATGTGTTTGGATGAATGTGTTTGAAGAACTCAATTGCATTTCATTTAGGCCTTTCTTTGACAACTTCCACCAAACGTGGCGTGTAAAGAGAGATGGAGGAAATGGAAACAACATTTTACCACTACAATCTTGCTTTGGTGATGATGCGGGACAACCCCAGAAAATTATGCTATTGACGTCCCATTATTTGAATAAGCAAACCTAGCCAAAATAGTGGTACTTGCACAATAAAGGAAAGGAAAGAAATTGAAAGATGCGATGCAGGGAACATTGAGCCTAATTCTAAAAATATAACCATTATCAGGGTGTTGAAGTATCAGCACACAACTAGGAACACAAAATAGCTATCCACCATCTTCTTGAAGTTGAACACTTGCTCAGGTGAATTATGCAGTCTCACTCGATAGTGGGGATCGATTGTTAGGAACCAGGAAGAATGAATTAGCAAAGATGATATGCTGAGCTAGACTATAGCAACCTCCTTTTGGATTATTCTCTTCCAAAAAACTTGATCTCCTAATTTAAAATTGGAAAAAAATCCAAAAAATAGAATTGTATGTGTCAAATTGATATGAATTGTGGCCTTACATGTACTTCAACAACGGGATAATATTGAAATGGTTTGTTTCCTACTGTGCTTAATTACCTTTTTGAACGATTCTGGAAATATCACGGGGCTTACAAGGTAGAATTATTACTTTTAGACTCATTGATGAATTTAATATCGTGCGCGCTATGCAGTAGTGAAGTTGTTTCTCCTTTCTTTTGGCAGGTCTTTAAAGGACTCTTGAATAAGTATGGTCCTGACAGAGTCATTGATATACCAATTATTGAGGTTGTTTTCATTCATCATCAATGTTCTATAATCTTACTTAATTCCCTTCCATTTTTAGTCACCGGAATATAATCAATTTGCATCCTTTATTTTAATTCTCTTTTAGCCATCATGACACGTAGATTTTTTATTCTAGCAATATCTTGAAATCTTTTCAGCCGCTCTGCAATACATTACATTATAACTGGTGATGTAATCTCAGGCACACGAAAAGAGAGCAATTTGATTGTTGC

>LNC_001075

CTTCTATGAAGTTCTTCACTTCTTCAGTGAAAGGACCGAATGTCCAAGCTTTTCCTTTCTCTGTTCTGCGACTCTCTCAGTTCGTTCTTCTATGAGTTTTTCTTTCACCATTCTCTCTGTTTGCGCTCTCCCGTGATTTCTATCTGCGATAACATTCCGTTCTAATGGATGTGTTGTTTTAGTTCCATAAATTAGGGTTTAGTTCTGCTCTAATATCGTTGTGAATGGCATTACGAAAACTCGAAGAGCATAGAGGAAGGCTATGTCAGCAAAATTTTCAAAGATAAACGTAGAACTCCTTCCATTGCTTGAGGGAAAAACACTGCGCCCCAACTGCAAGACAGCTGATGGAGATATGATGTGTTTGGATGAATGTGTTTGAAGAACTCAATTGCATTTCATTTAGGCCTTTCTTTGACAACTTCCACCAAACGTGGCGTGTAAAGAGAGATGGAGGAAATGGTTCATCTCTAGCACACAGATTCATTTCACAAATTGTCCCTTTTTGTTAAAATTAACTTGTGACCTTAACTAATTCCTTTGATTTTGCTAAATCTAAACAGGAAACAACATTTTACCACTACAATCTTGCTTTGGTGATGATGCGGGACAACCCCAGAAAATTATGCTATTGACGTCCCATTATTTGAATAAGCAAACCTAGCCAAAATAGTGGTACTTGCACAATAAAGGAAAGGAAAGAAATTGAAAGATGCGATGCAGGGAACATTGAGCCTAATTCTAAAAATATAACCATTATCAGGGTGTTGAAGTATCAGCACACAACTAGGAACACAAAATAGCTATCCACCATCTTCTTGAAGTTGAACACTTGCTCAGGTGAATTATGCAGTCTCACTCGATAGTGGGGATCGATTGTTAGGAACCAGGAAGAATGAATTAGCAAAGATGATATGCTGAGCTAGACTATAGCAACCTCCTTTTGGATTATTCTCTTCCAAAAAACTTGATCTCCTAATTTAAAATTGGAAAAAAATCCAAAAAATAGAATTGTATGTGTCAAATTGATATGAATTGTGGCCTTACATGTACTTCAACAACGGGATAATATTGAAATGGTTTGTTTCCTACTGTGCTTAATTACCTTTTTGAACGATTCTGGAAATATCACGGGGCTTACAAGGTCTTTAAAGGACTCTTGAATAAGTATGGTCCTGACAGAGTCATTGATATACCAATTATTGAGAACTCAATACACGACGACTACAAAAATAAGAAATTTTGAAGGTGGAAAACAATACACATCTTAAAGCTCTACCTACAGGCAACAGCTTTCAAACTGGAGGAGGCACCAAAAAAAAAACTTGTGGTTGAGTTGGCAGGTATAACAGACGGTCAAAACAGTTCAATTTACGGATAACCTGAGTTGAAGATACGAGTATTAGATAAATAGTTTATTAATGGTGTTATTTTGTTTTGGATCGGTATAATGTCTATGTGAAGGTGTTTCTTTGGTGTAAACTTAAGTTTTTGTTA

>LNC_001076

CTTCTATGAAGTTCTTCACTTCTTCAGTGAAAGGACCGAATGTCCAAGCTTTTCCTTTCTCTGTTCTGCGACTCTCTCAGTTCGTTCTTCTATGAGTTTTTCTTTCACCATTCTCTCTGTTTGCGCTCTCCCGTGATTTCTATCTGCGATAACATTCCGTTCTAATGGATGTGTTGTTTTAGTTCCATAAATTAGGGTTTAGTTCTGCTCTAATATCGTTGTGAATGGCATTACGAAAACTCGAAGAGCATAGAGGAAGGCTATGTCAGCAAAATTTTCAAAGATAAATACTATCTCCGTTGGACTACATTATAGTACGTCTGTCCCAAGTTTTGCTCCTGCTGTCTTTAAGCCTTTTAATGAAACAAGTTGATTGAAGAGATTCAACGAAGGTAACTTCGAAGCTTGAGGCATTTTCCCATTAGGTAAGGGGTTCTTGAAGTCTTCTTTTAGTAATGGCACCTCAAAATCTGCCTTCTTATGTTTAACTAAATTATTCTTAGCTGCTATGTGAGCTCCTGGTTCCATATGGTTTGTCGAAAAGCTCATTTGATTTGGAAAGTTCGGATAAAGGCTACGTATCCTCTTAAGTACATCATGTCTATCAAGAACTTCTTCCATGGAGCTTGCCATTCATTAGTTCTTGATTTTGGTGTTTGTACGAATTTTGCTTAGGATCTTCCGTGAATCTCATGTTCATGTAGACGTAGAACTCCTTCCATTGCTTGAGGGAAAAACACTGCGCCCCAACTGCAAGACAGCTGATGGAGATATGATGTGTTTGGATGAATGTGTTTGAAGAACTCAATTGCATTTCATTTAGGCCTTTCTTTGACAACTTCCACCAAACGTGGCGTGTAAAGAGAGATGGAGGAAATGGAAACAACATTTTACCACTACAATCTTGCTTTGGTGATGATGCGGGACAACCCCAGAAAATTATGCTATTGACGTCCCATTATTTGAATAAGCAAACCTAGCCAAAATAGTGGTACTTGCACAATAAAGGAAAGGAAAGAAATTGAAAGATGCGATGCAGGGAACATTGAGCCTAATTCTAAAAATATAACCATTATCAGGGTGTTGAAGTATCAGCACACAACTAGGAACACAAAATAGCTATCCACCATCTTCTTGAAGTTGAACACTTGCTCAGGTGAATTATGCAGTCTCACTCGATAGTGGGGATCGATTGTTAGGAACCAGGAAGAATGAATTAGCAAAGATGATATGCTGAGCTAGACTATAGCAACCTCCTTTTGGATTATTCTCTTCCAAAAAACTTGATCTCCTAATTTAAAATTGGAAAAAAATCCAAAAAATAGAATTGTATGTGTCAAATTGATATGAATTGTGGCCTTACATGTACTTCAACAACGGGATAATATTGAAATGGTTTGTTTCCTACTGTGCTTAATTACCTTTTTGAACGATTCTGGAAATATCACGGGGCTTACAAGGTCTTTAAAGGACTCTTGAATAAGTATGGTCCTGACAGAGTCATTGATATACCAATTATTGAGAACTCAATACACGACGACTACAAAAATAAGAAATTTTGAAGGTGGAAAACAATACACATCTTAAAGCTCTACCTACAGGCAACAGCTTTCAAACTGGAGGAGGCACCAAAAAAAAAACTTGTGGTTGAGTTGGCAGGTATAACAGACGGTCAAAACAGTTCAATTTACGGATAACCTGAGTTGAAGATACGAGTATTAGATAAATAGTTTATTAATGGTGTTATTTTGTTTTGGATCGGTATAATGTCTATGTGAAGGTGTTTCTTTGGTGTAAACTTAAGTTTTTGTTA

>LNC_001077

TGAAGTTCTTCACTTCTTCAGTGAAAGGACCGAATGTCCAAGCTTTTCCTTTCTCTGTTCTGCGACTCTCTCAGTTCGTTCTTCTATGAGTTTTTCTTTCACCATTCTCTCTGTTTGCGCTCTCCCGTGATTTCTATCTGCGATAACATTCCGTTCTAATGGATGTGTTGTTTTAGTTCCATAAATTAGGGTTTAGTTCTGCTCTAATATCGTTGTGAATGGCATTACGAAAACTCGAAGAGCATAGAGGAAGGCTATGTCAGCAAAATTTTCAAAGATAAATACTATCTCCGTTGGACTACATTATAGTACGTCTGTCCCAAGTTTTGCTCCTGCTGTCTTTAAGCCTTTTAATGAAACAAGTTGATTGAAGAGATTCAACGAAGGTAACTTCGAAGCTTGAGGCATTTTCCCATTAGCTGCTATGTGAGCTCCTGGTTCCATATGGTTTGTCGAAAAGCTCATTTGATTTGGAAAGTTCGGATAAAGGCTACGTATCCTCTTAAGTACATCATGTCTATCAAGAACTTCTTCCATGGAGCTTGCCATTCATTAGTTCTTGATTTTGGTGTTTGTACGAATTTTGCTTAGGATCTTCCGTGAATCTCATGTTCATGTAGACGTAGAACTCCTTCCATTGCTTGAGGGAAAAACACTGCGCCCCAACTGCAAGACAGCTGATGGAGATATGATGTGTTTGGATGAATGTGTTTGAAGAACTCAATTGCATTTCATTTAGGCCTTTCTTTGACAACTTCCACCAAACGTGGCGTGTAAAGAGAGATGGAGGAAATGGAAACAACATTTTACCACTACAATCTTGCTTTGGTGATGATGCGGGACAACCCCAGAAAATTATGCTATTGACGTCCCATTATTTGAATAAGCAAACCTAGCCAAAATAGTGGTACTTGCACAATAAAGGAAAGGAAAGAAATTGAAAGATGCGATGCAGGGAACATTGAGCCTAATTCTAAAAATATAACCATTATCAGGGTGTTGAAGTATCAGCACACAACTAGGAACACAAAATAGCTATCCACCATCTTCTTGAAGTTGAACACTTGCTCAGGTGAATTATGCAGTCTCACTCGATAGTGGGGATCGATTGTTAGGAACCAGGAAGAATGAATTAGCAAAGATGATATGCTGAGCTAGACTATAGCAACCTCCTTTTGGATTATTCTCTTCCAAAAAACTTGATCTCCTAATTTAAAATTGGAAAAAAATCCAAAAAATAGAATTGTATGTGTCAAATTGATATGAATTGTGGCCTTACATGTACTTCAACAACGGGATAATATTGAAATGGTTTGTTTCCTACTGTGCTTAATTACCTTTTTGAACGATTCTGGAAATATCACGGGGCTTACAAGGTCTTTAAAGGACTCTTGAATAAGTATGGTCCTGACAGAGTCATTGATATACCAATTATTGAGGTTGTTTTCATTCATCATCAATGTTCTATAATCTTACTTAATTCCCTTCCATTTTTAGTCACCGGAATATAATCAATTTGCATCCTTTATTTTAATTCTCTTTTAGCCATCATGACACGTAGATTTTTTATTCTAGCAATATCTTGAAATCTTTTCAGCCGCTCTGCAATACATTACATTATAACTGGTGATGTAATCTCAGGCACACGAAAAGAGAGCAATTTGATTGTTGC

>LNC_001078

TGAAGTTCTTCACTTCTTCAGTGAAAGGACCGAATGTCCAAGCTTTTCCTTTCTCTGTTCTGCGACTCTCTCAGTTCGTTCTTCTATGAGTTTTTCTTTCACCATTCTCTCTGTTTGCGCTCTCCCGTGATTTCTATCTGCGATAACATTCCGTTCTAATGGATGTGTTGTTTTAGTTCCATAAATTAGGGTTTAGTTCTGCTCTAATATCGTTGTGAATGGCATTACGAAAACTCGAAGAGCATAGAGGAAGGCTATGTCAGCAAAATTTTCAAAGATAAATACTATCTCCGTTGGACTACATTATAGTACGTCTGTCCCAAGTTTTGCTCCTGCTGTCTTTAAGCCTTTTAATGAAACAAGTTGATTGAAGAGATTCAACGAAGGTAACTTCGAAGCTTGAGGCATTTTCCCATTAGGATCTTCCGTGAATCTCATGTTCATGTAGACGTAGAACTCCTTCCATTGCTTGAGGGAAAAACACTGCGCCCCAACTGCAAGACAGCTGATGGAGATATGATGTGTTTGGATGAATGTGTTTGAAGAACTCAATTGCATTTCATTTAGGCCTTTCTTTGACAACTTCCACCAAACGTGGCGTGTAAAGAGAGATGGAGGAAATGGAAACAACATTTTACCACTACAATCTTGCTTTGGTGATGATGCGGGACAACCCCAGAAAATTATGCTATTGACGTCCCATTATTTGAATAAGCAAACCTAGCCAAAATAGTGGTACTTGCACAATAAAGGAAAGGAAAGAAATTGAAAGATGCGATGCAGGGAACATTGAGCCTAATTCTAAAAATATAACCATTATCAGGGTGTTGAAGTATCAGCACACAACTAGGAACACAAAATAGCTATCCACCATCTTCTTGAAGTTGAACACTTGCTCAGGTGAATTATGCAGTCTCACTCGATAGTGGGGATCGATTGTTAGGAACCAGGAAGAATGAATTAGCAAAGATGATATGCTGAGCTAGACTATAGCAACCTCCTTTTGGATTATTCTCTTCCAAAAAACTTGATCTCCTAATTTAAAATTGGAAAAAAATCCAAAAAATAGAATTGTATGTGTCAAATTGATATGAATTGTGGCCTTACATGTACTTCAACAACGGGATAATATTGAAATGGTTTGTTTCCTACTGTGCTTAATTACCTTTTTGAACGATTCTGGAAATATCACGGGGCTTACAAGGTAGAATTATTACTTTTAGACTCATTGATGAATTTAATATCGTGCGCGCTATGCAGTAGTGAAGTTGTTTCTCCTTTCTTTTGGCAGGTCTTTAAAGGACTCTTGAATAAGTATGGTCCTGACAGAGTCATTGATATACCAATTATTGAGGTTGTTTTCATTCATCATCAATGTTCTATAATCTTACTTAATTCCCTTCCATTTTTAGTCACCGGAATATAATCAATTTGCATCCTTTATTTTAATTCTCTTTTAGCCATCATGACACGTAGATTTTTTATTCTAGCAATATCTTGAAATCTTTTCAGCCGCTCTGCAATACATTACATTATAACTGGTGATGTAATCTCAGGCACACGAAAAGAGAGCAATTTGATTGTTGC

>LNC_001079

GAAGTTCTTCACTTCTTCAGTGAAAGGACCGAATGTCCAAGCTTTTCCTTTCTCTGTTCTGCGACTCTCTCAGTTCGTTCTTCTATGAGTTTTTCTTTCACCATTCTCTCTGTTTGCGCTCTCCCGTGATTTCTATCTGCGATAACATTCCGTTCTAATGGATGTGTTGTTTTAGTTCCATAAATTAGGGTTTAGTTCTGCTCTAATATCGTTGTGAATGGCATTACGAAAACTCGAAGAGCATAGAGGAAGGCTATGTCAGCAAAATTTTCAAAGATAAACGTAGAACTCCTTCCATTGCTTGAGGGAAAAACACTGCGCCCCAACTGCAAGACAGCTGATGGAGATATGATGTGTTTGGATGAATGTGTTTGAAGAACTCAATTGCATTTCATTTAGGCCTTTCTTTGACAACTTCCACCAAACGTGGCGTGTAAAGAGAGATGGAGGAAATGGTTCATCTCTAGCACACAGATTCATTTCACAAATTGTCCCTTTTTGTTAAAATTAACTTGTGACCTTAACTAATTCCTTTGATTTTGCTAAATCTAAACAGGAAACAACATTTTACCACTACAATCTTGCTTTGGTGATGATGCGGGACAACCCCAGAAAATTATGCTATTGACGTCCCATTATTTGAATAAGCAAACCTAGCCAAAATAGTGGTACTTGCACAATAAAGGAAAGGAAAGAAATTGAAAGATGCGATGCAGGGAACATTGAGCCTAATTCTAAAAATATAACCATTATCAGGGTGTTGAAGTATCAGCACACAACTAGGAACACAAAATAGCTATCCACCATCTTCTTGAAGTTGAACACTTGCTCAGGTGAATTATGCAGTCTCACTCGATAGTGGGGATCGATTGTTAGGAACCAGGAAGAATGAATTAGCAAAGATGATATGCTGAGCTAGACTATAGCAACCTCCTTTTGGATTATTCTCTTCCAAAAAACTTGATCTCCTAATTTAAAATTGGAAAAAAATCCAAAAAATAGAATTGTATGTGTCAAATTGATATGAATTGTGGCCTTACATGTACTTCAACAACGGGATAATATTGAAATGGTTTGTTTCCTACTGTGCTTAATTACCTTTTTGAACGATTCTGGAAATATCACGGGGCTTACAAGGTAGAATTATTACTTTTAGACTCATTGATGAATTTAATATCGTGCGCGCTATGCAGTAGTGAAGTTGTTTCTCCTTTCTTTTGGCAGGTCTTTAAAGGACTCTTGAATAAGTATGGTCCTGACAGAGTCATTGATATACCAATTATTGAGGTTGTTTTCATTCATCATCAATGTTCTATAATCTTACTTAATTCCCTTCCATTTTTAGTCACCGGAATATAATCAATTTGCATCCTTTATTTTAATTCTCTTTTAGCCATCATGACACGTAGATTTTTTATTCTAGCAATATCTTGAAATCTTTTCAGCCGCTCTGCAATACATTACATTATAACTGGTGATGTAATCTCAGGCACACGAAAAGAGAGCAATTTGATTGTTGC

>LNC_001080

TTCACTTCTTCAGTGAAAGGACCGAATGTCCAAGCTTTTCCTTTCTCTGTTCTGCGACTCTCTCAGTTCGTTCTTCTATGAGTTTTTCTTTCACCATTCTCTCTGTTTGCGCTCTCCCGTGATTTCTATCTGCGATAACATTCCGTTCTAATGGATGTGTTGTTTTAGTTCCATAAATTAGGGTTTAGTTCTGCTCTAATATCGTTGTGAATGGCATTACGAAAACTCGAAGAGCATAGAGGAAGGCTATGTCAGCAAAATTTTCAAAGATAAATACTATCTCCGTTGGACTACATTATAGTACGTCTGTCCCAAGTTTTGCTCCTGCTGTCTTTAAGCCTTTTAATGAAACAAGTTGATTGAAGAGATTCAACGAAGGTAACTTCGAAGCTTGAGGCATTTTCCCATTAGACGTAGAACTCCTTCCATTGCTTGAGGGAAAAACACTGCGCCCCAACTGCAAGACAGCTGATGGAGATATGATGTGTTTGGATGAATGTGTTTGAAGAACTCAATTGCATTTCATTTAGGCCTTTCTTTGACAACTTCCACCAAACGTGGCGTGTAAAGAGAGATGGAGGAAATGGAAACAACATTTTACCACTACAATCTTGCTTTGGTGATGATGCGGGACAACCCCAGAAAATTATGCTATTGACGTCCCATTATTTGAATAAGCAAACCTAGCCAAAATAGTGGTACTTGCACAATAAAGGAAAGGAAAGAAATTGAAAGATGCGATGCAGGGAACATTGAGCCTAATTCTAAAAATATAACCATTATCAGGGTGTTGAAGTATCAGCACACAACTAGGAACACAAAATAGCTATCCACCATCTTCTTGAAGTTGAACACTTGCTCAGGTGAATTATGCAGTCTCACTCGATAGTGGGGATCGATTGTTAGGAACCAGGAAGAATGAATTAGCAAAGATGATATGCTGAGCTAGACTATAGCAACCTCCTTTTGGATTATTCTCTTCCAAAAAACTTGATCTCCTAATTTAAAATTGGAAAAAAATCCAAAAAATAGAATTGTATGTGTCAAATTGATATGAATTGTGGCCTTACATGTACTTCAACAACGGGATAATATTGAAATGGTTTGTTTCCTACTGTGCTTAATTACCTTTTTGAACGATTCTGGAAATATCACGGGGCTTACAAGGTCTTTAAAGGACTCTTGAATAAGTATGGTCCTGACAGAGTCATTGATATACCAATTATTGAGGAAGTAACTTTCTTATTTTTGCGACTTTTGGTTACGGAAAAATATAAGTTTTTTTTTTGACGGAGATCATCCATCTTTGAAAGCCCAAAGGCCAAATGCGTAAACGTGTTGAACATGAG

>LNC_001081

TCATAATCTATTTATCTTCCTTTGCCTCAAAACATTGTTATCATATATATATGTTATTATTTTGTTTCTTTTGTTTACTACTCTTCACCATGCTACTATATCTTATGTGTCAATTTAGTTATCAAATTTTTTTTCCAGGTAAAATATTAATGACCCATGAAATTTATTCTTGAAAAGCTCGGTGTTTTCTTGATCTATTTCCAAAGTTATTATATAGATCTTAAGAGTATTGAATACTTATCTGTTAGATTGAAGCAAATTACTCTGAATTTTATTACATCTACATCTTTATCTATTAAACTTAAATTTGACCTTTCTATAGCAATCAGTTGATTAGTGCTTTGTTAATAGTAACTCTCTGATGGGATGTAATTTAAGCATTTTCCCTTGATTTTGTGGCAGAGCAACACTATTATATTATATATAGATATAGCACATTTGCATCATAAGAGCACCTAGACTATTCTACAAAATGAACTTGTAATTTTCTAAAAGTGGGAACAATCTAAGTCAAAAGTTGAATTACGAAGATACTTTTTGACTATTAATTTATTTTCTTAAGATCATTTAAAAAAAATATTTTTCCATTTAAAATCAACTTCATAACTACACCTCTTCATTTTCCTAACTTATCATCTTATGATCATAAATAAACAATAAATAATAAATAATAAGAATAAAAATAAAA

>LNC_001082

AAAACTTAAAAATGGAGCTCAAGGATCGTTTACAACATCAAAAGCGAGTTAGGGAGCAGATCGATTCTTACCTAAGTTGTTCGAGCTGTGTGGATTCAGTGGGATGGAAACGAACCGAACGCAGCTTCTCCAAGGCATCACTTCGTCTTCATCCTCCATGGAAGCTCAAGCTTGAAGATGACCAACTCCTTTTTCCTTTTTTTTGGTTGTTTCATTTTTGTAATTTGTTTTTGGACAG

>LNC_001083

TTTATATTAAGGTTTTAAGGAAAATGAGTATTAGACCAGTTTTACATGGTTGCGTTCTAGCACATTGGTACTTGGAGTGTCTATAGCACTGGCTATGTTAAGTTTTTCTGCCGTGCGGTGAGAGAATTGTTCCACCAATTGGTGTGGCATAAAATGTAGTCTGTTCCTTTTTTAAGAGGGTTTCAGAGTGCAGATTACTTACTTAGCATAAACAACACCAAGTGAGCTGTGCTTTTTTCGTTCTGTC

>LNC_001084

GTTTTGAAGTGCAAAAAAGTTTGAAAACATTCATTCGAAAATCAGCATTGTTTAAAGATGGGGGAGGTTTACAAGAATCCAAAGACTCAGCTACTTTCATTAGAGAAGCATACCTAAAATCGTAAGACTTTCGCTTCAGCTCAGAAGCTCGGGGGCTTTTGGGGTTGACCCAAAGCTTGCTTTTTGATGAAGAAACAACTTTTCTATCCGAGTATTGAGAATTATTGGGATTTGAGACCTGGGATTGTTCTTGAAGTGACACTTGGGTCGTTCGGATACAAGATTTGGAGTGAAGTTTGAGGGGGAAAGTGAATTTTTGGGGAGAATTGAAGTTGCAGAAGATATTGGATTCATAGAGAGGAGAAAGTGTAGAGCTTGGAGTAGACGCCATTTTCTTGGAGCAAAGCTTTAACGTTGCCGCCTGCCTTATCAACTATGGTGTTTGAGAGAAATGGAAGAGGTGATACCCCTTAAAAGTTTAGCATTTTGAACAATTTGCCCCCTAAACTTTTCAAATATTTATTTGAAAACTATTTCAAGTAAATGTGTCCCCATTTTTTTTCTTGACAAAATTTAGTTCCTTTAATATCATTATGAATTTGGTTCAGACGTAGGATACCTTTTCAGAGGTTGTCGCAACTCTAGAGGCTGGATCGAATCAAGCTTATGCAATTTCAGAATTTTCTTTGTTGAATGGTTTGTTCTTCCCGGTTGGAATAGATAGTTTAATTCGATGGAATCATCAAAGATTTGACCTTTTCGAGTTATATTTGTAACGCACTATAGGCCCGAGAAACAAAGAGAAGATGTATATGAACGAACAATAACTCTAGAGCATTTGGCGATCTCAAATGTGAAGTTGTCTGATGGAAA

>LNC_001085

AGTTTGTACATATTACATGATAGTTGTGAATATGTTGTATTCTGTGTACATGTTTGGCTGTTGGATGAGAAGGGGAGAAGATGGAAATGGCTTAGAATTTAGCCTTTCTAAGAGACCCTTGTCTTAAATCAATTCATCTATGCACTTTCCAGGCTCGCTGCCTTGGTTCCCGGCAGTTTGCGTATATGGGTGCTTACAACACAAACAATATACAGTTTTTTGCCTTTCTGTAGTTTTTCAGTA

>LNC_001086

AAAAAAATTAGGGAAAAAAATTTCAATTGATTTTTATATATTTTGATTAGTTTTTACCAGATCTGCTATTGAGTTTTCAAATAACTCGATTTTTCATCGAGCTAGGGTTTGTTGGGTTTGCCGACTTTATTTGTGAAATTAGGGTTAGGGTTTATAGGTGAATTTAGGGTTTTCTTTGATACAGACGTTTACATGGCTGCTGGAAGACATGGTGGTTACAGGGATAATGAATTTAGGGGGCGTGAAGCTGAGTTTGAGGTTTCACGGAGGAAGC

>LNC_001087

TGCCAAACTCATAGCCCTGTAAACTGTTCTGGGTTTGTGAATCCTAACATCAGATTTAAGTTCCTCTTTCAACCCATTCAGGAACACCCCTAAGAGACAGTGATCTGGCCAATTTGTAACCCTAGATGAACGCTTTGCAAATTCTTGACGATACTCCTGTACAGAACCTGTTTGCTGAATGTTGCAAAGGTGCTCGTCGGGATTTTGAAACTCTGCAGGACCATAATTTTCCTGCAATGCTTTAACAAGTTCTTCCCAATAAAGCAAAGTTCGCTCACGATTTATCCAAGAGAAGAGATCTAGAACATCCCCTTCCAAATACA

>LNC_001088

GAAATCAGGATGAATCGACGGATCAGAAGAGAGTTGATCTCGAATCTGCTTAGTATATGGATCAGCTTCAATTGCTTCTGTCAAATCACTGAAATCTAATGGGATAGGCATCACCAAAGATAAAAAATCAGCATGTTGAGGGCGACGAGATAAGGCATCAGCGCCTTTGTTTTCACTTCCTGACTTATAGACAATAGTGAAATCAAAAGGCAATAATTTAAACAACAAACGTTGTTGTTCAGCAGTAGTCACCCGCTGATGCAAGAGATACTTTAGGCAACAATGGTCAGTAGTGACAATAAAATGCCGACCCAAAAGATAATGCTTCCATTTTTGCAA

>LNC_001089

GAACATTTATAGAGATCTCCACAAGCCTGCTCTCGTAATTGAAGGACGCTTTGTATTAGTGGTGTAACAAGAGGAGTTGCTAAATTGTGTTCTCACATAAGAGGGGATGCAACTAAAGAGAATAATTGCTCATCAATCAACAAATAGTATGCCACTTTCATCACTGTATTAGAGGATTGACATCCTAATGGAAGAAGGGTGGAGGGCTGAATATGATTGTGTATGAGAAAAGATGTTATGGAACGATTTGTGAACTTCATATTTTTAGGTTTTGCTTTCGGTATAAAAGTGTTTCTTATTGTGAAGTGCCATAATTGCCCTTGACAGAAATAACAAATCTGTTTGTATTGGTTATCACCGTTAGTAGAAGGACTAAAATAATTGATTATAATTAATTAAAGGTTAAACCTGGGAAGTAAGATATCACAGGGATCAAAATCAAAATAAGGCAATAACACAAG

>LNC_001090

GTTGAATTGTAAAAGTTAAGTGTTTATAATATTACTTTCTAAAGCTCTTTCTCTCTCCATGTCTTTGTAGATTTTTAACTTCTCTTCAAATGAGAAAATTTAATCAATTCTTGATGAAATTTCAAGAGAAAAATCATAGAGGAGCTCATATTGGAGTGATTTTTGCTCAAATTATGTCAAATTTTGATTTGTGTTAGCCATTTTTTTATTCGAGGCAGAGGTATACTTTCTTTTCGTCCCTCTTCCATCTTCTTTTTACAGGCTTCGATCAAGTTTTTTATTTTTGAATTCTATTATTTTTATCTAATTTTTTGATTAAGGAAATTAGTGTTTTATCTGATTTGATATCTGAAACTTTTTCTTACAAAAAAATTATGAGCTCAAGATTAAGGTGGGTAAAATTGCAGTAACAATTAGTATTGGCTTTTCAACTTTAGAATTAATCATTTTCTGCAATATGAATTGTGAAATCTATGAATTAATCATAGTCTTCTTTATAATTTTGAATTGTTATTGGAGCTGAAAGAAGTTAAACAAGTGACAACCTAAATTGCTCTTTGTCTCTTCAAAAATGTCAATTAGAATCTCCTACAATCATGTTTGTCGACCAAGACGGCAAGACCTGTATGCTGTATTCATAGCTAGTCATAAACCTCAACCTATACTATGTTTCTCCTTAGTTACTCATGTAATTAAGAGCAAAATAAGACTTGGCGTATTGAGAGAAAGGTGG

>LNC_001091

TTTAGATCTACTTTTTCTCTCTTCAATTTTGCTTGTAATTTTTAAAAAAAATAGCATTTACTATGATTTTCTCTGTGTTTTGATTCATAACAACTGAGAAAATACGAAAAAAAATGCTAAATTAATTTATTTTTTCGTACTTATGGACACGCAGATTTGAATATTTTTTCATGAATTTATGCGTTTTCTCGTAAGTTCAACATTTTTTTTTTGTTGATAAATTTAAGTTTTTTATTTTTGTATTTTGCTCAGTGATGATTGATTTTGCTTTAACCACACTGAAATAAAGAAGTTTGATGAAGATTTGGATAAGACCTTTTTGGATTTTTAGTTGAAGATTGTTTACTCAATTTTTTTATTTCTTTGTTTAAAGGCTTTATGAATCTTTTTGATCCAATTTATTTTGGTGTAAAAGATGAAAGCATTTTGCAGAGAAAATTAAAATTGAGTGGGGATTTATTTATTTAATTCTTCAACTTAAAAATCCTTAATTTGGTCAGTCAATTTCTTTGTTAAACTTTTTCATGTGGACAAACTTTGGCCCTGGTCTGAGAGCAAATAAATAATTTTTTGAATTTTGTTTGATTTATGTATTTTTGTTATAGTTTATTGGAATCATAAGCAAAGTTTAAATTATCAAGAAATTATATTATATAAATTGGTACACCATTAAATTGGATTAGATTAGAAATAAATAAATTTTGTATGAATTTTAGAAACTGATTGAAATAAGCATAATGTTAATAGTTTAGTGATTGTTTTGTTAGTTTGATACAACATCAAAAACGTAGTGAGATAGTTTTCGAAAGACCCTTGATCCCAATTCATAGCGTATACCCAATGCAGTTATCAATAGACTCTTGGTTTAAGCAAATCTAATAATATTAATTAAAACAAAAACTAATTGAATATCGATAATGTTGAAAGTGTAAGTTATCAATTAATAATCATCATTCATATAAGGTAAACTTTCATTTAAAATGTAATAATGTAACAATTACAAGTAGAATAAATAACAATTTTGATCCTTAGACTGACTAATCAAGTAATTTTTTGGTATACGATCTTCAAAAATTACAAAAGCAACACGAAAACAGAATTATATTTTTTTCTTGTAACGATTTTTGCTTTTTTAATCGAGATTTATCAATGTCATAAGAAACTTTCGAGGAGTTGGTGCTGGTGCTGGTGCTGGTGCGGGGCTAGATGAGGCTTGATGAAACAAAGTGGTGAAAATTTTATCTGCAAGCACTGCATTTGCTGCATCTGTTGGATGAAATGCATCCCAAAATACATACTCTCTTCTATTATTGCATAGTTTTGAATTTGGCAAACATAATCCTCCAACACTTGTGTCTACATTGCAACATGATGTATTTGATATCTTGAAACCTAAAAAAACAAAAAACAAAAATTGTATTCAAAATT

>LNC_001092

CTATTTGATAGCCTTTGTATGTCAAAGATTGCAACACACCTGAATCGTCAAATATTTTTTAATATATATTTGTTGATTCAGGTTTGTATGCAAACTATGACATATTTTAAAGGATGATGTATACAGGTCTATTTCTTAATGGTGAGATTTGAATTTTAATCTTTTGTAATTTTTGTATTTTTGTTTTATTAATCGTTAGATCATATTATCGAGTCTTAAATCCACTCTATTTCAATTGACAGTACTCTCATTTTTCTAAACTACCGTTTTTTTATTTCTTTTTTGATGTTTTTATGTTCGGATTTTATTTTGAATTATGAGATATTCATTCAAAAAAATAATTAGTGACAGATAAATTTTATTGTCAAACAATAAATATTAATTGCTAATCTTAATTAATGATGGATTATCAAAAAAATGTTATGTTAGTTGGGTGGTCTTTAATTATTTGGGTTCATAGGTGAGATATTTTTTTTTTGAA

>LNC_001093

GAATCTACTATTTAGTCAAAAGCTTTAATGTTAGTTCAATCAATTGGGGTTTTGGAGAATAAATAGAAAAAAGAGAAGTGGTCGGTTAGATTCTTTTAAAATTTTGTGTCTGCGAGAGACTCCTTTGAAGAAGGAAGGTCATCTTTTTGTTTCCCTTGGTGAAATGGACCTGCTTTACTTTTCTTTCAGAGATTTTGAGGAAGCACATATAGAATGATATGTGATTTAGTGTTGATGCAATTAAATATTTATAAGATATTACAGTTCCTTTGTATTGTTATTGAGA

>LNC_001094

AGTTAAATGAACTATGTGCCCAGAAAGGTTATATAAAGAAGAAGAATGTCGACTTTCGTGAGAATGGTATAGTATATATTACAGTAGAGGTTGAAGCAAATGGTGCTATTCACAAGGACACACACTCGGGACGAAACGAAAAAATGGCCAAGAAGGTGGCTAGCAAAAAAGTGCTCAAGTCTCTCAAAGAATATCCATACAATGCATAGCAATAGTTGTTTCTTTCTGCTACTTCAGTTGACAAAATTTTAGGATATTTGTTTTGATTCTCAGTTTTAAAAATTGGGAAAAAGGACAAATATACCTATGTGCCCCTGCCCCTGCCGTCCAAAAATTAGAGCATATATATCTTTCACTCTAACAGAAGATCAAATAGGGACACCTGGCGCAATCTTATCCGTCGATCTGATATTTAATAAATATTGGATCGGTGAATAAGATTATGACACGTGTATGTTTGTTAGTATAAAGAGTATATATGTTTTAGTTTTTGGACGACAGGAACATCAAATGTCCAAAAGTATGACGGAGGGTATCTGGA

>LNC_001095

GAGCGGTTGAATAATGAATCTACTAATTAGTCAAAAGCTTTAATGCCAGTTCAATCTATTGGATGTTTGGAGAACAAATAGAAAAAAGAGAAGTGGTCGGTTAGATTCTTTAAACATTTTGTGTCTGCGAGAGACTCCTTTGAAGAAGGAAGGTTATCTTTTTGTTTCCCTGGGCGAAATGGACCTGCTTTACTTTTCTTTCAGAGATTTTGAGGAAGCACATATAGAATGATATGTGATTTAGTGTTGATGCAATTAAATATATATAAGATCTTACAGTTCCTTTGTATTGTTATTGAGCCTACTTTGGATTCATCAATAATTATATGAACTTCC

>LNC_001096

CACCAAACAACCCCTGAAGTGTTCCGTTCAGATTGTTCCTGTTCCCAGTTAAAACTTGACCCTGCCCCAAAACGCCTCTGGTTGAGCGGTCAAAGTTCGAACCGAAACCAAGAACGGATCCTAAAATCCGACTGATTTAGGTCCGCCAATTTCAAACGGCGCCGGTCTTCTTCTTCCGGCGAATCGACAGAGACAGCACCTTCCTCCACCATCTTCGACAAAATCGAGT

>LNC_001097

CTTGGATGGTGCCTTGCAAATAAGGGGCACCTCTATTTATACTACCTCCTAGGGACTAAAATGTAATTAATATTTACTTTACAAGTCCCTAAACTATTTACACTTTGGTCCCTCCCTAGAATCTTCTACTAATTCTAGGAAATTCTAAAGCTTTCCAAGAAAATATCTAGTCCCTCTTCTAGAATTCTCTACACATTCTAGAGAATTCTAAGGCTTTCTTGGAAACTATCTAGGACCTTCCATTGCCTTCTAAGTTATTCTAG

>LNC_001098

TACCAGTACCTCATAAAGGTAGTGAGGTTGTTTTTCAAAGACCCTCAGTTCAAGTACAACAAATTCAGATAATAAAGAAGAAAATAGTGAAGAAAACATAACAACTAGTAAGAGAAGTTATGCTACAAGAAAATCATAAGAACAAAAAAAAATAGTGTAGTAATGGAAACAGATGATAGTAGATATCAAAAGCAAGAACTACAAGAGTACGGCTAGTACAACGACAGTCCCCAACAAGCCTAAACTCTAAAAGATCTAGATAACACTCTACTACCTACTAATGTTCTACCCTAATACTCGACCTCCTCCACACCCTCCTATCTAAGATCATATCCTCGTTAATATGTAGTTGCGTCAAGTCCTGTCTAATCACATTTCCCCAATGCCTTTTCAGCTTACCTTTACCTTTTACTGTGCCTACTATATTCAACCTTTCACACTTCGGGGAGTCTGCGCATCTCCTTTTCATAGGTTCAAACCATCTCAATCTCGCTTCCCTTATCTTGTCCACCATGAACGCCACTCCTACCTTACCCCGGATTACCTCATTCCTAATTTTATCGCTCCTAGTATCTCCAGACATCCATCTCAACAACCTCATTTCCGC

>LNC_001099

CCACGAGCTAACCATGGAAACATCACAATTCTGATGTTTTTCTTTCTTTCTTCCATCAAGATTTGAGGGATTTTGATTAGTATAGGAAAAAGTAAAGTCCAAGCGGATATAACAAGAAAAGAAATCCTATTTCAGTAAGATTTGATTTGAATCGTAGTTTAGATTCAAGTTGAAAAAGGGACTTACCAAGTTGGCACGGAAAGGAAATCCTATTTCAGAAAGATTTGATCTGAATCGTAGTTCAGATTCAAGCTGAACAAACAGGAGTTACCACATTGGGCAGAAGACGATACTAAGAGAGAAGGCTTATTATCTAGCTGAAGGAAAGAGGTATACATACACATATATAAATAAAAATACTTGTTGAAGCTTAACCATCGATACAAATGAAGATCATAACGTGGCAAGCACAATGGGAAAATATGATTGCGTGTATGAATGGGATATAACGAGTTACGAAAGAGACATGGAAAAACAGGGTGGAAGAGGAAATGACGACAAGAACTGTGATGTGGATATTCGCGGTGAGAAACGACATTCGGGCGGAGACATGGTTAAGACAGGTGATGGATGCGCTTAGGATGGACGTGAACTGATCAAAGGTTGTGTCTGCATTACACCATGATGC

>LNC_001100

GATTATGATGATATTACTGGAAGCTTGGCTTGAACTATTCATAGACCATATAAGAAGATATATATGAATGGTTGAACTCTCATTTGTTCCCCCTCTATCTTTTTGATGTTTCACTTTGGTAAATTTGGTGAGATCAATAATAGATGTCACTAACTCTTTTACTTTTATTTCAAAAAAGATATTTGTTTTAGTTTAGATCTTTTTATTTGATGAGTACAAAACTAATACTAAATATCAGTACCAAGACATCATTTCACAAGTCTGCACCACGATTCTACACTGCTATGAACTTACCTATGTTTTCAACGAG

>LNC_001101

GATTATGATGATATTACTGGAAGCTTGGCTTGAACTATTCATAGACCATATAAGAAGATATATATGAATGGTTGAACTCTCATTTGTTCCCCCTCTATCTTTTTGATGTTTCACTTTGGTAAATTTGGTGAGATCAATAATAGATGTCACTAACTCTTTTACTTTTATTTCAAAAAAGATATTTGTTTTAGTTTAGATCTTTTTATTTGATGAGTACAAAACTAATACTAAATATCAGTACCAAGACATCATTTCACAAGTCTGCACCACGATTCTACACTGCTATGAACTTACCTATGTTTTCAACGAG

>LNC_001102

GGAAGCTGGTGATGATTATATGCTTCCACATCTTCTCAATTTGGTGAAGCAGATAAAATCATCATTATATTCGCTTGGGAAGATGTAGGGCTCTACATATACAGGGTTTTTAACGCGTCTAGTAAAAGTCGCATTGACCTACTAGCTTAAGGCAGAGGAATCTCATAAATTGAGCAGGCCAAAATATACTCAATTATTGAAAACTATAATATGATGTAATATC

>LNC_001103

GTTTGTCTTATCTCGCTGTTCCATTTCCTTTTGTTAAAACCCGTTCGTTTTAAATTGCGGTTGAATATTTCTTTAGAAAGCTTTAAACAACACGTTGTCATTTTGTCTTCTTGCTGCTTACAATCCTTCTAATCAGCCTCTTTTTAGGGCTTTATAAATTCATTCAATTTATTTCTTTTGTTTTTTTTAAAAAGGAAATTATATAGGAATTGCTCTATAATGATCAGAATACTTAGTTTGTTTTGATTTGGTCTAAATTTATTTTTTACTATGCCATTTCTCAATTTGCCAATTTTACAAGATTTGCTACTGCCCAAATATTGGTGTTGTGGATATATTCAATGCTACATGACTTTGTCCCATGATTCCCTCTCTTTGTTCCGTAATTGGTTTTAGTCAGTCAGATTAAGTAAAATTTTGTTTGAAGGGCTGTAATGTGGATTTTAAGTTTAGTTAATAATGAGGTTCGTCTGTTATCGCTTACTGTCTTTGGGTTCTTAGTTGTATCTTGGTGAGTGATATTTCTCCATACTCAATTACTCGATTTTTTGTTGCAATTATGCTTTAAGTGAG

>LNC_001104

CAGCAGCTTCCAAACATCCACAATACAATACATCATCCCATACGTATATACACAGTATCCCACCGCCCAACTTCGTCTTCCTCGCTGCAAATCAGCGATTCCAACAACACCCCGACCCCACTGCCCATGCTCGTTTTCTTCTTCCAGACGAAACCCAGAAGGCTAGTAAACGTCAACATCGACAGTCGCTCAGACCGGAAATCCCGCAGCAAATCGCGTCCTCCACTTCCCCTCTCCCCTCTTTTCCGTCGAAAATTGGTACAATGGAAAATTTATTTCTGATCTCTTCGAGATTTTGATTTTTCAAATTGGATTCGGATCCATCTTTTCAACCCGATTTCCCCTCTAAATCGACCCCAAATTCCCCTATATATAAACCCCCACTGCCTTCATCAGAGAGGGGGGTTAGGAGAAAATTTGAGAAGAGCTATAGAGTTGG

>LNC_001105

TCACCCTCTCACTATATTTTTTTTAGTAATGAAAGTAACCATAAGAAACGAGTAAACTTTGAAAATGAATTGGAAATTTGTTTCTTGTTTAGAGTTTCTTGTATGTGAACCTCTTCACTTTTGGAGGTCTTAGGACCATGAGATGCTTATAACCTGCAAAAGTAATTTTCTCTAAATAATAAAGACCCTTCTAAACCTTTTGTCTTAGTGAACCGTCTTAAGATCTAAGACTTTGACTTACTGTAGCTTTTCATTGGTCAAGGTTTGACTCTTTGCTGCTTGCCCGCTTCTACTTTTCTTGCTAAAATGTTATCAATTCTCCG

>LNC_001106

AAACTATCATTTTGTCCTTCAAAACACCTAGCATTCCTTTCTTTCCATAGAGTCCACCATATACAGGCTGGAATAGTCTTCCACCAATCTTCACTTTTCCCTCTGTTGCCAATTCTTGTCCAGCTATTAAGGAGCTCCATGGTTGTCTTTGGCATCACCCAGCTAACCCCTAGTATACACAAAAACATATTCCACAATTTTGAAGCTGCTTTACAGTGAATGAACAAATGATTATTTACTTCTGCTTTCTGCTCACACATAAAGCATCTTGAGCAAAACTGCATCCCCCTTTTTTGTAATGCCTCATGTGTAAGGCAAGCTTTTC

>LNC_001107

CGTCCTCCTTCCATTTCCTATTACAATTCTTGAGTTTCCCCACAATTTAGGCCACTGATTCCTGATTGTTCTCCATAGACCTACCCCATAAGGGCTTCTAACTTGCTTAGTGATCCATAAGCCTTCCATTGTGTATCTGGAAACTATAGCATCTTTCCACAAACCTTGTTCCCCTGAGGCAAACCTCCATAGCCACTTCAATAAGAGACTTTGATTCTGTACTTTGAGGTTCTTTATGCCCATTCCCCCTTCCTTTTTACTGTTGGTCACGACTTCCCATTTCACCAAGTGATACTTCTTTTCCCCCTCTCCATTTCCTTGCCATAAAAAATTCCTCCGGATGGAGTCTAGCTTCTCTAAGACATTCCCTGGCAAAGGAAATAAAGACATCATATAAGTTGGCATCGCATCAAGTACACTATTAATTAGGGTCAATCTTCCACCCAAAGATAGGTATTGACATCTCCAGTTTACCAACTTTTTTCCACACTTTTCCACCACTGCATTCCATATGTTCTTTGACTTACTCATTGCCCCGAGAGGCATCCCTAGGTAGGTGGTAGGCAGTTCTCCCACTTTACCTCCTAGAATATCAG

>LNC_001108

AAAGTTTCACGGTCAAGCTCGTAGTCACAGAACGCTGCCCCAAGAAAGGTAACCTCTTCACCTCTCCTACTTGTCTATTTCGGTGCAATATTTATGAAATTAGGAAATCACTGTTCTTGTTTGGATGTTGTTACTCGTGATGGAATGCTTGTGTTCTTATGTGGGATAGGCATTTGTATCTAGTCTTAATGCTGATTAAAAAGGGTTATTGTATGAATAAGCTTGTCACTTCTTTGTTTTCTGTATCCAAATTTGTCTCGTTACCTTCGTTTTTGAATACATATATGAATGTGGTGAACTCGCTTCCTTATATTTGAGTCTAGCTGAGTCTCGTTTGAGCCATGGTCGTGAGTTAAGTTCTCGTTCTTCTCTTTAAAGTGTATTCGATCCCAAATGATAAATAATTTTGGTTTGGATCATGTCGAGGCTGGTTTTCGCTGATTTGAGTACGCAACCGCTTCCATTCTTTCGGCTTGCATAGCTGTATATTGTAGGACCGCTTTGCTCTTTTTTTTTTAGTTTACTTTAGCTTGTTCTGGCCCTTGTTCGTGCTTTCAGAATTGTTGCATGCTCATATTTCTTTAATCTCTCTAAAGATCTTTGAATTTTTATCTTGTCAAAAGGTTTGATTTCCCTTAAGTATGTGCATCTGTAATATATGTTCTTGTCTGCAATGCTTTGCTAAAATTGGGTACTTTAAAATCATGTGGCTTTGATTTTTGTTCTTCACAAACTGTTAGAATGTGTCTTTGTCATTAAATATTTAN

>LNC_001109

AACTCCCACGGGTACATTGGACTATCAGTCTTGGTTTTGGCAGATCACAGATGTTAAGCAACCAAATAGACGTAAAAGTTAGTCTGTCTTTGATTTCGAGGGAGGATGGGTAAATCTGGTGAAAGTAATTGGCACAAATGACAAATGATGAACAAGCATATTAGTAAATTCTTTGTCTCACTAAAATGGGGATTCAGGCTATATTGTCTCATTGCAAGGCAATACTTCTAATGAAATAAACTTTATATCTATGAATGAATTGGGAAGACTAGTTATTTGCACAGATGGCAGAACCATGAACTAGCTATTAGTTAAATTTTTGTTTCTCTGAAGTGGGAAGTGAAGCTGTGTTGTCCCTCATTTAATTTAGGCACAATTATTTCTACTCTTTGCTCTAGTAGATTTCTTAGTTTGGAAATAAGTATTCTTCAGATCC

>LNC_001110

CCTCATAGTAGGGTTAGCTGAAAAAATTACAATCATGCCCCTATCTAGGGTTTTTATGGAAACACCTTATAAAGGAGTTAGAGATAGTAACAGTAGTAGTTTCTTTGTTTGTTTGTTTTGTCAAGCCCAACCTATAGTTGCAGCAGCTGCAAGTTGTTGTTCCTTTTTTGTGATTTCATTTTCACAAATATAGAATTACTTGCACTGGGTGCAACTGTAAGCAGGACTCGACAATTACCTTTTTAGCTATCCCAACATCATCTACAAGCTCAGAGGCTTCTGCTCCACACCTCCATCAGAGTAATTCCCAATTTCAGGGTCAACTTGGAGGTTTGAAGGTGGATGGATGCAAGCCCCTCAATTGCGAACAGCAAGTTCTCTCTCATAGTCATCATAATCATTGGCTGAATCAGATGGTTTAACAGGACCTTCTGATGGATTAACCTCTCCGATCTCCCTCAAAAATTTGGACACACTCTCAAGAATCTCAGACTCTGATACGGTGGAGGGTGGTATAATGGTGGGGATATCCAAAGGGCATATCGGAGAGTAAGACACCCCACTACCAATCTACATCTTCCTGACCATACCAGGACTAAGACCAGGAGGAAACAAGGGGTATGGAGGCAATTTTCCACCTCCATTGGCAGTTGATGCTGGGATGGGATGAACTGCAACTTGTAGATTTCCATGTGGAACAGAGGACAGCACAGGCATCGACCCTGGAAT

>LNC_001111

GAGATTCTTTTGACTTTAGATGGTACCAAAAATTAGTATTATGCATATGAATCACCTTCAACTACTTTTAGACAAATTTGAGGTCAACTTTGAGTAATCCCTGTAGTTTCAAATGGTATCTCAAATATTCTTAAGTATTAACAAGATTAGTGCATCTTGCATAAATTAAGGGACTAGAACTATTCATATCTTTATGTTTCAAGTGCCCTTACAACAAATAAAGTCTTTTTTCACACACTATAGAATAATTTAGATCATAGGACATTTTTGAAAGTCACTCACACTCAGAAAGATGTTCAATGAATAAGATTGAGATACCATATGTTTGGCCCTCATGTAAGTATCCACTAATGTGAGAACACAAAAAATGATATCAAA

>LNC_001112

TTAGCTCGTTTTATTTCCCCATCTTTCATTTACAACAATTTCAATCAACAAATAAAACTCATCCTCAATCCCCATTACTCCCCAACACAATTAGGAAAGTAGTCATGCGTAGTCTAAGAGTTTAACAAAGTTTAGAAGTCACTTGCCTCAAAACACTTCAACTAGTAACTCAAATTTGAGTTTTACCCTTCCGAGCACGCTCCAAATCACCACAATCTATTCATAAATGGAATCCCACAATCAATACGAATCTAACAACACCAATATCGTGAAATTTGGAGAACAAGGGTAAAATGGTCAAAAATCTCGTTCCGGAAAATATTCTCGAAATCTGGAAATTTTTAGTGAAAAATGTTCCCCAAGAAATTTAGAATCCAATAGTGAAAACCGTT

>LNC_001113

AGTCAGCTTGCTCCAATTTTTATTTGGGAAAAAAAATTCAAGTCATAAAACATAGCCTTTCAACTCTTAGGTAAAGCTTTGCCCAAATTAGAAAAAAAATCCCAATTTCTTGAAGAAGAAGAACGCCTTAGCTCCAAATCTGAAATTTTTCACCGAGCAAATTGAAGAATTGAAGAGTGGTTTCAAAATTAAAAGAACAAGATGCTTCAATTTAGAAGATGATTTCTTAAAGCTTCATCACATTTAAAGGATTTTTTTGGAATAGAGTTTATCAGGTAAATATTTCTATCTTTAAAAGTCAAAATTTATGGCATCTGGGTTTATTGCTTGCGTGTGGTTACATATTTAGCTTTAGGATTTTGATATTCTTGTGTTTGTTGATACGATTTTTTATTTTATTTTTGGGTGTCTAGCTTGTAGTTCTATTACCCACATCAGTTGATTCTTTGCCCAATGCATTCATCACTAAATAAGTATGAGAATTTCTTTTTTATTAGACTGATAAGCTGATTAGGGCTAAAATCGTCCATGATAATGTAGGTTTCATGTAATTTTTTTAAGCATTATGTTTTTGTTGCTTCGCCTTTATTAGTTTTTGAAGCTACAAACTTCTTAGATTGACTAGAAGAGATTCTATCGGTAAGAAAAAAGGAGAACATAACAATCAAGACTAAGTCACTAAGATAGTAGGCGTTGTTTACCGAAGACTAGAAAAATGATTGCAATATTGTCTGGTAATAATGGATTCTTGGTTTGCCGAAAGAATTTTATAAAACTGGACAGCAGTACCAGAGTGTCTAACATTGTTGTCAAAAATGTGTACACTTGCAAAAATACTTGTGAGACAGGAAAAATAGGAAAGGAAACATTACATATTATATATATTGGAGTGTCCTTGCTAGGTGAAGCTTTAGTATCTCTTGCTATGACATGTGATGATTTGCGTACGACGCTGTTATACCTTGAAAATAGAGGTAGGATGTGATGTTTCTGCTTACTTTTTCTCTTATAGTTATACTCTTGTTTCTTAAGATCCAAGCCATTGTATATGATCTTCCTCTTAGTTTCCTTATCCTCCTCTTCTGACAGCCTATGTCTAGAATTTTATATTTAATAACCAGTCTTATTTGCTATGGAAATTTTAAGTGATATTTGATCTATCATATGGAGAGCAAACCGAGGATGAGTTATACTATTATGTAAGGACACATGGAAGATGATAGACACTATAGTGTATAATAATGAAGGACGAGATATGCTACAATTTAATATTTCTCAATTACCCTAATTCTCAATATAGTATGCAGGGGACAAGTGGGAAGAAAGCCCGAACATATGCTTTCCTTAATCTAGAAGTAGCTAAGGGTACATATCATGTTGCATGCCATCTATGATTGTGAGGTCAGTACCTATGAATTGGTAATTCCGGGTGGTGTCTTTGTTTGATTAGTGTTATTAAAAGCGAGAGCTTTTTCGCTTAAAGCGAGAAGCAAGGCGAAGCGAAGTTGTATCACTTTTTTGCAGTGAAGCGAAGCTGTGGTAAAAGAGCGAGCGCTTCAGTCCCAGAAGTGAGAAGTGAGACTTCAGTAAAAGCGAGAAAAAGTTCGCTTTTCTCATTAAGCG

>LNC_001114

CAAGGCACTTTCCATTCCCTTCTGACGCATTTGGTAATGAGGAGAGAGTCGGTTTCTCCAATGATCACACTATGTCCATTATTTACACACCATTTTATGCCAAACAACATTGAAGCTGCTTCTGCCATGTTGGATGTTCCTGGTCCCAGAGGTATTGAGTAAGCACAAATAATTCTTCCTTCATTGTCTCTGACCACTCCCCCTCCTCCACATATACCACGTAAACAACTCCCATCACTATTTAACTTAACA

>LNC_001115

CCTTCCAAGCTAAACGTACTAAATCAATTAGCTTATTACTATTGGAAAATTCCATTCATTTCTTGAAAATATTTCCTACTCATATTAGCTATCTACGATAAAAGTAGAAGCAAGGTCTCCGCTTTTTTCAAGGAAAGTCTCAGTGCGTAAAGAGTCCTCATACAAGAAAAAACCCAGTGCCGTAGTCAGCCAGGTGAGTAACGAACAGGCCGTAGGCAGAGGAGGCCCCTTTCCCAATCAAAGTCTTTCCCCCTTAATCTGCATCCGAATATGGAGTAAGGATGAAATGAAATGAAGGAGTGGAGTAGTAAAGAAACTATGATGAAGTGTGTCAACAACATATTCCAAAATATGCTTAACAACAACAAGATGAATGCCAAGGTCGTACATGACGCGCTTAAATCGCTCTTTGCTATAGCTCGGAAAAAGGCCAAACCAACCCATGAAAGTAAGAAAGAAGGAAAGAACTAAATCCAATGCTATTGATTTACCGCGTAAGGATTTGCGCTCTCCAAGCAAGCATTAAGATCCTCTGTTCAATGGCATTAAGATCAAACATATAAGACGCACCAACAGATACTGTCACAGCTAATTTAAGTGCTAGTCTAGTAACTACGGTTATTGATTCAATTACCAGTCCTTTATCTTCAGTTAGAAGACCCTCAAGGCTGACGTTCTTCTTTTTGTTACCATGCTTCTACCCAAAGGTGATTAGTTACCAATGAAGACATGAATCGTCGAATGGAATCTGTACAATTTATCAATCTTGACTGTTGGCGGACGTATAACTATCGTCCGCAGTCAACCTTACTTCGGTACAAAGGATTGATGTGTCACGTTCCTAACCATAGGATTGCTCCACTAGAAAGGTCGGGCATTCATTACTACGTATATAATTACCAGCATCTCCGCTTTCCCTGCTCTGATATAAGTTGAAATTCTCAAAGTTGAGAGTAAAGTATAAGTAAAATGTCAGAGATTGCCTCCTCTTCTAGGGTCACTCTCTTCATTTATTCAATGCCTTGGAAGAAGAGGAGCGGCAAACAAAGAGAGCACTTGGCATTGGCTCATTTCATGCTACGATGCTGTCTCCCTATCTCTTGGTTGAAGCAGGGATGAGACCCTATAGTCTCTATCCAGTGCTCTTCCAGCTCGATATCAGTATAAGTATCAATCTCTTTCCTTTCCTGTGTATGAGTGGAATTTTGAGTAATCGATGAGACTAGTAAGGATTCTCCAATAGGTATAACCCTTGTCTTTGTTCTTTTTTAAATGCTTGAGGGACTTCTTTCAATGTCAATGCTTCGGGTACTACTAGGCACAACATAGCGAGAAGTCGTTCTGCTCTGTTCATCTACCCCTAACAATAACAAGCAAGAGGGGGTGCTAAAGTTCAAATCCGTAGTGATCTCTGCTGATGTATATAGTGTGTCACGTTAGGTAACTTGACTCATTTCCATCGATGAAATGAATCAATCTCGGTATGTCACGAATTCATGTTGTCCACGACTCTATCTGGGTTAGTCCAGGTTCGTTATCAAGTTCCAGTTCCAATTTAGATTTTCGTGTATATTGATGTGATTGTTCCGCAAGTCGACATGAAACTAACTTGCTAGCATGGAATTGCAACAATAAGGAATGAAGGTTCCGGTTGCAGTAAATGTCTCAAAGCTAGACTTGAAGCATGCTTCAAGTGGGAACTGTCTCATCCATCTGAACAGGACAACTGAATGAATGCCGCTGACCAGTGTGACCGGTCGATTAGTGGCCATGATTAAGACGCACATAAACCGAGCTATTCACGGGAAAAAAGGTCAGAGGGCCATATTGGAATCGTGGCCATCAACAAGCACTATGTCTTCCTTATTATAGGGATAATTGAAAGGAAGCTTAGTGAATATTGAAACTCTTACTTGATTACGGTGGGCAGGTTGTTGTATTCACAAGTGGATGGCGCTTGGACATACAGAGGGAATGGGTGATTCATCGAGCTGGAACTCTCGTCACTACGGTGAGACCAACTCCAGCCAGTATCCTAGCGGAATGTGGCACCGGTTCGTTTCATGGGAACACCTACATCCACAGCGGCGAGCCAGTACTCTCTTGCTCCAATGTATGCGACCCAATTCACTGGTATGAGAGGACCAATCCTCTCATTACTAAGGGTGAATCAAATCCACATATCGCACGTTGTTGGACCTTCTTTAGCCCCGATTGGCCTGTAACGAATATGAAATACATACTCACACCAAACAACACGTGTTAGAACATGCTGATTCACCAGGCAAGAGATGTCATCTGCCCGTTGGAAAGAAGCCGGGACTCATGCAAACAAAAAAGCGCCCTTACGCTCTAGTTTGATCGGGGGGCCGCAACTAAGATCAGAGGGATAAACTTTCCCGTTATCGGAACAAACATTTTATTTCCAGGGTTCCTCACTTGTTTTTTACTTTCTTTTTTACGAAAAAAAATTCCGGACGACCGGAGCGGAAAACCTCTCCCGACAATTGGAGAAGGAGAAAGCCATTCCTACGGGCCTGGAACTGTGATGAATGAAGTAAGAATTTCGGGCTAGGGCCTGAGAATTTA

>LNC_001116

GACCTCATTCATTCATTCTAACAACTCATTGCCCTATTTTCTCTAGATCTAGTCTATATAATGATAATGACCGAGACCCGTACATAAGATCGTTCTAGCTTATCTCCTTCTAAGGAAGTCTGAGCTCCTTCCCATGCTAAGAGCCTTTCTAAACTCTTGAACTTAAGAGAGGCTTCTTTTCAAGCTGATGAGTAGAAATTGAGTATAAAGAAAATGACAAAAGAAATCTCACACGGAGATCAGTGTACCCATAGATCTATATGAAAAATAGATATATGAAAATTCAAGAAATTCGTTTTCAAAAACCCGGGATTTCAGAGGCGAACGACGACATAAAGGTTCCCCAAGTCATCCATGGCGGCCGGGCAAGCTCTCCGTCGAGCTGCCGATTTCCCTCCTATCCTAATGACAAAACCAGATTCAGAGGTTGTTTCTCTTAAGACGAAAACGTACGATTGCAGTCACGAAGGGTTTCCACCCTACGTCATAATTTTAATGCTTTTTTGTTAAGTAACATGAGCACCTGGGGAGACCGGAGAGCTGTTGGCGGGGCAGAAGCACTCTTAACTGGGGGGAATGCTTTCTATTCGGCCTTCGATTCGATGATGCCTGTCTCGTTGTGGTAGAATAGTACTCCACCATCCGATACCAAACGCGTACGGAAACAAAAGAACGACCCATGTCCATCGTTCTTCTTCTCCATAGCGAAACCAAACCCAGAAACAAAGCAAATCGCGAACCAAATCCAATTCAAAACCCAACAAACTAGCCCATCGTTCGCCATAGACACGCTCCTCTCCCCTTTTTCTTCCAAAGCGCAAAAATCAATACCAAGAACCTTAATTTCGTCATCCAACTACACCGCCAATCACCATTAACAGTCTCTCCCTCCATATTCAAAGATTGCAAACCTCGAGCTTGATTTGTTGACTTTGGATTAGATAAAAATCTCACCCAAATATGATACGAATCGGAGCTATCACATCGAGTCCAGAATTTTAGGGAAACGGACAGAGGATTGAAATTTGAAGGTTGGAGAATAGTATTCTCTGAAATCGAACCCACGAAAGTTGGAGCCTCAGATTTTCAATTTTCTTTCGAGTTGTTTTGTTCATCTGTTGGTGGGGGTCAGTAGTAGTAGGAAGCTCTTCAAAGTTCGTTGTCTCAGGTTGCTGCTCCAGGAAAGGTAATATTCTCGTTTCTGCTTATCGCTCTTTCCTTTCAGATGAAGTGTGTTCTGTTTTTGTCACTGTTCGAATAGTATAATTCTTCCAGATTTCAAGATTTGATTAGTGACTTGTTACACCTCTTCATACTGTTTGTTGTATTCACTGCTTAAGTTTGGTGTATGGATTATTCGGAGATCATGCTCAATAAAGGTGTATCACTCATATCAGTACATATGTTTATCTGTTGGTTGTAGCTTTGAATTTCTAGTGTATATGTTGTTTTCGTCTTCATTTGGAATGAAGCTAAGACAAATTTTCTTATCCGTGTCTAAGTTGGTGTTTAACTGCATCATTCCTCCCTTCTCCATATGGCTTGAGATTCGTTTTCTCACTGCATAGAATGATATTTTGCTTGGAATTTCTGCCTTCTCCAGCTTAATAATTGTTGTTTAAATGGTTTGTTGTTTGCTGTTTGTAAGTCAAGTCAGTACAAATTTGTGTGTTCCTGTTTTAATCGGCTGCATCTTAGATATGTTGGAGTCGAATTTATTTTTTTTAGTTTACTTGAGGCTTGATTTTATTTGCATATCCTTTCATGTGTGTAGCATGTTGATACATGATCTCATATGAGCTTCCATCAACTCGAATTCGTCATGTTTATGGCTTCTCTGTTCTTTCTTTTTCTTTTAAGTAAAAAAAAATCAGCTTATTGTAGCTCTCTTAATTTGTAAGATCTTCATGATAATTGGCATTGATTATTGATTAGATGTATAACATATTGTTTCATTAGCAGACTCTCTTTATATTTCTAATTATTGTTTGAATGCTAAGTATTTGTTTTATAATAAGATGGTTCATTCCTATGATGTCTATGCATATTTCTTTACAGATTTTGTTGTCGTATATGTATGTGTTGGAGAATGCCCATAAGTCTAGGACTCTAATAATTAAGTGTTAAGGTTTTCTCTGTTTGTTAGCTTTGTTCCCTGTTTCTCTTAGTGCAGCTTGCTTGTCATTATTAAGTTTGAGGTGTTTGCTTTGTTGATATATATAGTTAGTTCATTTTGTCTTCTTAAATTCATCTGGAACACTGGTTTTGATGACCCTTTGAGTTTCGAGATACTTAAGCTATTCTCACCTCAATTAAAATCATGTCTCACTTTTTTTTTCTTCATCTCAAATGCTAATTTTTGTCTTTTCGTTTGTTGTGTGAACTCCATCTGAGTCTACTTTGGACTCCTCTCCTTGCATTTGTGAGTATCGAGCCAGCCATAGGTGTTCGAGGGTGTAAGGGAAGCAGATTACAGGTCTCGAAAAGCAGAAAAATGGGTTATATACATTCGATATACA

>LNC_001117

CTAATTGGTTCCACCAAAGGATTCTTTGGCATTTGAAAGTTGTGACTGTCCAAGAGGCAAGGCTGTAACTTTTATGCGATCCATTCTGGCGGGATATTGATAAATTAGGAATGGCAAGAAACAGGTGGTTTGGAGGAAGTGATGTGGATGAGATATTTGAGCTAGAACGTGGATTAGGCAAGGAAGCATGTCATGAAATCTGATGCTTTGTATGACCTATATGTGTGCAAGATAGTGTCAAACGAGTTTGAGATAAAGTGGGGGTTGTGTGATTAAAATATGGAAGGACAAGAAATTTTGTCTCAATCTATATAACCTGAGTTTTTCTTTCAAATTTTAGGTTTTATGGATTCCATGCATCTGTAGTTCCCTTTTTCTTCCTTAATTTTCGTTGAGATGGAATTTTGCACAAATGAAGAATAACCTCTTATCCCTTATTTCTTTTTGGTGGTACCCATGAGGTTCCTTTATGTGTAGAATGATTAGGTGCACATCTTTTTTGTAGGCTTTTTGATTTTTGGTATACTTCTTATAAACAAGAGTTCCATTCCCAC

>LNC_001118

ATCCGCCTCCTCAAAACGTTCCTTCCCTATGTGAAACTTGCATTTCTTCCTTGTAATTGGTGTCCATGTATATTATGGTTCAACTCCTTTCTGTAGCTTTCAAAGGTGAATGACCCAATTCTCATTTCCCGTTGGTGTGCTTTGTCCAAGAGCTTTGTTAACCAGTATGACACCGAGAAGCATATATGTTTCAAATTTGACTACAGTTGCTGCTCTTTGAATTCTGTTCTGACCCTGTAGCCCTCGACTCTTGGTAATCCTGTTTGTAAGCTGTCATAGCGTAGTATAGGTAGCTCTCCTTTTCTCGCCAATATTAAGCCCTGTTTTAATATGTATATATAATATCTGTGAGACTCTGTGATAGATCGATCACTGTGAATGATATCTATACTAATTGATAGTGTCCTTAATCTTTCTTGTCTTGATCCGCAAACTCATGACCTATTGCTTATCCATGTTTAGAATTTTCCTGCATTTCACTGGTAGATGATTAAACTGCTTGTATTCTTGCTTTTCTGTGTGATTGTATGCATTGTTAGCTAAAGCATCTGCTAGTTGATTACCCTTTCTAAAAATATGTTTCACTTGTATTCCATCCTGCTGAATCATATTAATAGCTTCTTCCATGACTTCACTCAGTTCCCAAGGAATTTTCCATTCCTTCAACAAAAAATTTCTAATTCCCTTGGAATCTGTTTCCATAATCACCTTGCTATATTGATAGTGCCTGCAATTCTTTAGAGCCTTAAGGACTGCAGCCACTTCCGCCATGTTATTTGTAGTAATCTCT

>LNC_001119

TAAAAATAAAATAACAATAAAATAAAAGGAGCTACAAAACCTGAAGTCACATTCGTGGTTGAAGAAAATAAAGGGAAAGAAAGTAAAACGATTGTATGGCAGAAACAGGGCAAAAAGAGGTTGAAAGCCTAGTGTAATGTCAAGGAGGGCAGAAAGTCACTAAACAGCACCCAAATGTACCACACCTGAGCCTGAGCCTACGTTACAAGCCAAGAAAGTCCTATAGTGATCCTAGAGTCTATTTTGGAGAGTCTGAGCAGTGAAAATAAGGGCAAGCTTATGGCATTGAGCACTAACTGTTTTTGAAATTACTTCTGAGTGTGAGTGTTGAATAAATCCTTG

>LNC_001120

CAAAAACAGCAAAAGGGACTAGTCCCAAAACGCCTAATCCAAAATCTGAAACAAGAAATGGAAACAAAAAACAAATAAAAAGGGGAAAGACAAAACCCTCCTATAGTCAAGACACCACTGTCTCTAAATCTTCCGTCATCAATGGTGGACCTCCGTCAACCTCTTCTCGACTCCGGCAGCTATGGTGGACCTACATCAAAGATCCTTGTGCAAATGATGAGATAGGAGTTAGACCTTACTCACTGTGGAACTAGAGACTCATATCATCGAGCTTGGATAGAGTACATCAAGACGTTAAAAATCTCACTAACGCCATGTATTCTTGTCCGTTGAAGACTTCTTCAGCTCAAAACATTGGTCCCTTTCCTGTGTGAGAGATCTATCTTCTTTGGAGCTTTCTCCAGATATCAGTGCCTTGTAGAAAACCATTTTCCTTAGATCTTCGCCTCTTGACAGATTGAGCTCCGAGGGAACTGATACCACTGTCTAAGAAGCTAGGATCTAAAGTCTATGTATATTTGTTAGGTGAGCAACCAGTAAGGAGCTCCTCACTATTGTCTGACCTGTATCTGTTATCAACATCTTCTAAATGCAGATGAGGCTCTTTCTCAAACTCCACCGTATCTAACACCACAGCATCCTGCCCTCCCAAGCTGTCTCCATTGAGTTGACCCGAGGTTAAAGCACAGCCTGGAGTGTCCGATGGATGGAGAAGGCATTGTCAGCTATCAAAACTCTATGTTTCTTCAATGGGGGTTTTCAGGATCGATTGCAG

>LNC_001121

CCCAAAACGCCTAATCCAAAATCTGAAACAAGAAATGGAAACAAAAAACAAATAAAAAGGGGAAAGACAAAACCCTCCTATAGTCAAGACACCACTGTCTCTAAATCTTCCGTCATCAATGGTGGACCTCCGTCAACCTCTTCTCGACTCCGGCAGCTATGGTGGACCTACATCAAAGATCCTTGTGCAAATGATGAGATAGGAGTTAGACCTTACTCACTGTGGAACTAGAGACTCATATCATCGAGCTTGGATAGAGTACATCAAGACGTTAAAAATCTCACTAACGCCATGTATTCTTGTCCGTTGAAGACTTCTTCAGCTCAAAACATTGGTCCCTTTCCTGTGTGAGAGATCTATCTTCTTTGGAGCTTTCTCCAGATATCAGTGCCTTGTAGAAAACCATTTTCCTTAGATCTTCGCCTCTTGACAGATTGAGCTCCGAGGGAACTGATACCACTGTCTAAGAAGCTAGGATCTAAAGTCTATGTATATTTGTTAGAGTATTAGACCAGGTATAGCATTGAATAGAGGTCTAAGATTAGCTGAACAAGTTGCATTCCACCACTTATACACTGTGTCCTTTATCTGAAGAAAAGGACCTTCTACACCAGCAGCTCTAGTAAAACTACTCCACATTTGATTAGCAGCAAGGCAGGAGACAAAGAGGTGTTGAAAACTTTCATTAGCCATCTGATCACAACAGCAACACACCATCTCCTCCTCAGTTTCAAATCTGGCTAGGACTTCACCAATAGGGATCCTTTTCATTGCTAATCTCCAGAAAAAGAAAGATACTTTAAAAGGTGAGCAACCAGTAAGGAGCTCCTCACTATTGTCTGACCTGTATCTGTTATCAACATCTTCTAAATGCAGATGAGGCTCTTTCTCAAACTCCACCGTATCTAACACCACAGCATCCTGCCCTCCCAAGCTGTCTCCATGTAAGCCTTGATAGTCCTTGTGAATTACTAATGCTCTATCCAAATTCTCAACTTCCTGAAGACCTGTGTCAAATTAGTTAGACGATCTGATTAGTTTATCCGTTGTTCTTTTTCGATTAGTGAATTTCCAATGGATTTTTTGTGATTAATATATAAGTTAGAGCTGGATAATTTTATATAAAATGTGTCAAGCCCATGTTGTAGATAATTAGAGGTTGTGAGCGTAAAAAATTGAAATTAATTATTTGAATGGTTATTTATATTTTGAAAATTGTCTATTAAAATTCTTTTTGTTTCTTTTTCTTTTATTTTTCATTTATCATTCTTTAAAAAATCATCTTTATGTTTCATATATTTTTCAACTTCTAGTTTTAGGATCCATTCGAACATAAAAATATTTATTTTTTTCGTAATAATATTTCAATTTATTTTAAAATCAACGTTTGGC

>LNC_001122

CAAAAACAGCAAAAGGGACTAGTCCCAAAACGCCTAATCCAAAATCTGAAACAAGAAATGGAAACAAAAAACAAATAAAAAGGGGAAAGACAAAACCCTCCTATAGTCAAGACACCACTGTCTCTAAATCTTCCGTCATCAATGGTGGACCTCCGTCAACCTCTTCTCGACTCCGGCAGCTATGGTGGACCTACATCAAAGATCCTTGTGCAAATGATGAGATAGGAGTTAGACCTTACTCACTGTGGAACTAGAGACTCATATCATCGAGCTTGGATAGAGTACATCAAGACGTTAAAAATCTCACTAACGCCATGTATTCTTGTCCGTTGAAGACTTCTTCAGCTCAAAACATTGGTCCCTTTCCTGTGTGAGAGATCTATCTTCTTTGGAGCTTTCTCCAGATATCAGTGCCTTGTAGAAAACCATTTTCCTTAGATCTTCGCCTCTTGACAGATTGAGCTCCGAGGGAACTGATACCACTGTCTAAGAAGCTAGGATCTAAAGTCTATGTATATTTGTTAGGTGAGCAACCAGTAAGGAGCTCCTCACTATTGTCTGACCTGTATCTGTTATCAACATCTTCTAAATGCAGATGAGGCTCTTTCTCAAACTCCACCGTATCTAACACCACAGCATCCTGCCCTCCCAAGCTGTCTCCATGTAAGCCTTGATAGTCCTTGTGAATTACTAATGCTCTATCCAAATTCTCAACTTCCTGAAGACCTGTGTCAAATTAGTTAGACGATCTGATTAGTTTATCCGTTGTTCTTTTTCGATTAGTGAATTTCCAATGGATTTTTTGTGATTAATATATAAGTTAGAGCTGGATAATTTTATATAAAATGTGTCAAGCCCATGTTGTAGATAATTAGAGGTTGTGAGCGTAAAAAATTGAAATTAATTATTTGAATGGTTATTTATATTTTGAAAATTGTCTATTAAAATTCTTTTTGTTTCTTTTTCTTTTATTTTTCATTTATCATTCTTTAAAAAATCATCTTTATGTTTCATATATTTTTCAACTTCTAGTTTTAGGATCCATTCGAACATAAAAATATTTATTTTTTTCGTAATAATATTTCAATTTATTTTAAAATCAACGTTTGGC

>LNC_001123

CCCTTTCCTAATAACTATAACCAATCTAATAAAAATCTGAACGACCCACCTCACCCCAGCCCATATGATAGAATTAGAGTTCAGTACTCCAAACTTTTCACCTCCTATTCCCTCATTGTCGGCAAAATTATCGTAGGTTCAGTCTCCACCACATCCATTTCCCCACTTTCTCGATAGAATCTAGCATAAATTAGGGAAATTATTGACTGAACAGTTATATCGAAGAGAGCCTTTAACCTAGATAACTACAAAACTTCAGTTGTTGGAGAAAAATCAGCAAAGGAACGCAGATAACGAGTGGTATGCATCTCGTAATACCTAGGTTTGTTAGCTATATTAAGGTCGTAGGTCTCTCACATTTTGATCGCAGAACTGAACTTTTTGTTCTTACTTTCAATCTCACCTGAATTTTTCTGGTCTTTGCTTAGTGTTATCATACCTAGACACACTCTTGAAGTTTCCTCTTCTTTAAGATGAAGAATTTGAAGATTAAAGAACATGATGTTGAAGAATTGTGGAGCCAGCTAAAGATAATGTTATATTTTATTATTTTATTGTTGGTGCGAGACGTTATTTGTACTTATGCTACAATTTGATTTTCTCAATTTTTAAGATTTGATAGATGGCGAA

>LNC_001124

CTTAGGACAGACATGCATCACGACTACATGACATCATTATTGCATTTGCATCGCATTTGACTTCATCTTTTTCTGTGGTGTATGGATTTTACCCATTTGCCCCTCTTCATGTGATATATGATCTATTTGCTCTTACATGAGGATATGAGGTTGACGTTGAGACACTGTTGGATATATCTGTTGATTATGAATTGTGTAGTATAGGTTGTTGGTTCGCTGTTAGAATGAAGTTTCGGTGGTTCGGTTGGGATTGAAAGGAGTTGTTTGTAGCTGCTAGTTTAGCTTAGTTTAGTGTTACTTGCGAGTACCTGTTGCTTTTGGTACTCACCCTTGCTTCTACACAGTTGTGTAGGTTG

>LNC_001125

GAAAAGCGATCAGATCAGAAGAGATGGTGGAGTTCTAGAAAAGCACAGTTAGAGTAGGTTTGGAGTGGTTGACTAGCCAATTCAATGTCATTTTTAAGACGACAAAGATGCCCGAGGAATGTAGGTGGAGCAAAATGGTCCTTTTGTTCAAGAACATGGGTGATATCCAGAATTGCAACAACTACATGGGTATTAAGTGGTTAAGTCACACTATGAAAGTTCGGGAGAGAATGGTGGAGATGAAGTTAAGGAGGGGTGTGTCCATTTCTCAGAATCAGTTTGGGTTCATGCCAGGGTGATCAACCATAGAAGCTATCCTTCTTGTGAGGAGAGTGATGGAGAAATATAGAGAAAGGAAGAGGAACTTACATATGGTGTTCATTGACCTTAAGAAGATCTACGACAAATACCGAGGGAAGTCTTGTGGCATGCCTGGAGGCAAGAGGTGTATAGTGGCCTACATTAGGGCAATAAAGGACATGTCTGGAGGAGCTAAAACGTGGTAAGAATGGTGGGAGGAGATTCAGAGCACTTTCCTATGGAGATGGGTTACACCCGG

>LNC_001126

AAAGAATCAGAAGAAGAACGAGAAACGTACAGCAGTAAAACCCTAACTGAGCTTCAAATCCTGCAAAATAGAACAAATTAACTGAACTCTTCTTCAAATCCCGTAAACGAAGGATGCACTACTTCACGTCTCTTCATCATGCTCCCAAAATTCTAACTATTTATTCCAGTTTACAGAAGCTTGAAATCACAAATCGTTACTTCCTTCTTCAGTTAAGAAAGAAGTTGGAATTATTTTGGGATTTTCTCTGTATG

>LNC_001127

CAAAATTATATATAATATAATATAAGAAGTAGAAAGAAAAGAATCAGAAGAAGAACGAGAAACGTACAGCAGTAAAACCCTAACTGAGCTTCAAATCCTGCAAAATAGAACAAATTAACTGAACTCTTCTTCAAATCCCGTAAACGAAGGATGCACTACTTCACGTCTCTTCATCATGCTCCCAAAATTCTAACTATTTATTCCAGTTTACAGAAGCTTGAAATCACAAATCGTTACTTCCTTCTTCAGTTAAGAAGTTGGAATTATTTTGGGATTTTCTCTGTATG

>LNC_001128

GCCTGCTACCACGTCCAAGCCTACCAAATGTACAAACACAGATGTCGAGTAAACCCATGCACCAAGGACTAAAGGAGCTTGATTTGACTGGAAACCTGCTTTCTGATTGGAGGGAAATTGCTGCAATCTGTAAAGAGTTACCAGCTCTTGTAACTCTGAACTTATCATACATCATCATGTCTCACGATATAAGTGGGATGCCCCTGCTAAATCACATCAAAGTTGTTGTTTTGAACCACACCGGTATAGGCTGGAAACAGGTCGAAATGCTTAAAGACTCTATCCCTCTTGTTGAAGAGCTCCATTTGATGGGAAATAAGCTGAGAGGAATAACGCCATTATCTTCAGATATTGTTCATGGATTTGATTCTCTCCGTCTTCTCAATTTGGAGAATAACTTCATAGCTGCCTGGGATGAAATCTTGAAGCTGTCACAGTTAAAAAGATTGGAACAGCTCTTTTTGAATAACAATTGTATCAGTCATATCTGGTACCCTGATCATAATCCATTATCTGAACCACCCAACAGTCATGAACTTCTTGGGGAAAGCTTTAGGCCTTTCCAGAATCTTCGTTGCCTTCTTGTGGGAGGGAACAAAATTGAAGACTTTTCTTCTATTGATACTCTTAATCTTTTTCCTAATTTGTTGGATATAAGGCTTTCTGAGAATCCAATAGCAGATCCAAGAAAAGGTGGTGTCCCCCGGTTTGTTTTGATTGCTCGTCTTGCAAAAGTTGAAATTTTAAATGGAAGCCAGGTTAGTCCTCGAGAGAGGAAAGACTCTGAAATTCGGTATGTCCGTTTGGTCATGTCTAAATGCCAGGATAACCCAGAGGAAATTAAGAAGCTTCACCCCAGATTTGCAGAGCTGAAAAAATTTCATGGGATTGAGGATGAAAAGGCTGCAACTGGAGCAACAGGTCCACAAAAAATGTCATCGAGCCTCATTTCTATTACCCTGAAGTGTGTTGCAGCATCAATTGGTGAAAAAGTCCCACTGACTAAAAAGCTGCCGGCAACAACAACAGTTGGCAAGCTGAAAATCCTATGTGAGAGTTTCTTCAAGATAAAATCTGTTAAACCAAAGTTGTTTCTTCAAGAAGAGGGTTCCCCATTACCAACACTGCTTGATGATGATATGGCATCCTTAATTGATTTTGGAGTTGGCAATGGATCAACAATTCTTGTGGAAGAAGAGTGTTAGAGGTCGTTTGGCACAGAGTAATAGAAGGAATAATTTATAAATTATGTATGGTATTATTTAGTATTATCTTTGGTAGAAATTTTTGGTCTATGTATAATTAATTCATGGATTAGTTATATACTCTACATGGTATTATAGCATGTATAACTTATACTTTTTATTTGGTGGTATAAGTAATACATAGGATTCAATACAATGAGATTAATTTATGTAAAATTAAAAATATCCCTCA

>LNC_001129

GGACCCCATTTCCCACAGTAGACATAAGAATTTTTTCCTATTAGATATGAAATAGCTCTTTATAGCCTCTATGGCAACTTCTCTTAAAAGCCTCGGTTGTCACCCCCCTAATCAAAAGACTTATCTTCATTTTCGTAAGGGAGACATCGGCACGACATTGTAGTAATATTATTATATTGAATAAGAATTGTAGTAGGGTATGGGGTGCTAAAGAGACATTACTGACTTGAAGCTCTGACCTCGGCTGTGAAAAGTTTTGCTCTTTCTTCCTTAGTAAGTTTATATAATTTGCCGAAACGTAGCTTTCATTCATATATAAAATTCGGAAAATAGTCAGACTAAACGAAAAAAAAAAGTCAGGCTTGAAGCACTGCTGGAACCCGTTTAAGGGTCGCTAACAAAAGGTGAAAGGAGGCCCTAGAGGAAGAATCTTTATACGAGTTCGAGGCGTGTAGCTTATAGTTACAGTTAGCCGACTCAATAGAAGAATGAAATTGGTGGGATCAGAGAGGGAAGTGACATCCAACTTGGGAAGCTCGGTACCACTAGTAAGAGGAGTGGCCAAAGGACGAGAATCAAGCATGTCTGCACGAGGCAAGAGATCTAGGCATATTTCTGCTGACGAAGGATAAGAGTGCGCCCCTGCCGATACACTTCCATACCAAGAAAGTAGTGGAGAAGGCCCAAGTCTTTCATATTTTAATGAGCATGCAAGAACGAAAGAAGAGCAAGTATTCCCTGTTGATCACTACCTGTGAGAAAAATATCATCGACATATAAAATAAGGTAGATACTATCCCCAGATGTGTGATGAATGAATAAAGAGGTATCACAGGCACTGCCACAGAACCCCTTTAGAGCACAATATAGTGCTGAAACAAGTGAAAAAAGCTCTGGGAGCTTGCTTCAGTCCATAAAGAGCTTTGTGAAGTCGGCAGACATATCCTGGATAAGCGGTATCCAG

>LNC_001130

CTCTAAGCCTAAGTATTCCTCAATGACCGATAGCGTACAAGTACCGTGAGGGAAAGGTGAAAAGAACCCTATTTAGGGAGTGCAATAGAGAACCTGATTTCCGATGCGAACAATCAGTCGAAGGAGCAGAGCTTAGAGCCTTTACTTTATGTAAAGCGCACTCACTCTAACGGCGTACCTTTTGCATGATGGGTCAGCGAGGAAATGGGAACAGCGGCTTAAGCCATTAGGTGTAGGCGCTTTTCTGAGGTGGAATCTTCTAGTTCTTCCTATTTGACCCGAAATCGATCGATCTAGCC

>LNC_001131

TGCCACTGAAATATTATTGCAGAGAACTGCATTGTTTGTAGCTAAATATTGAATGCCCATCTTCAGGGAGTTCTGAAAGTGTTGAGATGAAAATCTAGAAAAACAATTTGATACTTTGTTATTTTAGCATCACAACATCCCAACTGTAGAAGGACTAATGCAAGGCATGTAATGTGGGTATTCAAAATTAGATTCCCTCGGACAAGATTTTTCTGAATTGGCTCCGTGTGAGTTTTTCTTGAACAAGTGTCCTAGGAATTTTTTTTGTGTGCAATGCGGG

>LNC_001132

TCTGGTGAGCATCCGGTTTGAGGTCCGGCCTCCGTACTATCAGATTTTACAAATTGGGGTTGAGGTTCTGGTTCGTGTTCTCCATTCTTGGGTTCTCATATGCAATTCTCTTTAGTTATAGTTATTCTGTGTACTCGTCGGGCTTATGGGGGTCCGTTCGGGTTTTTATTTAACTTGCGCACGAGTATACCTTCTGGGCTTATGTGGCCCCAGTTAGGTGTAGTTAGCTTATAGATTACTTTAGTTAGTTCTTTGTACACTCGTGTGCATTCCCTTGTTAGTTAGATTTTAGTTCTTGACTTCGGTTTGTGTTATTCCTTCTTTTCATATTGCCTTTACTTTTCAAGTTCAGTCGGCCTATGATGCCTA

>LNC_001133

GTATAATTGGAAATTTTTGGCTTGCCTCGGTTCATGTGAATCATGTTTATTTTGTTACAATATGGTGATAGGCTTGAATAGATGTTAATTCATTTGTCCTCATATACGTTAAAAGTTCCTTTCCTTTATTTGGTGGAGTCTTAAATATTTTGTATGCATGGGTCTCCATACACATGTCCAGTATCTATGTTTGTTTCCACCTTGACAAATGCTTGACTCGTTACTTGTAGATGTTTAAAATTAATGTATTAATTCATTTTGTTATAGCTTATT

>LNC_001134

ATATAGTAAAAGTGCATATCCTTGAACTTGATTACTTATTCTGATGTGTTGACACCTTGGATTTCTATTTGTTGTCGGCCGGTTCTAATATTGCTATCTTAGGCGTTGATTGCTCATATATGGTTATTATAGATGGATGGCATATATGGCCGTTTTGAGTCAATAATTATGTCCTTGATGTTGTCGGCATATGGTTGTCCAAATGACTTGCATATGCATCAAATCTATGTGAAATGATTGAATATCCCTACATTTTATTACAATACGATTATGTTCTCTTGTTTAAGTTGTTCCTCCATATTTGTAGTTTATTCAAATTTGTAATTATTTGAGTTATCTTATGTAGCATTAAGTCATTTTAAAATATCTTT

>LNC_001135

TCTCTTCTCATTAGCCATGAACAATTTTGATTTGTTTAAATTATCATTCTAATTCTTATTGTTTGTTTCCTTGCATGAATCTCTTTGTACCGAGTCTGATTTGGGACTCCCTTTCCTCCTCGCATCGTCTTGTTGGATTGAATCCAACAGTTTCCCTCGAGTCAGCCCCCCTCCCCATTTCAAATCGTGGAGTAACGAAACAGGTTGAAGGCAGCAAATAGGACAGGAAATTGAAACTTAAGTGC

>LNC_001136

CTAACTTCGATTGGTTATAATTGTAATTCATTTAGCCTTCTTGAATTTTCAATTTTAGCCTATCATTTAATTCAATATTGGCAAGACCAAATTTTGGCCCAAAATGCAATTCCAATCTCCCAGCCCAATAATAATTCATTACAACAACAACATTACAGCAACAACAGCCAGCCCAATTCCATACCAAAGATTACCCGGCCCAAACCCATCACCCATAACAACCCAATTCTATACCAAAAACCCACCAAACAATACAATATACATACAGCAGTCCCTAATACAGCAAACAGTAGCCGAATGCCCCACCCCTGCGTTTTTCTCTTCTTCTCCGTGTGAACCAGACAGCAACAGGACGTGTTTTCTTCTCCCTCACGCGTCAACAAACATCAGCTCCCACACCCTGCTCTGCAACAACCGCCCATTTTAATCTTTTCCATCCGGATTTTAGGGGATTTTGGTTGAATTTCCATACAACAACAGCCTTTCCTTACCCTTGGGGAAAATTTGAATTGATTTGATTGGGGAATTCCGATTTTGCCCTAAGTTCCTATCCGTTTTCTTCCCCCTAAATCTGCCTTAATTTCTTGGCTATATATATTATTCACTATAAGGGGGCGAGAGAGGGGGGGTTGTTCTGGAGTTCGATTCTTTGACAATAGTCGTTTTACACTTAAGAAATTTCAAAACGATTTTCTCATTCTAAGTTCCAAGAAAAGCTTTGATCAAAGGTTGGTTCAAATTTCCTTGTCTATTGCTCGAGTCTAAACCGGTGTAAGAAGTCGTCGCTCTCTTTGGCTCGTTTGTTCTCAGTTTGCTGCATCGAATAAGGTAATTTCGCCTTCTCTTACGTCCTGTTTTCGATACGGTGTGCTATGTGTTCTATACTTGGTATGGAGTTTGTTATCAAGAATGTTTGGTTCCTTCGCTGTAAATCTTTGCTTTAGTATCGTCTTCCTGTGAATTATTTAGCAGTAAGCATAGCATGATTCAAGCATCAATGAAGTCATTTTAGTATTGTATGTTGGTCGGAATATTCGTTAGCTCGATTTTGTATCTATGAAACTAGTTGATTCGTTGCTTGGTGTTGCTCGAAATGACTTAAAACTCATTTGGTCTCAACTACGAGTGTATCGGTTGGGTCGTTTTGGATCATTCATTGTTCGGCTTTAGCTTTTTATTAAGTATGATGAGAATGTGACCATATTCTTGGTCGTTTGCCTTGGTATTTTTTATTTTGTGACGGTTTCTTGTTAATATTAGTTTGAATTCGTGTAGTAGATATATCTAGCCTCTGTTTTCGATTAGCTTGAGTGGTATATGTCTCTTTGGATTTCAACTCATGGCGGGATGCTTGTAATTCTACCATTGTAATGTGACGATTTAACTAAGGTTACTCTTATTTTCCAGAGAATAGTCATCACCATCTTTGTCCAAAACCTGAAAATATCCATTCCGAATTTTTAAATTATTGTTTGCCTTAGTTTAACTCGAGTGTTAATGTTCAACAGTGCTATGGACATCGTTTTATATTACACCATTATTTAACATGTTTGCTCATATTGCTCACTTTAGTTATTTGTTTGTAGTTATTTTCTTCCTTTGCTTATAATGAGTTAATTAGCTACTGCTTCAAAATTTTGTCTCTTTGGTACAACATAGCTGGTAGTTTTTAAAATTCCCATAGACATATCTAGTGGTGTTTATTAGATGAGGTGATTTTAGTCAAATGTGCAACATGTTTCATTATGCACTTTTTGGGTTATGTTAGTTATTGCTCATAGGTTTTATTGTCACGTATGTACACAAGCACATGCCCCTTTGTGTATGATTTCTCATTAGTTTACTATTAGTTTTTGTAGCCTCCTATCTTGTATTGAAATTTATGACATTTGGGTTTCAATTTGGTCTCATAGTGACTTGGGTGACATTTTTAGCTAAAAATAAATAAATAAAAATATTTTAGTTGCTTTCATTCTAATTAAGTTATGTCCTAATTGCTTACTTATTTCACATACTAATTTGGCTCCTTTCTTTGTCGCATAAACCCCTCCCGAGTCCATGATGGACTTATTTACCTTGCACATTTAAGTTGAGCCATAAAAGCTCAATCTTTGCTGTCCCTATTTCTCGAGTCAGCCCCCCTACATATTCGGAATCTGTTATAGTCCCTGGAGACCCAACATGCTGCTGGAAATTAATTCAAGAGTCCGAGAGATGGCCTGTACATGCTGTATATACATCAATATACACTAATATACATGTTTAGTGCAACAAATATGTGGCAAAAAATAAAGATGCAGGTTGGAAGCGTCGAATTGGGTTGTATACATTTAGTATACAGTTTGGTATGCAGGAAAAAAGGCTAAAATACAGCCATATACACTGATATACGATCAGTGTATACACTGATTTTTGGACGCGAAAATGGGTTGAAGCCCATCCTTTTGCACAGCAGGTGGCCAACAACAGAATTAACTCAATGGGCCAAAGTCCATTTTTGGCAAATAATAGATTTAGGACAGGTTGGGCCAAAAGCCCAAGTTGAATTCTCTTCCTTTATCATTATTTATTTGTATCAACTCTCTAACCTTCTATTTGTTTTCGATTTAATTTAATTAAATTCCCCAAAAGATGAACCATATTCTTTATGTTAGTTTTCTTTAAAAAATAATTCATTCTTATTAACTCTTATACTTATTCAAATATTATTATTTTTAGTCAAAACTTTCAC

>LNC_001137

AAAAACAATAAAATCATCCAACACAGCCCAACAATATCGAAAATAATGTAATAATGGCTATGAACCCAAAAACACAAGAACACAACAATGATTAAATACAGATAATCGGAGCATTATTTTTCAGTCAGATCTAAAAAATTGTCGAAATTACTGGAGCTTGGAGCTCTAATTCGTCGTTGTTGATTTCCTCCGGTCTACCGGAGTTAGGATTTCACGGTGATGGCTGGACACTGTTAGCGATGTGGGTCTGGATGAGAGAGCAGAGAGCAGAGTCAGTTTTCCATGTTTGACCAGTTCTTGGAATCAGAGAGGAAGACCTAGCTCTATCGATCAAAATAAAATAAAATAAAAAGGCCACCAGACTGAAGTTAGAGATACAGTTCATGTTTCAAGTAATTAAAATGAAATTTTCAGTTGGGAATGGTTGTATCAGGGATATTAATATATACCAAAAACTATACTAAATTATTAGTACTAAAAAAAAGGAACACATACCTCTTTGGAATCTTCTTATGCCTCGCATAGTTCACAAGGAACCAGCCACCAACATTGAACTTTATATCGAGCAGGTATGGGAAAAATTATAAATTGATGAATAGTAATGCCTATGTATCAACCTATGTCAGCTTAAGAACATTATTGGAATTTTTCGGAAGAACTTAAAGCTACGGAATCCACAAGTTTAAAGATGAAGATGAAGATGAACAAGAATGAAGATAGAACAAAAATGGGTTATATTATTGGGTGGGTCGAGTCGGGTTAAAATTGGGCAATTTAAGTAAAAATTGGTTAATTTGGGGATTTCCGTCAATTGAGGGGTATAAATGGTGAAATTAGTAACGACAAGGGTATAAATAACTTTGTTTTAACGGCAAGGGTATAGTCAACTAATAAACTAAAGTTGAGGGGTATTTTTGACCCTTTTC

>LNC_001138

CAGCAGCAGTCGCGAACTCGCAGCAGCAGCAGTCGCGACAGTCGACTTCAAGTTTCCAATCGCAGCAGCAGCAGTCGCGAACTCTAATTCCAGTCGAGGGATTGATTTCAAGTCTCCGGTCGCGAAATGAGGAGCAGCAGTCGCGACATAAGTTTGAAAAAATACCCAAAAAAAACCCTAATTTGATTATTTGGCTTTTTAATTATTAAAATTCAGACTTTTAAAAAAAAAGAAGAAAAAGGCGACGCCTATTGCATCGCCAATTCCACGTCACCCCTCGCCTTGGCTCGCCTTTTTGATCTCGCCACGCCTTGCCTGAAATAGGCGCAAACCCCTCGCCTCGCCTCGCCTTGTCGCCAAAGGCGAGAGGCGCTCGCCTTTAAGAACACTGGTCCATGGAGAGAAAGGTGAGACTTTGGTGTGTTGGGGGGAGTGGCTATATAGGTTGAAGGTGCAGAGAGGTTTCCAGTGAGAGAGAGTTATAGGATGGAGGTCAAGAGTTTTGCAATGGAGAAAATACGGC

>LNC_001139

AAAAAGGAATCGAGGGAGTAATTTGTTTTGAAATGATATTTCCGAGGACAGTAAATGACTTGGTCAAACTGAAAGGGCTTCTAAAATAAAATTGGCTTTTAAGTTTTATTTTACACGGTTGTTAAGAAATCATTAATAGATTGATCAACTTTACTATTTTGCCTTTTGTTCATATTAATTAGCCTCTTAAAAAGAAAATTTGGATTTTCAAAATCTGTTTAAACTTCCAAAGCCATATTAATTGTAGGGATAAAATAAAAATAAATTAC

>LNC_001140

TGATGATGAGATTGCGGTAGATGTTCAGTCGTATTTTGATAGAATGCAACAAGCTTGCAATTGATCTTTTAGGCTGACATGCTTTAGTTGGGCAAGTCCAAATATAGTTTTGAGGAAGTGGAAACACTGCGGAGATGAAGAAAGAGGCTGCAAGATCGACTATATATAATAACATTTTACATATGTTTTTCGTTTATTATATTAAGGCGTGTGAGCCTATTATGTTTTTA

>LNC_001141

TTTTAGTTGAGAGAGAGAGAGAGAGAAAGCTGCTCAATATCTATTCATTTATGCTCATTTTGACTGTTTAAAATTATTACTTCCACTGGTTATAGTAGGCAGAATAATGGCTAATCAAGAATTCGCATATCAGAAATGAAAATAAGAATGTTGAAATGAATGTGTGAGTGTATTAGATGATATAGGATTAAGAACGAAGATACTAGGATCAATATGGGAGTGACCTTGTGATGAACACGATAAGAGAAGCAAGATCGAGATGGTTCAGACAAATGAAGAGAAAATGTCCCGATGCTCCAGTGAGATGTTGACTATAGAGAGTCTGAGTAGAGGTACATACAAGCGAAAACGTATTGAAAAGAGGTGAAGACATAATTTGAAACTCAC

>LNC_001142

CCTCTTCTTTGATCAATCCTTAACATCAGTTCATATGCTATGTCTTCGCACCCCTGGAAAGCTGCCCCAAATTTTTTCCCTAATTTAATGACATTTGAATGTATCCACAATGTCGCTTTCTCCTTTGCTTCTTCCTCACTGATCGACTCGTCCACTTGTAATGGCTCTATTTCATTGTAATACATCATCTGCTTAGGGTTACTCTCCTGTGTTTCTTCTCCTTCCCAGGGTTCAATTTCCATGGAACTACTTCCAACTATGTTCTTTTCCCCTTGAACAATCTGTCTTTCCACGCACTGCATTATTCTTCCTTCTTTCTGCTTTTCCGTTGTGATTCTGATGGACTCCAGTTCGTCAGCAATGACGAGGAGATCCTGATTTTGGCCCGTATTCTTTAGGTGAGTATGGATATTACCTATGAAAGGCTCAGCTGTTTTAATTATTGTGGGCTTTGGTGATTTGGGCTTAAGGGGAGGCCCAGTACTCTGTCCCTCAACAAAACCTGACCTGCCTAGGTCTTTATCCATAATAATATTAAGTCTACTGCTGCTAGGTGTCTCCATCGGCCCCACCCATGTGGACCCATTCCCACGTGACACATCCCTGCTTCCATCCCATGTGCCTTCCACCTCCAGCTCCTCAATATCCATCGGATATTTCCCAGTCCTTCTTTTTCCTCTTTCGTGCTCTGTTTCTTTTTCTTTCAGAGAGGCAAACAGGCTGTCTACCTTCTTTCGGCTCTTGTCACTCTCATCTTGATGGTCGAGCCTGTACTCGTTGACCATTTTGTATCTCGCCGGAGCTTCTACCCAGATCGGTAGCGAGAACACTATGTCACCATCGGCGATCTCTACGAAGCGTGGTATTTCCTCGAGAGGTCCCTTCACCTTGATACGAGCCCATCTCATATGGTTCTTGAGAAGCGTTTCTTCTTCTGTCTCTAACCAGCCACCGCACTGGTCCCCTATTTTCTTCATTACTTTTTCCGACCAAAGCTGAAGGGGGAGCCCAAGAGCTCTTATCCAAAACCAGTGGAACTTTGCCTGAGACGGATAGGCCCCTGTTGTTGGAGACCACCATTCGAGTTTCAAGCTTATGCCCTGTCTCTTCCATTCTCCACCAAGAATGTGTTCAGCGATCTTTCGAGATTGGAATTCAAACAAAAATTGGATTCCATTCAAATCGAAGACTTGGAGGTTATGAACCCCTTTCCATGCCTGTTGTGCCCATCTCCTAACATCATTACGAGTGGGGATCTCTCTGCAGTCTGGAAAGCATCCCACTAGGCATCTGCTTAGGAGTTCGTTACCGCCTTTGGATTTGCAGGCCTGAAAAGAGTTAACCTCCTTTTGTATTTCTTGTTCCTTTGGAGCCCATCTATTCCTGTGTAATGCTTCTTTATAACTCCTTTCCCCTTCTGTATATCTGGATGTAATAGTCTCTTTGGCATTTAATGCAAGCTGTGTATTCGAATCATTTATGAAGCCTTCAATTTTCGTTGCAAGCCCCAACCACCCTTCATTAAAGTTGTTCTCAGGTAGAATTATTACTGATCTGGATGCACCATTTACTGCAATCATAGAGATGAATCTACCATATTTATTGAACTTCAAAGAGAAGTATAAATAGGTAACCAGATCTCTACCTCTCCATGATTTAAAAGCTTTCCCCCTTATGTCTGATGCCTCTCGCAACCTCTTGACTAACCATAACAATGCTGCTTTGCTAAGTGTCATCCTTCGCATGAGGAGCTTTGAGCTCTCCACCCAGTCGAACCAAGTTCCGTTAGTAGATTTTGTCTCGGTTAAGTCGTATGATTTACTCCCCAGCCTGAAATAA

>LNC_001143

GAAAAAGGCAACAACAATCATTGCCTTTTTCATCTATTAATCACTTCTTCAAGAGTGAAATATTTTCCAATTTCATCTTCTTCAATAAGCAATCATTGGTTTGAGTATCAAGTGGAAGTTTCAGTAGTGTGAAGTTCGAAGTTCGAGTATCCACTCATCGTCTAAATCGCTGCCTCTCCCAATCAGTTGAAAAGTGCGAGATACAAAATTTTAGCATTACTTGAAAAAATCATTGAGTACCACGTCAAATTCTGTTTTCACCTCATCCACAACTGGAGCATCTGTTGTTGCTCTACCAGGGCGGCGTCGACGGCAGCGGGACTACCTCCTACGATGCTTTGACGGCGGCAAAAGAACGAGTGAAAGTTGCTCGGTGCTGATGGAGTTTGGGAATGCGAATGAGGTGTTTTGTTTTGGTGGGGAAGGAAATTGAAGCTTTTAATTAATTGGATCTTGAAAAATGAAGAACTTGGCCAGAAGTTCTTTGAGAAATCCGAGAAATCTAGAGAAGATGGAGAAGTGTTTTGGGTTATAATTTGGGGCAGGTATTATCTTATTTGGGTGGTTTTAAAATTATTTTGGGTGGGTCTTCAAAATTATGGGTCAAAATTTAATAAATTTCGGATAGGCATTTTAGATTGAGACACGTGTCCCGCCGTTAGTAGAAAGGGTATATACGTTTCAAATTTTGGACGGCAAGGACATGAGAGTCCTAAAAGTATAACGAAGGGTATTTAAATACCATTTTCGATAAATTCGGGGGTATATTTGTCCTTTTTCCCTTTTAAAAAAGTGCTTCTGAAAATAAGCAGTTTTTAGCCGAATGCAAACAGGCTCTTAATAGTTTGATCCAAAAGTATCTTTGTTGTTAAAAGTTTGGGTTTAAAAATGCCCTTATGATTAATTAATGGATAAAAAAAATGCTATTTTCCTAATAAAATATAAACATTTTTTTTCCTTTTGAACATATTTTATTCCTGATAAAAATATTACAATTATAAAACTCAAATCTTATTTGTTTTCTTTTGAAATCATTTTTACTAATAAATAAGTAGGAAATAATTTATTTATTTATTTTTTCGTTAACAGTATCACTTTTATTAATGACAAAAAATATTTACAATCAAACCAGAGCAGTCTAGGACTATATATCTATATATTAAAGGCTAATAATAAATACATTTTGGACCAAACTATTGACAATAAAAATATATTTAGATCAATCTATTAACTTATGATATTTTATTCCCGTAATTTAAGTATTTTTGTTATTCCTTTTCGTTAATTTTATTTGACGTAGGGATATTGTCTTAAAACTTAGATTATTATTTCTGCATGATTTATTTGAACTATCTTGTGCTTCTTTAACGGTCAATTATTCAGGCAGACATAAAGGGAAATGACAATTTTAATCTCGATATTGTTGTATACTTTCAGTTTTAATCTATGTGTTATTTGACTAGGCACATTTAATTTTT

>LNC_001144

GTATATAAAATCATAGATTTGCGTGAAGCTCATAGCTCACTCTAAATTGAGTACATGTAAACAGATATTGTAAAGAAGTGAGAGCTATGCTACAATATATATAGGTTGTATATCATGATTTCGCAGTCTTCATATTCAAAGGTTTTATGATCGAGCTTGAAAATAAAGATTCTTTAAGGAGAGAGCGTTTTATCAGCAATTTTTTTAAATGGTCACATTTTTAGTTCTTGTAATTAAAAAAATAGTTATTATCTTGAAGTTTCAGAGTTCATGAGGACGAAGGAAGTAAGGCATATATGTCTCAAGACATTTGCAAAAGGTTTTCAGCTTTGATTTCTTATAATAAAGTATTCCTTTTGTTCCCATATTATTTGTCACTTTATAATATTGAAGTAGAGAATTATTTTTAAC

>LNC_001145

TCTAAACCCTTGTTAAGTGTATGGAATTCATGTATTTCCTTGTAATATGTGTTTAGGGTAATGAGGCTTGGTGATGGATTGACTTGTCCACATTGATTAATTCTAATGATGAAAAAGGGATAATAAAAGGCAATGTGATCAATTGTTTGTGTGATGTGTTGAGAATTGTTTGAAAGGCTTGTTGAATCATTGTTGATGTTGTATCCTGATTGTGTTGTTGTGAATTGTGCAATGTTATGAAAATGGTCATCTCTTCATTATTTGTGTGAACATGTCATTTGCATTGTTCTGAGATATGGTTGTGACAAGTGTTATG

>LNC_001146

TCAAACTATAGCTTATAACTTAAAAATAGAAGAAAAATTTAAGAATTTTCTCTAGGTCAAGTTGTTCAATATTTTCCACCATGATCCCACGGTCAGTCATGGCAGCTCAGCCCGGTGAGTTGTGGCGGTGGTTGGTGGCTCCGGTGGACGCCTTTTTCGCGATTCATAATTAGTTGAATCTTGAAGAAAGATTAATTTTTCATAATTAATCTTTAGCAGGGCAACTCCAATCCTTTGGCAATATTTCAAGTTTCTATTAATCATGCAGTGTTAATTGAGGAAGGTGGATTAGTTCCTTAATTACTTTG

>LNC_001147

CTTTTTTTGCTCTTCCCAAAAAGACTACTTCTTGGGATTTCAAAAATCTCCCCCTCAACTATTTTTTAGTACTTCCCTCTCTTTGATTCTTTCCATTTTCCTCAAGAAAAGGGAATTAAATTAACCCTAAATCTCCTCTGTCCCATTTCCTTACAAGAAGTTGTCAAAAGGGTTGAATTGATTTCTCTGATACTGCCGTTTTCTTGATTTTACAACTCTTTTTGACTAAAAGGGTATTTTAGAATCGCTTCAAAATTATTAAAAGACACCCAAGTTCACTTTTTTAGGTTCTTTGTTGTTTTGGGTCAGTTGTGAATGATCTG

>LNC_001148

CTATTATGTCATCCACAGTGAACTCAGGTCTGCTGTTATAGGCAAGTCTTCGACATGGGGCTTTACCGCTGATCCTATTTCATGTTTCCAGTGCAAGCCTCTTTTGTAAGTAGACAAACTCGATTTGTAAATATGTTTGGGACTCAACTAGTGGTTAGAGTATTGATTTTGTAAGATTTGTGTCCAGAAAATCAAATTAGAAAGATAACTCGCGATGATTGAATAAGCTCGAAGAAGTGGATTTGAATTTCAAATCGTGTCTTCTTTTTATGTGTGTGTGTGAGAAATTTG

>LNC_001149

CTACATACCATTTCACCAGGTTGAATCCCTTTTCTATCTTGTTCCCTTGCCATAAGAAGTTCCTTCTGAGTGCATCTAAAACCTTCACTATCCTACTAGGAATAGGAAAGAGGGACATCACATAAGTGGGTAAAGAATCCAGAACTGAGTTAATTAGTGTAACTCTACCTCCCAGAGATAGGTATTGAGATTTCCATATAGC

>LNC_001150

CACAACAAAAGCTCTTTTCAAAAAGTAGAACTAGTGAGATTCGAACATACGAGCACTTCCTCGTTCCAAGGAGGTGAAACCACTGGGCTATAACAACTTGTTATGTTAAGGGTGTCCAAAATATATTATTTAATCATTTCGTGTATCATTTTACTTGAATATACTGTATTTTTTTCCGACGAAGGGTGTCCAATTGGATACCCTG

>LNC_001151

CTCTCTCTCTCTCTCTTTGAATATCACTCTTCTTCTTCTTCTTCTTTTTTTCCCCTTAATCCTTCTATCTTCTTTCAAAAATGGAGGATCACAATGATGATGAAGATGTTGATGATCACATATTATTATCATGAAGTGACAATTTCTTTTGGGCAACTACATATCCTTTGGCAAATTTAAGCACCATGCTTAAGATTCAAGACTATTTATTTAATTTAGAGATATATTTGTGTTTTTGTTAAGAGGAATTTTCTTTTCTCCTTTTCTTGAAGTTTTCCAAATATAAATGCACATCATGGAGTTTATGTTATTCAAATACTATTCTCTAAGGTGCAAGTCTTTAATTAGTTATTTTCCTTGGTTTTTATGGATCTTGTTTGATGTATCATTTATAATAGACTTAGTAATAAAAGGAAATTAAT

>LNC_001152

TCCACAATGTTATAAAAGATAAGAATAAGAGGCTCTCAATCCATAAGATTAAGGACCAAGATGGCAATTGGGTGGAAGGTACCACTCAAGTTGCTACAGCTGCAGTCAATTACTTCTCCAACCTTTTCAAAGCTGAAGATATTGATGAGAATAACGATATTTTTAATGTGATAGATAGGGTGGTGACTCGAGAGGATAACATATCCCTCACCTCCCCTCCTTCTATCCAAGAACTTAAAGATGTTGTGTTCTCTATTGACCCTGATAGTGCCCCGGGACCTGATGGCCTAAGTGGGAAGTTCTATCAATCAGCCTGGCCTATTATTGCTTCAGATGTCCATGCTGCGATTAATTCCTTCTTTCAAGGTGCTACCCTTCCTAAATTCTTTACTCATACTTGTATTGTTATGCTTCCTAAG

>LNC_001153

TAGGGACAAGGGTCAAAAATACCCCTCAACTTTGTTTTATTGGTTGACTAAACCCTTACCGTTAAAAAGAAGTTAGTTTTACCCCTGCCGTCTACAAACTCATCATATGTACCCTCCAAATTAACGATTTGCCCCAAATCAATCTCAAATTCTCAATTTCGACCCGAATTACCCATTTAAAACTCATTTAGAATGAAATTCTTTTCTAATCTGATCCGACCCATATAAAACCCACCACCATTATAAACAAAAGTTAATTTAATTTTCAATTTTCTCAATCTTGTTGAGTCCACCAAAACTAAAAAGAATGGATTTCTCCCTTGTCATTGAATTTGTTGTAGTGTTACTCATTCTATAAATATTTTCAAAGCCATTGTTAAAAGCTTCTAAGGGTACTAATTTTTGAATTGGAGCCTGAAACTTGAAGAACCAACCATGGAAACTTCATAGCCATTAATGGAGCTTGGAGCTTGAGTTTGAATTTCGGATTTTACAAGCGAGTTCCAACTTCGAAAAATCAATAATTTCAACTTCTTCCCCAAAATACCCAAATCGAAAACATCC

>LNC_001154

GATCGGATTGGCCTAACCTATGGTGTACCGCCAGGTAGACTTACCAGTTTTGTTTTTTATTAGGGTGAATTTTGGGAATAGTGAGTGGAAAGGAATTTATTTGATATTAATTATCGAATTCTCTCAGTGAAAATTCGTCAAGGTCAAAAAGCACAACATTGAATATTATCTATATAGTTTTCTAGAGTAGAGATATAGTAGCATACTCCCCCTATACGAAGAAAGATAGAGAATATTTATTTCGAATCAAATTCAAAATTTATCTCGATATCAATATGATATAAATCTCTCAATTGAAGATCTATAGAGGGCAAGGAAGAAAAATTAGTAAAGAATAGAACTTTGAATATTGCTATTAATGGAAGTTGAAAAAGTTCCTCTTACAGTGATTTGTTACACATAAATTTATGTTCCGCATTGGAAGTACTATGCTCAGATGAACCTGCTGCTAAATAGATGCATTCGTATAATCTTCATACTTCTGCCGATTATAAGGCCAGGTAGTTTGCGCACACCTCGTTCATTTCATCGGATACCTGATACCTCTACAACATATGTACCAGCCCATCGGGTAACTTTGTCCGCCAAGGCTTAACAAATGAGAA

>LNC_001155

CAATTCCAAGTGACCCAACAGTAATATACGTACAACGGTCCTAAATACAACAAACAGTAGCCGACGCAGACCCGACCCTTGTACGTCGTTCTCCTTCTTCCTCATCGTGACCCGTACAAAACGAAACCCAGCATCGACCCGAATTCAGCAACTCATGTGAACCAGACAGCAGTACTACGCGACTTTTTCTCCTTCACACGTCAAAAAGACGGAAAGCTGCCATCGTCTTCATCGATGGAACAGACCTGCAGTTGCGTCCTGGAACAACCGCCTGTCAAGCCATCCACGCCCTCTTATCGTGCGGATTTTGGGGGATTTCGGTTGAAAATTTTCAAATTGGAGGTTGAACTCGAATTCCTATTCTACCCTTTTCCCCAATTTCGAATCCCAGTTCCTTTTCTATAAATACCTTCTTCTTGATTAAGGGGAGGATATTAAGAGTTAGCTAAGTGTTGAGAGGAAGAATTATATCTAAACAAAAAATCTTTGAAGTTGTTTGGATCAGTCTTGGCTCTTGCTCGTTTTGTCTTGTTCGTTCGAGTTTCTTCGAATCGCGTTTCGGTGGAAGAACCGCTGCCCATCTTAGCTCGTAGTCTTGGTTCACTGCTCTGAAACAGGTAAGCCCTTCCTCTTTCAGTTTTCTCTTTACAAAATTTCAAGCTTCATCTGCATATGTTTGAAGATTTTCTCTGCAATTTATTGTCTTAAGATTGGTTCTATTAGTCCTTTCTTTTCTTTCTTTTTCTTAGATTCAAAATCTTAAATTTTAAAATAAGGATGCAGTTGTTGGATGTTTTATTTTTCCAACAGTAGATTATTAGTTGATTTCCACATAATTTCTCACATCTTAGAGGTATTTTGATTGAGGTTTTGTTAATATATCTTTTTTTTCTAAAATATATGATCTAAATATGTCCCGTTATGATATCGTCTCTGTCTTGAGTGGTTATCTTGAATGAATTTTATTAGTCCTCTATCAGTTATGGATACTAAATGTTTGTTGTTATTATGAAATATATTGAACAATAGTATTATATTTGTTGGTTCTAGCACCTCAATGACGACGCCCCTGGTTGTTGATTTGTTTAGAGAGATGTAACTTACTCATGTTGCAGTGACAAGTTAAGCTTCGCTAAATTCTTTTTTTTTACATCTACACAGTGCTAAGGTTGCTTAATTGTCATCTTCTCGATAGTTTGGTTTCAATTGTCATGTTCTCTACACTTTGGTTGGTCGATTTGAGTTCCACAATCTAATCCTTCAAAACTTTTTAGCCTAGCTGGGTTTTGCTCTTCATTATTCATGTTTGGGTTGCTCGACTCGAAGTCATTCGGTTCATTTCACTTGTCATTGTTGGTATCATTTGAAATAGGTGTAGGAGTCTAAAAAATTAACCAATATGTTAGCTGTATATGGAATTATCTCATTATTTTCTGAGTGCAACCACTCGATTTGATCTTATTGTTGTATTTGAATTACCTTTTATTCTAAAAATCCCACAAACCAGTTGTCATGTTGATATGTTACTTAGTCCCTGCCATAGCTTTTTTTTTAGATCCATAGCTACGTGTTGCTCTACGCCATGATTTTTGCTTCTTGTTTAAGTTTTCCTAGATTCGTAGCATGTCTCAATATCTATAGTTTTGTCAAAGAATGTCTCTATCTTCATTTAACTTTTCGAAATTTCGAAATGATAGTTACGATAAAAGTTTGAAAGTGCTTAACTGTGCCTTGCTGCTAGAATAGATATGTAGGTGCTGAAAAATTGTGAATCATGTTTTGTTAAATGATAAATATTTCACTTTCATGAGAGCATGTCTTGCACTATTTTTTTTTATTTTTTTTTTCAAATTTCTCAAAGCTTAGTAGCTTTACTATTAGTTAGCATTTTTATTTGGAATAGAAGTTCATGATCTTTATACTTGAGTCACCTCAAGCTTAGATAATTTCTTGAAACCTATGTCTTGCATTTTAATTTTTTTTGAAATTCTCAAGTGTTGGTACATTTCTTCATACTTAAAATTTGCAAGAGTTGTTTAAACTAGTTATAATGAATTCGTATGTAATTATGAAATGATTAACATCCCTCTGTTCCAACTCTTAATATGGATAATCATGATTTGTTTTGTTAGTTTATCAAGTTAAGGCTATTAAGAGAATATATAGGTATCATGTTTAAGGCATTAATTTCATTCTAGATAATTACGTATTCTATTTAATGTTGTATGGCAATCTATGGGAGACTTAAGCAAATCATTTAGTGATTATTTTCATTCAAAATCGTGATTGTATCTTTGAATAGTCTCTTGCTAATCCTTATCATTTTGTTTTATGCATGACTCCCCTCATGAGCCCATCGAGACTCCTTCTTTGCATTTCGTTTGGTTGAAACCCAACAACTCTCTCGAGTACACCTCATCTATCGGAGGAGGAGTCAAAGGAAGCAAATATATAAATTGAAAGCCTGCAAAATGCAGCAACAATGGCAAAAAATGGGTTGCAAACCCACTTCATATTTTGTTGGCAGAATTGAGCCAAAAAGCTCAAGTTTAATTACTTGACTATTATGTTATTATGTCTTATCTATTCGGATGTTTTAGTACTAACATTTTATCAACTTAATTAGATTCCCCA

>LNC_001156

GCACCAAAACAACTTTTTTTTGTTTTCTCTGAAAACGCAAACACACGCAGACAACCAAAAAATCAGGTTATATTTGGAGGCTCCGATCAAAATTAAGCTCTTTTTCAGATTCAAATTCTTTTCTTTAGGAAATTCTCTACGGATTAAGGTCCAACTCTAGCACTTTATCTTATTTATTTGGGTCTTACATAGAGTTATTTAAATGCATATGAAATACAGATTGATTTAGTTTCGTGGCTTGTTATTGTATTGTTTAATTTTTAGGAAAAAGTATGTAATTTATTTTGCATTGTTTGATTTTTGGAATAACTTTATAATTTATTTGGTCTATTATTCATGTCATTCTACTAATATCTTTGCTTAGATTTAAAATAAATAAAACATAGTGGATTAAGATTGATCTTTAGGTGTTTTTTAGAATATTATTGTGAAAGATGAAAAAGAATTAG

>LNC_001157

TAACGGAAGCTCTTATTTTCATATTCTCATTCCTTACCTTAATTCTGAATCTATTTCTTGGAAGAAAATAAGTTTCTTGAAATTTTTCATCTCGAATTGTATTCCCACGAAAGGAATGGTGAAGTTGAAAACCTAATCCTTCAAATCTTTGTTGTGGAGTCGATAAATTATATGCCCTTTGGTTGAATCATAAGGACTTACTTCAATTTTGACTCTATCTCCTGGCAGTATCTGTATAAAACAAAGTTCCTTTGAGGTATCAACTAATGCGAAAGATATTAGACAACTCTCCCCCCTTTTTTTCTTTTTCGCAAATAGAAAATTTCGAATCCAATTTGCATATTATAAAGGATTACCAGATAT

>LNC_001158

TTATAAAGGTAGAGAGGGTATTTCCAATAGAGTATTTCGTGGTCAACTGACGGAAAATATTACTCCTTCCATTTCAATTTATATGGCGGTGTTTGATAGGAATGAAGTTTAAGAAAAAACTAAAGACTTTCGAATCTTATGGTTTAAAACCAAACACATTTGTGTGACTAGAAATCATCTCAACAACAACAACAACAACAACATATTGTCATACCCAGTGTAGTCCCAGCAAGTGGGATGACTATAAATCATCTCATTTAGGGTAAAGTGTGAAATTTAAAGTTAAATTGGTTATACATACAGAAAGTTGTCATTCGTTCAGGGACAAACTTAAAAAGAAAGTCTATCACATAAATGAGAAGTATG

>LNC_001159

AAAATTTATAAAAAGCTCTTCAAAATCTTTAGGATTTCAAGTTACCTTTTCAAAAAAAAATAAAAAATCTTTAGGATTTCAAGTTTGGATAGGTGAAGAAACTTAAAATATGGAAGTATGATGGGGAATAATAACTTTATTTACTATCCATAGATAAGTTGTAGACGAGGAAAATGAGACTCTCAAGGAGTCATATATGGGACTTTCTTGGAGCTTTAAGTATTGGCCGCCAGGTTCTAACATGAAAAAGGTAAAGGAACTGAATCTCTCTAATTCTTATTTTAATTTGCTTTGTTTTGAAATAAGAATTAAAAAAGAAACATCTTAACTATACAACCTGAAATTTTCAAGTAACGGTATTTCCCAAAAAAC

>LNC_001160

ATAGAACCCTCAGGGGTTGGCCTGCTGGCAATTGACTTGAGCCTTGGGGTTTGCTCCCTTTCAAGGTCTCAAGTTCGAAACCCACTAGGTGCAAACAATTTCTGAGGGCCATCGGACTGGGTAAAACCTGAATTAACCGTGGTGCACTTGCGGGAAACTCCTTGCCGAGGGCCTGTGCACCCCCGGGATTAGTCGGGGCTCAAAGAGACTCGGACACCCGGTGCAAATCAAAA

>LNC_001161

ACTAATTGAATTAAGCTGAATTTGGGCAAGCATGACGAGAATCTAACATTCAACCACAACCGATCTGATGACATGTAGAAGTGAAATGCATAATTAGCTGCAATTTAAGCAGGTCAATGCATTTGCATTATATATTGTTTTGTGTGGAGCGCTTTGTTATGGTGCTACAGAAAAGGATCAACATAGAATACGTTCTGTTCTCAGTCCACAGTCATTCAATCAAGTATGCCTCAATATTCA

>LNC_001162

TAAGGAGTCCACCCATCTCATTAACCACCGATGTGGGACTTTTGTCATTCTTTAACACCCCACCTCACGCCCAGTGCTTAGCATCTGGTGCGTGGACAATTTTGATTTTGGGGGCCCCAACATCGGGTGAGACGGGCCCTGCTCTGATACCATGTTAAATTAGGTCTTAGGCCTAACTCACACCCCAAAAGCTAGCTCAAAGCACCTAAGTCTTTAATCTTAAAATTATCTTGGAGAGTTTTCTTTGTTTCTACTATCATCTTGTGATCATTTCCTGTGATCAGTAGATCATCAACATATACTAATACCATCACCAAATTCTTTCCTTGATGCTTTGTGAATAGAGAGTAATCATAGTGGCTTTGCTGAAACCCAGAGTCGACTAAAGCCGTGGTTAATTTGATATTCCATTGCCTGCTAGCCTGCTTGAGTCCATAAAGTGACTTAGTGAGTTTACACACTGTCCCCCTCTCACTTTGATGGATAAAGCCCAGAGGTA

>LNC_001163

AAAAAATCTAACAAACAAAACCCACTTCAGATCCAGCCCAAAAACTAATCCCTATGAATACTTCCTTAAGTCTAGGGTTTTCTAAATCAGCCTCTTTTCATTCATCGCCTTCATCCCTAACCATCTCAAAGATGTTCCCTCTTTGGGATAACAAGAAGTTCTGCCTCGGCATGTTATGGATCAATGTTAAATTCTTCTCCTTGCATCCTTGTTTATCAACTAAATAAGAAAGAATTGAGATGGAGGAGGAAAGATTGGAGCGACTTCTTTTGTACGGCTTCAAAATATGAATAGAGATTTTGGCTTTAAAAATTAGTAAGAACGTTTTTCTGATACTATTACTTTATACTTTTTTGTATTGGTTGCAAATGTTTCCTATAATATTATTTTTTTCATACCTTAAACATGCACGTCTTATGTATCACTATGATTTAGAGAACATAATAGTCCTTATCCGTTATTATAACATGAATGGACTTATAATTAATTGGTTCAAGTGTTGAAAAGAAGATCAAGTATTATTGAACTCTGAGTTCCAAGAATTTTGATTTTCTTTATTATTTAAATAAGAGTCAACATTAACTCTTTCTAAATTCAGCTTCACAAAGTTATGTTGCTTCCTATTTAAAAAGTGAAGATCAGAATCCTTGTTAACCCATAGTTCAATAGCACCCAATATTCTCTAAAATGCTTAAACCAACACTAGTTCATCCATATTATAATGAATAAGAACAATTTTCTTCTCTAGTTCAAAAGAACACGTAAGATTGACATGTTTAAGACATGAAAATTTTTGTTATTGAAAACATTTGCAATTGACTCAGAAAAGTGTAAGTCGTAGTAGCAGAAAAATCAATTTTGCTAATATGTATCCATAGGCGAAACTCATAAACAGGCCTCTAAACTTGATGGATTTTTCCCCTTCGAGTACCTTCACTATGCTATTTTCCTATTGAATCTTGAACCACCCATAATTTGCTCCTTTTAAATATTGTTGGCTAATGTGGCACAAAATGTGATCTCATGTCGTTTGAGCGCGTGAAGACGTTTTGAAGGCGCTGATGTGGATCCCTGAACCACCCACCAACGATCTTCTTACCCTTTTAACCCTAATTTCTCCCTTTTTCTCTTATTTCTCCTCTTTTCTTTCTCTTCATCATTAAGTTGGAGCTACTCATCAAAATATATCTCTTCTTCATTGATTTCTTCATAATCACCAAGTTCCAATGTCGAAGTTATCTTGTTCATCGATTTCTTCTTCTTCAACTGCAAATGTGCTATGTCAATGTGGGGTTGTTGTGGAAATGAAGATCTCATGGACGCAATCCAACCCTGGACGTAGGTTCCTTTGTTGCAGAATTTCAAAGGTAATTCATTTTTACTGTCCAATATAGTAAAGAATCCTCTTATTTGTAATTCATTTTTTTCTTTAATTTTGTAGGCACGAGGTGGATGT

>LNC_001164

GGAAAAAGTATACAATCCTCTGACCATTTCTCTAACAGGTCTATTACTTCAAGGAGATAGGGGAGAATTGCAAGCATGAGATGCCGGAATGGAGCAGAAGCTTGAAATTCAAACAATCTATCAAATTTTTATATCGTTATACATCAGTTTACAGGCTAGCTTCCTGGTACGTATGAATGACAAGACTCAAATTGGACATAGAAGACGGTTTCCATGTTCTAAGGTATGAAACTTACCTTTAATATGTCTCTCATTTTTCTTGATTTTGGTCTCTGC

>LNC_001165

GGAAAAAGTATACAATCCTCTGACCATTTCTCTAACAGGTCTATTACTTCAAGGAGATAGGGGAGAATTGCAAGCATGAGATGCCGGAATGGAGCAGAAGCTTGAAATTCAAACAATCTATCAAATTTTTATATCGTTATACATCAGGCTAGCTTCCTGGTACGTATGAATGACAAGACTCAAATTGGACATAGAAGACGGTTTCCATGTTCTAAGGTATGAAACTTACCTTTAATATGTCTCTCATTTTTCTTGATTTTGGTCTCTGC

>LNC_001166

AAATTGGCCGATGAAGAAATTACCGTCGATTTCCAAGGCCTCCCATGTTAATAAGACGGGTTTTTATTTTTAAGTTACTATTAATTTATTTATTTTTTATTCATATTTATTTAATACTAACACTTTTACTAAGTGTCTTTACAGGTACCCGGAGCTGCTTCACATTGAAGCTATTCGAACAAGGTTCGAATAATATTTTGATTCATTATCGTATATTATTGACGATAGGCAAATCTAGGTTGCTTCTTATATTATTTTATTTTATTACTTGTGTATATTGTATATTTGTTTTATTTGTACATTATTTTATTTTCCTCCTCATTTTGAAAAAACGAATTCAGTCGGGATCCACAGTTGTGGATTTTGAAGGATGCCTAACACCTTTCCTTCGAAATAACTTGAACCCCTTACCTAGAATCTTGGTTTCGTAGATCACTTAATGGTTTCCTAGTTTTTTCTTAAAATTAGGTGGCGACTCTTTTAAAATTCGTTTATTCTTAAAATTCTTTCAAAATTTAATTAACTGATTATTTTTTGAGTCATCGCGACGTTTCGCCCTATTCGAATAGAGTAGGGATGTAAACAGAATGGCGACTCCGCTGGGGATACAGAGGTTTTAACCATTATGAGTATATTTTTTCAAATTGAGGAGTTGTTGTTTTATTATGTTATTCATCATATCTTGTATATTTTGTTGATTATTTGTATATTATACTTGTTATTTATTCATTATTTGTTTCAACTGTATATAAATTGTCGATGCATTTCATGATATTTCTGTTGTATGTATAGTATATTAAAGGACGGTTTTGCGGAACCGCAGTCGGACTTTTTTATACTCAACCCTTTTCTGGAAGGATCGACGATTTATTGGTACGTCATGCGTCCAATGGTTGTTGTGAATTCCAGCCTAAGTTGTCCACTTGGGTTTCCGGTCTTTCAAAAGCCACTCTTAGTCAACTAGCGTAGAGCAAAACCAAATCCC

>LNC_001167

AGAGCCTCTACGATCTTGGTAGAAAATCAAAGAGTTTCCGCTGCCGGCGGGTGGCTATTACCCATATATGCCGCCCGTCTGGCCAATGATTATGTTAATAAAAGTAGGACAAATGATACATGCATGAACCCCATGTTCAAGGGAAGAAACTTAGTCAGACAGGTCACCATGCATCAATTTTCGGTGACTTTCTAGGGCTGTTTTGTGTCAACCCCATATCCGTTGGTATTTGTGTAGCACCGTGCCATCATTCCGGTGACTACTTTTTCATATATAATTTGTGTAGCACCGTGCCATCCTTCCGGTGACTACCTTTTTTTTGCATATCTGATTTGCGTAGCACCGTGCATCTCTCCGGACTACTTTTCATATTTAATTTGTGTAGTACCGTGGATTTTTTCGAACTATTTTCTCATATCTGAGTTGCGTAGCATCATGTGTCTTTTTCAGTGACTCCTCAGATTTGATTTGCATAGTTCCATTCGTCTTTCCGGTGACTCCTCAAATCTGATTTGCATAGGGTTGTGCCATCATTCCAACCCTATCCGTACAATTTTTCTTTTTCAGTAGGGTCGTGCCATCATTCCAACCCTACCCATATTATTTCTCTTTTTCAATGGGGTCGTTCCATCAATTCGAACTATCCTATATAATTTCTATTTTCTAGTTGGGTCGTGACATCATTCCGACCCTACCCATATAATTTTATTTCTTAGATTGTGACCACTTTTAGCCATCAAATCTAATACTCCGACATATGAG

>LNC_001168

TAAGTTGCTTCTATTTATAGACTTTATCCACACATTAGACATGCTCATCTTTACACATTAAATGACTCATTTGCCCTCTAACTACCTTCTAACAGACTTAATGACAATTAACTAACTCTTAATACATCAGCTTGCATCTCTTGATTCAACACTCCTCCTCAAGCTGATGGCTTGAATATGTCTTTTAGTCCCAACTTGCTTATCAAGTATTCATGCTGAACTCTTCCTAACCCCTTTGTGAGTATATCTTCTAGTCGTTCTGTTGTAGATACGTGGTCTGTTCTAATCATGCCTTGACTGACTCTTTCTCTTACAAAATGGCAGTCGATATCTATGTGTTTGGTTCTCTCATGGAATATAGGATTAGCAGCAATTTGTATCGCAGCTTTGCTGTCACATTTCAAGTCTATAGGTTGTTGAATCTGAACTCCCAATTCTTTGAACAGCCCTGTCAACCACGTAAGTTCTGCAGTGCATGCTTCTAAATTCTGCTTCTGCTGAACTTCTGGACACAGTTTCTTGCTTGTTGGCTTTCCATGAAACTAAAGCTCCTCCAAACTTCACCAGATAACCTGTGACTAACCTCCTAGTTTCTAGACAAGCACCCCAATCTGAGTCACAGTAGGCTGATAGTTGATTACTGTTCTTAGCAGGCATGAATAACCCCAAACCAGGTGTACCTTTGATATATCTTACTACTCTTAGTGCAGCATCCATATGAGACTTCTTAGGGCAGTGCATCTATTGGCTGAGAACTTGCACTACAAATGCTATATCAGGTCTGGTCATAGTTAGGTATAGTAACCTTCCTACTAGTCTCTGATACATAGCAGGATCTTTTAGTTCATCATCATTAGTTGCTGCCTCATGCTGTATAGCTTTGTCATATACCACATATGTCAGTTTCAGATTGGTTTCAAGTGGAGTATTGGCTGGCTTTTTGCTCCACTTAAACCTGCTTCTGATATTAATTCTAGAGCATATTTTCTTTGACACAACACTATTCCCAGAAAGAATTTAAGTTCTCCAAGATCCTTCATTTTGAACATTTCTTGTTGATCTTTTCTAGCTTGATTTATCAGTTCTTGATTGTTCCCAGTAACTAGGAGATCATCCACATAGAAAAGAATGAACACCAATTTCCCTTATGTCTTTTTTGTGAATAATGAATAATCATAATGGCTTTGAGTGAATCCCATCTGGACAAGAGCATCAGTCAGTTTTCTATTCCATTGTCTTGGAGCTTGTTTTAGTCCGTATAATGATTTGTGAAGTTTGCAGACCTTCTTAGACTCCCCCTGTCCAGCAAACCCTTCAGGAATCTGAATATATACTTCCTCAAGAAGATCACCATTGAGGAAGGCATTGTGAACATCCATCTAATAAATGCACCACTGATTGGAGGCTACAAGAGCAATTAGGGACCTGACAGTGACCATCTTTGCCACTGGTGAGAAGGTTTCAGAATAATCTAACCCAACTTGTTGACTGAACCCCTTTGCCACTAAACAGGCCTTATATCTTTCAACTTCCCCTGAGGCTTTATACTTCACCTTATAGATCCATTTACAGCCAATAGGAGTTTTTCC

>LNC_001169

GATAGAACTGCACCAGCATCAATAAGCTCTCCATCAAACTCAAGGGCTTCTGAACAGCTTGATATAGCATCCTTCCCCCTACCAAGCCTCACCAAGACTATACACAAACCAAGATGAAGATTTACATTATGTGCAGAATGGTTGGGGTCCAACGCCAGGGCTGCTTTGATATAGCAACAGATAAAGGTCTGCACTTACAGCTTGTATGGATGGTTGTTACCTATTGTATTGTATTGTTAGTATAAATACAACATTTTATTTTAAAGACAACTCCATATCTAGGAGTTGACTCTAATTAAGAATTAA

>LNC_001170

TGAATTTGTATACAGATTATGATAGAACTGGTGAAATCAAGATGTATGACTATTTACCCTAAAAGGCTAGTGGTTAAATAGACCTCCAATAGAGCATTACTTGCTATGCATATTAAACATAATGTTGTGGATTAGGGATCAGAAAATCATGGATTTCAGTTAAGGATGTAATCTGATACCTTTCAAACCTCATTTCTTACTTATTATCCTTTCAAAGTTATGTGGGTATTATTTGGCGCAAGTAGGCTATCATATGGAGTTTTCATGAATATGTTTACCTCATGTACAACATTTCAACATTGGAAGGTTTTGCTTCCCTCAGCATAATTGGTGGAACATAT

>LNC_001171

ATTAAAAATAATAATAAACAACGATAGAATTAATAACAAAGTTTTGGTGCAGCTTTGCAAAGAATCGATAACCATGACACGCAAAACCTAGCACAAGCCACGGAGCTTTGGCTTCCCCTGTCGGTAGAGGCCTCAGTGGAATCACGTGGCATTATATTACTTGGGACATACATGCCAGCTACAACTTTTATTTTCTTCTTCTCTTATTTGGGCATATATAAGATGCTCATCTCATTCTATTCCTATTTTTTTCCC

>LNC_001172

CCGAACATAATTTCTGAAAACAGAGGAGTCCGCGTCCTGTCCGCGACGCGGAGAAGATTTTTGTTCACAGAGGGAACAAGTCCGCGACGCGGACGTGCTCCTCGCAAGCTGCCCGACTTTCTCCTTCACGTCTCCAACTCGAAATCCTCTAACCTCAAAGGTTCCAGCCCCAATTCACTTAGAAACATGAAATCCCTCAACATTCAATAG

>LNC_001173

CAACCTGTACAATTTAAAACCCCTTTTTGATTATGACAAACAAAGCAAGTATAACTATGCAAGTGTATCCATCTACTCACCCCATTCACTATATTGCTTATTGGAGAAACCGTTTTTGACCTACTTAGTTCTTTAGTAAATCGAAACAAATATATCTGCTGCTATTTGTGTGACATAATTCAGACGTCTAACATAAGTAAGGTTGACTTGTTTTTTAGTTATTTTACAGATGGGTATGGTATCTACCGATTCAATCAAATAAATCTATAAAAATGAAAATTATTTCTGGAATGATCATTCAGATCTTACTTTTGTACTTGTCAAGTTTTTAGTGTGTGGATCTTAAAGAAATATACTTAACAAAGGTATTTGCATATTTGTGAAGCTTA

>LNC_001174

AGCAACTGAAGTCCTTTGTGATAACAAATCTGCTGTTGCTATGGTAAAGAATCCAGTCTTTCATGGAAAGACTAAACACATTAAAATCAAATACCATTCCATTCGAGAAGCTGAAAGGGAAAATGAAGTTCATATTTTGTATTGTTGTGGTGAAGATCAACTGGCTGATATTTTTACCAAGGCGCTGCAGAAACAAAGATTTGAGCTTCTTAGAGCTAAGCTTGGAGTATCCAGCAGCACTTGCATCAAGGAGGAGTGATGAAGGTTGTTGTTTTTGTATGTGTTGCTAAGTGCTGTCATGTTAATACTTTTGTCTTGATATTTTTGGTATAACTTTTGAATTAATTTTAATACAAAGTTCTAGAATGTAGAAGATATTGTAACATGTGGTGGAGCTTGATTTAAGCACAAAATAGGGAGTCAAGTTTCCAGGATTTAAGGGATCTGTCACCAC

>LNC_001175

CATAGACCAACTTTTTTAATTAAATCAATACAAAAACTATTCTTCTCCCCAAAAATCAACCCAAACCATATCTTCAACAATCCCCAATTGAGAGATTCAATTACAATTTTGATTCAAAAGAAAAAAAGCCCTAAAAATGGATGTAAGAGAAAGATCAGAAGTTAAATTGAGATCACCATCAACAACAACAACAAAAGTGTTTATGAACAATCAGTATATGATGAATAATTCTAGAAGATCAGCAAATGGGAAAAAAGTGTTTTCAATTAGTTATTTCAGTTTGGAGTCATTTCTTCTGCTAATTTGTCTATCAGCATCATTGTTGCTATTGCCTTTGATTCTTCCACCATTACCACCACCACCATTTATGCTTCTATTACTTCCCATTTTTATTCTATCACTTCTTATGTTCTTGGCTTTTATGCCTTCTAATATCAAAAATGTTTCTTATAAATATGTATGATAAAATAATTCTGGGGCTAATTACTCTCTTTGGTTTCTTATTTGTTTACCTAACAAGCAAAATCTAGATTTCTTTATTTACATCTTTTGTTTTATCATAGTACTTTTGAGCAATGTAATCTCAGAGAAATTAAATGTGTATTACAGTAAATTTAGTTTTCTCTCTTTTATTTTGATCTCTATATTTGTAACATAGGCAAATCAACAACAAACATTGATGTTAAGCCGTTGTTTCGAATTTGAATTTTGAGTATATAAAGT

>LNC_001176

CAGGTAACGTGGGCTTGTAACCAAGTGGGGCATTAAAGTGGTTTGGGTGTTGATAAATGGGCTCAATAATGCGGACTCCGTTAGACCTCTCTACTCTTGACGGAATTGTGCACGCCACAGCTCTACATGAGCTGGTAGCATTAAAAAACAATTGTTATCTAGGGTTATGGTTCAAGAGGCCCCAAAAGTTGTGGGAAAAACCCACATCTTAGAGAGAGAATTCTTCGGATTTGATTAAGCGTGCCTATTGGGTTCAATTTAACATACAATCAC

>LNC_001177

CTTGAAGTTCATCGGTCTATCTTATAGGATCAACTAGTATCTCAGAGATGACATCTTTCTAGCAATTTCTGAAAAAAGTATACGCATCTTCATCTTTATATATAAACTCAACTACTCTTGTAAGATTAATTATACGCAAGAGCAAAACCCTTGGTATGATAGCCACATCCACTTTGAAGTATTCTTCATTCAAGTCCTTCCAATAATTTTCTATTTTGTTTCTTATATATAACAATTGTGCATGTAACTCAATGCTTCCTCAATCTCACGTTCAAAATGATAAGCTACACCAAGACATTGGAGTGTGTTGATGAGTTCAAGTTTTTCCAAAGATTTTGAAGGAGCCATCACTAGAATCTTTTTCACTTCTTGTTTTAGGTCTTCATGTTACTTCTCTTCAACTTCATTGGCTCCCTATTTGAGAATTAATATGATACTCATGATCATATGAATTTGTTAGGTATATTGGCGATATTAAAATTAGTAATTTATTTACCGGGAGATCAACATAGGCAAGAAAATGGTCTCCCCAGACAATAGGATGGTGACTGCTAAAGCGACGTCTAATTTTAACCTCATAATCTGCCGGAATGATTTGTG

>LNC_001178

CTAGCAGCCGCCTCTCTCTCTCTCTCATTCCCATCCGTCTCCGGTGAAAATCGCCACCTCCAAGAGGCAGCCTCTGGCCTCCAACTCACTCCCCTCGCCTCTTCTCCGTTCTCTCCTTCTCTCTGGCCAGCAGCGAAGAGGGCGAAGCAAACAGCTCCAGCTGCTCCGAGCAGCAACCACCAGCGCATCTCCGGCACTCGAGCAGCCACCAACGCAACGAGCACCACTGGCAGCTCAACCAAGCACTGCTCCGGACAGCAGACCGGCGAGCCCCTCTCCTCCCCTCTTCTCCTTTTCCGATTTTCTCCTCCAACGGCTCCGACTTTAGCGAGCAACGATGGTGCAACTCTGGCCAACAGCTATGAGAACAAGGAAATAAGCTAAGAGTTTCCGTAATTTTTCCGAATTTATCCTAGGATCTGTCCAGGTACGTCCCTCTAAATATTCATTTCGTGTCTTGTTAGTCCTGCATAAGGTAAAGCTTGGCAAATCTTTGATTACTACTTGTTCATATCTTTTCTGTCATTCTTGTTTTAGTTTGAGAACTTTGCTATCTATTTGCTTAAGATTAATTACTGTTATTTTGTTATTAGTATGCTTGACTGATCATTGCTCTCTATTTATGATAAATTAATCATTTTTGCTTCTCATTCAAATCATGGTAAATTAATTGGTTAATTGTATGCTCGATTTCAGAAATTAGTTGGAATAATTAGATGATTATTAGAAAGTGACTTAATTATGGGTAAATTGATTGTTAATTTTTTAGTAGTATGATTGGATTTTTGTTTGCTTACGGTAAATGTCAGCATGATTTATTGTCATTATTAAATTAAGGTTGGAAAGATTTCTTATTTTATTATATTTGGTAAAGAAGGATTAATTAAATGTTGGGCTAATTTACTCCCTCCGTTCAAGAAATTTAGTGCCGTAAATTGATTCTCCCTTTCCTTAAAAAAATAATTATTTAATTTCCTTGTGGCTAATTGTGTATAATCTATCATTAACTTTTTTCTTCTTAATTATAGTAATCCGATTTCTTACTTGTTTTATATTTGGTCAAAAAATAATTTTGTGAAAACTTTGGGCTCTCCTTTGAAATAAGGAAAATGTCATATTAATGGATTCCTTAAATTATTTATTTCCCTTATATGTGCCCCTCTTCTACTATATTATTGACCCCTCCTCCCTTAATTCTTGTCCAAAATAGATTCTTACTTGTTTATTTATGGTAATTTAGTATTAGGTGGTTATTTGACTTAGAAAATTAATACTTTTATGTGCCGGTCATTGCTCTTGGCTTACCGTTTTATAATTGATGATCCTTGTCTTATTTAGCTACTCTTATATTTGGTAAAAATAAGTTAAAAAATAAATCTGACTTTGACTCCTTAAAATAAAGAAAACATTGTAATTGATCCCTCTTCCCTTAATTACATACTAGACTGATTTCTTACTTGCTTATTTTAGGAAAATTAATTTTACAGATTTAGCTCTCTTTGTTAAGGAAATCTTTTAATCTGTAATTGCTCCTTATTAATTATTTTTCCTTTAGCATGATCCTTTCCTATCACTATAAAAGAGGACTCTCTCATTCTTTTGATCGGGGACAGACACACTTTTACACTCTCTGACCCACGGTGGTACACATTCATACATTTTACTCACGTCCTAACTTGCTCACATATTCTAATTCTAAGCAAAATAATATTGCTCTCTATACTATGAAAAAGAATTAAGATCTTTCGGTTAATTAAAGTTCATCTCTGTGTTGCTGTTTTGCTGTGTTCGGGTTATTCTCTGGATTAAGTTTGTCTCCATTGCTGATTTAATAAACTGCTTAATCGGTTAGTATATTTCTATACATTTAATTTTCTGTTTTGCATCAATATTATTTACTTGTATGCATAAATCCAAATGGAATTTAATAAATAAAGGAAGCATACGAGTCAGTTTTCTATTCCTTTCCTATAAGTGTTTACGTATGTGTGATTGGTTGTTACAATTTCTGTCTAAATATGTAAATGGTTGCTTTGTGTTGTTACTTCTAGTGTATTGTTCTTATCAATTAACAGGTTTCAAACTTGAAGTTTCAAGTTGAAGAATCGCCAAGAGACTAGATCATTTAATTTTATGTATTTTTGGATTTGTATTTATTACTTATTTCAGTTGTGTAATATTTGTATAGTACTCTAACTCTATATATAAATATAATTTGGGGATCGCCTAAAGGGGGAAGGGGAATTATGTGCTACTTGTTCATTTATTTTATTGTAACTTAGGTGTGTTTGAAAGCATGTTTATAAGTTTGTAACTCGTTTGCTAGTCCGTATTTTAACAAATCTATTTTAGATCTAACTAAAAATAAAACAAATATGTCATTGTCATTAATCATCTATTTAATATTTAAATTATAGTTTAAAAGTTAATCTTGTTTTAATCACTCACTTATAATATGTTATTGGTTGTCATTAACCACCACCTCAATTATTAACAAATAAATGATATATGTTTGTTCCTTGCTTCATTTATTTCACTAAGCTCCTTTATATGATAAATGAATAAAATTTGTATAGCGTCATAAATGTGTTTCATATTGAAATTTTATTTTGTCATCTTAGATACCATTCCTATGGGATCTAGATAATTTTGAATATATCATTTAAGTGTTGTAGGTTAAATAGTAATATATATTTAAGATCAGAAATTAATCCCAACTCTTCATTCAGAATTTATGCCTATAGGACTAAATCAAAATAATAATGTACTGTTGAATATTACCTACAACGAATCTATTTTTGACATTTAAAATGAGATAATAAATTGGTCAGTATGCTAATCTTTTTTTTAAAATAGTCTGTCGAGTTTACCAATTTAATCTGCCACTATTAAACAAATGCTATGACATTGCTATTTTCTACATTCGGGGTTAGAAAATCATGCCTATAGGTTTGATCGGATTTTTTTTATTTATTTAGAGTATCATGCCTATAGGTGCTATGCAAAGGTTTTGATCACCTAGAAATCATGTCCATAGGCATTCTAATAATTTCCAGCATGTTTAATAATATAAACAAAACTAATGTGAAATATTTTTTTGCCTATTGTAAACTGATTTCTGCGAATAAATTACCTGCTGTCATTTTAAAAAATAGAAGTAATAAATCATTCCCTGCACATTTTGAATTTGTCCTGTTGAATATAAATTTTATAAAATAATCCGCTATTGTTTTTAAATGTTTCTGCCACAGATTTCCCTCTTTTTAACTAATCTACTCTAATTAATTTGATCAATAAATAGTTAAAATCTGCGTCTTATAATAACTTTCTGCTGCTCTTTTAAGTATTCATGTTGTTGTATCGCCTGTTGTCAATACATGTTTGTATGCACACGCACTTAGCATGAATCCTTTCACTTTCTCTGTGTTTGCTAGCATGTCTAATAAAACCTAGAGGCCAATATGAGATTTACGTGCTAAGTGTTGTCCTATTTGTTTGACTTGGTGTCTAGGTGGGCCTATTATATTTTTTTTTAGTCTATACCCACCCTAATAGTAGTCTAATGCCTCTTGGACCTTAAGCGGGGACGATAGGCACCTTGTAGAGACGTTAAGCATGCGGTGGTGGGGTAGGGGTAGTTTAGGCCCTATGGAGATGATGACATCGACTCAGGCTTGAAGAGACAAACTTTGGTTTTGTCTGTCCCTACACGGAAGCCTATAAAAAAAACAAAGAAAGTCGAGTGCTGATCCTAAAGTCCTTCAGAGTCTCCTTTTCCTTTTCTCCCTTTACTTGTTATAATTATTTGGGGTAGTTTAATAAAAATAAAAAGTGTTTATTCTAT

>LNC_001179

CAGTTTAAGGGGCAAAGAGAAGCGCTTTTGCTACTGAGAAAGCGAACGGTCAGCGCGAAGGTTCAAGACTTAGCCGAGCGTTAGCGAAGCTAGATTCTCATAGCGAGGCGCTTCGAGTTAGCGAAGCGCTGTAGTAGCGCCGAAGCCCTATGTGCTATAATGCTGAGCCAAGGACGCTCCGCCTTCTCTATAGAAGCAGTCAACTGAGTTCTGAATGAATTAGCTCCTTGGTAATGGCTTAATCTATAGATAGAAAGCCCTGTGATGGGAAACTACCACGTTAGGTTTGGAGAGAGATGGGACCGGTTATATAATAGGGGAAGCAGATGCAAGCTTTTTCTTTCAATAGCCGGCCAAATGACTACAGGATCATCGGTCTACTC

>LNC_001180

AAAAAAATATCTGCAACTCTACCTAATAGAGGCTAAAGAAGCTCGACAGTGATAGCTCTCATCTTCACCATTCCTCAAACAACTAAACCCTAAAAATTCTTCATTCTACAAATTCAATCATCCCAATTTCGAATTTCCTTTTTTCTAACCCTTATTTCTCTTCCTTTCTACCCATGGACTTGGTTTATTCACCGATGGCCAAATGCATTTCAGCTCCGTTTCCTTCTTTCACTCATCGGCCACTTGCACTTTTTTCTAGGGTTTCCATTTCCCCC

>LNC_001181

AAAAGGGTAAGGCTCAAAATGTGTCAAGCAAGGTTTACTCATCTCACAAGGTTGACCACAAAAGAGGTATATTGTCAAATTGGCTCACTCTTCAAAATTTGTTGCGTAAGATCATTTCAAGTGCTTGCATCACTTAGTCAACTAGACCAACGAGGCAAATTCTAGGTGTCAGAACACACAATGTTCAAAGTAACCTCATCACACAAGGCATTGACACCAATATCAAACGAAACTCCACACTTTCTAGGTAGTGTGCAATGTTCATCACAAGACACTCATTCGATAACCGGCTAGTCACAACGAGATGTTAAAAGTTCACACAATGTCTATGCAACTTCATTGGACTCATCGTAGCCGCACATTCATTTCATTCAATTAAGAACACTATTTAAGTCATCGATATTGATCATAATTGATACTCAAATTTGCATAAGAACAACCACAATCAAGGCCCAAGCCTTTTCCC

>LNC_001182

CTAAAGCCTCCTTCAACCAGACAACCACAGCTAAAAACCAAACAAGCCCAAATCAGTAACTATTGATTCTGGTGTTTCTTCAACTATTTTCGAGGCCTAAAAACGAAGATTTGAAGTTTTCTTGTCATAAATCATGAAAAATTCAATGTTAATCTACAAATCTAAGTACAAGTAAATAATCTTGTCAGAACTGGTTAATATTTTTTTTATTTTTTTTCTGAACTGGTTAATATCGGATCTGAAAAGTTATGGAAGTTTTGTGACTCAAATCAAATGAATGTGGGATGATGTGGTTGTTATTGATTGATTTGAATCAATTTGTTTGTGTTATGGGTTGAATTAGTTCAAGATAAATCAATTTGGTGGTGTTTTGGGTTTGATTGGTTGAAGACTGCCAGCCATCACACAAAATTGTTGTTCAGGATGGAGGAGGTGATGATTGATGAAGATGATAAA

>LNC_001183

CTAGATAATCTAATTAGTACCTGGCGAATCTGTCTGCTTGATTCTTATGTATGGAGTGTTACATTTTTCTAAATTTATTATCTTCTTGGCTGTTGTTGGGACTTCATGGAAAGAATTGAACTCATAATTACCTGCAAAAACAAAAACTAGGTTAGCAAAAAAATCAGCAAGAGTATTTCCCTCCCTGAAGGAGTGTTGTACTCTTACTGATATAGAATTTCTGATTCTATTGATGGAGTTAACCTCCATTGTCACGCTCCAAGGTGACTCCCACTCTCCATTAAGAATATTAACCATTGACAAAGAGTCAGT

>LNC_001184

CAATGAATAGCCACTACAGGATGTGCTACTTCTTGAACTAAATCCTGAATATCCTGAACATCTTCCTCCACAGCCTCAGGCTGCTGGACATTGCTCCCACTACTTTGAGGTAGTGAAATATCTTGAGTTTGCTCTAGGGCAACCTCCTGCGCAGCCTCAGGTGCAATTTCTTGCCTATGGACATTTCTCCCACTACGTGGTC

>LNC_001185

GTCCACCAATTCATATATTATATGTAAGGGGGTAGGTGTAGGGTTTCTGATACATCTGGAAAAATTGAAACCAAGACTAGGAATTTTTAACAAACAGGATCAAGAATCATTACTCTTTCGTTTCGACACAAGAAAAAAGGATTTTTCTACACCCCTTTCTTGTGTCGAAGACTAGGAAGACAAATAATCCCTGCTAGAATTTTTTCTTCCCCGCATTTATCCCCCGCT

>LNC_001186

GCTGCAGCAGTCATGGGACTCAACAGACATAGTGTAAGAGGACTAGATGGAATGACATGTGATTTTTATCAAGACACTTGAAATATAGTGGGGCTAGACATCTTCAATATGGTTTAGTGACAAACACCTTTTCAGATATGAGGACCATATCCTTGAGTTATTTTGTCAACAAAATATTCTAAGAGTCTGAGAGAATCAAAGTATTGCTACTATATACTTGTACGGGAGCTGCTACTGAGTACTGAGTGAACGGGCGAAAGGAGATTGGAGAGAAATCCGTTCCACCTTCATCGCATATAAATGGCTTAGCGAACTCAAGAGCACCTTTGTCAGAGACTAGCTACTTTTCAACCGATATAGTTAGGTCATAGTTAGGTCGCAGTCCTATCAGGGGGCCGTATACACGTTTGCAACCTTTTCGTCGGCGATAACAATGTCGTCACCGAGAATGGCTTTTTGAAAACCTTTGCCCTCTATATACTTTATATACTTTCTCAGCCGCACACTAGCATACTAGCATATGGTGAGTTAGTTTAAATGCAGGCGTCAAACCATTTAGCCGGGTTGGGGGTTGGTGAAGTCGGTATTCGCGAAGGAAGAATGCTCTTCATCTCCTCTCGCCGTAATTATCTCTGGAAGTCGCCCTGAGGACCCCTTGACTCCTGCGGGCTACGTTCGAACGAAACGAGAGTAAGGCTTTCTCCACGAGAGAACAGACTGACTACTTGGAGAGGGGCGCCTAGTTAACGGAGTTCACGAGGAAGCGACTTTTGTTAGTGAAGCGAGGCCTCACACATCAAACCATATCTTTATCTTACTGAACCGAGTGAGGTATAGACAGATTATACCACAGCTTGTGTTGTGGCGAGACGAACGGACGAAGCCAGAGGCTCCCTTGCTTGCTTTTGCTTACCGGACAAGAAGGATTGCT

>LNC_001187

TTTTTAACTCTCTCTCCCATTTTTATTAAGAAAAAAATATATCATAAAACATTAAAAAATACTTTCCCCCCAAATCAGAACTGAAGTACTGAAGAACACAGTGAGTCCCCCAAATCGAGCTGAAAAGTTTTCTCTCCGTCAAACTTTCTCGCAGAATCACGGAAGAACACGCTGAATATCTGAAGAACTTCTCGCTGAATCGCTGAAGGTTTGTCAGGTTTCACTATTTCACATTGAAAGGCTGAAGAACACTATTTCACGATATCTTGCTGAAGAGCTGAAGGTTACGCCTCACAAATGAACACGCCTCACTAAAATGCTTCACTTGAGAAGATGGTGTCTTAAAGCTTCATCACATTTTTTTGGAATAGGGTTTATCAGGTAAGAAATTGTATCTTTGAAAGTCTAAATTTTAGCTTTAGGATTTTGATATTCTTGTGTCAGCATATACAATTTTTGTTGATTTTTGGAAGTCTAGCTCATAGTTGTATTACCCACATACGTTTTGATATTCGCCTGGACAGTTCTGAATGAGACATGCCTTACTCAGTATAATATGATGATAAGGAAAATTCAGCTATCCAACAGATGTAATATGTGTAAAAAGAAGGGGAAACAACAACATAATAGGCTGTCATTTGTCTTAATCTTGAAGCCTAGCTCTTGTTTACTTGTTCTCTTTTCCAATGGAGGTATGTTCATTGCTAAGGGTGGTAGTAAGGTTCTGGTGGAGCCTCATAGACATGGAGGCGTGTTCATTGCTAAGGGCAAGGAAGATGATCTTTGTACTAAGAATTGGTACCCGGTGAAGCCGTCTACAATGAAAAGAGAATCTCTGTTCAGGTTATAACCTTTCTTTTATCTTTATCCACCACTTACATCTAGAAGTATTATCATCACTAAGTCACATGTTTCTGCTACTTGCACCTTCTGGAGCTTAATTTATCACATTTTGCACTAATGTTGCAGTATCCTTAATCTCTAATTACTACATGCATAAATATTTTTTTGTTTTTTTTCCCGCCAAACTTGTTAAACGTTTAGAAGCCTTGTTAATGTTCTTAAAATTGGGAAGTCATTGTGTCTATGATCCTAAGCAATTTCTTAGAATGTTCAAGTTTTTAAAAGGGATTGTGATTTCGTAGTTCTATGTTTATGAGCTACTTAATGTATTATTGATATTCTTCAAGCTCAATCAGATATTTCCTTGTTTTGAATTTCTTTATCTTTCATTCTTTAGCCAAATGAGGTTTTTCATGTGTATCTCTTAACTTTACTATTTTGTTAACTTGCTGTAGTATCTCTTGATTACTTACGAGTTTTGGACTATCATGTATCTGCACGTTATATGTGCATTTTCCTTTTTGTTGGCACTTTGAATCATGCTCTTTTGTTTATTACAACATATATTTGCACCTTCATTTAGCTCGGGTACTTGTCCATTATTGTCGCTTACTGCAGATCAAGCTTGAGATTAAGATATTGTAGAATCATTGTGGTGAACCTAATATTGTGAAGTTACTCAATATTGTCAGAGATCAGCTGTCGAAGACCCCAAGTCTTATATTTGAATAGGTGAATAACACAGATTTTGAAGTGCTTTATCCAATTCTTTCTGACTTTGACGTTAGATATTACATCCATGAGATATTACAGTTGTTGCAGCCGAGGCGAAAGAAGTAGCAGTCTTTGCATCATCTTCAGAATCTTTCTCAAAGTCAAACATTAATTGCAGCATTGAGGAGAACCTTGTTCGATATCAAGCTGTTACATGTGCTTCCGAAAAGATGGCAATACGAATTCACGGGTAATGAAAGATCTTATACTTGAGCACAAGGTTGATTTAGTTCCATTTTTCCTTCAGTACTGAAGTAGGTTCTGCAAATTGGGCATGCATTTTGTGTTGGCATATTTTTATATTGAAAGAATTGCCTTTTGTTTTCTGTTATCACTGTGACTTCGAATCATTTTCTTTTGGCAATTATTTTGAAGTGAGTAATCTCAGGCCTGCTGCAAATTCACCCCAAACATTGCAAATTCACTGCTGTTTGCCAAAAGATCATATATTGAAACAATACTTTTTCTGAGACAAAACTTCTTATCTTGTTGGATATTGATTCCTGTTCAGTCATGCAAAGTACAAGTTACAGCTCCTGAAATTTTGACATTTATGCTAAAGTTAGACAATGCACTAGAAAGGCAGA

>LNC_001188

TTTTTAACTCTCTCTCCCATTTTTATTAAGAAAAAAATATATCATAAAACATTAAAAAATACTTTCCCCCCAAATCAGAACTGAAGTACTGAAGAACACAGTGAGTCCCCCAAATCGAGCTGAAAAGTTTTCTCTCCGTCAAACTTTCTCGCAGAATCACGGAAGAACACGCTGAATATCTGAAGAACTTCTCGCTGAATCGCTGAAGGTTTGTCAGGTTTCACTATTTCACATTGAAAGGCTGAAGAACACTATTTCACGATATCTTGCTGAAGAGCTGAAGGTTACGCCTCACAAATGAACACGCCTCACTAAAATGCTTCACTTGAGAAGATGGTGTCTTAAAGCTTCATCACATTTTTTTGGAATAGGGTTTATCAGGTAAGAAATTGTATCTTTGAAAGTCTAAATTTTAGCTTTAGGATTTTGATATTCTTGTGTCAGCATATACAATTTTTGTTGATTTTTGGAAGTCTAGCTCATAGTTGTATTACCCACATACGTTTTGATATTCGCCTGGACAGTTCTGAATGAGACATGCCTTACTCAGTATAATATGATGATAAGGAAAATTCAGCTATCCAACAGATGTAATATGTGTAAAAAGAAGGGGAAACAACAACATAATAGGCTGTCATTTGTCTTAATCTTGAAGCCTAGCTCTTGTTTACTTGTTCTCTTTTCCAATGGAGGTATGTTCATTGCTAAGGGTGGTAGTAAGGTTCTGGTGGAGCCTCATAGACATGGAGGCGTGTTCATTGCTAAGGGCAAGGAAGATGATCTTTGTACTAAGAATTGGTACCCGGTGAAGCCGTCTACAATGAAAAGAGAATCTCTGTTCAGATCAAGCTTGAGATTAAGATATTGTAGAATCATTGTGGTGAACCTAATATTGTGAAGTTACTCAATATTGTCAGAGATCAGCTGTCGAAGACCCCAAGTCTTATATTTGAATAGGTGAATAACACAGATTTTGAAGTGCTTTATCCAATTCTTTCTGACTTTGACGTTAGATATTACATCCATGAGATATTACAGTTGTTGCAGCCGAGGCGAAAGAAGTAGCAGTCTTTGCATCATCTTCAGAATCTTTCTCAAAGTCAAACATTAATTGCAGCATTGAGGAGAACCTTGTTCGATATCAAGCTGTTACATGTGCTTCCGAAAAGATGGCAATACGAATTCACGGTTGCCAAAATCACATCTGAGGCAGGTGCAGAATAAAGCAGAAGTTGAAGCAGCCAATACTGGAATGGCTTGGTGTATCCAGCACGACTGTAGCAGAATCATCCTTGAGGTTGACTCGGAGCAAGTGACCAAATGGTACAACAATAGCATTGCACCTCCATGGAGCATAACTGGCCTCATTCATCAACCTCATACTAATTTAATACAACTTCTACTACAATATACCAACATGAAATTGGCAATCAAACACAATCCTACAAGGACAACACTAAGACATTTAGCTGAAGCAGGTGATGGCTAATCTTTGGAGTCTATGAGTTGTGTGTACTTTTCACTTTGTTGTTGAGGTTTGTCACTAACAACAACCTGTTTTTCTTTGTTACTAGGATCATTTTTGTTAGCTGTAGCATTTTGTGTTTGAGGGTTGTCACTAACAACAACTTGTTTATGTTCATGTCTCAAAGGAATAACATTTTGACTGACTTGCTCATTTACAACAGGTTGTAAATGATTTTCAGAATTAGTGTTACCAACTTGACTAGGAGCATAGTTATGAGACTTACCTTGTATGCAGTAGAGTGTCTTGGTGTGGTTGGAGTTGATTTTCCTCTTTTTCTATGCTTTGTTTGGTGCGACTGCTATCCTGATTTACATGTCTGCATTAGAATATCTTTGGATATTTCTCCTGCTGCCCCTGTTATATATCTGCTGCAATCTGATATAATACAGTGCTGCCTGAACTCAAAAAATCTGCAGTCTGCAATCTGCAATCTGGAACTGCTGCAATATGCAATTTGGAATTTCTTGCCTCTTCTATAACTGCTGC

>LNC_001189

TCAGAACTGAAGTACTGAAGAACACAGTGAGTCCCCCAAATCGAGCTGAAAAGTTTTCTCTCCGTCAAACTTTCTCGCAGAATCACGGAAGAACACGCTGAATATCTGAAGAACTTCTCGCTGAATCGCTGAAGGTTTGTCAGGTTTCACTATTTCACATTGAAAGGCTGAAGAACACTATTTCACGATATCTTGCTGAAGAGCTGAAGGTTACGCCTCACAAATGAACACGCCTCACTAAAATGCTTCACTTGAGAAGATGGTGTCTTAAAGCTTCATCACATTTTTTTGGAATAGGGTTTATCAGGTATGTTCATTGCTAAGGGTGGTAGTAAGGTTCTGGTGGAGCCTCATAGACATGGAGGCGTGTTCATTGCTAAGGGCAAGGAAGATGATCTTTGTACTAAGAATTGGTACCCGGTGAAGCCGTCTACAATGAAAAGAGAATCTCTGTTCAGGTTATAACCTTTCTTTTATCTTTATCCACCACTTACATCTAGAAGTATTATCATCACTAAGTCACATGTTTCTGCTACTTGCACCTTCTGGAGCTTAATTTATCACATTTTGCACTAATGTTGCAGTATCCTTAATCTCTAATTACTACATGCATAAATATTTTTTTGTTTTTTTTCCCGCCAAACTTGTTAAACGTTTAGAAGCCTTGTTAATGTTCTTAAAATTGGGAAGTCATTGTGTCTATGATCCTAAGCAATTTCTTAGAATGTTCAAGTTTTTAAAAGGGATTGTGATTTCGTAGTTCTATGTTTATGAGCTACTTAATGTATTATTGATATTCTTCAAGCTCAATCAGATATTTCCTTGTTTTGAATTTCTTTATCTTTCATTCTTTAGCCAAATGAGGTTTTTCATGTGTATCTCTTAACTTTACTATTTTGTTAACTTGCTGTAGTATCTCTTGATTACTTACGAGTTTTGGACTATCATGTATCTGCACGTTATATGTGCATTTTCCTTTTTGTTGGCACTTTGAATCATGCTCTTTTGTTTATTACAACATATATTTGCACCTTCATTTAGCTCGGGTACTTGTCCATTATTGTCGCTTACTGCAGATCAAGCTTGAGATTAAGATATTGTAGAATCATTGTGGTGAACCTAATATTGTGAAGTTACTCAATATTGTCAGAGATCAGCTGTCGAAGACCCCAAGTCTTATATTTGAATAGGTGAATAACACAGATTTTGAAGTGCTTTATCCAATTCTTTCTGACTTTGACGTTAGATATTACATCCATGAGATATTACAGTTGTTGCAGCCGAGGCGAAAGAAGTAGCAGTCTTTGCATCATCTTCAGAATCTTTCTCAAAGTCAAACATTAATTGCAGCATTGAGGAGAACCTTGTTCGATATCAAGCTGTTACATGTGCTTCCGAAAAGATGGCAATACGAATTCACGGGTAATGAAAGATCTTATACTTGAGCACAAGGTTGATTTAGTTCCATTTTTCCTTCAGTACTGAAGTAGGTTCTGCAAATTGGGCATGCATTTTGTGTTGGCATATTTTTATATTGAAAGAATTGCCTTTTGTTTTCTGTTATCACTGTGACTTCGAATCATTTTCTTTTGGCAATTATTTTGAAGTGAGTAATCTCAGGCCTGCTGCAAATTCACCCCAAACATTGCAAATTCACTGCTGTTTGCCAAAAGATCATATATTGAAACAATACTTTTTCTGAGACAAAACTTCTTATCTTGTTGGATATTGATTCCTGTTCAGTCATGCAAAGTACAAGTTACAGCTCCTGAAATTTTGACATTTATGCTAAAGTTAGACAATGCACTAGAAAGGCAGA

>LNC_001190

AGTGTCTCCATCTACTTTTTCCCGTTTGGTGCTTCAAGGATATCAATGTCCGAAGGGCAGTAAAAGATTTCCCGTTTCAAGTAAAGTCTATCTTGTTTTCTCTGATTCAAATCACGTGAAGAACTCTGATTTGCAACGTTTGAATTTGAACTTTTGTTTTGACCAAGTTCAAATACATTTCCCGCTCAAGATTTCAACCCTAGAAAGTCTG

>LNC_001191

TATTTAGTCCATATTTGGACCATCAGATGGCCATTAAGCCAAAATTTTGGCCCAAATTCGTCAAGAAAGACCCAGCCCACCCCTCTTCTGAAAAACATCCAGACCCCTTCACGTTCTTCTTCATTCCACAAGGTAATCCAGCAAGAACAACAAGTCAAAACCTAAATTTCCAAATTTGCCGCCTCAAATTTCCCTTTTTCTACCTCATATTCATACAATCTGCATGCTTAGCACGAGCCTAAAGACACGCGGAGCTTCCATAGGCAACCATGGTGTGGTCCATTCCAATTTCCTTTTTCTTTTGAATTTCGTTTCAAATTCGTACTATTCCAGCTCACTGGAGCTTGATCCTCTAAGATGTTTTCAAAATTTGTTCCTCCCACATCGAAAAATTTCTCAGAATTTGGAGATTTTGGGTGCTATAAATACTCCCTCCCTCTTACTGTTCAACGAGGAGTTTTGGAGGCGAAGAAAAGAGCACCCCGCTAAGTTTTTTGGGAGTTCTTATTTGCGTCACTGAAATTCAGTTGATTCACGGCTATTTTGAGTCAGAGTGGTGGTGGAGTTCATCAGGTCCAGACTCGAGTTCCAGTCACTGCCCAGAACCAGGTAACTCTTTCCTTTCCTTTTCTATTTAGTTTAATTTTAGTCTATGTTGTTGTGTTATGTGTTAGAGCCGATTTAAGGCTAGTTATGTTTCCTAATATTTATATGTTTGATGAATACTTTTTCGCTGAATGCCTAAGTATTGGTGATTTTACAAGTATTTTAGCTATGTTCTTTTATTCCTGATGTGTTGTTTAAGTTTCCAATCCTAGCTTCTGAAGAAAGTTACTGCTTGTTTTTTTCCAGATTTTTCGTTTAGTAAAATGTGAAATCCTGTTTTGATTTAATAGCACATTTTGCTTGGTTGATTTTGTATCGCATCTGGTGATTGAAAACAACCTAAAAAATGCCTTACCCTTGCTAACTCGACTGAATAGTTACTCCTCTATTGTTCCTTAAGAATCTATCTCTTGCGCCATAAAATGGATAAAAATGTTAATACTTGGTTTAGTTTCTATCATTTTGGTCTCTATGAAATAGAGGTTTATTTATAAGTTTATAAAATCTGAGGTCTCAAATTTATCATTCAAGATTGTTTTGTATCATGTTTTTATCAGATTCTTAAGTCATATATGATTAAAATGCTGATGTGACTTGTTCGAGTTTAGTTGTTATTTATACTATGAACTTGTTTCTTTAGTTTAGTATGACACTTTTGATACTCTATTCTGTTCGTTTGTGGTCCAAGTGATTGAGCAATTGTATGTCGTATGTAGATTCAGTTTGAGTGCACTCAATCTCAATTCTACTTTTACATATGCTCCTTAAAGTGAACCCCTGCTTTCTTCTTTTTACATCCAATTCCCTTTTTCACAATGTGGGTATCTTGCTTGCTTCTTATGTTAAGGATTTTATTTGTTTGAAAACCATGAGTTGATCCTAGGTTTAGTTGAGCTCTAGATGAAGTTGAATTTAAATTCAACTTCTCTTAGGTTGCTCCTAAATCCCTTAGATCCTCATGTCACTCCTCTGAGCTATAGATCCTAAATTGTGTACATTGGCTTGGCTTGGAATTCGTCATATTTGCTGATTATTAGTTTAAATATTGGATGTTATATATGTGTTGTTCTATTTGGAAGCTCATATGTCATGTGTTTCTTTACTAATTGTGATCATTTATTGATTTGCTCAGTTTCTGAAATGGTAGAGCTTGTGCCTCACTTGTTTTGGATAATTTTCGTGTCAATTTAAATAAACTCAGCATGTTAGCCTTACAATGATTTCTTCCAAGTACATATTTTGAAGTTTTGTTATGCTCAGTTTACCTAGAGTTAGTATGGATCAATTATGCATCTAAGTGATATAATACATGTTAGTACATTGTTAATTTAACTTATTATTTACTCTTTGTTAAAATCAATTAAAGTGTGGTAATTTTGTGAGTTAGAGGCGAGTAATTTATAGCTAACATATGCTGATTGAAAGTTCAATCTATTTAATTAGACACAAGGCTATAAAATCAAGTGTTTTACTTTCTCTTTTTAGCAGCTTCTCCTATCTTTGTGCTTTTCTTTTATTAGTTTTTATTTCTGAGTTGTATTAGATAAAGATAAGAAGTTGCCTATTTGTTTTAATATTAGTTTTTTATGTTTCAATATAGACTAGAATACTATAATCTTGTTTAAGTTCATATACAACTTCTCCGTTCATATAATTTTATTTCTAAGTTCCTTCATTTCTCAAAATAATCTTCTTCGCCTTGTGCCTTGTTTAAGAAGACTGATTTGTGTCGTTTAGTATTGAATATTCTCGGCTCAGCTTTCCCTTTGGTTTTATGTTCTCTGTAATAATAATCCATTTATGTCTTCCTCTGTTTGTTTTAGCTTATTTTAAAAATCTCGCACTAAGTGTTTTTTTTTTAGTTGTTGATCATGCTTGTTCCTCATTTTGGTTATTAGTAGAAGGCCTACTCTTAAGATGGTAGTTAGATGTTGATAGGTTCTAAGTTTTTCCTGTAATGCTAAGTTAGCAGCTTAGCTTGATTTTTTTTTTATATTATTATTATTATTAATATTATTATTATTATTATTATTATTATTATTTTATGTCAGTTCTAATGTTACTTCCTTTCCTTTCTTTGCATGAACTCTCTTGTTCGAGTCCAATTGGACTCCCCATCCCTTGCATTTTCTTCTTTTCGAGTCAAACCTCCGCCAAGTTACGAAAGGGAAGCTGATATATTGGGTCAAGACCCAAAAAACCAGCAGCAACAGGGGCTCAAAAGAGCTGAAATGAGAGTGTTTTGGGTTCAAATTGGCCGAAATTCGAACACTTGATTTTTGCAGCAGTTTTAGCAGTCTCAAAAGGCTAGAAATAGCATTTTTCGGACTTAAAAAAGTTCGGAAATAGCACCAGCAGCAGCAAATAAGAAGTAGCAAGAGGACTTAAAATTGTCCAAGTTCTATTCGCCCTTTTATTCTCCTTATATTGTGATTTTGTGATAACTGCTGTTAATTTCTAACATTTATTTTGTTAATTTAGATGATAATTGCACTTAAGAACTTTTTATGTTCTTTCATAGTTATTGAAAAGCATTGAATCCATG

>LNC_001192

TTTCTTTCTCTCTTGGTACTAAGACAAACACTGAAAATGCTTTCTTTCTCTCTTGGTACTAAGACAAGTATTTTTGGTTGCCAACTCTTTGTTGTTGGTCTTGACTGCACAAATTCGACTTATGTTGCTGCTTTGCAACCTGGAACAATATCAGTCTTTACGTTCATACTTGCTTTACTCATGGGGTCAGTAAACATTCAATTCCTTTCATCTCTCGATTTTGTATCCTTGTTCCC

>LNC_001193

TAACAAAAACCTAATCTCTAAATTGTAAAATCTAGAAATCTATAAAACCTAAATCTAAAAAACACAAAATACCCTAAGTCCATAACCCTAAACCCTGAAAAACCCTAACAAACACTTAACCCTTAAAACTTTTAAGGAACCCAAAACCCCTATAATGCCCTAAATCATATATGCTCAACCCAAAATCACAAAAAAAAAGAAGCCCAAACCTCTAAACCTATAAAAACATAAAATAACATATCCTTAAAAGCTTGCAACACTAAAAATCTAGAAAAAACCCATAATCTAAAATACAAATCCTAAAAAAACTCTAAGCCTAAACCCTAAGAACTTAGATAAAAACCTTAACTGAAAAACCCAAACCCCTACAATACCCGTAGCTCTAAACACTACATACTAAAAATTTGTATACACAAA

>LNC_001194

AATTTATCTAGTTCCCACTTCAACTACACAGTATTCACATATCTAAATTCCTTCAACTACACATTATTCATATGATGTTTTGTGCTCATGTGGAAATTTTTTAACACTACACCTGATGGTTGAGTCATTGTTGTGGTCTATGCGGTGATGAAATTTGGTGGAAAACATGTTTAACGCTATATTCTACCATCTAAATGAAATTGATTGTTTTACACCAGGTTTTGCAAGGTTTTATTCAAGGTATGTTAAGGCAAAATTCTCTTTTTCATATCGCACGAAATCTTAAAATTTATAGATCATCAAATGAATTGCTTTACTATACTATTCCTAGAAAGACCCTCTGTGAAGGGCATTGAGAAGTGAGCTGATGTTATGCTTTGATTCTATCATATTGTTCTTATACTTGTATCCTTGTGAAGTGTGTTGCTGGTAAAGTTATCCTCCGTTAAGTTTCTTATTTCTTATATCTACTTGCGAGATATTGTCTTAAGATTAAGGCTAAAATATTGGGGAATTATCATGTTATACCCAGGGGAGTTGTTAAGTCATTCTTTCTTGTCAAAACCTTTTACAAAAATGTCAATTGAAAATGATACTAATATTAAATCTTGTAGTCTATTCTTTTTGTTAATTATATACTTGTCAAGAAACATATTTTCTAAGACGGACCATACTATTATCAATTTGGAGTCATTCATATAAATCAAGTTAAGCATGTTAGTATCTTTTTAAGTTTCTAGATGACTGATTTTCTAACTTGAAGTCGAGTCAAGAGCTTTGAATCCATTATGACTCTTTAAACCTAAATGGTTGTACGAAAGTTAAATGAGTTCATGAAAAGATTCTTTCAGCACCTTGAGTTCCTTTTGTATATCCTCATGTTTAAGTTACCTTTCTGAAATCGAGTTAGGTACTTAATAGGCTGTTCTAAAAGTATATGAGGTTCTATATGAGGCGTTTACATGTATGAAAGTGAATACAGAATTATGAGAACATGAGTTCCAACGGGTCAAGGTTGGCAACATGAGTATGGTAACATATATGCATTACGAATCCATGACATGACATGCTATGCAGTCTACTTCCGAGCTATAATCGGAAGGTATGAGGCCTATTGCCTATATTTATACGTATGAGACACAGATGACCACAAGTTGGCGAATATGATGCCGGTTCGCTACCGGAAGGCACCACCATTGCTAGCATTTGGTTGGGTATGTCATGCATTTGCATATATATATGTACTCACGACATACGATCAAATGAGCATATCATGCATATTTAATTAGTTGGAGGTCAGATGTCAGCCAGGGAGGCTATTAGAGTTCCAGTTTCAGGTGGTATGTCTATTACCCTTTCTATCACAGCTATTTATGACATTATTTTCCGCCTTACATACTAGTACATTTTCATTCGTACTGACATCCCATTGCCTGGGGATGCTGCATGTCGTGCTGCAGGTTTGAACAAATATCTTGATCTGCCTTTGCCACAGGGTTATCGTCTCAGTTGAGGATTGATAAGCTACATTCTTCCTGGAGCTGTTGGTTGTTTATAGATTTTACATACTTGAGTTTTGTATATAGTTATTGGCGTCGTTGGGGACTTACCCCAACCAGTTGAGTTGTAGTATTCGTAGAGACTTGTTG

>LNC_001195

AAAAAGGACCGATCCAATAGAAAAATACAATTGGTGATGCTACAGACAGGAGCTTAGAAGCTTTCTCATTTCTTCATCTAGCTGCCTTCGCCGTGAAACTGCCGATTCCGACTATTAAGAGTGCGAATGTCTTGTCCTCTGCCTCTATGTGCTCAAATCCTTTGAAATCCAAACTCTAGAGCTCTTACGCATCTCGAAAAAAGTGTAATCTCTCCTTCCATTCCCAGCGAAAGGTGTAGTCTCTTGGAGAAACTAAAAGCTTCAACTATGGAGCATCTATAGTTGACGGTCGAGCTCCCATTAAAGATCAAGTACATTTTAGTTAGCTTTCTTAGATCTAATTTCCCATGGGTTGAATCCCATAAAGGGGATTGATACACCATTTTCATTTAAATCCTCAAAAAATTGCTAATATGTATTGTTCCTGATCTCCGAGGCACACAGGAAACCATGGAAAGGCTTCTCGAGCTGGTTTGTTGACTATTCTTCGATTATCGCAACTCATTCTCATCAAGGATTAAGGTTGACTATGATCTCCACTTCCTTGCTTCTTCCCTTTTCTAGCAATCTTCATAATCCTTGGTGATAAGTCTGCTTTTGTGGCAATGTTCTCCAGAACTTCCACCAGTGCCATACAATGCTCCTGAAGCTGAGAATACTCTTCTTCTTCCTCCCTACTTGTACTCACCAAGCTTGCCTGATTGTTGCATAATTTGTCACCATGATGTTGCTGCTTATTGTTATCTGTTACAATTTCGTGTGACTGAAACGGGACCCTAGGACTTTGCTGATCCACCTCCTGCATATCTCTAACTTTTTCATCTACCTGGTTCATCAGGGTTGCTGAAAGTGGACTCACCATGCAAATCAGTCACAAAAGTTGTTGCCACCCATTCCTTTGTTGACTGATTATTTTGTTCACCCTTTTTAGCTTGTAATTCAGCATTATCATTATCAATGTTATGACAAGATTCTTCTTCTGAAAGGTGGTCTTAAGCTAGAACCTCAAATTTGTTCTCCACTCTGGTGGTTAGTTGAGGAGCAAAGACCCTTTTATCCCTAACAATATTCCATATACCTGAATCTCCAACTACTTTCCCACTTGACAGTATTTTTGCCTTCCCTTTCTGCATTTGGTGACCATAAGGAATCTGGCGTTCTAGGCTAATCTGCGGCTCTATCTTATCGTGCTGATAGTTATGATCTTCTTTTCTCCTCATCCTAACCTTACAATTTTCCTTGTTGTGACCTTGCACCTTGCAATCAAAGCAGTATTTAGGGACATAGTCATATTGGATTATAATCGTATTAGATCTTATTTCTCCAAAAGCAACATTCTCAATATTCATTTGCACTGCTTTAGGCAATTATCCTACAAGGTCTATCAAAACTTTCACCCTCGCACAACTAGGCCTTGTCTTGTTGATGGTAGCTTGGTCCAGCTGTATTGGTTTGCCCACTGTGGATGCAAGAGAAAATAAACACTCATTGACAAAAAAGGTAGGCAATAGATTTGGAAAAGATATCCATGCCTTGGTCATTGTTGTTTCTTCATTAACCTTAAATTTTGA

>LNC_001196

AAAAAGGACCGATCCAATAGAAAAATACAATTGGTGATGCTACAGACAGGAGCTTAGAAGCTTTCTCATTTCTTCATCTAGCTGCCTTCGCCGTGAAACTGCCGATTCCGACTATTAAGAGTGCGAATGTCTTGTCCTCTGCCTCTATGTGCTCAAATCCTTTGAAATCCAAACTCTAGAGCTCTTACGCATCTCGAAAAAAGTGTAATCTCTCCTTCCATTCCCAGCGAAAGGTGTAGTCTCTTGGAGAAACTAAAAGCTTCAACTATGGAGCATCTATAGTTGACGGTCGAGCTCCCATTAAAGATCAAGAAACCATGGAAAGGCTTCTCGAGCTGGTTTGTTGACTATTCTTCGATTATCGCAACTCATTCTCATCAAGGATTAAGGTTCATCAGGGTTGCTGAAAGTGGACTCACCATGCAAATCAGTCACAAAAGTTGTTGCCACCCATTCCTTTGTTGACTGATTATTTTGTTCACCCTTTTTAGCTTGTAATTCAGCATTATCATTATCAATGTTATGACAAGATTCTTCTTCTGAAAGGTGGTCTTAAGCTAGAACCTCAAATTTGTTCTCCACTCTGGTGGTTAGTTGAGGAGCAAAGACCCTTTTATCCCTAACAATATTCCATATACCTGAATCTCCAACTACTTTCCCACTTGACAGTATTTTTGCCTTCCCTTTCTGCATTTGGTGACCATAAGGAATCTGGCGTTCTAGGCTAATCTGCGGCTCTATCTTATCGTGCTGATAGTTATGATCTTCTTTTCTCCTCATCCTAACCTTACAATTTTCCTTGTTGTGACCTTGCACCTTGCAATCAAAGCAGTATTTAGGGACATAGTCATATTGGATTATAATCGTATTAGATCTTATTTCTCCAAAAGCAACATTCTCAATATTCATTTGCACTGCTTTAGGCAATTATCCTACAAGGTCTATCAAAACTTTCACCCTCGCACAACTAGGCCTTGTCTTGTTGATGGTAGCTTGGTCCAGCTGTATTGGTTTGCCCACTGTGGATGCAAGAGAAAATAAACACTCATTGACAAAAAAGGTAGGCAATAGATTTGGAAAAGATATCCATGCCTTGGTCATTGTTGTTTCTTCATTAACCTTAAATTTTGA

>LNC_001197

AAAATACAATTGGTGATGCTACAGACAGGAGCTTAGAAGCTTTCTCATTTCTTCATCTAGCTGCCTTCGCCGTGAAACTGCCGATTCCGACTATTAAGAGTGCGAATGTCTTGTCCTCTGCCTCTATGTGCTCAAATCCTTTGAAATCCAAACTCTAGAGCTCTTACGCATCTCGAAAAAAGTGTAATCTCTCCTTCCATTCCCAGCGAAAGGTGTAGTCTCTTGGAGAAACTAAAAGCTTCAACTATGGAGCATCTATAGTTGACGGTCGAGCTCCCATTAAAGATCAAGTACATTTTAGTTAGCTTTCTTAGATCTAATTTCCCATGGGTTGAATCCCATAAAGGGGATTGATACACCATTTTCATTTAAATCCTCAAAAAATTGCTAATATGTATTGTTCCTGATCTCCGAGGCACACAGGAAACCATGGAAAGGCTTCTCGAGCTGGTTTGTTGACTATTCTTCGATTATCGCAACTCATTCTCATCAAGGATTAAGGAACTTCCACCAGTGCCATACAATGCTCCTGAAGCTGAGAATACTCTTCTTCTTCCTCCCTACTTGTACTCACCAAGCTTGCCTGATTGTTGCATAATTTGTCACCATGATGTTGCTGCTTATTGTTATCTGTTACAATTTCGTGTGACTGAAACGGGACCCTAGGACTTTGCTGATCCACCTCCTGCATATCTCTAACTTTTTCATCTACCTGGTGGGCAATCTTTCCAATTTGTTCCCCTACAATCAGTTCTCGATTGGAAGGTTCATCAGGGTTGCTGAAAGTGGACTCACCATGCAAATCAGTCACAAAAGTTGTTGCCACCCATTCCTTTGTTGACTGATTATTTTGTTCACCCTTTTTAGCTTGTAATTCAGCATTATCATTATCAATGTTATGACAAGATTCTTCTTCTGAAAGGTGGTCTTAAGCTAGAACCTCAAATTTGTTCTCCACTCTGGTGGTTAGTTGAGGAGCAAAGACCCTTTTATCCCTAACAATATTCCATATACCTGAATCTCCAACTACTTTCCCACTTGACAGTATTTTTGCCTTCCCTTTCTGCATTTGGTGACCATAAGGAATCTGGCGTTCTAGGCTAATCTGCGGCTCTATCTTATCGTGCTGATAGTTATGATCTTCTTTTCTCCTCATCCTAACCTTACAATTTTCCTTGTTGTGACCTTGCACCTTGCAATCAAAGCAGTATTTAGGGACATAGTCATATTGGATTATAATCGTATTAGATCTTATTTCTCCAAAAGCAACATTCTCAATATTCATTTGCACTGCTTTAGGCAATTATCCTACAAGGTCTATCAAAACTTTCACCCTCGCACAACTAGGCCTTGTCTTGTTGATGGTAGCTTGGTCCAGCTGTATTGGTTTGCCCACTGTGGATGCAAGAGAAAATAAACACTCATTGACAAAAAAGGTAGGCAATAGATTTGGAAAAGATATCCATGCCTTGGTCATTGTTGTTTCTTCATTAACCTTAAATTTTGA

>LNC_001198

AAAATACAATTGGTGATGCTACAGACAGGAGCTTAGAAGCTTTCTCATTTCTTCATCTAGCTGCCTTCGCCGTGAAACTGCCGATTCCGACTATTAAGAGTGCGAATGTCTTGTCCTCTGCCTCTATGTGCTCAAATCCTTTGAAATCCAAACTCTAGAGCTCTTACGCATCTCGAAAAAAGTGTAATCTCTCCTTCCATTCCCAGCGAAAGGTGTAGTCTCTTGGAGAAACTAAAAGCTTCAACTATGGAGCATCTATAGTTGACGGTCGAGCTCCCATTAAAGATCAAGTACATTTTAGTTAGCTTTCTTAGATCTAATTTCCCATGGGTTGAATCCCATAAAGGGGATTGATACACCATTTTCATTTAAATCCTCAAAAAATTGCTAATATGTATTGTTCCTGATCTCCGAGGCACACAGGAAACCATGGAAAGGCTTCTCGAGCTGGTTTGTTGACTATTCTTCGATTATCGCAACTCATTCTCATCAAGGATTAAGGTTGACTATGATCTCCACTTCCTTGCTTCTTCCCTTTTCTAGCAATCTTCATAATCCTTGGTGATAAGTCTGCTTTTGTGGCAATGTTCTCCAGTTCATCAGGGTTGCTGAAAGTGGACTCACCATGCAAATCAGTCACAAAAGTTGTTGCCACCCATTCCTTTGTTGACTGATTATTTTGTTCACCCTTTTTAGCTTGTAATTCAGCATTATCATTATCAATGTTATGACAAGATTCTTCTTCTGAAAGGTGGTCTTAAGCTAGAACCTCAAATTTGTTCTCCACTCTGGTGGTTAGTTGAGGAGCAAAGACCCTTTTATCCCTAACAATATTCCATATACCTGAATCTCCAACTACTTTCCCACTTGACAGTATTTTTGCCTTCCCTTTCTGCATTTGGTGACCATAAGGAATCTGGCGTTCTAGGCTAATCTGCGGCTCTATCTTATCGTGCTGATAGTTATGATCTTCTTTTCTCCTCATCCTAACCTTACAATTTTCCTTGTTGTGACCTTGCACCTTGCAATCAAAGCAGTATTTAGGGACATAGTCATATTGGATTATAATCGTATTAGATCTTATTTCTCCAAAAGCAACATTCTCAATATTCATTTGCACTGCTTTAGGCAATTATCCTACAAGGTCTATCAAAACTTTCACCCTCGCACAACTAGGCCTTGTCTTGTTGATGGTAGCTTGGTCCAGCTGTATTGGTTTGCCCACTGTGGATGCAAGAGAAAATAAACACTCATTGACAAAAAAGGTAGGCAATAGATTTGGAAAAGATATCCATGCCTTGGTCATTGTTGTTTCTTCATTAACCTTAAATTTTGA

>LNC_001199

CACTTAAGTGTGCTTGAAGAGGTTGTATGGGCGGTCACTTCTTGTCTCACTCAAGTGTGTCTTAAGGTTATATTAGGTAGAGGTCCTATGGTAATGAAATGACATGTGTTCTTTTAAAGTAAAGCATGGGTTGTATATGGACATATGTTGATTATATGTATGGTGGGCTATGGTCTTATATGTTAATGTTGACTTGTATTATGTCTCTTATACTTGTAAAGGAAAGGTTTTATCAAATGATATGAAAGCATTACTTCCTTGAAAATGTCCTTTTTGGCATGTTTTTGCATGGTCTCCATACTTAGTACTTAATTGTGCTAA

>LNC_001200

TATTTCAATCGTGCCTGTTGGAGGAAAGCCCGTCTTTTTTTGCCTACTCCTGGTGCCTAAAGGCAAAGAGCTTCTTCAATAGCCAAGTCGAGCAGTCAAGCAACGGGTTGAAGTCAAAGACAAGACAAGTCCTACTCTCAAGCAAAAGGCTAAGAGCGCTTGTTCCACTTCTAGCTCGTTAGGGATAGGATTCTTAGGACCTCTGATGCCCTGAATCTCAGTCTTAATCAAGTAAGAAAGGGAACACTTTTAGCTACGGCAATGGGTATAGACCTCTTCCCTCCGCTGCTTACCCAATGGACCACTTAGAATAGACCGAATCGAAGGCGGTAAGCATTCTGTAATCATTCCAAGTTTTCCAGGCTTCCCATTCTCTTTGTATGAAAAGTAGGAGTAGGAAATGTCGTAGGTAAAGCC

>LNC_001201

AAACGAGCCCTAGCTCGACGGCGATTGATGAAGCTTCGTTAGAAGAGATCGACGAATCCTTAGCTAGCATTCTCTCCAACAAGGCGTTCAATTCTAATGTCATCAAACATTGTTATACCAACAGTCTTTAACAACTAAACATGAGCATAGTAAGACATTGGGGAATGGAAGTGCAAGAGTCTTGGTACAATATAAGGGTATGCAAGTCCAAGAGTTGTTTGGTAATTGCATTGTGTCCCTATTCTCGATGACAACAAATTAATGCAAGACGTTTGGAAAAAGCTCGTTGTCCTCTGTAATATGCATCCATCATCAGTAAATTTTCCATGCCTAGTATTGTTTCCAGTTGTGGTGCACACTGCCTTGTTGATATTCTTTTAAAACAGAAACAAATATATATATTTTTAAAACAGTTGGTGATATTCTTTTGCTACTAAGTGAAACACCAAGATATTGTATAGGAAGGCTGCCTCTAGAGAACCTAAAATAGACAAGATTTGTTGTTGCACATCATCAGATAATGTGATCCGTATAGCATTTTCGTGTGAGTTTTTTTCATTGCCCCTTTTCCTTTGCTACCTCCTTGGTTTGACTGAGTCATATCCTTTCGAGATTAATTTTTTCACATCATCAGATAATGTGATATGTATAGTATCTGTTACATCATGAGTACTAAGGAGGTAGTAAAGGAAAAGGGGCAATGAAAAAACTCACATGATAATGTGATATGTATAGTATCTGTTACACATCATGAGATGATGCAATCTGTAATCATTGTTGGCGTATTTGTTTGACTTTTTTAAGCTAGTTTTATTTTAGGTGGTATTTTTATTTAATATGGCTTCGACGAGCTATATTAATAGCAGTAAGAAGTGGAGTTAGGAGTAAAGATAGATTTTGAGGAGTAATATATGTCATATTGTTTGTGATATTGGTAACCTTATGTTTTGCCGTCATTGAAAGAATGCCATCACTCTTTTAACTAACAGGAGATACTTCCTAGTTGTCTCGCCATTCATGATATGAGAAACACTATTTGTGCAGATTAAGATGGAATTCGGTAATGCACTTCGGCTTATTGGTGTTACTATGGTTGGCATTGTTGGGTACTCTATTGTTGGCAGAATGTCATCGGTACATTCAGATGGCAACCACAGGAACACCAATAGGTCAAGGTGCATTGCCAGTGCATTACAAATTTCTATAGTGCATTATAACATTTAGTAATCGAATATTTGTCATAAAAAAAGGCATGTAAACATATTGCGCATACCAAACCCTCAGGGGTTGGCCTGCTGGCAATTGACTTGAGCCTTGGGGTTTGCTCCCTTTCAAGGTCTCAAGTTCGAAACCCACTGGGTGCAAACAATTTCTGAGGGCCATCGGACTGGGTAAAACCTGAATTAACCGTGGTGCACTTGCGGGAAACTCCTTGCCGAGGGCCTGTGCACCCCCGGGATTAGTCGGGTCTCAAAGAGACTCGGACACCCGGTGCAAATCAAAAAAAAAAAACATATTGCGCATACCAAGCTTGTATATCATTTTTTAGAGAGTTAACCTTCTTCCAATACCAACTTGAATCTAATGGAGGAGTGTGGTCTCATATATTCAAGTTATTTTTCATATGTACTCCATGAATCCATTTCACCCAAAGAATATCCCAATTGTCATCTTAATGTACTGATCCGTCGCATCTAGAAACAAATGTCTCTTGGTAGATATACGACAAGTAACCCTTGTGACTGAGTACCAAAGTTGTTTTGAGGCTATCCTATTGGTCATGGGTGAGTCAGTTGTACTTTCGTCATGCTATGCATTGGTTCATCCACATAGAAGCAATACATCCACTTTACATCTGCCACAATCTTACGATGAGTGGTCGTATTGCTTCTATGTGGATAAACATATGCAAGGCATGAAGAAAGTACAACAGACTCACCGAGGACTGATAGGATAGACTCAAAATAACTTTGGTACTGAGTCATAATGGTTACCTACCGTAAATCGACCCAAGAGACATCTGTTCCTAGAAACCTCCCAAACAATAGATTTGATATGATAGTGTTTAGGTAAACATTTATTATAGTGACAGTCTAAAAACTTTGCGCATCTTTGGTCATCACTTGTTTTTGGATTTGCTGAACGGTCTTGCTCAGGCTTTCTTGTTCAGCACTCTTCTCAGCTCCGACTCTGCCAGTGCCACTGTAAGTTTAGCTATTTGATCGGACATAGCAGCAATCTGAACATCGTCAAGTGCCTCGGCTTGAATTGACGATCCAATTCCTTCTAAGCCTGATTGGAGTCGTCTTACATTGTTTTGAGATCCTAGACCATAGACTCTACCCTTATATGTCCCCCCACCACTTTTTCTTTCCAGATCTGGGTGGAAAGTTCAGGTGTCATTTCGACCGAACTATCTAGATTCTCAGCGACCTAATGCTGAAATTCATTCTGCATATTAAATATAAAAATTGACATCAAGATATATACATATCATAAATATTCATGATTAATATATGTTATTCATATTAAATAACTTACAAAAGTTTGTTCGGCTCTTTCCTCCACCCACACATCCAGATCCTACTCATTTTCTTTCTTCCTGACATGAGTCTTCTTGAAAACCTCAGGTGCAATGGGAGTGCGTCCCAATTCTCTTTCCTATAAATAAAAAATTGAAATTT

>LNC_001202

GTTAGGATTAGTATAGTAAGCGGTGTTGTGTCCGCTCTGGCTCATCTAAGTAATAGGAGGTATTGCTTAGAGTTGGGATTAGTAGGTTAATCCTGGTTTGTAAACTTGCTTTTGCTTTTTGCTTGGAGAAGATTAGTGAAAGAAGTTTTGAAAAGTCCTGGCAGGCCAGGTCGTGGTTTTACTCCCTTGAGCAAGGAGGTTTTCACGTAAAGTTGTTGTGCAATCTTTACTTTCAGC

>LNC_001203

CAGCGTCTGTGCACATCAAAGTTCAGTTCTTCTCTTCAGCATTCTCCATGTGAATTAAGTTCATTGAACAAGGTATAAGTTACCCAAGAAAGTAGGAGTAGTGAGAGAGGTTTATTGGTTTTGATGAGAAAGGCAACAACTTGTTTGAGCTCGAATGGAGAGGGAAAATGGTGGAAGCAGGGTACCAGCTACTCCAGAAAGCAGCAAACACAAGCTCAAACAGATTATGACAAAGGAGGATTGAAAGGTATTCTCTACACATCTTTCACCTTTTCAATATTCCACGGGTTCTCTACTCGAGTTTGTGCTATTTTCATCTTTTTATCCAACAACATACCAAACTTATTTTTACTTGATATATGCTATGACGACTAAGCTATTATACCTTATTTGGTTGATGGTAAAAATTAAACTCGGGATAAATATATACCATCAACCAAATATGAGATGTCCAGCCTTATACCTCATACCAAATGATCAAACATATCAGCTCTTCAAGCTGTATTAATTTAACAAATAAATGCTTCACTCCATACTAAGTTGTTAATTATTTTGTCATTAGAAACATCACTGCTCCTGCGCTTAGATTGATGTAATTTCTTCTGAATTTTAAGAGCATAACTTGTGTGATCAAGGATACTGAATTTCTCATTTCATTTCTAAGTTTGCTGGTTATTCTGAGATTTCATTGTTTAAAATAGGCTATTTTGTGCTCTTTTGTTCTTGGGGTTTTCTCTAATTATTAAAAGCATAACTTGTGTGAATAGAAGATATTGACTTATCCATTGCTCATCTTCCTCTGAGTTCTTTCCCCTAAGTTCTTTTGAATTGAAAACACTTCATTGCAGCCATCAATCCAAGCTAAACTACGTTCATTTGTCTACACTGTATAGATAATATGTGTACATAGCCGGTCAAAATACAATTTCCTCATTTAATGGTGTTATGAAATTGTGCTTGGACAGGCAACTATAAAGAAATGTCTGTCCCAATCTTTCTTTGCAGATTGGAGGCCTTAGATATGTATTGTTGGGCAGGTGCTTTGTCATATATATATTAACACCTGATCAGTCTCTTCTATAAACTACTATGTATGCAAACTATAATGGTACATATTTAATCATTCCCTATTCGTCGGTATACATAGAATTTGATATCCTACCGTCTATTGTTTGACAATTTGGCGAAGCACCCACCCTGTATTTCTACCATTATGTATAAACACGTACTGCACTAAACTAGACTTTTGTGATAGTTATTTTATTTGATGCTGACAGTTTTACTACTTTCTTATCCCTCGCCTTGCATTTCATTTGACAACAATTGGTGTTGTCATAGAGCTGTTATAATGTTTTACTATTGATGCTAGAAGGAATGTTGTGAATGAAAAGATATCCTTCCTCAACTATGTTATATCCTCTTAAACAAGGAAGTTCTGATTTTATATCAAACAATACTAAATGGGTTCTGCAGATAAAAACAGGTCCATCCGTCTCCCAGACATTCTCTAACATGAGCAGTACCATGAATGTAGATAAGTATCTACACCATATTGCCATTTGTAAATTCTTTTGGATTTCACTTGACAATTGCTTCTCTTTAATTTTGACTAATTTTACCAGTGAAATATGTACTATGTTTTTGGAATATGGTTGGCATGTTGACGGCGTACACTTTTGCTCCCTACTTTTGGAAAATCATGACACTCACATCTCTCTCATTTCCAGTGATTTCACTACAGTTTACTAGCGCATTCCTAGACTAAGACTATGTCATTGAAGAAGCAAGCTCACTCTCATTCCAGGAATCAGAGGAAGGCATTTTGTCCGGTAATGAAGTTCGAAGTTTCCGGCTTGCTTTCTCTCCTCTTGCAGTTGGCAGTCTCAGTCAACCTTGTTTCGGGCCTTCTCTTCTACTCTATCTGCTTATTGGATTGGTTGTTATGTCAATGACACCAAAGGGATATTAAATGAATGGAATTGGGATATGGATGGAATATAATGAAATAGAGCCACTTTGAGGTTTCCTCTGGAATGAGGCATGCATGGAAGGGAGCCACTACGAAGAAGTTCCGAGAGTTACGAAGGAAGCTTCGAGCTCATATTGGNN

>LNC_001204

CTGTGCACATCAAAGTTCAGTTCTTCTCTTCAGCATTCTCCATGTGAATTAAGTTCATTGAACAAGGTATAAGTTACCCAAGAAAGTAGGAGTAGTGAGAGAGGTTTATTGGTTTTGATGAGAAAGGCAACAACTTGTTTGAGCTCGAATGGAGAGGGAAAATGGTGGAAGCAGGGTACCAGCTACTCCAGAAAGCAGCAAACACAAGCTCAAACAGATTATGACAAAGGAGGATTGAAAGATTGGAGGCCTTAGATATGTATTGTTGGGCAGGTGCTTTGTCATATATATATTAACACCTGATCAGTCTCTTCTATAAACTACTATGTATGCAAACTATAATGGTACATATTTAATCATTCCCTATTCGTCGGTATACATAGAATTTGATATCCTACCGTCTATTGTTTGACAATTTGGCGAAGCACCCACCCTGTATTTCTACCATTATGTATAAACACGTACTGCACTAAACTAGACTTTTGTGATAGTTATTTTATTTGATGCTGACAGTTTTACTACTTTCTTATCCCTCGCCTTGCATTTCATTTGACAACAATTGGTGTTGTCATAGAGCTGTTATAATGTTTTACTATTGATGCTAGAAGGAATGTTGTGAATGAAAAGATATCCTTCCTCAACTATGTTATATCCTCTTAAACAAGGAAGTTCTGATTTTATATCAAACAATACTAAATGGGTTCTGCAGATAAAAACAGGTCCATCCGTCTCCCAGACATTCTCTAACATGAGCAGTACCATGAATGTAGATAAGTATCTACACCATATTGCCATTTGTAAATTCTTTTGGATTTCACTTGACAATTGCTTCTCTTTAATTTTGACTAATTTTACCAGTGAAATATGTACTATGTTTTTGGAATATGGTTGGCATGTTGACGGCGTACACTTTTGCTCCCTACTTTTGGAAAATCATGACACTCACATCTCTCTCATTTCCAGTGATTTCACTACAGTTTACTAGCGCATTCCTAGACTAAGACTATGTCATTGAAGAAGCAAGCTCACTCTCATTCCAGGAATCAGAGGAAGGCATTTTGTCCGGTAATGAAGTTCGAAGTTTCCGGCTTGCTTTCTCTCCTCTTGCAGTTGGCAGTCTCAGTCAACCTTGTTTCGGGCCTTCTCTTCTACTCTATCTGCTTATTGGATTGGTTGTTATGTCAATGACACCAAAGGGATATTAAATGAATGGAATTGGGATATGGATGGAATATAATGAAATAGAGCCACTTTGAGGTTTCCTCTGGAATGAGGCATGCATGGAAGGGAGCCACTACGAAGAAGTTCCGAGAGTTACGAAGGAAGCTTCGAGCTCATATTGGNN

>LNC_001205

TTCAGTTCTTCTCTTCAGCATTCTCCATTGTAGGTGAATTAAGTTCATTGAACAAGGTATAAGTTACCCAAGAAAGTAGGAGTAGTGAGAGAGGTTTATTGGTTTTGATGAGAAAGGCAACAACTTGTTTGAGCTCGAATGGAGAGGGAAAATGGTGGAAGCAGGGTACCAGCTACTCCAGAAAGCAGCAAACACAAGCTCAAACAGATTATGACAAAGGAGGATTGAAAGGCAACTATAAAGAAATGTCTGTCCCAATCTTTCTTTGCAGATTGGAGGCCTTAGATATGTATTGTTGGGCAGGTGCTTTGTCATATATATATTAACACCTGATCAGTCTCTTCTATAAACTACTATGTATGCAAACTATAATGGTACATATTTAATCATTCCCTATTCGTCGGTATACATAGAATTTGATATCCTACCGTCTATTGTTTGACAATTTGGCGAAGCACCCACCCTGTATTTCTACCATTATGTATAAACACGTACTGCACTAAACTAGACTTTTGTGATAGTTATTTTATTTGATGCTGACAGTTTTACTACTTTCTTATCCCTCGCCTTGCATTTCATTTGACAACAATTGGTGTTGTCATAGAGCTGTTATAATGTTTTACTATTGATGCTAGAAGGAATGTTGTGAATGAAAAGATATCCTTCCTCAACTATGTTATATCCTCTTAAACAAGGAAGTTCTGATTTTATATCAAACAATACTAAATGGGTTCTGCAGATAAAAACAGGTCCATCCGTCTCCCAGACATTCTCTAACATGAGCAGTACCATGAATGTAGATAAGTATCTACACCATATTGCCATTTGTAAATTCTTTTGGATTTCACTTGACAATTGCTTCTCTTTAATTTTGACTAATTTTACCAGTGAAATATGTACTATGTTTTTGGAATATGGTTGGCATGTTGACGGCGTACACTTTTGCTCCCTACTTTTGGAAAATCATGACACTCACATCTCTCTCATTTCCAGTGATTTCACTACAGTTTACTAGCGCATTCCTAGACTAAGACTATGTCATTGAAGAAGCAAGCTCACTCTCATTCCAGGAATCAGAGGAAGGCATTTTGTCCGGTAATGAAGTTCGAAGTTTCCGGCTTGCTTTCTCTCCTCTTGCAGTTGGCAGTCTCAGTCAACCTTGTTTCGGGCCTTCTCTTCTACTCTATCTGCTTATTGGATTGGTTGTTATGTCAATGACACCAAAGGGATATTAAATGAATGGAATTGGGATATGGATGGAATATAATGAAATAGAGCCACTTTGAGGTTTCCTCTGGAATGAGGCATGCATGGAAGGGAGCCACTACGAAGAAGTTCCGAGAGTTACGAAGGAAGCTTCGAGCTCATATTGGNN

>LNC_001206

TGGTAACCCACGAGTAATTTCAAGCCTTTCTTTTGAGGAATTTAAGGTTGGTAGTCTCTTTCCCATTAGTTTTGCTTGTTGTTCAAGGATTGCGAACTTCAAAATATTTACATTACTCATTAAAAAGAACTTCAAAGCATTCACAAGGTTTTGTTATTACAATCATACAATGCTAATCTTTCTTCATAACATGATTATGTCAA

>LNC_001207

GATCATTTTTAAGACATTAACCTTCCATTAGATTTCGACAACCAAGGGTTTTCCTGGAAGGTTCTGATGGTGACTTTCACCAAAGTTGATATTATTTATTTTGGTGTATCTTTTGTGTTCATCTATAAATGTTAAGTTAAATTATTTGATGAGTTAAGTCCTTATATTCGATGTTGTTACATTTTGGAAAAATATATTGTTTTGTAACTGAGCCATATATATAGTTGCATTGAAGAATCAAAAATAAAAGAAGATGCACAACGAGACATCTCCAATTTGAAACTATGCATATCTACTGTATAGGTTACAGGTTACTGATGCATCAATATTTCTTAATTTATTTCAAGCTACACATATTACTTGGTAATGCTCGCAAAAGGGAGAGTAGTCGTGACTAATTAACTAACATGTATGTCTGCCACTAAGAG

>LNC_001208

CATATTCATATGCCCTCTAACTTGAACTTCCTTACTTCCCAGGGTTTCCACTGCCTAACTAGACTTCTCATTCATCGGAAGTAAGGGCTAAGAGTTATCATGAGTTAACTCCGGCTTATCTACTAAAGGCTGTAACTTTGAACTCAACTCTAAAGGAAGTGACTTCAGGGTTGGAACATCACAGTAGAGGCGGGCATAGAATCTATGAGTGTTTGGATTGAATCCCTT

>LNC_001209

TGACATCATATAAGAGGGTAATGCATCCATCACACTGTTAATTAAAGTCAATCTTCCTCCCAAGGACAAGTACTGACTCTTCCAGTTGGTGAGTCTCTTTTCACATTTCTCCAGGACCCCATTCCATATGCCTTTGGACTTGCTTTTAGCTCCCAAAGGCATACCTAGACAAGTAGTTGGAAGCTCTCCAACCTTGCCTCCTAGTGTTCAGGGAAAATCAGCTATCTCCAATATTAGTTGTCTTTTCACCCCTAGCGGCTACCTTGTGTTTAGTTGCTGGGTTCCTTCTATCTGATTCATTTCAGATGTTTGTCTTAGAAGATGAACCTAATGTGCCAGATGTAAATATTACAGAGAAAATGAAGGAACAATACTAAGAGGTAAGATGGAGTTTGATTTTGTGGGTGGACTGGTGGGCAAGTATGAGGATGATTTAAATTTGTATATTTCATGCTGGTGCTTCCAAATTACATTTTTGAGGAACTGAAACAAAAGTGTTTCCATGCAAAATGTAGTCTGATTAAGCTTCCACATTGACGGGTAAATGTTGCAGAATGCTTACACTTAGGCTTGCATCTGAGTACTCTTTTAGAACAGTGTGGCAGTCAAAGTGAAGTCCTAAACCTGC

>LNC_001210

TAGTAAGAGTTTCTCCTAAGTGGGGGAAATATGTGGTTAGATGATAAGGTCTTTAAGTGTGGTTGATGTTTATGAGTTAGGCTTGAAGGATGTTTTCATTACAAGAGGTTTAGTGAATTAAAGAGTTGGGTAAGAATGGTTTTGGTAAGATTGATGGTATTTTCATTGAGATTTTTATGAGATGTTAAGGTTGACCTTAGTTGGGTTTACCCTTAAGTGGGGATGGTATGTTGAGTAGTGAAGTTGTTGAGAAGTGTTGATATCCTTACCTACTTCTATATGTGCTTACTCAAGCCTAAAACAAAGAGTTCCTAGAAGCTTGGGTTATGTTTTGGAGTCTTATATGAAAAAGAGTTTTCCATGGTTTCCTTATGTCATGCATGTTTTAAATGAATTCTTGTTAAATGAGAAAATGAGTTTTCATAAATTTATGTTCCTCATGTTGATGTGCATCTATAATGATTTGCCTAAATGTTGCATGAGTTGACTTATGAGCATGTTTAAAAGTGTTTTGATGATGTTGCCT

>LNC_001211

GGTAGGCTTATGGCACCCTTTCATGTGTTAAATAATGTACCTTGAATCGGTAGGCTTATGGCACCCTTTCATGTGTGATGATGTCAAAGTAATGAACTATTCTATGGGAATGTAGGCTAAGCACCGAGCGGATTTGGTTAGATGGAAACTCCCCCGACAGTTAGGCATGAGTTTCAATGATCGTCTACCTTATCCCATAAACTATGTGCCCGCATAGGAAGCTAGTGGATCCATTAAGCTATATATACCGGTCTTCCTTAGGCAAGTAGACCACCTCTTTTCGGTGTGGGGACTCATGACACCGGATTCCATGACAAGCTCTCATGGTCTATGTCGGTTAAATGCTTACTTCCCATCATGTGAGATTTCATTATGGTTTCTTGATAGGTTCTACAAAGTGTAGGTAGTAGTATGGGATGCTACCTATACATTGCACGAGTAGGCTTGGAGAGGGTCATAGTGAGTTTCTCTAAATCCTAATGTATGTGACATGAATGTGCCCTAAATGGTTTCTTAAAGAATGTTGGTCTATTGAAGTTATGTTGTAAATGAAATGTCCTTATCTAAGGTAATCTTGGGGAACACTTAAGTATGCTTGAAGAG

>LNC_001212

TACCAATCTCCCACTTCTCCAAAAAGAAAACATAGAGTACTGCGTAAACTTTCCTCATTTCCATCTTTTTCTCTCTAGAACTGAACAAAAAATCTCAATTTTCTCAGCATCTACAGTCAATGGAGTGATGGTTAGAAAAAAATCAGGAATCGTTGGAGGAAATTCCGCAAGCACCTTCTCTTCCATGTTCTCTTTGAATAAAATCATCTAAGCAGAGATCTAATTACGTGCGTATCTCCAAGAACAACAAAATAGTCTTTCTCCTCTTACAGCTTGTTGTGACTTTGCTTCAATTGACATTATTCAGACCCTATCTTGGTGTTTCCATATCTTCCAGGTATGATTTTGTCATTATAACTATGAATTGATTTGTGGAATGCTTGATTTTGTTCGATTTAAGGTTTTTCTCAATTTTAGGTTTTTCCCTGGGTTTCATCTGGGTTTTTTTTGTTCCTTTTTTTTTGGTGTTCTTGAACTTATATACATATATATATTATTCATATTGATATTTTCACCATCCTTACTTGTTGTGTGTGCGAGTGGTCCACTTTGTGTACTTTTGTCGAGTTAGATAGTTGATGGAGGATCCACTTATAGTCATGTAACTTGAGCCAAACAATGAGTAATAGCATGAAAAATATCAATAACTTATGTACCGTAAATTTCAATTCTTAGTGGATGTAAGCTGTGTAGTAACGAAAGTTTAAGTTCATAAAAGAGGGCGAGTAACTCGAACACTAAACTGTTACCAACAAAAATTGTCAAACATTTCATTCTTCAGCTTTGAACATAAGTTTACAACTCTTATAGGACAAGAGGAAAAGAAAATGGAAAAAATTATAGCCTCTAATATGAACGGTTAGTTCCTCGTTGAACTGTTACTATCCCTGATCCACTATTTTGTGTCTCTGCTTAGAAATGATAATTGTACTAACACTAATCAATTGATTTTAAGGCTTCCCTAAGACATCCACATCACTTTTGTTAGATGTTTTACTAGTATTATTATCTATTGCATAATGTGTACCCTCTGTTTCTTTGTTTCTGCGCTGATGGACTATTGAAACTTGTATTATTGTCATAACTTGATATCTTCAGTTAGTGCCTACAAACTGCTATGTGTTTTTTAAAAAATAAAGTATACAACATGTTGTTGGCCTGCTTTACAATATGATAGAAAAGTGTTGTTGCACCTGGAGTGTTGCCGACTTAGTTGTTGCTAGCAACTGTTGTTAGGTGTTTAGCTGCTGCTTTACCTGCTAAGCTGTTGCTTTACCTGCTACTTTAGCTATTGCTAATAGATGATGAAGAATAGTAAAGGGTCCTTAGGTGTTTAGCTGCGAGTGTATAATAATAATATGATATTTACAAAATCTTCTTTTGTTAATTATTATATATTTAATAGTAAAATTCATAAAAGTTGAGAATTTATTCAATAGTGACATGTGTTAAAAGAAGAAATATAGCCTTTCGTCGTTACTACTCAACACTAGTGTTCTTTTATGTTTGACTCAAATATATGTTGGGTCAGCAAATGAGAATGAAACGACTACTTGTAAGCGTGATCCTCTATCCTCTCTCAATTCACCTATCACTTGATACGAATCAATCAAGAAGTAAAGAACTAATAAAATCCTTGAGTTGTACTGAACTAGTTACTGGGGACAATATTGTCCAAAAAAGGATAGAACTAAAACTAGCTTTAAGATGACGGCTTTGAGTTATTTGGGAGAATATATTCCATCAAACCACCTTATGTTTCTCTCATTTCTGAAGGCTCCAAAAATAGAAGCAAAGATCATCCACACGGTGTTCTTGATGGTCTTATCAACTTTCCAACGACTCCAACTCACATATGCCTCTATCTAAATCTGAGGTAACCCATCTCAATCCAAAAATGTTGAAAAATAAAATCCAAATGCCAAAATAAACTTATGTAAGTCATTCTGTCCGGAATACTTGTATCCTATTGGAAACTTCCACCTTGGCTAGCAATTCCAGCAAGCCCTCTAACTTCTAGGCTTGAGGGTGTATTCTTAAATGTACATTCCAGATATTGTTGCTCCAGTTTTTTGCAATTGAGAAGACCTTGTCAAGTTCCATATCATATAAACCAGGATAAGATGATGGCTTGGAGTTTTTTGGGACAATATTTTCCATCAAACCACCTCCTGTTTCTCTCATTCTGAAGTCTCCAAAAATAGTAGTAGGGATCATCAACAAGTTGTTCTTGATGGTCTTATCAAATTTCCAACAACTCCCAACTCACATATGCCTCTATCTAAACCTGAGGTAACCCATCTCAATCCAAAAATATTTAAAAATAAGCTCCAAATGCCAAAATAAACTTCAGTAAGTCATTCTGTCCGGAATACTTACATCACTGTTGGAAGCTTCCACCTTGATTAGCAATTCCAGCAAGCCATCTAGCTTCTAGGCTTGAGGGTGTCTTCCTAAATGTACATTCCATATGTTGTTGCTCCAGTTTTTGGCAATTGATAAGACCTTGTCAAGTTCCATATCATATAAACTAGGATAAGATGACGGCTTGGAGTTATTTGGGACAATATTTTCCATTAAACCACCTTATGTTTCTCTCATTTTGTAGTCTCCAAAAATAGCAGCAAGGACCATCAACAAGGTGTTCTTGATGTTCTTATCAACTTTCTAACGACTCCAAGTCACATATGCCCCTATCTAAATCTGAGTTAACCCGTCTCAATAAAAAAAATGTTGAAAAATAAGCTCCAAATTCCAAAATAAACTTTTATAAGTCATTCTGTCCGGAATACTTGCATCACTGTTGGAAGCCTCCCCCTTGGCTAGCAATTCCAGCAAGCCCTCTAACTCCTAGGCTCATTGAGGGTGTCTTCTTAAATGTACATTCCAGATGTTGTTGCTCCAGTTTTTTGCAATTGAGAAGACCTTGTCAAGTTCCATATCATATAAACCAGGATAAGGAGTTTCTACCAAGCCATTTATCTTTCCAAAATTTGACATGCTCTTAATTACTTGAATTGAACCGTACCTTTTGGAGAATTCATTACCCAGCTTAATAATATATTTCCAAAGTCCTACTCCATAGCGAGCATTAGAAATATAAAAAAATTCTTCACAAGGAATAAGGTAAAAAACTACTAATCTTAGCGTTTGAGGATTTGTTATTACTAATGATATATTAGGTATTAAAATTGGAGCCCCTTATGGTAATAACCGAAAGTAAACAGATCAGAGTTAGTAACAAAGGCCTAAAGATCAGGATAAGTCCTAAATGACAATCTACAAATTGTAAAGAGTTTCGATAATCTTAGCAAAGTAAGATAATCTCTGAACCTTTGAGCCTCCTATCTAAAAGAGTGAAACAATTTTAAGATACATATAAAGATAAAATGTAATTCTAAGTCTTTCTTAAGTGATTGATAGTAATTAAATTGACTAGTAATCTTAGCTAGCTAGTTAATGAGTTATTAAAATGATGAATATTTATTGTACTTACAAAAGAGAATAGTGATGCATCAAGTCTCTTAAATCTCTTAGCATTTTTAAAAAATAAATGCATCATCTAAGAATCACACCTTTCTTGATTTATACTATTTTCATGTCATCTTCACTGCACTACTCACTCAGAAACATGTGTTTACTATTCTATATATTATCCATATGTATTTCCATCATCTATCTCAAAAGTTATAACTGAAACATCGCTACAATGTTAAATATCTCTGGATTATATTTCACTTCCCAATAAGACATAATAGTTATTGACTTCTGTAAATATACATGAACAAAGTGTACATTTATAATTGGAGCAATCACTACACAAATAACTAATTAAAAATCACATAATTAGTTTTCTGAAACTTACCTAGAATCTTTTAGAAAGAGGTCTTCTTCAACATTTGAACAATAAATGGTTAGTTCTCACTTATGTTGGTGTATTTATAATATATTTTGTTGAATGAAGATTATGGCACCTAAACTTAGACCCAAAACTCCAAGAAACATACTAGGGACACGGCCCTTTACATTTGAAAGAAACTACAAGTCTATTGCAAATGTCTTAATAAAAGAAACTAGAATATAGAATCCTTATCCTCGACATATAAGGACATACCAAAGCCTTGAGAGAAACTCTAGTCCAAAGCTTCACTAAGGAAAGCACCTCACTTGCCAATACCTACACTTGTAGTAAAACATGGAAAAGAATGGAGTTAGCACAATTAAGTACTAAGTATGATGACTATGCAAAAACATGCAAAAAGGGACATTTATTTGAGGTATATGCTTTTCATGCTATTTTTTATAAAACTTCATAATACATGTATAAGAGACATATTACAAGTCACCATCCAGATATAAGATAATACTCACATAATATACATGCTCAACTAATACAAAACCATATATATCAAGTAAACCATTTCATCATCTAAGACCCTCCATCTAAGGTTATCCTAAGACCCACCTAAGTAAGACATGAAGGGGCCGCCCATACAACCTCTTCACACACACCTAAGTGTTCCTCAAGACTACCAAAGATAAGGACCTTTCCCTTTCAACATAAACCAATAAGTCAACACTCATTCTTAGAGACATTTAAGGAGACATTCATGCATATAGGGGACTTCACATAATAGTCATTAAAGACTTAGGGAACTCACTTTAGTACCTTCAAGGCCTACTCATGCCATGTGTAGATAGCATCCCATACTACTACCTACACGTTATAGAACCTATCAAGTAACTAGAGTGCACATCTCACATGATGGGAATAGTCATTAATCGACATAGACCACACTAGCTAGGCTTGGAATCTGGTGTCATAAGACCCCACACCGTGGAAGGTGTTCTACTTGCCAAGGGTAGAACTAGTAAACCACCCATGTAGGCATACGGTTTAGGGGATATCCAAAGACGTTCATTGTACTCTAACCTCATACTCAGAGAGAGCCACCATATTAACCATATCCACTCGGTGCTTAGCCTTATTTCGCGTAGAGTAATTTCATTCACATGTGAGTACTAGAGAAGGCGCCATCATTGGGCCTACTGACCCATAACACTCTACCAAGAACGGCACCACAAGGATCTACCGATCAAGGTTCATAAAGGGTAGCTCATTCACTTTCTTAGAGAGGGTTTTCTACCATTCCGGTCCACCAAATCTAAGTCTATAATTTCACACAAGAAGGAGGTCATCACCCTTAGGTTTACCCCTTCAAGGCCTTACATTCACATAGGGTCCTAGACTCATCATGAAGGGGCACCATGCTTAGGTCTAACTGACTCAAGTCATAGCCTAGAGTACCTCATTAGTCATTTATAGAAGGGCACCAAATTTCGGTCTACTGATCCTAGACGCTCATTCATTCATTCATTACTCAAGAAATGGATGCCTATGCCTACCAAGTCAAGGGATCACATTCAAGAAGCTTTCATGTAGCAAGGAGAGACAACTAGGTCTAACACATAAAGATACAACACCACCATATAGAGACTCTTCATAGTCTATCATCATCACATATGAATATTAAGAGAGAATGTACTTTCAATCACAACACAACGACATTTTTTACTAGATCACCTTGAGATCACATGAACACATTCACATTGTACATTCATATATACCATAACATAATAATCCTTCAATTTAGCATATAATTCCTTTAAGGTCGTAATCACAATACATACAATAAGTAAACAACTCCACCATAAGTGGATCAAACCATACAAAAGCAAATCCAAATACATCATGAATTAGGTCAACTTACCCTTCTCCAAAGACGTAACCACCATTTATCAATCCTCCAACAATTCATCATATAATGATATAGGTAACAATATAAATCAATAATACAAGTCAATCTAAACACAATTCCATCAATATACAATCATAATTATCAAACCCACTTCATAAGCCAAATTTAGGAATTTCTGAAGGTCATGGGTCCATGGAGTTTTTCACCTTAAAAAATCAATTAAACCATAATATAATCATAATAAATCTTTTGAAACCATTTAATGCAAAAACCCATGAGTTTGAGGAATAATTGGGGTTTTTCATAACTTTTGAAAATCTTGAAAACCATTTTGAGATTTGGCTCTAGGGAGAAAGCTAGCCCTAGATAAAGAGCCACCATACCTTAGTTGATGAATTCCCAAAGAAATTTTACGAAGAAAGCTTTGAATCCAAACCCAAGTTCCCAGTTCTTCTTCTTCCTTAAGTTGAGAGAGAAAACTTTGGAGGTTTTGATTTGGTTATTTGAGAATAATGTGTTAAAGGAAGGTTTTAAGACTTAAAACTAATTATATTAATGATATAAACCATTTAA

>LNC_001213

CCAACTATGGAGAATTGATGAATCGATGCTTTAAAGCTTGAGTTATGAGATGTATGGAAATCCAGAGATGTGTTGATGACAAACTAAGCTTGTGTATATGTTCATTCCATACGTGAGATTGCATTAGAGCCTAATGAGAATTTCCTATATAAAGTGTTGCATGACCATGCCCGGTCCGGGGGAAAAGAACCGGACAACCATGTGAGAGGTTTATATCTCACCACCTAGGATGCTTGGGGTGTGACCAACATCAACCATGTGAGAGGTTTATATCTCACCACCTAGGATGCTTGGGGTGTGACCAACATCAACCATGTGAGAGGTTTATATCTCACCACCTAGGATGCTTGGGGTGTGACCAACATCAACCATGTGAG

>LNC_001214

GTCACTATATTTTTCCTGTAGTTAAAAATCAATTTTTCACTAAATATTAGTAACGGAATAATATCCGGATGTTAAAAAGATTAATATCCGTAGTTAATAATACTAAAAAATTGAAGGTACTAATTAAGTTAAATAACTTCTCTCTTTAATTTTTCAATATTTAATAATTTTCCTATTTATTTTGCTCAACCACACACATCTCTTCGTGCACCCAGCCCCTCCCCTTCGTGCAGAAAACAGACTAGATAGTTGATGTTCATATTTCAGATTTCACTTTCTGGTAAGAGTCTTAGAAAATTCATCAAGCACGTGGAAGCACATCACTGGTGCAGGCAGAGAAAATTCATCAAAGCTTAGAGGAAATTGGGCAAACATGCTTATTTCCCTTCTATTAGAATATAATCAGCAAAGAAGAGATACACAATTGCACGTCACTTAAAGGAAAATCAGTTGATTGAGATACGCACATCTCTCTATTCAAACTTATAAAGAGGTAATCTGGGTCTCTTTGGTTTGATGGCAAAATAACAAAATCAGTCTTCTCTTCTTACTGCTTGTTGTGACCTTGCTGCCATTGATGTAATCGGACGCGATCTTGGAGTTTTCGGCTCTTCAAGTGGCATGTGTTTAAAGAAGAACTCCATTCATTTCTCGTTACCACTCATCACTTATATATCATCAAGCTTGCTCATCTATTGGACTACATATTCTTAGAGTGTTCTTCTCATCAAGCTCAAGAATTTGAATATGAAGAGTCTATCATTGAAGAAAGTATCGAGCAAGTTGAAGTTCAAGAGTACTTTTACTTTGATTAGAAATGTAGTTGAAAGACGATATCTTATTATTTATATATGTTTCATTTTTTCTCTTTTTAGGAGTTGATAAAGATCTGTCATATAACTTTTATGTAATATTTTTTAAAATTAGTTCTTTGTATTAACAATGAAGTTGTACATTCAATTACTAAGTATATTTTCTTGTGTTCCAATGTATGAAACAATTAATATTATTTGCTAGTATAAATTATTGTATAATAGAAAATGAAAATGAGTTTTCTAAAAGTCATTTACCAACGGCTTATCAAATAATGAAACGG

>LNC_001215

CTCAACCACACACATCTCTTCGTGCACCCAGCCCCTCCCCTTCGTGCAGAAAACAGACTAGATAGTTGATGTTCATATTTCAGATTTCACTTTCTGGTAAGAGTCTTAGAAAATTCATCAAGCACGTGGAAGCACATCACTGGTGCAGGCAGAGAAAATTCATCAAAGCTTAGAGGAAATTGGGCAAACATGCTTATTTCCCTTCTATTAGAATATAATCAGCAAAGAAGAGATACACAATTGCACGTCACTTAAAGGAAAATCAGTTGATTGAGATACGCACATCTCTCTATTCAAACTTATAAAGAGGTAATCTGGGTCTCTTTGGTTTGATGGCAAAATAACAAAATCAGTCTTCTCTTCTTACTGCTTGTTGTGACCTTGCTGCCATTGATGTAATCGGACGCGATCTTGGAGTTTTCGGCTCTTCAAGTGGCATGTGTTTAAAGAAGAACTCCATTCATTTCTCGTTACCACTCATCACTTATATATCATCAAGCTTGCTCATCTATTGGACTACATATTCTTAGAGTGTTCTTCTCATCAAGACAATATTTTGTATAGGAACACCCAGTTGGCCTTGACTTTTATCAAAATATGTATTTTGCAAGAGGAATATGGAATGATGCAGCTGCTAAAGTTTCTACTGAAGTTTGCAGTGATAATGAATTATAAGAATCAAGCTCAAGAATTTGAATATGAAGAGTCTATCATTGAAGAAAGTATCGAGCAAGTTGAAGTTCAAGAGTACTTTTACTTTGATTAGAAATGTAGTTGAAAGACGATATCTTATTATTTATATATGTTTCATTTTTTCTCTTTTTAGGAGTTGATAAAGATCTGTCATATAACTTTTATGTAATATTTTTTAAAATTAGTTCTTTGTATTAACAATGAAGTTGTACATTCAATTACTAAGTATATTTTCTTGTGTTCCAATGTATGAAACAATTAATATTATTTGCTAGTATAAATTATTGTATAATAGAAAATGAAAATGAGTTTTCTAAAAGTCATTTACCAACGGCTTATCAAATAATGAAACGG

>LNC_001216

TGTAAGTGTATTAATGAATTTGATTCTATGATACATCTTGTTAGGGAAGTGTGGGATTAGATACATGATCCTCCTTGATCACTGAGTTACTTCATTTTCTGAAGAAATCCTCTTAAATATGTGATGGGATCTCTTAGGAAGATGTATGTACACATGCCTATTGAGAATTTCATTATTGAATACCTGCATCAGGAGTCATCTGATTAAGAGCTTCGGGAACTAAAATTGAGAACAATAGAAAGATCCTCATTGGTGTTATACTCTTCAATTCATTTAGAAATCTTTAGTCTGAGTACATCAAAAGCAGATACTAGTAGTAAAATATAGAAGAAAGAGAGAGCTTAACTATGGCCTTAAGCTAACTGAATTTATTTACCAGAGAAAAATGTGATATACTAACTAAAGTAGAGCATGTTAAACTTAAACACTAAATAGAGTTAGTTTTTCAGTTCTTTGTGCTGAACCTACCAACAAAAAAATGTATTTTTGAAGGGGTAATCTTTTTTTGTTTAGTTATTTGTTTATGTACTGTAGCAAATTTTTGTACTGAACAGAGCAATTGGGTCATACAAGTAATTGGTTGTCATGTAAACTTTTGCTGGGAATTTGAATTGATTTGATGAAATTGTAGTGTCTTTAGTGGAAGACTACTGCACTATGTTAAGAAAAGTATAACCTGGACAACTATTTAACTTACTACCTTAAAGATAGATATGTCCATAAATATGATTTTCAGCAGCCCTTAAATACATTACTTTGAATGGACACCACTAACACTAAAGATCAGAAAAGCTGTGATTATGCAACTGTACATTCTAAAGAAAGAGCTAGATGTAGAATTATCAATGCTTCATTATGGTGAGTAATCTGGAAAGAGATGAATTCTAGGTGTTTTAAGAGCATACAGAGAGTGTGATATAATTGATGTTTATACTCATATGGACATAGTACAATGAATAGATGGAGTTCATCTGTAAATGAGGTATAACATGGAAAGCACTGCCAAAGAATGTATAAAACTAGAGAATGGAAGGCACTGCCAAAGAATGTCAAACAAATTCCTCTTGATCTCTCTGCTCATGCTCGGGAGACATCACTTAGTTTTTGTAGAGTCCATGAGAATTTTTATTTTAGTGATCTTGTAGAGAAGAGACTTCTAAGCCTTCGGCCTTCTTCTTTTTTAGTAAATAACAGAAATTATTACCAAAAGTGCCATGTACGAACAAGGAAAAAACAAGGCCTGAACAACCCCAACCTTGCTTATGAGAGAATCTAAACACTGAAACTAACTATAGTTTGTAATCTTGACAACCCAATGCACTAGCTAGTTTTGTCTTCTTACATCCTCTAATTGTGGATCTCCTAGACAATATTTTGTATAGGAACACCCAGTTGGCCTTGACTTTTATCAAAATATGTATTTTGCAAGAGGAATATGGAATGATGCAGCTGCTAAAGTTTCTACTGAAGTTTGCAGTGATAATGAATTATAAGAATCAAGCTCAAGAATTTGAATATGAAGAGTCTATCATTGAAGAAAGTATCGAGCAAGTTGAAGTTCAAGAGTACTTTTACTTTGATTAGAAATGTAGTTGAAAGACGATATCTTATTATTTATATATGTTTCATTTTTTCTCTTTTTAGGAGTTGATAAAGATCTGTCATATAACTTTTATGTAATATTTTTTAAAATTAGTTCTTTGTATTAACAATGAAGTTGTACATTCAATTACTAAGTATATTTTCTTGTGTTCCAATGTATGAAACAATTAATATTATTTGCTAGTATAAATTATTGTATAATAGAAAATGAAAATGAGTTTTCTAAAAGTCATTTACCAACGGCTTATCAAATAATGAAACGG

>LNC_001217

GGAGAGTTGAGGAATGGTGATTGTGGCTTGGGAAAAAGGGTAAGTGGACCTAACTTCATTGTTAAGGTAGAATTGATATAGAATTGTGATAGATTGATGTGTGATCCACTTATGGTGGAGGTGTTTACTTATTGAATGTATTGTGTTTATGGCCTTGGAGGCAATCATGTTGTGAATATAAAGGTGTTCATGTGATCTCAAGGTGATCTTGTTTAAAGTGTTATTGTATTGTGATTGAAAGTGTGTCCTCTATTATTGTTCATATGTGATGATGATGGAATGTGAAGGATCTTTATAATGTGATGTGTGTCTTGACTTGGTAGACTTAGGTGCCTCTTCTTGATACATTAGAAGCTTTGTTGAATATGAATCGATAGGATATGGCATTCCTTTCGGATGTAAGTGACGAAACCTGTTTTTGAGCTGGATAGTGTTTGGTATTTTAGGCATATCTCTTGCTCTAGAACTTAGTTTGCAGTGA

>LNC_001218

GAACAAGTTGAGGACCTTGATACATTGGTTGGACTGTTTCTTTGCTAAAGCACTGAGGTTTGTGTATAAACTTGTACTTGCACTCTTTTAACTTCTAGTATATCTGAAATTGGATCTTGGTACCTTGGTTACCTCTTGTCACTTTGCATTGTCGTGTTGGTATCCTTTGAGACTTTAAAGATGCTTGTGTAACTCGCCCACTTGTACGGATTGTTTGATCACTTGGCACTCTTGTTGGGACCTTGTCCTTCTTGTGGCTGTGACTTTGTCACTCTTGGCATGCCTCTTGATTCGGATCATTTGCCATCTTGACTCTGGATTTTTAGTTCGTCTTAAGTATGGAT

>LNC_001219

CGAGGTCCGGCCTCTATTGCCCAGATACTTATATAGAGCATCCGGTTTAAGGTCCGGCCTCTATTGCTCAGATACCTATATAGAGCATCCGGTTAGAGTTCCGGCCTCTTATACTTGTTACTTGTGATTGGTTACTTGGGTACTTCTGGGGAGCATCCGGTTCGAGGTCCGGCCTCTATTGCCCAGATACTTATATAGAGCATCCGGTTTAAGGTCCGGCCTCTATTGCTCAGATACCTATATAGAGCATCCGGTTAGAGTTCCGGCCTCTTATACTTGTTACTTGTGATTGGTTACTTGGGTACTTCTGGGGAGCATCCGGTTCGAGGTCCGGCCTTCGTACTGTCAGATTCTACTAATTAGGGTTGAGGTTCTG

>LNC_001220

CAGGAAACGAGGGTCCCGATCAGTCACAATAGACCTTGGAGGGCCATGAAGTCGAACAATTCCAATCACAAAAGCTTCTGCAACTGACTGAGCAGTGAACGAAGACGGCAATGCAATAAAGTGTCCATATTTTGACAATCGATCTACGACCGTCATGATAGTGGACTTACCTTTAGAACTAGGCAAGCAGGTGATGAAATCCATTGCGATATCCTCAAACACCATCGAAGGAATGGGTAGTGGTTGTAGCAGACCTGCAGGCAATTGATGGGAATCTTTCATTTGCTGGCAGACTTGGCAAGAGGCTACAT

>LNC_001221

ACTTAGATAGGTTAGATACTTCTTATATTAGGAGAATTATTGTAAAGGAGTAGTAAGGGAGTGAAACCCCGATCTAATCTTACACGTGCGAGGACAGTCCTCTGAATAGAGTCCATTGCATCCCACAATAATCGACGGGCAGCCCTCGTACATTTAGCATAAACCGCTGAAAACTCGAAAGTCTGTCCACTCTGAGTGGGAAGAGCCTTATTACCTTATTAATATGAGGGATGCAAGGAATTGATGCTACGAATGCCATAGCAAGTGAATCAGAATAAGCCCTAGCCCAGCTACTGATTGGAAAGTCTTATCGTCATCTGTATAAAGC

>LNC_001222

CACGGCGAAAAGCCAACCTTCTTTTTTTTGATTCGTTTGACGAGGGAATACCTTAAGTACTGAATTAATGAAATTACACCTATATCGCGAAAAAGAGCTGCTCTAGCTTTAGCACGCACCCTTTCTCCTATGAGGAAAGTTACTCTTTCCGATTCATGTCTGGCCCTTCCTGGGTCTGGATCACAGTATACTGGCGACTTTGTCCTTGAATTCATTCTATTTCATATCCACCGATCCGTGGAAGAGACCAAGGAAGAGTAAGATAGATTTAGGGCCGGCAGGCGGGTCTAGAATAGATTCCACAACGGGTCCTGACTTTTCAACAACAGAGGCGAAAACTTACTCTTTCAAGTCAAAGTCAAAGTCGGGTAGACTAAGATGGAGTTTATTTTACAGTTGTTCCAGGGATATGGGTATAGAGTGAATTCTATTCACTCTTTATGAAGTGGAGTGCACTCCTTATATCATAAATATAAAAGTCAGTATGGTGCCCCG

>LNC_001223

TTCGAATTTCAGAAAAAAAATCCACCCGTTTCCCCCTAAAAAATCAACCTTATTTTTCATTTATTTAAGATCGGTTCTCTATGTGTTGAAGGGAGGGAGAAGTTGATAGAGAAAAAGAATTACAAGTTATAAATGTCACACAAGAAATACACATTAGATATTCTTCTTATCCATTCCATGTGGGTTGCTGGAAAGTTGCCGAAATCCTAGGAGTTTTTCGATTTCGTGGTAGAAGAATCACTACCCTCAAGCTCGTCGTCTCAGTTCGCTGCCCGGAAAGAGGTGATTTCTCTTCTTAATTTTGGTTTATTTGGTTCTTCGCTGTTTCATGCTTTCATTTAAGTGTCCTTAATGGTTTTTAGTTTTTAATATCTAAATCAACTTGATCATTGTCTCTGTTATTTCGTAACTTGTTCTTGTTGGATTTATGAAGTTGTCTGTGATCAAATGTCATCCTTAATGTTATCGTTTGTTTATTTCATCCTAGTTTGGGTCACTTTTGCTATGAACATAATAGAAGCCACGTTAAGCTCAATTCAGTTTCATGGGATTGACTACACTTTCGTCTCCATTTAGCGCTATAACCTTCGATTTTGAATCTATGTTGTTAATGTTTTAGCTTAAATTTAGTCTCATCCTAGCCTACTGTGTGTGATTTTCGTTGGTTATTTGTTTAAGCTCATAGGCTGCCTCTTGAGTTTTACTTTTCATTTTACTCATTTTAGGTGTATTATAGGGTGTAAATCTAAGTATTCATGTTATGTTTATATTTCTCTGATCTGGGTTCTCTGATTATTAATGTCTCGTGAAGCTTTATTATGTTAAAGGATTGGTGTTTCAATCCTCCGAATATAGAATGCAGTTTATGCGTGATCGTCCTCTGATTTCACCTCGTTATTTTTTAGTTACCTAGTTTGGTTCAAACCCATATGTTTTGGATTCGTCCACATTATTTGACTTGTCTTATGATTCATGGTTTGAATCTGCTCCAAGTACTACTCTTATCTGCTAATTTAGTGTTTAATTTGAAGTCCTCATTGCTTATCTTGATCTATTTCTTTTAATCGATTCCCTGATTTATTTATCCTGAGTCCTCTTTTAAATTAAGTTTCGTTCAAATATTTGTCTAAATATGTGTTGGGTTAGTTGCAAAGATTTATTCATATGGATAGGTTGAAAATGGTTGGTCTGCTTACTTTAGATGTCAAAGGCTAGTAAGTTCATGTCTAAATAAATAATTCTTATTCGATCTTTACTGATGATGTTGTGCATGCGTACATTTTTGTAAAGTTCACTTGGTCATGAGTTAGGTTCTTATGTTGCCCTGAAGGTGGGTTATTTAGTCATAATATGTGATTTTAATGCACTAGTTTTCTTTGACAAAGAATGATAATGTTGCGGTTATATACTAATACTCCTTATTATTTTCCTATGCATGGTTTGTTCTCCAAATGAGTTCAATTGGACTCACGTTTCCCCTGCATCTTCATGTCGGGATGGAACCCAACAAAATTCTTTTATCGAGTCATCCAACCTTGAGAAATCGAAGAACAAGTCTTTGGAGTTGACGTACAGGTCCCGAAAAGCAGGAAAAT

>LNC_001224

TGCGCAGTACGCTCGACTTCAGCAATGGTCACCTAGAAAAGAACGAACCCATTCCCAAATTTTTGCCTCTTCAATTCCCCTTTTCTCCTTCAATTCGTACCTTTAAGCGTGCCTAGCACGAGCTCGAGGATAACTGGAGGTTTCCAGAACGTTCCCAGCAACATTATCCTCTCAATGTCCTTTCCTTTTGCCAATTTTCGTTCAAAAATGGTACGAATTTGTACACTACCTAGTTGGCAATCCTTATCCGCTTAAGAATTTTGAAAAAAATGCCCAATTCTCACATCGTTTCCCCCCTCAATTTCAACCCAAAAGTTGTCCTATATAAACTCCTCCCTTCTGCTGTTCATAGTGGCGTTTTGAGACCTGAGAGTACCCCTAAAAAGGTTTAGGAATTCTCGTCTTTGTAGTTTAAAATTTTGTGGTTGCTCGAGTTGAAAGAAGTGGAATCGTCTCCGTCAAACTCGCCGTCCAGTTCGCTGCCCCCTACCAGGTAATCTTTTCTTTATTTCAACATTTTCGTTGGCTCTTAATTATGTCTTATTCATTGAAATTTTGTTTGGCATTAGCTGTTTAAATATGTTTTCAGTAGCTTAAAAAATTGTAAAATTGCTTATCTTTGAATTCGACTAATTTATATGTGAGATGGCCAAGCCTTCCTCTCGGTTAATTAAATTTCAGCATTTTCTATTGTCTAAAGATAAGTTTAATCAGTGTTGAGTGCAGTTAGAGTTGTGATTTTTTTTTTGTATTCAAGCATTCCTAGTTGTTAGTTCGAAGTCTGTAACTTGCATTTATTTTTATTTGGAAATCTCTTGAATACCTAGTTTTCTCTTGATTGATTCACTTATCTAAGTGGTAACAAAATTTGAAATATGGAGTTGTACATTCCCATTCATGATCAGTGAAATGCTCTTCCCTTTTATCTAATCTTTTTATGTGGTTAAACATTCATTTACATGTTTGGGCTGTATGTATACCTTTGGATTTCACATATCAGTAGAGTATTATGCATTCCTTCCCTTGTTTACATTCCATCCAGTTCCCTTTTCACAATGTGGGTACCTTACTTGCTTCTTGTGTTAAGGATATTATTTGTTTTGGAATCATGAGTTGATCCTATGTTTAGTTGAGCTCTAGATGAAGTTGAGTTTTAGTTCAACTTCTCTTTGGTTGCTCCTAAATCCCTTAGATCCTCATGTCACTCCTTTGAGCTACAGATCCTAAATTGCGTACATTGGCTTGGTTTGGATTTTGTCCTATTTGTTGATTATTAGTTTAAATTTGGATGTTACATATCCGTTGTTCTATTTGAGAGTTCATATGTCTTGTAATCATGATCTTTTATTGGTTTGCTCATTTTCTTAAATGGTAGAGCTTGTTCTTCCCTTATTTTTATAATTTTCATGTCAGTTTAAAGAACTCAGCATGTCAGCCTGAAGTACTTTGAAGTCCTCGCTATGATCTGTTATTTTCGCTACCTTCAACATAATTAAGTTAATACATGTCATTTAGTCTTATGTGATTTTCGTGTTATGTTGCCTTGTTCATTCACAGTCCTACCAGATGTTTAAACTTAAAATCTAAAAAGAGATTTGAGTTTCGTACACTGAGTAGTGTTGGGTTTTGTCTTGGTGTGATACTTGTATGAATCTGTTTCAAGTTCCTTATTGGTTTGATCAAAGTTGTATTTAGTAGGAGGTTACTTGATAGCAAAATGGATTAAGAAGGCTTTGTTTAGTTGTTGCTTTATTTTAAGTTGTGGCTGCATATTGTCATTGTTGTTTTGCTTACACTAATTGCGAACTGCTTATGAAATGGTATGTCGTTCAAGCATTTTCGATTACAAAATGTGTTCCCCTGTTCATTTACTGATCGTAGGTGCAAAGATATCAACTCTTTTTTTCTTTAACAATTTGTTGTTAATCTAATCTCAAAATGACATAAATCTTCCAAGTGCTATCCTTGAATACTCTTAGATATTCTTTGTTGTAATTCTAGTTCAATAACTCTTGATAGGATTCTGTTTAAATTTAACCTGCTTTTGGTAGATTTCTTAATTGTTGTGTTTAAATATGGTCATATGTGTCATGTTTTAAATTAAATTCGTGTTGACATTAGCTTGTCCCTTAAGCAATGAGTTTATACTTACTTGATGTAGTTCTTTCTTTAGTTCATATTTGCGGGATGTCAAGTCTAAACTAAAATCTGGAAAATCTCTATCTTTATTTTATTTCGCTGATAGATAATTATAGTCAATTGTTCAGTATAATTTCAGTAGTTATAAGTATGTCACTTAGATTTTTGGAGCATACATGACATTGTTTGCATAGCCAGACTTCTCATACGTTGATTGATTGTTTTCAGCTTTGATTTAATATCATCTTAATAATGTTGTTAATCTTTCTCTCTTTTTCTTGGTGCATGAATACTTTCCCCTCGAGTCCTTGGACTCCTTGCTCCCTTGCATTCTCCTCTCTTTGAGTCAACTTCCATCAAGTCGTGACAGGGAAGCTAATATCATCGGGTTAAATTTCAAATCATTGGCAGCAGTTCAATAGCAGAGTTACAACACTCCCAAAGGCTGAAAGTTGGAAAATTTGGACATAAAAGAATCGAATTCGACTGAAGTAGCAGAAGCCCCAAAGGGCTGAAAATTTGAAGTTTTTGGACTTTCAAAAATAAAAGTCCAATGTTCAGCAGATTACGGCAATCTCGACAGCATTGGGAAACTGGAAGTCCGGATTATTCACTCTTGTTTGCCCATTTTCTTTGTTATTATTTAGATGACCCCTAATGTTTTTGTTTTTCATAACTTGGATGAATCCTTTGGAGGTTTTTAATGGCTTTCTATTTCGTCATCTTTAAGTATTCAATTTTTAAACTCTTTTAAGTTAGGCAATCTAAGC

>LNC_001225

CTTAAATCCTATAAACCCTAAAAACCATTTAAAACCCCTACAAAAGCCTAATCTCTAAATTGTAAAATCTAGAAATCTATAAACCCTAAATCTAAAAAACACAACATACCCTAAGCCCATAACCCTAAACCCTGAAAAACCCTAACAAACACTTAACCCTTAAAACCCCTAAGGAACCCAAAACCCCTATAAAGCTCAACCCTAAAACACAAAAAAAAAATAAACCCAAAACTCTAAACCTATAAAAACCTAAAAACCATAACCTAAAAAGATTGCAACACTAAAAATCTAGAAAAAACCCATAACCTAAAATCTTGATCCTAAAAAAACTCTAAGCCTAAACCCTAAGAACTTACATAAAAACCTTAACTTAAAAACCCAAAGCCCTAAATCATAAAATCCCATATAAAACTATTAACCTAAATCCCTAAACATAAAGAAAACCAAATCGCTAAACCCCAAACC

>LNC_001226

TGCCCGATGGGCAAGAAGCTAGGGCAGTTAAGGAGGGTTATATTGGGTGATTGTATCCGAACTTGAAAGACTTTCAGCGGAATCAGAATCAGATTGGATTGAAGGTAAGGACGGCGAAGCTGGAATTCTATGGTTTCGATTCTTCCTAAGTGATTAGGTCACTCCTCTTCTCTCCGATTGGGGAAGTCTCCAATTCCCTTACTTCGATTTCCAATGACTCAGTATAAAGCTCATTTGACTGTCGCGTAGGTAAAGTGTTTGCTGGTCCGCCGGGTGAGACCTCTTGTACTCCTTCTTTCGCTGATTAAGAGCCGGATGAGAATCCTCTCAAATCCTCGAATGAGAACTTCCCTTAAGGAA

>LNC_001227

CTAGTCTCCCCCCCTTTCCCCCTGTCCGTGTGTGGGCCACCCTTCTGAAAGGCGAAAAAGCGGGAGAACCCACTTAGACCCAATAGCGGGCAGCATGAAGCACCCTAAGATAGGGCACGCTCTGCTGTGTGCCATCCCGCTCGACCTGGCCCAAATACCTATTTAAGGTATTCTCAGAGAGAGCCGCCCCACCCCTAGTAGGGTGGTTAAATAGGAGGTTGCGGATTTCTGACCTCCGAGCCAGAACTATCTGGGGGGAAACCCATCCCCCACCAATGCGGCCTCGACCCTCTTTTCCAAGAGGAATTGCTTCTCCAGGATACCCAAAAAGAGTGGGAGGTACCTGGGCTCGTTCCTCCCGATAAAATAGAAGGACAGCCGGTGTTGAAGCTCCGCTCTCCTTTGGATA

>LNC_001228

TCTAAACGCAAAATTCGATCATTCAAGTCGGATGGCATTTATCTACGCTAGGATCCGACCTACATGAACTTATTTCTTCTCTTATGATATTTCTAGAAAGCGAAAAAAAGAAATAATGGGGAATAGAATACAAGTGTTGAGTACCAGCTTCTACTCTTGTGAACGGTGGGCCTGACCTCTTCCTTTCTATTTTAGAAAGTGAAAGTCCTTGCCGGTGTGACAGTATGAGTAGACGGTGCTCTTCTCTTTCCGGGTTCATAATCGGTGCTATTGAATCTGCAGTTAAGGGAATATCGCCAGTTCTTTGAAAAAGAGTTCCGCTTAGGAATCCCTTTGAGGGAGGTTTAGCCTAGCCTTCAGGTCCTATAAAAAAATGGAGCTTACCGGTTTATGGAATTGAGAAATAGATGTCCTACCAAGGCTGTATGGAATAGAATGAAGGGTTAGGGTTCTTTCGGGAAGAAGGACAAACCGGGCCCCTAGTGGTCCTTATCTTATCACGCCACACATGGAGACATCTCAAATAGGTAAATTGGTTTCGGA

>LNC_001229

TTTGGTATTGACTAAAATGTCATTCGGCTCCTCATCTATTGAACTCTCGGCTTACGCAATGATCGGATCGGAAGAACTCTTTTCAACCGCCCATGATTGAGACAAGCGTAAATGAGAAGAAATTCTTTTTCATAGTCTGTTAATTTTCTTCTTGTTGCATCTCGTCGATACGATAGGTTGATTTACCTTACTAGTCATATGCCCTAAAGGTTTTCTGTCTGGATGGTACTTTTCAACAGATAAGCGAATCGCGCTCAAGTGGTGGTTGACGAAATATCTAATCCTCGTTTTCCCTGTCTGGTGTGGTCTTCCGCTATCCCTTTCCGCTTCACGCAAATGCACTCTCCAAAGCAAGCGGGGTTCGTCGGTAGTAGCCTCCTAAATTTGTCGAATAGCTTCTTTCTGGCAAAGCTTACCAATCCTACACTCCTTTCTTCTATGTCTGGTGGAGCTATTCCCAGACAGCCTGAGAGCTGAGTGAGGACCTTATTTGACATGCTTTTTTCCTTCCAAAGCGAAATAATCACTCCTGTAGATTATTGCGCTTGATCTACATCCTACCTA

>LNC_001230

AAGTTTACATACCTAGGTCCTAAGTTTCTCATTACTCGGTGAAGTCTGATTTGAGCCCTGGTCAAACTGATCTCTCCGGCAGGCGGAGGATGGTCTCATATTAGCAATGTTTTGATTGCGCCTATCTGTCGTTGCCAATGAACCGATCACCAAGAGAAAGTATCTCTCTTAATCTTAAGGTGAAGCGCCTTTTAGCCAGCTGAAGGAAGGTCGCGTTAGCGCCCCTGCAATCAGAAAGCCTTTTGATAACCTTACTACTAATAGAAATGGGAAAGATGCGCTCAATCCCTTGCTTCTTGCTCCAACTTAGGAGTAGGGGCTGACGAGCTTTTTCCGCAG

>LNC_001231

ATGGAACATCCACGTTAGCTTCCGTTGCAGAAGATCGCATTTTTGTTGGAACTCTCTCCTTGTCGAAAAGTATTTAGAACGCTTCTCAGCTATCACAAAGAGGGAAATGTTGATCAATCGATCTGGAAAGACAATCCTGGTGAGAGCTTTTCATCATACCCTGCCGGGCTTGCTTTGCTCGGATGTTATTGCCTTTGCCCCCCACGCCAGCTTGC

>LNC_001232

GAATGACATTAAAATTGAACAACTGTGTAGGAGTACTAATAAGTTTTGTAAGGGTATAGTTTATTTAATTGAGTGAAATTAGGCCAGTAGTCTTTCTGAATGTGATAGCTGAGAACTGTGATTCTTCGCTCGATCAATTCTTGCCATCACGCTCTGGCTTTAGCATCTGCCAAGCACATCTAAGCAAAAAATAAAAAAAAGTGTCTGGATTTCAATTGGATACCCTTTTTCTCTGTTCCAGGTACAAGTCAAGTTGAATTTACAAAAAAAAATTCAGTGTTAGAAGTATTCAAATCCATAAATTCACTACGAAACAAGGCCGGAACCAATTTGAGACTTTGGAAAACTTCAAATAGTAAACTGAAACTATCAATCATATTAATATTACATCTATCTTCACATAGAAGTGTTGGAAAAGAATGCTATCTGAG

>LNC_001233

TCCGTTCATGGGGATATAGCATCGAGATAGATTCCATAAAGGGAAGGGTGAACTGCCTAGTAAAGAGAAAGGGCGGAAGGGGCCCTCGCTAGGAGTTAGAATTCAAAAAATAAAGAAGACGGTCACTCTACTATTATATACTACATTTAGACTTCTTCTATCAGTCCATTTAGGCATGATGATATGGTCTCTTTAGCTGCAAGGATGGTAGCCCGGGGGAAGCTAGTCTATCAGAAGGTAGTCTTGAGCAAGTGAGATTGGTTGAAGTTCAATATGCATCATCAACTGGAGAAGAAAGATAGGGTCGAATATGATTAAGACGAATATGTCGAGTTAGAGCACGGATTTCCATTCTTCTCTTACAGTTCCTCCACCGAACTCTAGTACAGAAGGAATGGAATAGCCGTGACGCGGTCTATCACCAAGCCCGCCTCTCTCTCCTTTCTTGCGGGCCATTACTCTACA

>LNC_001234

CAGATATACTCTCAACTTTCCCTGCTATCCTATCAACAGGTCAGTCAATATCTGTAGTGGAGGAAGAAGAGTAGTACCCTTTTTTCAGTTAGTCTTAATAGTTTAATAATTCAACTAGTGGTCTTCTTGCCTAAAAAAAGGAGTCAGCCCAACATGGGCAATGATAGACAGACTAAAGATTTACGCAGTCCTTGTGTGCTTGCTTTGTGCATCGGCACAGCAGAATTCGAAGCTGCTCGGCGTACTAGCCCTATAACGGAGTCAGACCCAAC

>LNC_001235

CTGAGTCGGGATGAGGGAATTGTTTCTATGCTTTTCTCAGATAATACGGGTTAAGAGTGAAGCTATCGCGCCTAAGGCAAAAGGCTCTGTTCGGCTCGATAGGTGGCAGGCTCAGAACAAGAGGATAAAGACAAGGGGGAAGTTTAGTTATTAAGGTAATGATAAGATAAAGAGAGGAAATATATATATATAACTATTATTTAGTTAGACTAGTGACCAATAGCTAGAGAAAGTGAAAGAAGTAGAATCAAGCCATTCATCTGAAAAAAGCTTTTTGCTTTATTTAAAACTTCTCGAACTAGAAAGAGAAGCCCTTTTCAGCAGGCTTCCAGGTTCGCAACTATACTTGTCTCTTACTTTCCCAGCGGAAAGCCAGATAGAACTTTGACTAGAGCAAAAACCCGGAGCTTGACTTTAACTGGAAGAGCAGAGCATAAAGCGCCTACTTGACGAGCGGGAG

>LNC_001236

GCCAAGATAGGGAATTGCTTCCGCAGAAAGGCAAGAAAATGCGCTCGAGCAGAAGGACTCACGCTCGGTGATTCAGGAAGACTGAAGAGCAGTCCCAGTTGGCAGCGATCCCAATCCTGCCTGGCGCGAGCCATAAAAGTAGAAATCCGCGAGGCACTCCCTATCTCTGCCCTTTCCATGGGGCACAGGCCTTAGGAACGTAGGAACTTGCTGGCTATTGAAACTATCCTAACTTTTTTAACGGGAACTGAGTTGACTTGCTAGGGGTAGGGCTGAGGATCCAGCATCCCAACCTGAATTAGAGGGGAGCTCCAATTCGAAGCGTGGGCTACTAATACTAAGGAAGGCCCCTTTCCATATCGGGGAGAGTCTGAAATAGTGTTTTGGAATTCATCGATATCGGAGTAAAAATTTTTCAGGTACTCTAACGGATAGAGCTTTTTCTGCGGCATATTGAGAGCCATATAGTCTCCCGCCAATCAAAAATCCCCTAACATTATCAGGTAGGCCCAGGATCTCTAACTGCGCGAATCGAATCAAAGCGTTCTCCAATAAGGGCATCATGAGATGTAGCGGTATCTCTCCTCAGTCTATGGTCTCGATTGTGGGTGGGAATATGAATGTTACTATTACTCTTTTTCAGCTTGAGAACCAATGAAAGCTGTGAAAAGAAAGAATGGCCTAGAGGGTTTATATGGATAGCAGTGCACTGAGGACGATGTGGAATAAATGAGGATATTTAATGTTTTATGTTTTCTAAAGTGTCAACATTCA

>LNC_001237

TTTCTCTTCTTTAAAACGTCCTTAAATTAAAGGCTTATAGAAAGCGCTTTAACGAATGACGTAAGATGATGCCCGAAATAAGTGAACTAACCTGGCATTCAATCTCTTTTGTCAGAGTTTTCGGGGTATTGCTTACAAAGACGCTTACACAAACTAGCCGAAAGCCCTATCTAAGAAAGTGGCTACTAGCTCAAGTGCTGGTGAAAACCTCGCTTTTTAACGATAAGAGGGTTTTCCAACTAAGCCGAAGATATAAGCCGACGGTCCAGGTACAAGAGAAGACTTTATAAGCCGCTTACTCGGAACTTGTGCTTTCCTATCCATACCGCTTTCCTTTATAGCAGCAAAGGAGAATGCGATATTGATTCAGTCGGAGGTGTACTACTTTTCTGATCAAATCAACCCACTACTCAACCGCCCACGTGTGTGTCTGAGTCAGTGCTGACAGTCGCCTTCATTGAGTTAGGTTAGTGTGCTTTGCATGCTTTTCTCGAAGCTCATATTGAAGCTCGTCGGTCAAGAAGTTGCATTCCGCTAATCTCACTTTGTTCGAGTGGCTTGGTCCTCTGACAAGGACATCTCTTTCTCTGCTTTAGTCCTATAGTCCATTAGGTCATGACATTTGTTTGATTAGACGATTATCAGGTGATCCAGAAGACCTAATCAATGAGATTTGGGGCTGTAATGTTCTTTATTACTTCTTGATTTCATGTTCTAACTCCCAGTAATAGAATGGGAGACCTACATTTGACACTTGCTATCTTGCTTGATGACTTTCTTTCATATTGGTTTCAGGTTCATACTTTGCTCCCTAATCCGGAAAGATCGTATGTCAATGTTCCCAGGGTAGGAACTTCCACTTTTCTTAGTAAGAGTATGGGCTTGGGCTTCGGTTCGGGACTCTG

>LNC_001238

CAGGTTTAAACAATCCACTTTCTGATTTTGGTTTTGTTTGAGTTTTGCTGCAAATGTCTGGATCACAATAAAAGGTGCAGGTTCAGGAATTTGCTCTTTCTTGGAGTTGTTCTGATGGTTCTGAAAGCTTTGATGGTTGTTGCCCGTATGGGCAGTGGTATTTTGACCTCCAGGTTTGCTCCTATTGGGAAGTTGGTTTTGCTTCTCAAAATTGCTGGAAATTTTGGGAAAATTGGAGTTGACAACAAGAGTGTTTTGCTTAACAGTGGTATTATTGCCCTGTTTTGTGTCCTGTTTAACATGATCATGATTCTCCTTTGGATTAAGGGTTGGTTAATTTTGGGATTTTAGGGCTAGTTTGAATTTGTGATTTTAAGACTAGTTTGAATTTTGAATTTAGGACTAGTTTGAATTATTATTGAGATTTTAGGACTTTTAATAGTTTGAATTTGAGATTTTAGCACTATAGTTTGATTTTTGAATTCTAGGAGTACTTTCAATTTGTGATTTTAAGGCTAGTTTGAATTTGGAATTTAGGACTAATAGATTGAATTATATTTGATATTTTAGGACTAATTAATAGTTTGAAGTTGGGACACTAGCTAGCTAGTTTGAATTTAGAATTTTAGGGATTTGAATTA

>LNC_001239

CGCCTTTGGTATACACAGGTCTTTGGGGCGAAAATGGGCCCAAAGTCCATCAGATTTAGCCCAAATTTGGGCATCCTCTTCGTTATTGCCATTATTTACTTATTTGTATTAATTCTCTAACCCATTTTATGTGATTTGGTTATTTAACTTAATTAATCCCCTAAAGATGAACCACTTTTGATTATGATAACTTATTTCGTCTCATTTAATTAGTTTTCTTT

>LNC_001240

GAACTTATAGGATACACATCTCGAAACTGTTGGTCTGTGATGCTGGCACCATTGAGGTCGAGTGTTTGTAAAGTATTGTAACCATCCAAAATAGCAATTTCGCAAGGATGATTAGTACGTTCTCCATATTTAAAGCATTTAAGATTTGGGGCCTGGACCACAGCAATCCAGATGTACTTGCAATTTGTTTATTCCTCGGCAATCGACTAGGGATAGATTCCTGATGGACGGGGATCTATCCATAAGTCTTTGCAATTGAGCATCTGAAATATAGACAGCATGATGCAAATGAAGAGTCTCAAGACAATCAAAACTTATGTTGTCAGCTTTTTAGCATCATAATAACAACATCAGGTAACTTAAGAAGGGAGGGCAAATGTCCAGTACTGTTTAACCGCCAATTCAATCCAAGTATCCAGGTGGGAATTCACTTTTGGATCACAATATGCAAGGATGAGCTCTTGAAGATGTAAGTTTTGTTCGATTATAATTGGTATTATAGTTTTGTGATTGATGGAATATGAGATTAGGTCTAGAAGACCAACAATAGTACCATCTCGCCACCTCTTTCAGGTCGAGGCTAGTGCGGCATAAGATTTGATGAATGATATGCACAGATGTAGATTTCAACCAGAGATACCACATGGAGGCGGCCATTCCCTCCTCAGGTGTCCCAGTACAACTGATAACATCTTTTAAGTTACGATGCC

>LNC_001241

TTCCAAATGACCCCACCCGATAACAACAACAACAATACGTACACAATACCCAAAACAGTACCCTGCGTCTTCCCCATTTCTAGATGCGACAAAATTTGAAACCCAGCAATTCCAATGCCATGTCCAGCAAAGGATCGTCCTTGTGACCTTAAGATTGAGCCGCGTGGCACAGCAAGGACATTGGAGTCGATCTGAGGCGTCAGTTCGCCCTTTCCCCTTTCGGAATTGGGCGAAATTTTCAGAGAAAGGTGTTTAGCCGGAAGGGTGAAAAGAATTTATGGCGTTTGAAATTTGAATGGATCCTATTCCTATCCGTTTCACCTTTTCCCCCCCAAGAAAAAATCCGAACCCTAGTTTCCTTCTATATATTCCTCCTTATTCATGTTAAAAAGGGGGGGGGGGAAGTTGGAATTTTTTAGGGAAAATACAGAGAGCAACTTAAGTAGAAGTAGTTGGATAATACTCCGGAAGAGAGAAAATCAACTCGTTTAGGATCCTTGTGTTATTTTCTTCTCTGTAACAAGTTTCCTTTGAATTCGAGGGAACTATTTGGGCATTCTTGCCCAGATTCTTGGCTACTGTTGGTGTTCCTAAGTTGGTGGTAAATCGCGGTCCTTCTAGGCTCGTCCCGTCTCAGTTCGTTGCCCCCGAAAAGGTAACTTCTTTTCGATCCCAGTTCCTTTAATTGTTATTTTGTTCTTAGGAACCGAAATGAGGCGTTCTCAGACGCCCTTGTTGATGCTGAGATTCTTATATGATGTGTTTGAGTCTAAGTCGTTTTTTCTTTCTTTTCGAAACGGGCATCATGTATCTTCATTATTGAAAGGATATGAGGCTTGTTGAAATTTGTTTTTCTCTAGTTGTGTTCTTCCCAAATTCGTGATCATCGTGTTGGTTTTAAATTATTGGTAATTGTTGTTCAATCTTCATGATGCCGTTATGTCCCTGCTTGTTAAAATAGGATTCCATTTTTTGTTATGTGTTTTGGAAGGGTCAAAGG

>LNC_001242

CAAATTGACTATTCATCTCGGAAACCATTAGCCCTGATATAAATTTGGACGCACTACTATTTTCAGGAAAGGGGCTTTGAAGAGAGAATGCAGAAATCGTGAGGGAAGCAAAATAATCAGCTAATATTCAGCTATGGCTTCCGATTCTCGTAAAGTGATTGTTCATTTAAGAGCAACTGGCGATGCTCCGATTCTTAAACAAGCCAAATTCAAGGTTTGTTTTCTTCCATCATATTCTCCTAATTCAGATTATCAGATAAATTGCTCCTGTTAAAATTCTAGGCTCTGTTAAGATCCAAAACCCTAGCTATATATTGCTGCATTGCCATTGTACTTTTTCCTTTGACTATGAGTTCTTAATACAACATAACATACCCAGTGTGGTTCCACAAATCGGGGACTGGGGAGGGTAGAGTGTACACAAAC

>LNC_001243

GAGCCATGATGGGAGGAGGAGTGAGAGGATTATGTGGAACAACTTCGTGCTTGACTTTTTCTTTGTAGAGTGCTTCCAGCTGGTGGAAATATGGGCAAGTTTTGGAATCTTCTGGTCTTTTTTTGTTGCTTTCTTTTACCTTCTTGAAGTACTTGTTGATGTTCTCCCATTTTTCCTTGCATCTCTTTGCATTTCTGTTGTATCCTATCTTTCGCATTCCAGATGAAATTTCTTCCCAGAGTGGTCCTTTTGGTCCATTTTCTTGGTATTTTAGATCTAGACAGGTTCGGAGGCTGATCAATGCTTC

>LNC_001244

AGAATGAATTGGCTAGCAGAATGGCCAAAAAACCCCAAACTATTAGAGGCAAGGCTAATGACAAAAAGGGTCTAAGAAGTCATGGTACTTACTCCCTCTGTTTCATCTTCGATTTACTTAAACTAATACTCATTATTATATAATTAAAGTTTAAAGTTACAATTTATGAGTGCAGTAATTTTAATAGCATCAATTGTTGAGATTTTTACTGGTAAAACAAAATACATACTAAAATACTTTAGCAGTACAAAAAGAAATATCTATTCATAATATCTTGAGATTGTTTCCTATTAAGCTATTCAAAGAAGGAAACAGTGTGATTGACTTGTCTTTTTGTTTACCTATTTTGATTAAAATAAAGATATTCTCCTTCACTCCACATTATCATTTTAC

>LNC_001245

CACCATAGAGGGATAACTTTCAATACCTCATCCTTAGAATTGAAACTGGCCACCCAAGGTTTAACAACATACTTAGTATTGATCATGTGAGGGCGAAAACTAAGTATTACATCTCTATCCGCTATTGATTGGAATTCACAACATAATAACCCTCATTGTGATAATTGACTTTAGCTTTCTGAGTGAATTTACCTTGTTCTCAAGGAATCTAGCTACAATTGCTATAGTTGGGGAATCCCAAATAACATATAGTATCATAGCACTTCTCCAAATTATAGATTCACTAGCTACCTACTCTTTGTCCTGTTGTCCTATTTTGACTCTATCCTTAAGCACAGTGGTATGAATTGAATCTATGTTGCTCGGACTCTTCAAAAACGTCAATGAGTGCATGTCGGATTCTCCAAAAGTAGTGTTTTTTTGAAGAATCTGACATAGATGCGGCATCGAAAGTGAAGAGTCCGTGCAACTTAGAATTGAATACAATTTTAGCATAAAAAATATGCAACATGCAAAATCCATATATATACTAATACTGAAAAAATCATTTTATGCAATATTTGCTAATAAGATTTCGTCAAATATTTTAAAATTATTTCAAGGGTATCATCCATTATAAATATGTGAGAAATATAACAATGACTCAAACAAGAATAAGTTGGAACGCTATTAAATATATTTGTACATTGTATGCAGAAGTAAAATGGTTGTTGCAATTTTTTGAACAATTACATTTAAAGTGCTTAAACAAACAAGAAAATAGTTTGTTATGATTATTTTTTTAATCATGGACCGCACATCTTACGGGCTCTAATACTAACAGTGTTGATAAAAGAAGACGATACATTCAATTAGGTTTAAATTATGACCTTTTCGCCATTAAGCTTTTTAACTCACCAAAAAATAGTGTAACATAAATTTATTTGTAAGATTAGAAGAAGACGACAAAAAATCCAACTAGGTTTAAATTGGACTTTTTTACCTGTTAAACTCGATTCACAAAGGCTATAATATGGCAGAGACTTCATTATAGTTATCGTGTGAATAAGGGAAGACGAAAGATTCAACTACAGGTTTACATTATGACCTTTTTTTGCCGCAAAATAGTGTAACATAAATTTACTTGTAAGATTAGTAGAAGAAGACAAAAAATATAACTAGGTTTAAATTGGACTTTTAACCTGTTAAACTCGATTCACAAAGGCTATAATATGGCAGAGACTTCGTTATAGTTATCGTGTGAATAAGGGAAGACGAAAGATTCAACTATAGGTTTACATTATGACCTTTTTTTGCCGCTAAATTTAACTCGCATAAATATAAGTATAAGATAATTAAATTATATATGTGACCG

>LNC_001246

TATTCTTCAATATTTCTTACCAAAATCATACAAAAACAATCCTTCATTTCATTTCCTTTTTTGAGAAATTTATTTAGAAAAATTGAAACAATGGGTTCAAATAATGTTGAATTAAATAAACCACATGCAGTTTGTATACCATATCCAGCCCAAGGCCATATTAATCCCATGTTAAAACTAGCCAAAATTCTCCATCACAAAGGCTTTCACATAACTTTTGTCAATACTGAATTTAACCATAGACGTCTTCTTAAGTCTCGTGGGCCCCATTCCCTCGACGGCCTATCGTCGTTCCGATTCGAGACTATTCCTGATGGACTCCCAACTTGTGATGCTGATGCAACACAAGACATACCTTCTCTATGTAAATCCACAACTGAAACTTGTTTAGCTCCTTTTAGAGATCTGCTTGCAAAGCTTAATGAGACTAGTAACACGTCGAACGTGCCACCTGTCACGTGCATCGTCTCCGATGGTATCATGAGCTTTACCTTAGCTGCTGCACAAGAAATTGGTGTCCCTGAAGTTCTGTTTTGGACAACTAGTGCTTGTGGTTTCTTAGGTTACATGCATTATACCACTCTTATTGAAAAAGGTTTTACTCCACTTAAAGATCCAAGTTACTTGACAAATGGATACCTAGAGACAACTTTGGATTGCATACCAGGCAT

>LNC_001247

GGAAATTGTATAGTCACATATTAAAGCTTTGGGAAAATATTGAAGGGCAACATAAAACTATCCTTAATAAGACCTTGAAGTGGAATTACTTGTTTTTTAATAACCGTGGTGTCCGAAAGATGCAAATAGCAGAGATGAGAATGTTGAGATGAATGTGGGCATACTAGGAGAAATAAAATCAATAAGGAAGATGTACAGGACAAGGTGGGTGTGGCCCTAGTGGTGGACAAGATGAGGAAGGTGATGTTGAGATAGTTCAAGACGTGTGAAGAGAAGATGCACGGATGTCCAGTGAGGAAGTGCAAAGGGTTGGTTGTAGTGGATTTTAGGAGAGGTAGAACAAAAAAGAATTAGGGAGATGTTAGTAGACAGGACATGACAACATACCAAGGATATGACCCTAGATAGAACAATATGTAGGTCGAGGATTTAAG

>LNC_001248

TATATATCACTATTTTATTTCCCTCTCAATATTTCTTCTCCAAAAAAATCAAATAGACTATAGTAGTTGTTAGATCTTTTGCTTTAATTATCATCAAACTTTCGATCTGCGTATATTCTGCTCTTTCCAATAACAAACATCAATAATCTTTTTCTTTTCTTTACTCTATAAAGGAGAAAAAGTCATATATATGGATAATATTGACTTGCTCAAATCCTCACCTTGTGAAGATCAAATGGAAATGTTCATGATGCAAATGGAAAATGAAATGCCAATGTTGGACTATAGTCCACGACGAAGCAATAACAACAACAACAATAATAATATTAATCGTAATAATTTCTTTGAAATTATCGATCATCATAATTCACCATCCACATTCTTGAACAATGTGCCATCATCTACCATTCCATTCAATGACTCTAGATCTCCTCAAATAATTCATCAAGAATCTTCAATAACCCTTCCTTTCTTGGAAAATTCGAGTCGTAGTAATGGGAAGTGGAGTACTTCTATTAAAGGTGACTATAAAGAACAAGTATCCATTGCCTCGCATTTGAGTACATTTCCTTCACACACATTGAAGCGAAATTCTATGGTGGCTATGAGAGAGATGATTTTTAGAATAGCTGCAATGCAACCAATTCAAATCGATCCATCATCCG

>LNC_001249

CTGAAAGTTGTTTCGGAAGTTGATCCGCCGATGGAAAAAGGGTTAAAAAACACTGTATTGGTAGTTTTAGATGGTGCATGCCAAATCTGCTTGAGTGATCTACCTATTAGTAATTCCTGAAAGCAAAATGAATGAAACTTTTGGCTTATTGATTTTGGATGACATACTACCTTGAAATTCTCTGAGAAGGTGATGAATTCCTAGAGTGCATGGAACTGTATGTGTCTACCTTGACAATGCCTTGGATCGTTTTTGCAGATTGTCACCTTTTTTTTTGGGTTTTAAATTGTAGTAGCACCACTTCTAAAGAGCCCGAGGGTGGATGTTGCTTTTTCTCTTGAGATCTTTGGCAGCTCAGGCTATTTATCTTTTTAACAATCGTCTCAGTCTATTTATGCATTCCTTGGTAGTAGCTACTATATACATTTTCTCTCTGCA

>LNC_001250

TTACGGTTTTGCGGAACCGCAGTCGGACTTTTTTATACTCAACCCTTTTCCGGAAGGATCGGCGATTTATTGGTACGTCATGCGTCCAATGGTTGTTGTGAATTCCAGCCTAAGTTGTCCACTTGGGTTTCCGGTCTTTCAAAAGCCACTCTTAGTCAACTAGCGTAGAGCAAAACCAAATCCCGATTTCTAGCGCATTTTGAACTAAGTCGACCTAACACCCAAGGCGTGTTGGGCCCATTAGAAGTCTACCTCGTGTCTATTTGGCCAATTGGTCAATAAAAGACAATCATATTTATGTGTTAAATTTGTTGGATGTTTACGTCATTTCGCAAAACAATGTCCCTTCGAGTAAGGAGTTTAAGAGTTTGTTTGAGTCAAATTAGGATAAAATTACAATTGCATCTGAAGACGTTTACATTTAGAGATGGATGCTTAGTATATGATTGTACCCCTGTGTGAGACCGATTATTTGTATCTTTTTTTTCCTATTATGTATACCCATTCAATTTATTTATGATGTTTGTATAGATTTTAATTTTTTGGGGATAATAGAATTTTGTCAAATGGTGTTATACATTCTTCAGATCGGTAGGCCTATCTTTGATAGAAAAGGGTCACATATTTGTATAGGATGCGCATTAATTGTTTATATGCTATGTGATATAATTGAGTTTGTTTCAATCAAAATAGTGCTTATGTGTTTAATTAATTTTATATTTATTTATTTATTTGTGATATTTTGCATTATTCATTATGAATATAAATTAACACTTTTATCTTTTGAGTTTATCTTATTTTTAAGCGAGAAATTGATCTAAAGAGAATTTAGAAGTCATTGTTCGTCCAGAATTGCAAGGGACAAGAGAATTCGCGAAGTTCAAGTGTTTATATCTCACAAGACATTAATATTCAGTCAAAAAGATAAATCAGGGGAGTCTTTGACTATAAGGTCACACTCATTTTTTATTTTATTTTTGTTATTTTTCATGTTGGCCCTGCTTTCACATATTTCTCATATTTTGCAGGGAAACCGTCCCCGACAACAGTCGGGGATGGTGATAATAACGTATAGTTCTTAGGAATCATTGGGAGTATACACAATTTGAAGAGACATCCAAAAGAGAATACAACAAGTGGTTATTGTAGAAATTCGGAGGTCACATTTAACAAACAGTATAAAGATCAACAGTTAAGAGGACGCAAAACATATTGGTGGAAACAGTCAGAAGACACTCTCTGACTAAAGATTTCAACGGCCCTATATTTACAAGTTATCACTCTAAGTTGGTTTAAATTTTTGAATAATTTTATACCCTATCT

>LNC_001251

TGTTTACATTTTATATCAAATTTTATCGATATAATTTCACTGAGTATATTGTTATTGTACTATTAAATTCATATTTTCTTTCTTTAATTCAGTAATTTTTTGTTCATTTAATCCAATTTACACATATTTTCCCTTGAAGGTAGAAAAGAAGTTCACTAATGTGTTTTTGTTTGGAGCAATGACCACATAATTGTTGTTATGCGATTTATAGGTCATAAATTTGAGTTATTGATACTTGCATATATAAATCACACCACCTTAAGGTACATTAATCCTTAAACCTTCATAAACACG

>LNC_001252

CACGATTCTTTCGCGAAGCGCTCTTTCCACTCCTGCCCCCGCAAGCAAAGAGGTTTCAAGATAGCAGGCGACCAAAAAGCTCCGAGCAAATCTTCTAGAGAGCGTACCCTTAGTTTCGACTCTTTCTCTATTCTAAAACGAAAAATAGAAAAAAGAAAAAGAAGCCAATTTTTTTTCTATGGACCCCTTCCACCAGATTACTTTACTGACTGTAATTCATTCAAAAAGAGTAAAGGAACTGGTTTCACAAAAGGGCTCTCAAAAGCGATCTGATACCTTCTTCCCTTGGTCTGGGAGGTAGGTTATGTAGGCGACAACTGTTCATAGGTTGCTCGATCAGGCCAAATAACCGATTAGTAGTATGCCTGAGATTGCCATAAGACCTTCTCCCTCTTGGAAACTGGCTAATCAAAAGGATTTTCCTTTTTTGAAATTTTCCGCTTTCTTGCCTTATTATGGGAGTACAATGTTTATGGATAGTATTAAGCATTGGTTCAAACTTTAACCTCACATCAGTGCCTAGGGCCTACTGAAGCGCTGCAGATCCTAGCAGAGTATCATGAATGAAGGCCTAAGTGATGCTCATCAGTGTCGGGACAGCGCCAAAAAAGGAATAGATAGGTGGTTCAGTATAGTTTTAAGTAAAAAA

>LNC_001253

CTTGGATGCCTTGCAAATGAATGGCACCACCTATTTATACTAACTCCTAGGGACTAAAGTACAATTTTAAATTACTTCCTAAGTCCCTAAACTTATTTACACTTTGGTCCTCTTTCTAGAGAATTCTACACTTTCTAAGATTTTCCCAAGACTTCTTAGAAAATATCTAGTCCCTCTCTAGAATTCTCTACATACTCTAGGACCTTCCAAAACCTTCTTGAAATATCCAAAGGGTTCTAGAGTCTTCCGGAAACCTCTCCACAACTCTAGACCCTTCCGCCCCTATC

>LNC_001254

GTGATTTTGGAGCGCGTGTGATTGGCCTACAGGTAGGCTACGGTTTTCCCTCTCTTAGATTGAGCTCGAGAATGTGAATGCATGTTGATTAGTTGGGATTTGGGTTGGGTAGTTATTGAATCCTACTTAGGTGTTAAGAAATCATGTTTTCGGCCTATTTCGGGAATTATCGGGTAACTGTGAGCATGCTTTGTGTTATTAACGGACCCTCCTCGCTATGTGGAGTACTTGCATGCTTAATTACTGTTTATTTGAAGCATGTTGGACCTTAGTTTAGGTTTGACTAGGGCTTGCCTCAGAAATACATTATTCGAATCCAATAGGCCTTAGTTTTGCCCCGACGTCGCTCGGCCGGCTTAGATCCCTGTAGACTGGTGTAGCAGACTTGAGTCTGATATTTTGGGCCTTAGTTAGGTGGTACTCTTTCTCCGACTATAGTTATCCTTATTTTCCCCGGTGTTACGGCTTCACGAGTTACATCGGCGACACTAGTTCCACTTCGCGATTTGAATTTGATTTTCAATTTGGTTCCAAGGATTTTCATTGATTGGCT

>LNC_001255

GTTAACTAAACATGCTCATAAGCCAGTAAGGGATACAGTATAAATAGGCTTTGGATAAATGAATTCTATGTTAGAGTTTCAATTAACAAATCCGAGTTAAGTGTTTCTCTGACAAATTCACCCTTTCTCAAACTGCTGATGGAATAAATAATCCAATGGAAGCTCCCTTTTAGGGGTATGAGAGAAGAAGTTTGTATGTTATTGGTAGATCAGGACAAATAATTTATTACTGCTCAAATGGTTGATTTGCAAGAATTTATGAATATGAAGATGACATTCCTAGAACTTATAATCTTTGTTTCTTTGATATAACTAAATTATGTGTGATTTCATTTTATTTTTTTTAAACGGGTAATTTCTATTCCTCAGCATTAAGGGCTATGCTGGCCACCTCC

>LNC_001256

GATATTCAAGATTTCCTTGATGAAAGACAATATTTGATATCTTCCTGCTCTCTTTGTGAATACCCAAAAATTATAAAATATTCTAAATACCTGCCTTTTTCAAGGGAATATATTACCTTTAAATAGGCATGAACTAGGATTTAGTGTAGAGTAATATCCAAAAAAAAAAAAAAAAACTAATTGAAATTGATCTAAATTAATTTAATTATATAAATAAATTAATTTAATTTATCTAAATATTAATGCGTGCCTTTTATCGCCTTGATTTGTATAAAAATAATAATCACTCGTATGTACAAACATGGTATTTTGGCTTGCTTATGTGGTTTTTCTGGGTCAAGTTATTTTCTGAGAATATTTTTAAGTTGAGTCACTTTACACCGACAAAAAAATAGTTTTTTTTATATAGAAAAAACTCCAAGTTCGATAAATATTTGAGAAATTAATTCAGAGAAATTTAACCAATCATTATG

>LNC_001257

AAAAGAAGATGTAAATTTTAAGCTAATTCGATATCACAAGGATGTCATATGATACAATAACAAATATGGACTAAAATTATTGTGGGTAATATTGTAGCATTACTATAACTTATTGTAATAATTTTTGGTTATAAATAGCCTTCCTTTCTATTTTATAATAAGACACATTATTTTTCTTTCAAACAACGTTCTCTCTCTCATAAGAAGTTTTGGTGAGAGTTTGCTCTCCTCTTTGTAGTCTTTAAAGTTGATTTGATAATAACATCAAATTTTAATTTTTACACTATTTTTTTCTTTACTAATTTATCTTTTTATTGCCTTTATTTCATATGGTTGGTACTAGCACCTATCTTTAATTAATTATTTGCTTTATTTAACGCATGATCTGCTCTCCCGCCTAATTTATTTATTACATATACATTTATCTTCTTGCATTATTCATTTAGTTATCATACTTTTATTTTATATTCTTCTTTCCCATATTTCACTTATATTACTTTATATATATAAAAATTTCATAAATTGGGCTTTTGAGATTTTTTATTTTGGACTTGAGTGGTGAACTCAAATTTATTTTAGAAGACCCACCAAAAATTGGGCTTTGAATTTTTCAGATTGGTTTGTTTATCACAGTCCACAACGTTGTTCACAACAATCGAAATGTGATTATTCTCCTGTTTTCTTCAATGCCTTCTCCGTCCCAAAATATTATTCTCAAAACAATTATGTTTATCAAAAAATTAATAAATATAAT

>LNC_001258

TTTCATTGTTACCTAATGATGAAGCAATATGATACAATGAAATTTAAATAACAATCAAAACAAACATTGCATTTAAACTAATAACACAATACAATAAGTAACAACCATACAAACAAGTTGTTATGATAAAAAAAAAAGTTTGCAATATATGAGAACTTCGTGACCATCTTTGCGTACTAAAACAATAATGTCCTAATATGTTCGCTTGATAATTCAAACCTACAATAACCTTCCGTGGTATAGTATATTTCTTGAGATTTTCATAAAAGATAAAGAAAGAACTACTTTTTCATTCCCCAGTATGACCTTGGTCGGCCATAGCGAACTTGGGGATTTGGCTATATATTCTAGTGTTTAGCTGAAATCTTCAACCATTGTTTAGCCAAGAAAGGAAGAATGAGACATCAGAAGGAAAAAATGGGATTTTCTGTAAAGGGTAGTTGCCCCCTCTCTCTCTGTTATGTCAATGATTTCTTTCTTCTCCATTCATTAAAGAAAACAACAGCCCTTTGATTAAAAAAAATCAAGAATTATCTACACATTTGGCAATAGAATTCCAAATATTTTTATACATATGACCCAACAAAGATAGCTGCTTTTTCGAGACTTCGGAACCTAAAAATGGGCTGTGATGAAGATTTGCTTCTTTCTGGCTAACAATCTACTTCATCGTGTTGAGAAGATGCGACCGGTATCTTGGCAGGGCAACCTGGTAAGGCATCAATACGCTAACCAGTGTATCTGCAGCCATGGCCATGTCGATTTCTCTTTGCATGTTTGTAGAACAGCAAAATCTGAGCCCTAAAGTGTACGGCAACCATTTGATAGTTGTTCTTGGGAATTGAGATACCCAATTGCGTTTTTCAGTTTCTCTTGAACACCCCATTTCAGATGCAGTGCTCTATCAAATTTTTAGCTCAAAAATGAGGTAATCGGAGTAAAAAAAGGCTTTTCAGGGTGGTTGGCCATCTCCGGTATGCCAGTGATTTTATTTAGCTCTAATCAACAGCCTTTTTAGCATATGTATGAGGTCTAGACACCTCTGCTGTGTCAGTTGAATGCTCCAACTTACAAAATGATCATCTAGACACCTCTAGGATTTATGTGTTACGTGTGTGCACGAGACACAATGGGGATGAGTTGGAGTGTTTAGTTGCCATTTGAGATCAAGTTGATGTGTCTAGATGTGCACTCTCAAAGTTGGAGTGCTTACTTGTCAGCCGAGACCAAGTTTGGGTGTGTTTATGTATTATGCCATGTATGTGTGTATTGTATGTCTGTTTCTGTCTATCTGTGACTACTTTGCTCCTGGGTGGCTGCATTTTCAGATAAAGCAGCAGCAAAAGCATAGAAAAGGGACAGTTAAGCAGGCTTGCAGGGTTTCTGGTTAAATTATCTCTTATAAATAAGAGTGTTCTATAGAAAAGTGAAGGTTCAGCCTCAATATTAAGAATGAGGCCGACAGAGAGAAAGGGAATTCTTGGAGGGTGGTTGCCCCCTCTGTTTGGTCTATGATTTCCCCATAAACAAAATCAACAACCCTCTTTTGGAAGTACTAGTACTGGTTTCTTGTTTTCTCTAAGTTCTGACCAAACATCCCTCTAATTTTAGCTTATGTTAGTGGACTATATATGCTCTTCCTTTTTCTGCTTCTGTATATACTATAGCTGATTTTTGCAAATATCCACTTTTATGTCCCTGTAAATACAAAGCTAGCTGTTTTTTGTAAGAGTACTAAACCTTATGAGGTGTTCTATTGATGTTGGTTAATCTGAAATAGGAGCAATCCGTGCAAAAACTTGAAAAATAATGGGATGTGATGATGGGTAGCTTCTTTCTGAACTATAATCTACTTTATTGCGTTGAGAAAATCCAACCCGTATCTTTGGCGGAGCGACATGGTATGGCATCCATATGCAAAGCTGTGTCTTGCAGCCATGGCATGTGTTGCTTTCTCTTTGCATATTCATATACAGCAAAATCTGAGCCCTAAATGTTTTGGCTAAAAATGAGGCAATCGGAGAAGAAAATGAGACTTTTCAGGGTGGTCCATCTCCAGTATGCTAATGATTTTTTTCTTAGCTCCAATCAATAACCCTTGTGTATCTGTGACTACTTTGCTCTTGCGTGCCTTCTTTTTGAGGCAAAGCAGCAGCAAAAGCATAGAAAAATGGAGGTAACCCAGTATGAAGAACGGAAGAGGGCCAAATACACCCCTCTACTATGAAGAATGAGGCCAACAGAAAGAGAAGGAAATTCCTGGAGGGTGGTTGCCCCCTCTGATTGGCCTATCATTTCTCCTTCAACCAAATCAACAACCCTCTTGAGATGTACTACTACTGTTTTCTTGTTTCTCCGAGGTTAGCAAACATACTCTGATCTTATCTTATGTTTGTTAAAAACTACTTGGAATTGTCCAAACGCCTACTTGTGGGGAAGTTCCCATCTTTCAGAAGTGGCTATGATGATAATGCTTATGGCAGAATTCTCACAGTTTAGCCCAGAAAGGAAGAATGAGATATCAGAGTGATGGTATTTTTTGTAAAGGGTGGTTGCCCCCTCTCTGCCATGTCCATGATTTCTTCTCCATTCATAAAAAACCAACAGCCCTTTCAGAAGAAAAAAAGAACAAAAATGTTGATTTTTTATATATATATATATATGCATGCAATGACAAAACAAGGTAGCTGCTTTTAATGGGTGATTTACAAGAATGTTGATTGATCTCAAAAAATGGGCTGTGATGATGATTTGATTCTTTCTGGGCTATATCTACTTCACTGCATTGAGAAGATCCGATCCGTATCTTGGCAGGGCGACCCGGTACGGCATCATCTGCAAACCGGTATCTGCAGCCATGGCGATGTCGCTTTCTCTTTGCATGTTTGTAGAACAGCAAAATCTGAGCCCTAAAGTGTACGGCAAAAGCATTTATATGTGTGTGTTAGAGAGTGTCTGTATTTTCAGATAAAGCAGCAGCAAAAACATTGAAAAGGGACAGTTAAGTGGACTTACAGGGTCTCTGGTTAAATTGTCTCTTATAAATCAGAGTGTTCTAGGGAAATCTTAGTAGCTCAGTAGGTTGGTTGGCTAATGGCTACTTGAACTTTCACCTTGTTGGTGAGGGTTCGATTCTCCACCTTGTAATCCCCTCCCCCTTTCCGCTTCTCCTAACCCCAGTTTTTCAAAAAAAATCAGAGTGTTCTAGGGAAATCTTAGTAGCTCAGTAGGTTGGTTGGCTAATGGCTACTTGAACTTTCACCTTGTTGGTGAGGGTTCGATTCTCCACCTTGTAATCCCCTCCCCCTTTCCGCTTCTCCTAACCCCAGTTTTTCAAAAAAAAAATCAGAGTGTTCTACAGAAAAATGGAGGTTCACCCCACTGTTTAGAATGAGGCCAACAGAGAAAAGGGAACTCTTAGAGGGTGGTTGCCCCTCTGTTTAGCCTATGATTTCCCCGTAAACAAAATCAACAACCCTTTTCATAAGTACTAGTACTGTTTCTTGTTTTCTCTAAACTTAGACCAAACATCCCTCTAACTTTAGCTTATGTTTGTGGGATTTCACTGGGTAGGTTGTTGTTTGTCAAAAGTTGCTCGAAATTGTCCAAACACCTACTTGTAGGGAAGTTTCCGTCTTTCAAATGAGGTTGTGATGATAATGCTTATGGAAGAATCCTAACCATAGTTTAACCCAGAAAGGAAGAATGAGACATCAGAGAGATAGGATTTTCTGTAAAGGGTGGTTGTCCTCTCTCTGTTATGTCCATGATTTTTCCTCTGTTCATAAAACACCAACAACCCTTTGGGAAAAAGAAAAGATCAACAATTGTGTAGGCATTTTGCCATAGATTCCATATATATGTATGTGTGTGTGTGTGTGTGTGTGTATAATATATATGTCTATGCTTTTTTCCTTTTTATGCTTGTGCTTCTGCAAGGTAGAATTGATACAACAAGATAGCTGTTTTATTAGGAGTTCAGAACCTAAATAAGGTGTTTAATTGATATTGATTGATCTAAAAATGGGCTGTGATGATGAGTTTCTTCTTTCTGGGCTAATAATCTACTGCATTGAGAAGATCTGACTCGTATCTTGGTGCGACATCATCTGCAAATCGGTGTCTTGCAGCCATGGCGTGTGTTGGTTTCTCTTTGCATGTTCATATACAGCAAACTCTGAGCCCTTAAGTGTATGGCAAGCATGTGATGGTCTTAAATGTATGGTTGTTCTTGGGAACTGAGATACCCACTTCGGTTTTTCAGTTTTTGTTGAACCCCATTTTCAGATGCAGTTCTCCATCAAATTTTATCTTAAAAATGAGGTAATCGGAGTGAAAAGGTGATTTTTCTGGGTGGTTGACCATCTCCGGAAAAGCAGCTGCAAAAGCATAGAAAAATGGAGGTTCAGCCCAGTATGAAGAATGAGGCCAACAGAGAAAAAGGGAATTCTTTGAGGGTGCCTCTGCTTGGCCTATGATTTCCCCATAAACAAAATTAACAACCCTCCTTAGAAGTACTAGTACTGTTTTTATTGTTTTCTCTAAATTCTTTGAGGGTAGTTGCCCTCTCTAATTGACCTATGATTTCTCTGACCAACCAAACCAACAATCCTCCGTTGGTATATGTTTCTATTGATGATTAGTTGATAGCAGCAGTAGTAATACTCCCTCTCTTCTAATTCACTTGTCTTGAACCTGTTCACTATTGATATGGAAAAGAATCCATCTTTCGAAGGAGGCAGTGATAATATTGCCAATTCTGGGCAAGGAGAAAATCCGACTCGTATCTTGGTGGGGCAAACCCAGATTATGTAACTGGTTGATTTTGCAGCCACGGTGTTGTCATCTTCTCTTTGCCCCATTTTACACAGATATGAGCCTCAACATCCAATGAGTTCCAGGATCCAAACCCCGAAATTCATTTGATGGAAACCTTGTTCATTTTAGCTCTAAAGGACAAGCCAATCGGAGGAAAATGGGCATTTTTACAGGGTGGTCGACCATCTCTGGTATGTCAATGATTTCTTTGCTCTAATCAACAATTCTTTTAGCTTTTGTATGTGGATGAAGTGCACAAATAGCCGCTTTTAGACCATTTCCAATGTCATTGGGTGTTGTAATTTCCTGTATTTTAGGACCCTGTTTAGAGAATAGCTGAAGTTTACAAAGTTGCAGGGACTTACAGTTTACAGAGTAATGGAAGCTTAGCCCAGTATGAAGAATGAGGCCAACAGGGATAGGGAAATTTTTTTGGAGGGTGGTTGTCCTCTCTGGCTGACCTATGATTTCTTGTTCAACCAAATCAACAACCCTCCATTGGTATATTTGTGAAACTTCCCAAAAATAAATAAATAAATCTGTTGCCATTGATGTAATGTTGCTAGCAGTAGTAGTAATGTCATACTCTCTCCATTTCAATATACTAGTCTGGAAAACTGTCCACAAATTGGTATAGAAAAGGTTCCATCTTTCTCAAAGACGCAGTGATGATATTGCTAATTCCGCGCTGAAAATCGGGGCATGGAGAAAACCCGACTCGTATCTTGGCAGGGCAAACCCGAATCACGTAACCCGTTCATTTTGCATCCATGGCGTTGTCGTCTTCTCTTTACCCCTTTTTTGCACCAATCTGAGCCTCAACTTCCAATGAGTCACTTCGAGTACGCCGGGATCGAAACCCAGAATCTCATTTGATGGAAACCCTGGTCCCTGTTCATTTTAGCTCGAAAACATGAGGCAGTCAGAGAAAAATGTGGATTTTTTCAGGGTGTTAGACCATCTCTGGTACGTCAATGATTTTTTCTTTGCTCTAATAAACAACCCTTTTAGCTTTGTTTGTGGACGAAGTGCAGAAATTGCTGTTTTAGACCAGTTCCAAGTTCATTGGGTGTAATTTCCTGGATTTACACTGTAATGGAAGCTCAGCCCAGTGTGAAGAATGAGACCAACAGTGAGAAAAAGGGAATTTCTTTGGAGGGTGGTTGCCCCCTCTGATTGGTCTATGATTTCTCCTTTAACCAAACAAACAACCCTCTGGAAAAGTAGAAACAACCAACAATATTTGTTGTCATTGCCTTCAATGGTGGTAATATGATGCTGATGTAAGTAATACTATTCAATTTTCTGATCATTCCGAGATAGCTTAGACCAAATGCTTTTGGTATGAAATACTCAGAGATCTGAAGGTCTCATTTGTTTCATTAATATTTCTTGTTAAAGTTATTCAAACGGACACTTATGGAAAAGTTTCCATATTTTCTGAAAGAGGCAGTGATGATATTGCTTATCTGGGCTGCTGTTTGGGGCATGGAGACTATTTGACCCGTATCTTGGCATGGCAAACCTGGATGGCTTGTGATCGAGTCTTCTCTTTCACATGCAACTGGTTCTTTTGCAGCCATGGTGTTGTCGCCTTCTCTTTGCCCCCATTTTTTGCTAGTCTGAGCCTCAATTTCCAACTAGTCCAACTGGACGTCCCAGGACTCAAAGAGTTCTGAACCTAAATGATGAAAAAATCTCTGCATGCACGCACACACATATCATGAGAAAATGTCCAAAATAGTCCCTTATTGAGGCAGTGATGATCTTTCAGTCTATCCTCTTGAGCTAGTTATTGAGCATTGAGGGTACCCGATGGCAACCTGGCGTGGCAATTACCCGGATGCCTGTGATTTTGTTGCATGCAACTTGGGGATATTTGCAGCCATGACATGGCAGGACTTTCTTTACCTGATCATGAATAGAAGAACTGAGCCTCAATTGTCACATTCATCTTACTCTGGATGTTTTAGTATGGAGGAAAATATTTTAGTGAAGCCGCCCTAAAGTGGCTAAATGGTGTCATATCTACAACGAAGACCTTTGACATGAGGACCATAATGAGGCTGTGATGATTTTTTGTCTTTACTCATCAGATGGTTGAGCCTTGCTATATCTTGGCGTGGCAGTTTTGTTGGGGTATTGTGAGATAGTGGAGCTATTTTTGTTTCTGTGTCTCACAAAGCATCCTTAAGCTTGCAGCCATAGTGGGGTTTAGCTTATGTATGAGAAAAGGCAAATCTGAGCCTCCCAAATCTCTGTTTGTGTTGGAAAAATGAGGCTTTTTATGGAAACTCGAGGAAGGTTGAGATTTTCCAAGGGTTTTTGCTCCCTTCTAAAGTTCAATTTAGCGAAGTGATTTCCCTATTCCTTTGTTACACAGGAAAGTACACCCAATGACCTTGATAATGGATGGTCTTGACCCCGCTTCCCCCATGGCAAACCTTCAAAGTCGAAGTCAGAAGTCAAAAGTTTCCATGGAGTAAGTGGGTCAAAAACCACACACATCTACAGGCCATTCTTGTAAAACACCCTACTTTAGTGCATTGAGAAAACCTGACCCGTATCTTGGCAGGGCGACCAGGTATGGCATCATTTGCAAACTGGTGTCAGCAGCCATGGCGTTGTCCCTTTCTCTTTGCATGTTCATGTACAACAAAATATAAGTCCTAAAGTCTGTGGTTAATATCTCAAATGGTCACTCAACTGATGGGGATTGGCATGGCAGCTTTAAGGCTCTGTGAGATATGGGAACTATATTGGTTGTCTCACTGGACTATTGATGCTTGCAGCCATGCGCCCACGTCCATTGTTTAGCTTTTTGAATGAGAAAAGGCAAATCTGAGCCTCCAAGTATCTCTGTGTTCAGTAAATGAGGCTTTTTATTGAACTCGAGAAAGGTAGTGATTTTCTGAGGGTGGTTATTCTCTTTTCTCTTACGTTCAATTTAGCCAATGAATTTTCTTATGCTTTTGTTAGACAGATATAGTTTAACAGCCCTTTCATCAGCAAAATGTTCTTGATGATGATGATGATTGCCATGTTTATGATAATAATAATATTAATAATAAGGGAACATACATTTTCCCGCCTTCAACTTGTGTAGAAAACCCCGAATCCTCAACCCTAAACTTTGTGAATGAGGCTGTGATGATATTCTCCAGGAAGTTTGAAAATCAGTTTTTTAAGCTGATTCATATCTTGGCTAGGCAAAGGCAGGGGAATTCATGAGTTTTGTTCTTACTTGCTCTGTCACAAAGGTATCTGCTTTTTGCAGCCATGGTGAATTAGCCCTTTTTTCTGATTGATTTTCACTCAAAATCTGAGCCTCTTTTCTGTTTAAAGTTTGGTTCTACAATATGTAGTCTGTTTTTTTCATCCTTCGTACGATCAGCACATATAGGAAGAGTCCCCTTTGAGAAAAAAACTTCTTATGTATCCATCTACACAATTTCCATAAATTGTTAGGTACTGATACGAAACAAGTTCAAAGGGAATGGAAGTGGATATTTAGGATCCATTTACTGTCTTTAACCAATTTGATATTGAAGCTGGTTGTTGCTGCATTTATCTACACTACTCAAACTTTCATGCCTTTTTAGATCCTGAATGCACCAAGTTTCCGAGGCAATGAAGATTATTTATAATGAAAATGAAAAGTGAATAAGATGAGAGTTGATTCATTGCCTGGCTTGGCTAAGCCCAATTGGAAGGTCTTGTAAGGCAATTGTTGCTTGCAACTGCAGCAGTGTTAACTCTGCTCTTCCTCCTCCATTTTGGATTAAATAAGCGAAAATCTGAGCCTCATTCTTAACATAGTTCATTTTCTTTTTTAAACTAGTCTTTCTGGAAAGAGATTTAGATTGCCATCATTATGGAACCATTGAATCAACAGCTTCGGCTGCCACTGCCAAAACCCTAAAGAAAAAATTCTTTTCTCGGTTGAGTACATGCATTTGTTATCTGATGAAGGTGAATCCATACCTAAACATGAGTATGTCAGCTAACAGTCCGTTCTACAGTTCATGTCGCGGCTGCAGGATCAGCGGCATTCTTTTCAGGTGAACAATGTTGCATTTCATCTTTGCTGTGCGGTGGACCGGTG

>LNC_001259

TCCAACACCTGCAGTACCCTCATCATAGTTTTGCCCAGGAATGAAGAATGAGACATCAGAGAGCTGGGATTTTCTGTAAAGGGCGTTTGGCCATAGATTCCAAATATATATATGCTTTTTCTGCTTGTGCTTCTGCAAGGTAGAAATGACACAACAAGGAACTGAGATACGCAGTTGGATTTTCAGCTTTTGTTGAACCCCATTTTTCAGATGCAGTTCTCCATCAAAATTTTATCTCAAAAATGAGGTTATCGGAGTAAAAAGGGATTTTTCAGGGTGGTTGACCATCTCCGGAAAAGCAGCTGCAAAAGCATAGAAAAATGGAGGTTCAGCCCAGTATGAAGAATGAGGCCAACAGAGAAAAAGGGAATTCTTTGAGGGTGCCTCTGCTTGGCCTATGATTTCCCCATAAACAAAATTAACAACCCTCCTTAGAAGTACTAGTACTGTTTTTATTGTTTTCTCTAAATTCTTTGAGGGTAGTTGCCCTCTCTAATTGACCTATGATTTCTCTGACCAACCAAACCAACAATCCTCCGTTGGTATATGTTTCTATTGATGATTAGTTGATAGCAGCAGTAGTAATACTCCCTCTCTTCTAATTCACTTGTCTTGAACCTGTTCACTATTGATATGGAAAAGAATCCATCTTTCGAAGGAGGCAGTGATAATATTGCCAATTCTGGGCAAGGAGAAAATCCGACTCGTATCTTGGTGGGGCAAACCCAGATTATGTAACTGGTTGATTTTGCAGCCACGGTGTTGTCATCTTCTCTTTGCCCCATTTTACACAGATATGAGCCTCAACATCCAATGAGTTCCAGGATCCAAACCCCGAAATTCATTTGATGGAAACCTTGTTCATTTTAGCTCTAAAGGACAAGCCAATCGGAGGAAAATGGGCATTTTTACAGGGTGGTCGACCATCTCTGGGACTTACAGTTTACAGAGTAATGGAAGCTTAGCCCAGTATGAAGAATGAGGCCAACAGGGATAGGGAAATTTTTTTGGAGGGTGGTTGTCCTCTCTGGCTGACCTATGATTTCTTGTTCAACCAAATCAACAACCCTCCATTGGTATATTTGTGAAACTTCCCAAAAATAAATAAATAAATCTGTTGCCATTGATGTAATGTTGCTAGCAGTAGTAGTAATGTCATACTCTCTCCATTTCAATATACTAGTCTGGAAAACTGTCCACAAATTGGTATAGAAAAGGTTCCATCTTTCTCAAAGACGCAGTGATGATATTGCTAATTCCGCGCTGAAAATCGGGGCATGGAGAAAACCCGACTCGTATCTTGGCAGGGCAAACCCGAATCACGTAACCCGTTCATTTTGCATCCATGGCGTTGTCGTCTTCTCTTTACCCCTTTTTTGCACCAATCTGAGCCTCAACTTCCAATGAGTCACTTCGAGTACGCCGGGATCGAAACCCAGAATCTCATTTGATGGAAACCCTGGTCCCTGTTCATTTTAGCTCGAAAACATGAGGCAGTCAGAGAAAAATGTGGATTTTTTCAGGGTGTTAGACCATCTCTGGTACGTCAATGATTTTTTCTTTGCTCTAATAAACAACCCTTTTAGCTTTGTTTGTGGACGAAGTGCAGAAATTGCTGTTTTAGACCAGTTCCAAGTTCATTGGGTGTAATTTCCTGGATTTACACTGTAATGGAAGCTCAGCCCAGTGTGAAGAATGAGACCAACAGTGAGAAAAAGGGAATTTCTTTGGAGGGTGGTTGCCCCCTCTGATTGGTCTATGATTTCTCCTTTAACCAAACAAACAACCCTCTGGAAAAGTAGAAACAACCAACAATATTTGTTGTCATTGCCTTCAATGGTGGTAATATGATGCTGATGTAAGTAATACTATTCAATTTTCTGATCATTCCGAGATAGCTTAGACCAAATGCTTTTGGTATGAAATACTCAGAGATCTGAAGGTCTCATTTGTTTCATTAATATTTCTTGTTAAAGTTATTCAAACGGACACTTATGGAAAAGTTTCCATATTTTCTGAAAGAGGCAGTGATGATATTGCTTATCTGGGCTGCTGTTTGGGGCATGGAGACTATTTGACCCGTATCTTGGCATGGCAAACCTGGATGGCTTGTGATCGAGTCTTCTCTTTCACATGCAACTGGTTCTTTTGCAGCCATGGTGTTGTCGCCTTCTCTTTGCCCCCATTTTTTGCTAGTCTGAGCCTCAATTTCCAACTAGTCCAACTGGACGTCCCAGGACTCAAAGAGTTCTGAACCTAAATGATGAAAAAATCTCTGCATGCACGCACACACATATCATGAGAAAATGTCCAAAATAGTCCCTTATTGAGGCAGTGATGATCTTTCAGTCTATCCTCTTGAGCTAGTTATTGAGCATTGAGGGTACCCGATGGCAACCTGGCGTGGCAATTACCCGGATGCCTGTGATTTTGTTGCATGCAACTTGGGGATATTTGCAGCCATGACATGGCAGGACTTTCTTTACCTGATCATGAATAGAAGAACTGAGCCTCAATTGTCACATTCATCTTACTCTGGATGTTTTAGTATGGAGGAAAATATTTTAGTGAAGCCGCCCTAAAGTGGCTAAATGGTGTCATATCTACAACGAAGACCTTTGACATGAGGACCATAATGAGGCTGTGATGATTTTTTGTCTTTACTCATCAGATGGTTGAGCCTTGCTATATCTTGGCGTGGCAGTTTTGTTGGGGTATTGTGAGATAGTGGAGCTATTTTTGTTTCTGTGTCTCACAAAGCATCCTTAAGCTTGCAGCCATAGTGGGGTTTAGCTTATGTATGAGAAAAGGCAAATCTGAGCCTCCCAAATCTCTGTTTGTGTTGGAAAAATGAGGCTTTTTATGGAAACTCGAGGAAGGTTGAGATTTTCCAAGGGTTTTTGCTCCCTTCTAAAGTTCAATTTAGCGAAGTGATTTCCCTATTCCTTTGTTACACAGGAAAGTACACCCAATGACCTTGATAATGGATGGTCTTGACCCCGCTTCCCCCATGGCAAACCTTCAAAGTCGAAGTCAGAAGTCAAAAGTTTCCATGGAGTAAGTGGGTCAAAAACCACACACATCTACAGGCCATTCTTGTAAAACACCCTACTTTAGTGCATTGAGAAAACCTGACCCGTATCTTGGCAGGGCGACCAGGTATGGCATCATTTGCAAACTGGTGTCAGCAGCCATGGCGTTGTCCCTTTCTCTTTGCATGTTCATGTACAACAAAATATAAGTCCTAAAGTCTGTGGTTAATATCTCAAATGGTCACTCAACTGATGGGGATTGGCATGGCAGCTTTAAGGCTCTGTGAGATATGGGAACTATATTGGTTGTCTCACTGGACTATTGATGCTTGCAGCCATGCGCCCACGTCCATTGTTTAGCTTTTTGAATGAGAAAAGGCAAATCTGAGCCTCCAAGTATCTCTGTGTTCAGTAAATGAGGCTTTTTATTGAACTCGAGAAAGGTAGTGATTTTCTGAGGGTGGTTATTCTCTTTTCTCTTACGTTCAATTTAGCCAATGAATTTTCTTATGCTTTTGTTAGACAGATATAGTTTAACAGCCCTTTCATCAGCAAAATGTTCTTGATGATGATGATGATTGCCATGTTTATGATAATAATAATATTAATAATAAGGGAACATACATTTTCCCGCCTTCAACTTGTGTAGAAAACCCCGAATCCTCAACCCTAAACTTTGTGAATGAGGCTGTGATGATATTCTCCAGGAAGTTTGAAAATCAGTTTTTTAAGCTGATTCATATCTTGGCTAGGCAAAGGCAGGGGAATTCATGAGTTTTGTTCTTACTTGCTCTGTCACAAAGGTATCTGCTTTTTGCAGCCATGGTGAATTAGCCCTTTTTTCTGATTGATTTTCACTCAAAATCTGAGCCTCTTTTCTGTTTAAAGTTTGGTTCTACAATATGTAGTCTGTTTTTTTCATCCTTCGTACGATCAGCACATATAGGAAGAGTCCCCTTTGAGAAAAAAACTTCTTATGTATCCATCTACACAATTTCCATAAATTGTTAGGTACTGATACGAAACAAGTTCAAAGGGAATGGAAGTGGATATTTAGGATCCATTTACTGTCTTTAACCAATTTGATATTGAAGCTGGTTGTTGCTGCATTTATCTACACTACTCAAACTTTCATGCCTTTTTAGATCCTGAATGCACCAAGTTTCCGAGGCAATGAAGATTATTTATAATGAAAATGAAAAGTGAATAAGATGAGAGTTGATTCATTGCCTGGCTTGGCTAAGCCCAATTGGAAGGTCTTGTAAGGCAATTGTTGCTTGCAACTGCAGCAGTGTTAACTCTGCTCTTCCTCCTCCATTTTGGATTAAATAAGCGAAAATCTGAGCCTCATTCTTAACATAGTTCATTTTCTTTTTTAAACTAGTCTTTCTGGAAAGAGATTTAGATTGCCATCATTATGGAACCATTGAATCAACAGCTTCGGCTGCCACTGCCAAAACCCTAAAGAAAAAATTCTTTTCTCGGTTGAGTACATGCATTTGTTATCTGATGAAGGTGAATCCATACCTAAACATGAGTATGTCAGCTAACAGTCCGTTCTACAGTTCATGTCGCGGCTGCAGGATCAGCGGCATTCTTTTCAGGTGAACAATGTTGCATTTCATCTTTGCTGTGCGGTGGACCGGTG

>LNC_001260

GCTCCAACACCTGCAGTACCCTCATCATAGTTTTGCCCAGGAATGAAGAATGAGACATCAGAGAGCTGGGATTTTCTGTAAAGGGCGTTTGGCCATAGATTCCAAATATATATATGCTTTTTCTGCTTGTGCTTCTGCAAGGTAGAAATGACACAACAAGGAACTGAGATACGCAGTTGGATTTTCAGCTTTTGTTGAACCCCATTTTTCAGATGCAGTTCTCCATCAAAATTTTATCTCAAAAATGAGGTTATCGGAGTAAAAAGGGATTTTTCAGGGTGGTTGACCATCTCCGGAAAAGCAGCTGCAAAAGCATAGAAAAATGGAGGTTCAGCCCAGTATGAAGAATGAGGCCAACAGAGAAAAAGGGAATTCTTTGAGGGTGCCTCTGCTTGGCCTATGATTTCCCCATAAACAAAATTAACAACCCTCCTTAGAAGTACTAGTACTGTTTTTATTGTTTTCTCTAAATTCTTTGAGGGTAGTTGCCCTCTCTAATTGACCTATGATTTCTCTGACCAACCAAACCAACAATCCTCCGTTGGTATATGTTTCTATTGATGATTAGTTGATAGCAGCAGTAGTAATACTCCCTCTCTTCTAATTCACTTGTCTTGAACCTGTTCACTATTGATATGGAAAAGAATCCATCTTTCGAAGGAGGCAGTGATAATATTGCCAATTCTGGGCAAGGAGAAAATCCGACTCGTATCTTGGTGGGGCAAACCCAGATTATGTAACTGGTTGATTTTGCAGCCACGGTGTTGTCATCTTCTCTTTGCCCCATTTTACACAGATATGAGCCTCAACATCCAATGAGTTCCAGGATCCAAACCCCGAAATTCATTTGATGGAAACCTTGTTCATTTTAGCTCTAAAGGACAAGCCAATCGGAGGAAAATGGGCATTTTTACAGGGTGGTCGACCATCTCTGGACCCTGTTTAGAGAATAGCTGAAGTTTACAAAGTTGCAGGGACTTACAGTTTACAGAGTAATGGAAGCTTAGCCCAGTATGAAGAATGAGGCCAACAGGGATAGGGAAATTTTTTTGGAGGGTGGTTGTCCTCTCTGGCTGACCTATGATTTCTTGTTCAACCAAATCAACAACCCTCCATTGGTATATTTGTGAAACTTCCCAAAAATAAATAAATAAATCTGTTGCCATTGATGTAATGTTGCTAGCAGTAGTAGTAATGTCATACTCTCTCCATTTCAATATACTAGTCTGGAAAACTGTCCACAAATTGGTATAGAAAAGGTTCCATCTTTCTCAAAGACGCAGTGATGATATTGCTAATTCCGCGCTGAAAATCGGGGCATGGAGAAAACCCGACTCGTATCTTGGCAGGGCAAACCCGAATCACGTAACCCGTTCATTTTGCATCCATGGCGTTGTCGTCTTCTCTTTACCCCTTTTTTGCACCAATCTGAGCCTCAACTTCCAATGAGTCACTTCGAGTACGCCGGGATCGAAACCCAGAATCTCATTTGATGGAAACCCTGGTCCCTGTTCATTTTAGCTCGAAAACATGAGGCAGTCAGAGAAAAATGTGGATTTTTTCAGGGTGTTAGACCATCTCTGGTACGTCAATGATTTTTTCTTTGCTCTAATAAACAACCCTTTTAGCTTTGTTTGTGGACGAAGTGCAGAAATTGCTGTTTTAGACCAGTTCCAAGTTCATTGGGTGTAATTTCCTGGATTTACACTGTAATGGAAGCTCAGCCCAGTGTGAAGAATGAGACCAACAGTGAGAAAAAGGGAATTTCTTTGGAGGGTGGTTGCCCCCTCTGATTGGTCTATGATTTCTCCTTTAACCAAACAAACAACCCTCTGGAAAAGTAGAAACAACCAACAATATTTGTTGTCATTGCCTTCAATGGTGGTAATATGATGCTGATGTAAGTAATACTATTCAATTTTCTGATCATTCCGAGATAGCTTAGACCAAATGCTTTTGGTATGAAATACTCAGAGATCTGAAGGTCTCATTTGTTTCATTAATATTTCTTGTTAAAGTTATTCAAACGGACACTTATGGAAAAGTTTCCATATTTTCTGAAAGAGGCAGTGATGATATTGCTTATCTGGGCTGCTGTTTGGGGCATGGAGACTATTTGACCCGTATCTTGGCATGGCAAACCTGGATGGCTTGTGATCGAGTCTTCTCTTTCACATGCAACTGGTTCTTTTGCAGCCATGGTGTTGTCGCCTTCTCTTTGCCCCCATTTTTTGCTAGTCTGAGCCTCAATTTCCAACTAGTCCAACTGGACGTCCCAGGACTCAAAGAGTTCTGAACCTAAATGATGAAAAAATCTCTGCATGCACGCACACACATATCATGAGAAAATGTCCAAAATAGTCCCTTATTGAGGCAGTGATGATCTTTCAGTCTATCCTCTTGAGCTAGTTATTGAGCATTGAGGGTACCCGATGGCAACCTGGCGTGGCAATTACCCGGATGCCTGTGATTTTGTTGCATGCAACTTGGGGATATTTGCAGCCATGACATGGCAGGACTTTCTTTACCTGATCATGAATAGAAGAACTGAGCCTCAATTGTCACATTCATCTTACTCTGGATGTTTTAGTATGGAGGAAAATATTTTAGTGAAGCCGCCCTAAAGTGGCTAAATGGTGTCATATCTACAACGAAGACCTTTGACATGAGGACCATAATGAGGCTGTGATGATTTTTTGTCTTTACTCATCAGATGGTTGAGCCTTGCTATATCTTGGCGTGGCAGTTTTGTTGGGGTATTGTGAGATAGTGGAGCTATTTTTGTTTCTGTGTCTCACAAAGCATCCTTAAGCTTGCAGCCATAGTGGGGTTTAGCTTATGTATGAGAAAAGGCAAATCTGAGCCTCCCAAATCTCTGTTTGTGTTGGAAAAATGAGGCTTTTTATGGAAACTCGAGGAAGGTTGAGATTTTCCAAGGGTTTTTGCTCCCTTCTAAAGTTCAATTTAGCGAAGTGATTTCCCTATTCCTTTGTTACACAGGAAAGTACACCCAATGACCTTGATAATGGATGGTCTTGACCCCGCTTCCCCCATGGCAAACCTTCAAAGTCGAAGTCAGAAGTCAAAAGTTTCCATGGAGTAAGTGGGTCAAAAACCACACACATCTACAGGCCATTCTTGTAAAACACCCTACTTTAGTGCATTGAGAAAACCTGACCCGTATCTTGGCAGGGCGACCAGGTATGGCATCATTTGCAAACTGGTGTCAGCAGCCATGGCGTTGTCCCTTTCTCTTTGCATGTTCATGTACAACAAAATATAAGTCCTAAAGTCTGTGGTTAATATCTCAAATGGTCACTCAACTGATGGGGATTGGCATGGCAGCTTTAAGGCTCTGTGAGATATGGGAACTATATTGGTTGTCTCACTGGACTATTGATGCTTGCAGCCATGCGCCCACGTCCATTGTTTAGCTTTTTGAATGAGAAAAGGCAAATCTGAGCCTCCAAGTATCTCTGTGTTCAGTAAATGAGGCTTTTTATTGAACTCGAGAAAGGTAGTGATTTTCTGAGGGTGGTTATTCTCTTTTCTCTTACGTTCAATTTAGCCAATGAATTTTCTTATGCTTTTGTTAGACAGATATAGTTTAACAGCCCTTTCATCAGCAAAATGTTCTTGATGATGATGATGATTGCCATGTTTATGATAATAATAATATTAATAATAAGGGAACATACATTTTCCCGCCTTCAACTTGTGTAGAAAACCCCGAATCCTCAACCCTAAACTTTGTGAATGAGGCTGTGATGATATTCTCCAGGAAGTTTGAAAATCAGTTTTTTAAGCTGATTCATATCTTGGCTAGGCAAAGGCAGGGGAATTCATGAGTTTTGTTCTTACTTGCTCTGTCACAAAGGTATCTGCTTTTTGCAGCCATGGTGAATTAGCCCTTTTTTCTGATTGATTTTCACTCAAAATCTGAGCCTCTTTTCTGTTTAAAGTTTGGTTCTACAATATGTAGTCTGTTTTTTTCATCCTTCGTACGATCAGCACATATAGGAAGAGTCCCCTTTGAGAAAAAAACTTCTTATGTATCCATCTACACAATTTCCATAAATTGTTAGGTACTGATACGAAACAAGTTCAAAGGGAATGGAAGTGGATATTTAGGATCCATTTACTGTCTTTAACCAATTTGATATTGAAGCTGGTTGTTGCTGCATTTATCTACACTACTCAAACTTTCATGCCTTTTTAGATCCTGAATGCACCAAGTTTCCGAGGCAATGAAGATTATTTATAATGAAAATGAAAAGTGAATAAGATGAGAGTTGATTCATTGCCTGGCTTGGCTAAGCCCAATTGGAAGGTCTTGTAAGGCAATTGTTGCTTGCAACTGCAGCAGTGTTAACTCTGCTCTTCCTCCTCCATTTTGGATTAAATAAGCGAAAATCTGAGCCTCATTCTTAACATAGTTCATTTTCTTTTTTAAACTAGTCTTTCTGGAAAGAGATTTAGATTGCCATCATTATGGAACCATTGAATCAACAGCTTCGGCTGCCACTGCCAAAACCCTAAAGAAAAAATTCTTTTCTCGGTTGAGTACATGCATTTGTTATCTGATGAAGGTGAATCCATACCTAAACATGAGTATGTCAGCTAACAGTCCGTTCTACAGTTCATGTCGCGGCTGCAGGATCAGCGGCATTCTTTTCAGGTGAACAATGTTGCATTTCATCTTTGCTGTGCGGTGGACCGGTG

>LNC_001261

GATGCCTGTTTGCTCCAACACCTGCAGGAACTGAGATACGCAGTTGGATTTTCAGCTTTTGTTGAACCCCATTTTTCAGATGCAGTTCTCCATCAAAATTTTATCTCAAAAATGAGGTTATCGGAGTAAAAAGGGATTTTTCAGGGTGGTTGACCATCTCCGGAAAAGCAGCTGCAAAAGCATAGAAAAATGGAGGTTCAGCCCAGTATGAAGAATGAGGCCAACAGAGAAAAAGGGAATTCTTTGAGGGTGCCTCTGCTTGGCCTATGATTTCCCCATAAACAAAATTAACAACCCTCCTTAGAAGTACTAGTACTGTTTTTATTGTTTTCTCTAAATTCTTTGAGGGTAGTTGCCCTCTCTAATTGACCTATGATTTCTCTGACCAACCAAACCAACAATCCTCCGTTGGTATATGTTTCTATTGATGATTAGTTGATAGCAGCAGTAGTAATACTCCCTCTCTTCTAATTCACTTGTCTTGAACCTGTTCACTATTGATATGGAAAAGAATCCATCTTTCGAAGGAGGCAGTGATAATATTGCCAATTCTGGGCAAGGAGAAAATCCGACTCGTATCTTGGTGGGGCAAACCCAGATTATGTAACTGGTTGATTTTGCAGCCACGGTGTTGTCATCTTCTCTTTGCCCCATTTTACACAGATATGAGCCTCAACATCCAATGAGTTCCAGGATCCAAACCCCGAAATTCATTTGATGGAAACCTTGTTCATTTTAGCTCTAAAGGACAAGCCAATCGGAGGAAAATGGGCATTTTTACAGGGTGGTCGACCATCTCTGGGACTTACAGTTTACAGAGTAATGGAAGCTTAGCCCAGTATGAAGAATGAGGCCAACAGGGATAGGGAAATTTTTTTGGAGGGTGGTTGTCCTCTCTGGCTGACCTATGATTTCTTGTTCAACCAAATCAACAACCCTCCATTGGTATATTTGTGAAACTTCCCAAAAATAAATAAATAAATCTGTTGCCATTGATGTAATGTTGCTAGCAGTAGTAGTAATGTCATACTCTCTCCATTTCAATATACTAGTCTGGAAAACTGTCCACAAATTGGTATAGAAAAGGTTCCATCTTTCTCAAAGACGCAGTGATGATATTGCTAATTCCGCGCTGAAAATCGGGGCATGGAGAAAACCCGACTCGTATCTTGGCAGGGCAAACCCGAATCACGTAACCCGTTCATTTTGCATCCATGGCGTTGTCGTCTTCTCTTTACCCCTTTTTTGCACCAATCTGAGCCTCAACTTCCAATGAGTCACTTCGAGTACGCCGGGATCGAAACCCAGAATCTCATTTGATGGAAACCCTGGTCCCTGTTCATTTTAGCTCGAAAACATGAGGCAGTCAGAGAAAAATGTGGATTTTTTCAGGGTGTTAGACCATCTCTGGTACGTCAATGATTTTTTCTTTGCTCTAATAAACAACCCTTTTAGCTTTGTTTGTGGACGAAGTGCAGAAATTGCTGTTTTAGACCAGTTCCAAGTTCATTGGGTGTAATTTCCTGGATTTACACTGTAATGGAAGCTCAGCCCAGTGTGAAGAATGAGACCAACAGTGAGAAAAAGGGAATTTCTTTGGAGGGTGGTTGCCCCCTCTGATTGGTCTATGATTTCTCCTTTAACCAAACAAACAACCCTCTGGAAAAGTAGAAACAACCAACAATATTTGTTGTCATTGCCTTCAATGGTGGTAATATGATGCTGATGTAAGTAATACTATTCAATTTTCTGATCATTCCGAGATAGCTTAGACCAAATGCTTTTGGTATGAAATACTCAGAGATCTGAAGGTCTCATTTGTTTCATTAATATTTCTTGTTAAAGTTATTCAAACGGACACTTATGGAAAAGTTTCCATATTTTCTGAAAGAGGCAGTGATGATATTGCTTATCTGGGCTGCTGTTTGGGGCATGGAGACTATTTGACCCGTATCTTGGCATGGCAAACCTGGATGGCTTGTGATCGAGTCTTCTCTTTCACATGCAACTGGTTCTTTTGCAGCCATGGTGTTGTCGCCTTCTCTTTGCCCCCATTTTTTGCTAGTCTGAGCCTCAATTTCCAACTAGTCCAACTGGACGTCCCAGGACTCAAAGAGTTCTGAACCTAAATGATGAAAAAATCTCTGCATGCACGCACACACATATCATGAGAAAATGTCCAAAATAGTCCCTTATTGAGGCAGTGATGATCTTTCAGTCTATCCTCTTGAGCTAGTTATTGAGCATTGAGGGTACCCGATGGCAACCTGGCGTGGCAATTACCCGGATGCCTGTGATTTTGTTGCATGCAACTTGGGGATATTTGCAGCCATGACATGGCAGGACTTTCTTTACCTGATCATGAATAGAAGAACTGAGCCTCAATTGTCACATTCATCTTACTCTGGATGTTTTAGTATGGAGGAAAATATTTTAGTGAAGCCGCCCTAAAGTGGCTAAATGGTGTCATATCTACAACGAAGACCTTTGACATGAGGACCATAATGAGGCTGTGATGATTTTTTGTCTTTACTCATCAGATGGTTGAGCCTTGCTATATCTTGGCGTGGCAGTTTTGTTGGGGTATTGTGAGATAGTGGAGCTATTTTTGTTTCTGTGTCTCACAAAGCATCCTTAAGCTTGCAGCCATAGTGGGGTTTAGCTTATGTATGAGAAAAGGCAAATCTGAGCCTCCCAAATCTCTGTTTGTGTTGGAAAAATGAGGCTTTTTATGGAAACTCGAGGAAGGAAAGTACACCCAATGACCTTGATAATGGATGGTCTTGACCCCGCTTCCCCCATGGCAAACCTTCAAAGTCGAAGTCAGAAGTCAAAAGTTTCCATGGAGTAAGTGGGTCAAAAACCACACACATCTACAGGCCATTCTTGTAAAACACCCTACTTTAGTGCATTGAGAAAACCTGACCCGTATCTTGGCAGGGCGACCAGGTATGGCATCATTTGCAAACTGGTGTCAGCAGCCATGGCGTTGTCCCTTTCTCTTTGCATGTTCATGTACAACAAAATATAAGTCCTAAAGTCTGTGGTTAATATCTCAAATGGTCACTCAACTGATGGGGATTGGCATGGCAGCTTTAAGGCTCTGTGAGATATGGGAACTATATTGGTTGTCTCACTGGACTATTGATGCTTGCAGCCATGCGCCCACGTCCATTGTTTAGCTTTTTGAATGAGAAAAGGCAAATCTGAGCCTCCAAGTATCTCTGTGTTCAGTAAATGAGGCTTTTTATTGAACTCGAGAAAGGTAGTGATTTTCTGAGGGTGGTTATTCTCTTTTCTCTTACGTTCAATTTAGCCAATGAATTTTCTTATGCTTTTGTTAGACAGATATAGTTTAACAGCCCTTTCATCAGCAAAATGTTCTTGATGATGATGATGATTGCCATGTTTATGATAATAATAATATTAATAATAAGGGAACATACATTTTCCCGCCTTCAACTTGTGTAGAAAACCCCGAATCCTCAACCCTAAACTTTGTGAATGAGGCTGTGATGATATTCTCCAGGAAGTTTGAAAATCAGTTTTTTAAGCTGATTCATATCTTGGCTAGGCAAAGGCAGGGGAATTCATGAGTTTTGTTCTTACTTGCTCTGTCACAAAGGTATCTGCTTTTTGCAGCCATGGTGAATTAGCCCTTTTTTCTGATTGATTTTCACTCAAAATCTGAGCCTCTTTTCTGTTTAAAGTTTGGTTCTACAATATGTAGTCTGTTTTTTTCATCCTTCGTACGATCAGCACATATAGGAAGAGTCCCCTTTGAGAAAAAAACTTCTTATGTATCCATCTACACAATTTCCATAAATTGTTAGGTACTGATACGAAACAAGTTCAAAGGGAATGGAAGTGGATATTTAGGATCCATTTACTGTCTTTAACCAATTTGATATTGAAGCTGGTTGTTGCTGCATTTATCTACACTACTCAAACTTTCATGCCTTTTTAGATCCTGAATGCACCAAGTTTCCGAGGCAATGAAGATTATTTATAATGAAAATGAAAAGTGAATAAGATGAGAGTTGATTCATTGCCTGGCTTGGCTAAGCCCAATTGGAAGGTCTTGTAAGGCAATTGTTGCTTGCAACTGCAGCAGTGTTAACTCTGCTCTTCCTCCTCCATTTTGGATTAAATAAGCGAAAATCTGAGCCTCATTCTTAACATAGTTCATTTTCTTTTTTAAACTAGTCTTTCTGGAAAGAGATTTAGATTGCCATCATTATGGAACCATTGAATCAACAGCTTCGGCTGCCACTGCCAAAACCCTAAAGAAAAAATTCTTTTCTCGGTTGAGTACATGCATTTGTTATCTGATGAAGGTGAATCCATACCTAAACATGAGTATGTCAGCTAACAGTCCGTTCTACAGTTCATGTCGCGGCTGCAGGATCAGCGGCATTCTTTTCAGGTGAACAATGTTGCATTTCATCTTTGCTGTGCGGTGGACCGGTG

>LNC_001262

CTTTGGTATGATGGAAAACCTTTTTCATGATAAAGTCAGCACTTCCTTACCATTGTATCCTCATGAGGAGAAATTTTAAGGTGAGAGCCAAGTATTCCTAAAAGAAGTCAGCCGGAAGGAGAAATGAGGTTAATTTCTTCTAAGGGTGGTTGTTCCTCTCTGTTGTGCCAATGATTTTCTGTAGCTACTACACCAACAGCCCTTGTTGATGCAATACTCAGAAGCAGAAGATGATCCATATATCTCAGTGAGTGTGTATTCTTAAGGAATTTGCACTGATGACCTTGTTTGAGGTAGTGATGATAATAATAGCACTTTCTGGGTTAACAAAATGGGGCATTGAATACAATCGGAGCCGTATCTTGGCCTGGAAAATCCCAAGGATGACCACTGACGTTCTCTCTTTTGCACCACCGGTGATTTTGCAGCCATGGTGTGGTTGCATTCTCTTTAAGGGGGTTGTTTGGTCATTGGATAGGAAATGAGTTGTTCCTGTTAGGAGGTTTTTTCGAAGGTGGTTGCCCCTCTCTGCAACTCTAGTGATTTCTCATATGAAAAGAAAATGAAATAAAAATCAAACCCCTTAATTCCCAGGGAAAAATTATCCACACCCTGTTGAGATTTTTGAATCAAATAGTCTTGCTATGTTATGGAGTTGAACTAAATTTTTCTCTCAAAGAAGAGAGAGAGAGAGAATTATGAGGCTTCAATGATACTTGACAAGAGAAATTACGAGACCCGTATCTTGGCGGGGCGAGCCGGAATGCAACCCGAGGTCTGCAGCCATGGCGATGTCTTCATTCTCTTGTTTTATCAACTGATGTCAGCTTCAGTGCTCTTGTTTGGATGGTTGTTACTAAAGTATTGTATTGTTATTAAGGGTGTCTTTGGTATGAAGGAAACTGTTTGTTTCATGAAGAATTTCATCAGTGTTTGGTTACTAATGTATCCCTCTGAGAAGAGGAAAGTTGGGAAATTTTCTGGTGAAAACTATGATTTTTCCTAAAGAAGTGTTGGCCCAGAAAGAAAATATAAGGTTATAGAGAGATGGGACTTTTCGTTAAGGGTGGTTGCCCCCTCTCTGTTGTACCAATGATTTTATGTTTAGCAACTAAACCAACAGCCCTTGTTCCTCAGACAAACATTAATCAACAACTATGATTATTGAAACATGTGCATGGGCCATGCAGTTTCTTGAATCTACAACTTAAGCCGTAGATCTCAGTGTGTGTGTGTATTTTTCCCTCCTTTTTGAGTAATTGTGCAATAGAGGCTGTGACGATGACAATGTTTTACTCTCTGGGCTACCAAAATTGGGCATTGAGGACAGTGTGACCCGTATCTTGGCTTGGCAAATTCCAGGTTGGTCTTCAATTGAGGCTCTCTCTCTTCCATGCAACTGGTGATTTTGCAGCCATAGCGTGGTTGCATCTCTTTGTCCAAATGCATTATCTGAGCCTCAAGTTTCTCAGAATCGAACTGGAGACCTCTTGTGCTTTGAAATACATTAAAGTTTGCTCTAACTACAAATAAAAAGAATGATGTATGACAGAAAGATGAGTACTTTATCTTCTTGATATAAGCCCATGATTTTATAGACAATAACCGAAGAGAGAAGGCTTGCAGAGAGGAGGAGTTTTTGAAGGGTGGTTGTCCCACTCTGTAACTTCTGTGATTTCTCATATGAAAAAGAAAAATCAAGCAACCTTTTATATCTCAGGGAATTATTGTTGACACCCAATTTTCGACTCAAATGGTCCTTTGTCTTATGGAGTTGAGCTAATTTTTTCTCACGAGGGAGAGGGAGGGAGGGAGAGAGAGAGAGAATTCCAAGGCTGTGATGATACTTTCTTCTTTGGGGCAAGACAAATTAAGAGATCCGTATCTTGGTGTGGCAACCCGGATGGCGACCCACTTTGTGTCATGCAACCGTTGGTTGCAGCCATGGCAATGTCTTCATTTTCTTGTCTCAACAACCGATCTGACCCTCAATTTTACTCTTATTCACATGTTTGGATGGTTGTTGCCTATTGTATTGTATTGCTATTCTAAAATGGTGTCTTTGGTATTAAGGAAAATGTTTTTCATGATGAAATCAGTGTTGGTTACTAAGGTGGCCCTCTGAGAAGAAGAAAGTTGGGAAATTTTCCAGTGAGTTCAAAAAGAGAAATGAGGTTAATTGAGAGAAGGGATTTTTTGTTAAGGGTGGTTGCCCCTCTCTGTTGTGCCAATGACTTTCCTTTTAGCAACTAAACCAACAGCCCTTGTTCATGAAATACTGAGACAAACATCAGTCAGCAGCTATGACAATTGAAACATATGCATGGATTTTCTTCAGTCTACAGCTTCAGCTGTAGATCTCAGTGTGGGTGTGATGATATTGCTGATCTGGGCTGATGTTTAGGGCATGGAGACAATATGACCCGTATCTTGGCATGGCAAACCCAGGTGGTTTGTGATTGAATCTTCTCTTTCACATGCTATTGGTTCCTTTGCAGCCATGGCGTTGTCGCATTCTCTTTGTTCCATTTACTGCAGATCTGAGCCTCAATTTCCTACTAGTCCTTCTGGACAGTCCAGGATACGAACCCAGGACCTGTGGTTTGGTGAACATCAATTGGGTTGTTCAGTTTTTCTTGAACACCTGATGGTGTTCTCCGTCAAATTTTTAGCTAAAAGAATGAGGCAGTTGGAGAAACCATCTTCATCACGCCTATGATTTCTATTATCTTTTAGCTCTAATCTATATTGAGGACCATTTTTGATCATTTTCTCTCCGTTTTCTGTTACTTCCGTACTCTGTTCCTCAGAGGCTGTGTTTTCCTGGTAAAGCAACAGCAAAAGCCTACAACAATCAATTGAAAAGGAAAATAATGGGGCTGTGATGATAATTTTAATGAGCAAATGGAATCAGGTATTGTTGATTCAGGCCCGTATCTTGGCAGGGCGACCCGTATTATAGGGTTTGCAGTCATGTCGGTTTCTAATAACCCAATACCTCAGATAAATTAAAGATCTGAGCCCCAGAAAGATGTACAAGGCCAATTAATAACCTTTTTTTTTCATAGGTATATTTTGGGGTGTTCATAGTTCGGTTTAGTTTTGACCAAAAAGAAGGCATTCTATTGCCATTGATGTGATGCTGATGTCAATCGTACTCCCCCTGTATCATTTTATTTGTCGTGTAACTGTCCGAATTGCTATGAAAAAAATTCCATCTTTCCAAAGCGGCTGTGATGATGTTGCTAATCTGGGTTGTTATTTGGGCGTAGAGAAAACTATCATGGCGGGGCAAACCCGGATCATGCAACCGGTTCAGTTTGCAGCCATGGTGTTGTCGTCTTCTCTAGGATCCAAGCCCAGAACCTCATTTGATGGAAACCCAGTTCACTTTATTTGCTCAAAAGGGTGAGGATTTTCCCTCCGATATGTCAATCATTTCTTCTCAGTTCTAACCAACAACCCTTTTAGTATTTGTATGTGGACG

>LNC_001263

AAAAAAAATTCAAACAACCCTTGAAGGTATAGACTAAAATGGTCCTTAATGTACGTAGTTGAGCTAAATTCATCTCTCAACGAAGAGATAGAGAGAGAAAGAGAGAATTTTGAGGCTGTAATTTGATACTTGCAAGAGAAACTAAGAGACCCATATCTTGGCGGGGCGACCCGGAACGTAACCCGATCAGCTGACCATCTCAGACAAGTA

>LNC_001264

AAAAAAAATTCAAACAACCCTTGAAGGTATAGACTAAAATGGTCCTTAATGTACGTAGTTGAGCTAAATTCATCTCTCAACGAAGAGATAGAGAGAGAAAGAGAGAATTTTGAGGCTGTAATTTGATACTTGCAAGAGAAACTAAGAGACCCATATCTTGGCGGGGCGACCCGGAACGTAACCCGATCTGACCATCTCAGACAAGTA

>LNC_001265

GGAGAGGTGGTTAAGTTTTGTGTGTGTGTGTGTTGGGGGGTGGGCTAGCAATTTAATCATGGAATGTCTATTGCCTGATCAAATGCAATTTTTTTTTTTTGAAAAAGATGTCCTAGAACATGTTATTGGATTAATGAGAGGTTCCTCTTTTCTTGTTCTGGAGATTGTGTGTCTGAACAATACTTTTGAGATTTTAAATAAGGCTGATACCAAGATTCTTTTTATATGTCTACTGATATGATGATATTTAAAAAAGATACTAACCTTACGGTTTTGAGATAGTAAGTTTTCTATCTAATGTCTGAAGCTTTGGATATTGGAGTTCCAATGGAATGGATATGACCTCTTTGCACTTTGGTTGAAAATTTGTAAACTTCTCGAGTCAGCTAACATCGGTTGCTGAACAATAATGTAATGCTTAGGAGCTTGACTACAGCTAAGAAGTTGTGGATTTAGTGGTTTTAGGTGAGAGGAAAATGTGACAGGTACGAGACAAGCAAGTTTCTCTTGTGGTATTTGTTTCAGTTTGCGGCTCAAACTTAACATATATCCATTTTCTGAAATTCAATTATGTGGTAAGAAAACTGTCATAGCTACATAATTATCTGATTATGTGATAGGAGACAACGAGTCACACACAATAGGGAAGATCAAAGCACCTAGTTTTGGGTGAAGACAAAAAAATGTTCGTTCGATAAATTCAATAATATTTTTGAATCAGATTAATGTATTTGTACATGTTAGAAATGGTTACCATTTCTTTCAATGATGCTCTGACTATGTAGAATTCAGGTTCACATAAGCTAAATCCTCAAAGGCAGAAAGCTTAGCTGTATAGGACTTGTGCAAAACCTAGGCTAAAAACGTTTAGCTCACAACTTTTAAACTATCCTCCATTAGAGTAGGAGTGAAGTTCTTGTGTATCAGCTAGCTTAAGGGATTCGTCTCATGTAGCTTCTGCATCTACTCTAGAAATATACCAAGTAAGACATCTTGGAAAGATTCGGGAATAGTATCTTCTATATTTCCCTCCTCGCTCTAACTCGGCCACAAGAGAGGACGGGC

>LNC_001266

TGGTTGTGAAGCGAGTGGCTGTCACGCACAGGTCATACAGTCTTTTGTTCATCACGGGCCGGCACGTGAAGGTGCGTGTCTGGCTGAATCGATTGGGTTTTAATTTACGGCATCGACACGTATTTCTGGTGAATCTCAGGTCTGTTCTGGTGTTAAAATAGATCTGGAAAGTCTGTTCGGTCACTTCTCAGGTCTGTTTTGGTGTTAAAATACGATCTGGAAAGTTTGTTCGTCACTCCCTGGTTCGTTTCTATTCTGCTACACTGAATCTGGTTTGTGATTTGTGGTTCTGTGGCTGCTAGCTTCTACTTCAGATCAGATAATTGGTGTTGTTTGGTCATAAGCTGCGGTTCAGAATTTCTTTCTCTGAACTACTTGCTTGGGTATTTGATTGTTCATTACTTGCTCTTGTCTACTGGT

>LNC_001267

TTTGGTTTGGTTCATAGAAGGCGGATTTGTGGTGTAGTATGCGTACTACGGTTACTAGTAGTACTCAAAGTCCATAGGATTTAGACCTGTCTGGCTCCCAAAAGAGGAGTTTGACAGTGGGAGGAGGGGCAACCCCCGCGGATTTGCTCCTCATTGTGGCTGGCTTAACAGAGCAGCGCTCCCAACTGATGGCCGGGGCGGCCATCAAAGACCAGTTCCCGTCCCAAGT

>LNC_001268

AGCCACTGACTTAACAGAGCAGCCCTCCCCGGGAGCTGACATAACAGTGAGAGTTGAGTTCCCGAGACTGAATCGGAAATCGGGTACTCGCCCCTCCTTGTTGTGGGGGGGCTAAGCGCTCTCACCCAATTGCCGAAGTTTGAGGCATGTTCGAAGAACAGTCCCAAAGTCAAACCACTCTAGATAAAAATAGATGGTTGTCAATTGGTTTGGACCATCATCT

>LNC_001269

TTCCGGCCAGCTACATCAAGATTCAAACTAACTCATTTCCATTATTGTTGTCCAAATGACCAAATCTTTGTTTATTCCTACAGACTATTATACTTTCTTTTTTCCCACATCGATGGGAGAAGGCAAATTCATTCATTTTATAAGAACTTAACTAAACAACAAATATTGCTGAGCTTAGTAACTTAAGCTTGTAACCCAAGTGGGAGCAATAAGGTGATTGGGACAATCAATTAAAGCCATTTGACAGATTTAGTGTGTGTTGATTTGGTTGGTTTCTTGGTGGTAAGCAGTATTATTCCAGCAGTAATAATACTGTGTGTCAATCCTTTAGAGAAGGGAGTTGACGTAGAGTGGGGGTAGATGAGGTGCCAAGAGGTGCACCTCCTGGGCTGACTTAACAGAGCAGCGCTCCCCGTTTGATAGCCCAGAAGTGCTGTCAAGACCAGAGACCAGTGCCGGCCAGGTAGCGTGCTTACTAAGATTTGTCAGGCTTACGTTGGTTGGACCATCATCT

>LNC_001270

TCTCGTGGATTTTCATAACCCTGCTGTGCAAAAATGGGATATGCACTTGAAATGGACGTCCGAGTTAAGATATATATCATGAGCGATACGGAAATAGATCGAGTAATCTGTTTCTTTAGCCAAGAAAAAGCATTTCTAGTTTGCATGGTCCAATCATTGATCGCGAAAATTTCTACAATAAATTGGGTAGGTCGCTAGTATAGTTCCCTAGCCACGATTCTGCTATTTGCTGGAATAATCTAGGATGAATCTTCCCGATGGTTAGAAATAGGAAGAGTAAATATGATTTTCAATACTTTGCTTATGTACAACTACAAAGTACCAAGCATTCTAATTGGATTAGATTAATATCAATGGATCTTTTATCATTCAGTTATTGAATCATAAATCAGTAAAATTGACGGAGACAGACTAGTTAAATAACGGAAAAGCCAATATTTAAAACATGTATCAAAAATTACTGGAAGGATGCAAACAAGAATAGTTATAGTTTGCTCATTCGCAACAACTCCAACCTCGTTATAGATAGAGCGTATAGCGAATTCCCAACCTGGGGGTGAATGATATCCGAGAAAAAACTCAGTTACTAGAAGAAGACACAAAGCTTTTGCTGTGTCACTTAAGTTATATAGGAATTCCTGAGCCCAAGAGTTAAGAATAACAAGTTTTTCCTTACCCAAAATTGAATACCCGCTTAGAATACCAAACCAGATGATATTTGTTGAGAAGTGCAAAATCGTATCCATACGATTCTCATTTTGTATCGTGATTAATTGGATCGTTTCTTTATGGATTCCTATCCCAAACTCTTCGAGATGTGTTTCCGAGTATTCCTTGATCATTTCGTCCAAGAAGAGGAGTTCCTCTAATTCTCTGAATTTTTCTAGAAGACTCTTTTCTTGAATATTATTCAAGACAATTTGGGATTGCCCAGTATTCCACCAATT

>LNC_001271

CTAATTTTTTTTTGATTTGCACCGGGTGTCCGAGTCTCTTTGAGCCCCGACTAATCCCGGGGGTGCACAGGCCCTCGGCAAGGAGTTTCCCGCAAGTGCACCACGGTTAATTCAGGTTTTACCCAGTCCGATGGCCCTCAGAAATTGTTTGCACCTAGTGGGTTTCGAACTTGAGACCTTGAAAGGGAGCAAACCCCAAGGCTCAAGTCAATTGCCAGCAGGCCAACCCCTGAGGGTTCT

>LNC_001272

AAGGAGTTTTTCCATGGTTTCCTTATGTCATGCATGTTTTAAATGAATTCTTATTAAATGAGAAAATGAGTTTTCATAAATTTATGTTCCTCATGTTGATGTGCATCTATAATGATTTGCCTAAATGTTGCATGAGTTGACTTATGAGCATGTTTAAAAGTGTTTTGATGATGTTGCCTAAAGTGCTTCTATGAGAAAGTTGCATAATGTGCATTTAGCATAAGTTGTCTATTTGCTTTATGAGGTGATAATGTGAGCATGCTTACTTGAGTTTTGGAGAAGGTTGCATGATGTGCTTTAATGAGAAAATTGCATATTGTGTCCTTTGATGTTATGATGAGCATGAGATGATTTGGAGAAGGAAGTACCTCCAATGAAATGAAATGAGAAATGAGAAAAGGTAAAGATGATGCATAAGGAGTTAGTAGATGTTGTGCCTACTTTCTTGAATTGCATGAAGTTGTGTGGGACTTTCTATGTGGAGTTGATGTTTCCTCCTATATTTATGCATTTGTATTGATGTTGTATGCATGTTGATGGGCTGTTTACTTGAGTCGTTACCCCTTATGTGTTTA

>LNC_001273

GTTATATGTGACTTGAATTTGTTGAATAAGTCATGTGATAACTGATATTGAGGGGATAGAAGAGCATGAGTAGGCTATGATTGATACAGACATTGATGTTGAGACAGATGATGTGTAATACTATGATGTGGTCGTGATATGGTTGTGATTGAGACAGGTGATGTGTAATACTATGATGTGGTCGTGATATGGTTGTGATTGAGACAGGTGATGTGTAATACTATGATGTGGTCGTGATATGGTTGTGATTGAGACAGGTGATGTGTAATACTATGATGTGGTCGTGATATGGTTGTGATTGAGACAGGTGATGTGTAATACTATGATGTGGTCGTGATATGGTTGTGATTGAGACAGGTGATGTGTAATACTATGATGTGGTCGTGATATGGTTGTGATTGAGACAGGTGATGTGTAATACTATGATGTGGTCGTGATATGGTTGTGATTGAGACAGATGATGTGTAATACTATGATGTGATCGTGATATGATTGTGATTGATGACATGTGCATATTTATTATTCATCCCATGTGTGAACTATCTGTTGCATGAGTTCTGAGACACTGATATGAGGATGGATGGATATGAGACACAGTT

>LNC_001274

GCTCCGATGAAGGTTTCTCCGGGCAACCTCCGAGCTAAGCTTTAACCATCTTCTTCATTGGCTTGAGTTTCCTAGCTCTTCGCTGTGACTATCGCCTTCCACCATTGAAGATATGTGCCTCTTAGCTCTCTGAATCGAAGATACATCAGGTACACTTCAATGTCGATTAATCTTTAATGTGTTAGCTAAAACCAGCCGCTCTAGTACAAAATTATACTATGTGAATCTCTTACTCCCAAGGCTTCTTCTGGTAATTTAGTAGGAATAAAACTTTAAATCTGTCCTCAATGTTCATC

>LNC_001275

TGTAAAGACATTTCTTTAAAACTTCTTTGGGAATACATAGTTCCCTAATAACTTTGAGAAATGAACTCAACTTTAAACTCTTGACTCTTTACTCTTTACTTGACTTGCAACTTGAGCCTTAGAATGAAGTTAAAACGTTCTATAAAGACTCTTGAAAAGCTTGGAGGACTTGCTTGAACTAACTTCTTAACTTCTAAGCTTGACTTCTAATTTCACTTGACTTCTAACTCGACTTGACTTGGAAACTA

>LNC_001276

ACTAGGGGGTTAGTGCCCGTGCAAGGCACGGGCAAATTTTGTATTGGGGTTGCATGGGCGAATTTTCTGTTGGCGTGAAAATAAAGAAGACGTGGTGATGTTTTAAATATATTACATTACTTTAGTGCAAGGTTATACAGTAAGCTTGCTAATGAAAAGGAAAAATGGGTGTTAGTGGTGCAGTTGCGGGTAAGGCTGTCGAAGTTGGATTCTGAATGGAGTTCTGGCAAAAAGAGTTGACTGGCCTTTGTGGCAAAGTAGAAACTGTACAAAGTGGTGG

>LNC_001277

GTGCTTAATGATGTATGGACTATGTAATGACTAGGAGAAGGTAGTTCTTCCTATGAACATGAGAAATGTTGATGTTAGTGTGTTATGACTAGGAGAAGGTAGTTCCTCCTTGAACATAACTAAACATGATAATGTCATTGCATACATTCATGTTTAAGTTGACAATTGGTTCATTGTGGTTTTACAATTATGTTGAGGTTTTTGAAATAAGGTGTAAGTGCATTGTTGCATCCTTATGTGGGCTGAAATTGCCATGTTTGTGTCTCTCATGTACGTTTCTTTAACATTCGGGGACGAATGTTCCCAAGGGGGAGATATTGAAACGACCCCAAAAATGCCCGAAAATGTGCT

>LNC_001278

CAATCATTTTCTAAACCCTAATTCATTTTTAGTCTCTTTGGTTGCCCCCAACTCTCAACTTTCTCTCCATTCATCCTTCAAACCATTGACAGCATGCCAAAGAAATCATTTGAGTATTTGACTCTGGATCTTTAAGTGAAAAGGAAACTCATGAACGACGGATCTATCTGACAGAAAAAGGGTTTTGAACTTCACGGTGTTCTTGTGGGTGATGATTGTGCTTGCACTTTACTACCGAACAAAATCTCTCTTTTCTTTGATCAGGAGCTAGAAAATATCCAGTTGGAGTATGAGTTTGAGTGCAAGGGTCAAACCTATGGATTTCCGGTCTACGTCTGTCAGGATTCACCCCACGGAGCCAGAAGCTCATGTTCAAAGTTCTTTAGCAGTTGCTTGGCATCCAGGTGTTATTCTAGGGTTGGTAACCGGTAGGTCGTAAGTGGGAAAAGTTGTTCGAAAAGAGTTGTCCAAGAGTAGGTGATGGTTGTTGTTTTTCAAGTTATGTAATGTGTGCATATTAATTGTCTCTATTGTAAAACTTGTACAATGCATGTTGGGGAAAACATAAGGGTTTGGATGTGAAATGATCAATTCTTGACAATCTCATGCAATTAAGCTGTTTTACTTACTTGTATTTACTAGTGTGATTGTATATTATTGTGAATGTGTTTGTGTGACTATTCGTTGACTCGGGTACCAAGCCTGGTGCAGGATATCCTAGGCCTTTCTCACGATTATTTCACCCAAATTAGGGTTTTTTTTCACAATTAACCTCTAAGGTAGTTCCCATAAATTCTATGACCTAAGAATTGAATTATCAAGCTAAGGGAGTAATTAAATTTCCTTTAACTATGATTACGATGAAAATAAGAGAAAATTCACCCTAGGTCACACTTGCTCTTCCCCAAAATGAAATTGCCAAACAATGGGTGGTTTTGGGAATTTTCTTGCATTAAATGCGACTAACGTCGCTATAACGGCCCAATCTCGCTATAGCGACATCGCTGTAGCGGGACCTTCCGCTATAACGAGAATTAAGTCCACCTCTATAGCATTGTTCACCCGCTATAGCCGCTTGCACGGGATAGAAATTCGAAAATTTCTCAAAAAGATCCCATTTTGCAACACACCTTCGAAACTTGATGTGTTAAACTAACACTTTCATGCCAAGTTCGATATATAAGAGTTTTACTTGCCTGAATTCACTTTTGCAAAGCCCTATGGAATCCCTGCGTCTTAGAAACAAGAAAAAACAAACTCACTTAGACATTTCACCCTATCCTTTTTTCGGATTGTCCTAAACCTTATCACACTCAAATTTCGTCCAAGCCTAACCTAGCTGAAATTTTTAATTCACTATTCCAAAAGTTTTGTTCCTCATTGACTTTCACTAGTAGCTCTCCCATGAAGACTGGAAGGGTCTTTCACACTACGACATTAGGAGAGTTCATATTACTAGACAGACTCTTCGTTACTTTATCCAATATTTGCTTCAAATACTTGCATTTACCTCTATTCTCATTTATAGTTTCTCGTGAATTTTGTTTTCTTTTAATCCTTTCATCTAGGGTAATTAACCCCCCCCCCTCCCCCTCATATGAACTCTATCTTGTGTATTAATAACAACTAATAAATACATTTATATGGGGACACAAACTAAAACCTATGAGAACTCACAAACTTACATGGATTAGGGAAAACAAGAGAAGTAGCACCAAATTTAATTTGGCGTTCAACTTTGTACAACATTATATATTACAGTTGAAAATAATATAACAAAAATGTATAAACAAATCTTAAGGCTGAGGCACGGATATCTTGCTCTCTTTAAGGAGATTCAAGCCCACTGCGGCATAATCTACACCGATCCAACAGTATCACTCTTGACTTGTCCCCTCCAGGATACAACAACCCAACAACGTGTACAACTCAAGAAACTCCGGATTAGTTGAAGAGTCCAAAGCTCCACATAAGAACACCTTCCTTCAACATATAAATATTCTCTTTTGTATATAGTGAATATAGTTGAAAATTAAACTTAGAATATTATTCTTTGTCTCAACTCTCTCTTTCACTACAACACTAATCTCAATATAAACACAATAAAATACTACTCATCTTTTCCACTCGATATTTTCTTGTACTTATCTTGTTTTTCCTTATCCCTCAACCCAAAGGAAGACCACACTATAGCTATTTATAGGTGATGAATTCTTCCTTGCAATGAATAGGAAGATAGTGGGTAGTAAATTTGCCAAATGAATGGCTCAAAAATGTTACATAAGTTACAAGACATAAAGATAGAATAATGGATGAAATGAAGAGTTGGTGGTTACATAAGTTACTAAAAACTAATGACCACTTTCTTCCACAATTGATGAGGGTAAAGAGGCACAAATATAATGTCCATCAATAGACATAACATGATATATAATGTGACATATAATATATTTTTATTAATATTAAAATGTCACACCAACAAATACATTTAGATGGGGACACAAACAAAAACCTATGAGAACTCACAAACTTACATGGATTAGGGAAAACAGGAGAAGTAGCACCAAATTTAATTTGGCGTTCAACTTTGCTACAACATTATATAGCAAATACAAACTTAATGTCGCTTGTTGTGTTCAAATAGGGTGGCTTAAGATCCTAAAAACTAACTACAACGTGGCTACTTATGGCGGATCGCACTGTGATGCGTATAATAGGTATTTTGTATCATGTGCTAGTGAAGGTTGAGACCTAAATATCAGGTTATTGTTTTTTATGTTTGGTTAGAAAGAAGAAAATATTGTCTCAAGAGCATTTAGGTAGAATCAGACAAATACTTTAACATTAGACTCGTACCTTACCATTTAACCCAAAACTAGACCCTGGTCCCTAACCTCAAACCTACCTATAACTAGATCCTCAAGACGATCATGCCCAAACACAAGATCCGACACTAACTCAAGACCTAATTTCTATCATAATCCCGACTGAGATTAAAGACCCAAATTCGAACCAGGATATGACCCGACACTTACTTAGGATGAGGTCAAGGCCGAGGTTGGATCTCAAGTTAGGAGTCAACTGCTCAGATCTCAAGTCAGGGTCAGTGGTCAAGAGTTGGGAATCAAGAGTCGAGTCTGAGGTCTCGAGTCTCAGGCTCAGGGTTAGGATTGGGGAATCGGGATCCATAGTTGAGTTTAATTAGGGTTGGGAGTTGGTACCCTTGTCAAGATCAATTCTGAAGTTAGGTCTTAAGTTGGGGAAGACAGGATTTATAAAATCATTCCACACTTAACCAGGAATAAGGTTGAATTAACTAAAAGAAAATTTCACTAAATTTCTTAAACTAGATGGATTTTATATCGTATATCAAGGAAAGAATTCAAATCAAGACGATCAAAAATGGAAAAATAAATTAGGGTTACAAGTGTTTTTGGGGTGGTGAGATGGGGATGGAAAAATAAAGATTGCAAAAATTTTAAATTATGATTTTTAGAATTGAGATAAAACATAGAGCTTGTTAAAAATAAACAAGGAAATGAGGGCACCTAATATGAAAGAGACTAAAATATATGATATCAAGAGAAATTTTAGTAGCTTAGTTAGTTGACCATCCAAATTTTTGTCTTGTTGTAAGAGTTTGATTCCCCACTTTGTCATTGATCCATCATTTCCCTTTTCAATAATAATAATAATAATAATAATAAATGGTATAAAATAAATCATACAAACAAGGAAACAAATCAATTGATGTTCAAAGAGGAAATATATAGTGTTACCCATTTAAATAGTTTCATCCATATTTTGCGATATCCTCATCTCCTTTCTTTTTTAGGACCAATTTATGAGAAAAAGTCCTCTCAGATTTTCATGTTTTTATCAAAACTTTACGGTAAGTGGATAAAGGAGAAAAAAAAGGAAGTGAAAAAGTGATTCAAATATGAAAAGGATACAGTGATTCACATACGAAATAATAGGGCATGAAGAAGGTCGTTAAGGTTTGTAGGATGAAAGAGATACAAAGAGGAGAGTTGGGAGCGATTGAAGGAAATTGAAATGGATTAGGGTTTAGAAAATAGTTATGAGCATTAAAAAAATAATGAAGCGGAGAAGTGTGGGCCATCCAGTTAAATTGAATGAATAACAGAGACTAAAAAGATCAAATGGGTTGCGGCCAAACAAAACATCCAGAACCCAACCCATTTGATCTTTTTAATCTCCGCAGTTAATTTAATTTAACCAGACCACCCACACATTTCCCTTTCACTATATATTTAACCCTTAACTATTTTCTAAACGCTAGTTCATTTCTATTTCCTTTGGTCGCCCCCAACTCTCCTTTTTCTTTCCCATTCATCCTACAAACCCTAACAACCTTCTTCAAGTTCTCATTATTTTGTATGTGATCGATATATTCTTTTCATTTTCAAACCTCCTTGTCACTTTTTTTTCTCCTCTGTCCATTTATTGTAAATATTAGATAACAAACATGAAGAATTGAGAAGACTTTTCATCATAAATTGGTCCAAAAAAGGAAAAGAGATGAAAATATTGAGAAACTTGAGTAAAATTATCTAAATACTAGTAAATTGGGTAACACTGTTTATTTCCTCTTTGAATGTTGATTGGTATGCTGTCATGTTTATATGATTTATTTTAATATCATTTTTTTACTTATTATTGATAAGAGAAGCGATGGAGGGGATTACAATGTGGAGAATCAGACTCTCACAAACAAAGCAAAATATTTAGATAGTCAATCAACTAATCTACTAAAATTTCCCTTAATACCGTCTATTTTAGTCTCTTCCATATTATGTATTCTCCATTCCTTGTTTAACTTTAACAGAATCAATGTTAAATCTCAACTCTAAAAATACTAATTAAATTTTTTTCTAATTTGTATTTTCCAATACCCCCGTCCCCCTCAGAAAAAACTTG

>LNC_001279

CAATCATTTTCTAAACCCTAATTCATTTTTAGTCTCTTTGGTTGCCCCCAACTCTCAACTTTCTCTCCATTCATCCTTCAAACCATTGACAGCATGCCAAAGAAATCATTTGAGTATTTGACTCTGGATCTTTAAGTGAAAAGGAAACTCATGAACGACGGATCTATCTGACAGAAAAAGGGTTTTGAACTTCACGGTGTTCTTGTGGGTGATGATTGTGCTTGCACTTTACTACCGAACAAAATCTCTCTTTTCTTTGATCAGATTCAAACCATGGAGCAAAGCTAGTAGTTCCGCATGATGGGCATTAACGACATTTACTTTGCAAGAAAATCCTACAATCCAACTGCCTAGATAGTCCCTTAAACTCCACCGGTTCCACCCATTTTCAAGTTTTGATTATCGGGACTAGGCAGGCCGTGGGCACTAACTACGAGTTTCCTAGCGAAGTGTATTGCCTTGATGAGCATAATTTTTGAATTTCTTTGTGAAGGTTTGGTCCAGTGATGATAATGCCATACTTATTCCATATATGCTTTGCATCTGGACACTCCATAAAAATATGAGTTACATTTTCTTCTTCATTTGTACAAGAG

>LNC_001280

TATTACCCCATTTTACTAGTATTTAATAGTTTCATCCATATTTTGTGATTTCCTCATCCCCTTCCTTTATTGGACCGACTTATGATAAAAAGGCCTCTCAGATCTTCATGGTTTTATCCAAACTGTATAGTAAATGGATAAAGGAAAAAGTCAACGAGTGGTTCAAAAAATGAAAAGAATATAGTGATTCACATACGAAATAATAGAGCTTGAAAAAGGTTATTAAGGTTTGTATATGAAAGAGAGACAAAGAGGAGAGTTGTGGGCGATTGAAGGAAATAGAAATTCATTAGCGTTTAAAAAATAGTTGTGAAAATTAAATAAATAGTGAAGGGGAGAAGTGTGGGTCGTCCAGTTGAATTGAATGAAAACCGGAGATTAAAGGATCAAATGGGATGTGGCCAAACAAAACAACCCAGAACCCAACCCATTTGATATTTTTAATCTCCGTTGTTTTTTCAATTTAACCAGAGGACACACACATCTTCCTTTCACTATTTATTTAATCCTAATAAAACTATTTTTTAAACCCTAATCCATTTCTAATTTCTTTGATCGCCCCCAACTCTGCATTTTCTTTCCCAGTCATCGTTCAAACCCTAACAACCTTCTTCAAACTCTCATAATTTTGTGTGTGAATCAGTGTATTCTTTCCATTTTTGAACCATTTTTTCACTTCTTTTTTCCCATTTATTCATTTACTATAATGCTTATAAATAAATATGAAGATTTGAGAGGACTTTTCATCATAAATTGATACAAGAAAGGAAAAGAGATGAAGAGATAGGGAAACTTGTATGAAACTATGCAACTACTACTAAAGTGGGGTAACACCATTTATTTCCTCTCTGAATGTTGATTGATATTTTTCATGTTTTTATGATTTATTTTAATATCACTTTTTAAATTATAATTGAAAAAGGAAACGATTGAGGTGATTACAATGTGGAGAATCGAACTCCCACCAATAAGACAAAAATTTAATATAGTCAATCAACTGAGCTACTAAAATACTTCTTAATACCGTCTATTTTAGTCTCTTCCATATTATGTGCCCTCATTTCCTTGTTTATTTTGATTTTTGAACATACTCTATGTTAATATCAACCTCAACTAGCATCTCTTGATTTAAAAAAAAAATCAAAGTCATTTCTTCATCATGTTTTTTATTGATCTCATTTATATCTCGTTTATATAGTCTATTCACATTTATACAATCCATTTTCCATCTTTAGAACTAAAGTTGTTCACTTGAAGAAAATGCATCCCCTACTTTCTCATCTAAAGGAAAAGCCACTATTTGCCTGGGCTCCTCTGCTCCTCCTCTATCTGTGTGTTGTTTTGTAGCTCTGTTAGGTTGTCGGCGTGGTCTTGTGATGGTTATATCTCAAAATAGAAATGGTTAAATAATGTATATCTTACCAAATGTGAATACTTAAATCTATAAAAGAAATTGAACTACTAGCTCATTACAAATTATTTTTAGTTTGTAGATTGAATAAATTAGATTTTATTTCATTTATTCGATGTTGAATTAATCCCTTATTCTTAGTAAGAAATATTTTTGGATCTAAGAAAAAAATTAAGTTTAAAAGACAAATTATTAGTTATTTTTTCTATTAAATCTGATACAACTACTAAAACAAGAATGATCTTTAAAACGTGAATCTTTTAAATAAAATAGAGTATTTGTAAGAGATAGTCCATTCAAATAATACAGAAGAAATGAACAGTACATCACTAAATAAAAGAGAAGAAAGAACGTACAACATGTTGACTTAATTTCAATTCCGAAGAATTGTTGAAAAAGATTCGTGCTTGAATTGAATTCCATCATCGCCATTGTCATCAAAGTCCTCTTCAGTGTTTTGAATGTCTTTCATCAAAGCCGCAATAGGTTGAGTTGTGTGATTGAATGGGAGGGATTGTTGAAGCGTTATATTTATAAGAGCGACAAGCGACTAGGGTTTTCTAAAACTTAGTTCAAACTTTTTAAAACTTAATTTTCTCACAAAAAACTTAGTTATCAATTTTTAACCCTTCATTATATATCTAGAAATAATAACATCCAGTAAATTTTAGAAAGTTAGGTTTTAAAATTTTAGTCGATTTATAAATATTGGCTAAAATTGCCACCTCTAATTGTGAGAAAATTAGGTTTTAAAAAGTTTGAACTAAGTTTTAGAAAACCCTAGTCGCTTGTCACTTTTATAAATATAACACTTCAACAATCCCTCTCATTCAATCACACAACTCAACCTATTGCGGCTTAGATGAAAGACATTCAAAACACTGAAGAGGACGTTGATGACAATGGTGATGATGGAATTCAATTCAAGTACGAATCCTTTTCAACAATTCTTCAGAATTGAAAATAAGTCAACATGTTATAAGTTCTTTCTTCTCTTTTATTTAGTGATGTACTGTTCATTTCTTCTGTATTACTTTAATGGACTATCTCTTACAAATACTCTATTTTATTTAAAAGATTC

>LNC_001281

NNAACATGCTCATATGTCGGAGTATTAGATTTGATGGCTAAAAGTGGTCACAATCTAAGAAATAAAATTATATGGGTAGGGTCGGAATGATGTCACGACCCAACTAGAAAATAGAAATTATATAGGATAGTTCGAATTGATGGAACGACCCCATTGAAAAAGAGAAATAATATGGGTAGGGTTGGAATGATGGCACGACCCTACTGAAAAAGAAAAATTGTACGGATAGGGTTGGAATGATGGCACAACCCTATGCAAATCAGATTTGAGGAGTCACCGGAAAGACGAATGGAACTATGCAAATCAAATCTGAGGAGTCACTGAAAAAGACACATGATGCTACGCAACTCAGATATGAGAAAATAGTTCGAAAAAATCCACGGTACTACACAAATTAAATATGAAAAGTAGTCCGGAGAGATGCACGGTGCTACGCAAATCAGATATGCAAAAAAAAGGTAGTCACCGGAAGGATGGCACGGTGCTACACAAATTATATATGAAAAAGTAGTCACCGGAATGATGGCACGGTGCTACACAAATACCAACGGATATGGGGTTGACACAAAACAGCCCTAGAAAGTCACCGAAAATTGATGCATGGTGACCTGTCTGACTAAGTTTCTTCCCTTGAACATGGGGTTCATGCATGTATCATTTGTCCTACTTTTATTAACATAATCATTGGCCAGACGGGCGGCATATATGGGTAATAGCCACCCGCCGGCAGCGGAAACTCTTTGATTTTCTACCAAGATCGTAGAGGCTCTGATACCATGT

>LNC_001282

TGATCAATTAATGTGGGGTGATCTGCCTAGTTTTGGTTTAGTAGAATGAATTATTATTATTTTTAATCTTTATTTATTTCATTTTTTTTTTCTCTTTGGAACTAGAAGGTTTTACAAGTTTTATTAAGGTTGAGTTGCGTAAAATATGTCGGGTTAAGGTCAAGTGTGTTCATTCCATTAAAATCGGTCCATGTTTCGCTATCTTCTCTCCATTTCGGATAATCTTTTTAATGCATAACTTCATCCGATTGAGTATTAGGTGTGCACCAAAATAAATGTTATCATCTCCTATGGTGTATGAATGTTTGTAAGGTTATTGTGAAAGATTGACCTTATTACTATTTTTACATACTTG

>LNC_001283

CTAATTCTCAAAATAGAAGTTTGTCCTAAAAGTTGCCCTATTCTCTTTAGATCACCACTACAATTGAGAATGAATGAAACCCAGCTGCCCTAGCTAATCTCAGCGATGCTTTGCCCCTTCAATGGATAGATGTGTATTCTTCTTTGTGCTATTGTTGTCTCCCCTAATCTCAACGACATTCTTCCATTAAATTGGTAAAGGTGTGTTCATTCTTCTGATCGAGTTGTTGCTGCACTGTTCGAAATATAATCCAACACAGCTTCGCTTGGGACAATATTTCAGGTAGTTCGTGTATGACTATTAGTTTCTGGAAGATTTCTACATTGGTGTTTCTATCATTGCTTAAGTTAGGTCTAGGTAATTAAAATTTAAGAATATGAGTGATACGCCATCATCAAATGCTGGTTCTCCTACTGGTCCTTGTACTCCAACCACTGCTAGTACTCCAGATGTCTCTATTGGGTCTACTAGTTGCTCATCAACATCTGCTCGGTTGGTCATCTCAGTTGTAGCGGAAAAATTCGTTCCTAATGGTCATTCTATCTCGAAGACCATCACAGAAACTTTCAAGGAACGACAAGATGCTACTGGCTACTCATGGAAGGATGTCAGTC

>LNC_001284

ATTTAACGTGGGCTTGTAACCAAGTGGGGCATTAAAGTTGTTTGGATGTTGATAAATGTTCCCAGTAATGCGGACTTCGTTGGACCTCTCTACTCTTGACAGAATTGTGCACGCCACAGCTCTACATGAGCTGGTAGCATTAAAAAAAATTGTTAACTGGGGTTAAGGTTCAAGAGGCCCCAGAAGTTGTGGGAAAAACTAGTGGTCTGAAAGGCGCCACCAGCCCCCATATCTTATAGAGAGGATTCTTCGGTTCTGATTAAGCGTGTCTATTGGGTTCAATTTAACATACAAC

>LNC_001285

GAAGGAACACGAAGATCAACAGCCTCACCAAAGAAGACTGAGTGACACGAGACAATCTGCACCAACTAAGGAGATAGATTGACTCACAATATGCACCAACTAAAGAAATAGATTGACTCAATCAATCCAAAAGATTGACTCAATCAGTTATCAATCCAAGAAGGAAAACAACACCAGGCAAGCAATCAAAGACAACAAGCAAAT

>LNC_001286

GGCCTAAAAACAAAAAAAGAACAATACAGGACTTAGACATTAGTATCAAATTAGGAAAGTGACAGGAAGTCTTCATCTTCTTCGGTGCTTTGCTCAAACATTGTTGGTGACTTGTCGATTTGTTCTCTATTGTTGATGGTTGCACTATGTTTCCTCCCGTAACAGCTTGCTGCTCCACCATTAATAAAGCTTATCAACTCAACTCGGCTGCTGATAGATCGATTCT

>LNC_001287

CGATCATCTCCTTCATATTCTGCCTTGAGATAATCCCAGATGGCTTTTGCTGATTTTAGAGACATGATGCGAGTGAAAATTGTTGAAGAGACCGCTGAAAAAAGGCATGCCTTTGCCTTAGATTTTCTGGTTTTCTTGTCCTTGTGATTTTTGAGTTGAGCCATTGTTAGGATTCCGGGTAGCGGAAGAACTTCAGAGTCTTCCTCGATTGCCTCCCAAAGATCTAAAGCTTCGAGATAAGCTTCCATCCTCACTGCCCACATGTGAAAATTTTCACCATTGAAGACAGGAGGTGCCA

>LNC_001288

GGCTGGGCTTGCGTAATGTAGTTGTAGTTAAGGCTGAAGTAGCGCTTTTTATTTTAGAAGATCTACTGAAGTACCAAAGGCGAACGGGGCTCCCAGGCCGGGACGTCTGGCAGAATGCTTGGCCTCCTGCGCTGGAAGCGAGACCCGAAGGGTGCATAAAGAAATATTTATCACTTTTGTCTTAATCTTAATAAAAAAAAGGACTTAAGCATCTTGGATTCAAATATTCAAACAGTCTTTCTTGTTATCGAAATCTAAAAATATATATATATATGGAAATAAATTGCGTCCAATAGGATTTGAACCTATACCAAAGGTTTAGAAGACCTCTGTCCTATCCATTAGACAATGGACGCTTTTCATTCTGATTTTTTTTTTTCGTATTTTAACTTTTTGTTTGAAAAAAAACCCAGAAAGAATCAGATATGAAAGAATTTTT

>LNC_001289

CAATCCTTCTTGTCCGGTAAGCAAAAGCAAGCAAGGGAGCCTCTGGCTTCGTCCGTTCGTCTCGCCACAACACAAGCTGTGGTATAATCTGTCTATACCTCACTCGGTTCAGTAAGATAAAGATATGGTTTGATGTGTGAGGCCTCGCTTCACTAACAAAAGTCGCTTCCTCGTGAACTCCGTTAACTAGGCGCCCCTCTCCAAGTAGTCAGTCTGTTCTCTCGTGGAGAAAGCCTTACTCTCGTTTCGTTCGAACGTAGCCCGCAGGAGTCAAGGGGTCCTCAGGGCGACTTCCAGAGATAATTACGGCGAGAGGAGATGAAGAGCATTCTTCCTTCGCGAATACCGACTTCACCAACCCCCAACCCGGCTAAATGGTTTGACGCCTGCATTTAAACTAACTCACCATATGCTAGTATGCTAGTGTGCGGCTGAGAAAGTATATAAAGTATATAGAGGGCAAAGGTTTTCAAAAAGCCATTCTCGGTGACGACATTGTTATCGCCGACGAAAAGGTTGCAAACGTGTATACGGCCCCCTGATAGGACTGCGACCTAACTATGACCTAACTATATCGGTTGAAAAGTAGCTAGTCTCTGACAAAGGTGCTCTTGAGTTCGCTAAGCCATTTATATGCGATGAAGGTGGAACGGATTTCTCTCCAATCTCCTTTCGCCCGTTCACTCAGTACTCAGTAGCAGCTCCCGTAC

>LNC_001290

GTAAATGTTGGGATCTTGCGATATAGATCAGTAGAGAAGTGCTTGATAATATGCATTCTTTGATCTTTTAACAGATGCTCTACAACTTAAAGCTAATTATTTCACTTCTTCGAATTTGATTTTATTATTTTAATTAGTTGAGTTGATATTTAGGGTCACTATATGGATTACACACTCCTTTTTTTGGTTTATTTGGCTGATTTGATTGCAGGTAGGGTGAGAATGTTGGCTTAAATATGATATGAGATTTATGCTTATACATTGCAAGTTTATTCTTTTGATGAATTCAACTGCCAACTCCCCAAGGAGGTCAAGGGGAATCTTGCTATTCTCACTTGCTGGTCAGGATGAGGATAACCCTTCTTTGTCAAATGCTTCCGAGATGTCTTTAGACTCTTCAACGTCTGAATATTATAGTTATTCTAGCATCTATAATCACGAGAAATGGACTTGAGGCTCACTGCATTAACATGGAAAGTGACCAACATGTTTGAGCAAGCTGTTAGTAGAAAATGCTCTATCAAAGATATCATTAACAATGAAAAGTTCTCTTGCCATTTTGGAGTTACTGACTTGAGGTAAACAAATGGACAAGAAAGGTACTTGAAGGCTGAAATGAGACGGGTTATATATTAAAAACAAAATACAGGGTAATAATTTATATGTTCTATCAATAGGTTTAGAATTGTATCTATGTCTCATGATACCTCTGATAAAGAGCGAACACTAACAGGAGCTTCAACACTTACCATCTGACCTAAGTAATCACATGTAGGGAGTCTACTCGAAATATTTTAGAACTGATAAGGAGAAGACGCCCATCTTAATTCTCATATCTACTTGTGTGACATTGTTACTCCCAAGAAATACTTGAAATTTGTAGTCAATAGATGATGCTTCTATATCAAAAAGTATTTTCTTTTTATTTTTGGGATAAATAAATTTGGTAGTAAGCAAATTTTCCGAGTCCAGTAAATGAAGAGCTTTAGCCAAAGTGTCCTCTCTTTTATGTAAGACATTATTTTTCTTCCTGGTTTTGCAGGAACGAACTTAGAGAAGGAAAGAAATTTGATGTTATTTTTCAATTCTTTGGTTTTCATGATTACATAGCAATGTTTTGTTTTTAAATTGGATTTTTAAAATCAACAATTCCAATAAAATCATGTGGAGGCACTTGGTCATGTTTTCTACAGCATTTTGGTGGCTAATCTTAATGGCAACATGGCTATTGTGATCAATTTGAAGAACATGTCTCTCAATTTTCTCTAGAGAAGCCTTTTAAGTTCGAAAAGAGAATTATCATATGAGCATAAGTTGAAATTTGTGTAATACACAAAGCAGATGCATTTGCTTTCTTACATATATGCAATAATATTATCAACTTCTTCATTGCAATGACATTGTACTACTTACTCACTAATATGAAAATCAATATATAGGACGGATACACATTTAATTTATAACTTTTCAGTTCTTGTACATAAGTTTGTAATTATGAACCAAGTATGAGAACATGAAGATAACTTTTGAATGTGCTACTAAAATAACTTGTGTGCATAATCAATTGTGTTGGGCCCGTGCTTAGCACGGGCTTGCTTATCTAGTATATATACAAACACAGACATACTTATACATACTTAAAAGCATGAACTCCCAAATTTGAATGTTGAATTACTATAATACTCTCAATTAAAAATACTCAATTTATTTTATTTATTAATTTAATTAATTTTATCATATTGAAAGTAGATACATTTTCAAAATAAAGGATTGTAACTTTTCAATGAGTTGCGAATTTTTCAAAGGGTTGTGACTTTTTG

>LNC_001291

TACGATACAACGCTAAGCTTCATAAACTCAAGTAGTCAGAGAAAACTGATAGCATTAAACTGACACATTATACTTAGTGTCGAGTCAGTCTGAGACATAATTGCATCCACATTTTGCAGCATGCTATTCATCACTCTTAGTGGTAATCTATGTAAGTATCAACAAAGAAAATAAACGCTTAAAGCTAATTAGAAAATTTCCTAACTTTTAATTAATAGCAAATGATTTTCATTCACATCGTCACATCCTTTATAGTTTAGCTAAATTCTTCTGCTTGCTGTGACTTTATTGCATTCCTCTTCTCCCTTAAGTCATCGACTTCATACCGGCCACTAAAATTTTTAATCAACTTTTATAATGATGATGATGCAGGGCAACTAAAACAGAAGTCTCCAAACTCGCATGGTAAGTTAGATGCTTAGGTAACGAGGGAAACCTAACACCACTAGGCTGCTTTTGAAGCTTTGGCCGGTGTAGTTTCTTAATCTTTCATGAAGATCATCTTTGGAATCGCAACATCCTTTAATAATACAGAATAAACAGCATGTAAATGTGATCCTTTACTTTATTCCAATTTGCTTAAGTTGCCAAGTTTAACATAGTCATTAGCTTCATGATTGTTCAACATCTAGCCTTGATCTATAGTAGTTCTAACCATGAAACTACTATTCTTCTTATAAGTAAATAAGTGTATCTAATCAAAGACAAGATAGTTGTCAAGAAGTAGAAGTGGGTAGTGGAGAATTAAATATGGAGAAGAAAAACTCCTTAGAACAATATATATACGGGCTGTTCTAGGTTACAACAAATGTATTTATAGGAAAAAAAAATTCATAGAGTGTCCCCTACATAAGGCCTTAATATCAGATTTTTGTTGACAGTAGCTTTGCTTCTAACAAGACAGGGGAGTTGTGGACGCTAAGAAAAGAAAGAAAGCAGTGTGGTGGAAAAAACTTTACATGATCTTCGTGCAATGTCCATTAATATAATGCTGCTAACACAAATCTTGAAGGATAAAAGTCGTGTATGTGCACGTTTGTTCTCCTTTTAAAAAATTGTGCTATGTTTTTCCTTTTTGGCACAATTTGAATTACTCTGTAGTAACTTTCTGGTTGAAAAGAAGGACTAACATGCACATACCAAAAAAAAATTCCTTCTAACTTGCACTTGATATCTCTTATTCTACTTGATTTTCCTAAAATCAAATACACACAAGCATAAAGATTAATGGTACATTGAGTTCTCTTCCTCTCAATGCAGTTTCATTGCTTTGGTTAATTTATATGAACTTGAGGAAAAGGAAAATGTAGAGTGAGTTTTTTCCTCTCTTTTTTTCCTGATGATTACTTATTGAGAATATGACTATCAATTGAAAAATCCTTGATAACTTCTGTCTGATATAAAGACTAATGATTTAAATTTATTTATTCTCTAATTTATTGGTCTTTAACATTGTACTTGATTATTGACTAGATATCGTTTAGAAGTGTTTATTAGTGGACTTACTAGCAAGTTCTACTTATGCAGAAATGAGGTGAATATGAATGTAGTAGTTTGTGACTCAGGACTGCACCCCTAAGAATTGCTCTTAAATGAAAGATTGCAGTTCTTCCTTTATGCAGTATGGATAAATGTTTCATCGTACGAAAGTCAAATCCTATACTTCTCTGTTTTTTTTTAAAAAAATTATACCTCTTTTTTTTGAAGAATCTGAAATTAACTATGACAAACATAAGTCACCAAGGATTGCTCTAGAAAGCTATGACCATCTTCTGAGAAAGGAATTATTAGATCCATTGTAATAGTTTAGGAAAGAATTGTTAAATATAATTAGGTTGGACTCTTCATTTACTAAACGAGATATTTCCTCGAACAATGGGATTGATCTAAAACACCATCAATACTTAAAGTTTGCATTTTAGGAATACCAAGAGAATCAACAACATCAAACACAACCTGACACCGATGATCACCTCACAATAAATGCTGGGATCTTGTGCTATAGATCTCACCTGAAATGAAAAATCTAAAACTTTTTTTTTTTATTATATAGAAGAGCATAACTTGTAAATGTTGGGATCTTGCGATATAGATCAGTAGAGAAGTGCTTGATAATATGCATTCTTTGATCTTTTAACAGATGCTCTACAACTTAAAGCTAATTATTTCACTTCTTCGAATTTGATTTTATTATTTTAATTAGTTGAGTTGATATTTAGGGTCACTATATGGATTACACACTCCTTTTTTTGGTTTATTTGGCTGATTTGATTGCAGGTAGGGTGAGAATGTTGGCTTAAATATGATATGAGATTTATGCTTATACATTGCAAGGTGTTGTCATCCTTTAAGTTTTCAGTTTATTCTTTTGATGAATTCAACTGCCAACTCCCCAAGGAGGTCAAGGGGAATCTTGCTATTCTCACTTGCTGGTCAGGTTGGTAATTCACTTTCACTGGTTTACTGCTTCTTTCTTTTTCTTGTTCAAAGTATCTTTTATAGTTTTGAATCGATATTTAAACTTAGGAATAACTTGTGCCTCCAACTAATGGAGGCACCATGTTAGTGATAATTTCTTCCCTTTAGCATGAGGATATTCCTAAGATGATTGAAGCACGAGATCAATTGTCTTTTGTACTCTATGTAATATGTATGTATTTGCTTTAGATTATGTTCATGTCTATCTGCAGATATATAAAGTATTTTTCTGCATTGGATGTTTAAAATCATAGCCTACTATTAAATTTACCTTAAACAATTCATCAATACAAGTTAATATGATTTGGCATGATCTCTATCTTTTGATATGTGATGAATTTTAATGAAATGGGACTTCATGTGGAGTTCAGGTTTTCTATTTCTCTCAACCTTTCCTGAAGTATGCCCTATCCTTAAGCAGTAATTGGGTTATCATTTATTTTGGTTCAATAAACTTTCAAACTTTCTGCTGAATATGTCCTCATTTTCATTCTCGGACATATTGAATTTTTATTTACCAAAAACGTTAGGTAGTGACTGTCAAGCTTGACTAATAGAGATGGTTCCCCTATAACAAACAAAGGTCCTGTGTGCTAAGTCCCGTAACTTAAAGTTTAAAGTTAGTGTACCAGATAGAGCTTAAATCTTACAAATTTAATAGGCAGAGCATATTAGTCACTTGATGTAATTCATTGTTTCTGAATCATTTTTTATTTCTCGGTAATTACTTATTATACATATGACTTGAAGTTAACATGCTTACTGATTTCTCTCTAGGATGAGGATAACCCTTCTTTGTCAAATGCTTCCGAGATGTCTTTAGACTCTTCAACGTCTGAATATTATAGTTATTCTAGCATCTATAATCACGAGAAATGGACTTGAGGCTCACTGCATTAACATGGAAAGTGACCAACATGTTTGAGCAAGCTGTTAGTAGAAAATGCTCTATCAAAGATATCATTAACAATGAAAAGTTCTCTTGCCATTTTGGAGTTACTGACTTGAGGTAAACAAATGGACAAGAAAGGTACTTGAAGGCTGAAATGAGACGGGTTATATATTAAAAACAAAATACAGGGTAATAATTTATATGTTCTATCAATAGGTTTAGAATTGTATCTATGTCTCATGATACCTCTGATAAAGAGCGAACACTAACAGGAGCTTCAACACTTACCATCTGACCTAAGTAATCACATGTAGGGAGTCTACTCGAAATATTTTAGAACTGATAAGGAGAAGACGCCCATCTTAATTCTCATATCTACTTGTGTGACATTGTTACTCCCAAGAAATACTTGAAATTTGTAGTCAATAGATGATGCTTCTATATCAAAAAGTATTTTCTTTTTATTTTTGGGATAAATAAATTTGGTAGTAAGCAAATTTTCCGAGTCCAGTAAATGAAGAGCTTTAGCCAAAGTGTCCTCTCTTTTATGTAAGACATTATTTTTCTTCCTGGTTTTGCAGGAACGAACTTAGAGAAGGAAAGAAATTTGATGTTATTTTTCAATTCTTTGGTTTTCATGATTACATAGCAATGTTTTGTTTTTAAATTGGATTTTTAAAATCAACAATTCCAATAAAATCATGTGGAGGCACTTGGTCATGTTTTCTACAGCATTTTGGTGGCTAATCTTAATGGCAACATGGCTATTGTGATCAATTTGAAGAACATGTCTCTCAATTTTCTCTAGAGAAGCCTTTTAAGTTCGAAAAGAGAATTATCATATGAGCATAAGTTGAAATTTGTGTAATACACAAAGCAGATGCATTTGCTTTCTTACATATATGCAATAATATTATCAACTTCTTCATTGCAATGACATTGTACTACTTACTCACTAATATGAAAATCAATATATAGGACGGATACACATTTAATTTATAACTTTTCAGTTCTTGTACATAAGTTTGTAATTATGAACCAAGTATGAGAACATGAAGATAACTTTTGAATGTGCTACTAAAATAACTTGTGTGCATAATCAATTGTGTTGGGCCCGTGCTTAGCACGGGCTTGCTTATCTAGTATATATACAAACACAGACATACTTATACATACTTAAAAGCATGAACTCCCAAATTTGAATGTTGAATTACTATAATACTCTCAATTAAAAATACTCAATTTATTTTATTTATTAATTTAATTAATTTTATCATATTGAAAGTAGATACATTTTCAAAATAAAGGATTGTAACTTTTCAATGAGTTGCGAATTTTTCAAAGGGTTGTGACTTTTTG

>LNC_001292

CTCACTCTCCGTCTCCACCATAAAATTTCATTACAATCTGAAAGTATCACCGATCTCCTTACTCCTCCATGGACAAATCCTCGAGGGGGGGCTACCCTCCATTGTTACCATCGAAACCAATCGCAAATCCTTCAAACGAATCTCGAATCATCCATTTGTATGAAAATTCTCTGAATTTTTTTAAAACAAGGGGATTGAAACTACATTAAAGGGTTAGTTTAATCAAGGTGGAAGAGTGTGTAAATTGATGGGAAACTACATCGGAGAAATTACAACTGGGGCAACTAAAACAGAAGTCTCCAAACTCGCATGGTAAGTTAGATGCTTAGGTAACGAGGGAAACCTAACACCACTAGGCTGCTTTTGAAGCTTTGGCCGGTGTAGTTTCTTAATCTTTCATGAAGATCATCTTTGGAATCGCAACATCCTTTAATAATACAGAATAAACAGCATGTAAATGTGATCCTTTACTTTATTCCAATTTGCTTAAGTTGCCAAGTTTAACATAGTCATTAGCTTCATGATTGTTCAACATCTAGCCTTGATCTATAGTAGTTCTAACCATGAAACTACTATTCTTCTTATAAGTAAATAAGTGTATCTAATCAAAGACAAGATAGTTGTCAAGAAGTAGAAGTGGGTAGTGGAGAATTAAATATGGAGAAGAAAAACTCCTTAGAACAATATATATACGGGCTGTTCTAGGTTACAACAAATGTATTTATAGGAAAAAAAAATTCATAGAGTGTCCCCTACATAAGGCCTTAATATCAGATTTTTGTTGACAGTAGCTTTGCTTCTAACAAGACAGGGGAGTTGTGGACGCTAAGAAAAGAAAGAAAGCAGTGTGGTGGAAAAAACTTTACATGATCTTCGTGCAATGTCCATTAATATAATGCTGCTAACACAAATCTTGAAGGATAAAAGTCGTGTATGTGCACGTTTGTTCTCCTTTTAAAAAATTGTGCTATGTTTTTCCTTTTTGGCACAATTTGAATTACTCTGTAGTAACTTTCTGGTTGAAAAGAAGGACTAACATGCACATACCAAAAAAAAATTCCTTCTAACTTGCACTTGATATCTCTTATTCTACTTGATTTTCCTAAAATCAAATACACACAAGCATAAAGATTAATGGTACATTGAGTTCTCTTCCTCTCAATGCAGTTTCATTGCTTTGGTTAATTTATATGAACTTGAGGAAAAGGAAAATGTAGAGTAGGGTGAGAATGTTGGCTTAAATATGATATGAGATTTATGCTTATACATTGCAAGTTTATTCTTTTGATGAATTCAACTGCCAACTCCCCAAGGAGGTCAAGGGGAATCTTGCTATTCTCACTTGCTGGTCAGGATGAGGATAACCCTTCTTTGTCAAATGCTTCCGAGATGTCTTTAGACTCTTCAACGTCTGAATATTATAGTTATTCTAGCATCTATAATCACGAGAAATGGACTTGAGGCTCACTGCATTAACATGGAAAGTGACCAACATGTTTGAGCAAGCTGTTAGTAGAAAATGCTCTATCAAAGATATCATTAACAATGAAAAGTTCTCTTGCCATTTTGGAGTTACTGACTTGAGGTAAACAAATGGACAAGAAAGGTACTTGAAGGCTGAAATGAGACGGGTTATATATTAAAAACAAAATACAGGGTAATAATTTATATGTTCTATCAATAGGTTTAGAATTGTATCTATGTCTCATGATACCTCTGATAAAGAGCGAACACTAACAGGAGCTTCAACACTTACCATCTGACCTAAGTAATCACATGTAGGGAGTCTACTCGAAATATTTTAGAACTGATAAGGAGAAGACGCCCATCTTAATTCTCATATCTACTTGTGTGACATTGTTACTCCCAAGAAATACTTGAAATTTGTAGTCAATAGATGATGCTTCTATATCAAAAAGTATTTTCTTTTTATTTTTGGGATAAATAAATTTGGTAGTAAGCAAATTTTCCGAGTCCAGTAAATGAAGAGCTTTAGCCAAAGTGTCCTCTCTTTTATGTAAGACATTATTTTTCTTCCTGGTTTTGCAGGAACGAACTTAGAGAAGGAAAGAAATTTGATGTTATTTTTCAATTCTTTGGTTTTCATGATTACATAGCAATGTTTTGTTTTTAAATTGGATTTTTAAAATCAACAATTCCAATAAAATCATGTGGAGGCACTTGGTCATGTTTTCTACAGCATTTTGGTGGCTAATCTTAATGGCAACATGGCTATTGTGATCAATTTGAAGAACATGTCTCTCAATTTTCTCTAGAGAAGCCTTTTAAGTTCGAAAAGAGAATTATCATATGAGCATAAGTTGAAATTTGTGTAATACACAAAGCAGATGCATTTGCTTTCTTACATATATGCAATAATATTATCAACTTCTTCATTGCAATGACATTGTACTACTTACTCACTAATATGAAAATCAATATATAGGACGGATACACATTTAATTTATAACTTTTCAGTTCTTGTACATAAGTTTGTAATTATGAACCAAGTATGAGAACATGAAGATAACTTTTGAATGTGCTACTAAAATAACTTGTGTGCATAATCAATTGTGTTGGGCCCGTGCTTAGCACGGGCTTGCTTATCTAGTATATATACAAACACAGACATACTTATACATACTTAAAAGCATGAACTCCCAAATTTGAATGTTGAATTACTATAATACTCTCAATTAAAAATACTCAATTTATTTTATTTATTAATTTAATTAATTTTATCATATTGAAAGTAGATACATTTTCAAAATAAAGGATTGTAACTTTTCAATGAGTTGCGAATTTTTCAAAGGGTTGTGACTTTTTG

>LNC_001293

CCTTACTCCTCCATGGACAAATCCTCGAGGGGGGGCTACCCTCCATTGTTACCATCGAAACCAATCGCAAATCCTTCAAACGAATCTCGAATCATCCATTTGTATGAAAATTCTCTGAATTTTTTTAAAACAAGGGGATTGAAACTACATTAAAGGGTTAGTTTAATCAAGGTGGAAGAGTGTGTAAATTGATGGGAAACTACATCGGAGAAATTACAACTGGGTAAGTATTAGTGTTTCTCCAATTTTGATGCTTTGTTTCAGATTTTTTTTGAAGTGTAACATGGTTGCTTCTTTGATGAAAATGATTGTTCTTTCATAAAGACAATAGATACTTATTTATTCAAATTTTCATTTAGCTTTCAGTTTGAATTAAACAAGAAGCAATTTGTAGCCAGTCTGAACCAACAACTTGAATAGGGATTCTTCTTCATGTTCTTTTTTCACTTGA

>LNC_001294

CACTTGTAAGACCCGTGAAAGCTTCTGTGGACTCCCCAGAACTCCTCAGCTTCCAGGAGAACCTTCACAGATCGTGAAGGATTGTATGGCACTTCCGCTAATTAATCCAACCATCCAACACGCGTACATGGAGCAAAATGTTGTGGCAGATGCACTTACTAAACAAGGATGTCGTCTTACAACTCTGCTAGGCTCTACAAATACCACCATATGCATGATAAATAACACTCATCTGCAAATTCTTCTGCAACACCTTGCTCTTTGTATGACCCTTTTGTTATAAATGTAATACAATATCCTTAACATAAAAAAAAATAAAAAAAAAT

>LNC_001295

CGAGAAGCCTAAGTTGTTCATGTCATCAATTTGAGCTTGAAATGTTTGGTAGTAATATTTTATTTTATTATTATTGACGTGTCTTGTATTAATAGATTATGAGAAGTATTAGGTTACGTCTCTATTTATTCATTCATGCCTAGTTATTAAATAATCACCTCTGCAATATTGGCTTATACTCTTAAATGTTCATCATGTGGGATTATATCTATTT

>LNC_001296

CATTCATCCCTCCCTCTAATACGATATGTAACGTCCTCGTTGATCTCACCATTACCTTGGATCGTACACCTAAGATACTTGAAACTTCCTCTCTTGGAGATGCTGTGAGTCTCGATCTTCACATGAGTTACGTCACTACACTCTACATTCTCCGTCTTAGTCCTGCTCAACCTGAACCCTTTAGACTTTAGGTTCTATCTCCAAACCTCCAGCTTATTGTTTACTCGTGTTGTGTGTCTTGTCAATCAGTACTATGTCATTCACAAATAGCATACAACCATACACCATAGTATCTCTCTTTGGTTACACCGCGTTAGTTCTCACCAAGACGAATAAAAACGGGCTAAGAGTTGATCCCTGGTGCAACATTATGACAAATGGAAAGTGCTCGAAGTTTCCTCTTCCATCCTCACCCGTGTCTTGGCTCCATCGTAGATGTCCTTAATCACCCTAATGTAAGCTACAATACACTTCTAGTTCCCAAACATCTCCAT

>LNC_001297

CAATAAAACAAGCTAGGTGTCTCTTTGGAGCCTTGGCACGAATTTAAAATTCTGAATTGTACATGCTTGCAAAATCTCAGATGAGTTGAGTCAGAATGAGGAGAGAACCAAGTTTCCTCGCCGGAACAAGTCCAGAAGTGGCACACACGCTCGGTGTCTGAAGAATCTCTCGACAGAAAAACTAGGATCTGATCGGCGCAAATAGATGCGTGCGATCTCCGTTTTCATCGTGGGTTGTTGGGGTTTTGCTTGCCAGAGGATGCTGGGTCTAGTGTTGGTGCTGGATATTTCACATCACCAACAAAAAGAAAGTTAGCTGCTGCCAGCTGCCAGAGAATACTGGGACAAATTCTCAGTTTACCGGAAAAATGTGCGGCAATGACAGCAATGGCACCGAAGATGAAATAAAGAGTTCCTATATCCTTGTGGTTAGTGGTATTGTCTTCCACAAGTCACTGGTAAATGCATAATATTCATGTAGGCTGTGATACTATATGGGAAAATTGAGAGGAACTCTGGATCATATTCATTCGGTACATATCCTAAAAAGGAAAGGAAAATCAGTAATGGTCTTATTACAATGACATACCTAGTGAAATTCGACAAGTGGGGTATAGGGAGGGTGGTGTGTACACACCCCTATCTATGGAGGTAGAGAGGCTGATTTTGATAGACCCTCG

>LNC_001298

GGAGTCTAGGGTAAAGAAAGAAAGGTAGAGTGGGCACACTTTTACTGTGCTTGGATTGGCATGGCTATCCCCCTTTGCTGGTTGAGGCTTGGCCTTTGACTCCGCTTGCCTCTACTTTTCATGTCAAGCGTACAAGTCTAGTTTAGTGGGATTTACTTCTAAAGACAGGAATTCTTTTTCATCTCCCCTGTCAAAGTCGACGTCTTCACCTGACTTCCTGAGCTTAGTTGATTCTTGAAGCAGTAGCTCT

>LNC_001299

GTTGGGGATGGGCGGGAGCTTAGTAGTTGCTTAGTCGTTCAAAAGTCAGACTGTCGTAAGCCCGACACTATTCTATTTACTCTCTCAAATGATGGGCTGGTTTATTTTGCTATAGGGGCCATCTCCTGTCATCCCTTACCTATGGTTCAGGAAAGGGATTGACCGGAATGATTGCAACGTGACACACCAACACCACTACTTAGAGGTGGAAAGGGGGGCGATCAGAGGATGACAGAGTAATAACGCCTTTCTCCCTCCTGACATCTATTGGGAGGGAACCTCCTTTCAGAGTGTGATTCGTCGTTTCCAATGCGGTTAATTGCTATTAGGCGTTTTAGGAAAGTTCCCCTCTTCCTATGCAGTTAGCTCGACTCCAGGTATAGCTCTTAATGGGCATGTAGCCGTAGGCGG

>LNC_001300

GGGATTCAATCCAAACACTCATAGATTCTATGCCCGCCTCTACTGTGATGTTCCAACCCTGAAGTCACTTCCTTTAGAGTTGAGTTCAAAGTTACAGCCTTTAGTAGATAAGCCGGAGTTAACTCATGATAACTCTTAGCCCTTACTTCCGATGAATGAGAAGTCTAGTTAGGCAGTGGAAACCCTGGGAAGTAAGGAAGTTCAAGTTAGAGGGCATATGAATATG

>LNC_001301

GTGGCAAACGGACCGGTTTTTGCCCGGTCCGGTCCGGTTCCGGTCCGGTTCCCGTACCGGTAAGACCGGTACCGGTTGAACCGGTAAGGAACCGGTCTTATAGGTACCGGAACCGGTACCGAAACGGAACCCGGATTTTTATCCGGTTGATCCGGTCCGGTAAGAACCGGTTCCTGTCCGGTAAGAACCGGTTCCGGTCCGGTTCAACGGTCAAAT

>LNC_001302

CACAAACAGAATGAAAAAAAAATAAAAAATTGCTCTGATTCTTGATTTTCTCTTCTGTTTTTATTCAACCCCAGAAGAAAAAAAAAGGTTCAAAACATTAGTAGAAACAACCCCTTAAGATCTGAAACTGAAACAAACTCTAAAGGACATGGTTTTTTTAGGAACAACAAAAAAAGATTGAATTTTTATTGTTTTAAGAGAGAGAGAAGTGATGTTTTTGTGTAAGATGATGAAAAATGAAGAGTGGTGGGTGAGGAAAAAGGGGGAAAAAGAAAGAAAATGGAGAGAGAATGGGTGAGAGAGACAGTAATGTTTGGGAATTTTGCTTGAGTTAAAGCTTTTTGAGCTCGCAAATCGAAAATGCCTGTGTTTTTATGCTGCTATCTCCTTCTTTGCTTCCCTCACTGTTCTTCCTTGTGTTTTTCTGTTTTCTTTGTTTTTTCCTCCTCCCTTTGTTACCGAAACAATAACTTATTCAGTGTAATCTCATTAAGTCATACGAAAGATAGAATGTAAAAAGGTC

>LNC_001303

ATTTGACTTCCCAAGGCCAAAGGGCAGTGATTGCATTTTGTGGGTATAGAAGCTTATATGCGACCTTGACTGAAAACTCCCCAGTCCTGCTATCTCCACATTTTAGTTGATCTGGTGCAAGGCCGTTGATTGTGGCATTGTGAAGTGCTCCCAAAAGGCTGATCAACTCTTCCATCTCCCAGTCTTGTATGTTTCTTCTGAGGTTGGGGTTCCATGAGTTTCCATCCCTGTGTTGGTGTATTGTAGCATCTGGCTGATTTGCTATCTGGAATATGTTGGGGTAGGCAACCTCTAGAGGGGTGTTGCCCAACCACTTATCTCTCCAGAAACTGATGTGTTGGCCATTCCCTATTATCAG

>LNC_001304

AAACGAAGGAATCTGAGCTTTATCAACATTGATAAGCTTTCTAGCTTGTGCAGTCAGCTGGTGGAACTCCTCAAAAGTGCCAGAAATCTCCTCATCAATGGCATAATTAGCTAGGCTATCTGCCACAGCATTTCCTTCCCTGTACACATGAGAAAATCACATTTTTTTAATCCCTCAACAATGTAATAAAATTTTCCTGACTATTGTATCAACAACCCAGGGTGCAGACACCTCTTGTATCATGATCAGTCTAGCAATGGTTGAATCAGTCTCAATCGCCACATTTGTGAAGCCTTTATGCACACAGATTTCAGCAGCTAACTGAATAGCCATTATCTCTGAATACATATTAGTTTGAACGGGTAGCAGC

>LNC_001305

ATTTATACCAATTTTCCCAGGGCAGTTTCCAGGAGATGGTCTTAGTGATCAGAATAGGTTTATATTTTTCTAAGAAGGTAACCAGGTCAGGCCAGAGGAAAGGAATATTTTTAAGCCAAGGGTACGTGCTCCTTGCTAGAAAATACAAGTTGTTATTCACTTCATGTATTACTCTAGTGAAACTCACCTTTCCACCATGCCTAATAGTGTTTCTCCTTTTCCATAATTCCCAAGTGATCATAGCTGGTACTGCATGTAAAATAGGTTTGAATTTCTCCCCACAATCT

>LNC_001306

AAACAACTTGTGAGCTGCTTCATTCTCTAGTTTCTGTTCAATCATGGTGCTTGTTATGTGTTCCTAGAGGCATGAAAAGCTGGATATGCAATAAAAGTTAGGAAAATATCAGAAGTTGTGGAAAGTTAAAAGTGACTTTCTGATGGATTGAGTGTGGGGAAGAGATTAAGACTTAGTGGTTGCTTTATAAGTGAAATTGAATTAAGGGATTTGAGTTTCATCATGTTTTTCAATTTCATACATTCAAAATCAAAATCTTTATGAGTGATTTCAGCCAAATTAATCGAAATATTCTACAAAGGTTTTTTGTTGAAGAAGATGAAGAATAAGAAATTGTCCAAAGGGTTTCTGGAAAAAAAA

>LNC_001307

CTGTCAACAAGCTGCCAGGTTCCGTTCTTCTCGATCATGTCTAGTTCATCTTGCATAGCTCTCCTCCATGCCTGAGAGTCTTGTGCTTCATCATAGCTTGTTGGTTCAGAGGTAACCAAGTTACACCGCTGATAGACATCTTTTAAGGATCGAGTTCCCCTCACTGGTACATCATCCACTAGTTCATCTTCTGAAAGTTGAGGCTGCTCTTTTGAAAATAAATCAAAATAAGAGGTCTTCTGATTTTTCCAATCCCAACCAGCTGCTTCATCAAACTTCACTTCTCGGCTGAGAATTAGTTTTTCTGATTTCAAATAGAATATCCTGTAACCTTTGGATGAACTATAGCCCATAAATATGCCTTTATGAGCCTTGTTGTCCAGCTTAGTCCTCTTTGTTTCTGGAACTCGATAATAACATATACACCCAAATATTCTGAGATAGTGTATTGATGGTTTGTTCCCATACCAAGCTTCATATGGAGTCATGTCCTGCAAGGCCTTAGTAGGCAATCTGTTCAGCAAATATATAGAGGTATTGACTGCCTTCGCCCAAAACTGATTTGGGATCTTTCTTTCGAGCAAAAGACATCTGGCCATCTCCATTACTGTCCTGTTCTTCCTTTCAGACACGCCATTTTGTTGAGGAGTGTATGGTAATGTCAATTGACGTTCAATACCTGTGCTATTATAAAACTCCACAAACTGAAAAAAAGTATATTCTCCTCCATTGTCTGACCTTAAAGCCTTAATACTAAGATTACATTGATTTTCTACTAAAGCTTTAAATTGTTTAAAAACATCAAAGACTTCACTCTTCAGTCTAATGAAATAAACCCAACACATTCTGGTGTAGTCATCAATAAAGAGGAGAAAGTATTTGTTACCACTCAATGAATGTGTTTTCATTGGGCCGCAAACATCCGTATGAATGAGCTGAAGTTTCTAGTTAGCTCTCCATACTTGATTTGCTTGAAATGGCAATTTTGTTTGCTTTCCCTGTTGGCAAGTTTCACAAACTTGAGCATTAGAAAGAAATTCAGACATATTTTCCACTAGCTCCTTCTTTTTCATCTCTGTGATGCTTCTCAGATTGAAGAGACCAAACCTTTTGTGCCATAGGTTTGTACATGTTTGTGAAGTAATAGTGTAAGCCTGCTCAGTTATTTTCTCCCAATCAACTGAAAACATTCTGTTGCTCATCTTGACACAAAATAATTCAACTCCAGAAGGGTCAGAAACAACACATTCACGATTCTTAAAATGCAGAGAATAATTATTTTCAAGCATTTGTCCAACACTCAACAAATTTTGACTCATATCAGGTGTGTACAAAACATCAGGAATAGTTTTGATACCTGATATTGTTGAGATAGACACTGTACCTCTGCCTTTGACTTCTACTGCCTCACCGTTTCCCACTTTTACTTTAGATTTGTAAGTATCATCGAGGAATTTGAACATTTCAGCATCATTGCACAAGTGATGTGTGCAACCACTGTCAAGAAGCCATGAATCAGAGGATTCATTGATTGAAAAGTATGAAACCGCAAAGAGTTGCTCCTCATGTGCATCCGCAGCTTCTGCTACTTGTGCTTGCAAAGAACCAGAGGCCTTTGCTCTTGCT

>LNC_001308

TTCGAATAAGTGCTCTTCAATTTATTTTTGGGTAAACTTCAAACGGACATATCTCTTAGCATATAAAGAGTTAGGTGGCCCATAAGATATCAAATCGAAGGTCTTCGGGTCCTTTTTCCAATGCCACCAAGTTTGCCTCAATAAGAGCTCTGAGTAAAAAGTTATGACTGATTTACTAGAGACATGCGAGCCTGACTATCGGGGCACCATACTGACTTTTATATTTATGATATAAGGAGTGCACTCCACTTCATAAAGAGTGAATAGAATTCACTCTATACCCATATCCCTGGAACAACTGTAAAATAAACTCCATCTTAGTCTACCCGACTTTGACTTTGACTTGAAAGAGTAAGTTTTCGCCTCTGTTGTTGAAAAGTCAGGACCCGTTGTGGAATCTATTCTAGACCCGCCTGCCGGCCCTAAATCTATCTTACTCTTCCTTGGTCTCTTCCACGGATCGGTGGATATGAAATAGAATGAATTCAAGGACAAAGTCGCCAGTATACTGTGATCCAGACCCAGGAAGGGCCAGACATGAATCGGAAAGAGTAACTTTCCTCATAGGAGAAAGGGTGCGTGCTAAAGCTAGAGCAGCTCTTTTTCGCGATATAGGTGTAATTTCATTAATTCAGTACTTAAGGTATTCCCTCGTCAAACGAATCAAAAAAAAGAAGGTTGGCTTTTCGCCGTGCT

>LNC_001309

GAAACAGTTAAGAACTCGTATGAATCGAACGAGAAGGAAGATCATAGGCGAGAAATCGCAGATTTGAAGCAAGAAATGGAAATAGCTAAAAAATTGTATGAACAACACACCCTAGAAATGAAAGAAAAGGCTACAGAAGCTCAACAAGAACTTGAGGAGAAGTTGAAGGAGGCCATGAGCCTTTTGACAGAATCAAGAAATAGGATAAAGGAACTTGAGACATTTGCTCAATCAAACTCTCACAGTTGGAAGAAGAAGGAGCATATTTACCAAATATTTACAGACTTCCAGCTGGGTGCACTGCGTGAACTGAAGTTTTCTTCTCAGTCAATAAGGCAAGAAGTGGTGAAAACTCAGCAGAGCTACGTGGAGGAATTCAATCAACTAGGTGCAAAAGTTAGAGCACTTGGACATGCAGCTGCAAACTACTCTGCACTCCTTGCTGAGAATCGCAAACTGCACAATGAGGTGCAGGAGCTAAAAGGAAATATCAGAGTATATTGTCGAATTAGGCCATTCTTTCGTGGACAAAAGGAAAAGCAATCAGTTGTTGAATACATTGGAGAAAATGGGGAGCTGATTATTGTAAATCCTTCCAAACAAGGAAAGGAAGGCCGCAGGTCTTTCAAGTTTAATAAGGTCTACAATCCAGCTGCAACACAAGCTGATGTATATTCAGATATTCAGCCATTAATACAGTCCGTG

>LNC_001310

ACTGATAAAAAAATTCCTAGCAGCCGCACTTTCTCCCTCAACTCTCTTATTTTCTTCCCTTCCATTTTCAGAAAAACAGCAAGGCTTGTCAACCATGGACAGAGGTAAGTTACTCCATTTGCATGCTACTTTCTTCTCAATCATGTGAAGCTTACAAGTCTCTCCAAAACCTACCCCAAAGTTCAATTTCTTTGACAAATTTCAAATTTGAAATTCAAAATCCCACCCAAACTAAGAACCCTAATTTTCTACTCCTATAATTATTAGCATTTGGAATACATTTGAAGAATATTTTAGCAGCCTTCTTCACCCAAAAGCTTGATAATACCTTTATGTTTATCTTAGCTTTGTTCTTCTCTGTTGGCACTAGCCACAACATAGACTTTGCATTTTAATTCACTCGAGCGAAACTCGTTTTCATTTGCAAAAGGAGTGTAGATCCGAGGCTCAAAGACGTCTTAGTTTACTGCTCCACAAAAGGAAAGGGAGTTGTGAAGCATTCAGAGGAAATAACTAAGATGGATGTTGATGGCTTTATAGGAATCCACTTGGAAGAATAAATAATTACGGAAATCCTGAGCAGGCTACCCGCGCAGTCTCTTTTTCGAGTCATATGTGTTTTAAAATCTTGGATGATTCCGAACCTTACTTTTTGATGAAGCATCTCAATCATGCCAAGAATAACCAAATTCTATAAAGAATTCATCTTCTTA

>LNC_001311

TCCTCTTCCAAAACAAAACATTAGCAGTGCAAATCAAAGCGTTAGGGTTACAATTTGCATCAAATCTGATCGCAGACCTACCATGAAACTTCCTTCTCACAAAACTAAGTTCAATATAACAAAAACATCTCAAAACTGAAAAATAAAGACACCCTAACTGATTCACTTTTCTCGACTCTAAATACAGTTGCCCTAACTGCGTCTATCAGTAGCCCTAGCGCTCAAAAAATAAATCTGATTTTGGGGGAAACACTCAAACTCAATCCTTTTGAAGAAGAGGGGGCGGATTGAGTCAACCATATAATCGGTGTGGGTGTTATCAACCCACTTGAATCGATGTATAATTGAGCTTAAGATGCAACAAGATATCTAGAGCTTCAGCATGTCCCTGCATTGTTGCACTATTGTTGATTGAATCTTGCCTCAAATTTGAATTTTTTATGACATCAAAGATCTCTTCCAAGTCATTAGAAGCAACCACTTCACCACTTAAAGTGAGGTAGCAACGTTAAGCGCGCTTCATTGTCTGCAAATGCGACAACAATGGAGAGATCACTCTTTAGCCAGAAGCTTGAAGCGAGTCGGCGGGAAGTTGCTTATGTTCTTCGTCAAACTCCATACACGGTTGGATTTCACTGCTATGTATCCGACTTGAGCACTTTCAGGTGAAACCTTGTCCTTCAGTTGTGTTGATTCGCCGATCTGGTACTTTTTCTTACATTAACTTGAGATTCTCTGTAGATTTTTGTGATTTGGTCTGCTCTTGTTAAGAAGCTAGTATGACTATTGTTGTTCTTCGGGTGGATTGTTCTTATTTACTCTGTCTTTTCCTTCTTTGTTGGGTTGTTTTATGCTTTTTCCTTGTGTTCTTACTTGTTGATTTTTAATTGATACATGAGCGGATTTTCAGGGGTCAGTTTTATGTATACCTTTCTCTGCCTCATACCTTGTTCATTTGGTTCTTGAGCAATAGGATGTGTATCTAATACTACACAACGTTTAAGTCCCAAATGGATAGATATGCTGCAAGAGTTTAAGGTATTGTTTTGTGTTGGTGACCTATGTTTTCCACTCAATGTGTATTGAGTTTGATGTTTACTGTCGTTGCATCTGTATAGTGGTAGCTGAATTGTCTTCTTGCCAAGTGCTTATGTATCTCTTATGAACATGGTCTGTTGTTGTATTTCCTGCGTACAGTTGGTAAAAAAACCTTTCTATGTGAATTCTCACTATCCATTTTACTCCAATTGAACTAATCTTGTTCCAATATTCAATAATTTAAGATTCTCTAACATAACAATATTTTTTCCTTTTTTACTGACATACAAACCTTGAAACAGACTCTTAGTAAAATATGGACCCTATGTATGTGTGCCCGACTATATTTTCTTCTTTGATGTGTACTACTATCTGTTGTGGCGTTTATTTTGTATAATTCATTTTGTTGTCTCTTTTTTTTACAATCCTGCATAGGATACATCCCTTTTAAGCTGAGGGTCTAACGAAAACAACCTCTCTACAATCTTAGGTATATTGTTTTTGTTGTTTGTTGTTTGTTGGTAAAATATATTCCCCTATTTATTGCATGCTTACAAATGTGTTTATCGGCATATGACCCTCCCAAAAATAGTTTGAGGGAGGTTAAGATTATATTGTCCTATGTTGCGCGGACTCTTCATATTTGCAGGCGAACCCGTCTCGACGCGACACTGGTGCGGGCGCGGGTGCGGGGTGCGTGTCGGATTCGGTCAATCCGATCCGGACACTTTGACCGAAGCCGAGAAAAGATTTGGGGAAGAAAAAATCAGTTTCCGGCTCCGGCTCCGGCGACGCAACGTAAAGAAGACAAAGTTGAAAAAGTAAAGAGAAGAGAAGAAAGAAGAGTTTTTTCTTCAAAAAATAAAGGAAATACAAAGTCTTTTTTTTAATCTTTCTTATATGTACACAATACATTTACCTTTGAAAAAAAGAAAGATCAAAAAGTCTTCAAAAAA

>LNC_001312

TCCTCTTCCAAAACAAAACATTAGCAGTGCAAATCAAAGCGTTAGGGTTACAATTTGCATCAAATCTGATCGCAGACCTACCATGAAACTTCCTTCTCACAAAACTAAGTTCAATATAACAAAAACATCTCAAAACTGAAAAATAAAGACACCCTAACTGATTCACTTTTCTCGACTCTAAATACAGTTGCCCTAACTGCGTCTATCAGTAGCCCTAGCGCTCAAAAAATAAATCTGATTTTGGGGGAAACACTCAAACTCAATCCTTTTGAAGAAGAGGGGGCGGATTGAGTCAACCATATAATCGGTGTGGGTGTTATCAACCCACTTGAATCGATGTATAATTGAGCTTAAGATGCAACAAGATATCTAGAGCTTCAGATCTCTTCCAAGTCATTAGAAGCAACCACTTCACCACTTAAAGTGAGGTAGCAACGTTAAGCGCGCTTCATTGTCTGCAAATGCGACAACAATGGAGAGATCACTCTTTAGCCAGAAGCTTGAAGCGAGTCGGCGGGAAGTTGCTTATGTTCTTCGTCAAACTCCATACACGGTTGGATTTCACTGCTATGTATCCGACTTGAGCACTTTCAGGTGAAACCTTGTCCTTCAGTTGTGTTGATTCGCCGATCTGGCGAACCCGTCTCGACGCGACACTGGTGCGGGCGCGGGTGCGGGGTGCGTGTCGGATTCGGTCAATCCGATCCGGACACTTTGACCGAAGCCGAGAAAAGATTTGGGGAAGAAAAAATCAGTTTCCGGCTCCGGCTCCGGCGACGCAACGTAAAGAAGACAAAGTTGAAAAAGTAAAGAGAAGAGAAGAAAGAAGAGTTTTTTCTTCAAAAAATAAAGGAAATACAAAGTCTTTTTTTTAATCTTTCTTATATGTACACAATACATTTACCTTTGAAAAAAAGAAAGATCAAAAAGTCTTCAAAAAA

>LNC_001313

CTTAACCCACCCTTCCTCTTCCAAAACAAAACATTAGCAGTGCAAATCAAAGCGTTAGGGTTACAATTTGCATCAAATCTGATCGCAGACCTACCATGAAACTTCCTTCTCACAAAACTAAGTTCAATATAACAAAAACATCTCAAAACTGAAAAATAAAGACACCCTAACTGATTCACTTTTCTCGACTCTAAATACAGTTGCCCTAACTGCGTCTATCAGTAGCCCTAGCGCTCAAAAAATAAATCTGATTTTGGGGGAAACACTCAAACTCAATCCTTTTGAAGAAGAGGGGGCGGATTGAGTCAACCATATAATCGGTGTGGGTGTTATCAACCCACTTGAATCGATGTATAATTGAGCTTAAGATGCAACAAGATATCTAGAGCTTCAGCATGTCCCTGCATTGTTGCACTATTGTTGATTGAATCTTGCCTCAAATTTGAATTTTTTATGACATCAAAGATCTCTTCCAAGTCATTAGAAGCAACCACTTCACCACTTAAAGTGAGGTAGCAACGTTAAGCGCGCTTCATTGTCTGCAAATGCGACAACAATGGAGAGATCACTCTTTAGCCAGAAGCTTGAAGCGAGTCGGCGGGAAGTTGCTTATGTTCTTCGTCAAACTCCATGTATTCTTCCTCCTTCTTCTCCTTGGTTGTTCTGCTGCTTCTTTTTCATTCTTCACAGTTTTCTCTGTTTCATGCTTTCATCACAGTTTTCTCTGTCTTTTTTTATCCTCTGTTTTAAAATTTATGTTATTTCTTCTTTTCACTATTTCAATGAATCAAGCACATAGGTTCTTGCTTCATACGTTAAGAAAAGTAAGACCTCGTTGCTATTTTAATTTGTGCTTTCATAAACATTGGTTATATAAAGCTATCTATTGAGTTTGTGGTAGCTGCTTGATGTTGGAGATTTGTTTTATTGAATTATGTGTTTCCAGTTCATATCTTGCTGAACAATTCATATGATAGTTTTTTCCAGTTCATATCTTGCTGAACAATTCATATGATTATTTTTTCCAGTTCATATCTTGCTGAACAATTCATATGATAGTTTTTCCAGTTCATATCTTGCTGAACAATTCATATGATAGTTTAATCCTTGTTTTTCTTAGATATCTCAAGAAAGTCTAGAATAGTGGTCTTAAGTGATCATTTTATGTTTTTGGACCAAGCATATAAAAAGAATATTGTATGTCGATTGATTTGTTCTTTCTTTTACTTGAACGTTCATGCCTCCTTATCATTTCTTTCGACTATTTTTTTCAATAGAAAATTTCTAAGTATTAGTTCATTGCTTGGAAAATATAGAACATTTAAATAGTTAATTACTTGAAAGTAACCTATTTTTAGACTTACTTAATTTTGGCCAGGTTTAGGTTCCAATGCGTGCCTTTGACTCAACTTACATTAAATATTAGTTTCAGTCATTTTAACCATTTTGTACTGTTGCATGCTTTATTGTTATGAATCAGGAATCAACAATTGAATGTTTCTCACAAGGAGTTTAATGTCTAATCTAGTTCACCCATGCTTGAAAAATACCATCTTTATTAAATTAAAGCTTTTCTACAAAGTCAGTACAATATCCTCTAAGAATGTCGTGATCTGGACTGGAAGACATGATGAATCTATCAGTAATTAGCTGAAAGCAATTTATTTGATATTTTCTGCCATAGAAACATGTAGTTGGATGATGGAGCTTGTCTAGTGCAAAAATTTAGGAAGGTTTCCCATTTCAAAGTTGTCCACAACGTGTGAAGTTGATATACATTTTCTTTATGGATACCTAAACATGATTTTTCCCTTCCTTTTTTTAAACCAAGGGCATTGGGTGTTGCCCATGACATTTTTATTGATTACTATTGAGTCTTCCATAAAAAGATAAATTAAATTCTAGATAATTTTAAAATAAAAATAATGTACTAGAGGGAAAAGACAGGTTTCTAAACATCCAGGATATTATAATGTTGTCCTTGCACAGAGATGATCTAATTCCAACAATCTGGTGCTGATTTTGTCGTTCTTCATGCCATTTCTGGCATATATCTTAGCTCCAAGTTACTCATCATTGTGCTGTTTCTTGTTCTCCTTTTTATTACCTCTATTTCACTGTCTTTCTTCTGCTAGACACGGTTGGATTTCACTGCTATGTATCCGACTTGAGCACTTTCAGGTGAAACCTTGTCCTTCAGTTGTGTTGATTCGCCGATCTGGTACTTTTTCTTACATTAACTTGAGATTCTCTGTAGATTTTTGTGATTTGGTCTGCTCTTGTTAAGAAGCTAGTATGACTATTGTTGTTCTTCGGGTGGATTGTTCTTATTTACTCTGTCTTTTCCTTCTTTGTTGGGTTGTTTTATGCTTTTTCCTTGTGTTCTTACTTGTTGATTTTTAATTGATACATGAGCGGATTTTCAGGGGTCAGTTTTATGTATACCTTTCTCTGCCTCATACCTTGTTCATTTGGTTCTTGAGCAATAGGATGTGTATCTAATACTACACAACGTTTAAGTCCCAAATGGATAGATATGCTGCAAGAGTTTAAGGTATTGTTTTGTGTTGGTGACCTATGTTTTCCACTCAATGTGTATTGAGTTTGATGTTTACTGTCGTTGCATCTGTATAGTGGTAGCTGAATTGTCTTCTTGCCAAGTGCTTATGTATCTCTTATGAACATGGTCTGTTGTTGTATTTCCTGCGTACAGTTGGTAAAAAAACCTTTCTATGTGAATTCTCACTATCCATTTTACTCCAATTGAACTAATCTTGTTCCAATATTCAATAATTTAAGATTCTCTAACATAACAATATTTTTTCCTTTTTTACTGACATACAAACCTTGAAACAGACTCTTAGTAAAATATGGACCCTATGTATGTGTGCCCGACTATATTTTCTTCTTTGATGTGTACTACTATCTGTTGTGGCGTTTATTTTGTATAATTCATTTTGTTGTCTCTTTTTTTTACAATCCTGCATAGGATACATCCCTTTTAAGCTGAGGGTCTAACGAAAACAACCTCTCTACAATCTTAGGTATATTGTTTTTGTTGTTTGTTGTTTGTTGGTAAAATATATTCCCCTATTTATTGCATGCTTACAAATGTGTTTATCGGCATATGACCCTCCCAAAAATAGTTTGAGGGAGGTTAAGATTATATTGTCCTATGTTGCGCGGACTCTTCATATTTGCAGGCGAACCCGTCTCGACGCGACACTGGTGCGGGCGCGGGTGCGGGGTGCGTGTCGGATTCGGTCAATCCGATCCGGACACTTTGACCGAAGCCGAGAAAAGATTTGGGGAAGAAAAAATCAGTTTCCGGCTCCGGCTCCGGCGACGCAACGTAAAGAAGACAAAGTTGAAAAAGTAAAGAGAAGAGAAGAAAGAAGAGTTTTTTCTTCAAAAAATAAAGGAAATACAAAGTCTTTTTTTTAATCTTTCTTATATGTACACAATACATTTACCTTTGAAAAAAAGAAAGATCAAAAAGTCTTCAAAAAA

>LNC_001314

CTTTTTCTTAACCCACCCTTCCTCTTCCAAAACAAAACATTAGCAGTGCAAATCAAAGCGTTAGGGTTACAATTTGCATCAAATCTGATCGCAGACCTACCATGAAACTTCCTTCTCACAAAACTAAGTTCAATATAACAAAAACATCTCAAAACTGAAAAATAAAGACACCCTAACTGATTCACTTTTCTCGACTCTAAATACAGTTGCCCTAACTGCGTCTATCAGTAGCCCTAGCGCTCAAAAAATAAATCTGATTTTGGGGGAAACACTCAAACTCAATCCTTTTGAAGAAGAGGGGGCGGATTGAGTCAACCATATAATCGGTGTGGGTGTTATCAACCCACTTGAATCGATGTATAATTGAGCTTAAGATGCAACAAGATATCTAGAGCTTCAGATCTCTTCCAAGTCATTAGAAGCAACCACTTCACCACTTAAAGTGAGGTAGCAACGTTAAGCGCGCTTCATTGTCTGCAAATGCGACAACAATGGAGAGATCACTCTTTAGCCAGAAGCTTGAAGCGAGTCGGCGGGAAGTTGCTTATGTTCTTCGTCAAACTCCATGTATTCTTCCTCCTTCTTCTCCTTGGTTGTTCTGCTGCTTCTTTTTCATTCTTCACAGTTTTCTCTGTTTCATGCTTTCATCACAGTTTTCTCTGTCTTTTTTTATCCTCTGTTTTAAAATTTATGTTATTTCTTCTTTTCACTATTTCAATGAATCAAGCACATAGGTTCTTGCTTCATACGTTAAGAAAAGTAAGACCTCGTTGCTATTTTAATTTGTGCTTTCATAAACATTGGTTATATAAAGCTATCTATTGAGTTTGTGGTAGCTGCTTGATGTTGGAGATTTGTTTTATTGAATTATGTGTTTCCAGTTCATATCTTGCTGAACAATTCATATGATAGTTTTTTCCAGTTCATATCTTGCTGAACAATTCATATGATTATTTTTTCCAGTTCATATCTTGCTGAACAATTCATATGATAGTTTTTCCAGTTCATATCTTGCTGAACAATTCATATGATAGTTTAATCCTTGTTTTTCTTAGATATCTCAAGAAAGTCTAGAATAGTGGTCTTAAGTGATCATTTTATGTTTTTGGACCAAGCATATAAAAAGAATATTGTATGTCGATTGATTTGTTCTTTCTTTTACTTGAACGTTCATGCCTCCTTATCATTTCTTTCGACTATTTTTTTCAATAGAAAATTTCTAAGTATTAGTTCATTGCTTGGAAAATATAGAACATTTAAATAGTTAATTACTTGAAAGTAACCTATTTTTAGACTTACTTAATTTTGGCCAGGTTTAGGTTCCAATGCGTGCCTTTGACTCAACTTACATTAAATATTAGTTTCAGTCATTTTAACCATTTTGTACTGTTGCATGCTTTATTGTTATGAATCAGGAATCAACAATTGAATGTTTCTCACAAGGAGTTTAATGTCTAATCTAGTTCACCCATGCTTGAAAAATACCATCTTTATTAAATTAAAGCTTTTCTACAAAGTCAGTACAATATCCTCTAAGAATGTCGTGATCTGGACTGGAAGACATGATGAATCTATCAGTAATTAGCTGAAAGCAATTTATTTGATATTTTCTGCCATAGAAACATGTAGTTGGATGATGGAGCTTGTCTAGTGCAAAAATTTAGGAAGGTTTCCCATTTCAAAGTTGTCCACAACGTGTGAAGTTGATATACATTTTCTTTATGGATACCTAAACATGATTTTTCCCTTCCTTTTTTTAAACCAAGGGCATTGGGTGTTGCCCATGACATTTTTATTGATTACTATTGAGTCTTCCATAAAAAGATAAATTAAATTCTAGATAATTTTAAAATAAAAATAATGTACTAGAGGGAAAAGACAGGTTTCTAAACATCCAGGATATTATAATGTTGTCCTTGCACAGAGATGATCTAATTCCAACAATCTGGTGCTGATTTTGTCGTTCTTCATGCCATTTCTGGCATATATCTTAGCTCCAAGTTACTCATCATTGTGCTGTTTCTTGTTCTCCTTTTTATTACCTCTATTTCACTGTCTTTCTTCTGCTAGACACGGTTGGATTTCACTGCTATGTATCCGACTTGAGCACTTTCAGGTGAAACCTTGTCCTTCAGTTGTGTTGATTCGCCGATCTGGTACTTTTTCTTACATTAACTTGAGATTCTCTGTAGATTTTTGTGATTTGGTCTGCTCTTGTTAAGAAGCTAGTATGACTATTGTTGTTCTTCGGGTGGATTGTTCTTATTTACTCTGTCTTTTCCTTCTTTGTTGGGTTGTTTTATGCTTTTTCCTTGTGTTCTTACTTGTTGATTTTTAATTGATACATGAGCGGATTTTCAGGGGTCAGTTTTATGTATACCTTTCTCTGCCTCATACCTTGTTCATTTGGTTCTTGAGCAATAGGATGTGTATCTAATACTACACAACGTTTAAGTCCCAAATGGATAGATATGCTGCAAGAGTTTAAGGTATTGTTTTGTGTTGGTGACCTATGTTTTCCACTCAATGTGTATTGAGTTTGATGTTTACTGTCGTTGCATCTGTATAGTGGTAGCTGAATTGTCTTCTTGCCAAGTGCTTATGTATCTCTTATGAACATGGTCTGTTGTTGTATTTCCTGCGTACAGTTGGTAAAAAAACCTTTCTATGTGAATTCTCACTATCCATTTTACTCCAATTGAACTAATCTTGTTCCAATATTCAATAATTTAAGATTCTCTAACATAACAATATTTTTTCCTTTTTTACTGACATACAAACCTTGAAACAGACTCTTAGTAAAATATGGACCCTATGTATGTGTGCCCGACTATATTTTCTTCTTTGATGTGTACTACTATCTGTTGTGGCGTTTATTTTGTATAATTCATTTTGTTGTCTCTTTTTTTTACAATCCTGCATAGGATACATCCCTTTTAAGCTGAGGGTCTAACGAAAACAACCTCTCTACAATCTTAGGTATATTGTTTTTGTTGTTTGTTGTTTGTTGGTAAAATATATTCCCCTATTTATTGCATGCTTACAAATGTGTTTATCGGCATATGACCCTCCCAAAAATAGTTTGAGGGAGGTTAAGATTATATTGTCCTATGTTGCGCGGACTCTTCATATTTGCAGGCGAACCCGTCTCGACGCGACACTGGTGCGGGCGCGGGTGCGGGGTGCGTGTCGGATTCGGTCAATCCGATCCGGACACTTTGACCGAAGCCGAGAAAAGATTTGGGGAAGAAAAAATCAGTTTCCGGCTCCGGCTCCGGCGACGCAACGTAAAGAAGACAAAGTTGAAAAAGTAAAGAGAAGAGAAGAAAGAAGAGTTTTTTCTTCAAAAAATAAAGGAAATACAAAGTCTTTTTTTTAATCTTTCTTATATGTACACAATACATTTACCTTTGAAAAAAAGAAAGATCAAAAAGTCTTCAAAAAA

>LNC_001315

TGGGGGTCTAGGGTTTTTAATTAAAGGTTCTGCTGAGAGATTGAGAGTTGAGAAGCGAGAGAGATTGAGATTGAGATTAAGAGTTGAGACGCGAGAGTGATCGAAAGGTTGCCTGGAGCGTCCACTGTTGGCATCACGCTTGTTGCCATCGATTGCCACTACTCATTTGTCTTCCTCGTCTTCATGGACAATTATGTTAAACAATCACAAATGACGTGATTAAGGAGGAATGCGAAGACACTACATCTGTTGCTTTTTAAATTTTATGTAAGTGCATAAAATATACAATATCATATTGTGTAGTACGAGTATATTTAACTGGCTTTTTAGTGGTAGAACTGTTTAAAATATTCGAGATGTGGTGTATTTTGTTGTGTTCTCTGTAATGATATTGGTTGTTTTGCTATTGATATGAAATTGAAATAATATAAATATATATATAATTTTTTTTTGTTAGAAAAAATTGGTATTAGTGGCGACTAATGTTGCTACTAAATTAAGGGTGGTAGCGACAATAGCTGATGTTGAAGGTAGAATGATTTGTGGCGACTTATTTTGCCACTAAATCTAAGGCTTTTGCAGCGACAAGGGTTGTTGCTGAAGCTTTAATCGTTATTGTGGCAAAAGAGCTCGCCACTAAAGGTCATACTATAGTGGCAACCCGACCAAATACCTATTTTGCGGTGGCATATTTTGTTACCTTTTGGTGGCGACAATAGTTGCCACAAATAAGGGTATTAAGTGGCGACGCGCTATTATCGTCACAAATAACATTTAGTTATGGAAGTACTTGCCACGGCGCTAATGTCGCCACTGATAATATATAGTGGCGACTTTCTAGCTATCTGTAGCGACATTTGCTGCCACTAAAAATACAGTTTCTTGTAGTGACCGTATGATTCAACTTTAAAATTAACCTATCTCGGGTTTTAGCTCACCTAATGGCACCCCTTGGCTCGCCAAGGGAACTAGGCGACACACCTAGTGGACCCTTGGCTCGCCTAATTTTGCAGAATGCTCCATAATAGAGCCATTCACTTGGGCGATAAGGGGGGCCTTTGGGTGATGCGCCAAGTGGGTTGGTGAGCCCCGACCTAGCTCCCCTAAAATTCCAGTAGCTAGGCCAAGGGGAGGCTACCCACTAGGTGAGGGAGGTTCATTTGGCTTGTCACCAATGGACTTGGGCGAGGTTTCTACCCTATCGCCGAATGGTCTAAAGGAAAAATTATTTTCCTTCATTCTTTCTTGGTAGCTTAGGCCCTTGCCCTCTTCCTAGACTTGTACCCCTTTCTTTTTATCTCTAGTTTAGTCTAAAAATTTCCGGAATGTTACACTAGCCCTCACGTTTAGCTCTAGGGTAGCCTACAAGACTCCGGATTGACAAATACTATGTCTCAAGTTGAAACTATTCTAGTCTAACATGCATGATTATTTTAGGTAAGTAAGCCTTTAACCTAGAATCATTCTATGTGACAGAACCTTTCATCTCCTTAGCATATTAATGTCTAAGTGCGTATATCAAAATATCCTTTGAACAACACAACAACATATCATAATCATAGACACTTCACTCTCAAGGTGTTCATAAAGCATTTACATAGGTTCTATAAGAACATGCATAATCTCATATTTCTATAGTTTAGGTCAAGTGGGTAATGTCCTTCCATAATAACTCTACCTATTAGTCTACCATTATCATACAAGTTTCACTTCTTTAAAGACGTAACATACATAATTCATCATATTGGCTTTACTTTCAAAACCCTAGTACTCAACACTCATGACTACAGACCTTACTCTCAAAGCTTTACTTCTCACAATTGTCATACATACCTAGATTACAAGGTATTCGAATCACATGCTTCATTCTTAAGTGAATCTACTCATAAATTATCATAAAGGTTCAACTTCAAGCCTTACTAGTCATGACCCATCATATATACTATATTCTAATTCAATTCATGACTACTTGTTTTTATTCTTGAACAATTACTATAGACAATCATCATTTCTAGAAGATCATCCCTAAGTCTTCAGTTTCCCTCTATAAGGCACAAACCCACATTACTTCAATGTCATCAATTAAACTAGGGTTAGC

>LNC_001316

TATGAATATATTTATTCATTTTCCCTGTAATATATGAACCATTCATTAATTTATAACTTAAACATTGTATTTGTTTATTGGGGGTCTAGGGTTTTTAATTAAAGGTTCTGCTGAGAGATTGAGAGTTGAGAAGCGAGAGAGATTGAGATTGAGATTAAGAGTTGAGACGCGAGAGTGATCGAAAGGTTGCCTGGAGCGTCCACTGTTGGCATCACGCTTGTTGCCATCGATTGCCACTACTCATTTGTCTTCCTCGTCTTCATTGGAATTGGACAACTACTTGGTTGATTCTTTTGTTTAGCTATTGCAGGAGCTCTTTTGAGTTGTGGGACAATTATGTTAAACAATCACAAATGACGTGATTAAGGAGGAATGCGAAGACACTACATCTGTTGCTTTTTAAATTTTATGTAAGTGCATAAAATATACAATATCATATTGTGTAGTACGAGTATATTTAACTGGCTTTTTAGTGGTAGAACTGTTTAAAATATTCGAGATGTGGTGTATTTTGTTGTGTTCTCTGTAATGATATTGGTTGTTTTGCTATTGATATGAAATTGAAATAATATAAATATATATATAATTTTTTTTTGTTAGAAAAAATTGGTATTAGTGGCGACTAATGTTGCTACTAAATTAAGGGTGGTAGCGACAATAGCTGATGTTGAAGGTAGAATGATTTGTGGCGACTTATTTTGCCACTAAATCTAAGGCTTTTGCAGCGACAAGGGTTGTTGCTGAAGCTTTAATCGTTATTGTGGCAAAAGAGCTCGCCACTAAAGGTCATACTATAGTGGCAACCCGACCAAATACCTATTTTGCGGTGGCATATTTTGTTACCTTTTGGTGGCGACAATAGTTGCCACAAATAAGGGTATTAAGTGGCGACGCGCTATTATCGTCACAAATAACATTTAGTTATGGAAGTACTTGCCACGGCGCTAATGTCGCCACTGATAATATATAGTGGCGACTTTCTAGCTATCTGTAGCGACATTTGCTGCCACTAAAAATACAGTTTCTTGTAGTGACCGTATGATTCAACTTTAAAATTAACCTATCTCGGGTTTTAGCTCACCTAATGGCACCCCTTGGCTCGCCAAGGGAACTAGGCGACACACCTAGTGGACCCTTGGCTCGCCTAATTTTGCAGAATGCTCCATAATAGAGCCATTCACTTGGGCGATAAGGGGGGCCTTTGGGTGATGCGCCAAGTGGGTTGGTGAGCCCCGACCTAGCTCCCCTAAAATTCCAGTAGCTAGGCCAAGGGGAGGCTACCCACTAGGTGAGGGAGGTTCATTTGGCTTGTCACCAATGGACTTGGGCGAGGTTTCTACCCTATCGCCGAATGGTCTAAAGGAAAAATTATTTTCCTTCATTCTTTCTTGGTAGCTTAGGCCCTTGCCCTCTTCCTAGACTTGTACCCCTTTCTTTTTATCTCTAGTTTAGTCTAAAAATTTCCGGAATGTTACACTAGCCCTCACGTTTAGCTCTAGGGTAGCCTACAAGACTCCGGATTGACAAATACTATGTCTCAAGTTGAAACTATTCTAGTCTAACATGCATGATTATTTTAGGTAAGTAAGCCTTTAACCTAGAATCATTCTATGTGACAGAACCTTTCATCTCCTTAGCATATTAATGTCTAAGTGCGTATATCAAAATATCCTTTGAACAACACAACAACATATCATAATCATAGACACTTCACTCTCAAGGTGTTCATAAAGCATTTACATAGGTTCTATAAGAACATGCATAATCTCATATTTCTATAGTTTAGGTCAAGTGGGTAATGTCCTTCCATAATAACTCTACCTATTAGTCTACCATTATCATACAAGTTTCACTTCTTTAAAGACGTAACATACATAATTCATCATATTGGCTTTACTTTCAAAACCCTAGTACTCAACACTCATGACTACAGACCTTACTCTCAAAGCTTTACTTCTCACAATTGTCATACATACCTAGATTACAAGGTATTCGAATCACATGCTTCATTCTTAAGTGAATCTACTCATAAATTATCATAAAGGTTCAACTTCAAGCCTTACTAGTCATGACCCATCATATATACTATATTCTAATTCAATTCATGACTACTTGTTTTTATTCTTGAACAATTACTATAGACAATCATCATTTCTAGAAGATCATCCCTAAGTCTTCAGTTTCCCTCTATAAGGCACAAACCCACATTACTTCAATGTCATCAATTAAACTAGGGTTAGC

>LNC_001317

CCTGTCTAAGCAGTCGCTCCATCTGCCAGGGAATCAATGCTCGATGTGTCACTCTGGAATTTAATAATGGTCCCCGGAGCCTTTTCAGATGATTCAACAGCAGCTGACTTACTAAGATTGTCCAAATCATGAGCCGCCGGAAAGGAGTGGATAGCTGTGAAGAATCCACGTCTCCATGAGGGAATAGTGGGGTCTCGGTGATTTGTTTTCTTGCTTGCTTTAGGTTTCTTGCCCTCATTATCTGTTTTGGGAGGATCTATCGCCTTTGTATTTTTCCTGAAATTTTTCGCTCTTGTTTGTCGGGGTGGCATCTCTTCCAGCTACTTTCAGTCTAGCCATTTCTGCAAAGCTTTGATATAAAGTTAGAAAGTATTCAGACAAAAGCAAGGCCGTTTGATTTCAGAATTACTTTGCCGAACCATTTGGCGAAGCGCTCAGCTACTTAGGCGGTGCGCCGAGTGTAGCGCACAAAGATTATTTAGGGTGCAGACAAAGGGCAAACACGTAGTCTTTTGGCAAGGCGCTGAATGCGTTTGGTGACATTGTGCGTCTCTCCG

>LNC_001318

TGTGACGGGAATACGGGTTAACCTAGATTATTGGGTACATGCTTTCGATAGAAGATTCATAAAATAATTGTGAGTAGGCTAAACTTTAAAGGGGATAAGCTCTCTCTCGAGCAACTTACCCCGTTTCAAATGCGTTCCTCTCGGACATCCACTTGCCGCATGAAGACCAGCCTACGCCTTAACCCACTCACTCTCTCGAGCTGAGTGTGTGGGAATGAGACTAGGATTCACTCTCTCGAGCTGAACCACATGTCGACCCACTCCCACCGGCCATCAATTTAGTAGTTTTAGTTTTACGACCTCTCTCTCGAGCAAGCCGA

>LNC_001319

AAGAGGTCTCACCCGGCGGACCAGCAAACACTTTACCTACGCGACAGTCAAATGAGCTTTATACTGAGTCATTGGAAATCGAAGTAAGGGAATTGGAGACTTCCCCAATCGGAGAGAAGAGGAGTGACCTAATCACTTAGGAAGAATCGAAACCATAGAATTCCAGCTTCGCCGTCCTTACCTTCAATCCAATCTGATTCTGATTCCGCTGAAAGTCTTTCAAGTTCGGATACAATCACCCAATATAACCCTCCTTAACTGCCCTAGCTTCTTGCCCATCGGGC

>LNC_001320

GGACAACCAGCTCACTTCATAAAACAAAATATTTACAACACAAAAGATATAGGAAAGTGGAAGGGCAAAATAGTAGCAAAAAAGAGAGTGGAACTTGTCTCAAATTCAGGCTTACTCCTTCCGTTCTTGTTCATCTCTCAAATGCCATTTTGATTCATCTTCAAGGGATAGGACTGCTCATGGCTACTGGTAAAACAACAACTTGTTGGTGTGGGTTACTGAATTAATAGTAATGGATAGATCATCTTCACTGTTAGCACTACATTGATACTCCACACTCCCGAAAATGGGTATCTAAAGGTTTTTCAAGACTCTGAATCCTCGGTTGTTCACTTATTGAGTTGGCATCCTTCCTTTGTGTAGGAAGTTCTTGCTTCCCAAAAGTTTGTAACACCCATTCCTTTCTAGACTTCACATGCTTACTCGGTTGATCAGTTATATCCTCATGCTAACTTGTTGATTCTTGATTCTCATCATTGTTTGATCTGATTGCTTGTGGATCATCCAACAAAGCTGCAAACCTGTTGTTGTTGAGCTCCAGTCTTCTCCACTCAGAGGTGAACCTACATGTTCTCGTGAGGGTGCACGTGCACCTGTTAACTTAGGAAAAATCAGGTATATATATGTATATTTTCATTATTCGAGTCTACATCATGGACATGGCCGACAGTCAAGAACCATTGTTGGTCCCCAAGCGAACACTCATCCTGGCTGACTACAAGTGGAAGACTAACTCAAACATATATAAGCTTAAAACTGAATTAAATTCATTAAACTCAAATACTCAACTTTAAAAAGTCTGAACAATACTTAACATATATACTCAGAGTTTGTAAAACAACTCAAAAGAAAGTAAATACAGAAAACTCCTCAACTTTGTCTATTTATGAAGCCTCTACTATTACAGAAGGATATTGGGACAAGACCCACGACATCTCAAAACTACTAAATGAAGGTAAAGGTAAAGTCCTTCGGAATACAAGAAAGCTCACCACAACTGACTGGAATACCACATGACCTCAACGAAATGCATATTGATGATCCTGAATACCTATATCTGCATCATGAAATGATGCAGGCCAAATGACGTCAGTACACGGAATGTACGAGCATGTAAGGAGAACTCTGCAACATAAGTATAAGCTCGATTTAATAAAAGGGAAGACATACTTACCTCGTCTCTACTCAATATGACTCAAGAATACACAACTTAACTCATGGATACTCAACTCAAGTCAAGTTAACTCAGGATACTCAACTCAACTTAGGATACTGTACTGAACTCAGGGTACTCAACTTTACTCAAGATACTCAACTCAAACTCAAGGAATAATCAACTCAAAATAAAAGCAACAATAAATGATGATATATACATGAGACTTTTAAAACAATAGATAATAACTCAAATGTATATGAAAATACAATAATAAACTCATTACTCTTATTTGGGATATTCTCTAACCGACAACCATCACTATGAGAAATGTGATGATACAACATCTCGCCCACGCTGCCAGAACTTTCCTATACCTTGCCGGAGTATAGGACATCCTCAACTAAGTGGATCCACTAAGTTATGCTTAAAAATCAATAAGGAATCATCTAAAACTTATGACCCTTTACCCATGTTGGCAGACATGGTTTATGAGGATTTTGAGTTGTTAGAACTAATCCTTATATCGATGCTCAATACTAGTCCCAATAATATATACTCATGCTCATATATTTAAAAACATAGCTCTTCCTCTGGTGTGAGATAATTGCTCAAACTACCCTCTTAAAAGAGGTAACTGCTCTTGAATTATCTTTTAAACTCAAAACTCATTTAGAAATCTTAGTTTCTCTTTTATCAATGCAAAAATTGATGCCTTTGGGAATACATAGTCCCCTTATACTCTTACTCTACTCATACTTGAAAACTCTTTCTCAAAAACATATCAAGATCATATTATAATATGATCATTGATTACTCGGGAAGTCATACTGCATAAACTTTGATGATACATCTCGATACCATACTGCTCATACATTGATGGCATAGTCGATTGTCATACTAATCATGTTTTAGAAATATCTTTTCTCAAAATTCATCTTTACTTTCTCAAAAACGCTATGTAAGATTCAAGTGTTGGGTTTAAAAAGTTTCTCAAGATTTATAACTCAATTCATATGGTTCATGCGGAATTATGGACATGAATGACTCAACTCAAGGATTTTAATAGCAATATGAAACTCAATACGCAGAACTCAAGACTCAAGAACACAAGGACTCACGAACTCGGAAATCATAACTCAATGATATTACTCCTCTCAAACATACTCAATTTATGGAGTTCATGCGGAATTATGGGCATGAACGATTCGGCTTAAGGATCTCAATAATAGTGTAAACTCATGAATACACTCTTCTCATTGTGATACTCATTTACTCAAGTCTTGAAACATATTATAAATAATAATAGAAGACTTCTCAAAAAGACTCAAAGGGACTTTTTCGAACTTTACTCTTAACTCAACCTTGAATTTCAATTATGAATTCAAGGATCATGATCATGATTTTGATATATAGGATTTCTCAATCATTAGATGTAGTTTGGATTGTAAAAATCAGGAAGAGAGAGAAGACACGTTTTAAAAGAATTCAGACGGGACGAACGACAGAAGAAGAACAGGGGAAGGCAACCCTGGCGCATGGCCACAACCCCTAATCTTCAAAACACCATTTCTGGTGCAGTGTAGGCATGGCGCGCCAGCCCCCCTGGCGCATCTTTGGTGCGTCGCGCCACCCCTCCCCTAGAAAATTCTGACGAATTTTTCTTCTCGATTTTTGGTCCTAATTCATCTAGAATCAATGGATTTCTTCCAAACTTCCTTAGATTCGTAAACCCAACATAAGTGAATGAATTCCTATTCAACTTTTCTCAAAAACAACCCTCAAACTCATAACCCTCACCTAAAGAAGTCATTAAACTCCAATAATAAAGATTGAAGAACGAAACTTCAAGAAAACTTATCAAGAACTCATTACACCAAATCTCATGGACGAATTCAATAAGGAAAATCATGATTGGTGTGAGGGAGAACTAACCCATCACTATGAAAGCTTACATACCTCGAAAGAGTTAAACTCTTGGCGAAATCCTCAAAGCTTGCAAGAATCTTGAATGCTCTTCTTTTCTCCTCTTGAACTCCTCTCAAAAACCCTAAGCGTGTTTAAGAATGACTAAAACTTATTCCAATCAGTTTAGACTCCTAAAATATTAACAAAAAACGAGTTAGGTTAGTGGGGGTAAGGAAAAGACCAAAATACCCCTCCTTAAAATGGATGGATTGACTTAATTAGACAAGCTGCACTCAAACATGCATATCTCCTTCATCCGAACTCAGAATTTAGCAAACTCGGCACCTCTGGAAAGAGGACTCAGAGATCTTCGATTTGATTGCTCATGGGCCACCTAAATCTTTATGTGCTGAAAGTTATGGTCGTTTGAAGTTGACCAAGAAATAATCTCATAGAGGAATTTGATTTGACGGAAAATTTCCAACTCAACTTTATGCTATGAAGTTTAGCAACCTTAACATCTAAGAAAATATCGAATCACTCGGAAAAAAACTTATTTACCATAAAGAATGACTCGAGTCTCAACTTAGAAATTTTTGGGACGTTATAATATCTCCCTCTTGGGATCATTCATCCTCGAATGACGGCTGAACAAAGAATGGAAAGGGGTTACTCATTCTATACTCAAGCCTAACTCAAAATCACCAAGTACTTAACATAAGGTGATTATTCTTGATCTAGATCACAAGATTTGTGTTCTATAACTTTGTAGAAAATCTCATCCCAAGAACACTACATTCAAGCCTAGAACCTTAACCACTCCATGGACTCCTACTAACCACTTCTGATAAGAATTCCTAATCCTCTCATATCTCTATCAAAAGAACTTTCATGAATTATTACTAGGAATTTGACTTATTACTTATCGTAAAACCTTCTCTCATTCTCCTTTCACATTCAAAACCTTAGATAGAATAAACACACCATCACTATCACAATAAAAAGCAACAATCTTCTTCTCTATCATCACACCGCTACTATCAACAATCCGACTCTCAAAAATAAAAGGGTAACTGAAGGAGCTTTAGGGTGAAAGGTCATAAGCATGATTCATTCGATATTTCTGATATAAGAATATGCATGGCATAACATTATGTTGCTAGCTATGTAGAGTAAATCATGAATTTGCAAGACTGGACATACTATGAGACATGAATGCTTATATGAATAGATATCATGAGATTGATACCCATAACTTCATGGATTAAGCTTGAGATTAATGGGCTAAAGTCCTTGCCCCTCATTTTGGAAACTAAGCATATATGTGAATCTCTCTGAAGTTTATGAGCATAGGTCATAAACACAATTCTGATATAAGAAACATCATTATGATGTTGTAATGACTGAACTTTGAGGAATACACTAGAGTATGAATGGATTGATATACCTCCTTAACTCTTTCTGAAGACATAACTATCTCATTCTCAACACTTGACTTGATTTCTCATCTTATCATCTCCTCCTTGGGTCTGAAGCTAACACTTGAAGGTTATGGATCTATTACTCAGGAAAGGCTTATCTTAGGCCTACTGAACTCTCCATAAACAGGTACTAAGACTCCTTCAATTCCTAGGGAGGACCTCTTTCGAAGATGCTTCTCTTACCTTCTTCTCATTGTCTTATAAGGATACCTCATCTAACTCTTTAAGGGAAAAACATTGCTTATATCAGCCTATTCCACATTGACAACTCAATTATATTATCTAGGCACACATGGAAAATCATTAACTTAATTGGAACCTCATCTGATTACATACCATCTCGGTACACCTCTTAATGCTCTTGTTACTCCTATGACTTAGCCATACTTCACTGCCCTTATCTAAAAGTTAGGGTCTCTCCCTTGTACCATTCATATTTATTGCAGCTAACATATCAATTTCTCCCCTCTCTATGTCAAGTCTACCAGATCAGATCTCGAGACCAACGTCATCACCCTCATGTTGCCATTTTTTTTATCATGACACTCATCTCAAATTCAAGCTTATTTTTTACTACTAAACCCAAATATTAATATGATTTTTCGCTT

>LNC_001321

TGGGTAAGAAGGCAGAAAGAAGTTCACACCAGGATTGAAGAAGCGGGTTTGATAGAACCAGGGTCAGCGAAAGACGATGGAAAAGGCTGTTTGTAAGCGTGATCCTCTATCCTCTCTCTTTCTATCATCAGAGAATCAATCTCGTAGCCTAGCTACGTAGCAGGCTCAGAGCCTTTTGACGAAGGAAGAACCGCAGCTATGGAATCTGCTGCTCTGGCCGCTGTTCTGTTACCCGATCTTGGTGGTGTAGTTATAGGAGGTGCCGTAGCTTTAAACTCTTTTTTCGAGATGAGAATCATAGACCTTTTTATTGCCCAAGCAAGGGAATCCATTTTCCTTCGACCTGACTTTCTGAAATGGGATTCCGAGAAGGTGGAGTTCAGTAACCCAGTTCAGTGATCGAGGAGAGGTTGAGAAGAAGACAATGAATGGAATGGTCCGAAAGTCGCTATAGAAGATCAAATGAGTCTTAGTGAAGCTAGTCCATTAATTAGATAGATCACATGTGCGTACATTATCTATAATAAGAAAAAACCTGGCTCCAGGTAGCGAAAGGGAGTAGCAAAGGGGAAATTCGTTAGGAGAGAAGACCAGGATTCCCTTGGTCCTTCTAAGGACCAGCAGCATACCTGGATTTACCGTCCATCCAGAGAATACCAGTCGTCCAGCTTAGAAACCATTGAACTAGCTGCCGGAGAAGGAGAACTTCAACTAAGAAAGAAGAAGAATAAGAAGAATTGCTTAAGTATGGCCATTTTGTGTTCGAATTCTAAACCGGCAAGGCGCTACTGGATGCTTCTGATCAATAGAACACGAAGAAGAGAAAATCGGATTATGTATGATGAGCTTGAGTCGAGTCACAAAAAGGGTTATGATGAGCTTGAGTCGAGTCACAAAAAAAGGTATGATGAGCTTGAGTCAATTATTTCCAAGATCGAATTCCAGCTTTTTCTTATGTAGTGTATTATTATTTTGCCTTTTTTCTACCCTTTCTATTC

>LNC_001322

CTATGAGTCACTGAAACGACGGCAGGAGTGCCCTTTTTCTATCAATAGAGGGAGCAAAAAAGGGGCTTTGCTCCCCTTTACAATATGAAGAAAGAAATAAGGGTCGAAGTTTAGACCGCTCACAGTAGTTCTACCTATAGAAAGGATCATGAAAGAGGCGATCAGAATGGTACTCGAATCCATTTAAGATCTCGAGTTTCCAGACACATCGCACTTCCGCCCGGGTCGAGGCTTCCACTCCGTCCTAAGACGGATCAAAGAAGAGTGGGGAACCTCTCGCAGGTTTTTGGTATTCGA

>LNC_001323

TTTTGACGGCTGCTCTGTGCTTGCCAGTATCTTTTGTTCTTCCGGCCAAGACCAATTCCGCCTAAGGTCATGATCGAGTCATGACGAGCTAGACTGATGACGCTCTTTCAAGGACAAAATGCCCTGGATATGAGTATGACTCTTTAGGGTAATACAGCAGATTATCCATGTGCAAATCGAGATGCGGTCCGGTTCAAGCATATCCAGTTTCATATCCATATCTCGCACATCCTGAT

>LNC_001324

GACGATTATCAGGTGATCCAGAAGACCTAATCAATGAGATTTGGGGCGGTAATGTTCTTTATTACTTCTTCATTTCATGTTCTAACTCCCAGTAATAGAATGGGAGACCTACACTTGACACTTGCTATCTTGCTTGATGACTTTCTTTCATATTGGTTTCTGGTTCATACTCTGCTCCCTAATCCGGAAAGATCATATGTCAATGCAATTGGCTACTAGCTCAAGTGCTGGTGAAAACCTCGCTCTTTAGCGATAGGAGGGTTTTCCAACTAAGCCAAAGATATAAGCCGACGGTCCAGGTACAAGAGAAGACTTTATAAGCCGCTTAGTCGGAACTTGTGCTTTCCTATCCATACCGCTTTCCTTCATAGCAGCAAAGGAGAACGCGATATTGATTCAGTCGGAGGTATACTACTTTTCTGATCAAATCAACCCACTACTCAACCGCCCACATGTGTGTCTGAGTCAGTGCTGGCAGTCGCCTTCATTGAGTTAGGTTAGTGTGCTTTGCATGCTTTTCTCGAAGCTCATATTGAAGCTCGTCGGTCAAGAAGT

>LNC_001325

ACTTTCTATTATATTAGTAAAGCGCTACCGCGCTACAACTAGCATAGAAGCCTGCGGAAAAAGCTCGTCAGCCCCTACTCCTAAGTTGGAGCAAGAAGCAAGGGATTGAGCGCATCTTTCCCATTTCTATTAGTAGTAAGGTTATCAAAAGGCTTTCTGATTGCAGGGGCGCTAACGCGACCTTCCTTCAGCTGGCTAAAAGGCGCTTCACCTTAAGATTAAGAGAGATACTTTCTCTTGGTGATCGGTTCATTGGCAACGACAGATAGGCGCAATCAAAACATTGCTAATATGAGACCATCCTCCGCCTGCCGGAGAGATCAGTTTGACCAGGGCTCAAATCAGACTTCACCGAGTAATGAGAAACTTAGGACCTAGGTATGTAAACTTGCTAGTCGAAAAGA

>LNC_001326

GCAAATTGAACCCACGGAACTCCCTTTGCTTTGTCTCTGGATTGACTCAATCAATGAGAACCACAGCGAAATGCTTCTGGCATGCCAAACAAGGTAAGCGATCGATTCTCTTTCCCAAAGTCAAATGCTACTGTACTGAGCCAAAGCTTCATGCCCTTCTTGAATTGTAATCATTGAGTTCAGATTCAGAGCTGACTGAATGGCTTCATCTTAATGAATAGAAGAATTCCCTCTTAGGGTTGGTTTAAGGGGGAACTTCTGTCAATT

>LNC_001327

CAAGCTGGCGTGGGGGGCAAAGGCAATAACATCCGAGCAAAGCAAGCCCGGCAGGGTATGATGAAAAGCTCTCACCAGGATTGTCTTTCCAGATCGATTGATCAACATTTCCCTCTTTGTGATAGCTGAGAAGCGTTCTAAATACTTTTCGACAAGGAGAGAGTTCCAACAAAAATGCGATCTTCTGCAACGGAAGCTAACGTGGATGTTCCATTGGCACCGGGTTTCGAAGGTCACGATCATCGGAGCAATCAACAGAGTGTTTCCTTCCGTAACAGAGGAAATCCCTTATTCTAAGGAAATATTTTTGGCAAGAAAGCCTGTCCTAGAAGGAAGCCTGCCCACAAGAACTCTGGTTAAGTGCCCTCCACCGCAACCGAC

>LNC_001328

TTTTTTTTTCCAGGGGTCCAGAAGGCTAAAAGGTGGGGGAGAAGGAAGCCGAACCTCTAATCGACCTGGACACATAAAGAATTCTCGGTGTCGAGAGAGATTTGAGATTCCGTAAGTAACTCAGTGAGTGCTTTCTAAGAAGGGCTTGGAAGAAGAAAATGAAATAGGAACAACCGCGCTGGTCGTAATAGATCGACTTTCATGCTAGTTCTTGCTCCAGCATGAAAGTTCCATTTCAGGGAAGGACGACGTACTATGATACTTTCCATTTTGTCGAGCC

>LNC_001329

CCATAACCAGAGCTCGGAGAAGGAAGAGATAAGAGGGCCCTATGGAGAATGTGGTCAGAAATCCATAATAGAGTCCGACCACAACGACCGAATTGATTATCTTCATGCATAAGGATCCTAGATTACCTAGTAGAAAAGATTGAAAAATAATCACAAACCTCGCCTAGCACCGAAAGAGATAGACTAGAAACGACATCTGTTATGTCAATGACACCAAAGGGATATTAAATGAATGGAATTGGGATATGGATGGAATATAATGAAATAGAGCCACTTTGAGGTTCCCTCTGAAATGAGGGATGTAAGGGAGCCACTACGAAGAAGTTCCGGGAGTTACGAAGGAAGCTTCGAGCTCATATTGTTCATGGGTTGAGAACGGGAATTGAATTCTATGAGATGGAATCTCAAGGAAGAAGCGGGAAGGAAAGGT

>LNC_001330

GCGTCTTCCCTCAGAAAGGAAGTAGTTATGGGGGGATTTATCCCTAAAAGCAAAGGCTAGGTAGAGGTCTCTTGGATAATAATATGATATATATTTCGCTAGAGGTAGGGCTCAGGCTTGTCCGCAGCAGGACTTATTGGGCTTTTTAAGGTAGACCAAGCCCTAGATAAGGTGAATCTTACTTTAGAGAATTAGACTAAATTGAGTCTAAGTATACTTAAAGAAGAAAGAGGGAGCTAACAATCTCCCCGAAGGGGGTGCAGGGAATCATCTTTTGAAACATGTAGCCTGAAAGAGGAGTTACCC

>LNC_001331

GTAGAGTAATGGCCCGCAAGAAAGGAGAGAGAGGCGGGCTTGGTGATAGACCGCGTCACGGCTATTCCATTCCTTCTGTACTAGAGTTCGGTGGAGGAACTGTAAGAGAAGAATGGAAATCCGTGCTCTAACTCGACATATTCGTCTTAATCATATTCGACCCTATCTTTCTTCTCCAGTTGATGATGCATATTGAACTTCAACCAATCTCACTTGCTCAAGACTACCTTCTGATAGACTAGCTTCCCCCGGGCTACCATCCTTGCAGCTAAAGAGACCATATCATCATGCCTAAA

>LNC_001332

ATAACATTGATTCAATTGGCATTGACATAATAGGAATATTTCATCTATTAAGGAGGATTCCCCGAATACCTGAAATAGAGATAATCATAGAAAATGTGAAATATTTGATAGGATCCATTTCGGGAACGTAGAGTGTAAGAGAAATTCAAAATGTGAAACAATACGTCAGCTCTTCTACTTGAAATATCCTTCAATTATGGAAGATATCTGATTTTTTATACTCATGTTTTCCCTTCCCATCGCGCTCCGCCGATTGTTGTGCTCGTGTCAACCAAGCCCTCAAGTGCCGTAAATTTACCCTGGGGACATTGGGTTTGTTGGCATTATAGGCAACTTTGCTGGCGAAGCGGTCAATAATCATTTCGACCATCTCACTTGGTGATGGAAGTTCACCCAAGTTGTGGTTCCACAACCTTCGAACTAGGCGTGCCTTCTCCGATTCAATCAGGGGGCTAATCTGTTCGGCCAAGCGATCTTT

>LNC_001333

CTAAGAGAGTGTGGTTGGGTCTGACTCCGTTATAGGGCTAGTACGCCGAGCAGCTTCGAATTCTGCTGTGCCGATGCACAAAGCAAGCACACAAGGACTGCGTAAATCTTTAGTCTGTCTATCATTGCCCATGTTGGGCTGACTCCTTTTTTTAGGCAAGAAGACCACTAGTTGAATTATTAAACTATTAAGACTAACTGAAAAAAGGGTACTACTCTTCTTCCTCCACTACAGATATTGAC

>LNC_001334

AGCCCTCGACCCCCCTAGGGTATGGCCTACCAGCCCGCAGATGTAGGAAGGCGGCCTGCTTGCTAACCAAACTGCGTAACCAAAGCGACTCCTTGTTGGTGGTTGATAGGTGTTGGTCCGATTCACCTATGTCAGTTCGAACTTGGCGGGTCGTTCGTTCGTGTGGAGAATCTCTATCGTTACTCAGGATTTCTCTGAGTTGATCCGATGCGTCATAGATAGAATGGAGTGCGCTATACGCCGACCCACGTTAAAAGCTTTCTTCTCTTATGAGATTTCGATTGAATAAACAATGGTTATTTCAAACACCGTCTACTGTCCCACAAACTGATCAGTGGGTTCATGCCGCACAGAAACCGAGACCTGAGAAGGAGGCTGTAGGAGAGAGTCTAGCATAACAGAAATCATCTAAGCTGACAAGGACCCAGAAAAGGAGGATGCTGAGGAAGAAGGTAATGGAATTAAAAATACAGACTGAAGCAGAGAAGAAACAGAATGTTGGTCTGATGGTAAAGTACCCAGCAGAAGGTAAGGCTGGGAAAGACCACAAGAAGCTGGAGAAGGGACAAGCCTCGTCAACGGAGTTTTCTGATGAGCTTTCTTTCAGAAGGCTTGGTTCCGGAAGACGACCTGATGGAGGAA

>LNC_001335

CCATTTGATTGATCGATTCAGAGTCCCGAACCGAAGCCCAAGCCCATACTCTTACTAAGAAAAGTGGAAGTTCCTACCCTGGGAACATTGACATACGATCTTTCCGGATTAGGGAGCAAAGTATGAACCTGAAACCAATATGAAAGAAAGTCATCAAGCAAGATAGCAAGTGTCAAATGTAGGTCTCCCATTCTATTACTGGGAGTTAGAACATGAAATCAAGAAGTAATAAAGAACATTACAGCCCCAAATCTCATTGATTAGGTCTTCTGGATCACCTGATAATCGTCTAATCAAACAAATGTCATGACCTAATGGACTATAGGACTAAAGCAGAGAAAGAGATGTCCTTGTCAGAGGACCAAGCCACTCGAACAAAGTGAGATTAGCGGAATGCAACTTCTTGACCGACGAGCTTCAATATGAGCTTCGAGAAAAGCATGCAAAGCACACTAACCTAACTCAATGAAGGCGACTGTCAGCACTGACTCAGACACACACGTGGGCGGTTGAGTAGTGGGTTGATTTGATCAGAAAAGTAGTACACCTCCGACTGAATCAATATCGCATTCTCCTTTGCTGCTATAAAGGAAAGCGGTATGGATAGGAAAGCACAAGTTCCGAGTAAGCGGCTTATAAAGTCTTCTCTTGTACCTGGACCGTCGGCTTATATCTTCGGCTTAGTTGGAAAACCCTCTTATCGTTAAAAAGCGAGGTTTTCACCAGCACTTGAGCTAGTAGCCACTTTCTTAGATAGGGCTTTCGGCTAGTTTGTGTAAGCGTCTTTGTAAGCAATACCCCGAAAACTCTGACAAAAGAGATTGAATGCCAGGTTAGTTCACTTATTTCGGGCATCATCTTACGTCATTCGTTAAAGCGCTTTCTATAAGCCTTTAATTTAAGGACGTTTTAAAGAAGAGAAAAAGTTCCGCATTTTCTGATTCTTGATAGCAGGAGAAATAGAGTCTTTTTTTGGCGACTGTAGCGGAGCCGAAGGAGATTCTTTCAATCGAGATTCGCCCAAAAAAAAGTACAAATAGCAGCCCTCGATCCCCC

>LNC_001336

TAAGGACTGAAGCAGCCACACCAGCATCTATCTGAGAAAGCAGTAATAGAGCACGCAACGAAAAGGGCAAGAGGAGCGTCCAGTCGAATCAGTTTCTCATTTTTCTGAAATTGACTTGGCCGGTTGTTGCTCCAACACTTGGGTATTAGCAGCCGTTTCCAGCTGTTGTTCCCCTCCCAAGAGCGGGTTCAGTTTACCGAGTTGTCCGAGCTCGAGTCAGGATACTATACTGCCTTTGAAAAGCACGGATGCCGTTACAAGTGTTCAAGATAGTTAAAGAGTTGAGGGATATTCCTCCCCATGAGAACCCACTACAAATAGTACCCTTTATGCCTGAGATTACTTGGTATTTAAGTGACTAGGTAATAAAACCAACTATTCTGCAATGAGTGAAAGGCTATCAGTGCTTCCAGAGCTGCCAACTCAGCTCTTTTTTATGTCATCTTCAGAGTCTTCCTTCCTAGCAGCCTATGTCTAGTGTCAAAAAAGGCATTAGCAAGGTCTTTCCACATGGACCTTGCTGAACTAGATTCCCTGCTAGCGAGAGCAGCTTAGAAAGATAAAGGAGCGCAAACAGGGGTTGTTTGAAAATGAACAGATAAGCTAAGGCACTACTTCTTTTCTGCCTACTAGCAATGCAACTACTCCTGAGAAATAGTCAAACTGAACTAGTTGAAGCTAAGGGCCATCGAAGACCAGATGATGGGTTACGAAGATATACAAGGAAAGAGAGGCTCTGGAATACCTTTCACTTTCAGTTAATCAGG

>LNC_001337

TTTAAGGAACGGTTGAACTCTTTTTTTCTTTTTCAAGCTGTCGATAGTCATGTTCTTATATTTCTTTACTGTTCCGTACTAAATTCCTTTGTTCACCAATCAACTGAAGAACCAATTAGTTGTAGCAGCACCTGCGACTGAAAAAGTAGCTCTGGAAAACATTCAAAAGAAGAGGTCTAGGGCTGGTTCCGGAAAGGGATCGAAGAGAATGTAGTTCATTCAAGGCTCAGACAGAATTAGTTAGAAGCACCTCTTTCTATTGATATTGACTTGATGTAATGCTCTCTTCTCTCGCCCGTAAACCATAGGAAACATCCGCAGCAGACATCTTCAAAAAAGCTGGTTGGAGTTTGGGCAGTTGGTCGGAAAGTACCTAGTTCAGTGACGCCTATTGCCTTCGTCTTATTCAAACGTTATTGGCAGAATGCCTTCATCTAGTCTTGAAGCAAGGAACCAATCGTCATGCTTAACATACACGTTTTGTAGAACTTATGCCCCTATTCTCTTTAAACTGAGAAAGAAGAAAGAAGATGGTTTCAGTAGGTAGGATGAAGTCACTCCGATAGGATGAAGTATAGGTCGATTGAAGTAGGGAAGGTCGATTAAGGAAGAGAATGTAGCTTCAAGAATGAGGCAAGGCCAGCCCTAAGTCTAAGTTGAAGTTATAGGGCTGGGAGGTATGAGTTG

>LNC_001338

ATCTACCTTCTTTGTGAGGTATTATTATTCTAATTCACATTATTCTTTAGGTAATAATCGCCTTATTTCTCTCTTATTTCTTTCTTGTAGTACTGTCGGTCTAAGGAGAGGAATGGAAGTAGTCGTCTTGCTTCTTTTCTTTGGATCCGCTTATGTCTCTACTTCGCTTGATTCTCATGCCAGTGGACTCCATGCTCTGTTTTTGGCCTTATTAGCTTGATTTCATAAGTGAGCATTTTAAATTGAATAGATAGTGAGAAAAGCGCCTTTCGTGATTCAAGTAGCAAGCTCGGATTCATGATTGAGAAAGAAATCTTCCAACCGCGGCTTTAGCTG

>LNC_001339

TAGGATAGTAGCTTCGGTCGATAGGGTAAGCGCTCATTTCGTTCTTGCTTGCTTTCATTCTGTTATGAGAGAGAGCTGCTATACGTCTATTCTCTTTAGACAAATGACTGTCCACTGGAAAATACAGAGTAGGATTCATCCGTGTGGTGAATGAAAGAACATTCATTAAATGAGTTGACGCTAAACCTCCTGCTCTTTCAGAGAGTAAAGGATGTATGCATTACTGGATTAATTCGAATTAATAGTAATAGAAACTAGGGTCCACGTAGACTATTCAGAGGAGGTATGCTAATCAATGATCTACTGATGTAGCTGGTGTTACTGAAGCTTTTGCTAGATATGCTATTGGCTTAACTAGCTTTAAACCCTCCCACCGGATAAAGGAATGGAATTTCAAGTACAAGTAGTCGTCTACACTTAAAAGCTTTCTTGTCTTCACTGATCAACATTTAGTTTGGCCTAAGATTTTCTAATTCCGACGCATCACTTACCTTTCTTCATCGTGGGTCCATAACCATTTTACTACCTATTTATGTGGTTGATATCTTGATCACTGGCATAATCACTCAGCCCTTGAGGATGTAGCTTCTACGGATTGAGGTTATTCCCCGCTAGAATGGGCCAGGCGGGATGGGGGAGCGGTCGAGCGTGCAAAGGCGCAAGTCGAGCAACGTATCTGAACTGAAATGTAAAAA

>LNC_001340

CAGTATTGACTGGTGTGCTTTGGTGTAACCGGGGGGCTGGAACCAAAGAAAGACTGTACCCGGCGTCCCTTCCTCTCATCAGGCAGCTATCTCGAGCTCATTGAACACTAACCTTCTTCTTTTTCTTTTCTAATCGTCGAAATTATCTCAATTGTGCCCCACTTCCATTGGAGCACTCTTTGATACGTGTTCTGGAATTCTCCAATTCAGTGGAGTGGGGCTTACCTTGAAGCGGATTCCAATGTCATAGCTTTTGC

>LNC_001341

CTTTTCTTTGTCTCGTCCACACCCAGGAAAGAAAAGTTCAGATCAGTAACAGCAGACTTCCAGTTCCTTGTTCCCATGAAAGCATGAGTGAACGGTCAATTATTGTTGAGAGGATTTGAAAGAGCCGAGTCTGTAAAAGGCTTATCGGATGGGTAAGAAAGACTTCGGTCTATCCAATTCAAGTTCAAGACCGGTTAAGAGAGCTACGTCTAGCAGGGTTGGGCTCATCATCCCAAATCGAAAATGAAAGGAATTGGTAGTGAAAGACCAGATCCTTTTTTTCATCAATTGTAGTATCTATGCGCTAATCCTTTTAGATTTACAATCCTATTTCTATTATAAGAAATGAGGTTTTAACCCACTTTAAAAGAGTCCTTAAGGTCATAAAGAGACCTTAAAAATGAGTTTTCTAGAGGTGAATTCTTTTTTCCGGTCATAGTATACGGCATTACCGGTCTTAGTTTTAGGAATCCCATCCCTAACGAGCTAGAAGTTAAATAAGTGCTCTTAGCCTTTTTCTTGACAGTAGGATTTGGGAAGCTCTTCCCTTCTGCTCGACTTGTCTATTGAATCAGCTCTTTTCCAGGAGGAGGCAAAAAGAAAAGCTTGTGTTAAGCATATCGCTTTTTTAGAATGATTTGATCCATCAATGGCAGCTATATGAGATAGCCTCAGTTCGTACCTTCGGAACAAGCAGAATAGGAACAGTGGACCTAAGCAAAGGAAAAGATGGACCGTAAAGAAGAAGGGGATTACCAAAGAAAATGCCCAAACAAGATAGGATCACTTCTAGCGGGACGGAACTCTACAGACTTGATGGACGGGCTTTCCTTTCAAAAGGCAACAAAGAAAAAGAAAGAGATATGTGGCAAAGAATGAATTGATAGAGGTGTGGAGTTTAACTGAGATAAAACAGAATCAAAAAGGGCTAGGAGCTCTGGCCGGTAACGAAACTAAAGCTTCACACGAGTCTTACTTCTTTTTTCCAACTTGAAAGGGGAATAAAGCATGATTGCGAACTGCAGATAAACTCGTTAGCGGGAATAACAACGACTAGCAGTACGATGAAATCTGATAAAAAAGCGGAGTAGGTAGTCTTCAAAAACTAGGGTTGCTTTCTCTGTTGGTTTAATTCGCCACTAGGTGGAATCAGAC

>LNC_001342

CGAAGATTATGAATTAATCAAGTTTGGGTACGTTTGGAAGTGTTACGATATGGGAGCAGGTTGGAACCTTTCAACTACACCTAGGTGGTTGTTTGGTCCTCCAACTCTCAGTTTTATATTGGTTGATCAATAGGTATTTGGTGGTCTTCACGGCAAGGATCTCCTTATCAGTCCTAAGGATTTACCATCCTTACGGAAAATGGATA

>LNC_001343

AGGAATTATGTGAATGAGTTTTATGTGAAGCTATATGTGAAGGAATGATGTGAATGAGTTTTATGTGAAGCTATATGTGAAGGAATGATGTGAATGAGTTTTATGTGAAGCTATATGTGAAGGAATTATGTGAATGAGTTTTATGTGAAGCTATATGTGAAGGAATTATGTGAATGAGTTTTATGTGAAGCTATATGTGAAGGAATTATGTGAATGAGTTTTATGTGAAGCTATATGTGAAGGAATGATGTGAATGAGTTTTATGTGAAGCTATATGTGAAGGAATGATGTGAATGAGTTTTATGTGAAGCTATATGTGAAGGAATGATGTGAATGAGTTTTATGTGAAGCTATATGTGAAGGAATGATGTGAATGAGTTTTATGTGAAGCTATATGTGAAGGAATGATGTGAATGAGTTTTATGTGAAGCTATATGTGAAGGAATGATGTGAATGAGTTTTATGTGAAGCTATATGTGAAGGAATGATGTGAATGAGTTTTATGTGAAGCTATATGTGAAGGAATGATGTGAATGAGTTTTATGTGAAGCTATATGTGAAGGAATGATGTGAATGAGTTTTATGTGAAGCTATATGTGAAGGAATGATGTGAATGAGTTTTATGTGAAGCTATATGTGAAGGTATGATGTGAATGAGTTTTATGTGATCTTGTGATGATCCTAT

>LNC_001344

GCAAAAATTGTGAAATTTTGATCAAGAGTTGACTTTGGTCAACAAACGAGGTTCCGGTGCTCGAAATGGAATTCCGAGGGCACCGTTGGATTCAGGGGGTGATTCTAAGTCTAGAATGAGTCTTGGTTGAATTTTTAGAGGCTCCAAGTGCATTTCGAGTTTTTGAGCTTAAAGTTAGTTTCTTGACGCCTTATCGGATACCGGGTCAAGGAGACCTCGAATTCAAATTCCGACGATTTCATTGAGTCCGAAATGTCATTTTCAAGCTAGTAGCATATTTGGTTTGTGTTCGGGAAGTGCCAAATGAGTTTTGGGTGAGCTTTTAAGCTTCTTGGATGCTTTGACACTATTTCAGCAAATTGTGGCAACTAAAGGGTTCATTATTAGTTTTAA

>LNC_001345

TGGCACTTTCACTTGCTAAATCCACCTTTTTCTCTAAACCTTCCAAAAAATGGCATTCGACTTCGTATACCCTTCTCTTCATTTTCTTCTTCTTCGTCTCTTCCAGTAGATAGAGTCCATCATCACAAAAGGAGGCAATCTGTACTAGTCTCCGCTCTCGAGCTCGGTGGACTCAAGATTTCCAAAGAAGCTTACCAAGGCATGACCCTAGATAGATAGGAAGACTGGAGGTGGAGGTTGAGGATTAAGGTAGAAGGTTAGTTGGTAGTCGGGCATTGCCTAGATCTCTTATTGGTATTTGTTGTTTCCTTTGCTCTAGTTACTTGTTCCATCTTAGTAGTTTTCTGCATGGCTTCTCCGCTATTTTACTTGTTCCATCTTAGTTGTTTTCTGCATGGCTTCTTCGCTATTGTATTTGCTTTACTTACTTAATTTTGATACTTACTCAAGCTAAGGGTCTATCGAAAACAACCTTTCTACCTTCACATGGTGAAGGTGAGGGTAAGGTTGTGTATACACCATCCTCCCCAGACCCCACTTGTGGTATTACACGGGGTATGTTGTTATTGTTGTAAGCTTGGCATTTTCTGTGGTAGTTTTGACTTTTGAGTTCCAGATATGGTTTTGTTTTTTGTTAAATTTTGAAAGACTGCTATAAGAGTATTAGCAGTCATCACGTTGACGGTAAACATCAATATGCAAGTGAGTATTACCTACAGTTGTAAATCAGATAATGGAAACACATATTTGATTCTTGTTTGACAGCAAATATCTCTGTGAGTGGCTACTTTAATTGTGAAATTTTATGCAATTGCCTTCCTTTCTCTCTAATAATTTTGTTTAAATTTGCTGTCAAAATATATACAACAAGAACCCAGGAGCTGCTACCAAAACCAATAATCTATTTATCCATCTCCGATTCTTCATTTAATCATCATTTTATGATTGCTCGATTTCAATTATCCGTCTTTTGCTTATGGGTAAAATCGCACCGATTATTTACATCAAATTAATAGATGCATGACCTTGACCAATTTTTTAAATAAAAAACACAAAATAGCCCATTTTGAATTTTAAAGTAGCTCATGTTACAAACAGAAAAATAGCCCATTATATCATACGTGTATCGAAGAACTGAACCAAATTAGAAAAAACCGC

>LNC_001346

CAAGTTTAGACTGCTGCATATTTTCTTTTAAGTTGATTAAAGTGTGGTTTTCTTGCGTGTTGGGTTTTGCTGCGTTTTGAGCCCCGAATTGTGTTCCATTTTGGAACACAAGTTCGGGGTATTGTTTAGGGTCTCTTGATGGAGTCAAATTCAGACAGTTCGGTGCGGATTCCTCGAGTCATGATGGCACCTACTATAACCTACCAGTAGGTAAGCCAACCCGTAACCCGAAACAACAAGTAATGGGTTTGAGGGCAGAAACTAAACAAGAGTAGGCAAAATTTAAGATAACAAT

>LNC_001347

CTTTGGTGTATTCACTCCAATCTTCATTTACATGACTAGGGATACTAAACTTCAAACGGTTTTGATCTTAGAATGTTAATCTCTCATATGGTATTTAGTGACATTGGAATGTTATAGTTTCCCCACTATTGGTGGCTATTCAAGTTTGAATTTTGATAGCTCATGTGTATTGAAATTTGCAGGTTCCTCGATCAGAGCTTTTCAAATTTTGAATATTCTCAACTTCACAATCACTCATTCTCAATTTCAAGAAGAATTTTAAAAGAATGTACTCTGTGCAGTTTCAATGTCTGGGCAGTCGTTCATCTGAAAATTTCAGAGTTCCTAATAAATTTCTTGTTTAGCTGGGTAGTTACTAAAAAACACACCAATTTTCACCAGGTCATTCCAACTTCAATTTAGTAAATTGTATTGACATATCAATGTTTCCACCAGAACCTATAAGAGGTACATGTTTGTGTTAAGCTGAAGAGTCC

>LNC_001348

ATATCATTAGGTTAGACATGCATCACTATACTTGACATTGCATTTCATTGCATTGCACTTCTTTATTATTGGTGAACTTGATCTTGTGTGTTGCTGATCTTGTGAGTGCCTTTCTATGGAACTTGTGACTGATGAATATTGAGCTTGTTGTTGAAGATATGCAATTGTTAGAGTGTTGTTGTTGAGGATATGAAACTATTGTTGTTGTTGAGGATATGAA

>LNC_001349

CAATTCTCTCCCTCTCTCTCTTTACTTTAGCTTTCTTATCATTTCCTTCTTGTTTATTTAAGTTTGTTCCACTTATTTATTTTTTATAAGACGTATCAGTGCGAGTCTCTTTCTATTCAAGTAAATATTTTGAAAGTCTCGAATATAAGAAAAAGTCTCAAATAATATCAAATCTTTCTAAATTTAAATTTCTTGATTTAGATATACCAAAAAAAAAAGCTATCTATTTTTGCTACTAGATGCTAAAATTTATCTTGATATAACGGATTTGACAGACATTAATCAAGATAAAAATCAGGCATCAAATCAAAATCGTGCCAAAGTAATGATATTTCTCCGACATCATCTTGATGAAGATTTAAAAATGAAATATCTCAATGTTTAAATTATTTTACAAGACAAGAGAATCTTTAACAGAAAGATATTTCATTTTTAAACCTTCGTCAAGGTGATGTAGGAAAAATATCATTGCTTTGGCACGATTTTGATTTGATGCCTGATTTTTATCTTGGATATTGTATGTCAGATCCATCACATCAAGATGAATTTTGGTATCTATTACCTAAGGTAGGTAGCTTTTTCCGATATATCCAAAGCAATAAATTTAAGTATAGAAAGATTTGATATTATTTAAGAAAGGAAAATTTATACCTTCAAAACTTTTAAAATATTTACTTGAATTGATAGAGATTCGTGTTAATAACATATTATAAAATAAATAAGAAAACAAACTTAAATAAACCAGAGGAAAAAATAAAAAAGCTAAAGTAAGTAGAAAGAGAGAAGGAGAGATTGTGTATATATTGTTGTTCTTTGTATATGAACAAAGTGGTTTTTTATAATCAAATATTGTTTCTTGGCCATTAAGAAGGAGGCTCATTGACTAAAAGAAGATAGGTGGTCTCACACATAGACATCCACTTTTATTTTACAACACTCTATCTTGAATGTCCATTTTAAAATAGAAATTATAGTGAAGGAACAAATACTATATATATTTTTTATTTTATTTTTTAATACGCATTGCATGCACCTTCACTAAAAACTTTATCGGAAAAATTCAGTGAGATTAAACCTTGGTTAAAGAAAAAAAAGTATAACGTGCATTCTACTGCACCTGATAAAGTCCTCACATTTTTATCTTGTATATCATCCTCAAAGGAGTTGACACTGATAAAGATTTGGTGTATAATCTGCAAGATTATCACTCGAACGAATTTGTTGTACATCAGTATCACCATTCTTCTGGAGATCATGTAACACCCCGAGAAATCTTTTGTCTTATCGATAAAAGTGTCGAGGAACAATTAAATAGCACAATAATAATTTAACTTATCATTTAGAAAAATAGTAGTCCTGGAAATAAGATGAATATTACTATGATAATATATAAATAATAATGCAGAATATTAATACTCAAGTTGTGTTGGACATAGTCCTAAAAAACTATTTCAACGGTGTAATATTTCCCTAAGATTAAATAAATTTTCTCTATTTAAATAGAGAATACATGAATCAGTAAATTAATTAACATCAACAAGTTATTAGTGGGAAAATAAAATAACAAAACTCAAATTATTATAATTAATTAACTTAAAAGGTGAAGACTAAAGAGAGAGGGGCAGAACATGAGGGAGAGTAAGAGAGTGACAGTTTTGTTACTGTTTTTTTCATAAAGGAAAGATAATTATGCAAAATTCATCATAGGACAGGTGTCAGGGAGGGTGTAAAAGAATGGCATACACTGCCAAGTGCTTCATTGTTTCCATTTTTCCAATAAAGAAGACAACTTGATAACTACAATTCCCTTATGGCTTCACAAATACTTTTTTTAATTAAAAAAAGTATTTGGAAATAAACCATGTTGCTTACAATTTTCC

>LNC_001350

GTTCATCCGTGCTATCTACTGCCTGCTCCACATTTTTCCTATCGCTGCCTTTTGCTCTTCATCAAATCCGGATCATCGTTTTTGGATCTCAGTTTTCCCTCAAATGTTTCTTCCATCTTGCTCTAATCCTTCGTTACAGTAAGTTTTGCTTTCCCTTCTTCCCCAATTTTTCGGTAATGTGTCCAGCTCTTGAAGAGAACCTATAATGGTAGTTTCTCCCATTATTCTTTCGATTCTTAGGTTTTCTTTCGATTCTTAG

>LNC_001351

CGGGAACGCTGCAAACGACCCGACCCCACTGCTTCATCTTCTTCCCTACGCGACTAGACCCAGCAAAACCAGCGAGCAAATCGCGTTGCAAACCCAGTAAAATCGCAGAAATTCAGCAGCAAATCACGACCTCTAGACTGACTCCTCCCTCTCCATTATCAATCCGTTGTAGATTGTTGAGATAGGACTATTGATAGCTCCTACAGCCTTATCCAGCGGCTTTCGGGGGCACCCTCTGAGGAACAAGTTTATTTTTACTCTCAAAGAACATAAGGTTTATAACCTAAAGGCTAATCTTCTATAAAAGTGATGTGTCGATTCTCCGCTTTCTTAAATCAAACTTGTGTTGGTGGAAGTTTCTTCGAAGTTAAGATCGTTGTCTCCCGTTTGCTGCCTCGAAGAAGGTAACATTCCGTTCATTCTTCTTTTCTTTCAGTTTATTTCATTTCAACGTTATATGAAATCTTTAGTGTATTGTTAATACGTTCTTGTGATCATCATGTTTGTCTCGTGTATTTAGCCATCTGAGTTTGTTCCCGTTTAAATTCTTATGTATGCTCTGATTTGCCCTAGTTATAATATTGAGCTTTCTAAATATGTTTAATTTCTCACCCCTTTCCCTTGGAATTGTTCTTTAGTTCAAGTATAGCTCATATGTTGATGAAATATTGTTGCCTTAATAATTTTTTACCATGAATCTACATTTGGATGCGATATGGCCCAAGTTAATGTGTATTGCTACTTGTTTTCCACCAAATGGTTTGTTACTACATTTAGCAATACAACATAAGCTCTTGCTTCGGTCATATTATATGTAGACCCATCTAACTTATAATGTCTGGACACTAGTGTAGTCTCGAGTATCTTTGGTTATGATTTTAACACATTCCAGTGTCAACTTCTAGATGCATTCGTGTGTAGTTCGGCAAACTCACCTTGATTCATGTCATCTCGATTTAAGCTCAGTCGGTGTTTGTCTGGTCGAGTCCTATTTCAAATGTCTATCGTTTGAATACTAATCACTATGCCTCTGTTTCCTATTTACTTCTTCCCTTAAATTTCATGTGCCATCCAGTTGTGATTCTTATCTCGTGATGTGTCGATGTCTTCATCTAAAGTCTTATTAATTTGCATCTCGCGTAATTTGTGTATCTTGTTATTGATTTTCCGAATGCCGACAAACTAGTTTGAATCATTTCTCTTAATATATTACATTTGTTGCGAATCTTTATGTTGTGAAGCATTAGGATCATTTGAATGCTAGAAATTGTATTGTTACTTTTCCAGATAGGTATTAGCAAAGTGCATCTAATTCCTTACCTCATGAGATATAATGCAACCACTTTCATACTTTATCAAAATTGTTTGGCCTTATCCTTGTGGGCTAACAGTAGCTTTAAAAATTGCTCAAGTGTATTCAATTCTATTCTCCTCTTAATCTTGTTGAATCATAAATGCTAAATCATAAAAATAGTTTGTTATATGTATTCGTTTGAGTGCCAATGGTATAATGTGATTTTTTAATCTATCGGGAAGTATGTTGATAGATCTGGGAGTTTAAATTGTTTAAATGTGTTTAGTTGATTAATTAAATGATACTAATTTGTTCACTTCTTCTTTTTCTTGTATGTAATATTATTCGAGTCCATTTTGAACTCCATCCCTGCATTTCGTTGAGATGAGATCTTTATTGAGTTGGCCACATCCTGTTCGAGTTACTGGAAAGTTAAATTACAGGTCTCGAAAAAGTCAAAAATGGGTCGTATACAAGATGTATACATCTGTATACACTGATATACCGGTCGAAAATGTGGGAATATAGGACTGAGGTGAGATATAGGTGTTCGGAAGCAAGAGATACATGAAAAGAGCTGATATATACGTTCATATACAGTAATATACATCAGGTGTATACAAAAATGGGTTTAAGATTAAACCCCAAAAATTTCAGCGAATTTGGCCCAAAAGGTGGCCCAAATTCAATTTCTCCCTTACTCTTTAATTATTTTATTGTGATTATTCATTGTTTGTTGTATAACTCTAACTTTATAATTTTTAATTAACTTAATTAGATTCTCCA

>LNC_001352

CAGACAGATCCCACCGAAATAAATTACTAACAAATTCACTACACCCCATCTTCATAAAAACCCACAGAATTCACCAATCTTTTTTGTTGTTTCATTTATTTTTATTTATAATAATTTTTTTGAGCTATAATTGCTTGTTGAGAAGAAGATCAAGAAGAAGAGGGGTGACTCCTCTTTTAGAATTAGAAAATGATGCTTGGTGTTTCTTCAAGTTTAATAGCTAGCAGTACTATTACTGCTACAGGTGAT

>LNC_001353

CTCTAATCACTCTGGGGAGAGATTTAGGTTCTTCAATTAGTCTTCCCAACCTTCTTCCTACTTAAGAAGTGAACTCCATCATTCTATGTGGTGCATGTTCCATTGTGAAAGCTTGTTTATCAGTCTCAAGTTGATCCCAGTTGATACTCTGACCAGTGGGTAATATGACTATTAACACATCAAAGCATGTAAACTACATCCTTCATCTTGTACTTGGAAATTGGAAAAGAAGGGTGTAGGACACTACGATGAGGGTTGGTGCTGTCTGAGACATATTTCAAACAGATGAACAAGAAGTTTCTTCTGAGGGGTGCCTATACTGCATAATATTCAACAAGGAAAAATG

>LNC_001354

CTTATTTGAGATTTTCGATAAGGAATTGCTAACATCTCAAAAAGGTTAATGTCTTACTTATAACATCTCTAAAGGGTTAATGTCTTATTTGAGATTTTCGATAAGTCTTATATCCTTGTTTTATATTCGTTGCTATCATTATTTTGCCTTCACCATGTCTACATGTTTTACTCATTTGGTGATCTATTGTAATTTTAAGGATATCTCATTTGAATTACCTTATTTATGCTTCTCAGTTGCATTCTCTAGATTGATACAGTAGGGTGCTCCTAAGTTTGGTGCTTTAAATTTTCATTATGTGTTCCCTTAATCACATTTGAATCAACGAGACTCTTATGTTTTTATCCTATTGTTTCTTTTGCATGTGAAATCTGTCCAAGTCCTC

>LNC_001355

GTGTTAACGAAGAAAATGTAAGAATCCAAAACAATGTTTCCTTAATTTCTTCTATTTGAATAGGGTTAAGATAAATAATTTTTTATTTATAAAAATTTGAGGCCCCCAAAAATTGAGGGCCTAAGGCCAATGCCTAAAATTTTATAGGCAAGAGCCGCCCCAGCATATTATTTGCAAATGTTGAAATATTACTTCCAAATCAATGTTTGTTTGTCAGTTGTCCATTATTTCAACTTGAACTTCAAATAGTGAGTTTGAAGTTGAAATAATCATCTGACCAAATTTTATCGAAATTTTACTCATTGGGGTTCTTTTTTTCTTTACTTTTTTAAGGAAGATTTCTCCTTGTACGCTTTGCAATTTATAATTCAACCCAACATTTATTACTACCTAACACTTTCTTCTTCTTTGCCTTTTTTTCTTCTCTTTCTAGAGGAGTTGGACTAGAGTGAAAGGATTGTAATATTAATTAGGAATATATTTTAAGCTTAGTTGTGCGGACTCTTCACTTTTGATGCCGCACCCGTCTTGGATTCTCCAAAAATACACTACTTTTGGCGAATCCGACACGCATCCGTTCACATTTTTGAAGAGTCCGAGAAACATAGATTTTAAGGCATGATAAAAGCACCGTTGTTTTTTTTTTTTTTGATAATCGAGAAATCCGTCTGTCACCCACCCGCCCTTTGGACCAATCACAGCCTTCTAAACTCGGTGGATAATGGGCCCGCCCCTCTACCTTTCTCCACTTAAATACCAGGCTTCGCTTTGCATGGTGTGGGGCTTGAACTTAAAGCACCGTTGTTTTAAATTGTCAAAGTTGGTTGTCCTGTAACAAAGTTTGATAAAGTTTGGGAACTTTTGATAGTTACTAGAAAATGTGGGGTATCAGTTATATATATTGGAATTACTCTAAGCATTAAATTTATTCCAAATATTTCCAGTTTCAAAAAAATTTATTCCAGATATTTTTGTAAAAAATGTTTCTTTTATTAAGAATTTTTGTAGAAAGTGTTATCCGAGCACAACTTCATTTCAAATCAAAACTTCGACGAAGTCAACAACATAGCCAGTATATTCCCACAAGTGGGGTCTGGGGAGGGTGGGGTGTACGTTGACCTTACCGTACCTTTGAGGGATAGAGAGACAAAACTTCAATGAAGTCAAGCATAGAGATACTCAAATTTTAGCTAAAAGAGATTATCCCTCATTTCATCTCCTTTGTCTTAAAAAAGCTCCGTAATTCTCTTTCCCTACTGTGGTCTGCTTAATCATATTTTTACTTGTTTCCCCACTTTCATCTTAAGAGTTACCTTTTATTCTGATAAGGTAATGATTTTTGTTGACCAATAGGACACTTCCTAGTATACAATTAGGGGCTGTTTGGGTTGAGAGATAAGGATGTTTAATCCTTGTTTAAATTTGGGTGTATTCCCTATACAACAACTTGTTTTCCGTATAAAACTCAGCATTACTAATATATTCTTGTATAACTAATATAGTGTTTGGTAGAAAACTTCGCACAAGTTATGCCAAATTTAGTTCTGAAAACCAAATGTTGTATTGTCAATGCTTATATAACTTGTATGGGAATACAAGTTACCTTTATAAAAAAAAACTTGTATGGGAAGATGTGTGTTATTTTATGCATGATAGAATGTGGAATAAGAATACATGTATTAGCAATACATGGATAATTAGTGGATAAGAGTACCTAAATAAAAGAATGCCCTTAAAGTAGATAATTTCCCTTTTATCAATCCATGCATTTGTTATTCACCACATAACAACCAATACTTTATTATTCATGTAAACAAACCTTACATTAGTATTAATCGCATAAACAATCCC

>LNC_001356

TATCAATAGGTTCAACTAGATCCAATAAATCCATAAAGAAGGTATGAATCATCAAGTGTTGTTGGATAGGTTTGAAGAAGAAGACAAGAGAAAAATACTTCAAGTTACTTTTGATGGGCAATCAATCTTTTTTAATTCATGCTTTAGTAATAGAAAGTTTGAGGCTGGTAACTCAACTTTCTTCTTGATTGACATATTTTTACATTATTTAAGCATCCTCATTTGTAATATCATCATGGAGTGTATTACAAACTCTGTGATGATCAGAAAATACTTAGTTCTCTCTAGCTCTTATTTTCTTCATGCTTGTTAGGTA

>LNC_001357

CAATTTGAAAGAAATGATCACTTTCCAACTAAGGGAATTAAGTGTGAAGTTTTATTTAATTTAATTACTCTTTTCGGGTAAGAGGTTGTTGATGAAAGATGAGCTTATTTTGACTAACACCTCCAATTCTTGAGTAACGGCCAAATTGAGTGAGAAGCTCTTCCTTCATCCAAGATTTTTGACGTTAATATTGTCAGTGAAGTCCTCTAATGATTGTGCCATTTTAACAGATAATGTTCACCTCACAAAATGGAATGGGGTTGTATGTACTTTCAGTTTTTCCTTTTCTTTTAAAATTGTCGATCAGAGTTGGTCATTCTGGTTGTTCCCC

>LNC_001358

AGTCTCGCTGCCCAGAAGAAGGTAAAATACTTCTAACTTTACTTTGTGTAATTCCTGTTAGTTTTAAGTATACCTTTCTTGATCATTGTATTGGTTATTATGTGTTAAGATTGGGTGATTTAGTAACAAAAATCTGTTTTGTCTTGTGGGAAGCTTAGTTTGTATGAAAGAACTCCTGATTCTTGGTTTAAGTGTTAGTTGATCAGCCTTCCTCTAGCTCGTTTCCAATGTCTATATTCACCTGCTCGCATCTCGTATTCAAGTTATATGACTAATCATCTCATTGAATGTCACTGCCCGACCTTCAGAACGAGCTTATATTTTGAGGTTGGTTGATTAATGTTTGGTATTACCTTGATTAAGCATTCTGTTGGAAAATCGCGTTGATCGAGTACGCTATAGTTCTTATTTGTGCTATTGCCTATTAGAGTTATATAAAACTGATTGTTTGTTGTAAGTTTTGGCAGAGTTGAGTTCACTTTCTTATCGTTTGTCAGATTGAGATTCGCATCCTTATCGTAGCTGCATTTTGATATCATTAAGATCTCGTTAAGTTCGAAAAGGGCACGAGGCTACCTCTTCCTTAAAGTCATATTTGTTAATCAGCACTGTAGGTTGCCATTCTGTTTTCACTATTTTTCGTTCTTAGCATATCAAAGTTCATGGTTATTCGAAATTCTATATTATTCATCTAAGTTGTGGTTTGGTTGCCTGTCGGTTCTATTTCGATTTATCTTAAATGTTGTTTTGTTCTATGTTCAGCATGGCAGATTCTACTTAAAATTTAATTCCAGCTTCTGTATAGCTGATTAGGTTCTTTGTTTAGCCCAAGCGTAAAAATCATAATCTAGTTGTTCCAACTTGTTTGCTACCTATTGTTTAGCTTTAGTCCTTGCTGACCACTGTTTAGTATAAAATGTGATTACTTTGAATAATTTTAGCTAGTACATGCGGAGAATTAGTTGAGTCTTAATTTGAATTAATGCTCCTAATTGTCCAATTTGTCCTCAGCCTTTAGTTGTGATTTAATTTTGAGTTTAAATGGGTGATATAACATTCAATTATCAATGTCTATGATATTCTACAATACATTGCTCCTACTGTGCTATTTCTTCCTTCCTTAGGCAGTAATAAAAATGTCAAATAATTTAGTAGTTGCATAACTTGTTCTTTGCGGTGGTTCAAATTTTAGTGTGTTTATTCATGAAGTTATCTCGTGTATTTTCTTTTCAACAATGGTTGTGAACAATTTCAAGTTAAATAAATTATCATTCTAATTGTTGTCATTTTTTTTATGCATGAACTACCTCGGTTTGAGTCCGTAGGACTCCCCTTTTTCTCTTGCATCTCGAGTTGGTTGAAACCCAACATTCCTCCTTCTTCGAGTCAACTTCACCATTGTTTCCAAGTTTTGGAAGCTGATACAAAATACTAGGTTTCAAAAGGCTGAAACATAGCTTGTATACACTGATATACGGCGATATACGCCAACATATATGGTAAAAAATGCAGCAGCCACAGGTTGAGGTCGGGAATAAAAAATGCAGGTGTCCGGAAGCAAGAAATACATGAAAAGGGGCCAAGAAATGACCGATATACACTGATATACATCGGCTGTATACCAAAAACGGGAATTGTGTTAAACTCCAAAAACAACAACGAAATTGGCCAAAAAGGGGCCCGAATTCAATTTCTCCCTTACTCTTTAATTATTTAATTGTGATTATTTATTGTTTGTTGTTTGACTCTAACTTTATAATTGTTAATTAACTTAGTTGAATTCCCCAAAAGACGAACTATTTCTCCGTGTTGTTGTATTTCTAACAAATCTCATTTTATC

>LNC_001359

GGAAAATTCTTTATCTTATTATGAGTAGAGAATAATTACTTATCTTATGGTAGACTAATGCTTCTATAGTCCCATTTGAGAAGTCACTTTTGTATATTATCAACTCCGGGAGAGTCCTCTCTCTCTTTTTCTTGCTTCATATTGTATTGGAGCCTAGCTTGGTAAGGACTTAAGTAGTTTTCTGCCAGAGTTTTCTCTCTTTTTTTTTCTTGCTTCATATTGTATTCAGCCTAGCTTTGTAGGGACTTGATCAGTTTCTGCCTACCTAGGTGTCTGCTGGCCTAAGTTTTCCTGTATTGTCATTATTTTACTAGTCTTCTTGTCTCTTTATGATGACTGGTCTAGGAGTATGACTCTTTTTTCCAATGACCGCTTTGGCATCAGTCTCCACCATTGCATTCTCTTCTGTTACTCTTGGGGCCCTTTATTTCTGCAGTACATACCACATTCATCTAGGACTGATATATCATTCTATGTCAACTAACCTGTCAAATCATCTCTGCACATCTAAATGTCTAGTTAAGAGTCTTTGTCTATTGACCTATTAAGTCAACCGTCCCTATTTATTCAAGGAGGACCACATTAGAAGTTATACAACTATGCTGTGCCATGTTATGAAACAATAAACACTTTCGGCATATTTGAGTTGACTATTCAGTTTTCACTTACAAACTCAACTTTTATTGTCACCTTGAGTTTGAGGCCCGTCGAAAGTCAACAGAATTAATTAAGTTATATCTTAAAATGTGTGTAGATTTAATGAACTTGATTATAGAATATTATGTAGGATATTTCATAGGTTATACTAGAAAGTTTTGTACTTAAAGGTAACAGAACAATTATTACCTTGTTTTACTTATATTAAGACTCTTATACAAGGAGTCTCTTGTATATTGTCAATAATCAATTTGTGATGACTTTTCTTTCCTCTTCTCTTATCTTGTTTTCAAAATGTAGTTGCGCACCACTCACCACTCACCGCTCTAAG

>LNC_001360

CTTCTTTTTATACTCTTCATTCTTCCCTTTTATTCTCTCTTTGTTTTATTCTTTTTTTATAATGAAATAAATATAACTCCTAAAGAAATTTATTTCACTTTAACCTAATTTTTCAAGTTGGGTTTCTAAGAATTCATTCAATCATCATATATTTTGTAGATGACTTAAGGGATTGAAAGTTCTACTAGTGCACATAAATTATTTTGGAGTTAACATATTGTGAGGTATTGCTAATGGAATTTGATGGACATTTGTATCAGCTTTCGGTTCGTTTGGATACTTGGATTTTCTACATACATATTTGCTGATATCAGGAAATTAGAGCTCTCAATTATCTTGCCTCTTTCAAGAAAATATTTATGATTTTGGTATTCTTATCCCTTCCCCTGATTTCGTCTCTGTTGGAGGTAGATCTGTTGTCAATTTTAATTCCAATTTTTGAGTGATTTTTGATTTTTGGTTAGTTTATCATGAAATTTTCTAATTGAATGTTTCTAATGGAGATTAGTTTAATGTTGAATTTTCTTATCCTAATCAATTTTCTACTTCATGTTCCTAATTGAGGCGAGTGTATTGTTGATTTTTCTACAAACTGTACTGCTGACTTTTTGTCAAAGAGGAGGCACAAATCAGATATAGTACACCATTTCTACACCTACCATCAGTTACCTACCTGTTAAAGAATGGCAAAAGTCCACATCGGTGGTTAATGAGATGGGTGGACTCCTTATAAGGCTTGGACAATCCTCCTCCCTTTGAGCTAGCTTTTG

>LNC_001361

TCTTCTTCTTTTTATACTCTTCATTCTTCCCTTTTATTCTCTCTTTGTTTTATTCTTTTTTTATAATGAAATAAATATAACTCCTAAAGAAATTTATTTCACTTTAACCTAATTTTTCAAGTTGGGTTTCTAAGAATTCATTCAATCATCATATATTTTGTAGATGACTTAAGGGATTGAAAGTTCTACTAGTGCACATAAATTATTTTGGAGTTAACATATTGTGAGGTATTGCTAATGGAATTTGATGGACATTTGTATCAGCTTTCGGCTGATATCAGGAAATTAGAGCTCTCAATTATCTTGCCTCTTTCAAGAAAATATTTATGATTTTGGTATTCTTATCCCTTCCCCTGATTTCGTCTCTGTTGGAGGTAGATCTGTTGTCAATTTTAATTCCAATTTTTGAGTGATTTTTGATTTTTGGTTAGTTTATCATGAAATTTTCTAATTGAATGTTTCTAATGGAGATTAGTTTAATGTTGAATTTTCTTATCCTAATCAATTTTCTACTTCATGTTCCTAATTGAGGCGAGTGTATTGTTGATTTTTCTACAAACTGTACTGCTGACTTTTTGTCAAAGAGGAGGCACAAATCAGATATAGTACACCATTTCTACACCTACCATCAGTTACCTACCTGTTAAAGAATGGCAAAAGTCCACATCGGTGGTTAATGAGATGGGTGGACTCCTTATAAGGCTTGGACAATCCTCCTCCCTTTGAGCTAGCTTTTG

>LNC_001362

CACAATAAAATTGTACAAAATGCATTCCAAATAGTACTATAAAATTAATTGTGGCACATTAACAAATAGACACGTCTATTATACGTGTCCTTTCTATCCATACAACAACCCACGTGTACCTTCCTTTTTCCAATTTATAATTACTTTTCTTCTTTTCTCCCTCAGTTTCTCCCTCTCTGTATCTTGTTATCTTCTTCTTTTTATACTCTTCATTCTTCCCTTTTATTCTCTCTTTGTTTTATTCTTTTTTTATAATGAAATAAATATAACTCCTAAAGAAATTTATTTCACTTTAACCTAATTTTTCAAGTTGGGTTTCTAAGAATTCATTCAATCATCATATATTTTGTAGATGACTTAAGGGATTGAAAGTTCTACTAGTGCACATAAATTATTTTGGAGTTAACATATTGTGAGGTATTGCTAATGGAATTTGATGGACATTTGTATCAGCTTTCGGTTCGTTTGGATACTTGGATTTTCTACATACATATTTGTATGTGTTTGATTTACGGGTTCTTTCCACTTTCAATTTTTTTCGTCGAAGTTTATGCATATGTTTAATTTTATAATCGAATTTCCCCATGTATCTTCAAGCTAAGCAAGTAAAGAAGAAGCCTGGATATATGTGGAACATTTCAAATTTCGAGATTCAAATAAGTTTATCATCAACTTCGTGGTACTTAATTTACGTCTTCGTGGAAGAGAAAACTAAATCCTAATTCAGAACATTATATCTGCCTATTATAAACATGTTACTGTATCACGACACTATTAATTATTAAAATGAGAGCTTGCAAAACTGATTTAGACCAAAGAGAGCTACTTCATTTTTATTTGTTTTAGCGACCTGGTGCAAATTAGAACAATATTGAGCATTATAACATTTTTTATTTTATTTTATATTATACCTTTTTAGAAGTGGGAGTTGTGCTAGGACGAACACAGCAAATTGTACGGTAACCAAAGACATTTCAAATTGTATTGTGAGGCCTCATAATTTTTGGCCCTTTGTGGCCCAATTTGATTGGGTATTAACACTTGGAGGCTCTTTTATTCCACGTGTGAGCAATTGATATTGCTTCAGGCTGATATCAGGAAATTAGAGCTCTCAATTATCTTGCCTCTTTCAAGAAAATATTTATGGTAAAATCTCTGCTTTTCTTTTTATAAGTTTTTAGTTTTCTTAATCTTCTCACTCTTTTGTTTTGATTTTACAGATTTTGGTATTCTTATCCCTTCCCCTGATTTCGTCTCTGTTGGAGGTAGATCTGTTGTCAATTTTAATTCCAATTTTTGAGTGATTTTTGATTTTTGGTTAGTTTATCATGAAATTTTCTAATTGAATGTTTCTAATGGAGATTAGTTTAATGTTGAATTTTCTTATCCTAATCAATTTTCTACTTCATGTTCCTAATTGAGGCGAGTGTATTGTTGATTTTTCTACAAACTGTACTGCTGACTTTTTGTCAAAGAGGAGGCACAAATCAGATATAGTACACCATTTCTACACCTACCATCAGTTACCTACCTGTTAAAGAATGGCAAAAGTCCACATCGGTGGTTAATGAGATGGGTGGACTCCTTATAAGGCTTGGACAATCCTCCTCCCTTTGAGCTAGCTTTTG

>LNC_001363

CAAAATTTTCTGTTTTTAACTAAAGCCCAATAATTCCAAGTTAGAGAAGCCCAATTAACACTTGGAAATTTCCCCTTTGGTGTATAAAAGTAAAAGGGAAGTGGAAACCCTAAAAAACAAAGCAGATCTTCTTCAATCAGCGTTGGCCAATACATTCTGAAAAAATCTCAAATCTTATTTTGAGGTAATTATCATGAAAATATGTATTGGAAATTGCTGTTTGTACTATAGTTTATAAGAAGATCTAATAGAAAAAACCTCAAAAGGGTTAGGCATCTCAGAAAGCCATTGTTTTGTTTTTCAATCAACTTTTGGCTCGCTATGGTGCCTACAAAATATATA

>LNC_001364

GCATTCATTCATGTAATATTTATTTATTTTTGCAATGATGTGCTTCAATATATGCGTTTTGATATCTAATGCCTGCCCCCTCCAACATGTTCTTCTGAGGCATCTTCTGTCATGTATGAATGGTCAGTTATTCTATGCTGTGAATGCGGTGCTTGATTCATGATATAAATAATTGGATAAGTGGTTGGTCTCTCCTCCATTTCTCATCTTTTTGGACATTTCCTGTTTCCTTATGCTGTATGTTTTTGTAATTCTAGCAGAAAGACAAGTCCTTGTAGTGGCTTAGACCATATTTGTTAATTTTAGTACACATCTTCTTTTTTCTTGGCTTTATGTTACTATCAAATGGTGCTGGAGAAGTATTTGGTCTTTCCTCCATTTCTCATTTTTTTGATGTTTTCTGTTTCCTA

>LNC_001365

GAAAATTGTTGTTTCCTCTTTATTTTGGGTTGCCTCTGTTGCATTGCAGCTATTAGTTGGTTTCTTTTTCTTTTTTCTCAGAAGACATTTAATTGTTCATTATGTTGTCAGCTTCTGATAATTGCTTGTTCGTTTAGTCATTGGACTCATCTATTTTTTGTTTTGGTTGGTCATTCTCTTATATTTTCTTTGCTTGCTGTTTTCTCATTAAATTTGAATTGGTCAAAGCAGAAACTTGAGGGTAATTGATTATCCCAACCCCCAAACCCCCCAATATTATGCAAAAATCCATATATTTTTAAATGTAAACGGTGCGTGTGTTTTGTGCATCCTGGTCCTGACCAGCATTATGGTTATGAGTTCTAATACAAGCTGGGGAGGAATCTGAGTAGAATCCAGGATCGTCTCCTTCAACCTATGGATTAATAATTCTGTGTAGGCTAGATTTGGGGTCTAGAATCAACTGGTGAAAATTTATTTTGAC

>LNC_001366

TGGAGCTGAGAATTGGCTTGCACACGATTCCCGTCCCAACCATGAACCCGTCGCTTCTGTAATCAGAGGATTAATTTAAAGGGGGTGGTTTACCCGGTGTTTTTCCCCCAGTTGTGCCAAACAAAAGAAACACCGATGCCTACGCGTGTGTTAGTTCTCAGAGTTGAATTATCTCCCGGCAGGTTAGAAGGTAGGTGGCCATTGACATGAAAATGTTCGATGGTCACTTTGTTCCTCGCTTGCCAAGTACCCCCA

>LNC_001367

ATTGTATTTAACTTTGTTCTGAAGACCCATCTCACATCAATGACTTTTCTATCTTCAGGTTTGTTGACTAACACCCAAGTTTTATTTTTCTCAATCATGGAGATCTCCTCCTCGATTGCTTTCTTTTATTTGGGGTCTTGAAGAGCTTCTACTGGTCTAGTCCCACTGTTTTTTTTTTTGACAAAGGTAACAGTTTTGTGTATTAATATTTAGCACTAAAGGCTGGGATCCCAAATTTACATCATGGAGCTACATACAATTGCGTGTACTTAAAACAAAACTAAACTAGATCCTACTTCATCTTACAAAGGACCTAGGATATCTCTAACCGCTATAGGATCATCTACATACTCCGAACTACACCAATAGCAAAAAGTGATTATGCAGTTCATTTTAATCCTGTGTAAAGGACTACTACTACTCTCAAAGCATCTCATATTTCTCTCCTTCCATATGGTCCACCAAATCACTGCTGGGACAATCCTC

>LNC_001368

TTCAATTTTATGTGTGTCCATCGTGTAGATATTTCATCCTCAATGCTCGTTTCAGTTCTGATTAGTGACTGATAATATTTACTAATATGGATTTACTTATCAAAAAAGTAATAGATTTAATATCATGATTGCAATTTCTTTTTGTTACTCTGCTTCGGTTTTCGCATGCATGTATCTCAAAACTCGAATTGAAGTAGTATCCTTTAGATATGCAAATAATACTAGGAGAATGTCTATTGTGATGTTTGAAAAGTAAACATAAGGAATCAAAGTCCTTCAACCTCTGTGACAATTTGAAACTGATAAATAGTAACCCAACTGCCAT

>LNC_001369

CACAAATTCAAACTAGTCCTAAAATCCCAAAATCTAACTAGTACTAAAATCCTAAATTCAAATTAGCTAGCTATTCCTAATCCCAAATTCAAACTATTAGTCCTAAAATCTCAATAATAATTCAAACTAACTCAAATAATACCCAAGTTCAAACAACTCCTTAAATACCCTAATTCAATCTAATCTTACAATTCCAAAATCAAACTAATTAACTTAAGAAATACCGAATTTCAAACAAATCCTAAGATACCCTAATTCAAACTAACCCTAACATTAAAATTTTCAATTCACAATCAACAAACACAAACTAACAATCAAATAATCCCTAATTCAAACTAACAATCAAATAATACCTAATTCAAACTAACAATCAAAAACCCTAAATCATTCACTTACTAACTAACAATCAATC

>LNC_001370

AAAGAATGTAACATCAGCGGACATAAGGTATCGATGAAGATCATGTGAATAACAACGATACCCTTTTTGGACTCGAGAGTAACCAAGAAAGACACATTTGAGGGCACGAGGAGCTAATTTATCTTTTCCTGGGGCTAAGTTATGAACAAAGCATGTGCTCCCAAAGACACGAGGAGGGATAGGATAGAGATGTGACTGAGGAAATAGTATGGAATGTGGAACCTGATTCTGAATAGAAGAAGAGGGCATTCTATTTATCAAATAACAAGACGTAAGAACTGCATCTCCCCAAAAACGCAGCGGAACATTGGATTCAAGGAGAAGAGTACGAGCGGTCTCAATAAGATGCCGATTTTTTCTTTCAGCTACACCGTTTTGTTGAGGGGTGTATGGACAAGTAGTCTGGTG

>LNC_001371

TCCATGGAAAGCCCTTTGAGTTAGCTACCTAACGCACTTCGTTTCACCTCATTCGGAGTTCGGACGAAGAAGTTATGCCCATTTTCGTAAAGTCCGTCCGGCAGAAAACGCAAATTCCAGTAGCTATTTTTACTGTTCATTACTGTTCACCCGCCCCATTATTTTTTCTAAGTGTTGGACTATTCTTCCAAATGACTTATATTATTTCCTAGGTACCATTAGTTCAATTCACTTCTCTAAAACATTTCCCTCACTTCTCAAATATTTC

>LNC_001372

AGCAAGAGTATTGTTGCTCCCACCAACAGGCTTCAACGGAAACGAAATCTCTATCCAATGACCATGGTGAACCACCATGGTAATAGCCAAATTGAATAAAAAAAATTGAGTTCGGAAGTTCGATTTTGATTTAACAGACCTTTTCCCGTGTAAGGAATATGCTACAATAACTAGACATCATAGACAAAAAGAGGCAACAAAAAGCAATGTAGGCAAATTATTATAAGAGTGTGGCTGAAGCGGCAACACATCCATGGCAATTTGTACATTTTCCAAAGTACAATTGTTGCAATAGAAGTCAATGCAACATGATTTTCACCTTCCACAACAACATTCAGTTCTGTTAAATTCAATTGCTAAAATGAAGCAAATGTTCATTAGTTTGTGAAAACGAGGGTTGGTAAATTTGTATTGTACACTAGTTTGTGTAATTAACGAAGCAGTTTTGATTTTATTGCTTTAGTTTTATTAATGTTTAGCATGAGTATGTAATCTTTG

>LNC_001373

TAATGTAAATATCGAACATCAGGTGGAAAATAATAATAAAAAAACAACCCAACACCTGCTCTCCTTTCCCATTCCAGTACAGCGAACCCAAACCGAAACTCTCCGTACGCAAATCGTACAAGACCGTTTCAGAACATTCTCGAAGGCTCCAGGTGTTGTCATCTGCCGTCCTTCCATCTCCAAATCAACGTTCTCCTTTCAAATCAGTTGGAGCTTTATCCAAAAGGAAGTTTGAATTTCAAATTCCATTCAATTCTCACCCGTTTCCCCCCATTTCGACCCTAGTCTCGACTTCACTGTTAAGGGGAGGATTTTTGACCATTGAAAAGATACAGAAAAAGTGATTTTCTCTTGAGAAATCAAAAGCTCTCCGAATCTCTGAATTTCAAATTTAAATCATTGTTCGTATTGTTTTCGTTTCCGGCGAAGTTAAGCTCGGAATCGTGGTGGAAGTATTGCTCTTCTCGTCCGTCCCAGATTTGCTGCGCTACAAGAGTTCCTTATCTTTGTCTGATTATCAATTTCTATT

>LNC_001374

TTTTCATTCTCCACTCTCCTTGAGAACAAACACCGCCCCTCTTTCCTTTTTCCAGTCGCCAGCACCACCCACACCCTCACCGGCGTCCCTGTCTCTTCGTCGGCGAAGTGTCGCCGGTGGACCACTTCGCTGCTCTACCCTCTCCTGCTCTTCGTTCCCTCTCAACCAGACAGCCACCAAATCGCCTCTCCCCTCCCTTCTTCTCCTTCCAGTAACTTCTCCACCAAAAGACTGGCGAGTATCGCCAGCTTCAGATGTCCCTTGTCCTCTCATCTCCGCCTGACACCAGCGAAGCGAGCATAACCGCATCACAACCTACTACAGAGAAAGTTCTGTACCTAAGCACCAGAGGCTCCTTATCGAAGGATCATTTTATGACTGTTTGGTGAGTGTTTGATACAACTTTTAGAAAGAATTGCAACACAGATCAAGTGATTACATCTCTCTACGTCAACATTTTATACAGATCTCCACAAGCCTGCTCTCGTAATTGAGTGATGCTTTGTATTAGTGGTATTAATCCTCTCTTCTCTTAATTTTTTTCAGCATCTCAGATACTATACTAATCCA

>LNC_001375

CAAAGATTTAGACATCATGATCATCGATGAACTTCCGAGCTATTTGCAAGTCTTTGAAGAAAGGCTAAAGAATCTGAAGGAAGAATTGTCCGAACAAGATTTGCAGACAACTTTTATTGGAAGGAGAAAGATATTGAAAACTGTGATTTGCATCATAGACGGGGAAGAGGTAGATGAAGATCCAAGAAAGGAGTTGAAGTCACAAGACCGAGTTGTGGATATCTTCATCAAGTCAAGAAAAAATTAGAATGCTTGAGAGTGATGACCTAAGTATAAAGGAGGGTGTTGAAAATAAACTTGATTAATCTCATGTACATGAACTCTATCATGTATTAGGAATTAATGTACTCATGTATGAATAGGTTTTGTTCATGAACTAGGAGAAGATGAAAATTGTTCATGAACTAGAGATTATTAGATTATGTACATACTAGAGAAAACCTATGCATACA

>LNC_001376

CCTTATCTAAGAAAGTTAAATGAGCTATCCTTATGAACCTTGATTGTCTATGACTAAGAAATGGGGCCTTTCTTGGGAAGAGGTACTATGAGTTGGTTGAACTTGAAAAGGAACCCTCTCTAAGTACATATGTGATTGAATACTCTATGGGAAATAAGGCTAGCACCGAGTGGATGTGGTTAGGAAGGTGGCCTAGTTGAGTATGAGATTAGGCACAATGAACTTCCTTGGACATCCCCTAAACCATGTGCCTACATGGGTTGCTTACTAGTTCTACCCTTGGCAAGTAGAACAACCTCCACG

>LNC_001377

TGCAACTCCAGCAAACAAGAACCAGATGAACTCAGCCCTAAATCCTCCGGATCGCCTCAAATTTCCCTTTTTCTCCTTCGATTCCGTACATTCAAGCGTGCTTAGCACGAGCACAACGACATTATCCTCTCAATTTCCCTTTTTCTTTTGCCAACTTTTGTTCAAAAATGGTACGAATTCGTACACTGCCTAGTTGGCAATCCTTATCCGCTCAAGGATTTTTGAAATTTCTCAAGTCCCACATCGAATTCCATCTTCAAATTCAACTCGTTATTTGTCCTATATAAACCCATTTCCTCTTACTGTTCAAGGGGAGTTTTTTTTTTTAGGAGGCGAAGAAAAGAGCACCCCGCTAAGTTTTTGGGAGTTCTTATTTTCGTTTCTGAAATTCTATTGATTTGCGGCTATTTTGAGTCAGAGTGGTGGTGGAGTTCGTCACGTTCACTCGAGTCCCAGTCGCTGCCCAGCACCAGGTAATTTTCTAGCTCTTTTCTTAGTCTTTTCTTTCTGTTGCATTGTAGAGTTGTTATTTGTTTAGTATTGTTATTTATCTCTTTTAGATTCAATGTGATTAGTGGTGTGAATGATAGGAAAAAGTCTTCATTATCCTGTTCTTTTAAAGTTAAGTCAATTTTGAATGAGTTTAAATATTTTTCTCTATTGATGGTTGTTTTCTTATGATTCAGAGAGGTGAATGTGATTCTCTCACTTAATCGAGTTCATAAATCTATATATATGCTTCTAGTTACTGTTATCTTTATTCGATTTCGAAATTCTTGTTATATAGTTGTTGTTCTCTGCCTTAAGTTGTCGTCTTCTGTATTAAGACTATTACCTCGATTTTTGTCGAAGCCTAATTTCTCCGACCTGCTTTATAAATGTAAAACTGCTGATTAGTTGGTTTTTAAGGTGAAATCTTGTTGGTGGTATGAGTTTAAGATTATTATTTTTTTTGTCTAGATGATGGGTGTTACATAAGCTTAAGTTTGTTTATGTGTTAGGATTAGCTGCTGATTCTCTCTTGTATTGATCATGGTTTAGTATATTTCTTATCTTTAAATTAGTTTAATTATTTTGCTCAGGGTATACACAAGGTTAATTCGTTTTTCCCCTTTAAGCAAGGATCAATGTCTCTTTAGTAGACTCCTGTAAGCATAAATGTGTTAGTGTGATTTGCCAATCAACAGATCCTTTGGCTCTCAATGCAAATCGAGAGACTGTTTGAGTGAGAAATAGCTTACATTTGGGGGCTGCTCTTTAGTACTCTCAAAAGAGTTAGATTGGGATGGATGAAAGCCCATATCATAACTTTTATACATCCTAAAATTTATTAGATTACTCTACTTGAATCCTTGTTTAAATTCGCCAAGTTATTTTGGCGTAAGGTTATTTTATATCTTCCTACTCTCTGCTTTCTTGTTTGTCCTTCGTGTACGCTAAATGAGATGGCTTAAAACTTTGATCACATTGAGTCTAAACTTGCTTATCTTTAAATTATGTATGAATTCGTTTCGCACTCTTTTTTTTTGGTTTAGTCCAAGGTTGTGTTCAAGTAGCAGTATACTTAATAGCAAATGGGTTTAGTTGTTGTTATGTAAATTTTTGGCTGCCTATCGTTGTTAGTTCCTCCATCTAGGACTGTTATTGGTTTATACTAGTAGTGAATTTCTTATTGTTTGTTGTAGATCATAAGTGGATCTATTCATTACCTTATCTTTTATTTAATTAGTTTTGGCATTTTACTTGTTTCTCATTTTATGTTAGTACTAGTATGGTCTCTTTTATTGAATGCATTGAAATAATGACGTGCTAATTGGCATTTGACTTTAGATTCCGTAAGGATAGGTGTTTGTCCTGATTTGTGTCTAATATCTTTGTGATCACTGTTAGTAGTACTTTCATTTCGTAACTTCAAGTGTTATATTTTGTATCTTGTTTAAGTTGGTTGTTTAGCAATTTAATGAATCCAGTTAATTGATTTATTTCATGAGACATCTAATCCTTATTTCTCTCATTTTCTTTTCATGAACTCTCTCCGAATGAGTCCAATGGACTCCCCTCTCTTGCATTTTTCTCTTCTTGAGTCAACCTCATCTAAATTGTGGAAGGGAAGCTAATGTTAATGGGTTTAAACCCAAATCAGTGGCATCAGCTAAGTAGCGGAGTAATAGCAATAAAAAATGGCCGGAAATTGAATTTTTGGATTTAAGAATCCAATTTGATTATTGCAGCAGTCCCAAATGGACTAAAATTGAGGATTTTTGGACTTAAAAAGGTCCAATTTTACACTATGGCAGCAGCAGCAGTAGCCTAAATGGCTGAAACGAAAATTGAAAATTGGACTTAAAAGAAAGTCCAACTTTAATTCAGCAACAGCAGCGCCTACCTTCGTTTGGACCAAAAGTCCAAATTCTTTTAAATTTTGTCATATTTTACCATGTCGGATGTGTTATTATTTATTATTTTATTTGTTGACTACTAACTTAGATGAATCCTTGGGAAGATATTTTGTTCAAG

>LNC_001378

TCATTACACGTGGGTCATAGGTGTCCTCCATGCCCACTTCCGTCCCTTTTATCAAAATCCATTCAATCTACCAAAGCTCAAAGCTTTCTTCATCTTCAACTTTCTCACACTCACACGTTGCTGCAATTCTAAGGGATCGAAGTGGAAATAAGAGTTCGAGCATTTATCTTTGCTCTCTTCTATCATTTGGTGGAAAGAGGATGTAAGAGACTATTAGTCACTTGCCCATTGGTTTTATGTGTTTCACCGCAACAAAGCATCTGGAAATTTCTCTATCATCATCTTTATTTCTGTTTGCCCTGCAATTCTAGTCAGTTTCTTCATTTTTTTCTCTGTCGAATGTACAATTTTCTTTATTCTTTGCTCATCACTGTACAAGACTGTTGGTAATGAGTATTTGTTAGTATTTCCACCCCAATTTATTCATGCATACTTAGTATTTTTCTGATTACTCATTTAGTCTGATTTTTACTTGTTGGAGTTCTAAACTCCATTCTTCTCTCTTGAGCCAATTATACTTTCTCTTATCACATTCAATCATTTAAGACACTCTAAATTCTGATATACTGCTTTCTTGAGTGTTTTATGTTGCCACTTTTTGCAAGCTTATTGCGTCAAATTTGTTTCATTTTTATGAGATAAGCTGTTATTAGTTTGTAGATATGGAGCCTCCTAAAATGATCTACTAAATTCATCGAGTTGTCCCAAGGGATCAAAACAGACAATTATTAATGGAATTCTTCTCCGCTATTAGGTTTCATTACTTTAGTTTGCTTTGCAACTCTTTTTCTATTATGATTACTCAAAGAAAATTAAGTATCAATATGTTACCCTTTCCTGTTGCTTCTATTGTTCGGAATCCTCAAGTTTGTTGTATTATTTTTTTGCGGATTGCCATATCCTAGACTACAATGTGTGTTCTTGACTATTAGTAGTTGATTTAAAAAAAATTATAATTACCTCAAGGTCTTTGTTGATTTTTTGTACAGATGGAGTTTCCCAAAGGAAAACCCTTTTGGATAGCCTCAAAGCTCTTGTGTAAATCCCTCAAATGACTCCATTATTGATATTGGTATTGATATTTATTAATTCTGAGTCAATCATACATGGGTCGTTCATGTGTGCAGTTTTTCTTTCATCCCCTCCTTAACTCTTAATTTCAAAATTTTCATCCATTTACTAGAACATGGTTCCGGACCTTTCTGCTTGGCCCTCTTTCAATTTGGATCATTCAAATTTGTGATTTATGTTAATGGCGGTCTTCATGTATTATGTTCTGTTTGGATAGAGTCAGGGCCTCTATCTAATATATTAGTGTGTATTATGTTTATAAATGTGTACTTTCTTTCCTCATATAATTTATTCCTCTCATTTGTTTGTTTATAGATGTGTACTTTCTTTTCTCCATCATTTATTCATCTAATATGTTTTACCAGAAATTTTAGCTATTACCTTGGGTCCTTTGCACCAGTTTTTTTAAAAAGTTGGGTTGAAGAATGATGTCCAACCCTTTAAAAAGGAAATCTCAACATCTATTTTGTCAATCATGCATCACAAAGTTGCAACGATAATTAAGAAAATCAAGATGACTACCACCAAAGTAGTAATGGGATGCTTTACTATTTCACCTTTAGGGATATCAACTTCTATAAATATGTAAATATAGACTCTGCATTTGCTATTTCACATTTTGCAATATCACCTTTTGTAAATATGTAAATACATATATAAACTGTGCAATCATAAGCGCAATGCATATAGAATGTGTGTAGTCGTTTACCATGAAATGTTCTTCAACAACTACTTTGTTCTGATTTTTTTAGCTGTTTCCTTACAAGTATTTTCGAACTATTGTTGGCGTCATATATATTGGGC

>LNC_001379

TTTTATCAAAATCCATTCAATCTACCAAAGCTCAAAGCTTTCTTCATCTTCAACTTTCTCACACTCACACGTTGCTGCAATTCTAAGGGATCGAAGTGGAAATAAGAGTTCGAGCATTTATCTTTGCTCTCTTCTATCATTTGGTGGAAAGAGGATGTAAGAGACTATTAGTCACTTGCCCATTGGTTTTATGTGTTTCACCGCAACAAAGCATCTGGAAATTTCTCTATCATCATCTTTATTTCTGTTTGCCCTGCAATTCTAATGGAGTTTCCCAAAGGAAAACCCTTTTGGATAGCCTCAAAGCTCTTGTGTAAATCCCTCAAATGACTCCATTATTGATATTGGTATTGATATTTATTAATTCTGAGTCAATCATACATGGGTCGTTCATGTGTGCAGTTTTTCTTTCATCCCCTCCTTAACTCTTAATTTCAAAATTTTCATCCATTTACTAGAACATGGTTCCGGACCTTTCTGCTTGGCCCTCTTTCAATTTGGATCATTCAAATTTGTGATTTATGTTAATGGCGGTCTTCATGTATTATGTTCTGTTTGGATAGAGTCAGGGCCTCTATCTAATATATTAGTGTGTATTATGTTTATAAATGTGTACTTTCTTTCCTCATATAATTTATTCCTCTCATTTGTTTGTTTATAGATGTGTACTTTCTTTTCTCCATCATTTATTCATCTAATATGTTTTACCAGAAATTTTAGCTATTACCTTGGGTCCTTTGCACCAGTTTTTTTAAAAAGTTGGGTTGAAGAATGATGTCCAACCCTTTAAAAAGGAAATCTCAACATCTATTTTGTCAATCATGCATCACAAAGTTGCAACGATAATTAAGAAAATCAAGATGACTACCACCAAAGTAGTAATGGGATGCTTTACTATTTCACCTTTAGGGATATCAACTTCTATAAATATGTAAATATAGACTCTGCATTTGCTATTTCACATTTTGCAATATCACCTTTTGTAAATATGTAAATACATATATAAACTGTGCAATCATAAGCGCAATGCATATAGAATGTGTGTAGTCGTTTACCATGAAATGTTCTTCAACAACTACTTTGTTCTGATTTTTTTAGCTGTTTCCTTACAAGTATTTTCGAACTATTGTTGGCGTCATATATATTGGGC

>LNC_001380

TGTAACTAGATCGATCGGAAGATTCACGAATCCATTTTGTTTAAGACAGATCATTTTTAGGAATGTCCTAGATTTCTCTCATTATGTCTATGATTGACTATAGTCTTTTATATATAAGCATGGCCTAATGCTCTTCTTTGGAGACTTTTTTACACTCTAGACATGAGAACACATTTTTACTTTCTCATTTGAGCATACTCTCAAAAGCACTAAAGATTCTGGCGTATTACTGAAAGAAGTTTCTTCATTTTGGCTACTACACGAGGTTTGGCTTCGAGCTTTCATATCAAGTTTTGCGTTGCTTAGTAACTTATCTACGTATCCGGTTAGTAATTCTTACTAGAATAAGTTTAACACTTGCATATAGTATAGTATTACTTTAAGGTTTTTTGCACAAATTCATATTAACTTTAAAGGAAGCGTCCAAGTGAGTTTTCTTTTACTTTTGCCTAGTATTAGATATGTTTCACCTTTTCATATCTTTTGATTGATATGTGAAGTCTTTGTTTTGAAAATTTAGATTATTAATTGTTATTTTATTTGACTACATGAATATAACTGTCTGCAATATTATTATAGTTTAATATGAAGTTTAAAGACATATTTAAGAGAACCCGTGGATGGGGCTTACCAGATGGAGTCAACTTTGCTTAAATTTTTATTCGTTTGTATTACTTGTTACTTTTGACTTGTAAAAATAATGTAACGAAACTCTACTCTCTGTATATATTAAATATTTGGGGACTATCTAAGGGGGAGGGGAACTAACGTGCTACTTGTGTTTATCTTCTGTCGTAATTTATGTGTGGCTTTAGTACATAAATATCCCTACGTGTGTTAACTATTTAAACATATAAAATCATGTGCCACTAACTCTCTGCTAGGACCCAATTTTAAATGTTAATTTAAAGGCAATTTCAGGGTAATTTCACAAACTCTGTTTAATTAAGAGTATTCTGCCTACTGTTATTATCATGAATATTACCTAATATTGTTTACTTTCACATATAGTATTTAAAGAAAAATGGTCTACTATCAGCATATTAATTGCTTAATCTTAGCATGGATTAATGGTCCACTACCAGTGTGTAATCAAGTCCTTCTTACATATACTTTTATAATTATGAAAATGTGCCACTATTGCTTCTTAGTAAAAACCTTTAATAAATCTATTACATGCTCCCCTCATTCCAATTGCTAATCAACTTGTTGTTGTGTTGTTGAAATAGTCTAAAAGCCTAACTAGTTATAACGTGCCCTTTGTTAGGTATCATAATTTAGGATTATCGTTTTGATTTTTATCACAACATATTTAAAATGGCCTTTGTCAAGTTTTGAGAATTACTCTGTATAAAACCATGTCTATATGAATTATGTAATATATTTATCATGTTAATTAATTAGATAT

>LNC_001381

ATATCATTACCCACTGTCCATTTGCCATGCTGAATTTTAGTCTTATCAAAATCAGAATTCACTCAAATTCCTCACTATATTAATTAGAATATTGGAATGTAATTACAACTTTAGAATCATAATTGCAATAATAAAGCAATTGAATTACAATGGAAATATTTAAATAATAAGTAACTAAGATAGAGATAAGAATTTGGATGAGAAGCTTTTAGACTCTAGAAGAAGAGGAGAGGAGCAAACATTTTTCTTCAATGTGCATAACTCTTTCGTGTTGACTCCAAGACCTATTTAACCAATTTATAATTGGGTGTGGATCCCAAATCAAACTCCCCAACATTTTAATCTTCCCAATAATCTTAAATGGATCAGTCTCATTTACCCCTTCTAGCCATAGGCCGTCATGTAGTCAGTTTGG

>LNC_001382

ACATATTCGAGTGTTCTGGTTGTAGTTTGGAAAATTTCTTCTCACATAATCAATTGTATATTAGAGTATTACAGTGCCTTTTTAAGAGCAAAACATGTTTATGATCTATCTCTCTTTAACCATTCAATATATTACACCTCTAAATAGGACCCTAATACAAACATGCAACAAGATTTGTAGTCTATAACTGAATTCAATGGTGGATTACTGCT

>LNC_001383

AATAAGTCCAAATCAGCGACTAGAGGAAACAATCAGAAAATGGTAAGTTAAACATCTGTTATCAATTTACTAGAGCAAGTATTAAGGTGCCAATCACAAAGGCCAATCAATAAGATAAAGAGTATGCGATTAAACATTATCATAATCAAATTCCAAAATTAATTCGATAATATTCTACACTATAATTAAGCAAGACAAACAATTTCAACATGGTGTAAAGTCAATTAAGGAATGAAAATATGCAAAGGTTAGTGGACAATAAAATTCAGATTAAAACCAATTTTTGAAAAGAATCAGTGATTAGCAAGTTAATACCAGCATAGGTTCAATAAGCTGTCAACCCAAAAGTTTATGCATACAACGGAACGAATTCCTATCATTCAAGCATCAAGCACATAAAAGCAATCATATGAAAAATATAATTTAAAAAGTAGGAACAACATAGGGTTAAATAGATGAAGTAATAATTGATCGAAAGAAAATACCGAGACGGATCTGTTGAGATCCGTCTTCCACCGATCCTAATTTCGGATTTCGCCGGAGCTCGCTTGGTCGGACTGTTGTTGCTGTTTCGCCGGCAGCTGCCTTGGAGTTGTTGTCGTCTTC

>LNC_001384

AGAGCGTAGCATGAGTACCAAAACAACAGGTACCCAGTAGGCATCATAGGCCGACTGAACTTGAAAAGTAAAGGCAATATGAAAAGAAGGAATAACACAAACCGAGGGTCAAGAGCGGAAATCTAAGTAACAATGGAATGCACACGAGTGTAGAAAGAACTAACTAAAGTAATCTATAAGCTAACTACACCTAACTGGACCCCCATAAGCCCAGAAGGTACACTCGTGCGCAAGTTAAATAAAAACCCGAACGGACCCCCATAAGCCCGACGAGTACACAGA

>LNC_001385

TTTCCTTCTTGTGTAGCTCCCTTAATCATTATCAACATCCAGCTGCCTCTGGATGTGATTTCTCATTGTTGACTCTATCCTCTATGTCTTCCAACTGATGTGAGACAATGTTATGCAACACCTGAACTGGAGAGACTGTATTGAAGATTGCTAGAGAAGTCTCATCTTTGTATGGAGTCTGACCACCTTGTATCTGAGAAACTACTGGATATATTCCTTCTTTCTTCTCTATGACTTGATTGGGATCATCAATTTGAAGAGGTGTCTTCTGGATACTGGAGTGTGGTTCCTCTTGACTTATCTTATCAAAATCGACTATAGTAGTTATAGAAGTGTTGTCATCTTTGTTCAATACATCATTCATAGCTGGACAATTGTTTGTAACACTATTATCTAAGGATTTGGCAAGTGCATTTAATTCCTCATTAATGTGGCTCTCATCCCTTTGCCCTGCATCCTGTACCTGTGAAATGTTTTTTGTGGAAGCGTAATATTTTGAAGTTTGCTTCCTATCACTTCCTCTCCCATGCTCAGCC

>LNC_001386

AGATTAGGTTAGCCTTAACCTTTTGACTAGTTGATTGCAGAAGGGTAGATGAGACTTGAGAGCAGTATATATAGATAGTTAATCTCCACAAATTGTTGACTCAATTAGCAAAGTCTGTATTAACATATAGGTTTGATTGTTAGTACTAGTAAAAAGGCAAGTGATCATGGGGCCAAGAGGACCTAGACCAGGAGGGCCACCTGGGTGGGGCCCTCCTCTAGGTCCGGGAGGACCACCGATGCCTGGACCAGGAGGACCACCTAGATTGGGTCCTCCTCAAAGTCCGGGAGGACCACCGATGGCAGGACCATGTGGCTTCTTTAGTAGTTTTTGCAACCCTATAGGCTCCTGCCTAAGCTTTTTTTTGCTGCTGCTGGTTGTTACAAGACTGTTTTGGTCACCCAATGGGTTCACCTGGC

>LNC_001387

AAACTTTCTTGTGAGGGTTAAAAAATAAATAAAAAATCAGAGAGATTTGAGTATATACCAAAATGATCATATTTGACCACTGTCAACTCTAGCACCGTTAACCAAAAAAAAAAAAACCGGAAGAAAACCCCAATCTGACGAGCTGTTTTCAGAACCCTAACCCTAACATTCAATTCTCGTTTGGAATTGGTTTGTGATTAGAGAATGGGAAAGAAGAAGAAGAGAGCGTCAGATAAGGTTTGGTGCTATTACTGTGATAGGGAATTTGATGATGAGAAGATATTGGTTCAGCACCAGAAAGCCAAACACTTCAAGTGCAATTTTTGCCATAGGAAGCTCTCCACTGCTAGCGGCATGGCCATTCACGTCCTTCAGGTTCACAAAGAGACTGTCAACAAGGTACCAAATGCCAAAGCAGGCAGAGAATCAACAGATATTGAAATATATGGAATGGAAGGAATCCCACCGGATGTGTTAGCTGCTCACTATGGAGAGGAAGAGGATGAGGCCCTGGCAAAGACTGCCAAATTAGACATCCTATCATCCCAGTATGTTGGTGGTGTGCTTCCAGGATTATTAGCTGCCGGTTATCCTCCCCAGGCAACTTTCGGCACAGTACCACCACTCTACAATCCTGCTGTTCCAATGCGTCCTGCCGGTTGGCCAGTTCCTCCTCGCCTCCATCCTTGGTATCCACAGTATCCTGCTGTCATGTTTCCACCTCCTGCACCTATGGGTTTGCTGCAACAACCACTGTTTCCTGTGCAGAATGTGAGGCCTCCAGTGCAAGCAACTGCACCGCCTATACTTCAACCATCACTACCTGTTGCTCCTCCAGGACTGCCTGTATCTACCCCACCTGTTCCTGTGTCTCAACCATTGTTTCCTGTGGTTCCTAACAACAATTTTGTTCAAAGTTTGCCAATGCTGACAGCAAGTGTGCCATTAAGCTCACCTGCTGAAGTGAACAACTCAATTGCCCCTAAAATGGGAAACTGTTCTCCTTCAACCATTGGCTATCAGGTTCCAGCCCCAACATTAGTAAATTTACATTCTTATGCCTCTGGCCCTAATACTGGTGGACCCTCAATTGGACCACCTCCTGTAATTACAAACAAAGCTCCAGCGTCAAATGAGGTCTACTTGGTTTGGGATGATGACGCTATGTCCATGGAGGAAAGACGAATGTCCTTGCCAAAGTACCAGGTGCATGATGAGACTAGCCAGATGACCTCAATAGATGCAGCAATAGACAGAAGAATCTCAGAAAGCAGGCTTGCTGGGAGTATGGCTTTCTAGACATTAGCAACATCTAGTGCTATAGTTTTCCTTTGATGCAGGGTGCTCGCAACTCCAGGAATACTACTCTTTGCGTATCAGTTTTGGATGTAAAATCCTTTTAAGAGCCGTTTATTTTTACCCTGTATTGGAGAAAGGAAGATAGAAGGGGAACATATATTTTTCTTTAGGGTGTGTTGGGTACAGATCGATCCTCTTCTCGTGTTCGGTTTGTCAAAACTTATGAACAATATTTTCTTTAGGAAGACACTCTTTAAAAAAAAGTAAAATGACTTTTCTAAATGAAAGTAGGGTATACAAG

>LNC_001388

AAACTTTCTTGTGAGGGTTAAAAAATAAATAAAAAATCAGAGAGATTTGAGTATATACCAAAATGATCATATTTGACCACTGTCAACTCTAGCACCGTTAACCAAAAAAAAAAAAACCGGAAGAAAACCCCAATCTGACGAGCTGTTTTCAGAACCCTAACCCTAACATTCAATTCTCGTTTGGAATTGGTTTGTGATTAGAGAATGGGAAAGAAGAAGAAGAGAGCGTCAGATAAGGTTTGGTGCTATTACTGTGATAGGGAATTTGATGATGAGAAGATATTGGTTCAGCACCAGAAAGCCAAACACTTCAAGTGCAATTTTTGCCATAGGAAGCTCTCCACTGCTAGCGGCATGGCCATTCACGTCCTTCAGGTTCACAAAGAGACTGTCAACAAGGTACCAAATGCCAAAGCAGGCAGAGAATCAACAGATATTGAAATATATGGAATGGAAGGAATCCCACCGGATGTGTTAGCTGCTCACTATGGAGAGGAAGAGGATGAGGCCCTGGCAAAGACTGCCAAATTAGACATCCTATCATCCCAGTATGTTGGTGGTGTGCTTCCAGGATTATTAGCTGCCGGTTATCCTCCCCAGGCAACTTTCGGCACAGTACCACCACTCTACAATCCTGCTGTTCCAATGCGTCCTGCCGGTTGGCCAGTTCCTCCTCGCCTCCATCCTTGGTATCCACAGTATCCTGCTGTCATGTTTCCACCTCCTGCACCTATGGGTTTGCTGCAACAACCACTGTTTCCTGTGCAGAATGTGAGGCCTCCAGTGCAAGCAACTGCACCGCCTATACTTCAACCATCACTACCTGTTGCTCCTCCAGGACTGCCTGTATCTACCCCACCTGTTCCTGTGTCTCAACCATTGTTTCCTGTGGTTCCTAACAACAATTTTGTTCAAAGTTTGCCAATGCTGACAGCAAGTGTGCCATTAAGCTCACCTGCTGAAGTGAACAACTCAATTGCCCCTAAAATGGGAAACTGTTCTCCTTCAACCATTGGCTATCAGGTTCCAGCCCCAACATTAGTAAATTTACATTCTTATGCCTCTGGCCCTAATACTGGTGGACCCTCAATTGGACCACCTCCTGTAATTACAAACAAAGCTCCAGCGTCAAATGAGGTCTACTTGGTTTGGGATGATGACGCTATGTCCATGGAGGAAAGACGAATGTCCTTGCCAAAGTACCAGGTGCATGATGAGACTAGCCAGGTAAGTTATCATTACCTAGAACTGGTATTCTTTCTTCTGGATATGGACTTGAGTTGTGCGCCTGTTGAGAGGTGTTGCATTCCATTTCTCAGATTCAGTTGTTTTACTATTCTTTCCTTGTCGTTAAGCAAGTTGTCCCCATTCTCCTTCCTCTTGCTGAAACTATCTAATAAACTCTTTGAAGAATACCTGATAATCCAATCAATCATACTTTATGTTTCGACCCCATTAGTTCATGGACTGTTTTATCTTTGGCCACTCTCCGGTTGGCAAGGAATCCCAATAAGTGTGGTCATCGAAAGATTCAAGTATGTATTTTCATTATCTTTTACTCGTAATTAAGTAATGATATTTCTGTCAAGGAGGTGGGCATCTACTCTGAGGAACAGGATTATGAATCGAGTGTATGTATTTGTTCTTGGAAATTTCATTTTGTGTTTATATCTTCGCCTGTGCTTGGCCTTGAAAAGATTTGGGTTTGGGTGAGCAAGCGTACACCTACCAGATGCCTTGTAAATTATATTGATTATACTATTGACATATGGTCAAACAGAGGTTCAATGTTGGCTCAGGTCAGATTCTTTTGCCGGGTTAGGAATCTCATTGTATCAGAAGCATCCTATGATTTTCCACTTGTTCGTAATTCTGAGTACTATCGGTATTTCGGTCTTAAGGACTGTTTGTGGTTTCTTCTTTCAGATGACCTCAATAGATGCAGCAATAGACAGAAGAATCTCAGAAAGCAGGCTTGCTGGGAGTATGGCTTTCTAGACATTAGCAACATCTAGTGCTATAGTTTTCCTTTGATGCAGGGTGCTCGCAACTCCAGGAATACTACTCTTTGCGTATCAGTTTTGGATGTAAAATCCTTTTAAGAGCCGTTTATTTTTACCCTGTATTGGAGAAAGGAAGATAGAAGGGGAACATATATTTTTCTTTAGGGTGTGTTGGGTACAGATCGATCCTCTTCTCGTGTTCGGTTTGTCAAAACTTATGAACAATATTTTCTTTAGGAAGACACTCTTTAAAAAAAAGTAAAATGACTTTTCTAAATGAAAGTAGGGTATACAAG

>LNC_001389

GTGGGGAATTACAGTTGGAGTTCTCAATTCGTTTCTTCTGCACGTGCTGACAACTAGAAAAAAAACAAATTACCAACAGGGCTTTCATTTGTGGTTGTGTTATCGTGTCCTTGCCCTGCTTATTGGCCCAGGCCACTCTTTCGTCACAGATCTTGCTCAGTATCTTTCTTAGTCTGCTCGTGACCCTTACTATCAACTTTTTGCATAACCAAGTGACTTTTCTTTATCACCCTCATTTCCTTCATTTTATGTACTTTTAGTAGTAACTATCATCCAATCCATCTTCAACTTCTGCATGAACATGCCCAGGCCACTCTTTCATCACAGATCTTGCTCAGCATCTTTCTTAGTCTGCTCGTGACCCTTACTAT

>LNC_001390

TACTCATTTTCTTTCTCTGGTCATTAATGTGCTCCATCCGTGGTTGTGGAAGTGTTGTATTAGCTAACAGAGATGTGAAATCCGGTGGAGGCGTTTGTAGTTCAAGATGAACTTTTTGTTGGGATTTAACCATGACACTCCTCCTGAAGGCTTTGAGGTATATCGACCTGCTGGATCGAGTCTGCACTTCCAGTGATTTATCGGTTTGAGATCCCCCACTTTTTGCTTGTCCTTTTTTGTGAGTTGAGGTAGCTCTAGGCTAATAAGCTTCAATACAACTAGATCGTCTCTTAGAAGACAAAATTCTTATCTGTACATATTATCGTTGTGTATTTCTGATTTGTAACTGCACTTAGCTTAGCTTATTGGGTTAATCCTCCTTGTTTTAATTCTCAATGACGGAATTTGTTGTTTGTCTATGTTAATGTTCCGTCTTCCTTTGCTTGGTAGATTGTAGAAAC

>LNC_001391

GTATCCTAATTTGAATTGTTAGTGTTGTTTGGCAGCTTTGATGTTACAAAAGTTCAAAAGATTATTAAGATATGAGCAAAGATGACATGTAGAAGTGGAGATTAAAAATGGCTACTTCAAATTGTTTAGCAAGAATTGAGCATGTTGTCGTCCCTGTTTCAAGTATAATTTATTCATAACATGGGTTTTCACATTACTACTACAACAATGATACTAAACCTATTTGTTCATGAAAAAATTATCTTTTTTTATTCTTCTGTAATGTACAGACTATATATATATATATATATATTGGTTTTTCAC

>LNC_001392

GGTGGCAAAAGAATACTACAGTTCTAAATCTACTTTATCTAGAAAGAATATTACAGTGAAAAATCTAAGTTTATGTTCATTTCTACTTCACAACAACATACCAGTGTTCATTTCTGCTTAAGATTTAATTATTTAATTATTCATAAGATGTGCAAGAGTAGGGCGACTAGACCAGATGAAAACCCAGTGGAATTTGAAAGAACATAGGCAGGGTGGTTTTGGAGAGGTTGATTGAGTTGCTTAATGTCATTTTTAAGGCAACAAAAATGCTTGAAGAGTGGAGGTGGAGTATAATGATTCCATAGTATAAGATCAAGGGTGATGTTAAAAATTGCAACAATTATAAGGGGATCAAGTTGTTGAGCCACCCTATGGAACTGTGGGAGAGAGTGGTGGAGTTGAAATGAGGAAGGGTGTGACTATTTTCGAGAACCAATTTGGATTCATGTCGGGTGTTCTATTCTAGAAGCCATTCATCTTGTGAGGAGACTGGTGGAGTAATATTGGGAGAGGAAGAAGGAGGGATTTACACATTGTGCTCATTGACCTAGAAAAGACATACAACAAAGTGCCTGGGGAGGTCCTAGGG

>LNC_001393

TACAATAGTTCTCAAATGACATACTAAAGGAAACTACCAAGTAAAGATACTCGTCCTCGAATCTTGCGGACTCACCAAATAGCAAGGAGAATGACCTATTCCAAGCTTGAACCAATAGAAGACCACCTTCAAGAACCAAACCCTACACTTATAGAAAATGTAGAAATATGGGTTAGTACAAGAATGCACCAAGTATGGTAGCCATGCATAAAAATCATTGAAACATGCAAAAAGGGACATTTTAGTTGAAACCATGCTATATGCCAATTTGGAGATCTTCTTTGAAAGAGTTGGAGAACATCACTCATAAGGCAACTCATATATAGTTCATCATAGCACAAAGGATAACAACATAATCATAAGCATAGTCTCCAATCATAGACATAGTGCAAATATCAATCATCATAAGACATAATGCACATACATAGTCAAATACCAAGATCATCTCATCATAGTAGAATCATCACATAAGTAACCCTAAGTTATACTTGTGCCATGCATAGATAAGATCCCATAACCCTATCTACACTAAGTACCACTTTAGGTCACCATGCTCATACTTGTCATACATTAATCTCATCCATAGTTAATACTCATAGACAAGGAAACATTCACATTCATTAGCTCATGTCAAGGAAAGTTAATCATAACAACAATCCAAATGGAGCATGCATTAATTCATAAATCATCATAATGTAA

>LNC_001394

ATTAGTATTACCTCTCTGTTCTCTCTTTCTCTCTACTGTTATCAGGTCGACCTTGGTCGGGTGGGGTAAGCTGAAGTTGAGCTAAACTGTGATTCTATCAACTTATTGAAGAAAAGGGGTGAAGAGTAGCTGAAAGTTTTTGATAGGCTTTTTGGTTAATGCATGTGGAGATTGTTGGGGAAAGTGTGGGATCATAATGGGGAAAAGGTGGGGTATCTTGAGAATAAGGGTGATGAATTGGTGGGAGTGTTTAGCGTTTCAGTTGGTTTTGAGAGTTGGAATTGAGGGTTTTGTGTAATTGAGTGGATAATTACAGGAAAGGATGTTGCTGTAGTACTACTACAATTTTCTTAGATTTTTTAAGGGAAGTGGCTTTATAAAATA

>LNC_001395

TCTCAACCATAATAAATAAAAAGATTGGAATTCTAACGGTTACATTCCCATAATTTGAAAGTCATCATACTAGGGCTATACCTATCAAACGTCTAAGACTAGCAAAACAAATAAAATAGTCCAATGTCTGGAAAGAAATGACATTTATTATCAAAAGATCTGTGGTAGCCTGATATGGAAGTAGCTCACCCTCAGATCTGAAATGTAACTGCGCTAGATGTGGTGCTGCGCAGAAGACATAGTAAATCATGAGATCTTCAAACATAGTGCACATAAAATACTATTTTTTTATTTGAAGTTGAAGATGACAAACCTTTTGAAGGATGAACGAGATTATAGACTTTCTCGAGAATAAAGAGG

>LNC_001396

TGGACATACTTTCTTTTTTCTTCTACTTTGGGGGAAAAGTTTTTTCATTCCTCTGTAAAATGAAAACCGAGCAACCTTCAATTTTATCCACTTATGCAAAGGGGGCCATAAAAATCTGATGTTAAAAATACCTATGGTCCCAATAGGTATCCTAGAGAGGTTTGGATATATCTTGAAGTGGTTGGTGTAGCATGGTGGATCAAGGTATTCAATGTCATATTAAAGAGTGGAAAGATGGCAAGTGGATTGTGAGAGAGTATAGGTTTCGATAGGTAAGAACGATGGATGATTAAAAGTTGTGAAAACTATTCTTATCAAAAGATATTGGAAATTGTGCAAACTATCATGGAATTAAAGTTTATGACTTATATGATGATCATTTGAGAAAGAATTATAGAGTTCAAACTTGAAATTTGACTGTCATGTGGATGTGTAAATTGTAATCATACAAGATTGGACAAGAATATGATCACATTCAGCAAAAGTTGCAAGTAGCACGTGTAGAGGTAATCCGAGA

>LNC_001397

CTTTTCCATCCCATTTATTAATAAAAAAGTACTTTGTACTGACTGACCTAATTGTGCATCTTCATCTTCACAGAAGGCTTTGGATGTCCGGTGAAACTTTAGTCATTCTTCCACCTTCTTCTTTCAATCGTTGATGTTTAGGGATGAAGCGTAGGAACTTCATATTCAAAATATCTTCCCAATTTCTCCATTTCTCATAGGGAATGTCGACAAAAAAAATTGAGAGCGTTGCATGCCTTGAAGGAAGGTTGCAGGTTCTATCCTTTTTGCTAAAAAGTTTGGTTCCCACTGTTTGGGTAAAAAATTGAAATTGAAGAGTTGCTTAAAAAAGGAAAGAGACATTCTTTTTGGGACTAAAAGGAAAGTAAGACAAATAAATTCTACCGGATGGAGCAATTCTTCTTTCTTTCTTTGGCAATGCCAACATAAATCGCCTTGGATTCCTTCGATTATTTGATGTATGTTTCTCCCTATCTCTTCTCAAATAGGATACCTTTTATCACATGTTTCTAACAATATATTAGCTACAAGTTACATGTCTTTTAAATAGACAAATATTTTTATCTAGCCTAACCGATGTTTTAACAACATGATGAAATTTTTATAAAGTCATATTCAATCTTCATTTGAGAGTATTTTTTTCTAGCAATATCTCAAAATCTTTTTAGCTTCTCAACAATACAGTACATTATAACTGGTGATGTCACCTAAGGCACATGAAAAGAGAGCAGTTTGATTGCTACACTCTTTACTGGTAAGTTTGATACTTGAGTTCTGTAGGAGTGATGAAACAACTTACTTATGTTATATTTCGGCTCCTCCATTTCATGTGCTAATTTACATGCAACCTATACTATAGTTAACCTAATTCAACTCTTTTTAAGCTACAATAAGAAGTTCTGCCATGAGTATACTTTACGATTTTCATTTTGTCTGGTGCTTATTTTGTGCTCTTTGTATAGAACATAGCTATTTGTGTAGTTTATATGTTATAGGACCAAAGAGTCAAAGTTACACTTCTGAAGTTTATGCGTTATAGGTTGGTGATAGGGTAAGAAATAATAGTACATGAATGATTATGAGATGATGGAAGCAAAAATTAGTAGTATGATACATGATATGTGTAATGTTTATGATTTGTAACTTATATTGATTGTTTCTCTTAGCTTTGATTTTACAGGAATTCCTCTGTTGTGTTATTATCAAGTCACGATATATGATTCATTTATCACATTATGACAAATTGAATTTATTAACTAGGACAGAGAATGCAACCGACTCTTCTCAGCATCTGTCACTTCTCTAGATATTAGACATCATATGAGAAATTCAAGAACAATGGACATCTCTATGTTTCCTTTTGAGAGTGATTGCGTCACCTC

>LNC_001398

CTTTTCCATCCCATTTATTAATAAAAAAGTACTTTGTACTGACTGACCTAATTGTGCATCTTCATCTTCACAGAAGGCTTTGGATGTCCGGTGAAACTTTAGTCATTCTTCCACCTTCTTCTTTCAATCGTTGATGTTTAGGGATGAAGCGTAGGAACTTCATATTCAAAATATCTTCCCAATTTCTCCATTTCTCATAGGGAATGTCGACAAAAAAAATTGAGAGCGTTGCATGCCTTGAAGGAAGGTTGCAGGTTCTATCCTTTTTGCTAAAAAGTTTGGTTCCCACTGTTTGGGTAAAAAATTGAAATTGAAGAGTTGCTTAAAAAAGGAAAGAGACATTCTTTTTGGGACTAAAAGGAAAGTAAGACAAATAAATTCTACCGGATGGAGCAATTCTTCTTTAGAATGCAACCGACTCTTCTCAGCATCTGTCACTTCTCTAGATATTAGACATCATATGAGAAATTCAAGAACAATGGACATCTCTATGTTTCCTTTTGAGAGTGATTGCGTCACCTC

>LNC_001399

TTCACAGAAGGCTTTGGATGTCCGGTGAAACTTTAGTCATTCTTCCACCTTCTTCTTTCAATCGTTGATGTTTAGGTTTTTTCCCACTATCCTCATAAGTTTTGTCTCGAAAATTTAAGTGGGATGAAGCGTAGGAACTTCATATTCAAAATATCTTCCCAATTTCTCCATTTCTCATAGGGAATGTCGACAAAAAAAATTGAGAGCGTTGCATGCCTTGAAGGAAGGTTGCAGGTTCTATCCTTTTTGCTAAAAAGTTTGGTTCCCACTGTTTGGGTTAGTTTCTGAACTATCTTCATGACTTCTCCCTGTTTTTCTCACTTTTTCAAAGTGTCAAATATCCTGTTGATGGATATCTGATGGTAGTTTATGGCTTGAGAGCCCTAAGACTTTTAATTGGTCTATCTAATTTCACTAATGTAATCGCTAGATAGTAATTTATAACAATGAATATACACTTCAATCATCTTAAAAGTCTTAAACAATTACCTGAACAAGTTGGTAATTTGTTTTAAACACAAAAACTATAATTAATGTTGATAATAGGGATGATCGAAACAGTATATTGGATACCATATATTCTAACTTAAAATGTCACAGTTGATTCGCTATTACAAGGTATTCAATAGTGTTTGTCTTAGCTGAAAATACATCTATTTACCTGTAACAAAAGCTCACGTTTTCTGTCTCGCTGTTTATATAATACTCTCCCTGT

>LNC_001400

GAGTCGATCAATAATGAAACCACCTTTGGCACAAATTTTGATTACTTTCAAGATGAAAAGGAGATAAGAGTGTTGCAAGTTAATTTGCCCATCATATGACATGGACTTGAGACTAAAATTGTTTCCTCTATCTGGTGTAACAATAATTTCATGGAGAAGATCATTTTTGTTCTCTTTAATGTGTTATTAGTTGCTAATTAAACCTTTGCCTTGGATATCTACTTAATTGAGGAAATAAGATGTCATAGTTTATTTCATAACAATATTTCTTAAATACATGGTACTTCCAGGAATACATGTTTGGTGCGCAAGACACTTCGATCTTGGAGTTGAGTATGAAAGTGAAAGTTAACAATATTTGGACTGTCGAGAGAACTACAAGGGAGTGAAAGTTGCAAGTGCTAATGATAGTGCTAAAATCATTGAGAAGATTCACTGTAGCAAAGGCAGAACAGAAATTGTGACTCTATGTTTCACCATGATGAGGAAATAGAAACCACGAGTATCATTGATGTTAGATTGTTTCTTCTATGTTTCACGATTGGTGAAGAAATAGAAACCACGAGTATTGTTGATGTTAGATTGTTCTTTTATTAATCTAAATCTAAAGACTTCAATTTTCTTCTCCTTTTTTATTTATTTATGGAGAACTTATATTTCATATTTGGATGAGACTTTAGTACTTTTTTATTTGGTGACTTGTTCCTGAATATTTTGGCAGCAGCCGTTTTTCTTTCTAAGCATATTCAGAAAATTTACACTTTTAAGTTTGATCATTTACTGCCATAGTTCATCTACTTGAGTTGGAAAATGACTATTCTCTTAGCTTCTCATATCATAGTATACTTGATCAAAGATAA

>LNC_001401

GCTCTTGTGGGCACATTTATCTTGTTATTTTCTTTCTATTTGTTTCAAACATAGCACCACCATTGACTCTTTTCTTATATTTATTATTATACCTTTTCAAAAAGAGGCATTTGTTGCCCTTGCAAAACTTAATAAGCATCTTTATGCTTGGTTGTGATGTATTGGTTTCTTTTCTTTATAATGAGAAATACTTGTGTGCATTGGGGATATGTTATTGATGTATCTGTTATTTACCTGATAGTAGTAGGTATACAAGTTAAAACATGTAAAAAGGGTTGGTTGGTTAATGAATATCTTTGTGTGATCAGCGAAGGGGAAATGGGAAATGGTACTGAGATCGAATTTCATCGCCACCCAATGGTTTTTT

>LNC_001402

GCTTAATATGCTGGAAGCGAAATGCTTTTAAAGTAAGACAAGGAAAAACAAGCTAAAATCAAAGGGACTTACCTTGGTTTCTTGTTGATTAGAACCATCTGGAGGAGAATCATTAGTTCGATTAGCCAAATTATTTTGCAAATCAATGGTACAGTCAATGTCTTCGACCACCAAGATGGACTTGTTAGCCGTGGCCACAAGCAGCCGTCTTAAATCAGAGTTTCTCACCACCTCAGTCAACTCCAAATCATATATATCAAAGTTCAAATAATTAGCCATGGCGGCGACCAAACTGGATTTTCCTGTCCCTGGAGGGCCATACATCAACTCTGAGTCCATTGCCAGAGTCTCAAAGGTTGCCGGGTGATCAAGATTAATCGCCTCCCATATATCATGTAAATTATAAATGCTTTGGTAATCCACAGTGTGAATCTTGATTGCCCTGTTCTCAAGT

>LNC_001403

TGCCACTTCCTGTACAGATGCAACAGAAAAAATAGGGAAAATAATTTGTGCAAGGCACAAAAGGTTGCATCGTACAGATGCTATATGTAAAAGCAAATGCTACCTTACCACTGTAAAAACTGATTAATTCTTCTAGAGATCAGCAGAAATTATATGATTAGCTGGAATTGCTGTTGTGCCACGTTACAGCAACAAATATGCCTCAATATCAAGTTAGTGGGAATGGGGTATATGAATCTTCTATATTCATTCCGCTCTTGTTGGGTCTGTTCATTGTTATACCCAACAATTTGTCTGTTTGGACAAATTTTAAGGGTTCTCTAAAACTAGAGATAGTGTCACATTCTATAGTTTTAATCCTGTAGGACTCATCC

>LNC_001404

TGTATGTTCCCTTATCAAGGACAATAATAATTATATTTGTGAAGTTGTGATGCTAGTATAAGGTTGATTCCGCCTGTGTTTGTTTTTGTTGGTTAATTGTGTTTACTGGTTTTAGTTGATTCCCTTTATCCCAATTTAGACGAAATTGTTTGACCTTCTTGAGCATCATAAAAAGAATGTTTTGATTGCATTGTTGTATTGGAGTTGGGAGATATGGATTTTGAAAAGTAATAATTTAATATTACCATATAATGTGCTGTTTAGAATAGCCCAGAAAACCC

>LNC_001405

AACTCTGTATTTTTCTGCACAAAAGGCCATCTCTCGAATACAGAAGCTTTCTTGATCAATTTATCCAGTATGTGATGCTATATCATTCCTGTGTTAGATTCTTTTATGATCTAGTGTTATTTTTTGTGGAGCGACACATCTATTTTCCCCGACATTGGAACCTGTTGCTGCTTCTTCATTGCCTTGCAGCTATCCATTTGGTGTTAAGATTTTGATGTAAATGAGGTCTGATAGCTGTTTTGAC

>LNC_001406

CTTGTCATTTCGATTGTTATTGTTGTATTAGTTTAGGACAGGCGGAAGAGTTGGGACTAAGGCCCAAAAGATAGAAAATACTTCTATTATGCTAGAATAGGACAGGTACAAATGACTTAAATGGGCCGAAGGCCCAAAACGAAAATGGGCCCAAATACTGGTCCAGGCGGACAGAAACCAGATTTGGGTCCAACCCTCTTTCCCTCTTTACTTTACTGTATTTGTTTTTGCTTGTTAGTATTTGTCGTTAATCTTAGGGTTGAAAAGTTCAAATCCCCGAAAGACGCTCATTTCGTTTTTAACAACTTAACTAAACCGATCATCTTTCAAAAGACTTAAAAGA

>LNC_001407

TGTTGTTGTTATTATTTCAGTTTTAGAGAAAGAAGAGTACGTATGCTTCTGGCATGCACTTTCTTTTTCTCATAGTCCCTTAAGCTACTGCTCTTCCACATGCCTTTTAATTAATGTAAACCAGGTTTAACACTGGGAAATATGAGCAACAATATAATAATCAGTTTGGTAAGTTCAAAGACTTGGATGGAAAAGAAAGGAGGATTTGCTTTCAGACTATATAAAATCATATTGTTATCAAATGTTTTTCCTTATACAAATTGCAGAACCTAATCTGGTTGCTGGGACACATGCCAGCCTT

>LNC_001408

ATAATTTTGAGTAATCTTCGATTTATGAGTTAATTTTTTTTTATTTAGAGTAATATTTAATGACAATTTGTTTAACACTTGCAATCAACACGAGAACTTACTAATTTTATTTAGTTTTTAAAAGAGGAGATAGACAGTATTCTTTTAGAGTAGGAAAGAGAAGTACTCAAAAGAGACCAAGGACAAAATTAGAGAAGGATAAATGAAGGAAAAGTAACTAAATACTCGCTCCGATCTAATTTTACATTTTATGGATTTTGAGATTCAAATAAAAAAAGATCTGAATATAAATCATATGATAATTTATATATGTAAATTTTATTTCAAA

>LNC_001409

ATTTAAATGTTTTTTCTATTTTTTTCTTCCTCTTTTTTCTTTCTTTAAGAACAACAACTTCCAATCCAAAACTAGCAGAATTTTTATAAACCCAATGTGTTTGTAAAAGCTTATTATTTAAAAATCAGACATACGGATTAAACAAGATGAAAGCAAGTAGTTAAAATATTTTCATATAATGCTTTATAAACCGATATTTATGAAGTTTGAACTAATTCTAAATTTAGAAAAATTTCTAGCTTTCATCCTTTTCAAAAGTGATTCAGTCAGAGATAATGAGCAAACAAAAGTTTAAAACTCCATGTGAGAGAAACAAGCAAATAACAGGAGGTATCAAATTAAAGATACGAAAATTGGGTTAGATTTATCAAAAATGGTTTTGAGCGTAAGGTTTATTTGTTTGATAAAAAAAAAGTTAGATATATTTTTAGGAATTTTTTCTACTTGTTTACTGAATATAATATAAATAAAAAATTTATTTTAAAAAAAGTCTTGCAGCGGCAAGAAAAAAATATTTTTGATATGTATATATCTAACACAAAACAAGAAATTTTTTTTGAAAGCGGACGTTAGGTGAAGTGGGT

>LNC_001410

GACCACGATAAGCTGATGTATCATACTTGTTCCTCTGGCACACAGCACACTTGTGTACAAAATTAGCTATATCTGATCGAATACCCTTCCAATAGAAATAGGCCATGACCTTCCTAGTAGTGGCATCAATGCCTGAATGCCCACCAGCAGGAGTAGAGTGCCATAGTGATAAGATTTTGGTTCTAAGGGAAGCATCATCACCAACAACTAATTTCCCTTTTCGTCTCAGCTGTCCATTAAGCCAAGTGAAATGCTTACGAGGTTGCACCAGCAAGGATTGAATAAGGACTTGTAATGCAAGATCCGTAGTCCAACTAACTTTGATCTCATCCCATAAATCAGCTTGGACGGAAGAGATCATCATGGACATAAGTTCTGCACCTTGGATCCTAGATAAAACATCTGCAACCAGG

>LNC_001411

TAAGTGAAGTTAAAAAGAAGCTGAACCAAGACTCAATGGTTCGCCTATCGGCTAAGAAATTGGAAATGACGAAGGATTACCGCTGGATGGGACTCAACACTCCCCTTCTATACTATCTCATATCTCACCTCAGCGCTAATAGAAGCTTCAAGCCCGAGTTATAGGAGCAACCATCGGAAGAAGGAAAAGTCAAGCCAAACAGGAAGCGAACTCAAAACGGGTCCGTCTGTCATGACTTCCAAAAAGAATAAAGGCTAGCTTCAAGAGAACAGCAACCATCCATTGCGAGAGAGATCCCAATTCCGGATATGCTGTTTTATGTCACTCGATGACTTGATTGGGACATTCTTGTTTTAATACGCGAAGATGGTGATGAATCGTTCTCATATATTGCCAGAATCTTTTCGATTGTTCTCTGGTTACGCTAGATTTTGATAAAAGAAAGGGGGCCCAGCGATACAAGAGCATTTTCCAAGCATAAAATCACGAAGCCGGCCCAAAAAGTGGGTGAGACTCGTCATTCTCCGCTTGCAAGTCAAGCTTTTCAAGAGAATCTTTCAGGATGTTCTGATGATCCTAATCAGAGCCAACAAGCAGGTCGAGCTGACATAGGCGGGAATTTGAACCCTTTTGGACTGGAGGGCCGGGTCAGAAGGACCGAGATTGATCTCAGATTGGGGTCAGTAGAGTTTCATTTATCGAATTTGCGGTCCAACCTTGCTAAATAATGAAGATGAATGGTGCTATATCCGCCTGGAGTCTACCATAAGATTTATACCGGGAAAGTGTTAGATAGAAATGTCTGCCTATTAAATCAATACCGGGATCTAACGAGGAAACTATAGCCCTCTCGAAGGAATCTTTCAGGGTGAAAAGCAAGATGTTTGACCTGCCGGGCGTAGCCTTGGTCACTTCTTTTTAGAAAAGCGGAATCAGAAGCTGACCACAGGAGTCGATATTCTGAAGCTGGCAAGCAGGATCCAACCTCAGAAATGGGGTTTCGCTTGACGGGGATCACTCTAAAGAAAGGAAGTCGTTTTGGCTGTTGTTATTCCAAATCACTTTCTTTATAAATGTCATAAAAGACAGGGGCTTTCTTGCTTCAGCATGTGAGGGAAATGCCCCGGGTACCGAAGTTAGCGGCTTTAGAGATAGGGGCGAGGGCGGCGGGACA

>LNC_001412

AAAAAATTGGGAATAAAGAAGGGAATTTGAAAAGGAGAGAGATTATGTGCAAAATAAAAAATACTAAAAAAGATGAAGATAGAACAGGCAAAATGGTAGAATTAAATGAAATAGAAATTCTCTCTCCTTATGGGTAAAAGAAAAGAAAAGAAAAGAAAAGGAGTGCACGAAAAACATTGAGAAGGTGGAGAGTATTATCTCTCTCTCTCTGACCCCCTCTTATATTCCTTTCTCCCTAACTTTCAACTCCAAAAACCAAAAGCAATCATCATGTTTTCTGAATCATCATCACCACCAAAACCTTACTACTTGTTTCACCGCCGTCATCATTGCTCCCACCACCAAAAATATTTCAAAATACCATCATTATTATCATCACCACCTTCAAATATTACTCGTCAGAAAAAACGTGGTCAACATGATGATGGTGATGTTTATCTTAATGATGATGATGAAGACGAAGATGAAGATATTAAAATGGAGAGTATATTAATTAGTTACTCTAATTGATTTGGATTATCTCTCCTGATCAATCTCGCAAGAGTTCTAGTGAAACTACTCCTTCTCTTTAAAGTCACTTTTTTCCTTTGTTGGGGAATCAAGCTTCCTGGTCCGATCCCTTTGCTATTTCAAGCAAAGTTATCACTTTTTACTACTAGATCTCGCTCCTTCCATACTCAAAACTTGAAGAAACGTTTTGCTTATTGTTATCTGCAGGTGGAGAGTTTGGCTTTACGTGTGGTTGGTTGGATTTTTGCCATTATGTTCATCATTACCCTCATAAGATACCAGCAAACTGAGTGTGTGGATCTATATATGTTGCTGGGATTGCTGTGAGGATCATAATGTAAAATACGTATATGAATATGGGATGCTTGAGTTAATATAAATTTGTAGATGGAGGATTAGGGATATAAGCTAGTACGATAAATACCTGCCCATACTCGTTGATCGTTTAAATTTGTTGAATGTGTGTGATTATCTCAATTAAAAGTTTAAGATGTTATAGAAAAATCCACTTTTTAAATTACTTATTACGTATGTTAATTTAGATATTCTTTCACATACTGATTTGATTCTTTTCATGAGTTAAGTATGTAGAAATTCTTTTGATAATAGATGATGTGAGACTCACACATGAACAATATAGCTATTTGATTACCTCGACTTAAAAAGCTTAAGTTATTAAAGAAATTGTAGTTTTAAGTTATTTAATGAGAAAAGTATCCAGTCTGGCAGTTCAAATGGAGGAAGTTTCGCAAATAGCCCCTAAAAATAACTCAATTAGGCTCCATAGTTATAATTTGCTAATTACGCTTAGTAGCTACCTGTTAAAGGGAGGATAGAGACAAGCAAGATTAGGAGAGGAAGGAGAGAGGTGAGCGAGATTGGGAGAGGAAGGAGAGGTGAGCAAGAGAGGGCGGAGAGTGGAGGGAGGTGAATTGTATTTGTATATCGGTCTAATTGTATATGTATATCTATTTGATATTTGTATATATGTAACTGGTATACATATGTGTATAGCTAAATGATAGCTATGTAGAGAGGAGAGAGGCGTACACAATTATATCTAAAAAGAAGCTAGCTAAAAAGAGGCTGTAAATAATAATCAATTCAAATTATAATTATTTTAGCTAATTAACTAGTATATATTTATTTATTCACGTAATTTTTACTCAAATGTTATAGCTTATAGCCCAACTCATAAAATGTTACAACGATTTTAGCCCTAAATCTTTTTTCTATTACAAAGTGGCTAAAGCTTCTTTCTCTTCTTCTTTTTTTTTTAAATAAAAAAAATAAAAATAAAAATTAGAAATCAAGAAAGAAAAATGGGGATGAGATTACAAGGCGAAGAATCAAATTCTCACCGTTCATCAATATAATATAGTCTAAATAATCATCAATATATATATTATAATAAGAAAAAAATCCTTTGGCTAGAAAATTTGGCCAGAATGGTAAAATAACATATTTCACACTATGCTATTTTACCTTTTTAAACATTTTTACCCTTTTAACCTTAATACATTTTAATTAAAAAATAGAAAAATAAATTAATTTATTTTAAAATAAAAAATATATTATAGACTTTTTAAATTTCTGGCCAAATTTTCTAGTCATTTAGCATTACCCTTATAATAATACTCATGCATAAATTATAATATTAGTGTTTCTTGTTCTCTAGTGTCTATTTAGTAGTGTGTCTTTTTTCAATAAAGTTGATGCCATTAAATTCAAAAAGTTTTGCTTTTAATGGTACAATGGAGAGAACCATATTGTTTTCCGAATGCTAAATGACCAAAAAAAATTTAGTCAAAATTTGATTAGAAATTTAAAATTATAATATCTTTTTTATTTTAAAATAAAATGATTTTTTTTCATTTTTTAATTAAAAGATATTAAAATTAAAAATATAAAAATATTTTAAAAAATAAAATAACATAGCTAAAATATCATTCTGCCCAAAAGAATTTTTCCTTGTTTTCCATATATATACCGTCATATCTTGGTAATTCCTTTTTCTGCTTCTTTCCCTTTTACGAGATGTGAGAGTACTGTTTGTTACGAGAACTTTTCAAATTCTTTTTGGATTTTTTTTTCATTTTGTTTGTAAATTAACTTTAAATTGTATTTGATTTGAAACGTTCAAAATTTTGTTATCACTTTTACAAAAAATAAATCCAAAATTATCCTGATTTTTTTATTCTCTAAAAAATTGTTGTTTCTATACCAAACACATTCTAAGGGATCATTTGGTTGGGAATAAGTTATTCCAGGATTAATTAGTTCAGGATAAATTATTTCATCATGTATATGAATTATCTTATTCCATCACTAAGATATAAATGGTGGATAAATAATTCAAGGATTACATAATATCTCCAACCAAACACAGGGTAAAATAATCCTGCATTTTATCTCGAAATTATTATATCTTATACCTCAAACTAATCGAC

>LNC_001413

AAATAACTTGGGGATCGCCTAAAGGGAGGGGAATTACGTGCTACTTGTTTATTTTATTTATTGACCATGCTTACAAGTTTGTGATTTGTTTGCTAGTCCACTTTTAACAAATACGTTTCAGATCCATTAAAATAAAGCAATAGGCCTTTGTCATTAGTCATCTATTTAATCCTTTAATCATTTTAAAAGTAGATTGTGTTTTAATTGCTTATTATAGTTTGTTATTAGATTAGCATTAATCACTTTCATAATTATTAAATAAGTAATATATGTTTGCTCCTTGTTTTCACGTAATTCAACTAAAACTCCCTTTTATAATAAATGAATAAGTTTCGAATATTGTCATAATATGTTTCATATTATTTTATCATCTTAGATACCATGCCTATAGGATCTGAGTAATTTTAGAAGTGTAGTGTGTATGCATTTAGGTGTGAACTTTATTACATATTAGATAAATGTCATGCCTATAGGGTTTAATAATTTGCTTAAATGAAATAGATGCTATGCCTATAGTTTTAATAATTTTCAGCATGCTTACACTTAGAGATCATGTCTAAGGCTCATTATATCTTATGCCTTCTGGGGATTAGAAATCATGCCTATTGGTGCAATGCAACTTTCTGATCACATAGAAATCATGGTCATAGGCATCGCTAATTTCCAGCATGTTTAAACAGTTTTTTTTAAAAATAATGTCCTACTTTAATTATCAATTAAATCAGTCACTGTTAACGAATGTCGCGACAATTATTGTCTATCATTTAAAATAAATCTAGCTAATTGCTATCAGCATATTATAACCAAATAAAATAAATTATGCATAATCTGATCCGCTCCAGTATGCCACTGCTTTAAATAATCTGCCTGACTGAAGCTGTTTAAAATATTATCTTTCTTGTATCGTGAGTGTTGTCCATCTATCTTTAAAATTAAATCAATAATTATTCTGCTTTATAACAATATTTATGCCTTTATTTAAAATCAATTGAAATCTAAAATCACTTCATGTAATAATTTGCCGCCACCGTTAAAATCAGTTAATTAATTATCTAGATACTTTAAGAACTGTCTCGCCTGTTTAACTCATTGTCGATGTCCTAATGCTCGTGTTCTGTATACACCACACACTAAGCATGAAACTTTTCATTTTTATATGTGTTTGTAGCATGTCTAAATTAAAATCTAGAGGCCAATATGAGACCCTACGTGCTAAATGCTCGTTCAAATTGTTTGATTTGATATCTAGGTGGGCCTATTATATTTTATTTTGTCTAAATTCACCCTAATAGTAGTCCAATGCCTCTTGGACCTTAAGCAGGGACGCTAGACACCTCTTAGAGACGAAAAGCATGCGGTGGCGGGGTAGGGGTAGTTTAGGCCCTATGGAGATGATGACATCGACTCGGTCTAAATGACAAACCTCGGTTTTGCCTGTCCCTACAAGAAATCGACCGAAATAAAGAAAGTCGAGTGCTGATCCTAAAAGTCCTTCAGAGTCTCCCTTTCCCTTTGTCCCTTTGTCCCTTTATCTGTTTATAATTATATTTATTTGGGGTAGTTTAATTAAATATAAAATGATATTTACTTATATATTTGTCTACTTTCTATCTTCTCGCTATCTCTTTAAATTTTATTTTGTTAGTTATTTAGTATTATGTTATCATCTACTTTTTGCATGCT

>LNC_001414

CAACAACAAATACCAGAAACGACCCCACCCCGTATGTTCTTCTTCTTTCTCTTTCACGCAGAAACGCGACCCAACTCGCGAAACCCAGCCAGAACGTGACCCGAATCCAGCGAGCCCAAGCAGCGCGACGTCAAAATACACGACGTCTTCACAGCCAGCAGACGGATTCCTCCAACGTTCACAAGCAGACGGAAAGCGATTCACGGCGTCTATGTACACGTAACAGCTTTATTCTCGTCGTCTTCTTCACCCAGTACGATGGTACTGTTCCAGTCGCGATTCCAGCGAAAAGCACCTGCAACAGTCGCATTTTCGTCCTTTTCCAGCTTAAGATAGAGACACGTCGACGCTCAAGGACATTGGAATCATCCTAGACGTCCACCTCGCCCTTCCTCTTTTTGAAATCTTTTGGATTTGAGCGAAAATAACTCTCCCCTGTTTGAGGATAAGAGGAGAATTTTGAATTTTTGAATTCGTTCCTTGGTTCTTACCCGTTTTACCCTCATCGTTTCCCCTCTATTACGAACCCTAATTTCCCCTATATAAACCTCCTCCTTATTCATTATTAGGGGAGATTTTTTGAGCAAAAAATATTTTGGAACAACTTAAGCAAAAACAATAAAAATACGTACTAAGCACATTTGAAACAGAGGAGAGATCAATGCAATTATAGTGTGGTTTCCTTGCTCATCCATAGGAGTCTTTATAGTCTCGTTTTGGTGGTAGAGGTTGTCTTTGTCAAACTCGTCCATCCAGTTCGCTACCTAGAACCAATTCGAGATGTGAATTTGGTTCTTTTACTTAGTCGAGTTCATAAATCTTTATAGGCGTTTCTAGTTACTATTATTTTCATTCGACTTCGAAATCCATGTTATGTAGTTGTTGATTTCTCTTCATTGCTTTCTTTAGATACATATGTTTAGAGTTCTAAGTTGTGCACTTCATTGCTTCAAGTTTGTTTGGACCGAGTGTTATGTTGAGCTTTGTTGTTCCCAAATTATTAGTTTTTTAGCTAATCTCTAGTATAGTTGAATCAATCGAGTTATTGTTGCTCCATCTAGCATTTCTTCATTATCATATGCTGTTTTGTTAAAATTTCAGATTCAACACAGTTTGATTATTTTCTTATGGTTAAAAGTTGTCCTAGCTCAGGCTGAGCTTTTCCCTTTCTTTTGTCTATTTAACAATCTATTTGTTATCTCTGATTTGAAATGAATATACACTAGTCTTGTGATCATCTGGTTTAAATGTGTAACTTCCTGAAACCTGCTAGACCTAGCTAAACATTGTGCCTCTAGTCTTGACAAATTCTTAAGTTCCCATCCCTAGAAATTTGCTGTATATAGACTAGGATGAATCATGTAGTTAGAGAAGGCTATTGGTGTTGCTCATCTAAGCTTAAGTTCTTTTTCTTGAGACTTTTTCCTTTTCGTGAAATATGAACTTGCCTATGATTAATAGCAAATTTTATTTATTTGATTTTGCATTACATCTGATTAGACTGAAAAAAGCCTAAAAAATGCACCATCCTTGCTAGCTCGACTGTCATGTTTGTGTTGAAAATAGCTTACCTTTGGGGTTGCTCTTTAGCATTCTCCAAAGAGCTAGGCTTGGATGACTGAAAGCTTATATCGCGGCTCTTTTGCATCCTAAAGTATATTGATTTACCATCCTCAGACCCCTGCTTGAATTTGAGTACAGATTTTCTTGGATTTACATTTAAATGCTACATGATAGTATCATCTTATTTTCTGCTCTCTGTTTGTCCCGAGGCTGGTTTTAAAGGTTCAACTTAGGTATGTATTAAGTGTTGGTCTGCTTACTTGAGTAGTTTGATTCTATTAGGGGTTGAATTTTAAATATTCGTTATGAAGTTTAACTTGAGACATAGAGCCTCGTTCACAACAAGTTTAGCATGCGTTGTTTTCCATTTTTTATTGATCAATTGCTTATTGTTTCCACTGCTTTGTTTAGCTTTGTTAGCTATTTTAAATTTAATCAATTTCTCTGTTTTTCCTCTCTTATTTGAATTTCTTGGGTCCCTTTGCACATGTTTAGTTCTAGATTTCTCATAGAACCTCTGATTGTATAAGTTTTAGTTGGATCTTTTGAGTTTGGTTTACATTAGGAGTGGATTTTTGGCTCCAAAGATATTGTTCCAAAGTAGTGTTTTGTTATTGTTTGTCCAATGCCTTTCTGTACATCAGTGGCTAAGCTAAATATAATTGAATTATGACTCCTCTGTTTGTTTTAGCTTATTTTTTTAAAAAATCTCTCGCACTAAGTGCTTAATTATTTTTTTTTAGTTGTTGATCATGCATGTTCCTTATTTTGGTTATTAGTAGAAGGTCTACTCTTAAGATGGTAGTTAGATGTTGATAGATTCTAATTTTTTTTTCCTGTAATGCTAAGTTAGCAGCTTAGCTTAATTTTTTGTTTTATTGTATTATTATTATTATTATTATTATTTTATGTTAGTTCTAACGTTACTTCCTTTCCTTTCTTCGCATGTGACATTCGAGTCCAATGGACTCCCCCCTTCTTGCATACTCCTCTCTTTTGAGTCAACCTCATCCGAGACGTGAAAGAGAAGCTAATATTAATGGGTTAAAACCCAAACCACTGGCAGCAGCTCAAATAGCAGAGTTATAACACTCCCAAAAGCTGAAAATTGGCAAAATTTGGACTTAAAAGAATCCAATTTAATCGAAGTAGTAGCAGCAGTCCCCAAAGGGACTAAATTGAGAATTCTTGGACTTTAACAAGTCCAATTTCCACGGCAGCAGCAAGGAGCATGATAGGCCGAGGGCCTAATTTCTCGTTATTTATTTTGTCATATTGTTTCGTGTTCTTATTATATTTTGGAAATGCTAACATTTTCTCCTTTTTGTTAACTTAGATGAATCCTTGGGGAAGATTTCTATTTTGTTACTCATTTTAAACTTCTATTTTGTCACTCATTTTTTTTAAGTATTTAAATTTTCAAAACCCCTTTTATTTAAGGCAAATTTAAAGCTCTTAGACTATCTTGAGAATTATCCGTATTCTTTCATAGTTAGTAATTTGCTTTGAATCCACGTTAATGAGATTGAGCTAACCATTTAGTTCAAAATCTATAACTTGGATAAATTATCAAGTTAAAAATGTTTTCGACGCTTTAACATTATAAATGTTCCATTACTTATCATGTTAGTTAAATCTTTATTTATTTTCATAAAGATTTTTTCACAAGTAATCTTAAGTTTTCTTTTACAATTAGCCAACTTTTAAATCTGTCGGTCAACCACGTGTTAGTGGATTCTTTAAGATGCTTAAACCCTTCCTCAGAGAATTATTTGAACCCTTACCCGACTCTAGTTATTTAAATTGTTTCCTCTCAAATATTCCCTTTTTAAATGGTTTTCTTAATTTTTCCTAAAAATTAAGTGGCGACTTCCAAATAACTCATTTTTCCAAAACATGTACAAAGTTGCCAAAGTTGCGAAATTGGTTTCGAAACCTTAGTTTTCGAGATCGGGTCGTAACAGAGACAATGATCTCTCTATATATGTATATGCTAGTAGGAAATAGTATAACAGGTACACGTTTATTTATTTAGCAGAAAAATAAACAAAAGTTTTATGAATTTTATTTATTGTGTGAAACTAAATTAAGAAAGAAAAGAAACTATGCGGGATGGAGCGGGACGGATTGAAAACACAATTTTTTGCTATGTAGGGCGGAGCGGGGCAGATTAGTATGTTTGCGGATCTATGCGGGTTTGTCCCACAACCGCCCCTCCCCGCCCAATTGCCAGCCCTAAATATACGTTGAGGGGTATATGACCATTTTCTCAGTTTTAATATCATAGTAAGAATATTCAAATCAGATTTTCCAATGATATTCATA

>LNC_001415

GTGAATTTGAATATGATTTTTTTTTTTAGAAATAAAATTCACCGCAAAATAAAGATTGTGATTCTATTGCTCACTGCCTCCTTCCAAGTGATCTGCTGTCCGCTAGTAGTTTATGAATCTGCCATATTTTTCATGATGTTCAGTGTGACAGATTTTGCCTCTGACAAACGTAAGAGTATATTTTTCCTCCATGATTGCTGCAAATCTTTTAGTTTTAGTTGATTTCATTTTCAGTTGCTCTTGCGGCGCAGTAATTTTCATTCGCGATTCATATATCAAGCTCTTGCTCCAGCTTATATGTCGCATTGAAAGTATGAAAAACGTATATAGTTCGATATCCCCGACTTTAGGCTCAGCGATTCTTCAACATTTATGTTTACCATGAATAACCTAGTTTCTATTACTAATTTTTGTAAGATATATGCGCTTTAGCTTTGGTAGTGTTAGAAGATGAGGAAGATGAAGGGGGGAATGGGTAAAGTTACATAGACCCGAGGATTGAGAATCAGAAATCATTTGAAAAGGTAGGGCCTGCTGTGAGTTTAAGTGAGGAAAATAAAGGAAATTGGGGTTATAATTGGATATGTGAACCTCTGTATAAGTGCTATGGATGATAGAGAATAGGGATACTTGCTACATGAGATGATTTTGGTTTGGGGGGTTTTGCAGAACAACTCCTGATGTTAGAAGTAGCTGCTATAAGATGATATTGGTTGGTATGGTTCAGAATTTGAACGCCATAGGAAATTACAGTTCCCTATTTAAGTATCATAAGATGAAAACTTACCAATTTGAAGTGCTTTGTTGCAGTTAGTAGCTCATATATGTGCCAAGGGAAATGTTGTTCGGACTCTTCAAAAATGTCAAGGGGTGCTTGTCAGATTCACCAAAAGTAGTGTATCTTTCCATTCAGTATATTGATTATTTTATTTCTTAGTAACTTTCTATTATCTTGTAGGTATCTCCATATAAGGAATTTAAGCACAACTGAGCTTGAGGAATATCAGTAGTTTCCTTCAAATTCTTATTCATATTAGCTGTGACCGGGAAAGTTCTGCAGAGGTTCTTTGGAAATCTGGAAGAATTTTATTTCCCTGATTTGGTTGAAGCTTGCCTAAGTATTGAGATTCTGTGCTTAAATGAGATAGAGGATACAGCACCAAGAAGAAGGAATCTTTCAAGAAGCAGGAGGGAGCTTTTCAATAGTTCATCTTCAAAAAATTGTTTATGGTTCAAGTTCATAACGACTCAGGTGTCTCTTCCTATAAAATATGTGAAAGAATGGAAACTGCTAAAAGTGAAAGAAAAATAGAGAAGGATAGATCAAAGGAATCTGGAAAAGAATATTTCACAGAAATTGACTGGTATCTGTTTTCTCTGGAGAGATTCAGTGCTTTTTGCCACTTTTTGACTTCACCAGATGAGGGAAGACACTCAAAATGTTGTGAGCTCTCAAGTCTTGCCTGGTTAAGATTCCCACCACTGGAGATACCTGAAAAAGGAGCATGGTTTGATGTTAGTAATGCTTTTATCACTTCTATTGTAAAATAACACTTCCACCTAGACTCTTCCTTAACAAGCTCTGATGCTACGATATGACTTTTTCTGTGTATCGTTAGACTATGATAGGAGGGAGATATTTGCATAAGATTAATAACTGAAATTTGCTTATAAGCTCACCCCTGCTGCTTGATATTTGGGTACAATGAGCATGTGGCGGAGCCCCACTTGCCTGAAAAGCACCATTGCCTTAGCCACTGACATGCTTTCTACCACAGTATAGGGGGTTGTGTTGGTCAAGGGATGCAA

>LNC_001416

TGAAAATATAAAAATAACTATTAAAAATAGTCCTATATGAATTATTCTGGTCTTTTATATATACCATTTTATTTAAATAGTCTTTAATGTATATATAAAGAGTATATTATTTTGATCCTTGCCCTAAAAACTAATTGAAAACATAAAAATAAGTATTAAATTTAATGGATAATGGAAGCTTCTGAAAATTTTGCTTCAATGGAGTTAAAAATGGATGAGGTGTGTAGTATTTTTCTTATGAAGATATAAAGCAGGAGAGAAAATGGCGAAAGAAAGAAGTTGAGGAGACGGGTCTGCGGTGGTAATGGTGAAAGAAAGCAGATTGAGGAAGAAGGGTTTGCCATCTATCAATTCTGTGGGACCCACGATTAAATCTAACCATTTTTATTTATTTTTTTGGTTAGTTTTAGGATTAAGGACTAAAATAATTGTCTTTTTATATATATTAAGGACTATTTAGATAAAGTGATATATATAAAGGATCAAAATAATTCAATGAAGGGCCATTTTGATCATTTTCTCAAGGAAGATATGTGATTTAAGTTTGTGTTTCCACATAAAATCCACATGTATTACTGCAAACTTTTTTGCTTTTCTTAGAAAAAAAATAGACATTTATTAGTTATATTTGGAGTTCGTAGTTACAGTCAAGGAGATTTGACTTATCATTATTTCATTATTTGGATATATTATTGCAATTTTTTGGTCTTCCTTT

>LNC_001417

TAAAAATAAGTATTAAATTTAATGGTGGGTCCCAACTCTTCTTCCTCCCTCTTTCTCCGCCATTGCTTCTCCTTCATCTTTTTTCTTTTGCTATTTCTTCTACGATTTCTGCTCCCAAAATCTTCTCATACACACAAAAGTTTCTTCAAATTTCAAAGAATATTCGATAATGGAAGCTTCTGAAAATTTTGCTTCAATGGAGTTAAAAATGGATGAGGTGTGTAGTATTTTTCTTATGAAGATATAAAGCAGGAGAGAAAATGGCGAAAGAAAGAAGTTGAGGAGACGGGTCTGCGGTGGTAATGGTGAAAGAAAGCAGATTGAGGAAGAAGGGTTTGCCATCTATCAATTCTGTGGGACCCACGATTAAATCTAACCATTTTTATTTATTTTTTTGGTTAGTTTTAGGATTAAGGACTAAAATAATTGTCTTTTTATATATATTAAGGACTATTTAGATAAAGTGATATATATAAAGGATCAAAATAATTCAATGAAGGGCCATTTTGATCATTTTCTCAAGGAAGATATGTGATTTAAGTTTGTGTTTCCACATAAAATCCACATGTATTACTGCAAACTTTTTTGCTTTTCTTAGAAAAAAAATAGACATTTATTAGTTATATTTGGAGTTCGTAGTTACAGTCAAGGAGATTTGACTTATCATTATTTCATTATTTGGATATATTATTGCAATTTTTTGGTCTTCCTTT

>LNC_001418

AAAAGTTTCTTCAAATTTCAAAGAATATTCGTAAGGATAATGGAAGCTTCTGAAAATTTTGCTTCAATGGAGTTAAAAATGGATGAGGTGTGTAGTATTTTTCTTATGAAGATATAAAGCAGGAGAGAAAATGGCGAAAGAAAGAAGTTGAGGAGACGGGTCTGCGGTGGTAATGGTGAAAGAAAGCAGATTGAGGAAGAAGGGTTTGCCATCTATCAATTCTGTGGGACCCACGATTAAATCTAACCATTTTTATTTATTTTTTTGGTTAGTTTTAGGATTAAGGACTAAAATAATTGTCTTTTTATATATATTAAGGACTATTTAGATAAAGTGATATATATAAAGGATCAAAATAATTCAATGAAGGGCCATTTTGATCATTTTCTCAAGGAAGATATGTGATTTAAGTTTGTGTTTCCACATAAAATCCACATGTATTACTGCAAACTTTTTTGCTTTTCTTAGAAAAAAAATAGACATTTATTAGTTATATTTGGAGTTCGTAGTTACAGTCAAGGAGATTTGACTTATCATTATTTCATTATTTGGATATATTATTGCAATTTTTTGGTCTTCCTTT

>LNC_001419

TGCATTTCATGATATTTCTGTTGTATGTATAGTATATTAAAGGACGGTTTTGCGGAACCGCAGTCGGACTTTTTTATACTCAACCCTTTTCCAGAAGGATCGGCGATTTATTGGTACGTCATGCGTCCAATGGTTGTTGTGAATTCCAGCCTAAGTTGTCCACTTGGGTTCCCGGTCTTTCAAAAGCCTCTCTTAGTCAACTAGCGTAGAGTGAAACCAAATCTCAATTTCTAGCGCATTTTGAACTAGGTCGACCTAACACTCAAGGCGTGTTAGGCCCATTAGAAGTCTAGCTTGTGTCTATTTGGCCAATTGATCAATAAAAGACAATTATTTGTATCTGTTTAAATTTGAAGGATGTATACGTCATTTGACAAAACAATGTTCCTTGGGTTAGGGAGTTTAAGAATTTGTTCGAGTCAAAGAATGATCAAATTACCATTGCATCTGAAGACGTTGACATTTAGAGATGGATGCTTAGTATAGGATTGTACCCTTGTGTGGGATCGATTTTTGTATCATTTTTCTCTATTATGTATACCCATTCAATTTATTTACGATGCTTGTATAGATTTTAATTTTTTGGGGACAATAGAATGTTGTCAAATGGCGTTATACATTTTTCAGATCGATAGGCTTACCCTTGGCACAAAACGGTCACATATTTATATAGAACGCAC

>LNC_001420

GGAAACTTGATAGCCATTTTAGTGTATTGAGATAGTGCAATGTCTTAGTGATTCCTGAAATATTTTGTATATTCTTGATTTGGGATTGTGATATAGGTGATTGATTTAGATGTGGTGCTGATGCTTATGTTACAGTTAGCTATGTATGATGATGTTATCATAATTACTTTCAGATATGTGAAGAGGTGAAATAACAGTGCTTCATTTCTATAAGTTTGTCGGTGCTTTTGTTTTTGTTGTGGTATGCTTGATTTACTATCTATATCTGCAGCGGTGATCATTCTCATGGATCTTTTACTTCCTTTAAATGTGTCTAAGGAAAGCATTTTACATGAAACTCTCAAAAGAATCTACCTGATTCTAATTTTTCCTTTATGAACCTTCTAAAAATGTTATTCTGAAATTGTTCCAAGTATTGAATTATATTTTCGGTGGC

>LNC_001421

AGAAAGGCCAAAATTTTGGGATTGTTCTTAGTTAAATGCACTTGTGGCTCTGACTGCTTTATCATTTGGCATAAGAGAGTGATGTTGTTTCACCATCATGCTGGGAACTTCTTCTGTTCAGTAAAGAATGAGAGCTTATCATCTTCCCTATTTAAGGAAAAGATAATCGAGATAGACACAAGAAAAACAAAGCAGAATTAGAAGGCAAACAGAAAAAAAGTTCAGTTTGGCATTTGGTAATTTATCGTTGCTATATCTGGAAAATCGTACTGGTGATCTATAGCTAAATGGTTAATGCATCAGTGATTCCCCCTTTTAATTAGCACTATATTCAGTAATCTATGCATCTCTACTTTCCTATGGAAGCTAGG

>LNC_001422

TTTCTTTTTCTGTAGGTGTTTAGACTTTAGTGTGGGGTGACAGTTTAGAAGGCTATTAAATCATTGGGTCTTTTTGTATTTTCTCTCTTCACAAGGAGTCTTCCACCCTAATTAGCTTCAAGCAGCTTAGCTCCCATGGGAGGTTTCCTGACATTTGTTGGCAAATACTTGCATGAACTGGCATCTCATGTTCTGTATAACCATTAAGATCTAGCATTAATTGGTATATGCTGGCCTTGAGATGGGAAATTACATTGCTACATTTTTCTTCTTATGATGTGATTCCCGTCTTCATCAAAGGAAAAATGAGAAGATTAAGTACAAGATGACATAATGTGTTTGATATAGCTGGTATAGCAGAGAGCTTATAAGAGATACATATCCATAACACCTCATCCAAAAAAAAGAAAGAGATATATATCCATAACATCAGAAGATCCTTGATCTATTTTTTCCCTTTGCACTGTGGTCTTCTCTTAATTCAAGGAATAAGAGTCATCTTACACATATATGTCATAATAATATTAATAGCCAAGTTCCTTAAGCTCCAAAAAGCAAGCCGAAGATCTTGAAGCTTCTTGGATATCCACTTGCCAAGGAAATTAGAAGAGCAATTTTTCATGAAGTATGTCGAAAGACCTCTACACATGTAAATGTCCAACTACAAATTGACTTCAATTTGGAAAAAGATTACTATTGATCAATTTAATATTGATTATATTTTGAAAAACTGTGGTTAATAGTGTATCCTACTTACTTCTGATGAATTTTTCATGATATGCAGCTTTTGTGCTTTAAATTTTATTTTAATATTTAAGCTATAAATGAGAGAAGAATGAAGAACCTGCAGCATATTGCATATTTGAACAGAGGAGAAAAAAGAAAAGGAGAGAGAATCTTATAGCATAGCAATCTAGCTGATTGGCCCTTTACTCATTTCTTCTATTGTTGTGAAAGGACTGTTTCTTCTCCTACTTCCC

>LNC_001423

CTTTCAAGAGAGGAAAAATATGAAATCGGATCTAATTCAACCCAAAAGTTAGTTGATGAGAGGGATTTACCAAGTTCATATAAGCAAATCATAAGTCTAACGCCAACAAATGTGAAACCGTAACCCACTCTAAAAGAATGTGATGCCAAAATTTAAGGGCTTTTCTACCGAGTGGTGGACCATATAATTAGGAATGAAGTTATACGGGACAAGGCGAGAGTTACATTTATGACATATATAAAGAGGAAGTAATCATTTTCTTGGGGAGCTTTTTGATCTTTGAAGTCTTGGAGGAGTATCTATAGCTAACTTGAGAAGGATTTAGAGATTTTCACCATAGAGTTTGCATCTTTCTTCTTTGTTTAATCTTTAACTATGTTTATTATTGCATTTCATCTTGTATTTTCTTTCTCCTGCATGAGTAGCTAGCCCATTAGCTGGGGTTGTTGACTCTTATTGCTTTTGTGTGATTGAATACTAGTTTATCTTGCTTATTATTACTTGGGTTGTGATTATCTACTTGTTAGATTCTTATTTGTGAATTTGGTGGTTGCAAACACTAGATCCCCCTACATATGCATTTCTTGCTTGGAAGAGTAGAAATTGTATTGAGTTTTTAATGAGTAGTAAGGAATCAAGTATATTGAGGAATCGATTGTGCTTGATTAATGAATTGAACATTTTCCCTACTGGTAAAAGTAGGAATTGGGAGATTCCTACTTGGATCTATCAATTGGGTCTTAATATTTTTTCTTGGGTTTGGAAGAACTTTTGGGGATTAGCTTCGTAGAGTTGGAAAATCCTGATAAGTAATTATCAATTGGATCATTTTCATTGATCCTATGTATTTGAGCAAGATACATGTGTTCACGAACTAGTTATGTCATATTGAGGCTACAACCCTGGGGTTCCTCTCTTAATTGAAAAACAAAACATTGTTATTGCACTCCAAATGTTCGATTAAATTCTTGGCTTAGAAAATAGTCTAGTTTTAGAATTAACTACTCAATCACCCTTTTATTTGAAGATAACTTAGGACTTTAGTGTAGGATTGAACGTTATTAGTAGGTAGCCAAGTGTGGTCATCTGTTAGGTGGGAGAGGCAAGGGGCTTGTCTCATATCCTCTTCTCCTTATTAGTAGTAGAATTTAGCAACCGTGTAGTTTCTTATTCTTCTATGTATTTCTATTATCTGTTGCTTCATTTTGCTTTGTATCTTGATATTTTGTTATCATTTTTTTCTTCTTGCAAGCCTTCTCTGAGTAACCTCCCTTCTGAGCCGAAGGTCTATTGGAAACAACCCATCTACCCCACAAAAGTTGCGTTAAGGTCTGTGTACATTTTACCCTTCTTAGACCCCACTTATGGAATTACATTGAGTATGTTGTTGTTGTTTGCCTTGGAAGTTAATCAAGGCTAATTTTGTTTGAAGGGCTATGCATGTGATTGCCTGAACCATTGCTGCTTGACTTTTGTTCTTGTTTCTTCTAGACATTGCTTTCCTCAGTGTGAAGTAGTTATGCAATTGTTTTTTTGACGTTCTGAGTGAAATATGAAAAAGCCTGAAATTTTGAGTTGAAAGTTTCCATTTTGTTCTTTTCACTATCGGAACTAATTTCTTTTTGTTTTGAGTTTGCCTTTTGGGGTTTTGAACCTGTACTTTGTTGACAGTTGAATTCCCTTACCGAATTGTTGCAAAGGCTACAATTTTTTTTCATAACAAGTATGGAAGTTTAAAGAAACCACATAATAGGTCTATGGCACCAATCTTAATTCAGTACGATATGATAAAACATCCTCTCCGCTATCTGTCACTACTTTGATGTCATCAGCTTCTTCCTCCATTCCCCCTTCAGGTAGATTTCTCCCCCAACCATAATCATCATAATTAAGTCCAAGGTTCTCCGCAAGCTCTATAATCTCTTGATTATCGTTGACAAAGTGGCTTGCTTTATTCCGACAACAATATGGATGTTGGATAATGATCTAATGTTTCTGGCAAAGGCGTCTCTGTCTTTCTGTTTAGTGTGTCGAAAGGAAATCGTTTTCTGTTAAAGAAACAAAGAGATTATTTTTACTACTAGAAAAATATTAGTGGTAGCAACAGTTTACTGTTTCCTTGTGCTCATGTGAAGTTGATTGTTTCAATGTTCTTTTTGGGCTGTCTGTTGTTCTTTAGAGTGTCATTTGTAGCTATCCTTTAAAAGGTTTCAAACAAAATTTTCGCTGGTGTCTAGTGTCTACAAATTTATTGTCTAAGAAGTGAACTTTCTCCTAAGATCAAAAGAGGTGAATAAAACGGGCCCCACTTGTGGGATTACACTGGGTATGTTGTTGTTGTTCTAGCAGCAATATATGTAGCGCTAACCTCAAACTTCTGTCAACCTCAAACTTTGATAATTTTCAGCGTATAAGACATGGTGCTGGCATTGTGAACTCTTACGAAGCTGTACTTCTTGTTTTTCGTTTTTTTGTCTCGCTTGTCTCATTAAAAGAGGAGATATCTCCCCTTTTCTTCTACCTGTCTCTGGTGGATCCTTTTATTTTCTTTTTGGAAATGCCCTGATTGAAAATCCAATGAACTGATGGTACCTCCGACACTGTGATCCATATGCATCCCTACGTTCCTTAACAATTTTTTCCGTGGGATTTAATAGATCAGGTAACATACATACAGTAGTCTTTGATTCCTCAAGTTGACATGGATAAGTTTTCTTATGTGGACAAATTTCAGTATCCTAGGTTTCTTGTTCCTACACTTGTGATCATTAGTGTATTTCAGTGTTAAACGCAAAACTACATCTCACTTCAAGGGAAATTTTCTCTGCTTGTGTTGGCGGTACTTTGTTATATCTTTTAGCTTCTCTGATTCCGTCCTAGATTTTCCTGGCCACTGTAGCATATGCATTGCCCTATATTCCTATCTTACACTTGAAAAGAGGTAGTTTAGACACTAACTTTTGTTAAAGTCTAAAAAAGCCTGTATTCACTGCACTTTATGTCATTTAGGATGATTAGAGGAATTTCCTTCTGGCCATGATTACTGTCCACTCTCTCCATGTTGTCTTGACAAGCGATAAGAGCATCTAAGGGAAGCTAAATGAGCACCAACCGTTTAAGGGTTCAATCTTTTCAGTCAAGTAGCATACTAAATATCAAGCAGTCTAATCATATATATATATATAAAAGAAAACCTTAGGCCCGCCTACGTGGCGCCACCACAAAC

>LNC_001424

GTTCCTACACTTGTGATCATTAGTGTATTTCAGTGTTTAATGCAAAACTACATCTCATTTCAAGGCAGTTTTTTTTTTGCTAGTGTTGGTGGTACTGTGTTATATCTTTTAGCTCTTCTGATTTAGTCTCATTTCCCTATAATCCTATCTTACATTTGTAAAGAGGTAGTTTAGCCACAAACTTTTGTTTAAGTCTAATGGCCTGTATTCACAGCACTTTACATCATTTAGGATGACTAAAGGAATTTCCTTCATGATTATTGTCCACTTCCCTTCATATTGTCTTGACAGAGCAATATAAGCATTGAAGGGAAGTCAAATGAACACCAGCCATTTAAGGTTTCCATCTTTTCAGTCAAGTAGCATACTAAATATCAAGTAGTCTATTTCTTACATAATCCAAGAATTGTATGAATGATTTTCCTTCAATAATTTCATATGCCAATACTTCTGCATAGTTATTTGGTGATAAACCACTTGTTTAACTTCTACTTTCTACTTTCTACTTGCTTTGATAGATAGACACCTATGATATATGTAAACAATAATTTGCCACTAATTATAGTTACCGTTGATCTATCATGTTTAAGTGCCCTCTCAAAAATCATAAAGTCTGGATTTTCAACTATTAATCTCACTCATTTCTTCACAACAGGGCCAGATCTATTCCCTTTTTCACTCGGTTTTTCTTTTTCTTATGTACATTAACTTTGTGACATCAATAATCTCTTTGCTGCCAACACTCATGTTTATGCTGGTTTAAATGTGTGGAATGCCCCAAAAAAAATGTATACTGTCTGTTTAGTTGTGTTAGGAGTCCAGTCCTCCCCACCCCTCCTACCTCTCCTTTTTCTTTATTTACTTTTAAAGATCTCAGAAGGGGAGTGCCTTATATATGTAACTTACTTCGCCGTCCAATTTTCTTCTCCTACTTTGTGATTGCTAGGTTCTCATAACTATGAATTTCTCAATAGACTGAGCTGCCTGCATCCAACATTTGCACCAGAAGAAAAAGCTGAAAATTTCTTATATATCCTGTGTTTGAAATTTCTGTGACATGATTCTCATTTCAGGAAAAAATGATACCTGACTCAGTTAAGCCTTTTCAGACATTGAGCTCACATTGTGTTCTCTGTTGATATTAATACTACTATTTTGTAAAGGTGCTGACTTGGACCTCTTTGTGTGTAAAATCTAGTCGAACCATCCATACCAGGAACACACATCAAAACACCTTCCTGCTCTAAAATTGCTTCATTAGCCTCTCCATTCAATACTCTGTCTCAACTAATGCACAACTCCCATCTTCAAATTGTCAAACCTTATTCCACTGTACTGAAAATGACTTGAGTTTCAACACACGCTAGAGTCCTTCCAAACTCCAAAATAACGAGTCTCACAGGGCGACTAGCTCCAAATTTGGAATTCATACCAGTACTTGCTCCTCTATATGTAATCCATAGTCAAACCACCATGCTGACGGCCTCACAACTCAATATATCAGCTACCACCATACATCCGATGAGCCAGTCTGATAGAACAACCTGCCCTATAGTAGAAAAACAAACTCTCTCAGTTTTCCTAGCAGAGGCATAATTGGTAATAGAAGACAATAAGGAAGCAACGGAACAAAAACTAAGCAAGAACAAAACAAAGGATCATACTATACCATTAAACATAATTGCCACACGATGCTGCAATATAGTATAAACCCATCCAGTTCTCCAAAATAAATCAGTGGATGCATTGACAAGTATAGTCAAACTAGATCAAAGATCACTATAAAGCTGCAACAAGAGAACTATTAGCTGCTATATTTATTTGGCTGAAACAATAACAGCATGGCCTGGAAAAAGATCATCGTCTACTTATTAGTAGTAGAATTTAGTAATTACGTAGTTTTGTTATTCTTCTATGTGTTCCTACAATCTGTTGCTTCATTTTGCTTTGCATTTTGATACTTCATTGTCATTTTTTTCTTCTTGCAACCCTTCTCTGAATAACTTTCCTTTTGAGCCTCAGGTCTGTTGGAAACAACCTCTCTACGACTCTATCCCACAAAGGTAACGGTAAGTGTTATCATAAAGACAGGATTTTCAGCTATTAATCTCACATATTTCTTCACCGCAGGGCCAGATGATGAAATTTGATGGTCTCAGGGAACTTGGTACTGAGGCTGCTGTTGAGCTGATCCTACACCAAGACTAGGCTTCATAACATGTTGACGAATTAAGGCCCCTGAACTGAGGGTTCGGTTAGTAAAAGGGACGACTTTGTCTCCCTGGAATATCGTGCAAAAAAGCTCAATCTGGGATTATTTTAGCCCACCCTCTATATGGGTCTAACTATATAATAGTACCTATTATCATAAGCAAGATAAAATAATTCTCATGGAATATCTCAGGATCATTATCCTTATCCTACCCCTGAATTTTTTTCTGAAACTAATCTGTTGTTGTACAACAGGCTGCTGGTAAGTGGTAAGTATACACAAATTTTAATCTGTATTTACTCATTTAGTTGGAAGGCAATATACATATTATATACTGTTGCCTCATCATATAAGGCCTAATTTCTGCAAAGGAATTTGCATCTCTATCTGGGAGATTGTACAAAAAAACTACATATATAGTACTCCCTCCGTCCCATTTTATATGTCACATTTTTATTTTGCACTTGCATTAAGAAATGATGTAACTTTACCCTTCTACCCTTATTATTTATTTTTTTGGAACTTAAGTTTTCAATTAATGAATATGAATTAATTTATTTTGATTAATTAATCTAACCAATGAACATGAATCATTTACTTAACTTTTCTAAGTTGGTCAAATGT

>LNC_001425

GATTTGAAATAAGTACATTTCGGTATCTGAAGGGTTGCCTAAAAGATACAGTCCCTTAGAGGGGCAATTATTTGCTACATTTGGATATCAGGCTCTGGTAAGTTATGTTTTGGCTACTTATGATGGTTGGAATCCTTGAATGTTAAGAAAATTATTGAGAAGGGGCTAGAGGTTTCAAAAGATAATTTGGAGCATAACTATGCATAGTTCTGGAATATGCACAATTTGAGAGTTGCATTACATGTTAGGAACCATGAATTGGGACTTCTCATTCCTGTAGGTAATTTTCTAAAGTTTCTTGCTATGACTTGTTTAAGGTAATAAGTATGATTTGAGGTTCAAATCCAGCGGAGGCAAAAATATTACTCCCTCCGATTTTGTTTGGGTTTGACTTGGCACATAGTTTAAGTCTTTAAGAAAGTAAAGAAGACTTTTGAATCTCGTGC

>LNC_001426

CGCCAAAAAGTTGTGGTTCTGGTTACCCTGTTAGTTTTTCTGTTAATGTTGGTAGCTGCTGTACTAATATGGATTGGGAGGGGAAGGAACCCTATTGATTCATCTGTTGTGGCTCGGGTGTATGTAGATCTCTTTGCCTTAGCAGTTCTTTTGCTGGGAGTGGCATTGGCATGCTATGGACTGGTATTGTTCTTGAAAATGAGCAAAGTGAGATCTGAAAGGGCTTCATCAGAAATGTGGAAGGCTTGGCAGTTGTTTCTGTTCTATGTTTCACTTCAAGTGCCACTGTCGCCGT

>LNC_001427

TATACATACAACTCTATTCTCTTCTTCACTACCGTATATTCTCACACACACGCTCTTCTCAAGCCGATGAAGATGAAGATGGGTCTGACCTTCAAATGACAATGTCGAAGATCACGCTGGCGAGAATAAAATTGCTGAGAAACAAGAGGGAGGTGGTTGTGAGGCAAATGAGGAGAGACAGACATTGGTATGCTTCTTGAATTCCGTCCGGATGCCACTGCTCGTAATCAGGTACTTTTTCCTCTGACTTAGGGTTGGGGGCTTAGTTGATTGATTGATTAATTGAACCTCGAAATCAACTCGTAGGAATGGGGAATTTGGTTTATCAGTTTATGGATCATTTAGATTGAAACATAGAATTTTGCTTTGGTTCAAGATTATAGCTTGCAATTGTTCACTGTCACAGCAAGGGTGTTTACCACCGAGATCGCTAATTGGAAAATCTGTTGTGAAGATCTATCTTAATCTTTTAACTATTGTTCCTTGTTCCCCAGCCTGAAAATTTGTGTATTTAACTTCTGACTTAGGGAGTGCAGTTTTTGTAGGGTATCAAGTTAGATGATTTGGACATCAGAAGACTAGCTGCAAGTATGTATCATTTAGAGAGTGTTAGAGGTTAATAATAGTAACAGTTGAGAGAGATTAAAAAATAATTTAGGAGAAGTGATTAGACGTAAGCTTAGATAAGAAGATATGAAGGCCAAGAAGTATATTAGAAAGTTAGTAGATATTCAAGCATTGTCACATATAAAAGGCAATTTTATTCTTCAATGTGTTTGTTACATCATCTGCTTTGTATGATGATTTTTATGTCATAATAATTTCTTGCAACCCTGCTTTGAAGACCTGTCTAAAACAATCCATGTGTAGAGTAAGGTTTGTGTTTCCCAGATCCTATTTGTGAGATTACATATGTTGTTGTTGTAACATAATCAAGGCAGATGATACATCAGTGAAGAAACACTGGGACAAGTGACTACATAAGGGATGATGTGCACAGTCAAACCTTAATTTCTAGCATAGCTAAGCAGAGAATTGTGTGTATTACAATGCAATATTAGACACCAACAACTACCAATAGTTATTCTCATGATCAATGCAAAACATGAGATTGTTCTTTTTCATTTTCCCTTTGTTTTGTGATTCTTCAACCTAAATCAACCTAGACATACATCTGTAGGACATATTTAGATACTATGGTTTGGCTAGCTTTTCAGGTACCTTGCATTTTGATTCGATCTTTTTCTTTTACAGAGAATGCTGTCAGTCAATATTGGAAAGGATTGGGCTGACTTGTGTCAACACATCTAGTTCTGTCTATCCATCCATTTATGGCATGTTCGACGAATCTCTGATATTTGTTAAATAAGGAATTATCCAACAAAACCAGAAATACAGTCTATAACAGCAATTTTGAGCTAATTACAACTTATCCCTAATGGTTTTCCCTAATGACAAGGTTGAGATACTTAATCCCAATTCATGGGAATTGTAGAGTCTTGTGTAGGTATTTTAGTTTTTTTTGGATGTGGTGAGGCTGCATTTTTCCACGTTTGTGGCTTTAGAAACTGTAAAAGAGAATGGATACTAAGTTTTTTACGTGGCTTGGGCTTTCCTTTGCTGTTGTTATGGCAATTTTTTTTCCCTCAGAATTTGGAGCTTGATTTTTGGGATCGTTTTTGGTTCTTTGTTTGTTGTTTTTGTCTTGTTCCTGCTGCTCTAATGGTCCTACAGAATCAATGGAGTTGCTTTTTGTTCTTTTGGTCATTTATGAGCAAATTAAATGTACTGATGTTGAATGTTTGTTTTTTTCTTTTTTGGGTAAACTATAGTTTGCCATCCTGCATTTTCATTTAGTTCAGGAGTGATCTTATCAGGGGATGGCTCTGTTTGGTTAGGATTTTTGATGTAGAAGGTATATTGTTTGGCTTATTTGGGGCATGAATGTACTAAGTGACCAAGTCTTCCACATACAGTGCATAGAGGTGTGGAAGGTTCATATTGGACTTTTTGTAAATGGATACCTATAAGAATATCTCTTGGAAGAGTTTTGGACAGTGGTGCTAGGATACAGAGTCTTGCGTATCGTCCTCTAGTGGTATTCATAGTCACTGAATCTATTTTTACTATTGTTCCTATTTGGTTGCCTATTCGTTGGAGGATGTTAAGGTCATAAATTTCTGTTGGGAGTTCCAGTAGTCTTATCCAAACCGTCGAGAAATTTATTTGAGCCATCGAGGGATTGAATTTTGGTTCCCATTTCCTAATGGAGATAAATTGCGAGCCGAGGAACAATGGTCCTTGATGAAGTATCTTCTGTTCCTCTCTTCTCTCTCCTCTCTCTTTCTCCAAGCCAACGGTAACTTGCTGGCTTTATAGTAATCTCCGGCGACCGCTTTCCGATCTCCTCTCAGTTCCCGGTAAGTCAAGATCTGCTCTCTCTCTTTCTCTCTCTCCTCTAGTCTACTAGCAGCTCAGGCGAAGCTGCGAACGGCGCCGGCCAGCGAACAGCAGCGA

>LNC_001428

TATACATACAACTCTATTCTCTTCTTCACTACCGTATATTCTCACACACACGCTCTTCTCAAGCCGATGAAGATGAAGATGGGTCTGACCTTCAAATGCAGACAATGTCGAAGATCACGCTGGCGAGAATAAAATTGCTGAGAAACAAGAGGGAGGTGGTTGTGAGGCAAATGAGGAGAGACAGACATTGGTATGCTTCTTGAATTCCGTCCGGATGCCACTGCTCGTAATCAGGTACTTTTTCCTCTGACTTAGGGTTGGGGGCTTAGTTGATTGATTGATTAATTGAACCTCGAAATCAACTCGTAGGAATGGGGAATTTGGTTTATCAGTTTATGGATCATTTAGATTGAAACATAGAATTTTGCTTTGGTTCAAGATTATAGCTTGCAATTGTTCACTGTCACAGCAAGGGTGTTTACCACCGAGATCGCTAATTGGAAAATCTGTTGTGAAGATCTATCTTAATCTTTTAACTATTGTTCCTTGTTCCCCAGCCTGAAAATTTGTGTATTTAACTTCTGACTTAGGGAGTGCAGTTTTTGTAGGGTATCAAGTTAGATGATTTGGACATCAGAAGACTAGCTGCAAGTATGTATCATTTAGAGAGTGTTAGAGGTTAATAATAGTAACAGTTGAGAGAGATTAAAAAATAATTTAGGAGAAGTGATTAGACGTAAGCTTAGATAAGAAGATATGAAGGCCAAGAAGTATATTAGAAAGTTAGTAGATATTCAAGCATTGTCACATATAAAAGGCAATTTTATTCTTCAATGTGTTTGTTACATCATCTGCTTTGTATGATGATTTTTATGTCATAATAATTTCTTGCAACCCTGCTTTGAAGACCTGTCTAAAACAATCCATGTGTAGAGTAAGGTTTGTGTTTCCCAGATCCTATTTGTGAGATTACATATGTTGTTGTTGTAACATAATCAAGGCAGATGATACATCAGTGAAGAAACACTGGGACAAGTGACTACATAAGGGATGATGTGCACAGTCAAACCTTAATTTCTAGCATAGCTAAGCAGAGAATTGTGTGTATTACAATGCAATATTAGACACCAACAACTACCAATAGTTATTCTCATGATCAATGCAAAACATGAGATTGTTCTTTTTCATTTTCCCTTTGTTTTGTGATTCTTCAACCTAAATCAACCTAGACATACATCTGTAGGACATATTTAGATACTATGGTTTGGCTAGCTTTTCAGGTACCTTGCATTTTGATTCGATCTTTTTCTTTTACAGAGAATGCTGTCAGTCAATATTGGAAAGGATTGGGCTGACTTGTGTCAACACATCTAGTTCTGTCTATCCATCCATTTATGGCATGTTCGACGAATCTCTGATATTTGTTAAATAAGGAATTATCCAACAAAACCAGAAATACAGTCTATAACAGCAATTTTGAGCTAATTACAACTTATCCCTAATGGTTTTCCCTAATGACAAGGTTGAGATACTTAATCCCAATTCATGGGAATTGTAGAGTCTTGTGTAGGTATTTTAGTTTTTTTTGGATGTGGTGAGGCTGCATTTTTCCACGTTTGTGGCTTTAGAAACTGTAAAAGAGAATGGATACTAAGTTTTTTACGTGGCTTGGGCTTTCCTTTGCTGTTGTTATGGCAATTTTTTTTCCCTCAGAATTTGGAGCTTGATTTTTGGGATCGTTTTTGGTTCTTTGTTTGTTGTTTTTGTCTTGTTCCTGCTGCTCTAATGGTCCTACAGAATCAATGGAGTTGCTTTTTGTTCTTTTGGTCATTTATGAGCAAATTAAATGTACTGATGTTGAATGTTTGTTTTTTTCTTTTTTGGGTAAACTATAGTTTGCCATCCTGCATTTTCATTTAGTTCAGGAGTGATCTTATCAGGGGATGGCTCTGTTTGGTTAGGATTTTTGATGTAGAAGGTATATTGTTTGGCTTATTTGGGGCATGAATGTACTAAGTGACCAAGTCTTCCACATACAGTGCATAGAGGTGTGGAAGGTTCATATTGGACTTTTTGTAAATGGATACCTATAAGAATATCTCTTGGAAGAGTTTTGGACAGTGGTGCTAGGATACAGAGTCTTGCGTATCGTCCTCTAGTGGTATTCATAGTCACTGAATCTATTTTTACTATTGTTCCTATTTGGTTGCCTATTCGTTGGAGGATGTTAAGGTCATAAATTTCTGTTGGGAGTTCCAGTAGTCTTATCCAAACCGTCGAGAAATTTATTTGAGCCATCGAGGGATTGAATTTTGGTTCCCATTTCCTAATGGAGATAAATTGCGAGCCGAGGAACAATGGTCCTTGATGAAGTATCTTCTGTTCCTCTCTTCTCTCTCCTCTCTCTTTCTCCAAGCCAACGGTAACTTGCTGGCTTTATAGTAATCTCCGGCGACCGCTTTCCGATCTCCTCTCAGTTCCCGGTAAGTCAAGATCTGCTCTCTCTCTTTCTCTCTCTCCTCTAGTCTACTAGCAGCTCAGGCGAAGCTGCGAACGGCGCCGGCCAGCGAACAGCAGCGA

>LNC_001429

CTCTATTCTCTTCTTCACTACCGTATATTCTCACACACACGCTCTTCTCAAGCCGATGAAGATGAAGATGGGTCTGACCTTCAAATGCAGACAATGTCGAAGATCACGCTGGCGAGAATAAAATTGCTGAGAAACAAGAGGGAGGTGGTTGTGAGGCAAATGAGGAGAGACAGACATTGGTATGCTTCTTGAATTCCGTCCGGATGCCACTGCTCGTAATCAGCTCGTAGGAATGGAGAATTTGGTATATCATGCCTGGTGGTTACTTTCAACAACTCATTGATGCAATTGCTCACTGTCACAACAATACCATCGAGATCTGAAGGTTTGCAATTGCTGATTGGAATATCTGTTGTGAAGACTATCTATCTTACTTAATCTTTAACTATTGTTCCTTGTTCCCCAGCCTGAAAATTTGCTTCTTGATTTCTAAGGGAACTTAAAAGTTTCTGACTTTGGGCATTTATTCAAATCTCTAACAGGGAGTCAGGGTCATACCACTTGTGGGACTCCAAATTATGTTGCACATGAGAGATGTGATGCATATGTAGTTTGAATTATTCAGTTACTTCTTAATAGTTCTTATGAGTGCATTCTTCTTGTTTTGCACATGCAACCTGTCATTCTTCATTTGATTGGCCTGCTATCCCCCCAAAACCTTTTTATTATTTATACTTTAGATTGCGAGCTAAGAGGCAAATATATACTTTTACGTTTGTAGTCCTGGTTTAGTAATTGGTTGTTATATTATTTTCCCATTCTGATGTCCACATTAATAGCACCATCTGATTTGTTATGTCTGCAAAGCTTGAATCAGAAAGAGGCAGG

>LNC_001430

AGGCACAACAGGCACACATCGTGCCTCGAGGAGAAGGGGCCTCGTAGAACGGTGCCTCAGGGTGAGTACAACCAAACATGCTTTGCTCGAGCTCGAGTACTATGCATTCTTTGAGGAAATTCCTTTAACGGTAATATGCTTTCCAATTTCCCTTTTCCTTTCTTCAACTTCCATTCGAAATGGTACAAATTCATACATATTCCTTGCTACTCCTTTTCACTGATCATCAATGGTTTTAGAGGTTAGAAAAAAGGGAACCACGTAAAGTTTTTTGGAGCTCTTGTTTCGATAGTTGAAACTTCATTATTTTGTGGCTATTTCGAGGTGGAAGCTGTCATGGTTAGCTGCTTGGCAAAAGGTAATCTTCTTTCTCCTTCCTTTCTTTTCTCGAGCATTGACTAGTTTCTAGCTTTGTTTCTTTTAGTTTATGATCCATGTCTGTTGTATTTCCTCATGTATTTAGTTAGTAGTTTAATTACATTTTCAACAGCTTATATTTGATGTTTCTGTCAACCAATATGAAAGTTTGAGTTTATTCTTAGTTTTTGTCGAGATGTATAAGCTTCCGTTGTTTATATGTTCATATAAAACCTGTGATAATTTGTAAAGGTTGGGCTTAATGAAGTCATCTCGGTTGAGTCTATTTGCTTAAGAATAACGTTGTCAAGCCGTCTCCCTTCAACACATCCGTTAATAAGAGTCTAAGTTTTTCTCTTGAAGCATTTGGTTTTTGTATAACTTTCCAGATAGACTTCCTGAAAGCTTTCAAATGCTCCCTTTTTATGTACGAATTTGTTTCTAAAAAATGACTATATGTCAATCAAGGAGGATACATACCATCGATTGATAGATATAAGAATGAATTGTTGTCTGCCTGCATATTTGAGATCTTATCTATTGGGATTTTCTTTACTTTGTTTTGGCTTCATTTCCAATTTCAGTCTCTTCTCAATGTATTTATGTAAGTTGGCATCTCTGTTTTATGTATTTCGAGATTGTCCAATGAACCAAATGATTGGGGATATATATTCTTGATCAGTTAAGGGCAAGATTCAGTTTTCTTTCTTAAGTCGTTATTGAAATGTCCATTTCTTTGTGCTAACAATTTCGAGCTTGCTGTGTATTTGAAATATGGGTGTCCTGTTTGAGT

>LNC_001431

AACTAGCTCGTCATGACTCGATCATGACCTTAGGCGGAATTGGTCTTGGCCGGAAGAACGAAAGATACCGGCAAGCACAGAGCAGCCGTCAAAAGGGGTTCCGTAGAGTGGTCTAACCCCAGGGTAATCCTTGGAGCTTTCTCCATGCCCGACAAAGAGAACTTGGTGTGAATCGGGGTAGCTAGCTACCAGCTCGTCTAACTAATGTACTGATGTAATTCCATGCAAATATGGCTACCCGGCGAACATGAGGTCCCACTCACTATTCATGGCCAGACAAAAAGGGACCACTAGCCTAGAGAATCAAATAGGGAATAGGCCCTGAGTTCACCCGCTTGCCCGAAGATGAAGGGGAAGAAAGGGATGCCAGGGACAGAGTAACCGGAGGGGGCATGAAATGAACCCGGAAGGGAAGCCACAGGAGCAGGAGTGGAGAGAAAGCGAAAGAGGAAAGGTGTTTTCCCATTCGATTGATCGATTCAGAGTCCCGAACCGAAGCCCAAGCCCATACTCTGACTAAGAAAAGTGGAAGTTCCTACCCTGGGAACATTGACATACGGG

>LNC_001432

CAAACATCTATGACTTGTTTTAGGCCACAAACTTCAAAGGTCTTTCTTTCTTTAACTCCATGCCAAGTCAAAAAACATCACATAAATTGAGACGGAGAGAGCATTACCTTTAGCTCAGTCACCTCCTTTCTTCTTCTTCTTTTATCCTCCATTTCCTTTTCTCCCTAGCAAAATAGTTTCACATTCTCTTAAAAAGACAATTTTCAATACTTAGCTCCAAATGAGTATATACTTACTCCCTTCATCCATTTTTACTTGTCCAGTATTGACTTGAAACACCCCTTAAAGAGCAATAAATAGAATGAAAATTTTATATATTACCCTTTGAATACAATTAATTCAATGTGTTTGAAAAATGCATAGGGAAACGATTTAGTTTATAATAAGGATAATTAGGAACTGAATGATAAATTATTACTTGATTTTCTAAACTGGACAAGTAAAAATTGATATCTATTTTTAGTATAGTGGACAAGTAAAAATTGAGAGAACGGCTATTATATGTCGCTTACGCAAGTTATGTGGAATAATAGTTATAGCAGAGGTTTATCATCATTTTTATACATACAATGCTAAGATTTTTCCAAATGAGGATGTAGCTAGATCGAATTGTTTTGACATCTTGTATTTCCACTGTGAGCTGTACAACTAGTTTTGACAACTCATGGAATGACATGACAAAAAAGACGTCTCAATTTTGACAAGAATAGCTTGAGAGATAAAATGACTATAAAGCGACAACTACCTTTAAAGAAAACTGACAACCACCTCAATCGCTTTAATAGTGTCCGTTTTGGCACCACAATAAATGCACCACAGGGTTGCAGAATCTTCTAGCAAAATTTATAAGCGGTACAGAGATGCCCCCATTTTACTCCATTTAGCTGAATGAGCCATCCAAATCTATTCTTATGCAAGAGATATACATAAACAGCAC

>LNC_001433

CAACTAATAAGGAAAGCATCACAGAGCATTCAGAAAATTAACACAAAGAATTATGATTCACCAAGAGGTTGTGTACCTATCACCTCCAAAGATCTGTAATGTGTTCATTCTTCATGGAATGACATAATTACATTTTTTAACCAGGGTGGATTGTACCACACTGTTAATTTTTCATCTGATAGCAGGTTCTGAAAGTTAGTTTAAGTTGCCAGTAACGTCTCTGAGGAATAGAAAAATGAAATGAAAGGTGATTCTTAAATTCAGTAGGGAAATGAACCACATCCAATATATGACAACATCTGTATTCCTTGCTTCCATCTACTCGATATGCTATAACTGCATGACTTACTGTTTGGTTAGGATTGAAAACACATGCTAGTCGCTGACAATGATTGAACCGTTTAAAGCCAAAAGAAAAGAAAGAATGATTTCATACAGTTACTGGGACATTTCTCTCATGTACAGTTTGATGTATGTTCCCTTATCAAGTAACCATTATTATGTATGCTTATTTATCTTAGTGTGGTATCTTAATTGTTAATTCATTGTCATGAATCTGCTCCTTGCTATTTTCTTTTGGCAACATATTTTTCACCTACGTGCAACTTACCCTTATTTTCCCACACTGTGAAAGTTTCACATCATTATCCTGAAAGTCAAGGATTATCTGTGCAGAGTAACATCTATCGTGTGTTCTTTTTCCTTTTCTCATTTCCTTCTGAAAGCATAAAACCCTAGCCCAGTAAGGTATAGAACAATAACGCCTCAGGCCCAAGCAAGTTGGGGTGAGGATTCATCATAAATTGACTAGATTTATGTTGATTATCACCAACCGCAATCCTCC

>LNC_001434

GAAATATCCTTCCCACATTCTTCCCTCTTCTATGGATTCTCTCACCCATGAATTGTGGTGTGTTAAATTGTAATGCAAGAAACAACTAAAGTAAGAATAGGGTTTAGAGTTTGTGGTTTGGTGGCTCAATTTTGTGGCTCTTGATAGTTTAAAGATGCACCTTTATTAGGGATGGAATTCATTTACAGAGTAGACTCCATAGTGAAAGTCAATTAAAGTTTATCAGGAGGATTCAGAATCTTTCTAACCAGAAAAAAGATATTGAAGCATGAAAGATCACCTTCAATACTGTTCATAGACAGTGTTAAACACAACAGTAGGCATATCAATCTAACTCTTTAACTCTTTTAACTTTGTCACCTGTTCTAAAAGCAATTTAGTGCTAGCATTTGGCATTTAGGCAAGGAAAAGCTTGATTAAATAGTGAGTGTCCCTGCACCCCATGGCCATGGAAGTTTTTCTTCAGAGTGAAGGGGTCTCATAAAAATCTTTTCTTCTGTTGGATAAACATAAAAAACTATCCAATTTGAATTCTAGACTTGCTGGAATTATGGTGGAAGAGCTACCAATCAAACAGAAAATTCCAGTCTGAATTTTTTATTAGCTAATGAAACCTTAATTAGCTTTAGATGTTG

>LNC_001435

GTGTTTTTTTTAAAAAGATGTTGATCTCAATCTCCCAACTGAACGCATGTAACAAGTTTTTAGGTCTGAAAATAAAGTCTTTTCTGAAGGAAAAATGGTTTCCAAATTCTTAAAAAAATACTAGGAATATTCCATATAACAGTAATCCATGCATTCCAATTTAACTGTTTCCTATTAAATGGATGTGTTGTGGTAGGCTTTGAAGTTGGCAGAATATAAAATATTTGCGGACCCTATGCTAGAGTTGAAATCAGGTGGTTAAGATATAAACTTCTCTTCATATAGAAATGGTTCCTACTCCCACCTTATATTTTGGTGACATGAAATCAAAAAGTATTTAATGATGTGTAGGAGGGGGTCTAGAAACAAGTCTTATAGGATTCACATTCTGAAACAAACAGCAGTTTTTTTTGTTTTTGTGTGCAAAAAATGCTCTTTCTTTTTGGCCTAGAAATATTTATTTTCACGTGAATTTGAGATGCC

>LNC_001436

AATTACACTCCTTCAATTCTTTCAAATTCATTGGATCCTTTTCCGCGTTCGAGAATCCCCCCCTTCTTCCACTCCGCCCCGAAGAGTAACTAGGACCAATTTAGTCACCTTTGAATGTTCCAATTGAACACTGTCCATTTTTATTATTCTCAAAGGATAAGATTATTCTCTTTACCAAACATATGCGGATCCAATCACGATCTTATATATAAGAAGAACAAAAGATCTTTCT

>LNC_001437

GCAACATATATCATTTACAAATGACTTAAAAAGCATTAAAAATTACAATAATTACCAATTTAAAATATTTAAAAGCTATATAATAAAATAGGTTGACTCTCAGAATTCTATCAGGCTCACATATATTGGGACAGAAAAAATAACACATATCATTTTAAAATTACCAAAAAAGTATTATAAATAATAATAATTAACAACTTAAAATATCTGAAAGACATTTTAAAAGGGTTAATTTTGTAATTTTATC

>LNC_001438

GTTTTGAACAATTCTGGGGTCCAAACAGAATCGGCGTCTTCCCGAACGCGAAGTCTGTCGCACCGCTATTCTCACCCGCCGTTGTCACCAGCGAATTATCTGCAACTTCCACCGAGTAATTTTCATGTTACATCTGTTCTATGGTGGTAAAGTGAAAGAAACTTCAGAATGAATTTCGAACTTGGATATTAATTTGTTATGATAAATGAAGATATGTCAGATTCAACGAGGCGGCGATAATGGATGAAGCTCTTTCATCTGATAATAGGACGCAGATAACCATTGGCTATTGGCTGCTGATGGAAGAAGGCGAAAGGTATGATGAACAACTTTAATGCTTTATCTACAGCAATATATGTAGCAACTTAATTTTATAATCTGGCATTGCAGGTGTAGCTTTTTTATCTTGCTTTCTATTTATCTCTTCATCTTATAATTGGGAATGCTTTACTTTAGAGTTGAGAAAAAGGTGCAACTCTCAATAAGAATTTCAGCTATTAGTGGATGCTATATAGATTGACCTCTTTTCCACGTGCTATTTCCCCTTACGAGGTGAACTGCTTATTGTGGATTTGTTCATAACTAGTCTTGCAGCGTGAATAAAAGAAGCTTTTTACTGCATATTGGTGGAGTCTGTCAAAGCAAATTTTCCTTTATTTTGAAGAATAATCATAAAACCTGCAATTTTAAAGTCTATTTTCGGGATAAAAAG

>LNC_001439

GTTTTGAACAATTCTGGGGTCCAAACAGAATCGGCGTCTTCCCGAACGCGAAGTCTGTCGCACCGCTATTCTCACCCGCCGTTGTCACCAGCGAATTATCTGCAACTTCCACCGAGTAATTTTCATGTTACATCTGTTCTATGGTGGTAAAGTGAAAGAAACTTCAGAATGAATTTCGAACTTGGATATTAATTTGTTATGATAAATGAAGATATGTCAGATTCAACGAGGCGGCGATAATGGATGAAGCTCTTTCATCTGATAATAGGACGCAGATAACCATTGGCTATTGGCTGCTGATGGAAGAAGGCGAAAGTAAAATGTGAAAGCATATGGACATTTGGGATTTTACATGTCTCAAAGCCACGATGCTCTGACGTGGGAAAAACAATTCAATTTCTACATAAGGTGCATTCCTATAGAT

>LNC_001440

GCTTTATATATACATAAAAGATTGTGGAATAGAATCACCTAAATCTTTACATTAAGAGAAAGAATCAGAGATATGATATTCTAGATTACCACAATTATACTAAGATCTTAGAGGATCATATCTTTGATGACAAGAATATCTTCTTGGAAATCCTCCATCAGCCAACAGTTAGTTTATTCTTAAGCACTTAGCTTTAATATGTCTGTGTGCTAGGGCCTAGTGATCTATGAAATGTGAGAAGAGCCATAAGGTCTAGGGTTCAAATCCCAGCGGAGGCAACAACACTAGATGCTTTATTCACATCTGCCAAAGTCTTGGTGGGCAGGCACTGTGTTAGTGGGAGGTAGTAGGTACCCAGTGAAATAGTCGAGGTGTCCACTAACAAACTCGGACACCACCGTCATCCAGGTTTTTTTTCTCTATATTAGTTCAGCAACTGGAAAGATACCATGTGTGTAGAACATTAACAAGCACTGCAGTAGTTCAAAGGTCAGAGAGCTAACCACTCCATTCTCTTCCTTTCTTTCTGCTTGTGAATGCTGCATAATGTCCTGG

>LNC_001441

GAGATGGTCTTCCGGATTTCCCAATGCAGAGACTTGAGTAAGTTTTCTACTTCCTTAGTTGTAGCAGAGTCATCGCGGACAATCAATTTACCTCCAGGTCTAACTATTCTGTCAACCTCTGCCATTACAGCAACAATTTTGCACCTACAAATGCCACATGAAAGTCATTAGTTAGTCCACTGACTACAATGGTTCTACTCCATAAGTAAACAAAACTACTGTAGTATTATATACCTCTTCTTCAACTGGGAGAAAAGATGATCAGCATGTAGAAGATCGTACGTCCTTGGGTATGTACTAAATGATTCACACCAATCATGATAAATCCCAAAAAGACCACGTTCATATATTATGGGAAGTGTATCTGGTGCATTGACATTCACCACATTCACAACCCACATTTTGAGATCTCTAAGTGCTGCAGCAAACCTGTTAATTAATAGGAGGAGCCTTTTGTTAGTATTCTTTTTTGGTGCATAAGCAAAGGTACAAGAGAGAAGTTAATCACCCTCCATATACTGCTCTCATGTCCATGGCATTTCTCACATTTGACCAGCTAATTCCTAGTCCTTTCATATACACCTTGCTTATCAAATGTTTCCAATGTTCTAAATCTGCTGCGAAGTCATCTGGTGCTGGTTTTCCATAAATTCCAACTTGTGATCGGTTTAGCCAGTATGGAGGTGTCTGCAGTCGCTCAGGCCACTCTACAGGCCACCGATATCCTCTTTCCGTTTCTTTACTTGGCACCCTATGCATACATGAATTGAAGGGGACATACCTGAAACACGTGTAATATTTCCGATCTCATTAAAAGTTGGTAAGTATTTCTAACAAATAAATATGTGGAAATTACAGTTATTCTTTGTAGATGTTGCAAATGGCATTGGCAGGCAGATTTCACTGAGTAAACAAAAAAATTGCCAGAGAACAATGAGCTTACCAGGCAGCATTGGGATCATCATCTTGTTTACACATTGGAGAGTTTGGTAGACGGGAGTTGCAGACCAGGCAAAGTAACCTCCGGGGCGAAGGACACGATTCAGTTCCAGGAGAAGAGCACCACCTTCATTGCAAATTTAAACTTTATTAAGGAATTCATATGCACAACTCAAAGGCAAAGTCAAAATGAGGACTTGAAAGTTTAAATAGTTCTAAAATATATTATTTCTAGGAAACACCTTCCTCATGCCAAGGGACTCTGCAGCGTGCACAATGCACAAGGTCAAAAACTCCACTTGGAAATGGCAATCTCTGAGTACCCATCACAGCAGATATTGCAGGTAATGCAATGCTCCATGGATAAACTGAGTACCTCCACCTGGGAAAGTAAGGAATTCACCAGAAACCTTAACCCAGTTTTGGTGCCCCTTCACCTCTGCTAACAATGTATGAGGCACATTATGGTACCATATCTGGATAAAAGCAGAAATTCTCATAAGATAAACATTCTCCCAATCCATATTAAGTACTAACAAAGTAGTTTAAGTAGACAACTACCTTA

>LNC_001442

CCTCTCCAGCCGCCTCCTCCCTCTTTTCCTTCCCTTTCGTTCTCTCCATTTTTCCGGTGAAACAGCCACCGGCGAGGCCGGCAGCCGCTGGCCTCCGTCGCTTCTCCCTCCAGCTTCTCTTTTCTCTCATTCCCCTCTCCTCACATCTGCTTCTCTGTCTCCTTTCTCCTCGTCTGACCAGCAGCTCCGACCAGGGAAGCAGCTCCAGCCGGACAGCAACCAGCAGCAGCTCCAGCCGGACAGCAACCAGCAGCAGGCAACACAACCAGTCCCTCCAGCCGACTCCAACCAGCAGCCAGGCGAACCAGCGCAGGCAGCAGCTCCGTGACAGCAACAGCTCGGCGAGAAAACAGCAGCAACAACTGCTCCATCCAGCAGCGACGGACGACAACGACAGCTGCTCCGGCCAGCAGACAGGCGAGCCAGCGCCAACAGCAACTCCACGAAGCAGCAACCAGCAGCCGGCGCAGCTACTCCAGCAACCAGCGCAATAACTCCGGCCAGCAACAACACCAACAACAACCAGCAGTTCCGGCGAAAGCCAAAAACTCTGGCAAACAGTAGCAATCAAAAAGCTACCCCAACTACGAGTTCCGGTGAATCCACAAAGCATTTCGATAAGGACAGATCCTTACAGATCTGTCCCAGGTTTCGAGCTTAGAGTTCAAGGTGTTGAAGAGTCTCCTAGAAGCGATTTCCAGACGAGACTATTATGTTTAAGTTTATTTGAATGGTGTAAATATTATCCACTTGCTTTTAGTTTGTAATGTTCGTATAACTCTAAAACTCTCTCTCTATATATATGAAATAACTTGGGGATCGTCTAAGGGGAGGGGGATTTTTACGTGTTACTTGTTATATTGTCACAACTTACTTGTTAGAAAGCATGCATAGAGGAGTGTGATTTAATTACATGCTCAATCCTCACTCTAAATCTATTTATAAAAAATAAAAAAAGATAAAAAAATAAATAAAATGAAAATTGATCTGGCCGGTATTTTTATTACTCTTAAATGTTGTCCAATTTGTAAAGCATATGTTATGTTGGCCGCTGTTTCTTAATGTCCATTAATTATGTGTTAAAATAAAATCTTTTTTTTTATAACACCTTTAGCATAATATTCATAACCTAAAGTGTTTTGACTTTTACATGGTTATCTAGTGTCGTGTTCAGTAAAATCACTTCCCTCTCTTATAAACACATGAAAAGAGGTAAAAAATAAATGAGTGTTCGGTCAATGATCTGTCTCACATTAGAAATAATTTAGAAGCCATGTTTAAATGTTATCCTAATAATCTGTCAGCATGTTAGTTTGTTTGGTTCAGGCTGTAATGAATAATTTTTGGTAATTCATTTAAGGGCATACTTTGGGCATAGACAGCATGTAGGATTTAATACATAATGTAATTTCCTATCTAAAAAATAAGTCCTACCTATAGGTGTCGCATATTAATGTTTGCATGTTAATATGTTAAAGCTTATGATTAATAATTTCAAACATGTTAATGAACTAAAAATCCCTATAGGAATTAAATAACTTGTCATATCTATGTGCACATATCTGAATATAATAAATTACATGCATGAGTCTTGTTGCATCTTTAAACACTTAAATATCTCACTGCCTGTAAAAATTTGCATAGTAATAATCATTGTTTTAAATTAATAGTGAATTGACCGCTCAAAGATTCCTTTGCGACTATCTGTTTTCTGTCACACAAAATTTTTTTTTATCAAATTAGCTACTCAGTAATTTCTGCATCTATTAAACCGACTGCTACTCAAAACAAATTTCACATTATAGTCTAATCTGCTACTGCATTAACATGCTATTTTAGCATAATCTGTCACTTTAGATTCGCGATCCTAATAAAATTTTCCTCAAAATAAATTTAGTTTTCTGCATTATGAATAATTGTCTTGCCATTTTTAATCAACTAATAACTAAATTCTTCCTGCATGCTTTATTAATAAAACAAATAAACTTTGCATTTTGTCATTACACTCTGTCCCTGTTAAATTATTTATCATTATTATGAAGCTTGTTTACGTGTTTATATGCATACACTTAAGCATGAGACTTTTCACTTTGTGTGTGTTTGCTAGCATGTTAAATAACCTAATCTAGAGGCCAAAATGAGACGTATGTGCTAAGTGCTCGTCCAAACTGTTTGAATTGGTGTCTAGGTGGGCCTATTATATTTTATTTTAGTCTAAAACCCTTCCTATTAGTCGTCCAAATGCCTCTTGGACCTTAAGCGGGGACGTTAGATACCTCTTACAGACGAAAGCATGCAGTGATGGGGTAGGGGTAGTTTAGGCCCTATGGAGATGATGACATCGACTCGGTCTTAATGACAAACCTTGGTTTTGTCTGTCCCTACAAGAAGCTGATCGAAATAAAGAAAGTCGAGTGCTGATCCTAAAGTCCTTCAGAGTCTCCCTTTCCCTTTCTCTTTTATATGTTTATTATAATTATTTGGGTAGTTTAATAAAAAATAAAACTTAATTATTTGTTTATCTACTTGTGTGAATTCTGTCTCCTCG

>LNC_001443

GAAAATCAAATTAATTTGGGGTTAAAACTAATCCCAATCTTATATTTCTCCGCCAACCTGACGAGCGACCAAACACATCTGAATTCTCATCTCTTAAATTTTCTCGTCAAAGTTTCACAATCCCAAACACTTCTCTTCTCGTGAAGTGTTATATGTTACGTTGGGTAGGGTTGGGTTGAAATTTGATTTGAGTACTCATCAGATCCTATGTGCATTGAATTCCTCTCCCATTTTCAGTTTGGATGGTTTTTGGGGGAAACAGAAGGTCAATTTCAGTGAGAAGTGGGAGGGCTCTGTAATTTCTTGATCTTGATAACATGGAGTCCAGGCGCTGTGCAAGAGAGGGAAATTAGCATCTTTTTTGTAATGGAGTTGTTGCAATGGATTACCCCTGTGGGCTTTCAGAAGGCCTTCCTTCATGGTTACATACCAAGTTTTGACTCGCTGGTTGATGACGTCATTGAGCATTACTCAAAATTTAAAGACTGCCTATGATGTCATCTCTTACAAACATAAACCCTGTTTACAGACCGCCCTGGAGTTGCTGAATGCCACAAAAGAAGTAGAGGGACTACTAGCTAGAAAAGGTGCGTCACCTGTTTCAACTTTTTCTTTGAATTAGCGTAAAAGTTCATCGCCGTGTTAACTGTTTAAAGGTTGATATGTGAATATTTTCCATTTATGGGAGTTGCTCCACAGTCAGAATGTGTTGAATTCGGTCATATGCTTATTTTTTCATGTTTTGCTCTTGTAGTTTAGGAAACACTTGGCCTCATTTAGCTTACTAAAGAGTCACGAGAACTGTCCATTACTGTGCACACAAGTTGATTAAGCATCCCGTTTAAGTAAATGCACAAATTTTTTGACACTATTGATGTACAAAAGTTAATAAGTATCATTTTAAAATAATTATACAATTTTTTTTGACCTTATTGATGCACAAAAGTTAATCAAGTACTGCTTTTAAAAAGCTGTATAAATTTTTTGACACGATTGTTGCTAAAACCTTAATTAAACACCCCAATCTCATTGATGTTTTCTATTGTTCATTCCTTACTTTTTTTGTTGCCTCTAGATCTTCATAACCATTTTAGAGTTGCAATTTACTCGACGAGATTTGAGATAAGCAACTTGAGATATAAGATCTGGGCATTCGGTAATTCGGTTCGGTTTTGATTTTTGAAATTGTTGTACTGAATACCGAACTGAATTAATTTGGTTTGGTTTGTGCTTTTTAAATTCGACTTGTTTTTTGTAATTCGGTTTTGAAATCACTGGTCGGCGCTCGCTGGAGGCTTAACTTGGAATTGAAATTTGGAAGAGAAGGCTAGAAGGCAAAAGCAGAAGGTGCGGCAGAAATTGGAAAAACAAAAATGAAAATCTAATGCTAACTTACTAGTTGCTGAGGGTAAAAAAGGAAATAAACTTAAAAGGCTTAATAGTATTAATAATTACATAATATTTATTATAGTATGTATATTAGTGTGTATATTTTGGTTAAACCAAAATATCAAAAATATGAAAAAAATATTATCAAAAACCGAACTGCAATATCAAAAATACAAAAATATGTTGCCGAAATCGAAATATCGAAAAATTTTGGTTTGGTTCGGTAACATGTTTTTGTGTTTTCGGTATTTCAGATCGGTTTTCGATAATATGTTCTCATATTTTTGGTATTTCAGTTTAATGGAAATATACACACTAATATATATATACAATAATTAATAAATATTATGTAATTATTAATACCACTAAGATTTTAAGTTTATTTTCTTTTTTGCCCTTAGCAATTAGTAAGTGAGAATTATGTTTTCATTTTTGTTTTTCCAATTTCCGCCTTCTGCTTCTGCCTTCTAGCCTTCTCTTCCAAATTCCAATTCCAAGTTTAAGCCTCTAGCGATCCCCGACCAGGGAAATAACCAGCGACCACGACCAGTGATTTTAAAATCGAATTTCATAAACCGAACCAAAGTACAAAAACTAAATCGAATTTAAAAAATTGAAATGAATTAATTCAATTTGGTGTTTGGTACAACAGTTCCAAAAATAAAAGAACCGAACTAAACGCCCAACCCTAGTTGTTTGTGCACAAAAGTCAATTAAGCACCACTTTTAAGTGACTGCGCAATTTTTTTTGCACTATTGGTGCACACAAGTTATAATTAAGCATCACTTTTAAGTAAAGACATATTTATTTTTTTACACAATTGGTGCACACAGGTAAATTTAGCGCACTTTTAAGTAATTACACAAGTTTTTTTTTTTTTGACTATTTTGATACACAAAAGTTAAATAAAACACTGATTTTAAGTGCACCGTTTTTTTCTCTCATAATATTGGTGCACAAATGTTAATTAAGCACCACTTTGAAGTGACTACACAATTCTTTTTGACACTATTGTTGCACCAAAGTTAATTAAGCACTTCTTTTAATTAGTGAACTACCCAATTATTTTTTCACATTGGTACATATAAGTTAATTGAGCATCGCTTTTAACTAACTACATAATATTTGACATTGTTGATGCACAAATGTTAATTAAGCTTTTTCTAAAAAATAGCTACACAATTATTTTGGCCCGAAATCTCAAATAAAAAAACTTTTACATTATACATCACATACAGAAAAGAGAAAAATAAAATTGGTAGAAAAATACCAAGATCAAGAACAACATAATTGAATTTCCCAGCGATGTGAGACTTAACAAACGAAAATAAAAATACATATACATTAATCCACAAGCTGGGGGATTTATCTATTTTTTTTTATAATAGCGAAGGTACTCTTCGTAAGTGTAAGGTGACCAAATCCACTCCATAAGTTCAAAATCCCAATTAGCTTTAGTAGCTAATGATGATCCTTCATCTGGAGGAGGAATTTCC

>LNC_001444

GGGATTTCACTTTTGGGATAACTGTGCAACTTGGAGAAACATTCAACTTTGTAATTCTCCTGATGAGACCAGAACAGTTCAGGATGGTGGTGGACTGCAGAAGCAATGGATTGATAGAGTAACATTGCTAACATTACATAATAGACAATTTCAAAGGACTGATACTTGTTGCATGATCTGGAATTATCTGTCGAAGCATTAAGTAGGCCCACCTCTACACTTGAAAGCAATAAAACAGACATTTGGAGGAGGGAGAAGATGAACATACTACAGGGATTTGTTCCATACAGCACGGAGTCGGTTTGAACTACTGCAGAAATGTTTTATACCACTCAATGGAAGATTGCATTACAAGGACCTGTTCTTACCACACATTGACTTTTAAAATTAAG

>LNC_001445

AGACAATTTAAACAAAGGAAAAAGTATACCATCCTCTGACCATTTCTCTAACAAGTCTATTACTTCAAGGAGATAGGGGAGAATTGCAAGCATGAGATGCCGGAATGGAGCAGAAGCTTGAAATTCAAACAATCTATCAAATTTTTATATCGTTATACATCAGACTAGCTTTCTGTTACATATGAATAACAAGGCTCAATCGGACATAGAAGACGGTCTCCATGTTCTAAGCGTCTGAGAAAAAAAGAAGAAGAAATGAAGAAGAATATGGAGAAATGATCTTGGCAAAAGGGTACTAACAGGAGAAGCTTTACAAAGAGTTTAATGGTTCTTTGTCAAAATCTTAGCTTGTGACAACAATGCAGGGAGGAATTTTGAATGGATGGATTTTTCTTTGCATCAACTATAGAATATTTATTTAGTTTTTGAGTTCCTCTTTATTTAACTAGAACTACAGTGATTACAATATTCAATTTTTATATGTTATGTATTATGTAATGACAATTAAAATGTTTTTGATACAGGTGTTCAAAAGTCATTTTTTCTGCATTGGAAATTAAAAAAAGGTCAAAACACAAATATACGTGTTGGGCCCGTGCTGGCACGGGCCATGCCCATCTAGT

>LNC_001446

AGACAATTTAAACAAAGGAAAAAGTATACCATCCTCTGACCATTTCTCTAACAAGTCTATTACTTCAAGGAGATAGGGGAGAATTGCAAGCATGAGATGCCGGAATGGAGCAGAAGCTTGAAATTCAAACAATCTATCAAATTTTTATATCGTTATACATCAGGAAATTCTACTTTAAAGTTTACAGACTAGCTTTCTGTTACATATGAATAACAAGGCTCAATCGGACATAGAAGACGGTCTCCATGTTCTAAGCGTCTGAGAAAAAAAGAAGAAGAAATGAAGAAGAATATGGAGAAATGATCTTGGCAAAAGGGTACTAACAGGAGAAGCTTTACAAAGAGTTTAATGGTTCTTTGTCAAAATCTTAGCTTGTGACAACAATGCAGGGAGGAATTTTGAATGGATGGATTTTTCTTTGCATCAACTATAGAATATTTATTTAGTTTTTGAGTTCCTCTTTATTTAACTAGAACTACAGTGATTACAATATTCAATTTTTATATGTTATGTATTATGTAATGACAATTAAAATGTTTTTGATACAGGTGTTCAAAAGTCATTTTTTCTGCATTGGAAATTAAAAAAAGGTCAAAACACAAATATACGTGTTGGGCCCGTGCTGGCACGGGCCATGCCCATCTAGT

>LNC_001447

NTGAAATTAAACCTTGTGCCCGGCTGTTATCTGCAAAAAATTTCGTCAACTGCTTATTGCTTTGGCTTCTATTCTTTGATTTAGTCCCTTGTTGGTATTTGTTAACTACAGAACTAAAGGAACAAGCTATTTGTTAACATTAGTAGAATGCTCTTGTTTTGCTTTCCAACAGCTTATGTTAGCCTGGAAACTGGAAGTGGAAAATCAATGTTTGGAATTTTACTCAGTCATCGTCACTGTATAATGATAACTAAGTGGTTGGATGCCTGCTAGATTAGATCCACAGAACTCAAGCGTACAAGTGATTCATACTTGGACTAGGAGTTCTCTTAGTACTGTTTTGTTGCTGCAGTTTCTTTTGATAATTTTTTTTGTCGTAAAGTTGCTCTATTAGGTGCCAGTTTCACTATTTTAACTCTGGTAAGTGATTCTTCTGCTTTTAAGAGTACGCCCATGGTTATCTAGTTATGTAAGATTTCAAGGCCTGCAAGTAATGCAATCTGAGATTGCGCATGTCAGGTTTCTGGGTATGGTGAGTCTATTGCTCATGTCTCAATTGTTCCAAAAAGAAAGTTATTTTTTTGTAATTATAGACAATTTAACTTGAGAAAATCTCTTTGTATCCAAATAAAATGATTTATAGTAATGCAAATGGTCAAGGAGTATTTTTATGTCACAAATATCACCTATCTCCCTTTTTTAAAAACGTGTGTCAACTCAAACAATGTCACCTGCAAGTTTTGGCATGCTTCTTTAAATTAAACATTAAGCGTCCTCGCAATTACCAAAATGAGTATTAAGTGAAATTGTTGAGTTGTACTTGAACTAACTTGGATTGAAATTTGAAACACACAAAAGCTAGTTTGGTTACTTCTATTACATCTTTTTTTTTTTTTTAATTTATAAAAC

>LNC_001448

CTCCATGTCTGCTTTCCACGAATCTTCATCTATTGAAAAGGAAAAAGGCGGAGCTATTTCTCTTCAATCTTTTCAAGTACTCTTCAATTTCAAGCGTACCAAAAATGAAGAGGCATAACACTTGAAGAGGCCCTACTACTAAAGTAATTCCACATGACAAGTTTCCGTTTCATTGTTAGAATGAATTTTTTTCCAGTTTAGAGAATATTAGTTTATTCATAGATGTATACGTGGTTATAGTGTCATTGTGTTATTTGGCAACTCTTCCCCTCTATGTGGGGAAAACTCAAATAACCATATTGGTTCCTTCAGAGATGTATAGTTATTAATTAATAACATTCAAGTAGCATGAGGTGGTTATAGTGTCACAATTATGTTATTGGCAAAATTGATGTATTTAAGCTATCATACATTTTTTAAGGAATTGTATCTTAGTCATGACATAATTAATCATGTTTGCTGAAAATATGTTGAAATCTTTTTGCTTGAGATTTCAGTCTCAATGTATGATTAGTTAACATTAGTTGAAGGAAAAGTTTCTTCGTTCAAAAGTAACTGCCATTCTAAATAAGGACTGTGTTGCATAGCTCGGAGGATATCAGTACGATCCTCTATTACTTGTCAACACATGCTTATTTGATATGTTTTAGTAATAAACACATCTTATCTGGAATCAACTTTCCCCTTTAAAAGCCAATCTTATCACTTGGAAAGTGAAATTGCACTCTCTACTTAGTTTAGTGACATAATTTATGCAAATAATACTCAACGCAACAGCCAAAAATCATTTGGAAACACGCTTGTCACAGAAGTATATTATCTAGTCTAGTTACAAGAGTAAGCAGACGAGAACTCCAATCCTATATGGTGATCATTTGTAAAAACCTTGAAATCAATTTATTATTACTAATAATAACCAAGTAAAAAC

>LNC_001449

GCTATTTCTCTTCAATCTTTTCAAGTACTCTTCAATTTCAAGCGTACCAAAAATGAAGAGGCATAACACTTGAAGAGGCCCTACTACTAAAGTAATTCCACATGACAAGTTTCCGTTTCATTGTTAGAATGAATTTTTTTCCAGTTTAGAGAATATTAGTTTATTCATAGATGTATACGTGGTTATAGTGTCATTGTGTTATTTGGCAACTCTTCCCCTCTATGTGGGGAAAACTCAAATAACCATATTGGTTCCTTCAGAGATGTATAGTTATTAATTAATAACATTCAAGTAGCATGAGGTGGTTATAGTGTCACAATTATGTTATTGGCAAAATTGATGTATTTAAGCTATCATACATTTTTTAAGGAATTGTATCTTAGTCATGACATAATTAATCATGTTTGCTGAAAATATGTTGAAATCTTTTTGCTTGAGATTTCAGTCTCAATGTATGATTAGTTAACATTAGTTGAAGGAAAAGTTTCTTCGTTCAAAAGTAACTGCCATTCTAAATAAGGACTGTGTTGCATAGCTCGGAGGATATCAGTACGATCCTCTATTACTTGTCAACACATGCTTATTTGATATGTTTTAGTAATAAACACATCTTATCTGGAATCAACTTTCCCCTTTAAAAGCCAATCTTATCACTTGGAAAGTGAAATTGCACTCTCTACTTAGTTTAGTGACATAATTTATGCAAATAATACTCAACGCAACAGCCAAAAATCATTTGGAAACACGCTTGTCACAGAAGTATATTATCTAGTCTAGTTACAAGAGTAAGCAGACGAGAACTCCAATCCTATATGGTGATCATTTGTAAAAACCTTGAAATCAATTTATTATTACTAATAATAACCAAGTAAAAAC

>LNC_001450

GTAATTTTTTCTTCTTCCTCTTCTTTTTTTTTAGATCTGATTTCTTGTGATAAGTGGTTCTCTTCAGAGCTTCATCGCATTTGTGCTGAACCGATTTATAAAGCTTTGGTTTTAGATTTAGTTTTATGGGGTGATGAAGTTTGAATTTGAGAAGACGCAATAAAAAATAAAACGAACTAAACTTCAATGTTGAGACACAACCCCCAACTAATTAGAGTTCTGCCAGTTAAGTGTTTTCCTAAAAATAATAAATATGTAGATTGTTAAGTAGAAATTGTCATCAGTTACCAAAGCGGTGGTCCACATAGTAACCTTTGTATTTAACGATATCTTTATTGATGTTCAAAGACTAATATTAGGATTAAATTAGAATACATTTTGTTAAGCCACCACAACTTGGCTATTTATATATGTAAATTGTAAAGGGAAGGAAAAGACACATTTTGCTTTTGATTAGCCATAGTTTTGAGGAGGTTGTATGAACATATAGGGAAAAGAATTACCGTTGTGCCTTTTAGGCAAGCTTCGTGTGATGAGTTTTGTACCTGAAACTTCGCAGGTAAAAGATTAGCAATTCAACTAATTCCATAAGCA

>LNC_001451

ATTTGTATTCCTGCAGTCCAATGTTGTTAGTGTCATTTTCCTATATATATTGCTTCATTATTGATGCTCTTCTTATTTTAACGTAATTCCAATGAGAAGTAGTATTTGTTTTGCTTAAAGAGGCTGAAGTGTCCATTCCTTCCATGATCAAGTGATTTTAAGTACTACCAGGCATCCTCCCACTTTCATCCATCTTTATTACGTTATGTTTCCATCACAATCAAAATGCATCAGTATAATCCTTGCACTAACAAAAATCTATTTGAATCTATCTAATTCTCAGCTCATCGATCAGTTTAATGCATATGCACCTATAAAAGGAATAAGTGTGTTTTATGTTCGTATAGTTTGATTGTTATATTAATTCCTCCTGTTCACATAAAAGACATTTTTGTTACACTCGAAAATTATGACTTACCTGGCTAAAATTTTGGATTCATATCGATGGGCACTTGATTGAGTAATTTCCAAGTTTGTTTTTTCTTCTACAAAATTCTTAGTGGCTAAATAGATGGTTCATCTGTATATGTATGATGCCACCTAATGACAATGTTGGACTACACTCCTCTCTTTTCTAAAAGAGAGTAGATTGTACATGTTAAGATCTGTTATATTTATTTAATTCCACTCTTAGTTGTG

>LNC_001452

CTTAAGACTTCCTCCTCAGTGAAGTTTTCCTGCAATCTCACCTTGTCTTCCACTGAAAGAGTGGGACAATTATTGAGCATGCTGGTAGGTCTCTAGTGAGTGGTCTCTGAATACAACTTTTGGTAGAAGTCAATAATGGCACTTTCTATTCTTGTAGACTTCTCGGTTAATTCGTCTTGTACCATAAGTTGGTCTATGTAGTTGAACCTTTATGAGTGTTTGCAACTTTATGAAAGAACTTGGTGTTCCTATTGCCTTCCTTTAACCATAAGGCCCTTGACTTCTGCCTCCACGCAACTTCTTCATTTTTAATCAGACCCTCAAATTCTAAGGTTAAAGCTGCTTTCTTGGATGATTCTTCTTCTGTGAGAGGTCTATATTCCAGTATCAAATCCATTTCAGCCAATTGTTCTAAGGTTTGTTTTCTATGGATCACTAGGTTGCCAGCCTCACTTCTGCTCCATTCCTTTAGTTTATGTTTGAGTGCCTTC

>LNC_001453

GGAAATTTGGAAAATTGATTGGGTAATGTTTCAACCTCAGTCGTCTCTTAGGCTATAATCCTAGAAAATAGCTAAAAGTTGAGAATCTCATCAGAGGGGACGATAAATTCATTCCAACCCCAGCTTCCGGCCGTCAGAGACTCCTGGAACTTGGATGGGGTGGTTTTGGAAGGTTGACTTGACCCCCAGAATTTAAAGAGCCCCTTATAGCAGGGAAGTTAGCACTTGCTGGCACCAGAATTCATTGAAAGATTCCAACTTCTGTTTGGTGCATAGCACTGTGTCAAGCTTCTGATGATGATGTGGCCTCTTAGTTGGTTGCTTGCTTAAGAATATCTAATCTTCTTTGGTGCTTGATTATGTGCAACACCCACAGAAATGCAAATGCAATGGTTGCCAAATAACGTCTAATGGTGAAACTCCTCGTCTTTTTGACGATGCTGGTAAATGCACAATGAATGTTTCTTACAGAAGCTCTGGTAAACTGGTGG

>LNC_001454

GTTAGGGTTCCCTATCACCATGTTAACGAGAGAGCTTGCAGGTTTCAACATAGGATGATTATTTTTTTTGCATTATTTGTAAATTTGATTGTTGGTTTGTATTTGAATTGCACTAGGAAATAGGATCTGTGGCTGCTAAAGCATGCATATGCCCAGACTCCTAATATATAAAAAGTGCATATACTGATTTTTATAGGGTACCGCTTTGCAGCTAGGAATTATCTCAGGTCTATGCTGCCTGTATCGCCCT

>LNC_001455

AAAAAAAGAAAAAAAAATAACATGTCAAATACCGTGACTTATAAAAACTTAGCCTGCTAGAAGTAATGAATTTGGGATATATACCTTCAAAGTAAATTTCGAAAATTTAACATCTTTAACCTACTTATTGACCAAATGAGTTATTGATGGCTATAACCAGGTGAAAATAAAAACTCTTGCTATAAACTGTGGCGCTTGCTCCTTGTGATGTCTTTCCTGCCTTCATCTCCAGTTATGGACTTCAACTTCTCTTTGTTCTCCACGCATGCTTATACATGCCACAACTAAAGTGACTATGCTAAGAATTGCTGTGTGTCCCTTTTGATTCTTCATGGCTATGATTATAGTCAAATATCAACTTAAGTGGGACTAGTTCTACTTTTGTAAGTCAGGTTCACTAAATATTGTCAGAATAGGAATACGTATATACAATCCTACTTAGAATAGGAACAGTAATCCTAGTTAGAAAAG

>LNC_001456

TAAACACTAACTCTCAAAACCTAGTCCACTTTCACTCCCAACTGCAGATCCACCCCTGACCCACCCCATCGGCGCCGATCCCCACCACCCATCCCCGACCGGCGCCACGCCACCTCTCCGACCGGCGCCACGCCGCCATCCCCCACCGCAGCCACCCTCCTTCGCAGCAGCAGAGGACGTCGCGGCAGCTGCAGAGGGAGTGACACCCAGCAGCAGGAGGTCGCGCGCCTCCCTCCCGACCGGCGTCGCCTCCATCTCGGCCAGCACCTCCATCCCCGACCTTCTTAGTGAAGGTGAATTTTTAGAACCTCCATAATAAATTACTTTTGTTTCTATTTAGTTGAAGTATCTCAAAATTAGAGTACTAATATACTTAGTTTTATTGATTGTTAGTTTGATTGATTGTTAGTTAGTAAGTGAATGATTTAGGGTTTTTGATTGTTAGTTTGAATTAGGTATTATTTGATTGTTAGTTTGAATTAGGGATTATTTGATTGTTAGTTTGTGTTTGTTGATTGTGAATTGAAAATTTTAATGTTAGGGTTAGTTTGAATTAGGGTATCTTAGGATTTGTTTGAAATTCGGTATTTCTTAAGTTAATTAGTTTGATTTTGGAATTGTAAGATTAGATTGAATTAGGGTATTTAAGGAGTTGTTTGAACTTGGGTATTATTTGAGTTAGTTTGAATTATTATTGAGATTTTAGGACTAATAGTTTGAATTTGGGATTAGGAATAGCTAGCTAATTTGAATTTAGGATTTTAGTACTAGTTAGATTTTGGGATTTTAGGACTAGTTTGAATTTGTGATT

>LNC_001457

AAACCCATTTCATAAGTCAAATTTAGGGTTTTGGTGAAAACATGGGTTCTTGAAGAAATTTCATATAAAATCATCTTAAATTCATAAATAAAACCGTAATTCATCAACATAATGACTTTGAAAACGTTTTACCAATGAACCCATGCCTAGAATAGAAAATAGAACTTTTGTTCTTTAGAAGACTTTTGAAACCCTTTTTGAATGGACTCCTTGAAAGGAAGGATAATCAAGGATCAAGGGTCACCATACCTTAGAGATGAATTCCCCACGAAATTGAAGAAGAAAGCACATGAAATCTTCAAGCCTAGCTCCAACTTGACCTTGAGCTTCAATGGTGTTCTAGAGAGAACCTTTTTGAAAGAGGGGTTTTGATTTTGAAGAATGAATTCTAAATGGAGGTTTAATTATATTAACATACTTAAAATACCTAAAAATACATAAAGCAATCGACCCATACCAAACAAGACTTAATATCAATTTACCAAAAGCTCCTTGCCCAAGCCGTGTCACTTGACAGCAACCCAGGTCCAATCGACGGGTCCCCCCATCCACGGACCGGGGATGGGTTGACGGACCGTAATGGTGTTCCGTAC

>LNC_001458

CGAGTCCCATGTTTTGTGGTTGTAAAGTTGGTTTGAGGAAAAGTTGGTAAAAAGTTGAGTTTTTGAGAAGTTGAGTTTTTATGAGTTAAAGTTGGTTAAAAAGTGCTTTTTCGAGTCATTTGGAGTTTCGGGACTCGGATCGGATTTCCGTCGATTCCGGCAGTTTTAGAATGTCGAAACGGGTCTGTGTGAAGTTACGGAGTTGAATTTGGAGTTGAAACGAAGAATTGAGGTCCTAAGTTGCTAAAGTTGTGAAATTTTGATAAAGAGTTGACTTTGGTCAACATATGAGGTTCCGAGGCTCAGATTGAAATTCCGAGAGTACCGTTGGTTTCGGGAGGCGATTCTAAGTCTAGAATGAGTCTTGGTTGAATTTTTAGACGCTCCGTTTGAGTTTCGAATTTTTT

>LNC_001459

CTCCATTCCAATCTTCATCAAGAATTCTCCAAAGTTGAGGTATGTGGGTTGATTGTGGAAACCTTCCTTTCCACAAGTCCAACCATTTATCCTCTATTTTACTTCAAGTTTATGGGTCCTTATGATCATTTATGAACTCTTGAATTATGGAATTACTACTACACATTCTATTATGGTCTATTTTTGCATATTATCATGAAATGGAATTATTATGTGATGTTTATGGTTTTCATGCTTCCATGATCAAATTATGAAGGTTGTTATATATGTATATATATATGTATGTTGTTGGTGGG

>LNC_001460

GAGGAAGCTCAACATGTAAAAAAGTTGATACAAGTTTATTTTGCTTCTTTCATCAAAGTTGGAGATAAGGTTGGTGCTTCTAAATTTCTAAAGTTCAAGTACAATTGTTTTCAATAATGTGTTTGTGTCCATGTAGTATTGATACTTAGCTTTAAATTTAGAAATAATATATTTGGTAAGCATAAGTACCAACACTAGTTGATCCTATTGATGACAAAATTGATTTTCTTGCAAGATCGATTTATA

>LNC_001461

CAAGAATACTTGGTTGGACGTAGTTTAGGATCGATGTAATTGAGACAACTAAGTAGAGGATGGGAAGATGTGAGGCCGGCCAAAGGTGGTTCTATATAGCTCTTACGCTTTTACAACTCGTACTATTTTATTTGAAAGTGAAATCGTTTTTTTTTTGGCATCATCCGGGATTATCAGTCCCGGTTGGAAAGTCTTCCAACTTTCCAATTAGGATTTTCCGAGCACACAAATTGATTTTATTTGAATAATAAGGGTAATTTGGTTGTTTGGTTAATTCTAAGGATGTCGAATGGAGCACTAACAAGGGTTGTGTTCGAAGGTCAATTGTGTTGTAGCTGTTAAAGGCTACTTCACTATTGGTCAAGAACTAATATCTTGTTATAATCTCTAGTTCTATCCCGATATCGGTTATCCTTCCCAGAAGCTTCGGCTAA

>LNC_001462

ATCCTCCTCCGCGTGGCTAATACAATTTTCTATCGAGGTTATTGGTGAGTCCTCCATTCCGGGGACGTTGCTTATGATCTTTTGCTATGTATAAGACTTTTCCTTTTTAGTTATGTTTTGACTATGAGGGTGAGCCCGGTAGTTTGTCTTGGCCCCCGCTAAGTACCCATGTTTAGAGGTGGTGTTGGACAAGTTGTGTATTATGCTTGAGCTTGACTCATCTATGGTATTTATGTATCAT

>LNC_001463

GAGAAGGGACGGATTTACCCCCGAACTTTAATAAATGGTACGTCTATGCCCTCCGTTATACTTTCCGTCCACATAAGCCCCTGCCGTCCAATTATAGGTACACACATACCCCTCTCACTAACGGCCCCCTCATTTCTTACACGTGTCTTAATCCTATTGAACTACCCGTTTGACCCTTTTTTTTAACCCATAACCCAACTATCCGCCCCATAACCCAATACCCACCCAATTTCCTCTAAAAGGAATCCTAATTAAAAGACCCAGAATATGATGTTGAAATTGTTGGTGGTGGTGCTGATGATGCCGG

>LNC_001464

GGTCAAGGGGCTAAGTGACTCTAGAAAGTCGTCTTTTGTATCGCGTCAAGAGAAATGACATAGATAAGATCATTGCTAAAAGAGTTTCATTGTCGAATTGATCTCGAAATTGGATCCCAATAGTAGCTGCTGCCCAAAGGTGCTTTATGAAAGCCGATCCATAGCAGTGAAAGTACGATTGATTCCGTCGATCGAACGAAAACCGCTTCTTGCTTTTTTGGCTTGACTACTCTGCTTGCTTAACACAAGTCTTATTTAACCTTCACGGACCACTTCACTACCCAGCAAGCTCCGGCTCGAAATCAAGAAGCAAGGGCGCAGCTGCTG

>LNC_001465

CTTTATGAAGTTGTCGAATGAAACTCTTGAAATTGTAAAGAAACTACTATCAAACGTTCTTAATTTGTTGGGTGTTTTCTTTATTTACAGGAAATTAATAAATCTGCTCTTTGCCATTGTTGAAATTTCCCAACACAGAGAAAAAGCAAATACAGTTTAGGAAAATGACAACATCCAGCTGATATGAATTTGAACGAAGTCTCACCTAAACTTCATATGGGACACTGAAGGATGCTCCATATGTAATGGAGGCATGCAAAAGTGTATTCCTCACACTTTCATCGATATTTGTTGGGATTTATCTTTGCTTTCTCCGCTACTTGCCCTGCTTCTTCCACTTCATTCATTGATTTCAGATAATATCTCCTTCAGATTTCTTGTGATTTGTCCTTAATCTATAATTTCCTCCGGCTAGCCTTTCTGCTTTCCTGCTTGTGTTTAATTTTGGTCATTAATAACCTTATTCTTGCTACTTCGGATATAAATTAATTGGTCTAGCTTTCACCAATTTTCCTACCTTCCTGTTGGTTAACACTATGACAAAACTGTTTTGAATTTTAGAGTTTCAACTTAAGTCATTGGAGCTGACTGGCCTTTGCATTCGATAAAAGACAAATGAACTTACTGCAACGACGAACAGTGTGCCTTCATTACCCCCTCGCTTGGAAACATGTTAGTACTCTCAAAATTTCTCACTTCATAGATCTAACCTGAAGTGAACTTGAAAGATTCAATTCTTTTTTTGACAAAAAGTCCCTCTTCTCATATTACAACGTCTAATTAAAATTGAATCCACCGGTGAGCAGCCTAGAGAGGAAAATAACGTTTCATTGAAGCCAAACTCTAGCTCCAAAATACAGAAGGTATTGCCCCCAAGGCCTAGCTCGAGTGGCAAAAGGTGGAGGATTTGTGACTTAGGTCGCAGGTTCAAGCCCCACACCATGCAAAGCGAAGCCCGGTATTTAAGTGGAGAAGGGTAGAGGGGCGGGCCCATTATTCACCGAGTTTAGAAGGCTGTGATTGGTCCAAAGGGCGGGTCACAGACGGATTTCTCGGTTATAAAAAAAAATAAAAAATAAAAAAAATACAGAAGGTATTCCACTCACTCTTCCCACTTTTACTTGTATGTTTGTGTGTGTTTGCCCCCTTTTCTGTTTGTCCTGACACATAATTATGCTTCTGCTCTGCCTTTACAAGTTATCATAATTGCAAACTTCC

>LNC_001466

CTTTGGTATACTAATTACTTAAATGTCGTCAAGGAATTGCGAGAATAGTATTATGTTGTTGTGGTTGTTTTTTTTTTTTTGAGTATATTTACAGTATGCCTACAACTACATAATACTTATTTTACATGTCTTTGATGAACCTAAAATTCAGTCGCTAATTCTATTTCTTCTTTGTTTGTCACATGTACACTCGGGAGCGCGTTTGGAGTTAGTATATGACTCCATCTCTGTCCAATTCAGAGAAGCGACCAATAGCCTACATTATTTGCTCGTGTCTAGC

>LNC_001467

GCACTCGTCGTATCCCACCAAGACAAGTCAGCCTAAATCCAATCATTTCGATAAAGTGCGGAAATAAAGCAAAGTTCAATAATAATTACTATTACGAAAACAAGTCAACTTAATTCAACCCCCAAAACCTGGTTGTCACGTGCACAAGCCTCTAATGTAAATACTAGAATTGAAATAAAATACGCAAGTCTCAAATGACGTTGTCTTTCAAGTAAAAA

>LNC_001468

CACGATTAAGCCCAATCCACTACCAAAATAAACCTAAAAGCAGGTCGTTGAGTGTCCAGACTACCGTGGATTTCCATTTATGTTTTCTGGTGGCTTCGTGAGATGGGTCAGTGGAGTTGAGCAGAGGAGTGAATATTGGCAGAGCTTTTATTGATTGATTTGGAAAATCTGAGAGGATTAGAAAACATATCAGGCTGTGTATGATAGCTCAGAGATCACCGGGCAACGGATCAAATCACCTCGTATTGTCAAGCCCAAGCGAGTTTTCTTAGAAGCTGATCTGAAGCACTAAGCTACCCAAATCAGTTAGGCCCCCTCTATACCCCCTTCTCTGGTTACTTGCATCATCATTCCCAACTATTGGTTAAATTGGGATCTAGCCAGCAAGCAGTTCCTCCGTGTCTGTGATAACACATTATTTCCACTGCCCATATTCATCCTTATTCCACTACTATAGACTTTGAATTCTGTTCTCTCTAATTGCCCAAGTTTGCCCATATGTTCTCAACCAAAAACTACATCTATTTTTGAATAAAATGCTTAACGAATTTTGTGTGTCGAG

>LNC_001469

ACTCAACCTCTTATTTACCATTTTCTATTGTTTGAATCTTGCTTAAGATTAAGTAGTAGTGTTTAGGTTTATTTCCCTTTTAGTAGTAATGTATTTGTTCTAGTTGTTTTCTTTAGTATCGTATTTCCATTCGAATCTGAAAGTGCTTGCTACAGACCTCTGCATCGTTTCCTTGGTTTGCTTTGTTTAAAAATTGTTTCAATCTTAAAAGGACAATGTTATTACATCAAATTAGATCTTAA

>LNC_001470

GTTTTATTTTGGAGCCATTTATGATGTTGGACCAGCCGGAGACTCACTTAATGTAATTGGAAAACATTTATGTCATATCTGGTTGATGGGATCGTGATGACTTGACTGGCAGCTTAATATGTCAGATTTGTCAGCTAGAAGGGCTTATTCTTTCCCCTCTCTTTTCGATTGTATTGTTTACTGTGGTTGTAGGTAAATTTGATCGAGTACCGAATATTTATTAGACCTCATAAATGTTATTCTTCAGTTTGATTATGTGTATAGGTTAAAATTGCTGAGACTATCAAGATTTAAGAAGATAAGGAAAAAACATCAATTCTGCTTGGCCTAGAGAAAATTCTCAGATGTAGACCTGTTTCAGAATTCTGGAAATAGAGAATTTATTGAAGTATCTGTAGGCGCAATAGCCTTGAGAAAACGTTCCTTTTAGAAATGTTATAGACTGTTATTACTTTTCGGTATAATTATTTTATAGACTGCTGT

>LNC_001471

GGTAGGGTTGGAATGATGGCACAACCCTACGCAAATCAGATTTGAGGAGTCACCGGAAAGACGAATGGAACTATGCAAATCAAATCTGAGGAGTCACTGAAAAGACACATGATGCTATGCAACTCAGATATGAGAAAATAGTTCGGAAAAATCCACGGTACTACACAAATTAAATATGAAAAGTAGTCCGGAGAGATGCACGGTGCTACGCAAATCAGATATGCAAAAA

>LNC_001472

CTCCAGTGCCCATATTTAACTAGAACTCAGAATCCTAATTCTAAAGGGAAAAGGGGAGAGGCGTTAGTTTTCTTATAAAAAGAAAACTAGCGTGTATTTTTCGGTTGAAGAAAAATAACGTGTGTTTGGTTCTCCTCTTTGGACTTTGAAGAAATCTCTATAATTAGGGATTATTCTAGCCCTAGATTGTTTAGTTTTCTATTCGATACTTGCCCACCCTAGTATTGAGAGAGTTCAGATGTGAACTTGGGATCACTGTAGAAGACCATGGCTATCTTGTAGATTCACTTGAAGGACTGTCTAACTTGGGGATTCGCTTGAAGGTATCTGTTCATGCTTCAAGAGGTATTTATCGAATTAAGTTTCAGTATGTTTATGTGTTCTAGCATGATTATGTGGTCTAGCATAAACAATATTGGTTATCTATTATTCATGTAAG

>LNC_001473

AAAAATTACTAACTATGTAAGTACATTTGTGCAACCAATGACTCAGGGAGTTGCTCTGTAATTATGTTATACAATTTATTTTTTTGAAATTACTAGGTTGTGTTCTTACTACCCAGTAATTGCCCAACCTATTAATTACACATTGATTTGTAAACACACCCTAAGAGTTAGAAGGCAGGATTTAGCCCAAGGCCTGCACGTATTAGAGAGAAGAGATAATAAGATCTAGCCTAAGGTCAAATGGCCATGAATGAAATATTCAAAAATGTCACTTGACAGACTGAATTTTTAGATGTCTCACACCATATGTTTTATGCTCGTATTGTATTTGGTATTCATGTTTCTTCATGGTTGTGGGATATCCGAGAATCATGTGCTTTTATCCCAAAAAGAAGTACCTTACTAGTATCTATATTGTTTGTTCTTGTAATATGACACAATGATGTTACTCTGCTCAACTTACTTCCAACTGGTACTAAGAAATTAGAAGGCTTAGTTAGCTCACATGTAGAAGTACTTGGCATCAATTGAATAATGGATGGACAATATTAACTTTTTCTAGTCAACTTTAAAATAGGGGTATTACTTTGTTGATTATGTCCAAG

>LNC_001474

GAAAAGATAAAAAATAAACAAAGAATAAAAAATAGTAAAAGAGAAAAGGAAACTATCCAAATCTGGTCTAGAAGAGACCAGATCTCTCCGTCTTCTTCAGCTGAGCAGCAGCCTCAAAGTCCTTCGATGGCCATCATGTCATTTCCTAGTACCTTCATATCCCTGAGCTTGAGCGGCTTCCATTTAGACATTTAACCTCTCACAAATGTCATGGAATCTT

>LNC_001475

GTCGACCATTACGAGCGATAGCGAAGCCAAGCCGTATAAAGGCGAGCAGCCCTTATAGCAATAGCAAACGGCCTACTTATAGCCTATCAATAGGTCAGTCAATATCAGTAGGGGTCCTCTTGTCCTCTCAACTCATATTTCCCACGTAGTTCGTCGGTTAAACCAACGATTCTCTTCTCAAAGTCAAAGCAATGAAAGAACCACCCCTTCCTGTTTTTTGTACTGTCAGATAGAAAGAAAATT

>LNC_001476

TGTGGGCCTTCATCGCCTTAAGGCAATCTATCCAAAGAGCCTACCCTATAATAGATTCAGCTAAAGCCGCACTTGGAAGATTTCTTTCTCAATCATGAATCCAAGCTTACTACTTGAATCACGAAAGGCGCTTTTCTCACTATCTATTCAATTTAAAATGCTCACTTATGAAATCAAGCTAATAAGGCCAAAAACAGAGCATGGAGTCCAC

>LNC_001477

CATATGATAGTTCCTAATCATTGGGGTTAATGGGATACTCAATTCATTCCCTAGCCCTGATTTACTATTTATTTATTTTCTCCCCTGGATATTATCTTTTCAAGAATACCTGTTTCCAGAGTTCACTTAATCCACAATTCAAATGGTGTTTCCAACTTCTAAATATCTTCATTATATGATATATTTATCATAGTGGAAGGAAAAAAGCAAGAAATTACCTCTTGAAGAAACCCTAGAAATTTTCAAGTTCTTGATGAACCTTGAGTTTTTGGTGAATTTTAATCCATATGAGTGTTACATGTAATAAATAGACGAGATCTATCATAAATGAATTAATGCCGTACCTCGATGTAGCATTAATGGTGAAGCCCTTCCTCTTCAAAAATCTGTCAAAGCTTCAATAAATGAGTTCCCATAATGAATTCTCAATCTTTTCCCTCACCGAAGTCCCGCCGGGCAAGACCCTTTTCTTTGGCGAAGGCCAGTTTCTAGTCACTTCCTCTCCTTGCAAGAATCCAATT

>LNC_001478

TTGCCATCTAAACCCCTGAATTCATCGAAACATAACATTTTGAACCCATTTTTTCCTACTTAATTGTGTGTGTATCACAAACGCTTGACACGTAATATTTAAAATAATTATAAAATAACAGGTATGATTATAAAATGACAAGTGTATATATAGCCACTCCAGCTCATCCTTTCCCTTTTATCATATTAGATTAATGTATCTGTACTTCTTTTTTCAATTTTTATTTGCCTCTGCCATAGCTATTCCCACTCCCACAAATCACAATCTCTTTCCATTTCTCTCTCTCATCTTCCTTGCATTTTTTCTTTCTCAACCTCTTCTACTATTTTCAATGAAGAAAAAATGTCGAAATGATTTATGCTTGTTCACAATCTCCTTGGAGCTCGAAGTTGATTTTGGATTCTATTGGGTTGCAATTTAAATGGTGACTGATAGCATTAAGGAAGAGTGGGGATCTGTTGGGTGGTCTGGTTGTTCACAATTCTCGCCGGAGTTTAGGGCTGGCGGCGTTGTAAAGGTCGAGGTTTATTGGTGGAGTTGGTTTGGGGTTTTTTGT

>LNC_001479

TTTCTGTGTACTCGTCGGGCTTATGGGGGTCCGTTCGGGTTTTTATTTAACTTGCGCACGAGTGTACCTTCTGGGCTTATGGGGGTCCAGTTAGGTGTAGTTAGCTTATAGATTACTTTAGTTAGTTCTTTCTACACTCGTGTGCATTCCATTGTTACTTAGATTTCCGCTCTTGACCCTCGGTTTGTGTTATTCCTTCTTTTCATATTGCCTTTACTTTTCAAGTTCAGTCG

>LNC_001480

TTTGAGACTGAGGGTCTAACAAACTACCTCCACTAGTTGAACTCAACGTCCTCTTCGAGGTTGTTTTGAGGTAGTCATCAATTTGCGTGTCAAACTACCGCAGATCCTTGCTTTTATTCCAAACTGCAGCTTACTTAGCTTTGCCCTTCTATTGCACTAAGAATTGTGTTTTCGTATTCTCTTTACTGTTCCCAACAACCCGGTGATCAAGTATTTCCTCAGTCTTTGCTTCAAACGATGTTGGGGCGAAGGAGGAGCCCTCTTTTACCTGTTTCTGTTCGGATCATCAACATCTTAAAAATACGGTTTCAGATAACTTACATGGAACGTGGGATGAATTTTCAATCTTTCCATCAACTTAAACTTATAAGAAACTTCACCTACCCTTTCAACTAGTTCAAATGGTTC

>LNC_001481

AAGTTGCACTATGTTGTTGAACAAAGTAAATTGAAAGAATATTCTCAGACAATACATATACACTTGCATTAAACCAATTGATACAGAGCTAATTTATTTCCTCGTCACCACTGCTAGGCAGTCAACACACTCACACTCAACTTCAGCTGTCTAAGTCCTTAGACTGTTTTCTTAGGTATCATAGTATAGTAACAACACAGTAGCGTACAGGAACAAGTCCCAACCTTTATTAATATCAAAGATAAACAAAGGACTAGGCTTCACGAATTTACTAAGGCATAGAACACACTCTACTGCCTTCGTCAAATTTCTGCCCAGAATTTAAAACTTCTGCACATAGGCAGTTACAAGCGGTTACAACTGCTCATTTGCTTGGCACATGGATTAATAGAAATCCTATTATGAAATTAACTAAGTATACTATAATATTCTAACAACTATACGACATTCAAATTTCAAATAAAGCAACCCA

>LNC_001482

GGTAACTTTATATTCACATCAAAACTCATCAGCAAAGCCTGATGAGGTGGGATTTACAGCCCCCAGGGGGTAATTTCAAAAGCCAAAAAACAGCCAAAACTTTACAATTGAGCTTTAAAATCCACTAAGCTATCTACCTCCCCCACCAAATCCTGTTTACACCAAAAATACAGCAGGCTCAAGCTATTCATCTTGATTTTCTGAGTGCTGTTAAGTTTCCCATCATGTGATCTTTCATTTCTTTCCTTCCACAATGTCCACCAGATGCAGGCTGGGATGCATTTCCACCAGTCCTCTTTGGATCTTCTTCTTCTGCCCACTCCTTCCCACTGTCTCAACAACTCTAAAGTAGTCTTGGGCATTACCCAGTTAACTCCAAGTATACATAAGAACATGC

>LNC_001483

CCTAAACTCCTAAATTTGCGAATGAAGTGGGTCTTTTGCTTATGATTCAAAGTTTATGAAATTGTGTTTGGATTGGGTTGTATTGGTGACTCTTATGATTTTCTACAACTATCTATTATGAATTGATGATGTATGGATGAATTGAGAGTTGTGGCCTTATGGGCAAGTTATGGATGAATTCTCTTGTGGTAAATAAAATGGTTGGGACTTGAGTGTTATTGATTATGGCTTTGGAGGATGGTAAGTTAGCTTAATCTCATGTTATTGATATAATTGTTCTTGCTTGGTTTGATCCACTTATGGTGGAGGTGTTTAATTATGGATCTTACTATGATTATGGCCTTGAAGGCATTATATGAGGAATTGAAGTGTTGAC

>LNC_001484

TTCAGACATGGTTTTGGATTATGATTCTCTGTCTATGTTCAGACGTGTTTTTACTTTTGTGGGTGGATGCCTAGTGGTGAATCGGATGGACCGATTCATGCTCTCTTGGCAGAATAGAATACTTGTGTGGGTGGGCCGATTCAGAGATAGTGGGTTCGATGTGGTATTGGGGTAGTGGAAGGTTTTCGGAAGTTCTACAAGTGTCATCTTGCTTTTGGAAAGGTATAGAGAGGCTACCGTATGTGACTACAGTTTTTGGCTATTTTGGTATTTATCTTGCCCCTGTATGCATGGTTCAACT

>LNC_001485

CGACTCTTGGGTGGTCATGATTATATTGGGAACTAATGACTTCATGATGAGTCGAAAGAAATGGGCAACAATAAGCCATGGATGAAGAAAGTTTTAACAAGTATTCTCATGTAAAGGCAATTAAGGATTTTAGCGAGAAGGTACGATTAAAAAGGAATCGGGTTGGTTTTGTCAAGATGTGGGTTGGCAAGGTAAGAGATGATTGGTTGTATCAACTTTTTAGCCATTGGAAGGACTTTTTGCATGGTTTGAGTTGTAGCAGTTAAATACTTGTGATTGTAGAAGAGTTCGATGAAGTTTCATTTGAGAAGTTTGACTTAAGGTAAAAGGTAATATGCG

>LNC_001486

AGTGTTATCAAAGGCGCGCTTTAAGCGCGCTTAAGCCCTAAAGCAAGGCTCAAAACGTGTTGAGCGCTTCGCCTCGCTTTATGTGCGCTTCAGTGTCGTCATCAAAGTTCTAAGAAAAACTTTTCCTTTTCAATGAGCCTCTTATGAAGAAGTGACACTAAACAATTGATATTTCACTTTATCATAAAAAAATTCAATTTCTTTGGTCATATATTTGTCATTCATGTTTATAATTATTAGTTTTGGACTAAACATACATATTTTATTTTTTCACCCTTTGCGCCTTTTTTCATTAAAGCCCACGCTTTATTTGCGCTTTGCGCTTAAAGCCCCCACGGAC

>LNC_001487

TCTTCGTTCTTGGGTTCGTATATGTAATTCTCTTTAGTTATAGTTATTCTGTGTACTCGTCGGGCTTATGGGGGTCCGTTCGGGTTTTTATTTAACTTGCGCACGAGTGTACCTTCTGGGCTTATGGGGGGTCCAGTTAGGTGTAGTTAGCTTATAGATTACTTTAGTTAGTTCTTTGTACACTCGTGTGCATTCCATTGTTACTTAGATTTTCGCTCTTGACCCTCGGTTTGTATTATTCCTTCTTTTCATATTGCCTTTATTTTTCAAGTTCAGTCGGCCTATGATGCCTACTGGGTACCGGTT

>LNC_001488

GTTTACCCACAGCAACAACTAAAGAGAACAAACATTCTTTGACAAAAAAAAGTGGGCAATAGGTTAGGGAAAGATATCCACGCCATGGCCAGCGATGTTTCTTCGTTAACATTAAACCTAGAATCATATATCAGTGTGCGCATTAGGTAAGAATACCCGTCTTTGCATTTGACATAGAAAGCTCCCTTTGAAATCAGATTAACAAAGTCCTCATGTTTTGTCAAACGAATGAGGATATGTTTATTACGAAAGAAGCCAATTTGACAATTACCCTTAACATCACATTGTTGAGGAATTATCCTCCTTAATTCTTCTAA

>LNC_001489

CTGCCCTTTATCTGCATCTATGACCTCAATCTCATTGCTATTCTGCTGCACTTCATCTCCTTTGATATGTTTCTGTATATTTGTTGTTCTTTGTTGCGCATGTTTATCAAGTACAATTATATCATTTTTCTTACCAAAGGACCTTGTGACCCAATCTTTAGAGGATTCCCCTTGCTGGAATATTCTTGCATCCTTATTAGTTTGCTTATTGCTACTGAT

>LNC_001490

ATCCGGAACCTCTTGGAGAGATCTTAATTCATATGTACCTGCAAAAAGAAAAACCAAGTTAGCAAAAAAATCAGCAAGAGTATTTCCCTCCCTTAAGGAGTGTTGTACTCTTACTGACATGGAATTCCTCAATCTATTGATGGAATTGACCTCCATTGTCACATTCCAAGGTATTTCCCACTCTCCCTTCAGCATATGTACCAAAGCCAACTAGTCAGTTTCAATTATAATGTTTGGAAAGTTGTTTTCCAAACAACATTGAA

>LNC_001491

CATTTTAGCCAGAAATTTGTTGATTTCTTCGAGTTTTGGGATACGAATCTACTGATGTAAGCTCTATTTGATTTTTCTGATTCTTTGATTTTGCTCTTCAAATTGTAATTTATATTTTCTTTCGTCTGGTGTATTTTGAATTTATTTTCTCGACTTATTTGTGTGTTTTACTTCACTGATTCTAGCTATCATGATTTCTTTTGTATGTATATGATTTAAGATTAGTTTTTGTTTTGGTATATTGATGGATTTTGAGAATAGTTTTGAATTTTGGTTCTATAATCACCAATTTTCTCAGCTACTTGAGGTGTAGTTTTGAACTGTGGTTCTTCCAGTTCCCGGATTTCTTATTTGACTCTGATAAGTCGTCGTCTTGGCCTAGGGTTTCTGCCTGGACTTGTTTCTGTGCTCTGCTCGAAATGACTCTACTTGGCAATGCTGAGTTGAGATAGAAAATGGAG

>LNC_001492

CAAAAAGCAATTAAAAGTGTAGGATGAATTTTTTTCCTTTCTAAGCAACAAACAGAAATACACAGTTGAGTAGGTACTCTTCGTTTTTCACAATCTCATCCAAACCCTTTCCCAACTTTGAGGGTTTCATTGTTCTGATTCACAAGCACAAAAATAAAATAAAATAAAAGTCTGAACTGTGTATTTCATTTGATTTTATGATGACAGAAGAGGGATTAGAGAGGGAAAAGATAAGAGAAAAGGTGAATCAACGTCTATGAACTCCATAAAATCAGGGTGGGTATTTCAGGACGATCTGTAAATCAACTTCGCGAATGGTTGAATTGTACTTAACTGTTATTAAAGTAACTCAAGTAATCAATCATTTCTATTATTGTGGAAGTTGTATGAGGTGAAGGTACGGATTTTTGTTTTCACTTTCTTATGTTATTTTGTATATGAAATATGGATCTTTGTGAAAATAGAACTTTGACTAGATTATAATGGAGGTTGTATTGGATTTTTCCGCTTTTGAAACAATTGAACTTTAGAAATTTAAATTTTCCTTATTTTCTTCCTCGAATTTCCATATTCTTCGCTTTTGAATGGTTACAAGGAAATGTAGTTACCAATCTTTTAGCACAATGAAACTTCTGCCAGAGATAAGTTAGAGGTATTAGCTTTAGTAACATAAGTTCTTATATTTCACTATGTGAGAAAATCATTGTATTAGTTGATTTCAGTTATAATATCATCATCCATGTGGATGCATGTTCTTAATGTTTTCTTCGGTGATTCCTCTCAATTTTTCTAGAAGGATAACAAGAATGATTTGATAATAGAGGATGAAGAAAGCATGAAAAAGGACGATAATAAAAGATCATAGACCATACACTTGGAATGAGGGGGCTTCCGAAGGGCACTGAAGATACAAAAGAGATGATCATATGTTTTGTTGACACTGGAATGAGTTGAAACTCCCAATCGAAGTTGTTGTAACTATAGAGAAACCACAAAAAGAGCTTTTCTTCTTGGCTTGTGATCAACTGTGATGTTTCACGGAGCTTTCCAGGTTATTTCCTTACTATTTTTACCCTTCTCATTATACTGATGGATTTACTATTCAGGTGAAGTGCACGTCTTCAAATTATTTACTGTCATTGAATGTATCATATACTTTCTTGGATTTTCTACTTAATGCAACATGAAAGAAGTATAAATTGGTCTCGGCTATGTGGTAAATCAGTTTGCTATAAATGTATATTCTATTTATATTGATCATGTGTAAGGTCTTGATAATGTGCATAATTTGT

>LNC_001493

GATTTTCTAAACTTTGTGATTCCTATAAAGCCCATACCTAAACACTTTCATTTTGTAAAATTCATCAACATTTTTAATTCTCTTTTCCAACTTTAGTGTTACAAAATTCTCTTTTTTCCATGGATAGCACACAACTTCATGTTGCTATAGTGTCAAGCCCTGGCATTGGACATTTAATCCCAGTACTAGTTATAGGCAATCGATTAGCTACTCATCATAATATTAAAATTACAATTCTTACTATCACAACCAACTCTTTATCACCCGAAATCGAATTCCTCAAGAAATTCAACG

>LNC_001494

TTTAAAATAACATATGTATTTTTACTTTTATATGAATTTTGTAAGCATAAAAATGGTATGAAATTTGTATATTGATATAACATATGTATAAAAGTTATTTGTACTTTTGTATGATATAATATTGACACAATAATTTATGATGAAACAGAAAAATATGCAATTCAAAAAGTGTCGGAGCAGAAATTATTGTCGCCTGGTTCAAAAAGACTGTAGGGAGACCACAA

>LNC_001495

CCAGGCTCCTCTCCATCACCTCCCCCACGTGCTCCCCCATCCCCTTCGTCAGAACAGCTCTGGTCGCCGGAAAAACGCCTTATCAGTCACTCAATGCTTTCAAATCCGCCTGAAAAGGCAATTCTTGGGGGATTTTTCTCCCCCTTCATCATTAGTCCTCCCGTTTTGGCTTTTCAAGAATTCAAATTTTGAATTCTTGAAGCCAATAGAGGTTGCGATTTTGGCTATAAAAAGGAGCCATTTGGTTCCTTCTAAGAACAGAAAAAAAAGGATCCGGAAATCAATAAAAAAACATCCAAGAATCGACGAAAAATATCCATCAACAAACTGAAAACATCCGGAAAATCATAGAGTTTCAGAAATAAACAAAAGGGTAACACAAAGCTTTTAGAGTTCGAAAGTTCTGTTCTGAATTCGAGTTTTGAGGAAGTTCGGAGTCGAAATCTCTTGACGTCAAAGCTCTTGTTTGCTGCTCCAAAAGAGGTACATTTTTGTTACTCTTTCGCTTTTATCTTGTTTGTTACGAATCAAACTGATCTGAGTTGTTGTATGTGAAATAGTGAACTGTTAATTTGAATTAGTGTCTTATTTTCGCCATGACTTATCTTATTCTGAAAACTAGCTGAATCACTGTTTCGTTTTTATTCAAAATAGTTTGAATGTTCATTGTTATAGAACCTCATTATGTTAGACAAATCTTTGTCCTATTATTCTTGCGCTTATTTTCGAGTACAAATCAATAGTTGCTGCCTGGATTAGTAGAGTTTAATTTGTTATGATCGTCTTGCAGAATGAATGATTCTTTCGTTTCTATTCATTTGGTTTTCCCTTTTGGTATTAGAGGTTGTCCAGGTTTAGTTTGGTTGTGTTGTTTTCCAGGAATTGTTGGTACTGTTTGTCGTTTGCTGTCAGATAGTATTATGACATGAACATGCCTCGTCTAAGTTATATTTGTCTAAACACATACAGTTAAGACCATTAGAGTCATCCTAATCTGTCTTGTTACGCCAATTTTTTTAGCTTTAGTTGATTGGTTCGCCTTTTAGCTTAAAATATTTAAGTTAAAAAGTTTGGAATGTGTTTGCTGCTCAAATATCTGCATGTTAAACATTTTTGACATGGATTGATGAATATTTGCACGTTAAAATCTTTCTCCCTGTATCAATCAAGTTTTGGGATGGTAAATATTTCCCCTGTTTTCAGTTTTAAAATGGTGGCATGATACTTTGTTATCGTTCTGCTCTAAATTGGTCTTAATATGAATTAGCACTCAAATGACTAAGCAGCCGCTCGACTTTCTTCCCCCTATTTGTTTTAGACTTTTGGTCTTAAAGTTAGTAATGAAGAAAAGAAATTTTAATTTTGCTCAGAACATGTGAAGTAACTTATTACCCGTAGCCTTTATCTGCCCATTTGGTGTGTTTCCAGTTGGTATTAGTTGTTATCTCTCTCCTCATGATATCTGGATGTACAAATTACAATAGAACATGAACAAATATGTGAATACAAATGCCTCTTTCTATTTACTAAAATGCACTACTTATTTGTTGAAAGTTGGCAAAATTCAAATTGTACATTGTTGTTGTCTCATCTTATTGTGAAGTACATAAGATTAATTTTTGGTCTGCTAGCTCATCCTAAGATTTTGCATTTTAGCTAGTTGTCTGTAAAATATGGAAACCCGCGGCTGCTTGTTTGTTAATATAAACTCGAGTTAGAATTGACCTATAAAGCGCAAGACATGTCTATATTTCTGCCTTTGAGTTTTTCAGCGATTTCGTGAGATGATTTGTGTTGCTCTCTGCTTTTAAGTGACATCCACGTTTAGGCCGATGTTTTGCATTTTAAAAATTTCATAAGTAATCTCATTGTTTCGAAAGTCAAATTCCATTTAAAGTTCGAGGGGCCTCGATTCTAAACTTTGTTTACGCCTTTCAAGGGTAGCCAAGCTTTGTCTAAATAATTTTTTTTATGTGGGAATGAACCATTTTCTGGCTTTAAAGATCAGTTTTTTCACCTGTTGCTGGTTCATCCTATACTATATAAAAAATGTTTTCTTCTTCTTATGTTTCATGGTGTATAGTAGATGCCACTGTGAAAGGTTATTATTCATTTGTTTATCTCCATTAATGGATTTTATGTTCATTAGTAAATATCTTTTATTTTGTTTTCTTTTCCTTCAGAAGCATGACATTGCTCCTTTGAGCTTATGCTTCTTAATTTGCTTCTATTTTTTTTCGTAGTCTTTTAAATTCCAAGTATGTAAGAATGTGATCATGTTTTGTCCTAGGTTTAGACCATATCATAATTATTCCTTCTAAATAATATTATTCTAGTCATTTTTGTAGTAGTCGTAACTTGCTTGAATTCTAATTTGGTTATATTCCACTGATTTGAAGTTTCTAATCTTTTTTTATTTTTTTATTTCACATGTACACTCGAAAGCGCATTTGGAGTTGTGATACGACTCCATTCATCTGTCCCAAATCAGATTTGTGCTCATCGATACATTATAAGCTCATGTCTAGCAGCTTTTATCGCGTCAATAGTGTCCAATGCTTCCTTCTTGCTTCAAAGTTTCATGTGCCTTATATACATATATGTGTTGCTAGTTTTGACTAACATTTTCCATATTTTCGTATTTATGTTTATGCTTGCTTTTATTTTGTTTGGTTTGGAACAACTAAGAACGATGGTAAAGTATAGTCGTTCACCCCCACATCCCTTAAGGTATTGGGTTTATTATAGTTATTTAGTTTAGAAAGACATTTTTCAACTAAATTTTATTTGTTACTTGACACATTTTGGTGGTTATGAAAACATATCTTTTAAAGAAGTTCACACGGAAATAATATTTTCAACTGTTGTGAATTTATTTTCAGATTTTTCTCTTTATAAACAAGTATTTAAAGTACAAGTTTTAGCAACTTAAAGTTTGATATTTTGGATCTCAAAAGTGCAGATTGTGGTGGTTCAAAGTGCAGATTTCAAGGTTAAATATATCACCTTTCTTGAGAGTTTAAGAGTGGTATATTGAGTCCCGAAAATGCAGCATTTTGAGCTCAAAAAGTGCAGTGTTTTGAGCCCAAAAAGTACAGTGTTTTGAGCTCAAAAGAGTGCCGAATTTGAGCTCAAAATGTGCATGATTTGAGCACAAAAAATGCAGATTTTTAGCTTAAAAAATTGCACCTTTAACAAGCTTTACAAAGC

>LNC_001496

TAGGAAGACCAAGATACACAAAGGATTAGTAATTGAGATTATGTAAGTTATCCAAGAGGATATTTCTATACATAAAAGGAATTCAAATTGGGGCTTTACGTTCGTAGAAATGATCTAGAAGTACTCCCCATGATTCTGATCCAGAGTATGTTCCTATCCACTGAGTAAGTAACTAACTATCAAGAACGAAGTAATCTTTTACTTTGGTTAAAGGCCCTTTTAC

>LNC_001497

TACACGGCAAGACCACGCCGTCTTGACGATTGCTCAAAAACGACATCAACAACAGCTTCAAATGACGACAAGTCAAGCTCCGACGCCCACTTTTCACCAACGGTCCCAAACGGAGATAGACGGAAAGCAATACACTGCGTTTCTTTTATCCTCGCAGTACTGTTCCAGTCGAGATTTTCGGCAAAAGTAGCTACTGGAGCAGCAAGCAGAATCGTACTCGCGGCGTTAGGATGGAACCACGTCGCGCCTAAGGACGAGTAAGTTGATTCCAGCCGTCTATCTCACCATTTCCACTTCCGGAATGGGTTAAATCTGCAGAGAAAGGGTGCCTCATCGAATTAGTGAAAGATTTTTAAATTTGAATTTGGATTGGGTCCAATTCCTATCCGGATTTTTTGATCCGTTTCCCCCCATTCCGAACCCTACTCTCCCTATATATATTTGCTTGTTGTTCACTGTTAAAAGGGGGTTTGAAAAATTAAATAAAGAAGGAGAAATATTGAACATAATTTTTTTATATAAGTAAGGAGCGAAAATTTACAAAAGATATACAAGGAAAATAAAGGTAGATTTTTAGGTCAATAGTGTTGATATTGCCAAAGTTTTCTGGGTCACCTGTTTTGGAATCTGAGTTTTCCGTTGAAATTTTCTGGGATTGGTGGTGGAAGCATCCTTTCAAGCTTGTCGTCCCAGAACGCTGCTCATAAAGGGGTAATTCCCTCCACCCTTTCTTGTTATTTACTCAAATTGTTCTTTTATTTTCTATTTCAGCTTAGAATTGTGATTATTTTCTCTTTGAATGTTGTAATGAAATTTTCAATATTTGGTTCGTTCTTTTAATATTCTCTTCGCATACGTTGCCATTATGTAGCTCTAGTAGGCTCAAATCTTGGCATTTTGGTTCTAGAATACAATAGAGTTCCTTTTATTGATTTATCCGTAGTGTTATATCAAATTTCTTTTATATTAGCCTGTGGCCTAATTCAAGTGATTCGATACTGAGATACCACTTGTCTGTTGTATTTGATTTGATTTGAGCGACTACTTTTAAAAGTTGAGTCTGCTAGTTATATGAAAGCTTGCTATAAAGAATATGAAAATTATTGTGTTTTCAATTTAAACTTTGAATTTACTTATCTTTTGTAATATCTCCTAGAAGACCTGGTAAACTCAAGCATTCATACAAGTTGGTTGAACTCACTGGAGTGTAGTTTTATGATTTAATACTAAGTAGTTGCATTTTTTTTTAACAAGTGATTGTGGGATACTATGTTGAATTCCATGAGATTTCAAGTTTGGTGTTATTTGTTTTTTTTTTACCTTCTTTAAATTAGCACATGCTTGATTGTAATGCTCACTGGCCAATTTCCCCTCTTTGAAATTGTAATATCTAGTTATGGATGTGGTAGCTAATCTAGGATAATTAAATTTATTGTGCTAAGGTTTGCCTTCTGGCAGTGATTCTATTTTCACAAATATGAAGCCACTTGTGTGTTTGTCTAATTCCATCGTAGCATTGATTTTCTCGGATGATAATAATGTTCCAATAGTCTGTTCAATTCTTGGAAAATGTGGGTTTTGTTCACTGCTCTATTTATAGAACATGTGGGTTTGATTGAATCATTTTGGCCTGATCAAAATTGCCTTCTTTTCCTCTTGGAATTTGTCACTTATTCACTCTAATTATCTAATCCATCAATTTATTTTCTTTAGCTCAAGATGTTAAGTTCTAATTCCCTTTGATGCATTTATTATTTTGTGATTGGGTTAATCATCATTAAGCTACAGTTTGAAGATATCATTATTGGGCACTAATCTTATTCTTATTTTTGTTTTGCATGTGTAAATCTGTTTGAGTTCGCCATGAACTCCTATTTCCTTATCCCATATTTCGTGAGAGCCATAAAGGCTCAAATTCCTCCTCCATCGAGTCAGCCATCTTGAAAGTTGACGTACAGGTCCCGGAGTCGAAAAAATATTGGAGCAGATTAGAGAATTGAAAATATTGGGCCAAAGGTCCACTTTTGATTCCAAAATTGTATTTTGTTATCTATTGGGACTAAGCCCATTTAGTCGCTATTCTTATTTTTTGTTAGAAGCCTAGGACAAGTGAAAAATTGGGCCTAAGGCCCAAAGAAAAATATCATTTTACGTTAGTATAGGATAGGTGCAGGTGACTCAAATGGGCCAAATGCCCAAAATGGAAATGGGCCCAAGTACTGGTCCAGGCGGACAGAAAAACCATACTTGGGCCCAAATCTCTTTCCCTCTTTGTCTTATCATGTTTATTTACTTGTTAGTATTTGCCGTTAATTTTAAGGTTGGAAAATTTAAATCCCCGAAAGACGTCCATTTTCGCTTTAAAAGAAAATTAACTAATTAGCCATCTCTTAACAAGATTTAATGAAAATAATTTTATAAGATAGCTCCCTTAGATAATATTTAAATATTTCCCTTTTTCCCCTTTAAAATGGTTCTAACTATAAAAATTGTTCAAATCAAATTAAGTTAAATTATTTTAAGCCAAATAAATTAATTGTCGGATAACTGTATTAGCGGATATTCTAGGTGCCTTAAAAACCTTCCTAGAATATTAATAGGAACCTCGAACCCCTCTTTAAATATTTTCAATCGATTTCCCTGTTTTAATCTTTTGAAAAATAAGTTTTCTTAATTTCCCTTTAAAAATTAAGTGGCAACTCTTAAACCAGTCAAAATTATATTTTTCTAATAAAACAAACGGCCCCACCCTGGAGGGAATAATCTCGTCATAGCGGCTTGCATGGAAACAAAGAGCTAAATTAAGAGTTTCATACCCCCATTTCACAACACACCTTCATCCTATGATACTCATAAACTACCCCTTCACGCTAAGATCAGTTTCGGGAGTTCTACTGGCCCAAATTCAATTCTGTAAAGTCGTACGAGTTTCTTAACGTCTTAGCTATCCACTCATGTAATCACATTCAATATTAAATCTCTCATTTGTTACCAGATTTTTCTATACCTCACTGACTCCAATTTCGT

>LNC_001498

CCTTCTCAATTTTACTTCTTCTTGGATAAAAGAAATCAAGTTTTTTTTGTGTGTGTATGCACAGACAATGGTGAAGATCATGGTGGCGAGGATAAAATTGCTGAGAAACAAGAGGGAGGTGGTTGTGAGGCAAATGAGGAAAGACAAACATTGGTATGCTTCTTGAATCCCATCCGGATGCCACCGCTGCTCGTATTCAGGGAGTCAGGGCTGACTATACCACTTGTGGGACTCTAAATTATGTTGCACTTGAGAGGTAAGATGCATATGTAGTTCGAATCATTCAGTTACTTCTTAATAGTTCTTAAGAGTGCATTCTTCTTGTTTTGTAAATGTAACCTGTCATCATTTGATTGGCCTGCTATCCCCTAAAACCCTTTTCTCATTTAAACTTTAGATTGTGAGCTAAGAGGCAAATATGTACTTTTACATTTGTAGTCTTGGTTTAGTAATTAGTTGTTGTTATTAGGAATCTTATTTTCCTTAAGTAGCTATATTATTTTCCCATTCCTGATGTCCTCCACATTAATAGCACCATCTATATTTGTTTTATCTGCAAAACTTGAATCAGAAAGAAGCTAAGCAGAGGGGTGGTTGTCTTCATTTTAATTCATTGATCCTTTT

>LNC_001499

TAAGCTTGGAACTTGAAGTTCTTCCAAGATCTTGTGGTATGTCCTCATATGTCGAGGACAAGACTTTTCTATTCTAATTTTATGATAAGGTTCTTGTAATAGACTTAATAGACTTCTTTTGATTGTAAAGGGCCGTGTCCCTACTTATGTTTTGTGATTGTGTCTAAGATGGCCATGTGAGACTTAGACTTCCGCTGTGTGTTTTTAAAGTTGTTTTAATGAAGTTTGAATGTGTGGATGTAAATAAGTTTTTCATTCCTCACTATTATCATTATTGAATGCAATGAATGACTATGTGGTTTGTATAAGACCCTTTCGGGGTCAAGTACGCCTCGTTACGAC

>LNC_001500

TGTTTCTAAAGATTGATTAAGATATTCTTCAAAATCATCACGGTTAGTAGAATTAAGACCAAGAAAATCTCGCATATAACTTGAAATTCGAGCTTTACTCTGAGGATTAGAAGTTTGACCAACTTCTTGAGTATTTCCTGTAGTTCTATATTTTTCATACAAAATTTTTGCTTCTACTTTTATGCTAGACTTACACATTTCACAAGTTGGATTTATTTCCGATAAAATTTCTAAGGTATCTTAAAAAGTGTCAACAAGTCCCTGCACACCTTGTAATTTATAGTCAGGATGAAGTAAAGTAGCAGTTAAAAAAATTTGAGGAATAGGAAAAAAATATTTTTAAAATTTTTCAATCATACCTTCAATTGCAACTCTAAATTTTTCAATTTTTTTGTATTTAGAAAATTGAATTGAAATAGC

>LNC_001501

GCTGTTTTCCCTATCTGGTTGAATTTTAGCTCAGGTTTTAGTGCCTTATAAATTTCTAAATGAAACACATACATCTATGTTGGAAATGAAATGCAACTGTGCTATTTTCCTTATCTGGTTGAATTTTAACTCGGATTTTAGTGCCATATAAATTTCTAAATGAAATGCCTACATCTATGTTGGAAGTGAAGAACATGGTATAATTTCCCCTAACCTGCCTTACTATGGCGTAAGCCTGTTAATTAGATTCTGTTTGAGGGCTTGGTTTTAAATCATCTCCCCATATATATATGTGAAATAGATTGTTGTGTTGTTGTGGAAGTTGGTGCTTTAAATCATAGGTGTGAAACAGATTGTTGTGGAAGTTGGTGTTTTCCAAGTAATAGTGATGTACTTGCATTTCTGATAATCTAAAC

>LNC_001502

CTAAATCTAAAGGATCACAAAAATAGACTTCTAAATCTAAAGGCTCACAACTTATATGTTCCAACGTCAAAATACTACATTCATATAAATTATCTAGCCAATACAAGACAATTAGAATAGCTACTTCAGCATAATAGAGCTCTGCACAAATACAAACACACACACACAAAATGTAGTTGCAGATTAAACACCATTTCAGCGCGTTAACATTTCACAATTTAACCACTATTTTCAGCAACAACAGGACAATTACATCATAACTAATATAAAGTGGCATAATGTGACATTCATGTTCAATGTTCACCACAAAAAAGAGTAAACTGAAAAGACATTAACTAAAAAGTATTGTCATAATTAGTAATTAAAACCCATGAAACATAACAAAAATATTTTTCAAC

>LNC_001503

AAACAGCAGCAGCAACAGCTCAAAATGCTGAAACATGAAGAGCAAAATTGGGTTAAAACCCATAAGGAATGGGGCAGCAGTTCGCAGCAATTTCAGCCCCCAAAATGGTCGAATATGGGCTGGAAATTTCAGTTTTATAATTCACTGTTTATGTTTATTTCCTCAGTATTAATTTCATCATGAATGCTAACATTTTTCTGACGTTTTTACTGACTTGGATGAATCCTCATAAGAGTTAAC

>LNC_001504

GTCGTCTTCCTCCCATCCTTGTCGCGACAGCAAAAGGCACCTGCAGCAGCCGCGTCCTCTGTCCTTGTAGCGTGGGATCGATCCACGTCGCGCCCAAGGACGAGTGATTCGATCCCACCCGTCCGTCCCCTTTTCCCCTTTTGGAATAGGGTTGAAATCTCAGAGAAAAAGGTGTCGTCCCAATTTGGTGAAAGGTTTTTCAGATTTGAATTTCGAATTGGGCCCTTTCCATTCGGATTTCAGCCCTTCCCCCTCAAAATCAAACCCTAGCTCCTTTCTATATAATACCTTACTGTTCCAATATCAGGAGGGGGGAGATTTTAGAGAATTTTTTTTTTTTAAAGGATAGAGTTAGAAAAGGGGAGTGAATACAGAGAGGTAGTTCTCTTGGCACAAAATTATACATTTGAGGACTTTCGAACTGTTCTTGAATCAGAAGGGAAAGAAGATTCTTGTACTCGAAGTTGGTTCAAATTCCTGGGTTGGTTGTTTGAATCGAAGGTCGAATTTAAGGTTGTGTGAAAGAAGTTGATGCTCGTCCGCTCGTTGTCTTAGCTCGCTGCCCCACAAACAAGTTTCACTTTGGGACTCAACGTCTTTGCTTACGGAGTTTGCATATCATTTACCGACCTATATGCTCGGAATCCATGTGTCATCCGTGATCTTACTGTGTCTAAGCAGAACATAGTTACCCCTTCATGAGGGCCGACAATGGCTTTTCTTGTTTATGCTCCTATTCTTGCTTGCACATATTTTCCCTTATGATTAACTTTATATCTATATTTTTGTGCAATTGCTTGTTAATACCTGCGTTATTTATTCATAGTCATGCTTCATTTATTGGTTTGCTTTGTCTAATAATAGACAACTTAGTTGGGATATTTTTTTAGATATAAAGGGAGGGTTTGAAAATGAGAAATTTTATAAGCTTCTTGCAAGTTTTCTCCACAACTTTGAAGTTCTGTCAGTTTGCCCTTTTTAAAAAAAAAAAAAAAAAGTTTTATTCTTGCAAAATAACTCCTTTCTTAAAACAAGAATCACGTTAGCTTTCAAAACCTCCCTTCAAATTTATTTTCACAAGTTAGATTTCACATGAAACGATTTTTCAAAATTGATTATTGTATACTTAGCATTTCAACATTTTTTTTACTCTTTTACACTTAGCCTAATTAATTAATTAAGTTTGGCCGGTTAACTGTTTTTAACAGATTTTAAAGGATGCCTTACCCCTTCCCTTTAATATAAATTAGAACCCCCACCTAGAATCTCACAAGGTTAAGTACACTTTACTATAGAGTTGATTTAACATAACTTTAGCAAAATAGGTGTCCTAATTCACCTTTAACATAATTAGGTGGCGACTCTTTAAAATAGTTTTAAGATTCACTAATATGTTGTACACCATTTGAACCTCGAATTAAAATGGGGTATAACACTATTTATTTGATTATTTGTATTTGACTAACCTTTTATTTGAATTGGCTTTATTTAATTAAATTCTCCAAAAG

>LNC_001505

CACCTCCACCCCACAAAGAGAAAGAAAATGCCCCACTTTCCTTATATACCTTAAAATGCTTCCTCAAATAGGTCATACCATTTGTTCTATCATATTTTTGCTTTTTAATTTCTTGAATCTATTTCGGTCCGTAAGTTCAGAACACATAAACTTCAAATCCTGATTCCGTCTCGTATTTTTGAATCTGTATGTTGGTTTTTGAGAGGTTAAAGCTGCCAGCATGATCTGGTCATCTAGCAGCTTCAATCACTCACCAACAATACACACAAAGGAAGGAAAAAAAAGAGCATCTAATTTTCACTTAATTCTCAATCACATTCATTTATCTCAGGTAATTATTTCTCTAATCTTTTTATTTTATGTAATTTTATTTTTTACTGTTGATTATCTTCTTCCCTTTTTTTTTTCTCTTCTCAGAATGTAACATTATATTTTATTTTCATCTAATACAATATATAACATATAATGATGAATAGTCAGGTGAATTCGGGACTCAAATTTTATGGATTTTAGAATTTAAATTTTATGGATTTAATCTTCAGGTGTTTAATGTCTATTATTCATTCAAACTTACTATTTATTTTAATTTTAATGGATTTTTCTAATATATAGAAATTCATGTTGTATTTTGAAATTATTGAATTCAGATGAATCTGTGATTGATGATGGTTTCTGATGACTTTAGATTTGAATTTGAACTTTAATTAGGCATGTGAAATTTCCAAATATATAAGGCAACAAATTATTTAGGGTTCTACTTAACTTTTTCTGTGAATTTTAATCCATTGTGTACCTTGTGTGGGTGTGGGACTGTGATTGTTTGTTTGTGTGATTTTCTATGCAGATAATACTCCTCGGTACACTATATTAAAATAGATATTTATTACCCCTGTCCCTAATTATTTGTCCACTTTTTCTTTTATAATTGTCCCTAATTACTTGTCCATTTTGACAAATCAACAAAGGACAAATTCTTTTTATCTATTATACCCTCAATTAAATGACTAATTATTTTGAAAAATGTAGAACTTTTCATCCACTTCATAATTAATAGGGGTAAAATGGTAAATTCACTATGTCATTAATTATTTGTGTGTTAATTCAAAAGTGGACAAGTAATTAGGGACAGAGGGAGTATTACTTATTCAGTAAAAATGAAAAATTTAATTTATTATTAATTAATAACTATAATCTTTTGCAATGAATTTTTTAAAATATTAAATTTATTATATTTAAAAGGATAATACAAAAAATTGTATTTTATTTATTTAAGGACTGTGTCATGCTAATACTGGACAAATAAAAATGAAAAAGTGTATCGATTGAGCCAAGTAATTAAAAAAAAAGTTTAAAACAGATTCCCAAGACACGAGAAAGATATGATATTGTGCTAGTAATTTTTATCTTATGGTATTCAATTACATAATGACAACTGTACTTTTGTTATATATGTGGAAATTATAATATAGTAATACCTAATTAATTAATGACATCATAAAAGTGTAGTGAATGGTGCACCTTATTTTTAAAAAAAAACTATTACAAATTCATTTGTGGTAGGTAAAACATTGATAAGATTTCCATTATTTAGTGCCTTGCTATTATTTACATATCACCACCTAACAATAACTATTTGAAGAATAAATACGTCCGCGTAAACTTGTCATTCTTGTGTTTTTTCAACTGTTTAATTAATTTCTTCATGTTTCCATTTTGGCATATTATTATTGAGTTTGAATTATATAAACATATATAATCATGTCACTTATAAGATGATTAAAAAAAATTATGTATAGGTAAACTTTTATTTATAAATATGGTGACTAGCATGTCGATGTCCCGTGGTCCCCTTGGAAAAAAATTCAACATCTTTTGGTTTAATTCATCGGTGACCCCATAAAGTTGACACGATATTTCACTTAGACACCTTAACTGAAATATCGATGTTTATTTTAAACACCTCAAACAATATATATAAATATGTCTCGACTCGAGTTTGAGTCTACAAATTGAAGAGGTTTATTTCTTCATTCGTGTTTGTGGTTTTTTGCCTTTTGACTTTGAATCGGCTTAAATTGTACGTGATAATTAATCACTTTAGTATTAAGGGAAAAATAACGAGCTTTTTACATAAATGTACCCCACAGCAAAAGATTTTTTCTAGCTCAATGGTCGTATGTATATGAAATGTATTTTTATTGTATAAAGAATGTATAATTGTGTATATATATTAACATACTTTGTTATATAGAGTAATATTTATATATTGTAGCAACTCATCTTCTTGAATGAGCTGTAATTATCTTTTCAATCCCAACCAGCTTAAATCTCCTTTAAACAATTCCAAATTTTAGATATGAGTTCATCTCAATATTTTGAATTTTTTGTTAATTCACTCAAAACAATTCACGTTTGTGGTAGGTCAAATTTTAAAATTTGAACTTCGAAGCTCTTCAATGAAAAAAAAGAAGTTCTTCAATGACAAATTTCAATGGAAACCAGCTCAAATCTTTCTCTAATTAACAACTCAAATTTTAGATATGAACTTCTTTTGATATCTCGATTCTTTTGGTATATTATTCACTCGAATCGATTCACGTGTTTGATAGAGAGTATAGAGGAAGGAAACAGAGAAAACAGATGAGGACGAAGGAGAAAAATAATCACATGGCTATATCTAGTAGTAAGTAATAAAATGATGATAGTTAAGTCATTTTTGGTTAAAACAATGATCGAAGGGTTATTTTGTTTGATTTACTCGAAAACAAATCATTAATTATTTCCCAAACCATTTAATTGGAGAAGAACTAAATCCTATCAAGGGTTGTGGCGAAATGATAAATAATTATTCATCATTAATCAGAGGTCTCAAGTTTGAGTCTCCTTACGTACAAAGTCGTCTTTATTAGAGAGCGCTTTACCCCTAATGTGTGACTTCCCGACACGAATCCAGATTTTATCGAACTCCACTGTAG

>LNC_001506

CAAAATTCATACTCCGAAGGGAGGAACAGCACAGAAACAGAGTGAACAAACTTGATTCTCTGGGAATCCGATTAACGACGTGAGCTTCATCTGAAATCATCCATTAGAACTTCTTCCCTGGTAATCCGATTACGACGGGGTAAACTCTCGATGGTATCGGTTTCGACATAGGAGTTGGTTCGATTGTATGGTTTGAAGTTCCGATCGAGGTTTAGTCGTCCGGTCAAGGTTCCAGGTCCTGGTCCTTGGCATTGCATATACTCAGTTTCATTGTTGTTGGGTCTTCGTTCATTTTGCTGTGTCGGCTCGATTCAATTTTGGAAAGGAATTTCAGCTATACAGAAGGTTCATTTCTAATCCTATAGTATTTAATTAGTGCTTGGATTTTAATAGATCTATGTGTTACGAATTGAAATGCTTGCCTTTGATTCTTCTGTGATATTTCTTCACGTAGTGTTCTTATCACTGTTAAATTTTGGATTGATATTGAATTGAGATTTTAAGTGTATTTGTTGTTAGACAAAGTAAAGGGATTGTAGTAAGTAATCTGAAACTAAATAAAGTGTTTGAGCTCAGGTTCATGGAATTGATCGTACCGTGGGTGTTGCTAAATATTGAATTCGGGTTTCAAGTAAAAGGAGTTTTGGGTTGTTACGGATGATAGATAGTGTTTGGGTTGGTAGGCAATTCTAGGTCTGTTTAATTGCATGTGTCTTTACTTGCTTTCAATAAAT

>LNC_001507

ATTTAGTTTCATTTTTTAGTCTGTGAGTGTGGGTTTATCTGGATTGTAATTTCATTTCATTTTTCAGTCTTCGAGTGTGGGTATATCTGGTTATCTAATTTGGAAAAGTTGTGCTCGACCTAAATCACATGTTATAGTTACACAAAACAAGACATCTGGTAGTCTTTATTTCCTGGGTATTAAGACTTAAGAGGCTCATTTCTTTGACTTTCATGATATGCTTTGAGATGCTAACTTTGAAACACTTGGCCTGTGTAAATGAACCTTATAGGTATTTACTAGCAGTAGTTTGATGGCTATATGCTTTTCGATATCTTTTTGTTTTCTCTTCCTCTTGCCTATTATTTTGCT

>LNC_001508

CTTTTTTCAGCCATTCTTGATTCTCTGTTACTATTTTGCCTATCTTGTAAAGTAAGTCTTGGGGCCCCTTTTTCCTTTTGAGGGTATCTTTCAAAGATAACCACTCAACCCACAAGCATAACTTTTTGTTTGTTGGTTCGTTTTGCCGATTCTTGAATTCTTGTCATTTGAGTGAGGACCCAGAAACTGATTTTGTTTTGTAGTGGTGAAATAATGATTGGTTAATGTGTTGTTATAGATGTAAACGGCACTTTAGAAGCAGAGTTTTCGTTAATCATATAAGATTCCTGCTATAAATGCAGTGTGTTTTGATACTGTGTGTATATGTATTCTATGGTTATTGCTTGGTTGTAGAAAGGGTATATGTATTCCATGGTTTTTTTGTTTCTGATAATGTTGTGTGTGGTGGAATTAAAAAAAGAATCTATTTTTGGACTCTGAACATGCTGTTGTCGAAAAGTTTAAACATGTTATGATTTCC

>LNC_001509

AAACAAACACATACACAACAACACAACAGCAAACAGTACGCTACCCCACCCGTGTAACTTGTTCTTCTTCCCAGCGAACAAACCCAGAAACTCAGCAACCCAAATCAGCAGCAATCGCGACCCATAAGCAGTCCAGAATTGCAAGTCCAGCAGAAAATTGAGCCCAGCTCTCGCGCATCCTCCTCCTCCTCCTCTCTCCTTATCGTTCGTACCAAAATCTTTGGAGAATCATTATGTGTCTCCATCTCCGCAACTCACTCGTCCTTGCTCGAAAATCAAGCGCCCAAAATTGTTTTCCGTTGGAGATTTGTACTAATTCCAGCTCCAACCTTATCTGTTAAGAGATTTTCGAATTTCGAATTCATAAAATCTTATCCAAATCTGCCCGTTTTCCCCTCTAAATCGAACCCTATTTTCCCCTGTATTTAAACCCCTCATTTACCTTCAGTAAGAGAGTTGGAAAAGATTGGGAAAAAAATTGGGATTTCAGTTGATACAGAGAGAAATACTTAGTAAGAAATAGCTATAGAAACCAAATACGAAATAGGTTCGAGTTTTGGTTTTCGACTTCCTTTAGATTTTTCGGTTTTCCAGTAGCTAAAGACTACATACAAGAATATATTTGTAAGAAGAAAGGGATAGCTATAGTTGTTAAATCTAGAAATTCCGAGTCCTAGAAATTTGTTTCGAAGCTTGCATCGGAGATTCTTCATAGTAGCTCTATTTGATTTCCATTCGTGGTGAAGTCGCTACTCCTCCGTTTGTTTTGGTCCCAGTCCGTTGCCTAGGAAAAGGTAACAACCTTGTCCCTATCTCGTTTTCGTTTCATTTCTTTCTTCTCGACTTTTATGTTCCTTGAATGTTCATGTCACTTAGATTATGCTTGAATATGGGTTGATTGATGATAGTCCTCCCTCTAATTGCTAGATATATTCATGTTATCATGTTCGGATTTAGTTTAATGCGATGCTTTCTATGTGGAGTACATTGAATACTCGTCATATTCTCTGTTTTCGTATTTTTTTTAGACAAATATGGGTTGTTCAACTTGTTTTCTTTATGTATCACTCTTCTTTTCTCTACAGATTTGATTATAGTTCTGATGATATTTGAGCGGTCGCTTATGATGGTTCCCTTACTGATTTTATTTGTATAAGAGTGTGATTAGTGCTAGTTTTAAGTAATCTTGTTGTTCTACATGTTAACAACTGATTGTGTTGCCTATACTTTATTTTCTTGTTTCGATTATTGCAAATCTGATTTGGGAGATGTGATTATAGCTTTGATAAGGTGTGGTGTTTTATATTCAGGTTTCATTTTAGCTCTTAATATCCTGTTTTTGCTCTTTCTTTTGAAACTAAAATTGACCTTTTGTGTTTAGTGTAGTTGTGTGATTTTCCCTAATGCAACCTAGTTATTTTGAGCATGGATTTTTGTTTTCAAAATTGTCCCTGCTCAGCTGCATGCGTTTCATATAGTGTGAACGAGTTTGATAAAAGTTTGATCAAAGTGTTCTTAGCATGATTTTTCAGATTTCATGTGCCCTGGTCATTTTATTCCTTAAAAGGGCTTAAAGTTAGTCGTAAGAAACTTCCCGATAATGTTAATATTTAGATTGAGTCGTTGTTTGAGTTTAAGTCATGGCTGCTCACTTCTTTTTGTTATGTTTGTTTGGGAGACTCACCTCTGTAGATGGTCTCATTTATAAATTTTATTTGTCCCATTTGTTCAAAAAGGTAGCATGTTTTTTTTTTGTTTCATAGCCACTGTATGTTATGAGTGAAACTTATTATGAGTGTTGTATTGTTCGTATATGCATAGTCTTCTTTGTTTTGACTAGTTCGAGTAGTTAAATAATAGCTTCATTTTATTTTTATTTGTCCTTTCATCAAGTTTATTATTTATTCCATGTCACTTTTCCTTGGCTAATTCTTATCATCATTTTCGTGGCATGTGAAATCTGTTCGAGTTCGCCATGAACTCCTATTTTTCTTTCCCTGCATTTCAAGAGATCCATAAAGGCTCAAACCCTTTCCTTCGAGTCGGCCAGCCTTTAGAATCGATGAACAGGTCAAAATCGGGATGAAACACATGCTGAAAAAATGGATTGCATACGTCTGTATACGTATAATATACAAGAACAGGGGCTGATATACATCAGTATACAAATGTATACAATTCTGGGAAACTCAAAAATGGGCTTCAAGCCCAAGATCTTTGTTGCCAGCAGGTCCAGGTAGCAAAAAATGGGCCAAAGCCCAATGAAATTACAGCAGACCCAAAAATGATAAGGAGCTGATTTTTGATGAGATGGGCCAAAACCCATCATTATTGACAAACTTTAGATAATAGGACATGGATAAAAGTGTTGGCAAGCCCAATTTCGAATTTGGGCAAAAAGCCCAAGTTCAATATTCTCTCCCTTTTCCTCTTTATTTATTTCATTGAATTAACTAACAATTTATTTTCACTTTGGTTTAACTTAATTAAATTCCCGAAGATAGTCATTTCTCTTTATATTCAAAAGTTTTATTTATTTTTCTTATATATATGTTTTATTTATTACTCAGCTCTATTTTAGCCTTAGTCGTGTTAACACTTAAGTTACTTCCCTTTCGAATGATCTCTTTTATTTTATTTCCCCTAAGAAATAGTTAATTAGAAAAGAAAAGAGTTCTTGTACTTAGTTAAAGGTTTAAAAATTAGTCAAAGTTATTTTTAGTCAAATCGTCTGTCAACTGCAATTTAGCGGACATTTCGAGGGCTTAACACCTTCTCGGAATGTACATTGAACTTGAACCCTTTTTCAATTGATTTTATCTGTTTGAATCTTTTGAAAACTTTAAGTTTTTTCTTGATTTTTACTAAAAAAAATTAAGTGGCGA

>LNC_001510

ATTACCTCACATATTGGTTTTTTGCGTCAAAATAATTGTTTTATGTTGCAAGATTTGAGAAGCTTAATCTTTAATTAAATACGTAATTTACTTTTGCTTTCAATTTATTTAAGATACGGTAAGAGGACCAGTTCCTTAACCCACTTATTTTTCCTCAGCTTTTTAATCCAATTTTTTGGCCCCAAAAAGCTTTAAACCAGCCCACACCCTTTATTCCACTATTTTCCTAATCCCCTTTACCTCACGCGCACACACACTCTTTTTGTTTCCTAGAAAGAACACACAGAACTCTCCTACACGCCCCCCGATCCCACCTCTTTCTTCTTCTTCTTCCTCAGCGAAAGCAACCAAACGAACCCAACTTCAAATCCTCAGATTCACCGCCTCAATTTTCCTCTTTTCTCCTTCAATTCGTACTCTCCGCGTGCATAGTACGAGCTCAAAGACGCCTCAAGCTTCCAGCATTGCATTCCTCAACGGTAATACCCTTATAATTTTCGATTTTCCTTTTTCAATTTCGTTCAAAATGGTACGAATTCGTACACTAACTAGTTGGCAATCCTTATATGTTAAGGGATTTTGAAAATTCTCAAAATCCCACATCGAAAATCTTTCCAAATTTCGTGAGATTTTAGGTCCTATAAATACCCCTCCCTCTTACTGTTCAAGGGAGGTTTTTTGGGAGGCGAAGAAAAGAGCACCCCGCTATGTTTTTTTGGGAGCTCTTATTTTCGTCACTGAAATTCAATTGATTTGCGGCTATTTTGAGTCGGGGTGGTGGTAGAGTTCGTCACATCCGGACTCCAGTTTCAGTCGCTGCCCAGAACCAGGTAACTCTTTCCTTTCCTTTTCTATTTAGTTTAATTTTAGTCTATGTTGTTGTGTTATGTGTTAGAGCCGATTTAAGGCTAGTATGTTTCCTAACATTTATATGTTGATGAATATTTTTGTTGAATGCCTAAGTATTGGTGATTTTACAAGTACATTAGCAATGTTTCTTTTATTCCCTGTGGGTTGTTTGAATTTCCAATCCTAGTTTCTGGAGAGAGTTACTGTTTTTTTTTCTTTCCAGATTTTTCTTTTAGTAAAAATGTGAAATCCTGTTTTGACTTAATAGCACATTTTGCTTGATCGATTCTGTATCGCATCTGGTGATTGAAAACAACCTAAAAAATGCCTTACCCTTGCTAACTCGACTGAATAGTCACTCCTCTATTGTTTCCTTAAGAATCTATCTCTTGCTCCATAAAATGGTTAAAATGTTAGTACTTGGTTTAGTTTCTGTCATTTTAGTCTCTGTGAAATAGGGGTTTATTTATAAGTTTATAAAATTTGAGGTCTCAAATTTATCGTTCAAGATTGTTTTGTATCACGTTTTTATCAAATTTCTCAAGTAATATATGATTAAAATGTTGCTGTGATTTGTTCGAGTTTAACTGTTGTTTATATTATGAACTTGTTTCTTTAGTTTAGTATGACACTTTTGATACTCTATTTAGTTCGTTTGTGGTCCAAGGGATTGAGCAATTGTATGTTATATGTAGATTCATTTGAGTGCACTCAATCTCAATTCTACTTTTACATATGCTCCTTAAAGTGAACCCCTGCTTTCTTCGTTTTACATCCAGTTCCCTTTTTTCACAATGTGGGTTTCTTACTTGCTTCTTATGTTAAGGATTTTATTTGTTCGGAAACCATGAGTTGATCCTAGGTTTAGTTGAGCTCTAGATGAAGTTGAATTTCTCTTTGGTTGCTCCTAAATCCTTTAGATCCTCATGTCACTCCTTGAGCTATAGATCCTAAATTGTGTACATTTGCTTGGCTTGGAATTCATCCTATTTGCTGATTATTAGTTTAAATATTGGATGTCATAGATGTGTTGTTCTATTCGGAAGCTCATATGTCATGTGCTGCTTTACTAGTCGTGATCTTTTGTTGATTCGCTCAATTTCTGAAATGGTAAAGCATGTTAGCCTTACAATGATTTCTTCCAAGTACAGTTTTTTGAAGTCCTGTTATGCTTAGTTTACCTAGAGTTAGTATGGATAAATTATGCATCTAAGTGATATAATATATGTTAGTACTTTGTTAATTTAGCTCATTGTTTACTCTTTGTTAAAATCAATCGAAGTGTGGTAATTTTGTGAGTTAGAGATGAGTAATTTATAGCTAGCAATGTGCTGATTGAAACTTGAATCTATGAGTTGGTTAATTAGACTTAAGGCTATAAAATCAAGTGTTTTATTTTTCTCTTCTTAGCATTCTCTCCTATCTTTGTGTTTTGCTCTTATTAGTTTTTATTTTTGAGTCACACTAGATAAAGCTAAGAAGTTGTCTGTTTGTTTTAATTTTAGTTTTGCATGTTTCAATATAGACTACAATACTATAATCTTGTTTAAGTTCACATACAACTTCTATGCATGTATAATTTACTTCTAAGTTCCTTCATTTCTCAAAATATTTTTCTTTGTCTTGTGTCTTGCTTAAGAAAGTTGATGTGCTGCTTAGTATTGAATGCTTTGGTTTTTATGTTCTCTGCAATGATCCCTTATTTTTTTTGCCTACAGATGACTTGTTTATTGACTTAGTTTTTCCTTTTTTTTTTAAAAAAAAAAAAAAGAAAATCTGTCCAGTTTACCATACTACTTAAGTTCTGAAAGCTGTGACTAATTAATGATCATGTGTTTCTGGTTTGGTTAGTTTTAACGATAATTTTAATTCGTTATGTTTTCCTTGGAGTTCATCTATACTAGCTAATTCTTTTTCTTATTATGCATGAACACGCTTCCATTGAGTTCACACGGACTCCCCTCTCTTGCATAGTCCTCTTCTTGAGTCAAACCTCCTCCGAGTCGTGAAAGGGAAGCTAGTATTATTGGGTTAAAACCCAAGCAAACCAGCAGCAACAGCAGCCTTGAAAAGGCAGGGAAACGAGGAGCAAAAATTGGGTTTAAAACCCAAGTAACGTGAGCAGATCGCAACTGCCCTAAAGGGCTGATATTTTAGGGATTTTTGACTTATTGATTCAATTATTATTGATGGCAGCTGCTCAGTAACAGGTCACAGCAGTTTCGAAGGGCTGAAACTTGCATTTTTCAGACTAAGAAAGTTTGAAAATTGCGCCAGCAACAACAATTAAGAAGCAGCGAATGGACTTAGATTTGTCCAAGTTCTCTTCGCACTTTTATTTTCCTTATTTTGTGATAATTGCTCTTAACTTCTAACATTTATTTTCTTAACTTAGATGAATCTTTGGAGGGATTTTTATTTCATATTTTGTCGCTTGTTTACTTTAAAATTTCCAAATTTCCAAAACCCATTTTAAGTTAGGTAATATGCTTATTAGAATAACTTGAGAACCTTTTATGTTCTTTCATAGTTATTGAAAAGCTTTGA

>LNC_001511

TTGGAATTACTAGTTGACTTTGAATTATAAAATCAACGTTGCCAATTTAATTTGTCTTTGTTAGTTTAAGTAGTGACTAAATCGAGAAGCTCTTATTTTTTGGCCCTATGCTTGTCCTACTATTCTTGAAAAGGAAGTATTGAATATTAGCCTAACCTCATGTATTTGATCTCTGCTCCCGACTTTGGACTACAATATTTCCCTTTGAGTTTGGAGCCTTTACATTAAATTTCTCTACTCATCATATATCCGGTTTAAGTCCTTAATTTGTTGAATGAATAATCGAATACCAATTAAAATCTTTTGTTGGTTTAGCATGAGTCCTGCAAATGACTAAAATTGCATAAGGTGTTCATCCCCTTCTTATTTTGTTTCTTACATTGTCCTCTTGCTTAAATGGGGTAAAATATATATTTTCCATAATGCTCATATTATTATCTTGGAATCATCGGG

>LNC_001512

CTTCAATTCTGCTTAACCTCATCCTTTGGTCTTCTTCGCTGGGTCTTCTTAGCTGACAATCTTGTTGCCACAACATCATTTTCATTTTGTCCATCACCAAATCAAAATTGAAATTGAGAAATCAAATCCGCTATCAAATTAATTTTTTTCAAATTAAAATTTCTTTTTGTGATTTTAGCATTGATTTTTGCTCTAAATCAACATGGGTTGTATTTGAAAAAAACATAAATTCTCTCTTCAGGCTCAGATGAGATAGTTTTTGGTTGGTTTGAACATTATTTTAGCCCCGAAATTGATCAGAAGTCACTTGAAATACGATTTTTTCTTTAACGTTGCATCATTTTTTCGGCGCTTCTCACCCCCGCCGACCAAGAAGGAGTAAAAATTTAAGGATGAAACAATAAATCTTTCATCGTCGCTATCGCAAAGGAAATCAGCTAGTTGTCAATTTTTGCCGGTGACCGA

>LNC_001513

TAGTAATCTTACCACAATTGATGTTTTATAACTTGCATGACTTTCACATGGTTTATAAAGTAAATGTGTTATCGAATGTTTTGAACTTGCATGTGGGACAAGTCTTTTACTGGATATGATACTGAAAAATAGTATTTTGACAGGTGATTGTTCAACTTTGAAATGATATGATTTGATAAGATTTAAATGCTCTGTTCGATTTTGAATTGTTTTTACTTATTACAAGAAAAATAAAAATGAGAGTAGACCTTGATGAATCCCGTAGCTAACGGCGGGTTCGTTAGACCTGGTGCACCTTGTAGTTCTGATTACTGATAAAGCCCTTGCTAGTGGAAAGGTAGAACTAGTATACTGTTACTGATATCCCTCGAGTAGGGATCTACTGATATGAATAACTAACTCATTTATGAGGAGTTCACTATTATTGGTTACTTCTTAAGGAGAAGACCACACTGATTACATGATTCATCCTTGGAAACCTCCN

>LNC_001514

GGGGTCCGTTTGGGTTTTTATTTAACTTGCGCACGAGTGTACCTTCTGGGCTTATGGGGGCCCAGTTAGGTGTAGTTAGCTTATAGATTACTTTAGTTAGTTCTTTGTACACTCGTGTGCATTCCATTGTTAGTTAGATTTTAGTTCTTGACTTCGGTTTGTGTTATTCCTTCTTTTCATATTGCCTTTACTTTTCAAGTTCAGTCGGCCTATGATGCCTACTGGGTACCTGTTGTTTTGGTACTCATGCTACGCTCTGCATCTATTTCGTGATTCAGGTCCGGGCACTAGTAGCCAGCGTTGATCGAGCTTGGAGCAGACTGATCCGGAGACGGGAGTGAGCACACGACGTTTTGTACTATTTCAGTCTCCATCTGTATATATAGACTTGTCTTTTACCTTTCGAGACAGTCCAGTCTCTGTTGTCCACTTTTGGGACTTGTACTCATTTTGTTAGTAGCTCTGTACTAGTGACTTCCAGGTTCTGGG

>LNC_001515

CCCGTATGTCAATGTTCCCAGGGTAGGAACTTCCACTTTTCTTAGTCAGAGTATGGGCTTGGGCTTCGGTTCGGGACTCTGAATCGATCAATCGAATGGGAAAACACCTTTCCTCTTTCGCTTTCTCTCCACTCCTGCTCCTGTGGCTTCCCTTCCGGGTTCATTTCATGCCCCCTCCGGTTACTCTGTCCCTGGCATCCCTTTCTTCCCCTTCATCTTCGGGCAAGCGGGTGAACTCAGGGCCTATTCCCTATTTGATTCTCTAGGCTAGTGGTCCCTTTTTGTCTGGCCATGAATAGTGAGTGGGACCTCATGTTCGCCGGGTAGCCATATTTGCATGGAATTACATCAGTACATTAGTTAGACGAGCTGGTAGCTAGCTACCCCGATTCACACCAAGTTCTCTTTGTCGGGCATGGAGAAAGCTCCAAGGATTACCCTGGGGTTAGACCACTCTACGGAACCCCTTTTGACGGCTGCTCTGTGCTTGCCGGTATCTTTCGTTCTTCCGGCCAAGACCAATTCCGCCTAAGGTCATGATCGAGTCATGACGAGCTAG

>LNC_001516

AAAAGTCCGAAAATGGGCTGTATGCAGTTTGTATACAACATTATACACTGATATACAGGTTCAGTGCAGCAAATATGTGGCCAAAACGTAAAAACATAGGGCTGAGGCGAAATACAGGTGTTCGGGAGCAAGAGATACATGAAAAGAGCTGATATATAAGCTCATATACACTAATATACATCAGGTGTATACAAAAACGGGTTTAGGTTTAAACCCCAAAAATTTCAGCGAATTTGGCCCAAAAGGAGGCCCAAATTCAATTTCTT

>LNC_001517

CAGAAATTCCAGACGCGTGAAAGAGCAGTACTCTGCTTTCCCTTGGCTTCTTCTTCAGACGTCCCTATCCCCATTAATGTTGCAACAGCAAGAAAGAAAGCACGCGTCTGTCTCACTTTCCCTTTCCGAAATGAGTCAGAAACTCAAAGAAAAGGTGTTTAGCCGGAAGGGTGAAAGGTTTTGGAGTTTTGAATTTTGAATTTGGCCTTTGAATTTGATTTTTTTTATCCCTTTCCCCCCAAAAAAAAAATCCGAACCCGAATTTTTCCCCTTTAAATGGTCTTATTCTTATTGGAGAAGGGGAGGAAATTTTTTTTAGGAGAAGCAGATAGAAAAATTTCCCAAAGCTGTAAAAGATCCCCAGAAACATACACTTCGGATATATATATTTTTTTCTGCTCGATTCTTTGTGGTTTGCAAAAAGATTAACCGGATTTCTCGAAAGTTCTTGTTGTGGTGTTAAGTTCGACGTTCCTCTGCTCGTCCTCGTCTCAGTTTTGCTGTCCGAAAAAAGCCATAGAGGCTCAAACCTCTTTTCGTCATTAAGTCAGCCAGACCATAGGTGTACAGGGAAGGATGTGAAGATGCAAGGGGGCTGAAAAACTGAATTTTCAGATCTCAACAAACCCAAAAGAGGGCAGTATACATGCTGTATACAAGTATATACACAAGTATACATAGCAAAAATATCGATATATAGGTTCAAAAATTAAATGCAGGTTGCAGGGGTCAAGATAGACCGTAGACACTCGGTATGCAGCCCGATATACACAAAAACGGATCAAAATATGGCTTATATACACCGATATACACCTTTTGTATACACAGGTGTTTGGGAGCGAAAATGGGCCGAAGGAGGCCCAAAACGCAGCAATGGGCCCAATTTCTTCTCTCCCTCTCTTTATCATTTGATTGCAATATTTTTGTTTTGTTTAACTCTAATTTTAAAATTTTGTTAATTAACTTAATTAAATTCCCAAAGATGAACCATAT

>LNC_001518

GTCAAAATACAGAAATTCCAGACGCGTGAAAGAGCAGTACTCTGCTTTCCCTTGGCTTCTTCTTCAGACGTCCCTATCCCCATTAATGTTGCAACAGCAAGAAAGAAAGCACGCGTCTGTCTCACTTTCCCTTTCCGAAATGAGTCAGAAACTCAAAGAAAAGGTGTTTAGCCGGAAGGGTGAAAGGTTTTGGAGTTTTGAATTTTGAATTTGGCCTTTGAATTTGATTTTTTTTATCCCTTTCCCCCCAAAAAAAAAATCCGAACCCGAATTTTTCCCCTTTAAATGGTCTTATTCTTATTGGAGAAGGGGAGGAAATTTTTTTTAGGAGAAGCAGATAGAAAAATTTCCCAAAGCTGTAAAAGATCCCCAGAAACATACACTTCGGATATATATATTTTTTTCTGCTCGATTCTTTGTGGTTTGCAAAAAGATTAACCGGATTTCTCGAAAGTTCTTGTTGTGGTGTTAAGTTCGACGTTCCTCTGCTCGTCCTCGTCTCAGTTTTGCTGTCCGAAAAAAGTCCATAATGGATTTCTTTCCTTTGCATTCCCGAATCGAGCCATAGAGGCTCAAACCTCTTTTCGTCATTAAGTCAGCCAGACCATAGGTGTACAGGGAAGGATGTGAAGATGCAAGGGGGCTGAAAAACTGAATTTTCAGATCTCAACAAACCCAAAAGAGGGCAGTATACATGCTGTATACAAGTATATACACAAGTATACATAGCAAAAATATCGATATATAGGTTCAAAAATTAAATGCAGGTTGCAGGGGTCAAGATAGACCGTAGACACTCGGTATGCAGCCCGATATACACAAAAACGGATCAAAATATGGCTTATATACACCGATATACACCTTTTGTATACACAGGTGTTTGGGAGCGAAAATGGGCCGAAGGAGGCCCAAAACGCAGCAATGGGCCCAATTTCTTCTCTCCCTCTCTTTATCATTTGATTGCAATATTTTTGTTTTGTTTAACTCTAATTTTAAAATTTTGTTAATTAACTTAATTAAATTCCCAAAGATGAACCATAT

>LNC_001519

TACAACGTCAAAATACAGAAATTCCAGACGCGTGAAAGAGCAGTACTCTGCTTTCCCTTGGCTTCTTCTTCAGACGTCCCTATCCCCATTAATGTTGCAACAGCAAGAAAGAAAGCACGCGTCTGTCTCACTTTCCCTTTCCGAAATGAGTCAGAAACTCAAAGAAAAGGTGTTTAGCCGGAAGGGTGAAAGGTTTTGGAGTTTTGAATTTTGAATTTGGCCTTTGAATTTGATTTTTTTTATCCCTTTCCCCCCAAAAAAAAAATCCGAACCCGAATTTTTCCCCTTTAAATGGTCTTATTCTTATTGGAGAAGGGGAGGAAATTTTTTTTAGGAGAAGCAGATAGAAAAATTTCCCAAAGCTGTAAAAGATCCCCAGAAACATACACTTCGGATATATATATTTTTTTCTGCTCGATTCTTTGTGGTTTGCAAAAAGATTAACCGGATTTCTCGAAAGTTCTTGTTGTGGTGTTAAGTTCGACGTTCCTCTGCTCGTCCTCGTCTCAGTTTTGCTGTCCGAAAAAAGAGGCTCAAACCTCTTTTCGTCATTAAGTCAGCCAGACCATAGGTGTACAGGGAAGGATGTGAAGATGCAAGGGGGCTGAAAAACTGAATTTTCAGATCTCAACAAACCCAAAAGAGGGCAGTATACATGCTGTATACAAGTATATACACAAGTATACATAGCAAAAATATCGATATATAGGTTCAAAAATTAAATGCAGGTTGCAGGGGTCAAGATAGACCGTAGACACTCGGTATGCAGCCCGATATACACAAAAACGGATCAAAATATGGCTTATATACACCGATATACACCTTTTGTATACACAGGTGTTTGGGAGCGAAAATGGGCCGAAGGAGGCCCAAAACGCAGCAATGGGCCCAATTTCTTCTCTCCCTCTCTTTATCATTTGATTGCAATATTTTTGTTTTGTTTAACTCTAATTTTAAAATTTTGTTAATTAACTTAATTAAATTCCCAAAGATGAACCATAT

>LNC_001520

TTGATGTGTTCAGGCCTGATGGTTGTAGATTTCGGCATTTGACGGATATCTGGGAAGTTAAAAACGCAAGTTTCAGTCGAAAGTTGAGGTACGTTAAGGCTACCTTCCTAAATGGATTATTGACAAAAGCATGTCTACCAAGTGTTTGAAAGAAGTTTTGAGGTTAAGTTCTTAGGGGACAAGCAGAGTGCTTAACTCTCCGTATTCTATCCTAGCACAGTTTTCAGATAAATAATTGCATTATGTT

>LNC_001521

GGTAAATCAGTAAGGGGTCGTTTAGTAGAGTGTATACAAATAATGCTAAACAGAGTGTATTAGTAATGCTTGCATTAGTAATGTTTGTATTAGTTATGCTTGCATTAGTTATACAATATATTATTTCTTATGCATTGTTTGGTTTGGTGTATTAAAAATAACATGTATTGTACAAATAACCCTTCTTTTTCTGTCCGGCATCTTTATGCCAATGCATTCCCTTGATGCATGTCAGATAACCGAGACTTGTGAAATTCTGG

>LNC_001522

CATTATTCGAATCCAATAGGTCTTAGTTTTGCCCCGACGTCGCTCGGCCGACTTAGATCCCTGTAGACTGGTGTAGCAGACTTGAGTCTGATAGTTTGGGCCTTAGTTAGGTGATACTCTTTCTCCGACTATAGTTATCCTTATTTTCCCCGATGTTACGGCTTCACGAGTTACATCGGCGACACTAGTTCTACTTCGCGATTTGAATTTGATTTTCGATTTGGTTCCAAGGTATTTT

>LNC_001523

AAAATAACTTGGCGCGCAGATGCAAACCCTAGTCCGAGCTCGTCTCTCCCTTACCTCTGTGCTCGAGTTTCTCGCCGAGTTGGACATAGAGAAATTGATGATATTAGAAGCCTTCAACATATTGGTTTCCTCATGGCTGAAACAGAATATATAACCATTGATTTTATTGAAGTCCCTTCATGCCCATTGCGTTCAAATATTGTGAAGTTGTTAGAGGTGGAACAGAGAAGGAAAGCAA

>LNC_001524

CTTCTGAAGTATTGAATTGAGGAATCATCATCATATTCAACAACAATTTGTTCAGGCATGGTTATCTGCAAGTAAATTAAGTGTCAGTTTGACAACATTTGCGGCCTACCTGGAAGAACTGACAACGAAAAGTAGTATACTTGGTTCAAGAAACACACAATGATACTATTGCACACTTGGCAACAAGTGGAATATCACTTTTGAACGCCATCTCACTTTCCAATATTTGGAAGTTTAAAGAGTAAAGATTCCAGTAAACTTCAAGTAGAATCTCTTGGTTCTGCTGGATGTCCTGAAGAATTCAACTGACATCTCCTATCTGTGGTAGTGATGCTTTTCAATCTAGAATTTCCCAAATCAGTGTTGGTTCGAGGCATAGCCCTTAACACAAGAAAGGTTCAATGTGGAAGACTGGCAAATCCAAGTAATATTTAGCACTATGTCATATGAAGAGCCCTTAATTGCACTTATCATATAGTGACTTATTTCAGATATCCGAGTGGTGCAAAATAACGGGTAGGGAAAGAGCCCCAAGTACCTACAATCCTTTAAGCTTTTACCAGTGCCTGGTAGCTGAGGGTGTCATCTTTTGTATTAGTCTTGTTGTAAGTCATATGTAACAGAGGGACAGCAAAGCTGAAAGAGTAGCTATATGCAGAATTACAGACAGGTTTAGAAATGGTTGATTATGAATTTCAGAAAATAAGATGCCCTGGGACCTCTTTAAGTGTAGATAATGATTTTGTAACCTCTGTAAAGGCAACGTTATGTTACTGTGGATGTGAATCATTTGGGTTGACCCATAGGCATGCCTAATTTTGTGACTTCACAACTTTTACTGTATCTAAAGAGTGATTGCAAATTACAGCGATTAAGAAACAAAAACTGCTGGAACAGTTTTAAATAAAGACCGACATTTCTACTGAATTCCGTTAGTAAATAACGAATGTACTTCAAGATTGGAATAGAGAAAGTGGTTGTTGCATGACATCTGCGCCTCTAGGAGGGAGAAGAAAAGGAGTTTTGCTATAGAACACCAATAATTT

>LNC_001525

CTTTATTGAGTCAGCCGGCTATAAAAATCAACGAACAGGTCCCGGAGCCTGAAAATATGCATCGAATCAGAGAGTTGAAGAAATTGGGCCAACAAGCCCACTTTTATTTTTAAAAATTGTATTTTGTTACTTTTGGGCCTAAGCCCTTTGCGTTTTAATTATCATTATTTGATAGTTGTTAGGACAGGTACCAAGAGAGAAATTGAGCCACAAACCCAATATTATTAGTCTAGGACATGTGTGGAAATGGGCCGAAGGCCCAAAACGAAAATGGGCCCAAATACTGGTCCAGGCGGACAGAAACCAGATTTGGGTCCAACCCTCTTTCCCTCTTTACTTCACTGTATTTGTTTTGCTTGTTAGTATTTGTCGTTAATCTTAGGGTTGAAAAGTTCAAATCCCCGAAAGACGCTCATTTCGTTTTTAACAACTTAACTAAGCCGATCATCTTTCAAAAGACTTAAAAG

>LNC_001526

ATGATTATGTCAAGTTGGAAAAACTCTCTATGTGTTGATTTATCTGTTTTCATTAGCTCTAAATTTGGCATGTTTTAATAGATTATATAATTTTCTTGTCATTCAAGTTTGGTCGAATAGATATGGCTTAGTTCTTGCTTCATGTTTGAATTACGTGGCAGATTCATGTATGGTTTTTTTTTTTATCGCACAAACTTGGTCATTCAAATCTTACCCTATGCTTGTATGATTTGCATCAGATTCTGTCCAGTTTGAGTTTTCTATTTAAACTTTCATGCTTTGAAATTTGTGATTTTCGGTTAAATTGTCTCATTTATTGGTAGTATGGTCTAAACTTGTCCTATATGAGGTTTAAATCATCGAATCGAGATTCACGTCAAGTTAAAGGATGCTTGTGTTTCCCATATTACTCACTTGAGCTTAGATTTATCCTTTCCGTGATAGCAGCAGGTTTTTTTTTTTTTCGGGTTTTAGTTTGAATCCTCTTATCATGGCAACTCGGGTTTCCAACAAATTTCTTGCTGTTTTGGTTGTCATATTTGAGCCATTTTTATGCCTTTGTTCGGCTTATTCCAAGTGAAAATAATTGCTTACCCCTTGTAGTTTGAAGTTATAAAATGTCCACTTCTAGTTCTTGTTTCTTCC

>LNC_001527

CTTTAACGTGTTTGGGAATAACACACCTCTCTTCAGAAAAATATCTAACGATAAAAATTAAAGATTACTTTCCACTATATCCTTTTGATCACTCCTAACTATTCTTTTAGAATATGTCAAGTAATTATGAGTCCAACAATTCTCTTAAACTCTAATTTGGTTTTACTTATTTTGAATTATGAAAAAAATCCTGAACAAATACTATCCATTGTTTGGTAGGCATACTTACAGTTCTAACTTCCGGGGTTTGGTCAGATAGGACCGGATCTGATTATAGCTTCGAGTTTAGGTACCATACTGCAACTGGATGGTGAAGGAGGGGCACAATAAACCTCCTGGCAAGAGGCCAAGGTTGCTTGCTAACTCTGAGTCACTTGTTGGAAGTGCAGCTTGAATGGGGGATATTTTATTGATCTTTTAATCGATCTCTCCAAATTTCCAGAGCCAACGGTACTACACGAAGCACCATTAACCAATTTTAAGCAATTGAGCCGTATTCTGCTGGGATCCGTGGAAAAGAGGAGAATCGAGCAGTAAAACGAAAAGGGTTTCTCTGGGTTTTAGTGTTGAAATTGGGGGTTTTCCTTTCTGCTTTGGCCGCCACATTATTTGAAGCACCCATTCATGTAAGGGACAAGCTATGTGCTAATAATACACGTTTGCCAAAGTCTAGTGGGAACAGGGCAGTGAGCACTGGAGATGACAACATTATCCATCGAGTTTCGAACAGTGCACTATTTGGCCAAGCTGAGACAAAGTTCTGATTTTTGGAAGAGCAGCCACAGCAACTCCAGGAATTCAACTATTTAGGCTTTTTTATCCCTTTTGACAAAAGTTCCCATGTTTCTCTGGTATGTCCTTCCATTAATTTCTTTTCCTTAGAGGAGTTGGTGAAATGTATGTCCTTAAAATTGTAGAATTTGACATTATCTTTGTGTCATATATATTATAAGAAAGGCTACTGTTTGTTTGTTCTTGTTTATTGTTGACGGAGCGTG

>LNC_001528

CAAGAAACGATAGAGAAGAAATTTTTGGGAAAAAGATCGGAAAAGAGGCCAAGAAAACCTCAAATTTTGTCCAACAATGGTGTTAGATGCAAATAGGAAGATGATGGCTTTACAAAGCCCTAATGTGACTGTCAGACCCTTTTCCAAGGGTCTCACGCTCCTAATAGGTGTGTGTCACACGCTCTTTAGTAGTAACTGCCACATAGGATGCCAAGTCAAAAAATGTGTTTAAAATATTGCGTTTTGATGAGTTCAGGGGTTCAGATGACAAATGAGTAAGTAGAAGGGTTCACAATACAAACACATACAAGTACAAGGGTCCAC

>LNC_001529

CATAAATGGTATGTCAATACCCTCCGTTATACTTTAAGGTTGTATATGCCCTTACCGTCATTCTTTTGGGTTGTATATATCCTTGAATCTAACGGATGGACACGTGACATCATCTAAATGATTTACCCGATTTTATTTATTTTTTACCCAAAAATTAAAAATCCACCCGACCCACAATCAAAATGGGTGGTGCCAAAATCCACTCTTCATTTTCATCAAAAATCATCCCTTTATTCTTCTCAACCCA

>LNC_001530

GCAAAATAGAAGAAAAAGGTTGGTGCTCAAGATTTAATCGATCAATAACAACTCCAGCATTCATCTTTACTTGGTAGTCACAGAGAATAGGAAGGAAGTTAATTGCTAGGAGTGCAATGGGATGATGGGAGGAATGAAAGAGACTGAAGAAATGTTAGATTTTGCACCAAAGCATTACATACGTAGCAATGGACTACATGAATACAATATTGGAACACCTTCTGAAATCCAATGTGAAGTATAGTTTTGTGCGAGACATGGGCAAAAAAATGAACTCTATTTAATCTCAAAACTATTTTCGATTCTTCTGCATGGCAGAAGTAAGGCTTAATTTCACTATTGAATAAACACGTGTGCTACACGTGTCCTTCTACATCTTTACTTTTCAATTATTTTCCTATGGTTCTTTCACCAATCTATCACTATGTTTTCCCCCATATAGAAACTTAGAATGGAAAAGAGAGCCAACGCAAAGTCACCATACTCTCTCCTTTCAATAGCCTGAAATTTGACTCTTAAACTTCTTCTGAGTGAGTTCTTTCTACCTTCTTCTTCCTCTGCTCGATTGATATGGACAAACATCGAAATAAGAAATACAAAGAGCCAATAAATTGGCTCGATTCTACCGAACATGAAGGGCGAATTACCTATTTCACCAAAACAGATGCGATAAATTTTGATTCGAATGTATACTATTTCCATGTACATCTTTCAAAACTTTTAGTTGAGGATCTACTTGATTCTTCATTCAATCAGTTATTCAAAGTTTTATGTGTGGTTTCATTTTGTAGCTTTCATCCTGGCTCAATACAAGGGTTTTTAATTGCTATTAAATAGGTCTATACATTTCTTTTATGCAGAACGCAAATTTTCCTAAAAATCTCAAATAAATTTTCATTGTTTACTCTGCAGATGTCTGATTTTGTTGCTA

>LNC_001531

TAGATGAGGGGCATCCAATACTGTGAGTGAGTTCTTTCAGTCACATCCATGGTTGAGGTCTCTTTCCCTTTTTTGCACCTTCTCTTTCACATTCGCTAATATTGCCTAAACTGCAATATTCATGGAGTTCGATTATAGGACATTACTGCTCATATCTTTAGCCTGTGGAGTTATGTTCCAACTTCCTTTTTGATGGAGATCAATTAGCAATCCGATGAACTTCATTTTGTTTTATGCCTTAACTTTCTACATTGTTATGAACACTTCTTTACAGCTTTGCAGGATCTTGTGGCTTTCATAGCACCACTCCAGCCTCTTTATGTTTTCATTCTTATTTTATCATTGTGGTCCCTTGTGCCTGTATGCGCGAAGTTAGTTCTCACTTTCACTGGGGAATTTCTAGTAGTTCCTATCTCCGCAGCCCAGAACCTGTCCAAG

>LNC_001532

GGAAATTAAAATATATAGCAGAAGTCCATCCACCACTCAACATCACCACCCACCAACCAAAAGAATTTAAAAGAAATTCAAGGGTGGAGTGTTCTCGTGATTTCTAATAGTGTGTCCAAGCGCAAAACCCCGAAAGAAGTGGCCGAGCGGTTGAAGAGTTGGAGTGACAACCTTGGTACCAAGATTAGAATGCCATAAGAGCGACACTAGGTGAAGTTTTCCTATCTGTCTAAGCTTTGGTGGGTAGAAATATCCTGTACGTGCACTAGCAGGAGGTTGGAGGTAATTGGTAAATCAGTTGAGGCACACACAAGTTGGCATGAACACCATGGTTATTAACTTAAAATTTTGTTCGAGAACAAGCAGTTATGTTTGAGGGAATGTCCCAAATTCTTATTAATCATACTTACCAAAAAAAGTGACGACCC

>LNC_001533

TTCCAATGTTGTCTTTTGTTGGTTGTACTTATTTTATAATCAGAACTCTCTTCTATCTATCTTAATGAGATTGTTAAAGAAAACCTCTTCTATCTATCTTAATGAGATTGTTAAAGAAAACCTGCATGACACTTGTTGTAAGCCGAAGCAAGTGCCTTTACTTAAGAACATAGAAACTAACCCGAGGATTCTTTCGATGTTGTCTAAAAATTATTGTGTGCTGTACATTTACATGTGGCACTTTGATCTGCTATATTATTTTTGGTTGATAGAAGAGCATAGTGCCAATCTTATCAGTCGTAGTCATATCGTGATGGGAGATAGTTAATTAATAAGTACATTTCCGTGTGAATCCATTCTGCTCGTACTATTGCA

>LNC_001534

CGGAATTTCAATGTCCACTCCATCTCTTCTTTACTGTTTTCTCTTTCTCTACAGACTTTAAATCTGTCGCTGAAGCTTTGCTGAAGATTTCTGTTTCTCTCCTACGAGCTGCGCAGCCTATAAATTCCTCTCTGAACGTTTCTTCAACATTTCGCTTTCTGTCGGATGACCTGCTCAGGAAATTCTCTCCAACTCAAACATACAAATACCACTGATACAGAACACCCTGCACAGTCTGCAGCAGCCTCTTCGTTATCCCAACCTTAGAGGGATTCTTTCAACTGTTGACGCACACTGTATATAGGAGCTACCCAGTCGACAACATTCCCAGGCATCCCTCACTTGCTCACAGGAGATTGCCCACAGCTAAAACATAGAAATAACGCTGATACAGAACAGTGTGCAGGCTGTGCATCTGACTCTTCATTATGACCACCTTAGAGGGATTGCTTCCACTCCTCACGCAAACCGTATATACCGGCTAGCCAGTCCACAAGATTGACACACATCCCACACGTTCTCACAGCATCATCTTCATTACCGTCACCTTAGAGCGATTGCTTCAACTGCCAACACAAATCGTATATACAGCCTACCGACTCCACAAGATTCACAGACATCCCACCCGTCATCACAGCTACAACATTCCAAGTACAGTGACAATTGTTATGTCACCCAGTTGTATTAGTCACTCCAGTACGAAAGTCTCTCATAATGGTACTACGACTAAATAATCGTACTTTATTTGTGCTGCTAATTTACTGCTTCTCTTACTGATTTTGTTTCCACTGTTTGCCCATCACTTTTCATTCAACTCTTTTAATTAATGTTTCAACTTTTTGCACTTTTCTTTTTTCCACGATAGTCCAAACTGTTAATAGCTATTCAAAATCATGAATTGCTAGCCATACCACTGTAGGGAAATAGTTGCTTCTAGGCTTATCCTTACTTTCTTTCATCAGCTTATCTTTTCTTTTTCCACAATTAAATGTGCTTACAATATAAATGGCATTTACATATTACACTCACATAGGACGCAAAAAACTACATAAGAAAAAGAAACACTATAATACAAAATACAACTTTCATCCTTTACTTTAGTTTTGTTTCCGAATGTCAATTCAAAGGAGAAAGCATTGTTTAGTTGTTGTTTCTTTTGTTACTTCCTATGTCCTGCATTCCTACACAAGGACAGAAAATTTCTACAAACTTGCCAACTTTTGTACTATCTTCAAATCCAAACTAACAAAATTTATTGTCTTTTCCCACATATTTTCTACAACGTATTCCTTTGTGTGTGAATCATACAAAAGGTACATCATTTGTTTCTGCTACATGTTTGCTGTTTATATATTTACCCCCACCAAATATATTTGAAAGCTGTATCCTTCCTTTGCATCTACTTCCACTGTAACTTGTTCCTATTAGTTTAACTTTCTGTTCATAACTCATTTTCTCTCTTACTTTACCTCCGCTAAAACGCCCTAGCAGTTTTACACATGACTGTACTTCCACGTTCATAAGACTGCTGTCTCTGTTTTTAGCTCTTCCTTATTTGGTTTTTTTATACACCATACAAAGTTTATTATGCTTCTGCAGTTTATATCTCATAGTCCCCAATAGTAACAACTACAACTGTAAAGTGCTATAGTTGTTCCACATACTCTCACAGACTTCAAATTAAGGTTCACCAAATTTTCAGTTCTCCAGTTTCTCTATTAATAATGGACAGTCACGGATTTTTCTCTTCTGTTTGTATCAGAAGTTCTTTTCAGACAGAATCAACGTGTGAGATAAAAGGTACTATATGACTACATTTACGTAGCAAATACAGTGCCCATGTTT

>LNC_001535

AAATGTCATTGTCTCTTCCTTCTATGCCAAAATAGTGAATTTTGAAGTTTATTCAGTAAATTCAATGATCCAAAGTTCTGTAATGCCAGTCATTTTAACTATTTTTTCGTTTGAATGGATTGTTGTCACTTTTTTCACGTTTTGAATGAATTGTTTCTTTTATTTTTATGTATGGTTAATAACCGAACCAAACCAAACCAAAATCGAAAACAACCAAACCGATAAATGTATATTATATTTGCTTTGGTTTGGTTTTGATAATTTTAAAACCGATTAAGTTGGTTTGGTTTTGATTTTAACCAATAACCGATCCAAACCAACCCGTGAACACCCCTACTAATGTATCCAACAACTGAATGCCGACTATGATCGTGAATAACTCCAGCAGCCTCACCTCTGAGTGAGTGAGTGAGTGTAGGTGCTCTGCTTTTTGTTTTTTGTTTGGGAGAGAAAAAAGAAGACGGTAAAATGCAGAAATTTCAAATATATGTGTGTAAAGAAGAGGAAAGCAGCCAACTTGGATTGTTTTTAGTCTTGCTTATACAGCCATTTCAGTATAAATTCCTATACATGTTGAATGAAAATCTGAATTTCTATCCTTGTCTTAGGTTTTCCTTCCTTCCTTCCACCGTCGCAACATTGAGTGCGTGTGTTACCTCTTTGTATGGTCTTTGACTAACTCTTTTCTCATAAGTTAACTTTTTTGAGGTTGAGTTAGAGAATGTTTTTGGGGGCATTTGCCCAAATGGCCAGACACTGAAAGTTTTATCTGCCAGCATGGAGTTCACAGTAATAGGAAAAAGTGACCTCAAAAAGGGCCACATTATGCATACAATCCAACTTTTGATGGCAGCATATTCATGGATTAAATCAACGATGATAATTTGGTTGATACATGGTCCAAAGCAAATATAGTATATTCTCCATACTATGCCTAAAATTATTAGTATAAATATATTAAATTAAATGCGATTTCTCACTTGGAATACTCGCAAACCATT

>LNC_001536

ATTTCTTTTCATCTTCTCTCTAGCAAATCCCTTTTTACCCTACGCTTGAGAGCTCCCTCTCTGGCTGAAGTTTGTCTCTTCGTTGCTACTCCTCTTTCTCAGCCATTCCATGGCTGTTGTAGCCATCGGATAGCCTCCTTCCGAGGTTATCCATTTCCCCAAACGCCACCTATATCACCCTTCTTAATCACAATGTCCAAAATAGTCCCACACAACTCATCTCTCAGATCTGAAGCTTCACACTTAGCCAGTCGAAAATCATGATTTGCCACCTGAAAACTCCATTGATAGTTGAGAAACCTAGTAACCTAAATCTCTATGGTGGGAGTTCGTAGCATGCTAAGGAGAATAAAACTCGAAGATAGTTGGCAGGAAAAAGTGGAAGCACAAAATGAGGAGGAAGAAGTGATGGATATAAGGAGCCAACAAACAATGTCTTGAAGAC

>LNC_001537

CAAGACCCTGTAATTTCAGTTGAAGCTTATAAGTTTTCTCTGTGGCTTACCCCTGCACTATTTGGAAGTGCAATTCTTAAGCCTCTATTCAGATATTTGCAGATTCAAAGTTTGATTCTTCCGATGCTCGTAACCGCCTTTCTGGTTCTATGTCTCCACATATCTCTGTCCTGGTTTTTCATATTTCAGTTGGGACTAGGAAAATCAGGTGCAGCTATAGCCTTTTGTTTTTCGATCTGGACTTTTGTAGCATTACTTGTGTTTTACATCAGTCGATCGAGTTCTTGTGAGAGGACTCGAACTTATCTGAATAAAGATGCCTTCCTTGCTGTCGGACAATTCTTTCGTTATGCAACTCCATCCGCTTTAATGGTTTGCCTTAAATGGTGGTCATTTGAGGTGCTCATTTTGCTATCTGGACTTTTACCAAATCCAAAACTTGAAACATCAGTACTATCAATATGCTTAACGATTT

>LNC_001538

TTACCCCTGCACTATTTGGAAGTGCAATTCTTAAGCCTCTATTCAGATATTTGCAGATTCAAAGTTTGATTCTTCCGATGCTCGTAACCGCCTTTCTGGTTCTATGTCTCCACATATCTCTGTCCTGGTTTTTCATATTTCAGTTGGGACTAGGAAAATCAGGTGCAGCTATAGCCTTTTGTTTTTCGATCTGGACTTTTGTAGCATTACTTGTGTTTTACATCAGTCGATCGAGTTCTTGTGAGAGGACTCGAACTTATCTGAATAAAGATGCCTTCCTTGCTGTCGGACAATTCTTTCGTTATGCAACTCCATCCGCTTTAATGGTTTGCCTTAAATGGTGGTCATTTGAGGTGCTCATTTTGCTATCTGGACTTTTACCAAATCCAAAACTTGAAACATCAG

>LNC_001539

CATGGACAAGCTGCTCATTTTAATGGGACAAGACCCTGTAATTTCAGTTGAAGCTTATAAGTTTTCTCTGTGGCTTACCCCTGCACTATTTGGAAGTGCAATTCTTAAGCCTCTATTCAGATATTTGCAGATTCAAAGTTTGATTCTTCCGATGCTCGTAACCGCCTTTCTGGTTCTATGTCTCCACATATCTCTGTCCTGGTTTTTCATATTTCAGTTGGGACTAGGAAAATCAGGTGCAGCTATAGCCTTTTGTTTTTCGATCTGGACTTTTGTAGCATTACTTGTGTTTTACATCAGTCGATCGAGTTCTTGTGAGAGGACTCGAACTTATCTGAATAAAGATGCCTTCCTTGCTGTCGGACAATTCTTTCGTTATGCAACTCCATCCGCTTTAATGGTTTGCCTTAAATGGTGGTCATTTGAGGTGCTCACTTTGCTATCTG

>LNC_001540

GAAGAATTGGTGTTAAGATGCTATGTTTTGTGACTTTTCCCCTATTTATCTTGCTAATAGGTTTGTTTGAGAAAGAAATTTAGCAGTCTGCTTCTGGTCATGTGAGATATTGTTGACCACGCCAATTGTTCATACTCAAAATGTTCTCTTTCTGGAAGACAATGCGACTATATGTGTAGAACTGGAAAGTGGCTTTTATCTGTTTCATCCATGCTTTTATGGTTTGTCTTTAGTTTTGCTGCTTATATGAGCATAACAAGCTTAGGAGGGTTTGAATGTAGGCA

>LNC_001541

CACTCACTCTTACACACGGAATACTCTCATACCACTCAGACATCTATATTGCTCTTACTTGAATAAAAATATATTGCTCACTTTAAAATTTAAGCTTTTTCCGGTAAATATAAAGGTTCCTCTCTGTGTTGCTGTTTTGCCGTGTTCGGGTCCAGTATCTGATTAAGTTTTATCTTCATTGCTGATAAATAATTCTGCATAATTGGTTAGTTTCCTTCCACTAGTTTAATTTTTGCATTGTTCATATTTTATGTATGCTTGAAATAATAGAGGAAGCATCCGGTTTAGTTCCCTTATCCCTTTCTTTCTCAATATGTGATTATATATTCTCCACGACTTGTTGGAATTCTTACGTGTCCATACGCTGCTTGTGTACATGAATTAGTGTGCCCAGGAGTTCTACCATTTTTGCTAATATCAAATTGTCTTTTTGTATTATCATTACTTGCTCCTATTTTGTTGCATGTTAACAGGTTTTGAACTCAAAGTTCAAGATATTGAAGAGTTGCCATTGAAGGAAAACATTAGACAAGCTAAATTTGTTTAATTTGGGTTTTATATATTATTTAATTTCTATTATTGTCTTGTAATAATTTGTAAACTCTAACTCTCTCTCATATATGTAATAATTTGGGGGATCGTCTAAGGGAGGGATGGGGATTTACG

>LNC_001542

CTAAATTTGTCCTTACGGCGTCGTTCCCGCTTATTTTATCCCTAAACTAGTCCTTACGGCATCGTTTCCGTTCCCGCTTATTTTAGCCCTAAACTCGTCCTTACGGCATCGTTCTCGTTCCCGCTTCCTCAACTGTTGTCCTCTTTTCATCCCTACGTATTCGTTGCAGTTCGCGCGTCTGCTTATCGTCTTTCGCCCGGCACATTGTTTTCTCATACGCAACAACAGGAAGACTGAGCGAGCATGGGTCGTCCTTCGCCGCGCGCCGCCTTTAACTCCATCACAGCTTGGCTCCTCTGCCTCTTGTCGGAAACACTAGCGGGACTGGGGTAGTAGATCGGAAGATGCATGTACTGTCTTTGTGCATCCTTTAATTAAAACTCTGGTTACGCCTATTCTCCATGTAATCCGCTCCAAAGAGTATTTGGATTATGAAACACTGCCGCCAGAAAGGATAATTACGTAATTTACTATGTCAATGAATTAAATGTTTTTGTGTATTTGCTGATTGGTTGTTTAGGAAGCAGGATGATAAAAAAGTGTTCGGAGGGGGAAAGTAACTCTGAAGTCTTTTGGAGGATTCACACAGTGGATAATGTAAAAGGCGTTATCAAAATTCCTTGAAGATGTAAAGAAGAAAAAAAGACTTCTTTGATAGGTGTTACTTCAGCTAAGAGTCTGTTGGTAACCTCTCTAGGTAGGTAGGGGTAAGGTCTGCATGCATTCTACCTTCCCCAAACCCTACCTGACGGTGGGTATGTTGTTGTTGTTTCATCCCTTGCTTTGGAAAAGGTATGCAAAGGCACATCTTTGTTACCCTCTAAGTCTAAGACATGTTAAATTGGTGTACATAGAAAAGACTCTTCTATTGATTACAAATTGGTTCGTAGGTTTTACTTTTATTTGGTTCCACATATGTGAGAGAACAAGATTTGAAAAAAAATATGTACTGAACATGAAACTGTTAACTGTCTTTCAACAATGAATTATGATGTTGATAAATGAGAAAGCAAAACTCAATCATGATCGATTGTGTCAGTTTACTTGGTTTGGTTCCACATATGTGAGGTGAGTTTGACTTAAAATAGAAGATACAAAGACAACAAGCTACTCTCTTTCTCTACAATGAATTAAGTAGATCATCTAACTGGAATGCTAAATAAATGTCCAATTGATGACCACAACATACTCCTTCAACCCCAAGAATGTCATGAAAACAAAAGGAAGTCAAAAGTAATCTTAATCTCCTGGAATAATTTTGTGAAATAATATTTTTAAACTGTTTCTAGCTTCCTGGAGACTTTCCTTTTCCCCTTCTTATCAAACCTGACCAAGAGCATTGATTGAGTTTAAAAAGAGATAATAACTGAAGTAGTGATATAGGAAACAAGGAAGATAGAAATAACCGAGAAAAATCAGGGGAGACAGAAGAAAGAGCAATATAAAATCCAATATATCTAACTTGGGGATCTTGACAATTTACATCACATCCATACTTAAATTCATCAAATTTCCTACCTAAGAGTTTGTAAAATCTTGTTTCTGCTGGATAAAAGCAGGAGTTCCAGCATGTAAAAATAGATATATGGGTGTCTACTGTTACCGTCACTACTAAATGTATCAAGACATAAAGACAAGGAAGGGGGGATGGCCTAGTAGAAGAACCCCCCTCCACCTCGAACAATGAAGTCAGGGATTTTCTTTTTAGAAGGTAGAAAGCTTTTCACTGGCATCAAACGAGCATTAAGTGGTGCTGGGCTATTTCATACATCAGCAGCGAAAGCTAAAAATAGAAATGGAAGAAAAGAGCTAAGGATCAAAATAGGTTGCCTATTATATATTCATCTATACATGAAGAGTTTCGAAGGTCTCTAGTATAGTCTCAGCCTCCCTTATGTATTTTACACCACCAAGTAAACAAAAGTAAATTGATAAACTTCTTAATACTCATTTTTTTATACTTATTATTTAATAATCCTATTTTATCTTTTACTATGTCTTTTATAGTAGGTATTCCACGTAGCCTGTTTTTCTTGTATATTTATCCTTCAAATTGTTGATGTATAACATTATGTAATGATGGAAAGCGATACAATCTACCAAAGTATTATATTCATTAAAACAATATAGTACGATACAATACAATACAACACATCATGAAACAATACGTATCAATCATCCAAACAAAGTGTTAAGTTTATATGGGGTTTCAAGAACTTTGCACTCAAATAAGAGTACAAACTTGAAGGATCTCTTTAATCAGTCAAGTTCTCAGCCCCGTCTTTCATCTATAGTGATCTCTCATGTGATCTCCCCTGCAACATGGAAGTCAACCTCAAATTTAACAACATACCCAATGTAATTCCACAAATGACCCGGGGAAGTGGGGGGAGGGTTAAGTATACGTCGACTTTACCAATAGTCTCACGACTTCAATAAAGCATTTCCTAATAGTTTGGAAGGTCAACTTCCATTGTTTTGTCTAATTGGCTGCTCAGTCATTTAACTTTACTTGGCTTACGCATATAATACAGGTGCTTGTGCATTAGAGATTTCTGATTCCACTGATCAGTGAGAGGACCAACGAGATTAGATTATAAGGGTAAATGCATGGGCTGTATGTTTGCGTTTACATTGTCATGCTTTTTTCTGGGGTTCCTTCCTGATAAAAGAAATCAGATACTTACTTTGTATCATACCCTATAAAATATCTCTCTGTGGAAATGGATAACAACAGATCGAATAGACCGATCAGTTTTAGTGTTTACATAAATAAGTACCAACTAGAATTAGAATGTTATCTTGCAGCCCATCTTGCTGCTTTGTTGTGGAATCTCATGATGAATTAGAA

>LNC_001543

CTAAATTTGTCCTTACGGCGTCGTTCCCGCTTATTTTATCCCTAAACTAGTCCTTACGGCATCGTTTCCGTTCCCGCTTATTTTAGCCCTAAACTCGTCCTTACGGCATCGTTCTCGTTCCCGCTTCCTCAACTGTTGTCCTCTTTTCATCCCTACGTATTCGTTGCAGTTCGCGCGTCTGCTTATCGTCTTTCGCCCGGCACATTGTTTTCTCATACGCAACAACAGGAAGACTGAGCGAGCATGGGTCGTCCTTCGCCGCGCGCCGCCTTTAACTCCATCACAGCTTGGCTCCTCTGCCTCTTGTCGGAAACACTAGCGGGACTGGGGTAGTAGATCGGAAGATGCATGTACTGTCTTTGTGCATCCTTTAATTAAAACTCTGGTTACGCCTATTCTCCATGTAATCCGCTCCAAAGAGTATTTGGATTATGAAACACTGCCGCCAGAAAGGATAATTACGTAATTTACTATGTCAATGAATTAAATGTTTTTGTGTATTTGCTGATTGGTTGTTTAGGAAGCAGGATGATAAAAAAGTGTTCGGAGGGGGAAAGTAACTCTGAAGTCTTTTGGAGGATTCACACAGTGGATAATGTAAAAGGCGTTATCAAAATTCCTTGAAGATGTAAAGAAGAAAAAAAGGTGCTTTTTGGGAATGTAAACTAGTAGGGGATCATTTATTTAGAGATTAGATCAGCTTGATACTCAGGGAACTTGCTGTGATTTCAAAGATGATGAAATAAGCCGACCTAAGGAAAAAACTAAACCATGTGTTGTGCTTCCGAATACCTTGGCTTCTTTGGTCTCTTTGGACTGTAGACTAGCTCACAAAGCTGATTAATGGATAATGAATAGAATGGTGCTTTTCTGGTGGAAATTATAATGGACTGTAACTTTCTTTTCCTAAAAAACTCCCATGAACCTAACTCATACTTTTAAGTAAAACAACTTCTACTCCCCCGTGATAGAAATCCCAAACTACTGGTAATTAACTCCATTCACTATTTACTTCTAGAATTATGGTCATTCCCTTGGATTAAGCAAGATACAAGGTTGAGGATTATGGTAGAATGTTGTAGATAGTAGAATGTTGTCTCTCTTTACTACAGGATAGGTTTGGTGCTCCGTACAAGGTTGGGGATTATGGTAGAATGTTGTAGATAGTTGAATGTTGTCTCTCTTTACTATAGGATAGGTTTGGTGCTCCGTACATGTTTCTCCTTACTTACTAGTAGCATTAGTACTAATCCCTTAGATTCTTGTCCTTCGATTTATGTAACTTGCCTGTTATTTCTTTGTGGC

>LNC_001544

CTTCTACAGAAAGTTGTTCCTAGTTGCATAGAATAATGATCATGGCATCAGAGCAAATTACTGACAGCTTAACCCTCCTTTCAATCAACACTTGCCCTAATAAAGTCCACTCTTCCTCAAGTTTTTCGTTACCGGTTTCTTCATAGAGACAGATTTTTTTTGCTCTTTAATCAGCAAAACATTTAAGTGCTAAGTTAGTCTAGTAACTCCCCCATTCTTCAGTCACCCTATTCATGATCGGTGTTCTCTTACTTCTCCTAAATCAAGCCCATAATACTGGAGGGGTGTTTTTTGTCGTGAACTAGAGTAATACCAAATAATTGGTTACTTGCAACGACTTGTTCTTGACTCTTGTAAGCTGTAATCAGTTTGTCATATTCTTGACTCTTGTAAGCTGTAATCAGTATATCACAATGCATGAGTAGGCCTGCTGAAGTGTCATATATTTTAATTCCTAAGTGTTAAGTTGTTATCAGTGTAGCTTTCTTATGGCCTAAAATTGCACATACAACAAGGTAACACAAAGAACAGTACTATCGGTAAAGCATAAACTTCTTCAACCCAGTTGTTTATCTGATGAACTTGCATATGCAATTTCTTGACCATAAACACTATGCATCCTATATTATGAGGTAAACAGACTTCCTTCATCCGAAAGGGATAACTCTTGGATATGAAATCCCCAAGCCCATGTGTTATAGTACATTCCAAATAATGGGTTTAGATTGCAATTGCTTGATTGTGGATACAGTCAGATACTAGTGGTTAGTGAACATCAGACTTGTCAATGATCACCCTTGATCTGGGCTGGTCCTCGGTGGAGGAAGAGAGAGATAATACTTGTGGGGGGGGGGGATTTCTTTTTTGGATAATTTGGTGATTCAACCCAAGTGTCTTAGTATAAATAGCGATGTTGTAAGTAAGTGTGTTGTTACAGGTACCTCTCAAGATGCAATTGTTGATTTATTCTAGTCATTTTTAAGCTCCATTCTTGATTCATTCCATAGTTTTTCTGGTCAACTCTGATCGAAATTTTATGACTTCTTGAACTCTGGAATTTATTTCTTCACTCCAAAATACAATGTAAAATCTTTTAGTTGTGCATTCAGTAATTATCCATGTCCCGTTGTCATTTCTGCAAATAATCCTGATTTCATGAAGGTGGAAG

>LNC_001545

CTCCATATTTCCTTAGGAGCATTAGTATTTTGTTTTATGTGGTTTGACCGAGTGCCTGCAACATTTTACTACTTCTGCCTTCATGTCTCTCTGTTCTGGTTTTCTTGATGATGTATTTTGTCCTTCTGGATTCCGCTCTCATGCTTTGAAGCGTATTATCCCTGAAAGAGAAATACAGCAGCCTCCTTTCCTTGCACATCATTCTTTTCTTGTATGCCCATAGCCCATTAAAAAAATAAGAAAAGATCA

>LNC_001546

GCAAGTGATAACACTTGTTACATTTGAAACTAGTGCTATTGCGATGAACTTAAGTGTGGTGAAACATCATGGCATTGGGCTCATGTGCTAACTTTGAATCCCCAAAACAATTAACTGAACATTTTCATCTTTCCAAGATGGGAATAGTTTAATGGGTTTACAGTTCTGATTTGTTGAGAAACCATGAGAAAATGCTTTACATGGTGATATACTGGCAACTTTTTAGTGCTTAAGTTTTATCAGTAATTTGTTTGCCTAGGAAGGGTCATCATTAAATCCAACAACACAAAAGACTTCCATTTGCACTGAGCAATTGATTAC

>LNC_001547

AGATTATAAAAAAAATGATAAGAGTAAAGTATGTAAAGTAAATATCAGAATATTGAAAGATTTTGATCTTCTTTCTATATAAGTACAGTAAATATTTTGCACTCATCCAACCAAACAATCTTTTGCCCCTCCATCTCCTTTCAATAATCCAAAATTCCCATAAACAAAACCTTCATCTTGTGAGAGAGAGATAATAGAGATGGATACACCACAGGTAGAGACCATGGACATCTCCGGCAAAAGTTATACCCCAATTGAAGATGAGCAGGATAAAACAGTTGACCAGTGTTGTTCTTGCTTTTACGACTGTATTGATAGCTTGTTGAACTACTTGTGCTGCTATGATTTCTGGCGTGCTTAGGGATACACATCATATAACTACTCTGGTAGTCTTATTTTTCCCAAAAGGGAACTTGTTAGATGAACCAGTTATGTTCTTTCGCTTTTGCACTGCTTAGATCAAAGTTATTTATCCATCTTATATTGTAATTAAAGTTCTAATGAGAATTTGAGATGGTCAATATCTTCTTCATTATCGTCTTTTTCCAATTAATTATATGGCTATGCTCCATAAGATAGTGTGTTCAACTAAAACCCCAAATATTGTTTTATGACCAATTTTGATATTCAAAGAGGCCATAGTAGAAGGGAAAAAAACCTTACATAAAAGCATGTTGAGACTCTGTATCAATAGACTCTTTTGTTCTAACCCTTCTCAAGACCCGTGCATAGCAGGAACTTTTGTGCATTGAGCTGACCTTAACGAGGCATCGGTTCTTTTAGCACTTTTTTTCTGATAAGATAAGGAGTGTTGGGACCGGTAGAGTGGTAAAAGCTTCAGTTGCGAGGCTCAATTGATAATAATGGAGGAAATCAGAAACCTGAGAGTCGAAGAATGGTCAGGAATTCTCTTGGATTAGTTTTAAAGTTGGTCAAATTTACTTTTCCATTAATTTTTGACTTCTGAAAGTGTTTGATAAATATAAAAAATAATTTAAAATAAATTAGGAAGGACTTGACAAGATAAAAAAAATAACTTAAAATAAGTCAAAAATCTAAAGTCATTTCAGTTTGACTCTTATTTTTTATTTAATTTTTTTTTTTTTTTAAGCCAATCCAAACGGCTCTTAGTAAT

>LNC_001548

CGTCAATGAAAAAGATTATAAAAAAAATGATAGAATAAAGTATGTGAAGTAAATATTTCCATATAAGTTGAAAGACAGAAAATTGAAAGACTTTGATCTTCTTTCCATATATTTACCCCTCATCCAACCAAATAAACGTCCCCCAATCAATGCCCTTTCCTTTATTTGCCCCTCCATCTCCTTTCAATAATCCAAAATTCCCATAAACAAAACCTTCATCTTGTGTGAGAGAGAAAGACAATAGAGATGGATCCACCACAGGTAGAGACCATGGACATCTCCAGCAAAAGTTATCCCCCAATTGAAGATGAGCAGGACAAAATAGTTGACCAGTGTTGTTCATGCTTTTACGACTGTACTGATAACTTGTTGGACTACTTGTGCTGCTATGATTTCTGGTGTTCTTAGGGATACACATTATATAACTACTCTGTAGTCGGGAACTTGTTAGATGCAATTATGTTCTTTTACTTTTGGTTAGATCAAAGTTATTTATCCATCTTATATTGTAATTAAAGTTCTAATGAGAATTTGAGCTTCTTCATTGTCATCTTTTTCCAATTAATTATATGGCTATGGTCCATAAAATGGTGTGTTCAACAACTAAAACCCCAAATATTGTTTTATGATATTCAAAGAGGCCATGGTTGAAGGGTAAAAAACCTCTTACATAAAAGCATGTTGAGACTGCATATAATAGTCAATAGACTCTTTTGTTCCAATCCTTGCGCAAAACAGGAGCTTTTGTGCATTGAGCTGACCTTAAAGAGGCGTCGTTCGTTTAGCACTTTTTCTGACAGGAAGTGTTGGGACCAGTAGAGTGGTAAAAGTTTCAGTTGCGAGGCTCAATTGATAATAATGGGGGAAATCAGAAACCAGAGAGTTGAAGAATGTCAG

>LNC_001549

GCCTTGGATGAAAAAGCTCTTTACAGCAGAGAATCAACTACATTAAGAGACTGCTACCTTGATTTAGGATCATTTCCTGAAGATCATAGAATTCATCCTGCTGCTCTTTTGGATATGTGGGTGGAACGATACAATCTAGATGAAGACGGCATCACTGCAATGGCCATCTTCGTTGAACTTTCTTCACAAAACCTGGTTAATCTCGCCCTTGCAAGACAGGATGCACGAGCAGTTCTTGGCTTACATAACTTGCATTACATACAGCAGCATGATATGCTGAGAGAACTGGTCATCCACCAGTGTGATGAGAAGCCGGTAGAAGAGAGAATGAGAATATATATCAACATCAAGGGGAATGACTTTCCTGGGTGGTGGTTTGAACAAAGACTTCAACCACTTCAAGCGGAGGTTTTGTCTATTGTCACAGATGAAGATTTCTCCTCTAACTGGGATGATGTGCGATTTCCTAAAGTTGAAGTACTTGTGTTGAACTTTGAAACAAGGACCTACAACTTACCCCTTTTTATGGAGCAGATGAGCCAATTGAAGACACTGATTGTGACAAACAATGGTTTCTTCCCAGCCAATTTGAATAACTTTCAGCTTTGTTCTCTGCTGAATCTTAAGAGAATCAACCTGGAACGCATCTCTGTTACATCCATTTTCACAGCCAATCTGCAGCTACCAAATTTGCGCAAAATCTCCCTGAACATTTGTGAAATTGGGGAGGCATTTGAGAACTCTGCTGCCAAGATGCCTTACATGTGGCCAAAACTCGTTGAGATGAATATCGAGTACTGCAGCGATTTAACAAAAGTACCAGCTGAAATATGTGATCTTGTTGATCTTAAAAGGCTCAGCATCTGTTATTGTCACGAACTGGTTGCCCTTCCGGAAGAGCTTGGGAGATTGACAAATTTGGAAATACTAAGGCTTCATTCTTGCACAAATGTGGCTGAGTTGCCTAAGTCTATAGCAAAACTTAATAAATTAAAATTGCTTGATGTATATGATTGTGTGGCTATGGATAACTTGCCATTGGAAGTGGATCAACTCTGTTCTTTACAGACTCTTTGCATGGGAAGTTCCCTGGGTATTGATAAGCTGCCCGATTCTCCGCAAAAACTAGTAAAGTTGGAAGATGTTGTATGTGATGAAGAAACAGCCTATTTATGGGAATACGTTAAGGAACATCTGAAGAATCTGAGAATCACTGTGATGGAGGAACATATCAATTTGAATTTGCTGCATAGATCATAATTTATCTGAGATGCTGCTGGAGCACTCACTATGGTGGAACTTGAATATGGGTGGATAAGGCAAAGCAAACACTGGAATAACTTGCAATTGGGTGGATAGTGCAAAGTAAACACTGGAATAACTTGATCAACAGTGACAAGTGATGGGTGTTTCTTGAGGCGACTTCCGGACTAGAACAAAACGAGCTGAAAGTAGCCAAAGGTGAGGGTTATAATAATTCCATCCAACATTGTTTCCAGTGTATTTGGAAAGATGCAGCTCAAGATTGGTTATTAGAAGCCGTCACTAGAGCTGGAATGCGGAAAAGCTAGTAGCACAAACAGACTGATGTAAGTTTAAACTTTACACTTTGTTTGGATGGTTGTTATCCATTATATTGTATTGTATTTTTATTTTAAATATAATGTTTGTTTTGATTGTTACTGTAAAATAATTATATTGTGTTGTTTAATCCATTGTTAGGCAATAATGAAAATATCGATTAGTGTGATTGCGTCA

>LNC_001550

CGGTGCTTCTCATTATAAATACTGATAGGTTGCTCCTTCATCTTTTGTCTGCGTCTTCTATCCAGTGCTTTTAGTTCTTCGTTTAATCTCGCCTCTTGCTCGATCATTTATTTCTGTTTTGTGCATTTTATACATGTTTTCGTATTTGAATTTCCTTTTTTGAACTGAATTTGGGGTTTTTGGTTCTTTGGAGGATTTGTATTTTTGAGGCCTTATTTTTTTTGGGTTTTAGGGTTGAGCCGCCTTGATTCAGCGTATGCTACCTCTTAATTCTACATTGATGATATGACACGAAAGAATCTTTGTTGTGGCTGTCAATCAGATAAAAAAAGGTAATGATGTTTGGCAAATGGCATTGCAGAAATAGATACATAAAGTCCTAAGGATCTGAAAGATGTTTTATTGCTTTAATGATTTTATGAACGTCGGAATAACAAGAATTGCTCCATTATGGAATGCTTGGTGTTTGTGGGCTGTGTTAAAAGTGCTGAGTTTCAGCGAATGGCAGGGCAGAAATAGGTACTAAAGTGCCCATGGATCAAGAGGGACCTCAAAGTTGAAAGGCTTAGCCAGGCTCTGGTAACTTCTTTCCAAAATTCAGGACCACAACTTCAATGTTCTTGCTGAGATAGAATCTACAATGCTACATTTTCTTTTAAACCATATCTTTAGAATATTGGTTAGGATTATCCTCCGAAACAATGATACAATCTTTAATGAGGCATAATACGTAAAACAAGTAAAATTATCATTAGTTAATATGGATATGACACAAATTCTTCTCATTTGCCTAAATGCTGGTGGGCAGAATAATTTGACACCTGTGCTTTTGAGAGGTAGTTGAGGGAGTCGAGCATTTTGGACACCACCTTGTGTGTGTATACGATATAATCTCGAGCAGCAAACTTGTACAAAGTTAGTTGATCATATACAAGAAATAAGATTACTATATCTGGATTTTGTTGACGAATGAACCAGCAATGTAGTTTGTGTTTTCACATTGAATTAGGAAATCACTAAAGGTTGTTTTATCGCTTTAATAATTTTATTGAGTCGGAATCACTAAAATTGCTCCACGGTGGATTGCTTGGCTTTTATGAGTTGAAGAAAAAGTGCTGACGTTCAGACAAATGGTAGACCAGAAATAGATTGCTAAAATTCCCTTGGAATCTGAGGGACCTCAAACTTGAAAGCTTAGCTGTGCTCAGGTGACTTAAGTGGAACCTACTCTTTTCACTTTTTCTCAGGACTCTACCTCCACAAAAGCAAAAAGTGCTACATTTTTGTATAAACTACTACTATAGTACTATATGCTTTTAAATGCTGGTTTAGATTAACCTCAGGTCCTTTGACAGTCAACTGATGCCTTTTAAAATGAGGCATCTTTGTTGAGGCACTGAAAAGTAATTGGACATGATTTAAAAGGGAGTATAAGATAGTAGAGCACCTTGAATATCCTGTTGTTAATTAAATTATATCAACTATATCTGGATCAGGAATATCTCAAACAATGAGTTGTTTGTCCATTACTCTGGCAGGAATAATATTGGCAGTGACACATTGATGAGTATTCTTTTTCTTTGCTAGTGAGTTGTGGGGTGGATAAAGGTTCTTTGTTCAACTAATAGCAGTGCAGAATTGGATACTAGAAGTACCCAATGATCAAAAAGGACCTAAAAGTGAATGTCCAAGCCATTCTTGGATGAAATTTCTTAGCCACAAAGTTCTTACCTCCTTACATTTTCATCGTATTCTTCGATAAGCTGGAACTTGCCGTGTTACTTTATCAATGACTACTTCACATCAAAAAAAGGCAAAAATATTGCATCCGTGCTTTCCTATCCTTGGAGAAGTGGTCCTTTTATTCTTTATCTGTGATGGGACATTCATTGTACTGAAGGAGAATCTGGGGAAGGCTCTGGACCAAAAGCAGCTATTGATGAACATTATGTATCAAATTCAAAAAGATATGTTCCAACAAAAAGATGACCACAGTGGAAAGCAGAAGTGAGCTTCAATTATATTGCACCAGCACACATTGTCATATACAACTGGGCAACCAGAGGTTGTTGGAGGATTCAGCAACAACCATGAAGCTGCTTCATCTTCATCTTCATCTTCTTCATCTACAGTCATATCATCTGCATTTTGAGTAAAACTCAAGAACCCATAATCCTCCGTGCTTTCCCCTGTGGGCCCACCAATCATCATAGAACCACTGCCAACGGTTTCGACAGCTGGAGGACCATAAAGGGTTCCTGGAAAGTGGAATCAAGAGAATTTAGAATTTAAGTTTGATGGGTTCAATCTTTAAATGTTCTTGCTACTTAATGCTATACATTTTGGAATTATGGTTCAAAAATCATATTTATTGAAATATTTGTTATTTTTTACATGTAGGAGTATAATTTTTGTGTGGCTACCTAAAATGGGCATAATTGGGACACGGTGGTGACGGCGCGCCAAGGGGTTGGCAGAGTGGATGTCAGTGTCGCAGGAGGCACAGAGTGAGGCAGCATCTGCCTTGCAAAGAAAGGCTGCAGGGGCACGTCCGCAGGCCTCGCAAACCCACACACGCTCGTGACGAGAAGCCAACAGATTTGCTGCATGCATGCGGGCGTCACAGCCGGCACACAAGAAGGTGGAATCTTCCCTGCAGTAAACGGTACATGCGGTGGAACGGCAACTGTCACACACCCTTGACCAGTTGTTGCTACTGCCATCAAAACCCCCACTGTTCTCCTTTTTCAACATTGAACTGATTTTTTTTTTCCCTTCTTGCTCTCTATTTTCTATTGTACTTTGAATACTGAACTTGAGCAGGTATGCTGTAAACTTATGGGGTGCTGTGAAATGTGAAAGTGGAGTAGAAGGAAAAATAACAGTAGGCAACTACAAAGTGGGAAGAGAATCCACAGGTCCACAACTCAATGTTCTTGTTGAGATAGAATCTACTCTTTTCACTTTATGGTATGACTCTTCATCCACAAAAGTTAGGAAGCTCTACATTGTCTTTACTGTATGTTTTAAAATATTGATCTGGATTAGCCTCCGAATGCATTCTTTGATGCATATAACATTGTTTCATAAATAAAACATCTACTTGCGTAGATAGTGATAGTTTAGGTGGGAAAGATTTTTTCTATTGGCTTCAAAAATGGCAATGAAAGAGGGTGATAACAGAAAGTGCACACCGGGAACAACTAATAGCTAGTTTAATCAGCGCCGTGGTGTATTTTAATACATTTATGGTGATATAGGAAAAGGGAACAACTCATAATTTGGGTGTGAAACTCGCGAAAAAAGTGATAGTTCAGGTGTTTTTTTAACCATTAACTCTAAAATTTAAGAAACCACTAAAACAACAGTATATTTAAACTAGCAACATAATACACTACAATGCAATAGGTAACAACCATCCAAACAAGCTATTTAGAGGAAAGTTCGCCAACTTCATGAACATTACTAACTTCGGAATGGAATGAACCATCAATAAATCTTTTGCCCTAAATAAAGTCTAGCAATGACAAGAAATAGGAATTTTCTGGGAAAAGAGTTTTTATAAGATCAATTTAGTATAGAAATTGGTCTCCTTTTGTCTTAACTGCTTTACTCAAGTCTCTTACCTCTAATTCACGTTTCCTGTATTCGCATCTTAAGAGTTTAAAGATTCAGGTTCAATATTTTCTCCAACTCAAAAATTGGATGTCCCTATAAAATATAGGAACACAATTATAAAATATTTTTGTCAGTTTTCATTAAATATTCCCTTAAGTTAGTTAAAAGTTCTAATGCAAATAAATTTGCAGGGGAAAAGTCATTTTAGGGAATTGAAATAACTTAAATATCTTTTAAAAAGCAAAATTGTAATTTTGCTTCTCAATTTACTATGAAATGAAATTAATTTATACCAAGTCTGGAATTTCATTTAACAGAAGTAATGACAGACCTTGAACCTTAAAGATGCGTTCTTCTTAATGCTTCATTTTCCTTTGCTTTGTAACCCAAGGATTAGAAAGGTTTTAATCAGAGTTATTATTCAATGGACTTCTTTATGAATTTTTTTAAATATCAACTTTAAGCTTGATTCTGTAACTGAGTGCATTTTACTAAAACCAGGAACTTCTGCAAAGTAGACTACATTAAGAAATAGCGAAGTAAATCATGGAATTTCTTCGAAGAAGAGTAGTGGAAGAAAAAAGTAGTGGAAGTTGCTTTTAATTTTAGTGACAACGAGTTGCAACCCTTCAATCTTATTATGATGTTATGAAGATCTAAAGCAAGTTTGGAGCGAACTGGTCGAAGAATCACTGAAGTGGGTTTAATTAAATTGCAAAAGATAATTAACGTTCATTATATTCATGAACTTGAAACTTTTTAAAATTTTAATTTTATAATGGAACACATCTATTTATATATTAATAGTATGTTGTTTTAAGAAGATTACTTCACATTGATGCGCGATAAATGCATGCCCAAAAATTAGTAATTTTATAAGCATATAGGTATCACAAGAATTGCTCCATGGTGGATTTCTTGGCATTTATAAACTGTGGAAAAAGTGCTAAGGCTCAGCAAATGGCAGAGCAGAAATAGATAGCTAAAGTTCCCTTGGAATCTGAGGGACCTAAAACTTGAAAGGCTTAGCTGTGCTCAGGTGACTTACGTGGAACCTACACTTTTCACTTTTTCTCAGGACTCTACCTCCACAAAAGTAGAAAGTTTTAGATTAGCCTCCAGGTCCTTTAACAGTCAACTGATACTTTTTAAAATGAGACATCTTTGTTGAAGTCCTGAAAAGTAATTGGACATGATTTAAAAGGGATAAGATAGTAGAGCACCTTGAATATTCTGTTGTGTGGATCAGGAATATCTCAAACAATTTGACTTGTTTGTCCTTTATTCTGGCACGAATAATATTGGCCGTGACACATTGATGCGTATTCTTTTCCTTTGCTAGTGAGTTGTGAGGTGGATAAAAGTTCTTCTGTCCAACGAATAGCAGTGCAGAATAGGATATTAAAAGTACTCAATGATCAAAAAGGACCTAAAAGTGGACATCCAAGCCATCTTGGATGAAATTTCTTAGCCACAAAGTTCTTACCTACACTTCCATTTTATTCCTTGATATGCAGGACAATTACTTCACATCAGAAAAGGTAAAAGTGCAATATGCTTTCCTTCTGCGTCTTGTTCAGGCAGTTGCATACTTTGAGTCCCCCGTATCAGCTTAAAAGTGATAAAAGGACATTGTGGTTTGATTACTGATACTGTTGGGGCCATGGAATACCTACTCATTTTATGCCTCATGGTTTACTGTGACTGGAAATCCAATGAACTGGATCACATGGATTGCTAAAAGATAGAAGATTATCAATTTATGTAGCTGCGGCAGAACTTGTTGGATTATGTAGCAATTTGTTAAAAGAGTACTTTGCGTTGTTTAGGCAGTGCTTAAGTGTAAGTCTGTGCCTAGAAAGACAGCCAACTAATTGCTTCTTAATAAGTGTAAGTAGTAATTTATAATGCCTGTAAGTTTTGTTTTGAAGAGGGCGATAATATACAATAATTATAAAACAACATAAATCCAATGTAATCCCACAAGTGGGGTTTGGGGTGGGTAAGATGTATGCAATCTTACCCCTAACTTTATGTGGTATAGAAGCTGTTTCCGATTGACCTTCGGGCTTCGGCAACCCTCCTCTCTTTATCTGGGCTTAGGACCAATAATTTCAACAAGCTCACATTGACA

>LNC_001551

AAAATACCCAGTTTTTCAAGCATTATTTTACTATATTCATCCACTACCACTTATAAAAATGAACTAAATTAACATCTTAAACTGCGTGTCCAGGTAAAATGGGATGGCGGAATAATAGTTATATATAGTTTCATTTCTTTCTAGTTAATCTGGGACTGGATAAGTATGTTACAGTTTTGAAAAAGTAGAACTAGGACAAGGGAAAATAGAGGAGATTAGTTGCCACGCGAGCAATTGCATTTTCTGCTACTGTTCTTGAATATGGACGACCTCTAATAGCTGGGGACTTCACTTACCATTTTTGGCTTGAGGTGACCATGGGGAGATTTTACATGTGATAAGTGTTTTTTTGTTAAATAAAGAAACCTTGCTCTTTCTGAAATTATCACTCTTATAGTCTTCTTTTATGAAGTAGTGTAGTTCATTGATTGAGATCTAGGGGACGCATAGAAAGTACAAATCTATCATGAAAATCGCCATGCCA

>LNC_001552

TTTTATTCCGTGAAATTTTTTGACTTCGGACACTAGAGTTTTCTCTAGGCTCCAATATAATTTGACAAAAAAAACCCTTTGCATTACATAAATTTTACTTTTAGTCGTAGCAATAATTATGGACGTTAAACTTCACTTATGGAGACAGACTAGACTTCCATGTTAGGAAGGAGTTGGCAACCATTATGCAATGGAATGGATATGCAAATGCACAAAAAGAAGAGGAGAGAGATGCAAATGCAAAGGGTATGGCAGACCCGAGAAAGACGGAATTAATTGTGACCGCCTGCTGATTTGTGCAATTATTACTAGTACTACGTACGTTTGTTATATATGCTTGTAACTTTGTTCTCTTTGTTTGTCTTTTATCCTACAA

>LNC_001553

CGGGGACTAAGTTTAAAGAATGACCTCAGAATAGGCCTACGGTATAACTAGTTCATTTTACGCTGATTTACAAAAATAAACACATGTGTGGATTGTATTTAGATGTTGGCCGCTTTTCATAATTAGACCTGTGGTGTCAAGTTCAAAAATGGCTCTCTGTTTTTTATGTTTTAGAAGAAGAAATTTGATGAGTGGAACTGTTAAACAGGATGGAGAAGATACAGTTGAAAAGATCAACCACTCGAGCCAAAGTGTTGGGCACGGTGGTGTCCATAGCTGGAGCATTCGTAGTAACTCTATACAAAGGCCCAAAAATATTAGTGCCTACCACGTCCACACCCAATTTACTACGTCAACCTCTCGGCTCATCTCAATCGAATTGGATGCTTGGTGGCCTTTTCCTCACAACTGAATATTTTTTAGTCCCAATGTGGTATATAGTTCAGACATGGATTATGAAGGTGTATCCAGCAGAAGTGACGGTAGTTTTCTTCTACAACTTGACTGTTAGCATCCTAGCTGGTATTGTAGGATTTTTATCTGAACCTGACTCCAACAAATGGATAATTAAACCGGATATCGCACTGGCCTCCATCCTCTGCTCTGGAATATTGGGTTCATCCCTAAATAATACAATTCACACCTGGGCTTTGCGCGTGAAAGGACCAGTATATGTTGCAATGTTTAAACCATTGTCCATCGCGATTGCAGTTGCCATGGGAGTCATTCTCTTAGGAGATACTCTTTATTTAGGAAGCATCCTTGGAGCAACGATAATTGCTATTGGATTCTATACGGTAATGTGGGGAAAGGCAAAAGAGATGCCTGAATATAATGATTCTAGTGATCTCGAGTCGTCTCCAGATCAAAAGTTCCCATTGTTACACAATTTCAAAAATGAAGGCATCTCAAACAAGTAAACAATTAGTTTGAAGGCAGAAAGGGATGTCACACAAAAGGCTGTAACTGCAAATCACGCCATATTTTATGAACAAAAATCGAGTATGGGGTTGTATAGGAAGAAACAAGATGCTGCTATGGGAAAAGAAGGAGCAGCAAATTGTTGGCATATATATATCCATCCTTTGTTCTGTACAGCTATTCTGGAAAAAGACTTGTTTACTACTGACATTTTATCATTTGGCTCCAAAGTACTGAGTCACAACAGAATGTTCCTTTTGTATCTCAGGAATGAACTAAAGCCTATTTTGGGGCCTTGCTAGTTAAT

>LNC_001554

TGGTAATAAAAACTTCTGCACAACTATACCTCAAACTATAAACTCAAAGTTCTAGGTCTTTCTTGGTTTTCTTCTTCAATATTGATGTGAAGAGATTGTTTTTGACCTTGTATTTGTGTTCAAGAAAATATGAGTTAGCTAATAGTTTGTGGGTTGTTGTTATATAATGGGAAAAGGGGGAAGAGTTGGAGAATCATTAAAGAAATGTTCCAAAAGTACATAAATTTGGGACAAAGACAAGCTAAGGCTGTTGGCTGGAAGAGTAGCAACTTTGAACATGTAGGGGACAAGAGAGGGACAAGCTCACCCACATAAATGGACTAACAC

>LNC_001555

TTACCATTCTTCTACTCTTTCCCGGAAGTCGATGACTATTCATAGCTATTACCTTGACACCAAAGAAGAGTTCGACCCAATGCTTTATTTCTGTCCTAGTTGATCCTGATTCGACATTAGAAGTATATTGATTTTTCCCCAATAACCGAATACTTTTGTTTGTAAATACTGCATATTTGATTCCATCCATAAATCTATTTTCTTCCCTATGAGTTATAGTCTCAATAAGAATGCTAGTTCTTACTGTTCATATATTATGATATGAATATACCACATCAATTCGTTATGTATGGATGATGAGATTCCATTGATACAGAGCCAATTCCAATAGACTTATTGGAGGGTCCCATTGGCGTGCATCCAGTAGGAATTGAACCTACGAATTCGCCAATTATGAGTTGGGCGCTTTAACCATTCAGCCATGGATGCTTAGCGGGGATCCTCGTACATGGTGAATACCAT

>LNC_001556

CTTTTTAATGATAACAAGAAGCTTTTAAAGAATTTTGCAGTGAACCTCCCATCACTCGGCGTCACTGTGCCGCTTCCGTCCGCCGCGAACCACCGTGCCCATCGCCTCTTTCTTACCGGCGCCATTCCCGATGCCTTCTTCCGGTGCGGCGGTTGATCCATCTATCAAGAGTTCATCATCGATACCTAATCCAGAGGTTGAAGAGGATGAAGACGAGGAATACGAAGAAATTGAGGTTGAAGAGGAGATTGAAGTTGAAGAAGAAATAGAGGTAGAAGAAGAAGAGGAAGAAGAAATGGAGGTAGAAGAAGAGGTAAAGGAAGAGGG

>LNC_001557

CATATTCTGATTCTTCCACATCTCTGCCTATTTTATCTGTTTTCCACTATTAGATGTATATCATGTTTAGTTAAAAGGTATTGTCCATGCTTATGGTTTCCTTGTTTAAGAATCATATGTTGCTAGCTATTGATTTTGTTTATTTCTTCTCATTTTCAGCATGTTTCTATAGTTTGTTGAGTATAGCATATGCTTCCTTTGTTTACTGTAATAATTTCAA

>LNC_001558

CCTAGTAGACTGTTTTTAGCTTTCACGTGTCGAGAGGGATAAAACCTTTAGCTTTAGCAAGTACTACTACTCCTATGTTTTCGGATAGTATAACCGATTATATGATGTTCTTTTCGCTCTTGGAGTAAGAAAAATATGTTTGAAAGGTGCGTAGCGGTGAAGTCAAGAGATTCCAGAATCTCTCTTTCCTGTGCTATCAAGAAG

>LNC_001559

GTTACAGTCACTCAATGGAGATGTTCGCCTTTGTTTGGAATGAAGATGGATGAACAGGCACTTGAGCCTGGAACTGATGAAATTCGGGGAAAACATGGAAGCGGTTACTATACTGGGGGGCAAGACATGACCGCGACTTAAGAAAAAAGTACCCTTGAAAAGACTAAAGGAACGGGGGAATGACTACCTGTAATAAAGCACAGGCTAATACGAGCCGACCAGAAGAAGCAAGGCTTAGATTACCAGATCCTGGACATAATCTTCCTAGAGATGGAGAGCCACTTGACTGTGAGGAAAGGGGCC

>LNC_001560

AAAATAGCCAAATTTTTAACGGCAGTTTCCCTCACTGTACTCTCTCCCATTTTCTAAAACTTCCCCATATTTTCCTCAAACTCCGTCCGGATCATTTGCCGTAGATATTATTTAGAGAAAAAATTGCTGATTTCTTCAAGTTTTGGCATACAAATTGTATGATAATGGCAGGATTTTTTGATTACTTCAATTTTTAATTTATGAATCTACTAATCTAAGATATGCCGAAGCCCAAACGTTACGAGAATTTACAGAACTCAGCATCTACTTCACAAGGTGTAGCACAATCTTCATCAGCAACTGAATCAGACC

>LNC_001561

GTACAGTGCTTTCGGCTATAGCAGACTGCTGTTCCGTCCTCGCCACTACGAGATTACTCCACGTCGACACTCAAGGACGAGAGGTCAATCGAGTCCGTCCATCTCCCTTTCCTCTTTCGGAATGGGGTGAATTTTTCAGTGAAAAGGTTGCCTCCCCAGAATTGTGTAAGATTTTCGATTTTGAATTTGGAATGGGCCTTGAACCCGAATTTTCGTC

>LNC_001562

TACAACTCCCATTCAACACGGATGATCTAGATCAGAAGATTTTCAAAACTTTCAATAATTTCTTACCATTCAAGGGAGAAGAAGTATGAAATAAATAAGTATTTTTAGAGCAGCAGAACGAGCACTGATCCTAATCTAGAATGAAAACAAGGCAATCCAAGACTGATTCAAAAATACAAAAGGAAATTGAAGAAAGACATTCAGATATGTCATTCCTTTTCTCTCTGTTTTTCTCTTTGCCCAGTTTCTCC

>LNC_001563

CAAGCTTGTAACTCAAGTGAGGGCATTAAGGTGATGTGAACAAAACTAAAACAAATGGTGAAGTCTCATGTGGGTGTTATATGGACAAGCATAGATACTGGCTTGTTGTTTAGCAGTAGGAAGAGTTTGGTTTAGAAGATGCTCATGTGTAGCACATACCTCTTGCTGTCTTCACAGAGCAGCGGCACTTCCTCTTGCTCTCTCAAAGACCACGTTGCTAACAAGTCCAAAATCCAATCCAAAATGTACTTCCCATAGTTTGGACCATCACCTT

>LNC_001564

GAGAATATTACTTTATATTCGCACTATTTTATATAAATAAAATACGTACTAGTGTTATTATATACGTTTACTCAATTATAATAAATATTTTTACTTAAATGTCCTCTATCAGAAAGTTTCAAAATTTAACTATTAATACTCAAAAATCAGACTTGTAAACCCAAATAGTTTTCTCCACCGGCGGCGAATTCCACCAGCAAATTGATTAATGAAATGCACGGAATCGGCGGCATCCATTATTGTCCGATTTGGCCCCGTTCAGCTAATTGATTTTCCGGCCGACTAATTTTGAGTGAGGTAATTTTCCCCTAATTTATATGATCTGTAAATTGAGTTACAGTGCTTTGACTCTGCGTAATGATAGCCCTAACTTCAATCTCTTGTTTCTTTGTTCAACTATTTATTGTTCTATATACCTACCTAACCTATCACCTTTTTGTGAGTTTCGTGCTTCAACTATCAGTCAGTTTGTACTAAAATACTCATGGATGTTAAGTTAGCCTTCACGGGGAACAAGTTAATATTTGGTTATCTCAATATCGATGTGTGTTTTGATAAAAGGATGGATAGTTTAGATAAGTAAAGAGAACCATTGATGATTGAGGTGTGAAACTTACAAAAAAGTGATGGTTTAGGTGTTCTTTTTTTACCATTAACTCCACAAAAAAGGAATATTAGTAATCAAAGTTTGAAACTTTCTACAACAGAAGAGCAGAAATACAATCAACATATGAGAATAAAATAATTATCATTCTGTATTGAGGTTTTAGCGCTTGGTGTAAAAGACTTCAATCTGAAGTCATTTTTGAAGTGCTTAGATTGAGATATAGTTGATTCATTGATTTTCCTACTATTCAAAGCAACCGTTTTACCGCCCGGGATCTAAGCATTTGAAATCTATATAGTTTCCAAATCCAAATCAGTCCATTCACAACAGGTAAGAGTAATTTTTTTTTTTGATAACCAAGAAATCTGTCTGTGAC

>LNC_001565

GTTTTCTCCACCGGCGGCGAATTCCACCAGCAAATTGATTAATGAAATGCACGGAATCGGCGGCATCCATTATTGTCCGATTTGGCCCCGTTCAGCTAATTGATTTTCCGGCCGACTAATTTTGAGTGAGGGGACAACTTGTTGGCCAAATAAGTCGCTGCAGCTTCATAAATTTTGTTGTTGACGAGGCCATCAAATTCGTCAATCACCATCGTTAGCTGATTTGAGAACTTGGTGAACATGTTTTTGAGTCCAAAGAAGAGATATGCATGCAGTTCCTTGGGGATGTAGTTGTGGATTATCGTATGAACCAACATGACTGTTGCAGCAACTGAGCCTACTCTCGATAAATCTGTCTTGGCAAGGGTTATTTTCGATTCTGCTGATGAAGAGGGCATGCTTCTTAAGGGCTTTTTGGCTAGTGGGTGTGTAACAAGTAAATTTTTATTCCTGAAAATGTAATAATCAAGACAAAAAAATAGCGACAAATATTTAATATTTGATTTCAATAATGTGCGTGGATCACAATCTTTATGAAATCTTAAATATAAAGACGTAATCCTTTTATTGTTATCATCATGACCAGTATTTTGGATGGCGAAAGACGCTAAAAGGCG

>LNC_001566

GCTAATTGATTTTCCGGCCGACTAATTTTGAGTGAGGGGACAACTTGTTGGCCAAATAAGTCGCTGCAGCTTCATAAATTTTGTTGTTGACGAGGCCATCAAATTCGTCAATCACCATCGTTAGCTGATTTGAGAACTTGGTGAACATGTTTTTGAGTCCAAAGAAGAGATATGCATGCAGTTCCTTGGGGATGTAGTTGTGGATTATCGTATGAACCAACATGACTGTTGCAGCAACTGAGCCTACTCTCGATAAATCTGTCTTGGCAAGGGTTATTTTCGATTCTGCTGATGAAGAGGGCATGCTTCTTAAGGGCTTTTTGGCTAGTGGGTGTGTAACAAGTAAATTTTTATTCCTGAAAATGTAATAATCAAGACAAAAAAATAGCGACAAATATTTAATATTTGATTTCAATAATGTGCGTGGATCACAATCTTTATGAAATCTTAAATATAAAGACGTAATCCTTTTATTGTTATCATCATGACCAGTATTTTGGATGGCGAAAGACGCTAAAAGGCG

>LNC_001567

TCTTCTCCTCACATCTTCTCTGTTAGCTACTCTCTGCAAACGTTTGAGATTTTGGGTATTTTTGGTGAGAATTGGATGGATTTTTTCGAGATCATCTTGCATTATTATCATCGATATAATAATTTGGTATATTTTATATTTGTTACTTTTCAATTAGTTTGAATATAATTTCTTGCTTGAATTTCAGGTACACGTTAGTAATTTAACCTTATTATTGCATGCATTTAGTTTCTTTGATTAATTTCGTCTAATCTTCATTTAGTGCTTGAGTTTCTCTTAAACGATTCATAAACTTTCTTTGATTAGAGAAGATGTGGAGAACTACTTCAGGTGCGTGTGTTGACTTAATTTTTGTTTCAACTTGAAATTCTTTTATGGAACTGAGCTATGAATCTATTGTTTATTTATATTCTTTTCACTAACCTGCTACTCTCTGCTCCCAATTTAAGTTGTCTCTTTAGTATGTGTACAAAGTGGATATATTAGGTAACTTTCTGAGATAAACACTTTTAAGTAGAAAAAAAAATGATGCTCGGAGTGAATTTTTTTTATAAGCAAATCTTGAAAAATCTTTGAGAAGTGTAGAGAAAAGCATTGTCTGCAACAAATTGAACAAATCAACTTAGAAACTACTTATGGTAGTGAAAGACTTGACCTTTTTGATGGGTCTTCCGTATTGCCTTCTCTCATCGTTAAAGGTGCCGCAAGCCACTTGACTAGAAGGAGTGGATGTTAGGCACGCACGTAAGACTCTGACTATGCACTTGCACTATCCCTGGGAATGGATTCTTCTTTGATTGGAGGAACGGGACATAAGAGAACCAGAACCATATCAGATTAGTTATTATTTTTCCTGCTCCTGGTGATTTCTTTCATCAACGATGATAGTTTCCTTCAAATTTGATGTTTATGCGTTTTTGCTTATTTTCGCCGCATATATAATTTGTTGCTGCTCTTTTAAATGAAGTGAACTAGCAGGAAGAATTCTCGGTGGTGGCTCTATCTGAAGCAATTTCCCCATAACTGACACTTGCAAATTTAGCAAATTCAAAACATTCACATTGCAAGTTTGTGACAATTCCAACCAATTAAAATACATTTCATAATTGGAAAACACTTCTTCTGCTATGCACCTGTGATATTTGATGAAATGTCTTGCTCATCTTATTTGGCAATAACTTATGGTGCTATTGGTCATATCTTAAAAGTTGTGCGTTTAATACTTGAGACAATAATTTGTTATGTTGATGAGCTTACTAATAACAATGTCTTGATGCAGAGTACAAAAGCATCTTATAGCGATGGTGACCCGATTGAAGTGCAATGATAAGGTCCACCACTTTATTCTTTCCTCGAGGCTCATGCTTGATTGGGGGGTTCCCCATTCTATGGATCAAATCCCTGCATTCATTTAACATATTTGTGATCTTATAACGAAGCAGATTATTGGTAAAGTACATGTATCAGATGGTCTATACATTCTTGATGAATGGGCGCCTCCATCAGTTGCTTGCTCCAGTATCGTC

>LNC_001568

AAAGCTCTAAGGTCCGTGAGGGCTTTAAGCTCAAAGCGCAAATAAAGCGTGGGCTTTAATGAAAAGAGGCGCAAAGGGAGAAAAGAACACAAATATATATGTTTAGTCCAAGACTAATAATTATTAACATGAATGACAAATATATGACCAAAAAAATTGAAAAAAAAAATGATAAAGTGAATTATCAATTATTTAGTGTCACTTCTTCATACGAGGCTCATTGGCAAGGAAACGTTTGCCTTAGATACTTGATGACGACACTGAAGCGCACATAAAGCGAGGCGAAGCGCTCAACACGTTTTGAGCCTCGCTTCAGGGCTTAAGCGCGCTTAAAGCGCACCTTTGATAAC

>LNC_001569

AAAGAAACAAACTCTATGGACAAAATTCTCTATTATTTTTTCTTCTTTAGTCATTTCTTTATCTATCTTCTCCAAAACTTGAGAGATATTTAGTTTTCTAGATATGAATAGTACCTTAAAAAAGGGCTAGCCCAAGAGTCCGCTAGACAGAGATAATCGGTTTGGTTTGATTTATCAGTTTTCTCTACACACCTTGTTAGAGATATGATGAAGAAGTTTTCTCTTTTGTGATGATGGATCAAGAAAAATTTCATATCTTTTTAGTCACATTCTCTATTATGGTGATCAAGAAGTGGAGTTTTTCTTTATTCTCAAGATGAAGGATAGCATATCTTAATATTATTATAAGTTTGGAAAGAGGCACACATTATGGAGCCAGGCAATTTTTTTTATTTCTGTTTTTTCTTCTTTTTTATTTTTTTGCCTTCTTATTTAGAGACTGATCAATCTCAAGCTCTATGTTGACTAATAATTCTTGAACATGAGCCAAGAGTTGTACTGAAGAAATTTTTTGCTGATAATTTTTATGTTGATTATTCTATTTGCTAATATTTTTTATCGAAAATGAATATGATCTTATTATATATGTACGTTTTATTTATTTATTTATTGTATAGATCCTCCTTTGGTTGTTGCCGATTACCGATATATTTATGTAATATGCAGGGAACGATCTATCGTTGAAGTTATGAGTTCGGTAAAATTGAGTAGTTGTGAATCAAATCTTGTATATCCTGTTTTAAGATATTTAGTGAATATACTTATAAATAATTTATCTAAAACTTGAAAAATAAAAGAGGTTGTTAGTTCAAAATTCATAAATTTAAAATTCTAGATCTGCAGAACGGAGGGTGTAACCTTTTTGCCCAATTTTTTAATGTGAATTGTATATTATATTAAATAGATCATTTCTTGAAATTTTTCAATGAGCCATCTTGAAGACAAGTACATATATCCTTGATTATTTAGCTTAATTTTGATAATGTAAATGAAGTCTTTAGGTCGCTTA

>LNC_001570

GTATTTGTGGTTCCACGATAGGGTGGGGTTCTTGTAATTGTAATCATGTGGGACATGTTCTAGGTATTGGTTAATTCATGTTATTGTGACTGTGAGTCTATGTGATTGTGATTGTTGTTATATGTGTTGGCTAATGTTTACTATGTTGTGGTTATTTGTTTACATTCATGATTTGGAACTGACAGAGTGATCCTACCAGTACACAGTACACTCTGGTTGTGTATTGATATTGCACCTATTTTTTTTTGTTGAGTACATAACATCTTTCAGGGGCTGATATGAGACCTCAGTTAAAGGATGTTTGACTTTGAATCCAAGGTGAGCTACTCTTCCAGACTGTCATGGATTCCTCTACATCTTCTTGCCTATCTTTTCAGACACAAACTTTAGATTATTAGTTTATGTTTCCTAGCTTTCAGGGTTGCATCCTCATTATTGACTAAATAGAGTTTTGCTACTACGATTTTCAGTTTCTAG

>LNC_001571

CTAGAGTATGATTGTGAGAAAAAAAACTGTCGAAGTATTCATTCAATTCTCTACAGAGTTTCAAGAACTTTCTTTAGAATTTAAGACTCAAATCAGCTGAAGTATTTAGTTATTTTTTATTATTGTTGTTGCATATCACCCACATCAATTCCAAATTGGTCTAAGACGCAAGCTTCAACAATGGTGGATTTCGAAGATATTATTATTTTTTGATTCGCAAAGCTGGATGATTTTTAGATCTTCAATCACATAGGTGTGTAAAGAGCCCACCATATGAAATTGATAGAAATTATGGGACAACATTTTGCATGCGGCATGATCTCAAACTACTTAATGGCGATGGACTAAGAGTACCTAACTTTGTGAGTATAACTATCTCAAGATTTGATCCATTTTCCCCTTCAACCTGTGATTTTCATTTCTTCATCCTATATATGCTTTTGTGTTTGATGCATTCTTTGCCGGTATATTTTGCTGAGCCAACATGAAAGTTTACTCACATTGTCCTACATAATTTGGACTACCAATAATGGAATCTATGTATTATAATAATAACTAAATCATGAGTCAGAAAAATTCTCTTTCAATCTATTTCTATCTGTCACCGACTCGTGAGCT

>LNC_001572

GAAAAAGTAAAGGACACTAGAAATTAAAAAATGAAAAAAATAAAAAACAAGAAGACAAAGAGACATTGGGAAAGTGCATCCACTTAGTACTCGTTCTTCTTCCATGGCTTCCATCAGTCACCGGAAAACCTCACAGATCTTGCAGGTGTGGGTTGCAATTATTTGGAGGTCCACAGATCTCACAGACTTCATGATTCAACACCAGTGAAGACTTCTTCAACTTGGAAGCTGCAACGGTTAACTGGATCTACAGGTTGAATTCCTGAGGAATTGATACCACTGTTCAAGACTTATAATTTTCGGAGTACTTCATTTATAGCACTGAGGCTGAAGATCGCAACCTTCCCTTACG

>LNC_001573

ATTCGTCTCCGTTCATCAGAGATCACTCTAGCCGGTGTCTCCACCCATATTTGCACTTTGAATGTCAAATCTCCATAGGTCAGCTCCACTGTGCTCGGAATCGACTCGCCATCTCCTCGCACCTTCAGCCTTGCCCATTTGAGGTGATTGCGTAATTTTGTCTCTTCTTCCGTACGGATCCAGCCTCCACAGAGATTTCTGATCTCACGGAAAACTTTTTGGGACCACAAATGGAGAGGGAGTCCTACTACTCTTATCCAGACTTGATTCGGTCTTTCAAATATGGCGTTTGAGGTTGGTGACCACCATTGAAGGCTGAATTTGTAAGATTTCCAGAACCAGTCCCCCCTTACTATGTGATCAGCAGCTGTTCTATTGGGGAATTCGAAGAGAAATCGATTCCCCCCAGATCGAAGATATTGATTCCATGATTGTGCCTCCAAGTGCTTGCGACCCATCGTCAGATCTCTG

>LNC_001574

CCGATGAAAATTTGAAGAAAAAAAAAAGAAGCTAAAACAAACCAAAGAAACCAAGAGGCAAACAGAGATCGAAAAAGAAATAAAAAAGTTGTAAGGACAAAGGTCCTTCTAGCTTTTGGAAACATCTTCTCCTTGACCCCTCTTTCTTCAGTGAGCTAATGGAGCTCCAAATCGAGTTTGAGCTCCCAACTGGGCATGAAGCTTCTCCCCCGATTGACACTACTAACTCAGCATCAGCGGGCTATAAAGGATCTTTTCAAGCTCAATTCGATGTCTCTAGGTGAACGAACACTCAAATAAAGGTATCTTCTATCAACAAGTATTTGACACAAGAGACATTCATGTAATCAACCTGACATCTACCTCGAACATAAAACTAATTGATCCAATTCACATCAGCTACATTGAAACAACCATTATGGGTTTTTTGGAGTATCCCAACGTCTCCTTCTAGCTCTGGATATTTAGTGTCTTCCGATCGACATATCTCTACATGTTTATAGTCATGCGACTGACTTATATCTTTAGACTTTAGTTTTTGTATCTCATTGTCCATACTATCCACTTTTGGTACAAAGTGTAGTAATGGCTTTGAGTATATCTTCAATTGTATTTTCTTTCTCAGTCTGAGTAGTTTTGTCTTGATAACTAATCTGCAATAACATTCTTATCAGTTTTTATTACTTCAATAGTAAATGTAAAATTTAATATATTTAATACCAATCTTC

>LNC_001575

GAAAATTTGAAGAAAAAAAAAAGAAGCTAAAACAAACCAAAGAAACCAAGAGGCAAACAGAGATCGAAAAAGAAATAAAAAAGTTGTAAGGACAAAGGTCCTTCTAGCTTTTGGAAACATCTTCTCCTTGACCCCTCTTTCTTCAGTGAGCTAATGGAGCTCCAAATCGAGTTTGAGCTCCCAACTGGGCATGAAGCTTCTCCCCCGATTGACACTACTAACTCAGCATCAGCGGGCTATAAAGGATCTTTTCAAGCTCAATTCGATGTCTCTAGGTGAACGAACACTCAAATAAAGATTTGACACAAGAGACATTCATGTAATCAACCTGACATCTACCTCGAACATAAAACTAATTGATCCAATTCACATCAGGTACACAATCTTTATGATACTAGTGCTGGACACGTGTGATGCACGTGTGTCCTGTGTTAATTTTTACAGACTTATTCTAATGATTTATAACATTCTATGAAACACATTCATGTAATGAGCAGTAAAAAACGTAATTATGGAACATATAATTAAATTCATTTTGAAAAATATTCTAAGTGTTTAAATATGTTGGTGACCAAAGGTAGTGAATACAAAAAGTGTGTTATAACACAAATGTTAATTTAGAGAAAACTGATTGAGAATGTGAAATAAATGCACATTTATAAGCATGATCAAAAGTAATACAATAAAATAAAAACAGAAACCAATTGACACTTACATACCAACCTTTAATAAAGCTTCGGATTTTGTGAAACTATGTCAAATGTCAAATAAAAATAAAAAGATAACTTAAACTTTCGTACAATCAAATCTGCAACCATCTTTCTTTTCGTTTTCTTAGCTACTCTCTTAATAATGGC

>LNC_001576

ACTCCCTCCGTCCCATTTTATATGATGTACTTTGATTTGGCACAAACTTTAAGAAAAATGGAAAGACTTTTAAAATTTGTGGTCCAAAATGAATGATAGAAATTTGTGTGGTTGTAAATCATTTCATTAAGGGTAAAAAAGACATTTTATAGGCAAATTGTTACTTAAAATAGAAATGTGTCATTCTTTTTGGGACTGACTAAAAAGGAAAGTAAGTCATATAAATTGGGACAGAGGG

>LNC_001577

GATCTTTTAATGATGTTCGTATGAATTACTTCATTTCTTGATATAATTGGTTGTTTACAAGTTATTGTTAGTCTTTGACTGTACAAAAGTTATTCATTTTGTTATGGAGTTTCAACTCATGTATATAGAGATGAATTTGGTAGAAGGCATAGGGGAGAGTTCCTCACCGTTGAGGAGTTTTGGGACTTGTGTAGTGTATGTTATAAAAAATGATGCGTATAACCTTCTTGTAGAGATTGGAAATGCTTAGGTCGTTTCCACTACTACCAATATAAAAATTATGGACTCTCCTTTTGAGTACGATATTGAGAAATAAAATCGACAGAATATCCGAATATGTAGCAAATTTGCTCTGTTTTCTCATGAGTACTCTTTTTGATAACCGATTTAGCACATGCGAAA

>LNC_001578

CTCTCTTCTTCCTCTCCAACATAGATCAGCCGCCTCTCCTCTCAACTCCTCCGGCGATGGCGAGTTTTCGACGAGAACTAGAGTCCGCCCTCTCCTCCTATCCTCTTCTCCTCTCTTCCTCTCTCCTCTTCTCCCTCGCTCCGTCTCTTTTTCTCCGGGCAGCAGCAACCGGAAACAGCAGCAGCCAGCACGAGGAGAAGCAGCAGCAACAGCAGCAGCTCCCGCCGGACAGCAACAGCAGCAGCTCCAGCCGGACACCGACCAGCAGCAAGCTCCAGCCGGACACCGACCAGCGCAAGAGCCATCGCCGACCAGCAGCAATCCGGCGTCCCTCTCATTTTTCTACGAGGACGAACAATGTATTTCGGCAAGCATCTCGAGGTTTTGAGTATATGAATGACGGATCTTTAACAGATCCGTCCATGTAAGTTTTATTTTATTCAACACTTTGTTCGTGACTTTTAAAATATTACGAGTTATTACAGCATCGTATAAAGTCATATTACTAATCCTTTTTGTTGATTCTTAGAAAGCAAGTCTAGGTTATGACTTTCTCTGTCTAAACTAGTGATCGTTTGCTTCTGTGATAATATTTGTGTTAAGTTTGTTAATTCATAATGCATGCTATAATAAGTTTGAAACCATGCCTGTGAGTTTCATTGTTTGATGAATTTCTTTGGATTGATCTCTTTCTTGTAATCAATTTAAGCCTTAATTAATGGAGAATTACTCTGTTCTGTTTCCCAAATTAGTCCGGCGGATTTCCTTGATTATTGTTGAAATATATGGGTGATTCTGTACGGACTTCGCCATAATCGTTGCTTAAAAAAGAGTAAACTTATCAAATCTCTAATTTCCTTAATTTGTTGAATTGAGTATGTAAACTGTGAATTAGTTTTACTATTGCTAAAATTGATTTGCTAGTGTCTTTATGGTAAGTCTTTGAATTAATAGGTAAGGCGTGTGATATTGATTTTATATGATGCAAATTTACGGGTCACTTCTTTTTGATATTCCTTTACCTTAACTAAATTAGGCAGATTTCTTGCTTATATTTGGTAGAGAATAAATTAAATTAAGTCTAATTTTGGCTCTCCTTTAAAATAAGGAAAAACTAATTCTAAATGTCTCCTTAATTATTTCTTTTCCTTTTATGTTGTGCCCTCTCTTTTGTATATAAATAAGGCTCATAATTCCTTATAACGGAGGACACTCTCATCCGGACCCTCTCACACACACGGTTGATCACACACCTCTAGACACTCTCACATCTACTTTGCTCTTACTT

>LNC_001579

AGCCGCAGCAGAGTGCTTTGCCCATGCTGCTATCCGCCGCCGAAGCGCTCAAGGGATTCGTGCTTGAATGTCGAGATCAGGGGACTCTATCCAATCCATGCGGCAAAATCTGTTTGTGGTGGAAAAAAGTCAACAATAAAGAAAAAAGAAATAAATAGAATATAGAAAATCATTGAATTTATTTGCTCCGATACAAGAGATCGGCCCCGAAGCAAGATATGGTGGGTGCCAGCAACTTTCACTTGTTAGGAGTAGGGGCTGAAAAGAGCTCTTTGTATAGGTTGGGTTTTCT

>LNC_001580

ACAGTGGTATTGGTTAGAAAATAAGGTATGTAGGGCGATTCGATTCCATATTCCTTGGCATGAAATCTGATACCTGCGATTAATGTCACTAAGTGAATTTCCTAAGTTCTATTCCTAGTAAAGGAAACATAGTGGCAACGTACGATATCCAAATAGCTAACAATATCCCCCCTCCTAAGTGTACATAATTACTTAGTGACACGTTCAGTACTCTATAGTCAATTGAAGTCAATTGTGTCATTTA

>LNC_001581

AGTTCGAGTTCAAAGACGGATCTCAACAAATCCGTCCTAGTTTGAACACAAAGTTCAAGATATGGAGAGTCGCCATGGGAGGGAAACAATAGACAAGCGGATTTCATTTAATTTACGTCTGTGTATTATTTAATCTTTATTATTTTTCTGTAATAATCTGTAAACTCGAACTCCATGGATTTATATGAAATACTTGGGGAATCGTCTAAGGGGGAGGGGATTACGTGCTAACTGTTTCTTCTTTTTTAGGCCTTATTTTATTATGTGATTGTTATAGATGATTTGTATATCACTAAAAAAAAAAACATACAGGCATATAGAAATATATCCTAATAATTGGCTTAAAATCAACCTTTGTTTATAACTTTATCTGATTGATCAATCTTGCATGAATACGTGTTAAAATATGCTATGAATCGTGAACTTGTAGAATCTTGTCTCACATGCTAATATTATTAAATTTTGGAAATTGACTAAACTATTAGACATCATGCCTATAGGTTTTTCTGAATCTTATTATAATTTTCAGCCTGTTCTATTTAGAAATCATGATCATAAGGTTTATTTTTCACTCTGCATATTATTCGGGGTTTAGAAAATCATGCCTATAGGTTTCGGAATATTATTATAACTTTATATAGAAATTATGTCTATAAGTTTAATAAATTTCAACATGTTCACTTAGAAACCATGTCTATAGGATCTAACTAATAAGACTAGTGTTAATAAGATTAAATAAAATTTCTTGTTAAAATCTTACTCTAATAATTTTCAGCATGTTCAAAAACTGAAATAAATTTGCTACATATTTTTTGTAAATCAAAATAAATATTAACTATTTCTAGCATGAAAAAAAATTAACTTACAGAACTACTTTTTGCACTATTAATGCTGAAATATTTCCTGCATGTTTAAGAGAAATCAGTACAACAATATTTTGATTAAAACCTGAGCTGTTTTAAATTAACAGTTGTGAATAAATTCAGACCACTTAGAAATCATGCCTATAGGTTGTAGACTTGATTTTACTTATTTATAGTATCATGCCTATAGGTGCTATGCAATATTTTTCAAACACCTAGAGATCATGTTCAAGGTATTATTAATTAATCTCCAGCATGCTAATCACTGAAATAATAATTTACTTCTGCCACTGTTTTCCAGCATGTTAAATAACCCAAATTCTGTTATGACTTTTCCGTCATTATCAAAGTACTGTGACATTATTATAATCTGCTACTAAATAAAGCTAATAAATCAGCTCTTGCACGTTTTAATTTTAAAATCTGCCCTTGCAAAATCATGCCCGAAATTAATTATTTTCCGCATTCGAATTGCCACTTAAATTAATTTCTGCACTAATAATACCATATGCTACTGTTCTAAAAAATATATATTTTCTGCATTTGTTACTCGCTTGTCCTAATTAAAAATTAGCTACTGTCTTTCTACATTTTAACAAATTGACTGCCATATTAAATTAATCTTTGCAATAATAATTAAATTTGTTGTTGTTTTAAAATATTTTTGCATGTGTGACCACTTTTCTGCTATATTTTAAAATCAATAAATAAAATAGGCTATGTCATAAATTTCTGTCCTTTTAAAATCAGTTGATAAATAAAATCTGTTTTTCTGTAATAATTTTTGCCAATCTCAATTAAATAATTTCATTGTTGTAATGCTTGTGTATACGCCACACACTTAGCATGCGACCTTTTCACCTTACTTGTGTTTGCTAGCATGTTAATAAACTAATCTAGAGGCCAACATGAGATTTACGTGCTAAAATGCTCGTCTTATTTGTTTGACTTGGTGTCTAGGTGGGCCTAATTAGGAGCCTCTATTTTTATTTTAGTCTAAAACCTTCATGTTAGCCGTCCAATGCCTCTTGGACCTTAAGTGGGTACGGTAGGCACCTTTTAGAGACGAAAATCATGCGA

>LNC_001582

GTTTAAATAAGTTTGGATTTTGTGTATATTTTCGATATAGGTTCGTGTGATGTAGTTATATGTCTTTTTCTCATTTTGGTTTTGCAAACTATCTGCGTATGTAGATGTTTGTGGTTCTATGGACATATGTCTAGGTGTCCTTTGGGTAAATGGTTTTTCGAATAGCTGGTTTAAGTTCGCATTTTTATGTTGTTTATCGCTAACTTGTTTGCTTGTACCTGCA

>LNC_001583

TCTTGCCACTGTTGTTGCAAGTTTTTGCAAATAACTTTTTGCCCTTTTCGCTTCCTTGTTTGATTTACTGCTTCTTCCTCTTTGTTTTTTCCTTTTGAATTTGACATGGTTCCGAATAATATTTTGCTACATTTTCTGTATGCATGTATTACTGTTTACTCCTTTTGATCCAGTTGTAATAATTTCGGGTGAAGATTGATGAACCAAAGGTGTTCGATGATATGCCTGAAACAACTAAATCATTAAGTAAGATTCTTACTGCGGCTGCTGGATTTGCTTGCAACAACACAGGTTGCTATTTGTGAATTTCGACGAACAATAGGAAAACAAAAGGATTTTGGAGCTCACATTTCTCCAGCTAAGGAAACAAAGGCTATTCTCTTCACTGACAAACGGAAAAAAATATTGAGCTTGTAGTAGGGGAAACAAAGCTGATGAAACTCGAGGTGTGAAAGTGTCACTCAACACCATCGACCTCAAGCAGCATGTTGATCTTCATCAGTTAATGCTTCCCTATCTCGAAAATTGTATCTATATTTCGGAGAAGTAGTGGTAAGGCCTGTGCACACTCTACCTTCTTCAGATTCCACTTTGTGAGATTTCACTAGTTCATTTCCTTTGTTACCACAACACCATCAAGTCTTCTGTCAAGGAAATAAGGTGGCAAATAAATTACATAAGCCAACGTCGGAATGTCACTGAATTGAGTAGTATAGGCAAATAGCAACCTATGAGACGACGCAACCTAATAACAAACACGGTTGGTCAACTATAAAGTTTCAGGTTAGGTAGTATACACTGAACATCTTACTTTATTATTTTCTCACCCTGTTAAATTGTAATCTTTATCCATTATACCTCAACATTTTGATTAATTAGATCGAAGCATATTAGTTCATGTGAGATGTTTGATTATTATGTTTGAATAAGGTTAATATGTTGATATTCAAACATATTAAGTTATTTGAGATGTTATTCTGTTTGTGCATTTCAAGTTCACTTTTTCCGACTTAATTTTGAATTTTTTTGAGTATTTGGTGTAAATATGAGATTCATATGAAAGGTATTCTCTTCTACTGTATGAAAGACTCTAAGACCCGATGAGTCATCTGCTTCTTCAGTTTAGTAGAAATTCTTTCATGCTATTGCATGAAACATTTGCCTGGTCTCTTATTGCTATCTGAACTAGCTTGAAAAGTATGGTTGCTCTCTTCTTTTCTGTTGATCCTTATCGAGAATTTCCTCCTTTTTCCACTCTGCCCTGAAGATTACCTATATAGGCTTCACAACCATCCCTGCTTGCATTTTTATAAAAAGATCAATAGTGGTTGTCAAGCATCTCCCCAATACATTTGTATTACCCACCAAACAAGAAAATATTTCTAACATATTTATTATATTTGGTTTCCAATTGTCTTTGGCAAGGCCAACAAGTTAAATTAATTGATGATTCCTCTGTGGTTCAGCTTGATTCTCTTATCTGAACCTTGAGTGAGATTTAATAACAATTTTCCAATTGTCGACTTTCATATATGTTTCTTATATTCTTCTTTGTCCTGCTTCATGTTTTCCAATTGCAGGGAGAATTCTGATTTTTGTTACTCTAGGTTGAAACTTAGTGTTCTTGAATCTCACAAATTTAATAGGTATAGCATATTAGTCATATGAATTGAAGCTAACATAATTATTGGTTACTCTTTAGGATGATGATAATCCTTCTTTGTCAAATGCTTACATGGCGTCCAAAAATTTTCAAATTCTGAATTTTATAGTTATTCTACCATCTATTATTGCGAGAGATGGGACTTGAGGATCACTGCATTAAACATGAGAAGTGGCTGACATGTTTGAGCCAGGTGTGGCACCAAATGCTATAACAAGGATGTCACTAACATTGAAGAGTTCTCTTACTATTTTGGAGCTATTAATGCAAGGTAAACAAATGGATAGGAAACGTGGCAGAAGGCTGAAATTAGATATGTTACATGTAAATTTCTTTTTTTCCAGATTAAGGACAATTATGTAATTTTCTTTTTCCAATTTCTATTTTTGCTTGCCGCATGTTTCCAAAGATCCACACAGTGGATCTAAAAACTTCCACATCTTTTATTCTCAAAATCCTACTTTTATGTTCCATGAAGAACAACAGCTTCATTTCATCCCAATTTTAGAAAGGTTTGCTCCTTACAACAACAAGGAAAGGGTACATCTCATTTCCCGTGTTTTCTCTTCATGGATATTGTGGGTTTTCTTTTCAATCTCAATTGTTGCTTCTTAGATTTGCTAAACTTTCTTACTGGTGGTGCGTTTTTGGCTTGATTTGCTGGGTTTATGTGTTATTCAATGTTTTCTTTTCTATCCTAAATTACCACCTTCTACTTGGAGTCAATTTTTATTTATTTTGTTTTTTAATTTATGTGTGAACGTCACTTGCTGGATTTGCACGCGAGCTGCTATTTGTTGGGAGTTTCGACGAACAATAGGAAAACAAAAGGTATAAGAATGACCCAGTTGCCCTAGAAAAATTTATGTGAAGCAAGATGTTTTGTTATTAGTGGACTGAAGTCTTTGGCATTAATGAAATGGTGAAGCATTGTTCACCAGTGTAGTGTGCACTGCAGACAATGTGGAGGTGACTTTTTGAGAAGGACATGTATTAGGTCTTAACCATAGAACACCCTTTCCTTTTGACATTCTGGTCTACCTTGTGTCATACATAGACCCAGTGTAGTTTTTGTTCACTATGCACCGTCTTTTGGTTATTCCCATATATGTATTTCTGTTCAGTATGGATAGAACGTTGCAGTAGATAAACTCACTACATTTCTTCTTTTGGTCTCTTGTGTTTGGTGTTCAAAAAGAACCTATAAACATTACTATCTAGACAGAATATTACTGAAGTGTCTAAAGTTATCAGGGCAGGTTTAACTAAAAGACAACTGAATAGAGATGTCAACTTAATTTGAAAACATGACTAATTCAAACGCGAAGTCAAGCTCTACCTTATGAGTAAAATGGTTATGGCATGAGGGCTG

>LNC_001584

TCTTGCCACTGTTGTTGCAAGTTTTTGCAAATAACTTTTTGCCCTTTTCGCTTCCTTGTTTGATTTACTGCTTCTTCCTCTTTGTTTTTTCCTTTTGAATTTGACATGGTTCCGAATAATATTTTGCTACATTTTCTGTATGCATGTATTACTGTTTACTCCTTTTGATCCAGTTGTAATAATTTCGGGTGAAGATTGATGAACCAAAGGTGTTCGATGATATGCCTGAAACAACTAAATCATTAAGTAAGATTCTTACTGCGGCTGCTGGATTTGCTTGCAACAACACAGGTTGCTATTTGTGAATTTCGACGAACAATAGGAAAACAAAAGGATTTTGGAGCTCACATTTCTCCAGCTAAGGAAACAAAGGCTATTCTCTTCACTGACAAACTTTACAGGGAAAAAAATATTGAGCTTGTAGTAGGGGAAACAAAGCTGATGAAACTCGAGGTGTGAAAGTGTCACTCAACACCATCGACCTCAAGCAGCATGTTGATCTTCATCAGTTAATGCTTCCCTATCTCGAAAATTGTATCTATATTTCGGAGAAGTAGTGGTAAGGCCTGTGCACACTCTACCTTCTTCAGATTCCACTTTGTGAGATTTCACTAGTTCATTTCCTTTGTTACCACAACACCATCAAGTCTTCTGTCAAGGAAATAAGGTGGCAAATAAATTACATAAGCCAACGTCGGAATGTCACTGAATTGAGTAGTATAGGCAAATAGCAACCTATGAGACGACGCAACCTAATAACAAACACGGTTGGTCAACTATAAAGTTTCAGGTTAGGTAGTATACACTGAACATCTTACTTTATTATTTTCTCACCCTGTTAAATTGTAATCTTTATCCATTATACCTCAACATTTTGATTAATTAGATCGAAGCATATTAGTTCATGTGAGATGTTTGATTATTATGTTTGAATAAGGTTAATATGTTGATATTCAAACATATTAAGTTATTTGAGATGTTATTCTGTTTGTGCATTTCAAGTTCACTTTTTCCGACTTAATTTTGAATTTTTTTGAGTATTTGGTGTAAATATGAGATTCATATGAAAGGTATTCTCTTCTACTGTATGAAAGACTCTAAGACCCGATGAGTCATCTGCTTCTTCAGTTTAGTAGAAATTCTTTCATGCTATTGCATGAAACATTTGCCTGGTCTCTTATTGCTATCTGAACTAGCTTGAAAAGTATGGTTGCTCTCTTCTTTTCTGTTGATCCTTATCGAGAATTTCCTCCTTTTTCCACTCTGCCCTGAAGATTACCTATATAGGCTTCACAACCATCCCTGCTTGCATTTTTATAAAAAGATCAATAGTGGTTGTCAAGCATCTCCCCAATACATTTGTATTACCCACCAAACAAGAAAATATTTCTAACATATTTATTATATTTGGTTTCCAATTGTCTTTGGCAAGGCCAACAAGTTAAATTAATTGATGATTCCTCTGTGGTTCAGCTTGATTCTCTTATCTGAACCTTGAGTGAGATTTAATAACAATTTTCCAATTGTCGACTTTCATATATGTTTCTTATATTCTTCTTTGTCCTGCTTCATGTTTTCCAATTGCAGGGAGAATTCTGATTTTTGTTACTCTAGGTTGAAACTTAGTGTTCTTGAATCTCACAAATTTAATAGGTATAGCATATTAGTCATATGAATTGAAGCTAACATAATTATTGGTTACTCTTTAGGATGATGATAATCCTTCTTTGTCAAATGCTTACATGGCGTCCAAAAATTTTCAAATTCTGAATTTTATAGTTATTCTACCATCTATTATTGCGAGAGATGGGACTTGAGGATCACTGCATTAAACATGAGAAGTGGCTGACATGTTTGAGCCAGGTGTGGCACCAAATGCTATAACAAGGATGTCACTAACATTGAAGAGTTCTCTTACTATTTTGGAGCTATTAATGCAAGGTAAACAAATGGATAGGAAACGTGGCAGAAGGCTGAAATTAGATATGTTACATGTAAATTTCTTTTTTTCCAGATTAAGGACAATTATGTAATTTTCTTTTTCCAATTTCTATTTTTGCTTGCCGCATGTTTCCAAAGATCCACACAGTGGATCTAAAAACTTCCACATCTTTTATTCTCAAAATCCTACTTTTATGTTCCATGAAGAACAACAGCTTCATTTCATCCCAATTTTAGAAAGGTTTGCTCCTTACAACAACAAGGAAAGGGTACATCTCATTTCCCGTGTTTTCTCTTCATGGATATTGTGGGTTTTCTTTTCAATCTCAATTGTTGCTTCTTAGATTTGCTAAACTTTCTTACTGGTGGTGCGTTTTTGGCTTGATTTGCTGGGTTTATGTGTTATTCAATGTTTTCTTTTCTATCCTAAATTACCACCTTCTACTTGGAGTCAATTTTTATTTATTTTGTTTTTTAATTTATGTGTGAACGTCACTTGCTGGATTTGCACGCGAGCTGCTATTTGTTGGGAGTTTCGACGAACAATAGGAAAACAAAAGGTATAAGAATGACCCAGTTGCCCTAGAAAAATTTATGTGAAGCAAGATGTTTTGTTATTAGTGGACTGAAGTCTTTGGCATTAATGAAATGGTGAAGCATTGTTCACCAGTGTAGTGTGCACTGCAGACAATGTGGAGGTGACTTTTTGAGAAGGACATGTATTAGGTCTTAACCATAGAACACCCTTTCCTTTTGACATTCTGGTCTACCTTGTGTCATACATAGACCCAGTGTAGTTTTTGTTCACTATGCACCGTCTTTTGGTTATTCCCATATATGTATTTCTGTTCAGTATGGATAGAACGTTGCAGTAGATAAACTCACTACATTTCTTCTTTTGGTCTCTTGTGTTTGGTGTTCAAAAAGAACCTATAAACATTACTATCTAGACAGAATATTACTGAAGTGTCTAAAGTTATCAGGGCAGGTTTAACTAAAAGACAACTGAATAGAGATGTCAACTTAATTTGAAAACATGACTAATTCAAACGCGAAGTCAAGCTCTACCTTATGAGTAAAATGGTTATGGCATGAGGGCTG

>LNC_001585

AAACTCTTCATCAAGTATTTATCTTTATTTGCTCCTGTTTGGCTTGGGATTTGATTTATCTATTTTTTTTGTCGATTTCTTTTTCAGAGGTTTCAATTTTGGTTCGAATTTTTCTCCCTGATCACTAATGGGTAGGTAGTATTGCTTTGACGATTTTGCCTCTTCTCTCCCAAAACAATTCAATGGCTGAAATTTTATGTTTTCGCGTCACTACTAATTTAGTAACTTTAGAAGATATGAATTCTGTAATGATTATTCTACAATTAGTTGTGTCTACTCATGCATGTTTTGTTTTGAATATTTGGTGTCAAATTTCTTGATTCCTAAAAGGACGTTTTTGGAGAGGTCAGCGAGGTTTTGGAGATCAGAGAAGATTGCAGTGGGTATTGTTCTAGGGTTTTTTGGTGTTCTCTTTCGTTCTCCATCATTGTTGGCCTTCGGATTTTTTTAAACTGATTTTGTATATGCATGTACTCATTCACTATTTTGTGTCAATTGCATAGTCCTTACTAAGAAGAATCGAACATGCTCTCTGAGTAAAATTTTCACAAGTTAGTAATGGATTCATGTTGTGTCAATTATTTTGATTGCCTTTCGATTAATTTGTAATCTAAATTATAGTTGGCTTTGCCCGTTTTAGTAAGGTAATTTATTCTAATTTCAAAGATTTAAGTTTACAGGGAAAAAAATATTGAGCTTGTAGTAGGGGAAACAAAGCTGATGAAACTCGAGGTGTGAAAGTGTCACTCAACACCATCGACCTCAAGCAGCATGTTGATCTTCATCAGTTAATGCTTCCCTATCTCGAAAATTGTATCTATATTTCGGAGAAGTAGTGGTAAGGCCTGTGCACACTCTACCTTCTTCAGATTCCACTTTGTGAGATTTCACTAGGATGATGATAATCCTTCTTTGTCAAATGCTTACATGGCGTCCAAAAATTTTCAAATTCTGAATTTTATAGTTATTCTACCATCTATTATTGCGAGAGATGGGACTTGAGGATCACTGCATTAAACATGAGAAGTGGCTGACATGTTTGAGCCAGGTGTGGCACCAAATGCTATAACAAGGATGTCACTAACATTGAAGAGTTCTCTTACTATTTTGGAGCTATTAATGCAAGGTAAACAAATGGATAGGAAACGTGGCAGAAGGCTGAAATTAGATATGTTACATGTAAATTTCTTTTTTTCCAGATTAAGGACAATTATGTAATTTTCTTTTTCCAATTTCTATTTTTGCTTGCCGCATGTTTCCAAAGATCCACACAGTGGATCTAAAAACTTCCACATCTTTTATTCTCAAAATCCTACTTTTATGTTCCATGAAGAACAACAGCTTCATTTCATCCCAATTTTAGAAAGGTTTGCTCCTTACAACAACAAGGAAAGGGTACATCTCATTTCCCGTGTTTTCTCTTCATGGATATTGTGGGTTTTCTTTTCAATCTCAATTGTTGCTTCTTAGATTTGCTAAACTTTCTTACTGGTGGTGCGTTTTTGGCTTGATTTGCTGGGTTTATGTGTTATTCAATGTTTTCTTTTCTATCCTAAATTACCACCTTCTACTTGGAGTCAATTTTTATTTATTTTGTTTTTTAATTTATGTGTGAACGTCACTTGCTGGATTTGCACGCGAGCTGCTATTTGTTGGGAGTTTCGACGAACAATAGGAAAACAAAAGGTATAAGAATGACCCAGTTGCCCTAGAAAAATTTATGTGAAGCAAGATGTTTTGTTATTAGTGGACTGAAGTCTTTGGCATTAATGAAATGGTGAAGCATTGTTCACCAGTGTAGTGTGCACTGCAGACAATGTGGAGGTGACTTTTTGAGAAGGACATGTATTAGGTCTTAACCATAGAACACCCTTTCCTTTTGACATTCTGGTCTACCTTGTGTCATACATAGACCCAGTGTAGTTTTTGTTCACTATGCACCGTCTTTTGGTTATTCCCATATATGTATTTCTGTTCAGTATGGATAGAACGTTGCAGTAGATAAACTCACTACATTTCTTCTTTTGGTCTCTTGTGTTTGGTGTTCAAAAAGAACCTATAAACATTACTATCTAGACAGAATATTACTGAAGTGTCTAAAGTTATCAGGGCAGGTTTAACTAAAAGACAACTGAATAGAGATGTCAACTTAATTTGAAAACATGACTAATTCAAACGCGAAGTCAAGCTCTACCTTATGAGTAAAATGGTTATGGCATGAGGGCTG

>LNC_001586

GTTTTCATATGCATAATAAATCTTTACAGCAATAACATCAATTTCCCAAAACAAATAGATAAGTATTGGAAAAGAAAATGATCAATATGCTATAGCTGAGAACAAAGAGCTTTTGCAAGTCTTTATTTTTGTCTTTTAAATGAGTTGATCTTTAATTTAAGTCAATAGCTAAAAGAACTTATACCTACATAAAATAAGTTAGTTTGTTGGATCTGGATCAATTTGTTGTCATTTAAGTGCAATTTGAACTGAGACAGACAGATCATTGTTCCCTAACTCTAAAGCTTTCAAGTAAGAACACTCCCCTATGCTTGCAGAGATACTTCCTGTTAGCTTATTGTCTGATAGATTAATGACTTCAAGAAGAGTTACCTTTAGTATTTCTAATTCATAAACAAAACTTCTAATCTTTGTTTTTAACTGTAGTAGTGTAAGTTTGGTTGCACTTTCATGACTATACCATCTTACTTTTCGTGGCCTACTATGAGATCATGTATCGCAAGGTGAAAGAAATTTAGACTCACATTTACTTTTAGTATTTTTTAATTCAAACACAAGACTTCCAACATTTAGCTTTAACTTGAGTAACTGTTTTGTTAGCTTTAACTAGAGTAATTGTTTCATGCATGTGTAAAGCACAATGCAGTTTCAAGATTCAATACTCTAAATCAAGAAATAGAAAGCTGGTAAAGATTAGCTTAAATGTTCATTCTACATCTATGAATGCTCTACGATATGCTATCCTAAGTTCTATGTTCTATCAATTGGCTTAAAATTGTATATATGTCTCGTGATATATGATAAAAGAGAGAACACTAACATAGACTTGAGCACTTAGCATCTGACTGTACCAAGTGAGGTTAATTACATGAAGGGGGTCTACTTGAAATATTTTTTTAACTGGCAAGAAGAAGACACCTTTCTTAATTCTCATATTTACTTGTGTGACATTGTTACTCCCAGGAAATACTCGAAAACTGTAGTCAATAGATGGTGCTTCTATATCAGCAAGTATTTTCTTTTTAAACATTTTTTAATGATAATTTT

>LNC_001587

TTATGATCTCTTTTGGGGTTTGTTGAGATACTTGTACTGTCATTGTTCCCTTTTTCTCTGTGAGACCATCAGTACTTACTAGCAGAATTTGTTGACTTAGATGGGATTGATTCTGGTTATCTGGTGGTTTTGATTTTCTCTCCGGGATTGTTGCTTCCATCTCCCTACTTTGAACTTGGTTCATGCCCCTAATGTTATTCTCTGATACAATTTCCTGGTCAATTTTTTCTCCTGTTATCAATGATTCTGATAGATTATTCCTCTCATTTTCCTCTTTTGTGGTGCTGTGATTTTCTCCCATGTTAACAGAGGATCCTTGGATAGATCCATTGGAGTTAGATTCATCCACAGAATCTTCTATATCTTCTACCCTATCACCCCATTTTGAATTGGACACTTCCCCTTTTTCCTTTGTCATAATTTCCTGATGGGTTTGCTGAAACTGAGAGTCGTCTTCCCCTTGTCGTGCTTTGTTTGTGTTTTCCCCTAGGACTACAGTGGTGTTGTTAATCTGGCCAAAAGATGCCTCCACCCAGGCTTTTGCACTTCCACCCTTGCCAACTG

>LNC_001588

TTGAAACGACCCCAAAAATGCCCGAAAATGTGCTTCGGAAACACCTATAATTGTAATTTCCATATATCTCAAAATCCGTGCATCATTTCGGAAATTCGACTTCATATTCTTATTCAGGGGGTCAAATTGAGTGGGAAATGAGTCTAACCCGAATTTTAGAACAACCGTATCAAAATTTGAAAATCGCAAAAAATGCGATTTTGAGGGTCAACTTTAAAGGGGCATATCTCTCAGCACACAAGGAACTGGAAGGAGCTCAAACTATCAAAATTAAAGCTCTGTGAGTTAGCTTTCCAACGCAATTTGTTTCACCTCATTCCGAGTTAGGATGAAGGAGTTATGACCATTTTCGTAAAACCTGTCCGGCAGAAAATGCAAATTCCAGTAGCCGAAAACACTGTTCACCCGCCTCAATTTTTTTTCTAAGTGTTGGGACGTTTTTATAAAAAGGGGACATATCCATACTTAGTCATTTAACTTCAAAACATCCAAAAACTCTCTCTAAACCCTCTCCAAAAATTCCACCAAGATCATCAACCAAATTCAAGGATCCCAAGCTTTAATTCCGGATTCAAGATTCAATCTCAAGCAATTCTCCATTCCAATCTTCATCAAGAATTCTCCAAAGTTGAGGTATGTGGGTTGATTGTGGAAACCTTCCTTTCCACAAGTCCAACCATTTATCCTCTATTTTACTTCAAGTTTATGGGTCCTTATGATCATTTATGAACTCTTGAATTATGGAATTATTACTACACATCCTATTATGGTCTATTTTTGTATATTATCATGAAATGGAATGATTATATGATGTTTATGGTTTTCATGCCTCAATGATCAAATTATGAAGGTTGTTATATATGTATATATATATGTATGTTGTTGGTGGGCTGTTTTACATGGATTTTGAAATTTGTTGGACTTAGTACTCTTGATACACATGATTTTATTTACATGAACAAGTAGCCCACCATATGTTTGATAAAATGCCATTTATAGAATTCTTATGAAATGGAAGGTCCAATGTACGTCCTATGAGTCGGATGATTATATAAGTATTGAAATAATGATGAAATGGATACATGTATATGATTTTCTCTATATACGTGTTGTGAGTCGACCAAGCCTAGTACGGGGGGTAGTAGGCCCGTATAACCATATCGAGGGGTTTGTACCTTGGTCCCTAGCTCGGGGGGTGGTAGGCCCGGGTAACCACATTGAGGGGTTTGTACCTCTTTCGCCTCTCCAAGGGGGTGGTAGGCCTTGGAAATACTATATTGATGGTCACTCACCACACTTGTGCTACGTCCCACTAAA

>LNC_001589

AAAAAACTAAAAACAACAGAATTGGAAGGCGTATCCCTTATGTACTTTTTCTTAGCCGGTGTGTTGATGATCCATAAGCCCTATTCACTCTTTTTTTCTTTTTTTTGCTTCTACGTCTCATGACGGGAGTGCTAGAGCCAAAAAATGCATTCTTTAGTCAAGCAATGAGAAAGATTGACCAATCTCATCATATGCTATTTCATATTCATATGCCCTCTAACTTGAACTTCCTTACTTCCCAG

>LNC_001590

TCAAAAGCGGAAACAGCTAGATCAACTGCCGAATCAGTCGAACTCGAAGCCTTCGCTCCTATGTCAATGGTTGGGATAGCTGGCTTACCTTCTTAGTTAGGACATTCAAGCTTCCAGTCTAATGACTGGACACGACCAACCCCGACTCTCTAAGCCCTCGTCTTTACAGGAATTGTCGCAGCTGAGCGTAGATGACTTCCCGCAGATTGGGCTACTTCTATTTATCAATATAGAAAGAAGATCCTAC

>LNC_001591

GAAAATCAAATGAAATTCTTTTCGTAAGGCAAGAAAGTCGCCTAAGGAAAGTAGAAGTCTATGCACACTAACCGAATCTTTGGGGAGGGATGGTGGTGAAAATTGGCCTTTTCCCTTCTCCTTTCTAAAGCAAAAAAGAATGTCATCATTCTGATCGTGACTTGAAAAAAGAAAGAAGAGAAGGAACTTGGAGTTGGCGCCCACATAACGGATTGCTTGCTTACCAAGCCATCCTGGCTTTTCGCTCCTCTCCTTACAGTCAAGTGGCTTTCACTCCTCCAGTTCTATTATTATTGACTTGACTTATTATTATAAAAACTACTCACTATCCAAATGAAAGACGATCGATTATCTACCCAAGAAGATAGAGAGTCCCACATACGAGAAAAGGTCACAAATAGAGTTGAACCAAGTAACATTGCAAAGGCATAATGATAGTAGGGTCGGGATATCCCCGCCCTCCGAACCGGACGTGAGGGTCTCCCCTCATCCGGCTCTCTGCAGGGGAATCTCCACTCACTGCTTCCCCTAATATCCTCCCTTTTCCACATCATGGGGGTTTACAGGAGATCCCAGAGGCTCGCTCGGAAAGGCTGCTATACCATACCCTTTGACTTAACTCTACTCTAGTAGTCACTAGACTCACTATAGTAGTCCGTCTGGCTGCTCTTGCTGAAATCATTACTCTTAATTCTCCCGTGCTCTAGCAATTGCGCTCCCTAGACCACTACCATGTAGTTAGGTAGGGACAATAAGTCCGTAGTGACGGGAATCTAGGAATGAATGGGGATCCCTATCAATAGAAAAAGAATATCTAATTAGAGTTTTCTCCCTTTCTCTCACTCAATAGATCTATCTGGTCTGATACGGCACAGTACAATACGAGACGATGGAATGCAATGGGATAGATGGTAGAGGGCTGCCTGCGCCCAAAAGCGATGATTCACTTGTCCCCTTGTCCATAGGGACCTCGTGGCATACAACCGAAACGACTCCCGCTAGATAGCCGCCCCTTTCTCTCTTTTTACAGCCTCGTGGACGGACGAAAGAAGGGAAGTTAC

>LNC_001592

TGGAAGTTGATGACTATTCATAGCTATTACCTTGACACCAAAGAAGAGTTCGACCCAATGCTTTATTTCTGTCCTAGTTGATCCTGATTCGACATTAGAAGTATATTGATTTTTCCCCAATAACCGAATACTTTTGTCTGTAAATACTGCATATTTGATTCCATCCATAAATCTATTTTCTTCCCTATGAGTTATAGTCTCAATAAGAATGCTAGTTCTTACTGTTCATATATTATGATATGAATATACCACATCAATTCGTTATGTATGGATGATGAGATTCCATTGATACAGAGCCA

>LNC_001593

TTTCAATGACCAAAAATTTGGTTTCACAATGCCCTGCTTCATTTTAGGTTTTCCAAATTTCTCCTCTGCTTCTCTCATTTCTTCAGTCTTTTCATGTTCACTGTCTTCTTCATTGACTCTTTCAAATTCAAGCTTCTTAATTGTATTTTGTGGTAAGTTTTTCTCGCTTTTTTCTTCCTTAGTTTTTTTTTTTTGCTTCAATATTTTTCTTGTGCAATTACTGATATTTTTAGAGAATATAAAGACATGAAGTTTACAGATGTGACCATTTTTATTTTCACTGATCCATTTGTGGAAAGGAAAATTGGATGAAAATGACTATAAACAAAATAATATTTAACAACCATCCAAACAAGTTGTTGATGAAGATGGAGATACTGTTGCTCAAATTTCGACATACATTCATCATCTGAATCAAATTTTGATATTTATTCTTTTAGGTTATTGTTCTCTATTGTATTTTCTCTATAAATAATATATGAATGTCAATAGTATTTTCTGATTTATTCATGCATCTTCACGAATAAATACTCAAGTTTCCTTAGTGCACCAAGGGTGTTTGATAATATGCCTACATCGAGATGGATCTTTGATTCAGCACTAAAGGTTGGTCAAGCTGTGAACTAATAATTATTTTTCCGTTCCTCGCTTCTTAGTGAACTTTTTTGTACTGTTGAAGGGAGAGAGGTAGAAAAGAGATATGCGATACAGAAATATCAAGACAAATAAATAAATGTCTTTTTTTCCTTTTCTTAAGTGAAGGAGAAAAGGAAAAAACATATGATTGTTATTTGTGGAATTTGAACCTTTCAATTCCAGGTAGAGTTTAAGTTATTCATAAAATGAATTCACCCTCAACTCCCTCTAATTCTATATAGAAGCAACATTACGTGCCTCATTATGAAAAATCATTTTTTTCCCATTTTATCACAGCTAAACTGGAAGGACCAAAGTTATTTTCTTGCTTCCTGGAATAAGAATACAAGAGGGTACTTGAATGTTTCTCATAATATCAAATAATTTAGAATAATTGAAGGATATTCTCTAGATTTGGTAACAACATTTGATGTTGGACCTAATTAATGTGCGGAATGATAGTTAAGTTATCCGTATGCGGTCATCATCAGTTGTATTGGGTTATGAATGCTAACGATATGAAATAATGCATTTATTGCAAATGATGTACCTTTACATTTGAGTTTTATACATGATCAATGTAAATATTTTTTGCACTATCAGGTTAGCAGATTTACATTAACCTTCTTGATAAAATGTGCTACCCACATGTAAAAATTATTTACGTTACGTTGACAATGTATATTATTTATTAAACTCATATGTTTTATTGTGCTTTCTATTCTGGCTTTGAAATTTGGGGCTGAGAAAAAGGTTGGGATGTTCATTGTTCAACTAATTTGAGATATTAGACGATGCTATTGATGTAATGAAATTGGAAGATGGTGTAATTCAAAACATGATAATTAATGGCTTGATGCGGTTGCATGTTTGACGAAATTTCCCGTGAAGGTAAGACTTGTGTGTTATGAAGAGAGATTAGTTACAAGAGTTGATTGTTTTGGCAGATGATTCAAGGTGCTAATGAAGTTGAACGGTGGGCTTAGGTGAATAGACATTTTCCCTTATAATATCTTTTTTCCATATGGCATCTTCCCCAAAAAAAATTGAGAAAGAATTTGAGTTGCCGAATTCTTTGATGCTGCCATGAAATTAGAAGTGATAATGTAATGCTGAAAATTCAGGGTAAGCTTTCAGTATTGATCTTTTTGTTGTTTGAACTACATTTTTGAAAAAAATCAGCTACTAATATAGTAAGTTGAGGTGGTATTCAAAGACAAATTGTTTAAATACAAAGTCAGAATAACAAGCAGGTTGAATCAGGCGAAAAGGTTGATTTCTGGCTCTCCAATTTCCAAGTCCTTTAAGGAACCATTCCACATTTTCTTAAGAACCTTTAAACTTTTGGCAATGGACCGTGATGTTGGTGTCGCATTTATTTTTAGCAGTTGTTCTTGGATAAAAGCTGGAACTTTAATATTGAAAATTCTGTTACTAGATTGAGTTCTAGAAGTCTGTCATATGATCTTATGCTTTCTCAAAGTTTTTGTTTCATGTGTTCATATTAGTTAGATACAATTGATAATGTTAAAAATATTTTTCATCTGATCAATCCAGAAAAAAATGAACATAAGAAATGTAGCAAATAAGTAATAATGTTGACGCTAGAATTTTTTTGAGAAATGAGTGAGTAATAAAATGTCCGGGTAATGGAAGAATCCTATAAGACTATTAATTTAACGCAGAAACAAACTCTTTTCTTTAATCTTGAGTTTGTTGATCAAGAATAGCTACTAACTCCTAAACTTTTTTATATCATACGTGAAATAGGAGTAGTAATTTGAGTATGCAATATAATAATATTGTGTTAAGAGTGCTGTCATATGCTTTTTATAGAGAAATATCAACTTCATGTTTTCTCTTTTATTTGTAGCATAATATTCTTTCATCAGAATTTTGTGTCATGGTCATTAATGGGTTCCTTCTTTTGCTTATTGCATTTGAAGGTTTGGCAATGAAATAGATGAAAAAAATGGAGGTTTTGCTTGATCCAAGTATGAGCTCTAAAATGTGAGACGAAGAGAAGCTGACATTTTTAGTATGTGCTCTTTCTATGTCCCCAAATTAAGAAGCATAAGGATAGGTAGAGCATGAGCTCTTGCAGACTAAGTTGTTGTATTATATGTTAAATGTAATAAAAGGATTTTCTTAGTGGTTTATACAACATCAATGTAATATTATGTTATTCAAACTTAAGGTCTGATTTGATTGTGAACTTTGGGCGGCCCAAAGTAAGAAATGAACTTTTTGTTTTAGAACTACCAACTTCCATATTTGAATGTGTAGCAGCCATTAAAAAGATATCGATTGAGTGCTTATAACTTTAAGGTGGAATAGAACTACAAAAAGTTAGGGTTGATGTTCATAAAATAAACAAATGGTGAAGCTTGCCAATTGTAAATCAAATATGTACAAATAGTTGAACTGGTCGGTACTGACAGGTACAACCTTTGGTATTTTTATACATATGTTTGGAAAACACTTGCCTAAATTTCCATTTTGCAAAAATAGGAGGATTGACAGTAATCTCAATTTCAATTGGTGGATAGTTATGAGAGTAAAAATTATTAAATGAGTTTAGATGATTAATATACAAAGTGAAAACGTGAGAGATCTACTATTGAGTTCTTGTCATATCAGAGTCAACAACTTTGTTCGAACTGATCGTTACACGCTTGTGAACAATTTTGTAAAAATAACCTAAAAAATAAAGAGTTACAAGTGACTATACAAACTCTTACATGGAACTCTGGTGAACAACTCATGCCTTTGATTTGCTTCACTATATTATGTTCGAATACCACTTGATTGTATGAAGAGCTCCTACTTTGTTTTACTCAATTTATTCTAGGATGTGAAAGTGAGTTTGCCTTATATGGGTCAATTTATGCATACTGAACGTCAACATCAGTTGTGGTATTAGGTGTATTAAACTTTATTGCTGAAATATTGTAAAGATGTACAATTTCTCAAATGGGTGGATTTGTTGATCTCCACAACCAACTATACAAGAATATTGGAAACAAATGGAATTGGGTATTTCAAAACAGGGAAGTCATATTTGTCGCGTTGGAAAATTCGTCGTTTTTGCTTATTTGTTTCTGGTGTGAAGGCTGAAAACTTTATCTAAACTATCATGTGTGTGTGAGAGTTTCTTACCTGAAATTCAATCATCGATTCACATAGGAGAAGTGAATTTTTGATGGTTGATAGGTATTTTGATAAATAGTTGGCGGTGTAGTTCAATAATGAAACTAAAATATAAGGACACTTTAGGAGTTCTTTTAACCATCAACTCTTTGTTTCTCATTTCTCGCAGAATGATTTTTTGTTATTCCCTGTTAATTAAATAGATAATTCCCATGAACCTATACGAATTTTTGAAATATAGTCTTCCAAACTACTTATTTTTGCTCCTCCAGGAGGTGAT

>LNC_001594

CAAAAATTTGGTTTCACAATGCCCTGCTTCATTTTAGGTTTTCCAAATTTCTCCTCTGCTTCTCTCATTTCTTCAGTCTTTTCATGTTCACTGTCTTCTTCATTGACTCTTTCAAATTCAAGCTTCTTAATTGTATTTTGTGATAATTAATGGCTTGATGCGGTTGCATGTTTGACGAAATTTCCCGTGAAGGTAAGACTTGTGTGTTATGAAGAGAGATTAGTTACAAGAGTTGATTGTTTTGGCAGATGATTCAAGGTGCTAATGAAGTTGAACGGTGGGCTTAGGTGAATAGACATTTTCCCTTATAATATCTTTTTTCCATATGGCATCTTCCCCAAAAAAAATTGAGAAAGAATTTGAGTTGCCGAATTCTTTGATGCTGCCATGAAATTAGAAGTGATAATGTAATGCTGAAAATTCAGGGTAAGCTTTCAGTATTGATCTTTTTGTTGTTTGAACTACATTTTTGAAAAAAATCAGCTACTAATATAGTAAGTTGAGGTGGTATTCAAAGACAAATTGTTTAAATACAAAGTCAGAATAACAAGCAGGTTGAATCAGGCGAAAAGGTTGATTTCTGGCTCTCCAATTTCCAAGTCCTTTAAGGAACCATTCCACATTTTCTTAAGAACCTTTAAACTTTTGGCAATGGACCGTGATGTTGGTGTCGCATTTATTTTTAGCAGTTGTTCTTGGATAAAAGCTGGAACTTTAATATTGAAAATTCTGTTACTAGATTGAGTTCTAGAAGTCTGTCATATGATCTTATGCTTTCTCAAAGTTTTTGTTTCATGTGTTCATATTAGTTAGATACAATTGATAATGTTAAAAATATTTTTCATCTGATCAATCCAGAAAAAAATGAACATAAGAAATGTAGCAAATAAGTAATAATGTTGACGCTAGAATTTTTTTGAGAAATGAGTGAGTAATAAAATGTCCGGGTAATGGAAGAATCCTATAAGACTATTAATTTAACGCAGAAACAAACTCTTTTCTTTAATCTTGAGTTTGTTGATCAAGAATAGCTACTAACTCCTAAACTTTTTTATATCATACGTGAAATAGGAGTAGTAATTTGAGTATGCAATATAATAATATTGTGTTAAGAGTGCTGTCATATGCTTTTTATAGAGAAATATCAACTTCATGTTTTCTCTTTTATTTGTAGCATAATATTCTTTCATCAGAATTTTGTGTCATGGTCATTAATGGGTTCCTTCTTTTGCTTATTGCATTTGAAGGTTTGGCAATGAAATAGATGAAAAAAATGGAGGTTTTGCTTGATCCAAGTATGAGCTCTAAAATGTGAGACGAAGAGAAGCTGACATTTTTAGTATGTGCTCTTTCTATGTCCCCAAATTAAGAAGCATAAGGATAGGTAGAGCATGAGCTCTTGCAGACTAAGTTGTTGTATTATATGTTAAATGTAATAAAAGGATTTTCTTAGTGGTTTATACAACATCAATGTAATATTATGTTATTCAAACTTAAGGTCTGATTTGATTGTGAACTTTGGGCGGCCCAAAGTAAGAAATGAACTTTTTGTTTTAGAACTACCAACTTCCATATTTGAATGTGTAGCAGCCATTAAAAAGATATCGATTGAGTGCTTATAACTTTAAGGTGGAATAGAACTACAAAAAGTTAGGGTTGATGTTCATAAAATAAACAAATGGTGAAGCTTGCCAATTGTAAATCAAATATGTACAAATAGTTGAACTGGTCGGTACTGACAGGTACAACCTTTGGTATTTTTATACATATGTTTGGAAAACACTTGCCTAAATTTCCATTTTGCAAAAATAGGAGGATTGACAGTAATCTCAATTTCAATTGGTGGATAGTTATGAGAGTAAAAATTATTAAATGAGTTTAGATGATTAATATACAAAGTGAAAACGTGAGAGATCTACTATTGAGTTCTTGTCATATCAGAGTCAACAACTTTGTTCGAACTGATCGTTACACGCTTGTGAACAATTTTGTAAAAATAACCTAAAAAATAAAGAGTTACAAGTGACTATACAAACTCTTACATGGAACTCTGGTGAACAACTCATGCCTTTGATTTGCTTCACTATATTATGTTCGAATACCACTTGATTGTATGAAGAGCTCCTACTTTGTTTTACTCAATTTATTCTAGGATGTGAAAGTGAGTTTGCCTTATATGGGTCAATTTATGCATACTGAACGTCAACATCAGTTGTGGTATTAGGTGTATTAAACTTTATTGCTGAAATATTGTAAAGATGTACAATTTCTCAAATGGGTGGATTTGTTGATCTCCACAACCAACTATACAAGAATATTGGAAACAAATGGAATTGGGTATTTCAAAACAGGGAAGTCATATTTGTCGCGTTGGAAAATTCGTCGTTTTTGCTTATTTGTTTCTGGTGTGAAGGCTGAAAACTTTATCTAAACTATCATGTGTGTGTGAGAGTTTCTTACCTGAAATTCAATCATCGATTCACATAGGAGAAGTGAATTTTTGATGGTTGATAGGTATTTTGATAAATAGTTGGCGGTGTAGTTCAATAATGAAACTAAAATATAAGGACACTTTAGGAGTTCTTTTAACCATCAACTCTTTGTTTCTCATTTCTCGCAGAATGATTTTTTGTTATTCCCTGTTAATTAAATAGATAATTCCCATGAACCTATACGAATTTTTGAAATATAGTCTTCCAAACTACTTATTTTTGCTCCTCCAGGAGGTGAT

>LNC_001595

AGAGTTTCCAAATCCTTGGTAAAATGCCTTGACTTTGTATTAATATATACTATGTGTACTATTTAACTCCTCTGTGATTCAAAAACGTTTGATACACTCGCCAATTAGAACTTTTGGATCCTTACTCGCTTTAACAATTGTATTGCTGAGTGAGGTAAGTGACGTCAAACCAAGGAAAATGATTGATTTCAGATTCTCTAATTTTCAAGCCTTCAGGTACAATTTCACACCCTTTTCAAGCTTTTAAGATTTGTAGGAACTGTAGTATTCTATTTTGGATGATGTTGTATTAGTATTTAGTAGTTATTCTTAGATAGAAGTTATGCTTTTAGTATGATCTAAATTTCTTCTGTTATTTTTGTATTGTTTGAATTGCAACTTCTGATATATTAGCAGACTGGCAGACGATACCAAAACACCATAGTTTATCATATTCATAATTACAATTCTGCAATTGAAAGGATGATCATACAGTAGGAGACTAATGATGTTTGGAAATGATTTAGGAACAACATATAGTGAGTTGATGTTCTCCATGGTACATTGGATTGTTCAGAAGTTGCGACACCAATAGAATATGAATGATAAACTGTAAATGCAGAATGTTCCTAACAACTTTGAGCATTCTCCCTTTTTTTTCTTACTTTTTGATTGTTTTGTTTGGGTGCAATTTTTTTGAGTTGTGCCAACAACTCCTATGCTATGATTTTTTGAATGAAGAAGATTAGCTACTACGAAGCGAACTCCGGCATCCTCTACATATGATAAACAATGACATGC

>LNC_001596

CAGGATTTCCTACGGTACTCTCTCTTCTTCTTGCTTTCCAAAAAACCACCGTTTCCATGTTTCCATCTCCTTTCTTAGATTCTTTGGTCTTTCTCCAACTATGATTGATCGATCTCCCACCTTGGTATGACTGAGTTTTACCAGCTCACAAAGATGTGAATCCAGTGGAGGTGTTTTTAGTTCTTGGTGAACTTTTGTTGGGATTGAACCAATGTACTCCTTCCGGGAGCTCGAGGATCACTCCCCTGAGGCGAGTTGCTGAATCAGTTCGGAGACCCCCTTTTACTGGTCCTTTTCTGTAAGCTGGAACAGTTTAAACTAGGAGGCTTTGTCCCAATTAGGTAGCTTTCATGTAATCCAACCTGTAATACATAAATATGACAACTATGTTTTTCTGATTTTTGCATCATCGTTATCAGCATGTTTGCACTATCCTTCTGGTCTTGATTCGCAATGCTGGTATTTGCTGCTTGTCTATATTGATGATTTTCCTGCCTCTGCTAGATAGATCATAAAAGTTATGTGCATGTATTGTCCCTGCTTGCTCAATCTCTAGATTAGCTAGATAGTCTGCCAAGGAATTAGCCTCTCTGAATATATGTTTGATATTGACTTGTTTCCTTGTTATGATCTTTTGTATCTCTTTTATCTTTTTTGCATACTCTCACGGGATTTTCCACTGCCTAGTTAATATGTTCTTAATCGTTAAGGAGCC

>LNC_001597

CTTTAAGAACAAAAATGCTGCATGTATGCATGCTGTAAACATTGGTCAATGCAGGAAGAGCTGTTAAAACACCAAGAACGAAGGATGAAAAGAAGAAATCAAAGCCAAGAGGTGAACAACATCAAAGGTAATTTGGTAGATGATCAAGTAATGGTATAATTTGATTTCTTCATGAATGGATAGATTCAACATCCGTGCTAGCGTTACTGATTAGCCTTGTACATTCCAAAAGAAGAAAAGAGTCACTCTAACAGCAGGGCAAGAAATCTTTTGAGGTTTTATTTATGGGATCACATGATCCTGCACCATTGTCTATTTAATCTTAAGAAGACGAGCAAGCTCAATAAGGTTGAAGAAGGCTTCGGCCTTTTCCTATGCGATCCATTCATCTATGAATTTGTTAGGGGTAAAAACTTAGTTTTGTTCTGCTATATGTAAACTTGATTTCTTTGCCCAACTTATATGATGAAACTATTTTTTTTTCTCTATTTAGAGGAATGATTCCAACCACCATTCACGTACTATTCCTCATCAGTCGTCATGAGGAAGAGCAATTGAGAATTCTCATTCCAGAAATACACTGACAAGAACTTAGAGTTTGTTTCTTCAAAATGCAGATTTCAGCTTCCCTTGTATCAATGAGTTTCAGTTTTAACTTTGTTATCTAGGTGTGTTTGGTATGATGGAAAAATGTACCTGTTTAGTTGGAGAGAGAACTTTTAACCATTTACTAAGGATTTTAAATAATATTAGCAACTGGATTCATAAAAACAACATAGCAAACCAAATATTGTTTGATGGAATTTTTTAATTTAAAGTTGATATATTGTCTAAGTTTTGGTCAAAGTTGTGATATATAAAGTGAAG

>LNC_001598

AAAGAAATTACTGAGTAGAAGGATCAACTAGATGTAGTCCTCGACTTTCCAATCATTGAGATTTCTTCTGGGTGTTATGGACCATCTCTGGTCTTGTTTGAAATTGGCAATCAGTGCATTCTGAGAAATGGAACAATGGTAGAGAATAGGGAAGTGTTTGGGAGAGATCCCGAGCCTTGGATCATGCCAAAACCTGATTTCCCTTCGGTCAAGAATCATAATGAATAATGAGAAAGCATCCATGTCACCTTCCCTAGATTTGGTTATCTGGCAAAAACAACTAGCCTCCCTTCTCCTCAATAAGATCATAATCACTAGATTATTCCGCCTGAAGGCTGCAATTGAGGATTGTTTCATATGCCTTTCAATGTCATGAACCATTTTACTCACCAGGATTTGATCTATTAAATAGGATCTGAACGTGTTCCAGACTATTGACAGTCCCTTCAACAACAACTACATACCCAATGAAATCCCAGAGGTGGGAAATTGACAGTCCC

>LNC_001599

AGGAAAGGAAAAATCTCGTTTGTAAGATGCCGATGAAGAGACAATTGGTTTGCCGTGGCAGCAGTTTTCATGTGTGGACATTGACGGTCAGAGGTATTTGGTGGACGTGAAAGACGTAGAGATATGCTCTGCATATGAGATGTAAAAAGTTAGCAATTCATGTAAGTAGGCTGCTTAATTTTTCATTAGTGGGAAATCTGTGCTTGGAAATTTTGGAGGATGCAATCTAGTCAAATTCATTTGTGTAGGCACATTTCCCCTCTTAGATTTCGAAAAAAAATATGTAGTGGTTTAGTTATTGAAATGAAAAGTTATTTCTGCACTTTTATCTCTCTGGCCGATTAGGTAAATAGTCAAAGATGACACTGCTCTCGGTGCGTCGAATAACAAGACACTGTCCATTGAGTTGGGATTTTACCTGGATACTACAACGACGTCTCGGGAGGGTAGTGTATGTAGACTTGACTCTGGAGTTATAGAGACCTTTTTACTTGGGTATAAGAAAATACAAACCAAAAATAGCAATACTTTGTTAACAAAATATATGCTATCTGGTAAAACATACAATTGGCATACAAATCACACAATTATATTGTTGTATGTGTTTTGTATGTTGTCTGCATGTATCCTGTATCTCGTTTGTGCGCTGTATGTCCATTGCTCATTGAAGTTTATGGTTCTTCTTTGGAGTTTCAATTTGGAAATCCAACTAAAATCAACTCTAATTTTCACCAAACTACCTTAAAATTGATGTATAAACTCCAAATCATGCAACAATCAATCCAAAGAAATGACAATTTTGCTATACCTGATTTTTGAATTCAAAGTTTTTTTTTTAATGATCGTCTATGGAGCTTTAAATGGAATTCACTAATTTGTACACATGATCAGTCATAGGGTGGTGAGAAAGAGGGAAGACAGAAGAAATATTGTTATTAAATATACG

>LNC_001600

TAGGGATCGCAACTCAACTAACCAATATACTCAGAGATGTAGGAGAAGATGATTGTGTGTGGCAGGCTTCCACCGATCTTCATCACTTTCACGTTTCATCTTCATATCATTTCACATCAACACAAAAGACGAATTTCCTCAATGTTTCATCTCCTCAAGCTTTACATCTTCAATTACTTCAAGTATTGCTGCAATTTTAAGGTGTTTATGCTACTGCAGTAGGTTTTGATCGGGGTTGCAGGGTTATTATAGGCGAGGGGCTGCATATTTGACAATTTAGAAGTCAATTACGCACTCGAAGATTTTCAACCGGTTGGTGT

>LNC_001601

GGAAAAGATGATGGAAGAAAAATAAATGAAACCAAGTTTAATGTCTTCATAAGAGTGATCCGATAGCTGCCAAATAGACCCAAAACAACTGAAACTACGACTCAGCCACAAAAGCTGGAGGTTGAAGCCGTGTTGACAGCAGAATCCAAGGCACAACAATAGTCTTTGAGGTTATTGTTTTTAATTTTTTACATCTTCATGCAAGAGTAAGATAACTTTCACTTTTTACCCAAATGATCAGAATTAGCTAAAATGAAACTGAAGGTAGTACTTGCAGCTTCCGAGAGTCGGCTACCAATAAGGCACGAATAGAAATGACATTTTGCTCCTCTTCTTTTCGGGAGTTGGTTATATTATATTAGATTGTGAGTGGATGAACTTGAAGCAAGTTGTGATTTTGGTCTCTTCATTAAAGAGTTTGATAATCGATGATTTGCCTTCTTTTACTAAGTGGCTCCACTAATGATCTAAATAGCTGTGAAATAAAGATTGATGCAACAAGCCTTAGTTTCTTT

>LNC_001602

ATGGCCTTGAAGGCATAGTATGAGAAATTGAAGTGTTATCATGACATTGTATGAATGAGAGTTCAATGAAGATAATGTGATCATGTTATGAATGTAAAGGTGTTGTTGTAGGTTGTTCACTGATTTGGTTGCATAATTACTACCCTATTTTCCATGCTCTTTGATTTCATTACATGCCTTCAAGGTGTTTGACAAAATGTCCAACTATGGAGAATTGATGAATCGATGCTTTAAAGTTTGAGTTATGAGATGTATGGAAATCCAGAGATGTGTTGATGACACTCATGAATGTTGATGATATAATATGAAGGATTCTTCAATATAAAGTATACCTTGAATTGGTAGGGCTTATGCATCATTTTCTTGTATAAATGATTATATTGTTGATTGTTGAATCCTTGGATCGGTAGGCTTATGGCACCCTTCCACACATGCTTATAATGTACCTCGGATCGGCAGGCTTATGGCACCCTTCCATTAATGATAAGTAATGAACCTTGGAATCGGCAGGCTTATGGCACCCTTTCCATAAAGGTTAATGATGAGACTTGAATCGGCAGGCCCATGGCACCCTTTCAAGAGGAGTTAATGAGCTTTCCTAAATGTACCTTGAATCGGCAGGCTTATGGCACCCTTTCCATAAAGGTTAATGATGAGACTTGAATCGGCAGGCCCATGGCACCCTTTCAAGAGGAGTTAATGAGCTTTCCTAAATGTACCTTGAATCGGCAGGCTTATGGCACCCTTTCCATAAAGGTTAATGATGAGACTTGAATCGGCAGGCCCATGGCACCCTTTCAAGAGGAGTTAATGAGCTTTCCTAAATGTACCTTGAATCGGCAGGCTTATGGCACCCTTTCCATAAAGGTTAATGATGAGACTTGAATCGGCAGGCCCATGGCACCCTTTCAAGAGGAGTTAATGAGCTTTCCTAAATGTACCTTGAATCGGCAGGCTTATGGCACCCTTTCCATAAAGGTTAATGATGAGACTTGAATCGGCAGGCCCATGGCACCCTTTCAAGAGGAGTTAATGAGCTTTCCTAAATGTACCTTGAATCGGCAGGCTTATGGCACCCTTTCATGTGTTAAATAATGTACCTTGAATCGGTAGGCTTATGGCACCCTTTCATGTGT

>LNC_001603

TAAAGACCTCCATCTCCAAACGTTTTGCTCTGTCCTCTTGTCCTATTCTCAAATTTGCACTATGGTGGAGTTTACACCTCCGTTCTATCTCAGGTAAGCATCCATCAATCTTGGCGCCATTTGCAAAGTATGCCGACCGTCAAACACCTCCATCTCCGACCGTCTGTTCTCCCCTCCTTTCCTCTTGTTCTTAGCAAGTCTACGATCTCGGGTGAGCATACTTTTCAGTGGTGTTTTCTAGAAATTTCTCTCCAAAATTTTATTGTGTCTTCTAGCTTCTTTGATTTTGATTCCCTAATTCTACTTTGAAATTCTTTTGTGCTTGACACCAAATTTGGGTATGTTTCGTATTTCATTATTTTACCCTTTCTATCTATTTCTTTATTTTTTTTATTCATGTTGGTGTAGTCAAGGGTCTGTGTTTGAGAAGATTTGATGACAAAGTTTGGATTTTTCCTGTTGAAAATGAATTGAAAAATTTGTGGATTATATTTGGGTTTGTAGTTTTCCTTTTTGCTGTTGGGTTAAGTAATGGTATGTACTTGAACAATTCTCCCGTTGAGCTAACCTTTTGGGTTGAGTTAGACCAACTGTTATATTCTTTACGGATTTTATGGGATTGATAAATTCTTGAACATATTAATAAAAGCTTTGTAGTTTGGCTATGTAGGATTAATAAACATTCTAATTTCTTATTTTCAATCTGTAAAGGTCTGATTCTAGATATACATTTCTTTTTTTTCCGTAGTTGTAGGGACAAAAGATTGTTCCTTTTTGATTTGGCAAGAAATAATTACAAAATTCACCACAATAATATTTCTAATATGATCATGAATATCTTTCTTCAAACCTTTAGCTAAGAGGTTTCTTGATATTTATAGTTTCCTTGAAAATAATCTAAAATATATATTGCTCGGACTCTACAACAGTGTGGGCACACCCATCTCAGATCCTTAAAAATAAGTTACTTTTAGTGAATCCGCCATGTACATAGATTTTGTTTTTCCAATCAGCAATCATTGTTTAACTTGCAAAATCATAGTATTAGCCTCTCTTATACTTTTTGTTAAATTATGGATCCTTACATAGGAGGCAGGGCACTCAGTATTGAGATTTTGAAGGAGTGATTGAAGAATCGAATAGACTTTACTATTTAGACTTTTGAACGAGAAACATAAACAATCGGTAATAAAAAACTATTATGAGAGGTGGTTGTTATAAAAAGGTCGAATTGTTTACAACAACATAGATTTTCAGTTCTCATCACATAGATCAAAATAACCCCATACTTTTGGAATCTGTGGACAACAACATAAATATTTTTATGACTCATGTTGTGATCTTCATAGTAAATACGACTTTAGCTTTCCAGATTTATTGGCGTACATTCTTTGATAATAACGACAAGTTCTCCTTTTGTTAGTATGCCTCAAATTGATCCCAACTTTTACACTGGTTGAATTAGGTTGTGCCCAAATTGATGTGCTGATGAATATATCCCAACCTTAGCCACCAAAGTTTGATCTTACATTTTATGGTCAAAATTTGACACCCCCACTCTTCATGGAATAAATACTTATTACTGCCAAACTAATTTTTGCTTTATCTTTTGAGGAAATAGGTGTTGCATACACATTACATTCTCCAGACCCTACTTGTGGAATTACAATGGCTGAAAATAATCCTGTTGAGGTCTCTAATAATGTTGTTCTACAAAGGCTTCTTCTGAGTTGAAGTAATAGGATTTGCAGTGATGATTTTCACTGCAGCAACTTCATTCGCTTTCATTTGTTGGGAGATTAATCAGATGCTTTTCTGTCACACACACTTGGGATTTTCGCTTTGGATTAGTCTTGAAGACACATATATGCACCCAATCCCATTTAATAACCTTCATAATTTTATAGAAACTGTATCTTCTTTATGAATCTGACAGTCTTTTCAATAGTGGAAACATAATTGAGCCCATTGTCACGGACTTAGAGTGATGGCTAAAGTTCCGTGTGGCCCTTGACGGTTTACTCACTAATCTTTCAAGCTTCTTGTAAACTCAAGGAAGCCTTTAGGACTTGGTTTGCTAAGAGAATGTTTAAGGAAAGTTTTGGAAAGAACAAAGAGAGAGCTTTTGAAGAAGGCTTTGTATTGAGCTTTGGAAGATTGCTTACAACTTGCTTGGTGCCTTTGCAAATGGTGGCACCTCTATTTATACTACTCTCTAAGGGCTAAAGTGTAATTAATATTTACATGGGAGGTCCCTAAGTTATCTACACATTGGTCCCCTTTCTAGAGAATTCTACATGATCTAGAATTCTCCAAGGCCTTCTTGAATTGTCTAAGGTATTCTAGAGTTTTCTCCTTAACTCTAGAAACCTCTACGCCTCTTTGGATGCAAAATTTGCCTATGTTGGTGGCGGGACGTCACATGCTCCCCCACCTGATGATGCGACGACCGCGGCGCATTGCTGCAAAAACTCTCGGACTTGGTCTTTGAACTGCCACAAGTCTTCATATCTCTCCCACGTGGCCTCCTCCGGTGATTGTCCCTTCCAATGTACAAGGAACATGGCGCTGGCTTGTTGACCCCGCTTCCGTTTGGCTTGATAATCTATGATGGCTTCGATCTCCCGGTCATGCGAGGTAGTGACAGTAATGGGAGCCCGTTGTGATCGGTTCCGGCTAGTGTCTTCCTTGTCCTCATGATAAGGTTTGAGGACGCTTGCATGAAATACCGGATGGATTTTGAAGTGTGGAGGCAACTCCACCTTATATGAGATCTTGCCCACTTTGGCAATGATCTTGAACGGACCCTCATATTTTCGCACTAAGTTTTGATGAACGCCCCTTAGTGCCTTGAACTGTCTTGGATTGAACTTCACCAAGACCATGTCGCCTTCTTTGAAGTTCGTGGGACGCCTTTTGCGGTCGGCAAATTTTTTCATCTTGCTTGCTGCCTTGTCCAGATAGGACTTGGCAGTGTCAAGCTGCTCTTCCCATCCTTTGGCAAGGTGGTAAGCTCCCAAACTCTTCCCTTCGAAAGCGGCTGGCAAAGAATGCGGAGTTTGGGGCTGTTGGCCTGTCGCCAACTCAAATGGTGTGCGCCCGGTGGACTCACTCCTCTGCAAGTTATATGAGAATTGCGCCATGTCTAGGAGTCTTGCCTAATCCTTCTGATGCGCACTAACATAGTGCCTCAGGTAGCACTCCAGCAGGGCGTTGACGCGTTCGGTCTGGCCGTCGGTCTGCGGGTGGAAGCTTGTGGAGAAATGTAGTTTTGTACCAAGTACCTCGAACAGCTCCCTCCAAAAGTTCCCAGTGAAGCGGGGGTCTCGGTCGCTAATGATATGTCTGGGCAGCCCCCAATACTTCACCACATTTTTGAAGAACAATCGAGCGGCCTCTTTTGCGGTGCATCCGGCTGTGGCGGGTATGAAAGTAGCATACTTTGAAAACCTGTCCACCACGACCATGATAGTCCCATACCCGTCGGACTTAGGTAGGGAAGTGATGAAGTCCATGGTCACGCTCTCCCATGGACGCTCCGCTACGGGTAGTGGCTCCAGTAGTCCTCCCGGGTGTCTTTGCTCCACCTTGTCTTGTTGGCACACAAGACAAGTCTGCACGTAGCACTCTATATCGTCCCGCATGCGTGGCCAGAAATAGATAGCCTCGATCAGCGCCCTTGTTCTTCGCTGCCTAGGATGCCCGGCCCACGGTGTGTCGTGGCTTTCTTTGATGATCCGCCGCCTGATAGTCCCGAACTTTGGCACGTAGACCCTTCGACCGAATGTGAACAAAAGGCCATCTTCTGCCCAAAAGCGCCTTGTCTTGCCTTGGGTGGCCAACTCCATCAACTTCTTGGCCTCGGGATCATGTTGCATGCCATCTTTGATCGCATCTTGAATATCACAATGGACCGTTGTGATGGCAGCAAGCTCGGCCTTCCTACTTAGCGCGTCGGCCACGACATTGCCCCTTCCTGGCTTATACTCCAAGGTGTAGTCAAACTCGGCTAGAAAGTCCTGCCACCTAGCTTGTTTCGGAGTGATCTTCTTTTGTGACTGAAAATAGCTAGTGGCCACGTTGTCTGTCTTGACTACGAACTTGGAGCCTAGTAGGTAGTGTCTCCACGTGCGCAGGCAATGTACGATGGCTGTCATCTCTTTCTCCTGAACCGTGTACCGCCGTTCGGTCTCGTTCAACTTGCGGCTCTCGAATGCTATGGGGTGCCTATCCTGCATTAAGACTCCTCCAATGGCAAAGTCCGAAGCATCCGTATGCACTTCGAAGGTTTTCGAAAAGTCGGGCAGCGTCAAGACCGGTTCCTCTGTCACCGCGGCCTTGAGACCTTCGAAGGCTTGTTGGCACTCCGTGCTCCAAACCCACGGCTTATTTTTCTTCAACAACTCGGTCAGTGGAGCGGCTTTTGCGGAGTAACCCCTTATGAACCTGCGATAATAATTCGCAAGTCCAAGGAACGATCGTAGCTCGGTCACCTTAGTGGGCACCTCCCACTCTTGGATTGCTCGTATTTTGGCCTCGTCCATCCGTAGTTCGCCCTGGCTGATGATGTGACCCAAGAAATGTACTTCATGTTGGGCGAACTCACACTTCTCCCGCTTGATGTATAGCTGGTTCTCCCGTAAGACTTGGAAGACCTTCCTCAGATGCTCCACGTGCTCCTCCAAGGTGTTGCTGTAAATGACAATATCGTCCAAGTAGACCACTACGAACTGATCCAAGTAGGGATGGAAGATCTTGTTCATCAGCGTGCAGAAAGTGGCGGGTGCGTTGGTTAAGCCAAAAGGCATCACCAACCACTCGAACGCTCCGTATATGGTTACGCACGCTGTCTTTGGTTCGTCCCCTTCCGCGATACGCACTTGGTAGTAGCCTTTTCTGAGGTCCACCTTGGTGAAATACTTGGCCTGCCCAAGTCTATCGAACAAATCTGCAATAAGCGGGATAGGGTATTTGTTTTTAATAGTTACCTTATTGAGCGCCCGGTAGTCGATGCATAGACGTAAGGATCCGTCCTTCTTCTTTTGGAATAGTACTGGTGCGCCATAAGGTGCCTTGGATGGGCGGATGTGACCGGCCTCGAGGAGCTCCCTTAGCTGCTTCCGTAGCTCCTCTAACTCAGGTGGAGCCATGCGGTAGGGTGCATGTGCGGGCGGTTTGGCTCCAACTTCCAGCTCTATCTTGTGGTCTACCTCGCGCCTCGGAGGTAGAGTCTTCGGCAGCTCGTCTGGCATCACGTCCTTGTTCTCTTCAAGGACCTTCTCTATGATCGGTGGCAAGGTCTCCCTAGCACCATTGTCTTCACCCAAACTTGCGATGGTGGCTAGGAACGTCGGTTCTCCTTTC

>LNC_001604

TTTAAGTGACACTTTTTCATCAAAGAATACAATTCTTTTGAACGAGTAATATAATGTTGTGGTTTTATACTAGTCATATCATATACTAGTAAAGAGAGACTCAACGACCACAATATCAAATATACTCGAAGAATTTTGTGGGTCTAACATAATACAAGAATGTCATTAAATTTCATATCTTGTACTTTTAAATTTAAATTAGATAGTAAGTCTAATTTTGTCTTTATCATAAGTAAAGAACCCCCCACATTTTAAATATGTTCTCAAAAATATTTTTCAAGTATTTGAAATTATTTTTCACATATTTTTTTCCATGCTTTCATATTGTATACCATC

>LNC_001605

CTTGAACAAAAATTGTTCTGCAAAATGAAAGGCCCCATCTGAAATTTCCAGTGGAACTTTAGCTACCCTAGGCCTTTGCATGTACTTCGACAAGAAAGGGAAGAAAAGAAATTGAAAATTCTGTTATTTTGATATTGGAAACATCTTAATTCCAAAATTCCAAGCATTTTCAGCTCTCTCTGTTAGTTCGGTGCATCTACCAGTGATAATGGTCAAATGGGTCAATTTGCTGATCTCCACAACCAACTCCACAAGAATGTTGGAAAGAAATAAAGATATTTCAAGTCTTTGAGAAGGTGATGGAAGAAAAATAGTATCTGGCATGGCACGACAATTGAAGGAGTCACATACTTCTATTTTGTAGCATCACGGCATCTATTTGGTTCACAAAAGACATTATGACCAACAAAAAATTGTTTGAGTATTAAAATAAACTCCAGAGAATTCCGAGGTCTCTCATCTGATGTTAGAACTTCATGTGGCCAATTCAAAGATGTGGGCATTTGCATTTACTTTGAGGAGAAAGTAAAGTTGATGAAACTTCATACCCAGAAACCTCTCAGTTTCTCAGAGAAATTGTTATATAGCAGATAGATTTGGCACACCTATATTGGAACAATACAATATGATGAACATGGTTATAAAGGAGCAACAAATATTTCACTATGTCAACTGCTTCAGAACTTGAGCTAATAAAGCATACAATGATGTTCAAGTTAGTACTTATGTGAATCATGTTGTCTTTACTTCTAATTTATGCTTGACATTATAATCTATTTTTTAACCG

>LNC_001606

AGAGAATAGACGTATAGCAGCTCTCTCTCATAACAGAATGAAAGCAAGCAAGAACGAAATGAGCGCTTACCCTATCGACCGAAGCTACTATCCTAAAATCAGAAACTCCTAAATTAAAGAACTACAGGAAAGGTAATATACTGAGCTAGCCTCCCGTTATGCACCAATGTTTGAAGAGGACCGTTAACCTTTACCAAAAGAAAGAAGAGCTATTGGTCAGTTTCCTATAGTTGACCGATAGAGCTCGACCGATTGAGACAGGGCAGATGCGACCGATGGAAGGGATCTAATTTGAAGAAGGAAGTCATTTGTGGACTGATTCCGACCCTTTACCAGAAGACAAGAGATTCGCGGTATGGCTTCAGAAGAGGTATTCATTATACAGATATCGTTTAAC

>LNC_001607

CCTACATGATACTCCCCCTGAAGATGAGTAGTAAAATAGAGTTGCGACTCAAAGAAAGTTAGGTCCATTGTGACAATTATCTTCCTAGCATGTGGATCATAACACTTATATCCCTTTTGACTTGGGGCATATCCTACAAAAATACACTTCTTAGCCCGCGGTTCAAGTTTACTACGATTATGAACATGAACAAATGTTGTACAACCAAAAACTCTCAGAGGCAGATCTGTAGTTAACCTAGAACTAGGAAAACTTTCTTTGAAAACTTTAAAAGGCGTCTTAAATTCAAGAGGGCGAGATGGCATCCTATTAATAAGGTAAGTGGCAGTCAAGAGAGCCTCTCCCCAAAGGTGTTGTGGAACTTTACTAGTAAACATTAAAGCTCTCGTTACCTCTAAAAGGTGTCTATTCTTTCTTTCGGCGATACCATTTTGTTGAGGTGTATCTGGACAAGAGCTTTGGTGTACTACCCCAGTTTTTCTGAAAAAACTTCCTAATTGTTCATTAAAAAATTCACGGCCATTGTCACTCCGAAAAATCTTGATTTTCTCATTGAACTGTGTTTCCACCATGACATAGAAAGTTTCAAACACATTTTTAACCTCAGATTTGCCCTTCAATAAAAAAACCCAACTCAATCTAGTATGATCGTC

>LNC_001608

CATAGCTGTTGTGGGGACACATTATTGAAAGATGAATTTAAGAGATTGTATAGAATTTCTTAAGAGGTTTACAGAGTGGAGAGACATTTTGGGACTTGAAAAATTTATAATCATGTAAATGAATAATTCATAATCATGCAATCAATTCTGTACCGGATCAAAAGGGTTGTCTGATTTATAGTTCTAGTCAGGCCTCTCTATAACAACATTTTATTATAGCTGCCAAGTTTTCTTGAA

>LNC_001609

CCAGAATGGAGTTTAATAGCAGAAGAATAATATGCTTAAATGAATAATTTTCTTTGCAGTTATATTTCAGTATTTTATGCATACATATATACACTTCTAATTGCTTCATTGGTTGTATTTCAAAATATATGAAGATTTCTCTGTATCTTGTACAAATAACTTTAGCATGCTGGTCCATCTAATGCCACAAGTGAACCCAGGTTCTCCTGGGTTGACTATTTGCATAAATAATTGGACATTGACTTTGAAAACCAACTCAAGCTTATTGGTGAACATGCTTGGTATCAGTGTTTAGGTTTATACATATTCAAGGTATTGCTATCCTGATATACAAATGAAGTTATCACATGCAAAGCTCAATATATGGTAAAATAGGGAAATTCTGGTTTTTAGAAGGAATGAGTTTGGGGATTGCCCATTCTTTATGGGATTTTACTTATTAGCAAGTATAGGAATTTGTTACCCGTTCCTGATACATTCATTGTTTTGGTTCTTTTGTTTGAAATATTCTTGTAAATCTACTTCTTAATGAAAATTGACTGCAAAAGACATGCTATCCCTCCCAAGGACCAACTATAGAGGCAAATAAGTGGGCGTTTGGCCATGTTTCATTGAGAAATTTGAAGAAAATTGGAGTTGTGGTCACCATGAATACAAATTGGAGTTGTTTGTCTTT

>LNC_001610

TTTCAAGTCAAGTCAAAAGTAAAGAGTTCAAGTAAAGTAAGGAGTAATGAGTCGAGTCCATTTCTCCAAAGTTAATAGGGAACTATGTACTTCCCAAAGAAGTTTCTAAATGTTTTTACATTTAAGTAAGAAAGGAATCATGTTTTCCAAAAGAGCCTTAGGGCTAGTTTTCAAGAAAAGGAACATCGTTTCCAAGTGAGCCTTTGGGCTAGATTTTGAGTAATTATCTCATTTAAAGAAAGATGTGTTTAAAACACTTATGAGTCAAAAGCATTTTGGGAGTAGTATCGAGCACCGATTTGGAGACGCGAGTTCATATTAAACTCAACTCTCCATAAGAACCATGTAGCCAACATGGGAGTTAACGGGTCATACTTTTTAGATGAACCCGTAAAGTTAAGCTAGTGGATCCACTTAGTAAAGTTAGCTTCCTATATCAAAACCAGGTATAGGATGGTCCTCAGGCAACGTGAGGTAAAACGTTGTATCATTAGCTTCCTATATCACGGCAAGGTATAGGATGGTCCTCGGGCAACCTGAGGTAAAACGTTGTATCAC

>LNC_001611

TAGTAATCAAGAAAGACGGGATCTAAAGGCACGCAATTCTTGATCTTTTTAGAATTCTGAAGGACATCGATCTTTCGCCTTGCGCAAGGATATCTCTCTTTTCTTAGACTCCATAAGGAGAGAATAGTCCACTTGGGTGGAAGGGGAGGCCTGGTTTTATTTTACCTGGGCCAAAGACTGTCCCTTAAGCGGAATGGAGCAAGGAGTTATCCACTAAATGATAGAAAGGCTACCTTCTCAAGGAGGAAGACTTCTTTCAATATAAAGCAAGGTGTAAGCAAGCTTCTTTCCGACCAGACTGACCTGGCAATTATCTTCCTATAGATAGCTCCTTGCTTATAAAAAGATTTATAATGAGAATCCGCTGAAGCTTATTCAGGATTGATCTTTCTTATCCGAGAGTCTTATCAAACTCTCTGCCACTGTAGGTGCGACACAGGTAGTAGTGACTGAAAGAGCTGATGCGGCATGATCGCGATTATAAGCGTCCTATTTAGTTTAGGGATTTAGGGACTAGGCCTGTTGAGGAACTGAGATGATTCATCTTAGTCTTAGTATGCCGGGTATGTCTTTGGCCAAGCCCAAGGATTGACAAATCTATAGTGGCGGGTCTTATCTTCTTCTGGGCGAGCTGCGGAGCAACTTTTTCTTTCTTTCATTCCATCTGTCTTGGTTGATTGGTTAAAGAGAGCGCTTTGATAAGACTGTTCCGCAG

>LNC_001612

TAGCCGGAACTCTTTCGTAGGCAGCTCTCGAGGCAGTGGCTTGAAACTAATCTCCCGCAGTCACCCACTATGATCGGAGCTGAGATGATTCATACTAGCTTGGTTAGGTCAATATATGTACCATCGCTTCTATTTGATGTAATATAGTATAGAGGCTGAAGGTGAGAAATCATCAATTGAAGTAGATGCAGCTTCCCTGGCTTAAAGGACGGAACTCCAGCAAGAAATCTATTGAAGAGTTGGTGCTGGGTTAGCCGAAAATCAAAGTTTATCCGGTCCCTGAAAAATTAGCTTTTTATGATTACATCGGCAATAATCCAGAAAGTGGATCTATTTCGTTTTTTCTCTTTTAAGAAAAGGAAAATCTTATTTCCCGGATCATTGTACATTTCAATTTTAATTAGGCATTCTGTGTCCAACTCTAAGCAGTCCTTACCTACATATGCATCTGATCATATATGTATATTATCTATTCCAAC

>LNC_001613

TGGGGACACGAGTTCATATTAACTCAACTCTCCATAAGAACCATGCGCCAACATGGGACTTGACGTATCATTCTTTTTAGATGATCACGTAAGATTAGGCTAGTGGATCCACTAAGCAAAAGTAAGGTCCTATATCGATGGCAAGGTATAGGATGGTCCTTTGGCAACGTGAGGTAAAACGTTGTATCATCACTAAGGCTCGTAGTGGTGGTTGTCGGTTAAAGAACCTCCCA

>LNC_001614

CATAAATATTCTTATCTTTTTTCAATTCAACCAGATCACTCCCCGTAACACCTCGGATTCAAAAGATAAAAAAAATTCTGTCTTTCCAAAAATCTGGTGCCAGGACAGATGGAGTTACTAACGGACCGTTAGTGGTATCACGGTTCGTAGTTGAAAAGAATTTCAGTTTTGATCGAAGAAACTCACTGTTACTGTTATTGATCAAAGCAAAAGCTTCATCGATTCACCCAAAGCTTCGAGGTTTGTCTGTTACTGACCGGAGCTTCAGAGAGTAAAAAATATTCAGACCAAGCTCCGAGAAGAAAAGTTCAAAATCTAAGCTTTGAGAAGAAACCTTCTAAATATATCGATGATTTGACTTTTTAAAATTTTGGCTTTGATAGATATATTTATTTGGATTTTGATAGATATAGGGTAACTTGAGTAAATGTTAATGAGGGCTGGGTAAATATTTATTTGGATTTTTATAGATATAGGGTAACTTGGGTAAATGTTAATGAGGGTCGGGTAAATATTTATATGTGGACAATCACACCAGCCATCTTTTCACTGTTGATAGCGTGTGTATAATGATTGAAGTCTTTGGACGATATCATATAAAAAATCAAGTTCAATGTATATTTAAATCTTGTTGTAGTTAAGATGTTCATTGAATAAAGAGTTACAAGTTCAGAGGGTATTTAACACTTCTCTCTTTTATGTATTGAACGCAAAGTTGATTGACCACTCATTAAATTTATTAAAACATGTAATGTAACGATCCACAAGGTCGTTTTGTTCTTTTAGATTCATTTATCCAGTTTGTACCTTTCTCTATTTTGTTTATGACCTGCGAGAATAGGAAACATGATTTTCAC

>LNC_001615

GGTGATAAAGAGTAGTTGTATCTATTTGACATTTTAGACTTCTATATTTTTGGAATTGTTTAATTTGATCGTATTTTAGTTTATTTTGAAGTTGGATGAAGTTTATTTTGAAATAGTTTGTATATGTTGAAAATATTGTGTTGTTTTGGTTGGATGAAGTTTATTTGGATTTGGGTTGTTTTGGTATAAATATGCAGGTGGGCAACTAAAAATTGCAGCAATTTGCTGCCTGTTTTGTAG

>LNC_001616

TTCTGTGCATGATTTTCCCCATGTTTATGACATGTCGGTTACCATAGGTATAGCTGAGTGGTTAGTGGAAGTCTGTAGAAGCAAATTTTCTTTTCCAATTACTGCAATTTTCTTCAATTTGCACTTTGTTATTGTTTATTTTGAGGGTGCCTAATGTGAATGGAATTGTATTTCTACCTGTTGTTAGGTAAGTTAGCTGCTGTCTGCTGTGCTGCTTAGTGATGTTGGTGTCAGATGATAAGAGTTCTTTTCTCTTCATGCCTGGAAAGTCTATTGCAGACCAAGTACCTGATAAGAACGCAGCAAAATTCTTCTTAAGAAAATTTAAGGATTTGTATCGGGACTCTTGGAATGCACGATAGTGCCATTTGACCTCCAAATTAAAACTTCAACTCAATTGAACTAAAGAGAGGATGATGTTGTTTGAACTAAACAGAGAATGATGTCTAAAATAATATGCCACCACAAGTAAAAAGTTGATGTCACTATAACTAATCAATTATGCTATGTGGTAATTGCAC

>LNC_001617

ATATGACAAAGTGAAGTTGGTTTTGTACATAAAATATTGACAATGATTACCTTGCATCTTAGACAAGTAATTTCATTGATATTTTGTAATAGTACCCATGAATATTTTTTCCACCGATTCTTATGTCACTAATACCTACCATGCTAAACTAAGTGGTGCAATTATAAAATTAACTAATCGTTTGATTAATTAAACAAATCCACCATTCATGGTAGAGTAAAA

>LNC_001618

CCAAAATAAAAGAATTTCAGCTTGACAACTTTGAGTAAAGGAAAATGTGAAATTTGAGTCAATTTCTAAAGAAGAGGGTTGTGCATTGCCGGAATGTTTATACACATTCGTTTGATAATAGTAATTTTGAGATTTTGATGTTAATGTGGCCATAACGTGAAAGTCTCCAAATAGTATAGAATTGAAGTGGATGGTGTTATCCAAGGACCATATTGCG

>LNC_001619

TGTGGTCCTTTGACAAAAGTGCTTCGTTTGGTGGATGGGGAGAAAAAACCACCAATGGGCTATATTTATGAAGCAATGGATAGAGCCAAAGAAACTATTGAACGGGGTTTTCGTGGAATTAAGAAGCAATATGAGAAAGTGTTTGAAATTATTGATGCAAGGTGGTCAGACCAACTCCATCGGCCTTTGCATGCTGCAGGCCATGTTAA

>LNC_001620

TGAATGAGGGCCGGAGGATCTGTAACAGAGCATGCTGTGAAGGAATTTGAAGAGGTAAGATAGTAAAGGCCTTGTGATTCATGTCCTGTGCCAATCGTCTGTCCCGTACTGCGGTCCTGCATGAGAAAAAAATCATCAAAAAAGGTTATGCCACAATGTAGGGCACGTGTCAAACGACTAACAGATGCCAAATTAAAAGGAGAACCAGG

>LNC_001621

CAAACGTGAGCCCTAATTCTAATTCGTACATGCAGCTGCGCCTGGTCCAAAAGAGCAGCATCGTTCTTCCTCTTTGAGAAAATTTCAGAGATCTTTAACATTATGCAGAGCTTAGTGTCTCCATTTTCGTGCAAGTGTTTCACCTCTCTCTTCTGCAAAAAGCCAGCGCCCTCTTCCCCTTCCTGATTCACTCTTTCTTCTTTAATTTAGTCAACAAAAAGCCAAAATACTTTTGACATCTCTGATGAAGAATCACATGCACAATTTTGAATTCAAAACGGCTCTTTTACTTCTTCAAATCCCGCCCAAACTTGAGCCAAGAACCCTAGTTCATGGCTATAAATACCAACATATTCCACTCGTTTTAGGGGGACTTTTTGGATCTTGATAATAGCCTTGATATTCATTCTTATGAAAAGAAGCTCTTCTGCCCCGATAGCGTCTTGAACTATTGTCTTTTGCTCTCAAAATTACCTTTTGTATTCTGTTGCGATCTCAGCCAAATCTCTGGTTGTAAGTTTGCTCGAGTTAGTCGTGGTTTGAGATTACCTTCGATTGAAGCACCTCGTTCACTGCCCCGCAACAGGTGATATTCGCCTCCTCCTCCTCTCTGTTTTCTTCGTTACTTAATCAAAATATTTTTCTTGTTACACTATTAGAATGCTATATATATCAAAAGTGATTTATGCTTAGCATAACTGCAGTAAAAGTGATATTTGCATTCCCTTCTCTATGTTTTATTTTGTTTCGTTTTTGTTGCTGTTGATTGGTAAATTGTTGGGCTTATTTTGAGAAACTTATGAATCGAGAATAACTCTTTGCTTTCATTTTGACCTCTGCTCTCGTTTTAGCCACAATAAGTGGATCCTTCTGTGTATATATCTTTGTTTAGTTGGAATCATTTAGAGGTCCAAAATCGTATTCTTTTTTATTTAGCCACTTTGAATTAAAAGAACGACGGGAAGAGATAGTCATATGAAATCAGTTTGTCTTTCTGTGTGAACCGTTAATTTGGTGTCTCTGTATAATGAGAAATGTGCATCTCGTTTTTCTTATATAGAAGTTTTGTTCAAATTAGATTTATTATTGTTTACTTAGTGTGAAATTTTGAGCAGATTCTGTACATTCTTAGTATAAAAAATGTAAAGAGTTACTGTTATAGGCTTTGTTTTAAGATCTGGACTTTGCCAGATATAAGCTTAGGTTTTTGGTCTCTTTATTAGTCAAATCATGTGCACATATTTTATTTGTCTTAGTACAAATAATAGCAGGTAATAACATTTCAAAGTCTTGGTTACTTCATCTATTTGGATGGTGTATTTGTTGTGTGACTTTATTCGTTTGTGATTTATTTATCGTTTAAAGTTGATTGTTTCACTAATTTCCTTACCCTCTTTTTATTTGCATGAACACTTCGGGAGCTAAATGGAGTCTTCATGAAAGACTCTCACCGCCGTATAGTCTCATTCCATTGCTAGACTATGAAAATGTGCACCCACTCCTTCACGTTGCATCCCTTGCCTCCTCAAAACTCATATGAGTGAGACAATGAGGAAATACAAAAATGCTCTACTATATTTTAGTGGTTTTGTGTTATATTTTGCTTCAAATGATTGTTGTGTTTTGCTTGTCTTATGTTTGATATTATTATGTGCAATCGCTTATTTAATTTATCTTTAGATTATTACTTTAGAATATCTATGCCTTTAGATGGGGTGA

>LNC_001622

TTTTGACTAATTATTTGGTAATTCAAAAACAAATATAGACACACAAAATGATGTGCCTTCACAGGAGAGTAACTGGTCATGGCAAAGTAAGCTGCAACAGTGTAAGTTTCACTAAGCTTTATGAAGAGTCCCTGTGAGCAATTCGATTGTAACATCTTGTATCAAGCTTCAGCTGTGTAAGTTTCACTAAGCTTTGTGAAGAGTCCCTGTGAACAATTCGATTGTAACATCTTGTATCAAGCTTCAGCTGTGGATTTATGACACCCGACTAACGTTGAAGAAGTGCAGAACTTAGTTGTGGAGTACTGTGTCTCAAATTATGCAATGAAGGAACTCATATCTGTTAAAAGATTTCTAATTTCTGTCGAAGGCGAGGAGCTACCTGTAAGACATTAGAAATAAGCTAACTGGAATTAATCTTTAAAAGTGTTAAGAAAGCTCACAGTCTTCTCCAATGGAAGCTGTGGCTATGGAGTATTTGAGAATAAAGGAGAGCAGGGATGAAAAGTAAACCAACTGCAATATTATTTCAACCTTCTTGTTCATATTACATTCTATGTATTAGTATTTATACAGATACTAAAAC

>LNC_001623

TTTTGGTCTCAAGTTCGTTGTCTAGCTGTTGATTTGGCTCGTTGCTTCTGTTTCTGGTTTTAATATGGTTGTATGAGTTTTCGATAGATATACATTATTTTCTTCTGTTTGTGTTTTAAAATTACTCGAATGTATGTGTATGGACGCCGGAGGGCCGATCATTCCTCTCTTGTTCGCTGCTTGTATTTCCCCTATGTCTTAGCTTGATTTTCAAAGTAACGTCTTCATAGCTTGAGTATGAAATCAGTTCATAATTTATCCATTTA

>LNC_001624

CTTTGCCCTAAACCTAGACTATAAAACAATAAGAGGACCTAATGAAGGATCAAGAAAAATCAGCCGCAACACTCATCTTCTTCACCTCAGACGAGTGCTGAGAAATGATATAGTTTAGTCTTCCCTCACCATTAATAAACCTCCACCATTTCAGCTAAGCACCCTAAAACACCCATCAAAACCCCAACATAACCTCATCGTCAAACACCCAAAATACCAACCTTTCTTCGCCGTGTAAACTCCATGTTCTTTCTATAGTTTTAGGTCTATTGAGGGTTCCAGTTCGATCAAGGTTTTTTTCCCGATCAGCCAAGTTTCAGAAGACATCATCTTGACATAAAGGTCCATTTTTTACACTACAAGAAAACAGTCGTTTAGCTACAAACGCA

>LNC_001625

CTATACATAAATAAATGCATGCCAATAGAGAGTATCCCCATCACTATTTCCCTTAGTCATTCACAAACTTGTCTCTCTCATTTTTTTTCTTTTGGTTCATCACAACAGAGCCTTCCATGCTTTTATAGAGCATACCTTCTCAGTTTAGAGACGGGATAAACCACTAGAGTAGCGGCATATCTTGTGACTTTTCATGCTAGCTGTGACCTTTAGATATCTCCTTACATTATCTATGGGATTGCTTTTTCTAGGGTCAGAAAATGAGGTAACTTTTTTATCTTTTTACAATTTTATTTTACTAAAGCAACTCCTCTGGAAAAATATTTTCGTTAGATTCCCTTAAGAAAAAGTTACTGAAAGGAGATGCTGCATGATTTTTATGCCTTTGAAGTGTCTCATTTGATTCAAGGACATTTAAACAGCATATCCTTCATCATATTCCTTATTTTCTTGTTCCTTTTACTTCTTTTTTTGTCAAATTTTCATTTCTATGTTACGTTTATTGAATTTTTTGCTTTTCATGTGGCCTTTTGTTTTTTCGAATTCCAGTAAATCAAGGGTTCTACTGCTTCCTTTGTCTGAGTTTAACATAATTAAAGTTCGAAGAGACCAAGATTGTTATATTCTCTCATATTACTTAAGTGTGTTGTCATTTATCAAATGAAGTTCAGTTCTGGATGTTATAAAAGAAAACATGGACTAACAGTTGATAAAAATTGTTTTCGACAGACAAACAATTACTAATTAATTATTTTATTTTCTTCCACCAGTGCCAGATTTCTCTCGCACCAAAAGTTCTAATACCTGATGTACCTACATCACAATTAAAAGACATTATCCTATTATTTACTAAGTACTACGATATACTCTTATGTCTTTTAAGAAAAAAAACTAGAACAACTTTTCTTAGCAGATTAAAGAAAAAAGCTTGACACATAAATTGAAATAGAGGGAGTAATATAAAAAAGTTGATAAGTTTTTAGAACTAGAAACTCGGCACGTGCGTTTCACAAGTATGTTGAGTCATGTAGTACACGGTTTCTTATTGATTAATAATTATTTGAAAAAACAAAGCACAAGTTCTGTTAATGATTCATGTGAGTTGTCGTATGATTACTTGTTTGTTTGTGACACTCATAGGCATAATCTCCACCATTAAGCAATGCACGTGCCACATGGCAATACGGGGAATCTGATGAAAATATTTGTTCAAATGAGCTTTCACGAAATGTTATTGTAAATGATAAGAAAAGTTATGCTCATTTTATGACCCTAGAATAATCCATAGGCCACACGGCATAAAAATAGCAGGATATGCCAATTCTCAATTGTCCTATTCCATCTCGTAAACTAAGAAGGAATGCTCTATAAAAGGTTGAAAGGTAACCAAGAAGCAGAAAAATGAGAAAGAATAGTCGGTGAGTGATAATAATAATAATAATCTTGAATTTATAAGCAAATCAGTGATAGGGCTACTCTCTATTGGCATACGGTTATATGTTCATCACATATTTCAGCTGATACGCCAAAGGAGAGCTGCCCTGTTACTGTCTCTGCTTAGTGTAATTCTGATGCAAAAGTTGTTAAACCACTGCTTATGTACCGTATCCGCCAGTGGTAAGTCCTACATCTGAATTATTCTTCTCTAAAAACTGAAACTATATTTTTCGTAAGGCATAAGTACCCCCAAGACTATGACCGAAATCCCATAGACACGCCTTAACTAAACTAAGGTCATGTTACCGCCTGAACTTATTTTTTTTGTAATTTTGTACACCTTTTGTCTTACGTGACACACTCCGTGACTCCATGCAATTGAGGCGCGTGGAAGTTATTTAAATGCCACGTAAGCCAAAAAGGTGCACAAAATTACAAAAAAATGGGTTCATGAGGGTAATAGGACCTTAGTTTAGTTAAGATAGTCTCTGAGATTTCGGTCATAGTCTAGGGGATACTTCTACCTTTTCCCTATTTTTTATAGTTCTACATTTGTATTATTCTGCACAAATATTCATTTCCTCCATGACAGTACGCCCAATTTCCTTTTTAGTCAGTCTCAAAATATCTATATTTAGTAGAATTCAACTTTAAAATGCTTATTTTATCCTTAATAAAATGATTATATTCACACAAACATTTATGATTTATCTTATAGGATAAGTTTCAAAAGTCTTTCTTTTTAAACTCCATGTCAAGTCACACTACATCACATAAATTTGGACGAGGGAGTATTACTATATCTCAAGTAAAGGTTCCTGGATAATGTAATACTAATCCTTGAAAAAATAGTATGAGGGGCTTAGTGTAACCTCTTAATTTCAACACAGATTCACAGAACATTGCCATTCATGACAAAAAATTGAATCTATCATATATGTAATTCCAATGCTTGGAAATGGCCACATATCCAACAGGAAAGGTCATTTTGCATTTCGTTGAAGCTGATGAAATGCATAGGCGAATGGCCCTTCATGCATCCGGTTTTAATGAATGAAATATATGGATATTACTCTTCATTTCTCCTAAAATCCTAACTCAAACACTAAGACAACCTCTACTCTTGTTGTAACACCTCGGGTCCAAGGAGATGAGAAATTTTAGTTTGTTCACAGGCATGCATGGTGTCAGCACAGATGACACCACCCACAATCCGTAGGTGGGACGATGGTCCGTAAGTGGGGTCCTTCCATTGGGGTCCAAGATTCAGGGGTCTCTGATCCCACCCACAGCCGATCAGTACGGTCCGTGGTTGGACCTACCGATCGTAGGTCTGGCCGTTGGTGAGGGTCGACAATTATTTGGAAAGAAAGGGCTTTTAGATCAACTTCAAATGGTCATATCTTTTAGTTAAAAATGAATTAGGTAGCCCTTAACCTATCAAATTAAAGATAATTGAATCCTATTTTCAATGCCACTGAGTTTGCTAAAATCCAAGCTTGAAGTAAAAAGTTACGCTTGTTTCAGTAGAGCCCTATTGGCAGGGCCACAACTATGACCCAGTTGACGGGCAGTGGTTCTAATGACGGCCCATAAGTCCCACCGTGAAGGGCCTTTGTCAACTTTTAAGTCAGGGTCATTTTGGTCTTTTCCCCTATGCACTGGGCACTTCAACTATGTCGTTTGATGCCTAAACTACGTTGTTTTACTCAGTTTAAGCCTAATAATCTAGTGAGACAATACCCACAACCCTCATTCTCCAAAAATCACCCAACTTAGAGCAAAAAGAAGAGGAAAAGTTGCCAGTTTCTCCCAGGAACAAGCAGAAGTGTTCTTCGAAGTTTATCCCCGAAATCCAAAGGATTTCTCTGTAGATTTCGTCACCAGGTATGTGGGATTTCACTAGTGGGTTCCTTTCACCCATTAGGTCCCTAGAATTCAGTTAGTTCTTCATATTCTCATAAACTTTTCAGACCTAGGGTTTCTAGATTTTCAAGATTTATTGTGATTATGGTTCCTTTGATCTAAATAACTTATTTCTTGATAATTTTAGTATAATACATGCATAGCACTCAGAACCCTAGCTTGTGTAAATTTATCCTAGTTCGTGAATTATACTTGCTAGGCCAACCAGACAGAAATTCATGCTTCAGATTTTGAATGTTATATTCTCAGTATATAAATGTTGTATTCTCAGTTAGCATGTGAGTTTTGAGCTATTCAGTATCTAGTGCATTTTAGTTTCATGAGTATTCAGTTGGGAGTAGACTTAGCACCGACTTAGACTAAGGTTCAGTGTACCCTCAAAAGTCCTAGAACTACGTGCTTGCGTAGGTTTGTAAGTTCCCCAGTTGGGAATCAGTTTTAGTGATCACGCCACAGTCATGCCTCTATACTCCTGGCAAGGCATATTGGGTCCTCTCGATGGGCGTATACATTGGACTCCACGTGTAACATCCGACAATTTGAAATAGCTTTGAAGGGGCCTAGAATTGGAAATTTTCATTTTTTGAAAGAATTTGAAAATCTGGAATTTTTTTTTGGCTAAGTATGGAAAATGTGAATTTTTGGCCAACTTTGAACAGCC

>LNC_001626

GTTAGTTTTGAGTCTCTCATCCATGTATATGTATATATATGTAAATATTTTATTTTGAGACTCGAGTTGCCCTTTGGGCCATATTTTTATCAGTTAAGTTGTTTATGTCTTTGCATGGCATTGAGTTATTCTGTTGAGTTAGGTTTCCGCTGAGTTAAGAAAGCCAGGCCAAGGGTTCGCCTGGGGCCAGCAATGGTCTCCGGGTGCCGGTCCCGCCTAGGGTGTAGGCTCGGGGCGTGACACCATGTTTAGCTCACGTCGTTTATGTTGGTTAATAGTAGCTCCCACAGTCGACTCTTAGTGCATTGACCCGGTTTTCAACAGTTACTTCAGTATACAGTTCAACATGTTATCTTCATGATTAGTACTTAGTCATTGCATTCAGTTTAGTTTTTAAATTATACTTCAGTACCTATCTATATCCCTATTCAGATTATATCATTGCTCAGTTATGCATTGTTCAGCTTATATACTTTGTTCAACTTAGCATATCTATATCTTGCATGCTCAGTACATTCCAAGTACTGATGCATACTTTGCGCTACATTGTTTCATGATGTAGGTTTAGGCACTTAGCTTCCAGATCACGCTTAGATCAATTCTCGATTCGACTTCAATAGCTTCAGTGGTGAGTCCTCATACTTCAAGGACAATAGACATGCCTTTTATTTTAGTCCTTACTTTTAGTTTTCAGTTTTGTTAGAGTTAGCTAGGGGCATGTCCCACCAACTCAACTCAGTTAGAGGCTTTTCAAACATAGTAGTAGATTCAGCTCGAGTTGTTAAGTGTTGTTTCAGTCATCACTAAATTCTTATTATATTTCACATATTCAGACTAGTTGGGTTTTTCCCCATTTCAGATATCCACTATTTTTAGCTTCCGCATAGGACATGTTTTACTCAGTTATTTCTAGTATGCTTGATATATGTCAGACTCAAGGTTAGCTTGGGGTCACTCGTGATCCTAGGACCCGTGTTATGTCTAGGGGTAGCCTCGGGACGTGACATACACTTGTGTTCAGGGGATTCAAAACCTATATATATATATATATAATAATAAATTAAAAAAATTCCTTCTATATAATGTAACTTTTTGCCAAGGTGGCTTGGGTGAACACCCTCATCCCCTTTAGATGCCCCTAAAACAGATACTCAAATAATAGCTGAGGATATCATGGCAAATTGAGGATTGGGATGCTTAAGTAGTAATGTGACTGGCTCAAAAGACAAAGAGCAGGAACCAGCTGGACAATATTGTGAGTTGGATTTGCCGTTGTTGTTTACTATGTTTGGAATGCAAGGAACAAAAGAAGGTTTCAAGTTGATAAAATGGAACGAGGAGGATAGAGTTCGAGACATAATCATGATGATACATATTGCTAGTCAAGAAAACTGTTAATGGAATTTTATACTAGATAACTATGATAGCTACTCTTATTAGGTTCATGTAAGAATGTTGGCTAATGAGTTACTCTGAGAGGCTATTTTTGTTTAGACCTGACTCTAGAGTACAATGATCAGGTGGTGTAATATTTCCAATTGGGTGAATACATTTTTTTACGTTGAACAATAAAAAACACCGTTTGAAATTATTGTCTACTTGTCTTTGATATCTATTTCTAATAAGAAAAGATGTCCAATGAAACACTTTTGATGGTGACTCACAAATTGTTCAAGGAAAAATTGTGCTTTATGGTTGAAGACTTGACAATGGTATTCACATATTGAGATTACATAAATTTGAGTTTTTGATGCCCTTCGTTGTCTGGAGAAGCAAATCACTAC

>LNC_001627

CAAAAGTTACAAAGTGATATATCTAAAGGTACTTGAATATTTTCCAAAGTTTCAAATATACAGTTCAAACTCGAGGAATATTTATTCTGGAGTTTGAATTAGTTAAGTTTATAATATTGGTTCGAAATAATGTCTTCTCCAGAAGACTCTAGCCCTGTTAATTATGATATCGGGGAGCTCAACATTGATTCGGAAAAGGCCAACTGGTTCAGCTGCAGCTCAAAGACCATTTTACAGTTGTAACATTAGACTTGTAGTTTTATTCTGAGCCATTTTATGTG

>LNC_001628

CACTGATAACAAGCTTTGTCTGGTATGGTCATGGATAATTGTGAAAGCATTTATGCGAATTATTATTTTGATGTACGACAGTCTGAGAAGTGAGAACAATTTATGTTCCCAGCTTCTCCTTATGAGTGGAATTGTTAGGGGCACTGTGGGCAGATTGTTTGGTGTACATAGAGTAGTTGCTTGCAATTGTTCAGTTCTCTGACTGTTTAATTCTGGGTGTTGTTTGACCCCATTTCTTCTTTAGTAATTGTGACGAAAAGTTAGTGTGGTTTTTTTAATCACAGTAAAACAATCCCGAATCAATGCATGGTGTACTGTAATAAGAAGCTAGTCTACCTTTTCAGCACAATTACTAAAGAAGTAATGGCCTCACCATTTCTTCTGGGGCCATATTTGTTTGGTTGTGATAATGTTCTATCTTGTTAAATTACAACAACAACAATTACACCTCAATTCCAAGCTAGTTGGGTTTGGCTGTATGAATCCTCATCATCATAAATGCTTTGTTTGGGT

>LNC_001629

AGCTTATACCGAAACTCAATCGGTGAAGAAACTTTCAACTCAACTTCGTGCTAGAGGATTCTTATGACCTTAATTCATCTCCAAAACTCTTCCCACACTAAAGAATTGAACTTAACACCTTTGCACAATTAGGAATCACCCAGTTCGGGCCTATACACATACGAAGAATGGTTTGGGTCTTAGCTTAAAAAATTTTGGGGTGTTACAATTAAGTCCAGGTTTCTAGATTGTCTACATTTAGGTACCTCAATCCCATCTAATCTCACTTCAACTTCACTCCTATATTGCAGGTTACTTGCAGTGGATGTATTCTAACTTACTCCTACCTATCCTATATTACTTGCTAAAGTGCTTCTCTATAGCTCGAGCTTTGATTGAAGCCTCACTAGTTTCGTTGTTTAACACAATATCGTCTGGTGACTGTCATGGCAGTATGACACCTCTTTCTAATGAGCATATTAATGGCATCCCTTCTTTTTGGCTATTGTTCTGGCAACTCGTCCAGTGATGATTCCAACCATTGTTTGGCACTTTCCAGCA

>LNC_001630

GAAATGGGGAAATTAGTGTCAAGCTTGGATAGAATACACTTGAGACGTTATACTTCTCACTAACGCCATGTATTCTTGTCCATCAAAGACTTCTTTAGCTCAACCCATCTTGGTCCAGCTCATGTGTGAAAGACCTGTTCCAGGTGGAAATCTGCAGTTCTCATTCCGATAAACTGAGCTCTGAGGGAACTAATACCACTGTCTAAAAAACCAAGATCTAAAGTCCATAGGCTTCTGTTAGTCTGTAAGGATATTCATGTTCTCAGCATCCTCAGAATTAAGAAGAAGGTCCTCCTCAAACTCCAACATAGCTATAACCACAACACCATGTCCACCTACACTGTCTCCATGTCAGTATGAAGAACCAGAGTTTTATTTAAATCCTCAAACTCCTGAGAAGGCATCACATTGTTGGCAATCATCTCCCCTGATTCATCTCCTTCATGCATGATTTCTACATTTTTGTCCTTGGTGTTTTCCATGTTATTTGCTTCACTGTTCTTAAAGCCTTGATTACCTGCTGTGATTGTATTTTTGTAGAAGGAATTTGTAGTCCATTCTTTGGTATTCATCTTCTCCCCCTCCACTTTATGTTTTTCTCCATCATCCTGCTCTTCATTAAGAGCATTAAATGAATTGTTGGTCGCAATATCATCCTTCTTTATTTCCATGCCTGCAGTAGTAACCTCCATATCTGTGTCTCTGATGAATCTTCTGTTGGTCGGGTTCCACTATTTTTTAAGGAACCTTGGTGATATGATCACTTTTTTATCCTTTCCTCCCATAGCTGTATTCTGTTCTTGTTTTCTCAATTCAGGGTGGAGGATTCTACATTCTTCCTCGTTGTGACCTTGTAATTTACACCTCCTACAATACTTGGGTAGCATGTCATACATTACTTTCACCTTTTCCACTCTTGACGAAGTTTTTTCTTCAATCTCAATCTCCAGTTCCATATATTTAGGCAGATCTTCTAATAAGTCCAG

>LNC_001631

TCTGGCCAGCTTCTTCTCACACTTCTCTATCACACCATTCCAAATTTCTAAGGACTTGGATTTTGCTCCCAATGGCATACCCAAATATGTTGTAGGTAGAGTACCTACTTCACCCCCTAGAATTGAAGCTAACAAGTCTATGTTGTTGACCTCATTGATAGTATACATAGAACTTTTAATCCAGTTAATGTGCAACCCGGATACCCCTTCAAACAGCACCAGGATCACCCTTAAGTATTTCAGTTGGTCTTCCTCAGCTCCACAAAAAATCAGTGTATCATCTGCATACTGAAGATGTGTCACCTCCAAGCTTTCTGTATTTTCTCTAGCCACATCAAAACCTTTTATCCATCCTCTCATATTTGCTGTCTTGAGCATATTATTCAGCCCCTCCATAGTAATTAAGAACAAGAAAGGGGACAATGGATCACCCTGCCTGAGTCCTCTTTGAGTTTGAAAAAACCCTGCAGGGGACCCATTAATCAGAACAGAGAAGCTAACTGTAGAAATGCAGAAGCTAATCCAGTTCAACCATTTTTGTCCAAATCCCATCATCTCTAAAATCTTCAGAAGGTACTTCC

>LNC_001632

TCTATCTTCTCTGATATTTGCTTGAAAATTTTAATTTTAACATTTAGATATCTTGTTCTTTTGTATATCCAATTTTTTTCTAAAATTAAGCTTAACAAATGATAAGGATAATGAACTCGATTTAATTTACTTCCGAAAAAGGAAAATAGTTTATCGATTGACCAATATGCCCCTGATTCCACTTCCCCAGAATTTTTGCCCTATTTTACACACAGGTGTTCTGTGAGAAGCAACAAATTCGAGCATAGCCAAAAATGGAAGAAGAATGAGGCGACAGAGAGTTTGGGATTTTCTGTAAAAGGGTGGTTGGCCTTCTCTGCTTTGCCAGTGATTTCTCCATTAATGGAACAAAAAGCCAACAACCCTTTTAAATGAACAAGAAAAACAAACATTATTAATATTTAATTCTTCCAAACACACCCTAATTATACTTTTCTTCCTCTTTCTCCTTCTCCTCTCTTTGTGCTGCTGAATGCTGATGTTGTAACGGGGGCTATGACGATTGATTGCTTCCTTTCTGGGCTATGTTCAATCACGGCATTGAGGAAAATCCGACCCGTATCTTGGCAGGGCGACCCGGTTAAGGCTTTGAATAGCAAACTGGTGTCTGCAGCCATGGCGGCGTCGCTTTTCTCTTTTGCTTTGTTAAGAAATACAGCAAATCTGAGCCCTTAATATCCCAATTCGTGGTCTTGAATGGTGAGTCCATTCTCGGGTCGGAAAACCCATTACGGGTTTGAGACGAAGAATGAGGCATCGGAGAAATTGGGATTTTCTGTGAAGGGTGGTTGCCCATCTCATTTTTGCCAGTGATTTCGCCATTGCTAACCAAAAAAAACCAACAGCCCTTTAAAATAAACAAAATAAATAAATAAAAAATTGGATGAACTTCGTCCGTACACACCCCTATACTCTTGTTGTTGATGATGATGTTGTGAAGGGGCTGTGATGATTGCTTGCTTCCTTTTTGGGCTTTGTTCAATCACGGCATTGAGAAGATCCGACCCGTATCTTGGCAGGGCGACCTGGTTAAGGCTTTGAATAGCAAATTAGTGTCACTGCAGCCATGGCGTTGTCGCTCTTCTCCATCGCATAATTCAAATACAGCAAATCTGAGCCCTCAATAATTTGTTTCATGGTTTTGGATGGCGAGTTTTATCGGGTCGGAAAACCTATTATGGGTTTGCGTTGAAATTTCAGCTCTGAAGAATGAGGCATACAACAAACGGGAAATTCAAAGAATGAAAAAAACAAATAATTTCCCCAAACATGGAAAAGGTTAGCCTAGAAAGAAAGAAAGAAATGAGCCAAAATAGAGAGTTTGGATGCTTGTTACCTTATTGTTTTTGTTTTTGCGTATAATTTAGTTTGTCTTATTTTATTCTTTACCATACCTATTTAAAGTAGTGTTTATCCCCAAATTTGATACCCTTTTCTATATCTTGATAGTCCAAAAATTCCGAGGTTATGAAGATATCTTAAAAAAGACTGAAAAACATTTGAGTTAATTCCTAGTCCTGGCATGGCAAAGACAACTTGGAAGGTCTGTGAAGGAGGATGAGAAATAATAAATTTCTTGTAGTCCCTTCTCACGAGGCAACCAATCTTTGCAGCCATGCCTGTTTTAGCTCTGCTCTTTGATTAACTAGGCGAAAATCTGAGCCTCCTACCTCAGTTTAACTTAGTTTCATGATTTGATCACTGAAACAGTATCTGTTAGGGGCTTTCTTGAATTGATCTAAAAAATCAGTTATTGTTTTTCAATCTTGAAATTGATTTTATTTTTTCAAAAATGTTGTGGGATAATTTGGTTTTCATGAAAAGAGTGGCAAACAATGGCATGGTCTTGAGTCTTGATGCCTTCATGTCCCCACTAACTTATTTCTGAAATTTGGTGACTGGTCCCAAGCAAGAGTCGATGATAGCAAACACAGGTGATGGTGAATGTTGATAGGAAAGTAAAACATTAATTTCTGAAATTTGATGACTGGTCCCAAACAAGAGTCGATGATAGCAAACACAGGTGACGGTGAATGTTGATAGGAAAGTAGAACTACTTTTAGCTTCAACTGACAAGTATATCTATAGTTTGGTCAGTAATAAACTTGGCATATTGGTCTTTCTTATTCATTATCACTATCTTAATGTAAAGTAATACTAATTTCTATGCATCATGAGTATATCTCTTACAAAGAATCCCTCAAATTGCCAATGCATGGTGAATTCTGTATTGTTCTAGCGCTCATTTGTAAGTCAGACTGTCCTCGAGTCTCACTGGACTTTTACTTGACATGCTCAAAGCCTCTTGTTGCAAGGTCTTTAGCATCTTGAAGACAGTTACAGAACTTTTGCCTTACAATTCCAAGAATTCTGGGAGGTCAATCTTTCTCTTGCTCTTTCTTGCGGTGCCTTTTGAGATTCTTCACTAAAAAATCACTGGACATTTAGTTGCAACAGCATATGATATTTGGAACATGTTATCTCTATTTCTGGAATAGGCCGGGTCAAGCCTAACAGCATCAAGGAAGCATATGAAAGCTGGTGTTCCTGGGAAATTGAAAAATCCATCTAAAAGACTCTACTGGAGATAAAGAGAAGGAACTCTTTCATACCATGGTCTGCTGTGGCTGTAAAAAGTTAAACTCTATGCATGAAGTTGTCATTTGCATAATGATACACATAGTATTTCAGGGCATGGAGAACTTATTAACAATGAGATGAGGATAGTTCAATGAAAGATAAGATGACCAAGTTGATGATTAGTTGAAAGATTCAGCAAAAAAAGGTAAAACCAGCTTCTGAAGATTAACTGCAGCACATCAAAGTATCAACTCTTTCATGTGAGATAGAGAATTGGTTTATAGAGTGGCAGGGCTCCTTGAGTAAATGTGCAACGAGTAGAGTAAGATTTTTTCTGGTACAAAAGTGGCAGTGCTGAGCCCACGGAGTAACAACTTAATTCAATGCCGTTGGTGTTTCTACAATTACTCGTATATATCTAGGAAATGACTTTTAGGCGTGAAATCGACATGTATACTGATTGAAGAATTAATGCAGCAGCTTCATTTAAGATTCAACGAAGTCCATAGGTATACCAAACATCTTTTGTTGATTTGATTGATAATTTGTACTCCTGTATATTAATTTTTGATTATCTCATTTTGTTAAAAATGTCATGTAAGAGTCACAAGTTAAGTTAAAAATACGATATTGTGC

>LNC_001633

TTTTGTATATCCAATTTTTTTCTAAAATTAAGCTTAACAAATGATAAGGATAATGAACTCGATTTAATTTACTTCCGAAAAAGGAAAATAGTTTATCGATTGACCAATATGCCCCTGATTCCACTTCCCCAGAATTTTTGCCCTATTTTACACACAGGTGTTCTGTGAGAAGCAACAAATTCGAGCATAGCCAAAAATGGAAGAAGAATGAGGCGACAGAGAGTTTGGGATTTTCTGTAAAAGGGTGGTTGGCCTTCTCTGCTTTGCCAGTGATTTCTCCATTAATGGAACAAAAAGCCAACAACCCTTTTAAATGAACAAGAAAAACAAACATTATTAATATTTAATTCTTCCAAACACACCCTAATTATACTTTTCTTCCTCTTTCTCCTTCTCCTCTCTTTGTGCTGCTGAATGCTGATGTTGTAACGGGGGCTATGACGATTGATTGCTTCCTTTCTGGGCTATGTTCAATCACGGCATTGAGGAAAATCCGACCCGTATCTTGGCAGGGCGACCCGGTTAAGGCTTTGAATAGCAAACTGGTGTCTGCAGCCATGGCGGCGTCGCTTTTCTCTTTTGCTTTGTTAAGAAATACAGCAAATCTGAGCCCTTAATATCCCAATTCGTGGTCTTGAATGGTGAGTCCATTCTCGGGTCGGAAAACCCATTACGGGTTTGAGACGAAGAATGAGGCATCGGAGAAATTGGGATTTTCTGTGAAGGGTGGTTGCCCATCTCATTTTTGCCAGTGATTTCGCCATTGCTAACCAAAAAAAACCAACAGCCCTTTAAAATAAACAAAATAAATAAATAAAAAATTGGATGAACTTCGTCCGTACACACCCCTATACTCTTGTTGTTGATGATGATGTTGTGAAGGGGCTGTGATGATTGCTTGCTTCCTTTTTGGGCTTTGTTCAATCACGGCATTGAGAAGATCCGACCCGTATCTTGGCAGGGCGACCTGGTTAAGGCTTTGAATAGCAAATTAGTGTCACTGCAGCCATGGCGTTGTCGCTCTTCTCCATCGCATAATTCAAATACAGCAAATCTGAGCCCTCAATAATTTGTTTCATGGTTTTGGATGGCGAGTTTTATCGGGTCGGAAAACCTATTATGGGTTTGCGTTGAAATTTCAGCTCTGAAGAATGAGGCATACAACAAACGGGAAATTCAAAGAATGAAAAAAACAAATAATTTCCCCAAACATGGAAAAGGTTAGCCTAGAAAGAAAGAAAGAAATGAGCCAAAATAGAGAGTTTGGATGCTTGTTACCTTATTGTTTTTGTTTTTGCGTATAATTTAGTTTGTCTTATTTTATTCTTTACCATACCTATTTAAAGTAGTGTTTATCCCCAAATTTGATACCCTTTTCTATATCTTGATAGTCCAAAAATTCCGAGGTTATGAAGATATCTTAAAAAAGACTGAAAAACATTTGAGTTAATTCCTAGTCCTGGCATGGCAAAGACAACTTGGAAGGTCTGTGAAGGAGGATGAGAAATAATAAATTTCTTGTAGTCCCTTCTCACGAGGCAACCAATCTTTGCAGCCATGCCTGTTTTAGCTCTGCTCTTTGATTAACTAGGCGAAAATCTGAGCCTCCTACCTCAGTTTAACTTAGTTTCATGATTTGATCACTGAAACAGTATCTGTTAGGGGCTTTCTTGAATTGATCTAAAAAATCAGTTATTGTTTTTCAATCTTGAAATTGATTTTATTTTTTCAAAAATGTTGTGGGATAATTTGGTTTTCATGAAAAGAGTGGCAAACAATGGCATGGTCTTGAGTCTTGATGCCTTCATGTCCCCACTAACTTATTTCTGAAATTTGGTGACTGGTCCCAAGCAAGAGTCGATGATAGCAAACACAGGTGATGGTGAATGTTGATAGGAAAGTAAAACATTAATTTCTGAAATTTGATGACTGGTCCCAAACAAGAGTCGATGATAGCAAACACAGGTGACGGTGAATGTTGATAGGAAAGTAGAACTACTTTTAGCTTCAACTGACAAGTATATCTATAGTTTGGTCAGTAATAAACTTGGCATATTGGTCTTTCTTATTCATTATCACTATCTTAATGTAAAGTAATACTAATTTCTATGCATCATGAGTATATCTCTTACAAAGAATCCCTCAAATTGCCAATGCATGGTGAATTCTGTATTGTTCTAGCGCTCATTTGTAAGTCAGACTGTCCTCGAGTCTCACTGGACTTTTACTTGACATGCTCAAAGCCTCTTGTTGCAAGGTCTTTAGCATCTTGAAGACAGTTACAGAACTTTTGCCTTACAATTCCAAGAATTCTGGGAGGTCAATCTTTCTCTTGCTCTTTCTTGCGGTGCCTTTTGAGATTCTTCACTAAAAAATCACTGGACATTTAGTTGCAACAGCATATGATATTTGGAACATGTTATCTCTATTTCTGGAATAGGCCGGGTCAAGCCTAACAGCATCAAGGAAGCATATGAAAGCTGGTGTTCCTGGGAAATTGAAAAATCCATCTAAAAGACTCTACTGGAGATAAAGAGAAGGAACTCTTTCATACCATGGTCTGCTGTGGCTGTAAAAAGTTAAACTCTATGCATGAAGTTGTCATTTGCATAATGATACACATAGTATTTCAGGGCATGGAGAACTTATTAACAATGGAGGAGGAGATTTAACAGCCTGTTTAGGTTGCTGCTACCATAATTTTTTTTCTTTATGCTTCGCCGGAGTGTTCAATGCTGCCAGTAGGGATGGTGGAAGTGGCGGTGGTTAGTAGGATTGACAGCTTTAAAGAGGTGATGATGATTTTGTCAATGCTATTTCGATACCTTGTCAAGTTTCACATGTCTATTTTAGTGGATGCTTTTAAGAATATAACAGTAGAAGTATATACCGGATTGTTGCACCAGATAATCTTATTTAAAGAATTTCAAGGCATTGAAGTACCACCTAGCTACCACTTATGCTAAGATCCAGAGGAGAACTATCTTTGGATTACACTCTAGCCCTTTTCCCAGACACTTGATCTCTTATGTTGTTGATGAAGCATTGAAGATGAAAATCCTTAGTGAAATGTCTCTTTTGTTTTGTTTTTTTTCTTCTTTTCAAATTAGTGCAGAGATGAGGATAGTTCAATGAAAGATAAGATGACCAAGTTGATGATTAGTTGAAAGATTCAGCAAAAAAAGGTAAAACCAGCTTCTGAAGATTAACTGCAGCACATCAAAGTATCAACTCTTTCATGTGAGATAGAGAATTGGTTTATAGAGTGGCAGGGCTCCTTGAGTAAATGTGCAACGAGTAGAGTAAGATTTTTTCTGGTACAAAAGTGGCAGTGCTGAGCCCACGGAGTAACAACTTAATTCAATGCCGTTGGTGTTTCTACAATTACTCGTATATATCTAGGAAATGACTTTTAGGCGTGAAATCGACATGTATACTGATTGAAGAATTAATGCAGCAGCTTCATTTAAGATTCAACGAAGTCCATAGGTATACCAAACATCTTTTGTTGATTTGATTGATAATTTGTACTCCTGTATATTAATTTTTGATTATCTCATTTTGTTAAAAATGTCATGTAAGAGTCACAAGTTAAGTTAAAAATACGATATTGTGC

>LNC_001634

CAAAAACAATACATACACATACAACCAACCCCACCCACGTGCTCTTCTTCTTCACATCACGATGAAACCCAGAACAGTCCAGCAACGCGACTGGACCAAACGCGAAGAACAGCGAGTTCACGGAACCGGTAGCTTTCTGTGTCTTCTTCCTCTGCCAGAGCAGTTCGAACAACCTTTCTCTGCAACAGCCGCCCAATCCCTCCGTCCAGATTTTGTGGGATTTGGTTGAAATATCGCACAAACCAGCCTTTTCTTGGTCCTTGGGAAGTTTTGAATTTTGAGAATTTGTGGGGAATCTCGATTTTACCCTTGTTCTTATTCAAAGCCCCTTCCATTTTCCCCCTCAAAAGGAACCCGAATTTTCCCCTATAAATACATCTTTTCTTGTCATTTTTAGGGGGGTTTTTTTTTGGTTGAAAACTTTTAGAGCAAAAGGTATTGTAAAGCAATTAGCTGCAACATATATATATAGAAATACATTTCAAAGAAAACCAGAGGAGACACTTATTTTTCTTTAGTGTTTGGGTTGATATTTCCGCTCAAGCATCTTGCTTGTTGTTTGTGTTCGATTCCTAACGTTGTGAAGGTGTCGTTCTGCTCGTTTCCTGTCCAGTTTGCTGCACCTTAAAAGGTAACCCCTGTTTTTTTTAGTTCATATTTTGGTTATATTCAATATGCTGGTATGTTAACTGTTGAGTTATTTGAAATGACAGAAATTTGGGTTGTTTCGATTTTGTTTGGTCTCAAGTCATCAGCATAGCTTTTTCTGTTTAGTCAATCATTTTTTTTTTCACTCCTTTCCCGTGTGAATTCGGTTTCCTTCTTCGATAATGGTTTGTTCGCTTTCGGATATTGATAAGAGTTGAGGTTTCTTTTTGTCTACTATTTCACCTTTAGTGTTGATTGTGACTTGAATATGTATGTAGATGTTGGAGGGTTATTTTCTAATTGTTATTACTACTCTTGCTGTCTTAGCTTTTATAATATAGTTGTGATCAATTTTTTGAAATGACTGTATTAGTCTTAGTTTGCTCTTGTTTAGTATGAATCTGGGATAATGTTTGGTTTTCGACGTCAGTCTTTGTTTTTTTCTTCCATCTCAAATTTGATGAATGAATCAAGACGTCAAGATTTTGAGTTCACTTACTATCCATATTGTTGTTTGTCTTGAATCTCTAGTTGTTTATTTCGATCATGTTCACTCGCATCCATACCCCCTTTGAGGGTCTTGAAACTTGCATTCTCCCCATACCTCTTTTGAATCTGATCTAGAATATTATTTTCTATTATGTAAAGGTTCGGTGAATATAGCTTGCACGTGACAAATTAGTCGAGTTTCATATTTGGTTTGAGTTAAAAAATCTTTCCAAAATGCAGTGGCGTCCTATTCTTGTATGTGCTTATTGAGCGCATGAGCTGTTTATTTTCTTCTCTTTTGATGGTTTCTTTTGATTGAATCTCATTTTAGAAAGATTATAGTCTTAAGGTTTAAATTTTAATCCCTTTTTGTTTAGCTTTAAATACATGCCTTAGTACGTGAAAAATTGTGCTTAAGTTATTTGTATGTTTTGCCGCCTATGCTTTTTCCTATTTCTTTATTTTAAGTTGTGAAAATTGCTAAGAATCCATCACTTAGTCACGTCTAAAATTGGTGCAAAGCAGTAGCATTATTCTGTGAATTGGGTCTACTACTTTGCTTATGTGCAAAAAATGCTCAAGTTTAGTCCATTAACACATGAGTGCATTATTGCCTTTGTATAGTTTCATATAGCATTTTTTTAAAAAAACTTAATCACATTAGCCTCAATAGTCCGCTCCATTTTTTACTAATAATTGCTTCCTTATCATTATTTATGTCTAAGTAATCATGTCTAAATCTACTTCATTTAATCACTATTAGTTTTATTGTTTAGTTATCACTACCTTTTGGTGTTTCATCTTGTTATGTGAAGTTAGTTTAATTTAAATGAAAGTTCATTAAGTATTGTATGCATTCCTTTGTCATATTCGCGTAACATTGGCAGTGAACTAATCACATGCCTATTTTGTCTTATTTCTTTTTTCATGTATCTTGGTTGTATTGCATAAATTTAAAGTGTCCTCCATGACTAATTCCTATCTTGTTTCTTTTTCCGTGGTTTTTTCTATGTTTGAATCCGCCTTGGGTCCTCATGGACTTCTCTACTCCCCTTTGCATTTCGATTTGGGCCGTAAAGGCCCAAACTTGCTCTTTTTGCTCGAGTTGGCCCTTACCGGATCTGCCAAGCTGGTGTACAGGTTGAAAGCAGCAAGATACGGGCTGGAAATTAAGTTTTCATATTTCGGAAACTTGAGAATAAGCTGAAATACAGCTTGTATACAGTGGTATACGTCAATATACACGACAAAAACAGTGAGATACAGGTCTAAAACGCAGGAATACAGGGCTAAGGCAAAATCTAGGTGTTCGGAAGCAAGATATACACGAAAAACACCTATACACACGCCGATATACACTGATATACAGTCAGTGTATACAAAAAGAGGATTGGGTTAAAACCCAAAAACTTGTAGCAAAATTGGCCCAAAAAGGGGCCCAATTTTATTCTCTTTTACTCTCCTAATTATTTTGATTACGTATGACTAACTTCATTTATCTCTAACCTTAATTTATTAATAACTTAATTAGATTCCCAAAAGACG

>LNC_001635

CTGTTTTGGTCCTACTGAAAGAAATAAGACGCTCTCCTCTCTCTCCTTCATCCACATCTCTCTTCGTTAATTCGATCAACAAGAAGCAAAAATACTTTTGAAATCTCTTATGAATTCTGCCCAAAGTTGCAATTAGAAGAACCTTAGATCGTCACTATAAATAGCAGGTCTTGCATTCATTTTTGTGGACGCCTTTTGCCCCGCACCACTACACCTGAAACCACTGTTTTATGCTCGTCTGAAGAAAGTTTTAATTAAGGAGAAGCTTCGTGTAGGCGTTGGACTCGCTCTCGTTCACTGTCCCGCAACAGGTGACATTAAACTGTTATTCTCTATCTTAAGAAAAATCATGACTTGCCATTAATGTTTCTTACTTATCTATAATTATATGTGACTACAAAATATACTCGGTTCTTGACTTCTTGTTCGAGCTCTGTATAGATCTGAAGCCATATCCCGTCATTTATTTAAGAAATGATTTCGTTTCGTGATCAAATCTTCACTTGAAATCAGTTTGTCGGTCTAAGTTAAATGTTTATTTGATGTCTTTATATGATAATTTTTTTGCAGCTCATGTATTCATAGGTAAAAGTATGTTAAAAAGAGATAAGTGTTGTTCTTGCTTAGTTCGAAATGTTATGCAGGTTACATTTTAAAAAAAAATTAGTGTGAAAAAAACCATG

>LNC_001636

GAAATGTCTAATGAGCAAGTTTTGTTGAAAGTTTTAAACCATTCCAAAAAAGGGGAACATGAACTTTTACTAAAGATGGATAAGTTTTCAAACGCTTGGATGAAGGTTTCTTGATCTACTTTGGTTTTATTACTTTCCTGATTTGTTGCAAAATATGAGGGATTTTATTTTCAAGATACATATCACAAGTATTTTATCAAGTATTTATAACATATTCCTTTTAAATTTGCTCCTTTAATTTTTAACTAATTAACCTAAGTTTTGGCCA

>LNC_001637

TTTCAAAGAAACCCAGCCCACCAACAATACGTACAAGTTACAACAATACCAAACAGACCCACCGCCCCCACTTCGTTCTTCTTCCCAGCGAGAACACAGGCGCGAAGAACCAGAAACCGAACCAGTCCAGCAATAGCGAACCGATCGAGCCCAGCTCGCGCGTGTCCGTGCTCTCTCAGCCCCGTCCTTATCCTCTCGTACAGAACCGTTGGAGATTCGTTCAACGTGTCGCCATTTCATGTGCTCGAAATCGTGCGGCTCCTATCAATTTTCCGTTGCCCTCCAACGTTCTCCTTAAGAAGAATTCGAATTTCAAATTCAAAATGGTTCCGATTCCCCCCATTTCGAACCCTCATCTCCCCTCTATAAAAGGACCCCCATCTTCTATATTTATGGGGTTGGTGATTTCTTTAGTTTTTGTCTTCAAAACGACACAAGAGACAGTGAGTAAAGAGAAGAAGAACGGAAAGGTTACAAAACATAGATAGGCAAAAGATTTCGGTTGATAATATCCCTGTAGCTGGGTCTGCTGTTTTGGAAATCAATTTTCCGCCGGAATCTCATCGTGGTGATGTTAGAGCTCGCTCCAATTCGCTTGTCTCGTCTCAGCTCGCTGCCCGGAAAGAGGATGAACTCTCTCGGACTGAGTCAATTGGACTCCACATGATCTCCCTTCCATCTCGAAATAGGACAAGGAGGCCCAATGCTTCCCTTTATCGAGTCAACCGAGTCAATGTAATCGAAGAATAGGTTCTCTAAAGTTGATGTACAGGTGTTCGGAGCGGATTAAAAGGTTGGATATGCACAATATACAACGATATACATCAGCGGTATATGTTGTATAGCATTGAAAATGGGCTAAATAGCCTCAATTTCAGCTTTGTATTTTGTTGCTTTGGGCCTAAGCCCCTTTTTGTCATTATTATTATTATTATTGTTATTATTATTATTATTATGTGTTAGTTATTAGGACAGGTACAAAATGCAAGAATTGGGCCTAAAACGCAACAGAGAAATTAGCTTATCTTAGTTTCTTTAGGACAGGTGTAAATGGACCATAGGTCCAAAGCTAAATTCAGGCCCAAGTGTTGGTCCAGGCGGACAGATCCAGACTTGGGCCCAAATTTTCTTAAATATCTATTTAACTTTTATTATGTGTTATTTGTTGAATTGATTTTAAGGTTTCAGAAAATTCAATTCCCCAAAGGACACCCGTTTTGAGTTAACTACTTAATATTAAGTAAAATAATCTATTTCCTACTTGTTTTAAAAAATTATATTTAGTATGTTTTACTTGAGTAATATTTCCCCTTTCTCCCCTAAGAGAAAGTTTTAAGTTTTATGTTGTTCATTTCTTCAATTTTGCTCAAGTATGATATTTTTTTTATTAGCCAAGTAGTTAATTATCGGATAACCGCATGTTAGCGGGCGTTTCGAGTGTTTTCAAACCCTTCTTGAAATGTTAATATGAACCCTGAACTCTTTAAATGTTTTCAATTGATTTTTGTTTTAATCTTTTGAAAAATAATGGTTTATGTTCTTTTTTAAATAAAAGTATGTCCAATTTCTCTTGTAGTTTCTTACGTATTACTTGGACAAGTCCTATGATAATTGTACTAAGAATGCTTTCAAAATGTGCCAAACAATCAACGATAATTTTTTTCCAATGGTTTGTGAACTATAATTTCATTACTACAGGTGAAAAACAAAATTGATCCTTTTGAAGAAAGTGCCCCGTAATAAAGCTGATGGAAGTGCACAATCATCTTT

>LNC_001638

CGAGAACACAGGCGCGAAGAACCAGAAACCGAACCAGTCCAGCAATAGCGAACCGATCGAGCCCAGCTCGCGCGTGTCCGTGCTCTCTCAGCCCCGTCCTTATCCTCTCGTACAGAACCGTTGGAGATTCGTTCAACGTGTCGCCATTTCATGTGCTCGAAATCGTGCGGCTCCTATCAATTTTCCGTTGCCCTCCAACGTTCTCCTTAAGAAGAATTCGAATTTCAAATTCAAAATGGTTCCGATTCCCCCCATTTCGAACCCTCATCTCCCCTCTATAAAAGGACCCCCATCTTCTATATTTATGGGGTTGGTGATTTCTTTAGTTTTTGTCTTCAAAACGACACAAGAGACAGTGAGTAAAGAGAAGAAGAACGGAAAGGTTACAAAACATAGATAGGCAAAAGATTTCGGTTGATAATATCCCTGTAGCTGGGTCTGCTGTTTTGGAAATCAATTTTCCGCCGGAATCTCATCGTGGTGATGTTAGAGCTCGCTCCAATTCGCTTGTCTCGTCTCAGCTCGCTGCCCGGAAAGAGGATGAACTCTCTCGGACTGAGTCAATTGGACTCCACATGATCTCCCTTCCATCTCGAAATAGGACAAGGAGGCCCAATGCTTCCCTTTATCGAGTCAACCGAGTCAATGTAATCGAAGAATAGGTTCTCTAAAGTTGATGTACAGGTGTTCGGAGCGGATTAAAAGGTGAAAAACAAAATTGATCCTTTTGAAGAAAGTGCCCCGTAATAAAGCTGATGGAAGTGCACAATCATCTTT

>LNC_001639

GTTTGAAATCACAAACATTTCATTTATAATAAAGAATATGGAGATATGTAATTTATCAATGCAACGCCTTAGTATATCTTGATGTCTTGTTTATGAACTATGGTTTGCTCATTTGATTATAAGAATTTAGAAAATTTTGATAGTTTACATGGGGTTTTCATGTTCATGAGCCAAAAAAAAAAATACGACTTAAGAAATCTATATTGAATGTCCAAACAGAACTTCATCTTTAAGTTCAAATGAATGTGATTTTAATTCATGTCCAAATGGAGCCTAAAGTTGAAGTGTTAGTGCTGCTACTGCTTGTTGTATCTGTTGGTTGCCTTTATGGTTCGTTAAGCTTTAAGTTGTTGAATACAAAGAGTGAAAAGTAATTCATAGTCATCATAAATCATTTCTCCTTTATGTTTTTCCAATAGTTTAGAAACTATGGTTTTAGAATACTTAATCTGCTTAGAAGACACATTTAATAATAGTGGAAAAAATATAACACGTGCAGATTGGTTCATAAAGCTGACTCTGACTTGTTTGGAATGATGTTTTAGTTAACAGGTCTTGAATCGTTTGAGCTTGACTGACCATAAATCGTTTTAGTTAACACCTAATCTCTTGTACTTCCCATTATGTCTTTCTCTAGACTGTTTTTGTCTTGAGTCGGGGGTGTATCGGAAAATGCCTCTCTACCTCAACTCTAAGGTAGAGGTAAGGTTTGCCTACACTCTTTTCCCTTGATACCATTGTGTTGGATGACATTGGATATGTTGTTGTATAAAGGAAAACCTTGCTTTCATCAAAATGAAGTGCTATCTTTTCTGTTCTTTTAACTCTATACTAAACAAGAGTTACAAGAGGGAACACTGATATCTTATTAGTATTTCTTTGATTAGTGGATCTCTGATAATTTTTAATGAGGAGTTTAGGTTTGTTTCCATTTAACCTCATCTTCCCTTTTTTTAATGCCAAAATGATCTGTTTAAATACGTTACATTTCAGTTAAAATTTTGCCTTGAGATGTCAGGTGATAGTCCAGCATGTGTTAGTTTTCATCTTTATAATTCAACTTTTAAATATTTGGAGTTGGGCACACAAAAGAGACCCTTAAACTTGCACTGTAGTCTAGAAAAACTGCATATGATTTTTTCCCTTCAGAGACCAATTCAAGATATATTCAGGTCCAACTTCAATAAGTCTGTTCTTGATCATGGATTTATACCTGACTGCCCTTTGT

>LNC_001640

GAATCTGCAGATATTTTTGGAGGATGAAGAATTTCTCTCCTCTGCCTTCACTTCTGAATAGGAGGGAAGACATGGTTGTAAATGGTCTAGAAGAACTTTTTGATTGACTGAGGCCCTCTGTAGATGCCAAATGGGATTTCTGGTCTGGCGAGAAGTCTGTTGAATGAAGATTGATCATTTGTTATAATTCCCTGCTAGTTGTCAATGCCCTTTGTTTTGTATTTAGTTGCTCTAAGGTTCTCTGTGTAATTTACTAAATTTGTTCTTAGAGAGATACAATGCTCTGTTCCATGTCTTTAATTCTGCATCTTTTTCCAGTTTTATAATAATTGAAATTATTCTCTTTAGTTCACATGTTTATCTACCATAATGTAGTGTGTGCAATCTCTCATGCGTGTATAGATGAGCCATTTGTGAATCCTTTTTGACTTCCCCACCCATGAAGAAACTTTCTGAAAATCTGGTACAAAAGAAGTTGAGATTTCTCC

>LNC_001641

GCGACCATGACGAACACCTGGGGGCAAGAGAACATTCTTAGAATATAGATAGGCAGAAGACATTACGCTAGCCATAAACGCTTTCCTTCAATGACCGATGAACAGACGAACCAAGCGAGCTCAATGCAAAGAACTCGAGTGAGGGGATCTGGTTACTTTGTATAATATGTCTTACGAGTAGGCAATGCTAATGGAAGACGGAAGTCAGACAAGGCGATGACTATTGACATAGGCTATAGATTTGGTAATTCATATTGTGAAAGTACTTAACTTCAGATTCAATAAAGATCTGGAATAAGACACGAAAGGGCAATGCGGAATAGCTAGTCTAGAGCTAGAGAAAGATTTTGACTTTATCAATTAATATCCTACAGCTTGCATTCGTCTTACACAACCGCCCCAGGAAAGTAATTATCATTAAGAGGGAGAACTTTGATATCTAACTACGACAGCTATGGGGCACGAAGGGTTTAAGAATAAGAACAAG

>LNC_001642

AAAAGATAATACAAGCTTTAATAAATAAAAGTTTTACATAATCTCTTTGCTGTGACACAGCAGGTTTTTGCCCACATCGAAACAAAAGAAAACAAAACTGAAAAATCCCATCTGGAGCAAACTCCAGCGACGGTGGCTGCCTCTTCTCTCTAAATTGACACTTCTTCAACCTCAGATTCACTCAAAAAAACTCCGTCAATCGCCATTTTCTTTGTTGATTATACCTATTACTTCGATCAATTTGTCACCGCCGATACTTTCCCCGGTGTTTTTATTGTTTCAATCATCGTCTTTTTTGATAAAGACTAGATTTTTACGGAAAAGGGGGAAAATCGATTTGAGTGAATTTCCGTGAGTTGAAAATCTTGAGGGATGGATCTGTTGATGATAAAGATCATGGTTTGATTGATTGTTGATGAAAACTGGGTGAATGAGGATGGGATTAGAATCTTCTGGTGAAATTCCTCGTGTACCGGAAGATTAATGTGTTACATGAGGGTGCTATTTGATCCGATGATGCCCTTGGTTTAGTTTTTTTGTTTCTGTTGTGAGTTGTAATATTAATAGTTAGTAAGCGGAGGTATGTTCATGGATGAGGCTAGTACTAGCTCTAAGGCCGTTGATTCCTCGG

>LNC_001643

AGCATATTTTGAAACTTTTAGAGGCTACAATGTGTTTGCTAAGAAGACTTTGATACTAGTGACAGTTTGAGAGTAAGGTAAATGGCTGGTGTAGTTATGATGTTTGTTGGTATTAAGAGTGTTAGCTTCAATGGAGTAATGTGAGAAGTATGTGGGTTTGATAAATTGAGTATTTGTAAGATCGTAATGTGGACCAGACTGGTTGATTGTGGGCGGCTAAAAATTTAGAGATGGAGTCAACTGAATGTAAATGTTTTATATGGGTTTGGAAGATATATTTAGTGTTATTCAACCTTGGTTCCGTATATTCTTATGTGACCACCTTCTTTGTACTGGACATTATTTGGAGTATGATTCACTGCTTATGCTACGCGTGTTTCCACATCTATGGGTGAATCCCTAGTGGTGGATCGGGTGTACCGATCCTGTGATTTTTCTCTTAGTATGGTGGCA

>LNC_001644

TTTCTCTATTTTCTAACCAATTTATTTACTGGGTCGGGTCAGATTGTGCGTTGTAAATGCCCACGTGGCGAATACCAATTGGCTAAGTCTCCCCGATGACGATTCTTCCTCCTTATTCACCTTCTCCCATGGCTACCAAATTTATTTTCTTCAGGTACTCATGTATGAAATTGTGAATAGCTAAGGATTCAGGATCTTCCCAACGATACAGAATCTTCACAGGAATTAGAGGAGATGATGAGGTTACAAACTTATGCGGGGCTTAGCTTAATAGCTACACTGGCTGTTATTTATCATGCTTTTAATTGCAGAGGACAATTCTACCCAACAATGGTTTATCTGTCGACGTCCAAGATCAGTCTTGTGCTTCTTCTCAATATGGGTCTTGTTGTCATGTGCATTTTGTGGCAGATTACAAAGAAAATTTTTCTTGGTCCCCTTCGAGAGGCTGAGGTGGAGAGGCTGAACGAGCAGTCTTGGCGGGAACTCATGGAAATACTTTTTGCTATCACAATTTTCCGGCAGGACTTCTCTGTGACCTTTCTTGCAATGGTAACTGCGCTCTTGCTTATCAAGGGTTTGCACTGGTTGGCTCAAAAGAGAGTTGAGTACATTGAAACAACTCCTACTGTTACTAGATTGTCCCACATTCGCATAGTATCCTTCCTGGGCTTTCTCCTCTTCATAGACAGTCTCTTTTTGTACGACTCCATGAACTATTTGATACAGACAAGACAGGCATCTGTTTCTCTCTTCTTTGCCTTTGAGTACATGATACTTGCAACAACAACTGTCTCAACTTTTGTAAAATATGTCTTCTACGTAAGTGACATGCTTATGGAAGGACAATGGGAGAATAAGCCTGTCTATACTTTCTACTTAGAACTTATCCGAGACTTGCTCCACTTGTCTCTTTACTTGTGCTTCTTCATGGTGATTTTCATCGGTCATATAATGGGCTGTCTTTGCTCTGACTCAAATGCGCCACATTTAACAATAAAG

>LNC_001645

TATTGATTATTTGCTTTGCATATTTATTAGACCCTTCCTCATCCTACAGATGTGAAAAGCTGAATCATACCTCTCTATAATCATTTTTCAGCCTACTTTTAGTGACAAGTAATTTTGGAACTTTATACAGGCAGGGTAAAATTAGATAGGATTTCATTTTGGAAATGAGCAAGAGGGAGTTTCAGCTTTTTTATACATGCTTCGTCCACACTCTAGGCAATCTGTTACTGTACTACTCTTTTATGCAATTTAGAGCTGTTTTGGTTTTTGTTCTTAAATTCTCTGGATAAGTCAACTCCTGTTTTCTCTCTTCGTCCCTGTGTTGCTATTATTATTGGATGGCTACATTGAACCTTCATTTCTTGTACTTACCAAATTAATATCTTTCTTAAAAAGAAAATTCTGTTAGCATATTACATGGTACAGAAGTTACATCTTTACCTCCGGGATTCTTTTTTCCTATTCCAATTTTTACGAAACTGCAATAAGTAAGTTGTGCTTCTTGCATACTCCTCAATGAACATTTAAGAAAGACCCATTTCAAGTGTCATCATATTAGTTTGGAAAGTCAAATTTAAATCTGTTTGAGATAAAGTGAGCATGAAACTGTAAGATGCTAACAAGAGACGTTGTTTGCAGATTCACAATTCATTTTTAGATGCCTTTATGCATTGTGATGTAATCTCTAGTCTGATTGTGGGCCATTTCCTATACATCCATTATTTGGGTCAATTTCTTAGGAACTATTGGAC

>LNC_001646

CCTCACCCCAATTTCTCCTTCTCCAACACTCTTCCCACCATTGACCTTAAACTTTCTTCTCTTACAAAATCAACCACCACACCCATTTTTTTTCTTCGAAATCATCGGTCATACACTCTTTTTTTCTTCTTCAACTCACTCTCCTCTTTGCAACAAATCAAAACCCGTCATTAATTTCTTTTTTCATCCCCGTTTTTTTAAATTCTCAGTGTTTTTTATTTTAAAAGTTGAAACTATTGTTTGTGTTAGTTAGATTCAAAAAGGAGGTATGATATGCATTAACGTAAAGGGTGATGGTGGGTATTGAAAACTCCATTGATAAGCTTGACGAATAACAAATGGGTTTTATGAATTTGTGAAATTAATTCAAATTTGTGAAGGGAGCTCGTTGGATTTTAATTCTTCATTTTTTGACTTGAAATTTAAACTAGAAGTTCATAAACACATATCCTACGAGCCACAATCACAAATTTGAATTAATTTCACAAATTCATAAGACCCATTTGTTGCTCTTCTAGTTCTCTCAAGCTTTTCAACGGAGTTTTCAACATATTCAATACCACCATCACCCTTTACGTTAATCAAAATCATACATTCTCTTTAAATCCGACACAAACAATAATTTCAACTTTTAAAATAAAACACTAAAAAATTAGAAAAATCGGGCTGAAAAAAAAAGAAATTAAGCGCGGGATTTGATTTGTTGCAAAGAGGGGAGTGGGTTGAAGAAGAAAAAGAGTATATGACGGGTGATTAGAAAAATGTGGTGGGTGATTTTGTTCAAGAAGAAAGTTTAGGGTTATGGGTGGGAAGTGTGACGGAAAAGAAGAGA

>LNC_001647

CGCAATATATTTTCCGCAGTTGAAACCCTACTTTTCCGCCGCATTCCCAAAATTCACATTTTCCTACTTCCTCAAACACCAAAATGAGGCTATTACTCTTTACCTTGTAAGGATAAAGTCTGCGCGCACACTAACCTCCTAAACCTACTTGTGGGATCATACTTGGTATGTTGCTGCACTTTCTTCTCTATGGTGGTAGGGGCTGTTTGGTTGCTGTTTAGATTTATGCAGGTACTAGTTATGCATGGTTTAATTATGTAGGGTTTAAGAGTGGGGTTTAGTTATGTAGATATTAGTTATGCAGGGTTTAATTATGTGAGTATTAGTTATGTTGAGATTACATCATATGAATTATTAACTATTGAATATTAAATTTAATATATATTATTAATATATTTCAAGTACATTATATTATCCTATTTTATATAATGTAAATTTCTATCAATATGCTGCATGTGTTGTTTATACATTTGAGTATACC

>LNC_001648

CTTTTCCGCCGCATTCCCAAAATTCACATTTTCCTACTTCCTCAAACACCAAAATGGCTATTACTCTTTACCTTGTAAGGATAAAGTCTGCGCGCACACTAACCTCCTAAACCTACTTGTGGGATCATACTTGGTATGTTGCTGCACTTTCTTCTCTATGGTGGTAGGGGCTGTTTGGTTGCTGTTTAGATTTATGCAGGTACTAGTTATGCATGGTTTAATTATGTAGGGTTTAAGAGTGGGGTTTAGTTATGTAGATATTAGTTATGCAGGGTTTAATTATGTGAGTATTAGTTATGTTGAGATTACATCATATGAATTATTAACTATTGAATATTAAATTTAATATATATTATTAATATATTTCAAGTACATTATATTATCCTATTTTATATAATGTAAATTTCTATCAATATGCTGCATGTGTTGTTTATACATTTGAGTATACC

>LNC_001649

TCAAACACCAAAATGGTGAGCTCGTTGTTATTTCTCTTTGTTCCCTCCTGGGTTCTGGGTTCTGGGTTCTCTCTTGTGTAACCATGTTTCTTTACTATAATGAAATATGTGTAGCTGATATTGCTAATTTAGTTTATTGTTCATCATGTAGTAGTGGGTTCGGCGTCTAGCAGTTATGCATTTACCTCATTGTTCATGTAGTAGAGGGTTCTGGATTAATCTGTTATTTCAGAATCGTAGTTTTGGTTATTTTCCTTTAACGAACTGTTTTTCAGTCGGTTCGAAACAAGGGGTAAGGTCCTACACACTATCGTCCCCAGACCTCACTTTGTGTGATTACACCAGTATGTTGTTGTAGTTCAGGTGATTTTGGTAATTAACAAAGGCGAGTGAAAATGCTTTGATAAGTTTTATAGTGTTAGGCTCCGCTAGCTCGAGTAGTAAGCACCCTTTACTTCCAACCTTGAGGTTATGAGTTCGAGTTACTAAGAGACCAAAGTGGGCGAGCTACTAGGGAGGAAAAAAGGATAAGCTCTGTAAAGGGGAAGTAAATGGTAAACTAGAGTACTCAAATTTGTTTTATTTTGTTTGGTAGTGCATCTTTTTTTCTTCAATTTGGTGTAGGGGAAGGGAAAATGGGGAGGGGATTAAGTTACCAGTTTCATAAAAAACAAGGGAGGAGATTACAAGGTGGAAAATTGAATCCTCACCAACAAATTGAAAATTTAGGTAGTCATCTAGCTGATCTACTAAGATTTCCCTTTAAACGCAGGCTGTTGGGTTCATTTGTAGTGTAATAATGCTGTTGTATTGTAGCTTAACGGTTCTTACAACACTATCTATCACCAACAGAAGTATGAAATCTTCCACTTGTGTATGTATCTGTTTGTTACTGCTTTATTGTGGTGCAGAATGCCATTTTGCAAGTGTCGGTGATGATTGTTTTAGTTACTAGTTTGAATCTGTTGGGCGGATACAATTGTAGTGGTTCGCGGTATATACTATTGGGTGATTTGACTAGAACAGTGCAGTTGTACTTGTTCAGAATCCAATGATGAATGCTTATATTGCAAGTGCCGAATGACACTCCAACCAATGTTGGATGTGTGATTTGAAAAAGGTTTCTGAGGATTCTTTTCCCTTGTTTCAAATCATCAACTACACTTAATCCTTAATTACTTTATGTGCCAATTGTCCCTCATCGAATAAATAGAGAAATGCTAATGGATTTTTAGGCTATAAATAGGTTCTCCCACTATCAAACTAGTCTTTTGGGTTGGGCTCTCCTGTTATTCCCATTTGGTCTAAAAATTGTTATCAATGTTTTGATAAGATGAAAGGAATAACAGGAACAAGGAGGTTAGAAGTAGCTTAGGAGGCTCGTGGAAATCTAAAGGAGAAGTTATGTGAGGTTTGGAACTGAACCGTTGGAGGATATTGTAGAGAGTTTCACCCAACCTTGAGATTGTGGATTTGAGTCACCAAGAGAGCAAAGTGAGGGTCTGCAAAGGGGAAGTAAATGAGGCTATTACTCTTTACCTTGTAAGGATAAAGTCTGCGCGCACACTAACCTCCTAAACCTACTTGTGGGATCATACTTGGTATGTTGCTGCACTTTCTTCTCTATGGTGGTAGGGGCTGTTTGGTTGCTGTTTAGATTTATGCAGGTACTAGTTATGCATGGTTTAATTATGTAGGGTTTAAGAGTGGGGTTTAGTTATGTAGATATTAGTTATGCAGGGTTTAATTATGTGAGTATTAGTTATGTTGAGATTACATCATATGAATTATTAACTATTGAATATTAAATTTAATATATATTATTAATATATTTCAAGTACATTATATTATCCTATTTTATATAATGTAAATTTCTATCAATATGCTGCATGTGTTGTTTATACATTTGAGTATACC

>LNC_001650

GAAAATGTACTGTCATGTTGGTTGAAGATTATACATGCAGACTGACTGAATTCTCATTTCTTGACATGCAATGGAGGTAGACGAGTAATCTTTTGTAAAGTTTCTTTCGATGTAAAAAAAGTTATTCCCGAATAGTGTCTGTCTTTGTTTTATGATTTAGAGCATAACTACTTCTGCAATTCCTTTGACAGTGTATTTTAGTCTTTCTACATTACTGCAAAGCAAGAACATAGCCATAAATCTGCTGTTACTGATGTTTGAAGTGTTTTTCTCTTTGAATTTGCAGTGTGGACTATTTGGAGGACTTGATTTTTTCCTTCTTTGTAAGAGATGAAAAAACTTTTGCCTCTGGTCATGAAGGTGGAGGGATTATACCTTTTCAGGCACTTCAGTAGCACCATTTGGAAAAATAAAATCTAAAGTTCAGTACTTTTTTTTCTATCATCTAGCAAGCATCAATTTGAAAACCAAGTGTAATGGAAAATGATAAAGGAAACTTGTTTAACTGTCAAACTATGTGGATTGAGATATTACCTAACTGTTCTTCAACATTAAGTTACTTGGAAGTTGTTATATGAATCTTTTATTAACCATTGGCTTCTCTATATGTGGACCTATATTGCTCGGGCTCTTCAAAAATGTCAACAAGTGCGTGTTGAATTCGCCAAAAATAGTGTATTTTTGGTGAATCCGATACGGGTGCGGCATCGAAAGTGAAGAGTTCGCGCTCATTCACAAATAAAAAAATTGCAAAAAAGTAAAAAAGTTCTCTGTTTCTTC

>LNC_001651

CTTGCCACATGGAACATACTTTCAAGAAAGAGCTAGCACATCCTTTCATTTACTGTACCACTTCTCCAAGAAAAAATTTAGCTTCTTTAACACATCTTTGAAGACTAATTTAAAAGGCACACTTTTAAAAACAATTCTTTGAAATAAGCAGTAATTTACTTATATATTCATCTTACAAATAGTTTCTAAAATCATCAGTTGTGGAACTTGTTAATTGTATTTGATATGAATCACCGAAAAGATTTTTAAAAAGAGACATGAAACAAAAAAGAGAAATCACGAATTGAAGGTTTTTCAGTTAAGCAAATTATATATAATCAATAATATCATGACAATTGACAATTGACAAGAATAAAGAATTTCAATCTTTTTGGTGTTTATGTAAAAAAAATCAATACGGAGGAATTGTTTCAAAACAG

>LNC_001652

GCGAAAATTTGTGACCTGAGAAAAGGGATTAGGATTTGATTAGGTACTTCCAACACTATTGAAGAACATGTGAGATTCTATGACATGGAAGCTCATAAAATCAGGAGAAAGAAAGCTAAAGTTAGTTTCCCCCATGAAGACGACCACTAATACAGTTGTTCAGAGGCTTTGCTTACGAATTTTATGATAGTTGTCTTTCATATTCCGAATCCACCTCTAATAAAAATATCCATCCCTTAAAAATATAATACCCTCCAGAATGTTCAAACAGGAAGTGCATCATTCTCTACTATTGAAAAAAATTATGTAGCAAACTTTATT

>LNC_001653

TTCTGAGGTAATTCTTTGTTATTTTTTCTTCACTGTTCTTTTATTTTATTTGGTTGAATGGTTTCATATTTTTTTCTTTCAAGTTTTCTCTTCATCTTTTTATATATAATTTCTTTGAATGGGTTTATGAAGTTTGGGTGTTAATTAAGAATACGTATGATTGAAAAGAGCTGCAATAAGCAAAGGAGAAAAAGAAGATGGGGTTCCTACTTATGTATTTGACAACTCTATGGGTTTTTGAAGTTTTGGTGCTTATTAGTTCTATATATAGGTTGACAAAGATTTCAATTTTATGTTGGTCAAAATTCAATGAGAATCAAAATTAGGGCAAAATTTGAGTTACAAACCTACCAAAAATTTAGTGCAGACGAGCAAACTTAAAAGAACAACCTTCAAAATCTAGAGAGGAGAGTATTTATGAAGGAATTCGTATGTTGTTCGATTAGGTAAGTTATTGAATGTGCAGAGAAATAAAGTAATTTTTTTGCAGGGATTGAAGTGGAAATAAGAGTTCAAGCATCTATCTTTGCTCTCTTCTATCCTTTGGTAGAAAAAGGATACCAGGGAAAATATTTTCACTCTATTTTTAGCTCTCATCGAATTTGCAGATTTTTGAAGTGGTCCGTTCACATGTGTTAAAGTATTTTATTTTATGTAGATGATTGGTCTCTAGCGTGAAACTCTCCAGGGATTAAAAAAGAAATTTGACTTTTCCCTTACACCGCACTTGCCCATTGGTTTTTATGTGTTTCACCGTAACAAAGCATCTAGAAATTTCTCTATTATTATCTTTGTTTCTGTTTGCCCTGCAATTCTGGTCAGTTTTTTCATTTTCTTCTCTGTCGAATGTACAATTTTCTCTTATTTTTTGCTCATCACTGTACAATATTGTTGGTAATGGGTATTTGTTATTGTTTCCACCCCTTTGGCCAGTTTCTTAGTGAATTTATTCATGCATATTTAGTACATTTCTGATTACTCATTTCTTCGCTGGGTAGTCTGATATTTACTTGGAGTTTCAAACTCCATTCTTCTCTCTTGATCCAATTACACATTCTCTCATCACATTCTATCGTTTAAGACTGTCTCTCAATTTTGATATACTGCTTTCTCGAGTGTTTTATGTTACAATTTTTTACAAGCTATTTCCTTAAATCTGTTCATTTTTATGAGAGAAGTTGTTATTAGTTTGTAGATGTAGCTTCCTAAAACGATCTACTAAATTCCTTGAGTTGTTTCAAGGGATCGGTGAGTTATTAATGGAATTCCTTCCCCGCTCTTAGGTTCCGTTACTTCAGTTTGTTTTGTAACTCTTTTTCTCTTATGATTATTCAAAGAAAATTAAGGACCAATATGTTACCCTTACCTTTTGTTTCTATTGTTCAGGAAGCCTCAAGTTTGTTGTACTAAATTTTCACAGATTGCCGGATCCTAGACTACAATATGCATTCTTGACTTGTAATTGATTATAAATTCTTTATAATTACCTCACGTTTTTTGTTGATTGTTTGTATAGAGGGAGTTCCCCACAGGAGAACCCTTCTGGATAGCCTCAAATCCTTTGTATAAGTCCTTCAAATGACTCTGTTATTGGTATTAGTATTGATATTTATTAATTCTGAGTCAATCATACGTCGGCCATTCATTTGTGTAGTTTTTCTTTCATCCCCTCTTTGAATCTTAATTTTAGATTTTTCATCCATTTACTTTGATTTGTAGAAACAAATTGCTCTTGCATTCGCAATAACTTTCATATGCGAGCAAAGTAATGTTCATCTGCTGAAGCTTCAATTTTGGACAAGTTATAAGTTGTAGGTTGTTACGTTGTATTGATGATTAATTGACTATTACCTTCCATTTTCCTACTTCCTCTTATGACTGTTCGGGCCAAGTGAATCATCAAGGGTACTGATTTGTCCCCGGTGTAAGCAATCTTGAATTTACCCAACTTCGATTGAAAATTTTAATGTGAGAGTTCACAATTTTTGTTCTTAGTTATTTTTTCCATCTTTATAATGTAATTGTTACTATATTTATGGTTGTCTTGTAATTATGATATACTTCTATAAATTAGGGTATTGGTTCTTGGTTCGGCTTCTATTTAATTTCACATGACTAAATATTCAAAAAAATTGTTTATCCCTAAACTTGGCCATATAGGCAATAAAAACCTATACTACTGAGTGTTTCATAAGTTCACTCACAATAATTATAGTAATGAGTTTGTTTAACGTTTATCATTCTCAAAGATAGTTGAAGATGGATACATGAAGTAGTTGATTCTACTACCAAGTAAAGCCTTTTGTTATAATGCTTGTTGCAATTTCATGTTTAATGATACTTCACTTTCAGAATGAGTTGTTCACCTTTCAATTATTGGTCCATGATCATTAGCAATTGTTCCTCTTCACCTCTTATGTATCAATTTATGTGTTTCTCTGTTCTGATTCTTTTATAGATGTGGGTTACGTTGTCTACCTTTTCGATTGAGTTTATTAAAGCTTTATCTTTTAAATTTTTTTATTTCCCATGTTAAATGGGGTTGTATGCTGATCTAATGAAATCTTATAATGAGGTTGTGAAGTTATAGACATTTTCCGAGTACATGTTGAAGAACATGGTTCCAAACCTTTTTACTTGGCCCTCTTTCAATTTGAATAATTCAAATTTGTGATTTATGTTAGTAGCGGTCCTCATGTAATATGTTCTGTTTGGATAGAATCGGGGCGTTTATCTAATATATATTCTTAGATCTTAAATTAATATATAACTGTGTGTATTATGTTTATATATAATTTTGTATTTTCTTTTCTATTGTCATTTATTCCTCTCAATTGTTTGTTTATA

>LNC_001654

CCTAGACTGCGCCTTAGACTAGGAAGAGATAGAAAAAACATTTTCCCTTCCCCAGAACGCCTCTCCATACACTCTCCTCTCCTCTCCCATCAAGTTTGCTTCTTCCCCAGGCCCCTTTCACGCTCATCTCCCATCCCTCTGCCAAAGAAACAGCCATGAACCACCGGAAAACAACAACATCCATTGGTTTTCAACATATCTCCCTACCCTCCATCAATCATATCCTTCTCATGTCAACTTTACGTCTCTTCCATCGTCTTTTCTGAACCACGGCAGGCCTAAAAAGCCACACTACCGCCGCTGCTACGACTGAAAAGGCGAAAGGCATGAAGAACCATCGGATTTGGGGGGTAGTCGTCCCTTTAATCTGATGGGTGTAAGCAGTGGCGGAGCCAGGATTTTCGTGGAGGGGGTTCAACATATAAAGAAGTAAATAAAAAAATGGAGCAAAACGAATCGGGGAAGAAAAAAATTATCCTTAATCTGTTAAATTTAATTTGCCTTGTCAAGCCGCGTTTAAC

>LNC_001655

AGATGCTAAGCACTGGGCGTGAGGTGGGGTGTTAAAGAATGACAAAAAGTCCCACATCGGTGGTTAATGAGATGGGTGGACTCCTTATAAGGCTTGGGCAATCCTCCTCCCTTTGAGCTAGCTTTTGGGGTGTGAGTTAGGCCTAAGACCTAATTTAACATGGTATCAGAGCAGGGCCCGTCTCACCCGATGTTGGGACCCCCAAAATCAAAATTGCCCACGCACCAGATGCTAAGCACTGGGCGTGAGGTGGGGTGTTAAAGAATGACAAAAAGTCCCACATCGGTGGTTAATGAGATGGGTGGACTCCTTATAAGGCTTAGGCAATCCTCCTCCCTTTGAGCTAGCTT

>LNC_001656

TGACGACGATAGCGGCAAAAGCACGCGTCCTCCGTCCTTGCAGCTACGAGATGGAGACACGTAGCACCTCAAGGACGGGTGATTTGATCCCATCCGTCCGCCCCCTTTACCTTTTCGGAATGAGCCCAAAACCTCAAGGAAAAGGTGTTTGTTTGAATTGGGTAAAGAATTCAATTTTTGAATTTGGAATTCGTCCAAAAATCTACCCGATTTCCCCCCCTTTTTTCCATCCCAAAATCTACCCTAAGTTTCCCCTATATAACGCCTTTGATGTTCATTTGCAAGGGGGGATTTTTTTCGGAGTTAAAAATATTTTTGTGTTAGAGAATCATTAGAGAAAAGAAAAGAAAATACTAAGAGGAAGTTGCTGGAATTTGGGAGTACATACAGAAAACTAGAATCAAAGAGTGAGAATTTCGGTTTGCTAGAGTCTTTGCTCGTTTTCTCTGGTTTGTTGTCGGTGTTCGTGGTCGTGTGGTGGAAGAGCTCGTTCCAGCTCGGCTGTCCCAAGTTCGCTGCTCCCCGAAAGTTGCCTTGAGTCCACGAGACTTTTCCCTTGCATTTGGAATCGGATTGAAATCCAACACCGTTATCGAGTCAATCATAATGGTGTACAGGTTGGAAATTGACAATACTGGCTGGAAGTTGAATTCTCATGTTCCGGAAACTCGAAAACAAGCTGAAATACAACTTGTATACAGATGTATACATGAGTATACAGTCGAAAAATAACAACATACAGGGAAAAACGTGAGAACTACAGGTGGAAAAGGCGAAAATACAGAATTGAAACAAAATTACAGGTCCGAGAGTCTAAAAATTAGCGATATATATGGTGTATACCTCGGATATACACTTCATGTATATCATGATCAATGAAGATGGAAATGGGTCAAAAGCCCAATGAGTTGTTATTTTTGCAGATTTAGGAATTTGTTTGGCCCAAATTCAATATACTTCCTTTCCCTCTCTTATTTATTTTATTGTATTTGACTAACACTTTGATTATCTTATGATTCAATTTAATTAAATTCCCCCGAGAATGGTCATTTCATTTTATTTTCGAATTAAAGTATATATTATTTTATTACTTAGTTTTTTATTTTAATATTAGGCTTGTTGACATTTTATGAACTATTCCCCTTTAGAAAATCC

>LNC_001657

CAACAGTAGATTTACCTGCAGGAAGGGAGACAAGCTCCCAAGTACCACTCTTATGTAAAGCAGACATCTCATCAACCATAGCCTGCCTCCATCCAGAATGAGAAAGTGCTTCACCTGTAGTCTTAGGAATGGAAATAGAGGACAAAGACGACACAAAGGCATAATGGGGTGATGATAGACGATGATAACTTAAGAAGGTATAATGTGGGTTAGTATTTCGAGTGGATCGTATACCTTTTTGAAGTGCAAGCGGTTGGCTAGGAGGAGGCAAGTCCGCAGTAGGAGCAGGGTCCGGCGCATGACATGAATCATCTGGGACTAGGGTTGGACGTGGACGGCGATGATAAGTTAGTAGTGGTGGCACTACAACTGGAGATGTAGAAGTAACTGTAGATTCCTCAAAGGTTGGCACAGGTAAAACTTGAGGTATGGGTAAGACCATAGATACATCAGGATGATCAGAAGATGTATAGTAAGGTTGAGACTCAAATAATGTAACATCAGCGGACATAAGATATCGATGAAGATCATGTGAATAACAACGATACCCTTTTTGGACTCGAGAGTAACCAAGAAAGACACATTTGAGGGCACGAGGAGCTAATTTATCTTTTCCTGGGGTAAAGTTATGAACAAAGCATGTGCTCCCAAAGACACGAGGGGGGATATGATAGAGATGTGACTGAGGAAACAGTATGGAATGTGGAACCTGATTCTGAATAGAGAAGAATAGAAGAAGAGGGCATTCTATTTATCAAATAACAAGACGTAAGAACTGCATCTCCCCAAAACCGCAGCGGAATATTGGATTCAAGGAGAAGAGTACGAGCAGTCTCAATAAGATGCCGATTTTTTCTTTCAGCTACACCGTTTTGTTGAGGGGTGTATGGACAAGTAGTTTGGTGAATAACGCCTTGGTGAGTCATAAACTCCTGAAATTGGGAGGATAAGTATTCTAAGGCATTATCACTACGAAAAGTACGAATAGAAACACCAAACTGATTTTGTATTTCAGCAAAGAAACTTTTGAAAATAGAAAATAACTCAGAAAACCCAAGTACATCTTGAATAATCATCAATGAAATTAACAAAATAACGAAATCCTAAGGTGGAACTGACTCTACTAGGACCCCAAATGTCAGAATGAACTAATGAAAAAATAGACTCTGAACGACCCTCAGTACTACGAGAAAACGTAGCACGAGTGTGTTTTCCAAGTTGACATAACTCATAATCTAATGTGGATAAACTAGACAAACTAGGCACCATCTTTTGCAGTTTGGATAGACTCGGATGTCCCAAACGTTTGTGAATTAGATCTGGGGAATCTGTAACGGAGCATGTTGTTAAGGAATTTAGAGGTAAGATAGTAAAGGCCTTGTGATTCATGTCTTGTTCCAATCATCTGTCCCGTACTGCGGTCCTGCATGAGAAAAAAATCATCGAAAAAAGTTATGCATTAATGTAGGGCTTTTGTCAAACGACTAACAGATGCTAGATTAAAAGGAGAACCAGGGACATAAAGAACAGAGTCTAGGTTGACAGAAGATAGGGGTTTGGCTTTTCCAACCCCTTTTGGTTTTGTTTGGATCCCATGGGCTAAAGTAATAGCTGGAAGAGATTGTGAATAAACAATATTGAATAAAAGTGATTTATTACCAGAGATATGATCAGATTCCCCAGAGTCCATGACCCATGTTCCAAGAGTATTAGATCGCGACACACAAGCAAAAGAATTACCCGCAACAGACACGTTTGCTTATTTGCACGATACTGAAGGAACTTATCATATTTTGTCTGAGCAATATGAGCATTATTGGATGGTTGATTAGGTTGAGCACCATATGCTACTGGTGGAGATGTTTGTTTACTTGTGCGATTTCGAAGGAACTCATTATATTCCTTTAGAACAATAGGATCATAACTGGGTGGTGGACCATGCAAAATATAACATATGACACGAGTGTGTCCAGGCTTATGACAATGACTACATTTGGATCGAGGTTTTACCAAAACGCCTCCCCTTCGCCGATTCTCCATAGACTGATATGTACGCTTTTCAAAGGTTTGAGATGCGAGAATAGAGGAGTCAACAATGGGTGATGAAACTACTTTGTGACTGGGAGGTGCCGCAAGACAAAGGAGACGAGAGAATAATTCATCGATTGTAGGAACTGTAGGGCTAGCTAAAATTTTATCACGCACAAAATCATGATCAAGAGGAAGTCCAGCAAGAGTAGGAACTAGAAACAATGTTTGTCTGTTTTCTTGTTGTTTTTCCACATTTGTAGTAACTGGCATCAACGTGTCGAATTCCTCCATGATTGCCTGTACCTGTCCCAAGTAAGTAGACATATCAGATTCTTGTTTCTTTAAGTTGGTCAATCGAGATATCACATCATAGAATCGAGATATGTCATTAGTGTATAAAGCACGAGCCTTTTCCCAAACTGTATAACACGTCTGGAATGGGTGAAACTAGGGCATCAATTTGGAATCAATAGAACGCCATAGGAGACTACATAATTGAGTATCTACTTTCTCCCATTGTGCTTTGACTTTTGCATCTTCCTCACTAGTCTTTTCCTGTACATCTACCACATAAGTCTTGTTCGTTAAGTGATCTTGGACACCTTGACCCTTGCACCACAGCTCAACCGAGGAAGCCCAAGATAAATAGTTTGAACTTCCCAATAAAAGTTTTGAAGTGATTATAGGGCTAGAACTTCCAATCCCCATATTTTTAGAACCAAAAACATCACACCCAAAAGACATTATGCTAAATTTGTCTTGGAAACAACAACAAAAAAAAACTTGATTATTGAGATCTGATTGTCGGAAAATTCAAATCTCGAAGAAAAAAAATCGCCGAAAACAGCCTGGAAATAGCCGGAACCCTAATTTCGGCGATCGGATCTGGTCAAAAATAGTATGGGCCTGGTCGGAATGGCTGGACGACCCCAACTGTCAAAAAAATTCATCAGAAAAGCCCTCACGCACCGGCGCGTGAGCCAGATCTCGCCGGAAACTACCACCTCCGATCGGCGCGTGAGAGTGCCTGGGGTGATGTTTTCCGGTGGTTCTGGCTGGGGTTTGGTCGCAGGAGCTTTTCGAGCCTCGTGGTGGTGTTGGTTTTCGCACAACACCCACAGAAAGAAAAAAGAATAACAAAATCAGACCCAACAATCACCGGAAATTGACATAGACATACGGTGACTTGTTTGTTCTCACCAATGCTCTGATACCATGTGAGAAATATATGAGAAAAATATTATTGAATTGTGTATCTAAACTGTTACATTGAG

>LNC_001658

GACAAAATGTGAAATCTACAACTACAGTTGAAGATCTAATTGGAGAGAAAAAGATGTGAGCTGTAAACTGATTTCATTGATTAGAAGAAGTGATCTGTACAATGCACTATGTACATACATATATACTGCTACTACTAAACTCACATCCTATCACAATCTTACACCTGTCAATAATAACAGAATTCAGTTACATCTAATAATAACTAACTGCTAAACTAAGACTGCTAAACTAAGCTGTAACAAATCAGTAACAAACCAGTGAGGTCAAGTTACAC

>LNC_001659

ACCTTACCCACTTACCTCGTGGAGGTAGAGAGGCTGTTTCCGGAAGACTCTCGGCTCAAGTAATGCAAATCAAAGCAGTTTGAAAAGGAAAATACAGAAGTAAAGAGGACATATCAAATAATAGGGAAATCATTACATAGCCTTTAGCAAACAAGGAATAATAACAACAACCAAATAATGGGATAACTGAGCACAGGAAATAACAAGTAGTAATAGAAATCTAGGATAAGAGACTACAAGAGTACTGCTACATATCTTATTATTAACTATAAGATGGCAGACCTGGTCATATTTTTGTTAAGAGAAAATGTATTCATGCTCTTTGCAACTATCATTGTGAATCATCTATCATTAGAAACACCTTCTACTGTACCTAGTTAGTTGGAGCAACCTAACCCCTGTACATAATGTTGGTTCATATCTAGATTTTATCAATTCACTTTCTTAGTTTCTTTAACTTGGGCCATATTTTTGCTTCTTTTCATTAGCTCCCTGGTTAGAATGGAGCATTTTGTAATGGAGCTAACAGTTATATTGGCTATTTTTGTATCTTTGCATCTATGGAACATCTTGATGCCATCTATGAAACATCTTACTTCAACAACAACATACCCAGTGAAATCCCACTATGAAACATCTTTCTTCATCTGAAAATATATATATCCCTTGACATACTTCATAGGCTCATCACCATTGAACTAGAACTAGTGGTTGATTTTTTTGTAACTGCACTCTTTATGACTCGGCTCAATCTGATCTAATGCGTAAACGACAGAACTGCAGACTTGCAGCAATCTATTTCAATTTTATTGTTGTACTTGGTAATATGTACTCTTTATATCCC

>LNC_001660

CAAGAGCCTCACGAGATTTCTGTGTTGTAATCTGGCAATTAAGACAACTTCATTCTTGAACTCTTTCAGTCCCTGACCGGACGTTCTAGAGAGCCTCTTGATTGCAATTGCTATGCCATTGGCTAGAGTACCCTGGAATGTTATACACAACACTAAATAACCCAAAAATCATGAAAAATTGACCCCCTCTAGCAACTTAGATTCACTAGTCTCTAGACTAAGATTTAAGGACTTCCTAGGTAATTGGTTATGACAAATGATAGGAATCGTTACCTTGTACACTGGACCAAATCCACCTTCTCCAAGCTTGTTTTCTTCAGAAAATTTTTGTGTAGCAGCTCGAATGAGGTCTAGTTTTACTACTGGAAATTCTCTGGATCTCCCCTGCTTTTCTGAGCCAAAATCATCATTCGCATCAAGCGTTTCTCCTATCATGTCAAGCAATTGAATTTCTCGACTCTTTTCATTCCTCCCTGTAAAGATCCAACTTTTGATTGGTTAGCTGAAACTACAATTATTTTCTTTTCAATGCATAATACCAAAAGAAAAATTACCCTTCTCTATTCGTCTTCTTCTTTTTACAAGGTAAAAGCAAGTACCTGCCAGTACAACAGCTAAAATTGCTAGTATAACACCTATAATGATACTTATAAGCTTTGTCTTTGAAGTATTTTTGCCTGCAAAAAAAAATAGTTGGTCAATTAAAAGCTGAATTAGACCGTTACTTCGTGAAGTGTTTAAGTGTGCGTGACTATCTTCTCTACAAGTTGGAGAAATGATAATTGATACATGCACAATGATCATTCAGCCATAACTCCTATTAGTAAATGATTTTCTTTGAGGGTGTGCATCAAAATTTAAACGACATTCGATATTGACCATGACACCATGTAGTATATGATTATGATTAAAAGCTTTTAATGTGAAAAATAGTGAGAAAAGGTGCCTTCAAATAGATTTCCTGGGAAATTTGGCTTTTAATGTAATGAACTCATACCTTCGTCTTCATTCCCCTGTCTGGTCGATGGCGGTACACTGGCATAGAGGAATGGATATAGC

>LNC_001661

GTAGTATTGTACTTAAAAAGAAGGGAAAAGAAAAGATGATGAATAGCATATAGGAATATCTCTCTTTGATCTCGTGTACACACAAATCAGAATGCATCACTTGTCATACCTGGATTTAATTATGTTTCATTTTCGTTTGTTGTTGTAACCACCATTGTACAGTAGTGTAGTTTTAATAAAAGTTAAGTTTCTCTTTGGAGATCCCATAAAAGGAAATTGTTTAGTGAAACTGTCTAGCCTGTTTGGCCAAAGCTTCTGGGAACCAAAAATGCTTATTTTAGTGAGTTGAAGTGTTTGGCCAAGTTTTTAAGGAAAAAGTTAGTGCTTTTGACTAGTAGCAGAGGCTGAAAAAAGTAGCTTCTTTCTGGTAGCTTGGCCAAACGCAAATTGATGCTCTAATATTGACAAAAGTGCTTTTCAAATTGATTAGCTAAAAACAAATTGCTACTTACCAAGTACTTTCCGAAAAACACTTTTCAGTATAAGCAGATTCTGAAGGCTTGGACAAACATTCCATATGTGTTTTTTGTCACGACTTTCCCAAATCCAATTGATTTGGGCATCGGCT

>LNC_001662

TAAGTCTTCAGTTCTTTTATATCTGCTGCTTTTATGCTTGTGTGACTAGTTAATCTTAAGTTTTGTGGTCTGTCGTTAAGCCTTATTTCGCTCCTGCTTTCCCATCATTATACCTGGTCAACTTTCACATATTCTAATTTAGGAACTTAAGCAGTTAAGATTAGCTCTTAACTAGCTTTTGCGCCAATTTTATGAAAGCCAGGATCACCTGGCTTGTCTAGTTTAAAGGGTTTGGTGCTAGTTTTTGATACGATTAAACATAATCATTGAATGGGCATCAGGAAGATGCCAAATTACAAAAGGTGTGACTAGTTAGCTCTAGTACAGAAGGTCAAAAAAAAACATTTCAGAACTACTACACTAGTACAAGTAGCCTAAGCTTGCTAAGGAGCTAACAAAATCCAAAAAACATGTCGACGCTATTGACAGGTGAAATAAAATCCAATTGAGCAGATTAACTAAACATTTAGTCTTTAGAGAGTGGGTAGGAATTGAAATTCCATCAAAGCATCTGCGATTCCTTTCATTCCAGATAACCCAAAAAATGATTGCTGGGATCATCCTCCAGATTTTCTTGATGGCTTTGTC

>LNC_001663

GTTTTTTCCTGGAGAGCTGCATAACAGTGCATACCTGTTGAACTTTCTCCAACCCACTGGAAAAGAAATGATTGTGGTATTATTTGAAACAACATTAGTAAGTGGTGGATAAGCTATTAGCATTTATTATGTAGATTAATGAGAAGAGAGGAAAAAATGTAGATTCTAGTTGAAAGACCTTTTGTCTTGAATGGTGGACTTATCTGGGGGTGGGGGTGGAGGTGCAAGTGCAAGCATTGAGATAGTACTGCACATTATGAAGCGGGTATCATGTCCAAACTTCCTCCCTATAACGAGCTATTATCAAAGCCATAACCTATTTAACATAAGAAGAAGTTTATGAACAGTTTCCACAATTAGGTACCAAATGAACAGTACAAATAGAGATGATTATTCTCTTTAATTCCTTCTGCACAGATTTGCTGTACATCTGTCTCTTTTCTACCAATTATGTTTGAGTCATGCAGACTTCATGCACCCATAACCCAAGAAGAAAAGACCACCTTTTATGGTCTGT

>LNC_001664

GATGCTTATGGTCGATGGTACCAGATGAAATTAAGTCAGAATTTATGAATTATTTTGCTGGGTAACAATCCCTCGAGGCATTTTAACCCATGATGTGATTATTGAGGTGCTAAATCGACTTCGTTGATCTTTGTCGTACTTTTGAATTGTTGACTGGAGTCATTTCATATCGGATTCTCGATCAATTATGATGTTCAAGCGTACTTTCTACTACATTCACAAGCAAAATTTTATTACGGAGTTTCCTTCTCTGTAAGATTGTAGGCGCGACGAAATAGATTATTAGCATGTGGACAAAGGAAGACACGCAAAACTTGTGACAACTTGGTATGAAGTTTGTTTTGTATTTTACAATTGCTCACTCCCTTGAAAATGAAAAATTGAACTAAAAACAGTTTATTGAAATTAATTTCTCTACCTTGCATGAATAAGGTTTGCATATACATTA

>LNC_001665

TTGGCTTCTTCTTATGTTCCTTCCTTGCTCAATGTCCACTCAAAAATAGAGAATTTATTCGAAAACAAAAAAACTTCTAGAAAAGATCATAAATTATATTTATTAAAAACTACTCATTTTCCAAAATACAACTGCTAAAAATTATTCTTCGCTTCAAATGTCTGACCAAATCGCCAGTTAGCAGGAGCAACATTATCAGATTGAACCCATTTGCCATCGCTAGTTTGTACTTGGAAAGACAAGCTTTGTCCTGCCAACTGCTGAGAAATCTGCCAATTTTGTCCCCAATTCCGCAACATCTGTGTCCATCCAGTCTTAGATCCTTTAATTTTGACACTGACAACATCTCCAACACCTCCCACATTGAAAACAAGAACAAGAATCCAATTAGGATTCCCTTTGATCTCAAACTTGAGACCTCCTTTTTTGTGGCAAGTGACTCTTCTGTAAACAACAGGGACAACCCCTGCTTTGTACTCGGCAATCTTTAAGAACATAGGCAATGACAGATCAAAGTGTTGTTGTGGAGGATTGCACCAAACGTCTGTAGTTTTTGTGTAATTTGGAGGGCAGAAATTGGTGGCAGTTATAGTTATGACTTGGTCTGGATGGCATGCTTTAGGAGCATTGACACACTTTATTTGAAAGCAAGCTCCACAGGTAGATCCATTGTTAAAGAGTTCTGTGCTTAGTGCCGCTGTTTCCAGGCCATACCCTTGCTTGAATAAGTCTCCA

>LNC_001666

CAAAATCTTCCTGATAGGGTACAATGTCTTTCACTATGATCATTGATTTAATTAATATTTTTTCCGCATTCTCGTCATCAATCCACTAGCTCCACATCCCTTGGAGTTTTGTTGCTAATTATGTACTGGCACTAGCAGCAGCAGAAATAGAGCCACATAAAGAGATAATTGCAAAAAGAACTGTCTCTAAATTGCTACCTCGAATTCAATATATGCAGTCCATTTTGCATGTAGGCCAATTATGCCAATTGTCAAGAAATGTTGTTCGTTCCATGTGTCTCTCTCCATAGTTAGAGATAGAGAGATGCTCATATGTAGTTAGTTCAGTGTTGTGAATGTAATGGTAATTGTTTAAGGTCAGGTTGTAGTGTAC

>LNC_001667

CACGAAACCATCCAAAAGCACAATAAATACATCATTTTGACCCGTTTCAAACCCATCAGTTTTGTCACGCAGGTTGGGCGCATCAAGGAATAAAAAAACAACATCCAACCAATGTTAAAACGCCATGTGGCATTCCCAATTTGAATTAGCTTCTTCATTAAATAGATCACCTTCCCAAAATTCTCCATTGTTAGCCATTGGAGATCTGCAATTTCTCATTTTAATTACTTTTTTTTTGTTTAAAATCTGTGAAATCAATTTCCTGTGATTAGGTTTTGAGGGAGAGATGTCAAATGCAAGACAAACGAAATACGCATCAAACAATGTAAAATAAACTAACGACTAATCACTACAAAATTCACTTACAATGTAAAATAAACTTTTTTTCAAAGATTTTTCAAATAACTAACCTATGAGCCTTCACTTCTTCGTTCCGACACCACTATTCAACAAATTTCAACCAGCAATGTTACCAACTAGCTTCGCCTCATGAAGTTTCTTAGGGTTTTG

>LNC_001668

CTTTTACATCTTCCTCACTAGTCATTGCCTTTACATCTACCACATAAGCCTTGTTCGTTAAGTGATCTTGGACACCTTGACCCTTGCACCACAGCTCAACCGAGGAAGCCCAAGATAAATAGTTTGAACTTCCCAATAAAAGTTCTGAAGTGATTATAGGGCTAGAACTTCCAATCCCCATATTTTTAGAACCAAAAACATCACACCCAAAAGACATTATGCTAAATTTGTCTTGGAAACAAC

>LNC_001669

AGCGTACAACACACCAAACGACCCAGCTTCTTCTTCACGCTGCAAAACCAAGCGAAACCCAGAAAATGAGCCAGCAGTCCAGACGCGAGCCCGAGCCTAGCAGCAACGCAATTCCTTTTCTCTCTCTCCTCGCGACGTGCCCATCTCCGTACACGAATTCTCGTACGAAGCCAGGTGTTTGCATCTCAGGTGCTCTTCGTCCTTCGCTCAAAATCAACGCTTCTCCATTCAAATTCGTACACTAATCAGTTGGCAATCCTTATCTGTTAAGGGATTTTGAAAATTTTCAAAATTCCACATCGAAAATCTTTCCAAATTTCAGAGATTTTAGTCCCTATAAATAAGGTTTCTTCTCACTGTTCAAAGAGAGGATTTTTGAGAACGGAGAGCACCCAGGTTTTGGGAGCTTTTAGTTCATAGTCAATTTTTTTATCTCCTCTCGGCTATTCCTAGTCACATTTTTGGCATTGGAATTCGTTCTTTCAAGCTCGAGTCCCAGTTGCTGCTCCAGGAAAGGTAAATCATTTTATTTGCTTAATTAAATTTGTTGTTTACTTTGCCGGTAGATTTGCTTTCTGCTTTGTTTGTAGATTGTCTCTTTAGCATTGTTCTTTTATATTGATTTAGTTCCTTTGGATGGTGCATCTTTTCTATTATTTTAATCTATTTTGAACTGTATTAAGCTGTTCGTTTTGCCATTAAGAAGGATTAAGCATGTTTGTCTATACTCTCGTAATTTCTAAATATGCCTTATTTCTAAATCTTTGGCTGAAGATGATGGCTTTTGAGACTTGTGTTTTAGGATGGTCTTATTATGAGTATTCTGTGGGTTTTTCTTTAGATGTGCAACAACTTCAACCCTCTATTTTCCCATTGTAAAGTTAAACCCTTCCCCTCTATTCTTGAGTTCATTTTTGTAGACAATTGGTTACTTACAACTGTTTTTTTTTTTTGGATTCGATCTCCCATGCTTTTTTAGGGGCAAATTCTCAATGTTGTTAAGTGCTGCAGTCTGATTTTTGTGGAAAGTTTAGCTTGACTCTTTTTGTTCTTTATTACTAAGTGTCAATCATGTCCAAGTTAATGTGTTGCATTCTAAAAAGCTCATGTCTTGCCTCATTATTTGGAGGTCAATCAACTTTTGTTTACTGCTTAGTTTCGTAAGTTTTAGCATTATGTTTTCTCCTAGGCTATTTGGCATAGCTAAAGTTTTATCCTAGAATTTCGAGTTTAACTTGATATGAATCCGAAAATTATCGCTTTGTTTCCCTTATATCTTCTTTGCTGTTGAAAAAAAAAAATTCCAACACTCTCCAAATTCCCTTGCTTTTTGGCTGCTTTATTTTCAGCTTTTGATGTAGAGTCTTGGATGAATTTTGATATTGTTGTGTCCTAATATATTAGTCATTATAAGTTTTTTTTTTTTTATCCTTTCATAAATTCAATGTAGGTGAAGAGCATATGCCTTAAGTTTTTACTCAATTGTTTTGCCAATTATTTCACTTAGAAGTTAACCCGGTTTGTTAATAAAAAATTTTTAGCTTTGTCTTTGTTTATATGTGCATTTGCTACTTGAACATTTTGAGATGCAATTTTTTTTAAAATCTAATTCTTTAGTTGTCACTTTTAGCACTCACCGTGCGTAGATATCAAAACAAATGTGCGACAGTTTCCTAAACATTTAATCTATTTTATTTCTTTTGCCATAGGGTACCAATGTGTTGATTGTTGTTTAAATGGTCCTTATGACGTGATTGGTTATTAGGAAGAGACCCTTTGATTGTCATTTCTTTATTCACTATGAGAAGTACATCTTGATCATCAAATATTCTTAATTCTAATTGGTTTTATGTTATAATTTACTGGTCTTGTGATTAATTCTAATTTGTTAATAATAGCTCTTTGGACAGTAATGGGTCTGTTGGTTAAATTTTGATGAAGGATTGTTGAATTGATTAACCTTGAGCTTTATATCTTAGTTTATTAAGAAGAAAAAAAATTGTTCGTGTACTAATCGTTTTTTTTTCTAATTTTCTTTGCATGAACTCACCTGTTTGAGTCCATTCGGACTTTGCATTCCCTTCTTTTTTGAGTCAAACCTCCGCCAAGTTACGAAAGGGAAGCTGATATATAGGGTTGAGACCCAAAAACCAGCAGCAACAGTAGGTCAAAAGGAGCTTAAACGAGGAGCAAAATTGGGTTAAAACCCTTAACCGATGAAGCAACGGATTTAACATTTCAACCCCCAAAATGGTCAAATAAGGGCTGGAAATTTCAGTTATTTACTTTATATCCTCATTATTATTTTTGTTTATTGTGAATGCTAACATTTTCTTCTTATATCTAACTTAGATGAATCCTTGGGAG

>LNC_001670

GACAAATCCTAGGCCTCCAAGATTGTTATATATAATAGGACTTGCATAAAACAAGAGAAAACAGCAACTCAACTCCTATTAGTTGTGATTTGTGAAGCACAAGAAGATGTGGAGAAAGAAAAAAAAGAAGGATGTTGATGTACCACTCGAAGAAATGGCGATCACTAGGTCACACCCTTTGGTGCATCTGATGTGAGTCGATACCAAGGGCGAAAAAAAGGCAAGTCATATAGGCAAAGGGAAGGCAACGCCTTTCTTCCAGGATTTTAATTCCTCTTTTTTTTTCTTGTATTTTCATACTGTATTACTTTCCATCATGGATAAGTTGGGGTTTTCAAGTTTCACATGTATAATGATGTTTTAAGCCTTTTAGCTTTGGATTGAACTACTGTTCAACCAAATATCCCTGTATTGTGTTTCTTTTCAAGTAATACTGCCCTATTCATACTTTCCTATTTTATCCAATGTGTTCTACGATCTACTAATCAGTGGCGGCGGATTCATGATTTTTACTAATAGGATGAGGTTCAATTGCTCAAACGGTTACACCATTGAAACAATTCAA

>LNC_001671

CACTGATAGTTGCTGATGTTTAGGGAAACCATAAGACCCTTGAGGCCGTAAATGTTTTAAAATTGCAAGTGAATGCCAGAGTTGTAGATGGTAGTGAGCAATCTATGTTCACAACTGCCAGCCTAGTGCTACACAATTTACTGCATAAGCAAACTTACCAAATTTCCTTCTGAAGAGCTTCACAGAAGCTTTTGATGCCAGTCCCTGAACTACTGGATAGTGAAAAGGAACCTTCTACTCTGCTGTGGGCTTGGAAAATTTCTTGCTGGGTTGATTTTCCCTTCCTTACAAAAACTCGAC

>LNC_001672

ATGTCTACGGACTCATTTGCTTATGAATATTGTATACTTGATAGATTGGAAACGTTCTGATGCATTGAGGAAAGGAAAAACATTGGGGAATTAGCTTGCTTGACTTCGGTTCTTCAGTGAATGTAGATTATGATTTATTCTATTTGATAGAGAGACTCTTAATAGTGATTGATAGTCATTAAGTAATTTTATGAAATTTCTATATACTTAATTGTTGTGGTTTGGATGATCATGTGTTGTTTGTGATTGATCTGTAATCTCAAGACCATGAAATCCATAAGATTGAACTTATTCTATTAAAGTGATGCCTTGAATAAAGAAGATTTGGTGAAATAGTGTTAATGAAGTGGAAAGTGATGAAAAAATGATGAGTTAACTATGTTGTTGGATCGGGTGCCCCGAGGTGTCACGATAGTTAACTCATCATTATTTTCATCACTTAACACTTCATTAACACTTTCACCAAGCCTTCTTTATTCAAGGCATCACTTCAATAAAATAAGTTCAAGATTATGGATTTTGCGGTCTTGGGATTACACACCAATCACAAACAACACATGAATCATCTAAACCACAATAATTACGTACATAGAAAATTTCACAGAATTACTCGATGACTATCAGTCACTATTAAGAGTCTCTCTATCACATAGAATAAACCATAACCTTTCTTTATCGAAGTAAAGCAAGCTAATTCCCCAATTTTTTTCTTTCCTTAATGCCTCAGAACATTTCTAATCTATCAAGTATACAATATTCATAAGCAAATGAGTCCGTAGACATCCATATTACTATATATCTAGCTTAGACTCAAAAACTCATCCAAATCTATAATCAAATTCCTAAATTTTGATACCAACTAATATATCAAGTCTCATTCGTTCCTAAATATTCAAGCCTAAGGTTAAGTCTCAATTCGTCCAAATAACATAACTCTAATGGAATTCATATAACACAATACCCTTAATATTTAATTATTTATCTCAATATATCAAAACTTAATTTATAATATGATAATAATACTGAAATCACTTAGTTACAGAAACACGGTCTGCCAAACCTAAACTATTTACAATGAATGATATACTACATAACTCAATCCTAATGATTGAATTATCTAATGGCAATCATCATTCAACTGATAGCCGTCAACATATTGATCAAAGACATAGTAGTAACACATTGCAGTGCATCAATTCAAATTACCTTCTCTCCTATCTAATATATTAATATTATCCAACCCATATACATAAAACGAAGATTTAGGGGCGTCTTGATTTACCAGATTCTCGATCTTTTCCTCTCCTGGTCGCTAAGTTTCTCTCTTTTCTTTTCTACAAATTATTTACGACCCTAATTATTATACATGATTAATTATAAAAGTAGTGAAAGTGGTCCACTACTAATTTCAATGCTATTTTATTAGAATTACCCCATTAATTATATAATTGTAGTTACGAATAGTCAGAAATACTCATTTAGAATATATTGGAAGTTATTTATGAAATAAGAAACTCTAGTACTTAAAATGACCTAATGGGTCGTTACAGAGTCTGTCTCTTGAAGGGGAATGACTGAGCCAGTTGTCGTGCCAAAACGGTAGCTTTCTAACATCACCAACGCATTCTGTGTTAAAAGGCCAAAGTTCGAAGAAGAACTACTCTCCTTGCGCCGTTCATTCTAATTCTTTCTTGAGCTACAATGAGATCTCTCTATTAAGCCTGAGTAGACTTTCCTCTTCCTTCCTTTCAATGATTGTGCTCTATTTCACTAAACTGGAAGTCTTTTTTCCCTGCTGCGTTGCCGTTGAAACTCTCTCACTTTATCAATCTTCTATCCCATTTGAAAGGGGCATAAATAAAGGCATGGTGTAGGCGGGAAAATACACTGCTAGTGAAATGGAACTAATACCTTTTAAAAAAAAAAATCGAGGGTCTTTCTTTTCTTGTGAATAATATTTCATTTTTTAAAATTCATTTTAAATTTTACCATTATCAAAACTCGAGATGTATTTTAAGAGGCATTAACTTAAAAGAGTGATTAGGGAAAAATACTTGCAGAAGAATTGAAAATAACATGTAGTATCCACCTGAGAATGATGTTGAATATTACATGATTGTTCAGTTCCCACTGTGTTTATACTTGTCGGGTAAATTAAGCTTGGTTAGGTGATCGGGACTCTAGTAGTAGGATATTTGATGTCAAATCTAGTTCACAGCTTATCAGATTTTAAGAGCGAATATTACCAAATACTAGACAAAATTC

>LNC_001673

TCCAAAAGACAGGTGTATTTTGTGAATTTGTAATGAGTAGTTTTCTGTTGGTTTGTGAGTGTGTTTAGTATTTATTAAGGTGCTAACCCAAGGCAATATTGCTCATTAGGCACGTAGCAAAAAAGAGAGCTATTTATTTTACTAATATAAAACTCTATCTTTTTCTCAAAAAATGATGAACAATCAAACTCACCTTTTGGTATGGCTTCCAGTTTTTCATTTATTCAAGTTGTTTATTAGTAGTATTATTTTTAATTAATACAAACTTACGTTGAAAGCTGCATTTATTTTTTTACATTTTCTTACTAAAAGAATGTTAGTGCTAAAAAATATTCTTGCAGGGAACTTTCAGACTTCCATGGTTCTACTTTTGTGAAGTCCAAGTTTATTCCAAGTCTGTCCGGTAATTTTCTACAAAAAATAAATAAATTAATTAGTAGCCAAATTTTCTTGATTCACATACATGAAAATGGATGTTGTTTTAAGTAGCTAGACAATAAAAATCTGTCAAGGTTTTATCAAAAGATCATGTTATGCTACGAGCGATTGATTTTGTAGCTAAATCATTTTTTTAATATATGTTTTCAAAGTTTTTTATGTCTTTATTTTACGTTTTACAAACCAAATCTTTTGTGCTTTGATGTGCACAACAGCACAAGTACTTC

>LNC_001674

CAAGAAAACACGACGACGCCGTATTAAAGACGCAGGAATAATATGGCGGGAAAACCTTTAAGGCTTTTCAATGTGATACAGTGTTAGGGTTCTTCAGTCGAAGCGAATTGTCGCATTCAAGAAGATGCCGATTTACAAAATTAGGGGAATCGATGTCGATTTCCCTTACGAGGCTTACGATTGCCAGATCGCTTACATGGAAAAAGTTATTCAGTCCCTTCAAAATAGATCTAACGCATTGCTTGAGAGTCCAACTGGGACTGGGAAAACCTTGTGTCTTCTTTGTGCCACATTGGCTTGGAGGAAGAGTTTGGGGGGTTTCTCAGTCCGGAAGAGTGGAAGAAGAGATCATATTTCCAGTAGCCAGCAGTCTGATGAGTCTTCTCAATCTGAATCTTCAACATTACCGAGTATTGTATATGCATCACGCACACATAGCCAAATTCGACAAGTGGTGAAAGAGTTAAAGAGGACCAACTATAGGCCCAAAATGGTAGTCTTAGGGGTCTCGGGAACAACTATGCATTCATGAAGAAGTGAGTCAGCTGCGTGGAAAAACACAGACAAATGCCTGCCATGCACTTTGCAAAAAACACAAAAAACGTTATTGTGCCCATTTTTCTCGTGTTGCAGGTATATTAAGCATATCTGCTTGTACTCAAACTCTTCATGGTTGAGCTGGATTCTGTTTTTGGTTATTGATTCACTTATAAATGTTCCTAGTGTCATTTATGCTTCAGCCTTCCCACATCCCAGGTGTATTCTTCTCCTAAGGAGGTTATTGCACTCTTTATTTTATTAGAAAGTACATTTACCTGCATGGTCAGCTAAAAAATTTAGGCTCTCGTTTTCCTGCTCATATCCCTTCTCAATCCCTATGGAATTATGTAAATAGAAGATAAAAGACCACATCTTGCATCCAAGGGTGTGACCATGATCAATGAAAATGACAGGGCCATGGGCAACTAGGGTTTAAATCCCAACAAAGTAAAAAAATAATCCAGGTGTTTCTTTCCATTTGCCTAATCTTTGTGGACAAAGTTAGCTGGTACTTCGTACTTTTGCTGGTGGGAGGTAACAAGTTTAAATTTTACGCTCATAGGACCACTTCATGCTTTTAATTTTATTGGAGAAACAAATAAGCAAAGATGCAAATTTACTCGGTGGAACATGTGGTATGTGATACTGCTAGGAATTTATTTAGCTTTCTAGTTTATAAAGGTGCTGTATGTCAGTATGTCAAACACTTTTGATACTAAGAAAGTATGTATATCCAAATATATAATGATTGGACATATCAAGACCCCTCATAATACTAGTCACTATGCACTTGTTTTTTTGCTTGATCATACTTTATTCGGTAAACTCAGAATCAATTATTAATTTATCATTTGATCCTTGTATTGTTGCATCATCTGTGTATGATCTCTGTGATAAGGAGACAAGCATCTGCACCTTGAATATTTAGGCAGATAACTCTGAAGAACACTGAAAAAATTGTCCTATTTAGCTAAATTAAATAACTGGTTTAATTGCTTGTTTGGTTAATTATTACTTCTTCTGTCCTATAACATATGTCAGCTATATGCTCTTTGGTATTTTGACTTCTTGAACATCATAAATAGTTGACTCCTTGTATAGTTTATGTTTCCTTTCTCTATCTCATCATGATTACCGTTTCTTTTTGTCCGTGTATGTACTTTGTGATCTGGAGACAAAATGTGTACCTTGATCTTTTAAGAAGATATTCTTTAAGACCTTAGGCCAAATGTACTCGTTAGCTGGATGAAATGACCGGTTTATTGCACATATGACTAAACTTCTTCCTCCTCTGTCCTTTAATGCATGTCTGCTATATGACTTCTTTGATATTTCTTGGAGGATCATGAATAGTTGATTGCTTGTATAAATAACCTGTAC

>LNC_001675

GTCAGCATTGTGGCTCATTATACCAAACAGTTTAAATGGGATCGCAGAGGCATTCAGCGCGATCGGCTCCACAGAGTTCTATTATTCGGAGCTCCCAAAGAGTATGTCAAGTATTGCATCTGCTCTTTTAGGACTGGGAATGGCAGTGGCAAGTCTTTTAGCAAGTGTGATTTTGAATGCTGTGGATAAGTACACCAAAGGAGAAGGGACAGAAAGTTGGGTTTCAAGCAATATCAACAAGGGACACTATGAGTATTACTACTGGCTTCTTGCTCTATTGACAGGTTTTAATCTGCTTTATTTTGTGGCTTGTTGCTGGCAATATGGACCTTCTGCTGATGTTGACATCACTAAGAGAATGATGGAGCTTAGTGATGATGATGATCATCTTTCATAGTAGAATGACTCAGGCAAACTCAACTGACAAAACTCAGGTAAACTCGGTGAAATGCGCCAAATTATGCTAGTTTACCACAGTCAAATTATAAATTGAGATACTAATATATACTTATGAAGCTATAAAGCTTATGAAAAAGATGTGTGGTCTTCAGTTATTGATTTGATGCCTGTTTATGGTTTGGAGGTGAGCAAGGGTTGGAACAACTCACCTTCCCCATAGCATTCCGAGGCAGCGAATCGAGTAGGTATCTGTTACATAAGTTGAACGACAAACATTAGTTTAGCAGGCATATCTTGGTACTCGAGTATTACATTTATCTTGCATAAGAACCAAATCACAAAACAATACTTTCTGTCCCAAGTTATAGTTAATCAATATCAACAGAGTAAGCGCATAAATTCCTTATCCCTGGAATGGAAGCAGAAAGAAGTCA

>LNC_001676

AAGAATATACATTTAAAATATATATTTTTTGGAGGGCTAAAGGCTTCACCTTTTCAATTATAGAGAAGAATAATTAATTTTATAGAAGAACGTAAAAAAGGAAGAGAAGTTACAATATTTTTAGTGATGTCATAAATTTTAAGTGAATAGAAGTTGGAAGCTTTAACTCATTTGGCTATTCCATATTTTATCCGTAATTTACCCATATTTTTATGGGTTGAGTATGGGTATCAACCCATATTTTACCAATATAAAAAATCACTCACCCAACCCATTAAAATATGGTCGGGTTACCAAAATATGGGCTCGT

>LNC_001677

TCATCTTGGTCTCGACCTTGTTGAGCTCTCCCTGATCGAAAGTTCCTATTTTGCTGCCTCTTCGATCGTCTCCGACTCCCTAAAAAAGAAGATTGGTGACCTAGATGGTCAGGGTCTTTCCTTACCTTAGCTGAGCCTTATGTCCCTTCAACATTCCTATTCATCTTGCCTTGTCTTGGGCACCGCAGTGGACTTATTATCCTATATATAATATCTACTCTAAAGATTAAGAGGATAGAGGGTCTCCCTTCCATAGATTCGATTGGGATTGATTCTCAGGATCGAGCCCTTATCAGAGGCGTGCCTACCGACACCTGTCAAAGTCTTCGGCTTGCACGACGTCTCCTTCCATTTGCTCCAGCTATGAAAGAAAAGAATGGGCGCAACACTTTATTCTCTTCTACCGCTCTTTGTTTACTTCTGGCCTTCCCGAACTCCTACACGGGGGGAGTCAGATCTTGGAGTAAGCTCCCTTCCATGGGCAATCTACTCAAGAGTTTAGGCCCCTAATTAGGTCTATGTCTGGGCTGCTGCCGTACTCAAAGGCGAGGGGCTCCAAAAGTGGATGCGATCGGCCTCAGAGGAGAGGGGAGGCAACTTGGGATTCTAGGCAAAGTGCTTTTTTTGTATATAGGCCTTCGTATAGGATAGGATTTTCATTCCTCCCGCCAAAGCTAGAAAAGAAAGGAGCTCTCTCTTTTTTCCAGCCTTCTTTTTATTTTATTTTAAAGGCATATAGTGCGTTCTCGCTTTCAGGGGGAATTCAAGTAAAAATCTAGTACAAGGAGGAAGCGAAAGGCTAGCTACCAGGTACGAAACAGAGTATGACTATAATAAATCATAGAGCAGTGGGGAGCATGCCCATCCCATTAGATATCATTTGAGCTTCCCCTTTCCTAAGATCCTGGACGTGGTACTGACGCATGAACTTGGTTACTGGTTTGACCGGTTTGTTGGCAAAAGGAAAGGTCTGTTCATTTGTCCAGAAATATGTTTTCGGCCTATCTATCGATTGAACCAGGGGCTGGGCTCCTGTTGACAGACATAAGAAGTGGGATTCAGAATAGGGGAAGAGCAGCTCTACTCA

>LNC_001678

AGTATTTTCTTTTAAAAGAATATAAACTTGTGGACCAATTTTTGTATTTGAAAAATGTAAAATGGTGATATCAAATTCCCAATCAAACTTTTTGGAAAATTTGAGATGTCATCCCAAATCATGAGATGGAATTGCAAGTTCAAAATCAGATTTTATCAAATGACTCAAATCACGAGATGAAATCACATAACCAAAGCTACTCAGTGAAATAGTATAAATTGTTGCTCATGAGGAGCTGAAAAGTGGTTATTTGTTGAGTTTTAGCAAGTGATTTTCCCTTCCTTGTTTCAAGCACTCACTCAGGTCTCAGGTACTAGCAACTCTCTGACTCTTATGTTAAACACCCTCAAGAATTATGAACACACCTCTTGGCAACTAGTGAACATGAATAAATCTTGCTATTTGGTGGCTCCAAACACATATGCCGATACAAATTGAGGAATTAAATCATCCATTGACGTAACTGGTAAAGTTGGACTTGGACGTGACATGTGACCAAAAGGTGTAGAAATAGCCTTTTGTAGAAATGCAGGGTAAGGACTAAGGACCCTTGCAGTTCGGCCCTTTCCTGAATCCCACACATAGTGGGAGCTTAGTGCACCGAATTGCTTTATATTGGCTTCAACAACAGCAACTTTCCTTTTATATATCTTGGATATCCAAACTAGTTGTGAAGAAAAAAAGTGGCATACTTTAGTGGCCAAGATCACTGGAAAGTTACAAGGAAAATTATTGTCTTATAGGGGCAGAGGTGCTCTTATTAAAGCAATTTTACAAGCCATCTATCCCTACTTACACTATCTCTGTGATTGATCCCCCTAAAACAATGAAGAAGCAAATTGTGAGTTACGTCTAACTTTTCCGGCTCGCTTAGTTTGCTATGTCAGTTTTAAAAGGATAAGTTGTGTTGGAATTGATAAAGTTCTATGGAATTCATAATTGCTCAAAAGGAC

>LNC_001679

GGAAAAGGAAAGCATCTGCTTAGACAAAACCTTCCTCAAAGATGCATACTCTCATGTTTGATCGAACTTGTGGCGCCACACTTTCTTGTTTTTGGAAGCTTAACCGATCTGGCTAAGTACAAAGTATGAAAATGAGTTGAAATCAAGGTCTTTTAATCTCTCAGTTGAAAGTGTGCCCTGCGTGTGAATTGAAATTCCAGTGGCTTCAAATGTGTGACCAGATGTATAACTTATTTTGCCTCATTCGAATTGAGCTCTTTTTATTTGAAAAAAATAGCTCTGCCCTTTGTATTGACGTATTTTTTATCTGCAAAAATATAAACTGTCTCTCCAGTAGGCTATTAGTCTTGTTGTAAATTGGACCGAAGTGAACTCGTGTGAATTCTCGCTATGTTAGAAATCTGAATTTGAATTCTTCCATATCGTATTCACTTATCATGATGTTGGCTAGTGTCTAAAAAAGGCCTTTGATCAAATGATAAAAAGAAAATTCCCCTTCCTTGATGTTGTGTTGCTGTGAAATCCTTTTAGTTTTGATCCTTAAGTTTGGCACTAGTTGTTTTTCAAGGTTTAGGATTTTCCTGGGTAGTTCGGTGAAAATGGAAAAACTCAATCTACCTTCCCTATCCAAAGCTAGATTAGCCATGTAATCCGCCAATGTATTCCCTTCCCTATAAACATGTTGGATCCTAACTTGACATTGAAGTGCTAGAGCCTTGATATCACTTACTAGAATAGCAATATGCCAAGGCACTTCCCAAATTCCGGCTAGTATCTTCACCAAACTTAAATAATCTGTTTTGATGCATACCTTATCGAGACATATGGCAACACAGTGATAAATTGCTTCTTTGATTGCAATTGCCTCTGCCTCAATGTTGGTGCATTCATTGATTTCTTCCCCTTGAGCGTAGATTAGATCCCCTTTATGATTTCTCAAGCAAAACCCATAATCACTTCTACCTGGATTACCCCTTGATGCTCCATCAGTGTTGCATTTTAGGGTACCTAACTCAGGTGGTTTCCAATCTCCATATGTCATTGCTAACTCTATAAATCAGACTGTTTGTAACTCATCCTTCTTCCATGTTTGATACTACTCCTTCTTTTCCATAAATACCATAGAATAATTGTTGGTACTGCAGTGATAATAGGTCTTAGCTTTAGGATTATGGCAACACCAACATTGAGATACAAGTTGAATTCCAATATTTTGCAAGACTTCATCTATTGGAATTCTAAACTTCCATAGTCTCCAAAGGAAAAATGACATTTTGAAAGGCAATTTGTTAATCCACATATGCTTAAAATTGTCTTCCTTTTCCTCTTTCAGTCTTATGTATCCCCAAGCACTCTTCACAGAGAATCTCCCCGTTGTTTCCAGCATCCACTTTGCCTGGTCAATATCACTACTAATATCTGGAGGTGGTATGTTTCTAATAATATGCTGCACATACTCCTCTGGCATACAATCCCTTCGCTCATGTATATTCCATCCTTCCATCCCTGCGAAAGTGTTGACCTCTATTTCCTCTTCCTCGGCTTCTGGTAAGACATGATAAAGTGATCCCAAGCCTGTCCAATTGTCGTACCAGATGTATTTCACTAACTTCTATCACATTTCAAAAAAAAGAAATAATTTAGTACTTCTTATTCATATTTTTATAGCATATAGAATAAAATTTTGGGGCCTTCAATTTGGGGGTCTAATTGTTTTTTTTTTTTTTCACCTGGGGTCTAATTGTCTAAAACACTTGATTTAGTGGATTTCCCATTCAACCGACACTGACAACATATAATTTGCGGAAGACTTAGAATATTTTTTCTCTATCTCAACACTCTCTTTCACTACACTAATCTCAATATAAACACGATATAATACTTTTCATCTTTTACCCTCTATGTTTTCTTATATATAAAATAATGAAAGACCTTCACTATTTATAGAAAGATTATAGGGTAATAAATGAAATGTTGCACAATCACATTTCACATGATTAAATATTGAAATGCATTTTGCTCCAACACATTACATAAGAATACTGCGCAAAATTAAAGGTAAAAGAACACAAACTCACTCATCCTCTGCATCAGTAAAAAGCACAAAACAAAGGATAGAAGCACTGAACTCATTCTGTTTCAAGATTTGCCGGTAATCAAATGAAATAGTGATATTTATTGCAACCATCTAGAATTTCTGAAAACTGAATCATGCACAAATCAAGTGCTAAAAAAAACAGAAGCAATCTTTACATGAATCCAAAAATTTAGCAAACAAGTGATTGATTTGTAACAAGAGATTCTGAGAAAATGCTCAACATGTAAGAAGTTGAATCTTGATCTTCTGTAGCATCCCTACAACATCTTTCATGTTTGTCCTTCTTGCCGGAGATTCAGC

>LNC_001680

TGGGGATTCGAACCTGCGCACATGGGCTTCACAAGTGAGCCTCCATCCGCTGGGCCGCAGTGGCTGATTGAGTCAAGGGTGTCCAAAGTATTAAATTTAAGCATTTTGAACAGCATTTTACTTAAGTGTACGGTATAATTTTCCGATAAAGGGTGTCCAATTGGACACCCTCAAGACACTGTAGCTCCGCCCCTGCCCTTATCGATGAATGATGAATGCGATGATTCGTATCAGCTTTACTGCAGATCTGTATTGTGTCCATAGTCTTCATGCTGTTGCAAATGTTGTGATTTGGGAAGCAATATCTGCATGTAATTTTGTTAATTTTGAATGACTTAGAATTAAAGAACAGATAAATAGAGAATTGAGCCTATGATTTGCAGAGGCAAAAACATCCATTTCTTAGTGTTGCCAATGTTAACAGAACGAATATGTATCTTTGTCCTGCAAAATTGAGCGTCAACGGAAAGAGACAGTATTATGCATTTTAAAAAGTAAATAGATGCTGTTTAGTGGTTGTGTTTCCATCCTACATTACGAAGGTGCAAAGTTTCAATCTCACCAATGGTGTTCTTTTCTTTATCTTCCTCTCCTTATCTATTTAACGAAGATTACTTTCCTTGAAATAGCATTTAGGATAAACATGATATTAAGAAAACATACACGGAAAGAAGTAGGGAAGTAGCACACAAGTTTACATGTTTTGTTCCATGGATATGATGAGTGCTTTGCTCTTTGCATGTTTTTTCCAGAAGAGTTGCCAAGAAAATTTATTGCTTTGACCATACAGAGGATATCGAAAATGCCTAAAACTTAGATTGTACAAATGATGAATTTGAGCTAGTGGTACAGTAGATTCGTGCTAATTTGAGGTTTCTAATTCTTTGTTCTGATTGATTTTTTCCTTTTATTGTACCTGTAGAGGAAAATAAGGCTCGCCAACAAGTATGTAATCAAGAATGGAGCTTAACTTTGTTCAGAAAATGAGGCAATGCATGGAAATTTCTAAAGAACAACTTCAGGAATGAAGTGTTTGCATGTAATGTCTGTTTCTCCACGGCAATGTCTCCCGTGTTTTCTTCTCCTCATCCCCTACTTATTCTGAGATTGAAGAAGAAATCAGAGACTAGTACTGCAGATAAAGTACAAAAGAAAGCTCATGATGTGAAGACAATGACTAAAGACACGATGGATTCTATAGAGGTTAGGTTATATTGTTCCCTCTGTTCTGATTTATGTGGGCACGAGCTTTAAAAAGGAAGGAATTGACTATTAGTACTTCAATTAGTTTGAAAAATGATTCCTTAATGGTAGTGGTGAAATTGTTTTTTTGTTTGTTTGTAATTCCCTTTTAAATTGCTTAATTGAACAAGTAAAAGAAAAAGTTAATTTTTCTAAATTGACAAGTAAAAGGGGTTGAAGTAAAAAAGAGGAGTTTTTTGTGAGGATTTATATTTATAGTCAAAAAGATTAAAAAAGTGATTATGACTAGTTGATGATCTTAGCATGGAATCAAGTGTAAAATATAACTGGTGAAATTTGTCGGTAACTGATAAAGGGTTTGTACAAATTAATTAGGTAAATGTCCAATTGTGCAAAAGTCACATACTTAGTAGATTTTTTTAATTTTTTTTTGGTTTGAGCGAGGTGTTTCAATTAAAATTTGTTGTCTGTCAACTAAATTTATGTTTGGACAAGCATTCGTTTGCCTTGTTCAAAGAATTGAGCTCCCTTCTCTTATTTGACTATGATTTACAAAACAAAACTATCACGCATCAATTCTAAACAAGAAATTACATTCAAAAAATAAAAATGAGCTAAATTCAAGTGACGAGTTTGTAGCTAATTTTTTTTTTGTAGTGTAACTTTTGCTTGGGATTGATGCAAAATAGTTTTGTTTTGGAGCAAAGTCAAATAAGAGAAGAGAACCCAATTCTTACAATTAAAAGAGAACAAGGCAACAAATAAATTCTTGTGCAAACATGAATTTTAGTTGATAAAGGTAAAAAAAAAATTCATTGAAACACCTTACTCAAACCCCCCAAAAAAGTGAATTCTACTATGTGACTTTTGCACAGTTGCACAATTACCTAATCAATTTGTACAAATTCTCTAATAATTACCGACAAATTTCACCATTTATTTTTTACACTTGATTCCACGCTAAGATCACAAACTAGTCATGATCACTTCTTAAATCTTTTTAACTGTAAATATAAATTCTCACAAAAAAAGAACCTTCTCTTTTACTTACATCATCCCCTTTTACTCGTCAATTTATAAAAAATAGTGTCTTGCCAGTATTTTTAAATTATTATGTAGTTTTTTCTTAGTGTTAGAAATTCTCCGCATTTTCTAAATGATCATATAGTTTCTGACTAGACCAAAAGACTAGTACCAAGTAGTGGACCTAATGTAAATGTATCATCAATATTAAAACTTTAACCACAGCTAATTGGTAGCGCTTACATAGATTGTTTTTTCTTATTGCATGTTATTTTTTGGTAAGTCTATGTGTTTTTAAAGAGATCGTATGTATTTGAAAACAACTTCATTTCATGTGATTTTATGCCTACCTCATTCTTTTGAACACATACAAATTGGGTTTTCTTCTTCTTTTCACTATTCCATGATCCTCTTTTAAAAAATATTAATTCTCCACTCCGGAGTTAATAAACAAAGGAAATTTCTAGGAATAAAATTGAAGTGTCCAAGCATACAAGGTCCTAAGATTATCATCATAAACTTCTTTAAAAATATTCATTGAAAACACTTTTAGAAATGTTCTCAAATAGTTAAGAATTTAAATTCAAATATTTTTTAAAATTTGGCAAACAAATCAATTTCTTAAATAGATAACTACCTGACACCTTAAAGTTTTTTCTTTTAAACTTCTCTCAATATTCTTCTTCAGGAGAACTTCTAAAATTGTCTGATGACTTTAAGAGTCAAAGGCATAAGAAGCAAATAAATTATTTCTGCATTGTACCAAACGTATAAAAGGAAAATTCTTAAAAGGAAAAAAAAGTGTAATAAGAATTAATTGATTAAAAAAGAAAAAGCTTTAAATCTTAAAGAAAATCTTAGTTGTGGAGACGAAACAAGAATACTTTTTATTACTATGAATTCTTTTGTGCATATGGTTTTTATTCTATTTATTAACCAAATTATCACTCATCCTTCAATTTACTGCATTGTTTACCTGCGATACTTTTTTTCTGTACACACTTTATAATGATTTTTTTTTTCCATTTTAACAATGAAAATGTTGATTTCTTT

>LNC_001681

TATGCCAAGGACAATATAGACTAGCATCAGCGGCGTTTTTTGTTACTGGTTATATTCAAGATAAAAAGTGTATGGGCAAAACCGTCACACATGCTGGCACAGGGTGTGTGGAAGCCTTGGATGCTGATCATTACTTGCAAGAGCTCGATGACAATGAAGTAGAGGAAAATAAGGCTCGCCAACAAGTATGTAATCAAGAATGGAGCTTAACTGTCAGTCCCCTGAATTTTTATAGCATCTGAAAGTTGACTTTATCTGCATTGTTCATTATAGTATTCCGTTCAGGTTCATCTTTAAGTTCGTTTGTTCTCAGGGAGCTTGCACTGCTGCCCCTGCTATTGGCTATCAATTGTCAAGTCCCAGTTTATGAGTACAAATCAAAAGAAACAATTTTCAAAGACTTCATCCTGTCCTTAACAGGATGTTTTACCTAAAGTGGCCTGGCAGCTAAAGCTTTACAGCGCACAATTTCTGCCCTTGCAAGTTTTAGTGATTTCAACGATAATATGCTTATGCATGTTAGCTTAAGGGAAGAACATGCCAGAATAAGCTGAATACTCATAAGTGATCCCATGATCGTAACATTTAGTAGTTGTTTTGGTTACGATATCCAGTGTGAATTTGTTTCCAGAATATTTTTGGTCATTGAGATATCTTTCTGCAACTTTGTGCATGATTTTATCAGTATTCAAGGGAGAAATGCAACTTAAAATTCTAATGTGAAATGTGAGAGCTTTATGCAGGATTACGAAGAAAGTGAAACTCTTCAAGGTGAAGATTTACACAAGTTTTCAGCAGAAACACCTTAAAAGTTTGGTGATAGGCAGGCTAAAGGAGAAATTAAGGATTTTTACTTTTATCATACTAGATAGGGGGCACAACGGGGTTCATCCAAACCCCCTTTTCCGGAAAAATATACTTCTTTTTTTATGTTTATAGAGATAGATATTGAATTCCCTTGTCTTCTTCGTGTCCTTACTTCTATATATATTAAACCCTTTTACTGAAAATCCTGGTTCTGCCACCATCTTGTAGTTGTTCAGAAAATGAGGCAATGCATGGAAATTTCTAAAGAACAACTTCAGGAATGAAGTGTTTGCATGTAATGTCTGTTTCTCCACGGCAATGTCTCCCGTGTTTTCTTCTCCTCATCCCCTACTTATTCTGAGATTGAAGAAGAAATCAGAGACTAGTACTGCAGATAAAGTACAAAAGAAAGCTCATGATGTGAAGACAATGACTAAAGACACGATGGATTCTATAGAGGTTAGGTTATATTGTTCCCTCTGTTCTGATTTATGTGGGCACGAGCTTTAAAAAGGAAGGAATTGACTATTAGTACTTCAATTAGTTTGAAAAATGATTCCTTAATGGTAGTGGTGAAATTGTTTTTTTGTTTGTTTGTAATTCCCTTTTAAATTGCTTAATTGAACAAGTAAAAGAAAAAGTTAATTTTTCTAAATTGACAAGTAAAAGGGGTTGAAGTAAAAAAGAGGAGTTTTTTGTGAGGATTTATATTTATAGTCAAAAAGATTAAAAAAGTGATTATGACTAGTTGATGATCTTAGCATGGAATCAAGTGTAAAATATAACTGGTGAAATTTGTCGGTAACTGATAAAGGGTTTGTACAAATTAATTAGGTAAATGTCCAATTGTGCAAAAGTCACATACTTAGTAGATTTTTTTAATTTTTTTTTGGTTTGAGCGAGGTGTTTCAATTAAAATTTGTTGTCTGTCAACTAAATTTATGTTTGGACAAGCATTCGTTTGCCTTGTTCAAAGAATTGAGCTCCCTTCTCTTATTTGACTATGATTTACAAAACAAAACTATCACGCATCAATTCTAAACAAGAAATTACATTCAAAAAATAAAAATGAGCTAAATTCAAGTGACGAGTTTGTAGCTAATTTTTTTTTTGTAGTGTAACTTTTGCTTGGGATTGATGCAAAATAGTTTTGTTTTGGAGCAAAGTCAAATAAGAGAAGAGAACCCAATTCTTACAATTAAAAGAGAACAAGGCAACAAATAAATTCTTGTGCAAACATGAATTTTAGTTGATAAAGGTAAAAAAAAAATTCATTGAAACACCTTACTCAAACCCCCCAAAAAAGTGAATTCTACTATGTGACTTTTGCACAGTTGCACAATTACCTAATCAATTTGTACAAATTCTCTAATAATTACCGACAAATTTCACCATTTATTTTTTACACTTGATTCCACGCTAAGATCACAAACTAGTCATGATCACTTCTTAAATCTTTTTAACTGTAAATATAAATTCTCACAAAAAAAGAACCTTCTCTTTTACTTACATCATCCCCTTTTACTCGTCAATTTATAAAAAATAGTGTCTTGCCAGTATTTTTAAATTATTATGTAGTTTTTTCTTAGTGTTAGAAATTCTCCGCATTTTCTAAATGATCATATAGTTTCTGACTAGACCAAAAGACTAGTACCAAGTAGTGGACCTAATGTAAATGTATCATCAATATTAAAACTTTAACCACAGCTAATTGGTAGCGCTTACATAGATTGTTTTTTCTTATTGCATGTTATTTTTTGGTAAGTCTATGTGTTTTTAAAGAGATCGTATGTATTTGAAAACAACTTCATTTCATGTGATTTTATGCCTACCTCATTCTTTTGAACACATACAAATTGGGTTTTCTTCTTCTTTTCACTATTCCATGATCCTCTTTTAAAAAATATTAATTCTCCACTCCGGAGTTAATAAACAAAGGAAATTTCTAGGAATAAAATTGAAGTGTCCAAGCATACAAGGTCCTAAGATTATCATCATAAACTTCTTTAAAAATATTCATTGAAAACACTTTTAGAAATGTTCTCAAATAGTTAAGAATTTAAATTCAAATATTTTTTAAAATTTGGCAAACAAATCAATTTCTTAAATAGATAACTACCTGACACCTTAAAGTTTTTTCTTTTAAACTTCTCTCAATATTCTTCTTCAGGAGAACTTCTAAAATTGTCTGATGACTTTAAGAGTCAAAGGCATAAGAAGCAAATAAATTATTTCTGCATTGTACCAAACGTATAAAAGGAAAATTCTTAAAAGGAAAAAAAAGTGTAATAAGAATTAATTGATTAAAAAAGAAAAAGCTTTAAATCTTAAAGAAAATCTTAGTTGTGGAGACGAAACAAGAATACTTTTTATTACTATGAATTCTTTTGTGCATATGGTTTTTATTCTATTTATTAACCAAATTATCACTCATCCTTCAATTTACTGCATTGTTTACCTGCGATACTTTTTTTCTGTACACACTTTATAATGATTTTTTTTTTCCATTTTAACAATGAAAATGTTGATTTCTTT

>LNC_001682

CTCCATTTGACCGTCCAAGACCAACTACAACAGCAAAAAAAAAAAAACTGGAAATCTGAAAATGAGATTCAGCTAGTTCTAAAGCTCATATTATTTGGCGCTTCGAATTCAGCTAAGTTTCTTCAAACTCTCCTCATCCTCATCCTTTTCTCATTCATCTAGATCGATGGGTTGAGCTTACAAGAAGCTAATACCACCATCACATATACATAAATGAGAATCAGATCAGTATATACTAATTTCGACAACTTTGTTTTAAGTTATCACTCTAATCAGCCAGACGCCAATCAACACAACAGGAGTCGAGGTAAACTTGCGCGGAATTTGGATTCTAT

>LNC_001683

GGCCCAAAGGCCCCTTTCCTCACAGTCAAGTGGCTCTCCATCTCTAGGAAGATTATGTCCAGGATCTGGTAATCTAAGCCTTGCTTCTTCTGGTCGGCTCGTATTAGCCTGTGCTTTATTACAGGTAGTCATTCCCCCGTTCCTTTAGTCTTTTCAAGGGTACTTTTTTCTTAAGTCGCGGTCATGTCTTGCCCCCCAGTATAGTAACCGCTTCCATGTTTTCCCCGAATTTCATCAGTTCCAGGCTCAAGTGCCTGTTCATCCATCTTCATTCCAAACAAAGGCGAACATCTCCATTGAGTGACTGTAAC

>LNC_001684

CAAAAGTCAAGAGCTGCAAACCCAGAAATCGCGGACAGCAGCCAAAATCGCGAGCAGTACCAAACCCGTACGTCACCCATCTGCAAACCCATAACCCTAGCAACTTAATCGATCACTTCGTCGTCCCCTTTCCCCCCTTTTCACGAAATTCAGCTTTTTCCCATTCCCTTTACTGTGGCTTCGAAAATAGCAGAAGAAATTCAAATATTTGTTTGAATTCTTAATGGATTTTCCCCCCAAAACGATTTTTCCTTCTTCTATATATTCTCTCTTTCAATCACTGTTTAGGGGGGGAATTTTTGAAGTGGTAATATTAGATTGATGAATAATATCAAAAACCACAAAGTCAAGTCTAGATTTCTTGGAATTTTTCTTCGTCTCATCCGAAGCTTTTAGCGGAGTACATCAGAATTCTCGAAGTTCATCTCTTCTTTGGTGTGGAAGTTGCTTCATAGTCAAGCGTCCTAGTCTCGCTGCTCCGAAAGAGGTGAAAGAAATTCCGAAGACATTTGTATCCATTGATCATTATTTGAAGTCATACATTACTCCACTAGTTGAAGAA

>LNC_001685

TATTTTTAGACTCTAGGCATCTGGAGAACAAGTGCAGTAGATTCACCCTCCCCCTCACAAACAATTAATGATAGTATGACTTAAAAGAAAATGATCCACCTTTCCTCGACCCCACCTCCACAAACCTGGCTGTTATGTGATAAATTTTGTTTATAAAGCATTTTCAACACACTCCAGGATGTGGTTACCTCCTAGTTGTGCAAACTTCTCCTAAACTTCATGCCCCAGAGAGCTCTTGCTTGTGATCTCTGGATCTGCTGTACTGTTATCTTTTGCTTGCCACAGAAGCTATTTTTTTTGTTCCAGACCCTACATGTAATGTAAAAGTCTTCCTACTCCTTCATGATATTCTTCTGTTAGTACAGTTGAATGTGGTGTGGTTAGTACATACTTCTCTGTACACAGTTTCATACTTTCTTGTTATTAGTTGTCTCAATGAAGTGTGCTC

>LNC_001686

GAGAGAAAGAAGAGAAGGAAGAGGAGAAAGAGAAGAGCAATCAAGGAGATCATCGTGGATTTTTCGTCGGGGGTGATCCCTATCAAGGTATGTGAGTCTTTAGTGTGGGTTTATCCTTCCCCCCCACATACCAAGCTCGTTTTTATGATTTGAGAAGAGATTTTTAGTTGTTGTTGAAGTTGTTGAAGTTGTTAGTTGTTGTTGATGTTGTTAGTTGTGTTGTTGAAGTTATTGTTGGTTGTGGGACAGGATTGGGCTGTGTATTTGAGTTGTAATTTATTGTATGTTGAGAGATTGATGATCCTTAGTGTTGGTTGGTTTATACCCCCACACACCAACTCGTTTTTAATTCCAAGAAAATATTTATTTTGGTTGTTGAAGTTGTTGG

>LNC_001687

TTCATATCAACAACATTACGCGTCAAATTCCAACGATTTAATTCACGGAAGCAAGCGGAGGCAGACGGAAAAGCAGTAGCAAGCAAGCAGTACTCTGCATTTTTCTTGTCTTCTTCCTCAATGTCCCTTTCCAATTTGTACCTGCAGCAGAGCACCAAGCCACGTCTCAGTCCTTGTAGCCATGAGATTACTCCACGTCGCGCCCAAGGACGAATGAATCGATCCCGGGCGCCCATCTCGCCCTTTCCTCTTTCGGAATGGGTTGAATTTGCAGGAAAAAGTATGTCTTTCTTTAATGGTGAAAGATTTTGAAGTTTGAATTTGGGGTGGGTCTCATTCCTATTCGAATTCTATCCGTTTTCCCCCCAAATCCGAACCCTAATTTTGCCTATATATATTCTACCCTTATTCACTGTAAAGGGTTGGAGAAAGAGTGGGGAGTTTTCTTGAGCAATTGATTTTTACACTTGAGAAATTCGTAATGCAGTTTTAGTTTTTGGTTTTGAAAGAGTCTTTGTTCGAAGTCAGTTCGGATATTCCGGTTATTTGCTCGAGTTTTGAAGTGGTGTAAGAGGTCGCTGCCCATTCTAGCTCGATCTGTCTCGGTTTTGCTGCACCTTTAAAGGTAACACTCACTTCGTTTCTACGTTTTGCTCGTAATGTGTTGTTAGTTGACTTTCGAATGTGTGAGCTCCTTACATGCTAGGATTTGGAATGTCTTTGATACACTACTGTTGGTTGCAAATCAGTGATAGGATTTGTTTGAAAGTCCATAGTTGTTGTTTGTTTTCATTTTCGTTGAAGTCTGATGTTTCCCCCCTCCCGAGTTCTTGTTCTCATTGTTGGAATCTTTTATTTCGCTCTTATCTCGGCTAGTGAGCTGTGAAAGTTGTGGTTCAGTCGTGAAACTCGAAATCATCTTCTACAGGTGATTTCGACTTACATGACCTAATATATAGGTTGTTTTCTACATTTATTATTTGCAAATTAAAAGTTGTGATTTATCTGTTGCTTAGTGCATCATTAATTTTCTTTATTTAATAGAATCGGCCGTATATTCCATGTAGATAAGCTAGTTAGTTCAAGTTGAGGAATATCCGTTTGTGTCGGCTGTGCTGTTCCATCCTCTTTACTGTTTCTTTGTGTTCCCGGATGTCCCTTCTAATTGTGTCGCTTCATGATTGTAGCTTTCCAATAAAGTTTAGCCTAATCTTGTATGATTCACCCCTGTTTTCGCGAGTAGTTTGAGTGATGTCTATATCTCATTGGTTCCTATTCATGATATTATGTTCGCATCTCATCTATTGCAATGCATCGATTTACCTAAAGTTCACGTTTACTTTCCAGAAAATAATTTTCTTGTCCTTGTCTAATATGAAAATCTGACATGAACTTATTTGATACTTAGCCTTGGTACTGGTTATTTCTTATCTATGAGCTTTAATGTGATGGCCTTTGCAAATTTTAATCAACACGTCATAAAATTCCTTGTTGAATTTTCAAAGTTGGCTGATATTGTTTAGTCAATTGTGCTTTTACTTATTTTAGCATTTCTCATTTTTTTTATTTAAGTCTCGATAGGTTGAGAGTGTATGTGCTTTCACTCAATTTCAGTATGTGCTTACAGTTGTGTAACACCCCAGAAATTTTTTCGAACTAAGACTTGAACCATCCTTCGTTGTGAGTAGAATTTTACCGAGGAATTAAAATTTATTAAGCGTTAAGGTCACTAGATGTAGCACCTTGAGTTCCAAAAAGAATTAAATTGGAATTAGCAAAATCTGAAATTTTACAAGTTAAGCTAAGTATGAGTTTTGGGTTGACTTCAAACGACCATAACTCTTAGCTCAGGATGATTTAGGCGTTCTACCAGTTACCGTAGGAAAGAACTTTGAATTATCTTTCCAACGCCGTCAAGTTTGCTAAGTTTCGAGTTTGGATGAGTGAGATATGGCCTTTTGAAGTTAGGCTGCCCAGTTAAGGAAAGTTAGCCGGAAATAGTAAGGGGTATTTTGGTCTTTTCCTTACCCAATCAGATTTAATTCGTTTTTAGTAATATTTTAAGGGTCTAATACTATTAGGTTCAGTTTTGTAATCCTAGTTTACGCCTAGGGTTTTAGTTGAGAGAAAAGAAGA

>LNC_001688

GTCAAAGAAAAGGATCTTACTCAGTGCTATAAATGAAGTACTCCGAAAATTATAAGTCTTGAACAGTGGTATCAATTCCTCAGGAATTCAACCTGTTGAGATGAATAATTGGAGGAAGTAAATCAAATATAAACTGATGTACTATAAGATGAAGGGCAATATTGCACAAATGATGAACGAATTCACTTTTACCTGTAGATCCAGTTAACCGTTGCAGCTTCCAAGTTGAAGAAGTCTTCACTGGTGTTGAATCATGAAGTCTGTGAGATCTGTGGACCTCCAAATAATTGCAACCCACACCTGCAAGATCTGTGAGGTTTTCCGGTGACTGATGGAAGCCATGGAAGAAGAACGAGTACTAAGTGGATGCACTTTCCCAATGTCTCTTTGTCTTCTTGTTTTTTATTTTTTTCATTTTTTAATTTCTAG

>LNC_001689

AGGCACCCTTCCAGGGGGATTTAATGAGCTATCCTTATGAACCTTGATTGGCCATGACTAAGAAATGGGGCCTTTCTTGGGAAGAGGTACTATGAGTTGGTTGAACTAGAAAAGGAACCTTCTCTAAGTACATATGTGATTGAATACTCTATGGGAAACAAGGCTAGCACCGAGTGGATGTGGTTAGGAAGGTGGCCTAGTTGAGTATGAGATTAGGCACAATGAACTTCCTTGGACATCCCCTAAACCATGTGCCTACATGGGTTGCTTACTAGTTCTACCTTTGGCAAGTAGAACAACCTCCACGGAGTAGGATAGACTCCGAATTCCATACGTAGCTAGTATGGTCTATGTCGGTTATGCCTATTCCCATCATGTGGGATGTGCACTCTAGTTATTGGATAGGTTCTACAACGTGTAGGTAGTAGTATGGGATGCTATCTACACATGGCACGAGTAGGCTTTGAAGATACTAAAGTGAGTTCCCTAAGTCTAACGACTATTATGTGAAAGTCCCCTTATGTGCTTGAATGTCTCCAACATGGTTTCTTAAAGAATGTTGGTCTATTGAAGTTATGTTGTAAATGAAATGTCCTTATCTAAGGTAATCTTGGGGAACACTTAAGTGTGCTTGAAGAGGTTGTATGGGCGGTCACTTCTTGTCTCACTCAAGTGTGTCTTAAGGTTATATTAGGTAGAGGTCTTATGGTAATGAAATGACATGTGTTCTTTTAAAGTTAAGCATGGATTGTATATGGACATATGTTGAGTATATGTATGGTTGGCTATGGTCTTATATGTTAATGTTGACTTGTATTATGTCTCTTATACTTGTAA

>LNC_001690

CACTTCTGAATTTCTCCAAAAATGAATTAGTTGCCTGACCACTTTGTACAGTTTCTGCCATGATAACGACACCAGGTCACTTTTTTTGATGCATTTCAAAATCCAACTTCAACAGCTCTACGTGCAACTATAACATTAACATCTATTTTTTCTCTTTCACATCAAGCTTTCTGCAAGGACACCTTCTTTATTTCCACATCAATATCAAACTCACTGGCTTAACATTTCAAGTGAGACACATATATGAAGATTGCATATTAAGTACATAACAACTGTGACACATAGCTATATATATCAAAACTATATATTAGGTGTTGGAGTCAAATTTTATAACATAGGTATGTACATTTTATTTACATAGGCAAAAGGAAAGTGGTCTGTTATGGCTATGCTTGCTGCAATGATAAAAAATGGCTCCAATCATATTGTAAAATGTAGGCGACATGACTTTTCACTGATGAAAAAAATTAAAATACAACAGTTAGTTGAAGTGTTGAATGTAGGAAGTAGCAGTTCTGAAAGGGTAAAATGGTATAGTTTATGGAGAAGTTTATATACCTAGTTATAGTGTTTTGGAGGGGGAGTTTTCAGCTCTAGAACATGCATGTGATGGGAAGTGGCCTCTTGCTTGTCTTTTTTGTTTGTGACAGGATTTTCAATCTTCCTTTTGCTGCTTCCTGTTGTATCCATTGG

>LNC_001691

ACACCTAAACTAAATTCCGTTTTAATTACCCCCGAACTTGTTTATTCCTCCGTCGCGTACACCTTTTTCGTCCACCTGCTCCTATTGTGACTACACGCGCGCGACAGCTGGCAAAAGTGGTGATGTGGATTTCCACCTGGATAAATAATAGTCACCTAGATAAATTAATATTGCAAAAAATAAATTTCTTAAATTTAAAATTCATTGTTTAAAAGTTATTTTTGAATAAGGGCTGATTTCGCGGAACTCCTTCTGGATTTGGGTTTTTCTAGACGTGAAGTTGGTCGATTAAATTTCAAATTTGTCTGAAATTCTTACCAAGTAAGTGTTAATCTTTGTTTCCTTTAATTTTCATTTACATTTTGCC

>LNC_001692

AATTTTGCCTTGATAGAAAAGACTTGATGTTATTTGATTTAAGTTTATTCTGATGGCTATTACCTGTAGCTAGTAATTCTGAGACTGTTTAAACTGTTGATTCCAATAACAAATTGCAAGTTATGTCTTGTGGTGATTCCGTCACCGTCGTGTCTAGTATCTCAGATTTTCTTCATTGTTATGACTTATGACCACTGGCGCAACTTCATCTTCTTTTCTCTGTTTGATCATGGTATGATATACTTTGTTGCAATAATTTTCCCCTATTCCCAGTATTTGCTGCTATTTATCTTAGCTATGTCGTTTGGGACGGATTTGAGACTGATTCACTGCTACATTTGGGCAGATCACAGGATGCATTTTGACCAAAATTTGTTACACTTGGGTGTTTGATATCTGTTATATTTTGAAAAATGTAGCTAACTGAAAC

>LNC_001693

TTAGCGACGAATTAAATTGGGTTTATCGAAAAGTTTCGTAGCTATCCTGCTTTTATTTTTCAAGTCACATGTTGGTCTACTTTATATAAACAGACTTCTATAACTGTGCATAAAGGTTATTTTTGTAGATCACTCTATATAATAGAAGCACCTATAATCCTATATAACAATAAGACACAAAAACCAGGGACATAAATTGAATAAGAATTGAACATATAAACACATTGAATATTCTACAGAATTATCTTACTTATCTATGCTTTTATCTAACTGAACAAGTTGCTTGATGTGTTTCAATTATAAGGTAAAAACAGTAAAGTAAAAAACACATTACTTAACGTGGAAACACTCGGCTTAAAAGGTATAAAAAACCACGACTTGTTCCACAACCCAATTTACTCCCCAACTTCAATAGAACAATGAGCCTCAACAAACAGGATTACAAACCTATGGAATGTACGAAATTATCAATTAAGCACTATCTAGCACAAACCTCCGAGGCTTGTGCTCCCAAGTCTTCGAGTTGCCCTCAACTTGAAGACTATGATCCTATTTTAGTTTATAACTTACTGAACTATCAATACATCAAGACACAAA

>LNC_001694

GACATCGTGATGTAAGTCTGTTATTCCTTGCATTATAGGTGTTGCTGCGTCTTGAGATCCTAATTGCCATGGTTCCGCTGCATCACAAGGAGCAATTGTGAGGAATAGCCATTCTAGAACAATCATTTGGTTTGGTTCATTCTTTGACTGCTCTGCTCCCCCCCAAAAAAAAAGAGACTGAATTTCTTCCACCTTCCCTGTACGAGTA

>LNC_001695

TTTTGAGGTGTAATTCTACTATTTTTTAAAAATATTTTTAGCGTCTAGTGCGAATTTTTGTGTTGCATATTTTAAGATTTGATGGAACTTTCTTGTTGTTTATGATTACATATTTTTCCTCACTGTTTTCGTCAAATCTAACAAAACATAAATGGTTAAATATTTGACGAAGACTAAAAGTGTTACTTTTGGACAAACTAAAAGGACCATAATTGTTCAGTGATTCTTAGAGGACTATTTTAAACCTACCACTAAACATACATGTCCATTTTTGTCATTCTCTCCTAAACTCAAATTGTGAATGGATACAAGTTTAGACCTTTGAATTTCCC

>LNC_001696

CAAATCCAAAATTTCAACTCACTCTATGCCCAAACATAATTCTAGAAGTGTCACGTCTTCCCTCCCTCTTGTTCCAACAACTATGAATCGATTTGCAATTCTTCCTGATTCCAGGTTGTGCTTGCTACTCGACTATTCTCCAAAACAGAACAACCTTCAGTTCTCCTCCTTCAACTTTAAAGAATACTTAGGAGCGTCCTCTTTCAGCGTCATCACTAAGTGTGAGATTTCATTAGTACGTGACCGTTAGGAATCATGGTTACGCTAGGCTTCCATGCCTCCAACTCTGCCATTTGGACTCACAAGGACGATTTAATATATCAGCCCTTCCAATATCAATTCAATGAACAAGGTAAGTAGTCTAATTCATTTGTTGTGGTATGTTAAATGCTATTTCTAACTTCATCGTAATTTCACAGCTAACATAATGACAATTTAGATATTTTGTCCACCTTTAAAGGTATCGAAACATCTAGATAACTATGGTCTACTCGCGTTCGTTTCTCTTCTGCATTTCTCTTTCCCGAGTAGTTCCTCCATGGCAACGCTTCAGATTTAACTCAGTTACTCAGCTTCTCCCCATTTCTACTACTTGTGCAAAAATAATCATAAGTCTCTTGTAAACTCCTTTGCTCTTATCAGTTAAGTTTCTGTTGTTACCTACAATTTAGATATGAAGATACATTTGATCCACTGCTGATTGATATTTGTTCTTTCAGAATTACTACTGCTAGATAGAATAATGTCCAGCTATTAGTAACAGGTCTATTCAGGCGATGTTGGCATTTCTTTTGACATTCAGTTTTAGTTTTCTGCTTGTACAATTCGTTAAATGCAATATGCTATTCCAGAGTACTTGTTCCCGATCTCCTCCTCCAAACTTTTCACGATCTCTACTCTGAGTTTTTTCCTTATTCTCTTATGTCTTCTTACTATTTCATGTATGATTCTCTCTCAAATATTGCTCGAATCTTTTTTTTTTTTTATTCTCGGCCTTCTTTTCTTTCTTCATGTATTTGAGTTCTTTTAACAATTTTCGATTTCATGACTGAATTCCTTGTTGTTATTGTTCTTGGTGATATTCTTATGCCTATAGAAGGAAGGAATTTGAAACTTTTTATTGAAAAAATAGCCTTTGTTACTTTAATATGATTTTTTTGAGCTATTTTCAGCATAATCTTTATTGAAAGTTCTCTTCTCACCCTTTTGTTTCAGCCAAAAGATCTTAGAAATTTAAAATAGAGAAAAAATAGCAAAATATAGTGAACTATAATTTTCTTTCAACTTTCCTTTTTCTTTTGGTTGCTATGTTTTTTTTTTTTCTTGGAAGGTATGTTGTGTGTAAAATGTCTATTAGGATTAGATATCAATGAGTACTGATTTCATGTGCATTAGACTTCTATTTGAATTTTTTTAAAAAGTCCCTTCAAATATTTTGTTAACACCCAATCTAATAATTTTTATGGATGAATAAAGTAACTCTTTTTTTCATCTCCTGTCAATTTTTTTCATTCACTCTTCTTTGTTAATATATTTGACAAAAAACATCATAGCTATAGATTAATGATAATTTTATAGTTATAGATGATCATGTTTTTGAAATACTCTTCTCAAAGTTATTGCATTTGTTCTTTCTGAATGTAGAACAATTTGATTAGTGTTATCCGTATTGTTTGACTTAATAGTAATCATCTTAGTAGAGAGATGGTCAGTGTTGGTTTTCCACTGTTTTGGTTCGATAAGAAAGAAATTTGAATTTACAATGCTTCTCCTTGAAAAGATGAACCAACCCCCGTCTTTTTACTCCCAAACACACAAAGTTGTTGATGGAGCTCTTCTTAGTCTCAGCTGAGAACTTTGATTGTATGCATTCACAGAGTCTGTCAAGAACAAGATATTGATAATTACATGTAAGTTTGACCTTTGCACAATTAATGTTTTCTTGAACAGAAATAAGTGAGGAGGTGTTCATTGAAAACTTTCGTCATAATTTTGCATTAGTCTATTAGTTTGATTTTTTCCCCCTCAAACTATATGATTGTGAATTTCTGAAAGCTGAATGAATAATGAATAACAGTTTCTTGGTTTCTGATCTCTTCTATTATGATAAATTGATAATATGGGTGACAATAGGATTGTACATATTTCAGGTAAAATCAATAAATCGAATCAAAAAATGTTATTGATTTATCGATATTGTGTTTTTGATGTAATGGTGCAGAACTATCTAGCGTTATTGGACTTTAGGGTTATCGGTTTAGGACTCGATTTGTCCTATTCCCCAAGGAGTTTTTTGGTGTGAGGTATAAGGAATTCATGGCTATTTTATCTTGCATTTGGTTGGAGGTATTAGGCAGTTCTGAGACTATTTATCTCACACTTTATACCTTAGTGATGAGATAAGTTATTCCATATATATGGTGGAATAACTTATCCCAGGATAACTTAACTAGGGATAATTAATTCTGGGATAACTTGTTTCCAACCAAACAACCCTCAGCGATTTAACCTATAACCCGATAAACTTATTATAATAAAAAATTCGATGAAATTATAAGTTAATGCTTCCAAATAAGCTAATCAGACATAGCTTCCTAATGTTGTGGGTTCTATTTCACCTATAATTAACATCTACCATAAAGAGCTTCTTTTAATGATAAGATCTAGAATACCAGTAACCTCGATAATTTTAAAATGACTATCCAATATTAATCTGTATGTATCACTATATGATCCATTACTAACTTAATGATAAGAAATTCTTACAAGTTATCTTTTGAGTGAGGTTCACATATATGTGAGTTGAAGAGTTCTCTACCAGCACTTTCTATTACTGGACAACCATTTTTTTTATCGTAGCAATATGGTTATCTATGGTGTATTCTTGATTCTTATATTGATTTTACTGATTTGCAATAATTTCAGAGAAATTAGATGCCCTC

>LNC_001697

TCCTATTTGAGATAACTTATTAAATTCCTCAACGGTATCTTCAACCACTGTCTCCCCAAATCTCACACAAAGTTCCTCCTTATATTCAGCCAAAGTAACTACTCCTCTATCCAAAAGAAGTGAATTATACCATGTTTCATCAAGTCCATTCAAATAGAGTGCAGCTCCCTTTACCTTTTGATTATCAACCGTTCTGTATAGGTTAAAGTACTTTTCACACTTACGCAACCATACCTTAGGTTCGTTCCCTTCGAAGCTTGGTAGTCCCATTTAGGAGGTTGAATTTGGCAATCTGGCCCTACCTTCCTGTATGGGATTTGGTAGCAACCCATCTCTAACTGGAAAAACACCTTCAGGAGCTCTGACTGGTGTTCTGTCTAGAGCTGTCAAACAATCCAAAATAAGCTCCAAGGTTCCCTTGATTCCTGTTTGAGTTTCCTGTAAGTCGTAGTGAGATCTCCCAAAATTTTGTCATGACTCAACAACTTATCGTCTAATAGCTTCATCTAG

>LNC_001698

CATTTGAGCCCACATAAATCTTCCCCTTCCTTCTCAATACTCCCGAGTAAAACTGCCACAAGTTAGGACTATTAGTATCCACTGTCAACTGTGTAATGAGCTCCACAGCCATAGGATCACCTTCATAGCTGGTTGCAACCTCGTGGACCCACAATGGAACTGCTACACTGATGGCATGCAACTGGCCTTCTTCGCTCAAGTGGTTCTCCTCTTGTTGGTTCGAAATTGTATCAGCTTCACTCAAGCTAATCCCTTCATGCTGCTTTGAGAGTGCATCAGCCACTCTATTCTCTGCCCCTTTCTTATACTGCACCTCATAATCTAACCCCAACAATTTGGTCAGACCTCTTTGTTGCACTGCTATAGTAACC

>LNC_001699

CGTCTCCTAAAATATCACCCTAGTAACTCGTTCGACTTTAAACATATTCCACATCATGCACCATAGCCTATATTCTCTCATGGTTTATGAATAATCAATTTGACTTGACACATATTTCAAATTATGCACCGTATTATGTGTTGTGTCAGAACCTGACCTCAATTCAACAATTATCGAACTGAGATTTAATAAAACAGTAGAGTTTTTTG

>LNC_001700

CCTTCATAACTTTCTCCCACTTTTTGTTTCCTAATGCTTCAGACAAATTTTTGGGGATAGGAATTGTGTGGAGGTTACAGAGAAAAGTTTTATGTTTTGGGGATAGATTTTGATATGTCACGAAAAGGCTCAAAGGGTGTTGTGTGCAAGTTCTGGTTCCTTTTCTGAGGGCAATGGAAAGGTCAAGATCATTAGGTTGGTTAGGTTCTTCATGATCAGTTTGATTTGGTGTTTCTGATGGTTCAACAGGTGCAGGATGGGTACTAGGAGCAG

>LNC_001701

TTTATAGTCATATTGTGTATGAATGATGGTGTGAACTAAGTACTTGAGCACAAGAAGACATAATGTAGGGATATAATCATATATAAGGAGCACAAGGGTTCTAACGGAGGATAGTGTGTACGTAGAACTTACCCCTACCTTGTGGGGTATAGATGTTGTTTTCAATAGGCCCATAATGCTAGTAAAAACATTACAATATAGTTCTAAAAAGAAAAATAACGATAGAGAAAAAGCCAAAGACAAAATGCTAAAGAAAGCATGACAAAACAATTTATTTTAATCCGGTAACATTTTGTATTCAAGTTGGTTCTTAGGTAGTACTAAAAAACCATATTTACAAGAATGAGATTAGATAAAACCTAAATGTTTTCTAGCACATCTAGGATTGATTCTGTAGCCCAGAATACAATTTAGTACACCAAAGCAAAATAACAAAATACAATTTAGCTTAATATTTTGTACTGAATTGCACCTATCCTCAAAACATCTATCATTTCTTTCTTTCCAAATTGTCCACCATATACATGAAGGGATAATCCTCCATCTCTCTCTGATACTAGTTCCAGCTCCAGCTTCCTCCCAACTTAAAAGAGTGTCAACAATCTTGCTTAGAATTGCCCATGCTACGCCCCTAAGATTGATAAAAATCTTCCTTAGCTAATTAATGATACTACAATGTAGGAACAAGTGATAACATGTAAGTTGTTAGTGCATCTAAAACTGCATTTACTAGTGTGAGTCTTTCCCAGAGATAAATACGGAGACTTCCACCTAGTCAATTTTTTCTCACACTTTTCCAGTACTAAGTCCCATATCGTTTGGATTTTGACTTTGTTCCCTGAGCCATACCTAAGTAGATAGTGGGAAGGCTTCCCACTTCACCTCCCAATATTATGGCTAGGTGCTCCATATTGGGCACATGGTTTATGGGGTATATGTAACTCTCTCTCCAATTAATTTGAAGACCTGAAATGTCTTCAAACAGGATTAGAATCACTCTTAAATACTTTAGCTACTCTTCTTCTACATCACAAAAAACTAGGGTGTCATCTGCATATTGAAGGTCGGTGATCTTCATACTTA

>LNC_001702

GAAATCAGGCCTCCTTGTGAACCCAAATGAACTTCTCCATTCTCTAACCTTGTCCAAAAAACCTTTAGATGTCAACCACCAACTTTGGAATCTAAAGTAGGACTTTGTTAGTTCCCAGCTACCACATTGAAGTGCACTAGGCACATTGTTAGATGTCAACATCTGCAAGGTATTTGTTTGATATCTCTGAAACACTTGTCCCATTCAGAAGAAAAAAGGATCCTATCTATTCTTGAAGTAGCCTCTTGGTTGTCAATTCTAAACCATGTATATGACCGCCTTCAAGCTGTGTGTCGATCAACTCCATACGCTCTATGAAATTTGAAAATTCTTGCATTGCTGAGGATCTCTTGTTGCAGTTCCTTTTCTCAAGTTGGAGTCCACAAACACTGAAGTCACCACAAATTGCCGAAAGACCCTCC

>LNC_001703

GAGTATCTTATCCTTGAGTACTTCTGAGTTGAGTAAGTTTGAGTAGTTTTGAGTACCCTTGAGTATTCCTTGAGTTGAGTAAGTTTGAGAAGAGGTAAGTATGTTTCATTTCTATCAAGTTTCAAGCTTATGTTTATGTTTTAGAATTCCTCTTGCATGCTCGTACATTCCACGTACTGAGCCATTTGGCCTGCATCTTCTCATGGATGCAGACACAGGTATTCAG

>LNC_001704

AAAAAAAAAGAATTTCAAAAAGGTGCTCTTATGCTTAAGCTTAAATTCTGGAATATTTAGATGTAGAATTGAAGCCACTTTGTAATTTCAAAGAAATTCAAGATGCATAGAGCCTGAGGAGAAAAGATGGCTGGAAAGAAAAAAAGGGGGTGAGGGATAATGATATGCCAACTATTTGTTAAGTGTATAGAGTAGCAAAATAGGAGTCAAGATGGGGGGCTAGGTAGGAAGTGAACTGCATAATTACATCATCTGTGAAGTGTTCTCCAAATGTTTGCACAAAAAGATACGTATAATTACACTCATGTATTATTGCTTTGATTTTCTTGATAAAACCATCAAGAAAATCTGGAGAATGGTCCCAACTCCCATCAGTACTTTTGGTGTGTATTCACGGAACGATATTGTTCTATGTTTTGATGGTCTACTACAAACCACTCTTTGAAAGCTAGGGGCCTATG

>LNC_001705

CACCCTGGGAAGTAAGGAAGTTCAAGTTAGAGGGCATATGAATATGAAATAGCATATGATGAGATTGGTCAATCTTTCTCATTGCTTGACTAAAGAATGCATTTTTTGGCTCTAGCACTCCCGTCATGAGACGTAGAAGCAAAAAAAAGAAAAAAAGAGTGAATAGGGCTTATGGATCATCAACACACCGGCTAAGAAAAAGTACATAAGGGATACGCCTTCCAATT

>LNC_001706

ACAATACATGTATCCTAAGAGTAATACATTTAGCTTCTAAATTCATAAACTAAGATAATTAATTCATGAATCACAAAAATCTAAGAAAACCTTTCCTTTTCCAACCCAAGGTTCAATTTTCAACAGTATGCTGTAGGACCTGGAGCAGACCACTTTTTGTATAGGAATTTTGGGTTTCACGATATTAAGGTTGATCTTTAAATTACTAATAAAACTAGAGGATCTGTTATTATATTATGTTTCTAATCTCGAAACTAATTTAGCTTATTAATATGAATTGCAGTCTCTCCAGCAGTTTATCTGGTTTTTATTGAATTATAGGTTTTCATGTCCTCATAAATGTTTTCCATAGCTTACCTTGTGCACCTTCCTCATTTTTATCAGACTAGAAGACCTTCCTTCTTCTTTTTCCGGTCACGATCTTCACTCTCTTAGTTTTACAAGGAAGCAAGGGAGTGCCTTTTATTCTGTTTAATGATAGCGAGTGCATCACTGGATGCTTGTTCCTAACTGAAGA

>LNC_001707

CTTTATAGTCGATTGTTGCTTTGTCTACAAAAGAGGCAGAATATATGGCAGCAACGGAGGCAGTGAAAGAAGCTATCTAGTTGAAAGGTTTGGTGGCAGAATTGAGTTTGGTTCAGCTGGAATCAACTCTAAAATGTGATAGTCGAAGTGCTATTCATTTGATTAAAAATCAAAGATTTCATGAGCGCACCTAACACATTGATGTCAAATTCCATTTCATTCGAGATGTCATTCAAGAGGGAGCTATCAAGGTCGAGAAGGCTATCACGGACGATAACGCTGCAGACATGGTAACCAAGATAGTCCCGCTTGCCAAGTTTGCATATTGCAAGGACTTGGCGGGAGTATGCATCAACTGATGCAACTCTGGGATAGCAACTGCTAGGTGGAGCTAGTGGTTCAACGAAGGTTTAATTATTCTTGTTTCTTACAACGAGATTGCCCAGTAAGCTTAGAAGTTTTGGCCGAAGTTGTTCATATGCATGCTCGGAATGCATACCAAGGTGGAGATTGAAAGAGTTGGTTTGGTCCTTGTGGGATCAAACCCAATGGAAGAAAATTCCATGTGCCAATTTACTTGGCTAGGAATTAAACTTGGAGATCAAGCTAAAATTTGTGGAAGACAAATTTTGTATTTCTTTTCTTTGTGCTTGGAGCCCAAGTTTGGCCACTTATAAAAGGATGGTCATTCTTCATTGTTAAAATCATCCTAAACTTATAGCAAAATACATTAGAAGAGTAGAGAATTAGTAGAGCAATTCTCTTAGATGTATTTGGGATCTCTTCCCTTTCTTTGTTAATATACA

>LNC_001708

AAGATGCTATTTTTCCAGGGAGTCCGACTCTTCAATCGAAAAAAAAGACGGTCGTGTGCGAGTCGGCTGGGAAAACACAAGCTTAACACGTCTTTCTTTATCTTTTTCATATCTGAATGTCTTTGTCGAGTTGGTTGGCATCATTCGCTCCTTTCTGTCTTTTCATTGTCCGCCTCCGCCCGAACAGATACGCACTAACCCACCTGAAATAAGGTA

>LNC_001709

AATATCATCAACGTAAACAACCAAATAGATACATAGACTTGGTGCAGAATGACGATAAAACACAGAGTGATCAGCTCCACTACGAGTCATGCCAAACTCCTGAATTACTGTGCTGAACTTCCCAAACCAAGCTCGAGGAGACTGTTTCAGACCATAGAGTGACCTACGCAATCGACATACAAGGCTACTAGACTCCCCCTGAGCAACAAAACCAGGTGGTTGCTCCATATAGACTTCTTCCTCAAGATCACCATGCAGAAAAGCATTCTTAATGTCCAACTGATGAAGAGGCCAATGACGAACGGCAGCCATAGATAGAAAAAGAC

>LNC_001710

ATTTGTTCTTATGATTTGTATGATGAATGTAAAACAACTAAATACCAGCAGTTGTAGGTATTGGGTCAGTTTTTCACTGTTGGATATGTGCAAGGAAGAACATTGTATCAAATGGACACTGGGCAGATTCCTTATGGACCATTCAGCACTTGGTTTAGGTAGTCTGAGGTCACCTTCTCATGTCAAGTATAAGCACCTGATCATACTAACAACAACAGCAACATACCCAGTGTAGTCCAAATATAAGCACCTGATCATGGGACTGTTTCTGTCATGTGCCAGAAACATGTTGAAAGCGCAACCGGTTAGATGTT

>LNC_001711

GAAGAAGTCAAGAACGATCAAGAACTTATATTAATTTCATTTCACTTGATAATATTTCAATTGTAATCATAACCCGACAAGTATCCATTCACTCTTGCTTACGACTAAAGCTTTCTCTTTCATCCTCGGAGCCTTCAGAATATCAAAGAAATACCCACTTTCTTCATCCTTTCAACTAGCTTACAAGTCCAGCGCCAACACAACTGTGATCTTTAATTGATACATAGCCGCTGGGAATCGTGGTTCCTCAAGGCTACCATGCCTCCAACTCTGTCATTTGGACTAACAATGACGATTCAATATTGGTTCAATGTACAAGGAGTTGATTTTTTTAGGCTCTAAAAAGCATTTGGTCTACCTCAATGCCAACAAATGATGTGCAACAATTCTCCAAGGTAAGTTCCATTCAAGTTATTTTGAAAATTTTGGGTTAGAATGTTGTGGCATGCTTAAATATCAACAAATCTATCTGTAAGATAACATATATCGTGTTTGAAAATTGATGCTCATGTCGGGGATGTGAATGATCTTGGCATTGGTTACCCGAATAACAATTATGTGTAGTTATGTCGAGATGTAAAGTAGATGATGCTAACATCTAAACTCTTCTTGCGGGAATCTATAATATGTAGTTGTGAATTATTCTAGGAGTAAAAATTTGAATTACATTTGATAGTTGTGAATTCTTCTAAGAGAAAATTTGAATTACGTTCGATATGATAGAAATTAGAGGGGAACAAAGTCGTTTAATTTAGAAATGATATGAGGCTCTTGTGTACTTTTTTTTTTTTGGTTTCCTACCCGATGTCCGGTATCCACATTGGAGTCCGACTAAATCCGAATTCGCACCGAAAAGTCCCACTGAGGGCAAAGAGGCTCTTGTGTACTTAATATGCCTTTATCAGACACAGAAATGAAGATAGAGACTCATGCCTAGGAGAAAACATAACATTCTTACTACTATTATTTCTAGTTTGTAACCTTGTAAGTTGTTATTTATAATTTCAGAAGGCATAGAAAATATATACATAGATTTGGCCTCAGTGTTGTCACATTGTCTTCCATTGTAGGTGTCTTTGTAATCTAGAGCAAAGATGATTGAGCCAAGTTCAACAAGTGCTTACCATGGTTGTCACG

>LNC_001712

CACTTTTTCTCTTCTTTCTCACCAATCTCTCCAAATCCGAACTCTAATCATGGATCCAAACATCAAGAAGAAGTCAAGAACGATCAAGAACTTATATTAATTTCATTTCACTTGATAATATTTCAATTGTAATCATAACCCGACAAGTATCCATTCACTCTTGCTTACGACTAAAGCTTTCTCTTTCATCCTCGGAGCCTTCAGAATATCAAAGAAATACCCACTTTCTTCATCCTTTCAACTAGCTTACAAGTCCAGAAGTAGATCATCGGCTATTCTCCTAAACAGAACAACCTTCTATTCTCTTTCTTCAGCGCCAACACAACTGTGATCTTTAATTGATACATAGCCGCTGGGAATCGTGGTTCCTCAAGGCTACCATGCCTCCAACTCTGTCATTTGGACTAACAATGACGATTCAATATTGGTTCAATGTACAAGGAGTTGATTTTTTTAGGCTCTAAAAAGCATTTGGTCTACCTCAATGCCAACAAATGATGTGCAACAATTCTCCAAGGTAAGTTCCATTCAAGTTATTTTGAAAATTTTGGGTTAGAATGTTGTGGCATGCTTAAATATCAACAAATCTATCTGTAAGATAACATATATCGTGTTTGAAAATTGATGCTCATGTCGGGGATGTGAATGATCTTGGCATTGGTTACCCGAATAACAATTATGTGTAGTTATGTCGAGATGTAAAGTAGATGATGCTAACATCTAAACTCTTCTTGCGGGAATCTATAATATGTAGTTGTGAATTATTCTAGGAGTAAAAATTTGAATTACATTTGATAGTTGTGAATTCTTCTAAGAGAAAATTTGAATTACGTTCGATATGATAGAAATTAGAGGGGAACAAAGTCGTTTAATTTAGAAATGATATGAGGCTCTTGTGTACTTTTTTTTTTTTGGTTTCCTACCCGATGTCCGGTATCCACATTGGAGTCCGACTAAATCCGAATTCGCACCGAAAAGTCCCACTGAGGGCAAAGAGGCTCTTGTGTACTTAATATGCCTTTATCAGACACAGAAATGAAGATAGAGACTCATGCCTAGGAGAAAACATAACATTCTTACTACTATTATTTCTAGTTTGTAACCTTGTAAGTTGTTATTTATAATTTCAGAAGGCATAGAAAATATATACATAGATTTGGCCTCAGTGTTGTCACATTGTCTTCCATTGTAGGTGTCTTTGTAATCTAGAGCAAAGATGATTGAGCCAAGTTCAACAAGTGCTTACCATGGTTGTCACG

>LNC_001713

AACTTATCCGAGTTCACAGGTGTCCTTGCTTTCCCTTAATAGTCTGAGGGGAACAGAATGGCAGAAGCGAAGTGAAACTATGAACAGACGACTCGATCCTTGACCTCTTATTATAGGCAATAGCGAAGGCTCGACTTATCAATATCATAAATATCATAAGAGAAGAAAAGCAAGTCAAGCAGTATGCCTGGTTCACCAGGTTCTACCACTGCAGGGCCCAATCATAATCAAAAGCGGAAAAGAGAGGATTTGCAAGAAAAAGTCGAATAGACTG

>LNC_001714

TTTGAAGAGGTTCTTGGAATTTAGCCAACGCAAGAGCTTTCTCTACCTCATGACCTTCAATTTCAGCATATGAGTGATTAAGATCTTCACCAGATCTGACACCTGCACGACCCACACCTATATCACTAAATTCATTATTAGAGACTGTGAAGACTGCGGTGTGCTTGGCTGATTTGTTGTGCTTTGTGCTGCTTGTAATTTTGGTTCCTCATTGTGCTGCCCTTCACTCCGATGACCTATATTATTCTGACCCACT

>LNC_001715

GTTCGACGCCGGAAGTAATCCCTGTTGATGTTGAAGAACCAAGATATGAGAGGGTAGAGTTTGGATAAATTTCACCTTCAACATTCACAAGTCTCACGAATGCCATGTAATTTATTCTAATAGAAGACATATTCAACTCTACCATGCTATTCATGGCCATCCACACAAAGATAAGAAACCACCTCCATTCTCTTGAACCATAATAAACAATATTGAGCTGAAACTTTCTTGACATTTCTATTTCACAGCAGGTTGAGCTAATAACCACTACTTGAGATCAAGAAATGGC

>LNC_001716

GTAAATGCCTGGCTATAAGCCTAGAAGCATCTTTCAATTTTCAATTATTTTTTTAAAAAAATAATAATAATTTCACTCCATGATATTTTCAAGTTGGAGACTGAAAGTCTGAAACTGTCTCTTTCATTCTGGGAGCTTCCAGAGTTCTTCCATTGGAGGACACACATACTCTATAAACGTTCGTTTCATTTTCCAAGAGAAGAGAGGAGTACTATGCCAATTCTCCTTAAATAAATTTAAAGAATTTCTTGCAATTTAAGGAGTTGCAGGGAGCTATAATGATTTCTTCAGCTTTCCAAACAATTCTTGGATACAGTGGGCTGGTGCCAGTATTACTTTGGTTAAATGGGATGGAATAGGACATGAGCTTACTCTCTCATCAGTATTTGTAGTGTTGTCTGTTTATCAGCATACAAGATTTATGAATACATACTTTTTTGTGGAAGTGATATCAAGTTTGAATCTACATTGTTTGTTTACTTTTTCTTATCAAATGTTGTAAGAACTTTTAGTTTCTTATTTTACTTAAACTTTCATACAAACAACAAAGGGCTATTGGCCTTCCTCCATACCTAAGAGGCAATTTTGTAGTATATTTGTAAATATAAATTTGCATTCCTTATAATTTTGTAATACAAATAATTTGTTGGGCCCGTGCATAGCACGGGCTGCACTGATCTAGTATAAGAGACTAACTCAAGCAAGGTCGTCATGCTTGCTTGCCGACCAAGGGGCACTCTCGAAATATCAGAAATTAAAAGCTTTGAGTATCTTAAGCAAAATTCGGTTAATAATTGTACTTGTTATAAAATAAAGACTAAGTAATAAAATTAAACTAATAACAACCAAGAAGAGAAGAATGTAGTAGAGAGATAAAATAGGAAATTTTTGTGTGTTTTTCCATACAAGTTACAATGTCTTTTATATAGGCATAAGAAGGGTATAATGGACATTCATTAATCCATTTACAACACTCCCCCTTGGATGTCCATGTAAAATAAAATTCTTAGTAAAGTAACAAGTATAAATAGTTGTTACTAATACGCATTACTTGCTGCCTCATTAAAAACCTTATCAGGAAAACCTAGTGGGATAAAACCTTGATTAAGGAAAAAAGAGTACAGCGCGTATTTTACTCCCCTGATGAAAATCACGATTTAGTTATCCAAGTTTTCTCATTCCAATCTTGTATACCATCTTCTCGAAAGTTGCGATTGGCAAAGACTTAGTGAATAAACCAGCCACATTATCACTCGAACGAATCTGATGTACGTTGATATCACCATTCTTCTGGAGCTCATGTGTGTATAGAAGAACTTCGGTGAAATGTGTTTCGTTCTATCTCCTTTTT

>LNC_001717

GTAAATGCCTGGCTATAAGCCTAGAAGCATCTTTCAATTTTCAATTATTTTTTTAAAAAAATAATAATAATTTCACTCCATGATATTTTCAAGTTGGAGACTGAAAGTCTGAAACTGTCTCTTTCATTCTGGGAGCTTCCAGAGTTCTTCCATTGGAGGACACACATACTCTATAAACGTTCGTTTCATTTTCCAAGAGAAGAGAGGAGTACTATGCCAATTCTCCTTAAATAAATTTAAAGAATTTCTTGCAATTTAAGAATTTCAAACAAATCATAAAGGAGTTGCAGGGAGCTATAATGATTTCTTCAGCTTTCCAAACAATTCTTGGATACAGTGGGCTGGTGCCAGTATTACTTTGGTTAAATGGGATGGAATAGGACATGAGCTTACTCTCTCATCAGTATTTGTAGTGTTGTCTGTTTATCAGCATACAAGATTTATGAATACATACTTTTTTGTGGAAGTGATATCAAGGTAATATGCTCTGACCTTAGTCTGCTATGGACTTTTATGTGTTTTTGGTTGCTTGTTATAGTGGAAATTCCTTTATGTAGTTTTTGGCATTTTTTGGGTTCTTTTTTGGTGGAGATCTTTGTCTGATACTTACTTTTACCTAATTTAGCAAGCTTATAAAATAATAGCATGTGATTTTATATGCTTTCGCTTACTCCGTTGTCTATTACGTTCTTCATGTTTTCTATTGGATATCTATCAAACTTTAAGTGGTTGGGCTCCTGCTAGAGAGCATCACAGCAAAGGCAGTCGCTTTCTTGAATAAATGTTATGAAAGCACCATTTCTCAAGGCAATATGGTAATGCCATTATCTATGAGCAGCAATATCTATAGATGCATACCAATTTTTTAAATGTTTCTCCCACAAAATATTCAGTTGAGCATCTTTCGAAGAACTGCGGTCAGCCATACTGCAAATGCAGTTGGTAGAACCCGTTCGACGATGCATTTCAAAGTAACTCGGTAAGAAATTCTTCTCATCCTTTCCGCAACTCTCTATGAGTTTAGTTGAACAATTTTTTTTTAAAAGTTAAACAAGTAACTGATAGCATTCCTACTCTTTTCTGAACCACCACGAATCCAAAAAAAACTAAAAGCTAAGCCTATATATGTCATGTACAGGCAGGTAGTGGTAGAAACTAAAAGAGTTAATGTTGCTGAACTACTTCTTTTTTTAAAATTTTTTTAACCCTTAACTCTATGGGCCTGATTTCTATAATTAAAATGGTAAATGGAGAAGATTGTTGCTTGAAAGTTGTTACTGAATGAATTTACTACTACTGAATACTCCAGATGCCTAAATCAAGTATGCTGCATCTTATATTTCAATTTTTTTTTCTTTATACATGCAAGATATTTTCTACTTTGGCTATGTCATGGTACAAGTTCTAACACTTCATTGTGTCGTGAATGTGTATACTAAGAGAATCACTAATTATTCCACCTCCTTTCACCTTTTGGAGTTGGTGGAAGGTGTTGAGTTCAAACATCAAAAGGTACATATATATTTAACATTCAAAGTTTTAAATGTAAGAGAGCGACTTTGATGAGATTAATATATTAGATGTCGACAAATGTATTTTAGGATGGATAAGATGGTTAATATATATTGTTAGTCATTTGACAAGCTATTCTCAATATAAAATATTGGGCCATCAGATAGTCGCAAACTTAGCTTAGCACTTTTTATATGTCAAATGATAATTTGTATATGTGCAGGAGAAAAAATCATGTCTGATGGTTCTAAGAATCGACTGAACAATGTTAACAAAGAATAAGATTAACATTTATACTTATATCATCACTTTTTTCATTTCAACTTTTTTATATTTTGTTCTTTTAATTAGATTTTACAGTTGTGTTTGTTTCTTTAAGGGTATTTTCATGAATTTAAGGTTGTACAATAGAGATCAATGGTAACTTGGAGCAGGTTTATCTCTATTGAATGAATTTTATGATTATTCATCTTCATTTCTTCTATTATAGCTAAACTTACTGAAATACGTATAATGTATTTTGATATTGATATTACAAAAATCAGGAAGGGAATTTATCAATGAAAAATTATTTAAAAAGTTGCCTAAAGTATCAACATTTCCTAATGGATTAGCCGCATAAGGCAGCTGCATAATCTTTAACCCAATTGAATGTACCTCATTTAGGACTTGATTAATCTAGAGTCTTCACTTTAATTGATCCACCAGTCTTGCTATATGTTACACTACTACATGTAAGTGTGGTACATTCTTTGTGATTGGATTTGATGGGAAATCTTTTCTTTTCGACTTAAGTTTGATGCTTATTGTATTTCCAAATTTATCTTTTAGGTTTTTATTTATTTATTCCTTGAAACATTTTATCTCTGCATCATTTGGAACCTCTTCCTCTTATTAGCACCACATTAAATTTGAAAATTCTTCTGACCCTTAGTTATTCAAGTTTTTTGATGAACTTTGTGTAGATTTTTCAATATTAGATATTTGTATAGTTCTTTTATTACTTTTTTGAGGCAACATGTATAGTTTTAATCAATAATCTTGCATGCTTTCATGATTTTGCTATGGATACCTTGACAAAATTAGTTGTAGGAACAAATGTTTCTATTGTAGTTATATTTAATGCGGACCCTAACGACTATTCTTTTCTTTACTTCTTTAACAGTAATAATCTATTCACATTATATTTTCTACCGTGATTATGATTGTCTTTTCGCTATCTGAACCTTCATATGCTCTTCAATTCAATTCTTTTTGTTATAATGTTTATACATTAGAAGCTCAGATCTTAGATGTGTATCTGAGTTTTTTATTCCTTTTCTTTTTCTTTTTTTAGTGTATGACACCTTGCATTAGATTAATATACTTGGATGTTGTCACTATTATTGATCTAATGAACCTAAGGAGCAAGTACTAAGATCCTTTTTAATTTTAGGGACAATACTGAATCAAATTGGAAGTGCAAAATACACGTAATCTTAGATTAACTCATGAAGAGTACAGAAAAAGCTGCA

>LNC_001718

ACATACTCTATAAACGTTCGTTTCATTTTCCAAGAGAAGAGAGGAGTACTATGCCAATTCTCCTTAAATAAATTTAAAGAATTTCTTGCAATTTAAGGTATGGCTTCTTCTCAATCTCCGCTCAAATCTTCTTCTCGAAATTTCTTAGTTGATGCTGTAGATTGGAATTTTGGGTTGAGAAAACCTGAAACTGAAATTTTGGGTTGAGGAGAAGTAGGATTTTCCTTGCTATTTTCTCAGTTGTTATTGATGTTGTTTTGCTAGCAGACAAACAAGGATTTCTTCCTAAGTTATTATAATCTCTTCAGATTTCAAGATACTCTTTCAATTCTCTTCATCTCCGCAGTTGAATGGAGTTGCAGGGAGCTATAATGATTTCTTCAGCTTTCCAAACAATTCTTGGATACAGTGGGCTGGTGCCAGTATTACTTTG

>LNC_001719

CTCACATCCACACGTTCCAAATTATTGCGACCTTCTCCCTCCAAACAACCAAAGAACATTAGCAGGAAGAAGAAGAGTAGTTAAAAATGGAATCACATTCTAAGTTTTTTGGAGTTAAGCAAAGAGATTAGGAGGCAGACAACATAAAATAGTAGAAGTAAAAGAAGAAATGTGTAAGGAGGAAAAACGAGTGGATAGAAGTAGTGTCTATAGCCAAGTTTTACTGTAGCTGCAAAAGATTGGATGTAGTAACTGTTGATTTGGATTTCCTCTACGGAGTTGTTCAACTACTTTTGCAAACTCCATTCCTGGTCTCTCAAACTCAACAAGATGCCAGTGGCAGAGAGCAAATGGAGTCAATAGTTTGAAGGATAGATTTTCTAAGGGTTCCATGCCAGCTGTATGTATGCAAGAGATAATTCAGGGCGACCTGTGAATCCAGAAACCTTGGAGACACTAAGATGATAAATTGGAGCCACTGCCTGGTGACAATTGAATCCTCAAAATAGCATCTTCTCAATTAACTACTTTGTCAAATACAAACTCTAGCATAAGCAGTAGTAGCAGGAGTAGCAAGCAAGACGACAAGGATGGTTTCTCATTAAGGAACATGTTTTGGTCTAATCTTGAGTAGCTATAAAATTCAAGTGGTGATCTGAAGACCTAACTGTGTTTAGGCAATGTTGATTGTAAATAAATATCAACATCCGTACAATTTTAAATAATGATAGATGCATGGTACAATGTACACATCTATGCAGTGTAAAATCATTTGAAATTCCAAAGGTCACAACTTGAATCTTGATTGAGTACTGATAAAAATATAATTGCAACATCCCATAAACTTTCTTTACGGAAAAGTAAGAAGAAGAGAAATGCAATTATGTATTATACATTACCAG

>LNC_001720

CTGGTGAGCTTCTCCCTCCAAGGAGTTGTTCTTCTCACATCCACACGTTCCAAATTATTGCGACCTTCTCCCTCCAAACAACCAAAGAACATTAGCAGGAAGAAGAAGAGTAGTTAAAAATGGAATCACATTCTAAGTTTTTTGGAGTTAAGCAAAGAGATTAGGAGGCAGACAACATAAAATAGTAGAAGTAAAAGAAGAAATGTGTAAGGAGGAAAAACGAGATGCCAGTGGCAGAGAGCAAATGGAGTCAATAGTTTGAAGGATAGATTTTCTAAGGGTTCCATGCCAGCTGTATGTATGCAAGAGATAATTCAGGGCGACCTGTGAATCCAGAAACCTTGGAGACACTAAGATGATAAATTGGAGCCACTGCCTGGTGACAATTGAATCCTCAAAATAGCATCTTCTCAATTAACTACTTTGTCAAATACAAACTCTAGCATAAGCAGTAGTAGCAGGAGTAGCAAGCAAGACGACAAGGATGGTTTCTCATTAAGGAACATGTTTTGGTCTAATCTTGAGTAGCTATAAAATTCAAGTGGTGATCTGAAGACCTAACTGTGTTTAGGCAATGTTGATTGTAAATAAATATCAACATCCGTACAATTTTAAATAATGATAGATGCATGGTACAATGTACACATCTATGCAGTGTAAAATCATTTGAAATTCCAAAGGTCACAACTTGAATCTTGATTGAGTACTGATAAAAATATAATTGCAACATCCCATAAACTTTCTTTACGGAAAAGTAAGAAGAAGAGAAATGCAATTATGTATTATACATTACCAG

>LNC_001721

CTCACATCCACACGTTCCAAATTATTGCGACCTTCTCCCTCCAAACAACCAAAGAACATTAGCAGGAAGAAGAAGAGTAGTTAAAAATGGAATCACATTCTAAGTTTTTTGGAGTTAAGCAAAGAGATTAGGAGGCAGACAACATAAAATAGTAGAAGTAAAAGAAGAAATGTGTAAGGAGGAAAAACGAGTGGATAGAAGTAGTGTCTATAGCCAAGTTTTACTGTAGCTGCAAAAGATTGGATGTAGTAACTGTTGATTTGGATTTCCTCTACGGAGTTGTTCAACTACTTTTGCAAACTCCATTCCTGGTCTCTCAAACTCAACAAGGTAAAATATCAGTTAATTATCTTTATGTTGGCATTATTGTGTAAAGCAAGGTTTAGTTTGTATTCGTCTCCTGGATCCTCTGGCTTAGTTGATTCTAATATGGCAGAGCTGAATGGACTTTAGAAATTTGGGGCGTTTTAGGTTGGCAATATTACTTCTGAAATGATGAAAGTCAAAATAAATTGATATAGAAAAATCAGTTCTTGATTCCGGAATGAATCGAGTTGAGAATAGTAATGTGGGTTATATAAGGTGTGAATGATGTGTTTGGGAAAAATGAATACAATGTGTGTTCTTTTAACAAGTTTAACTGATGAACAAGTTTCTATGTCCTTTGAGAGTGTGATGGTCTTTGATTATATTGTATATCAACTGAATACTCTTGTATCTTCATAGAGATTAATTAGTTTTAGTTTCTACATATAGAATAAACGCTTGTGGTTCGGACCTTCTATGGACTATGGGCATAGCAGAAGCTTG

>LNC_001722

GGCATAATACATAAAGAAGACCTTTAACTTGACATCAATTCACAACTATGACCCTAAACTTTGCCAATGCACAAGTAGGCACTTTAACTATCACAAATTTAAACAATTAAACACTTGTATATCCCAGGCAAAGTGCGTGATATACAAATGCTTCTACATGGCTGCCAACTAATTAAAATAAATACACGTGAATTTTTCAATTAAAAAACTGAAAATAAAAACAAAATAAATATCAATTAACAACTCTGACCCTATGCTTATAACGGAGAACAACGTTTTCCTCTTTCTTCTCTACAAAATCCTAACCTAAGTTCCCACATTTCATCCTTCGAATCAAAGGTAAAATTGTTCAAGAAATTGTGAAGAAAGAGAGGAAAATGGCAAAAAGCTGTTGAAGAAATTAGAAAGAAGAGAGTAAAGTGAGATTTTGATTTAAAAAAGGATATATA

>LNC_001723

CCCGCCCTCCGGGATAGAAAAAGAAAAAAATCCTAAGTGGCTTCTTGTTTGGGGTATCTGAACCGTATTCTTGTTCGGATGCTTGAATCGAACTCATCTGTTTATTTACGGCAAACCTTGACCATCGTTTCCAAGGGAAATTGATTGAATGCACGATCACGAGCCATTCCCACCGACCCATCATGAGGTCACATCTCATGAGTCTATCGTGCCAAAACTACTAATGCTAAGAATGCTTCGCCCTGACTAGCTAGTCTGGGAAGGTAGGTAAGTAAGAGGCGGATCTAGGGCTTCTTCGACTTCTTATTCATAGTTGAGATACTCCCAACCTCCACCAGCAAGCAGCACCGGAGTTAATCGTAACGTTTCGATCTGGGGGTCAGGAGCAGAGGTTGTAGTATCGTAGCCAATGCTCTTTGATCACCGTCACGAGATTTGACTATAGCTAATCAATCTCCTGCTTTCATGGAGTTTGGAATCTTAATGCTATACTTAGAGCGGATCTAGAAATCCCCAGGTAGTGGTAGTTGGCTTGGTGAAATCCGATAATCGATTGGCTTTAGAAAGGCGAAAGGCGGCTTGCCCAACCTAGACATTGTAACTGAAAGTCCTGCCTACTTCTCTTCTAGGCTTAGGGTAGTCCTAGACTACCTTCTTCTTTGCTGGCTTGGGAAAGACCCGCGGGCTATGGATCCCACTAATAGAATGGG

>LNC_001724

TGATTACATGTATGGTGGGCTATGGTCTTATATGTTAATGTTGACTTGTATTATGTCTCTTATACTTGTAAAGGAAAGGTTTTATCAAATTGATATGAAAGCATTTATTCTTTGAAAATGTCCTTTTTAGCATGTTTGTGCATGGTCTCCATACTTAGTACTTAATTGTGCTAACCCCTTTCTTTCCCTTTTTAACTAAAGTGTAGGGATTTGGAGATGGTGACATGTCATGGCTACTTGATAGGTTTCTAAGTGTTGGAGATGAAGACTTGGTGAGTCCTCATAGATTCGAGGACAATACCCTACATGTTCCTTTTGATGTATTTCTAGTATTGTTCAAGTTGTGTATGGGATTAGTCCCAAATTTTGTACTCTAAAGTATTAGATGGTTTTGAGACATCATGTATAAAGTCTAGAAAGTCTTCC

>LNC_001725

TGACGAACTAATTATAGAAATATCTCTCTATCTCTAAAGTTCACTAATTATTTGACTGTTGGCGGGTCTCTTTGTATGTGTTGTCCGGAAAGAGGAGGACTCAATGATTATTCGTTCGCCGGAACCAGAAGTTAAAATTTTGGTAGATAGGGATCCCGTAAAAACTTCTTTCGAGGAATGGGCCAGACCGGGTCATTTCTCAAGAACAATAGTCG

>LNC_001726

CAACGACACCGCTACAACATGGAGCTTGCATATCCCTCACATGGAATTGATCATATATATTTCTTTGACACTACACCTATTCTCTTTTCCTATTCCCTATTTCTAGATACGTGTCCGGATAAAACGAGACATTATAGTTAATTAGTTGTCTAGCGCATTACTTACCGTTATGCTTACTTATTTGTTTGGTTTGCTGTTATGTTACAAGAATTTGC

>LNC_001727

CAGAAAACCTCCCTAACCTAATCCCCTAAAAAAGATCAGCCACTCCCTTTCAATTTCTCTCTATTCGATTTCAGAAAATTTCCAAAATGCTTTCGGTAAAAGCAGGACTATGTTCTTCCATTTTTGTGCAAAATTGTAGTGCTTTGAAATTCTCTCTTTCATATTCAACTGATCAATCAACACAAGAAGCCAAAAGACTTTTTGAAAATATGATTTTTTTAATGTAGAAAATCACTTTAGCAACTTCAAACGACTCATTTTGGGACTCCAAGATCTACCCGTGACCTCCCTACTCTGTAAAATTTGAATTTAAAAAATAAAAAATTTGTCTTACCATTCTTTTCCTATAACTACTTCCTCATCTTCAACAAAGGAGAAACTTTTCTTAATCATAAGAACATCATACATGGTGGAAGTTATTTATAACGTTTAGCTATCACCATCCATGCCAAAATTCTTGTATTTTCTGTTTGCTTTTATCGAACTCATTAGGTTCGAGGGGTGAATCTGATCATAACAAGAGAGTAGATTCTACGCCGACGTCCTAGTTCACTACACTACAAAAGGTACGAAGACTTGATGTTTGGGTACAAGAAGTGGCATATCACCAAAAGGAACCAAGAAGGATGGACATAACAAAATTTCTTTTACTTTATTTTAAAAGTACTTTACGTTGTATTCATTCGACATGTTTTGTACCTTATTTTGATTTTTTTTTATTTAATTAATCAACAGTAATGGGATGACATATTATGACAAATATATTCCTCAGATTTACGTTAAGCCTACCTCCGACACAAAGAGGTCACAAGCATATTAAGACGCGTTTGCATGCTATCTTTACAATATATTTGAATTTACTTGATTAATTGTTTACATGTTATTCTTAC

>LNC_001728

AGAAATAAAAGGTCCCGGTAGAAAAGACTAGAACCTCCTGCCGTTCATCCTCAACTCCAACACAGTGAAAATTCTGGCCAGACCTTAACCAGATGGACAACCCCATAGTTGAGATGGGGAAGCTTGGATGATATTCACTACGGCCTTTTACAGGTTTTACAAATGACATGAATCTAACTCCATTGAAGACAACTTCAGCTCCCAAATTGGCTTCGACCACAACACCTACAGATAAGGCCTTCTTATGTCAATTAAACTATGAATTGATCATTTTTTCAATGACTTATGCTAATAATTTATTGTTCTTGAATTGAGATTTACTTATTAATTTGGGAATGTATTTAAATAAAAACTAAAATCACTGTAAGAGAATTTGAAAAGCTTGAAGAACACCCAAATCTATGAGAATTAAAATTATTTCCCCTTAGGGTGTGTTTGGTATGAAGGAAAACGTAAAATATTTTCCAATTTTCTCATGTTTGGTTGGGTAAAATGATTTTCAAATCAACTCATTTTACTCAAATTTAAGGAAAATGACTTCCCTTACAAAATTAAAGAAAATATTTTCC

>LNC_001729

TTGACAACTATTAGAAAGTTCACTAATAAAACATTGTTTATATCTTAGTAAAGGTACAAAATTATCATTTCGTTAGTTGCCAACTGATATTTTCATGATGGGAAGACTTCTATTTTTGTGTAAATTCTATCCTTCTAAGCTTCACTAGCATGGGGAAATGTAGTACATGTTGTTTCTTATCTTTCAACTTATTGGTTTGCTGATGAACAGAAACTCCCACCTTTTTTGTTTTCTAAAGTGTCATTGGACTGTCTTGGCATTCATATGGCATCATTAAGTGGCATATAGGTTTAATCAATTAGAAAGCCAGTTAAGTCAGACAATGAAATGATTGACTACAAAATACATTTCTGATCTGAGTACTAATCTAATGGACCTCTGTCCTCTTTGTACTTGAGTTATTGCACGATTAATAGAATCCTGTTTTCTGAATCATGTTTGCAC

>LNC_001730

AATACTCGAAAAAGGACTACGAGTCACCTTCTTTGAGCAATATTATATTTTTCAGAGAGAAGAATGACCAAAAGTAGACCTGACCTGAACAAGTACATCCGACCTTTCGTCTTGATTATGAATCAAGAAAGACGGGATCTAAAGGCACGCAATTCTTGATCTTTTTAGAATTCTGAAGGACATCGATCTTTCGCCTTGTGCAAGGATATCTCTCTTA

>LNC_001731

CTCAAATATTGATGCACAAGTTTGCTTGAATTATCTTACCTACACAAGTTTACTGAGATAAGCAGACTTTTGGGCATCCCAGTGACAGAAAACAGAAGTTTGAAAGCTACAGGACACAAACAAGCATGATAAGAAATATGTAATCTAATTTTGTTATATAGGATTTATATACTAACTAATTAAACATTTAGAACTACTCATCATATATGACATTTCATTGATTATGTTGGTTCCAATGTAACAAACGATAGATTCAAGTCATTCAACTTTGTTAAAGATTATAAGCGTTGAACATCTAACTTAATTCATAGTGCCAAGAAACTTAAAACACCATGCTAAAAATCTAGCTGGAAGTTCCTCCTCCTCTCAATTGAGGACTTTGTCGAATAATAGGCACTTGAAGATTTGGTAGGTCATACGTTTCATTTATCATAAGAGACTTAATAAAATCCTTTTAAAATTCTTGTTATGCACCATGAGGATCGGCAGAAAATTATGAGATAGTGCCCTACTTGTGTCTAGTAGACCGATACAGGTCTCCAAGTCTACTGAACTCTTTAAATGTTAAAGGCTAGGTGTTTGACATATGACACTCAAGACTCCAAATACAATTTATATGTGCATCTTAAACTTTGATGTCATACTTCTCCAGGTAGTATTCTTAATTAGTGTTATCAAAGGCGT

>LNC_001732

NCATCCAGAACAAAAATTCTATCCCAAAATGTACATTCTTTAACCCACCACCCCATTAGTTCTTTATGGACCATTGGATTCTTGAATAGCTTAAGCTACTACAACTTCTTTAAGCTTGTCCTCTATTGTGTATGATAATATGGAAGCAGCCATGTGTTGTTGCAAACTAACATGACCATCTGCCTGTATTTGTTTGCAATGACAAGACATTACTAGTAGCAACCACTCTGCTTTCATTGCTTCGATCTTTCCATAGTTGTTTGGTAGCTGGATAGAGTTATGTGAGTATTAGTTATGCAGGTATTACTTATGTTGGTTTTAGTTATGCAGGTATTACTTATGCGGGTATTAGTTATGCAGGTATTAGTCAAGCAAATAGTCTGGACATGAATTTTTGAAATAAAACCAAGACTATAAATTGATTTTGCCATCTACTTTGCCCTTTTCATAACCATTCCATATCCTCCCTAAATAACTCTCATAGACCATTTAGGTCTACCTGTTCAGAAAATAAGAGATTTTCCATAAATCCCCAATATTTCGATCAAGGCAGTTGCCGAATCATATTACATAGTTCTGGCAACAACAAAAGCTTAAAAAATTAGCAACTTACCCCATCATCACATCAAAACTTGGTAGAGTTCATAGCAGTAAAACTGGTATGGTATGGTGTGAAGTTTTGTCCAAATGTAAGCAGAATGATGTCCAAAACTAGCTAACATTTTTGTCTGACAATCATGGCTTTGTTGTTTTATCAACAAAAATATGACGAGGAAAACTCTCCCTCAACAAAGTTCCAGTAGATAACAACTATTTGGCTACAATTAAGATTATTCAGCAGATGTGTAATGACTTATTCCGTCATTATAGTTGTTT

>LNC_001733

TGTGTCTGTCTTTCTTTCTCATTCCTCTTTTTGTGTGACTAATAGGGTCTACTAGATGAATCTAATATTGTTTATCTTCCTATCACATATCTTGAACTTAGTCGTCCAATAACAAGAATAGTCCAAAGGTCTGGAGACATTAAGGGTCACCTTTGTAAACAACCGTCTCTCCCAATAAGGGGAACAAACAAATGGTTGGAAAAGCCAAGAAAGAGGGGGTTATGTCATTGTGAGGAATTCTTCGTGCACCTATGTGATAGGTAATATAGTTTTTTTCTGAAAAGAGTACTACTCTGCACTTTAACAACATGAGAGTGTTACTGTTAGGACACGTATCTTCCTATACCGAGCAAGATATATTAATTGACAGAGCGAATAGATTCAGGCAACAAAATATAGCCCAAGAGCAGCTCCAGGCAACTAGAAGGATTCAGATTTGTCTCAAAAAAAAAAAAGTAGCCTAGAGATTTCTAAAGATAAAACCTCCAAGTTTCTAACAACTCAAAGATCCTTAAAGGTTCTTGGAAAATATTCTTGGGTGAAATACTTAAAGCTTTATAAGCTCGAGCATGTTTCAGCAAGTGTTATAGTTCTGGTAGCATACAGACGGTGAACGTGGTTAATTACTTGGTATAATAATGTACTATTCAAAATACCAGTAGGACAACACCAAAGACTTGAGGCGATTCACTAAGTTATTATGGATTGTTTTTGTCTAGACAGTTGAGAATGAGTAACTTAATAAGGAATTTTAAGGAATTTGTTCAAAATGATTCGTTGGAACTAAGATATGACTCAGGATTATTCAATTAGCTAGATATGCTCCCTATTTATTAACTAATGGAGATTTTTTTGAAAGGATGGTTAGCCTCTTAGACTGCGCAGTAACCTCGCACACAAATGCTTTACCTACTTTGAGGTCGTTGACATTGCTAAGCACATTTTGAAAAACATGTGAAGTTATAAAAAGAGCAACCATGGATTCATGCTAGGTTGAAACTGGAAAAAAAAAAAGAGAGAGAGAAGGAAATCTCAGGCTTTAGAAAGTAGTGATGTTATACCATGATGAGGGTTCAAGATTTACTAAAGGGCATGAGGGAGGAGATATTTATTCTAATTTAGAAATGGTATTGGTAGTGACATAATTTAAAGATGTATTTTTGTGGATTTGCCAAGACCTACTAGCACGATAAATAGATTTTTCTGTTGACTTGGTGTGGAGTGCAACCCTTTTGATGCCCCTATATCGTATGACACCGAGGGAGTTCAGAAGAGATAAGTAAATAATTGTAGGTCATATTAAATAAAGGTTCATCAGACTGAAAGTATCACTGGTGTGCTTCGACTACATACTTTTGAACAAGTAACGACATAATAAATCCTAGTTGTCTGGAATAGATTTCATACATAATTGGTTACAACGAGTCATCCTCTTGTCGGAAAATCAATCCCCAGTTTGGGTTATTATCAACTTACAATCAAAGAAAGATGATATTTCTAAGACCACCTCCAATTTTGAGTGCAAATATTATGAGTTCCCTGTGATATCTCTCGGACTGGTCAATGTTCAATGTGCATTCGTGGACTTAATAAATAGGGTGTTCAAGACATTCTGTACAGATTGTTTAATAGTGTTTGCAGATGATATTTCTGTATACTCTCATAGTCGAGAAAAACATAAAGATGAGGAGGAATTGGTTACAAACACTGTGAGGCATCGACACTATGTAAATGCTAAGTACAATTGTGACTAGACTCAATAGCATTGCTTGGGTATGTTGTATCTAATGATGGGATTTTGGCGACACCTAAGTTGGAAAACGTAGTTCAGAAATGATACAAGCCCACTACTCTTACAGAGAATAACAACTTCTTGATTTTAACAGGCTTTGTGCATAGTTTCTTTGGAATAACACTACTCTAGAAATTACAACTCCACTTAATTACCAACTACAAAAAAAAAAGATGAAGTTTCAGTAAACAGAGGAATATGAATAGATGCTTTTAGAAGCTCAGAATATATATGTTTGATCACCACAACAGTGTTAGCCTTCCTGTCAAGTTTTGGAGGATTCATGATGCTCTGTGATGTTTCGAGGGTGGGATTATGACGTGTTCTCATTAAGAAAGATCGTGTGACAGTTATACTTCGAGATATCTAGATAAGCATGAGCAGGACCGCCCTCTCATAATTTGAAGGTGGTTACAATGGTATTTGCGCTAAAGTTCGGAAGCACTCTTATACAATGAAACCAATGCGAATTATACAAATCGCAAGAACCTGAGATGTAAGTTTAAAAAAAAGAAGAAGATTTGATCCTTAAATTTACACGGATGGACAAAACTACTCTGTCTATGTCAAATTTTACTATTCGACGTCATCTGGGAAACAAAGTTGTTTAGATAGATGCTTTGAAAAGGAAATCCATAGGTTATATGGAACATAATGTTCACGTGAAATGACTTTTAGCTGGTGAAGCAGTGGAAGTGTTTGGAGAGTCCAGATAATATAACAACAGGCTTCTTTGTAGGACAGGCAGGCTGGACGAGGCAGGTGAAAATTGCCTAAGTGTGGCTATTTATACAAGGGAAAATAGCTCATGTGAAAGGACTTTTAGCCTAGAATTTGCACATACTCAGGGATACAAGTATCAAATTAGTGTGAGAAACTCAGAGACACTATTTGCTTATGTTCAGCTCAAGTCTTTATTGGTAACACATATCAAGGCCAACTAATACGAGGATGAGAAGTTCTGTGAATATCGAAATGAGATTCTAGCGGGTAAGGACAAGGATACGACTTTTGATTGTTATGTGGTTTCCCATTTAACATAGATGGTTTAAGGCAGGTTATTCTTGAGGAAGTCATAACTCTAACTACACTATAGGTTCGGGATCACCAAGAAGTAATATTTCCTTAGAGAATTATATTGATAGGACAAAAAAAATGTTGCTATCCTTGTTTCTAGTTGTTTGACTGGTTAACATGTTAAAGTTGAGCATCAACGACCCTTAAGATCAATACAACAACTCGGGATCTCTAGAGTGAATATGAAAGTGTTACCATGGATTTTGTGACCCATTTAACATGAACTCCTAGAGGTTACGACTCTGTATGGTTAGTTGTAAGTCGACTCAATCGGGATATAACGGAGGAATATAGTAGAGATATGCTTGAATGGAATTTTTGACTCTATGAAGTTCCATTATTGTTTATATTGGACGGTAAATCACAATGTACAACATATTTTTGAAATTTGTTTCAGAAAACCTTGACTACATTTCTAGAGGATATTCTAAAATCTTTCGTTCTTAATGTAATTGGAGGTAGTTGAGATGCTTATCTAGCATTGGCTGAAATTGCCTATCACAAAAAATCACACTACACAATAGATGGATCCATATGAAGAATTATGATAGGTGACGTCTTTCTCCTATTAGAAAGTCTGATGTTGGAGACATTAATGTATTGGGACCAGACTTGGTATTGAAAGCTTGGAAAACGTTCAGTTAGTCAGACAAAAGTTGCTCGCAATTCAAGTAGGTTAAAGGTTTATACATAAAAGAGGAAAGGAAATTTAGAGTACATAGTTGGGGACAAAACCTTCCTAAGAGTCCATCAAGGAATAGCGTGAAGTGATTTGAAAAGAAGAAGAAGAAGAGCAAATTTGAGCTCAAAATTTGCAAGACCATATAAAATTTCTAGACCAGAGAACTCAGGTGGTATTAAAGAGGATTTCATTAGCTAGAGAGTGTGATTCGGTAACACCTGTCTTTCCACTG

>LNC_001734

CTCAATTGCTTCCTTAAAACTTTTTGAGCGAGCTACATTGAATGGGATCCCATTATCATAAAAGAAGCGAGCTACTTTTTGAATCGTCATTGCTCTTGCTTCTTTATCACACGAGTCTTTGATATTTGTTTGCCTAAGTGTTCCACGCTTTTGTAGAAACATATCCATAGGTCCCTTTCCTTTGGCCAGTTTCTTATTAGAGCCACTAGGAAAGCTTCCTCCGCCTCTTTTCTTATGATTGATCTCTTGGACTTCATCTTCTCCACCAACATTGTCTTGATATTCAAACTCATCAAATTCGGGAAACTCGTTAGTATAGACTTCCTTTCTTATTTTTTTCTCCTCCATGTAATTTTTCAGTTCCTCTCTAACCTCCTCTGGGCATTTTTTACACTTCGCTGC

>LNC_001735

AAAAGTTCAGCTGTCAAAAAGATACATTTTCTCTCTCCTTTGGGCTATTTATAACTTCCCCAAAAATTAGTTCGAAATTCCATGTGCTACCTTATCGGTTACATTAGTTGGGCTCGCCTACATGATATGGTGAATCGATGTTCTGTTCTTCAGCTCACCCTTTCTTGGTTTCAAGCTTCAAGTGTTGGAACCTCAGTTGGCGAACTCGATTGAGTCAGCCTTTCACAATCAACAAATCGCCGATTACCACCTTTACTCACCCATTCAGCCTTCATTTTAACTGTCACCTTTGGCGATAGAGCGGTTAGCTCGCCCAACTACTTTGGTGAATCGTCGTTTACACTTTGCATCGTTGACCTACCTTGAATACTTTCAGAAGTGAACAATGTTACTTTAGGCGAGGTCCGAACCTATTGGGCGATCCCCCCATTGTAATAGGCGATCCTCAGCGGGGCGTTTACCCTTTCTTTAATGTTTTTGGC

>LNC_001736

CAAAAGTCAATCTATATTCTTGGCCTTGCTTTCGTCAATTTTCTTTTATTCATCCAATCTATTATTTCCTTTTCCCTTTATTTCACAAGTAGAAAAATATTACTTTAGGTACGCCGAAGTTTTATATTATTTGAGCCAAAAGTGTTGCATTGTTTGAGCCAATGATTATACACTGCTCATGGCGAAGACTTTACACACCTTCTTAAGACAGACTTTTCATGATTTGAGCTGAAGGCATCATGTTGTCTGAGCCAAAGACTTTGCACCATCTGCGGCAAAGACTTCACATGTTTTAAGCTGATGACATCATATCATCCGAACCAAAGACTTTACACTGTCTAAGGCAAAGACGTTATGTGTTTTAAGATGATGGAATCATGCCATCCGAGCCAAGGACTTTACATCGTCTAAGGCAAAGACTCTACATGATTTAAGCCGAAAGCATCACATCATCTTGAGTCAAAGATTTTACATCGCTTAGACTATGCATAATTCGCACTCAAAGAAACAATTATTCATTATCTGGATTCATAACATTGATTATTGAATTACTAACTTAAGTGCTAATAAAACACTTGTCCTCTTTTTTTTTGTGTAGTTTGAATCATGGTTTACTTCTCTTCTTCCAAGTCGCGATACCTTGTAATAGTGAAGTGGAGACCAAATTGCTTGTAGGTACGCCATTAGGTGGTCAGTATGCTTCTCTGTGAACTTCCTGGTTTAGATGATTGGACTCTTAAGATTTTAATTTGGACTATATTAAATTTAAAGGTTATGTG

>LNC_001737

CTCATTTATCATGCTGGACACATTAACTTCAAAGAATATACTTAAAATTGGAAAAAATATGTTCAAGAAATCGTTGGGATAGGGCACAGGTTTATTGTATTTGTTGTCTTTAAAAGTTGTCACAGCACTCTCCAAGTTTGACAAATTAATTGGCATGTCGATGTAGTGCCAGCACTAGACTTTAGAACATTGAAAATGGATGTGTCCTTTCATATCACACAATTGCTCTGGTGAAATTTGACCTTCAT

>LNC_001738

CAAAAAATTGTTCTTATCTATAAGGTCACAGAGAAGGAATTAAGGATATGTTTATTGATAAAGCACCAGAAGTGGATGACTTCCTTATTGAATTCTACACTAGCCTATGAAACACTGTTAAAGCAGATGTGATCTTGTGTGTACAAGAATTTTTCCACTCAGTTGAAGCTTGTCGGAGACGATGGTGGAACTTCGACGCCGATTAGATCGAGTTTTGGACATCTCAAGAGCTTCAAGTTGTCACAATAATGGTGCCGCAGAGGACATCATTTCCAATCACTAACATTCATCAGATTATCTTGCCAACATTGGGAAAAGAAACCAAATGTTCAATTAGTTGATTCTCTGCTTTGTATGGTTAAGGCTCTTATCACAATGGATAGAGAAGGCACACCAAACTTCAGGGTCAAACTTTGAAGGAATCAATTTGTTATTAATGATAGATGATAATGTACTTCCACTAGTCTTAACTACTTATTTTAGCCTCATTTTGTATAGATGAATACTCTCATTCTAATATGGATATTTATATTCATTCTAACAACTCTCATTTATTAGTTACATTCTTCCTTTTCATAGTGAGGCTATGGTTCATGTCCTCATCACTGTACTACTTTTGTTTGATATACCTACAGGTTAGGGTCATGCCTAAATCCCAAAAAACCACCTAGTGTGGAGTGTTAAAATAAAATAAAATGTTTATTACTGCCATGAATATTTTTTTGTTCTCTGAAGTTGGGTAGCGATTTGAACTTTATTTTTATCCGATGTATTTGTACTACTTCTTACATATTCATGGATCCTTTCTAATTTGTAACAGCATCACATTGAACTTGTTTCAATTTTTCACAACATTTCTCAAATACATGATATTTTTGAAAATTTGAAACATCGTAGTAAAAGATGCTTCAAAACTTAATTTATGATTACATTTCTTAAATACGTGATACTTTCAGAAATACAAATTTGTTGCTCAAGATTCTCTGATCTTATAGCCAAGCATGATAGTGGAAGTTCACAAAAGTTGATTTGTCGAGGGTCCCAAGTCCTAGTGGATTTTCTTATTATTGTTGATAGAAATGTTGTCCGTACCTTTATGATTCCTCTCTGTGTATATTTTCGTGAAAATATAGTATGATAATTGATAGATAATGAAATTTGTTTATTAAAATAACTGAGAAAATTTCAAAAGGTAAAAAAAAAATCCTAGAGTTGGTACTAAATGTACTTCAGGTCTCTTTTCATTTTGAGGCCTAAGAAGCTAGACATTGCACAAAAATATTATACTAAATATTTAAAATATAGATTTTTTTAAAATAAAATATTTCATAATTTTATTGGTCAAAATTATTATCTAAAAGTTAAAGTTCAAAGGAAGAATTATCTTTTTTTTC

>LNC_001739

CTACTTCACTAGGAAGACGCGGTACTCATTAGCGAGCGTCTGGTGACCAACGATTCCGGATTCCCCTGCCTTTTTGCCATAGTCTACACTTGTCCGTCCAAATGGTCGACCTTGCCATCAAGTTGGGTCACTCGATCCTTGAGTGTTTCCTTAACAACAGTCGCCGCTGACAACACTCCACTGCTATCGTCATGTACATGGTACAACAAGAAGAGAATTAGCAGAAATCGATAGGTTTTTACCCAAAACTCCGCTCTGATACCTCCTAAAGAAAAATAGGCACGCGCACGGGTAGAGAGTCTGCCTGAGTCGTGCGGCCCCAGGTAACGCCTAGTTTAGTGTTGCACCTGGGTTCCGCCTTCTCTTTGTCGAGTTTTGCTTCTGATCTCTCTCTGAGTCTCTAGTTCGTTACCTTCTACTACCGATCTTCTCCCTCTGCTGCTACTGGAGCTACTGGATATCGACCTAAGAGATACTAGACCGGGTGCGAGAGGAAGGGATGCTGCTATTGATGGACCGGTGCTACCTCTGGATCTAAGAATCATGGAAGACGTAATTGAAATATGCACCTGGAATTAAATCACATTGGTAGAAAGAGCCGGAAAGGAAAAAGAGGAGGAAAGAAGCAAGGGACTGAGCGACGAAGCGAGGTTTGGAACCCTTCAATTACAAAGTGGAG

>LNC_001740

GCTGAACATCATTTGGATGGGCTCGGATCCTTTGCATGATTTCATATACCTTTGACGGGCTTTGAATTTGGACAGATCTGGATGGCCTTCTTGCATGGACTTGGGCTTGCTTTGATGATGGGCAGGCCTTTTCTTCGCATAGGCCTTCCCTATAGTACTGGGGCTAACGCGCTAGGTCGAGCGGTCGCCGAAGCTTGCTTGTTAGTATAAAACTAACGAAACCTCAAACCAACCAATATCAAGACTACTCGGGCATACTTAGCTTTTCCTCCTAAGATAAGAGCTTTCCACCAAGCTTGTCACTTTTATAAACCTCTTCACTAATTACCTAGTAACTTTTAAAAATGAATTTCCACTAGGGTAAAGTAAAATAAAGGGAAGATGAAAAAACGACTACCCTAGCCTAGTAAGTAGTTGTCTATTTCGCGTGCCCTTCCTGTACTGACTTATACTGACTTACTGTATACCGCTGTTAGTGGACTGGTCTATTGCTGTAGTATGCTGCTTACCAGTTATGCCGAAG

>LNC_001741

GAAAGAACATTATTTCGCGGATTCTTGCCATCACTGCTGGAAAACCCATTTTTCTGTAGGCATCCATCGGGTCCGAGATTATATACTGCTGCAGAAACTGAATCGAGGGGGTTGGTTGAAAGGTAAGGATAGCGTGATTGGCCGATTGGTTAGAAGGTAAGGATAGCACGATTAACTAGTTTCGGGTCTCTTGGATCATGGGCCAGAGCTACTTGGGTTTAGCCCGCTGAACATCATTTGGATGGGCTCGGATCCTTTGCATGATTTCATAT

>LNC_001742

TGCCACCTGTCAATTTTATATAATTTCCTAATCTTTGTTTTTATTTATGTTTTTTTTTTAGTTTTAATTCTTTATTTTTTCTTTCTTCGCTTAACCCATCCGTTGGTCTTCTTCGTTGTGGAGTGGCCGCCATTGCTACAACACCATTTACAACACTAAATCAAAATTAAAATTGGAAAATCAAATCCTCTTTCAAAATTTATTGTTTTCAAATTAAAATTTCTATCAAAGTCTTCTTTTTGTGTATTTTGCCTTTGATTTTTTGCTCTAAATCAACTTGGGTTGTATTTGAAAAAAACATTGAAATAACGCCGACAATTCTATCTTCAAAGTCGCCATCTTCTTCCCGGAGATTCTCACCCCAAACGGTTAAGGAGGAGGAGGAGAAATCTGAGGATGAAACAACAAATTTGTTGTTGCTTCCGTTATCAATGAGATTTTTTTGTCAGTGATTTTTCTTTTATGTACTGAGATTGTGTTATTTAGTTGCAATTAAATCTCTCCGTCGAAATCCAATCTAAATGAAAAACGAAAAGAGAGC

>LNC_001743

TGCAGATATTTCAATTCCAGCGAATGATGACGCCATATCCTCAATCCGATTAATTCTTAACAAATTAGTATTCGCAATTTGTGAGGGTCGTTCTAGCTATATAAGAAATCCTTGATTAATAATAACATAATAAGATAAATAACTTACTTCAGAAATCCCTAGAAATCCCTTATGGAATCGCTTACTATTTCTGAATTTCAAAAAATAGAA

>LNC_001744

CGAACTCTATGAGATTGAATCTCAAGGAAGAAGCGGGAAGGAAAGGTTGGTTGCACGTTTTCTGGGAGTAATGCCTCCTTTCTCTCTCCCCCAAGGACTCAGTCTCTCGGGTATCTATCAACATATAGAGATCTTGCCTTTGTCGCTGCATAGAGTGATCTTCTTCAATCTCCGAGTTCGAAGGGTAGAACAAGGGAAGGTCAAGGCTTTCCTCTCATTACTCTTTCCACGGCAAAGTCAGCATCTTA

>LNC_001745

AGAAAAAGGATGTCTTCCCTGATTTGTGAAAGAATTTTGGCATTTTGAATTTGGAATTGGGCCTTGGTTTGCCCGATTTTGTCCTTTTTCCCAATTATTCTGAACTCTAATTTCTTCTATATATTCTTGGATTATTCATTTGTAAAGGGGGGAACTTTGGTTTTGAAATTTTAGAGTTAAAAATACATAGAATAGCTTAAGAGGAAATAGCTAGAATACCTACTAACTACTCAAGGAAAAGAAAGGAGATATTAGCTTTGAGTGTTTGGGTTTGATATTCCTACCTAGATTTCTTAACCACTGTTCGCGTTCGGAATTGCTTAGTGGTGAAGTCCCCCCTGCTCGTCACCGTCCCCAGTTCTGCTGCACTCTTAAAGTCTTTGGAGGCTCCCCCTCTTCGCATGCCGTGGATTGGGCCTCCCTAGCCCGATTCTATATTTTCCATCGATTCTAGGATCTTGAGACAAATGCAGGGGGTTGGAACTAAAGTTTTCATATTCCGGAAAGCCCAAGGAAAGGGTTGTACACTTGCTGTATACAGCAATATACACTAGTATACATGGAGGATTTTAATATGCAGGTCCAAAAATTAATACAGGAGCTCGAGGAGCTGCATTAGGATGTATACATTGAGTGTACAGTCTGATATACCGTAAAAAATCCCAAAATACGGTCCATAAACAATAATATACCTTTGTTGTATACACAGGTGCTTGGGAGCGACAATGGACCCAAAAAGGTCCGAAAAAATGGCTCGGCAGCAGAATTTTGATCGAATAGGCCCAAAATCATGTCAGAAATTTTTAGAATAGGACAGGTTCAACAAATTTGGGGAATTTGGGCCAAAGGCCCCAGTTCAATACTCTTTTCCTTGCTCTTATTTATTCTGTTGTACTTGACTAACATTTGATTATCTTTTGGTTTAATTTAATTAAATTCCCG

>LNC_001746

CTGAGCTTAGTAACGTGAGCTTGTAACCCAAGTGGGAGCATTAAGATGATCGGGACGTTCAAATCTTCAGCCCTCGACAAATAGTGAGTTCATAATAAGTGCATCATGTTTACGGTGGGAGTAAAATAAAGTAATTTTATTAGGCTGGCTTAACAGAGCAGCAGTCTCACCAATGGCCCGAGTTTGGGCAAAGTTTCAAGTCAAACCACTAGAAATGTCAGGTCATTCGTTGAATTGGACCATCA

>LNC_001747

AGGCTACGGTTTCCCTCTATTAGATTGAGCTCGAGAATGTGAATGCACGTTGATTAGTTGGGATTTGGGTTGGGTAGTTATTGAATCTTGCATAGGTGTTAAGAAATCATGTTTTCGGCCAATTTCAGGAATTATCGAGTAACTGTGAGCATGCTTTGTGTTATTAATGGACCCTCCTCGCGATGTGGAGTGCTTTCATGCTTAATTCTTGTCTATTTGAAGCATTTGGGCCTTAGTTTAGGTTTGACTAGGGCTTGCCTTAGAAATACATGATTCGGATCCGATAGGCCTTAGTTTTGTCCCCGACGTCGCTCGACCGACTTAGATCCCTGTAGACTGGTGTAGCAGACTTGAGTCTGATAGATTGGGCCTTAGCTAGGCGATACGCTTGCTCCGACTTTAGTTATCCTTATTTTCCCCGATGTTACGGCTTCACGAGTTACGTCGGCGACACTAATTCTACTTCGCGATTTGAATTTGGTTC

>LNC_001748

TGTGTAATGGAGCTCTTGAAATTGTAGAAAAACTGTCATCAAAATAGGTTTTTACTTTTTGTCGTTCTTAATTTCTTTGGTTTTATCTTAATTTTGTCTCTTCAAGTAAATCTGCTCTTTGTCATTCCTGAAATTTTTCACCACAATAAAAAATTACAACACGGAGAAAGAGCAAATACAGTTCAAGTTTCCTCATCTTTTACCTCCAATTGACACACAACAATAATGTAGCTTCATCTCCTTATTATGAGTTTCCACAACATTTTCCTCTGTCAGACAACTATCCAAAAGGTCAATGCGGTTATGATCTGAACAAGGTTAGTGATATCAACTCAACAACTTTGTATACATATGTGTTTTTTGAGGCATCAAATGCTAGCATTATATATGGCTCAATTTTATGTTTACATTCAGCATATGCATGTTTGCACTTAACTCTGTATGCTGTTACTCAGTAATTGGCACCACATTGTTATATGCTAAGCATACATGTGAAAGTTGCTCTTCTAAGTACCTGCAGATACCATCTAGCCTGCCTATTCTCCC

>LNC_001749

GGGTTGTGGTGGCGTAATCTGTAGGAAGCATGAAGATGGAGGCCACCGTCAATGTGAAGAGTGAGCATGGTTCTGAATTGCAGGATATAATCTGAGCGTCTCAGTTAATGATGCATGGAGATACTGCATTTTATCCAGAGAGGGTGCTTGCACATCATATAGAAGAACCAGGAAAGTGTGCTAGCTGTAGTGTCTTTGCCAGCAATTATAAAGCTAAGGATTATGTCCTTAAGCAAATGATCGATGATTGTTCCAGCAACAGGATGGTACTTTCCCTTGTTGAAGACAATTACTTTGAACACAAATGCTGCTACTGCTAAAATGACACAAAATGCTATTATTGTGAGACTAGTAGGAATAGATAGAACATCCATCTCTTTCCTCTCCTCTTTTCAGTGTGAATGACAATGTTAAGCTGCTTGAGAATGACTTTACCATATATAAAATAATGTAAATATAAATATTTGAGATTGAGACCCCCTTATTAAAGAATGACGTAACATACTAAC

>LNC_001750

GCGTAATCTGTAGGAAGCATGAAGATGGAGGCCACCGTCAATGTGAAGAGTGAGCATGGTTCTGTATGTAACTCTTCTTTCTTCATCACTCAGCTTAAATCTAAAGGTGCCCAGCAACACTGCTGAAAAGATCTTCATCTGTCTGAATTGCAGGATATAATCTGAGCGTCTCAGTTAATGATGCATGGAGATACTGCATTTTATCCAGAGAGGGTGCTTGCACATCATATAGAAGAACCAGGAAAGTGTGCTAGCTGTAGTGTCTTTGCCAGCAATTATAAAGCTAAGGATTATGTCCTTAAGAAGAGCAAACCAGAGTTCCATACTTGAATATCTGTTGCTTGATTTGAGGTCACAGCTTCAGATACTTTGTGAGCAAGTTTTACCGCACTAATTCTGAAAACTGCACTACTAAAGTCCC

>LNC_001751

CGGGTTGTGGTGGCGTAATCTGTAGGAAGCATGAAGATGGAGGCCACCGTCAATGTGAAGAGTGAGCATGGTTCTGAATTGCAGGATATAATCTGAGCGTCTCAGTTAATGATGCATGGAGATACTGCATTTTATCCAGAGAGGGTGCTTGCACATCATATAGAAGAACCAGGAAAGTGTGCTAGCTGTAGTGTCTTTGCCAGCAATTATAAAGCTAAGGATTATGTCCTTAAGAAGAGCAAACCAGAGTTCCATACTTGAATATCTGTTGCTTGATTTGAGGTCACAGCTTCAGATACTTTGTGAGCAAGTTTTACCGCACTAATTCTGAAAACTGCACTACTAAAGTCCC

>LNC_001752

GCAATTACACATTCTGACTGGAGCTTATATTCATGTTCACAAGTATTCTGGACGACTTCATCTCTATTTAAGCACAATTATTCATAGAGTTATTGTTCACTTCAAACTCCTCATTGACTTGAACTCTCTTGACTTAGATTTCTAAGACTCCTTCTTCCATTCAAACTCCTTGCTTCTTTAGACTCTTTCTTCAAATCAAATTCATTGATTCTTTTGTATTGTAGCGAACTCCAACTCTTCAATTCAGCAGTAACTTGAAGAGTTCTACTTCAATAGAGCTTGCTATGATCAATGAGGACAGAC

>LNC_001753

ATACACATTGAGGTAAATACAAACAATTCTCTTCAAGGATACAACAAAGCCCTTTGTTTATACATGCAGATTTTTCATAGGCTACTTCTAGAGAGTCTTTATATTTATAGAACTTAAGAGATGACTCTTCATAAGAGTCATGTTCTTTCATGAACATATGACTTTTCATAAGAGTCGTGTTCTTTCATGAACATATGACTTTTCATAAAGGTCATGTTCTTTCTTGAACAAAAGACTTTTCATAATAGTCACGTTCTTTCACGAACAGATGTATCTGTTCAATTTAAGACCTCTATGTATTACAAAATACTCAACACTTGCTACTTCCACTACCATCACCCCTAGTAATGCATCATTTATGAGAGTTCGATCATTTGAAGAAAAGGCACTGAATTACACAAAGTGTCCCTAATGCTATGATGTTGACAATCAAGTCAGCAACATTTTTGAATTTAAAATATTAAAGATTATTTGGATCATTGTTTCAATATATCTCAGCCTTCAGGGCTAGCTTGCTTCCACTAGTAGCCAAGGGGAGACCTTAAGACATGGTATCGAGGTGAAGGTATGGAGGTGCGACAATGCAGCCCAATCTAGTCTTAGGAGGAGTGGGACTTTGGAAGGTACAGGAGAGAGAAAGGACATAGCTTAATGGAGGAGTTAGAATCATCTACTCGATTCCTTGTGAGAAGATTAAGGACTAGGAGCTTTTTTTTTTTTTGCTCATCTGAGTGTAAATATGAGGGACTACACCCTTCGTGTGGTTTGTTATTAATACACAAAAATGTTACCTGTTCAAAAAAAAAAAGACTTTCACCAATTTTTTGAGTGAGAAGTGGATGTTGGTGAGAAAAGGAAGTTGAGTTCAAGTTTGGAGCACATGGCATAAGCCCAAGATCTCGAGATGACTCTAAAAGCTAACGAGATGATTCTACTTATAAACCAGCATTCCTCCGAGCAAGGGAAAATCTTTCAAAAATCCATGTGGAAATTCTTTTTAATGTGGGTGACGTTGAGATTCGATCTCAGAATCTCTATCTGTTTTGATACCATGTTGAAGTCTGTGACCATGTCATCTAAAAGTTTAAGTTTAAGATGTTAGAGAGGGC

>LNC_001754

CAAATATGTTCTCAGCATATTTTCCCATGTGTCTTGTAATTTAGTCATTATGTATTTCCTTCTATTGTACTGCTTTGAGCTCTCGAGTTTAATTATCAATTTGATCAAGCTAGTAGTTTGTTTGGTTCTTGGTTGGATTGAGAATATTTGGGAGTAGTTACGGTGAGTCTTAAATTAAAAATCAGGAGCATTATTTAATGAACTTATGTGGCAGGGTTTTACTTCTTTTAATTTTTCTTATCAAGTTCCCTGATTATATGTGTGTTCATAAATTGAGTTGAGGAGTTGAACTATGCTTTGTCTTAAAAATGGGTCTTGATGAGCATCTTAAATGTAACCTTTCTAATGCATTTTCCTACCCTTTTAACTCGAAAACATCTACTTAGTTGTAT

>LNC_001755

CCGGCGAGAAATCAGCGTGAAATGCAGACTCGTCACATCAATCGCATCTCCAAGCAGCGCCAACAGTGCTCGCCCATACTCTTCGTCTTCCTTTTCTGCTTCAACAGTATGATTTGTTCAAAAAGCAGTGCCAGATGGAGTAATCTCGGCGCTTCACTCATCCTGGCATCAAGATCAATGGATACAATTCTCAATCCGTTGAGAATTGGTACTATTCTAGTTGTTTCCGTCTTCCAAGATTTCGAATTTCAAAATTTGAAATTTATCCAATCCACCGAGTTTCCCCACTAAATCGTCCATTTTCCCC

>LNC_001756

CGAATCAAGGCCTAAAGTTGGTTCGTCGAGCAAGAAGAGAGAAGGATCAACAAGGATTTCATACCCTATGCTAGTTCTTTTTCTCTCTCCCCCAGATATGCCTTTAATAAGTCCTCCACCTATTCTCGTGTGACGACATCTATGATTGTGAAATTAAGGAATGAACAGTTGCAACAATCATGTTCAATTTCTTTTGACAGTTTTAGATTGTGGTGCTTTGTAATCAAGGGCGAGAAGGAAGTTCATTGACAGACCGAAGACAAGCCGATTTGACCGTTCGACAGCTACCGTTGGAGAGCTTTCAACCGGTTTTGTACCTATACTCCATTCCTAGCTGAAAGAACCTATTCGATAACAGGGTCCAATATCCGATTTGAGGAGAGAGTGGTACCGTACTGGCTTGAAAGCGGCTTTCCTCACTCCCTGGCTGACTACAGGCATGACTACAGAAGAGATGGAATTCCTACCAGACCAGAGAGAGGTGATTCTCTAACAAAGGAAAGAAAGGAATGACTATTTATATCAAGAGACAGGCTTGCTAACCTTCGGCCATAGTGACTAGTGACAAGAGGAGTTGCTTAAGGACCTACGAAGAAAGTGACCGAAGCGGAGTCAATTCATATTAAAACAAGAGGAGAGAATTCAATGTTAATCGCCATCTCCTTGGCACACGTGACGGATCATACTTTCTTGCCTTAGACCACTATCGAGGCTATGAGTTTTCATAGGGCAGATGATCCTTACCCGTGAAGGTAGTTTACTTGCTTACTTGTTAAAGTAAGGCAGAAAGAGTACCTGTTGTTAACAAAACGGAGTCTCAAAAGCTCATTTATCGCAGTGGTCTCAAAAACCCAATTATCGTATTTAAGAAAAAGAGGTCTATAAAAGTCACTTAAGAAAGATGGCGCCTTTGCGAGGATGTCTTGAATCAGCCAATGCCAAGGCAACAAGGCTAGCATCAAACCTACCTGCCGAGAACCCTCCAACATTCCTACCAAGGTTCCCATCCCCGATGACAGAAGAAGGACGTAACCGGTGTTAAGGTTAGAAGGGTAACTGCTCTCGTAGTCGTAGCCGCTCTTGGGACTGATTCCTTAAGGAAATGGAATTCTTCCAAAATTCCCATGGAAGGTGTAGATGTTTAGGCTTGCCCCTGCTGCTGCTAGAGAATAGGTCGTCACTTTCTTTGCGGTTGATGCGGTTAAATCAACTAGTAGAAGGAAGGTGGCACAGTTAATCAACTGAATCCGCGCTTAGAAAGAATAACTTTGAAGCCACGTTTAGAATGGTCAGCACCTTTTGAGAAGAATTGCTCTTTGGAGAGCTAAATATAAGGGCGATGAAAGTTGTAAGCTGTAGAAAGGAGATTTTTCATGTTCATAGACACTTTGAAACTCATTGTGGAAATTCACTCTTTTATTCTGAGTGCGCAGAATGGTGCTTTGCGGCTACTATGGGTCTTCAAAAGGAACCGGGAACGGGGCAAAGACATCCTAAGAAGTTTGCCTATGGACTCTACCTCAGCGACCTTCTTCCGTACTTTCACGAGAAAGCAAGCTATCGACCTTATCTCCGTTAGTTTTTCAAGATCAGTGAACCCAGATGTCAACATCTCTATGGGGCCTACTCTAGTCTTTTATCTGCGGAGAGATGGTCGTTTACATGCTTACTTACTAAAGTACGGGAAAGAAATAGCTTACAAGAACAAGTAAGAGAGTTGACTTGCTTACTTTAACAAGTAAGGGAACTGAGGTTGCTTACTAAAGTAAGGGATTGTTGACCCTTTCAATCTTGTTTGTACCCACGGGGCACATTGAACACCAACTCAACTAAAAAGAGAAAAAAGGGTACTCACTCGTCGGTAAGGGAAAGGCTAGGCTTTTCTTACGCTAATTCTTCTCTTATTATATTTACTAGATTTACTATTGCACTGAGCCCTATTCTATCTCGGGTAACCTGATTACGGCTTTTCCGACTAGGCGCCCTACCTAAGCCTTACTTCCCGTCCGACTAGCATGGTGTCATATCACTCTTGAAAGAACACGAGATCTCGGTCAATCAGAGCAATTTCCTGGGGAAAGGGGCAGAACCATCCCCACCTTATCAAGACAATTTGGTACCTGTTTTCAAACCCATCTTTCGTTGAAAAATCAAACCATTCAGCTAGGGCCGGATAGTAGTCTGAAACCGACTTCGGCTTGGGCCGTGTTCTCTAAAACCCGATTAATTAACTGGGCCAGATTCCCCTGCAACATACCCTTTCTCAGAACAAGATTCCGATTCTGATTTTGATTCCTCTTCTTCCCCCGTCCACCGGAACTGGGTTTGTTTGGCAACAACCAAGCGCTAACGTCTGAAAAGGACGAAGAACGGACTCGCTCAACCACCGGAAGAAGGAAATAGAGGGAGAACAGCATTGATTGAGAGGGAGGACAACCCTTTCTTGATTAGCTAGGAATAGACATCGAGATTCACCTACGAAAGAAAGATTCTTAAGCGCTAACGTAACTCCCCGGGTATAGAAGAGAGGTTTGGTTGCTCACTAAAAGTACAGGCCGGGTCCAAACCAAATTGTGAAAGACCCCTTTTCCCTTATGTCACTCGGGAATGGGGAAACTTATGTGAAACTTTCCCTCCCCACACCTTAAATAATCATATACCGAGATCTCGTATTCGATCAATAAAGAACGAGGACTAGCTCACCAGTAAAGGAGAGGGGCGGACTAACTTGACCGGTAGAAGGTGAGAATAGTCAATGAATCGGAAGAGGAATGTTGATCGCCTTAGTTTATGGTTCCGTTCCCCTGTTCATTAATAGGGGCTCCCGAATCCGACTTATTTGAGGAACAAAGTAGCTCGACCAACGAGAGAGGAACAACTGGCTAATTTCACACTTACCTATGAGCTATGAGAAAAGAGGTTTCTGGAACAGGTAGTGGGTACCGGCTCGCCGAAGACTCATTCTTTCCTCGGGGATGACTTCTTGCTGACCTAAGCTCAAAGAGGTGAGAACTCTCAATCGAATGTGATCGACACGACAGTCCCATCCTTCTGCTTTCCTTTGTATGAAATGAGGGCCCCCCTATCACTTAAAGGCAGCCTCCTAACCCGCGGAACCTGCTTGCCCGTTGGTTAGCTGCATCTGTTCGGATGGCGGTACCGATTCTAAGTCGTTCTTGTGCTCTAAAAGACTTGCCTCATAAGAGAAAAAAATGACCTGCGGGAACTCACTCTCCTGCGCTAGTTAATCAGATGCCATCCGAGTCGCCACCAATCATGAATCATGATGGGGAGATTGGTCATCTTCTAGCTTACTCTGCTAGAACTACTATAACTTCCACGGCTGCTACTAAGCTAATAAGATCGAGGGGCCGTAGGCGGTGATTCAAAAATGTTCATTTTATTTGATAAGTAATCAAGGAGCTAATAGTATATAGCACTCAGATGAGGCTTAGTGAAAAACTAAACTGATTCAAGTTAGCCCAACCAAAGGACTAATCCAGCTACAAATGAAAGAGTAAAGCGCATTATCTTCATTCCACTTCCAATTAAATGTGTTACGCACAGTGACATTTGCCAATCCTGTTCCTGTTAGGAATCAATTTGATAAAAAATATACAGCCACGAGCTCTCTCTCTTTCTTATCCCGTCTCTTAAGGAAAAGCGCCTCGAGGATATCAATCATAATTCTAATTAATATAGTAGACAAAACCAACGATTAGCCCTGTCTCTTGCTGTTCTTCCATTCCCGACACCAAGCCAAGTCCGTGCTCTTTTTTAGTAGTCTTTGTAAATATCCTTTGGGATT

>LNC_001757

TCCTTTGTTCCCCATAGCAACTGGGGATCAAAAAGCACCAAATTTGATGACTTATTAAGGGAAAGACGAAGTAAGAGCATGCTATTGGAGACGTTGCAACATATGTCGGGAAGGCCTCCGTTGATTGTGTACGAACAAAATACGAATCCAAAGGCAGGGTAAGAAAGGGTTTAGCTAATGGAGATATTAACTCTTCCGGGAACCAGTAACGCGTAAACCATTTCAAACCAAGACCAATCAATTT

>LNC_001758

CTTTTTATAATAGCTCTATTTTATACTCTACTTTTCTCGTATGTTTATCATTCAAATTATATATGTGACATTATGTAACAATAGAAAATAATGCAATCTATCAAACCATTGCTATAACAATCTGTAAGTGAAGGTACATATGGTATATGGGGAGGGAAAAAAAGATACACATATTATATAGGAACAACCAGTAGAGACTTTTCTTTCTGTAGAGTAACACCAAACTTTTCTTTCATATCTAACTGCTTTGGATCCTCATCATTGGGTAGTGACCACTTAAAGTGATGGATCAAACTAGCTAAAATCAAGTGAACTTCTTGTCTAGCAAAGGGTAAGTCAGGGCATATTCTCCTCCCTGCACCAAATGGTATGAACTCAAAATATTGACCCCTCAAGTCTACATTCGAGTCAAGAAACCACTCAGCCCGGAAACATAATGCATCTTCCCATGTTTTAGAGTCGCGCCCTATTGCCCAA

>LNC_001759

CAACTCAAAGAAAGTTAAGAAAACCAAAAAAGGAAAGGGAAAACTAAACTAGAACTGATCTTCTTCTTGCTTGAGATTCACCTGCCATGGAGGACGAAGAAGCAACGTATTCCTTTTAGAGAAATTGATTCATATATGGCTGCAACTAGCTGAGTTTGGATCGTTTCCACTCAGTACACTCTAAATCTCACGAATGACATGGAAACGACTCCCATTGAATAACTCTTCAACACAGCTTGAGCAACGCAGATTGAGCCAGAAACTAACTAATACCACTGCATAGGCCAGGTAAATCCTATCCATTTTGCTAAAAGATCGTTCTTGTAAATCATTGCAGCACTGAAGATTC

>LNC_001760

TTGAATTTCATCACCCCTCACCATCTTGAAACAATCGTCTTCCCTCTTCACTATGTCAATCGATGCACCACAAATCGTATATAGCAATTTCATCCATCACTCTCCACAATCAAGAACTATGCACCAAAAATCTTCCAAATCAAAGCAAAATTCAAGCTTGAATACGCTTAATATAGTGACACTCAACTAATTTTCTTTAAGAAATTGTAATACTAAGGAAATTTCTAATCCTATATTCAATATCCAACAGATCTATGGAAAAAAAAATTCAACTGATTTTAACAACTAGACAAATTCCTGCAAATCTGAAAATGCAGCGCCATTCTTCATCTTCTTCATCCCGTTGCTCTGAATAATTGATGCAAAATCGCCTTAAAACTTCTTTAAATCAAGCAAAATTTTAGATATAAGCT

>LNC_001761

CTCAAATCTTCTTGGCTGTGCATATCTGGGTAGCATCCTTTTTGTCAATTTACACTAACAAGGTGGTAATGGATTCCAAAACAATTGTTGTAGTGTCCAATTTTACATCCTCAATCGAAAGAAAGGTTCAGACAGTGTTTCTATTAGAAACTATAGGATGGAATCATTGCATTAATTATAGCCGGACTGGTGTTGCTGCTCCGAGGGGTTAATGGATATGATCATGGCCATTGGTGTCACTACCTCAGCTGTGTTCAAACCTCTAAATGGACAGTAACTAGAGATGCTAGCACAAAGCCTGTAGAGCAGTAGTTAAATCATTTGTTTCTTTTAGCATGTTTTCTTCTTTCTAGTTTTTACCACTAAATATCTAGTTTCATTACTAGTATTTCTTTTGTCTTTGTTCTGCTAATTTCCT

>LNC_001762

CATTGTTTAGTTTGATCCAGCTGAAGCATTTAATCTTCATAGGTAGATTGGTCTTCCATATTAACTTCCATGGCCATCTAGCAGGTGCTCGACTTTGTGAGCATAGTTGTCTATAACATTCTTCACTGTGAAATGAGCCTTTGCTTCCCCATCTTAACCTATGTGGTTCACTTTCATCAATACTGTGAGCTTCCACCTTTGCTAACATTTCCATTTAACTCCCATTACATAAAAATTAAAATCCTAGGTATTCCCTTTAGTCCAACTCTCAATGGTTTATGAAATAATAATTATCCCCAAGCATCCCAATAACTACTCTGCACTCAAGTCACCTCATGTTGCTTGGGT

>LNC_001763

AAAGAGGCCATCGATTGTTACTAAGATCCTCTTTGAAAACAGAGAAATATTAAATATAATTCTATATTGGATAGAATTTACCAGCCAATTTTAACTTTGATATTTATAGAAAGAAAATTAAGTAATCAATTTGCATAAGCTGCCGTTCAAGTAATAGAAATGCACTAAATGAGGTGAATTTGAGAAAATGACGGACCTCACACAGTGTACTTGGTCCACATAAATACTCTTTTTGACTGTCACTCTTCATATCACGTGGTCTATAGTTCAG

>LNC_001764

GAATTCTTAGATTTTTTTTGGGGAAAGTTCTAAGCTTTATGCAAATATTTTAGTTTTCTGCTAAATTAGTCATACTATATCCATTTATCGCAGCCAACTCAATTTACAGCTTTTGACTTGTACTAATATTGTTACCTAGTTGATTGAATCTCAACCCATTTAACAACAGCTAGAAAGCCTTGCTGGTATAATGAACTTCCAATCATTCAACCAGAAACATATTTACATACTAAGTAATTAACTTGAAGTGATTA

>LNC_001765

GTCTGGTCCAGTAAATAGTTTATCAACAATAGGTTATCCATGACTTAACAAACATGGTAATGTGTATTCAATTACTTTGTAATCTACCTCTATATTCTCTGTCTAACTCTTTCAAGATTCCTTCTATGGAAGATAGACAATTGTTAGGTCTATAGTGATTATGCCCTTTCGACCAAGATTGAAATTGTATTGTTCGGATTGATGACTAAATTGATGACTAATGGAATGAGGGCTCATCTTCGTTCACTCTGCGAAAATC

>LNC_001766

AACTGAGAAACTATGGAAGTATTTCATTAACTTGAGAGGTATTACATGAACCATGCTAGAAATACTGCAGAAGTCCTAGCATGTTGGAATAGAGATGGAAATCAGTCAGGACACAAGGAGAGATAGAAGATTGTCCCTGCCTACATTTGGTGGACAATATGGAGGGAGACAAATGAGAGATGTTTTGAGAACAACGTCCCTTTCCAAAGCATTAAAATGAATTGTTTAGTACCCAAGGATCCGGGGGACATCACCTATTCTTAGATAGTTTATGAGGGAGATAGGTGTTTAGACTCTTATTGACAATCCCTCTATACTTTTGATGTAATTACTACAGGTGTTCACTATTGTCTATTGGTGTACTTTATAATACTAGTTAACTTTCTCAAAATAAAAAAATCTCAATTTCCCTCCCAAATTTTTCC

>LNC_001767

CTAAATTAGACATACTATATCCATGCGGGGAGATTTTATCACAGCCAACTTAATTTACAGCTTTTGACTTGTACTAATATTGTCACCTAGTCGATTGAATCTCTACCCATTTAACAACAGCTAGCAAGCCTTGCTGTTATAATGAACTTCCAATTATTAAGCCAGAAGCATATTTACATACTAAGTACTTAACTTGAGGTAATTATAGGTGAGAAACTAGTAGTTGTTATTGAAGACTAGGAAGACCCTGTTAAACATCTAAAAGCCCAAGGACCGTCAAAACATATTATTGTCAAAAAATGTCTACGTAATTCAAAAAAACTTTCCTTCCTACATAGGTCTAACCCTTCGAGAAGTTCAGTAAGGAAACAAACAATTTAAGGAAAGAGAACAGAAGAAGTTACAAACAAGACTACCGTATATCTTTAGTTCTATATGTATATGTTTGCCTACAGGTGGTCTGAGTTAAGAAGAACGAGAACAGTGAAGGTTAGAGATTAGGATCAGAAATGCATAAGTAGCATATGAAGTTTAGTCTCTGATAAA

>LNC_001768

TTTTAATGATATTATGGACAACTCGGAAAAGGTAGGAGGTAGAAGTAGAGAAGTAGCTTCTTTTAAAGACTTTAAGAATTTCTTGTGGAACTTAGGGGCAGTTGATTTAGGATTTAAAGGGAAGCCTTGGACTTGGTGGTGTTTCAGGGAGAATGATGGAATTATCCAAGAGAGATTAGATCGAGCCATTGTATCTCCCGGATGGCGTTTAGAATTCCCTTTGGCCCAAATAATTCATTTGAATAGTG

>LNC_001769

ATAAAACCTCACCAAATGAAACCATAACCTTTCTACGTCAATATGCAATTAATTCATAGTGCTGGTGGATTATTCCAAAGGGATCGCATTGATCCTGTGTCCCAAATTAATTAACTGGGGTCTATGGGATCATGCGATCTCTTCGGAATTCTCCATATTACTGGCTCTAATTAATCAACGACTGATCGAGGATCTAGTATCAATAAATTCAGAACGAGTATGATTTATATAGTTCGAATTGAACTCCTTGCTAATCGTACAAATACTCCGAGGTGAGATTAATTTCATAATTTCTTCACTTGAGCACTCATATGTGTACTATTGTTATTTTGTGTAATCTAATTAAAAGTATTCCTCCTAATTCTTTTCACTTTTTTCTAATTAAATTAAAAATTCACATACGGTTTTTCATTTTCAAAAGTTGTTCTATAATTTTGAACTGTGAGTACTTGCTGTTCATCATCGTTTTTCTTAAAATAATTTCAGCCTTATACAATTCCATATTTTATTTTTTAACATAAATTGACTTTATTCTTTTCATTATATTCTTGTCACCCTGGTTTATCTGTCAATATAAACAAATTTTTATACTACTCATTTTTTATTGTTATAGAACATAGTCTATATATATGCAACTTGATTTTATTAACAATCAAAGAAATATTGTCAGAATACTTAACAATAGCTTCATCCTCTTGCATTCTAAGAGATTCAAAATCTCTTTTCAAATTCAAAATTTGTATTTGCCTGACTCGGACAATTTCTTGATATTCTTTTTTTTTTAACTTTTCCCAAGCTTCTTTTGTCGTCTCACAAACAATGATTTTAGAAAAAATCGAATCCGCAACTGAATTTTGATTTACAATTTTGACTTTGAATTTTTTAATTTTTTCATTCGAATGACATTTAATTTGAGTGAAGGTAGAATTTGCAGGGAGAGGTTGTAAGGCTATATCTTTCATTATTCAAAGAGAGACTATTGCTTGCCATTATTTTTGGTTAAAAATTGGTTTAGAAAAGTCTGTGCAATGAAAAAAGCACGAGTTAAAAAAGGGTTATATTAATTTAATATATTAAAATTAAAATTTAGATATTTAATTTTACTACGTGAAAATTACTATAATTGGAATTCTTATCATATAGTGGAGAAAGTATAACTTAAAATTTTGGCCAGACTTTATATAGTTTGACTCTCAAAATTCAAAGCGTGACAAATATTTTGAGTGAATTTTGGGGTAAGATTGATTCATTAAAGACATGATTATAAAGAATAAACGGGCGAGTTAATTGAGAGGGTGGATGTTACACACCAACGTAGAGCCATTGTTTCAAGAAGATCTTGGTGACTTCACCATTTCATGCACTATTGGGGTGTTCCACTTTGTTACAACCCTTTATGACCTATAGAGAAAAGTATCAACTTGATGTTTAACATCATTTTTAAGAATTTAGTTTTA

>LNC_001770

GTTGCTGTTTACGATGATGATAATGCTGTAGACCTTTGTCATTTCTGAACTTTTAAAAGGAAAGAAAGACCTATATCATTTCTGATTATGTCAAGATTTAGTCAGATTCAGGAATGCCTTCAACTTTTTTGATTAGTAATGTGATCTCAGGACATCTGAGTGTATGCAGTTGAAAGGGAAATAAACTACTCTCGTGCATCCTTCTATAAAGCGCTTTTTTTAATTGCATTGCTCCGACATTTTCAGAAAATCTATCACAAGGGACCATAACATTTACATTTCTCCTGCAATCTTGCTTGCTTAGTTTGAAGTATCAAAAGGTACTATGAGTCCACATACTCTTGATTCATTTGTATCTGATAAGTCACGTATCTTCCATACTTACTCAAGGAGGAAGAAACAAAGAAAGATAGGTGAAGATAATGATCTACATCTTCTCAAATTTGTGGAGGTTTCACACATAGAGGATTGGGCAGTTTCTTACGTTATTAGCAGTTATTTGGCAATTCCTTATGGTTATTGTCAAGAGGACTACACAGAAGGATAAACCAATATCACAGCCTCAGTCCCTGAGAATGGAAGTGAAGAAGGAAGGAATTTGGGGAAAAACAAGCAATCGATTCCATTACAAGTGTGTGTATATTTATATAAGGAGGAAACAAAAATATCTGACTAGTAACTACCTTACAGCTGGCAATGAGCTGGATACTGCAGCTGCCACTAACT

>LNC_001771

CAAAAAAGAACCAAAACCAAAAGAAATATAGCAAACAAAAGGCAAATAGTTCTGGGTGAAGAACAAATCAATCCTTTCTGGGTTCAAATTGGGAAGGACCCAAAGTGTGTAACAAGTCAAACTGGTAAATATAGCAAACAAAAGGCAAATAGTTCTGGGTGAAGAACAAATCAATCCTTTCTGGGTTCAAATTGGGAAGGACCCAAAGTGTGTAACAAGTCAAACTGAATTAGGTAGGATTAGCAGATCTTTGTTAGGTAGAAAACAGGGAAAAAATCTCGTTTTTATCGCTGCAAATATGAACGTCAG

>LNC_001772

CAACATCACAGCGGCAACAACGACTCCCAAACGGCAACACACGGAAATCATCACGGAGTCGAGATGGAGACAAACGGAAAGCATTCCGCGTGGTCTTCTCGCCCAGACAGTACACTCGTACAGCAGGAGCAGCCTGCAGAATCATCCTCGCAGCCACGAGATCGCTCCACATCGTCGCTCCAAGGACGTTGAATAGATCCCAGCCGTTCGTCTCACCCTTTCCTCTTCGGGAATGGGTTGAAATTTCAGAAAAAGGGTGTCTCCCCTGATTTGTGAAAGGATTTGAATTTTAAATGGCCTCTTGTCCTATCCGGATTTTGGGATCTCCTTCCCCCCATTTTTCCAAACGGGAATTTCCCTCCTATATATGGTCATGTATTATTCACTAAGAGGGGAGGTTCTTCTAGGTTGTAAAATTGAGGGTCAAAAGGCGTGGTTTTTTTTAGTAGTGAACAAAAATTTCTATTTTCTTCTTAGCGTTTCTTCCTTAGTGCTGAAAATTCTGTCGGAATCTTCGGAGTTCTCCGGAGTCGCTGGTGAAATCTCGCTATCCTCTGCTCGTCTCACCCCCTGGTTCGCTGCCCTGGAGAAGTCAAAATCGGACTCAAAATATCTTCCCCTTGCATTCCTAATGAGCTGTAGAGGCTCCATTCTTCTCTATCGAGTCAGCCAAAGTAACGAAACAGGTCCTTGGAGGCAAAATGCAGGTGGAATGTGGAGATATAAGTCCCCAAAGTCCAGAAAACGAGTTGTATATACAATGTATACACTGGTATACAGGTCTAAAATATCAAAAACCAGAAATACACGTTGCAAGGGACGAAAAGGAATGTGTACATCTGGAATACGGCCCGATATACATGAAAACGAGCCTAAATACGGCCCGTATACAGTGATATACGTAAGGAATATACACAGATTTTCCCAAAGAAAAAACAGGCCCAAAGGCCTTAGACAATTTCAGCAGGTCCAAGATGTGGGAAATTTGGGCCAAAGGCCCAAAAATTTGATTATTTCCTTTCCCTCCTTTGTTATTTCATTATATTTGACTAACGATTTTGATTATCTTATGATTTAACTTAATTAAATTCCTCAAG

>LNC_001773

ATCCCCACTCATCCCCGACCGGCGCCACGCCACCATCCCCGACCGGCGCCTCGCCGCCATCCCCGACCGCAGCCACCCACCGTCGCAGCAGCAGAGGACGTCGCGGCAGCTGCAGAGGAAGTGACGCCCAGCAGAGGAAGTCACGCCCAGCCTCCATTCCGACCGGTGCTGCCTCCATCTCGACCAGCACCTCCATCCCCGACCTTCTTAGTGAAGGTGAATTTTTAG

>LNC_001774

TCTTAAACCCTTCGTGCCCCATAGCTGTCGTAGTTAGATATCAAAGTTCTCCCTCTTAATGATAATTACTTTCCTGGGGCGGTTGTGTAAGACGAATGCAAGCTGTAGGATATTAATTGATAAAGTCAAAATCTTTCTCTAGCTCTAGACTAGCTATTCCGCATTGCCCTTTCGTGTCTTATTCCAGATCTTTATTGAATCTGAAGTTAAGTACTTTCACAATATGAATTACCAAATCTATAGCCTATGTCAATAGTCATCGCCTTGTCTGACTTCCGTCTTCCATTAGCATTGCCTACTCGTAAGACATATTATACAAAGTAACCAGATCCCCTCACTCGAGTTCTTTGCATTGAGCTCGCTTGGTTCGTCTGTTCATCGGTCATTGAAGGAAAGCGTTTATGGCTAGCGTAATGTCTTCTGCCTATCTATATTCTAAGAATGTTCTCTTGCCCCCAGGTGTTCGTCATGGTCGCGG

>LNC_001775

AAAAATATGCAGCAGATCGAGAAATTGAAAAGATTGGGTCAAAGGCCCACTCTTAATTCCAAAGAATTGTATTTTGTTACTTTGGGCCTAAGCCCTTTATCATTTTAATTGTTATTTATGTGTTAGTCTAGGACAGGTGGAAGAATTGGGCCTAAGGCCCAAAGAGAAAATATTTTAGTATGTTAGAATAGGACAGGTACAAATGATTTTGATGGGCCGAGGGCCCAAAATGAAAACAGGCCCAAGTACTGG

>LNC_001776

ATTATTTTCATGTTTTGAGATTAAGTGCAAGTACTTAATTTTATTTGCCCATCAGAATAAGAGAGATGTAATTCTTAACCAGCAAAATATGCTCTAGTTACACTTGTAGAATTTTCAGATTATCTGAATAGTGTTTTAGCCAAGAGATACGAGTTGCTTGATGAATTGATTTATTGGAATTATGTGATGAAATTGGAGTGAAATGAATCAGTTTCTCTTAATTTATGTAATGCATAGTTTAAATGAATTTGGTCTATTTCCTAATTGGGCCTTTTCCTATGAGGATCAGTTGCAAAATGTACATAGAGGTCTTTGTCAACAACTTTCCCGTATGTTGTGAAAGGGAAGACAAGTTTTGGAGCTGAC

>LNC_001777

TACTTCTTCTGACACATTGCATCTAAAATTTCATCCACACAAAGTTCTCTCCTTCAAAATCTTCTCTCCTCGAATACACATCTAAAATCTTCTTCTTCATGGATCTTCAGCGATCCAGATTTTTCATGGATCTTGAAGTTTTGTTCCAGAATCCAGGTAAAAAAATTCTCTCCTCTTTATGGATACACAGATCTAAAATCTCCCCTTCTTCGTGGATACATAGATCCAGAAAAGAAAAAAAACCAATCTTGAATCACTGGTTATGTTGTTGTAATTTCAATCTTTGAGTAATCAATGTTTGTTTTTATAGTTGAAGCAATCAAGATTCAATTTTTATTGCTTTTTAAAGTTACTTCCAGATCTTGTTTGTTTTATAGATATAAGCTTAAAAACTGAAATCCAATTTCATTGCTCTTAGACACGCCCTTAAGTTCTAACCATATCTGTATCATTCTATTGGGCATAGTCTGTTCCAGTATAAACAGTTTGGCTTCAGGTTTCTCCTCATCTAGTGCTTCTCCTAAAGCTAATCTAGGCTTCTCCTAAAGGTTTTTTCTATGTTCATCTCTACAGGTTTTTCATGTTATCACAAACCTGGATCTGAAACTTTGTTCCTTTTCATGGATACAGATCTAATATATCTCCCACTCTTCAAGGATACATGATTCATAGATCATAATGAATCAGCACAAGAATTACGGTTTATGAAGAAGTGAAGAAAAATGACAGAAGTCAACACCAACAACATGCTGCCCCCAGCATATTATTTCAAGCGGATCATCCTTTGAATCCACACAAATTAGCAAGAACCTCATTCTGCAGAAAAAATCAGAGTAGGAACACTAACCTCTCTTACTGCTGTGACCTCTGTATGAGCTGTAGGGTCAGTATGCTTCAAACCCATCTTATGGCAGCTAACAACTACTTCATTATTGCAAACAACATTATTTGTCGTTTGTCATAAATTAGATATCTCTACTTAAGCATCATTTGAGAAGAAAACTACTCAAGCACTGCAATATCAAACAAATTCAAGCCCAATGAATGGGATGATGTACAAATTTCAACAAGATCTGAGTAGATTGAACACATTGCACTGGAAAGTTAAAAGCATTAATCCAAAGACATCACGACAGAATTACCGGATAAAGCATAGGTTTTGTTATTACTTCATTGAAAATCTCCCATCATAATCCTGATAACTACTTCATAGAAACATCCCCATCAATCTTGGCCACTAGACAGTGGTATTGTTCCCTCCCTTGCCACCATTTTTTCCTTGCTTGTCATTCTTTCCTCCCTTGGTAGCTGCTGCTTGAACATCTTTGTCATGTCGTGCCTGAAAGTACATGTATAAAAAACAGGGAAGTTCTATAATCCAATAAAACAATTGCATCATTACTGTACCTTAGTTCCTTTCTTGCACTTGGAAACATGGTCCATCAAAATACTCACCCAGCCTGCTTCAGTTGGAACAGTAAATCCGCATGTTGCACAAACATTACTCATATCTGAAACAGGAAATTTGTACAATTTGTCCATTCATCGAGGAAAGAAAAAAATTACATCCTGGAACTAGGAATATGAATGTAAACAGCAAATGCTCCCAGTACAAAAGCATTAGGTTAATCTTTTTTCCCCCAATTTATGGGACTCCCAGTTTACCATTTAAATAAGTAAAGATCTATGGAACTCCCAATTTACCATTTGAATCAATTGACTACAATCAAGCTTAAATAGATCAATTGAACTCCCAATTTACCATTTAAATCAATCGAAC

>LNC_001778

TTTAGCCCTAAGTCTTTACTTTAACTTTGTACTTCTTCTGACACATTGCATCTAAAATTTCATCCACACAAAGTTCTCTCCTTCAAAATCTTCTCTCCTCGAATACACATCTAAAATCTTCTTCTTCATGGATCTTCAGCGATCCAGATTTTTCATGGATCTTGAAGTTTTGTTCCAGAATCCAGATCTAATATATCTCCCACTCTTCAAGGATACATGATTCATAGATCATAATGAATCAGCACAAGAATTACGGTTTATGAAGAAGTGAAGAAAAATGACAGAAGTCAACACCAACAACATGCTGCCCCCAGCATATTATTTCAAGCGGATCATCCTTTGAATCCACACAAATTAGCAAGAACCTCATTCTGCAGAAAAAATCAGAGTAGGAACACTAACCTCTCTTACTGCTGTGACCTCTGTATGAGCTGTAGGGTCAGTATGCTTCAAACCCATCTTATGGCAGCTAACAACTACTTCATTATTGCAAACAACATTATTTGTCGTTTGTCATAAATTAGATATCTCTACTTAAGCATCATTTGAGAAGAAAACTACTCAAGCACTGCAATATCAAACAAATTCAAGCCCAATGAATGGGATGATGTACAAATTTCAACAAGATCTGAGTAGATTGAACACATTGCACTGGAAAGTTAAAAGCATTAATCCAAAGACATCACGACAGAATTACCGGATAAAGCATAGGTTTTGTTATTACTTCATTGAAAATCTCCCATCATAATCCTGATAACTACTTCATAGAAACATCCCCATCAATCTTGGCCACTAGACAGTGGTATTGTTCCCTCCCTTGCCACCATTTTTTCCTTGCTTGTCATTCTTTCCTCCCTTGGTAGCTGCTGCTTGAACATCTTTGTCATGTCGTGCCTGAAAGTACATGTATAAAAAACAGGGAAGTTCTATAATCCAATAAAACAATTGCATCATTACTGTACCTTAGTTCCTTTCTTGCACTTGGAAACATGGTCCATCAAAATACTCACCCAGCCTGCTTCAGTTGGAACAGTAAATCCGCATGTTGCACAAACATTACTCATATCTGAAACAGGAAATTTGTACAATTTGTCCATTCATCGAGGAAAGAAAAAAATTACATCCTGGAACTAGGAATATGAATGTAAACAGCAAATGCTCCCAGTACAAAAGCATTAGGTTAATCTTTTTTCCCCCAATTTATGGGACTCCCAGTTTACCATTTAAATAAGTAAAGATCTATGGAACTCCCAATTTACCATTTGAATCAATTGACTACAATCAAGCTTAAATAGATCAATTGAACTCCCAATTTACCATTTAAATCAATCGAAC

>LNC_001779

GACACCCCTTTAGCCCTAAGTCTTTACTTTAACTTTGTACTTCTTCTGACACATTGCATCTAAAATTTCATCCACACAAAGTTCTCTCCTTCAAAATCTTCTCTCCTCGAATACACATCTAAAATCTTCTTCTTCATGGATCTTCAGCGATCCAGATTTTTCATGGATCTTGAAGTTTTGTTCCAGAATCCAGGTTTTTCATGTTATCACAAACCTGGATCTGAAACTTTGTTCCTTTTCATGGATACAGATCTAATATATCTCCCACTCTTCAAGGATACATGATTCATAGATCATAATGAATCAGCACAAGAATTACGGTTTATGAAGAAGTGAAGAAAAATGACAGAAGTCAACACCAACAACATGCTGCCCCCAGCATATTATTTCAAGCGGATCATCCTTTGAATCCACACAAATTAGCAAGAACCTCATTCTGCAGAAAAAATCAGAGTAGGAACACTAACCTCTCTTACTGCTGTGACCTCTGTATGAGCTGTAGGGTCAGTATGCTTCAAACCCATCTTATGGCAGCTAACAACTACTTCATTATTGCAAACAACATTATTTGTCGTTTGTCATAAATTAGATATCTCTACTTAAGCATCATTTGAGAAGAAAACTACTCAAGCACTGCAATATCAAACAAATTCAAGCCCAATGAATGGGATGATGTACAAATTTCAACAAGATCTGAGTAGATTGAACACATTGCACTGGAAAGTTAAAAGCATTAATCCAAAGACATCACGACAGAATTACCGGATAAAGCATAGGTTTTGTTATTACTTCATTGAAAATCTCCCATCATAATCCTGATAACTACTTCATAGAAACATCCCCATCAATCTTGGCCACTAGACAGTGGTATTGTTCCCTCCCTTGCCACCATTTTTTCCTTGCTTGTCATTCTTTCCTCCCTTGGTAGCTGCTGCTTGAACATCTTTGTCATGTCGTGCCTGAAAGTACATGTATAAAAAACAGGGAAGTTCTATAATCCAATAAAACAATTGCATCATTACTGTACCTTAGTTCCTTTCTTGCACTTGGAAACATGGTCCATCAAAATACTCACCCAGCCTGCTTCAGTTGGAACAGTAAATCCGCATGTTGCACAAACATTACTCATATCTGAAACAGGAAATTTGTACAATTTGTCCATTCATCGAGGAAAGAAAAAAATTACATCCTGGAACTAGGAATATGAATGTAAACAGCAAATGCTCCCAGTACAAAAGCATTAGGTTAATCTTTTTTCCCCCAATTTATGGGACTCCCAGTTTACCATTTAAATAAGTAAAGATCTATGGAACTCCCAATTTACCATTTGAATCAATTGACTACAATCAAGCTTAAATAGATCAATTGAACTCCCAATTTACCATTTAAATCAATCGAAC

>LNC_001780

AAAAGAAGAAGTCATTTCAATCTTCTATATATAAAAATAATTTTTTTATTATATATAGAAGTGAACGTTCTCTTATGAAAAGGACATAGAATTAAAAATAAATTTTTAAAGGACCGGAGAACAAAATCTTCCGTTTTCTTCGATGTGCTGCTTATCTGAGTAAACAATTTGTACATGTGAGACCAATTAAGGTAATACAATATTCTTTATGACTAGTAAACATACTAATTAATGTATTCTAGATTTCATTAGTGAGGAGGACTAGAGATTTATTTCCTTGTGATTTCCTATTAAAAGGAGTATATTTTTCAATCTTAATTATTCCAGGTGGTCATATTTGATTTTGACCAATATAATCGAAGCATCGTTTTATCCATTTCAGCAGCAATCCATGTACAAATTAAAAGTTGCGTTCAATATTCAAATTGTTTTATCCGCTTTATTATATGCTTGTATCCATTTATGTCTTTTACTGACATTCTTCTTTCTCTTTTCCCAATAAAAATAATGGAATCATTTACAGATTCGTTTTCTTATTTCTATGCACGGGAGGGGGAAGACTGAAAGACAAAAAAGAAGAAGCAATGAGTGCCACAGACTCACAGGGCCCAAGTAATCCCAAAAGGCCAAAACTTAAAGAGGCAAAAGGACATGACAAAGCAGAAGCAAGTTCATTCTTCCCATGCTGACATTTCTTTTTGACATTTTGAATCAAACATTGATTAGCACTACAAAAAATACCAACTAATTTCAGGGGGGCGTTTGATGGTGGAGATGGGGGATACAATGGGTGGAGGTGAAGATGAGGTGCCTTAGAGGATGGGGAAGACACAATCAATGTAAACATGTTTTTCCTACTTCAAATAAGGGAGTCATTTTCCTAATTGTTAAGCAACTTCTTTTTTTTCCTGAAAATTCTTTTTCTCCATACCGAACACACCATAA

>LNC_001781

TTAAAGCTTGAAGCTTTGTCCATCAATGGTGGTTGTATTTTCTCAAACCCTTTCAACAATAACAAATCGATTTGAGCTTGAAATTTTCGATTTGAATGTAGACCCACTTTAAAGCTTGAAGCTTTGTCCATCAATGGTGGTAGTATTTTCTCAACCCCTTTCAACAAATAATAAATTGATTTGAACCAAAAAAAAAATGAAATTGATTTCTTGATTTTGAT

>LNC_001782

AGAAAGGGTTGGGCCACATCGGAGCCCACCCTACAAACACATTATCACTGCTTTGCTAAACTGCCAACTACGCCTTAAAATTGGGAATTGATCAAGGGAAAAGCTATGTGAAGTAAAACTAAGGTAGTCGCCATAAGGCGACTTTTCTTTTTTTCGGTATGTCGCTCCGCGAGCAAGGAGTGCCACGCACGAGCGGAGCAAAAAGAAAGCAAGGGAAACTCTTCTCTTTTTTGGGGAGAAATCCTTTGATTGCGTATTGAATATAGATCCATGTCTTTC

>LNC_001783

CTGAGATGTGGAAATGAACTGTTAGTAAGGGTTATTATTGGTTTGTCAACATGTATTTATTCAATGAATGGTAGATGGCATCTTTTGCCTTTTTATTAACAGGACTTAGTGACTGTTTGGTAGTAGTTCTCCATGCTAAAACTTTTAATCATGTTGTTCTGCCACCAAAACGAGGAGGCGAGCTCCATCTCTTAACAAGTTATTCTACAGCAAATCTACCC

>LNC_001784

CAAAGACACCCTTAGTGACCATTGATATTTATGTAAAGAGAGAAAAGAAGATAGAGAAACTCTTGTTGAGTTGAGTGATTAACGACATACATGAGAGTTCTTTGTATATGAGTTGGAGCAAATAAATAAGACAGCTTCCCTAGTTCTTCTGATTCTTAATAATTGTAAGATAACGAAAAATCTCTATGTAATGTTGTTAATGGTTTTCTTGACTTTTAATATGTTCCCTCAAAGAATCTAATCCAGTCATGAGACAAACTGATTTTGCAC

>LNC_001785

GAGTTCCAAGCCTTTATGCTCCGTACAAGTGCTGCATACAAGTTCCGGCCAGGATGATTGAGAAAGATTCAACCAATTTGAACCGCTCACATCACAATAGTAGTAGCGTAAAGACCGTAAGTCGGGGGCGGCCATAACATAAGGCTATTACTTTCACACCCCTCTCTCTCTCTGAGAGAGTGATCCGCTGCGTGAGCAACCCGA

>LNC_001786

CATCAACTCCAATCTCTCAACTCTCGCCTCACGTCTGTTCAACAGTCAACTCTCATCTCTCATCTCTCAGTTCGTCGTCTCTCAACTCTAATCTCTGACTCTCGCCGAGTGCTCGCAGCCCGCATCGCTTCTCACTGGCCAATGCTCAGTGCTCGCTCGCGACTCACGGCAGTAGCAGAGCAGTGTAGCACTCTAGAAGTTGAAGAGCAAAATCTGTAAGCATTGTTAGAGATTCTTGAAGTGAAACAACAAAAGAAATTGTTAGTTACACATTAACAACAAAATCTGATGAAAACCCATGGAAAGAATATGAAAGACATGTATTGCGTCCTTTTTTAATCATTGAATGCAAGTATGCAACTATGTGATTATTTGTGTGATTTGTGAACTGTAATGTACTGCGTCTTTCTGTAATTAAGTTATTTATAGGCTAGTCTGTTGACATTTGTGTTAAATAAGTATTCATGAAGTAGTGTTGAGTCACGAGTGTCTAATGTCTATCATCTTATTACATCTGTAATGGCTATTTTCCGGTGCATTTGTATGTAGAGGACATCAAAGCATTTGATTCTAATGAGGCTTACGGTAAATTAATGACTACTATCCATCCTTGATAAGTATAAATAAATTTTCATTCTTTAATTTTTGC

>LNC_001787

AAAATAAGTTGAAGTTCCTGCACTTGTGATCCAACAGTTTTACTATCTGACATTTTATAGTCTAAAAACTTTGCGACCACAAACTTTTTCAAGCATGCATCTTCTGTCTTATATTTCTTCTCAAGTGCATCCCACAATTCTTTCGAAGTTGTCATTGCACTATAGACATTATATAAGTCATCTTCTAACGCACTCAAAATGTAGCCCTTACATAGGAAGTCTGCCTGTTTCCATGCTTCAATAATCATGAATTTTTTTCTGTCTGGCATTTCAGCAGAAGGCACTAGAGTTACTTCATTGGTAAATTTCTGCAGACCAAGAGTGGTAAGCCAGAAAAATACCCGTTGCTGCCATCCTTTGAAATTCACACCAGAGAATTTTCCCGGTTTCTCTGCTGGTGGTACAGCAGTTCGGGTTGGTGCAGCAACAGCCACCGTAACATTGTCATTAGC

>LNC_001788

CAGTAAGCAAAAGTAAACACGAAGAACTATGTCAACAACTTCAAAAGCACAAGCAAGAAAAATGCTATGATGTTTTATAATAGCTTCATCAGAGTGATAAATATTCAGTAACTCGAATCAAGAGTGTGTGCGTGAGCGTGCAATTGCAAGTTTCCTCCCAATCATCAGTAACTCATCATCATTGCTACTTGAGACAAAGATGGAATGTTCCCAGCATTGTAGAGAAGCAAACGGTTCAAGAGGCTGTTACAAGGATCTCATCACAAGCAGCATAGGTACCACTTTGCATTGCCATAATGTTTTCTTCTTAACCTTATTATAAATAATTTTGGGTCTTTGAGAAGCAACCAATTTGATGCACAAAGCTAACAACGAACAAACAGAAGCCACTAGTTGTCCTGCTTATGTAGGAGAGCTGTTTCTAACCTGTGATACCTTTCACTTGAAGTAGCAAATTTGTCGGGCAACTGAATTTATGATCCAGGATTTCTTCAAGCCTTCAAACATCAAGTCCCTCCCACAAAATGAAAGAAAAATATGTTAGTGATGCAACTAGCAAAGTTATTCAGAAGTGTCATTGACGCACGGTAT

>LNC_001789

TCTGCATCTCTGCCCATTCTGTCCATTGAACCTCCGTTAATGATTAGGTGTTTTGGTGAGTTATAGCAGTGTCAATGGGAGTTTCAGATGTTGAACAAGAAGGCTGATTTTGCTCCGGTGGTTATGGGTCTTCTATGGTGGTTATGTGATGATTTTGCTTCGGTGGCGATTTTGCTCCGATGGCTTGCTTCTCCTAATTCTATCCATAAATTTCTTTTTTCTATATTGAGATCTGGGTTTGATTGTGCGTGGTCTTATTGTTCGAGCCTTAAAAATCTACCATAAAATCCAATATTAAGAAAACATTGTCTGCTCATATTAGTTTTGAGATTTGGGTTTGACTTGTGCTTTCTGAGCTTCATACTCATTTTTCAAAGAGGAAAAAAATTAGGAGAAGGGAGATTGTTTTGTTAGTCTATATAGCACCATAACAATTTAAGAGTGTTCAACTAAGCTATAGAAGTGGAATTTGTTGAATTTGTGTAGCTTAAGCATTTTAGGCGGAAACTATCAATGAATTTTTTTTTGATTTTTGTATGCTGTATTTACTCGATCTGTAGTAGCTTGCCCAAATTATCAATTGAAATTGAAGTTAAAGGAAGAAGGAAAGTGATTCAATCGGAT

>LNC_001790

TATGAAATAGATCATTTATACCCTTCGTTACATTTTGGGATCAAAAATGCCCCTGCTGTTATCCTAAGAGACCACAAATACCCTCAAGAGTTAACACCCCAAATTTTTGTTGACGTGGCAAGCCACGTGGGACTAATCCTTCCACCTAAGCGTTGCCAACTAGGATTCACTACAAAAGAACTAGACCTTTAGCGGCAACAACAGTCGCCACAAAAGCCCAGAAAATCGTCA

>LNC_001791

CAACCAGCAGCCCATCCCTCGCTTAACCCAATTTTTTTTCATTAACCAATTCGACCCGGCCCACTTTCTTATTCCTAGGAAACCCAGAATGCTCCAATGTTCTCGAACAACTATTCCCAAGCGGCGTTAATGCAACAATACAACAACAATTCAAACGGCGAACTCACGGAAAGCAATACATGGCGTCTACGTGGGTACAACACAGTACACTGCATTTTTCTCGTCGTCTTCCTCTTTCCCCCGACAAACAGTACGCATAGTACTGTTCTAGCACCGATTCCAGCAGGCAGCTTCGTCCTTCGCAGCCAGGTGGAGTGATCTCCTCGTCCCTTTGTCCTCGCCTCAAGATCGAACGGCCCTTGTTGTTTAGCCGTTGGAATTTGTTAAAAAATTGGTGATTTTCGCACATCAAGCAGCAACTTTTCTGTTCTTGGAAAAGAAGTTCAAATTTCAAAACGGGGATTCCAAGTTTACCCTCATCTTTTCCCCCCAAATCTGCCCTAGCTCCCCCTCTATATAAACTCACTTTTTCTTCATTGTTAAAGGGGGATTTTCTCAGTTTTGGGCTTGAAAAAGAAACCACACACAAGAGAACAGTGAAAAATATATATATATATATATATATAAGAAAGGAGTTGAAATTTACCAAGCCTAGACAGCTTATATTAGACCTAGATGATAAGGAAATAGAATCGACATACATAGGACATTTTGAGACATGTTCTGAGATTGAGAGTATTTGGAGCCATACTTTCTCGGTTTTTCTTCTCACGAATCTGTTGTAGTCTCCTTGGGGTTGTGGAATCGCTCTCAAGCTCGTTTCGTTCCAGTCGCTGCCCAAAACCAGGTAATCTTCCCTCCCTTTCTTTATTCAGCTATGCTATTTCTTCTTTAGTTAATTGGTAGTGATGGTTTTTTTTTGCAGAGGCTATAAATTTGTATTTGTCCTGTTGTGTCATAATAACATGCTTACGTTCATTTTTTTTTTGTTATGTTTCTTGTTTCTTTAATTTTCTCATCATGTTTAAATTTTGTTTAACTTCCCTGAGATTTTTTTTTCTAGCTATTAATATGCATATGAGTTCAATAACTTTTTATTTCATCTTGGGCTACTTCGTATAGGTCCTTCATGTTGATTTTGGGTGATCTTTTAGTTTCAGTTTTGTTGTTCCATGGGTAGAGTAAATTTCTTTTCATGTGTGTGCTTCTAGTTGTGGTTTCCTGTAAGTTGGAATAGCAACTATAGAATGGGGCAGTGACTATTCCTTAGTATTATCTTCATGACCATTTGATTTGTTGTCTGTTATCTCTCTATCTTAACTGAGTTCTTGAACCTGAAGTGCATGCTCCTTAGTATATTTACCGTTTTATCCTCCCGTGCCCTTTTAAATTAATGTTGTACAACTGATGGTTTCCTTCCTTCTCGTTGTCCTCTTTAAATACATATAATTAAGAATATAAGTCGTGTACTCCTCCGCATTGAAATGTTTGGTCTTAAAATGATGTCTTTAACTTGTCGGTTCTTAAATTTCTCATCATTTGTATTGCTAAAGTCTGTCTCTGTATGCCGAAACTGATTTTTTTTTTTTTAGTATGTCTGTCTTGGTGGAAATCGAATATTGATAAAACATAGATTGATTTTCTCTAAGTTTCCTTCAGTTGCATTCCTTTAAATTCTTGGCTGCTGACGACTATTAGTTTATATTGCTTGAAGTTAATCATTGTTGCTAATATCTTATAGTTAAAGCTAGTATCTTAAAGTTAAAGTTGAGTTGTGATTAATGGAAACAACCATGTGGTCAACTGATTTCCTACTTACCTTGTTTGATCTTTATTGTGGTTGGACATGTAGTTAATGTTTGTTGTTCACTTATGCCTTATTAAGTTCACTTGCTAATCTTTCTTCTTCTTTGGACTGCGCATGAACTCTCTCGTTGAGTCCAATTTGGACTCCCATCCCTTGCATACTCCTCTTCTTGAGTCAACCCCCATGTCGTTGAATGGAAAGCCATGTTATTGGGTTAAATACCCAAGAAGACAGCAGCAACAGCAGTAGCCTAAAGGGCTAAAAGAATAGCATTTCTTGGACTGAAAAAGTCTGAAAATGGCACCAGCTGAAGCAGAGTTGCAACAGCCTCAAAAGCCTAAAAATTGGAGAGCAAAAATTGGGTTAAAACCCATAAATAATAGAGCAGCAGAGGCAGAATAGCAGCAGTCCCAAATGACTGAAATTGGAATTTTTTTTGGACTTTAAGAGGTCCCATTGGAACGGCAACAACAACATCAACCAAAAGGCTTAGGCTTGGGCTGAAATAAATTTCAGCAGCCACCTCGTTTCCTTCATTCCCTTATTCTTACCTTTTCATGGTTATATATCTTTAGTATATTTGATTGCGGATGCTAATAACTTTAAAATCTTTTATTGACCTACATGAATCCTTGGAGGGGAGATTTTATTCCACTATTTCTTTACTTATTTACTTTAAAGTTTTCAAATTTTGAAACCCGTTTAAGTTAGTTAATCCAAGCTTGTAGAATGACTTAAGAACCTTTTATGTTCTTTCATAGTTATTGAAAAGCTTTGAATCCATGTTTATGAGTTTGAACTAACTACTTAGTTCAAATTTTTGAATAGGATAAAATTGTTAAGTTTCTTAAAATAAATTAGTTGTCTTCTAATTTAACTTAGAACTTTGTATGAATGACTTTAGGATCTTTAATATCCTTTCATAGTTGTTAAACAAGGTTCTTTAATCCATGTTTATGAGTTTGAATTAATCGTATGATTCAAAACATGACAAATGGATAAAAGTTTAAGTTAGGTTTATTCTTGAGATTTTTTTAGAAATATCAAAAGATTTTATCCAAGTTTCAAACAAGATTTTTACAAGTAACCCCCTTTTACAAAATTAGCCAAATTTTTAAATCCGTCGGTGAGCCGCGTGTTAGCGGATTTTCTAAGATGCCTAAACCCTTCCTTAGAGAATTATTTGAACCCTTACCCG

>LNC_001792

TTGAAGTTTAGGGAGAATTAGTTTCGTAAGTCACTTATAAATTATTTTAGTTTTATTGTCTTTCTATAGAAATCTTCATTTTTACACATAAGAGATATGAAAAGGTCATTTTCTTCTATTCAATTTGTAGCCAAGAGATCTCATTTTTTCTATAAAAAAAGGTTGATATAGTTACTACCATAAACATTACTCTTCCCAGTTTCAAAATAAAATTGAATGGTCTAAGACTGATAGCTTCCTATTGTTATCATCGTCTTTATATTTCTTTTGACCTTTGGGTGTGGCTGTATTCATTCATAGCAGAAGTGTCCAAGAAATATAGGGAGGAAAGATTAAATGAAACAACAAAAAG

>LNC_001793

TAAAGATCACGGTCTTTGACTTTGAAAACTTGTAATACGTCACAAATACTCTCTGCCTCTGCCTGAAACTGTTCTTTGTATGATCAAGAAAATCAAGAAAACTGTTAGGTCTCTGACTTGTGAGGAATCATTAATTTGGGTAAACTATGCTATGCACATTTCCTACAGTACAAAAAATTACTGAAATTTTTTAGGGGAACAACTTAAATTGGACAACTCTCATCCAGTACTTCGGGGATATGGTGCTATTATGTGACTGGTTCTCTCGTATTATCACATCAAAGTTCATGCTTCCTCTTAGGTTTGTATATGTGCTTTAGTGGCCTTTAACTACTATCCTTCAATAACCAAACCTGTTGTCTTGGATTTGATCATTAGACATCTCAAAAACTCTTCCCGGC

>LNC_001794

GTGCAAATGCCAAATTAAGCAAACAGTACACGAGAAAAGCACAAACACCCTGAAAAAAGTTGTGAATTTCAAACTCAAAATTTGAGAAAAAAATGCAATTAACGTGAGTGGTTTATGTATGCCAATTTTGTCCCTAAATTTGCTCCCAAACACAAACCCCATTTGTATATAATCAGCATTTGTTGTGTCTGAGGTTGCATTTTCTTCCTAGGATGTTATTTATTTTTGGGGTTCTTGAAAGGGCAAATTTTTATACAAGTTTCTTGAGGTTTTGAGACTGAGAGTGAATGCTCCTTCCCAGAAAGAAATTGAAGATGGGCTAAAAGATTGTGGGAATAGCCTCATCAACTTTCCTCATTCTACTGAAGAGCTCATCAGTCTTCTTGATGTTCATTTGTTGTTGGAAGATGTCGTCGCTCTACAAGAATGTGATGGACCTTCATCCGTAGAAGCATTTCATTTTTCAGATATAAAAGTAATCATTTGAAGGACTGTTTTAAAGTACAAAATGTTTTTCTCTTAATAATACAATGAGGTTGGTCCAATTTTAAAACTGTATTAAAAAGAACATCATTTCTTAGATTCTTTTCTGCAATATTACTTTCCTATTCTAAATCTTAAACTCTATCATAAAGTTAAAAATTTAGTCAAATGCATAAAATATCAAATATTCAATAACTTAAACTGTCTCTCCCCACCTTTCCCTTTGTTTTTGTTTAACTGTTTTAGGCTGCTTGCATGTGTTTGTCAACTATACTTGTTGAGAGTTGAAATAGCAAGGATCTAGAAAGGTATAAAATAGAGATACTTCATAAGCTTCTTCGTTTGTCTCTCCTCCCTTCTTGCTACACGTCCAGGTTCATCGTTGGTGCTGCTGGAAGATGTTGTCGCTCTACAAGAATGTGATGGACCTTCATCCGTACGCTCAACCTTCAGCCTCTTAGCAGCCTGATCTGGGAATGACTTATGGGAGATTTGGATTTGTTATTATCAGGAGCTAGTTGAATTTCTTTGGCTGCAGCATCGTCACTCCTTTTAAAATGAAACCTTGCCAAGCAATGTTGATGTATAAATTTCATAGAAATAACATAATTGGATGTGTGCAATGCATGGTTCGTCAAGCAGGGTTGGGAGTTAAAACTTGTATAGTACGGATCCAATTATGTTATCTTTGAAATCTATATATCGGCATTCCTTGGCAGGGTTTTTTGTTAAAGAGAGTGAAGATGCAGCCAAAGAAACTTAACCATCTCCTGATAATAACAAATTCAAAACTCCCAGTGAACCATCCTCAGACCAGGCTGCTTAGAGGCGGA

>LNC_001795

GCATCCTCTTAGTTTTTTTAATTTTTTTAATTTTATGAAGTAAGTAGATTTATTGTTGGCATGAAGATATTAGTGTACAAAAGAGATGCTGTCAAATTCATTACAATTGTCTATGCTACAAAAAGGTTATACAAAAATATTTAGAATGTTAGCCCAGTCCCCAATGAGGCAAAAGACTATTTTATGTGCAACTTCCTCTTAGGCTTGTATATGTGCTTTAGTATCCTTCAATAACTGAACCCATTGTCTTGGATTTGATCATTAGATATCTCAAGCAGTTCTCGTTATCTATTCATCTTTCTATCATTTACACTCCAAATTGTTCTCCCTTATGAATATGAACTATGTCAATGCATTTCTACGGTTCAAGATACCAAGACTCGCTTCAATATGGCGGTGCCTGACAGAGTTAAATAATTCCATGTTGCCATTATTTTTCTTGAAATTTAAAGAACGAGAAGGTAACTCATGAGGATTTCCTGTTGATACATATAACTGTATTTGTTATGTTACTTATGTAGATCATATGATTATTTAGTTACTTTGATGGGATTATTTCTATTAGTAGCTGC

>LNC_001796

CCAGCCCATAATTCTCATAATACATCAACAATTCCAAACAACATTACAACATCAACACCAAATATCCAACGCCTGCCAAACCGACCCCATCCCTGTACCAACGTCTTCTTCCCCATAAATCCAGCAGCAAATCGCGGCTGACCAAACTCGGAATTCGCGCCAACAGTGCTCTCCCATACGCAAGAATCAACCTTTTCCCTGATTTTGAGCAAGGATTTGAGGATCTTGGTACAAATTCAAATTTCAAATTCGACCGTTTTTCCCCTAAATCGACCCCAAATTTCCTCTATATAAACCTCCATCTTCTTTAGTGTTTGGGGGGACGAAAAAGGCTAGAGAAAATATTGAAACCTAACTAGCATTAGAGAGGAAGAAACCGACTTAAACCGTATAGTTTTTGAAAGCATAGAGAAACCGAAAATTCTGATTTCGTTCAGTCTTTGCTCTAGATTGTTTTGTTTGTTCATCCAAATTCTCGGAATCGTACCTTAGTGGTGGTGAAGTCGTTTCAAGCTTGTTGTCCCACGTCGTTGCCTCGAAGAAGGTAACCTTTCCTCCTCTTGAATTTGTTTTACCATTTCGGCTAAATTCAGATGTATGTTATGTGAATTTAATTCACTGTGTGATTTAATTTGGTAGTTTGGTGGTGCCTATATATGATCTGCTTCTGCTTCTTTATGAAAGATATGGTTATGTATTGTTTTAAAGTGGTATAAAATGGCAGTTTGAGATCTTACCACTAGCTATTGTTGCTTTCCCTTGTTCTTCACGGTGTAAGGCATATTACTAAGATACAAATCGATTTAAATGAAGCTATAGTAATCTTGGTTGCTTGTCTTTTATTTCTTTCTTTCTTTTCTTATTGTTGTAGATTCTCATTAATTCTAGCTTATTCCCATGAAGTGTAAGCTCTACTTTCGTTTTAGTTGGAATGTTGTTCACATTATTTCATGTTTTCGACTCCCGATTTAAGATTGTTTTAGCTTACTGCTTTACTCTTGTTTATGGTCTCTAAGATTTCTTACTCATTCCATGTCTTTGGGTTTAGCTATCATTTCATTACTAATATACATCACTCCATTTATTAGTATATATTTGAACCTGTTAGTCTAATAGTTATTAAAATGCATTTGTTGTTCTTAGTGACCATGCTAAATTTTGAAATTCATGTTAGCCATCTCATGTTAGCATTCAAATCAATGTGTTTTACTTCCTTCATTTAGTTTGTCATTGTTGGGTTTGTTTATTTATTATGGATTTTCTTCACATTTATGGGTAAGTCTCTTTCCAAAAAATGACCATATCAACTCTATGAATTGTCATTAGTTTAATACAATTATATCTCATTAATCCAAGTGCATTATCTTTATGTTGAAAATATTATTTCTTACTTCATCATCTTCATCCGATGCATATGAACTATATTTAAATTTGTCTTCTTATAAGCTTAGAAAGTCACTGACCACTTAGCTAGAATTTAGATTTGTAATTTCAAATGCTTAGTTTCCTTAAATGATTAACGTTGCTATAAGTTAGATACTAATTTGTTTTTTTTCTTTTCTTTTCGTTTGTTGTACATGTGAAAACTCCATTCGAATCTGTTCCGGACTCTTTTCTTGCATTTCTTTGAGTTGAGTTTCTTCGAGTCAGCCTCTTCGTTGTTCGAGTCAAGGGAACCGATGAACAGGGATTAAAAACTGATTTACAAGTCTTGGAAAAGACCAGAATGGACTGTGCACATGCCGATATACACTATGATACACTGGTATACAGCCGTACACAACTGATATACAGGTCTAGGTGCTGAAATATAGATATAGACTGCGGTTATACAGGTCGAAGACAAGAAATACAGGTCTCGGGGGTTAAAACGGAGTTATATACACTGATATACGGGCAGTATACACTTTGTATGCAAAAAAAAAAAAAAATGGGCCCAAAAGCCCCAAGAAAATTCTGGCAGCTTTTTTGGAAGATAATTGGGCCAAAGCCCATCTAATTTTGAGCAGCTTTTATAGTAGTTTAGGACAGGGAAATGGGGCCAAGGTCCAAGATCAAAAAGGCCCAAATCTCCATCTCTCACTCTCTCTATTGAATTTATTAATTTGTCTAACTCTTACTTGCTTATTTGTTAATTAATCTTTGGTCTAACTTAATCGAATTCCCCAAAAGACAAACTATTTCTCTGTGTTGTTTTATTTCTAACAAATCTCATTTTATCAACTCTTGCATTATCATTTATAATCTTTAGTCAAAACCTTTTTTTTATTTAGTTTAAAAATAGTTTTTTC

>LNC_001797

AAAACAATGACACAAAGTCACAAAATATGTATCACTTATTTAGAATTAATTTGAGTAAATTTTCAAATTATATTATATAAGATTAATTAAATATTTCAAAACTATACCCAAAAAAAAAAACTAATTCCTCATTTTCTGTTATAGGACATGTTGACTCACACACTACATATGAGTCTTGTTTGAAATAAGTCATAAGTTATAAATTCGTTCAAATAAGTTCAATATTGATAAAGTAGCTATAAAAAGTGAAGGAATACTTACTAATATAGCAAAACAAAATATGTAATTTAGAGATAAGTAGTTAAGTACTATTAAAAAGACCAAAAAAAAAAAAAGCGAGAGGAAGGACCGAAGGAATATAATTGAAGAAGAACACGGCAGTGAAGCAATGAAGAAAACAAAAGAAGTTAGGTCACAGTGATTACTGATTAGTGGCATGGAACTGTGGAAGTGTCAGCTGCCATTTTATTTTTCCTACTAGCTTTTGTGAGTTTGTCTGTGAATATGAACATGTAAGTAATTAAAGTAAGTGGTCAGAATTTGTTCTGACATTTGGTCAGTTTTCTTAGAATTTCGTGATATCTACTACCATCACTTACCCACCTTTTTATCTCTTGATTCTATGGTTAATTACCACGGACACAAATTAAAAAATATCAATCCAATATTTTAATTTATTTAAATTTGTATTACAAATTACAATTTACAACTATTAGTAGAGTGCATAGTTTACGCAGCCACCTTCTAGGAAGGGTTCTGTTTCAACTAAGCCTCAAATGTAATACTAATAGTTGTATAGTCCCGTAAATTCTTTCTCTAACTCAGCTATAGATTGACCGTAATCAACTACTGGTCTTGGTAAAGCTCGTTGACGATCTTGAAGTTCCATGCCATCGTCACTACCACATCCAATGGCGGAATCTATGAAAAGTTCAAATTGACTATCCAACTTTGGAAGTCCTTGATTTCTCCGCTCAGCATTTATCCAATCTCTAAACAACACCGATATCTCCAAACTNN

>LNC_001798

CGGCGACATCCACACCCAACAGCACCTCCGACCACCAGTAACAATCTACAAACACCTTCCATGTTCAGCTTGTAATCATCTGGACATCAAAAATTCTCACAGATATCATGATTCACCACCAATGAAGACTTCTTCAACATGAATGTGACATAGTTATTCTAGAATTGAACCCCAAAGGAATTGACACCACTGTTAAAGAAAACAATTTTTCGGCTGCCAAATAAGGGTGGATCTACGTTCAAGCTTGGAAGTGCCACGACACTCGATGGTCTCGATTGAAACACTGTATATATAAGAATTTGTATAAATATATAACATGTCACCCAGAGTTGCACCTAAAGACTCAAAAGAGCCAACGAGCCAAAGCTATTGCTATTGACAAAACTCGATCATATAACCTATTTAGTCTATCCAATGTACATATAGGAATCACACTAATGTTCAAAGGATATGGTCATGTGGCATCCGGAACTTCAAAACTCTGGATCCATCTCTAATATTGGAGATGGGTTCATCCATACCTCCTTCGGCAG

>LNC_001799

CTCAGCTCCATCTGCTGCGCCGGCGACATCCACACCCAACAGCACCTCCGACCACCAGTAACAATCTACAAACACCTTCCATGTTCAGCTTGTAATCATCTGGACATCAAAAATTCTCACAGATATCATGATTCACCACCAATGAAGACTTCTTCAACATGAATGTGACATAGTTATTCTAGAGCAAGTGATTGAACCCCAAAGGAATTGACACCACTGTTAAAGAAAACAATTTTTCGGCTGCCAAATAAGGGTGGATCTACGTTCAAGCTTGGAAGTGCCACGACACTCGATGGTCTCGATTGAAACACTGTATATATAAGAATTTGTATAAATATATAACATGTCACCCAGAGTTGCACCTAAAGACTCAAAAGAGCCAACGAGCCAAAGCTATTGCTATTGACAAAACTCGATCATATAACCTATTTAGTCTATCCAATGTACATATAGGAATCACACTAATGTTCAAAGGATATGGTCATGTGGCATCCGGAACTTCAAAACTCTGGATCCATCTCTAATATTGGAGATGGGTTCATCCATACCTCCTTCGGCAG

>LNC_001800

CTCAGCTCCATCTGCTGCGCCGGCGACATCCACACCCAACAGCACCTCCGACCACCAGTAACAATCTACAAACACCTTCCATGTTCAGCTTGTAATCATCTGGACATCAAAAATTCTCACAGATATCATGATTCACCACCAATGAAGACTTCTTCAACATGAATATTGAACCCCAAAGGAATTGACACCACTGTTAAAGAAAACAATTTTTCGGCTGCCAAATAAGGGTGGATCTACGTTCAAGCTTGGAAGTGCCACGACACTCGATGGTCTCGATTGAAACACTGTATATATAAGAATTTGTATAAATATATAACATGTCACCCAGAGTTGCACCTAAAGACTCAAAAGAGCCAACGAGCCAAAGCTATTGCTATTGACAAAACTCGATCATATAACCTATTTAGTCTATCCAATGTACATATAGGAATCACACTAATGTTCAAAGGATATGGTCATGTGGCATCCGGAACTTCAAAACTCTGGATCCATCTCTAATATTGGAGATGGGTTCATCCATACCTCCTTCGGCAG

>LNC_001801

AATTATGGCCAGTAATGGTCTTGAATCCACCAGGTATTTCTTGCATCATTTAGAAATTAGAACGTATCCTACTGATATCTGGATACTGAATAGCAGAAATATCCTCTACGAAAAGCAAAGCATTAATAATGATCAGCGTTTTAGAACGGCGTGCTTTTTATGCGATAACTTTTAGGTCTGACAGAATGTCCCCTAAAGAGGTCTCTGGATATTCACTGTCTATGACCATTTCATTGGTTGATGAAATATGAAAATGGAATAGAGGAACTGGAAGACCCTATCATTCGTGTTCCTTCAAGGGCAGTTCTATATCAAGCCTAAATGTTATCCAACATTTTGACTGTTTTATTTATAGTATGCAAGTCTTTTGCTACCTGTCCCTGTCTCTAATACCTATACCTATGCTAAC

>LNC_001802

CAGAGAGGGGCCTGCAATAGTAACATATATCATCATTTATAAAAATAAATTTTACATGTGATATTTCACATCGATAATATTAATCAATAGACGGTTGTGTTTGAGACTCTGAGTCTTTGGTCACTTGGACTCAACCTGGATTCTTTCCAAGTGTCATGTGTGGATTGTAGAACACTCATCATATGTTTTGATTGGAGAAAATTATATAATGTAATAAAAAACATGTCTCTTTCAGTTTTACTTTTTTACAGTCCATTTAAAAAAAAAACTAATTAATTTGATAACTTTTGAATTTCAATATTTAATGTGACATGTGTTTATGATAGTAAGTTTAAAATACATTTTGATATATTATGTATCTTTAGTTTAAAAATATTAAATTTGAAAAAACTTCTTTATTTCCTTAATTTTTGTATTCAGTTAAATTAAAATAAATAAACTAAAAACGAGGGTGTAATATTTGACTATTTATAGTCACTTATAAAGAAGAAAGGAAAAGTGTGTTTTTAAACAAAATTTAACTCAACAAATCTTATGTTGCAACTAAAATATATGATGATTGACATGAATTTGATAATTGTAATATAAAAACCCATGAATCAAAAAAAATCTATGTAAAATTATATGCAATAATCATTTAAAATAAATATCTACTGTCTTACATATAATAACAAGTTTTAAATAATGAAAATTTTACTTTTATCTTGTCTCCACATCCGCAAATCAAAACTTAACTATAAAGGGAATAAAGGTGGATGGGAAATATATACAACTCTCTCAATGACTTAAATTAAATCACTTGGTTTATATATATACGATCATTTAAGTAAATTGATACTCTTGCTGTCTTAAAATAAGTGTCTCGTTAATAAAATAATGTTGTCCCGAAATAAGTTTTATCTTAAAAATTTAAGATAAAAAATAGTATTTTTTTTCAACTATATTTTTAAATTTCATATCCTTAAAAATGTTCAACTTTCAAAAAAAAACATTTATTAAATCCGAATAGTTTTATAAACTACACATTATAGTTATTAAGTTCTTAATAAACATGATTTTTGCTAAGATAACACTTATTTTGGTAGAGAGAAGCACTCCTCAAGATCCAAATGCTAATGCTTTGTATATTTGTTGTAACATGCTTTATATAGATTATATATAGAGATATATAGTATAATTACAATTAGGTTAGATTAATTATTTGTTTATATATAATTAAAATCAATCCTGATTTTTAGTTTATGTAAGTACTTCTTTTTTTTTTGCTTTTTCATTCCGTGTTCGATACTCATAATGAAACTCGAATCTGGGTAAGGTCGATTCAGGGGTAGCACTCTCTACCTGGAATTCTTTCATATCTTGGGCTTGAAACTGAAACATTTATTTGATGGAGGAGTAACCTTATCCACCGCATCACATCCTCTTTTGGGGATATATCTAGTGTACTTAATTACATTAGTAAACTAAAGAAACCTTGCAATTTTAACAAAGCTACAAGAAGATGTGACAAATTAATTAGATCCTAATTAATTCTCTTCAGGTCTAATTATATGCATATTTTCTACATATTTGAGGAGAAACTTAGGAATAAAAAAATGTTGGTCACAAAAAAAATAAAAGATAAATCATTTGGGAATGTCACCTATGAGGATATGTTTTATTTAATTTCTTGTTCCCCACTTTTGTGCTTCTCCTTTTTTGGTTCATTTAACCTATCCATTACCTTTCTCCTTTTATTTCTTAGTAACTTTCTTCTTTTAACCTTTTATGCTCATTAAGGAATCATTTTCAGGTACTATATATAAGTCACTACCTTTATTTCTTGGTTCTCAAAGCCACCCATATGTTTATCCTCATCTATTAATAACCTTTTATGAGTGCTATATTAACCGGAGGTGGTGTGGGGGTTTGACATGACCCCTATCCATCCCAAAGTCCATGTACGGAGGAGGACATAAACCTTTCATGTTAATCAATTTAAACTATGATATTAAGAAAATTGAAGAAATTATTCCAATTTGATGCCTTTAATTTCTCTAACATCTTGTTTTGCCTAGATTAGTCAATGACATAATTGATTCAATAGTATACAACTATACATCCTATATCAATGAAC

>LNC_001803

GTCGATTTCTCTACTCTTTAAAATCGGCCCTGGTTTGAAGGTGTCGTGCATCTATTTATAATAGAAGAATGAGTGATTTTGTTCGAACCCTAAGGTATTATGGCCTATCTACTCGATTCTCGATGGCTAACGGCTCAAGCAAAGCTATAAGCTAGAAATTGTACAAAATATGAAAGAAATGAAGATGGAATATTATTAGGGCATGATTTAAGGAGTGCAGTTTTGATGCAGAACAATGCAAGTAATGAAAACAATTAATAAACAGCCGAGCCGGTCATTTTTGTATGTAGCCTCTCCCCAGGCGCGGCGCTCTCTCAGGTTAGGGTTCTTCAGGCGGCTTCAAATTAGCAGAACCAAAAAATGGTACTCTAATTAATTAGATGAACTTATCTATCATCTGTAGCATAGTTTAGCATCTGATTCTTTCTTGTTTTTTTCTCATCACATCAGAAAAATAGAGTTCCAAATTAAATTCTATAGGCATATATTTTCAGGGTTCTTTTTTTCTCCAATTACAGTTTCAGATGCTCCTCCTTTACCTTTTACACCAATACTGTCCAATATCTGTATTGGAGCGAGATAGATTCAATTTTAAATTTTGGATCTCAATTATCCCATCACAACTCATGTTAGTGTCAAACGGATCTGAAATGCTTTAGGCATTTATTCCATTTTCACCCTTCCCCATGGAATCTGAGCCCTAGTTTTATCTTTCTTCTAGGGTTAGAAGAAAATATCAGAGAAGTTCATGGTACAAAGAGGGTGCAGGTAGAGCACGTGTATATACAGAAACCCTAGTGAGGAAGAAGCAATATGATAGGTCAAAAACCCTAAATTTGTGCTGTAGGTGCCTGCAGCTAAGCTAATTCCACCTTATAGGATGGGGGACCAAATATGGTATGCCCGGGCCACCATAGGTTCAAAATTTTGTCGCCGTGGGTCGGATAGTACACTGAGAAACATACACTCCTCTTCTTTCCCACAATTCATTAATTTTGACTGCAAATATGCTCTGTACTCTGTACTATGGTCATTTACCACTTGGGTGGTTTCAAAAAGGCGGATCCAAAATTTAAAGATAATTAGACATACCTAAAAGCAGATGCACGTTAGTTGAAGATGCTTGTATGAAGATTTTATTAAGGTACCCATAGAACTGATGTATACTAACATAAAAATTTAAATAGAAGGGCATAGTTGGATGAAGCTCACCTAGTGGACGCGTGGTTTGAGCATCACTTTGCATACGTTAGGTTGTCTGTTTAAAATCACAACTTGGATTGCGTGCCCTTGAGGGATGCTTTTTTGTTGAAGTCAATTGGTATATGTGCTTCTTCCATGTGGGTCTGCCTCTAAATGCGACACTATCATGCTTCTTGCTTTTAATTCAATTTTTTTCTAAATAAAAAATTCTTAGCATCATATATATGTTCACTTTATTTATTTTTTTAAATCTAAGTATTGAGAAATTGGAATCTTCACTAATGCCTCCTTTAAAGAAGGTGAATTTTATTGGGATTTAAAAAAATAAAATTAATAGTAATTTCCAACTCCAGCTAATTAAACCAAGTAATTGGATTTTAGAATCCATATCATGTCTGTAAAGTTCTTCCAAGTCAAATATTACTGCTATTACTAGGATTTACTTTACCATATTTAGGTCGGT

>LNC_001804

ATTTGATGACAGCTATATTGATGATTTTTAAGTATCTGTTTTTTCTTGATAAATATATTCTTGATAACTATTTAGTAAAATGGTAATTCCTGCATCCTTGGTTACAAATTTCTAATCTAATTTTGTTGCAAAATCTCAACTTCTAAAGGCTTCTTCTAGCTGTTTCTGGAAACTTTCCCTGCATAGAACAGTTGCTTTAGTTTCCAGTTCCTTTGTGAGGAGTGAGAACCAAGAGTTGGACTAAAAGGTAAAAGATGGTGCCTCTCAATAGTTGTTTCATTACTAGTTATCTTTTAGTAAAACTATAGTTTGTTAGCTAGTGAGGTTGTATATAACAGGTCAATTACTATATAATGTGATGTCTGCTTCAATTTGGAACGAAGGGACAGCTCCACGGTAAAAAC

>LNC_001805

TTTAAATGTTAACTAGTGCAAGTGAGATCAAATAAGAATTGAAATATTTGTGAAGGATAAAGATGTCGTCATTTTGGTGGAAAACATTTTTCAGGTCTCCTTGAGCATAACATTTTCCATTATACCAAGTAAACCCTATTTCATATCACTTGCTTCATTCATTATTTAGACAGGTTTGTCTGGTATTGTGAAATGTTTTCCATGGAAAATATTTTACCTTGTGAGGTCATTTCTAGTGTTTTGTCGGGTATAAAGTAGGTGAAATATGATAAATACTAGAAGTAGTATAGTTTATAAGGATAACAGTTTGGTGATGATTTAAAAGTTGGTTATAGTGTGTAAATGTTGATTGAAGTATGCATCTTCTCTTCCCATCTATGAATGCACCTATTTCACATGAGAGCAAATGAACCCGAAAATATTATTCCAGAACTTTCGGCTCATCCACAATATCATTAGTCACATAATTTTCAAATGAGTTGGCTTGAAATGTAAAATCTTTGTCCACACATGAATTTAACTTTCTTCCAGCAAAACTGATTCGTTGCCTTACTGTGCAAAAAAAGTTATTTCTCTAAAATTTAAGAACCATTATCAAGTCGAACTTTGCTAATTTGGCACGTCTTGACAATATTATACTTGAAAGGAAATTTTCTCTCAAAATTAAAGGGTCC

>LNC_001806

CAAAATTTGATTCTCTTTAATTTTTCAGTTCAGGATTTGGATGGATCAACTCTGTAGTTGCCGAAGCAACATGCTGGAGAGACGACTTCAGCTTTCGAACACGGTAAAATATTCTTGAATTTTTTTTGGCCTTGTGCCTACAAAGTTTTTTAACAATCCATTATAATTAGATTAATAAAAAGATTTCGGAACCCTTGCTATGAGTGAAATGGTGGGAATCCTTGTTTGATTTTTTTGAGTATCCTCCTTCCTTGGATTAGTACTGTGACACATATATCGGAAGTTCAGTTTGAAATTTGAATGAGATATATCTTTTCCTTTTTTTTTTTTTCCATATTCCAAAATTTTAGTAATCCCTTTATATTCGAAGCAGGAGTAGCTTGTTAAGAAGAGCAAGTGCTATTGTTTTGCTAATATATAGAGAGTGTACACTCCATTCATCATTTAAAGCAGCGGGATAGTTCTGGCTTCTACTTAGGTAGTGGTGTTGTTGTCCTTTACATTGGCATAGCTGCTTTGCAAATTATATTGCTATAGAAATATGCATGGCCCAACTAGATTGTTCTATGATTTGTTACAGAGCATGAACCTACATCCTTGCTTGGATCTACCGGGATTAAAGACTTTCAGAGCATTTGCAGTTATAGCCTTATAGGTGCTGAAGTCCTTGGGCTCTGTAACTTTGCAAACATTGCTTATACTGGATAGGATTCATCAAGGTCACCAAGAGTGTGGATATTGCCACTTAAAGTCGCTAATTTCTGTTGTGATCTTGTTGAAATGGCATATACATTAGGGAATTGAAGAAGAAACTAAGGCCATGGTGGCAGAAATTGAGGATGAGGCTAATGATGAAGGTTAGATGTTCACTCAACCCAGAAAACTGAGTGATCGAGTTCCACAGCCTTCTGCTAATGAAGCAGCTGCCGGCTTTGATAATGGATGTCTTATGACTTAGGACCACACAAGGAGCCAGAGTGATCACTCTTCATGTGGTAGTATGTGCTCAAGAACCTGACTCTGGTGCGCTCAGTTACTGCGGTGAACCGAAGCTTGGC

>LNC_001807

CGAGGATGATATTTGTTTCCTACTTCCAGTTTTGTAGCCATTTTCTAGAATTTTTGACCTACCAAACATTGGAAAGTGGAAAGCATTTTCCATACCAAACGCACCCGTACATGTTCTTTTTTAGTTTAGAAGTTGCTCCTGCACGTGATTGAGCATGATTTATGACCAAGTATTCTGTCAACAGGTCCATCCTGCTGAAGCTGTTGATACCTGAGGTGAATGAGAACCTTAACTGGATTGTGGGATCACCTTAGGCGGTAGAATGCAAGTTCATTTTATCCTGTGTTTGCTTAGGGGGGTATTGGCTAGTTGTGGGATTATTTATCCCACCATTTATTCTATAATAATGTAACAAGTTATCTCCACATATATGTTGGGATAATTTATCCCAACTTTGTTTATCCACTGTAATGTTTAAGGAAAAATGTAGTTTAGTTATACCTTTTGGTTTGTTTGGTTGTAACATGCGACAGTTCTTCAAGTTAGTTCAGTCAAATGAGCAATATCGCATTACATTCCATCTCTGCTTGTCTATTTTATCCATGTCTCTCTAGATATACATCGATCTTAACCGTTCTACTCTTAGTGTTTTAAACTGTTTGCTCACTGTCATCTTACGTGTTAATGCTTTTGCAGGCTGTTGGTGTTAAATCTTTGCTGAAAATGCTTGCAAGTCCATGTTTAAGGTTTACTCAGATTAGTGATTTACC

>LNC_001808

CATAGCTGTTGGTGTTGAAGTCATATTTAGTTTAATCCTTTTATTTCAGTTTTAGTTCTAGTTGGACAAATTCAGGTAGAAGTTAAGTTGTAGAGAGTAGAGAAGAGGGAAACAAAGAGATTGATAGGTGTCAAAGACTGTGATACTCTCATCAGCAATACCATACCACCTGTTCGTCTCCAATTACTTCCGTGTTGCAGCACCGCACCGCTCCTCTTCCCTTTTATTCTCCATCTGCCGCTTCTCCACTCATCACCATGATCTCTTCCTTCTATGGCTTCCGATTATTCATCCTCTCCTATCACCCTTAACGACCTTAATCCAAAGGTTTTGAAATGCGCATATGCTATCCGCGGAGAAATTGTCACCCATGCTCAGACATTACAGCAAGACCTCACGGAGAATCCAGGCTCTCATCCCTTTAATGAGATCTTATACTGCAATATTGGGAATCCTCATTCCCTGGGTCAGAAGCCTATTTCTTTCTTTAGGGAGGTCCTTGCGTTATGTGACCATCCACTTCTTTTGGACAGAAGTGAAACAAAAGCTCTGTTCAGGGATGAAGTTTGTTTATCAACCTTGGTGCACCTTGTAGTTCAATGAAACAGAT

>LNC_001809

AAAAAACCCAATTCTTTCAACCCATACATTACAACAACAACAAATACCAGAAACGACCCCACCCCCGTATGTTCTTCTTCTTTCTCTTTCACGCAGAAACGTGACCCAACTCGCGAAACCCAGCCAGAACGTGACCCGAATCCAGCGAGCCCAAGCAGCGCGACGTCAAAATGCACGACGTCTTCACAGCCAGCAGACGGAAGCATCAACAATATTTCCTCCAACGTTCACAAGCGGACGGAAAGCGATTCACGGCGTTTATGTACGCGTAACAATTTTATTCTCGTCGCCTTCTTCACCCAGTACGATGGTACTGTTCCAGTCGCGATTCCAGCGAAAAGCACCTGCAACAGCCGCATTTTTGTCCTTTTTCAGCTTAAGATGGAGACACGTCGACGTTCAAGGACATTGGAATCATCCTAGACGTCCACCTCGCCCTTCCTCTTTTTGAAATCTTTTGGATTTGAGCGAAAATAAGCTCTCCCCTGTTTGAGGATAAGAGGAGAATTTTGAATTTTGAATTCGTTCCTTGGTTCTTACCCGTTTTACCCTCATCTTTTCCCCTCTATTACGAACCCTAATTTCCCCTATATAAACCTCCTCCTTATTCATTCATAGGGGGGATTTTTTTTGAGCAAAAAAAATATTTGGAACAACTTAAGCAAAAACAGTAAAAATACGTACTAAGCACATTCGAAACAGAGGAGAGATCAGTGCGATTATAGTGTGGATTCCTTGCTCATTCATAGGAGTCCTCATAGTCTCGTTTTGGTGGTAGAGGTTGTCATCGTCAAACTCGTCCATCCGGTTCGCTGCCCAGAACCAAGTAATCTTCTTGCTCTTTCTTTAATCCTTTCTCTCTGTTGCATTGCAGAATTGTTGTTTGTTTTAGTATTAATTATTTTTTAGATTCAATGTGATTAGTGTAATGGTTATGTGAACGACAACAAAAGTTTTTATTATTCAATTTTAAATGAGTTTATTAAATATTTTGCTCTACTGATGGTTTTTTCTTCTTCTTATATAGTTCAAGATGTGAATTTGGTTCTTTTACTTAGTCGAGTTCATAAGTCTTTATAGGCGTTTCTAGTTGCTATTATTTTCATTCGACTTTGAAATCCATGTTATATAGTTGTTGATTTCTCTTCATTGCTTTCTTTAGATACATATGTTTAGAGTTGTAAGTTGTGTACTTCACTGCCTCAAGTTTGTTTGGACCGAGTGTTATGCTGAACTTTGTTGTGCCCAAATTATTAGTTTTTTAGCTAATCTCTAGTATAGTTTGAATCAATCGAGTTATTGTTGTTCCATCTAGCATTTCTTCATTATCATATGTTGTTTGTTAAAATTTCAGATTCAACACAGTTTGATTATCTTTCTTATGGTTAAAAGTTGTCCTAGCTCAGGCTGAGCTTTTCCCTTTCTTTTGTCTATTTAACAATCTGTTTGCTATCTCTGATTTAAAATGAATATACACTAGTCTTGTGATCATCTGGTTTAAATGTGTAACTTCCTGAAACCTGCCAGACCTAGCTAAACATTGAGTCTTGACAAATTTTTAAGTTTCCATGCCTAGAAATTTACTGTATATAGACTAGGATGAATCATGTAGTTAGAGAAGGCTATTGGTGTTGTTGATCTAAGCTTAAGTTCTTTTTCTTAAGACTTTTCCTTTTCGTGAAATATGAACTTGTCTATGATAAATAGCAAATTTTACTTATTTGGTTTTGCATTACATCTGATTAGACAGAAAAAAGCCTAAAAAATGCATCATTCTTGCTAGCTCGACTGTCATGTTTGTGTTGAAAATAGCTTACCTTTGGGGTTGCTCTTTAGCATTCTCCAAAGAGCTAGGCTTGGATGAATGAAAGCTTATATCGTGGCTCTTATGCATCCTAAAGTATATTGATTTACCATCCTCAGACCCCTACTTGAATTTGAGTATAGAGTTTCTTGGATTTACATTTAAATGCTACATGATAGTATTATCTTATTTTCTGCTCTCTGTTTTGTCCCGAGGCTGGTTTTAAAGGTTCAACTTAGGTATGGTATTAAGTGTTGGTCTGCTTACCTGAGTAGTTTGATTCTATTAGGGGTTGAATTTTAAATATTCGTTATGAAGTTTAACTTGAGACCTAGAGCCTCGTTCACAACAAGTTTAGCATGCGTTGTTTTCCATTTTTCATTGATCAATTGCTTATTGTTCCCACTGCTTTGTTTAGCTTTGTTAGCTATTTTAAATTGAATCAATTTCTCTGTTTTTCCTCTCTTATTTGAATTTCTTGGGTCCCTTTGCACATGTTTAGTTCTAGATTTCTCATAGAACCTCTGATGGTATAAGTTTTAGTTGGATCTTTTGAGTTTGGTTTACATTATGAGTGGATTTTTGGCTCCAAAGATATTGTTCCAAAGTAGTGTTCTGTTATTGTTTATCCAATGTTTTTCTGCACATCAGTAGCTAAGCTAAATATTATGACTCCTCTGTTTGTTTTAGCTTATTTTAAAAATAAAAAAAAATCTCGCACTAAGTGTTTAATTTTTTTTAGTTGTTAATCATGCATGTTCCTTATTTTGGTTATTAGTAGAAGGCCTACTCTTAAGATGGTAGTTAGATGTTGATAGATTCTAAGTTTTTTTTTCTGTAATGCTAAGTTAGCAGCTTAGCTTGATTTTTTATTTTATTGTACTATTATTATTATTATTTTATGTTAGTTCTAACGTTACTTCCTTTCCTTTCTTCGCATGTGACATTCATTTGAGTCCAATGGACTCCCCCCTTCTTGCATACTCCTCTCTTTTGAGTCAATCTCATCCGAGACGTGAAACAGAAGCTAATATTAACGGGTTAAAATCCAAACCACGGGCAGCAGCTCTATAGCAGAGTTACGACACTCCCAAAAGCTGAAAATTGGAAAATTTGGACTTAAAAGAGTCTCATTTAACCAGCAGGATGGCAGCAGCCCCAAAGGATTGAAAATTGGATTCTTTTTTGGACTTAAAAAGTCCAATTAGATCGGCAATAATAACACCATGGCAGCAGCAAAGAGAATTTAGGCCAACGGCCTAGATTCTTATTATATACTATCTCGATTTATGCCCTTAATTTGTTTTGGAAATGCTAACTTTTATTAACTTTTCTCATTAATTTAGATGAATCATTGGGGAAGTTTTTTCTATTTTGTTACTCATTTTACTCAAGCATTTAAATTTTCAAAACCATTTTTTTTAGTTAGACAATCTAAGCTTTAAAGCTAACTTGAGAATTATCCATATTCTTTCATAGTTAGTATTTAGCTTTCAAATCCATGCCAAAGTAATTGTGTTGATTATTCGACTCAAAGCCTTGACTCGGATAAATTGTTAAGTTAAAATTTTATTTTGACACTTTAGCATTTGAGTTATTTCATACTTGGAATAAGATGGATGTATAAGTATAAAAAGACTTGTTATTTTAAATAAGATTTTTCACAAGTAATCTTAAGTTTTTCCTTAAAATTAGCCAACTTTTAAATCCGTCGGTCAACCGCGTGTTAGCGGATTCTCTAAGATGCCTAAACCCTTCCTTAGAGAATTAATTGAACCCTTACCCGACTCTGGTTTATTCAAATGTTTTCTTTCAAAAATTATCCCTTTTAAATGGTTTTCTTAATTTTTCTTAAAAAATTAAGTGGCGACTTCCAAATTATAAATTTTTCCAAAACTTAACAAAATTGCCAAAAAGTTGCGAAATTGGTTCCGAAACCTTAGTTTTCGAAATCGGATCGTAACATCTAACATAGTTGTTTCTTTATCTTATTCAATTAATAGAAGAAATAGGAAGAAAAATGTTGCTATTCTCTC

>LNC_001810

CCCTTAGTGCGGGTATGGGGCCCTTCGTCAGCAGTGGTTCGTTTAGCAAGCAGTCTTATTGCTTGAAAGCACACACAGCACACAGTAGGAGAGTTCGAGTGCGAAAGCAGTGTACTCATGCCCCTGGGATAAGGTATGCTTTTAGTGCGTTATCTAACTTTAAGAGTAGGAGTAGCAAGCACTTACTTTCTTTAAGACGCTAACTTCCACAGCAGCTAAGAATTATCTCAGGTTTTCCGTTAGTGCGCTCCGGAAAGAAGGAGCAAAGGTGAAAAAGCTCAAGTGATTTTCCCTTAACTACTGTCAGGCCAGGGAAGATAACTGCATACCCTAGGGAATACAACTCCATAAGAGCGAGAAAGTACGGGGATAGAAAGAATCACACGATTCCCTATCGTGATAGAAGCAACAATAGGATAGGAGTGCACATGTTCACAACTGGACTCTTCTCTGACTAGAAGAGCTGCGCCTAAGACTCTGAAGCGAAGGGAGGTGGTCCCGAGCCTTGCCTATATGGCTATATAGCGTCAGCGTTGTT

>LNC_001811

CGATACTTCATCACATTCTCTCATCCACTATAGGTTCTTCATCTTGCAGAGTTTCCCAGACGAAAAATTGGAAAACTTCCCGATTCTCCAAGAACTATATTTTTATGAAGTTTTTCATCTCCCATAAGTATTGGCTTTTCATATTCAAATCGTCTCCGAGTATTACTGCAATTTTGAGGAATTTTAATATTTTCATTGGTGAATAAGGACCGACAAGCAGAAGATATTGAAGTTGATTCTTGAAAACTTTATTCACTATATCTCATTTGGCTGGTAGTGAATCTTCTAAGTTCTAACCCTCATTCACATGTTCCATTTAATCTTAGCTTTATAAATTTATGATATGGCATGCATAAAAGTCACTCGAGAATGTTTTTTACTGCAAATCTCAAAACTATTTAAGGAGAACATATTAGTGACTAATATGATTAATTTTGGGATAATTTAAAAGGAAAGAAAAGTAATATAATTTCCCGCACAAGACAATAATTGCATATTCTTTGAAGTAAATGACCGAATCATCGATAAGAACAACGAGCAATAATATTTAAATATTTTATTTCTAACTGCACAAGTTCATGGTGAACATATTATGTCGTTAAACCTTCAATTGATAGGGTTAATATTGTTCTATTTTTGTTGGTAAAATTTTAAATATGTGACATAATGTATTGTAGACATGGTGTATTTGAAGATGCAGGATTATGTGCTTGATCATATCCATCACAAATTAAAAAATGAACCAAACTTCAATATAATAGATAGTGTTTACAGGTCCCCGAGTGAGATTCAGCAGGAAAGATTGAAAAATCTGAGCTCTATTTTAGAATCTCCCGCAAAGAAGAACTCAGGGACGGAAATAAGCTCCTAAAGAATGCACATTGTTATTCTCTTTCCCAGATTGTTCTACTCAGCAAGCTGTTTACAAAGGGAAGGTTAGCCAGATGAAGATGCTTGGAGGATGAGAAGTGTGGACTACACATCAAGGATAATGAGTATGCTGAACATCTCTTTTTTATGTGCCCTTATTTAGTGCAAGTCTGGAATACCATGCTAATATAACTGCAAATTCAAAGACAAGCTCAAGGATGGAAGTGGGATGAATTAGAAAATTACAAACTTCAAAGACAATAGTTTACATGCAGAAGTTTTTAGAATGACATTAGCTGCGGGAGTTTATATGGCGAAAAAGAAATTAGAGGATCTTCCAAACAGAGAACAAAAGGCCATAAATCCTCAACATGTTGTTAATTCAAGATACTCATACTAGAGGAGTTAGTGAAAGCAACATTGTTTAGAAAGTTATTGGCTATCTGGTAATGTTTCATTGTGAAATTTGAAAAAAAAAAAAAAATTTGTTTTCAAAGTGAAAAATGATATATGAAATTTGGAATTGTGTTTGGTCATGAATATAAATTGGAGTTGTTTTTGACTTTTTGTGAGTAATTGAAGCGCAAGTGTATAGACAAGTTTAGCATATAAATTGATCCATTCTCATCTATTTTTATCTGTGAATTTCAATAATCGAAAGTGAAGTGAAAAAATTTAATGGTTGAATGGCAGATCTAAATGCTATTATAGCTCCAACAGATATCAGTTATACAAGAATCACAGTGATCATAACTGTTGAGGATTAAGCTTTGTGGAGCTTACACATCCATCATTCATATGTATACTCATGTCAGATATTTTCATAGTTACTGCAACTTCCGTT

>LNC_001812

TCCATCGATACTTCATCACATTCTCTCATCCACTATAGGTTCTTCATCTTGCAGAGTTTCCCAGACGAAAAATTGGAAAACTTCCCGATTCTCCAAGAACTATATTTTTATGAAGTTTTTCATCTCCCATAAGTATTGGCTTTTCATATTCAAATCGTCTCCGAGTATTACTGCAATTTTGAGGTCCCCGAGTGAGATTCAGCAGGAAAGATTGAAAAATCTGAGCTCTATTTTAGAATCTCCCGCAAAGAAGAACTCAGGGACGGAAATAAGCTCCTAAAGAATGCACATTGTTATTCTCTTTCCCAGATTGTTCTACTCAGTTCACAAATACTTTGAGGAAGTTGGTAGGTGAGAAATGGATGTACTAAGATCAGATAAGTTTACTATCAGCAAACTCTTCAGCTAAAAAGAGCTTACCAGAAGGTTAATTGGACAAGGCTGATTTACAACAAAATATACCCTCCTAAATGGTCAACTTATCCTATATAGCAAGCTGTTTACAAAGGGAAGGTTAGCCAGATGAAGATGCTTGGAGGATGAGAAGTGTGGACTACACATCAAGGATAATGAGTATGCTGAACATCTCTTTTTTATGTGCCCTTATTTAGTGCAAGTCTGGAATACCATGCTAATATAACTGCAAATTCAAAGACAAGCTCAAGGATGGAAGTGGGATGAATTAGAAAATTACAAACTTCAAAGACAATAGTTTACATGCAGAAGTTTTTAGAATGACATTAGCTGCGGGAGTTTATATGGCGAAAAAGAAATTAGAGGATCTTCCAAACAGAGAACAAAAGGCCATAAATCCTCAACATGTTGTTAATTCAAGATACTCATACTAGAGGAGTTAGTGAAAGCAACATTGTTTAGAAAGTTATTGGCTATCTGGTAATGTTTCATTGTGAAATTTGAAAAAAAAAAAAAAATTTGTTTTCAAAGTGAAAAATGATATATGAAATTTGGAATTGTGTTTGGTCATGAATATAAATTGGAGTTGTTTTTGACTTTTTGTGAGTAATTGAAGCGCAAGTGTATAGACAAGTTTAGCATATAAATTGATCCATTCTCATCTATTTTTATCTGTGAATTTCAATAATCGAAAGTGAAGTGAAAAAATTTAATGGTTGAATGGCAGATCTAAATGCTATTATAGCTCCAACAGATATCAGTTATACAAGAATCACAGTGATCATAACTGTTGAGGATTAAGCTTTGTGGAGCTTACACATCCATCATTCATATGTATACTCATGTCAGATATTTTCATAGTTACTGCAACTTCCGTT

>LNC_001813

TCCATCGATACTTCATCACATTCTCTCATCCACTATAGGTTCTTCATCTTGCAGAGTTTCCCAGACGAAAAATTGGAAAACTTCCCGATTCTCCAAGAACTATATTTTTATGAAGTTTTTCATCTCCCATAAGTATTGGCTTTTCATATTCAAATCGTCTCCGAGTATTACTGCAATTTTGAGGTCCCCGAGTGAGATTCAGCAGGAAAGATTGAAAAATCTGAGCTCTATTTTAGAATCTCCCGCAAAGAAGAACTCAGGGACGGAAATAAGCTCCTAAAGAATGCACATTGTTATTCTCTTTCCCAGATTGTTCTACTCAGCAAGCTGTTTACAAAGGGAAGGTTAGCCAGATGAAGATGCTTGGAGGATGAGAAGTGTGGACTACACATCAAGGATAATGAGTATGCTGAACATCTCTTTTTTATGTGCCCTTATTTAGTGCAAGTCTGGAATACCATGCTAATATAACTGCAAATTCAAAGACAAGCTCAAGGATGGAAGTGGGATGAATTAGAAAATTACAAACTTCAAAGACAATAGTTTACATGCAGAAGTTTTTAGAATGACATTAGCTGCGGGAGTTTATATGGCGAAAAAGAAATTAGAGGATCTTCCAAACAGAGAACAAAAGGCCATAAATCCTCAACATGTTGTTAATTCAAGATACTCATACTAGAGGAGTTAGTGAAAGCAACATTGTTTAGAAAGTTATTGGCTATCTGGTAATGTTTCATTGTGAAATTTGAAAAAAAAAAAAAAATTTGTTTTCAAAGTGAAAAATGATATATGAAATTTGGAATTGTGTTTGGTCATGAATATAAATTGGAGTTGTTTTTGACTTTTTGTGAGTAATTGAAGCGCAAGTGTATAGACAAGTTTAGCATATAAATTGATCCATTCTCATCTATTTTTATCTGTGAATTTCAATAATCGAAAGTGAAGTGAAAAAATTTAATGGTTGAATGGCAGATCTAAATGCTATTATAGCTCCAACAGATATCAGTTATACAAGAATCACAGTGATCATAACTGTTGAGGATTAAGCTTTGTGGAGCTTACACATCCATCATTCATATGTATACTCATGTCAGATATTTTCATAGTTACTGCAACTTCCGTT

>LNC_001814

TCCATCGATACTTCATCACATTCTCTCATCCACTATAGGTTCTTCATCTTGCAGAGTTTCCCAGACGAAAAATTGGAAAACTTCCCGATTCTCCAAGAACTATATTTTTATGAAGTTTTTCATCTCCCATAAGTATTGGCTTTTCATATTCAAATCGTCTCCGAGTATTACTGCAATTTTGAGGTCCCCGAGTGAGATTCAGCAGGAAAGATTGAAAAATCTGAGCTCTATTTTAGAATCTCCCGCAAAGAAGAACTCAGGGACGGAAATAAGCTCCTAAAGAATGCACATTGTTATTCTCTTTCCCAGATTGTTCTACTCAGGTTGTTGTTTTGCCTTTCTGGTTAGTTGTTCCTTCAATTTATTGGAGCCTAAGTTGTTTTGGTGGTAAATATTGTAGATTATAAAAAATGGTATTTTTAACTCAGATAACACTTTCAAATATCTTTAACTTTTGGAGCCTGATTGTTTCGTTAGCTGATGAATATGTAGATTAGAATGGTTCAGTCTTTTGAGCTGCATGTTGAATTTTTAGTCAAATGTCTTGATGGATCACTAGCTAATTGTTGAAATTCATCCGACTCATAATTTTGATTGGGGACAAAATATAGTATCATAGTTTTGCCAAAATTAATGAACTGTTTGGAGGCGAAAAGAGTGATTCTTTTCGTTGGACTACAAATTGACCATTTTCATATATTTTTATCTGTTATCATCGAAGTTCACAAATACTTTGAGGAAGTTGGTAGGTGAGAAATGGATGTACTAAGATCAGATAAGTTTACTATCAGCAAACTCTTCAGCTAAAAAGAGCTTACCAGAAGGTTAATTGGACAAGGCTGATTTACAACAAAATATACCCTCCTAAATGGTCAACTTATCCTATATAGCAAGCTGTTTACAAAGGGAAGGTTAGCCAGATGAAGATGCTTGGAGGATGAGAAGTGTGGACTACACATCAAGGATAATGAGTATGCTGAACATCTCTTTTTTATGTGCCCTTATTTAGTGCAAGTCTGGAATACCATGCTAATATAACTGCAAATTCAAAGACAAGCTCAAGGATGGAAGTGGGATGAATTAGAAAATTACAAACTTCAAAGACAATAGTTTACATGCAGAAGTTTTTAGAATGACATTAGCTGCGGGAGTTTATATGGCGAAAAAGAAATTAGAGGATCTTCCAAACAGAGAACAAAAGGCCATAAATCCTCAACATGTTGTTAATTCAAGATACTCATACTAGAGGAGTTAGTGAAAGCAACATTGTTTAGAAAGTTATTGGCTATCTGGTAATGTTTCATTGTGAAATTTGAAAAAAAAAAAAAAATTTGTTTTCAAAGTGAAAAATGATATATGAAATTTGGAATTGTGTTTGGTCATGAATATAAATTGGAGTTGTTTTTGACTTTTTGTGAGTAATTGAAGCGCAAGTGTATAGACAAGTTTAGCATATAAATTGATCCATTCTCATCTATTTTTATCTGTGAATTTCAATAATCGAAAGTGAAGTGAAAAAATTTAATGGTTGAATGGCAGATCTAAATGCTATTATAGCTCCAACAGATATCAGTTATACAAGAATCACAGTGATCATAACTGTTGAGGATTAAGCTTTGTGGAGCTTACACATCCATCATTCATATGTATACTCATGTCAGATATTTTCATAGTTACTGCAACTTCCGTT

>LNC_001815

CACAATTTGAAGGAGCTTGACCAAAATTCTTCTTCCCCAGTCCAGGCATTCCAACAGTTGATAACATCTAGATCTGCCAATCCTTTGTATTTTTGTTCTTCTGTTTTTCACATGACATCTTCAAAACCCACTATGAGCTTCATTATGAGCTATGAAATGGTGAGTTTTGATGATGTCATGCCTAAAATAGGATTAATGAAAGTATTAGTAAAAAGGTGTTCAAATCTGGAGGTTATGTTAAGCTGGTGGTATGCTTTGACTGGGAAAGATGACTTAGGTCAAAATAAATATATCCAGATCTGGCACAATCTTGTGTTTGCTAATGGTATACGAAGGAAAACTGTATCTGACAATGAAGGTGACCAAAGATATTAGTAAGAAGTAAC

>LNC_001816

CACAATTTGAAGGAGCTTGACCAAAATTCTTCTTCCCCAGTCCAGGCATTCCAACAGTTGATAACATCTAGATCTGCCAATCCTTTGTATTTTTGTTCTTCTGTTTTTCACATGACATCTTCAAAACCCACTATGAGCTTCATTATGAGCTATGAAATGGTGAGTTTTGATGATGTCATGCCTAAAATAGGATTAATGAAAGTATTAGTAAAAAGGTGTTCAAATCTGGAGGTTATGTTAAGCTGGTGGTATGCTTTGACTGGGAAAGATGACTTAGGTCAAAATAAATATATCCAGATCTGGCACAATCTTGTGTTTGCTAATGGCTTAACATTGAACATGAA

>LNC_001817

CACAATTTGAAGGAGCTTGACCAAAATTCTTCTTCCCCAGTCCAGGCATTCCAACAGTTGATAACATCTAGATCTGCCAATCCTTTGTATTTTTGTTCTTCTGTTTTTCACATGACATCTTCAAAACCCACTATGAGCTTCATTATGAGCTATGAAATGGTGAGTTTTGATGATGTCATGCCTAAAATAGGATTAATGAAAGTATTAGTAAAAAGGTGTTCAAATCTGGAGGTTATGTTAAGCTGGTGGTATGCTTTGACTGGGAAAGATGACTTAGGTCAAAATAAATATATCCAGATCTGGCACAATCTTGTGTTTGCTAATGGTAGAAGAAAATGGAAGCTTGTACAACACCAAATATAGGTAACAATGATGGGAGTCTCAAGATATTCATGTTGTTTTCCTC

>LNC_001818

AAAAAAGGTAGTTACTTCAGCTACCAGAGTTGGACCTTATTCACACTTGTAAGATATACTATCATTGCTCTTGAAGGTAGCTAGATCATGGGAATCTTAGTAGTCCGAACTTTTCCTTGTTGGTGAGGGTTTAATTTTCAACCTTGTAACTCTCTCTGTTCCCCTTCCCGCTGTGGTTTGACATAATTAGTTTTCTGGTGTTTTGAGAAGTGTCTACAAATTGTTTTGATGTTTTAACCAAGCATGGAGAAATTGCTTCAACAACAACAACATACCCTGTGAAATCCCATTAAGTGGGGTCAGCGGAGGGTAGAATGTACACAGACCTTACTACTA

>LNC_001819

TGAATTCCTTTCCTTCCAAACAGCCCACCAGATAGCCCCCGGGATAATTCTCCATCTACCTCTATCCTTGGCATGTACACCTGCTTCCTCCCAGCATAATAATGCCTCAGCCACCTTCCTGGGCATTGTCCAGGAGATGCCTTTGAGGCTTAGGAATAATCTCCACAGATGATGGGTGTACTTGCAATGTAAAAATATATGGTTAACAGTCTCAGATGTTTCACCACATAGGAAGCACCTAGAACACAGGGTTCTCCCCCTCTTCATAACATTGTCCTGTGTAAGGACGGCTTCCTTAGCCATTGACCAGACAAAAAAGGAGACTTTGTGAGGGATTCTGCTCTTCCATATTTGCTTCCAAGGCCAACTTGAGCACTGTTGATCTGTCTGATCCAT

>LNC_001820

TTCATCTCCATGTTCCGGTAACAACTCTTAGATCTCCAACAAACAACTACTAATGGCAACCTAAAGAACTGTTAGCTAAGTTTTCTTCTTTTCTAGCATTATTTTGTTTTTCCAACCCCTTTCTCTGTCCCTATTTTATCATATTTTTCTGTGTTTGTTCCGTATTAATTCATCCATACAGATTGGATTTTGGTGAATTTTATATGCTAATTATCCAGATCTGTCCGGAACGTTTGGAATTCCCACCAATCGTTGCCCGTTCTCTCCGGTAAAACATCAATGTTTGTTTCTGCCTGTGAACCTTTTGTGTTGATTATTATATATTATTGTTACCTTATCATTCTTATTATTATTGGCATGGCATATTGCTTTGCTGAGATTAGAATTTCAATAATATCATATTATATCTTGTTTGTTGTTTGTTTTTATTTATTTTTTATTTTCTTTTACTTCTTGTTTTTAATTACTGTTGCTAATTTTATTTTATTTTAAAGATAATGTACATTCACATGTTCCGATATAGAGGTAATAAGTTTTGGTTTGCTACTATTTTACAT

>LNC_001821

GTTGTTGTTGATGTTGTTAGTTGTTGTTGATGTTGTTAGTTGTGTTGTTGAAGTTATTGTTGGTTGTGGGACAGGATTGGGCTGTGTATTTGAGTTGTGATCTATTGTATGTTGAGAGATTGATGATCCTTAGTGTTGGTTGGTTCATACCCCCACACACCAACTCGTTTTTAATTCCAAGAGAAGATTTATTGGTTGTTGAAGTTGTAGTGGTGTTGTGTTGAAGTTCTTGCTGGTTTAGTACAGATTTCTGGCTGTGATTTTTGAGTTGAATCTATTGTATATTAA

>LNC_001822

CTAACATTCACAAAAGACAAGGACATGGCGTCTACACAGCAATCCCAAACGGCGTGAACAACAATTCCAAAACGACCCCAAACGGCGTGAACAGTAGACGGCGAATTCAACGAAAATTCACAGCGAAGAACGAGTACGTACTCAGTACTCGTTGTCTTCTTCCTCTTTGGCCTCGACAATCCGTACGTGGGCAGTGCTAGGGGGAGCAATCTCCTTCCTCGAGCGTCCTTGATTCGAAATCGGACGGCTCTCCTCAACAATCCGTTGCAAATTTGTTGAATTTGGTACTATTGCCAGCCGTTTGAGCCTTATCTCCCAAGGACCCTTTTTCCCTCTCGATTTCCCCCCAAAAATCAGCCTAGTCTCCCTCTATATAAACGCTTTTCTTTTTGGAAGAAAACTTGGGAAAAAAGTTGGAAAAGGTTGGCAAGAGAAATCATATAAAGAAACTTTTCTCAAATCTGTAAGCATCATCCAGAACTTACACTTTAGATACATATTTTTTTTCTTCTCGTTCTTCGTGAGTTGCGGAGGAATTAGTCGGATTTCTCAAAGTTCTCATTTGTGGTGTTAAGCTCGCTCGATTTAAAGCTCGTTTGTTCCGGTTGCTGCTCATAAAAAGGTAATTACTGCCCTTGGTAACACTCTAACCTCCGTCCCTTGCCCTCTCTCTCTTTTTTGAATCATGGATACATCATTTTGATACTATCTGAAATGGCATGTTGTGCCTTGGTATAGCAGGAATATTTCAATATATGTTGTTTTCTGTTTGTCGTAGATGATTTTGGTACGTTTCAAAATGATATCGAACTTCGTTTATTTCATGAGTGTGTTATCAATCAGTCTGCTTTGCTTATATTCCGTTCTGTCTTTGATTATCATCTTCTCCTGGTTTGTTGTTGTGTTGTTCATTTCGAATCAGTCCTAGCATGATTTATCAAATTAGTATGTCCCAGTTGTTGTTTACCATTCTGTATTACCACAAGTTTATTTGGCGTGAGTTTAGTTTGTTGTTCTTTGTCTCGGGTCAATTAAAGTATAGTGTGGCTGCCTTTGCTAATCGTGATATGTAATTCACTTATGCATTTTTGAAATGGTCTGTTAACTTTCTGTTAAGCACTTAGCAAACTTGATTTTGCTTATCCGTTTCTCTGTTTATTATATAGCATGTAATCCCTCCGCCCTTGATTGGTCGCTATGACAAAAAATAAGACTGTAGCTTCTTGTGATTAATATATGGTTAGATCTTTATATTTTGGTAATGCATCCTTATATTGATTGAGTTCTTTGTGGTTAGAATGATCGTCAGCCTAGATGTAGACAGAGGTTTCAGTTTTGCTGCTTCATGTGATTGTCTAGGGTCTCTAGTTATGCATGAAAAGACAATGTTTCTTCTCATTGCCTTTAGTTTAGATTTTCATTATAAGAAGTGTCTCTTCATGCCTAGTTCTGTTAAGTTCATCTACATGGTTTTAATTTTAAATAGTTTGCCCACAATCAAGAATAATTAACTTTGACTAGTGACTACTGGGCCTTCTTAATTGCTCCTTTAAAGTTGTTCTTTTTTGGGTTAATTACTTGAGTAAATCCCTTTTAAAAAATAATTACTTATTTTAAATATACTCTTTAATTATTACCATTTATAGCAATATATAACAATACAATGTATTTTATTAAATTGTGTTTCTCTCGCTCTTTTTTTCTCTCTCTCGCACTCTTTCTTCCTTTCTTTCTCTCTACAAACTTTTTTATGAGCTCTTTTTCTATCTCTCCAATCCTTGGCTGATCAATGATGTAGCAAACGAAAGAGCATTTTATTATCTGTAAATTGAATAAAACTTTGTATCAATAATGAATTAAACAAGTAATCCAATCGTAACAAATAAACCAATAAACTGCAAAATGAATTAAACTTGTATTAAAAGTGTATTACACAAATATTAAAGTTGAATTAGAAGAGAACATTAAGCATGAATAAAAAAACGTAACTAATGAAAGTATTATCCAAAATTAAATTTGCATTAAAAATGTATTATACAAAATTAAAATTGAATTAGAAGAGAGAATTAAGAATGAATGAAAAGTGTAAGTAACGAAAGACAAGACAATAATCTATAGATTGAATTAAACTTGTATTATGTTCACATTAAGGCCGGTGTGATCTGTGATTTGCAAATTATACAGATTTGTCTTATGTGAAATATTAAACATTAAGCCCTTATTGTATGTTAGGTTGGAAAAATTATCTAAATACACAACGTATTTTATTGTATTTTCTAAAATTTTCTACCATTTGAATTTTTTTACAAAAATCTCTATTATCTCCTATTTCCAGATACATCACTTTACGTGATGTATCAAGGCGTTTGAGTGATGTATCCGAAATGAACACAATGTATCGGGATGTATTCGAAACCGGGATGCGATGTGTTGGGACATTTTGGGATGTATCCGGATCCCACCATATTTAAGAGATTTTTGTAAATTGAAAAAAAGTAGGGATAAATATAATTAGCTTTCAACACTATAAAATTTGTGTAAAATTTTCTTTTTATTTTTGCTTAGTAATAGAGCTATCTGTCCTAAGGATAGTCAAACGAATATTAATCGAAAAAATTACATGAGATATATAGGAAAAATATTGTTCTAGATTATTTTTTTAAATCCATATAAAGTGAATTATTCAGGTATAATACATTCATTAAGAAAAAATATTTTAAAATATAAATAAAATGTAGGTTTTCACTATTGCCCTTTTCTCATAATATGTGTATTTACTAATTTTTTTAGTGGGTAAAAATTGGATTGAAGATCCTTACTACTAAAAATTCGGTGAACATTTTGACAATTTTTATCTGACTTTAATTTATATCCTTTTAAAATTGTGTTTCGAAGTCCGACTGAATATGATTATGATTAGAAATTCGGTCCACACAAATTAAAAATTAGTACAAGTTTAAACTATTATTTTTAATATGAGATTATATTTTAAAGGTATAAAAGTTGTTCAATATTTAAATGATATTTTGATCAACCAACATTTATATTCATGTTGTTGTAACTTTGATGTTTTAATGATTTTCACAAACTATGAGACCTGGTCCCATGAGTCGAGCATGTATCTCTTGTGAAATGGGGAACAACTGTAAAACGTGATTGGTGAGCACGTGAAAAGAGGACAGTATACTCAGCCAATGAAAGTACACTATGTTGTTTTATTCAGAGGATAATATATTCTTATTAACTTGAAACAACCTAGTCAAAACAATTGAGCATAATTGAAAAAGTTGTCTTTTGTCTCTCTCAAGCATAACACAAGCTTTCAAGATCTACAAGGGACCTGATAGAGTTATCAAGTGTTTCGTTTATTGTGATTAAAAGATTGTATCTTGTGCGTTTCAACTTGTAAGATCCATTCCTAATCAATAAAGGAATAGATCGTTTCTTTACTACTTTAACAAACAATAAGCATAGTTAGAGTTAATAGTGTAGTATGCGATTGAAGTCTATATTAACTAGAGTCAATTGATACAATTGGCAATAGAGCTATTGCTTTGACACTTTCTGCAATAGGGTGTTCTAGGCTGTAGGATTAAGAGTTTAATTCGTACATGGTTGATTAGAGTCCGTATTCACTTGTTCAGTTGAGGAATCAGCTACTATATGACCTCGCCTTCTGTACCAGTTACTAATTTGGGCGTTCTGCAAATTATATATGATGGTGAAAACAAACAAATAGATAATTCAAAATGAACCATGTAGGGGTGAAGATGTAAGAAATTAAATTAATTACCTCAGCTGCTATATCTTCCTTAATCCCAATGTCTGAATCTCCAACGCTTAACTGCAAATAAAACAAATTACTAATGAGGCGAAGCAATATATGAAAGAAAACACTAGTAATCAGATGAAAATGCATTGATACTCTCCCGCAATCTCAGAAACTTATTTCTTCTATGACATAATACATAAATATATCATTTAACTTGGCTTCAGCTCGTATCTATGTCTTGTAGACATTTCAACTTGTATAAATTGAACAAGTAAACATATGCGTCCTACGTGACATAATACACCTGTAGAGATGCAAACAGAGGCTACGAACAGCCTTTTATTGACATCCAACAGGAAGTGCTTATACCAGT

>LNC_001823

AAAGTTTTGGCCTTACCAAAAGATGTGTCAATTGGAGTAGGACAAAAAACTATCACATGTGGTGGGTTATGGAATGCCGAAGGAGGTGGGGAGATAAAATTAATTGTTTTTTGTTATAAAACCAAAGTGAATAAGACAAGAATATTAAAGATGAAAATAATAGTACAGCAAAACCTCTCTAAATTAATATAGATTGAACTATAAATTATTATTATTTTATAAAGGTATTATTTAATCGATAAATTAATATTTATTAGTTTGAAAAGTGTTTCGACATCGGAAAAATTATTCAAGAATGAAGTCTCATAGAATTTGAAAGAATCCACTTGTAAAAACATCGTTCATGAACTTAAGATCATGATTAATGATTTCGGTTACCGCAACAAAATGGACGTTAATAACTTATTGGATGGGTGAAAATGATACAAGTTAAGAGGTCAAGAGCTTAGAACAAATTGTGGATATCATTAGAAACACAATGTTGATGATACAATACATTTGGAACCAGTTACACGTAAGGAAACACTTGCATCTAGAACTCTTCACAATTTTATGGTGCAATTAGAAAAGACAATACTGAAACTTTTGCATGCAATAAGAAATTTTAACTGATATAATATTTGTTTTTTCATAACAAGTAAAATAAAGAGACTTTTTGAAGAATCTTTTATTTCTTTAATATATAACCATATGAATTCTCGCTTATTTACACATTATAATTGAATTGAAATGGCCAACCGATTATGACAATTAATATTTGTTTTGGTAAACCTAAGACAAATTTGTATAATTTGCAAATCACATACCGACCTTAATGTGAACATTCCACTGCTAGTCTAATCAAAGTCTAGATTCGAAATTTATTAGTTTAAGCTTACGAGTATTTTGAATTATTAAAACAAAGTTTTTCTACTCGAAATGACAAATATATTTGCAAATGGATAAGCATTTGTCAGTTGATATTTTCGGGTTAATTTAAGACCGAGGCGATTAATAATAGATTTTAAATATTTTCTTTATCTAAATTTTGTGGAATCGAGTGAAAGATGGAGTTATATTTCATTATATTTTTTTTGCATATAATAATAGGAATAATGTTC

>LNC_001824

CATAATTCCATGAAGCCTTGTCCTCTAGCTTTAATGTGGGTCATTTGGAGAGAGAAAAATAGTAGATCTTTTGTAGGAGTGGAGATGAACTTTGCTTAATTGAGGAGTACACTCCGAGCCCTTATTTTCCTTTGGTGCACCCATGTAGTTCCTGAATGTGTAGAGGATTGGGTGGCTGTTGGTGAGGACCATATTTTGTAGGTCTCTCCTTTTTTGGTATACCTCTTGTATACGGCCATGTTGACCTTGTTATTATCAATGAAAACTTCAACTTGATCAATAAAATAATTCCATGAAGC

>LNC_001825

GAATTGAGAAGATCTATCAATTTGTCTCTTCCATTTATGTCCTGTAACTGACATCAAAGAGCTAGACAGTGAATTACTTGATCCTCATCAACTCTCCTTCCAACAAAGACCCTTTTGTGTCTCTTAACTCCGAAACTGCCCACCAAATGGCTTCAGAATGGTATTCCTCTGTGCCACTGGAAGAGTGAATCTTTTGTAGTTTCTGGCATGCACCACTCATCCACTATACATTTAAGTACATGCATAATACCTGGATCCTTATCAAAGAGAGATAGAGATAGAGAGAGAGAACTAGCATCTTTGCGGGCTTTCTTCACTAATGTTATCTGTCGCAATAGCAATACACCTCTACTTTTGTAAGCCGTACTGAGTCAGACAGGACTATGTGCCTCCAACCAAACACTGCACTTTGTGGGATGCTTTATTTCTCAACTCTGTTTCTTTTTCCAACATGTGGTTGCTCTTGGCCTTTCCCTACTTGTGTCTTGTTGTGACTTGTGATTGTAGTCATATCTTAGGTATATAGCAGCTGTAGGGGGTTTTCAATTCCAGAATGTCAATTGCTCTGTATCACCTTTATCCAC

>LNC_001826

GTTTATTGACTTCTTTGGTGATGACTATCGTAGTTTAGAGTAAGCAAGAGCTGGAACTTTTGCTGAGATGATCATTTTTTGGCTTGGATATGGTTAATTTGGCTACAGGGTTGTGCTAGAAATTTTAATTGCATTTTGTATAGTAACATTGCTAGCAAAATTTAACTACAATGATCTGTCTGTGACATAGTTTGCCACTATTTCCCCCCTCTATCTATTAAAGCTTATTGTCTTCATTATTCA

>LNC_001827

TTTTTTTGAGAAGGTAACATTTGTATTTCTATTTCCAAAAAATCAAAACCTGCCACAACATGTTACAGATTCCTAATTACAGATTAAAGAGAGTATAACAGAAGATCTTCAATCCTATGACAAAATTTACAAAAATTACAGGGATCCTAAGTCATTCATAACTGATTCTAGATCTTCCATATACTCCTGTTTACACCAAAAATGAAATAACACAAGACAATTCATCTTCATCTTCTGAATAGTGTTGCTCTTGTTCTGATGACATCTAAGGTTCCTTTCTTTCCACGTTGACCACCAAATACAAGCTGGGATGATTTTCCATCTCTCCCTATGTCCTGAGATGTTGGCATAACTACTCC

>LNC_001828

GTTTTATTATTTTTGTTATACATGTGAGCACTATTGAGGATTCCATTTGGATTCCAATCCCACTTGCGTTTCGAATGGGCCACGGAAGCCCAGTTGCATCCGCTCGAGTCAGCCTCCATTTTGCTCAGGTCCACGGGAGTAAATTACAGCGTATAAATCTGAAAAAATGGAATTGCAGGTCCTAAAGTCTAGGGGAATGGATCGTATACAATCTGTATACACTTGTTATACAGTAATATACAGGCCAGATTATCGGAAATACAGGTTGAAA

>LNC_001829

TTGGTATTAAGATGCTAGTCGTTAGATTCGCTGGTTCAACGGGGTATCAAAATGATGTACTGTTCGATTGGTTTCGACTTTGAATATGCTATATGTATAAGTTTGATTGGTTATCCTCTCTATCGTTTTATTCATTATGATAGACTCTCATTTATATTAGCTCAATCTCATGCTATAGTTAAATAAAATCTCTGCTTTTACATACTTGAGTGCTGTCCATGTCTTGTTATTGTTTTGTTTCTTCTGGTCTCAAAGCATGGTGTCATTATAACTCGCTTGTTTCCATGATCCACAACCTCGATGCTAAAACCTCTCATCCTTTCCATGAAAAACATTGTTTCCTGACCATGTGAGTGTATTGGCATCTACATTCTATTTTATTATGTCAAATAAAGTTTATCTTCATGATAAGTATCGACTATTTGTACGATGTGATTAACGAGCTCAATAGTCTAAGTGTAAGATTGACCTCCTTGGCTTTCTCTGTTAAGTATTTCATTTCAAGCTTGTTGATAGTTTCATTATTGTGCAACGGTAGATGATTTCTAGCATTTAAAGTTGTCCAGCTTCAGTTTTGTGTAGCATTAATCATGTTTGAGAGTATGAGTAAGTGCTCTAAATTTGTTTGTTTGTTTATTAGTATCTAATGTAGTCCTTATATTTTTGCTAAAAAAGAAACAATAAAAAATCAATAAATAAATATGCTTTACTTTTCACATG

>LNC_001830

CCGTGGTCCAGCTTACCTGTGATCTGGTTTTAGCTACAGCACCCGGTTAGAGTCCGTGGTCCAGCTTACTTATGTCCAGGCTTAGCTACAGTACCCGGTTAGAGTCCGTGGTCCAGCTTACCTGTGATCTGGTTTTAGCTACAGTACCCGGTTAGAGTCCGTGGTCCAGCTTACTTATGTCCAGGCTTAGCTACAGTACCCGGTTAGAGTCCGTGG

>LNC_001831

TGCAGATGGAGCTGATTGGCAAGAAGAGGTTTACCAACAGTCTTGACATTTCTGCAATTGACTTAATGAAAATTTGTAATGCAGATCAAGTCTATGAAGGAGAAGTATTTATCAGATATCCATGATGAATACCAGAATATTGCTTCTAAAGTGCAGCAGGTATGTCATCAACATCTTATTCCACTATTATCATTTCTGGAAGCATGCTGGATTTACTAGAGTTTGATATAATTTGAGATGTTAATAGTTCTCAGAAGTCATTCTTCTTATGGGGACATCACTTGCAATCATATATTTTGTCTTCTTCTTGTCCTAAAACCGTTGCCTTCTAAGTGCTTCATCAACAACAACAACATACCTAGTGTAATCCCACAGTTTGGGGTTGGGGAAGGTGGTGGGTAGGCAAACCTTATCCCTATCTTGTGAAGGCAAAGAGGTTGTTTCCAAAAGATCC

>LNC_001832

TGCTTCTAAAGTCTGGCAGGTTTGTTTTTTAGCTAAATTAGCAGATCCCAAGCGAACCATAGTCCTTGATATAATTACTGTTACTGGATTTTAGTCTTAACTCTTCTCTTTTACTTCCTTCAATCATCATAATGTCCCACCTATTGATGCTTTCGGACGTCTTCTTAGGACATGTCTATACCTTCTTAGGTGAGAGAGCCATCTTAGACGACGTCTCATTTTAACCTTTATGTGACACTGTTATATAGACATGATTTTGATGTTAATCTTTTTTCCTTCACTGAAAGGGAT

>LNC_001833

ATAATAGAGAAGAATAGTAACAAGAACAAATACTACGATAATTGAAGGAAAGAAAACAACAAGTGGTAATAAAGATTAGTAATAATACTAACGGTGAGAAAAATTAGACAATGCTCAACTAGCTACTAGCCTTATACCATGATCTCCGACCTTCATGTCCTTCTATCTAGGGTCATTTTCTTGGCTACAATTGTGTTATGTTCTTTGTAATCATCTCTTCCCAATATTTCTTAGGCCTACCTCTACCTCTCGTTATATCCACCATATTCATATCCAACCTCTCAAACCTTCTTACTGGAGCATCGGAGCATCTCCTCTTCACATGCCCAAAGCCTCTCAACCTCGCTTTCCTCACGTGTCCACCACGGAGGGCACCCCCACCTTGTCCCTTATTTTCTCATTTTTTGGTTATATCTCTTCTAGTATGCCCACACATCTAGAGGCAGCGGGAAGAGGTACAATAGTACAGTAGTTC

>LNC_001834

ATCACTTGTTCATGATTAATTACCTTAAGATCCCATTTGTCCTTAGATTTTTGTTAACTATATGTTATTTTGCTATTTTATGATCAAAGCTTAAATAATCTTAATCCCATTTTTGTGCTTGCCGGTTCATTTGATTATTTTGATAACTTGTTTGAGGGTCATTTAAGCCATGATCCATATCCTAACACTTTAAATCCCTGTAGTAAATTGATATGGCTGCAAATTAGATGCTAATGATTTCTAATTATTTCCCTATGCATGAACCGGTTCAAATCGAGTCCATTGGACTCTTCCTTTCACCCTTGTCGAGTCAAAA

>LNC_001835

ACAAAAGTAGGATTTAGCGGCAATTATTTAGTGGCAATGATAAAATTGCCACTAAATAGTATTTTAAAGCCAACATTTACCGGCAATTAACCTTAAGCCACTAAAAAGTAACCCTTAATATCACTTTCCTTTTCTCTTTTTTCCTCAAATTTTATTTCATTCTCAAATTCCCAAACTATTTGATCTCTCTTTCTCCATTCCATCTGAAGCATAGGGTTGATTTGAAGCTTCCTTCCATGGCTACGATGGATTTGATCTGAAGCACAAACCAATTTTTGTTTCTTTTTGCTCGATCTGAAGCACACAGCAAAAGATTGCAGAGTACCCTGGCACTGAAAGAGATTGCAGAGTACCCTGACACTGAAAGAGAGTACCCTGGCACTTGTCTTAAGCTTACGTTATTTATTTACGTGATTGTTTTAATGTGTTATCTTAAAGTTGCTTTTGATACTATTGTAGTGAGCTGTGGAAATCTCCCTTCTTTAACACTATCACAGATATACTGAAAACCTCCCTTCTTTCTGCATCTGTTATAGGCCATGTTTCTGGGAGGAAGAACATTGGGATTATGTCAGTGGTATACTCATTCAAATGGTGAGATCCTTCAAGGAAGGAATGACACACTGATCCAAAAAGCTGCAACATTTTGCATCAGGGAAAAAACAGTGGCTTAATTGCCTTATTTTCTCCATTGTTGAACATATTTTTTCTATTTTGATAATTCATAGTTTCTTTTACCTCTACGAGATAAGAAAGTGAATGGATTAAGATTGTTTGTTCTTATTTTAAGTGAATGACATTGTTTTGAGTTTGGTAATTCTTCTTTATTACCTTTGAACTTATTTTTCTTGTAAATTTTTATTTTTGGTGTTATAAAAGAGGGTTAAATATGTGAATTTAGGTGAATTTATGATCAGTTTTTAAGGCGTTACAATAATTGAGTGACAATTATTATCTTTCCCGGCAATAGAAAAAGCCATAAATATGCTTGTTTCTAAGGTGGCTTTAATGGCAATAGTAAAG

>LNC_001836

TGTAGATATTTTGTTAGTTAATTGTTTTTATTTTTATTACATAAAAATAGTTTAAATAAAAAGTTGTAAAAGGGAAGTGATGGCAACATGTCCTAAATTTGGTTTTCACCAAGAAATCATATCTCATTCTTGGTCAAAAATAAATTAAGGATTTGTCACCATCACTCTCTTTTCCAACTTTTATTCAAAATCTGTTGTTTTGGTAATAAAAAAAATCAAAAAACTAACAAA

>LNC_001837

CTCATCACGTGTTTGTGTACAAATTACCTAAAAGGCAATTAGGGTCAAATCTTTCTATAATAGCAGTATTTGTTTAAAAGTTTTATGATTATATTATAGTGAGATATTGCTATATATGTATATTAGTCTTTAACGTTTAGATCTTATTTAGATACTATAAACAAAAGTATGTAAAATTAGAAAGGATGTGAATGTAAAGAATGGCAAACACTTATAGTCTTTCCCCGTGAAGTTATGGCCATGTACCATTACACAATATATTTTTTTTTATAAATATTTGTATTTGAGTTTTACTATGTTTATTTAATGATGAATTAATTAAGGC

>LNC_001838

CCCTCCTCTCCTTGTTTGCTGCTACCAGTAACCAAACCAACAACAGCCCAAAACCACAACCTAACCATATATTTTTTGGCTTTGTTTAATTAATTGTTGATTTGTTGTCTTAATATTTTGGGTTAAATTTTAATGATTATTGTTATTTTTTAGTGAATGTGTTTATATTTTTTGCAATTTTTAGATCTTTTATATGGTTAGCTACTATTTGTTCATTACTTCATCATTGTTGCTCTATTTTTTTAGTTTTAATTGTTGAATTTTTTAATGTTATTTATCAATTATTTTCGTCGATTTGAACAAATGACCAATGAAAAAATCTCTCTGTTGATTTTTGAGGTCTTCTCATTTTTAAAGACGGAAATTTTAGTTCAAGTTTATATAACAGATCATACATTACAAGTTAAAATTGATTGATGAAGAATTTTTCATCTATTTCTAAGGTCTTCTATCTTAATAAAACTTACGGGGTTACTTATTCCATTTTAGAATAGTCTCCTGTAAAGTATAAGTCTGTTGTTGGGCATCCGATAATAGATTGGAAAACCAAATAGTAGATATTGAATCCCTCTAATTTTGATTACTATTATTTTGATGCATTTTTTATTTGATTCATGAAAAATAAATTGTACAAGGCTACAAGCATTACACATATTCATGTATTATGATGGTTGAAATTGTTTCTTTTTCATACCCAAATGTAGGACAATTAAAAATCAATAGTGTAGATATTTTGTTAGTTAATTTTTATTTTATTTTATTACATAAATAGAGTTTAAATAAAATGTTGTAAAAGGAAAGTGATGTCAACATGTCCTAAATTTGGTTTTCACCAAGAAATCATATCTCATTCTTGGTCAAAAATAAATTAAGGATTTGTTGCCATCACCCCCTTTTCCAACTTTTATTTAAAATCTGTTGTTTGGGTAATAAAAAAAATCAAAAAATTAACCAACAAAATATTCACATCGTTGATTTTTAATTATCCTACATCTAAGAACAATTTGAATTTATCATAATTACATGAATAAGTGTTATACTTATAGCCTTGTAAAATTAATTTTTCATGTATAGAAAAGTAGTTAACCGCAAGCAAACACTTAAAGAAAAACAGTAAAAAAATAATCCCTACAAGAAAAAATATAATTAAAAAATAATCTTTCGGAGGTTAGGTTTTAAAAATATATTTTCTTAACAAAATATAATTGACCTTCAGAGTTAAAAAAAAAAGAAGACCTTTAGAGGTCGATTTTTTAATAGCAATATCCACAATGTAATTATTAAGAAAGTTTTTGTAATGAAGATTGAAGAGATTATACTCTTATAGTTTAATATTTTTTTTTATATTAGTTTCACATATATAGGTCAATTTTGACCAAACCCTATTATTATGTTTTATGTATCTTTAGTATATCTTTTTATTATATGATTTATTGTATTTCAAAGTTTATAATTATTGATCTCCGCAGGACATTTTTTTATTTCGATCCCAACAAACAAGTTTTACAAGAAATATTTTATATTGACGGTGTCCTCCTCACTCTCCAACCACATCAAATCTTCACTCCCACAAAGTTTACTTTATATTTATGGTGGATCGTTTTGTATATCTTTGTTGCTCATCATGTGTTTGTGTACAAATTACCTAAAAGGCAATTAGGGTTAAATCTTTCTATAATAGCAACATTTGTTTAAAAGTTTTATGATTATATTATAGTGAGATATTTCTATATATGTATATTAATATTTAACGTTTAGATCTTATTTAGATATTATAAACAAAAGTGTGTGAAATTAGAAAGGATGTGAATGTAAAGAATGACAAGCACTTATAGTCTTTTCTCGTGAAGTTATGGCCATGCACCATTACACAATATATATAT

>LNC_001839

CTGCTACTCTTCTTCTTCAGAAAATAGCATCCAGCAGCCCAGCAGAAACGAACACGCAAGCCAGCAGAAGACAGCAACTTTGTTGTCTCCTTCCTCCGATGTCCCTTTCAAATTGTACAGTAGCAGTAGGGCACGCGTCCTGCCCCTTTCCTCTTTCGGAATGAGTTGAAATTGTGAGAAAAGAGTGTTTGTCCAAATTGGTGAAAGAGTTTTTGTTTTTGAATTTCGAATTTGGGCCTTGGTCTGCGGGATTTTCGTTTTTCCCCCCCATTTTTTGAATCCTTTTCCCTTCTATATAATGGCTCTTATCACTGTTGAGCAGGGGGAATTTTTGGAAGATTTTTTTTTTAGTTGAAAATTTGGAGTCAAGAAAAAGCAAAAGATACATAGATTTTTTAAAAAGAAAAATACAAACTTAAGAAATCAAGCGTTATAGAGTTTATTCAAGCGTCGATCTCTTTTTCCGAGACATTCTTGCTACTCGTCGGAGCTCTCGGAGTTTGTGTGTGGTGGTGGAAGCTCTTTCGTGGTCAGTTCGTTAATGTCCTAGTTGCTGCCCAGGAAAAGGTAACATTTCCCCCTTTATAATATCCCTCCTTTCCATTAAGACATGACGTTACTAGCTGCGTTTGTTCTCTGTTATTCACTACCGAGCTGTGGGTGTTTTTATTAAGCGACTTTAGTGCCTACTCTTAGTGCTGATTCAAAGTTTTCTTTTTGCTGTTTTGTAGTCGATGCTTCTTTGTTTCGAATTTGTTTTATCGCTTGCTTCTCGTTTATTTGTCTTGATGTTTGAACTCGTATCCCCTTGTTTTTCGTTGTCTTAAACATCCGGGTTTATTCACTTCGTTACTGTTAATGTTTTTTGCTTTCCTTTTGAAATGAGGGCAGAGCCTAAAAAGTAAAAAATAAAAATCTTTGAGTATGAAGTTGTATATTGTCTTAGCTATTGTTTGATGGGCTGTAGGAGTTGGTTTTTATCCATTTTCGGGAACCATGACGTTCTTACTGTCCTGCAGGTCATGTTTGAATTAAGTTTATGTCTCTATTTTGCTTAAATTTTAGTCGAAATCGCTTACCATTTGTAATCTAGTTCCAATTGCGAGCTTTATAGAATACTTAAGACTATACCATGTGGTCTTTCTCACTGATCAGATTGTTGTCTGGTATACCCCCTTTACACTTTAAGTTGTCACTTGTCCATTGTAATGCCATCTGCCATTGCTACATCTATGTTTGCTTGCTTTACCAAGATTATTTTCTTTCATACGTATGTATTCTTGGTTCTCTCCAAGTTTGGACTTTCCACAATTTAGTATTAGGATTTTGTTGATCATACTAGTTTTGATTACTTCCTTGTTTAGCCTTATTTTTACCCTTAAACTTCAATGGTTTTTTTTTTTTTTTGTTGAGTTTGTCACTGTTAAGGGGCCAAATGTCTGTTGATTAATATTCAGTAGCTCATCTTTTGTATTCTTAGAAGTTCTAACAGTAGACCTTGGCAGTGGTCCCCTTTTGGTCTTGAGATAATTAAGTTGACTTTAATTTTCAATTAAGTTATGTCTCATTTGCTTCCTTATCTTCGAATGCTAATTTTGTTTTCTTTCTTTTGTTGCGTGAACCTTTCCCCGAGCCCGTGGTGGACTCCTTCTCTCTTGCATCCTCGAGATGAGCCGTAGAGGCTTAATTTATTTATTTTTTCTCCATCGAGTCGGCCCTCCAGCAAGCAGGGACTGATGTACAGGTCTTGGAGAAGTAAAAAATCGGACTGTATACATGACCTATACAGCTGTATACACAAGTATACACGGAAAAACAGTGATATACAAGTCCAAAAATTGAAACAGGTGTTCTGGAAGATGAATTAGGTTGTATACATTCGGTATACAGTCTTATATACAAACAAAATGGGCTGGGTTCATACGATATACACTGATATACAACCAGTGTATACAAAAACGGGATTGAGCCTAAGCCTAAAATCAGCAGCGAATTTGGCCCAAGAAGGGCCCAAATTCAATTTCTCCCTTACTCTTTAATTATTTAATTAT

>LNC_001840

TGTGCTCTCAAATGGGGAACGAGCATTGAGTAAATAATTATTGCATGATCAATTAGCATATGTACCCTTTGCTCAATATCCACAATTTGTTTTCTCCTTTTTCAATATCCAGGTGCTAACGACCTTGGTATTCCAAAGGCTCAACTTGAGGCCCATTAGATATCTTTACAGCAACAAATTGAGAGGCAAGGAAGTACATATTAAATGAACTCAATGGCACTGCGAGTAAACACCAAGCATGTCGTGAAATGTGACATACAACTCATATGGAGTCGAATTCTTTTGATACTGCAATTGATTTAGCTGTGCCTACGC

>LNC_001841

GTTGAATCAAGAATTTGGATTATATTTTTATCCTATTTACGTGTGTTTTGTAGGTTCATTATAGAAAACAAGTTCAAGCTCTCTTTCTCCTATATAAAATAACTGCAATGAATCATATTGTTACAATATGTTTTGTCGTGCAATCTATGCTAAACTCCTTCCAGTTTCTATGAATTCTTCCAAAGTAAGTAGAGTTATTGTCATTCTCTTATCTTTATTATATATAGTAACATACAAC

>LNC_001842

ATCTAATGGTCGTAGACCACCTTCTTGTGACTATGTTGAAGAATTCTTTCCCCCCTTTGAGTTCTCGAGTGAAAGGGTCCAAGCCATAGGAAAATATCCAGTTTCTCTCGCAACATATCTAGTTTATCCTGAAGCAGCATTTCTCGAAGTAGCATTCCCATAAGTAGAGGATGAAACCCTTGCTTGTTTATCCCGACGAGGGTTTATGAATCACTCAAACGAGCCTTTCTAGCCGCATCTGAATCGGCATCCCTATCCATATGTTTATGCGCAGGGGCATCCAAATCCGAATCTGTAGAATCTATTGGGGAATCCCCATCCGCACCCGAATTGGCTAAATCTAGTATAGTGGGTCTTGTTCCGATGCGGGAAAAAATCTTTTTAAAAA

>LNC_001843

GGAGAAAATACCTTAGTATATTTTGGGCCGTTCAAATAGAAAAAACTCAGATATTTTGAGGGAAAAGGTGAACCCATATCATATGTAAAGCCTAGATATTGTCAAAATTTGTACTTCTGGTTCTGGTGTAGAGTGAACTCACCTATGTAAGTTTCTCATAGCCAATCTCCAGCCTAGAGAATGGATGATGGTTGTCATATGCACTAAACGGCTTCTTGAAGGGGGGGGGGGGTG

>LNC_001844

TACTAGGGTAGTAAATCCAGCCTAGTAATTACATAACTTAGTAATTACATTGACCTGTTTGTCAATCTTTCGGTGATTAGGTTAGAAAAGATCTTGTACACCACTCCAATTATGTTGATTGGTCTGAAATCTTTTAATTTCATTGCCTCAGGTCTTGTTGACCAATATCAATTAGAAAGGTGTACAAATTGAGATTGGTAGTACCCGAAGCTTTTCTTTTGAATTTGTTAAAGGACCATGATGCTGCTTGCTTGTACTCTCCCATTTCTAGTTCCTTTAACAAAATTATAATTATTAAGGTTTTGGCCAATAATGACAAATTAAAGTAATTCTCCAATCTTGCAATAGTATCAGTTTACTATTTTGGGAGGAAAATTCAGATTTACTACTTTTGTAAGTAAATTTCAAAATATTCCTTTGTAATTATTTTACCCCGTTTGGAAGTGTGTGGTGATAACTTCACAACCAACATTCTTTTATTAGTGTGACAATAATCATCATCATCGTGTAAACATCAACGTGTATAAAACATAATCAAGTTATGCATGAACCATGATATTCATATTTTAATTAATTCATCACACTCATCCGTAGATTTCATATAATGAAATCAAGTTCTTAGTTTCATAATATTTATCCATGATGGCAGAGTATCGTACTTATAATGTGTTGTAAAATAATAAACAAAAGGAAGAAAATTGTAGAGAAACGTTGAGAGAAGGTAGAAAGCTACATATGTTGTACATATTTATAGAATCTTGTAGGTAACGAGACTACAATTGATTATTTGTACATATTGTATGGTGTTAAAGGATCTAAACGTGTAACAATGTAAGAGAGTAAAAGCTCTAAAACTGTAAAGTATCCTTACATGCTTGTTGAATATGAAGCATTGTTGATCGTTAGGGAAGGAGTGTTTGCTTGAGGTTAGATTATTAAGAAGGGTATTTTAGTAATTTAACTTTTAAGTTGGGATTCCTACTTTTAATATAATATGAATATGAATAAATATAGATTAAAAAGATGGGACATATTTGTATAGATACTCATTCTTAGACAAATACATTTAGATGGCTACTAGAATGTATCTATATACTTGAAATAATATCTGCCACTGTCCCCCAAAATTTGCCTGAAATTTTAATGTTGTAAGCAATCAACTTCTTTATTTTGTGCTATCCTCAATTTTGTTTTATTTGTAGCACCTTCATATTCTTTAGCTCAGATGGGAAACATAAATGATTCTGATTGGCAAGAAGAGGTTTATCAAAAGGTTCAATTATCAGTATACTTAACCATCCAATTCGTTATTTTGTTATTATTCAAGTGAAACAGTTTCTCCTTGTGAGTAAGTTGAATCTCATGCTTGTTAAACTTCAACACGTGTTGCAGAATGCGTCCCAACTTAACAAGCATGAGTTCAACTTGCTCACTTGGAGAAACTGTTTTTGCTTGAGAAGCACGTAAGTTTCTTTCTTAGTGGAAAATGGCCGCATAAGCCTACTTCTATATTACCCGAGCAACTTCCTCAGCCTTCCATGCATACAAGTTAGGCTTCGATGATCAACCCGGGCACACTTTGGCTGTTTTGAAATCTTGGATACACCTTTGAG

>LNC_001845

TAAATGATTCTGATTGGCAAGAAGAGGTTTATCAAAAGAATGCGTCCCAACTTAACAAGCATGAGTTCAACTTGCTCACTTGGAGAAACTGTTTTTGCTTGAGAAGCACGTAAGTTTCTTTCTTAGTGGAAAATGGCCGCATAAGCCTACTTCTATATTACCCGAGCAACTTCCTCAGCCTTCCATGCATACAAGTTAGGTATGCCGTATGAACTTGTTTCGATAATTCAATTGTTTTCACCTTGTTGAAGTTGCTTTTCTGCTTGTGTTAAAATACATCTACATTTCCTATAATTGTTTGTACAAAACCTTGTCATGCAACAATCTGAAATTTGGCGGTTGTTTTCTTCGGTGGGGGGTGGGGCTTTGATAGCTTTGGACTTGTGAAAATTGTCCAAACATAAATCTCTTAAGTGATATATGATCTCTCTTGTTTCTTTTGATTCATTCTAAGATCTCATGTGTTTGAATATGATTATATAAAGGTTGAAAATTTGAGTAGAAGGTTAGTAAGCAATTGATTGTCATACATCTAGTAGAACATCAGAGCTCAAATTCCTTAATCGTTATTGTCCAGGCTTCGATGATCAACCCGGGCACACTTTGGCTGTTTTGAAATCTTGGATACACCTTTGAG

>LNC_001846

GAAAGTTTTACTCCGTCAACCTTAAGAAGAGTAGTGTTTGTGGGTTCCCTTGTCATAAATAAAAAAGAAAAGGAAATGATGAAGGGTAGAGGGTGGACCCATGATCTGATTAGTTTCTAACTCTGCGCCATTGACCACGGGGATTCTTGGCTATAAAAAAGGGTAAAAATAGTGAACAACAACAATGACATAGATGCTTTTATTTTATTGGACCACTATCAATTCTTTATTCATGGAAAGAGTACAACAACATACTTAGTGTAATTCTACTAGTGGTGCCTGTGGTGTTGGCTCAAAGAAGTGGTATAGATTTGTAAAATTAATTGTAATTACAATATTATCCAACACAGAGTGCACTTTTGAATGGTAACCAAAAATATTGTGTTGGAGATTCATACTTTTATAATAGTATAAGATAAAAAGATGGAAATATTAGCATATAACCAAATACCCAATCAATACCCCAATGTAAAGTGGAAACTTAACTTTCTTAGACAAGTACATTTAGAGGGAAGTTAAAATGTATTAACATACCTAAAATATTTAACACTGTCCCAAATTTGCCTGAAATTTTAA

>LNC_001847

CAAAAGGTTCAATTATCAGTATACTTAACCATCCAATTCGTTATTTTGTTATTATTCAAGTGAAACAGTTTCTCCTTGTGAGTAAGTTGAATCTCATGCTTGTTAAACTTCAACACGTGTTGCAGAATGCGTCCCAACTTAACAAGCATGAGTTCAACTTGCTCACTTGGAGAAACTGTTTTTGCTTGAGAAGCACGTAAGTTTCTTTCTTAGTGGAAAATGGCCGCATAAGCCTACTTCTATATTGCCCGAGCAACTTCCTCAGCCTTCCATGCATACAACAACTTAGGCTTCGATGATCAACCAGG

>LNC_001848

TTGAAAAAGGGTAATTAGTCATAAATTGTTGGTCTGATGCATAGAAGTGTTGTTGAATACAATATGTCATACCATTGACCAACACCCAAAGTTTTGTTCTCATAAGCAGTGGTTCAGGTATTTATTGAAGGTATTCAATATCAAATTAGCTTGGATATAGATCTGCATTATCACGATGAATTGTCTTGATTACACAATCTAAAAACTATGTTGTTAACTCAAGCAAAGAACTTTGTGAATGTCAAACTGCATATTTACAATAAATGCACATGTGGTCATCTTATAGATGCATCTATTTAAGATATCACATGATAGGTGAACATGCCGATACTGACCTTTTAAATTTTTCAGGTTTTAGTGGATTCAATTCAAATATTAATTGGTCTAATCAACTTATCATGAGAACAAACAACATAAAAGAATTTTGAAGAATTTTGTAGTTCAATATATTTTGCATACTTAATATGCACATCTTCAGGATTCCAAAATTATTCATGATTAACATCTAAATATTTTTTTAATCCAAGCGGTAATTTTATATTATTGTCACTTCCTTTAGGAATGTGTATGAATTGTTAATTTGTTCAGGCAAATTAATTAAACATACCGTAGAAAGCATTCACCAAGATTTATTTCCTTCCGCCTTAAAATGATACTTAAATAAAGTCATTCAAGATGTTTTTATGTACGACAAGTACATATTGCAATGACTTCCATACCACAAAAATAACATGTATATGCTTTGTTATTTTATCTCTTCAGGAGACAAGTTGTGGTATTTCTTTCATTCATTTCGGGAAATGAATCCTGATTATTTGAATGTGCTTGAAATACAACATTCATCAATAGCTTACTCCCTTCGTCCCATTTTATGTGAGGCAGTTTGACACGATACGGAGTTTAAGAAAGAAATGTAGACTTTTAAAACTTGTGGTCCAAGATGAATGATAGAAATTTGTGTCCCTTAAATCATTTCATTAAGGGTAAAATAGACATTTTATAGTTAAATTGTCACTTAATATAGAAATGTGTCATTCTTTTTTAGACAGACTAAATATGAAAGTAAGTCACATAAATTGGGACAAAGGGAGTATTATTTTTCTCAAGCACAATAAGACACTTCTTCAAAGTATTTCTCAGACATTTTTCAAATTACTTTTGCTTATGGAGAAAATTTTGAAGTTTTACTATTTGAAGAATGTGAGTTCAAAATTTTACCACTTCCGGAGTAAATTTTGAAGTTCTACTACTCCAGGAGTAAAGTACACAGTTCTACTACTTCGGGAGTAGATATTAGATTCTTACTACTTGGGGAGTTGGTGGCAAAGTGTTTACTACTTCTGGAGAGAATTTTAGGTTTATGTCACGCCATAAACTACTCCTAGACATGATTGGCACCCAACACAAACCACTGACCAGGTCAACCCAATTTCCATACCTGAACCATTAATTCACAACATTTGATGAATTAAATAAGTATTCAAACAGTATGATAAATAAATATGAACACTTAGGGTCGGTTTGAAAGCCACTAGGTAATTGAAATTGGTGTCATTACTCATTACTAGGGTAGTAAATCCAGCCTAGTAATTACATAACCTAGTA

>LNC_001849

TAAAAATCAAGGTATAAAACCATTGCATGTTTGAGTTTTCCTCCAGAGTTCTACTCTCTGTGTGTGACCAAATGTTAAGCTTTTTCAGCAAGTGGAAACTTCACTCTTGGAGTTTTTGCATGTCTGCACTTTCTTTCACCATGAAAATTAGCAACATGTATATATTTCTGTCTGGATTCTTAACAGGGTTGCTATTGTATATTCCATTGTTTCTTTTGTTATAATAGATATTGAATACTCATCT

>LNC_001850

CTTTATCACATTCTACACTATTCTTCGTGAAGTTGTGTAATGGAGCTCTTGAAATTGTATCAAAATAGGTTTTTACTTTTTATCGTTCTTAATTTCTTTGGTGTTATCTTTATTTTGTCTCTTCAAGTAAATCTGCTCTTTGTCATTCCTGAAATTTTCCACCACAATAAAAAATCACAACACGGATAAAGAGCAAATACAATTCAGGTTTCCTCATCTTTTACCTCCAATTGACACACAACAATAATGTAGCTTCATCTCCTTATTATGAGTTTCCACAACATTTTCCTCTGTCAGACAACTAT

>LNC_001851

CTTACACTTCCCAAAGCGTGAAAAACCCCACTCCCACCCCAACCAAATAAAATTATCAAAAAACCCTCTTCCTCAAACACCACGATGCCTTCCTCTATGGTGGAGCTTTGAACTCCCATTCTTCATTGCAATGACTTGCCTTCTCTGAGGGCGATTTGAACTTGGACACCCACTCTACACATCTGGTGAATTTGTCTTGGCTTAGGGTTTGTTATCCTTCTTGTCAAGGCAAGTAATGCATCCTGCAATGTGCCACCTCTTTCAAATATGATCAACTGTATTTCCAACACCTGCTGTCCACATATTCAAAACTTGTCTCCAGGTTTTACAGTCGAGGGTCCAACAATCTACTGGCTTGAGTTTGTCAAGAGCATCTAAATCACCGTAACTAACTCTTGACATTCATGTAAATCCAAAAAATTTGGAAGCAAGACGTCAGCTTTGGGACTCCGTTATTGATTCTATCAAGAATTTTAAATTTGAAGAACCGGCCTGCATCTGATCAGAGTGAAGTGGAAGTATGAGCAACTGAAAATGAAAGAACAAGTTGAAGCACAAGACAAACCTACTGGTTTATGTTTTAGGAATTATTTACAGATATTCTCATTTATACATATTACATTTTATTCTCTTTTGTAAGGAGTTGATATTTAATGTCTTATAATAAGGATAGAAAATTTTATGCAATGCAACACTACACTTCTTTGTAATTTACTATGAAATTTTATATGTATAATCACAATGTTGATTATTTGGTCACTAGTTTTTTATAGTGATCAATTATCAAAAATCGGATGTCACTGATAGTTGATAGATAATTAGCAAAGGTAAAATCTGGTTGCAAAAAGCACTATCAATGATCAGATTTGTCATCGTAAAAAGAATTTTAACGACCAAAATGACAACTAACGAAGGG

>LNC_001852

TTACACTTCCCAAAGCGTGAAAAACCCCACTCCCACCCCAACCAAATAAAATTATCAAAAAACCCTCTTCCTCAAACACCACGATGCCTTCCTCTATGGTGGAGCTTTGAACTCCCATTCTTCATTGCAATGACTTGCCTTCTCTGAGGGCGATTTGAACTTGGACACCCACTCTACACATCTGGTGAATTTGTCTTGGCTTAGGGTTTGTTATCCTTCTTGTCAAGATCAAGAATTTTAAATTTGAAGAACCGGCCTGCATCTGATCAGAGTGAAGTGGAAGTATGAGCAACTGAAAATGAAAGAACAAGTTGAAGCACAAGACAAACCTACTGGTTTATGTTTTAGGAATTATTTACAGATATTCTCATTTATACATATTACATTTTATTCTCTTTTGTAAGGAGTTGATATTTAATGTCTTATAATAAGGATAGAAAATTTTATGCAATGCAACACTACACTTCTTTGTAATTTACTATGAAATTTTATATGTATAATCACAATGTTGATTATTTGGTCACTAGTTTTTTATAGTGATCAATTATCAAAAATCGGATGTCACTGATAGTTGATAGATAATTAGCAAAGGTAAAATCTGGTTGCAAAAAGCACTATCAATGATCAGATTTGTCATCGTAAAAAGAATTTTAACGACCAAAATGACAACTAACGAAGGG

>LNC_001853

AAAGCGTGAAAAACCCCACTCCCACCCCAACCAAATAAAATTATCAAAAAACCCTCTTCCTCAAACACCACGATGCCTTCCTCTATGGTGGAGCTTTGAACTCCCATTCTTCATTGCAATGACTTGCCTTCTCTGAGGGCGATTTGAACTTGGACACCCACTCTACACATCTGGTGAATTTGTCTTGGCTTAGGGTTTGTTATCCTTCTTGTCAAGGTTTTACAGTCGAGGGTCCAACAATCTACTGGCTTGAGTTTGTCAAGAGCATCTAAATCACCGTAACTAACTCTTGACATTCATGTAAATCCAAAAAATTTGGAAGCAAGACGTCAGCTTTGGGACTCCGTTATTGATTCTATCAAGAATTTTAAATTTGAAGAACCGGCCTGCATCTGATCAGAGTGAAGTGGAAGTATGAGCAACTGAAAATGAAAGAACAAGTTGAAGCACAAGACAAACCTACTGGTTTATGTTTTAGGAATTATTTACAGATATTCTCATTTATACATATTACATTTTATTCTCTTTTGTAAGGAGTTGATATTTAATGTCTTATAATAAGGATAGAAAATTTTATGCAATGCAACACTACACTTCTTTGTAATTTACTATGAAATTTTATATGTATAATCACAATGTTGATTATTTGGTCACTAGTTTTTTATAGTGATCAATTATCAAAAATCGGATGTCACTGATAGTTGATAGATAATTAGCAAAGGTAAAATCTGGTTGCAAAAAGCACTATCAATGATCAGATTTGTCATCGTAAAAAGAATTTTAACGACCAAAATGACAACTAACGAAGGG

>LNC_001854

GGATAATTTATTGTTATGAAGTGATGTTGGACAGTGATACTGTGAAAGTGTATCATCAGCACCTATGGTTACTTAATAAGGATATATTTATAGCTTTCAGAAATTGATCCTTTCATAAATAATTTAATGTATCTGTTGAACTTTGATTGTCTTCTTAGTTCCACAATAAAACATATTTTATGCTATGAGTGCTTGATCTGTGGACTATAATTTTCATTGTTACTACTTATTTACCACACTACTCCTGGATTGGATATGTATGTGAGCTTACCAGACAGATCTTTAAGCTTTTATTATTTACCATCATCACATTTGATTTTATTGGTCATGCTAAACATAAATGGACAATAGAGAAAATTATTTGCTTCAAGTTTGAAGGAACCAAATAAGAATTAGACTGTTTGTCCTTTGGCTTCCTTAGCATTAGCAGAAGCTTAGGAGTTTTTTTGTGTGAGGTATAAGATATAATAATCTCGGGATGAAATATAGAATTATTTTATCCTGTGTTTGGCTGGAGGTATTAGGTAGTCCTTTGGTTATTTATCCCACTATTTATACCACAGTGATGTGTGATAAGTTATCTCACATATATGGTGGAATAACTATCAGAGGATAACTTATTCCGGTATAATTAATCCTTGGATAACATATTCCCAACCAAACGACCCTTAGTGTACACCTTAATGCAAACTCTATCAGATGGTTGGAACTATAATTTCTTGAGAATATGTAATCTTTATTCAATAGATTCAAGATGTTATAATTGCTATATGATAATGTGATGTCAAGTTATGTTCTTAGCTAGTTCATTTACTGAAGATCAAGGATACTGTTGTTAGCATTTGAAGTATCATGTGTGCAGTAGGTTCACTGGTTTTAAAAAAATCAATTTAAAGATTAAAACTGCTAGAAAAATGTCTACTCTGTAGTCTTCTACACTTAAGAAATATAAAGTAAGTTTTCAAAAGGAAGAACGAAGTTAGTATAAAAAAAAATTATAATGTGATGTACATGAAGTAGAGTGACACTTCTTATCTTTCAAAATGAATATTGTCAAAATAAGATATTTGTTAGACCACCAGGTAGTAGTTACAGTGATTCCACCACACACATGCTCTCTTCCTCTTTAATTAATTAGTAGAGAATAGTTACTTTAAAATACATGCTGCAAATTCATAATGATTTAATTTTAGCACGTGCATGTTTTTTTACTTTGCACATTAAGATTTTTTTTGGTCGAGTAAATATTATTAACAACATGCAATCCTTTATTTTCGGGTGAGTTTCTCTTAGTCATACATTTTATGGTCGAAGATGACTGCACAATTCTTTTTATATAGGCAAGTAATGCATCCTGCAATGTGCCACCTCTTTCAAATATGATCAACTGTATTTCCAACACCTGCTGTCCACATATTCAAAACTTGTCTCCAGGTTTTACAGTCGAGGGTCCAACAATCTACTGGCTTGAGTTTGTCAAGAGCATCTAAATCACCGTAACTAACTCTTGACATTCATGTAAATCCAAAAAATTTGGAAGCAAGACGTCAGCTTTGGGACTCCGTTATTGATTCTATCAAGAATTTTAAATTTGAAGAACCGGCCTGCATCTGATCAGAGTGAAGTGGAAGTATGAGCAACTGAAAATGAAAGAACAAGTTGAAGCACAAGACAAACCTACTGGTTTATGTTTTAGGAATTATTTACAGATATTCTCATTTATACATATTACATTTTATTCTCTTTTGTAAGGAGTTGATATTTAATGTCTTATAATAAGGATAGAAAATTTTATGCAATGCAACACTACACTTCTTTGTAATTTACTATGAAATTTTATATGTATAATCACAATGTTGATTATTTGGTCACTAGTTTTTTATAGTGATCAATTATCAAAAATCGGATGTCACTGATAGTTGATAGATAATTAGCAAAGGTAAAATCTGGTTGCAAAAAGCACTATCAATGATCAGATTTGTCATCGTAAAAAGAATTTTAACGACCAAAATGACAACTAACGAAGGG

>LNC_001855

GGAGGCTTGCCTCTGGAGTCTTGTGTGATAAGAAAATTCCACCTAGACTTAAAGGTAAGTTTTACAGAATGGTAGTTAGACCGGCCTTGTTGTATGGAGCGGAGTGTTGGCCAGTCAAGAACTCACATGTCCAGAAAATGCATGTTGCGGAGATGAGGATGTTAAGATGGATGTGTGGGCACACTAGGAGTGATAAGATTAGGAATGAAGTTATCCGGGAGAAGGTGGGAGTGGCCTCTGTGGTGGACAAGCTGAGAGAAGCGAGACTGAGATGGTTTGGACATGTGAGGAGACGGTGCGCAGACGCACCAGTGAGGAGGTGTGAGGGGCTGGTAGTAGAGGGTACGCGGAGGGGTAGAGGTAGGCCTAAGAAGTATTGGGGAGAGGTGATTAGACAGGACTTGGCTCAGCTTCACATTACCGAAGACATGACTCTAGATAGGAAAGCGTGGAGGTCACGTATTAAGGTTGTAGGTTAGTAGGGCTAGAGTGTTGTCCTC

>LNC_001856

TCTTCTCCAAGGTTTAACATGAAGAGCTCTGACCAACAACCGCCGGAGAGATCGAAATCCAGCTTCCCAATGTCGCATCTCCTCCATTTCACCTGCTACCAGCTTTCTCCAAGAGATTGGATATGTTGAGGAAGCAAATAGGAGCTATCAACAATCGAATACACCACTAACAACTTTCGAGTCTTGGTTTTGTATCACTTCAGACACCTCTATGCTCTCACCAGGGACTTCAGCAACATTTTGGAGATCAATTTCCTTGTCTGGTACAACCCTCTTATCCATAGAAGATGATCCAACAACTTCGACTTTACCAAAACATTTATTGATCCAATCTTTAGTAGTAATTTGTTGTTGACTTTGCCCATTCTTAACTTTGTTTTCATTTTGTTGAACTGCATCATTGTTGGATTCATTTTTACTACATAAATTGTCATTTGCTCGTTCTTGATTAGGCAATCCAGTGGCAGATTCTTTGCTTAGTGCCTCAAAACTATTCTCTCCCATATGCTTTTCAAGATTCTCAGAGCTTTGGACTTTATTTTTAAAATGTTTTTTATGCTGGACAACTTTCCAATTTCCGAGATCACCAACCACTTTACCACTAGAGAGAATCTTAACTTTGCCTGTTTGTAACTTGTGTATTCTAGTCTCTTGAACTCTATCATTGTG

>LNC_001857

ATGGGTTGAATCCCACAAAGAGATTAATACCACCATCTAAATTTTAGACCTTTGAATAAAACAACTAACTCCATTTTGGTTATATGATTTATGTGCCTGCAAAACCTTTTGGAGAGATTGGATATGTTGAGGAAGCAAATAGGAGCTATCAACAATCGAATACACCACTAACAACTTTCGAGTCTTGGTTTTGTATCACTTCAGACACCTCTATGCTCTCACCAGGGACTTCAGCAACATTTTGGAGATCAATTTCCTTGTCTGGTCTACCATTACCTTTACTCTTGCACAACTTGGCCTAGTCTTACTGATCGTTGCTAGGTCCAATTGAACTTGATTTCCAACTGCTAATGCTAAATAGAAGAGGTACTCCTTAACAAAGAAAGTGGGCAGAAGATTGGGAAAAGAAATCCATGCCATAGCTTTTGTTGTTTCTTCATTGATCTTAAATCTAGTATCATATAGTAGTGTACGCATCAAGTAAGAATACCCATCTTTAAACGTGATATAGAATGCTCCTTTTGAAACTAGATCTATAAAATCTTTTTGGTGGTTGAGACGAATACGGATGTGTCGATTGCGAAATAAACCAATCTGAAACCCTCTTTTCACATCACATTGTTGTGGTATAATCCTCCTTAATTCGTATAGATCAGACAATTCATAGGTGAATTTTTCAATGACCGCATATG

>LNC_001858

GTGAAGAAGGAAATCAAGCAGACAGCTTAATGGAAATAAGTTAATATAATATTAATATAATTTATTTGCAAATAAGGAAAGAAGAACTTACCTGCTGGTCCAAGTGTCACGCAGCAACGTCAAAAGAAGTTACACGCACAAAATTGGAAGCTACACGCGGAGAAAAAGGAATCCAATTCCTTTTGGTTTTGGATTCCTAATTTAATTTGGACTCAATTATTTTATTTAGGGTTTCCTACATCTATAAATAGTGCATAGAAACAATTAGAAGAAGCCAAGAACCAAGATACAAGACACAATATAAATTTTCTTCTAGCTTAGGAACTTTGGAAGAGCCGCCGTGGAGGCCGGAGAATTGTGGTTTATTCTTTCTTCTCCTTTATTATTTATTTGTATTCGTGATTAATAAAAGATAGTTTAGCTA

>LNC_001859

TTCTCATGGGTAAGGATAAGGTAATGGAATTGATAGAGAAATTCATAGACCTAAATAGGATAGGAGAATTGATAAGGGGAATAGAGATGATGATAGAGATCATACTGAGAAACAGAAGAATTCCGTACGGGTACAACTATTATTTGAACGAAGTGCAAAAAATGCGATCTTTGTTTATAATAGAACAAACACTAATACCTTAATTGAATCGGTCA

>LNC_001860

CCCCTAACAACTAACCGAGTTTTAAGTCTCTCCACAGAACCATTAGAGTTGAGTTTGACCTTGTAAACCCATTTATTAGGTAAAGCCTTCTTTCCCGGGGGTAATGAGACAACTTCCCAGGTGTCATTAGTGATAAGAGCTTCTAATTCTCTTGTCATAGCCTCTTGCCAATATGGATATAAACTGGCGTGGGAAAAACTTATAGGTTCTTTAATTTGTGAAAGAGAATTGATATATTGTTGATTAGACGTGGATAAAGCATTAAAAGAAAATGGTGTGACAGAAATAGGAGAAGAAAAACAAGTATCATATACATTAGAAAAATGTACACTATTGCAAATGTAATCTTCTAAATATGCCGGAGTCTTGATAGATCTAGTAGATTTTCTCAAGGTAATAGGATTGATTTGTGAAGGAAGAGTGGGTGTAGAAGGTACTTGAGAAAGTTGATCCGGAGGAAATGAAGTACTATGTGGAGGAGAGGATATAGAATGATAATCATGATCAG

>LNC_001861

AGACAAATCAAAAGAAACAGTTACAACAATACTACGATCCAAACGTACAGCAATACCAAAATAGTAACCGACCCTGTATTGTTCTTCTTCCTTGCGATAACAAACCCAGGAAATCGTGACCCAATCCAGGAAATCGCGAGCCCAGTGGATCTAAACCCGAACCAGCAGCCCATCCTCTCGTGCGTCGTCCTCTCCTCTCCGTACAAAGCAATCCAGGTGTTGCAATCTCAATGCTCGATTGTCCTTCTCGAGAAATCAGATGGCTCCCTTCATTTTTTCATTGAAGAATTGTACAAGATGGTACTATTTCCATCAACTTTATCCTTTAGAGGAGAATTTGAAATTCGAATGGGAATTCATCCAAAAACCCACCTGATTTCCCCTTTTTTCAGCCCTATTCTCCTTTATTCTTCTTTATTGTTAGAGGTTGAAGAAAAGGTTGGGAATTTCAATTGATATAAAGAGAACTGCCTAGCAAGAAATAGCTATAGAAATTACATACGAAATACGTTCGAAATAGATAGGAGGTTCAGTTCGATCTCACTGTTGTGGTCTCGTTCATCGAGACTTTTGGAGTTTCATTGTGGTGGTGATAGCGCTCGTTTCAAGCTCATCCTCCTGGTCTCGCTGCTCCAAAAAAAGGTAATTATTTTCCCTTTATTAAGTTTCTGCTTGTGTTGTTACTGTGTGTTATTGATTACCTTATTTCTTAATGTTCTACCTGACTCTTCTTAAATGTTTTCAATGCTCCGTCTTAATTTTATGCAGAGCTTGACGCGTTAGCATTCAGAGTTTGGTTTGTTTTCTATTAGATTTGTATAATCGTGGTTTTTGTCTTAAACTCTGCTTGGTCATAAAGTTCGACTCCTTCACTGTGTGTGGTGATATAGGTGTAATTGTAGTCATTTCTCTTTTGTTTCAATACACTGTTTCAAACTCTTTATCAATATTTATGGTTTAACTATTCTGCTTGAGAACTAGATAGTTTCAAGTTATATATGTGGGATTCATGCTTCAGATAATTGTTTGCTAGCCATGTCAATAATACTGCACAGCCATGGCTAAGCATCATCCATGGATGCAATACGTGAGAGAGAGAGACTGAAGAAAAAGAGGGCAATTTGGGGAAAATCTCTTAACTGATTTTCATTATGAGAAGAGCGTATAATTATACAGTGAAGGAACAGTAAATCTAACTAACAATTGAACTAACATATTAACAGAACACTAACAGTATACTAACTACCCTGACTAATTAATCTGACAAGACTAACTAACTAACAAACTTATTAGACTAGTACATAATTAGCTTAATCACAATACCCCTCCCCCCTCAAGTTGGAGGTGTGGAGAACATTGACAACTTGCTCAATGTTGTAGATTGTTTGATCCTAGTCAAAGTCTTGGTTAGTATATCTCCAAGTTGCTTATCAGAGTTTATGTGATGTAGGGAAATAAGTCCAGTTTGAAGCAGATTCCTTACAAAGTGGCAATCAAGTTCAATATGTTTGGTTCGTTCATGAAACACTGGATTTCGAGCAATATGAAAAGTTGATTGACTGTCACAAAATACCTCAATGGGTGTTGGCAGTGGCACTGTAAGTTCTGTCAATAATCTGCTAAGCCAAATCAACTTACCTGCAACTTTTCTTAGAGCCCTATACTCTGCCTCTACTGAAGACAAAGATACAGTCTCTTGTTTCTTGGATTTCCAGCTCAGTGGACTATTGCCTAACAGCACAATATAGCCACTCACTAACTTCCTGGAGTCAGGGCATGCAGCCCAGTCAGAGTCACAATAGGCCTTGATATTGTATGACTGGTCTTTGGACATAAAAATTCCTAGAGTGGGATCAGCCTTGAGATACCTCAATAAATGGAATGTTGCCTGCAAGTGTGGCTCTCCAGGATCCTGCATAAATTGACTAAGCTTTACTGCAGGGTCCAGTGGAGATGTGCAGCTTCTCATCCCTGCAACCTTGTAATTCTTCAGCATGTCTAGGAAAAATTTCCTTTGTGAGATGATAAGTCCATCATCTTTATAAAGCACTTCCATCCCAAGAAAATAGTGTAACTTTCCCAAGTCCTTAATCCTAAATTTATCATGGAGAAAGGTCTTGAGTTCTTCAATTTCAAAAACACTTGTTCCTGTCAAAATCACATCATCTACATATACAACAACAAAAATGGTGGAGGTTGTAGACTTTTTGTGAAACAATGAATAATCATTCAGTGAGTGTAACCTTTTGAATACAAAAGCCTCAACCAATTTGACATACCACTGCCTGCTGGCTTGTTTCAGGCCATACAGGGACTTATTTAGTTTGCATACCATCCCTAACTTATGCACTACCAAGCCGGGTGGAACTTCTATATATATACCTCCTCATGTAGATCCCCATGAAGGAATGCATTGTTTACATCAAGCTAAAAAATGTTCCAGCCCTTTTTAACAGCTATGGCTAATAGAGCCCTCACAGTTGTCATCTTTATTACTGGTGAGAAAGTTTATATATAATCAATCCTAGATTCTTGAGTGTAGCCTTTCACTACAAGTCTAGCTTTGAATCTTTCAACAGTCCCATCTGCCTTAAGTTTCACTTTATAAACCCACATGCATCATATGGCCTTCTTGCCACTAGGTAAATCAACTAGATCACAAGTATGATTTGCATGCAATGCCTCAAATTCATGTGTCATGTCTTGCTGCCAGGCAGGATTCATAGTTGCCTCCCCATATAAGGAAGGTTCATCATCATTGCTGATGTTTCTCACAAGAGTTTGACTCTCATCTGCCAAGACCTCAGGTCGTATATGTTGATGTTTGGAAAATGTAGCATTGAGGGAGATGTTTGAATTTTGCAGTTAGTTAGGTGCATTAGCACTAGGTGCAGTTGTCATATTCTTAGATAAGACATAATCTTGTAAGTATGTGGGCAGTTTATGTGATCTAGATGGTCTAGTGGGGGTCGTAGGTTCAGTTATGTGGATAGAATTAGGAGGTTGAATGGTGGTAGATAAATTGGTTGAATGGTTAGGTGAAGTGTGAAGTAGAGTGGTACTTGCATTAGCTATATTATTTTCAGTGTTGCATATTTTGTGTTCCAATTTCAGTATGTACAGTGTCCATGAAGGGCACAGTATGTAAAACAGAAGGAAAAGAATACATCAGAAAGTGCAACAAAAGGAAAAATGGATTCCTTAAAAACTACATCCCTGGAGATATGTATTTTTCTTGTTGGCACAGTATGTAAAACAGAAGGAAAAGAAGATACATCAGAAAGTGCAGCAAAAGGAAAAATGGATTCCTTAAAAACTACATCCCTGGAGATATGTATTTTTATTGTAGCCAGGCTTAGAACTTTGTATCCTTTTGATCCAAAAGGGTAACCTATGAAGATGTGAGGTGTTGTTCTAGGTTGAAATTTGTCCATGTGAGACTTTGGAAAGGTAGGATAACAAAGACAACCAAAGGTACTCATGTGGGAGTAAGTTGGTTTAGTTTTATACAACAATTCATATGGACATTTCCCCATCATGTGTGAAGTAGGTAGTCTGTAAATAATATATGTGGCACACAAAATGCATTCCCCCCAGTACTTTAGTGGTAATTTCGATTGGAAAAGAAGAGCCCTGGCAGTTTCTAGCAAGTATTTGTGTTTTCTTTCCACCACCCCATTTTGTTGTGGTGTGTAAGGACAATTTCTTTCATGAATGATTCCCTTAGACTTAAGAAAAGTTGAGGTTTCTGAGTTTACAAATTCAAGTCCATTATCAATTCTTATAGTCTGGACAGAGACATTGAATTGGTTTTCAATCATGGACATGAAGTTTTTGATGACTTCCAAGGTGTTACTTTTACATGTCAATAACTGAGTCCATGAACACCTACTATAGTCATCAACAAGGGTAATGAAATATCTATAGTTGTCATGTGTCATCCTATGGTAGGGACCCCAAAGATCTATATGCAACAATTTAAAAGGTTTGGTAGATGTGTTGGTTCTGTGTGGAAAGGGTAGTCTTTCTTGTCTTGCCATTGTACAAATGGTACAAAGAAAAGGTTGCTTAGTGGAAAAGTTCTTAGGTATGGATTTGATTTCCCTTATCTTCAGAAAAGGTACATGTCCAAGCCTATTGTGCCATAATACATTTACATCATTGCAAGAATGAGTCATAAAAGAAGCAGAATTCTGAGATTTTTTATTGAATGAAGCACAATGTTGTACAAGAGATGCAAGAAAAGGAACAGCTTTAACAGATGTATGATATGTGATTAATGCAATGTACTCAATTCCTCCCATAGCCTCTTCATTCTAGTATAGTAAGTGGTGATATATAATGTACCTTATGACAACTCACTGATTTCTCGTTGAACTTGATACAACTTAGCACCGTTTGTTTGATCATATCGATCATCCAATTCCTTCCATAATTCGAATGAGTTGGGGACATACTCCACACTGTCTGTGATCTCCCTAGCTAGAGAGTTGAGAATCTAGGAAGTAACCATATCATAACATCTCACCCATTGGCGAAAGAGTGATGTTCCTGGATCGGGTTGTTTGCAATCTCCATTGATAAAGCCCAGCTTATTTTTCACTGACAAAACTCGAAGAACACCTCATTCCATGACCTATAACCGACTCCATCAAAGGGAACTTGTACCAGCGAAGCACCTAGATTATCTGAAGGATGCATGTACAAAGGAGACTAATGTCGATACTACCAACACTGTTATTGTTTACAGAATTCTAAGTTGATTGAGATCCACTTTCATTCATCATTACAACACAATTCAACAGAAATCAAAACACACAATAAAATTCGAAGTCGATCTAAGACCCAGCAACAAACCAGTAAAAGACGAAAAAGAAAAATTGATCCAAAATTCGATCAATTGGGGACCAAATCTGGAACGTGAACCAGTAAAAAAAATGCAATATATGATGAACGTGAGGATCTAAGACGATGCACAGAAGTAATCGCTGAAATGAAATACCTCGTGCTCTGATACCATGTCAATAATACTGCACAACCATGGCTAAGCACCATCCATGGATGCAATACGTGAGAGAGAGATTGGAGAAGAAGAGAG

>LNC_001862

CAAACCCAGGAAATCGTGACCCAATCCAGGAAATCGCGAGCCCAGTGGATCTAAACCCGAACCAGCAGCCCATCCTCTCGTGCGTCGTCCTCTCCTCTCCGTACAAAGCAATCCAGGTGTTGCAATCTCAATGCTCGATTGTCCTTCTCGAGAAATCAGATGGCTCCCTTCATTTTTTCATTGAAGAATTGTACAAGATGGTACTATTTCCATCAACTTTATCCTTTAGAGGAGAATTTGAAATTCGAATGGGAATTCATCCAAAAACCCACCTGATTTCCCCTTTTTTCAGCCCTATTCTCCTTTATTCTTCTTTATTGTTAGAGGTTGAAGAAAAGGTTGGGAATTTCAATTGATATAAAGAGAACTGCCTAGCAAGAAATAGCTATAGAAATTACATACGAAATACGTTCGAAATAGATAGGAGGTTCAGTTCGATCTCACTGTTGTGGTCTCGTTCATCGAGACTTTTGGAGTTTCATTGTGGTGGTGATAGCGCTCGTTTCAAGCTCATCCTCCTGGTCTCGCTGCTCCAAAAAAAGGTAATTATTTTCCCTTTATTAAGTTTCTGCTTGTGTTGTTACTGTGTGTTATTGATTACCTTATTTCTTAATGTTCTACCTGACTCTTCTTAAATGTTTTCAATGCTCCGTCTTAATTTTATGCAGAGCTTGACGCGTTAGCATTCAGAGTTTGGTTTGTTTTCTATTAGATTTGTATAATCGTGGTTTTTGTCTTAAACTCTGCTTGGTCATAAAGTTCGACTCCTTCACTGTGTGTGGTGATATAGGTGTAATTGTAGTCATTTCTCTTTTGTTTCAATACACTGTTTCAAACTCTTTATCAATATTTATGGTTTAACTATTCTGCTTGAGAACTAGATAGTTTCAAGTTATATATGTGGGATTCATGCTTCAGATAATTGTTTGCTAGCCATGTCAATAATACTGCACAGCCATGGCTAAGCATCATCCATGGATGCAATACGTGAGAGAGAGAGACTGAAGAAAAAGAGGGCAATTTGGGGAAAATCTCTTAACTGATTTTCATTATGAGAAGAGCGTATAATTATACAGTGAAGGAACAGTAAATCTAACTAACAATTGAACTAACATATTAACAGAACACTAACAGTATACTAACTACCCTGACTAATTAATCTGACAAGACTAACTAACTAACAAACTTATTAGACTAGTACATAATTAGCTTAATCACAATACCCCTCCCCCCTCAAGTTGGAGGTGTGGAGAACATTGACAACTTGCTCAATGTTGTAGATTGTTTGATCCTAGTCAAAGTCTTGGTTAGTATATCTCCAAGTTGCTTATCAGAGTTTATGTGATGTAGGGAAATAAGTCCAGTTTGAAGCAGATTCCTTACAAAGTGGCAATCAAGTTCAATATGTTTGGTTCGTTCATGAAACACTGGATTTCGAGCAATATGAAAAGTTGATTGACTGTCACAAAATACCTCAATGGGTGTTGGCAGTGGCACTGTAAGTTCTGTCAATAATCTGCTAAGCCAAATCAACTTACCTGCAACTTTTCTTAGAGCCCTATACTCTGCCTCTACTGAAGACAAAGATACAGTCTCTTGTTTCTTGGATTTCCAGCTCAGTGGACTATTGCCTAACAGCACAATATAGCCACTCACTAACTTCCTGGAGTCAGGGCATGCAGCCCAGTCAGAGTCACAATAGGCCTTGATATTGTATGACTGGTCTTTGGACATAAAAATTCCTAGAGTGGGATCAGCCTTGAGATACCTCAATAAATGGAATGTTGCCTGCAAGTGTGGCTCTCCAGGATCCTGCATAAATTGACTAAGGGTCCAGTGGAGATGTGCAGCTTCTCATCCCTGCAACCTTGTAATTCTTCAGCATGTCTAGGAAAAATTTCCTTTGTGAGATGATAAGTCCATCATCTTTATAAAGCACTTCCATCCCAAGAAAATAGTGTAACTTTCCCAAGTCCTTAATCCTAAATTTATCATGGAGAAAGGTCTTGAGTTCTTCAATTTCAAAAACACTTGTTCCTGTCAAAATCACATCATCTACATATACAACAACAAAAATGGTGGAGGTTGTAGACTTTTTGTGAAACAATGAATAATCATTCAGTGAGTGTAACCTTTTGAATACAAAAGCCTCAACCAATTTGACATACCACTGCCTGCTGGCTTGTTTCAGGCCATACAGGGACTTATTTAGTTTGCATACCATCCCTAACTTATGCACTACCAAGCCGGGTGGAACTTCTATATATATACCTCCTCATGTAGATCCCCATGAAGGAATGCATTGTTTACATCAAGCTAAAAAATGTTCCAGCCCTTTTTAACAGCTATGGCTAATAGAGCCCTCACAGTTGTCATCTTTATTACTGGTGAGAAAGTTTATATATAATCAATCCTAGATTCTTGAGTGTAGCCTTTCACTACAAGTCTAGCTTTGAATCTTTCAACAGTCCCATCTGCCTTAAGTTTCACTTTATAAACCCACATGCATCATATGGCCTTCTTGCCACTAGGTAAATCAACTAGATCACAAGTATGATTTGCATGCAATGCCTCAAATTCATGTGTCATGTCTTGCTGCCAGGCAGGATTCATAGTTGCCTCCCCATATAAGGAAGGTTCATCATCATTGCTGATGTTTCTCACAAGAGTTTGACTCTCATCTGCCAAGACCTCAGGTCGTATATGTTGATGTTTGGAAAATGTAGCATTGAGGGAGATGTTTGAATTTTGCAGTTAGTTAGGTGCATTAGCACTAGGTGCAGTTGTCATATTCTTAGATAAGACATAATCTTGTAAGTATGTGGGCAGTTTATGTGATCTAGATGGTCTAGTGGGGGTCGTAGGTTCAGTTATGTGGATAGAATTAGGAGGTTGAATGGTGGTAGATAAATTGGTTGAATGGTTAGGTGAAGTGTGAAGTAGAGTGGTACTTGCATTAGCTATATTATTTTCAGTGTTGCATATTTTGTGTTCCAATTTCAGTATGTACAGTGTCCATGAAGGGCACAGTATGTAAAACAGAAGGAAAAGAATACATCAGAAAGTGCAACAAAAGGAAAAATGGATTCCTTAAAAACTACATCCCTGGAGATATGTATTTTTCTTGTTGGCACAGTATGTAAAACAGAAGGAAAAGAAGATACATCAGAAAGTGCAGCAAAAGGAAAAATGGATTCCTTAAAAACTACATCCCTGGAGATATGTATTTTTATTGTAGCCAGGCTTAGAACTTTGTATCCTTTTGATCCAAAAGGGTAACCTATGAAGATGTGAGGTGTTGTTCTAGGTTGAAATTTGTCCATGTGAGACTTTGGAAAGGTAGGATAACAAAGACAACCAAAGGTACTCATGTGGGAGTAAGTTGGTTTAGTTTTATACAACAATTCATATGGACATTTCCCCATCATGTGTGAAGTAGGTAGTCTGTAAATAATATATGTGGCACACAAAATGCATTCCCCCCAGTACTTTAGTGGTAATTTCGATTGGAAAAGAAGAGCCCTGGCAGTTTCTAGCAAGTATTTGTGTTTTCTTTCCACCACCCCATTTTGTTGTGGTGTGTAAGGACAATTTCTTTCATGAATGATTCCCTTAGACTTAAGAAAAGTTGAGGTTTCTGAGTTTACAAATTCAAGTCCATTATCAATTCTTATAGTCTGGACAGAGACATTGAATTGGTTTTCAATCATGGACATGAAGTTTTTGATGACTTCCAAGGTGTTACTTTTACATGTCAATAACTGAGTCCATGAACACCTACTATAGTCATCAACAAGGGTAATGAAATATCTATAGTTGTCATGTGTCATCCTATGGTAGGGACCCCAAAGATCTATATGCAACAATTTAAAAGGTTTGGTAGATGTGTTGGTTCTGTGTGGAAAGGGTAGTCTTTCTTGTCTTGCCATTGTACAAATGGTACAAAGAAAAGGTTGCTTAGTGGAAAAGTTCTTAGGTATGGATTTGATTTCCCTTATCTTCAGAAAAGGTACATGTCCAAGCCTATTGTGCCATAATACATTTACATCATTGCAAGAATGAGTCATAAAAGAAGCAGAATTCTGAGATTTTTTATTGAATGAAGCACAATGTTGTACAAGAGATGCAAGAAAAGGAACAGCTTTAACAGATGTATGATATGTGATTAATGCAATGTACTCAATTCCTCCCATAGCCTCTTCATTCTAGTATAGTAAGTGGTGATATATAATGTACCTTATGACAACTCACTGATTTCTCGTTGAACTTGATACAACTTAGCACCGTTTGTTTGATCATATCGATCATCCAATTCCTTCCATAATTCGAATGAGTTGGGGACATACTCCACACTGTCTGTGATCTCCCTAGCTAGAGAGTTGAGAATCTAGGAAGTAACCATATCATAACATCTCACCCATTGGCGAAAGAGTGATGTTCCTGGATCGGGTTGTTTGCAATCTCCATTGATAAAGCCCAGCTTATTTTTCACTGACAAAACTCGAAGAACACCTCATTCCATGACCTATAACCGACTCCATCAAAGGGAACTTGTACCAGCGAAGCACCTAGATTATCTGAAGGATGCATGTACAAAGGAGACTAATGTCGATACTACCAACACTGTTATTGTTTACAGAATTCTAAGTTGATTGAGATCCACTTTCATTCATCATTACAACACAATTCAACAGAAATCAAAACACACAATAAAATTCGAAGTCGATCTAAGACCCAGCAACAAACCAGTAAAAGACGAAAAAGAAAAATTGATCCAAAATTCGATCAATTGGGGACCAAATCTGGAACGTGAACCAGTAAAAAAAATGCAATATATGATGAACGTGAGGATCTAAGACGATGCACAGAAGTAATCGCTGAAATGAAATACCTCGTGCTCTGATACCATGTCAATAATACTGCACAACCATGGCTAAGCACCATCCATGGATGCAATACGTGAGAGAGAGATTGGAGAAGAAGAGAG

>LNC_001863

TTTGAATCTAAATAATACAATTGGATTTACTTTTGAAAGTTCTTTTTTTATTTTTTTTATAAATCCAATACTTTTGATAAACCATTTTTTGGTTTCTTTTGAAACTCTTCTAAATAATTTTGTTTTTCCTTTGAAAATTTTTAGAGTTAGAAAATATTTCTTTTTGAATTTTCCAATTTTTTTTTCGAGTTCCTTAAAAATGGGCTCAAAAAAGGAAGGGCGCTTTCGGGGAGAACCAAAGGGAAGTTCAGCTTCCATTCCCCAAACTGTTAAAAAACAAAAATCATCTTTTTGTTTTTGCTTTTTCATTAGCTCTCCGCGGGAGGGGTACAGTTTAGATATATGCCAAGGTTTCAGACAAAAAGGAAATAAAATTTTTATCTGAATCCCGTCTCTCAACCAATCTTTGGGAAATTCTGTTTCTGATAATTGAACACCATTATAAGTACATTTAATCTGCATTTCTCTATTCCATTCCTGCAAATCTTCAGACCATTCAGGAAGTTGCAAGAATAACATACGCCCGAGATTTTTGGCTATTATCAATGAAGGTAATAGAATATATTTTCGAAGAATTGATTGAGTTATTAACATGTAACCTCTTATTATTTGTGCAAAAGGAATGTTATCCCAGGCTTCTTCGATCGCTATCCGTGCTTGTTCCTTTCTTTTGTTATCCTCTTTTTTGTCCTTTTCTTTTTTCTCTTCTCTTTTTGTTTGTTCTTCTCTAGACTCTAGAATCTTGAATTCTCCCTCTTTACCTGTCCAATTTCGAAAAATTGGTTTAATTAGTTCAGAGATATCAAAAGAAAAAAGACGTAGGGGGGTTATTCTGTCGACAAAAAGGGGGGAATGTACATTTGCTTGAAATAGTTTCGAAATAAATGTTTTGCGCCTTTGAGCCCGCATAGAGCCTTTAATTATACCTCGCCGAAAATCTGATTGTTGCGAATAGCTTATTAAAGCCACTTCCTCCTCAGGATCGTTAGTCGCGTTGTTGTCAGTAAAAATAACTACACGTTTGGCTTTTCTTGAACGAAGTTGATGATCAATTGATGCGCGATCTTGAAATTCACCCATTTGTTGGTCCAATTCGGTGATTAATTTATGGGACCAGCGAGGTACTTTTTTACTGATTTCTTTTATTCCAATCGATTTTTTTCCCGATTTTGTCTCATTAGGATCAATTGCATTAAATACAAATTTTACAAATTTCGCTGATGATCAATTGATGCGCGATCTTGAAATTCACCCATTTGTTGGTCCAATTCGGTGATTAATTTATGGGACCAGCGAGGTACTTTTTTACTGATTTCTTTTATTCCAATCGATTTTTTTCCCGATTTTGTCTCATTAGGATCAATTGCATTGAATACAAATTTTACAAATTTCGTTCTTTTTTCTGAATTCACTCTTCTCTGTTCTTGGTCTGAAAATAAAGAAAGACCCTTCAAATTTAAACTCGATTTTGGTTCGTTACCTAATTCATTGATTAAAGTTAAGAACTCGTCAATTTTTGTTGATAATGGTTTTTTATCAATCGTATACGCTTTTTGTTCAAATTCTTGGTAATCCATATTCGGAAGAAAGATAGTATGAATCCTATTTAGTCTAACTCTCTTTTCCCAATTTTCTAGCAAAGTATTGTTTATGATTGAAGATGAAACCCCCTTTTTTATTGTTCCTCGATATGGTCCATTTAACAAAGGATCATACATTTTAGGCACGTATTCTTTTTTAGTATCATCATTACACAATCTAGTCCTTGTTTCGAGTATATCCATAGAAAGAGATTCCGTGGCTAGAATTTCAAGTCGATTTAAAAATTCCTTATTCAGATTATTACTTTTTTCTTTGTTGGTAGAAACCCACTGAGTGTCCAGTTCATTAGGGAGCGTTTTTTGGAGTGACAATAGGGGTATCTTTCTTTTTATCATTTTCCAAAAAGTTGATAAACTTGGCGGGTATGTAAAAGATATTCTTTGTTTTCCATCACTTTTACATGTGTCAAAAAAATATTGTGACATCTCTGTTCTTACGGCCTGCTCAAATCGATTATTCTTTATGTAACGAAATGGTCGATTCCAT

>LNC_001864

TTTTCATTTAGTCCCCAAATTTCTCAAGGCTTCTCTACAGTTCTACTTCTATCTCTTCTCTGTAAAAAAATTGATTCAATCCTTTGCTCCAAATTGCTGAAGTTCTCATGAAGAAGTGTCTAAGCATTCCTTGAAGAAGTGCCTAAGGATGACACAAGCAAACCCTGCAAGGTAACTTCCTTCTTGGGTTATAAGGTTGGAATGAACCACATTGTGAGAGAAGTTGAAAATTTTGTATCAAGTAAAATCACTCTTTCATTTATAGCATTTCAGTTTAGTATTCATATTTTCAGCATAGATATTATCATTTAGACTCTGGCTTCAACAAATTGTACAAAAAGCTATCAAATTTGTACAAAAGAAGGCTAAAGGAGGATAGAGCCGAGGATTTACTGGAAAGCTTAAAGAAAAAAAAAGAACAATAAAAAGAGTAAAGAGGGAGACGTGATCTTCGTTCAAAAAAAGTATTGTTGCCTCAAAGTTTCCATCTTTCATAGTTTTGTCTATGAATAAGTGAATGGGCATATAGAAGATACTCCAACTAATTTGGGATTGTGGCACATTTGGTTGATTATACTCACCTAATTAAATACAAGTAATTAACCTTAATGTATTATATCCAATCTTCAAACCCATTAACTATCTTTTTAAATGTTTGATTAAAATATTTGCATTCTGAGCTATTTTAGTTTTAAGCTTCATGGGCAAAATTTATTGATCACTCAAATGGCCCCTAAATTGATTTGCATCTTGCTCTTCTCTTTGACGAACTACTTGATAACTTTTAGTCTTTAGATATGCAAGAGGAGTGAGTCCATTTTTGTGAGACTGACCTTTTCATTCAGTTTTCACCTATATTGTTCGGACTCTTCAAAAATGGCAATGGGTGCATTTTAGATTCTCCAAAAGTAGTGTATATTTGAAGAATGTGAAACGGGTGCGACATTGAAGGGTAGATAAACAACTTCAATTTTAATATGCTTTTTGCTTCAGCAGTCCTTGCCAATCTAACATGACTGTTTTCTTTGCTTTCAGATAATTGAGATAGGCAACACTCATTTGCTTGGAGTATCAAGTTTTGTAGGATTCTTTTTCATAATTTCTCAGAAATATGTAATTCTCAACGGATTATCTACTTGTGTCCTTGGAATCTTTTCTTCTTTCTAATTTCCTTTTGTATTGAAAATAGGTGCAACTTGAACCAACTTTTTTTTTATCTCTGGGAATAGGAAAATGGGAGTATTACATGGTGTCCGCTTCGTAACTTCACAGACACTCGTCAAATTTTATTTTTGAGAATTGGCAAGAGAGAGTTACATGTGAATGAGTGTAAAATTAACTGACGAGGATATAACAAGACATGTTGAGATGGTTCGGAAAGCATTCCAAAGAATTCCAAGTAAGTAGCCCTCGATACAAAGTTGCTTAAAACTGTCCATAAATCCTCTCTTGCTTTTGAGCCCCTCTATGTTTCATTTCGCCTGAGATTGTTTTTCAATGTAGCAACAACTCTTTAGATCAACAAAAAATGTTGGACACAGAGATGTTTGCTTTCCGAGCTGAGATCAATCAGCTTCTCAGTCTTATTATCAACACTTTCTACAGCTACAAGGAGATCTTTATCCGTCAACTCAACAACAGTTCTTCAGTTGCAAGTCTAGAGTGTTTTTTTTTGTGTTCTCTTTGTGTATTGTTACGTCATCATCTAAGATACTGTAGTTTAACTACACGCTCTCTATGATTTTGAAATTTGACTATTTTAGAGCAATCTTAGTGTTTGTTATTGTTTTCGTAGATAGCCTAATGTGGTTTTCTAGAGTACTGTGGTGCCATCGATTTATATGATTTTTAAATTTGATTATTTAAGAGCAATCTGAGGACTTTTATTGTATTTATGAATAGCTTAATTTGATCATCTAGGATACTTTATAGACAGCATTCTTAGTGGTTTTGTAATTTGATTATTATTTTTCTTGAGGTTGTTTATATGTGTTGTCTTCGTGGATAGTTTAATGTGATCTTTTAGGATATTGTAGTGTCATCTCTCTCTATGATTTTGCAATTTGATTATTTATAGAACAGTTGAGTTTCTATTTGAGGGTTTTTTATCTGTATTGTCTTCGTATCTGCTAGGATACTAAACTGTCAAGGCTCGCAGAGATTTTGAAATGTAATTATTTTAGAGCAGTATGAGTTTTTTTTTTTTTGAGGTTTTTTATCTGTATTATATTTGTGGACAGCTTGATGTGATCGTGTAGGTCACTGTGGTTTCATTGCCCTCTGTAATTTTGAAATTTGATTAGTTTAGAGCAACTATGTGACAGTTCATATATTAGTCAGATATCATCTTGAAACTTAGTTAATTAGAGTGAGCTTGATTTGTGGTTTGTGTCTTCTAGATCTCTACTCTGAACATAGATATTATCTATAGTGTTAGTATATCATTTTGCAGATAATATATTGTAGTTTCATCGCCCTCTGTGACTTTGAAATTAGAGTAATTTAGAGCAGTTGTGTCATATCACACAGTCCATATATCAGGC

>LNC_001865

GGCTTCTCTACAGTTCTACTTCTATCTCTTCTCTGTAAAAAAATTGATTCAATCCTTTGCTCCAAATTGCTGAAGTTCTCATGAAGAAGTGTCTAAGCATTCCTTGAAGAAGTGCCTAAGGATGACACAAGCAAACCCTGCAAGATAATTGAGATAGGCAACACTCATTTGCTTGGAGTATCAAGTTTTGTAGGATTCTTTTTCATAATTTCTCAGAAATATGAAAATGGGAGTATTACATGGTGTCCGCTTCGTAACTTCACAGACACTCGTCAAATTTTATTTTTGAGAATTGGCAAGAGAGAGTTACATGTGAATGAGTGTAAAATTAACTGACGAGGATATAACAAGACATGTTGAGATGGTTCGGAAAGCATTCCAAAGAATTCCAAGTAAGTAGCCCTCGATACAAAGTTGCTTAAAACTGTCCATAAATCCTCTCTTGCTTTTGAGCCCCTCTATGTTTCATTTCGCCTGAGATTGTTTTTCAATGTAGCAACAACTCTTTAGATCAACAAAAAATGTTGGACACAGAGATGTTTGCTTTCCGAGCTGAGATCAATCAGCTTCTCAGTCTTATTATCAACACTTTCTACAGCTACAAGGAGATCTTTATCCGTCAACTCAACAACAGTTCTTCAGTTGCAAGTCTAGAGTGTTTTTTTTTGTGTTCTCTTTGTGTATTGTTACGTCATCATCTAAGATACTGTAGTTTAACTACACGCTCTCTATGATTTTGAAATTTGACTATTTTAGAGCAATCTTAGTGTTTGTTATTGTTTTCGTAGATAGCCTAATGTGGTTTTCTAGAGTACTGTGGTGCCATCGATTTATATGATTTTTAAATTTGATTATTTAAGAGCAATCTGAGGACTTTTATTGTATTTATGAATAGCTTAATTTGATCATCTAGGATACTTTATAGACAGCATTCTTAGTGGTTTTGTAATTTGATTATTATTTTTCTTGAGGTTGTTTATATGTGTTGTCTTCGTGGATAGTTTAATGTGATCTTTTAGGATATTGTAGTGTCATCTCTCTCTATGATTTTGCAATTTGATTATTTATAGAACAGTTGAGTTTCTATTTGAGGGTTTTTTATCTGTATTGTCTTCGTATCTGCTAGGATACTAAACTGTCAAGGCTCGCAGAGATTTTGAAATGTAATTATTTTAGAGCAGTATGAGTTTTTTTTTTTTTGAGGTTTTTTATCTGTATTATATTTGTGGACAGCTTGATGTGATCGTGTAGGTCACTGTGGTTTCATTGCCCTCTGTAATTTTGAAATTTGATTAGTTTAGAGCAACTATGTGACAGTTCATATATTAGTCAGATATCATCTTGAAACTTAGTTAATTAGAGTGAGCTTGATTTGTGGTTTGTGTCTTCTAGATCTCTACTCTGAACATAGATATTATCTATAGTGTTAGTATATCATTTTGCAGATAATATATTGTAGTTTCATCGCCCTCTGTGACTTTGAAATTAGAGTAATTTAGAGCAGTTGTGTCATATCACACAGTCCATATATCAGGC

>LNC_001866

TCAATAATCGCCTAATTACCCTTCAAAATCTTCATTTCTACATCATTTCATGCTTTTTCTCTCGAGATTGATGAAATTCCACTCAAAATCATTATCAATTCAATGCTCCAAAAAGTTGGTCAATTTTGTGGATTAAATTTTTCGATGCAGTTAGTTTAGTTTAGTTGGGTAGAAATATTTAAAATGAAGATTTTGTAAGATTTTTGTACTATTCGATGTTTATTGTAGATTCGATAGCCAAAGCTTTATCAGATCTGAAAAAGGAAGGTAGATGTGATACCTAACTATTTAAATTTTTTGTTGATGCTTAAATCTGCATCTCCTATTTTAAAAATCTTGAAGATACAAAGGAAGTGGCAGTGGTGTTTTACAACGAAATTGATGGTTGATGGGTCTGTTCTCACATAGTGGAGTAACAATAATTATTTTCGATTAATTTTTAATTGAGCTAATGTGAGACGTATGTGATTTGAAGGTAAGAGGGGGCTTAATGATGGAAGATCGATGAGGACGGAGACTTGGTGGTAGATGAGTGAAGTTCACATGGTGGATCATAGGTGTTGCCGCCGCACTACCCATGTTGTTGTTGGTGTTTCTTCTTCGTTATAAAGAGAAAGAAGAAAAGGAATGTTAAAAGTATAAAAATAAAATAAAAGGAAAC

>LNC_001867

AGTTGAAAATACGATTATAGATTGGGGAGACTTTTGACCATTTTCTCTAAGAGAAAACTAGATGAACTTGGATAAAAAGAACCATTATCACAAGTTAAAAAATTGAATCAGAATAAATAAAATAGCAATTCTTCTAGTTGTCAAAGAAAGCTCTATAGCCAACATATAATTTGGCTAGTTTTCAATAACTGATCAATACTTTGGTGTTTGGTTCAAAATTTGAAATTTTATTTAGGCCAAATCCATCGGTGGCCCTCTAAAGTTGGCACCAAGTTTCACTTAGACACCTTAATTGGGCGATGTTCATTTTAGA

>LNC_001868

GGGAAAAAGGTGGGAAAAAAATAGACGTTGGAGAGGCAGAAAATATACAGAGAGAAATGGAGTCGATATAAGTGAAAGGATACATAGAATTGCATAGTCTTTGAGACAAAATAGACTCTAAAATCAACAAATATTATAAAGAGTATTCAGTTTTCAGCTTCTTCTTTTGAACACGACTTTCCCGCTGCTCGTTGGAACTTTTGAAGTTTGTGACTGGTGGTGGAAGAGCTCGTCCTTGCTCGTTCT

>LNC_001869

TTCCTCCAGCGAGGCAGCGAGCCCAGGCGAAGCAACAAGCTCCAGCAGCTCCGATCGACGAAGCCCACCCCACCAGGCAGCAACCCCGACAACCAGCGTGCACCGGCGAGAACGGTCAGCAGTCGGCGCTGCTCCATCGCCTTTCCTTTTATCCTTTTTTGTAGCGAGGACAACAACAACAAACCATGAGCGGACCAGGTCTTGGTCGACACCAAGTTCAAGAATTCGATTCGCTAAAGATGGATTTTTCCAGATCCGTCCGGGTACATTTCTTTAAATTATTATATCATATTTTAGCAACTTTGTTCTTCTTTTCATTTATACAAGTTGGCTTTGATTTTCTGATGTATAAAATGTATATGCATGCTCCATTTTGGTCTAAAATGGAATTATTTGTTATTTATGATCTTAATCATGTCCTCATAATTTTCTGATTTTGTGTATTGTTAAAATAATATGATTTGACTTGGAATGTCAATATATCTCTTGTTATCTGTTTCTCTATAAATAGTAGTATATTCGTGATTTTAATATTATTGGAATTGCTAATTCTTGATTTAAGTTGATAAGCTGGAGTGATAGCTGATGCCCTTAATTGATTTTTTCCTTAGCTAAATATAGTCATTTTGCTATAATGCGGTCCGACTTCAACTCTTAAAATGGGATTTTGTGATTTGTGACTTGCCCTTAATTACTGTGTTGGGATTGTTACTTGTTTGTATATGAAAAAATATGAGAAGCTGTATATTTGGAACTTGATTTGAGCTATGTGATATTCCCCTTTCCTTAAAATAATTATTTATTACCTTGTGGCTGATTGTGAATAATTCTCTATTAACTAACTACTTTTTTCTTAATTATAGTAGGCCGATTCCTTGCCTGTCTTCTACATGGTAAAATTTATTAAAATATTGGACTGATTTTACTCTCTTTGAATAAGGAAAATACTAATTTATTTGTCCCCCTAATTTATTCCTTTCCTTTAAGTGTTGTGCCCTCTCATCCAGTATATAAGCATGCCCCCTTTACCCTTTAGGACACACGCAAAAACCATCAGAAATAGCAAAAAACACTCTCATGCTTGAATACTCCAACGCTAAATCACGCTTACACACTTTAATAGTAAAAGAATACTCTCTACTCTTGGAAAGAAAATAAGAACTCTAGAAAAGCTTGGGATCATTCTTTGTGGTATTGTTGTGTTGTTGTTTCGGTGGATATCTCTTTAAAACACACCATTGCTGATAATTATTTCTGCTTGATTGGTTAGTAATTACTTCATTATTTTAAATTTCGTCTTGCATAATCTATTATGAATATTGTGTGTTTGAAATAAATAAAGGAAGCGTTCAGGTCAGTCTTCTTACCCTTGTTTCTATGCGTGACTTTATACTCGCGTGATTAGTTGGAATATGTTTGTGTGTCTGTTCCTGCTCGTAAGTATGAAATAATTAATGTCATGTGTGTTGGTTGATAACTAGTATTATTATCTTGTTGTCTTC

>LNC_001870

TGAAAACCTAAGCCTATCTTGCCACTCATCTCCCTCAACCTGAGAATCCGCAAGATTTTATGCTCAAATCTCTGCCACCCCTATCAATCATCTGTGAACATCAATAGTCCCCTAGCTTCTCTAAAATGACTGAATTCAAACGTTCACACTTCCAAATCTCTTGAAAACACAAAATATAATTCAAAATCTGCGATTCAGATTCAAATGGCTCTTAAACTTTCTCAAAACTACACCCTTTGGAAAATCTGTCTAGAGGGAGTCGATAGCGGAGTATAAGATTCAAAGCACTAGTTTGCTGCCCTAGAGAAGTTTCAGGTACTCACGATAGCAGACGTTTAGTAAGATCTCTTTTCATCAGCAGTTGGTGAGACCTCCTTGCTTCCGAACTGGCATGGCTATAACTGATCAAGATATATAGGTAAGGAATGAAAAAATCTGAGTTGAATAAG

>LNC_001871

GCCTATCTTGCCACTCATCTCCCTCAACCTGAGAATCCGCAAGATTTTATGCTCAAATCTCTGCCACCCCTATCAATCATCTGTGAACATCAATAGTCCCCTAGCTTCTCTAAAATGACTGAATTCAAACGTTCACACTTCCAAATCTCTTGAAAACACAAAATATAATTCAAAATCTGCGATTCAGATTCAAATGGCTCTTAAACTTTCTCAAAACTACACCCTTTGGAAAATCTGTCTAGAGGGAGTCGATAGCGGAGTATAAGATTCAAAGCACTAGTTTGCTGCCCTAGAGAAGTTTCAGGTACTCACGATAGCAGACGTTTAGTAAGATCTCTTTTCATCAGCAGTTGGTGAGACCTCCTTGCTTCCGAACTGGCATGGCTATAACTGATCAAGATATATAGGCATTTCGAAAGTGGATAACTC

>LNC_001872

TGCTTTGTTAGATTTGGCATGTAAAAAGAGCCAGCCACCATCTTCGTCTTGAAATTCTCATTAATAAAACTTGAGACATTACTACTCTCATCCCAAAAGCTTAGATCCTATCCATATCCATCTCCTCTATAACATCTACCTCATCTATATCCATCTCCTCTATAGCAATTTAGGAAACTAGTAAAGAAGTGACAGAGCCGAAAATTTATATAAGGGGATTCAAAAAGAATGACCAGAGTACTATTGCGCTTAGGATTTGAACTTGTGACCTAAAGCAATAATTGAATTTCCTTCACAAAAACAATAAAATCTTGATGGATTTCTTCATGGGAATTCACTAGTTCATATATAATGAAAAAAAATAAATCATTTCCCATAATAGCGCAAAACGCCAAACATTTTATAGACATTGAATAACACCTTGACATACTAGGTAATCTCTCAGTTTCATTTTGTTTGACCAATATGACTTGGCACGGAG

>LNC_001873

GTTCCGACACGGTAACATTAAAGGAAAAAGATACTGAATTAACTTAATGTACTCAATCTCAATGAACGCATTTCCCAAACGAGTGTGCTGTGGAGGCATGAGTCCTCGTGTGTGTGCTTGATATTGTGATTGATTACGTACTTGCTTCAACTGTCACTTGTTCATGTTGTTGTTTCTTATCACCTGCTAAGTGCTATAGTTGACTTTATACTATTATTCATTGTATATTAGTTTCTATTTTGGGTTGGCCGATGATACCTACTCAGTACATGTTGCCCC

>LNC_001874

CTAAGGCATACTTGATCTGGATTGAGCAACTTCCATACCTAGAAAATACTTCATGCTGCCGATGGAATTGGTGAAAGAGATGTTGCTTCAAATTAGTGATACCATTTTGTTTATTGCCTGTGATAATGATGTTGTCAACATAAATCACCAAATAAATATATAGATTCGGTGTAGAATGTTGATAAAACATAGTGATCAACTCCACAATGAGTCATGCCAAAATCTTGAATTACTGTGTAGAACTTTCCAAACTAAGCTCGAGGAGACTGTTTAAAATTGTAGAGTGACGTGCACAATCGACACACAAGGCTTCTAAGTTCCCCTTAAGCAACATAAGTAGGTAGTTACTCCATATAGACTTTTCCCTCAAGATCACCATGTAGAAAAACATTCTTAATGGCACCCAAGGGTGTGGTCAATGAAGTGGTTGAGAGCCATGAGGTCTCAGATTTACACAGTAATTCAAGAAGGAGAGGTGTTGTCTCAACCATATAAGCTCACATGTAGGTAGTGCCATAGCTCGATATTTTGCCTTTGCACTTGATCTAGCTACGATAACTTACTTTTTACTTTTCCAAGATATTAGATTGCCTCCAATTAAGAACACAATAACCTAAAGTGGACGGTCTGTCAGAAGGAGATCCTGCCCAATTTGCATCAGAATATCCGACAATGTTGGTGTGTCCCTTATCTTCATACAATAAACCTTGTCCTGGAGAGCTTTTGATATATCGCAGGATGCGAATCACTGCATTCCAACGACTATCACAAGGTGATTGCAAAAATTGGCTAACTACACTAATAACAAATGAGATGTTTGGTCGTGTTATCGTAAGATAGTTGAGTTTGCCAACAAGTCTCCAATATCTGCTACGATCTTTTAGAGGCTCCCCCTGTCTTGGAATAAGCTTAGTATTTGGATCCATTGGACTGTCCACCTGTTTGCAGCCCAACATACCAGCATCTTCCAAAATGTCCAAGGCATATTTCCTTTGAGTAATAGCAATACCATGGCCAGATTGTGCCACTTCAATGCCTAGAAAGTATTTCAACTTGCCCAAATCCTTAGTCTGAAAGTGACTTGAGAGGTGTTATTTGAGTTGAGCAATACCTTCATAATCATCTCCT

>LNC_001875

GCTTTGAGACGATCAATAGCTCCATCCGAACCTACCTTAATGGTGAATACCTAACGACAACCAACTGTGGTTTTGCCTTCAGGTAAAGGAACAAGCTCCCAAGTACCATTATCATGCAAAGCAGTCATCTCCTCAATCATAACTTGACGCCAACCTGGATGAACCAGTGCTTCTGCCACAGTCTTAGGAACTTTGACCGATGATAAGCTAGTAAGGAAGGCATGATATGAAGGTGACAAACGATGATAACTTAAATAAGAGTAAACAGGAATTTGATTGGCAGTAGTTCTAGTATCTTCGCGAAGAGCAATGGGAAGATCATCGGATAAGTGCTCGACCGAAGCAGAAGACATTGTTGGAGCAGGTAATGAGTTACCAAGATTGTTTTGTGCACCTGTATCAGTTAGAAGTACCAACTGGATTAGCAAGTTCAACAGGAGGCTGAGGTCGTCGGGAATAGACCTTAAGAGTTGGAGGTGGAGCTGGGGTTGGAATAGTTGGAATGGAAAGAACTGCTATGGCCTTTTGATTTTCCGTATTATGAGAAAGATGCGGAGAAGTCTCGAAAAAAGTAACATCTGCAGAGATCAAGTACTTATTGGTAGAAGGATCAAAGCATTTATAACTTTTTTGTAGATGAGAATACCCAAGAAAGACACATTTAAAAGCCTTGTGCTAAAGTTTATCTTTTCCCGGGGTAAGGTTATGAACAAAACAGGTGCAACCAAACACACGTGGAGGAAGAGGAAACAAATTTTGGTCCGGATAAAGGACTGAGTGAGGACTCTGGTACTGCAAAACAGATGAAGGCATGTGATTGATTAAATAACAAGCCGTAATAACAGCATCACCCAAAAAACGTATAGGGACATGATTGTGAATGAGCAATGTTCGAGCTGTTTCAATTAGATGCATGTTCTTTTGCTCAGCAACTCCATTTTGCTGAGGAGTATGTTCACAAGAAGACAAGTGCAATATGTCTTGGGATGACATAAAATAAGAG

>LNC_001876

GAGAAAAGTGCGAGCAGTCTCAATGAGATGTCTATTCTTCCTTTTTGTTGAGTGGTGTACGAACAAGATGTCTAGTGAATAATTCCTTAGTGAATCATAAATTCCTGAAACTGGTAGGATAAGTATTCTAAGGTATTGTTACTACGAAAAGTATGAATCGAACAGCAAATTGATTTTGTATTTCAGGAAAACTTTTGGATATAGAAAATCACTCAGAATGATCTTGCATTAAGAAAATCCAACTACATCTTGAATAATCATTAATGAGACTAACAAACTAACAAAATCCTAAGGTTGGCTGATTCTAGTAGGACCCCAAATATTAGAATGAACTAATGAAAAAATAGACAGATCTGGTTTTGTGGTGTTTCCGTGGTCACCGGCAGAGATAGAGAAAGAAAGTGTTGCAGAGAGGTTCTTGCATTCGCCGGAAAAGAAAGAAGAAGACCAGAGTTTCTTGCATTCGCTGGAAATGAAAGAGAGAAGAAGAAGAAGACCAAAGCAGTAGTTTCCTTATTCAGTCGTAGTCGCCGGCCGGTTTTATTACTCTGAGGAGTTTGCCGGAGATCGTTCTTGCCGAAGGTTGCTGGGAGATTGCAGGTTACCCAGCGGTTGTCATATATTGAAGAAGAATTCTCATCTTTGTTTTATCTTTTATTTTTTTCCCCTGTTTTATTTCCCATTTTATGTTACTCAGCTTGTTTTGTTATCTCTTAGAACAGACGTGGTGGAAAAACATGGGACAGGATTATGGCCTTACAATGCAAATAATGTTCCCTTCCCCTTTTTCTCTTTCAACTTCTTGCTTGATTAGCAAATCTTTCACTCCTGTGAGTACACTTTTGCTGTAACACCAG

>LNC_001877

AACAATGATTTCTTAAGCATTTTGAGCACATCTTTGTTTTTTCTCCCCATGGAGAGAATGTTGAATCAAAGTTTATGTCTTTAGGAATGAATTCAACTGACAGAAACGCTAGAGGAATAACTAATTGAAGTTAGATTACACTTACAGTTCCTCTCGACTTAAACGCCTAATAACTTTCTGACTGTTCTCAAATTGACTATGATATTTCAGATTGATAATGACAACATCATGCTGATAAAATCATGCTTGAATGTATTTTCTTTGAGTATTCCATGCTTGATCTATATTTAGCTGACTAGATGGCTGCCTTAGCAAATCTTAACATTCCTTGAATTAGGTTCACAACTGATTGACCCCGAGCGTATTTGCTTAAACTTGATCTCTGTTCAAATTTCTAGCTTTATTTCAGATTAGATGGGAGTTCATCTATGTATGTAAATACATATTTTAAAAGGAAAATAATTTCCTTGTTTTTGCA

>LNC_001878

TCTTATTTCATTTCTTAACCTCTAGTTTTTCTCCTCTTGATACACTCTTGCTTCATACACTCAACCAAGCTTTGAATTGGAAGATCCAAACGAGTTTTGGCCTCTCCTCCAACAAAAATTAGGGGTTCAAATTGATGTTGTTTTGGATCGAAGGGTTACTTTTTCCTCTTCTTGCTATTAATCATGGGTTCTTCTTCTACATTGCTTCAGCCCAAAACCCAGGTATATTTTTTTACATCTTTCTTTTTCAATTTTTTGAAAATCTATTCCTCTTTTTGTTTCCTTAGATGCTTTATTTTTAAAATTAGATGATTATATGGGTAGTAAAAGTTGTAAGCTTTATTTCATGAAAACCTTTTGTGGTTTTAGACAATCTTAGATGAACTTTCGAATTTATTTTCTTTTTTCTTTGGGAGTGGATGATATTAACATGTCGAGAGATGATTTTGTGATTTTTTTTTTCTTTTTTGAGAGATGGGAGATGGGTTTACTAGTAGGGCTGATTTCTTGATCCCAAGTATTTTAGTCAGAGATAAGGCTTTGGGATGCAGTCTAGGTTTTTTTCTGCATCAAATCATTACCAGTATAGGAAGATATTATAAGCAGTTATTTGTTCACTTGTTGTGTGTTTCTTGGTTATGCTATATGTAGTGTATATATTGTGTATTGTGCAAATCATCAATCTTGAACAAAATGTGGTTAACATCACTACTTGTGTATGCTTTCAAAATGTAAATTTCATGTTGACACACGACGTGAGAATATTGAATATATTGTGTATGTGTTCATTACATTTTTTGTTGGTTGTTGTTGTCTGGTTAAATTTCTTAACAGAATATTATGTGGTTAAATCAATGTAATTTTGCATCTGAAACCTCATCTGTTGGATTGCTCATTTACCATTCATTGAAGCAGTGTCATAACTTCTATAGCATTATGGTTAATCATTTTTCTTATGAAAAAGGAAACCCACAGCCGCTCTTCTTTGGGTGCGCACAGGTTAAAATGTCTGCTCCTATGCAATAGCTTGCAAATCACACAGGAGAGGTAACCCGCACTAGGCAAGTCCGGTGTGACGAGCTCAACCCAGAAGGCAAACCCCTTGCTTTTGCTGGCAAGGGGTTTCGAAAGGATGTTGAATCTTTGACATAAACGATTGGAAGGTGGTGTTGGGCACATCCTCAATGACGTAACTTTTCAGCCAGTTGGTTCATTTATCTCTTAGTCTATCTAATAGATATTGGAGTATCCCCAAGGTTTGTTTGTTAGTATTAATTGTTGTTAGGTACTCTGATGAGCATGTGTCTTTATTCGATAATCTATCTAGCATTAATACAAAAAGGAATAGAAACCCCTTCCTACATCTTAAAACATGTACCTCTTTTATTGTATTGTGTTGTAGCTTGTCTGACGACAACGATCTAACATATGCCCACCTTTTTAGCAATTTTAGATCTAATGCCTATCACAATTTGATATACTTTGAATAGTGTGTGTAGTGTGAATATTAAGAGTTCGGTGGAGTGTGCTAGGAGTTTAGCTTCTAGTTAGCCATTTAGAAAATGGTTTATTTGTTTGATACTTCCATAAGGTTTATTTTACTCATTTCTTGGATACATTCATAAGGTAAAAAGAGCTTTATTTTACTCATTTTTGGAGTTCCTTTTTAGTTGAATGCATCAACTATTTCAACCATTTGAATCTGCATTGCACAAGGTTTGTACAGATTGATTGGTGATAGGTTGCAAGAAATTTTTTACAGAGAGAACAACAATGGTAATTTTTACAGAGAGAACAACAAAAATAATTTTTCCAAAGGCTGCATGAGAGCTACACGGAGTGGGTGGACACAATTTTTATGAAGTCATGGAATTACCACTTTTGAAGGTGATCAAAACCGATATAACAGAACCAAAACAAAAATTTGAAAGATAGCAAGTAATTTATATTTTTATCAGGAGAAAAACTAGCAACAAGTACAAATGATATCAATATATGATATACTATGATTTATACCAGCATTTATTGTTGAACCCTTATTTTGTTAGTATTTATGGTGATGAGTTTTGTTCATAATTCTTGAAAGCATTTGAAGTTTTCAATGTGTTCCGTAACTCTCTTATGTGTTCAACTTGAAAAGACTTTTTATTTCACCTTTTTTTTTTCTTCTCTATAAATTGGATCTTCTTAATCAACACAAGGAGTAGAGTAGCAAAGGCAGAGTTCTTTTGTTAGCACATGTTGTCATGCACTTAGATGTATCTTCCTTAATTAAAATATCCTCTTCAATTGTTGCTTTTGTAACAAGACCGAAATCTAGATGTTTTCTCTATTGTAAGTCCAACTAATAGTGAGGTTTTCAACTATTCATATCACTTGGGGATTTGTAATCTTTGTAAATTTGTTGAGAACTGCATTTCTTGTGGGAATAAAATTAAATGCATTATATTTTGTAGTTGTTGAGTCTTTGTAAGCAGAGAGTCTATCTTACTTTTATGAAGTATCTAATACTCCTGCAAGCCTTGAGTTAGCAGCATCTATTGCACTCAAGGTTAGTATCTTCAC

>LNC_001879

TCTTATTTCATTTCTTAACCTCTAGTTTTTCTCCTCTTGATACACTCTTGCTTCATACACTCAACCAAGCTTTGAATTGGAAGATCCAAACGAGTTTTGGCCTCTCCTCCAACAAAAATTAGGGGTTCAAATTGTAAGCTAAGTCTACTATAGTTTTATTTGTATCACTGTATCTATGTAATGGTTTTGGTTCCTCATATCAAGCTCTTCAATTGCACATATGTAAAAGGATGTTGTTTTGGATCGAAGGGTTACTTTTTCCTCTTCTTGCTATTAATCATGGGTTCTTCTTCTACATTGCTTCAGCCCAAAACCCAGCCGCTCTTCTTTGGGTGCGCACAGGTTAAAATGTCTGCTCCTATGCAATAGCTTGCAAATCACACAGGAGAGGTAACCCGCACTAGGCAAGTCCGGTGTGACGAGCTCAACCCAGAAGGCAAACCCCTTGCTTTTGCTGGCAAGGGGTTTCGAAAGGATGTTGAATCTTTGACATAAACGATTGGAAGGTGGTGTTGGGCACATCCTCAATGACGTAACTTTTCAGCCAGTTGGTTCATTTATCTCTTAGTCTATCTAATAGATATTGGAGTATCCCCAAGGTTTGTTTGTTAGTATTAATTGTTGTTAGGTACTCTGATGAGCATGTGTCTTTATTCGATAATCTATCTAGCATTAATACAAAAAGGAATAGAAACCCCTTCCTACATCTTAAAACATGTACCTCTTTTATTGTATTGTGTTGTAGCTTGTCTGACGACAACGATCTAACATATGCCCACCTTTTTAGCAATTTTAGATCTAATGCCTATCACAATTTGATATACTTTGAATAGTGTGTGTAGTGTGAATATTAAGAGTTCGGTGGAGTGTGCTAGGAGTTTAGCTTCTAGTTAGCCATTTAGAAAATGGTTTATTTGTTTGATACTTCCATAAGGTTTATTTTACTCATTTCTTGGATACATTCATAAGGTAAAAAGAGCTTTATTTTACTCATTTTTGGAGTTCCTTTTTAGTTGAATGCATCAACTATTTCAACCATTTGAATCTGCATTGCACAAGGTTTGTACAGATTGATTGGTGATAGGTTGCAAGAAATTTTTTACAGAGAGAACAACAATGGTAATTTTTACAGAGAGAACAACAAAAATAATTTTTCCAAAGGCTGCATGAGAGCTACACGGAGTGGGTGGACACAATTTTTATGAAGTCATGGAATTACCACTTTTGAAGGTGATCAAAACCGATATAACAGAACCAAAACAAAAATTTGAAAGATAGCAAGTAATTTATATTTTTATCAGGAGAAAAACTAGCAACAAGTACAAATGATATCAATATATGATATACTATGATTTATACCAGCATTTATTGTTGAACCCTTATTTTGTTAGTATTTATGGTGATGAGTTTTGTTCATAATTCTTGAAAGCATTTGAAGTTTTCAATGTGTTCCGTAACTCTCTTATGTGTTCAACTTGAAAAGACTTTTTATTTCACCTTTTTTTTTTCTTCTCTATAAATTGGATCTTCTTAATCAACACAAGGAGTAGAGTAGCAAAGGCAGAGTTCTTTTGTTAGCACATGTTGTCATGCACTTAGATGTATCTTCCTTAATTAAAATATCCTCTTCAATTGTTGCTTTTGTAACAAGACCGAAATCTAGATGTTTTCTCTATTGTAAGTCCAACTAATAGTGAGGTTTTCAACTATTCATATCACTTGGGGATTTGTAATCTTTGTAAATTTGTTGAGAACTGCATTTCTTGTGGGAATAAAATTAAATGCATTATATTTTGTAGTTGTTGAGTCTTTGTAAGCAGAGAGTCTATCTTACTTTTATGAAGTATCTAATACTCCTGCAAGCCTTGAGTTAGCAGCATCTATTGCACTCAAGGTTAGTATCTTCAC

>LNC_001880

CATTTCTTAACCTCTAGTTTTTCTCCTCTTGATACACTCTTGCTTCATACACTCAACCAAGCTTTGAATTGGAAGATCCAAACGAGTTTTGGCCTCTCCTCCAACAAAAATTAGGGGTTCAAATTGTAAGCTAAGTCTACTATAGTTTTATTTGTATCACTGTATCTATGTAATGGTTTTGGTTCCTCATATCAAGCTCTTCAATTGCACATATGTAAAAGGATGTTGTTTTGGATCGAAGGGTTACTTTTTCCTCTTCTTGCTATTAATCATGGGTTCTTCTTCTACATTGCTTCAGCCCAAAACCCAGGAAACCCACAGCCGCTCTTCTTTGGGTGCGCACAGGTTAAAATGTCTGCTCCTATGCAATAGCTTGCAAATCACACAGGAGAGGTAACCCGCACTAGGCAAGTCCGGTGTGACGAGCTCAACCCAGAAGGCAAACCCCTTGCTTTTGCTGGCAAGGGGTTTCGAAAGGATGTTGAATCTTTGACATAAACGATTGGAAGGTGGTGTTGGGCACATCCTCAATGACGTAACTTTTCAGCCAGTTGGTTCATTTATCTCTTAGTCTATCTAATAGATATTGGAGTATCCCCAAGGTTTGTTTGTTAGTATTAATTGTTGTTAGGTACTCTGATGAGCATGTGTCTTTATTCGATAATCTATCTAGCATTAATACAAAAAGGAATAGAAACCCCTTCCTACATCTTAAAACATGTACCTCTTTTATTGTATTGTGTTGTAGCTTGTCTGACGACAACGATCTAACATATGCCCACCTTTTTAGCAATTTTAGATCTAATGCCTATCACAATTTGATATACTTTGAATAGTGTGTGTAGTGTGAATATTAAGAGTTCGGTGGAGTGTGCTAGGAGTTTAGCTTCTAGTTAGCCATTTAGAAAATGGTTTATTTGTTTGATACTTCCATAAGGTTTATTTTACTCATTTCTTGGATACATTCATAAGGTAAAAAGAGCTTTATTTTACTCATTTTTGGAGTTCCTTTTTAGTTGAATGCATCAACTATTTCAACCATTTGAATCTGCATTGCACAAGGTTTGTACAGATTGATTGGTGATAGGTTGCAAGAAATTTTTTACAGAGAGAACAACAATGGTAATTTTTACAGAGAGAACAACAAAAATAATTTTTCCAAAGGCTGCATGAGAGCTACACGGAGTGGGTGGACACAATTTTTATGAAGTCATGGAATTACCACTTTTGAAGGTGATCAAAACCGATATAACAGAACCAAAACAAAAATTTGAAAGATAGCAAGTAATTTATATTTTTATCAGGAGAAAAACTAGCAACAAGTACAAATGATATCAATATATGATATACTATGATTTATACCAGCATTTATTGTTGAACCCTTATTTTGTTAGTATTTATGGTGATGAGTTTTGTTCATAATTCTTGAAAGCATTTGAAGTTTTCAATGTGTTCCGTAACTCTCTTATGTGTTCAACTTGAAAAGACTTTTTATTTCACCTTTTTTTTTTCTTCTCTATAAATTGGATCTTCTTAATCAACACAAGGAGTAGAGTAGCAAAGGCAGAGTTCTTTTGTTAGCACATGTTGTCATGCACTTAGATGTATCTTCCTTAATTAAAATATCCTCTTCAATTGTTGCTTTTGTAACAAGACCGAAATCTAGATGTTTTCTCTATTGTAAGTCCAACTAATAGTGAGGTTTTCAACTATTCATATCACTTGGGGATTTGTAATCTTTGTAAATTTGTTGAGAACTGCATTTCTTGTGGGAATAAAATTAAATGCATTATATTTTGTAGTTGTTGAGTCTTTGTAAGCAGAGAGTCTATCTTACTTTTATGAAGTATCTAATACTCCTGCAAGCCTTGAGTTAGCAGCATCTATTGCACTCAAGGTTAGTATCTTCAC

>LNC_001881

CATTTCTTAACCTCTAGTTTTTCTCCTCTTGATACACTCTTGCTTCATACACTCAACCAAGCTTTGAATTGGAAGATCCAAACGAGTTTTGGCCTCTCCTCCAACAAAAATTAGGGGTTCAAATTGTAAGCTAAGTCTACTATAGTTTTATTTGTATCACTGTATCTATGTAATGGTTTTGGTTCCTCATATCAAGCTCTTCAATTGCACATATGTAAAAGGATGTTGTTTTGGATCGAAGGGTTACTTTTTCCTCTTCTTGCTATTAATCATGGGTTCTTCTTCTACATTGCTTCAGCCCAAAACCCAGGTATATTTTTTTACATCTTTCTTTTTCAATTTTTTGAAAATCTATTCCTCTTTTTGTTTCCTTAGATGCTTTATTTTTAAAATTAGATGATTATATGGGTAGTAAAAGTTGTAAGCTTTATTTCATGAAAACCTTTTGTGGTTTTAGACAATCTTAGATGAACTTTCGAATTTATTTTCTTTTTTCTTTGGGAGTGGATGATATTAACATGTCGAGAGATGATTTTGTGATTTTTTTTTTCTTTTTTGAGAGATGGGAGATGGGTTTACTAGTAGGGCTGATTTCTTGATCCCAAGTATTTTAGTCAGAGATAAGGCTTTGGGATGCAGTCTAGGTTTTTTTCTGCATCAAATCATTACCAGTATAGGAAGATATTATAAGCAGTTATTTGTTCACTTGTTGTGTGTTTCTTGGTTATGCTATATGTAGTGTATATATTGTGTATTGTGCAAATCATCAATCTTGAACAAAATGTGGTTAACATCACTACTTGTGTATGCTTTCAAAATGTAAATTTCATGTTGACACACGACGTGAGAATATTGAATATATTGTGTATGTGTTCATTACATTTTTTGTTGGTTGTTGTTGTCTGGTTAAATTTCTTAACAGAATATTATGTGGTTAAATCAATGTAATTTTGCATCTGAAACCTCATCTGTTGGATTGCTCATTTACCATTCATTGAAGCAGTGTCATAACTTCTATAGCATTATGGTTAATCATTTTTCTTATGAAAAAGGAAACCCACAGCCGCTCTTCTTTGGGTGCGCACAGGTTAAAATGTCTGCTCCTATGCAATAGCTTGCAAATCACACAGGAGAGGTAACCCGCACTAGGCAAGTCCGGTGTGACGAGCTCAACCCAGAAGGCAAACCCCTTGCTTTTGCTGGCAAGGGGTTTCGAAAGGATGTTGAATCTTTGACATAAACGATTGGAAGGTGGTGTTGGGCACATCCTCAATGACGTAACTTTTCAGCCAGTTGGTTCATTTATCTCTTAGTCTATCTAATAGATATTGGAGTATCCCCAAGACAGGTTGTGGAAGGTGGAAATCAAGACTAGTTGCTAGAGAAGTGCTTTTGCAATGGTATTTTGAGAAGCAACCTCTATTAATTTTGTTAATATTTAGTACATCTTAGTTGGATTTACAAGTTGGATAATTTTTACTTAATATTTTTTTTCCTTAAGGGTGTTGAGATATTATCTCATGAAGGTCATTTTAGATGTTCTTAATTATTATGAC

>LNC_001882

TTGTAGGTCTACAGATCTCACAGATTTCATGATTCAACACCAGTGAAGACTTCTTCAACTTGGAAGCTGCAACAGTTAGCTGGATCTAGAGGTTGAATTCCTGAGGAATTGATACCACTGTTCAAGACTTATAATTTTCGGAGTACTTCATTTATAGCACTGAGTAAGATCCTTTTCTTTGACTGTATTACTATGTTTCTCCTTTGATATCAATGTTG

>LNC_001883

TTCTTCGGAGCGGAAAAGGACGTGGCCAAAACATATAAAGATCGGCGTAGTCGCTCATAGGGACATATCTATCCGGATAGAGGATAGTCTAGGCCGATTCATAGATAGATCTCTCTCCATATAGATAGGTATCTCCGTGGATAGAGATAAAGATAGGGATCCATCTAGATCTTGATTCACTATTTCCATATTTTTTCTTTTTTCTGATTGATTGCCTTTTTTTTGACG

>LNC_001884

CATGACGCTACGATTCATAAGAAAATCTTCTTGCATTGGCAATTGTTATAATTTTTTTCTTACTACTTATATTCTTTATTCATATGGATTAGTGAAGGTGGGAAACAACATTACGTACACTTACCCTCTCCAGACCCCACTTTGTGCCACTATACATGGTCCTGATGTTTTCGTGAAGACCACGAATAAAGGAGACTGATAATGACTAGCGGGGCTATCCCTTGAAGTAAATACATTACATGGTAGTACCAAGCAAGCAATTGGTATGTAATAGACACATCCTCTTTCTGTTGTTTTGTGTAATAAGTTTTTATTAACTATGCCTCTGAAATTGAAGATAAGATTTTGAACATGAATTTGGTATTCCACATTCTAATTTTGTGGTGCTGGTGGTTTTGGATTAGCTTGTTATTACTCTATGTGGATGAATTTATCTCATAAACATACTAATAATCTTGAGTTTATACTGCCTGATTTTCAAGA

>LNC_001885

TCATATTCACCTGCCAAGGCATGACATAAATAGTAGACAGTCAGCTCTCTTACAGCCAGTTACTGCGATTGATTGGGGAGAGAGTGCTAGTCTAACTGCCGGTACGGGTATGCTAATACATAGAGGATCTTCCAGTCCTAGAATAAGAAAGTAGCTTGTCTGCCGTACTGGCTGCTTCATTGAATGTGTCGATCTGCATTCTATATAAGGAGTTGATTTGCATCCTTGTCTGGCAGTTAGCTAAGTGAGAAGCT

>LNC_001886

CTAAAACTTTTGAATGTAAAGAAAGAGTAAATGAAAGTTGCAGGCTGATCATTACATTATGATTACTTAGGAAAAAAACATACAGTAGAAAACTTATTTGACTGAAATGTTTAACTATATTGTCCACATGTAATCAAAAACCAATTAGACAACAGAAAATTTTGAGTTTTTCATTCAAGATTTCCTATTCTGGAAGGAGACCGAGACATCCAATTAAAAAGCTTCTTGCATGAAATATCTATTGCACAACTTCTATTATTTGTTATAGCTTGAACCTCGAAGAAAAGAGTGAACTATAGAAGTCTTATACTCCTGTGTGTAATGAAGTTGTTCTTATGTTTTCCATCTTTGCTCAACTTTTTTTTTTTAACTTTCTTCCTCCTTTTCATCTCAACTTGGAATTTGATGAAAGGTAAGTTCCTAATTTGTAGCGGTGAGTGATAACTTGTCCAATGTAATTTGGAATTATCAAGATGAATTTAGTGTGATGCTAAGTAAGAACACCATTGGATGTTGAGAGCAATAACAATTGACATCCATCATCCCTAACCTTCTCCCTTAATTCGTTCCCCATTAGTAAGGATTGATTTGGTAGATGAATGGTCTTGTAAACTAATGGAATGAAAAGTAATCATCATGGAATACAAAACAACAAACAATTAAGGCTGCTTAATTAAGATAAGAAAACCCACAAATTGATGGAATACAAGTAATTACTTAATAATTTGATGGGGGTAATTTTATTGTTAGGAGAGGATAAATTGGTCATTCAATATCTGAAGGGTATTTTTTAAATCCAAATTTCAACTTAAGATGGTCCCACTTTTAGTATAGTATGATACTTTTAGTATAGTATGATATGATGATTATCAAGAGCTCTTTCTTTCAATCAATAGTCTTCTTCATATGCGTGGATGCCCTAGTGAGGATGTGCGAGATGTTAGATATAGTAGGTACGAGGAGAGGTAGAGTTAGGCTAGATAAATGTTGGGGAGAAATGATTAGACATGGACATGGTGCTCCTTCAAATTACCGAGTACATGACCTTAGATAAGAGGATGTGAAGTACGTGTATTAGGGTAGAAGGTTAGTTGATAGTGGAGTGTGGTCTTGCGTTTTGATATTGCAACTAGCCTGGCCTGTTGCATGAGAACTAGTTATAAAATCTGCCAACTTTAGTTTGAGCTTATTTTATTGTTATTTCTTCAACAAAAGCAATTCTCTTTCATGTGGACCTTCCTTAAATGACATTGAACCTTCTATAATAGTTTGTAGTACTGAACAAGCGGAGTGGGTTATTGGTATGGGAATTGATAGGGTTAAATGTGCTGGGGTCATGCAAATACATAGG

>LNC_001887

AATAAATGTGCATAAGTTTCAATAATACCTTTTGTATGCCTTATGTCATCATAAATGATCTTGATATCCATCAATATGGATAAAAGATAAATTAGTCAAATTAATATCTTATTTATGATTTCTTAAGTATTGTGTAAAATGGAAAGTGATCAGTTAATATGGGATAGAGGGAGTACTTTCTGATTCTTACCTGATTAATTTTTATGCCCACAAACCTGGCCTCGGAAATTAAAATATTTTGAGCACTAGTTTTTATGGTCCATTGGGCAATTTAAGTTACCATGGGGAAAGGATCCCTCCAGAAAAATTTGTTCCATTGGTCAATTTAAGTTGCCATGGGGAAAGGATCCCCTCCAGTAAATTTTGGTCCATTGGCCAATTTAAGTTAGCACAAGGAAAGGATCCCTGCAGTAAATTTTGCTCCTTTACTCTTTACTGGAGTTATGAATGTGTGCCATGTTTTAAAAATAACTCTTAAACGTTTGAAATAAATGCATCTTAACACAGTAGACAAATTATATAATAATTTTATTCTTAATACATTGCTCCC

>LNC_001888

TTTCCTTTCTAGGGTCATGACCTCGGTGAGCTGAAGATAAGTCATGTCATATCTAATCACGTCACCATAATTCTTCCTTGGGTTACCTTTACCTCTCCTTAGACCTAATCACCGCAAACCTCTCACACCTACTCATTGGGGCATCTGTACTCCTATGTACATGTCCAACCATCTCAATCTTACTTCCTAAATCTTGTCCATAACTGAAGTCACTCCTACCTTACCTTGTATATCTTCGTTCATAATCTTATTTCTCCTAGTGTGTCTACACATCCATCTAAGCATCCTCATCTCCGCTACTCATCTTTTGTATGTGTGAATTTCTTACTTGCCAACACTTTGCCCCAGACAACGTAGTTGGTCTAACAGCCACTCTATAGAACTTACCTTTGAGTCTTGGTGGCACCTTCTTATCACATAGTACACCGGATGCGAGCTTCCATTTTAC

>LNC_001889

CAACCATAAAATCCAAAGAGGATGATGAAATTTAATACGTATTATTGAGTTAGTTAGGTTTGATGGGAATAGAAAAATTGCAAACGCTTCAGGTTTTATGTAAAAAGGATCAAGGAGATCCTCCCTCAGTTAGATCAATGGATACTAGAGCTTGTAAGGAACATAAGTATTTCAGTTTTCTTTATTTTATCAAACTTCCATTAAGGATGTATAGACGATACTATATGGTAAGGATCTTTGTTTTATTCATCAGTCATTATTGGGAGAAAACCAAGAGGTCTCTGGCCTTTCCTGATTCACTTTGTCTTTGATCAATTTCAACACTTATTGTTCTTTCCTGTTTGAAATCATTTCTCTCTGCTCCTCTTTTTTTTACTTTGGGTTTACTGTAGTAAATCCATTGATAAAAATGTGTGATTTCTGTAAACAAGAGGTATGATAAATAGAGTTTAGGGACAAGCATTGAGATTCATAAAGTCCATCATTTATTTGTACCAATGGATCTCATGGGCTTAAACATGTCTAGAAAACAATAGTTTTCTCAAGATTTGGTTGAAAGTCTCATTCCTTGTCTTAAAATTTTTAGTTAAGACTTGGGAAATATATCGAGGCATTAGATAGGAACTGAATGTTCTGTACAGGGTTATGTCACGTCAGTAACAGTCCTGATCATTTATAGATGGATTCCAAACTTTTTCTCTGAAGTTGGCCGTTGTTCTCTTGTATAATCTATCTTATGAAATATCCAAATGGTAAAGTTAAATGGATAACAGATTGTTCTGTTTGGTGGAAGTTCTTTCAGGATATTCACTAAAAGGGTGACAGGACGTTGAATTTGGAATAGAGGGAGTTGGAAGCTTTACTTAGTGTTCGGTATCCAAATTGGAGCCCAATTAAATTCGGATTCGCGCCGTAAAGTTCCACATTGAGGGGTAAAGCGCTC

>LNC_001890

CATCACCCATGTTGAAGAAGGAAAGAGAGAGTTAGCAAAAGATTTACACAGACTGGCACGCCTGGGAGTCAGACTTTCAGATTCCGCAGAAGGAGGAATAGCGGTGACGAGCAGGGCCGAGTCATCACTAGTGTCAGAAGTGAAAGAGAAACAAGATCGAGATCCCATTTTGCTTGAACTAAAGGCAAATGTTCAAAAGCAAAGAGTATTAGCTTTTGAACAAGGGGGAGATGGTGTTTTGAGATATCGAAGTAGATTGTGTGTGCCTATGATAGATGGACTCCAAGAGAAAGTTATGGAGGAAGCTCACAGCTCCAGATATTCTATTCATCCGGGTTCCACCAAGATGTATCGTGATTTGCGTGAAGTTTATTGGTGGAATGGCATGAAGAAGGACATTGCTGAATTTGTTGCTAAGTGTCCAAACTGCCAACAAGTGAAAGTCGAGCACCAAAGGCCCGGTGGTTTGGCTCAGAGGATAGAACTTCCGGAATGGAAGT

>LNC_001891

CTAGTCTCTTTGCGGAGCAGTGATGAAGGGAGTGGCAGCGAGGTCCTTCATGTTGGTCTGTGATTGCCATCAAGATTGTTTTCTGGAGGTCCGGCCATAGCGGAAATAGAGAGAGCTAAACAATTTGAGAGAAAGGAGATGAAACAGGCGTTGAATGAAGACAAAGTGGGATCCACTTATTTTCTTTTTAAATTTCATGCTATTTACGCGCCTAGAACTTTTTTATTTGCCACGTCTGCCATTAAAGTGGTATTTAATTCACTTGAAAGTTTTTAAGGGGTACTTAAACGTCCGTAAAGTTATAGTGCTAAAGTGAGTTTTGCGGACAAGTTTGAGGGGGATTTTATGTATTTCCTCTTTCTTAAGTGATGTGTCAAATTAATACTGAACAAGTAAAGATGAACAGAAGGAGTAGAAACTAAATTTCCTCTATAGAGAGATTCTAGAACTAAAGTAATTATAACAAGCGTAGTTTAAAGGAAAAAGGAAAAAAATTATTATTAAA

>LNC_001892

AATGTACTCTTACCTAAATCGGTGCTCATTACTACTCCCAAATTAGTCAATATCAATTATGCTCAATAACCCTTTTTAGGGAAATGACCTTTACTTTACTTTACTGAAATGTTTTTCAGAAAACAATTTATGCTCTTTTTGAAATAGTGCCTCTTTTGTGAATGTAAAATAAAACATTTGGAACCATTTATGTTCCTTTAAAACCCTTTTCAATGATGCTTTAAAATTAC

>LNC_001893

AAATGGACTGTGCTTTCACCATTCCTAATCTTTGCTTCTTTATCCTCTAAAAAAAATTGTCACCAATTACTACATAGTATTGCACAATATCTTAATTTATTTCGTAAACTAACATATCATATATATAACTGCTCAATTTTTGTTTGTTTATTACTATAATTTTTCTGCCCAAACTCGGAGACGAACGTTTTGATTCCAATTTCTCATAGATTTGCTCTTGCGAACAAAATGGATTCGGCGTTCCCGATTCACACACTTGTGGCTAGGTTCCTAGAGGATTGTGAAGGGAAGCTTTGGTGAGTATCATTGTATTCGAGGATTGGATCACCCTATCTCTTTTATGTCTTTCTCTTATGTTTGAGACTATTGTATGGGCTGCATCCCAAGATTTCTATTTCTTATGTTTAGATGGTTTGAGACAATGTCATGTTAATCTTCCGCTTGCTTTTATAAAGGACTTTGATTGTACTGAACTTTTTTAATTCTCGTTCATTTCTAATACTTTATGTTATGATATGCTAAGTGGCTTATATGAGGCCTCTTGGGGTCTTATACGGCTTGTTACATCTAGGGGTACCCTTGGGTCGTGACATTTTAATTTTTCTTTTGTTTACTTGGTTTGCTTCTTTGTTTCACTATTCTCCGTCTTTAATCCTTATAGCATGCTAAAATTAGTTGTTTTGCTATGTGCCTTTTTCACTTGCCACGTATTCGACAATTTGTACAGAAGAAAGAAGGAAGGAATAGTCATCATTGATTGAGTACAATTAGTAATCTGTATTGCTTAGTTGAGCTAGATTTTCGCTTCAGAATGATTATTAGGTCGTAATTGGTCCTTGGCTGCTATTTGTTGATTCAAGATAGAGCATAATCAACCTCTCCCCCCGTTAACACTTCTAACATTGTCGATTTTAGATAGAGTACGGTAACCTTCAACCCCATTGAAGAGTGGAAAATCTCTTCATATGATATTATAGTTGTACAATCATAGTACCATAGGGATGGTAAGAGTTCATAAGAGCCCG

>LNC_001894

GGAGGATTCCCCGAATACCTGAAATAGAGATAATCATAGAAAATGTGAAATATATGATAGGATCCATTTCGGGAACGTAGAATTTAAGAGAAATTCAAATGTGAAACAATACGTCAGCTCTTCTACTTGAAATATCCTTCAATTATGGAAGAAATCGTATTTTTTATACTCATGTTTTCCCTTCCCATCGCGCTCCGCCGATTGTTGTGCTCGTGTCAACC

>LNC_001895

TAGAGAATCTTCTCCTTCAGAGTCAAGGCAGTTTGAACTGAACCCCAGCTAGTATTGAGATTGAGAGCCGTAAAGAGTCCGGAGATCAGTCATTCGACTTAGGAATCCCGTCCAACTTCAGATTAGGTAAGATAAGGTAGGAAAGCTACAAACCAGTCTTAATTCCTGCAGACTAAGATTCAGTTTCAAGTCCCAAGGGATTCAATCCAAACACTCATAGATTCTATC

>LNC_001896

ATCAGTAATGGAAAGGGTGGCTAAAATGGATAGGTCTCTTTTACCCGGGGATCTAACTGGATACCTTGAAAAGCTCTCATGAGATCTCGTTGGCATGCCCTACCAAATGAAAAGGAAGTCGCAGGAGCAAGTTAAGAGTTAGAATTCCCCGACTCCGTTCCTATTTATTAGTCTTCTTTCTACTTCAACATCAACTTCGTTAGCTAACTAATTGATCCTCGCCCATATGTGGCTGCCTGTTAGGAGGATTATGTTGGACCTATGCGATCTCGTTACAACGTTCTTTTTTTTACAGAGATTTCTGGTGAAAACTTCAATCTTGAAGCATGGGAGCTTCATAAGATGTGCATGAACAACTGCAGGCACGACCTGTCGTCGAGACTCCTGCC

>LNC_001897

GGAGGACGTTGCAGATGTCGGGGGCGGACACGACTTTTTCTAAGGCTAGAAGACCACTGGAAGACACCCAGGACTATAGCATGTCGTGAAAGAAGCACACTGGACGGGCGTGCTTCGACCGTGTCTCCGGGGCACAGTTGGATGTAGATATCATGAACGCGAGGTGCAGGATACCAGGCCGAGAAACCCGGGCAACCATTCCCCTATGTGCCAATCGCTAGCGCATCCAGGGCCCCGGCACCCAGCTCAGCTGGAGAGGCCTAGCCTGATCTGAGAAGTGCTAATTCGGTTCGGATAGACTTCATTCAAGAATGCCACCGCCCCGGGAATCAATAAGAAAACAAGTGCTAAGTTTCAGATCAGTGAATAAACTGAAAGGAAAGAAGAGACCTTTCTCGCTCAGCGCCTAAAGCGCACTATGCTCCGCTTCAACAAATGAAACTTTTCGG

>LNC_001898

AGTAACCATTGACTCTATTTATTATTATGGTAAATCAGTGTATCAAGATGTCAATCTGAGATCTTATTTCGGTTCGATACGTCCACCTACGAGACTCACCTTTGGCTTTCGTCTCGGTAGGTGTATTATTATACATTTTCCCAAAAGAACATTCATTCATTTCTTTCTTCCCCGTCGACCACGACGACTGAAACGACGCAAAAAATCCAGACCCGTAAAGGAGAAGGGCCGGTGGGGGGCATTTGGGAAAGTCAGGCCGATCGGGTGTCTTCATTCAAGCGACGGG

>LNC_001899

AGTGGAAAAAGCTCTGGGAGCTTGCTTCAGTCCATAAAGAGCTTTGTGAAGTCGGCAGACATATCCTGGATGAGCGGTATCCAGAAAACCAGGAGGTTGAGACATATAGACTTCCTCCTCAAGAGTTCCGTGTAAAAAGGCATTACAGACCTCCAGTTGCTTAAGAACCCAATTATATGTGCAAGCGAGATATTTTATAATACTTAGAGTAGTTGGTTTGACAACAAGACGTGAAGGTATCAGAAAAGTAAATACCTGCCTGCTGATGGAAGCCTTTCTCCATAAAGTATGCCACTGGAGGGGTCCTTCCCTGCAATGTATGTTGGAGGGTCAGCTTCTACTTACTTCCTTCTAGAAGCGAAGGTTGGCAAGGAAGACCCATTCATGTTCTATTCTATGCCATGCCGCAAATTCAGTATACCAATGTTAGCGAATCAGATTCTTCAAAAGAAGAGTCTGCAGGGGCAGCAAATATAGTTTCCAAATAAGTCAGAATCTTAGCGGAAGCTCTTCCTTTGCCAGCTATTCCTATGTCAGCTACTACTTTGTCAGAGGCTAAGTTCCCTATATCTTCCGGGAAGGGAGTAGAAGAAGAAGCCCTCGTGACTCGGCTGCATAAATTAGCATATCATC

>LNC_001900

TCGATGACGAGGGACGAAAAGAGAATCCGCTTTCATCGGAATATCCGATTCAAAGCAAGAGACGAAGAGAGAATAAGTTCATTTCGGGTCACAGAAAGGATCGGATCGAAGGAGAAAGGTTAGGCACTTCCTAAAGTCATTTCAATAAGAGGTTGCTTCTTGCATCTTAGCTAATTCAACTATTTCATTGATCCAAGGATGAATTTCCCTTAGCTCTTAAGCTTGCTAGAAAGCCGAAAGCCTTTTAGTAAACAAGTACGGATTTTTAGACGGGCATCTTAGAATAAGAAAGGATTGTTCCTAATCCGGAATTTGCAACACAACATAAGTAGAAGCATGCTATGCTAGCATAGAATAGTTGTACCGAGTGAAAACTTCTTTTCTTAGGATGAACAGTATAAAGATCTTCTTCGTCGATAACAAGACTTTCCTTTCTTCATAAAGGAAGAGAACCGGAAAG

>LNC_001901

GCCAATTCAAAGTATTTTCAAAGTTCTTCAAAAATCCTAAAATCCTAATTTCGATTCTAGCATGGGTTCTTGCATAAAAAGGGTTTTAAATGATGATTTACATTGTTATTAATGATTATTAATGGTGTTTAGAAAGATATCCCCATGAACCCATGTTTTCCCTAAATTCCTAAATTTGCTTATGAAGTGGGTCTTTTGCTTATGATTCAAAGTTTATTAAATTGTGTTTGGATTGGATTGTATTAGTGACTCTTATGATTTTCTACATCTATCTATGATGAATTGATGATGTATGGATGAATTGAAAGTTGTGGTCTTATGGGCAAGTTATGGATGAATTCTCTTGTGGTAAGAAAAATGGTTGGGACTTGAGTGTTGTTGATTATGGC

>LNC_001902

TCTCTCAAAACCCCATGGAAGCTCAAGGTGAAAGATGGAGCTAGGGTGGATGGTGGCTACTGATTTCTTCTCCAATTTCGTTTGGTCCTTGAAGATTAAGGTATGGGGACTCTTCATCTTTGATTCCTTCTTCAATCAAAGAGTCAATCCAAAAGATTTCAAAAGTTGTTCAAAAACTCAAAATCCTAATTTTGATTCTAGCATGGGTTCTTGCATTAAATGTTTTTAATGATGATTTATGTTATTATTGATGGTTTTAAGAAAGGAAATTCCATGAACCCATTGGTTTGCAAGTTTCCTAAAGTTGGCTTATGAAGTGGGGTTATTGTTTATGATTGAATGATGATAGAATTATGCTTAAATTGAGTTGAATAGTTGATTTCTATTATTATCTATGTTTATCTATTGTGAATTATTGGAGAGTTGAGGAATGGTGATTGTGGCTTTGGAAAAGGGTAAGTTGACCTATTTTATGAAAAATGTTGAATTAGTAGGGAATTGAGTAATGTTGGTTATGGCTTTGGAAAATGGTAAGATGGCTTAATCTCTTAGTATTGATATAATTGTTATTGGTTGGTTTGATCCACCTTTGGTGGAGGTGGTTATTTGTTGTATTATTGTGAATTGAGGTCATGAAGGGCATTATGTGAATGTTATTATATTGTTGTATTATTATACACATATGAGGCTATATATAAAGTTTTATGTGAAGCTATATGTGAAGGAATGATGTGAATGAGTTTTATGTGAAGCTATATGTGAAGGAATGATGTGAATGAGTTTTATGTGAAGCTATATGTGAAGGAATGATGTGAATGAGTTTTATGTGAAGCTATATGTGAAGGAATGATGTGAATGAGTTTTATGTGAAGCTATATGTGAAGGAATGATGTGAATGAGTTTTATGTGAAGCTATATGTGAAGGAATGATGTGAATGAGTTTTATGTGAAGCTATATGTGAAGGAATGATGTGAATGAGTTTTATGTGAAGCTATATGTGAAGGAATGATGTGAATGAGTTTTATGTGAAGCTATATGTGAAGGAATGATGTGAATGAGTTTTATGTGAAGCTATATGTGAAGGAATGATGTGAATGAGTTTTATGTGAAGCTATATGTGAAGGAATGGTGTGAATGAGTTTTATGTGATCTTGTGATGATCCTATGTAAAGTGGATAGCATTGTGAGTTAAAGTATGATTTCTCATGATGTATATATGAAATGATGTTGGATTATGAAGTATCCTTAAATGAAAATGTTGTGACTTGGTTGGTAGACTAAAGTGTCCCTCCTTGTTACTTAAAGTCTTGAATGCATGAATGTGTGAAATGGGCAAAACTAAGAAATGGTGCCCTTTCATAAGTCTAGCTTTCCTAAGTAAATGTAAAACCATGAAATCGGTAGACCTAATGGTGGTAGCCCTTCTCATGTGTGAAATGATGAACCTTGGATCGGTAGGCCTAAGGCACCCTTCTAGGGGGATTTAATGAGCTATCCTTATGAACCTTGATTGGCCATGACTAAGAAATGGGGCCTTTCTTGGGAAGAGGTACTATGAGTTGGTTGAACTAGAAAAGGAACCTTCTCTAAGTACATATGTGATTGAATACTCTATGGGAAACAAGGCTAGCACCGAGTGGATGTGGTTAGGAAGGTGGCCTAGTTGAGTATGAGATTAGGCACAATGAACTTCCTTGGACATCCCCTAAACCATGTGCCTACATGGGTTGCTTACTAGTTCTACCTTTGGCAAGTAGAACAACCTCCACGGAGTAGGATAGACTCCGGATTCCATACCTAGCTAGTATGGTCTATGTCGGTTATGCCTATTCCCATCATGTGGGATGTGCACTCTAGTTATTGGATAGGTTCTACAACGTGTAGGTAGTAGTATGGGATGCTATCTACACATGGCACGAGTAGGCTTTGAAGATACTAAAGTGAGTTCCCTAAGTCTAACGACTATTATGTGAAAGTCCCCTTATGTGCTTGAATGTCTCCAAAAATGGTTTCTTAAAGAATGTTGGTCTATTGATGTTATGTTGTAAAGGAAATGTCCTTATCTAAGGTAATCTTGAGGAACACTTAAGTGTGCTTGAAGAGGTTGTATGGGCGGTCACTTCTTGTCTTACTCAAGTGTGTCTTAAGGTTATATTAGGTAGAGGTCCTATGGTAATGAAATGGCATGTGTTCCTTTAAATTTAAGCATGGGTTGTATATGAATATATGTTGAGTATGTATACGGTTGGCTATGGTTTTATATGTGAACATTGACTTAGTATGTTATATTGTAATGGTAAATTTCCTTATCTATGGTAACCCTTAGGAATACTTAGGTGTATATGAAGAGATTGTATGGGCGGTCACTTCATGTTTCACTTAAGTGTGGCTTTGGGTAACTTGAGATAGAGGTCAAGA

>LNC_001903

TGTACAGGGGATTCAAACCAACTTTTCTGTTTGCTGCTGAAACTAATGCTGAGCAAAGGGAAAAAAACATGACCATCAAGTTCAGAGTTTATTCTCTCAACAGTTTTCGTCCTCTCTTATTGATCCAAGTTCAGCTCCTCTGAATTGCAAGGCTCTTATGGTGTTAATTAAGGCACACAAGAAGAAGACCGAACCAACAAATCTCATGGTTTCAAGTGTGTAATGCTTGCAAAGAGATGGAAGTGTTTGATTCAGAAATGTCTGTCCATGAATTTGCGCTAAAGGTCATGTTAAAGGGTAAATACCTATTGAAAATACACTAGTTGGAATGTATAGAAAAGTTAGAAACGTG

>LNC_001904

CCAAAAGTGGTGATAAGGGTTGACATGGTCTAGAGTTGTCTTGATCCAACTTTAGTGAAAAAGAGAAAGGTTTCTTGGTCAAAACTCCTCTAAGTTCCTCTAAGGTTTAACCAAGAAAGGATGTCTACCCGTAAGTCTCAAAGAGTGAGTGGTAGTGGATCTTAAGGGTTCCGGTATGTGTTTTATGTTTTATTGAATTGCGGAAATTCCATGGGCCAAAGATAGAGGAAGATCCTCTAGAGTTTATAAAGGAGTTATCTAAGGTGATGAACCTTGTAGGAGTAATCTTAGTAATACTTGTCTTTCGTGACTTGATAAAGAGAATATCGCAAACTTTTTTTGTGTCGTCCTTAATATTCATGGGTTTAGTTATGGCCATATGTGAGTGATTAGTATAACTCAACCTAGTCCTTATGAGTTTTATGATGTGTTGGATTTTGACGAATAGTGATTCTTAAGTGGTACCTAGAGATGTTGAGTGATTAAGCTCCTTTAAAAGAGGGTAGGAGTGAGATCCTCATTGGTTTGAGAATCATGTTGTGTAATGAGATAGTTTAGGCTTACCCCAATGCGGGATAATGTGCTTAGATGGTAAGGTTATTGAGTGTCCTTGATGTTTATGAGATAGGTTTGCTTGAATTCTCACCTTATGTGGTTTAGTGAATTGGAGCAATCTAGACCCGGAGTGGTAGATTCATATGAGATCTTGAGTGATATCCTAGTGACGGCCTTAGAAAAGGATGCTACTAGGGTTGACTTGGAAGAATTTTTGGGATGAAGGTACCGGTATGTGGTAATTTCATATGAGAGTAAGTGCTATGTTGATTTCCGGTACTACCTTATCTTGTATGATGCCTTATGAGGCTATGAGGCTTAGAGTTCTCCGGATGAGTTAATAGAACCTTTTTCGGATTTTACCCCGGTTAATGGTTTAAAGTTAGAAAAAAAAGGGGTTTGTGAAGAATATCCCTTACCTTGTTCCCCTAGGAATAC

>LNC_001905

AAAACACTCTCCAAGCCCTCTCCAAATTCCACCAAGATCTTCAACCAAATTCAAACATCTCAACCTCTAATTCCGGATTCAAGACTCAATCTCAAGCTTCAATTCTCCATTCCAATCTTCATCAAGGATTCTCCAAAGTTGAGGTATGTGGGTTGATTGTGGAAACCTTCCTTTCCACAAGTCCAACCATTTATCCTCTATTTTACTTCAAGTTTATGGGTCCTTATGATCATTCATGAACTCTTGAATTATGGAATTACTACTACACATCCTATTATGGTCTATTTTTGCATATTATCATGAAATGGAATTATTATGTGATGTTATGGTTTTCATGCTTCCATGATCAAATTATGAAGGTTGATATATATATGTATGTTGTTGGTGGGCTGATTTATATGTATTTCGGAATTG

>LNC_001906

CTGGGCAGGGACCTATTCCTAAGCCATTTTCAGTAGACAGACAGGGAAAAGGACTAAAAACATTCTTCGCAAGATGAGAGCAAGCATTAGATGAAGCCGACTTTGTCCATCTCGCATATCAACTTCCTCTATCGATAGCGTAAAGCCGCCCAACACAGAGAACGAACATGGTCGGTAGGTAATTAATCTTCGTTTGGATAGCAGTACGGTACACGCATCAAGA

>LNC_001907

CGCCAATTACCAGAAGCACTTGAAAGGGTTTTCTTGAACTTTCTTTATTTCTCCTCTACAGCTTTCTGTCCTATTTTCCCTCTTTAACTCCACTTTTTTGCTCCATTTTGTAAAGAGTTTTGTTTAAACTTTGTTGCGGGTGATCTACCGGACGGCCGTATGTTGCATGTACAGAATTCCTATGTTGAATCCTCTTCGTCATTCCTATGATCTGATTTTCCTTATTATTGGAACCTGTTGGACGTAGCTTTCTAAAAACTGATTTCGTAGCTGATATTTCGTTGCATACATAATAAAAGAAAGGATGAAGAGTGCATATTTGAATTGGTACCGTATGTTGGTTTAGGAATCTGGTGGCGTTGATATTATCCAAATAATCTTGGTTTGATCATATTGGGCAACAAGAGCCTTGATAATCTGTTCATATACAAGATCCAGACAAAGCCAGAGAGTTGAGAGAAACACACACACACACACAAAGATGAAGAAGAAGAATAAGGTAGGAAGATTACAAGGTATGGAATCAAACCGTTCCAATAGAGATCTCATCACCGATGCTCCTCATAAGGTACTATCCCTAAAATTATTGTGGATCAACTACTGATACAGAATCACGTTACTCATAGAGGCTATATTGCTCGAGGTCATGAAATTTGTCCACAACTAATCAATATCGAGGTACCTCGACCTATTTCTATGCCATGATTTCTTGAAATACGAAAGATTGGCTACAAAGTTTCGGACTCCAACTGAAACACCTTACCATTATTAAAATAGGATATGCTTATGATCTAAATGAAGACTCAAAACATTTGTTACATTTTTGGTGTATTATGTCTATTACAACTACGCTTGTAGACAAGTAGACGGGGATTCCTTATTGCATTTTCATATCATCCAGAGGCGTTGCTCTTAAATTTATTTTTTGAGTATGAGATAGATGTTGCTCTTTTGTTCCTGGGTAAGCTTGACAACCCCATCCCAAACAAGGTACCCTTTGCAACCGCCTTTTCAGATAGGGCATTTCATTCATCGGAAATCAAATTCCTCTCTTCTCTTTAATGACTTGATTTTTTTTTTCAAATTTCAAAATTCCTTGTTCTTATTCTTCGCTAAATGTGTGAAATAGACCAAATCCTTATGCCAACCTATGCCTTGAGTTCCTCATCCCCTACAATAGGTCCAAGAATAACAACAAGGTACTGATCTACACTAGAATTGAGCAGAGGGTATGCTCATTTTCCA

>LNC_001908

GTTTTCTTGAACTTTCTTTATTTCTCCTCTACAGCTTTCTGTCCTATTTTCCCTCTTTAACTCCACTTTTTTGCTCCATTTTGTAAAGAGTTTTGTTTAAACTTTGTTGCGGGTGATCTACCGGACGGCCGTATGTTGCATGTACAGAATTCCTATGTTGAATCCTCTTCGTCATTCCTATGATCTGATTTTCCTTATTATTGGAACCTGTTGGACGTAGCTTTCTAAAAACTGATTTCGTAGCTGATATTTCGTTGCATACATAATAAAAGAAAGGATGAAGAGTGCATATTTGAATTGGTACCGTATGTTGGTTTAGGAATCTGGTGGCGTTGATATTATCCAAATAATCTTGGTTTGATCATATTGGGCAACAAGAGCCTTGATAATCTGTTCATATACAAGATCCAGACAAAGCCAGAGAGTTGAGAGAAACACACACACACACACAAAGATGAAGAAGAAGAATAAGGTAGGAAGATTACAAGGTATGGAATCAAACCGTTCCAATAGAGATCTCATCACCGATGCTCCTCATAAGGTACTATCCCTAAAATTATTGTGGATCAACTACTGATACAGAATCACGTTACTCATAGAGGCTATATTGCTCGAGGTCATGAAATTTGTCCACAACTAATCAATATCGAGGTCAGCTACCTTCAAGTGACTAACAGGGATATTCGATTGTATTATGGTATAGATCATGCACAAATATCATAGACAAATTTTCTCCTGCTAGACTTCTGTTTAACTATTTTTCCTCTTCATTCTCTTCTTAAGTTAGAACACATTCTTACTCTCATTTTTTGATGAATTCCTTTTATTCTATGTCATTGTACGAATTAATTTCTTATATATCTTCATTTACTACGTGAAAAATTTATGAAGAAGTTGACAAAATTAAGAGTAAAAGCCATAATTTGGAGAACACCCTCAATGGCTTTGACTTATTACTATTATATTGATTGTTACGTATGCATATACCAAGCAAAAATTAGTAAATGAATATGAAATCGGATTATAGATGCTATGATGAGAGATGAGAGAAGCTAATAAGAAGTTCAAATTTTTTTAGGCATGATTGAAAAACATCATCAATTTTCAGTGCATTCATGTATATTTGATTACTAATTCACTCTTTTTTTTCCTTGTGAAGGAAATTTACATGAAGACACAATTGTAGATTTTATAATGTCAACAATCGCACTGTAGATGGTCATTATATTTTTTCATATCATTATTTTTATTACACACCTTTCTTGCATGTACCATTAATGTGTGCATTGTATGAACTCTTATCATTTCTCATTAAACTTCCTTTGTGGAGAGGGAGATTACTTAGTAAATGCAGTGAGAATGATAACATTGTGTCACACTGTGTATATCTAGGAGTCACAAAATTGCAGATGTACACTGATAAGTATCTGTACTTACTGATTTTTGATTATTTTTTTCTGCATTTGTCTCTTTTATTATATATGTATAATGTTTGAAGCTTTGCATACATGCATCTCCAACTTCCTAGATTGACTGATATTTATATGTTTTTCTCAGGTACCTCGACCTATTTCTATGCCATGATTTCTTGAAATACGAAAGATTGGCTACAAAGTTTCGGACTCCAACTGAAACACCTTACCATTATTAAAATAGGATATGCTTATGATCTAAATGAAGACTCAAAACATTTGTTACATTTTTGGTGTATTATGTCTATTACAACTACGCTTGTAGACAAGTAGACGGGGATTCCTTATTGCATTTTCATATCATCCAGAGGCGTTGCTCTTAAATTTATTTTTTGAGTATGAGATAGATGTTGCTCTTTTGTTCCTGGGTAAGCTTGACAACCCCATCCCAAACAAGGTACCCTTTGCAACCGCCTTTTCAGATAGGGCATTTCATTCATCGGAAATCAAATTCCTCTCTTCTCTTTAATGACTTGATTTTTTTTTTCAAATTTCAAAATTCCTTGTTCTTATTCTTCGCTAAATGTGTGAAATAGACCAAATCCTTATGCCAACCTATGCCTTGAGTTCCTCATCCCCTACAATAGGTCCAAGAATAACAACAAGGTACTGATCTACACTAGAATTGAGCAGAGGGTATGCTCATTTTCCA

>LNC_001909

AAATAATCTTGGTTTGATCATATTGGGTAAAGTTTTCTCATTTTCATACCATCTTTATGTGTTTATCATTTCTTGAAGCTTTTTAGGAATACACATATCGTCTTACAATGGTGTCACAAAGGAGTGATAGAGAAATAGGTGTCATAATTCGTTATGATCTGAAAGATTTGAGCTCATACAATTGGCTTTTCGTTATTCTTTTATGTTTTATCCATGTCATGAAGCTATTCCCTGAAATTTGTAAAGAGAAGACAAAGGTGCATCTTATATGTGTTGAAGTTACTCATGAAGAAGTTATATGTTTTGCATGCACCAAATTGATCCAAATATCCTTAACACAATATGTCACTTTTGACTGCAGGGTGTTGAAGTATCAACACACAAGTAGGAACACAAAATAGCTATCCACCATCTTTTTGAATTTGAACACTTGCTCAAGTGCATTACTATTTTTTCCCTGCTGCTTCAAGTAAAATTAAGAAATTATTTTAGAAGGTTAACGAGTTAATGAAACTGATTAATCTATACTTATTAATCTTAGCTTCTGGGAGTTTTTTTATTGATGAGTTGTAATATCACAATAATCAACTTTAATTGACTTGTATAGCCCATAGTGCAGAATATTATGTAGATATGACTAGGTTAATGTTTTAGAAAGTTAATTCTCTAAAATATTGTGAATGTGCGTGCTTGCTTCTTGCTTGAAAATTTGAAGAATTCCTTTTTGCTGGTTGTCTTTAAAATTCTTATTGTCTACCTCCCAAAATATTTACTAACTGGACTAAGCATTAGTATGGAGCTTATTAGCTTTTAACATAAGTGCAGATAGAGAAAAAAGTGAGGCCAAACTTAGTAAACGGCTATTTAGAGCATTTGTATGTATAGAGCTATAGGAGGCATTATGAACCTGCTAAAAAAATATCATATTTGTGAAAATGTGTTGTTTTCTTCTCAGTTTATATGCAATAGTTGAGAAAATGTTACTCAAACCTCATTTTGCAGGCAACAAGAGCCTTGATAATCTGTTCATATACAAGATCCAGACAAAGCCAGAGAGTTGAGAGAAACACACACACACACACAAAGATGAAGAAGAAGAATAAGGTAGGAAGATTACAAGGTATGGAATCAAACCGTTCCAATAGAGATCTCATCACCGATGCTCCTCATAAGGTACTATCCCTAAAATTATTGTGGATCAACTACTGATACAGAATCACGTTACTCATAGAGGCTATATTGCTCGAGGTCATGAAATTTGTCCACAACTAATCAATATCGAGGTACCTCGACCTATTTCTATGCCATGATTTCTTGAAATACGAAAGATTGGCTACAAAGTTTCGGACTCCAACTGAAACACCTTACCATTATTAAAATAGGATATGCTTATGATCTAAATGAAGACTCAAAACATTTGTTACATTTTTGGTGTATTATGTCTATTACAACTACGCTTGTAGACAAGTAGACGGGGATTCCTTATTGCATTTTCATATCATCCAGAGGCGTTGCTCTTAAATTTATTTTTTGAGTATGAGATAGATGTTGCTCTTTTGTTCCTGGGTAAGCTTGACAACCCCATCCCAAACAAGGTACCCTTTGCAACCGCCTTTTCAGATAGGGCATTTCATTCATCGGAAATCAAATTCCTCTCTTCTCTTTAATGACTTGATTTTTTTTTTCAAATTTCAAAATTCCTTGTTCTTATTCTTCGCTAAATGTGTGAAATAGACCAAATCCTTATGCCAACCTATGCCTTGAGTTCCTCATCCCCTACAATAGGTCCAAGAATAACAACAAGGTACTGATCTACACTAGAATTGAGCAGAGGGTATGCTCATTTTCCA

>LNC_001910

GTGAACTTGATCTTGTGTGTTGCTGATCTTGTGAGTGCCTTTCTATGGAACATGTGACTGATGAATATTAAGCTTGTTGTTGAAGATATGCAATTGTTAGAGTGTTGTTGTTGAGGATATGAAACTATTGTTGTTGTTGAGGATATGAAATTGTTAGAGTGTTGTGTTGAGCTATGTGCTTTGTAAACTGTGAACTGTTAGGTTGGGCTGGTTTTATACAGATTGTAGTTGTGAAGGTTCGGTTGGGGTGTAAGGAGTACTTGTATTCTATCCCCTTAGCTCGTGTTTAGAGGTTTACTTGCTGAGTACCGT

>LNC_001911

CTTTCGAATACAGGAGTAAATGCCATATTTACGTGATAAATGGATTTCTGCCTCTTCTTTTCATGTTGTTGCTTGTCTGCTGCAAAGGAAACAGGGTCGGTCTCTCTACATATTGTCCCTTTTTTTTCTTTCTTTTTTGTCCTTTTTTCATGGACGTGTGCGGCTCTGAGTCAATGATGAAGGGGTTGATCTCTGCACATCAGCAATGAATTTTTTTTAGTGATTTAACTTACTATCTTCAAAAATTGATTATCCAGGTGTTATTTTGGAGATGAGTTATAGAGAGGGCATGAAAAGTAAATCGTTAGAGAGGAAACCTTGATAGGCTAGTAGACAGTTTTAATTTTTGGCGTAGACAAGAGTACATTGAAGACACAGAGTCTCTATTACATGATTTAGAATCACTATAGACTA

>LNC_001912

CCTGTATTCACTTATTGCTTGATATTAGTGAAAACAGTTGGAAATCCTTGATAGCTCTATCAGGTCCCTTGCGGTGTGCATGTTAAAGCTTGAGTTCTTCCGAGACAATATCACACTTTTTGAATGAATAATATTTGTTGTGTTGAATGTATCAATCAACCAAGGATGTATTATCTGTTGAACAAACAACCTCGTTCATTCTCATTGGAGGAGTACACCGTCCTTCTCACCAACCACTAACAAGACAGCTTTACAGTTGTTCACCATTCATACTTGGACAAAGGCTATTCTACCTTGGACCAGGTCCCAACCTTTGTGAGAATCATCAAAACACTGAAATACTACATATGGC

>LNC_001913

GTGTGCTATACGGTGCTTCTCACCATTTATACTCCATTTTACATCGCCTTCATCAGATCTGGTCGTCTCTTTTATATTCTTCTAAGAGGATTTTGGTCCACTATTCACTTGTTTTAGCATGAATTATATTATTGGAGCGTTCAAGCCAGCATGCAACATCTCAATCACGTTTGGTGATGGAAAATCCCGCAAACAGGTAAGTTATTAGTCTCTTAGAACTTATCATGCTTATAACATTTGTCAATGAAGATGATGCCCTTGGTGGAGATTGATGAAATAATCTACCGTTACGTGTAGCAACTTCAGCTCGTGAATGTGCTTATTATTTTTATTCTATTACCTGATCAGGAGATTTCGTCACGTTAGTAGAAACACTTTCGTCTTCAGTGATGTAAGACAGCCAGTAGGCAGTAATTGCTCAATATCAACTTCACAAACTGGATGGGAAAAAGTAGCTGAGATACTATATATTCTTTGTTTTGGCAAATAGAAGCTTGTTGTTCCATGATATATCCCCCTTGATGATCTATATGCTGCTAATTGGTCTTTTTCCTTAATATAAATTAATCAATTGTGTATCAAACCTCAACCTTATTGGTTGCTGTCTATGAAGTTTCATTTAATCTACTTTGGTACACTATTGTTATATGACAAACCTATCCCCTCCGGCCCTCCCCTAAACAACAAAAACTATTTTTGCATGACAGTCAATGTAATTATCAGAAAA

>LNC_001914

TGTGCTGGTTGGTAGTTTTGGTAGGGCAATAATAGTCTTGAAGATAAGCAGGGGTTCTATGATTTTTAGTAGACTTTCTTGGGACATGAGTTTCATCAATGAGGTTTGGAATGTGGTCCAGGGTGTCCCCAGTGGGAATGTACCACAATGTCATGTGAATGTACAGAGTTGTTATGTGAAGAACTAGTGTGTGTGATACACTTTCTTGATCACTTGAATGAGGTGCAAGCACAAACATATCATCATAATCAATAGGATGGGAAGAAGGATTTGAATGATGAACAGGAATTGAACTCAAGAACTTATAAATCAAATTAAATAAAGCTTCTTTGGGAGAATATGCAAATGGAAAAACATTCTCATGAAACATTACATCTCTTGAAATGTGTGACCTTTTAGTAGCTAGGCTTAGTACCTTGTATCATTTTGTTCCAAATGGGTACCCAACGAAAATGTGATGTGTTGTCCTAGGTTCAAATCTGTATCTATGGACTTTTGGTATTGTTGGGTAGCAAAGGCATCCAAAACTTTTCAAGAAGGAATAAAGAGGTTTTCTCCCATAAAAGACTTGAAAGGGACATTTATCCTTCAGATGGGTAGTAGGAAGTTTGTTTATGTATGAGTGGCTATCAAAATGCACTCTCCCTAATATCTTTGGGGTAAATGTGATTGGAACATTAGGGCTCTGTGTTATGAATCTTTAGGGAAGGATTGATTCACAGGGAGATATATAAATTCAACTCGTTAAAGGGAGGC

>LNC_001915

TTTTGGAGTTCGTTTTAAGGCAAGTGGCTTCCAAACTTTGTAAAACTCTTAGACTACGCATGACTACTTTCCTAATTGTGTTGGGGAGTAATGGGGATTGAGGATGAGTTTTATTTGTTGATTGAAATTGTTGTAAATGAAAGATGGGGAATAAAACGAGCTAAGTGTGTTATATGTGACTTGAATTTGTTGAATAAGTCATGTGATAACTGATATTGAGGGGATAGAAGAGCATGAGTAGGCTATGATTGATACAGACATTGATGTTGAGACAGATGATGTGTAATACTATGATGTGATCGTAATATGATTGTGATTGATGACATGTGCATATTCATTATTCATCTCATGTGTGAACTATCTGTTGCATGAGTTCTGAGACACTGATATGAGGATGGATGGATATGAGACACAGTTGAGACTAGCTCCGGCTAGCGATTTGGATGCCGATGGGATCTGGTTCCGGCGGTGATACATGGTCCATGTGTGGCCCCCATGGGTTCTGATTTGAGTATTCAGCGCGGACTGATTACGTCAACAGATGTGTATCGTAGGACAGACATGCATCACGACTACATGACATCATTATTGCATTTGCATCGCATTTCACTTCATCTTTGTCTGTGGTGTGTGGATTTTACCTATTTGCCCCTCTTCATGTGATATATGATCTATTTGCTCTTATATGAGGATATGAGGTTGATTTTGAGACACTGTTGGATATATATGTTGATTATGAATTGTGTAGTATAGGTTGTTGGTTCGCTGTTAGAATGAAGTTTCGGTGGTTCGGTTGGGATTGAAAGGAGTTGTTTGTAGCTGC

>LNC_001916

AAAATAAATTCCTTTTTTTGAGCTCCAATTGGACAAATTTTCTTTGACTTTTGAAGACTTGCCTACAGAAAACAGCGAATTACTTTCTGCCAGGCTCAGGAATTCTACTTTCTATTACTTTCTATTTTTTTTTATTTTTTTCTGAAAGTAGCGCAAGGCGAGCCGAGGCTGCTGGACCACCAGATTGTTGGACTGGCACCACAGACGAGAAGCTTTTACGTATTACGGAAAAGCAACTCAACCGAATCGCTGAGGAAAGAAATGCCCTAGCC

>LNC_001917

CCTAGTTCTGTTAGAGTAAGAGCTCTTGCTGGAATGCCTTCACCACCAGATATTGCATCAAGAGCTTCTATTGCATCAACTTTTATTGAACACTATACAATTTAGAAAAGAAGGAGCTTTGCCGTCGAGAGAAAAAAAAGGCAATTACTTCACTTTACTCGAGCTAATCAGGCTTGATGTCGGGAATAGAAGAAGATCTGATTCACCAATAGGCGAAGAAGCAATATATATTTCCAATATCTACTATTATCTTCTCACCTCATTCAGTCTTGATGGCAGATAGTTCAAATGTACGCTCGATTGCTAGTAGTTTGAGGGAGAGTTTGAAAGCTTTGCTTTGATTCAATAGGTAATAGAGAGCAAAG

>LNC_001918

TGCATCAATAAGAAGGAAGAGCGGGATGCGGCATTACTAGTACCAACTGGAACGGAATGGGATGACTGAGAATACCACATTCCTCTTTGTTCTTCTTCTGGTCCGTAAAAAAAGCAAGAATGTAGAGTGGAAGGAAGGAAAGTGACTTCGTTTAGGTGCATAGCAAGGTAGTCCAATAGAGCTGGTAAACTAAGCTAATAGCAGGCATTCCGAAAGCAGAATTGAAACCCTTGGAGAAGGGTGGCTGGGTGCTTTAAATAAGAAGATTAAAAAAAATGCGTATGTAAAAATTATAAGGTGTTTTGTCTTCTCGATCGAGATTGGGTTAGGG

>LNC_001919

CGTGCCAGTTGGAGGAAAGCCCGTCTTTTTTTGCCTACTCCTGGTGCCTAAAGGCAAAGAGCTTCTTCAATAGCCAAGTCGAGTAGTCAAGCAGCGGGTTGAAGTCAAAGACAAGACAAGTCCTACTCTCAAGCAAAAGGCTAAGAGCGCTTGTTCCACTTCTACCTCGTTAGGGATGGAATTCCTAAGACCTCTCCTTGACCCTTTAGGTTTTTCAAGAAGTAGACACGGGCAGGGAAATTCCTAGCTTGGTTCTTCGTCTCAGTGCTCTCTCGGCGGCCAGCTCGTCAAAGTACCAAACCCTGGCTTTCTACCCTGAACATTGGGTATCTTCTTCTGGCCCCTTGGGGGCAAGTGAGAGCACTAGATCGAATACACAGGATTGTCCATGTTTTTTACCTTAGCTCTACTTCTCTATTTTTCCTTTTTACGATTGTTTGCTTCCAAAGGCTTACGCGGG

>LNC_001920

GCATTAGATAAAAGAGTTTTTTTTTTAGAAATCCAAAATTTCCTCATAAACATGCTTCAAAAAGCATAACCCCAATTTAAGTTCGGAAATAGTAAAATTACAGAAAAACACAAAATCCTATGAAGAAATGTTTGAAATAGCACAAAATTTCATAAAGCACAAAATTTTGAAATTATACTTCCAAAATTTAGGGATCTTACTGAATGAGCTCTGAGATCCAAATCCCTAGCTTTGCAAGATTTGGTTGGGTTCTCTGCTACATTCGAATACTTTACCATGTCTGTTGGGAGTTCATTTTTTAAAGAAAAGTTGAGAGTAAAAAAAGTTTCTCTAGAAAGTTCCGTATTTTCTTCCGAAAGTTGGAGGAGGACGCAACAACGGCAGATGAGGACTATGATATGGACGATCGGAGGGTTACACTAGCATGCACAACATAATTTATCTATGGCTCAAGGAATTGATCGGGTACGTGGAAGCCTAGAAAGAATGGGATAATCATTTTTGGATGTTCGAAGGGCTCCCAAGTTCAAGCAAAAAGTCTACTAGAGAAGTGTAAGAATAATATACAGTAGTTAAGCTAGATAAATCGAAGATTGATATATTTATATATATTTAAGTGAAGAGCCTTTTAACCGTTCATTTATAAAAAGTTAAAAGGTTGGGTCATGTTAGGTGACAAATTTTTTATTTGAGCGAGATCTTTTTATTTTTAAAGTTTTAGATTATATCAGAATAGAAAATAATTAATTCTCATATTTCTTAAAGTGAACTAGGAAAATAATAATCAACACAAGTCAAGTGTAAAATGTAATATTAATTGATTAATTGCGTATCATGAAAAGTTTTTGGTTTGGTTTGTCAGGTAATATCCTCTTGGAAGAGCTTTCATGGTCCTTTCCTGATTTGTCTTTCCTATGTTCTTCAAATTCCTTATTCTTCTTGGCATCCTCTTTGTTTTTCTTTTGTGATTCGATCATACTGAACATATACTGTGAAATCAAATACAAAAGGCAAGATCAGCCAATTGTTTAAATTTCCAACAAGTACAAAGTTGGAAAACAAATGAAATTATAGAAATCAGTAAAAGAAAATACTTAGGCTTATATACACATATGCCTTTGAAGATACATTAAACATATCTCAATCATCATTTTTTTTTGAAATCAGTAACTTACATATCTCAATCATCATTGTATATATTGTATCTCATAAATTGAAAATGATAAAAAACTAACCGTAGATATATATCAAATATAGCTTTGAGATTCAAGAGAAAAAAAATAGAGGGATGAATATAAGGGAGGGGGACTAGTTTTACATCAAATACATGGCAATACATAGTCTTGGCACCAAAATTTGATCTAAGTCCTAAACCAAGTCCCAACATCAGCATAACCTAGTTGTAGATCCAGCTTCCTTCCACTATCCTCCATCATAAAATTATCTCGTCTTCTCTGAAGTTCTTTTTATCGCCAATAACTCAAGTTCCCCGAGAGCAATTTTTACCACAAGTGTCCTGTTTTTTACCACTGTATATGTTTTTGTACCCTTTATGTTATATGTGAACACTTGCAATGAATTATTGTATTTGAATGAGTCCTATACATATTAATACACAAAAACAATAGCAACATACCTA

>LNC_001921

GTTTCATTATCTCTTTCCTTCTGTCTCTTCCAGTTTAAAAGAGAGAATGAGACTGTATGATTCTTCCACATCAGGCAAAGAAGAGCACAAAAATTGTATTACAATTGATGATTCTTTCACATCGGGAATTCTCTCAGTCCTAACATGAAGCAAAGAACTTCAATTATGGAAATCTTTGCTTTTTGGTAACAACTTCATCAACCTACCACACCAGGAGATTAAGAGCATAATGACTTAATTGGAGGTGTGCTTATGTGATACAAAGAAACAAATAAGTAAGACTTACAATTGTGTGGTCTTATTCTCCTCGGAATCAACAGATGCTTACATCAGAAAAGGAACACTTATATCTTCTTATTTTCTGCGGGTTTGAATTAC

>LNC_001922

ATCTGCTCTTATCGTTAATTAGGTATGCCTCCAGATCTGAATGTATACCCTAGGCCAAGGGGTGCGATCCCCCGAGCAGCTCTTCATACTATGGGGTTATATAATCTCCTTGACCTTTCGATTGAGGGTATTCAGCAGTGGGCCTACCTTAAGGTAGGAGGTGATTGAAAAAGGGGAAGCCGGTCCCCTTACATAGCCATAATAGTAAGTTTTTCGAAGTAGCTGAACAATTGAATCGGCTGGTATAGAATCAGCTGCACATTAATCTTCCTCTATTC

>LNC_001923

TTTTTTTGGAAAGAAAATATAAGGTCGGTCGAAAAACTCATCCATATTAAGAATAGTTTGCTGAAAGTCTAGCAGAAAATATTCAAAGTAATTAGACGGTATACTCGGAGAAAAGAGGAGAAAAAAGCTTGAGCTTGGTTTCATTCTCCCGTTCATTCTGTTAAGATCTGTCTAAATCTTATTTTTTGTGGCGGAAGCTCGTCGCTATCTAGCTCGTTCTCGTCTCAAGTTTGCTGCCCGAAAGAGGTAAACTCCTCTTAGCTTTCTGTTTAGGTCCTAGTTTCCTCGTTGATTTTGTGTGCGTTATTAAGATGTTGATGTTGAGGATTTGAAATCTAAAAATTGTGGTTCTAACATATCTTTTGCCTAAAAATGTTGAGTTGAAATTAGCCGCTGGTTGATGCCTTTACTGGCTATGAAATGCTTTCGTTAATGTTGGAAACAAATCTTGTCCATAGTCGTCGACCTAAATTTCTTTGAGATTCTTAATTCTGTGTTTTATTATGAGGTGATTCTATTTATGTTTAAGCTGAAATTCTAGTGACAATATACTAAATCATGGTTCAATTTAGCTAAAAGTTTTCCAATTAATGCAAAGGTTTAAGTTTAAGAATTTGCTATTCCATTCCGAACCATTAGCATAAATCTCTATCAAATGTCATTCTTAATCTCTTGAGACTGTTTGATTACTTACCGAGGTCAAGACTATGATATGTTAAGGATAAAGGCAAACTGAGCTCGTCATGCTGTGAATAATGTGTCTAGATTTTTTTTAATGTTTATTTTTTTATTTTATTTTTTATTTATTTATTTTGAAAATAGCGTGACTTAGTTGTTAAAATGAGAGTCATATAATGTGTATTTGGTAGTTGCTAGCTTACTTCTTTTGCAATGTGACTATCTTTATTTTCTTTTATACTTTTTATTGTTCATACCATGAGTTCTTAGATTTTTCTAGTATTATGAGTTTTCTGCATCTCTTTATGAATGACTATTTTTCTAAAATTTGTTCAAGTATTCAAAAGCTCAAATACAACTTGTGCATATCTTATCCATAACCTTTTTTTTGTTATTATTATTATTATTATTTTATTTTTTCTTCTCTCTAAATGATGTCAAGTTTGTATTTGTCATTTATTACTTGTTAGCTTAATGTTTTTTCACTCATTAATGCCACTTGCCAATAATCTTGAGTGCTACATTTTCTTTTAATTCATATTAATATTATGTTTGATCTTTTGATGTAAGACATTGAGCTAGTACTCTCCTTTGCATTATATAATTGGCTCGATTTTATATTTGTTGTCTAGTAGTATGGTTTGGCAGTGACGGCTATAATTATAACTATCATTGTTTTATTTATTTATTTATTATTTTATTTTTTGTATGAACTATTCGGATTGAGTCCAAACGGGATTCCCTTTTACATCACCTTGCATTCTAGTTGGGCCAAACCCAACGTCCCTCTATCGAGTCAACTAGGTTCATTGAAAGCTGATGGCCAGAGGAAGCTATCCCAGGTCTTAGAAGCGAAATCACAAGTTCTTAAAGCCGAAAATAATGCTGCAGATCAGAAAGTTGAAGAGATTTGGCCCAAAAGGCCCATCCTTATTAATAATTGTATTTTATTATCTTGGGCCTAGGTTTCCTTGTATTTCAATTATTATTATCATTTTGTTAGAATTTAGGACAGGTGGAAGATGGGCCGTATAAAGAAACAAGTAAAAATAATAATATTTAATTTAGTCTAGGACAGGTACAAAGGTGGCGAAATTGGGCCAAAGGCCCAAAACGAAAATAGGCCCAAGTGCTGGCCCAGGCGAACAGATCCAGACTTGGGCCCGAATTTTATTTCCCTATTTACTTGACTGATGTTATGTGCTTATTTGTTAAAAAATAATATTAAGGTTTCAAAACCTCAAATCCCCAAAAGACACCCATTTTAAATTAATTACTTAACTAAATTATTCATCTTTACTTTGACTAAAAAAATAA

>LNC_001924

AATTAAGTGATTCAAAAGGCAAACTTCAAGAGAATTTCGAGGGGAATCTAGCAGTGGTCTCAGAAACGCGAGGGGAGGCTTCATTTGAGGTAAGAAATCGATTTTTAGCCACTGCCAGGGTGATTTCCGGATGGTTTTGTTGTTGAATTTTGAAATGTGAGATCTTGATCATCGTAAGTCCGTTTTAAGTCATTCTTGGGGCTAACTTGTAAAGTTTTTCGCAAGGATCGTCGTGGTATAATCTGTTTGGGTTGAAAGGGTTTCGTTTTTCCGAAAATTGGTGATCAAGGGGCTGCCCTTTTTAATTATTGTGGCTGATTTGCGGTGTTGTAGGCATCTGTTTGTGCTAAAATTTTGGGATATTAGTTTAGTAAGGTAGTTGGGACGTTTCCACGGATTCGATTTTGAGATTCTAAGTGTGGGCCCCACAATCCCGTTTTAGACCCGATTTTGGGTCCGTCTCCAAAAATTATAATTTTAGTGTCAATATATTCGTAATGACAAGGTGATTAACCTTTTGTTAGTGGGGAAGCATTTGGAGACCGTTCGAAGAGGAAAAGATCCGGTACCGTGAGTCGGAGCACGCGTGATCGGCTTTCAGGTAGGCTATGGCTTTCTCTCGTGAGATTGAGCATGTTTAGGCAATTGTTTATTGATTTGCTTGAGTATGGAGGGGTTCGGGTGTCGAGCATGCTAAATTTCTATAATTCCTGCCTTAGACCTTATTTCGGGAAAGTGTTGGATTATGACAGCATGTCTCTTAATATTATTGAATATCCATATCTTGTGGTGTATATGATAATATTGATTTGAAGTATGTTTGGCCTTAGTCTAGGCTTAGACTAGTGTTGCCATAGACTTGCGAGATTTCGGATGTTGTTGGGCAGTAGAACTTTTTCCGACGTCGTTTCGGACGGTCAGCTCCTTGTAGACTGATATAGCAGACTTGAGTCTGATAGTCTGAGCCTTAGCTAGGAGGTAACTCAGCTTATGACCTTGTATCCATAATGCACTATTTTTCTATCGGCTATAGTACCCGGTTAAAGTCCGTGGTCTAGCTTACTCACTATTGGATACTTCCTCGGCGATTTGGGGTATATGAGAGTTGGACTGAAATGATTATTTATGGTAATCGGTTTGGTGATATTTCCCGCGACGTATGGTTGGATATTGATTCTTAGCTACAGTACCCGGTTAAAGTCCGTGGTCCAGCTTACTTATCTCCAGGCTTAGCTACAGTACCCGGTTAGAGTCCGTGGTCCAGCTTACCTGTGATCTGGTTTTAGCTACAGTACCCGGTTAGAGTCCGTGGTCCAGCTTACCTGTGATCTGGTTTTAGCTACAGTACCCGGTTAGAGTCCGTG

>LNC_001925

TAGGAGTAGGGGCTGACGAGCTTTTTCCGCAGGCTGCTATGCTAGTTGTAGCGCGATAGCGCTTTACTAATATAATAGAAAGTCAAGTTAGTGTTATGGTGCAGCGGAAGGCCCCCTCTCCTTACAGTCAAGTGGCTGAACGCCCCTCGAATCTTCCTTGAGTTGACGTCACTGGGACATCTACCGCTTGGATCCTGCCTTGTTAGTCATCCGTAGAGCTAAAGGAAATTTTTGAAGCCATTCCCA

>LNC_001926

TGCTAATTGTCCCAGGTACGCTATTCCTCTTTGTCTCTAATATTATGGAATGACATTACATGAGTTTATCATTGATTCTACTTATTTACGAGACTTGTCTTACAAAATTTTCAATGAAAGGTGTACTTCATGAAGAGTCTCTTATTACTTATCCAAAAAAGATATAGGTCATTTAGAGTTGGTTCTGAAAATATTATTGTTATTCTAAAGAAGTTAAATTTGTTTAGAGTATTAATTTTGACTATTTCTGTTTCCTGGAGTTATTGTCTAAAAATGACCAGAGATATCTTAGATTATTTTTTGTAGAGTAGAGCTTAACATTACTTTAGATATAGTGAGAATAATTTTAAAATATCCTATGTTGCTTGGACTCTTCAAAAATGTTAATGGGTGCGCGCTGAATTCTCTAAAAATAGTGTATTTTTGGAGAATCCAACATGGGTGCATCATCAAAATTGAAGAGTCCGCACACTTCACCTCTATTACTTTTCTCTTTTTTTTACTTTCAATTTTCAAGTAATACTTTTTAGGTGCGCTTTAGAAAACAGATTTAATAGTAAAATAGTAAAATTTTGCATGATAAATTGGCTAGATTTCGTTTTTCTCTGAAATTAGTTGGTTTGAGTTTTTCTTTTACTTGTTTCAACCTTTTCCTTTGTGCTATGTGTAATTGCAGGCATAACTCATAAGCTAAGTTGCACAAAAATCGCAATCTTGAATATTCATCTGTATTTTATATTTTTCAAGCTAGCAAAATTTACTGTTAAGAGACTTTCTTCTGCAATCATTTGCGTGAGAGTTTAGCTGGCTCCATATGCTACATTTTCCTCACTTTGTCGTGAAACATTTGGTATTTTGGTACTAGGTAACAGGTAAAACTTCATCTGTATGCACAATTTTAAATGCAATGTCCATTGCAAGCAGATCGATTATCTAGCATGAGTGGAGTAATCTGACTCTCCCTATGGATGAAAACATTAATAATATACATGTGTGCTGTCTTAAAATTGAAAATGAGTGTTGGGATGCACATGTTTATTTTACTGCTATAATTTCACCTTACCTGGTTATGTAAAGTTGAAGGTGCAACTAATTTGTATTTTCATTGGAGTTGATGTTGTGAAGTTTGTTAATGTCGTATTAATATTTTATAGTATTCACTTTGATTTTTGATCGAAAAGGCCTAACACATGATAATTTTCATACACTTTGACAATGAGGAAGAAAATAAGTCTTTTATGATGAGCTTGAGCAAGGGAAGATGGAAACGGTTGATCACATCCTCTTCACTGTTTGCTCCAATATTTCCCGGCGAGATCTACTGTTCCAAAATTTAATCATGCTAGAG

>LNC_001927

CTTATGTTGACTTATGTTTATATGAGGCTTATGTTGGAGACCATGCTATGATTATGATGATTATGTTATGACTATGTTATTATCTCTTATGCTTATGTCTTATGTTGACTAACCATTTTGAGATGCTCTTATGATGTTGTGCTAGCTTTCATACTTGGTACATTTTGTACTAACGCATATTGCCTACATTCTAATCAAATATTCTAATCAAATGTAGGGTTGAGGAGATTGAGTTGCTTTGAACGCTAGGTTTCCAAGGATCTAGAAGAGTTGAAGAATTGGTGAGTCCTCATAGCTTCGAGGACAAACCCTTATTTCCTTTATGTCATTTCTTTATGTTTTTAGACTTTGTATGTGCTGCGTCCCAAGAGTTTTATTCAAGAGTTATGTTAGATGGTTTGAGACAAAGGTTTTAGTAAAGACTTCCGCTTGCTTGTATGAAATTTGACTTTGATTCATGTAAATTTTTTAAATTGTTTCGTATTTTGTTTCTATTACCTATGTTATGATGCTAAGGGCTTATATGAGTTCCCTTCGGGGTCAAGTATGTCATGTTACGTCTAAGGGGTACCCCTGAGTCGTGACACATCCTTTAAGATCGATCCATTAAAAATGATTAACCGGCTAAACTTAATTAATTAATTAGATTAAGTGAAAAATCAGTGATGAGAATAAAATTTGACTAAGAAGGAAATTTAGTATTTAAAAGTGGAAAGTAGTAATTTTTTGTATTTGAAAATAATGTGGATTTAGAAAAGTTTTACACTTGGACACATGAACCA

>LNC_001928

TCACTATTCAACCCATGCCCCATAGAGGACAGAAATCCTAAACTTAAATCCCTAACGCCCCACGCAGCTCATCTCTCTAGTCTCAACTCTCGTCAGTCACTCTCAGGCCAGCCGGCGCTGCCGCTCAGTTCTCGCTGAGTGCTCGCAGGCGTCGCGCAGCCCGCTACTCACTGGCCAGTGGCCAGTGGCCACTGCCCACTGCTCACGCTCGCGAGTCGCGCCTCGAAGCGGGATAGGCTGCAGGGATACAATGTTCTCCACCTAAAAGTTATCAAGACTCAAGCTTATTTGATATTTTGTAGTGAAAATCTTAATTCCACTGCTATTTCGCAACTATTCTTTTTCATGTTCCAATTTACTCAGATTCATAATCTTTATTTTGATGCTAAATTAATAATGGAATCTAATAAACTTGTGGAAAACAACACGAGTAGCACTCTACGAGCTCAGATTAGAGTTTTTTTTATTCATTGAATGCAACTATGTGATTATTTGTGTGATCTGTGAACTGTAAATTGTAATGTACTGCGTCTTTCTGTAATTAAGTTGCAACTCTGTTGACATTTGTGTTAAATGAGTATTCATGAAGTAGTGTTGAGTCATGAGTGTCTATCATCCCTAGAATCAGTTTTGTTTGAATATTTAAGTCAATGACATGGTAAGTTGCAAGTTGAATGCTTGTCGAAATTTGAAGGCAAAAGCCAAACTGTATGGCATAGATTGCTGGAGTTTGGAGAGTATATCGTTGAGTGTTGATTGTTGTACAGTTAGTGATCATTTGAACAGCAGCACAGCCCACAGGTGACGAGGTATTGAGGTGAAAACTTACCATTTGCACAAATTTGATATTTGAACAGGTAAAATTATGCCCTTTTGAGTTTAGTGATGGCCTAATTGGGCAGGCTGCAGGTCACAACTCACAGGTGAAGTTGCAAATTATTCCTTTTCTTCTATTTATAAGGTGGCTCTGCCCCTGGACATGAAGATGCATGATATGAAGTACCCACGTATAGTGATGAGTAAACTGCATTGTATACCTCCTTCTCTTCCCTTCCTACCCCTATAGGTGGGGGTGAGAGTTCATGGTTTAAGCATAGAGAAAAGAATATGTCTACTTGCCGCAAAAGAGTCCTTTAATGCACATGCCATTTTTATATAAAAAATATAAGAGTTACACATAACTGCTCCATGAAAAAGCACAACAATAGGATGACCTTAACAAGGCTCAGACTGTGGAGTTCAATTACTACTACTCTTACCCATTTTTAGGCGTACGTATGAATACACTAACATTTTAATTTCAAGTCAAAGAACCTGCTAAATTCTACCCAAAAAAAAAACATTTCTCTAAGATTATAGGGCTAGGTTTACTTTTTGCAAGAAGGAAAGAGAAGAATTTAGAACAAAAAGAGAGAGTGTGTGCGGTCCTTAGGGTAAGTTTGTTAACAAAGACGGACAAATACACAGAAGTGTCTTCTCTCTTTCTCTCTCTCGTTCAAAAAGCCATCAAAACCATTCCTTCAGCCTCAATTTTTTTAATACTAAACCATTCTTCAAAAAAGTTCCTAAAACAAATCTTCCAAAACAGAAAAGCACACACATCAATTGTTTTCAAAACAGTGTTTTTCATATCCAAATGCCATATTTATCTATCTTTCAAATCTAAAAAACAAAAAAAAGGATAGTGGCACATGCATCACATGCATATACCAATATATACTCTTCTAGGAGAAGGGGGATGAAAGTAACTACCTTTATCTTGATGGCACAAGACCCTTGACGAAAAATCTGTGGAAGGTCCTTGTAGTTGATACCAAACTGCTGAAAGAGTAATTCAGTTTGCTCCTGCTTTTGTGTCCCCTGCTTTTGTCGAACACTATTAAGCCTCAAGTCTAGGGTTCTTTGAGTCTAACCATAAATTTGAAAAATGATGTGTTTAAATGCCTTCAAAAGTAAGTAGATCCAAAATATAAAAATGCAGTTAATCC

>LNC_001929

TCAACCCATGCCCCATAGAGGACAGAAATCCTAAACTTAAATCCCTAACGCCCCACGCAGCTCATCTCTCTAGTCTCAACTCTCGTCAGTCACTCTCAGGCCAGCCGGCGCTGCCGCTCAGTTCTCGCTGAGTGCTCGCAGGCGTCGCGCAGCCCGCTACTCACTGGCCAGTGGCCAGTGGCCACTGCCCACTGCTCACGCTCGCGAGTCGCGCCTCGAAGGTAAAATTATGCCCTTTTGAGTTTAGTGATGGCCTAATTGGGCAGGCTGCAGGTCACAACTCACAGGTATTGACATTTTTGAAAGGAGTTCAACACATAAATTCCAGGTAACAAACTGTAGACGAGTAATCCATAGGAGATCATAGATATGTATGCAGAGCTATCACTCCAGAAACCTTTGCTGGGAGCTTTAATACTTTTTTCTTCACATAATAGTCATTTTCTAAGACCTTCTCTAATAAGCATCTTGCTTTGTGCAAATGAAGTCACTAAATGCATATAACTGCGTACCTTCTGTAGCTACTAGCATATGAAAACCACCTGCTACTATATCTGGCAAGTTTGGCCTGGACCAGGACTGACACACTTGGCCTCTGGCCCAGGGAGATGGTCTGGTTCACGAACCGGACCCTTTCTTAAGAGGCCGGTCTGGGCCAGTAAATTCTGAAGAGTGGTGGACCTGACACCAACTCCTGATTCCTAACCACTATGTGATCTGGTAGTGGACCAATCCGAG

>LNC_001930

TCAACCCATGCCCCATAGAGGACAGAAATCCTAAACTTAAATCCCTAACGCCCCACGCAGCTCATCTCTCTAGTCTCAACTCTCGTCAGTCACTCTCAGGCCAGCCGGCGCTGCCGCTCAGTTCTCGCTGAGTGCTCGCAGGCGTCGCGCAGCCCGCTACTCACTGGCCAGTGGCCAGTGGCCACTGCCCACTGCTCACGCTCGCGAGTCGCGCCTCGAAGGTAAAATTATGCCCTTTTGAGTTTAGTGATGGCCTAATTGGGCAGGCTGCAGGTCACAACTCACAGTGCAGGTATTGACATTTTTGAAAGGAGTTCAACACATAAATTCCAGGTAACAAACTGTAGACGAGTAATCCATAGGAGATCATAGATATGTATGCAGAGCTATCACTCCAGAAACCTTTGCTGGGAGCTTTAATACTTTTTTCTTCACATAATAGTCATTTTCTAAGACCTTCTCTAATAAGCATCTTGCTTTGTGCAAATGAAGTCACTAAATGCATATAACTGCGTACCTTCTGTAGCTACTAGCATATGAAAACCACCTGCTACTATATCTGGCAAGTTTGGCCTGGACCAGGACTGACACACTTGGCCTCTGGCCCAGGGAGATGGTCTGGTTCACGAACCGGACCCTTTCTTAAGAGGCCGGTCTGGGCCAGTAAATTCTGAAGAGTGGTGGACCTGACACCAACTCCTGATTCCTAACCACTATGTGATCTGGTAGTGGACCAATCCGAG

>LNC_001931

AGCTAATCCAAATGGGCACTTAAACCTGAAACCTCGTGGTTTTCAATCCACTTCATTGACCACTAGGTCACTCCCTTGGGTGCTAAGTATGGATTTTATCCGAGTCATTATTAGAAATCATTGTGAGCTTTGATACTTGCGGACTTTATTTCACTTGAAGTTTAGTTGACGTTGATCATTTCTAGTTATAAACTATGGGTTGGGTCTTTTAGCGATTATTCAGAAATTCGTCTGAAATTCTACTATGCTGGACACTTTTCACTGAAATTCAATTAATTTCTGTTCTTTTTTCATTGTAATAGGTCATTGTACAATTAGGTGGCTATGTTGGAAATATTCTGAGTTTCTGCCAATACTGTACTCATTTAACCTTCAGCCCACCTTCTATATTCCTTTGTTTCGTTTGGTTGTTTGGTGTGATTTCTGAAGGCTTGATTGTGAATTTTCTCCATCACTGGGATTTCTATTGCAATTTGCGCCTTTAAGTCTTATTTCTAAGAAGTTTTGATTTGACTCCATCCATGGTTGCTGACACCCTGCTATCAGAGCTTTCAAAATATAGTGTTCCAATAGTCAGATGCACTAAAGCTAATGCTGATTCGCAGAGTCACTCTTCTTCAATTATAGACCACAACAAAGATGGCGGTAAGACTTGAATTGGCCTAATTTGAAGTTTTTAATTGAATGCCAAGTTTTGAGACATCATAGTTACCATTATGGCAAAAAGAATGACATCTCTCTTTATCTCATAAAAATACTTCGAACTTCTCATTTATCCTTAATGAGATGATTTAAAGCCACAAAAATTTTAATGGCTTGGCTTGTTTTAGACCACAAGTTCAATAAATCTTTCTTTTTCTCTTAAATTTTGTGCCTAGTCAAACACCTTCATATAAATTGGAACGGAGGGGATAGTATTTATCTAATCATTTAGTTCCTTCTGTCATTAGTTAACATTATCGGTCACTTCAGTTTCCTGACTTATATTCTTCATTTGATGATCTCTGTTATTAGTTTACATTAATTGCTGTCATCATCATTCATCTCCAGAAGCTTTATCTGTGCTGTTCTGCGATCATTCCTTTAGCATAGCCGCTAACCGAAACCTCAGACTCTCTGATATCCCAGGTTCTGTTTGTTTTAGTCATATTGTCAAGCATGTCACTGGCCTTAGTGAAAGTGTGAGGCATCATAGAACCTCCAACAATATTATATGTCATAGCTTTGTTCATAAGTTCAATGACCTCTAGGACACCTCCAAAAGGACATCATCTGGCAACCTATGATTTGGACATCACTTCAGCTTCTTCTTGAACCGAAGCCAAGTTTCATGCAAGGCTTCCCCTGATAGCTCTGTTCGTAGAGTTCAATGTTTCTGCCTAATTGTATCTATGTGGATAATAGGTGGACCAACATATATTTGCTATAAAACATCAAAATATAGCTATATTGTGTACAATAACAACAATAGCATACTTAGTGTAATTTCACGTGTGGGATCTGGGGAGAATAGGATGTAAGCAAACCTTACCCATACCTTTGTGGGTAGAGAGGTTATTTCCGATATACCCTTGCCTTCAAAAAAAAAAATGTAATCTAAACTAGGTAGAAAGAAAAATAGTGATTGCAGAATAACAATATAAACAAAAGCAAACAACTAAACTAATAGTAAAATAGAAAGATAAGATACTATGTGAATACTAGGTAAGACTGCTAAAAAAGAAGAGAAAATGAGCTCTTTGCCTCCCCCATGTAAATGTACTACAACGCTCGGCTATCTACTATCCTTCTTCCCTAATATCCGACCTCCAAACATTTATATTTAGGATCATGTGTTCAGTAAGTTGAAGATATACCATGTCCTGTTTAATCACCTTTCCCTGGTTTTTCTTTGGACTACCTCTACTTTTTCTCTATACCTGCTATAGTCAAACTTTCACACGCGCAACTCTTCTTCATATGTACAAATCTTTTTAGTCTCACTTTCTCATTTATTCTCAATGGAGACCATTTCCACCGTCTCATGTATAACTTCATTCCTAATATTGTTTCTCCTAATATGTCCATACATACGTCTCAACATTCTCATCTCCTCTATCTTCGTATTCTAAACATGTGGATTCTTAACAGATCATTTCTTTGCTCAATACAACAACATAGGTCTTAACAAACTATGTAGAACTTACCTTTAAACTTTGGTGACATATTTTTATCACATAGTACCCTAAACTTGAATCACTCACCCCTCTCTAATACGATGAAAAACATCATCATCAATCTTTTCATTTGCTTTGATTATAGACCCAATGATTCGAAACTTTCTTTCTTGGGTATGCCTTGTGAATCAATCTTTACTTCCACCATTGTTTCTTGCGATTTGTCACTAAACTTGCATTTAAGGTATTGTTTCTTAGTGCGGCTCAACCTTAAACCTTTAGATTTCAGAATTTGTCACCAAATATCCAACCAATCGTTA

>LNC_001932

CACAAATCTATTCCAAATTTCATTTAAAAAAAAATTACAACAACATCAAAAATGGCTATTGTTAACCAAAAAACACATTGGTACTTAATCATTTCTATCATTTTCTTCTTCTTCTTGATCACTTGTCAAGCAAGGAATTTGCAAGTTGATAATTATGAATTAGTTCAAAAGAGTGGCAACCAACAAATTGTTATAAGCAATAAAGAGAAATCAAAGTATGAAGATTCAGTAGCAATAGACATAGATTATACTCCTGCTAGAAAGAAACCTCCTATTCATAATTAGAATTTGGAGAATTATCGAGTGACGAATCTAAAATTTAGAGTCAGTCGATTATGTTTCTGATATTATAAGTGTTATTATTTTTTGTTACACGTATATATACC

>LNC_001933

GAACGATGTGTGCTGCTGCATTCTATGCTCGATAGGAAAATGATTTAAGTTCAGAAAATTCTTATTAAGATGACAGAACACCATTAACTATTTTAATGTACTAGGAGTATTAATTTAAGGGAGAGAAAATTCCGTAAGAGTTTGGGGGCATCACTTAATTAATATAATTAACATATGATGCATATTAAATTTTTGGAGTGTAGTCTAGGAGCCCGTTGAAATTAGAAATTTTTGAGTACCCATCAGAAGACATCAAGAAGATTTAAGGAAAAAAATTACTGCAACATGATCAATCAATGGTGGAGTCTGCAAGGTTGGGATCTGATTTATAGAAGAGCTCTAAATGATTGGGAAATTGAGAGTTTTAACAGAATGTTGCAGTTACTGGGATCTTTTGGTGGTACATCCGATTGCCATGACAGACTTAGATGGAAAATGAATAACAAAGGGATTTTTTAAAAGTAAAATCAGTTTACTGGAACCTGAATTAGAGAGGCCCT

>LNC_001934

ATTTTTTCAACTTACTGCTCATTAAGGATCTAAAGTAACTCCACTATAACTTATATGAGCACAGCATGAACAAAAAGCACAAAAACTCAACTAGACAATGCATAGATTGTCTTCTGTTATATAGTTTAGAAAAGGGAAAGAGATAAAATCAGGAGTCAAATCACTAGAGGGGAGATTCTAAGGGGTCGTTTGGTACAAAGATTAATAACGCAGGGATTATTGTTTATCAAATGTTTGGTTCATTGCTTCCCACCTAATCTTGTGTTTGGTTTAAAACTCTACAAAAAGCTCTTTTCCAATTATATCCTTGAATTATTACGTGATTTTCTATTCTATTTCATATAATTGGGTCAAGGTAGATAACTACTGGACAATATTATTTTTTTGTGGCCTTCTAACTAGCAAGGAAAAGAAC

>LNC_001935

TATAAATTGAAATGGAGGGAGTACTAAATAATACCATACATAATACATGAACTATTTCTCCTAATACACTCTACCAAACGATTCCTAAATGTTCAGAGAGTTGCAACTTGAATTTTTTTGTTGGTTTTAAATTGGAGATGAACGGCTAGAATTGCATCTTGTCTCACCCTTTTTTATGTTACACTCCTTTTTGCAATTATAAACCTTAAGATGTCCTTTCCATCTCACCCAGTTTCCTCATTGAATTGGAAAAGAAGAAAAACAAAGAAGTCTCTCATAGTTGAAGCGTTTTAAAAGCCATTGCTTCGACATTTAAAGTGATGAAGTGTTGCGCTGCCAGG

>LNC_001936

CCTGTATTTCATCCATTGGCAAGTTCTGTCCGTTCTTGGCCTATTAGTTGGTTGAGCATATGCAGATTGAAACAGACTCACATAAATGCATAGGATGGGACAGTTCATCAGTTAGTTTCCAAGTTTGTTTCTATTGAAGTCTCAAACCATACAGTGAATTTTCCTGATCACGCAGAAAGAAACTTAATTGCTAGGATGAAATTATTTACACCAAGACATGCATTACTGAGGAATTATATAATAAAGCCTTTCTTTGAACCTTTTTCATCATCACTCTAGTAGAGCCTGCACATTAAAAAAGGGGAAAAGGGTCAATTTACGTGTGTACAACTCAAAGCAACTTTGCCTCCGTTAAACTTTTGGTTTAATATTCCCCTTGTTAGGAAAGACTCTCATTTTTCTCTTGACCTCTATTGGAGCTTTAACCTGTGGGTAATTTAAGCATTGTCGAAAGTTGAAGGATAAATTTGGACCTTTTTCTTGATGAATTTGGACACGC

>LNC_001937

TTGCAACCAAACACGCCCTACTATTAAAATTGAGTTAATCATTCATTTTACAATGTTACTAGTGTAGTAGAAAGAATTTTCAGCTTGTTTTATATCTTTGATATATTAGTAGGAAGATAAATATAGAAAAAAATGAGGTTAAAAAGTTTCTTTCTTGTACTCTCTAGAAGTTAACTACTTCCTGCGCAACATGTGCTATAAAAAGGGGACAAAAAAGGCCAGAATGAAACAATTGAAAGCTCTTGGTTTATTTGTTTATTGTCCATCTAGTTAAGAATCTTAGTTGAGGAGATTATCAAGAACAGAGGTCATATCAAGCTTAGGTGGTCCAAAAGTGTGTAATGCTTTAAACACCAAGCTAGAGTCTTTCAAGTTGGTATCCCTATTTATCATGTGACGTTATTGGTTCTAGGTACTATCATGCTGGCTTTGAAATGACATGGTTTTTATTGAGTGATTTACTTCATAATTCGTGATGTGCACACACATGTATATATTTGGATAGATGTGTACGTGTGAGCTGGAGCACAAGCGTAAC

>LNC_001938

TAATCACTTGTTCATCGTAGTGGCATTTGCTCTTATTACATCTATGTTTGCTTTCTTACCAAGTTTATTTTTTTTATACATATGTATGTATTTTGGGTGTTGGTTTCACTTGCTCAAATCTTGTCATGTGTTTATTTCCAACAAACTAAATCCTAAGCATGCTTTCTTTCTTTCTTTACTAGCTTAAGCAGTGATAGTTCTGATGCTTTAAATATCTTTACTGTTAACTTGAACTCTCTACATGGTTATAAGTTGTTAGCTTAGCTAGATAGTGTTTTGTGTTGGTTCTAACTTTAGTTCCCTTTTCTTTTTCATATGAACCTTCTTGTTCGAGTCCAATTGGACTCCCCTCTCTTGCATTCGATCCTCTTTGAGTCGACCTCCATCAAGTTTTGAAAGGAAGCTAATTTTATTGGGTTAAAACCCAAA

>LNC_001939

GTTTTAGCCATTGCTAGAAGTTGTGGAGTTAATTGTAAGAAGCAGTTATGAAAAGGTATGTAACCATTTAGACTTTGTTACTTACTGTATTAGTTTTTTTGAGCTTATTTTACAAATTTATTTCTTAATTAAACTTTATTTGTAATCTTTGTTTGTTGGGTATTAATCCTTCCCTCTTCTTCCGGTAAATTGTTTTGTATATGAGATGTAGGTGAGACGAAGAGAAGCCGACATTTTTAGTATGTGCTCTTTCTACGTCCCCAGATTAAGAAGCATAAGGATAGGTAGAGCATGAGCTCTTGAAGACTAAGTTGTTGTATTATATGTTAAATGTAATAAAAGGATTTTCTTAGTGGTTTATACAACATCAAGGTAATATTATGTTATTCAAACTTAAGGTCTGATTTGATTGTGAACTGTGGGCGGCCCAAAGTAAGAAATGAACTTTTTG

>LNC_001940

CTGAACTATCTTCATGACTTCTCCCTGTTTTTCTCACTTTTTCTAAAGTGTCAAATATCCTATTGATGGATATCTGATGGTAGTTTATGGCTTGAGAGCCCTAAGACTTTTAATTTGTCTCTCTAATTTCACTAATGTAATCACTAGATAGTAATTTACAGCAATGAATATACACTTCAATCATCTTAAAGGTCTTAAACAATTACCTGAACAAGTTGGTAATTTGTTTTAAACACAAAAACTATAATTAATGTTGATAATAGGGATGATCGAAACAGTATATTAGATACCATATATTCTAACTTAAAAGGTCACAGTTGATTCACTATTACAGGTATTCAATAGTGTTTGTCTTAGCTGAAAATACATCTATTTACCTGTAACAAAAGCTCACGTTTTCTGTCTCACTGTTTATATAATACTCCCCTTGATTCAATTTGTTTGTCCTATTTTGACTTGACATGAAGTTTAAAAAAATATAAAAGAATTTTGAATCTTGTGGTCTTAAATTAAAAGTTATGTCAAATGGACCAAAATGTCATCTAGTCTTGTGTTTTAAAACATTTCAGGTAAAAAATTGAAATTGAAGAGTTGCTTAAAAGAGGAAAGAGACATTCTTTTTGGGACTAAAAGGAAAGTAAGACAAATAAATTCTACCGGATGGAGCAATTCTTCTTTCTCTGGTCAAAATAGAAGAATAAGATTGGTGTTCAAAATTTAATCGATAAACTGGGACACCAAAGCACCGCTAAAAAACGGTCATTGTGGAGTCTCCGTTGGTCAAAGAAAGCCGCGAGTTTCAATCAAAATTGAAAAGTGTATGCTAAGGTTGAAGAGGAAGCGCCGAAAGATGAGATAGAGATTGAAGTAGAAGCACATTCCGCAGTTCTCAACGGTATATCTCTGCTTATCGTATTCTTTAAGCTTATGTTGTTTAAGTTTTAGTTTGAAAATTTCATTAACATTTGAGTTTTGATTTTGTTTTAATTGCACCGAATTCAAATTTTGGTATCCAATTTTGGCTATATATGAATTTATGCTTCTTATTATTCGTATATTTTGCCATTCCTCTTTATAATGTACTTTGCTTTGAATTGTCTGCATTTATATATTTGTCAAAATATGTTTTATTTGAGCTAAGCCTCTTTTGGAAACTGGCTCATGAGTTATTTATATTATAGTATTATTTATGTTATTCATTTTAGTCTTTATATCATGTGCCAATCTTTTGCCGCTTTGGACTAGATTGTTTGTCCAATTAAGAGTTAAATTGATCGATGTTTGATCATTTTCGAAATGCACACATTGCTTAGGTTGTTTGATAAAAAAAAAAACTTGGTGCATTAACATGCTTTATGCTGATACTGTACGGTCCATTTTAGATGGCAATTTAAATTTGCTTCCATGCCACAATGAAAACTTGTTTGAAGTTCCTATTAGAGACGCTACAAAACTTGGATATTATTTGAAGTTTGAACTGAAATTTTAGTAAAGTCCAGTTTTTTACCAGAGGCATTTTCATGCTAAGTGAGCATCTATGTAGTTAACCTTGAACTTGCTTGTGAGAAGATTGGGGTGTAATACGTAGTAGTTGAATTATAAATTCAAGAAATATTTTGCAACTGGACCTTAGTTTGCTTTTTATCAGGATGGGAATATGAGATCTCTTCGGCAATGAATGGACATATAGTTACATGTAGCGAATGTAATATTGTTTTGTATAAGATGACATGACATTGTTGTTCTAGATTAACATGTAGTTGTGTGCTATGAGATGGCCTAGAACTAGACGGGTGAAAGTTTGATCGTTTTTCTTGTGGAAACATAGATATTATACCCTTTGCCTTTGGTACTGTAATGGGAGGGCTTAGAAAGTTCACTTTGCCCTTCAATTTACAGCACAAGAGCTATTTTTTATGGCCTCATGTGCTCATTTTATCTACAGAGTAATAAAAAGTTATATTCTATGGGTTTTGCATTTGAAGTGGCTGTTGCATTGTATTATGTTTGTGTATTCCCAAGCAAACAAATCAAGTTTGTCCTTATTTTTTTTAGTTAATTCTTTGTTTCTATTTTTATTGGCTTAGTTAATGTCTCGAAGTTACCCCTAGCACTTTGGTGTAGTTGAAAAGACCGCAACATATGTAATGTGTGAGTTAAGTGCGTGTCATGACTTTAGACCCTAATGTGAATAAAAGCTGGGTATTTAAGTGAAGGGTAGATAGAGAAGCACACTCATTATTGATCGGCAAAAAATATTTGCAATGTTTCTATTCTTATGTTGTCCCAATCTCTGTCTCTCTCTCATTCTCCATGATTCAGGTTCAAGAGTGTGTCGAGAAGGATGATTTGAGGTGGTGAAATTTCACATTTGACAACTTTGGAGATAATTTTTGTTCAGATCATACATTTCTGGCCTTTTCCTTCAAGTTTTTGGGTTTATTTGTTTTTTGCCTTGAGGTACTGTTTTAATTTACTTTTAATGTATTTTTTGTTTAGAAATTTTCATGCAAGTTGGAGAAAGTATCTTGAAGGTGAGTGGTTCCTTGCAAGAGACTATTGGAGTGTAATTGGGGCTCAAAATCAAAATCAGAATACCAAATTATGTTTTCTGGTGAATTACAGAGAAACACTTCCGTCAGTATAAAAAAAGAATTTTTGTCTGAGGTACCCTGAACCCTCTCCATATATTGTTTGTTTGAAATGGAAAAGGTTGGGCTTGGGACTACAACTCCTTCTCAGTTGGAACAATGAATTCAGGAATACAAGTTTGGTGCGCAAGACACTTCGATCTTGGAGCTGAATATGAAAGTGAAAGTTAACAATATTTGGAATGTCGAGAGAACTACAAGGGAGTGAAAGTTGTAAGTGCTAATGATAGTGCTAAAATCATTGAGAAGATTCACTGTAGCAAAGGCAGAACAGAAATTGTGACTCTATGTTTCACCATGATGAGGAAATAGAAACCACGAGTATCATTGATGTTAGATTGTTTCTTCTATGTTTCACGATTGGTGAGGAAATAGAAACCACGAGTATTGTTGATGTTAGATTGTTCTTTTATTAATCTAAATCTAAAGACTTCAATTTTCTTCACCTTTTTTATTTATTTATGGAGAACTTATATTTCATATTTGGATGGGACTTTAGTACTTTTTTATTTGGTGACTTGTTCCTGAATATTTTGGC

>LNC_001941

TCTTCATGACTTCTCCCTGTTTTTCTCACTTTTTCTAAAGTGTCAAATATCCTATTGATGGATATCTGATGGTAGTTTATGGCTTGAGAGCCCTAAGACTTTTAATTTGTCTCTCTAATTTCACTAATGTAATCACTAGATAGTAATTTACAGCAATGAATATACACTTCAATCATCTTAAAGGTCTTAAACAATTACCTGAACAAGTTGGTAATTTGTTTTAAACACAAAAACTATAATTAATGTTGATAATAGGGATGATCGAAACAGTATATTAGATACCATATATTCTAACTTAAAAGGTCACAGTTGATTCACTATTACAGGTATTCAATAGTGTTTGTCTTAGCTGAAAATACATCTATTTACCTGTAACAAAAGCTCACGTTTTCTGTCTCACTGTTTATATAATACTCCCCTTGATTCAATTTGTTTGTCCTATTTTGACTTGACATGAAGTTTAAAAAAATATAAAAGAATTTTGAATCTTGTGGTCTTAAATTAAAAGTTATGTCAAATGGACCAAAATGTCATCTAGTCTTGTGTTTTAAAACATTTCAGGTAAAAAATTGAAATTGAAGAGTTGCTTAAAAGAGGAAAGAGACATTCTTTTTGGGACTAAAAGGAAAGTAAGACAAATAAATTCTACCGGATGGAGCAATTCTTCTTTTCTTCACATTGGAAGAGACAGTAATGTCGACAACATTGTTTCGTCTTTCACAGCTCTGGTCAAAATAGAAGAATAAGATTGGTGTTCAAAATTTAATCGATAAACTGGGACACCAAAGCACCGCTAAAAAACGGTCATTGTGGAGTCTCCGTTGGTCAAAGAAAGCCGCGAGTTTCAATCAAAATTGTGTATGCTAAGGTTGAAGAGGAAGCGCCGAAAGATGAGATAGAGATTGAAGTAGAAGCACATTCCGCAGTTCTCAACGGTATATCTCTGCTTATCGTATTCTTTAAGCTTATGTTGTTTAAGTTTTAGTTTGAAAATTTCATTAACATTTGAGTTTTGATTTTGTTTTAATTGCACCGAATTCAAATTTTGGTATCCAATTTTGGCTATATATGAATTTATGCTTCTTATTATTCGTATATTTTGCCATTCCTCTTTATAATGTACTTTGCTTTGAATTGTCTGCATTTATATATTTGTCAAAATATGTTTTATTTGAGCTAAGCCTCTTTTGGAAACTGGCTCATGAGTTATTTATATTATAGTATTATTTATGTTATTCATTTTAGTCTTTATATCATGTGCCAATCTTTTGCCGCTTTGGACTAGATTGTTTGTCCAATTAAGAGTTAAATTGATCGATGTTTGATCATTTTCGAAATGCACACATTGCTTAGGTTGTTTGATAAAAAAAAAAACTTGGTGCATTAACATGCTTTATGCTGATACTGTACGGTCCATTTTAGATGGCAATTTAAATTTGCTTCCATGCCACAATGAAAACTTGTTTGAAGTTCCTATTAGAGACGCTACAAAACTTGGATATTATTTGAAGTTTGAACTGAAATTTTAGTAAAGTCCAGTTTTTTACCAGAGGCATTTTCATGCTAAGTGAGCATCTATGTAGTTAACCTTGAACTTGCTTGTGAGAAGATTGGGGTGTAATACGTAGTAGTTGAATTATAAATTCAAGAAATATTTTGCAACTGGACCTTAGTTTGCTTTTTATCAGGATGGGAATATGAGATCTCTTCGGCAATGAATGGACATATAGTTACATGTAGCGAATGTAATATTGTTTTGTATAAGATGACATGACATTGTTGTTCTAGATTAACATGTAGTTGTGTGCTATGAGATGGCCTAGAACTAGACGGGTGAAAGTTTGATCGTTTTTCTTGTGGAAACATAGATATTATACCCTTTGCCTTTGGTACTGTAATGGGAGGGCTTAGAAAGTTCACTTTGCCCTTCAATTTACAGCACAAGAGCTATTTTTTATGGCCTCATGTGCTCATTTTATCTACAGAGTAATAAAAAGTTATATTCTATGGGTTTTGCATTTGAAGTGGCTGTTGCATTGTATTATGTTTGTGTATTCCCAAGCAAACAAATCAAGTTTGTCCTTATTTTTTTTAGTTAATTCTTTGTTTCTATTTTTATTGGCTTAGTTAATGTCTCGAAGTTACCCCTAGCACTTTGGTGTAGTTGAAAAGACCGCAACATATGTAATGTGTGAGTTAAGTGCGTGTCATGACTTTAGACCCTAATGTGAATAAAAGCTGGGTATTTAAGTGAAGGGTAGATAGAGAAGCACACTCATTATTGATCGGCAAAAAATATTTGCAATGTTTCTATTCTTATGTTGTCCCAATCTCTGTCTCTCTCTCATTCTCCATGATTCAGGTTCAAGAGTGTGTCGAGAAGGATGATTTGAGGTGGTGAAATTTCACATTTGACAACTTTGGAGATAATTTTTGTTCAGATCATACATTTCTGGCCTTTTCCTTCAAGTTTTTGGGTTTATTTGTTTTTTGCCTTGAGGTACTGTTTTAATTTACTTTTAATGTATTTTTTGTTTAGAAATTTTCATGCAAGTTGGAGAAAGTATCTTGAAGGTGAGTGGTTCCTTGCAAGAGACTATTGGAGTGTAATTGGGGCTCAAAATCAAAATCAGAATACCAAATTATGTTTTCTGGTGAATTACAGAGAAACACTTCCGTCAGTATAAAAAAAGAATTTTTGTCTGAGGTACCCTGAACCCTCTCCATATATTGTTTGTTTGAAATGGAAAAGGTTGGGCTTGGGACTACAACTCCTTCTCAGTTGGAACAATGAATTCAGGAATACAAGTTTGGTGCGCAAGACACTTCGATCTTGGAGCTGAATATGAAAGTGAAAGTTAACAATATTTGGAATGTCGAGAGAACTACAAGGGAGTGAAAGTTGTAAGTGCTAATGATAGTGCTAAAATCATTGAGAAGATTCACTGTAGCAAAGGCAGAACAGAAATTGTGACTCTATGTTTCACCATGATGAGGAAATAGAAACCACGAGTATCATTGATGTTAGATTGTTTCTTCTATGTTTCACGATTGGTGAGGAAATAGAAACCACGAGTATTGTTGATGTTAGATTGTTCTTTTATTAATCTAAATCTAAAGACTTCAATTTTCTTCACCTTTTTTATTTATTTATGGAGAACTTATATTTCATATTTGGATGGGACTTTAGTACTTTTTTATTTGGTGACTTGTTCCTGAATATTTTGGC

>LNC_001942

CTTCTCCCTGTTTTTCTCACTTTTTCTAAAGTGTCAAATATCCTATTGATGGATATCTGATGGTAGTTTATGGCTTGAGAGCCCTAAGACTTTTAATTTGTCTCTCTAATTTCACTAATGTAATCACTAGATAGTAATTTACAGCAATGAATATACACTTCAATCATCTTAAAGGTCTTAAACAATTACCTGAACAAGTTGGTAATTTGTTTTAAACACAAAAACTATAATTAATGTTGATAATAGGGATGATCGAAACAGTATATTAGATACCATATATTCTAACTTAAAAGGTCACAGTTGATTCACTATTACAGGTATTCAATAGTGTTTGTCTTAGCTGAAAATACATCTATTTACCTGTAACAAAAGCTCACGTTTTCTGTCTCACTGTTTATATAATACTCCCCTTGATTCAATTTGTTTGTCCTATTTTGACTTGACATGAAGTTTAAAAAAATATAAAAGAATTTTGAATCTTGTGGTCTTAAATTAAAAGTTATGTCAAATGGACCAAAATGTCATCTAGTCTTGTGTTTTAAAACATTTCAGGTAAAAAATTGAAATTGAAGAGTTGCTTAAAAGAGGAAAGAGACATTCTTTTTGGGACTAAAAGGAAAGTAAGACAAATAAATTCTACCGGATGGAGCAATTCTTCTTTGTAAGTATCACTTATGTATTAATAACTGTTATGTTATTTATTTGAAGATAAAGAGGCATGAGGCATAAGGGAGATTTCATAAACTTACTCTAATTTAGAGAGGAATCATAACTTTAGTTATTTTGTTACCAATAGTCTTAGAGATTGAACAAAAGAATTTATAAAATGATGATGATGATAAGTCGAGATAATATTTTTTGGATATCTGAGTGCTTTTTATGGAAGAGGAAGAAATTATTGTGATAAAATTTGACATCTTATTTCTAATATATAGAGACGTGGTAACACAAAAAGAGGAAAAAAATATAACTCTTTGAAAAGCTTGAATCTTTAAGCATATTCTTGGCAACTTTTCTAACTGATTCAATTAACTCTTGAAATGTTCCAATTGTTGAAAAAATAAATAAGGGAAGAAAAGGTCAAAAATTTACTATTATTTAAGTCCGGAATTGTTGTGTAAGATCGGTGAGTGGGCTTCCATGTTGTTTGTCCTCTGTGGAAGTTCTACCCTCAAGTTTCCACGTTAAGCTCATGTTCTGTAGAATAGAAACTAGAAAGACATAATTGGATATAGTTGGTCATTTCTGTATTCTATGGTGATACGAAGCTTACATTTTAATCTGCTTTTTATTCACCTTGATTCAGTATTTTCACATTGACAGGTGATATGATATGCTCTGCTTTTATTTCTACAAAATCATTCATCCATAATTGTATTTAAGGTCATTTGAATTTTCGTGGAGTTGATGTGAAAAATGTAACAATATCATAGAGGAAAGAACATGCTGATGAAAACACCTCTGCAGTATGAGCTTAGTTTTCTTAAGAATCTCCCCGATACATTTGTGTTTTCTAAGACATTTAGAATATTTCGTTTGAAGCTTTCTTTGGCAATGCCAAAATAAATCGCCTTGGATTCCTTCGTTTATTTGATGTATGTTTCTCCCTATCTCTTCTCAAATAGGATACCTTTTATCACATGTTTCTAACAATATATTAGCTACAAGTTACATGTCTTTTAAATAGACAAATATTTTTATCTAGCTTAACTGATGTTTTAACAACATGATGAAATTTTTATAAAGTCATATTCAATCTTCATTTGAAAGTATTTTTTTCTAGAAATATCTCGAAATCTTTTCAGCTTCTCAACAATACAATACATTATAACTGGTGATGTCACCTAAGGCACATGAAAAGAGAGCAGTTTGATTGCTACACTCTTTACTGGTAAGTTTGATACTTGAGTTCTGTAGGAGTGATGAAACAACTTACTTATGTTATATTTCGGCTCCTCCATTTCATGTACTAACTTACATGCAACCTATACTATAGTTTACCTAATTCAACTCTTTTTAAGTTACAATAAGAAGCTCTGCCATGAGTATACTTTACTATTTTCATTTTGTTTGGTGCTTATTTTGTGCTCTTTGTATAGAACATAGCTATTTGTGTAGTTTATATGTTCTAGGACCAAATAGTCAAAGTTACACTTCTGAAGTTTATACGTTATAGGTTGGTGATAGGGTAAGAAATAATAGTACATGAATGATTATGAGATGATGGAAGCAAAAATTAATAGTATGATACATGATATGTGTAATGTTTTATGATTTTAGTAACTTATATTGATTGTTTCTCTTAGCTTTGCTTTTACAGGAATTCCTCTGTTGTGTTATTATCAAGTCACGATATATGATTCATTTATGACATTATGACAAATTGAATTTATTAACTAGGACAGAGAATGCAACCGACTCTTCTCGACATCTGTCACTTCACTAGATATTAGACATCTTCACTAGATATTAGACATCAGATGAGAAATTCAAGAACAATGGACATCTCTATGTTTCCTTTTGAGAGTGATTGCGTCACCTCACTTTAGTTTCATGTTTTTTCGTTAGAGAAGATCCAATTATTACCAAATCAAAATTGGATTTATATGAGCAAAAGGTGTTTGTCTTTGTGCTTCTGCTGCAATTGTGAGTGCTACTTTCATTTTATCAATTTCCGCTGAATCTGATTGGATAAGTTCAATTTTAATAGGTACTACTTATGTCATTTTGTTTTGGATATGTGGAATGCCTATTATTACCTACGAAGACATTCTAACTTAAGTACTTATTCATTGTACTTTTACATTAGTGTAGTAGTGGTGGCTTTATGTTTTCCTTTCAACTTTACCCTGTGTTACAAAGTATCCATCACATTAAAATGTTAAATGTTGGGCCCGTGCTCAGCACTACAAATTGTACAAAATATAATGTGAGTGGTACTTTTACTTGGACTGTTGTTTACACGTGTGCACTATCCATAACCAAACCCTCTCCTCTTTCAATGACATGTGGATTCCACCTTCACTGCTCAATTAATTTCTACTATCTTTCATACCCATTTCTTGCTTCTTTCAGTCTTCACATTGGAAGAGACAGTAATGTCGACAACATTGTTTCGTCTTTCACAGCTCTGGTCAAAATAGAAGAATAAGATTGGTGTTCAAAATTTAATCGATAAACTGGGACACCAAAGCACCGCTAAAAAACGGTCATTGTGGAGTCTCCGTTGGTCAAAGAAAGCCGCGAGTTTCAATCAAAATTGTGTATGCTAAGGTTGAAGAGGAAGCGCCGAAAGATGAGATAGAGATTGAAGTAGAAGCACATTCCGCAGTTCTCAACGGTATATCTCTGCTTATCGTATTCTTTAAGCTTATGTTGTTTAAGTTTTAGTTTGAAAATTTCATTAACATTTGAGTTTTGATTTTGTTTTAATTGCACCGAATTCAAATTTTGGTATCCAATTTTGGCTATATATGAATTTATGCTTCTTATTATTCGTATATTTTGCCATTCCTCTTTATAATGTACTTTGCTTTGAATTGTCTGCATTTATATATTTGTCAAAATATGTTTTATTTGAGCTAAGCCTCTTTTGGAAACTGGCTCATGAGTTATTTATATTATAGTATTATTTATGTTATTCATTTTAGTCTTTATATCATGTGCCAATCTTTTGCCGCTTTGGACTAGATTGTTTGTCCAATTAAGAGTTAAATTGATCGATGTTTGATCATTTTCGAAATGCACACATTGCTTAGGTTGTTTGATAAAAAAAAAAACTTGGTGCATTAACATGCTTTATGCTGATACTGTACGGTCCATTTTAGATGGCAATTTAAATTTGCTTCCATGCCACAATGAAAACTTGTTTGAAGTTCCTATTAGAGACGCTACAAAACTTGGATATTATTTGAAGTTTGAACTGAAATTTTAGTAAAGTCCAGTTTTTTACCAGAGGCATTTTCATGCTAAGTGAGCATCTATGTAGTTAACCTTGAACTTGCTTGTGAGAAGATTGGGGTGTAATACGTAGTAGTTGAATTATAAATTCAAGAAATATTTTGCAACTGGACCTTAGTTTGCTTTTTATCAGGATGGGAATATGAGATCTCTTCGGCAATGAATGGACATATAGTTACATGTAGCGAATGTAATATTGTTTTGTATAAGATGACATGACATTGTTGTTCTAGATTAACATGTAGTTGTGTGCTATGAGATGGCCTAGAACTAGACGGGTGAAAGTTTGATCGTTTTTCTTGTGGAAACATAGATATTATACCCTTTGCCTTTGGTACTGTAATGGGAGGGCTTAGAAAGTTCACTTTGCCCTTCAATTTACAGCACAAGAGCTATTTTTTATGGCCTCATGTGCTCATTTTATCTACAGAGTAATAAAAAGTTATATTCTATGGGTTTTGCATTTGAAGTGGCTGTTGCATTGTATTATGTTTGTGTATTCCCAAGCAAACAAATCAAGTTTGTCCTTATTTTTTTTAGTTAATTCTTTGTTTCTATTTTTATTGGCTTAGTTAATGTCTCGAAGTTACCCCTAGCACTTTGGTGTAGTTGAAAAGACCGCAACATATGTAATGTGTGAGTTAAGTGCGTGTCATGACTTTAGACCCTAATGTGAATAAAAGCTGGGTATTTAAGTGAAGGGTAGATAGAGAAGCACACTCATTATTGATCGGCAAAAAATATTTGCAATGTTTCTATTCTTATGTTGTCCCAATCTCTGTCTCTCTCTCATTCTCCATGATTCAGGTTCAAGAGTGTGTCGAGAAGGATGATTTGAGGTGGTGAAATTTCACATTTGACAACTTTGGAGATAATTTTTGTTCAGATCATACATTTCTGGCCTTTTCCTTCAAGTTTTTGGGTTTATTTGTTTTTTGCCTTGAGGTACTGTTTTAATTTACTTTTAATGTATTTTTTGTTTAGAAATTTTCATGCAAGTTGGAGAAAGTATCTTGAAGGTGAGTGGTTCCTTGCAAGAGACTATTGGAGTGTAATTGGGGCTCAAAATCAAAATCAGAATACCAAATTATGTTTTCTGGTGAATTACAGAGAAACACTTCCGTCAGTATAAAAAAAGAATTTTTGTCTGAGGTACCCTGAACCCTCTCCATATATTGTTTGTTTGAAATGGAAAAGGTTGGGCTTGGGACTACAACTCCTTCTCAGTTGGAACAATGAATTCAGGAATACAAGTTTGGTGCGCAAGACACTTCGATCTTGGAGCTGAATATGAAAGTGAAAGTTAACAATATTTGGAATGTCGAGAGAACTACAAGGGAGTGAAAGTTGTAAGTGCTAATGATAGTGCTAAAATCATTGAGAAGATTCACTGTAGCAAAGGCAGAACAGAAATTGTGACTCTATGTTTCACCATGATGAGGAAATAGAAACCACGAGTATCATTGATGTTAGATTGTTTCTTCTATGTTTCACGATTGGTGAGGAAATAGAAACCACGAGTATTGTTGATGTTAGATTGTTCTTTTATTAATCTAAATCTAAAGACTTCAATTTTCTTCACCTTTTTTATTTATTTATGGAGAACTTATATTTCATATTTGGATGGGACTTTAGTACTTTTTTATTTGGTGACTTGTTCCTGAATATTTTGGC

>LNC_001943

CTTCTCCCTGTTTTTCTCACTTTTTCTAAAGTGTCAAATATCCTATTGATGGATATCTGATGGTAGTTTATGGCTTGAGAGCCCTAAGACTTTTAATTTGTCTCTCTAATTTCACTAATGTAATCACTAGATAGTAATTTACAGCAATGAATATACACTTCAATCATCTTAAAGGTCTTAAACAATTACCTGAACAAGTTGGTAATTTGTTTTAAACACAAAAACTATAATTAATGTTGATAATAGGGATGATCGAAACAGTATATTAGATACCATATATTCTAACTTAAAAGGTCACAGTTGATTCACTATTACAGGTATTCAATAGTGTTTGTCTTAGCTGAAAATACATCTATTTACCTGTAACAAAAGCTCACGTTTTCTGTCTCACTGTTTATATAATACTCCCCTTGATTCAATTTGTTTGTCCTATTTTGACTTGACATGAAGTTTAAAAAAATATAAAAGAATTTTGAATCTTGTGGTCTTAAATTAAAAGTTATGTCAAATGGACCAAAATGTCATCTAGTCTTGTGTTTTAAAACATTTCAGGTAAAAAATTGAAATTGAAGAGTTGCTTAAAAGAGGAAAGAGACATTCTTTTTGGGACTAAAAGGAAAGTAAGACAAATAAATTCTACCGGATGGAGCAATTCTTCTTTGTAAGTATCACTTATGTATTAATAACTGTTATGTTATTTATTTGAAGATAAAGAGGCATGAGGCATAAGGGAGATTTCATAAACTTACTCTAATTTAGAGAGGAATCATAACTTTAGTTATTTTGTTACCAATAGTCTTAGAGATTGAACAAAAGAATTTATAAAATGATGATGATGATAAGTCGAGATAATATTTTTTGGATATCTGAGTGCTTTTTATGGAAGAGGAAGAAATTATTGTGATAAAATTTGACATCTTATTTCTAATATATAGAGACGTGGTAACACAAAAAGAGGAAAAAAATATAACTCTTTGAAAAGCTTGAATCTTTAAGCATATTCTTGGCAACTTTTCTAACTGATTCAATTAACTCTTGAAATGTTCCAATTGTTGAAAAAATAAATAAGGGAAGAAAAGGTCAAAAATTTACTATTATTTAAGTCCGGAATTGTTGTGTAAGATCGGTGAGTGGGCTTCCATGTTGTTTGTCCTCTGTGGAAGTTCTACCCTCAAGTTTCCACGTTAAGCTCATGTTCTGTAGAATAGAAACTAGAAAGACATAATTGGATATAGTTGGTCATTTCTGTATTCTATGGTGATACGAAGCTTACATTTTAATCTGCTTTTTATTCACCTTGATTCAGTATTTTCACATTGACAGGTGATATGATATGCTCTGCTTTTATTTCTACAAAATCATTCATCCATAATTGTATTTAAGGTCATTTGAATTTTCGTGGAGTTGATGTGAAAAATGTAACAATATCATAGAGGAAAGAACATGCTGATGAAAACACCTCTGCAGTATGAGCTTAGTTTTCTTAAGAATCTCCCCGATACATTTGTGTTTTCTAAGACATTTAGAATATTTCGTTTGAAGCTTTCTTTGGCAATGCCAAAATAAATCGCCTTGGATTCCTTCGTTTATTTGATGTATGTTTCTCCCTATCTCTTCTCAAATAGGATACCTTTTATCACATGTTTCTAACAATATATTAGCTACAAGTTACATGTCTTTTAAATAGACAAATATTTTTATCTAGCTTAACTGATGTTTTAACAACATGATGAAATTTTTATAAAGTCATATTCAATCTTCATTTGAAAGTATTTTTTTCTAGAAATATCTCGAAATCTTTTCAGCTTCTCAACAATACAATACATTATAACTGGTGATGTCACCTAAGGCACATGAAAAGAGAGCAGTTTGATTGCTACACTCTTTACTGGTAAGTTTGATACTTGAGTTCTGTAGGAGTGATGAAACAACTTACTTATGTTATATTTCGGCTCCTCCATTTCATGTACTAACTTACATGCAACCTATACTATAGTTTACCTAATTCAACTCTTTTTAAGTTACAATAAGAAGCTCTGCCATGAGTATACTTTACTATTTTCATTTTGTTTGGTGCTTATTTTGTGCTCTTTGTATAGAACATAGCTATTTGTGTAGTTTATATGTTCTAGGACCAAATAGTCAAAGTTACACTTCTGAAGTTTATACGTTATAGGTTGGTGATAGGGTAAGAAATAATAGTACATGAATGATTATGAGATGATGGAAGCAAAAATTAATAGTATGATACATGATATGTGTAATGTTTTATGATTTTAGTAACTTATATTGATTGTTTCTCTTAGCTTTGCTTTTACAGGAATTCCTCTGTTGTGTTATTATCAAGTCACGATATATGATTCATTTATGACATTATGACAAATTGAATTTATTAACTAGGACAGAGAATGCAACCGACTCTTCTCGACATCTGTCACTTCACTAGATATTAGACATCTTCACTAGATATTAGACATCAGATGAGAAATTCAAGAACAATGGACATCTCTATGTTTCCTTTTGAGAGTGATTGCGTCACCTCACTTTAGTTTCATGTTTTTTCGTTAGAGAAGATCCAATTATTACCAAATCAAAATTGGATTTATATGAGCAAAAGGTGTTTGTCTTTGTGCTTCTGCTGCAATTGTGAGTGCTACTTTCATTTTATCAATTTCCGCTGAATCTGATTGGATAAGTTCAATTTTAATAGGTACTACTTATGTCATTTTGTTTTGGATATGTGGAATGCCTATTATTACCTACGAAGACATTCTAACTTAAGTACTTATTCATTGTACTTTTACATTAGTGTAGTAGTGGTGGCTTTATGTTTTCCTTTCAACTTTACCCTGTGTTACAAAGTATCCATCACATTAAAATGTTAAATGTTGGGCCCGTGCTCAGCACTACAAATTGTACAAAATATAATGTGAGTGGTACTTTTACTTGGACTGTTGTTTACACGTGTGCACTATCCATAACCAAACCCTCTCCTCTTTCAATGACATGTGGATTCCACCTTCACTGCTCAATTAATTTCTACTATCTTTCATACCCATTTCTTGCTTCTTTCAGTCTTCACATTGGAAGAGACAGTAATGTCGACAACATTGTTTCGTCTTTCACAGCTCTGGTCAAAATAGAAGAATAAGATTGGTGTTCAAAATTTAATCGATAAACTGGGACACCAAAGCACCGCTAAAAAACGGTCATTGTGGAGTCTCCGTTGGTCAAAGAAAGCCGCGAGTTTCAATCAAAATTGAAAAGTGTATGCTAAGGTTGAAGAGGAAGCGCCGAAAGATGAGATAGAGATTGAAGTAGAAGCACATTCCGCAGTTCTCAACGGTATATCTCTGCTTATCGTATTCTTTAAGCTTATGTTGTTTAAGTTTTAGTTTGAAAATTTCATTAACATTTGAGTTTTGATTTTGTTTTAATTGCACCGAATTCAAATTTTGGTATCCAATTTTGGCTATATATGAATTTATGCTTCTTATTATTCGTATATTTTGCCATTCCTCTTTATAATGTACTTTGCTTTGAATTGTCTGCATTTATATATTTGTCAAAATATGTTTTATTTGAGCTAAGCCTCTTTTGGAAACTGGCTCATGAGTTATTTATATTATAGTATTATTTATGTTATTCATTTTAGTCTTTATATCATGTGCCAATCTTTTGCCGCTTTGGACTAGATTGTTTGTCCAATTAAGAGTTAAATTGATCGATGTTTGATCATTTTCGAAATGCACACATTGCTTAGGTTGTTTGATAAAAAAAAAAACTTGGTGCATTAACATGCTTTATGCTGATACTGTACGGTCCATTTTAGATGGCAATTTAAATTTGCTTCCATGCCACAATGAAAACTTGTTTGAAGTTCCTATTAGAGACGCTACAAAACTTGGATATTATTTGAAGTTTGAACTGAAATTTTAGTAAAGTCCAGTTTTTTACCAGAGGCATTTTCATGCTAAGTGAGCATCTATGTAGTTAACCTTGAACTTGCTTGTGAGAAGATTGGGGTGTAATACGTAGTAGTTGAATTATAAATTCAAGAAATATTTTGCAACTGGACCTTAGTTTGCTTTTTATCAGGATGGGAATATGAGATCTCTTCGGCAATGAATGGACATATAGTTACATGTAGCGAATGTAATATTGTTTTGTATAAGATGACATGACATTGTTGTTCTAGATTAACATGTAGTTGTGTGCTATGAGATGGCCTAGAACTAGACGGGTGAAAGTTTGATCGTTTTTCTTGTGGAAACATAGATATTATACCCTTTGCCTTTGGTACTGTAATGGGAGGGCTTAGAAAGTTCACTTTGCCCTTCAATTTACAGCACAAGAGCTATTTTTTATGGCCTCATGTGCTCATTTTATCTACAGAGTAATAAAAAGTTATATTCTATGGGTTTTGCATTTGAAGTGGCTGTTGCATTGTATTATGTTTGTGTATTCCCAAGCAAACAAATCAAGTTTGTCCTTATTTTTTTTAGTTAATTCTTTGTTTCTATTTTTATTGGCTTAGTTAATGTCTCGAAGTTACCCCTAGCACTTTGGTGTAGTTGAAAAGACCGCAACATATGTAATGTGTGAGTTAAGTGCGTGTCATGACTTTAGACCCTAATGTGAATAAAAGCTGGGTATTTAAGTGAAGGGTAGATAGAGAAGCACACTCATTATTGATCGGCAAAAAATATTTGCAATGTTTCTATTCTTATGTTGTCCCAATCTCTGTCTCTCTCTCATTCTCCATGATTCAGGTTCAAGAGTGTGTCGAGAAGGATGATTTGAGGTGGTGAAATTTCACATTTGACAACTTTGGAGATAATTTTTGTTCAGATCATACATTTCTGGCCTTTTCCTTCAAGTTTTTGGGTTTATTTGTTTTTTGCCTTGAGGTACTGTTTTAATTTACTTTTAATGTATTTTTTGTTTAGAAATTTTCATGCAAGTTGGAGAAAGTATCTTGAAGGTGAGTGGTTCCTTGCAAGAGACTATTGGAGTGTAATTGGGGCTCAAAATCAAAATCAGAATACCAAATTATGTTTTCTGGTGAATTACAGAGAAACACTTCCGTCAGTATAAAAAAAGAATTTTTGTCTGAGGTACCCTGAACCCTCTCCATATATTGTTTGTTTGAAATGGAAAAGGTTGGGCTTGGGACTACAACTCCTTCTCAGTTGGAACAATGAATTCAGGAATACAAGTTTGGTGCGCAAGACACTTCGATCTTGGAGCTGAATATGAAAGTGAAAGTTAACAATATTTGGAATGTCGAGAGAACTACAAGGGAGTGAAAGTTGTAAGTGCTAATGATAGTGCTAAAATCATTGAGAAGATTCACTGTAGCAAAGGCAGAACAGAAATTGTGACTCTATGTTTCACCATGATGAGGAAATAGAAACCACGAGTATCATTGATGTTAGATTGTTTCTTCTATGTTTCACGATTGGTGAGGAAATAGAAACCACGAGTATTGTTGATGTTAGATTGTTCTTTTATTAATCTAAATCTAAAGACTTCAATTTTCTTCACCTTTTTTATTTATTTATGGAGAACTTATATTTCATATTTGGATGGGACTTTAGTACTTTTTTATTTGGTGACTTGTTCCTGAATATTTTGGC

>LNC_001944

AAGGAAAGTAAGACAAATAAATTCTACCGGATGGAGCAATTCTTCTTTCTCTGGTCAAAATAGAAGAATAAGATTGGTGTTCAAAATTTAATCGATAAACTGGGACACCAAAGCACCGCTAAAAAACGGTCATTGTGGAGTCTCCGTTGGTCAAAGAAAGCCGCGAGTTTCAATCAAAATTGTGTATGCTAAGGTTGAAGAGGAAGCGCCGAAAGATGAGATAGAGATTGAAGTAGAAGCACATTCCGCAGTTCTCAACGGTATATCTCTGCTTATCGTATTCTTTAAGCTTATGTTGTTTAAGTTTTAGTTTGAAAATTTCATTAACATTTGAGTTTTGATTTTGTTTTAATTGCACCGAATTCAAATTTTGGTATCCAATTTTGGCTATATATGAATTTATGCTTCTTATTATTCGTATATTTTGCCATTCCTCTTTATAATGTACTTTGCTTTGAATTGTCTGCATTTATATATTTGTCAAAATATGTTTTATTTGAGCTAAGCCTCTTTTGGAAACTGGCTCATGAGTTATTTATATTATAGTATTATTTATGTTATTCATTTTAGTCTTTATATCATGTGCCAATCTTTTGCCGCTTTGGACTAGATTGTTTGTCCAATTAAGAGTTAAATTGATCGATGTTTGATCATTTTCGAAATGCACACATTGCTTAGGTTGTTTGATAAAAAAAAAAACTTGGTGCATTAACATGCTTTATGCTGATACTGTACGGTCCATTTTAGATGGCAATTTAAATTTGCTTCCATGCCACAATGAAAACTTGTTTGAAGTTCCTATTAGAGACGCTACAAAACTTGGATATTATTTGAAGTTTGAACTGAAATTTTAGTAAAGTCCAGTTTTTTACCAGAGGCATTTTCATGCTAAGTGAGCATCTATGTAGTTAACCTTGAACTTGCTTGTGAGAAGATTGGGGTGTAATACGTAGTAGTTGAATTATAAATTCAAGAAATATTTTGCAACTGGACCTTAGTTTGCTTTTTATCAGGATGGGAATATGAGATCTCTTCGGCAATGAATGGACATATAGTTACATGTAGCGAATGTAATATTGTTTTGTATAAGATGACATGACATTGTTGTTCTAGATTAACATGTAGTTGTGTGCTATGAGATGGCCTAGAACTAGACGGGTGAAAGTTTGATCGTTTTTCTTGTGGAAACATAGATATTATACCCTTTGCCTTTGGTACTGTAATGGGAGGGCTTAGAAAGTTCACTTTGCCCTTCAATTTACAGCACAAGAGCTATTTTTTATGGCCTCATGTGCTCATTTTATCTACAGAGTAATAAAAAGTTATATTCTATGGGTTTTGCATTTGAAGTGGCTGTTGCATTGTATTATGTTTGTGTATTCCCAAGCAAACAAATCAAGTTTGTCCTTATTTTTTTTAGTTAATTCTTTGTTTCTATTTTTATTGGCTTAGTTAATGTCTCGAAGTTACCCCTAGCACTTTGGTGTAGTTGAAAAGACCGCAACATATGTAATGTGTGAGTTAAGTGCGTGTCATGACTTTAGACCCTAATGTGAATAAAAGCTGGGTATTTAAGTGAAGGGTAGATAGAGAAGCACACTCATTATTGATCGGCAAAAAATATTTGCAATGTTTCTATTCTTATGTTGTCCCAATCTCTGTCTCTCTCTCATTCTCCATGATTCAGGTTCAAGAGTGTGTCGAGAAGGATGATTTGAGGTGGTGAAATTTCACATTTGACAACTTTGGAGATAATTTTTGTTCAGATCATACATTTCTGGCCTTTTCCTTCAAGTTTTTGGGTTTATTTGTTTTTTGCCTTGAGGTACTGTTTTAATTTACTTTTAATGTATTTTTTGTTTAGAAATTTTCATGCAAGTTGGAGAAAGTATCTTGAAGGTGAGTGGTTCCTTGCAAGAGACTATTGGAGTGTAATTGGGGCTCAAAATCAAAATCAGAATACCAAATTATGTTTTCTGGTGAATTACAGAGAAACACTTCCGTCAGTATAAAAAAAGAATTTTTGTCTGAGGTACCCTGAACCCTCTCCATATATTGTTTGTTTGAAATGGAAAAGGTTGGGCTTGGGACTACAACTCCTTCTCAGTTGGAACAATGAATTCAGGAATACAAGTTTGGTGCGCAAGACACTTCGATCTTGGAGCTGAATATGAAAGTGAAAGTTAACAATATTTGGAATGTCGAGAGAACTACAAGGGAGTGAAAGTTGTAAGTGCTAATGATAGTGCTAAAATCATTGAGAAGATTCACTGTAGCAAAGGCAGAACAGAAATTGTGACTCTATGTTTCACCATGATGAGGAAATAGAAACCACGAGTATCATTGATGTTAGATTGTTTCTTCTATGTTTCACGATTGGTGAGGAAATAGAAACCACGAGTATTGTTGATGTTAGATTGTTCTTTTATTAATCTAAATCTAAAGACTTCAATTTTCTTCACCTTTTTTATTTATTTATGGAGAACTTATATTTCATATTTGGATGGGACTTTAGTACTTTTTTATTTGGTGACTTGTTCCTGAATATTTTGGC

>LNC_001945

TCTTGAAATGTTCCAATTGTTGAAAAAATAAATAAGGGAAGAAAAGGTCAAAAATTTACTATTATTTAAGTCCGGAATTGTTGTGTAAGATCGGTGAGTGGGCTTCCATGTTGTTTGTCCTCTGTGGAAGTTCTACCCTCAAGTTTCCACGTTAAGCTCATGTTCTGTAGAATAGAAACTAGAAAGACATAATTGGATATAGTTGGTCATTTCTGTATTCTATGGTGATACGAAGCTTACATTTTAATCTGCTTTTTATTCACCTTGATTCAGTATTTTCACATTGACAGGTGATATGATATGCTCTGCTTTTATTTCTACAAAATCATTCATCCATAATTGTATTTAAGGTCATTTGAATTTTCGTGGAGTTGATGTGAAAAATGTAACAATATCATAGAGGAAAGAACATGCTGATGAAAACACCTCTGCAGTATGAGCTTAGTTTTCTTAAGAATCTCCCCGATACATTTGTGTTTTCTAAGACATTTAGAATATTTCGTTTGAAGCTTTCTTTGGCAATGCCAAAATAAATCGCCTTGGATTCCTTCGTTTATTTGATGTATGTTTCTCCCTATCTCTTCTCAAATAGGATACCTTTTATCACATGTTTCTAACAATATATTAGCTACAAGTTACATGTCTTTTAAATAGACAAATATTTTTATCTAGCTTAACTGATGTTTTAACAACATGATGAAATTTTTATAAAGTCATATTCAATCTTCATTTGAAAGTATTTTTTTCTAGAAATATCTCGAAATCTTTTCAGCTTCTCAACAATACAATACATTATAACTGGTGATGTCACCTAAGGCACATGAAAAGAGAGCAGTTTGATTGCTACACTCTTTACTGCTCTGGTCAAAATAGAAGAATAAGATTGGTGTTCAAAATTTAATCGATAAACTGGGACACCAAAGCACCGCTAAAAAACGGTCATTGTGGAGTCTCCGTTGGTCAAAGAAAGCCGCGAGTTTCAATCAAAATTGAAAAGTGTATGCTAAGGTTGAAGAGGAAGCGCCGAAAGATGAGATAGAGATTGAAGTAGAAGCACATTCCGCAGTTCTCAACGGTATATCTCTGCTTATCGTATTCTTTAAGCTTATGTTGTTTAAGTTTTAGTTTGAAAATTTCATTAACATTTGAGTTTTGATTTTGTTTTAATTGCACCGAATTCAAATTTTGGTATCCAATTTTGGCTATATATGAATTTATGCTTCTTATTATTCGTATATTTTGCCATTCCTCTTTATAATGTACTTTGCTTTGAATTGTCTGCATTTATATATTTGTCAAAATATGTTTTATTTGAGCTAAGCCTCTTTTGGAAACTGGCTCATGAGTTATTTATATTATAGTATTATTTATGTTATTCATTTTAGTCTTTATATCATGTGCCAATCTTTTGCCGCTTTGGACTAGATTGTTTGTCCAATTAAGAGTTAAATTGATCGATGTTTGATCATTTTCGAAATGCACACATTGCTTAGGTTGTTTGATAAAAAAAAAAACTTGGTGCATTAACATGCTTTATGCTGATACTGTACGGTCCATTTTAGATGGCAATTTAAATTTGCTTCCATGCCACAATGAAAACTTGTTTGAAGTTCCTATTAGAGACGCTACAAAACTTGGATATTATTTGAAGTTTGAACTGAAATTTTAGTAAAGTCCAGTTTTTTACCAGAGGCATTTTCATGCTAAGTGAGCATCTATGTAGTTAACCTTGAACTTGCTTGTGAGAAGATTGGGGTGTAATACGTAGTAGTTGAATTATAAATTCAAGAAATATTTTGCAACTGGACCTTAGTTTGCTTTTTATCAGGATGGGAATATGAGATCTCTTCGGCAATGAATGGACATATAGTTACATGTAGCGAATGTAATATTGTTTTGTATAAGATGACATGACATTGTTGTTCTAGATTAACATGTAGTTGTGTGCTATGAGATGGCCTAGAACTAGACGGGTGAAAGTTTGATCGTTTTTCTTGTGGAAACATAGATATTATACCCTTTGCCTTTGGTACTGTAATGGGAGGGCTTAGAAAGTTCACTTTGCCCTTCAATTTACAGCACAAGAGCTATTTTTTATGGCCTCATGTGCTCATTTTATCTACAGAGTAATAAAAAGTTATATTCTATGGGTTTTGCATTTGAAGTGGCTGTTGCATTGTATTATGTTTGTGTATTCCCAAGCAAACAAATCAAGTTTGTCCTTATTTTTTTTAGTTAATTCTTTGTTTCTATTTTTATTGGCTTAGTTAATGTCTCGAAGTTACCCCTAGCACTTTGGTGTAGTTGAAAAGACCGCAACATATGTAATGTGTGAGTTAAGTGCGTGTCATGACTTTAGACCCTAATGTGAATAAAAGCTGGGTATTTAAGTGAAGGGTAGATAGAGAAGCACACTCATTATTGATCGGCAAAAAATATTTGCAATGTTTCTATTCTTATGTTGTCCCAATCTCTGTCTCTCTCTCATTCTCCATGATTCAGGTTCAAGAGTGTGTCGAGAAGGATGATTTGAGGTGGTGAAATTTCACATTTGACAACTTTGGAGATAATTTTTGTTCAGATCATACATTTCTGGCCTTTTCCTTCAAGTTTTTGGGTTTATTTGTTTTTTGCCTTGAGGTACTGTTTTAATTTACTTTTAATGTATTTTTTGTTTAGAAATTTTCATGCAAGTTGGAGAAAGTATCTTGAAGGTGAGTGGTTCCTTGCAAGAGACTATTGGAGTGTAATTGGGGCTCAAAATCAAAATCAGAATACCAAATTATGTTTTCTGGTGAATTACAGAGAAACACTTCCGTCAGTATAAAAAAAGAATTTTTGTCTGAGGTACCCTGAACCCTCTCCATATATTGTTTGTTTGAAATGGAAAAGGTTGGGCTTGGGACTACAACTCCTTCTCAGTTGGAACAATGAATTCAGGTGTACTTGTGATGTCTCCTTCGATACAAACATTTGTTTTGGTTATATGTTCTCTAAAAGTATTTAAATATGAATAGTTCTAATACACAATGTATTTAGGTATTTTGCACATATTATAGATTCAAGTATCTATTGAGCACATTGATGGCTAAATAAATAGAGATGATATTTAAGGATTGTTTAGTAAAAGGTATTAGAGAAAATAGTGCATGTATTAGCTTTGGTACTATTTAATACTTTGTATGGTAGTGTTTGTTTGATATCACATTGTGCAGATCCTAAATAAAGAATCATTCCCTTTACAAATGTACTTCAGTTTCTATATTCAGAGAGTCAATCAATAATGAAACCACCTTTGGCACAAATTTTGATTACTTTCGAGATGAAAAAGAGATAAGAGTGTTGCAAGTTAATTTGCCCATCATATGACATGGACTTGAGACTAAAATTGTTTCCTCTATCTCGTGTAACAATAATTTCATGGAGAAGATCATTTTTGTTCTCTTTAATGTGTTATTAGTTGCTAATTAAAC

>LNC_001946

AAATGTATCGAAAACAAATCAAATTGTTCTTAAAGTTGATGGATTTGGGGGTAAATTGGTACAGGGTTATTTCCTTAGAAACATATTTACTACTCTATTATCATTACTCACACATATACTACTTTATTCTTCATTAAAAAAAGTGTACCATATTTTATTAGGTAAGCAAAAATAATTGATCTCTATGGTAATTTTTTGGAGGATTAGAAATAATTTCCGATCATCACCAATTCAGAGTGAGAGCTAAGATAACATGAATTTTGTAGCACCATATTTGTCCATTTTCACTCATTCCGTAATTCTTTTATGGTCAATTTCGTCATTTTATTTAGTTTTTTTTTTTCTTACGGCACAAGAAAAGTGTATGCTAAGGTTGAAGAGGAAGCGCCGAAAGATGAGATAGAGATTGAAGTAGAAGCACATTCCGCAGTTCTCAACGGTATATCTCTGCTTATCGTATTCTTTAAGCTTATGTTGTTTAAGTTTTAGTTTGAAAATTTCATTAACATTTGAGTTTTGATTTTGTTTTAATTGCACCGAATTCAAATTTTGGTATCCAATTTTGGCTATATATGAATTTATGCTTCTTATTATTCGTATATTTTGCCATTCCTCTTTATAATGTACTTTGCTTTGAATTGTCTGCATTTATATATTTGTCAAAATATGTTTTATTTGAGCTAAGCCTCTTTTGGAAACTGGCTCATGAGTTATTTATATTATAGTATTATTTATGTTATTCATTTTAGTCTTTATATCATGTGCCAATCTTTTGCCGCTTTGGACTAGATTGTTTGTCCAATTAAGAGTTAAATTGATCGATGTTTGATCATTTTCGAAATGCACACATTGCTTAGGTTGTTTGATAAAAAAAAAAACTTGGTGCATTAACATGCTTTATGCTGATACTGTACGGTCCATTTTAGATGGCAATTTAAATTTGCTTCCATGCCACAATGAAAACTTGTTTGAAGTTCCTATTAGAGACGCTACAAAACTTGGATATTATTTGAAGTTTGAACTGAAATTTTAGTAAAGTCCAGTTTTTTACCAGAGGCATTTTCATGCTAAGTGAGCATCTATGTAGTTAACCTTGAACTTGCTTGTGAGAAGATTGGGGTGTAATACGTAGTAGTTGAATTATAAATTCAAGAAATATTTTGCAACTGGACCTTAGTTTGCTTTTTATCAGGATGGGAATATGAGATCTCTTCGGCAATGAATGGACATATAGTTACATGTAGCGAATGTAATATTGTTTTGTATAAGATGACATGACATTGTTGTTCTAGATTAACATGTAGTTGTGTGCTATGAGATGGCCTAGAACTAGACGGGTGAAAGTTTGATCGTTTTTCTTGTGGAAACATAGATATTATACCCTTTGCCTTTGGTACTGTAATGGGAGGGCTTAGAAAGTTCACTTTGCCCTTCAATTTACAGCACAAGAGCTATTTTTTATGGCCTCATGTGCTCATTTTATCTACAGAGTAATAAAAAGTTATATTCTATGGGTTTTGCATTTGAAGTGGCTGTTGCATTGTATTATGTTTGTGTATTCCCAAGCAAACAAATCAAGTTTGTCCTTATTTTTTTTAGTTAATTCTTTGTTTCTATTTTTATTGGCTTAGTTAATGTCTCGAAGTTACCCCTAGCACTTTGGTGTAGTTGAAAAGACCGCAACATATGTAATGTGTGAGTTAAGTGCGTGTCATGACTTTAGACCCTAATGTGAATAAAAGCTGGGTATTTAAGTGAAGGGTAGATAGAGAAGCACACTCATTATTGATCGGCAAAAAATATTTGCAATGTTTCTATTCTTATGTTGTCCCAATCTCTGTCTCTCTCTCATTCTCCATGATTCAGGTTCAAGAGTGTGTCGAGAAGGATGATTTGAGGTGGTGAAATTTCACATTTGACAACTTTGGAGATAATTTTTGTTCAGATCATACATTTCTGGCCTTTTCCTTCAAGTTTTTGGGTTTATTTGTTTTTTGCCTTGAGGTACTGTTTTAATTTACTTTTAATGTATTTTTTGTTTAGAAATTTTCATGCAAGTTGGAGAAAGTATCTTGAAGGTGAGTGGTTCCTTGCAAGAGACTATTGGAGTGTAATTGGGGCTCAAAATCAAAATCAGAATACCAAATTATGTTTTCTGGTGAATTACAGAGAAACACTTCCGTCAGTATAAAAAAAGAATTTTTGTCTGAGGTACCCTGAACCCTCTCCATATATTGTTTGTTTGAAATGGAAAAGGTTGGGCTTGGGACTACAACTCCTTCTCAGTTGGAACAATGAATTCAGGTGTACTTGTGATGTCTCCTTCGATACAAACATTTGTTTTGGTTATATGTTCTCTAAAAGTATTTAAATATGAATAGTTCTAATACACAATGTATTTAGGTATTTTGCACATATTATAGATTCAAGTATCTATTGAGCACATTGATGGCTAAATAAATAGAGATGATATTTAAGGATTGTTTAGTAAAAGGTATTAGAGAAAATAGTGCATGTATTAGCTTTGGTACTATTTAATACTTTGTATGGTAGTGTTTGTTTGATATCACATTGTGCAGATCCTAAATAAAGAATCATTCCCTTTACAAATGTACTTCAGTTTCTATATTCAGAGAGTCAATCAATAATGAAACCACCTTTGGCACAAATTTTGATTACTTTCGAGATGAAAAAGAGATAAGAGTGTTGCAAGTTAATTTGCCCATCATATGACATGGACTTGAGACTAAAATTGTTTCCTCTATCTCGTGTAACAATAATTTCATGGAGAAGATCATTTTTGTTCTCTTTAATGTGTTATTAGTTGCTAATTAAAC

>LNC_001947

ATTTGGGGGTAAATTGGTACAGGGTTATTTCCTTAGAAACATATTTACTACTCTATTATCATTACTCACACATATACTACTTTATTCTTCATTAAAAAAAGTGTACCATATTTTATTAGGTAAGCAAAAATAATTGATCTCTATGGTAATTTTTTGGAGGATTAGAAATAATTTCCGATCATCACCAATTCAGAGTGAGAGCTAAGATAACATGAATTTTGTAGCACCATATTTGTCCATTTTCACTCATTCCGTAATTCTTTTATGGTCAATTTCGTCATTTTATTTAGTTTTTTTTTTTCTTACGGCACAAGAAAAGTGTATGCTAAGGTTGAAGAGGAAGCGCCGAAAGATGAGATAGAGATTGAAGTAGAAGCACATTCCGCAGTTCTCAACGGTATATCTCTGCTTATCGTATTCTTTAAGCTTATGTTGTTTAAGTTTTAGTTTGAAAATTTCATTAACATTTGAGTTTTGATTTTGTTTTAATTGCACCGAATTCAAATTTTGGTATCCAATTTTGGCTATATATGAATTTATGCTTCTTATTATTCGTATATTTTGCCATTCCTCTTTATAATGTACTTTGCTTTGAATTGTCTGCATTTATATATTTGTCAAAATATGTTTTATTTGAGCTAAGCCTCTTTTGGAAACTGGCTCATGAGTTATTTATATTATAGTATTATTTATGTTATTCATTTTAGTCTTTATATCATGTGCCAATCTTTTGCCGCTTTGGACTAGATTGTTTGTCCAATTAAGAGTTAAATTGATCGATGTTTGATCATTTTCGAAATGCACACATTGCTTAGGTTGTTTGATAAAAAAAAAAACTTGGTGCATTAACATGCTTTATGCTGATACTGTACGGTCCATTTTAGATGGCAATTTAAATTTGCTTCCATGCCACAATGAAAACTTGTTTGAAGTTCCTATTAGAGACGCTACAAAACTTGGATATTATTTGAAGTTTGAACTGAAATTTTAGTAAAGTCCAGTTTTTTACCAGAGGCATTTTCATGCTAAGTGAGCATCTATGTAGTTAACCTTGAACTTGCTTGTGAGAAGATTGGGGTGTAATACGTAGTAGTTGAATTATAAATTCAAGAAATATTTTGCAACTGGACCTTAGTTTGCTTTTTATCAGGATGGGAATATGAGATCTCTTCGGCAATGAATGGACATATAGTTACATGTAGCGAATGTAATATTGTTTTGTATAAGATGACATGACATTGTTGTTCTAGATTAACATGTAGTTGTGTGCTATGAGATGGCCTAGAACTAGACGGGTGAAAGTTTGATCGTTTTTCTTGTGGAAACATAGATATTATACCCTTTGCCTTTGGTACTGTAATGGGAGGGCTTAGAAAGTTCACTTTGCCCTTCAATTTACAGCACAAGAGCTATTTTTTATGGCCTCATGTGCTCATTTTATCTACAGAGTAATAAAAAGTTATATTCTATGGGTTTTGCATTTGAAGTGGCTGTTGCATTGTATTATGTTTGTGTATTCCCAAGCAAACAAATCAAGTTTGTCCTTATTTTTTTTAGTTAATTCTTTGTTTCTATTTTTATTGGCTTAGTTAATGTCTCGAAGTTACCCCTAGCACTTTGGTGTAGTTGAAAAGACCGCAACATATGTAATGTGTGAGTTAAGTGCGTGTCATGACTTTAGACCCTAATGTGAATAAAAGCTGGGTATTTAAGTGAAGGGTAGATAGAGAAGCACACTCATTATTGATCGGCAAAAAATATTTGCAATGTTTCTATTCTTATGTTGTCCCAATCTCTGTCTCTCTCTCATTCTCCATGATTCAGGTTCAAGAGTGTGTCGAGAAGGATGATTTGAGGTGGTGAAATTTCACATTTGACAACTTTGGAGATAATTTTTGTTCAGATCATACATTTCTGGCCTTTTCCTTCAAGTTTTTGGGTTTATTTGTTTTTTGCCTTGAGGTACTGTTTTAATTTACTTTTAATGTATTTTTTGTTTAGAAATTTTCATGCAAGTTGGAGAAAGTATCTTGAAGGTGAGTGGTTCCTTGCAAGAGACTATTGGAGTGTAATTGGGGCTCAAAATCAAAATCAGAATACCAAATTATGTTTTCTGGTGAATTACAGAGAAACACTTCCGTCAGTATAAAAAAAGAATTTTTGTCTGAGGTACCCTGAACCCTCTCCATATATTGTTTGTTTGAAATGGAAAAGGTTGGGCTTGGGACTACAACTCCTTCTCAGTTGGAACAATGAATTCAGGAATACAAGTTTGGTGCGCAAGACACTTCGATCTTGGAGCTGAATATGAAAGTGAAAGTTAACAATATTTGGAATGTCGAGAGAACTACAAGGGAGTGAAAGTTGTAAGTGCTAATGATAGTGCTAAAATCATTGAGAAGATTCACTGTAGCAAAGGCAGAACAGAAATTGTGACTCTATGTTTCACCATGATGAGGAAATAGAAACCACGAGTATCATTGATGTTAGATTGTTTCTTCTATGTTTCACGATTGGTGAGGAAATAGAAACCACGAGTATTGTTGATGTTAGATTGTTCTTTTATTAATCTAAATCTAAAGACTTCAATTTTCTTCACCTTTTTTATTTATTTATGGAGAACTTATATTTCATATTTGGATGGGACTTTAGTACTTTTTTATTTGGTGACTTGTTCCTGAATATTTTGGC

>LNC_001948

CATCTCTGATTGCTTGAGTTTGAAGGTCAGATCGGGTTGACTGGTAAAGCTTGCATTATGCTTGGCCCCATTTTTTAAATAAATAAATGAGCATTATTGGACCTGAAAGTGTTAGGTGTAGAGCCTGACTTGTTCACTAAAAGCAAAGATCAAACTGAAGTAGGTGGGCTATTGCTTTATCATAAGCTAAGAGCGGACATCCCAACTCCTTTACATTTCTTAAACTTGATTATAGGATATTAAGTGCATGCCTACTGTTAACTTTTTTCTAAGAAAAAGTAAGGGGCATGCCTACTGTTAAACTTTAAATGCCCTAAAATATTATAGGGAGGACTCTACCCATGGATATCATGAAACTGTATACAATTCACAGAAGTAATGCAACATTTTATAAAACTAAGCTTTTTGCAACTCCCTTCTATTTTGCTTTGCTTCCCAAAAAGTGTCAGTTTCTTTCGCTTCTAGATATCCCAGTATGCAGCCGAAATTGACACGATGAGTTCTTAGATTTATTATCTAAATCCTCTTGTGGCCAAAATCCTCCAACTGGAAATTTTTTAAATGTGTCAAACAAGAACCACTTTTAAAGATGAGCCCGAGAAAAGCAACAAAACTGAAGAAAAAAAAAAAAAAGACAACAAAAAACCATCTTTGACTAGTGACCCAGTGAACTTAGGAATCCACATAAAGATGGTAAACTAAACCTTTGGCCTCACCGAGTTGTAAGCATGTGACTATTCCTCAACCTTCACCTTAGCAGGAGTGAGTAACTTGCCATTTATGGCTTAGTTCCTGGAGAGGCAATACAGAAACATACTCATCATTATTATAGCACAACCAAATTTGTTTGAGAAATATGTTACATCTGTCTGTCCAAAAAGTGCTTATGATACTTTCCTTTTGAGATGTCCCACAAGTTGTTC

>LNC_001949

GAAAACTTGCAAGTACAAAAGGAGAAAGAGGAGGGGGTGGGTGGGGTATGATGAAAAGAATGTCTCAGAATAAGAGAGTGCCCCAACGAGACAAAGCTGCCTTTCCAGTTCTGATCAACAAATTAATTACGGAAAATCTGTTGCTTGTCGATACTCTAAAAGCAGTGTATTTTTGGAATTAAGAGGAGTTGTATAAACTAAAATGACAAAAGAAGTGTAGGAAAGTGAGGAAGTAGTCAACTGTTGAGGTATTTCAACAACACCACTCAATCAATCCAATATCTCTTTTTCCATTTTTACCTTGTCGTAATTGCGGCACTTCTTCTTCATCAGCTTATAAGGAGAAAGAGTTTGTTATTTGAAGAGGGGGGGGGGGTATTTGGAGTCTTTTAGCCACAGTTCGAAGCCCTGGCGTATCTCTTTTAGCCACGGTTCAAGGCCCCAACATATTTTTTTGGCACGGTTCGAGGCCCCA

>LNC_001950

AATTTTTTGGAAGGATAGCACCTTGAAAGAATGAAATGATTGCTGCATGGAGGTCCGCTGCTATGATTTGCCAGGTTGATTGGTAGAATTTTCCCTTTAGTCCGTCAGGCCCAGGGGCGCTGTCTGGATCAATTGAAAAAATAGTGTCTTTGATCTCTTGGAGGGTTGGCAAGCTGTTAAGGTTGTTGTTGTCTTCAATGGTGACAACCCTTTCTACGAGATTAAAAATATTACTATCTTCCTCAGTGGAATCAGCTTTGAACAGATTGGTGAAAAAATTGACTGCAGCATCAACAACTTGAGTAGTTCCCTCTACCCAATTACCCTCTTGATCTTTGATTTTGTGTATTGAGAGCTTTTTTCTTTTATCCTTGATTACCCTGTGGAAGAAAGCAGAGTTAGTATCACCATCTTCAAGCCATTTAATCCTAGACTTTTGGCTTAGGACTTTTTCTTGAATTTTCAGGAATCTAGTGAATTCAGCCTTAGCCTTAGAAAAGTTTATCCTGTTCTCCGGAGTATTATTGTTGATGGAGTTCTCTTCCAGGTCTTTAATAATATTTTCTAGTTTCTTAGGTTCTTCATGGAAGTCTCCATAAGCTATTCTAGACCATGCACTAAGAGCTTTGATAGTGGCCTTGAGTTTTTTATGAAGGACTTGTAGGGCGTTTTCTTCTTGATCTTGAGACCAAGCTTTCTGTACAACACCGA

>LNC_001951

CTTTTTCTTGTTGAGATTCTTTGTTTTTTCAGAGTTAACCACCTCAACAAGTTGACTGGTAATCTGATCATCTAACTTAACTTCCAAATACGCAGTTTCCTTGTTGTGTTTCACCTTCATAGTGTTGTTTTTGATGATTTGAAGGTGTTGTGCTTTCCTTTCGTTTCTTTCAGCTTCTTTTTCCTTTCTTTTAGTAGGGGTTCTCAAAGTGTTGCTCCTGGAACAATTCATTTCTGCTGGTATCGTTTCATTGTGAGTAGTAACTTTCATATCCTC

>LNC_001952

CACTTGTAAGAATATCACTTGTAAGAATATTTGCACTAGTTCCATCTTCATCACTAGTTCTATCTTCATCTTCAGAAGAAACATAACCAAAGGTACGAAGATGATTCAAGTATCCAATATCATAAAAACTATATCTTTCAAATGCAAGCTCAAAATGTTGTGCTACATCAAGCATCGAATATGTAGAGTTCCACCTAGTAGGAACATCTAAACACAGAGATTTCTTACAAGCTATCAATTGAGATTCACAACATTCTTTGAACTTTCTAAGTCTTGCGGGAGATTGTTTTATGTACTTCACGGCTTGTCTCACTAGTTTGATAGACTTACCAATTTCCTTCAACCCATCTTGCACAAT

>LNC_001953

CCTCAGACACCCAACAAATTTCCAAAAAAATTTCTCCATTAGAGACAGACAAAATTCCTTGTTTCCTTCTTCCCCACTGCCTCTGTAATCTCCATTGCCGGCTTCCCTAATAAACCAATGCAACTCTCATTTGTCACTTTTCTATATCCCTGAAACCCAACTCAAAAATTCCCAATTCTTTCGTTATTTTCTTTCTTTTCTTCAATTCTCGTTCTGTAATTCAGGAGTGAACCATTTGATCTTTCTATACTTTTTTG

>LNC_001954

CTCAGCCCAAAAAAAGAACTCAAAATCCCCAAATCAATTCACTCCCCAAACCTATTTTCTTCTCCTTCTTCTCTGATGTTTGTCGGTCCAGCAAAGAATCAAATTAACTTGATGTATTTTGTATTTGGTATCTTATTTAAAACTCTTAAAAATAAGTTCTATCTTGGATATGTTAAGTAGGAGCCTATTTGAATACAACTTATGTCTTTATGGAATTATAATCTCATAATAGATGAAAAATCATGATAGCACCCCAAAATCAAAGCTCGTTTGCGCCTCAGTCGTGACTGGTAAGCTTGATTTAGAGTTTTGAGGAAAAGAGAAGCTCAATTGTCAAGTGAGAACCTGATTTTTTTATTTCAAAAACAATATACAGTTCTCATGGAGGAAATATGGATGAAGTAATTTACTTGTCTATGATATTTCTTCTTTCCAAGTAAAAGTATTGTTTTGCTATTTTCTACTGAAAGATTTCTATTTCAGATGAATTAATTCTCTGTTACGCTAAAGATTGGAGAAGATCGAAGAAGATGGAGAAGAAACTAGGGTTTTGGGAGTGAATTGATTTGGG

>LNC_001955

CCCACCTCTGTACCAGCGTCTTCCTCCAGCGAAGAACACAACCAGACGCCAGACGAAATCCCAGCAGAAATTGCGCTGCAAACTCGAAAATCTCGCCTAACTACCCTGCAGAACCCATGCCCTAGCAGCCTCAACGTCAAACCCTCACGTCCCCTTTCCCCCTTTTTCAGGGATTTGCAAATCCTTTCCACAATTTGGCTTTGAAAGAGCAAAGGGAGTCGAATTTAAAATTTTAAAAATCTGCCCGTTTCCTCCCCAATTTCTAGTCCTAAATCTTCTTCTATATAGTCATCTCATTTAGTCACTGATTGAGAGGAGATAGAGATTAGGGCCGGGTACCACCGAAGCACACAAATCTTTTATTAAGAAATAGGAGTCTTTGGATAGTAAAAAGAGTTTTCCAGAGGGATACGTCCTTTTCTTTTGTTTGTTGTTTTCGAGTTGTTGGTTCATTGGAGTTCGCCTGAGCTCGTCTCATTTGTGGTCGTAGATAGCTCGCTTCTGCTCGTTCGTCTCAAAACGCTGCACTCCAAAAGTCCTCTTGGACTTCATTTGTCTTCCCTCCGCACTTTGAGAGGGCCATAAAGGCCCAAAATTCTTTTATCGAGTCTGCCAACCTTCAAAAATCGAAGAACAAGTCTTTGGAGTTGACGTACAGGTCCCAGAAAGCAGAAAAATGGACTGTATACATCCGATATACACTAATATACACCCGATGTATACAAAAACGGGTTCATAATTAAACCCCCAAAAATTGCAACAGGCCCAAGAAGGGCCCAAATTTAATCTCTCCTTTACTCTTAAATTGTTTAATTGTGATTATTTTTTATGTGTTCTAACTTTAGAATTATCAATTTACTTAATTTGATTCCCCAAAAGATAAGTTATTTTTTTGCGTTGGTTGACTTTTAATATTTTTTTTTATTTTATTAATCAAATAGGTTGATCAAATTATACTTTCTAATTAAGTTAAACCTTTTATTCTTAAGATATATTATTTAACGCCTCT

>LNC_001956

GCCCAAACAAAATGACCCCTGCGTTCTCTTTCTTCTTCACCAACACGTGAATGCACTGAAAAACCCAGAAGTGTCAACAACTTGCAAACGTCAACAACGGCTGCTCTCACCAAACTGTTCCAACAGCTCCCACATCCTTTCTCTGCAACAACCGCCCATTTTGATCTTTTCCATCCGGATTTTAGGGGATTTTGGTTGAATTTCCATACAACAACAGCTTTTCCTCTTGGGGAAAAATTTGAATTGATTTGATTGGGGAATTCCGATTTTGCCCCAAGTTCCTATCCGTTTTTTTCTCCCAAATCTGCCCTAATTTTCTAGCTATATATACTCTTTTCTTATTCACTGTATGGGGCAGAGAGGGATTTTGGTGGAGTTTGAGTTTAGTTTGGAAGCAATAGTCGTTTTACAATTGAAAAATTTCAAGCGATTCTCTAATCCTAGGTTCCAAGGAAATATCTGTTTGTAGTCGGTGGTGAACTTCTTGGCTCGCTATTAGTATTTGAAATCTTGTGGTAGAAAAATCACTGTCCAAAGCTCGTTGTTGTCTTAGTTTCGCTGCACTTCGAAAGGTCGAAAAGTGTGAAAATATAGGTCTAAGGTGAAATACAGGGTTTGGAAGCAGGAGATACACGGAAAGAGCCGATATACATGCCGATATACACTGATATACATCAGTTGTATACCAAAAACGGGTTTTGGTTAAACCCCAAAAAGTAGCAACGAATTTGGCCCAAGAAAGGCCCAAATTCAACTTTCTCTCCGTTACTCTTAAATTATTTAATTATAATTATTTATTGTTTGTTGTTTATTAACCCTAACTTTATAATTGTTCATTAACTTAGTTAATTCCCCAAAAGACGAACT

>LNC_001957

CTTCTGAAGAAGCTCATTGACAAGGAAAAATATGTCTTAGAACCTTGATGACGACACTGAAGCGCACATAAAGTGAGGCGAAGAGCTCAACACGTTTTGAGCCTTGCTTGAGGGCTTAAGCACGCCTTTGACAACACTGGCTCGAGATAGAGATGTGGCCATATGGAGGTTGCAACCCACTTATTATGTACTAAATTCAAGGTGATTTTAGAATTTACTGGTTCTGCACTCTGTCCCTCTACACCATTCACTGGCAAGTGTCGGCAAGGTGCTATTTCCTTGAACCTTATGTTTTCAGCTATGATCAAGTCTTGGAGTTTAGTAGCTAAAAAC

>LNC_001958

AAGAATAGTCCCTTGTTAAGTCTTGCATTGAACAGAAAAAGAAGAGAAAAATCAGGACTTTTTTCAAGAATAGTTTGTGTGATGAACATTGTTAATGAGTAAGTGTTGTTCCACAACCCAACAAAGGAGTGAAGATCAACAACCACACACCATCTTTGTTTTTTTCTAGCTTCCCTCCAAAACATATTCATCCATGAAAAAAACCAATAATTGATGATAATAATCTCAAACTTGGAATGAGTCTTTTCAATATATAAGACGAAAGAAGTAAAGTTGCTTTCTTGGTAATTACATGATTTTGATGCACAGGATTGTTTATGAATTCTGGAGTTGCTGAATTTTGCAAGTTGTCAGCAGATTTTTATGTTTGTTGCTGAATCTGGA

>LNC_001959

CCTCCCCATCTTTGTCCAAAGTCTAAAAATATCCTTCTGATTTTTTAACTTGTTGTGTAACTTAGTTATTCACTTGAACGTTAATATTCAACAGTGATATGAACATTGTTTTGTATTATGCCATTGTTTAACATATTTGCTTATGTTGCCTAGTTTAGTTATTTAAATGTGGTTATTTTCTTCCTTTGCCTAGTTTAGTCATACATACATATACGTGGGCATGTGCTTTGGAAGTTCCATGTGGCTTGATTTTCATTACCTTAGTATAAATTTTATTACCTCCTATTTTGTGTTGAATTTAGTGACCTTTTGGTCTTAATTTGGTCTAACAGTGACTTTAGTAATATTTGCATCTCAAAAAATATTTAAGCCTTTTTTTTTTTTTTTAAAGTTACGTCTTAATTGTTCCCTTAATTCGTACACTAATTTTTATACTTGTTTTTCGTTGCATATGAAATTCCATTCGAGTTCACTTTGGACTCCACTCTTGCATATCCTTGAGTCGTTTCGAGTCATCCTCATATATTCGGTGAAAAAGAAGCTGGAACAGGCCCCGGAAGTCCAAAAAGCAGGCTGTATACACGTTATACACATTCAATATACACTATATGCAGATGTATACACTTATATGCAGGTCAAAAATGCAAAAATACAGGGCTAAGGCAAAATATAGGTGTTCGGAAGCAAGAGATACATGAAAAGAGCCTATTCACACGCTGATATACACTGATATACATCAGCCGTATACCAAAAACGGGAATTGGGTTAAACCCAAAAATTCCAACAAATTTGGCCCAAAAGGAGGCCCAAATTCAATTTCTCTCTTACTCTTTAATTATTTAATTGTGATTATTTATTGTTTGTCATTTAACTCTAACTTTATAATTTTTAATTAACTTAATTAGATTCCCC

>LNC_001960

GTCTAAAAATATCCTTCTGATTTTTTAACTTGTTGTGTAACTTAGTTATTCACTTGAACGTTAATATTCAACAGTGATATGAACATTGTTTTGTATTATGCCATTGTTTAACATATTTGCTTATGTTGCCTAGTTTAGTTATTTAAATGTGGTTATTTTCTTCCTTTGCCTATAATGAGTTAATTGGCTATTGTCTTCAATTTTGATCATTTGATATGACAAACCGGCAACCTTTAAGTCTCCATAGATAAAGCTAGTTTCGTATTGTATGAGGTGATGTTAACTCTTTAATGTATCTTTGGATTGCATCAATTATTGCTTATAAATTTTAGTCATATATACATATATGTGGGCATGTGCTTTGGAAGTTCCATGTGGCTTGATTTTCATTACCTTAGTATAAATTTTATTACCTCCTATTTTGTGTTGAATTTAGTGACCTTTTGGTCTTAATTTGGTCTAAAAGTGACTGTAGTAATATTTGCCTCTCAAAAAGTATTTAAGCCACTTTCATCTTGATTTTAAGTTACGTCTTAATTGTTCCCTTAATTCGTACACTAATTTTTATACTCGCTTTTCGTTGCATATGAAATTCCATTCGAGTTCACTTTGGACTCCACTCTTGCATATCCTTGAGTCGTTTCGAGTCATCCTCATATATTCGGTGAAAAAGAAGCTGAAACAGGCCCCGGAAGTCCAAAAAGCAGGCTGTATACACGTTATATACATTCAATGTACGCTATATGCAGCTGTATACACTCATATACAGGTCAAAAACGTAAAAGTACAGGGCTAAGGTGAAATATAGATGTTCGGAAGCAAAAGATACGTGAAAAGAGCCTATGCATACGCCGATATACACTGATATACATCAAGTGTATACAAAAACGGGAATTGGGTTAAGCCCAATAGTGGCAGCAAATTTGGCCCAAAAAGGGCCCAAATTCATTTCTCCTTTACTTTTAAATTATTTAATTGTCTTTATTTATTGTTTGTTTTTATTATCATTAACTCTAACTTTAGAATTGTTAATTAACTTAATCGAATTCCTCAAAAGACAAACTATTTCTCTGTGTTGTTTTATTTCTAACAAATTTCATTTTTATCAAATCTTGC

>LNC_001961

CTGCTGCTGGTTTCCAAACCATTTCGTTGATAGCCATTGAACTGGAGCTGAAGTTTTATGTATTAGTTGTTGCCACTTTAGAAATATAAGAAAAAAACTAGCTCGAGAAGTTGAAATCCCCGAGTCAGGGATTTGTTCCGAATGAGTACTGTTTCAATCGATTTTTGTTGCTGATTCCAGTCTGTTTCGTGGTTACCCATTGAGTTTGGAGAGGAACAATTTGAAGAAGAAGAAAATTCGCCGTTAAAGCTCCATCGAAGCTCAAGTTCGAAAAATTGGATTGCCGCAAACAAGCTTCGTTTCGGCTTTAATATTATTGCAAAAGATTAGGAACGTCTTGGAGAGCTCATATCTCAAATTTGGGATAATTTGGTGGAGGATTCGGCCGACTTTCAGCTGC

>LNC_001962

CTTTTTGTTCCTAGCTTTAAGGTAAAGCAGGTAAGTGAAATTTTTATATGACCTTTTCCTTAAAGATGACATCTTCCATCAAATCATGCTACACTGTCGCTGCACAGTCAACTGCACAATGTCATATAGGTTGCTAGTACTTCTGCTATTATAAACTCTGCTTGTGCACCTAACATTATTTATTTCATAGGTAAGCATTGTGCCTATCTTTATAACAGTGACTTTGGAGCCATTTGTCC

>LNC_001963

GAATACCAAGCTTTCCCGAGCACGTGCCCCATTTTTGTTGCTGATATCATGCCTTGCTAACTAATGTCTTGTTAACGACTCAACTAGCTTTCTGATGGAGCAGACATTGAGTGATTTGACACATTTGGTCCCTGTAGGTCTTATACTATCATTTCTCCTCAACTTGGCAAAGTTTGCCACCTTTGTTATCTTGTCTCTTTTGAGTTCAACTGCCATCATGCAAAAGAACCGCTTGAATTGAAAGGCTGTTGTCTCACCTAATTAGTTATAGGAAATGGTCGAGGAACACCTTAAAGGATTTACAAGGG

>LNC_001964

CGAGTGAATTGATTGAGGTTGTTTCTCTCTATATTTTGTACTCTCATATTTATAGTGGATTGCTCATCTCCTTTGTGGACGTAGGTCGATTGACCGAACCACGTTAAATCTGTCTCTTTTGGTGTGTTTCTCGTTTGTCTTCTTACTCGTGGTCTTTCGAGGTTTGCTTTGCTAGCTTCCGCATTACACCTGCTTATTTCGATC

>LNC_001965

GGTTCTGGTTTGATGTTCTTCGTTCTTGGGTTCTTTTATGCAATTCTCTTTAGTTATAGTTATTCTGTGTACTCGTCGGGCTTATGGGGGTCCGTTCGGGTTTTTATTTAACTTGCGCACGAGTGTACCTTCTGGGCTTATGGGGGCCCAGTTAGGTGTAGTTAGCTTATAGATTACTTTAGTTAGTTCTTTCTACACTCGTGTGCATTCCATTGTTAGTTAGATTTTAGTTCTTGACCCTCGGTTTGTATTATTCCTTCTTTTCATATTGCCTTTACTTTTCAAGTTCAGTCGGCCTATGATGCCTACTGG

>LNC_001966

GTATTTTTGTTGCTGGTGACAAATTACATTTTCTATACAAATGTTCAGGATGCTCATGATCGGTGTTAGCTGTGATCATCTTAAAGATGAAAGGAATCCACATGTCTAAGTGTAGCAGTTGAAGTTGATTCCAGGTCGAGATTTGTTATGCCGAGTAGAGATTTCAATATAAGATCAAATCTCAATGAGTTCCAAGGGTAGTGGTTTGACCATAAACATCTTACTGACTTGATTTTTTATTCATCTTAAAATGGACAAATAATATTAAATTTCCCAATCAGTAAATACTTTGTTCTAGTGTCTTTATTTGCTTTATGGTCTTGAAAGTGTTCAAGTACATAGCACACACCTATACAGTGCATGACTTCATGCTTATACTTGTGAAGTTGTAGCTGATATTTGTAGATCTGTTGTTGCTGTTAGCTGATTGCTTATCAAACTGATCCTGCTTTTTGGTGTTGATGGTTTGCTTTGATGTGTTTAATTGGATCCCTCTGCATGTGCCTTCTGAAGGAGCTTAAGCTGTGTGTTTATGTTGTACCGCGTCTCCTCTGATATGCACCAATCTGGTTTCTTTTTCATCGAGATGTGTGCACATTATCAATTTGGAGATTTGATCGATTCCTGTGAGATGATGTTAATTTATCAAACATAACCTTCTTTGACTCCATCTTTAAGTGTCTGGATGCGTGTATACCCACCCTCTTCATCATCTACATTTTTGCAATTAGTTTATAGAAGTTACATAAGAACTTCCCAATGGCA

>LNC_001967

CAACTATTCCTTCGAATCCGAGTAAAAATCCTGATGATGTGATGTACCCTATTGGCCCTGATTCTGCTGCTCTTGCACCTTCTGCTTTTTCACCTGTTTCCTCTGCTGTTTGTTGTGTTTCTCTTGCAACATATCTGAACATTGCAGTACTGCTGCTAGTAGTCAACTTATTTTGTTCGAGTTAGACATCTGGTATTCGAAATACACTGATTCAATTGGAGGGGGTATGAGCGCTCCCTACCAAAGTAGTCGGCTTAATTATTTGTTATTTGAGAAGTTGTATAATATCATCAACTGCGTTGTTCAGCACTATAACATGCTGTTTCAATCTTGTATAAGTTGATTCAGTCTGAAATTCATTTCTTTTACAAG

>LNC_001968

GAAAAAGAGAAGCAGCTTCCACCATTGAAATCGATTGTTAATCATAAGAGTTTATGAGTTTTATAAATTATTTATTTTTTATTTGTTATTAAAAATGCCTCTTCTTTCAGCTACAAGGGCAGAGATCTGAGATCGATTTCTTGTAGCTTTGTTCTGGAGGCATATATTTATTTCAATTCGAAGGTTTATTTATGCCCAGATTTTGTTTTTCTGGGTTACTTCGTCTTGGTACATTTGTTCTTTGAACTATAACATTTTTATTTGCTATTTCTAAGCTACCGCCGTAGAAAATAAT

>LNC_001969

GCTTTTGCTCCTAACATTGCTTCTTAAAAGCTCATTAATGGCTGGAAATTTATTTGTTCATTAGGAATTGAAATGGGTTTTGGTGAATTCATTACTTTTTTTTTTATACATTTGGTATGTAAGATCAGATTAAATGGGTTATGGGTCGGATTTAATTGATAAATTGTTTTTAAAGGGATAATTCGGGTCGAAGTTGAGAATTTGGAATTGATTTGGGGAAATCGTTAATTTGGAGGGTACATATGATGAGTTTGTAGACGGCAGGGGTAAAACTAACCTCGTTTTAACGGTAAGGGTTTAGTCAACCAATAAAACAAAGTTGAGGGGTATTTTTGACCATTTTCCCTTTAT

>LNC_001970

GTGAATTAGACATGATTCCTTTGTCCATAATAAGAAGTGGTGGGTCAATTTTCATAAATTTCATCGTAAATGTGAAATACTTATACAAATAAAAATGCGGGAGAGATAAAAAAGATGCAGGGTCGTTTGTCTGCTTGGCTAGTCAAGCATGGGCTAATTCATAGATCTTTGGGCTTTGATTACCAAGGAATTGAGACTTTACAAATAAAGCTC

>LNC_001971

GGGGAGGACTGCAGAATAGTAGTTAATGTATTGTTATTACGTAAATAAATTTTCTATAGTGTCTGGATCACATAAGTCAGAGCTCCTAAATTTTGAAGAAAGAAGAATCAATTTTCTTCCTTTCTTGATTACCTTCTTTGTGTATGTAGAAGACTGAATCCATTCAATTCCTAAGAAGGATTACTAATTCTTTTAACTTTTCAAAATATCCTTCATCTCAACCACTTATGTGTCATCTTTTCGACCTTGCTTTTGTAGGAGCAAGTGAGAAAGATTGAAAGGTAGCACCAGTGTATTAATTTGTTTTCAATAGCCATGTTGATCCCCATGTGGTGGTGCTTTTGTTGCTCCTTATTACAGTAGTGGCCTCTGAAGGATCAGAACCACTATTTTTTAGCTGGTAGTCCTGTCTAACCTAATTGAGATATATAGAGGATATGTGAAAATTGAGATAGGGGAAATAAAAAGGA

>LNC_001972

CACACACTGTAGTTCCCGTTGTTCGTATCAGCTTGCGCACACATCCCACCAACACATGTACCGGGTCATACTATCCACCAAGGTTGGGACAGATGGAAAGAAAATGATGTTTTTGTTTTTATCTCCGCTGAGAATTGAATAGTTCTGGACCCACTTCAGTCACACCTTGGGTGCAAAAAGTTGAGTTTTTCCTGGTTGTACTGTAATTTGTATAGTAAAGAGAAACAAAAGCTTCAATGGGAAAAAGAAAGACGAAGGTGAAGACAAAGTCTTTTGTGACTTTGTGAGCATTCGCTAGACAACAAGTCTAAGTCCCTAGAGATGATTTTGGACCTCAAGAATTCCAAGTCTTCAGTCAAAAGTTCATGTTCTCATACTGTCATGTCAGCTTGAAAATCTTGATTAATACACAAATTTTCAAGATTTTTACTGTCCATATCGAAGAAATCAATGGACTGATACTCATCAGGAAGGTTGTTGTTGATTGCTACTTGAAGGAGCTGGTTATTAACATCATCAATGTTGTTAGAATGCTGCAGATGCTCGTGGCTCGGGTTAGTAAAAAACTTGCCATGATCACTTCCACAGCTGTCCCATAGCTCGAGCCAGCATTATC

>LNC_001973

TAAAAACATTATAACTGGGAATATATTTTTTATCCAAAACTTAAAAAGAAGCAATTAATGCAAATGATCAAATTTAGAGTTGCCCAAAATGTTGCAACTCAGAAGCACAAACGGCAAACCTCCCGTCGGCGTCGAATATCTCTTCGTTTTGCCCAATCGCCATATCCAGCAGTAGTAATATGAACTTTCTGATCAATGTCGACAATGAATCGGAACTCAGAAGCACAATTTTCTTCCTTTTGCTCAATCGCCAAATCCAACAATAGGAGTATGAACAAGGCCTTCACATTCTGATCAAAGTAAGTTCAATTTCACTTGCATTATACATCTTCATTTTCAGATTCTCTCCATATATGTGTGTTCGAGTGCTTATACCTTCTTGACCTCAATGAATTTATGAGTCTTATGAGTTATGTTGGTGAAGTTCAATGAATTTTATTTGAAATTCTTGCGGAAACGCCTGGGGTTTACCTTTGCTCAGCTTAATTTTGGTTTTCGTTAATTTCACCCTAAGATATTGATTATACTTTGTAATTTCTCTGCATATTTCTAAGGGGATGGTATAGTACTGAAGAAGCTATAACTCTAATTATTATGTCATGACTCTAATTTCTAAGGGGGCAGTATAGTGCTGAAGAAGTTATAACTCTAATTTTGTTAAATTGTTCGAATTGTGTAATCATCAGTTATTGACATTGGGAAGCGACCCAAAGTGATATTCAATTCGTGACTTCAAATTTGAATTGCTTTCCTGTTAACCGTGTAAAAGAAGCTTTTGGTAATTGTGCTCCAAGATGCACTATAAATTGCTATTTTCCATTTGGTTACTATTGTATTTAGAGAGTTGATTTCCCACATAATCACTCAACTAATTATTATTATCTCATAAAGTCACATCTCTTCTTGATTATTCCAATGTTTTTCAATAAAGAAAATGACTTTCTGAGATAATAAAATTTAGTTATGTGGCCATATTAGTTCGAACTCTCTACACTAATGACTATTCAACTTTACAGTCACGGATTGCCTTCTAATAATGGGCCTTTGATATTAGAGAAGCTTTGCTATTATTGATAATCTGATACTAGTTGTGAGTGTTAAACATGCATACTATCTGTTTGATGGAAGCTAAGGTGATAATGCCAGCTCCAAGAATCAATATGCTCTATTAACAACTGAATCCTCTGCAGTACTATAGTTACCA

>LNC_001974

TGGCGTAACTGGTAAATTTGCCTCCATGTGACTGGAGGTTATATGAGCTATGTATATTACATTACCCTGTTGATTTTTTGGTGCTTCCTGATTTTATATGCTTCTTCACTTGTTTGCCAGAAATTAACTTAGTAGGCAAGGAAGCATGTTAACTGTAGACATGCTTGGTATATTGAGTCTGAGATGCCTATTGGTACTTCTTGACTGCACTATGCAACTTCTAGAAAGATTGGCTTTGTTCTTCAAGTCCAGAATAAATTGACTGGATATTATCTGCATTTTATTTACTGTGATCAGTGTTATCGTTGCTGCCTTGGATGATTGTGGATTGACTAGAATATATGTTGGTTAACTGCGTATTGCGGCTCTCCTCTTTAATCCGGCTGAAAATAGAACAGTAGTAGTATGAGGGTCTGACTAGGGTTGAGTATACACAAATATCACCCCTACCTTGTAAGGTAAAGAGACTGTTCTAATAGACCTTCGGCTCAA

>LNC_001975

CTCAAATGAAACACTCATAATGCATCCAAAGCTTGTAAACTCTGCTTCACTACTTGTACTCTGTCTTTTTCACTCTTCCCAATTCACCTCCCTTACAGCTAAGCTTCTTCCTTCAATTGAATTCTCCAGTTCATAGCCCACCAATCCAACAAACAACTCATTTCTCTTAGATCTCTCATTGGGATTTTCTCACAAACAGCTCTTCTTGGTTCTTTACCCCACCCCCCAAACCCTCCCCCCA

>LNC_001976

GTTGATCTAAGTCTCTTATTCTGTTTTTCTGATTTGACTTTTCTTCCTTTCTGGTTGTCCCTAATATTATAAAGTTACTTTGACTCTGTTCTGCAAGAAGAACAGTGCTACTTCCAATACAAAAACTGCTATTTTAGCATCTATGTTTGCCTCATGTAGTTGGTCTGATCTCTTTTGCTGTGAAGTAACTATTCACTGTCATATGGAAGGAGTTTGCTGTGAGAAGAGTATAAAGCATCAAATGGAAAACTATACTTCATTCTGGTCCTTGTCTTGATATTTTCTTTCTTCAAAAGTCGTATCTAGGGAATAGGGATCATATATTCTTTGTTTGTCCCTTCTAACCTGTGTAGTTTTCTATCTAAATTATACAATAAACCAGTGAGAATCATCCCAGATACCATTCATAAGACTTAGCTTTCTGCTGCATAGGGTTATCTCTTAGAAATTTTAACCTTCCTGTAACCATTCACAATATTATTTGTTCAACCTTTTCAATATTATGGAGCTCAATGTTATATGAGACTCACTTCTTTCAAAAGCTCACCTTCAAAACTCTACAGAAGTTCTAAGACACCCATCCTTTTCTATTCTTACCTATTTACCACATTTTTCTTGTGTCTTAATAGTGG

>LNC_001977

AAAAAACATCATCAAAATCTACAACTTTTTCTTTTCTTACATCCCACATATTTATAATGTCTAGTAATTCCATAGTCCCAAAAAAATATTTTCCACAAGGAAACAAGAAATTTAGTACTATTTCTAGTTATGGAAATGATAATATTGAAGATGATTTTCAAGAAGAAATTATTCAAGAGGAAGATGAAGAAAGATGTGTAAGTAGCAAAATCTACTTGAAGTCAACAGGGCCAATTATGAACAAAGATGTAGCACTAAGAAGAATACGTCACAGGAAGCAACTCAACAAATTCAAATCTTTTCTTGCTTTTCCTAATTTTAAAACTTCTTCCAAAAATGATAGTAATATTAGTACTAATTCTCAAATCATTAGATGGGTCGATGATGCCTTTGCTGCACCTTAGTCTGATTAATTCAGATTCGCGAGCATCCTTATGAAAGGAGGAGTAAAGTGCTTCAAATGTAGCGTTGTAAATATATGACTTGGTAGTACTTCATTTGTATTTATTTTTTTTCGTTTTTTTTTTGTAAGTGTAGATTTATGAACAAATTTTAATTTGTTCTTCTTTGTTGTATCAAATAATGAAGTCGTGGCT

>LNC_001978

TCTCTTTCCTCTACGTCCAACATCCTCTTTTCTCCAGATCTCTTCTAAGGAGTTTATCTTCTTCAATTTTCATCTTCAGATCTGTGTAGATCGTTCCTTGATCTACGTTTTGGATATGGATTTGCCGTTGTTCTGTATCGGAGCAGCGATTAGCCGGACGAATGGCGTGACTGTTCGATGTTTGTCGACTCTGGCCGACGGTTGACGAAGAGATGGTAAATCTGATGCTTTTTTGTGCTGGTTTGAGGTAGATGAGCATCGTGGAGCATCAGATCTGTTCGATCTTCATAGATTTGTGGAGGTTGGAGCTAACGTTTCAGCAACCGAGGTGTTATCCCCGCGATTGTTCGCAAGAGATTTATTGTCTTCTTACATGTTGCAG

>LNC_001979

AGCTTTTGGGGTGTGAGTTAGGCCCAAGACCTAATTTAACATGGTATCAGAGCAGGGCTCATCTCACCCGATGTTGGGGACCCCAAAATCAAAATTGCCCACGCACCAGATGCTAAGCACTGGGCGTGAGGTGGGGTGTTAAAGAATGACAAAAGTCCCACATCGGTGGTTAATGAGATGGGTGGACTCCTTATAAGGCTTGGGCAATCCTCCTCCCTTTGAG

>LNC_001980

GTTTGTTTTCTCATCATCAGGCGTGCTGCCTAGGGTTTGCTTCTTTAATTTCAACAAGATTGTATTGTTGTCTCTAATGGCTTTCCAAGTCGGAAGGGCTTTGCTGGGAGCATCAACAACACAAATCAGGGGATACTGCCAGAAAGTTTCATCCACTGCCGAACTCGATCGCTCATTTCGTCGTACTTGTATTATGTTTGGTGCTGGAGCCGTAACGGCTTGTAATATCCTCTATTGCACGTTTCACACTAAACTGAATGAAGTCTGGGAACAGGAGCGTGAGCGTGATCAGCAATGGAAGTGGGACACTCGTTATTGAATTGTTTTTAGTAGTATGTGTGTATTTATCAGCCTTTTGGTTAAGATCCAACTGTAATGTGTACTTATCTATCTATGAAGAAGTTAAGTTGATGTTCTACTTACAAATTTCCTGAAGTTTATTGGAATGTGGAAGGAACTGAATTTCGTTGTCATCACAATATGTTTTAGTCTTGTGGTCATCAG

>LNC_001981

CTCATCATCAGGCCTGCTGCCTAGGGTTTGGTTCTTTAATTTCAACAAGATTGTATTGTTGTCCGCTAATGGCTTTCCTATTCGGAAGGGCTGGTAGGGCTTTGCTGGGAGCATCAACAACACAAATCAGGGGATACTGCCAGAAAGTTTCATCCACTGCCGAACTCGATCGCTCATTTCGTCGGACTAGTATTATGTTTGGTGCTGGGGCCGTAACGGCTTGTAATATCCTCTATTTCACGGTTCACACTAAACTGAATGAAATCCGGGAACAGGAGCGTGAGCGTGATAAGCAATGGAAGTGGGACACTCGTTATTGAATTGTTTTTAGTAGTATGTGTGTATTTATCAGC

>LNC_001982

CGTGCTGCCTAGGGTTTGCTTCTTTAATTTCAACAAGATTGTATTGTTGTCTCTAATGGCTTTCCAAGTCGGAAGGGCTTTGCTGGGAGCATCAACAACACAAATCAGGGGATACTGCCAGAAAGTTTCATCCACTGCCGAACTCGATCGCTCATTTCGTCGTACTTGTATTATGTTTGGTGCTGGAGCCGTAACGGCTTGTAATATCCTCTATTGCACGTTTCACACTAAACTGAATGAAGTCTGGGAACAGGAGCGTGAGCGTGATAAGCAATGGAAGTGGGACACTCGTTATTGAATTGTTTTTAGTAGTATGTGTGTATTTATCAGC

>LNC_001983

TTAAGAAATATAGCTAAAAGTGCTAGCATAAGGAAGAGATTCTATGGATGGAAAGAGGCCCTTCCTTGCTTTCAAGGTTTCGGCATTAAAAGTCAAAGGATTCTTTGTTTAAGTTTAATAATAAAGTAAAGGAAATCGGCCTGCTCTTCTCTCTTTTCTCTATTGAACTATGGAAAGCACTTTATAGTAGCGACACGGATTCACCACAACTTATGACTTCGCCAATGGGGCAAACGAAGGCTAACCCGTTCTGGTTGTTATGACTGGCAAGAATCGGTAGGTTCTTTCAGTGCGCTTTGGTCCTTCTTACAGAGACTATCCTTTGTATAAGTTATGATCCATCCAAGGAATGAGCTTCCCCAGAGTAAAATCCCGTGGACTACAAGGAAAAGATAGAATCTCGCCCGGCAACAGCAGCTGATTCAATAGCAGATGGTCTTGGAAAGAACCTTTCATTCGATGCTTCTGATTCAACTCCCAGACAACCAAGGAAGTATGCGCGAAGATAGGGTTAGCCCCTGCGGCAAGCATTATCCGGGAACGAGAAT

>LNC_001984

CCCACACCAACCAAAAAGGTGTAAAAGGAACTACCTCGCACCATTTTGTGACTTTTGCTTATGATAGGGACAATATAATGAACAACTCTCCCTATGCTGTGCTTTATTTCATGAGGAAGATTAACTATGCACAAGCAAACTGCAGTCTCTTCTAAATATGATACTGCAACATGTATATTGTTTTTATTATGTTCCATTAGTTAGTATGTGCCAATAGTTCAACTCGACATCATACTGATGTGTATTGTACATATTCTGAATATTTCTAATAGTACATATTATAATATATGCTATTATCTATCTTCTATAAACACCCTAACCCTTAATTCATTAACATAACAACACTTTTACAAGCATAATACTTTGACTATTGGGCCCGTGCTTGCCACGGGCCTCCCTACCCTAG

>LNC_001985

GGCAAAGTGTGGAGCTACTCGGAATTCCGGTAGAGATGGCTTCGTCGGCGAGTGTGCTACGGTGGCTTAAACCCTATCTTTGTCAATTTTGTTTGGGCATTTGGGGTTAGCAGCTGCAGCTGGCTTCTAAGCTTGTAGGAGCTCTTAGCTTACCAGAATCAACAAATGAACTTCCGGCCACAACTAGTACAATGAAACAACGATACAGGAAAATAGATTTGAAATCTGAAACTAGAAGTCTAAAAGGCGTTCAGAAGATAGTAGAAGGAAATGAGATTATTCAGAATCACAAATAACTTCCTACCCACATTCTCACACTTCTATACCAATTCCATAACTAACTCTTGCCTTGCTCACATGACTTTCTTTCAGCCCAAAAGTAATTGTCCAACCTTTTAGGTGGAATTACACTTCTAGTACTCCGACACGGTTTGCTAATGCAAAAATCATCACTTGTTGCATCCTCTTTATTATCCAAATTAGCAAATGTATGAACTGAGTTCACAACATTACTCCCCTTCTGAAAATCAACCTGTTGTCACTCCCCGAGCCTACACCCTGGGTGGGATCGGCACCCGGAGACCATTGCTGGCTCCAAGCGAACCCTTGGCCTGGCTTTCTTAACTCAGCGGAAACCTGACTCAACAGAATAACTCAATGCCATGCAAAGACATAAAACAACTTAACTGATAAAATCTGGCCCAAAAAGGCAACTCGAGTCTCAAAATAGAATATTTACATATATACA

>LNC_001986

AAAACAAAATGGAAGGTTCAGCTGAAAAGACACAAAATTGCAGCTGAGAAGTAGAAAGTTCACGAACACCATCTCAATACGTCCGATCAGCCATTGATGTGTCTATCAAAGCTCAAGGTAGCTGGGGAACCATCACTGGAGAGACCCCAATTTCTGATCCTTCTTCTATTGTGAGTGTGTGCTTTTCCCCCTCAAACAAGAGAAAAAACACCAGGAGCAAATTTCTGAGATGTACGAATCGTTCAGCAACTCTCGTCTGTTGTATCTCCTTCACTTCGCCACACCTACCAGCTTAGCTTGGAGATGTGCAGCTCGATTAGCAACTTCAAATGAAACCACTTGAATCATCAAGATGAAACGTGAATGCACACTAAGTATTTAGACAAGAACAAATTCTACCAGTAACTCATGTGTCTTGGCACCATACCATTTCAACACCTCAAGCTCTCACCAGGTGCATGATAAACTTACCAATCGAACATATTTCCAACATCATGAGCATAATGTTGGAACATCAAATCTCTAGAAATTGAAGGCTGGTTCGAGGTTATAGATTAAATGGAAAAAGGAAGGTCATCGGTATGTCTACTAATACTTTTACATGCTCCAAATTAATACTTTCTTCATTATTATTTTTGTCATGTTATGCTTTTCGAGAGTTAATTTGACTAATTTTCATAGCTAAATTAGATTATATTCATTCAATATTTAAAATAAAATTTTAGATATTCAAAAACTATGCCGAAAAATACTATAAATTGCAATTTTTTAAAATATCAATATGATAAAAAAAAATACATCTTAAAATATTAGTCAAAATCTATATCGTTTAAGTCTTAAAAAGAAAACTATGACAACTAAAAAGGAATGGAAGGAATAGTGATTAAGCTGGTTACCTTTCTAGCAATTACTAGTTTTACATATTTTTGTAGGGAAAAGGCATAGATTTCTCCCTGAACTTGTTTCGAAAAGTCAGTTACACGCTTAATCTATTACAGTGACCTATTACACACCTTTAATACTCAAAAGTGAATTTATTACCACCATTAGACGTGTAATACCACTCTCACATTATATGGTGTATTACACTCGCTACCACATCAGCACTACATCAGCATCATGTCATAAATTATAATTTTTTCTTTTCTTTTCTTTATTAATTAACTTTTCTTTTGTTAACTATATTTTACTCTAATTAATCCCTAATTAATTACTTTAATTCTCTAATAATTACATCTTCTTCATCACTAAATCCCACCCATTTCAATTCTCATTTCATCAGAAAAATCATATCCGACCACCTTCACTATTGATTTTGTTCTTCACCACCACTATATCACTTTCAATTCTTCTTCTTCTTTGTCACCCAACACCAAATATCACCACCACCATTATTAATATCGATTTTTTCTTCATCACCATCTTCAATTACCTCTTTAATTTTTGTCACCCACCACCTATCACCACCAAACCCATAAAGTTGCTATCACCACTAAACTCAAAAATTGATCAACAACAAAAATTTCTTTAACAAAGTCAAATAAATGACAAAGATAGAAACAATCAACCGAAACTTTTCTAGATAAACTTTAATTTCAAATTGACGCACCTATTTTTTGTTTTATTTTTTTGTCACGTCAGTTTTAGAGGGTAAGAAAATTTACTTTTGAGTAGTATAAGTGTGTAATAGGTCACTATGATAGTTTAAGCGTGTAACTGACTTTTCGGAACAAGTTCAAGGAGGAATTGATGCCTTTTCCCTATTTTTGTAACAATACTATTGGTATATTTTACATGGCAATGCTAACGCCTATTCGAATATATAGAGGCATATATAGTCATTTTGCGGGAAAAAGATTCGGTCAAGTACTACTATTCCCTCAAGTAATATTTAATTACCTCTTAGATTAATGTCATGGGGATCATAAAATAATTTATGGTTGAGATTCATTTTAAAACTTACACTTAACAAAAAGGTCAAAAATAGTAAAAGCAAAGCTAACAATATCCATTAAAATATTTGTGACCATTGACTCTTGAAAAGGTTTGTCG

>LNC_001987

TAAAAACAAAATGGAAGGTTCAGCTGAAAAGACACAAAATTGCAGCTGAGAAGTAGAAAGTTCACGAACACCATCTCAATACGTCCGATCAGCCATTGATGTGTCTATCAAAGCTCAAGGTAGCTGGGGAACCATCACTGGAGAGACCCCAATTTCTGATCCTTCTTCTATTGTGAGTGTGTGCTTTTCCCCCTCAAACAAGAGAAAAAACACCAGGAGCAAATTTCTGAGATGTACGAATCGTTCAGCAACTCTCGTCTGTTGTATCTCCTTCACTTCGCCACACCTACCAGCTTAGCTTGGAGATGTGCAGCTCGATTAGCAACTTCAAATGAAACCACTTGAATCATCAAGATGAAACGTGAATGCACACTAAGTATTTAGACAAGAACAAATTCTACCAGTAACTCATGTGTCTTGGCACCATACCATTTCAACACCTCAAGCTCTCACCAGGTGCATGATAAACTTACCAATCGAACATATTTCCAACATCATGAGCATAATGGTAACTATTCTCTTGTTCAAATACATTGTGCTCATTATTAACTCGAATTACAAGGATATTGGCAAAATATGATGATACGCTAGACTTCCCTGCTTGTCTAATTCTGATCCTGCTTTTCTAACTTGAAATTGAAATTGATAGACCTTATGTTGGACCTTCCTGCACTGAATTGTATTTTATTGAACCTACATATAGAAGTTGTATAGATTCAATTCGTTTGTCTCCTTCTTATGTGAGGAGTTCCACTTTTATCCAAGTTTAGGATTCTTCGTCCTTCCATAGGAATGTCTTGAAAATGGCTAAAGTGATAATCACCTGCAAAATCAAAAACTAGGTTAGCAAAATAATCCGCAAGAGTATTTCCTTCTCTAAAGGTGTGTTGTACACGTGCTGATAAGGATCTTCTCAAGTAGTTTATGGAATTGACCTCCAAAGTCATACGCCAAGGGACATTCCACCCTCCTTCCAAAATTTGTACCATGGCCATAGAATCTGTTTCAATAATGACATGTGATAACAGATGCTCCTTACAAAAAAAACAATCCTTCTCTAATATCCCTTGCTTCAGCTACCAAATTAGAGGTATTATGTATTTTGAAATCCTTAGCCACAACAATGAACCAGAGTAATCTCTAATACAGAAGGCTGCAGATCTAGGTCTGGGATTACCCCTAGATGCTCCATCAGTATTACATTTCACCCAATTAATAGGGGGAGGAATCCATCTCACAATATTAATGCTATAGGAATTATTGTGCCTTTGTAACTCGGTAATTATATCCGGCCAATTGTTTCTTCCAAAATCTCTTTTGAATCTGATTTTAATCACCTTCATAATAATGTCATTAATCTCCCAAATAACCTTACTTTCTGAGAATGACCCACCATGTAAAATAGTGTTTCTTCTCTTCCACAGGCACCACAAAATGATAATGGGCACAACTTGAAAAATCACTTGAATTCTATAGATACCTTCTGAAGACCACCACCTTCTCATAGTCTGTTTCAAATTAAGCATAGGTCCTAGGAGACCAGCAACTCTGAAATAATGATTCCAAATTCTGGCAGCAACCTCACCTGTTAGAAAAAGATGTTCTATAGTCTCCTCTACTGGAATAGAACAACACCTGCAATTCTGAGATATGGTAGGATTCCAAAAGTGCATTAAACTTGCTACTGGAACTTTACCATTCCAAATTCTCCTAGCTAGAAAGGATAATTTGAATGGAAATCCTTTAATCCATAATAGTTTCAAGTCCTCATTCTTCTCAGCTTTTTGTCTCAACAACTCTCAAGCACTTTTAACACTAAACTTAATTCCGTGTGGCCTGTACTCGACTTAGTCCACCAAGGTTTATCACCCTGATCACTTGGCTGCACAAAAAACATGTTAAGCTTGATATGATCCACAACAAACTCAGGTAAAGCATCAAAATCCCAACCCTCTTCTGTTAAGATTCTCCAACGTCTCTCATAGGATGACAAGTATGCACATTAGACTGGTGCAAGTATAAAGGACCAAGATTGGTCCAGTTGTCATACCAAATATTAGAAGTACCCCCTCTAGGTTCCCACCATAAGTGATGTTCTAAATCTTTTCTATTCTCCAACATGTGTTTCCAAACCTGAGAACCCCCTTTAAAAATTTGAACCATTTCTCGATTTATGTGTGTCATCCTTGCATAGGGGCCATGCTAATCTTCTCTGTATCGTTCCAATTTTATCGAAATAGAGAAAATTCAGTTGGGAGCGTCACCCCCAAATGGGCCCTTGCAGTGCGCAATGCTCCAATGTGGACTCCGAACACCGGGTGAAAAACCCATAAAAAGGAGAGATTATTAAAATATTCTTGATAGAACTAACTATTAGTAAGTTTGGTTTTTGAAGAAATTAATTCTTATTATCACTTAAACTGAATAGAAAAATAGTTGACTATTTAATTTTTTTAATATAAATATAAAATTGTAAGAACATGGTATGTGAGCTATTGACCAACTTAACCTTTTGTCTTTTTTCTCTGTTTGTATGGCTATATAGATTCAAGGTAGCCTTAATTAGCATTTAAGTTTTATTGTGAGCTCTCTCAGAATATATCACATTCATCATTTCCTTGTTAATTTTCATGGTTTTAACAGCTTGGAAGTAAAGAAAAAGGTAAACTATTTTTTCTTTCTATTTCATTTTTTTACATGTGATATTTGAATTCAAAGGCAGAGAGAGTAATATTCCCTTTTTTGTTTTTATTTGTGGATTACATACATACATAAGAAACAGCAAAATGGTTTATATTTTTGACTTGATAATTATAGAAGGTATATGTTGGATATATAGAATAATCTGTAGGAGTTTCTATCATTTTTGCTTACAATCTTAAGGTTGAAGCCGAGAGCATGTTTATCATCCTAGCAAGTGGGCTACCTACTGGAGCTTCAACCAAGTACTACCGGTCAATTCCACTTTGGGGCTATCCCTGACTTTACCATTATTATAGGGGTATGGGGTTCGAGACAAAGAAAGATCAAATGTACTTTAGTAAGAGTATAATGGATATAGTGAGTCTACTTTCTTACTGATACAATCTCCCGCTGTGAAGTAGGAGATTTTCACCGACAGAGCGCAATTGTCCATAATTAAGCATCAATATATAGTAGTTGAAAGAATTTATTTCCTAGCATCAAAATTAGTATCCGAC

>LNC_001988

GCCATTTCTAGCCAAGAACCGTCCTTGCAGCTGCGAGATGAGACCACGTCGCACCTGAGGACGAATAAATTGATCTCGAGCGTCCATCTCGCCCTTTCCTCTTCGGGAATGGGCTGAATTTGCAGAAAAATTCATTCGAACTCGAGATAGCTGCTCAGATTTCTGTCCAGACTCCCTATTTGTTGTCCGGGGTCAAGTTCGTGTAGTGGTGAAGTCGTTGCTAGCTCTTGGTTCCTGTTCGCTGCCCGAAAAAAGGTAACACTACTCTCTCCCAATAACATTTTCTTCATCTCTTTCCTTATCTCAATATTTGATGTTGGAAGATCTCTGTTCCGAGGATGGCCTACTAATATATGTAGTTGGGTTTATGGTGATGTTGATGCGATGCATATACTCCTATGTGATTTCGTGTCTTGGTTAGGTGAGATATTAGTAAAGCTATCTTGGGATATATATTGATCGTTATATTCGCTAACTTGATTTTGAGTCTTTAATTAGCGGAATCCCTGCCTCACGTTGCTTGAGAAGGTTAGATTCACATTTGGTTTAAATGATGAGTGAATAGGTTGCGTCTTTTGGGCCTCGCACAGTTTGTTCTTATTTTTGTTCATATGATATAATGTTAGTCGTTAATTTGATGTCCATACCTATTTTGGCTTAACTCGTGATGTTGTGGTCATAGCTCCGTCTTTGCAAAGTTTCGGCTTAGCTAGAGTGATCCTTGCCTTTTCCAGAAAACAATTTTCCTGCCTTTGTTTAAATCTGTAGATATTATTTCTAGTTCTTACTTGTGGATTAGTCGAGTTATTCATTTGGTGTCATTGTCCAACCATGTTGTTTTCTACGGACATTATCTATGCTAGTTTATGCTATTGTTTAGCATATTTAGTTTGAATAGCGATGTCATTTTGCTTAGGCCTTTAGATTGTAGCCATTTTCCTCTTTTTGTTTCAAGTGAGTTAGCTTGACCCTTTAGCATGATAAGTTGTTAGCTTGGGGTCTCCATGGATAAACCGGGTATCGATTATTGCATAAAGTGTGATGTTTGTCTAGTGTGCAACATGCCTCCGCTCGAATTTTCTTTATGTTCATTACATTTAAGTAGGCTATCTCAATATTACGATTGCATAAAGCTCGATGTCACATTTTATGTTAGATTTTGTCGTTTAATTGCTCAGATTGATTTAAGTCGAGTATTCCGTATATTTCATTGTCAACCTATCTTCGGACGTGTCAATTTATGTCTAGTTTTGGATTATTTTCTCTTTTCCAGCATGTTGCCAGTAGTCAAGTATAGCATGAAGTCCATTTGATTGTTTCTTTGCCCCACTCATTTTTATAGATTTAATTTAAATGCATTTAGCTGCAATACTTGTTTGTTTTCATGTTCATTAAATTGGTCCAAGATTCTGTCCATATTGTGTGTTGCGGTAGTGCTTCCTGGGCTTATTTTCTAATATTTATTTGAAAGGCATGTGCAAGTTTGCATTTGGTCGAACGGAAAATAAAAGCAATATGGGTTTCCTTTCTGTTTAAATCAGTCGAGGTATTAAGTTTTTTTTAGGGTGATTATTCTCGATTGTTTTGTTTCAGCTAACCCTTTTATTTATTATTTGATTCCCCTTTTGAGATTTGGAATGGTCAGATGTCTTGGCGCATATTTTTGTCTCTTTTAAATTTCCTTTAGTCAGCGTCACCAATCCAAATGCATTACTTTCTTTATTTTTTTTATTTTTATTTTTATTTTTTTTAACTTGTTTAACTTTGCTCGGACTCTTAAGGTCTGTAAGATCGTCCCCTTTGAATACTTATTCATTGTTTTTGCCCTCTTTCTTGTATGTGAAATCTGGCCAAGTTCACCATGAACTCATCTTCCTCCTTTGCATTTCGAGAAAGCCATAAAGGCTTATATCACTTCCTTCCAAGTCAGCCAAATCATAAATCGGTGAACAGGTCCCACGGTCGAAAGAAGAAGCAACAGATCGACAAATCGAGAAAAATTAAAAATTTGGGCCAAAGGCCCACCCTTGATTCAAAAAATTGTATTTTGTTGTTTTGGGCCTAAGCCCACTTATTTGCTATACTTGTTAGGCTAGGGCAGGTGCAGGTGACTCGAATGGGCCAAAAGCCCAAAACGAAAATGGGCCCAAGTATTGGTCCAGGCGAACAGAAAAACCAAACTTGGGCCCGAGTCTCTTTCCCTCTCTATTTTTGTGTTTGTGTGCTTGCTAATGTTTGCTGCTAATCTTGAGGTCGAAAAGTTCAAATCCCCGAAAGATGCTCATTTCGTTTTTAACAACTTAACTAAACCGGTCGTCTTTCAAAAGACTTAAAAGAATAAATTTTGCGAATTGTCTCACTTAGATGGTAGTTCCATATTTCTCTTTCCCCCTTTTTAAAATAGTTCTAACTATAGAATAATTCAATTTTGCAATTTAAGTCAAGTTAAAATTATTTCCCCCCTTTTTAAAATAGTTCTAACTATAGAATAATTCAATTTTGCAATTTAAGTCAAGTTAAAA

>LNC_001989

TTATAAGGTAGGAAGAATAACTTGAATAACTTATCTCATTACTCAACAAATACAATGATATAAATACATAAGGAGACTAAGTTAACTATGGTAACTAGATAGCCAAATCCCTATAAATCAGCTATGCTAATATATACACAATCACTAATATATACACAATCATATACAAATCTGTAGTGATTCTGTAACACTCCCCCTCAAGCTGGAGAATAGATATTGATCATGCCCAGCTTGTTACAAAGATAATCAACTCGAGTTCCAGTCAACGCTTTTGTGAACAAATCAGCTAGTTGTTCTGTTGTCTTCACATAGCCAGTGGAAATCAGATTTTCCTGAATCTTCTCACGAATAAAATGACAATCGACCTCAATATGTTTAGTTCTTTCATGATACACTGGATTTGAGGCAATATGAAGAGCAGCTTGATTATCACACCAGAGTTTTGCTGGTGTATGATGCTTTAGGCCAATTTCCTTTAAAAGATTATGTATCCACATAATCTCACACGTAGACTGTGACATAGCTCTGTACTCAGATTCAGCACTAGATCGAGATACAACACTTTGCTTCTTACTCCTCCATGAAACTAGGTTCCCTCCAAAAAAGACACAATAACCTGTTGTCGATCTTCTATCAATCTTGGATCCGGCATAGTCAGCATCTGCAAAGCACTCAATACGAGTATGACTGTGATTGCTATACAGTATACCAAGTCCAGGAGCTCCTTTTAAGTAACATAGAATTTGTTCCAAAGCTTCCCAATGTTTGATTGTAGGCATAGACATGAACTGGCTAACAACACTTACCGCAAAAGCAATATCTGGACGAGTCACAGTAAGATAGTTTAACTTCCCAACTAACCTTCTGTATTTTTCTGGATCATCAAAAGGATCACCATCATCTTTCAAAAGATTCACATTAGGAACCATTGGAGTACTACAAGGTTTGGCTGCTAATTTTCCTGTGTCTCTAAGTAGGTCAAGAATATACTTTCTCTGAGACAAAAATATTCCCTTCTTGCTTCTATTTACTTCTACTCCCAAAAAGTACTTCAACTGGCCTAAATCCTTTGTACGAAACCTAGTATGTAGGAAAGACTTGAGAGAAGAGATCCCTACATAATCACTTCCTGTAATGACAATGTCATCAACATATACAACAAGGAGAATAATGCCAGTTGTTGATTGTTGGTAGAAGACAGAGTGATCACATTTGCTCATTTTTAACCCAAAATCCTGAACTACCTCACTGAACTTACCAAACCAAGCCCGAGGACTTTGCTTCAAGCCATACAAAGACTTCTTCAAATGACAAACTTTCTCATACTCCCCCTGAGCAACAAAACCTGGTGGTTGCTCCATATACACTTCCTCTTGAAGATCACCATGTAGAAACGCATTCTTGATATCCAACTGATGCAAAGGCCAATTCTCAGAAGCAGCTAGAGAAATGAACAAGCGAACAGAAGTGAGTTTGGCAACCGGGGAGAATGTGTCAGAATAATCTACTCCATATGTCTGAGCATACCCTTTAGCCACAAGTCTAGCCTTAAGTCTTGCTATGGAACCATCTGGATTAACTTTAACTGTGAAGACCCACTTACATCCCACTGGTTTCTT

>LNC_001990

CTCGAATTGGACGGTTCTCCTCTTCAATCCGTTGGAATTTGTACAAGAGTGGAACTGGCAGCCTTATCTCTTCAGGAGGGAGAGATTTTGAATTTCAAATTCGAATTCACCCAATTCAACCCGAAATTCCCCCCTAATGCCGTGCCTTAATTTCCTCCTATAATACCCCTCTTCCATTGATCGTGGGGTTGGATTTTTACTGATAGGAAGTAGCGGTTAGCAAGAGATAGATAGTGAATATACTTGAAAATACATTCCAAAATAGAGAAGCTAACTCGTTTAGGATCTTAGTTTCGTTTCTTTTGGTGTCGTCCATTGTCCTCTCAAGGTCATTTGAACTCGAGGTAGTTGTTCGATTCTGCCCAGGTTCCCTGTTTGTTGTTCATATTCAAGTTTGTGTGGTGGTGGAAGCTCATCCTCTCTAGCTCGTCCGGTCCCAGTCGCTGCTCGTTAAGAGGTAAATAACTCATGCTCCTTTACTCATTCTGTTTCTAGTAAATGGTGATGTGTTCTTTGATGGATTCTGAAATGCTCTTACTTGAAGTGTTCGACTCTTAAATGGTTCTTTCTTTCTTTATCCTCATCTTATTTTCATCAAGATTGGAGCGTGGGTTTCGTTTTCAAGATGATTTAATGTCTATCTTGAAGTCAATTTCGTTTCATGGTATTCTCGACTTGTGGCTGTTATGTCAGCTTCAAATTTTGGTTTATTAGTTTGTTGATAGCATTGATACCACCTTATTCTTATTTGTTGAAATATTGTTTACTGTTTTGTTATGTGTTTGAAGGTGCCAAGTGAATTATGCGGTTGCATATTTAACTTCTTATGGGCATAAGTTGAATTTTTGGGATTGTGTTCTTTAAAGCATGTTTAAAATCCAGTTCAGGAAAATTTCAGTTCGCTTGTTTCGCATTATGTGTAGTTCAAATATGCTTGCACTTAGCAGATTTTTAAGTAATAATATTGAATAAAATTAGTTCATCATCAAGCCTAGTTCATGTTCATTCATAGTATCATTTTTTCTAAAACCCCATTGTAGTGTTAGTATTCATGTGATATTTATTGGGTTCGTTTGAATGTATTAGGCAGTGTTTGTTGATGCCTTATTATGAAAGTCTCGTGTATGTGGGTTATTACTTTGGCATGATCTTCACACTTGAAATAAGCATGCCTATCCCTCCTCTCTCCTTTTATAAAGTTGCAGGATGATGAACTCTTGTTCCTATTCATGCCCAAAATAGTGATAGTTCTTTGGGGTGTTTTACTCTAATTCAGTCATTGGAGTGAAATTTTGTTGCCTTGATTTTTTGGGAAGCTTCCTTAATTGCATATCTTGTTCCAATCGAGTAGATAGTGAATAGTTAGTACGTTAAGAATTATTTGGGTATTAAGTTTGATCATTATTGACTTAGTTGTTTTGTTGCTTACTAATAGCCTTAAATGCTCATGTAAATAATGTCTTATCATGTGTAATTTGGATTATGCCTTGCCATAACTTGCCTTTTTTGATGACTTAAGGTCTTGAAGCTATAGTACATGTGCTTATTTAATGCTTTATTTTAGTGCTCCTTTATTTATATTGAGTCAATGGGTAGTTGCTCATATGTTTTTATTTCTTATGTTTGCATGTGAATACCATTGAGTCTATTGGACTCCTTTGTCTTTATCCACATTTCGGATTGGCCGTGAAGGTCCAAATCATCAATTCCTTTGAATCTGCCTCGTCAAAGATGTGAAAATTGAAGTACAGGGTTGGAGATCAATTTTACAGGTGTCCGAGAGTCAAGAGGAGGCTGTATACATGATGTATACAACAGAATACGTGGAATATACAGTGATATACACTCACTGTATACACCAAATGTAGCAGCGAAAATGGGCCCAAAAGACCCCAAAATTTAGGTTTTATATTGTGTTATGATTGGGCCAAAGGCCCACATTTTATTTGGTTATTATTTTGTTAGTTTAGGACAGGTATCAAGTATTGGGCCAAAAACTCAAAATAGGCCCAAGTACGGCCCAAATCATCCGTTAAAAGTTTAGTTCATCTGTGTCATTCCCATTTGAGAAAAGGCTCATCCATGCCATTATTTGTCAACTGAAATGGCATAGATAAGCCAAACTTTTAACAGATGACATAGATGATTTTTTTCTAAAGTTCAATGGCATATAATTTGAACCTTTTTCCTTTTTAAAAAATGATTTAATTAATGATGTAGATGAATCATAATTGACCACGTGTAAAAAATAATTTTACAAAATTAAAATTGTTTTTAGTTAACAAATAATGACATATATGAGCCAAACTTTTTCAAAGGCGAAATTCATACCCCCATCTGAGATTCACAATTTTAATACAAAACAATTCATTCATTTAGTGCATGTAGTACACATGTTTTGTCAACCATCAGAGCAATGTTATCCGTTAAAAGTTTAGCTCATTTGTGTCATTTTTATTTGAGAAACGGAAAAGGGCCAAAAATGCCCTTCTACTATTCGAAAAGGATTAAAAATACCCCTCATCCATCTATTGGGC

>LNC_001991

AAACAGCTGGAAACGGCTGCTAATACCCAAGTGTTGGAGCAACAACCGGCCAAGTCAATTTCAGAAAAATGAGAAACTGATTCGACTGGACGCTCCTCTTGCCTTTTTCGTTGCGTGCTCTATTACTGCTTTCTCAGATAGATGCTGGTGTGGCTGCTTCAGTACAATTTGCATTACCCTCCTAGAAAGCATTCCTTGTTCTGACCGCAACACCATATCTTGACCCTTTTCTCGAATTGGTCCATTTCGAGTTGGCCCATTCCCCTTCGACCGGTCGTAAAAGGAGAAGAACTGAGAGAGTATGCTATGCGCAGACTGACTTGCTGCTTGCTTTCTTTGAGGAATTTTGGAGTGAAAGGCGATGCCAGTCGATTGAGACTCTGATTCCTACATACCAGACTTTGATCAATCATACATGATAGGAGAGAAATTGTCTGATTAAGAAAGAA

>LNC_001992

GTTCTTTCCATAGTGCTTGTTCACTGGATGCTAGTCTCCATAGCCATTTCATCATAAGGCTTTGGTTCTGAATCTTCAAATTCCTTACCCCAAGACCTCCTTTTTGTTTGCTCCCAATCAATTTATCCCACTTAACCAAGTGGAATTTCTTAGTGTCACTATTTCCTTCCCATAGGAAATTCCTCCTTATTGCATCAAGTTTGTTAATGACCCCATCTGGGATAGGAAAGATAGACATCATGTAGGTAGGCATGGAGTCGAGAACACTATTGATGAGAGTGAGTCTCCCGCCGAGGGAGAGATATTGACTCTTCCAGTTCACCAATCTTTTTTCACACTTCTCAACCACCTCATTCCAGATTCCTGAAGACTTGCTTTCTGCTCCTAGTGGCATGCCCAAATATACAGTTGGTAATTCACCAGTCCTCCCCCCAAGTGTACCAGCCAGAGAGGTTAGATTGGTGACCTCATTCACTGGGTATAGGAAGCTTTTTTCCCAATTGATATGTAGTCCTGATATGGCTTCAAATAGCACGAATATAACTCTGAGAACCATCAGTTGGTTTCTGTTGGCATCACAGAAAACAAGAGTATCATCAGCATATTGTAAGTGTGTAATCTCCATGTCACAACCCCCCCTATTTGATACTCTGAAGCCCCTCAACCATAGTTTCTCTTGAGCAATCTTCAATATATCATTAAGTCCCTCCATAGCTATAATGAATAAGAAAGGAGACAGCGGGTCTCCCTGTCTTAATCCTCTCTGTGAAGAGAAGAAACCTGTAGGAGAGCCATTGATGAGAACAGAATATTTCACAGTACTGATACAAAACTTTATCCATCTCATCCATTTACCACCAAAACCTCTTTTGGATAAGGTCTCCAGCAAAAAATTCCAGTTAAGATGGTCATATGCTTTCTGAATGTCCAGCTTGCATAAAATGCCTGGCACCTTGCTTCTAATCCTAGAATCCACACATTCATTAGCCATCAAAATAGCATCCATGATCTGTCTTCCTTTAATGAAAGCAAGTTGTTGTGTGTCCACTAGCTTGTGCATAACCTTTTTAAGCCTCTCAGTAAGCACCTTGGAGATTATTTTATACACACTGCCAATTAAGCTAATGGGTCTAAAATCTGTGAGTTTCTTTGCACCCATCTTTTTGGGGATCAATGCCACATAGGTGGAGTTGAAGCTTTTCTCAAAGAAACATCTCTCATGGAAATTTTTTACTGCAGCAATGACTTCCTCTCCAACAACCTCCCAACAGTGGGCATAGAAGGCCATAGTGTAACCATCTGGACCAGGAGCTTTGTCTCCAGCACAAAGCTTTACACACTCCCATATCTCTTGGTTCTCAAATTCCCCCTGTAGCATCCTATTGTCTTCTTCAGAAATTGTGTGCCCTTCCCTGCAGCCACCAGAGGGTCTCCAAGTCTCAGTTTCAGAATATAGCTTTTGGTAGAAATCTATGATTTCTTTTTTTATTAACTCTGGATCTTTGATTGTTTCACCATCCACATTTAATTCGTCAATGTTATTGTATCTCCGGTGAGAGTTAGCAGTTTTGTGAAAGAACTTTGTATTCTTATCCCCCTGCTTCAACCAATTAACCCTAGATCTTTGTCGCCAAGATATCTCCTCAAACTTTGCTAAATTTTCAAACTCTATAAGGAGATCAGCCTTCAGTTTAATCTCATCTTCATGAAGAGCTCTTTGATCTTGCACTTCTTCTAATTCTGCAATTTGATTGAGAACACTCTGTTTCTGAGAGGCTAAGTTTCCTTGAGATGTTTTACTCCATTCCCTCAACTTATCCTTAAGAGCTTTCAGTTTGAAAGCTAGGATAAAGTCTGGCCTCCCTTCACATACTATAGAGCTCCACCATTCCTTCACTCTATCCAGGAAACCTTCTGTCTGTAGCCACCAATTTTCAAATTTGAAATAGGACCTCACAGGTTTCCAATTCCCACTTTGAAGCATTATGGGATTATGGTCAGAGGTAACCTTGTGCAATAATGTTTGTTTGATGTTTCTGAACACTTCATCCCAATCATCAGACAAAAGAAATCTATCTAATCTGGATGTTGTGTCATGCCTGTCCCCTTTCCTCCAAGTATACTTGCCCCCTACTAGGTGGGGATCCAACAATCCCATGTCTTCAATAAAGTCAGATAGATCTGTCATTGCTCTAGATATCCTGGAGCAATTCTTTTTTTTCAGATGGGAATCTTACTGTGTTAAAATCTCCGCAAACCACCCATGGGCCATCAAATAAACCTCTAGCTGAGCCAACTTCCCACCAGACTTCCTCTCTTTCTTCCCTACTATTTGGAGCATAAATGCCTGTGAGAAACCAAATTAAATCTTGGTTTATCCCAATGAGCTTACATGTAATTGAGTAAGATCCTAGGCTGCTTACTTCCCCTGACCACACTCTCCCATCCCACATAATTAAAATACCACCTCTAGTTCCACTGGCTTCCAGTTGGCAATATTTAACCCATCTGTTGGCCCATAAATCTTTCACGAAGCTCCCAATCTCTCCCTCAATTTTCGTCTCTTGAAAGCAATAAATGTCAGCTTTCCAACTATGAATTAAGTTCTTGATTATACACCTTTTACTCGCCTGGTTCAAACCCCTCACATTCCATGATATGATGTTGACTATCATTGATCACCTGAGATGGTATATCCCCCCCTTACTTCTTGTTCCATTGCTTCTGAAAATGCTGCCACATTCCAAGCTCTTCAGTTCTTTTGCCCCTTTGACTGCTGAAGTCAATTTAGCAATTTCCTTCCCCTTATCATTGTTGATCTGCCTCATACTGTCGATCTTCATAAATAGCATTGTTGCTTCCTCTTTGCATCCCTGATTGTCAACTCCAAAATCTTTGGC

>LNC_001993

CGACATTGATCAGCCCATTGCTACATTGCCTAGTAAATTGTGCTTGGACTACCTTCAACTCTATCCTGACATTGTCAATTTGTTGCCTCACATTCTTGAATTCTCTGTTATTCTGCTTCTTGAGTAATGGCTTGAGTGCCCTTAGCTTAAGCCATACATTCTTCATACTCTATTGGTCCTTTTGTTATCTCCATACTTCATTGACTATAGGAAAGAATTCTTCATGATCAACTAGGACATTGAAGAATCTGAACGGAGTCTTGTTAACTTGGGGTGCTGTGTGTAATTTAAGAACCATGGGGGAGTGGTCAGAGATCATGGGCAAGTCATACTCAACCTGCACATACATATCCCCAGTTCATCATCCAGTCAAAATTTCCAAGAGCCCTATCCAGTCTACTCCATATTCTATTAGCCCCTGGTTGCTTGTTTGTCCAGGTGAAAAATTCTTCTTTCTAAGATAGTTCATTTAGGGATAAGCGTTGTACAGAGGC

>LNC_001994

CACCATTGGATCAATATATATTATATTCAAATTTTCCCAACTTAGGATCCTAAAAGAATGTGCAGTAAGAGTTATACAGAGTGAGGGATCCAAGCAAGTATGATATTGAACATTTCTTCAAGATAATAGCTACTCCTTCATGTGAATCTTATTTGAAGCAAATGAATATCTAAAGGGAGAGCAGTTAAAGTTATGAAAATGTTATTTCTTATGTGAGAATGGTAGTTTTGGCTCTTTTTAGAAGGTCAGGGGAAGGTGAGATTGTAC

>LNC_001995

TTTTAAAAAGATTTTTTCCCGCATCGGAACAAGACCCACTATACTAGATTTAGCCAATTCGGGTGCGGATGGGGATTCCCCAATAGATTCTACAGATTCGGATTTGGATGCCCCTGCGCATAAACATATGGATAGGGATGCCGATTCAGATGCGGCTAGAAAGGCTCGTTTGAGTGATTCATAAACCCTCGTCGGGATAAACAAGCAAGGGTTTCATCCTCTACTTATGGGAATGCTACTTCGAGAAATGCTGCTTCAGGATAAACTAGATATGTTGCGAGAGAAACTGGATATTTTCCTATGGCTTGGACCCTTTCACTCGAGAACTCAAAGGGGGGAAAGAATTCTTCAACATAGTCACAAGAAGGTGGTCTACGACCATTAGATTCCTC

>LNC_001996

GTTACGTTCAGCATCTACAGAATGCAAGATTGAAGATCTTTTACAAGAAATGCAAAATCTTGAAATAGTTGAAAAAGAATATCCGGACACTACTTTGAGCATTGAGTAATTTAGAGCTCAAGCTAAGCTAAAGAAGATGATAATTGATCAATTTGGAGCTACTAATTTTTTTTTTGGTTTATTAGGAAAAATTCTCAGTTGCCATAAGTCCACATCATGCAAAATTCAATATGTTATATATCTATCCAAGACAACTCTGCTAGACTGCTAGTATTGATTTTCCACTACATATATAAATTGCTTTCTCTTTTTAGAAATAAGTCTTCTAACAACTTAATGAGTTTTTTTAATCCTCTTTCAATTGTTTTATGACTAGTATTGCTTAATTAAATTCACAATAGTTTTTCTATGGATTGTTTATGTACCATGTATTTGTGTCTATCGAGATGCATATGCCCTCAACAAATTATATACTTTCAAACCGAAATAACCAAAAAAATTGAACCAAACCGAACCAAACCAACAATAACCAAACCGACGGTTATTTTTTTGATT

>LNC_001997

AAGAATTTGGCATTCTTGATGTCCAATTTTGGACAGCCTCAGCTTGTGGTTTTATGGGATACCTTCAATTTGATGAACTAAGAAGAAGAGGGATTATCCCGTTTAAAGATGACAGTTTCATGGAGGATGGTACACTGGATACAATTATCGATGGGATTCCAGGAATGAGAAACATTAGGTTGAAGGATTTGCAAAGCTGTATCAGGACTACTGATCCACATGATATTCTGTTAAACTATTTAAGTGATGAAGCACAAAATTGCTTGAAATCTTCTGCAATAATTATCAACACATTCACTGAACTAGAACGTGAAGTCTATCTCCAAATGCCTACTCAATTAATAATGATGGATCTTTCATGCATGTTGGAAAAAGTAAGTCATAGACTTTAGGTTTTTAATCTTCTGTCTGCTGTAAAGTTCTCATCATATTTTTGAGACAAGCAAAATGAAATGGCACATACAAAGCATGACTGCTCTGTTAGCTGTTTTAGATCAAAACATGGCTATAAGTTATATGAGCATGTTATCACAAGAGGCTGTGCTGCTTTTCAACAAATCCATTCTAAGAAGTTTTCACAAATTCAATTCCATGAGTTTTCATCAAGTGAACTCATTTAACATTTCCATATTCTACTATTGT

>LNC_001998

TAGGGTTTAAACGACGTATCTTAGGGGTTAAACGAAATAGGAAAAGACCAAAAGACCCCTGAATTAACTGCTGTCGGAGTCATCGACGGATTGGACTGACGGGCCGTCAATCAATCGACGGACCGTCGATTGGGTCCGTAGGTCGAGTCTGCCCGACAGGGCTTCACTAAAACGAGCATAACTTTTTACTCGGAGGTCGGAATTTAGCAAACTCGGTGGCGTTGGAAAGATAATTCAATTATCTATCATACCAT

>LNC_001999

CATGTCTCTTCTTTCATTTTTTTCTGGAAAAGTAGCGTATTTTGTCAACGATAAGCAGAGGGTAGCTACTACATCTTCACACCGATGTCATCTCAATCACGTGGCTCCAGCAAATCTTATCCTAAGGGCTTCAAACGTTTGGATTTGAAGGCTTACCCTCGTCTTTGTATTCGAGCATAGATCTTCGACCGAAAACGCCCCAGTTCGTTGCCCCGAAATAGGTCAGTGACATCTAGTTTCTTTTTTATTTTATTTTCATGACATGTGGACGAATTTGTGTTTTATTGTGTTATGGACTTACTGTTAGTTTCATTTCAGTACTTTTGTGTTTTGGCATCATGATGAATGAATGTAACTTAGCTGGTCTTTGATTTTAATATTCACTTTTTGTTCATTTCTTTTTATAAAATCATATGTTAAAGTATTTTGAAGATTGTTTTTTTTTTTAGAAATCATGTTTCCAAGACTCATTTTTAGGTAGTAAACTCTTTGGAAACTATGCTTTGTTTGAAGTTTTGACAAATGAGTAAAATACTTATCAGCATACTTAAGTGATGTTAACCAAATATATATCCCTCTGTTTTGAGATATTGTTCAGCCTATACAAATATGTATTCCTTCGTTTATGTTTGTCGGAGTATTGCCTTTTACTAGATTAAGGCACAAATAAAATGCCCTAAACTTTGTATTTCGATAGCCAACTTGTAATGTGTTCCTCTTTTCCTTTTTTTTCTTTGTCATCATGACGAAAATGTTGTCTCTGCTCACTTTTAGCTATACTAAAGGTGATGTTTAAAATGTATTTGGGCTTAGCTCAAATCTTTTAGATATCCAAAGTCACGTTTATTTTACATTTTTAACTGTCATATAATGGTTTTGTGATCAAATCTTCTGTTCTTATAAATTAATGGCATTTGGTATCCTTATCTGGCAATAGATTTTATGTTATGATGCCTCTATGTGTTCCCTCTGTTGAATGGAAAACCTACTGTCAATTGCTTGTGAATCTTTAAACTTCTTTCCTATTATTTTTTAGCAAATATGTGTACATAAGCTTCCTTCAATTTTTCTAAATAAATGACATGTAAATCTCTGAATATGTTATTTTAAAAAATGTTAGTTTTTAAGTTTCTTTCCTATTATGTCAACATATATAGTATATCTGTTAAATCTTTCTTATAAGTTTTAAATAGATGGCTTGTTTTAATTATCTTCATGCCTTAAAATGTAACTTTGTTATAGATTAAGTTGGATAAACTTGAGAGTTAGTTTTATCTTGTAGACTAGTTACTTTGAGTTATGTTAATAAGTCGACTTATCCTATGCTTACATATTTTCCATTCCGGATAGTATTTTGGTTGTACGACTTTACTAGCTTCATGTTTGGTTTATTTGATATAGTCTTAAAGTGGATTTGTCTCCCTAATCTCTTTATTTTGTTCTTTATTTGCATGAACACTTCCGGAGCTATTTGGAGTCTCAATAAAAGACTCTCACCGTGCAAGAGGGCTCATTTAAGGACTCTACCAAAATTAGAGTCTCATGGACTCGACACCATCATACCCGCACATGAAGTCGATATCTTAGACTATCCAAATAGCAAG

>LNC_002000

GCTTACCCTCGTCTTCGTATTCGAGCATAGATCTTCGACCGAAAACGCCCCAGTTCGCTGCCCCGAAATAGGTCAGTGACATCTAGTTTCTTTTTTATTTTATTTTCATGAGATATGGACGAATTTGTGTTTTAATGTGTTATGGACTTACTGTTAGTTTCATTTCAGTACTTTTGTGTTTTGGCATCATGATGAATGAATGTAACTTAGCTGATCTTTGATTTTAATATTCACTGTTTGTTCATTTCTTTTGTTAAAATAAGATGTTAAAGTATTTTGAAGATTGTTTTATTTTTTTTTAGAAATCATGTTTCCAAGACTCATTTTTAGGTAGTAAACTCTTTGGAAACTATGCTTTGTTTGAAGTTTGACAAATGAGTAAAATACTTATTAGCATACTTAAGTGATGTTTAACCAAATATATATCCCTCTGTTTTGAGATAATGTTAAGCCTATAAAAATATGTATTGCTTTGTTTATGTTTGTCGGAGTATAGCCTTTTACTAGATTAAGGCGCAAATAAAATGTCCTAAACTTTGTATTTCGATAGCCAACTTGTAATGTGTTCCTCTTTTCCTCTGTTTTCTTTGTCATCATGATGAAAATGTTGTCTCTGGTCACTTTTAGCTATACTAAATGTGATGTTTAAAATGTATTTGGGCTTAGCTCAAATCTTTTAGATATCCAAAGCCATGTTTATTTTACATTTTTAGCCATCATATAATGGTTTTGTAATCAAATCTTCCGTTCTTATAAATTAATGGCATTTGGTATCCTTATCTGGCAATAGATTTTATGTTATGATGCCTCTATTTGTTCCCTCTGTTGAATGGAAAACCTACTGTCAATTGCTTGTGAATCTTTAAACTTCTTTCCTATTATTTTTTAGCAAATATGTGTACATAAGCTTCCTTCAATTTTTCTAAATAAATGACATGTAAATCTCTGAATATGTTATTTTAAAAAATGTTAGTTTTTAAGTTTCTTTCCTATTATGTCAACATATATAGTATATCTGTTAAATCTTTCTTATAAGTTTTAAATAGATGGCTTGTTTTAATTATCTTCATGCCTTAAAATGTAACTTTGTTATAGATTAAGTTGGATAAACTTGAGAGTTAGTTTTATCTTGTAGACTAGTTACTTTGAGTTATGTTAATAAGTCGACTTATCCTATGCTTACATATTTTCCATTCCGGATAGTATTTTGGTTGTACGACTTTACTAGCTTCATGTTTGGTTTATTTGATATAGTCTTAAAGTGGATTTGTCTCCCTAATCTCTTTATTTTGTTCTTTATTTGCATGAACACTTCCGGAGCTATTTGGAGTCTCAATAAAAGACTCTCACCGTGCAAGAGGGCTCATTTAAGGACTCTACCAAAATTAGAGTCTCATGGACTCGACACCATCATACCCGCACATGAAGTCGATATCTTAGACTATCCAAATAGCAAG

>LNC_002001

AATTCCACTCCCCCACCCCTTCGAAAAGATGCGTCCAGCAGAACGTGTCTTCCAACTCCTCTGCTAGAAATAGAGATACCTACGAGAGTGAATAACATAAATTCTTAATCAGAGTGACTCCTAATTTCCAATGCAGAACTGATTTGTTGGAGAAAAATCAGCAAAACAAACCACAATAATGGTGGTACCGTTCCTAAAATCCAGAAGTCACCTCTTTCTTTTTACTTCAAATTTCACTTGAGTTTTGCTG

>LNC_002002

GAAAAAAGGGCGTGGGTTTAGCCTCTCTGTCTAACGAAGGAAGCTGGGAAGAGAAAGATCCTTCACACAAGGCTGTTAAGTAAAACGAGAAGGAGGAAACAAGATTGATCGAATGAAGCTTTACGAATTTCTTGAGGTGACAAGTTTGGTATGTCAACACGAGTACGGTGAGTAGTAACCTCAACACAATTTGTATTTCACAAATTTCATGGTCTTTACTTGGTTTATAAAGTAAATGTGTTACCGACTATTTTGAACTTGCATGTGGGACATATCTTTTACTTGGTATGATACTATAAAGATTATTTGAGATGAACTTATTCTTACAAGATATGATTATTGTTATTCAAAGGGTTTTACACTTATTTGAGAAATAGTATTTTAACATGTGACTGTTAAACTTTGAAATAATATGATTTGATAAGATTTAAATGATCTATTCGATTTTGAGATGCTTCTACTGATTACAATAAAAGTTTAAATGGGAGTACACCTTGATGAATCCCGTAGCTAACTGCTGGTTCGTTATACCTGGTGTACTTTGTAGTTATAGATTATCGATAAAGCCCTTGCTAGTGGGAAGGTAGAACTAGCATACCGTTACCTATATCCCTCGAGGAGGGATCTACCGATATGAATACTTAACTCCTTTATGAGGAGTTTGCCGTTATTGGTTAGTTCTTAATTAGAAG

>LNC_002003

TTCAAACATTGGGATCTAAAGCTACTCGTTTTCCCTAAAAGCTTTGAAGCTATCACACCTAGTTTTAAAGTCTTGTTCTTCGCAATTTTGGATTTGAACGGGCTTAACCTCGTCTTCGTATTCGAGAGTAGATCTTCGACCGAAAACGCCCCAAGTTCACTGCTCCAAAATAGCACAAAGAGTCTCGTTTTGAGACTCAACCTCATCATCGCTATACCCGAAGATGACATCTTTCTCTTCATTGTCACTCATCTACTGCTCTTGTCTTTTATATTACTTCCAA

>LNC_002004

AATAACAACTAAATACAAAAGATCACTCGATCAGGTCCCTTGTTGCATATGAGAAATATTTCTTCGAGAGAATGGATTTTGCCATGTAATTCATCCAATGAAAAAGCTAGGTTGTATCCCAGGTCAAAGTAACTATTATAAGATGGAAAAAACATAGTGCAATGCTATTGGCCAAGAGTACTGATCCTTTGTAATCGCGCACCAATAAGCACTGATGTTGTGGCAGTGGTTGCAGCTTTTACAGTTGTTCTTTTTGCATTGTCTGAACCTTCTCAGTTTTCTCATGGGACATAGTGCTATACTAGGTCTCGTAGTTTGTTATATGATCAAAACATTAGGATAACTTCAGAATAGCAATCCATCCATCACCACTTTTCAAAGCTAGCGTGGCTCTAGGTCCTCGTCCTAAAAAATGGACAACTTTAGCATGTTGCTCTGTTTCATAGTAACTTTTGGAATCAAAAAATATAACTGCCTTTGAATTTCTAAGGCCATAAAATCCATCTTCCAATTTGAACACTACATAAGTTAAATAACTAGTTCTTTTCAACGACATTCGAGTGTCAACTTTTCCTCGGTTATCTAGCCAACTGATCCAATTAAGTTTAGCCACTTCAGAAAATC

>LNC_002005

ATTGGTGGATACTAGCCATCTTGCAATCTCCTCTAATCTCACCAGGTTGCCCAGACTGTGTATCACCCATCTATATCATCCCTTCAACAGAAGCCTTAAAAAAATCTTGATTAGTACTGAACTTCTAACATATTCACTTGTAAGAGAGTATGTGAAAAGTGTTTGATCAGAATTCAAGAACCCCTGCCCATTTACCAAGTCTTTAAAATATTGAATTCACTTAACAGTGTGTATTAATTTTCCTTAAGGTAAGATATTGACACTACTTTACTTGGATACCCTATTAGGGATCGCATTTATGTGATTCTAATTATTGTATTTTCCCCAAAGATATTGAGTGGCGATACAATTCCTAAGGGACTTCCTTTTTCGCTAGACTCCATGTGGCTTCTGTTGTTCACCTCACTCTACTCCACGAGGTAATGAATGTTACAAGTTACTTTGGAGCCACCCTTGGAAGAGTCGCTAACCGGATTGGTGGTTCTCAATTTATTCTG

>LNC_002006

TGATGTTCACAATTGTAATCTATGGTTCAAGATTTAAATTTTTCTTCGGTTATAGATCTTTTGAAATTGAGGGCTTAGGTTATAATCCTTGTTTAGTAGAATTGGGGCATAATTGTGACTAACGTATAGTTACTAAATACATCAACATTAATACATTTCTTGGCCAAAACATATGCATACCTTTGCGTAATTGTGAAAGTTCCCCTTCATGGGTCTCAATAAAGTTTCTTGCATAGGAAATTACCAAATCAATAAATGCATAACATATGTTTTCTGTAAAGAGTGAATATTTTATTAGTCTAGTTACTTCCTCAATAAAAAAATGAGAGAAACAATTTCATTACTTTCTATTCATTCCTTCTTAATTGTTGATCCGACATCCAATTTCCACA

>LNC_002007

TGGTTGCACTACGTTTCCTCATGTAACAACTTGCTGCTTCACCATTAATGAAGCTTATCAACTCAACTCGGCTGTTGATAGATCGATTCACAGGTGAATCCGCTCATTGGTACTGGTTACAAAGATTGCTTTAGCTGCTGTGATTCTTATCAGATGTGTTATGGTCTCAGGTACATTGTTTGCTCATATTTCAATACTCTTCAATTGTACTGATTCTTGTAGTACTTGAATTGCTATAACCTGCAGATGATATTTAATCACTAAGCACCAAGATTAATGTGTCTTGGTTTTATATCATTTGAAACACCTCAATCTCTCACCAGGTGCATGATAAGTTTAACCAACAGAACTTAGTTCAGACAACATTATGTACTTAGTCAATTAGTATCATCTGGCAGATTTTAGAATTTCAAGAAAAACCTGATTCTGCACTGTTAAGACCATGATTAGATTGTTGTGCATTTCCTTATCCTCAATTGTGGCGTGCTCTATTTATCCAAATTAATAATCTTCTTTGCTGTGCTTGGCATTTCCTGGAAAGTTTTGAATTCAAAGTTACCTGCAAAATGAAAAACCATGTTAGCTAAATAGTCCGCAAGAGTATTCCCCTCCCTCAAGGAGTGTTGTACTCTTGTTGTAATGGACTCCATCATTTTCCTTATAGAATTGACCTCCAATGTCACATTCCAAGGAATTTTCCATAGACCATTGATTATGTTCACTGCTGCAAGAGAGTCAGATTCTACAATTAACTTTGGAATATGATTTTCCAAACAAAATTGTAAGCCTGATCTTATAGCAATCATTTCAGCCACCAAATTAGTAGAATTAGGAAGCATGTTTCCTTGTGCACCAATTATATCCCCATGATGGTTTCTGACACAAAACGCAGCAGAACTTGGGCCTGGGTTACCTCTAGATGCCCCATCAGTGTTGCATTTCCACCACCCACAAGGTGGTGGA

>LNC_002008

GTCAGACTATTAAAGCTCTTTGAACTTATCAACCTAGAAGGAGTATACTCTATTACTCCTATTGAGTATTAATAGCTTAGTTATGCTAAGATTATTTTTTATTAGTACTCCCTCAGTTTTTATTTAGCTGTCATTTTTTTTAATTAAAAGGAGGTCTAGTTGACCATTTATTATAAAAGCCCAAAATTAGCCCAAAAGTACTACACCACTAACTTAAACAAAAACAACAAATAAAACAGAAAAGGAAAGAAGACCAAAAATTAAGAGAAAATGGAAAGACCAGCCGCCATTCTTCAGACCTTCATCTAGCAAAACAGCCATGGCGGACAGCGGAAGACGACGATTTGAATCCCTTCTGTCTACTAGAGTTGACTTCGTAGGTGCCAGTGAGTCTTGATTGTTTTACAAAGGCATTCAAACCCCTCACAAATGCCATAAATCAATCAAATGAAGACTTCTTCAACTCCTAGCACAACCAGTCATGTCTTGCACAGCCAAGGTTGAGCTCAACTTGGAGCTAATACCACTGTCTAAGCCAGAAGAATCCATAGATCCATTTATTAACTAATCGAATCCATGGTCATGGGATCGAACAACGGAGATCATTCTTCAAGATGACACTTGGAAAGACGTGATTAATTGGAACACAATACCATGTCAATTTCTCTGTAGAGGTGTTCCCAAAGAAGTTGGAGGTTCAGAATCTAATTTTTTTTCTTAATGGTTGAACCAAATGACAGTATAGCTGCAGTTAAAGCGTATATTCTGTGGGAATAAAACAACAACTAATTTGGCAACCACATTTGTTGAAATTACTTACCACAAATGTGAAAATTGGCAAGCCACAAATGTGAAAGTTGGTAGCCACATTTGTGGAAGTTTGATAGAGTTTTGGAAGTCAAAGTTTGGTAGAATTTTGGTAGAGTTTGGCAGCCAAACTTTGTGAAAATTGGCTACCACATTTATGTCAAAGTTGTTTACCTACAAATTTGAATTGTTAGTTTCCATTGATGTCATTAGTGACATCAAACGGACATAATTTTTCTTATAAATAGAGCACTTATGCTCATTTGTAAGGACACACCAAAAATAGAGAAACAATAGAGAGTAGAGAGAGTAATCCTTCGTAGTTTGTGAGAAAATAGTGTGAAAGTAATATAATTGTGAGTTGTAAGAAATAAAAGAGTGGTGAGTAGTAGTCTTT

>LNC_002009

GTCAGACTATTAAAGCTCTTTGAACTTATCAACCTAGAAGGAGTATACTCTATTACTCCTATTGAGTATTAATAGCTTAGTTATGCTAAGATTATTTTTTATTAGTACTCCCTCAGTTTTTATTTAGCTGTCATTTTTTTTAATTAAAAGGAGGTCTAGTTGACCATTTATTATAAAAGCCCAAAATTAGCCCAAAAGTACTACACCACTAACTTAAACAAAAACAACAAATAAAACAGAAAAGGAAAGAAGACCAAAAATTAAGAGAAAATGGAAAGACCAGCCGCCATTCTTCAGACCTTCATCTAGCAAAACAGCCATGGCGGACAGCGGAAGACGACGATTTGAATCCCTTCTGTCTACTAGAGTTGACTTCGTAGGTGCCAGTGAGTCTTGATTGTTTTACAAAGGCATTCAAACCCCTCACAAATGCCATAAATCAATCAAATGAAGACTTCTTCAACTCCTAGCACAACCAGTCATGTCTTGCACAGCCAAGGTTGAGCTCAACTTGGAGCTAATACCACTGTCTAAGCCAGAAGAATCCATAGATCCATTTATTAACTAATCGAGCTAGCATAGCAGACAATAACGCGAAATCCAGGAAAGGGAGGGGGAATGAATGCCAAATTTGAAGCAAACCGAAAAGTGAGTTCTTGTGGAGTGAGTCTAGTGCTTCCCTTTAACCTAGAAGCACTCAGTGAGCCTTCAACAATGTCCTTCCTGGCCTCTTATTTGAGTTGACCGAAGGCGGCGGGTGGACGAACATCTTGCAATAAAGAATGTTCTCCTACTCACAAACAGCGACTCCTGTTGGTGCGATCAACCACGAAAAGTCAGTAATAAAGAATATGTGCTCCTCGCTCCCGCTATGGTTCGATATGATATGGTTTGATTGGTCCAACCTTTAGAAACAACAAGGGTAAAATAGATATAAAAGATCCATGGTCATGGGATCGAACAACGGAGATCATTCTTCAAGATGACACTTGGAAAGACGTGATTAATTGGAACACAATACCATGTCAATTTCTCTGTAGAGGTGTTCCCAAAGAAGTTGGAGGTTCAGAATCTAATTTTTTTTCTTAATGGTTGAACCAAATGACAGTATAGCTGCAGTTAAAGCGTATATTCTGTGGGAATAAAACAACAACTAATTTGGCAACCACATTTGTTGAAATTACTTACCACAAATGTGAAAATTGGCAAGCCACAAATGTGAAAGTTGGTAGCCACATTTGTGGAAGTTTGATAGAGTTTTGGAAGTCAAAGTTTGGTAGAATTTTGGTAGAGTTTGGCAGCCAAACTTTGTGAAAATTGGCTACCACATTTATGTCAAAGTTGTTTACCTACAAATTTGAATTGTTAGTTTCCATTGATGTCATTAGTGACATCAAACGGACATAATTTTTCTTATAAATAGAGCACTTATGCTCATTTGTAAGGACACACCAAAAATAGAGAAACAATAGAGAGTAGAGAGAGTAATCCTTCGTAGTTTGTGAGAAAATAGTGTGAAAGTAATATAATTGTGAGTTGTAAGAAATAAAAGAGTGGTGAGTAGTAGTCTTT

>LNC_002010

CTCTTCCTCTCCTCTCCAAGCAGACCCGCCACCCCCTCTCTCTTCTCTCTTACCTGCTCCGGCGAAGCTCGCTCCGGCCAGCAGCTGGCGAGTGCCAGATGACACCAGCTCCAACAGCAGCAGCGCCAGCCGCTTCGGCCAGTACAAACAACAGCCAGTAGCCGCGAAAGCAACTGGGCAGCAACCATCGCTGCTCCACGCCACCAGTCACCCACGTCTCCGACAAAAAAACGGCAACCACTTTGACATATTTTGGTTTTGTTTAAGACGGATCTTAATAGATTCGTCCCAGGTACAAATTCCTTTACTTTTGAGACCAAATTTTAAGCATGTCACTATGAAAATTGTAAAATGTGAGATGGGTGCACCTTTTCGTTTTAGATTTCGAATTCTTAATATATATGTGGGCTGATTTGTTCTCCAAGTTCATGCTTTACTCTTGAGATCATGTTAAAACATTTGAACTTCCTTAATGCTTACTGATTTGTATTAACTTGGATTTCCTAAATCACTGTTTGTTTATAAAATTCCATTCCTTAACCGATTGTGATGCCCATTGTATCTATGGTAATAACTTTGGTCCTCCGCTAAAACGGATAGTTAATGTGTTAATTTGTATTTTCATTGTTATCACCTTGTGGCTGTCCATTGAATGGATTCGTGATTTGCTGAAAATGGTAGAATAATTATAATTTTGGACCTTAATTATCTCCCTTCTTAATGGTAATTCCTTTGTAATGATATGGTATTTGATTAATGGGTCCGGACATGTTTTGTGGCTCTTGGTAAGGTAAACTTTGCTTTACTAATTCACTTTTGGGTTGATTGATCATATCTGCCGTATTTAAAGTTGATATGAATCATGTTATATTGTAGTTATATTCTGTTGACTTTACCTTTTCTGTATTAAGGAAAAAATAAAAAGAAATTCCATAATTGTTGCTTATTGATTCTTGTTCTCTTAATAATTAACTTGGTAAGATCGTGTAGTGTATGAGAATATTGAATATTGATTTAAGCTGTATGTTATTCCTTTCCCTTAAATATTAACTTTTGTTTGTAATGTGTCATCTAAAATGAATTTTACTAATCGACTTTCTTGCCTTAGTGAAAGTTAGGCAGATTTTGTGGGTTATTTATGGTAAGAATATTATTCTGTAGTGAAAAAGAAAGGGATTGTGTACTCCCTTTGTTTAAGGAAAACTTTTTTCAATTGACTCCTAAAATTATTTCTTTCCTTGTATTATGTGCCTTTTTTGCTACTATATAAAGTGGCCCTTAGTCTCATTATTACGGACTCACGTTGACATATTCTAAGAAAAAAAAAAACTCTCTACTCTTAAAAGAATTAAGATTTCGATAAACATTTAAAAGAGCTATCTTTATTCGATTTTGCTGTGTTCGGGTCTAGTAGTGGATCAAGTTTGTCTTCATTGCTGTTAAACATATATTCTGCTTAACCGGTTAGTTGTTTCTCTATTAGTTTAATTTCCGTTTTGAATTGTTTCAATTGTTGTTGTATGTTTGAATGGCGGGAAGCCTCCGAGTCAGTCTTCTTATCCTTTCTTGCTTTGTGTGATTATATATTCTCCACAACTTGTTAAGTTCTTACATGCCATATGTTACTTGTGTACATGAAATAATTAAATGTGCGTACTATATAATTATTTATCATCTTGCTATCATCTATTATGTGGTGTTACTTGCTCGTATTTTTCGTTGTACTTTAACAGGGTTTTGAACTTAAAAGGTTCAAGAGTCGCCTCAAAGAGATTTTCGGACAAGATTCTTATGTTCAAGTTTATCTTAATGCTGGAAATACTGTTTATTTATTCTTTGAACTTGTAATATTTTGTATAACTCTAACTCTCTCTATATATGATAACTTGGGGAATCGTCCAAGGGGGAGGGGGATATACGTGCTAATTGTTCCTTTTCCTTTTAGCGTAGTTTATTTTTTGGTATGTGCGGCATGTCGGAATCACTTAGCAATCGACCTAATTTCAACCCTTCGCTCATGCAACTTTGCCTTATTGTTAAAATCAACCATTATGTATTAATTGTCTTATAATTTGAATATTTGCTTTATTTGGATTAGACTTTATTCACCTCAGCATGTTAATAAAATGGGCCCATAAGAATTTACCAAAATGTGCCTAACAAATTTAGTATGTCAATAGTTAGCAAATTAATCTTGATCAACTGCCAGCATGTTTAGAAAATTAAATCTGCAATCTGTCTAGAAAGTAATCTTAATTCAATAGGTCAATTAATTAGATTGTAATGGCTGATTGGTTGTTAATAAATTCAGATTACTTAGAAATGCATGCCTATAGGATTTAAATGTTTATTAATGTGTTATTCATGTATAGAAACCATGTCTATAAGTTGTGTTCTTTATTTAGAAATCATGTGCATAGGCATTCAGATATTTTATTTGGTATGTGAATTAATGAGTTTACCAATTAGTTGTAATGGTTGCTTTATGTGAATAAATCCAAACCATCTAGAAATCATGCCTATAGGATTAAAACAATAAAATCATTGATGTTAACTAATTAGTTTAAATAATTTCTGCATATTTTAGTAATTTAAGCCTGTCAGCTAATAAAAATATTTTAGGTTGTTAATAAAATTTCAGCATGTTAATCACTTAGTTAGCTTTTCATAGGCTTCTAATAAATTTTTAGTATTTTTTTTTTGTCTTTTATGAATAATCTCTACTTAAAATAAGTAATTTCTATCTAAAATAAATTAATGTCTTCTTCTGCATGTTAACTGTGACATTAAATTTATCTGTCATTTAAAGTTGTCCAAAATAATCAATTTGTTGCATCGTAATGAACTGGTTTGTCACTTAAAAAATAAATTAATTTTACAGCATGTTAAAATCCTAATTGCTTAAAATTTAGTCTGCCCTAATTGATCTTTTTTTTCTCGTTTAGTTATTTATGCATCTAATGAATTGACTGTTGCTTAATAATCTGATCTTCACTGCTCTAAAAGACATTTCCTGCCTCCATGATTGCTTTCTGCCACTTGTATAATTGCTTCCTGTATCTAATGTTCCAAAAATAATTATTGCTATAATATGTTGTATGCCACACGCCTAGCATGAGACTTTTTCACAATTATATGTGTTTGCAAGCATGCTAATAAAATCTAGAGGCTAATATGAGACTCTATGTGCTAAATGCTAGTCCAAATTATTTGGCTTGGTGTCTAGGTGGGCCTATTAGGAGTTTATTTCAGTCTAAACCCTTCCTAATAGTAGTTCAATGCCTCTTGGACCTTAAGCGGGGATGCTAGGC

>LNC_002011

TTCAACTAAGCCTCAAATGTAATACTAATAGTTGTATAGTCCCGTAAATCCTTTCTCTAACTCAGCTATAGATTGACCGTAATCAACTACTGGTCTTGGTAAAGCTCGTTGACGATCTTGAAGTTCCATGCCATCGTCACTACCACATCCAATGGCTGAATCTATGAAAAGTTCAAATTGACTATCCAACTTTGGAAGTCCTTGATTTCTCCGCTCAGTATTTATCCAATCTTTAAACAACACCGATATCTCCAAACTGTCTTCTGCTAACGAATATCTGTGGTCTCTAATTTGAAATCTTGCCGCGCTGAAAGCTGCCTCCGAAGCAACTGATGATCCTTGAATTGCAAGCACGTCCTTCACCATCCTACTAAGTTTTGGAAATTGAGTTCCACGAGCC

>LNC_002012

GTGCCATTTTTTGTGCAAAAGGTATGCTTTTCTAGAGCCGCCTCCATCTCTTCGTTTCCACTTTTGTGCTTGGAGAAAATTCGTATCAAAAGCACGTTGGACGAGATCATCTGTTTGTGAAATCTCTGATCAGCCCAGAGAGGAAAATCCGTGGCCTCCCTGCTGCTACAATTTTGTCCGTTACAAACTCCAATCTGTGACTCTTCAAGGGTCAAGCTTTTGAATTTCAAAATCTCAAGAAGCCCTAATTTTCCCCTATAAATACTACTCTATATCTTCAGCCAAAGGGGAGACAAATACACACAAGAACATCACCATACAGGGTCGAAATTACACATAGCATTTAGCTACCATCATCCTTGTTGTAACTCTTGCATTTTTTGTTTACTTTTTTTTTAACTCATTTGGTTTGAAGTTCGAATCTGTTCATAACGAGAGAGTAAATTCAAAGCCGACGACCTCGGTTCACTGCCCTACAAACGGTAATCTCAAACTTTGTTCTTTAAATTTTTAAACTCGATTGTTCAATCTGTTTAAAATTCAGGTTTAGAATTTATTAT

>LNC_002013

TTTGATTAATCATCATACATACATAACAGTACTTATAATAAAGCTAAATTGAAGTTTACCAGGAATTTTACATGGTCCAACAATTTCTCAAACACGTCTGTAAATAACTCTAATATTAGAACTCTAAAACTATTCAAATTTATTTAAGCTCTAGTTATATGAGACTGGCAAAACTAGATAATCAAACTTGGGATGGAAGGAGAAACAATGTCCGTGAATGTAGATTTTTTTAGGAGTCTAGACTCTCTCTTGACTCACTTGAGCCTTTATCTCAATATGAAACTGACACTCTGAAACATAGAGTTCATTCCAACAATCTTTGTATGGAAACAAGAAGACATACTGATTCCCAAGTAATTTGTGACATTCATCAGTCATCTTACCCAAGAGTCAGTTGTATGGAAGTTACTGAGCACTCTTCTGCATTTATTTTACTTAAAGACCGTCTGTGTTTGAAAGATATACAAATACACCACTGGATAAAACAAACAAGAAATCTCAGAATTAACAAGTGGAGCTTAGGTATAAATAGCCATGCTCTATTGGAGAACCATTCAGGTCAACAAGGCAGAAGCAAGAACATTCAGTCACTCCTGCCAATGTTATGTATGCACATATGAAAATAGAAAGGCTGCATGAAGTAGTAGCATCAAGTCAAACCATTTTACCTTTGAAGTATGGAAACCACTAACATGCGCACATAGATAAGGACTCAACTTCTGACCTCCTCGAGCGCACAACATTTCAGAAACTAATTTCAGCATGCTCCCATAAAAAAGAAACCTTTAATCAATAAGACAGTATTAGTTATCTATAATGTGTTTAAATTTCATTAAACATAAGTACCCAACACTAGTTGATCCTATTGGTGACAAAATTGATTTTTTTGCAAGATCAATTTGTGTCCATCTAGTGTTGACACTTGGCTTTAAATTTAGAAATAATATATTTGTTAAGCATAAGTACCAACACTAGTTGATCCTATTGATGACAAAATTGATTTTCTTGCAAGATCGATTTGTGTCCATCTAGTGTTGATACTAAGCTTTAAATTAGGAATACTGTGTTTGATAAGCATAAGTACC

>LNC_002014

TCAGAAGCTCAGCCTCGTAGGTATCTATCATATGCTGGGACTTCTTCTTCGCGGGTTATGCATTTTTCTAGCTACAGATTTTTGGTGGACGAACAATAAGTTGGTCATCAAGCTCTCGTCGCATCAGTTGAATACTTAAAAGGAGAAGATATAGAAGAAGATATGGAAACATCAGAAGAAGATGAGGACTTTGATGATGATGAGGACTTATATGATACCGA

>LNC_002015

TTGAATCTACATTCGATGAGAGTTCATTAAGTATATTAGCATCTCCACTTCATTTTGTCATAAATATCATGCTTAAGTAGCTGTTTTAGCCTTTAAAGGGTAAGAAAAATGCTTTGGACTTGGAGATCTATTTGTTAGAGCTCAACTTGTATCCTCCCTAATACTAACCTGCAGACTCACTTCCAGATAATTCAACTTCGAAAAGTATGTAGTCATCCTTACCTTTTTGGTGGTATAGAATCGGAACCATATGAAGAGGGA

>LNC_002016

GCTGAAGCTAAGTCTCTCTGTCACAGATGAACTGAAGCTAAGTCTCTTTGTCACGCTTTTCTCTCAGATTTCTCGGTGAAGAACACGATGAGGAACATGATTTCCGTCATAATTCTCACTGAAGAACACGATTTTGTTGGTATAGGGTTTATCAGGTAAGAAATTCTATCTTTGAAAGTCTAAATTTTAGCTTTAGGGTTTTGATATTCTTGTGTTTTTTATACGATTTTTGGGTGTCTAGCTCGCATTTTAGCTTCATCAGTTGATTCTTTGCCCTTTCTATTCTACATGTGAAGGTTAAATGATGTTTTTCCAACTTCCTTGCTTTATATCTGATTCAACTTGCCTGCTTTATATCTGTATTCAACTTTAGCCTTAAAATCTTATTCAACTTGCCTGCTTTAGATCGTGTTAACAGTTTGTCTACCTAATTTTAGAACTTGGAAATTAAACTGAATAGCTATAGGGTCTTGTTAGTCGAATCATATCAATTTCACTCTAAAAAGTCATTAGTTTTTGAAAGCGAAGCAGAATCGTCGTGCAAGCCATTGTCTTGTTTAGAACCTTGTGTCTGCAGGTTATAGAGTAAATTTCACTTTGTCTTGGAATGATATCATGTTGAGTAGATAGGTCTACATAATGTTAAGCTAATTGATATCTG

>LNC_002017

AAGCCGAGGAGGAAAGAATAATCTTCCAAGCGGACAGAGACCTAAATTTCCATTAGATTCATTCCTAAGCTTGCTTTGTTGCAGCAAGATGATCAGTCCGAGAGTGCTGGAGAGAAGAGAAAGCGGTAAAAACCTCTCTTATTCGGTCACCGAGAAGTCGGACGACTCTTCAGTAACCCGGGGTGATCCGACCCCTTCGACGCTTTTTTCGTTGTATACCCCCTCCATCCTTCGGAGGTGGAAGAAAGGGTACTCACATTTTAATACATAGTAGGCCCCAGAACGCTAAAAGGTGGGGGAACAAGAGTTGTCACGATAGAAAAGAGAAAAAAAGAAATGACTATAAGGAACCAACGGCTCTCTCTTCTTAAACAACCTATATCCTCCACACTTAATCAGCATTTGATAGATTATCCAACCCCAAGCAATCTTAGTTATTGGTGGGGGTTCGGTTCGTTAGCGGGTATTTGTTTAGTCATTCAGATAGTGACTGGCGTTTTTTTAGCTATGCATTACACACCTCATGTGGATCTAGCTTTCAACAGCGTGGAACACATTATGAGAGATGTTGAAGGGGGCTGGTTGCTCCGTTATATGCATGCTAATGGGGCAAG

>LNC_002018

AAAAAAGGCAATCAATCAGAAAAAAGAAAAAATATGGAAATAGTGAATCAAGATCTAGATGGATCCCTATCTTTATCTCTATCCACGGAGATACCTATCTATATGGAGAGAGATCTATCTATGAATCGGCCTAGACTATCCTCTATCCGGATAGATATGTCCCTATGAGCGACTACGCCGATCTTTATATGTTTTGGCCACGTCCTTTTCCGCTCCGA

>LNC_002019

ACTAAAATTCCCAAAGAAAACAGTATTGGGCTTGTAGTAGGGACAACAAGGCGGATGGAACTCGAGATGAGCAAGATGAGAAGTACATCACTCGATACCATTAAGAGAGAGAGTTGAATTTGAGAGTATAGAAGGTCCCTGAGATCGAGCAGTAGGTAATCTAACGTCGTTTTGCTCGATTATTGCCATTTTTCGATTAGATTATTGTTGTTATTTAACGGAAATCAGTTCATGCATATGAATAATATTGGGTGCTTCAAAATGGTGATGAGTATGCATAGAATATTTCAGATTTGTTTCTTGTATGGTGTCTCGAGTCTGTTTTTAATGCTAATAATGATTGATGGTGGGAAAATTCTGCACATAATTTCAGAGTACACAAACTCTTGTTGTTTCTTCTGATTGGGAAAGAATAGGGGTTTGGGAATTTTTAGGAAAGATTTATTTTATTTCTATTCAAGTACAAGTAGACTTCAACTGAGCTCAGAAAGTATAAAATATCAACTCTTATATAAAGAGTGTTCATCCATGTATATACTCAAAAGACAAGAATATGTATGCCATCAACTTAGTGGACCAGATGATAAATCATAAGACTTATTATTTCAGAACTTTCAAGACATTATGATAGAAGGAAAAGTGTTGATAAAATTGAATGTCAAATGGTTTCTTTCTCGTGATAATTGATATCAATTGGTGGAGAAAAAACAAGCCTAACCTATAAGTGCAACTTTTGTTGGATGATAAGGATTACTATTTTGGAAGTCTATTACTAACAAATAGAAAGAATGTACTTCGTACACAACTTCCAGATTGACAATAGTTAACAAGTAAAGATTGTGCTATTGCAATAGTAGCATTTATCAAAGGGTACTATTTGATTCTTTAACTCTCATTATTTGGATGTTTTTATTATTGTTACTTGAATTTAGTGTTCAAATTCTATTATCGATCTTCATATTTTGTGATGTATGAACTCATGTTCATTTTGTATTATCATGTCAACTGCTTCTCCTAGCATATGATAAACGAAGAAGATATTCACAAACCCAAATGATACTACTTTAATTTGGTTGTTTTTTCTATTTATTGCTTGTGACTCATATATTATGAGATATGGTTGTAATGTGTCTAAAGCTTGAGATTCTGAAAGTTACTAGCAATGAAAAAAGTTTATGAAAAGCTTTTGAAGTACCCTAACCACAAACATTTTGTTTGACACGACATCTACGAATATGCTTACCATGACCCTTGGACAAGTGATTTGCAGCGTCTTTGGACCATGGTACTTTGTATAACCAAGAGTAAATAAGAGAGAGAGAGATGATTATGAGACACCGAAGAGGTAGGAAATTTATTTATTTTTGAATGTCACATGTCTATATTCCTTCCTGTCTGTCTTTCTATCTTTTTCATTTATTATGCTATTTTGTAATATTAGAGTTCAATGTTTGCCTTACAGATGATGCATGATATTGATAGATTGAGTTTCATTATTCGTTTCATTTCAGTATCCCCACTCCTCCATCCATTGACATCAGTTTATCATAGGAATGAAATATAGGCATTGATTGTAAAACATTATATGTATCTTCTTTTTGCTCTTTCTGCATATTTATTTTTGGTAGATTCTGTATATGGACATGGTAATTTGTTCAAACATTTTTATGGAATTTAGATTAGCACAGAATGTAATAGAGGCTGACCATTAATCACATTGAATTGACATTTGGGGATAACAGAGGGACAAACTTTGATCCTAACTTCTAAGTGGCTCAGCAGAGGAAACAAAAAGGAGAATGGAGGAGAAATCAGAGCTGAAGATTCCTGTAATTCCATGGCGACTCTGGATTAACTTTAAGACCGTATTCGTCTCTTTCTAGATGTATGGAAAAAAAGGTGACTCAATTAATCATATTTTCCCAAAGCAGACTTGTAAACATTGATAATAAGAAATGCCTCTTTCTGTTTTCTCCGTAGACTAATGTTGTACTGTTATTATACGAGAAAATCAATAATACAGTTAAGTATTGATATTTTGTTATGACGCTAAAAAGATCACATCAAAATTCAGA

>LNC_002020

TTCAGATTAAAATCTCTGCTTTTCTTCTTATAAGTTTTTAGTTTACTTAATCTTCTCACTCTTTTGTTTAGATTTTTCAGATTTTGATTTTCTTATTTTTCTCACCCCTTTTCCCCCTGATTTTCGTTTTTATTAGAGGTAGATCTGTTGTCAATTTTGGATCCAATTCTTGGGTGATTTTCTGATTTTTTTCTTAGTTTGTTCTGAAATTTTCTACTTGCATGTTCCTAACGGAGATTAGTTTAATGTTGAATTTTCTTTTGCCAATCAATTTTCTACTTGCATATTTTTAATAGAGGTGAGTTTATTGTGGATTTTTCTACTTGCATGTTTCTAATCGAGTTTAGATTTTGTTTCTATGTTCTCTAATTTTCTTCATTTCATCGTCGTCTTTTTAAATTTTTGCTTTTCCGTAATGACTCTGTTCTACTTTCATTTGCCAATAATTGAATGTAACAACCTTTTACTTTAGAGAAATCAGAATATGCAACAAATATAACTACCCAGATACTCATAAAGACTCTGCTATGTCAATGAATTTTCCGTTGGTGCTCTTATATTCTGTCTCTTTACTTGCTGGTAATTAATAAACCTGCTCTGAAGGTTACACCAAAAAAGGAACAAATATAACAAAGCAGGTACTAATCCTTTTTGAAATTCACATTTTGAATTGTTTTTTTATTGGGAAGATCCAACCATGGATTCCATGAAGGACTTCCATCTAAGCTCGATGTTCCCTGTAACTCATCTTTTTAATTTCTTCCTTTCCTTTCTTCTCCAAGTACATGCAACGACCTATGGTAAAAAATTAACAAATTTAAAAAATGCCCACATCAAGAACAAAGCGGTTGAAACACAACATTTCAATTATTTTTTGGATTCTTATGTGTGCGTATCATCAATTTTATCTATTTTGCTTATGCATGATTCAAAATATAAATAGATACGTATATAACTCTGGATCTTTTCTTTAATGCACACACTGCATCTAAGGTGCTTTCTGTGCTGATAGTTTAGTTCACTTTAATTTAATTTTCCCGATAATCTTACTTCTGTTACATCTACCTGCTCTGGGGTTGAAACGCGAAGAAGTGGAAGGAGTTGCTATTTATGTTTTTTGAGGTTTTAAAGCAATCGTTCTTTAGTTATTGCACTCTCATTATCTGTAATCGTCACTTGTTCTTTTGGGTTGTTTCCTATTGGGTTTCAATATGCATGTACCAATTTTTTTGATATGCTTATTCAATATTCATGTTTGAATTGAGATATTCAGGTGCAATCTTATGAGATGTGTTGCAAATTCTGTGTTCTATCGTTAAAATTTATATTGCATGATTAACTTAATTTAAGAATTGGTGCAACAAAGGTGTTTGATAGTATGTTCTAACCAACTAAAAGACTTTTTCATTTCTTTTCCCTACAGATACCATTCCTCACTTATACATTTGATATTTACATGATTTTTGTAGTGTGGGGTGATTATTATCAGAAAGAACAAGCAAATTTGAAGGGATGTGTAGTGATGGATCATTGTAAAATGGAGCAAAACAGGGCAATGTGAGACTCAGAGCCTGTCTCTTCTTCTTTATGTGGGATATGGTATGTTTAAAATCCTTCGGACATCAATCACTACAACTTTAGAAAAAGAGAAACGTTGTATTTTTCATATATTTTTAAAAAAACATAGACAGGGAGAGCGAGAGAAAGAGGTATGAGCAAGGTACAGTGAAATTACTTCATTCCCAGTGTTCCAGAATGATATGCGCTCGAGGATCACGCCTCTAAAACAGTATTGGGCTTGTAGTAGGGACAACAAGGCGGATGGAACTCGAGATGAGCAAGATGAGAAGTACATCACTCGATACCATTAAGAGAGAGAGAGAGAGTTGAATTTGAGAGTATAGAAGGTCCCTGAGATCGAGCAGTAGGTAATCTAACGTCGTTTTGCTCGATTATTGCCATTTTTCGATTAGATTATTGTTGTTATTTAACGGAAATCAGTTCATGCATATGAATAATATTGGGTGCTTCAAAATGGTGATGAGTATGCATAGAATATTTCAGATTTGTTTCTTGTATGGTGTCTCGAGTCTGTTTTTAATGCTAATAATGATTGATGGTGGGAAAATTCTGCACATAATTTCAGAGTACACAAACTCTTGTTGTTTCTTCTGATTGGGAAAGAATAGGGGTTTGGGAATTTTTAGGAAAGATTTATTTTATTTCTATTCAAGTACAAGTAGACTTCAACTGAGCTCAGAAAGTATAAAATATCAACTCTTATATAAAGAGTGTTCATCCATGTATATACTCAAAAGACAAGAATATGTATGCCATCAACTTAGTGGACCAGATGATAAATCATAAGACTTATTATTTCAGAACTTTCAAGACATTATGATAGAAGGAAAAGTGTTGATAAAATTGAATGTCAAATGGTTTCTTTCTCGTGATAATTGATATCAATTGGTGGAGAAAAAACAAGCCTAACCTATAAGTGCAACTTTTGTTGGATGATAAGGATTACTATTTTGGAAGTCTATTACTAACAAATAGAAAGAATGTACTTCGTACACAACTTCCAGATTGACAATAGTTAACAAGTAAAGATTGTGCTATTGCAATAGTAGCATTTATCAAAGGGTACTATTTGATTCTTTAACTCTCATTATTTGGATGTTTTTATTATTGTTACTTGAATTTAGTGTTCAAATTCTATTATCGATCTTCATATTTTGTGATGTATGAACTCATGTTCATTTTGTATTATCATGTCAACTGCTTCTCCTAGCATATGATAAACGAAGAAGATATTCACAAACCCAAATGATACTACTTTAATTTGGTTGTTTTTTCTATTTATTGCTTGTGACTCATATATTATGAGATATGGTTGTAATGTGTCTAAAGCTTGAGATTCTGAAAGTTACTAGCAATGAAAAAAGTTTATGAAAAGCTTTTGAAGTACCCTAACCACAAACATTTTGTTTGACACGACATCTACGAATATGCTTACCATGACCCTTGGACAAGTGATTTGCAGCGTCTTTGGACCATGGTACTTTGTATAACCAAGAGTAAATAAGAGAGAGAGAGATGATTATGAGACACCGAAGAGGTAGGAAATTTATTTATTTTTGAATGTCACATGTCTATATTCCTTCCTGTCTGTCTTTCTATCTTTTTCATTTATTATGCTATTTTGTAATATTAGAGTTCAATGTTTGCCTTACAGATGATGCATGATATTGATAGATTGAGTTTCATTATTCGTTTCATTTCAGTATCCCCACTCCTCCATCCATTGACATCAGTTTATCATAGGAATGAAATATAGGCATTGATTGTAAAACATTATATGTATCTTCTTTTTGCTCTTTCTGCATATTTATTTTTGGTAGATTCTGTATATGGACATGGTAATTTGTTCAAACATTTTTATGGAATTTAGATTAGCACAGAATGTAATAGAGGCTGACCATTAATCACATTGAATTGACATTTGGGGATAACAGAGGGACAAACTTTGATCCTAACTTCTAAGTGGCTCAGCAGAGGAAACAAAAAGGAGAATGGAGGAGAAATCAGAGCTGAAGATTCCTGTAATTCCATGGCGACTCTGGATTAACTTTAAGACCGTATTCGTCTCTTTCTAGATGTATGGAAAAAAAGGTGACTCAATTAATCATATTTTCCCAAAGCAGACTTGTAAACATTGATAATAAGAAATGCCTCTTTCTGTTTTCTCCGTAGACTAATGTTGTACTGTTATTATACGAGAAAATCAATAATACAGTTAAGTATTGATATTTTGTTATGACGCTAAAAAGATCACATCAAAATTCAGA

>LNC_002021

GAAAGTTAAAACTGGTAAGTTTTTGGCCTTCAGATTAAAATCTCTGCTTTTCTTCTTATAAGTTTTTAGTTTACTTAATCTTCTCACTCTTTTGTTTAGATTTTTCAGATTTTGATTTTCTTATTTTTCTCACCCCTTTTCCCCCTGATTTTCGTTTTTATTAGAGGTAGATCTGTTGTCAATTTTGGATCCAATTCTTGGGTGATTTTCTGATTTTTTTCTTAGTTTGTTCTGAAATTTTCTACTTGCATGTTCCTAACGGAGATTAGTTTAATGTTGAATTTTCTTTTGCCAATCAATTTTCTACTTGCATATTTTTAATAGAGGTGAGTTTATTGTGGATTTTTCTACTTGCATGTTTCTAATCGAGTTTAGATTTTGTTTCTATGTTCTCTAATTTTCTTCATTTCATCGTCGTCTTTTTAAATTTTTGCTTTTCCGTAATGACTCTGTTCTACTTTCATTTGCCAATAATTGAATGTAACAACCTTTTACTTTAGAGAAATCAGAATATGCAACAAATATAACTACCCAGATACTCATAAAGACTCTGCTATGTCAATGAATTTTCCGTTGGTGCTCTTATATTCTGTCTCTTTACTTGCTGGTAATTAATAAACCTGCTCTGAAGGTTACACCAAAAAAGGAACAAATATAACAAAGCAGGTACTAATCCTTTTTGAAATTCACATTTTGAATTGTTTTTTTATTGGGAAGATCCAACCATGGATTCCATGAAGGACTTCCATCTAAGCTCGATGTTCCCTGTAACTCATCTTTTTAATTTCTTCCTTTCCTTTCTTCTCCAAGTACATGCAACGACCTATGGTAAAAAATTAACAAATTTAAAAAATGCCCACATCAAGAACAAAGCGGTTGAAACACAACATTTCAATTATTTTTTGGATTCTTATGTGTGCGTATCATCAATTTTATCTATTTTGCTTATGCATGATTCAAAATATAAATAGATACGTATATAACTCTGGATCTTTTCTTTAATGCACACACTGCATCTAAGGTGCTTTCTGTGCTGATAGTTTAGTTCACTTTAATTTAATTTTCCCGATAATCTTACTTCTGTTACATCTACCTGCTCTGGGGTTGAAACGCGAAGAAGTGGAAGGAGTTGCTATTTATGTTTTTTGAGGTTTTAAAGCAATCGTTCTTTAGTTATTGCACTCTCATTATCTGTAATCGTCACTTGTTCTTTTGGGTTGTTTCCTATTGGGTTTCAATATGCATGTACCAATTTTTTTGATATGCTTATTCAATATTCATGTTTGAATTGAGATATTCAGGTGCAATCTTATGAGATGTGTTGCAAATTCTGTGTTCTATCGTTAAAATTTATATTGCATGATTAACTTAATTTAAGAATTGGTGCAACAAAGGTGTTTGATAGTATGTTCTAACCAACTAAAAGACTTTTTCATTTCTTTTCCCTACAGATACCATTCCTCACTTATACATTTGATATTTACATGATTTTTGTAGTGTGGGGTGATTATTATCAGAAAGAACAAGCAAATTTGAAGGGATGTGTAGTGATGGATCATTGTAAAATGGAGCAAAACAGGGCAATGTGAGACTCAGAGCCTGTCTCTTCTTCTTTATGTGGGATATGGTATGTTTAAAATCCTTCGGACATCAATCACTACAACTTTAGAAAAAGAGAAACGTTGTATTTTTCATATATTTTTAAAAAAACATAGACAGGGAGAGCGAGAGAAAGAGGTATGAGCAAGGTACAGTGAAATTACTTCATTCCCAGTGTTCCAGAATGATATGCGCTCGAGGATCACGCCTCTGTGAGCTTCTCTTTTCTATATTTATTCATATTTACTCTAGACTGTACATCACTTCATGTACTTAAATAATGATTGCATTGGTATTAATCTACAAGGTGTGGTCATCTAGAAAGTGAGTACTAAATACCTATGGAAAATAAAATACTCATTCGCTTTTTCGTTACATGGTTACAGATGGGAAAATCAAGAGTAGGGATTAAGATCTAGAGGGTTGTTGTGATTAATTATATTTACTAAGAGCCACAAAATCAGTATCTTTTTATGTTCATTATGGTTGTGTTGACACATTATTGGACTTAGTCCAGAGAGCTAATGTATCTCTCAAGAAAAGATTGGAAACTACATATAAGATGTAGATTCTCTTAATGGTGTGAGGTCTTTCGAGAAAAACCTTACAATCATGGCAGTCACAATCATTTACAAGCTTTATGCCAATTTGAACTTTATTACAGTGGACTTTTGTGTTTGGAGTTCATAGTTGCTCATGTGAAGATGAAACATCTTCTTTCCCTCGTAGGCTTCTTTTCACTTTAAATTAAAATTCATTCACTGACTTTTAAATTCTTGGAAAGTATTGTTTCAATATGTTGATAAAGTTCTTCAATCATAACCAAGCTTGGGCTTGACTTTTTGGGCTGACACATTAAGGTTATTTGCGTCAGTCGCATAATCCTCAGGGGTATTGGGAAGAACTCAATAAAAATATAGACATGCTATGTGACTAAAATTCCCAAAGTAGGGACAACAAGGCGGATGGAACTCGAGATGAGCAAGATGAGAAGTACATCACTCGATACCATTAAGGGACAAACTTTGATCCTAACTTCTAAGTGGCTCAGCAGAGGAAACAAAAAGGAGAATGGAGGAGAAATCAGAGCTGAAGATTCCTGTAATTCCATGGCGACTCTGGATTAACTTTAAGACCGTATTCGTCTCTTTCTAGATGTATGGAAAAAAAGGTGACTCAATTAATCATATTTTCCCAAAGCAGACTTGTAAACATTGATAATAAGAAATGCCTCTTTCTGTTTTCTCCGTAGACTAATGTTGTACTGTTATTATACGAGAAAATCAATAATACAGTTAAGTATTGATATTTTGTTATGACGCTAAAAAGATCACATCAAAATTCAGA

>LNC_002022

CTAAGAGTAATTAGAGCTCTCAATTCAATCTCTTTCAAGAGAAAGTTAAAACTGTGTGGGGTGATTATTATCAGAAAGAACAAGCAAATTTGAAGGGATGTGTAGTGATGGATCATTGTAAAATGGAGCAAAACAGGGCAATGTGAGACTCAGAGCCTGTCTCTTCTTCTTTATGTGGGATATGGTATGTTTAAAATCCTTCGGACATCAATCACTACAACTTTAGAAAAAGAGAAACGTTGTATTTTTCATATATTTTTAAAAAAACATAGACAGGGAGAGCGAGAGAAAGAGGTATGAGCAAGGTACAGTGAAATTACTTCATTCCCAGTGTTCCAGAATGATATGCGCTCGAGGATCACGCCTCTAAAACAGTATTGGGCTTGTAGTAGGGACAACAAGGCGGATGGAACTCGAGATGAGCAAGATGAGAAGTACATCACTCGATACCATTAAGAGAGAGAGAGAGAGTTGAATTTGAGAGTATAGAAGGTCCCTGAGATCGAGCAGTAGGTAATCTAACGTCGTTTTGCTCGATTATTGCCATTTTTCGATTAGATTATTGTTGTTATTTAACGGAAATCAGTTCATGCATATGAATAATATTGGGTGCTTCAAAATGGTGATGAGTATGCATAGAATATTTCAGATTTGTTTCTTGTATGGTGTCTCGAGTCTGTTTTTAATGCTAATAATGATTGATGGTGGGAAAATTCTGCACATAATTTCAGAGTACACAAACTCTTGTTGTTTCTTCTGATTGGGAAAGAATAGGGGTTTGGGAATTTTTAGGAAAGATTTATTTTATTTCTATTCAAGTACAAGTAGACTTCAACTGAGCTCAGAAAGTATAAAATATCAACTCTTATATAAAGAGTGTTCATCCATGTATATACTCAAAAGACAAGAATATGTATGCCATCAACTTAGTGGACCAGATGATAAATCATAAGACTTATTATTTCAGAACTTTCAAGACATTATGATAGAAGGAAAAGTGTTGATAAAATTGAATGTCAAATGGTTTCTTTCTCGTGATAATTGATATCAATTGGTGGAGAAAAAACAAGCCTAACCTATAAGTGCAACTTTTGTTGGATGATAAGGATTACTATTTTGGAAGTCTATTACTAACAAATAGAAAGAATGTACTTCGTACACAACTTCCAGATTGACAATAGTTAACAAGTAAAGATTGTGCTATTGCAATAGTAGCATTTATCAAAGGGTACTATTTGATTCTTTAACTCTCATTATTTGGATGTTTTTATTATTGTTACTTGAATTTAGTGTTCAAATTCTATTATCGATCTTCATATTTTGTGATGTATGAACTCATGTTCATTTTGTATTATCATGTCAACTGCTTCTCCTAGCATATGATAAACGAAGAAGATATTCACAAACCCAAATGATACTACTTTAATTTGGTTGTTTTTTCTATTTATTGCTTGTGACTCATATATTATGAGATATGGTTGTAATGTGTCTAAAGCTTGAGATTCTGAAAGTTACTAGCAATGAAAAAAGTTTATGAAAAGCTTTTGAAGTACCCTAACCACAAACATTTTGTTTGACACGACATCTACGAATATGCTTACCATGACCCTTGGACAAGTGATTTGCAGCGTCTTTGGACCATGGTACTTTGTATAACCAAGAGTAAATAAGAGAGAGAGAGATGATTATGAGACACCGAAGAGAGGGACAAACTTTGATCCTAACTTCTAAGTGGCTCAGCAGAGGAAACAAAAAGGAGAATGGAGGAGAAATCAGAGCTGAAGATTCCTGTAATTCCATGGCGACTCTGGATTAACTTTAAGACCGTATTCGTCTCTTTCTAGATGTATGGAAAAAAAGGTGACTCAATTAATCATATTTTCCCAAAGCAGACTTGTAAACATTGATAATAAGAAATGCCTCTTTCTGTTTTCTCCGTAGACTAATGTTGTACTGTTATTATACGAGAAAATCAATAATACAGTTAAGTATTGATATTTTGTTATGACGCTAAAAAGATCACATCAAAATTCAGA

>LNC_002023

AAAATCTCTGCTTTTCTTCTTATAAGTTTTTAGTTTACTTAATCTTCTCACTCTTTTGTTTAGATTTTTCAGATTTTGATTTTCTTATTTTTCTCACCCCTTTTCCCCCTGATTTTCGTTTTTATTAGAGGTAGATCTGTTGTCAATTTTGGATCCAATTCTTGGGTGATTTTCTGATTTTTTTCTTAGTTTGTTCTGAAATTTTCTACTTGCATGTTCCTAACGGAGATTAGTTTAATGTTGAATTTTCTTTTGCCAATCAATTTTCTACTTGCATATTTTTAATAGAGGTGAGTTTATTGTGGATTTTTCTACTTGCATGTTTCTAATCGAGTTTAGATTTTGTTTCTATGTTCTCTAATTTTCTTCATTTCATCGTCGTCTTTTTAAATTTTTGCTTTTCCGTAATGACTCTGTTCTACTTTCATTTGCCAATAATTGAATGTAACAACCTTTTACTTTAGAGAAATCAGAATATGCAACAAATATAACTACCCAGATACTCATAAAGACTCTGCTATGTCAATGAATTTTCCGTTGGTGCTCTTATATTCTGTCTCTTTACTTGCTGGTAATTAATAAACCTGCTCTGAAGGTTACACCAAAAAAGGAACAAATATAACAAAGCAGGTACTAATCCTTTTTGAAATTCACATTTTGAATTGTTTTTTTATTGGGAAGATCCAACCATGGATTCCATGAAGGACTTCCATCTAAGCTCGATGTTCCCTGTAACTCATCTTTTTAATTTCTTCCTTTCCTTTCTTCTCCAAGTACATGCAACGACCTATGGTAAAAAATTAACAAATTTAAAAAATGCCCACATCAAGAACAAAGCGGTTGAAACACAACATTTCAATTATTTTTTGGATTCTTATGTGTGCGTATCATCAATTTTATCTATTTTGCTTATGCATGATTCAAAATATAAATAGATACGTATATAACTCTGGATCTTTTCTTTAATGCACACACTGCATCTAAGGTGCTTTCTGTGCTGATAGTTTAGTTCACTTTAATTTAATTTTCCCGATAATCTTACTTCTGTTACATCTACCTGCTCTGGGGTTGAAACGCGAAGAAGTGGAAGGAGTTGCTATTTATGTTTTTTGAGTGTGGGGTGATTATTATCAGAAAGAACAAGCAAATTTGAAGGGATGTGTAGTGATGGATCATTGTAAAATGGAGCAAAACAGGGCAATGTGAGACTCAGAGCCTGTCTCTTCTTCTTTATGTGGGATATGGTATGTTTAAAATCCTTCGGACATCAATCACTACAACTTTAGAAAAAGAGAAACGTTGTATTTTTCATATATTTTTAAAAAAACATAGACAGGGAGAGCGAGAGAAAGAGGTATGAGCAAGGTACAGTGAAATTACTTCATTCCCAGTGTTCCAGAATGATATGCGCTCGAGGATCACGCCTCTGTGAGCTTCTCTTTTCTATATTTATTCATATTTACTCTAGACTGTACATCACTTCATGTACTTAAATAATGATTGCATTGGTATTAATCTACAAGGTGTGGTCATCTAGAAAGTGAGTACTAAATACCTATGGAAAATAAAATACTCATTCGCTTTTTCGTTACATGGTTACAGATGGGAAAATCAAGAGTAGGGATTAAGATCTAGAGGGTTGTTGTGATTAATTATATTTACTAAGAGCCACAAAATCAGTATCTTTTTATGTTCATTATGGTTGTGTTGACACATTATTGGACTTAGTCCAGAGAGCTAATGTATCTCTCAAGAAAAGATTGGAAACTACATATAAGATGTAGATTCTCTTAATGGTGTGAGGTCTTTCGAGAAAAACCTTACAATCATGGCAGTCACAATCATTTACAAGCTTTATGCCAATTTGAACTTTATTACAGTGGACTTTTGTGTTTGGAGTTCATAGTTGCTCATGTGAAGATGAAACATCTTCTTTCCCTCGTAGGCTTCTTTTCACTTTAAATTAAAATTCATTCACTGACTTTTAAATTCTTGGAAAGTATTGTTTCAATATGTTGATAAAGTTCTTCAATCA

>LNC_002024

ATCACGAATACCATGAATTCATATTTAATGAAGACTTCTTCTACCACTAAATTGGGGGTTCCTTTTTTTACCGATGGGCAACATCAAATTCTCAGGGATTGATCTTCAACTCTAAGACATAGTTAGGTTTCCATTTCTTCATTCTTAACAGGTTGAGCTCCAGAGGAACTAATACCACTGCCAAAGAAATCTGATCTGAAATCTAATTATGTCATATTGGAGATGCCGACTTCAGGTCTTGCATAGGAGAATCTTTCTCTGGATAAGGAATAATGGGGAGATACGCTTTGTGCTCCATTGTTGTACAATCTGAAGACTCTTGAGGGCTTGTAGATATCATCTGTAGTGGTATAGGGTACTCAACATTAGCAATCACAGTGGACTGCTGGACACTGGCACATATTAATTGTTCCCCATCCTTACTATCTACTTCTTTATTACTACTGATTTGGATGCTTTGATTAATCATTAGTTGTTTGGTCTCTTGAGCTAACTTCTCATCCTTGAGATTTGTAGTCTTGTCCACCTTTTCTATCTTTGATTGCTCACCCTGAATATCCTCATGTGTGCTGTATATATTGCTGCTAATTGATTTGTTATCCTGAATCTCTTGAGCAGTAGAGGATACTTCATAGTATCAATTTGTTTTTTCATTGGTATTTTGATTTGGCTTTGTTGTACCTCCACCTTGTCTGATGTTTGCATTGAGACTGGCTCATAATTTTCCACAACTATTTGAACTTGTTGTGTCTCATCAGCCAGACAGTCAAAAGCATTGACAGTCGCCACTATATTTTTACCATTGGATTCTGCTTCTTTAGTCCTATCCATGATGAAAACTTTTTTAATTGGACACCATTTTTTGATGAACTTTTTATGTCTCTAGTTAATCTTCTGATATTGATCTTGAGCCTGACGTTGAGGTAGCTCCCCTTCCTCTTGAACCTTTTTTTCTCTAGCATATTTTAATTCAGGATGAATTGTTCTGCATTCTTCTTCATTGTGTCCTTGCAGCTTGCAGGTGCGACAATACTTAGGTAAAAAGTCATAATGGATTTTGATCTTCTCAATCCTTGCACTTTTCTTTCCTGGACTAATCACTTCTATCTCCATCCACTCCGGAATATTTGCAATAAGGTCTACCTGAACTTTGACTCTCGCACAACTTGGGTGTGTCTTATTAATTGTTGCCAAGTCCAATTGCACCGGCTTCCCCACAGCCGATGCTAATGAGAACAAAGTTTCTTTGCCATAAAAAGTGGGCAACAGTTGCGGGAAATAAATCCAAGACATTGCTTGAGTAGTCTCCTCC

>LNC_002025

CCTAGCCCAAAAATTAAAGCAAAACAAAAAATATAGGCAAAGAGACAGGTCATCTGCATAATTAGGAAAGTAGGTAGAAAGCTCCACCCCTTCATCTTCCTCAAACGAAATAACCGTAGATTTGATCTACTGTTAACTTGGTTTGGAAAAATCCTCCTCCGCTTCGATGTTCATCCTCAACTATCAGCTCAAAGCTCCATCTCTAAGTTTCCATGTCTTAATAGTTAGAGCTACATATCAGAATCGGTGGATCTATTGTTTGGATACTCGTACCATTAACACCCCAAGGTCTCACTGGGTGTAGGTGTTTGCATCCAAAGAAATCACTTCTAACAATAGCAAACTTTGTAAACATGGTCAATGTTCTACATCCCTATTCAAACCATCTGCAACATTTTCCAGATTTACTAACAGAAACACTAAGATCACAAACTACCAGATGTTGATGCCCATAAACAGGACTTTGTTCACCCCCTGAGTTGGCAGGAGATTATTGCTTGTGATGCTTGCTGGTAGTGTTGCTTGCTTCTCCTTTCCATGACTATGGAATGAACTGGATATCCATTCCTTGGTGGACATTTGATTCTCAAATATTTCCCCTTGCTTATTAATTCCTTGAGCTTTAGATATTGAAGTAATATTTGTATTATTAACAGGAATGTGATCATCCCCTTCTTCTGTAAGTGCTTCAAAGGGGTTATCATTGGTAATCACTTTATCTGGTAATATAACTTTCTCACTAAGATAACCCTTGTTAATGAACCTTCTGTTGGTTGGGTTCCATTGATGCCTTTGGTAAGTGTTTCTCCCTGCTTGTTTCCTTGATGTGGTGGTCTCCACTTTTTCGTTATGATCATTAACGTCTTTTTCCTCATTTTCTAGTTGCACCTGTTTAAGTTCATGATGAAGTACCCTACATTCTTGCTCATTGTGACCTTGTAACATGCACTTCTTGCAATATTTAGGTAAAAAATCGTACTTAATTTTCACCTGTTCAACCCTTGATTCCTTTGTGGTGGATTTAACAATCTCCGTCTCCACACACTTAGGTAAATCAGCCAAAAGGTCCACTTGCACCTTGACCTTAGCACAATTAGGTCTTGTTTTATTTACCGTTGCCAAATCCAAATGAATCGGTTTCCCTACTGCAGATGCGAGTGGAAATAGTGACTCTTTCATAAAGAAAGTTGGCTTGAGGTCAGGGAAAGCGATCCATGCCATTGCTTGAGATGTTTCCTCTTCAATCTTGAATTTAGCATCATAGATTAGGGGTCTCATTTGATAGGAATAACCATCCTTATCATTGATATAGTAGATGTTCTTGGACATACCATTAACGAAATCCTTAATCAAGTTGAATCTCATAAGAATGTGATGATTTCTCAAGAAACCAATCCTGCAATCTCCTTTCACTTTGAGTTATATAGGGATTTAGATGCGAAGATCCTCTAATTTAGGCCATCCATAGGAAATTTTCCCTATTGTTGCATATTGTAACTCTTTAATTACAATCATTCGATCCACTTCTTCCTCATTCCACATAACTCGAGGAATACCATTGATGTAGTTCAACTTTTTAATAGGAATAGGTTCAATTGATTGCTTACTCTGTGTCTTTGGAGCATTAATCGTTAAAGGTTTCAGAAGTTTGACATAAGGTTCCAAAGGTGATGGGGTTTCTTTGGCAGGCGAAGAAACTCTAGGCAAAATAGGCTCGATAGGGGTTGTGGTAATGAAGGAAAATTGTTGTCGGTAGTAGTGAAGGGTGACTGGTCAGCCATAAGAACAGGCTGACCAGTGGCCTGATTGACCATTTTCGGCAAATATTAGGGTTCAATTGAAGTTGACGGCATAAGTCGCTTTTGCTTCAGTTTCAACAAAAAAATTTAATCGTAAGTGAATATTCATTTGGTTCTCATATAATATGGATTTTGTAGCTTATGTTTGTTTGTATTTTTTTTAGGCAGAAACATATTTTAATAAAAGATGGTTGTTATTTTATTTATTTTAAAATATTGTGTTGATCATGCTTATACAAACCCATTTGCAGTTCTACTTCATGTTCTTGCACACATATTTTTAGTCTTTTTAGTCACTATTATCTCAATTCTCAAGTATAAGGCAGCTCTGTCATTTTTCATTATTCATGCTTCATAGAAATAATATTTCACTTCTAGTTTAAATTTGATGGGTTTGAGATTTAGCTTATACTCAAAATATATTTGTTATTGGTTTTAAAAGTC

>LNC_002026

CCTAGCCCAAAAATTAAAGCAAAACAAAAAATATAGGCAAAGAGACAGGTCATCTGCATAATTAGGAAAGTAGGTAGAAAGCTCCACCCCTTCATCTTCCTCAAACGAAATAACCGTAGATTTGATCTACTGTTAACTTGGTTTGGAAAAATCCTCCTCCGCTTCGATGTTCATCCTCAACTATCAGCTCAAAGCTCCATCTCTAAGTTTCCATGTCTTAATAGTTAGAGCTACATATCAGAATCGGTGGATCTATTGTTTGGATACTCGTACCATTAACACCCCAAGGTCTCACTGGGTGTAGGTGTTTGCATCCAAAGAAATCACTTCTAACAATAGCAAACTTTGTAAACATGGTCAATGTTCTACATCCCTATTCAAACCATCTGCAACATTTTCCAGATTTACTAACAGAAACACTAAGATCACAAACTACCAGATGTTGATGCCCATAAACAGGTGCTTCCAACATCTATAGACTTTGTTCACCCCCTGAGTTGGCAGGAGATTATTGCTTGTGATGCTTGCTGGTAGTGTTGCTTGCTTCTCCTTTCCATGACTATGGAATGAACTGGATATCCATTCCTTGGTGGACATTTGATTCTCAAATATTTCCCCTTGCTTATTAATTCCTTGAGCTTTAGATATTGAAGTAATATTTGTATTATTAACAGGAATGTGATCATCCCCTTCTTCTGTAAGTGCTTCAAAGGGGTTATCATTGGTAATCACTTTATCTGGTAATATAACTTTCTCACTAAGATAACCCTTGTTAATGAACCTTCTGTTGGTTGGGTTCCATTGATGCCTTTGGTAAGTGTTTCTCCCTGCTTGTTTCCTTGATGTGGTGGTCTCCACTTTTTCGTTATGATCATTAACGTCTTTTTCCTCATTTTCTAGTTGCACCTGTTTAAGTTCATGATGAAGTACCCTACATTCTTGCTCATTGTGACCTTGTAACATGCACTTCTTGCAATATTTAGGTAAAAAATCGTACTTAATTTTCACCTGTTCAACCCTTGATTCCTTTGTGGTGGATTTAACAATCTCCGTCTCCACACACTTAGGTAAATCAGCCAAAAGGTCCACTTGCACCTTGACCTTAGCACAATTAGGTCTTGTTTTATTTACCGTTGCCAAATCCAAATGAATCGGTTTCCCTACTGCAGATGCGAGTGGAAATAGTGACTCTTTCATAAAGAAAGTTGGCTTGAGGTCAGGGAAAGCGATCCATGCCATTGCTTGAGATGTTTCCTCTTCAATCTTGAATTTAGCATCATAGATTAGGGGTCTCATTTGATAGGAATAACCATCCTTATCATTGATATAGTAGATGTTCTTGGACATACCATTAACGAAATCCTTAATCAAGTTGAATCTCATAAGAATGTGATGATTTCTCAAGAAACCAATCCTGCAATCTCCTTTCACTTTGAGTTATATAGGGATTTAGATGCGAAGATCCTCTAATTTAGGCCATCCATAGGAAATTTTCCCTATTGTTGCATATTGTAACTCTTTAATTACAATCATTCGATCCACTTCTTCCTCATTCCACATAACTCGAGGAATACCATTGATGTAGTTCAACTTTTTAATAGGAATAGGTTCAATTGATTGCTTACTCTGTGTCTTTGGAGCATTAATCGTTAAAGGTTTCAGAAGTTTGACATAAGGTTCCAAAGGTGATGGGGTTTCTTTGGCAGGCGAAGAAACTCTAGGCAAAATAGGCTCGATAGGGGTTGTGGTAATGAAGGAAAATTGTTGTCGGTAGTAGTGAAGGGTGACTGGTCAGCCATAAGAACAGGCTGACCAGTGGCCTGATTGACCATTTTCGGCAAATATTAGGGTTCAATTGAAGTTGACGGCATAAGTCGCTTTTGCTTCAGTTTCAACAAAAAAATTTAATCGTAAGTGAATATTCATTTGGTTCTCATATAATATGGATTTTGTAGCTTATGTTTGTTTGTATTTTTTTTAGGCAGAAACATATTTTAATAAAAGATGGTTGTTATTTTATTTATTTTAAAATATTGTGTTGATCATGCTTATACAAACCCATTTGCAGTTCTACTTCATGTTCTTGCACACATATTTTTAGTCTTTTTAGTCACTATTATCTCAATTCTCAAGTATAAGGCAGCTCTGTCATTTTTCATTATTCATGCTTCATAGAAATAATATTTCACTTCTAGTTTAAATTTGATGGGTTTGAGATTTAGCTTATACTCAAAATATATTTGTTATTGGTTTTAAAAGTC

>LNC_002027

CGAGCTCTCTGGTTTGATGATTCTTCTTTGAAGTGTTGCATGAGGCGAACACCATGCTTACATCTCTCTCAGCTGTGGTAATATTTTCCTTTAATTAATTTCGTGGATAAATTTTCAGTAGATTCGTATTGGTGTGTTACATATCTACAAATTGATACTTCATAATTAATGATTTTGGATAACTATTATTTGACACTGTTTCTGGTTAATTTGTAATGCACTTGTGCTTCAGAAAACTACTGACCGAAATTTCCTTACAAACTAATTGTTACAAAATTTTTATTTTTATTTTAATATTTCCTTTTTACATCTTATGTTATTTACAAAATAGTACTAAAAATCAATATAAAACATTATAAATTTGGTTAACTTCTGAAATTTTACCGCATTGCATTAACCACTTAAAATATTTGAAAACATATTAAAATGTAATCAACTTTCGAAATTTCACATGTCTCACATAAATTGGGATAACATATATTGTTTGAAAATTACGATTTAGTTCAAAATTTCACATGTGCCGTGTAAGTTGGGACAGAGTGAGTAGCATATATTATTTGAAAATTACGTTAAAAATACTATAAATCACAACTTAAAAATATTAACATATTAAAAATTTGATTAACTCTCAAAATTTCACATGTGTGGACAAAGAAAATAACATATATTATTTAAAAAATTACATTAATTAAGAGCACTATAAGTCACAATAATTAACAACTTAAGATAATTAAAAAGACACACTAAAAGTTTTACTCTATATTATAAGAATGAATGTAGAGGAACTTAGGGTCATCCGGTCAATTGACCAGTTAGCCCTTTTCCTCAACTTCCTATATTTAAGAGTGAATGTTTGAGCAGCTTAGGATTAGTCCGTCAATTGACAGATCTCCCCTTCCTCAAATTTCC

>LNC_002028

CAACATGTCCCTTTTAATGTCATAATCTTCGGGTATTTTACATATGGTTATATAACTATTTCTTGTTTTCACAAAAGTCACTTAACTATCTTTTCTAACACAAGTCATTAAACTTAAAGATACTAACTAAAAAGTCACATAGCCCGAAAATGCTAAACTTTCCAATGCAACTCCTTTTTACTTTTAAAAAAAAAAAAATACGAATTGAAATTCAGTTAAAGCTTTGACCTGACTTGACCCAAAATCCAAAAATCAAACCCATATGAAGAAAGTAAAATATCAAAAGAACTAGTAAAATTACGCGCGCCTCGCGCAGGAATTTAAGATCACAGAATTTATAAACTAATACTTAAAACCTATCAGTCATTAAAACTAAAACACTATATTATCTTACATACAAGATTACGTAGTAACGAACATTAATAATGTAAAGAAAAATGATAATTATAATATCTTATCATTTATTCTTCAATCAATCATCTTTAATTTGATTTTATAATTTATCCTTTTGCTTCCAGGAACTGTTACTATTTGATAGTTTTGGGGAATCAAATGAACATCAACCTGAGATATTAAATTTTCTAATAAAT

>LNC_002029

TTTATTTCAAATATTTTTCACTTTATTTGGAATTTATCAAGTTGAAATTGAAGATGGATTTGGTTGTACTTTTTGCGAAGAAGGGAAGAGAACATTTTGTTTGGAATTTATAGAGATGGAGTTGGAACTTGGAATATAGGTTTGGAGCACTTTTCCTAATTTAGAATCCAGCTCCAAGTTGGATTTGGAAATTTTATAACTTAACACTGATTTTGAAATAAAGTGAAAAAGTATTCCAGAATAAATGTGAATAATTCTCATGGCCAAACAACTCCTCTGCACATAAGCATTACACTTTCA

>LNC_002030

TTTGTAGTATATTTAATATAAGCTTCGATGGAGAGACAATTTTGTTTATGTACATATTTGGGTTTATTAGCAGCCGCTCGTTTACAAATGTGCCATATTTAATCTGTCCGTTTTTATGGTTATTGTTGAATTGAAATAACCAGAAAAGAAAAAAGAAACTGTCTCGCAGGTAGCAAGTTGGTTCATATTAATTTTGGAAGTTTGAAGCTCTTTCATTTTAGCATATAGTTCATCATATACTATTTGCATACCAATAGCATTAGTACTAGTCTCGTAGTTTCTTGTCCTTCGATTTATGTTGCAGCCTATTGTTTCTTTGCTTTGGTTATGGCATTATTTTGTTGTTGCTGTTCCTTTATCTGAATGCCATGTGATGATTTCTCTATTATATTCTATGTCTGCTTTACTTCTGTAAATTTTTCAAAAAACTTTAAACTGCTTTGATATGTGTTACTTGAACCGAGAGTTTTTTTTGTAAGCAGCC

>LNC_002031

GCTTTGTATAGCTAGCATCAAGAAGATCAGTTACAAGAGATATGCTAGCTCCAACGACAGTAGGCCTTTAAGCATAGAGAGCTAATCAAAAAACAAAAGAGGGCTATTGAGCCAGGCTTAGAGAGCCAATGAAATCTACAAAAGAACCTACATCATTTACTGGAGCATAATAAGTCCAATTAAAGAGCAGTAACAGACATCTACCCTTGAGAAGTGAGATAGGAGTTGAAATTCCATCAAAACATCTTCTATTCCTTTCTATCCAAACACACTAAAAATGCAACTAAGGATCATTTTCCAGATGTTCTTGATGGCTTTGCTGATTCTCTAATAATTCCAGCTTTCAACTGCTTCCTTTATAGTC

>LNC_002032

CTTGACGTTCTGGGTTACGGGTTGGCTTACCTATTGGTAGGGTATAGTAGGTGCCATCATGACTCGAGAAATCGGGTCGTGACAACCTTTGAACAAAGTATAATCACCTCTTTATTTACATTTTTTTAAAAGTGACCCTTTAGCATTATTGATTGGTTTGTTTCAATTCTTATTTATTTAGAAAGCATGTTTTGGAGTTGTGATTTTTCCTTTAATCCTTGGCATGTAATTTATAATAACCATCATGTAGTCCTTTTTGTGTGTGTTAAAGAGCTCATTCTGTACTTGTTTGCGTTCATGAAGATCAGTAACTCTTCTTATTCAGTTTCCTTTCTTATTTTGCTCAATGTTTTCTAGTTTCTTATCCTTGTTTTCGTTGCATGTGAAACTCCATTCGAGTACCTTGTGGACTTCCTCCCTCCTTGCATATCGAGTTAGGCCATGGAGGCCCCCATTTTTGCCTCTTATCGAGTCTGCCGAGGCAATGGGATTGAAGAATAGGGCTGGAAAATTTTAATTTGCAGGTTTCGGAGCCGAAAATACACTGATATACAGTGTGATACAACGATATACACTCACTGTATACGCAGTA

>LNC_002033

AAAAGAAATAAAGAAACCAAAATACAATGAAAGAAAATTCAACTTTTCCAAAACGATCCTTCATCTTCATCTTCAGACTAGGAAGCTCATCGTTGCTTTCTTCAAATCGCTGGGAAAGCCTCACCTTCACAAACTTATCGCCATATCATAGTAATCGTGTAACGCTACCAAGTACGGGTTGAAATCATTCAGACATCTAAAATCTCACAGACGTCATGATTCAAGCACCATTGAAGACTTCTTCTACTTGGGACTTGGGAGTTGCAACTATTAACTGAATGTAGAGGTCTTGAATCTGGAATTATCCTTCGACGAGCTCCAACAAAAAAACTCAAACTAAGTACCCCAACTCCTATAATCTTCAAGTTTGTTTGTTGATTCATAGCTTTTAATTATGTATCTCAACATGTTGATTTCCAATTGGAATTAATATCATTGTTAAAGACATACAATTATCAACTATGTTTCAACTATAAATTTGAAAAATATCTCTTTTTTATTGTTTCTAATGAATCTCTAGC

>LNC_002034

CTTGGTTGACACGAGCACAACAATCGGCGGAGCGCGATGGGAAGGGAAAACATGAGTATAAAAAATACGATTTCTTCCATAATTGAAGGATATTTCAAGTAGAAGAGCTGACGTATTGTTTCACATTTGAATTTCTCTTAAATTCTACGTTCCCGAAATGGATCCTATCATATATTTCACATTTTCTATGATTATCTCTATTTCAGGTATTCGGGGAATCCTCCTTA

>LNC_002035

GATAGAATCTATGAGTGTTTGGATTGAATCCCTTGGGACTTGAAACTGAATCTTAGTCTGCAGGAATTAAGACTGGTTTGTAGCTTTCCTACCTTATCTTACCTAATCTGAAGTTGGACGGGATTCCTAAGTCGAATGACTGATCTCCGGACTCTTTACGGCTCTCAATCTCAATACTAGCTGGGGTTCAGTTCAAACTGCCTTGACTCTGAAGGAGAAGATTCTCTAGT

>LNC_002036

GGCAGGAGTCTCGACGACAGGTCGTGCCTGCAGTTGTTCATGCACATCTTATGAAGCTCCCATGCTTCAAGATTGAAGTTTTCACCAGAAATCTCTGTAAAAAAAAGAACGTTGTAACGAGATCGCATAGGTCCAACATAATCCTCCTAACAGGCAGCCACATATGGGCGAGGATCAATTAGTTAGCTAACGAAGTTGATGTTGAAGTAGAAAGAAGACTAATAAATAGGAACGGAGTCGGGGAATTCTAACTCTTAACTTGCTCCTGCGACTTCCTTTTCATTTGGTAGGGCATGCCAACGAGATCTCATGAGAGCTTTTCAAGGTATCCAGTTAGATCCCCGGGTAAAAGAGACCTATCCATTTTAGCCACCCTTTCCATTACTGATGTAGTTGCTCTTGCT

>LNC_002037

CGAAACTTAAGTTTGAAGGGTTGTAGAATTTTGACGCGGCGGTGACGTGGCAGAACCACGTGACGACACGTGGCATAAGAGTGATATCTGGAAATTTCCAGATTTTAAGAACGAAATTCGTCCAATTTTTTTGTTCAACTTCAACTTGAGATTAATCCTTCATTTTGAGTCCAAATTAAGTGATTCAAAAGGCAAACTTCAAGAGAATTTCGAGGGGAATCTAGCGGTGATCTCGGAAACGCGAGGGGCGGCTTCGTTTGAGGTAAGAAATCAATTTTAGCCACTGCCAGTGTGATTTCCGGATGGTTTTGTTATTGAATTTTGAAATGTGAGATCTTGATCATCGTAAGTCCGTTTTAAGTCATTCTTGGGGCTAACTTGTAAAGTTTTTCGCAAGGATCGTCGTGGTATAATCTGTTTGGGTTGAAAGGGTTTCGTTTTTCCGAAAATTGGTGATCAAGGGGCTGCCCTTTTTAATTATTGTGGTTGATTTGCGGTGTTGTTGGCATCTGTTTGTGCTAAAATTTTGGGATATTAGTTTAGTAAGGTAGTTGGGACGTTTCCACGGATTCGATTTTGAGATTCTAAGTGTGGGCCCCACAATCCCGTTTTAGACCCGATTTTGGGTCCGTCTCCGAAAAATATAATTTTAGTGTCAATTTATTCGTAATGACAAGGTGATTAACCTTTTGTTAGTGGGGAAGCATTTGGAGACCGTTCG

>LNC_002038

GCCATCTGAATCAATTCTGAACGAACAGTATCTTTATCTGGCTTTGTTATTATTACAGTGTTATCTGCACCCAGCCAGTAAGTAGAGATATGGAGAGAGGGGACAACTTTGTCGCGGACCTTTCTTTGCTTTCTTTTATGAACTCCACTGGTCGTCCATGGATTATAGGGGCTTTCCATTGTATATAAATACACTTTCGTATCCTACTCTGCAAATCCCATTTTCTTTAAGCATATAGAAACC

>LNC_002039

GGAGAAAGGAATGATGTCCCGGCTTGGGCTGGGCACCTATCAGCCAACTATTCCTTTTTGGAATGGAAAGAAGAAAGGATGGTCGTAGATCACTGATGTCGAATTCACGAAAAAGATAATATGTGAAAGAATCGTCAGATTATGGGTTCCCGGAGTAGACGAAATGGAGAATGAATGAAATAGAATAGTCTGGCAGAATCGTATGATAGGGAAAGTGCTCCGCCTCCCACTTGTCTGGGTTATCGGGCTGGGTAACGTAACTATGAATTTCCATCAATGTCGGGATAGACAGAGGTCTAGGATAGTACGATGTCGGAGTGAGACCGGGGTACAGTAACGAAGTTAGACCGAAATGAACGACTCAATTCCTTTAGAAGAAAGGAGAGAGAAAGAACAACCAATTCTATGAAACAATATCTTATTTACTAGGAAAAAATATCTTATTTACTAGAATCGGTTGCTACTAG

>LNC_002040

TTCCGGTTCTCTTCCTTTATGAAGAAAGGAAAGTCTTGTTATCGACGAAGAAGATCTTTATACTGTTCATCCTAAGAAAAGAAGTTTTCACTCGGTACAACTATTCTATGCTAGCATAGCATGCTTCTACTTATGTTGTGTTGCAAATTCCGGATTAGGAACAATCCTTTCTTATTCTAAGATGCCCGTCTAAAAATCCGTACTTGTTTACTAAAAGGCTTTCGGCTTTCTAGCAAGCTTAAGAGCTAAGGGAAATTCATCCTTGGATCAATGAAATAGTTGAATTAGCTAAGATGCAAGAAGCAACCTCTTATTGAAATGACTTTAGGAAGTGCCTAACCTTTCTCCTTCGATCCGATCCTTTCTGTGACCCGAAATGAACTTATTCTCTCTTCGTCTCTTGCTTTGAATCGGATATTCCGATGAAAGCGGATTCTCTTTTCGTCCC

>LNC_002041

TTATCTTATTTATAGAAATAGCACTCGATCAAAGGGGAGCATAAGCAAGTTCAGTGGTAGAAACCTCTGTAATCGATGGGAACCTCGAAGCTGTCTGGGGCAGGAGAAAACTCATATTAAGCACTGCCCCAGCATCAACTGGTACAAGGAGGAGGCACGATAGCGAAGATCAATCCATCTCAATCTAACAAAAGCATTGCCTTGGG

>LNC_002042

GAAGAAATTAACGTATAATATTTCTTTTGCCTTTGCAGCTTAAGAGTGATCAAGATATCCTAAAAACTTTTAGGTGAAGCACTATGTTCTGCTTGTACCGGCGAGGACATGCGACTTCTGCTGAAATTATTGTTCACTACACCGTAGGCAATGTGGAGGTGACTTTTATGGAGATGTACATGTTTTGGGTCTTAACTGTAAA

>LNC_002043

CAACTATAAACCAAGGCGGTGGTTCCTCTCATTGTCTTTTTGGCTCACCTCTCAATGATTGAATAGCAAGAATTATTTAGAAAACAGGTGCAATCATTCGGTTCAATGATTGATACTCGAGGGGAATTTGGGCACCAAACTGGGGAGATGAGAAAGCCTAAACTAGTAGACCTAAGAATGTATGCTTGGAAATCAGGAAACTCGATGGTTTTCACTTGGCTACCAGACTCCATGAAACCTGCTACATGTTACCATATAGCATGTGAAAAGTGAAATGATAAAGTTTTGTGACAGCAAGGGAATACTATATAGCATGTGAAAAGTGAAATGATAAAGTTTTGTGACGGCAAGGGAAATGGTAATCTTGGTGATGAAGTAGAAAAC

>LNC_002044

CTGCACAAGATTAAAATGAACTGCATTATCACTTTTTGCTATTTGTGTAGTTCAGAGTATATCGATGACCTTGTGGCTATTGTAGACATTCTAGGGTCCTTATAAAGGTGAAAGGAGGATCTAGGGATAGTTCTCTTTTTTCTTCTTTCTTTCTTTATCTTTTGTTTATGCTCGACACACGTGTGGAGGGAGGATATGATGTAAATTTTTGGATACCAACTTTGAGCTGATGATTGATATACAAATGTTACCTTTATAAAAAAAAATTAATAGAAATGTTTCTGAAGTTCCCTTTGAGAACTTCTCCTTATTTGTCCTGAGCAACTCAAATGGAACATGTGCTCCCTTGGTCAGTGTTGTTCTCGTTATTTTATGACGTCTCTGATAACAGTTATCACTTGCTGGAATTTACATGGTGGTTAGATGAAATGTCAAGAGATGTGTTAGGGGCTTATTAGTCTGAGTGTCAGGTGTTCTTGCTTATCCTGCAATTGGTAATTAGAAATTGCGGTAAGTTAATTCTTGCTTCAGTGAGAGTCTAAATCCCAATGTTTATGATGAGTTAAGGCATTGCAGTCTTGCAGCGTGG

>LNC_002045

CTTTGACATAAGGGGTTTTTTAGTCTTGTCTATGGCATATTTAGTGATTACGAGGGTGGTTTTGGGGTTCGTCAGATTTGCACATAATTGTTCCTTTCTTTCAATTCTTGCAGCCATTGAACCACAGGGCAGTGGATTATTCTTTTCATGAAGCGGCCGTGTTTATCCGTTTGTGTTGAGGTTTCTTTAGTAGAGTCTTTTATGAGTATTTGAGATTTTTGATTCTGTTTTCTTTCCTTATGGATCCCTTTTCTCTGTCAAAACTATTGTTGTATGGATGTTTTTTGACTAAGATAATATTTTCTTACAAGTGTGTGTTGTGTTCTGCTGCCATGAAGATTTTAATCCAGACTCTTTGTTCTTCAATGATCCAACAGATGAGTTCTCTCCTCATTATGGTTATAGTCACTTGATAAGTACTCGGTTTGTGGATCTATCTCTCCATAAAGTGCTGCTCTACTTCTGGTCCTCTTGCTACATATATTCTTTGCTGCCATTTTTTGTTGGATGGATGTGTAGACTATATGGTGAAAATATTAGAGGTTAGAATTTGGAGATTTCAAAGGTTGATACGCAGTATAGTATCTCTGTACCCTCTTGGTCTCTTTGGTTCTTGCATTGCAATCTGGATATATTGGTCAAGGCTTCAACAAAGAAGGGTTTTTAAATCTTGTCTAAATCTCATCTCTAGTGATTTCTTAGTTGGTGTGGGTTTCGTCAACGTTGGTCTTGATTGTACCCTTCTTTTTGTGCCTGCAGCCATTGGGTCTTACAGAGCAGTGAATTATTCTTTTCATGGAGTGGCGTTGTTTGTCCATACATGTTGAGGTTGCCTTAGTTCAGGCTTTTATGAGTACTGGGATTACAACTAAAATAGTTTTTGTTTTTACTACTGTGTCATTTTACTGTCATTTTTGTTTTTTAGTCCAGATGCTTAGTCTCTT

>LNC_002046

TTTTTGTGCTTGCACTCCAAGATAGATATTGTCAGGCTTCGACTAATGGGGTTTTTCAATATCTCTTGCGTCTCTAGTGATTTCTTAGGTGATAAGGGTTTTGTCAGCTTTGCACTTAACTGTTCCCTTCTTTCTGTGCTTGCAGCCTCCTTTCTGTGCTTGCAGTTGGGTCTAACCGAGCAGTGAACTATCCTTTTCATGGAGGGGCTTTGTTTGGACATTTGTGTTGATGTTCTTCTACTTCAGCCATTGAACCATAGGGCAGTGGATTATTCTTTTCATGAAGCGGCCGTGTTTATCCGTTTGTGTTGAGGTTTCTTTAGTAGAGTCTTTCATGAGTATTGAGATTTTCGATTCTGTTTTCTTTCCTTATAGGTCCCTTTTCTCTGTCAAAACTATTGTTGTATGGATGTTATTTTGACTAAGATAATATTTTCTTGCAAGTGTGTATTGTGTTCTGCTGCCATGAAGCTTTTAATCCAGACAATTTGTTCTTCAATGATCCA

>LNC_002047

TTTGCCAATTTATTGATTCGTTGTTGGTGTTTGTTTGATCAGGTATGGGTGATAATGATAGGAGAATGATTATGGGTACCTTTGCAGTGCATTTATTTTCTTGTGTACCTTCGATTGATTTCCAATGTTTTTTTTAGAAATTGGAAGCTGGTAGCCTTTCTAGATGCAGTTTTGGGGGAATCAAGTTTTGCTTCCAGGTTTTGGGAAATTCTGAGGTTTTTGTTGTGAGAAACCGTGTGTTTCCTATTTACTACTCTGAAATATAGCAATTTCCTTTTATTGAATCAGAATACGAGTGGAAGTGGAAATTTGGATCTTGAGATGAATCAGTCCCCTCATCTTTTCTTGTTACAACCATAGAGTGGACCTATCAGTTTTGGATTTGATATTTTGACTTCCATCTTTAATGCTAATTTTGTTATCATGTTTCTCCTCGCTCAACTTATTTGGTTTGGTCATCATTGGATGTCATTCTTAGCTGTCGAGACTACATGGACGGGCATCTAGGTCCACGTTGGTCTACTGGAATAAGTGTCTTGCTTCAATGTTTGTCTTGGGAAAGATGCAGCAGTTCCCAGGTTTTTGACTATAGGTCGAACTATATGGTGGCAGTATTAGAGGTTAAAGATTTGGAGATTTCAAAGGTTGATGTCCAGTATGCCATTGGGTCTTACAGAGCAGTAAAATATTCTTTTGATGGAGAGGTTTTGTTTGTCCAATTTCTGTGGAGGTTTCCATGGTTCAGGCTTGTATGAGTACTGAGATTTGCAACTAATTTTTTTTCAACTAGTGTGTGTCATATTCTATCATCATTTTTGGTTTTTCGTTTAGGTGCTTATTTTCTTCAATTTTCCAACTGATGAGATTCCTGATGGTTGATGGTTATGGTTTTGTGGACACCTAACAAGTAATCAATTTTGGATCTTTTGATTCGTTATTGGTGTTTGGTTGAAGGTCTGGGTAATAATGATAGGAGAATGATTATGGGTACCTTTCCAGTGCAAATATTTAATTTGTTCCCATTTTCTTGGTGTACCTTCGATTGATTTCCAATGTTTTTATTTTAGAAATTGG

>LNC_002048

TCTTCTTCAATTCTCGTGGGTTTTCAACTTAGAGGTATGGGGATCCTTTATCCTTGATTATTCTTCCTTTCAAGGAGCTCAATTCAAATGATTCAAAGTTCTTCCAAAAACACAAAAATCCTAATTTCGATTCTAAGCATGGGTTCTTGCATTAAATGATTTTTTTTGATGATTTATGTTATTAATGGTGTCTATTGATGGTTTTAAGAAAGAAATTTCCATGAACCCATGTCTTGGCAAGTTTCCTAAAGTTGGCTTATGAAGTGGGTTTATTATTTATGATTGAATGATGATGGAAGTATGCTTGGATTGAGTTGAATAGTTGACTTCTATTATTATCTATGCTTATCTATTGTGAATTGTTGGAGAGTTGAGGAATGGTGATTGTGGCTTGGGAAAAAGGGTAAGTGGACCTAACTTCATTGTTAAGGTAGAATTGATATAGAATTGTGATAGATTGATGTGTGATCCACTTATGGTGGAGGTGTTTACTTATTGAATGTATTGTGTTTATGGCCTTGGAGGCATTATATGAGGAATTGAAGTGTTGACATGATATCATATATGTGAATGTGATGTGAAGGACTTGAATGAGAATCAAATGAAGTAAAGGCAATCATGTTGTGAATATAAAGGTGTTCATGTGATCTCAAGGTGATCTTGTTTAAAGTGTTATTGTATTGTGATTGAAAGTGTGTCCTCTATTATTGTTCATATGTGATGATGATGGAATGTGAAGGATCTTTATAATGTGATGTGTGTCTTGACTTGGTAGACTTAGGTGCCTCTTCTTGATACATTAGAAGCTTTGTTGAATATGAATCGATAGGATATGGCATTCCTTT

>LNC_002049

CTCTCTTTGTTTCAATCTAGCTCTAGGGTTTCTCATTTTTTTTCTTTTCAACTGCTGAACTGATTTCTTCTCCGATAAGTCTATACGAAGAATTAAGAAAATAATTCATGTAATTCTTTTCTTAATTACAAAGAATTAAGGCTGGTAAAGATTGGGACTGATCTATTCCCACTAATTCATGTCATCATCTTCTCTGCACTGTCCGTGCCTTTTCTAGTCTACTTCGAATCCATCTTTTATTTCTTCTAGCATTTCGTTCATTTGTCTTCTTCGAAATTGACGTGCTCGCAAGGATAAAATTGTTGAAAATACATCTTAGAGAGCCGTATCTTTATTATTGAAAGGCGTCGCTGGCGGATCTAGAACAGAACTCAAGGATGTGATTTATAACATCGACAACCATATTTCCGTGTTTGGGATTCTTGGGTTCACAATGCGGTGGCACTTCTAAGTTCTGCGCCTAAATTGGTTGGTTTGCTGTCTGAGCTTGGTGAAGCCTTTAGCGGCTTGGTAACAAAGTTAATCCCCTGCTACAAGATATGCGCATTATGAAATTCCACTAACTACAGCAGGATGAACATGCATAATTCAACAATCCAGCAGTACAAGTTTTATCGGAAGAAGGTTTTTTTTAAATTGCTTGTAGAATTAGAATACAATGACTTTACTTATATGTATAATTTTTTTGACTATTAGTAGAGATATGCAAAAGTATTTAGTTTTGTAGGAAGTGGTAAATATCTAGTCCATCAATCTTATATATTGATGTTTGCAAATTCATATAATTTTTAGTTTAAATATTTGTGTAAATTTTTTAAAAGAAATGTGTAATGTGTATATAACAACTACAAAAAAATTATTGCTAAAGGTGTATGGAAAATATTTGTTTTATTGCAATAAAAATTATCGTTGCTAACAGATTAATTACTAATTTTTTGCAACAACTAATATTGTTGGTAAATAGTTTAACTTGTGGCAACAATAGATTTTGTTGTGATAACTTTTATAAGCCACAACTATACAAATGTAAGGCAACAAGTTTTTTGGTTGCATTAAGTCATTTTTGGCAACAATAAATTTTGTTGTTGCAAAAACTTTTCCC

>LNC_002050

CTCTCTTTGTTTCAATCTAGCTCTAGGGTTTCTCATTTTTTTTCTTTTCAACTGCTGAACTGATTTCTTCTCCGATAAGTCTATACGAAGAATTAAGAAAATAATTCATGTAATTCTTTTCTTAATTACAAAGAATTAAGGCTGGTAAAGATTGGGACTGATCTATTCCCACTAATTCATGTCATCATCTTCTCTGCACTGTCCGTGCCTTTTCTAGTCTACTTCGAATCCATCTTTTATTTCTTCTAGCATTTCGTTCATTTGTCTTCTTCGAAATTGACGTGCTCGCAAGGTAAGGATTTCATTTGGAATGGAAGGATTCATGTATTGCTGGTCATATGAGTCGATTTTCTACCATGCGTCGCTGGCGGATCTAGAACAGAACTCAAGGATGTGATTTATAACATCGACAACCATATTTCCGTGTTTGGGATTCTTGGGTTCACAATGCGGTGGCACTTCTAAGTTCTGCGCCTAAATTGGTTGGTTTGCTGTCTGAGCTTGGTGAAGCCTTTAGCGGCTTGGTAACAAAGTTAATCCCCTGCTACAAGATATGCGCATTATGAAATTCCACTAACTACAGCAGGATGAACATGCATAATTCAACAATCCAGCAGTACAAGTTTTATCGGAAGAAGGTTTTTTTTAAATTGCTTGTAGAATTAGAATACAATGACTTTACTTATATGTATAATTTTTTTGACTATTAGTAGAGATATGCAAAAGTATTTAGTTTTGTAGGAAGTGGTAAATATCTAGTCCATCAATCTTATATATTGATGTTTGCAAATTCATATAATTTTTAGTTTAAATATTTGTGTAAATTTTTTAAAAGAAATGTGTAATGTGTATATAACAACTACAAAAAAATTATTGCTAAAGGTGTATGGAAAATATTTGTTTTATTGCAATAAAAATTATCGTTGCTAACAGATTAATTACTAATTTTTTGCAACAACTAATATTGTTGGTAAATAGTTTAACTTGTGGCAACAATAGATTTTGTTGTGATAACTTTTATAAGCCACAACTATACAAATGTAAGGCAACAAGTTTTTTGGTTGCATTAAGTCATTTTTGGCAACAATAAATTTTGTTGTTGCAAAAACTTTTCCC

>LNC_002051

ATTTTCCCTCTCTTTGTTTCAATCTAGCTCTAGGGTTTCTCATTTTTTTTCTTTTCAACTGCTGAACTGATTTCTTCTCCGATAAGTCTATACGAAGAATTAAGAAAATAATTCATGTAATTCTTTTCTTAATTACAAAGAATTAAGGCTGGTAAAGATTGGGACTGATCTATTCCCACTAATTCATGTCATCATCTTCTCTGCACTGTCCGTGCCTTTTCTAGTCTACTTCGAATCCATCTTTTATTTCTTCTAGCATTTCGTTCATTTGTCTTCTTCGAAATTGACGTGCTCGCAAGGCGTCGCTGGCGGATCTAGAACAGAACTCAAGGATGTGATTTATAACATCGACAACCATATTTCCGTGTTTGGGATTCTTGGGTTCACAATGCGGTGGCACTTCTAAGTTCTGCGCCTAAATTGGTTGGTTTGCTGTCTGAGCTTGGTGAAGCCTTTAGCGGCTTGGTAACAAAGTTAATCCCCTGCTACAAGATATGCGCATTATGAAATTCCACTAACTACAGGATGAACATGCATAATTCAACAATCCAGCAGTACAAGTTTTATCGGAAGAAGGTTTTTTTTAAATTGCTTGTAGAATTAGAATACAATGACTTTACTTATATGTATAATTTTTTTGACTATTAGTAGAGATATGCAAAAGTATTTAGTTTTGTAGGAAGTGGTAAATATCTAGTCCATCAATCTTATATATTGATGTTTGCAAATTCATATAATTTTTAGTTTAAATATTTGTGTAAATTTTTTAAAAGAAATGTGTAATGTGTATATAACAACTACAAAAAAATTATTGCTAAAGGTGTATGGAAAATATTTGTTTTATTGCAATAAAAATTATCGTTGCTAACAGATTAATTACTAATTTTTTGCAACAACTAATATTGTTGGTAAATAGTTTAACTTGTGGCAACAATAGATTTTGTTGTGATAACTTTTATAAGCCACAACTATACAAATGTAAGGCAACAAGTTTTTTGGTTGCATTAAGTCATTTTTGGCAACAATAAATTTTGTTGTTGCAAAAACTTTTCCC

>LNC_002052

GCAATTTTCCCTCTCTTTGTTTCAATCTAGCTCTAGGGTTTCTCATTTTTTTTCTTTTCAACTGCTGAACTGATTTCTTCTCCGATAAGTCTATACGAAGAATTAAGAAAATAATTCATGTAATTCTTTTCTTAATTACAAAGAATTAAGGCTGGTAAAGATTGGGACTGATCTATTCCCACTAATTCATGTCATCATCTTCTCTGCACTGTCCGTGCCTTTTCTAGTCTACTTCGAATCCATCTTTTATTTCTTCTAGCATTTCGTTCATTTGTCTTCTTCGAAATTGACGTGCTCGCAAGGATAAAATTGTTGAAAATACATCTTAGAGAGCCGTATCTTTATTATTGAAAGGCGTCGCTGGCGGATCTAGAACAGAACTCAAGGATGTGATTTATAACATCGACAACCATATTTCCGTGTTTGGGATTCTTGGGTTCACAATGCGGTGGCACTTCTAAGTTCTGCGCCTAAATTGGTTGGTTTGCTGTCTGAGCTTGGTGAAGCCTTTAGCGGCTTGGTAACAAAGTTAATCCCCTGCTACAAGATATGCGCATTATGAAATTCCACTAACTACAGGATGAACATGCATAATTCAACAATCCAGCAGTACAAGTTTTATCGGAAGAAGGTTTTTTTTAAATTGCTTGTAGAATTAGAATACAATGACTTTACTTATATGTATAATTTTTTTGACTATTAGTAGAGATATGCAAAAGTATTTAGTTTTGTAGGAAGTGGTAAATATCTAGTCCATCAATCTTATATATTGATGTTTGCAAATTCATATAATTTTTAGTTTAAATATTTGTGTAAATTTTTTAAAAGAAATGTGTAATGTGTATATAACAACTACAAAAAAATTATTGCTAAAGGTGTATGGAAAATATTTGTTTTATTGCAATAAAAATTATCGTTGCTAACAGATTAATTACTAATTTTTTGCAACAACTAATATTGTTGGTAAATAGTTTAACTTGTGGCAACAATAGATTTTGTTGTGATAACTTTTATAAGCCACAACTATACAAATGTAAGGCAACAAGTTTTTTGGTTGCATTAAGTCATTTTTGGCAACAATAAATTTTGTTGTTGCAAAAACTTTTCCC

>LNC_002053

CTCTCTTTGTTTCAATCTAGCTCTAGGGTTTCTCATTTTTTTTCTTTTCAACTGCTGAACTGATTTCTTCTCCGATAAGTCTATACGAAGAATTAAGAAAATAATTCATGTAATTCTTTTCTTAATTACAAAGAATTAAGGCTGGTAAAGATTGGGACTGATCTATTCCCACTAATTCATGTCATCATCTTCTCTGCACTGTCCGTGCCTTTTCTAGTCTACTTCGAATCCATCTTTTATTTCTTCTAGCATTTCGTTCATTTGTCTTCTTCGAAATTGACGTGCTCGCAAGGATAAAATTGTTGAAAATACATCTTAGAGAGCCGTATCTTTATTATTGAAAGGCGTCGCTGGCGGATCTAGAACAGAACTCAAGGATGTGATTTATAACATCGACAACCATATTTCCGTGTTTGGGATTCTTGGGTTCACAATGCGGTGGCACTTCTAAGTTCTGCGCCTAAATTGGTTGGTTTGCTGTCTGAGCTTGGTGAAGCCTTTAGCGGCTTGGTAACAAAGTTAATCCCCTGCTACAAGATATGCGCATTATGAAATTCCACTAACTACAGGTTAGTCACAATTATGCTATTCAAACCTTAGGTTGCTGTGGAAGATACTTGTCTAAGTGCTTCTTGCCAAATGTCAAATTGTAGTTACATTGTGAATCAACATGTACATTTAGACTTGCTTGGTTGTGGTTCCCTTCAAATGGTTTTGGGGTTCTTTGGTGACAGTCTGTATAGTTAGTTACGCATGCTTTGTTAATGATACAAAAATGCTCAGTTGAAAGGATAAAATCGTGAAAGTTTAGAAATAATTGATTGGAGTAGTGTAACTGACAAGTATAGTTAAAACAGTGAGAATTGACTCCGATGAAATTAGTTGTTGCTCTCCTAGTTAGAAACTGAATGCAGTTTTTTATATGATAGTTTGATCTTGGAATGGGATTGCACTTGTCAACATATTACTTCCATTTTTCTCTATTAAGATTAACGGATTGTTCTATATGTACATACTAAAAGGTAATAATTAGTTAGATTTATTTCTTGTTTTGTTCTTTTGCAATTAAATCTTAGTTGGTCCCTCCTATTTTGTCCTTTGTGATTATCCAGATTACATAATATGCTAAGAGCCACTCTTGCATCCAATCTATGTCTAGATAACACTTAAAGCTAATAATCAATATTTCTAGTCACTCTGATTTTCATGATGGTTAATTCTTGTATTGATGAGCATGTTGTGATAATCAAAAACTCTAGTTTAAGAGAACTAATGAAATAAATGCTTCCACATGTAAATCATGTCTTTAAG

>LNC_002054

NNGAATTAATGAGGTTTGCGCATTTCCATAAAAACTTGTGTTTTTGAAGAAAGATGAGTTGTATGAGTTGTTTCCCTCATGTTTTAGTGCATTGGTTATAAGTGATCTATTGTACTTCATGAAAATGAGGTATGAGCATGTCTTAGTAAGGAGTTGAGAAGTTCCTCCTTGAAAGAGTGCTATTGAACATGGAAACTTGCATATGTGCTTAATGATGTATGGACTATGTAATGACTAGGAGAAGGTAGTTCCTCCTATGAACATGAGAAATGTTGATGTTGGTGTGTTATGACTAGGAGAAGGTAGTTCCTCC

>LNC_002055

GGGAGGAAGTAGTTCTCATTTCAGCCTAGATTTAAAACTAATACAAGACGATTATACTCATGTTGGTGAAACTTTTTTTATTTCACGTCCCAATATTTTAGAATTTAATTCTGAATATAGTAGACCATGAGACCTTAGATGGTTTGCTAATTTTGAGGAAGAGTTGGAGGAAGAAGTAGAAATAGAAAACCAAGACTTAGCGGACACCCTACTAGTCTGGCCACAAAATTACCTCAATAAAATGATCTCCCTCTAATACTTGTGTTTGTCTTTACTGAAAACTCTTTTGACCTCTAGGGGAGAACAGTTAGTGCGGTCATTATATGTGTTAATTTCCCATATTAAGAAGTTGTGTTTAAAAACCTAAGGTGACCTTGGGGATTTAGTT

>LNC_002056

GCTTGTTTAAGATTGAGGTAACGCAAAAACAACTAAGTCTAACTTCAAATACCTGAATTTAGTACTTGATGGCTAATGGTCATGGTTGGTACTAAGAGCATAGTCATGCATGTGAATAGTGTTGAGCATACCTCTCCGTTTTTGAAAGGTTGAGCATGTTGTGAATGATTTTGTGACAACTCTTACAACTCTTTTTGAGACTTTTGATTCGTTAGCGGTTAAAATGTTCAATGCTACTTGTATTTTGAAAAGGAAGTAAGATTTTGAATCATCTTGATTGATTTGCATGCCATGTGTGGTG

>LNC_002057

GACACTTCTTTCTTGTGAGGGCATGTAAGTTGAAAAAGTGGTGATTTTGAAAATTCCTAATTGAGTCAAGTGAGCATGTGAAAGAGTTAATGAGTTGAGTCATTTCTTGAGTTTGAGTTGTTGTCACTTAAATGATTCTTGGTTTTTGAAAATGTTTCATGAGAATGAGAGGATTTCTTTTGCGAAAAGTTTGCATGTGGACTATTTGTTAGTACCAACCGAAGCCATTACCTTTGTGCTTAATTGAGAAAAGAAATTGATTTGTGGTGTGTCTTGAAATAAGA

>LNC_002058

CTGGACTCCTCTATTCATCTATCAATCTTGTTTATGAATGTTATGATGGCCAGGCATAGCCGAAACTCTTTCGTAGGCAGCTCTTGAGGCAGTGGCTTGAAACTAATCTCCCGCAGTCACCCACCATGATCGGAGCTGAGATGATTCATACTAGCTTGGTTAGGTCAATACCTGTACCATCGCTTCTATTTGATGTAATATAGTATAGAGGCTGAAGGTGAGAAATCATCAATTAAAGTAGATGCAGCTTCCCTGGCTTAAAGGACGGAACTCCAGCAAGAAATCTATTGAAGAGTTGGTGCTGGGTTAGCCAAAAATAAAAGTTTATCCGGTCCCTGAAAAATTTGCTTTTTATGATTACATCGGCAATAATCCAGAAAGTGGATCTATTTCGTTTTTTTCTCTTTTAAGAAAAGGAAAATCTTATTTCCCGGATCATTGTACATTTCAATTTGAATTAGGCATTCTGTGTCCAACTCTAAGAAGTCCTTACCTACATTTGCATCTGATCATATATGTATATTATCTATTCCAACAAACAAATAATACAAAAGAACGAGGTTTGGATGCGCTTCTGTCCTTAAAGGGAGTTCGCCGAGGGTGAGAACGAGAATCGAATCGATTGAATCACAAAGATCTATTCACTTATATAGAAGAAGAATCGAAAAAGGAGCGGATGGTTCATGTAGCAGTGGAAGAGTCAGTCGCCTTTTATGAAGTCTGGGCTTTCGAGATCGAATCGTAGCCAAAGTCTTTTTCCAGGTTTAGAAAGACTGGTTCGAAAGGACCAGGAAAGATCAGCTGTTCGCTCTTCTTCAAGGAGTGAGGGAGCAAAAGAGAGTAATGACATCGTACCGTGCCCCTACCCTAGAAAGGGTAATGTGATTAGCTTGCTTGAACGAATCCGCTCTCTTTGCTGTGAACAAGAGGAAGCTCTGCAGATGAAGCAGGCGACGGGTAAATGCTTTTTTTGAGTTCATACCAGGATCCAGGGAAAGGCTTTTTGCTCACCCCGCGAAAGGCGCATGGGTTGACTCTATTTCTTTCTATGAGTTAGTAGGTGAGAAGGAAAGACTAGTGCTTAGAAATCCGGCTGACATTGAACTTAAACTAGCTGCTGCCATTGAAAGAGAAGAGAGCTCCGTGACTTCCGGGCTTAGCTCTTTTCCGGTGCAGATGAATAAACCGGTATTTTCTCTTTCAGTTTCTGCTCCGGGGAAAGAAGTTTAATTTGCTTTGGTAATTTTGTACGTATTCTAAATTAAAAGATTCCCGTGAGAGGGCTGAAAAGGAAGCTCGAACGAAGTGAGAGGGTGGATGTAATTCAGCTCGACCTACAGGGAGAGGGAAATTCTTTTTATTGCGTTCCCGGCTCTCGGCTCTCTCTTTCCCTTTATCCCGTGCCTGGGGAATATATGAATGTCTTTCTCCCGGCTTACTACGATTTCCGGGTGGGAAGAAAGTCATTGCCAGCTCTTTGAACGAATACGCTCTTGGGCCGCAGGAAGAGCATCTCTCTCTGTCTATTCAAAGATCCGAGACTGCTAGTGTTGTTGGTGACATTGGTTCCCCGTCCCTACTCTCCTATCTCTCTCACCCTGGTTGGGCTCTGGCTTAGTGTGATTAAACTGTGAATCGAGTCAGGAAAAAGAAGTCCAGCTCCAGGCTTTAAAGAAGAGAAGAAAGACCTGGCTTCAAGGGAAATGCTTTAGGAAAGAAAGATCCCAAGCTTATGCTGAATTGAGAGTGGACTTGCCTTGGTCTTCTGTGAGTTCCAGAGATTCAGATCAAGGCAGTTCAGTTCTCCGAGGCTAATTCAATTCTTTCTTGCTGTGAAAGAAAGCACTGCTGAAGAGGGACCTACTTTCAATGCTAGTTTTGAGTCATTTAGTCTTAGTCCTACTTTTATCTCTACGACTTCTGTTACATCTCATGAAATTCCGTCTTCTAAGCCAGAGCCAGGTCAGTCTAAATGGAAGTAGACCAACATAGGAAGAAATGTGAAAGTAGGGGCTGCTAAGCGCCCAGACCCAGAATTCTGTGAAAATGGAGTTCTTGGTACCGGTCAAGCAGAAATAATATAAATAGCGAGCCAGTACTATATTTATAGATTGGAAAGAAGGGACTCTCCAAAGAGAGGCCCGAGCGGAATGAAGTTGTGCTAAATGAACGAGTTGTCCTAAATGCCCCTTGTTAGCTGTCGCAGGAAGTGGTGAGGGCTCAGGAAGTGAAGCAAACTCGACCAAAGGACAAGTCTGTTCCTGCTTCGTTTTCGGTTCCCGATTCAATCTTCACCTTTCCTGAATGAAGAAAGAGAGTGAAAGAGAAGATATCAGTGCAATAGGTACAGAGGTACGAGAAACTGTCTTCGTTCGGTTCGGCAGAAGAAAGCCGAGAAAACAAACAAATAGGAAGAGCACTATGCAAGCTTATAATTAAGCAAATATATAAAAGCTATCACCACCTCAAGCAGTAAAACAAAGTCGTTATGGCTATGTTACTAACATAGACAACCATGAATGGTATTCATTGACAGGAGTGACCGCTTTCTAGTCGTAGTTGGTACCGCCCCTGTTCACTTCAGCATGCAAGGAACACCTTCGGGAAGAATCAAAGTTTTTCTCCTCTGCAAAGCTATCAATGCTTTATTCAAGATCTTTAGCAGTCGATCGTTCATTCTCCCTCCCCAATGGCTTTCAAAAGCTCGACATAAAAGAATAAAGAAAATAGGAAAAAAGAGTACGGGGATATTTAGCTATTTAGCTATTTAGCCTTAGAGAAAGGTGACTTGGTAAGACCAGAATCTGGAACAGAGATTGGATGAAGAATTGCTTTTTCTTTAGCGGGAATGGCGCCACCTTTTTATTTAGCAACTGCCGCCCCGCTTAATAGGAATAGGATGCACTTACCGACCGGTGAAAGGAAAGAGCTGGGTCAGCCATCGACAAAGGAATACCGAACTCTACCCGTGTGAGGCCTCGTAGCCAGGGAGAACCATAGAATCGGAGGGTTATATTATAACATCGGTGGAAAGGTCGAGAGGGCACCTAAAAATAGCTTTAGAAGCCAGATTAGGAAAGTCTTTCCCGAGTTCGAGCAAATGCAATGCGACATCCAGGCAAAAAAGGGCTGGGGTCCGATCTGCGCATACATCACTAAAGAAGATAAGAATCCACATATATGGGGGAACACGTCAAAGGCCCATGTTATGGAAGTAGCAGAGGCTTATGAGGGAAAGAAAAAAACAGGGGGTGCTCCTCTCCTTACAGTCAAGTGGCTTTCCGCTCCTCTACAACTACCTTTCGCCCAACATGATCACGAACGAAGACAGAGAATTGCGT

>LNC_002059

TTGGAATTAAGAAATAAAAATTAGCTCACTAATGTATTGTAATCTCTACATAACTAAACCATGATAATTGGAAATTTTTATTTTGAGCGAGGTACGACCAACAAAACTTGTATGAGGATCCATTAGCAAGTTGACACGTGGATTGTGTGGGCACAAATTGTCCTTTAAAGCTGCAACTAAAATCAAAGAGGAGAGAGAGTGTAAAGTTCAATTGGAAATTGGAAGAAAATCGTGGGGATCCGTGTCAAATACAATTAAATAAACTAAAATGGTGTATTTAAAGTAAAAAAGTGCACGATAAAAAAATAATGAAACGAACGAAAAGTTATGTTAT

>LNC_002060

TTTGTTTCTTTATATTGCACTTGTTTTAAATTGCTTTATATTGAAATGAGTTCAGTTAAATTGAGTTGAGTATACAGAGTTGAGTACCCAGAGTCGAATAACATATCTTTGAGTTGAGTATCTTATCCTTGAGTACTTCTGAGTTGAGTAAGTTTGAGTAGTTTTGAGTATCCTTGAGTATTCCTTGAGTTGAGTAAGTTTGAGAAGAGGTAAGTATGTTTCCTTTTTATCAAGTTTCAATCTTATGTTTATGTTTTAGAATTCCCCTTACATGCTCGTAC

>LNC_002061

GTTTCAAGACAAAATAAAATATAGCATCAATTGGACTCATTATAGTTGGTCATGAACTAAGTCTCTAGGGTTAGGCTAAAGTCTATTCTTCTTGGGCCTCATTAGAGTTGATTGGGTAGGCTTCTTCCATGACTATTATTTGTGCTTGAAATAGATTGCTTTCGAGGAAAGACACCACCTCAAGAAGCTAATGCCAAGGTTTTGAACTCCTAGAAAGCCATTGACAAGATCACAAGCTAAGTAACTTCAGGAAGAACTTATGGACTTTTAGTTCAAAAAAAGAAATGTCTTACGGTGAATATGAGTTAAAAACCTTAGAAGACACGTTCAAGAGCTCTAATATATTTTAAGCCCAAAACTT

>LNC_002062

ATTAGTAGAGTCAAACAAGCAATTATAAGGATAACTACTCAAACTCTAGGCCCAAGACCAAATATATAAGATTACATCAATTAGATTCTTATTAAATTATCAAGTTGATAGTGAAATAAGTCTTCTAGGAACTGGCTTTTAACCTTTTCTTCCTTGGCCTCATTGTTGTTGGGTCAACTTCTTCCATTTCTTCTATTTGAGCTTGAGATATATTAAAGCTCTTGGACCTGTCTTCTAAGGTTATAAGTCATCTTCCACCATTAGAATTTCATTATTTGAATTGAAGTCCAGGAAGTTTTCTTGAAGTTCCTTAGCTTGTAATCTCGTCAATGGCCTTCTAGAAGATTCCAAAGCTTGACCTTGAACTTCTTGAATTGGTGTCTTCCCTTGGTGGCTATCAATCTCCCGTGAAATTTCCCTCTAAAATTGGGACCCACGTTAAAAAGAGAAAGATCAACTACTTAAAAGTACCGCTAAATTGGTAGTTACTGGGAAGGTTTAACTTGTAGAAATTATTTCTTTTCTTCTGAAGAACTTCAAATGACAAATACCGTCTTAGGTGCAACTTGGATTTCGTTTGCTAGGAAACCTCACCTTGCACATATGCAC

>LNC_002063

TCTGTTCCCTCTCTCAAAAGTCAAAAACCCCTCTTCTATCCTTGCATAAGATGGAGTGTTACCGTAGTCTCTCTTCCCACCTCTGTATCTTTAACAATCCATCAGGTTCTTTTTCTCTCTGCTAGACTTCATGCATTTTCCGTGCAAACCAGTGAAGATGCTTGTATAAGCAAGAAGAACTATGGAAATGAAAGACACAAAAGAGCTTTTAGAAAAACAATATTAGGAGACATTCGGTAAAAAGGATCAGATCTACCCTAAAACTATGGATTCTTCTCAAAATTATTGGGAGTGTTCGATATGTGCTATCAATGAAAGGTTTATTTCATAGGTATCAGATGCAGGCACAAATAAGATTGACTAAAAATAAAAGGAAGTTTGTGCAAAGATGAGGTGAAAGACCCCGATGATGAAGTATGCAAACAAAATCTTGTAAAGATGAAACGAATACATCAAGAACCCTGAAATGATGGATGGTCTTGAAGACTCACATAACCAAACATCAAGAGTGAATCAGTGGAGAGACACTAAGGACGTGAGTATGTTTTTGGTAATAACAGGACATTAACAAGGAATATATGGTGTGCTTGAGCTCTACATTTAATTACATCTCACAACATATAAGTTGTGGAAGGTGGAAGTCAAGATTAGCTGCTAAAGTAATGCATCTACAATGGTATTTTGAGAAGCAATCTCCATTTTATTGTCTTAGTTTTGTTAATGTTTAGTATATCTCTCTTAGACTTTTAAGTTGGATAAATTTTACCTAATTATATAATGAGATATCATCTCATTAATGTCATTTGTATTGCTCTTAATTATTATATGAAAGTGTGTAATTTTTTTTAGTTATCTTGCTCCTTTAGTATATTGTTGTTTTTA

>LNC_002064

CCCAAATCCTCTGTTCCCTCTCTCAAAAGTCAAAAACCCCTCTTCTATCCTTGCATAAGATGGAGTGTTACCGTAGTCTCTCTTCCCACCTCTGTATCTTTAACAATCCATCAGGTTCTTTTTCTCTCTGCTAGACTTCATGCATTTTCCGTGCAAACCAGTGAAGATGCTTGTATAAGCAAGAAGAACTATGGAAATGAAAGACACAAAAGAGCTTTTAGAAAAACAATATTAGGAGACATTCGGTAAAAAGGATCAGATCTACCCTAAAACTATGGATTCTTCTCAAAATTATTGGGAGTGTTCGATATGTGCTATCAATGAAAGGTTTATTTCATAGGTATCAGATGCAGGCACAAATAAGATTGACTAAAAATAAAAGGAAGTTTGTGCAAAGATGAGGTGAAAGACCCCGATGATGAAGTATGCAAACAAAATCTTGTAAAGATGAAACGAATACATCAAGAACCCTGAAATGATGGATGGTCTTGAAGACTCACATAACCAAACATCAAGAGTGAATCAGTGGAGAGACACTAAGGACGTTGTGGAAGGTGGAAGTCAAGATTAGCTGCTAAAGTAATGCATCTACAATGGTATTTTGAGAAGCAATCTCCATTTTATTGTCTTAGTTTTGTTAATGTTTAGTATATCTCTCTTAGACTTTTAAGTTGGATAAATTTTACCTAATTATATAATGAGATATCATCTCATTAATGTCATTTGTATTGCTCTTAATTATTATATGAAAGTGTGTAATTTTTTTTAGTTATCTTGCTCCTTTAGTATATTGTTGTTTTTA

>LNC_002065

CCCAAATCCTCTGTTCCCTCTCTCAAAAGTCAAAAACCCCTCTTCTATCCTTGCATAAGATGGAGTGTTACCGTAGTCTCTCTTCCCACCTCTGTATCTTTAACAATCCATCAGGTTCTTTTTCTCTCTGCTAGACTTCATGCATTTTCCGTGCAAACCAGTGAAGATGCTTGTATAAGCAAGAAGAACTATGGAAATGAAAGACACAAAAGAGCTTTTAGAAAAACAATATTAGGAGACATTCGGTAAAAAGGATCAGATCTACCCTAAAACTATGGATTCTTCTCAAAATTATTGGGAGTGTTCGATATGTGCTATCAATGAAAGGTTTATTTCATAGGTATCAGATGCAGGCACAAATAAGATTGACTAAAAATAAAAGGAAGTTTGTGCAAAGATGAGGTGAAAGACCCCGATGATGAAGTATGCAAACAAAATCTTGTAAAGATGAAACGAATACATCAAGAACCCTGAAATGATGGATGGTCTTGAAGACTCACATAACCAAACATCAAGAGTGAATCAGTGGAGAGACACTAAGGACGTGAGTATGTTTTTGGTAATAACAGGACATTAACAAGGAATATATGGTGTGCTTGAGCTCTACATTTAATTACATCTCACAACATATAAGTAAGAAATTCCTATATTCAGAAACTGACAAGCATAATATTTCTGGATATAGAAAGGGTGTGCTTGGCTGATTTGCTTGTCATTTTTCTACTGATTCCACCTAGTCGACAATGATAAAGTTCACATTATACCTCCAATGTGTTCTGTTTAATTTATTATCCTAAAGTTCTATATTGCATAACTTGGGGGACAAATGAAGTAAGAGTAAAAATTACAAAGTGCTAACTTCTAATAGTTAACTTGTTTGGCATGAACTTGGTTGGAAATTTGTGCCTAATGAAGGCAGGTGCCAAACAACAGAATTCTTCAATTAGTATTTTTGATTAGTATTACTGATATAGCTTCAGTAACGAAAAGAATACTCTATAGTATACCTATTTGTTGTGTGATCTCTATCTAAAAATGTGGAGCCTGCGCATATCGTTGCTCTATTATTTACTTCCACAAAATAAAACTAAAAGAAGATCTCACTTGTGAAATTATATTAGCTATGTTATTGCATGTCACTAGAGGAAGTTGGTTGTTTGGAGTGTCTCCCTTTGGAGGAAGGAAATGTCTTATCTCTCAGTTTCTTTCCAAGTCTTTGTTGTCTACTTCTGCCTCTTTTGATAGTAGATGTACCGTCGTAATCAAACGTATCTTTGAGATGAGGTTGAGTACGGTCTTCTTCATTGTTCAAGTTAGAGTCGGTGAGCTCCATGTGACTGTGACTCTGTTCAGCAGCGTCATCATCTGATTGCAGCTTCTGTTTTTCACCATGTGTAGCAACTATGAAATCATCCGGAGAAGAGTCTGGTTATTATTGTTATCAAATGCTTTCTTACTGTAAGTTTGTGCATTTTCTTCCTCATTGTTCAACTTTGATTTGGTAAGTACCACGCCACTGTTTTTTTCTGATTGTAGTATATGTTGTTCACCTTGAGAAGTAATCATATCTTCTGTCAACTCATCTATCAGTTGCCCTCCTTCTTGGATTTTAGCTTCAGCACAAACATTTTTTGAGACACTTGAATCTTTTTTTTTTTTTGATAAAGATAATGTTGACACTTAAATCAGTGTTGACGTTATCCTCTCTTTTTCCATGCGGTTCCTCATCGTGAATATTAGTTGTTTGGTCAGTATAGACAGGGGCTGTTTTTCCTCTTTGTTTCTGGCACTTTTTTCTCAATTTGCTTCTACCTCTATCGGGAGAAATACCATATCGGTTAATTACTTCCTAAGGTAAGATTGTGCATGTACATTTTCTTCGTCTACATCTGATGTGATAAGCCTATTGTTCCTATGACTTTGTCCAGTGGTTTCCTCATCAGATAGTATGATTTTCTCATCTTCATATGTTATAGGTATATCTTCATTCAGCTCAACATATAATTGGATACCTTTTTGGTTTTTGATCTCTGTAGGCAAGCTTTTAGAAACTTCCTGGTCAGATAGAATGTTCTCTCCTCTACATCATCATAATGTTCTTGATCTTCATTTATCTTGCTGGCTTGTTGATCAACATCCTTAGAGTTGGCCTCATTCAGAATGGCATTGTCTTGTTGTGCATAGTCTACAGTGTCATCCTTCAACCTGTTATTCCGTCTTTGTTCATATTCAATATGTTGGTTTATGCTCGTTTCTTCTTCTGGAGTAGCTCTCTCAGGATTTTTCCTTTTGGAGTTTTTGTTGGCAGTTTCTATTGGCATAGGAGGTTTGAAAGTGACCTTGCCTTTCTTTTTAGGCAATTTTTCTCTTTCTTATTTTTCTTCTTCTTTTTGCCGATTCTAGCAGTCACAGTAGATTTCTCATCTTCTTTTTTTTGGTGCTCACTTATATACTCTGATGAAGTGTTACTTGTTCTGCTTCAATATTGTTTCCACTTGCATTTTGTTTCTCAATGTGCTCCTCATCCTTTTCATTATTAGTTTCCTCCTTTTAGCTTCCCACTTTTAATAATATATGATATATGATAGCTTTCCACTTTTAATAATATATGATGATGTATGTAATCAAATCTTATATTTTTTTCTATTACACAGGTTGTGGAAGGTGGAAGTCAAGATTAGCTGCTAAAGTAATGCATCTACAATGGTATTTTGAGAAGCAATCTCCATTTTATTGTCTTAGTTTTGTTAATGTTTAGTATATCTCTCTTAGACTTTTAAGTTGGATAAATTTTACCTAATTATATAATGAGATATCATCTCATTAATGTCATTTGTATTGCTCTTAATTATTATATGAAAGTGTGTAATTTTTTTTAGTTATCTTGCTCCTTTAGTATATTGTTGTTTTTA

>LNC_002066

TGGGGTGTGAGTTAGGCCTAAGACCTAATTTAACATGGTATCAGAGCAGGGCCCGTCTCACCCGATGTTGGGACCCCCAAAATCAAAATTGTCCACGCACCAGATGCTAAGCACTGGGCGTGAGGTGGGGTGTTAAAGAATGACAAAAGTCCCACATCGGTGATTAATGAGATGGGTGGACTCCTTATAAGGCTTGGGCAATCCTCCTCCCTTTGAGCTAGCTTTTGGGGTGTGAGTTAGGCCTAAGACCTAATTTAAC

>LNC_002067

GATGTATGAAGGGAAAAAAAGAGGATTTACCTTATGAACTTCCTTTTTTGCATGATTCAGATTCATTCTAATTGGGTTCTTTTGTCTGTCCTCTATCAGATTATACTTCATTCTTTCAGTATTATTTCACAGGTTGACAGCAACTACGGCAGGAGTTGTTAATAAGTTCTTTATTACATCTTTGATTATGTGGGCAACTCCAATTGCAATTTTGTATGCCTTTAACACAACTTAATTCTTGTGTGAGCTTTCCCTAATTTTTCTTTTTCGGAAAAGCTTCCCTGAATTTTTCCGATACAAATTTTACTTGATAAAAGATTTTATCATATGTGCCATAAGTTGATAGCCATTCTAAAGTAGCTATTGTTTGGTTTTAGATAACTAATATGTTTTCTTCTAACTAATCATTATTCAAAAACCTTGGACTGTGCCTTTCTTTTCATTCTTATGCTTGAGAACCTTGCCCTTTTCTTTTTTGTGCTTTTACATTTGATAAGAGTGGTATTTCGTTCGTATTCACATCAATTTGTGGAACATGAGAAAAAGTGTGACAGATTTGTGCCCATTAATTGCTCAAGTATGCACTGCCCTGCAGTTTTTGTTGCTTGAAATGATGTATCCAAAGGAACCAAAGTCCTTTCACAATTTTATCTGAGTTCCAGGGACAATTATTATAT

>LNC_002068

CTCCGATGTGACAGTCGCCGGAGAATCCTCCCAAAGTGACGATCAATCCTTCCAAATACTCACCTCCTCTTGATATGAGCTCTGTTCGATATCGATCCAGATCTCAAGTTATGCGCAAGAATTTTGGGGACACACTTCAGTCACCATCCAGATTTGAAGACAAGAGCTATTGAGATAAGTTTTACCTTTATAATTTAATTACTCATCTTTGAGATATGTTTTGATACTCGGTCCTTGATTATTCTCCTCAACAGGTTGAACCCCACCGAGATTAATACCACTGTTTAAGTCGACTAATCTCCGACCTTGCATCGCTCACCCTTTTCCAGGAGACTAAGTTGCTCCACCTTATGAGACACCAAAGCATGCAGAGCTTCCATTGGTGTTGATTTGCTAGTATTGAAATTTGTAATACTTGCATTTGTGCACTCTTGCACCTCTTAGTTCTGCTGGAATTGGATCTGCACCTCACCTTCCGCCACCATCTGCAAAGGTTCCTAGTGCATCATCACAGGCAGTCCCTACTCCAGTAACAGCCTTCACAACTGCATCATATACTACTACTGCTCCTCTATCTATTTGTTGTACCACATTCTGCACTAGTGTTCCAC

>LNC_002069

CTTCTCCGATGTGACAGTCGCCGGAGAATCCTCCCAAAGTGACGATCAATCCTTCCAAATACTCACCTCCTCTTGATATGAGCTCTGTTCGATATCGATCCAGATCTCAAGTTATGCGCAAGAATTTTGGGGACACACTTCAGTCACCATCCAGATTTGAAGACAAGAGCTATTGAGAGGTTGAACCCCACCGAGATTAATACCACTGTTTAAGTCGACTAATCTCCGACCTTGCATCGCTCACCCTTTTCCAGGAGACTAAGTTGCTCCACCTTATGAGACACCAAAGCATGCAGAGCTTCCATTGGTGTTGATTTGCTAGTATTGAAATTTGTAATACTTGCATTTGTGCACTCTTGCACCTCTTAGTTCTGCTGGAATTGGATCTGCACCTCACCTTCCGCCACCATCTGCAAAGGTGCATCATCACAGGCAGTCCCTACTCCAGTAACAGCCTTCACAACTGCATCATATACTACTACTGCTCCTCTATCTATTTGTTGTACCACATTCTGCACTAGTGTTCCAC

>LNC_002070

ATTTTTATTATTCTTCTTTTTTTGAGTTTGCCACTGTTCTTCCTACTGATTTGGCTCTTCTTCTTGGTAAATCTCTCTTCTGGCATATTCTATATTAGACTTCAGCAAACCTAGGAAGAATGTGAAAGTTTTAGTTTGACAGTCATATGAACAGTTTATTGTATGAACAACAAACCTATAATTGCAGGTAAGTATTGTACTCCCAACAAACCTAGGAAAAATGATACCTAGTTATGGTATGACATTATTTGTTTCATTTCAAGAACCAAGTAAATTCCGACATATAATGCAAGACGTCTTATTTGTTAAGTCAGCTTGGTCACTTTGCCTAGTTGACTTCTTTTATCCAGCAATTTAGATGCATCATGTACAGGTCTCGGAGAAGAACCTGTAATGATGTACCATTACCTTTGTCTTCATTGTTCCGTTAGATTCACCTTTTGTCTCTCTTCCTCTTCTCTTTTGC

>LNC_002071

CCCATCTTGAGTTAGAAACTAAAGAGAACTTCAACTACGGCGATACAATTTGCAGATAGGCCGATGAGTGCTTCAGATTGGAAATCATCTGAGGCATTGAAGAAAGGAATATCATTGGTGAATTAGCTTGCTTGACTTCGACTCGTCAGTGGAGCGAGTGTATCAGTGATTTCGACTCGCCCTCAACTCTAGCCAGTCTCCAGCACATCAGGTTCCAGGGTGAGCTATTCATTCCAGCTCGTGTTGGATTCTCTTGGTCATGGCATGATGTCCTTAGTTTTCGGACATAAACTATTTTATTCATTTATTTTGGTGTCTTTAATACTCTTAGACTTAGTATTTGAAGATTAGATGTCCTCGATGTGATGACTTTCAGATTTTGGGGATAATAAGTGTTAGACTTTAGAGGTTTATTTGATTGGTTACTTTAATAAGTTTTGAGCTTCCACATTATTGTTGTTATTCGTAGTTGAATTATTGATATATGTTAGGGTTTGGATTCCTTGGATCGCCCACTTAGG

>LNC_002072

GCAAAGCTTTCAAACTCTCCCTCAAACTACTAGCAATCGAGCGTACATTTGAACTATCTGCCATCAAGACTGAATGAGGTGAGAAGATAATAGTAGATATTGGAAATATATATTGCTTCTTCGCCTATTGGTGAATCAGATCTTCTTCTATTCCCGACATCAAGCCTGATTAGCTCGAGTAAAGTGAAGTAATTGCCTTTTTTTTCTCTCGACGGCAAAGCTCCTTCTTTTCTAAATTGTATAGTGTTCAATAAAAGTTGATGCAATAGAAGCTCTTGATGCAATATCTGGTGGTGAAGGCATTCCAGCAAGAGCTCTTACTCTAACAGAACTAGGAC

>LNC_002073

CAATCTCGATCGAGAAGACAAAACACCTTATAATTTTTACATACGCATTTTTTTTAATCTTCTTATTTAAAGCACCCAGCCACCCTTCTCCAAGGGTTTCAATTCTGCTTTCGGAATGCCTGCTATTAGCTTAGTTTACCAGCTCTATTGGACTACCTTGCTATGCACCTAAACGAAGTCACTTTCCTTCCTTCCACTCTACATTCTTGCTTTTTTTACGGACCAGAAGAAGAACAAAGAGGAATGTGGTATTCTCAGTCATCCCATTCCGTTCCAGTTGGTACTAGTAATGCCGCATCCCGCTCTTCCTTCTTATTGATGCACAG

>LNC_002074

GCGTAAGCCTTTGGAAGCAAACAATCGTAAAAAGGAAAAATAGAGAAGTAGAGCTAAGGTAAAAAACATGGACAATCCTGTGTATTCGATCTAGTGCTCTCACTTGCCCCCAAGGGGCCAGAAGAAGATACCCAATGTTCAGGGTAGAAAGCCAGGGTTTGGTACTTTGACGAGCTGGCCGCCGAGAGAGCACTGAGACGAAGAACCAAGCTAGGAATTTCCCTGCCCGTGTCTACTTCTTGAAAAACCTAAAGGGTCAAGGAGAGGTCTTAGGAATTCCATCCCTAACGAGGTAGAAGTGGAACAAGCGCTCTTAGCCTTTTGCTTGAGAGTAGGACTTGTCTTGTCTTTGACTTCAACCCGCTGCTTGACTACTCGACTTGGCTATTGAAGAAGCTCTTTGCCTTTAGGCACCAGGAGTAGGCAAAAAAAGACGGGCTTTCCTCCAACTGGCACGA

>LNC_002075

GAATAGAGGAAGATTAATGTGCAGCTGATTCTATACCAGCCGATTCAATTGTTCAGCTACTTCGAAAAACTTACTATTATGGCTATGTAAGGGGACCGGCTTCCCCTTTTTCAATCACCTCCTACCTTAAGGTAGGCCCACTGCTGAATACCCTCAATCGAAAGGTCAAGGAGATTATATAACCCCATAGTATGAAGAGCTGCTCGGGGGATCGCACCCCTTGGCCTAGGGTATACATTCAGATCTGGAGGCATACCTAATTAACGATAAGAGCAGA

>LNC_002076

TTGTCTCTAAAAGTATATCTATTGGGACGAGATTATATTCTAGTCCTTATTTGTATAGTCTATTGCGAAGGTATTAAATTTGGTCCCGAAAAAACTTATAACTAGTCCTAAATGGTAATTGAAGGTCTTTGAGCAGGACATATTTCCCCCAAATTTAATACTAAATTATTAAAATAGAAAAATTGTTTCAACAAAAACAAAGGCGCCAAACTTTTTACATTGAAAGTGAAAAATAAAAAAAAGAGCGCTTTGTCTTCTCAAAAACACATTGCGCAGCCGGTCACAAAGTTGCCCTATTCTCTCTTCAGTGCAACGTCTCCATTAGATTCTTTATCTCCAATCGAAATTCTCTACCCTAATCTCAACGCTTTCACTGTACTTTTGTCGCAGCTCAGCCCTAAAATCAAAGCAAGTACCATCGTCAAAATTTCATCGCAGCAGCAGAGTTACAGGTATGCTCTTCTTCCCATTGAGTTGCTCCATTTTTTTTTCCATAGGTTACTTTGATGACGTTTTTTCTTGTTATATTGAAGTTGCTCTTCTTCTTCC

>LNC_002077

TTCGGGTTTATTGTTATTTTGAGATATTAGCCATTAGATAGGATGGGGCTGCTAGATGAAGAGCTTGTCTTCTGACTTTGGATGTTCAATTTCGTGTTCCCTTATCAGATACTGTTATACATACATACATATGTAATGCACACTGTTGAATTCCATTCATATGATTCTATTAAGTTTGTCTATTTAGCCCCAAAATGGAACAATTTCCAATTTTCACTTTTTCCGTTGGTCAAATATTTTGG

>LNC_002078

ATTGCCTCAAATCCAATGTATCATGAACGGACAAAACATATTGAGGTTGATTGTCACTTTGTCCGTGAGAAGATTCAAGAGAACGTGATTTCTACTGGCTATGTGAAGACAGGAGAGCAATTGGGTGATATATTCACGAAGGCTTTGAATGGAACTCGGGTGGAATATCTTTGTAACAAGTTGGGCATGATTAATATCTATGCTCCAACTTGAGGGGGAGTGTTACAAAATGTACATAATCATATCTAATCATAATATTCATAG

>LNC_002079

ATGGGAATGGCTTCAAAAATTTCCTTTAGCTCTACGGATGACTAACAAGGCAGGATCCAAGCGGTAGATGTCCCAGTGACGTCAACTCAAGGAAGATTCGAGGGGCGTTCAGCCACTTGACTGTAAGGAGAGGGGGCCTTCCGCTGCACCATAACACTAACTTGACTTTCTATTATATTAGTAAAGCGCTATCGCGCTACAACTAGCATAGCAGCCTGCGGAAAAAGCTCGTCAGCCCCTACTCCTAAGTTAGAGCAAGAAGCAAGGGATTGAGCGCATCTTTCCCCTTTCTATTAGTAGTAAGGTTATCAAAAGGCTTTCTGATTG

>LNC_002080

AAAAACTCTCATTACCCACTTTCCTCTCTTTCTAGGTTTTTACCCCAATTTCTTCCTTTCTCTCTCTTGAGTTTTTCACCATTGTTCTCTCCTTTTCTTGTGTCTCTCTTGAGTATTTCACCATTGTTTTCTTTTTTTCTTGTCTAAGAAAATCACCTCTTCTACTTTCCTCTAATTTGTTCAAGAAATCTCACAATTCCTTCAAAATATTTTTCACCCACTGATAAAGGTAAATACATTGTCACCAAGTATCTTTTGATTTTCAAAATTGGGTGGTGAAGATTTCGAAAAACTTGTTGAAGACCAAGTTGAGGAAGAACAATTTCCTATTAGTTTGTTTTGTATTTATAGTTTTTTTTAGAATTTTGTATCCTTTCTCAATTAATATTCAAATCTGTATGTATGCTATTAATATTTATATTCATTCAAAATATCATGTTGCATAGATATGAATCTTATATTTGTTCCTAATTTGAATTCATC

>LNC_002081

TTTGTCTTCTCACTAAGATGACCACCATATATATGTTTTTGTTTTCAAGCTACTTATGTGGAGTTGAGTAAAATGGATTATAAGATACAAAATATCCCTCTTGATTTAGTCAAGAAGACAAAGAAAAGAGAGATGTAAAATAATCTTTGTTTCTTCATTTGAGATGAAGTTGCTCATGATTTTTCTAAGCTGACCAGAAACTTCAACGTGCAGCAAAATACTATCCATTATTGTGTCTTTCTTCTAATCTACATATGTAACCAAATGGAAAGAGTTCTTTTCTCAAAAGGATTTAAGTGTGCCTCCA

>LNC_002082

TGCTTCTTGAACACTCTTTGAACAATTGCATGATAACTTTGTATGTGATTCAACTAGAATCGTCTTTTCTGTTAACTACAATATACAAATCTCTTCCTCAGCAGTTGGTTTGCTAGATCATGTCCAATTTGTCTTTGATTTTAGATCATCATCTCAGCAATTTTTTTTGGATTCCCGACACCAACAATCTGTCATTATTTTAAACTTGACTGTTACATTGTCCTAATGCTGACTGCTGAGGAACTGTATATTATATAACCAGTCTCTAACAGTGAAGGTAATTCAATTATGCTCAGGGATTC

>LNC_002083

GCATTCTATAGTGGGAATTACCCTTTATCCAGTAGCAGCTGGGAGGCACCTTGCTGGCGAATTTGGACCGACATGGGAAAACAGCTCTTGTAGAAGAGGAAAACTTGGGAGTTACATTTCAGAAAGCTTGAAACAAGAATCTGATCAAACCAATTTGAGTGGGTTCTTGAAAAATTGTTGCGACATTTGGGAATGGCGAACAACAGGTATGAATACGTG

>LNC_002084

TGATGATCTGCAAATTTTGGCATCATTTTATTTGTTCATTTGCCCAACTTACTTTTCCAACTGAGCTGAAGCCTGGTAAAACTAAGTGAAGTGTTGTTGTTTAAAAGAGAAATGTACATTAGAGGAGATGTTCAATGTTGTACAATATTCTTTTGTTGATAAGCAATTTAAGTACGTGGATGGGAGTTGTTTATTTTTGTGAGTTGCCTGAGGTGATCCAACTTATTATCTTCTCAGAAATCTTAGTAGCTAGTTGTCTTTATTTCATGATGTGACCATTTACATTTAGGGGTCATTTGATAGAGTGTATTAGAAGAAAG

>LNC_002085

CTGATTTTGCTTCGAATTTGGAATTGCTCAATAATTTTATTCTGTAAAAATGGACAAAGATAGGTCTAAACAAGTAAACCACATTAAGCAAAATTTGACACGCTCATGGTGAAATGTCGATAAAAGTGTTAGCTTTGTGCTGGCACACTCTTTTGGTCTTCCCCCTTGTGTGATCATTCCATAGGATACCTAATTAGTTTATCTATGTCGTGAAGCTTTGGACACTGTAAGAGGAATTTAAAAGGAAACCACAAACAAAGTTGCTCTAGAGAGTTGACACATTCTCAATTCCAAGATGGTTCAAGCTCGCCAAGAAAGACTTTTTCTATCCTTTACATCAAATAATAAATTAGAGACTTTCTACTATCCCAAGATCGATAATTAAGGTTGGAGGACTCCACTAAAGAGGCCACTTCGAAAGAGAAAGATGATTTTAGATAGAAAATCATGGTTATGCTAGAAAGCTTTTTGGAAATTGGTTTAGTTACAAAAGCACAAGACCAC

>LNC_002086

CTGGATAAACACTACTCGAGATGTCTGTCTCAAAAAAATATAAAAACTAGTTGAGAGGTATTGTTCCTGAGTCTTTAGGTAAGCTTCGACGCCTGCAAGGACTGACGTTGAATGGAAACAAGTTATCAGGAAAGATTACATCTTCTATTGGTAACTTAACATCTTTGTCTTCTTTGCACATTGAGGACAATGAATTAGAAGGAAACATACCTCCGGAGTTCGGGCAGTGCATCAGTTTATCAGTGCTAAACCTTTTTAGTCTGAACAATTCGTTGACCGGTTCCTTGCCAG

>LNC_002087

CTCGGTGGCGTTAGGAAAGAGGAAACAAAGACCTTTCATAGGATAGGTAATGGCCAACCTAATTCGTTTTGAGCTAAAAGATATGACCATTTAAAGTTGACCCTTACAACTCACTAGTTCAAGTGACTAGTTTCCGGACGTCACGGTCATTTCTTGACGTTTGACTTCAAATCTCTTTAAACTAAACTTCAAAAGCCATTTCTCATCATTTCATCAAGTTCTCAACTCCAAACTCACATGTGAGGCTCTAGTTTCACTAGTTCGTTTTTAGGGTCGTT

>LNC_002088

CAAGAATTGTGTCCTTGTCAGCATAGGGACAAGTATCATGAGCTTGGTAGCGCATTGGAAGGATGTGTTCATTGGATGAAGATTATAAAATCTTGTTGTTATTTATAGAGAAGGTTATATTTGCCTATGATGGTTATAGGCATAACCCTAGGTGTACTTATACTGATGTTGAAACTGACCCGCTTTATATGAAGATTATAAAATCTTGAGTTACATAAAATCCCATCTTCAACATCAGTACAAGTAGGCCTAGGGTTATGCATATAACCACCATAGGCAAGTCTACCCATCTCTATAAATAACAAGATTTTATTGTCTTCGTGGACATGAAAACACCCTTCCAATGCACTACCAAGCTGATGATACCTGTCCTTATCTTTACAAGGACACAATTCCTTCATACCAACTTTCTCCATATAATCAAAAACTCAAATTTCCA

>LNC_002089

TTTTATGAAGGGGTGAGGGGGGTAGCAATATCTCTATTCCATTGGTCTTACAGACTCAATTTTGTATGTGGACATGTTTATTTTGAGTTTTCCAACATGATGGGTTTAGGCTCTTGATAATCTTAGCAGATATTTTTAGGATAAGTTGTTAACCTATTCAATAGAATGTCTATGATTTCTCAAATAAGATGCCCTTATATAAGACTTTTATGTTTATCTGATTGATACCAACTTTATACAAAATATTCAGCCTTTCTCAAATTCAAATAAGGATCTAAATGTCGAAGAAAATGAGAGGTAGACAACTAGGATTGTATAAGACTAGTATGTTAGCCTTTTCCCTTTATTAATTGGTGAAATGGAGAGGTGTAGGATAGTTTAAAACTGTACTTTCATTTTTTAGTTTAACAAGTTGAGTTAGCAGTGAAGAGATGGATGTATTAAACTCTTCTTTATTGTGTATTTCATTCTTTATTTCTTCTTTCTGCTGCTAAATCAGATTTTAGGAGCAAAAGTCATGATACTTGAGGCCTTATCCAGTAACAACTGACTGATTATCGTAATCTCTCTGGGACACCATATGAAACACTTGCAGCCCTAAACCTCTTTAGGGTGATATGCATCCATTCTTTTATCCCTTTTATACCTAATTGCTCTCAAAGTGTATATCTGATTGACAAGCGTCAATCTTTTATAATTGGTTAATATGTCATAGACATGATCGAGAAAGGGGTGTTCCGTGCACTAAAGCTGGGCTATGCGTGGGATCCGAGGAAGGGCTGGACAACAGGGTCTAATCCAAAAAATTCATGTTGAACATGGAGCTGCTATAGTGATGCTCAAGGCCAACTGTGGTGAATTAAATTACTTTTAGGATGTAAACTTACAAATTATATTTAAACAATTTTCTACTAACATGATATATCAGGTATCTTTTGCAGGTATTGAAGTGTTCACTTGAGCAAGTCAAGATACAAGTTGTCAAGTTTCTTATAATTTTTCTACTGTTAGGTAAATAATTTATATCTTTTGCTGAGGATGTTAGATTTCTTGAAGGATTGTGTAAACAATACAATGACCTACTAAAAGTTTTTTCTTTCCTACTCAAGGTTAATTTCCACTAATAATTGATGTTGAAACATAATTTAGGGACAATTATGTTACTACCGTTAAACAATTGTCGCAAAAAACTTTACTTGTTGCAGTGAGTAGGTGTTATAAAAATTGTGCATTAGTTAGAGCTTGCAGCTTGGCTGAGGCTGATTGAAACAAAAGAAACAGAAAAAGATTACCAAACCATGGGTTTGCAATAGTTTTGATCATGCCATTCTGAATTTTTCCCATATAAAGTGTCAATCTATCTCTATATGTTTTGTTCTTTCATGGATTTTGGGTTTGTAGCTTTACTGTCAATGTTCACTTGAACTGGTAGAACTGACTTGAGCTCCAATTTCCTTAAGTATCCCAGAATTCATGTTAGCTTAACTACAGTAGACGATGCTAAGCTTCTGTATTCAGTTTCAACTGAACTTCTAGATGCTATACTTTGCTTTTTTTATTTCCAAGAGATTACAGAGTCACCTAGTTTGATTAAAAAGCCTATCACAATTTTTCTGGAGTTTGGACATGCTGTCTTGTTCTTTGGTGCTGCTTGATATGAGTATTCCCCGCTGTTCATTTTGAAGGCTTGATGTAGTGCTGTCTTGGTTTCTTTAATTAGTTGTAAGTTACTACCAGTGATAAGCATGCCATCCACATATACTAGGATGACTATCAGATCATTTCCAACTTTCGTCGAGACTAAAAAGTGATCATCTTGGCTTTGTTTGAACCCAGATTGCTTCCAATAACTTTGTATTCCATTGTCTTGGGGCATGTTTTAGGCCATACAAGGATTTTTAAGAAGCTTGCATACTGGTCTTGTGTCCCTTAGCTCTTAAAACCTTATAGAAGATCCATGTAGACCTCATCACGAAGATCTACTTAAAGGAAAACATTGTATACATTTTGGGTTGATCACTTGATACCATTTTGATTACCTCTGGTAATCAATGCCCTCTTGTTGATTGTATCCCTTTGCTACTAATCTAGCCTTGAACCTTTTAATTCCACCTGCGTCTCTATATTTTATCCTAAAGTCCCATTTGCAGCCAATGCCAGTAGGCCTCTTTCCTTCGGGTAGAGGAACTATTTTCCGAGTGTTGTTACTTTCTAGAGCAGCTATTTTACCTTCATTGCATCCACCTACCTAGGATCTGAGACACCTTCACTATAAGATGATGGTTCTACCTTGGTTGAACTAGCAGCAGCGAAAGCTCGATAAGCAGAAGAGAGAATGATCATAGTGAATGTAATTTGCAATGATGGAAGGTCTATTGTGACACCCCAACTTAAGATAAAGAAATCTCACATCGGCAGAACACATGAGAGATGTTGGGTATATAAGTAAGCAGACTGTGC

>LNC_002090

TGAATTTATATGTGGGTCAGTGGACCGATTAACTCTTAATATTTGGAGGAGTAAAAGTTCATATAACTGTAGATCAAAGGGAAATATATTATAGTATATGATTTATTTTCTTAATAAGTTACCATAAATGAACAAAACATCATTCTACTTGCATTCACTTCACAGAGAACAAATTATGGGAGTGATGAATTGGGGAATGACAAACGGAAGAGGAAGATAGAGAAATTAGTCAAGGATGATGGAAACAACAACATCATTGATCATGATGGAAAGCATATGGTTTTGTACGTTGAAAAATTGCAAGAGTTAAGAGTTAGAGAAGCTTAACAAAGAGGCGAGTGGTTAGTTGTTGAAAGTTGCACAAAAGTACAATCGGATCCCCTTGTTAGTAGTTAATAACAGGGGGTATGAAAGTAAGGGGGTAGCCGCTTGTAAATAGTTTCCCTTTTTGGAGTACAAGTGCTCTTTCCCCAAAATTAGGTATCCAAGTATAGGCCATTTAACTTTTTGCTATAATTCGTTGGATCTTAGTGATATTATAAGTTTAGCCCATTTTAGTTTTGTATCGTATTAAGGATACAAAACAAAATGGTAATGTTGAACAAGTTTGCACATGACTCTTGAATGCTGACGAAGAACATTTTGTTTTCTTGTTTCTTTTAATGTTGGTTTACCAGTCAATTTGCTCTCATCAATGCCATGATTATTGTGATGAAGATTGAAATAATGAAAGCACAATTCTAGAATAGTTGGTTCTCCTCTTTTTCTTCAACTTACATGGATTTTGGTTCTCCATTTCTCTGGCTCCGATCAATAATTATTTGTGGATATTGCACGTGTCATACGGTATTTCAATCAATAATGTTTGATTCTGCAGCTAATTTTTTTTAATGATTCCGGAAATTCTGTAATATACATCACTTTCACTCCAAGGAATTTTTTTTATGAAGGGGTGAGGGGGGTAGCAATATCTCTATTCCATTGGTCTTACAGACTCAATTTTGTATGTGGACATGTTTATTTTGAGTTTTCCAACATGATGGGTTTAGGCTCTTGATAATCTTAGCAGATATTTTTAGGATAAGTTGTTAACCTATTCAATAGAATGTCTATGATTTCTCAAATAAGATGCCCTTATATAAGACTTTTATGTTTATCTGATTGATACCAACTTTATACAAAATATTCAGCCTTTCTCAAATTCAAATAAGGATCTAAATGTCGAAGAAAATGAGAGGTAGACAACTAGGATTGTATAAGACTAGTATGTTAGCCTTTTCCCTTTATTAATTGGTGAAATGGAGAGGTGTAGGATAGTTTAAAACTGTACTTTCATTTTTTAGTTTAACAAGTTGAGTTAGCAGTGAAGAGATGGATGTATTAAACTCTTCTTTATTGTGTATTTCATTCTTTATTTCTTCTTTCTGCTGCTAAATCAGATTTTAGGAGCAAAAGTCATGATACTTGAGGCCTTATCCAGTAACAACTGACTGATTATCGTAATCTCTCTGGGACACCATATGAAACACTTGCAGCCCTAAACCTCTTTAGGGTGATATGCATCCATTCTTTTATCCCTTTTATGTAAGTTAACCTACTTTAATAATCAAGTTAATTTGCTATTTCAGTTTTACCGTTGCGTGGTTTCTTTTGTTGAGTTGCATTTTACTCTATGATATTTTGATGTCAGTCATGTAAATTTTCTGTGTGAGTGTGTTAACATGGCTGGAAGTTTGATGCGACAACTCTTTTCCTTTTCATATTTGAATTTAATTTAGTTAGAGAAGAACAAAAATAGAAAGAGCTTTTCTATCGCATCCAATCCTGAATGTTCGTATGCATTTTAAGAAAAAAAGTCTTGTCGTCTAATTTTTCTCACTTTGCTAACAGTTGGCAGTATAACTTAGTTATAGTTTGATCAATGACTTGTAGGTTCATAAACAGATATGTTACCTCTGTTATCAGTTCTTTGTCTGACAACTTATTAAGTTTTTATTTTTAACAGACCTAATTGCTCTCAAAGTGTATATCTGATTGACAAGCGTCAATCTTTTATAATTGGTTAATATGTCATAGACATGATCGAGAAAGGGGTGTTCCGTGCACTAAAGCTGGGCTATGCGTGGGATCCGAGGAAGGGCTGGACAACAGGGTCTAATCCAAAAAATTCATGTTGAACATGGAGCTGCTATAGTGATGCTCAAGGCCAACTGTGGTATTGAAGTGTTCACTTGAGCAAGTCAAGATACAAGTTGTCAAGTTTCTTATAATTTTTCTACTGTTAGGTAAATAATTTATATCTTTTGCTGAGGATGTTAGATTTCTTGAAGGATTGTGTAAACAATACAATGACCTACTAAAAGTTTTTTCTTTCCTACTCAAGGTTAATTTCCACTAATAATTGATGTTGAAACATAATTTAGGGACAATTATGTTACTACCGTTAAACAATTGTCGCAAAAAACTTTACTTGTTGCAGTGAGTAGGTGTTATAAAAATTGTGCATTAGTTAGAGCTTGCAGCTTGGCTGAGGCTGATTGAAACAAAAGAAACAGAAAAAGATTACCAAACCATGGGTTTGCAATAGTTTTGATCATGCCATTCTGAATTTTTCCCATATAAAGTGTCAATCTATCTCTATATGTTTTGTTCTTTCATGGATTTTGGGTTTGTAGCTTTACTGTCAATGTTCACTTGAACTGGTAGAACTGACTTGAGCTCCAATTTCCTTAAGTATCCCAGAATTCATGTTAGCTTAACTACAGTAGACGATGCTAAGCTTCTGTATTCAGTTTCAACTGAACTTCTAGATGCTATACTTTGCTTTTTTTATTTCCAAGAGATTACAGAGTCACCTAGTTTGATTAAAAAGCCTATCACAATTTTTCTGGAGTTTGGACATGCTGTCTTGTTCTTTGGTGCTGCTTGATATGAGTATTCCCCGCTGTTCATTTTGAAGGCTTGATGTAGTGCTGTCTTGGTTTCTTTAATTAGTTGTAAGTTACTACCAGTGATAAGCATGCCATCCACATATACTAGGATGACTATCAGATCATTTCCAACTTTCGTCGAGACTAAAAAGTGATCATCTTGGCTTTGTTTGAACCCAGATTGCTTCCAATAACTTTGTATTCCATTGTCTTGGGGCATGTTTTAGGCCATACAAGGATTTTTAAGAAGCTTGCATACTGGTCTTGTGTCCCTTAGCTCTTAAAACCTTATAGAAGATCCATGTAGACCTCATCACGAAGATCTACTTAAAGGAAAACATTGTATACATTTTGGGTTGATCACTTGATACCATTTTGATTACCTCTGGTAATCAATGCCCTCTTGTTGATTGTATCCCTTTGCTACTAATCTAGCCTTGAACCTTTTAATTCCACCTGCGTCTCTATATTTTATCCTAAAGTCCCATTTGCAGCCAATGCCAGTAGGCCTCTTTCCTTCGGGTAGAGGAACTATTTTCCGAGTGTTGTTACTTTCTAGAGCAGCTATTTTACCTTCATTGCATCCACCTACCTAGGATCTGAGACACCTTCACTATAAGATGATGGTTCTACCTTGGTTGAACTAGCAGCAGCGAAAGCTCGATAAGCAGAAGAGAGAATGATCATAGTGAATGTAATTTGCAATGATGGAAGGTCTATTGTGACACCCCAACTTAAGATAAAGAAATCTCACATCGGCAGAACACATGAGAGATGTTGGGTATATAAGTAAGCAGACTGTGC

>LNC_002091

TGCACTTTGATGTTAGATTTTTCTCCGCACAAACATAGGGGATAGATCTATTGATTGTCTGTAATGGTGGTAAAAAACCTCTTTTTCTCCTACACTGTTTGAATACTGGATCTCGGGTGGTTCGATTTGTATTTGGTGTTTCTATTGATTGATGTTTTTTGTGTTATATAAGCCAACAAAAGTTGGAGCATATATGCTACTATTATGAGTCAAGAACATGTTGCTTCTCTGCGATACTCATTTCAAACTCTTTCAGGCTTGAAAGTGTCATTTCTCCTCGCCGTGTCTGTATGTTTTAATTGTATATTGCAACACCTTCTTGGTGTTGGTTATGCATATATCTTCTTTGAAATACTAAAATCTATTCTCTTGCTAATCAGATGAGGTAATTTATGCTATTATTGTGTTTGCCGAAATTCCCCACTGAAAAAAGATTTGTGTGTCGTGAAGAGAGCTTAGTACCAAGAGTTGGCCGCTTTAAAAAATGTTTGAAGTCTAATGAAATTGAAGTATGGGCTTCCCTCCGCTTATTGTTATCTTTTGCTTTTTATCTAAAATGTTTTGTTCCCGTGTGGAGAAGGGGTTACCAGTTAAATGTTTTTAGAATTTTATTGATCTTTTGTAATGGTAATTCATTTACTTGCATGTGTAATTCTACAGTAGCATTACTACTATTTATCTAACTTTTTTGTCGAAGTGAAGCGATGTTCTGTTTCAGAAAAAAATTGGCAGTCAACACTCTCTGCATTAAGAAACATTCGGTGGAAACCATTTTGATCCGTGGAGATAAAATTTTGACACTCAAGTCAAGATTGAATCAAATAGCAAGGGATCATGAAATTACTTGATCTCTCGAACTCTTTAGCGTTTGTGAATAGTAAGAAGAACGGAGTAAATGTTGCTAGCAATGCTCTTGTGGAAGTTCCCAGCTGCACCCCAGGAAGCTATGATTGTACACTATAACTTGCATTAGCTATGCGTCCTAGGCTCGATGTGTGAAAGTACCTGTCATGGAATCTTAAGACACTGAAATGGCTGAATTGAGAATCTCCGAGTACTTTCAATTGAAAGAGGTACTCTACGATTTTGCAGTGTATGAAGTTGAGAAGTCCTACATATTTTGATACATATAACAAGTGAGATTTTGAGGCCAAATATGTTTGAGTCACAGGAGATTGGTCAAAAAGTTGCAAAATTATTGAAGAAGTATATTTTGCTCTTATATTTCTTTCATGGCACGAATTGGAGTAGGTAGTTGCATCCTACATGTTCTGTGTTGGATTTCGCCTCATGAATCAACAATCGACTTCCCCATGTGTAAGGTAAATACATTTTGATTATAATATGTTTGTGATTACGTATGAATGAAGCCGGAAGGAAAATAAGGAATCAAAACAAAGGGCCCAGATAAGAGAGATCACCACTAGTGGTCTGGCTAAACACAAAGCCTTAAGAATACGTACATTCGACTACACAAAGCCTTAAGAATACGTACATTCGTCAAATGAACTTTACATAAGCCTCCTTATAGGTGAATTGATACTCAGAAGGTTGAGATGTAGGTATCTTTATGAGAAAACAAGATATTAAGAGAATACAAAAATTACCCGTTCGTCTACTTTCTCAAACAGTTGTAAGGTGTCCATAAGTACAGAACATGTTTGAGGAAGAACATTGCATTAAACTATTATAAAAGCCTTACAAATTGTGCTCTACATTTTGCCTCGATTGAATAAGCATAGTCTCTTCCTCTTTCTACTAAACTAAGGGCCTGTTTGGCCATGCGATATGGTATCATGATATGGTATTATGATATTAAATTATGAGATGAAATTGAAGGTTTGTTTGGACATGCGATATGAAATTTTTGTATTGTATATTTTCTCATAAACATAAAAATCTCATAAGTTGTAAAACTATTAAAATAGCCCCAATTGTTTATTCAATCTTATCAAATAAACAAAAAATTATAAAATCGCATATTAAATTATTACAAAGTTATTTGTTCTCC

>LNC_002092

AATCAGTTGTAATGACCCTAAAGGTCATTTTTGGAAATTTTCATAAAATGACTGTTTTACCCCTCCCGATAGTTGCCCCGAGTCCTAATTAATGTACTTTCGAAGTTGGTTTGAAGGAAAATTGATGAAAAGTTGAGTTTTTGGAAAGTTGAGTTTTTAAGAGTTAAAGTTTGGTTAAAAGTGCTATTTCGAGTCATTTGGAGTTTCGAGACTCGAATCGGATTTTCGTCGATTCTAGCAGTTTTAGAATGTCGAAACGGGTCTATGAGAATTTACGGAGTCGAATTTGGAGTTAAAACGAAGATTTGAGGTCCTAAGTTGCAAAAATTGTGAAATTTTGATCAAGATTTGACTTTGGTCAACAAACGAGGTTCCGGTGCTCGAAATGGAATTCCGAGGGCACCGTTGGATTCAGGGGGTGATTCTAAGTCTAGAATGAGTCTTGGTTGAATTTTTAGAGGCCCCAAGTGCGTTTCGAGTTTTTGAGCCTAAAGTTAGTTTCTTGACGCCTTATCGGATACCGGGTCAAGGAGACCTCGAATTCAAATTCCGACGATTTCATTGAGTCCGAAATGTCATTTTCAAG

>LNC_002093

TTTTCTTGTGTGGGATACTCAGGTTAGATTTCAATTTCTTTTAGTTTTCTATTATAGGGTTCTGTTATCTGTTCAACTTTTATGGTTTTTTTGCATTGAACTTAGGGTTTCTTCTATGATTTGATGTTGAAGTTTTTCTATTTTGTTTAGTTTGTAATTTTTTTTTGGAATTGGTGTGTATTTACTTTTGCATGGTCCCTAAATTATGTTTTTACCTGCAAAATTGCTGATGTAGAGGAATCATTGGGTGGCTAATGAAAAGCAGATGAAATAGGAAATGGGTTCGTTTTCTTTTGATTGTTTTGTTTCTCAATAGTGTAAAGACAAGTAAAAGATCAAAGATATGTGCTCTTCTTCATTTTTCTCTTTTTCTTTTTGCGTTACAGTTTGAAGTGCAACCTGTTTTGGCTTGGGAAACTGATAAGAGAGGGTAATCCAATAGGGGTGCGGATTATTCGGATTAATACGTCTTGTACAGTGTGTTGGAAAATATATA

>LNC_002094

CTAGCTTTTGGGGTGTGAGTTAGGCCTAAGACTTAATTTAACATGGTATCATAGCAGGGCCCGTCTCACCCGATGTTGGGACCCCTAAAATCAAAATTGCCCACTCACCAGATGCTAAGCATTGGGCGTGAGGTGGGGTGTTAAAAGAATGACAAAAGTCCCACATCGATGGTTAATGAGATGGGTGGACTCCTTATAAGGCTTGGGCAATCCTCCTCCCTTTGAGCTAGCT

>LNC_002095

CCAACAACACCAAACCGACCCAATTCCCCCGACCCGACCCATTTTGATCAGCAATAGAAACCCAACAGTGTCTCTTTCAACACTCCAACGTTACAGCAACAATAATACAGCAGCTACGCAGCTCACATCAGCAGCACACGCAACACCAGACGACCCATACGCGGCTTCTTCTCCTTCACAAGTCAAAATAGACGGAAAGCTTCCACCGTCTTCATCGATGGAACAACAGGAACAGACCTGCAGTTGCGTCCTGCAGCAGCCGCCTGTCAAGCCATACACCCCCTCTTGTCGTGCGGATTTTGGGGGATTTCGGTTGAATTCTTGTACCAAGGACAACTTTTCCCCTTCCTTTGGGAAGAAAATTTGAATTCTTCTTCGGAATCTCACTTGTGGTGGTGGATGCTTCATCATTAAGCTCGTCGTCTTCAGTCGCTGCCCTGAGAAAGGTAACACTTCTTCCTCGACAACATTCTCTTCATTTCTTTTCATATTTCGATCTTTCGGTGTTAAAAATCTCTGTTTTTGTGGCTGGTTGGTGGATGTGTGTTGCTAAGTTCATAATGTTCTTTAAAGTTGATACATGATCCCATGTGATTCCGTGACTTGGCTGAGTTAAAACTTTGGAAATTTGTATGATGTGGTTTCTTTGTTTCTCTATTCAAGTTTGCTTTGTTATTTTTTCCTCTTGTATGCTTGCTTGAATGCGGCTAGATTTGTATTGTTTCGTTGTTCTCGTTGTCTTAAACATCAAAGAGTGCATCAACTTGATTATTGTTAATGGCGTTTTCTATTCGAAAGTAGGGGCAGATCTTAAACCAATTGTTTGTCGTTTTATCTATTGTCTGTTGATGTTAGCAGTTGATTTTCTCCATATATTTTTTGAATCCTAGGATTGTCTTACTGTCCAACATACTTTGTTCGGGTTGAGTCCATGTTTCTATTCGGTTTAAAGTTTAGTCAAAATGCTTACCATTTGTAGTTGAACTTTGTAGAATGTGAGTTTTTCACAGATTACTTGAGAATGCATAGTGTAATCTTGGTCATTGTTTGGTTTTGTTTCCATAAGGTCTGTCGTAGTTCCCTTTCTAAATGGTTTACATTCCTTAAAGTTTTGGTGTTTAGATAAATAGCAGTAGGGGTGAGAATTGGATTTTCCATCTAGGTTCTCTTTGTCATAATTTGTTCATCTCTTTCTCTTCTAACATAAAGCAGCATGTCGTCCTCTTCTCTTGGCTTTTGATTAAAATTCACAGTGTATGCATATTTTAGTCTTAATGTATGTTTCTTTTAAATGTTGATTCATAGATTTGGCTTGAAAGTTAGATTGTTATTTTTTTCAAGTTGTTGGGATCATGTTATTTAAAAGATGTTTTTATCTAAATGTGTGAATAATTTCCTTCCATTTCAAGTATCTACTCCCTCCGTCCACAATTGTTTGGCAGGTATACTAAAAATAGATGTCCAAAATTAATTGTCAATTCAAACAATCAGTCACTTTTTTTCCAATTCTGCCCTTAATAATTACTCCATTTTACTAACTACTGATTTCAAAAAGTACTGACCATTTTAAAGTTACAAGACCACTTTAATTTGAAAAGATTACTTTTACAATTTTTAAAAATGCAAACCATTTTAAAGTTACAAAAAAACTTTAAAAATACCAAACCATTTTAAAGTTACAATAAAACTTTTAGTAATGCCCTTAGTTAGCTTAAGTAAATACTCTATAAATTAAGTACAGTTTTTGAAGAGTCATTAGATTAAGCACTAGTATTCCATTATAAACATGGACACCATATTCTAAATTTGAAAAATGAAAAAATTCATCCACGAAGAAAGCGGTAAAATTTTTCATTGAGTAGTGTAGAGTTATCTGGGTGGACCTTTGGTATTCTTCATACATATTTAATGATAGTGTAGTAGTAGTTCAATTGAAAAGTTATGTAAACTTTTGATATTCTTTGTATAGATTTAATAGTTTATTAATGAAGAGCAATTTTGATAATGATTGATAGTAGGGTTATATTAGTCAAATTATACCTTGTATTAAATTTTCCTTGAGGGGTGTACATGATCTTACCATGACAAACAATTGTGGACGGAGGGAGTAAATGATTAAGCATGAGATGGGTATTAATTTCTCAAGATGAAATTATTAGAAAGAGTATGAATAACCCAGTGAGCATCAATGATGCTTTTGATGCCGATATATTCTGTGTTGATACTTGTAGTAATTTGTAAGTGTCTTGAATGAGTTATATCACAATTATCTCTAATTGAATACATAATTATATATATGAATTGTTCAAAACCCTTCACTAATTCCTTTTCCCCTTTATTTTTGATACATTTGACATCAATCCGAGTCCATTTTGGACTCCATCCCTGCATTTCGTTGAATTGAGATCCTTATCGAGTCAACCTTCATCGTTGTTCAAGTTTTGAAAGCAGATGGGCAGGGGTACAGGGCTGTAGTCGAAATACAAGCCTCCAAAGTG

>LNC_002096

GGTGGTTTGAGACATCTTTATACCTTTTCTCATATCTTCAACCACTATTTTAACATATATCTATCAAATTGAATATAATAAATCATTAACTTATTCAAACTACTATTATATATCTAATAAATTCAATTATAATAACTCATTCAAACCTTTTCTCTTAAAGCCTTGAAAGTCAAATGTACTTCATAGCAAATGGGACAGAGGGAGCTTATATCCAAGGCGCCAATGTATAATGCTCTCTTTTTTGTTGAACCAGTGGAAGTGGGAACTGGACTTACTAAGAACTGGCAAGTGATAGAAGCTAATAGCTTAGTAAATTATCTTTAGGCGGATGATATTTTTTCTCCTTATGAAATTTGACTTTAATCTTCTCCAAGGATTCAAATTCTGAGGTCAGGTTAGCTTAGAATGGTAAGTTGGTATTGTTGGCTCATGTAATACCTGACCATGTCTGGGATTTGCCCCATGTACCACGTAAGACCATTAAATTCTCAATTAAGTCATCCAAAAGTTATTTATCATGCCTAAAGTCCTAAACTGGAAGGCAA

>LNC_002097

ATTCGAGTCGTGAAAACCGTCTTTTGGCAGAAATGCACGGTAAGGCTATGTACAATAGATCCTTGTGGTTCAGCCCTTCCCAAATTCCATGCATAGCGGGAGCTTAGTGCATCGGGATGCCCTTTTAATGTTGTTAGGAGATGGACCGATTCGTCCTCCAAATAGTGCCATTAGTGATGCTTATCCAAATCCCTTGCCCCTCAAAGCAGTGCCCTTTTGGTGTCCATGGCCACCATAGAAACTAATTAAGTGATGCTAAACTTCACTTCTCTTAGAACATGTTTGGCCAAGCTTCTTGGAGGCGGAAACACTTTTTTTTTTTTTTAGATTTGAGGTTTTGGCCTACAAAAAAAGTGATTGTAAGGAGTGGCAGAAATAATTTTTGCTATTGGGAAAGAAACCTATGTTGTTCAGACTCTCCAAAAATGTTGCCGTACTCGTGTTGAATTTTCCAAAAATACACTACTTTTGAAGTATCCGACACGCAC

>LNC_002098

CCCTATTTTGTAAGAGTAAAGAGATTGTTTGCAATAGAACTTTCACTTAATATTTGAGCAAAATGTAATATTCTAGGTAAGTGGTCAAGAGGATGATGATCTCAGCATATGATATTAAATTATTTAAGAGCTAGTCTAGGTATTTTATCTCTTTATAAACATGTTAGGCAAACCATTTAGCATATTCTTGGTTTGTCAAGGAAGGGAGAAAGTTATTTGGGAACTAAAGCAATGTTATCTGGATTACTACTCTGGGTCTGGGCGTTCGATGCAGGTTAGAAATTCAGTCAAAATGCTTAAAGGATAAAGTGCTCACTTCTCAGATATCTGTCAATATTCTTCATCAATGGCTTTTGGACTAAAAGTATAGAGGCCAGGAAGTATTAATTTGTCAAAATTAAGTCTGTTTCTACATGTTGTAAAACTTCATTAATATCAGTTACTAGTAGGTCCTTTTGTAGAGATTAAGTGATCATTGAAAGCTAGCTAGCTACTTTTTTTTAAAAAAAAAAATATATTTCAGCTTCATGTATTTATGATTGGTCTTTTTGCTAAAATGTAAAGATAATTCCG

>LNC_002099

GAGCAAGACGCGTGCTCGGTACCAATTTTACTGTCATCTTCTTCGCGTACCTCTCCGATAGTACTGTTCCAGCCGTGATTTTCGGCAAAAAGCATCTGTTGTAGCAGCGCTGGACCGTCCTTGGGGCAGTGAGATCGCTCCACGTTGCGCCTAAGGACAATTGAACCGATCCCTTTCCTCTTCCGAAATGGGTTGAAATTTCAAAAAAAATGTGTCTCCCCTGAATTGCAAAAGAAATTGGGAGTTTGAATTTCAAATTGGTCCATTGTCTTATCCGATTTCATTTGTTTCCCCCCAAAATTCCAAACCCTAGTTTTCTTCTATAAATGCCTCCTCTTTCTCCATTGTTAGGGTTGGGGGAGAACAAAGTTGGAAAAATTGAATGGAGCGACAGTGAGAAAGAAACTAAAGAAAGAGTTGTTATTTTACGTTTAGGACGATAGTGTTCGATAAAATTATGGTTTGTTCGCTTGGGCTATTCAAGTTTTCCGTTGGAGTCTCTCTCTTGTGTGGTTGGAAGAAACTTCGTCTCTGCTCATTGAGTCCCGTTTGCTGCCCCGGAAAAGGTGAAACTCTTTTCTAATTTTTAAATCGTTTTGATTGGTGTTTTCGTTTTGCGAACTGAAGTGGCCTGTTGTTTTC

>LNC_002100

GGATAAGGTAGTTATGGAGAAAAAAAGGGTTGGCATTAGAAATCTACGGATGCACAATAATAGTTTTCTTATAAAGTGGCTATGGAGGTACCGAAAGGAAGACAATTGTTTATGAGGGTAATTTTCTACAAGTATGGGAATCAAAGTCCTTGGTACATAGGTTATGTGCTCTCCCTCATGGACAATGAGTTTGGAAACATATCCGCAGCTTATGACCCACCTTTGAAGCCAATACAAATATCAAGTTAGGAAATGAGATGAAAATAAGATTTTGG

>LNC_002101

TTTGAATGGGAAGGACTGCTATGGCCTTCTGATTTTCCGTACTAGGAGAAAAATACAGAGAAGTTTTGGAAAAAGTATCATCGAGATGAAGTACTTATTGGTAGAGGGATCAAAACGTTTGTAACTTTTTTGTAGATGAGAATACTCGAGAAAGACACATTTAAAAACCTTATGGTGAAGTTATCTTTTCGAGGGGTAAGGTTATGAACAAAACAGGGGCAACCAAACACACACGGATGAGGGGGAATAAATTTTGGTCCGGATAGAGGAATGAGTGAGGACTCTGGTACTACAAAACAGATGAAGGCATGCGATTGATTAAATAACAAGCAGTAATAACGACATCACCCCAAAAATGTAAAGGGGCATGATTATGAATAAGCAATGTTCGAGCTGTTTCAATAAGATGTTTGTTCTTTCGCTCAACAACTCCGTTTTGTTGAGGAGTATGTGCACAAGAAGAGAAGTGCAATACACCCTGGGATGACATAAAAGAAGAGAGAGAAGTAGAGAAGTATTTCGGGCATTATCAGTGATTAACTTCT

>LNC_002102

GAAAATATAATGATTAGGTAGAAGAATAACTAGAGGATTTCTCCGCCCAAACATCAATGCCTGAGATGGAGGGATAATAAAAAGTGCTATTGCTTTTGCATTACATAGAAAGAAAGACTACTCTATCCCAAACATGATATATTGAAAATAACGTATCTTAGAGTGCTTTAAGTTACAACGGACCTACCCATGCCATATATTAGTGAAGTAGTCTCAAGAATTACACAAATTTAGGTAGTAGTTCTTTTTTGTATGCTTAATGATTTAAATTTTAAAAGTCTAAATAAATTCCTCTTCAGTTGGCCACTCATTTAGTTTGATTGGTTTTTGCTAATTGACCTTGCTTTTAGTTTTTGAATTTTAA

>LNC_002103

ACGCCTGAGCCAGCAAACCAGAAAATAAACCCAGCTTCAGCGATCCAGCAGCCTAGGGACCCGACCAGTCGCGCGTTCCCCTTATCTCCCCTACCAGACCGTTTCGAGAATCGACCAGGTATCGAAATCTCGCACCTCACTCGTCCTTCCCTTGGAACCAAACGATCCTTCATTCAATTTCATTTGAATTGGTACGATTCATGCTCACCCCAAGCTTTATCCAGTCAAGAATTTTCAAATTACTCCCAGTCCCACATCGATTCTAGGCTGTATTTTTTCAGATTTCTCAACCCCTATAAATAGCCTCCTTCTTACTGTTCAAAGGAGACGTTTTGAGACCTGAGAGTACCCTATAAGGTTTGGAAATTCTCAGTTCAATAGTTTAAAGTTTTTGTGGTTTTTCGAGTTAAAGAGGTGGAATCGTCTCTGTTGAACTCGTTTTTCGGTTCGCTGCCCAGAACCAGACGCATGAACTCTTCCATTGAGTCCACTTGGACTTCCCATCTCTTGCATATTCTTCTTTTCGAGGCAACCTCTACCAAGTTGAGAGAGAAGTAAATACATTGGGTTAAAGCTGCCTGAAAACCAGCAGCACCAGCAACTTCGAAAGGCCGAAAAACAAGGAGCGAAATTGGGTTAAAACCCAAAAATCAGAAGCAGCAGCTAAAGGGGTTATGAAGGTGGAATTTTGGACTTAAAAAGTCCAATTTGAACTTGCAACAACAGCGATACAAAGAAAAATTTATGGACTCAAAAGTCCGAAAACACAGATGCTACATAGAAGAAAAGGAGGCAACGGCTGAATTTGGACCAAAAGTCCAGACTTTTCTTTGTATTACTTCTCTGCCTTCCTTGTTATTATGACATCTAACCCTTTTTATTAACTTGTTATTACCTTGGATGAATCCTCATAAGAGTTGACGCTATTTATCAATTTTTATTTCAAAAGGGTGGTTTGCCTAATTATGTATCAACACAGTTTGTTCTTGGGCAACTTAAGCTTATAGAATAACTTGAGAACCATTCATGTTTTTTCATAGTTACTAAAAAGCTTTGAATTAACGTTAATGAATTTGAACTATTTCTTTGGTTTAAAATCTTTAACTCGGATAAATTATCAAGTTACAAATGTTT

>LNC_002104

GGTTGTTAGATTATTGGAAACCCTAATTGGAATTGGTAAAGATTGAATCTGTTTGATGAATTTTTTGATTGGTAGGGTTCAATCTGGTATTTAATCTGGAAAGGTGTTGGGGGTGATTTAGTTGGGTGATAGTTGTTGTTAGCTGTATTATTTATGTACTGAGTGTATTTTTGTCACATTGGTTGGATTTGTTTTTGATGCATCATTCATTCTGTGGAGTTGGGTACTGTTTGTTGCATTACTTGCTGTATATTTTATTGTGGGGAAATGAAAAGATAGGACTGTTTATGTGTTGTTTTGATGAGTTTGTAATGGTGGATTAATAGATGTGAATGATTGAGGCTAGGGTTTGTTAAGGGAATATGGGTAGATCTTTTGGAGGGCTATGAGAGAATGTTGACATACATAATTTTTATGCATTTGGATATAGTTCATATACAGATTGGAAAAATCATGTGTAATATAGGAACGAATTGCACCCCAAGGGGAATGGGTGTGGCCTAGTGGTTAGTGAAGTAGGTTTGAGAACATTGAGGCCTCAGGTTCGAATCCCAATTGAGGTGTCCCATTTGTCTGATACGTGGTGCTGGTGGGAGGTGGTTACTCTTGGAATTAGTCGAGGTGTAAAAAAAAGAGAGGAGAAATATAGGCACCAATTTTGAAATGAACTGATAACTTTCTTTGGAATTTGTGCAATCTCAGTAGCCCATTTTCCTTCCTCTTAGTTCTGTAGTTGTTTTTCCTCGTATTTGGTTGTTGAGGCTCATTCGTAATTGCATGGTGTTTCATAGGATTTGAACTTGCAGATATTTTTAGGGCTTGAGCTTGATGATGATTTTTCAGTGTCTTTTTGCCTTCTCTTTATTGTTTCATTGATTATGACACAGTTAATCTTTGAGCTACTGTAACAATCAAGATTCAAGAGTTTAACTCCCCGATCATAGCATGC

>LNC_002105

CCTCTGTCCCAATTTATATGACTTACTTTCCTTTTTAGTCAGTCCCAAAAAGAATGACACATTTCTATTTTAAGTAACAATTTAGCTATAAAATGTCTATTTTACCCTTAATGAAATGATTTACAGCCACACAAATTTCTATCATTCATTTTGGACCACAAGTTTTAAAAGTCTTCCTTCCTTTCTTAAACTCCGTGCCGAGTCAAAGTACCT

>LNC_002106

TTTCAAAAGACTTAAAAGAATAAATTTTGCGAATTTTCTCACTTAGATAGTAGTTCCATATTTCTCTTTCCCTTTTTAAATAGTTCTAACTATAGAATAATTCAATTTTGCAATTTAAGTTGAGTTAAAATTATTTTAGCCAAATAAATTAATTGTCGGATAACCGCATTAGCGGATATTCTAGGTGCCTTAAAAACCTTCCTAGAATATTAATAGGAACCCCG

>LNC_002107

GCTTTATTTGTAAAGTCTCAATTCCTTGGTAATCAAAGCCCAAAGATCTATGAATTAGCCCATGCTTGACTAGCCAAGCAGACAAACGACCCTGCATCTTTTTTATCTCTCCCGCATTTTTATTTGTATAAGTATTTCACATTTACGATGAAATTTATGAAAATTGACCCACCACTTCTTATTATGGACAAAGGAATCATGTCTAATTC

>LNC_002108

GTCTTTTTCATATTTATCTCAACCACAAAAACCCAAATAACCAAAACTAGATTAGCTATAAAAACCACACATACATTCAGACTGAATTGGGAAAAGTCCATCATCCTTCCCTTCTTTAATTCTGGCATTTTTGCAGAAAACTGTAAAGCAAATTGCCAGAATGAAAGAAAGAAGAAAAATCTCATCCCACCTTCGTCATCATTTGTCAGATTCACTGCAGACAGATATGACCCAAACTTCAAAGCTAAACTCCCCCATGCCAACTTGCTCCCACTTTACATTCTTTCTTTTGCAACTTGCCATTTTTAACGTTGTGGTGTATTGGTCGGACTGCTTTCACTCTTAAGTCTTAATTACTCCGTCCATCTTATTTTGTGTGAGATAGTTTGACTCGGCATGGAGTTTAAGAAAGAAAGAAAGACTTTTAAAACTTGTGATTCAAAATGAA

>LNC_002109

TGTGAAATGGATTGAAAAGGAGTCTCTGTTAGGAGAGATTCTGCAAAAAATGTCGGCAAGCATTTTTCTTTTAGAAAATTTGGCGTTCTGAAATTTCAAAGATTCGTTTTGTCATCTTAGATTGATGTCTTCGAGTTCCAGCATAGAAATTTTCCTGCATGTGGATCACTTTTTTTGTTTCTCTTCTTTTGTATCTTGTCAAAAGGAAGCTGCCACTGTAATTACACCAAGCACAGGAGTGCACAATGACTTGGGAGGTCCCTCATTTAGGGAATTTGCTGAAGGGAGTGAATAAGATGCTGTCAGTTTATCTTAGATTCAAGTTCCACTTTTGACTACTGACTTTTAAGGCAACGATTAAATGCAAGATTTTC

>LNC_002110

TGTCACACTACCCGATCATGTAAGAGGAGGTGATTGACATCTTCACCCAAATTTTGCACATAAAGCAAGGAAGTAAGGGATAAAAGGTGTGCCATGTGCCTATCACTAGATTTGTCCTATGATTTTGTATGTATTAGATTATGAAAGAATGTAATTGAATAGTTAGGAAACAAGCCAAACGATTTAGTGATATAAGATTAAAGGGATAACGTGGAACATAAAGAAGATAATTTAAAATTAAAGAGGGTTAGGGCTTTTCAAGAGCAACTCTAGTTGACTTTGGGATCCTTTTGCGAAGGTGAATGGACAATGAGATCAACTTTAGTTGCCTTGGTTTGGAAACTGTTATTAAATAATGGTTGGTAAAAGAGATTTATCACCAGAAATACAG

>LNC_002111

GTTTGGAATACATGGAACAACTCAAAACAATGTTTGATCAAGTTTATTTTGGATACTCATTAATGAAGCTGGAAAAAAACTTTTTTTGATCAAGTAATAAGATTTTATAAACAAAGGGCATAAAACACATGCATACAAGAAGTATACCAAAACATAGAAAATCTTACAAAAAACGCACTCCTTTCATCCCATTTTATATGAGTTAGTTTGACTTGAGTCACTTGACATAGAATTTAAGAAAGAAAGATTTTTG

>LNC_002112

AGTAGATGCTGCAAGCTGACTGTCTTAAGTTACTAGGCTGTGATGAGATCGAGAGAAGGCAAACAACAGATCAAGATGTAGATCTGTTGTCAATTTGGGATCCAATTCTTGAGTTACTTCACTGCAGTAGTAGTAGTTCTTTTCATCTTTTTCCATCACGTATTTGTCATTTCAAGCTGAGGATAGTTCTAGATCGATGTAAATGGACTCGTATTAGCATTAAAAAAGTATTTGGTTTCTTTTCTTCATGTTTGTGTAATTGTAAATAAAGTGAGAATATAAATGACTTCTTAATTTTTGTTGTACATATTATCGTTCACACCCCTTAATAGAACTGTCG

>LNC_002113

GGAGGCATCCTGTAAGGATCATGTAAGTAGATGCTGCAAGCTGACTGTCTTAAGTTACTAGGCTGTGATGAGATCGAGAGAAGGCAAACAACAGATCAAGTTACTTCACTGCAGTAGTAGTAGTTCTTTTCATCTTTTTCCATCACGTATTTGTCATTTCAAGCTGAGGATAGTTCTAGATCGATGTAAATGGACTCGTATTAGCATTAAAAAAGTATTTGGTTTCTTTTCTTCATGTTTGTGTAATTGTAAATAAAGTGAGAATATAAATGACTTCTTAATTTTTGTTGTACATATTATCGTTCACACCCCTTAATAGAACTGTCG

>LNC_002114

ATGCATCATCATGATAAATTAGAGAGATATAATTCCTTTGATAACAATATGTCTACTAGAAGTTTTATATTCATGTTAGCAGTTGTTCCTGATTTCGACATGCATTATGTCTTTTTGATGTATTTTGTGTGGATTAACAACTAATTTGAGGCGGATAATGCACCTAATGTATTTGATAATATGTTTGTCAGAGATGGAATCTAAACTTTTGTATTCTGTTGATTTTGAATGAAACATGTAAAACAATACACAAATTCGTTGGGCAAGTTTTCTCAATTGCCTCTTTCCCTTCTTAGTGTGTAATCTTGAGCTTAAAGTTATCTAATACTCATATTTTTAGATTGTAATTGAGGATTCTTATAATAGTTCTTGAGATTCTCTTTTGCTAATTTAGGAACATGACAACAAATAT

>LNC_002115

ACTGCAGTAACTCTAGATGATCCATTTTTTCTCTTGAGTCAGGCCAGTTTCCTCAGAATTTGTTCGTTCATGCAACTTTAAGAACCTTACTGCACCACATGGAGATGTGAGAATCATCTTCATTGTTATCCAGGTGCTAGTCAAAATGGAACCTAATCTCCCCCTCTTTTGTGTCTATTTGTCTTGAACTGAGAATCTTGACACAAAGGACATTGTTAAATTATGTCTTGTATTCTCACTGTTTGAAATGGCTGCTGCTGC

>LNC_002116

GTTTCAATTAGTTAAATATTAAAAAATAACTAAATTGAGAATTATAATATTTAAATGTACACAAAAATATATTACCAATTTGAGAAAAACCATGTAACCAGTTTCGAGTAGCAACAAGTGTTTGGACATTTTCATGGTGGATGCAACTTCTATACTTAGTGATTTCCTAAAAAACTTTAAATAGATTTTGAGTTTGGATCTCTTTAATTTTAAATTTTAATATTATATTATATTTTTTAATTAATTTGTATTAGCCCACGGGCCGGCCCTACCTATATTTCTCA

>LNC_002117

TTTCCTTGTTAGCATTTCCCCTTTTTTCTTTATTTCGCTCCATTTCTGGACAGTTGACCCAAATTACAAGAAACTCTACTTTGAGGCTAATCTTCCCATTCAGTCTCCATCTCCGGCAGTCGCCTCTTCCTCGTAACGCTAGGGCTCCTTATTCTGGGCAACTCCGTTCCCATATCAGAAGGCTATAGTCTCTTGGATTTTGGTTTCCAAAGTTTGCCATACCACACCTTAAAGCAGCTAATGGAAATTTGAAGTCACTGTG

>LNC_002118

TATAGACGAAAGTATAGTTGTAAAAGTATACCAGCTAAGACGAAAGTATAGTTGTAAATTTATACTAGCTATAGACGAAAGTATAGTTGTAAAAGTATACTAGCTATAGATGAAAGTATAGTTGTAAAAGTATACTAGCTATAGTATCTAACTTGGCCCCTGGATTTGCTTCAATGGCAAAGATAGTATAGCGCGAGATGTGTGGTAGGCGCACTTCAGTGGTCAAATCCTATTGGTTGTGAAGTCTGATGTACAAGAGGAGAAACTAGATGGCGAACCTATTATCCACCAAATTTCAGAACTGATATCAGAAAATTTCATGGTTATAACAAAAAAGAATCTAACTTATGCTACTTATTATTGGATGGTGGCTCCTCCTTCACATTAGTGTTTAACTGTGAG

>LNC_002119

TTCCGCAAATGGTAAAGCAGTCGGTGATTTCGCCGGAGTTGGAGACGTTATCAGCCGGTGAGGAGGAGTCCGGTGATGAAGTTGGAGATGGAGAGGGTTGGGTCGATTAGGAGGACCCGAGTTTGATTCTTCATTCTGGAAATACTTGGCAAGGAAAACTTTGGCATGTGGAGCTACAACGAAAGCAGCAGAAATTTCTCCCTTCTCAAAAGCTTTAGGGTAGTCACTGATTGAACTGATCTTCTTGATGTTCTCTGGCTTGAATTGCAAATTTACTAGATATTTG

>LNC_002120

GCACATAATTTTCAGACCCAAAACGAAGATGGCAAATTTCATGAGTTCAATCTTATTGATGGTGCTATCGCAGCTAACAATCCGACCTTTATTGCTATAAGTGAAGTGACCAAACAAATATGCGAACAAAACCCATATTTCAAAAATTTCGACGAAATGACGCCCACTAATTATGGGAAATTTTTGGTTATCTCCATTGGGACTGGATCAGCAAAATTGGAACATAAATATGATGCTAAAATTGCATCAAATTGGGGTATTTTTGGATGGCTAACTGGTGGAGGCTCAAATCCAATTATTGATGCATTTGCTGATGCGAGTGATGATATGGTTGATTATCATATTTCTGTTGTTTTTCAATCTATCTCTTCTCAAAAACAATATCTTCGGATTCAGGTACTATATATTCCACTCCTAAATATTCTTTCAATTTTAAAAATATAACAAACTTTCCCTTTTTAGTTTATTTTTAAAAAAAAGATCCTTTCAATTTTTTTAGCAACACTTTAATTTCAGTTTTCTAGGCGACATGTTTAATGCCACAAGATTAAAGAGTATTTTGGTACATTTGACATAACTTTAATTTAGGACCACAAGATTGAAACGTTTCTTTATTTTCTTAAATTTTGTGTCAAGTCAAACTAAATCATTCTTTTTGAAACGGAGAGAGTATCAAATGTGCTTGATCAGAAACAATAAGGACCAAAATCAAAATTTGTTAATATTTGAGGGATTAAAAGTGCTAATTTCCTAATTGAAATTGTTTCTAAATATTTGAAGTTATTGAAAAAGAAACATTTTAAACGAAGCCTTGTTATTTCGATTACTCGAGTATTCGTCAAATGAGAAATTCAAGAAGAGATAAATGATAGCTTAGAAATATGCTCTTATGATCTAGGTATAATACTTGAGGGTGAAGCGGTTTTTTTATAAAGGCAAATTCATATTCAGAATTTGATTTTGAGACGTATAGAGTATCTATCACTTTATTGAAATGCTAGATATTACT

>LNC_002121

AACCAACCCCACCCACACGCTCTTCTTCTTCACAGCACGATGAAACCCAGAACAGTCCAGCAACGCAACTGGACCGAACGCGAAGAACAGCGAGTTCACGGAACCAGCAGCTTTCTCCGTCTTCTTCCTCTGCCAGAGCAGTTCGAACAACCTTTCTCTGCAACAGCCGCCCAATCCCTCCGTCCAGATTTCGTGGGATTTAGTTGAAATATCGCACAAACCAGCCTTTTCTTGGTCCTTGGGAAGTTTTGAATTTTGGGAATTCGTGGGGAATCTCGATTTTACCCTTGTTCTTATTCAAAGTCCCTTCCGTTTTCCCCCTCAAAAGGAACCCGAATTTTCAATACATCTTTTCTTGTCATTTTTAGGGGTGGAGTTTTTTTTGGTTGAAAACTTTTAGAGCAAAAGGTATTGAAAAGCAATTAGCTGCAATATATATATAGAAATACATTTCAAAGAAAACCAGAGGAGGCACTTATTTTTCTTTAGTGTTTGGGTTGATATTTCCGCTCAAGCATCTTGCTTGTTGTTTGTGTTCGATTCCTAACGTGGTGAAGGTGTCGTTCTGCTCGTTTCCTGTCCAATTTGCTGCACCTTAAAAGGCCCAAATTTGCTCTTCTTACTCGAGTCAGCCCCTTTGGCAAGTTGTGAGACTGACGTACAGGTTGAGGTTGATGAACGTGGGCTGGAAATTTAATTTGCAAGCCTCCAAAAGCTCAAAAAATAGCTGTATACCTGCTGTATACCGCAATATACACTGCTGTACAGCAATTGTACACATGATATACAGGGCAGAAAAGTGAAATGCAGGTTGCAAGGGGTCAAAATAGACTGTATATCCTCAGTATACAGTCCGATATACACGAAAAATGGGTCAAAACCCAAGACGATCAGCAGCTGAAGGTCCAAAGTCGGTCCAGGCAAATAGAACCTGAATTTTAACTATTTGAATATTTAATTAACTTAATTAAATCCTCAAAAGATGAACCATATGCTTTATGTTAGATATATTTCAATAAAGTTTCTTTTTTATCAACTCTTGTACCATTATTGAAAACTTTTACTTGATTTTCGATTAATTTAAAATAGTTTACATAATTTAATTGACTAAATGGGTCTAAATTATTAGTATTTCTTTTAAAAAAAGAGACTCAAATAAAAATTC

>LNC_002122

ACGTGAGGTGGGGCCCTAAGTCGGTGGATTGTTACATATATCTTCTTCTTCGATCTTTTTCTTCATTTTTAAGTTAGAAACCAGAGAAATTAGAGAAACCCATTTCTAGGATCAAGTTAAGTATAGATTTTCTCGACTTAAGTCCTTGACGAAAGTTGTTCTACGCGTTGGGCTCATCATCGTGGTATAATTTGTTTTATCGATTGCGTAGCATATCACATTTTAGAGTTGGGACAGCAGGGGTTTGAATATATTTGAAGGTTTTGAGGTGAATTACGAACCTAAGATCGAGTATCGAGGGAGTTATGAGTAAAAGTCGTTAGGCCACGTGAATTTAGCGGATAAAAGGTATGTTAAGGCTATTCCCTACTTAC

>LNC_002123

CACCATTTGAGGTCATGCTCCATATACACTACATCCATTTCTCATCTCCCTCTAAAAATTCTATTTACATACATAATAAGAAGAAGAAAATCAAGATTACCAAAAAAATGGGAACTAAGTAAATTGGATAAAAATTTATATTTTCCCTCTGAGGAGATCGTCATAAATAAACTCTTTTTAAGCTGTAACTAGTTATTCTCTCTTATGACGGATATAGCTATCTTTTCTTTTTAACTTTTTCAAAATTAGTAAGTTTTTCAAGCATTGTTTTATCATTCTATTTTTAAAATAAAAGGGTAAAAGTTGAACACAAAGATAGAAAATAAAAATCTTACAAATGTTCAATTAAAATTTAAAGAAACTTTTGTAAGTATGAAAGATTTTTGGC

>LNC_002124
[truncated: 890,042 more chars]
