# Supplementary material for: Genome-Wide Analysis of Long Non-Coding RNAs in Potato and Their Potential Role in Tuber Sprouting Process
Source: Int J Mol Sci. 2017 Dec 29;19(1):101. doi: 10.3390/ijms19010101 (PMC5796051; doi:10.3390/ijms19010101)
Supplement: Supplementary file 1 [file ijms-19-00101-s001.zip › Supplementary Materials/Supplementary Figure S2.doc]

**Supplementary Figure S2.** Hierarchical clustering analysis of differentially expressed lncRNAs target genes shown in Supplementary Table S3. The colour scale indicates the log_10_ (FPKM + 1) values. Red and blue indicate high and low expression of genes, respectively.
